# Immunosignature Analysis of Myalgic Encephalomyelitis/Chronic Fatigue Syndrome (ME/CFS)

**DOI:** 10.1007/s12035-018-1354-8

**Published:** 2018-10-08

**Authors:** Oliver P. Günther, Jennifer L. Gardy, Phillip Stafford, Øystein Fluge, Olav Mella, Patrick Tang, Ruth R. Miller, Shoshana M. Parker, Stephen A. Johnston, David M. Patrick

**Affiliations:** 1 Günther Analytics, Vancouver, BC Canada; 2 British Columbia Centre for Disease Control, Vancouver, BC Canada; 3 School of Population and Public Health, University of British Columbia, Vancouver, BC Canada; 4 Biodesign Institute, Arizona State University, Phoenix, AZ USA; 5 Department of Oncology and Medical Physics, Haukeland University Hospital, Bergen, Norway; 6 Sidra Medical and Research Centre, Doha, Qatar; 7 Centre for Health Evaluation and Outcome Sciences, St. Paul’s Hospital, Vancouver, BC Canada

**Keywords:** Myalgic encephalomyelitis/chronic fatigue syndrome, Immunosignatures, Peptides, Diagnosis

## Abstract

A random-sequence peptide microarray can interrogate serum antibodies in a broad, unbiased fashion to generate disease-specific immunosignatures. This approach has been applied to cancer detection, diagnosis of infections, and interrogation of vaccine response. We hypothesized that there is an immunosignature specific to ME/CFS and that this could aid in the diagnosis. We studied two subject groups meeting the Canadian Consensus Definition of ME/CFS. ME/CFS (*n* = 25) and matched control (*n* = 25) sera were obtained from a Canadian study. ME/CFS (*n* = 25) sera were obtained from phase 1/2 Norwegian trials (NCT01156909). Sera from six healthy controls from the USA were included in the analysis. Canadian cases and controls were tested for a disease immunosignature. By combining results from unsupervised and supervised analyses, a candidate immunosignature with 654 peptides was able to differentiate ME/CFS from controls. The immunosignature was tested and further refined using the Norwegian and USA samples. This resulted in a 256-peptide immunosignature with the ability to separate ME/CFS cases from controls in the international data sets. We were able to identify a 256-peptide signature that separates ME/CFS samples from healthy controls, suggesting that the hit-and-run hypothesis of immune dysfunction merits further investigation. By extending testing of both our signature and one previously reported in the literature to larger cohorts, and further interrogating the specific peptides we and others have identified, we may deepen our understanding of the origins of ME/CFS and work towards a clinically meaningful diagnostic biomarker.

**Electronic supplementary material:**

The online version of this article (10.1007/s12035-018-1354-8) contains supplementary material, which is available to authorized users.

## Introduction

Myalgic encephalomyelitis/chronic fatigue syndrome (ME/CFS) is a complex syndrome whose symptoms include extreme fatigue that fails to improve with rest [1–4] and for which a specific cause remains elusive. Various working etiological hypotheses have been proposed, implicating roles for viruses, bacteria, environmental triggers, immune dysregulation, and mitochondrial dysfunction, but because the biological pathways leading to the syndrome remain poorly defined, there are no reliable biomarker-based tests for ME/CFS and diagnosis is on clinical grounds. Some have concluded that ME/CFS is a functional somatic disorder and that biomarkers may not be forthcoming, but such writing predates next-generation sequencing—a powerful tool for etiological agent discovery—and the observation of metabolic differences between cases and controls [5–7].

Mounting evidence suggests a role for autoimmunity or dysregulated inflammation [8], including the epidemiological over-representation of females, aberrant cytokine expression in early disease and in cerebrospinal fluid [9, 10], NK cell dysfunction [11] and the observation that autoantibodies directed against some beta-adrenergic and M-acetylcholine receptors are elevated in ME/CFS cases [12]. Unfortunately, encouraging phase 1 and 2 clinical trials of B cell depletion using rituximab [13–15] has been followed by reports of a negative phase 3 trial (not yet published) [16, 17]. The search for specific immunological biomarkers could benefit from a high-throughput platform able to interrogate a broad spectrum of antibody expression.

One prevalent etiological hypothesis is that of “hit and run,” whereby a pathogen or other immunological insult experienced by a subject may be gone, but leaves behind physiological disequilibrium [18]. In our initial studies, we employed metagenomics for pathogen discovery and RNA-seq to explore differential gene expression—both techniques that would pick up the “hit” but perhaps not the “run.” Indeed, we did not identify important differences between ME/CFS and healthy participants that would explain pathophysiology [19–21]. However, if the hit and run hypothesis were true, we might expect a difference in the adaptive immune profile—the “run”—of ME/CFS cases vs healthy controls and other disease groups.

To explore this hypothesis, we turned to an immunosignature assay (ISA) that employs a microarray of thousands of random-sequence peptides to interrogate antibodies in a broad and unbiased fashion. ISA technology has been applied to cancer detection, diagnosis of infections, and interrogation of vaccine response [22–25]. We hypothesized that ISA might identify differences in the adaptive immune history of ME/CFS cases vs healthy controls from our own case-control study, but also that an ME/CFS immunosignature may be generalizable to people with ME/CFS from different parts of the globe, such as those participating in the Norwegian clinical trials of rituximab treatment for B cell depletion [14, 15].

## Methods

### Canadian Complex Chronic Disease Study Samples

The Complex Chronic Disease Study has been described elsewhere [19], and includes 25 ME/CFS subjects meeting the 2003 Canadian Consensus Definition for ME/CFS (all of whom suffered post-exertional malaise with extreme fatigue severity scores) [26], and 25 age- and sex-matched healthy controls. The protocol was approved by the University of British Columbia’s IRB (H11-01998) and all subjects gave written informed consent to participate. Upon consent, serum was collected at baseline for serological tests and stored at − 20 °C until thawed for use. Samples were diluted 1:1 with reagent-grade glycerol plus 0.025% sodium azide to prevent freeze-thaw cycle damage.

### Norwegian Rituximab Study Samples

A pilot study and two phase-2 studies of B cell depletion using the monoclonal anti-CD20 antibody rituximab for treatment of ME/CFS have been previously described [13–15]. In the present analysis, we used pretreatment sera from 25 individuals drawn from the pilot study and the KTS-2-2010 single-center, open-label, one-armed phase II study (NCT01156909) [16], in which subjects received rituximab (500 mg/m^2^) infusions 2 weeks apart, followed by maintenance rituximab infusions after 3, 6, 10, and 15 months, and with follow-up for 36 months. The study was approved by the Regional Ethical Committee in Norway, no 2010/1318-4 and by the National Medicines Agency, and all subjects gave written consent to participate.

In this study, subjects improving according to the predefined criteria in the protocol were characterized as responders. For the analyses in this manuscript, we used only the pretreatment samples from subjects in the trial. The biobanked samples were aliquoted before freezing at − 80 °C. Samples of 100 μl were diluted to a final concentration of 50% glycerol for transport to the testing laboratory. Samples were shipped at − 20 °C, at which temperature they were kept throughout.

### American Healthy Control Samples

Non-affected control samples were obtained from Clinical Testing Solutions (Tempe, AZ), a national blood testing laboratory. Samples were stored at − 20 °C until use. Healthy samples were obtained from multiple locations throughout the continental US and consisted of blood donors who were negative for the presence of infection. We selected samples based on age (30–62 years of age) but not gender, race, or geography.

### Laboratory Methods

Deidentified samples were received and kept frozen at − 20 °C until use. The immunosignature arrays were synthesized and completed as described previously but used 125,000 peptides rather than 330,000 [22]. Peptides were 12 amino acids long and were composed of 16 amino acids, excluding threonine, methionine, isoleucine, and cysteine. Microarray slides were blocked with 1 mM PBS, 3% bovine serum albumin, 0.05% Tween 20, 0.014% mercaptohexanol for 1 h at 25 °C in a darkened humidified chamber, then sera were diluted in 3% bovine serum albumin, 1 mM PBS, 0.05% Tween 20 pH 7.2 to a 1:500 dilution for mouse and human sera, and allowed to bind for 1 h at 37 °C at 20 RPM rotation. Slides were washed 3 × 5’ with 1 mM Tris-buffered saline, 0.05% Tween 20 pH 7.2 followed by three washes with distilled water. Once incubation was completed, the slides were dried by centrifugation at 2400*g*×10’ and scanned by an Innopsys (Carbonne, France) Innoscan 910 0.5 um 2-color scanner. The images were stored as 16-bit uncompressed TIFF’s, aligned using GenePix Pro 6.0 (Molecular Devices, Santa Clara, CA), and stored in a local relational database prior to analysis. Analysis was done using R (CRAN) and GeneSpring 7.3.1 (Agilent, Mt. View, CA). Serum antibodies were detected by labeled secondary antibody. Labels included either Alexafluor 555 or 647. Secondary antibodies were incubated at a concentration of 5 nM for 1 h at RT. Single-color experiments were performed exclusively, but dye choice depends on availability, usually either Innova Biosciences (Cambridge, UK), Life Technologies (Madison, WI), or Jackson Labs (Bar Harbor, MA).

### Data Preprocessing

For each sample, data for 122,926 peptide abundances were available, ranging in value from 0 to 65,535 where 65,535 represented the upper detection limit of the 16-bit digitizer. Samples were typically run in duplicates and data processing included control peptide averaging as well as replicate sample testing for outliers before replicate samples were merged (Online Resource 1). In cases where one of the replicates was an outlier sample, the corresponding replicate pair was removed from the analyses (*n* = 8). Replicates that passed the outlier testing (*n* = 78) were merged by calculating the arithmetic mean of peptide abundances for each of the 122,926 peptides. Samples run as singletons were removed (*n* = 2) except the six American Healthy Control group samples that were all run as singletons. Each sample was then median-centered by dividing each peptide abundance by the median value over all peptides for the corresponding sample, followed by a log2-transformation of the data. The median normalization and log2-transformation put the median peptide value for each of the processed samples at zero.

### Data Partitioning

**Fig. 1:**
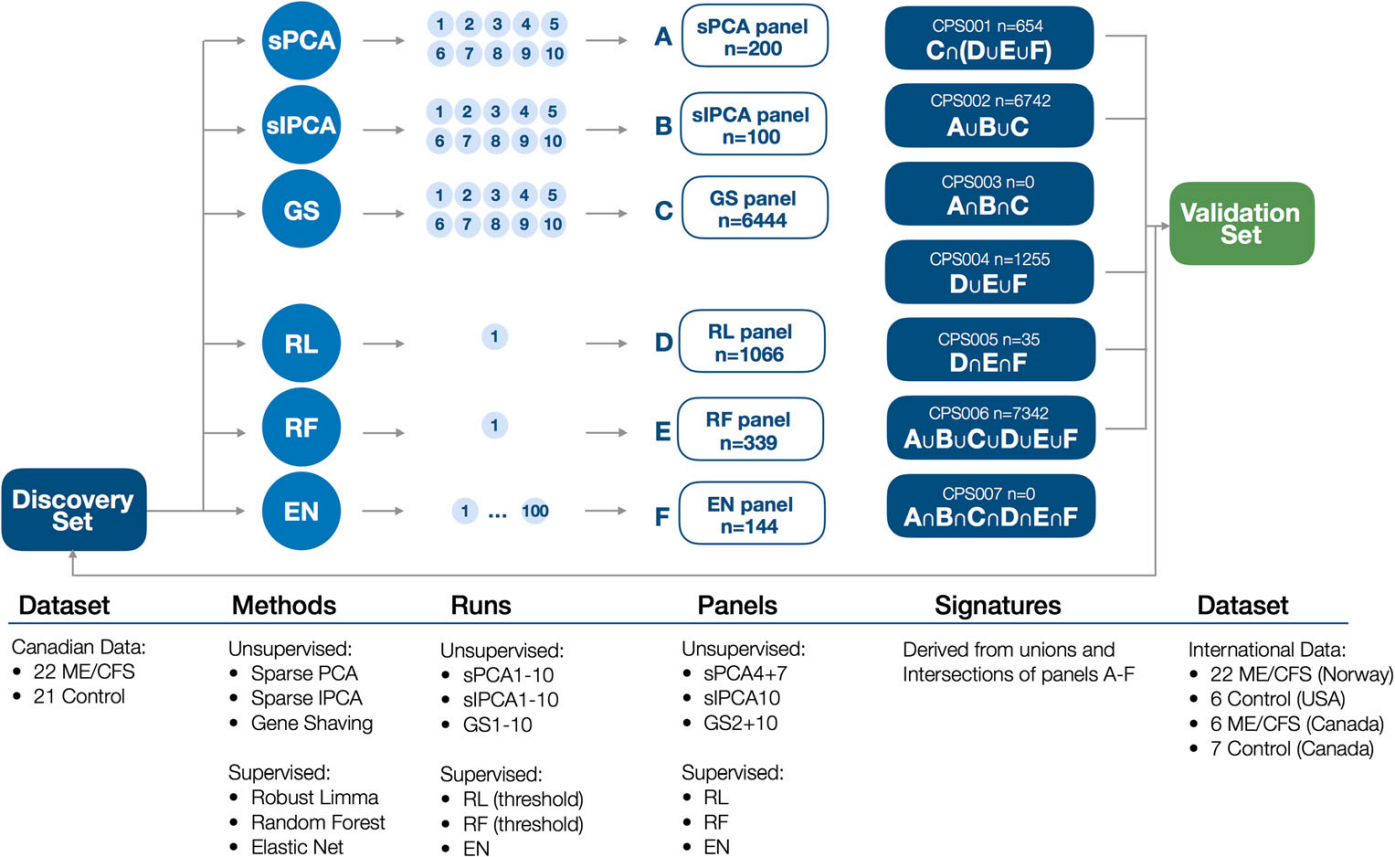
Analysis overview

After replicates were processed, the 84 samples from the three data sets—Canadian, Norwegian, and American—were used to create two data partitions: one for immunosignature discovery and one for immunosignature validation (Fig. 1). The data partition used in the discovery analysis (“Discovery Set”) comprised all Canadian ME/CFS (*n* = 22) and control (*n* = 21) samples. The validation data partition (“Validation Set”) included all Norwegian ME/CFS samples (*n* = 22), USA control samples (*n* = 6), and a subset of randomly selected Canadian ME/CFS (*n* = 6) and control (*n* = 7) samples, and was intended to evaluate each immunosignature’s potential to distinguish ME/CFS cases from healthy controls. Samples in the Validation Set were run in an immunoassay experiment separate from the samples in the Discovery Set. The Canadian and USA control samples were included in the Validation Set to compensate for the lack of Norwegian control samples and still being able to characterize the immunosignatures’ ability to distinguish cases from controls. Canadian cases were included to confirm separation of Canadian cases and controls in the Validation Set.

### Discovery Analyses

We performed all analyses using scripts implemented in R version 3.3.2 [27]. In the Discovery Analysis, we derived robust candidate peptide signatures based on unsupervised and supervised univariate and multivariate analysis methods as shown in Fig. 1, including PCA, hierarchical clustering, gene shaving, elastic net, and random forest [28–32]. The unsupervised analyses were carried out blind to group status while the supervised analyses used group status directly in the supervising vector.

Three unsupervised and three supervised analyses were run on the full 122,926 peptide dataset to select peptides best able to discriminate ME/CSF from control samples. Amongst the unsupervised methods, we set our sparse PCA (sPCA) and sparse IPCA (sIPCA) analyses to select 100 peptide features, while the gene shaving (GS) method automatically selected features. Each of these three unsupervised methods were instructed to return ten lists (sPCA1-sPCA10, sIPCA1-sIPCA10, and GS1-GS10). For each set of ten lists, the ability to separate ME/CFS cases from controls was reviewed, and subsets of peptide lists were selected and combined into three panels of peptide features: sPCA_panel, sIPCA_panel, and GS_panel.

The supervised methods used different feature selection approaches. Robust limma (RL) used a threshold for the adjusted *p* value and returned all peptides at or below the threshold. Random forest (RF) used internal bootstrapping to calculate feature importance measures that indicated how classification performance of the random forest was affected when the respective peptides were excluded from the analysis, and a threshold was chosen for a minimum required “Mean Decrease Gini” value to select peptides. Elastic net (EN) used internal cross-validation for parameter estimation and automatically performed feature selection. The list of peptides selected by this method was determined by a frequency-based approach that returned all peptides that were observed in at least 10% of elastic net panels over 100 runs, where each run was based on 39 samples with two ME/CFS cases and two control samples removed at random in each run. The supervised methods returned three panels of peptide features: RL_panel, RF_panel, and EN_panel.

The six panels (A–F) were then combined using different intersections and unions (Fig. 1) to define seven candidate peptide signatures (CPS): CPS001–CPS007. To characterize the predictive ability of these signatures, we calculated the area under the receiver-operating-characteristic curve (AUC) from signature scores defined by the mean signed log2 median-centered peptide abundance where the sign was determined by the sign of principal component 1 of the signature and known group labels [33].

### Validation Analyses and Signature Refinement

The seven candidate peptide signatures (CPS001–CPS007) were evaluated using the Validation Set, with the results used to select the most robust discovery signature. This signature was then further refined based on the ability of its individual peptides to separate samples from four different comparisons based on two-sample *t* tests (assuming unequal variances) using the limma package in R [34]. Our four comparisons were (i) 22 Canadian ME/CFS vs 21 Canadian control samples in the Discovery Set, (ii) six Canadian ME/CFS vs seven Canadian control samples in the Validation Set, (iii) 22 Norwegian ME/CFS vs seven Canadian control samples in the Validation Set, and (iv) 22 Norwegian ME/CFS vs six US control samples in the Validation Set. The final refined signature—CPS0001A—included only those peptide features whose *p* values for each of the four comparisons were less than 0.05. AUC values were derived from absolute values of peptide weights for principle component 1 (PC1) based on a PCA of peptide-standardized data (122,926 peptides). Higher AUC values indicated a stronger contribution of the tested signature to the separation of samples along PC1. In addition, PCA and hierarchical clustering approaches based on proposed signature were used to cluster validation samples in a blinded fashion, without the use of group status.

## Results

### Discovery Analysis for Candidate Peptide Signature Selection

In the discovery phase of our analysis, we used multiple statistical approaches to find sets of peptides that differentiated ME/CFS samples from healthy controls in our Discovery Set of 43 Canadian samples. Supervised and unsupervised approaches yielded 33 peptide lists—three from supervised approaches and 30 from unsupervised approaches, with varying degrees of overlap (Supplementary Table 1 in Online Resource 1). When we calculated area under the curve (AUC) values for each of the 33 lists (Supplementary Table 2 in Online Resource 1), reflecting their ability to differentiate case and control samples, AUCs were highest for the lists derived from the supervised robust limma, random forest, and elastic net methods. Interestingly, two of the gene shaving lists (GS2 and GS10) were strongly correlated with RL_panel and RF_panel, and displayed the largest AUCs of all of the peptide lists from the unsupervised analyses.

**Fig. 2:**
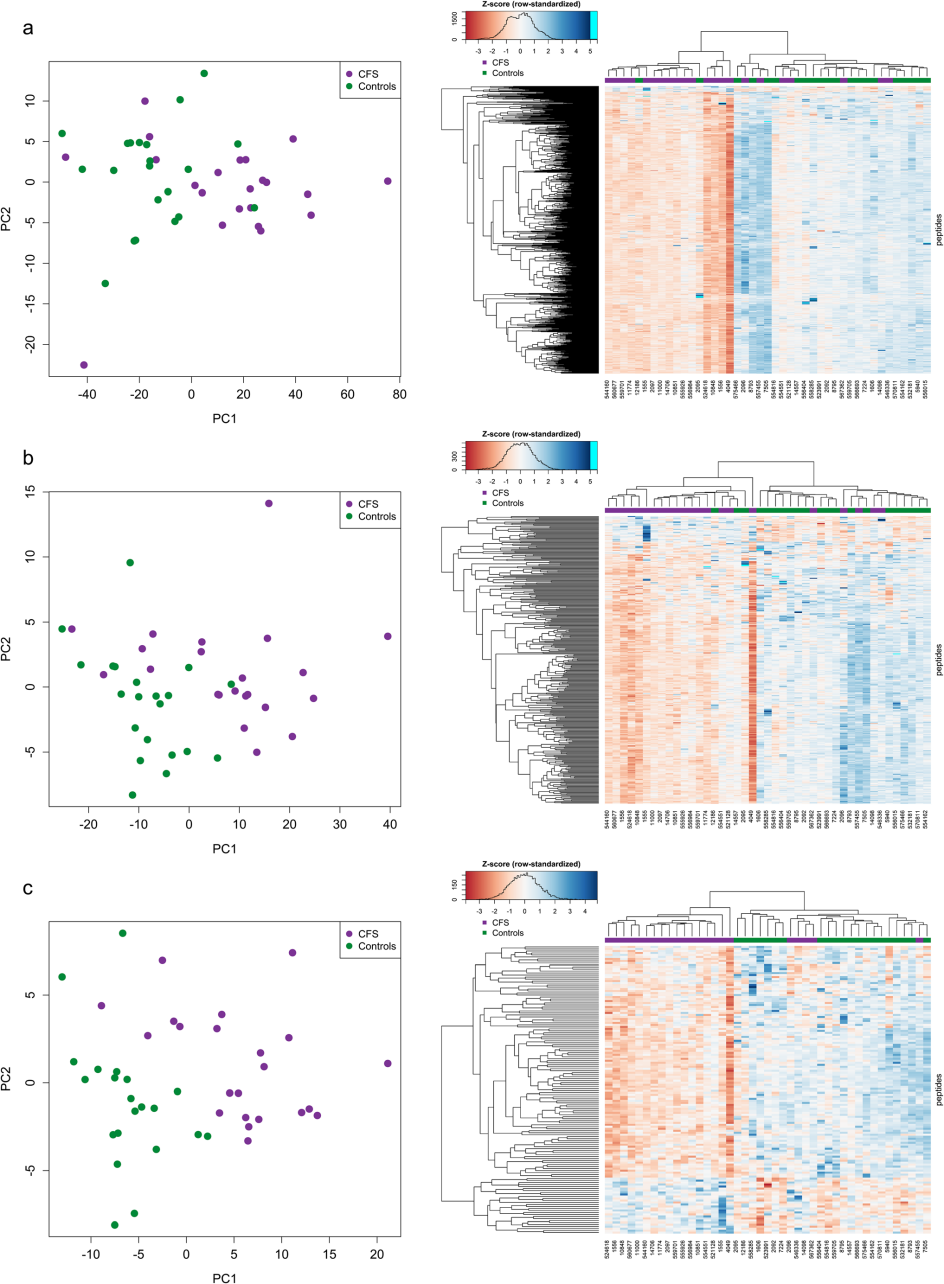
PCA projection (PC2 vs PC1) and unsupervised clustering (heatmap) results for the three peptide panels derived from supervised analyses on the Discovery Set of 43 Canadian ME/CFS and control samples (**a** RL_panel, **b** RF_panel, and **c** EN_panel). PCA plots and heatmaps are based on row-standardized data (Z-scores), where for each peptide, abundances had their mean value subtracted and were divided by the standard deviation

Considering only the three peptide lists resulting from the supervised approaches—RL_panel (1066 peptides from robust limma), RF_panel (339 peptides from random forest), and EN_panel (144 peptides from elastic net)—PCA projections and heatmaps show a visible, albeit imperfect, separation of ME/CFS samples and healthy controls (Fig. 2). Along PC1, five of 22 case samples cluster with controls, while two of 21 control samples cluster with cases across all three signatures. One ME/CFS sample consistently displayed the largest PC1 value and is prominent in the heatmaps. Of the three panels, EN_panel was best able to separate ME/CFS samples from healthy controls in the PCA projection (Fig. 2c). It also had the largest AUC of all 33 peptide lists in (Supplementary Table 2 in Online Resource 1).

Selected peptide lists from the unsupervised approaches were combined into three peptide panels—sPCA_panel (200 peptides from sparse PCA panels sPCA4 and sPCA7), sIPCA_panel (100 peptides from sparse IPCA panel sIPCA10), and GS_panel (6444 peptides from gene shaving panels GS2 and GS10)—and were also analyzed with PCA projections and heatmaps. While sPCA_panel and sIPCA_panel performed poorly (Supplementary Figure 6 in Online Resource 1), GS_panel returned an AUC of 0.75, indicating an ability to differentiate cases from controls (Supplementary Figure 7 in Online Resource 1).

As the final step in deriving candidate peptide signatures, we combined the six peptide panels (A–F) in various combinations (Fig. 1). Having demonstrated that the panels resulting from the three supervised methods (RL_panel, RF_panel, and EN_panel) and the panel resulting from gene shaving (GS_panel) resulted in the best classification performance on PCA projections and heatmaps, we created CPS001 from the intersection of GS_panel and the union of peptides from the supervised methods (*n* = 654 peptides). The intersection of GS_panel with the intersection of peptides from the supervised methods was considered as an alternative definition for CPS001 but it produced a small panel of only eight peptides and was dropped in favor of the larger, presumably more robust panel that was also better suited for panel refinement in the validation phase. Signatures CPS002–CPS007 were defined as: the union of all peptides in the unsupervised panels (CPS002, *n* = 6742 peptides), the intersection of all peptides in the unsupervised panels (CPS003, *n* = 0 peptides), the union of all peptides in the supervised panels (CPS004, *n* = 1255 peptides), the intersection of all peptides in the supervised panels (CPS005, *n* = 35 peptides), the union of all peptides from all six panels (CPS006, *n* = 7342 peptides), and the intersection of all peptides from all six panels (CPS007, *n* = 0 peptides).

### Validation Analysis

**Fig. 3:**
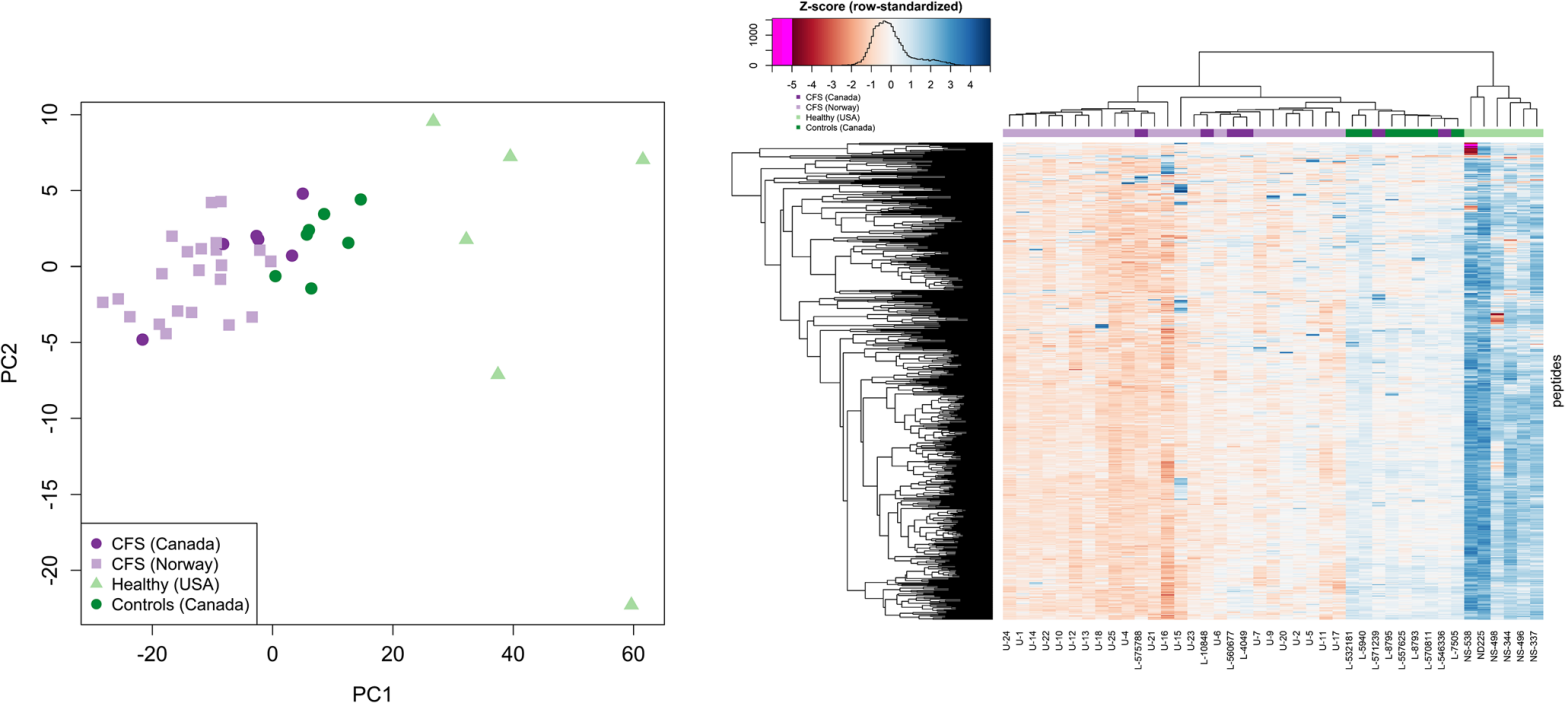
PCA projection and heatmap for candidate peptide signature CPS001 in the Validation Set

Excluding the empty candidate peptide signature panels CPS003 and CPS007 left us with five panels to evaluate in a series of validation analyses. Using the Validation Set, comprising 28 ME-CFS cases (22 from the Norwegian dataset and six from the Canadian data) and 13 healthy controls (six from the US dataset and seven from the Canadian data), we examined PCA plots, heatmaps, and AUCs for each signature. All five candidate signatures displayed a similar ability to separate ME/CFS and Healthy/Controls in PCA plots, but heatmaps and AUC values indicated that not all peptides in a signature contributed equally strongly to the separation. CPS001 showed the strongest validation performance (Fig. 3, Table 1, Supplementary Figure 9 in Online Resource 1), with an AUC of 0.82—similar to its AUC of 0.80 in the Discovery Set—while the other four signatures had AUCs below 0.75 upon validation. AUCs in the validation step were noticeably lower than those in the discovery step for the supervised signatures CPS004 and CPS005 (Table 1), suggesting that some over-fitting occurred in the supervised analyses.

**Table 1:** Area under the curve (AUC) values. Values are given for candidate peptide signatures in the Discovery Set comprising Canadian ME/CFS cases and controls, and the Validation Set VD0001 comprising Norwegian and Canadian ME/CFS cases and American and Canadian controls. Signatures CPS003 and CPS007 with zero peptides are excluded in the table

Signature (peptides)	AUC in Discovery Set	AUC in Validation Set
CPS001 (654)	0.80	0.82
CPS002 (6742)	0.75	0.74
CPS004 (1255)	0.83	0.74
CPS005 (35)	0.93	0.60
CPS006 (7342)	0.76	0.73

### Signature Refinement

Given the strong performance of CPS001, we next refined it using a peptide-by-peptide statistical analysis aimed at identifying those peptides within the signature best capable of differentiating cases from controls in the Discovery and Validation Sets. This returned a list of 256 peptides that defined signature CPS001A.

**Fig. 4:**
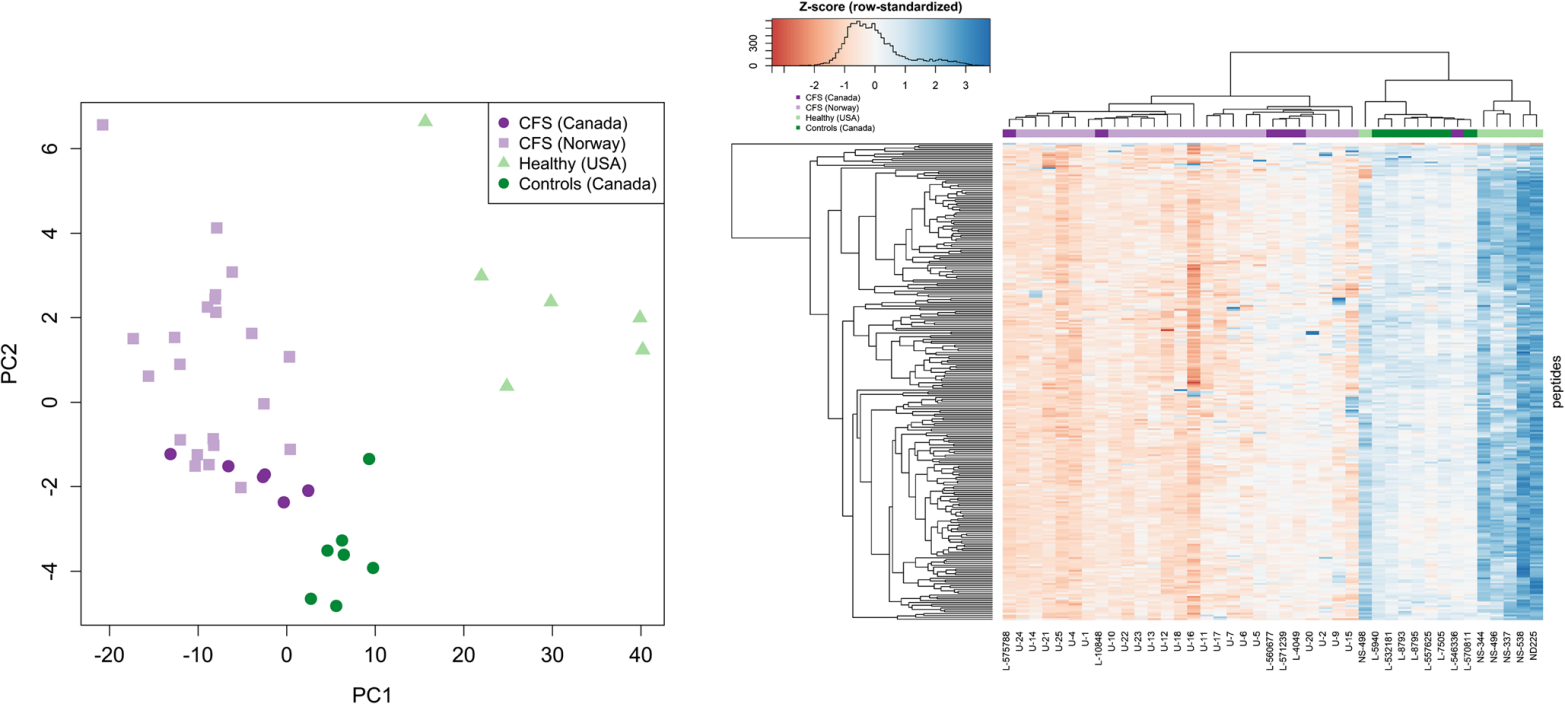
PCA projections and heatmaps for the refined signature CPS001A in the Validation Set

PCA projection and heatmap results for the refined signature CPS001A (Fig. 4) in the Validation Set show a clear separation of the Norwegian ME/CFS, Canadian ME/CFS, and Canadian control samples vs the American controls along PC1, but also a separation of all ME/CFS cases and Canadian controls along PC2.

## Discussion

If the “hit and run” hypothesis for ME/CFS is true, it may be possible to identify an immunosignature that clearly delineates ME/CFS cases from healthy controls based on their antibody repertoire. We therefore used a peptide-based assay to query the immune repertoire of participants in a Canadian ME/CFS case-control study. We then used a second dataset comprising ME/CFS cases from a Norwegian study and controls from an American biobank to validate the candidate ME/CFS-associated immunosignatures.

To avoid over-fitting and obtain a robust classification signature, we combined supervised and unsupervised approaches to identify candidate peptide lists, panels, and ultimately signatures. In the discovery phase of our analysis, we found that supervised methods generally outperformed unsupervised methods in discriminating the two participant groups. This is not surprising, given that unsupervised methods are not instructed to find features that separate specific groups but rather identify groups of samples that cluster together for any reason. Supervised methods, with their reliance on sample labelling, can perform poorly on heterogenous datasets, and although we have previously observed some heterogeneity in our study participants [19], the present analysis yielded good separation between cases and controls.

Using heatmaps, PCA plots, and AUC values, we evaluated five candidate peptide signatures and selected CPS001 as our best candidate for an ME/CFE classifier, given its reasonable size, its roots in both supervised and unsupervised analyses, and its high AUC (0.82) in the Validation Set analysis. CPS001 was further refined by selecting a subset of 256 peptides optimally separating ME/CFS cases and controls in the Discovery and Validation Sets—CPS001A. These 256 peptides are positioned throughout a phylogenetic tree derived from the complete 125 k-peptide dataset (Supplementary Figure 11 in Online Resource 1), indicating the signature is comprised of diverse peptides and is not reflecting any underlying bias in the design of the array. However, this diversity does not exclude the possibility that these peptides represent immune response to a common antigen—the genetic distance between peptides as represented in a phylogenetic tree does not reflect the physical conformation of epitopes bound by common antibodies.

In order to make a valid interpretation of the sequence evaluation of selected peptides, it should be noted that the peptide microarray was created using sequences selected from random space. All possible peptide sequences were created in memory, then compared to each other to select the broadest coverage of 3mer, 4mer and 5mer space, while reducing redundancy as much as possible. When the library of peptides was compared by BLASTP to the latest UniProt UP000005640 proteome consisting of 73,112 sequences, there were 7,051,312 unique hits covering 32.5% of UP000005640. Ninety-eight percent of these hits were 2mer or 3mer perfect matches. Thus, the library is not designed to exclude sequences of natural origin, and any alignments would be by pure chance. At this stage of development, the immunosignature assay is not designed or optimized to allow accurate inference back to source proteins.

A recent publication using the same immunosignature platform proposed a 25-peptide ME/CFS signature [35]. There was no direct overlap between this signature and CPS001A, and little overlap with our other candidate signatures (Online Resource 1). The exception was peptide LRVVWLSGVASG, which was found in four of our five candidate signatures, and which might be a good candidate for further biological exploration, as well as a set of similar peptides (EFRAKQWNSVAL, HVVWRVSGVALG, GWKNHRVLSGLS, RLRHLQSWVGVL, VQWWRPALGVAL, LRVVWLSGVASG, WGAVKVGVALSG, and WPRLHLSGVALG)—many containing a VAL or VAS motif—found in CPS002 and CPS006.

While our results suggest that it may one day be possible to use an immunosignature assay to diagnose certain cases of ME/CFS, the present study has a number of limitations. Importantly, the heterogenous nature of ME/CFS clinical presentation and the variance natural present amongst control samples means that group labels in the Discovery and Validation Sets are not based on any gold standard. Samples with the same group label might differ in certain aspects of disease or health, while some samples might represent transition stages between health and illness, disease variations, or diseases with a similar phenotype but a different underlying cause. We attempted to address this as best as possible by rigorous discovery analysis and validation; however, separation of cases and controls was not perfect for any of the supervised methods. Even the best research case definitions are often subjective and—in the absence of clear biomarkers—any group of ME/CFS cases likely comprise a heterogeneous set of pathologies.

Additionally, there is some variation in assay performance depending on the origin of the samples. While this likely reflects sample handling rather than geographic differences, it underscores the fact that should an immunosignature assay for ME/CFS or any other condition come to market, extensive clinical validation and proficiency testing will be required. Over the last 5 years, the immunosignature platform has evolved substantially. Current methods of in situ synthesis reduced the variability across manufactured lots. Synthesis efficiency has resulted in better sensitivity and specificity. The commercial manufacturer of immunosignature microarrays uses advanced robotics and automation which yields great improvements in consistency. In the context of MS/CFS, current manufacturing methods would support time-course experiments in single individuals which enhances the ability to identify potential immune fluctuations that presage or follow changes in symptoms.

In conclusion, despite a small sample size, we were able to identify a 256-peptide signature that clearly separates ME/CFS samples from healthy controls, suggesting that the hit-and-run hypothesis of immune dysfunction merits further investigation. By extending testing of both our signature and one previously reported in the literature to larger cohorts, and by further interrogating the specific peptides we and others have identified, we may deepen our understanding of the origins of myalgic encephalomyelitis/chronic fatigue syndrome and work towards a clinically meaningful diagnostic biomarker.

## Electronic Supplementary Material

ESM 1 (PDF 3052 kb)

ESM 2 (XLSX 40 kb)

ESM 3 (XLSX 268 kb)

ESM 4 (XLSX 19 kb)

ESM 5 (PDF 982 kb)

ESM 6 (PDF 385 kb)

ESM 7 (PDF 19 kb)

ESM 8 (PDF 19 kb)

ESM 9 (PDF 14873 kb)

ESM 10 (PDF 16 kb)

ESM 11 (PDF 16 kb)

ESM 12 (PDF 12693 kb)
