# Supplementary material for: Immunosignature Analysis of Myalgic Encephalomyelitis/Chronic Fatigue Syndrome (ME/CFS)
Source: Mol Neurobiol. 2018 Oct 8;56(6):4249–57. doi: 10.1007/s12035-018-1354-8 (PMC6505503; doi:10.1007/s12035-018-1354-8)
Supplement: Supplementary file 1 — (PDF 3052 kb) [file 12035_2018_1354_MOESM1_ESM.pdf]

Oliver P. Günther, Jennifer L. Gardy, Phillip Stafford, Oystein Fluge, Olav Mella, Patrick Tang, Ruth R. Miller, Shoshana M. Parker, Stephen A. Johnston, David M. Patrick

Online Resource 1: Supplementary Materials

This supplementary section contains additional information on data processing, replicate outlier detection, exploration of discovery and validation data sets, ME/CFS peptide signature discovery analysis, and exploration of an alternate ME/CFS signature.

Replicate Outlier Detection

Singleton samples (not run in replicates) were generally excluded from the analysis because previous analyses with other ISA datasets suggested that singleton samples show different characteristics compared to samples that were averaged over duplicates. The exception were six healthy control samples from the USA. Rather than excluding all samples in this group, they were included in the validation cohort. For samples that were run in triplicates, the two most correlated replicates were used for averaging.

Replicate outlier detection was based on Pearson and Spearman correlation coefficients that were derived from non-normalized and non-log-transformed replicate data, where peptides with at least one upper limit detection or zero value were removed[1]. The Pearson correlation coefficient has a direct relation to the commonly used R^2-value in linear regression, while Spearman correlation is based on ranks and provides a more robust estimate of correlation. Correlations were combined into an eight-criteria test, four each for Pearson and Spearman, involving median and 75% quantile for sample-specific and overall correlations. Duplicate-correlation was required to be above the correlation with other samples, ideally highest of all correlations but at least above 75% quantile in one duplicate and not below the median in the other duplicate. Samples were removed when duplicate correlation was below the median correlation or below median overall correlation for one of the two duplicates.

The above criteria were used together with visual inspection of correlation boxplots, peptide scatter, and distributions to determine outlier replicate samples. Additional criteria were applied in borderline cases, including a preference for a similar correlation distribution of duplicates with other samples (“parallel boxes” in correlation boxplots), similar peptide distributions for duplicates, and a preference for elongated shapes (along the diagonal) versus rounded data clouds in peptide scatter plots for duplicates. In addition, similarly derived plots and correlations based on all 122,926 peptides were consulted.

A stricter set of outlier definition rules was applied to the analysis samples in the Discovery Set than in the Validation Set, as outlier samples in the Discovery Set would impact the discovery of proposed signatures, while outlier samples in the validation data sets would have a lesser impact in validation analyses. Nine samples in the Validation Set were flagged as borderline but were included in the validation analyses (n=7 for Norwegian ME/CFS cases, n=2 for Canadian ME/CFS cases).

Exploratory Analyses

The Discovery and Validation Sets were explored with PCA and hierarchical clustering. Supplementary Figure 1 shows the percent variance explained by the first 10 principal components (PCs) for each of the datasets, while Supplementary Figures 2 and 3 show PC scatter plots for the first three components and heatmaps for each of the datasets separately. The PCA and scatter plots were based on all 122,926 peptides, while the heatmaps used the top 1% of peptides that displayed the largest absolute PC1 loadings. No apparent sample outliers were identified in the exploratory analysis and all 84 samples were used in the discovery and validation analyses.

The percent variance explained by the first principle component (PC1) is 25.10% in the Discovery Set and 29.48% in the Validation Set. The PC2 proportion of variance was more than half that of PC1 in the Discovery Set and about one-third that of PC1 in the Validation Set.

[1] The reduced data sets sizes were 85,987 peptides in 99 samples for the Discovery Set and 111,876 peptides in 94 samples for the Validation Set.

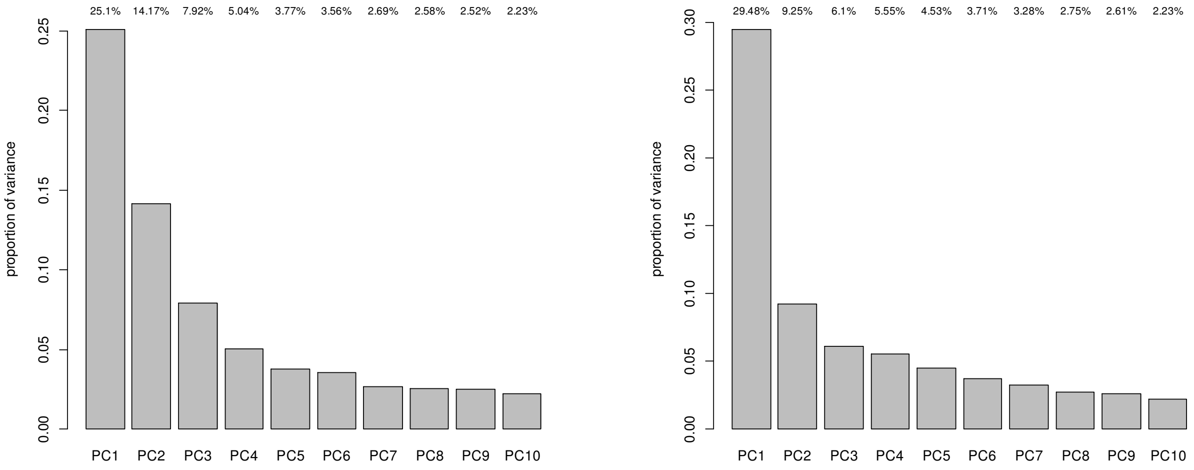

Supplementary Figure 1. Shown is the proportion of variance explained by the first ten principle components for a PCA based on 122,926 standardized peptides in the 43 Discovery Set samples (left) and 41 Validation Set samples (right).

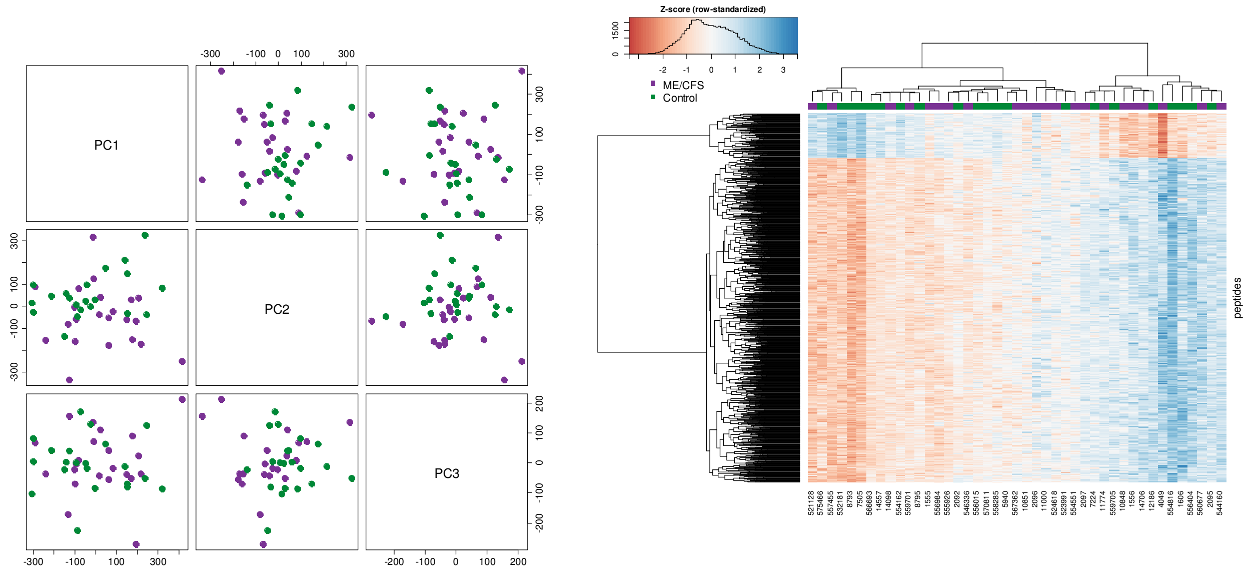

Supplementary Figure 2. Exploration analysis of the Discovery Set. Shown on the left is a scatter plot of sample scores for the first three principle components from a PCA on standardized, median-centered and log2-transformed 43-sample by 122,926-peptide Discovery Set. The heatmap on the right is based on 1,230 peptides with the largest absolute PC1 loadings.

The exploration analysis of the Discovery Set (Supplementary Figure 2) does not show any apparent clustering of ME/CFS vs Controls, though the 1,230 peptides in the heatmap (peptides with top 1% absolute PC1 loadings) clearly separate the samples into two groups with a transition zone in between, with each group representing a mix of ME/CFS samples and controls.

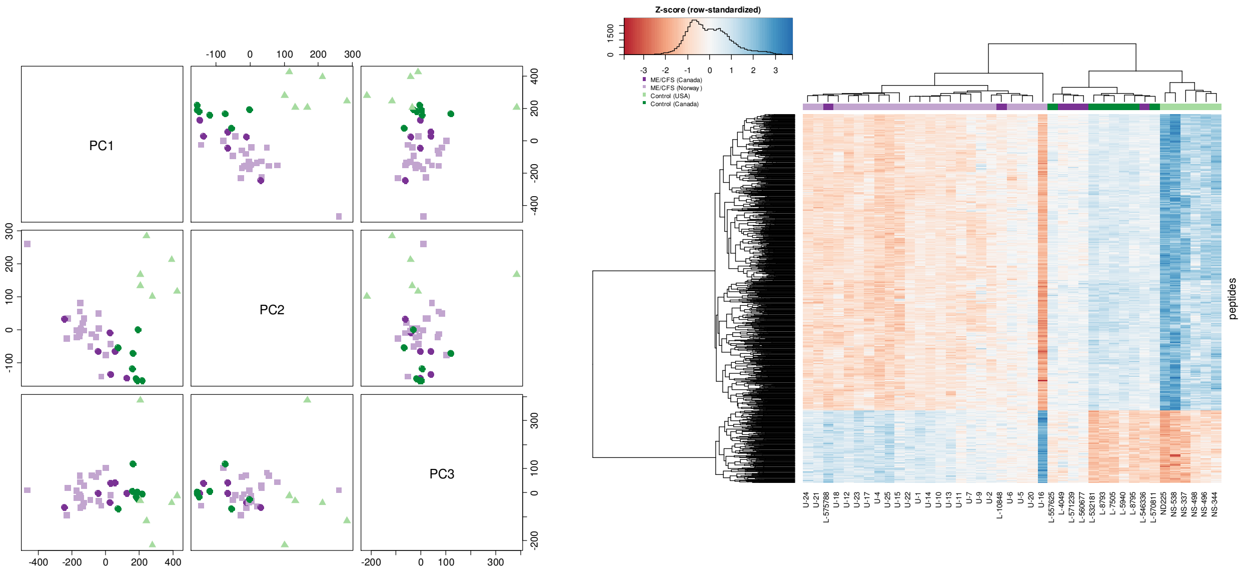

Supplementary Figure 3. Exploration analysis of the Validation Set. Shown on the left is a scatter plot of sample scores for the first three principle components from a PCA on the standardized, median-centered and log2-transformed 41-sample by 122,926-peptide Validation Set. The heatmap on the right is based on 1,230 peptides with the largest absolute PC1 loadings.

The exploration analysis of the Validation Set displayed visible clustering of ME/CFS and control samples along the first principle component (Supplementary Figure 3). This is an interesting result, as all 122,926 peptides were used for the PCA, and other, stronger signals could have been detected on PC1, as was the case for the Discovery Set. One of the Norwegian ME/CFS samples (U-16) stands out in the heatmap (column with the brightest red and blue colors) and in the PCA scatter plots, but it was kept in the analysis because it showed sufficiently high duplicate-correlation in the processing phase and had a perfect outlier testing score. One can see a group of Norwegian and Canadian ME/CFS samples that surround sample U-16 in the heatmap and that 'bridge' ME/CFS samples on one side and controls on the other. One of the Canadian Controls (L-557625) is included in this group.

Discovery Analyses

All discovery analyses used the Discovery Set, which included 22 ME/CFS and 21 controls from the Canadian Complex Chronic Disease Study.

A threshold of 0.05 applied to adjusted p-values from the robust limma analysis returned 1,066 peptides (RL_panel). The random forest analysis was based on 10,000 trees (*ntree*) and 350 randomly selected peptides at each split (*mtry*)[2]. The importance measures *MeanDecreaseGini* and *MeanDecreaseAccuracy* were determined for each peptide and were used to select peptides to define RF_panel. Supplementary Figure 4 shows a scatter plot of the two variables for all 122,926 peptides used in the analysis. Higher values indicate that a peptide was 'important' for classification and 339 peptides with *MeanDecreaseGini* above a threshold of 0.005 were selected (shown in red) to define RF_panel. The threshold was chosen in part to ensure that the *MeanDecreaseAccuracy* values were positive for most of the selected peptides. With a stricter threshold of 0.020, five peptides in the upper right corner of the plot would have been selected (LRSWFKLSGLSG, PRFRWLDGVASG, RYFQRWVNLSAL, WLRRLSFEYNHG and YKQSQRLRPYWL).

[2] Based on the default setting for classification in the randomForest-package: *mtry*=sqrt(number of variables).

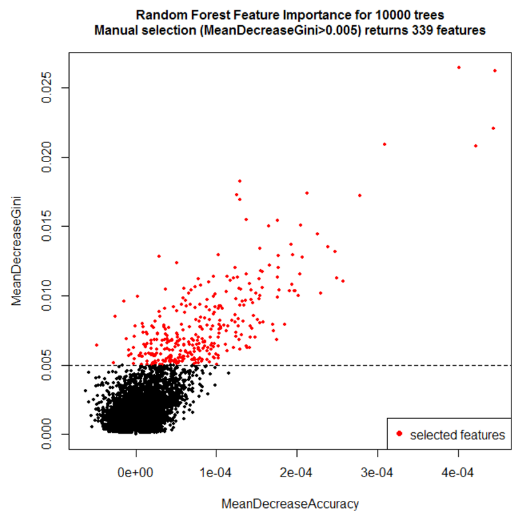

Supplementary Figure 4. Scatter plot of importance measures *MeanDecreaseGini* and *MeanDecreaseAccuracy* for a random forest analysis based on 10,000 trees. Shown in red are 339 peptides (RF_panel) with a *MeanDecreaseGini* above 0.005.

The elastic net supervised analysis was run with alpha=0.1 and the lambda parameter was estimated by internal cross-validation and a one-standard-error-criteria. One hundred elastic net runs were performed, each with two randomly selected ME/CFS and two randomly selected controls removed, producing a set of 100 panels. There were 833 peptides in the union of the 100 individual panels, and the 144 peptides that were observed in at least 10 of the 100 panels (10%) defined EN_panel. Supplementary Figure 5 shows the peptide inclusion matrix over 100 runs and the observed peptide frequency for the selected peptides.

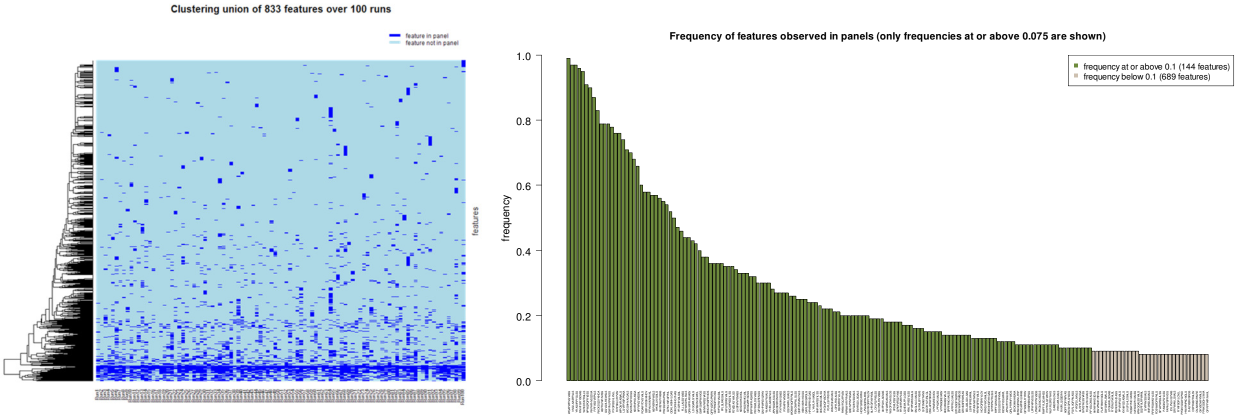

Supplementary Figure 5. Shown are peptide inclusion matrix (left) and frequency plot (right) derived from a set of 100 elastic net runs. The inclusion matrix shows 833 peptides (rows) in the union of panels over all 100 runs. Each column summarizes one elastic net run and indicates if a particular peptide was observed (dark blue) or not (light blue). The frequency plot shows in green all 144 peptides selected for EN_panel that were observed in a least 10% of panels over all 100 runs.

In addition to the three supervised analysis panels, Supplementary Table ***1*** summarizes concordances for 30 peptide lists resulting from unsupervised analyses (10x sPCA, 10x sIPCA and 10 gene shaving clusters). A fixed panel size of 100 peptides was set for sPCA and sIPCA analyses, while the gene shaving algorithm automatically selected an optimal cluster size. As expected for orthogonal PCA components, the overlap between the 10 sPCA peptide lists with each other was zero. There was almost no overlap between the sIPCA lists and very little overlap between the sPCA and sIPCA lists.

	RL_panel	RF_panel	EN_panel	sPC1	sPC2	sPC3	sPC4	sPC5	sPC6	sPC7	sPC8	sPC9	sPC10	sIPC1	sIPC2	sIPC3	sIPC4	sIPC5	sIPC6	sIPC7	sIPC8	sIPC9	sIPC10	GS1	GS2	GS3	GS4	GS5	GS6	GS7	GS8	GS9	GS10
**RL_panel**	**1066**	206	73	0	0	0	1	0	0	0	2	0	0	1	3	0	0	0	0	0	0	0	0	0	495	0	1	0	14	3	0	0	175
**RF_panel**	206	**339**	50	0	0	0	0	0	0	0	1	0	0	1	1	0	0	0	0	0	0	0	0	0	103	2	0	2	7	2	2	0	56
**EN_panel**	73	50	**144**	0	0	0	0	0	0	0	0	0	0	1	1	0	0	0	0	0	0	0	0	0	6	0	0	0	4	0	0	1	18
**sPC1 (sparse PCA)**	0	0	0	**100**	0	0	0	0	0	0	0	0	0	0	0	0	0	0	0	0	0	0	0	100	0	0	0	0	0	0	0	0	0
**sPC2 (sparse PCA)**	0	0	0	0	**100**	0	0	0	0	0	0	0	0	0	0	0	0	0	0	0	0	0	0	0	0	0	0	100	0	0	0	0	0
**sPC3 (sparse PCA)**	0	0	0	0	0	**100**	0	0	0	0	0	0	0	0	0	0	0	0	0	0	0	0	0	0	0	0	0	0	0	0	0	0	0
**sPC4 (sparse PCA)**	1	0	0	0	0	0	**100**	0	0	0	0	0	0	2	0	0	4	0	0	0	0	0	0	0	0	0	42	0	0	2	1	0	1
**sPC5 (sparse PCA)**	0	0	0	0	0	0	0	**100**	0	0	0	0	0	0	0	5	0	0	1	0	0	0	0	0	0	0	0	0	0	0	73	0	11
**sPC6 (sparse PCA)**	0	0	0	0	0	0	0	0	**100**	0	0	0	0	0	3	0	0	0	0	0	1	0	0	0	0	0	0	0	0	0	0	0	0
**sPC7 (sparse PCA)**	0	0	0	0	0	0	0	0	0	**100**	0	0	0	0	0	0	0	0	0	1	0	0	0	0	0	0	0	0	0	1	0	43	0
**sPC8 (sparse PCA)**	2	1	0	0	0	0	0	0	0	0	**100**	0	0	0	0	0	0	0	0	0	0	0	0	0	0	0	0	0	0	0	0	0	0
**sPC9 (sparse PCA)**	0	0	0	0	0	0	0	0	0	0	0	**100**	0	0	0	0	0	0	0	1	0	0	0	0	0	0	0	0	0	0	0	0	0
**sPC10 (sparse PCA)**	0	0	0	0	0	0	0	0	0	0	0	0	**100**	1	0	0	0	0	0	0	0	0	0	0	0	0	0	0	0	0	0	0	0
**sIPC1 (sparse IPCA)**	1	1	1	0	0	0	2	0	0	0	0	0	1	**100**	0	0	0	0	0	0	0	0	0	0	0	0	2	0	0	0	0	8	0
**sIPC2 (sparse IPCA)**	3	1	1	0	0	0	0	0	3	0	0	0	0	0	**100**	0	0	0	0	0	0	1	0	0	0	0	0	0	0	0	0	0	19
**sIPC3 (sparse IPCA)**	0	0	0	0	0	0	0	5	0	0	0	0	0	0	0	**100**	0	0	0	0	0	0	0	0	0	0	2	0	0	0	15	1	0
**sIPC4 (sparse IPCA)**	0	0	0	0	0	0	4	0	0	0	0	0	0	0	0	0	**100**	0	0	0	0	0	0	0	0	0	0	0	0	23	1	0	0
**sIPC5 (sparse IPCA)**	0	0	0	0	0	0	0	0	0	0	0	0	0	0	0	0	0	**100**	0	0	0	0	0	0	0	0	0	0	0	19	0	6	1
**sIPC6 (sparse IPCA)**	0	0	0	0	0	0	0	1	0	0	0	0	0	0	0	0	0	0	**100**	0	0	0	0	0	0	0	0	0	1	0	2	3	1
**sIPC7 (sparse IPCA)**	0	0	0	0	0	0	0	0	0	1	0	1	0	0	0	0	0	0	0	**100**	0	0	0	0	0	0	0	15	0	0	0	11	0
**sIPC8 (sparse IPCA)**	0	0	0	0	0	0	0	0	1	0	0	0	0	0	0	0	0	0	0	0	**100**	0	0	0	0	0	0	1	0	0	3	0	1
**sIPC9 (sparse IPCA)**	0	0	0	0	0	0	0	0	0	0	0	0	0	0	1	0	0	0	0	0	0	**100**	0	0	0	0	0	0	0	0	0	0	8
**sIPC10 (sparse IPCA)**	0	0	0	0	0	0	0	0	0	0	0	0	0	0	0	0	0	0	0	0	0	0	**100**	0	0	0	0	0	2	0	0	5	1
**GS1 (Gene Shaving)**	0	0	0	100	0	0	0	0	0	0	0	0	0	0	0	0	0	0	0	0	0	0	0	**5207**	0	0	20	0	0	28	0	164	0
**GS2 (Gene Shaving)**	495	103	6	0	0	0	0	0	0	0	0	0	0	0	0	0	0	0	0	0	0	0	0	0	**4217**	0	0	6	0	10	0	5	261
**GS3 (Gene Shaving)**	0	2	0	0	0	0	0	0	0	0	0	0	0	0	0	0	0	0	0	0	0	0	0	0	0	**2239**	1	0	0	3	3	0	10
**GS4 (Gene Shaving)**	1	0	0	0	0	0	42	0	0	0	0	0	0	2	0	2	0	0	0	0	0	0	0	20	0	1	**2239**	0	0	2	7	104	4
**GS5 (Gene Shaving)**	0	2	0	0	100	0	0	0	0	0	0	0	0	0	0	0	0	0	0	15	1	0	0	0	6	0	0	**1813**	1	5	0	37	12
**GS6 (Gene Shaving)**	14	7	4	0	0	0	0	0	0	0	0	0	0	0	0	0	0	0	1	0	0	0	2	0	0	0	0	1	**2015**	1	0	0	9
**GS7 (Gene Shaving)**	3	2	0	0	0	0	2	0	0	1	0	0	0	0	0	0	23	19	0	0	0	0	0	28	10	3	2	5	1	**2239**	0	33	3
**GS8 (Gene Shaving)**	0	2	0	0	0	0	1	73	0	0	0	0	0	0	0	15	1	0	2	0	3	0	0	0	0	3	7	0	0	0	**1631**	0	41
**GS9 (Gene Shaving)**	0	0	1	0	0	0	0	0	0	43	0	0	0	8	0	1	0	6	3	11	0	0	5	164	5	0	104	37	0	33	0	**2765**	1
**GS10 (Gene Shaving)**	175	56	18	0	0	0	1	11	0	0	0	0	0	0	19	0	0	1	1	0	1	8	1	0	261	10	4	12	9	3	41	1	**2488**

Supplementary Table 1. The table shows concordances for 33 peptide lists that were derived in the discovery analysis. The first three rows in the upper left represent panels from supervised analyses while the following three blocks of ten peptide lists each (sparse PCA, sparse IPCA and gene shaving) were derived with unsupervised approaches. An excel spreadsheet version of the table is provided in Online Resource 2.

The 10 gene shaving clusters contained between 1,631 to 5,207 peptides, and two of the ten clusters were supersets of the sPCA lists, including Gene Shaving Cluster 1 (GS1) which contained all 100 peptides from PC1 (and interestingly did not overlap with any of the other panels, including the supervised panels), and Gene Shaving Cluster 5 which contained all 100 peptides from PC2 as well as 15 peptides from sIPCA7. There was a fair amount of overlap between the supervised signatures on one side and Gene Shaving clusters 2 and 10 on the other side. These two Gene Shaving Clusters also showed high correlation with the supervised panels and displayed the highest AUCs of any of the unsupervised panels, as shown in Supplementary Table ***2***. The union of Gene Shaving Clusters 2 and 10 contained 6,444 peptides that defined signature GS_panel.

	Size	AUC	RL_panel	RF_panel	EN_panel	sPC1	sPC2	sPC3	sPC4	sPC5	sPC6	sPC7	sPC8	sPC9	sPC10	sIPC1	sIPC2	sIPC3	sIPC4	sIPC5	sIPC6	sIPC7	sIPC8	sIPC9	sIPC10	GS1	GS2	GS3	GS4	GS5	GS6	GS7	GS8	GS9	GS10
**RL_panel**	1066	0.82	1.00	0.98	0.87	0.46	0.31	0.20	0.75	0.09	-0.14	-0.80	-0.66	0.32	0.04	0.32	0.31	0.10	0.17	0.14	0.12	-0.06	0.03	0.00	0.76	0.48	0.96	0.27	0.15	0.42	0.68	-0.09	0.11	0.39	0.91
**RF_panel**	339	0.85	0.98	1.00	0.91	0.39	0.35	0.16	0.72	0.09	-0.13	-0.77	-0.59	0.28	0.02	0.31	0.35	0.08	0.15	0.13	0.13	-0.01	0.05	-0.09	0.74	0.41	0.93	0.33	0.09	0.46	0.69	-0.04	0.12	0.32	0.88
**EN_panel**	144	0.97	0.87	0.91	1.00	0.29	0.22	-0.03	0.63	0.13	0.00	-0.60	-0.46	0.19	-0.02	0.42	0.47	-0.01	0.11	0.11	0.14	0.02	-0.08	-0.07	0.56	0.29	0.77	0.35	0.10	0.36	0.64	0.04	0.09	0.23	0.74
**sPC1 (sparse PCA)**	100	0.55	0.46	0.39	0.29	1.00	-0.11	0.15	0.44	0.10	-0.04	-0.52	-0.85	0.34	0.03	0.37	-0.20	0.21	0.01	0.27	0.22	-0.20	-0.10	0.56	0.56	0.98	0.50	-0.35	0.72	-0.13	0.32	-0.53	-0.06	0.92	0.57
**sPC2 (sparse PCA)**	100	0.57	0.31	0.35	0.22	-0.11	1.00	0.17	0.01	0.04	0.11	-0.26	0.05	0.11	0.25	-0.14	0.17	-0.29	-0.11	0.12	0.13	0.58	0.49	-0.60	0.30	-0.07	0.42	0.17	-0.21	0.97	0.17	0.01	0.01	-0.18	0.23
**sPC3 (sparse PCA)**	100	0.51	0.20	0.16	-0.03	0.15	0.17	1.00	0.17	0.21	-0.07	-0.21	-0.21	0.15	0.19	-0.29	-0.28	0.03	-0.18	-0.32	-0.09	-0.32	0.56	-0.16	0.24	0.12	0.27	-0.29	-0.24	0.16	0.16	-0.52	-0.31	0.00	0.27
**sPC4 (sparse PCA)**	100	0.68	0.75	0.72	0.63	0.44	0.01	0.17	1.00	0.17	-0.19	-0.67	-0.53	0.16	0.17	0.44	0.16	0.30	0.41	0.02	-0.19	-0.14	-0.02	0.05	0.67	0.39	0.69	0.32	0.06	0.15	0.67	0.02	0.01	0.31	0.72
**sPC5 (sparse PCA)**	100	0.56	0.09	0.09	0.13	0.10	0.04	0.21	0.17	1.00	0.03	-0.09	-0.12	0.01	0.26	0.03	0.01	-0.48	0.00	-0.18	-0.43	-0.07	0.26	0.25	0.16	0.08	0.04	-0.09	0.05	0.02	0.21	-0.14	-0.89	0.08	0.30
**sPC6 (sparse PCA)**	100	0.51	-0.14	-0.13	0.00	-0.04	0.11	-0.07	-0.19	0.03	1.00	0.12	0.17	-0.23	-0.01	0.04	0.52	-0.01	-0.01	-0.02	0.25	0.32	0.46	0.09	-0.35	-0.05	-0.17	-0.05	0.05	0.08	-0.05	-0.04	-0.03	-0.01	-0.12
**sPC7 (sparse PCA)**	100	0.33	-0.80	-0.77	-0.60	-0.52	-0.26	-0.21	-0.67	-0.09	0.12	1.00	0.63	-0.33	-0.09	-0.12	-0.25	-0.22	-0.17	-0.12	0.05	-0.05	-0.06	-0.10	-0.75	-0.54	-0.82	-0.26	-0.13	-0.33	-0.74	0.10	-0.11	-0.43	-0.82
**sPC8 (sparse PCA)**	100	0.37	-0.66	-0.59	-0.46	-0.85	0.05	-0.21	-0.53	-0.12	0.17	0.63	1.00	-0.41	0.07	-0.32	0.07	-0.08	-0.06	-0.30	-0.20	0.33	0.12	-0.49	-0.62	-0.86	-0.68	0.22	-0.59	0.04	-0.40	0.42	0.04	-0.82	-0.75
**sPC9 (sparse PCA)**	100	0.55	0.32	0.28	0.19	0.34	0.11	0.15	0.16	0.01	-0.23	-0.33	-0.41	1.00	0.28	0.29	-0.14	0.18	-0.33	0.46	-0.01	-0.33	-0.08	0.06	0.37	0.40	0.39	-0.24	0.09	0.10	0.16	-0.22	0.03	0.33	0.35
**sPC10 (sparse PCA)**	100	0.48	0.04	0.02	-0.02	0.03	0.25	0.19	0.17	0.26	-0.01	-0.09	0.07	0.28	1.00	0.19	-0.07	-0.01	-0.18	-0.03	-0.17	0.06	0.22	-0.09	0.21	0.01	0.10	-0.04	-0.14	0.25	0.16	-0.17	-0.23	0.01	0.12
**sIPC1 (sparse IPCA)**	100	0.63	0.32	0.31	0.42	0.37	-0.14	-0.29	0.44	0.03	0.04	-0.12	-0.32	0.29	0.19	1.00	0.14	0.04	0.04	0.26	0.25	-0.10	-0.25	0.14	0.23	0.34	0.29	-0.03	0.23	-0.05	0.14	-0.18	0.06	0.35	0.25
**sIPC2 (sparse IPCA)**	100	0.69	0.31	0.35	0.47	-0.20	0.17	-0.28	0.16	0.01	0.52	-0.25	0.07	-0.14	-0.07	0.14	1.00	-0.04	0.33	0.21	-0.09	0.26	0.24	-0.02	-0.06	-0.19	0.21	0.51	-0.19	0.26	0.33	0.43	0.22	-0.18	0.29
**sIPC3 (sparse IPCA)**	100	0.47	0.10	0.08	-0.01	0.21	-0.29	0.03	0.30	-0.48	-0.01	-0.22	-0.08	0.18	-0.01	0.04	-0.04	1.00	0.00	0.01	0.02	-0.21	-0.05	0.06	0.03	0.19	0.09	0.08	-0.12	-0.24	0.17	-0.04	0.56	0.13	0.11
**sIPC4 (sparse IPCA)**	100	0.49	0.17	0.15	0.11	0.01	-0.11	-0.18	0.41	0.00	-0.01	-0.17	-0.06	-0.33	-0.18	0.04	0.33	0.00	1.00	0.15	-0.26	0.08	-0.01	0.06	0.06	-0.07	0.11	0.46	0.01	-0.04	0.02	0.52	0.07	-0.11	0.11
**sIPC5 (sparse IPCA)**	100	0.52	0.14	0.13	0.11	0.27	0.12	-0.32	0.02	-0.18	-0.02	-0.12	-0.30	0.46	-0.03	0.26	0.21	0.01	0.15	1.00	-0.07	-0.02	-0.13	0.18	0.13	0.31	0.19	-0.15	0.36	0.11	-0.18	0.19	0.26	0.28	0.15
**sIPC6 (sparse IPCA)**	100	0.57	0.12	0.13	0.14	0.22	0.13	-0.09	-0.19	-0.43	0.25	0.05	-0.20	-0.01	-0.17	0.25	-0.09	0.02	-0.26	-0.07	1.00	0.14	-0.06	-0.07	-0.07	0.24	0.17	-0.23	0.30	0.10	-0.21	-0.40	0.31	0.28	-0.03
**sIPC7 (sparse IPCA)**	100	0.47	-0.06	-0.01	0.02	-0.20	0.58	-0.32	-0.14	-0.07	0.32	-0.05	0.33	-0.33	0.06	-0.10	0.26	-0.21	0.08	-0.02	0.14	1.00	0.14	-0.27	0.03	-0.17	-0.01	0.38	-0.01	0.55	0.11	0.23	0.15	-0.15	-0.16
**sIPC8 (sparse IPCA)**	100	0.47	0.03	0.05	-0.08	-0.10	0.49	0.56	-0.02	0.26	0.46	-0.06	0.12	-0.08	0.22	-0.25	0.24	-0.05	-0.01	-0.13	-0.06	0.14	1.00	-0.28	-0.16	-0.14	0.12	-0.13	-0.39	0.43	0.01	-0.23	-0.30	-0.24	0.12
**sIPC9 (sparse IPCA)**	100	0.44	0.00	-0.09	-0.07	0.56	-0.60	-0.16	0.05	0.25	0.09	-0.10	-0.49	0.06	-0.09	0.14	-0.02	0.06	0.06	0.18	-0.07	-0.27	-0.28	1.00	0.03	0.60	-0.07	-0.35	0.70	-0.65	0.04	-0.24	-0.20	0.71	0.20
**sIPC10 (sparse IPCA)**	100	0.63	0.76	0.74	0.56	0.56	0.30	0.24	0.67	0.16	-0.35	-0.75	-0.62	0.37	0.21	0.23	-0.06	0.03	0.06	0.13	-0.07	0.03	-0.16	0.03	1.00	0.59	0.77	0.20	0.27	0.38	0.72	-0.16	0.00	0.51	0.75
**GS1 (Gene Shaving)**	5207	0.54	0.48	0.41	0.29	0.98	-0.07	0.12	0.39	0.08	-0.05	-0.54	-0.86	0.40	0.01	0.34	-0.19	0.19	-0.07	0.31	0.24	-0.17	-0.14	0.60	0.59	1.00	0.52	-0.38	0.76	-0.10	0.35	-0.53	-0.03	0.96	0.59
**GS2 (Gene Shaving)**	4217	0.75	0.96	0.93	0.77	0.50	0.42	0.27	0.69	0.04	-0.17	-0.82	-0.68	0.39	0.10	0.29	0.21	0.09	0.11	0.19	0.17	-0.01	0.12	-0.07	0.77	0.52	1.00	0.18	0.13	0.50	0.62	-0.20	0.12	0.41	0.89
**GS3 (Gene Shaving)**	2239	0.62	0.27	0.33	0.35	-0.35	0.17	-0.29	0.32	-0.09	-0.05	-0.26	0.22	-0.24	-0.04	-0.03	0.51	0.08	0.46	-0.15	-0.23	0.38	-0.13	-0.35	0.20	-0.38	0.18	1.00	-0.40	0.30	0.42	0.63	0.35	-0.39	0.17
**GS4 (Gene Shaving)**	2239	0.51	0.15	0.09	0.10	0.72	-0.21	-0.24	0.06	0.05	0.05	-0.13	-0.59	0.09	-0.14	0.23	-0.19	-0.12	0.01	0.36	0.30	-0.01	-0.39	0.70	0.27	0.76	0.13	-0.40	1.00	-0.28	-0.02	-0.27	-0.06	0.85	0.19
**GS5 (Gene Shaving)**	1813	0.63	0.42	0.46	0.36	-0.13	0.97	0.16	0.15	0.02	0.08	-0.33	0.04	0.10	0.25	-0.05	0.26	-0.24	-0.04	0.11	0.10	0.55	0.43	-0.65	0.38	-0.10	0.50	0.30	-0.28	1.00	0.29	0.10	0.07	-0.22	0.32
**GS6 (Gene Shaving)**	2015	0.71	0.68	0.69	0.64	0.32	0.17	0.16	0.67	0.21	-0.05	-0.74	-0.40	0.16	0.16	0.14	0.33	0.17	0.02	-0.18	-0.21	0.11	0.01	0.04	0.72	0.35	0.62	0.42	-0.02	0.29	1.00	0.00	-0.02	0.29	0.71
**GS7 (Gene Shaving)**	2239	0.52	-0.09	-0.04	0.04	-0.53	0.01	-0.52	0.02	-0.14	-0.04	0.10	0.42	-0.22	-0.17	-0.18	0.43	-0.04	0.52	0.19	-0.40	0.23	-0.23	-0.24	-0.16	-0.53	-0.20	0.63	-0.27	0.10	0.00	1.00	0.27	-0.52	-0.19
**GS8 (Gene Shaving)**	1631	0.50	0.11	0.12	0.09	-0.06	0.01	-0.31	0.01	-0.89	-0.03	-0.11	0.04	0.03	-0.23	0.06	0.22	0.56	0.07	0.26	0.31	0.15	-0.30	-0.20	0.00	-0.03	0.12	0.35	-0.06	0.07	-0.02	0.27	1.00	-0.05	-0.05
**GS9 (Gene Shaving)**	2765	0.51	0.39	0.32	0.23	0.92	-0.18	0.00	0.31	0.08	-0.01	-0.43	-0.82	0.33	0.01	0.35	-0.18	0.13	-0.11	0.28	0.28	-0.15	-0.24	0.71	0.51	0.96	0.41	-0.39	0.85	-0.22	0.29	-0.52	-0.05	1.00	0.50
**GS10 (Gene Shaving)**	2488	0.74	0.91	0.88	0.74	0.57	0.23	0.27	0.72	0.30	-0.12	-0.82	-0.75	0.35	0.12	0.25	0.29	0.11	0.11	0.15	-0.03	-0.16	0.12	0.20	0.75	0.59	0.89	0.17	0.19	0.32	0.71	-0.19	-0.05	0.50	1.00

Supplementary Table 2. Shown are panel sizes, AUCs and panel-correlations determined by Spearman correlation derived from mean signed peptide in each panel. Correlations are color-coded from red (-1) to blue (+1), while AUC values are color-coded from red (0) to green (1) with intermediate values shown in yellow. An excel spreadsheet version of the table is provided in Online Resource 2.

The definition of the unsupervised panels sPCA_panel from sparse PCA results, and sIPCA_panel from sparse IPCA results was not as clear and involved visual inspection of PCA plots and heatmaps to identify patterns of separation, as well as panel concordances and correlations in Supplementary Table *1* and Supplementary Table *2*. sPCA_panel was defined as the union of sPCA4 and sPCA7. sPCA4 had 42 of 100 peptides in common with GS4 while sPCA7 had 43 of 100 peptides in common with GS9. Sparse IPCA panel sIPCA10 was selected as signature sIPCA_panel. It had the highest correlation with the supervised panels of any of the ten sIPCA panels, and displayed higher correlation coefficients with panels sPCA4, GS2, GS6 and GS10.

Heatmaps for the final unsupervised panels sPCA_panel, sIPCA_panel, and GS_panel are shown in Supplementary Figures 6 and 7. The sPCA_panel and sIPCA_panel showed little separation between groups and had low AUC (0.49 and 0.63, respectively). In Supplementary Figure 6 (left), one can see two ME/CFS samples (4049 and 10848) that display high values (dark blue and turquoise values) in two different sets of peptides in sPCA_Panel that each correspond to the 100 peptides selected on sPC4 and sPC7 respectively. It is likely that the merging of the two sPCA panels into one panel and the use of mean signed peptide score in the calculation of the AUC contributed to the lower AUC for this panel. The AUC for sPCA4 and sPCA7 individually were 0.68 and 0.67 (sPC7 had an AUC of 0.33 in Supplementary Table ***2*** because of a reversed PCA sign but was converted to 1-0.33=0.67).

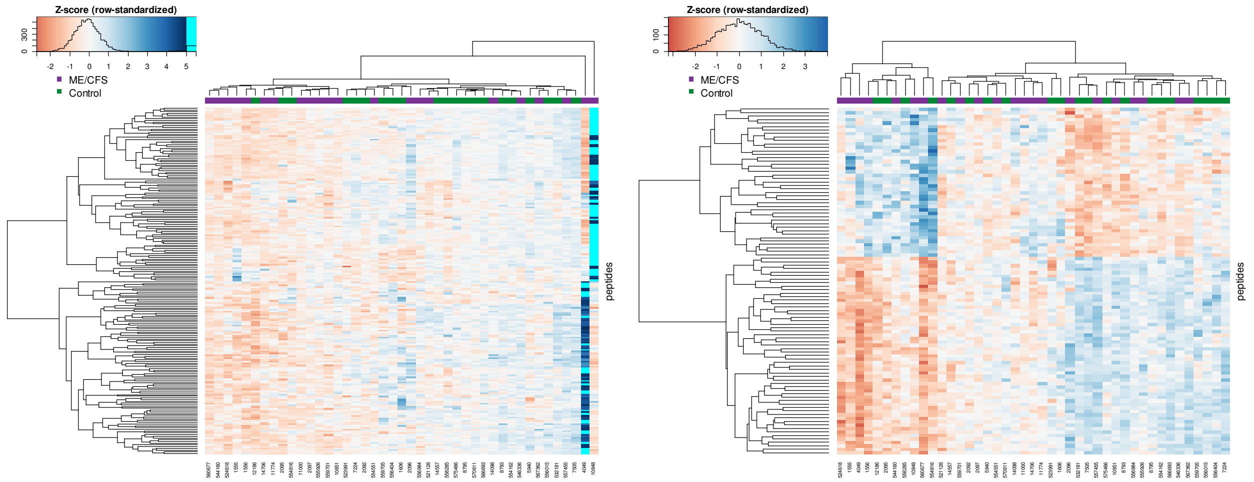

Supplementary Figure 6. Shown are heatmaps for unsupervised panels sPCA_panel (left), which represents the union of sparse PCA panels sPCA4 and sPCA7, and sIPCA_panel (right), which was defined by the 100-peptide sparse IPCA10 panel.

The sIPCA_panel shows an interesting heatmap pattern (Supplementary Figure 6, right) but without clear clustering of sample groups. On the other hand, the gene shaving-derived panel GS_panel (Supplementary Figure 7) has an AUC of 0.75 and shows a clearer separation of ME/CFS samples and controls. ME/CFS sample 4049 stands out in the heatmap and displays the lowest peptide abundance values for almost all 6,444 peptides in this signature.

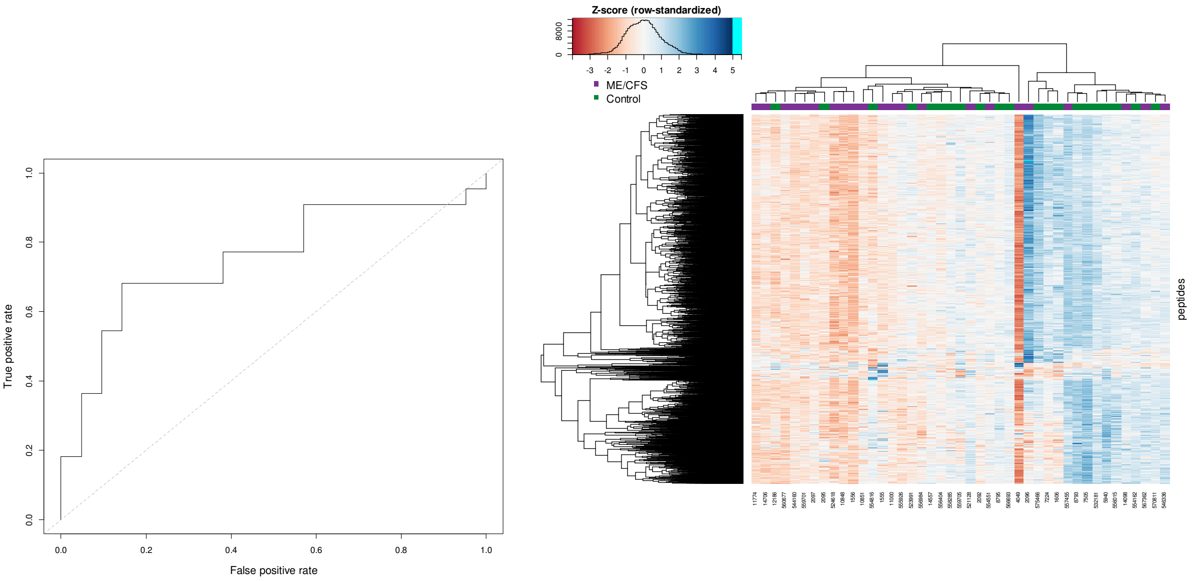

Supplementary Figure 7. Shown are the ROC curve, and heatmap for the combination of Gene Shaving Clusters GS2 and GS10, whose union of 6,444 peptides defined GS_panel. The ROC curve is based on the mean signed peptide score and corresponds to a discovery AUC value of 0.75.

There was very little overlap between the three supervised panels and either of the sPCA or sIPCA panels, but almost half (495) of the 1,066 RL_panel peptides were included in GS2. GS2 also included 103 of 339 peptides in the random forest panel RF_panel, but only six of 144 peptides in the elastic net panel EN_panel. GS10 contained 175 of 1,066 peptides in RL_panel, 56 of 339 peptides in RF_panel, and 18 of 144 peptides in EN_panel, while also having multiple peptides in common with sPCA5 (11 of 100 peptides), sIPCA2 (19 of 100 peptides) and sIPCA9 (eight of 100 peptides). This overlap was reassuring and was used to define the proposed candidate peptide signature CPS001 as the intersection of the union of supervised panels (CPS004) and GS_panel. The selection of CPS001 as the most robust signature was further supported by additional testing of the signature in subsets of the Validation Set (Supplementary Figure 9).

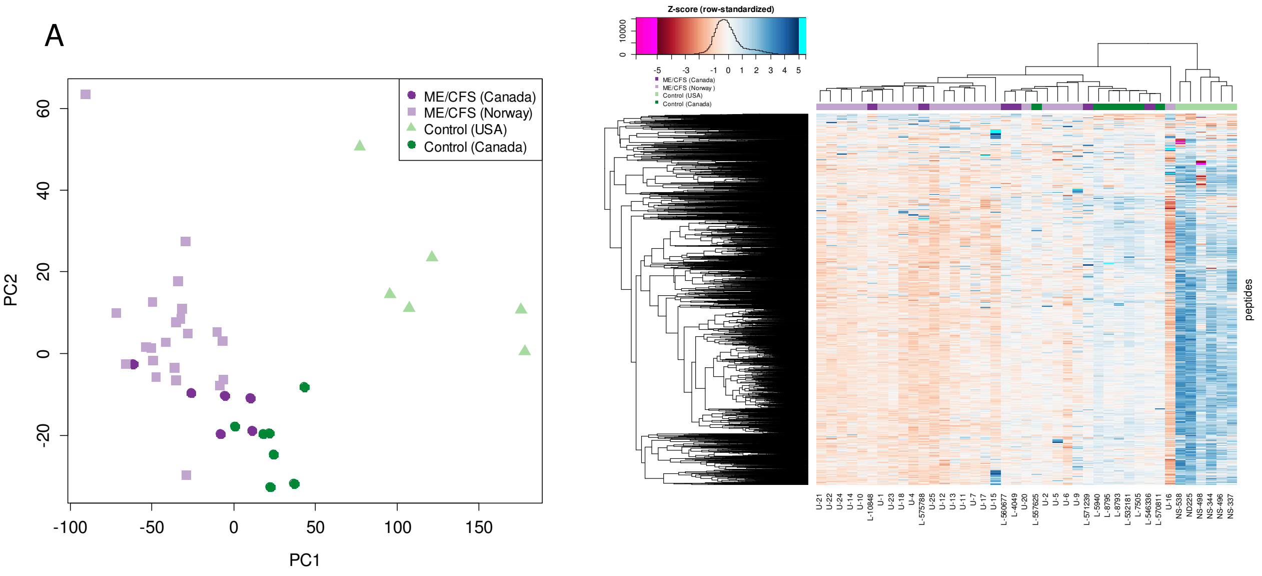

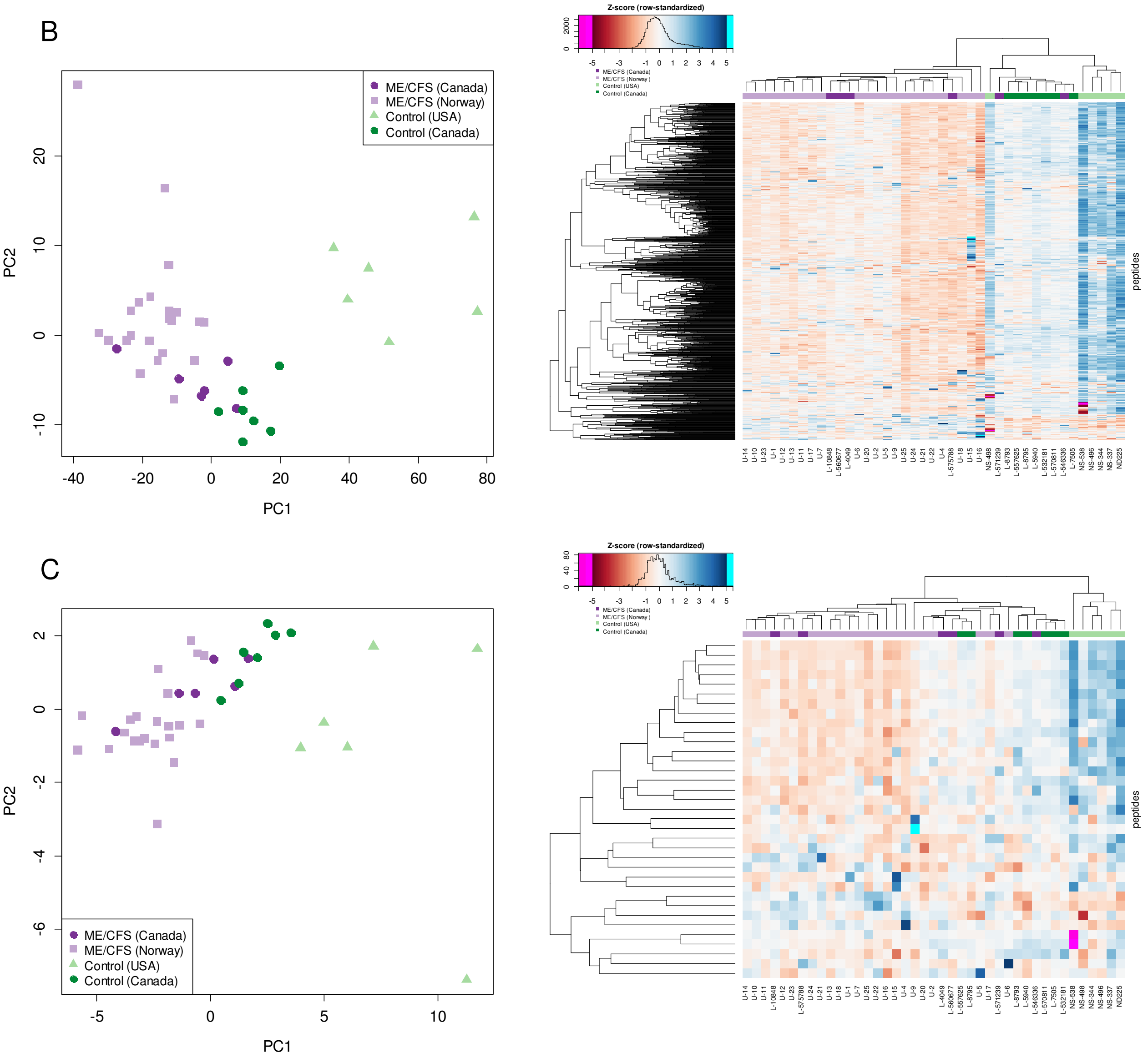
B
ME/CFS (Canada)
ME/CFS (Norway)
Control (USA)
Control (Canada)
PC1
PC2
Z-score (row-standardized)
peptides
C
ME/CFS (Canada)
ME/CFS (Norway)
Control (USA)
Control (Canada)
PC1
PC2
Z-score (row-standardized)
peptides

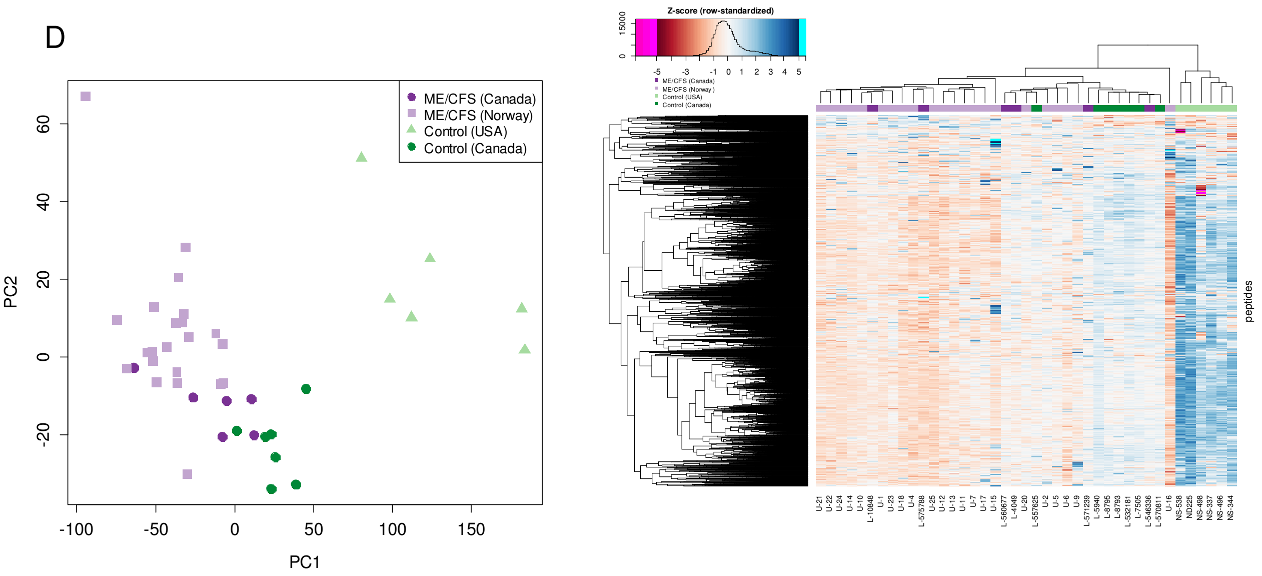

Supplementary Figure 8. PCA projection and heatmap for candidate peptide signatures CPS002 (A), CPS004 (B), CPS005 (C) and CPS006 (D) in the Validation Set.

Across the five CPS validation analyses, all candidate peptide signatures showed separation of Canadian ME/CFS case samples and control samples, with the exception of one ME/CFS sample (L-546336) that was consistently found in the controls group, and one control sample (L-557625) that was consistently found close to the ME/CFS samples. The intersection of the supervised signatures (CPS005; 35 peptides) showed the poorest separation of cases and controls. When Norwegian ME/CFS samples were included in the analysis, the ME/CFS samples L-546336 and L-571239 were consistently grouped with Canadian controls, and several of the signatures confirmed that control sample L-557625 was in a transition zone between ME/CFS samples and controls, though this was not observed for CPS001 (Supplementary Figure 9B). When the six American control samples were included, they clearly separated from the other samples and were closer to Canadian controls than to Canadian or Norwegian ME/CFS case samples. Control sample L-557625 now clustered with the other controls, but ME/CFS samples L-546336 and L-571239 still consistently grouped with controls. This can be seen in Figure 4 in the main text for signature CPS001, and Supplementary Figure 8 for signatures CPS002 (A), CPS004 (B), CPS005 (C) and CPS006 (D).

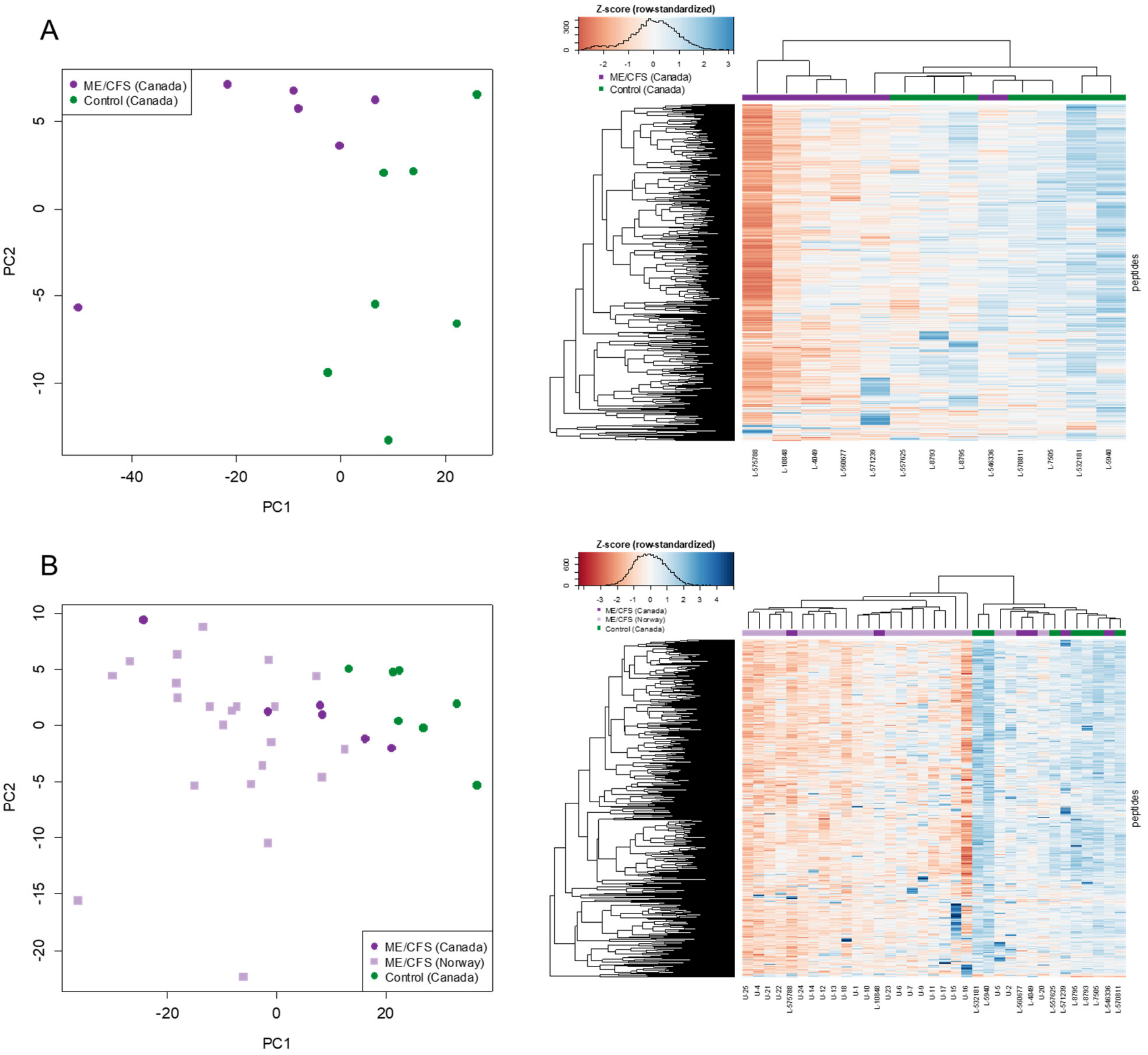

Supplementary Figure 9. Shown are PCAs and heatmaps of candidate proposed signature CPS001 in subsets of 13 (A) and 35 (B) samples from the Validation Set. The 13 samples in (A) represent Canadian ME/CFS cases (n=6) and controls (n=7), while (B) shows Norwegian ME/CFS cases (n=22), Canadian cases (n=6) and Canadian controls (n=7).

Validation of an alternate ME/CFS immunosignature

We also tested a recently published, 25-peptide signature discovered using the same immunosignature assay platform but a different cohort of ME/CFS cases and controls. When we applied this signature to both our Discovery and Validation Sets, we found moderate separation of cases and controls in both sets, though the corresponding heatmaps show a fair amount of variation (Supplementary Figure 10).

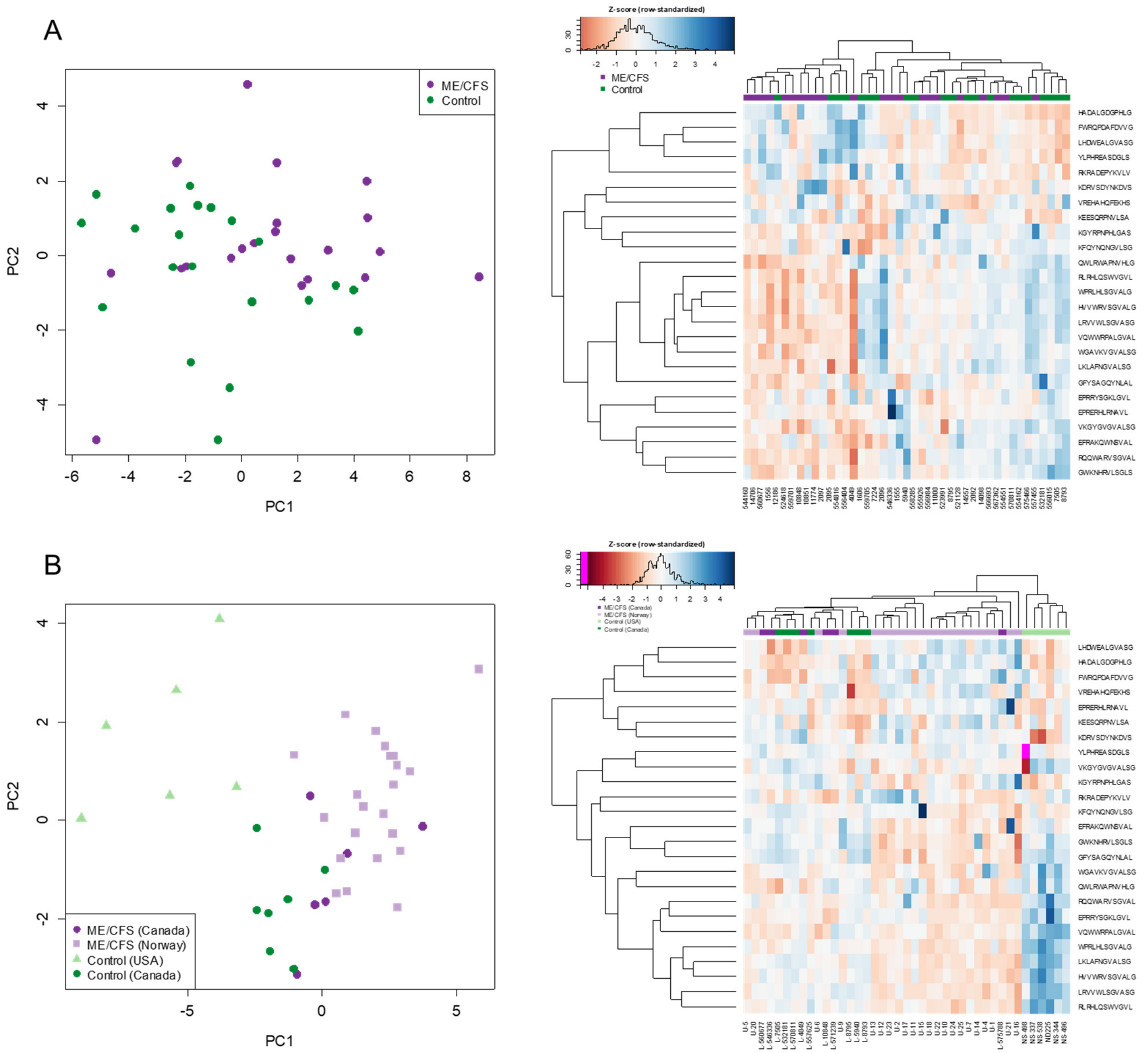

Supplementary Figure 10. PCA projections and heatmaps for a 25-peptide, alternate ME/CFS signature in the Discovery Set (A) and Validation Set (B).

Examining the peptide lists, we found no overlap between the 25 peptides in the previously-described signature and our refined peptide signature CPS001A. When we examined our other signatures, we found peptide LRVVWLSGVASG in multiple panels, including CPS001, and we found that signatures CPS002 and CPS006 shared an additional seven peptides (EFRAKQWNSVAL, HVVWRVSGVALG, GWKNHRVLSGLS, RLRHLQSWVGVL, VQWWRPALGVAL, LRVVWLSGVASG, WGAVKVGVALSG and WPRLHLSGVALG) with the previously-described signature.

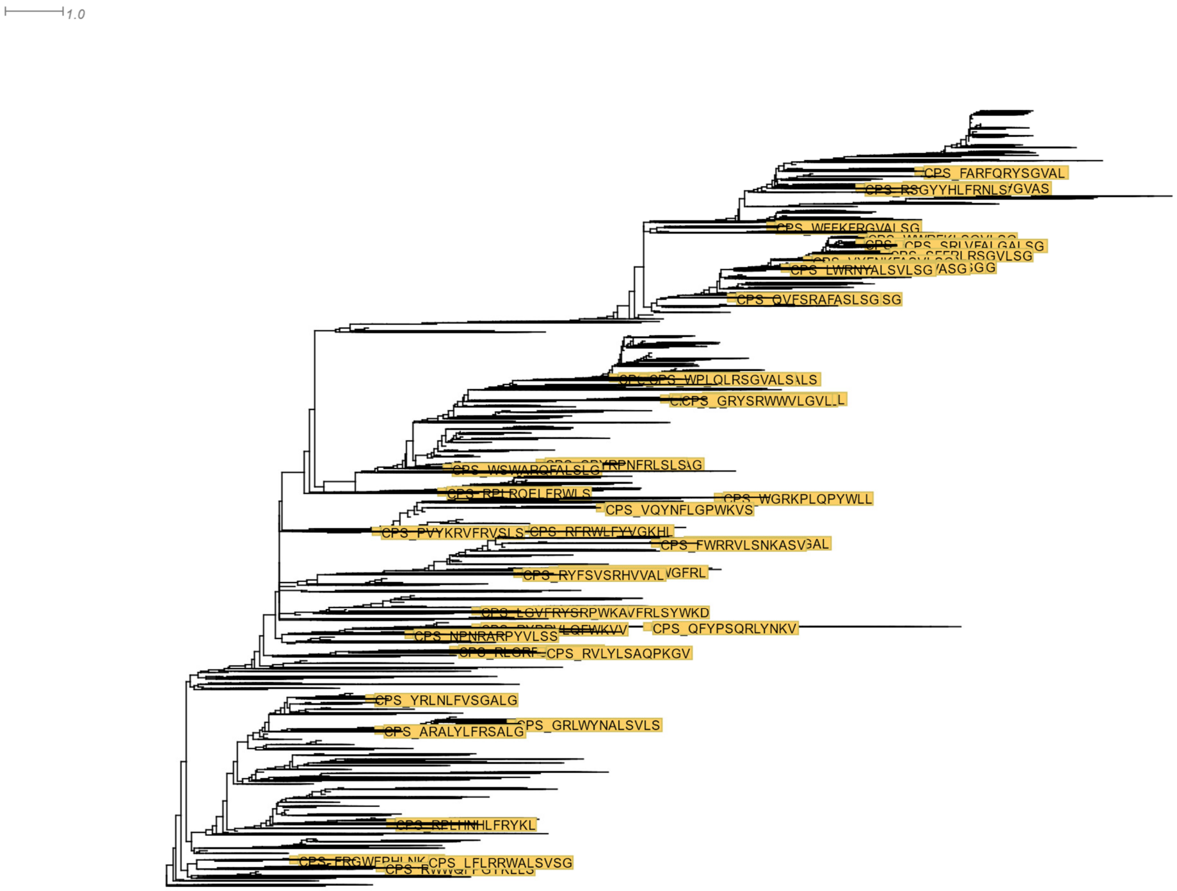

Supplementary Figure 11. Phylogenetic tree of the complete peptide array, with signature CPS001A peptides highlighted in yellow.

Additional materials, including sample correlation and scatter plots, box plots and spreadsheets versions of concordance and correlation tables, is provided in separate Online Resources as summarized in the table below:

Description	Location
Concordances, correlations and summary of discovery signatures, including three supervised, 30 unsupervised, and five candidate peptide signatures.	ESM_2.xlsx
Summary of 654 peptides in CPS001 and subset of 256 peptides in CPS001A, including p-values and fold-changes from t-tests for four comparisons.	ESM_3.xlsx
Concordance of proposed candidate signatures including refined CPS001A, main supervised and unsupervised signatures, and Lombardi et al signature.	ESM_4.xlsx
Boxplots for 654 peptides in signature CPS001 across 84 samples in eight groups in discovery and validation data sets.	ESM_5.pdf
Boxplots for 256 peptides in signature CPS001A across 84 samples in eight groups in discovery and validation data sets.	ESM_6.pdf
Correlation (Pearson) of duplicate sample pairs for 99 samples and 85,987 peptides in the Canadian discovery analysis data set (AD001).	ESM_7.pdf
Correlation (Spearman) of duplicate sample pairs for 99 samples and 85,987 peptides in the Canadian discovery analysis data set (AD001).	ESM_8.pdf
Summary of peptide scatter and distribution for duplicate samples in the Canadian discovery analysis data set (AD001).	ESM_9.pdf
Correlation (Pearson) of duplicate sample pairs for 94 samples and 111,876 peptides in the International validation analysis data set (VD001).	ESM_10.pdf
Correlation (Spearman) of duplicate sample pairs for 94 samples and 111,876 peptides in the International validation analysis data set (VD001).	ESM_11.pdf
Summary of peptide scatter and distribution for duplicate samples in the International validation analysis data set (VD001).	ESM_12.pdf
