# Supplementary material for: Immunosignature Analysis of Myalgic Encephalomyelitis/Chronic Fatigue Syndrome (ME/CFS)
Source: Mol Neurobiol. 2018 Oct 8;56(6):4249–57. doi: 10.1007/s12035-018-1354-8 (PMC6505503; doi:10.1007/s12035-018-1354-8)
Supplement: Supplementary file 5 — (PDF 982 kb) [file 12035_2018_1354_MOESM5_ESM.pdf]

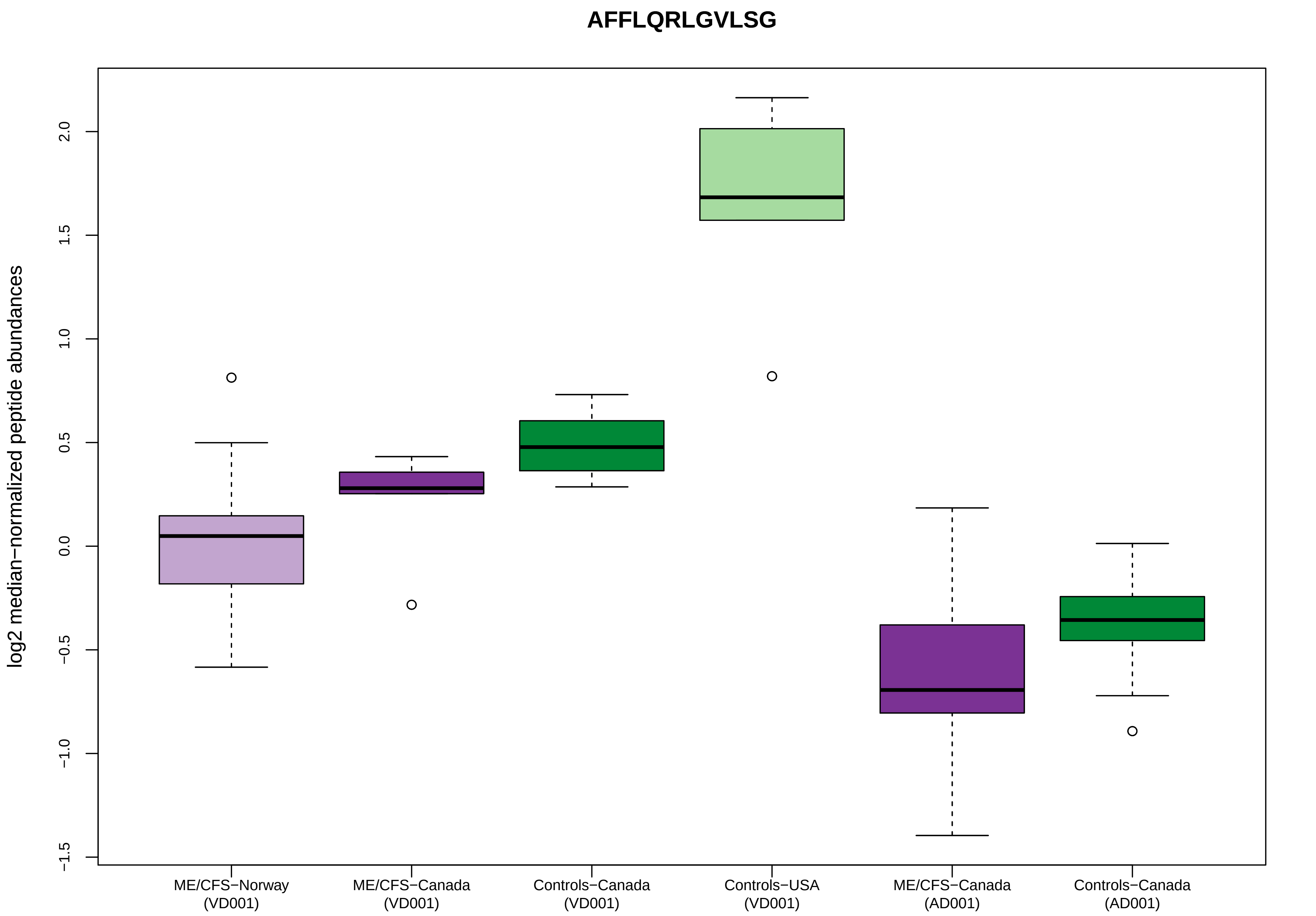

AFFLQRLGVLSG
log2 median−normalized peptide abundances
2.0
1.5
1.0
0.5
0.0
−0.5
−1.0
−1.5
ME/CFS−Norway (VD001)
ME/CFS−Canada (VD001)
Controls−Canada (VD001)
Controls−USA (VD001)
ME/CFS−Canada (AD001)
Controls−Canada (AD001)

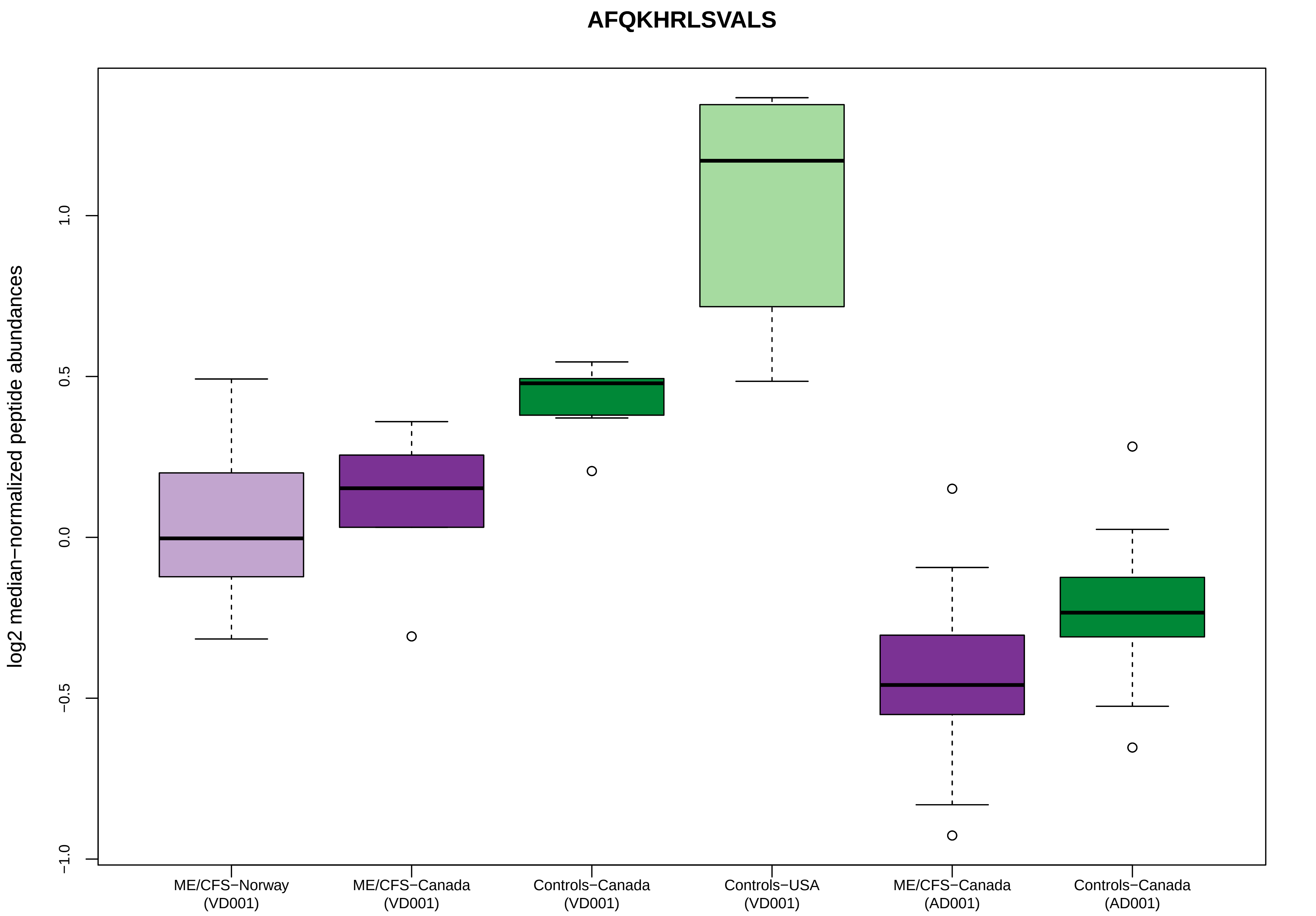
AFQKHRLSVALS
log2 median−normalized peptide abundances
1.0
0.5
0.0
−0.5
−1.0
ME/CFS−Norway (VD001)
ME/CFS−Canada (VD001)
Controls−Canada (VD001)
Controls−USA (VD001)
ME/CFS−Canada (AD001)
Controls−Canada (AD001)

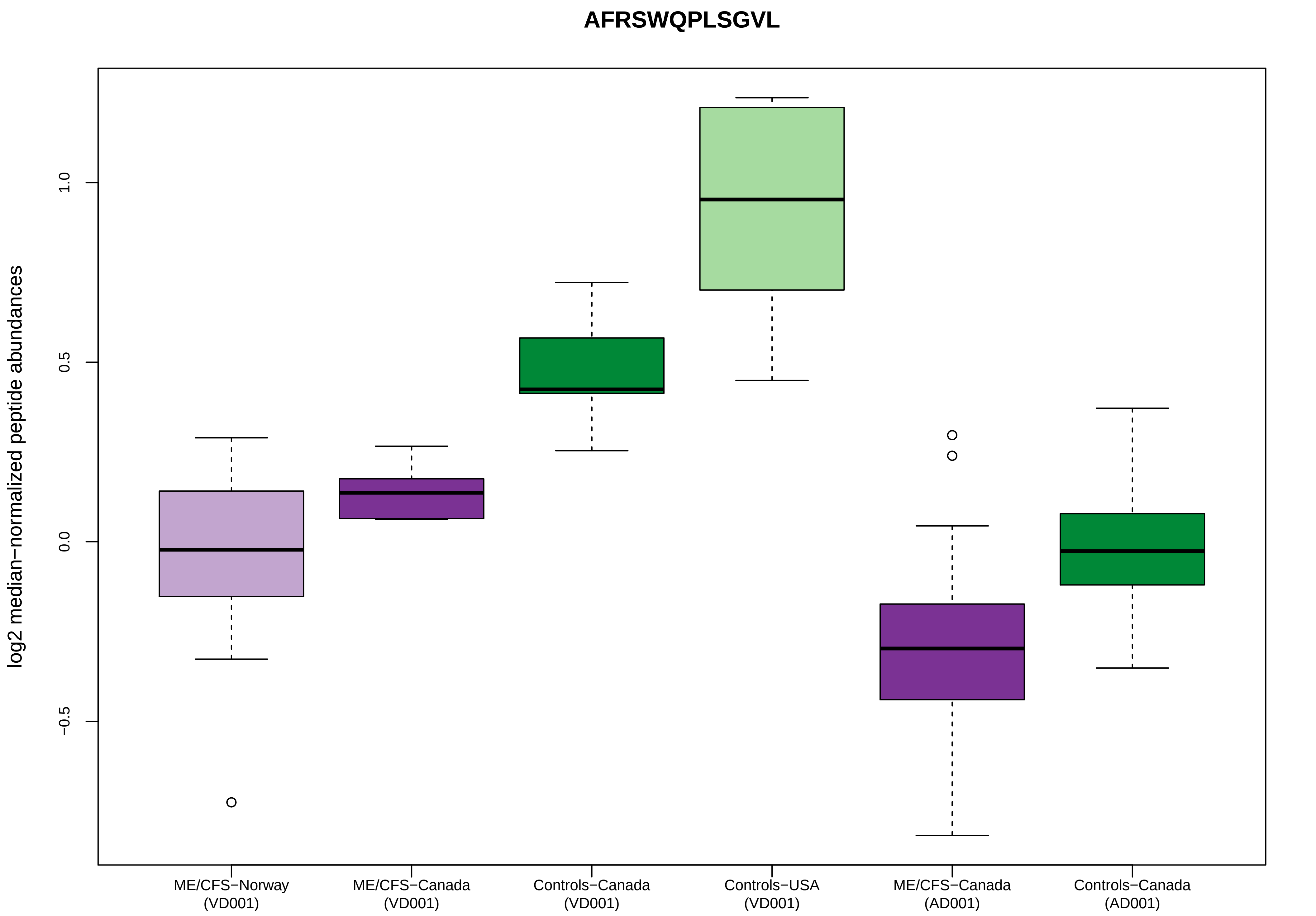

AFRSWQPLSGVL
log2 median−normalized peptide abundances
1.0
0.5
0.0
−0.5
ME/CFS−Norway (VD001)
ME/CFS−Canada (VD001)
Controls−Canada (VD001)
Controls−USA (VD001)
ME/CFS−Canada (AD001)
Controls−Canada (AD001)

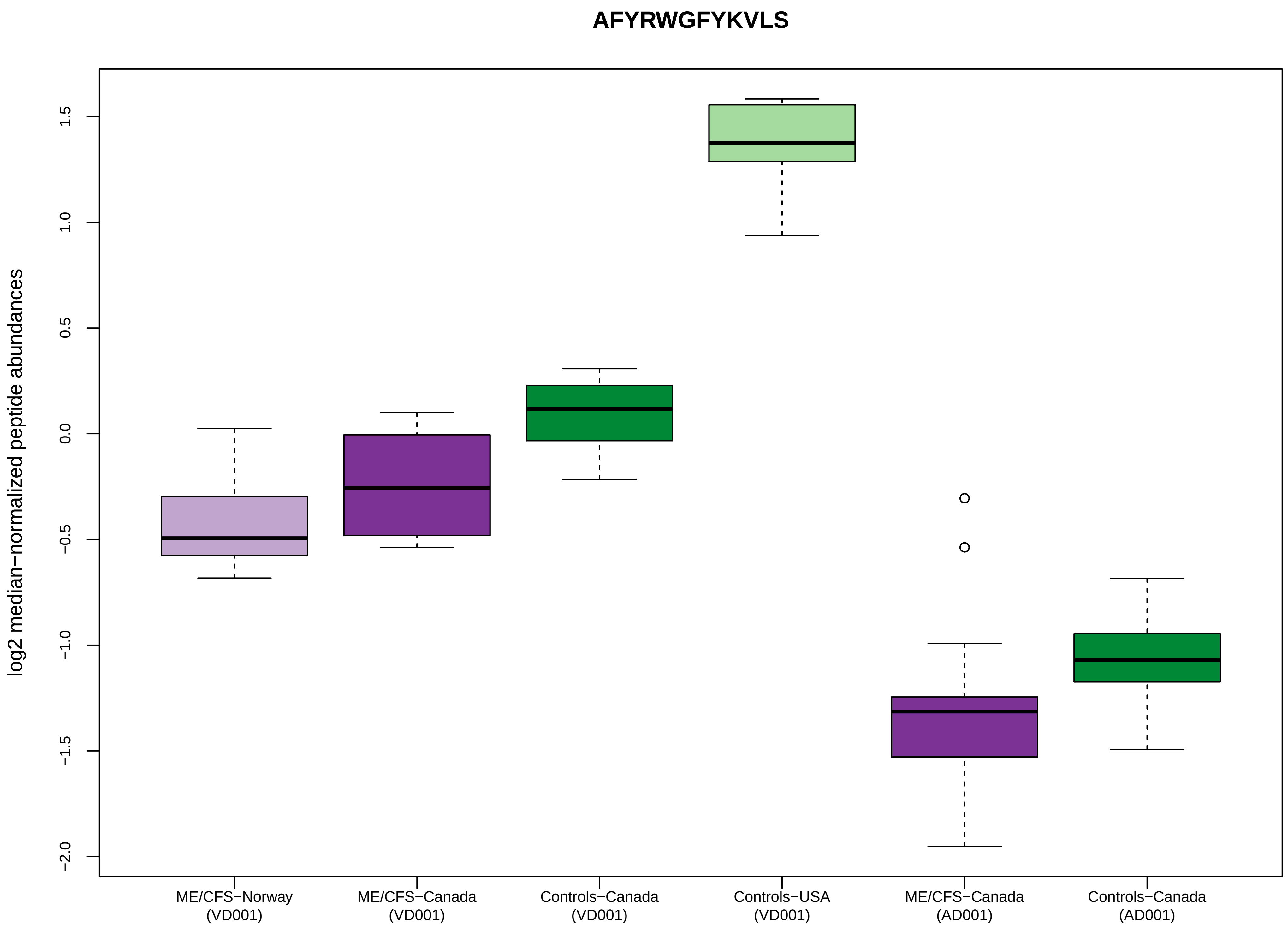
AFYRWGFYKVLS
log2 median−normalized peptide abundances
1.5
1.0
0.5
0.0
−0.5
−1.0
−1.5
−2.0
ME/CFS−Norway (VD001)
ME/CFS−Canada (VD001)
Controls−Canada (VD001)
Controls−USA (VD001)
ME/CFS−Canada (AD001)
Controls−Canada (AD001)

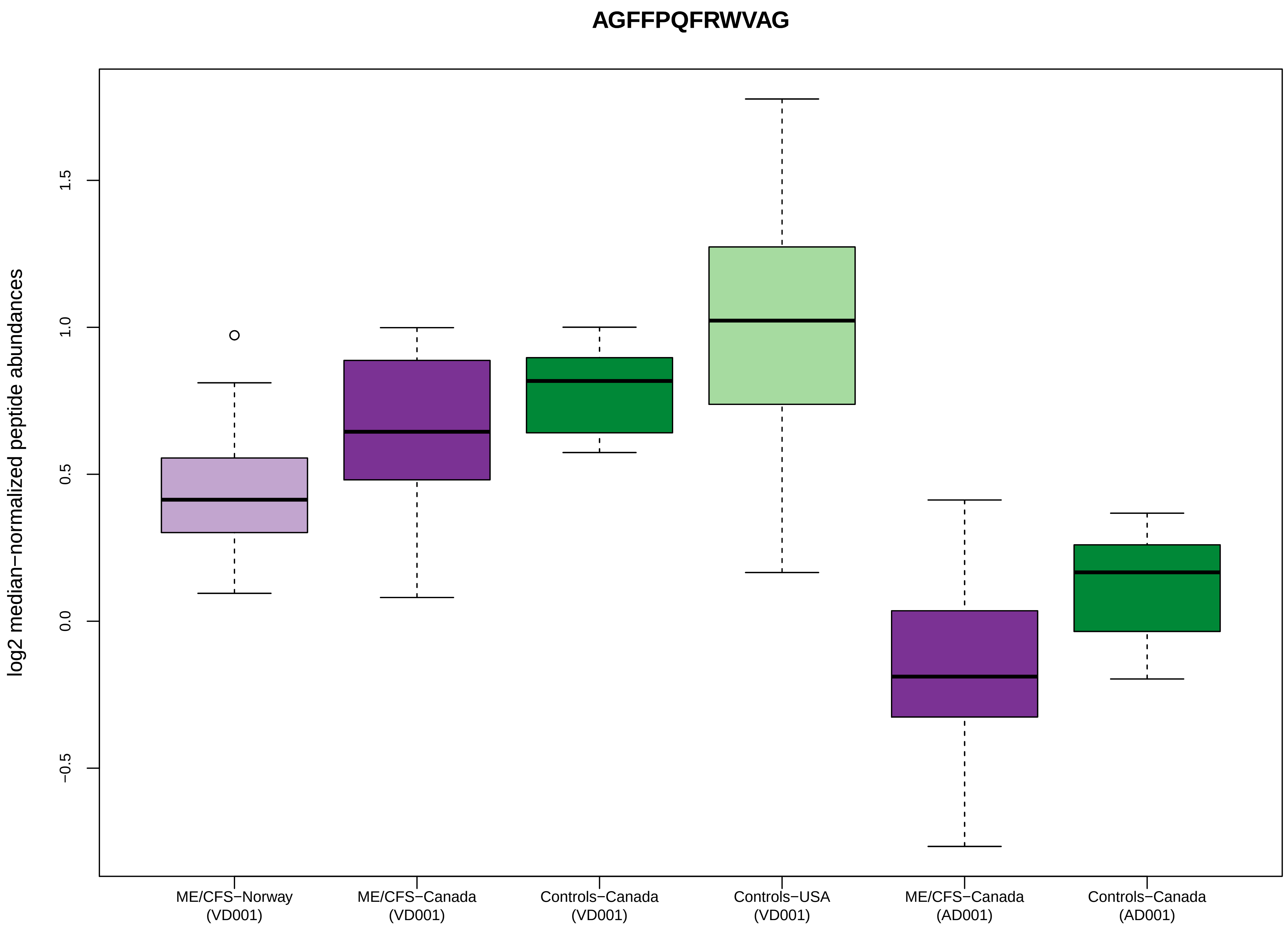

AGFFPQFRWVAG
log2 median−normalized peptide abundances
−0.5
0.0
0.5
1.0
1.5
ME/CFS−Norway (VD001)
ME/CFS−Canada (VD001)
Controls−Canada (VD001)
Controls−USA (VD001)
ME/CFS−Canada (AD001)
Controls−Canada (AD001)

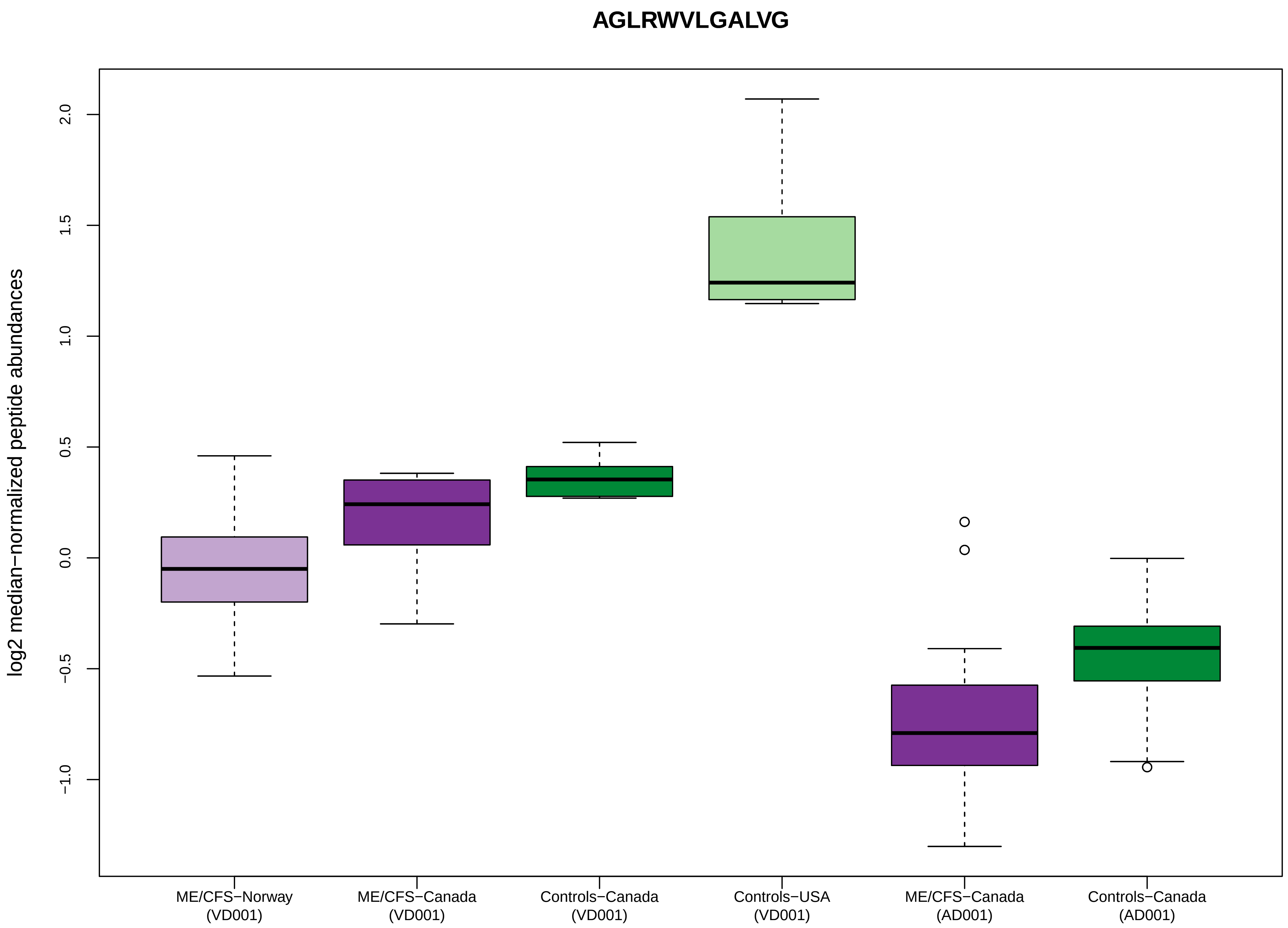

AGLRWVLGALVG
log2 median−normalized peptide abundances
2.0
1.5
1.0
0.5
0.0
−0.5
−1.0
ME/CFS−Norway (VD001)
ME/CFS−Canada (VD001)
Controls−Canada (VD001)
Controls−USA (VD001)
ME/CFS−Canada (AD001)
Controls−Canada (AD001)

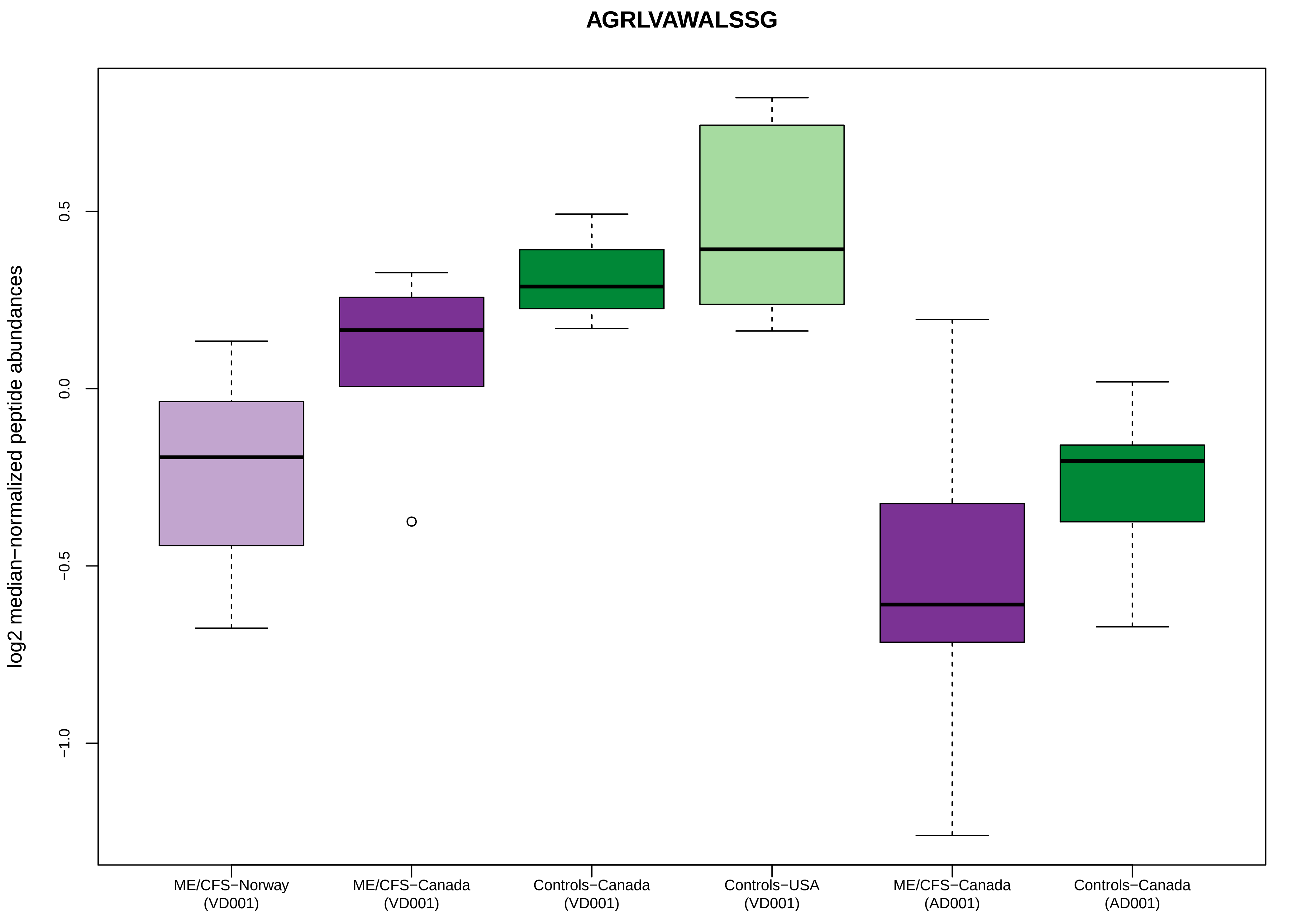

AGRLVAWALSSG
log2 median−normalized peptide abundances
0.5
0.0
−0.5
−1.0
ME/CFS−Norway (VD001)
ME/CFS−Canada (VD001)
Controls−Canada (VD001)
Controls−USA (VD001)
ME/CFS−Canada (AD001)
Controls−Canada (AD001)

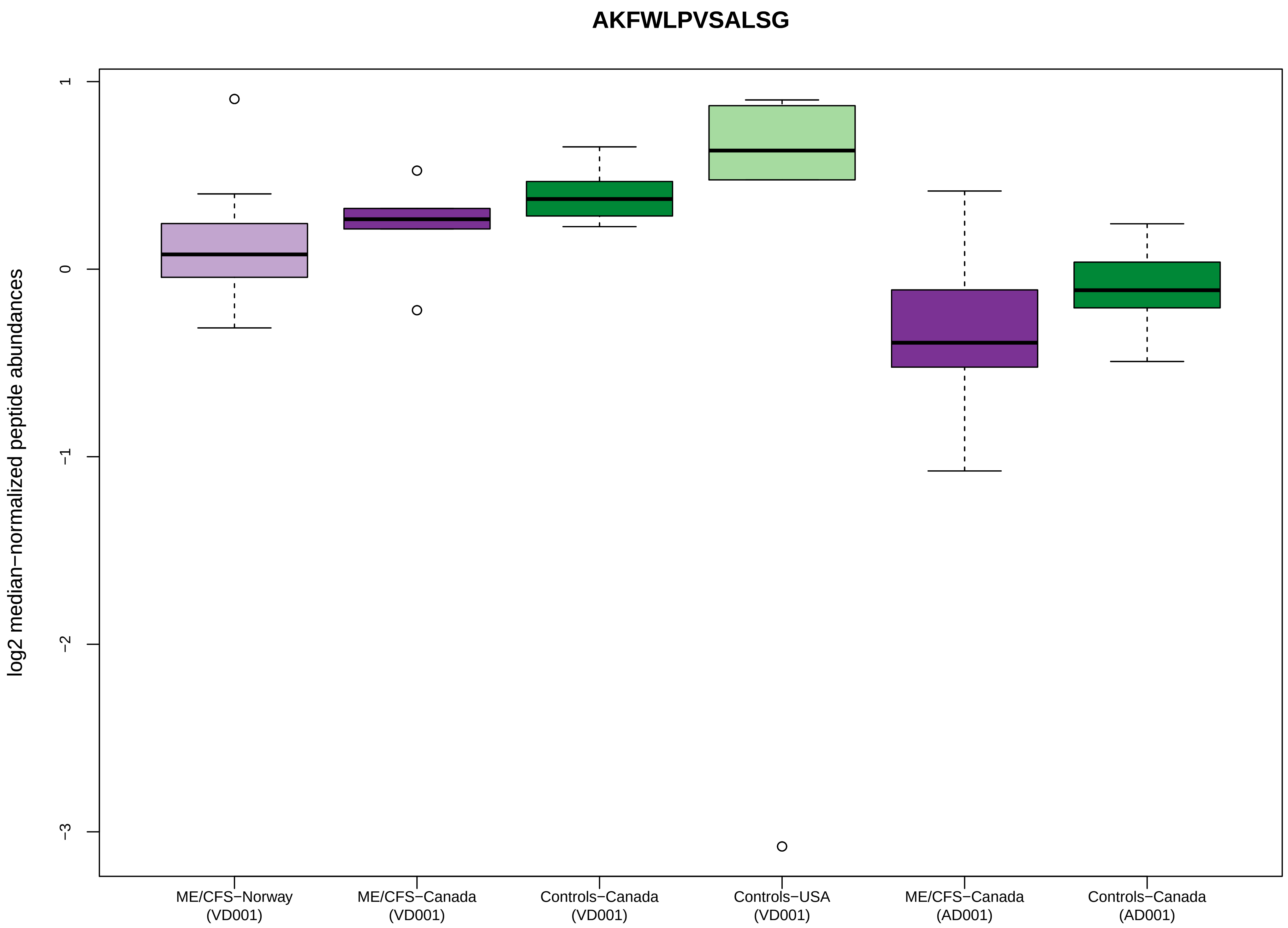
AKFWLPVSALSG
log2 median−normalized peptide abundances
1
0
−1
−2
−3
ME/CFS−Norway (VD001)
ME/CFS−Canada (VD001)
Controls−Canada (VD001)
Controls−USA (VD001)
ME/CFS−Canada (AD001)
Controls−Canada (AD001)

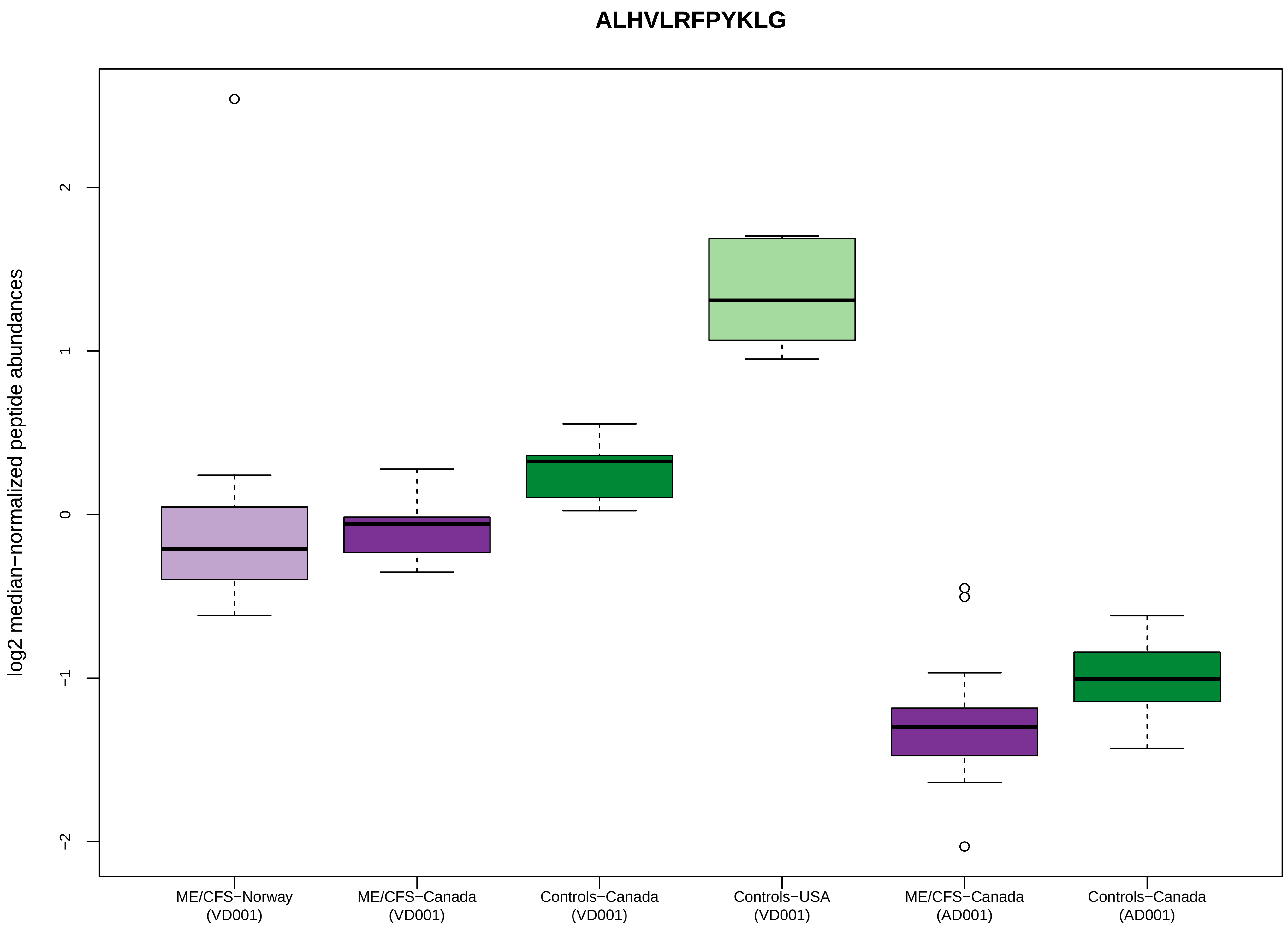

ALHVLRFPYKLG
log2 median−normalized peptide abundances
2
1
0
−1
−2
ME/CFS−Norway (VD001)
ME/CFS−Canada (VD001)
Controls−Canada (VD001)
Controls−USA (VD001)
ME/CFS−Canada (AD001)
Controls−Canada (AD001)

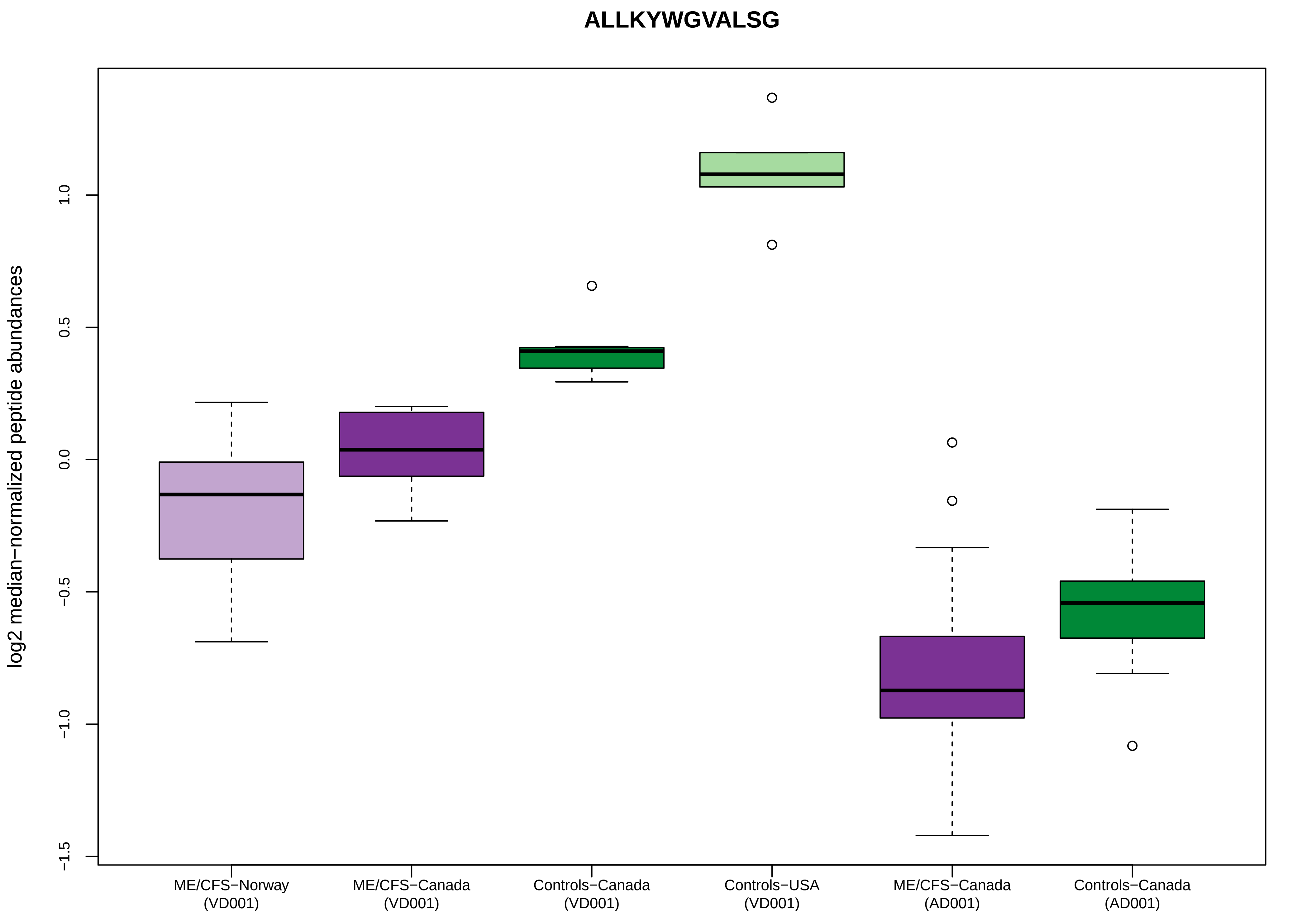
ALLKYWGVALSG
log2 median−normalized peptide abundances
1.0
0.5
0.0
−0.5
−1.0
−1.5
ME/CFS−Norway (VD001)
ME/CFS−Canada (VD001)
Controls−Canada (VD001)
Controls−USA (VD001)
ME/CFS−Canada (AD001)
Controls−Canada (AD001)

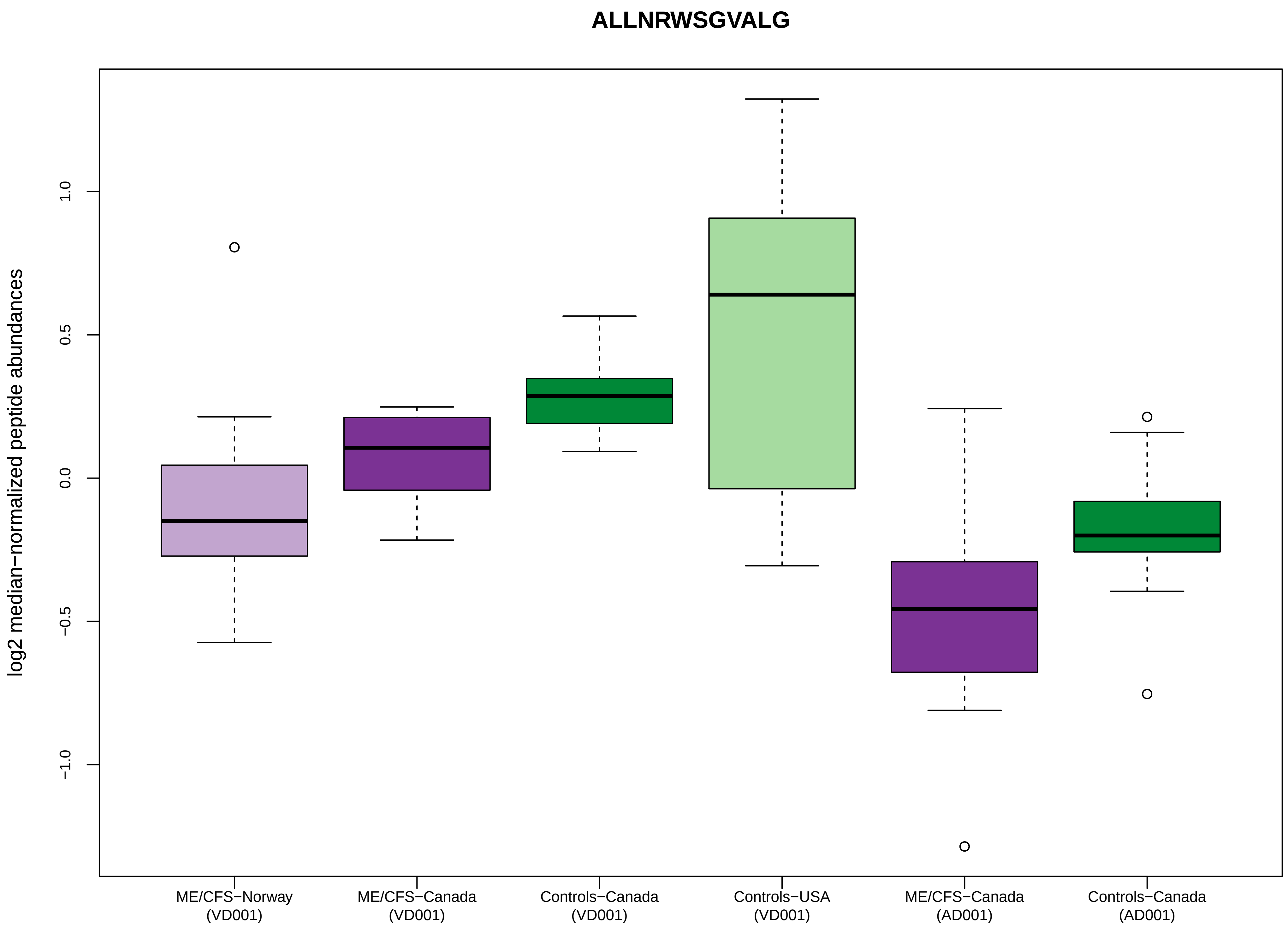
ALLNRWSGVALG
log2 median−normalized peptide abundances
1.0
0.5
0.0
−0.5
−1.0
ME/CFS−Norway (VD001)
ME/CFS−Canada (VD001)
Controls−Canada (VD001)
Controls−USA (VD001)
ME/CFS−Canada (AD001)
Controls−Canada (AD001)

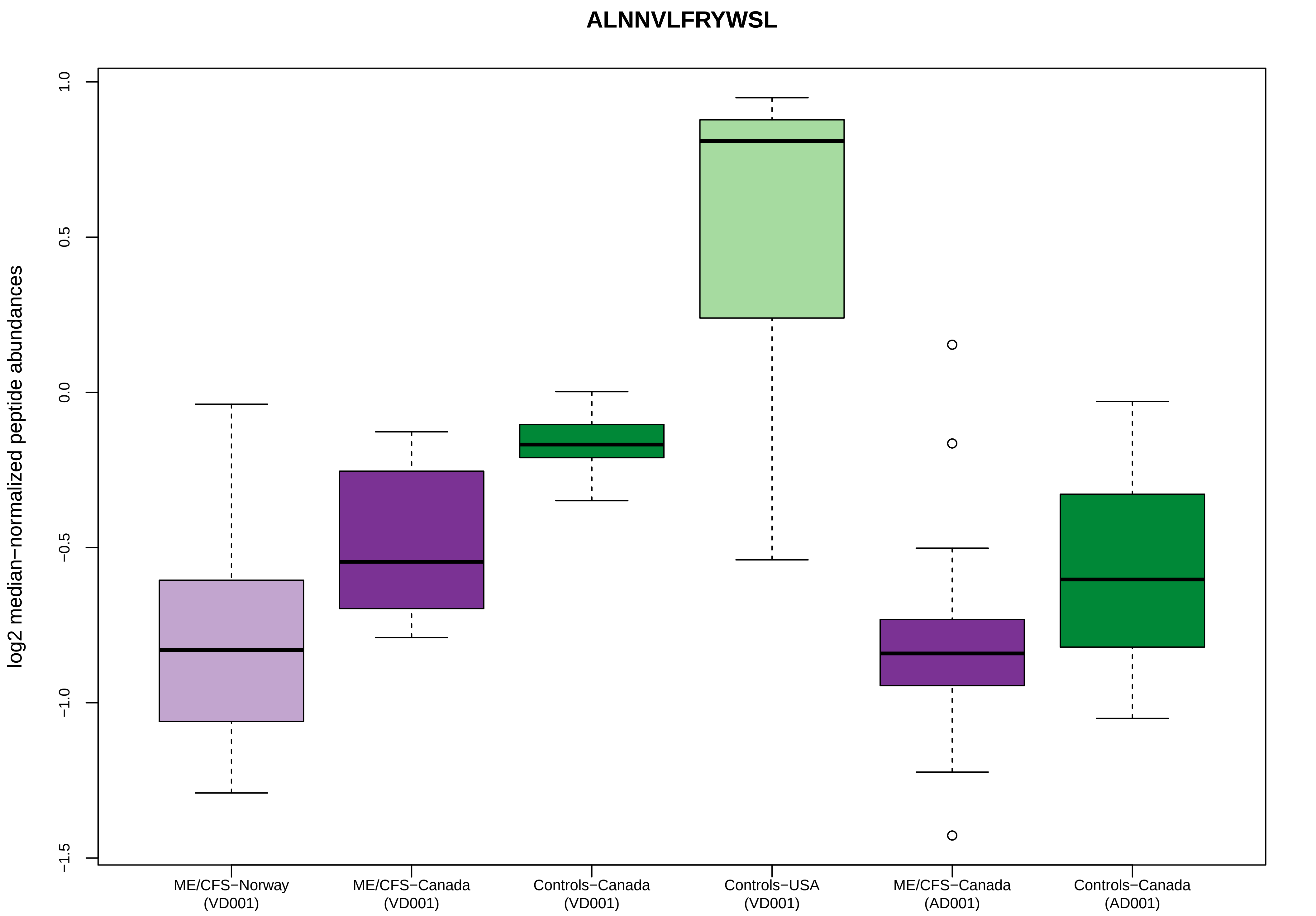

ALNNVLFRYWSL
log2 median−normalized peptide abundances
1.0
0.5
0.0
−0.5
−1.0
−1.5
ME/CFS−Norway (VD001)
ME/CFS−Canada (VD001)
Controls−Canada (VD001)
Controls−USA (VD001)
ME/CFS−Canada (AD001)
Controls−Canada (AD001)

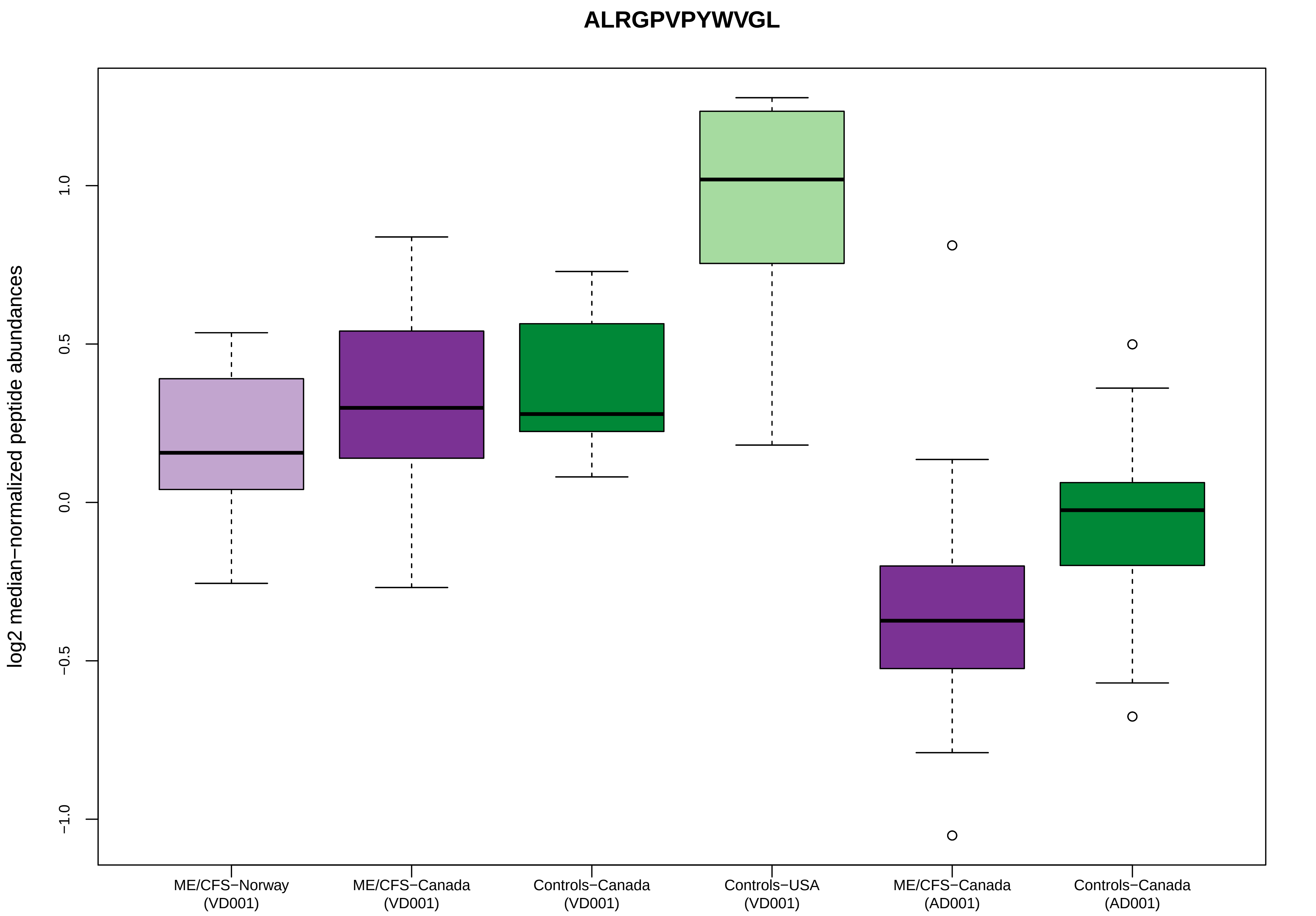
ALRGPVPYWVGL
log2 median−normalized peptide abundances
1.0
0.5
0.0
−0.5
−1.0
ME/CFS−Norway (VD001)
ME/CFS−Canada (VD001)
Controls−Canada (VD001)
Controls−USA (VD001)
ME/CFS−Canada (AD001)
Controls−Canada (AD001)

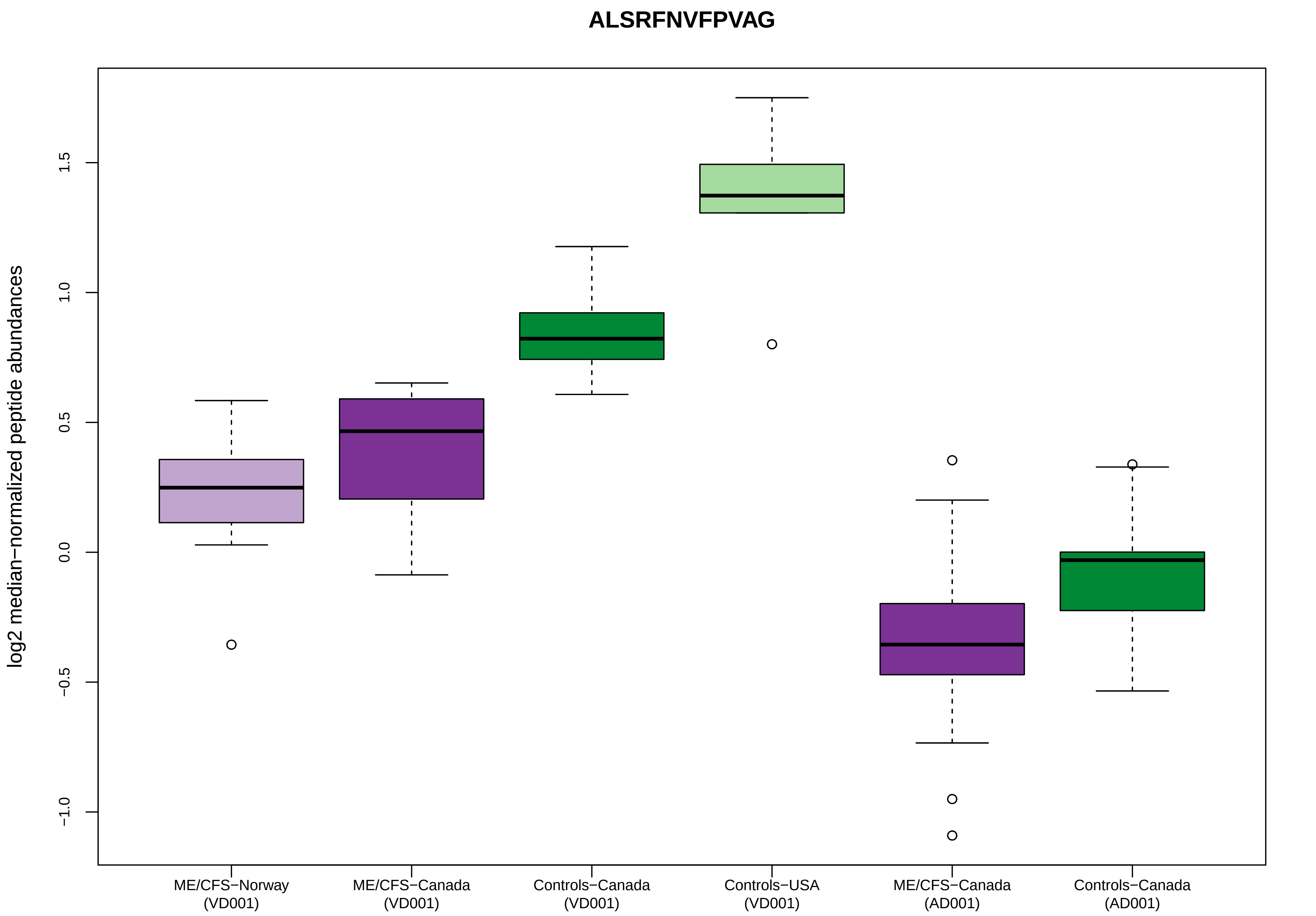
ALSRFNVFPVAG
log2 median−normalized peptide abundances
1.5
1.0
0.5
0.0
−0.5
−1.0
ME/CFS−Norway (VD001)
ME/CFS−Canada (VD001)
Controls−Canada (VD001)
Controls−USA (VD001)
ME/CFS−Canada (AD001)
Controls−Canada (AD001)

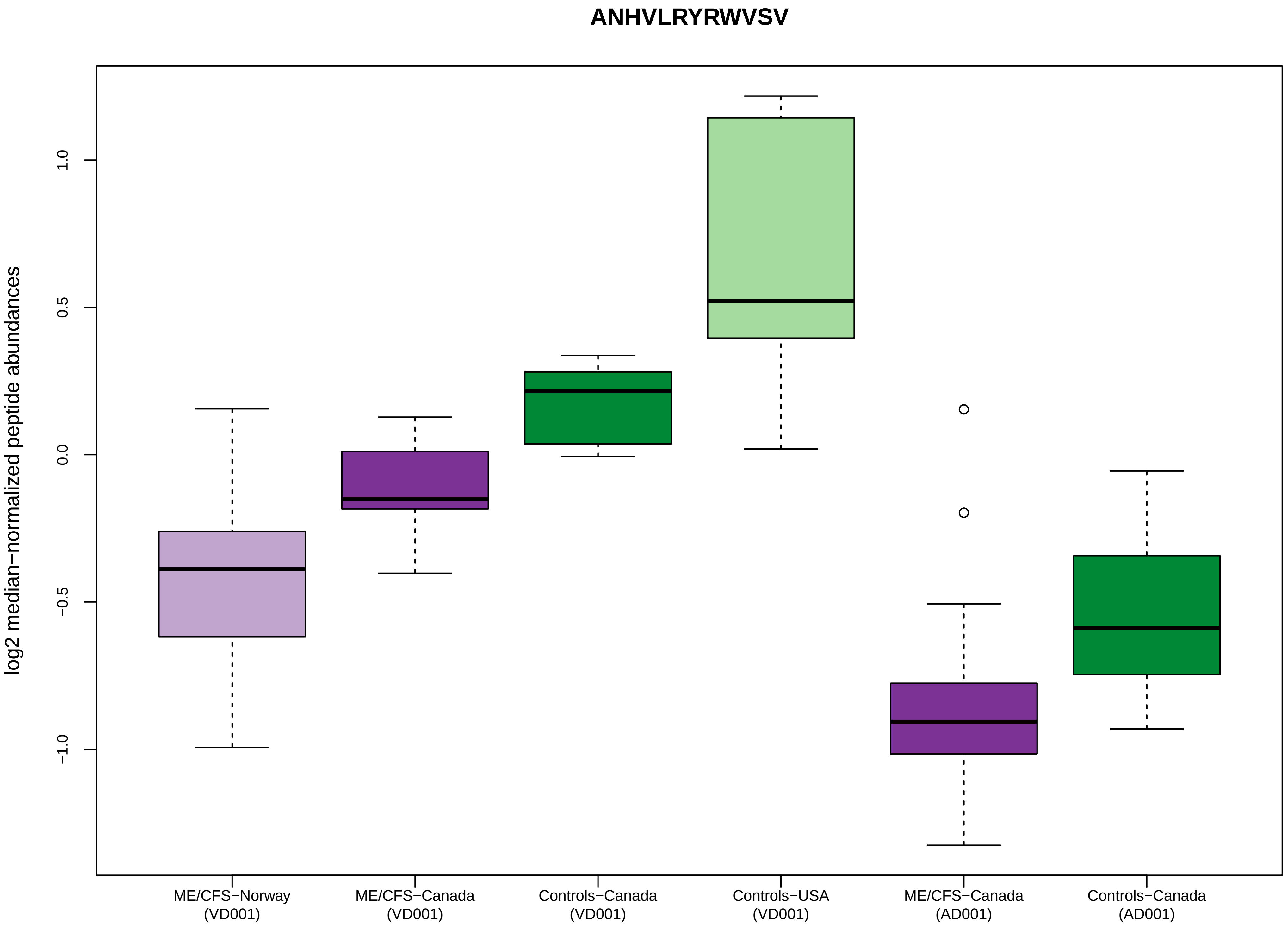
ANHVLRYRWVSV
log2 median−normalized peptide abundances
1.0
0.5
0.0
−0.5
−1.0
ME/CFS−Norway (VD001)
ME/CFS−Canada (VD001)
Controls−Canada (VD001)
Controls−USA (VD001)
ME/CFS−Canada (AD001)
Controls−Canada (AD001)

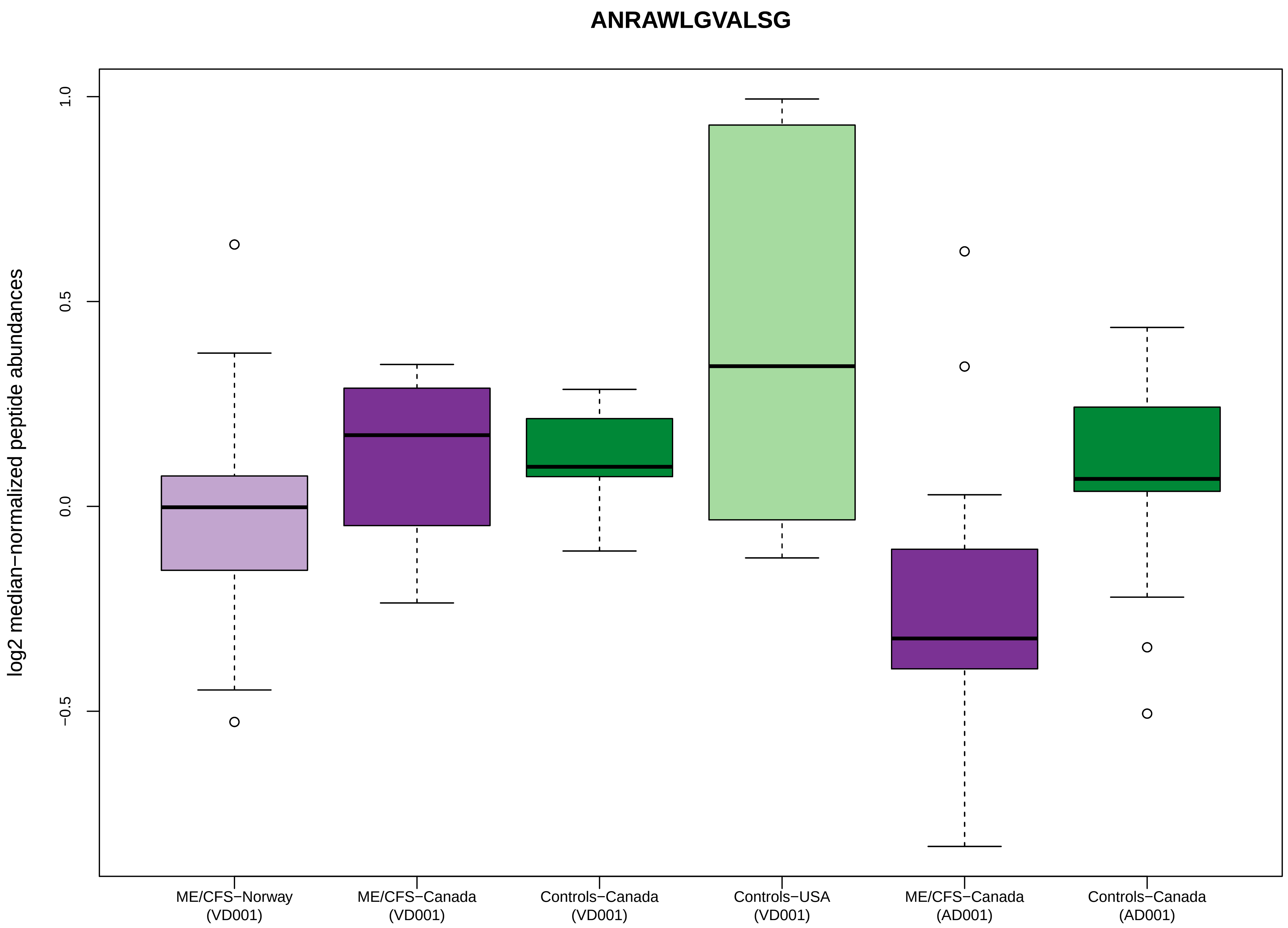

ANRAWLGVALSG
log2 median−normalized peptide abundances
1.0
0.5
0.0
−0.5
ME/CFS−Norway (VD001)
ME/CFS−Canada (VD001)
Controls−Canada (VD001)
Controls−USA (VD001)
ME/CFS−Canada (AD001)
Controls−Canada (AD001)

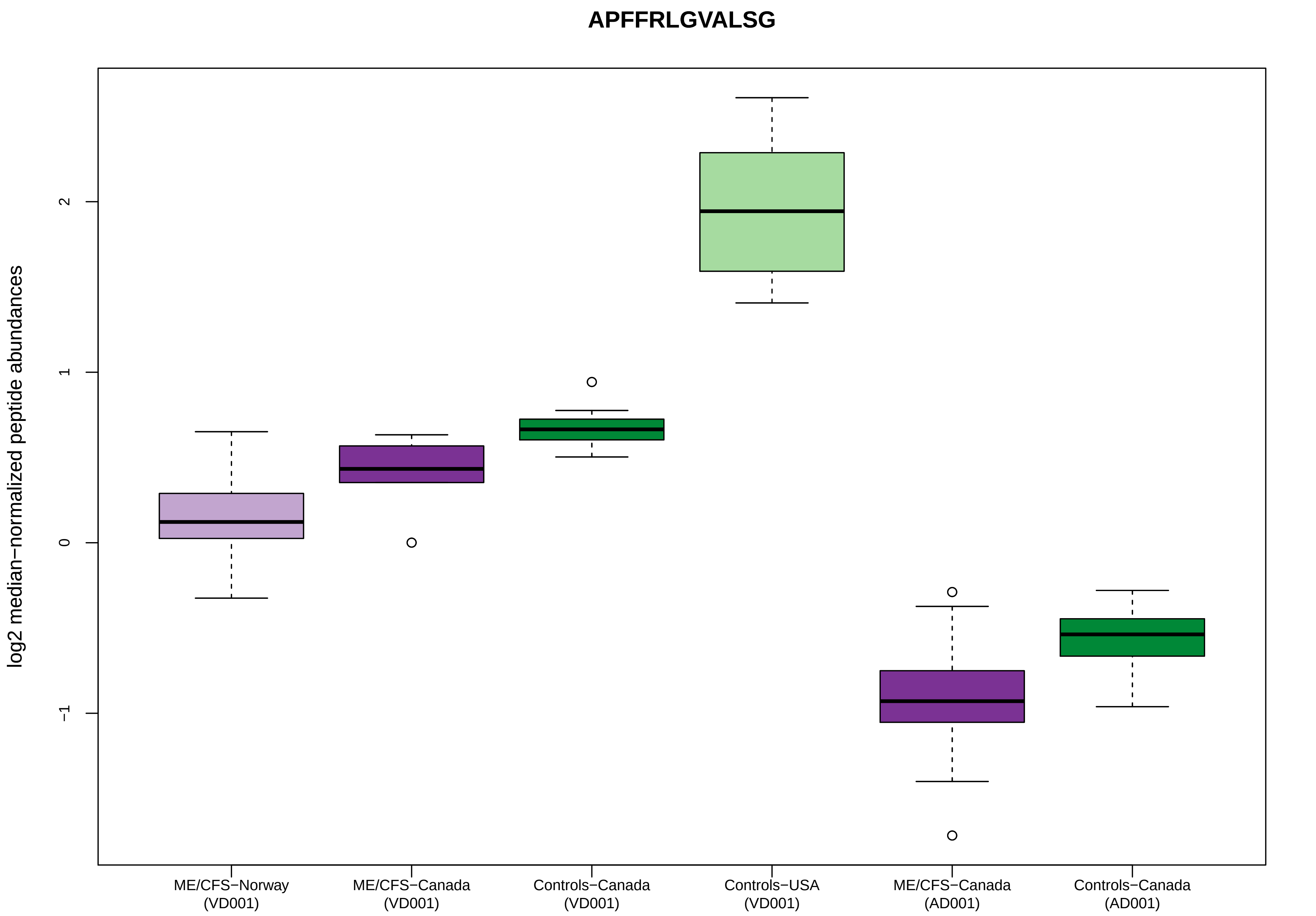
APFFRLGVALSG
log2 median−normalized peptide abundances
2
1
0
−1
ME/CFS−Norway (VD001)
ME/CFS−Canada (VD001)
Controls−Canada (VD001)
Controls−USA (VD001)
ME/CFS−Canada (AD001)
Controls−Canada (AD001)

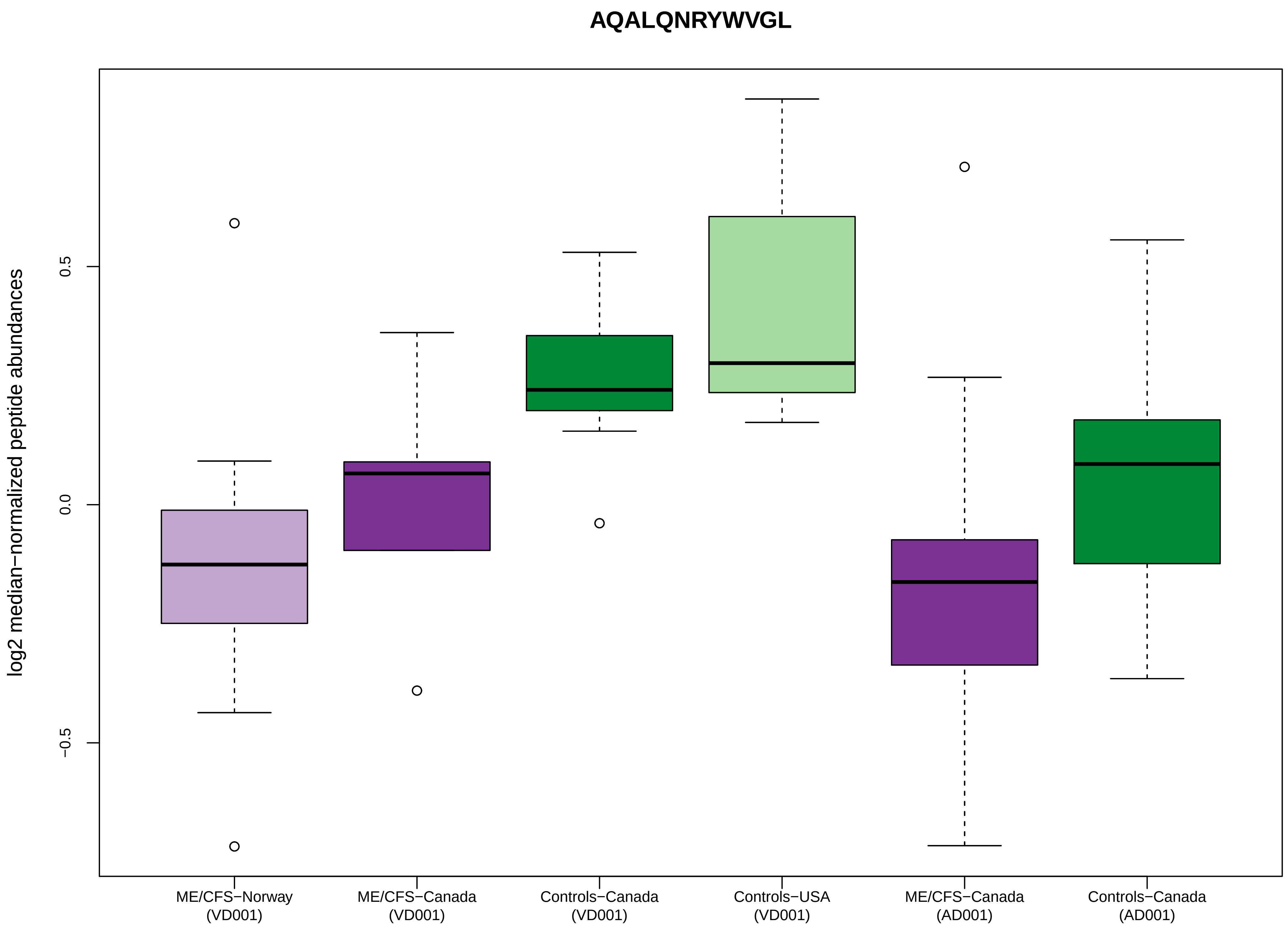

AQALQNRYWVGL
log2 median−normalized peptide abundances
0.5
0.0
−0.5
ME/CFS−Norway (VD001)
ME/CFS−Canada (VD001)
Controls−Canada (VD001)
Controls−USA (VD001)
ME/CFS−Canada (AD001)
Controls−Canada (AD001)

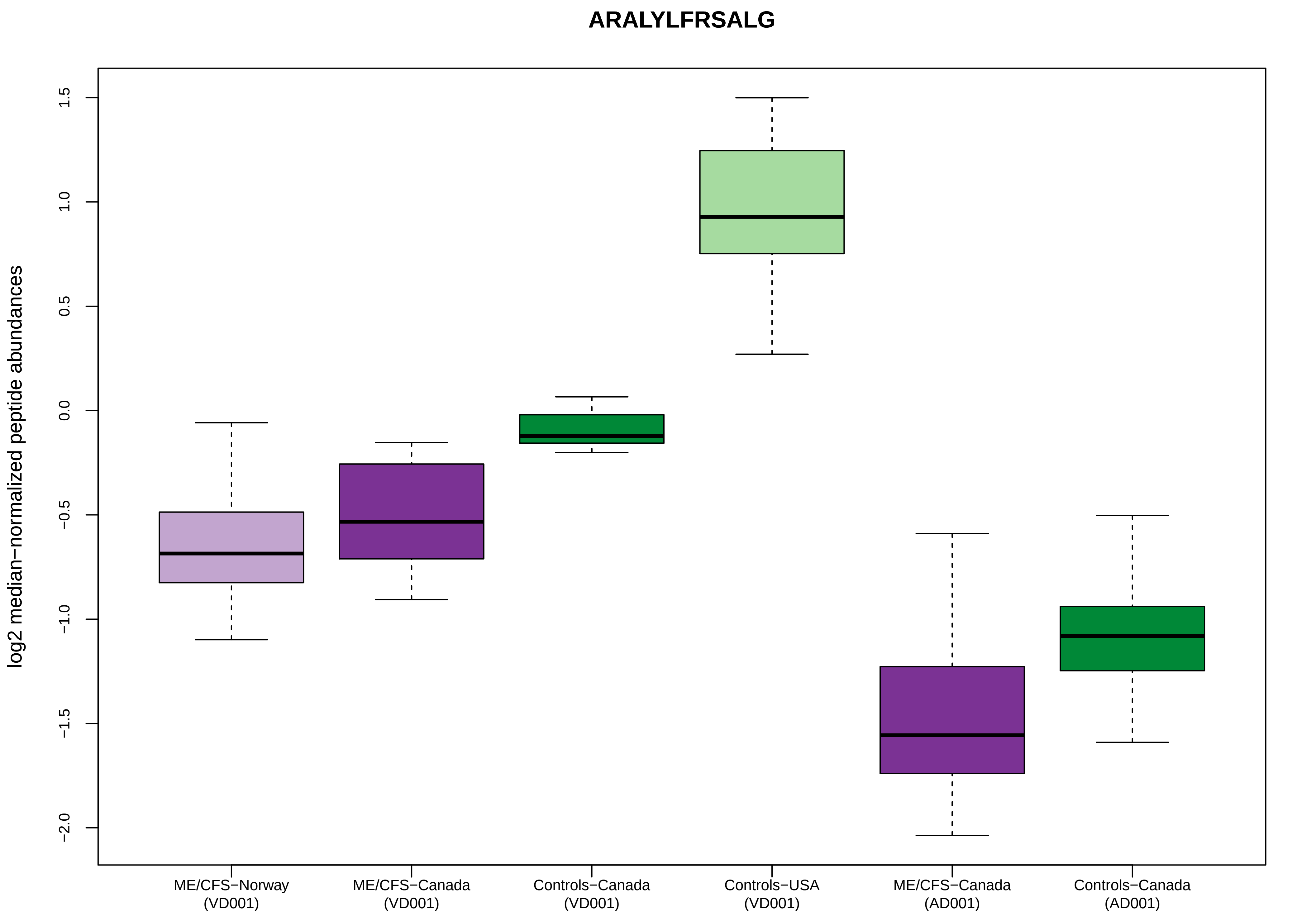
ARALYLFRSALG
log2 median−normalized peptide abundances
1.5
1.0
0.5
0.0
−0.5
−1.0
−1.5
−2.0
ME/CFS−Norway (VD001)
ME/CFS−Canada (VD001)
Controls−Canada (VD001)
Controls−USA (VD001)
ME/CFS−Canada (AD001)
Controls−Canada (AD001)

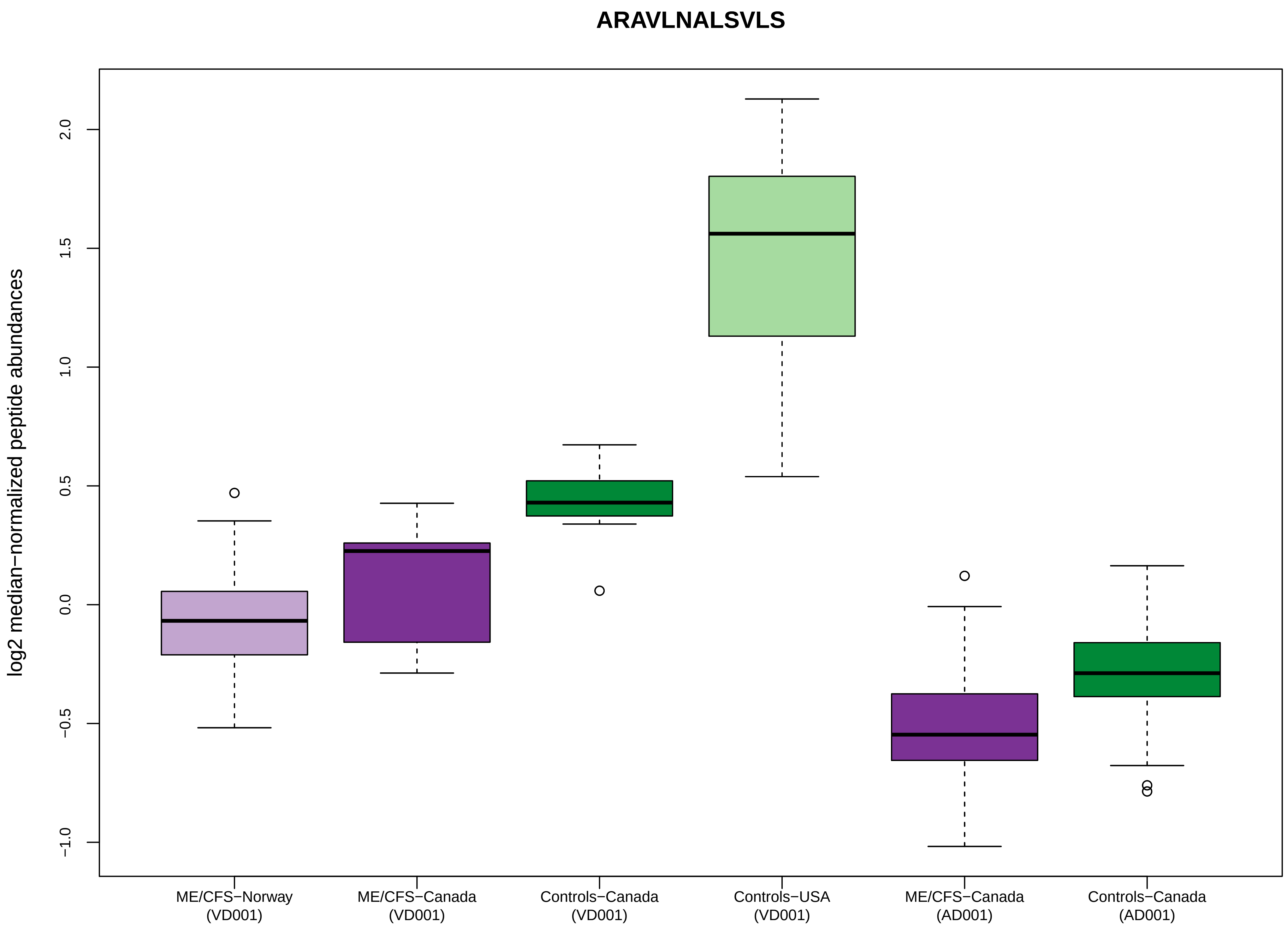
ARAVLNALSVLS
log2 median−normalized peptide abundances
2.0
1.5
1.0
0.5
0.0
−0.5
−1.0
ME/CFS−Norway (VD001)
ME/CFS−Canada (VD001)
Controls−Canada (VD001)
Controls−USA (VD001)
ME/CFS−Canada (AD001)
Controls−Canada (AD001)

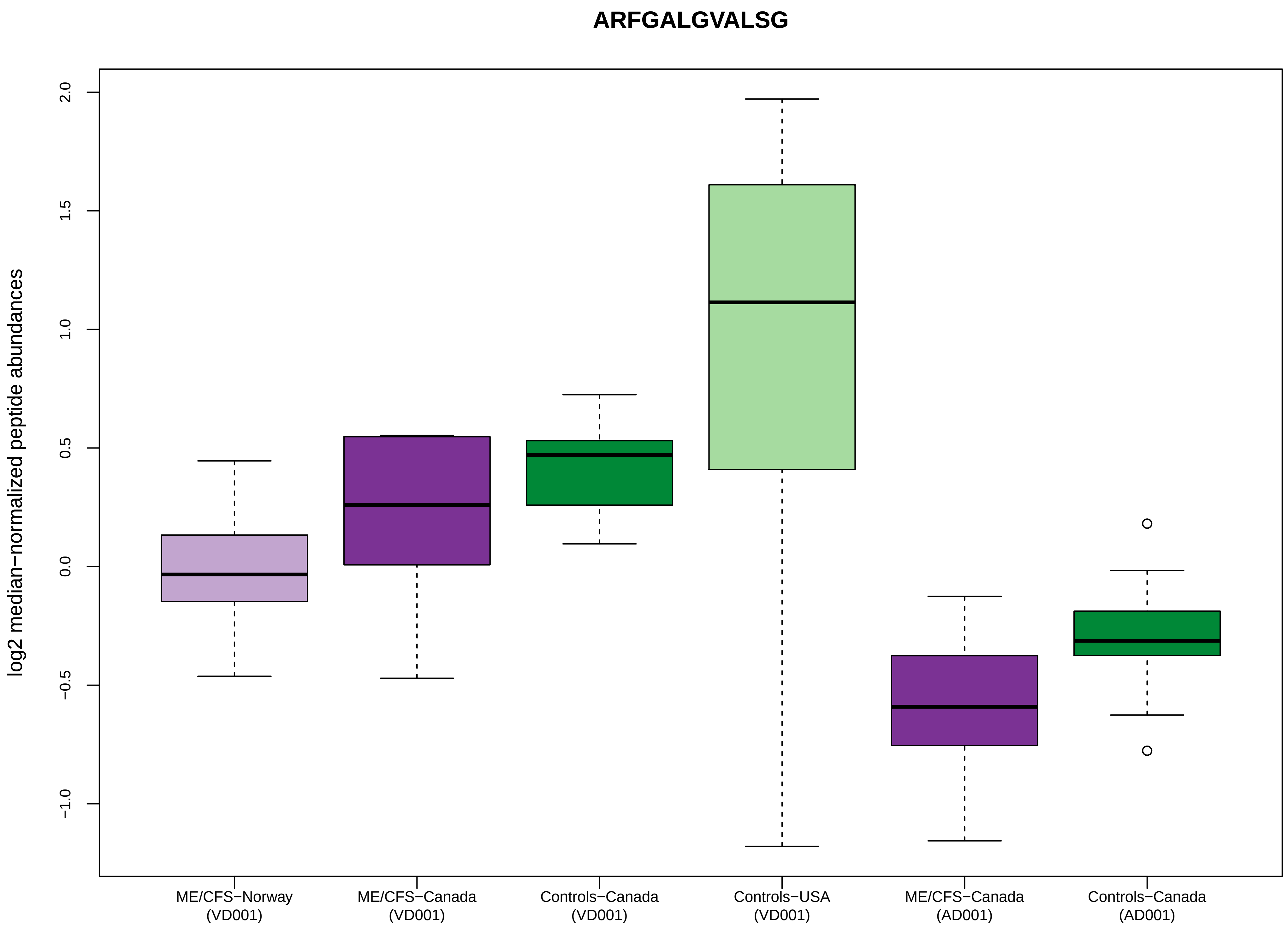

ARFGALGVALSG
log2 median−normalized peptide abundances
2.0
1.5
1.0
0.5
0.0
−0.5
−1.0
ME/CFS−Norway (VD001)
ME/CFS−Canada (VD001)
Controls−Canada (VD001)
Controls−USA (VD001)
ME/CFS−Canada (AD001)
Controls−Canada (AD001)

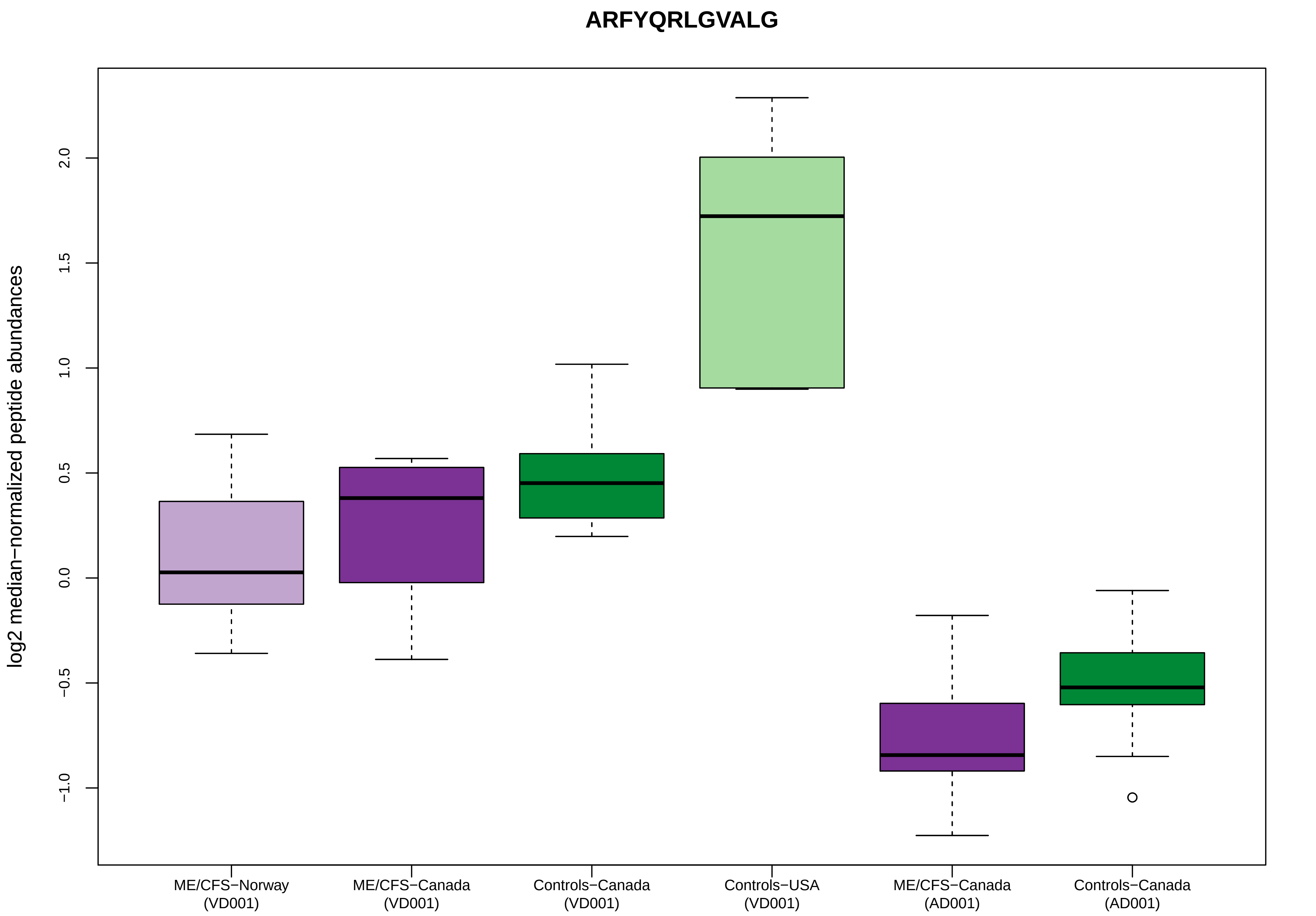
ARFYQRLGVALG
log2 median−normalized peptide abundances
−1.0
−0.5
0.0
0.5
1.0
1.5
2.0
ME/CFS−Norway (VD001)
ME/CFS−Canada (VD001)
Controls−Canada (VD001)
Controls−USA (VD001)
ME/CFS−Canada (AD001)
Controls−Canada (AD001)

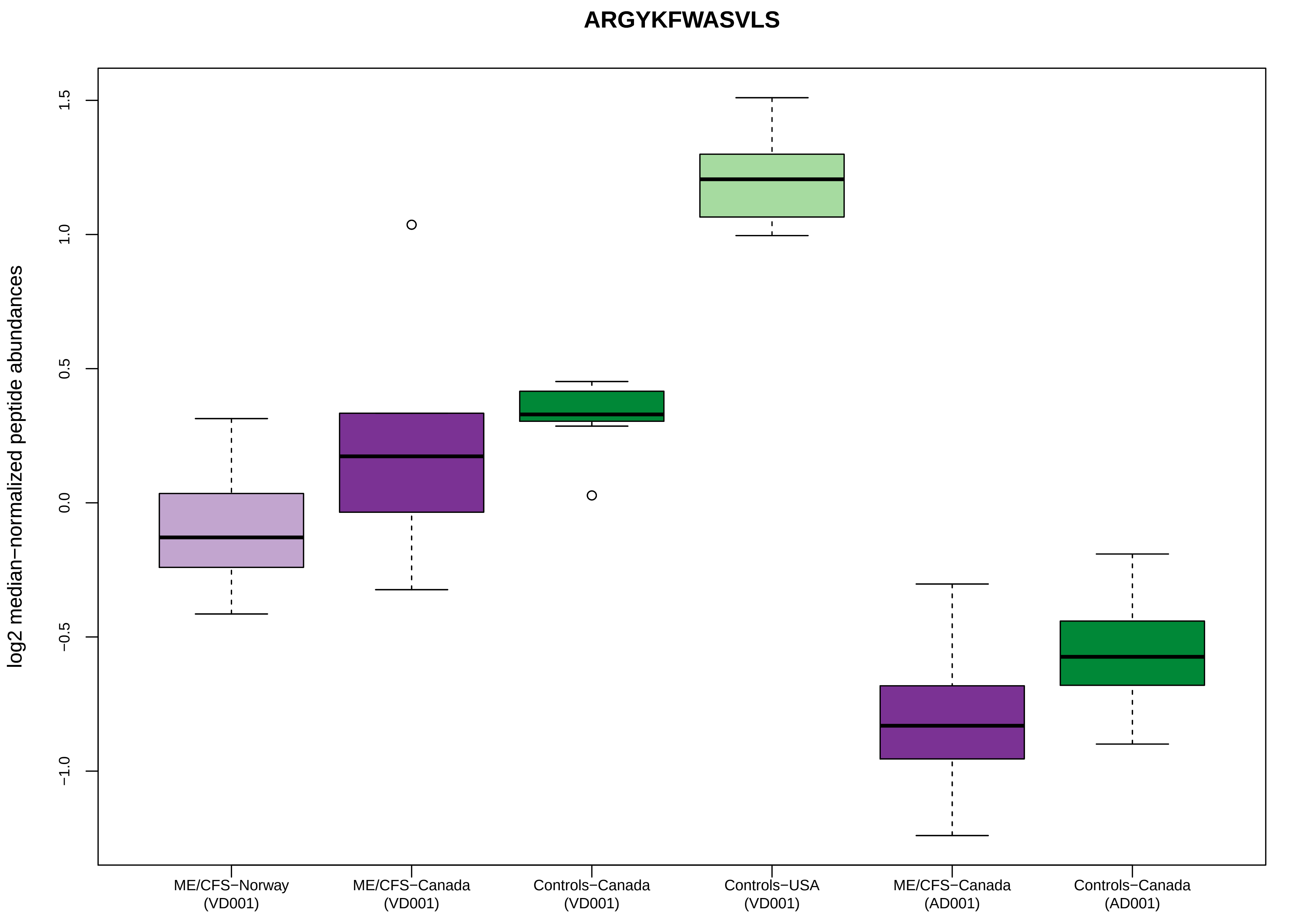

ARGYKFWASVLS
log2 median−normalized peptide abundances
1.5
1.0
0.5
0.0
−0.5
−1.0
ME/CFS−Norway (VD001)
ME/CFS−Canada (VD001)
Controls−Canada (VD001)
Controls−USA (VD001)
ME/CFS−Canada (AD001)
Controls−Canada (AD001)

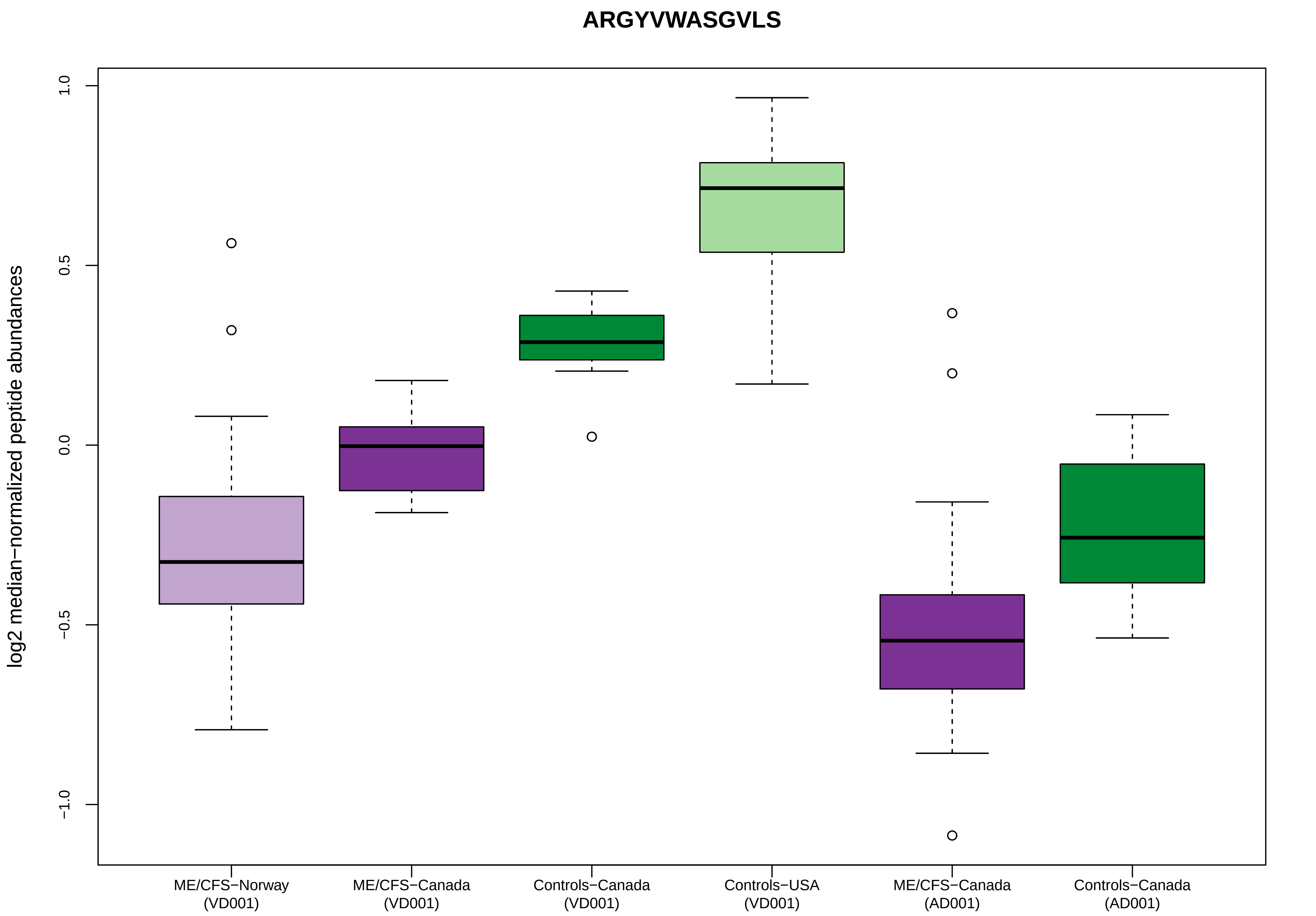
ARGYVWASGVLS
log2 median−normalized peptide abundances
1.0
0.5
0.0
−0.5
−1.0
ME/CFS−Norway (VD001)
ME/CFS−Canada (VD001)
Controls−Canada (VD001)
Controls−USA (VD001)
ME/CFS−Canada (AD001)
Controls−Canada (AD001)

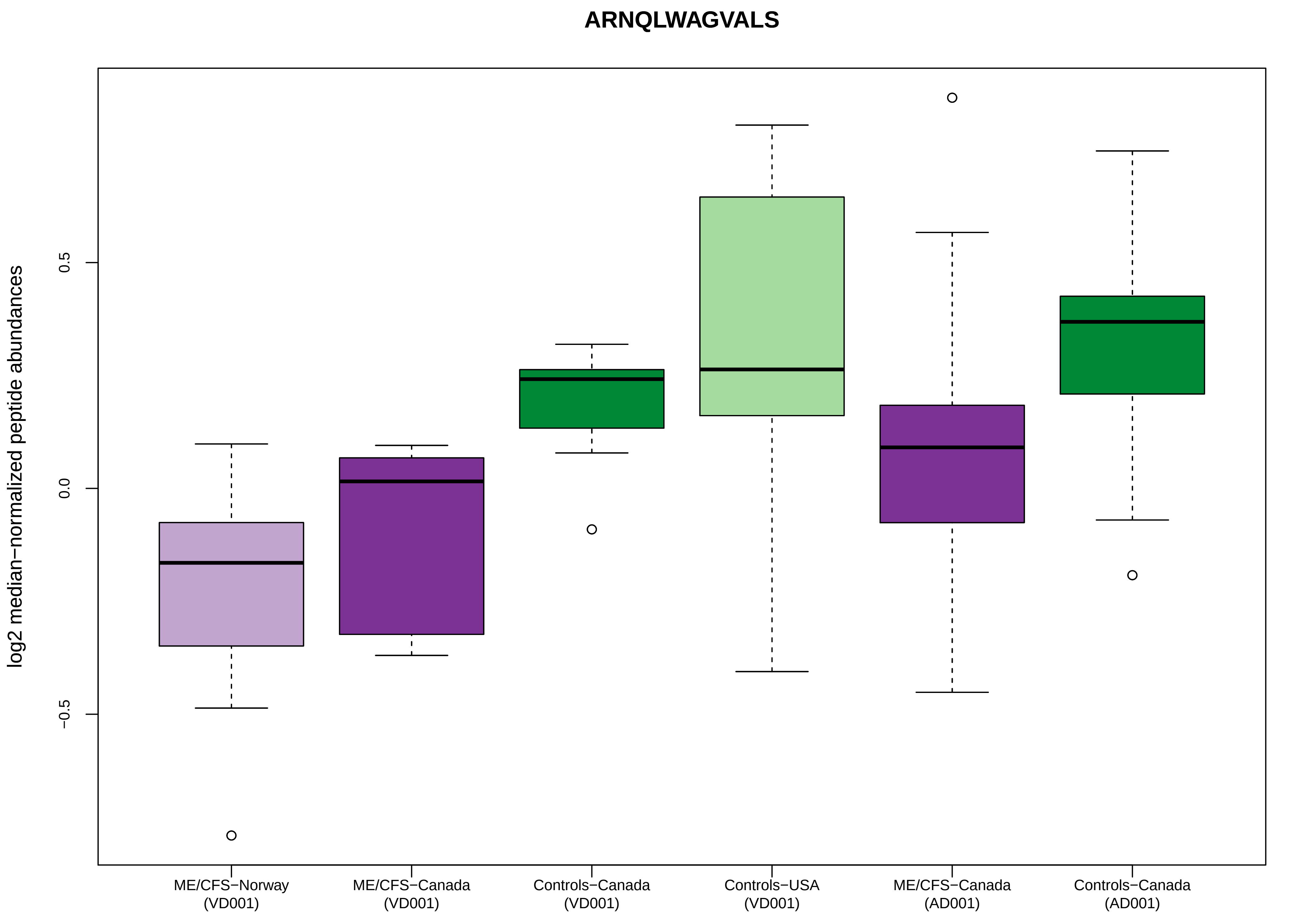
ARNQLWAGVALS
log2 median−normalized peptide abundances
0.5
0.0
−0.5
ME/CFS−Norway (VD001)
ME/CFS−Canada (VD001)
Controls−Canada (VD001)
Controls−USA (VD001)
ME/CFS−Canada (AD001)
Controls−Canada (AD001)

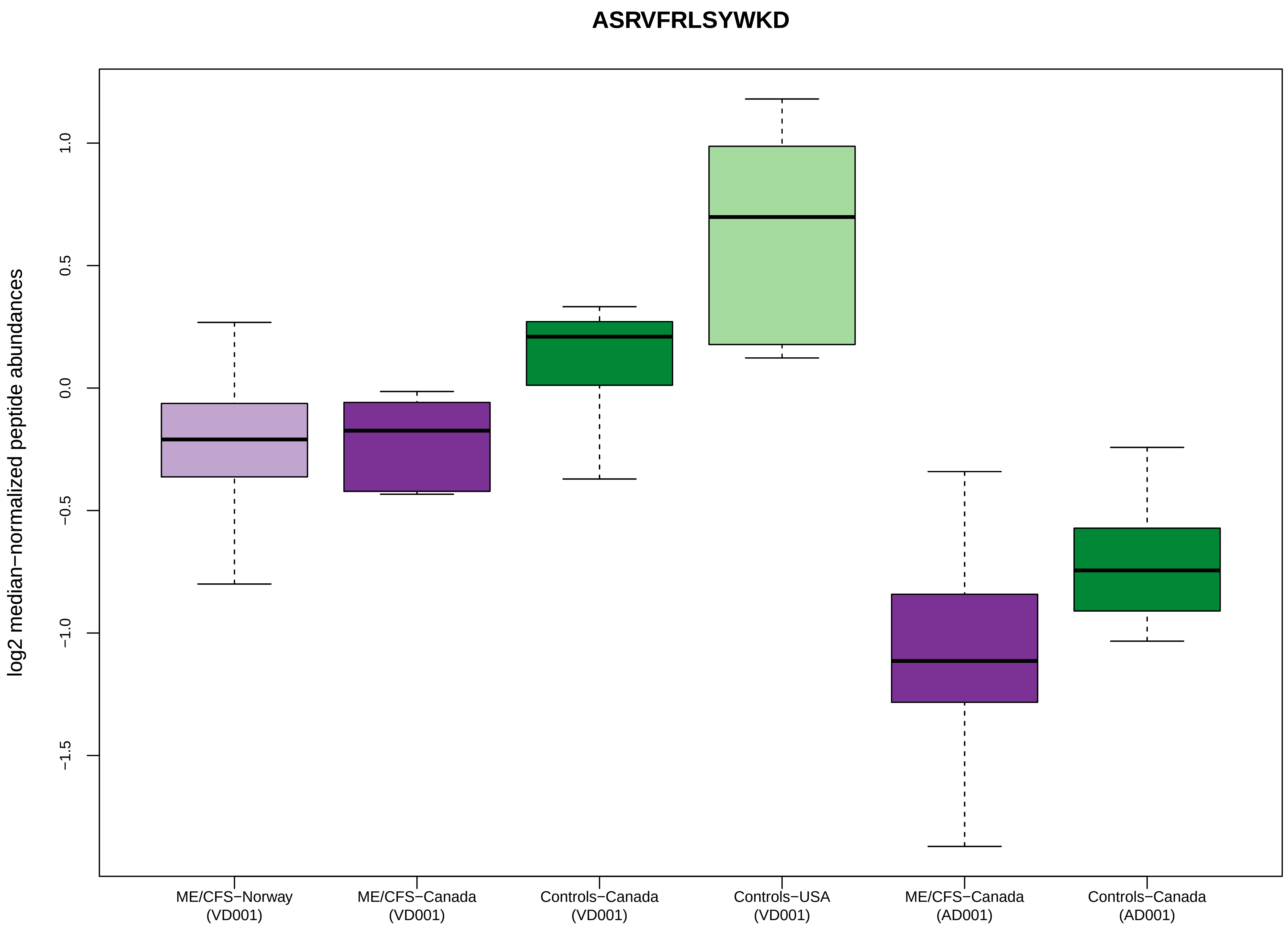

ASRVFRLSYWKD
log2 median−normalized peptide abundances
1.0
0.5
0.0
−0.5
−1.0
−1.5
ME/CFS−Norway (VD001)
ME/CFS−Canada (VD001)
Controls−Canada (VD001)
Controls−USA (VD001)
ME/CFS−Canada (AD001)
Controls−Canada (AD001)

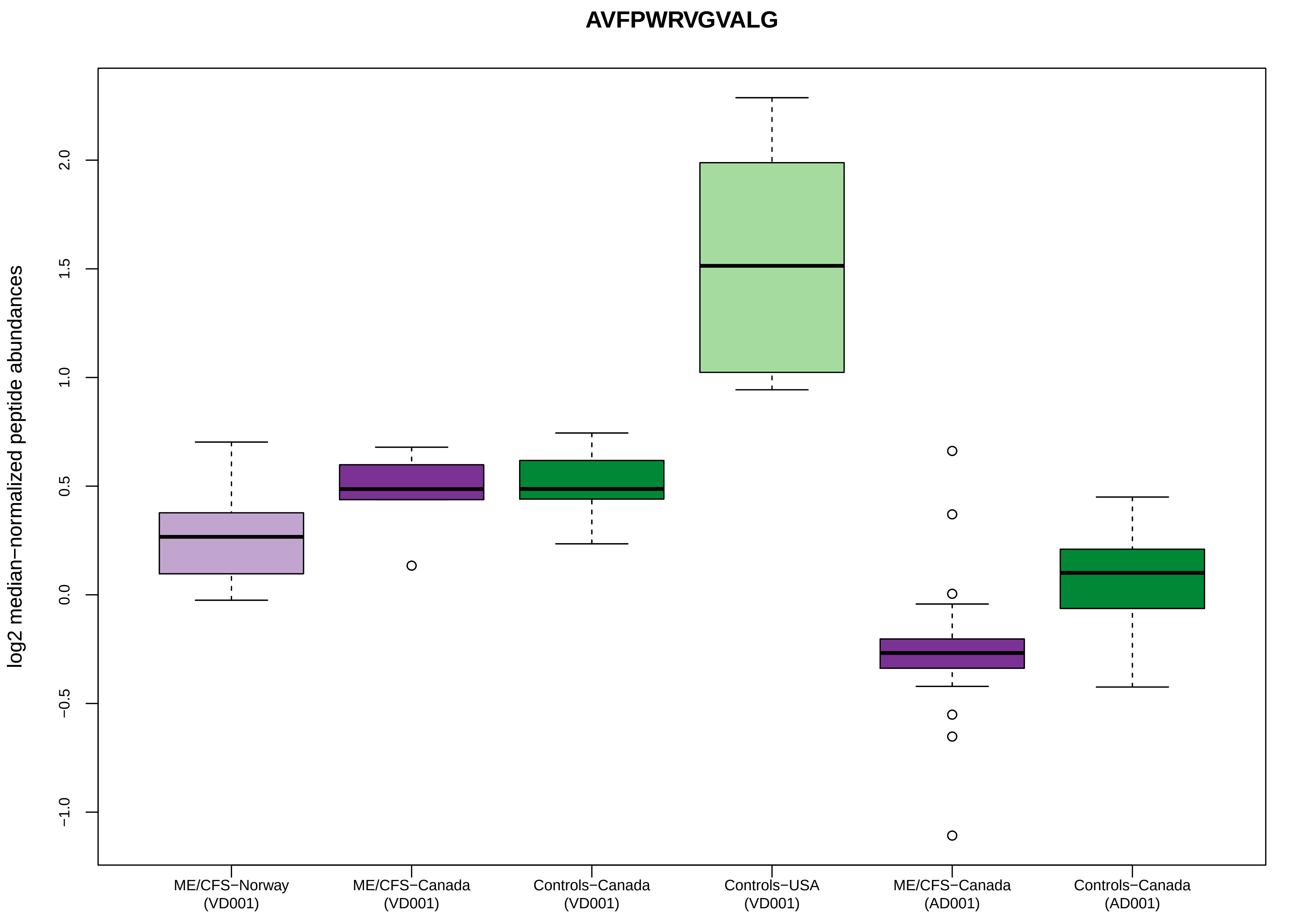
AVFPWRVGVALG
log2 median−normalized peptide abundances
2.0
1.5
1.0
0.5
0.0
−0.5
−1.0
ME/CFS−Norway (VD001)
ME/CFS−Canada (VD001)
Controls−Canada (VD001)
Controls−USA (VD001)
ME/CFS−Canada (AD001)
Controls−Canada (AD001)

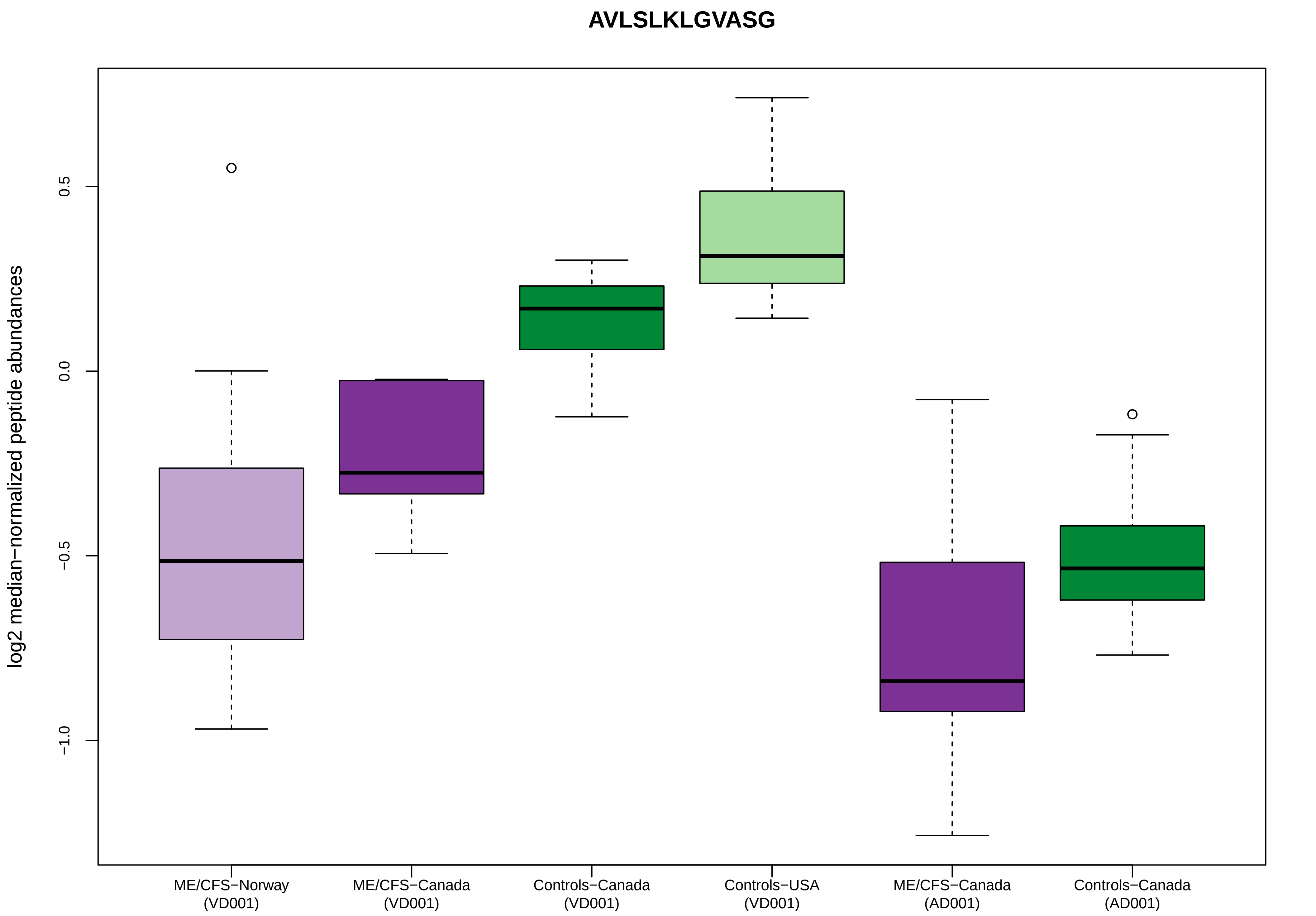
AVLSLKLGVASG
log2 median−normalized peptide abundances
0.5
0.0
−0.5
−1.0
ME/CFS−Norway (VD001)
ME/CFS−Canada (VD001)
Controls−Canada (VD001)
Controls−USA (VD001)
ME/CFS−Canada (AD001)
Controls−Canada (AD001)

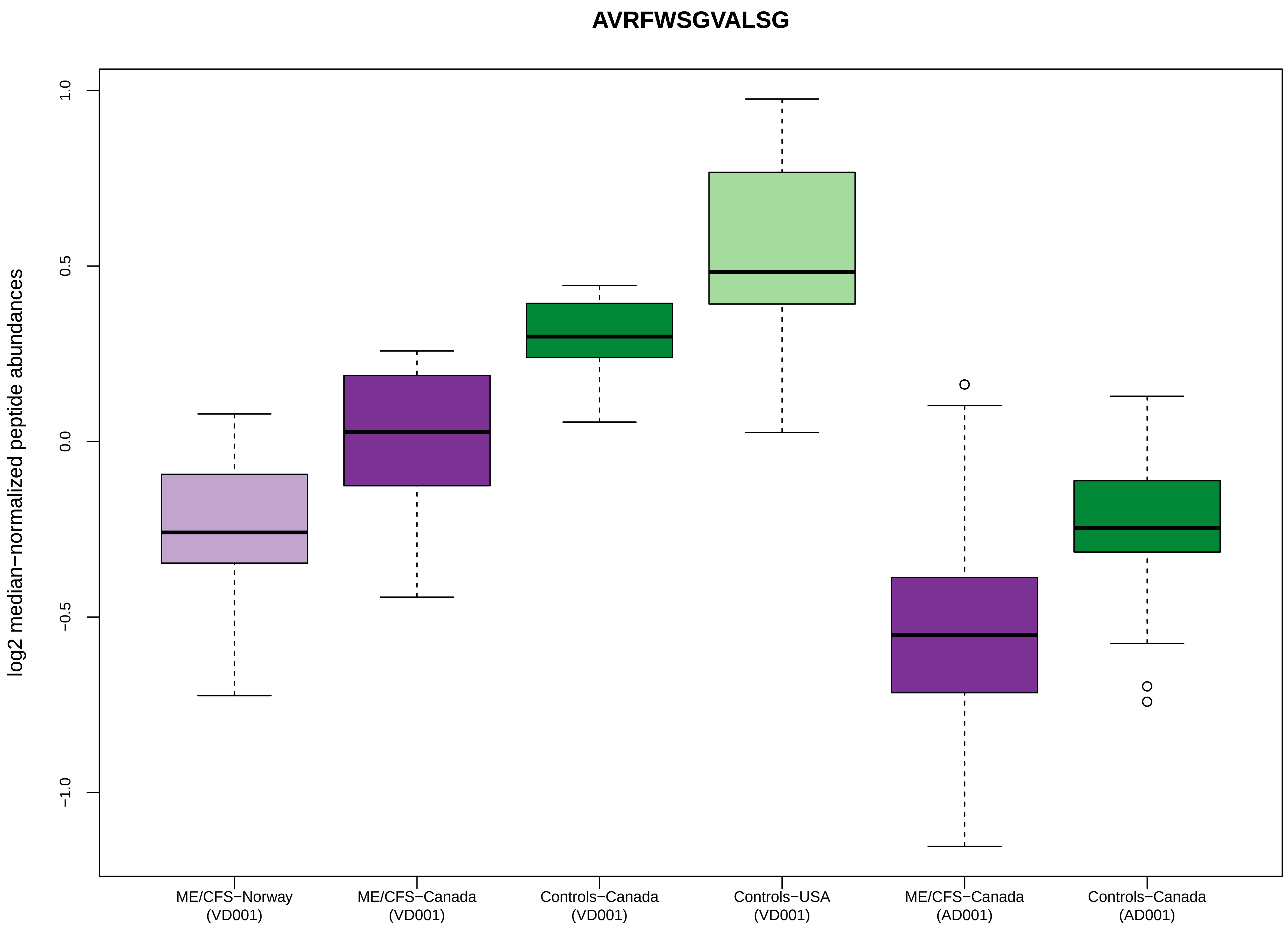

AVRFWSGVALSG
log2 median−normalized peptide abundances
1.0
0.5
0.0
−0.5
−1.0
ME/CFS−Norway (VD001)
ME/CFS−Canada (VD001)
Controls−Canada (VD001)
Controls−USA (VD001)
ME/CFS−Canada (AD001)
Controls−Canada (AD001)

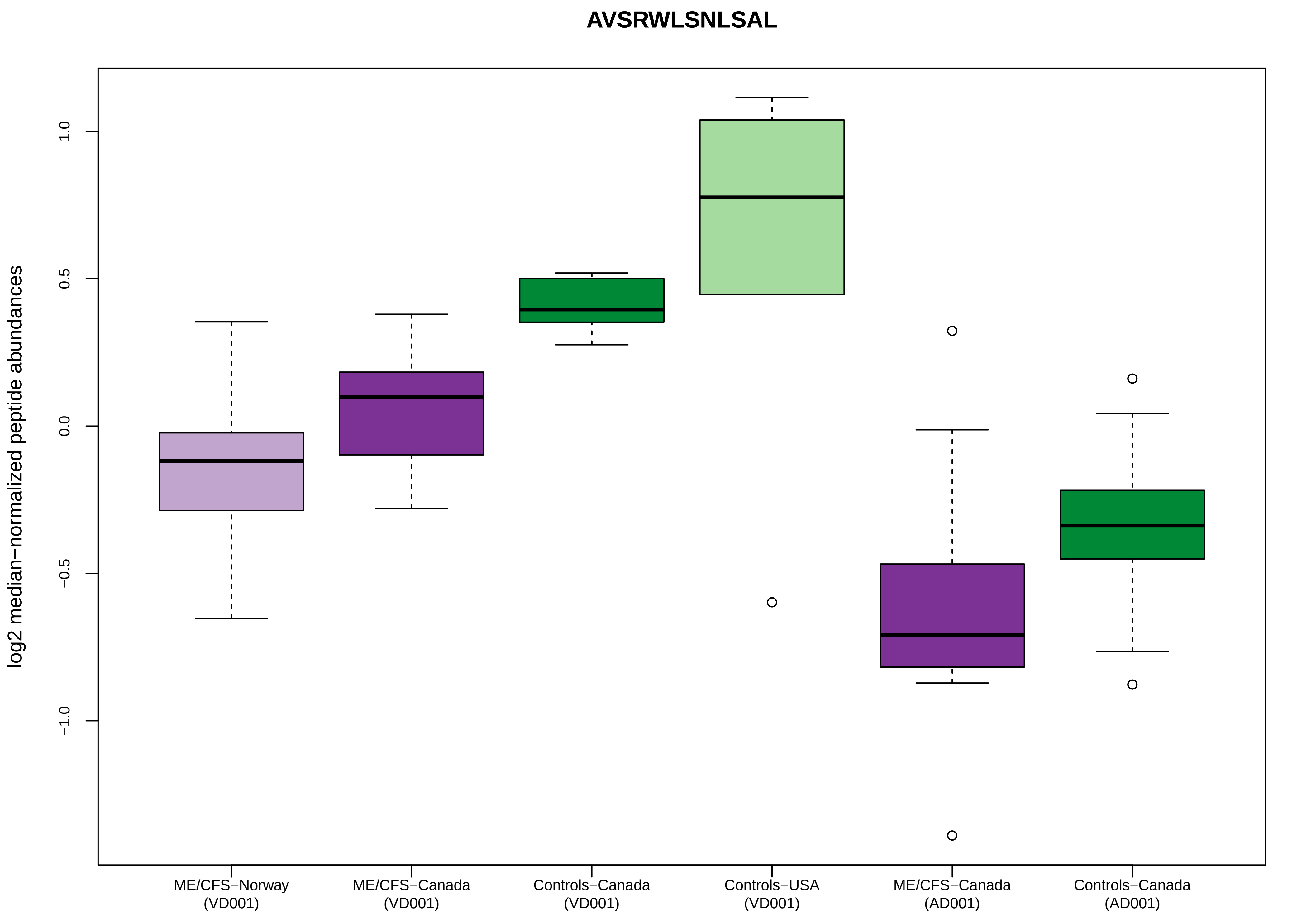
AVSRWLSNLSAL
log2 median−normalized peptide abundances
1.0
0.5
0.0
−0.5
−1.0
ME/CFS−Norway
(VD001)
ME/CFS−Canada
(VD001)
Controls−Canada
(VD001)
Controls−USA
(VD001)
ME/CFS−Canada
(AD001)
Controls−Canada
(AD001)

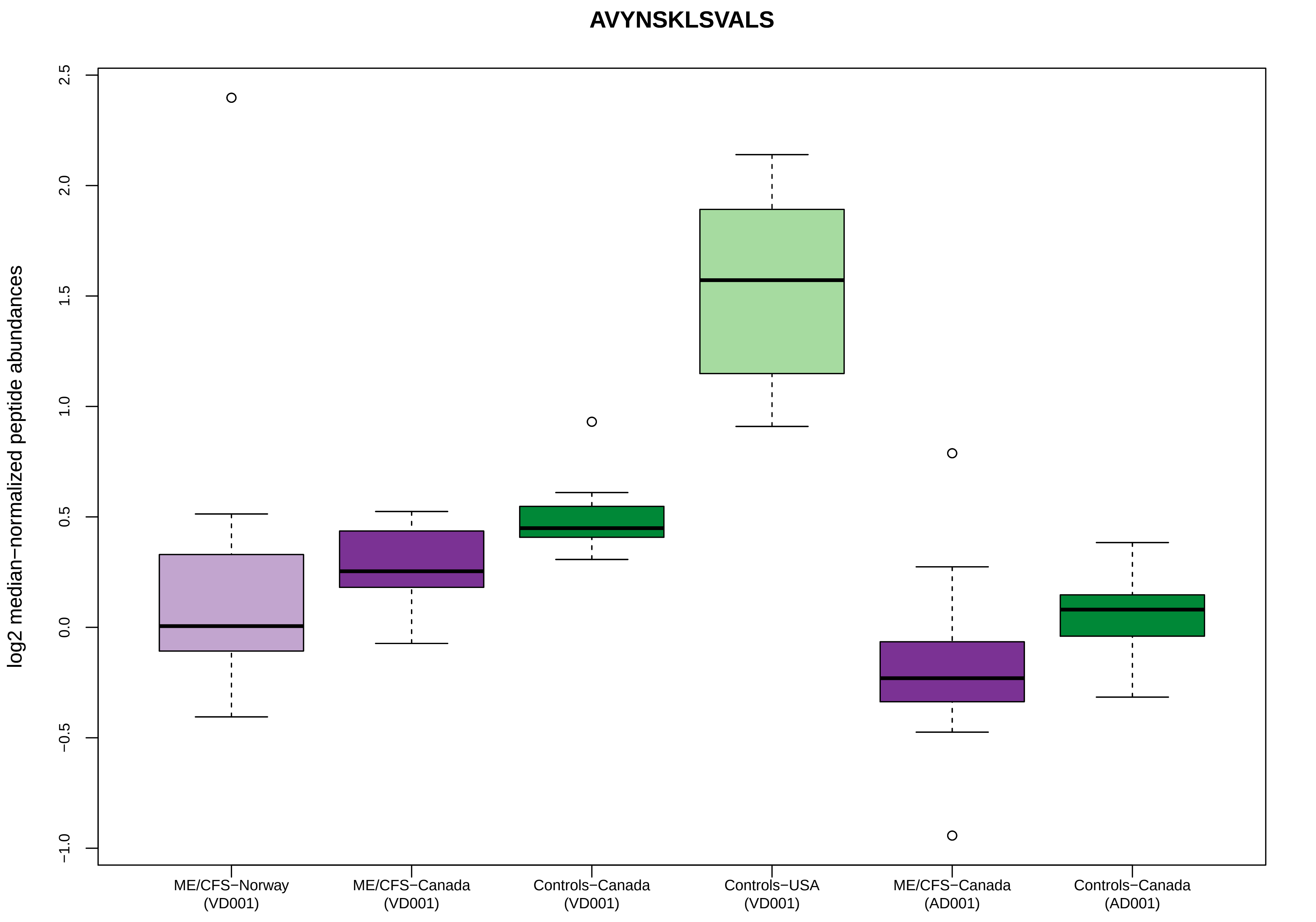
AVYNSKLSVALS
log2 median−normalized peptide abundances
2.5
2.0
1.5
1.0
0.5
0.0
−0.5
−1.0
ME/CFS−Norway (VD001)
ME/CFS−Canada (VD001)
Controls−Canada (VD001)
Controls−USA (VD001)
ME/CFS−Canada (AD001)
Controls−Canada (AD001)

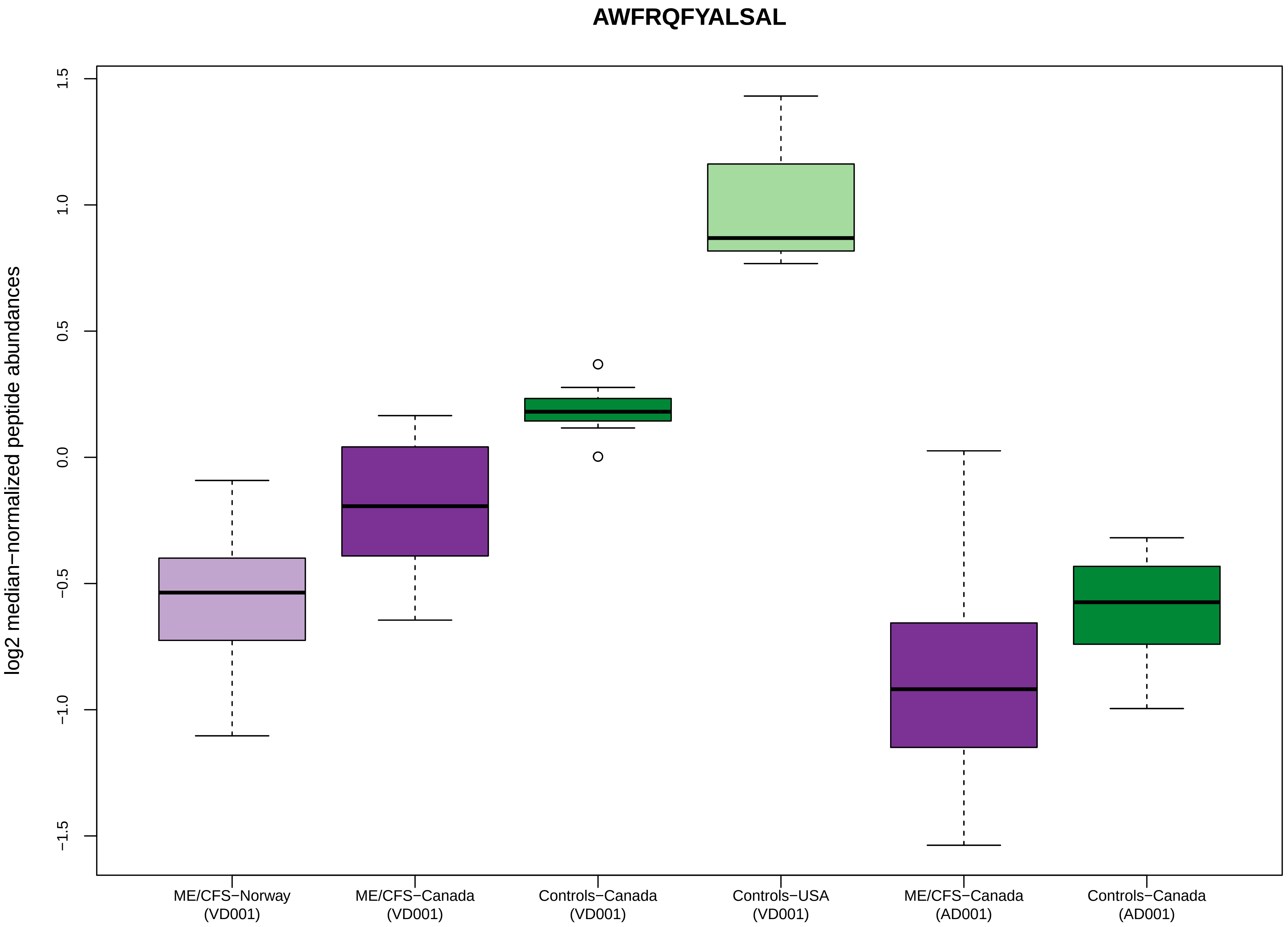

AWFRQFYALSAL
log2 median−normalized peptide abundances
1.5
1.0
0.5
0.0
−0.5
−1.0
−1.5
ME/CFS−Norway (VD001)
ME/CFS−Canada (VD001)
Controls−Canada (VD001)
Controls−USA (VD001)
ME/CFS−Canada (AD001)
Controls−Canada (AD001)

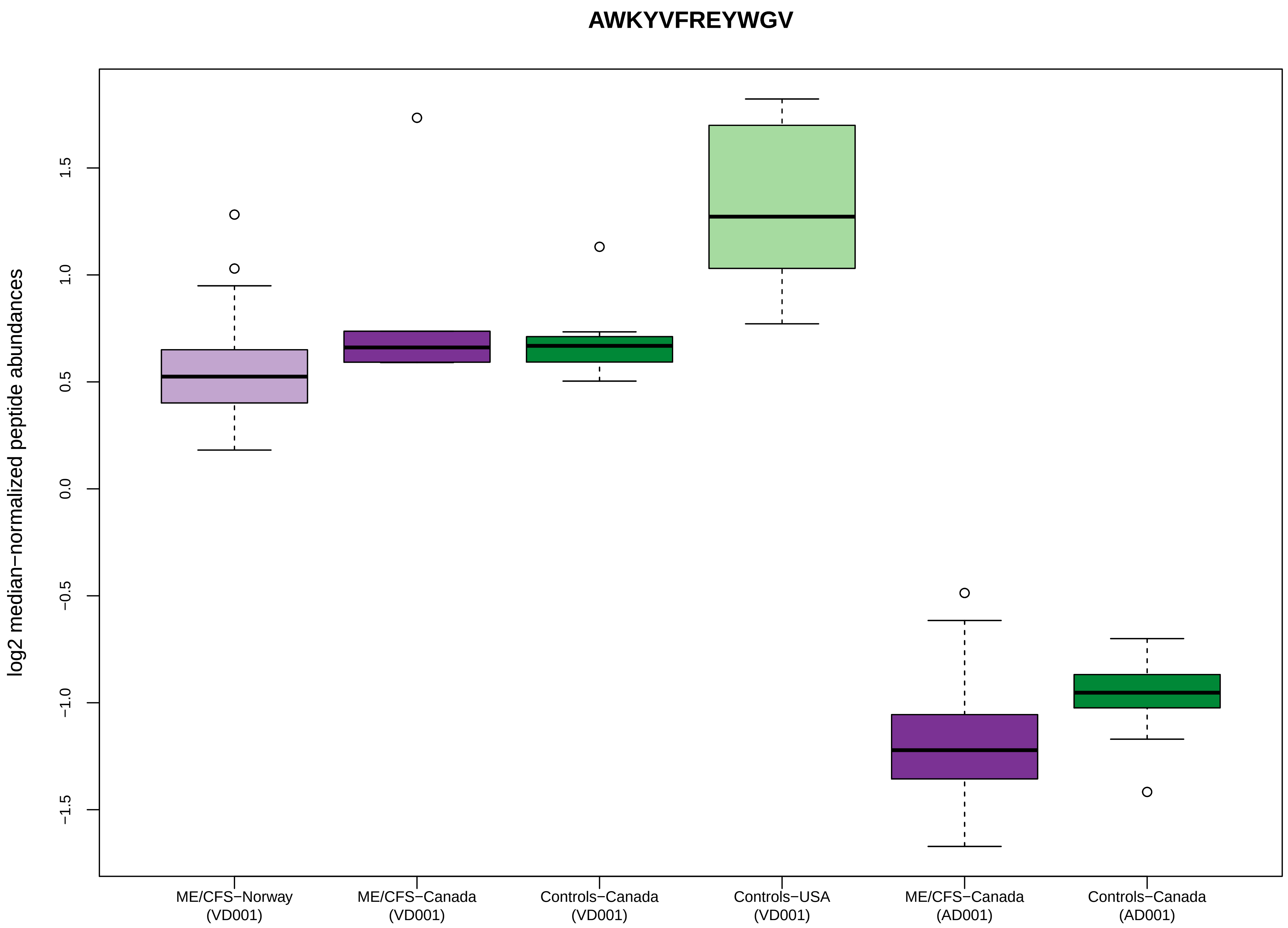
AWKYVFREYWGV
log2 median−normalized peptide abundances
1.5
1.0
0.5
0.0
−0.5
−1.0
−1.5
ME/CFS−Norway (VD001)
ME/CFS−Canada (VD001)
Controls−Canada (VD001)
Controls−USA (VD001)
ME/CFS−Canada (AD001)
Controls−Canada (AD001)

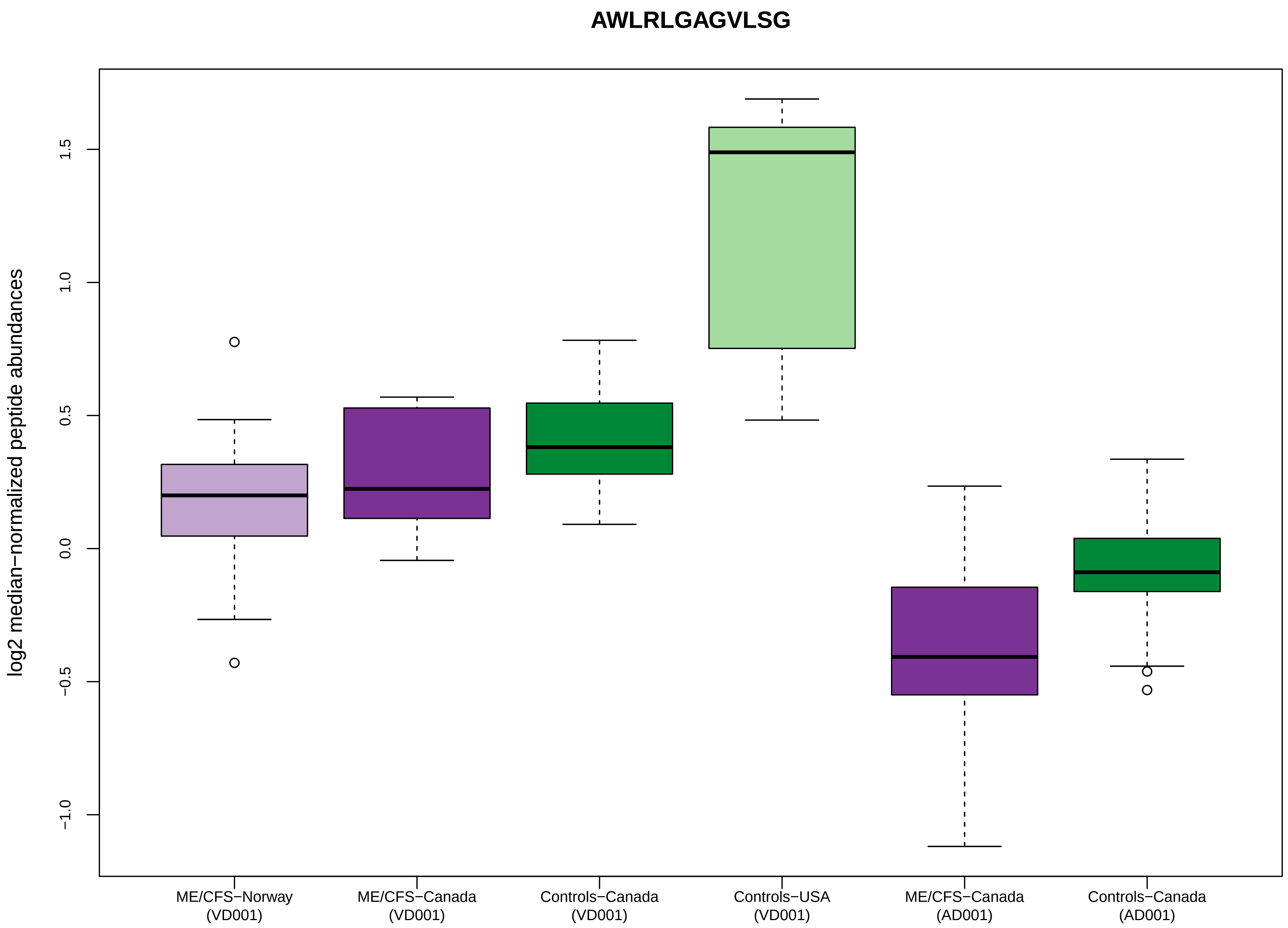

AWLRLGAGVLSG
log2 median−normalized peptide abundances
1.5
1.0
0.5
0.0
−0.5
−1.0
ME/CFS−Norway (VD001)
ME/CFS−Canada (VD001)
Controls−Canada (VD001)
Controls−USA (VD001)
ME/CFS−Canada (AD001)
Controls−Canada (AD001)

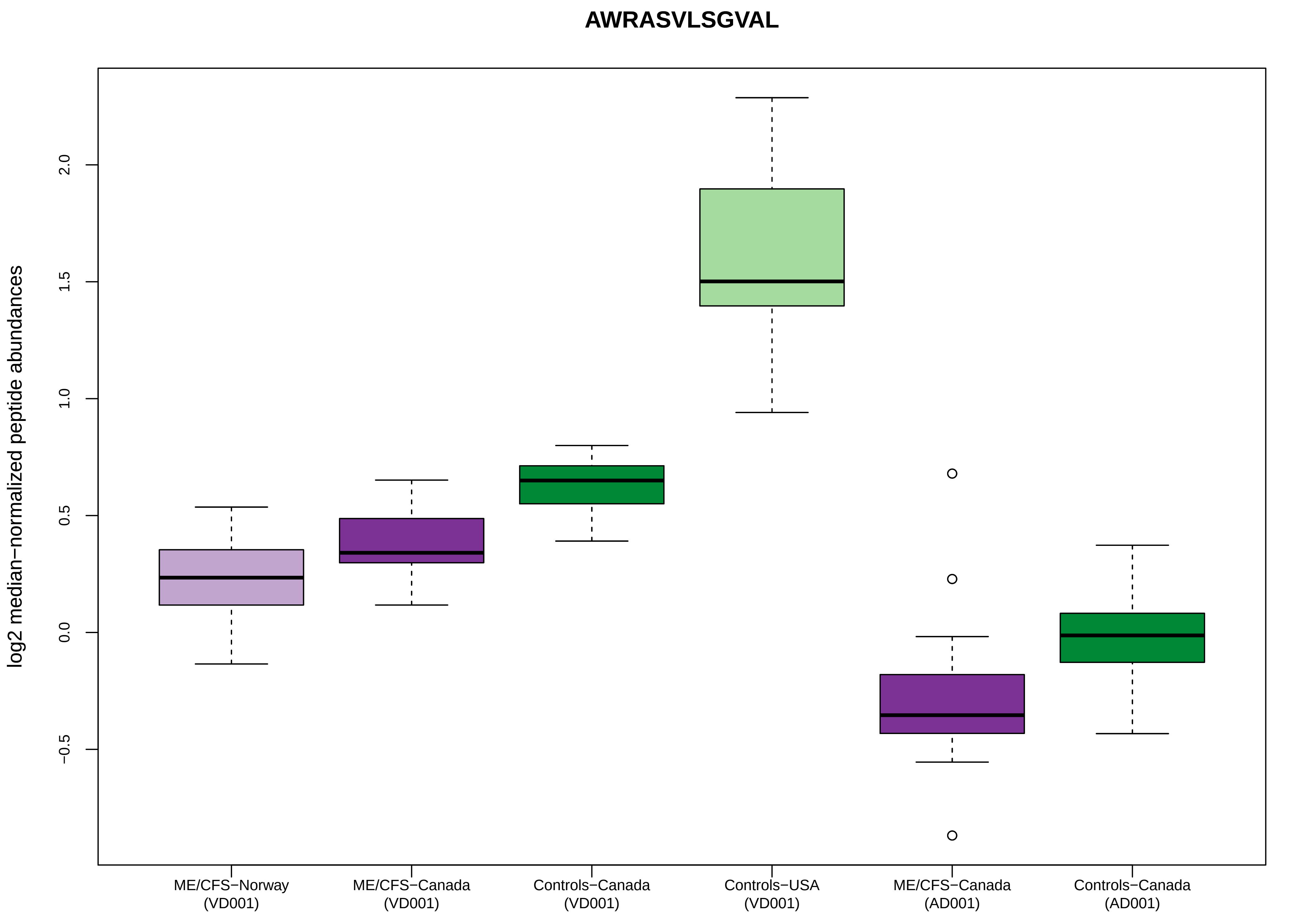
AWRASVLSGVAL
log2 median−normalized peptide abundances
−0.5
0.0
0.5
1.0
1.5
2.0
ME/CFS−Norway (VD001)
ME/CFS−Canada (VD001)
Controls−Canada (VD001)
Controls−USA (VD001)
ME/CFS−Canada (AD001)
Controls−Canada (AD001)

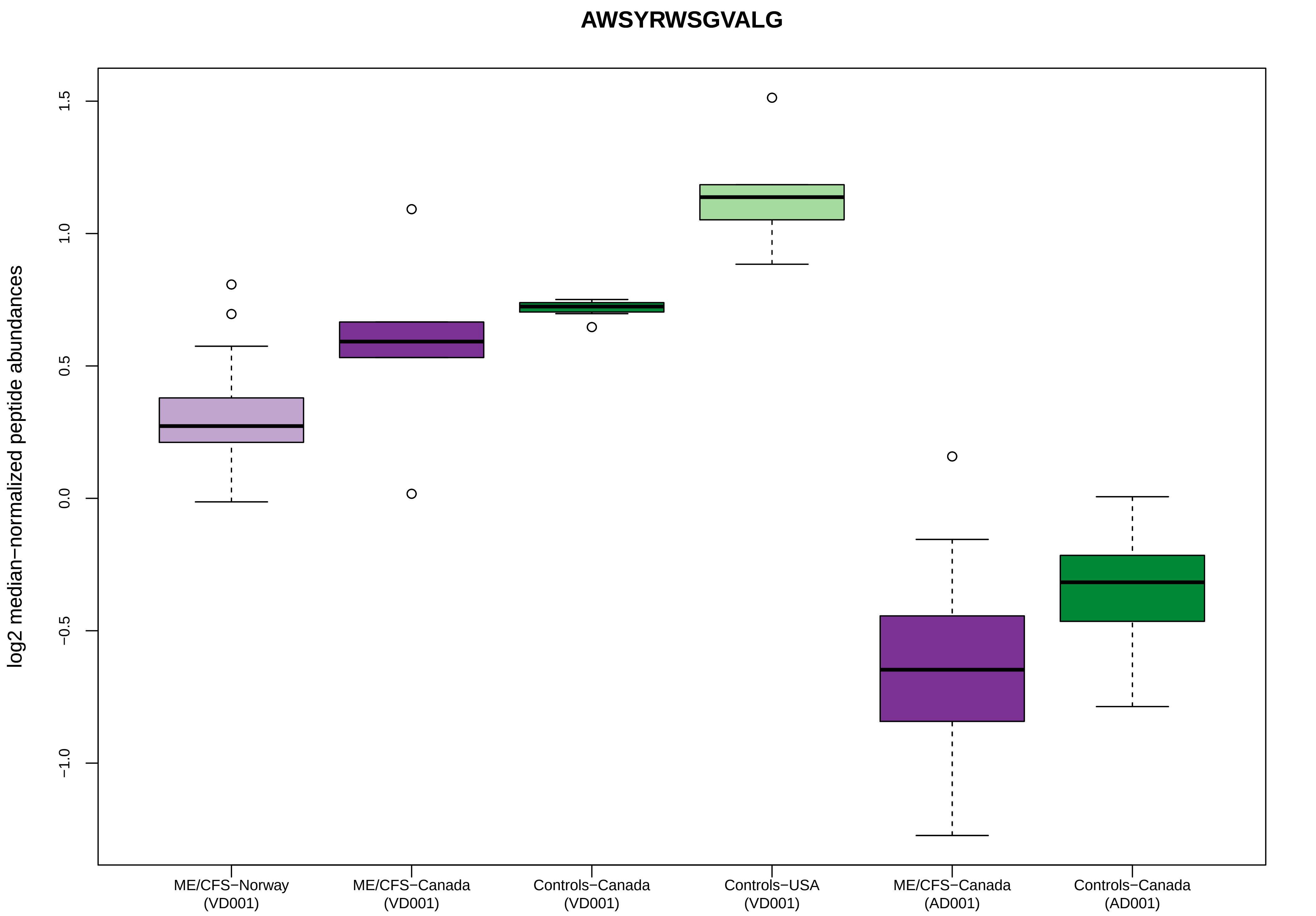
AWSYRWSGVALG
log2 median−normalized peptide abundances
1.5
1.0
0.5
0.0
−0.5
−1.0
ME/CFS−Norway (VD001)
ME/CFS−Canada (VD001)
Controls−Canada (VD001)
Controls−USA (VD001)
ME/CFS−Canada (AD001)
Controls−Canada (AD001)

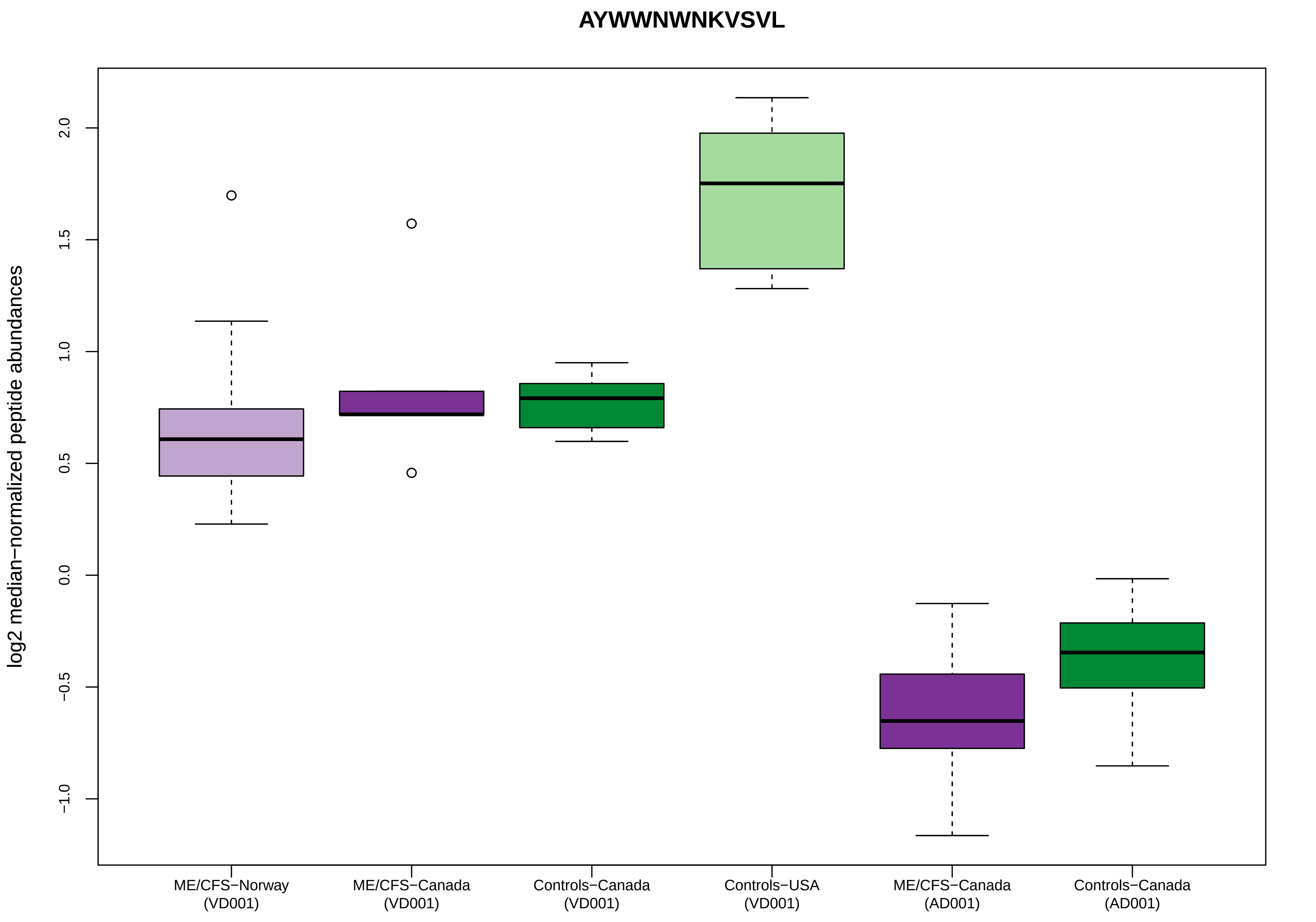

AYWWNWNKVSVL
log2 median−normalized peptide abundances
2.0
1.5
1.0
0.5
0.0
−0.5
−1.0
ME/CFS−Norway (VD001)
ME/CFS−Canada (VD001)
Controls−Canada (VD001)
Controls−USA (VD001)
ME/CFS−Canada (AD001)
Controls−Canada (AD001)

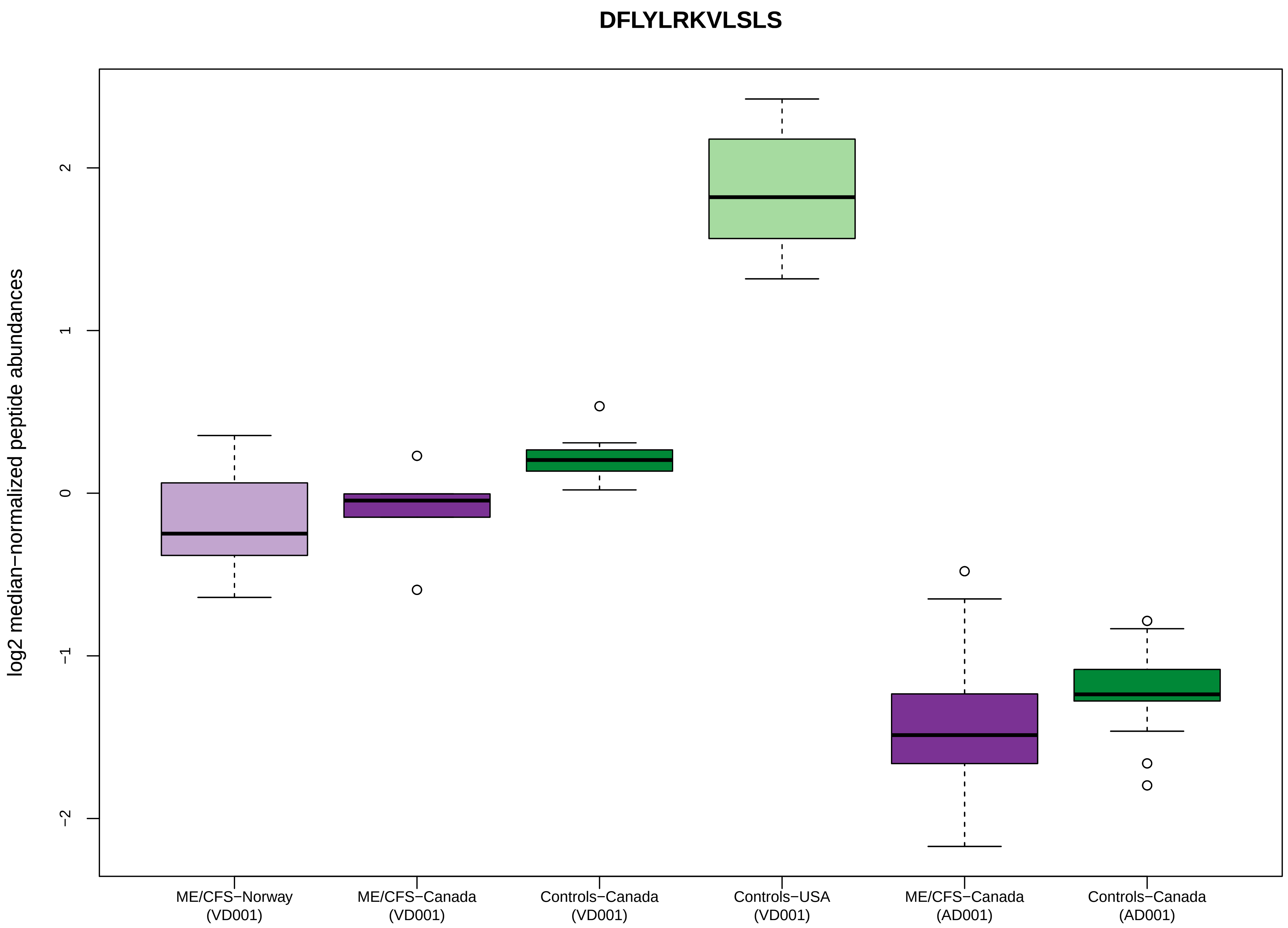
DFLYLRKVLSLS
log2 median−normalized peptide abundances
2
1
0
−1
−2
ME/CFS−Norway (VD001)
ME/CFS−Canada (VD001)
Controls−Canada (VD001)
Controls−USA (VD001)
ME/CFS−Canada (AD001)
Controls−Canada (AD001)

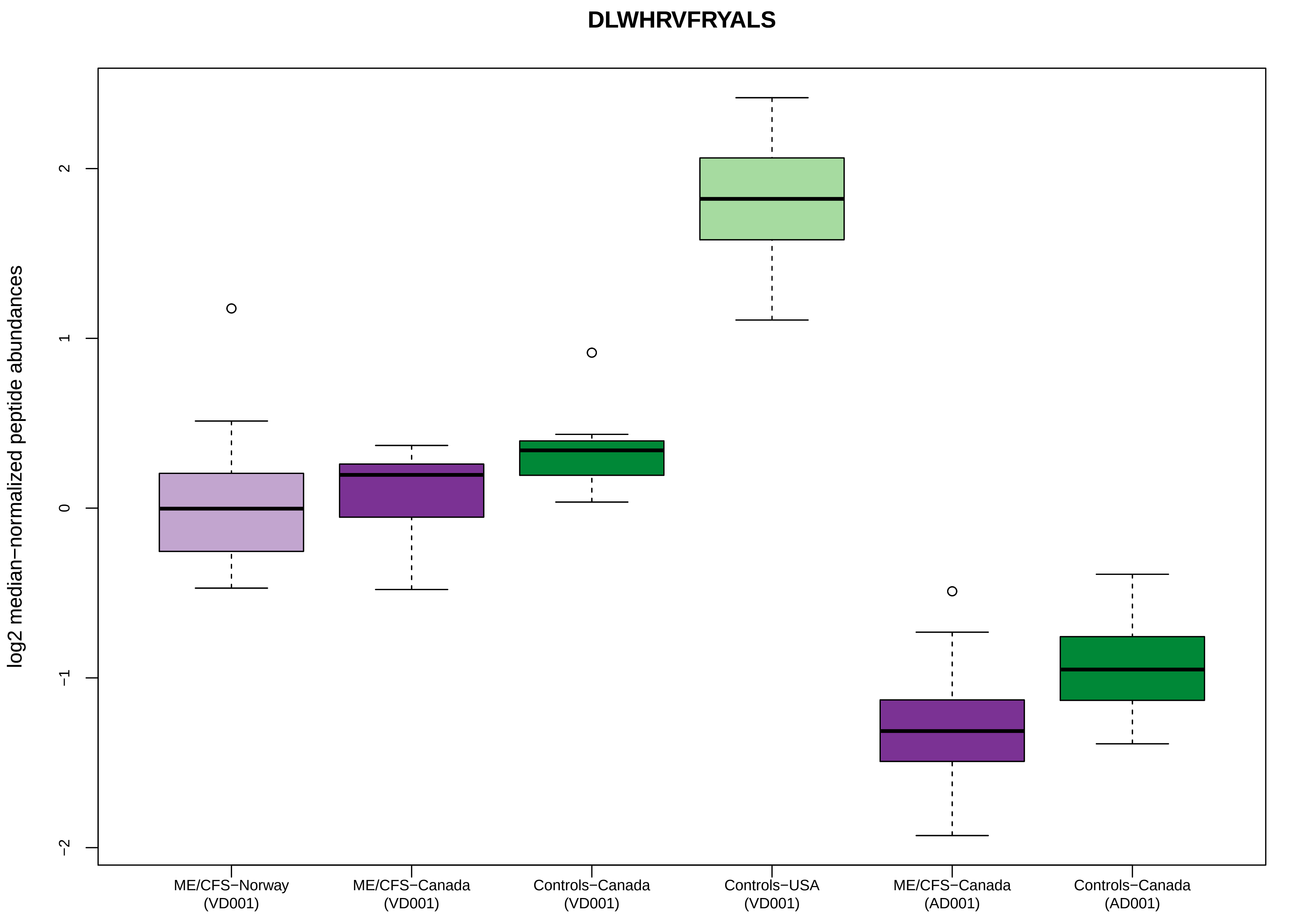
DLWHRVFRYALS
log2 median−normalized peptide abundances
2
1
0
−1
−2
ME/CFS−Norway (VD001)
ME/CFS−Canada (VD001)
Controls−Canada (VD001)
Controls−USA (VD001)
ME/CFS−Canada (AD001)
Controls−Canada (AD001)

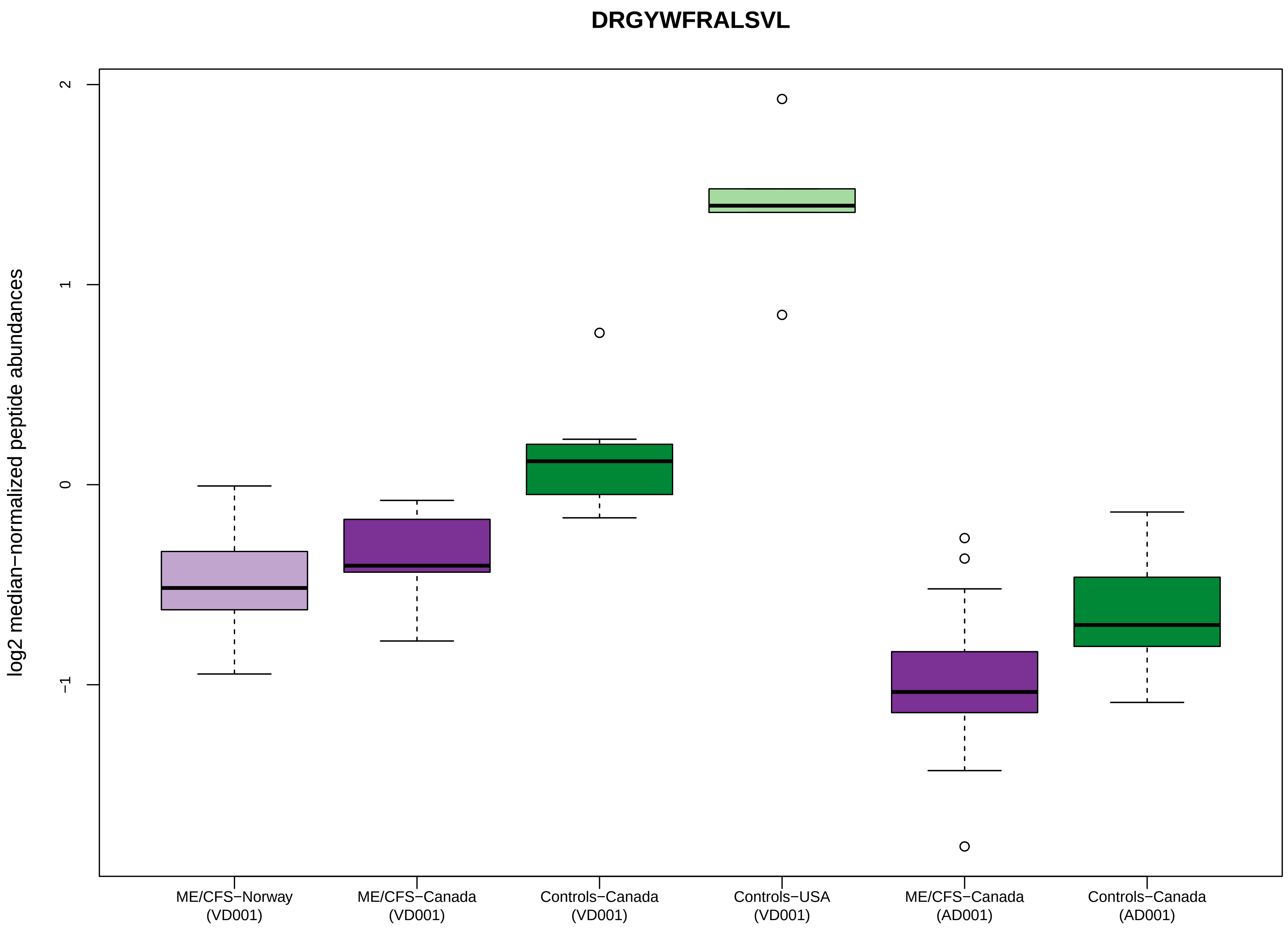

DRGYWFRALSVL
log2 median−normalized peptide abundances
2
1
0
−1
ME/CFS−Norway (VD001)
ME/CFS−Canada (VD001)
Controls−Canada (VD001)
Controls−USA (VD001)
ME/CFS−Canada (AD001)
Controls−Canada (AD001)

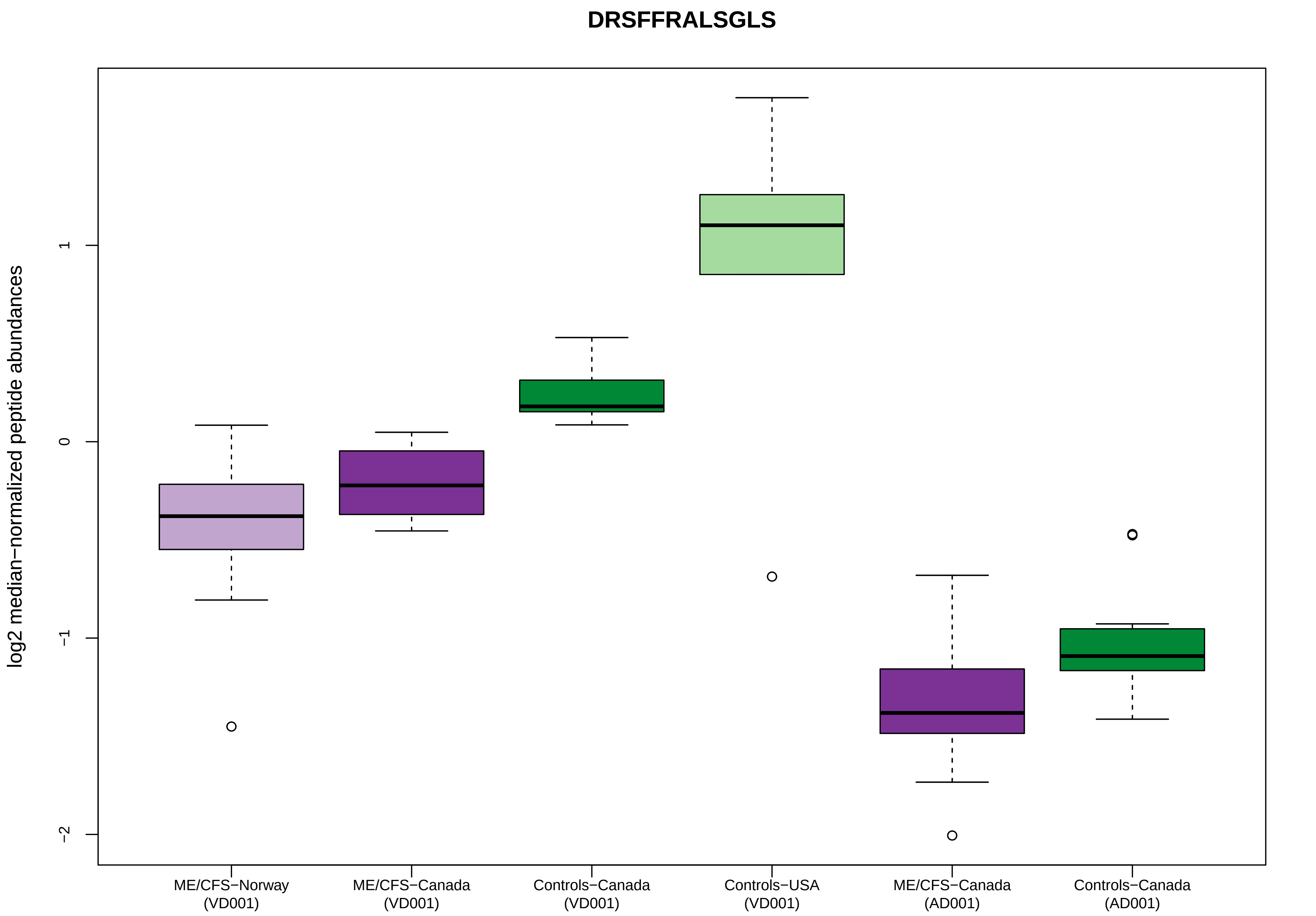

DRSFFRALSGLS
log2 median−normalized peptide abundances
−2
−1
0
1
ME/CFS−Norway (VD001)
ME/CFS−Canada (VD001)
Controls−Canada (VD001)
Controls−USA (VD001)
ME/CFS−Canada (AD001)
Controls−Canada (AD001)

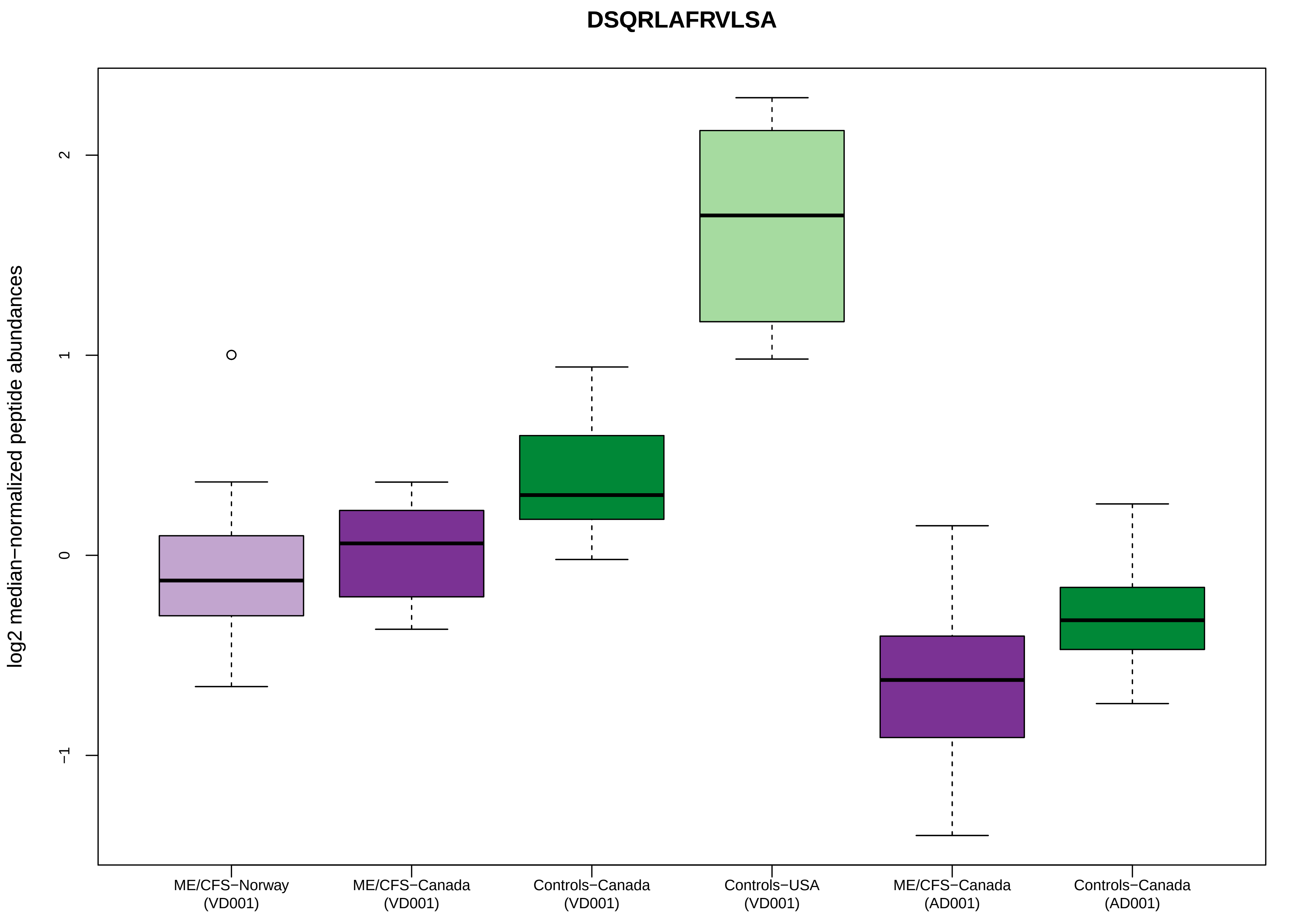

DSQRLAFRVLSA
log2 median−normalized peptide abundances
2
1
0
−1
ME/CFS−Norway (VD001)
ME/CFS−Canada (VD001)
Controls−Canada (VD001)
Controls−USA (VD001)
ME/CFS−Canada (AD001)
Controls−Canada (AD001)

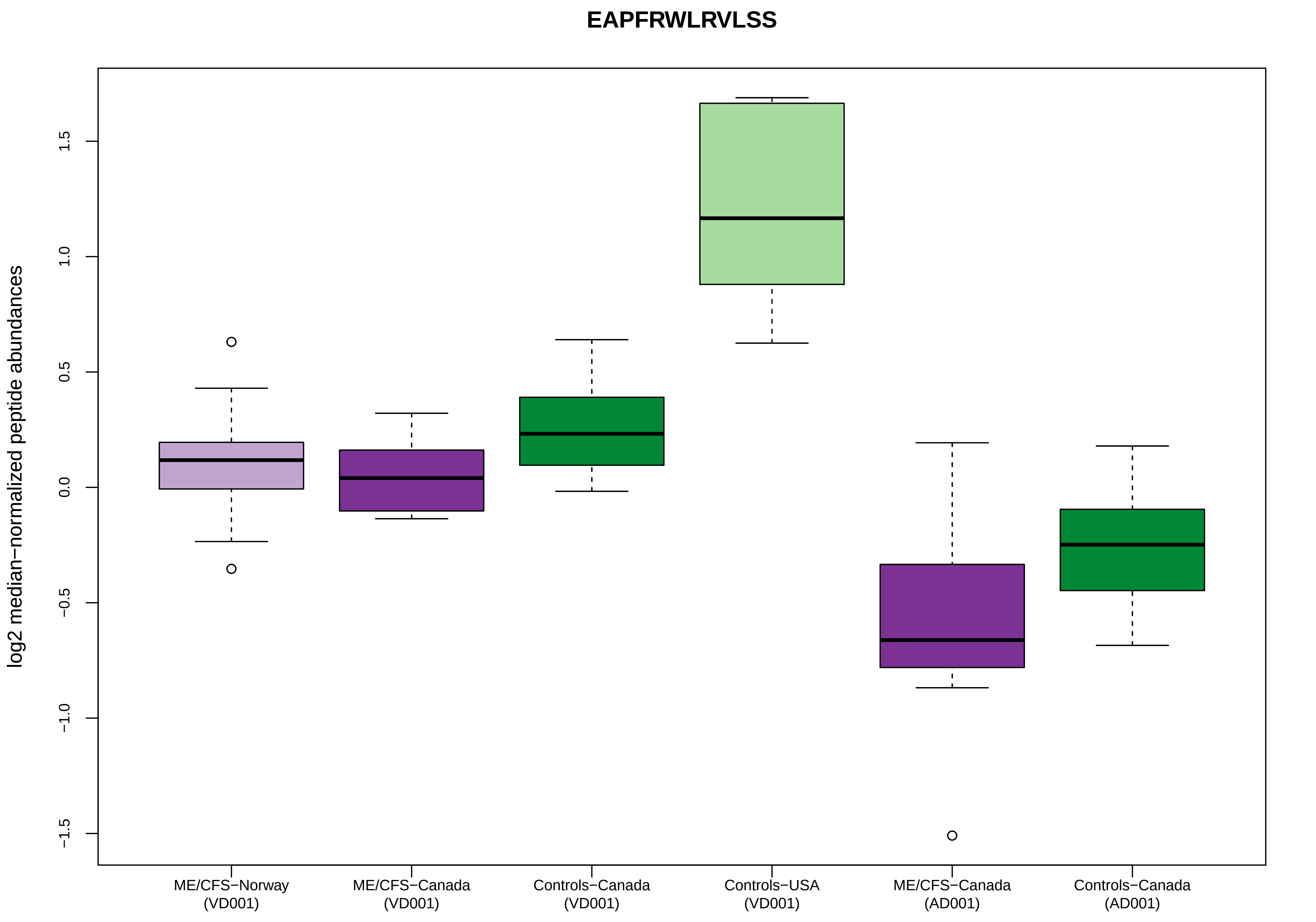

EAPFRWLRVLSS
log2 median−normalized peptide abundances
1.5
1.0
0.5
0.0
−0.5
−1.0
−1.5
ME/CFS−Norway (VD001)
ME/CFS−Canada (VD001)
Controls−Canada (VD001)
Controls−USA (VD001)
ME/CFS−Canada (AD001)
Controls−Canada (AD001)

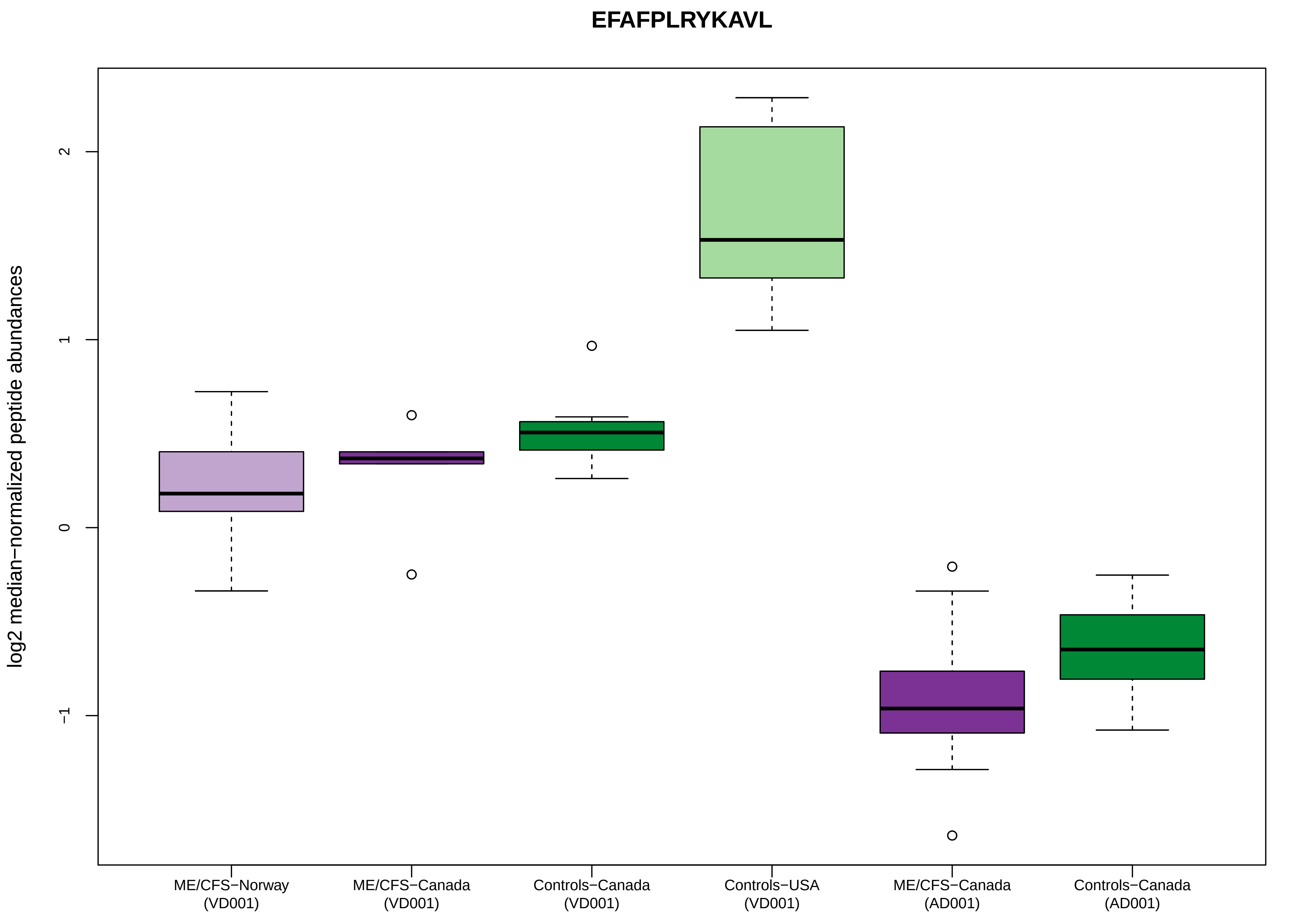

EFAFPLRYKAVL
log2 median−normalized peptide abundances
2
1
0
−1
ME/CFS−Norway (VD001)
ME/CFS−Canada (VD001)
Controls−Canada (VD001)
Controls−USA (VD001)
ME/CFS−Canada (AD001)
Controls−Canada (AD001)

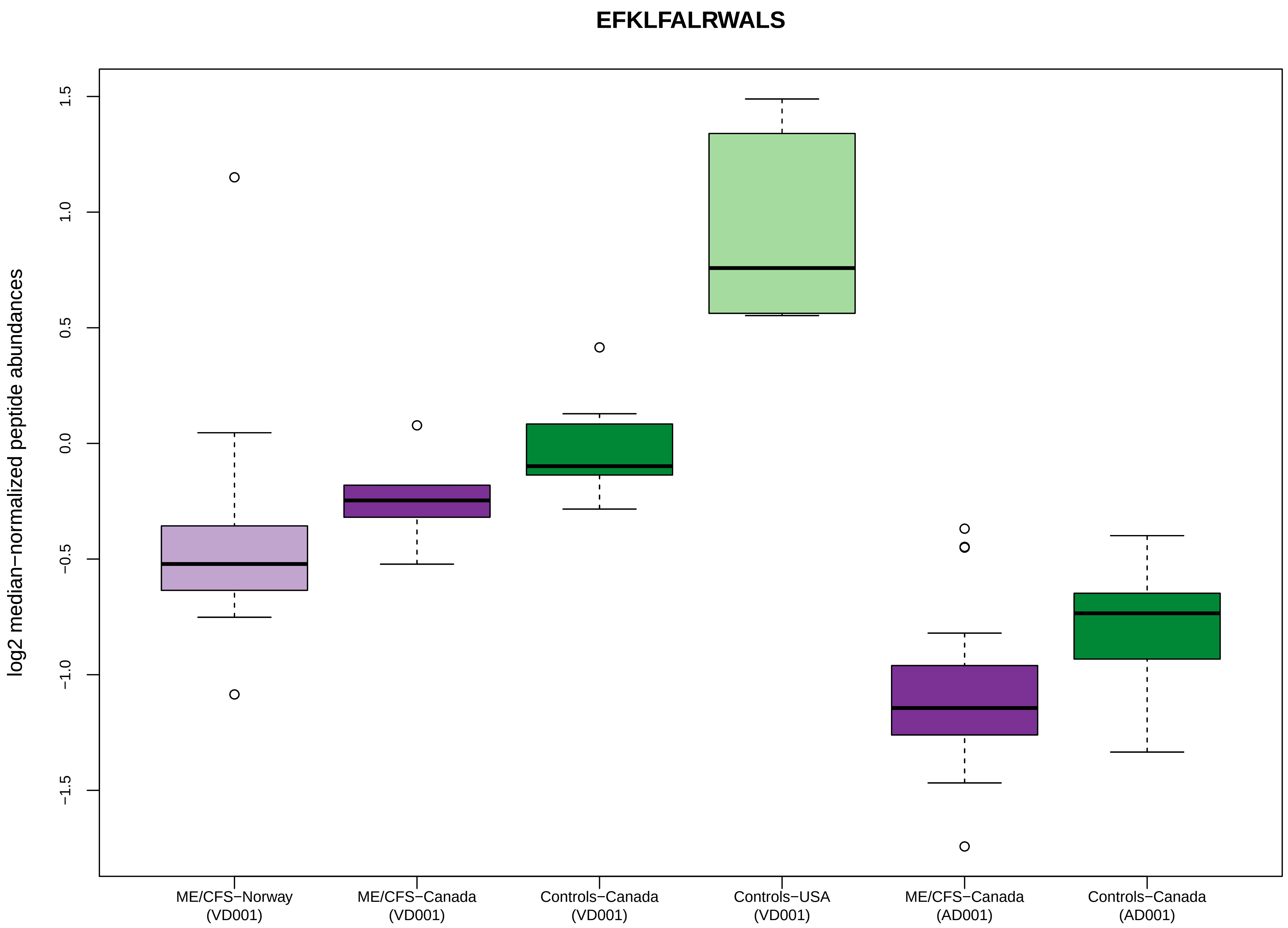

EFKLFALRWALS
log2 median−normalized peptide abundances
1.5
1.0
0.5
0.0
−0.5
−1.0
−1.5
ME/CFS−Norway (VD001)
ME/CFS−Canada (VD001)
Controls−Canada (VD001)
Controls−USA (VD001)
ME/CFS−Canada (AD001)
Controls−Canada (AD001)

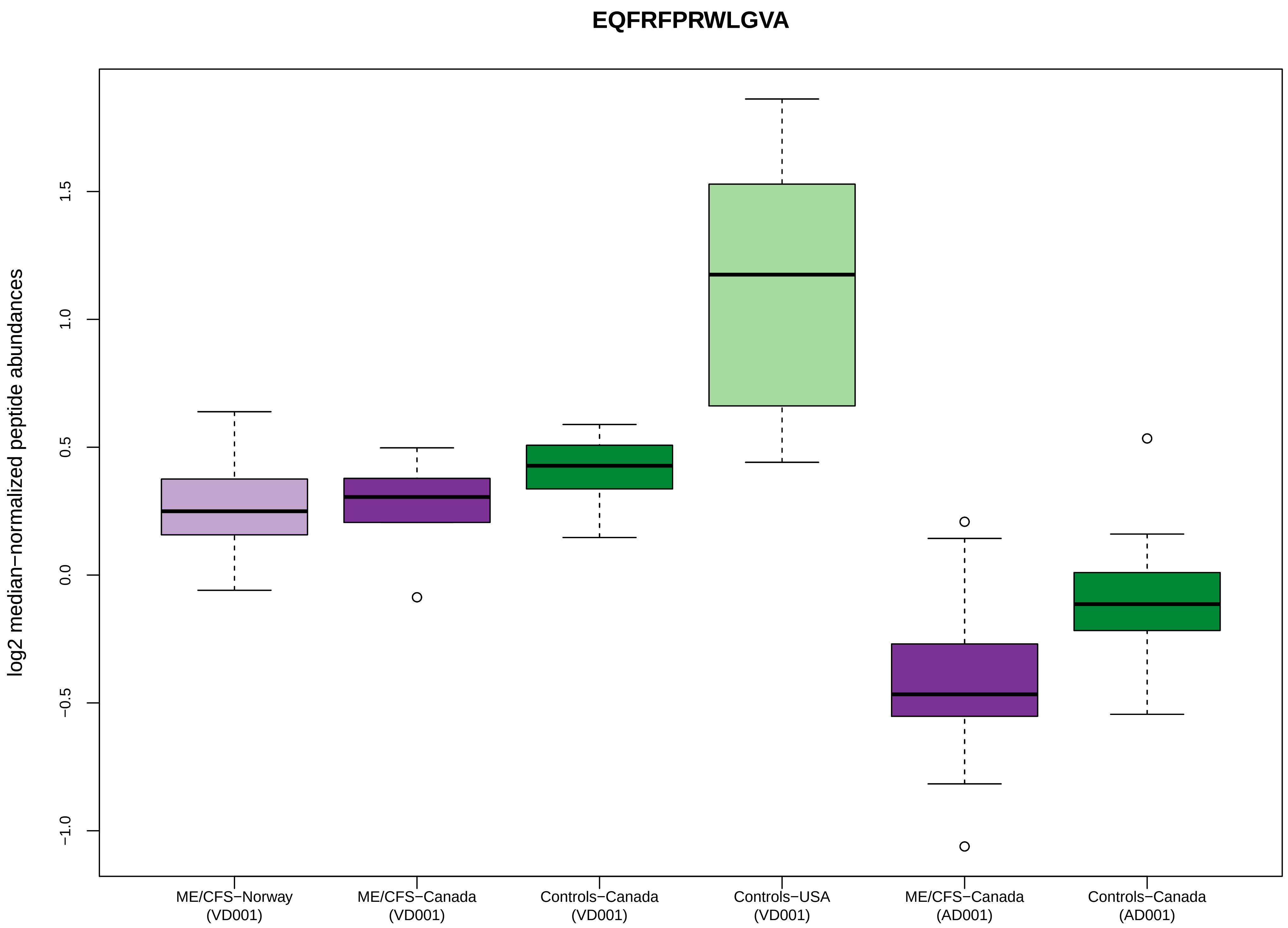
EQFRFPRWLGVA
log2 median−normalized peptide abundances
−1.0
−0.5
0.0
0.5
1.0
1.5
ME/CFS−Norway (VD001)
ME/CFS−Canada (VD001)
Controls−Canada (VD001)
Controls−USA (VD001)
ME/CFS−Canada (AD001)
Controls−Canada (AD001)

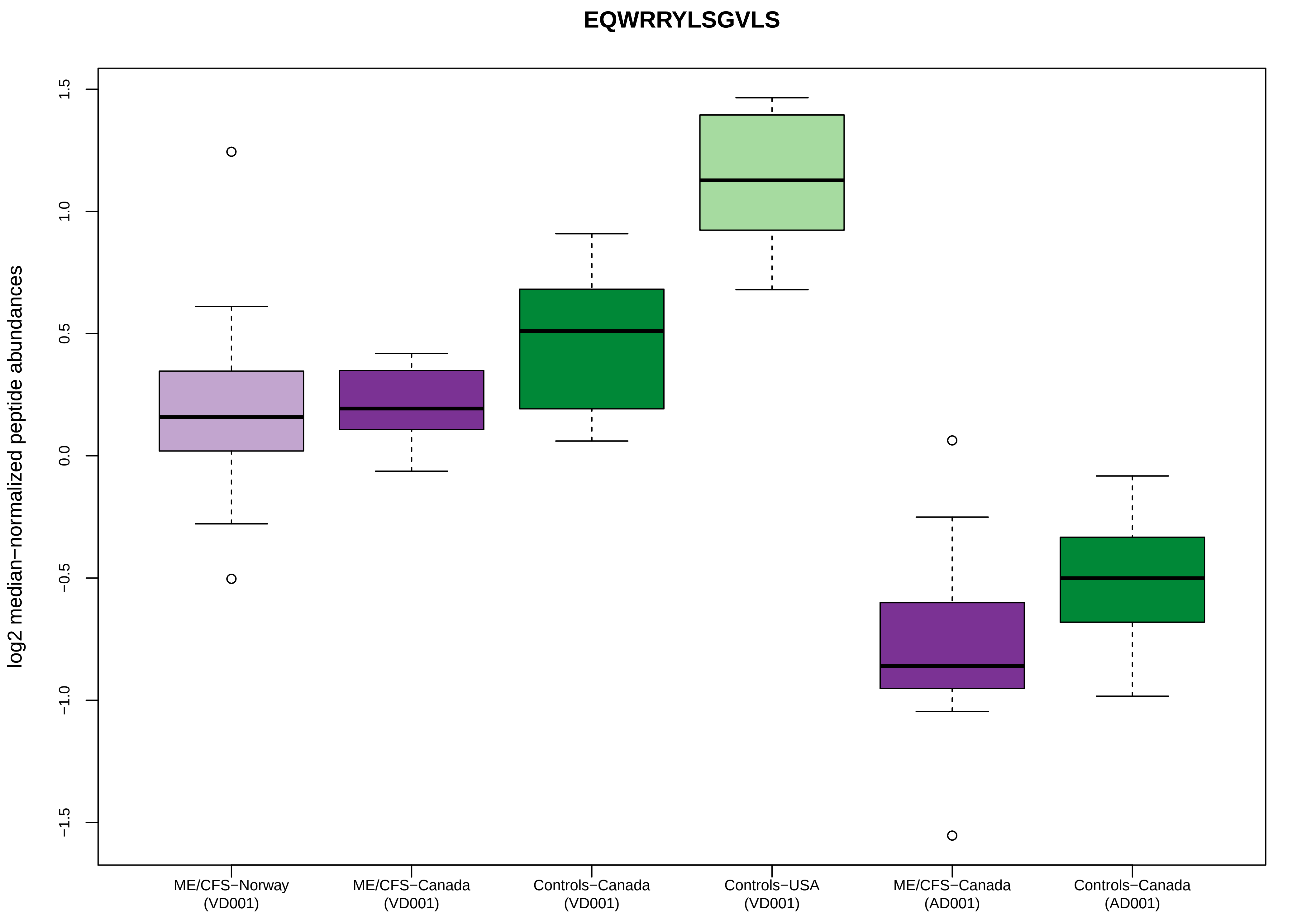

EQWRRYLSGVLS
log2 median−normalized peptide abundances
1.5
1.0
0.5
0.0
−0.5
−1.0
−1.5
ME/CFS−Norway (VD001)
ME/CFS−Canada (VD001)
Controls−Canada (VD001)
Controls−USA (VD001)
ME/CFS−Canada (AD001)
Controls−Canada (AD001)

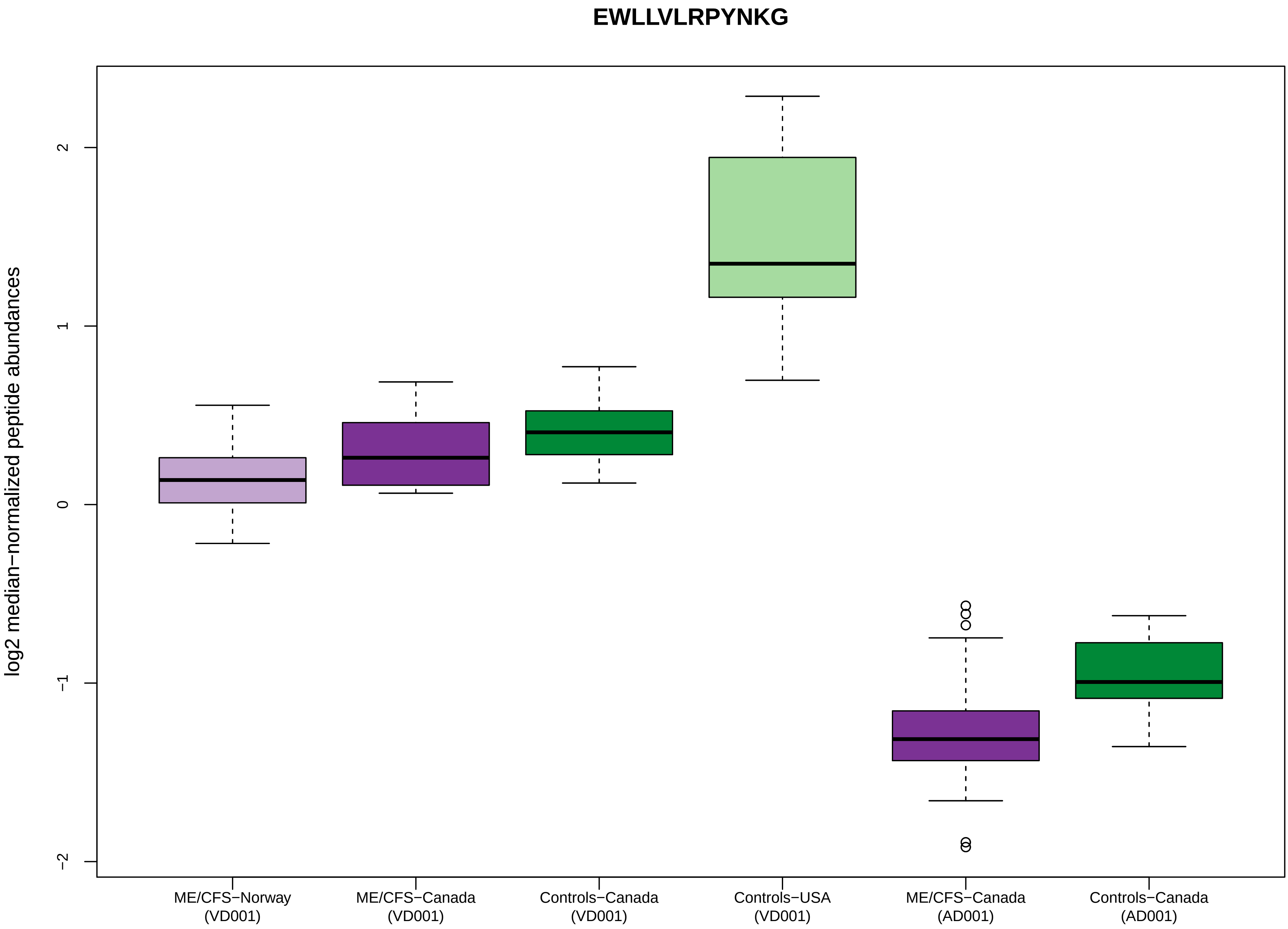
EWLLVLRPYNKG
log2 median−normalized peptide abundances
2
1
0
−1
−2
ME/CFS−Norway (VD001)
ME/CFS−Canada (VD001)
Controls−Canada (VD001)
Controls−USA (VD001)
ME/CFS−Canada (AD001)
Controls−Canada (AD001)

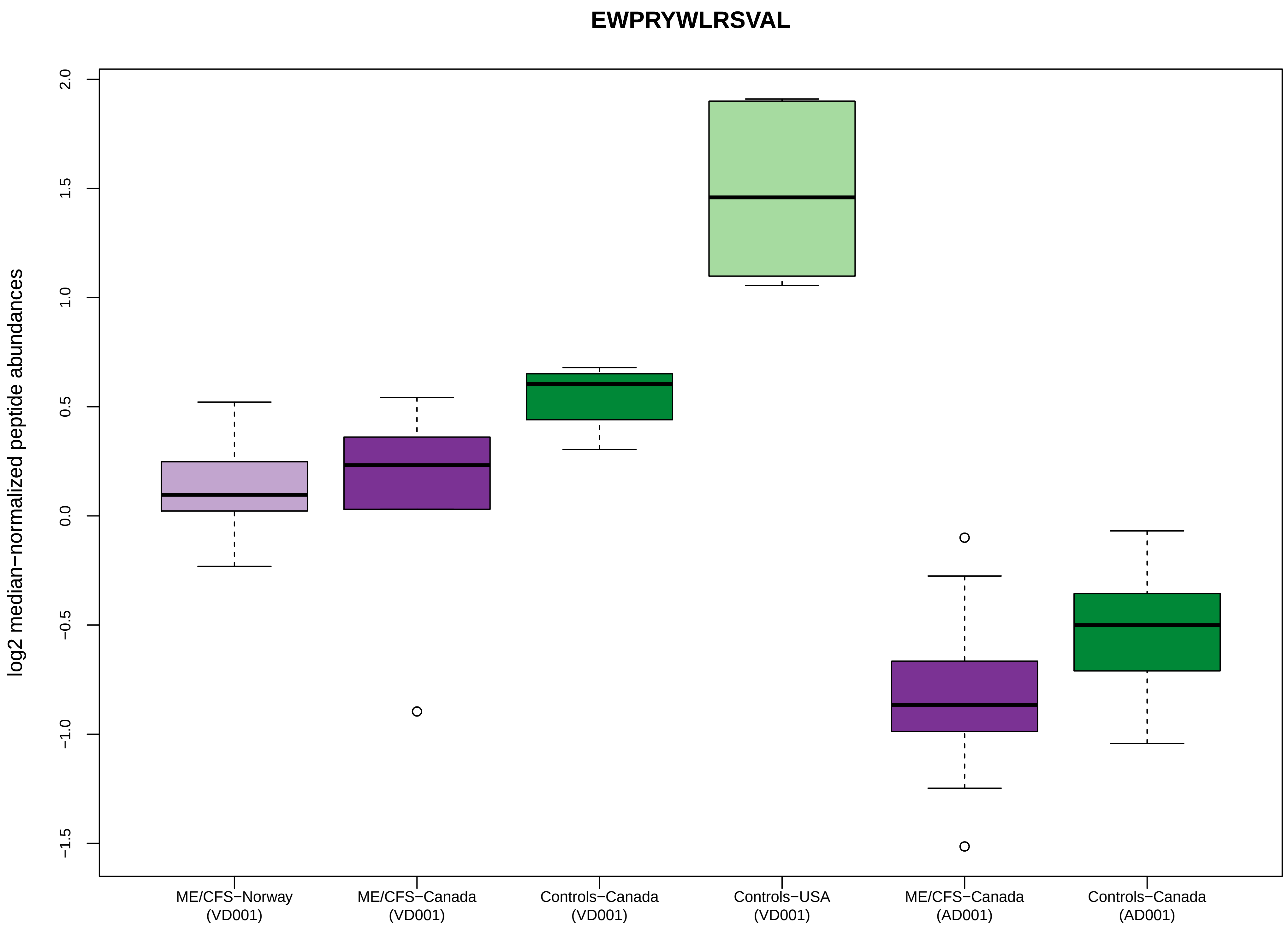

EWPRYWLRSVAL
log2 median−normalized peptide abundances
2.0
1.5
1.0
0.5
0.0
−0.5
−1.0
−1.5
ME/CFS−Norway (VD001)
ME/CFS−Canada (VD001)
Controls−Canada (VD001)
Controls−USA (VD001)
ME/CFS−Canada (AD001)
Controls−Canada (AD001)

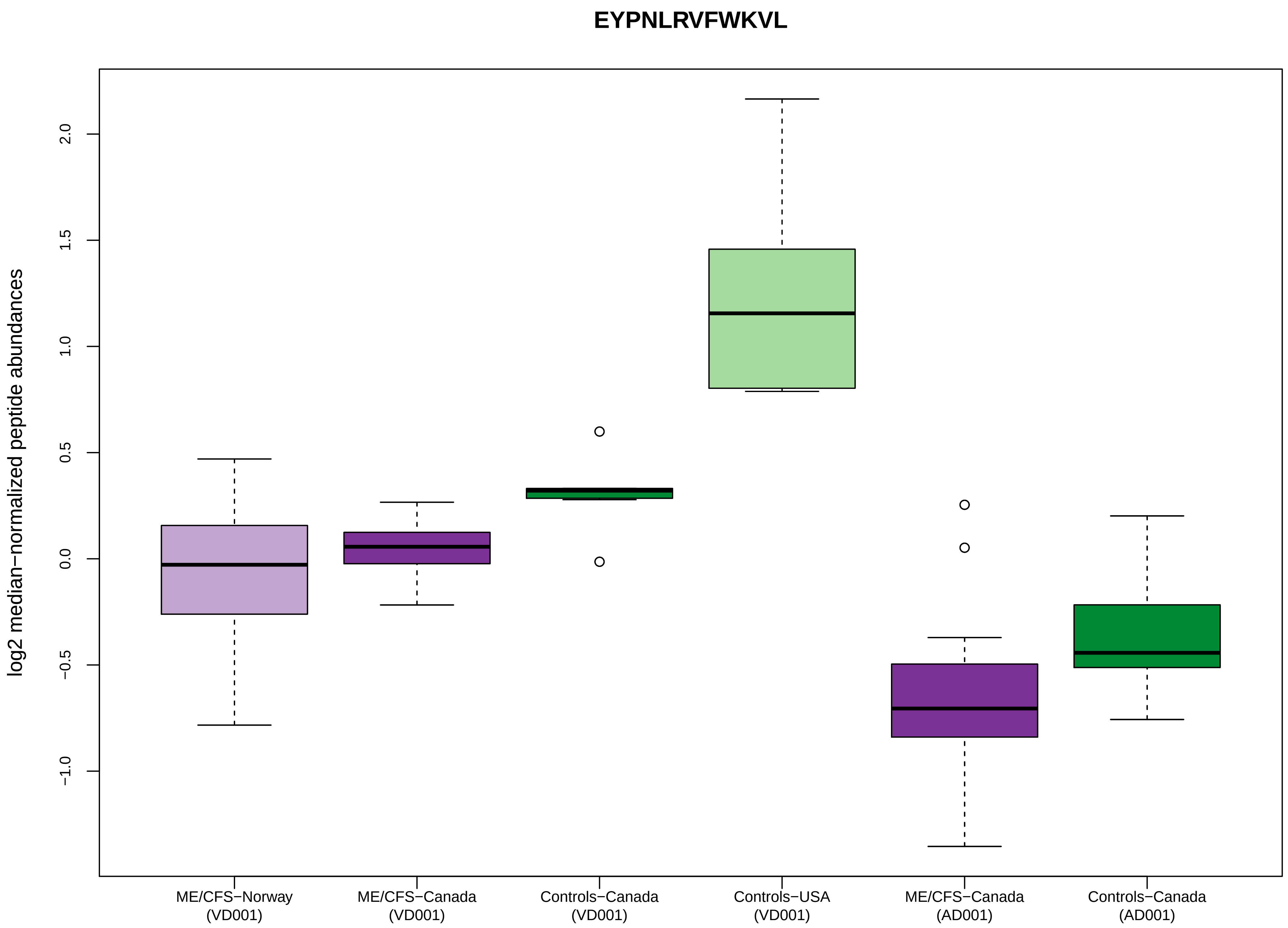

EYPNLRVFWKVL
log2 median−normalized peptide abundances
2.0
1.5
1.0
0.5
0.0
−0.5
−1.0
ME/CFS−Norway (VD001)
ME/CFS−Canada (VD001)
Controls−Canada (VD001)
Controls−USA (VD001)
ME/CFS−Canada (AD001)
Controls−Canada (AD001)

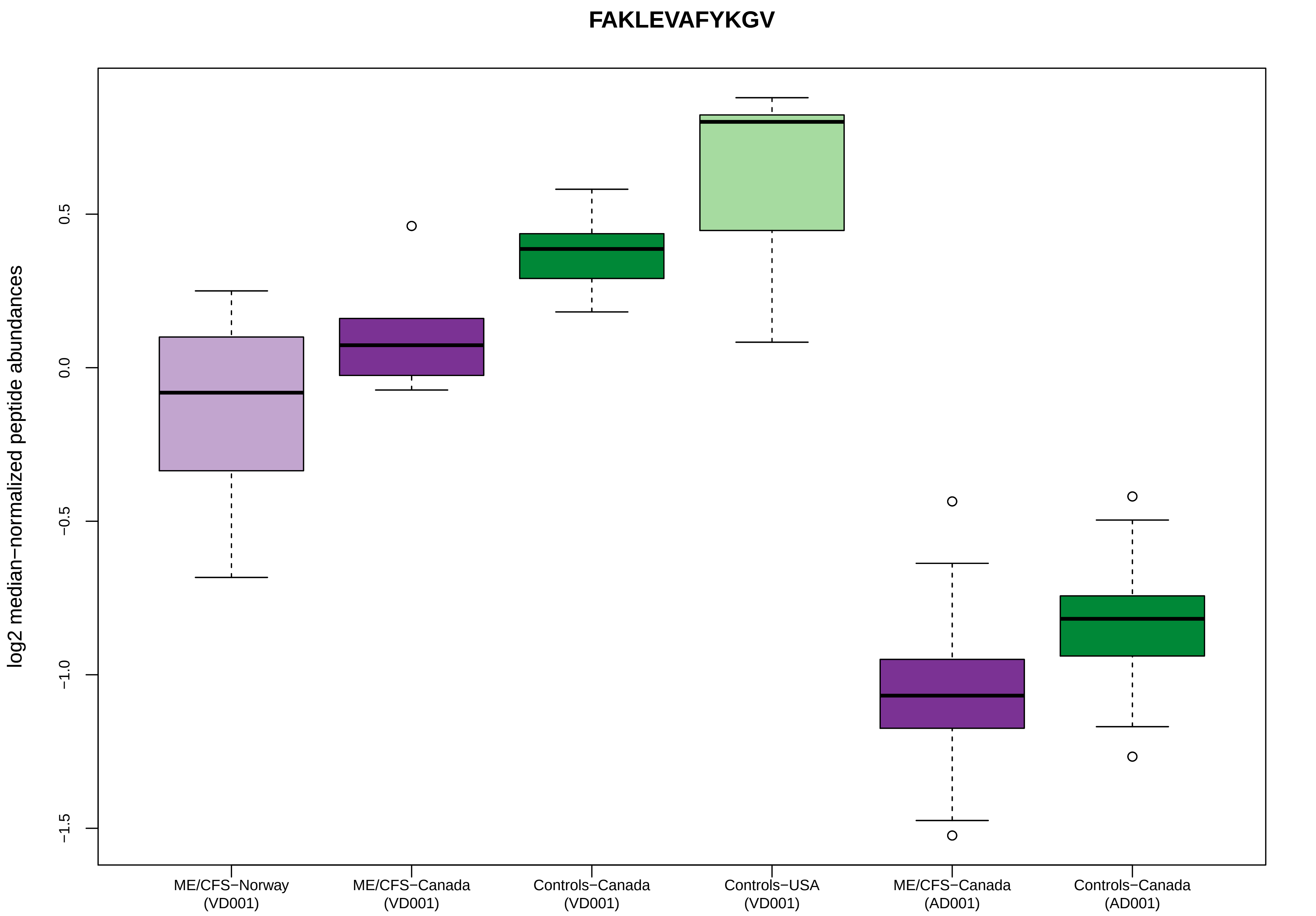

FAKLEVAFYKGV
log2 median−normalized peptide abundances
0.5
0.0
−0.5
−1.0
−1.5
ME/CFS−Norway (VD001)
ME/CFS−Canada (VD001)
Controls−Canada (VD001)
Controls−USA (VD001)
ME/CFS−Canada (AD001)
Controls−Canada (AD001)

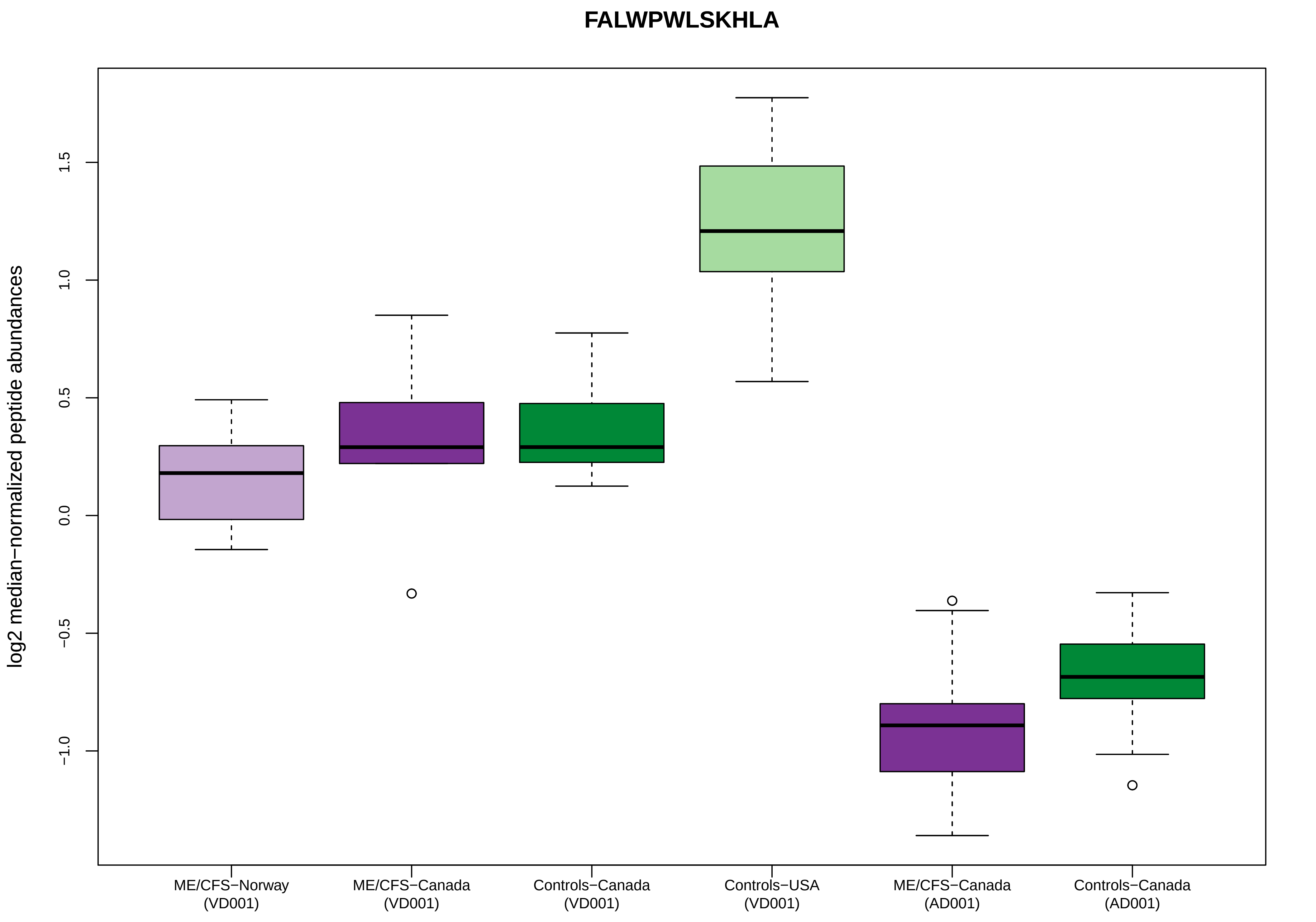

FALWPWLSKHLA
log2 median−normalized peptide abundances
1.5
1.0
0.5
0.0
−0.5
−1.0
ME/CFS−Norway (VD001)
ME/CFS−Canada (VD001)
Controls−Canada (VD001)
Controls−USA (VD001)
ME/CFS−Canada (AD001)
Controls−Canada (AD001)

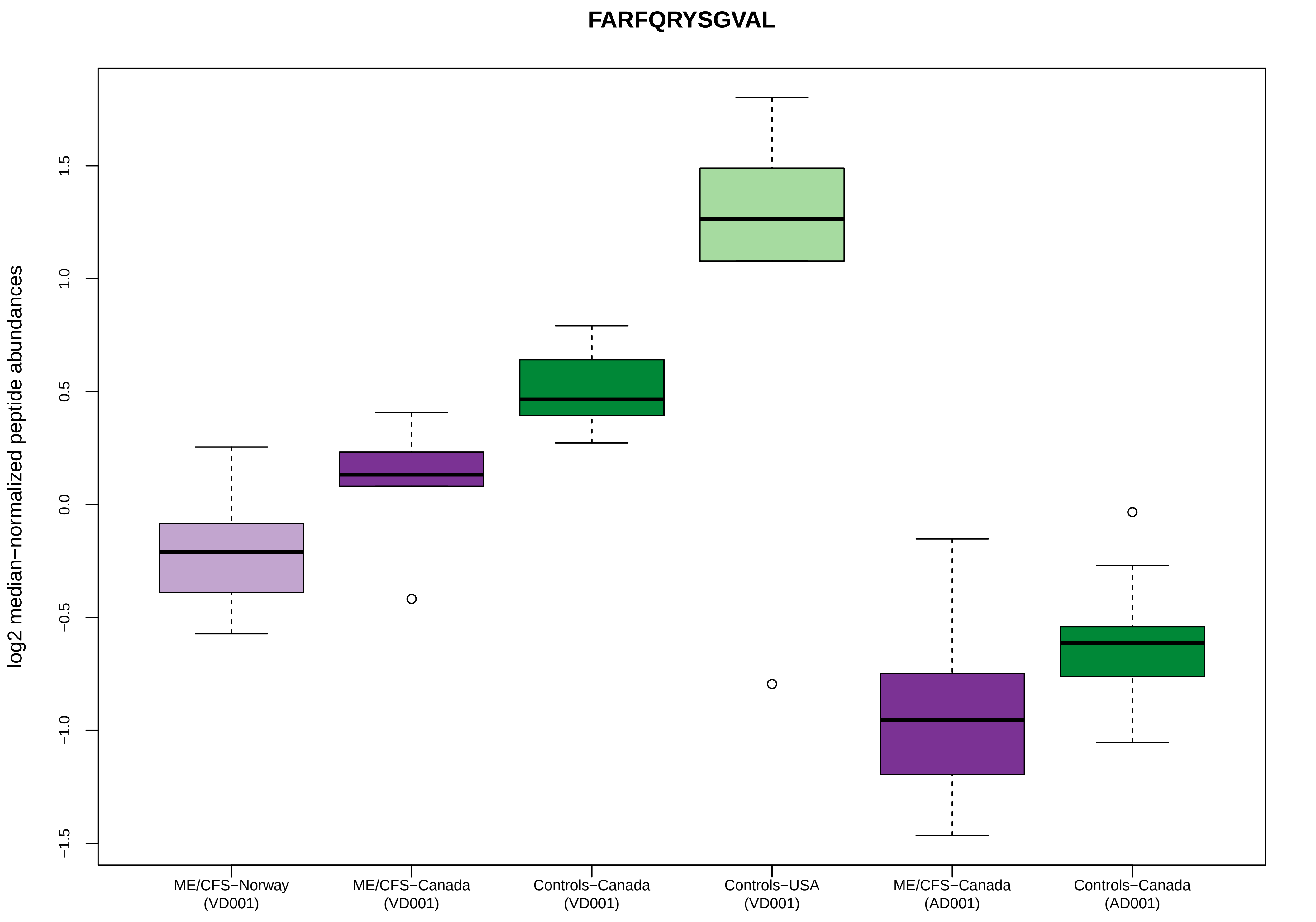
FARFQRYSGVAL
log2 median−normalized peptide abundances
1.5
1.0
0.5
0.0
−0.5
−1.0
−1.5
ME/CFS−Norway (VD001)
ME/CFS−Canada (VD001)
Controls−Canada (VD001)
Controls−USA (VD001)
ME/CFS−Canada (AD001)
Controls−Canada (AD001)

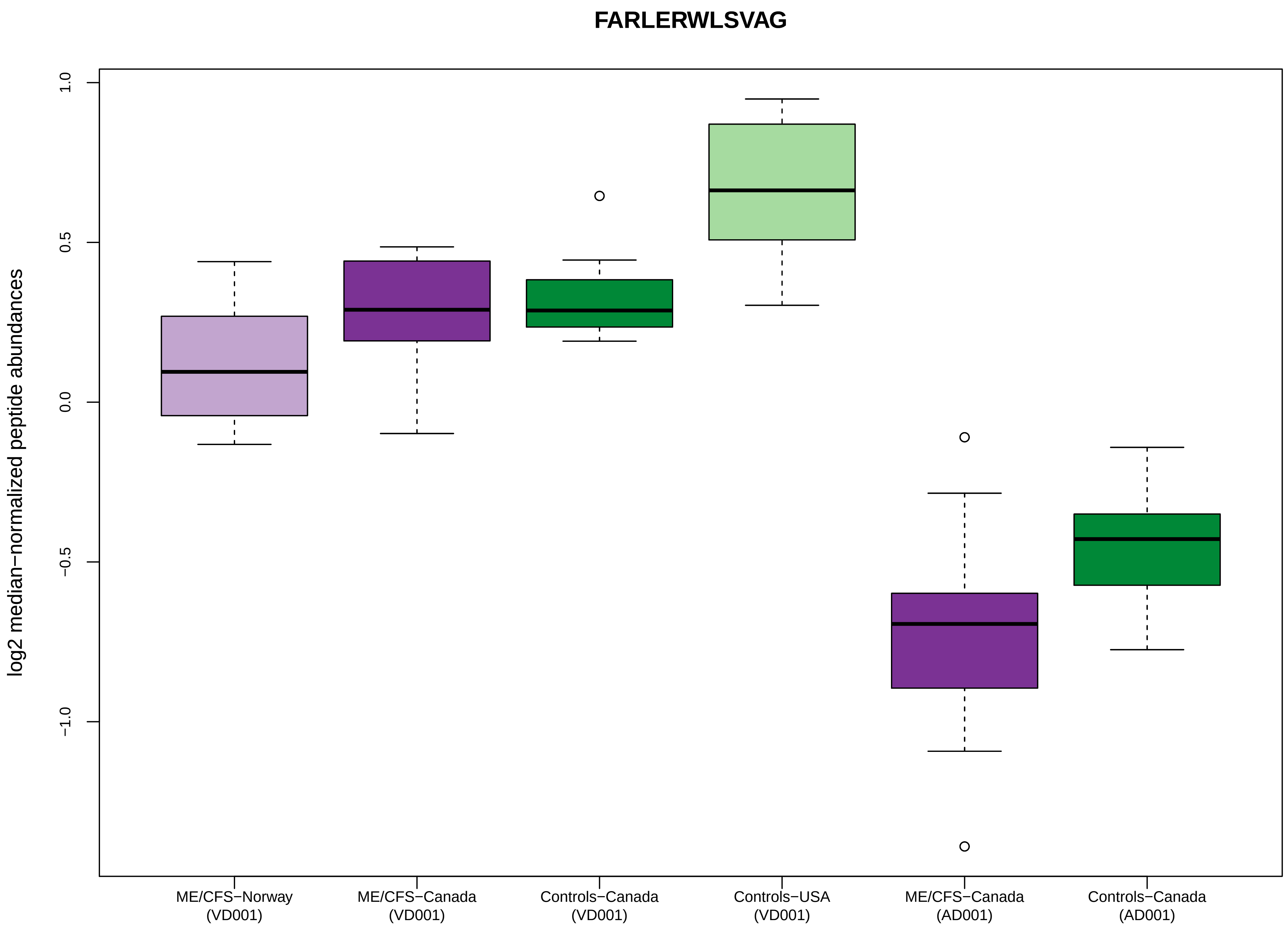
FARLERWLSVAG
log2 median−normalized peptide abundances
1.0
0.5
0.0
−0.5
−1.0
ME/CFS−Norway (VD001)
ME/CFS−Canada (VD001)
Controls−Canada (VD001)
Controls−USA (VD001)
ME/CFS−Canada (AD001)
Controls−Canada (AD001)

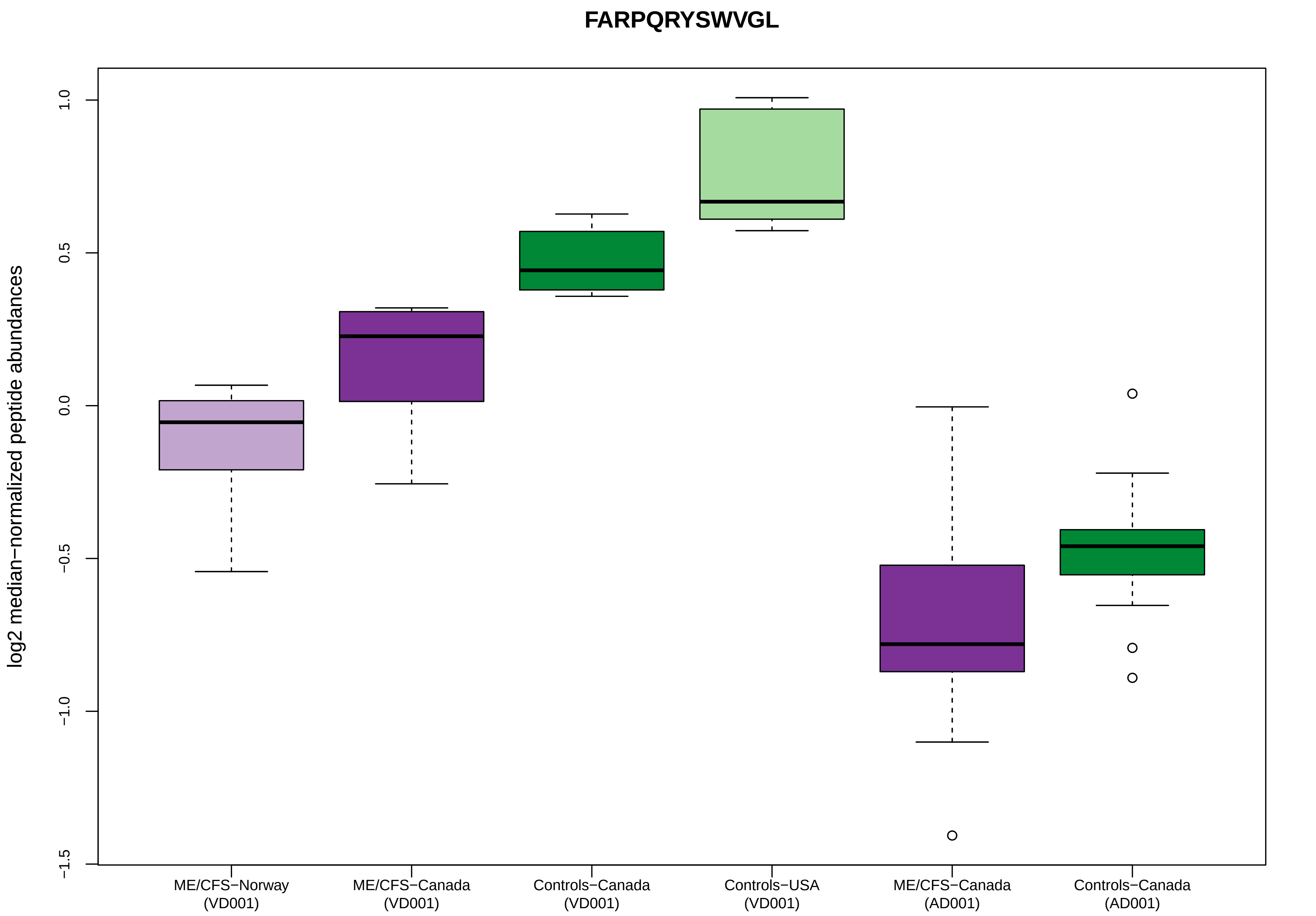
FARPQRYSWVGL
log2 median−normalized peptide abundances
1.0
0.5
0.0
−0.5
−1.0
−1.5
ME/CFS−Norway (VD001)
ME/CFS−Canada (VD001)
Controls−Canada (VD001)
Controls−USA (VD001)
ME/CFS−Canada (AD001)
Controls−Canada (AD001)

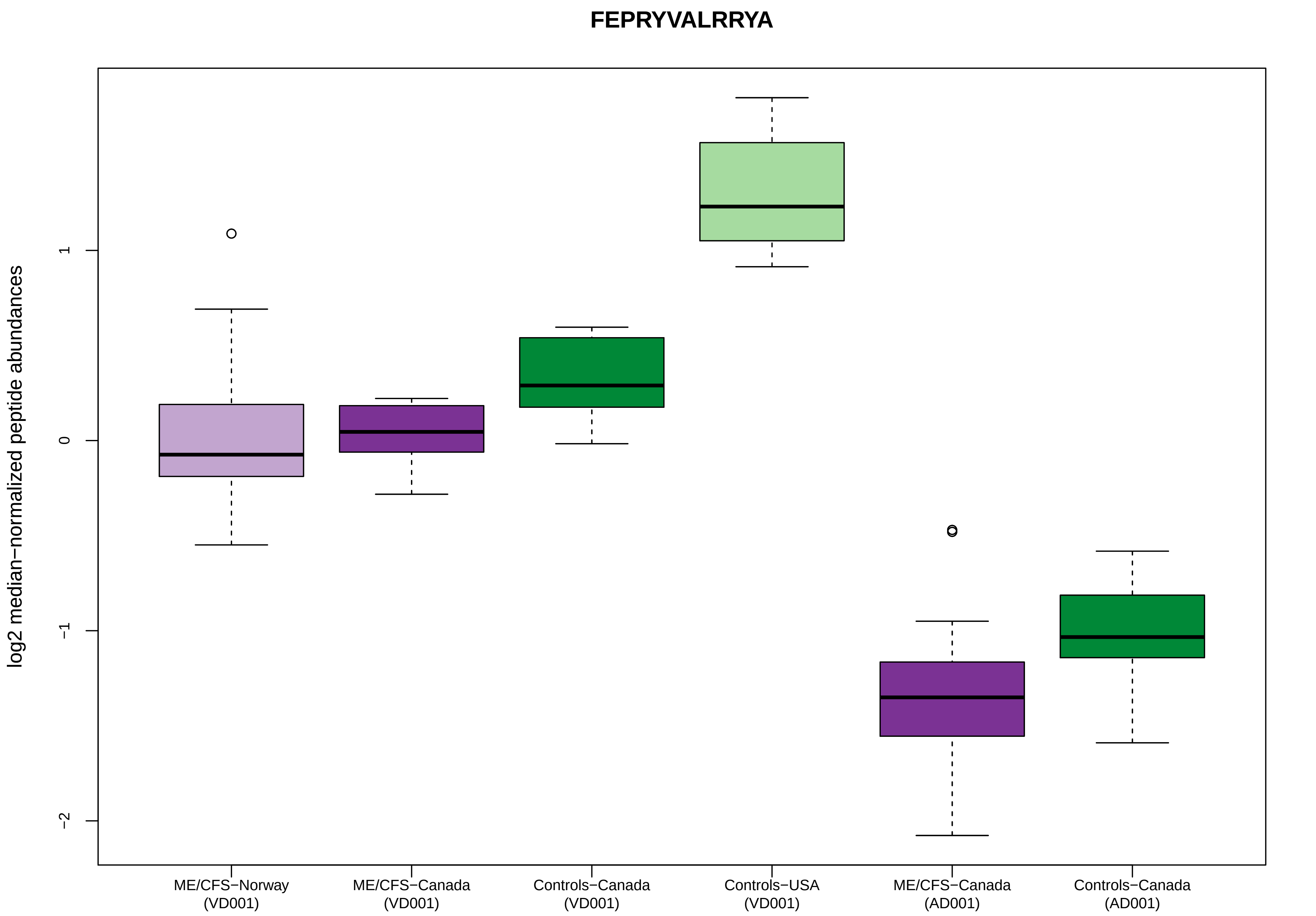

FEPRYVALRRYA
log2 median−normalized peptide abundances
1
0
−1
−2
ME/CFS−Norway (VD001)
ME/CFS−Canada (VD001)
Controls−Canada (VD001)
Controls−USA (VD001)
ME/CFS−Canada (AD001)
Controls−Canada (AD001)

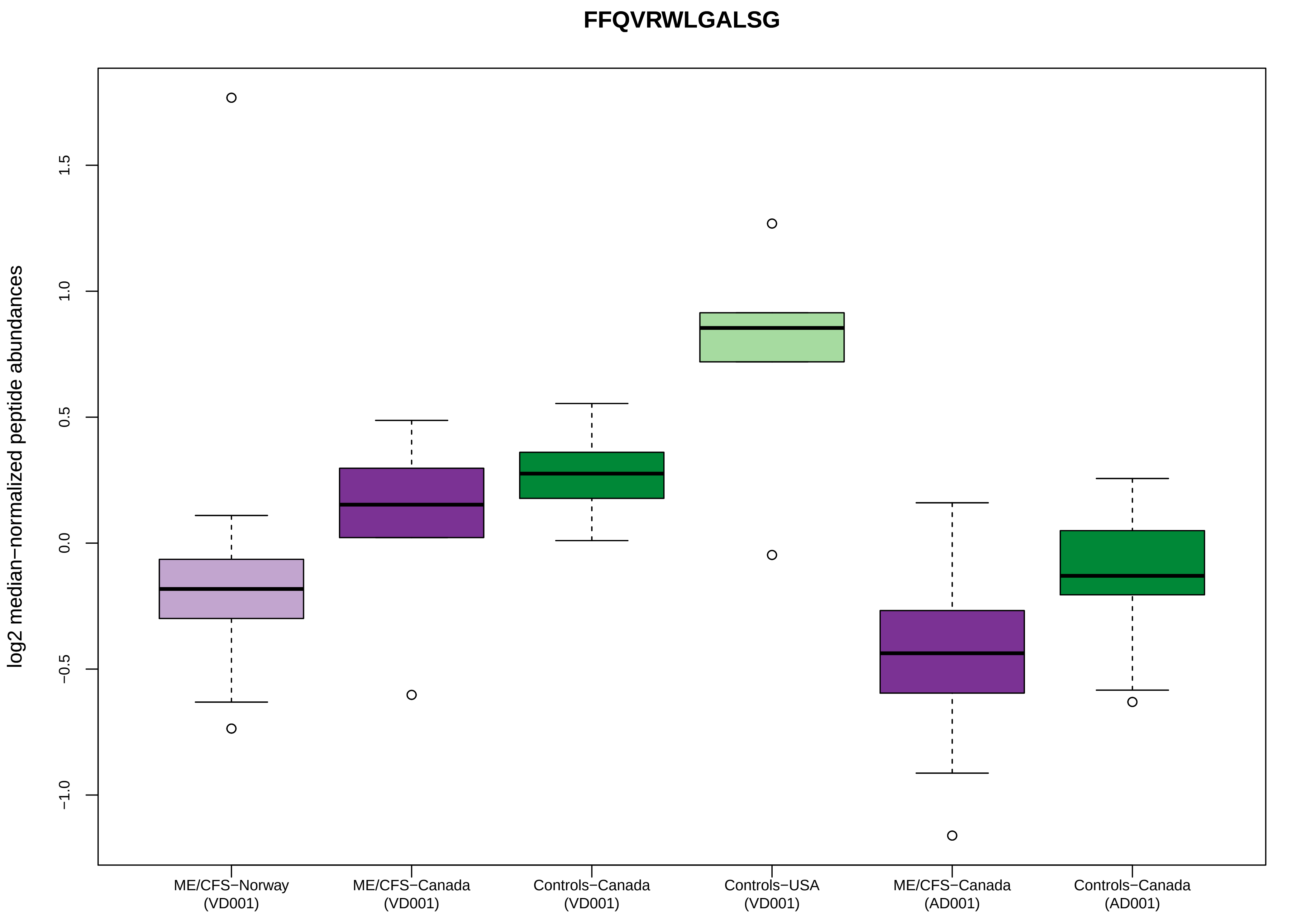
FFQVRWLGALSG
log2 median−normalized peptide abundances
1.5
1.0
0.5
0.0
−0.5
−1.0
ME/CFS−Norway (VD001)
ME/CFS−Canada (VD001)
Controls−Canada (VD001)
Controls−USA (VD001)
ME/CFS−Canada (AD001)
Controls−Canada (AD001)

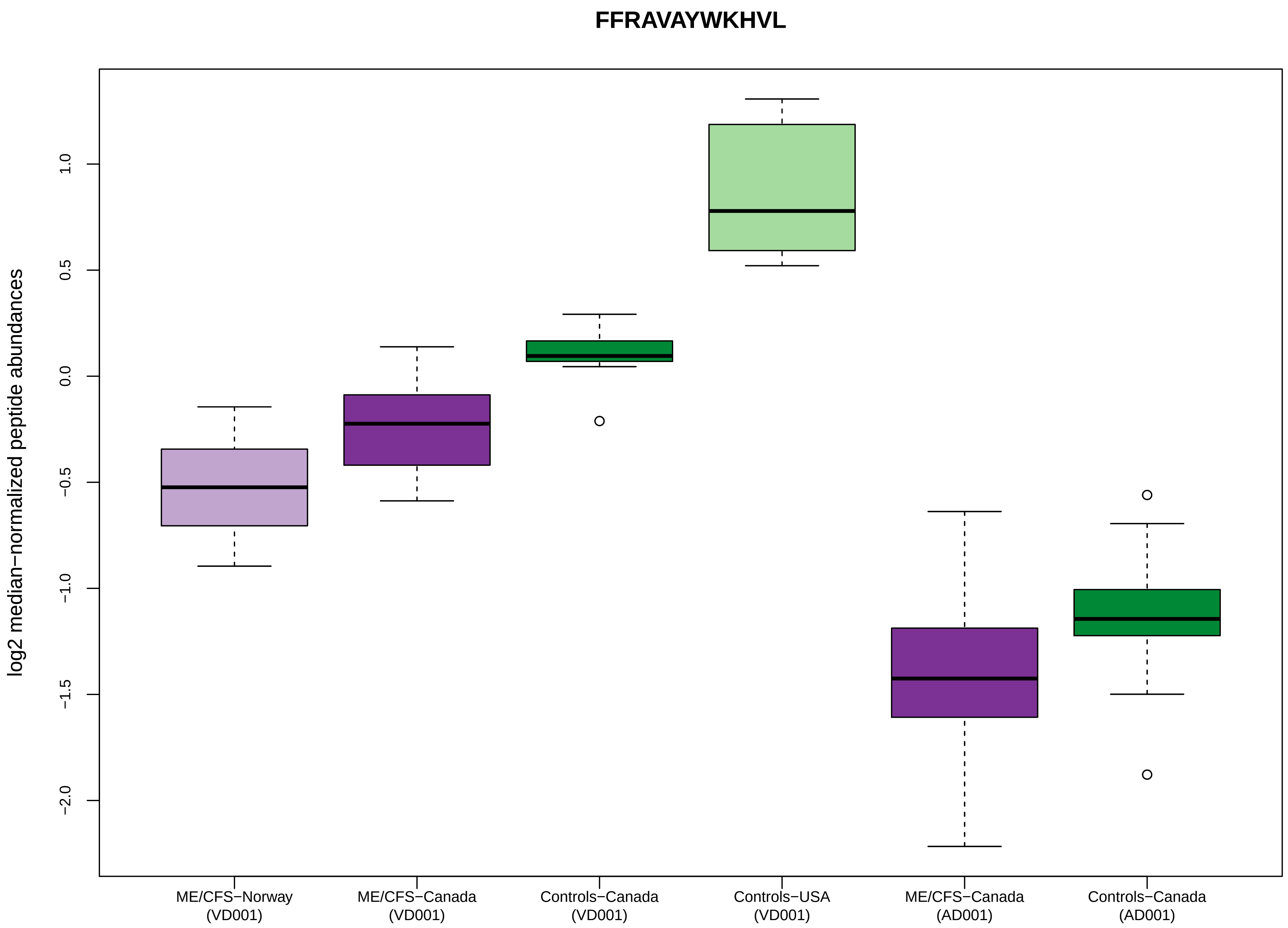

FFRAVAYWKHVL
log2 median−normalized peptide abundances
1.0
0.5
0.0
−0.5
−1.0
−1.5
−2.0
ME/CFS−Norway (VD001)
ME/CFS−Canada (VD001)
Controls−Canada (VD001)
Controls−USA (VD001)
ME/CFS−Canada (AD001)
Controls−Canada (AD001)

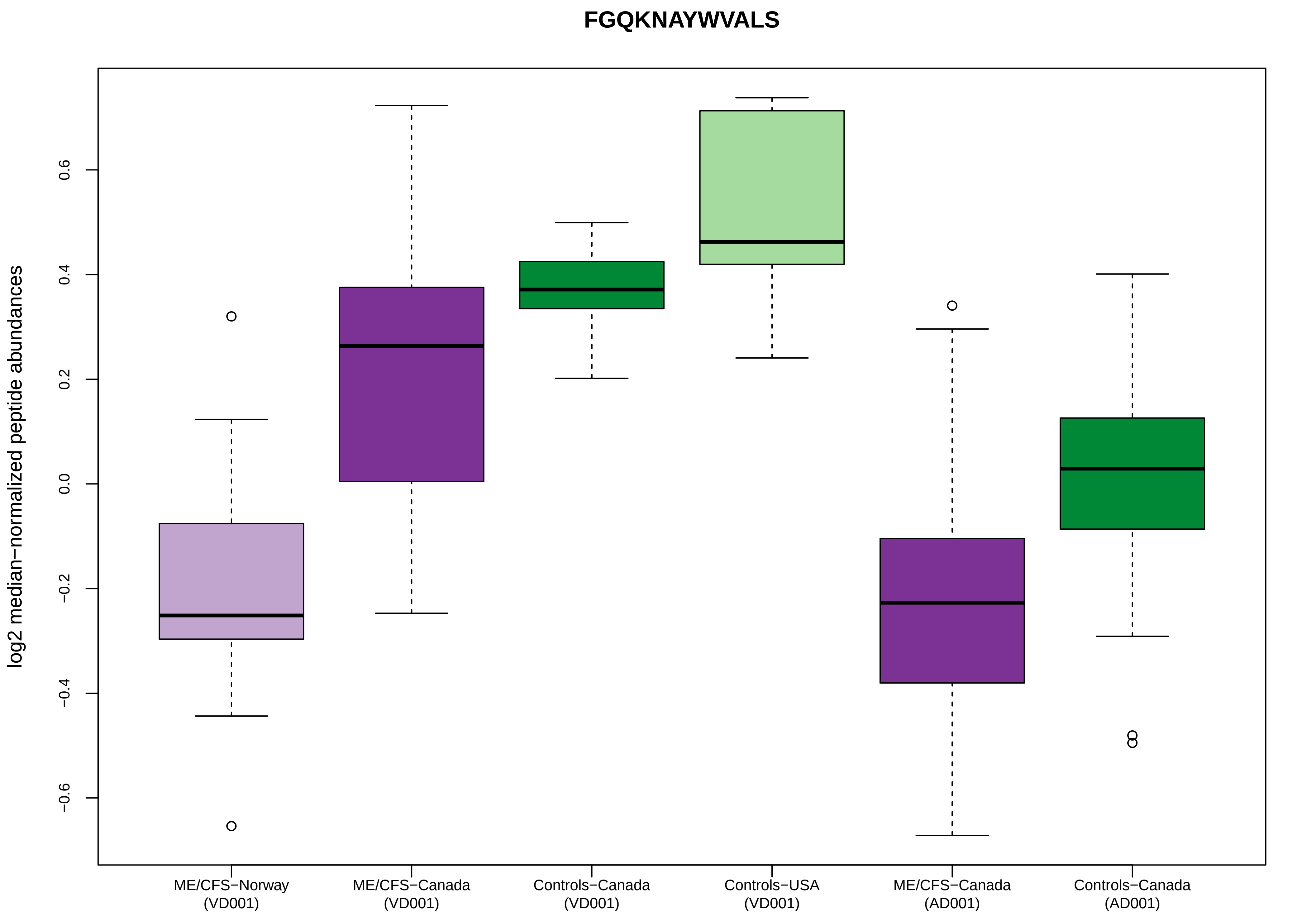
FGQKNAYWVALS
log2 median−normalized peptide abundances
0.6
0.4
0.2
0.0
−0.2
−0.4
−0.6
ME/CFS−Norway (VD001)
ME/CFS−Canada (VD001)
Controls−Canada (VD001)
Controls−USA (VD001)
ME/CFS−Canada (AD001)
Controls−Canada (AD001)

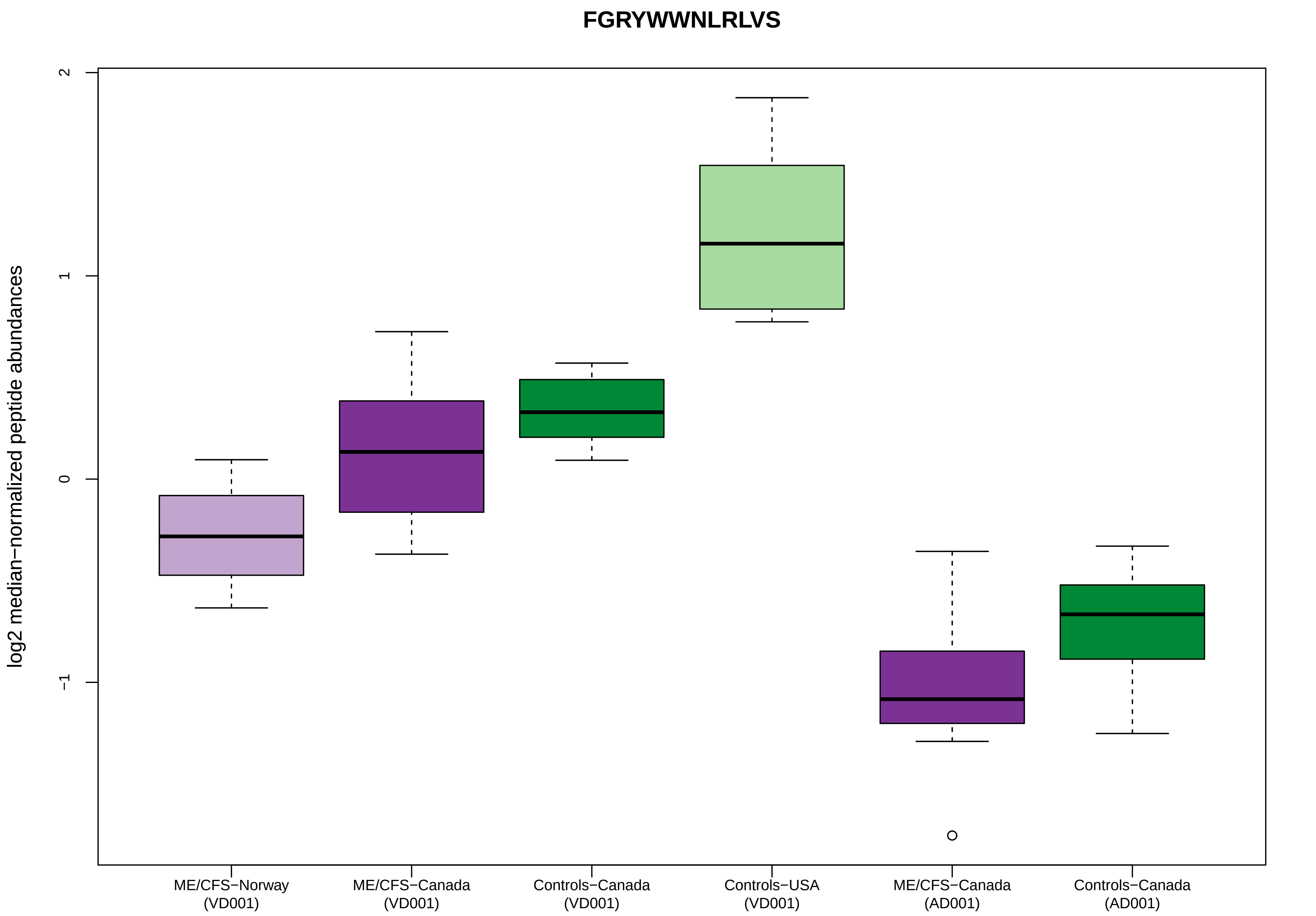
FGRYWWNLRLVS
log2 median−normalized peptide abundances
2
1
0
−1
ME/CFS−Norway (VD001)
ME/CFS−Canada (VD001)
Controls−Canada (VD001)
Controls−USA (VD001)
ME/CFS−Canada (AD001)
Controls−Canada (AD001)

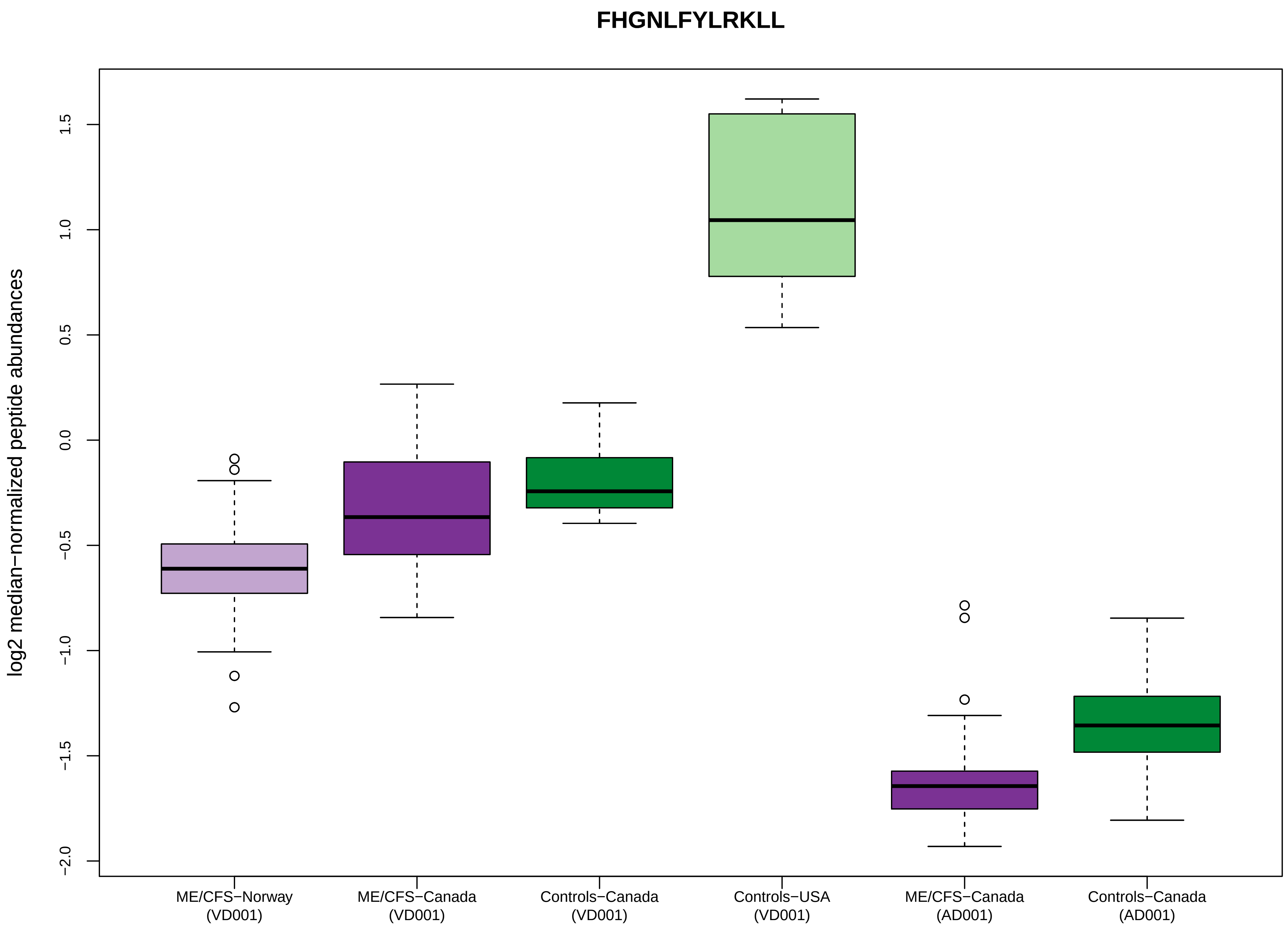

FHGNLFYLRKLL
log2 median−normalized peptide abundances
1.5
1.0
0.5
0.0
−0.5
−1.0
−1.5
−2.0
ME/CFS−Norway (VD001)
ME/CFS−Canada (VD001)
Controls−Canada (VD001)
Controls−USA (VD001)
ME/CFS−Canada (AD001)
Controls−Canada (AD001)

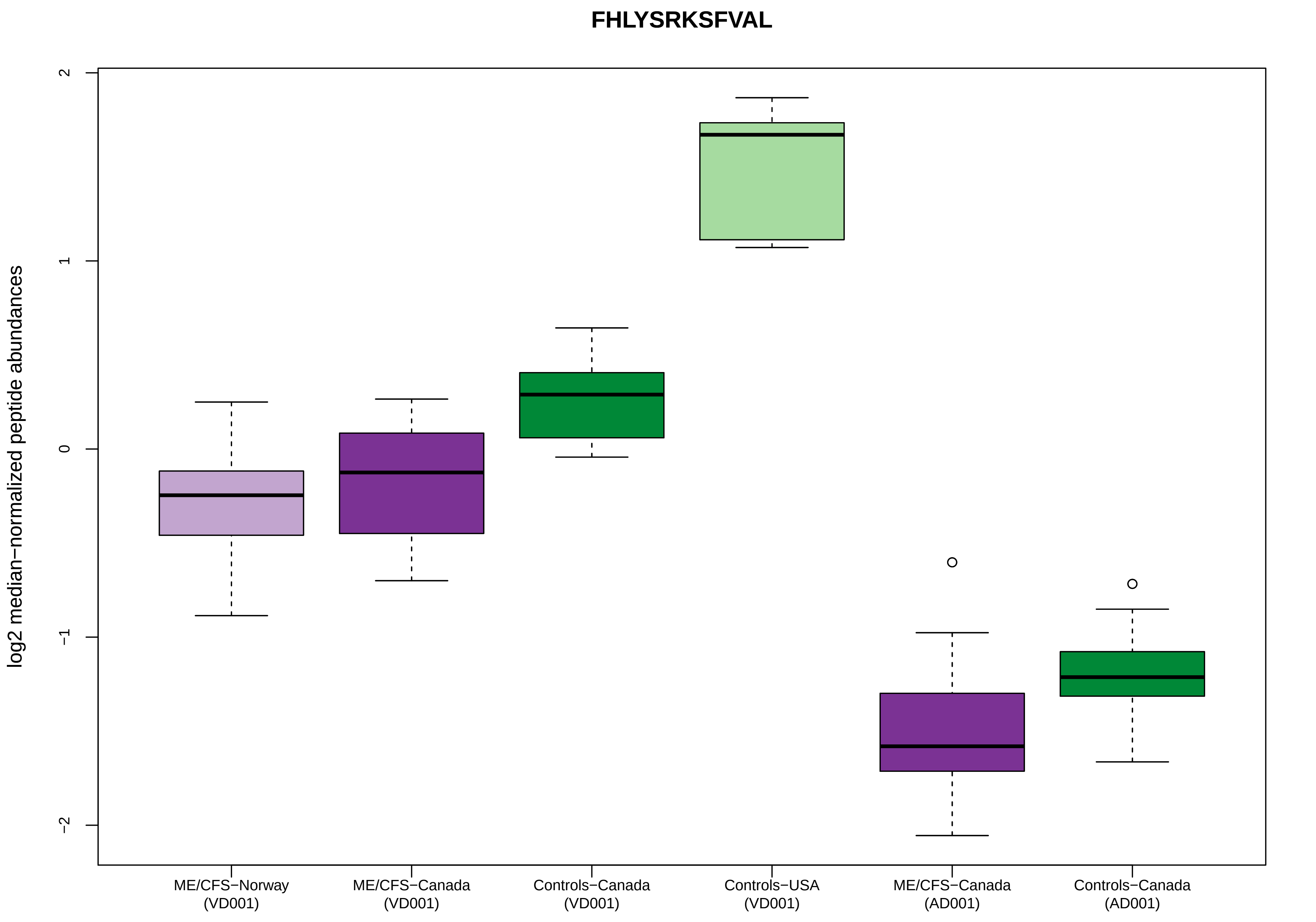

FHLYSRKSFVAL
log2 median−normalized peptide abundances
2
1
0
−1
−2
ME/CFS−Norway (VD001)
ME/CFS−Canada (VD001)
Controls−Canada (VD001)
Controls−USA (VD001)
ME/CFS−Canada (AD001)
Controls−Canada (AD001)

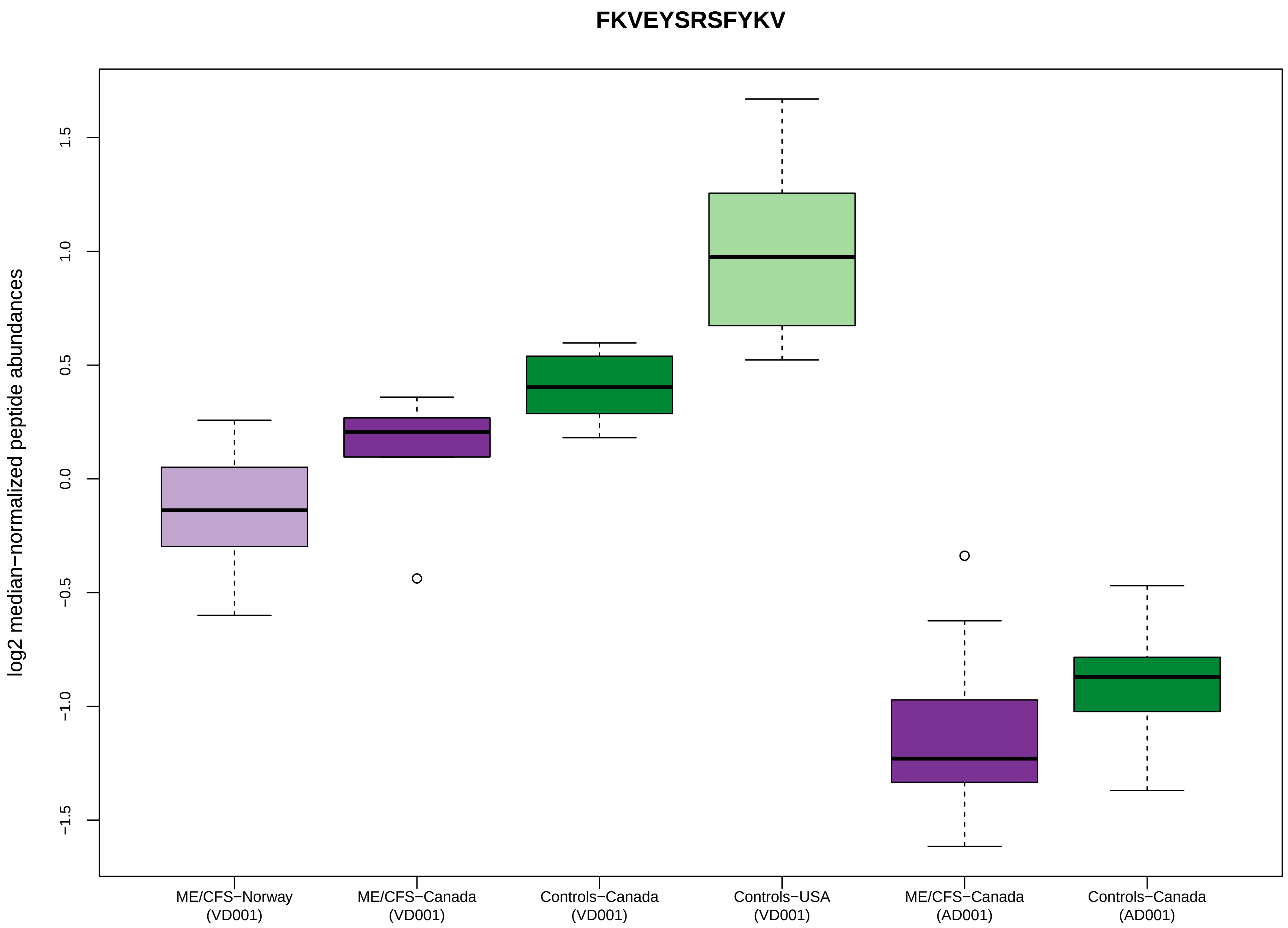
FKVEYSRSFYKV
log2 median−normalized peptide abundances
1.5
1.0
0.5
0.0
−0.5
−1.0
−1.5
ME/CFS−Norway (VD001)
ME/CFS−Canada (VD001)
Controls−Canada (VD001)
Controls−USA (VD001)
ME/CFS−Canada (AD001)
Controls−Canada (AD001)

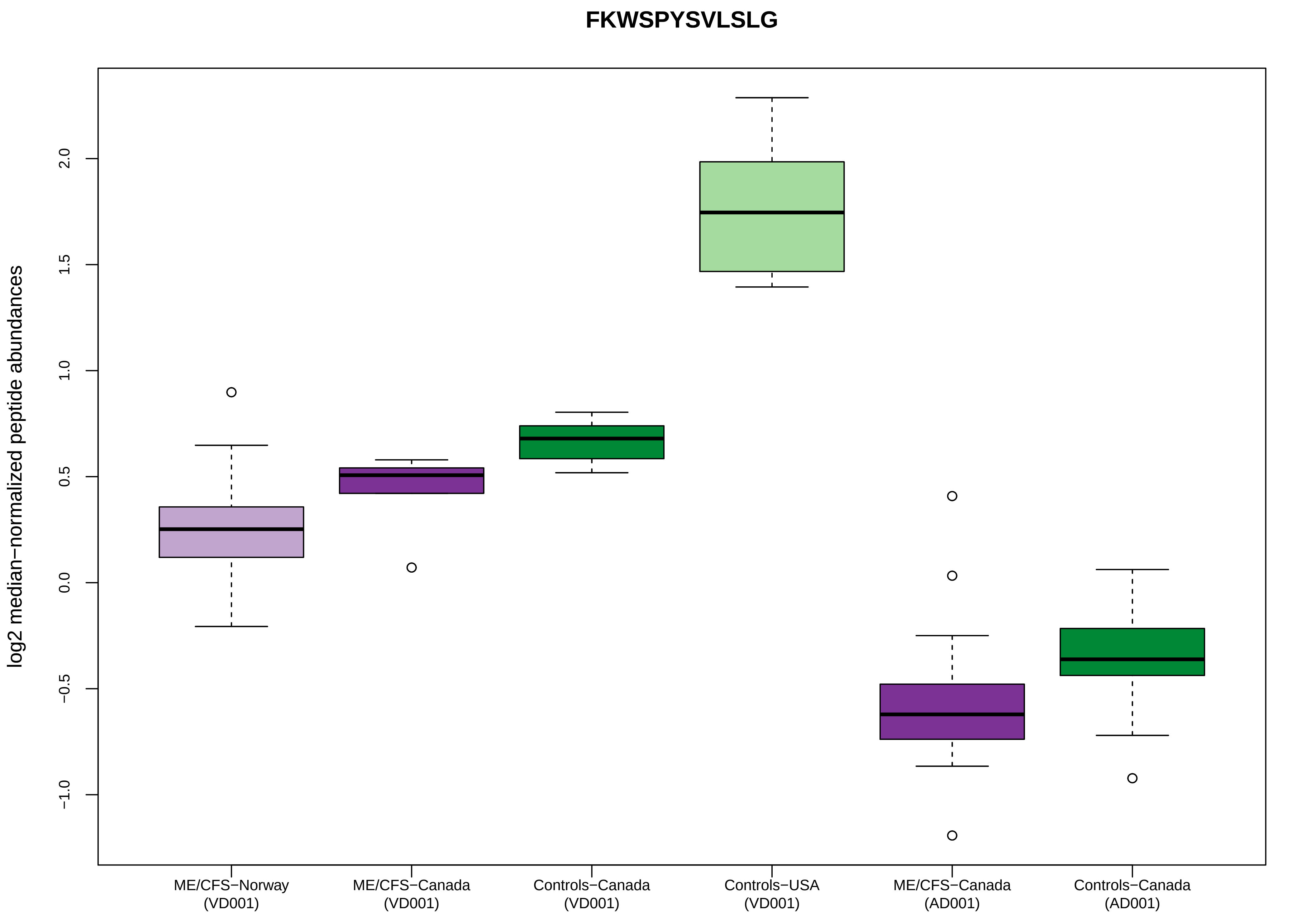
FKWSPYSVLSLG
log2 median−normalized peptide abundances
−1.0
−0.5
0.0
0.5
1.0
1.5
2.0
ME/CFS−Norway (VD001)
ME/CFS−Canada (VD001)
Controls−Canada (VD001)
Controls−USA (VD001)
ME/CFS−Canada (AD001)
Controls−Canada (AD001)

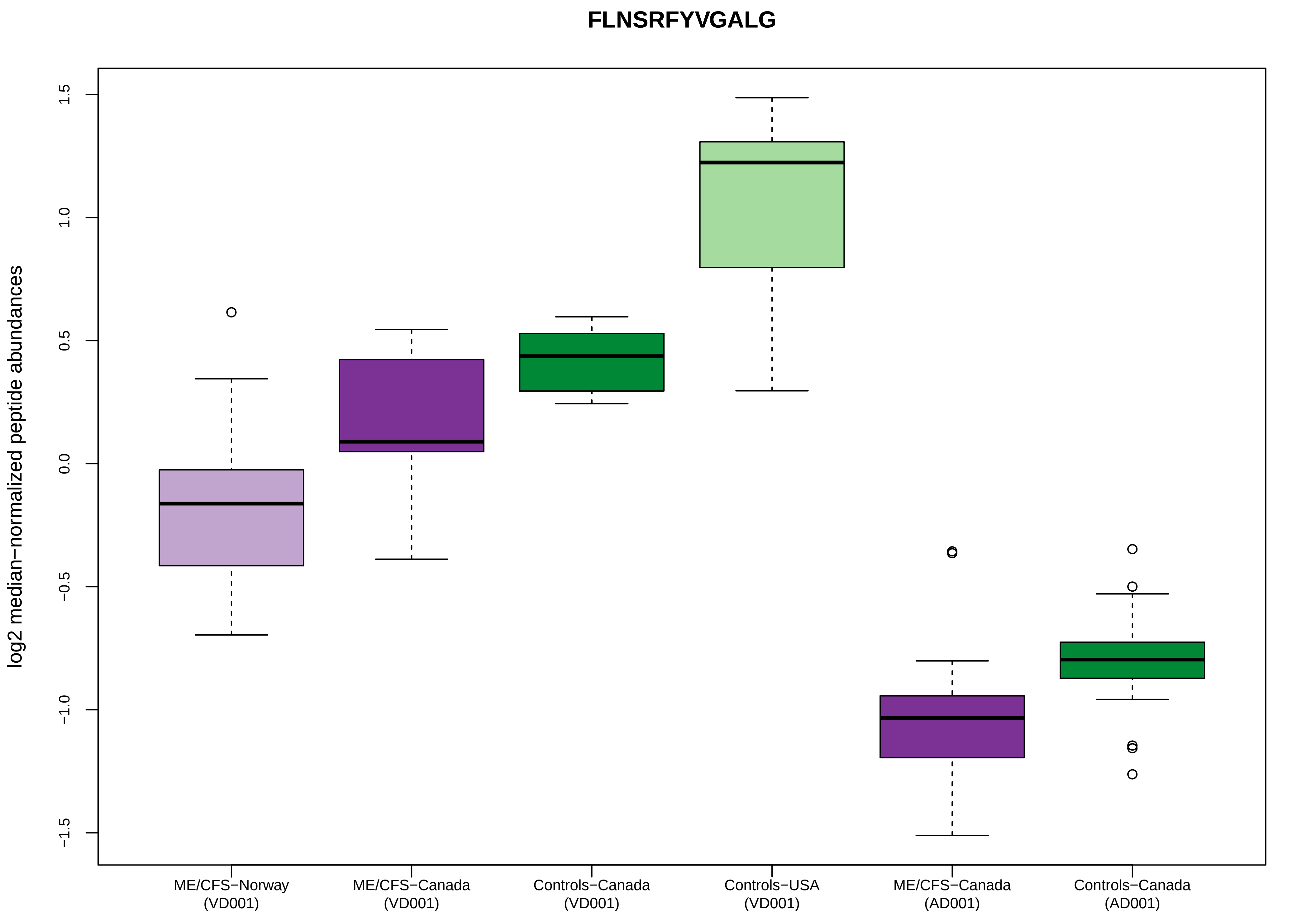

FLNSRFYVGALG
log2 median−normalized peptide abundances
1.5
1.0
0.5
0.0
−0.5
−1.0
−1.5
ME/CFS−Norway (VD001)
ME/CFS−Canada (VD001)
Controls−Canada (VD001)
Controls−USA (VD001)
ME/CFS−Canada (AD001)
Controls−Canada (AD001)

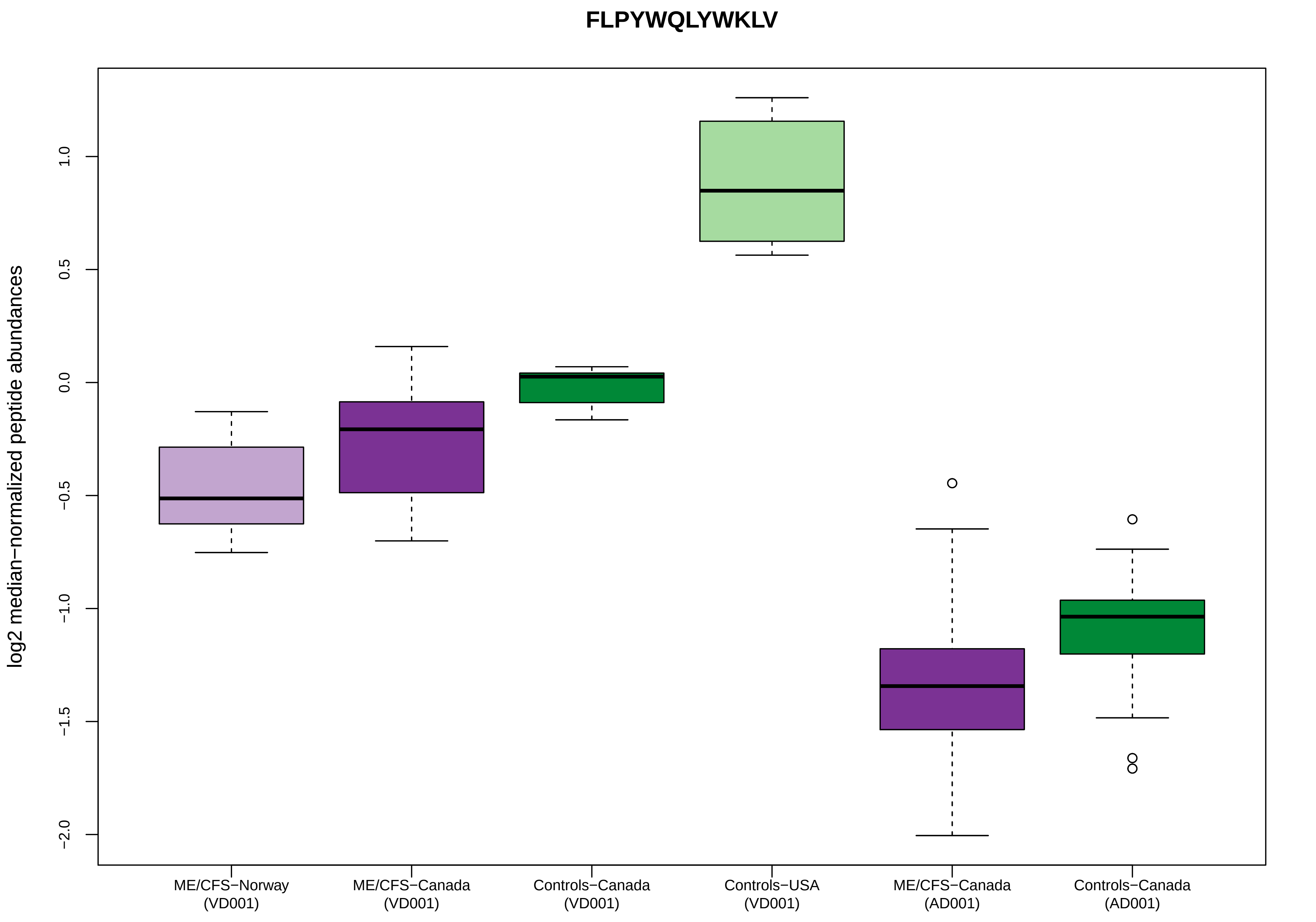
FLPYWQLYWKLV
log2 median−normalized peptide abundances
1.0
0.5
0.0
−0.5
−1.0
−1.5
−2.0
ME/CFS−Norway (VD001)
ME/CFS−Canada (VD001)
Controls−Canada (VD001)
Controls−USA (VD001)
ME/CFS−Canada (AD001)
Controls−Canada (AD001)

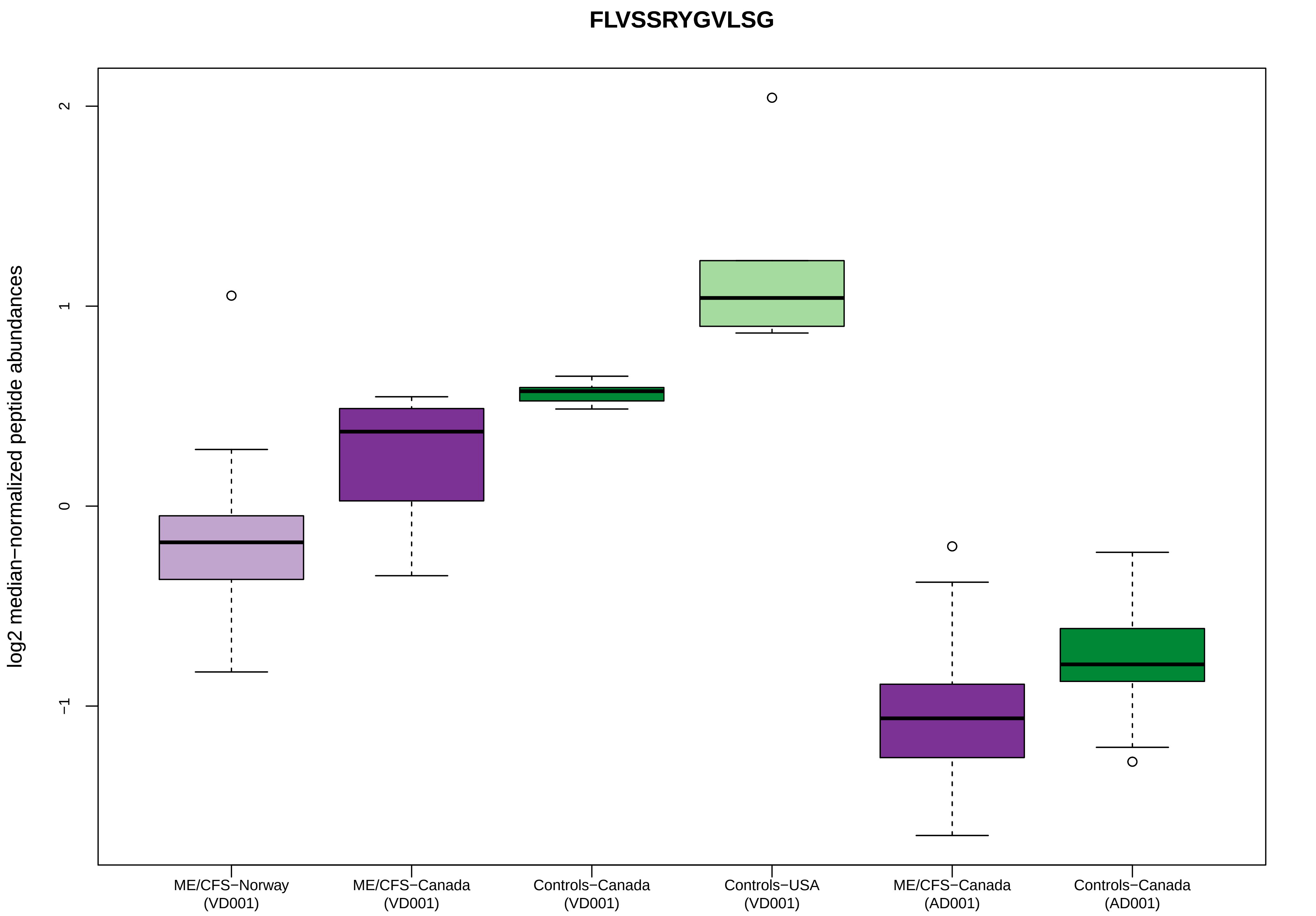
FLVSSRYGVLSG
log2 median−normalized peptide abundances
2
1
0
−1
ME/CFS−Norway (VD001)
ME/CFS−Canada (VD001)
Controls−Canada (VD001)
Controls−USA (VD001)
ME/CFS−Canada (AD001)
Controls−Canada (AD001)

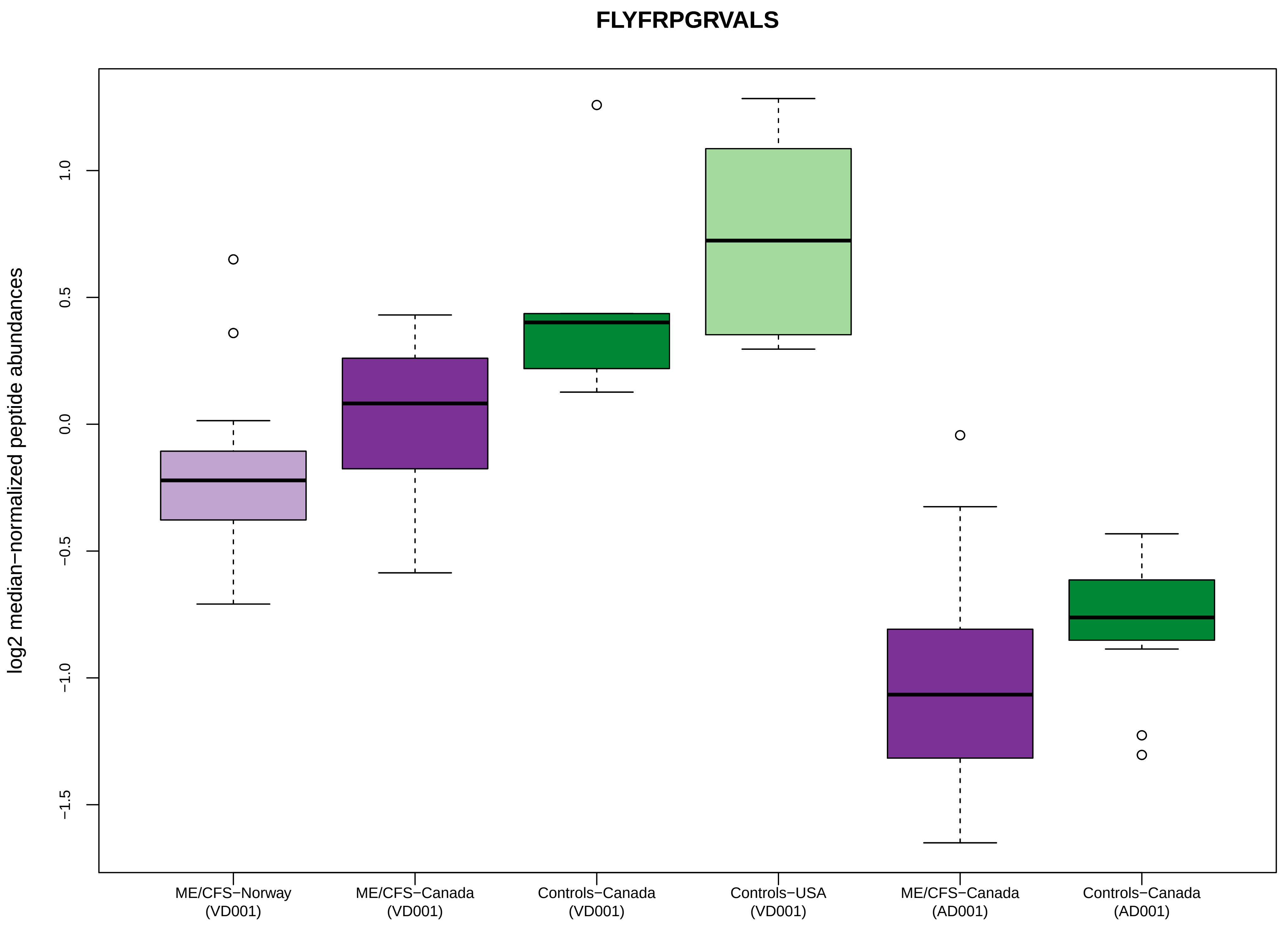
FLYFRPGRVALS
log2 median−normalized peptide abundances
1.0
0.5
0.0
−0.5
−1.0
−1.5
ME/CFS−Norway (VD001)
ME/CFS−Canada (VD001)
Controls−Canada (VD001)
Controls−USA (VD001)
ME/CFS−Canada (AD001)
Controls−Canada (AD001)

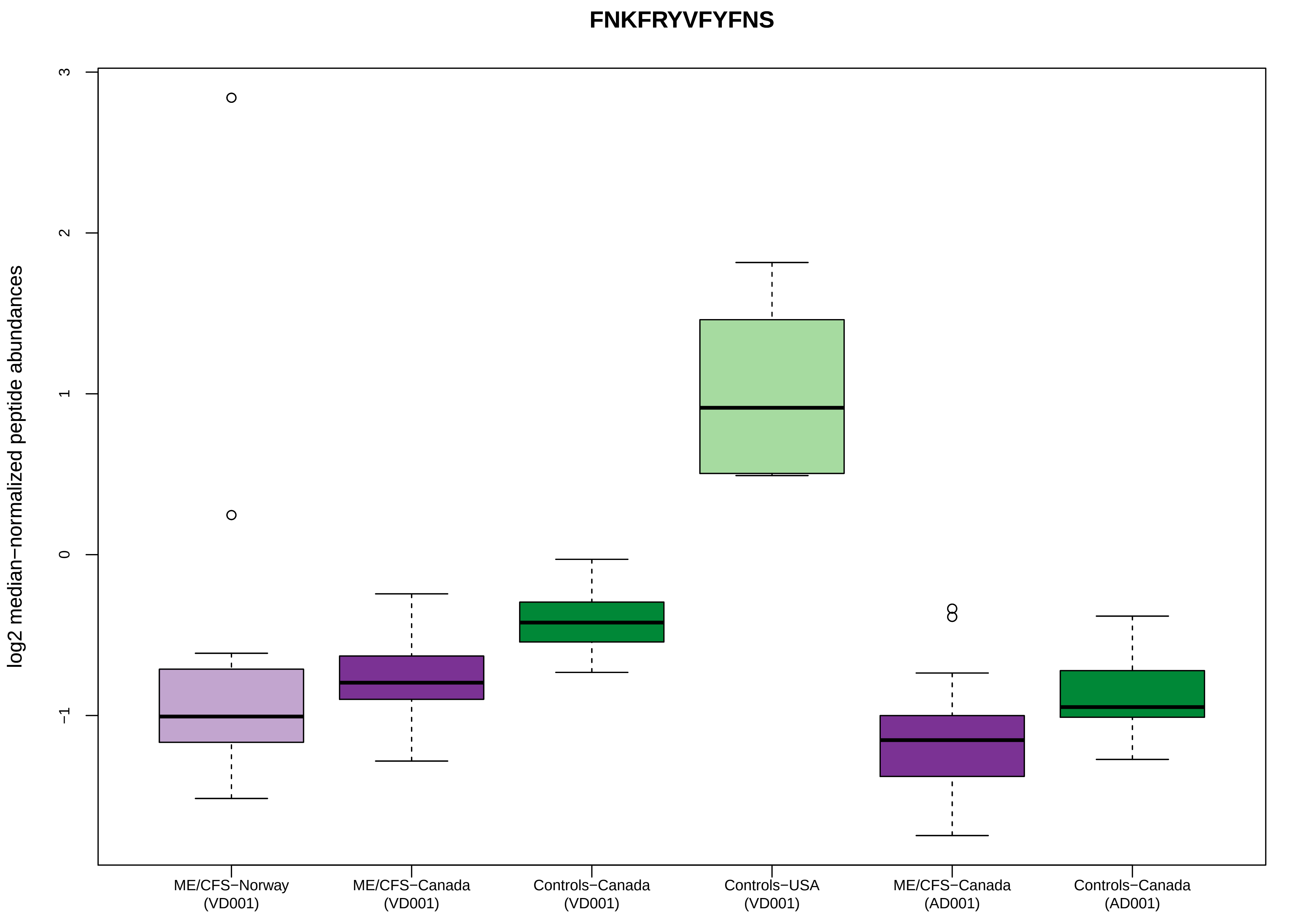
FNKFRYVFYFNS
log2 median−normalized peptide abundances
3
2
1
0
−1
ME/CFS−Norway (VD001)
ME/CFS−Canada (VD001)
Controls−Canada (VD001)
Controls−USA (VD001)
ME/CFS−Canada (AD001)
Controls−Canada (AD001)

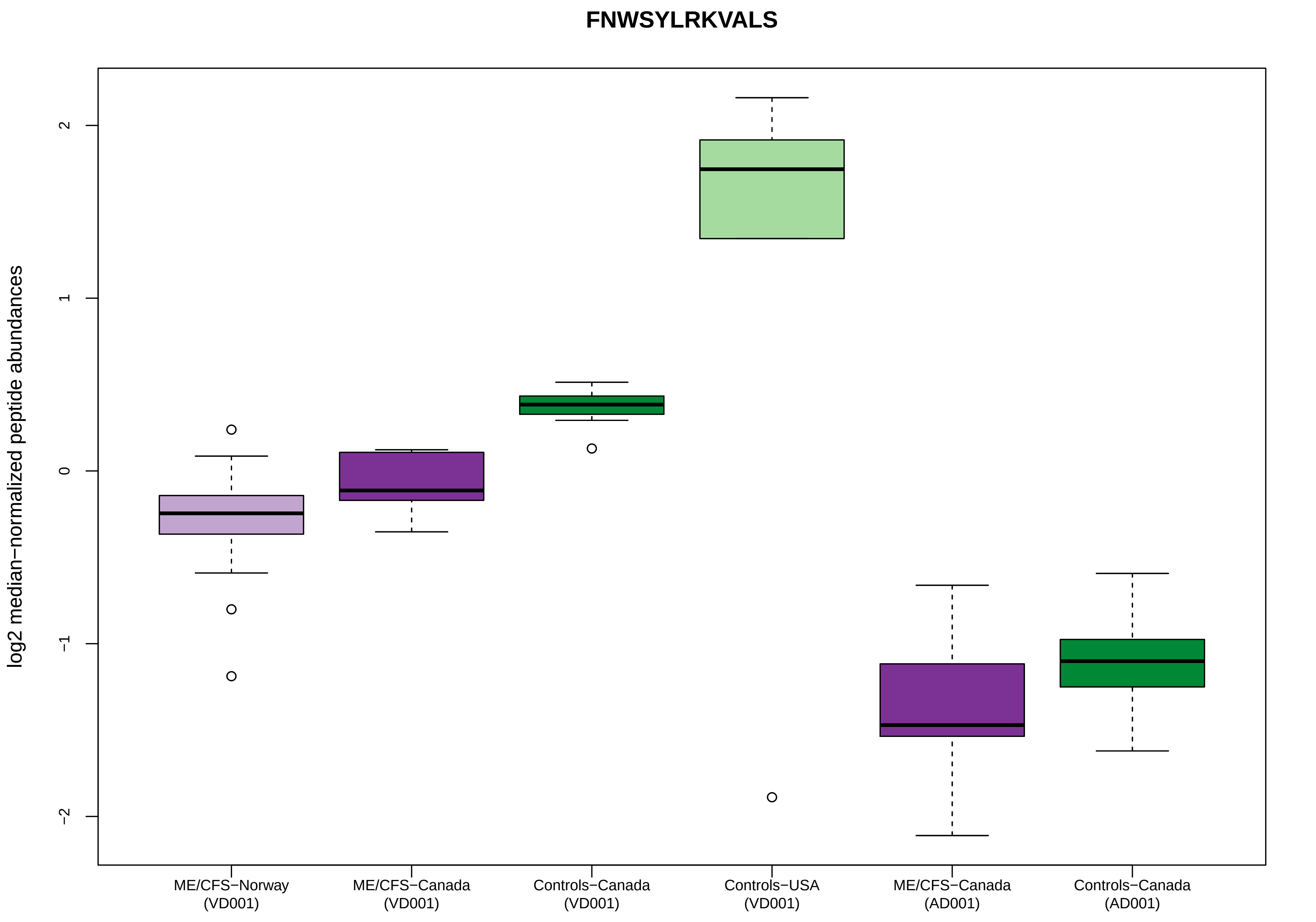
FNWSYLRKVALS
log2 median−normalized peptide abundances
2
1
0
−1
−2
ME/CFS−Norway
(VD001)
ME/CFS−Canada
(VD001)
Controls−Canada
(VD001)
Controls−USA
(VD001)
ME/CFS−Canada
(AD001)
Controls−Canada
(AD001)

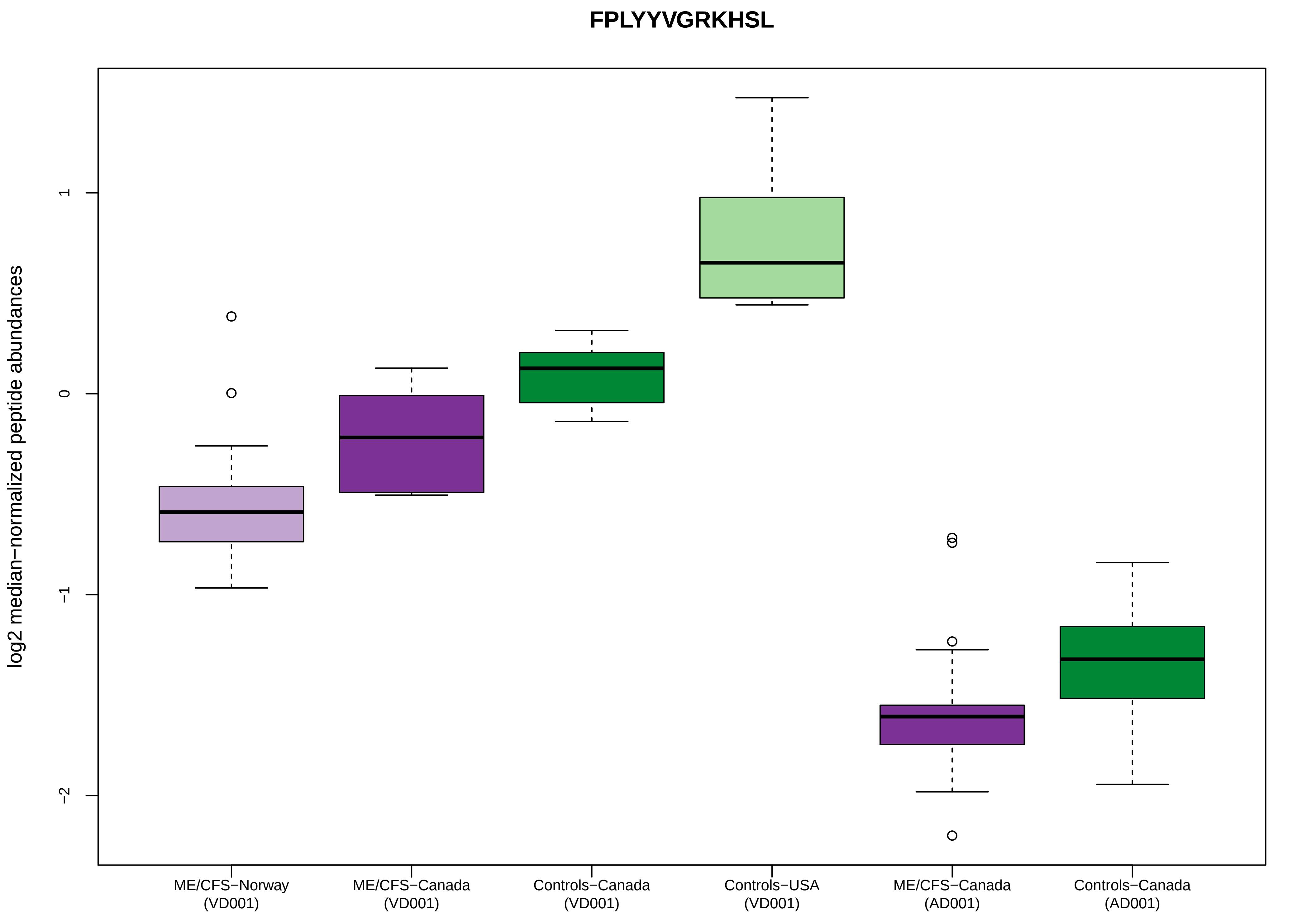
FPLYYVGRKHSL
log2 median−normalized peptide abundances
1
0
−1
−2
ME/CFS−Norway (VD001)
ME/CFS−Canada (VD001)
Controls−Canada (VD001)
Controls−USA (VD001)
ME/CFS−Canada (AD001)
Controls−Canada (AD001)

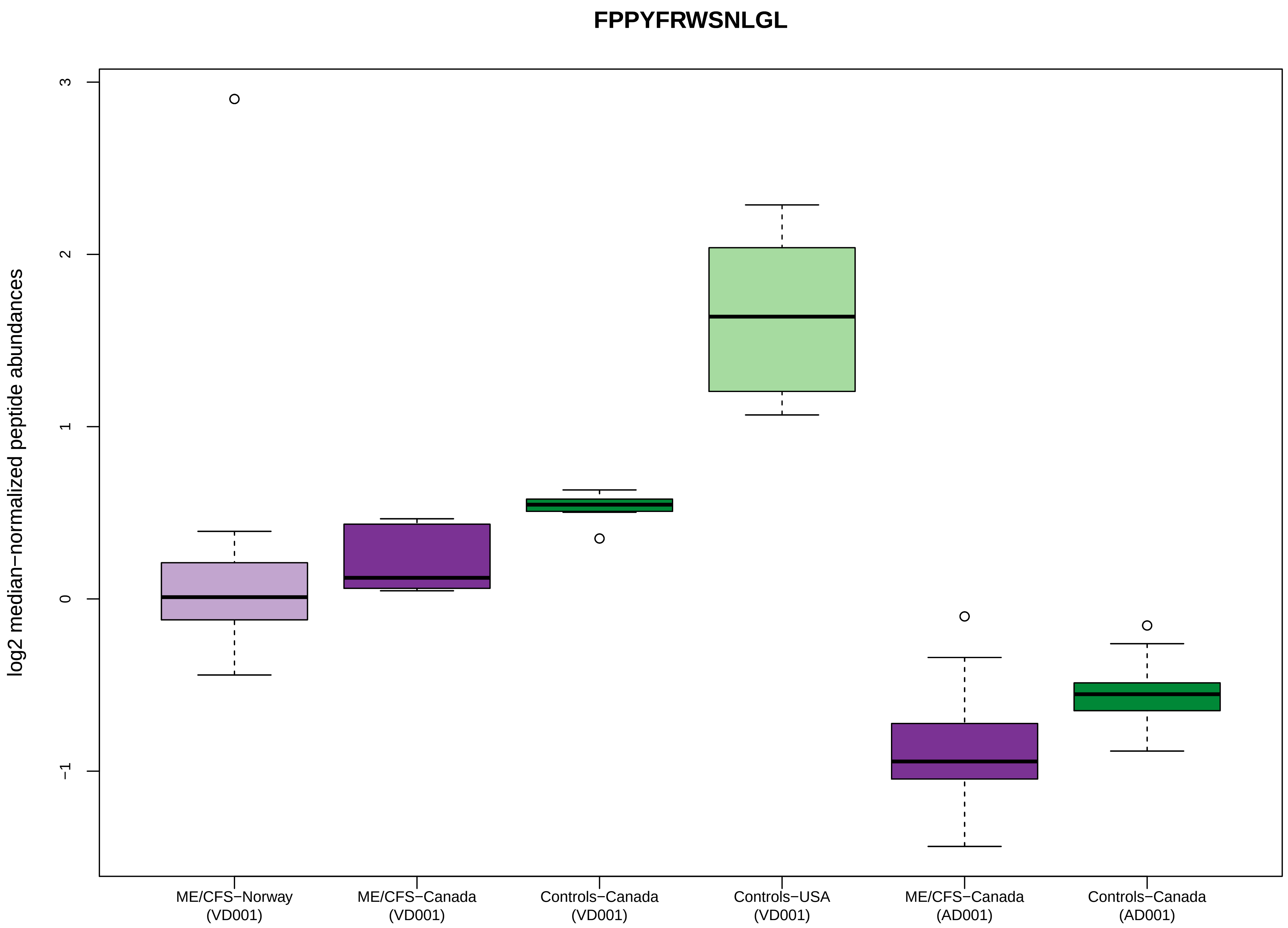

FPPYFRWSNLGL
log2 median−normalized peptide abundances
3
2
1
0
−1
ME/CFS−Norway (VD001)
ME/CFS−Canada (VD001)
Controls−Canada (VD001)
Controls−USA (VD001)
ME/CFS−Canada (AD001)
Controls−Canada (AD001)

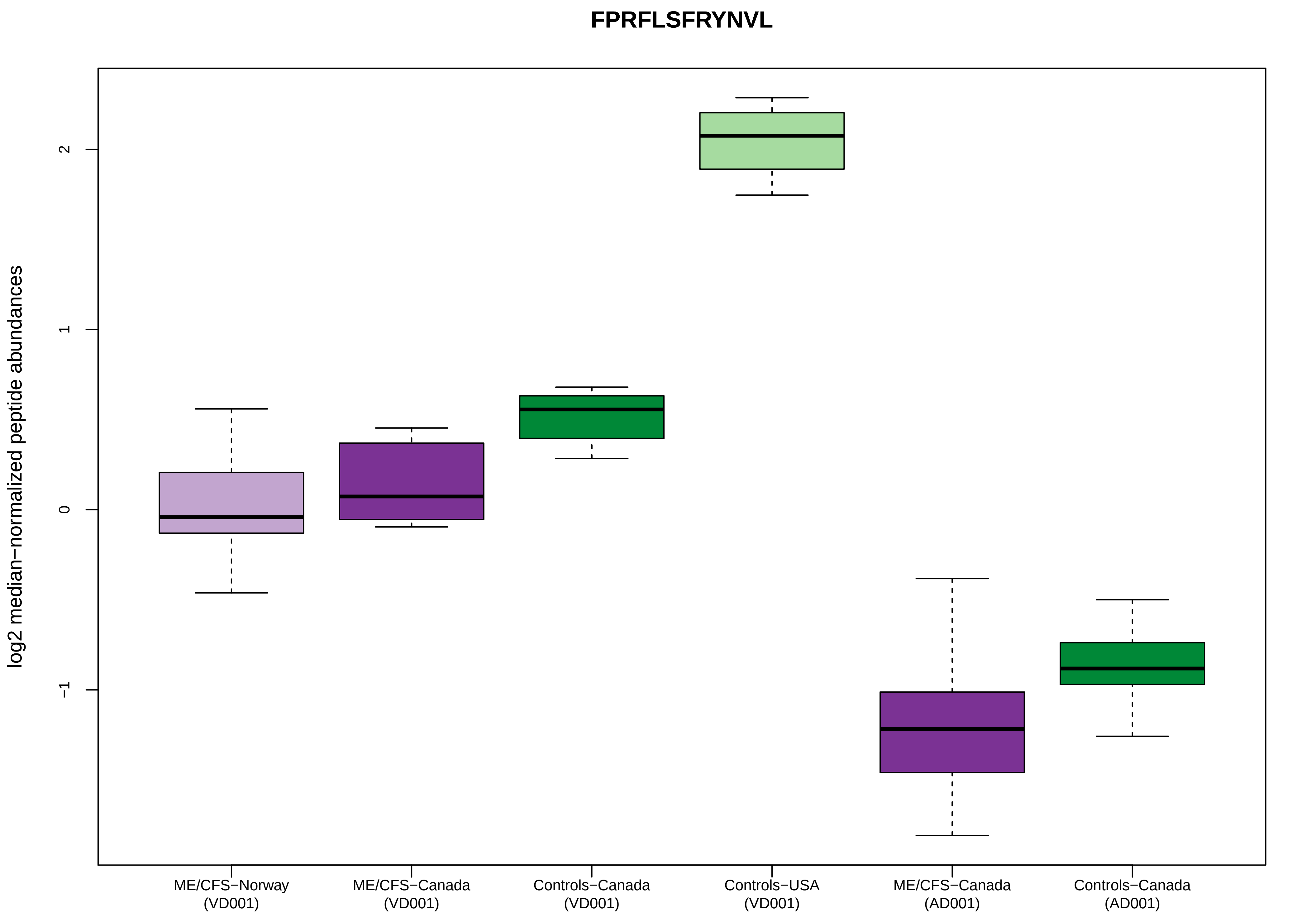
FPRFLSFRYNVL
log2 median−normalized peptide abundances
2
1
0
−1
ME/CFS−Norway (VD001)
ME/CFS−Canada (VD001)
Controls−Canada (VD001)
Controls−USA (VD001)
ME/CFS−Canada (AD001)
Controls−Canada (AD001)

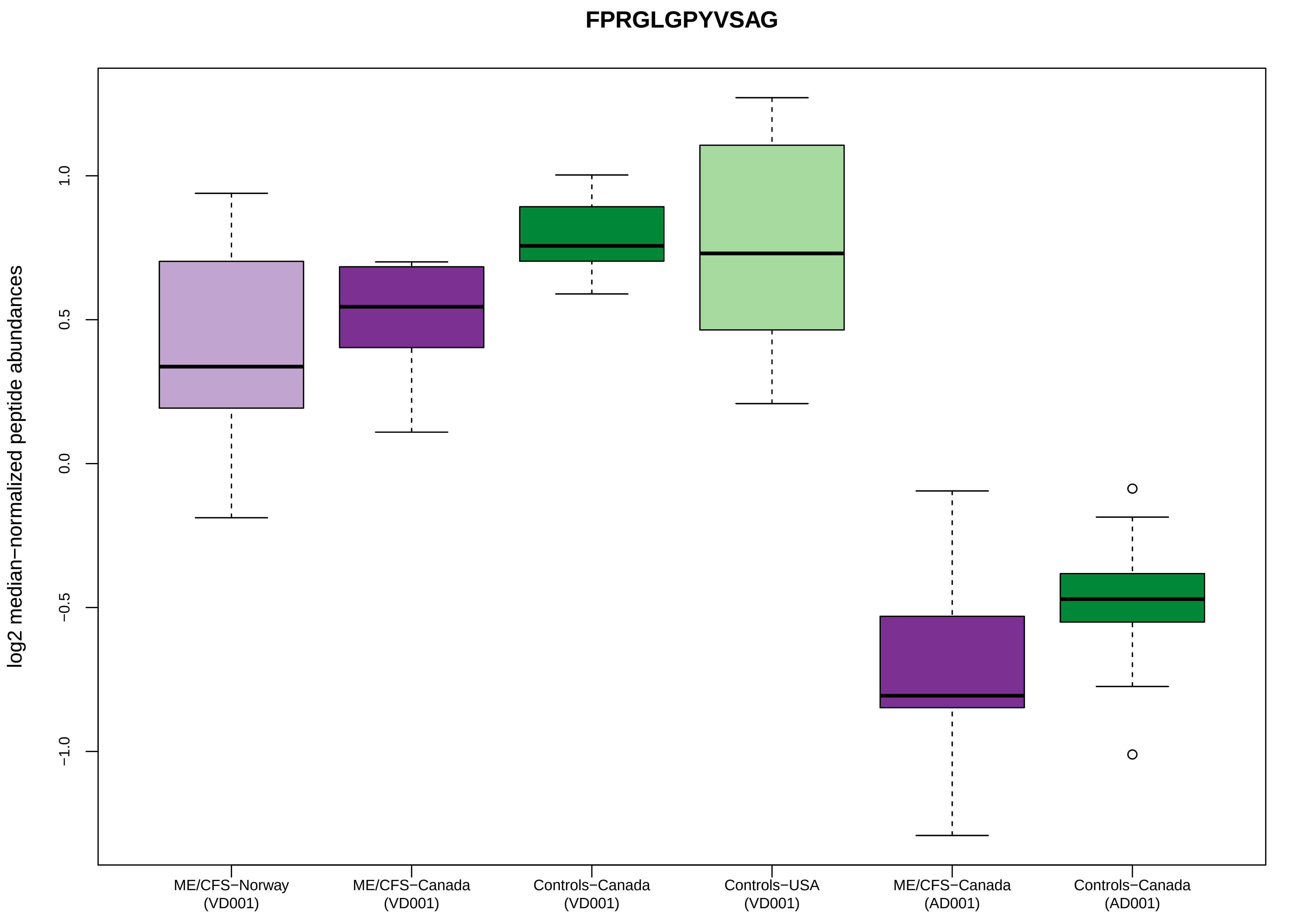

FPRGLGPYVSAG
log2 median−normalized peptide abundances
1.0
0.5
0.0
−0.5
−1.0
ME/CFS−Norway (VD001)
ME/CFS−Canada (VD001)
Controls−Canada (VD001)
Controls−USA (VD001)
ME/CFS−Canada (AD001)
Controls−Canada (AD001)

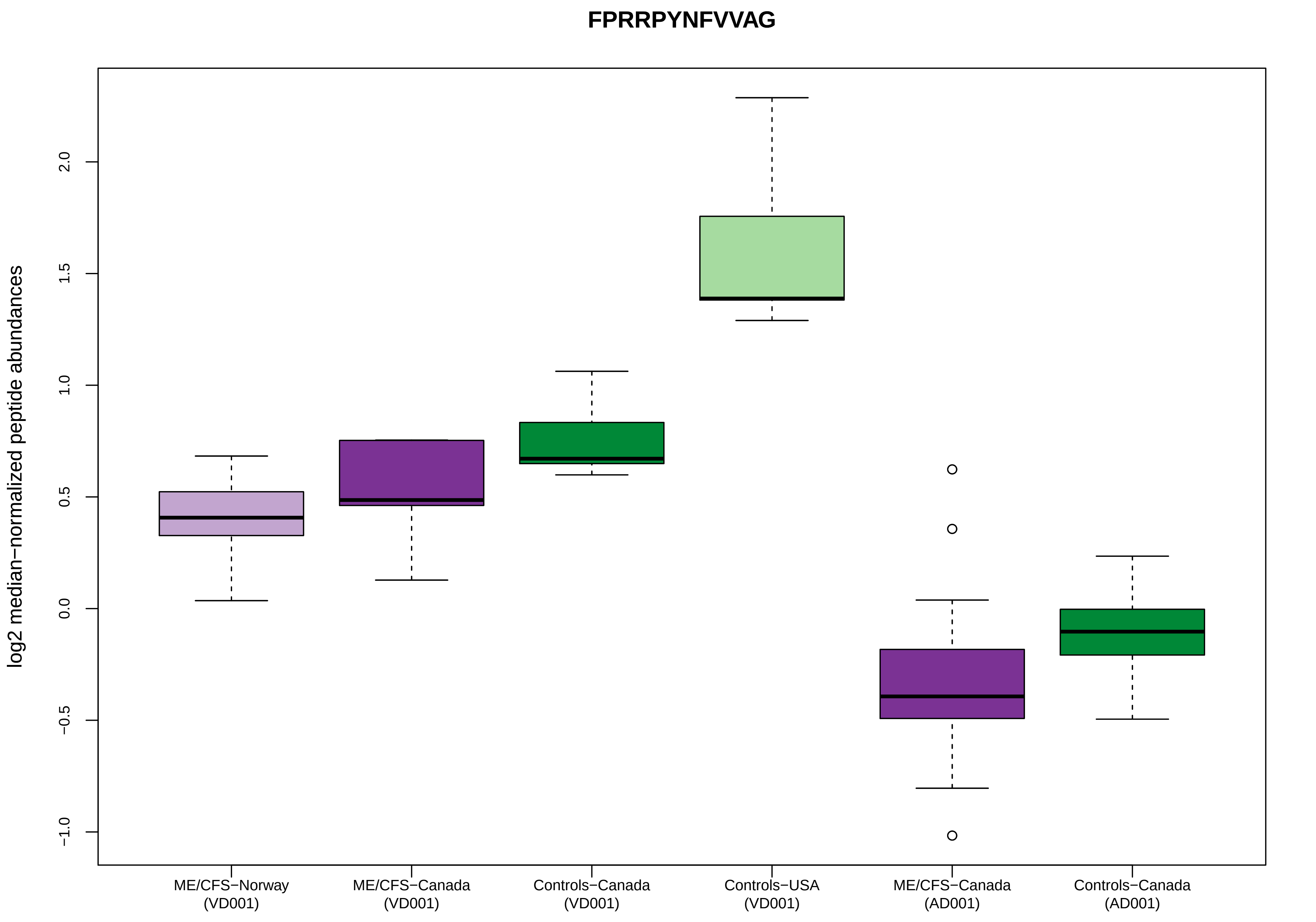
FPRRPYNFVVAG
log2 median−normalized peptide abundances
2.0
1.5
1.0
0.5
0.0
−0.5
−1.0
ME/CFS−Norway (VD001)
ME/CFS−Canada (VD001)
Controls−Canada (VD001)
Controls−USA (VD001)
ME/CFS−Canada (AD001)
Controls−Canada (AD001)

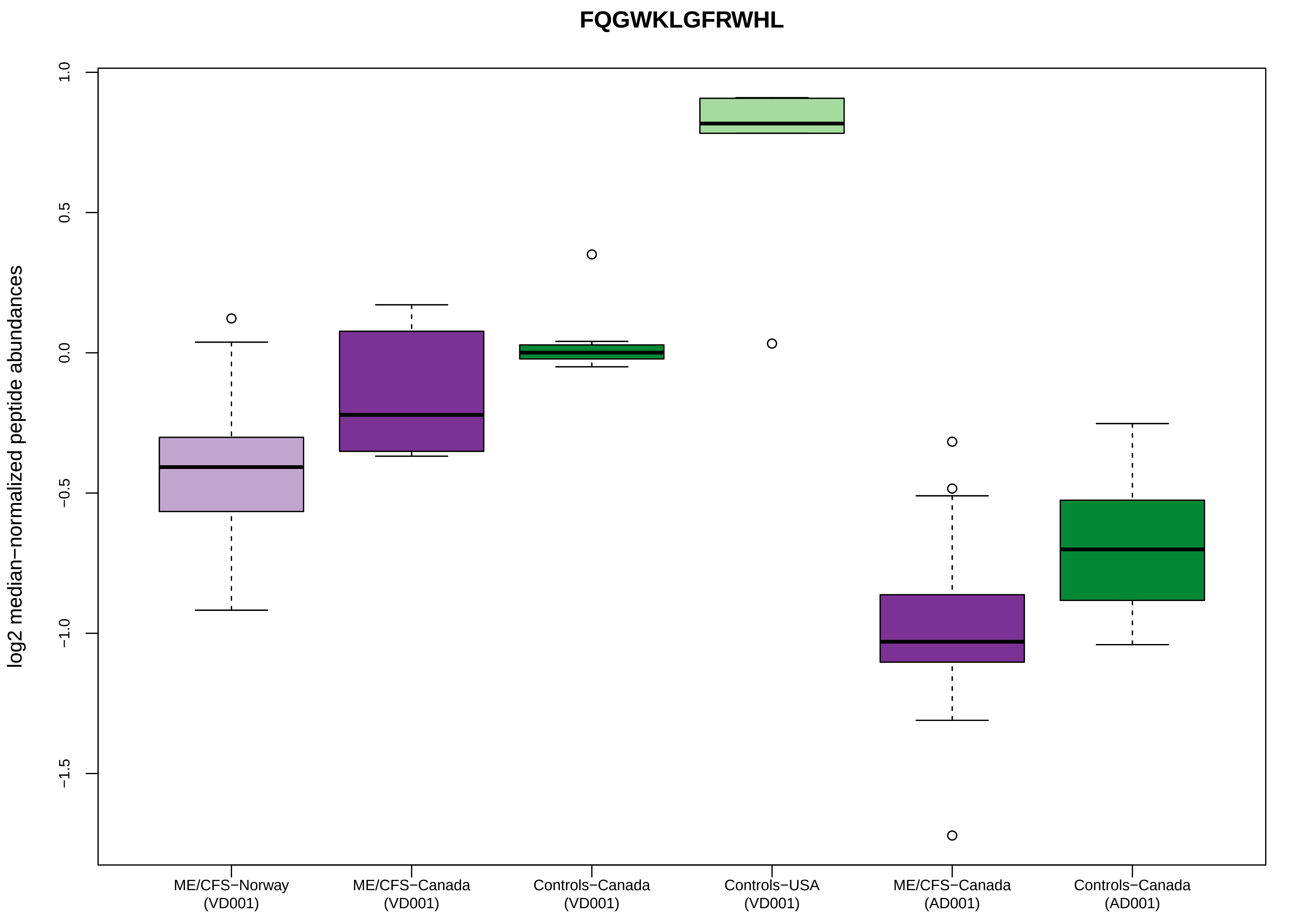
FQGWKLGFRWHL
log2 median−normalized peptide abundances
1.0
0.5
0.0
−0.5
−1.0
−1.5
ME/CFS−Norway (VD001)
ME/CFS−Canada (VD001)
Controls−Canada (VD001)
Controls−USA (VD001)
ME/CFS−Canada (AD001)
Controls−Canada (AD001)

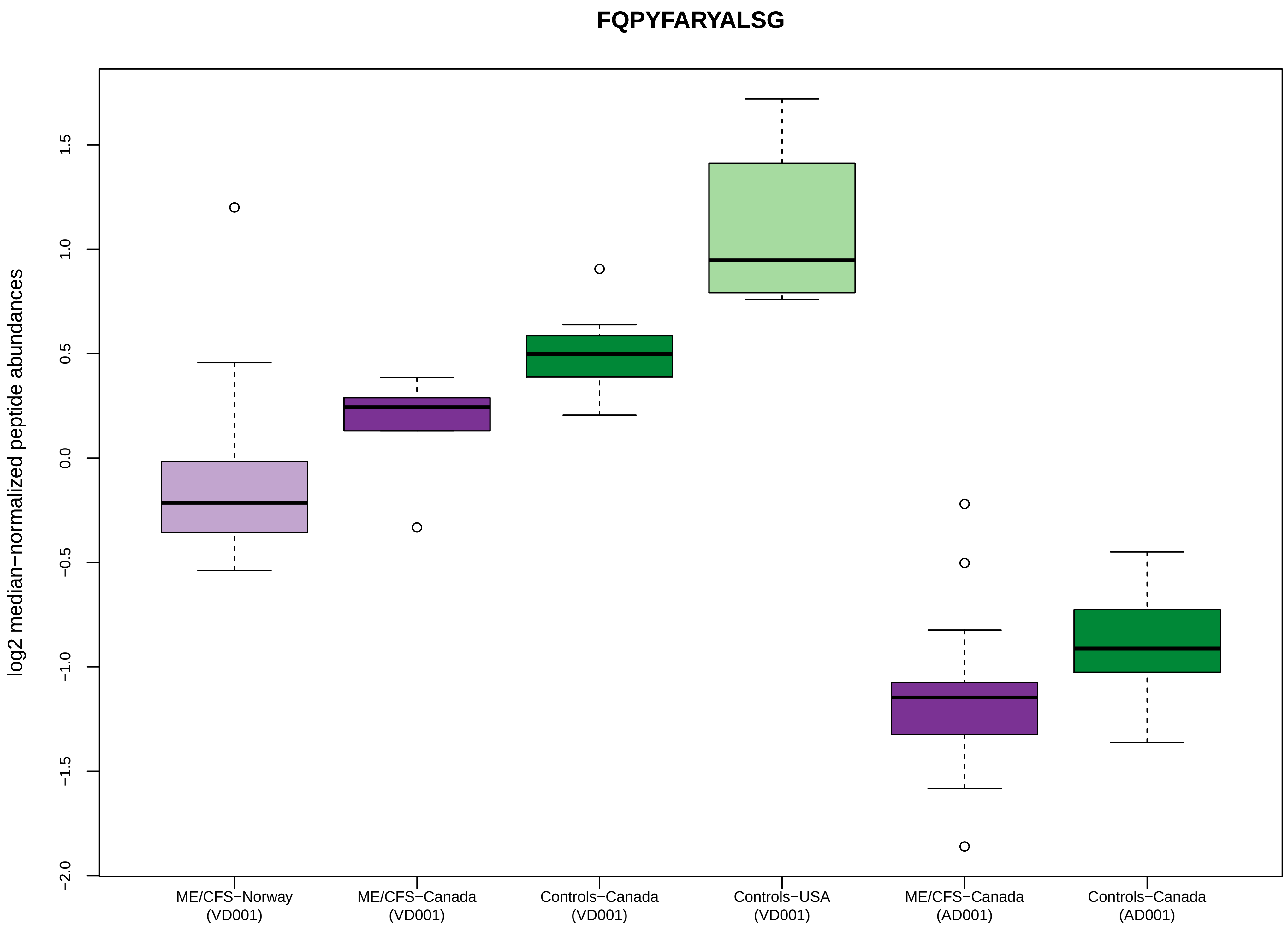
FQPYFARYALSG
log2 median−normalized peptide abundances
1.5
1.0
0.5
0.0
−0.5
−1.0
−1.5
−2.0
ME/CFS−Norway (VD001)
ME/CFS−Canada (VD001)
Controls−Canada (VD001)
Controls−USA (VD001)
ME/CFS−Canada (AD001)
Controls−Canada (AD001)

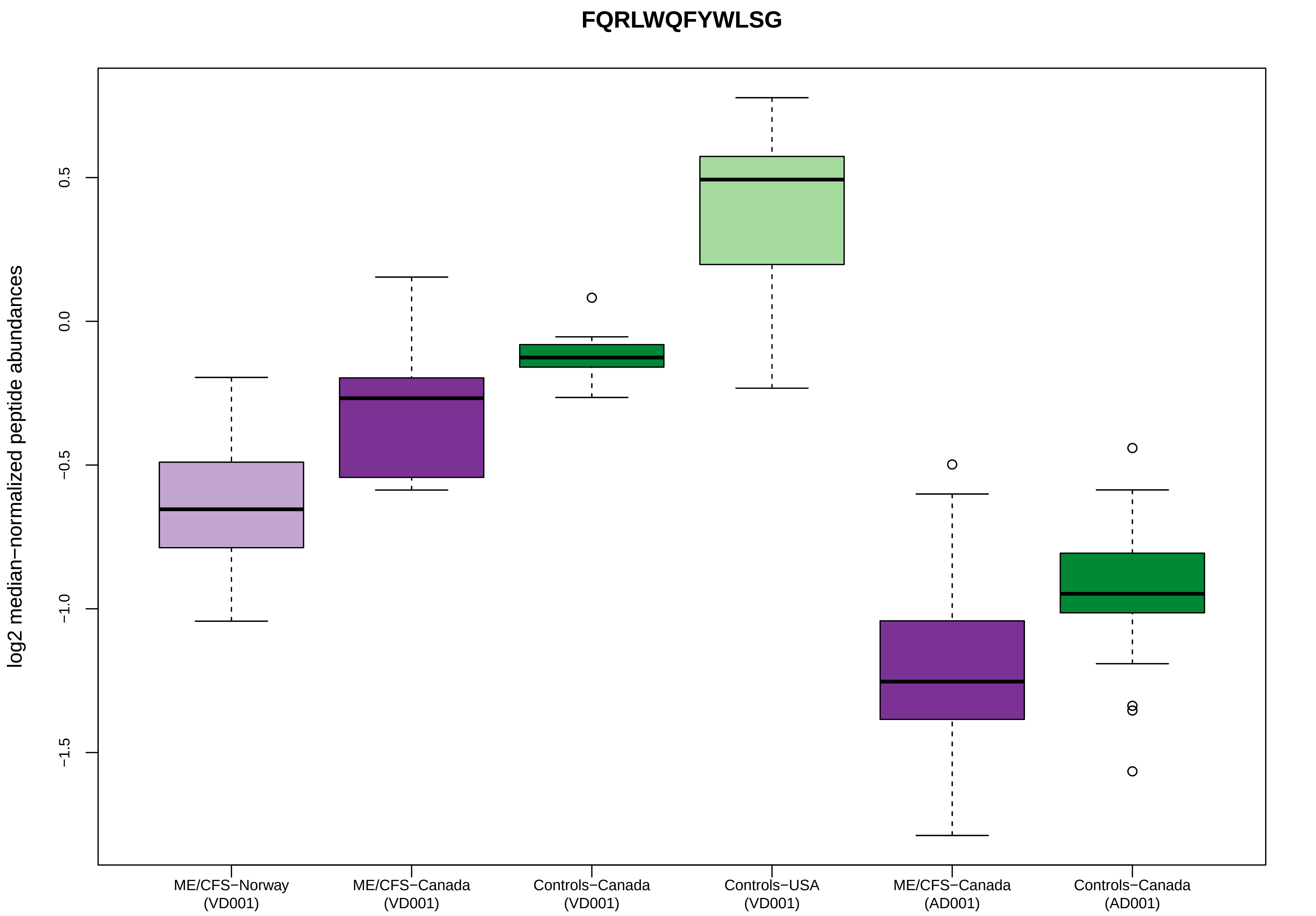
FQRLWQFYWLSG
log2 median−normalized peptide abundances
0.5
0.0
−0.5
−1.0
−1.5
ME/CFS−Norway (VD001)
ME/CFS−Canada (VD001)
Controls−Canada (VD001)
Controls−USA (VD001)
ME/CFS−Canada (AD001)
Controls−Canada (AD001)

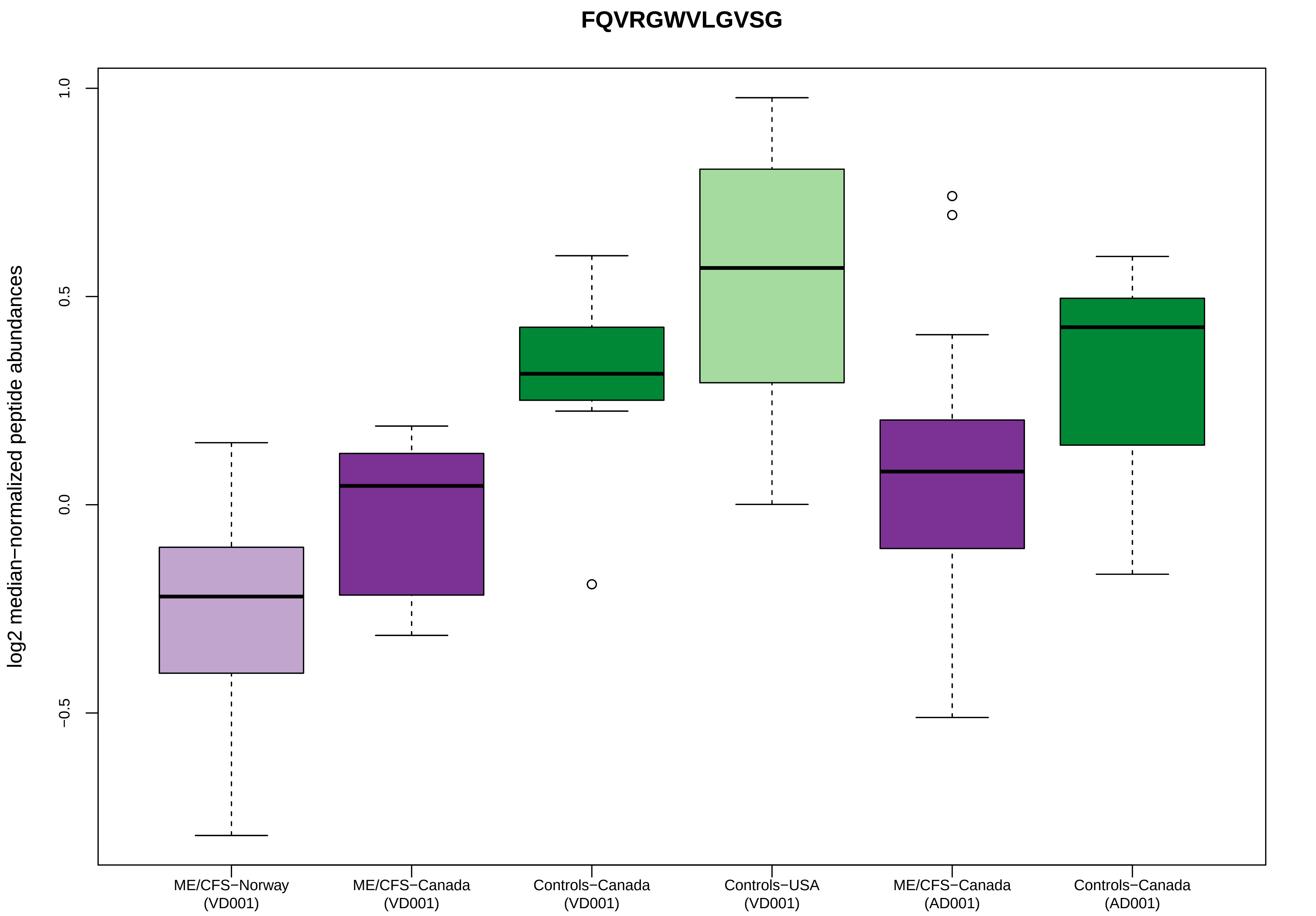
FQVRGWVLGVSG
log2 median−normalized peptide abundances
1.0
0.5
0.0
−0.5
ME/CFS−Norway (VD001)
ME/CFS−Canada (VD001)
Controls−Canada (VD001)
Controls−USA (VD001)
ME/CFS−Canada (AD001)
Controls−Canada (AD001)

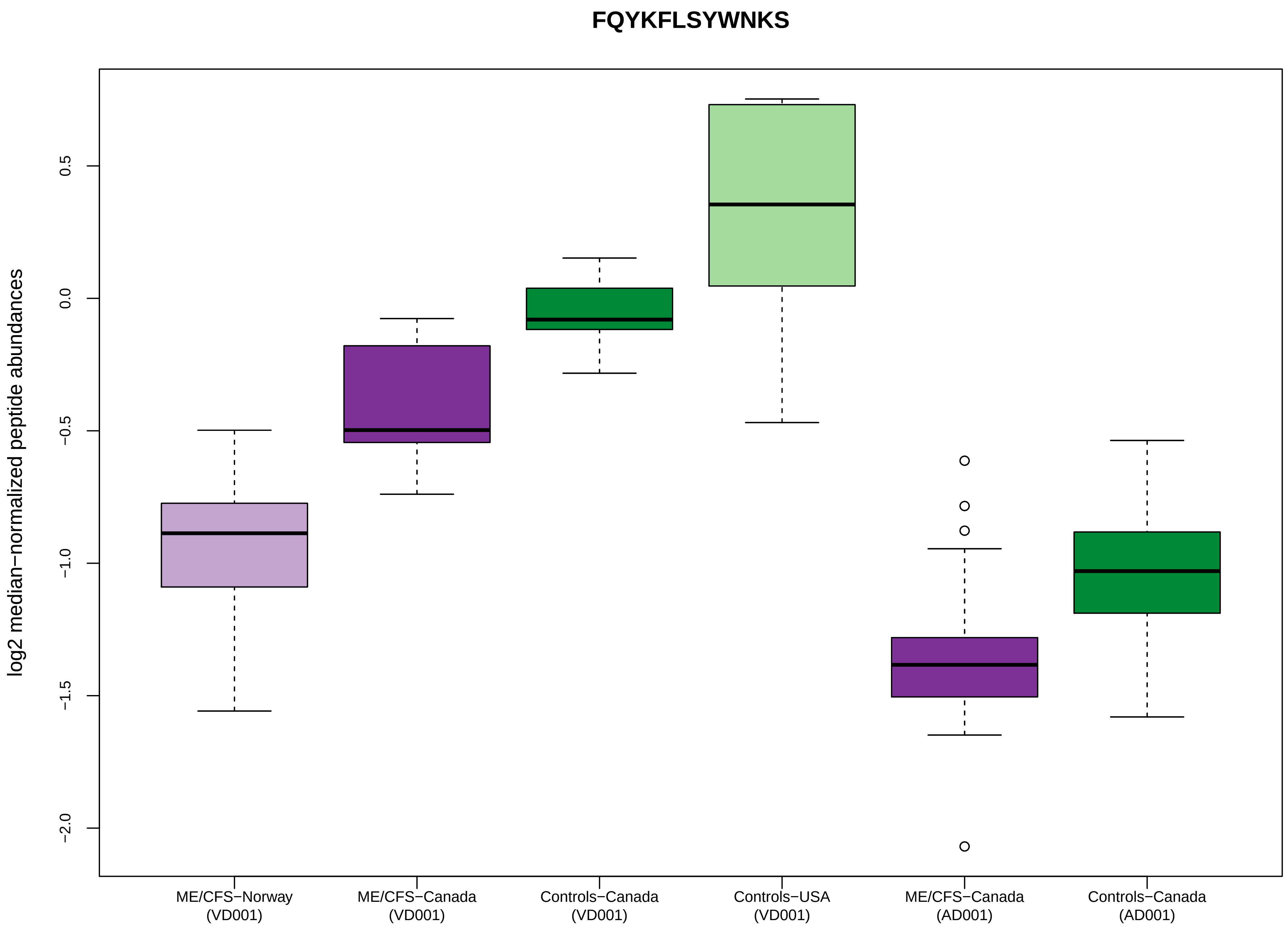

FQYKFLSYWNKS
log2 median−normalized peptide abundances
0.5
0.0
−0.5
−1.0
−1.5
−2.0
ME/CFS−Norway (VD001)
ME/CFS−Canada (VD001)
Controls−Canada (VD001)
Controls−USA (VD001)
ME/CFS−Canada (AD001)
Controls−Canada (AD001)

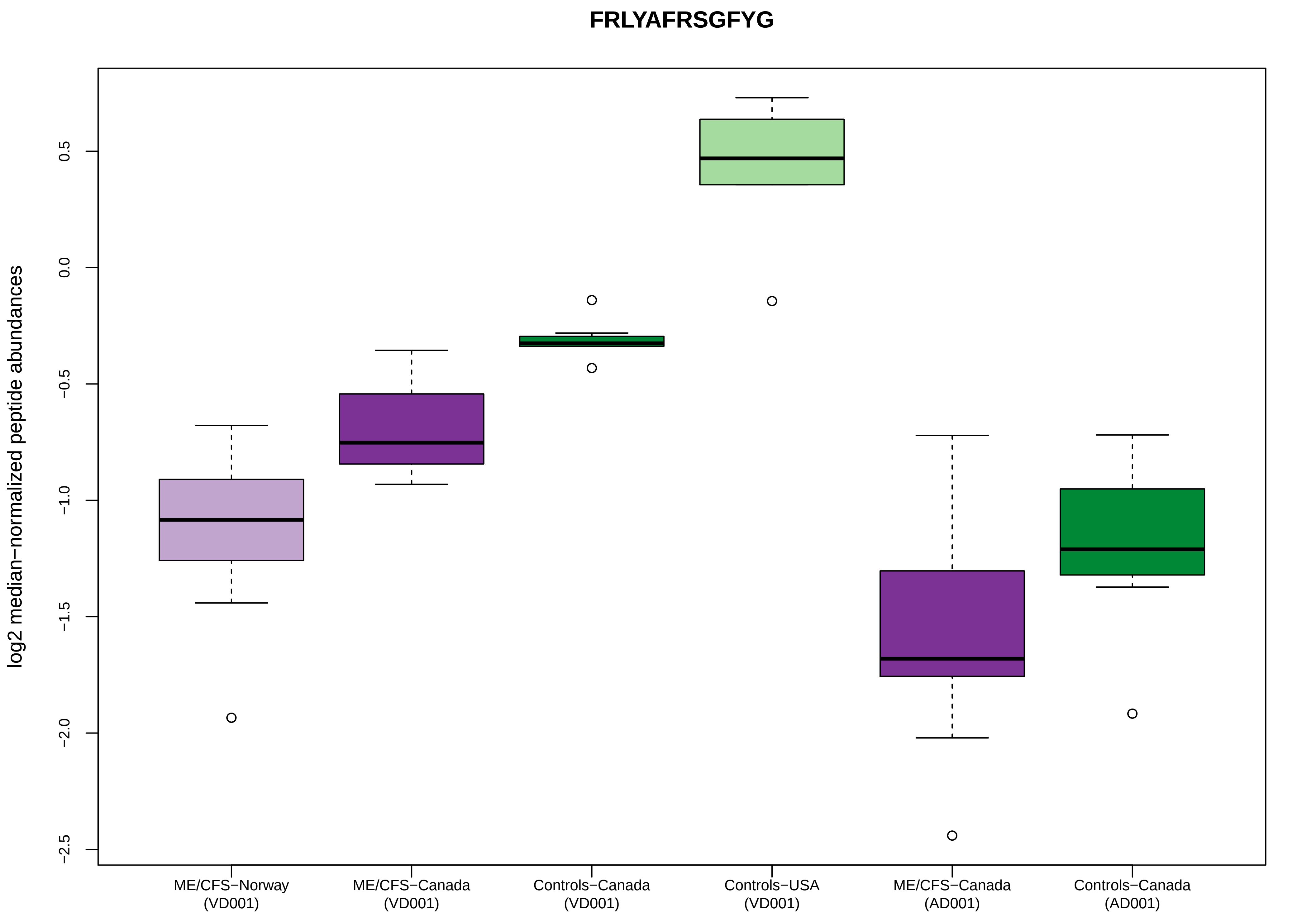
FRLYAFRSGFYG
log2 median−normalized peptide abundances
0.5
0.0
−0.5
−1.0
−1.5
−2.0
−2.5
ME/CFS−Norway (VD001)
ME/CFS−Canada (VD001)
Controls−Canada (VD001)
Controls−USA (VD001)
ME/CFS−Canada (AD001)
Controls−Canada (AD001)

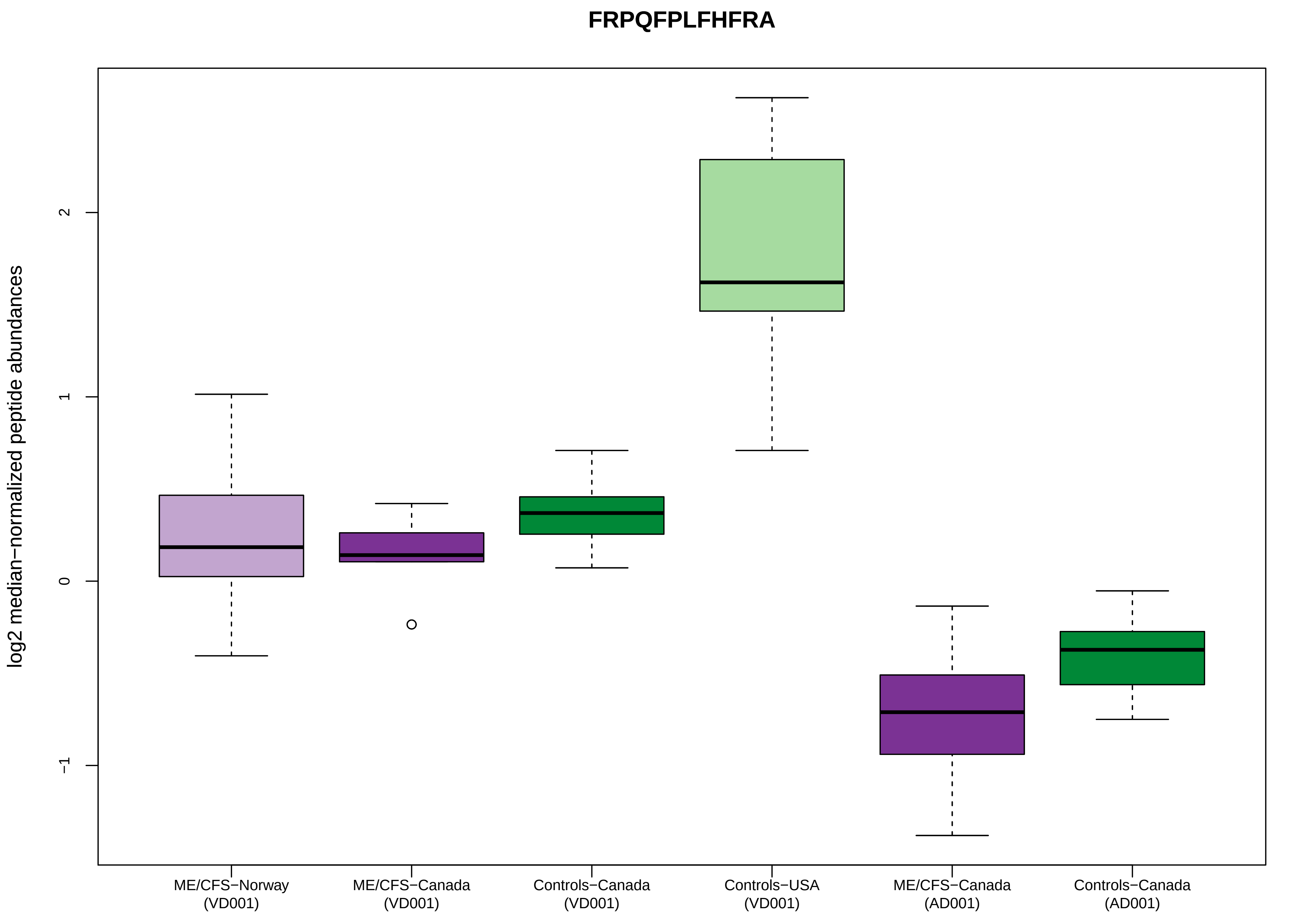
FRPQFPLFHFRA
log2 median−normalized peptide abundances
−1
0
1
2
ME/CFS−Norway (VD001)
ME/CFS−Canada (VD001)
Controls−Canada (VD001)
Controls−USA (VD001)
ME/CFS−Canada (AD001)
Controls−Canada (AD001)

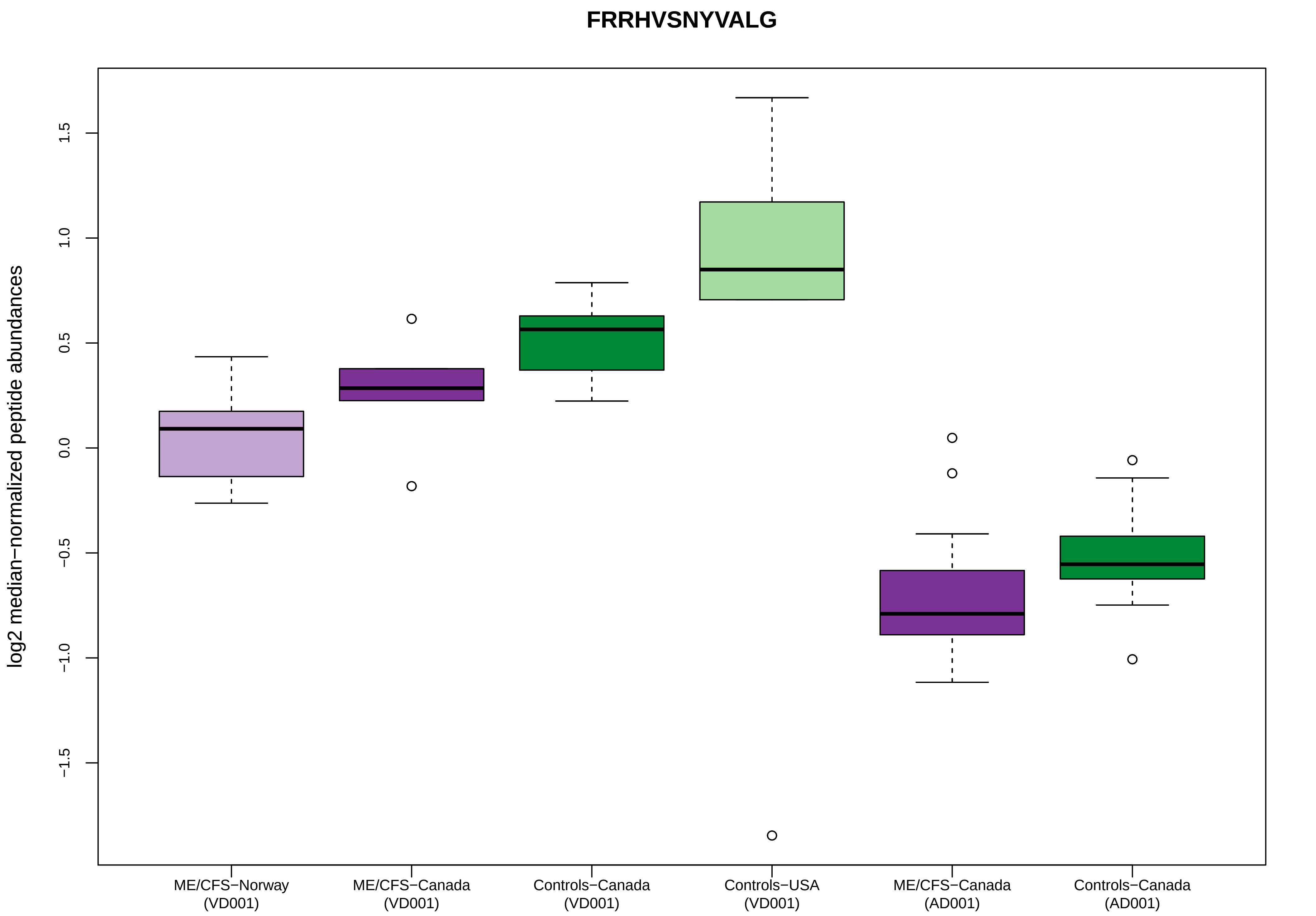

FRRHVSNYVALG
log2 median−normalized peptide abundances
1.5
1.0
0.5
0.0
−0.5
−1.0
−1.5
ME/CFS−Norway (VD001)
ME/CFS−Canada (VD001)
Controls−Canada (VD001)
Controls−USA (VD001)
ME/CFS−Canada (AD001)
Controls−Canada (AD001)

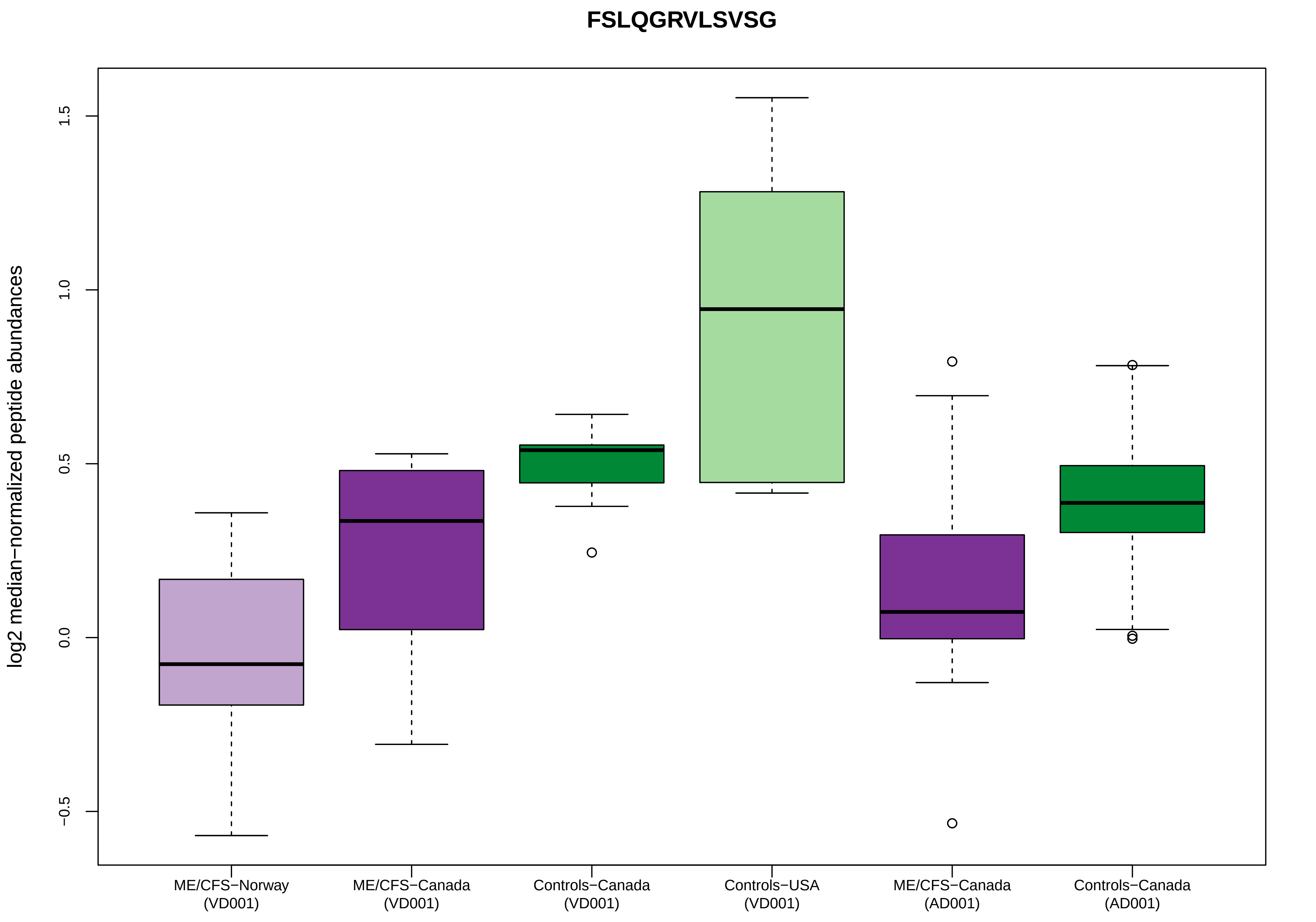
FSLQGRVLSVSG
log2 median−normalized peptide abundances
1.5
1.0
0.5
0.0
−0.5
ME/CFS−Norway (VD001)
ME/CFS−Canada (VD001)
Controls−Canada (VD001)
Controls−USA (VD001)
ME/CFS−Canada (AD001)
Controls−Canada (AD001)

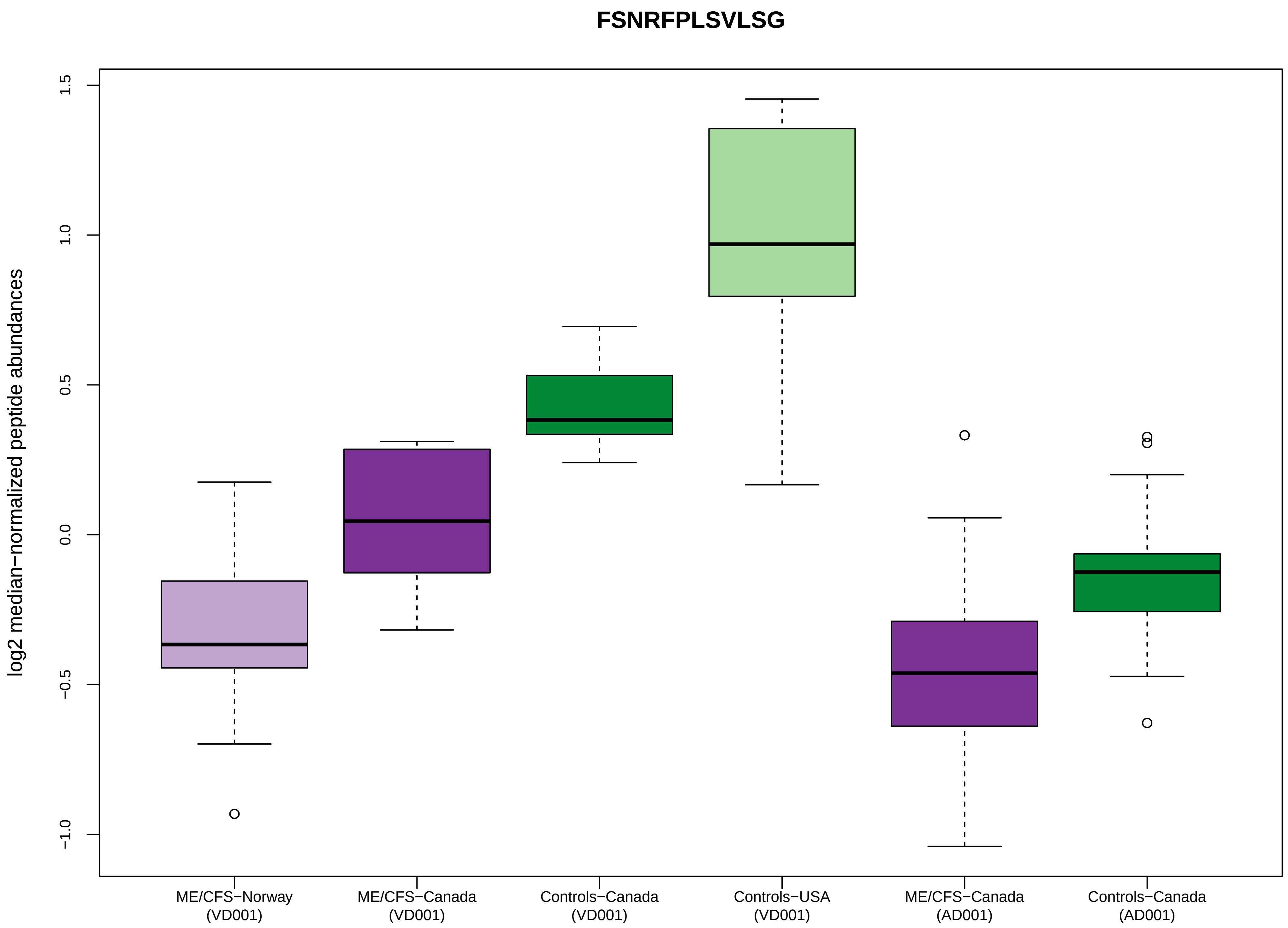
FSNRFPLSVLSG
log2 median−normalized peptide abundances
1.5
1.0
0.5
0.0
−0.5
−1.0
ME/CFS−Norway (VD001)
ME/CFS−Canada (VD001)
Controls−Canada (VD001)
Controls−USA (VD001)
ME/CFS−Canada (AD001)
Controls−Canada (AD001)

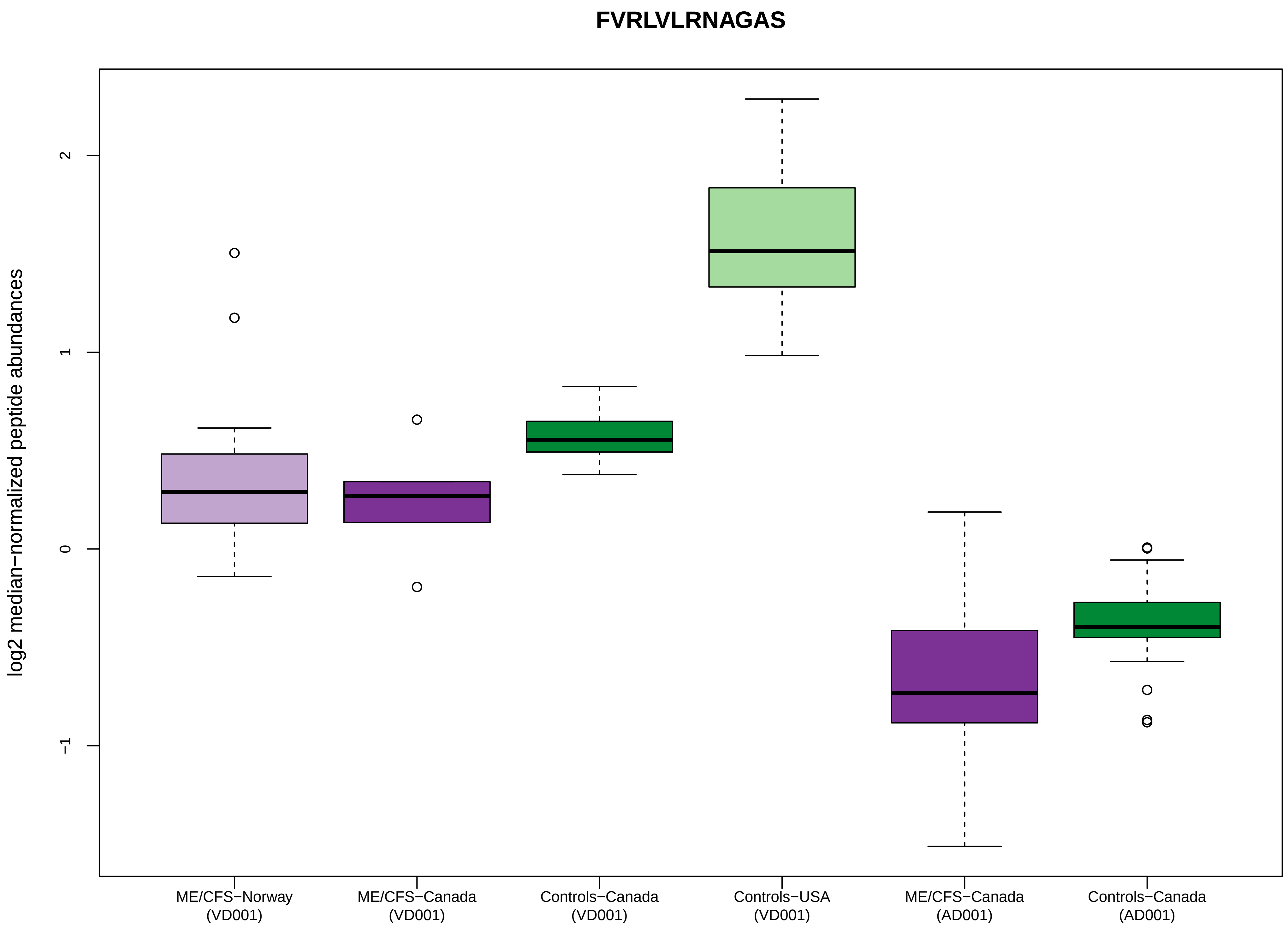

FVRLVLRNAGAS
log2 median−normalized peptide abundances
2
1
0
−1
ME/CFS−Norway
(VD001)
ME/CFS−Canada
(VD001)
Controls−Canada
(VD001)
Controls−USA
(VD001)
ME/CFS−Canada
(AD001)
Controls−Canada
(AD001)

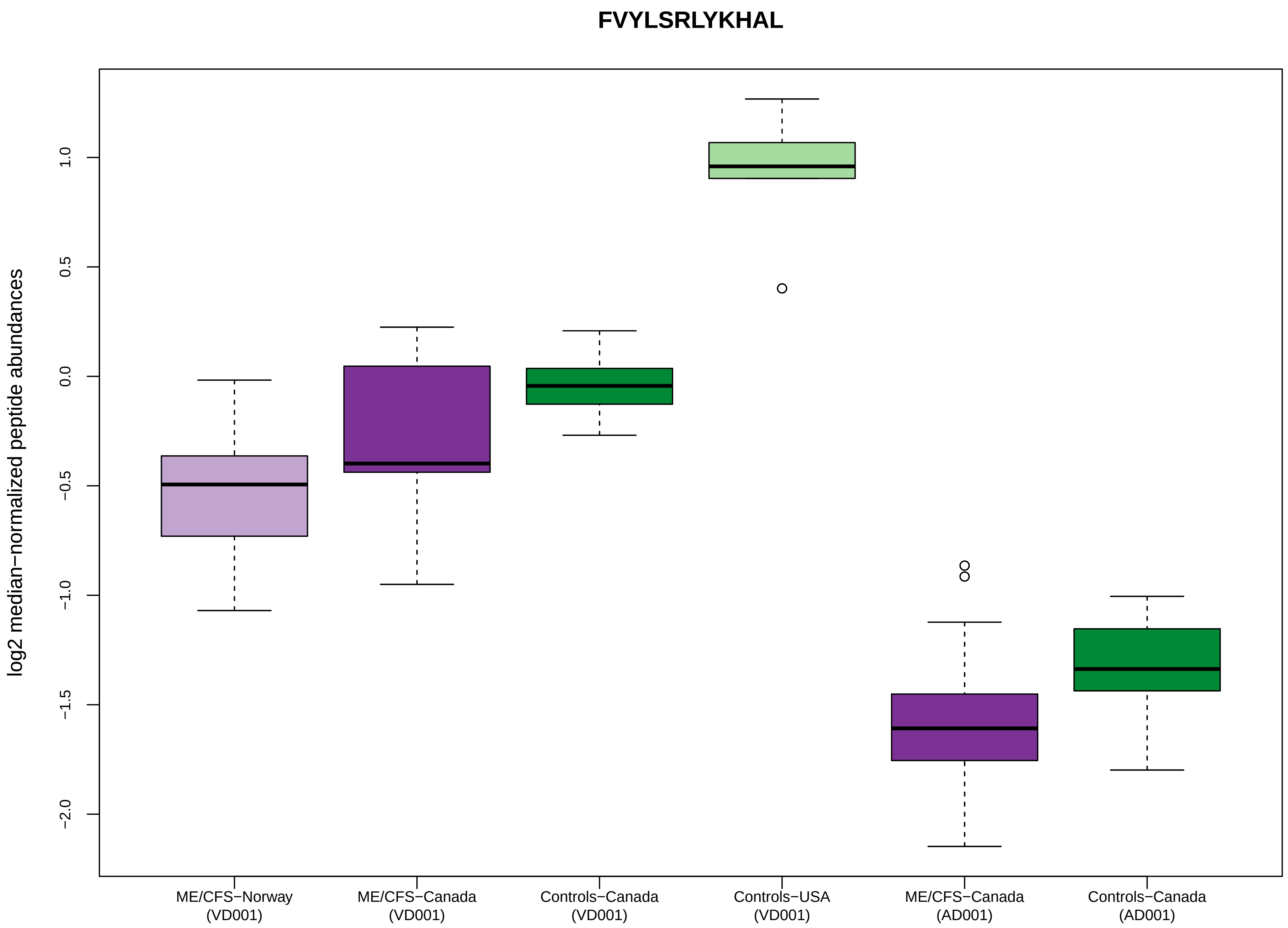

FVYLSRLYKHAL
log2 median−normalized peptide abundances
1.0
0.5
0.0
−0.5
−1.0
−1.5
−2.0
ME/CFS−Norway (VD001)
ME/CFS−Canada (VD001)
Controls−Canada (VD001)
Controls−USA (VD001)
ME/CFS−Canada (AD001)
Controls−Canada (AD001)

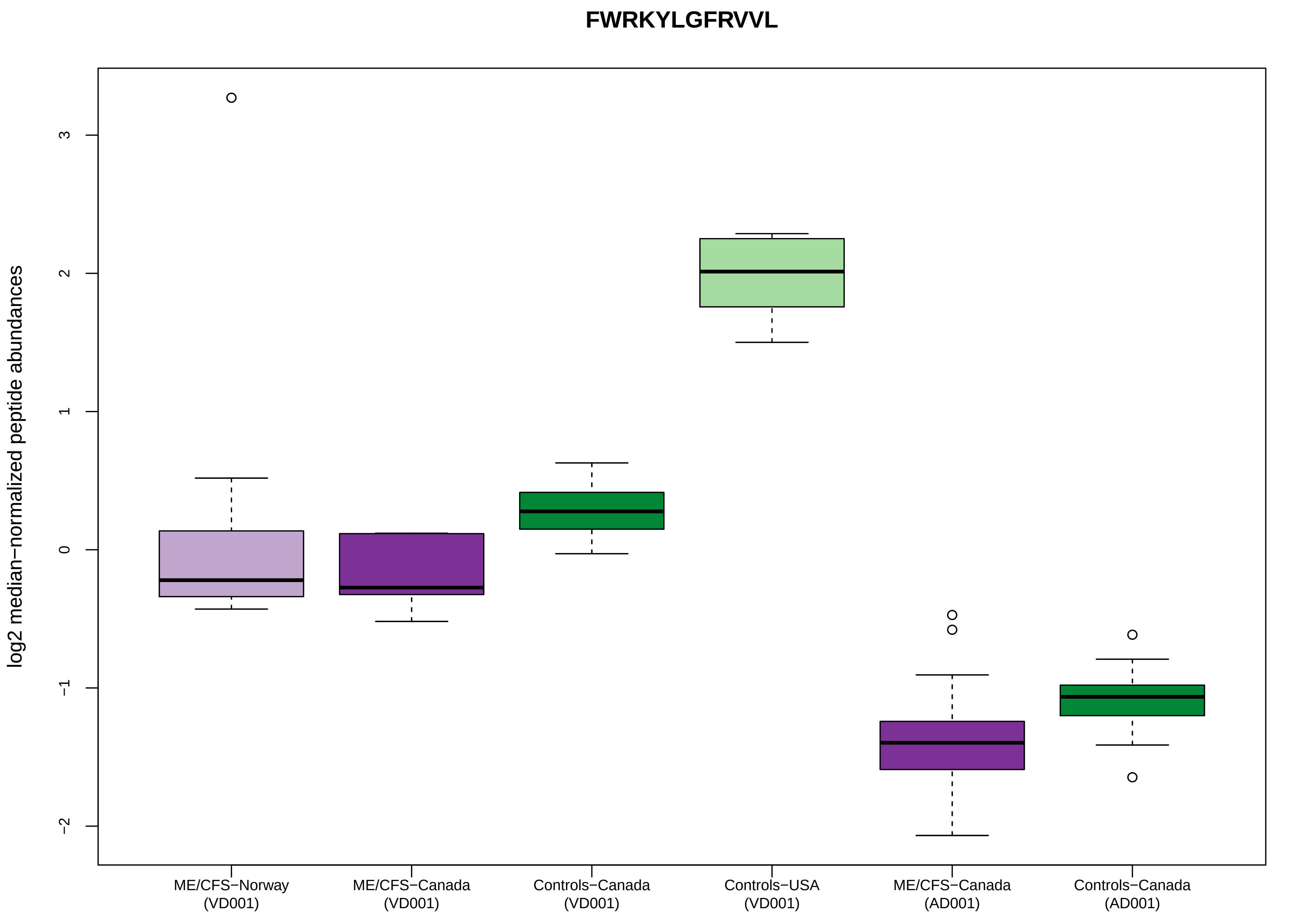
FWRKYLGFRVVL
log2 median−normalized peptide abundances
3
2
1
0
−1
−2
ME/CFS−Norway (VD001)
ME/CFS−Canada (VD001)
Controls−Canada (VD001)
Controls−USA (VD001)
ME/CFS−Canada (AD001)
Controls−Canada (AD001)

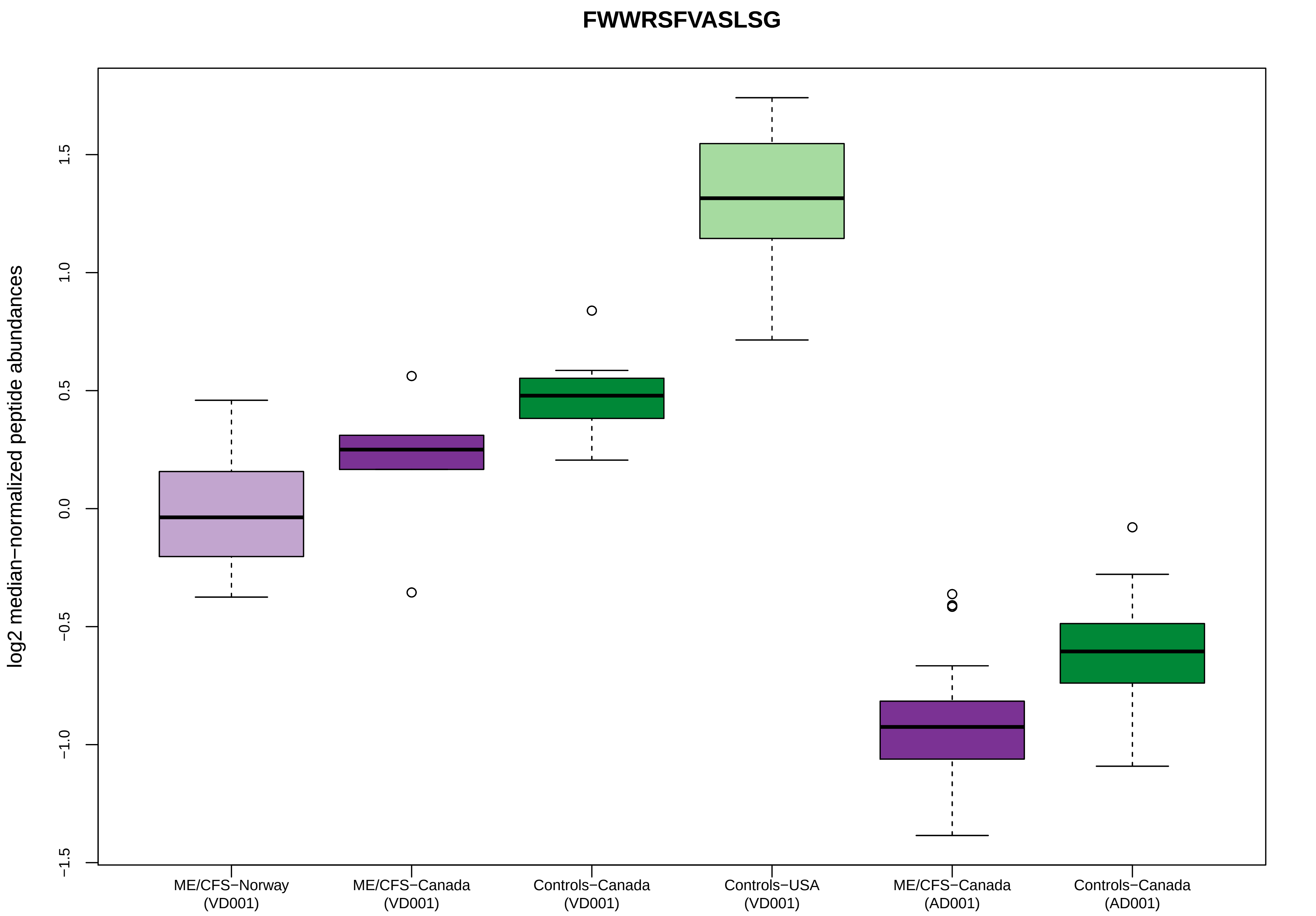
FWWRSFVASLSG
log2 median−normalized peptide abundances
1.5
1.0
0.5
0.0
−0.5
−1.0
−1.5
ME/CFS−Norway (VD001)
ME/CFS−Canada (VD001)
Controls−Canada (VD001)
Controls−USA (VD001)
ME/CFS−Canada (AD001)
Controls−Canada (AD001)

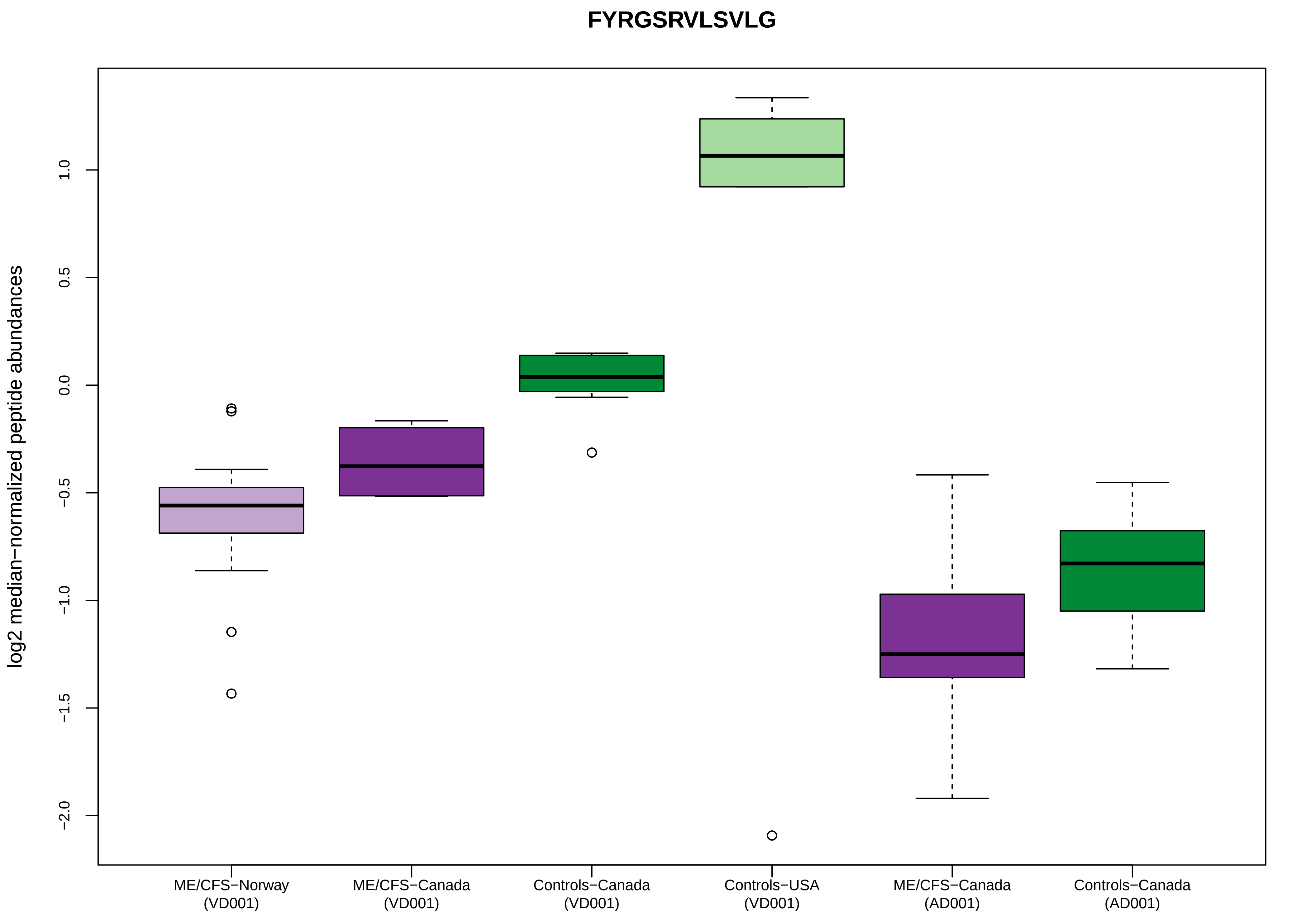
FYRGSRVLSVLG
log2 median−normalized peptide abundances
1.0
0.5
0.0
−0.5
−1.0
−1.5
−2.0
ME/CFS−Norway (VD001)
ME/CFS−Canada (VD001)
Controls−Canada (VD001)
Controls−USA (VD001)
ME/CFS−Canada (AD001)
Controls−Canada (AD001)

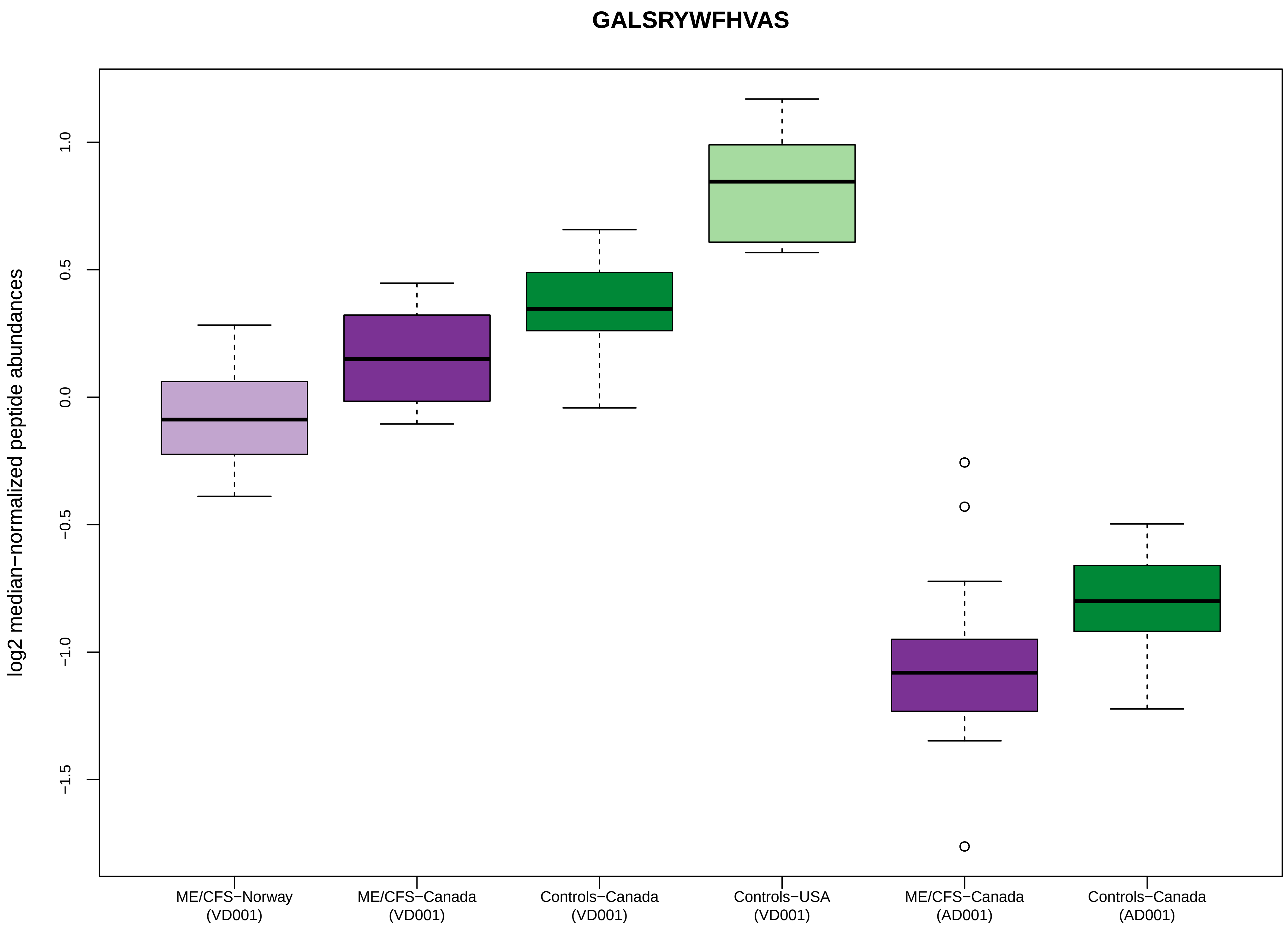

GALSRYWFHVAS
log2 median−normalized peptide abundances
1.0
0.5
0.0
−0.5
−1.0
−1.5
ME/CFS−Norway (VD001)
ME/CFS−Canada (VD001)
Controls−Canada (VD001)
Controls−USA (VD001)
ME/CFS−Canada (AD001)
Controls−Canada (AD001)

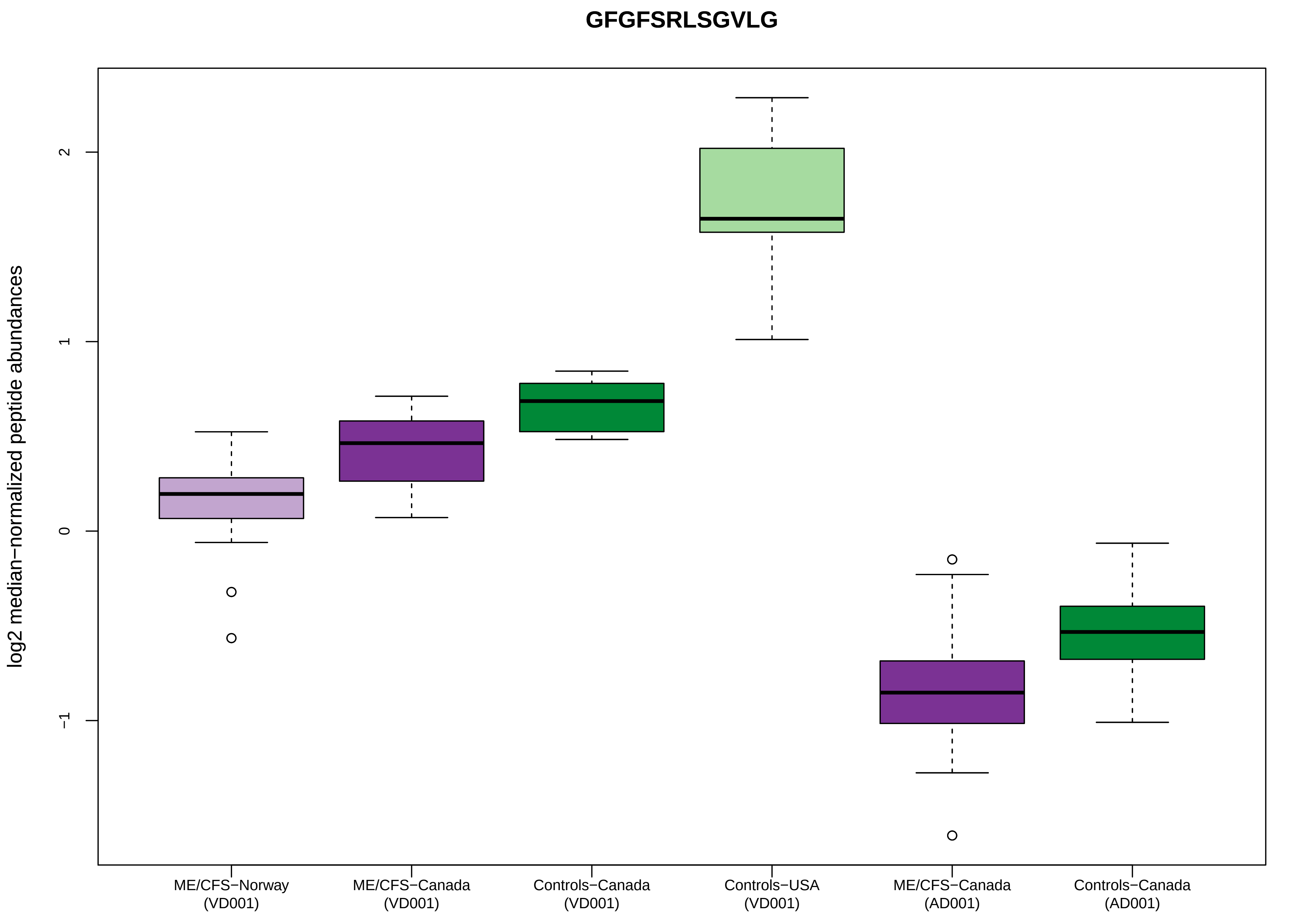
GFGFSRLSGVLG
log2 median−normalized peptide abundances
2
1
0
−1
ME/CFS−Norway (VD001)
ME/CFS−Canada (VD001)
Controls−Canada (VD001)
Controls−USA (VD001)
ME/CFS−Canada (AD001)
Controls−Canada (AD001)

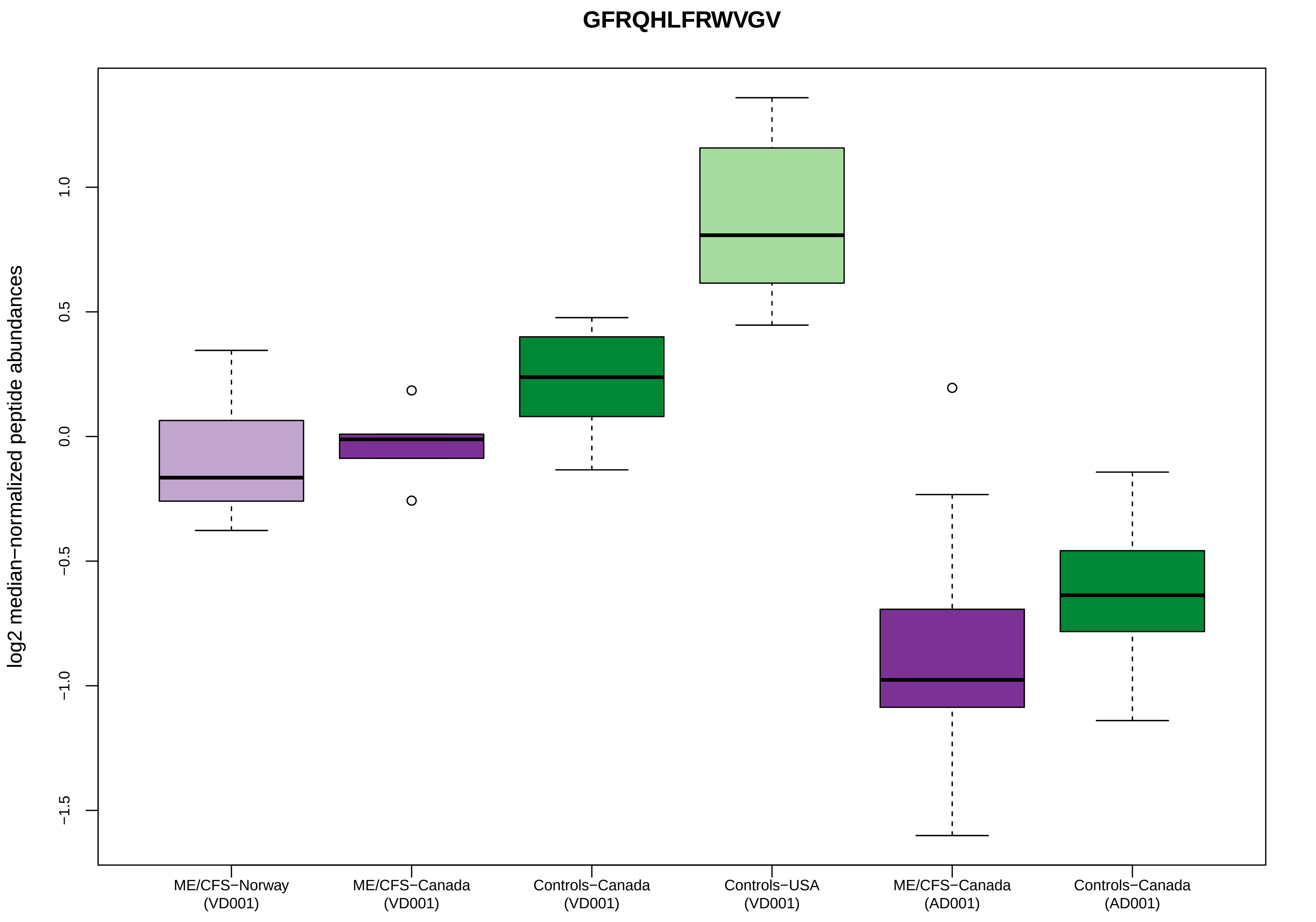
GFRQHLFRWVGV
log2 median−normalized peptide abundances
1.0
0.5
0.0
−0.5
−1.0
−1.5
ME/CFS−Norway (VD001)
ME/CFS−Canada (VD001)
Controls−Canada (VD001)
Controls−USA (VD001)
ME/CFS−Canada (AD001)
Controls−Canada (AD001)

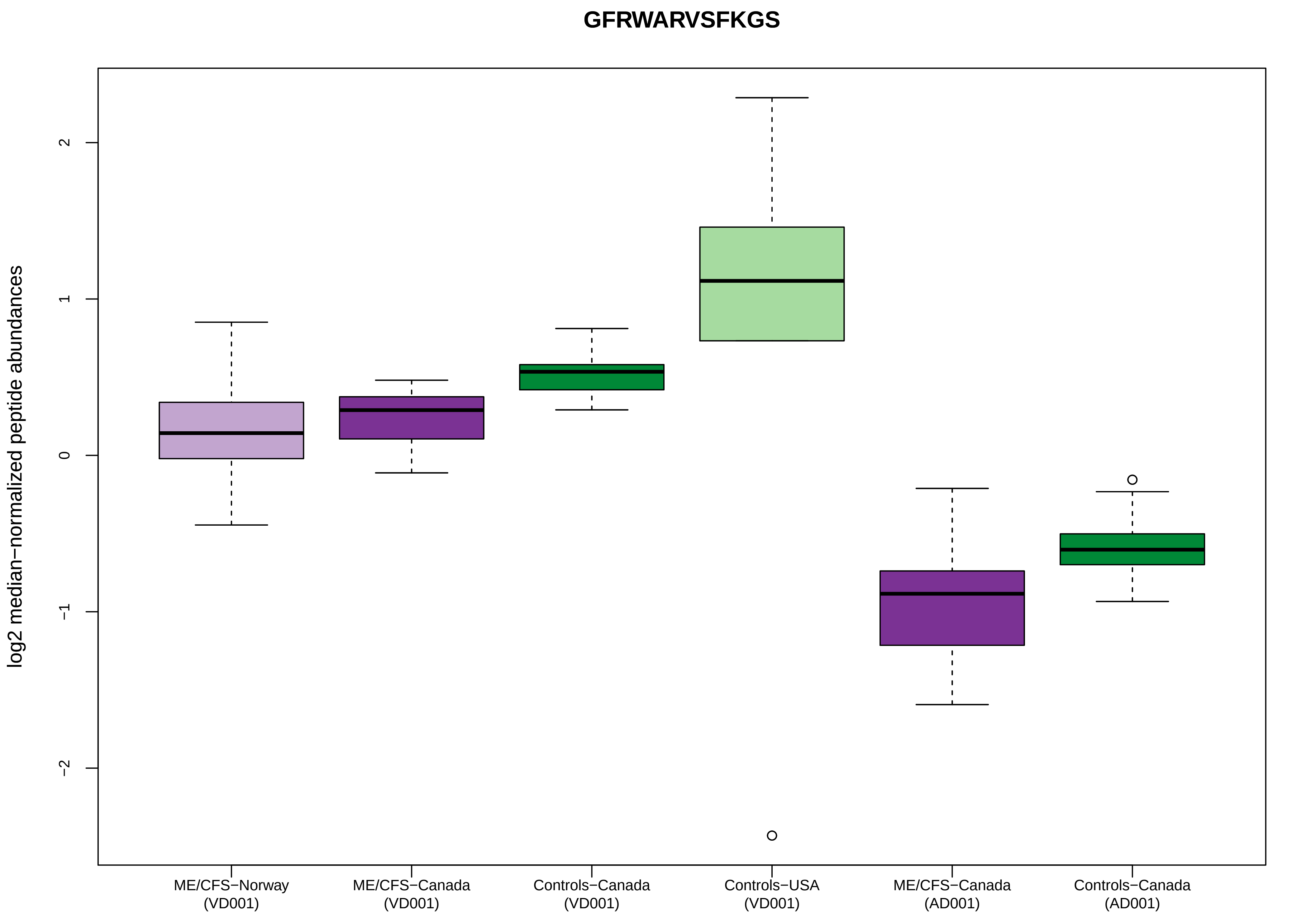
GFRWARVSFKGS
log2 median−normalized peptide abundances
2
1
0
−1
−2
ME/CFS−Norway (VD001)
ME/CFS−Canada (VD001)
Controls−Canada (VD001)
Controls−USA (VD001)
ME/CFS−Canada (AD001)
Controls−Canada (AD001)

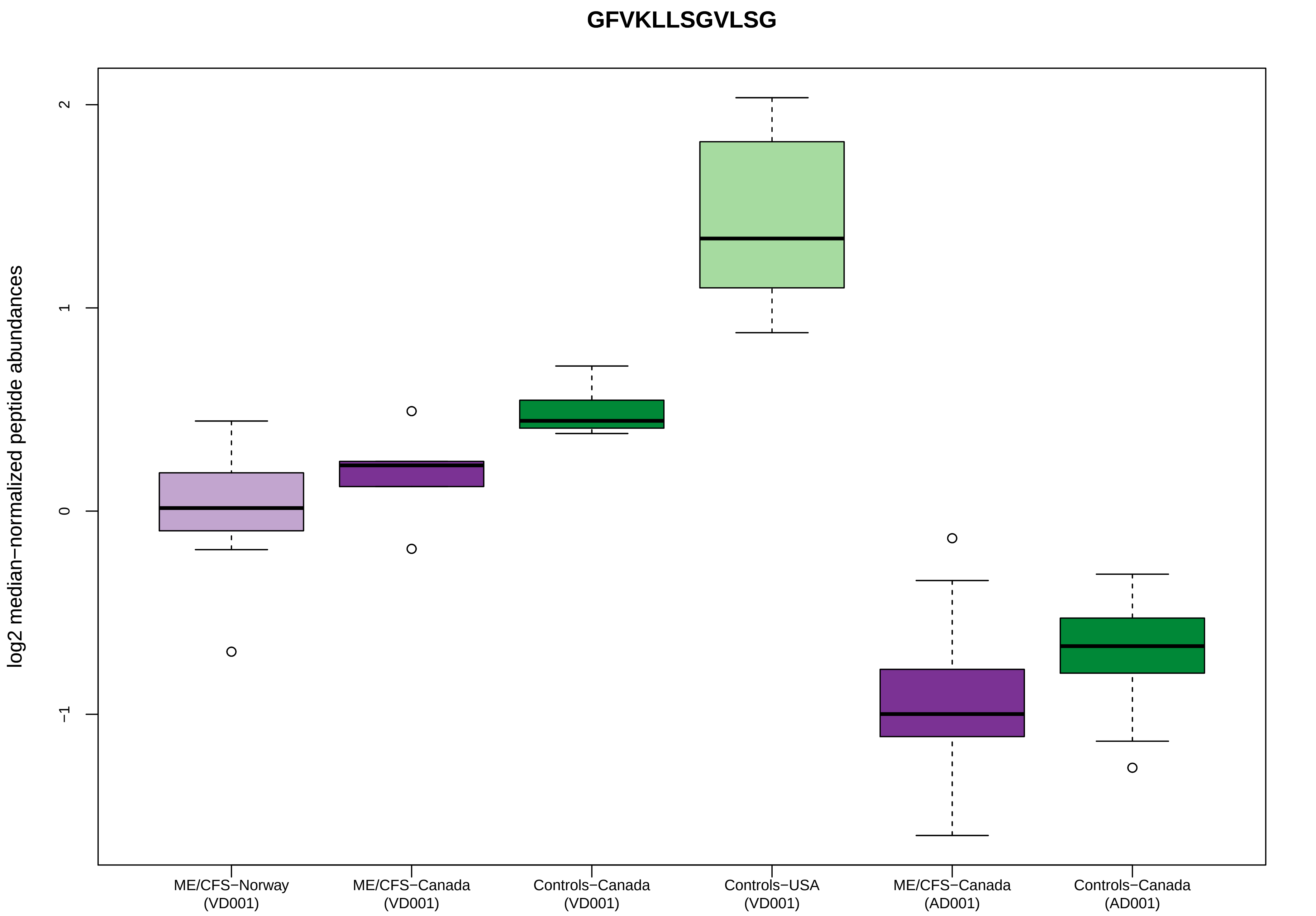
GFVKLLSGVLSG
log2 median−normalized peptide abundances
2
1
0
−1
ME/CFS−Norway (VD001)
ME/CFS−Canada (VD001)
Controls−Canada (VD001)
Controls−USA (VD001)
ME/CFS−Canada (AD001)
Controls−Canada (AD001)

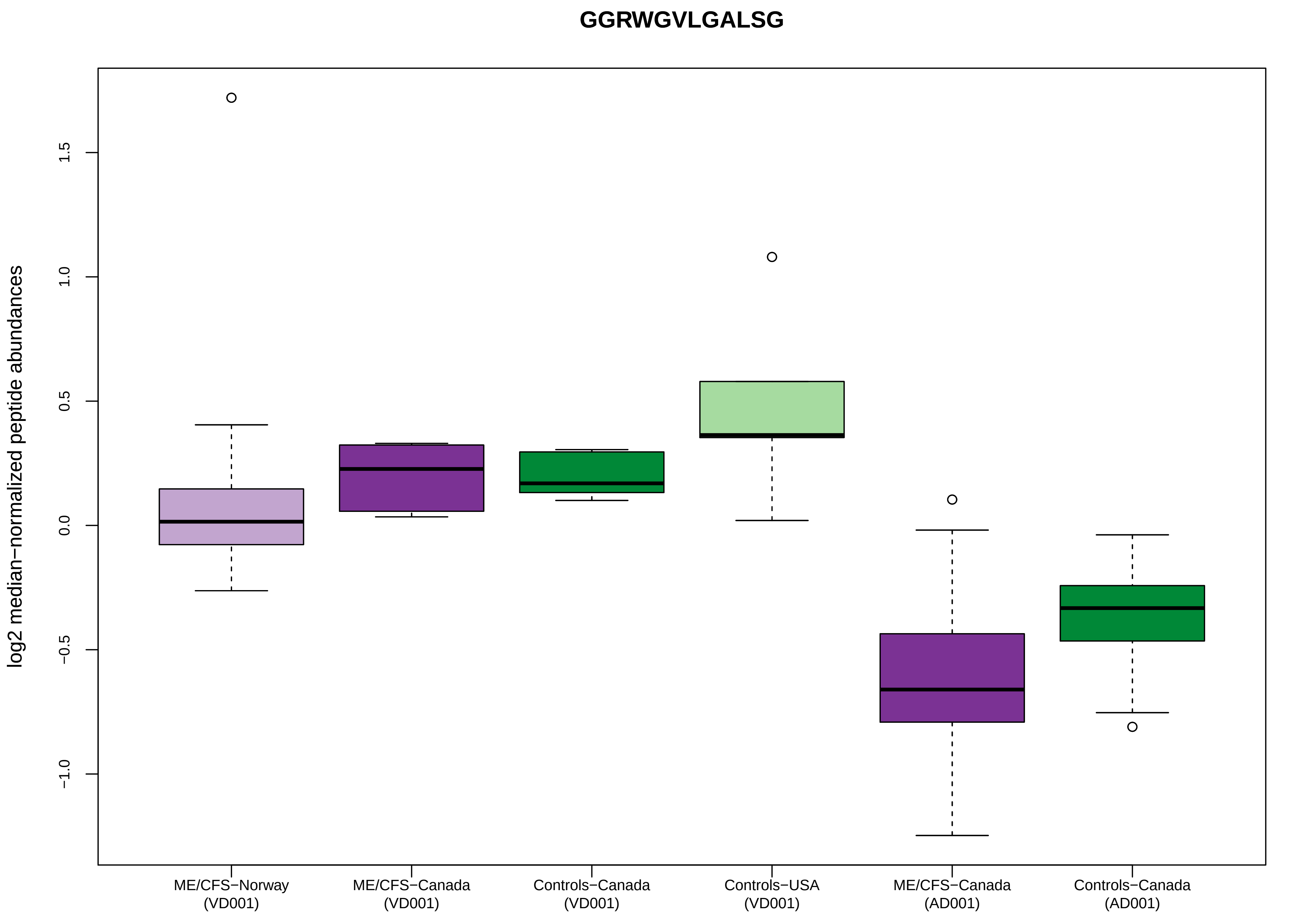

GGRWGVLGALSG
log2 median−normalized peptide abundances
1.5
1.0
0.5
0.0
−0.5
−1.0
ME/CFS−Norway (VD001)
ME/CFS−Canada (VD001)
Controls−Canada (VD001)
Controls−USA (VD001)
ME/CFS−Canada (AD001)
Controls−Canada (AD001)

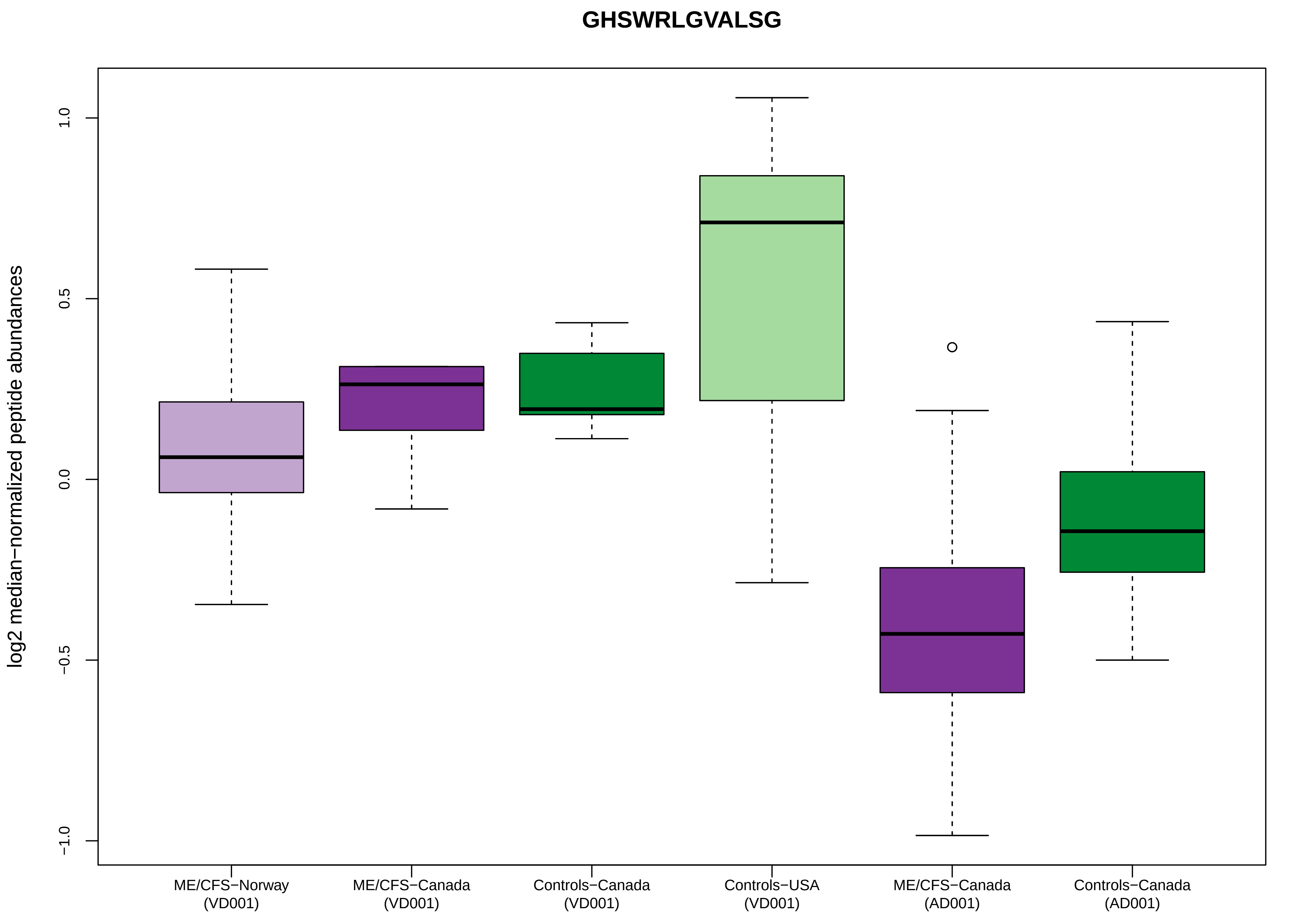

GHSWRLGVALSG
log2 median−normalized peptide abundances
1.0
0.5
0.0
−0.5
−1.0
ME/CFS−Norway (VD001)
ME/CFS−Canada (VD001)
Controls−Canada (VD001)
Controls−USA (VD001)
ME/CFS−Canada (AD001)
Controls−Canada (AD001)

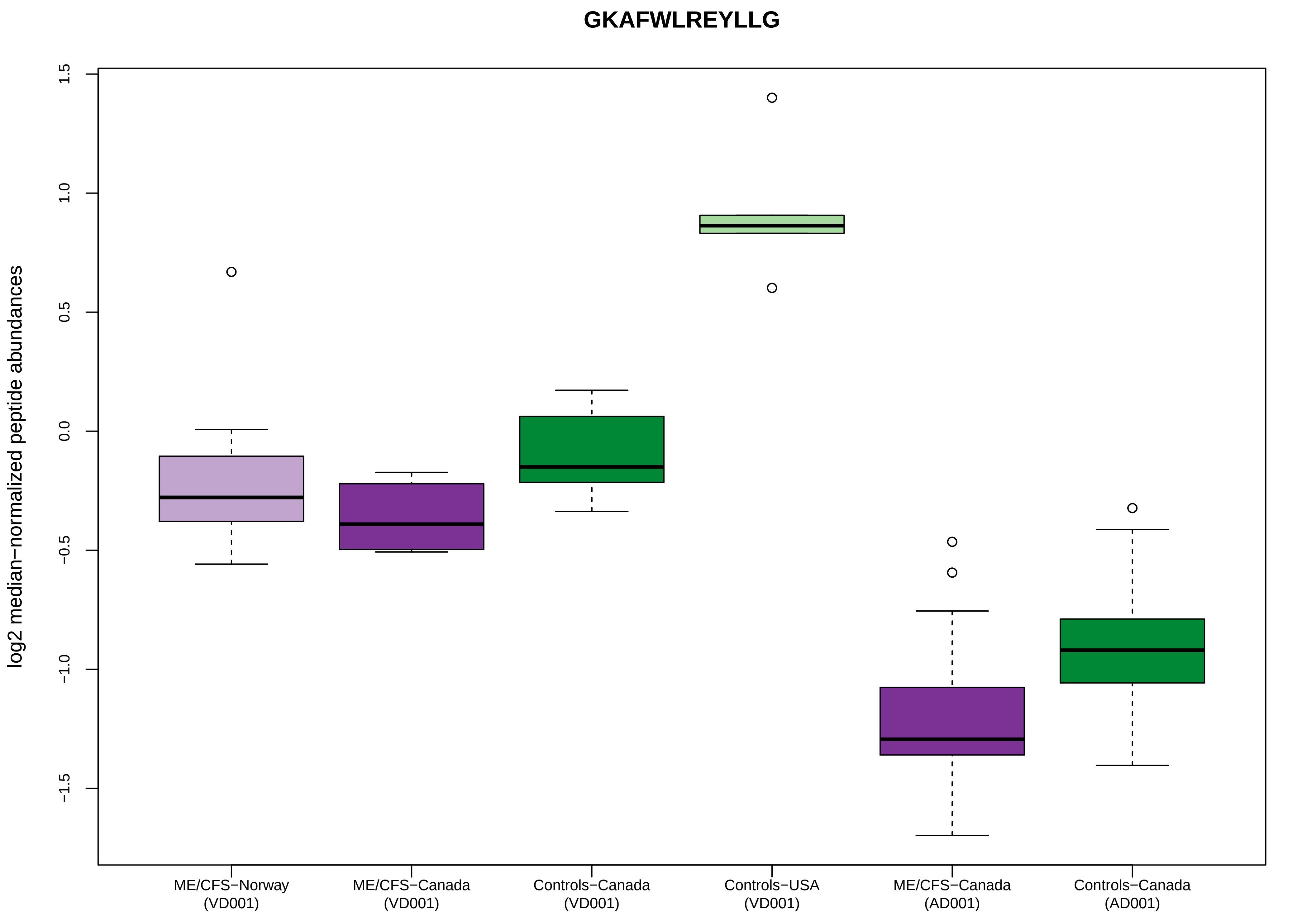
GKAFWLREYLLG
log2 median−normalized peptide abundances
1.5
1.0
0.5
0.0
−0.5
−1.0
−1.5
ME/CFS−Norway (VD001)
ME/CFS−Canada (VD001)
Controls−Canada (VD001)
Controls−USA (VD001)
ME/CFS−Canada (AD001)
Controls−Canada (AD001)

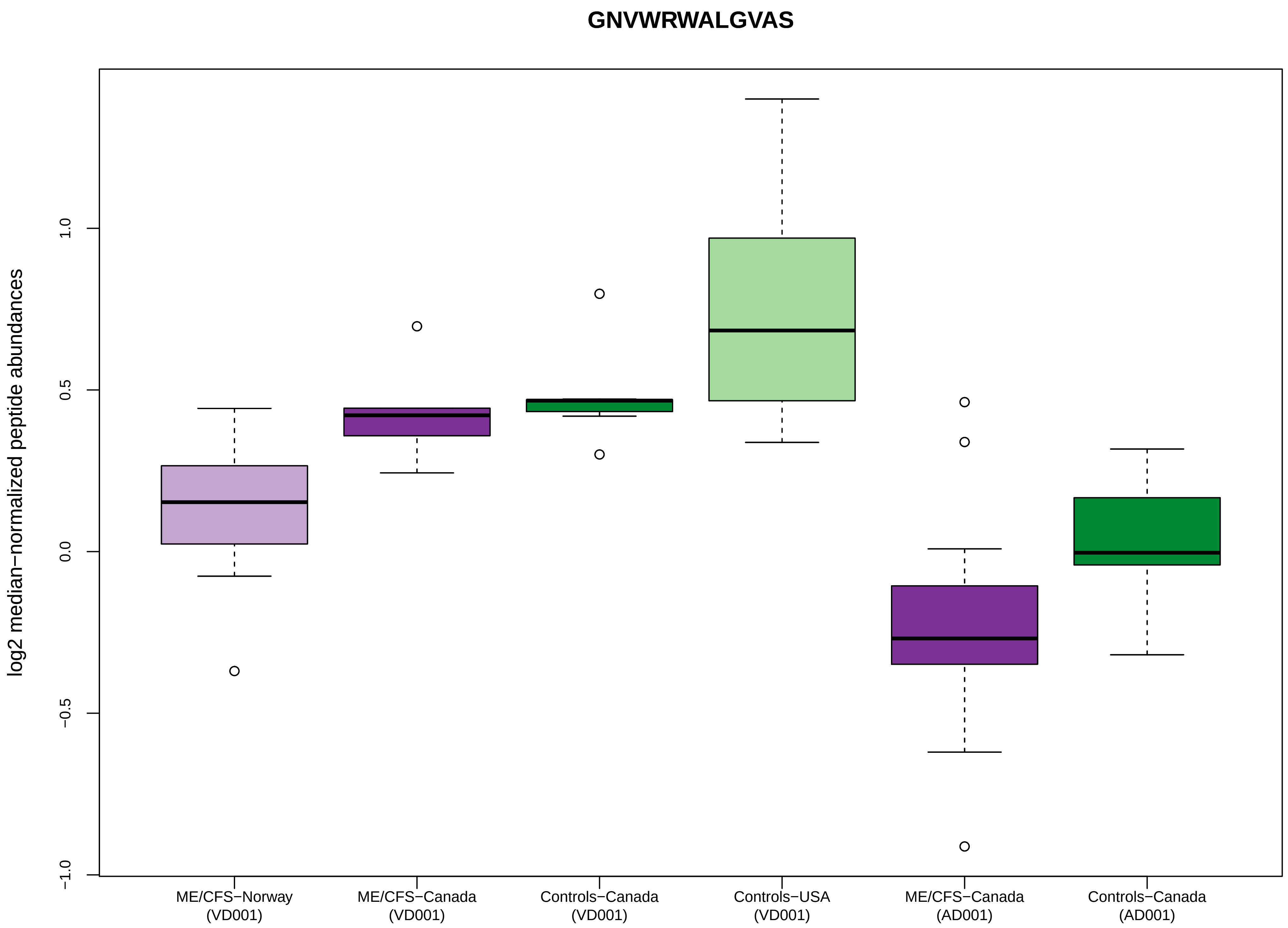

GNVWRWALGVAS
log2 median−normalized peptide abundances
1.0
0.5
0.0
−0.5
−1.0
ME/CFS−Norway (VD001)
ME/CFS−Canada (VD001)
Controls−Canada (VD001)
Controls−USA (VD001)
ME/CFS−Canada (AD001)
Controls−Canada (AD001)

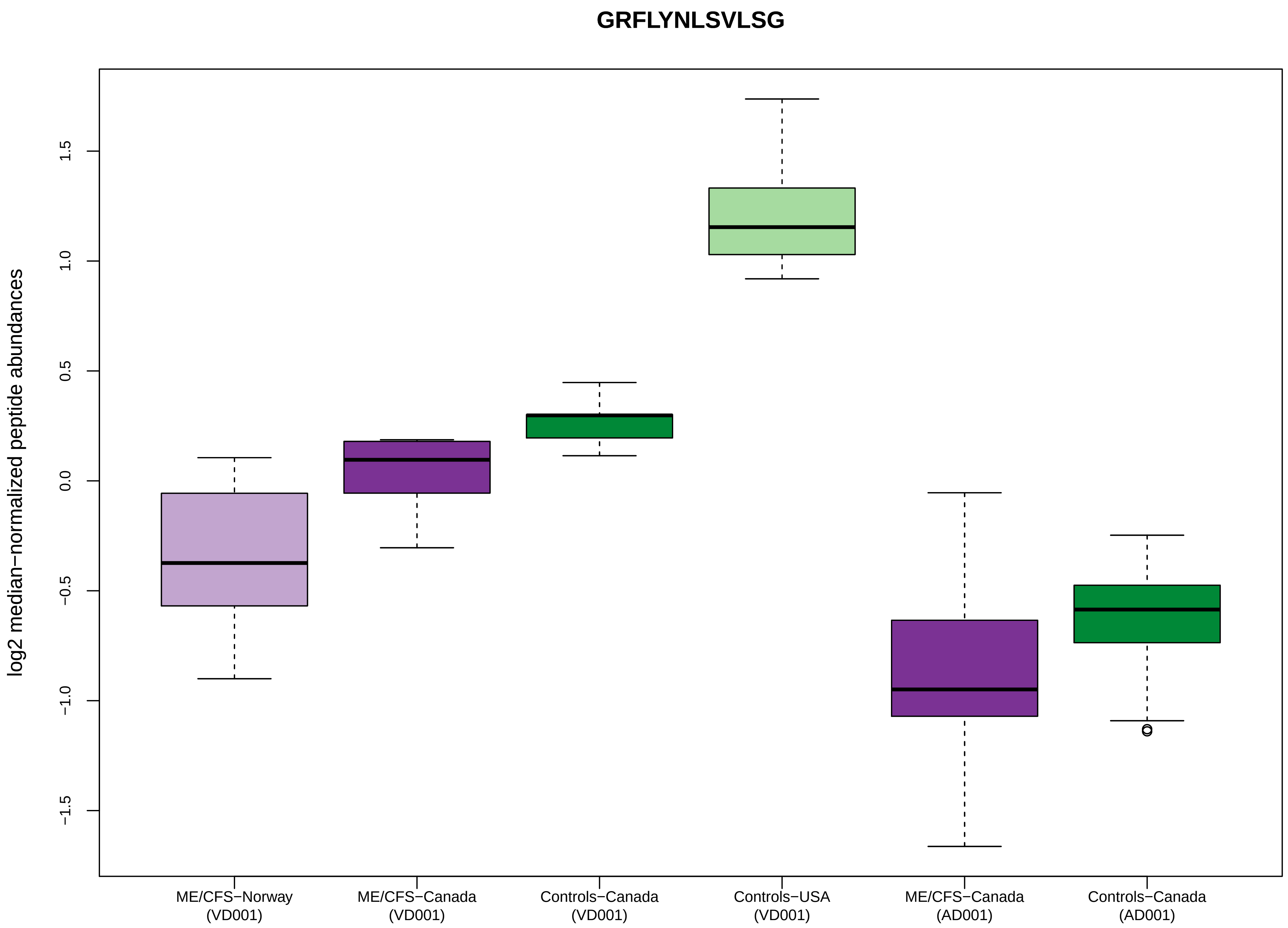
GRFLYNLSVLSG
log2 median−normalized peptide abundances
1.5
1.0
0.5
0.0
−0.5
−1.0
−1.5
ME/CFS−Norway (VD001)
ME/CFS−Canada (VD001)
Controls−Canada (VD001)
Controls−USA (VD001)
ME/CFS−Canada (AD001)
Controls−Canada (AD001)

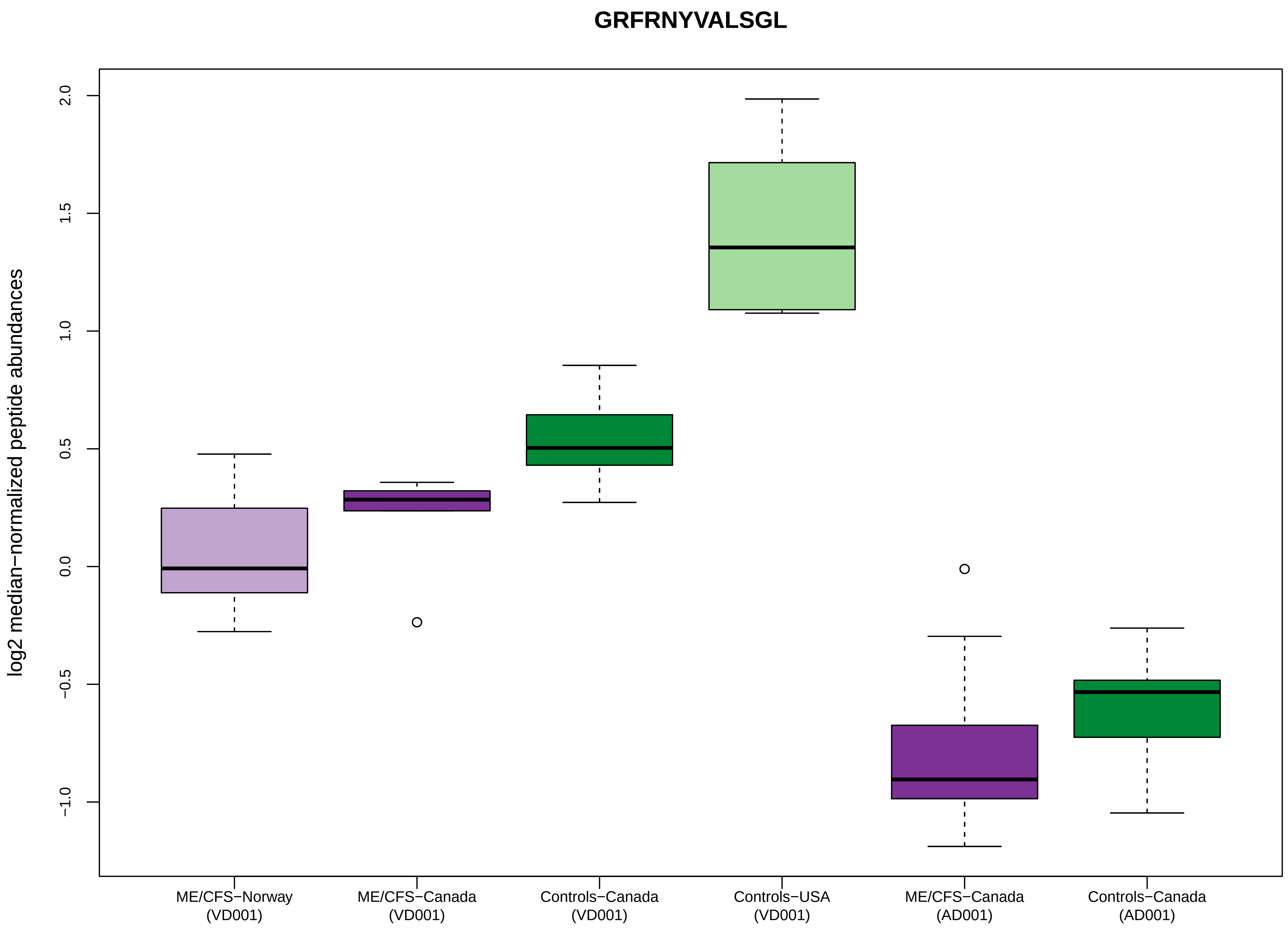
GRFRNYVALSGL
log2 median−normalized peptide abundances
2.0
1.5
1.0
0.5
0.0
−0.5
−1.0
ME/CFS−Norway (VD001)
ME/CFS−Canada (VD001)
Controls−Canada (VD001)
Controls−USA (VD001)
ME/CFS−Canada (AD001)
Controls−Canada (AD001)

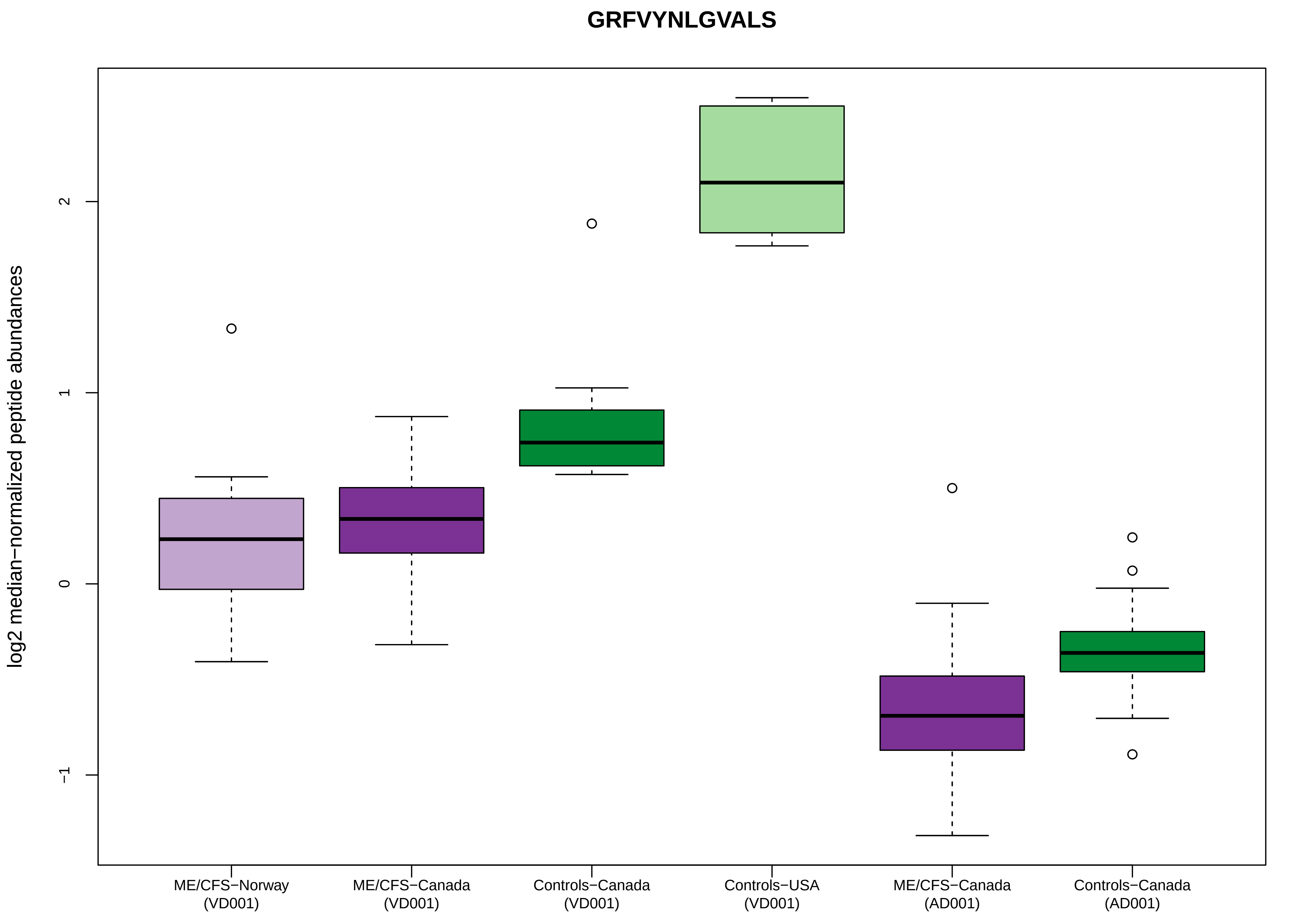
GRFVYNLGVALS
log2 median−normalized peptide abundances
−1
0
1
2
ME/CFS−Norway (VD001)
ME/CFS−Canada (VD001)
Controls−Canada (VD001)
Controls−USA (VD001)
ME/CFS−Canada (AD001)
Controls−Canada (AD001)

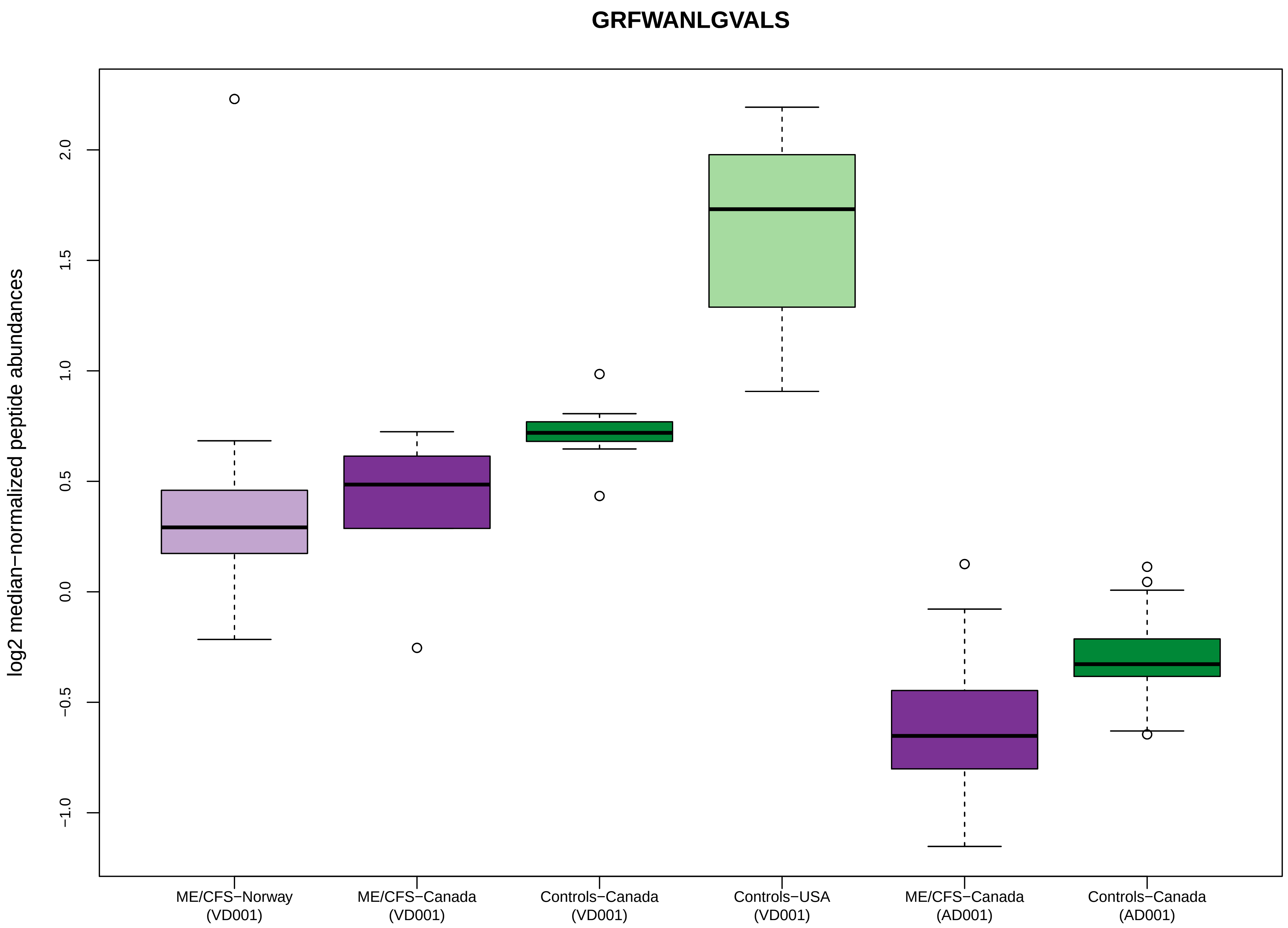
GRFWANLGVALS
log2 median−normalized peptide abundances
2.0
1.5
1.0
0.5
0.0
−0.5
−1.0
ME/CFS−Norway (VD001)
ME/CFS−Canada (VD001)
Controls−Canada (VD001)
Controls−USA (VD001)
ME/CFS−Canada (AD001)
Controls−Canada (AD001)

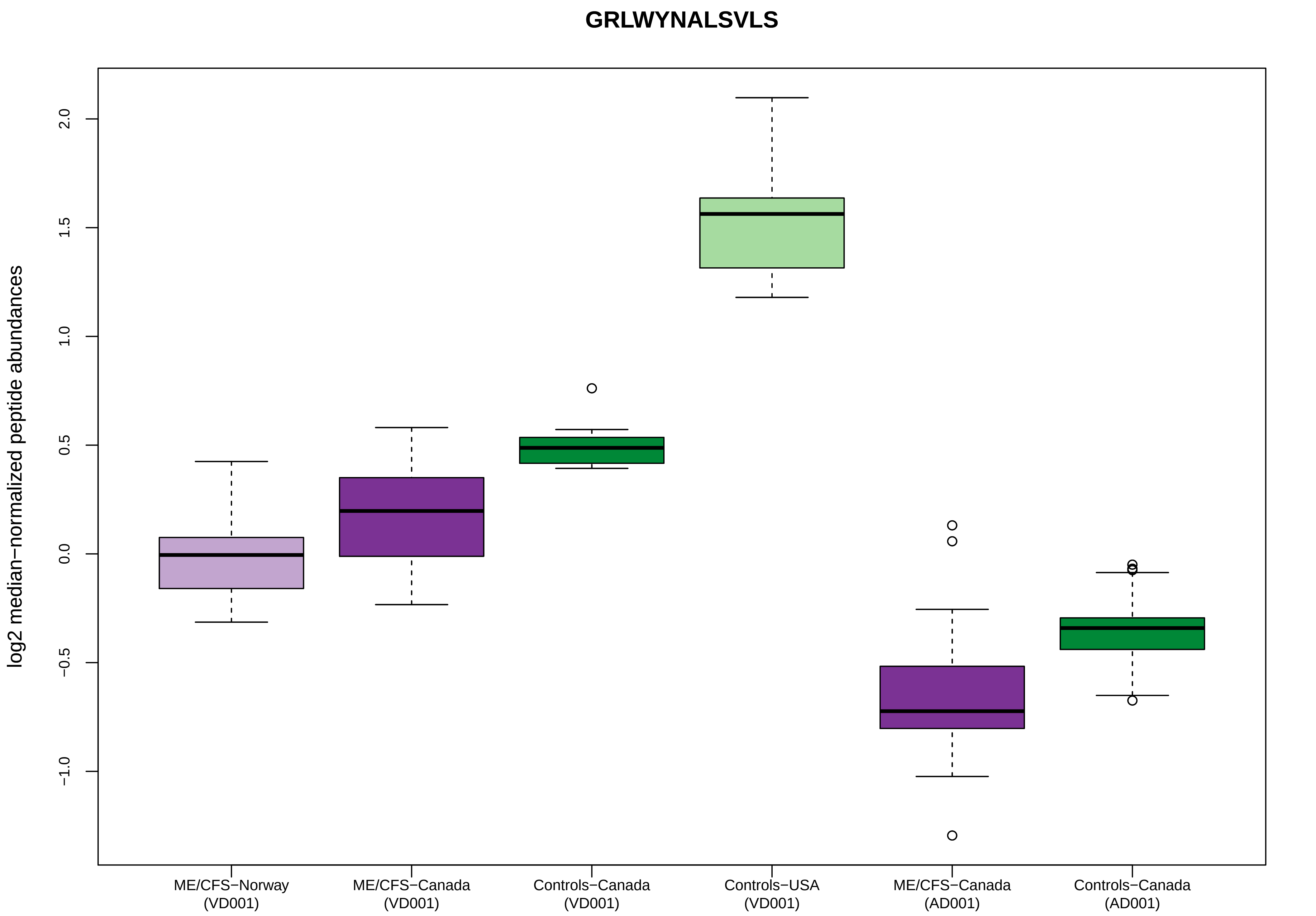

GRLWYNALSVLS
log2 median−normalized peptide abundances
2.0
1.5
1.0
0.5
0.0
−0.5
−1.0
ME/CFS−Norway (VD001)
ME/CFS−Canada (VD001)
Controls−Canada (VD001)
Controls−USA (VD001)
ME/CFS−Canada (AD001)
Controls−Canada (AD001)

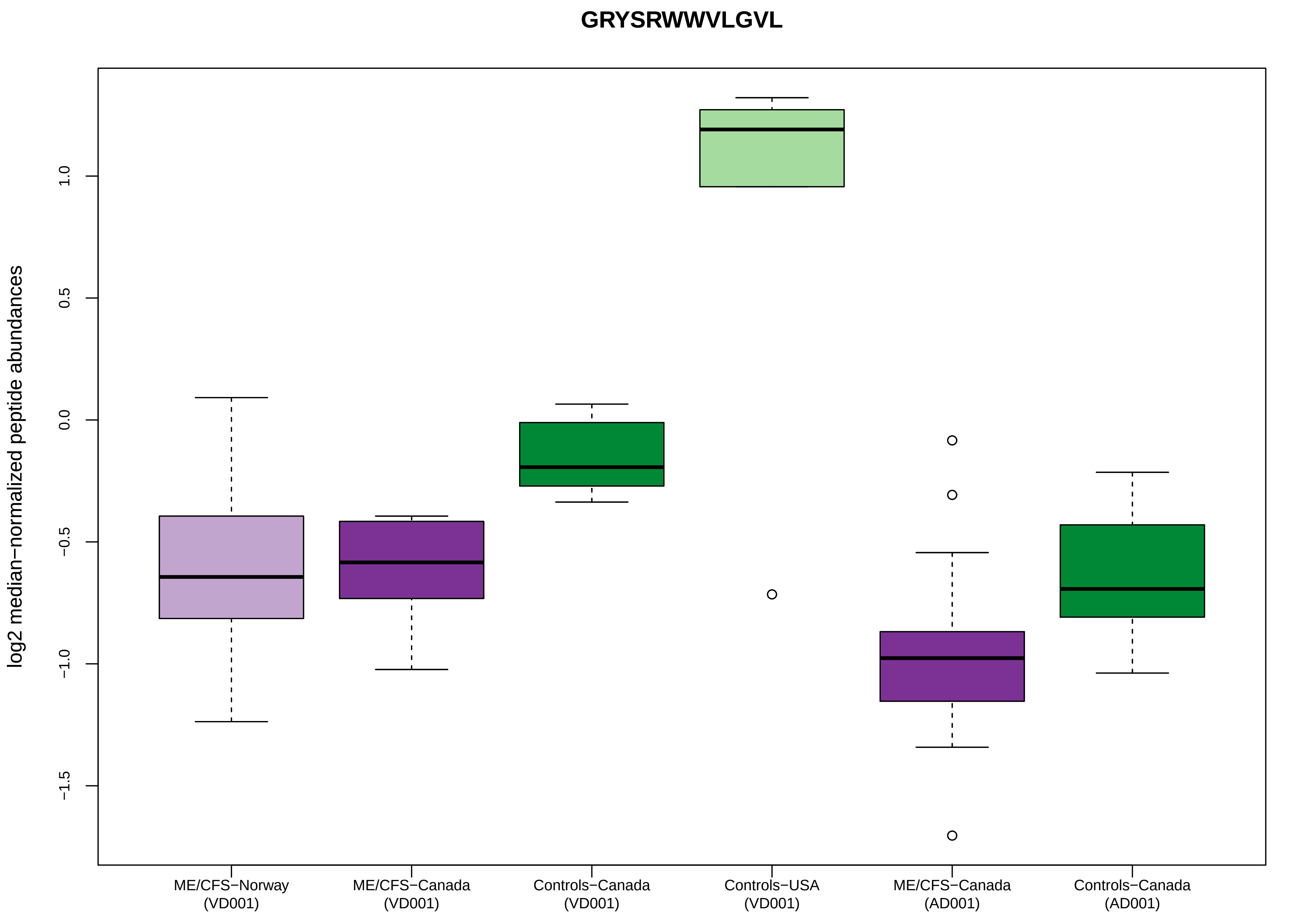
GRYSRWWVLGVL
log2 median−normalized peptide abundances
1.0
0.5
0.0
−0.5
−1.0
−1.5
ME/CFS−Norway (VD001)
ME/CFS−Canada (VD001)
Controls−Canada (VD001)
Controls−USA (VD001)
ME/CFS−Canada (AD001)
Controls−Canada (AD001)

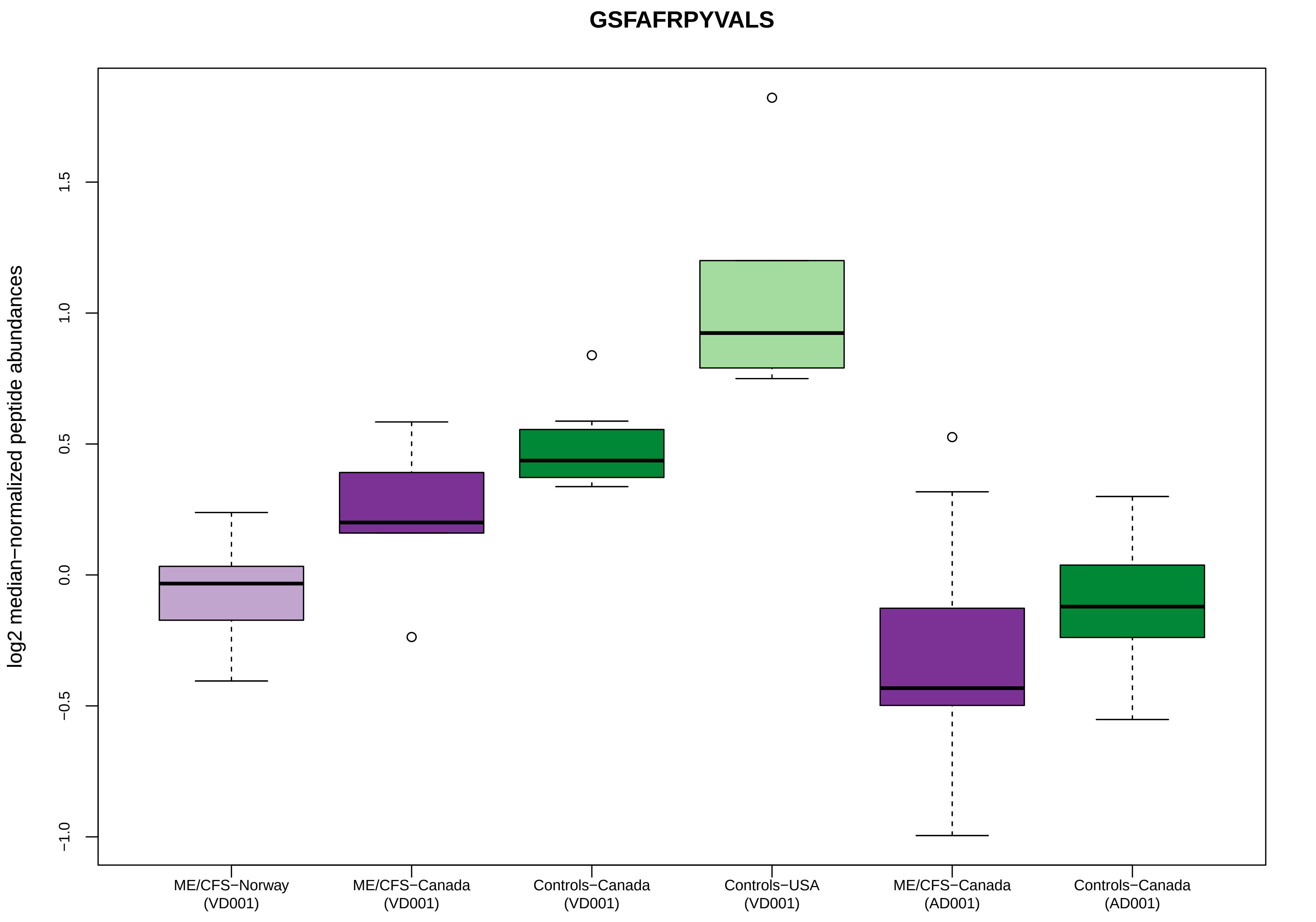
GSFAFRPYVALS
log2 median−normalized peptide abundances
−1.0
−0.5
0.0
0.5
1.0
1.5
ME/CFS−Norway (VD001)
ME/CFS−Canada (VD001)
Controls−Canada (VD001)
Controls−USA (VD001)
ME/CFS−Canada (AD001)
Controls−Canada (AD001)

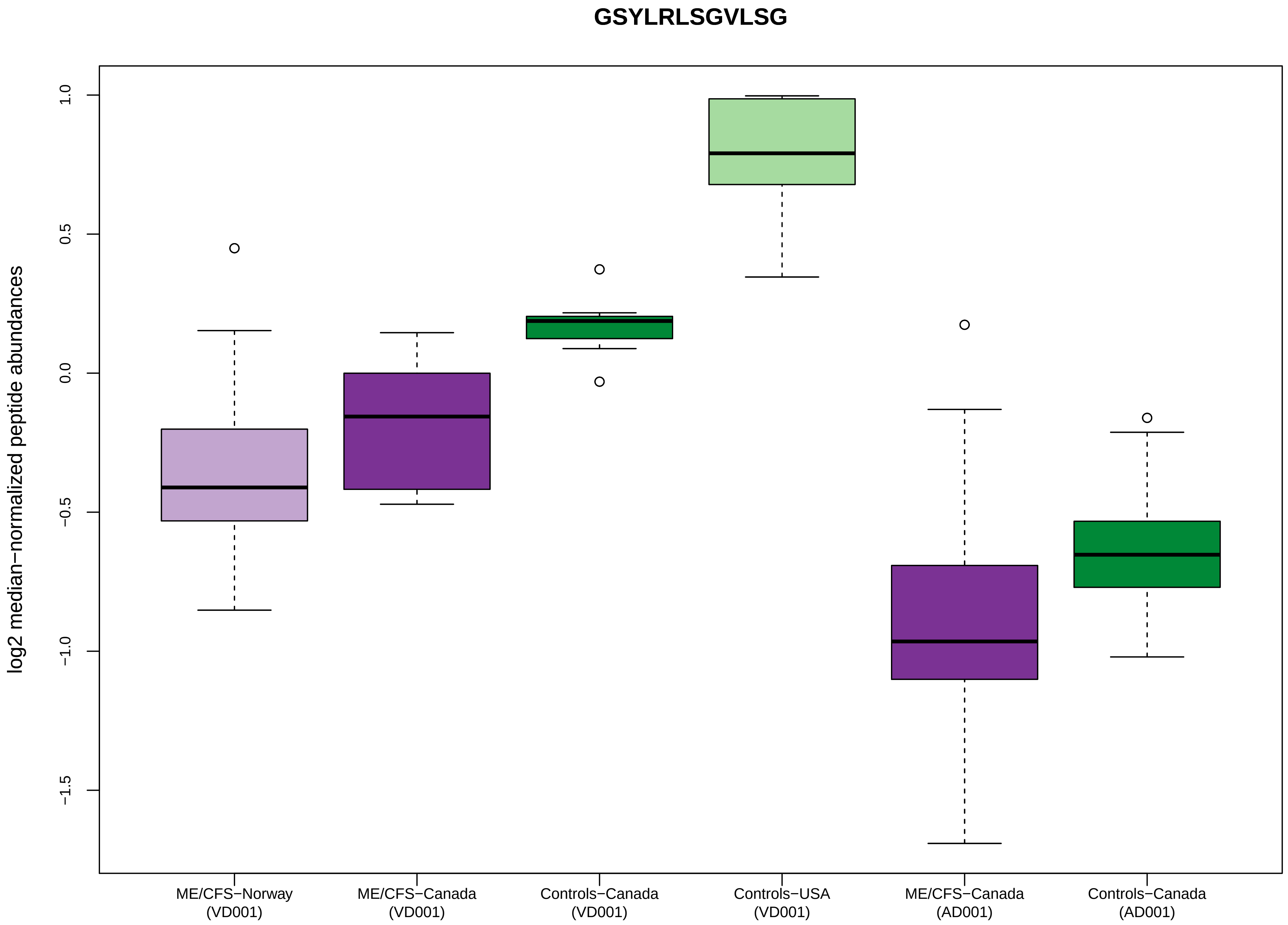
GSYLRLSGVLSG
log2 median−normalized peptide abundances
1.0
0.5
0.0
−0.5
−1.0
−1.5
ME/CFS−Norway (VD001)
ME/CFS−Canada (VD001)
Controls−Canada (VD001)
Controls−USA (VD001)
ME/CFS−Canada (AD001)
Controls−Canada (AD001)

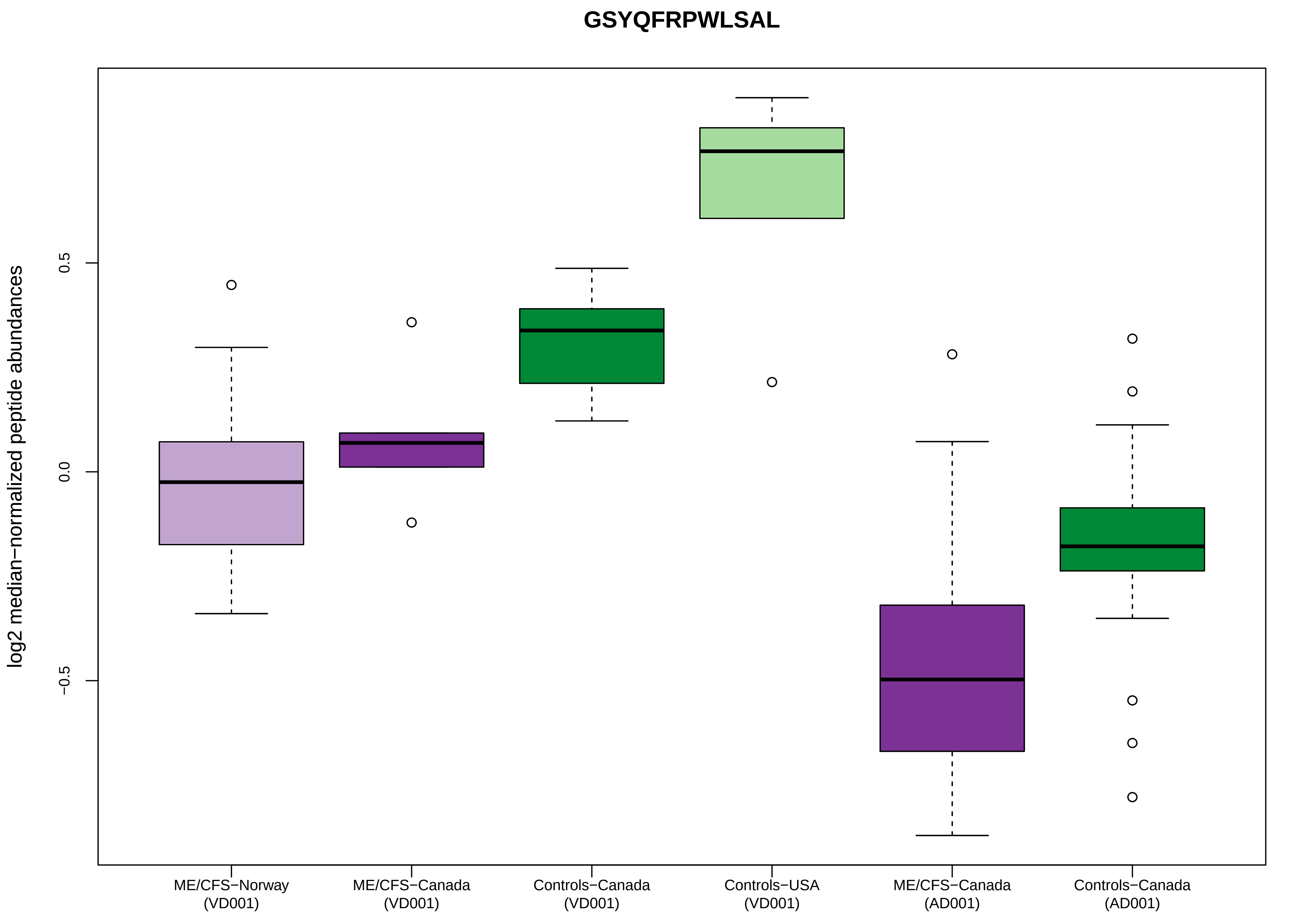

GSYQFRPWLSAL
log2 median−normalized peptide abundances
0.5
0.0
−0.5
ME/CFS−Norway (VD001)
ME/CFS−Canada (VD001)
Controls−Canada (VD001)
Controls−USA (VD001)
ME/CFS−Canada (AD001)
Controls−Canada (AD001)

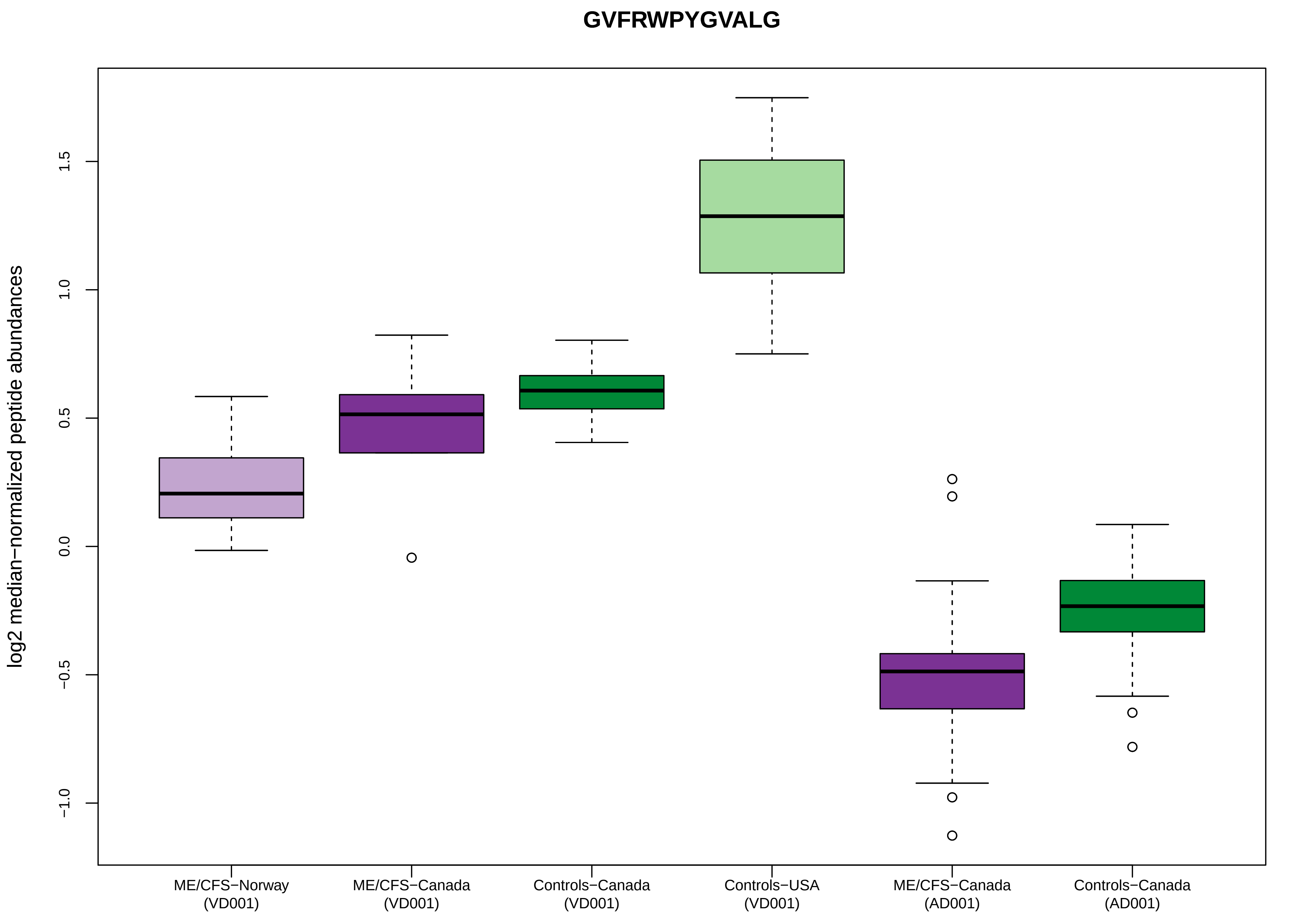
GVFRWPYGVALG
log2 median−normalized peptide abundances
1.5
1.0
0.5
0.0
−0.5
−1.0
ME/CFS−Norway (VD001)
ME/CFS−Canada (VD001)
Controls−Canada (VD001)
Controls−USA (VD001)
ME/CFS−Canada (AD001)
Controls−Canada (AD001)

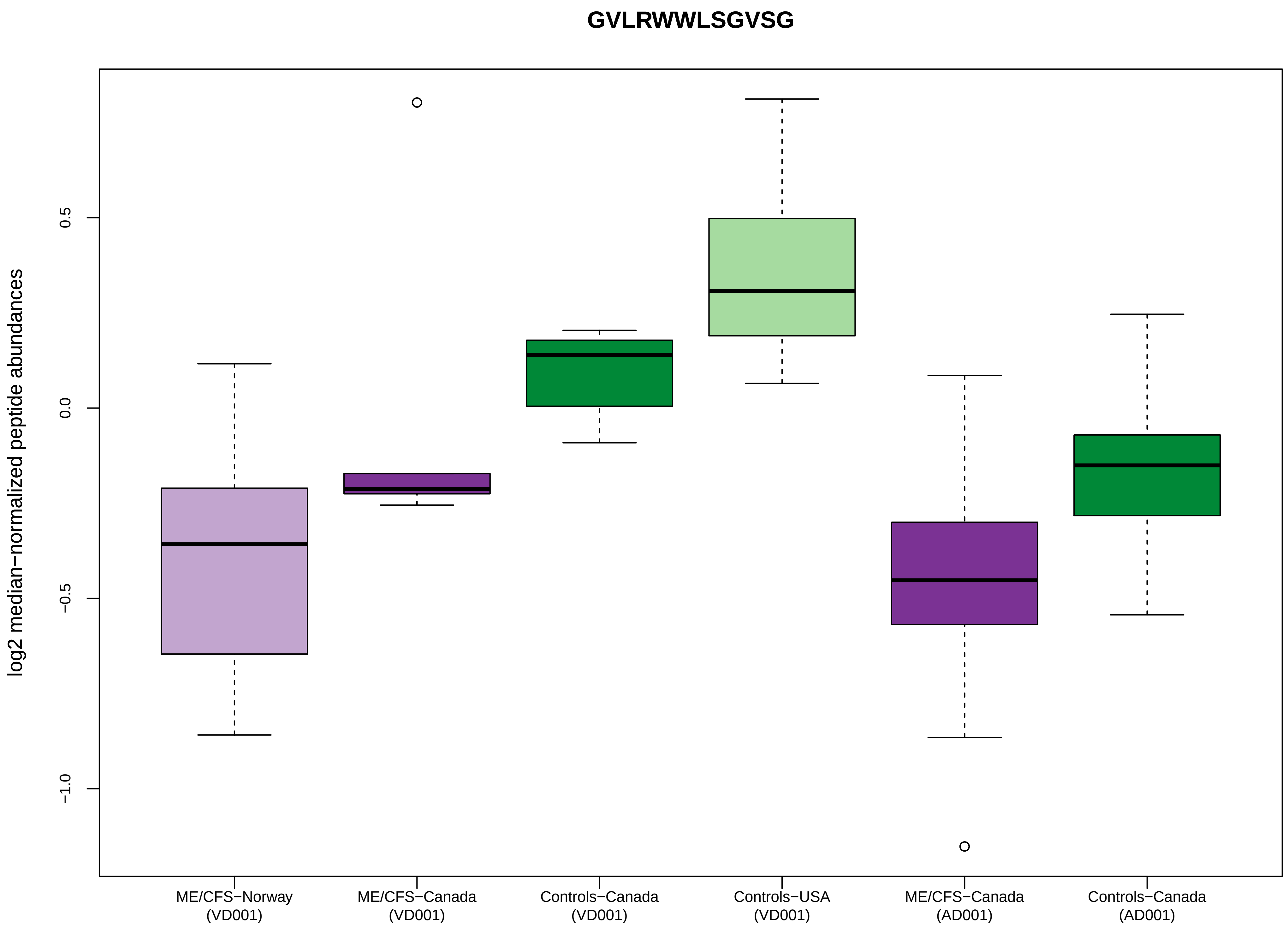
GVLRWWLSGVSG
log2 median−normalized peptide abundances
0.5
0.0
−0.5
−1.0
ME/CFS−Norway (VD001)
ME/CFS−Canada (VD001)
Controls−Canada (VD001)
Controls−USA (VD001)
ME/CFS−Canada (AD001)
Controls−Canada (AD001)

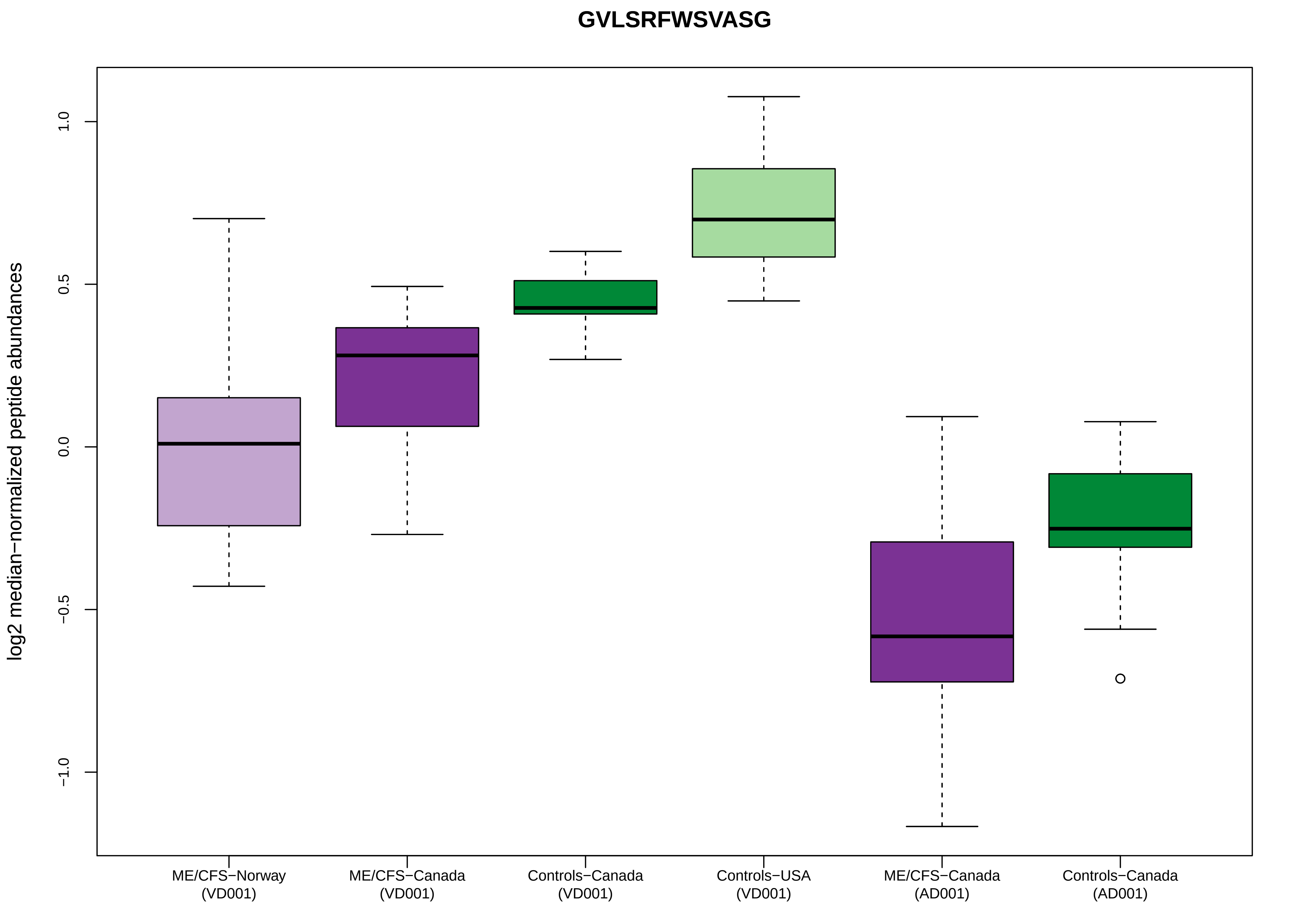

GVLSRFWSVASG
log2 median−normalized peptide abundances
1.0
0.5
0.0
−0.5
−1.0
ME/CFS−Norway (VD001)
ME/CFS−Canada (VD001)
Controls−Canada (VD001)
Controls−USA (VD001)
ME/CFS−Canada (AD001)
Controls−Canada (AD001)

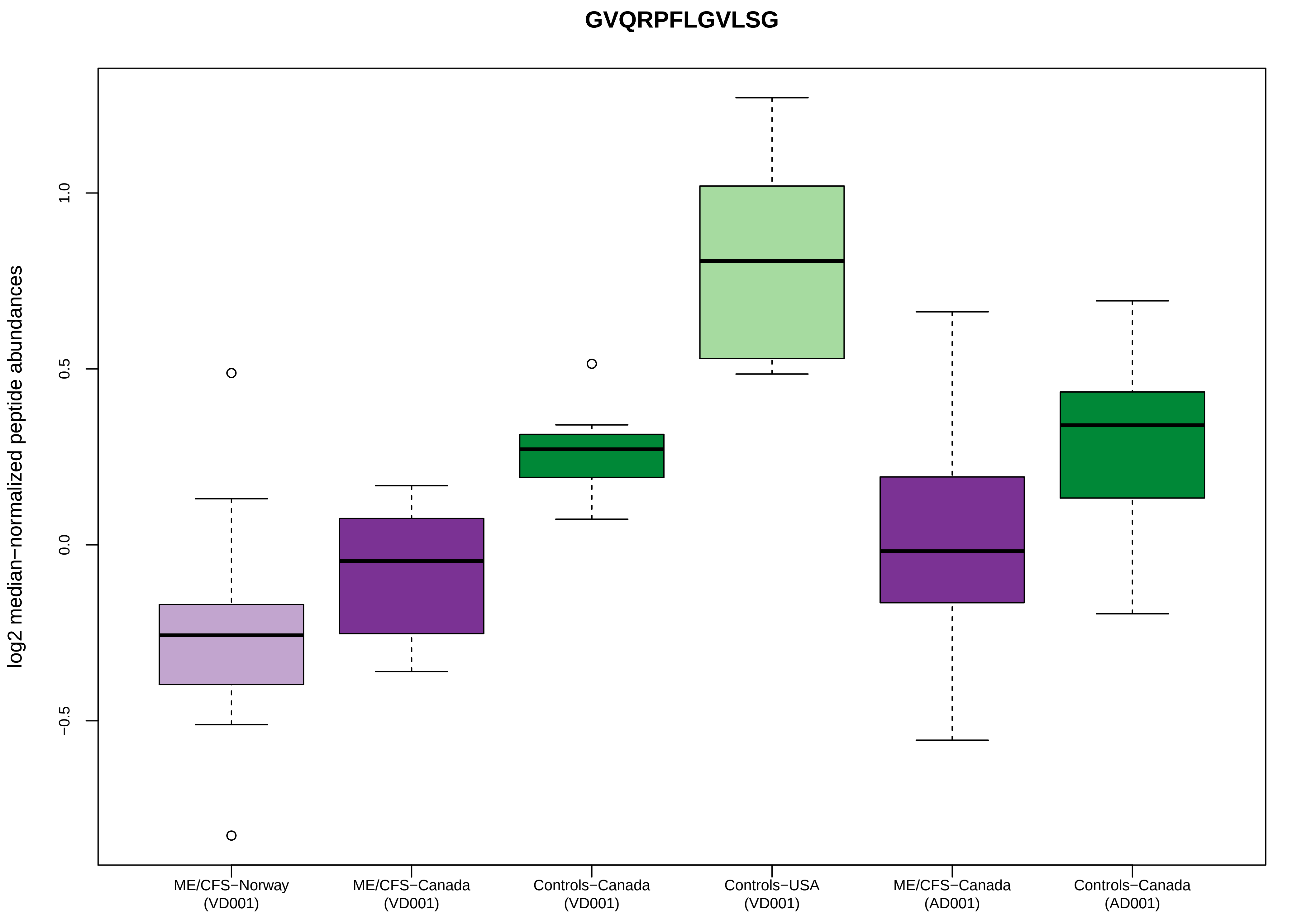

GVQRPFLGVLSG
log2 median−normalized peptide abundances
−0.5
0.0
0.5
1.0
ME/CFS−Norway (VD001)
ME/CFS−Canada (VD001)
Controls−Canada (VD001)
Controls−USA (VD001)
ME/CFS−Canada (AD001)
Controls−Canada (AD001)

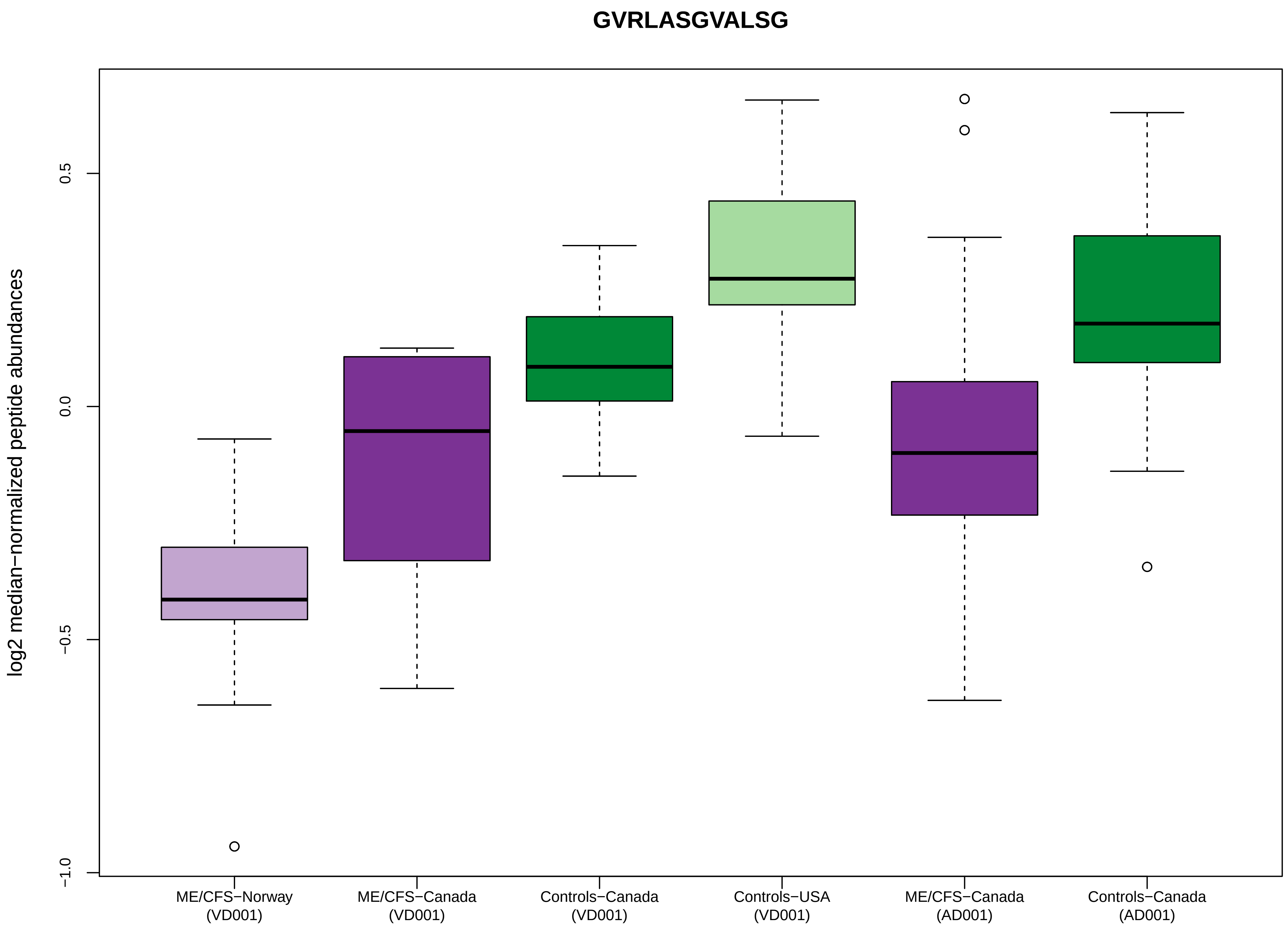

GVRLASGVALSG
log2 median−normalized peptide abundances
0.5
0.0
−0.5
−1.0
ME/CFS−Norway (VD001)
ME/CFS−Canada (VD001)
Controls−Canada (VD001)
Controls−USA (VD001)
ME/CFS−Canada (AD001)
Controls−Canada (AD001)

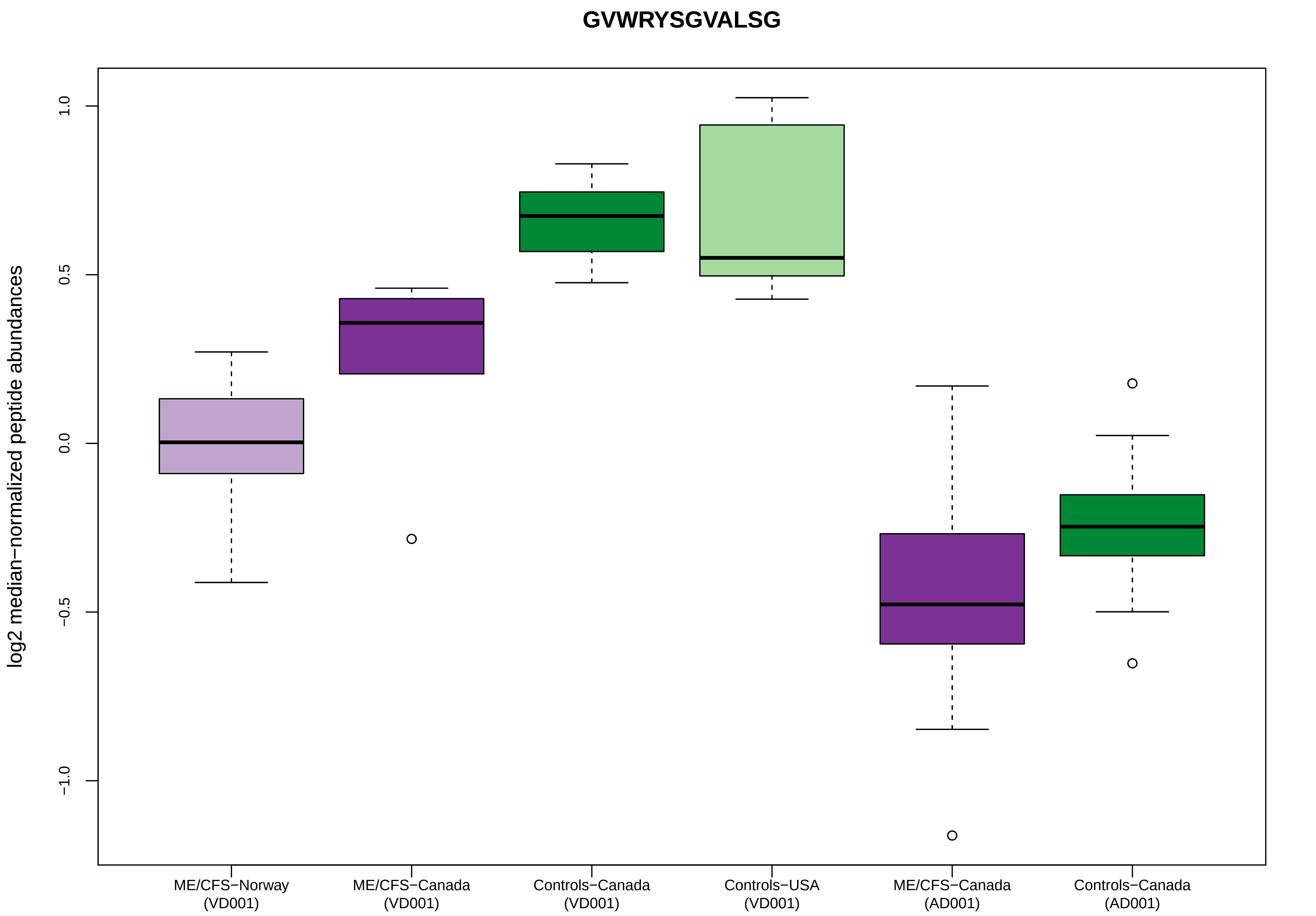

GVWRYSGVALSG
log2 median−normalized peptide abundances
1.0
0.5
0.0
−0.5
−1.0
ME/CFS−Norway (VD001)
ME/CFS−Canada (VD001)
Controls−Canada (VD001)
Controls−USA (VD001)
ME/CFS−Canada (AD001)
Controls−Canada (AD001)

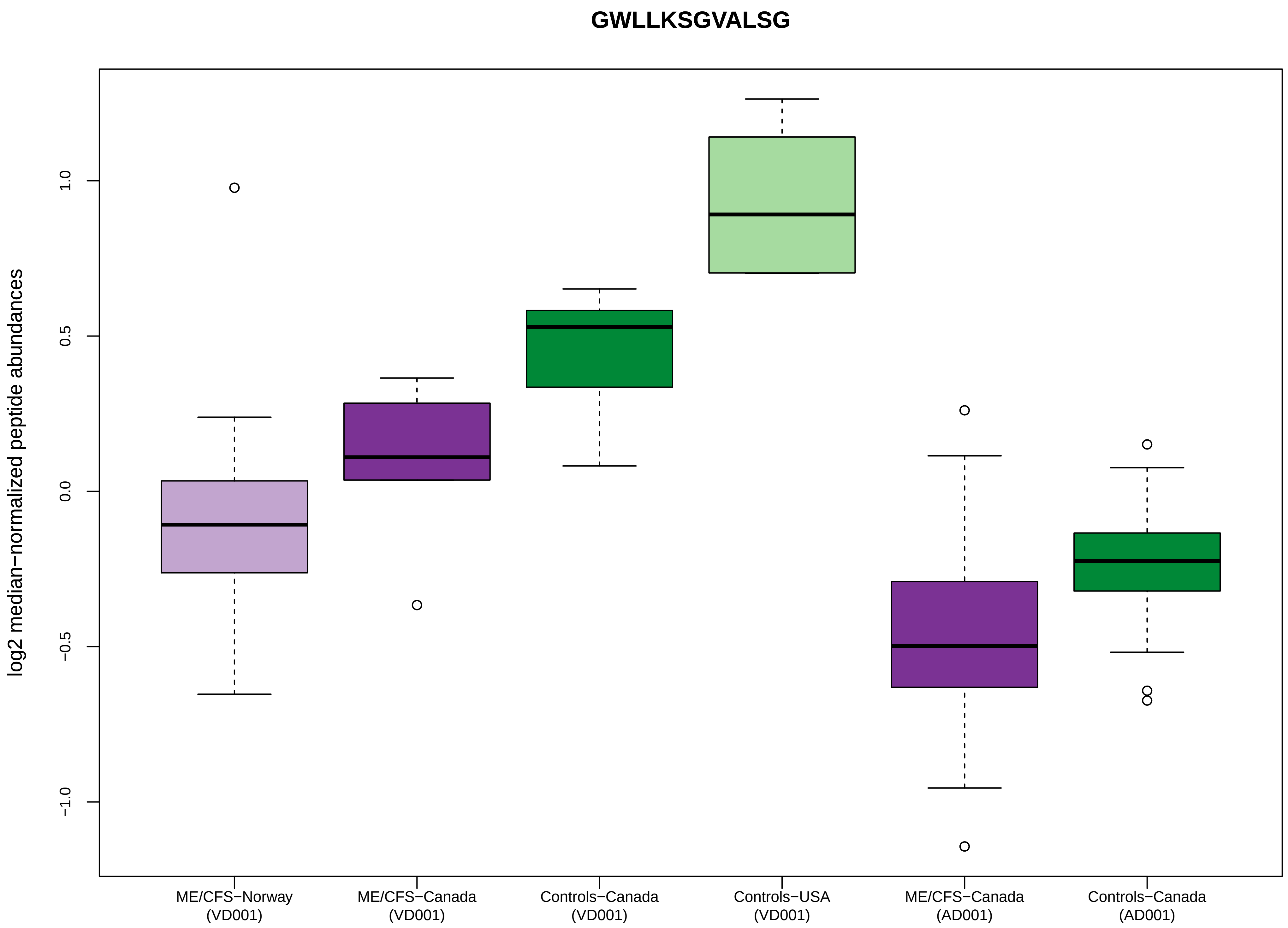

GWLLKSGVALSG
log2 median−normalized peptide abundances
1.0
0.5
0.0
−0.5
−1.0
ME/CFS−Norway (VD001)
ME/CFS−Canada (VD001)
Controls−Canada (VD001)
Controls−USA (VD001)
ME/CFS−Canada (AD001)
Controls−Canada (AD001)

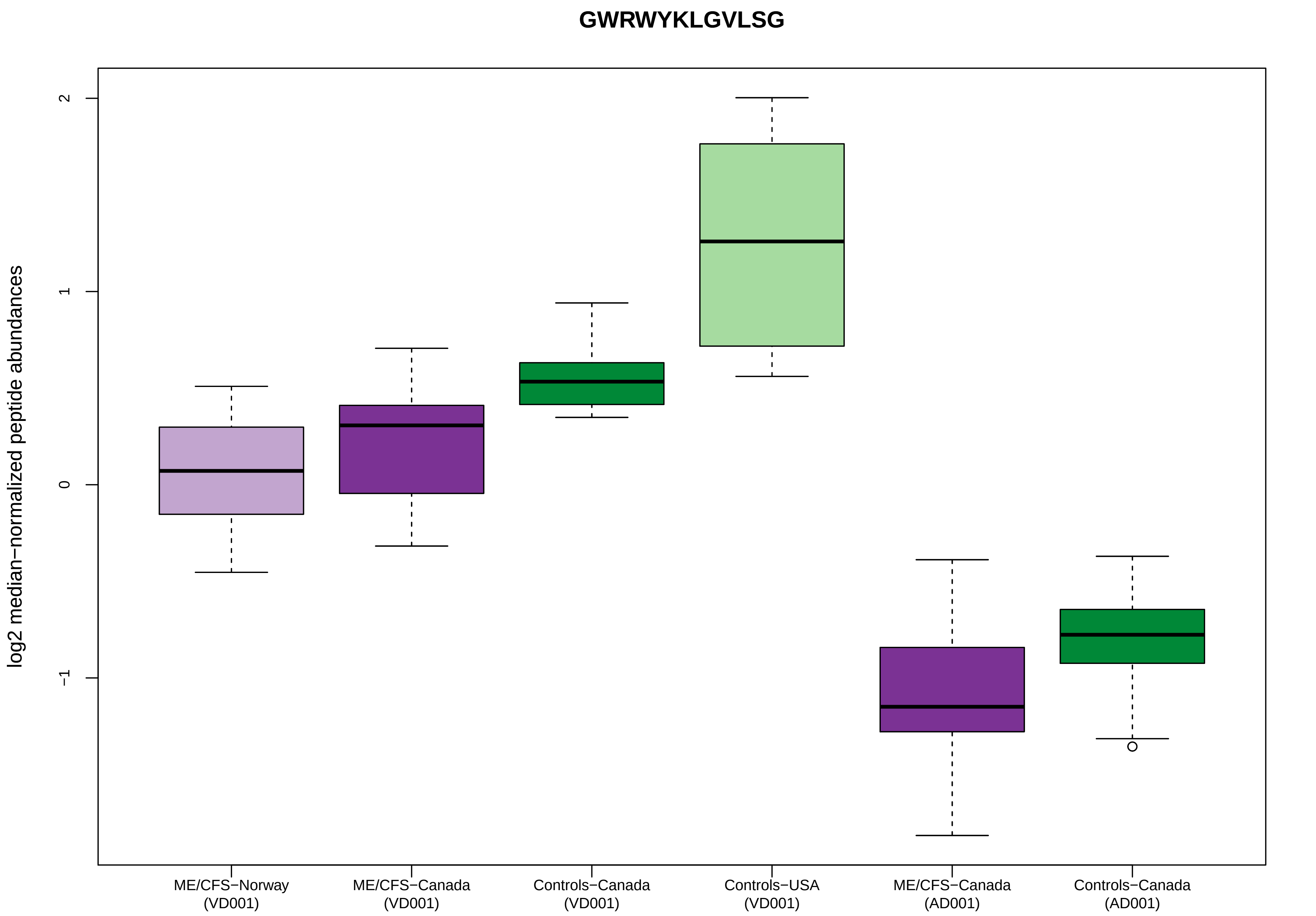

GWRWYKLGVLSG
log2 median−normalized peptide abundances
2
1
0
−1
ME/CFS−Norway (VD001)
ME/CFS−Canada (VD001)
Controls−Canada (VD001)
Controls−USA (VD001)
ME/CFS−Canada (AD001)
Controls−Canada (AD001)

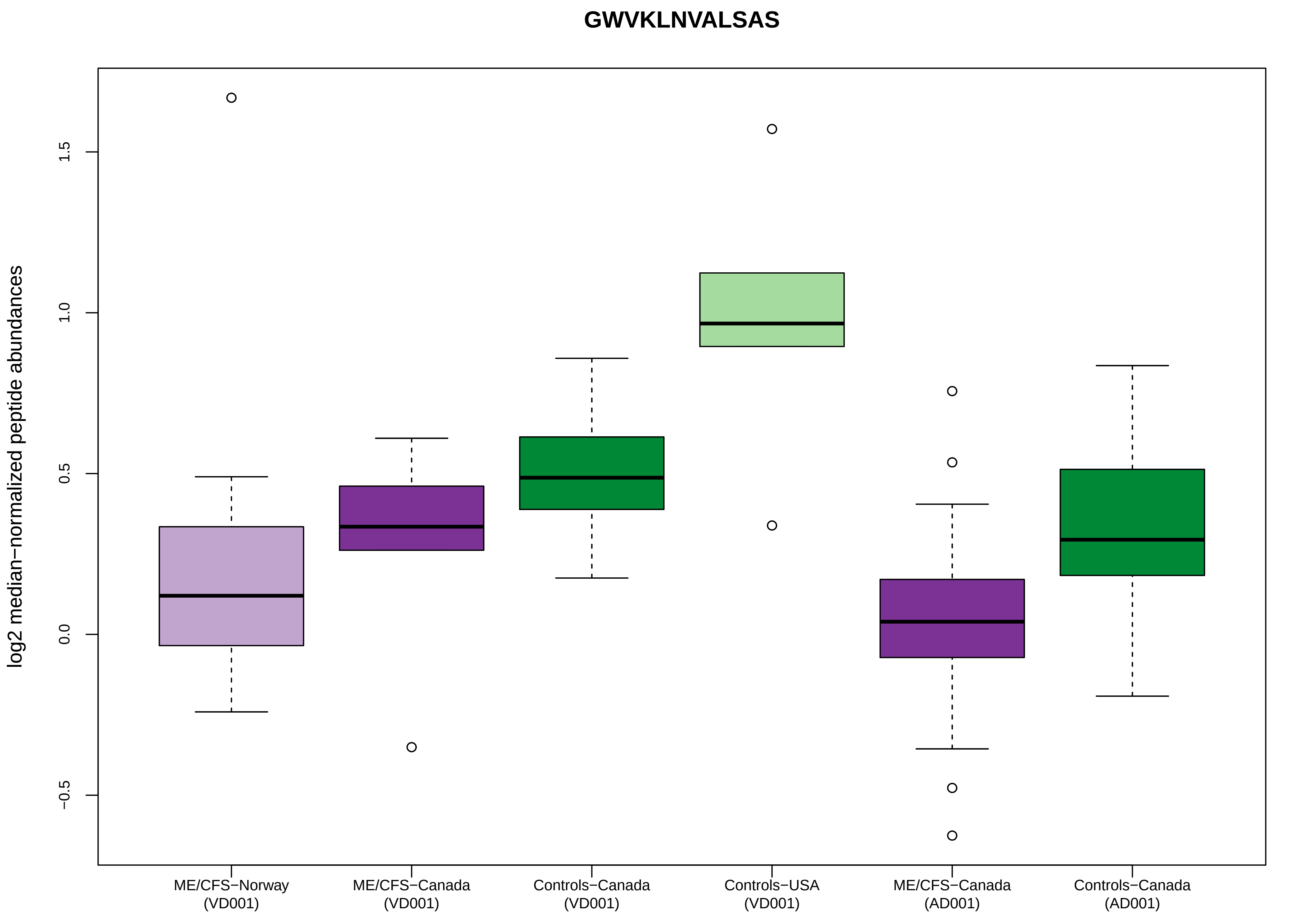

GWVKLNVALSAS
log2 median−normalized peptide abundances
−0.5
0.0
0.5
1.0
1.5
ME/CFS−Norway (VD001)
ME/CFS−Canada (VD001)
Controls−Canada (VD001)
Controls−USA (VD001)
ME/CFS−Canada (AD001)
Controls−Canada (AD001)

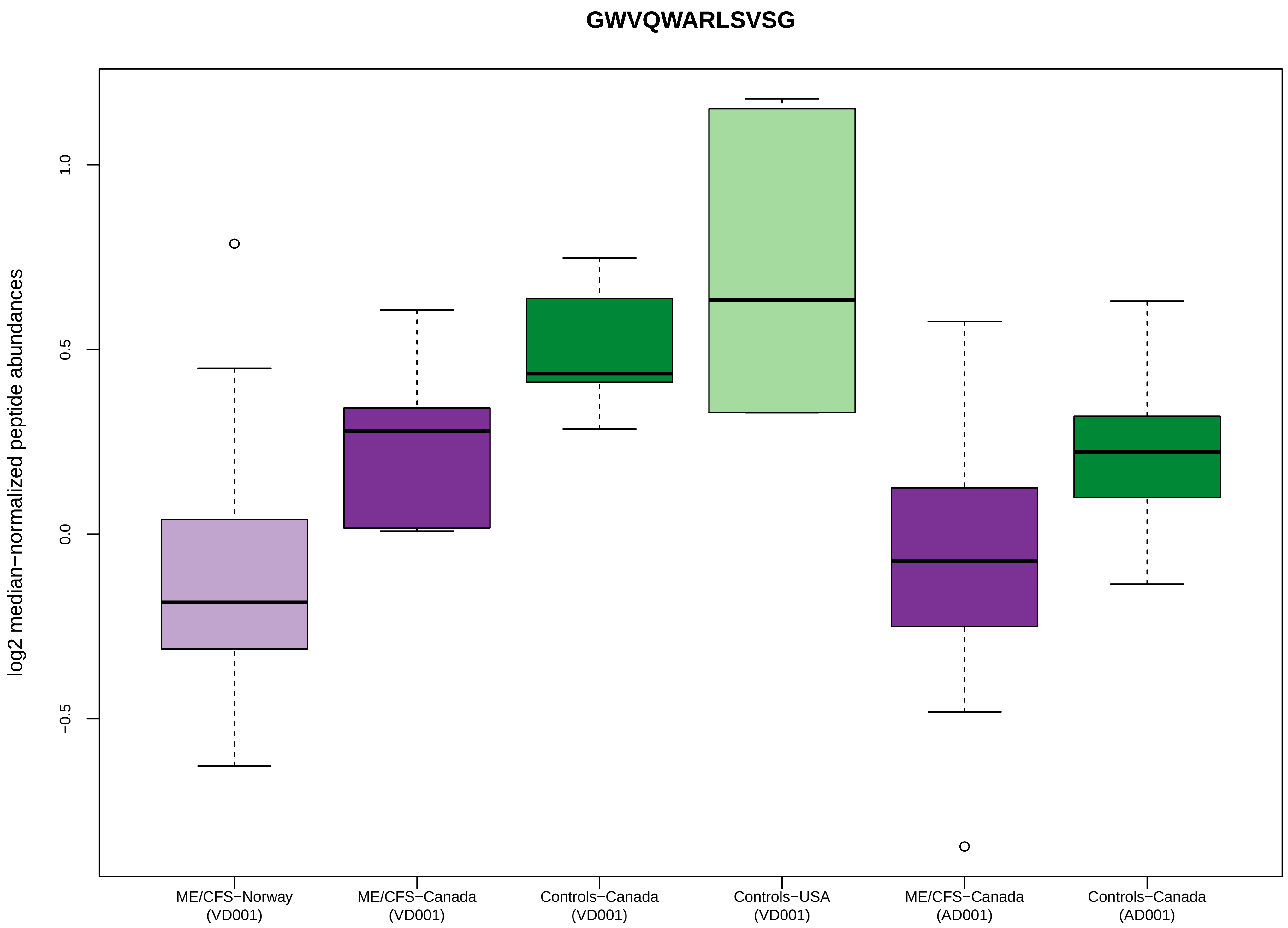

GWVQWARLSVSG
log2 median−normalized peptide abundances
1.0
0.5
0.0
−0.5
ME/CFS−Norway (VD001)
ME/CFS−Canada (VD001)
Controls−Canada (VD001)
Controls−USA (VD001)
ME/CFS−Canada (AD001)
Controls−Canada (AD001)

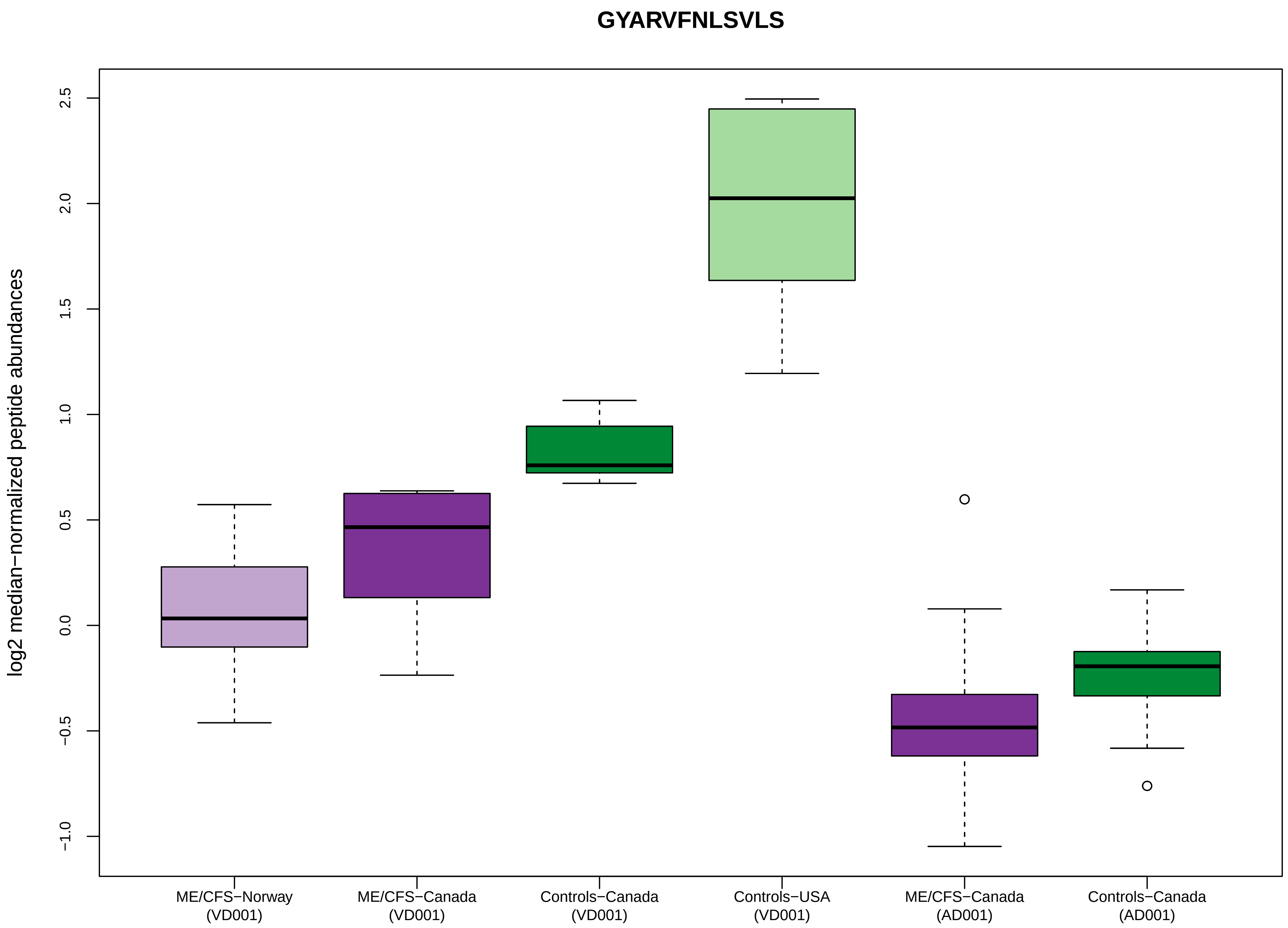
GYARVFNLSVLS
log2 median−normalized peptide abundances
2.5
2.0
1.5
1.0
0.5
0.0
−0.5
−1.0
ME/CFS−Norway
(VD001)
ME/CFS−Canada
(VD001)
Controls−Canada
(VD001)
Controls−USA
(VD001)
ME/CFS−Canada
(AD001)
Controls−Canada
(AD001)

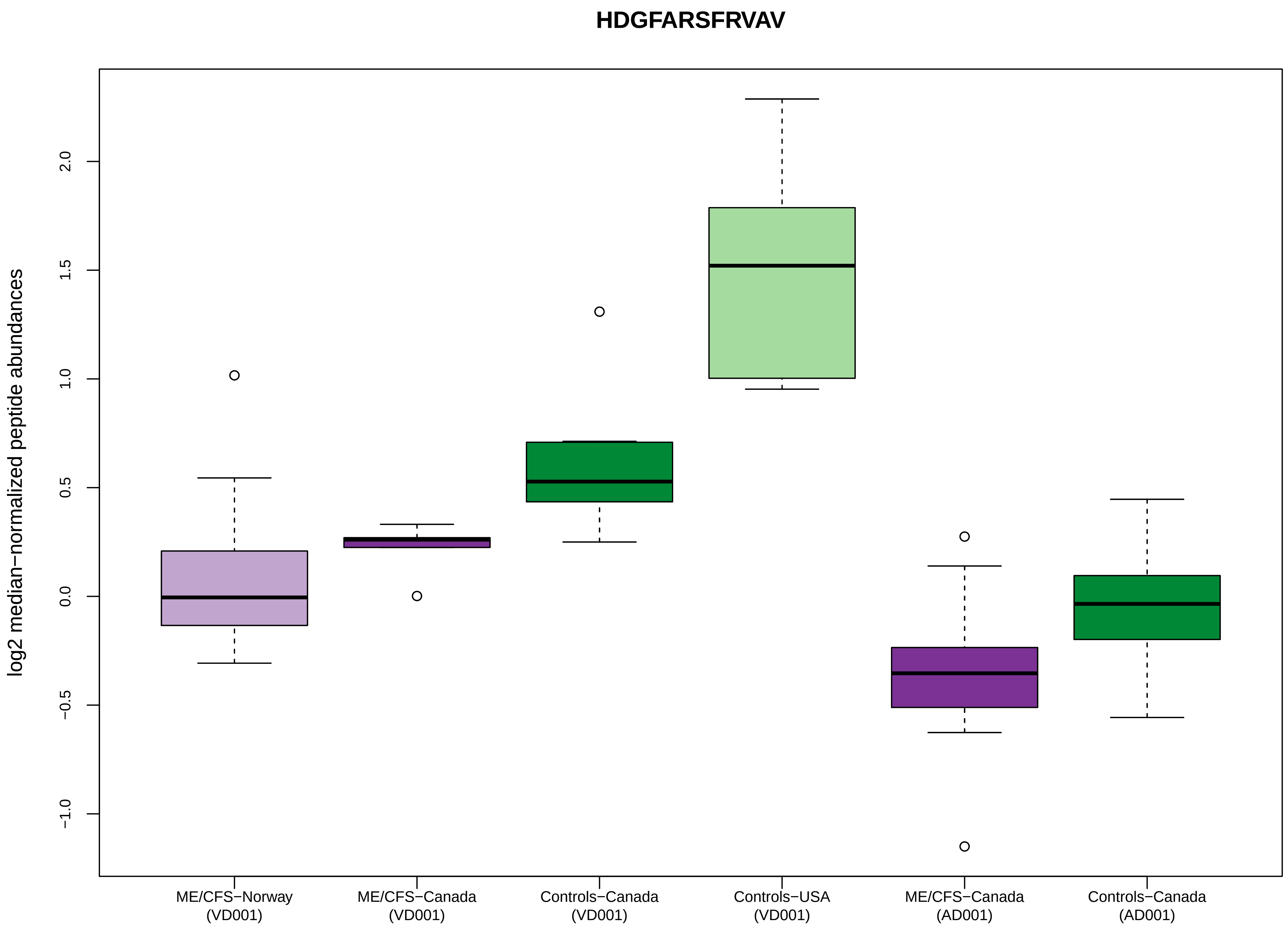
HDGFARSFRVAV
log2 median−normalized peptide abundances
2.0
1.5
1.0
0.5
0.0
−0.5
−1.0
ME/CFS−Norway (VD001)
ME/CFS−Canada (VD001)
Controls−Canada (VD001)
Controls−USA (VD001)
ME/CFS−Canada (AD001)
Controls−Canada (AD001)

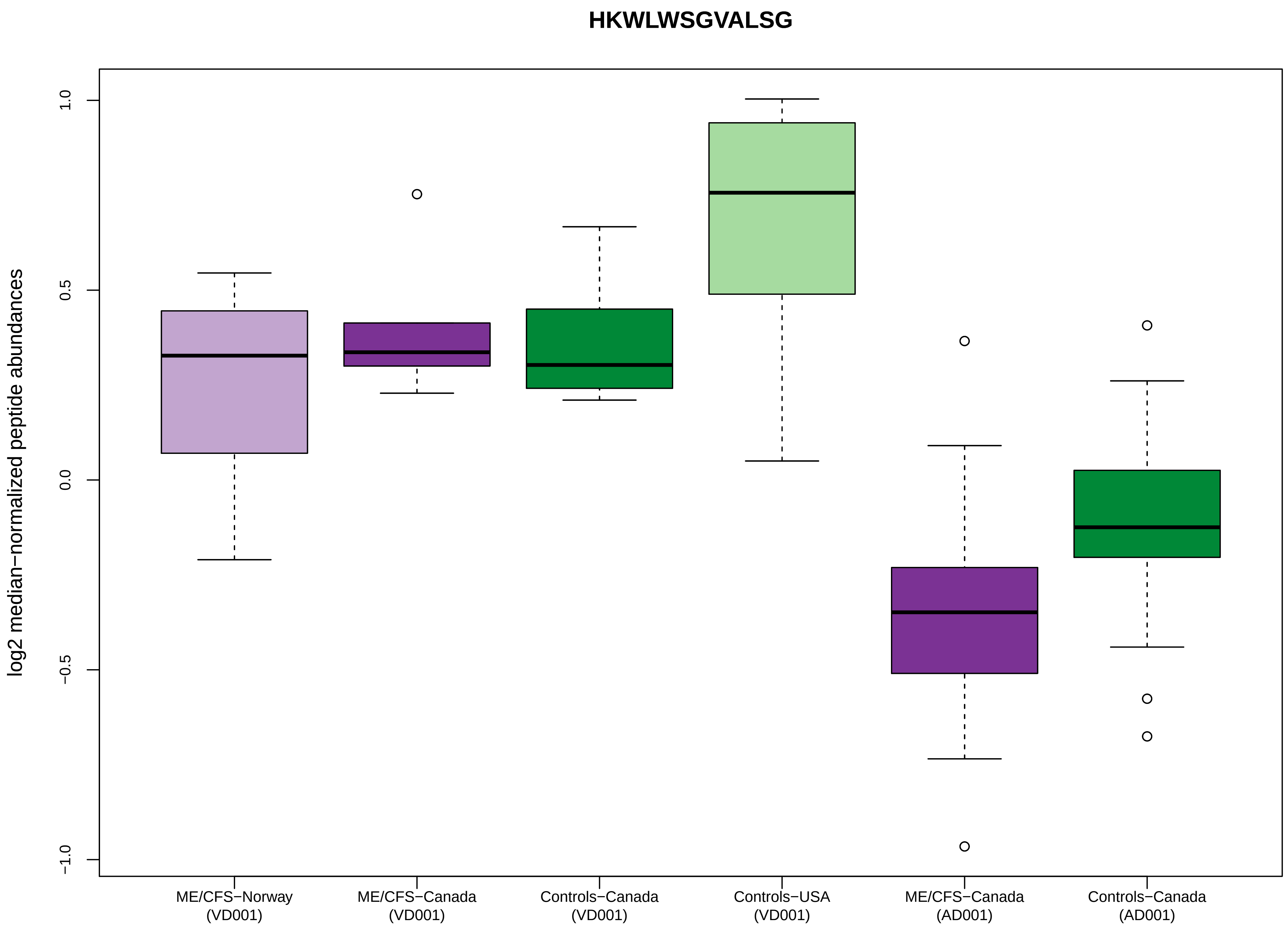
HKWLWSGVALSG
log2 median−normalized peptide abundances
1.0
0.5
0.0
−0.5
−1.0
ME/CFS−Norway (VD001)
ME/CFS−Canada (VD001)
Controls−Canada (VD001)
Controls−USA (VD001)
ME/CFS−Canada (AD001)
Controls−Canada (AD001)

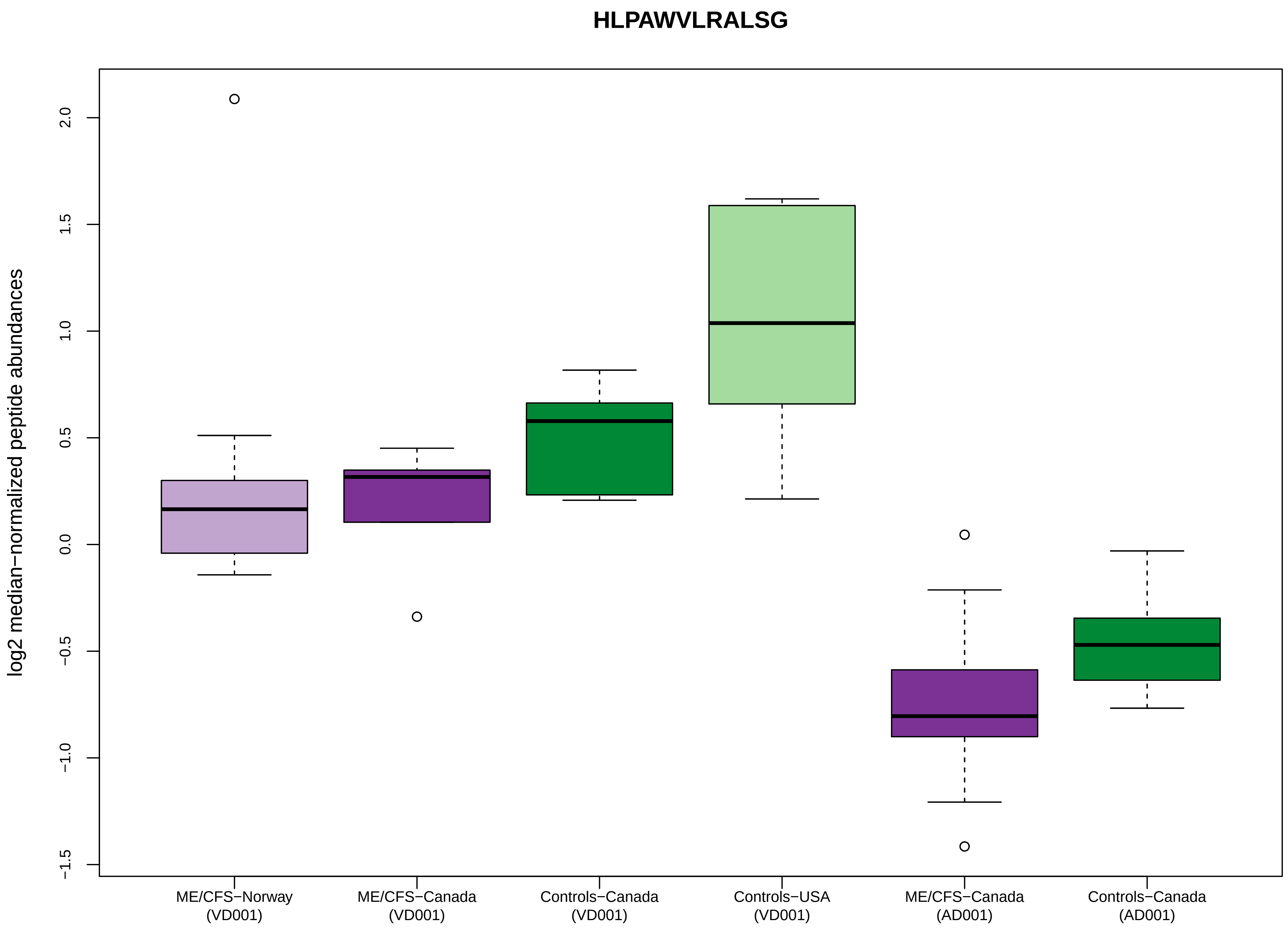

HLPAWVLRALSG
log2 median−normalized peptide abundances
2.0
1.5
1.0
0.5
0.0
−0.5
−1.0
−1.5
ME/CFS−Norway (VD001)
ME/CFS−Canada (VD001)
Controls−Canada (VD001)
Controls−USA (VD001)
ME/CFS−Canada (AD001)
Controls−Canada (AD001)

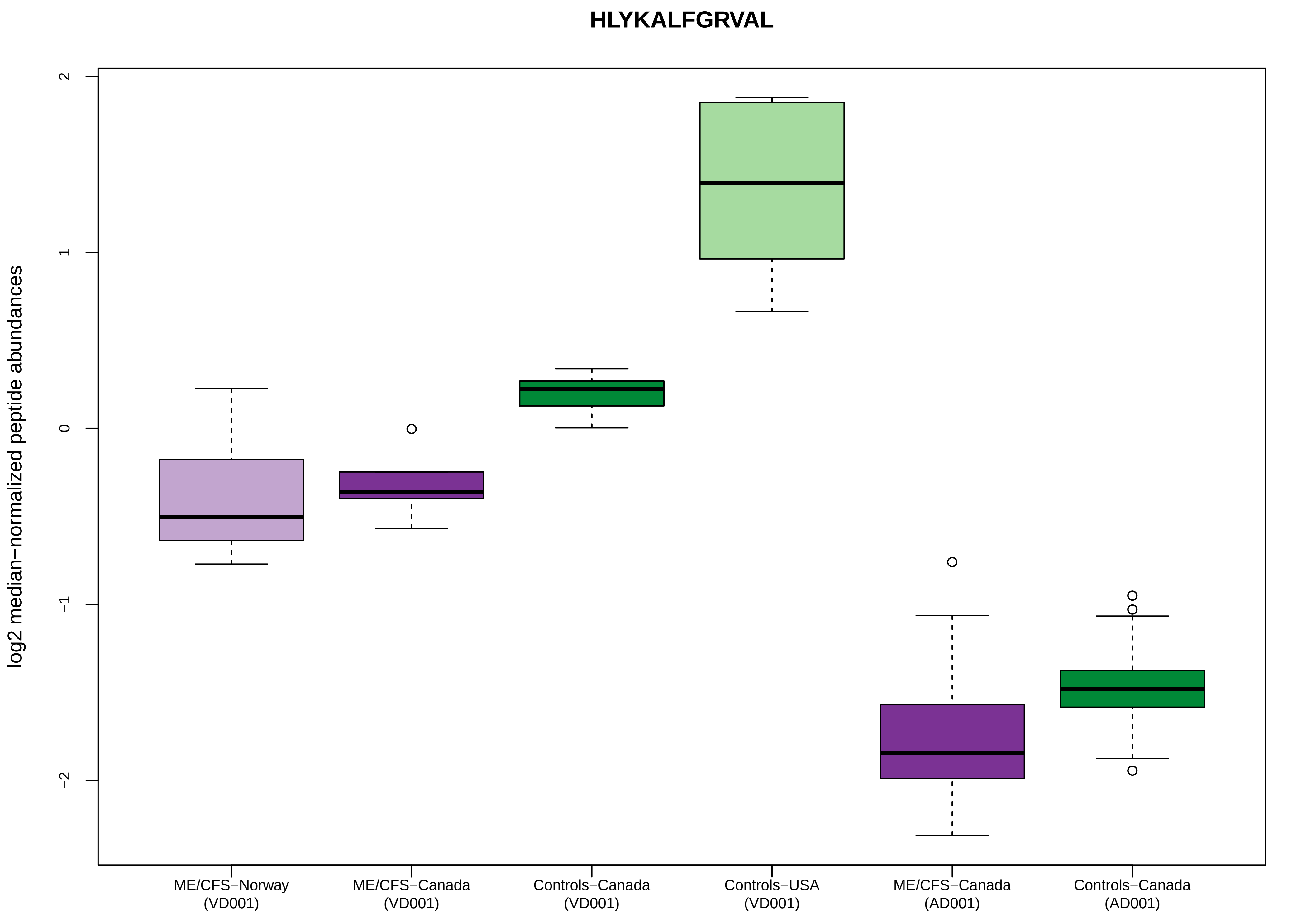
HLYKALFGRVAL
log2 median−normalized peptide abundances
2
1
0
−1
−2
ME/CFS−Norway (VD001)
ME/CFS−Canada (VD001)
Controls−Canada (VD001)
Controls−USA (VD001)
ME/CFS−Canada (AD001)
Controls−Canada (AD001)

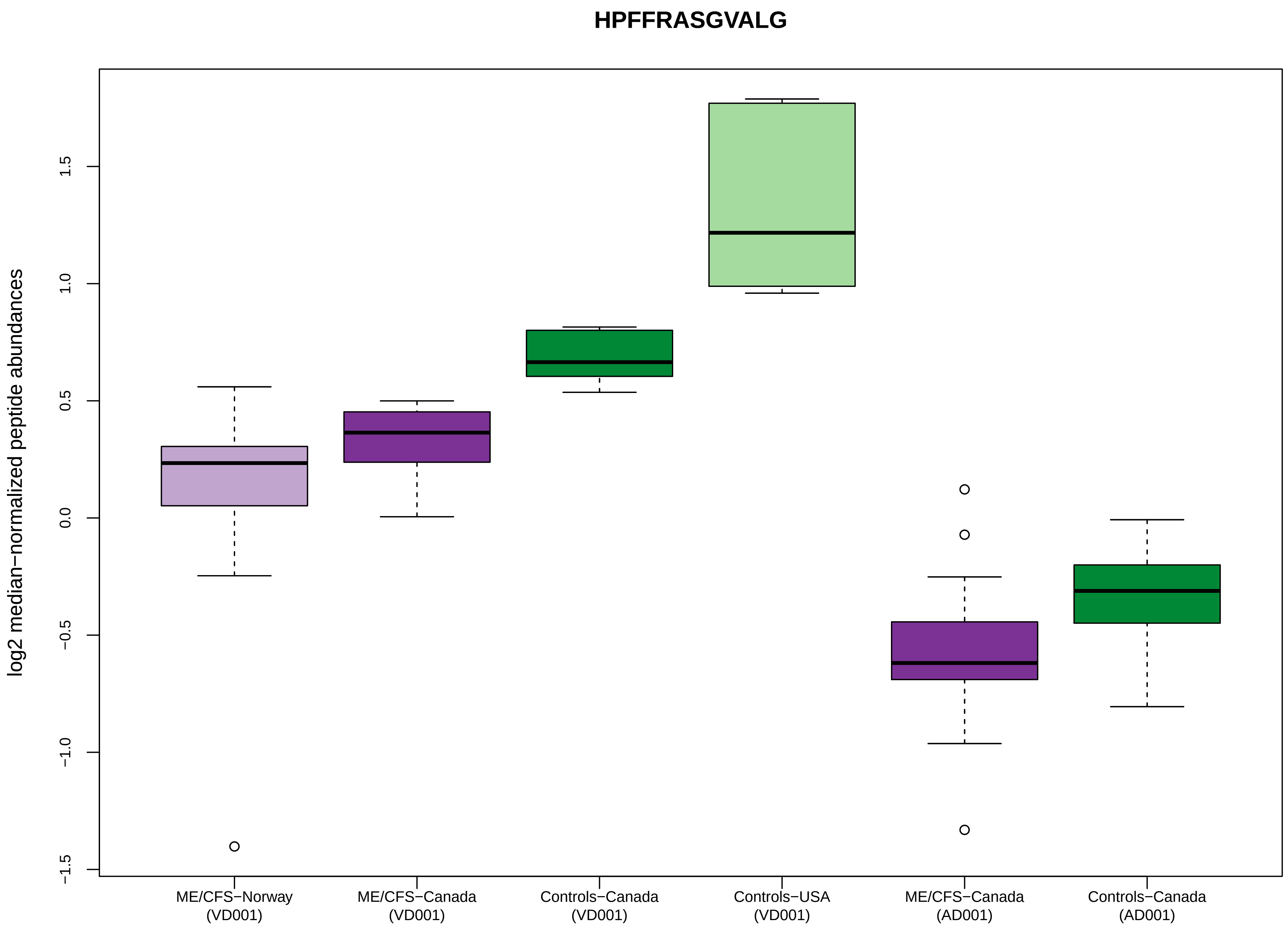
HPFFRASGVALG
log2 median−normalized peptide abundances
1.5
1.0
0.5
0.0
−0.5
−1.0
−1.5
ME/CFS−Norway (VD001)
ME/CFS−Canada (VD001)
Controls−Canada (VD001)
Controls−USA (VD001)
ME/CFS−Canada (AD001)
Controls−Canada (AD001)

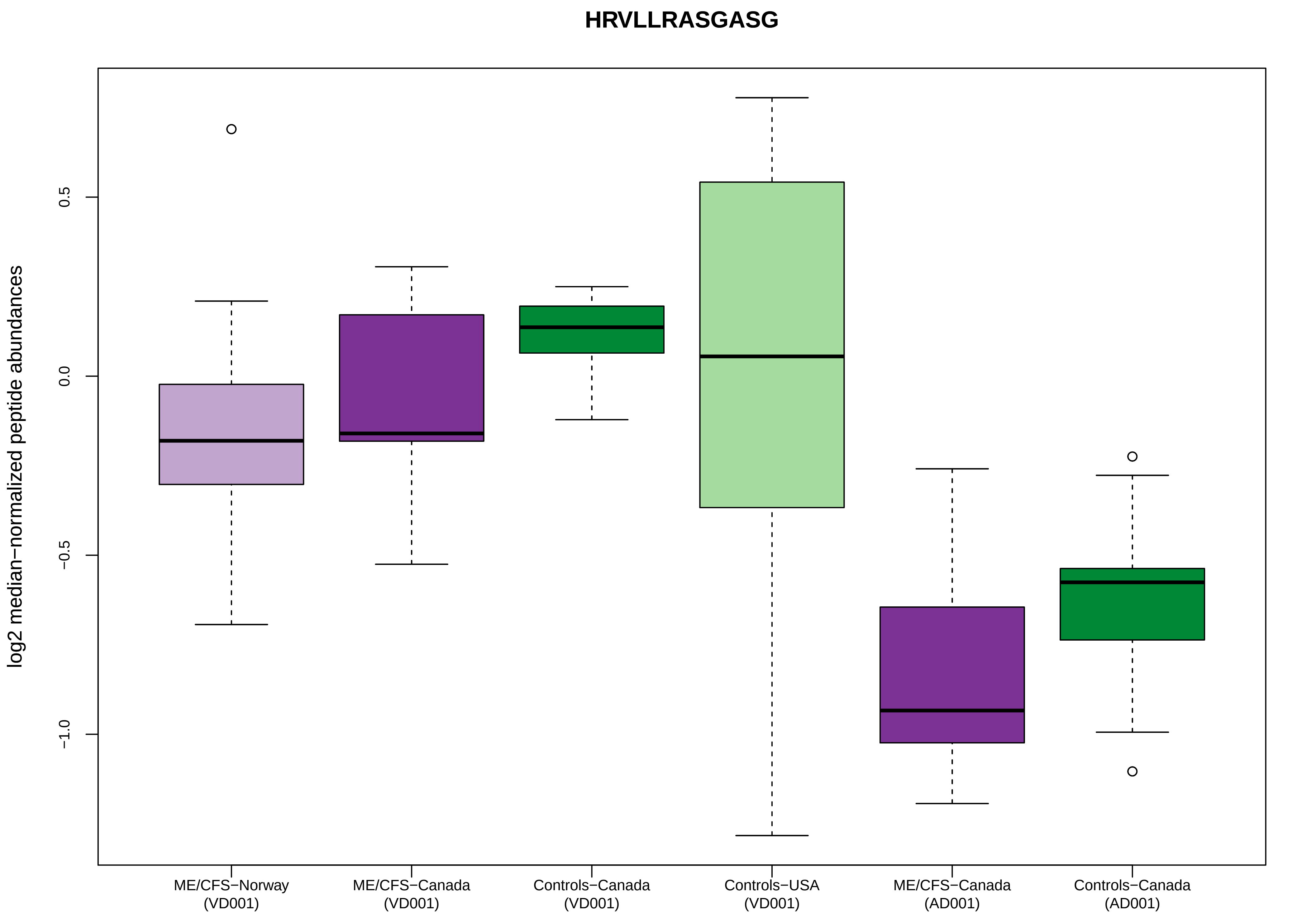
HRVLLRASGASG
log2 median−normalized peptide abundances
0.5
0.0
−0.5
−1.0
ME/CFS−Norway (VD001)
ME/CFS−Canada (VD001)
Controls−Canada (VD001)
Controls−USA (VD001)
ME/CFS−Canada (AD001)
Controls−Canada (AD001)

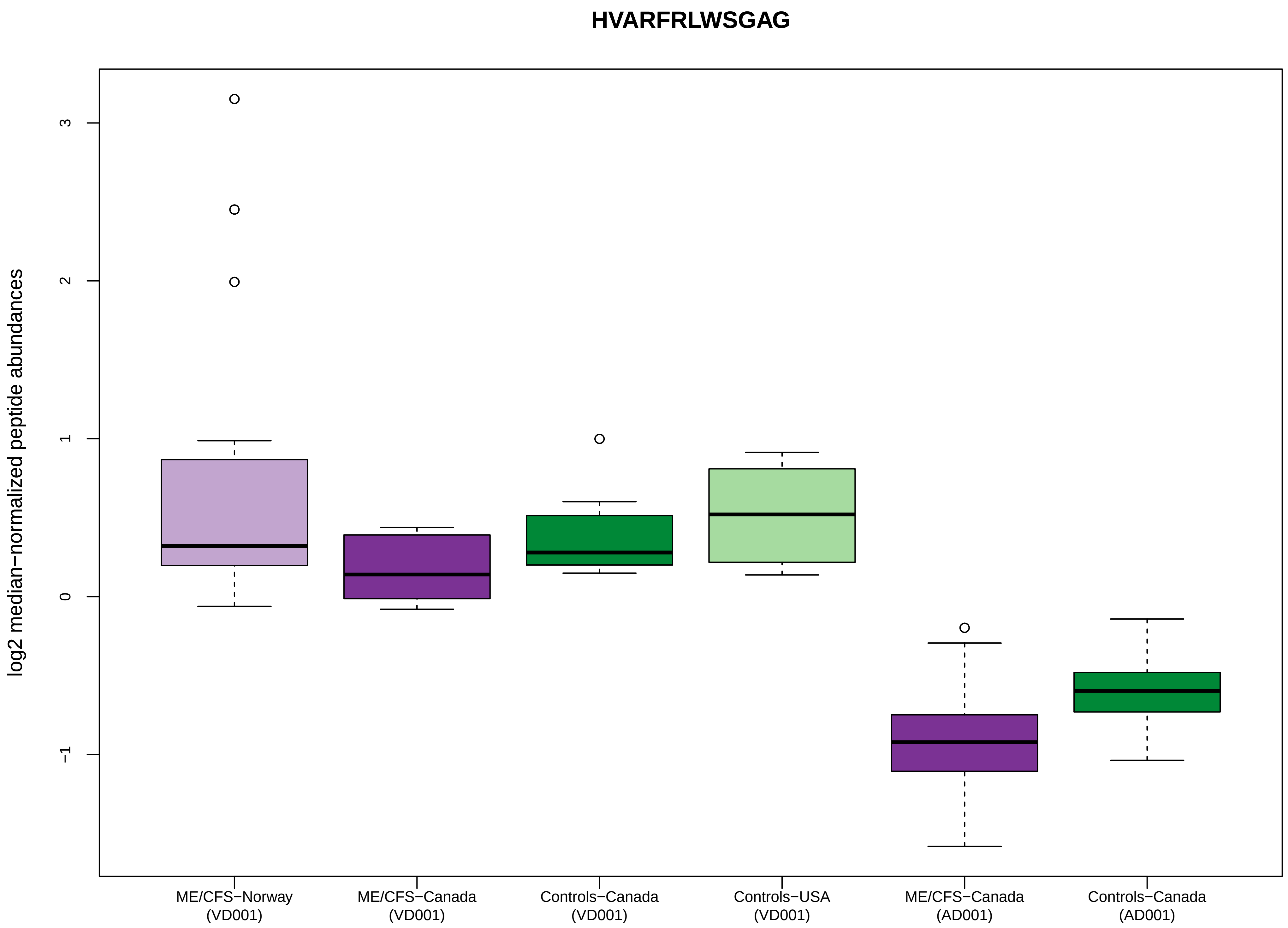
HVARFRLWSGAG
log2 median−normalized peptide abundances
3
2
1
0
−1
ME/CFS−Norway (VD001)
ME/CFS−Canada (VD001)
Controls−Canada (VD001)
Controls−USA (VD001)
ME/CFS−Canada (AD001)
Controls−Canada (AD001)

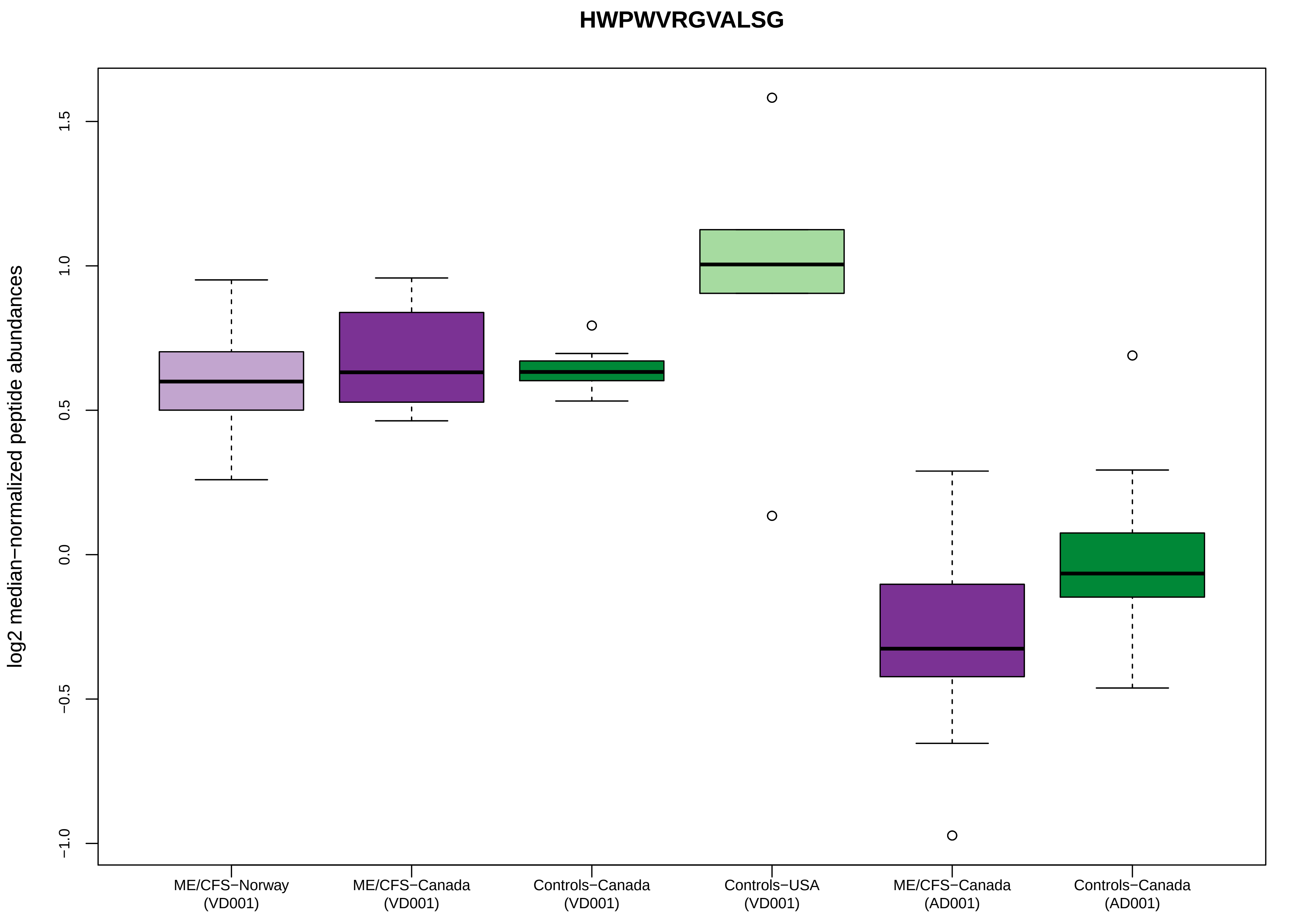
HWPWVRGVALSG
log2 median−normalized peptide abundances
−1.0
−0.5
0.0
0.5
1.0
1.5
ME/CFS−Norway (VD001)
ME/CFS−Canada (VD001)
Controls−Canada (VD001)
Controls−USA (VD001)
ME/CFS−Canada (AD001)
Controls−Canada (AD001)

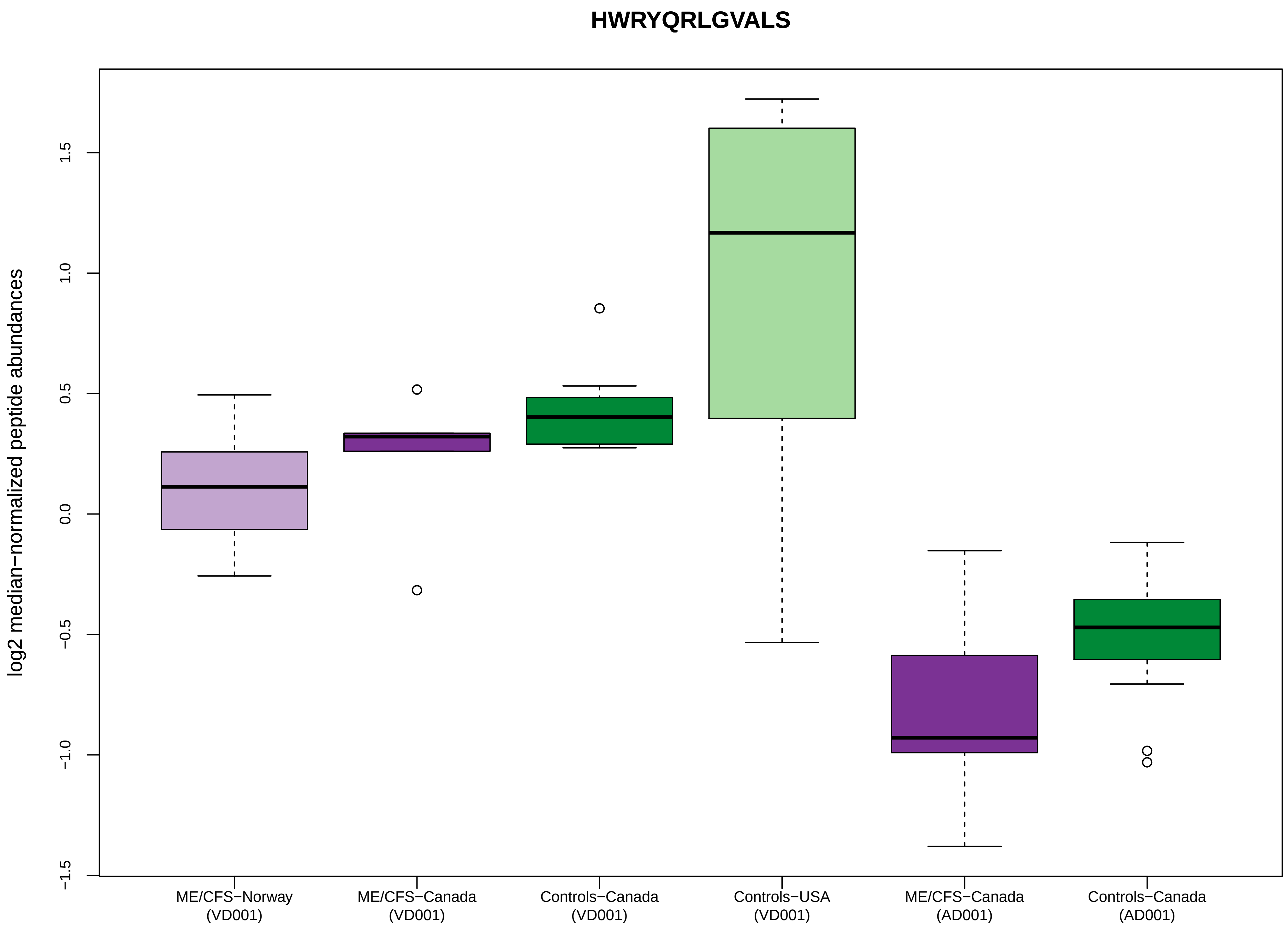
HWRYQRLGVALS
log2 median−normalized peptide abundances
1.5
1.0
0.5
0.0
−0.5
−1.0
−1.5
ME/CFS−Norway (VD001)
ME/CFS−Canada (VD001)
Controls−Canada (VD001)
Controls−USA (VD001)
ME/CFS−Canada (AD001)
Controls−Canada (AD001)

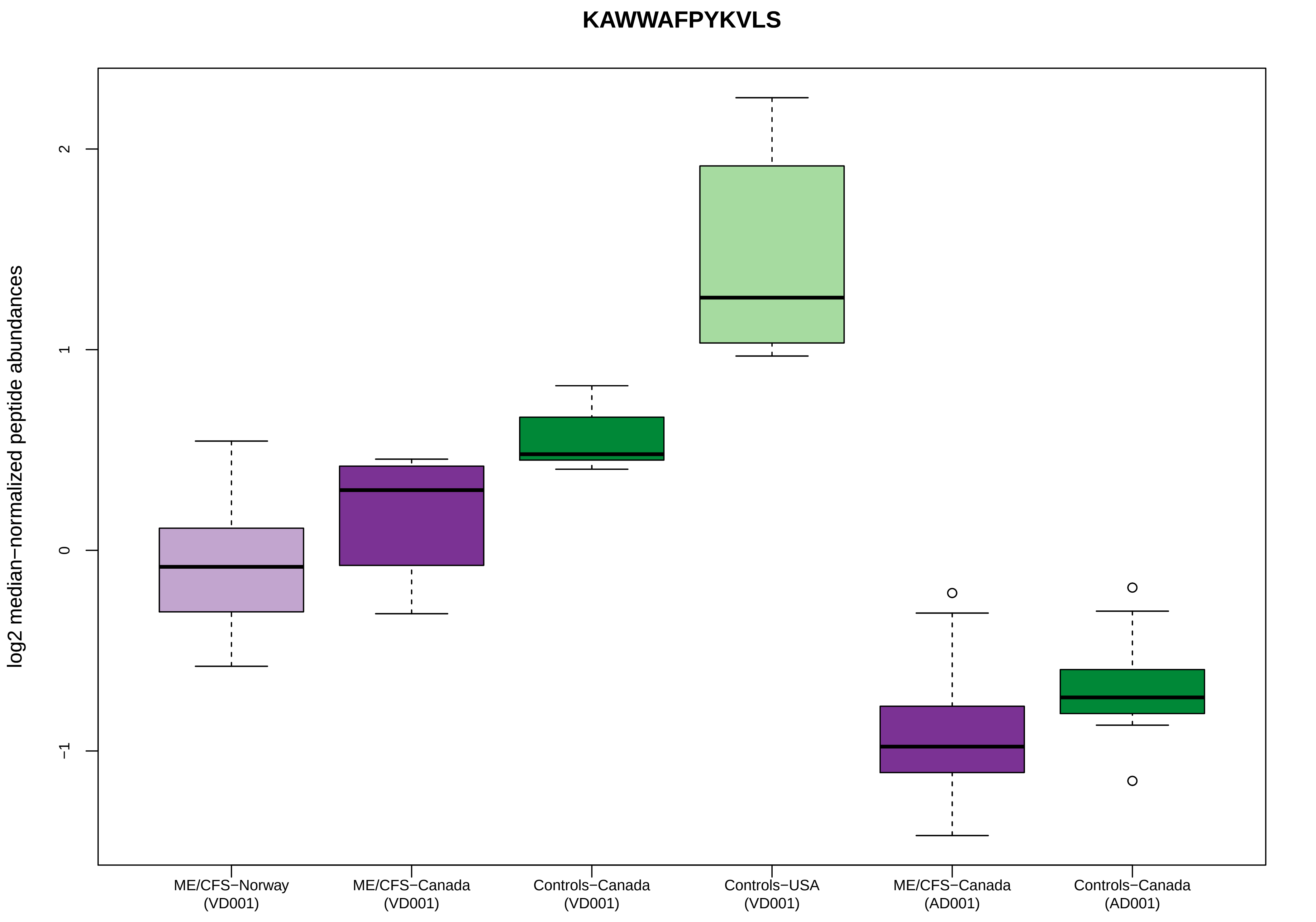
KAWWAFPYKVLS
log2 median−normalized peptide abundances
2
1
0
−1
ME/CFS−Norway (VD001)
ME/CFS−Canada (VD001)
Controls−Canada (VD001)
Controls−USA (VD001)
ME/CFS−Canada (AD001)
Controls−Canada (AD001)

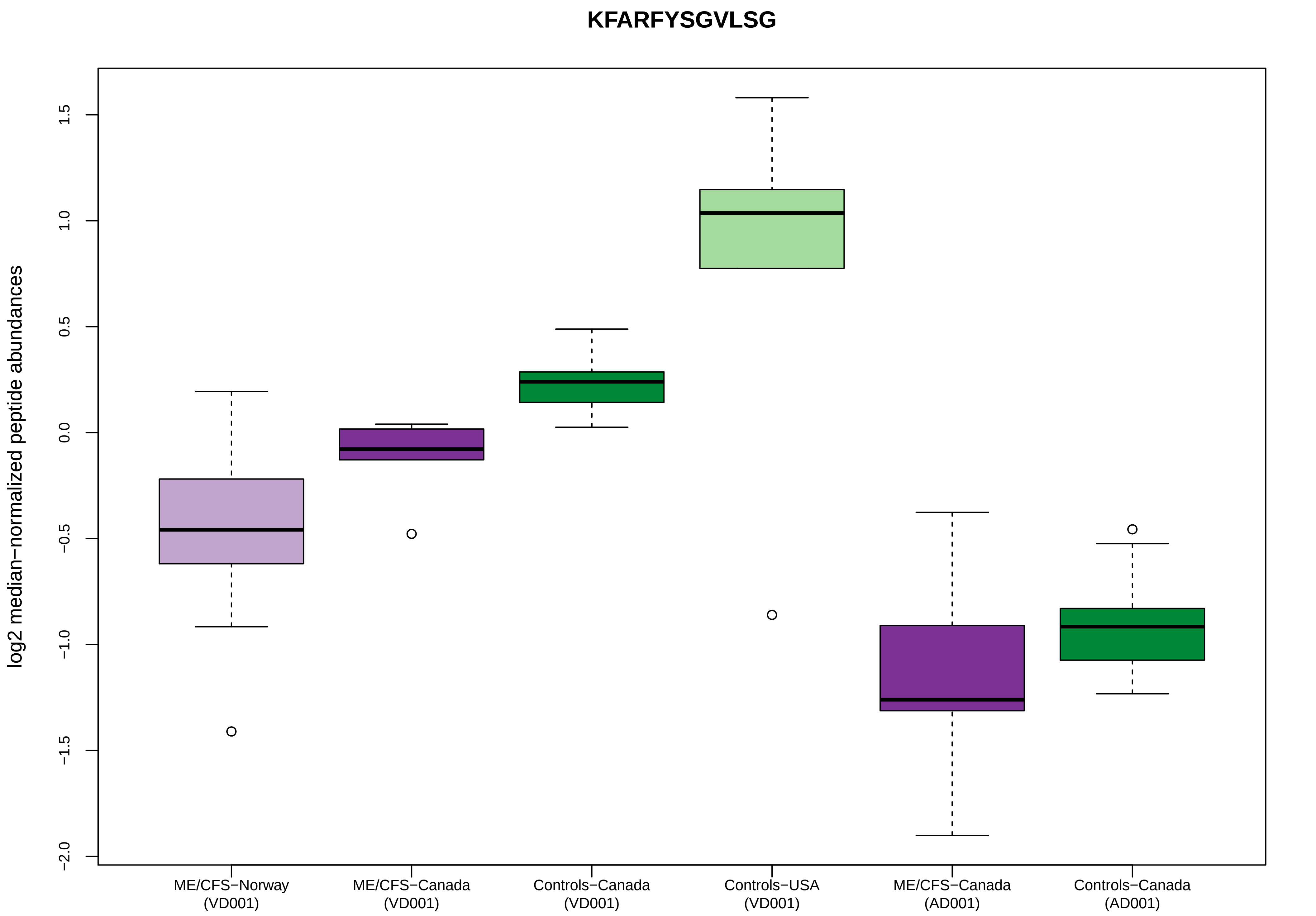

KFARFYSGVLSG
log2 median−normalized peptide abundances
1.5
1.0
0.5
0.0
−0.5
−1.0
−1.5
−2.0
ME/CFS−Norway (VD001)
ME/CFS−Canada (VD001)
Controls−Canada (VD001)
Controls−USA (VD001)
ME/CFS−Canada (AD001)
Controls−Canada (AD001)

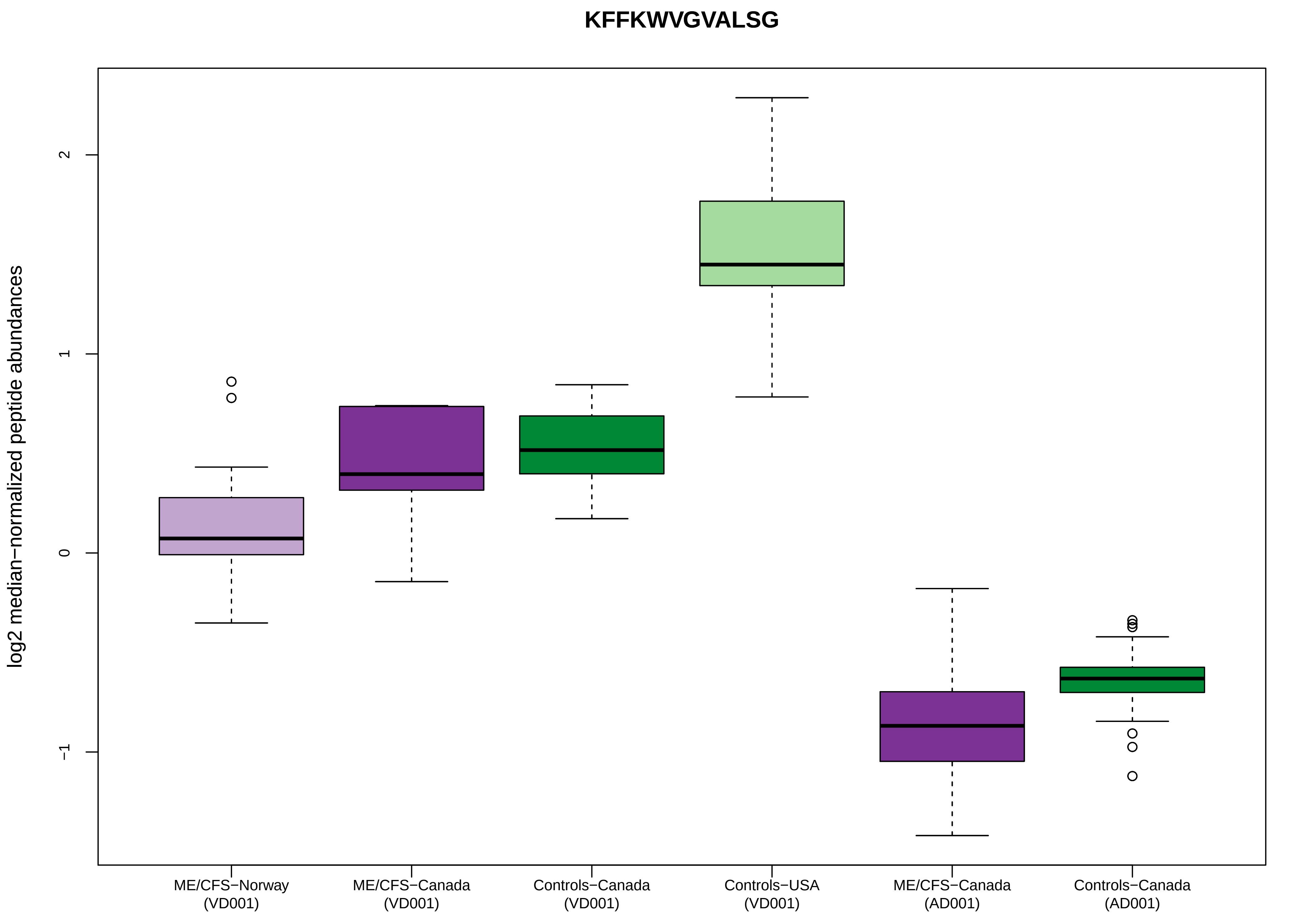

KFFKWVGVALSG
log2 median−normalized peptide abundances
2
1
0
−1
ME/CFS−Norway (VD001)
ME/CFS−Canada (VD001)
Controls−Canada (VD001)
Controls−USA (VD001)
ME/CFS−Canada (AD001)
Controls−Canada (AD001)

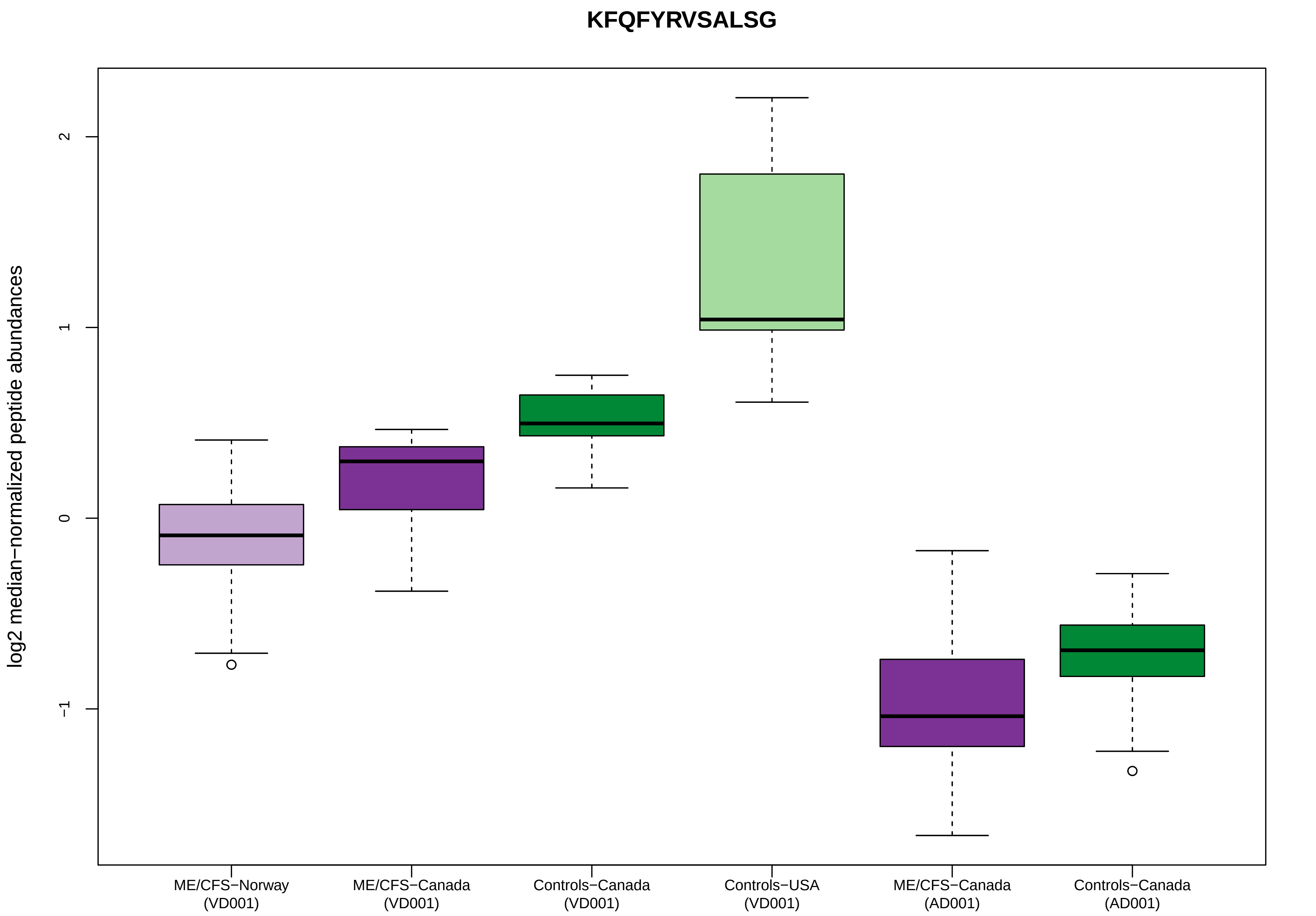
KFQFYRVSALSG
log2 median−normalized peptide abundances
2
1
0
−1
ME/CFS−Norway (VD001)
ME/CFS−Canada (VD001)
Controls−Canada (VD001)
Controls−USA (VD001)
ME/CFS−Canada (AD001)
Controls−Canada (AD001)

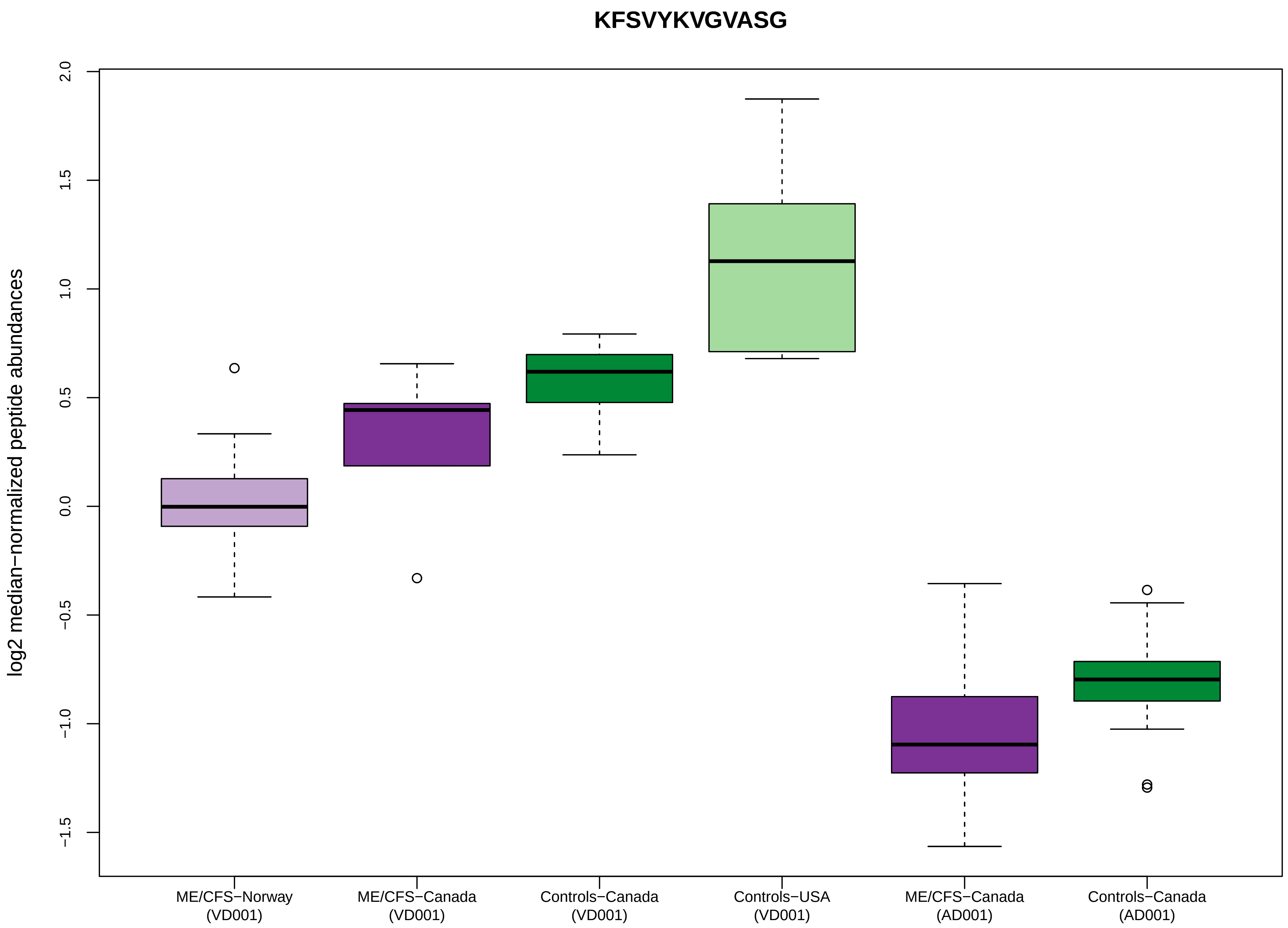
KFSVYKVGVASG
log2 median−normalized peptide abundances
2.0
1.5
1.0
0.5
0.0
−0.5
−1.0
−1.5
ME/CFS−Norway (VD001)
ME/CFS−Canada (VD001)
Controls−Canada (VD001)
Controls−USA (VD001)
ME/CFS−Canada (AD001)
Controls−Canada (AD001)

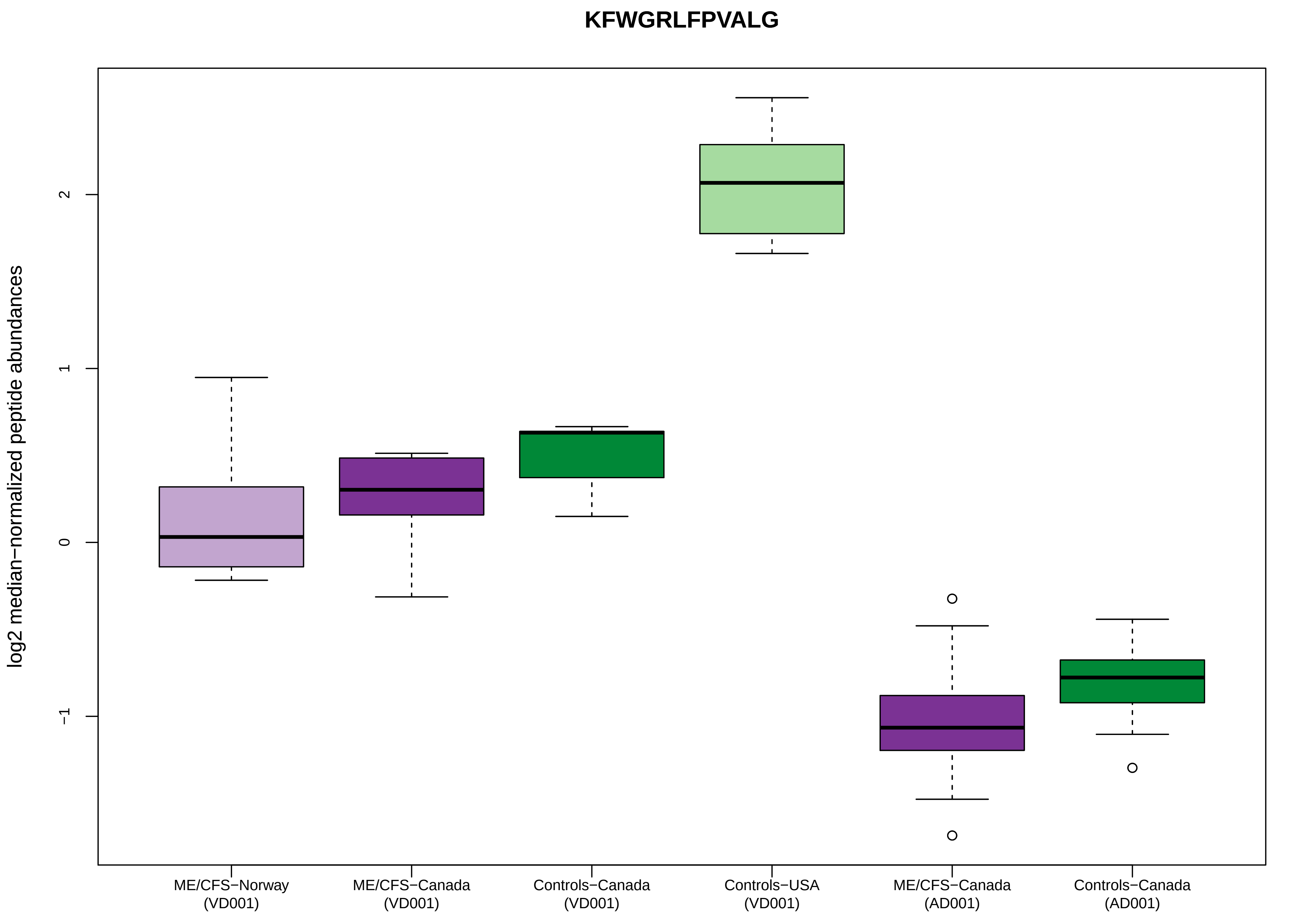

KFWGRLFPVALG
log2 median−normalized peptide abundances
2
1
0
−1
ME/CFS−Norway (VD001)
ME/CFS−Canada (VD001)
Controls−Canada (VD001)
Controls−USA (VD001)
ME/CFS−Canada (AD001)
Controls−Canada (AD001)

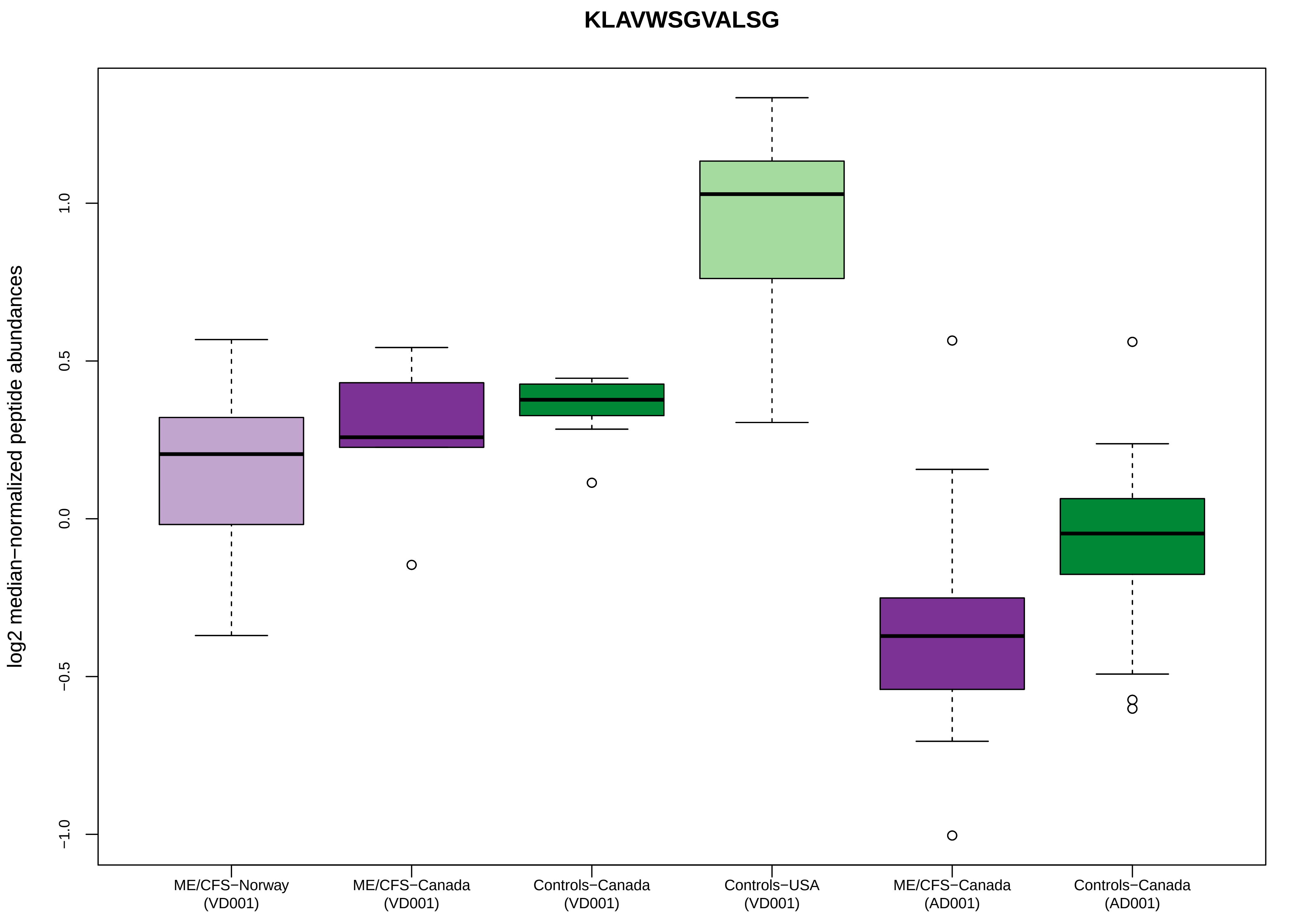
KLAVWSGVALSG
log2 median−normalized peptide abundances
1.0
0.5
0.0
−0.5
−1.0
ME/CFS−Norway (VD001)
ME/CFS−Canada (VD001)
Controls−Canada (VD001)
Controls−USA (VD001)
ME/CFS−Canada (AD001)
Controls−Canada (AD001)

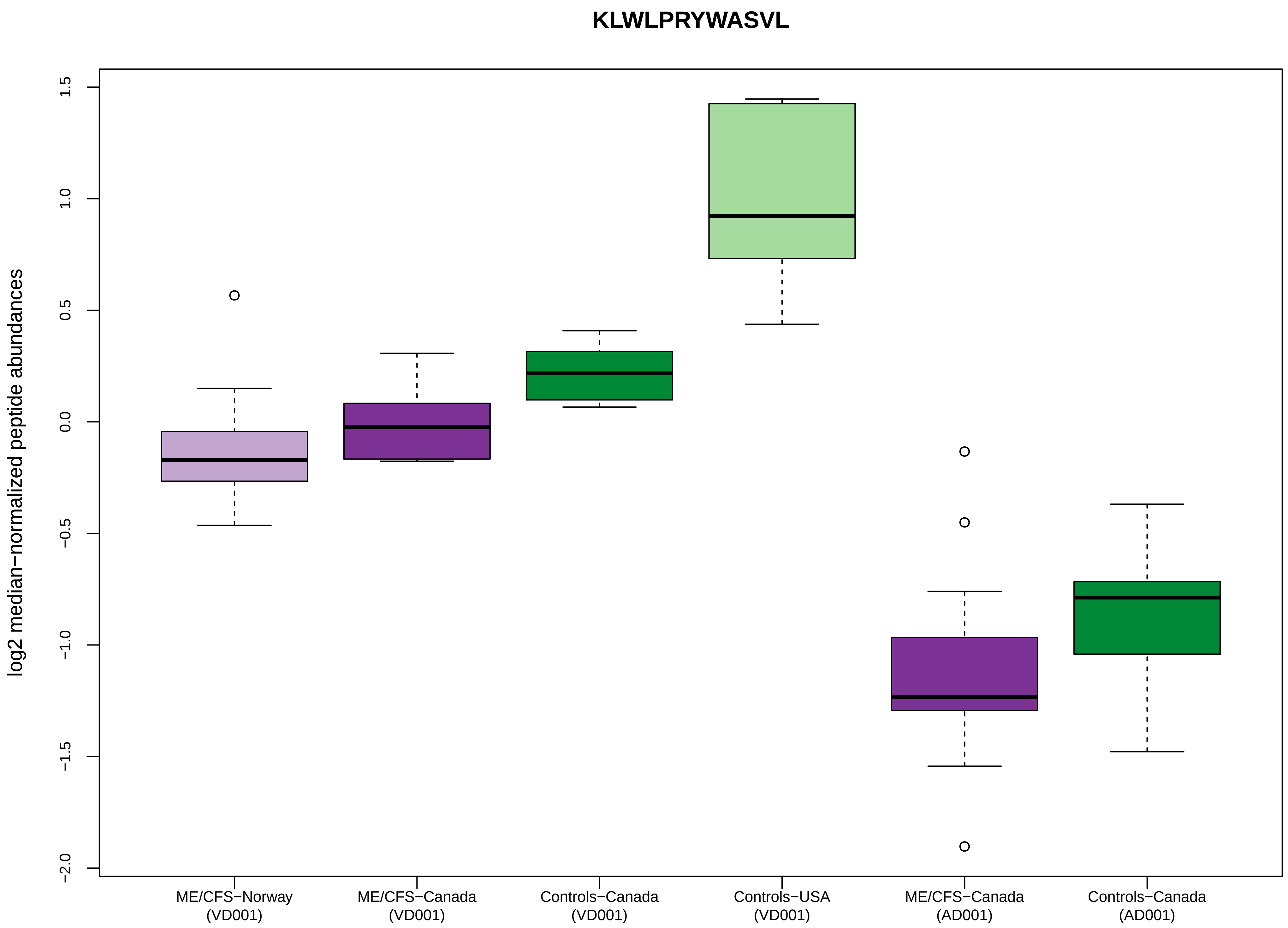
KLWLPRYWASVL
log2 median−normalized peptide abundances
1.5
1.0
0.5
0.0
−0.5
−1.0
−1.5
−2.0
ME/CFS−Norway (VD001)
ME/CFS−Canada (VD001)
Controls−Canada (VD001)
Controls−USA (VD001)
ME/CFS−Canada (AD001)
Controls−Canada (AD001)

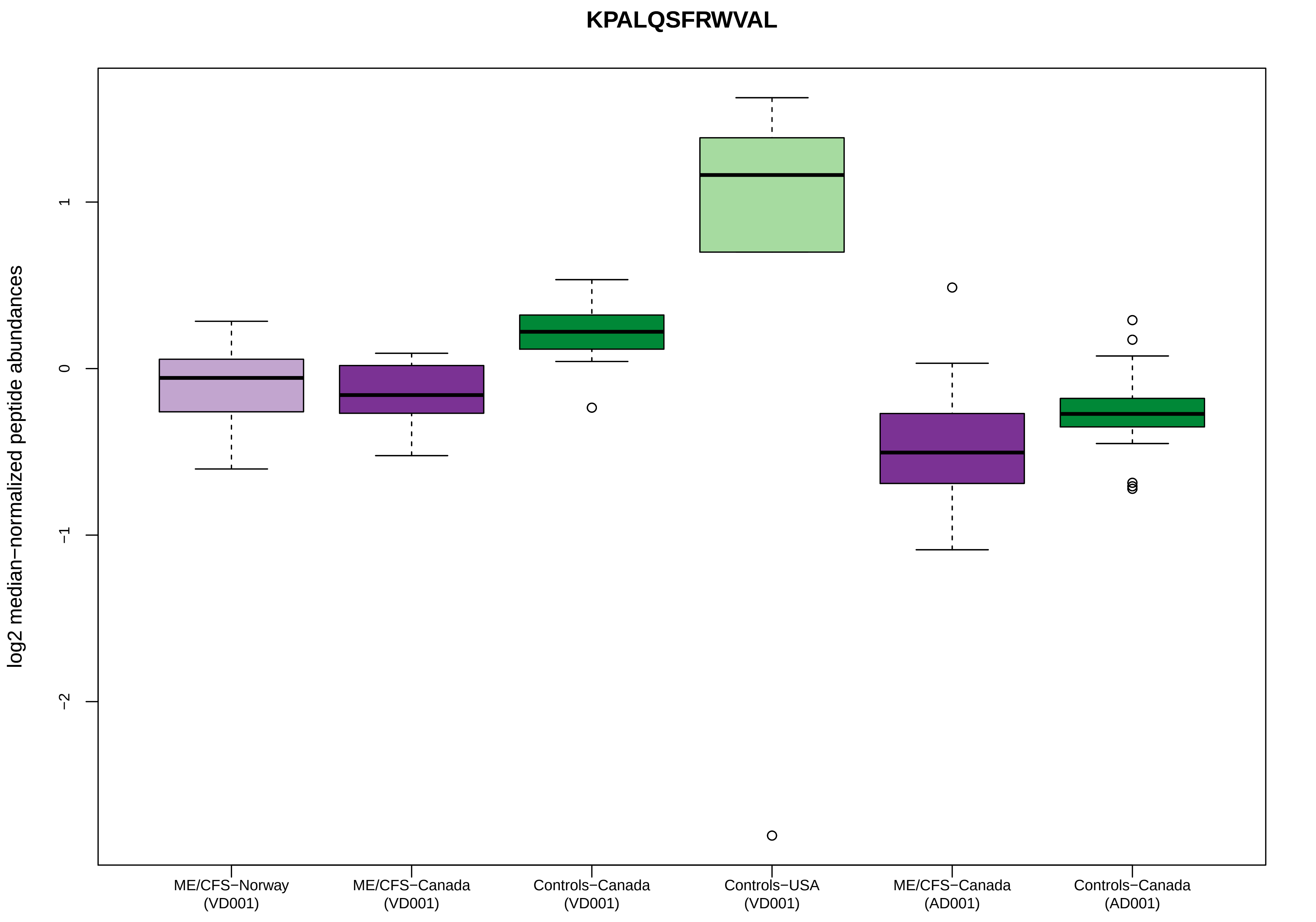

KPALQSFRWVAL
log2 median−normalized peptide abundances
1
0
−1
−2
ME/CFS−Norway (VD001)
ME/CFS−Canada (VD001)
Controls−Canada (VD001)
Controls−USA (VD001)
ME/CFS−Canada (AD001)
Controls−Canada (AD001)

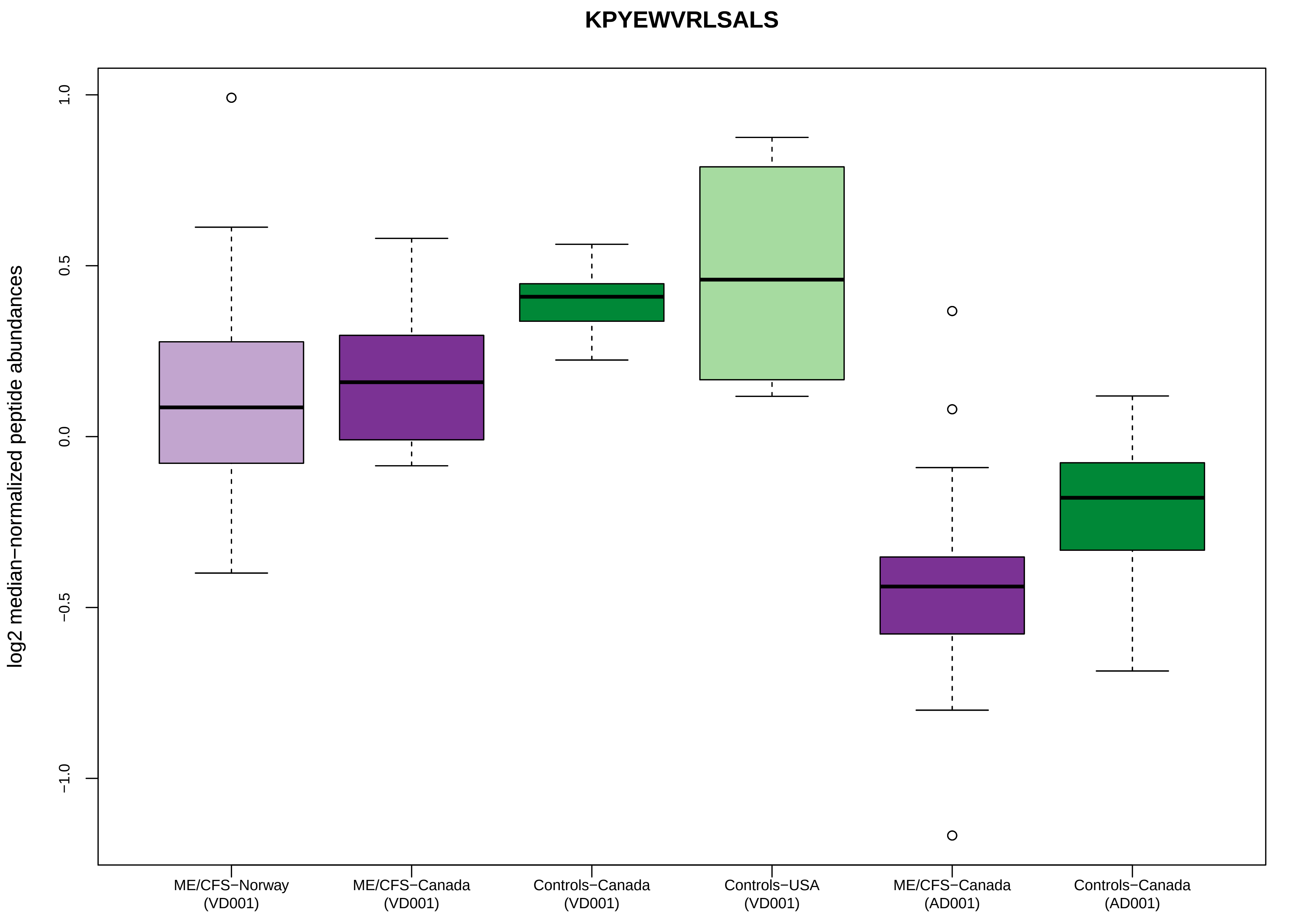

KPYEWVRLSALS
log2 median−normalized peptide abundances
1.0
0.5
0.0
−0.5
−1.0
ME/CFS−Norway (VD001)
ME/CFS−Canada (VD001)
Controls−Canada (VD001)
Controls−USA (VD001)
ME/CFS−Canada (AD001)
Controls−Canada (AD001)

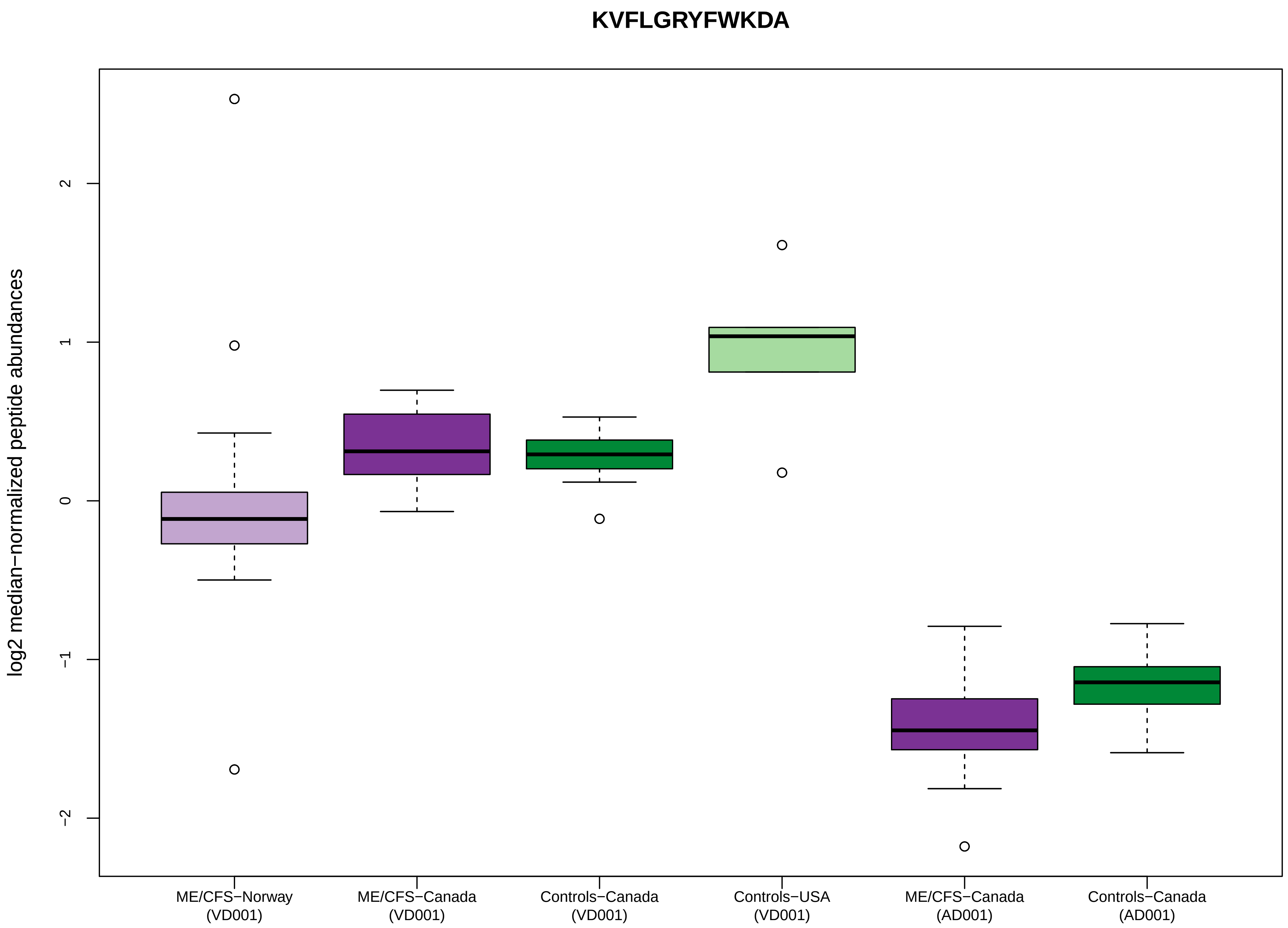
KVFLGRYFWKDA
log2 median−normalized peptide abundances
2
1
0
−1
−2
ME/CFS−Norway (VD001)
ME/CFS−Canada (VD001)
Controls−Canada (VD001)
Controls−USA (VD001)
ME/CFS−Canada (AD001)
Controls−Canada (AD001)

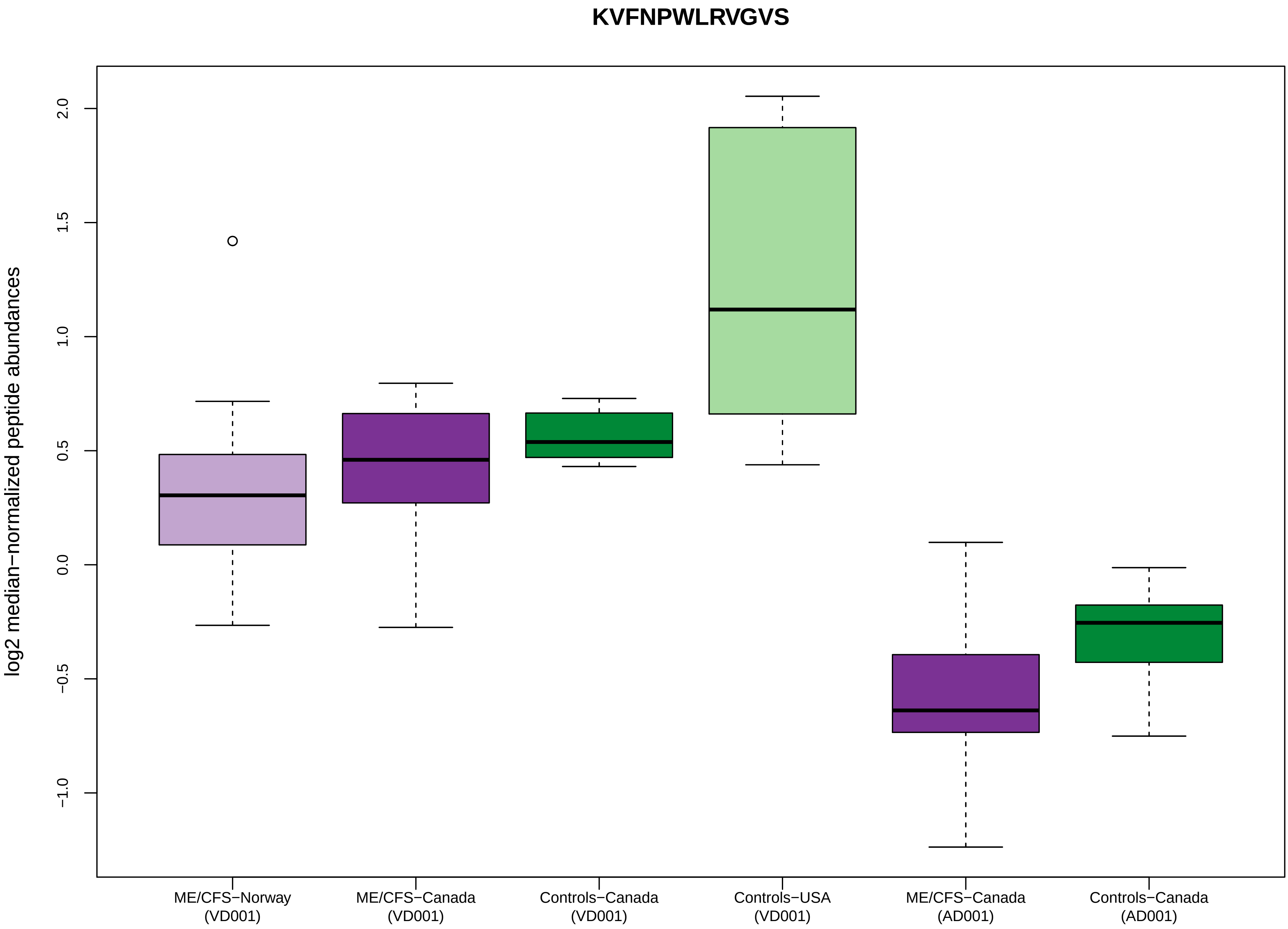
KVFNPWLRVGVS
log2 median−normalized peptide abundances
2.0
1.5
1.0
0.5
0.0
−0.5
−1.0
ME/CFS−Norway (VD001)
ME/CFS−Canada (VD001)
Controls−Canada (VD001)
Controls−USA (VD001)
ME/CFS−Canada (AD001)
Controls−Canada (AD001)

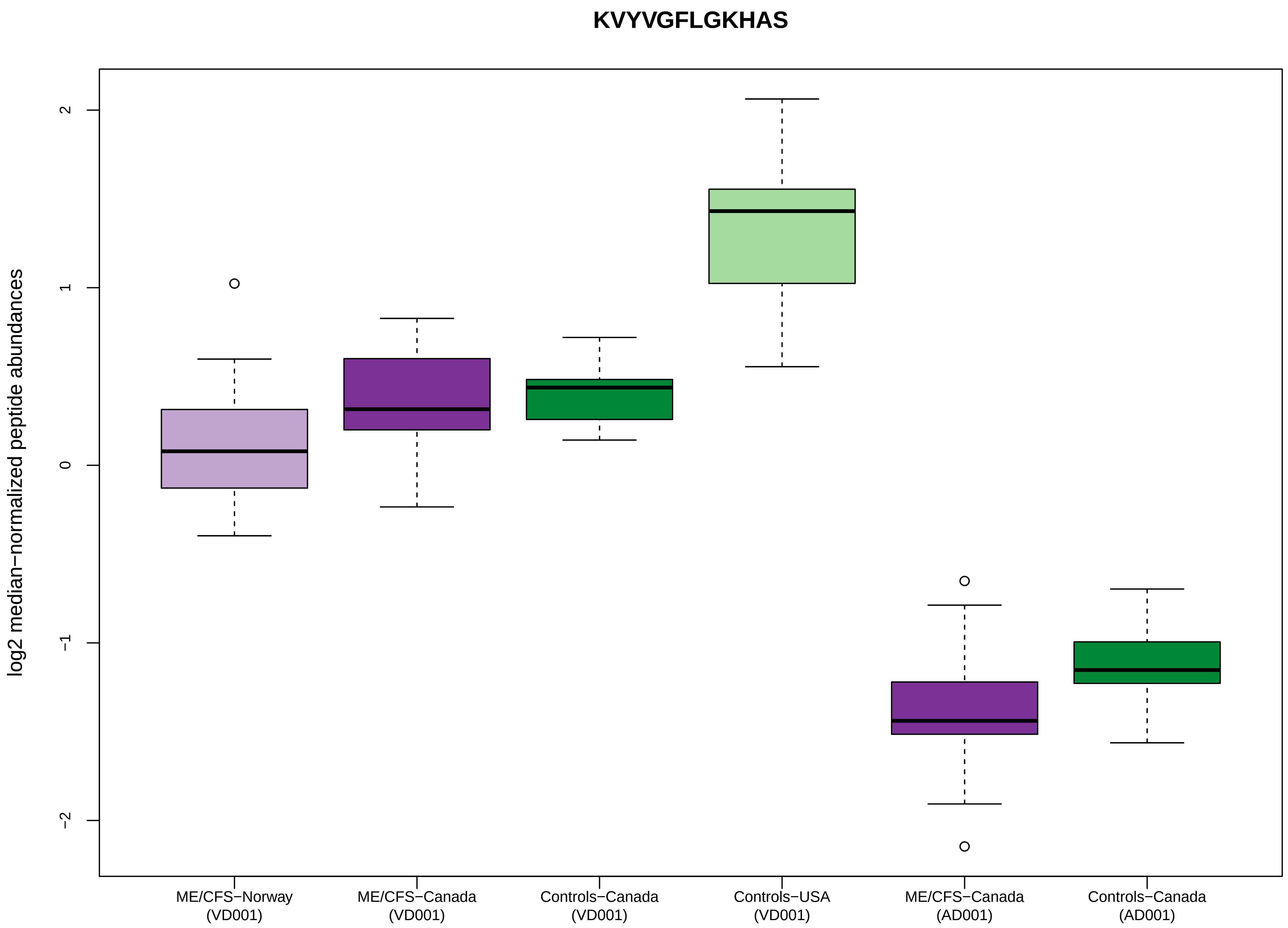
KVYVGFLGKHAS
log2 median−normalized peptide abundances
2
1
0
−1
−2
ME/CFS−Norway (VD001)
ME/CFS−Canada (VD001)
Controls−Canada (VD001)
Controls−USA (VD001)
ME/CFS−Canada (AD001)
Controls−Canada (AD001)

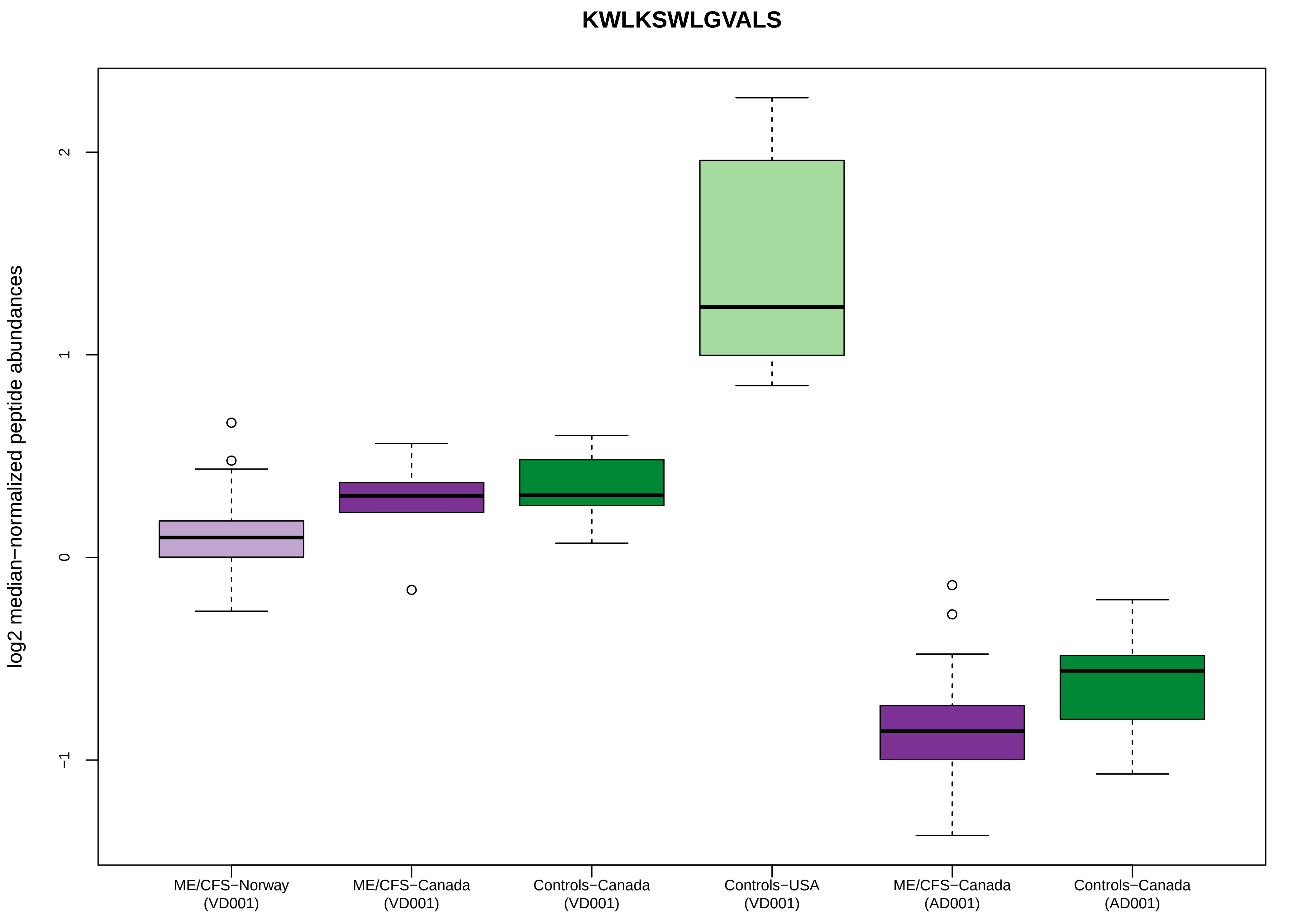
KWLKSWLGVALS
log2 median−normalized peptide abundances
2
1
0
−1
ME/CFS−Norway (VD001)
ME/CFS−Canada (VD001)
Controls−Canada (VD001)
Controls−USA (VD001)
ME/CFS−Canada (AD001)
Controls−Canada (AD001)

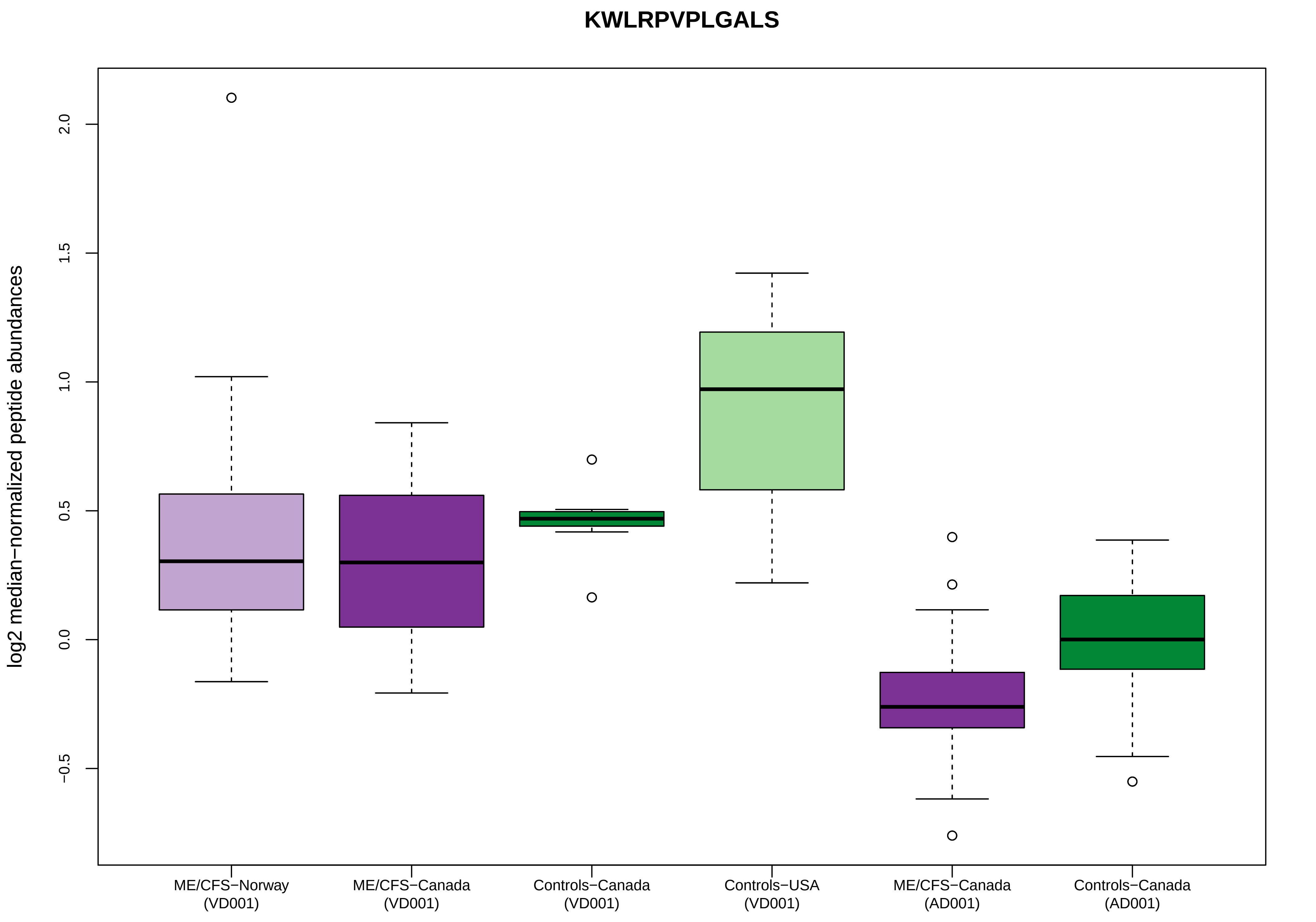

KWLRPVPLGALS
log2 median−normalized peptide abundances
2.0
1.5
1.0
0.5
0.0
−0.5
ME/CFS−Norway (VD001)
ME/CFS−Canada (VD001)
Controls−Canada (VD001)
Controls−USA (VD001)
ME/CFS−Canada (AD001)
Controls−Canada (AD001)

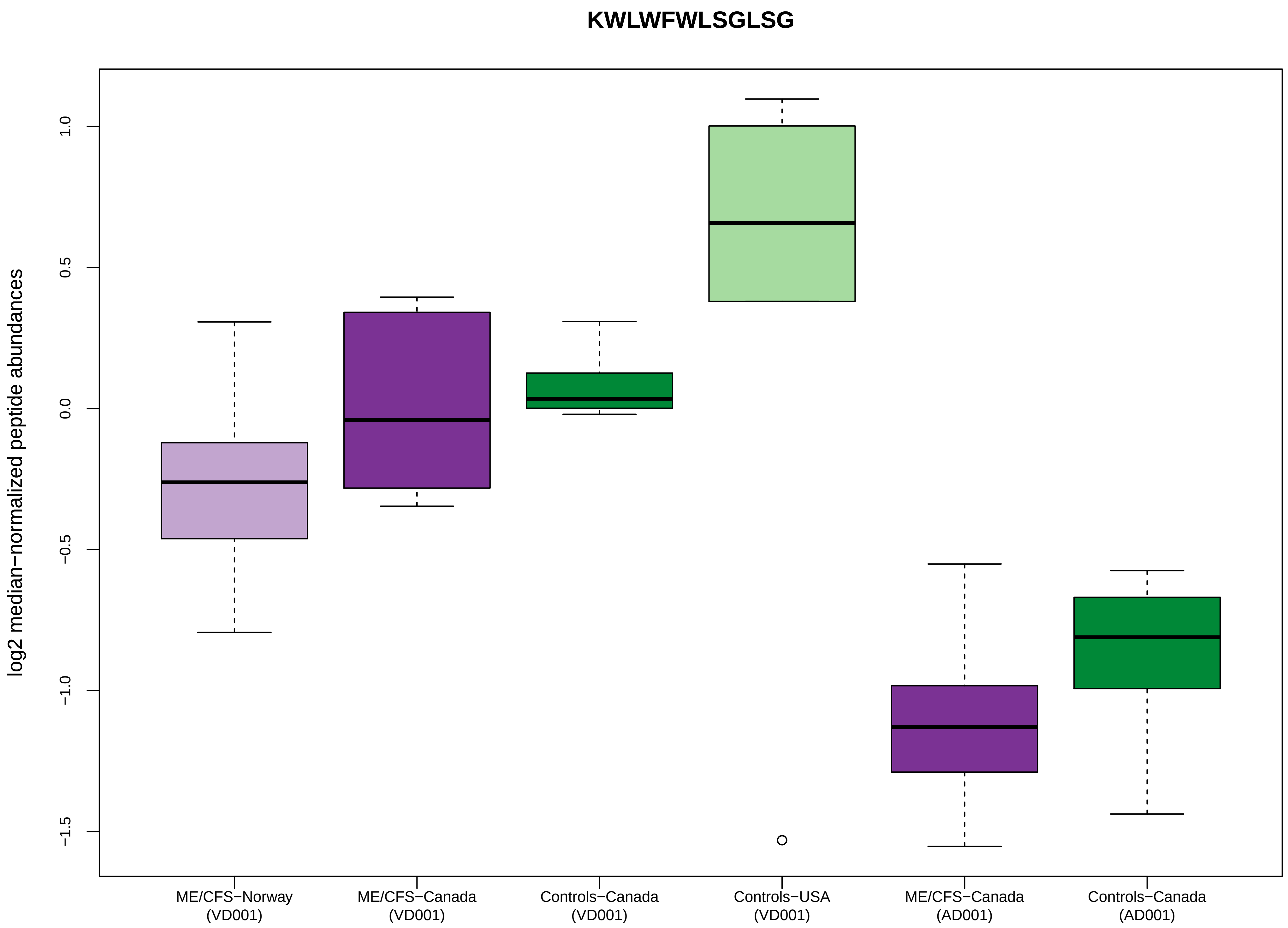

KWLWFWLSGLSG
log2 median−normalized peptide abundances
1.0
0.5
0.0
−0.5
−1.0
−1.5
ME/CFS−Norway (VD001)
ME/CFS−Canada (VD001)
Controls−Canada (VD001)
Controls−USA (VD001)
ME/CFS−Canada (AD001)
Controls−Canada (AD001)

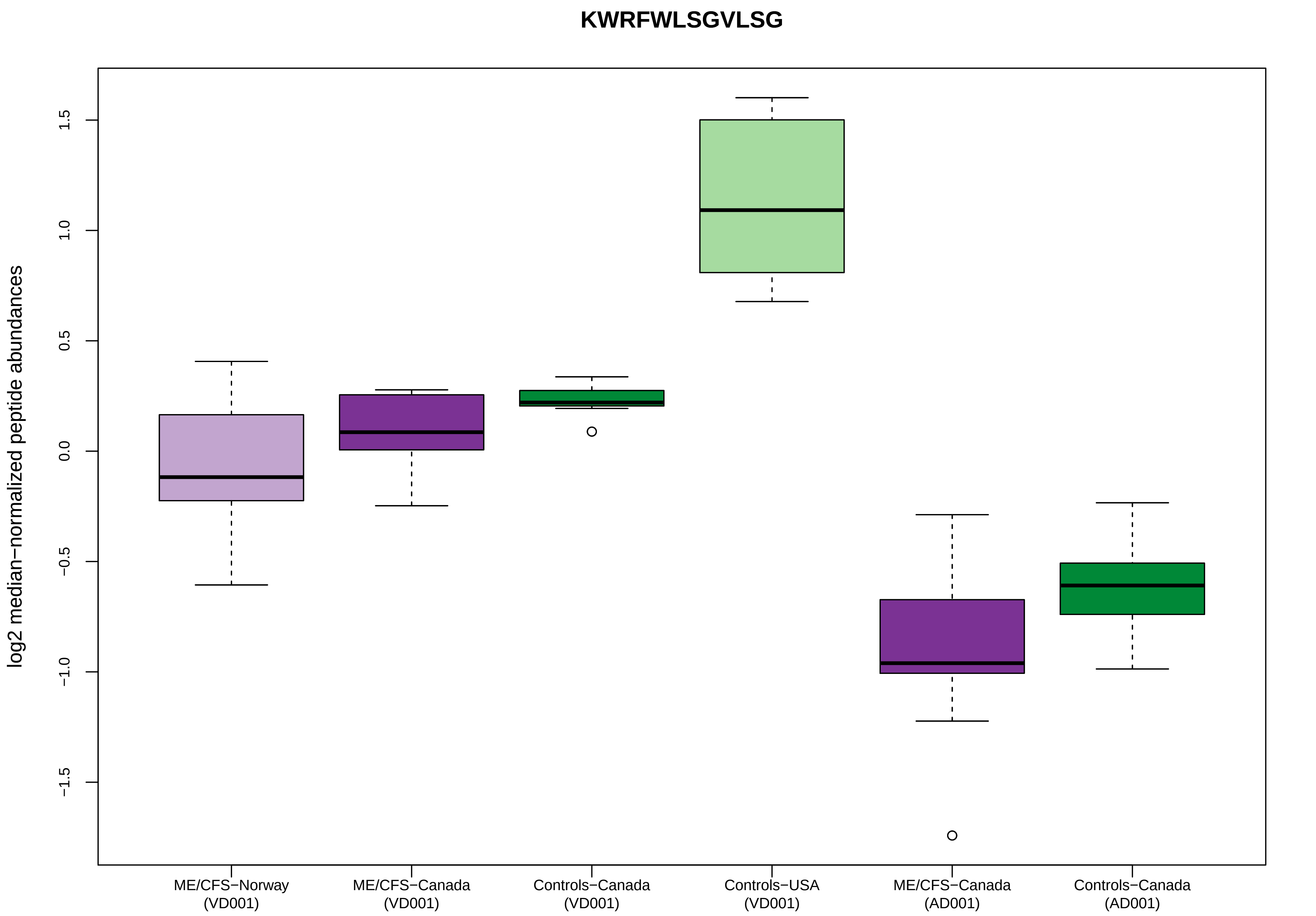

KWRFWLSGVLSG
log2 median−normalized peptide abundances
1.5
1.0
0.5
0.0
−0.5
−1.0
−1.5
ME/CFS−Norway (VD001)
ME/CFS−Canada (VD001)
Controls−Canada (VD001)
Controls−USA (VD001)
ME/CFS−Canada (AD001)
Controls−Canada (AD001)

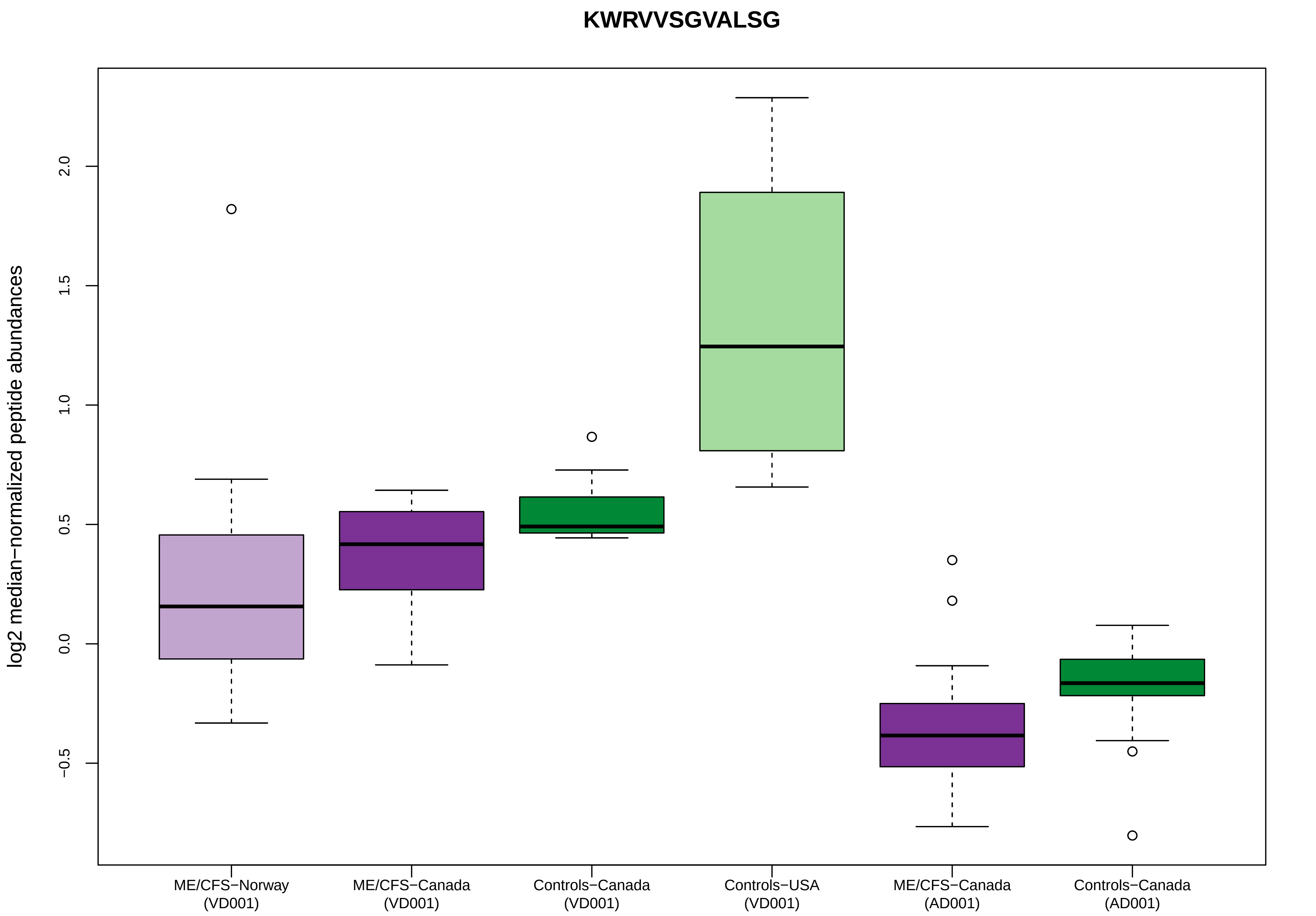

KWRVVSGVALSG
log2 median−normalized peptide abundances
−0.5
0.0
0.5
1.0
1.5
2.0
ME/CFS−Norway (VD001)
ME/CFS−Canada (VD001)
Controls−Canada (VD001)
Controls−USA (VD001)
ME/CFS−Canada (AD001)
Controls−Canada (AD001)

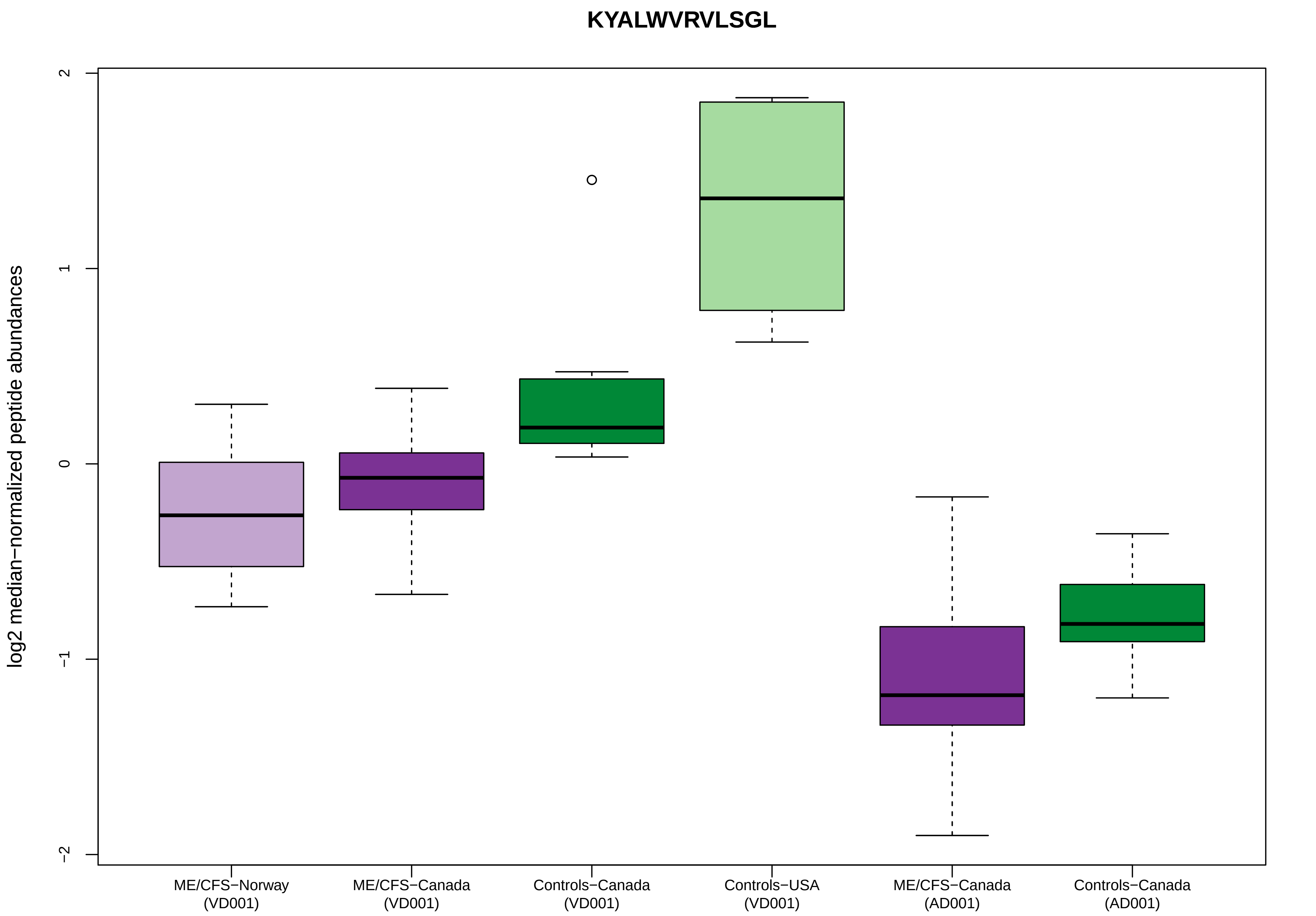

KYALWVRVLSGL
log2 median−normalized peptide abundances
2
1
0
−1
−2
ME/CFS−Norway (VD001)
ME/CFS−Canada (VD001)
Controls−Canada (VD001)
Controls−USA (VD001)
ME/CFS−Canada (AD001)
Controls−Canada (AD001)

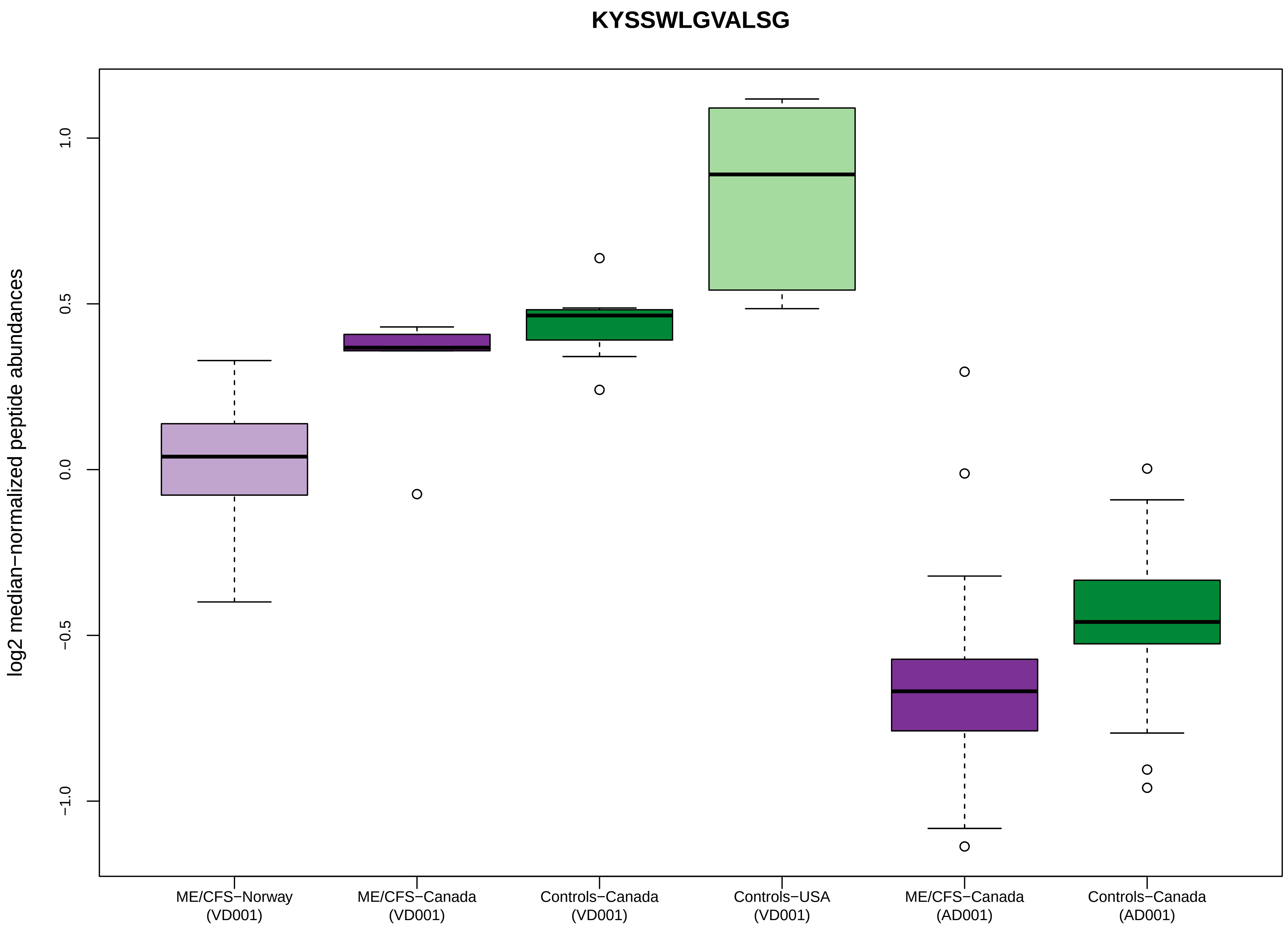
KYSSWLGVALSG
log2 median−normalized peptide abundances
1.0
0.5
0.0
−0.5
−1.0
ME/CFS−Norway (VD001)
ME/CFS−Canada (VD001)
Controls−Canada (VD001)
Controls−USA (VD001)
ME/CFS−Canada (AD001)
Controls−Canada (AD001)

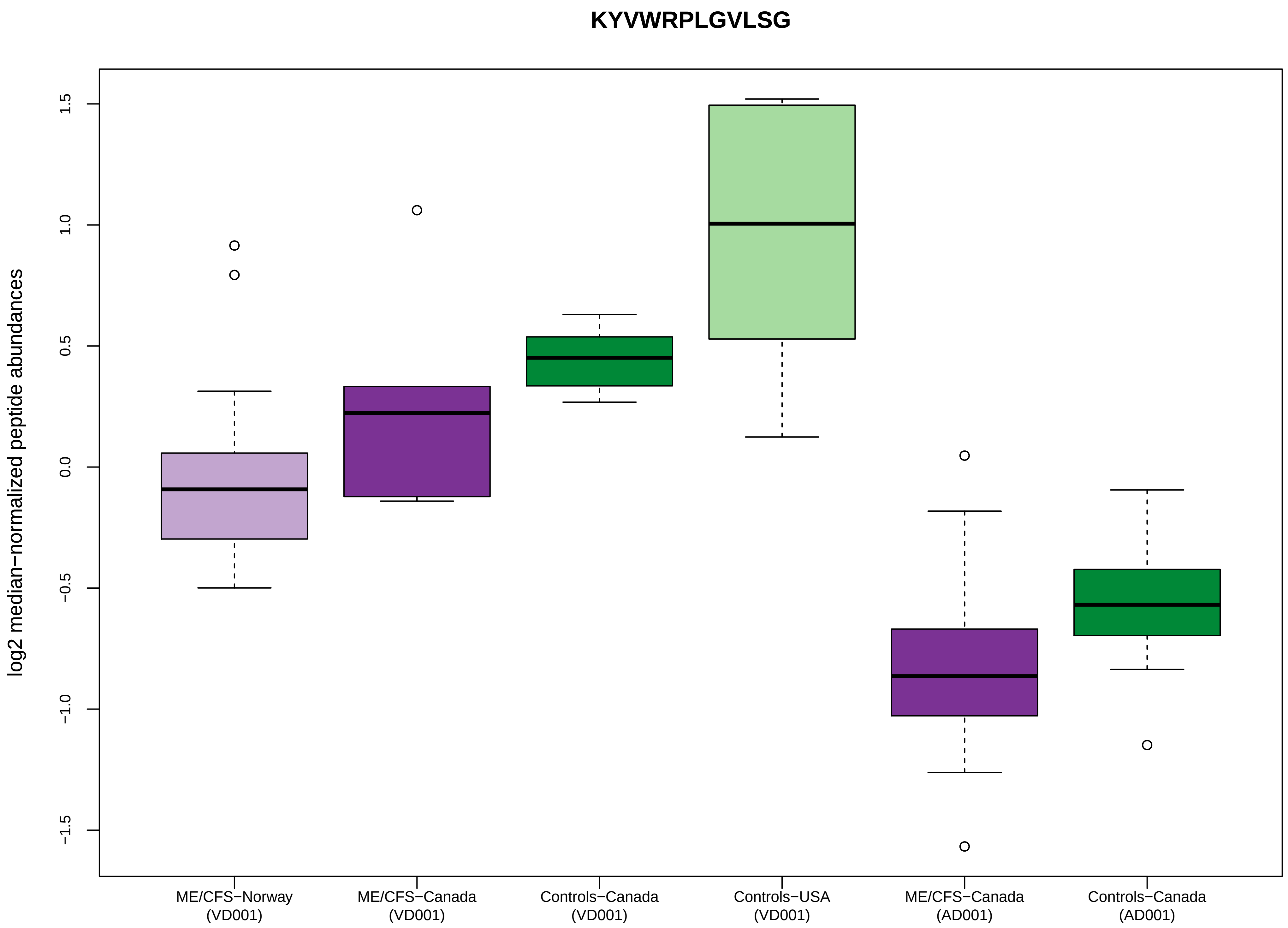

KYVWRPLGVLSG
log2 median−normalized peptide abundances
1.5
1.0
0.5
0.0
−0.5
−1.0
−1.5
ME/CFS−Norway (VD001)
ME/CFS−Canada (VD001)
Controls−Canada (VD001)
Controls−USA (VD001)
ME/CFS−Canada (AD001)
Controls−Canada (AD001)

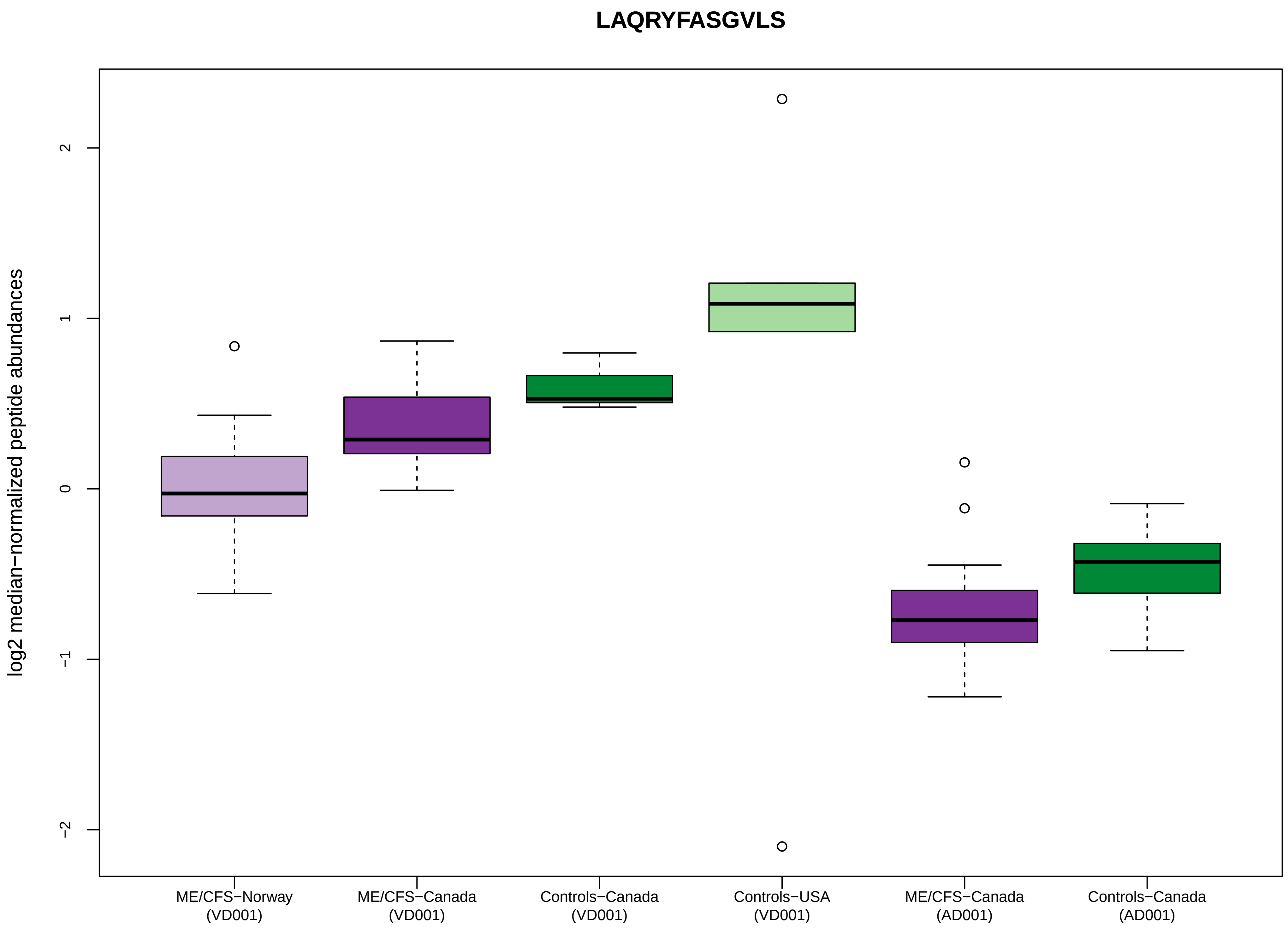
LAQRYFASGVLS
log2 median−normalized peptide abundances
2
1
0
−1
−2
ME/CFS−Norway (VD001)
ME/CFS−Canada (VD001)
Controls−Canada (VD001)
Controls−USA (VD001)
ME/CFS−Canada (AD001)
Controls−Canada (AD001)

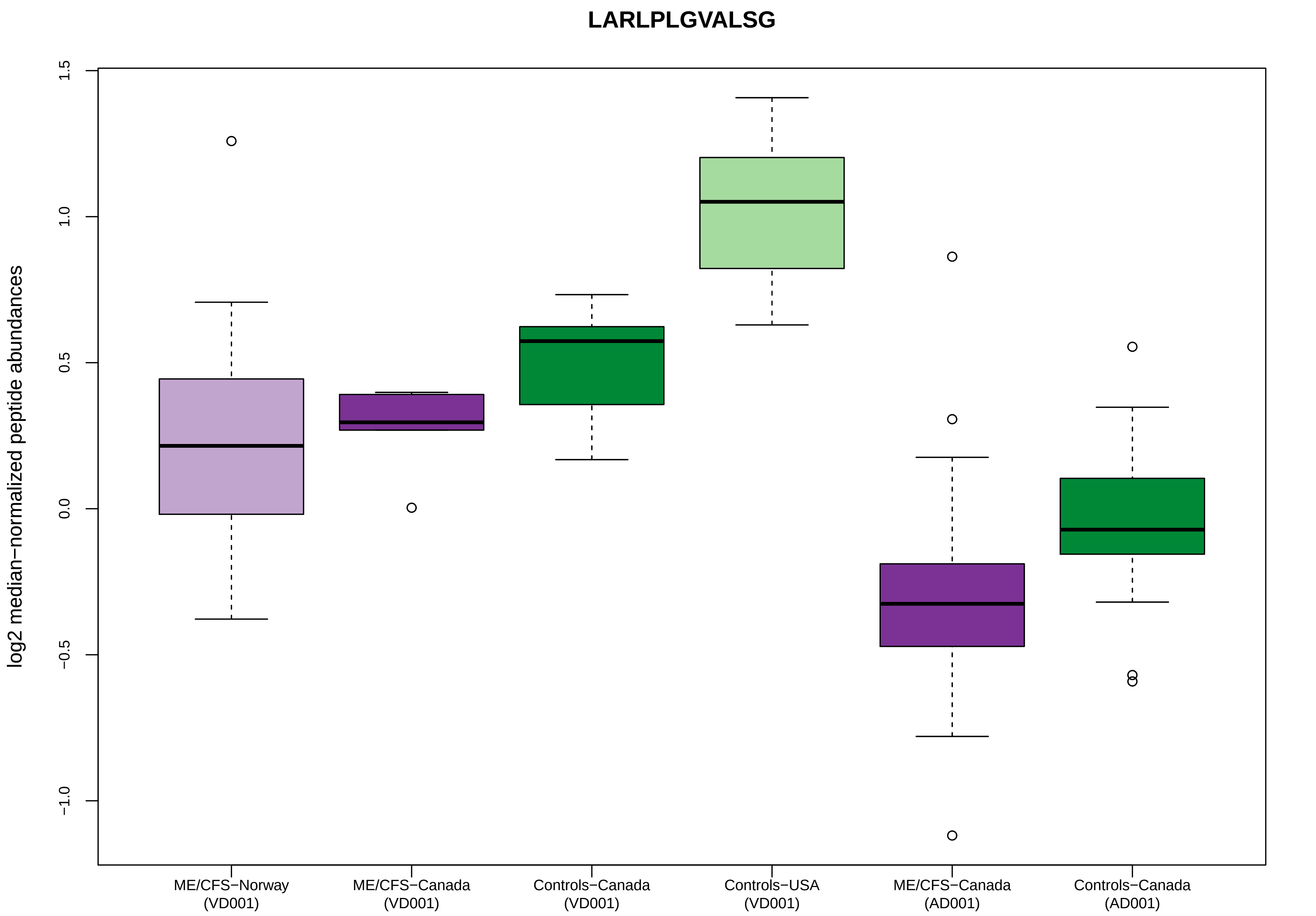

LARLPLGVALSG
log2 median−normalized peptide abundances
1.5
1.0
0.5
0.0
−0.5
−1.0
ME/CFS−Norway (VD001)
ME/CFS−Canada (VD001)
Controls−Canada (VD001)
Controls−USA (VD001)
ME/CFS−Canada (AD001)
Controls−Canada (AD001)

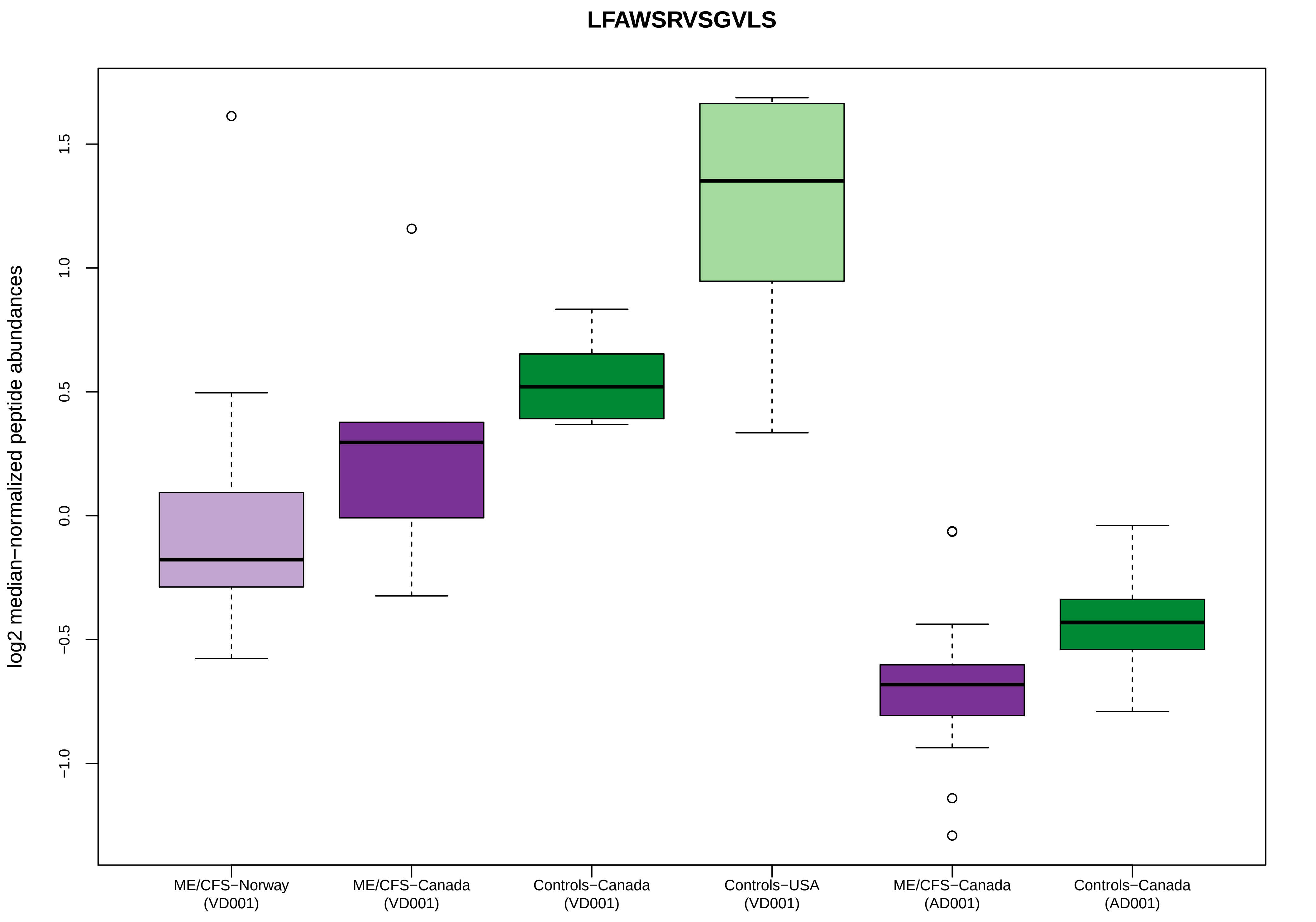
LFAWSRVSGVLS
log2 median−normalized peptide abundances
1.5
1.0
0.5
0.0
−0.5
−1.0
ME/CFS−Norway (VD001)
ME/CFS−Canada (VD001)
Controls−Canada (VD001)
Controls−USA (VD001)
ME/CFS−Canada (AD001)
Controls−Canada (AD001)

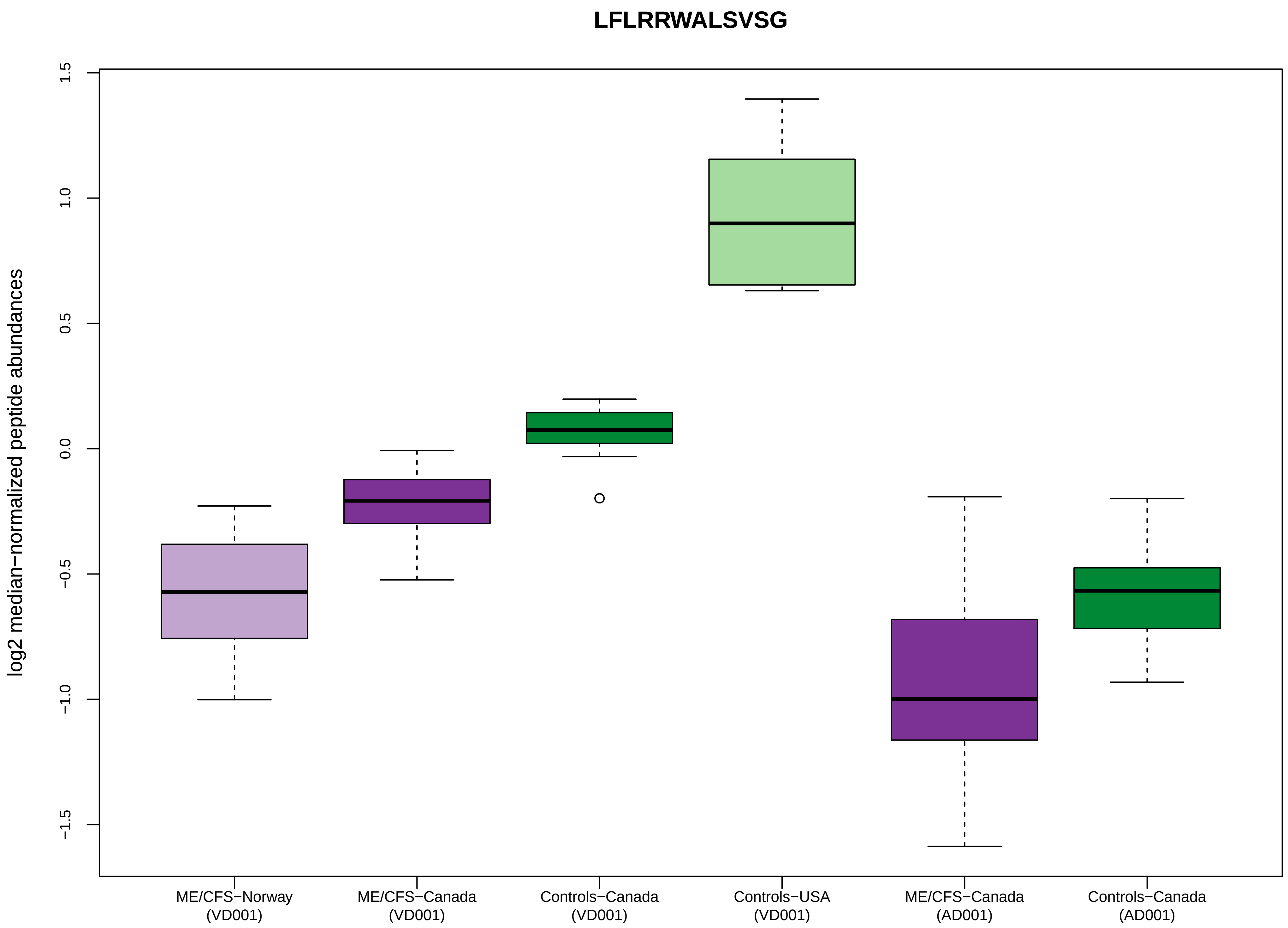
LFLRRWALSVSG
log2 median−normalized peptide abundances
1.5
1.0
0.5
0.0
−0.5
−1.0
−1.5
ME/CFS−Norway (VD001)
ME/CFS−Canada (VD001)
Controls−Canada (VD001)
Controls−USA (VD001)
ME/CFS−Canada (AD001)
Controls−Canada (AD001)

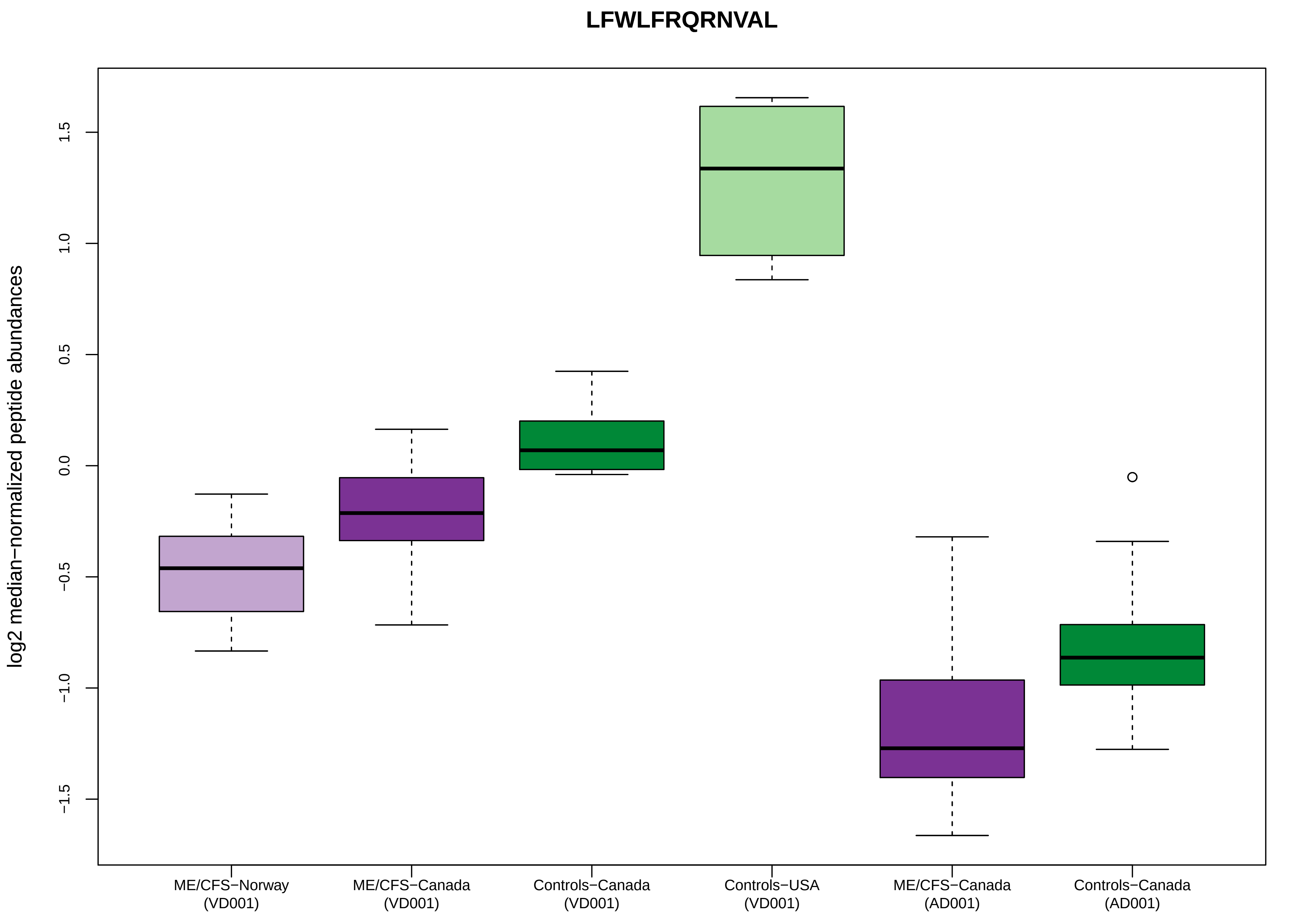
LFWLFRQRNVAL
log2 median−normalized peptide abundances
1.5
1.0
0.5
0.0
−0.5
−1.0
−1.5
ME/CFS−Norway (VD001)
ME/CFS−Canada (VD001)
Controls−Canada (VD001)
Controls−USA (VD001)
ME/CFS−Canada (AD001)
Controls−Canada (AD001)

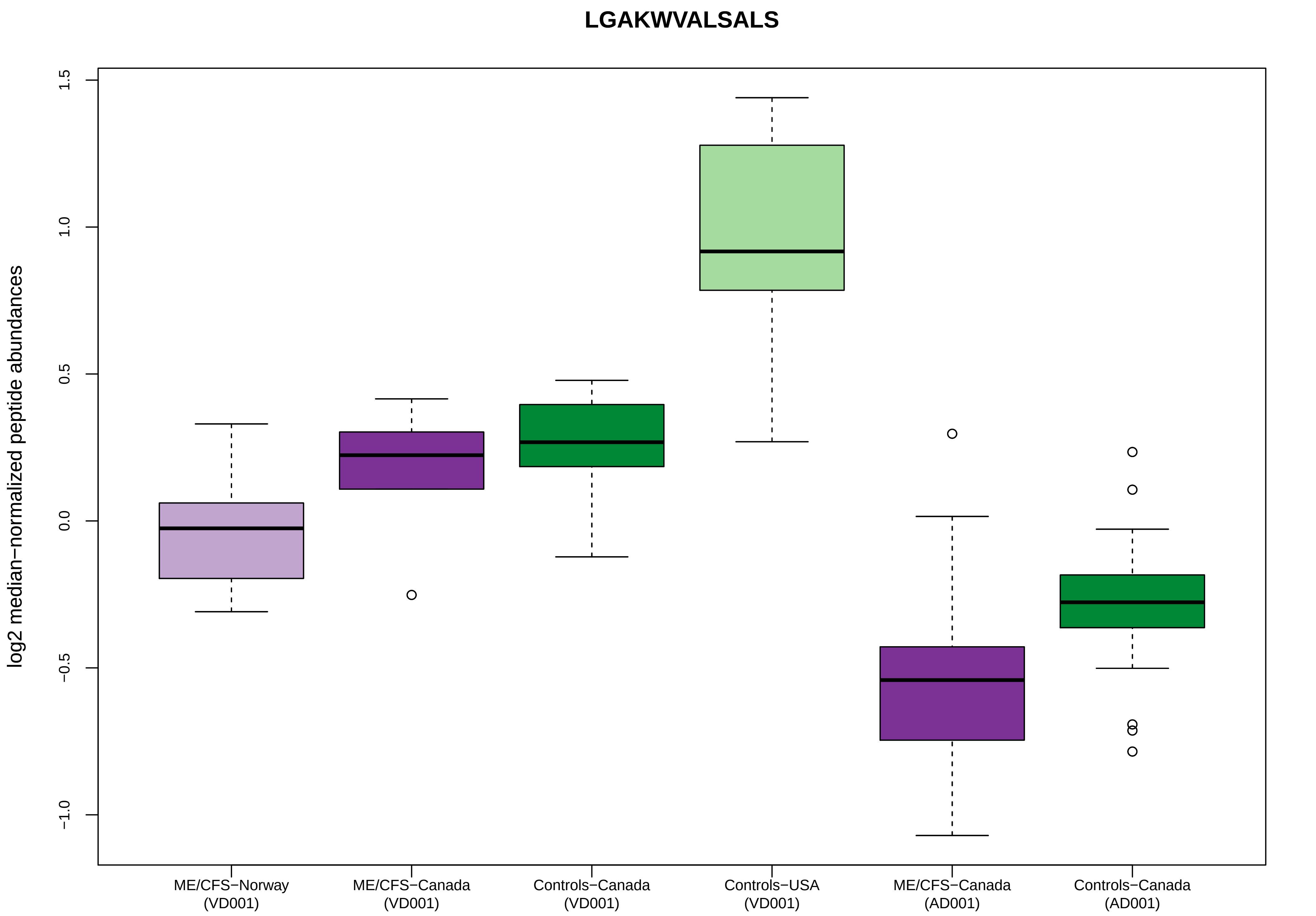

LGAKWVALSALS
log2 median−normalized peptide abundances
1.5
1.0
0.5
0.0
−0.5
−1.0
ME/CFS−Norway (VD001)
ME/CFS−Canada (VD001)
Controls−Canada (VD001)
Controls−USA (VD001)
ME/CFS−Canada (AD001)
Controls−Canada (AD001)

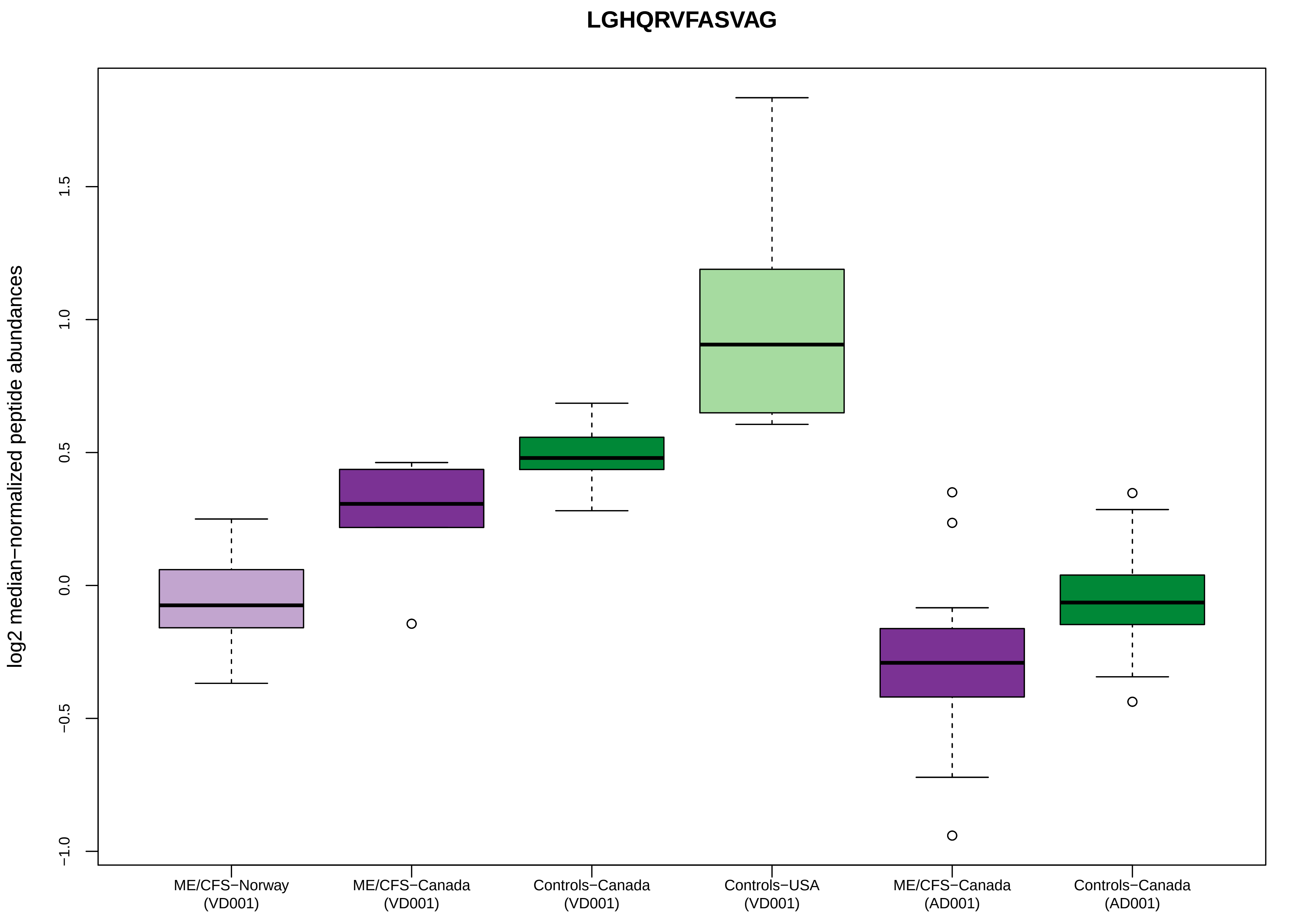
LGHQRVFASVAG
log2 median−normalized peptide abundances
1.5
1.0
0.5
0.0
−0.5
−1.0
ME/CFS−Norway (VD001)
ME/CFS−Canada (VD001)
Controls−Canada (VD001)
Controls−USA (VD001)
ME/CFS−Canada (AD001)
Controls−Canada (AD001)

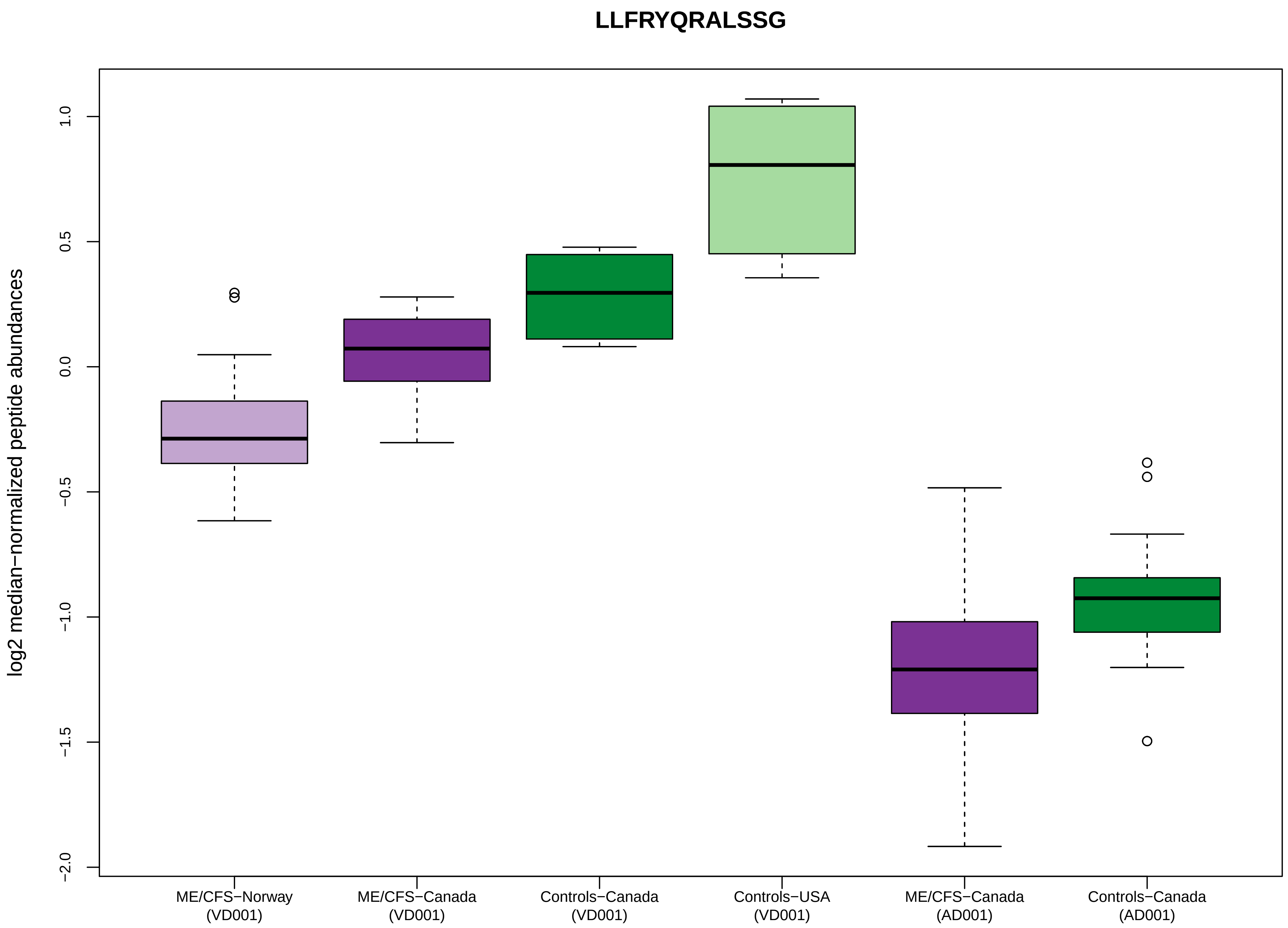

LLFRYQRALSSG
log2 median−normalized peptide abundances
1.0
0.5
0.0
−0.5
−1.0
−1.5
−2.0
ME/CFS−Norway (VD001)
ME/CFS−Canada (VD001)
Controls−Canada (VD001)
Controls−USA (VD001)
ME/CFS−Canada (AD001)
Controls−Canada (AD001)

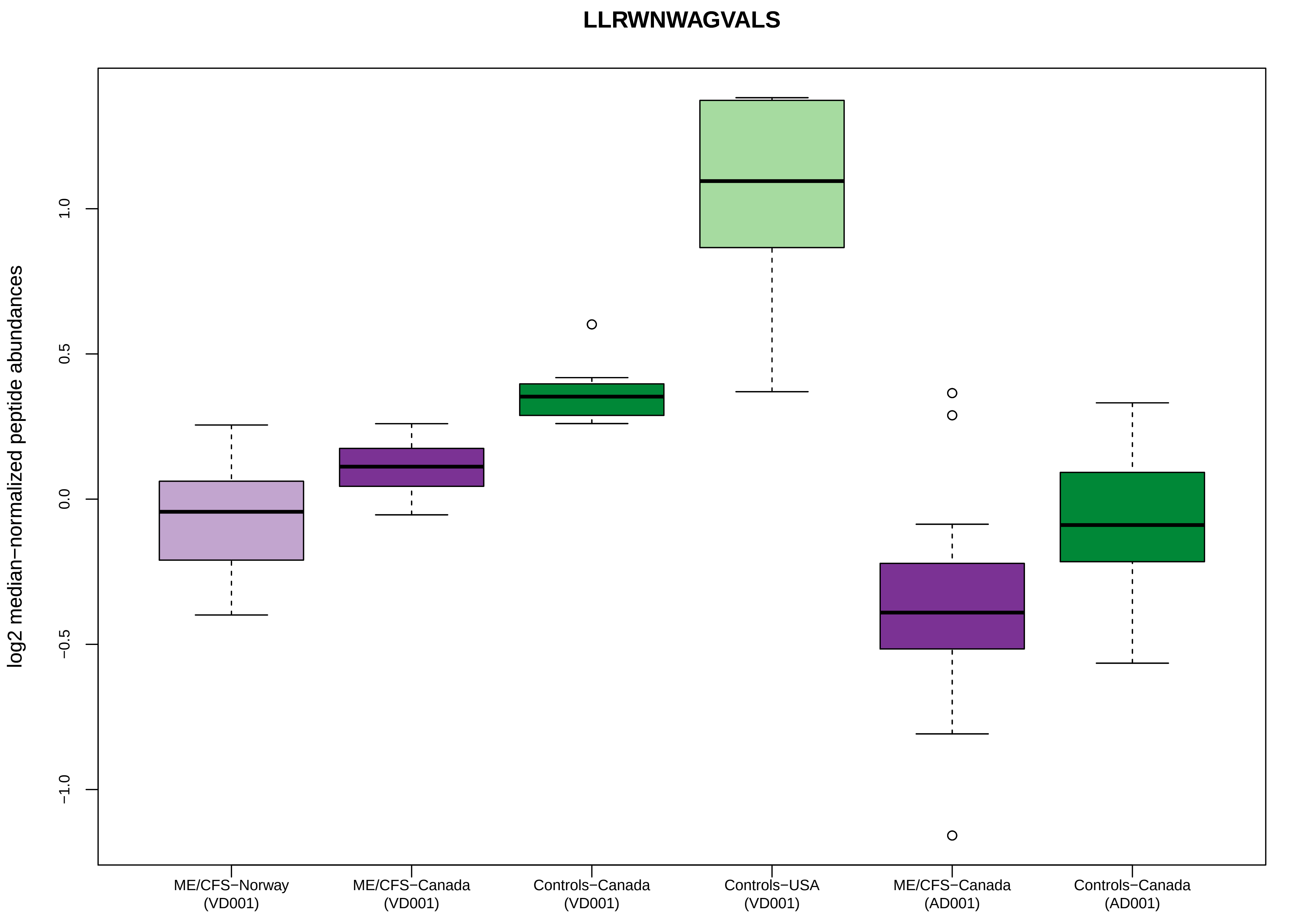

LLRWNWAGVALS
log2 median−normalized peptide abundances
1.0
0.5
0.0
−0.5
−1.0
ME/CFS−Norway (VD001)
ME/CFS−Canada (VD001)
Controls−Canada (VD001)
Controls−USA (VD001)
ME/CFS−Canada (AD001)
Controls−Canada (AD001)

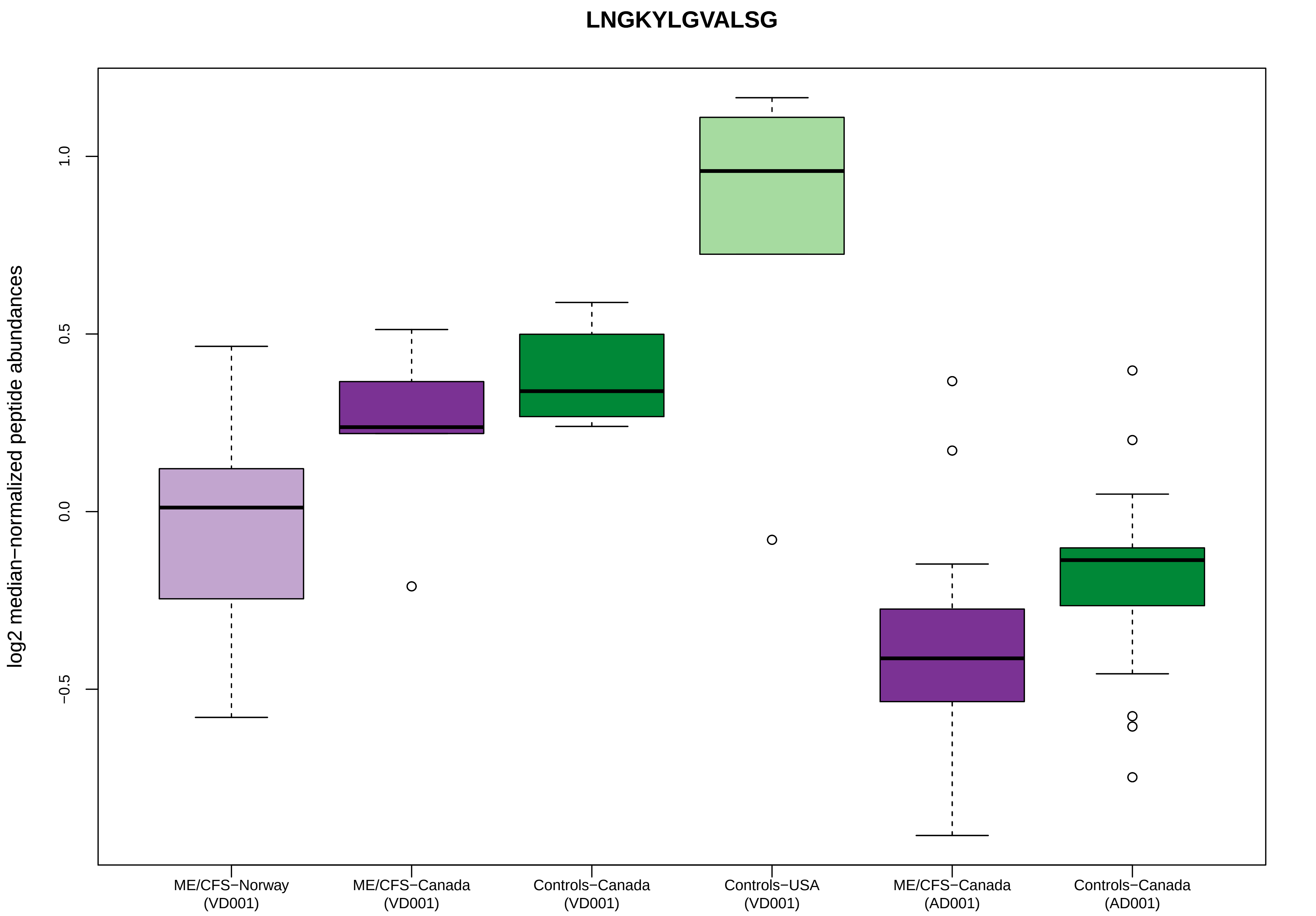
LNGKYLGVALSG
log2 median−normalized peptide abundances
1.0
0.5
0.0
−0.5
ME/CFS−Norway (VD001)
ME/CFS−Canada (VD001)
Controls−Canada (VD001)
Controls−USA (VD001)
ME/CFS−Canada (AD001)
Controls−Canada (AD001)

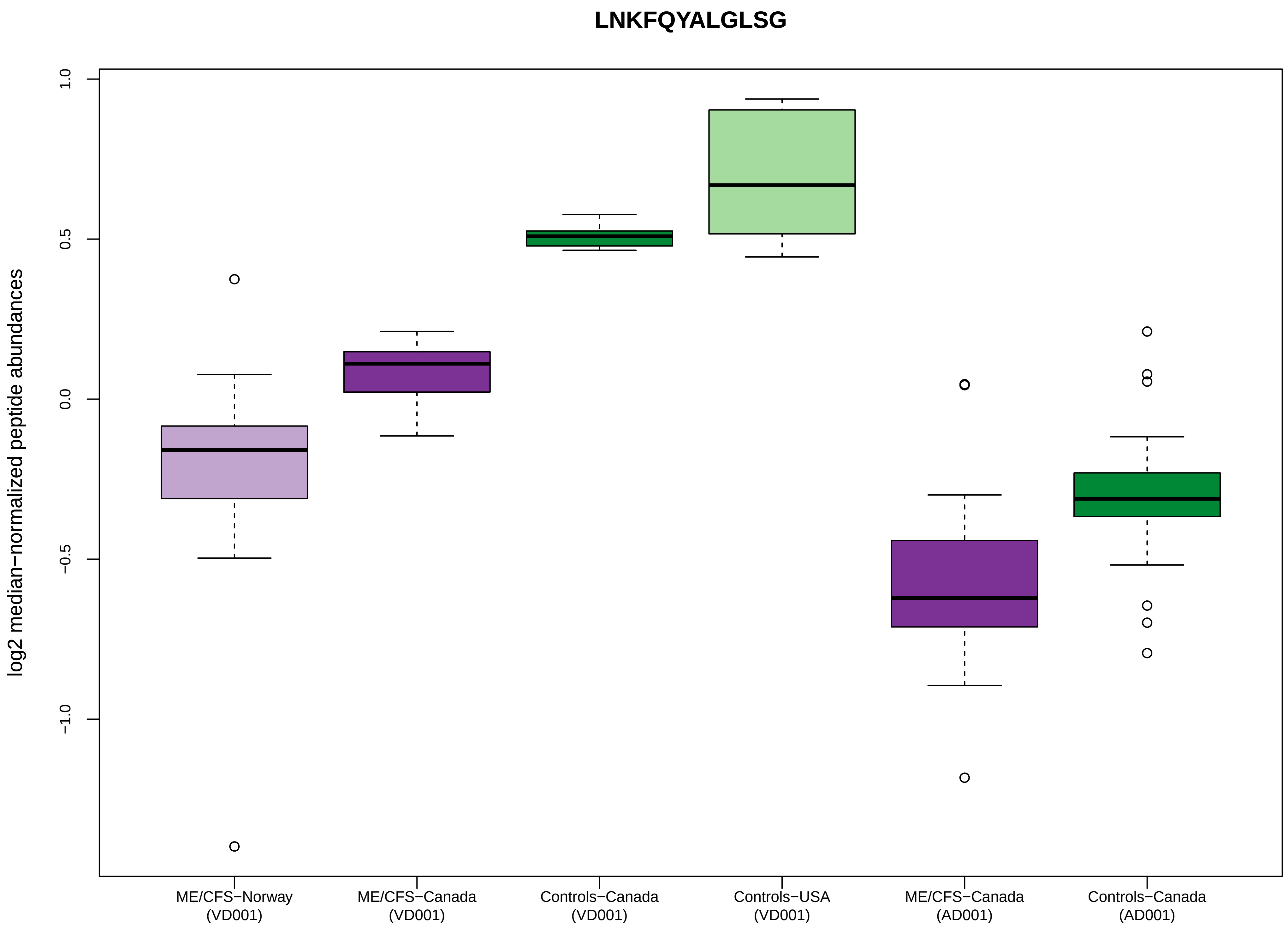

LNKFQYALGLSG
log2 median−normalized peptide abundances
1.0
0.5
0.0
−0.5
−1.0
ME/CFS−Norway (VD001)
ME/CFS−Canada (VD001)
Controls−Canada (VD001)
Controls−USA (VD001)
ME/CFS−Canada (AD001)
Controls−Canada (AD001)

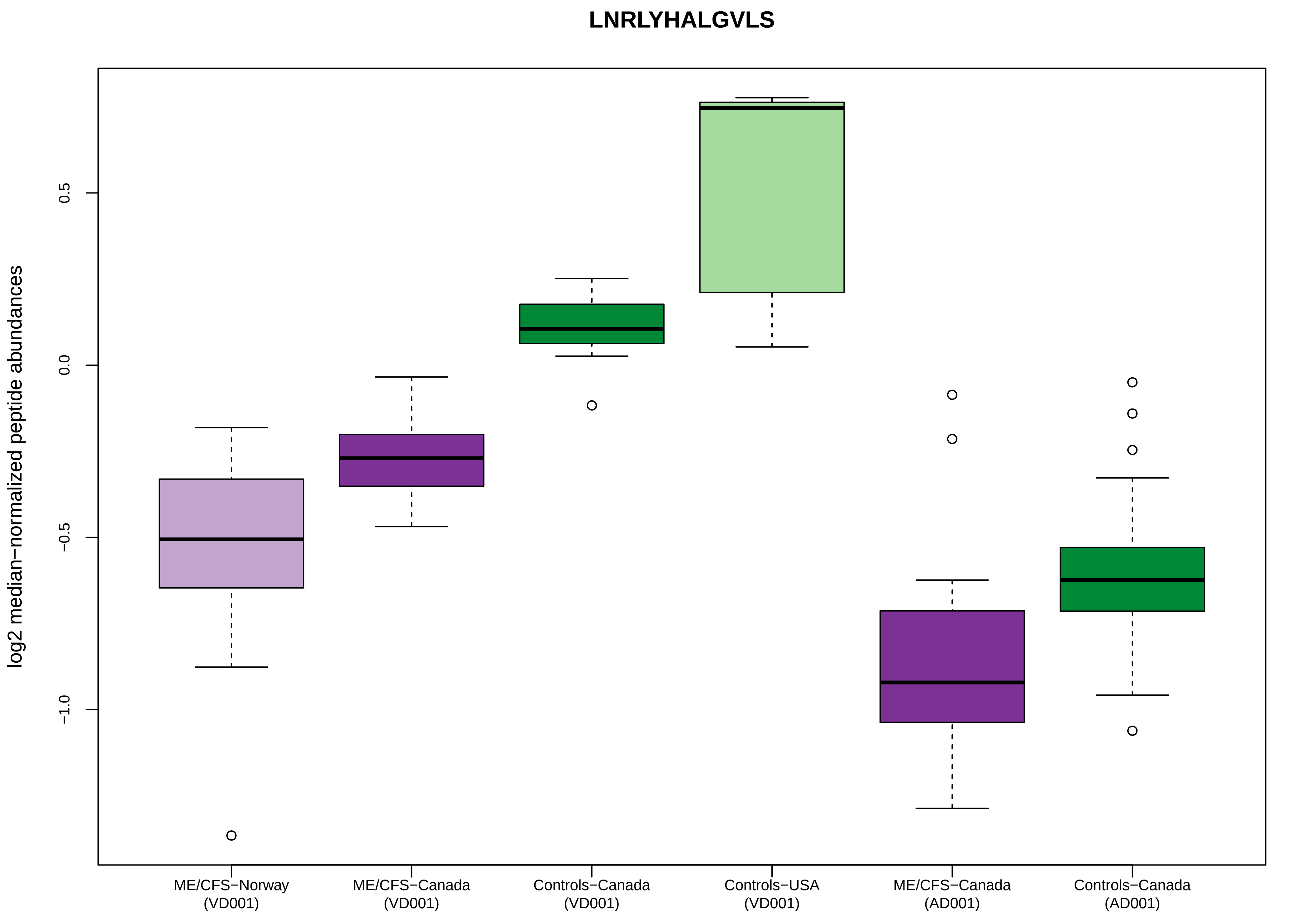

LNRLYHALGVLS
log2 median−normalized peptide abundances
0.5
0.0
−0.5
−1.0
ME/CFS−Norway (VD001)
ME/CFS−Canada (VD001)
Controls−Canada (VD001)
Controls−USA (VD001)
ME/CFS−Canada (AD001)
Controls−Canada (AD001)

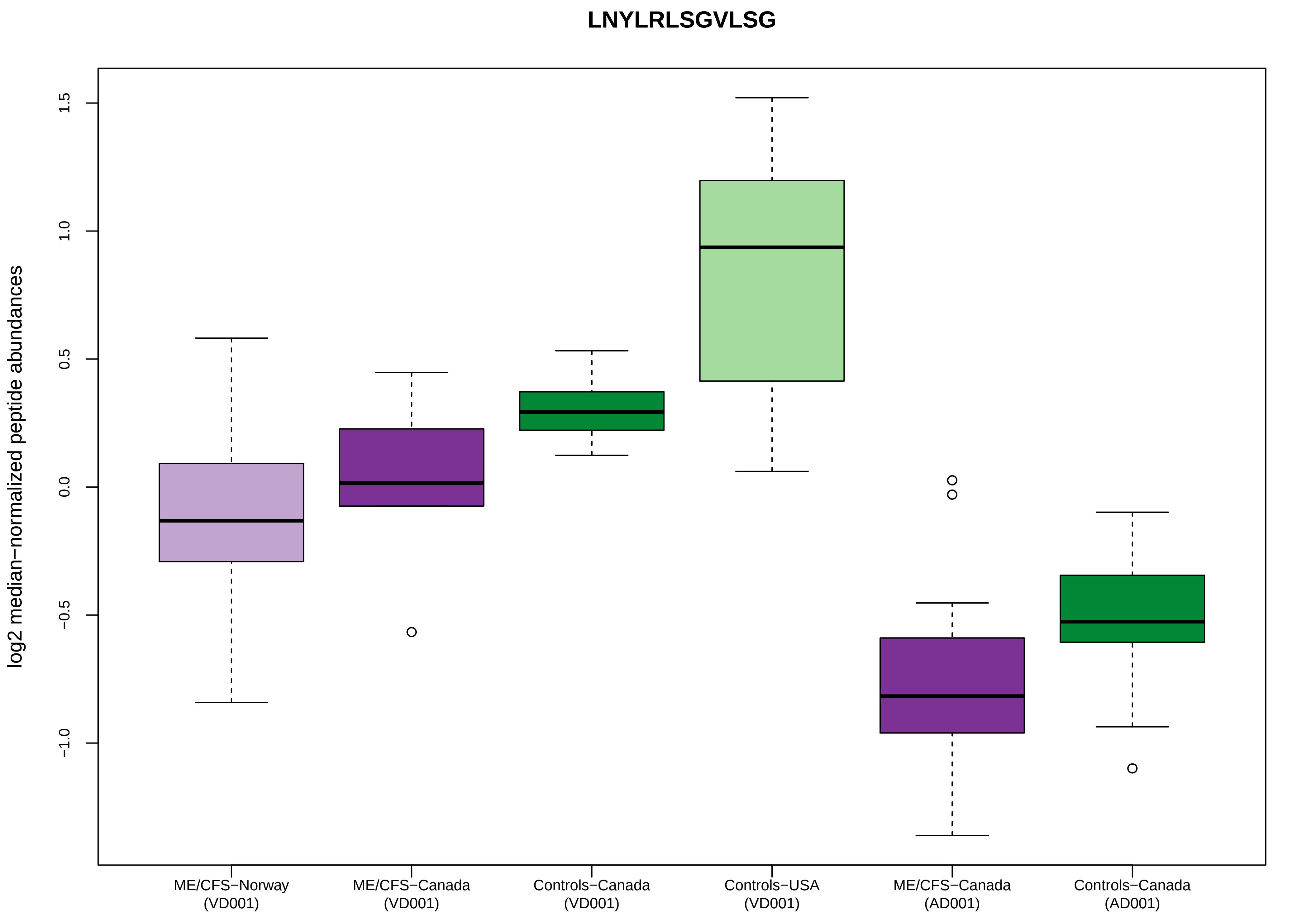
LNYLRLSGVLSG
log2 median−normalized peptide abundances
1.5
1.0
0.5
0.0
−0.5
−1.0
ME/CFS−Norway (VD001)
ME/CFS−Canada (VD001)
Controls−Canada (VD001)
Controls−USA (VD001)
ME/CFS−Canada (AD001)
Controls−Canada (AD001)

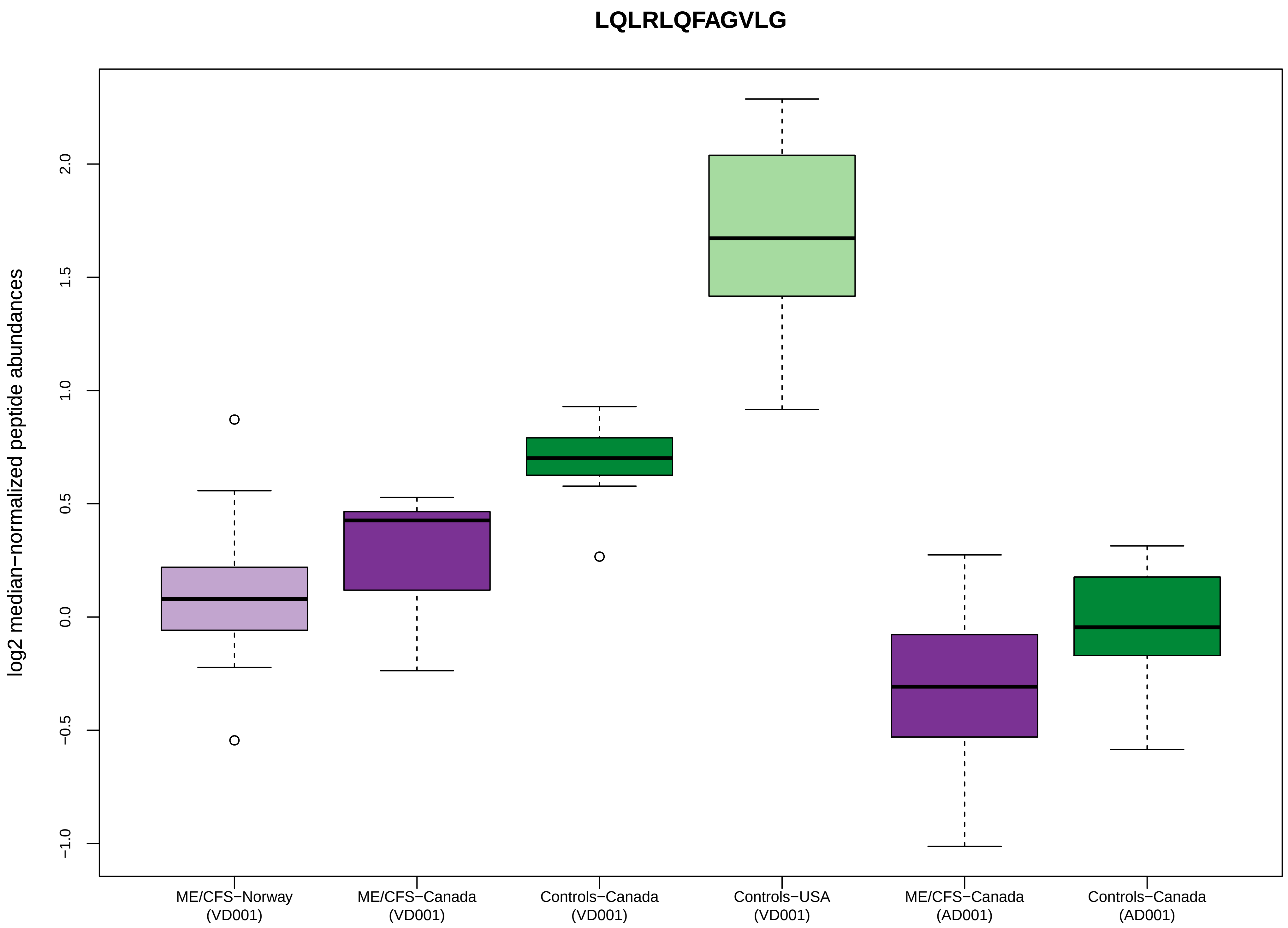
LQLRLQFAGVLG
log2 median−normalized peptide abundances
−1.0
−0.5
0.0
0.5
1.0
1.5
2.0
ME/CFS−Norway (VD001)
ME/CFS−Canada (VD001)
Controls−Canada (VD001)
Controls−USA (VD001)
ME/CFS−Canada (AD001)
Controls−Canada (AD001)

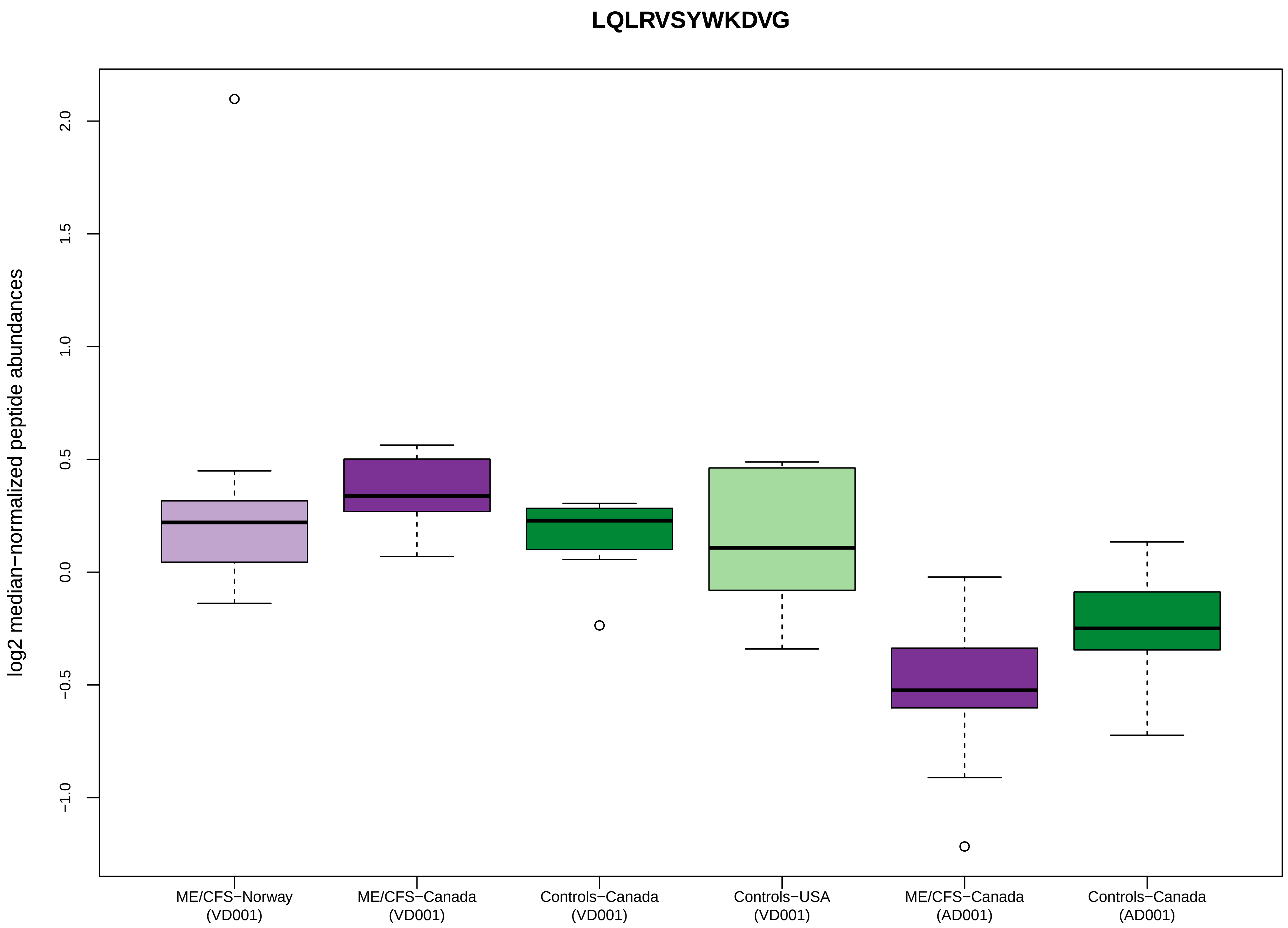

LQLRVSYWKDVG
log2 median−normalized peptide abundances
2.0
1.5
1.0
0.5
0.0
−0.5
−1.0
ME/CFS−Norway (VD001)
ME/CFS−Canada (VD001)
Controls−Canada (VD001)
Controls−USA (VD001)
ME/CFS−Canada (AD001)
Controls−Canada (AD001)

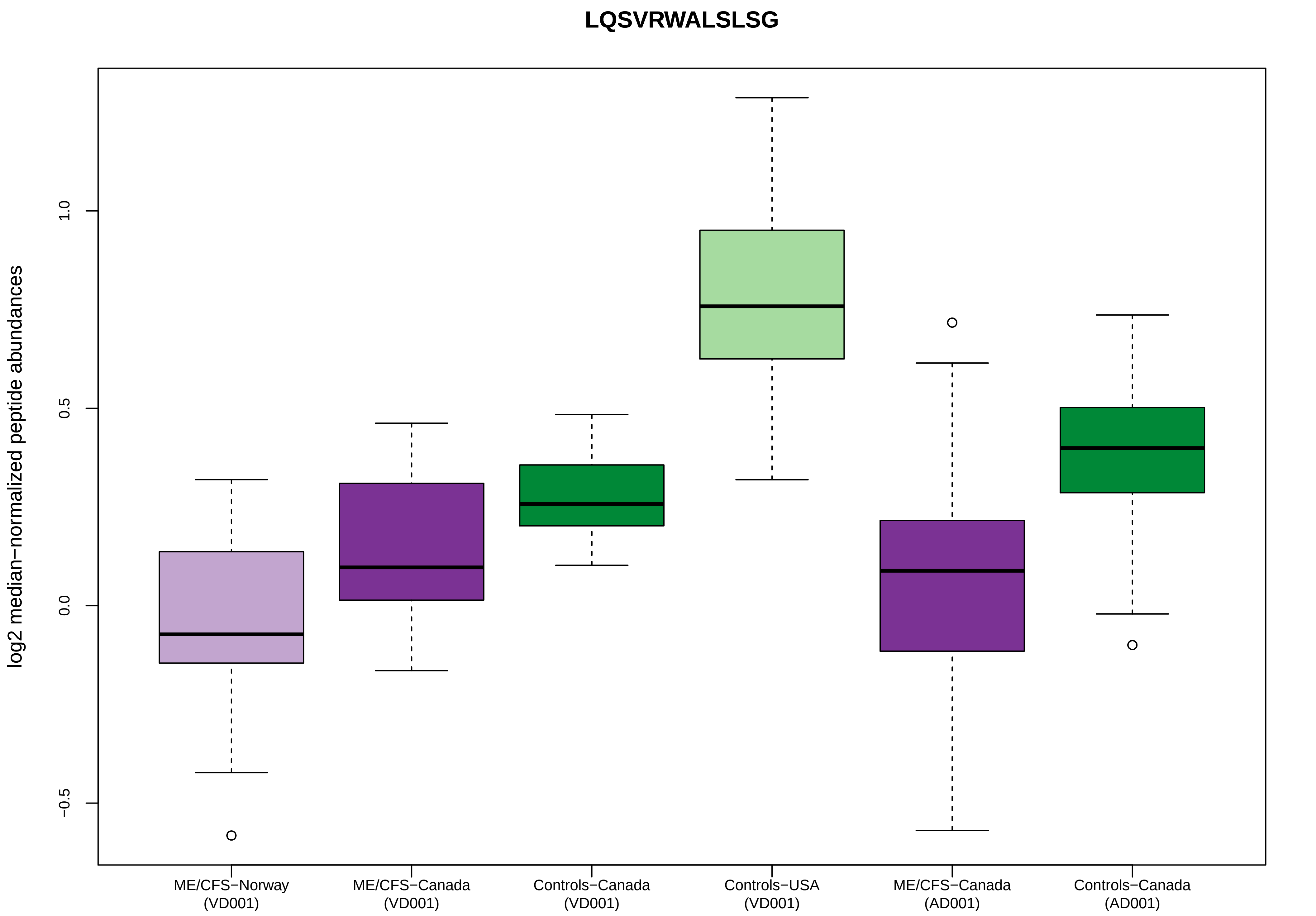

LQSVRWALSLSG
log2 median−normalized peptide abundances
1.0
0.5
0.0
−0.5
ME/CFS−Norway (VD001)
ME/CFS−Canada (VD001)
Controls−Canada (VD001)
Controls−USA (VD001)
ME/CFS−Canada (AD001)
Controls−Canada (AD001)

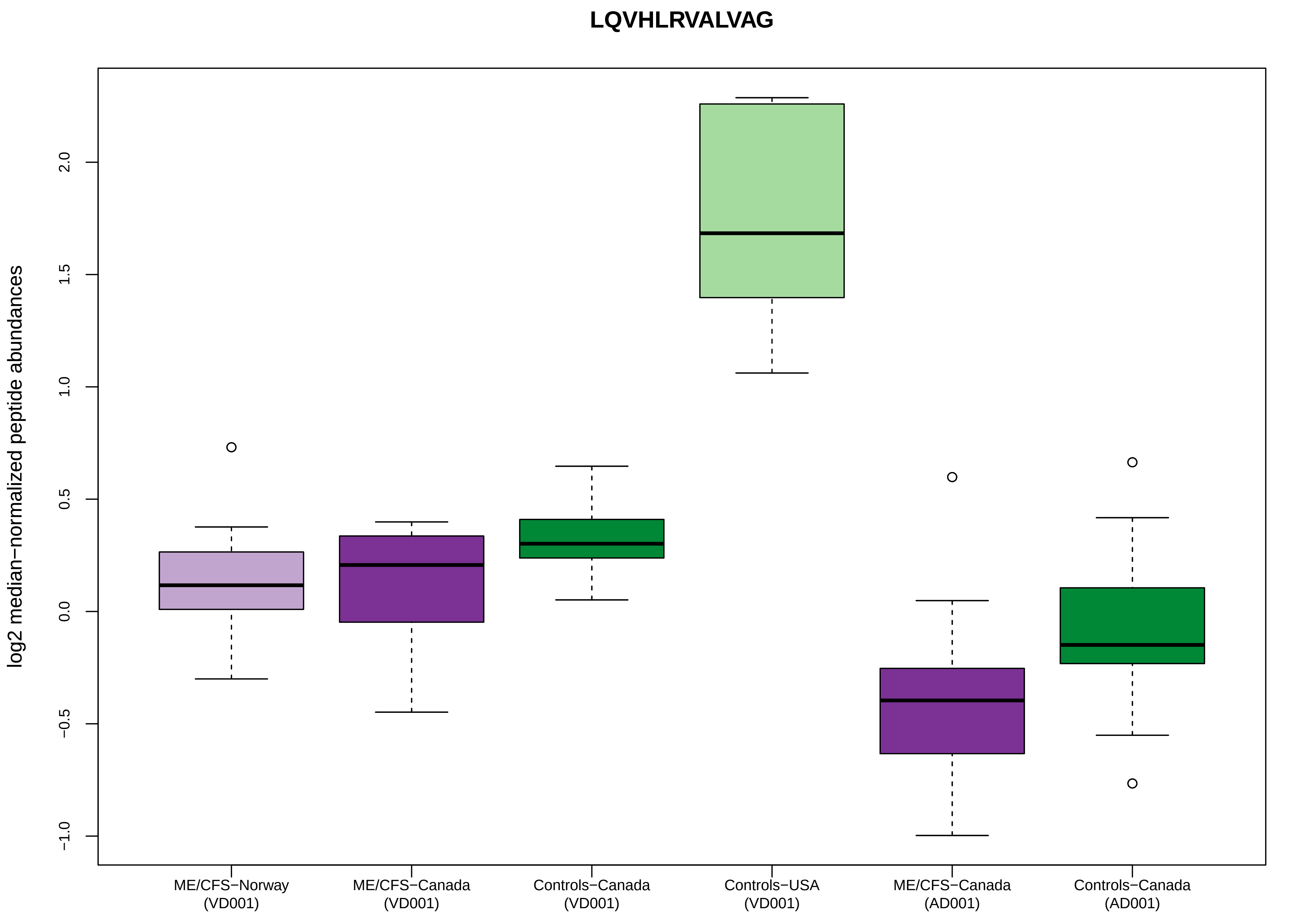
LQVHLRVALVAG
log2 median−normalized peptide abundances
2.0
1.5
1.0
0.5
0.0
−0.5
−1.0
ME/CFS−Norway (VD001)
ME/CFS−Canada (VD001)
Controls−Canada (VD001)
Controls−USA (VD001)
ME/CFS−Canada (AD001)
Controls−Canada (AD001)

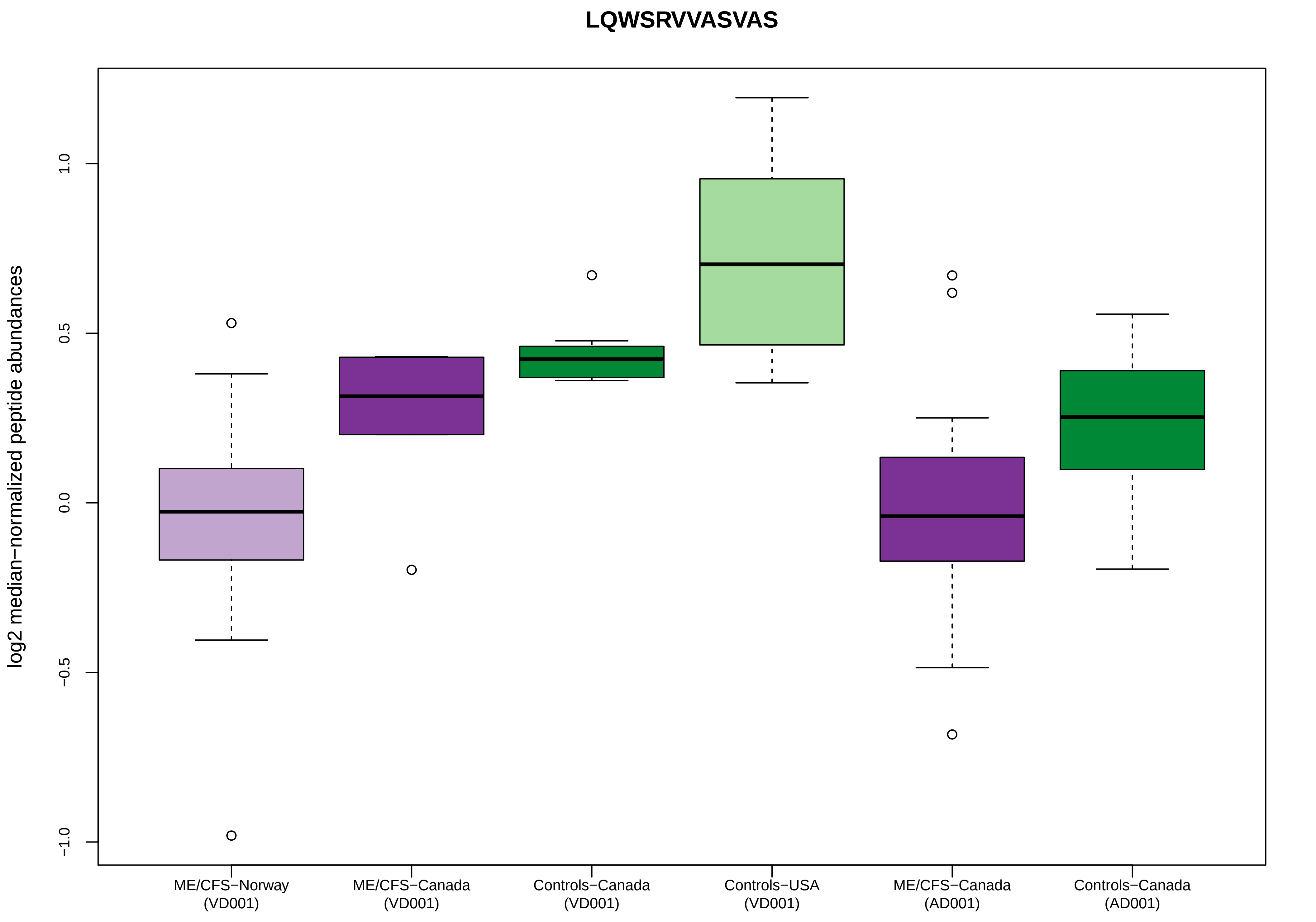

LQWSRVVASVAS
log2 median−normalized peptide abundances
1.0
0.5
0.0
−0.5
−1.0
ME/CFS−Norway (VD001)
ME/CFS−Canada (VD001)
Controls−Canada (VD001)
Controls−USA (VD001)
ME/CFS−Canada (AD001)
Controls−Canada (AD001)

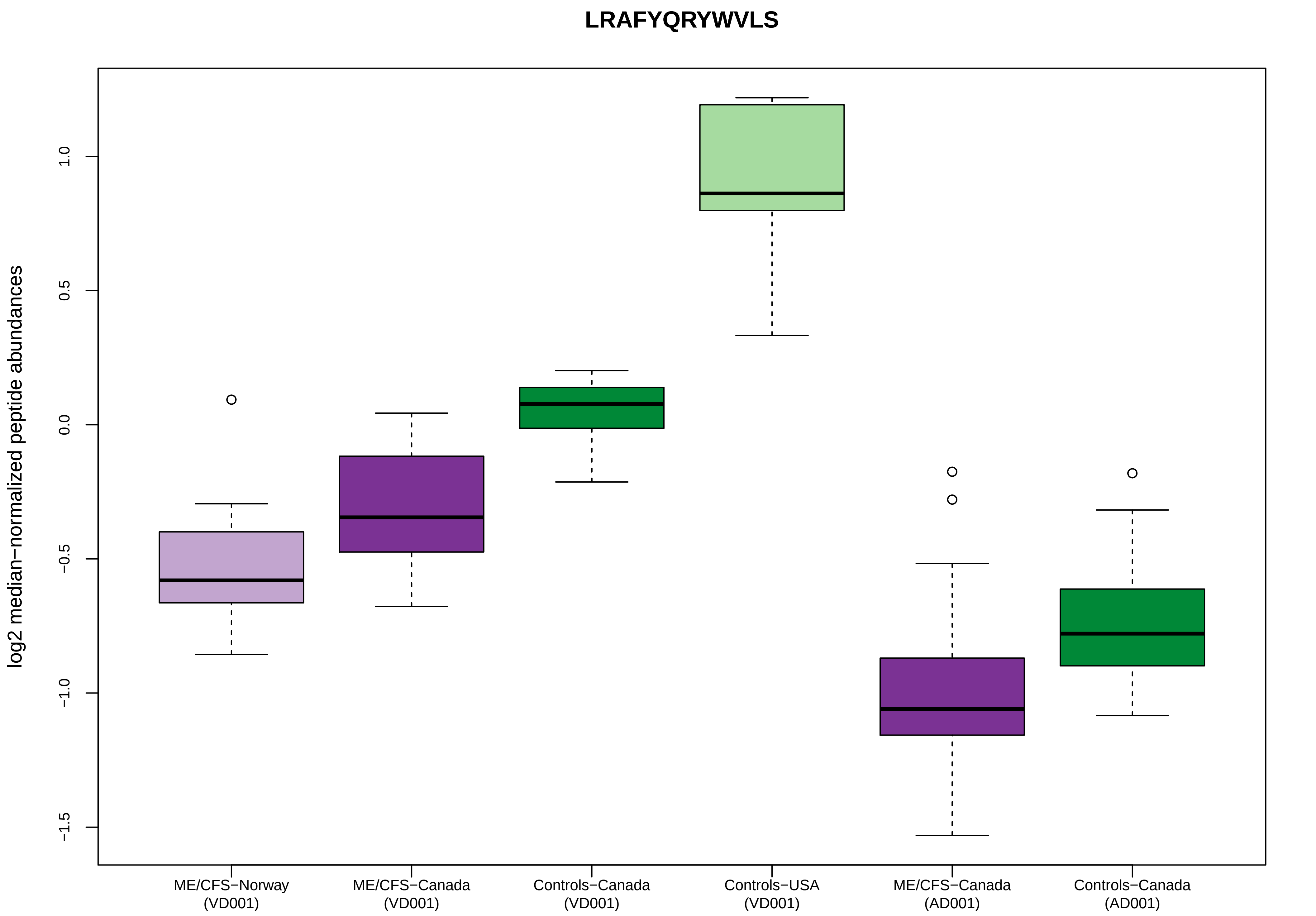

LRAFYQRYWVLS
log2 median−normalized peptide abundances
1.0
0.5
0.0
−0.5
−1.0
−1.5
ME/CFS−Norway (VD001)
ME/CFS−Canada (VD001)
Controls−Canada (VD001)
Controls−USA (VD001)
ME/CFS−Canada (AD001)
Controls−Canada (AD001)

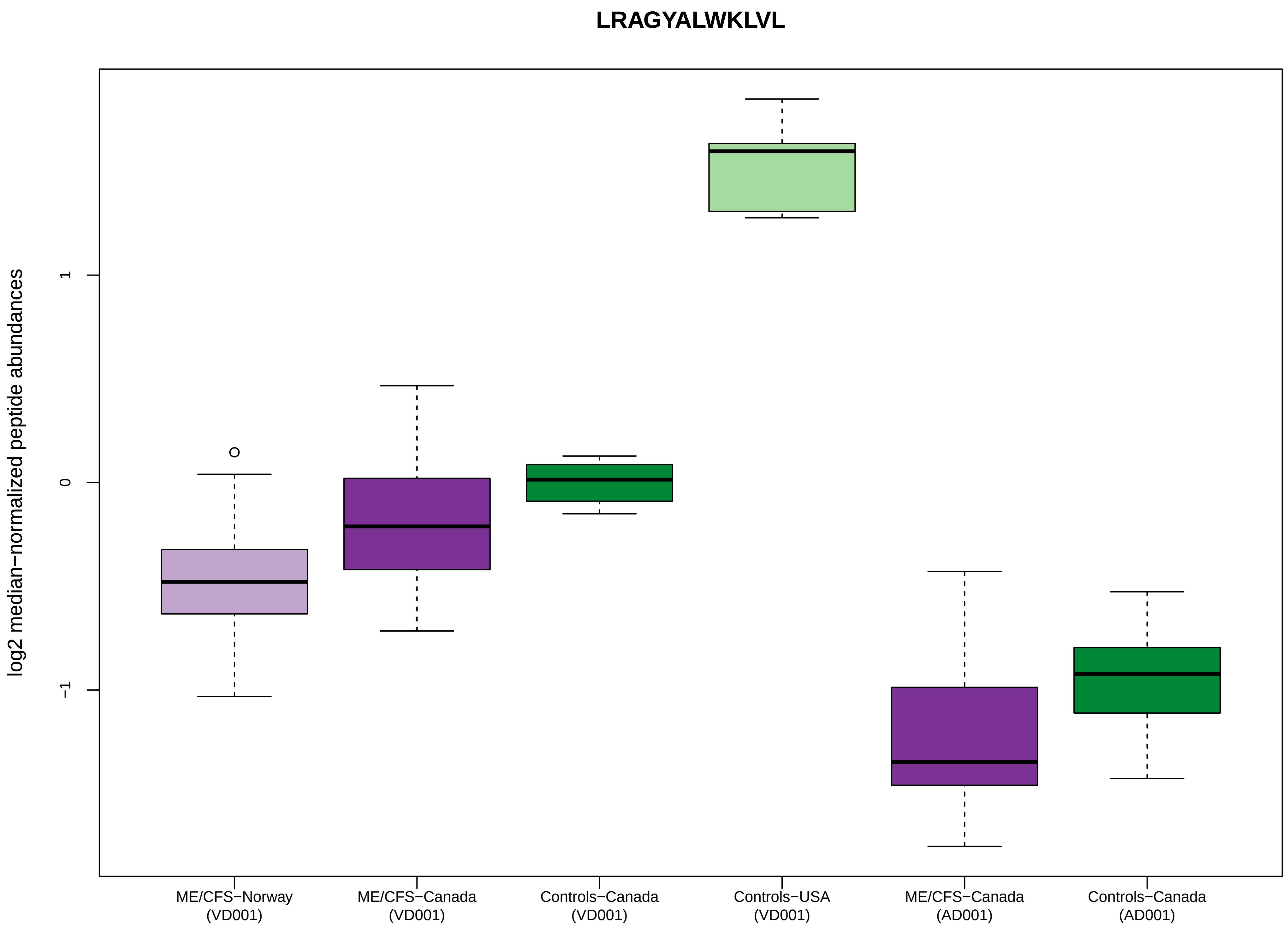

LRAGYALWKLVL
log2 median−normalized peptide abundances
1
0
−1
ME/CFS−Norway (VD001)
ME/CFS−Canada (VD001)
Controls−Canada (VD001)
Controls−USA (VD001)
ME/CFS−Canada (AD001)
Controls−Canada (AD001)

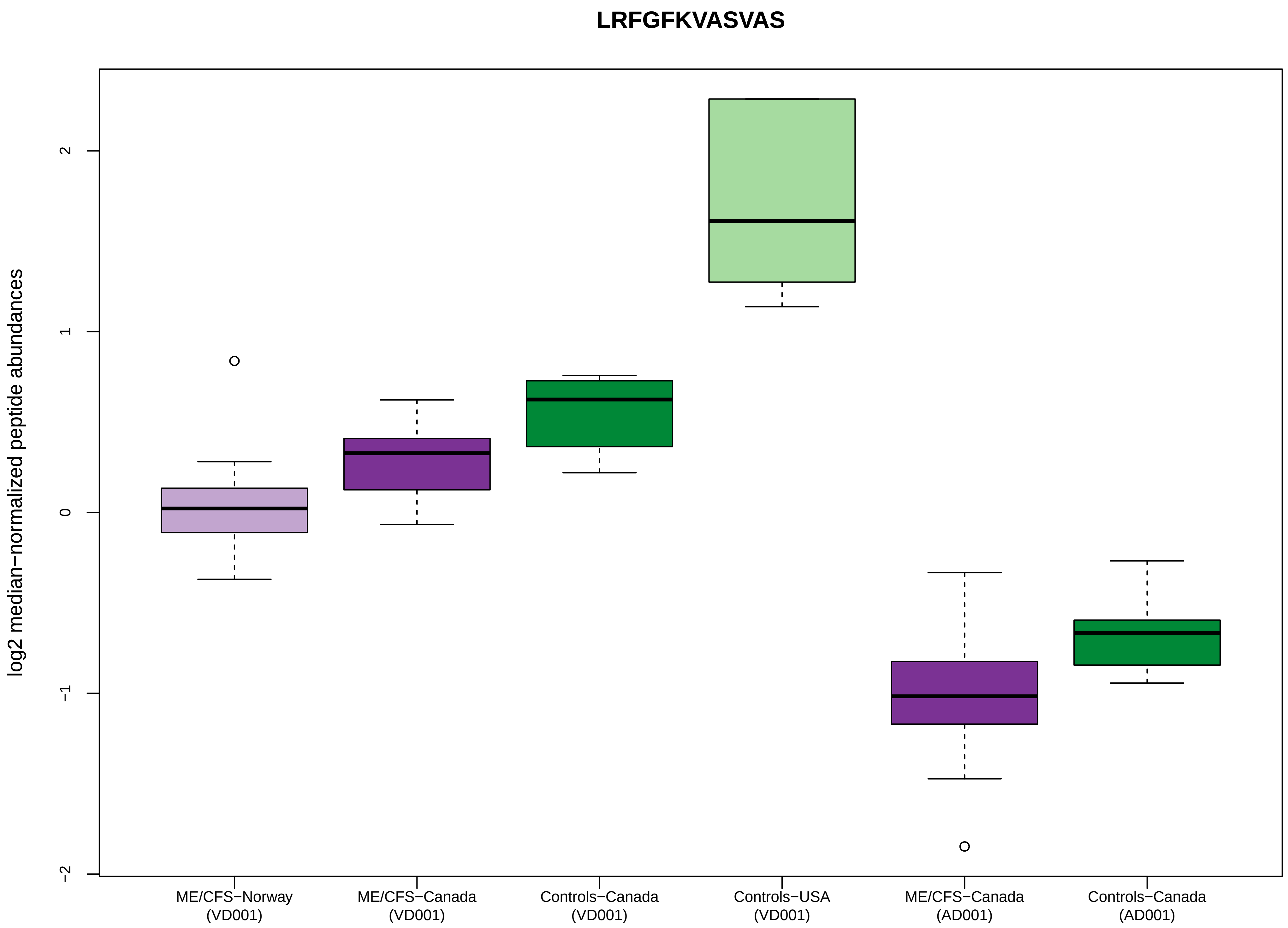

LRFGFKVASVAS
log2 median−normalized peptide abundances
2
1
0
−1
−2
ME/CFS−Norway (VD001)
ME/CFS−Canada (VD001)
Controls−Canada (VD001)
Controls−USA (VD001)
ME/CFS−Canada (AD001)
Controls−Canada (AD001)

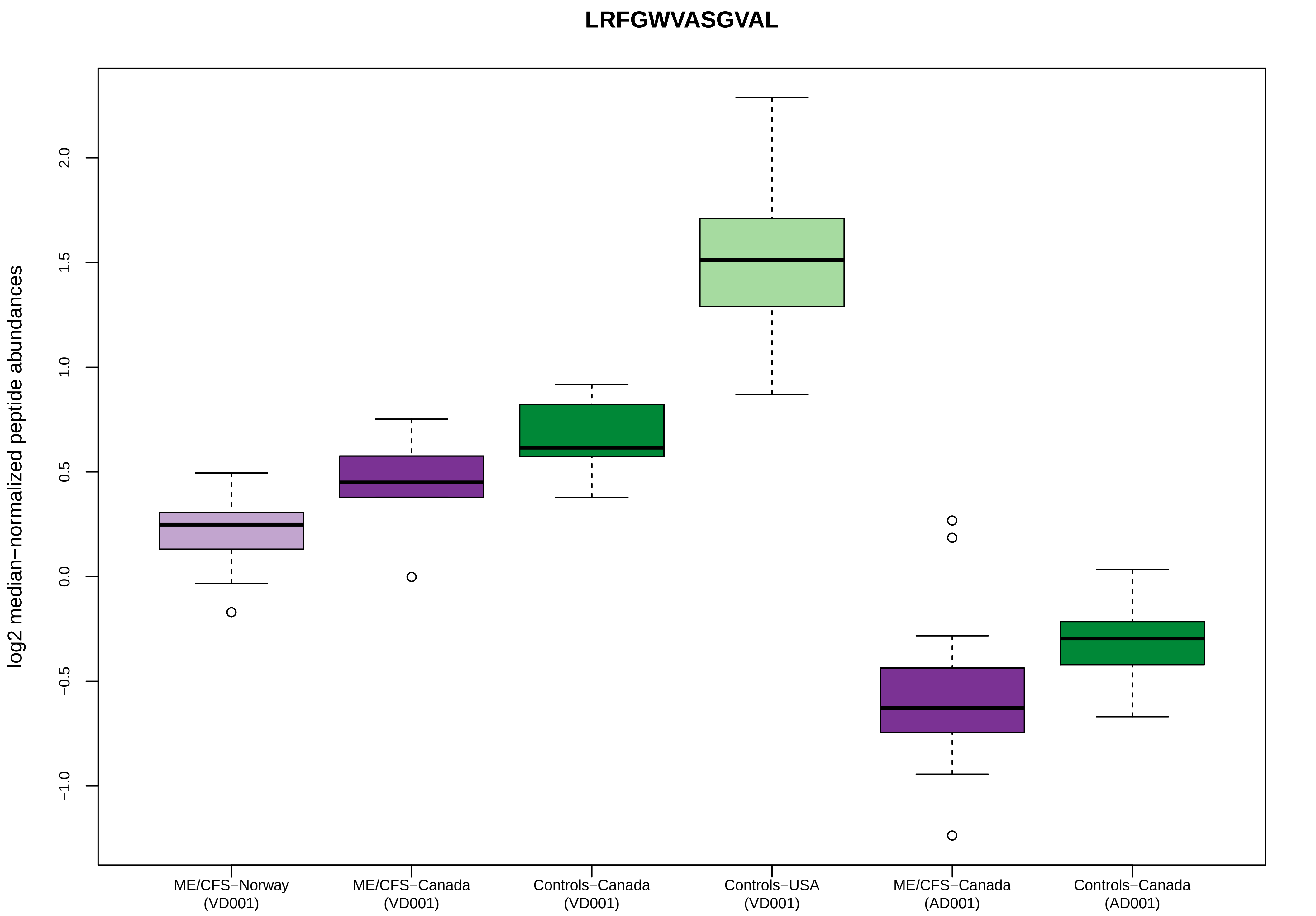

LRFGWVASGVAL
log2 median−normalized peptide abundances
2.0
1.5
1.0
0.5
0.0
−0.5
−1.0
ME/CFS−Norway (VD001)
ME/CFS−Canada (VD001)
Controls−Canada (VD001)
Controls−USA (VD001)
ME/CFS−Canada (AD001)
Controls−Canada (AD001)

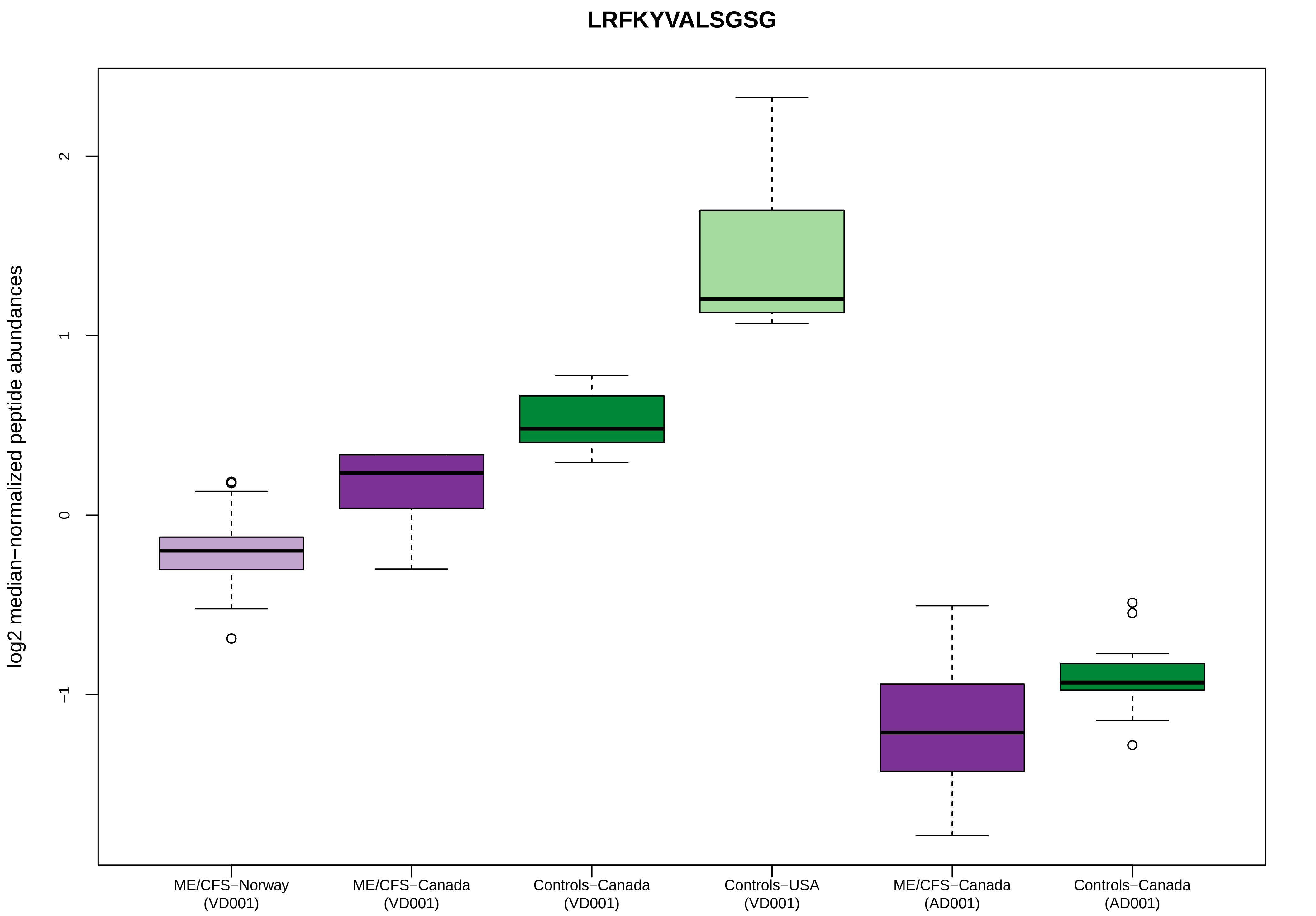
LRFKYVALSGSG
log2 median−normalized peptide abundances
2
1
0
−1
ME/CFS−Norway (VD001)
ME/CFS−Canada (VD001)
Controls−Canada (VD001)
Controls−USA (VD001)
ME/CFS−Canada (AD001)
Controls−Canada (AD001)

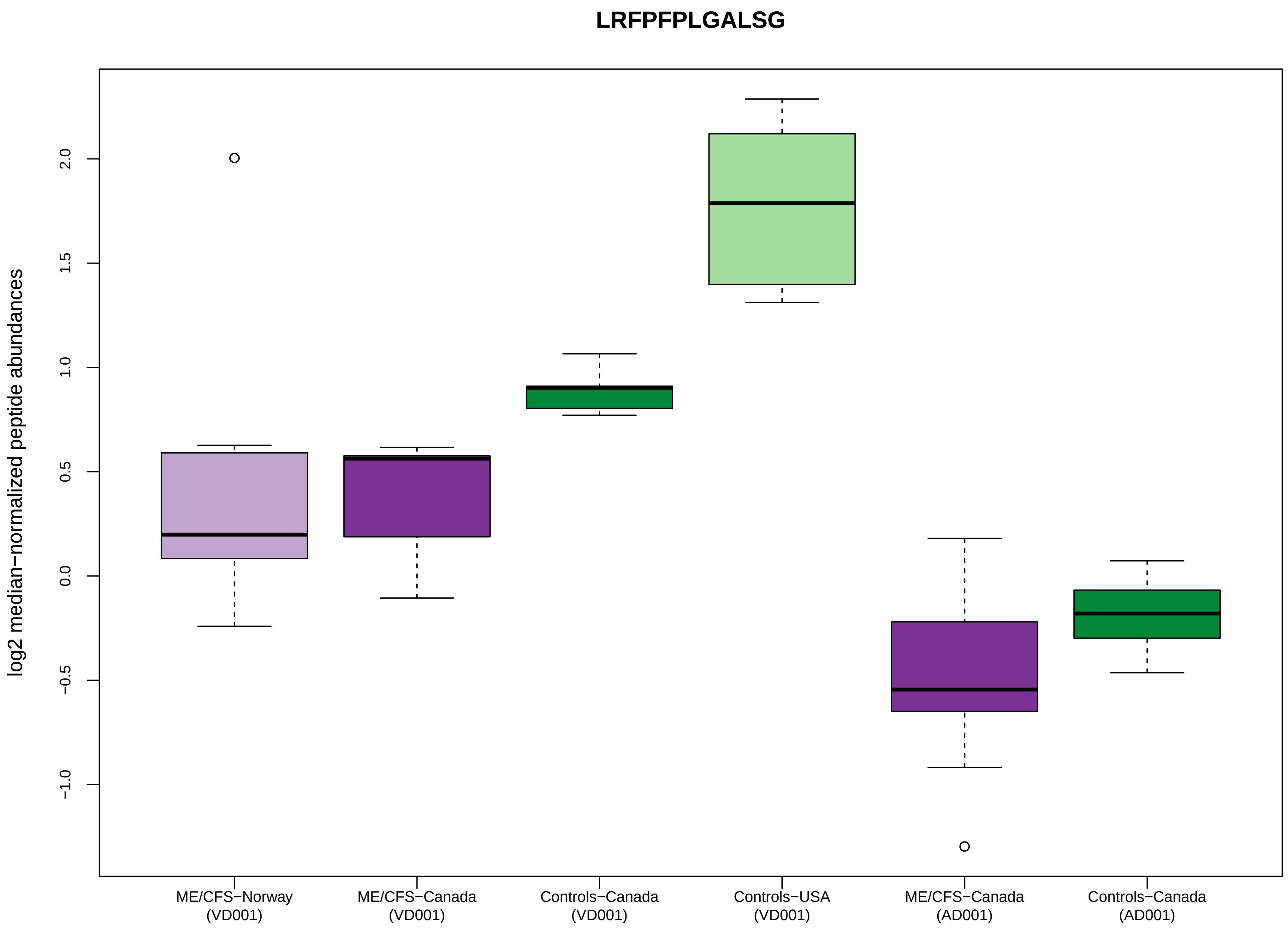

LRFPFPLGALSG
log2 median−normalized peptide abundances
2.0
1.5
1.0
0.5
0.0
−0.5
−1.0
ME/CFS−Norway (VD001)
ME/CFS−Canada (VD001)
Controls−Canada (VD001)
Controls−USA (VD001)
ME/CFS−Canada (AD001)
Controls−Canada (AD001)

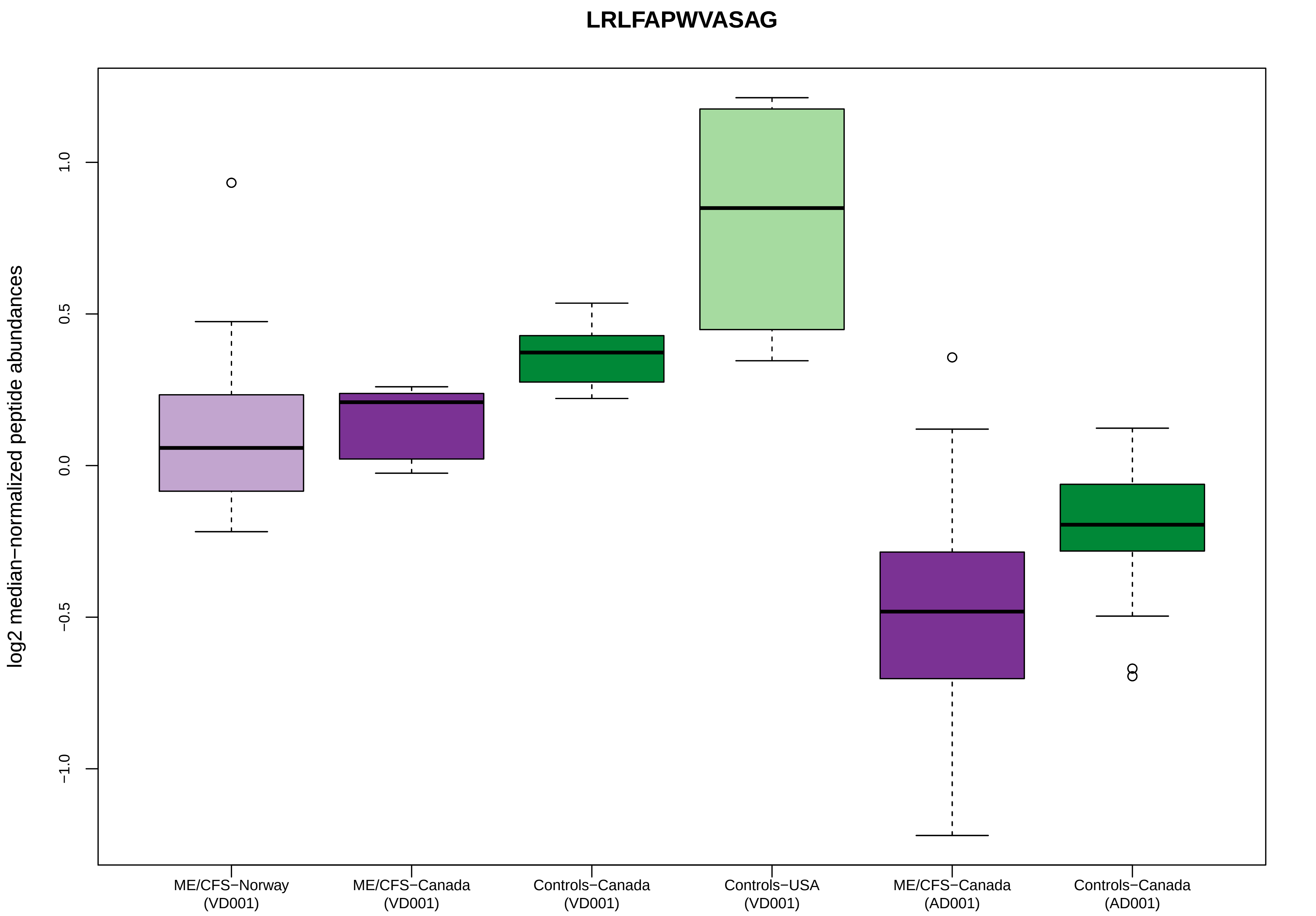

LRLFAPWVASAG
log2 median−normalized peptide abundances
1.0
0.5
0.0
−0.5
−1.0
ME/CFS−Norway (VD001)
ME/CFS−Canada (VD001)
Controls−Canada (VD001)
Controls−USA (VD001)
ME/CFS−Canada (AD001)
Controls−Canada (AD001)

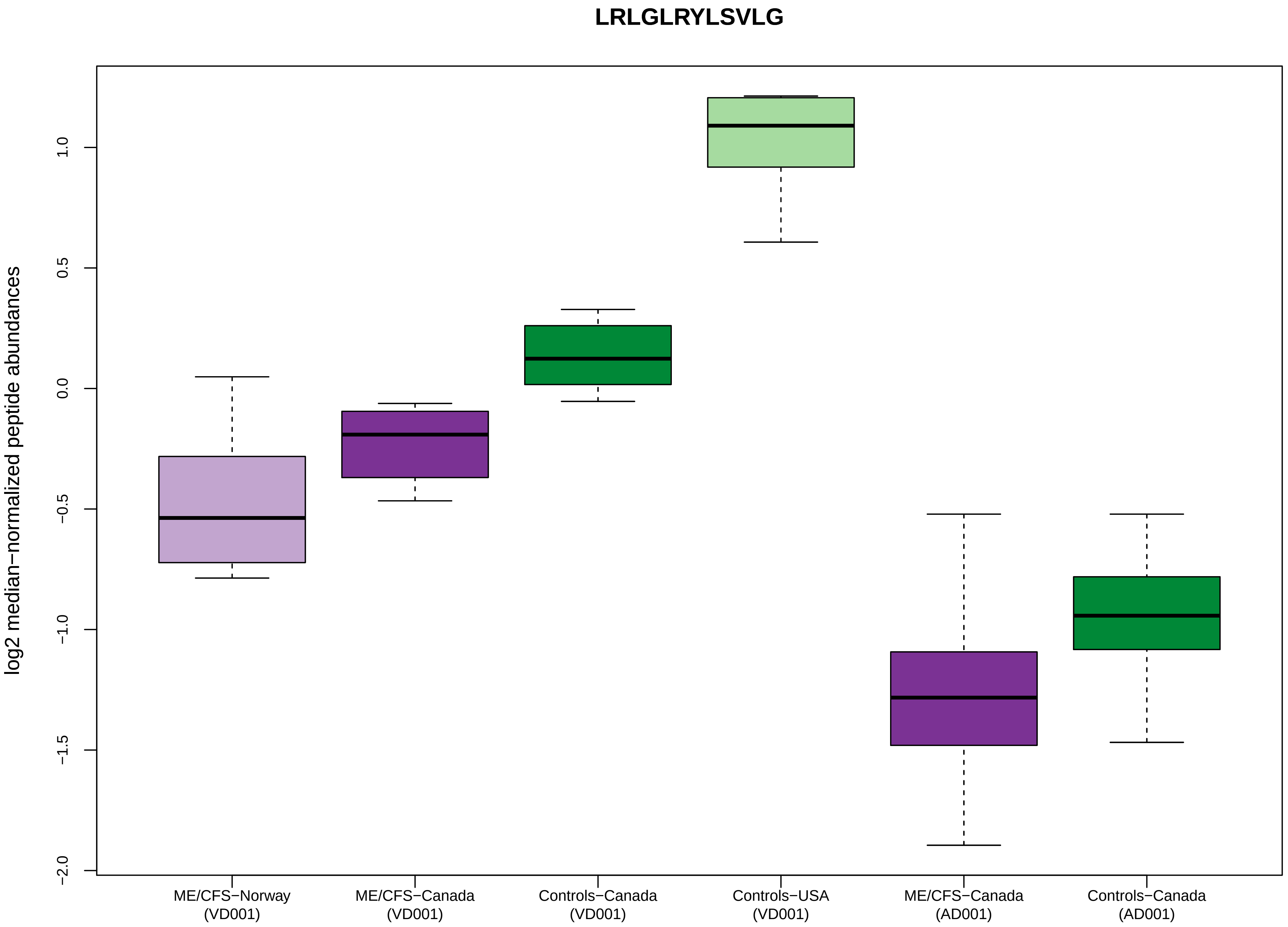

LRLGLRYLSVLG
log2 median−normalized peptide abundances
1.0
0.5
0.0
−0.5
−1.0
−1.5
−2.0
ME/CFS−Norway (VD001)
ME/CFS−Canada (VD001)
Controls−Canada (VD001)
Controls−USA (VD001)
ME/CFS−Canada (AD001)
Controls−Canada (AD001)

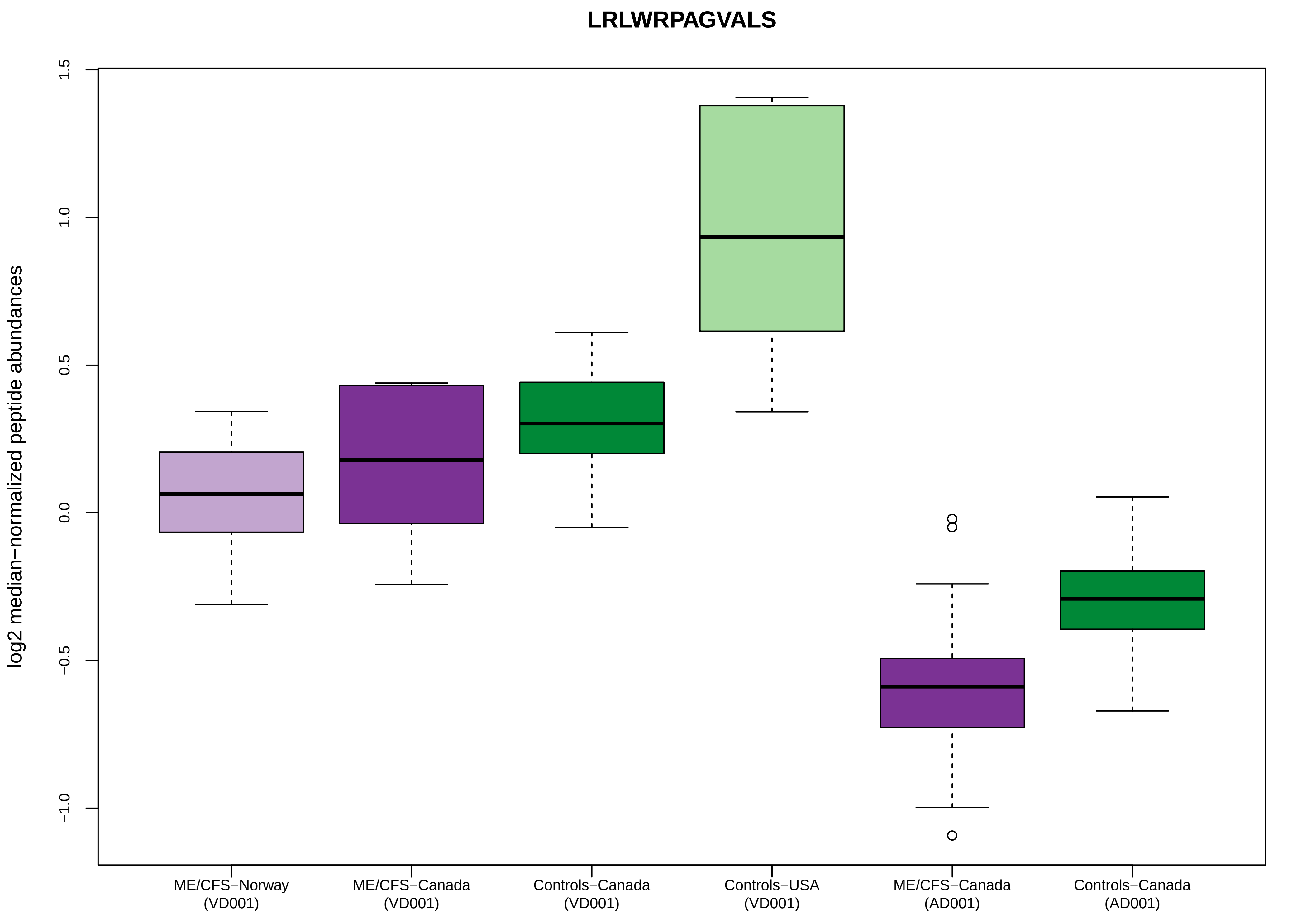
LRLWRPAGVALS
log2 median−normalized peptide abundances
1.5
1.0
0.5
0.0
−0.5
−1.0
ME/CFS−Norway (VD001)
ME/CFS−Canada (VD001)
Controls−Canada (VD001)
Controls−USA (VD001)
ME/CFS−Canada (AD001)
Controls−Canada (AD001)

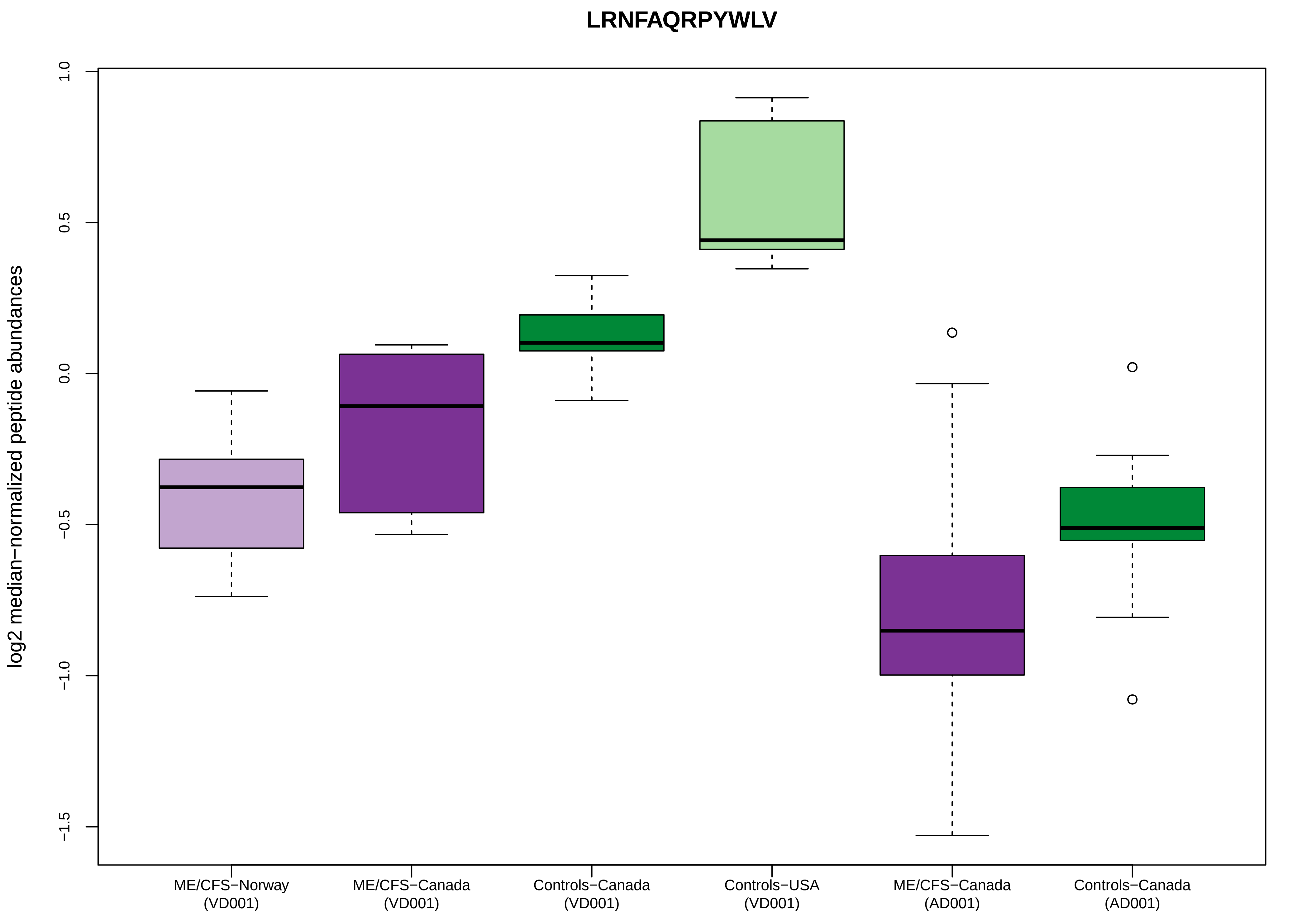
LRNFAQRPYWLV
log2 median−normalized peptide abundances
1.0
0.5
0.0
−0.5
−1.0
−1.5
ME/CFS−Norway (VD001)
ME/CFS−Canada (VD001)
Controls−Canada (VD001)
Controls−USA (VD001)
ME/CFS−Canada (AD001)
Controls−Canada (AD001)

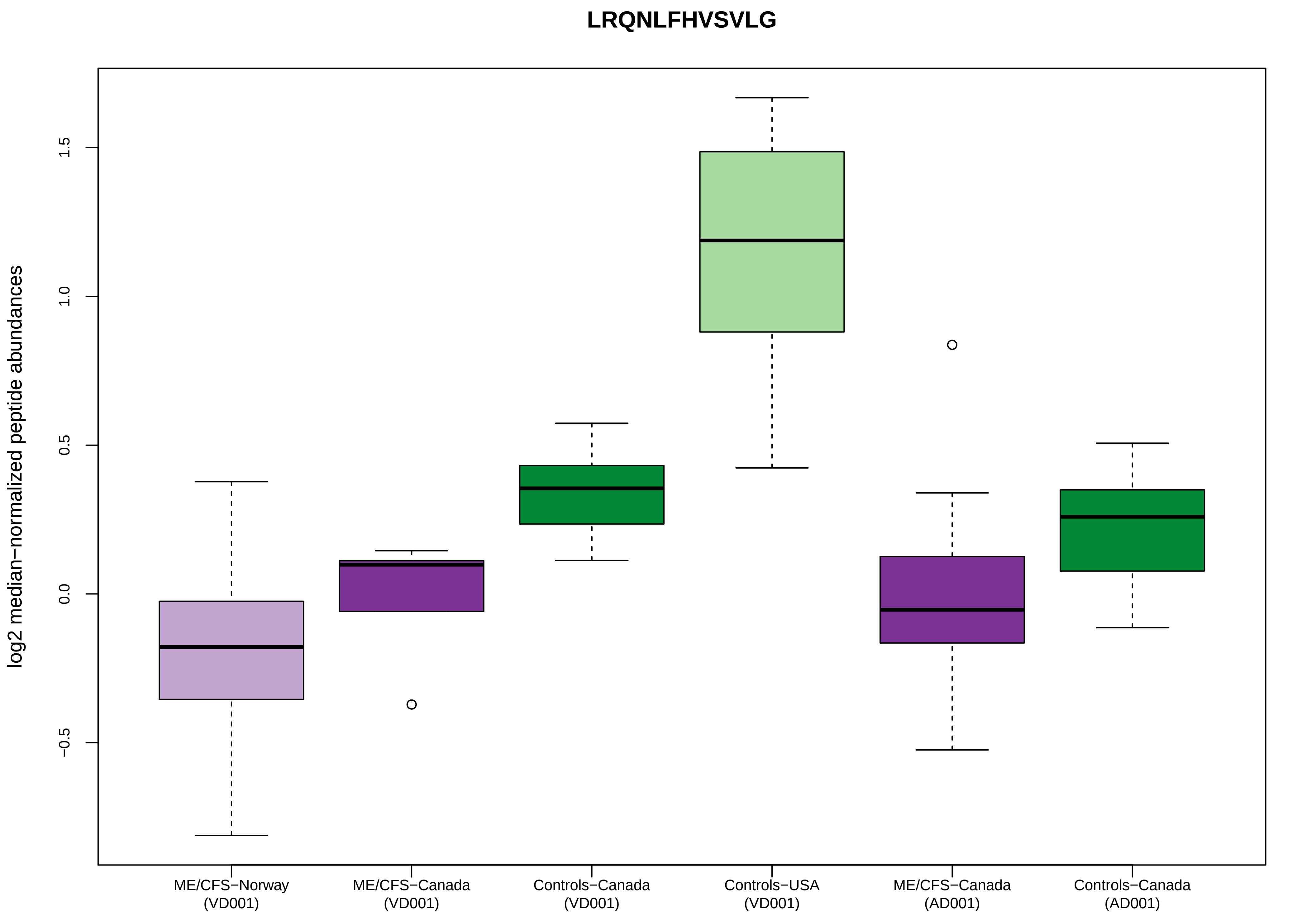
LRQNLFHVSVLG
log2 median−normalized peptide abundances
1.5
1.0
0.5
0.0
−0.5
ME/CFS−Norway (VD001)
ME/CFS−Canada (VD001)
Controls−Canada (VD001)
Controls−USA (VD001)
ME/CFS−Canada (AD001)
Controls−Canada (AD001)

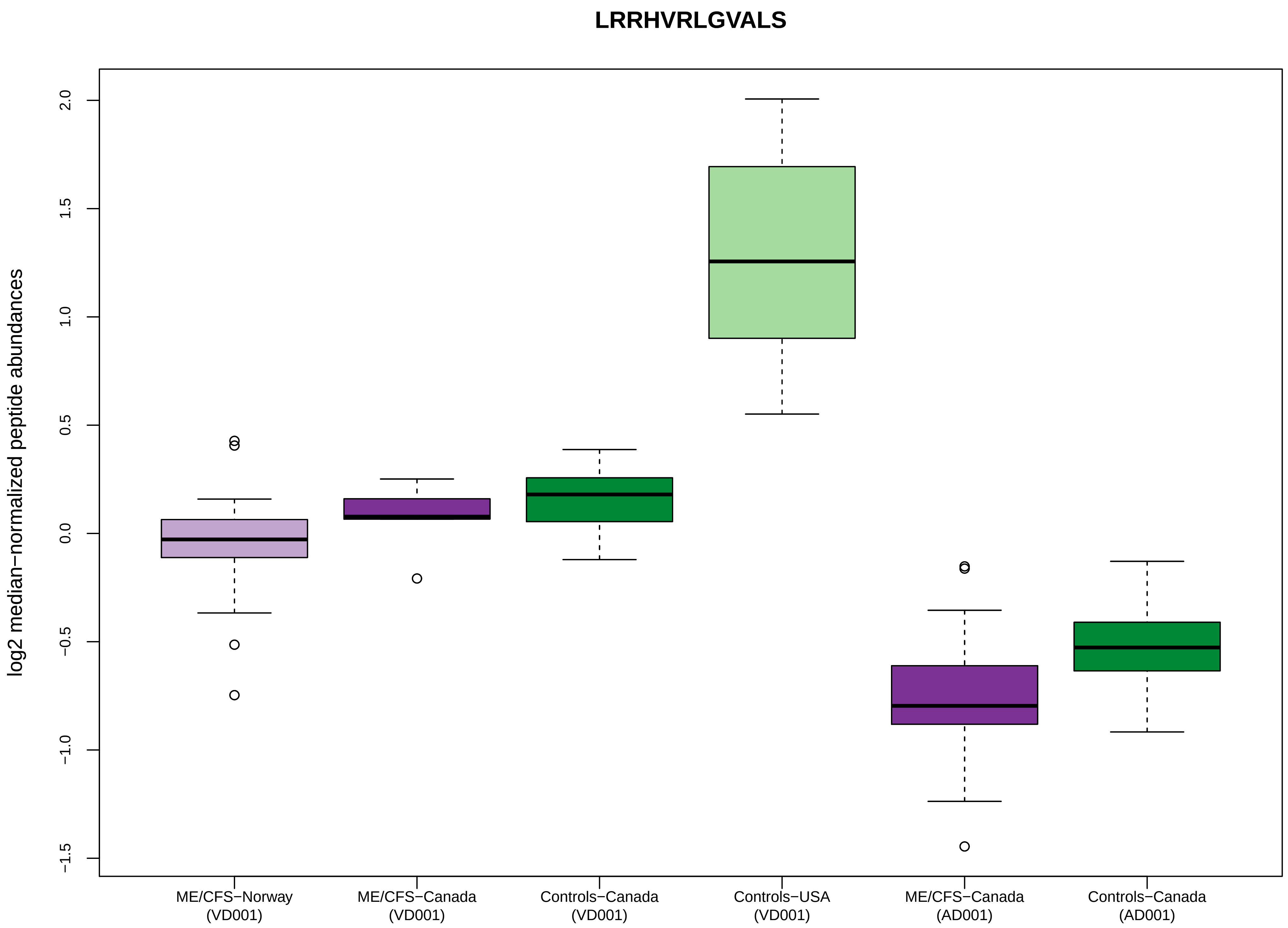

LRRHVRLGVALS
log2 median−normalized peptide abundances
2.0
1.5
1.0
0.5
0.0
−0.5
−1.0
−1.5
ME/CFS−Norway (VD001)
ME/CFS−Canada (VD001)
Controls−Canada (VD001)
Controls−USA (VD001)
ME/CFS−Canada (AD001)
Controls−Canada (AD001)

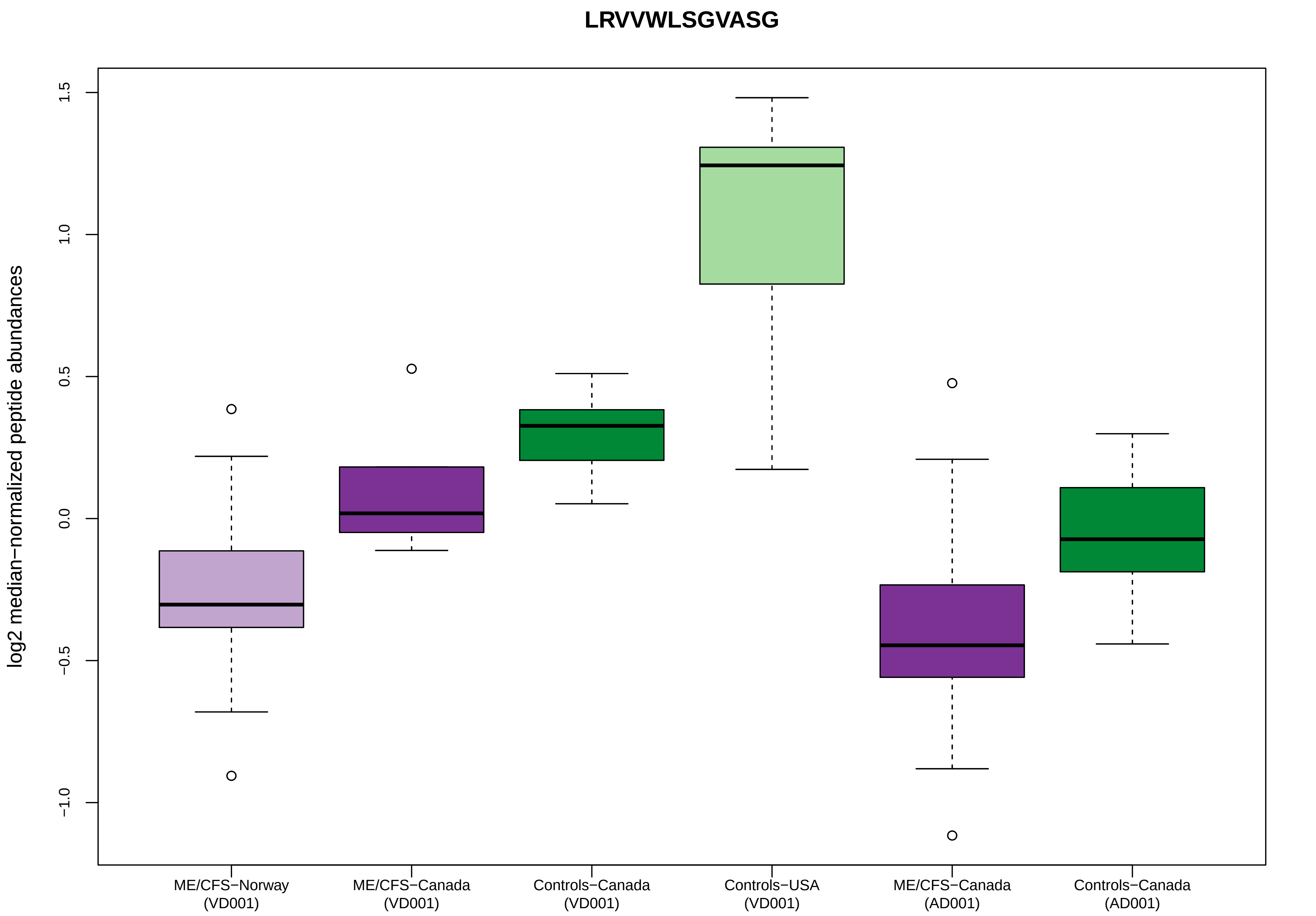
LRVVWLSGVASG
log2 median−normalized peptide abundances
1.5
1.0
0.5
0.0
−0.5
−1.0
ME/CFS−Norway (VD001)
ME/CFS−Canada (VD001)
Controls−Canada (VD001)
Controls−USA (VD001)
ME/CFS−Canada (AD001)
Controls−Canada (AD001)

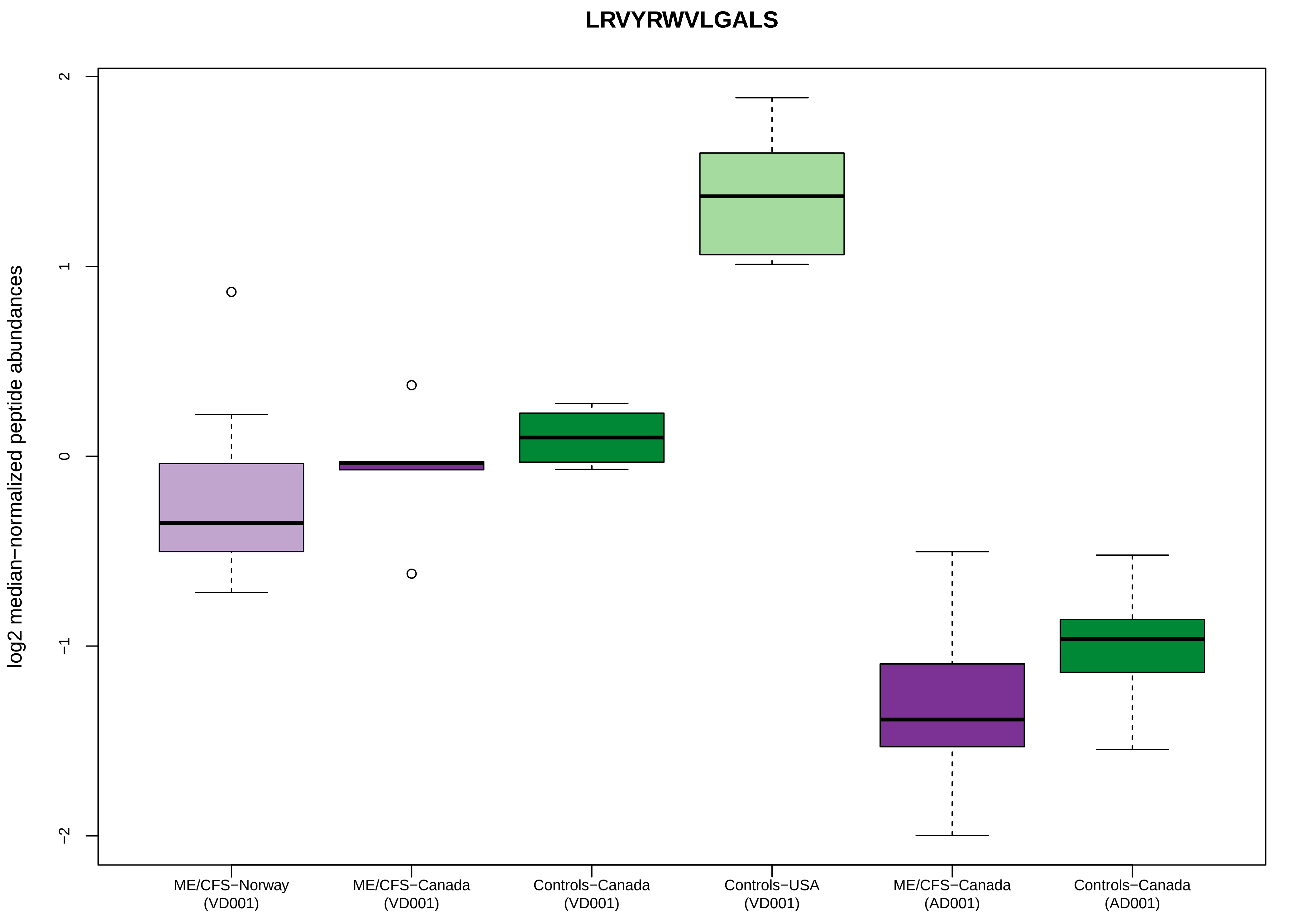
LRVYRWVLGALS
log2 median−normalized peptide abundances
2
1
0
−1
−2
ME/CFS−Norway (VD001)
ME/CFS−Canada (VD001)
Controls−Canada (VD001)
Controls−USA (VD001)
ME/CFS−Canada (AD001)
Controls−Canada (AD001)

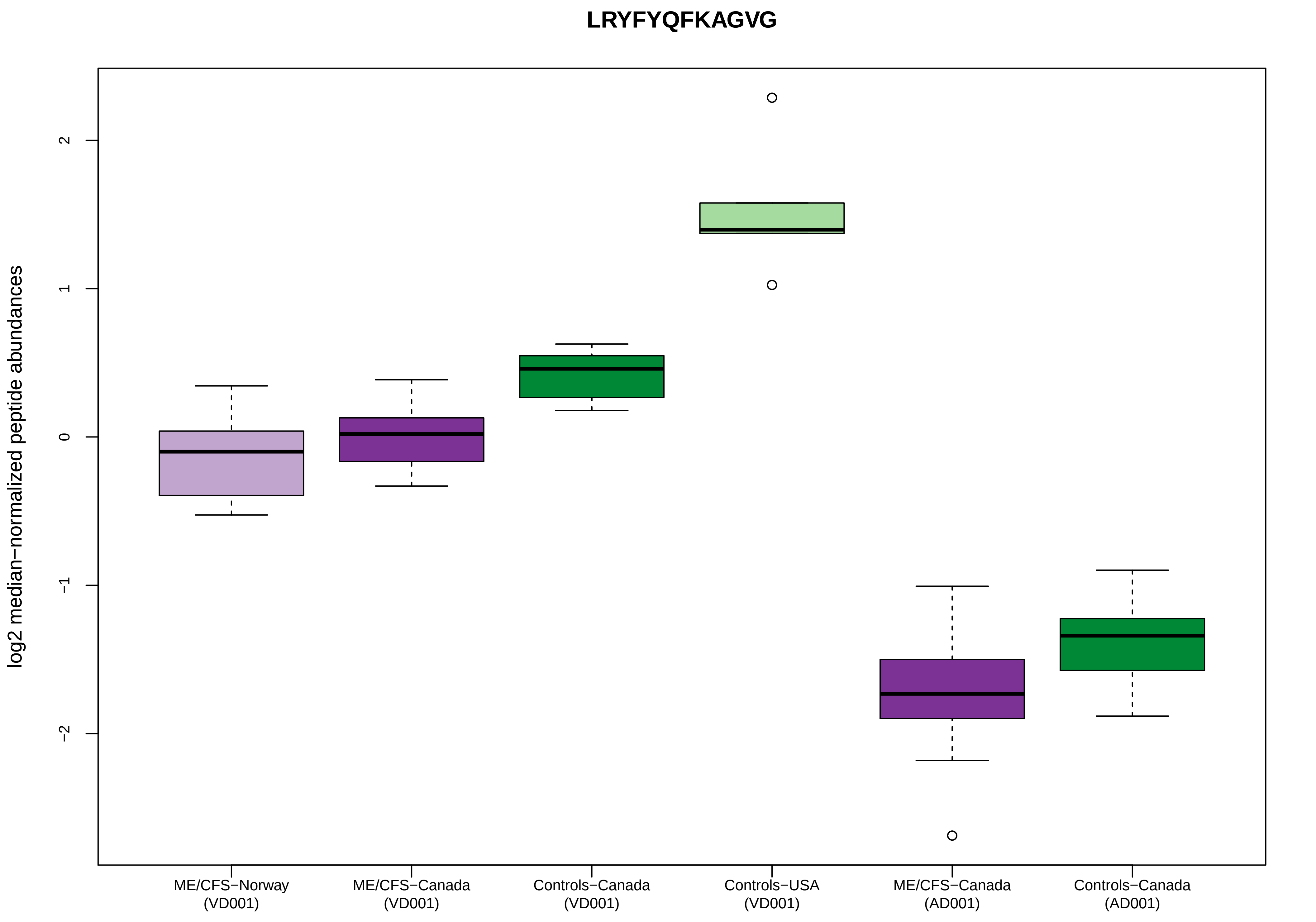

LRYFYQFKAGVG
log2 median−normalized peptide abundances
2
1
0
−1
−2
ME/CFS−Norway (VD001)
ME/CFS−Canada (VD001)
Controls−Canada (VD001)
Controls−USA (VD001)
ME/CFS−Canada (AD001)
Controls−Canada (AD001)

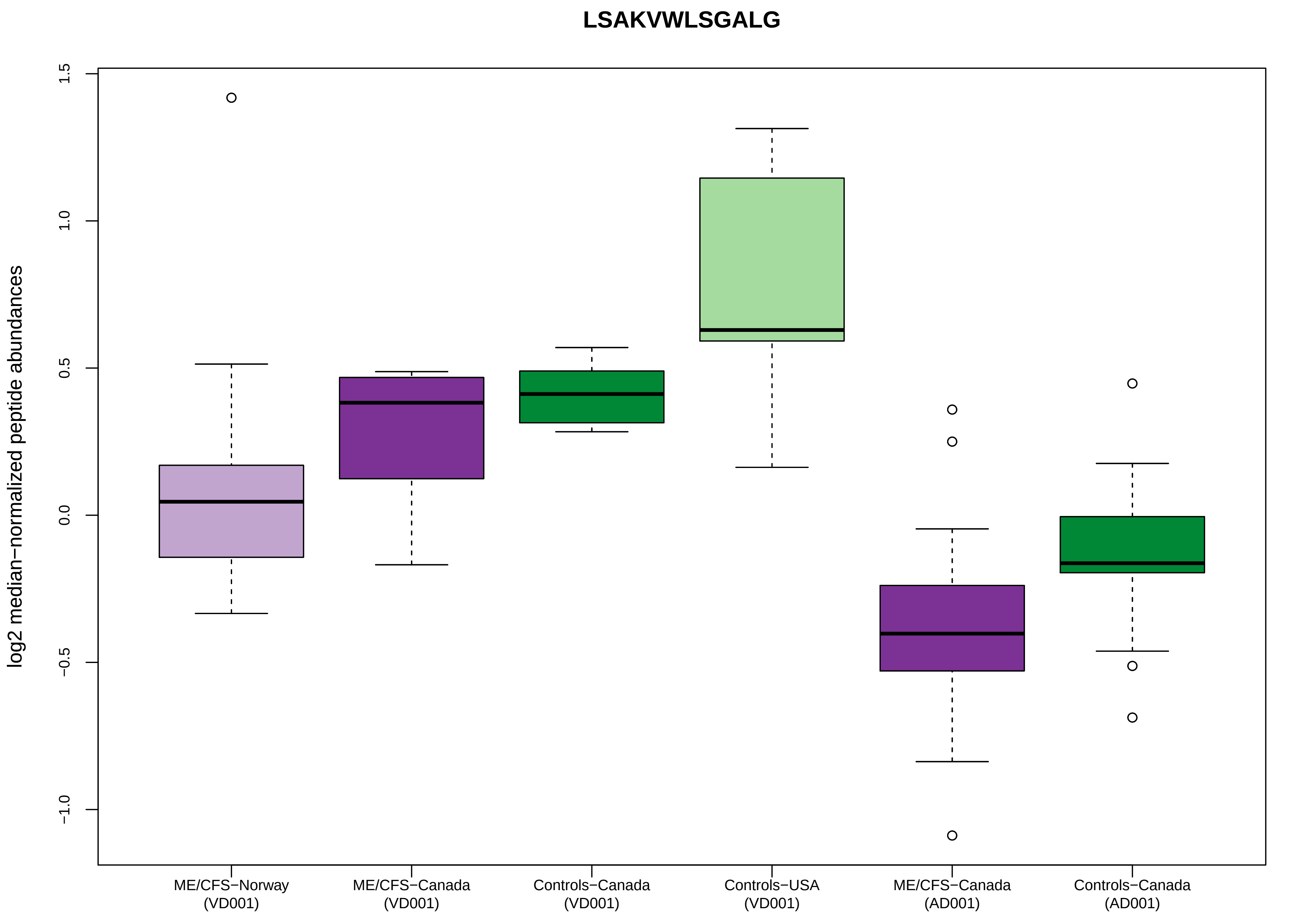

LSAKVWLSGALG
log2 median−normalized peptide abundances
1.5
1.0
0.5
0.0
−0.5
−1.0
ME/CFS−Norway (VD001)
ME/CFS−Canada (VD001)
Controls−Canada (VD001)
Controls−USA (VD001)
ME/CFS−Canada (AD001)
Controls−Canada (AD001)

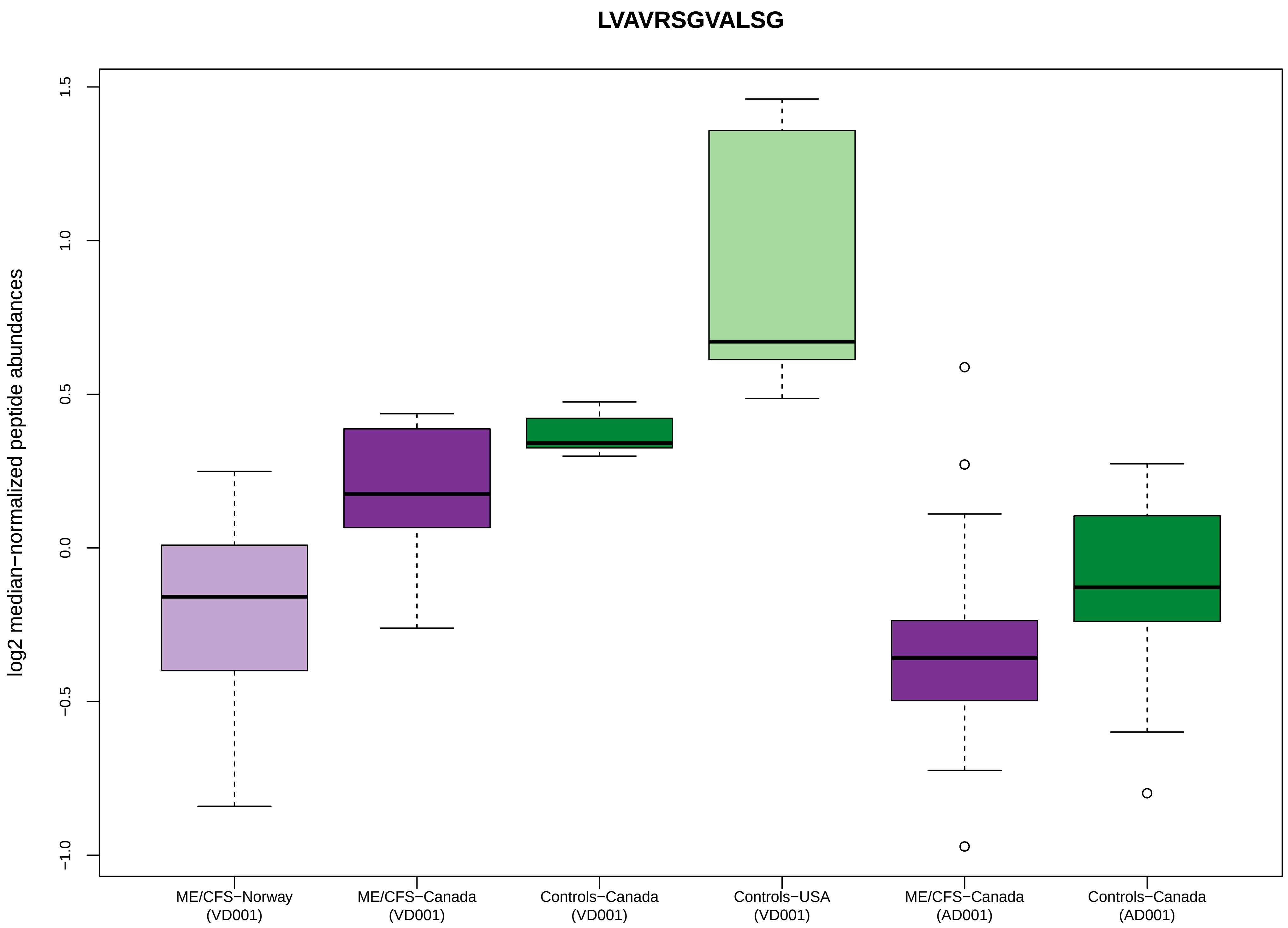
LVAVRSGVALSG
log2 median−normalized peptide abundances
1.5
1.0
0.5
0.0
−0.5
−1.0
ME/CFS−Norway (VD001)
ME/CFS−Canada (VD001)
Controls−Canada (VD001)
Controls−USA (VD001)
ME/CFS−Canada (AD001)
Controls−Canada (AD001)

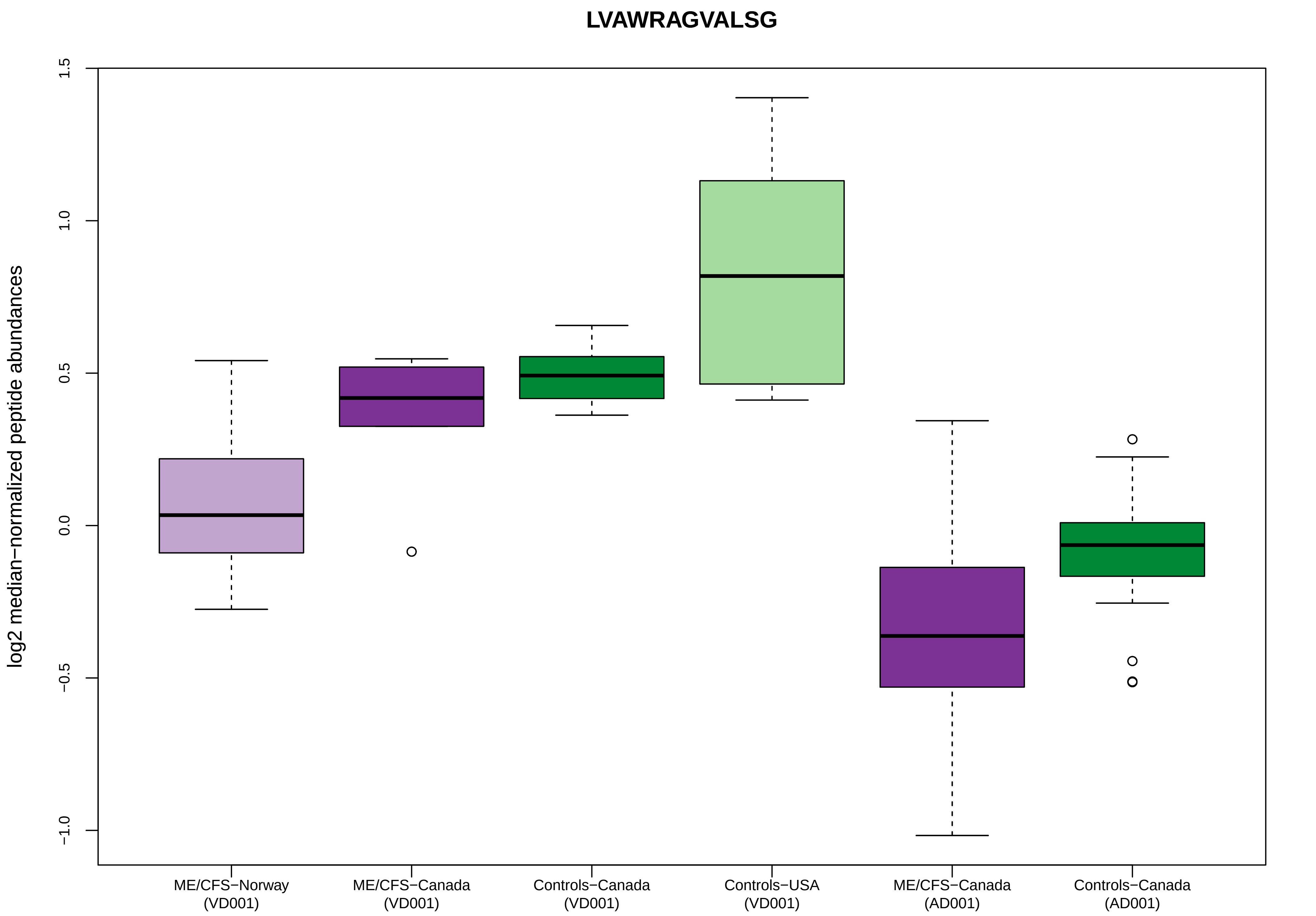
LVAWRAGVALSG
log2 median−normalized peptide abundances
1.5
1.0
0.5
0.0
−0.5
−1.0
ME/CFS−Norway (VD001)
ME/CFS−Canada (VD001)
Controls−Canada (VD001)
Controls−USA (VD001)
ME/CFS−Canada (AD001)
Controls−Canada (AD001)

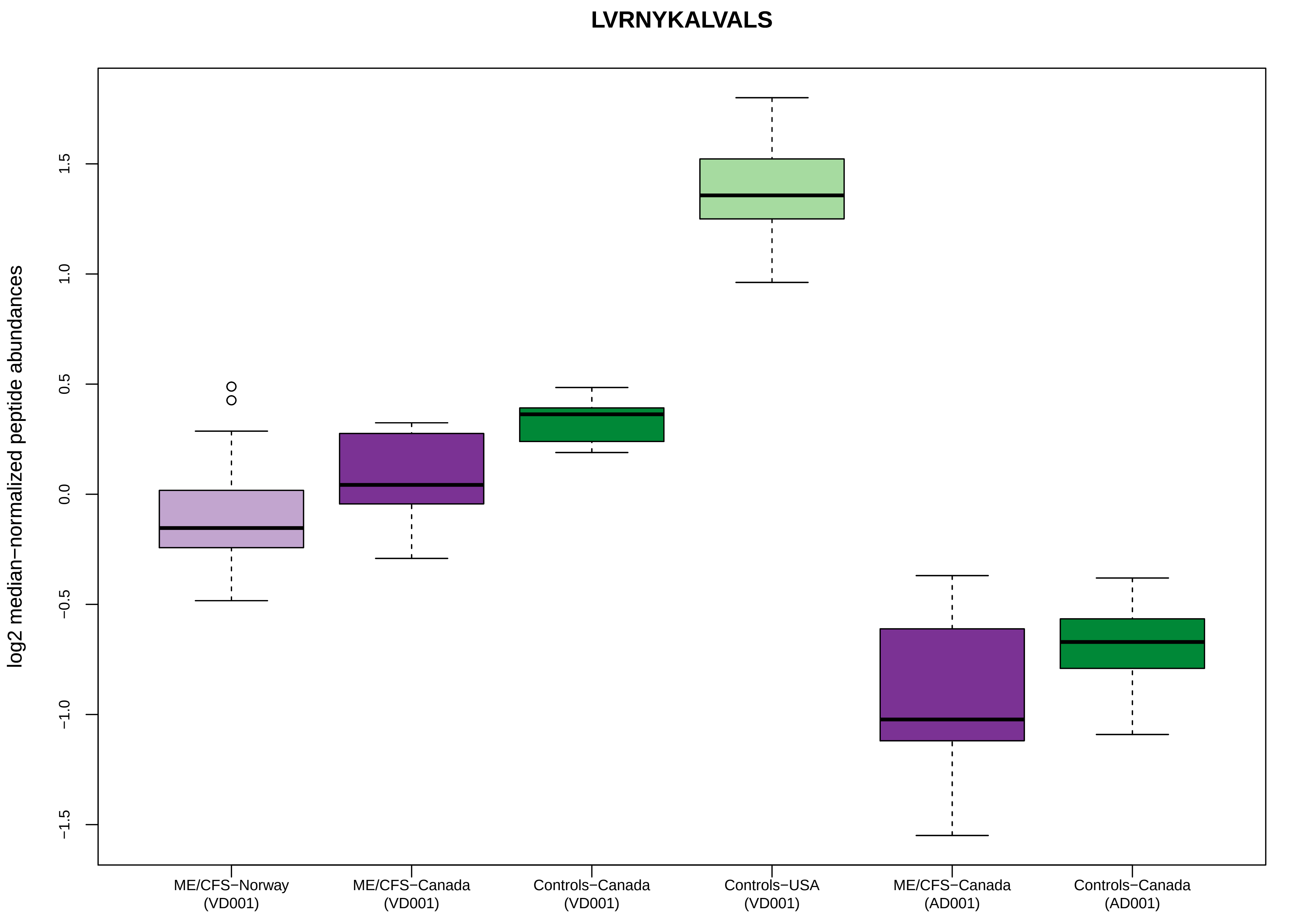
LVRNYKALVALS
log2 median−normalized peptide abundances
1.5
1.0
0.5
0.0
−0.5
−1.0
−1.5
ME/CFS−Norway (VD001)
ME/CFS−Canada (VD001)
Controls−Canada (VD001)
Controls−USA (VD001)
ME/CFS−Canada (AD001)
Controls−Canada (AD001)

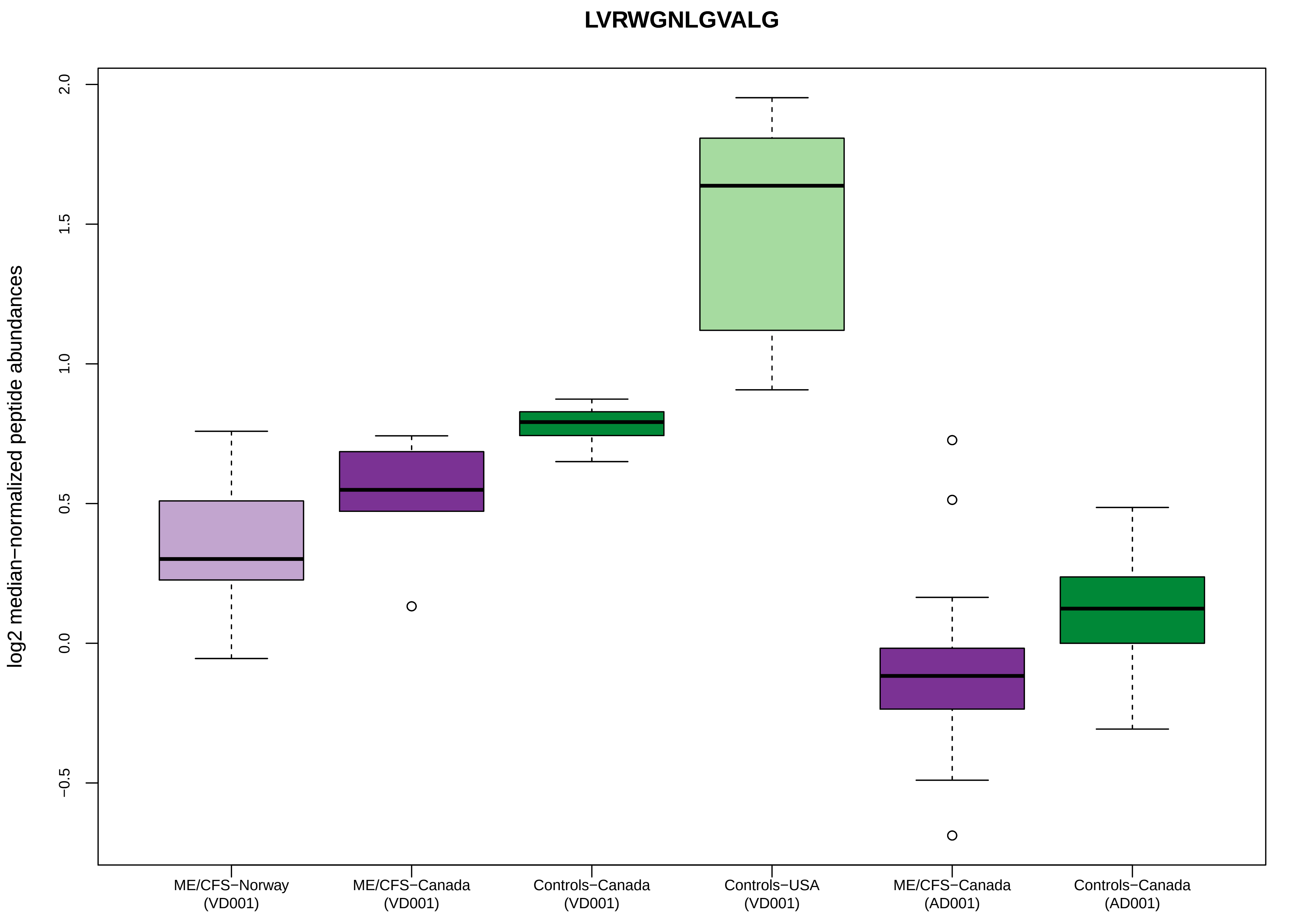

LVRWGNLGVALG
log2 median−normalized peptide abundances
2.0
1.5
1.0
0.5
0.0
−0.5
ME/CFS−Norway (VD001)
ME/CFS−Canada (VD001)
Controls−Canada (VD001)
Controls−USA (VD001)
ME/CFS−Canada (AD001)
Controls−Canada (AD001)

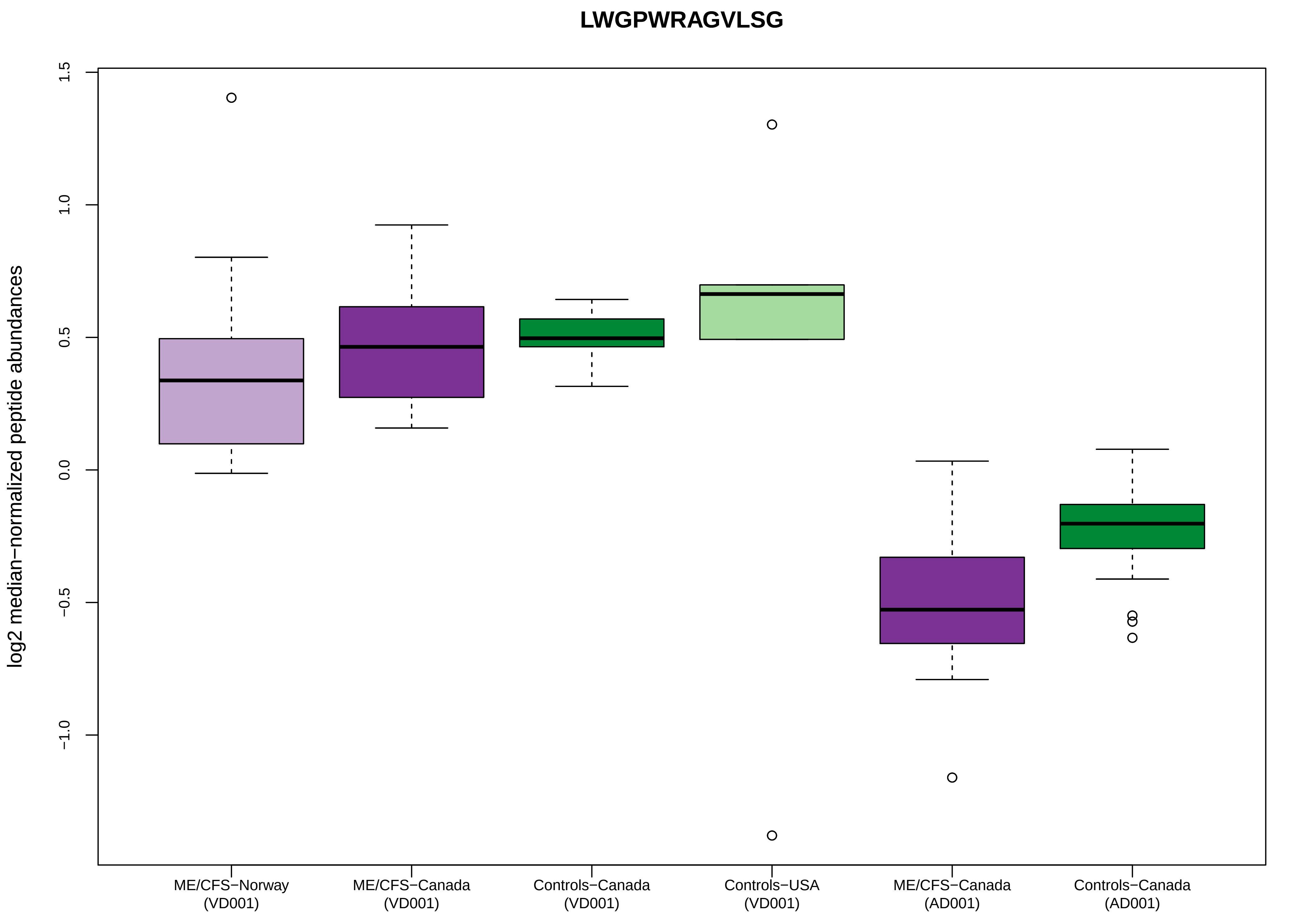

LWGPWRAGVLSG
log2 median−normalized peptide abundances
1.5
1.0
0.5
0.0
−0.5
−1.0
ME/CFS−Norway (VD001)
ME/CFS−Canada (VD001)
Controls−Canada (VD001)
Controls−USA (VD001)
ME/CFS−Canada (AD001)
Controls−Canada (AD001)

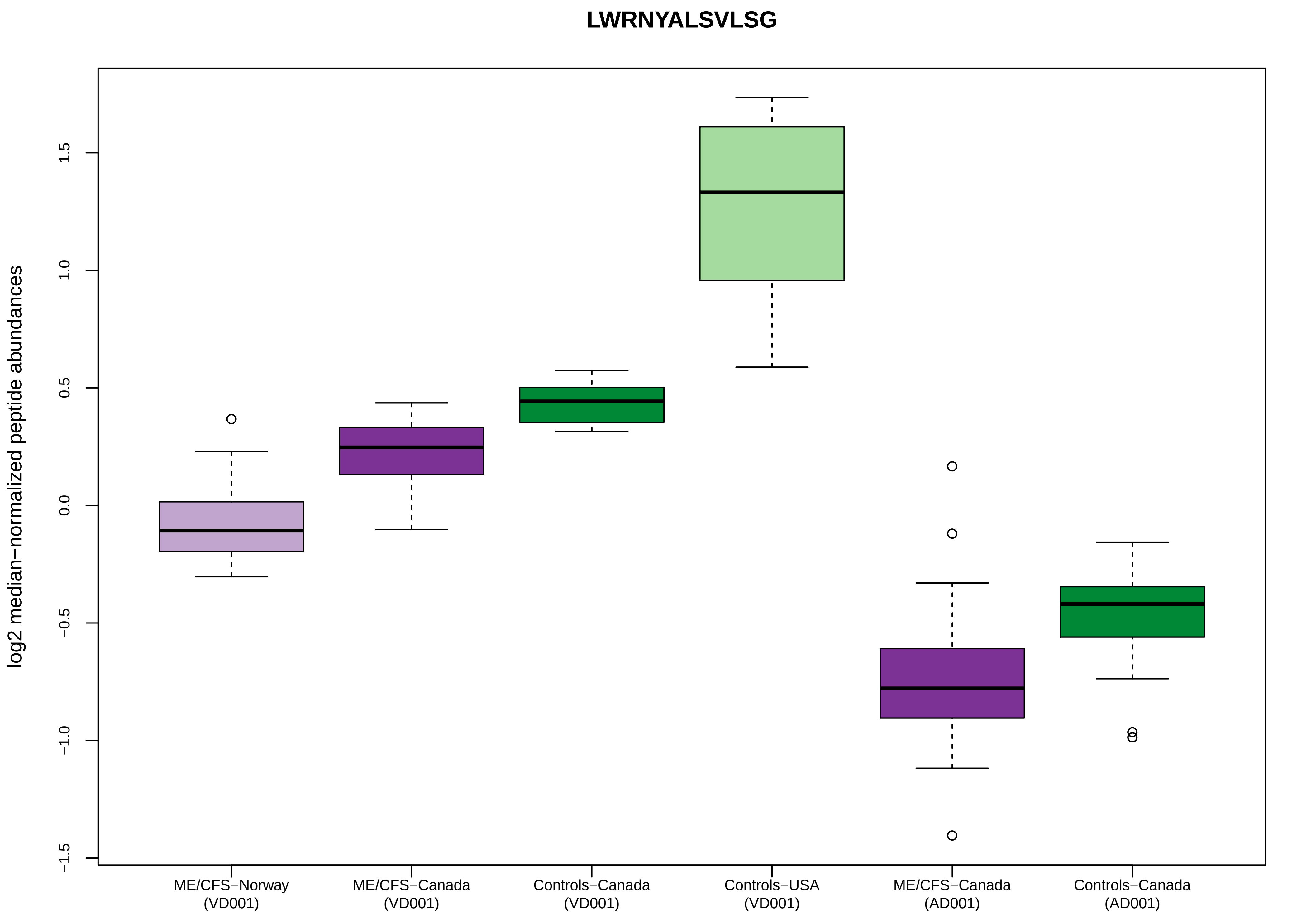
LWRNYALSVLSG
log2 median−normalized peptide abundances
1.5
1.0
0.5
0.0
−0.5
−1.0
−1.5
ME/CFS−Norway (VD001)
ME/CFS−Canada (VD001)
Controls−Canada (VD001)
Controls−USA (VD001)
ME/CFS−Canada (AD001)
Controls−Canada (AD001)

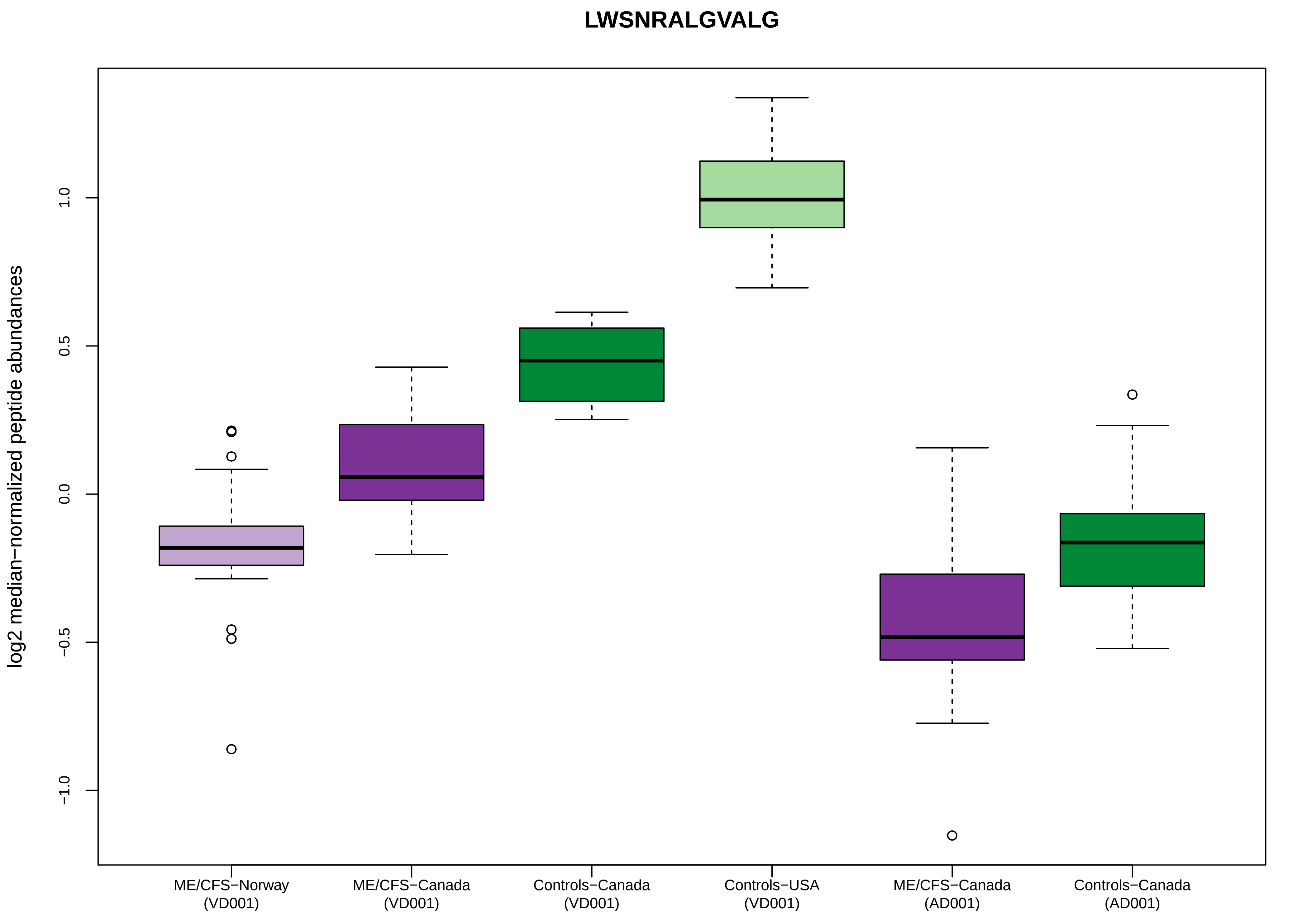

LWSNRALGVALG
log2 median−normalized peptide abundances
1.0
0.5
0.0
−0.5
−1.0
ME/CFS−Norway (VD001)
ME/CFS−Canada (VD001)
Controls−Canada (VD001)
Controls−USA (VD001)
ME/CFS−Canada (AD001)
Controls−Canada (AD001)

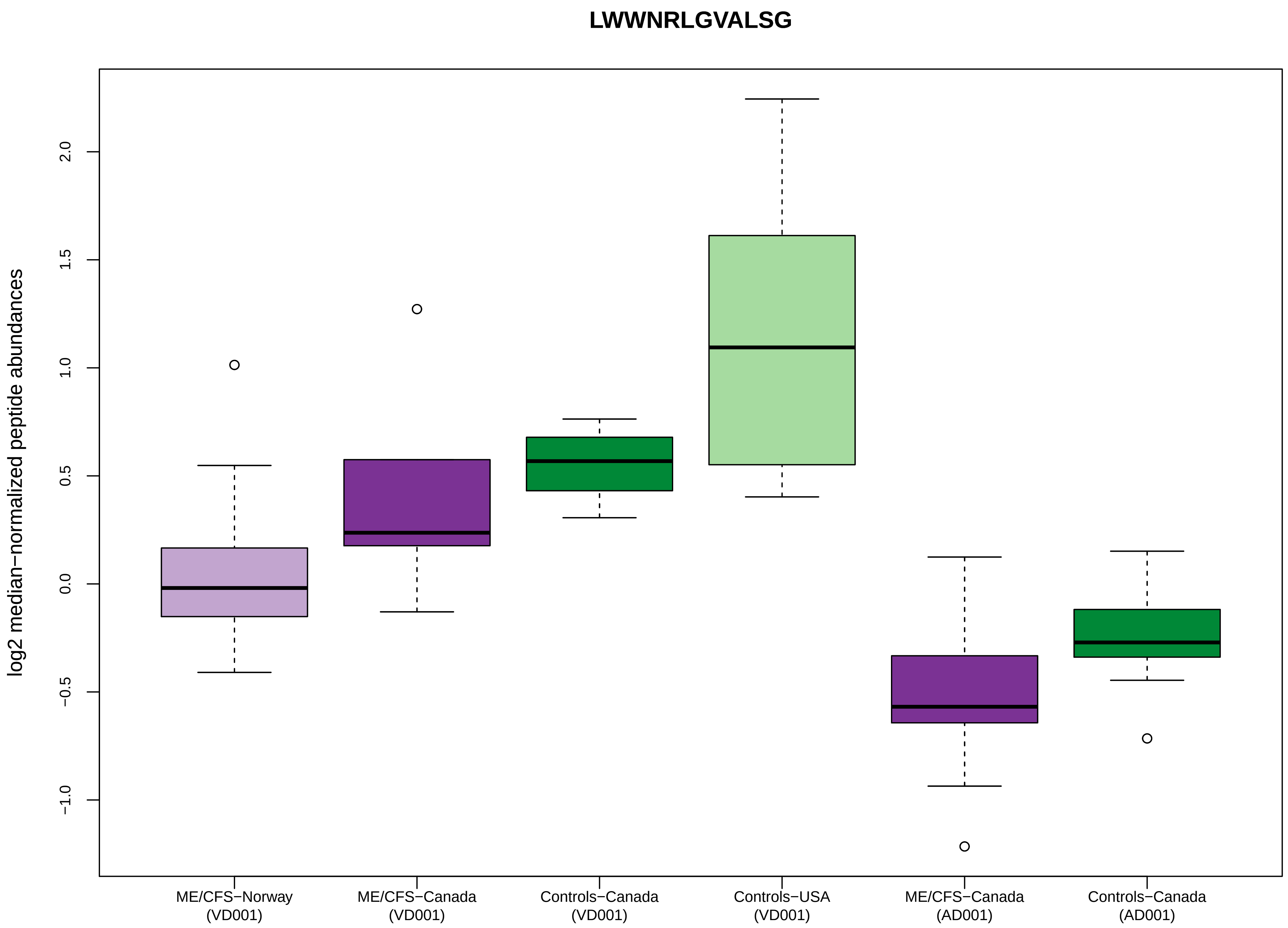
LWWNRLGVALSG
log2 median−normalized peptide abundances
2.0
1.5
1.0
0.5
0.0
−0.5
−1.0
ME/CFS−Norway (VD001)
ME/CFS−Canada (VD001)
Controls−Canada (VD001)
Controls−USA (VD001)
ME/CFS−Canada (AD001)
Controls−Canada (AD001)

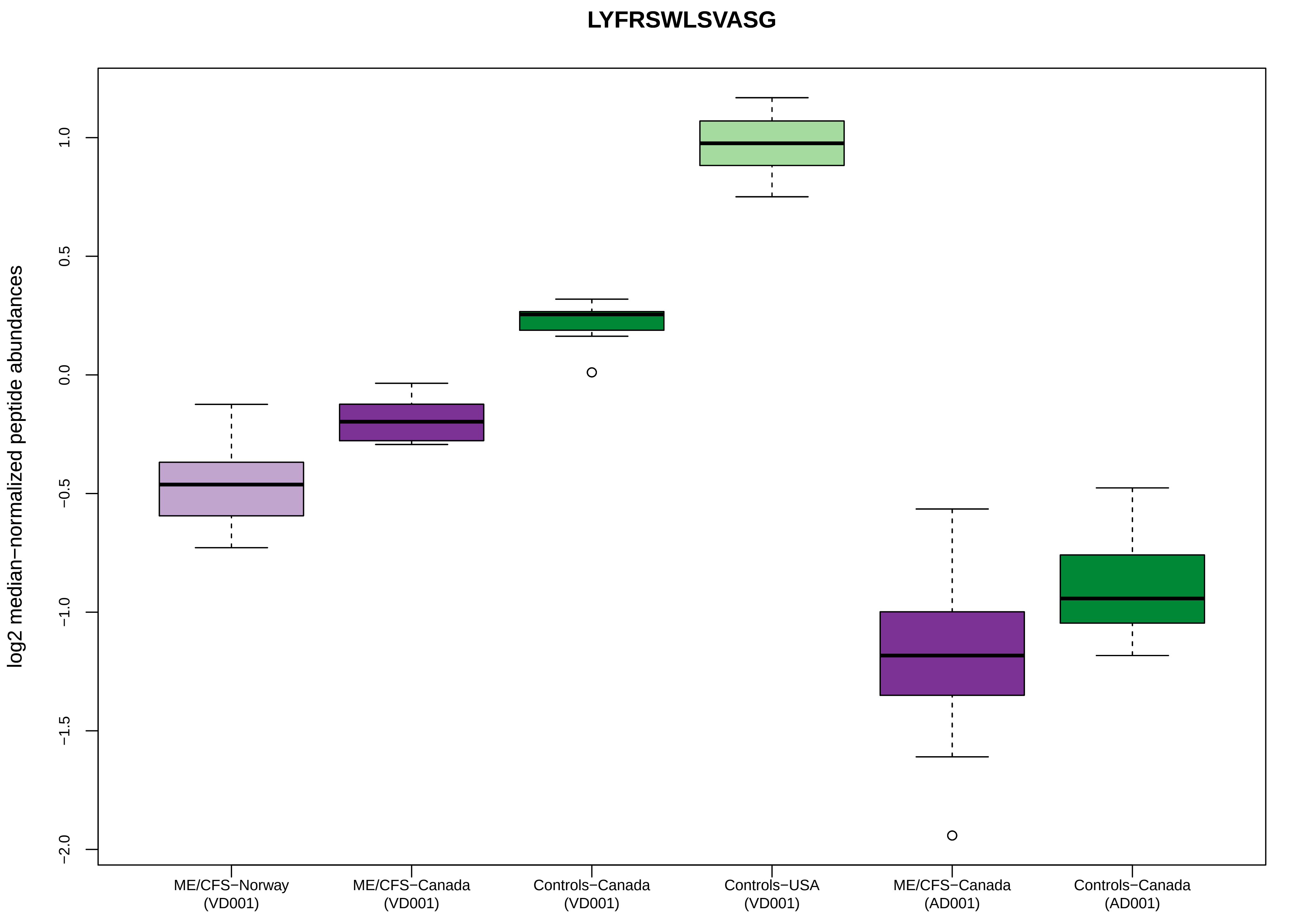

LYFRSWLSVASG
log2 median−normalized peptide abundances
1.0
0.5
0.0
−0.5
−1.0
−1.5
−2.0
ME/CFS−Norway (VD001)
ME/CFS−Canada (VD001)
Controls−Canada (VD001)
Controls−USA (VD001)
ME/CFS−Canada (AD001)
Controls−Canada (AD001)

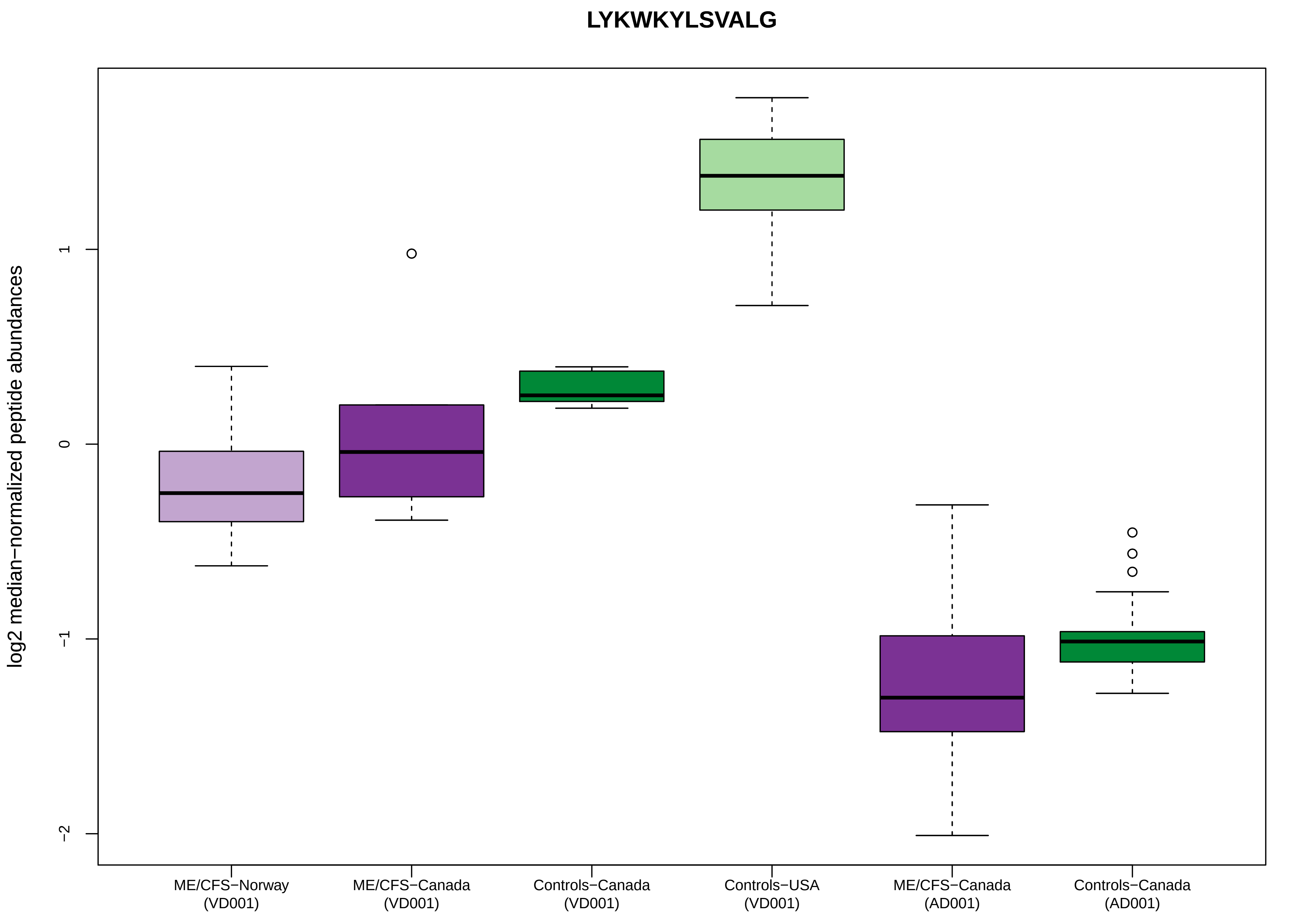

LYKWKYLSVALG
log2 median−normalized peptide abundances
1
0
−1
−2
ME/CFS−Norway (VD001)
ME/CFS−Canada (VD001)
Controls−Canada (VD001)
Controls−USA (VD001)
ME/CFS−Canada (AD001)
Controls−Canada (AD001)

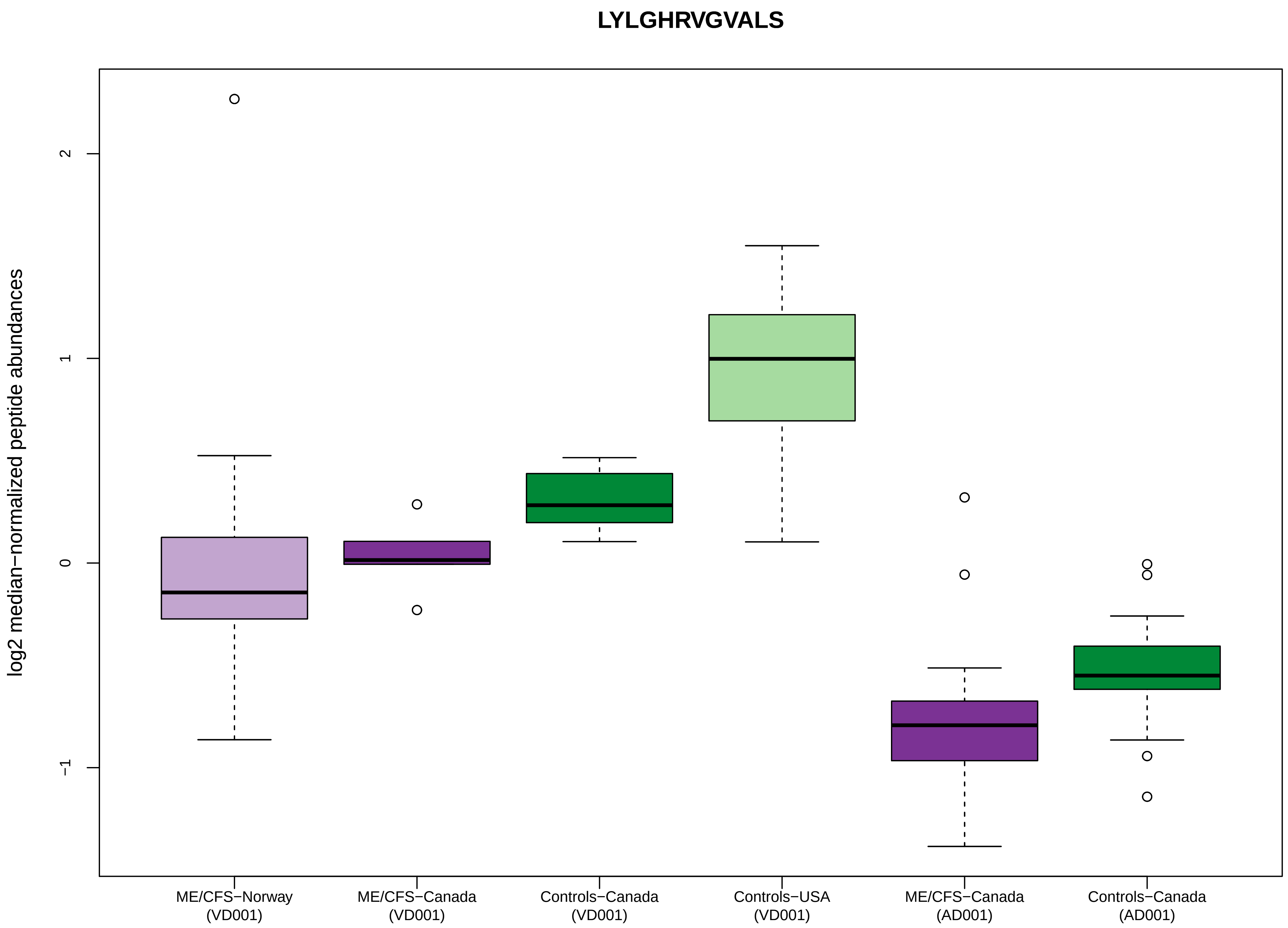
LYLGHRVGVALS
log2 median−normalized peptide abundances
2
1
0
−1
ME/CFS−Norway (VD001)
ME/CFS−Canada (VD001)
Controls−Canada (VD001)
Controls−USA (VD001)
ME/CFS−Canada (AD001)
Controls−Canada (AD001)

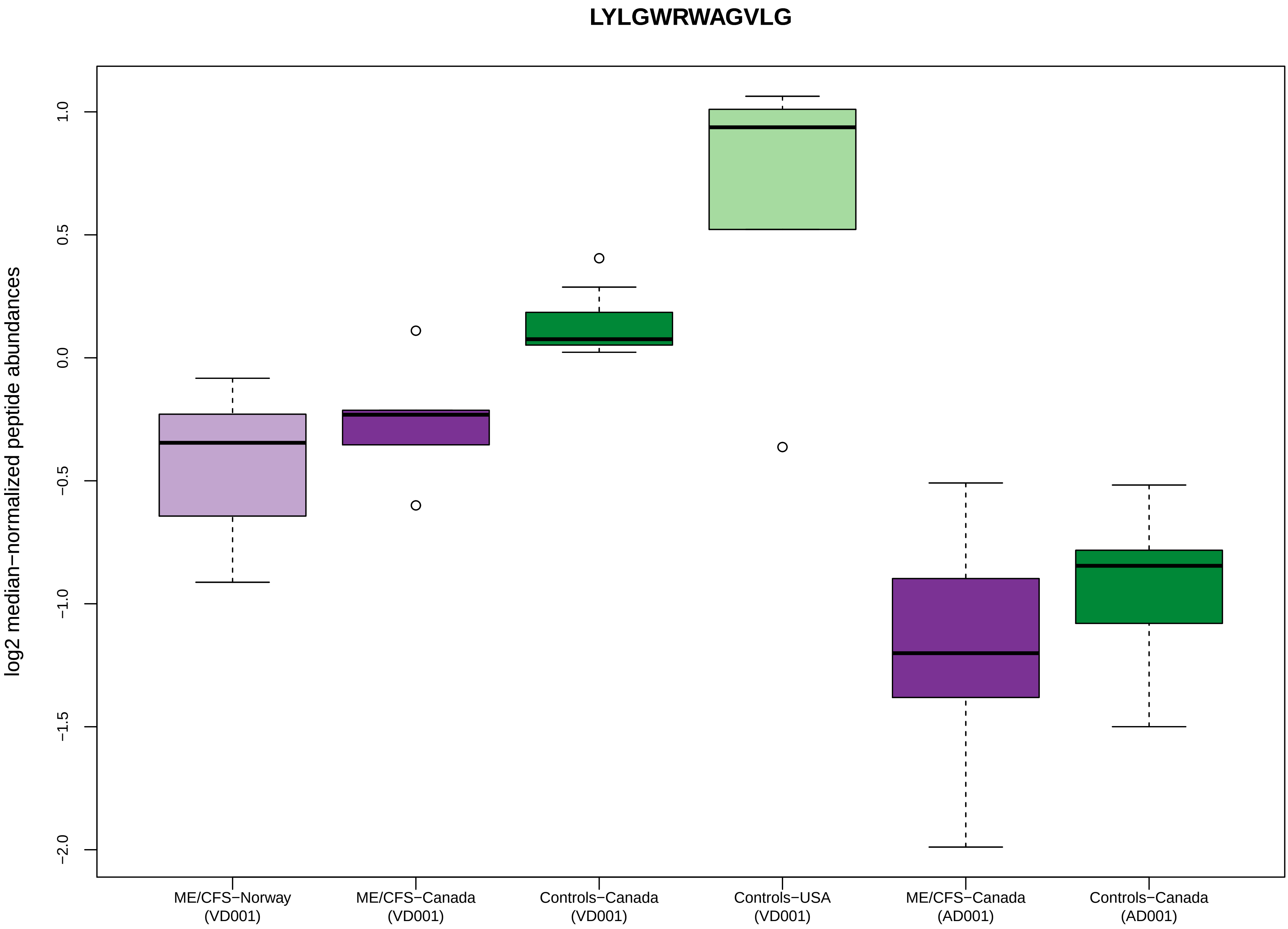

LYLGWRWAGVLG
log2 median−normalized peptide abundances
1.0
0.5
0.0
−0.5
−1.0
−1.5
−2.0
ME/CFS−Norway (VD001)
ME/CFS−Canada (VD001)
Controls−Canada (VD001)
Controls−USA (VD001)
ME/CFS−Canada (AD001)
Controls−Canada (AD001)

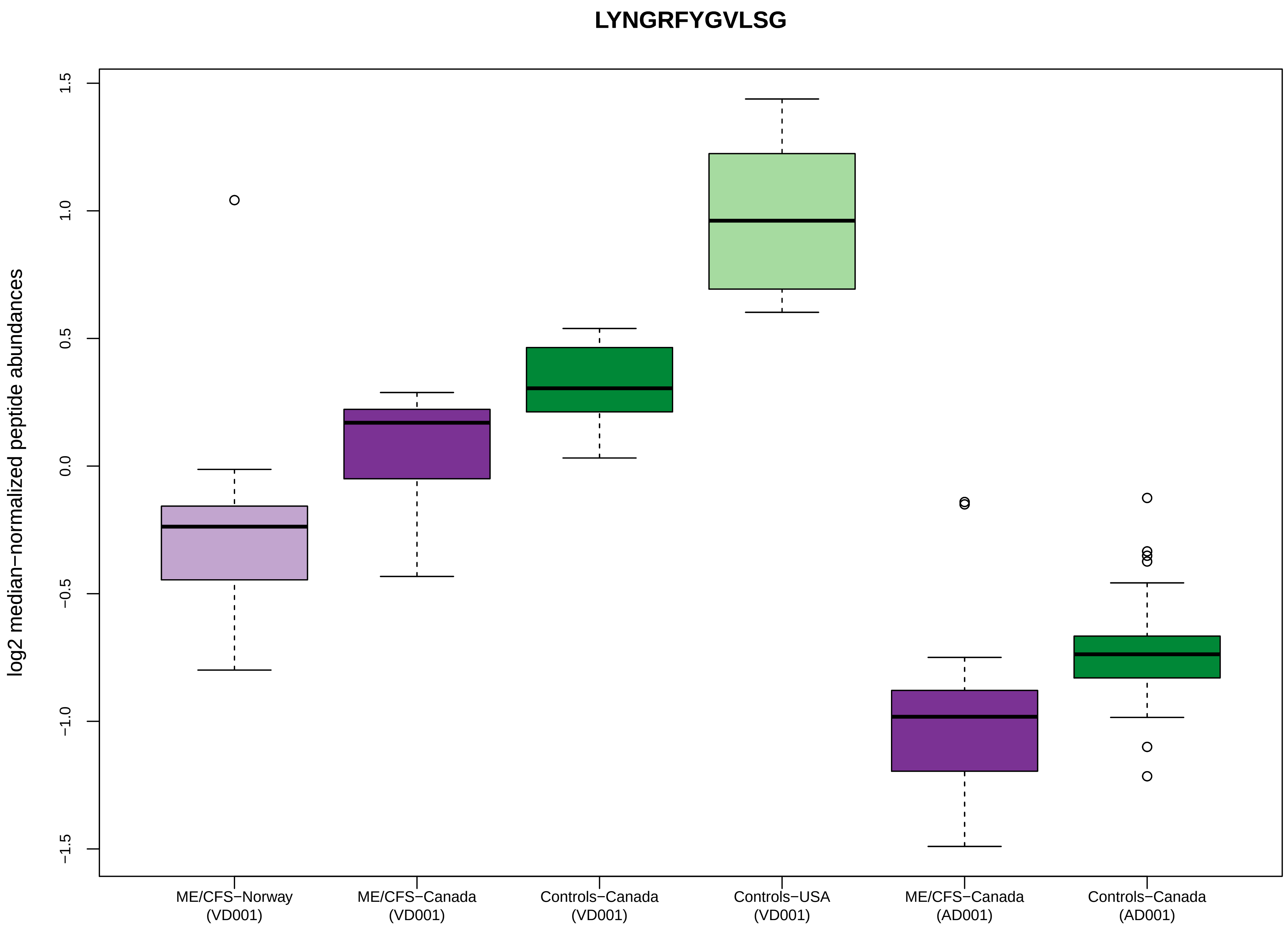

LYNGRFYGVLSG
log2 median−normalized peptide abundances
1.5
1.0
0.5
0.0
−0.5
−1.0
−1.5
ME/CFS−Norway (VD001)
ME/CFS−Canada (VD001)
Controls−Canada (VD001)
Controls−USA (VD001)
ME/CFS−Canada (AD001)
Controls−Canada (AD001)

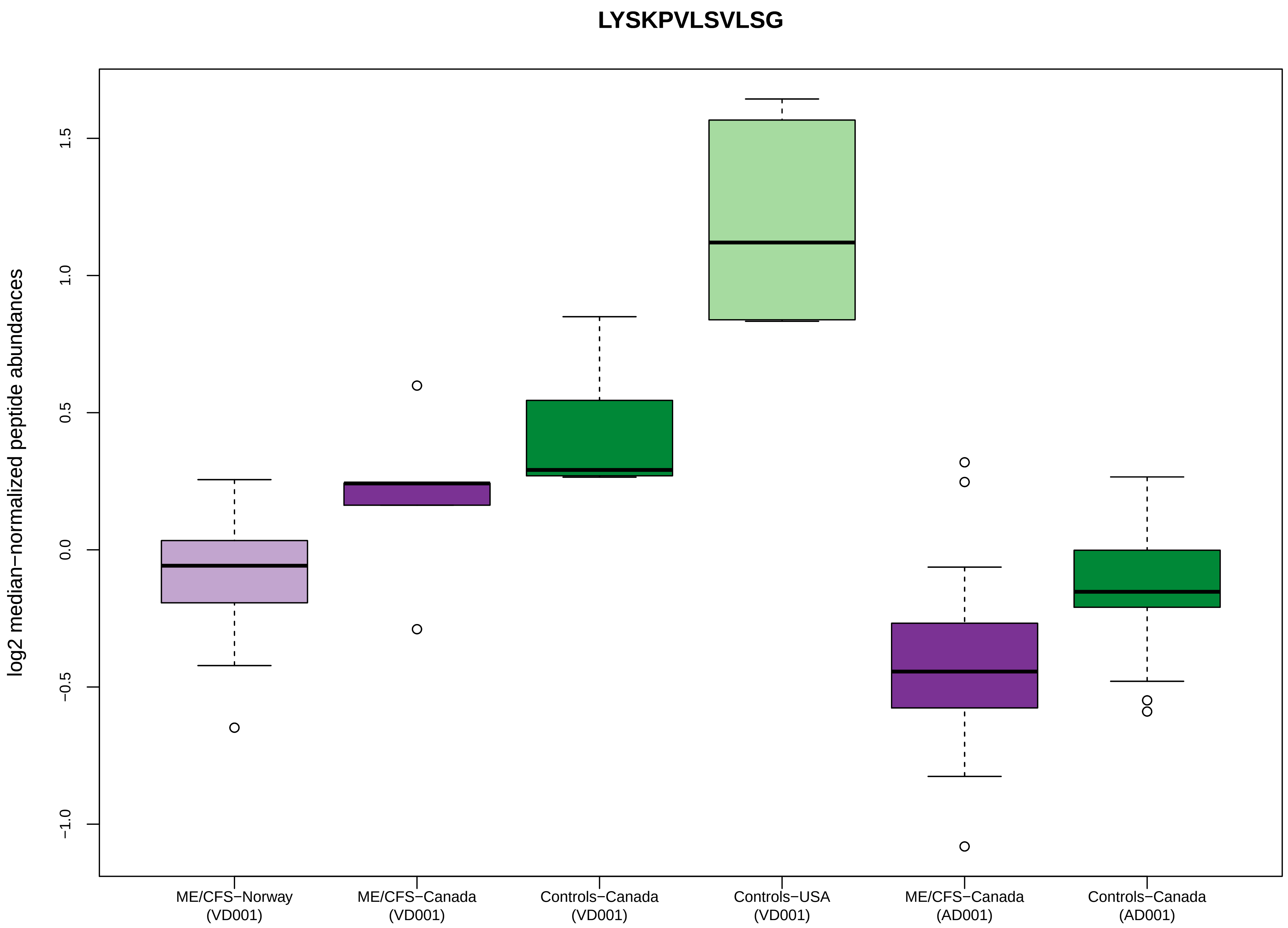

LYSKPVLSVLSG
log2 median−normalized peptide abundances
1.5
1.0
0.5
0.0
−0.5
−1.0
ME/CFS−Norway (VD001)
ME/CFS−Canada (VD001)
Controls−Canada (VD001)
Controls−USA (VD001)
ME/CFS−Canada (AD001)
Controls−Canada (AD001)

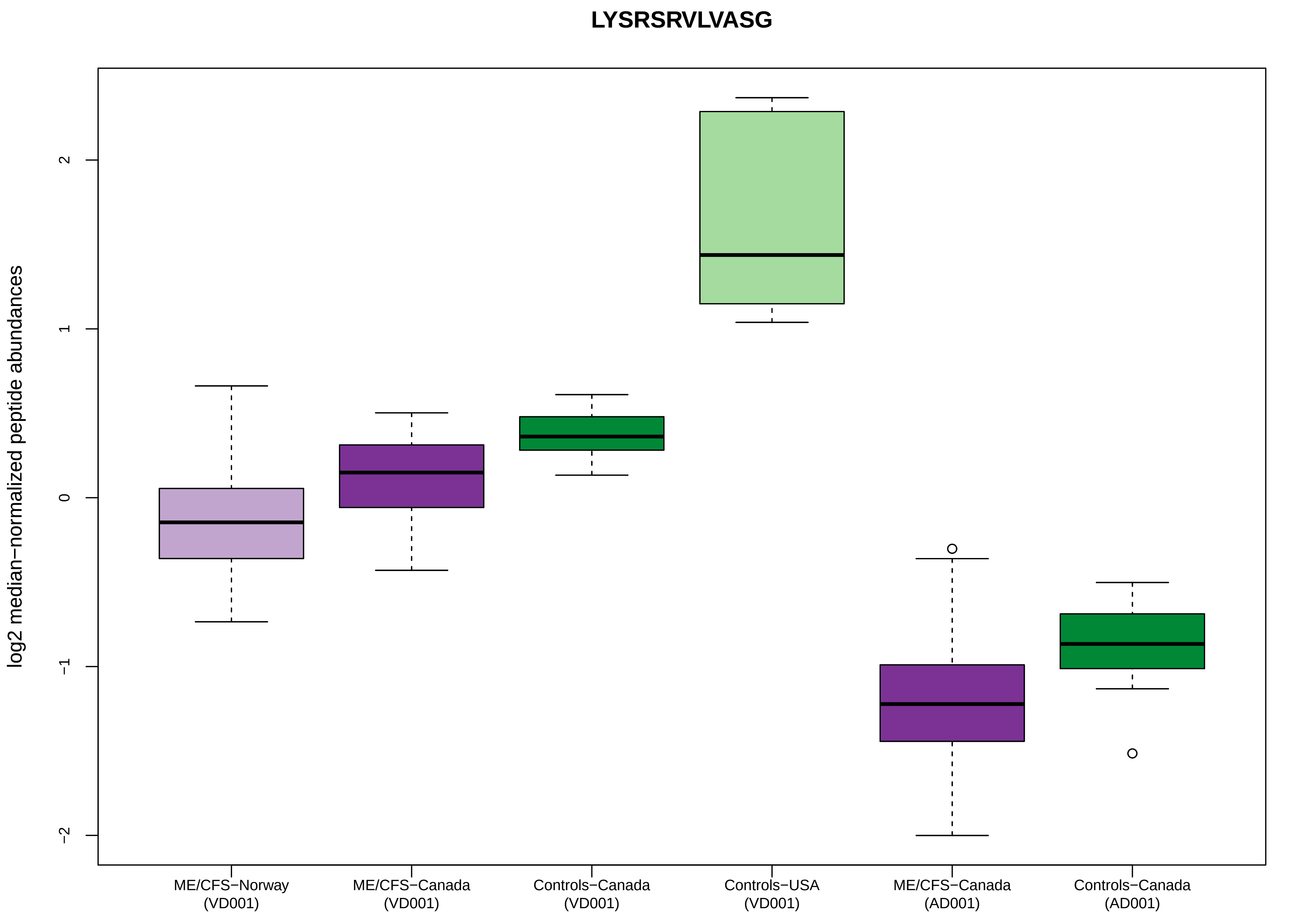
LYSRSRVLVASG
log2 median−normalized peptide abundances
2
1
0
−1
−2
ME/CFS−Norway (VD001)
ME/CFS−Canada (VD001)
Controls−Canada (VD001)
Controls−USA (VD001)
ME/CFS−Canada (AD001)
Controls−Canada (AD001)

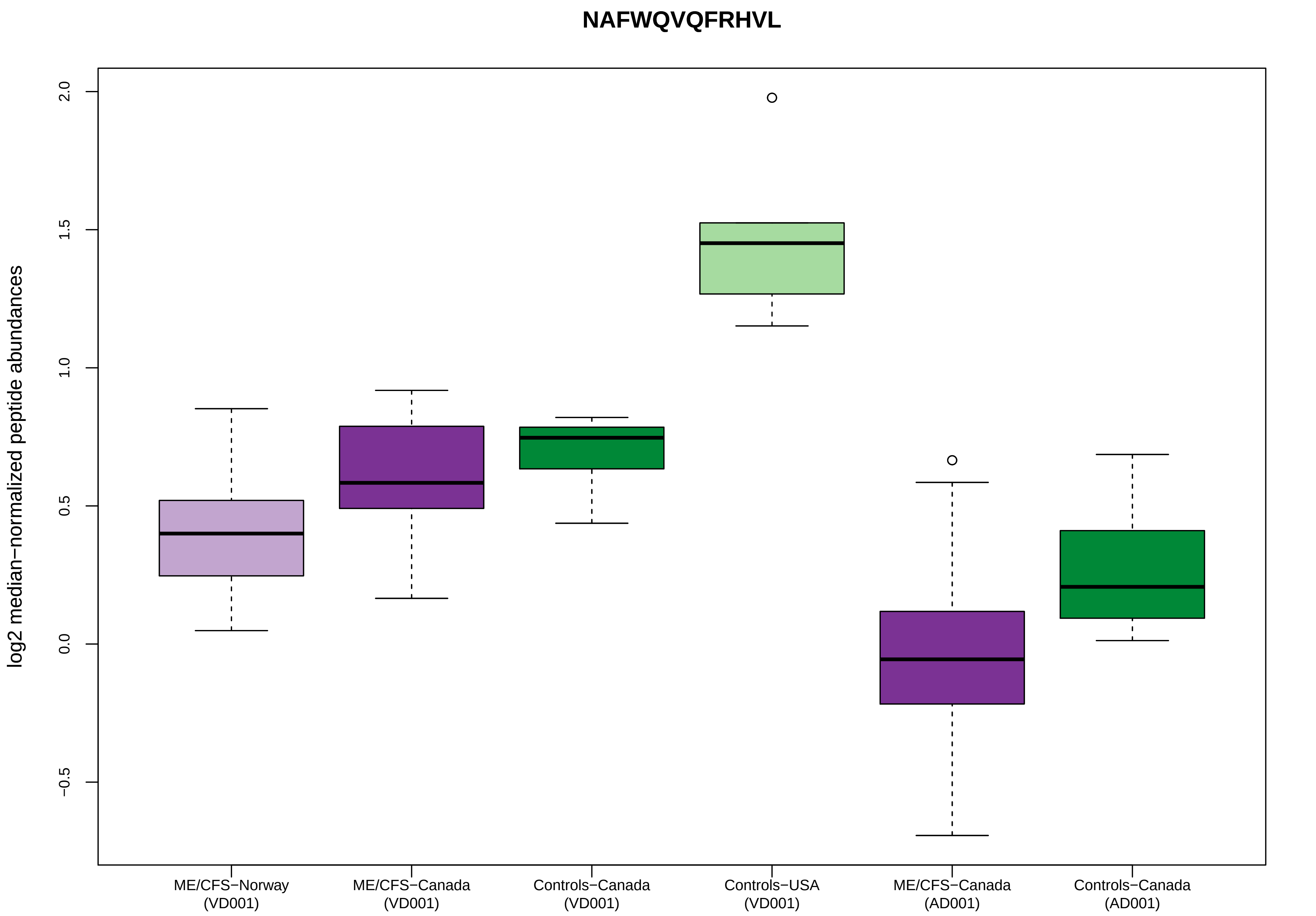

NAFWQVQFRHVL
log2 median−normalized peptide abundances
2.0
1.5
1.0
0.5
0.0
−0.5
ME/CFS−Norway (VD001)
ME/CFS−Canada (VD001)
Controls−Canada (VD001)
Controls−USA (VD001)
ME/CFS−Canada (AD001)
Controls−Canada (AD001)

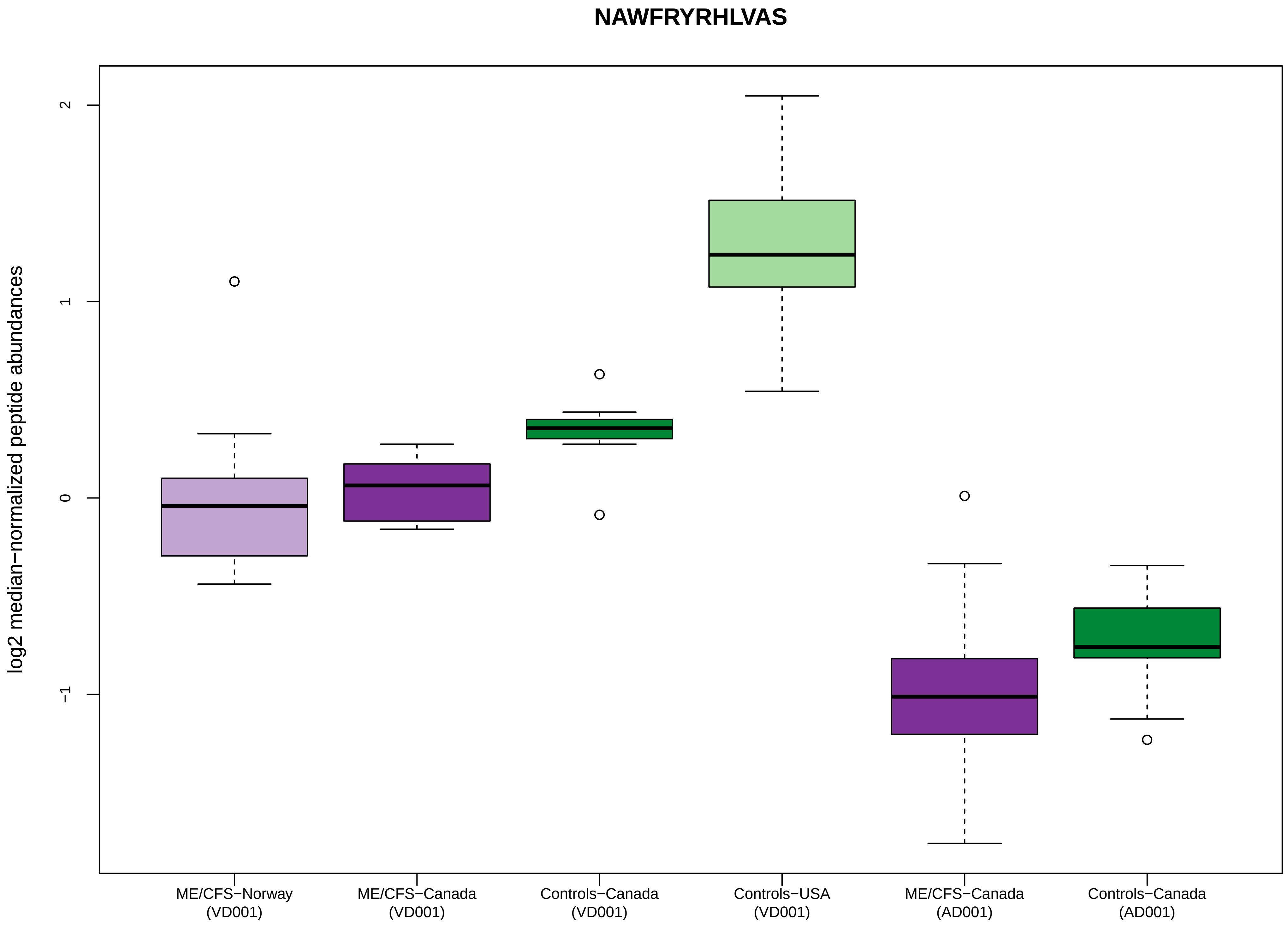

NAWFRYRHLVAS
log2 median−normalized peptide abundances
2
1
0
−1
ME/CFS−Norway (VD001)
ME/CFS−Canada (VD001)
Controls−Canada (VD001)
Controls−USA (VD001)
ME/CFS−Canada (AD001)
Controls−Canada (AD001)

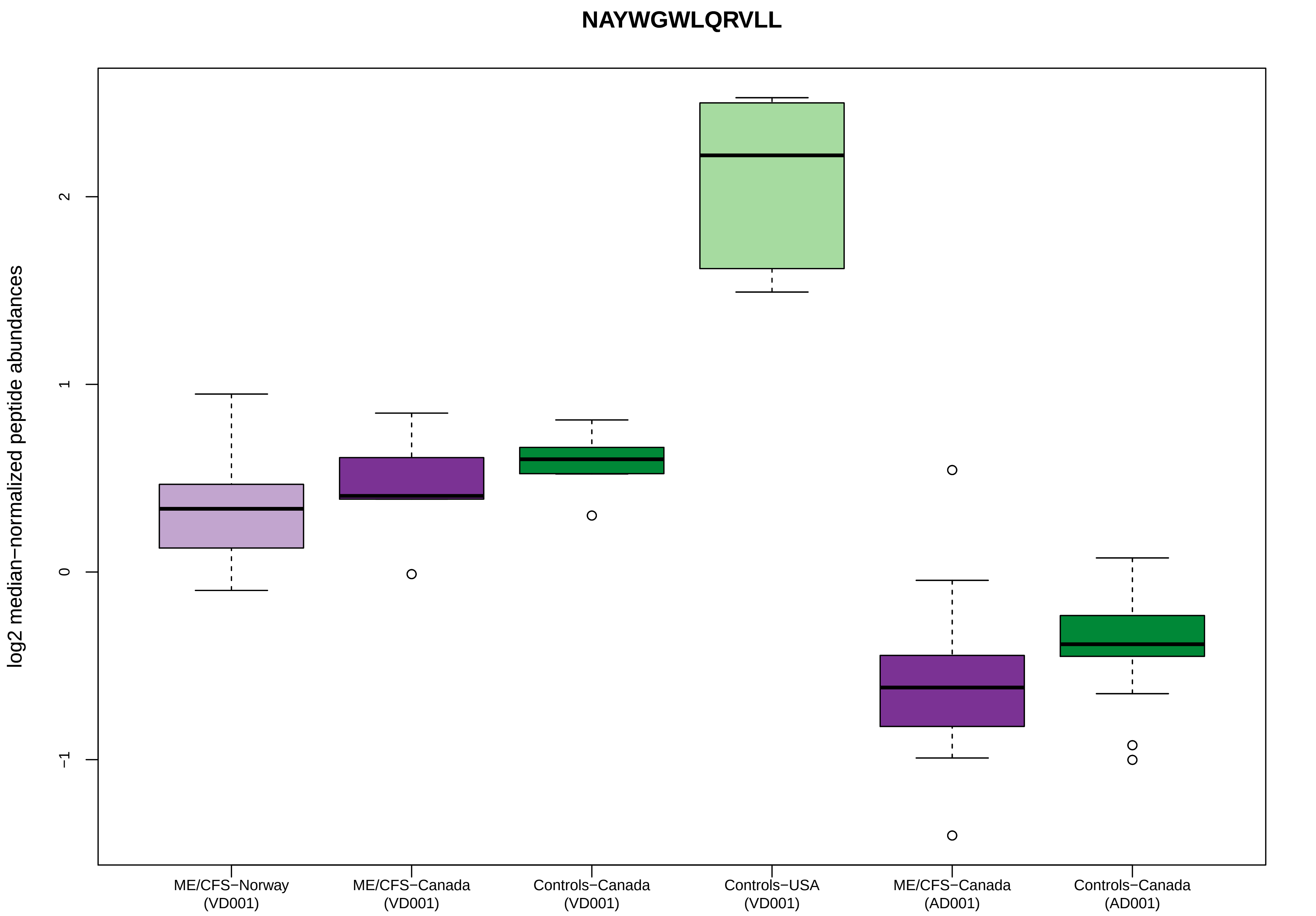

NAYWGWLQRVLL
log2 median−normalized peptide abundances
2
1
0
−1
ME/CFS−Norway (VD001)
ME/CFS−Canada (VD001)
Controls−Canada (VD001)
Controls−USA (VD001)
ME/CFS−Canada (AD001)
Controls−Canada (AD001)

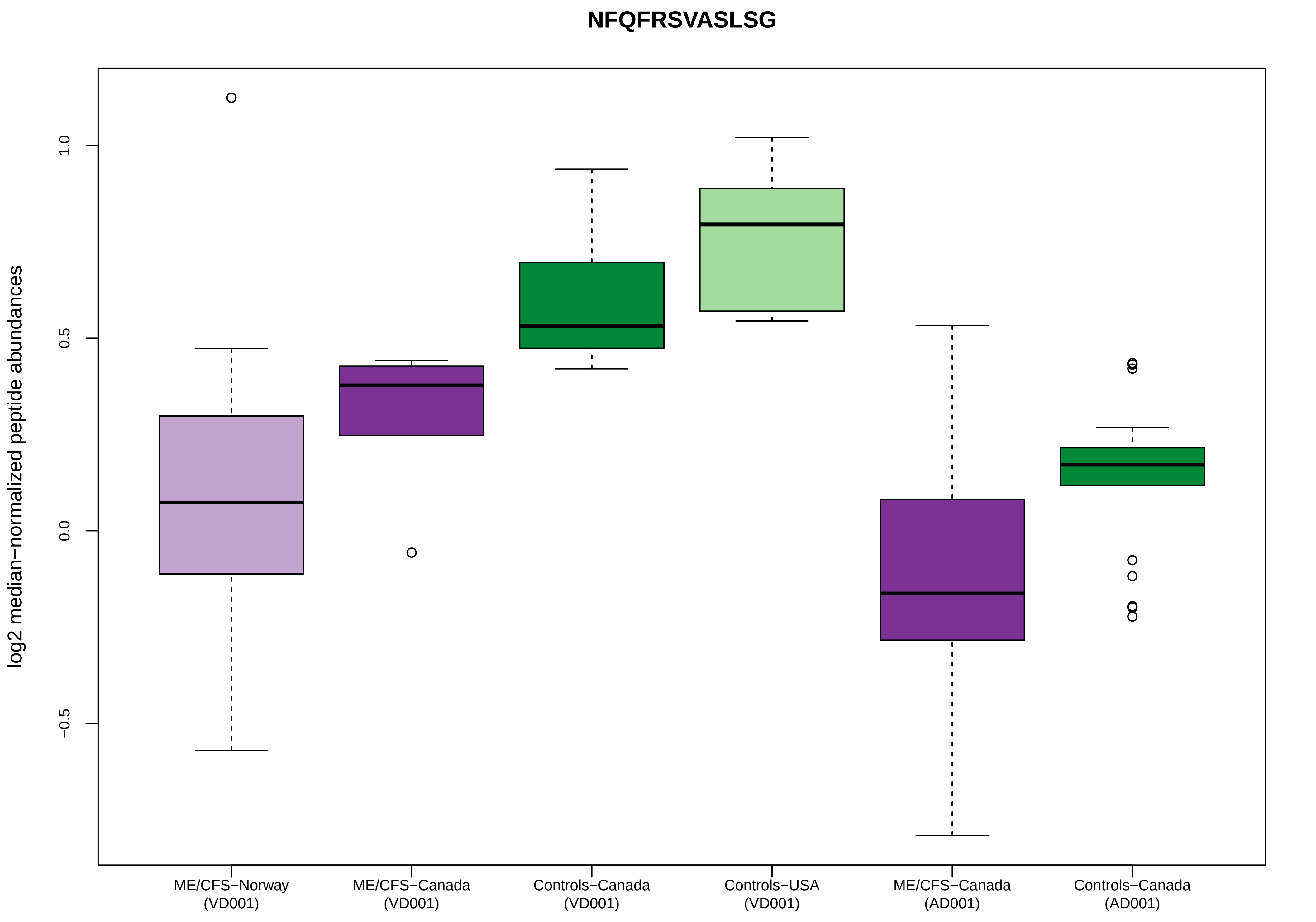
NFQFRSVASLSG
log2 median−normalized peptide abundances
1.0
0.5
0.0
−0.5
ME/CFS−Norway (VD001)
ME/CFS−Canada (VD001)
Controls−Canada (VD001)
Controls−USA (VD001)
ME/CFS−Canada (AD001)
Controls−Canada (AD001)

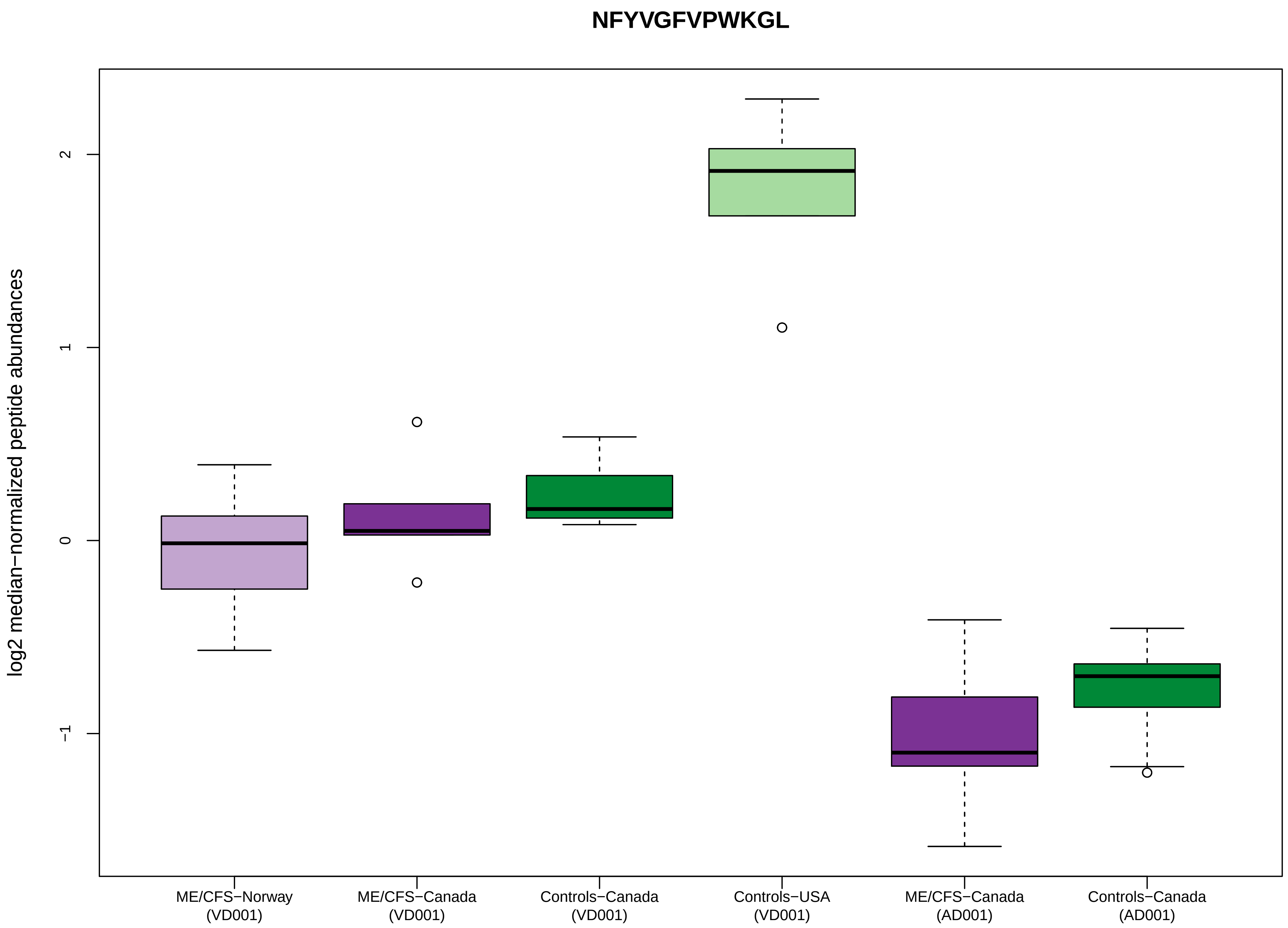
NFYVGFVPWKGL
log2 median−normalized peptide abundances
2
1
0
−1
ME/CFS−Norway (VD001)
ME/CFS−Canada (VD001)
Controls−Canada (VD001)
Controls−USA (VD001)
ME/CFS−Canada (AD001)
Controls−Canada (AD001)

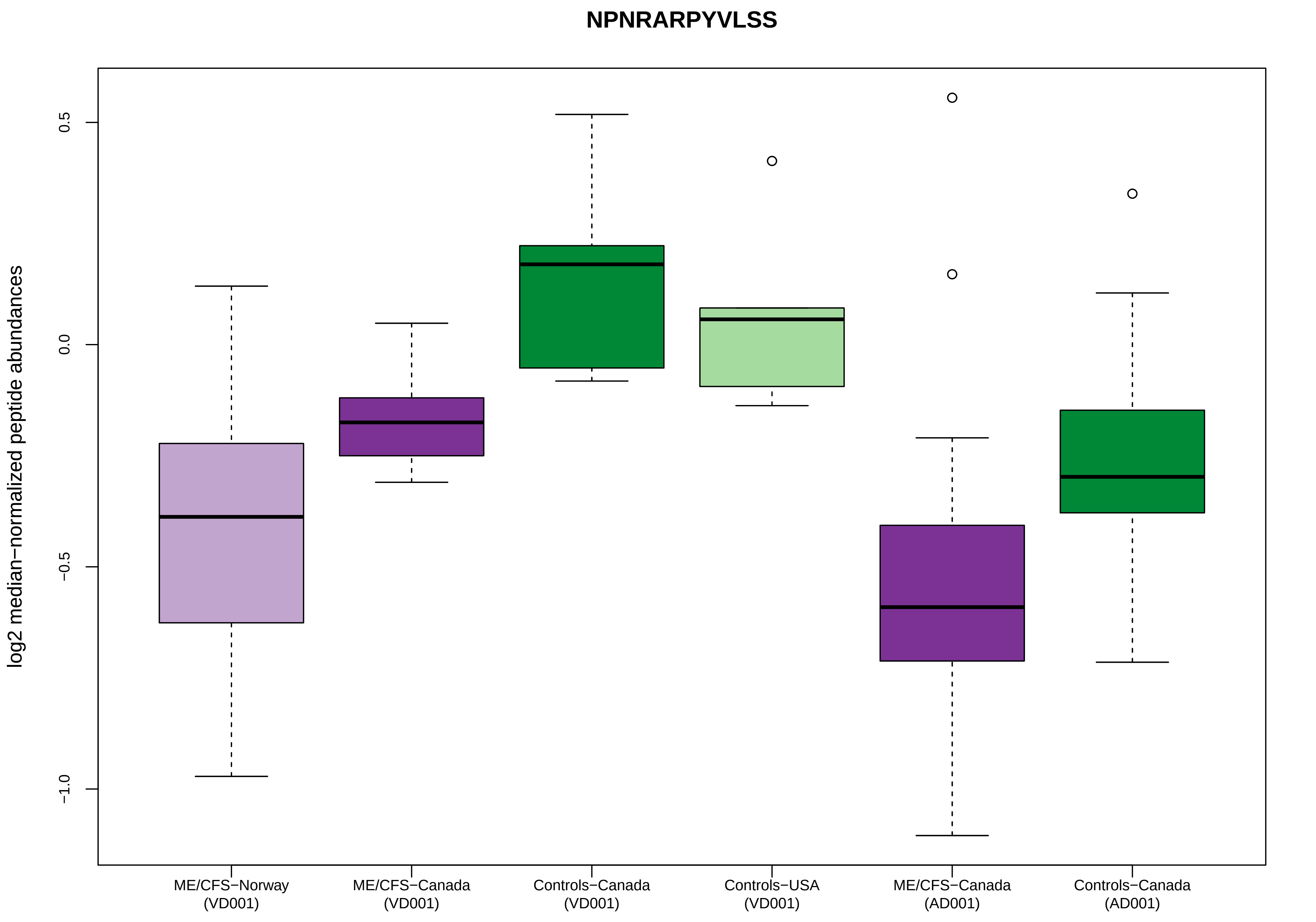
NPNRARPYVLSS
log2 median−normalized peptide abundances
0.5
0.0
−0.5
−1.0
ME/CFS−Norway (VD001)
ME/CFS−Canada (VD001)
Controls−Canada (VD001)
Controls−USA (VD001)
ME/CFS−Canada (AD001)
Controls−Canada (AD001)

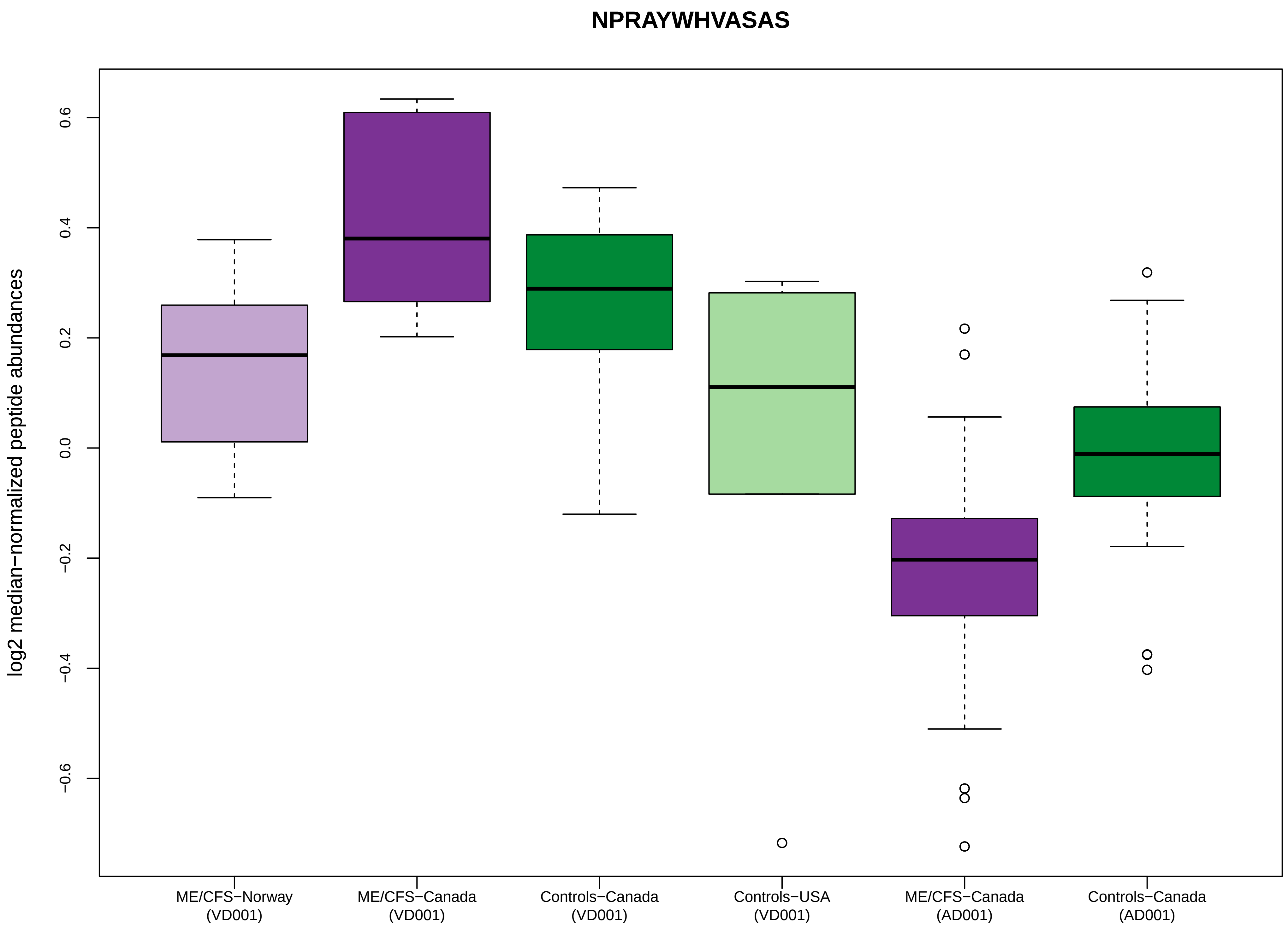
NPRAYWHVASAS
log2 median−normalized peptide abundances
0.6
0.4
0.2
0.0
−0.2
−0.4
−0.6
ME/CFS−Norway (VD001)
ME/CFS−Canada (VD001)
Controls−Canada (VD001)
Controls−USA (VD001)
ME/CFS−Canada (AD001)
Controls−Canada (AD001)

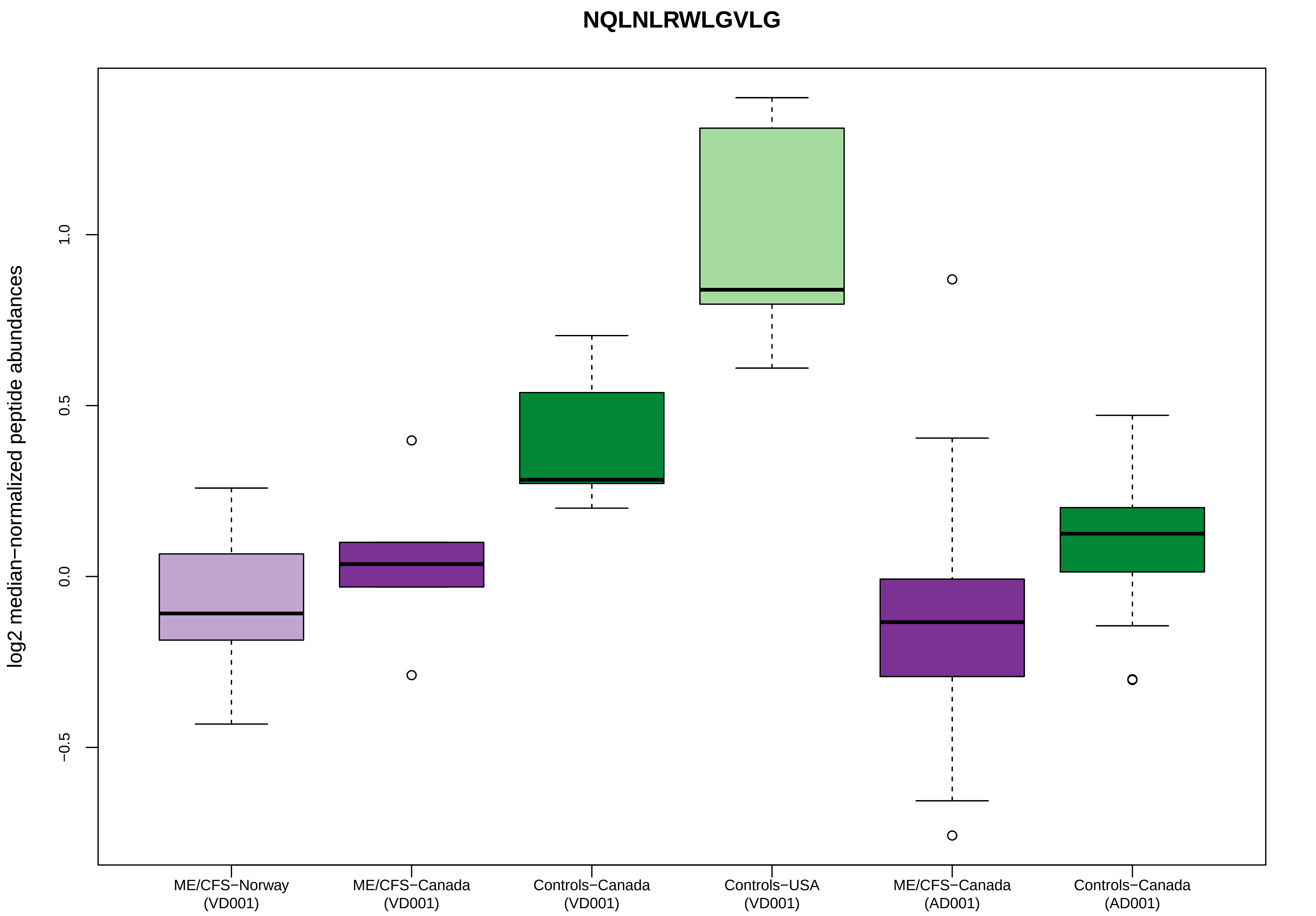
NQLNLRWLGVLG
log2 median−normalized peptide abundances
1.0
0.5
0.0
−0.5
ME/CFS−Norway (VD001)
ME/CFS−Canada (VD001)
Controls−Canada (VD001)
Controls−USA (VD001)
ME/CFS−Canada (AD001)
Controls−Canada (AD001)

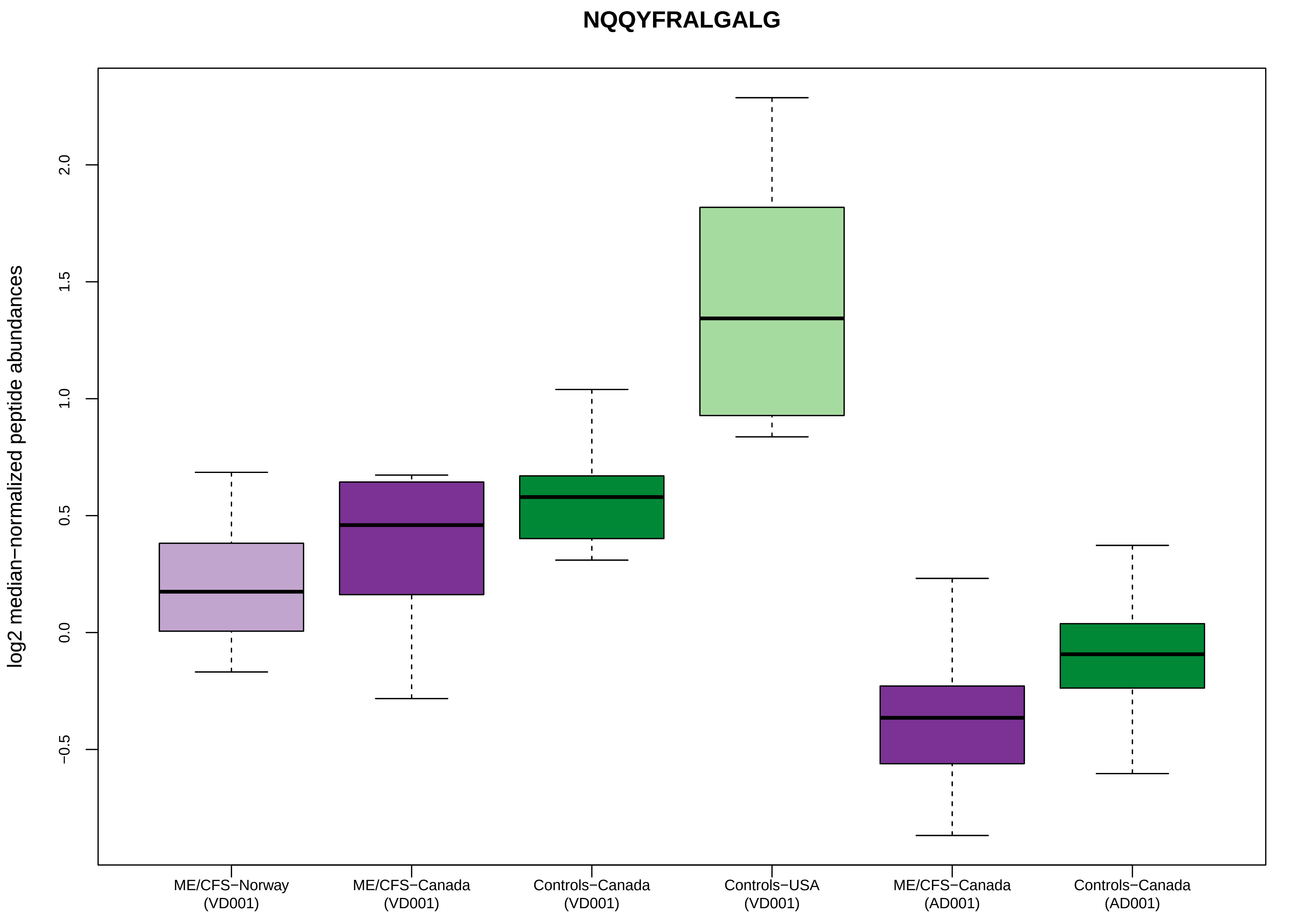

NQQYFRALGALG
log2 median−normalized peptide abundances
−0.5
0.0
0.5
1.0
1.5
2.0
ME/CFS−Norway (VD001)
ME/CFS−Canada (VD001)
Controls−Canada (VD001)
Controls−USA (VD001)
ME/CFS−Canada (AD001)
Controls−Canada (AD001)

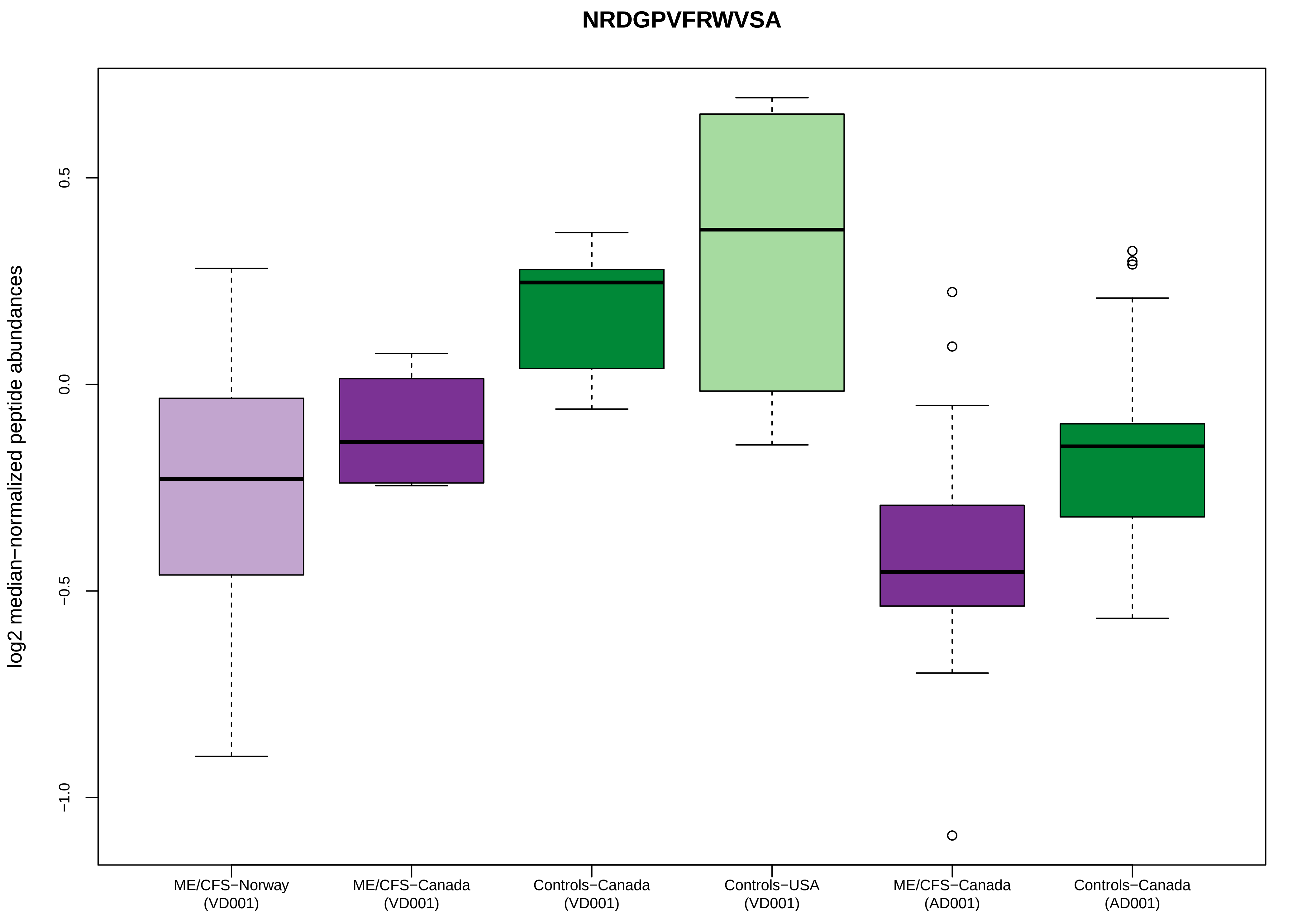
NRDGPVFRWVSA
log2 median−normalized peptide abundances
0.5
0.0
−0.5
−1.0
ME/CFS−Norway (VD001)
ME/CFS−Canada (VD001)
Controls−Canada (VD001)
Controls−USA (VD001)
ME/CFS−Canada (AD001)
Controls−Canada (AD001)

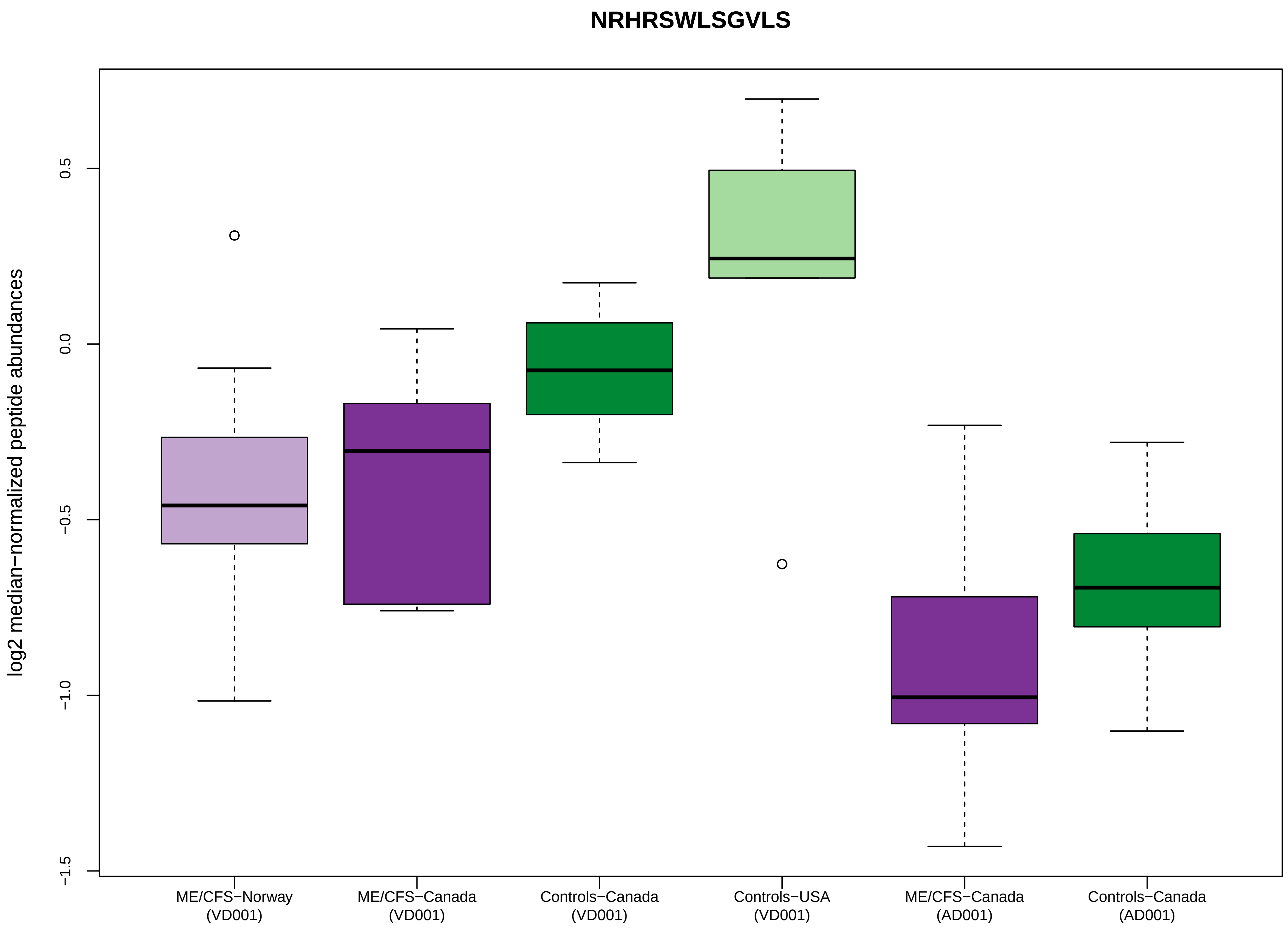
NRHRSWLSGVLS
log2 median−normalized peptide abundances
0.5
0.0
−0.5
−1.0
−1.5
ME/CFS−Norway (VD001)
ME/CFS−Canada (VD001)
Controls−Canada (VD001)
Controls−USA (VD001)
ME/CFS−Canada (AD001)
Controls−Canada (AD001)

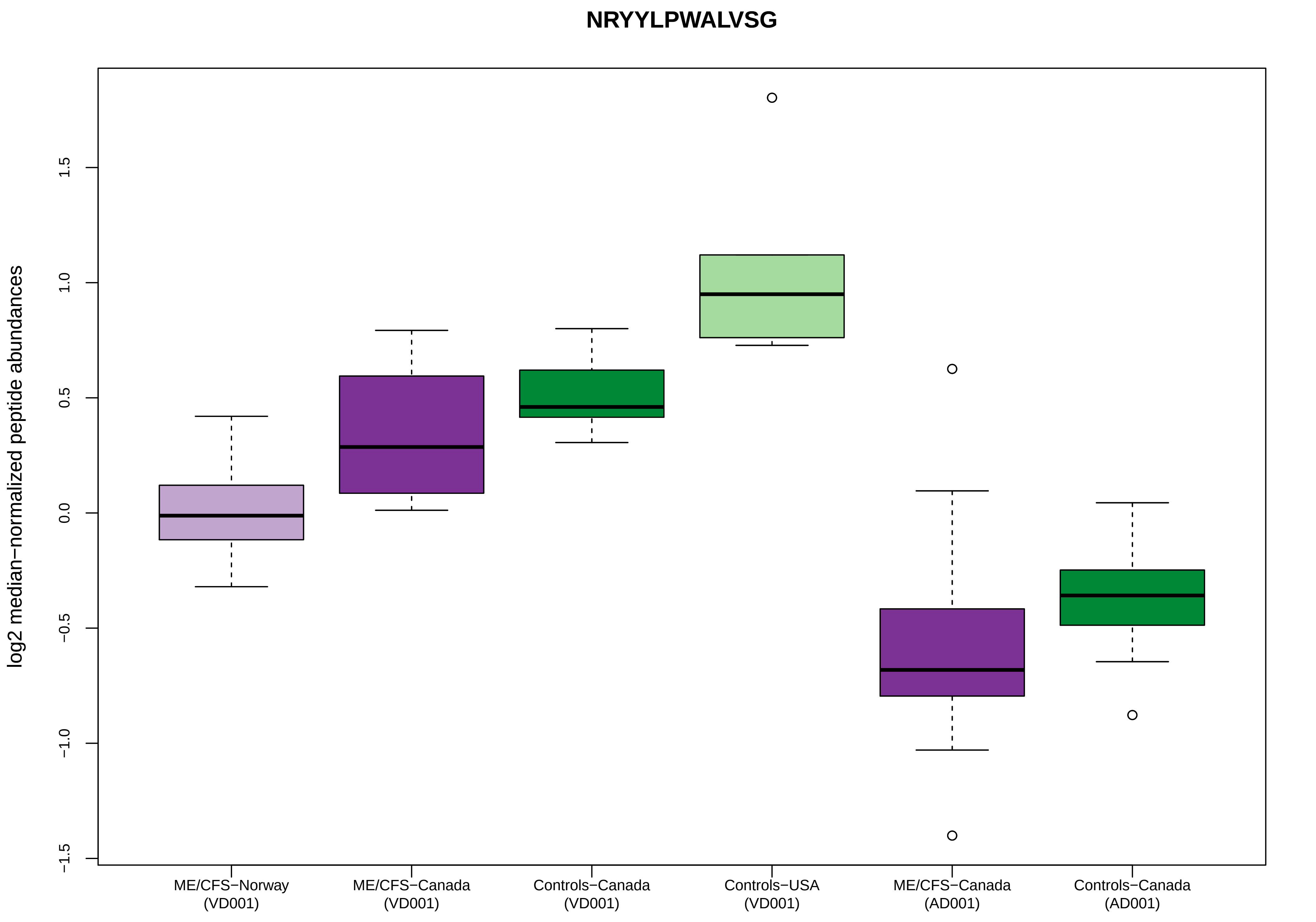

NRYYLPWALVSG
log2 median−normalized peptide abundances
1.5
1.0
0.5
0.0
−0.5
−1.0
−1.5
ME/CFS−Norway (VD001)
ME/CFS−Canada (VD001)
Controls−Canada (VD001)
Controls−USA (VD001)
ME/CFS−Canada (AD001)
Controls−Canada (AD001)

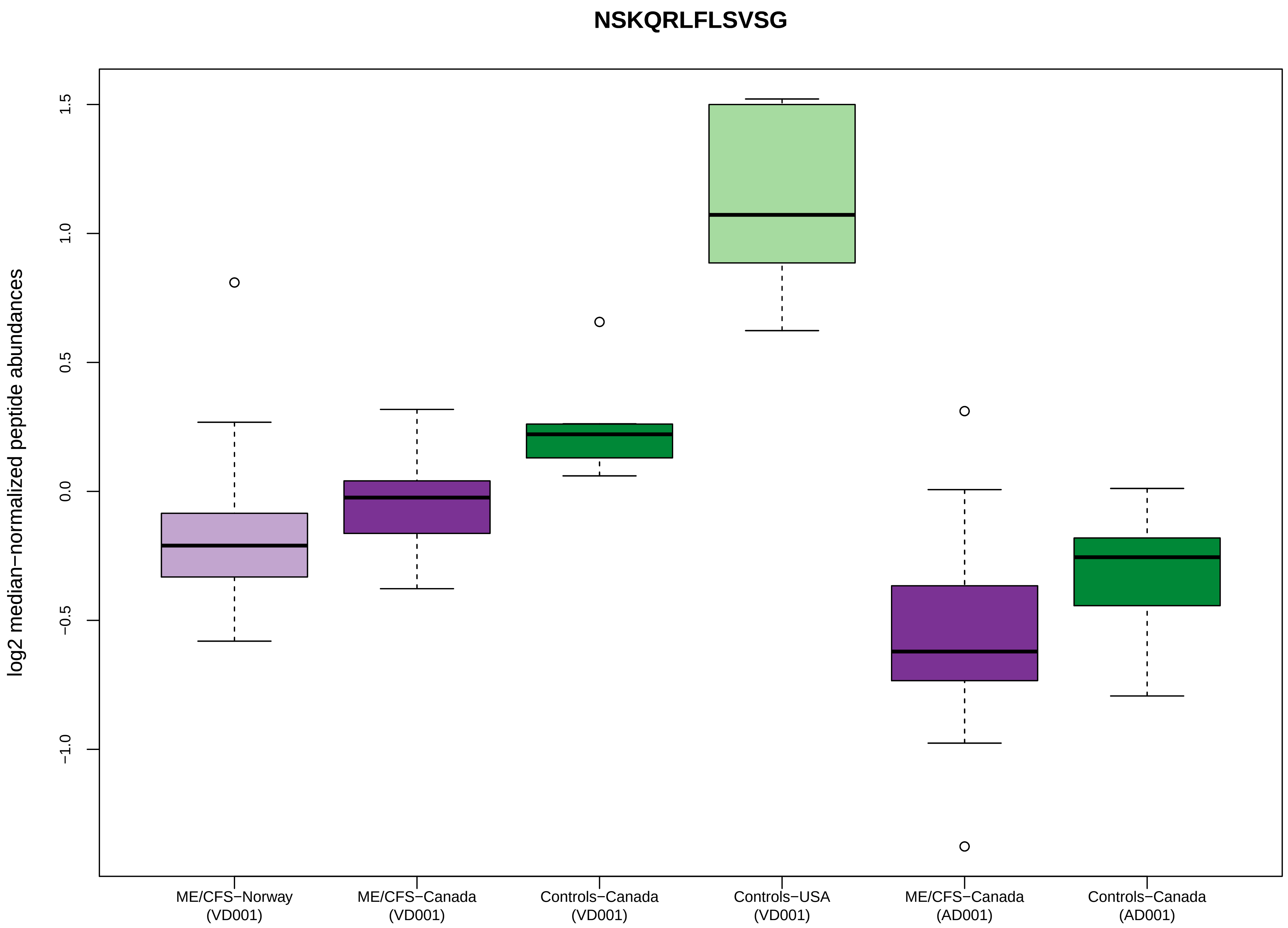

NSKQRLFLSVSG
log2 median−normalized peptide abundances
1.5
1.0
0.5
0.0
−0.5
−1.0
ME/CFS−Norway (VD001)
ME/CFS−Canada (VD001)
Controls−Canada (VD001)
Controls−USA (VD001)
ME/CFS−Canada (AD001)
Controls−Canada (AD001)

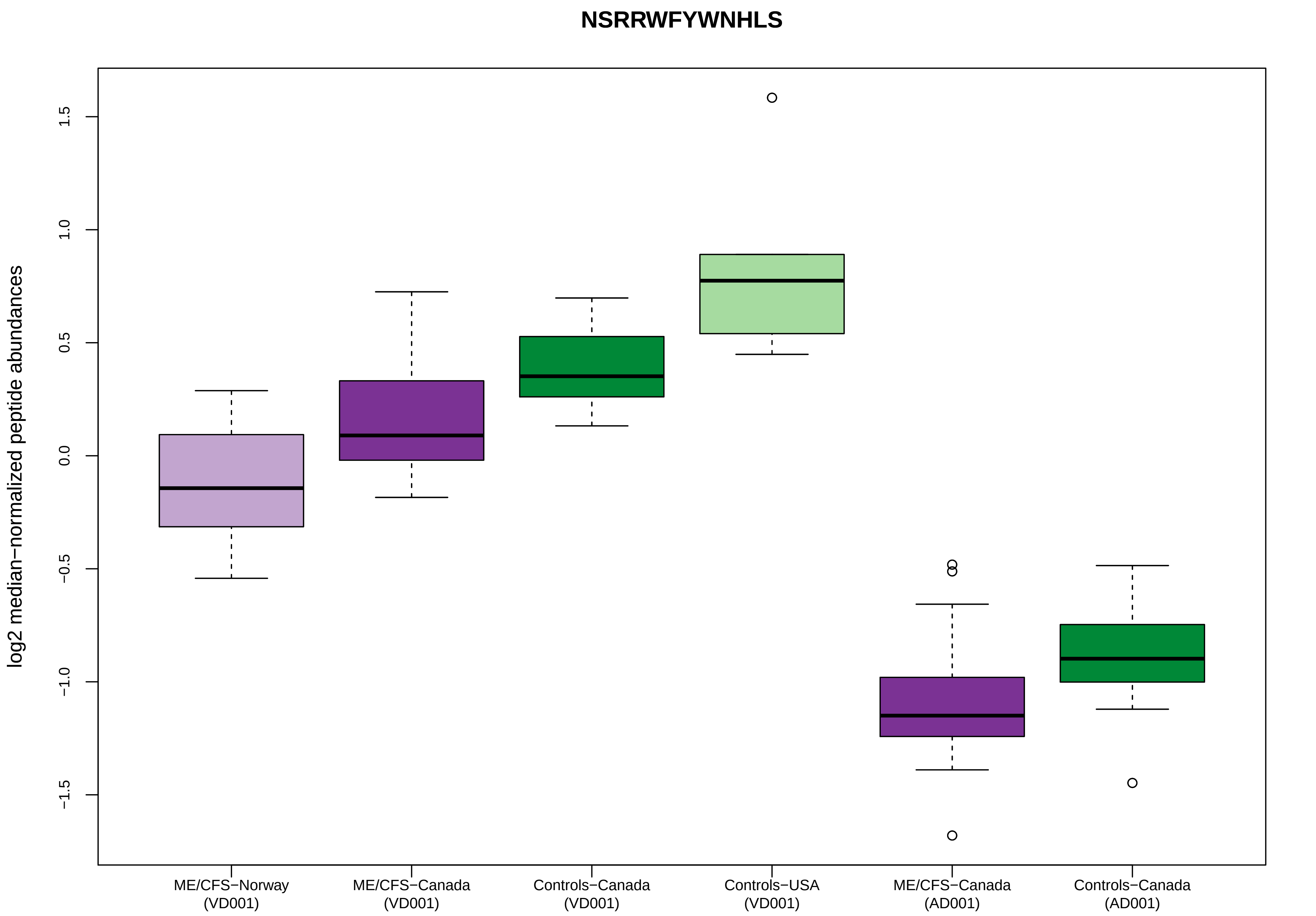

NSRRWFYWNHLS
log2 median−normalized peptide abundances
1.5
1.0
0.5
0.0
−0.5
−1.0
−1.5
ME/CFS−Norway (VD001)
ME/CFS−Canada (VD001)
Controls−Canada (VD001)
Controls−USA (VD001)
ME/CFS−Canada (AD001)
Controls−Canada (AD001)

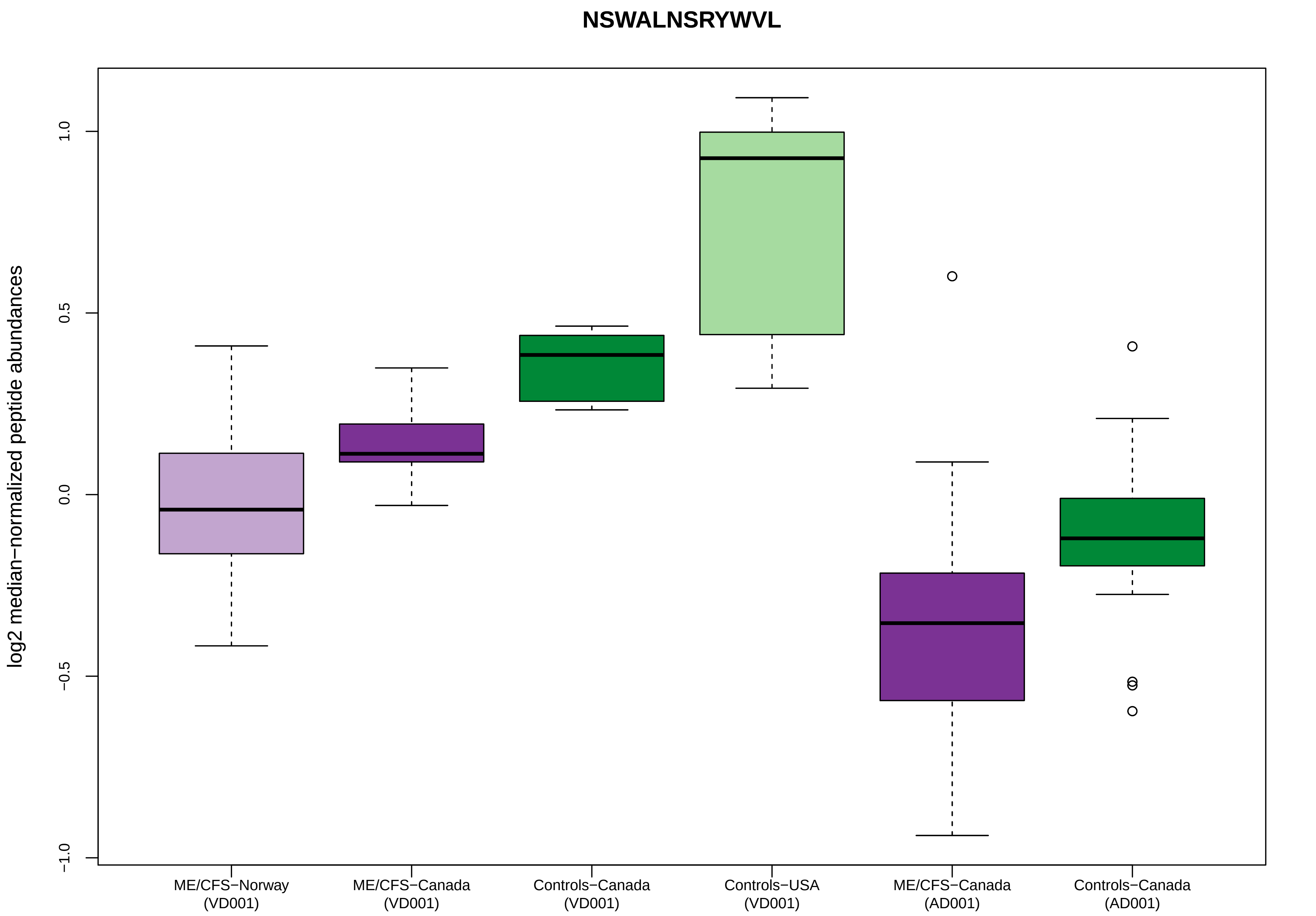
NSWALNSRYWVL
log2 median−normalized peptide abundances
1.0
0.5
0.0
−0.5
−1.0
ME/CFS−Norway (VD001)
ME/CFS−Canada (VD001)
Controls−Canada (VD001)
Controls−USA (VD001)
ME/CFS−Canada (AD001)
Controls−Canada (AD001)

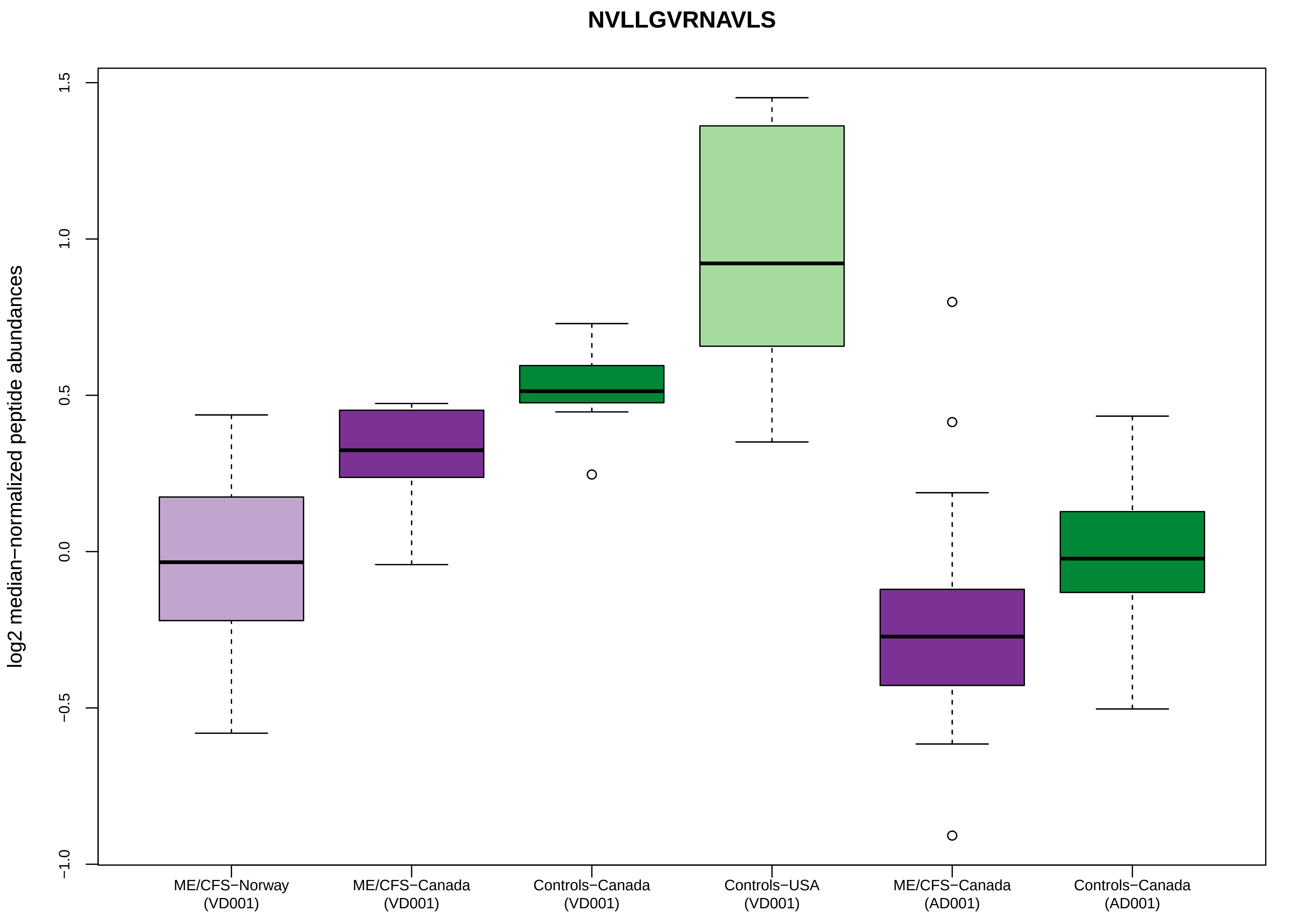
NVLLGVRNAVLS
log2 median−normalized peptide abundances
1.5
1.0
0.5
0.0
−0.5
−1.0
ME/CFS−Norway (VD001)
ME/CFS−Canada (VD001)
Controls−Canada (VD001)
Controls−USA (VD001)
ME/CFS−Canada (AD001)
Controls−Canada (AD001)

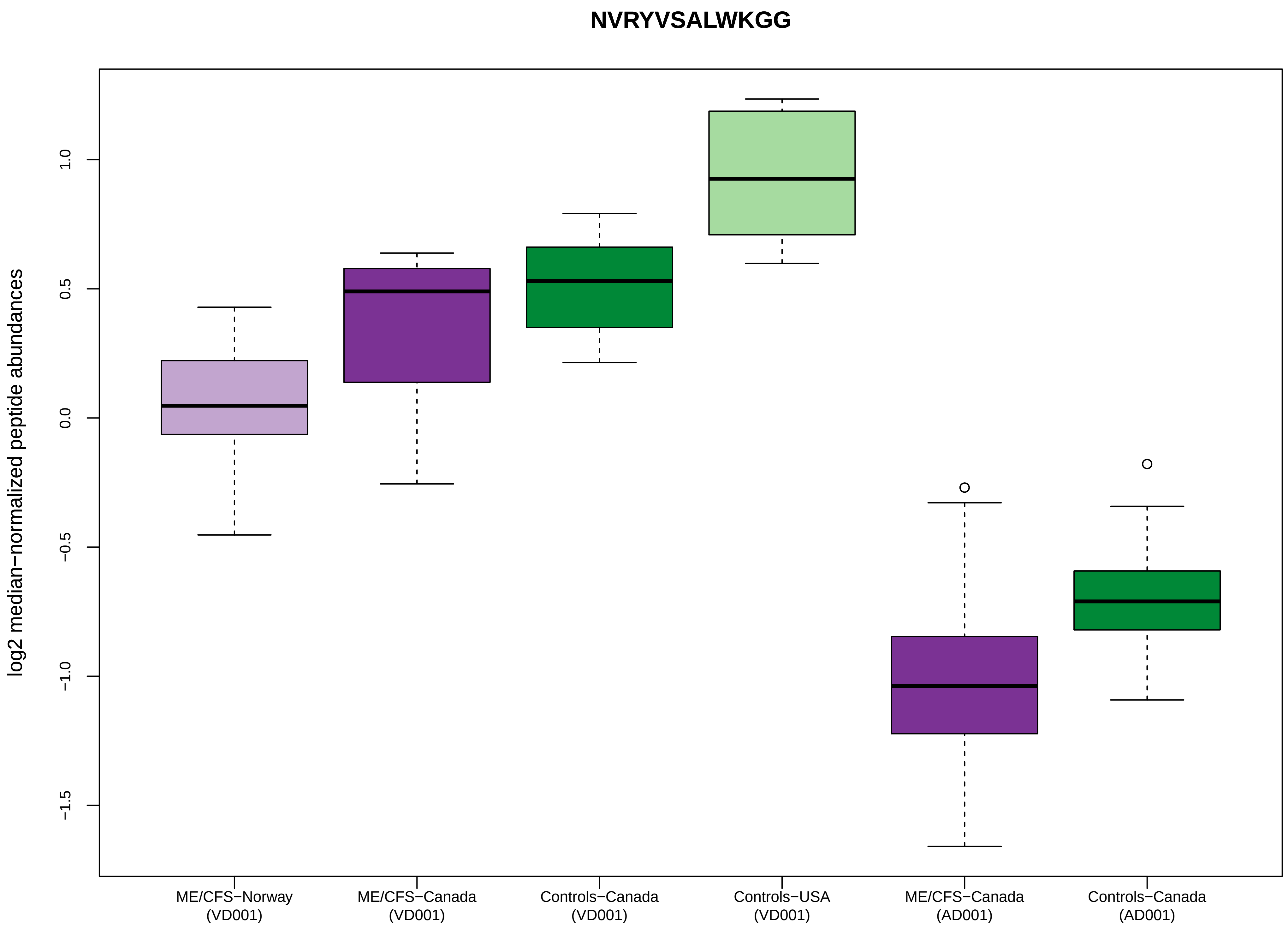
NVRYVSALWKGG
log2 median−normalized peptide abundances
1.0
0.5
0.0
−0.5
−1.0
−1.5
ME/CFS−Norway (VD001)
ME/CFS−Canada (VD001)
Controls−Canada (VD001)
Controls−USA (VD001)
ME/CFS−Canada (AD001)
Controls−Canada (AD001)

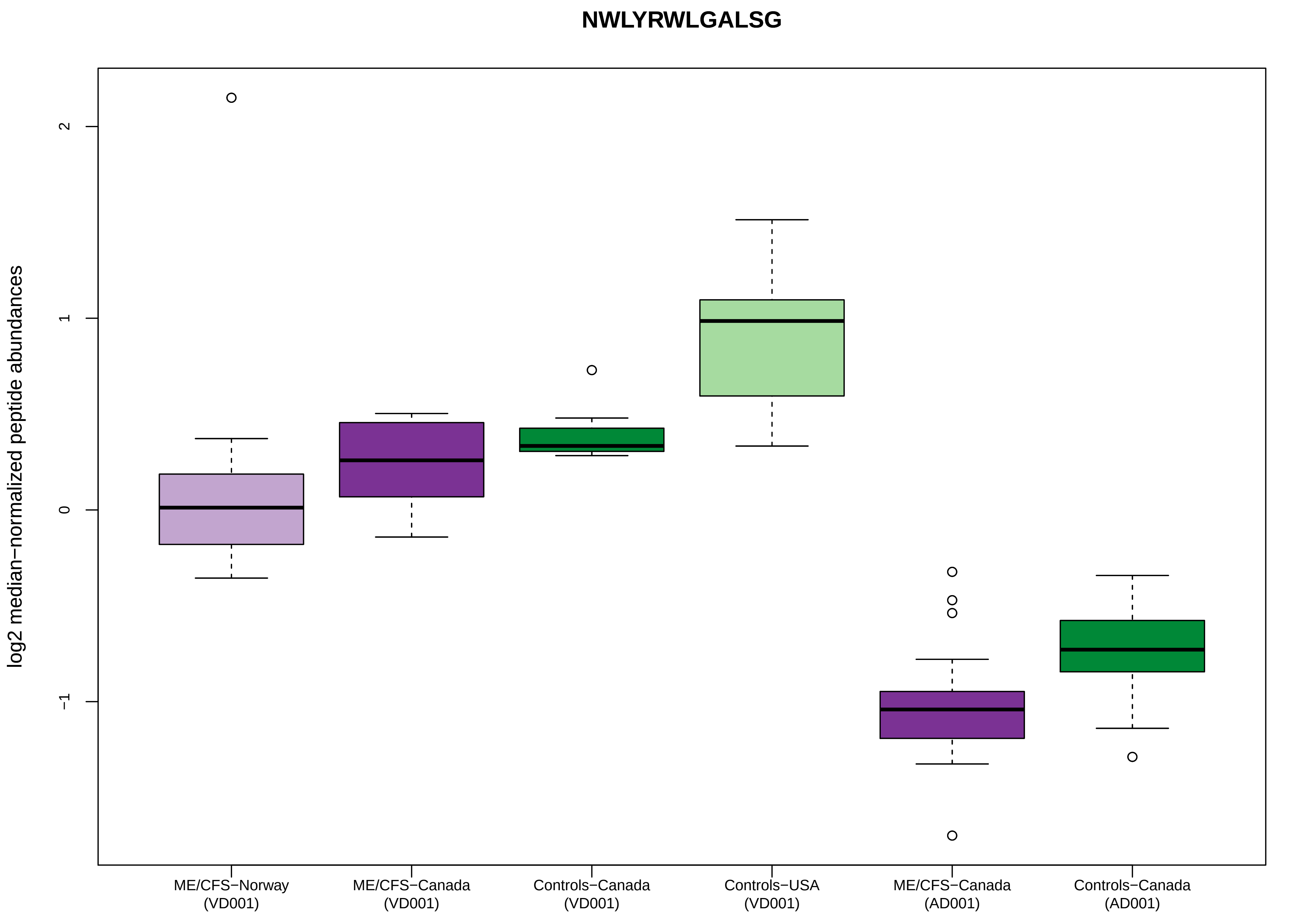

NWLYRWLGALSG
log2 median−normalized peptide abundances
2
1
0
−1
ME/CFS−Norway (VD001)
ME/CFS−Canada (VD001)
Controls−Canada (VD001)
Controls−USA (VD001)
ME/CFS−Canada (AD001)
Controls−Canada (AD001)

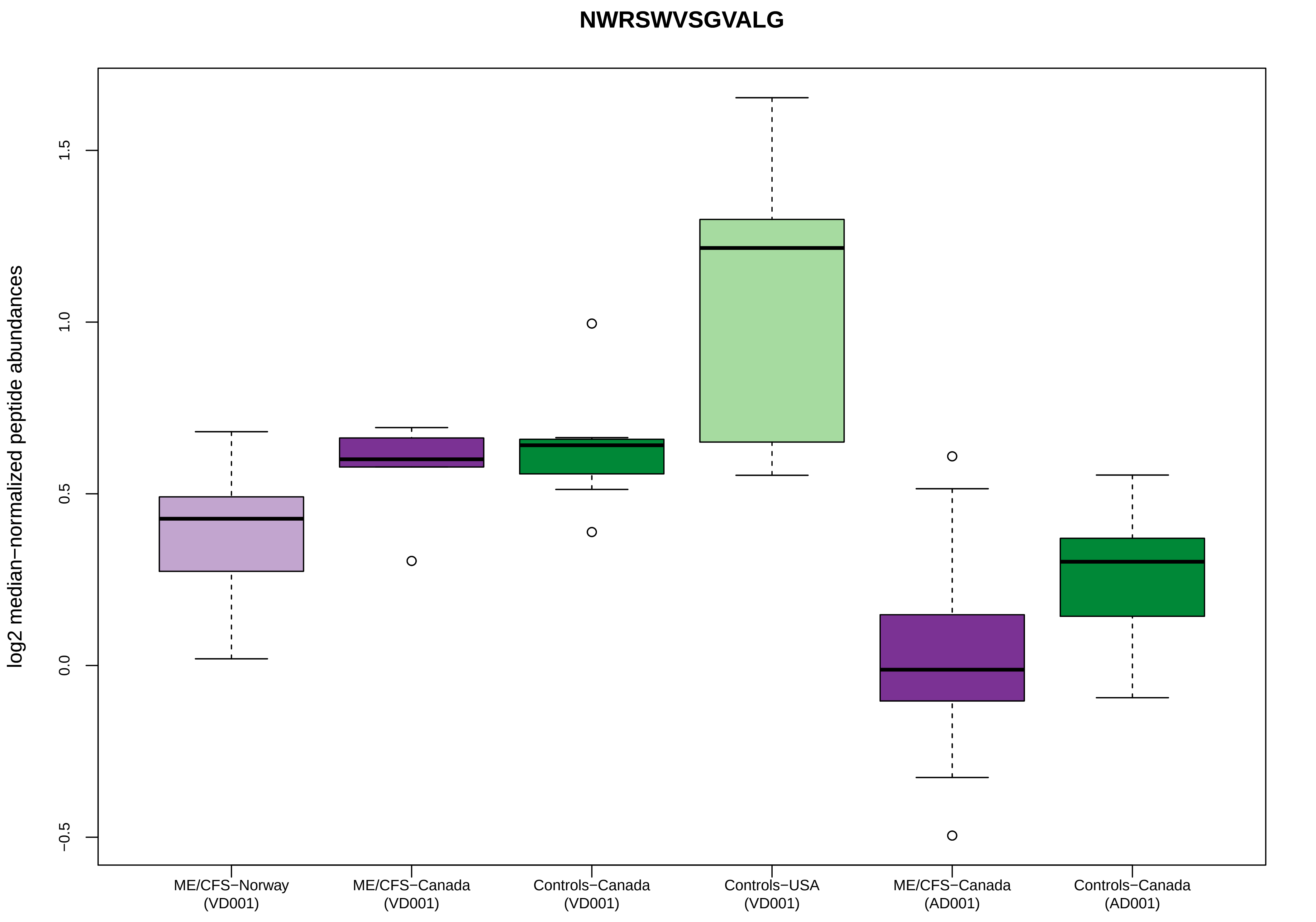
NWRSWVSGVALG
log2 median−normalized peptide abundances
−0.5
0.0
0.5
1.0
1.5
ME/CFS−Norway (VD001)
ME/CFS−Canada (VD001)
Controls−Canada (VD001)
Controls−USA (VD001)
ME/CFS−Canada (AD001)
Controls−Canada (AD001)

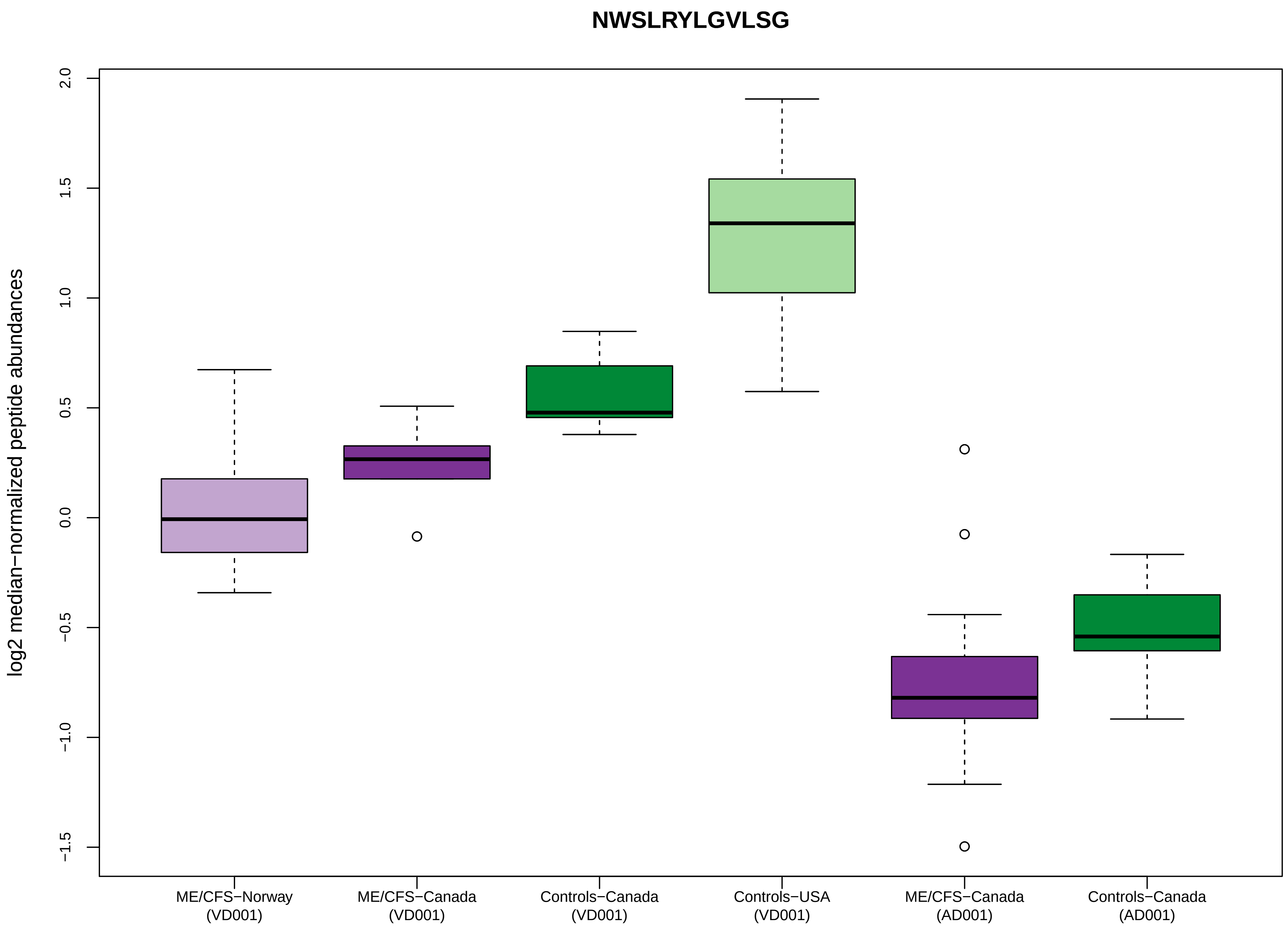

NWSLRYLGVLSG
log2 median−normalized peptide abundances
2.0
1.5
1.0
0.5
0.0
−0.5
−1.0
−1.5
ME/CFS−Norway (VD001)
ME/CFS−Canada (VD001)
Controls−Canada (VD001)
Controls−USA (VD001)
ME/CFS−Canada (AD001)
Controls−Canada (AD001)

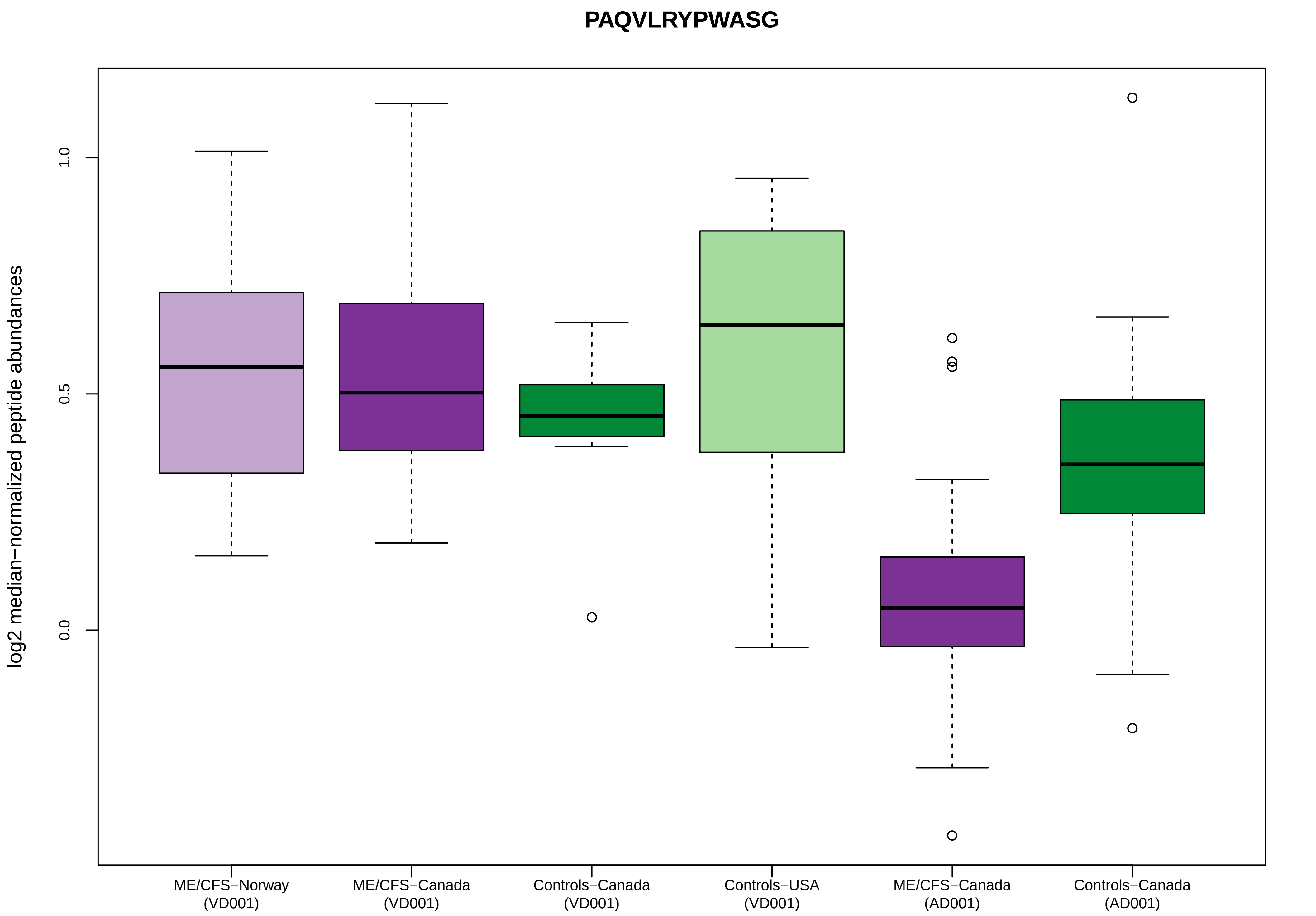

PAQVLRYPWASG
log2 median−normalized peptide abundances
1.0
0.5
0.0
ME/CFS−Norway (VD001)
ME/CFS−Canada (VD001)
Controls−Canada (VD001)
Controls−USA (VD001)
ME/CFS−Canada (AD001)
Controls−Canada (AD001)

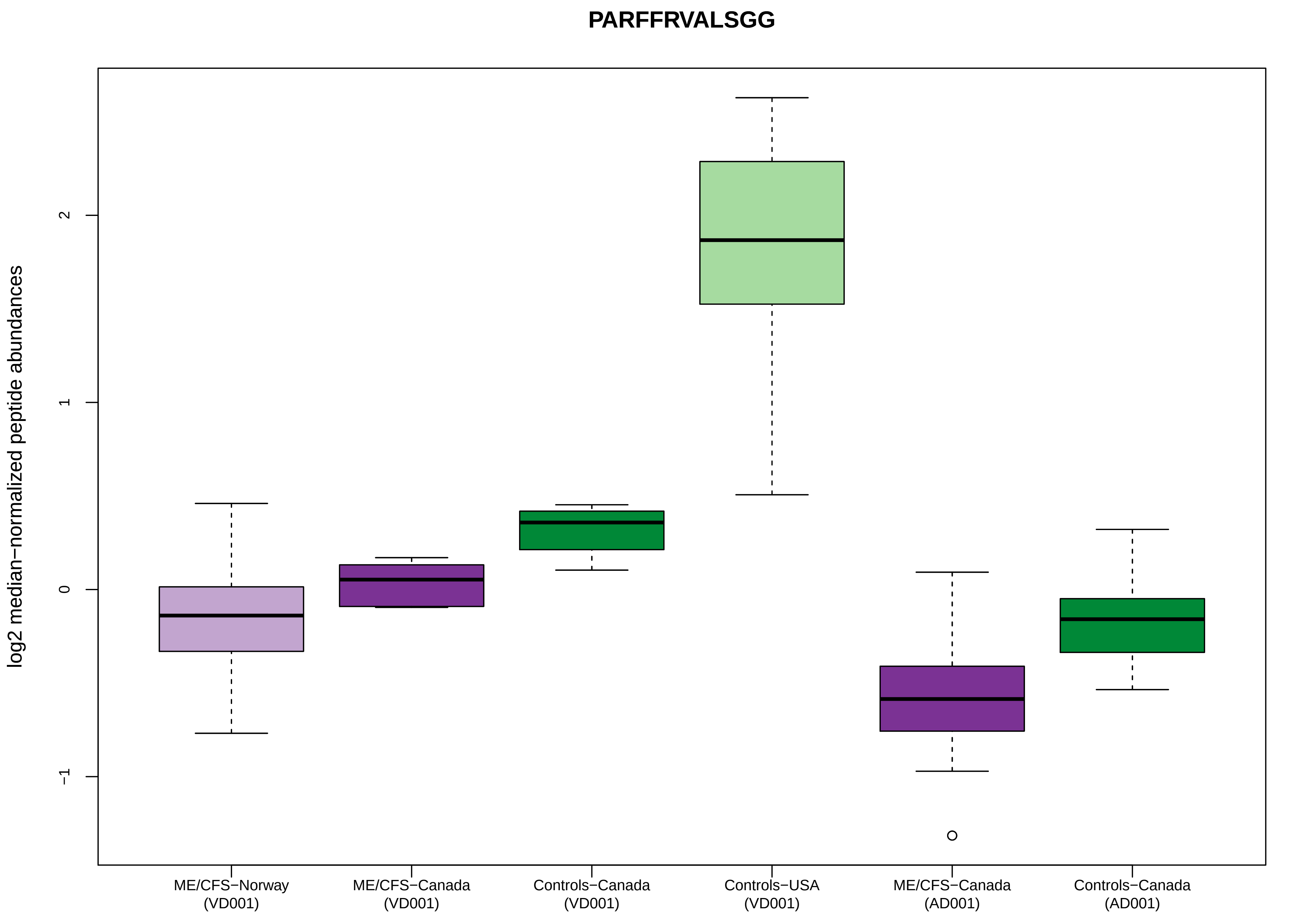
PARFFRVALSGG
log2 median−normalized peptide abundances
−1
0
1
2
ME/CFS−Norway (VD001)
ME/CFS−Canada (VD001)
Controls−Canada (VD001)
Controls−USA (VD001)
ME/CFS−Canada (AD001)
Controls−Canada (AD001)

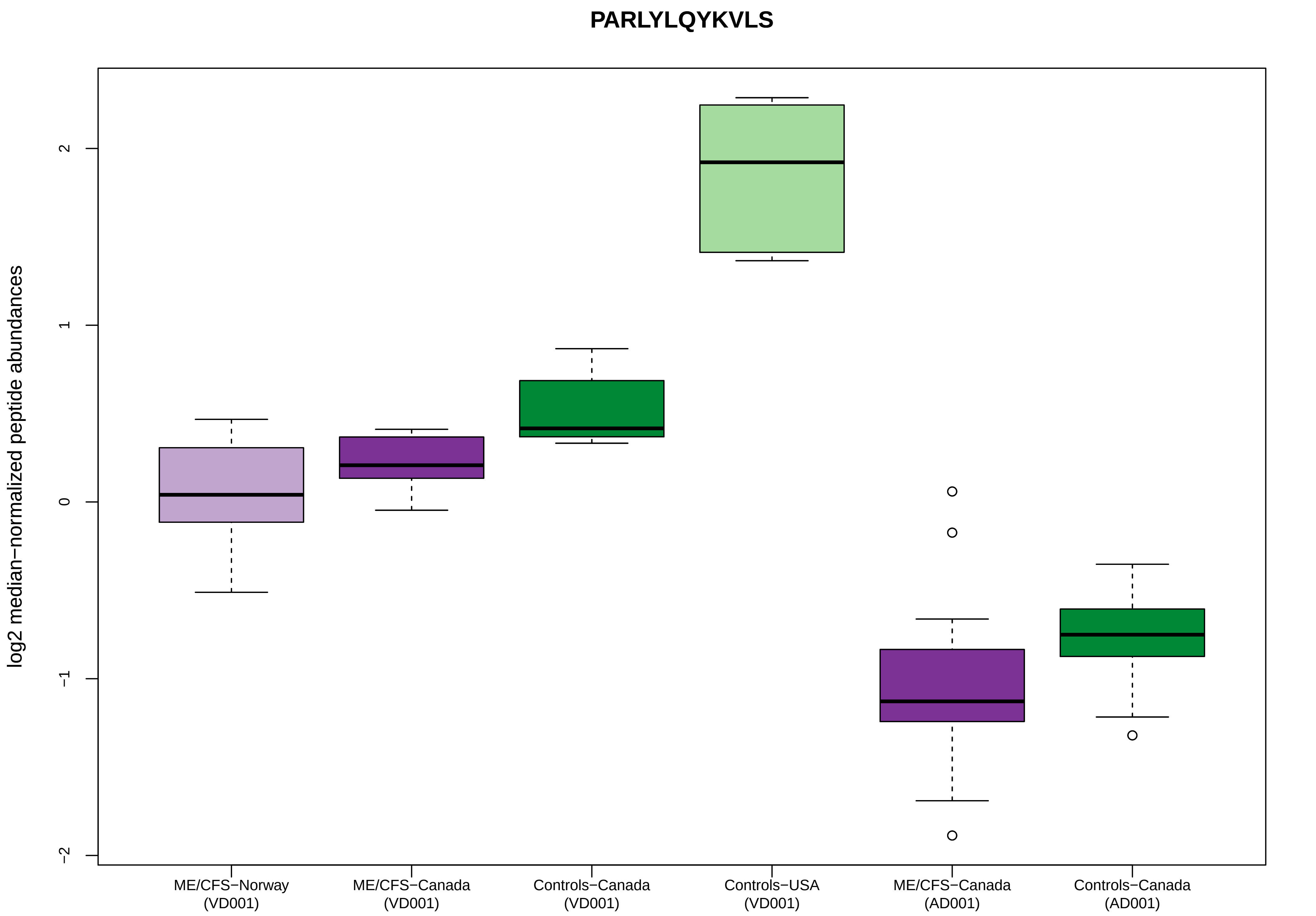
PARLYLQYKVLS
log2 median−normalized peptide abundances
2
1
0
−1
−2
ME/CFS−Norway (VD001)
ME/CFS−Canada (VD001)
Controls−Canada (VD001)
Controls−USA (VD001)
ME/CFS−Canada (AD001)
Controls−Canada (AD001)

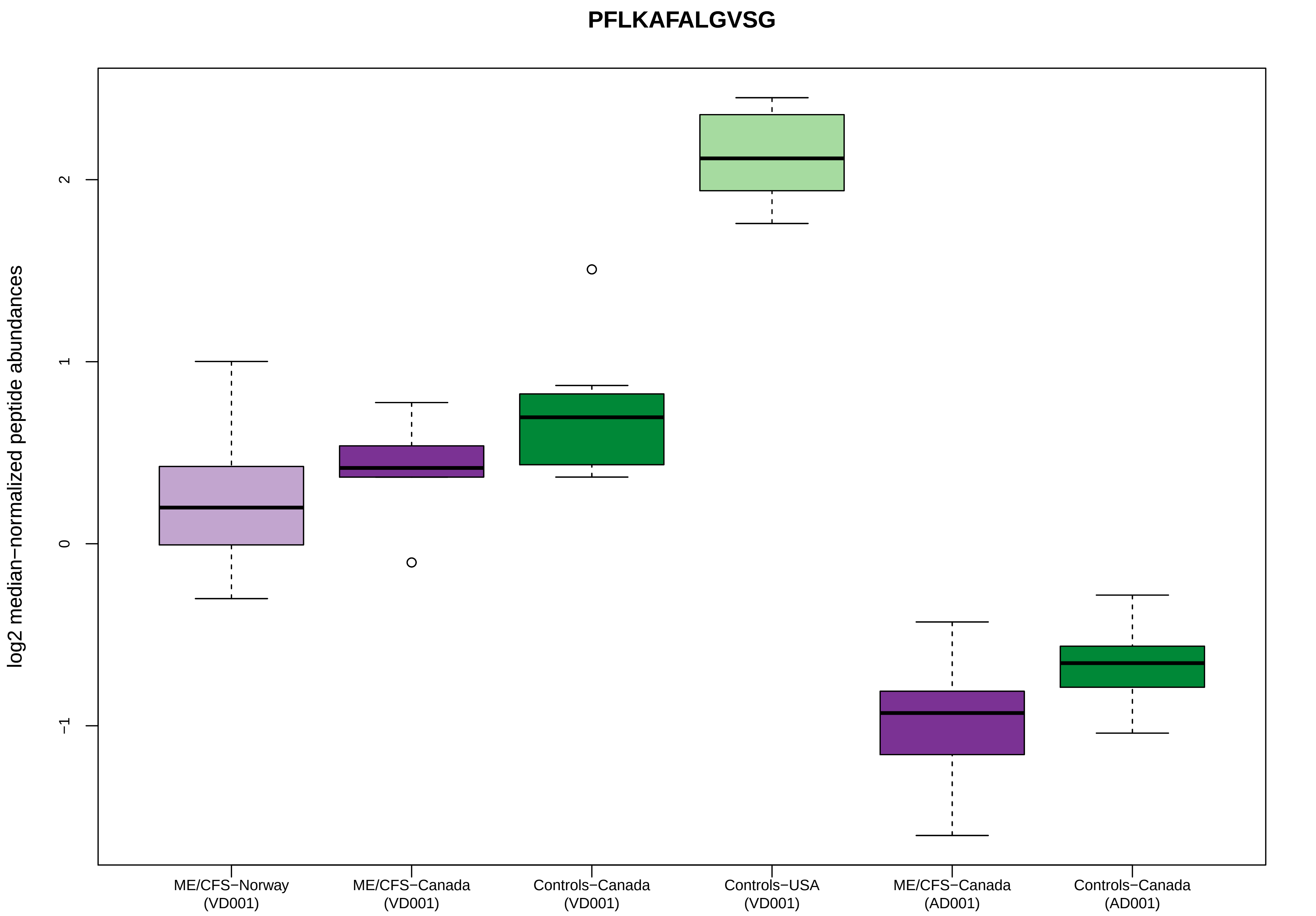
PFLKAFALGVSG
log2 median−normalized peptide abundances
2
1
0
−1
ME/CFS−Norway (VD001)
ME/CFS−Canada (VD001)
Controls−Canada (VD001)
Controls−USA (VD001)
ME/CFS−Canada (AD001)
Controls−Canada (AD001)

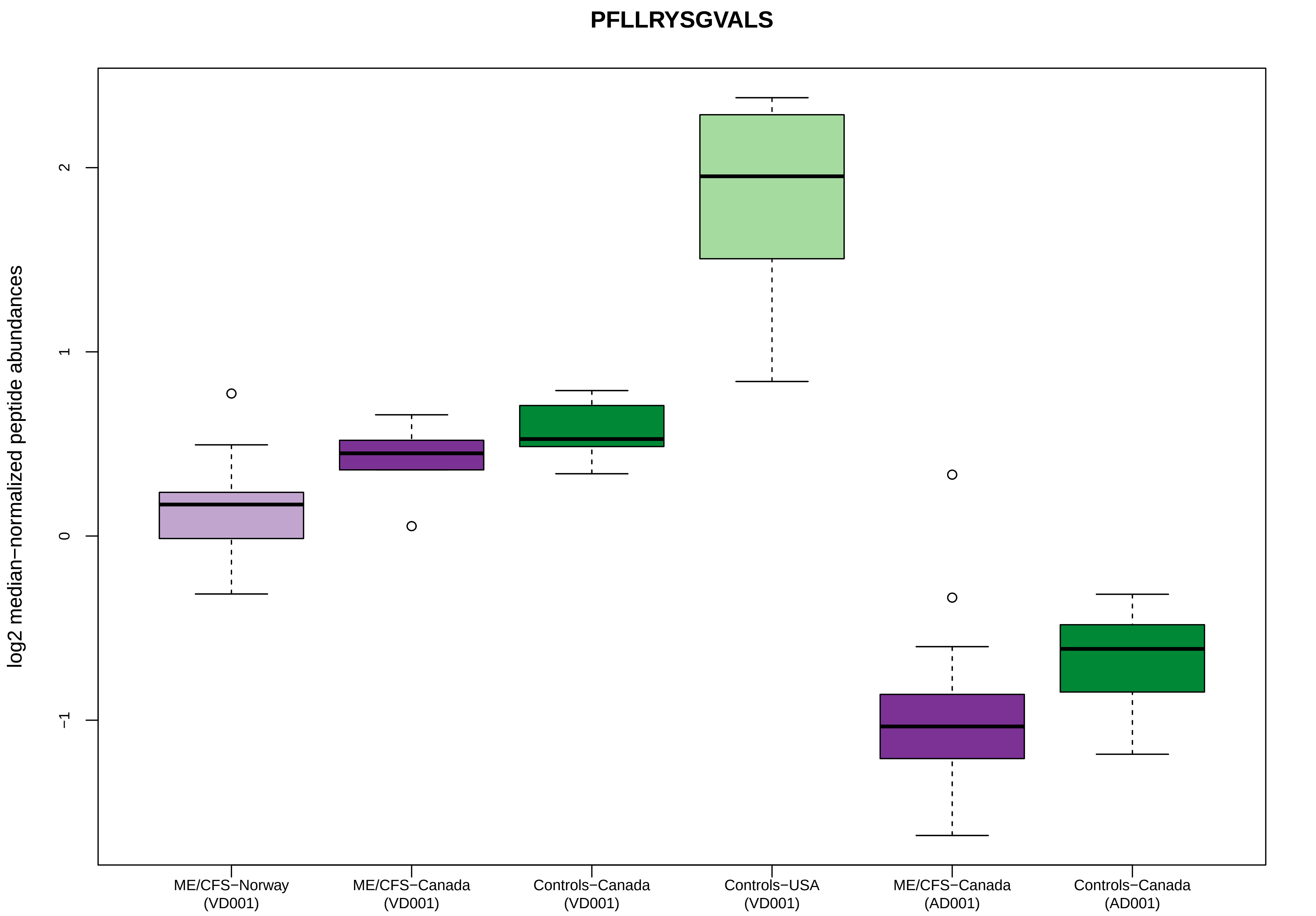
PFLLRYSGVALS
log2 median−normalized peptide abundances
2
1
0
−1
ME/CFS−Norway (VD001)
ME/CFS−Canada (VD001)
Controls−Canada (VD001)
Controls−USA (VD001)
ME/CFS−Canada (AD001)
Controls−Canada (AD001)

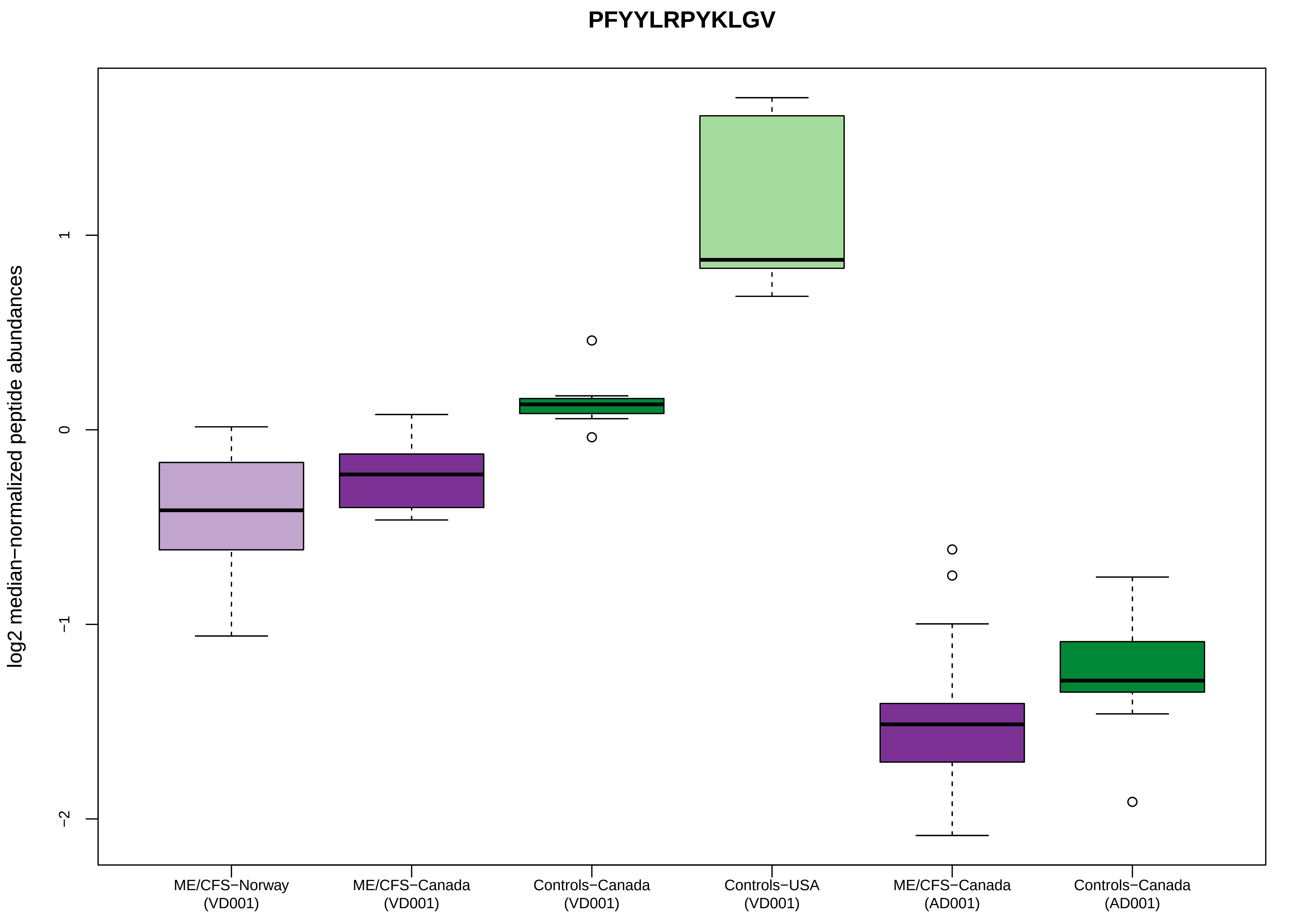
PFYYLRPYKLGV
log2 median−normalized peptide abundances
1
0
−1
−2
ME/CFS−Norway (VD001)
ME/CFS−Canada (VD001)
Controls−Canada (VD001)
Controls−USA (VD001)
ME/CFS−Canada (AD001)
Controls−Canada (AD001)

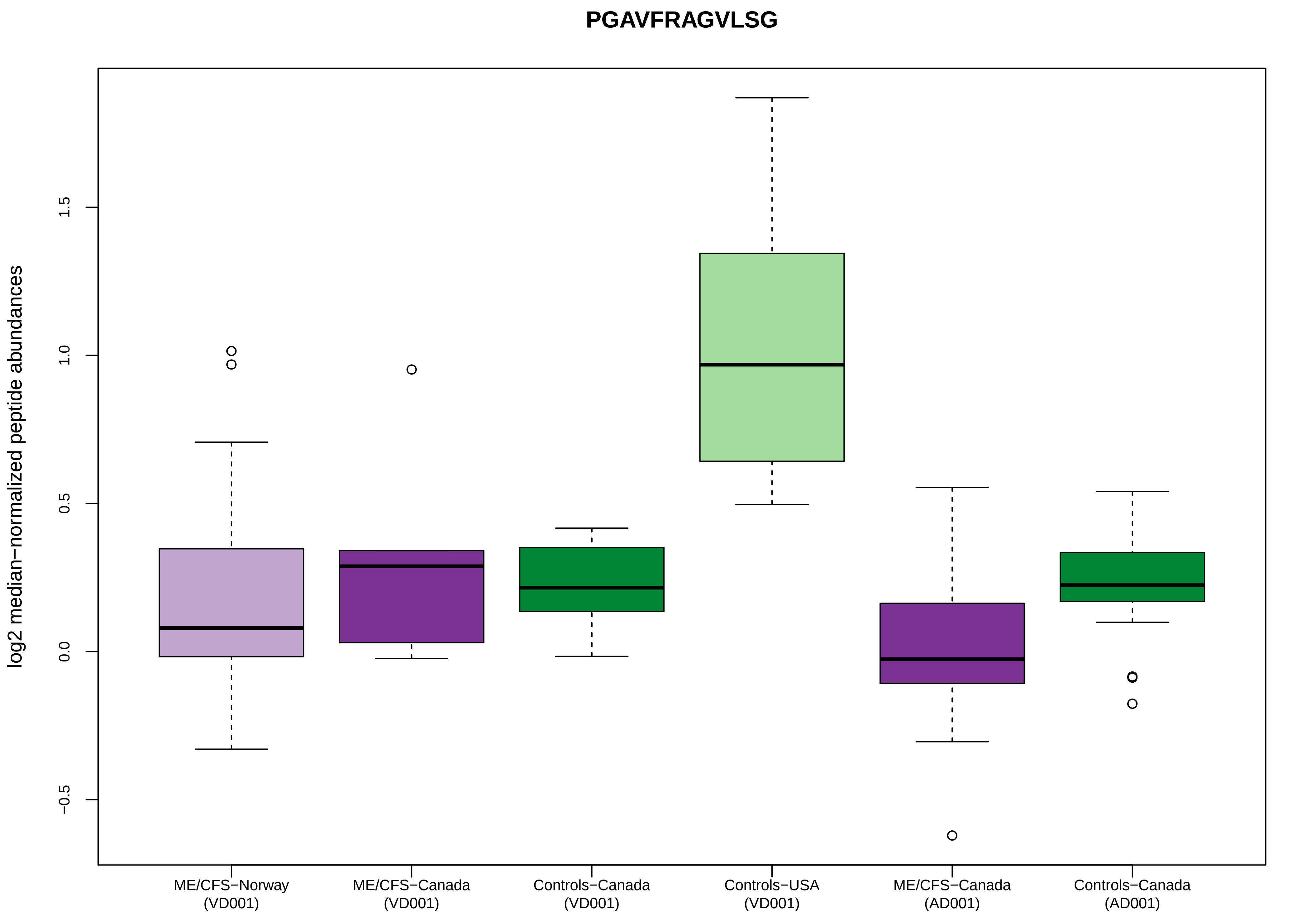
PGAVFRAGVLSG
log2 median−normalized peptide abundances
−0.5
0.0
0.5
1.0
1.5
ME/CFS−Norway (VD001)
ME/CFS−Canada (VD001)
Controls−Canada (VD001)
Controls−USA (VD001)
ME/CFS−Canada (AD001)
Controls−Canada (AD001)

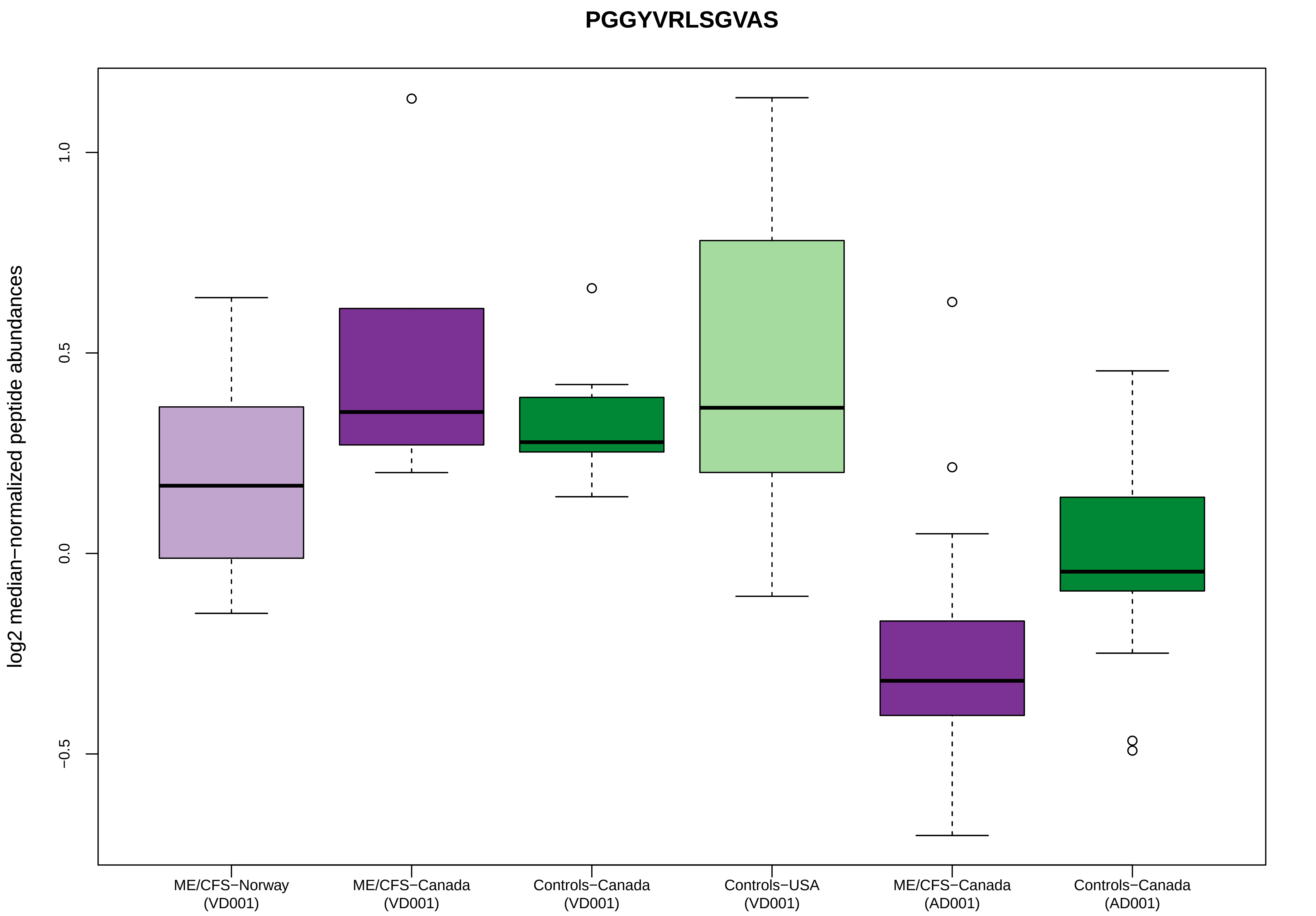

PGGYVRLSGVAS
log2 median−normalized peptide abundances
1.0
0.5
0.0
−0.5
ME/CFS−Norway (VD001)
ME/CFS−Canada (VD001)
Controls−Canada (VD001)
Controls−USA (VD001)
ME/CFS−Canada (AD001)
Controls−Canada (AD001)

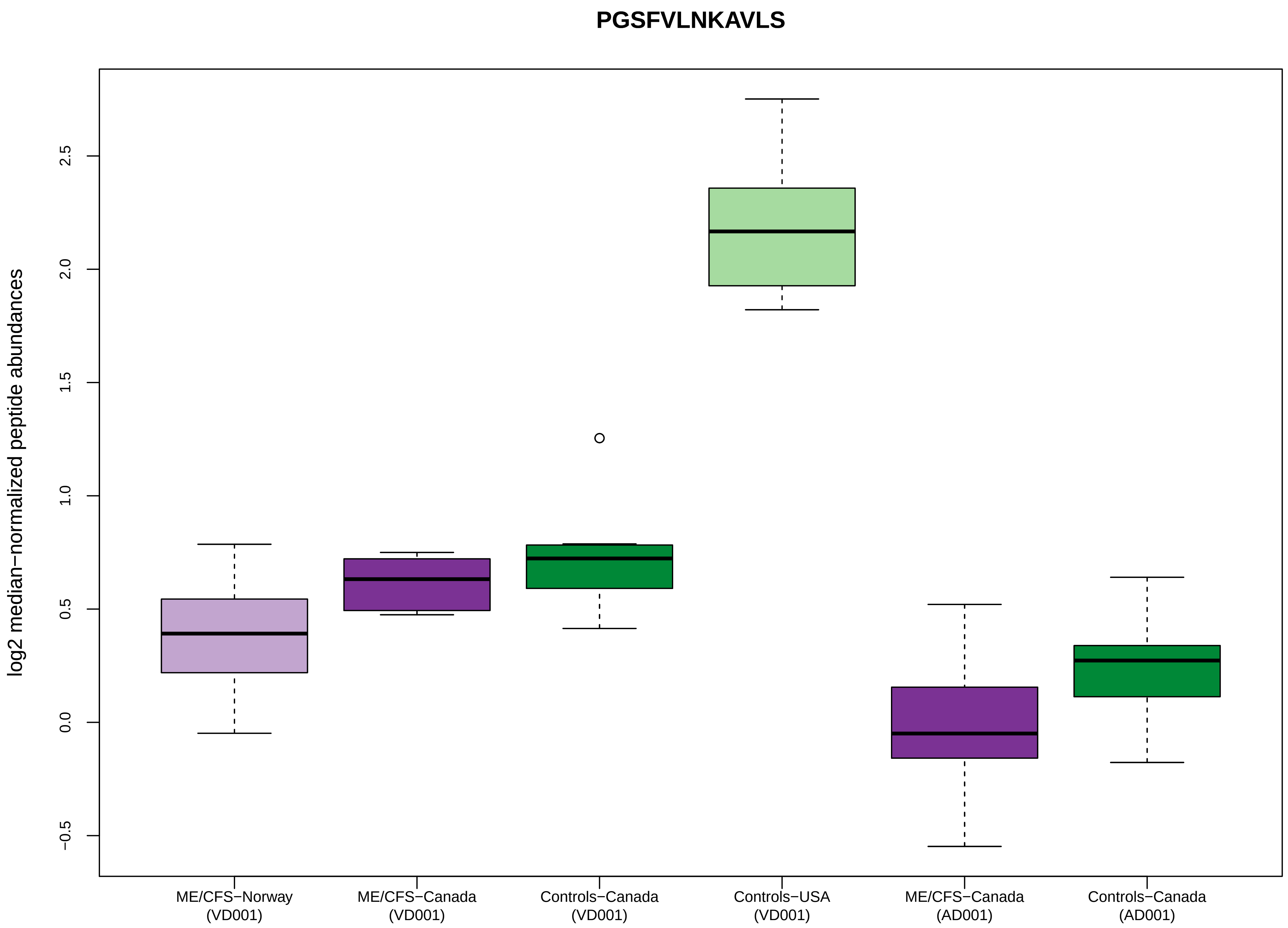
PGSFVLNKAVLS
log2 median−normalized peptide abundances
2.5
2.0
1.5
1.0
0.5
0.0
−0.5
ME/CFS−Norway (VD001)
ME/CFS−Canada (VD001)
Controls−Canada (VD001)
Controls−USA (VD001)
ME/CFS−Canada (AD001)
Controls−Canada (AD001)

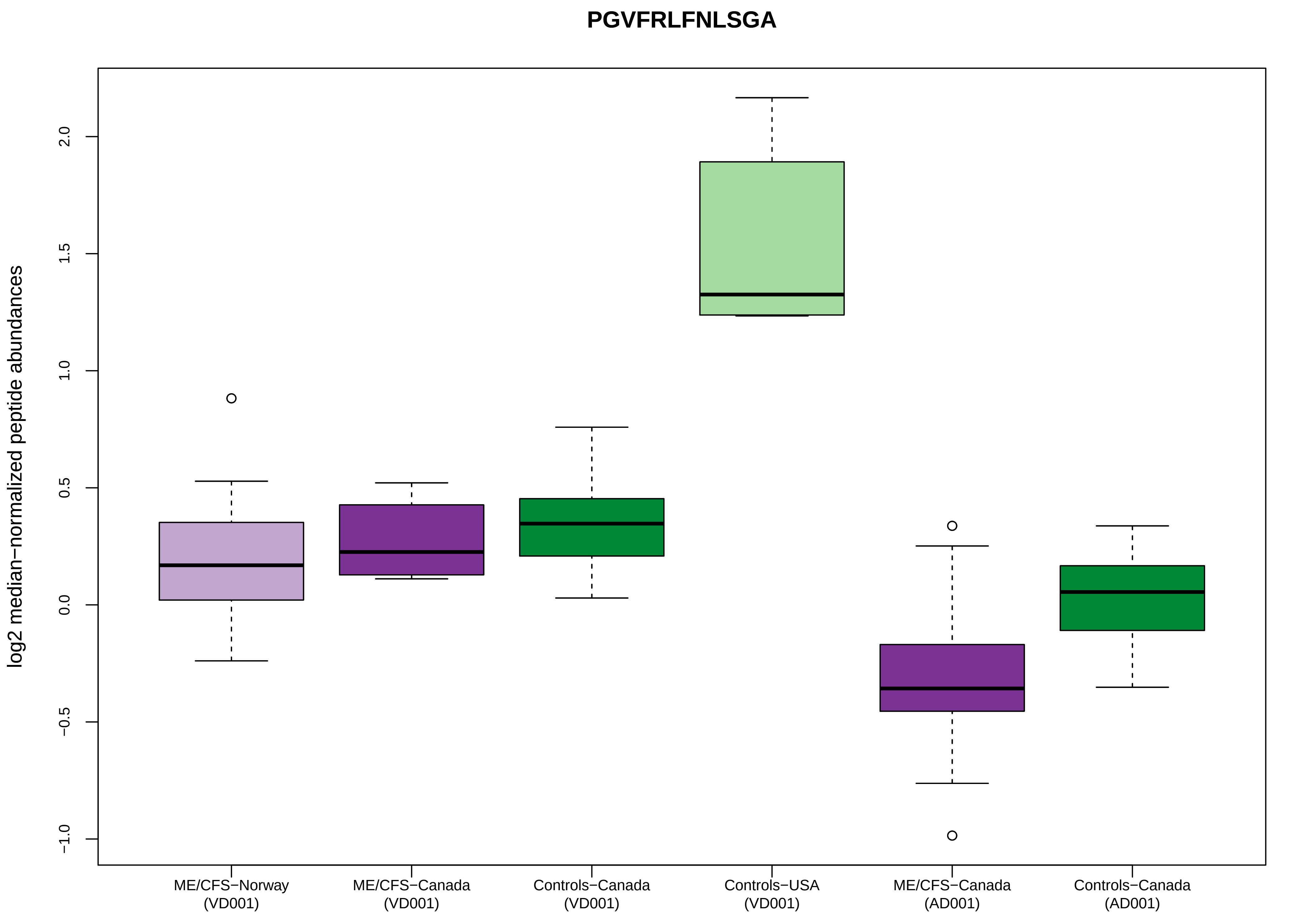

PGVFRLFNLSGA
log2 median−normalized peptide abundances
2.0
1.5
1.0
0.5
0.0
−0.5
−1.0
ME/CFS−Norway (VD001)
ME/CFS−Canada (VD001)
Controls−Canada (VD001)
Controls−USA (VD001)
ME/CFS−Canada (AD001)
Controls−Canada (AD001)

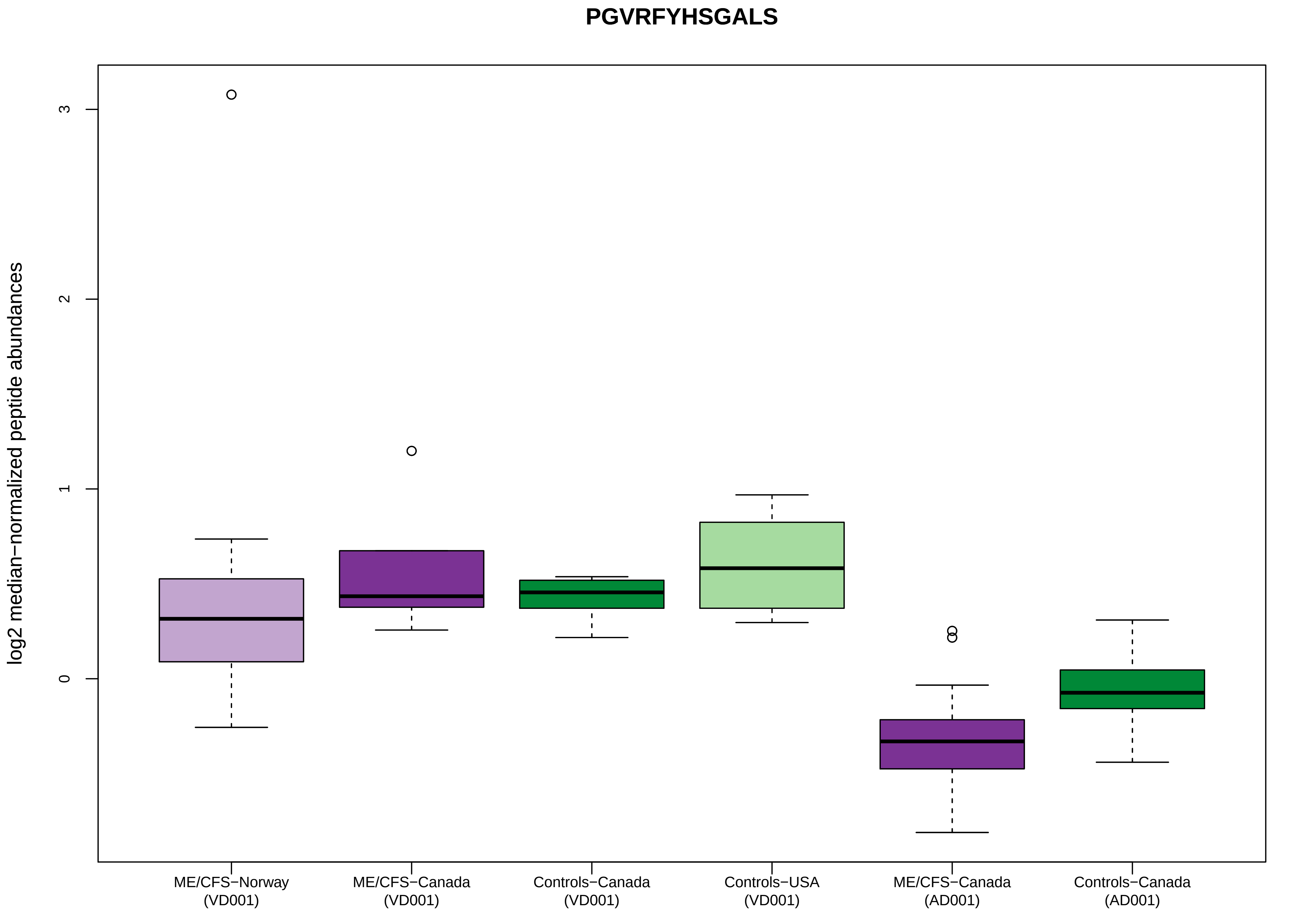

PGVRFYHSGALS
log2 median−normalized peptide abundances
0
1
2
3
ME/CFS−Norway
(VD001)
ME/CFS−Canada
(VD001)
Controls−Canada
(VD001)
Controls−USA
(VD001)
ME/CFS−Canada
(AD001)
Controls−Canada
(AD001)

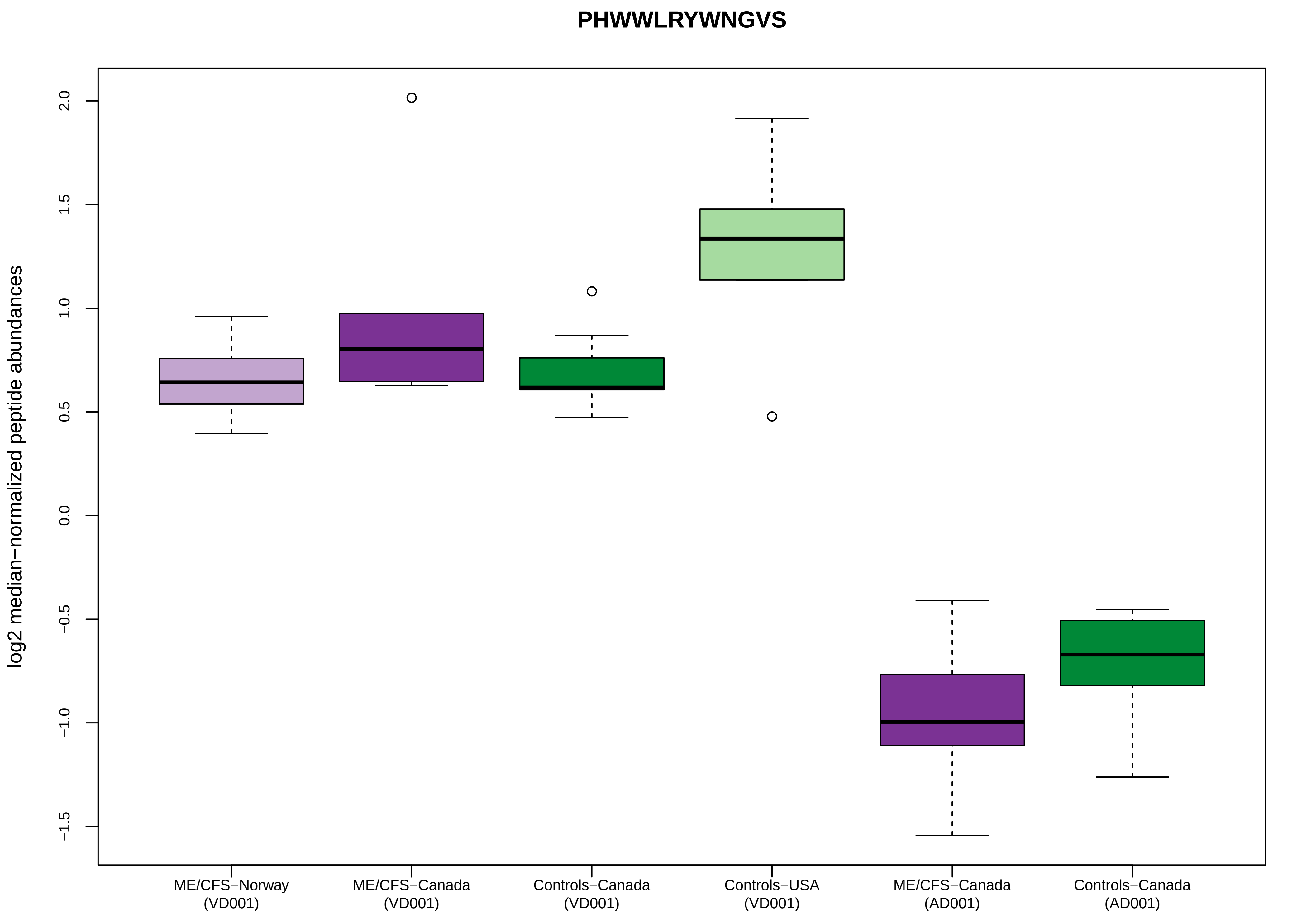

PHWWLRYWNGVS
log2 median−normalized peptide abundances
2.0
1.5
1.0
0.5
0.0
−0.5
−1.0
−1.5
ME/CFS−Norway (VD001)
ME/CFS−Canada (VD001)
Controls−Canada (VD001)
Controls−USA (VD001)
ME/CFS−Canada (AD001)
Controls−Canada (AD001)

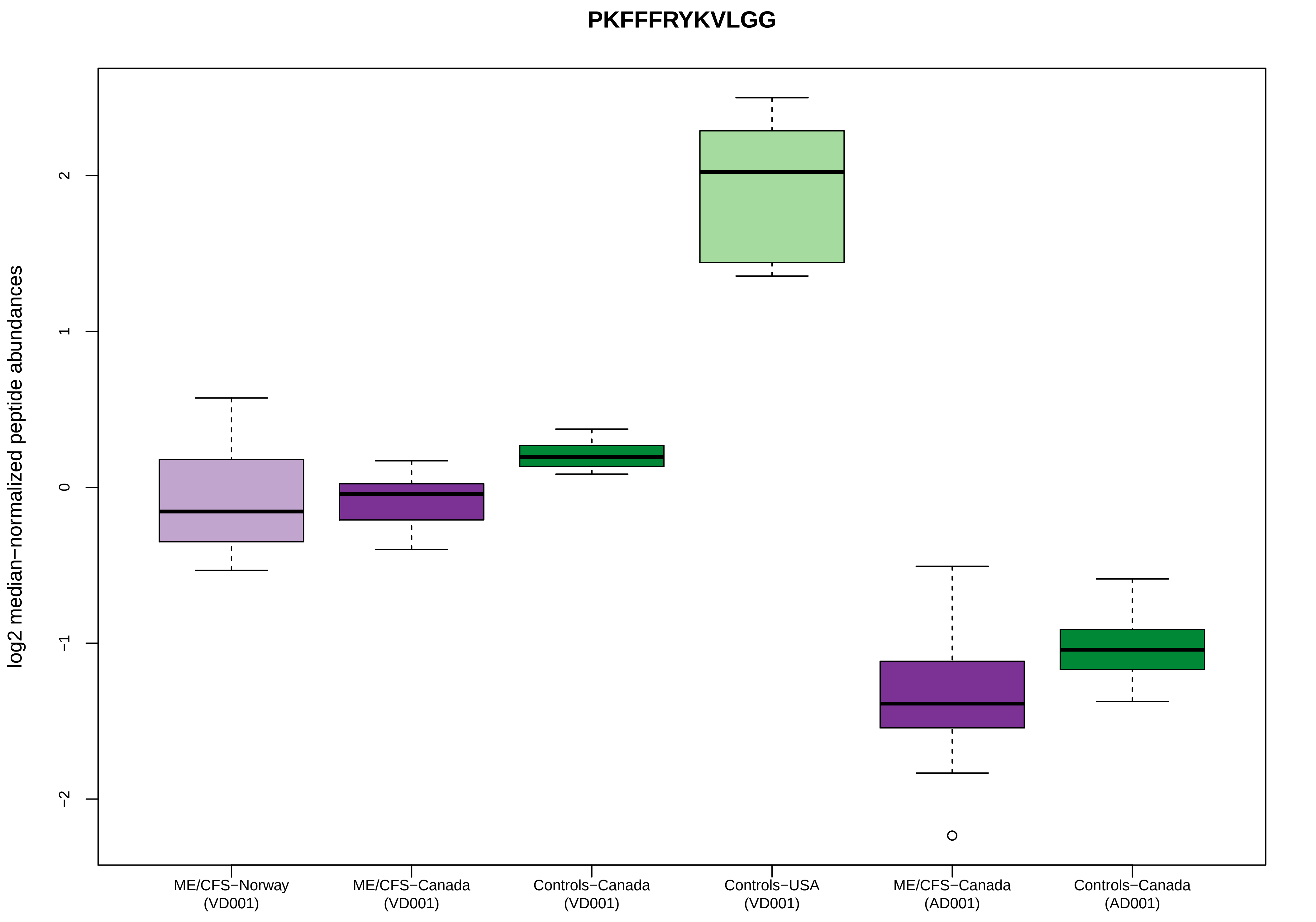

PKFFFRYKVLGG
log2 median−normalized peptide abundances
2
1
0
−1
−2
ME/CFS−Norway (VD001)
ME/CFS−Canada (VD001)
Controls−Canada (VD001)
Controls−USA (VD001)
ME/CFS−Canada (AD001)
Controls−Canada (AD001)

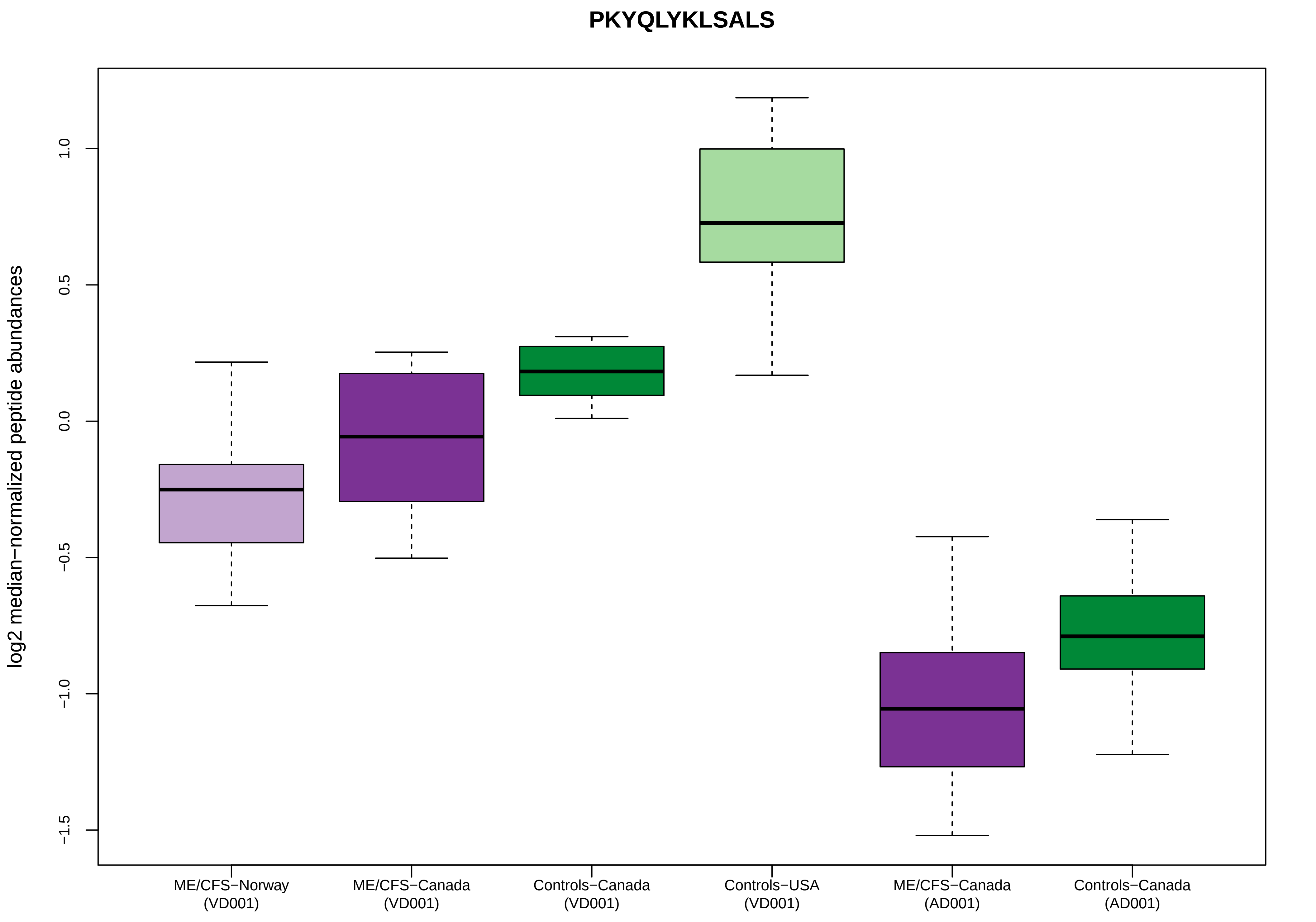

PKYQLYKLSALS
log2 median−normalized peptide abundances
1.0
0.5
0.0
−0.5
−1.0
−1.5
ME/CFS−Norway (VD001)
ME/CFS−Canada (VD001)
Controls−Canada (VD001)
Controls−USA (VD001)
ME/CFS−Canada (AD001)
Controls−Canada (AD001)

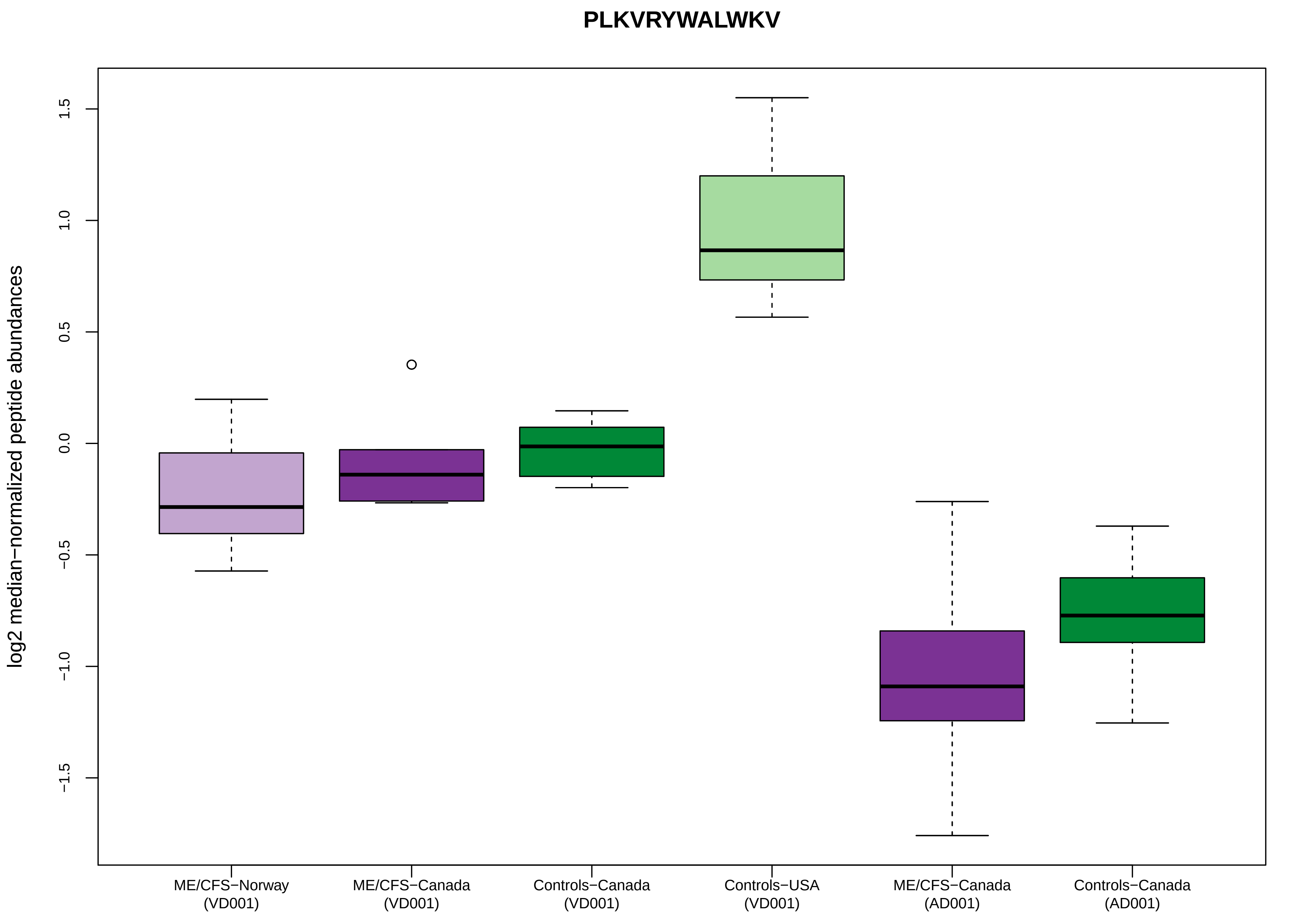

PLKVRYWALWKV
log2 median−normalized peptide abundances
1.5
1.0
0.5
0.0
−0.5
−1.0
−1.5
ME/CFS−Norway (VD001)
ME/CFS−Canada (VD001)
Controls−Canada (VD001)
Controls−USA (VD001)
ME/CFS−Canada (AD001)
Controls−Canada (AD001)

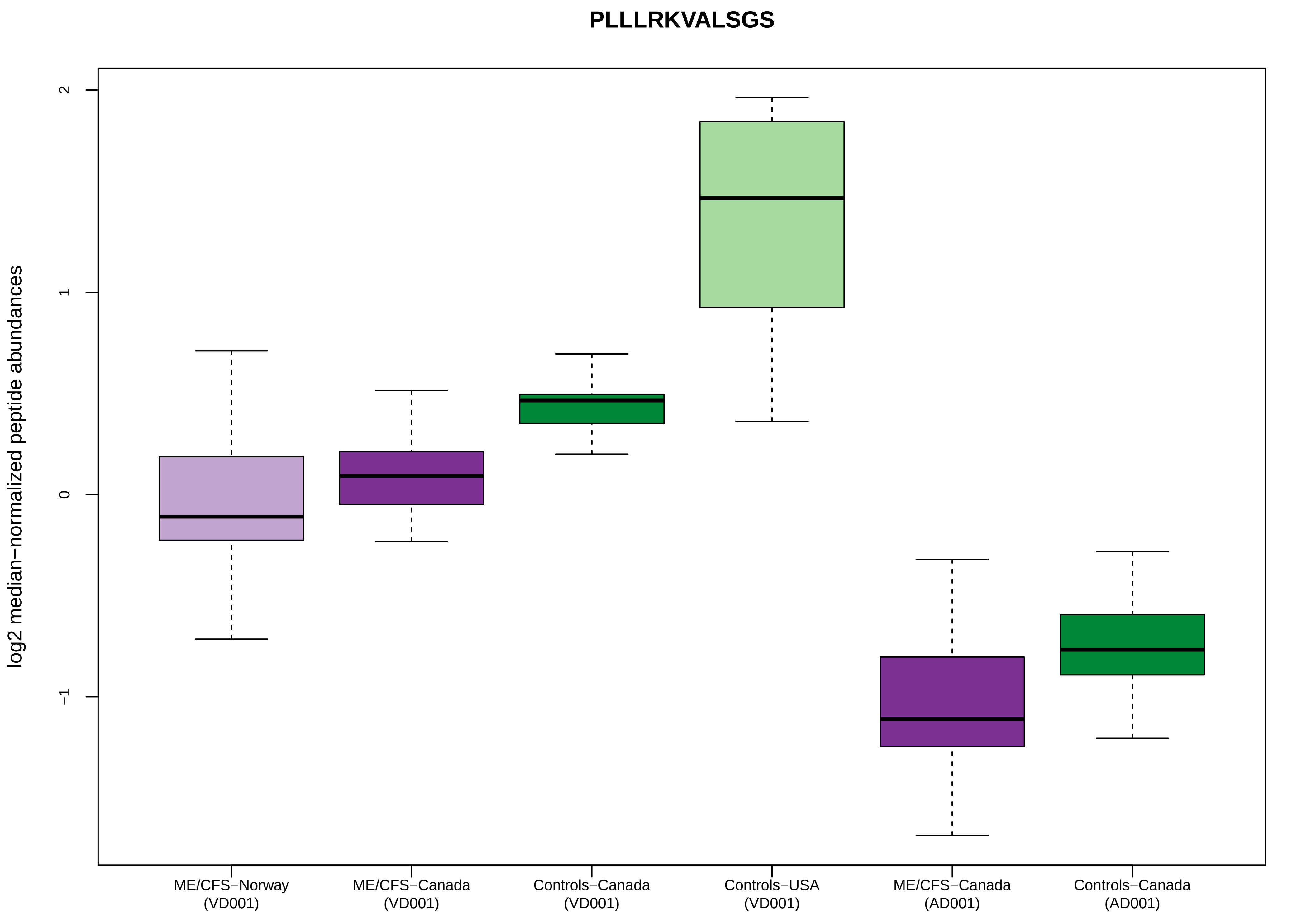
PLLLRKVALSGS
log2 median−normalized peptide abundances
2
1
0
−1
ME/CFS−Norway (VD001)
ME/CFS−Canada (VD001)
Controls−Canada (VD001)
Controls−USA (VD001)
ME/CFS−Canada (AD001)
Controls−Canada (AD001)

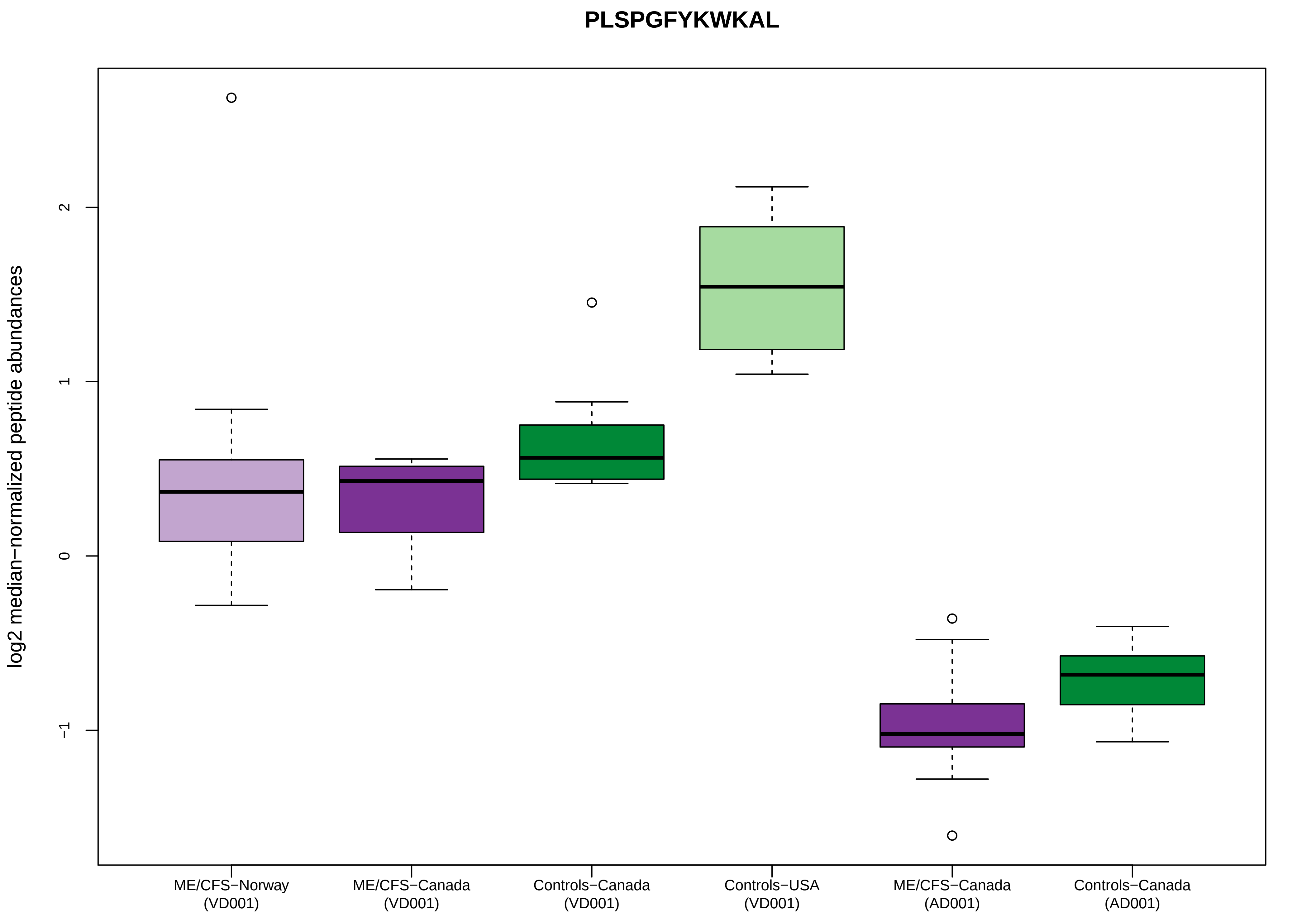
PLSPGFYKWKAL
log2 median−normalized peptide abundances
−1
0
1
2
ME/CFS−Norway (VD001)
ME/CFS−Canada (VD001)
Controls−Canada (VD001)
Controls−USA (VD001)
ME/CFS−Canada (AD001)
Controls−Canada (AD001)

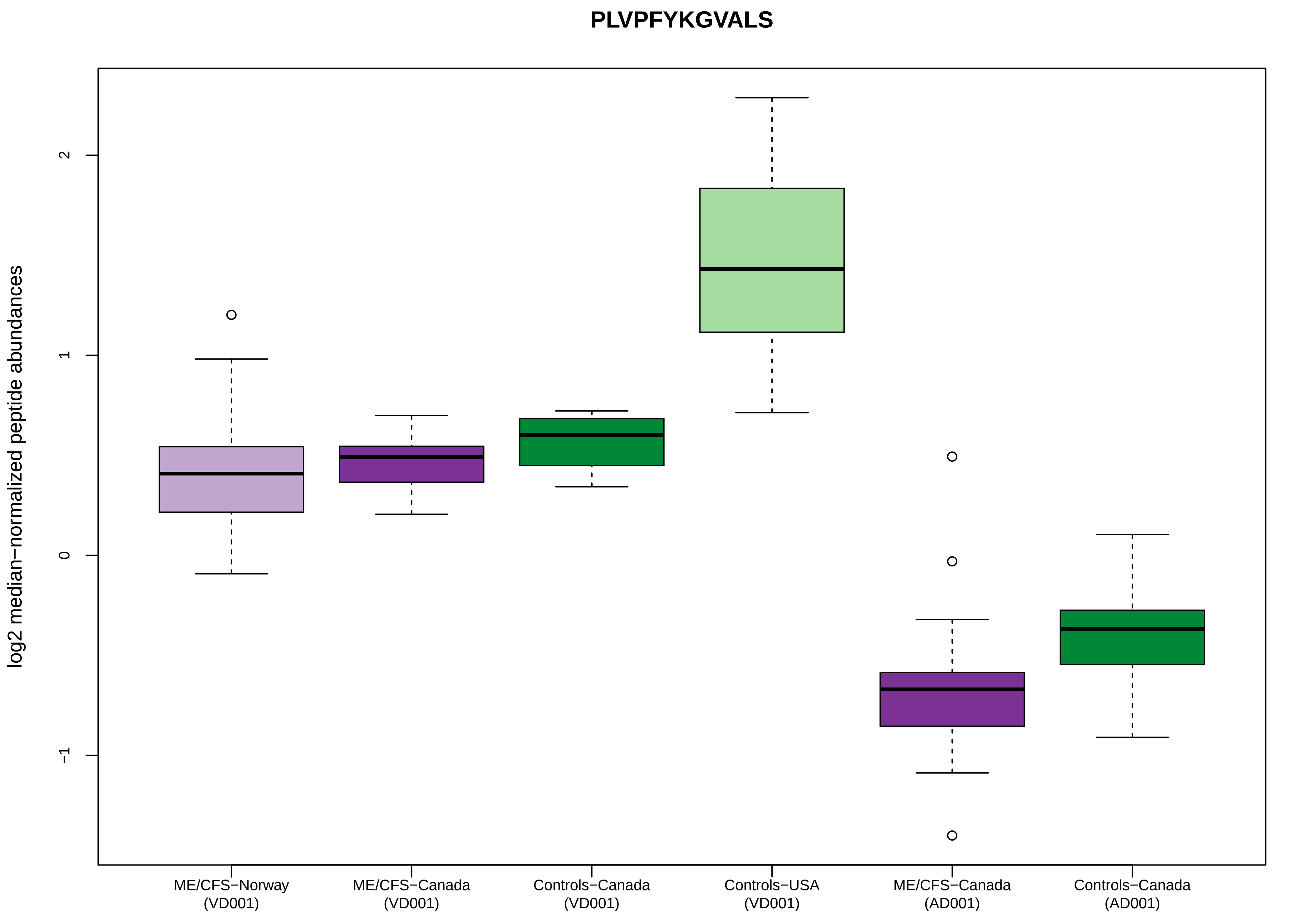
PLVPFYKGVALS
log2 median−normalized peptide abundances
2
1
0
−1
ME/CFS−Norway (VD001)
ME/CFS−Canada (VD001)
Controls−Canada (VD001)
Controls−USA (VD001)
ME/CFS−Canada (AD001)
Controls−Canada (AD001)

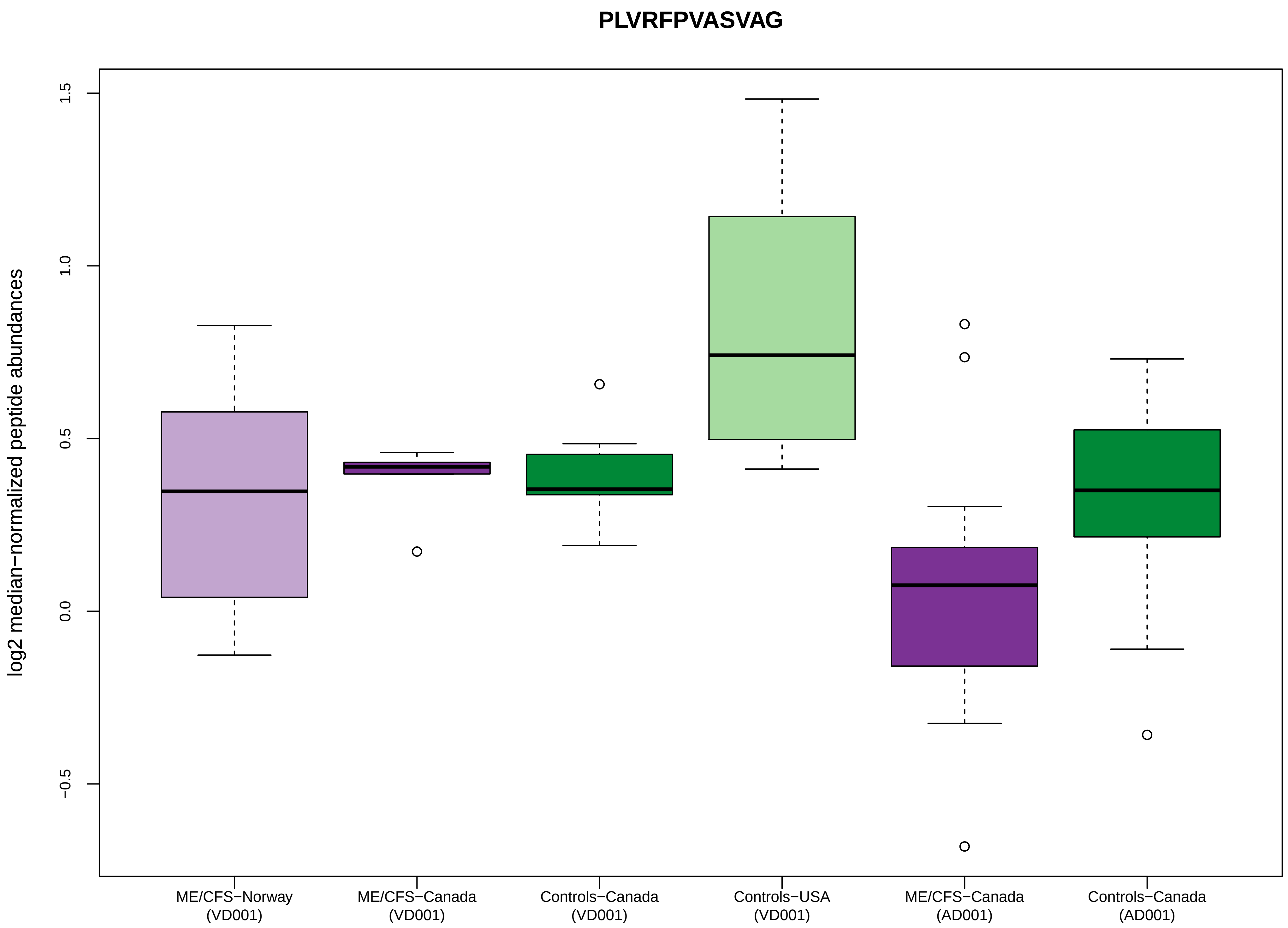

PLVRFPVASVAG
log2 median−normalized peptide abundances
1.5
1.0
0.5
0.0
−0.5
ME/CFS−Norway (VD001)
ME/CFS−Canada (VD001)
Controls−Canada (VD001)
Controls−USA (VD001)
ME/CFS−Canada (AD001)
Controls−Canada (AD001)

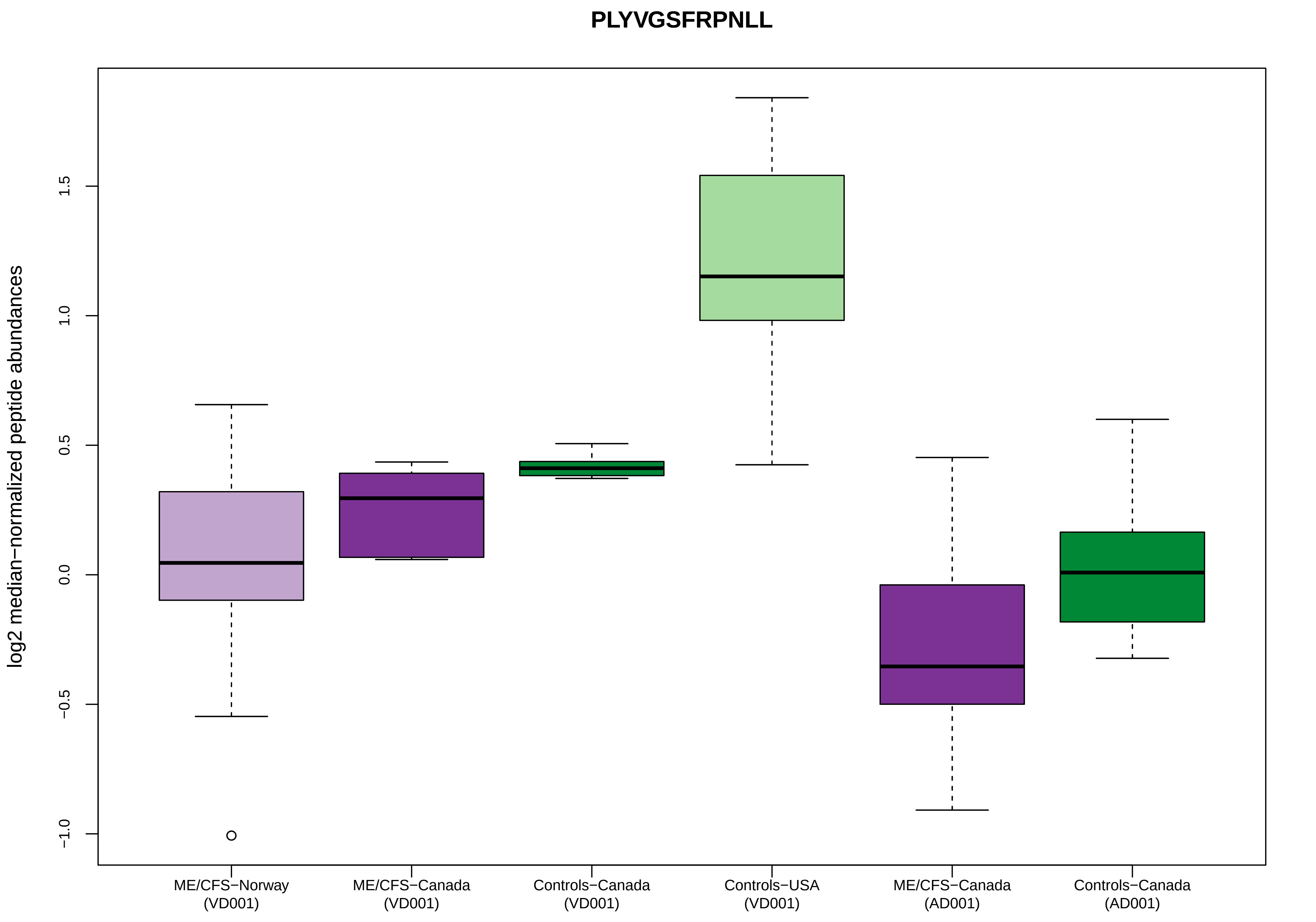
PLYVGSFRPNLL
log2 median−normalized peptide abundances
−1.0
−0.5
0.0
0.5
1.0
1.5
ME/CFS−Norway (VD001)
ME/CFS−Canada (VD001)
Controls−Canada (VD001)
Controls−USA (VD001)
ME/CFS−Canada (AD001)
Controls−Canada (AD001)

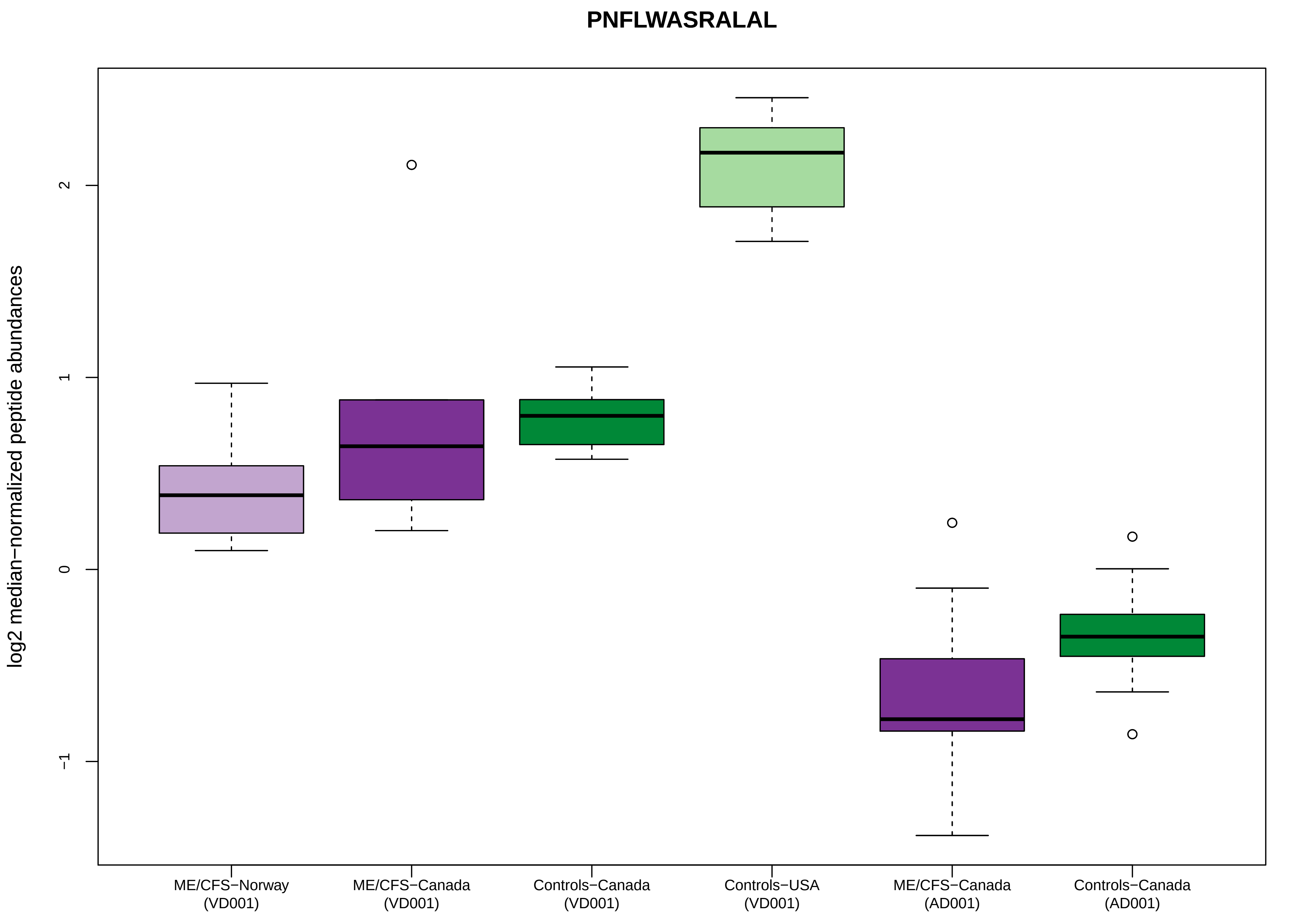
PNFLWASRALAL
log2 median−normalized peptide abundances
−1
0
1
2
ME/CFS−Norway
(VD001)
ME/CFS−Canada
(VD001)
Controls−Canada
(VD001)
Controls−USA
(VD001)
ME/CFS−Canada
(AD001)
Controls−Canada
(AD001)

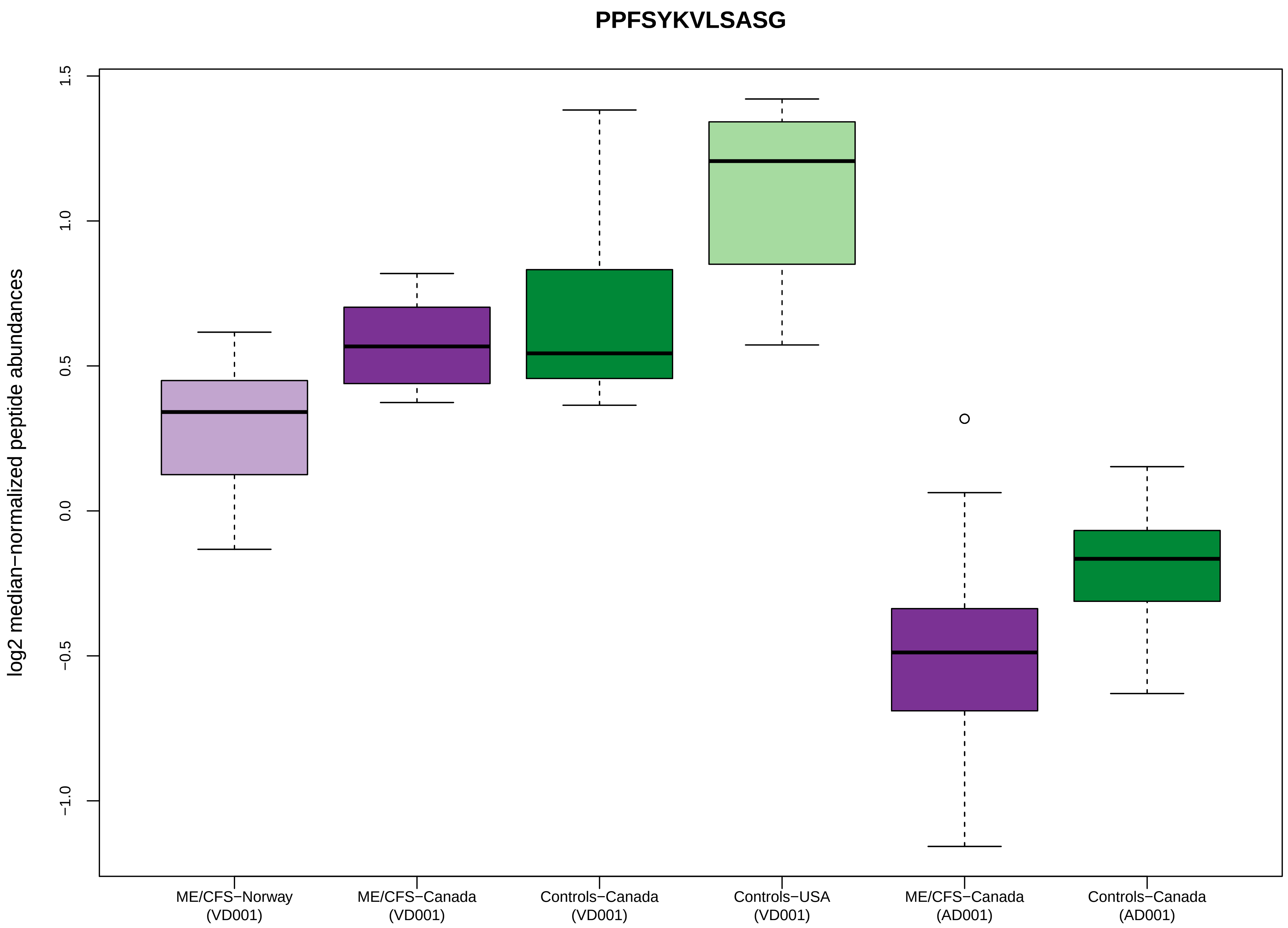

PPFSYKVLSASG
log2 median−normalized peptide abundances
1.5
1.0
0.5
0.0
−0.5
−1.0
ME/CFS−Norway (VD001)
ME/CFS−Canada (VD001)
Controls−Canada (VD001)
Controls−USA (VD001)
ME/CFS−Canada (AD001)
Controls−Canada (AD001)

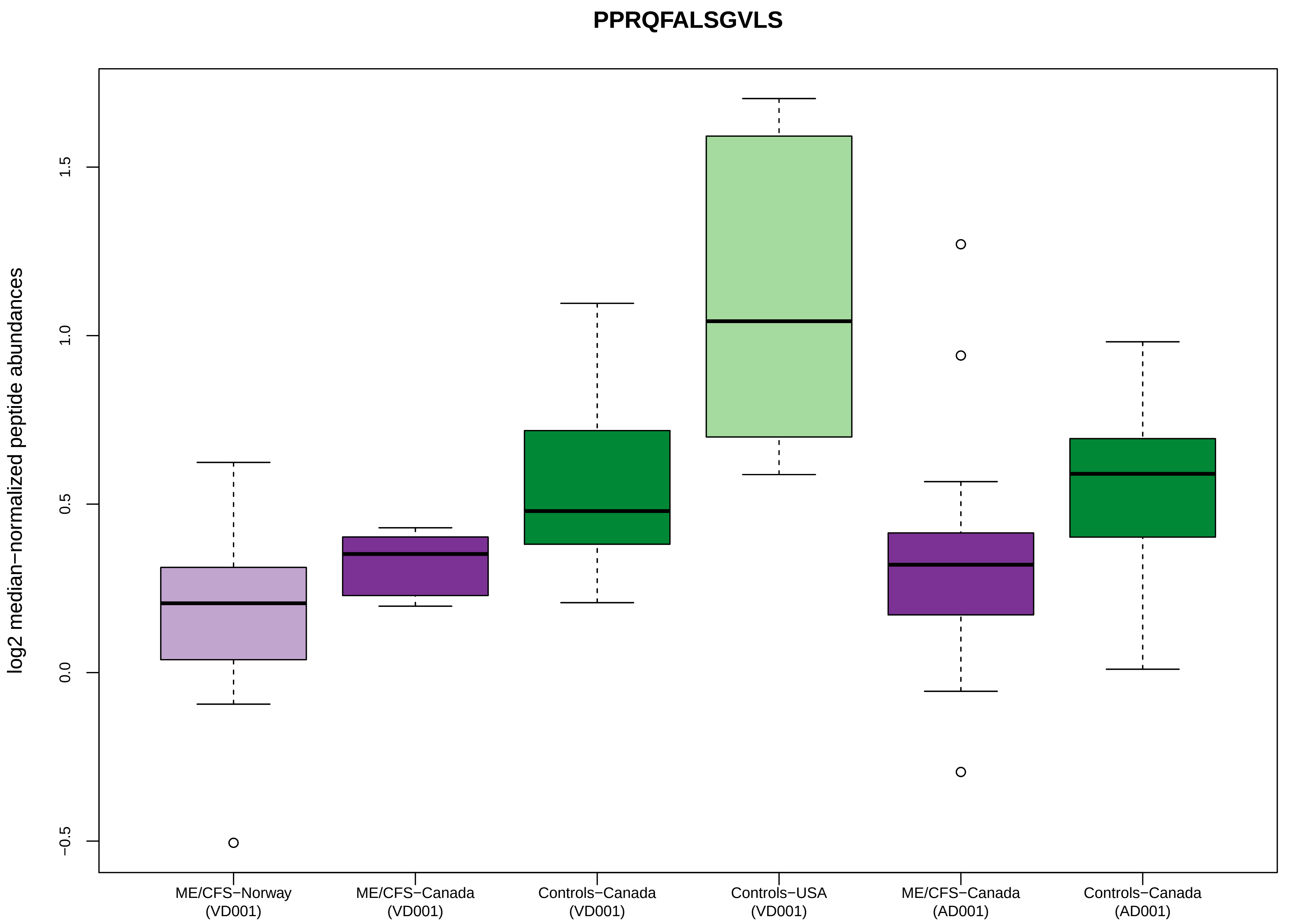
PPRQFALSGVLS
log2 median−normalized peptide abundances
−0.5
0.0
0.5
1.0
1.5
ME/CFS−Norway (VD001)
ME/CFS−Canada (VD001)
Controls−Canada (VD001)
Controls−USA (VD001)
ME/CFS−Canada (AD001)
Controls−Canada (AD001)

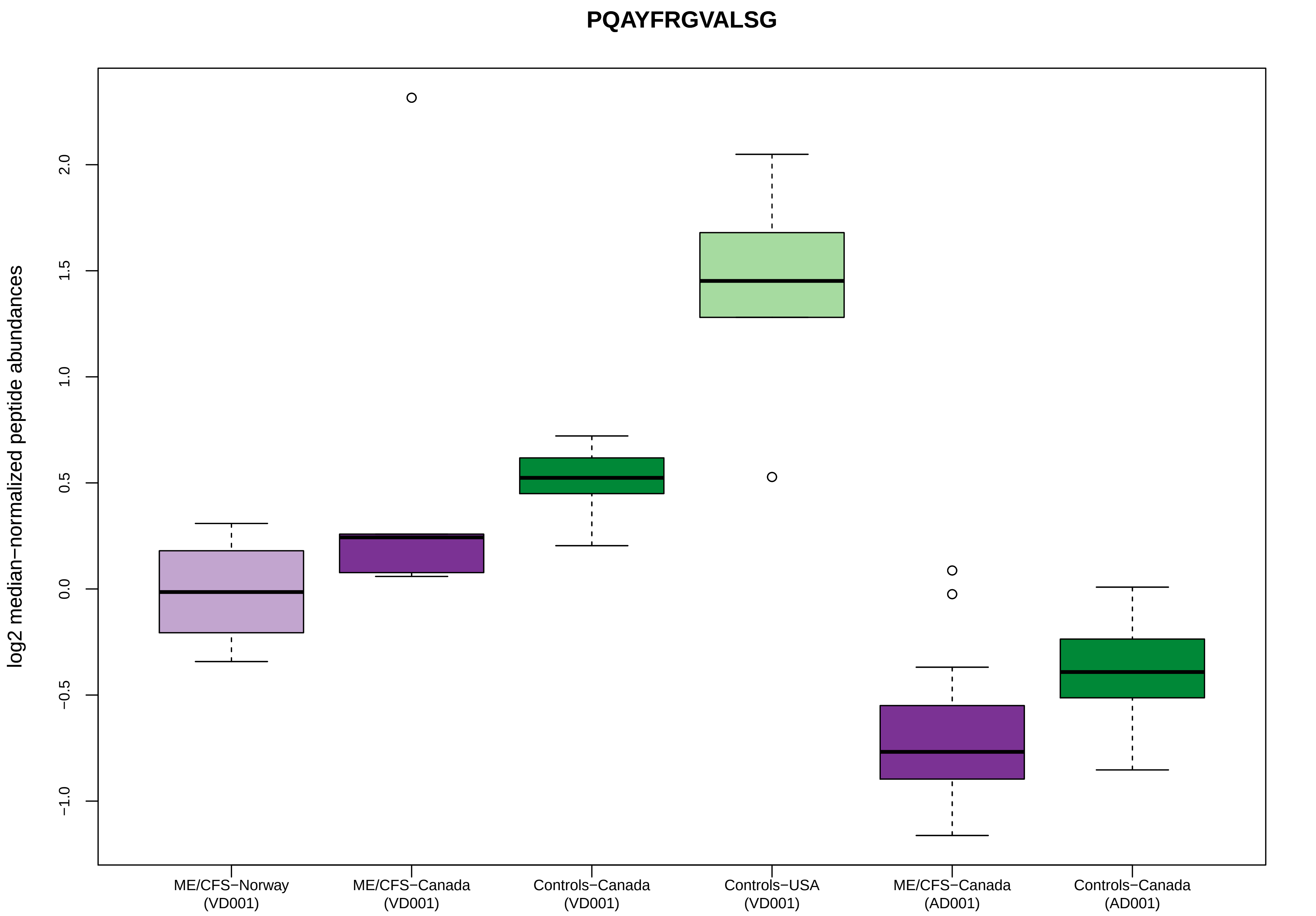
PQAYFRGVALSG
log2 median−normalized peptide abundances
2.0
1.5
1.0
0.5
0.0
−0.5
−1.0
ME/CFS−Norway (VD001)
ME/CFS−Canada (VD001)
Controls−Canada (VD001)
Controls−USA (VD001)
ME/CFS−Canada (AD001)
Controls−Canada (AD001)

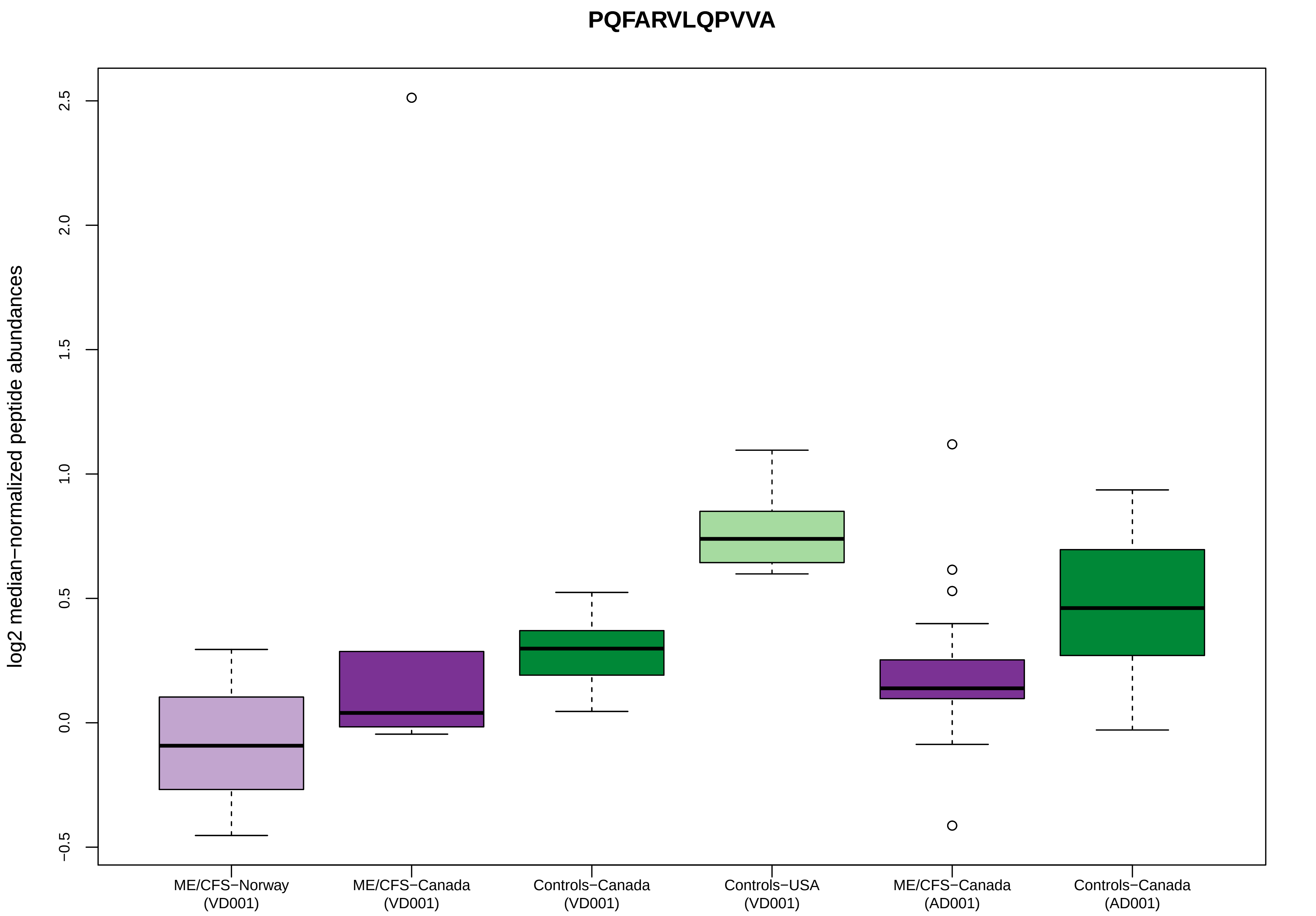

PQFARVLQPVVA
log2 median−normalized peptide abundances
−0.5
0.0
0.5
1.0
1.5
2.0
2.5
ME/CFS−Norway (VD001)
ME/CFS−Canada (VD001)
Controls−Canada (VD001)
Controls−USA (VD001)
ME/CFS−Canada (AD001)
Controls−Canada (AD001)

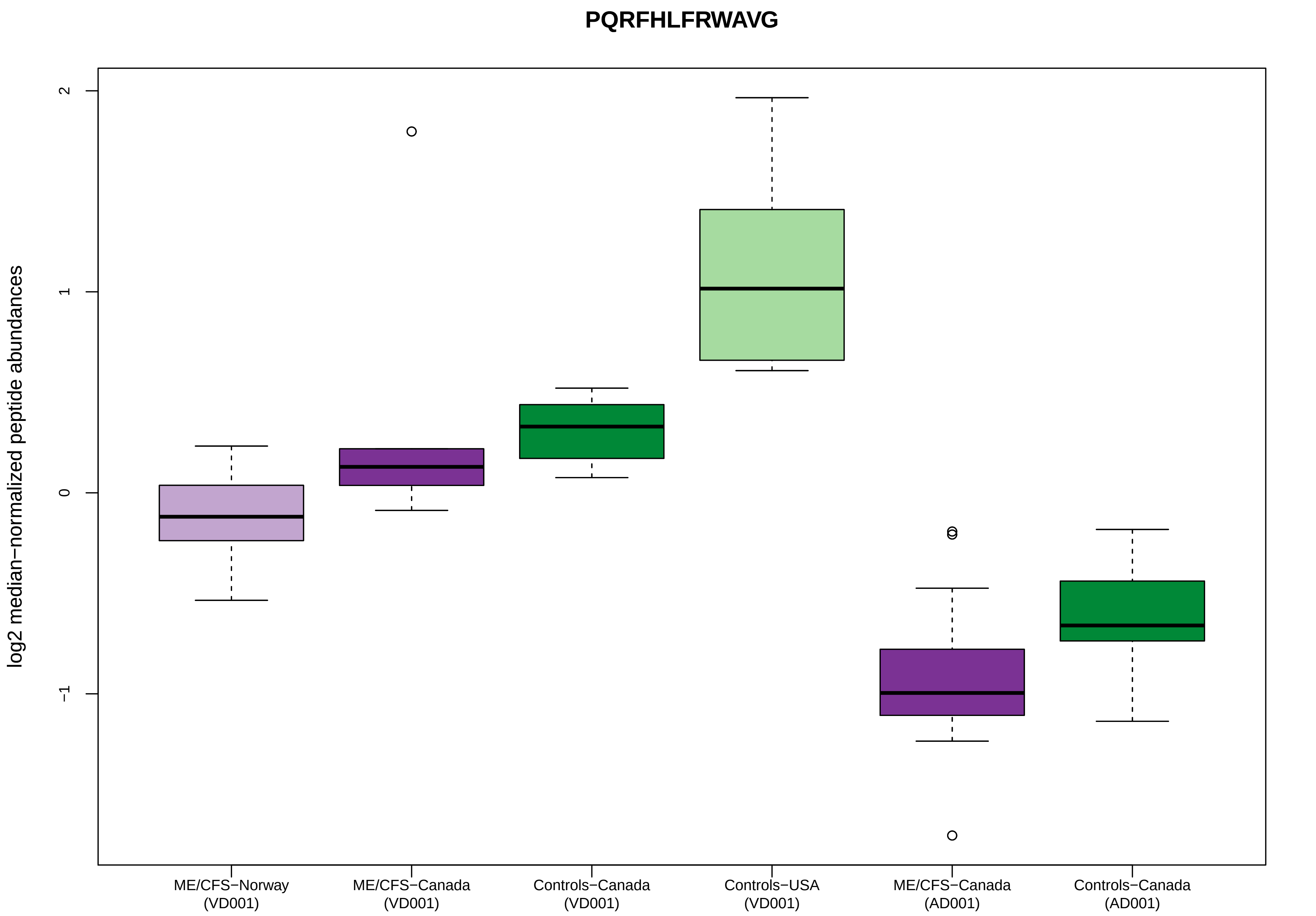
PQRFHLFRWAVG
log2 median−normalized peptide abundances
2
1
0
−1
ME/CFS−Norway (VD001)
ME/CFS−Canada (VD001)
Controls−Canada (VD001)
Controls−USA (VD001)
ME/CFS−Canada (AD001)
Controls−Canada (AD001)

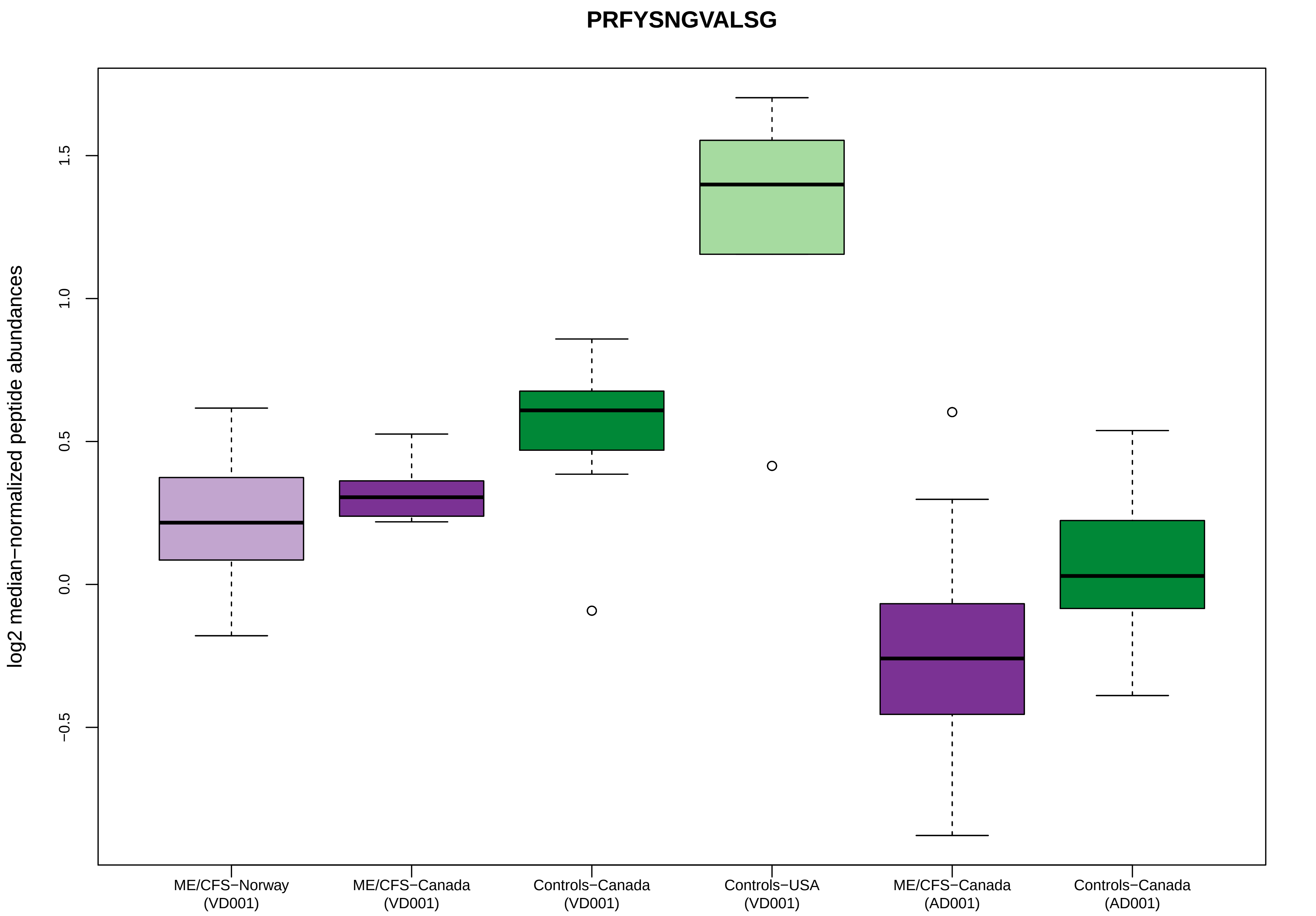
PRFYSNGVALSG
log2 median−normalized peptide abundances
−0.5
0.0
0.5
1.0
1.5
ME/CFS−Norway (VD001)
ME/CFS−Canada (VD001)
Controls−Canada (VD001)
Controls−USA (VD001)
ME/CFS−Canada (AD001)
Controls−Canada (AD001)

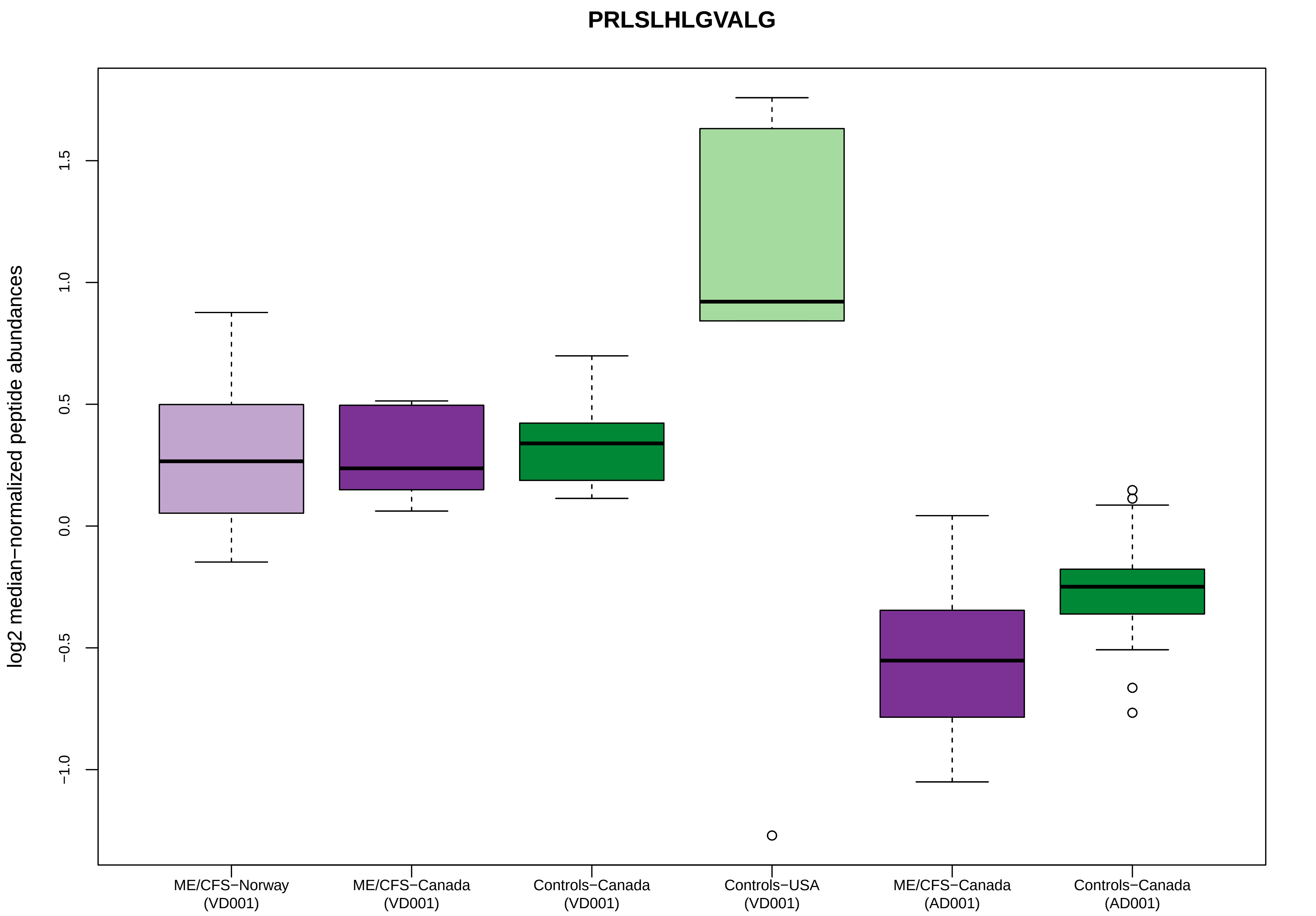

PRLSLHLGVALG
log2 median−normalized peptide abundances
1.5
1.0
0.5
0.0
−0.5
−1.0
ME/CFS−Norway (VD001)
ME/CFS−Canada (VD001)
Controls−Canada (VD001)
Controls−USA (VD001)
ME/CFS−Canada (AD001)
Controls−Canada (AD001)

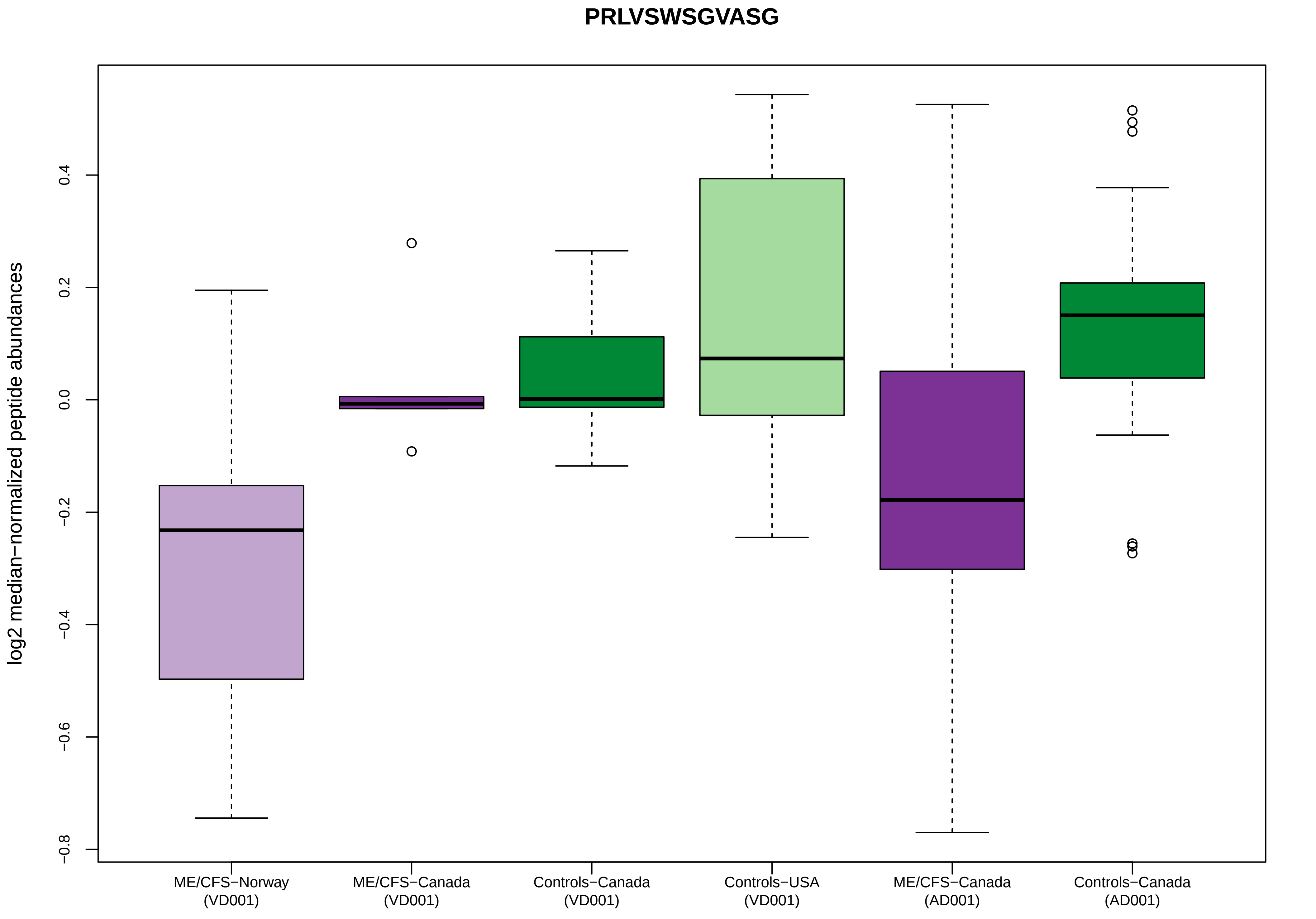

PRLVSWSGVASG
log2 median−normalized peptide abundances
0.4
0.2
0.0
−0.2
−0.4
−0.6
−0.8
ME/CFS−Norway (VD001)
ME/CFS−Canada (VD001)
Controls−Canada (VD001)
Controls−USA (VD001)
ME/CFS−Canada (AD001)
Controls−Canada (AD001)

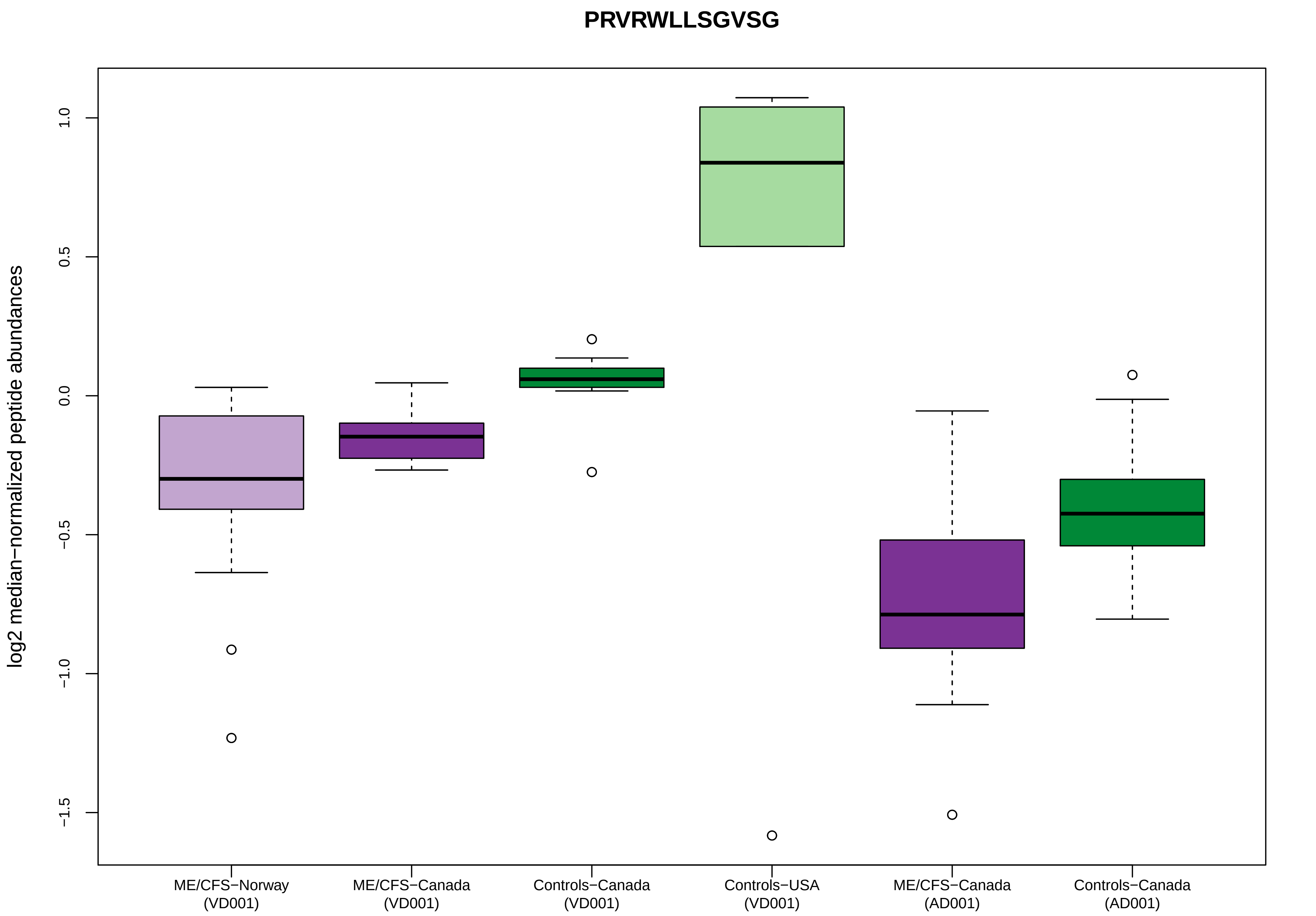

PRVRWLLSGVSG
log2 median−normalized peptide abundances
1.0
0.5
0.0
−0.5
−1.0
−1.5
ME/CFS−Norway (VD001)
ME/CFS−Canada (VD001)
Controls−Canada (VD001)
Controls−USA (VD001)
ME/CFS−Canada (AD001)
Controls−Canada (AD001)

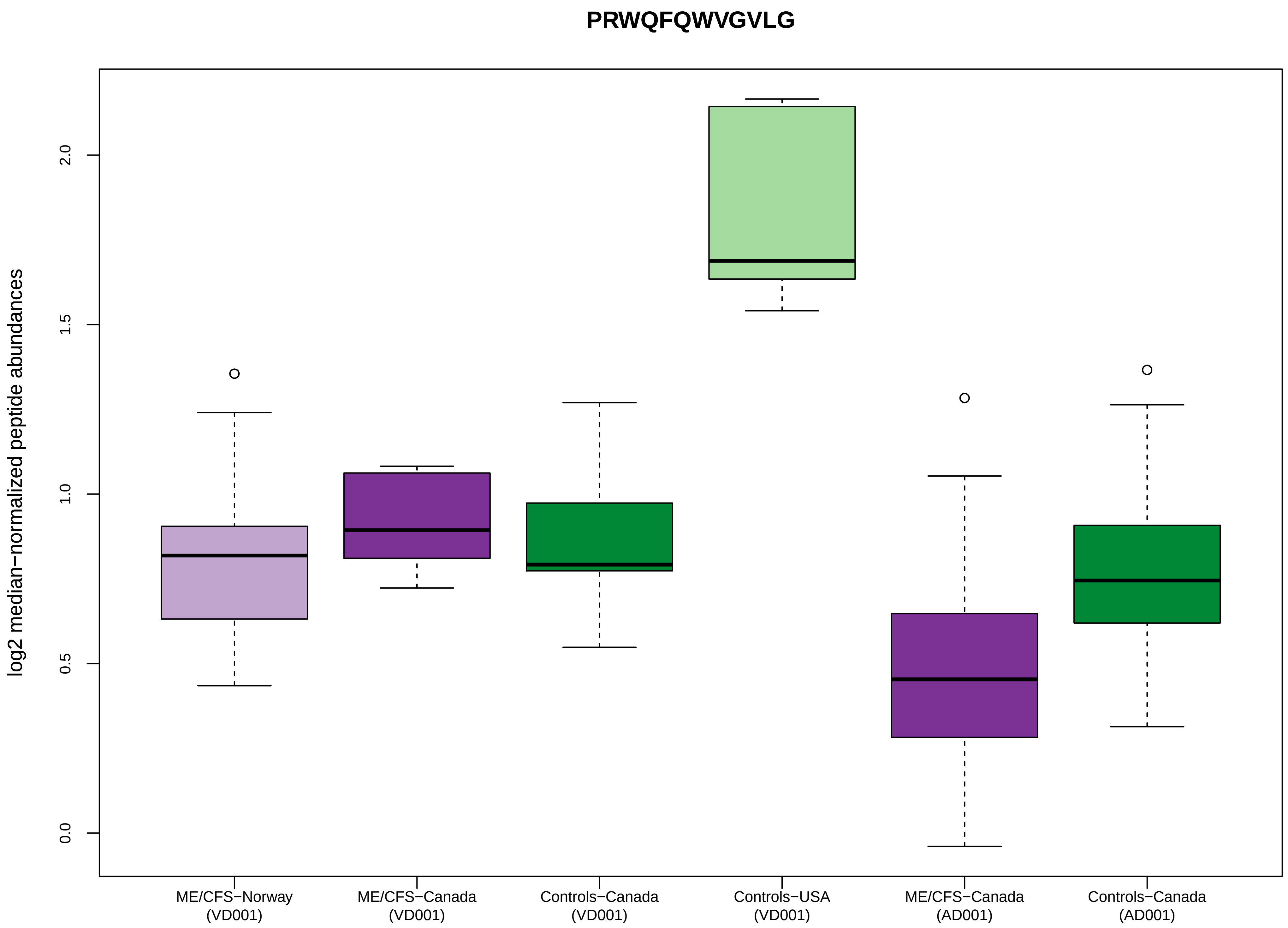
PRWQFQWVGVLG
log2 median−normalized peptide abundances
0.0
0.5
1.0
1.5
2.0
ME/CFS−Norway (VD001)
ME/CFS−Canada (VD001)
Controls−Canada (VD001)
Controls−USA (VD001)
ME/CFS−Canada (AD001)
Controls−Canada (AD001)

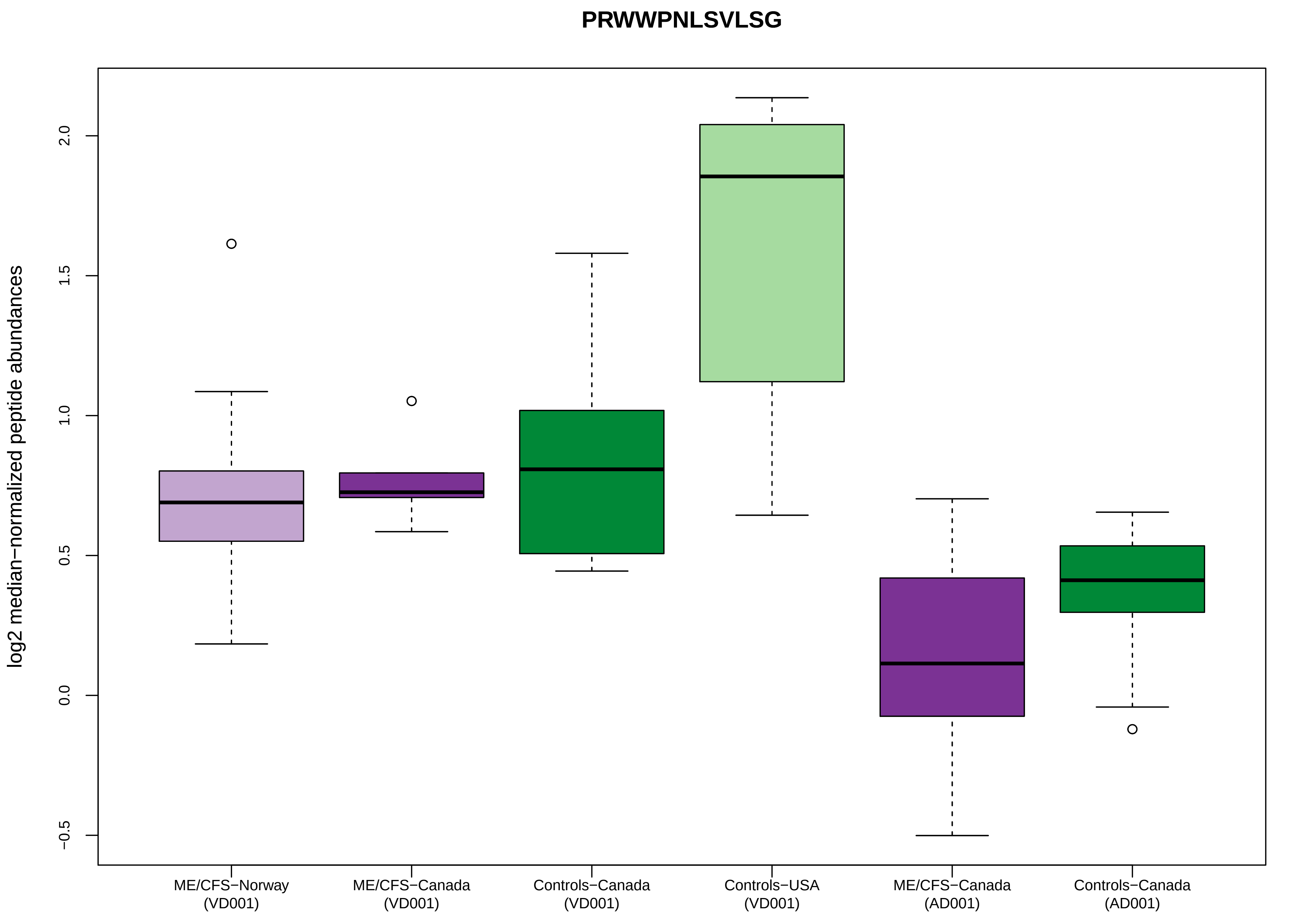
PRWWPNLSVLSG
log2 median−normalized peptide abundances
−0.5
0.0
0.5
1.0
1.5
2.0
ME/CFS−Norway (VD001)
ME/CFS−Canada (VD001)
Controls−Canada (VD001)
Controls−USA (VD001)
ME/CFS−Canada (AD001)
Controls−Canada (AD001)

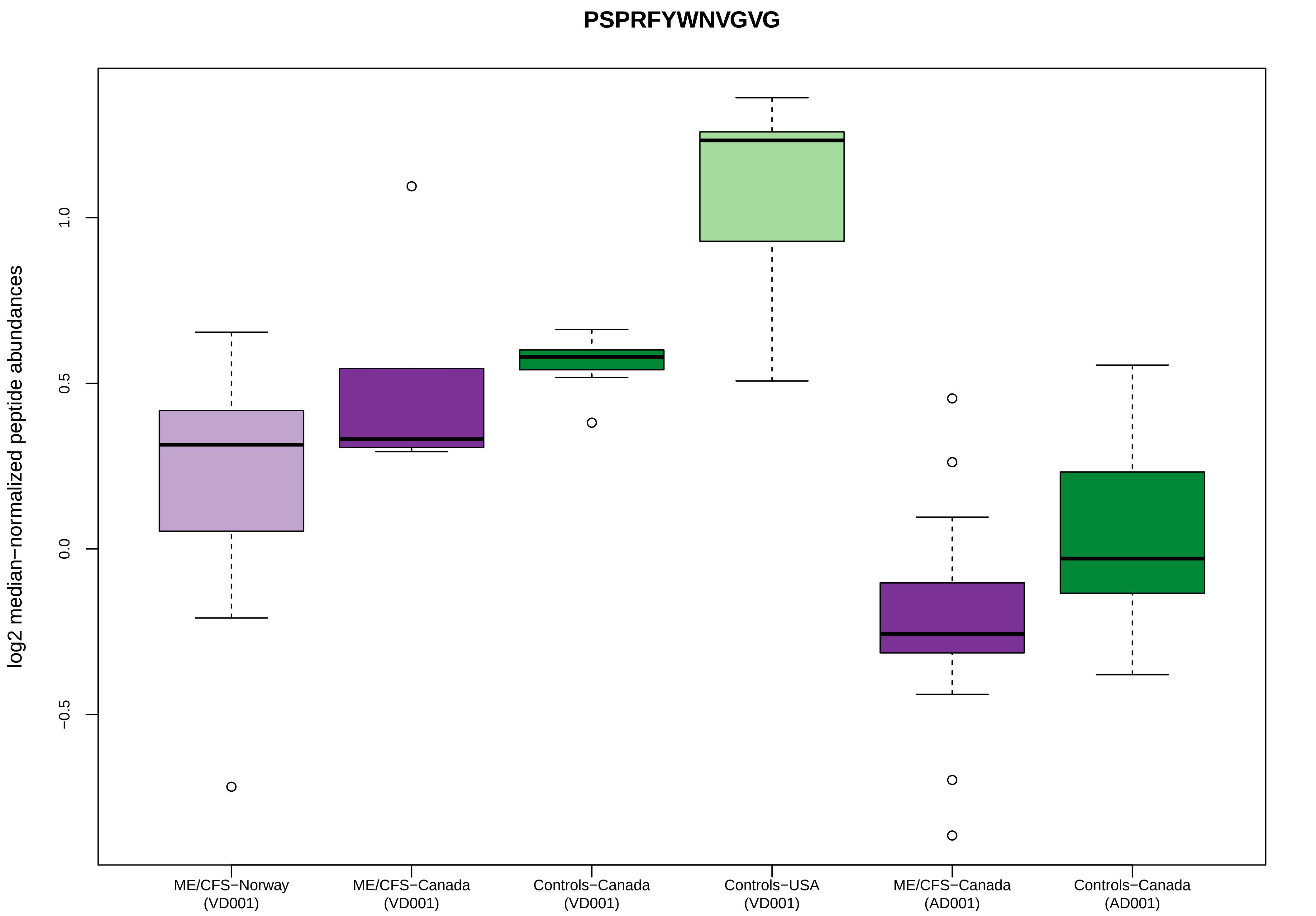
PSPRFYWNVGVG
log2 median−normalized peptide abundances
1.0
0.5
0.0
−0.5
ME/CFS−Norway (VD001)
ME/CFS−Canada (VD001)
Controls−Canada (VD001)
Controls−USA (VD001)
ME/CFS−Canada (AD001)
Controls−Canada (AD001)

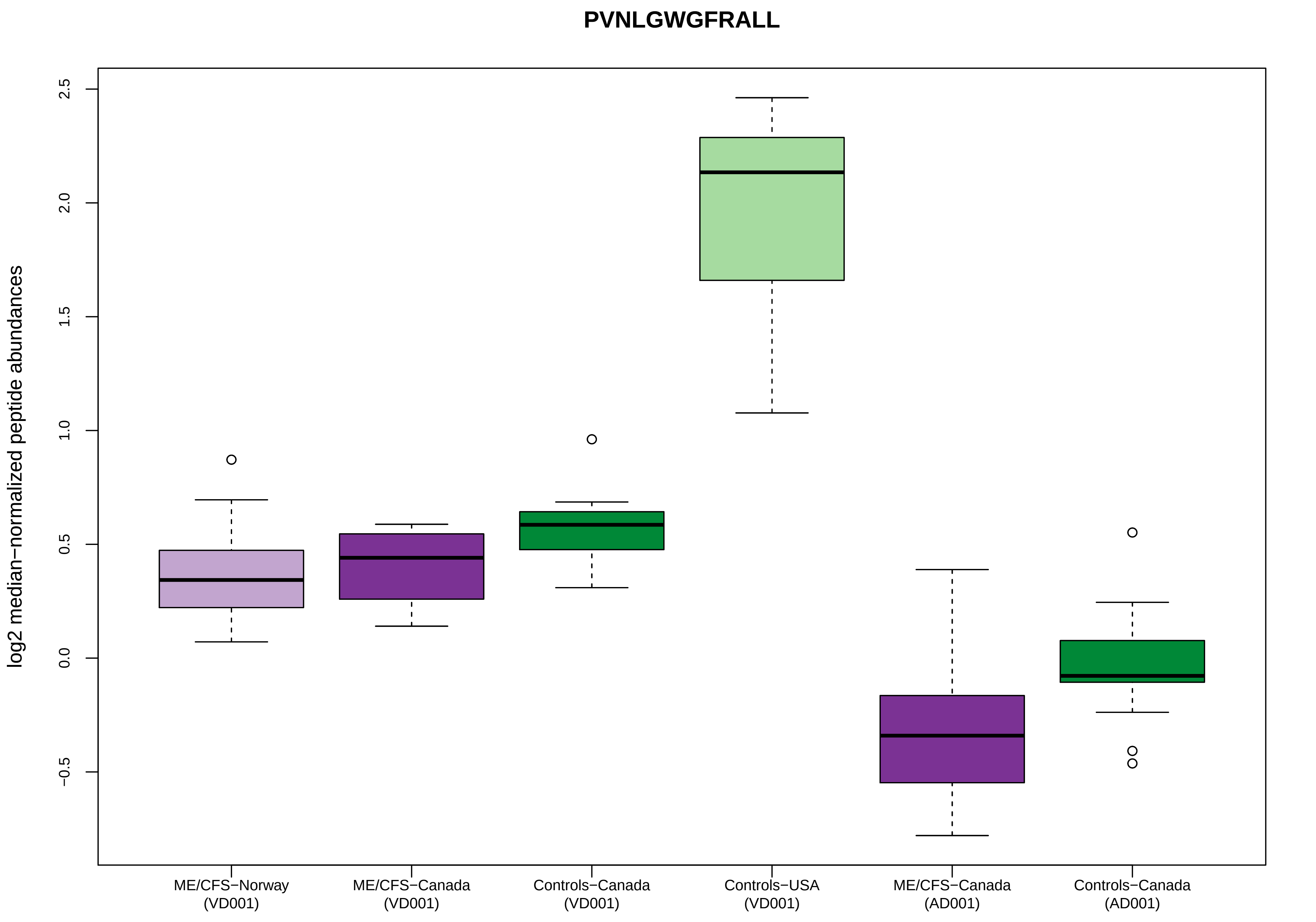

PVNLGWGFRALL
log2 median−normalized peptide abundances
2.5
2.0
1.5
1.0
0.5
0.0
−0.5
ME/CFS−Norway (VD001)
ME/CFS−Canada (VD001)
Controls−Canada (VD001)
Controls−USA (VD001)
ME/CFS−Canada (AD001)
Controls−Canada (AD001)

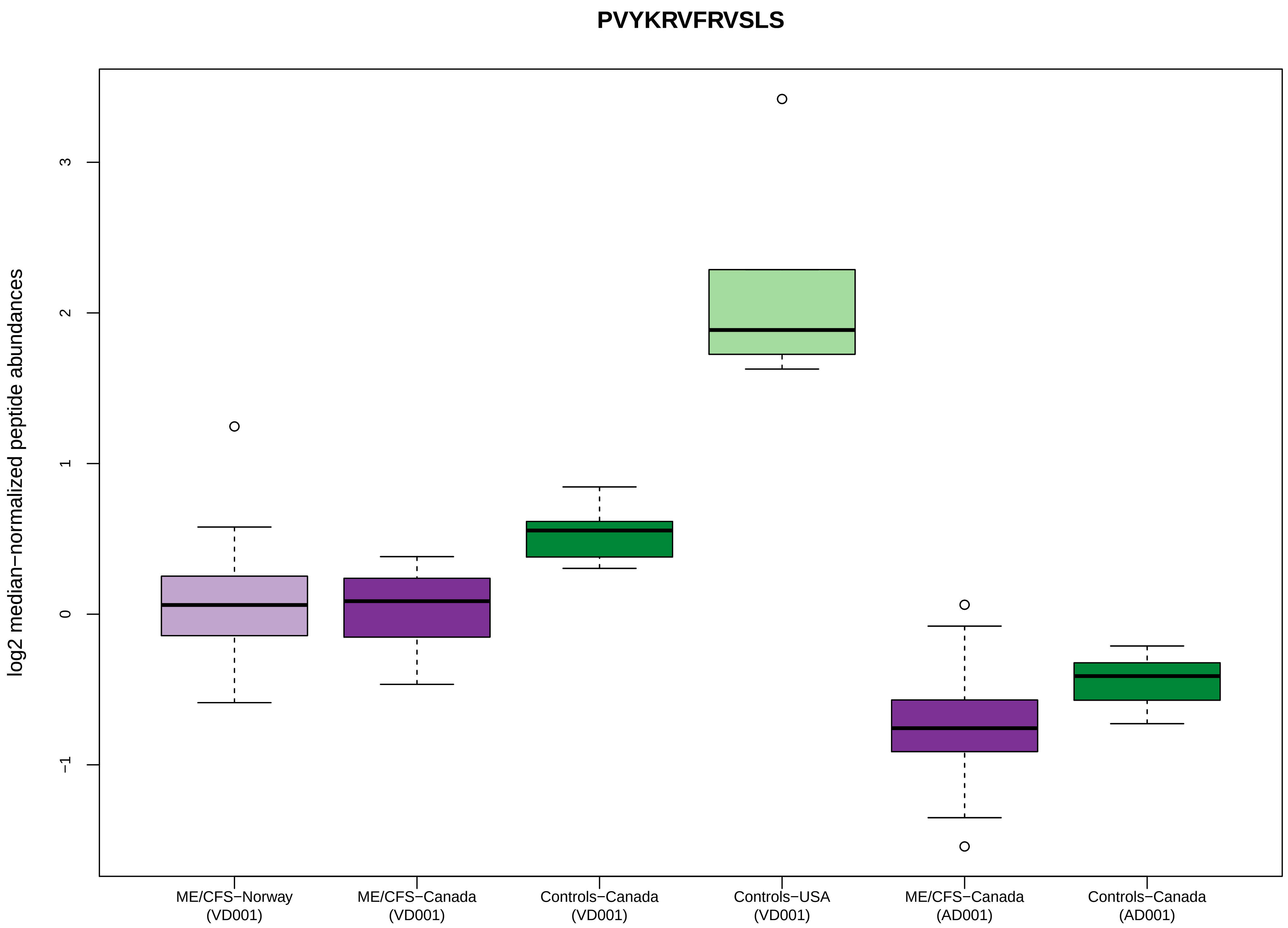
PVYKRVFRVSLS
log2 median−normalized peptide abundances
−1
0
1
2
3
ME/CFS−Norway (VD001)
ME/CFS−Canada (VD001)
Controls−Canada (VD001)
Controls−USA (VD001)
ME/CFS−Canada (AD001)
Controls−Canada (AD001)

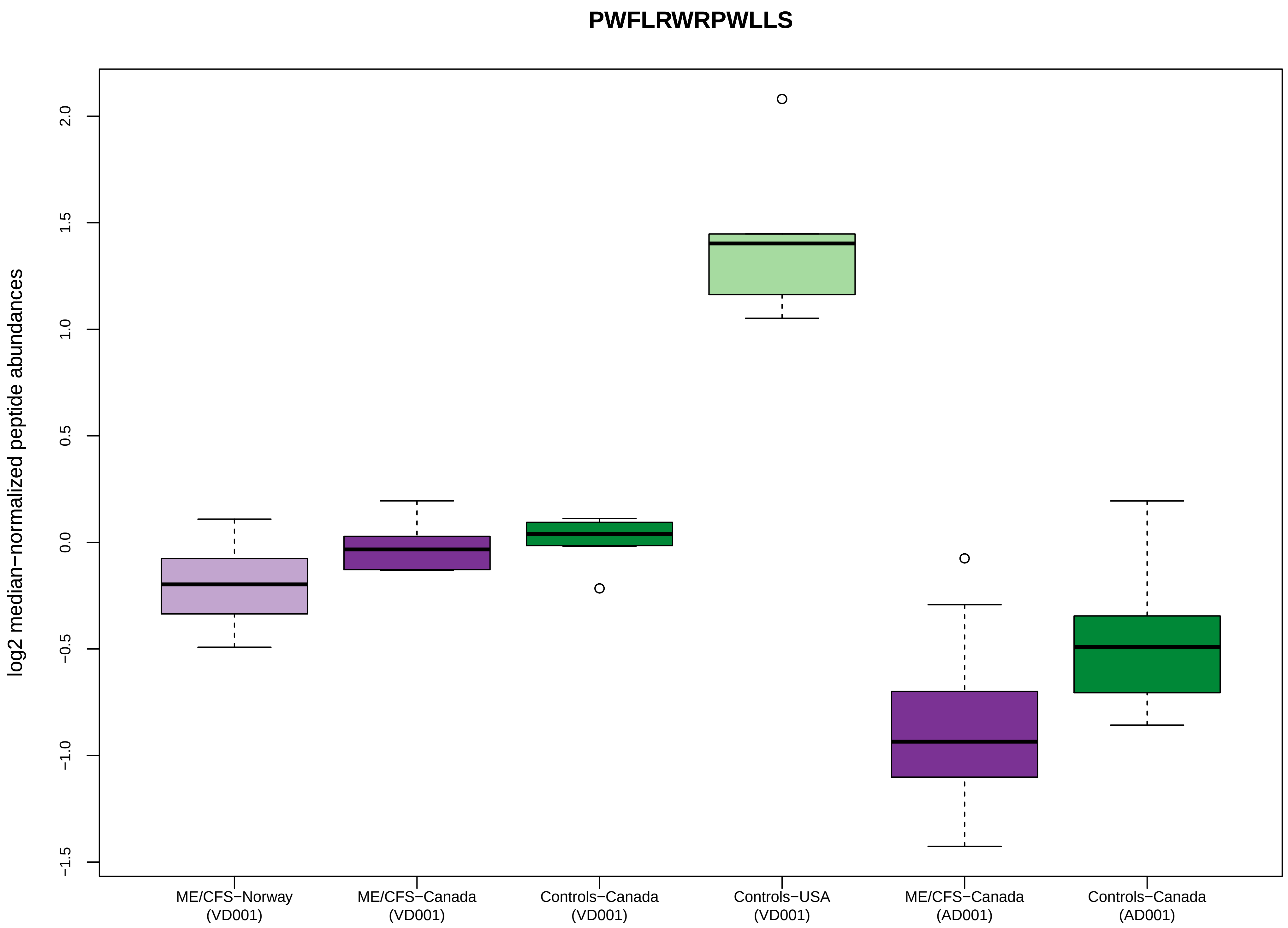

PWFLRWRPWLLS
log2 median−normalized peptide abundances
2.0
1.5
1.0
0.5
0.0
−0.5
−1.0
−1.5
ME/CFS−Norway (VD001)
ME/CFS−Canada (VD001)
Controls−Canada (VD001)
Controls−USA (VD001)
ME/CFS−Canada (AD001)
Controls−Canada (AD001)

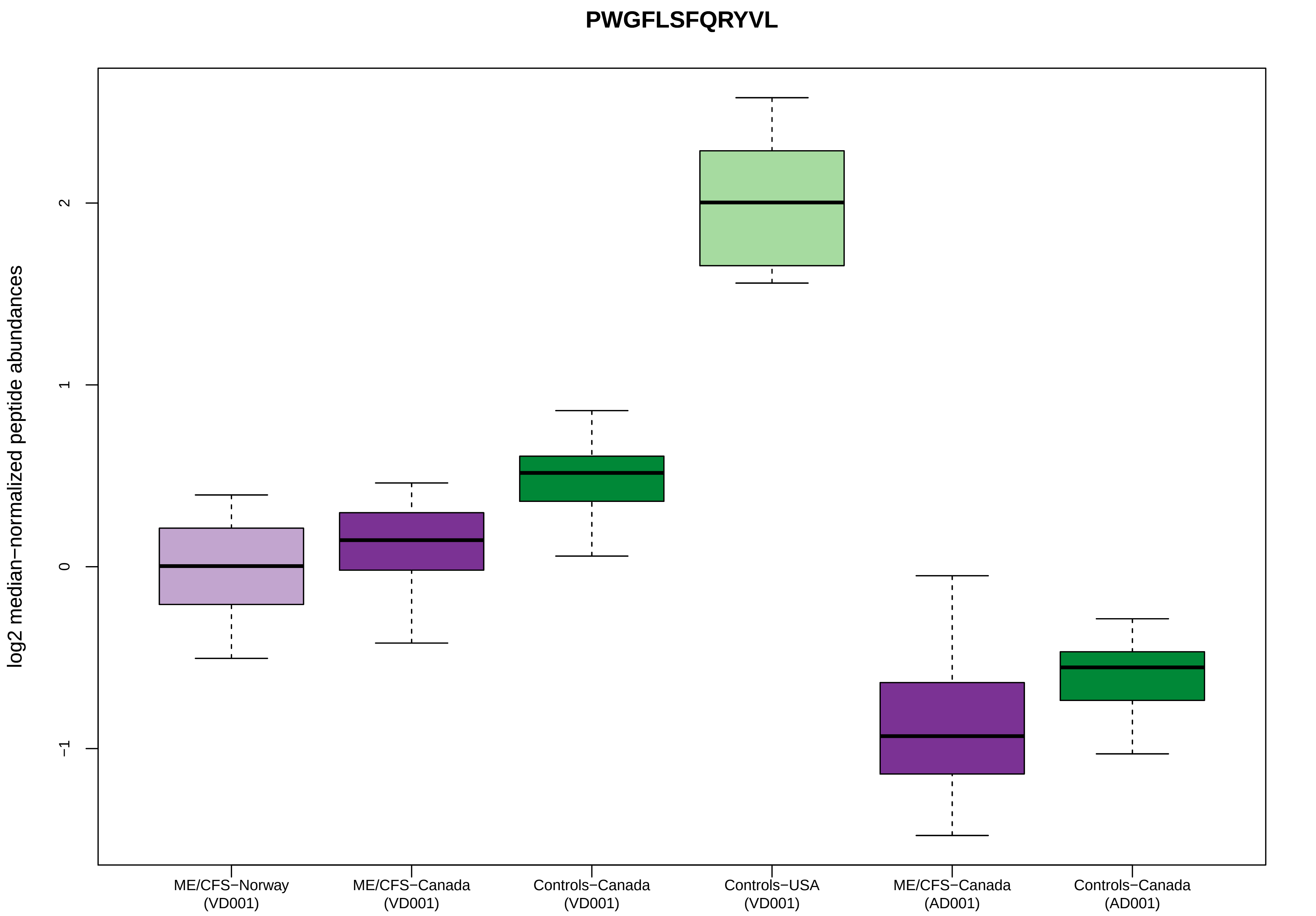
PWGFLSFQRYVL
log2 median−normalized peptide abundances
2
1
0
−1
ME/CFS−Norway (VD001)
ME/CFS−Canada (VD001)
Controls−Canada (VD001)
Controls−USA (VD001)
ME/CFS−Canada (AD001)
Controls−Canada (AD001)

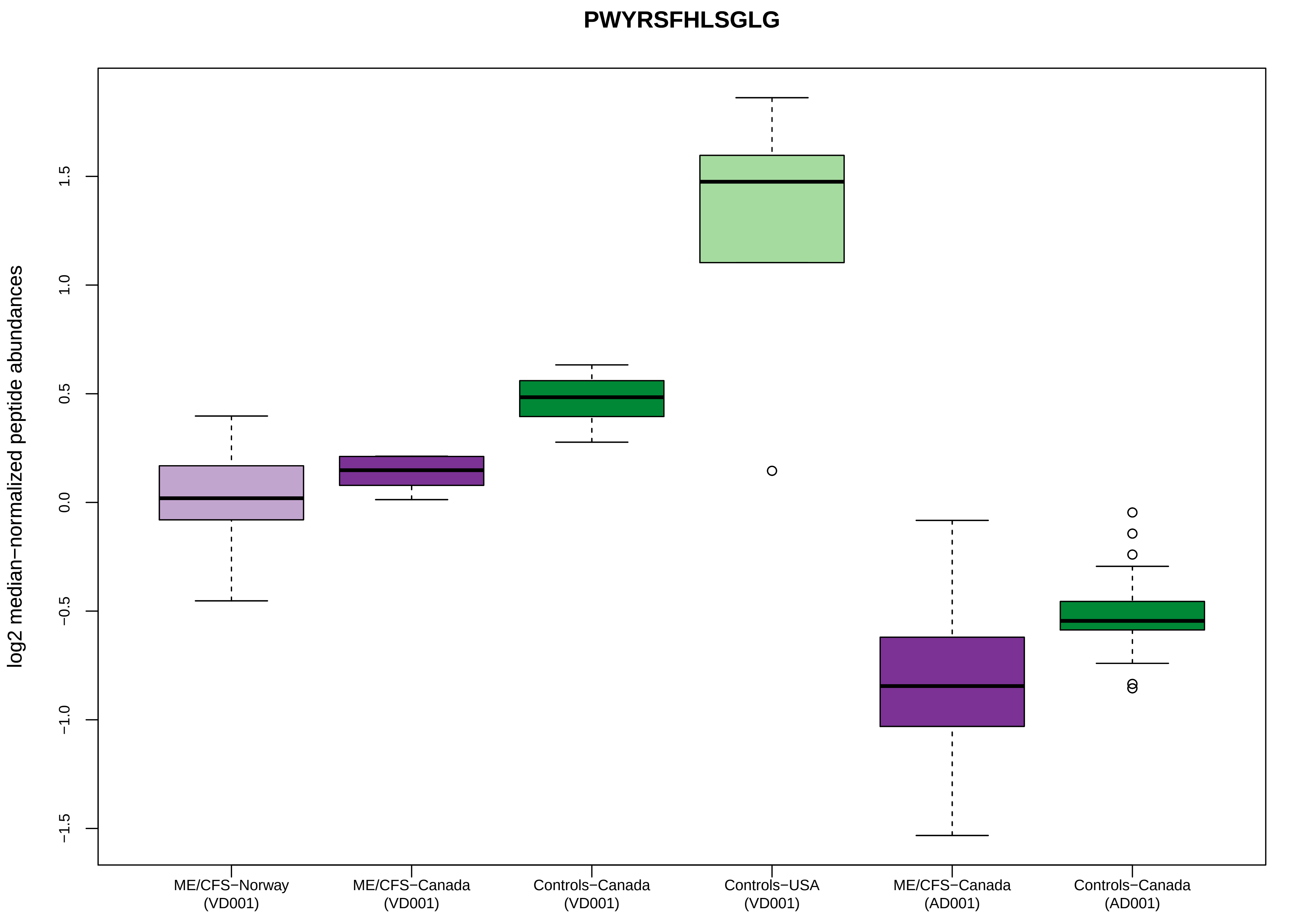

PWYRSFHLSGLG
log2 median−normalized peptide abundances
1.5
1.0
0.5
0.0
−0.5
−1.0
−1.5
ME/CFS−Norway (VD001)
ME/CFS−Canada (VD001)
Controls−Canada (VD001)
Controls−USA (VD001)
ME/CFS−Canada (AD001)
Controls−Canada (AD001)

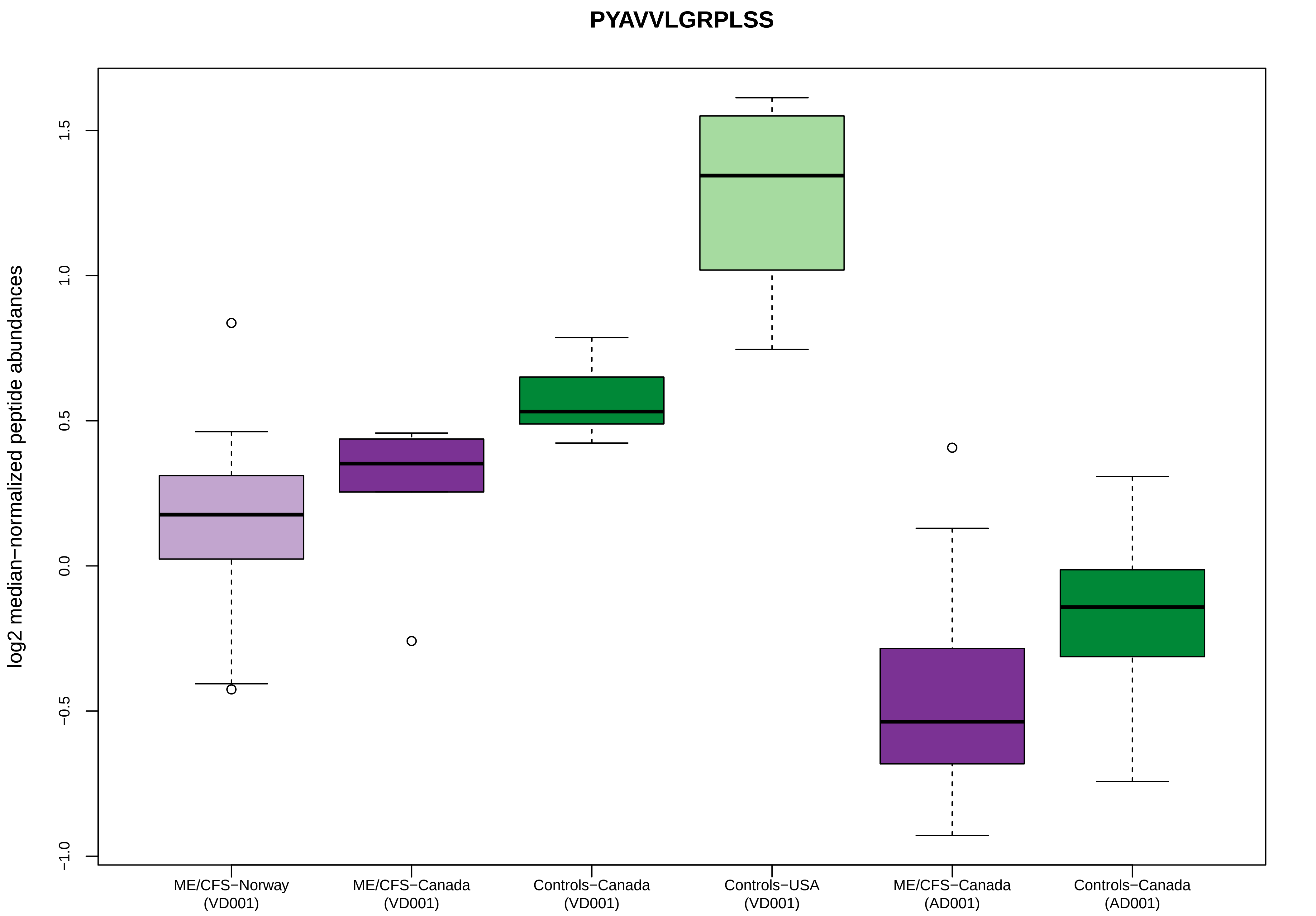

PYAVVLGRPLSS
log2 median−normalized peptide abundances
1.5
1.0
0.5
0.0
−0.5
−1.0
ME/CFS−Norway (VD001)
ME/CFS−Canada (VD001)
Controls−Canada (VD001)
Controls−USA (VD001)
ME/CFS−Canada (AD001)
Controls−Canada (AD001)

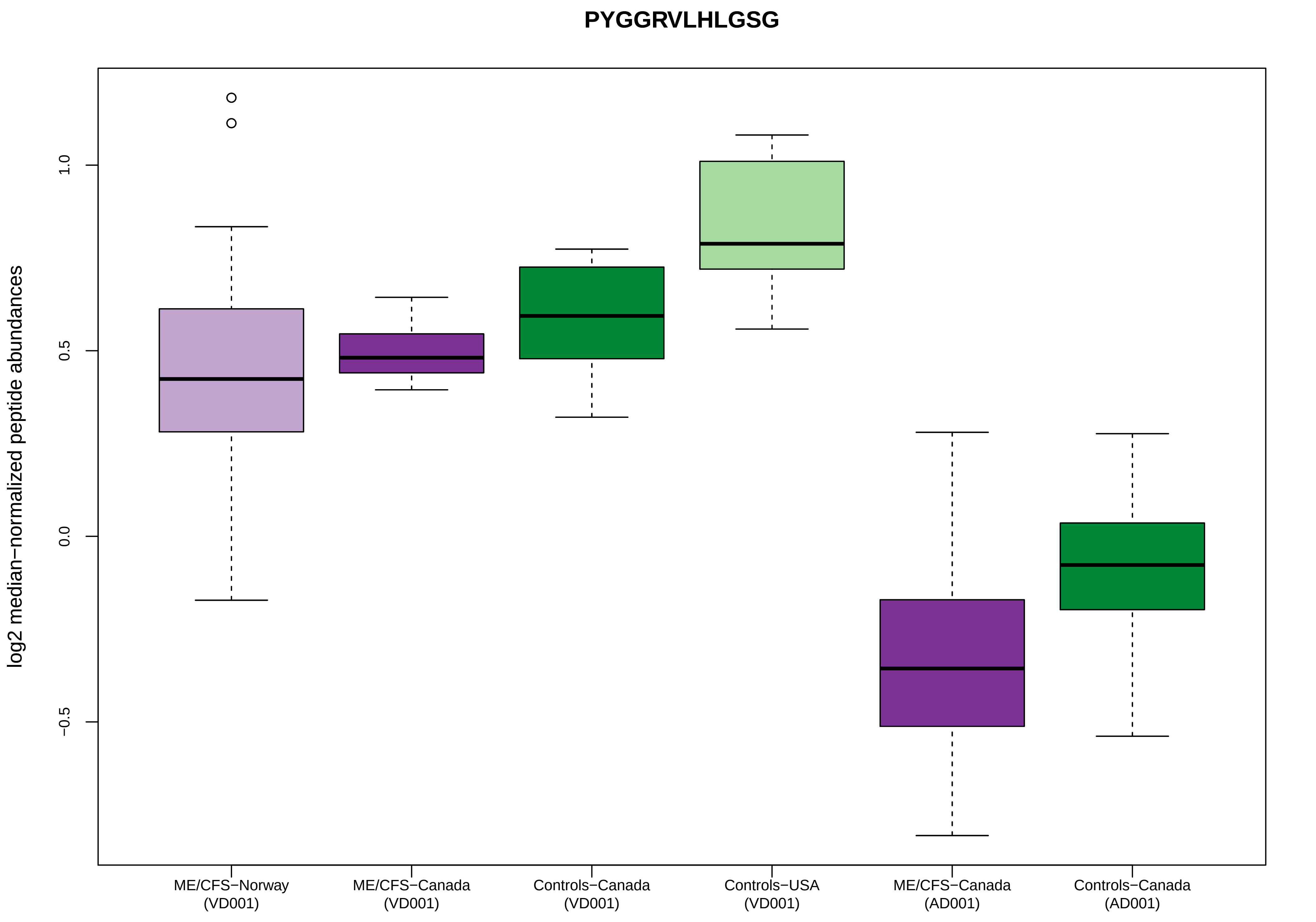

PYGGRVLHLGSG
log2 median−normalized peptide abundances
1.0
0.5
0.0
−0.5
ME/CFS−Norway (VD001)
ME/CFS−Canada (VD001)
Controls−Canada (VD001)
Controls−USA (VD001)
ME/CFS−Canada (AD001)
Controls−Canada (AD001)

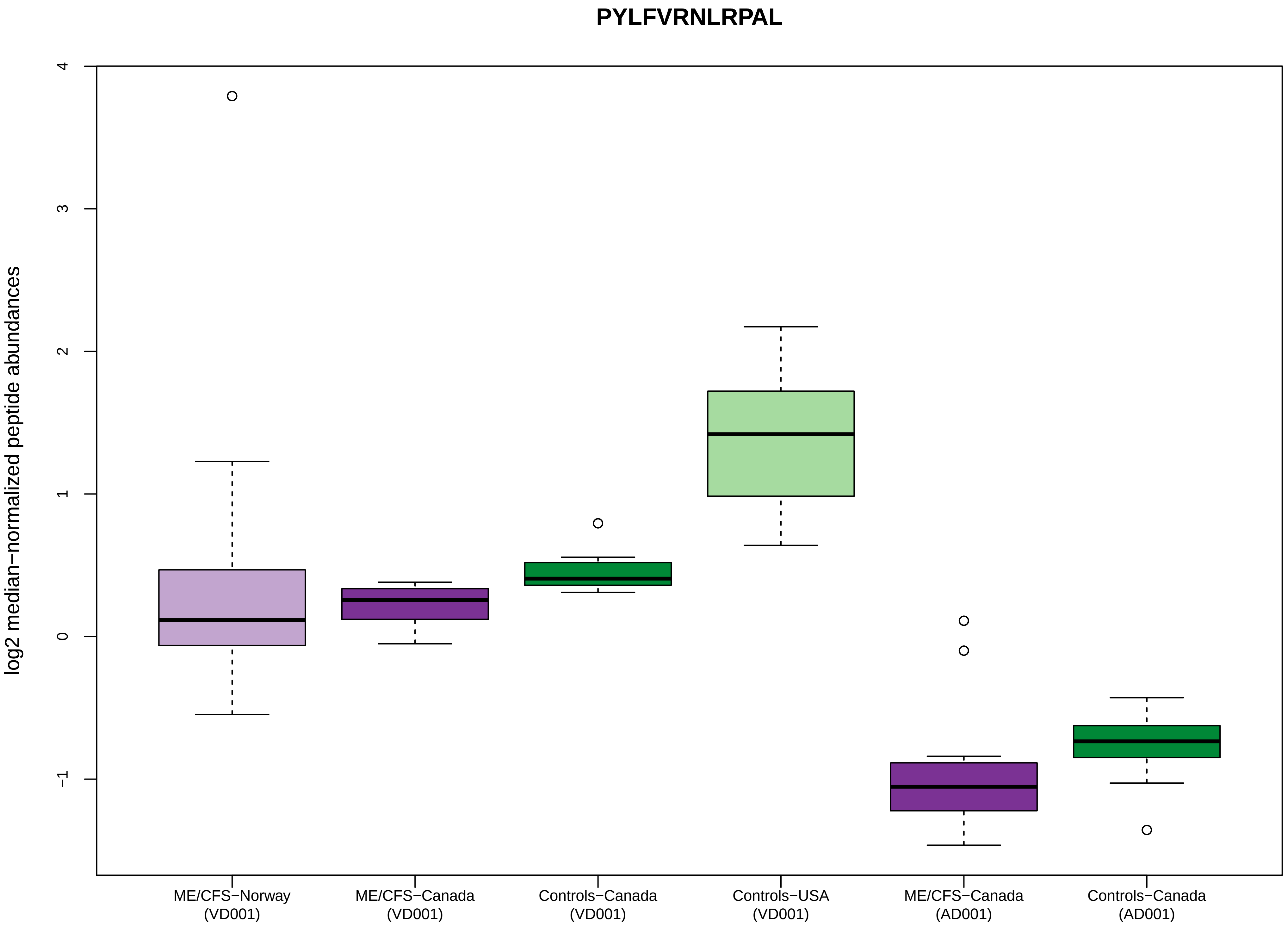
PYLFVRNLRPAL
log2 median−normalized peptide abundances
4
3
2
1
0
−1
ME/CFS−Norway (VD001)
ME/CFS−Canada (VD001)
Controls−Canada (VD001)
Controls−USA (VD001)
ME/CFS−Canada (AD001)
Controls−Canada (AD001)

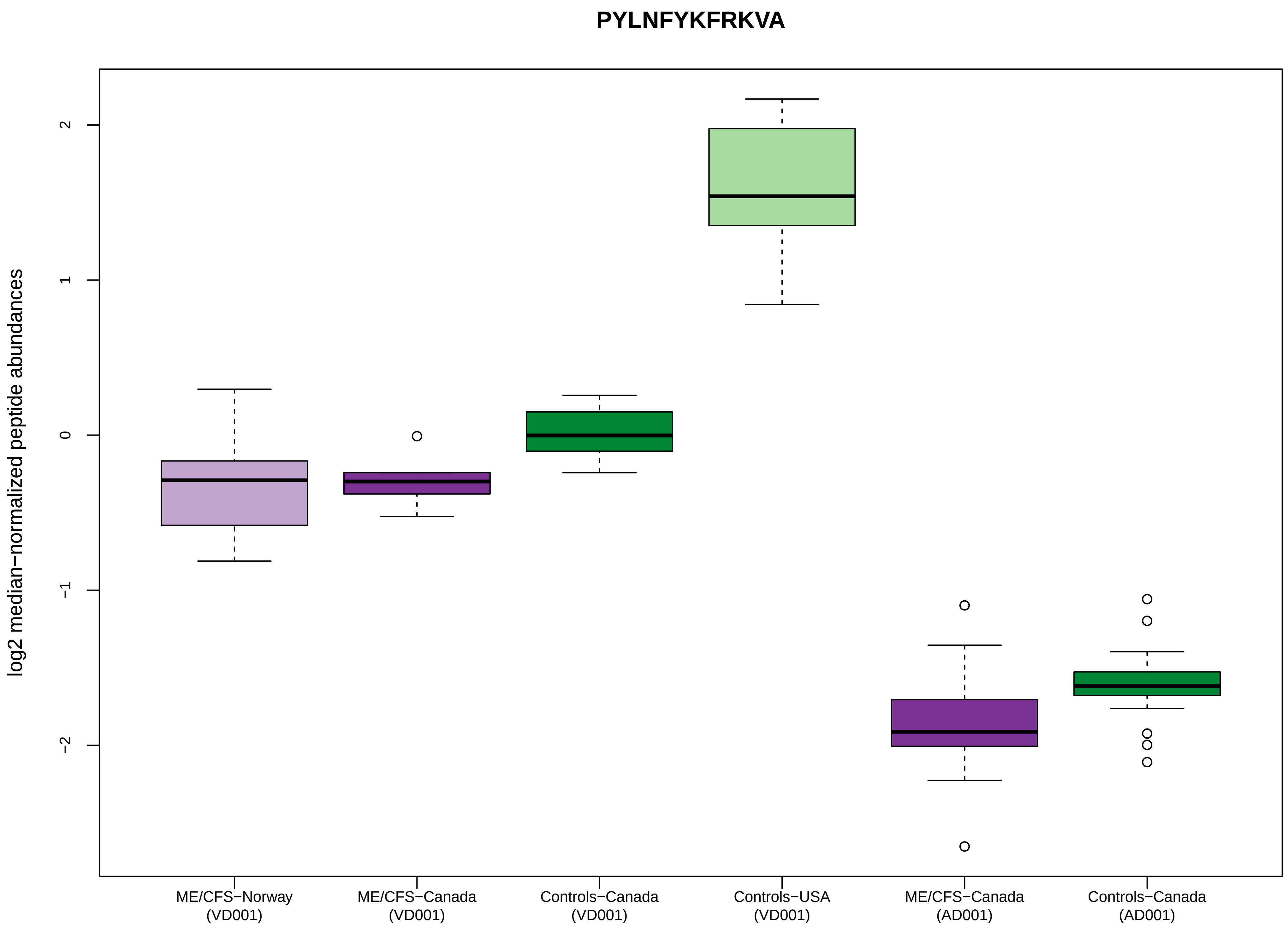
PYLNFYKFRKVA
log2 median−normalized peptide abundances
2
1
0
−1
−2
ME/CFS−Norway
(VD001)
ME/CFS−Canada
(VD001)
Controls−Canada
(VD001)
Controls−USA
(VD001)
ME/CFS−Canada
(AD001)
Controls−Canada
(AD001)

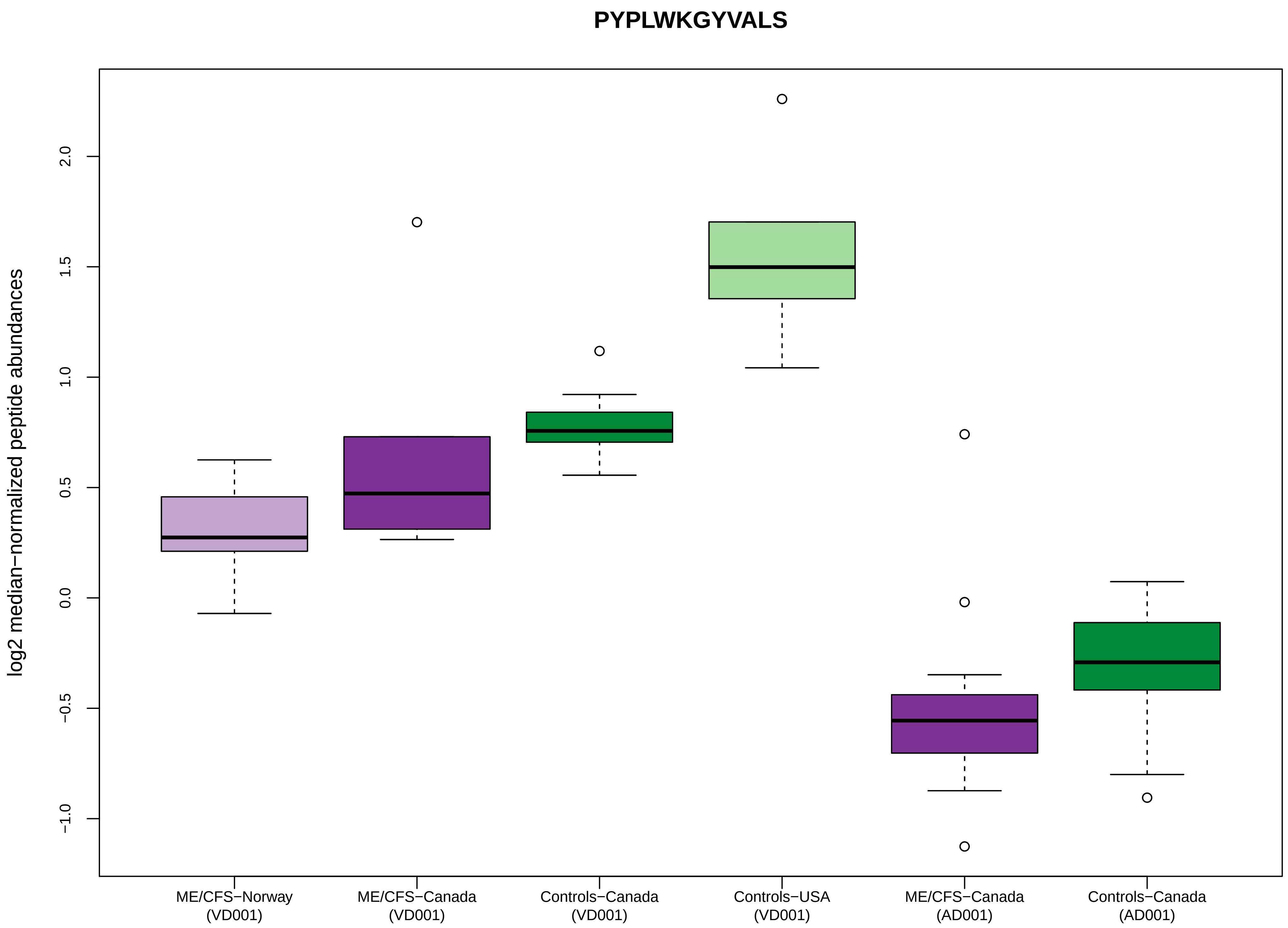

PYPLWKGYVALS
log2 median−normalized peptide abundances
2.0
1.5
1.0
0.5
0.0
−0.5
−1.0
ME/CFS−Norway (VD001)
ME/CFS−Canada (VD001)
Controls−Canada (VD001)
Controls−USA (VD001)
ME/CFS−Canada (AD001)
Controls−Canada (AD001)

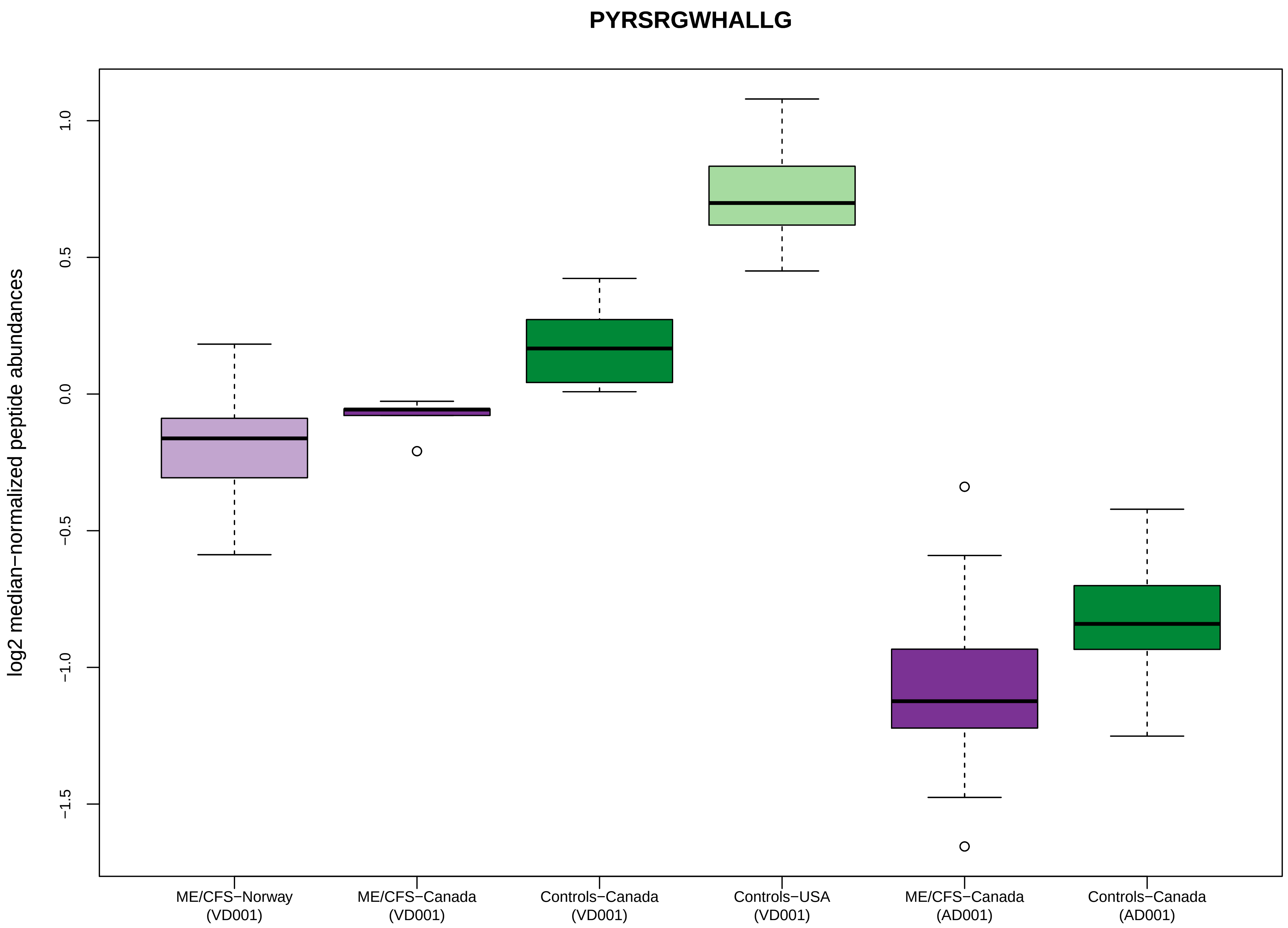

PYRSRGWHALLG
log2 median−normalized peptide abundances
1.0
0.5
0.0
−0.5
−1.0
−1.5
ME/CFS−Norway (VD001)
ME/CFS−Canada (VD001)
Controls−Canada (VD001)
Controls−USA (VD001)
ME/CFS−Canada (AD001)
Controls−Canada (AD001)

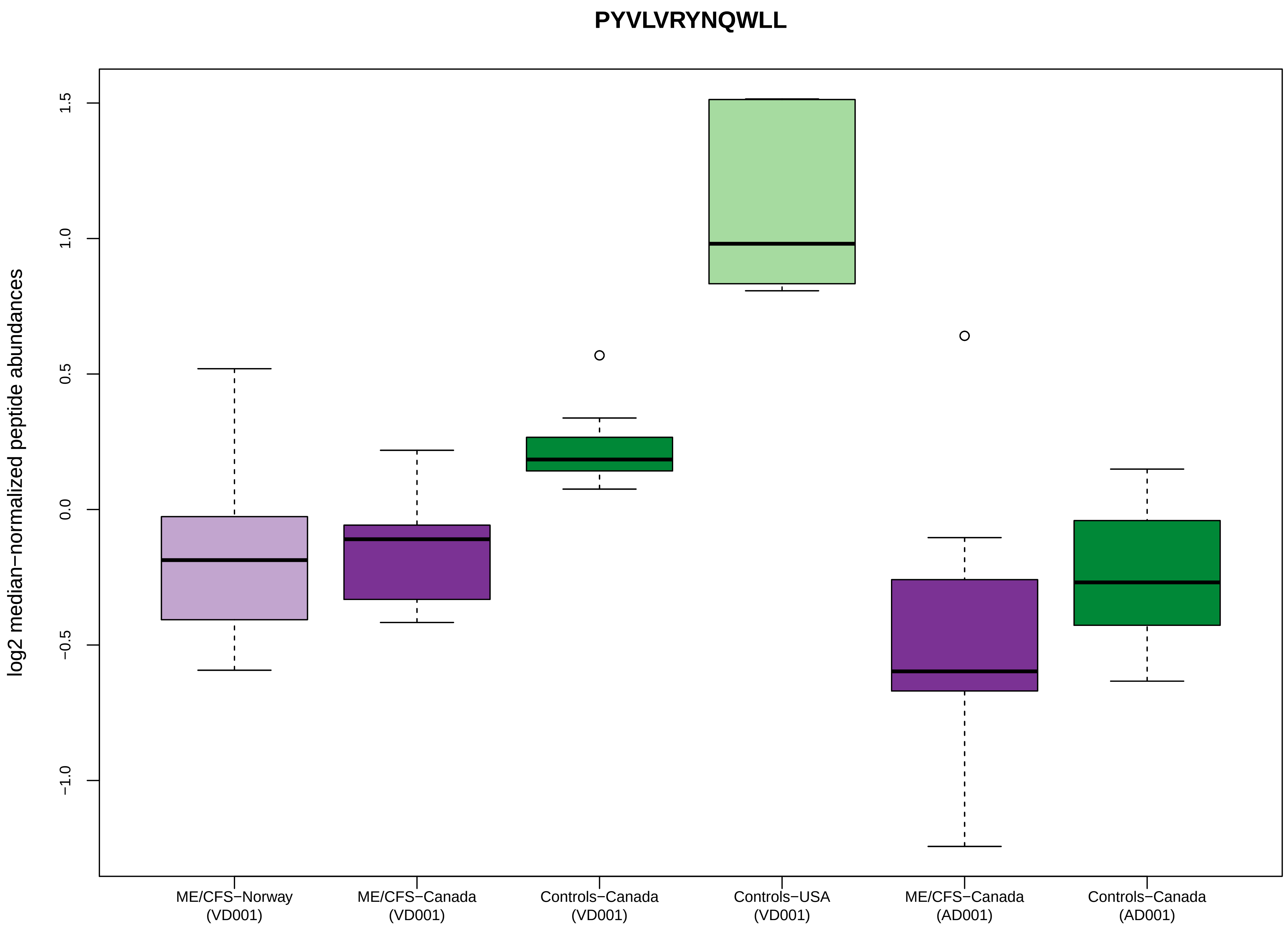
PYVLVRYNQWLL
log2 median−normalized peptide abundances
1.5
1.0
0.5
0.0
−0.5
−1.0
ME/CFS−Norway (VD001)
ME/CFS−Canada (VD001)
Controls−Canada (VD001)
Controls−USA (VD001)
ME/CFS−Canada (AD001)
Controls−Canada (AD001)

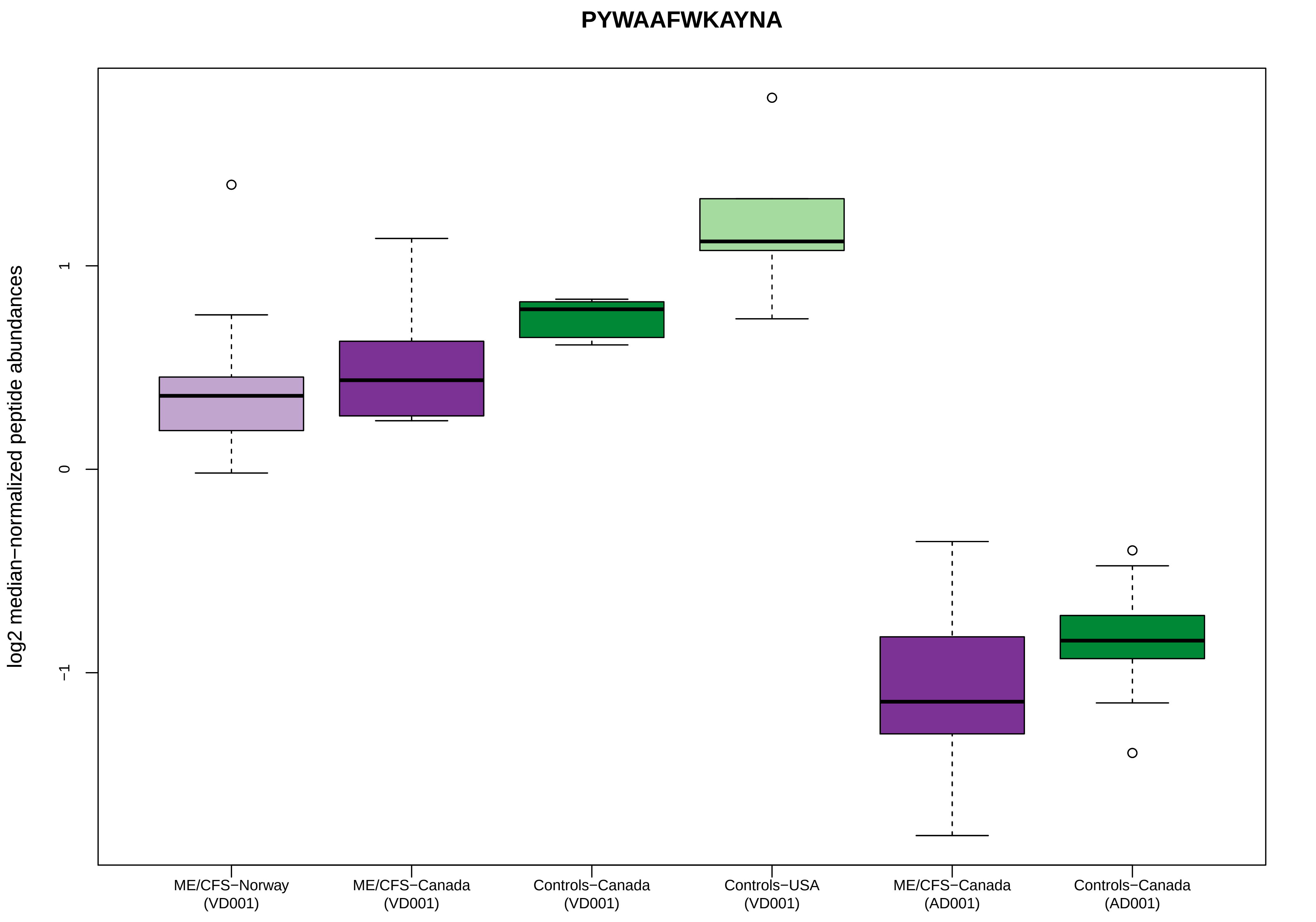

PYWAAFWKAYNA
log2 median−normalized peptide abundances
1
0
−1
ME/CFS−Norway (VD001)
ME/CFS−Canada (VD001)
Controls−Canada (VD001)
Controls−USA (VD001)
ME/CFS−Canada (AD001)
Controls−Canada (AD001)

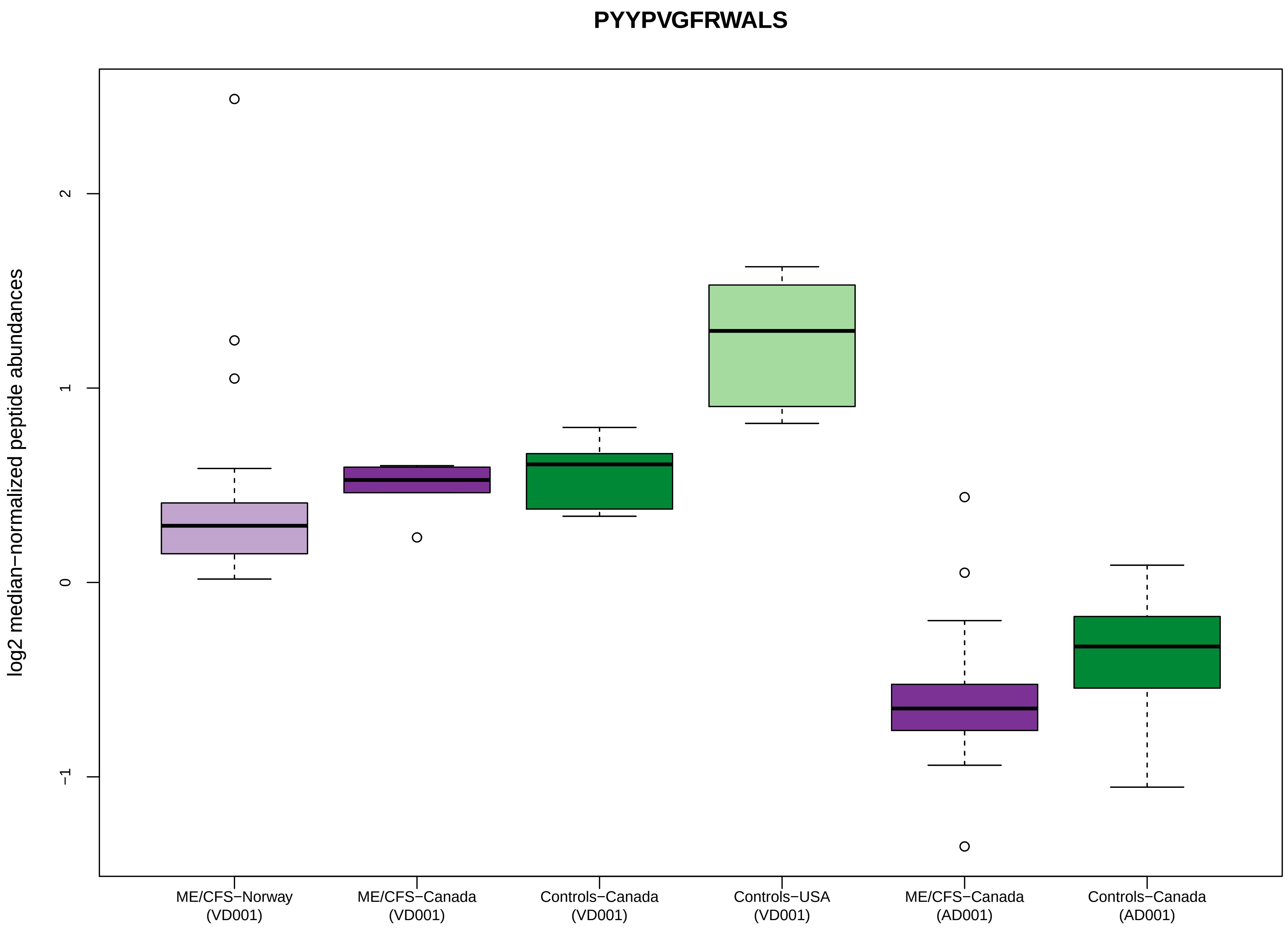
PYYPVGFRWALS
log2 median−normalized peptide abundances
2
1
0
−1
ME/CFS−Norway (VD001)
ME/CFS−Canada (VD001)
Controls−Canada (VD001)
Controls−USA (VD001)
ME/CFS−Canada (AD001)
Controls−Canada (AD001)

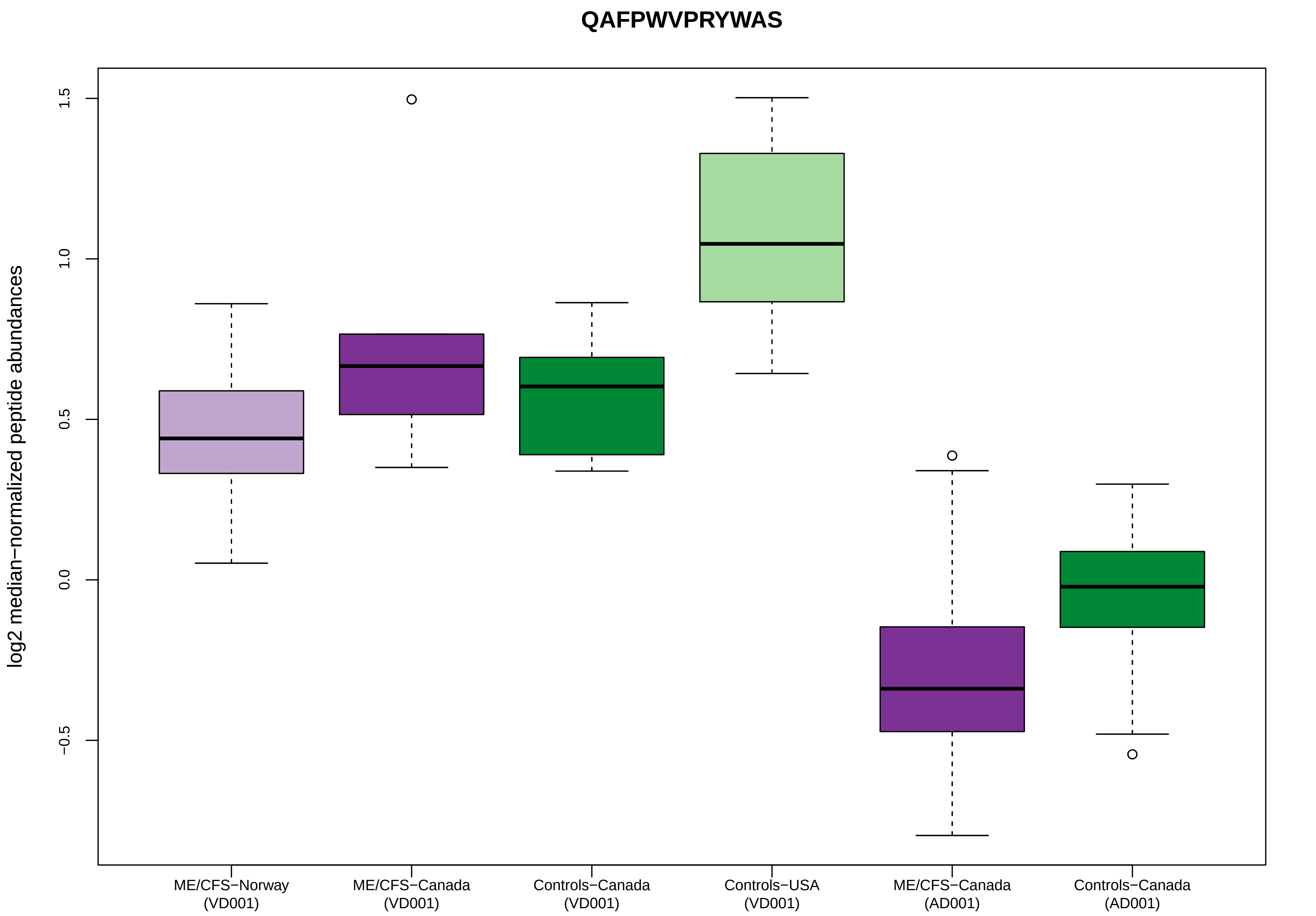
QAFPWVPRYWAS
log2 median−normalized peptide abundances
1.5
1.0
0.5
0.0
−0.5
ME/CFS−Norway (VD001)
ME/CFS−Canada (VD001)
Controls−Canada (VD001)
Controls−USA (VD001)
ME/CFS−Canada (AD001)
Controls−Canada (AD001)

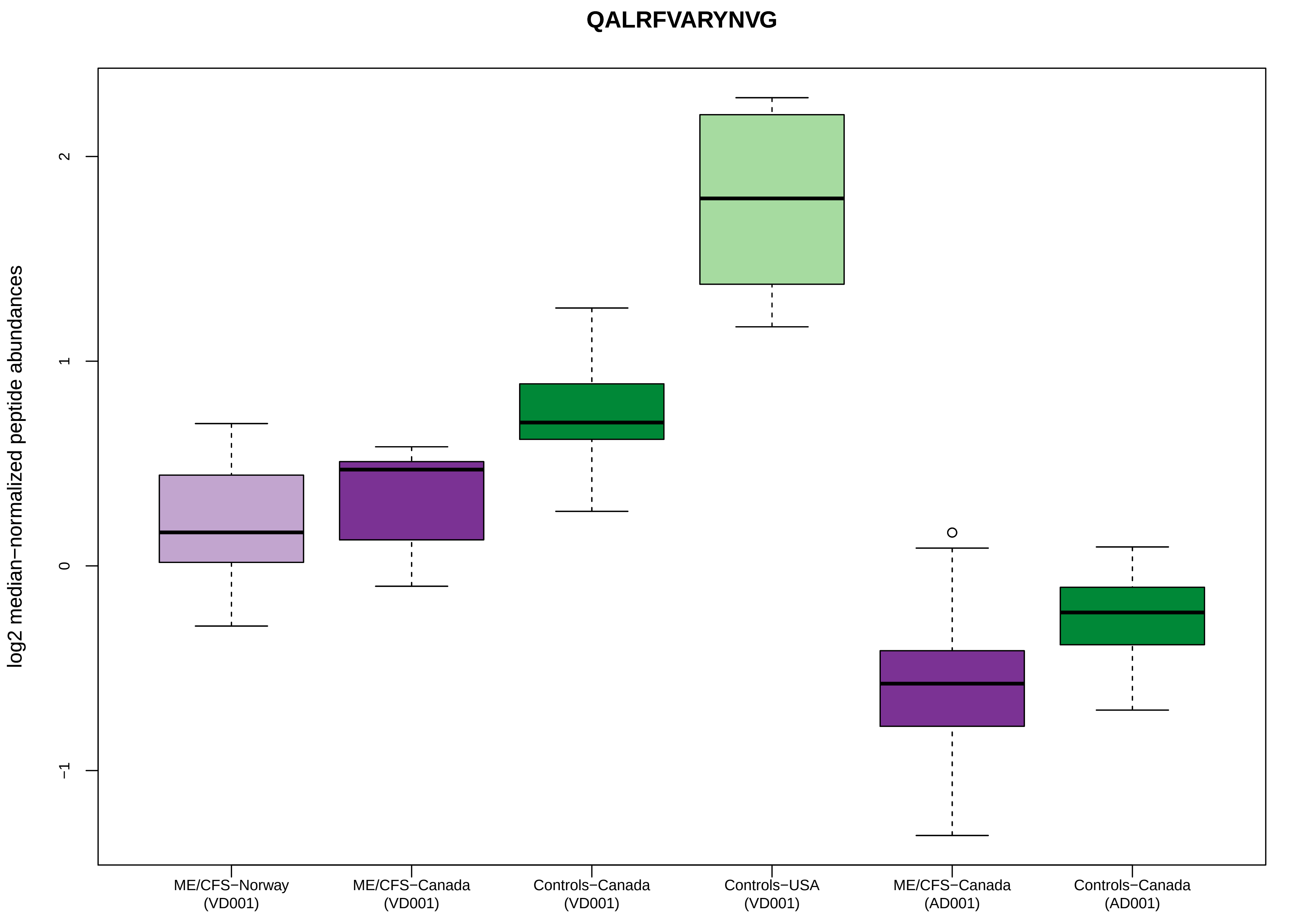

QALRFVARYNVG
log2 median−normalized peptide abundances
2
1
0
−1
ME/CFS−Norway (VD001)
ME/CFS−Canada (VD001)
Controls−Canada (VD001)
Controls−USA (VD001)
ME/CFS−Canada (AD001)
Controls−Canada (AD001)

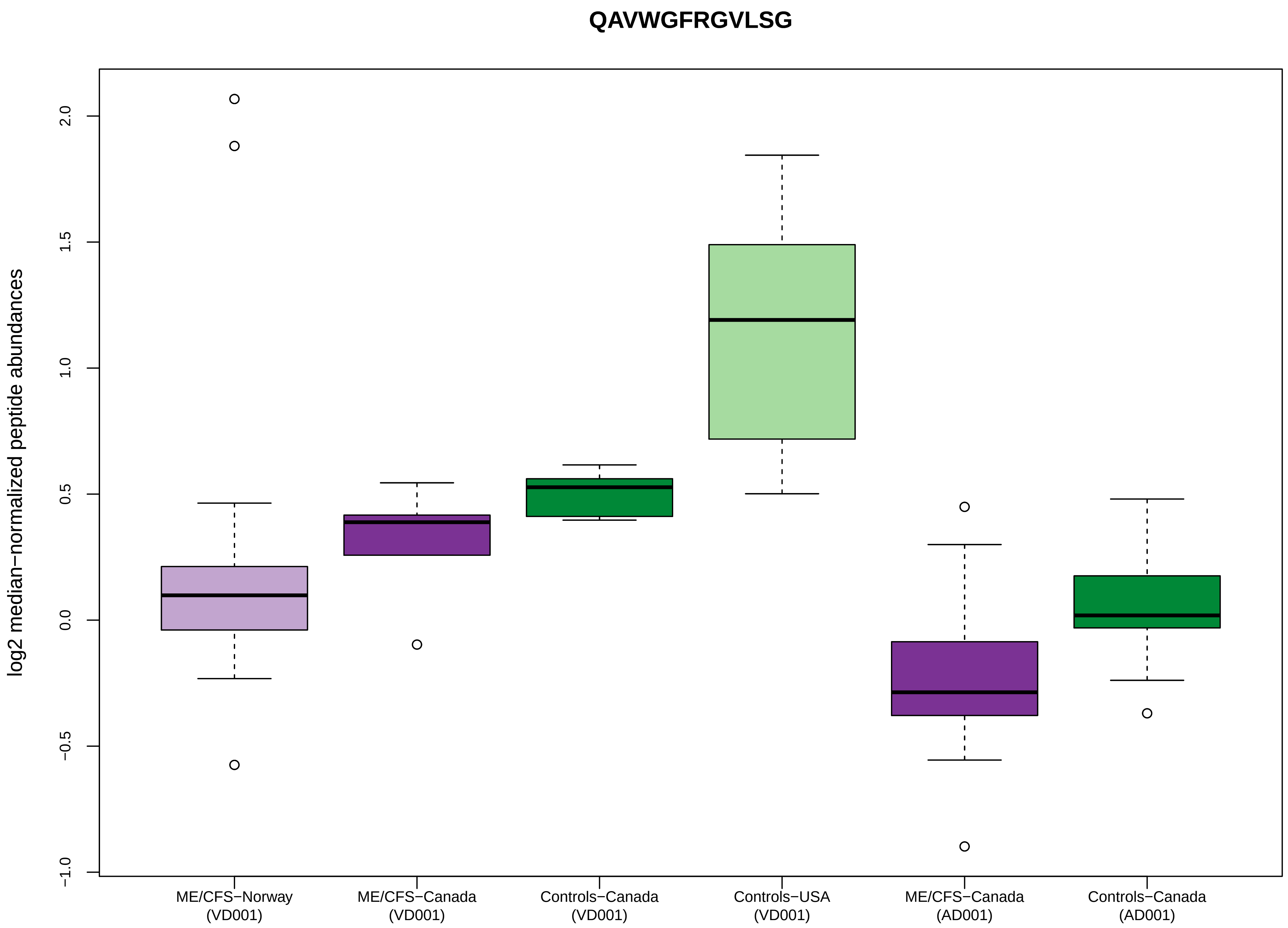
QAVWGFRGVLSG
log2 median−normalized peptide abundances
2.0
1.5
1.0
0.5
0.0
−0.5
−1.0
ME/CFS−Norway (VD001)
ME/CFS−Canada (VD001)
Controls−Canada (VD001)
Controls−USA (VD001)
ME/CFS−Canada (AD001)
Controls−Canada (AD001)

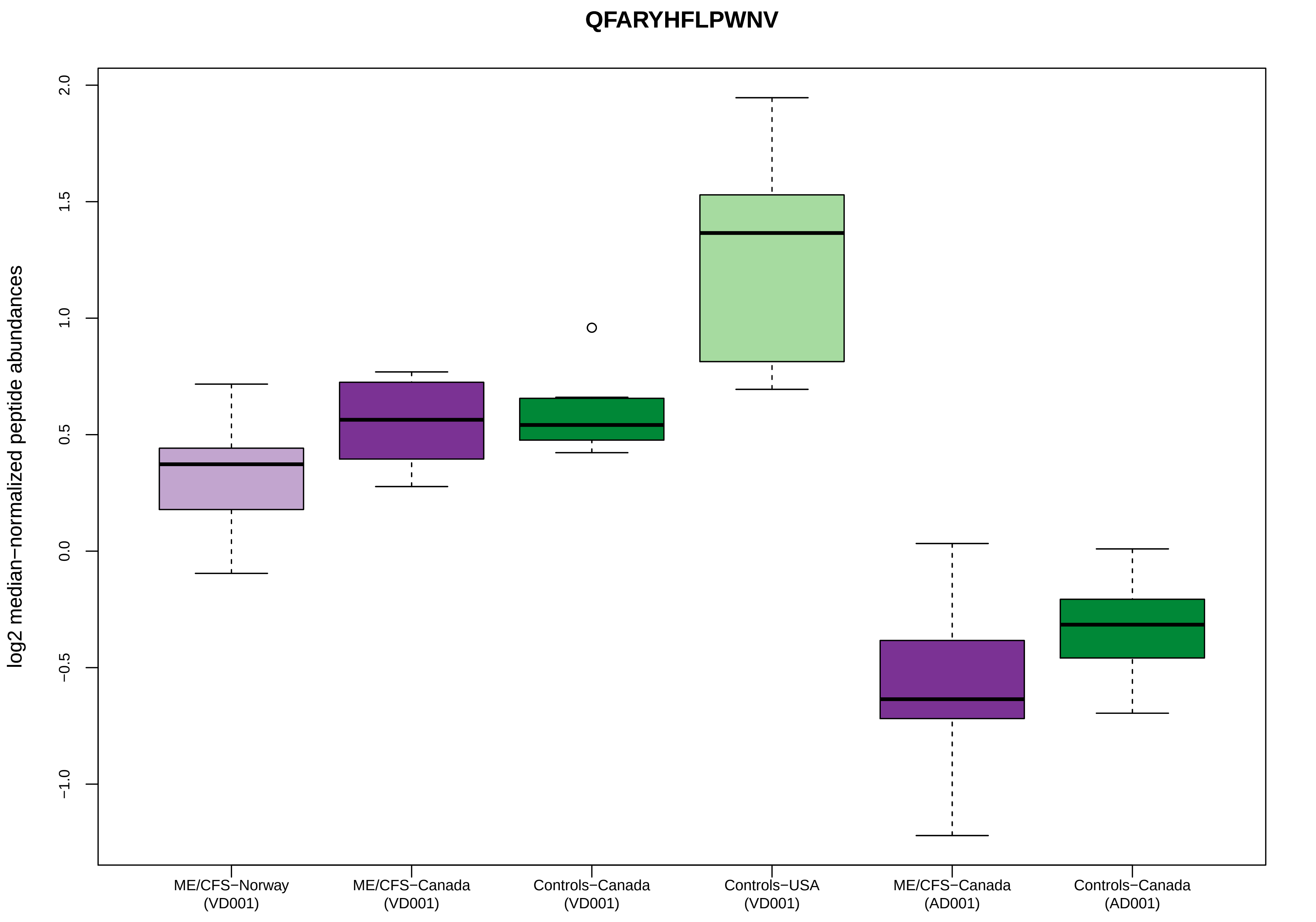
QFARYHFLPWNV
log2 median−normalized peptide abundances
2.0
1.5
1.0
0.5
0.0
−0.5
−1.0
ME/CFS−Norway (VD001)
ME/CFS−Canada (VD001)
Controls−Canada (VD001)
Controls−USA (VD001)
ME/CFS−Canada (AD001)
Controls−Canada (AD001)

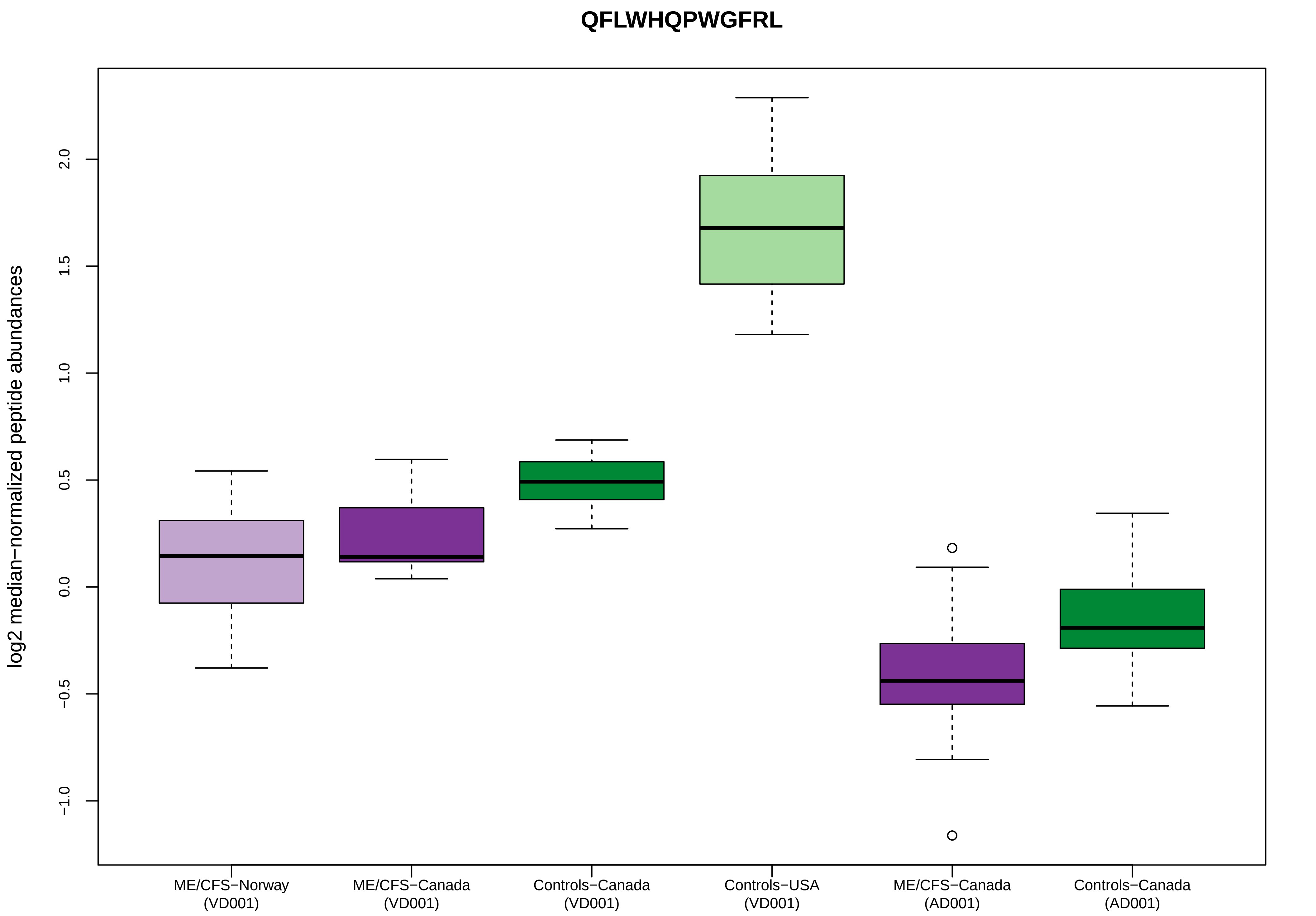
QFLWHQPWGFRL
log2 median−normalized peptide abundances
−1.0
−0.5
0.0
0.5
1.0
1.5
2.0
ME/CFS−Norway (VD001)
ME/CFS−Canada (VD001)
Controls−Canada (VD001)
Controls−USA (VD001)
ME/CFS−Canada (AD001)
Controls−Canada (AD001)

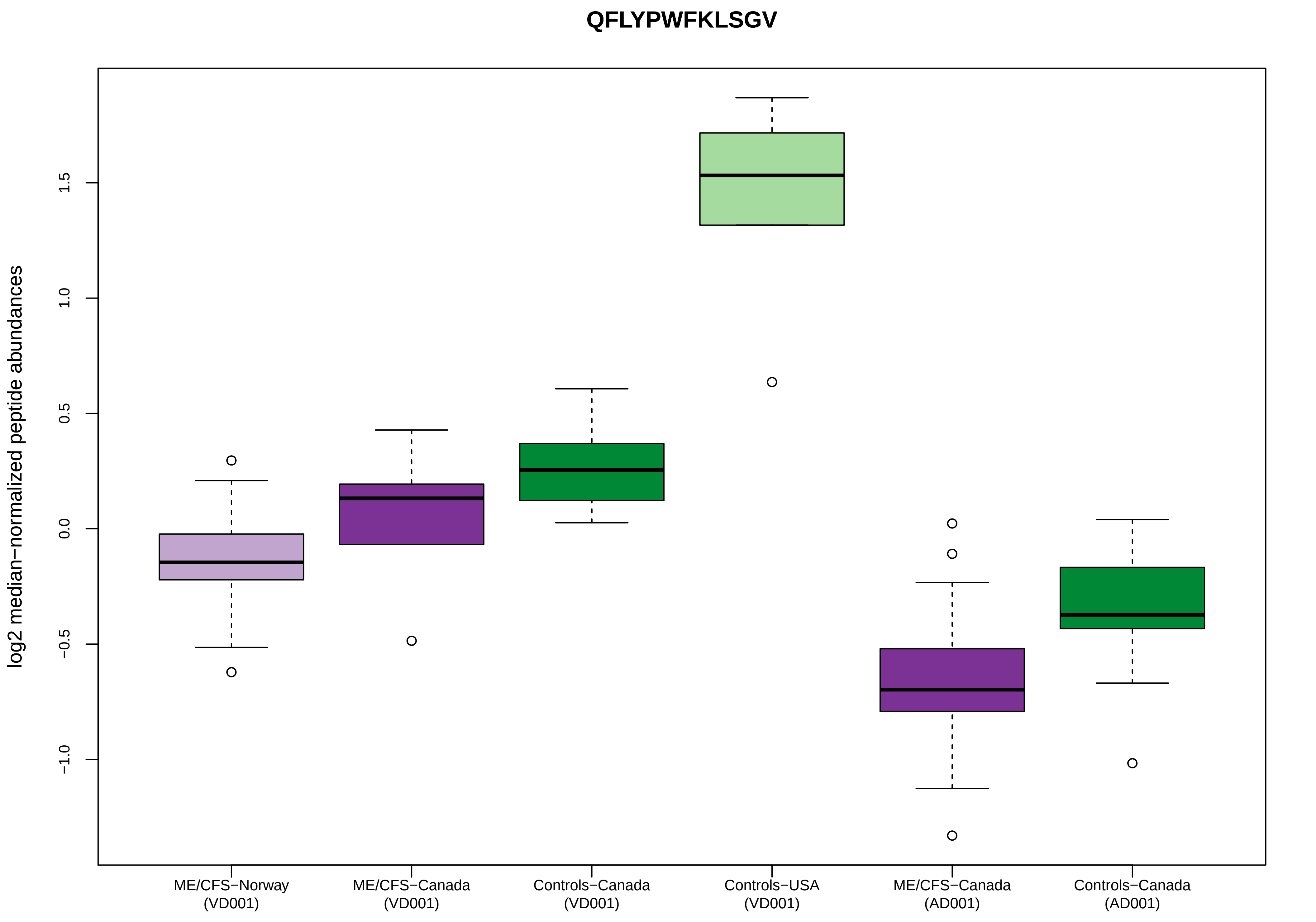
QFLYPWFKLSGV
log2 median−normalized peptide abundances
1.5
1.0
0.5
0.0
−0.5
−1.0
ME/CFS−Norway (VD001)
ME/CFS−Canada (VD001)
Controls−Canada (VD001)
Controls−USA (VD001)
ME/CFS−Canada (AD001)
Controls−Canada (AD001)

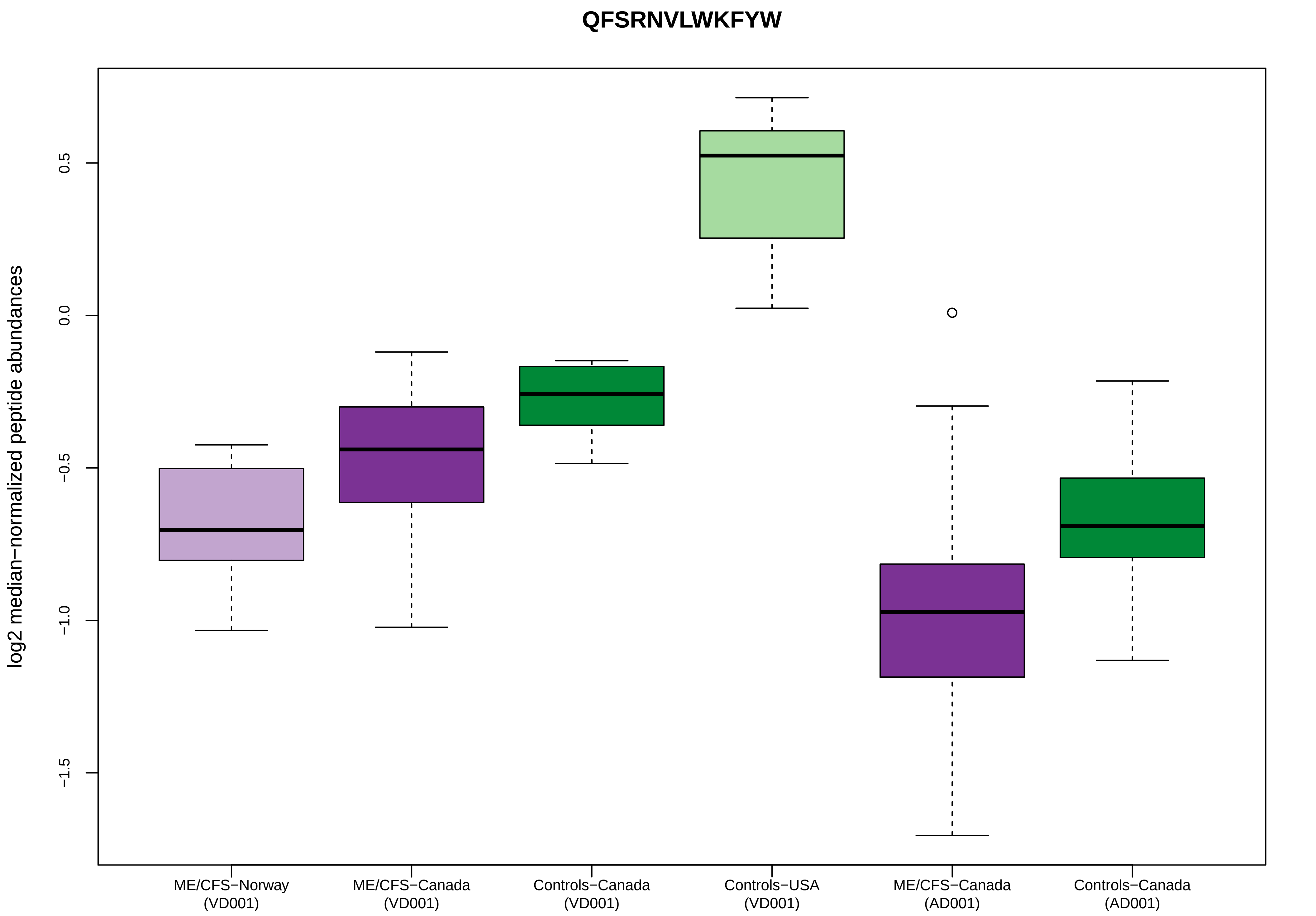

QFSRNVLWKFYW
log2 median−normalized peptide abundances
0.5
0.0
−0.5
−1.0
−1.5
ME/CFS−Norway (VD001)
ME/CFS−Canada (VD001)
Controls−Canada (VD001)
Controls−USA (VD001)
ME/CFS−Canada (AD001)
Controls−Canada (AD001)

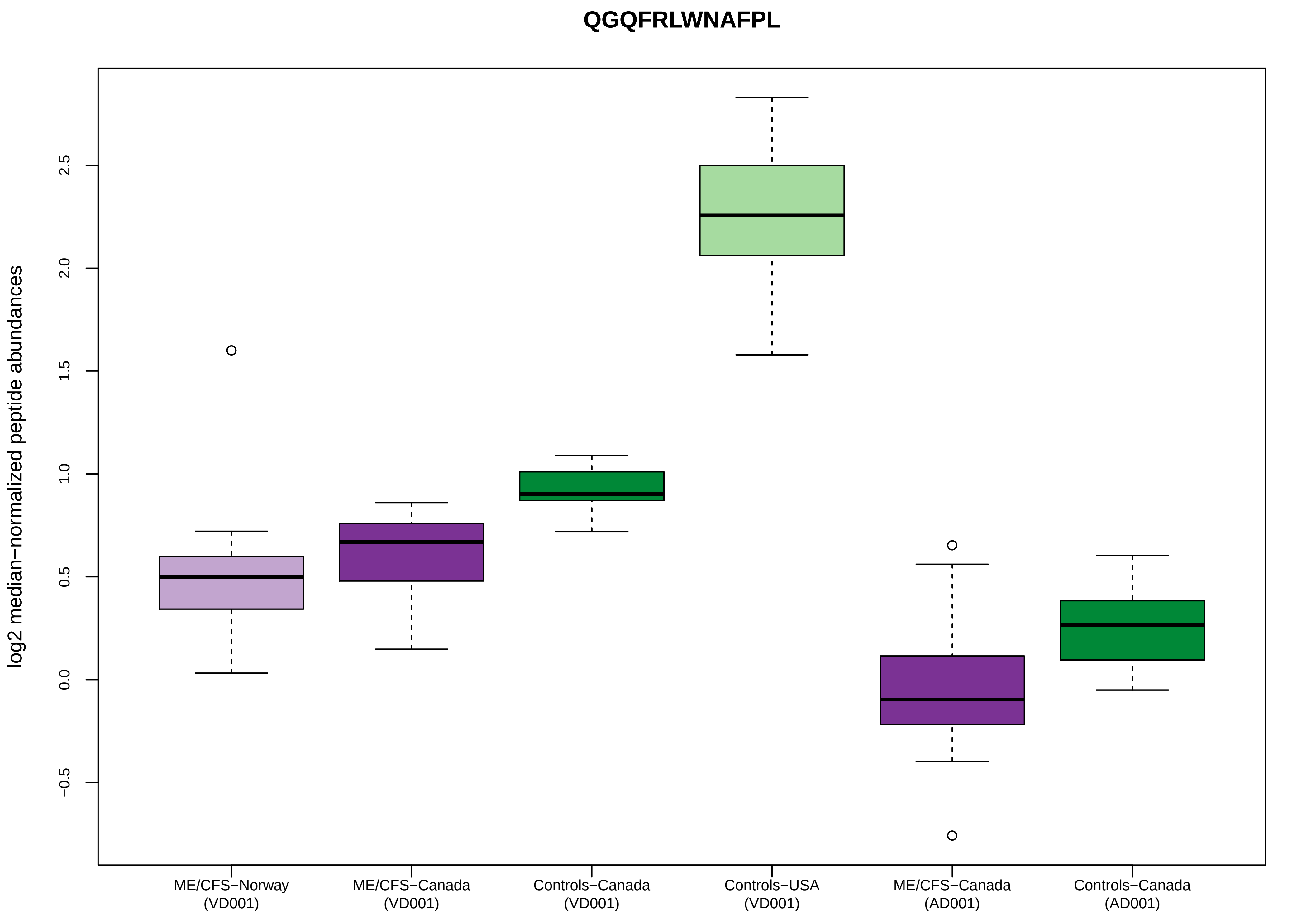
QGQFRLWNAFPL
log2 median−normalized peptide abundances
−0.5
0.0
0.5
1.0
1.5
2.0
2.5
ME/CFS−Norway (VD001)
ME/CFS−Canada (VD001)
Controls−Canada (VD001)
Controls−USA (VD001)
ME/CFS−Canada (AD001)
Controls−Canada (AD001)

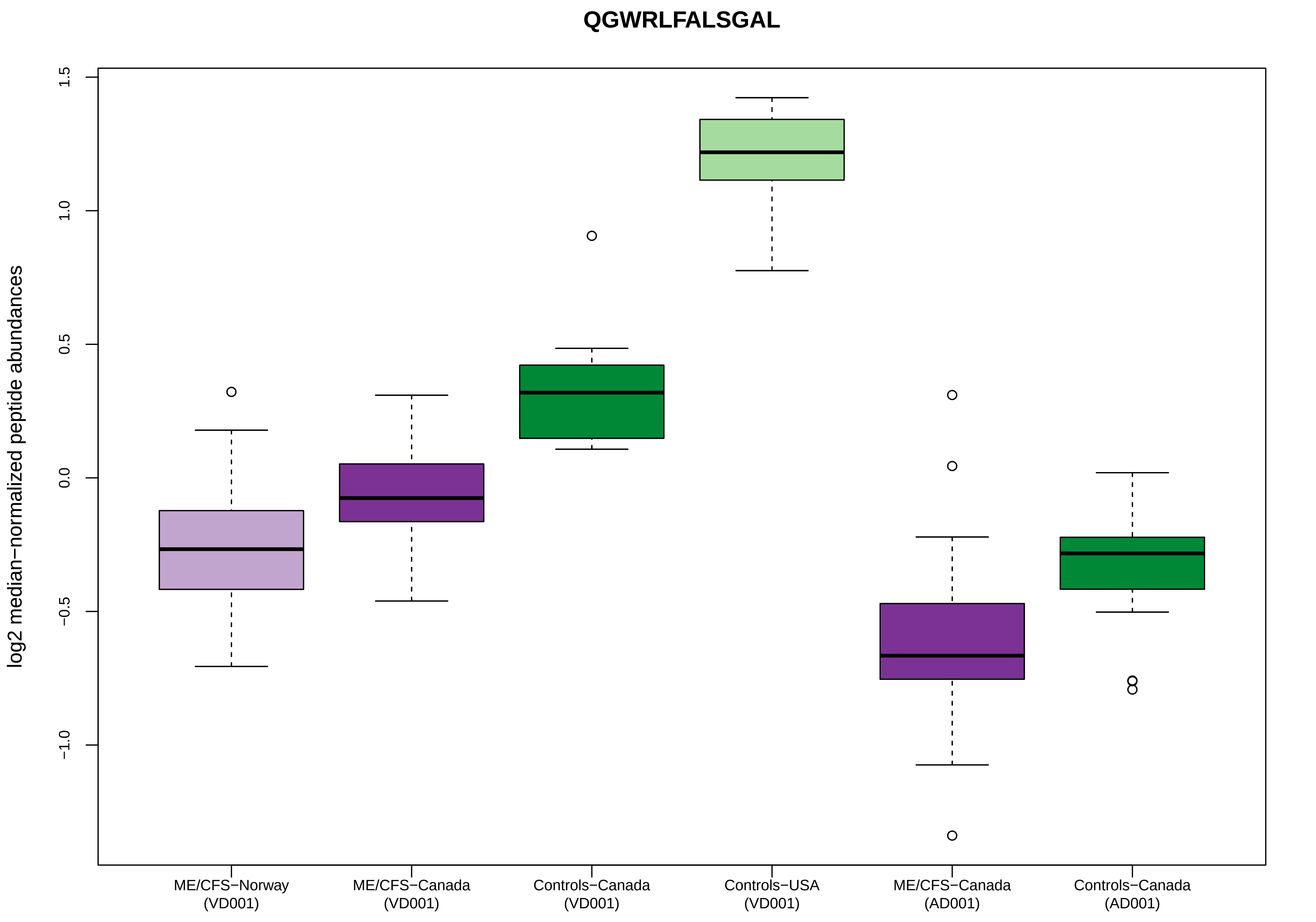
QGWRLFALSGAL
log2 median−normalized peptide abundances
1.5
1.0
0.5
0.0
−0.5
−1.0
ME/CFS−Norway (VD001)
ME/CFS−Canada (VD001)
Controls−Canada (VD001)
Controls−USA (VD001)
ME/CFS−Canada (AD001)
Controls−Canada (AD001)

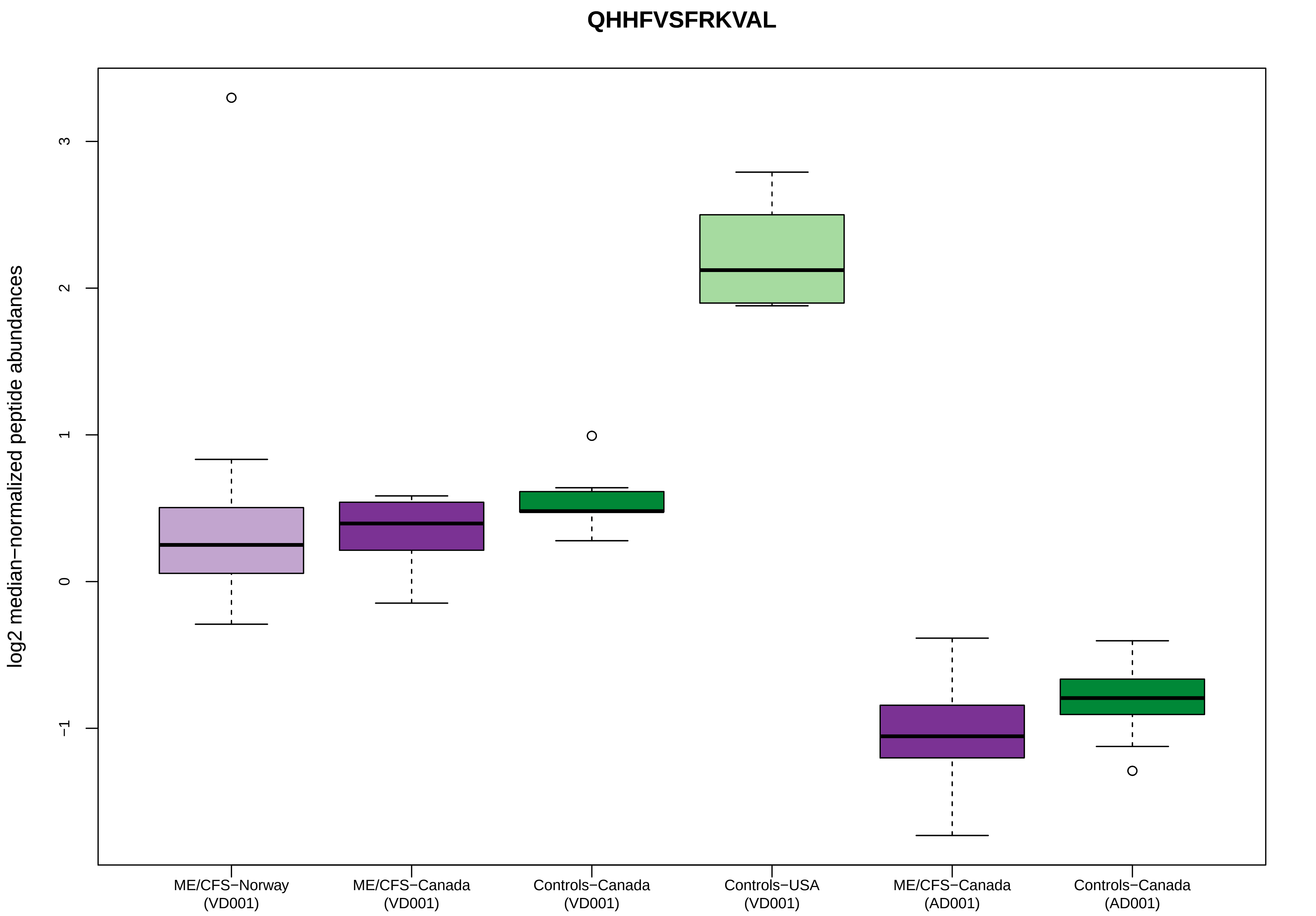
QHHFVSFRKVAL
log2 median−normalized peptide abundances
3
2
1
0
−1
ME/CFS−Norway (VD001)
ME/CFS−Canada (VD001)
Controls−Canada (VD001)
Controls−USA (VD001)
ME/CFS−Canada (AD001)
Controls−Canada (AD001)

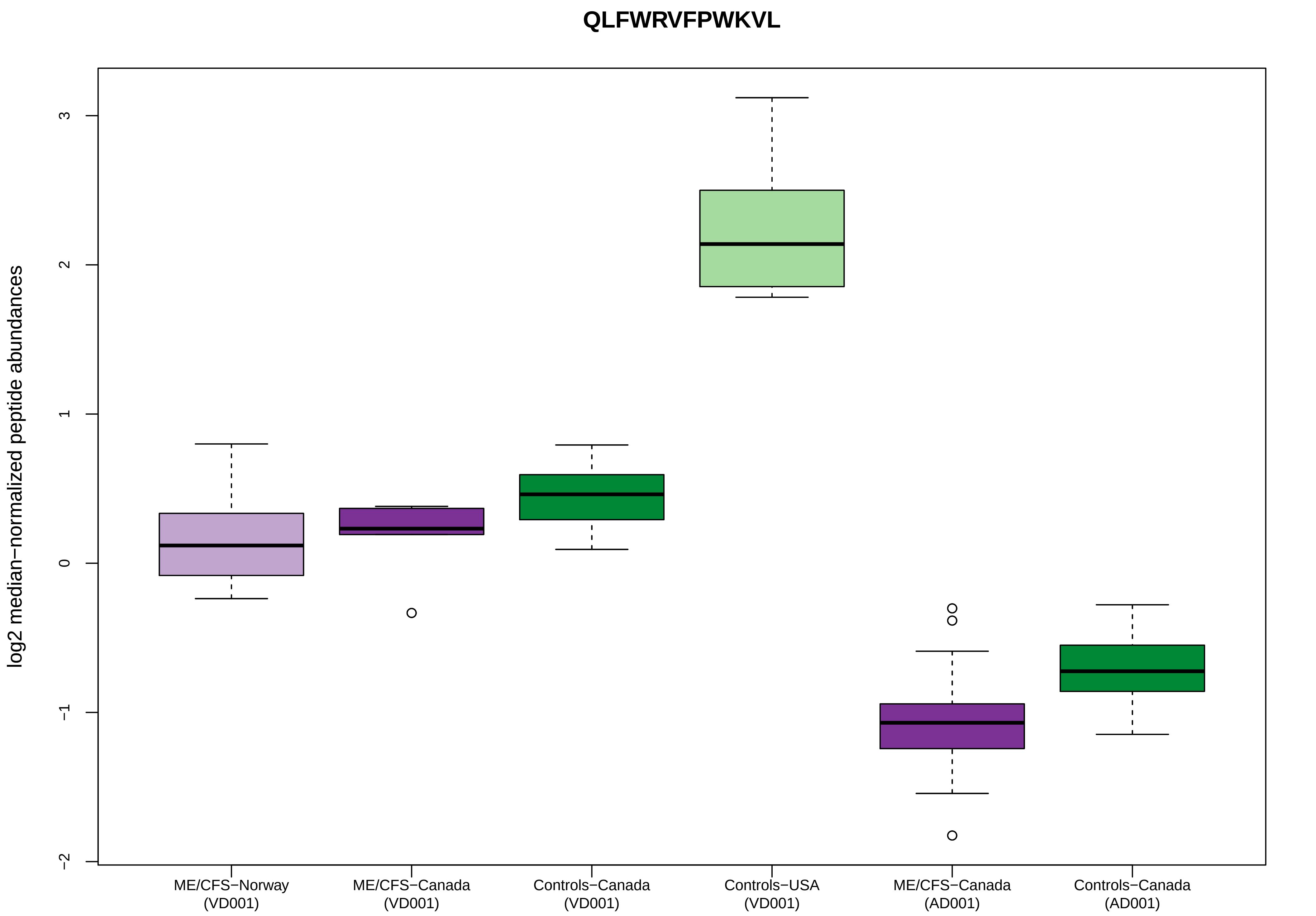
QLFWRVFPWKVL
log2 median−normalized peptide abundances
3
2
1
0
−1
−2
ME/CFS−Norway (VD001)
ME/CFS−Canada (VD001)
Controls−Canada (VD001)
Controls−USA (VD001)
ME/CFS−Canada (AD001)
Controls−Canada (AD001)

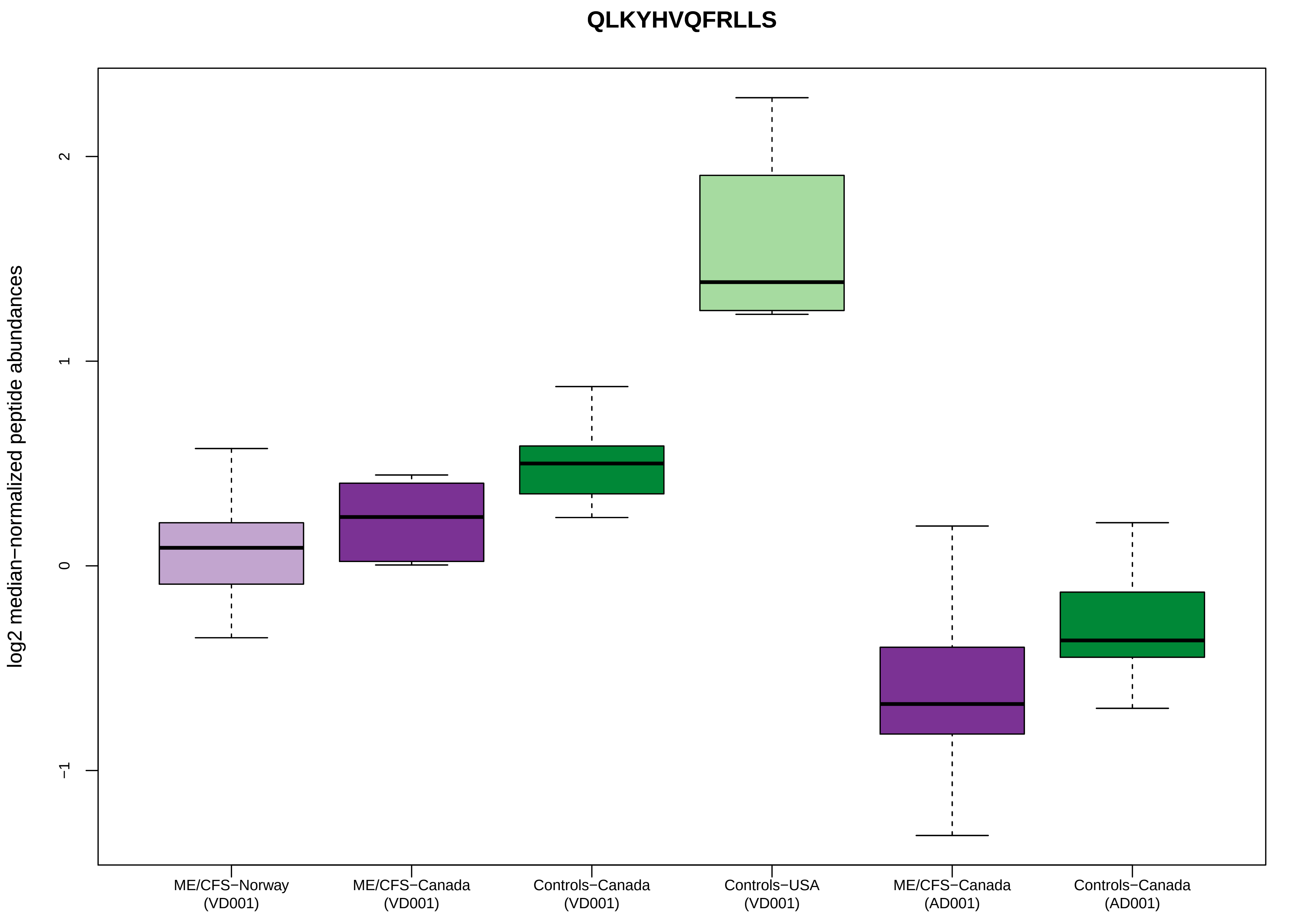
QLKYHVQFRLLS
log2 median−normalized peptide abundances
2
1
0
−1
ME/CFS−Norway (VD001)
ME/CFS−Canada (VD001)
Controls−Canada (VD001)
Controls−USA (VD001)
ME/CFS−Canada (AD001)
Controls−Canada (AD001)

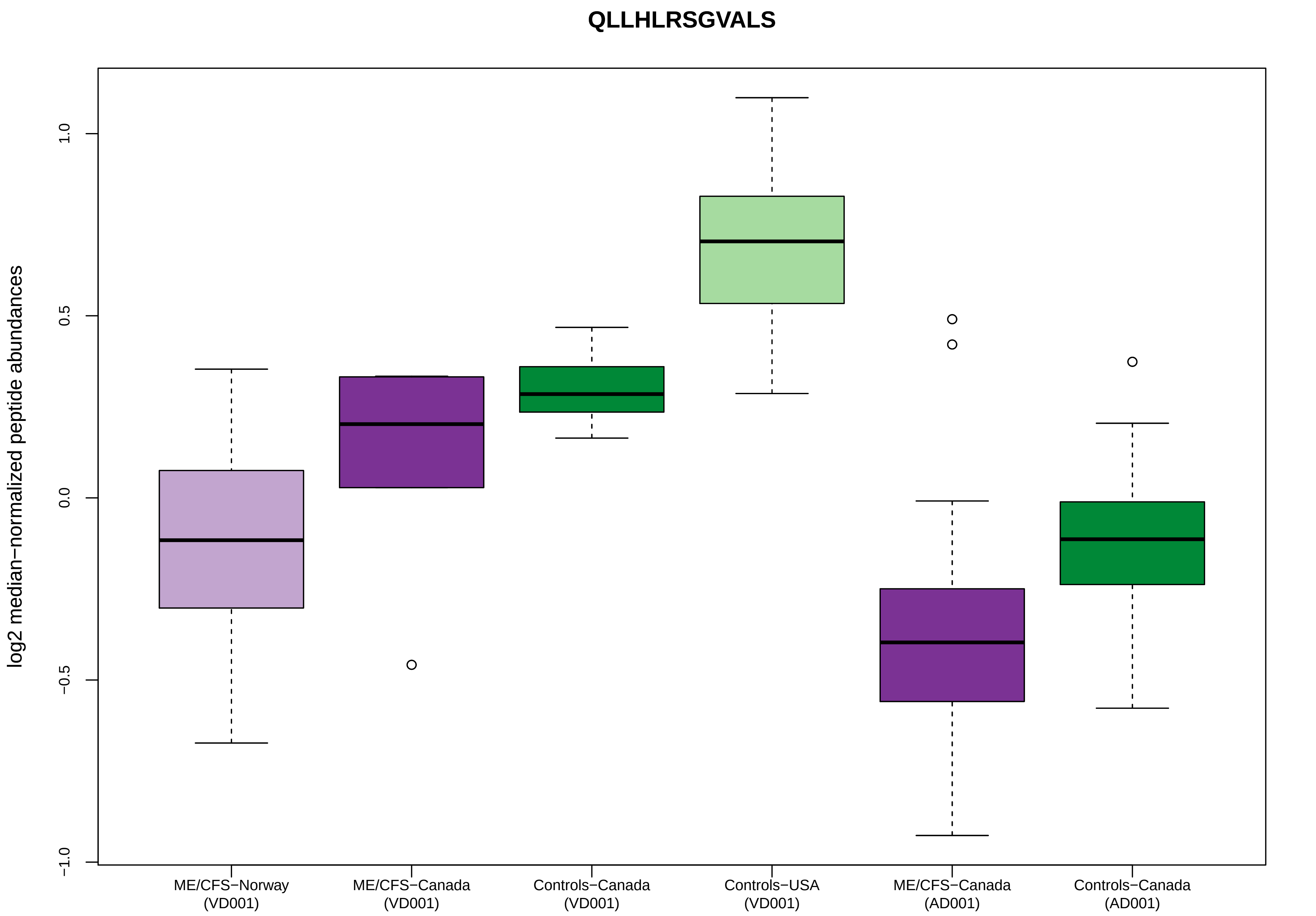
QLLHLRSGVALS
log2 median−normalized peptide abundances
1.0
0.5
0.0
−0.5
−1.0
ME/CFS−Norway (VD001)
ME/CFS−Canada (VD001)
Controls−Canada (VD001)
Controls−USA (VD001)
ME/CFS−Canada (AD001)
Controls−Canada (AD001)

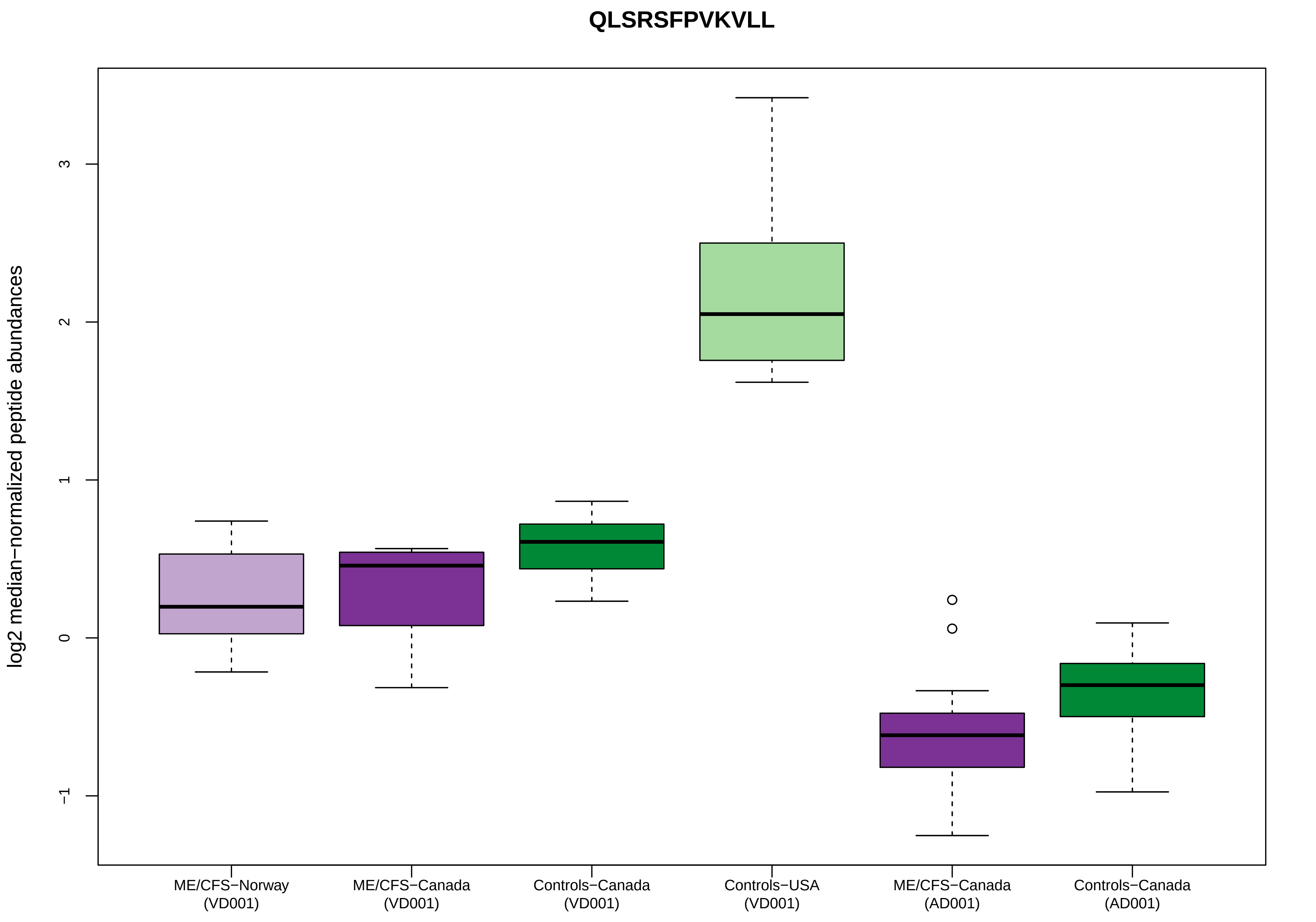
QLSRSFPVKVLL
log2 median−normalized peptide abundances
−1
0
1
2
3
ME/CFS−Norway (VD001)
ME/CFS−Canada (VD001)
Controls−Canada (VD001)
Controls−USA (VD001)
ME/CFS−Canada (AD001)
Controls−Canada (AD001)

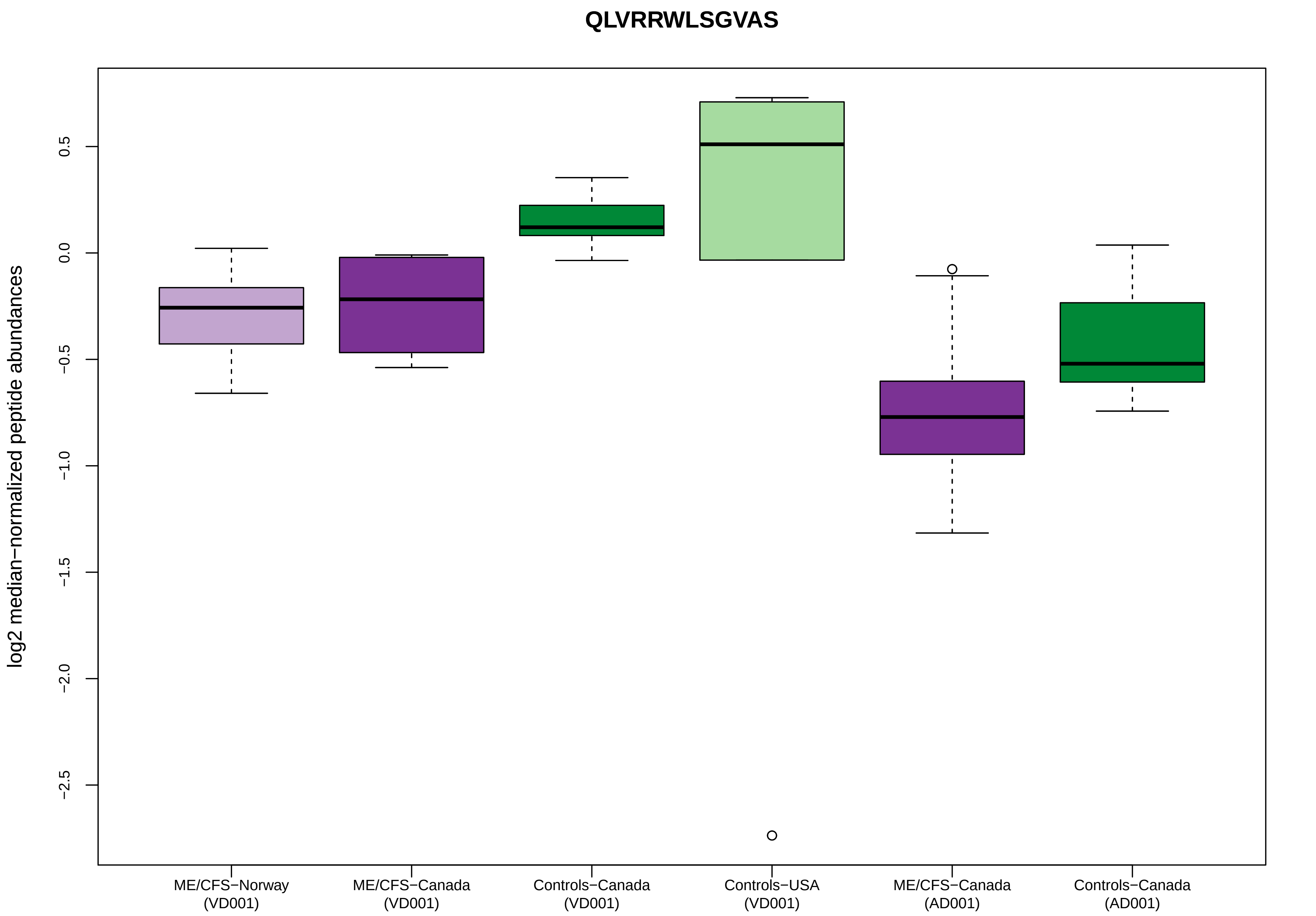
QLVRRWLSGVAS
log2 median−normalized peptide abundances
0.5
0.0
−0.5
−1.0
−1.5
−2.0
−2.5
ME/CFS−Norway (VD001)
ME/CFS−Canada (VD001)
Controls−Canada (VD001)
Controls−USA (VD001)
ME/CFS−Canada (AD001)
Controls−Canada (AD001)

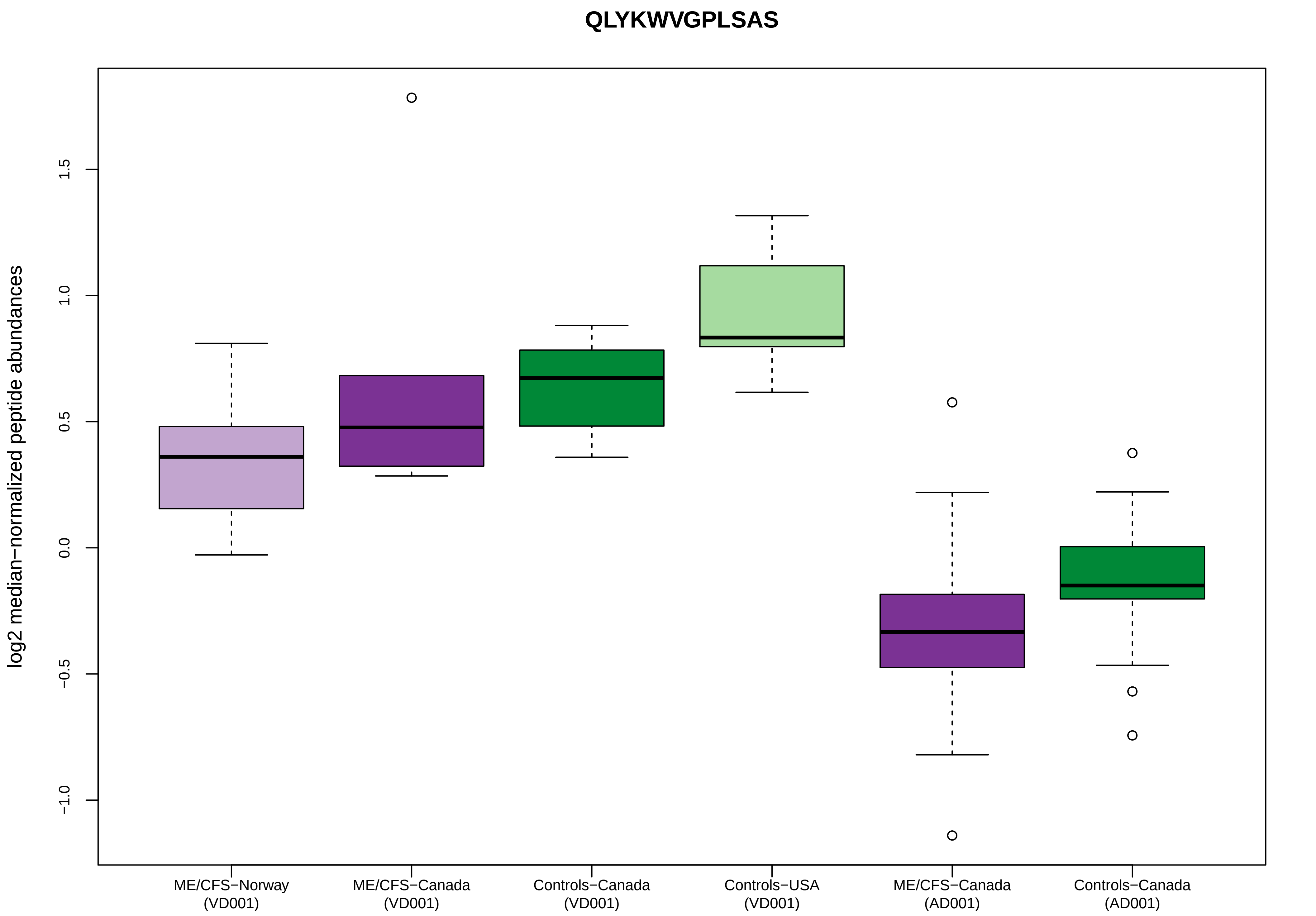

QLYKWVGPLSAS
log2 median−normalized peptide abundances
1.5
1.0
0.5
0.0
−0.5
−1.0
ME/CFS−Norway (VD001)
ME/CFS−Canada (VD001)
Controls−Canada (VD001)
Controls−USA (VD001)
ME/CFS−Canada (AD001)
Controls−Canada (AD001)

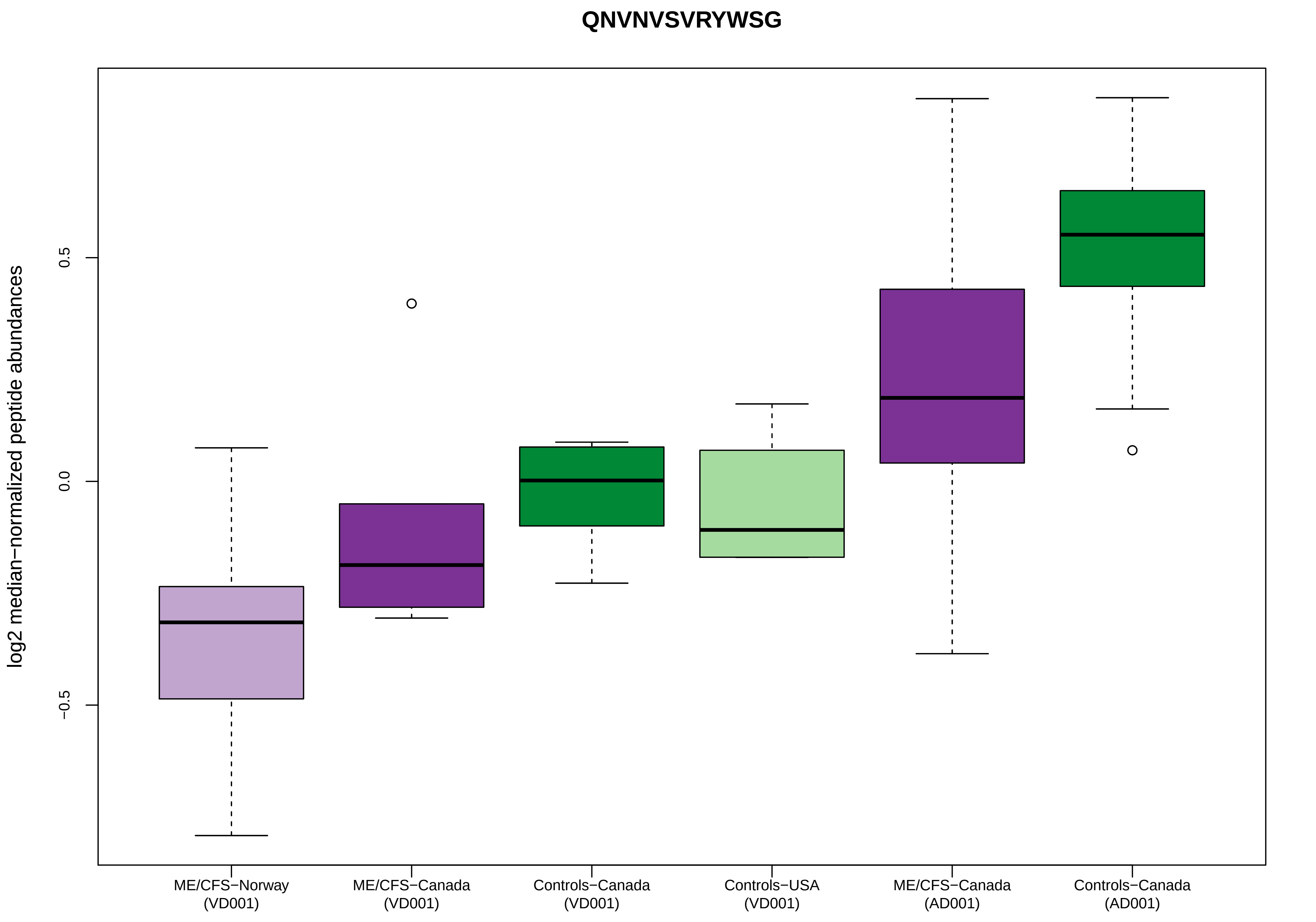

QNVNVSVRYWSG
log2 median−normalized peptide abundances
0.5
0.0
−0.5
ME/CFS−Norway (VD001)
ME/CFS−Canada (VD001)
Controls−Canada (VD001)
Controls−USA (VD001)
ME/CFS−Canada (AD001)
Controls−Canada (AD001)

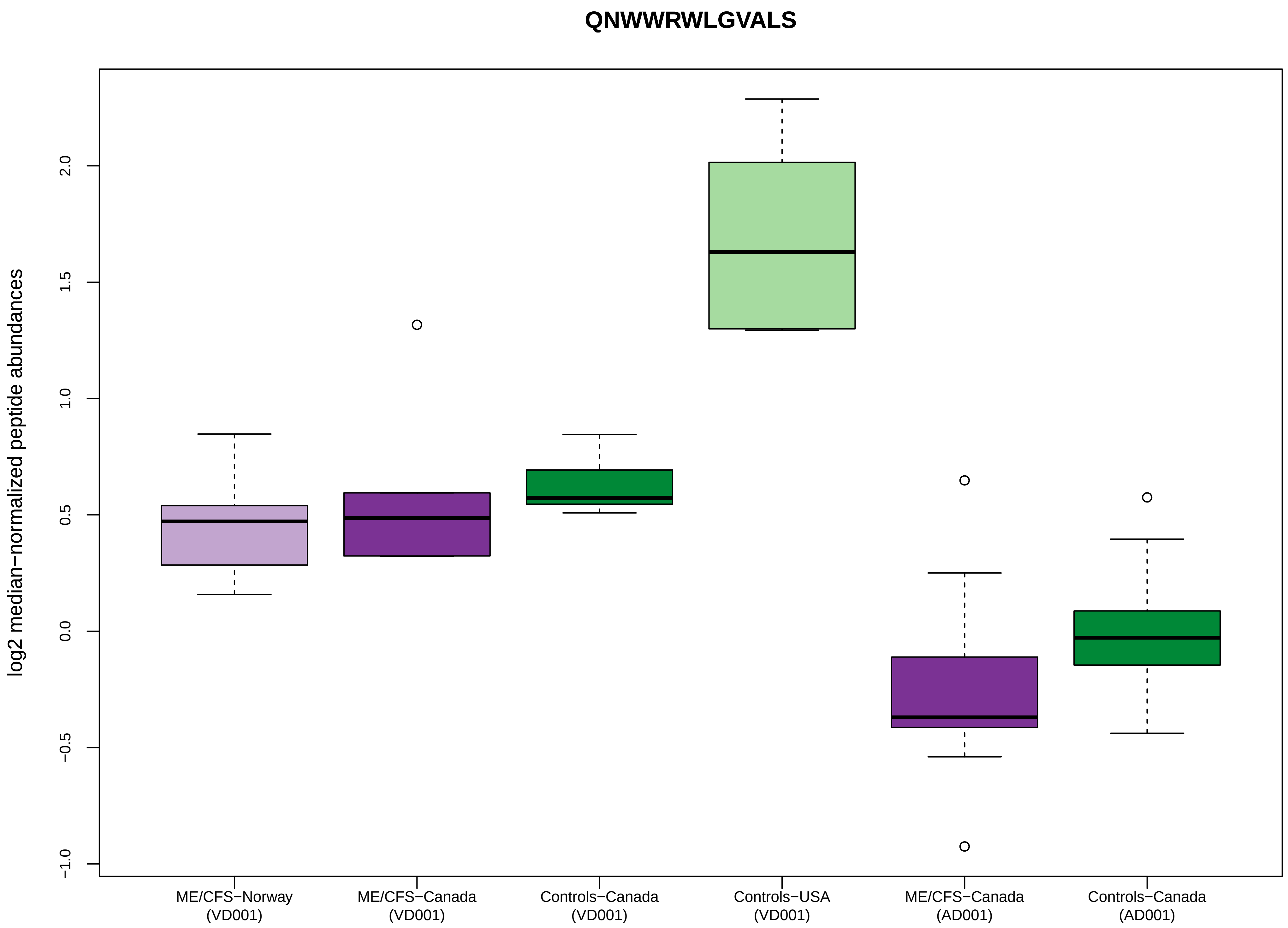

QNWWRWLGVALS
log2 median−normalized peptide abundances
2.0
1.5
1.0
0.5
0.0
−0.5
−1.0
ME/CFS−Norway (VD001)
ME/CFS−Canada (VD001)
Controls−Canada (VD001)
Controls−USA (VD001)
ME/CFS−Canada (AD001)
Controls−Canada (AD001)

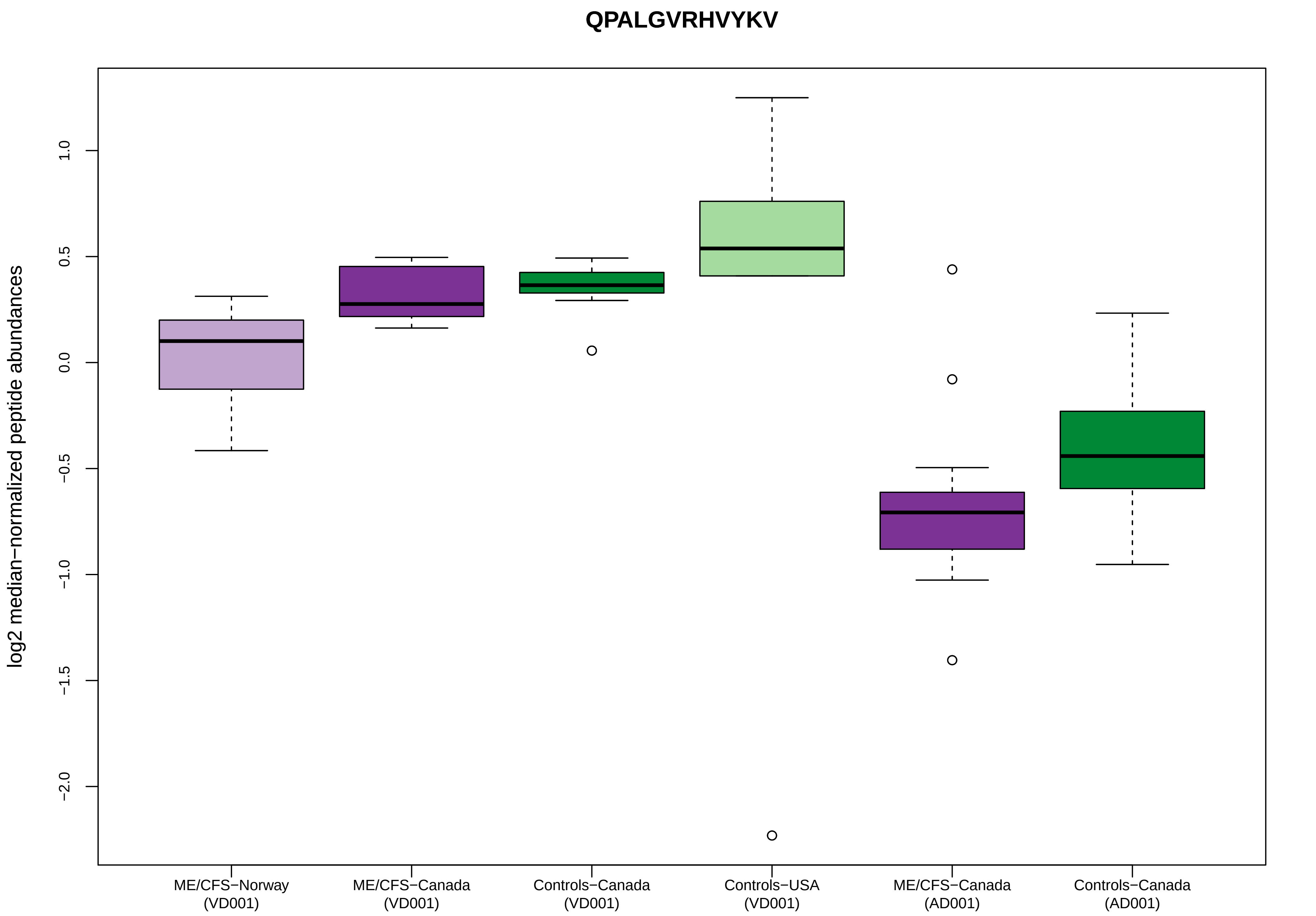

QPALGVRHVYKV
log2 median−normalized peptide abundances
1.0
0.5
0.0
−0.5
−1.0
−1.5
−2.0
ME/CFS−Norway (VD001)
ME/CFS−Canada (VD001)
Controls−Canada (VD001)
Controls−USA (VD001)
ME/CFS−Canada (AD001)
Controls−Canada (AD001)

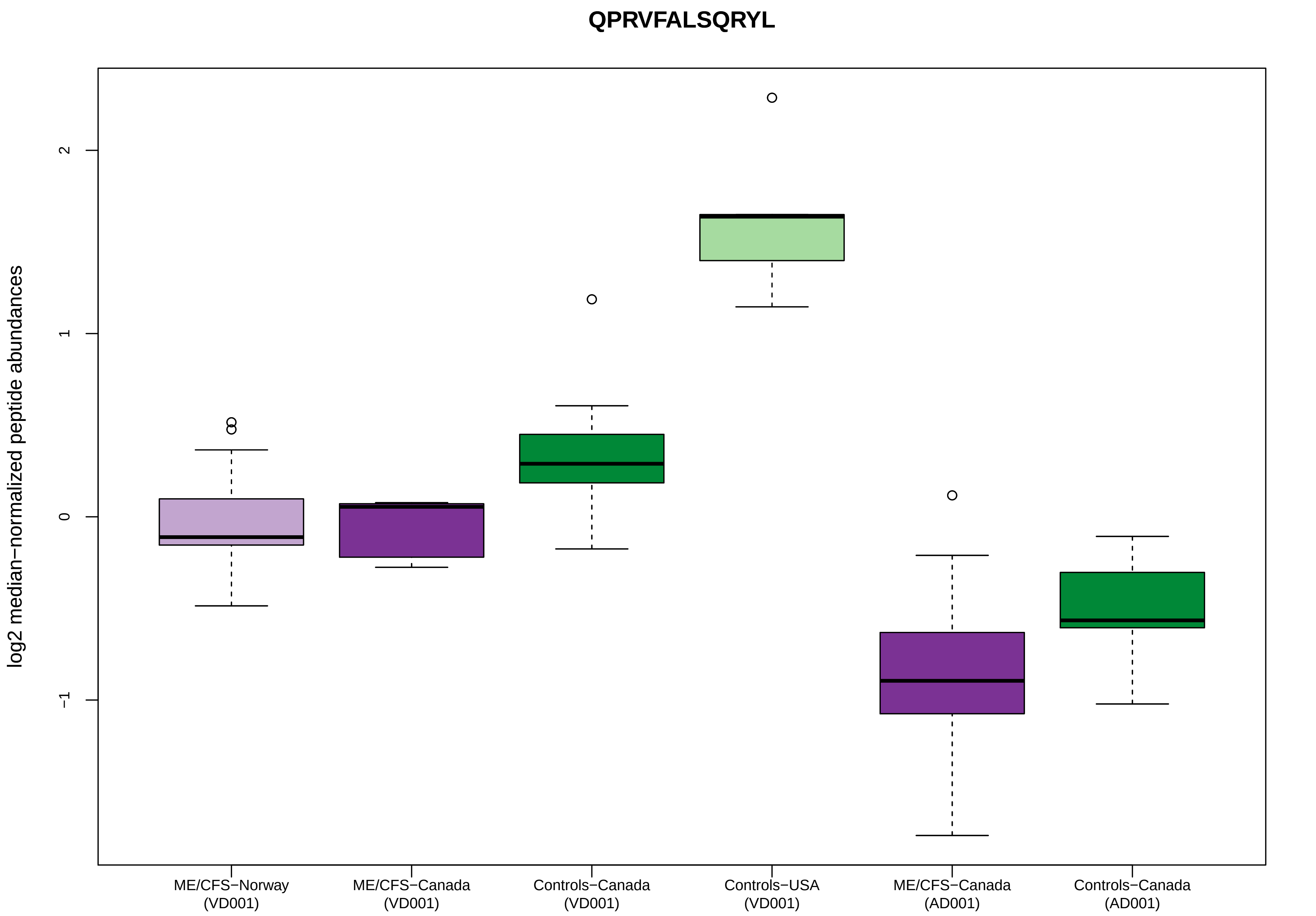

QPRVFALSQRYL
log2 median−normalized peptide abundances
2
1
0
−1
ME/CFS−Norway (VD001)
ME/CFS−Canada (VD001)
Controls−Canada (VD001)
Controls−USA (VD001)
ME/CFS−Canada (AD001)
Controls−Canada (AD001)

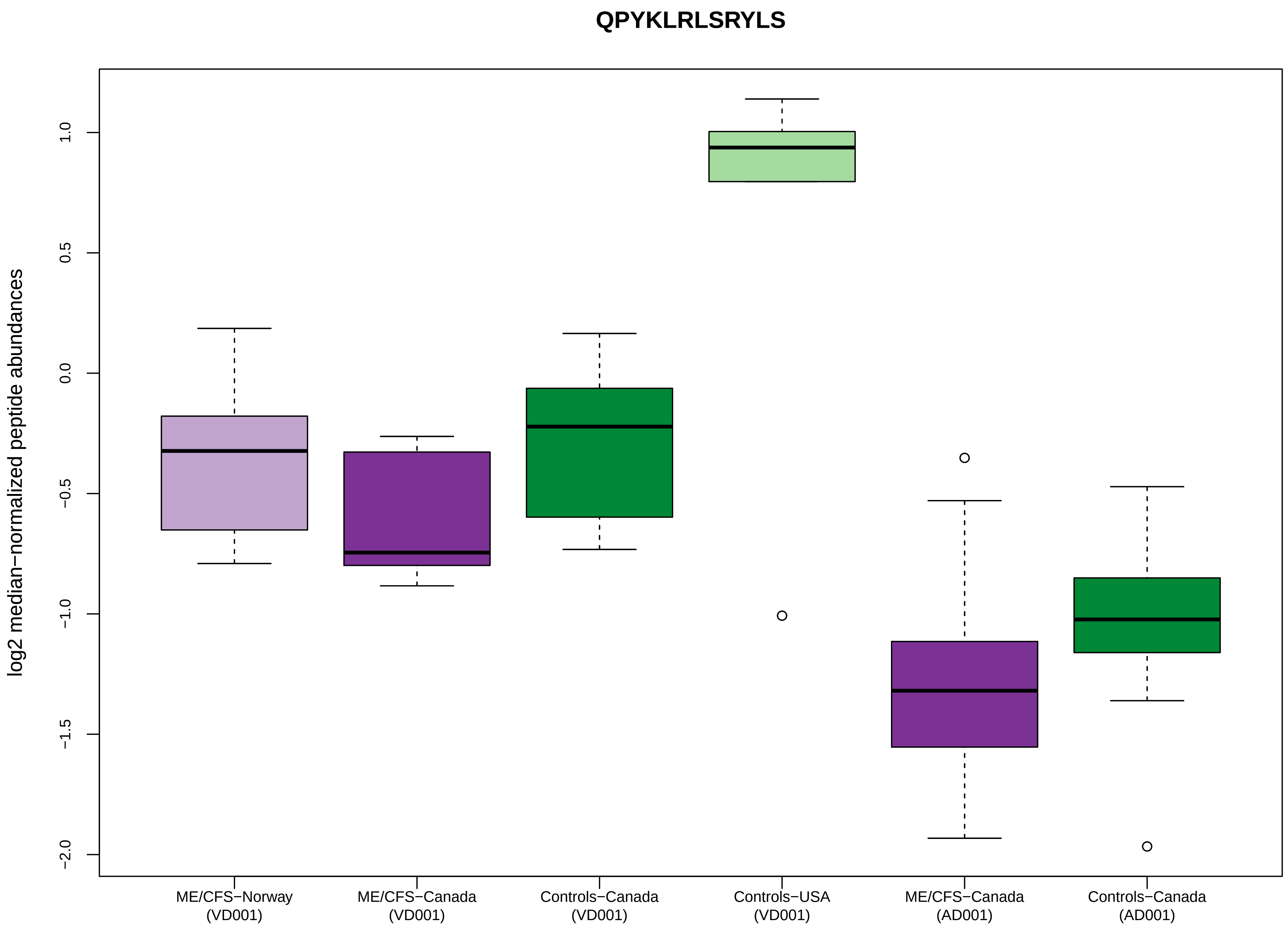

QPYKLRLSRYLS
log2 median−normalized peptide abundances
1.0
0.5
0.0
−0.5
−1.0
−1.5
−2.0
ME/CFS−Norway (VD001)
ME/CFS−Canada (VD001)
Controls−Canada (VD001)
Controls−USA (VD001)
ME/CFS−Canada (AD001)
Controls−Canada (AD001)

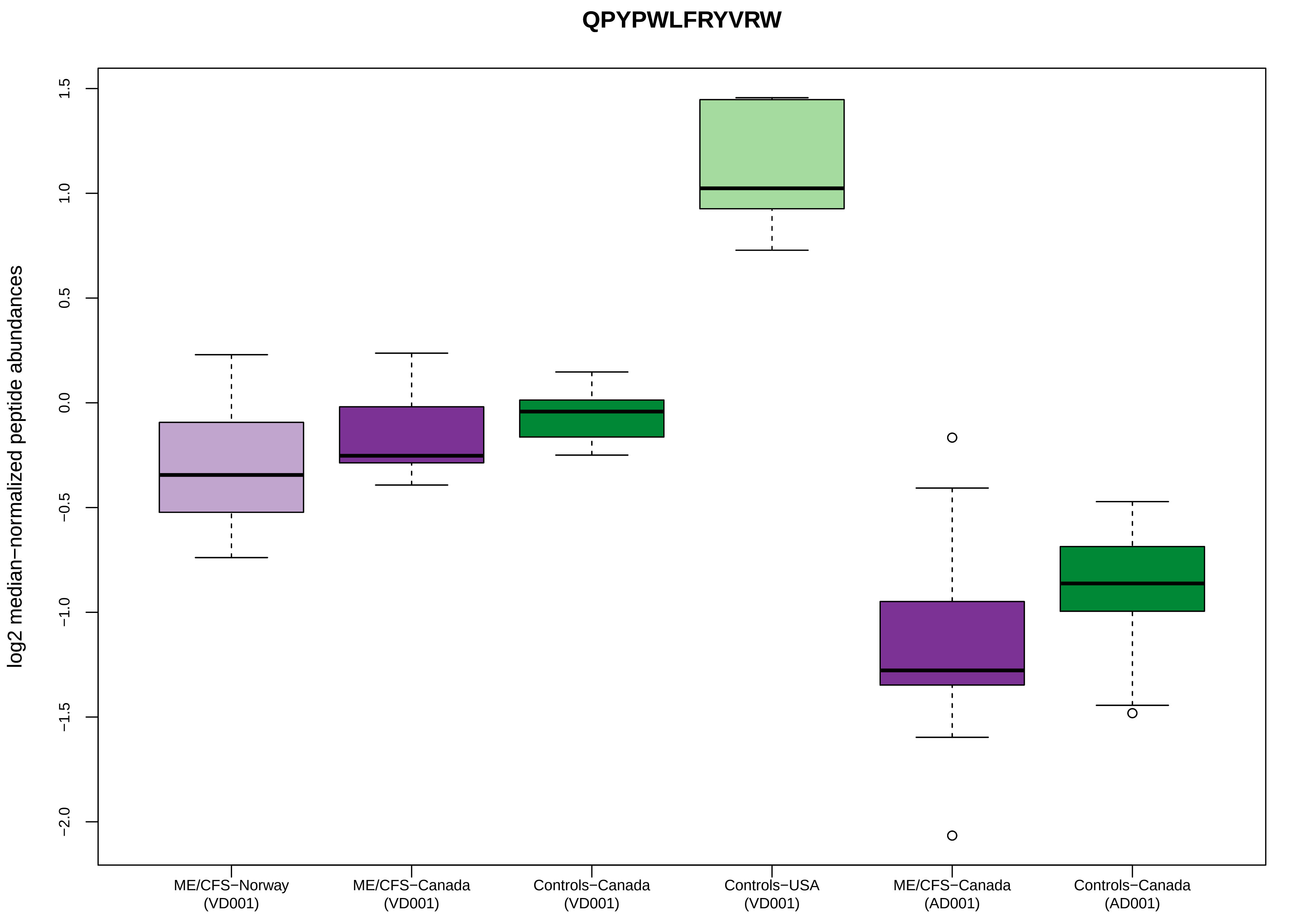
QPYPWLFRYVRW
log2 median−normalized peptide abundances
1.5
1.0
0.5
0.0
−0.5
−1.0
−1.5
−2.0
ME/CFS−Norway (VD001)
ME/CFS−Canada (VD001)
Controls−Canada (VD001)
Controls−USA (VD001)
ME/CFS−Canada (AD001)
Controls−Canada (AD001)

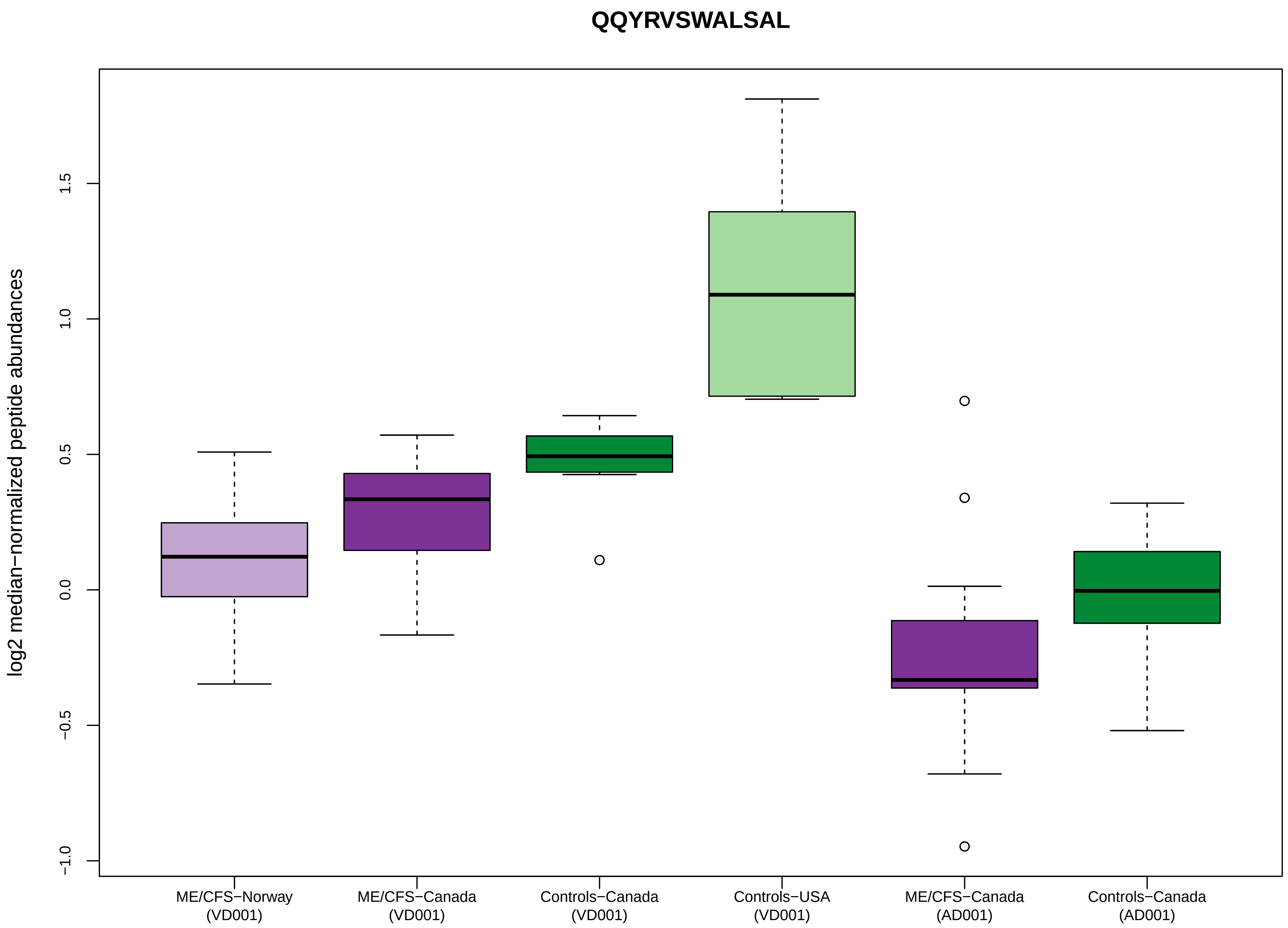
QQYRVSWALSAL
log2 median−normalized peptide abundances
1.5
1.0
0.5
0.0
−0.5
−1.0
ME/CFS−Norway (VD001)
ME/CFS−Canada (VD001)
Controls−Canada (VD001)
Controls−USA (VD001)
ME/CFS−Canada (AD001)
Controls−Canada (AD001)

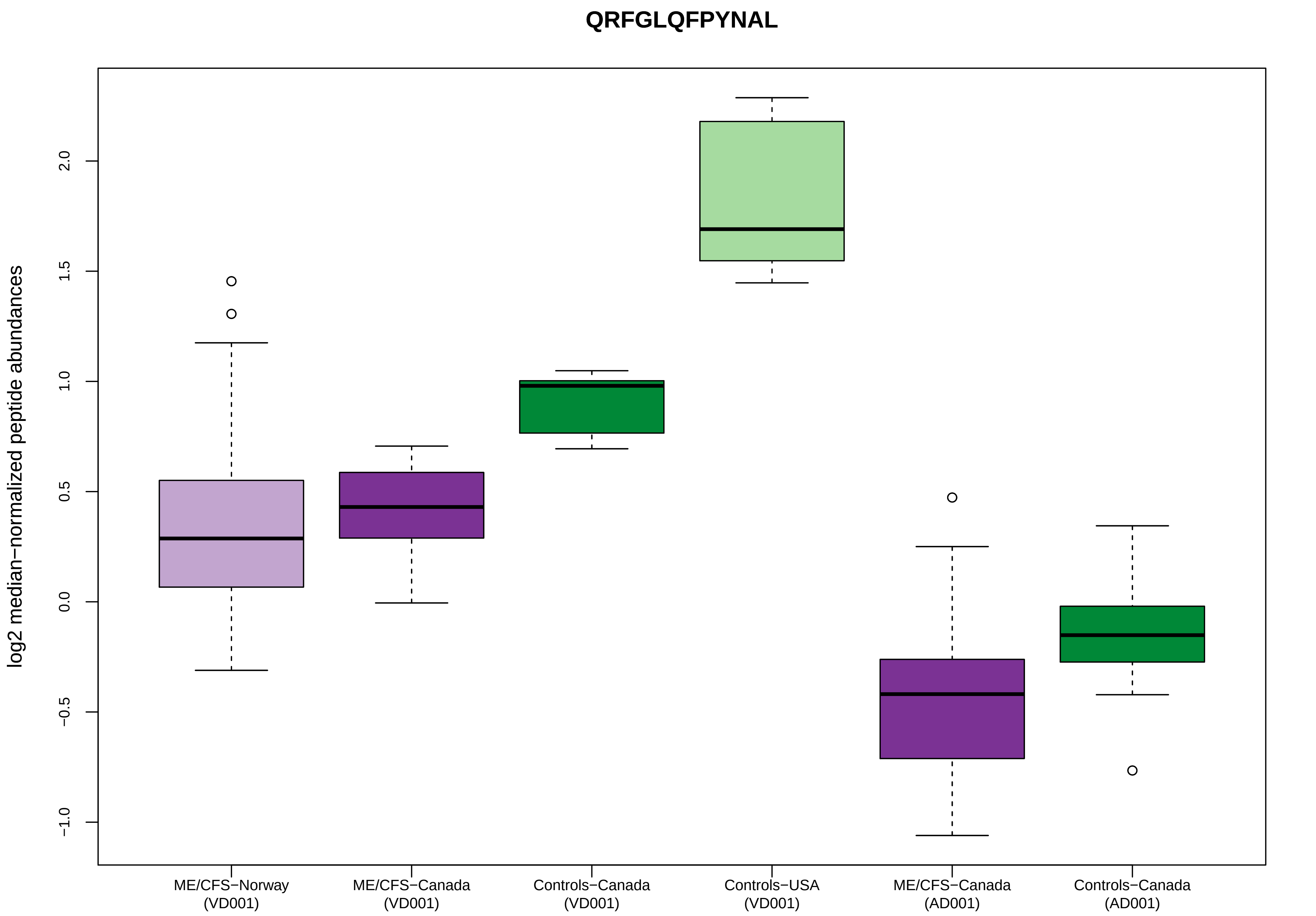
QRFGLQFPYNAL
log2 median−normalized peptide abundances
2.0
1.5
1.0
0.5
0.0
−0.5
−1.0
ME/CFS−Norway (VD001)
ME/CFS−Canada (VD001)
Controls−Canada (VD001)
Controls−USA (VD001)
ME/CFS−Canada (AD001)
Controls−Canada (AD001)

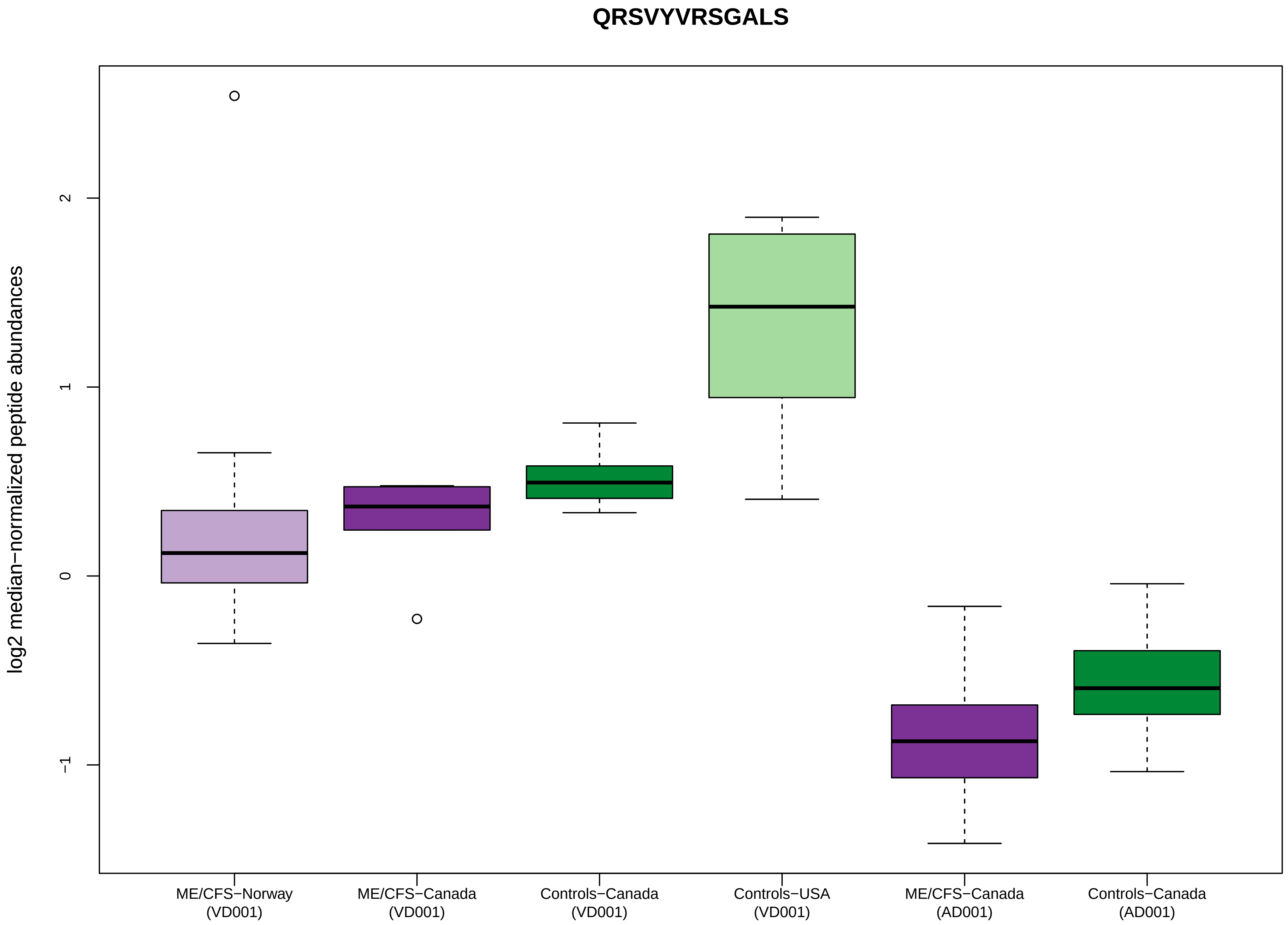

QRSVYVRSGALS
log2 median−normalized peptide abundances
2
1
0
−1
ME/CFS−Norway
(VD001)
ME/CFS−Canada
(VD001)
Controls−Canada
(VD001)
Controls−USA
(VD001)
ME/CFS−Canada
(AD001)
Controls−Canada
(AD001)

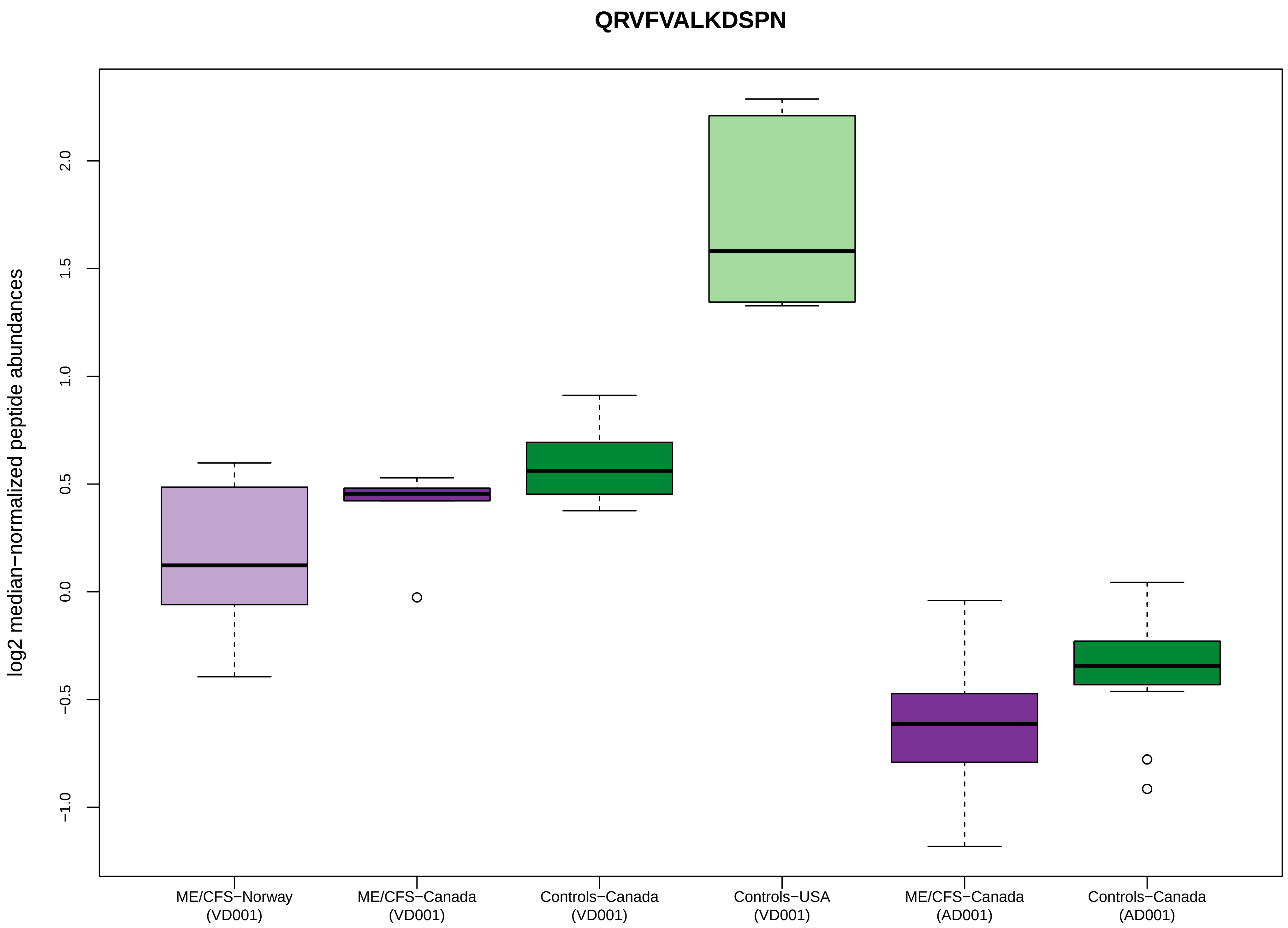

QRVFVALKDSPN
log2 median−normalized peptide abundances
2.0
1.5
1.0
0.5
0.0
−0.5
−1.0
ME/CFS−Norway (VD001)
ME/CFS−Canada (VD001)
Controls−Canada (VD001)
Controls−USA (VD001)
ME/CFS−Canada (AD001)
Controls−Canada (AD001)

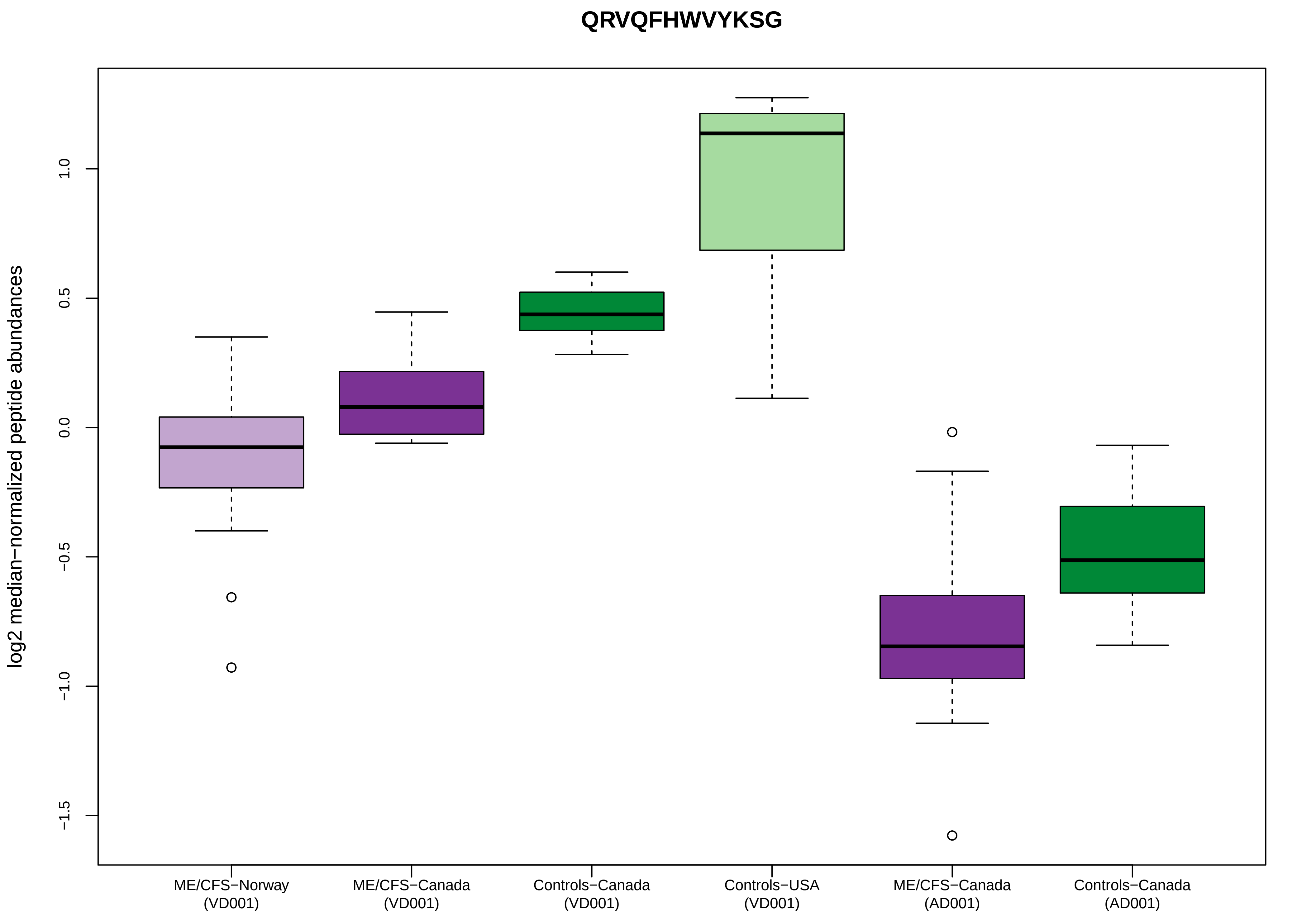

QRVQFHWVYKSG
log2 median−normalized peptide abundances
1.0
0.5
0.0
−0.5
−1.0
−1.5
ME/CFS−Norway (VD001)
ME/CFS−Canada (VD001)
Controls−Canada (VD001)
Controls−USA (VD001)
ME/CFS−Canada (AD001)
Controls−Canada (AD001)

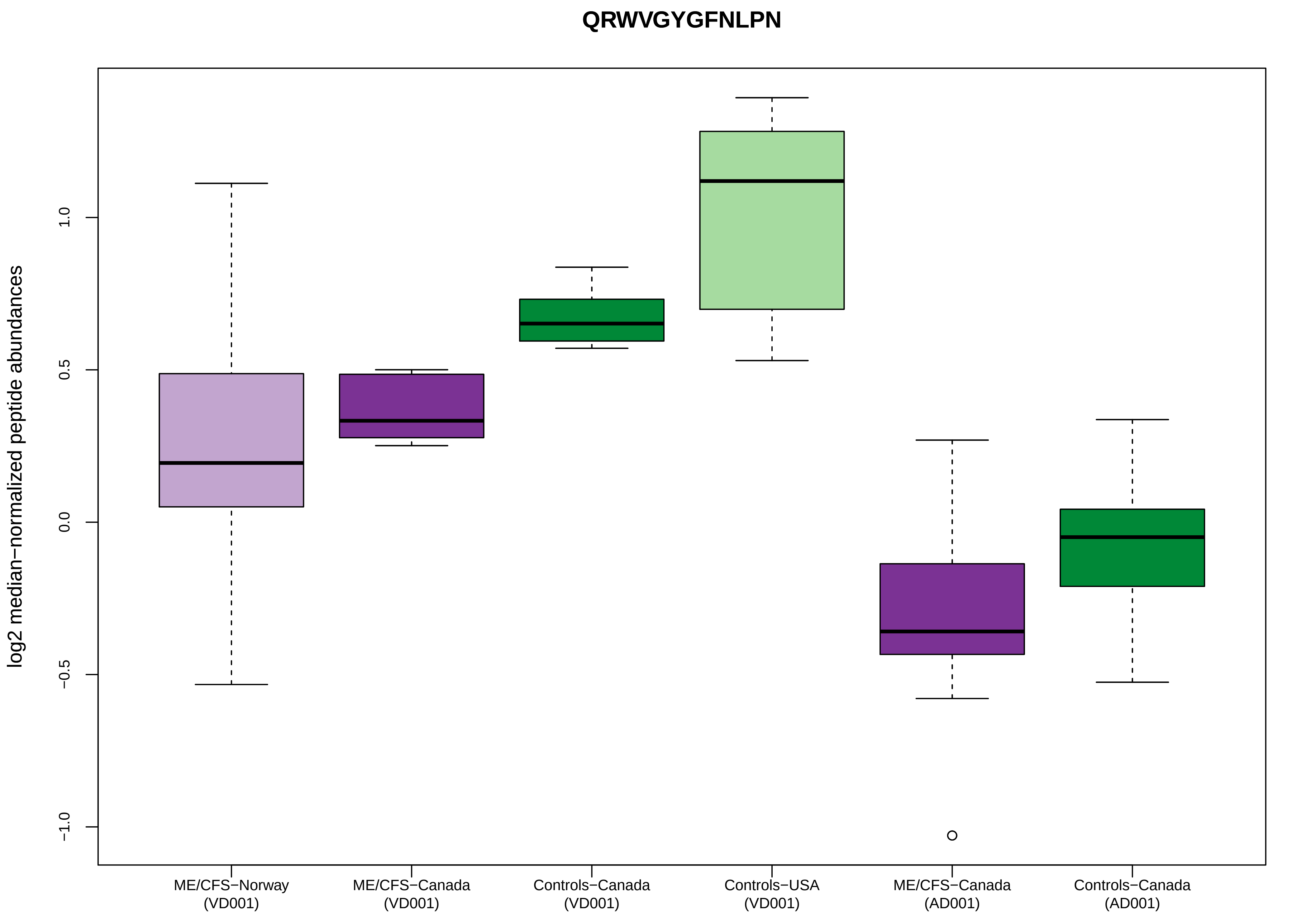
QRWVGYGFNLPN
log2 median−normalized peptide abundances
1.0
0.5
0.0
−0.5
−1.0
ME/CFS−Norway (VD001)
ME/CFS−Canada (VD001)
Controls−Canada (VD001)
Controls−USA (VD001)
ME/CFS−Canada (AD001)
Controls−Canada (AD001)

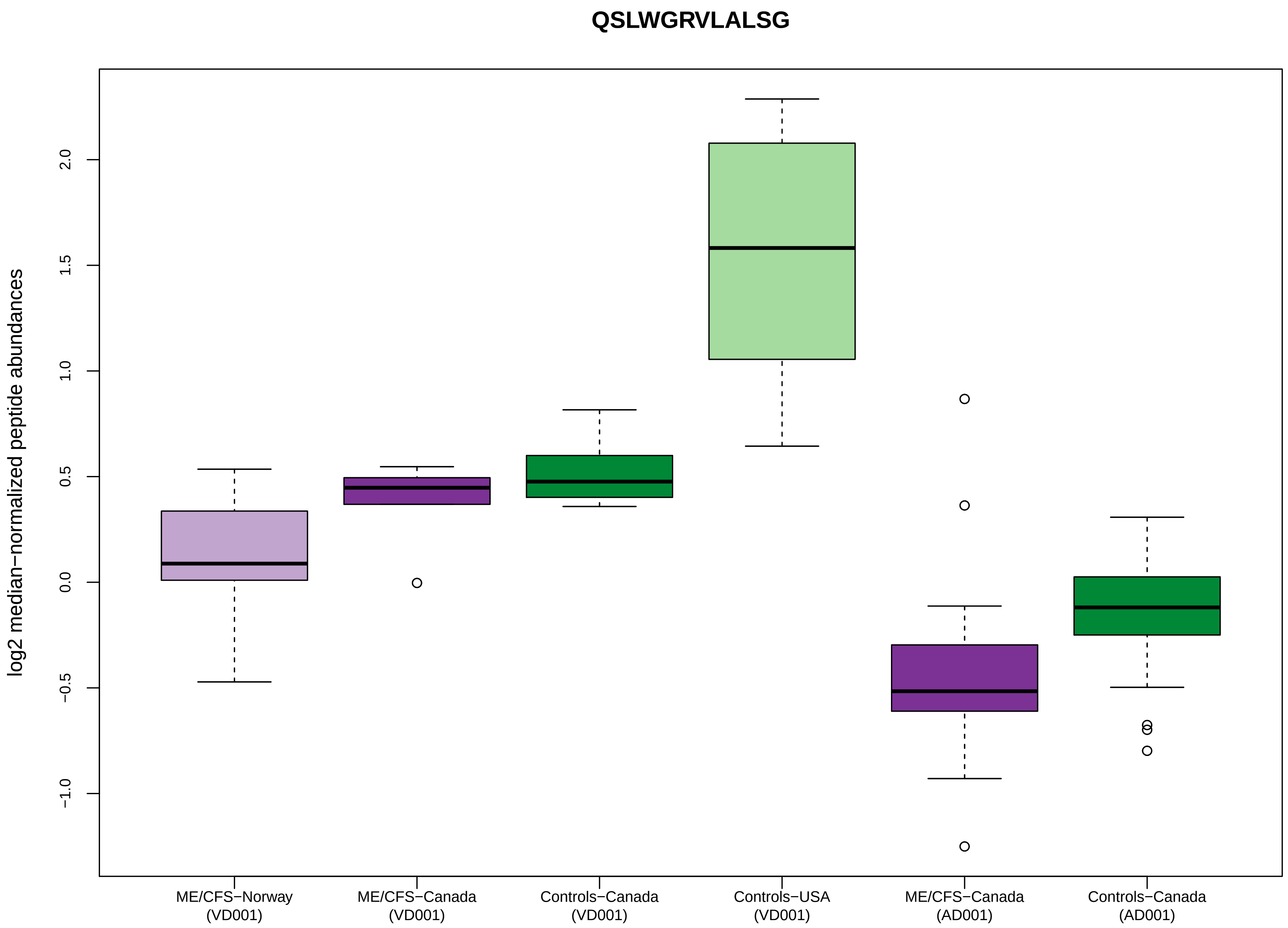

QSLWGRVLALSG
log2 median−normalized peptide abundances
2.0
1.5
1.0
0.5
0.0
−0.5
−1.0
ME/CFS−Norway (VD001)
ME/CFS−Canada (VD001)
Controls−Canada (VD001)
Controls−USA (VD001)
ME/CFS−Canada (AD001)
Controls−Canada (AD001)

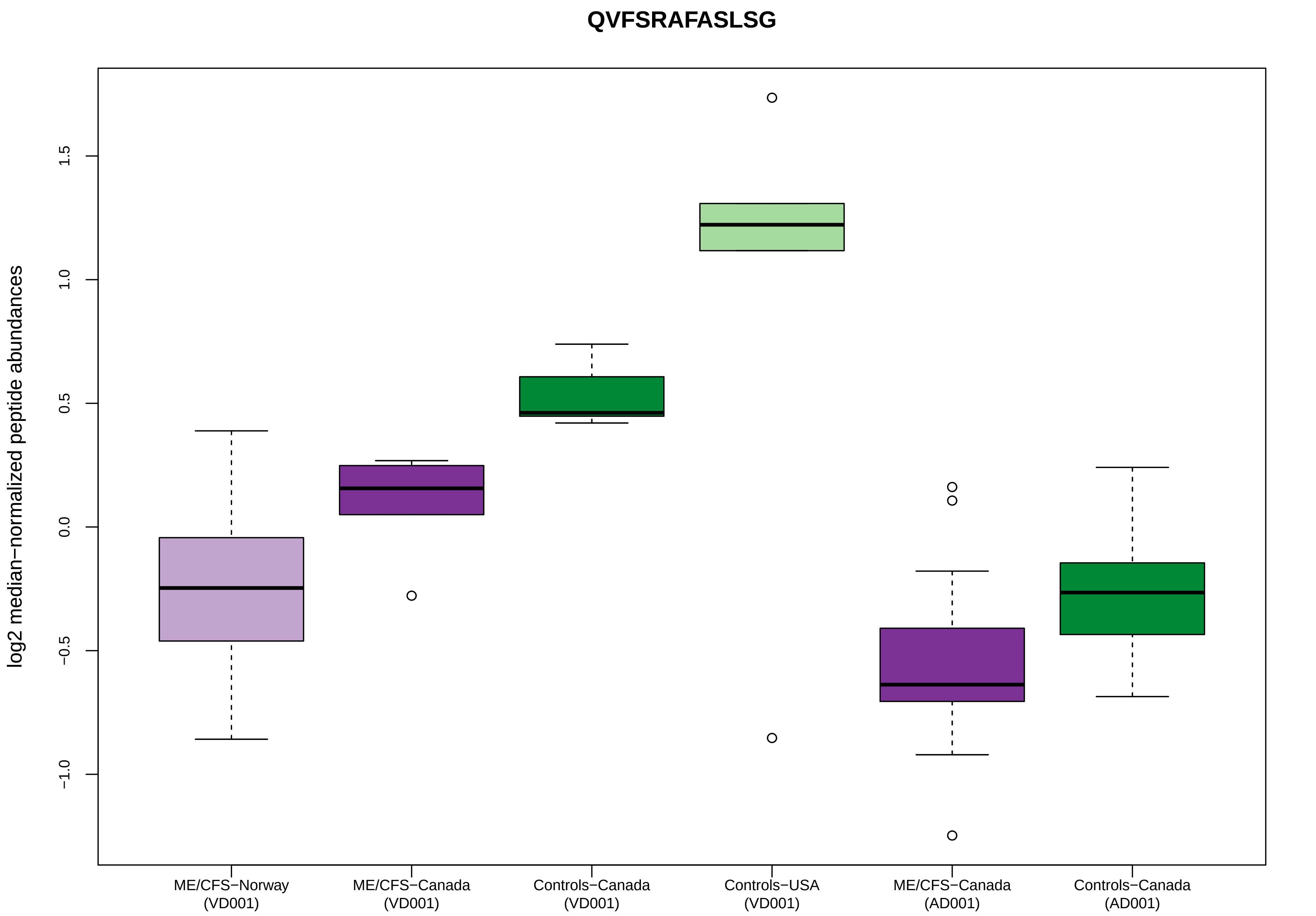

QVFSRAFASLSG
log2 median−normalized peptide abundances
1.5
1.0
0.5
0.0
−0.5
−1.0
ME/CFS−Norway (VD001)
ME/CFS−Canada (VD001)
Controls−Canada (VD001)
Controls−USA (VD001)
ME/CFS−Canada (AD001)
Controls−Canada (AD001)

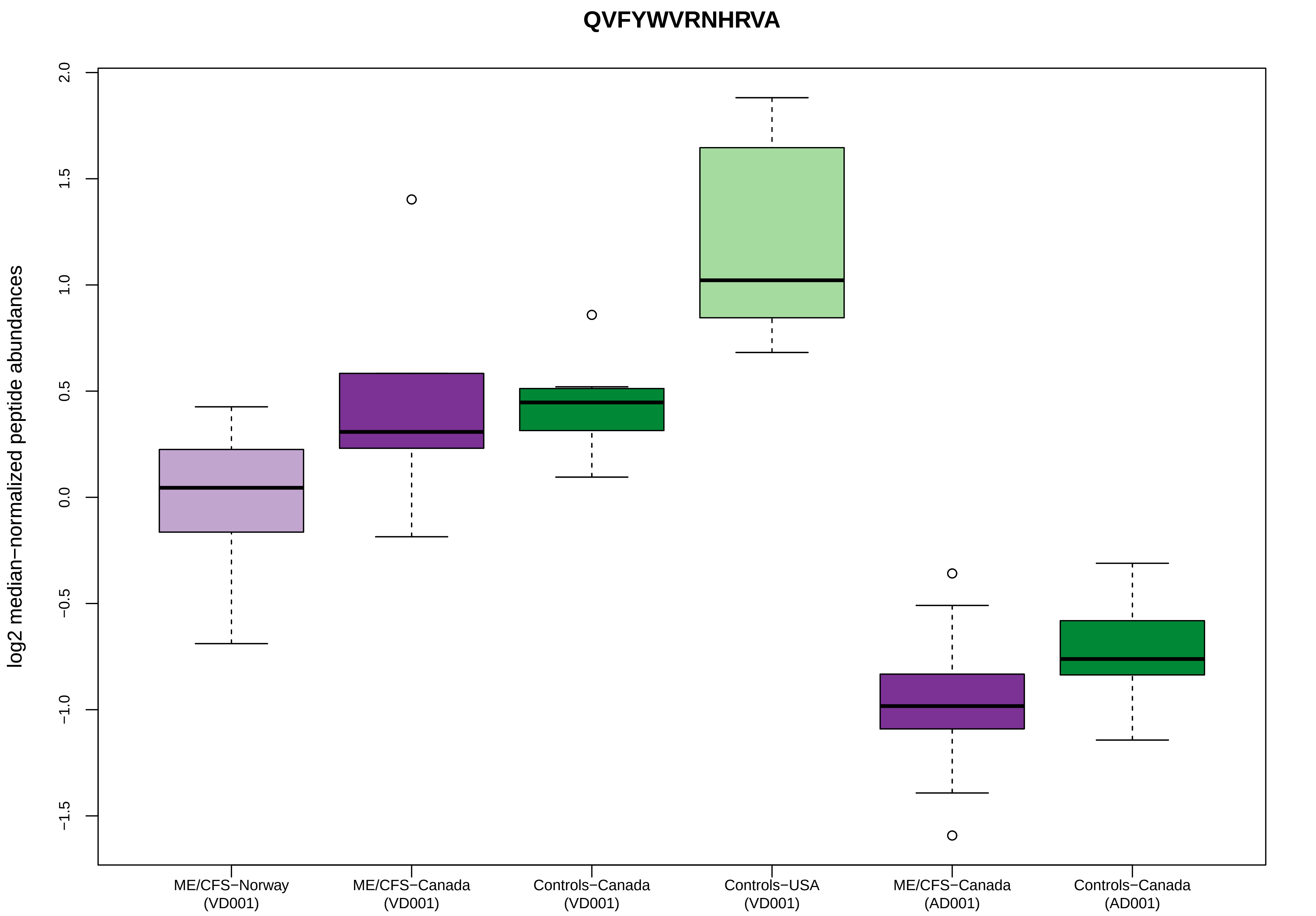

QVFYWVRNHRVA
log2 median−normalized peptide abundances
2.0
1.5
1.0
0.5
0.0
−0.5
−1.0
−1.5
ME/CFS−Norway (VD001)
ME/CFS−Canada (VD001)
Controls−Canada (VD001)
Controls−USA (VD001)
ME/CFS−Canada (AD001)
Controls−Canada (AD001)

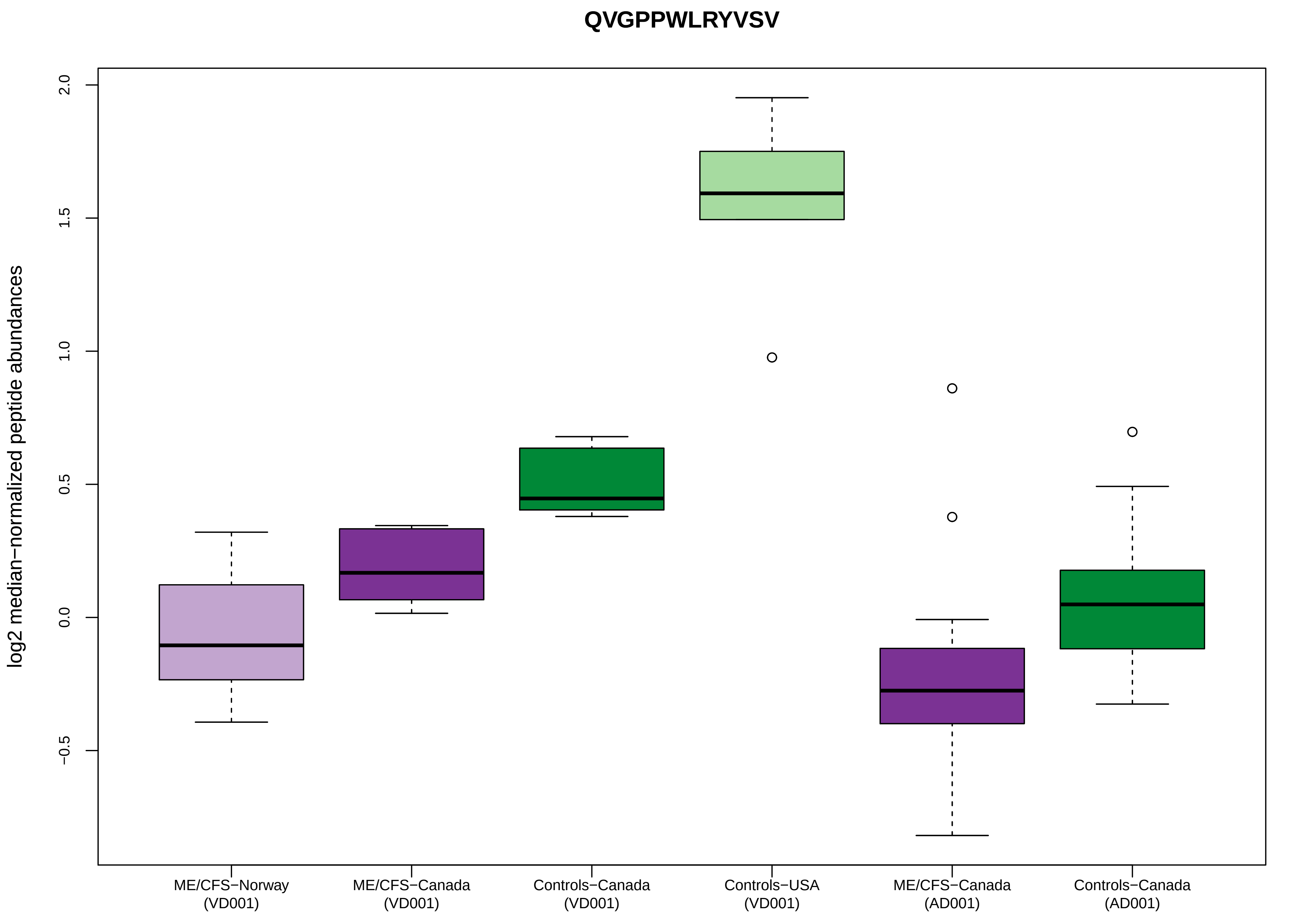
QVGPPWLRYVSV
log2 median−normalized peptide abundances
2.0
1.5
1.0
0.5
0.0
−0.5
ME/CFS−Norway (VD001)
ME/CFS−Canada (VD001)
Controls−Canada (VD001)
Controls−USA (VD001)
ME/CFS−Canada (AD001)
Controls−Canada (AD001)

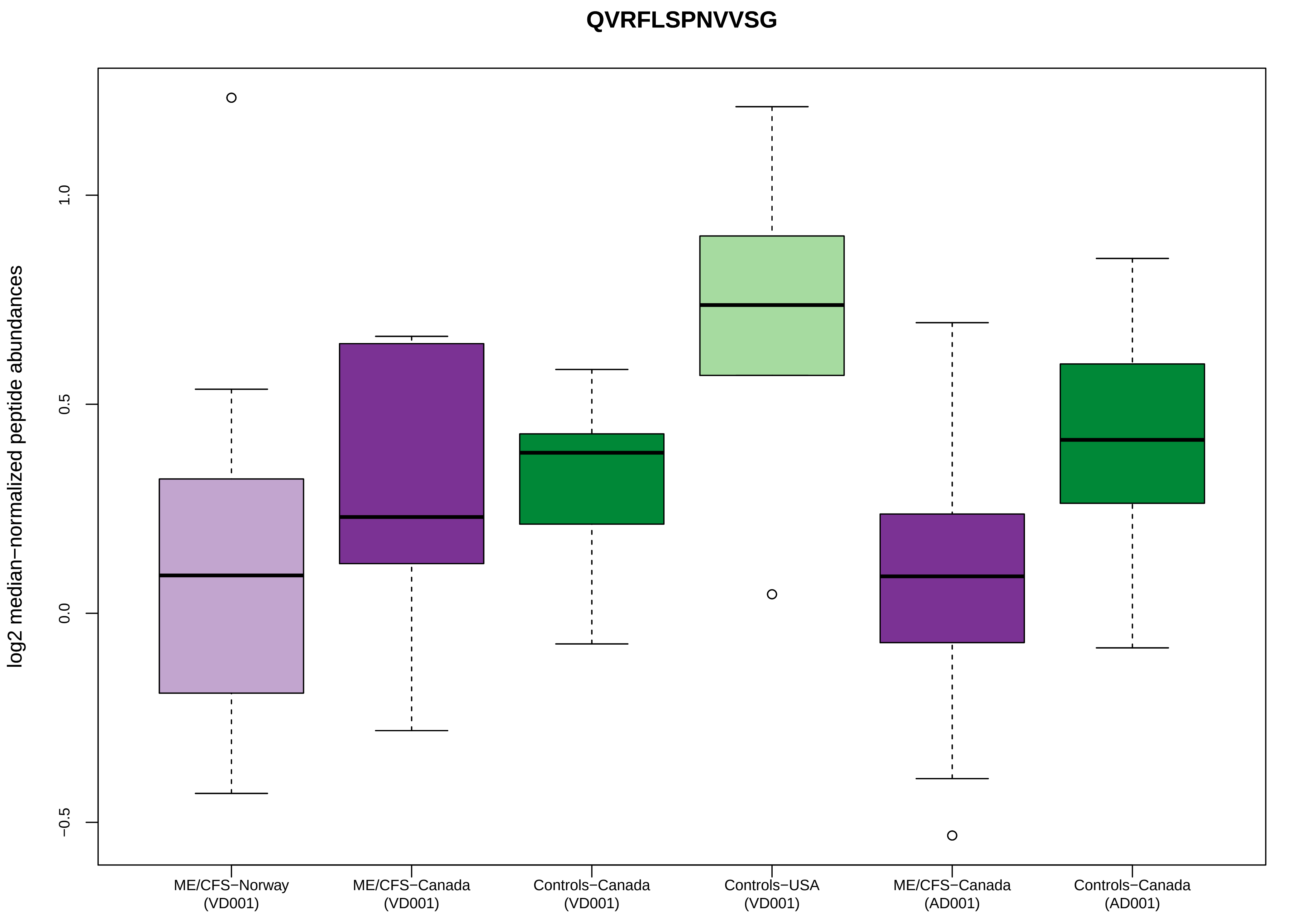
QVRFLSPNVVSG
log2 median−normalized peptide abundances
−0.5
0.0
0.5
1.0
ME/CFS−Norway (VD001)
ME/CFS−Canada (VD001)
Controls−Canada (VD001)
Controls−USA (VD001)
ME/CFS−Canada (AD001)
Controls−Canada (AD001)

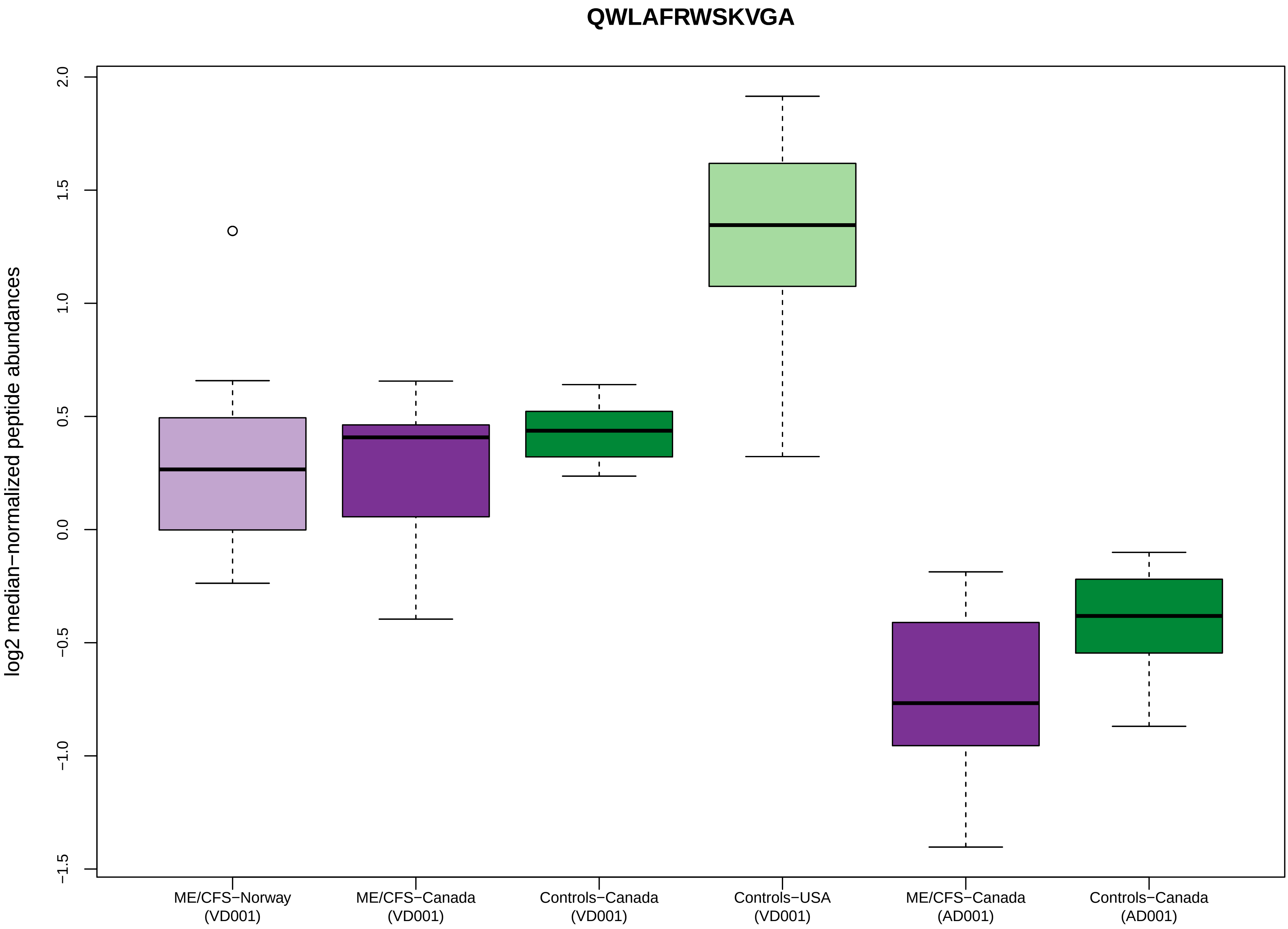

QWLAFRWSKVGA
log2 median−normalized peptide abundances
2.0
1.5
1.0
0.5
0.0
−0.5
−1.0
−1.5
ME/CFS−Norway (VD001)
ME/CFS−Canada (VD001)
Controls−Canada (VD001)
Controls−USA (VD001)
ME/CFS−Canada (AD001)
Controls−Canada (AD001)

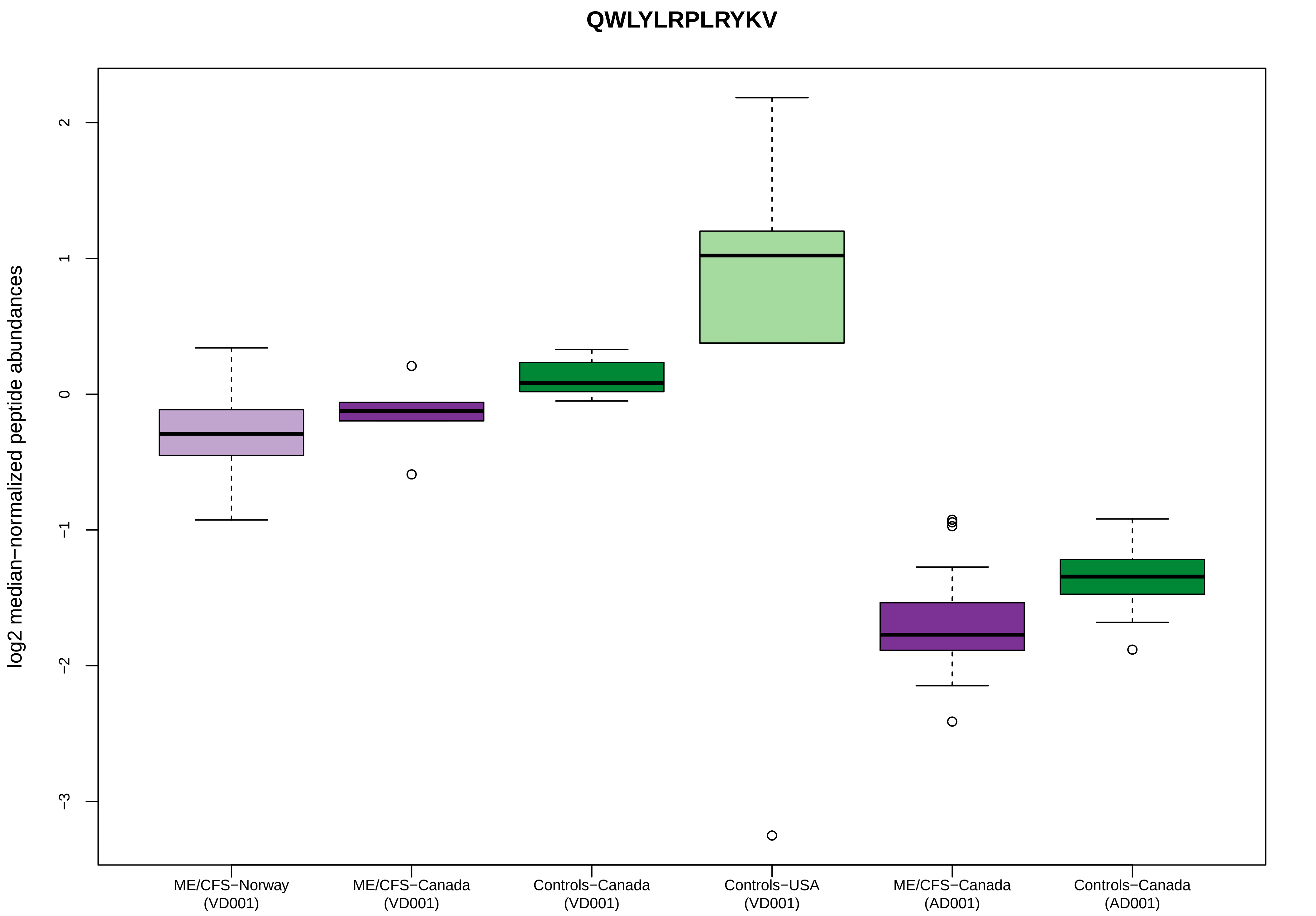

QWLYLRPLRYKV
log2 median−normalized peptide abundances
2
1
0
−1
−2
−3
ME/CFS−Norway (VD001)
ME/CFS−Canada (VD001)
Controls−Canada (VD001)
Controls−USA (VD001)
ME/CFS−Canada (AD001)
Controls−Canada (AD001)

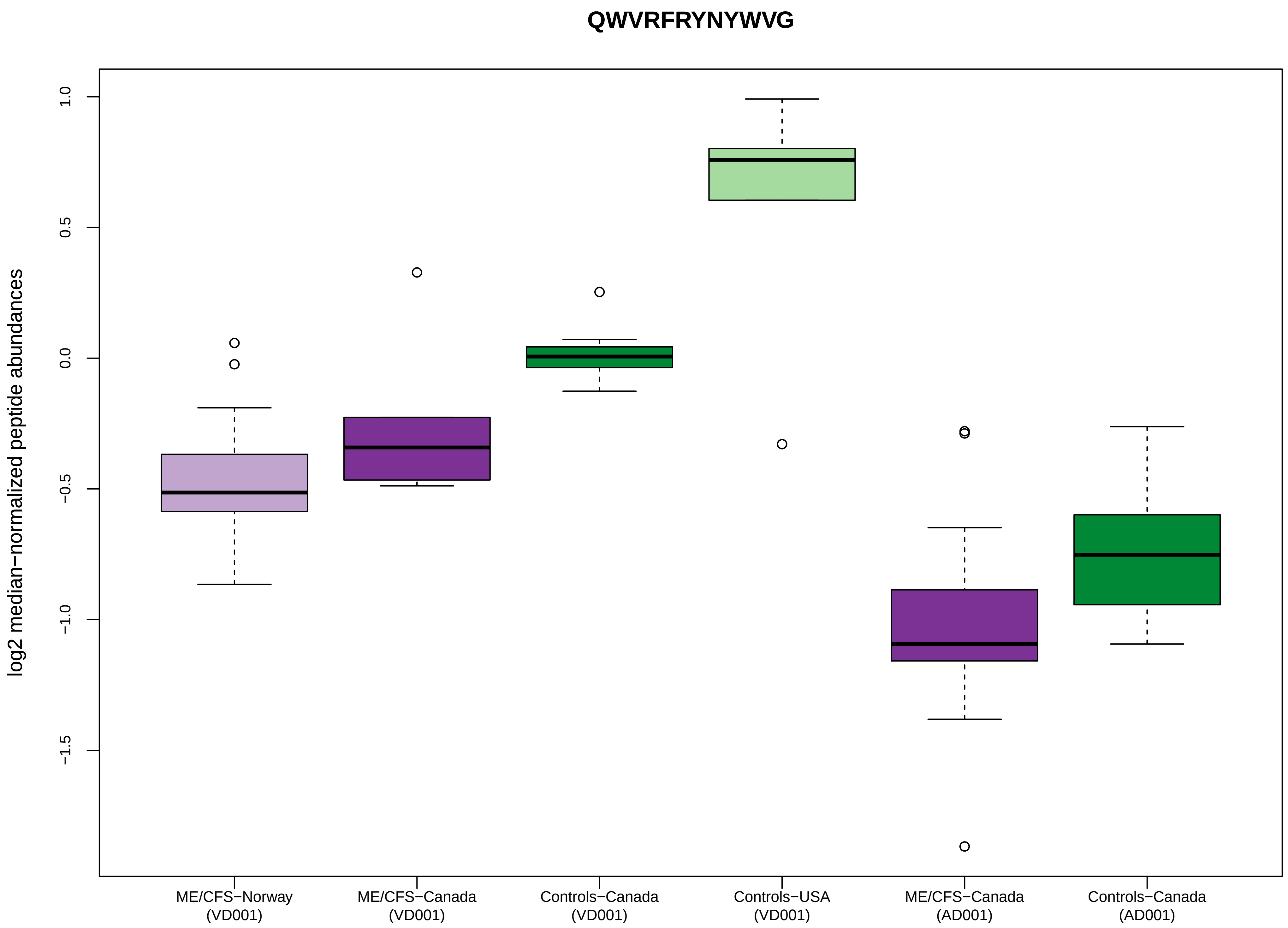

QWVRFRYNYWVG
log2 median−normalized peptide abundances
1.0
0.5
0.0
−0.5
−1.0
−1.5
ME/CFS−Norway (VD001)
ME/CFS−Canada (VD001)
Controls−Canada (VD001)
Controls−USA (VD001)
ME/CFS−Canada (AD001)
Controls−Canada (AD001)

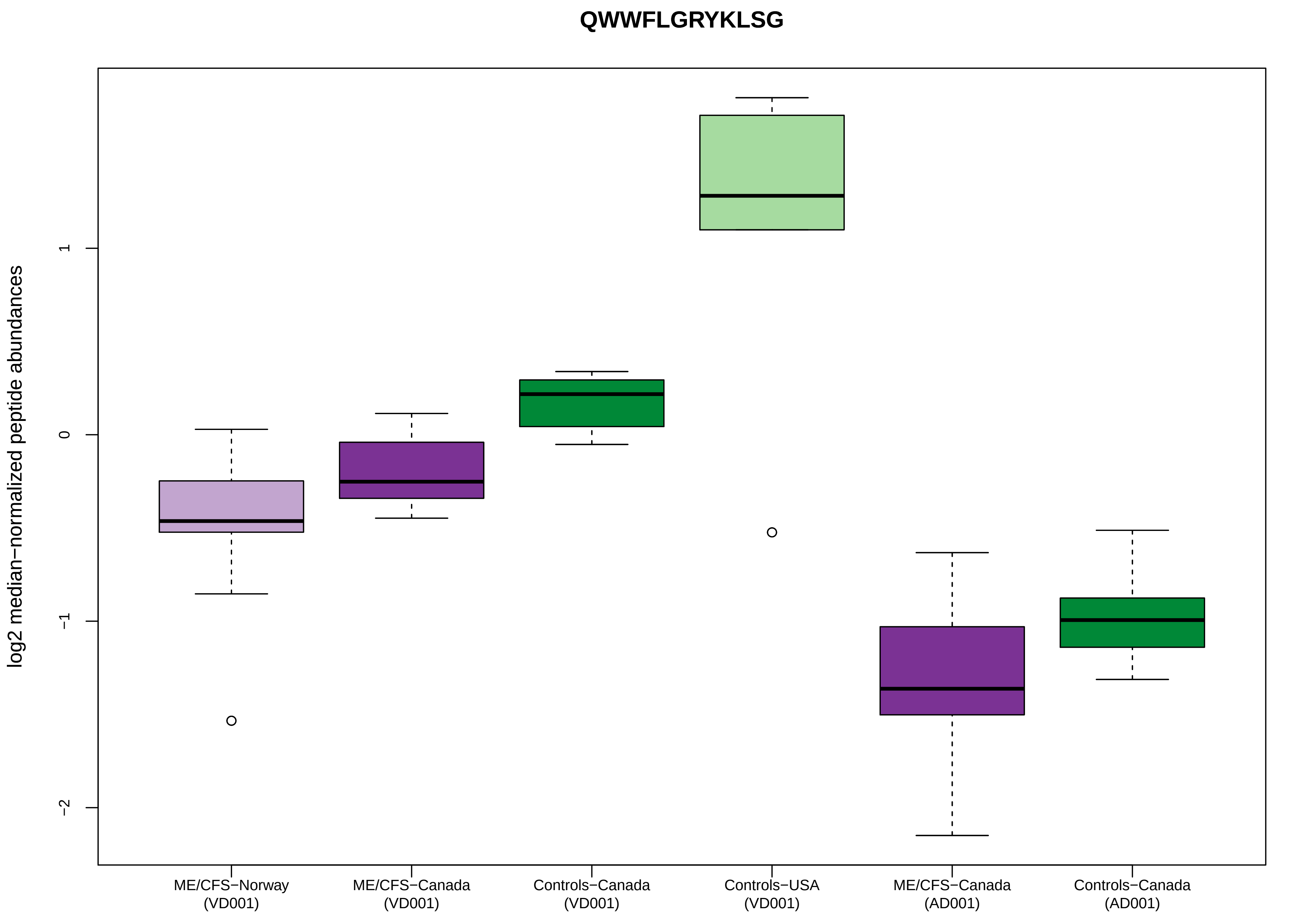
QWWFLGRYKLSG
log2 median−normalized peptide abundances
1
0
−1
−2
ME/CFS−Norway (VD001)
ME/CFS−Canada (VD001)
Controls−Canada (VD001)
Controls−USA (VD001)
ME/CFS−Canada (AD001)
Controls−Canada (AD001)

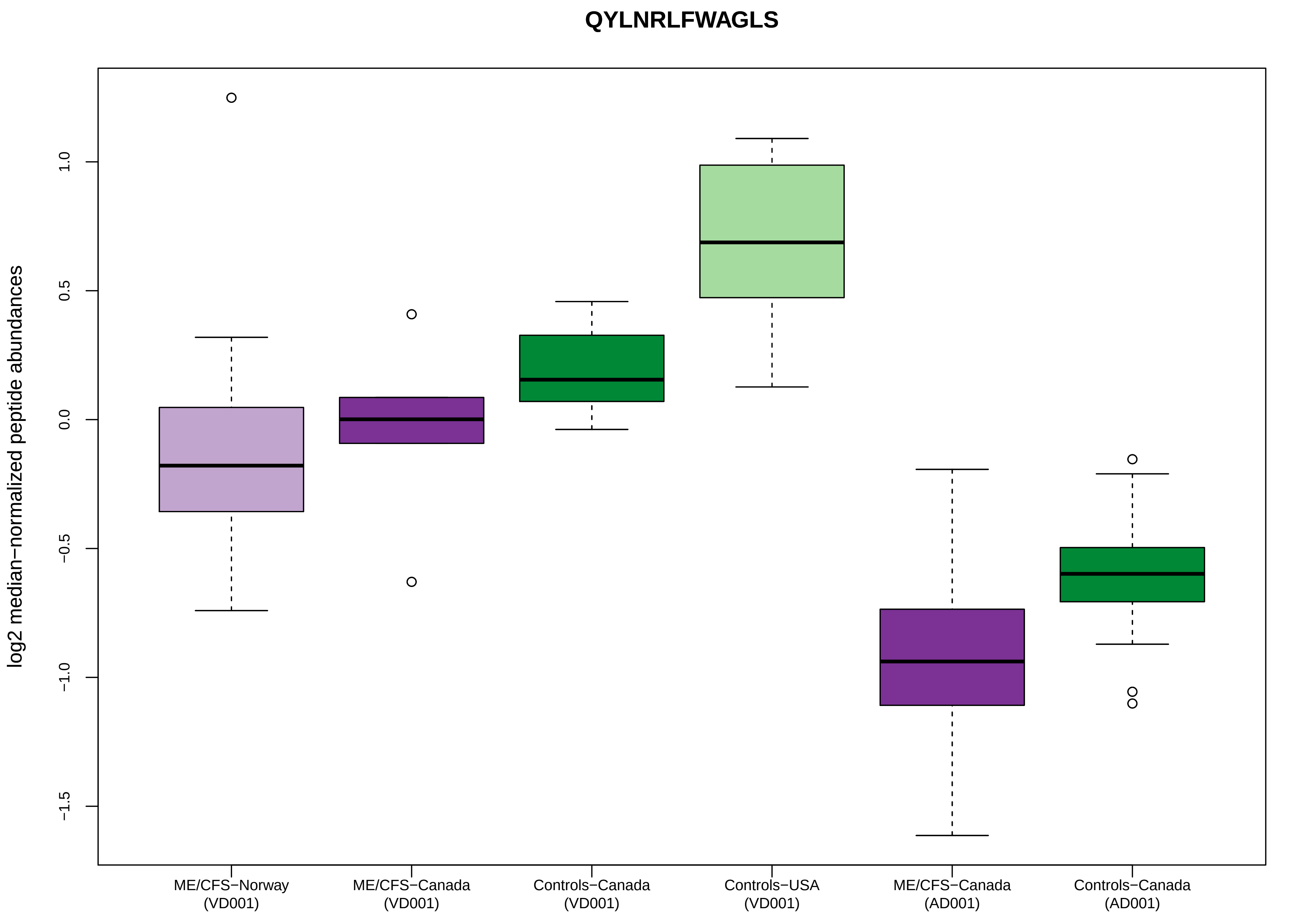
QYLNRLFWAGLS
log2 median−normalized peptide abundances
1.0
0.5
0.0
−0.5
−1.0
−1.5
ME/CFS−Norway (VD001)
ME/CFS−Canada (VD001)
Controls−Canada (VD001)
Controls−USA (VD001)
ME/CFS−Canada (AD001)
Controls−Canada (AD001)

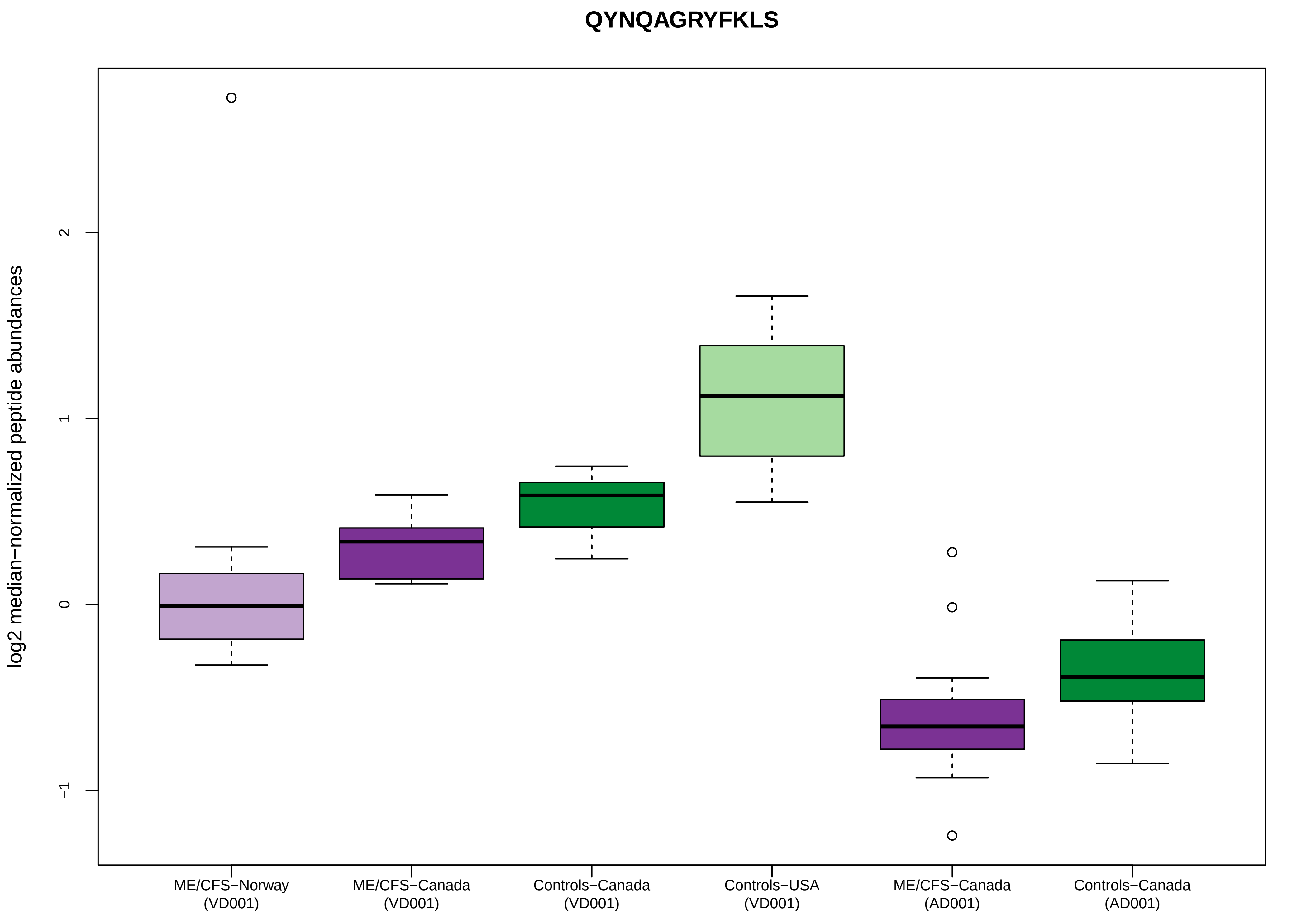
QYNQAGRYFKLS
log2 median−normalized peptide abundances
2
1
0
−1
ME/CFS−Norway (VD001)
ME/CFS−Canada (VD001)
Controls−Canada (VD001)
Controls−USA (VD001)
ME/CFS−Canada (AD001)
Controls−Canada (AD001)

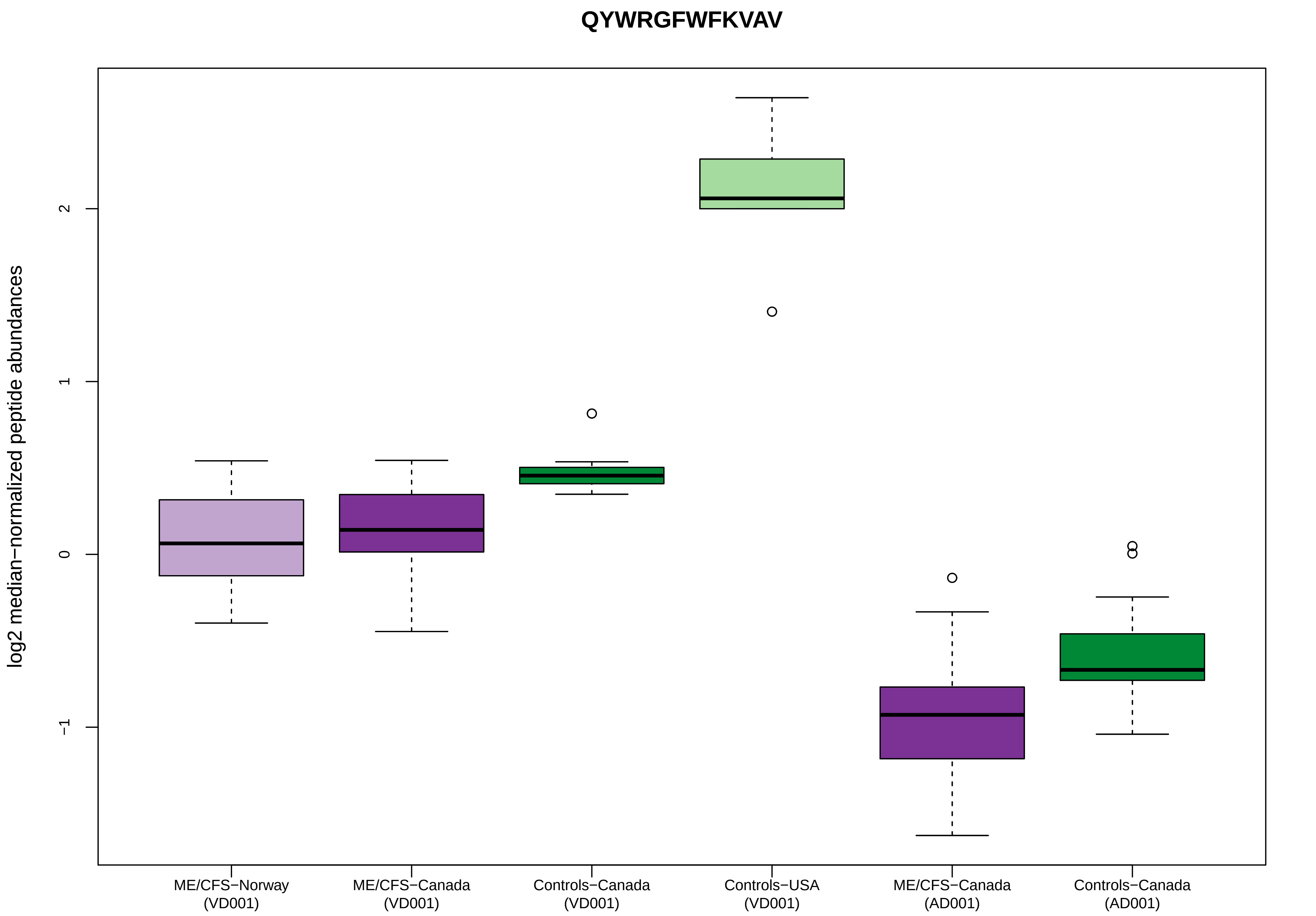
QYWRGFWFKVAV
log2 median−normalized peptide abundances
2
1
0
−1
ME/CFS−Norway (VD001)
ME/CFS−Canada (VD001)
Controls−Canada (VD001)
Controls−USA (VD001)
ME/CFS−Canada (AD001)
Controls−Canada (AD001)

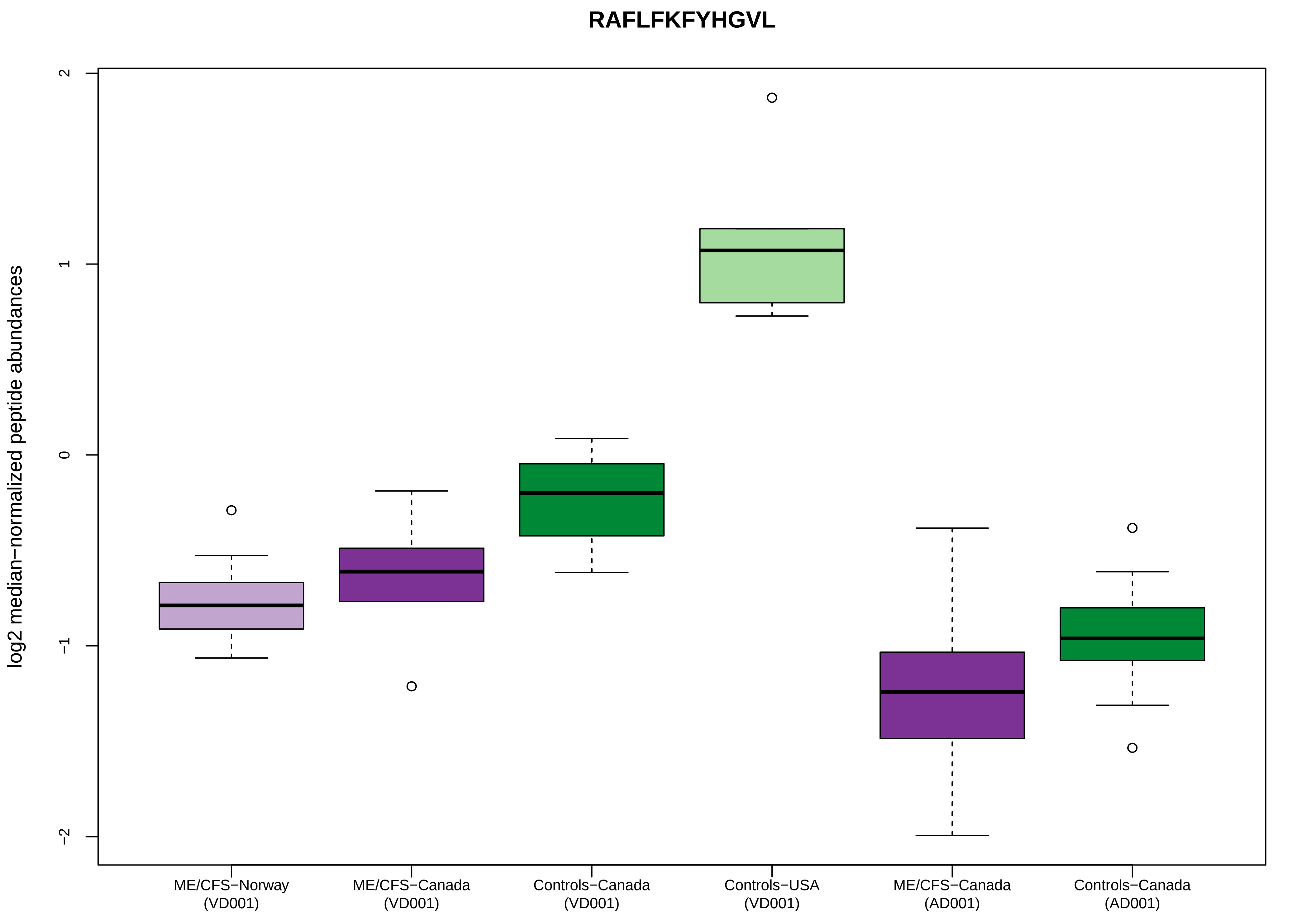

RAFLFKFYHGVL
log2 median−normalized peptide abundances
2
1
0
−1
−2
ME/CFS−Norway (VD001)
ME/CFS−Canada (VD001)
Controls−Canada (VD001)
Controls−USA (VD001)
ME/CFS−Canada (AD001)
Controls−Canada (AD001)

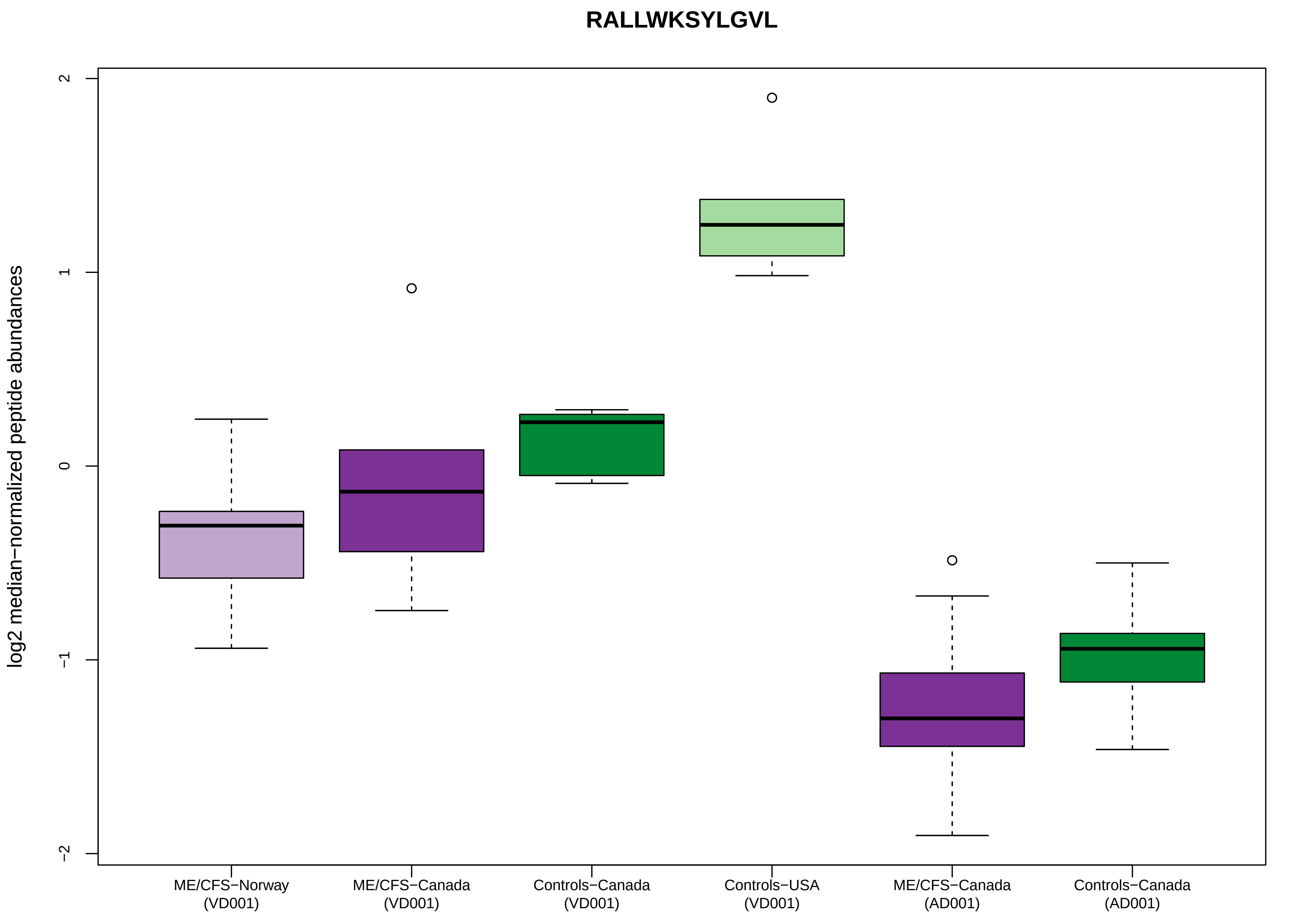
RALLWKSYLGVL
log2 median−normalized peptide abundances
2
1
0
−1
−2
ME/CFS−Norway (VD001)
ME/CFS−Canada (VD001)
Controls−Canada (VD001)
Controls−USA (VD001)
ME/CFS−Canada (AD001)
Controls−Canada (AD001)

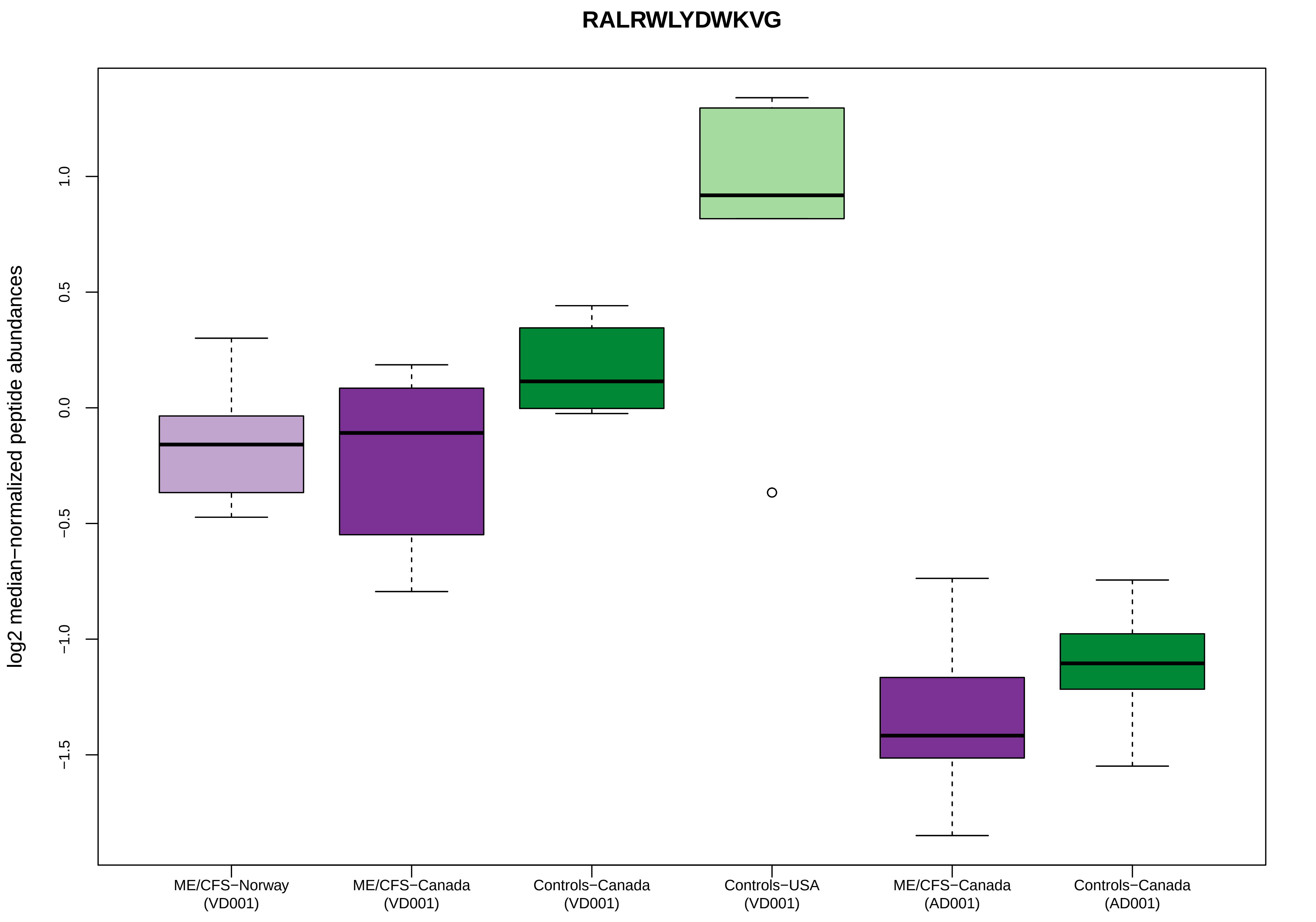

RALRWLYDWKVG
log2 median−normalized peptide abundances
1.0
0.5
0.0
−0.5
−1.0
−1.5
ME/CFS−Norway (VD001)
ME/CFS−Canada (VD001)
Controls−Canada (VD001)
Controls−USA (VD001)
ME/CFS−Canada (AD001)
Controls−Canada (AD001)

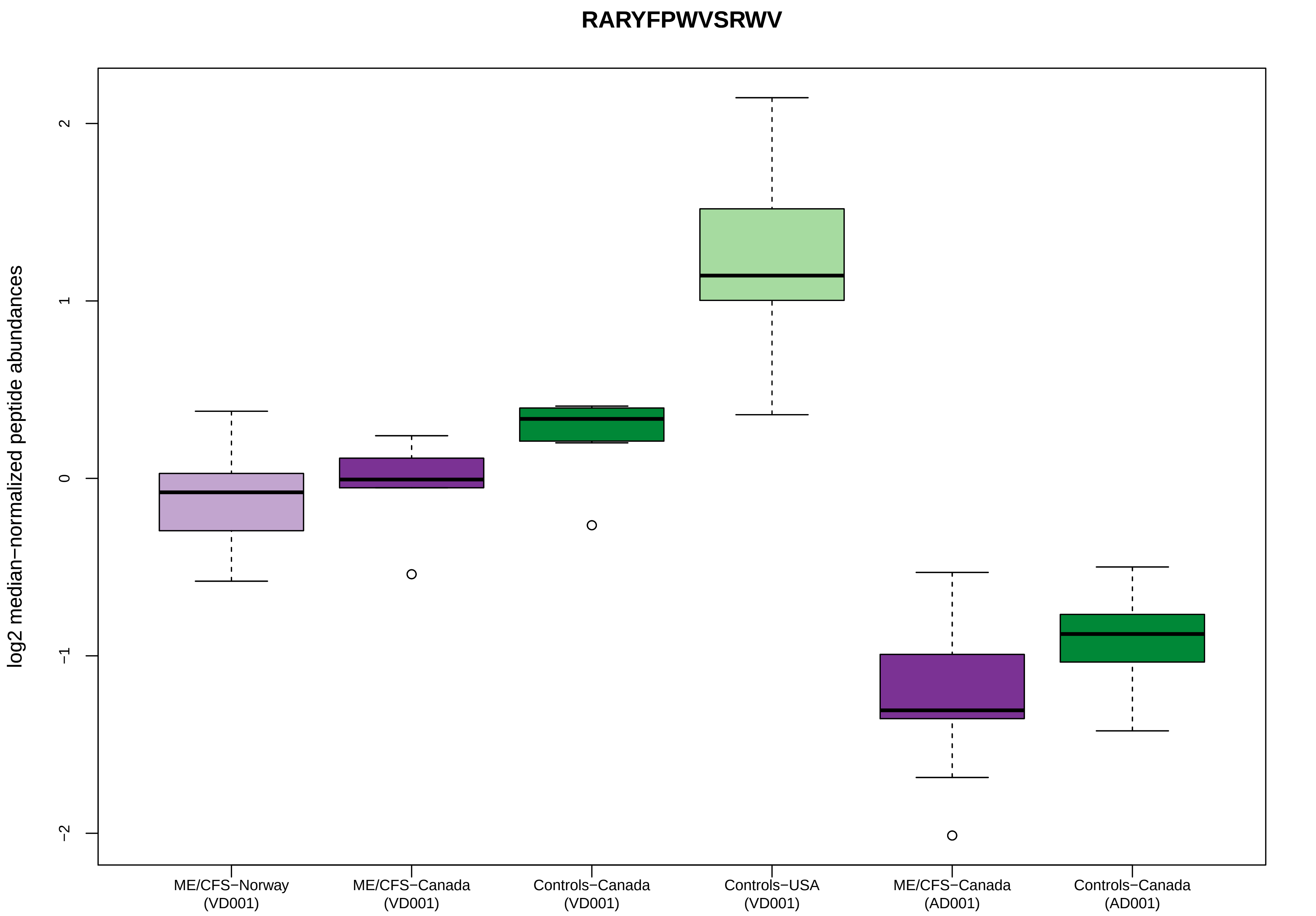
RARYFPWVSRWV
log2 median−normalized peptide abundances
2
1
0
−1
−2
ME/CFS−Norway (VD001)
ME/CFS−Canada (VD001)
Controls−Canada (VD001)
Controls−USA (VD001)
ME/CFS−Canada (AD001)
Controls−Canada (AD001)

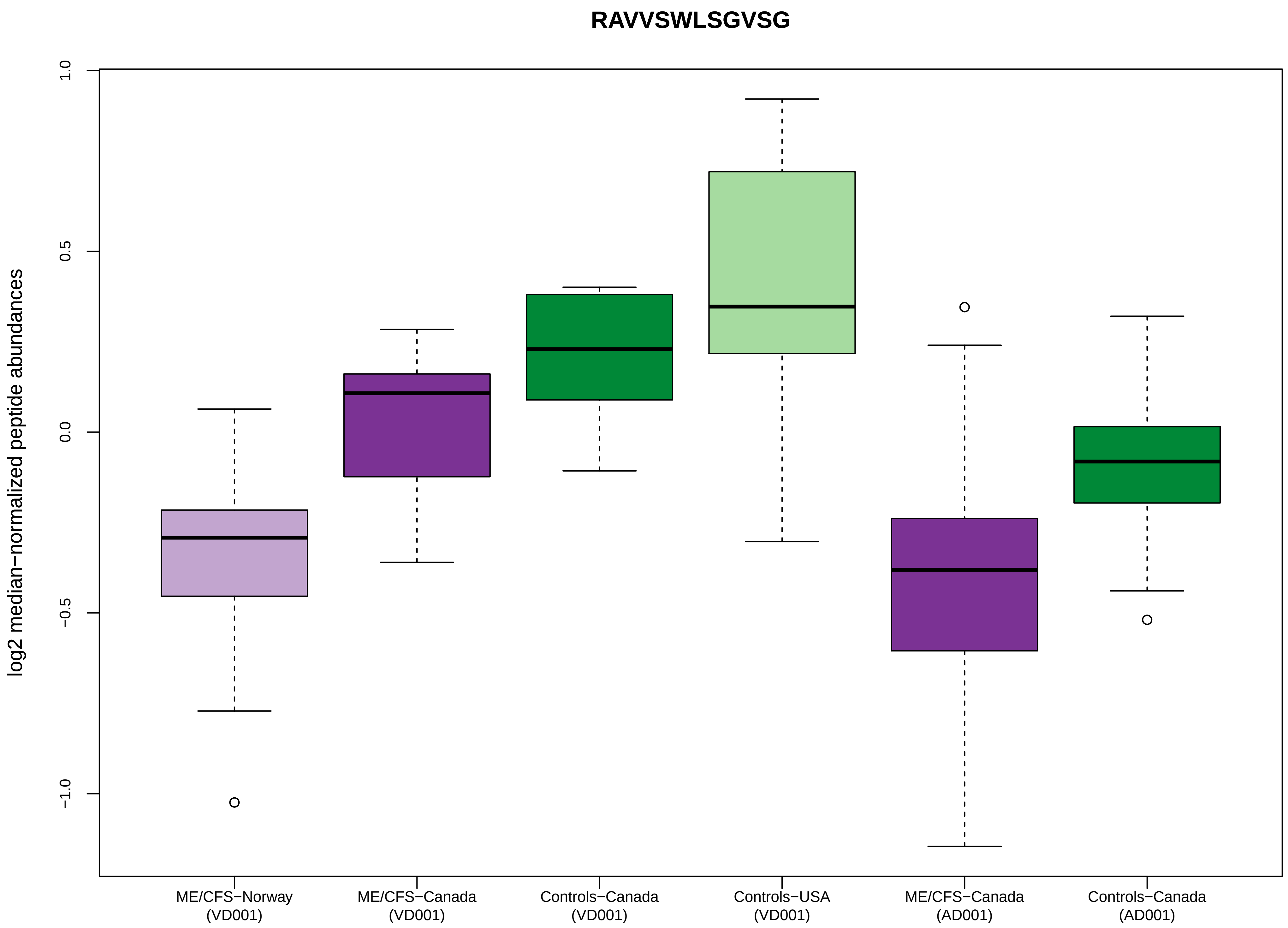

RAVVSWLSGVSG
log2 median−normalized peptide abundances
1.0
0.5
0.0
−0.5
−1.0
ME/CFS−Norway (VD001)
ME/CFS−Canada (VD001)
Controls−Canada (VD001)
Controls−USA (VD001)
ME/CFS−Canada (AD001)
Controls−Canada (AD001)

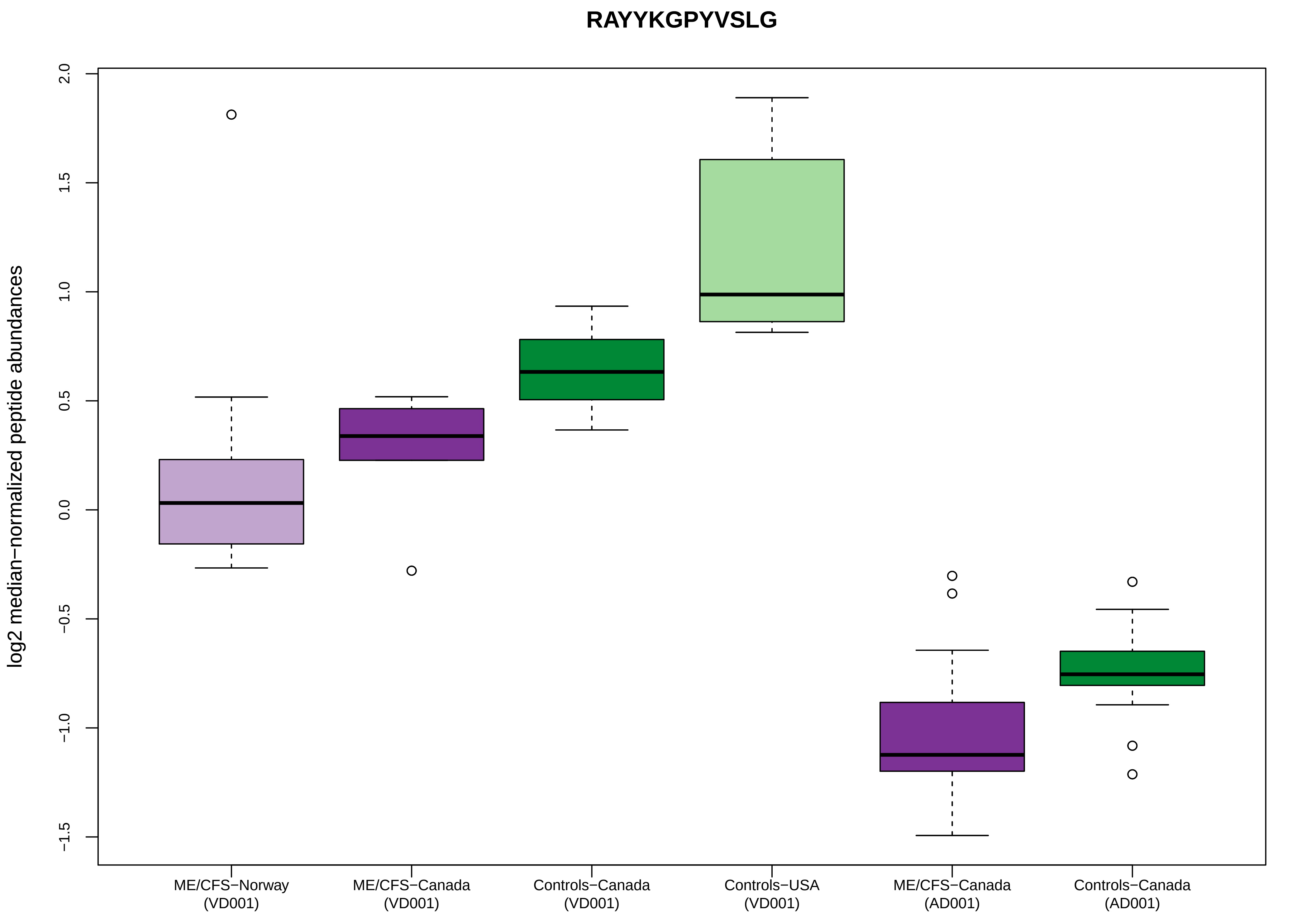

RAYYKGPYVSLG
log2 median−normalized peptide abundances
2.0
1.5
1.0
0.5
0.0
−0.5
−1.0
−1.5
ME/CFS−Norway (VD001)
ME/CFS−Canada (VD001)
Controls−Canada (VD001)
Controls−USA (VD001)
ME/CFS−Canada (AD001)
Controls−Canada (AD001)

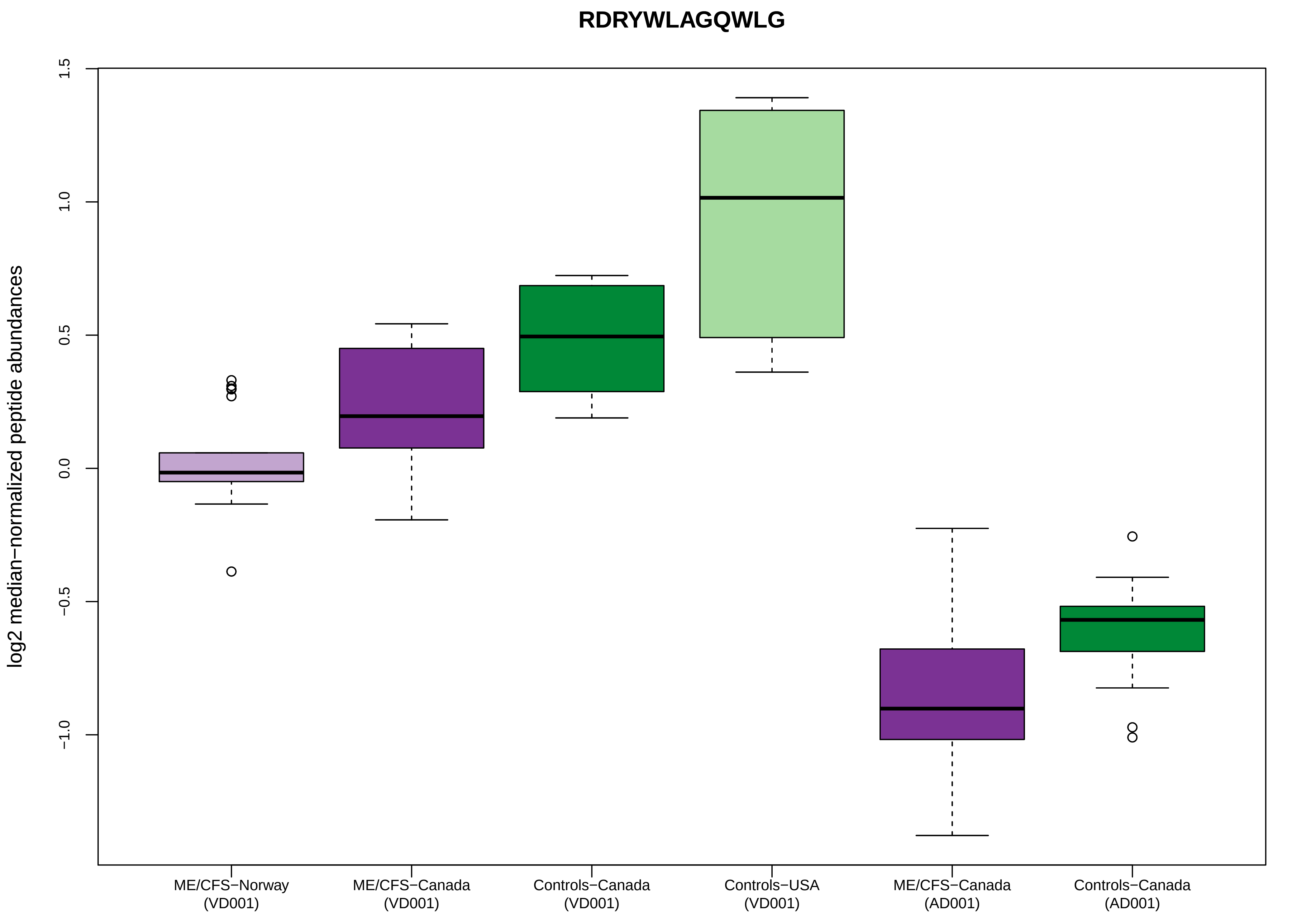

RDRYWLAGQWLG
log2 median−normalized peptide abundances
1.5
1.0
0.5
0.0
−0.5
−1.0
ME/CFS−Norway (VD001)
ME/CFS−Canada (VD001)
Controls−Canada (VD001)
Controls−USA (VD001)
ME/CFS−Canada (AD001)
Controls−Canada (AD001)

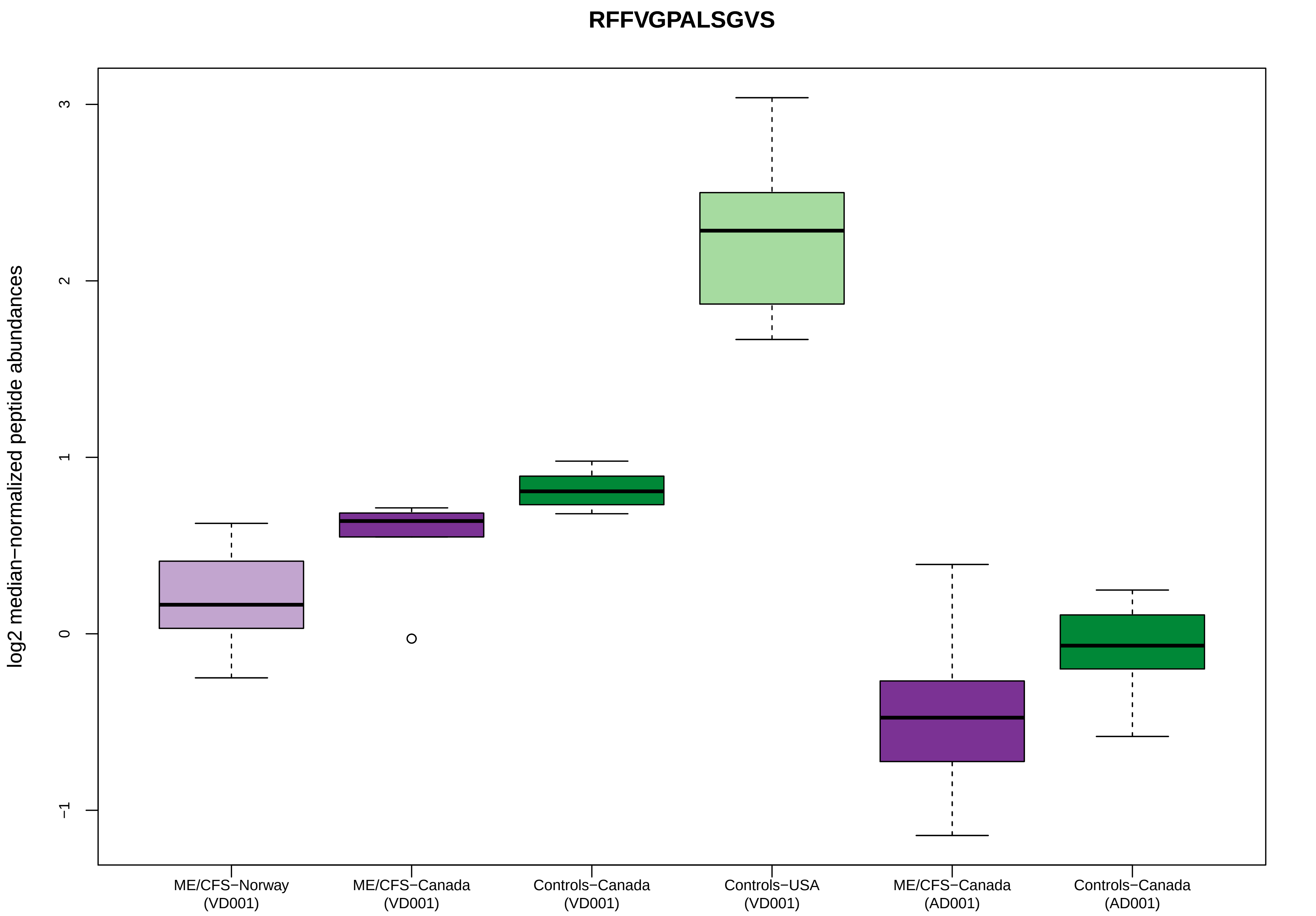
RFFVGPALSGVS
log2 median−normalized peptide abundances
3
2
1
0
−1
ME/CFS−Norway (VD001)
ME/CFS−Canada (VD001)
Controls−Canada (VD001)
Controls−USA (VD001)
ME/CFS−Canada (AD001)
Controls−Canada (AD001)

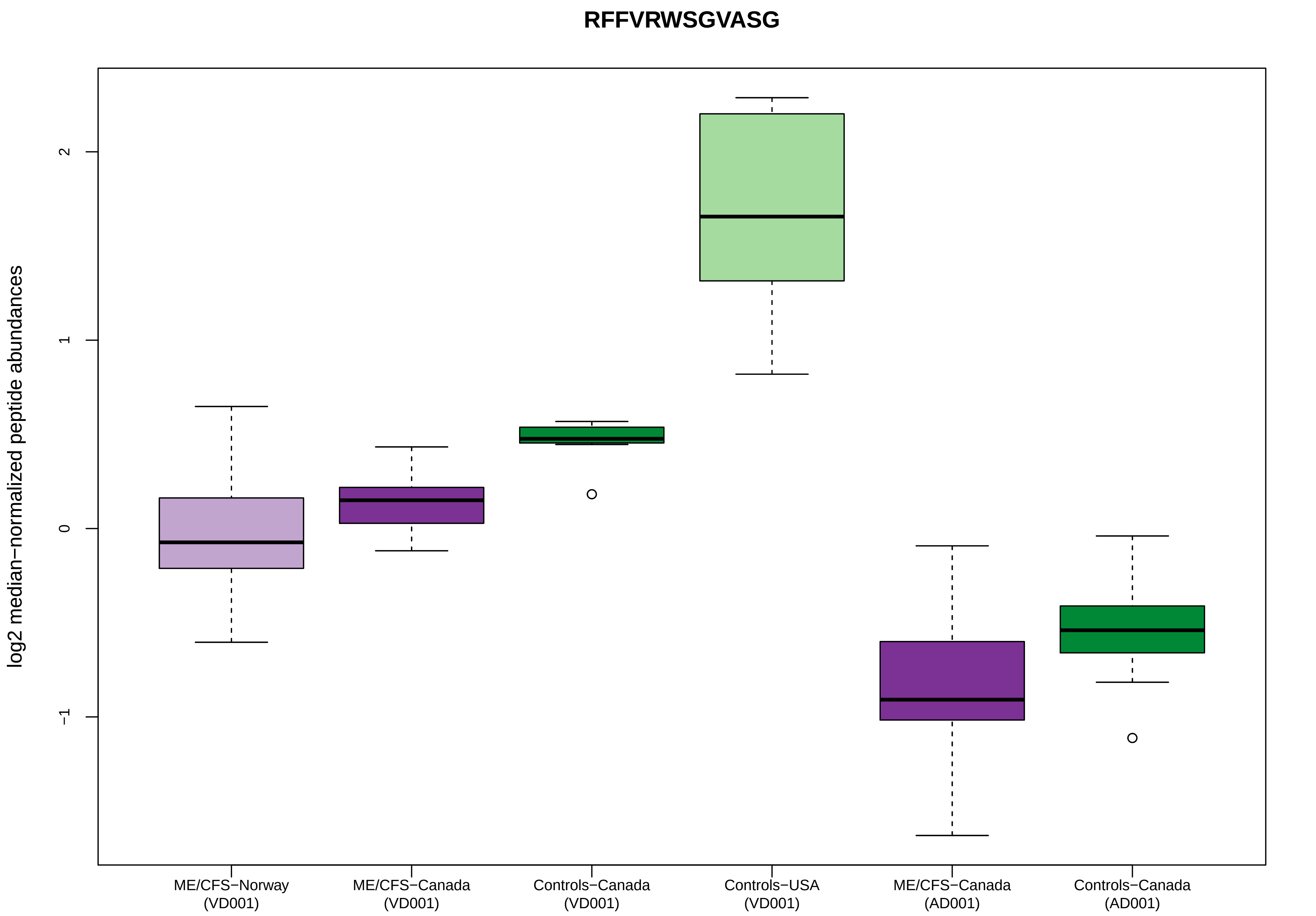

RFFVRWSGVASG
log2 median−normalized peptide abundances
2
1
0
−1
ME/CFS−Norway (VD001)
ME/CFS−Canada (VD001)
Controls−Canada (VD001)
Controls−USA (VD001)
ME/CFS−Canada (AD001)
Controls−Canada (AD001)

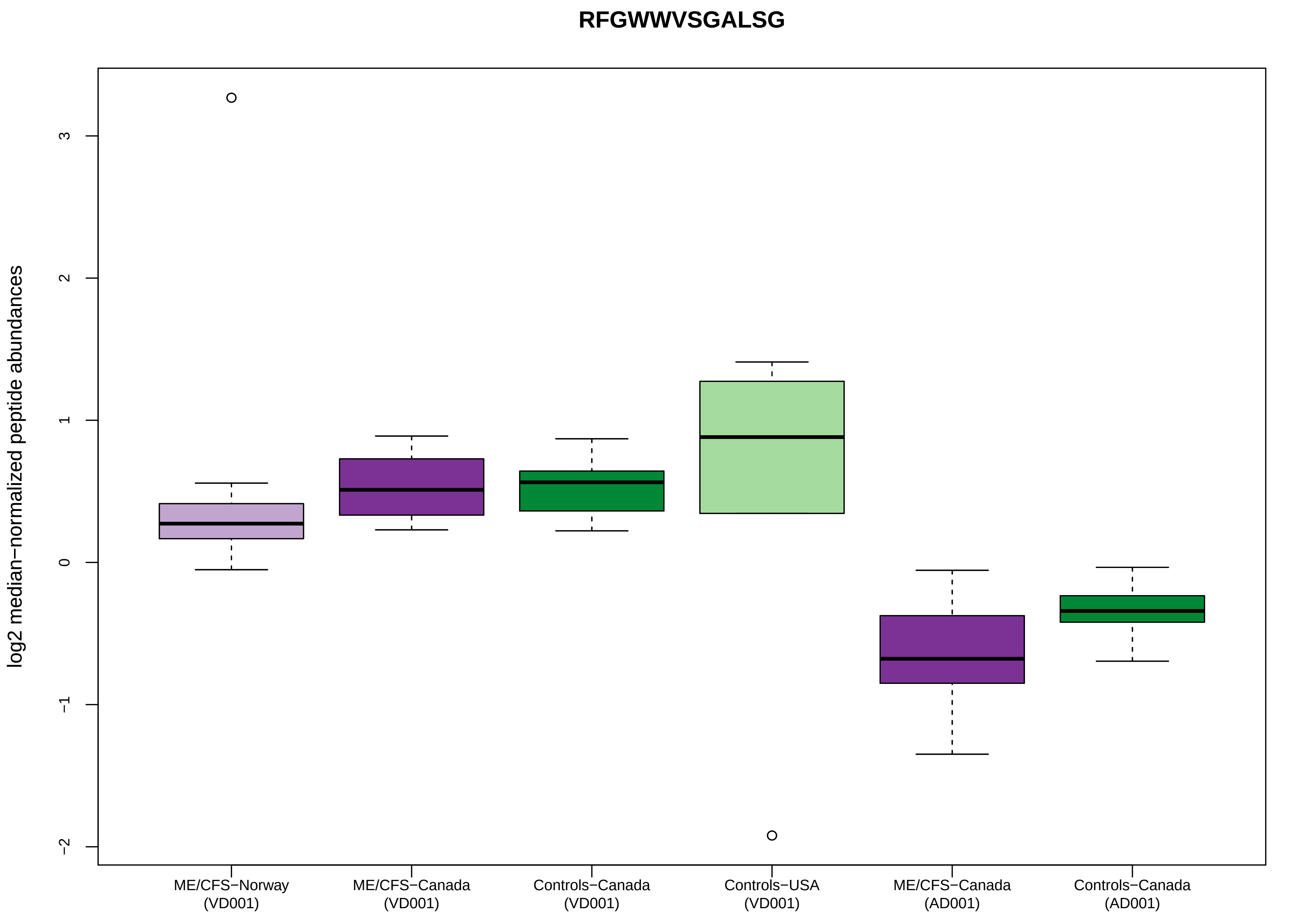
RFGWWVSGALSG
log2 median−normalized peptide abundances
3
2
1
0
−1
−2
ME/CFS−Norway (VD001)
ME/CFS−Canada (VD001)
Controls−Canada (VD001)
Controls−USA (VD001)
ME/CFS−Canada (AD001)
Controls−Canada (AD001)

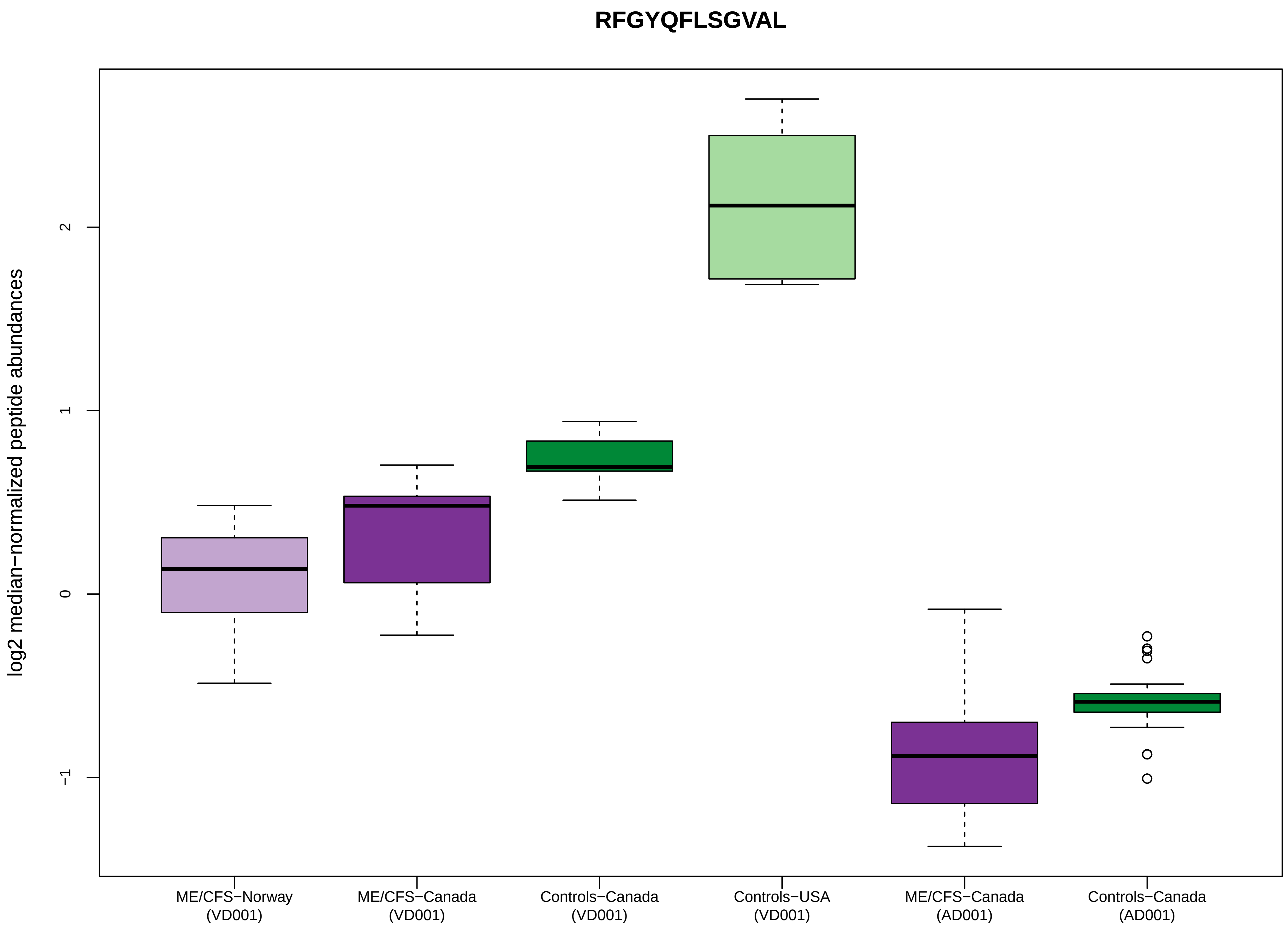

RFGYQFLSGVAL
log2 median−normalized peptide abundances
2
1
0
−1
ME/CFS−Norway (VD001)
ME/CFS−Canada (VD001)
Controls−Canada (VD001)
Controls−USA (VD001)
ME/CFS−Canada (AD001)
Controls−Canada (AD001)

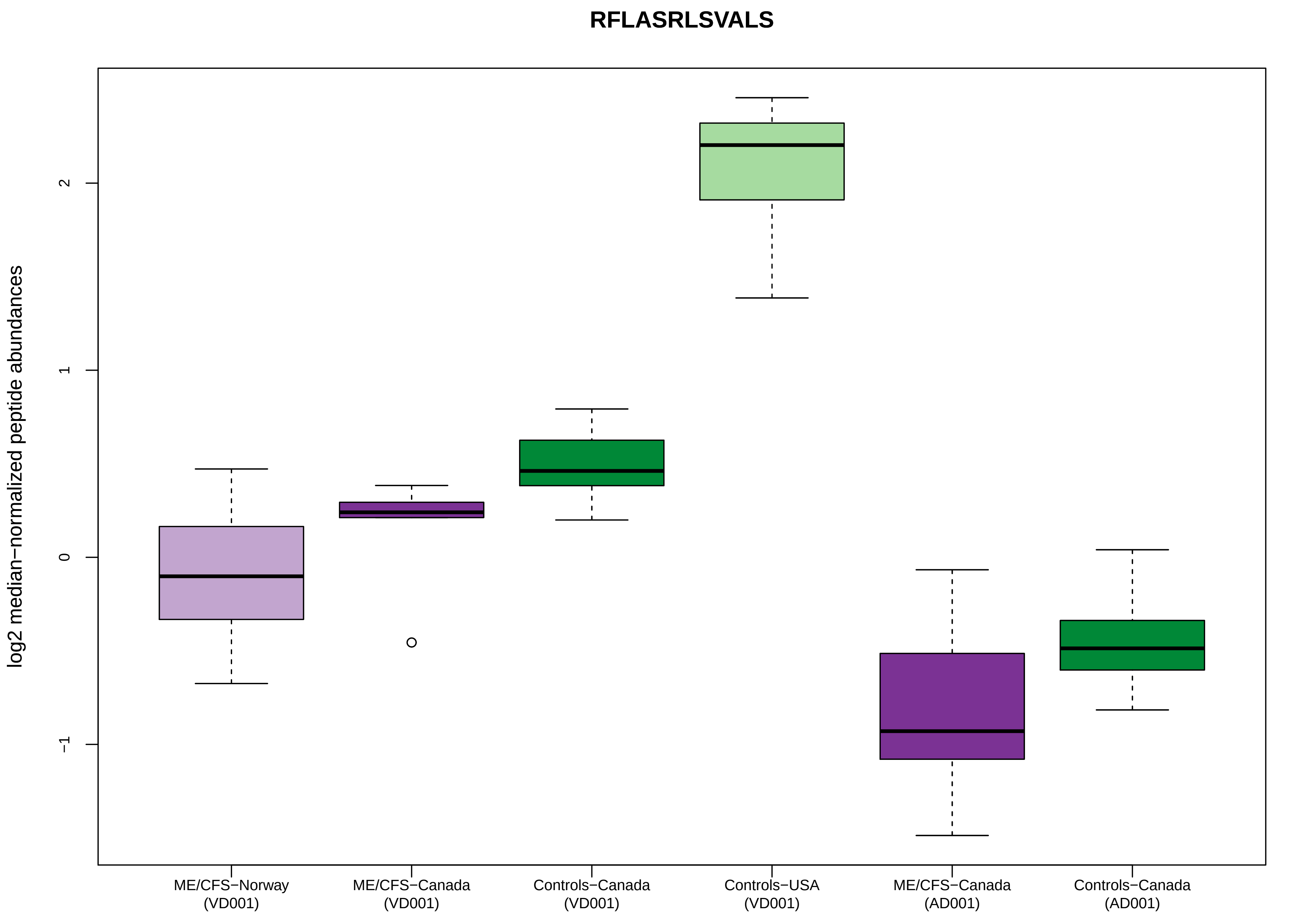
RFLASRLSVALS
log2 median−normalized peptide abundances
2
1
0
−1
ME/CFS−Norway (VD001)
ME/CFS−Canada (VD001)
Controls−Canada (VD001)
Controls−USA (VD001)
ME/CFS−Canada (AD001)
Controls−Canada (AD001)

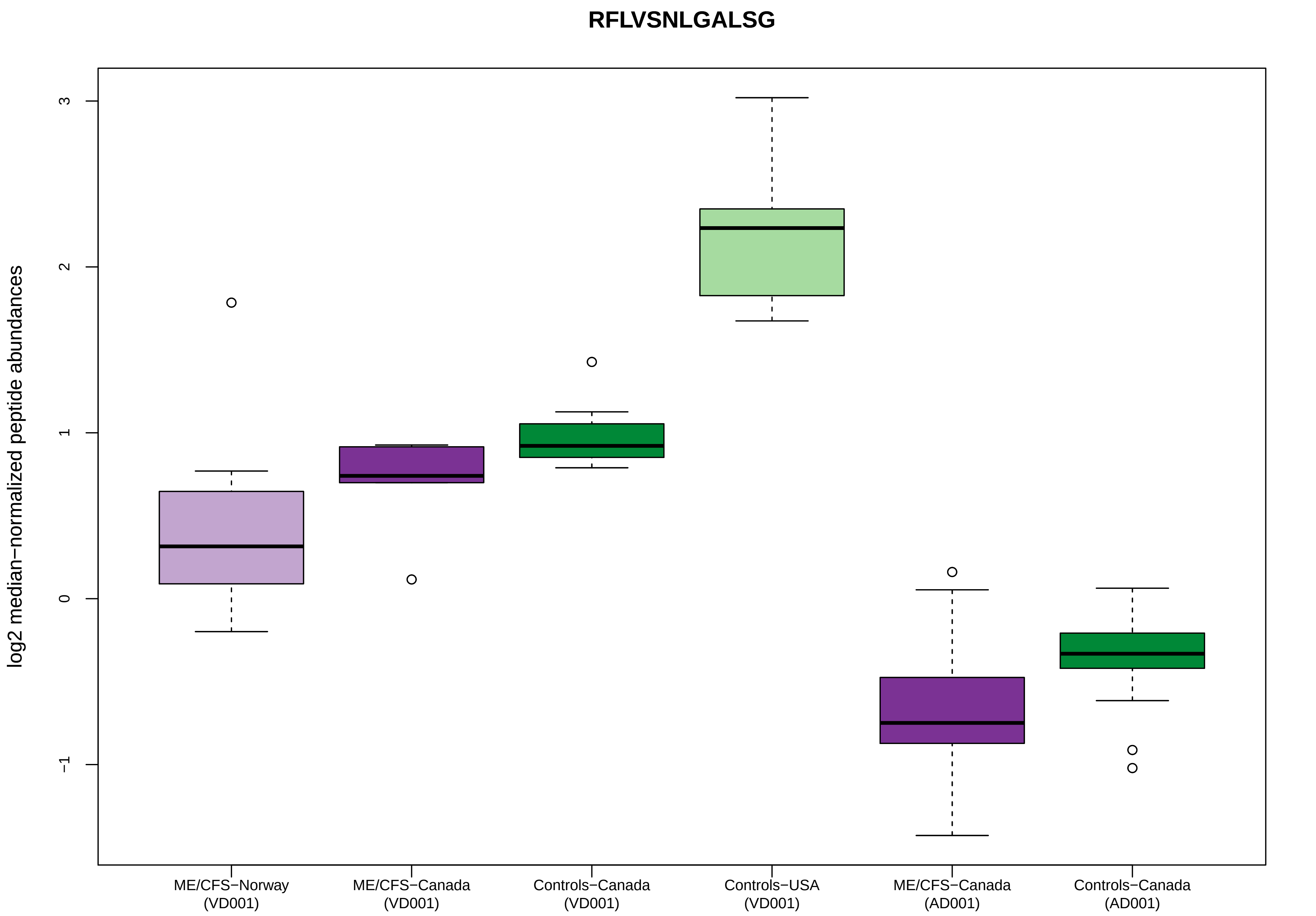
RFLVSNLGALSG
log2 median−normalized peptide abundances
3
2
1
0
−1
ME/CFS−Norway (VD001)
ME/CFS−Canada (VD001)
Controls−Canada (VD001)
Controls−USA (VD001)
ME/CFS−Canada (AD001)
Controls−Canada (AD001)

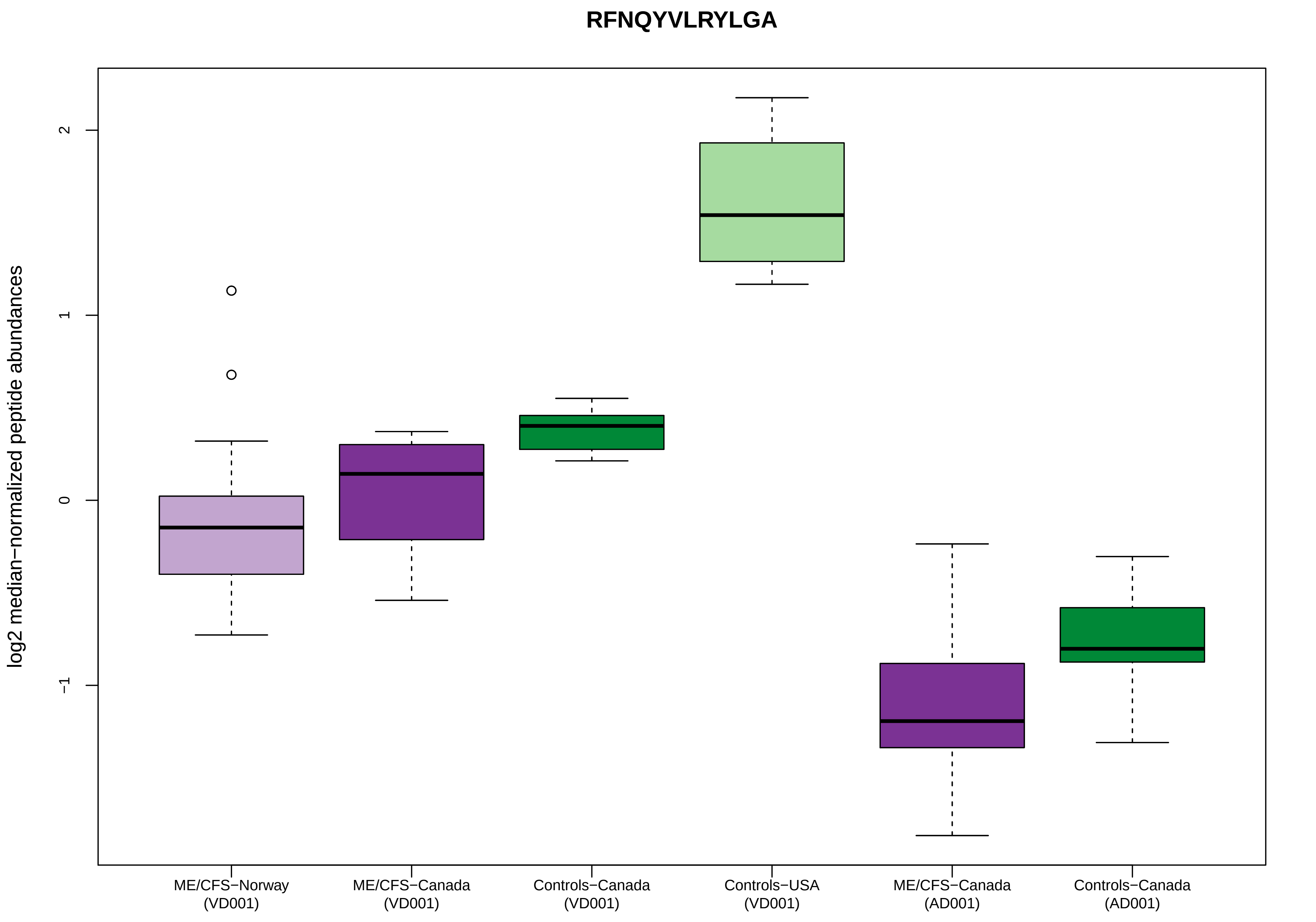

RFNQYVLRYLGA
log2 median−normalized peptide abundances
2
1
0
−1
ME/CFS−Norway (VD001)
ME/CFS−Canada (VD001)
Controls−Canada (VD001)
Controls−USA (VD001)
ME/CFS−Canada (AD001)
Controls−Canada (AD001)

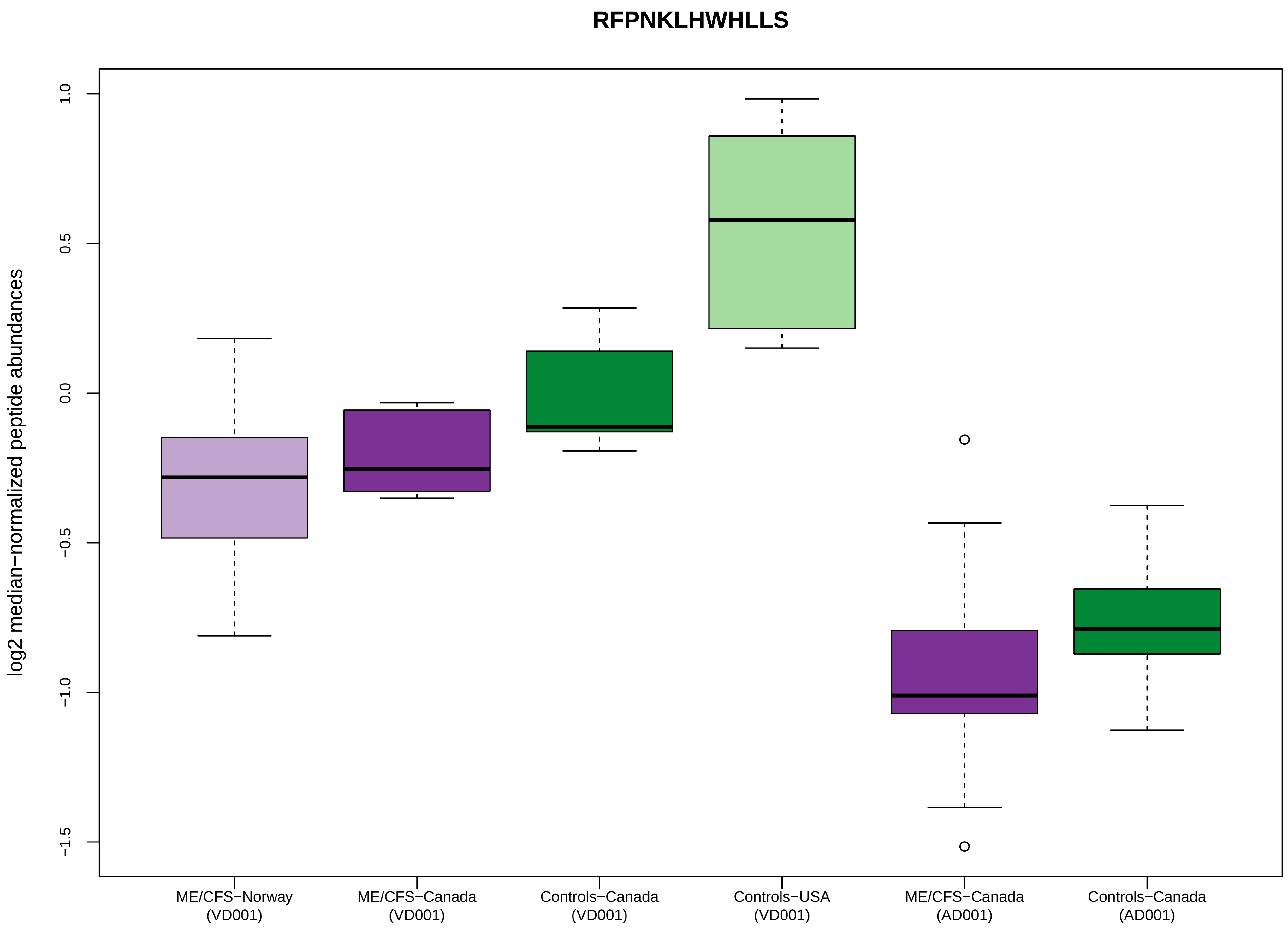

RFPNKLHWHLLS
log2 median−normalized peptide abundances
1.0
0.5
0.0
−0.5
−1.0
−1.5
ME/CFS−Norway (VD001)
ME/CFS−Canada (VD001)
Controls−Canada (VD001)
Controls−USA (VD001)
ME/CFS−Canada (AD001)
Controls−Canada (AD001)

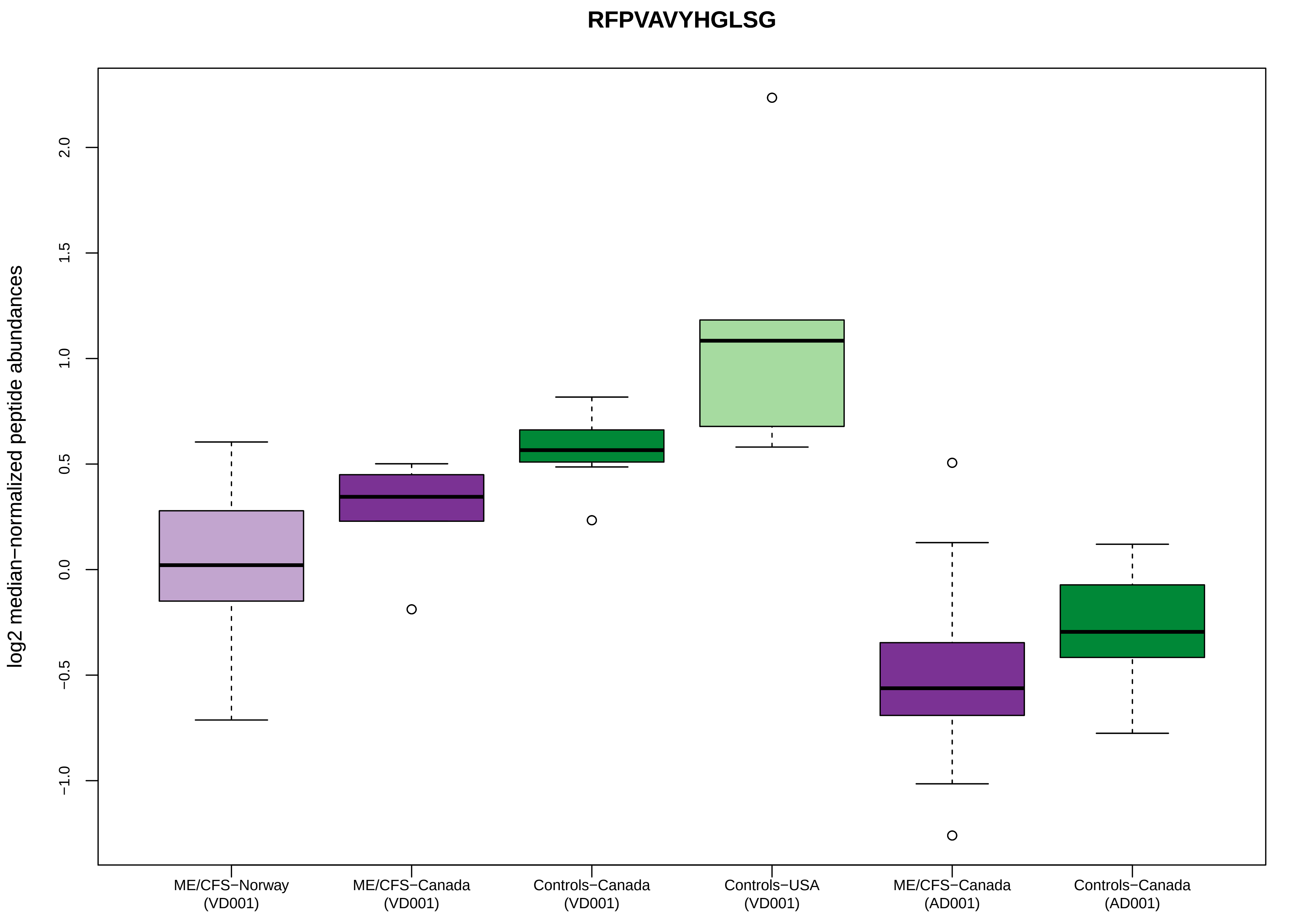

RFPVAVYHGLSG
log2 median−normalized peptide abundances
2.0
1.5
1.0
0.5
0.0
−0.5
−1.0
ME/CFS−Norway (VD001)
ME/CFS−Canada (VD001)
Controls−Canada (VD001)
Controls−USA (VD001)
ME/CFS−Canada (AD001)
Controls−Canada (AD001)

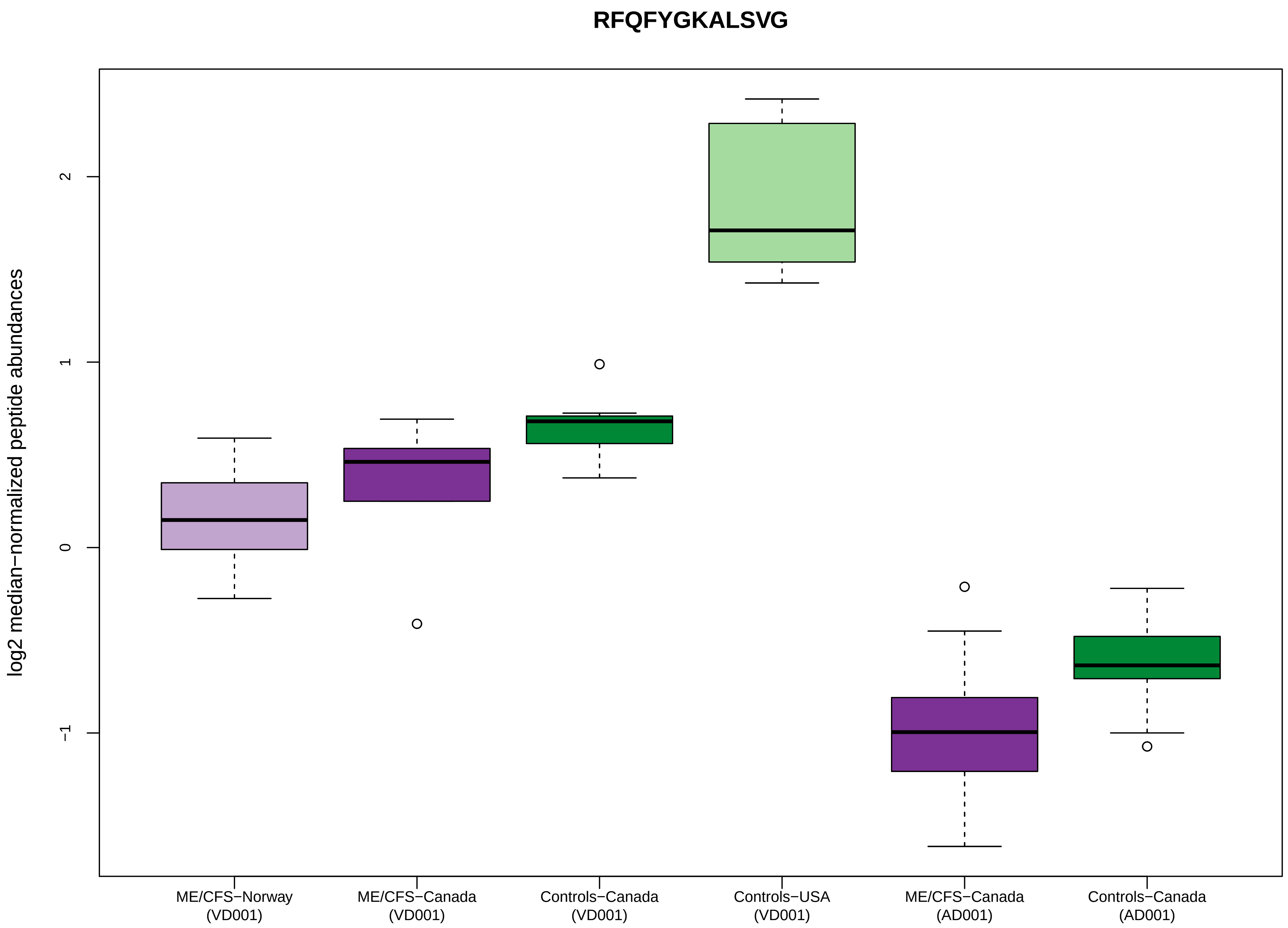
RFQFYGKALSVG
log2 median−normalized peptide abundances
2
1
0
−1
ME/CFS−Norway (VD001)
ME/CFS−Canada (VD001)
Controls−Canada (VD001)
Controls−USA (VD001)
ME/CFS−Canada (AD001)
Controls−Canada (AD001)

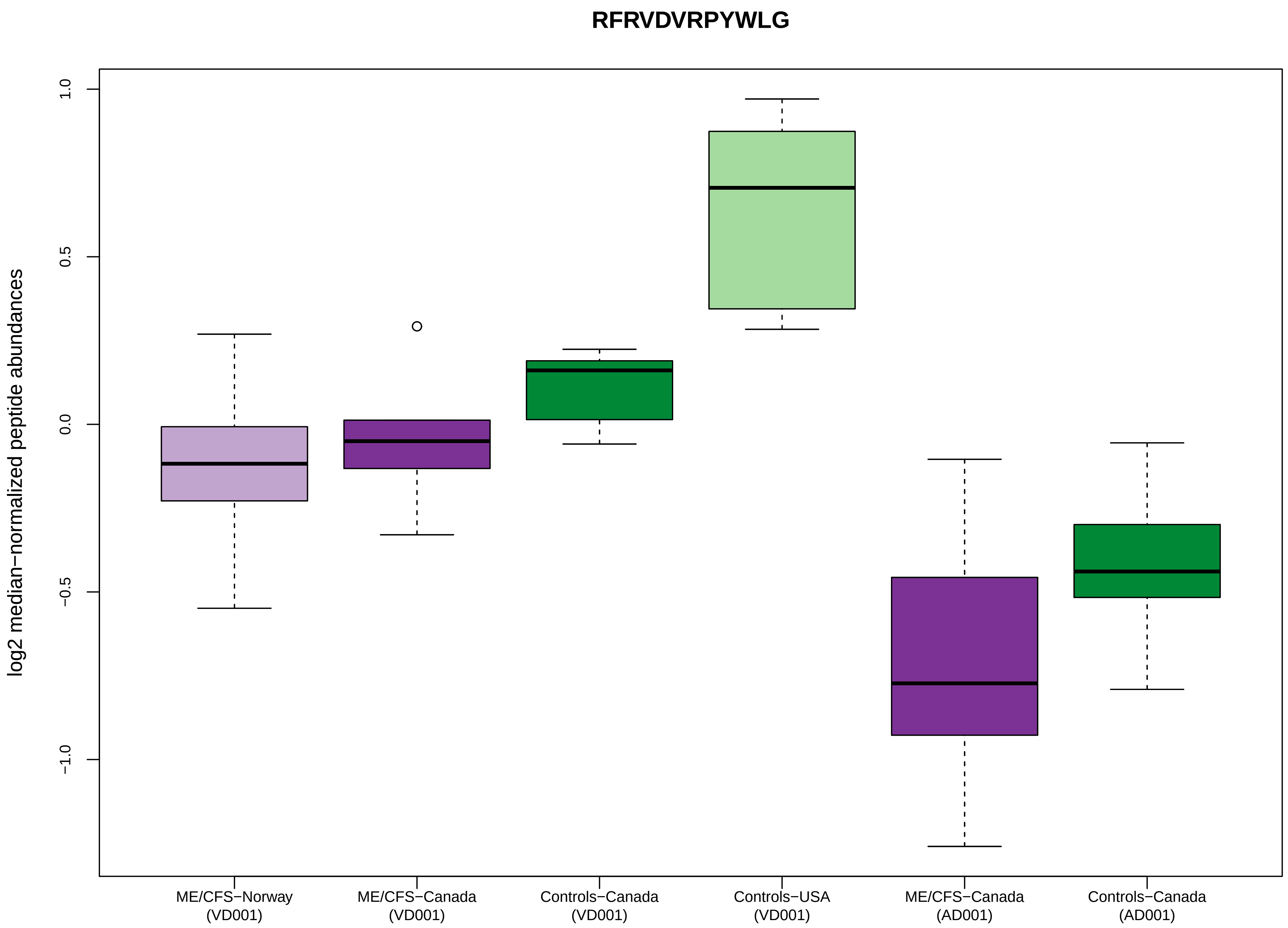

RFRVDVRPYWLG
log2 median−normalized peptide abundances
1.0
0.5
0.0
−0.5
−1.0
ME/CFS−Norway (VD001)
ME/CFS−Canada (VD001)
Controls−Canada (VD001)
Controls−USA (VD001)
ME/CFS−Canada (AD001)
Controls−Canada (AD001)

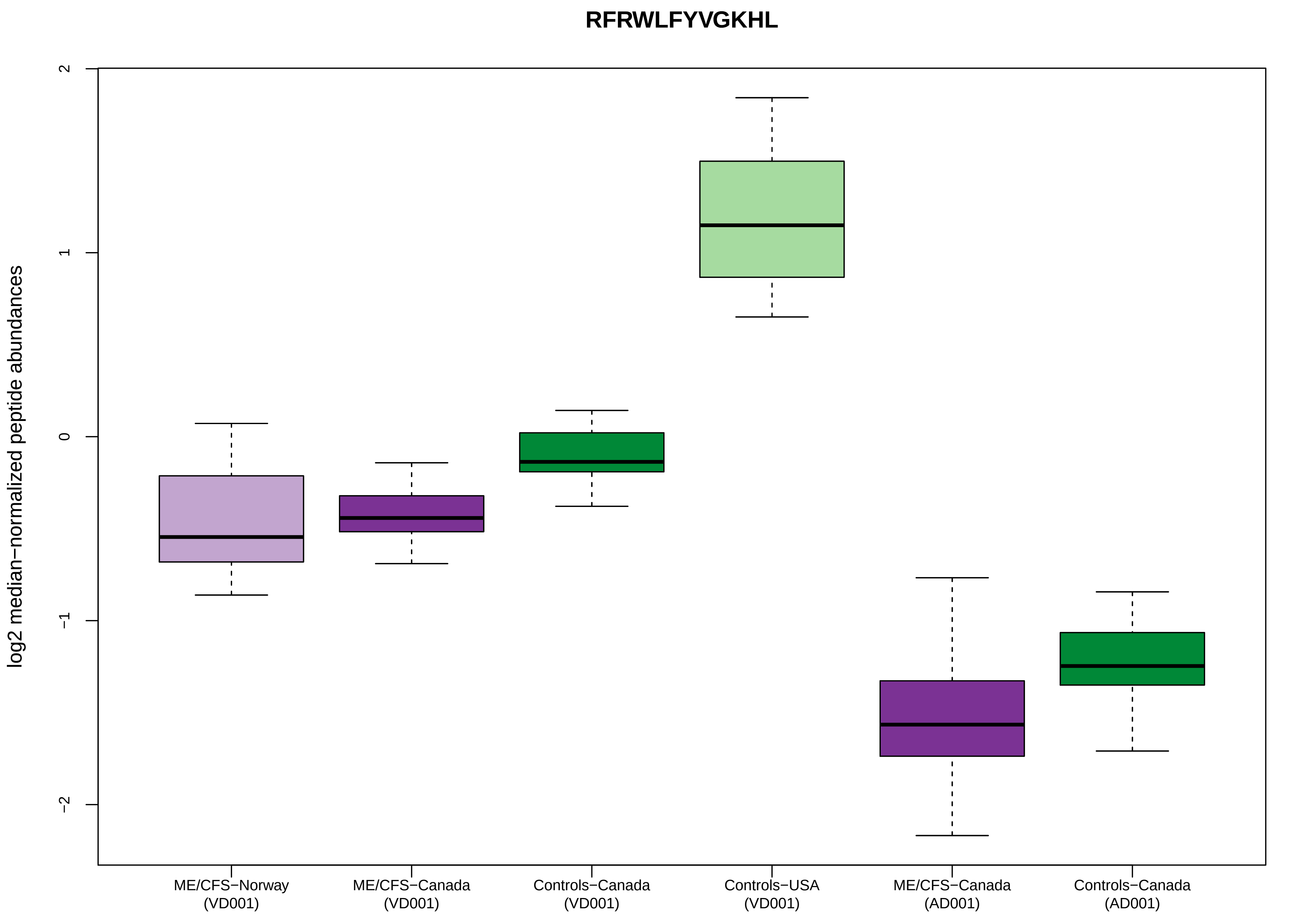
RFRWLFYVGKHL
log2 median−normalized peptide abundances
2
1
0
−1
−2
ME/CFS−Norway
(VD001)
ME/CFS−Canada
(VD001)
Controls−Canada
(VD001)
Controls−USA
(VD001)
ME/CFS−Canada
(AD001)
Controls−Canada
(AD001)

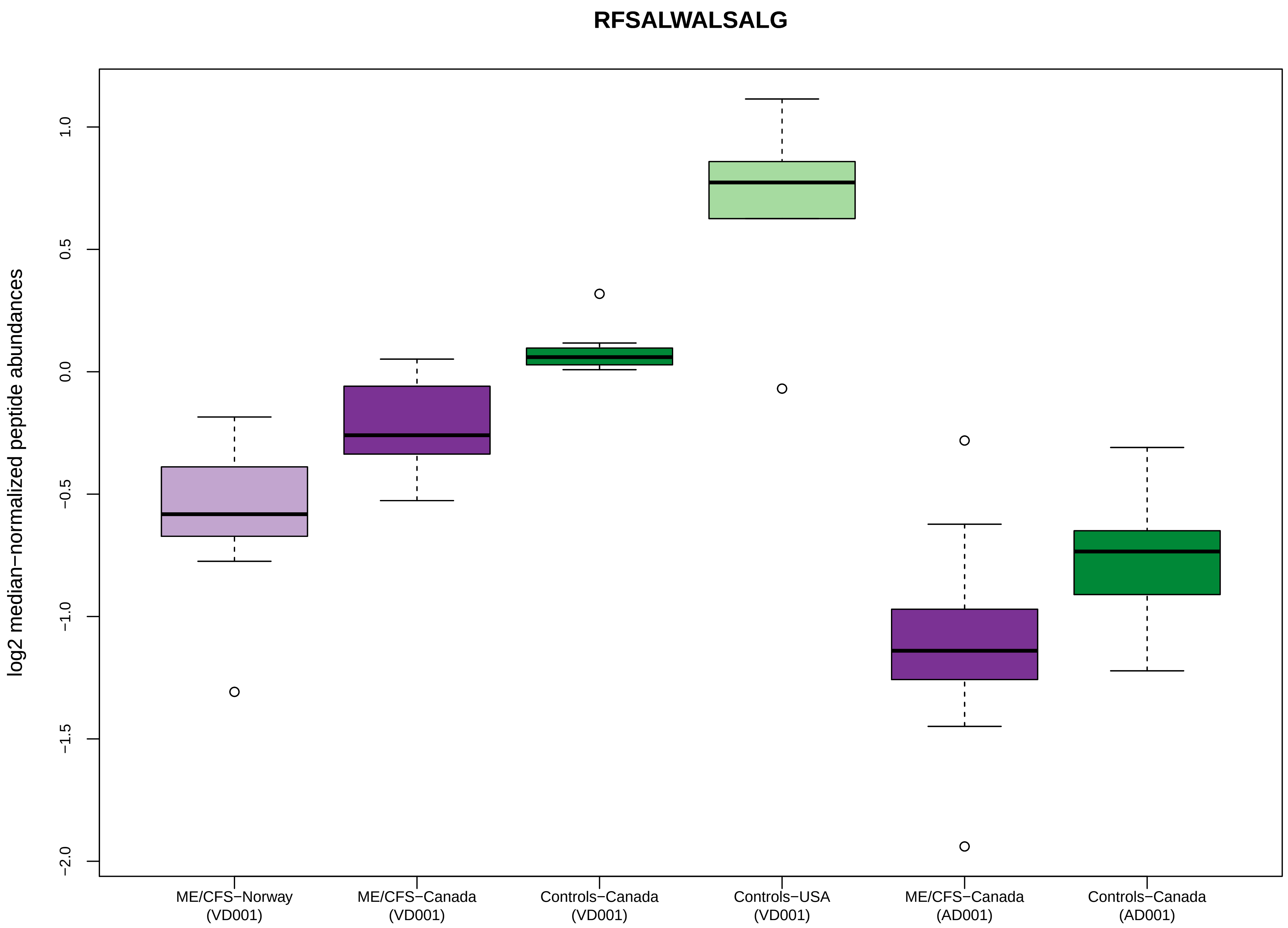

RFSALWALSALG
log2 median−normalized peptide abundances
1.0
0.5
0.0
−0.5
−1.0
−1.5
−2.0
ME/CFS−Norway (VD001)
ME/CFS−Canada (VD001)
Controls−Canada (VD001)
Controls−USA (VD001)
ME/CFS−Canada (AD001)
Controls−Canada (AD001)

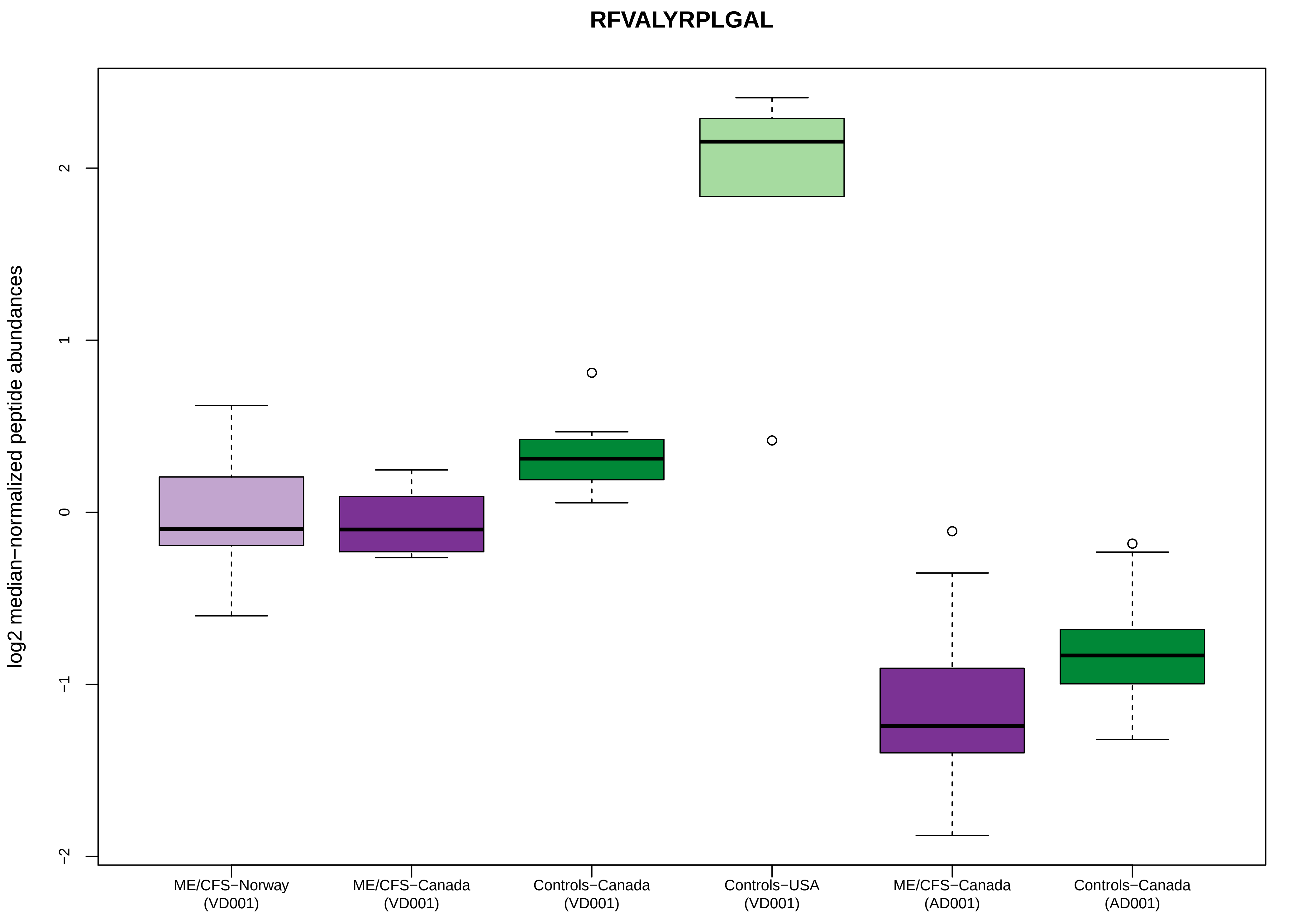
RFVALYRPLGAL
log2 median−normalized peptide abundances
2
1
0
−1
−2
ME/CFS−Norway (VD001)
ME/CFS−Canada (VD001)
Controls−Canada (VD001)
Controls−USA (VD001)
ME/CFS−Canada (AD001)
Controls−Canada (AD001)

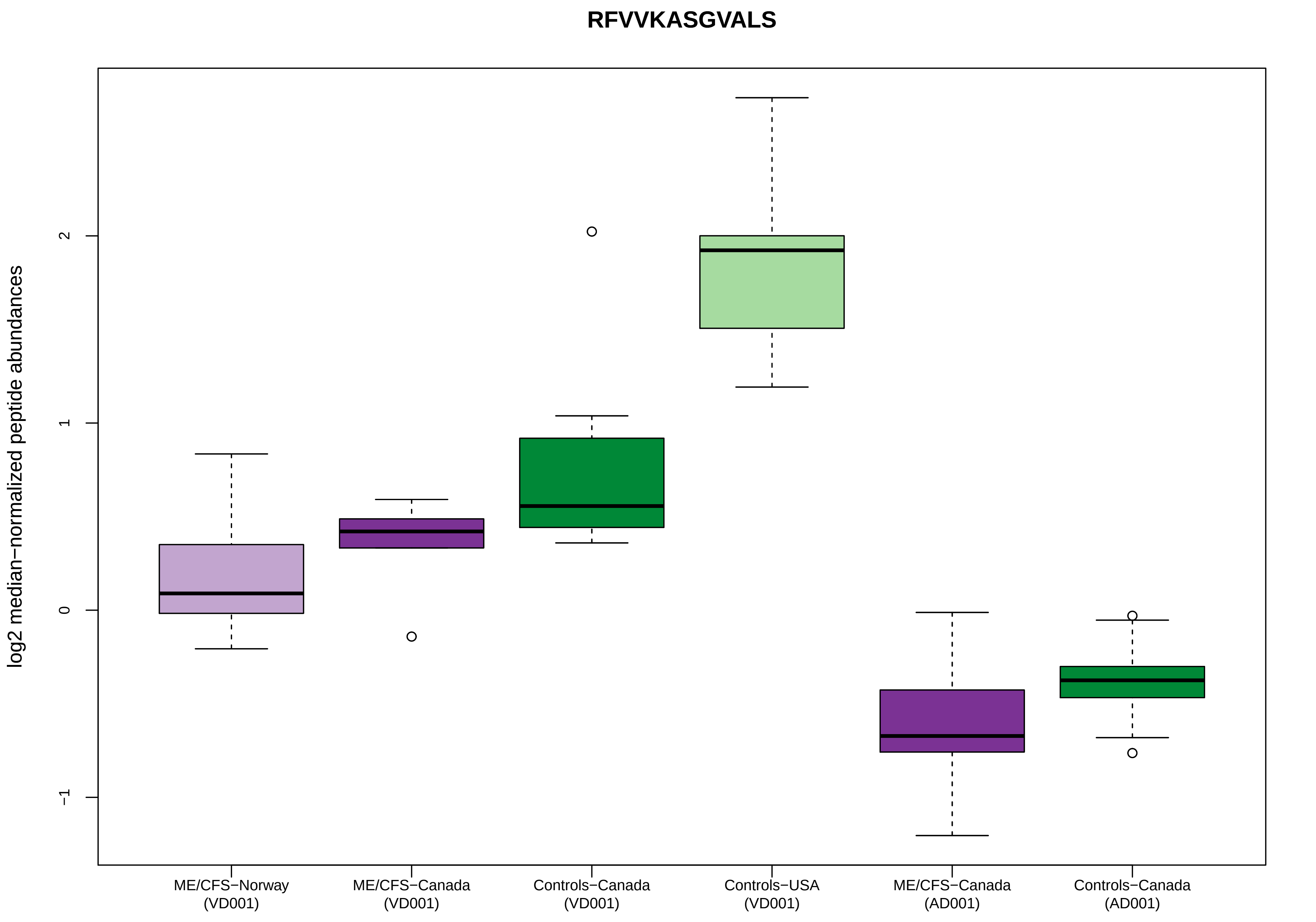
RFVVKASGVALS
log2 median−normalized peptide abundances
2
1
0
−1
ME/CFS−Norway (VD001)
ME/CFS−Canada (VD001)
Controls−Canada (VD001)
Controls−USA (VD001)
ME/CFS−Canada (AD001)
Controls−Canada (AD001)

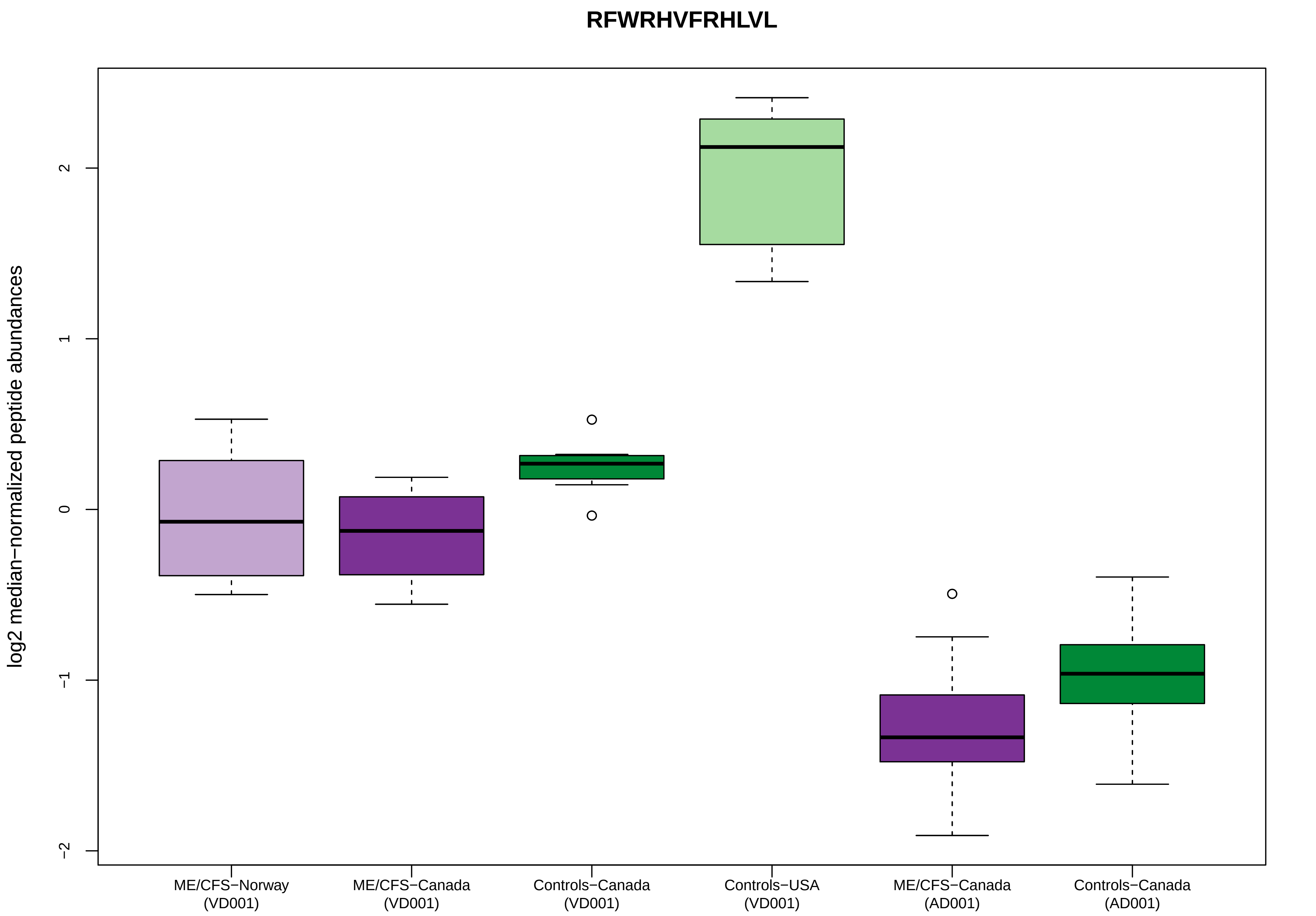

RFWRHVFRHLVL
log2 median−normalized peptide abundances
2
1
0
−1
−2
ME/CFS−Norway (VD001)
ME/CFS−Canada (VD001)
Controls−Canada (VD001)
Controls−USA (VD001)
ME/CFS−Canada (AD001)
Controls−Canada (AD001)

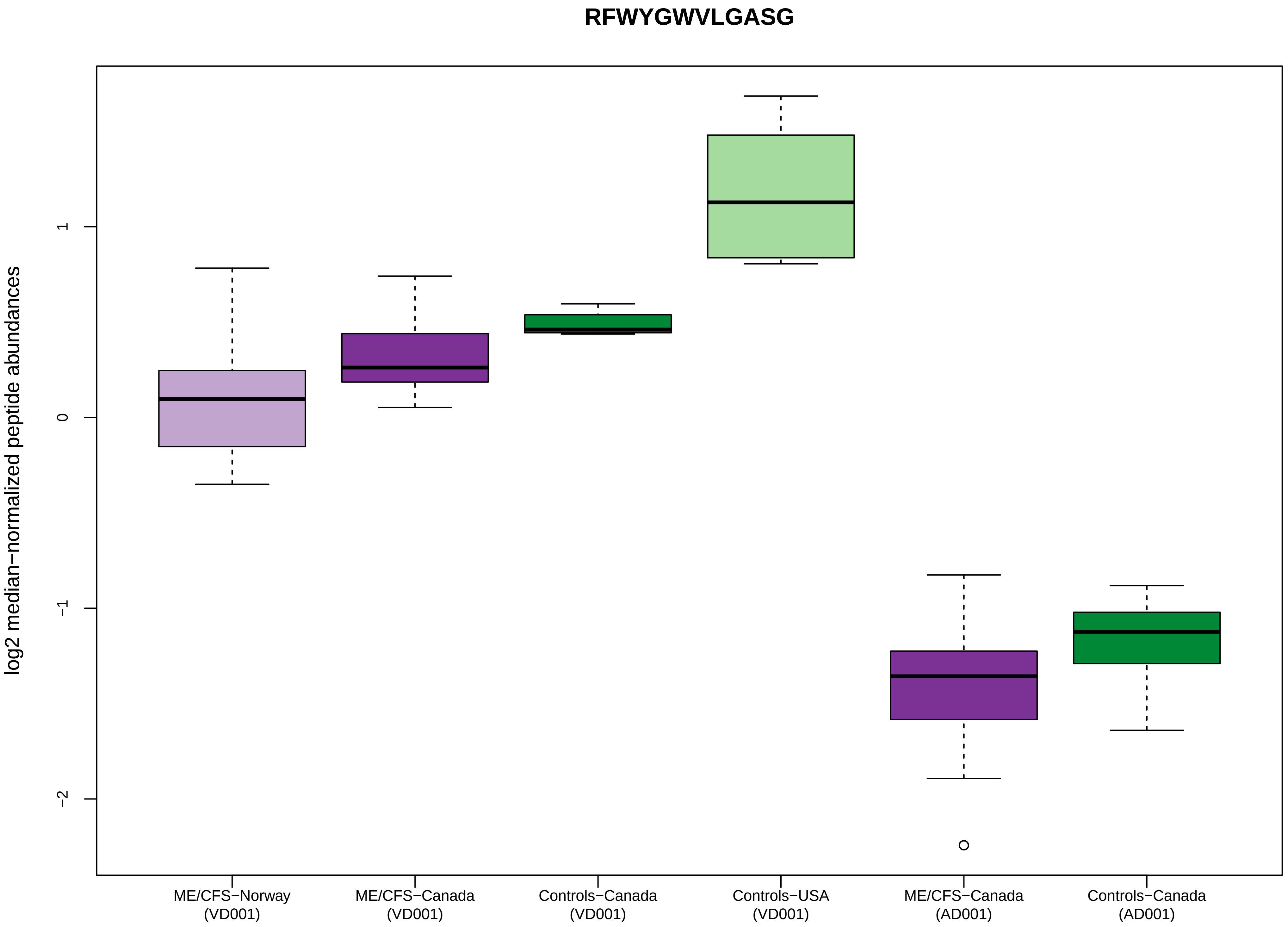

RFWYGWVLGASG
log2 median−normalized peptide abundances
1
0
−1
−2
ME/CFS−Norway (VD001)
ME/CFS−Canada (VD001)
Controls−Canada (VD001)
Controls−USA (VD001)
ME/CFS−Canada (AD001)
Controls−Canada (AD001)

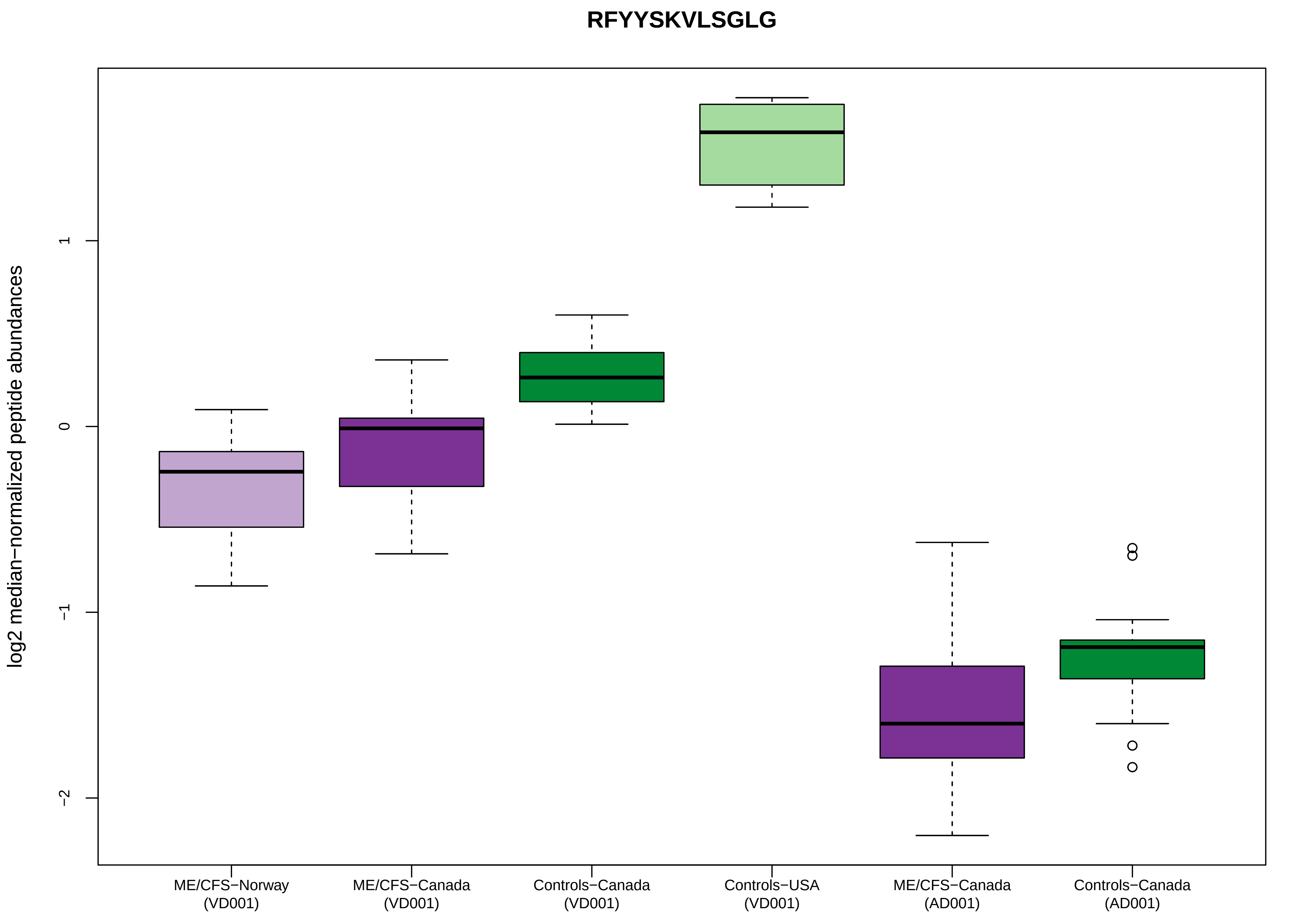

RFYYSKVLSGLG
log2 median−normalized peptide abundances
1
0
−1
−2
ME/CFS−Norway (VD001)
ME/CFS−Canada (VD001)
Controls−Canada (VD001)
Controls−USA (VD001)
ME/CFS−Canada (AD001)
Controls−Canada (AD001)

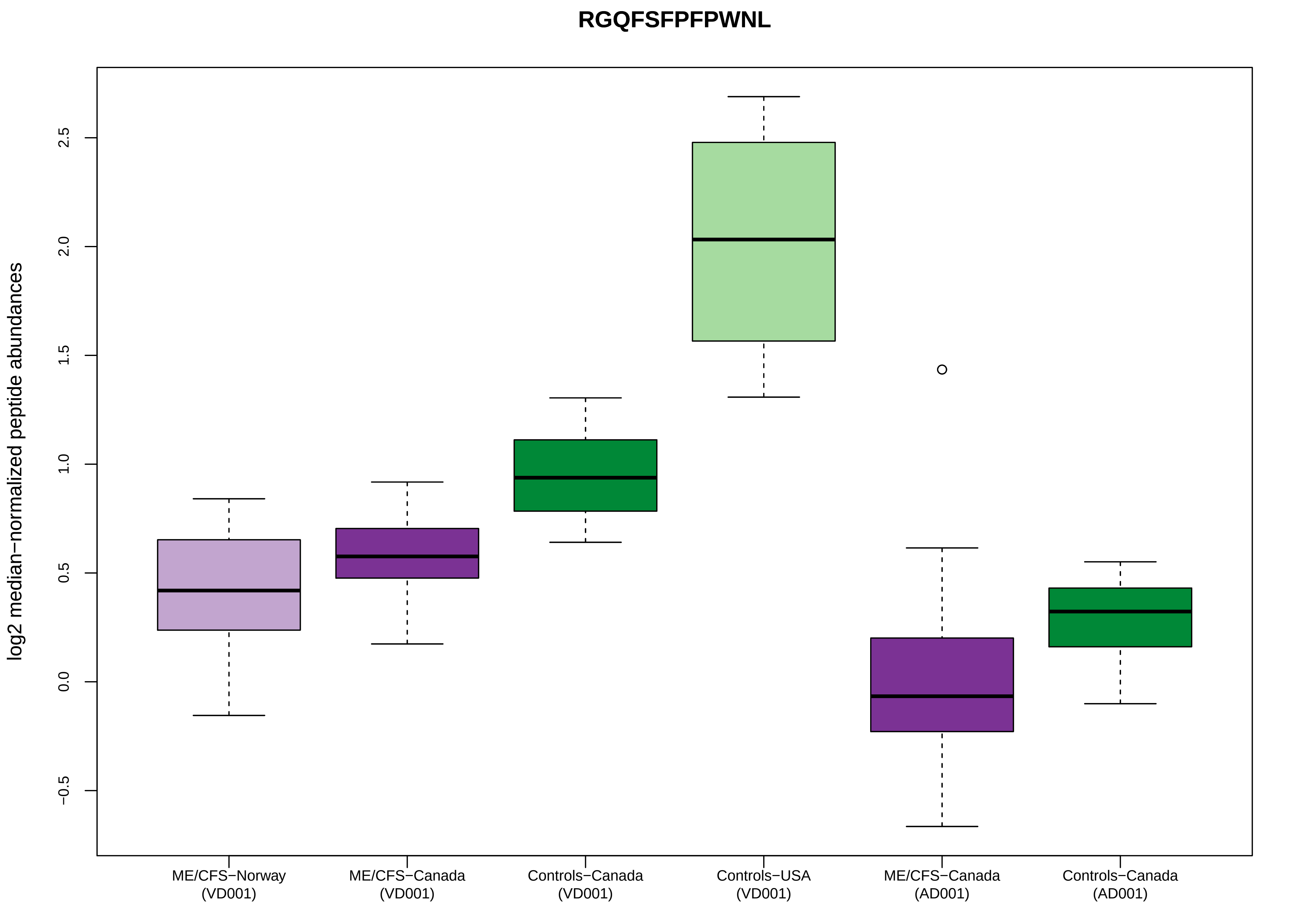
RGQFSFPFPWNL
log2 median−normalized peptide abundances
2.5
2.0
1.5
1.0
0.5
0.0
−0.5
ME/CFS−Norway (VD001)
ME/CFS−Canada (VD001)
Controls−Canada (VD001)
Controls−USA (VD001)
ME/CFS−Canada (AD001)
Controls−Canada (AD001)

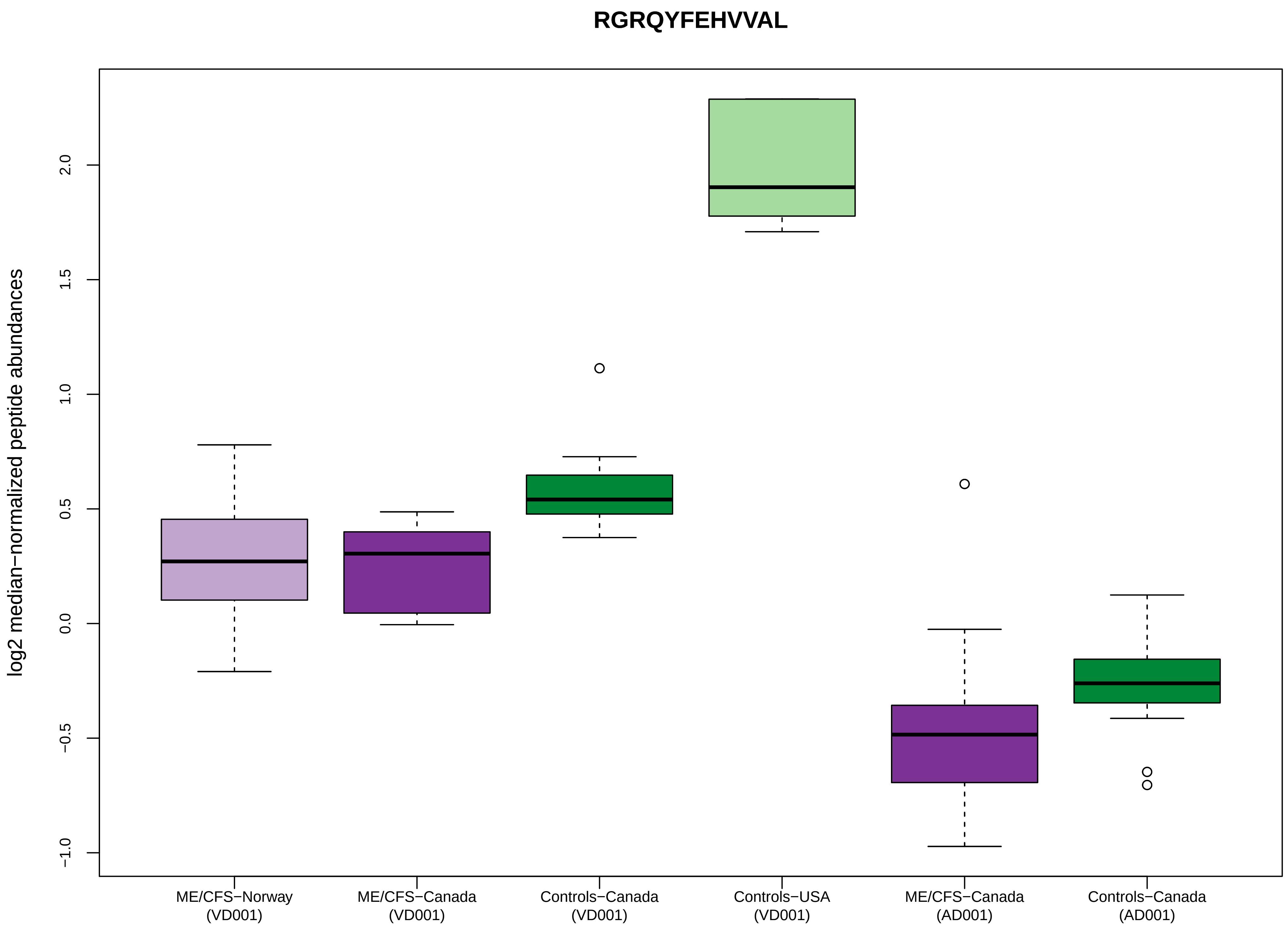
RGRQYFEHVVAL
log2 median−normalized peptide abundances
−1.0
−0.5
0.0
0.5
1.0
1.5
2.0
ME/CFS−Norway (VD001)
ME/CFS−Canada (VD001)
Controls−Canada (VD001)
Controls−USA (VD001)
ME/CFS−Canada (AD001)
Controls−Canada (AD001)

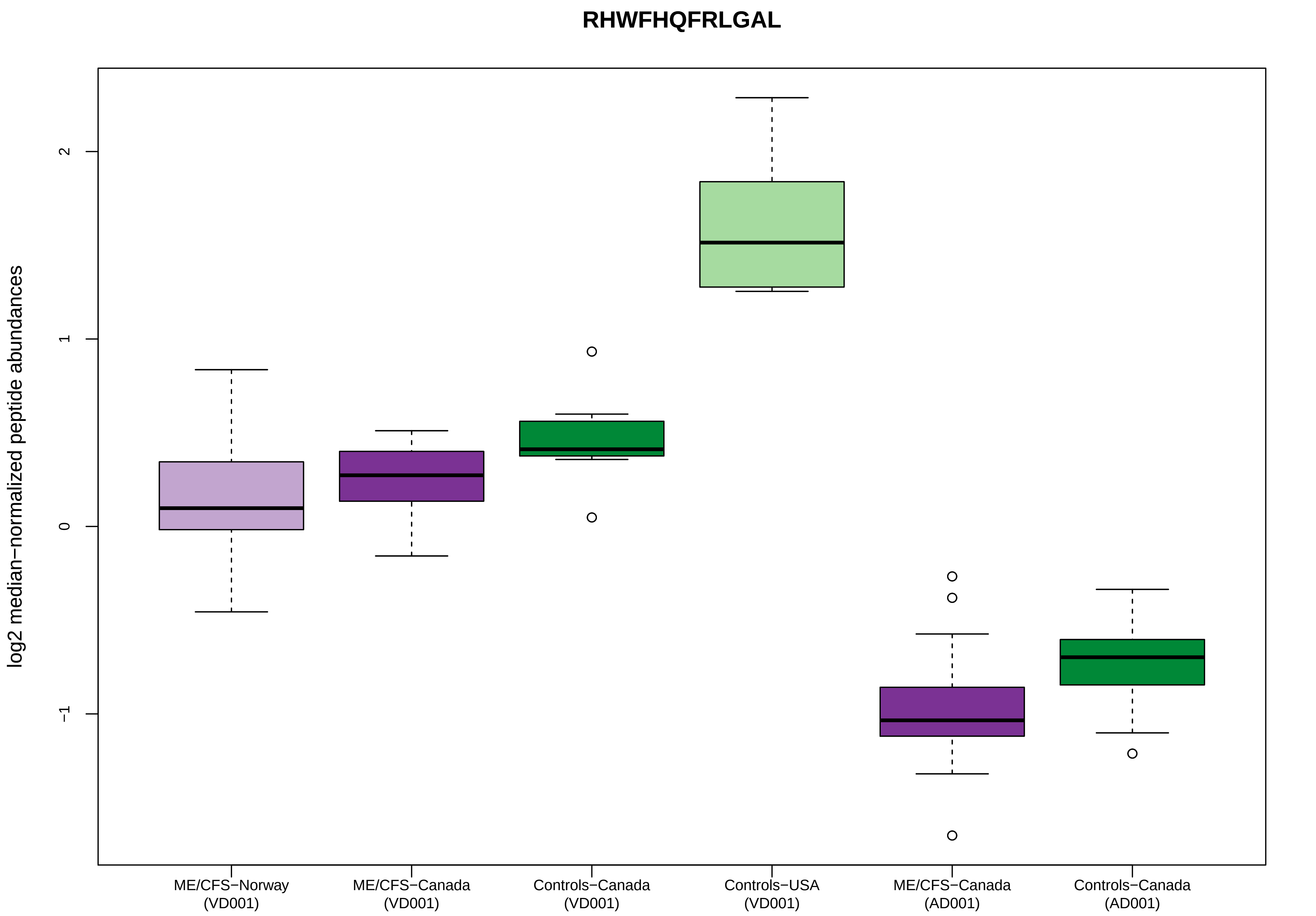

RHWFHQFRLGAL
log2 median−normalized peptide abundances
2
1
0
−1
ME/CFS−Norway (VD001)
ME/CFS−Canada (VD001)
Controls−Canada (VD001)
Controls−USA (VD001)
ME/CFS−Canada (AD001)
Controls−Canada (AD001)

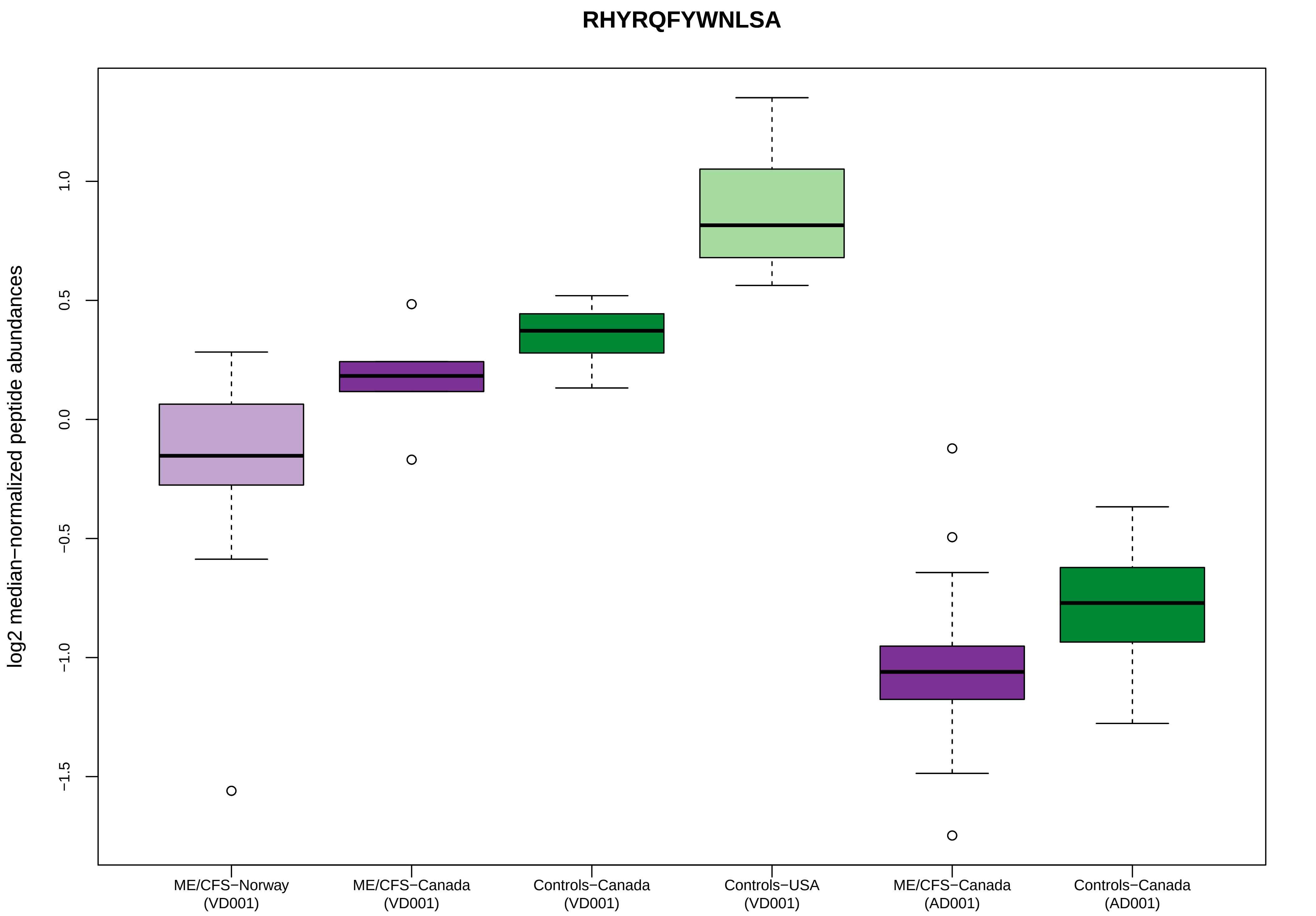

RHYRQFYWNLSA
log2 median−normalized peptide abundances
1.0
0.5
0.0
−0.5
−1.0
−1.5
ME/CFS−Norway (VD001)
ME/CFS−Canada (VD001)
Controls−Canada (VD001)
Controls−USA (VD001)
ME/CFS−Canada (AD001)
Controls−Canada (AD001)

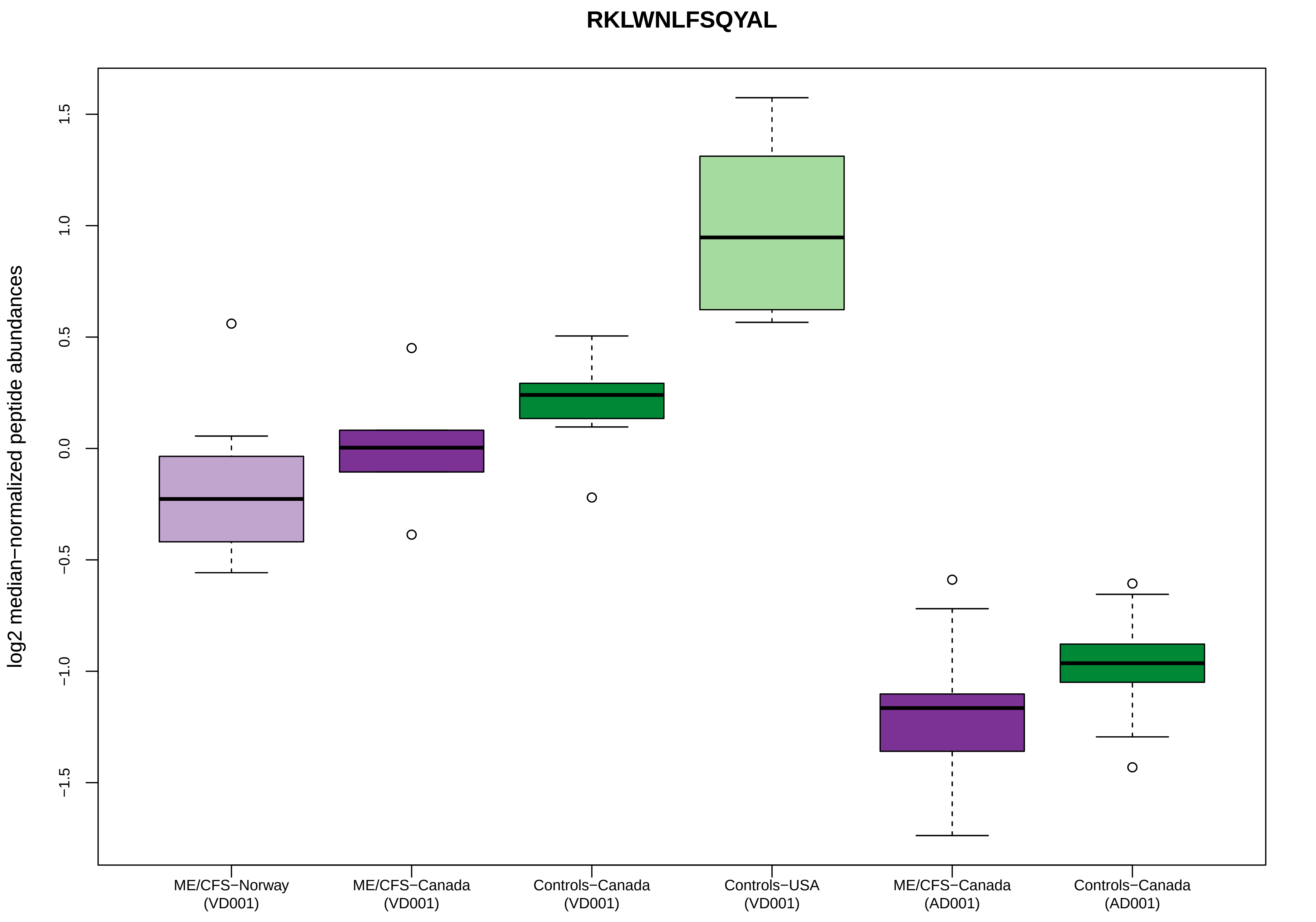
RKLWNLFSQYAL
log2 median−normalized peptide abundances
1.5
1.0
0.5
0.0
−0.5
−1.0
−1.5
ME/CFS−Norway (VD001)
ME/CFS−Canada (VD001)
Controls−Canada (VD001)
Controls−USA (VD001)
ME/CFS−Canada (AD001)
Controls−Canada (AD001)

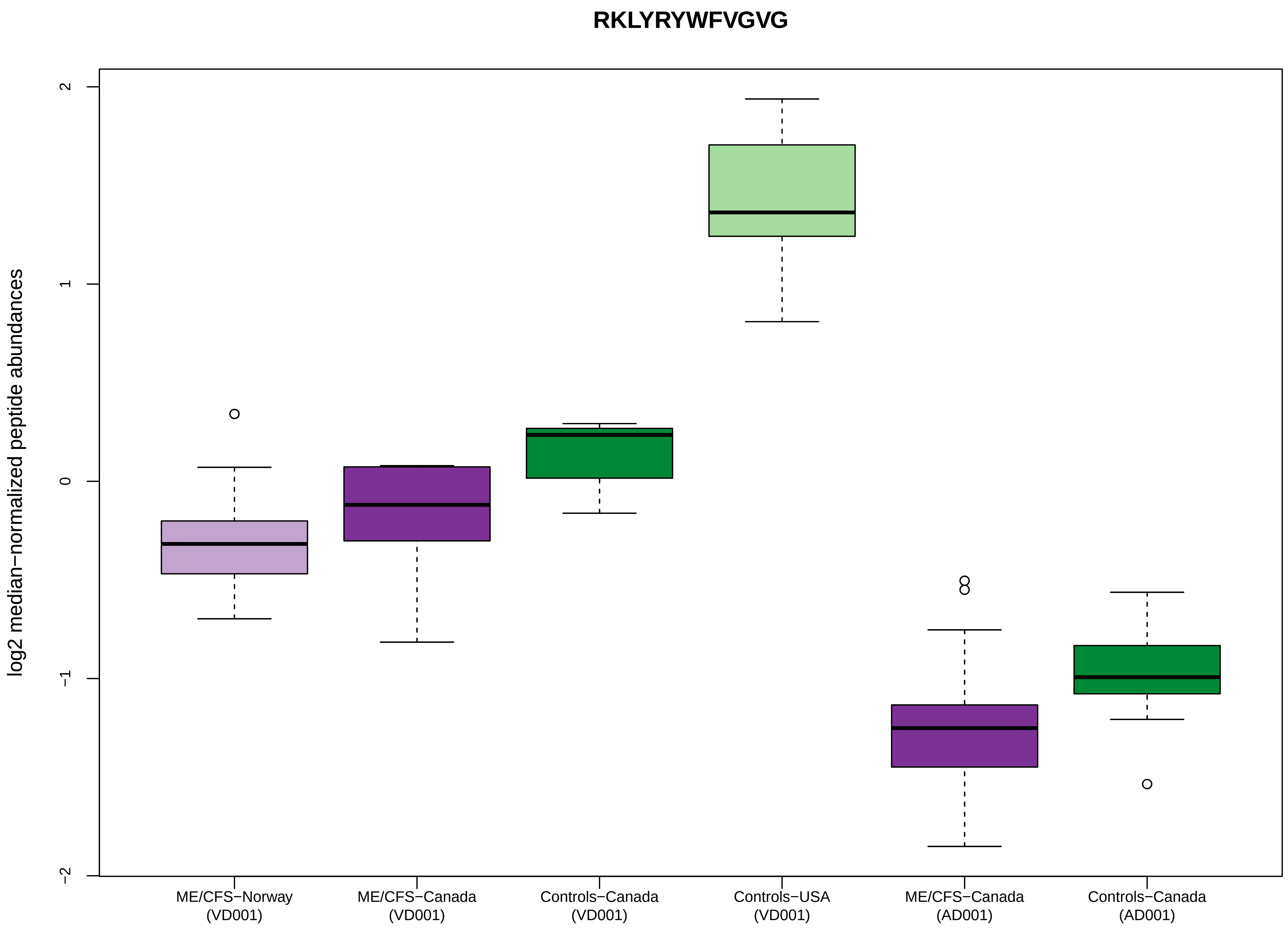

RKLYRYWFVGVG
log2 median−normalized peptide abundances
2
1
0
−1
−2
ME/CFS−Norway (VD001)
ME/CFS−Canada (VD001)
Controls−Canada (VD001)
Controls−USA (VD001)
ME/CFS−Canada (AD001)
Controls−Canada (AD001)

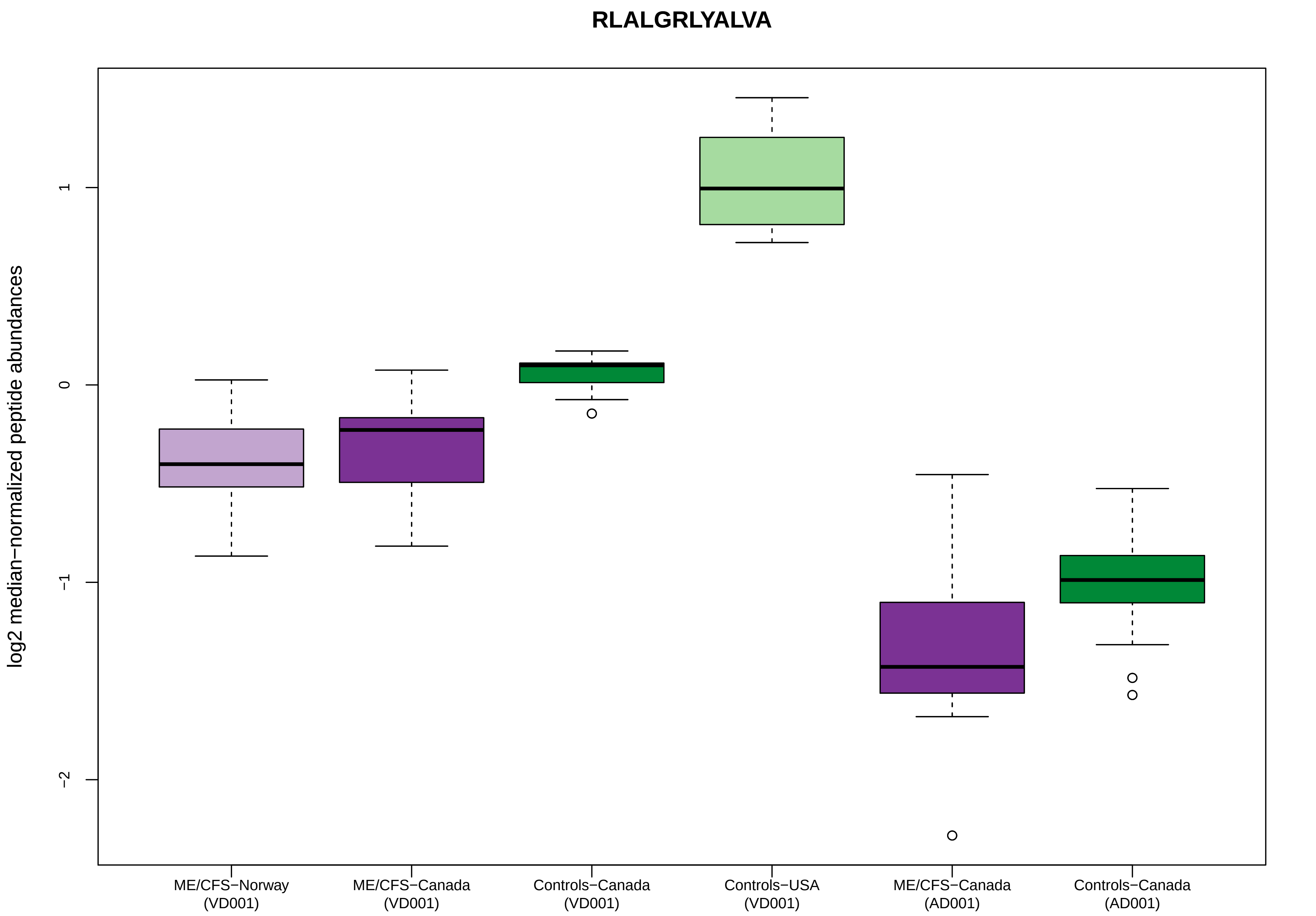
RLALGRLYALVA
log2 median−normalized peptide abundances
1
0
−1
−2
ME/CFS−Norway (VD001)
ME/CFS−Canada (VD001)
Controls−Canada (VD001)
Controls−USA (VD001)
ME/CFS−Canada (AD001)
Controls−Canada (AD001)

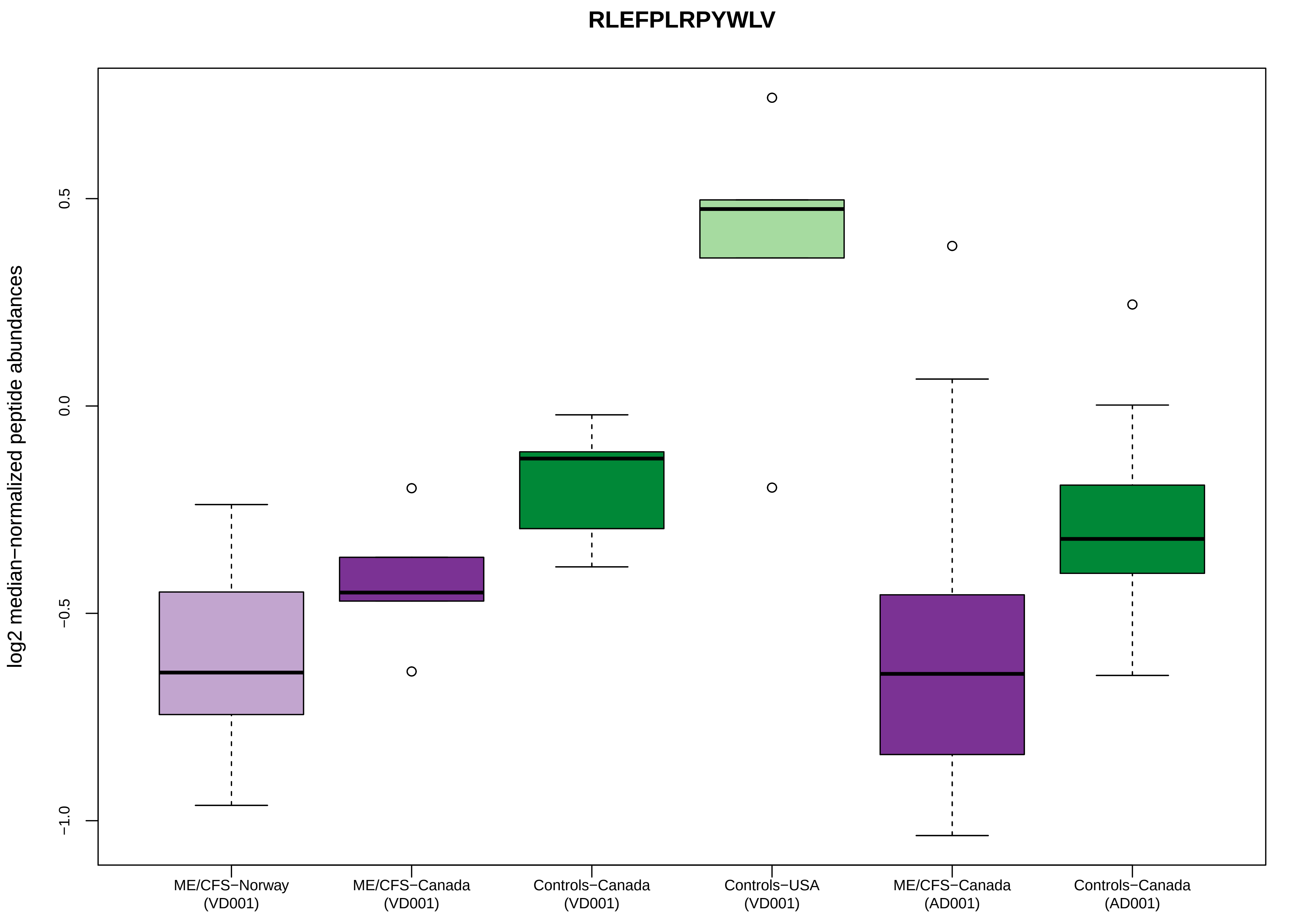
RLEFPLRPYWLV
log2 median−normalized peptide abundances
0.5
0.0
−0.5
−1.0
ME/CFS−Norway (VD001)
ME/CFS−Canada (VD001)
Controls−Canada (VD001)
Controls−USA (VD001)
ME/CFS−Canada (AD001)
Controls−Canada (AD001)

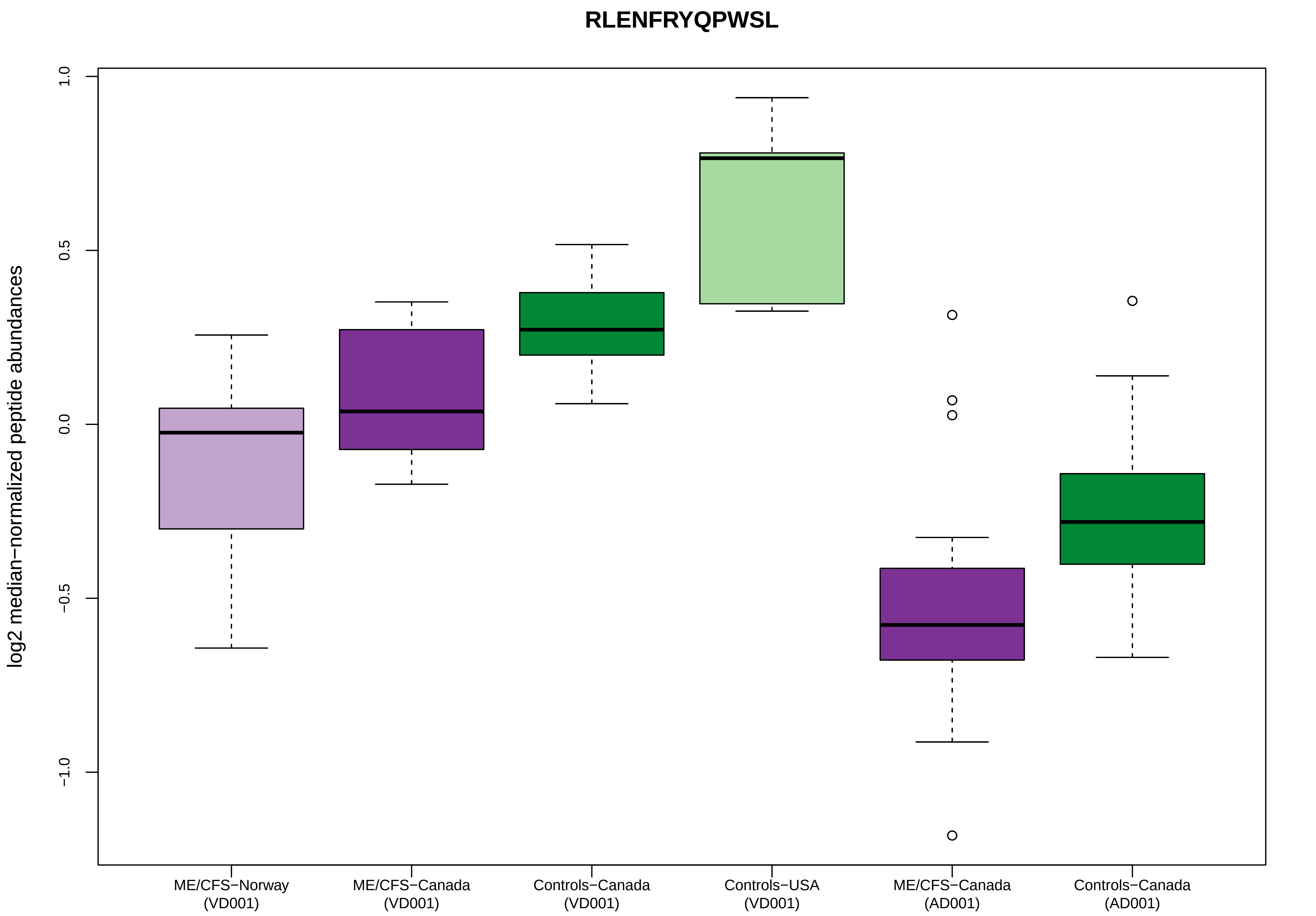

RLENFRYQPWSL
log2 median−normalized peptide abundances
1.0
0.5
0.0
−0.5
−1.0
ME/CFS−Norway (VD001)
ME/CFS−Canada (VD001)
Controls−Canada (VD001)
Controls−USA (VD001)
ME/CFS−Canada (AD001)
Controls−Canada (AD001)

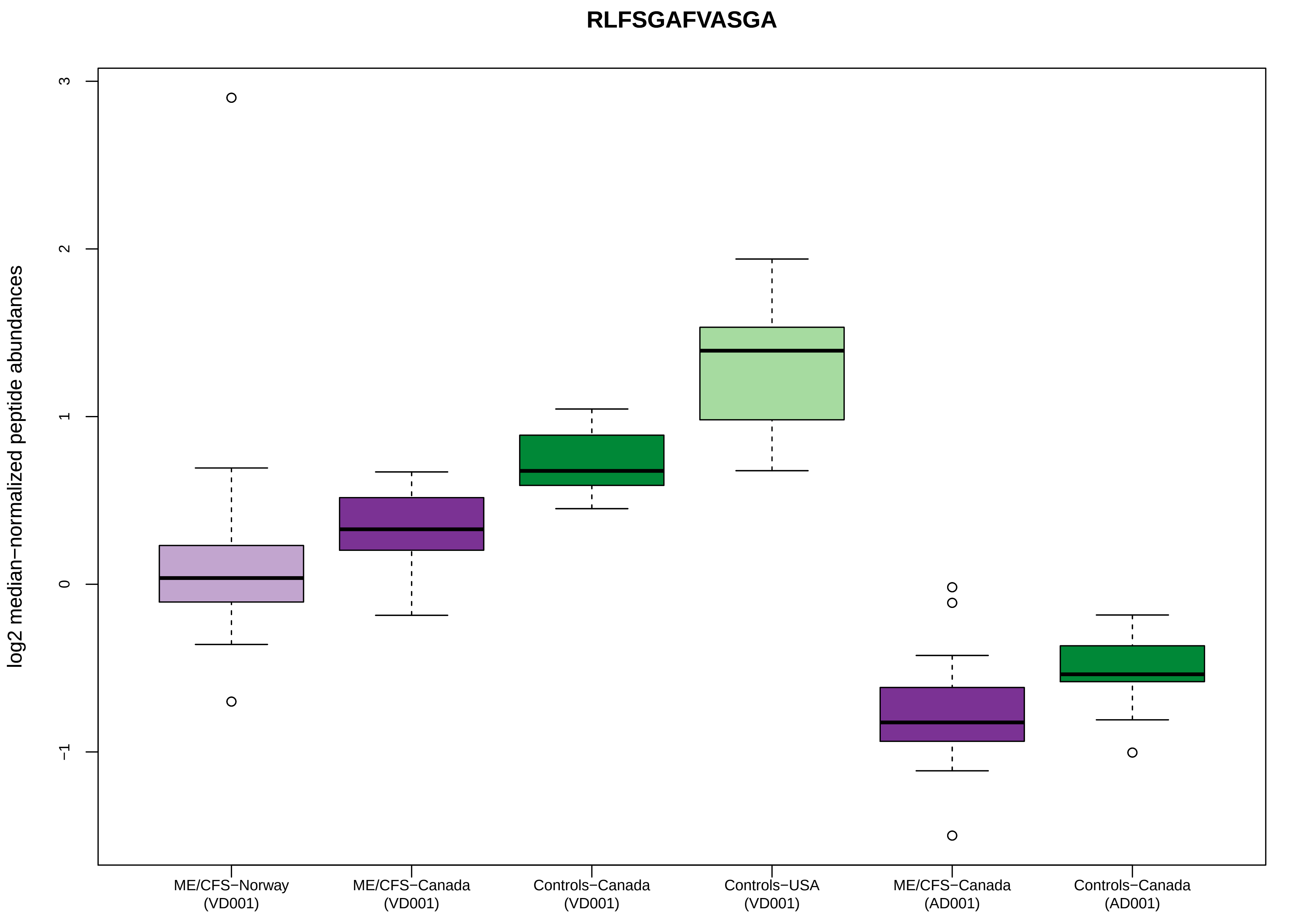

RLFSGAFVASGA
log2 median−normalized peptide abundances
3
2
1
0
−1
ME/CFS−Norway (VD001)
ME/CFS−Canada (VD001)
Controls−Canada (VD001)
Controls−USA (VD001)
ME/CFS−Canada (AD001)
Controls−Canada (AD001)

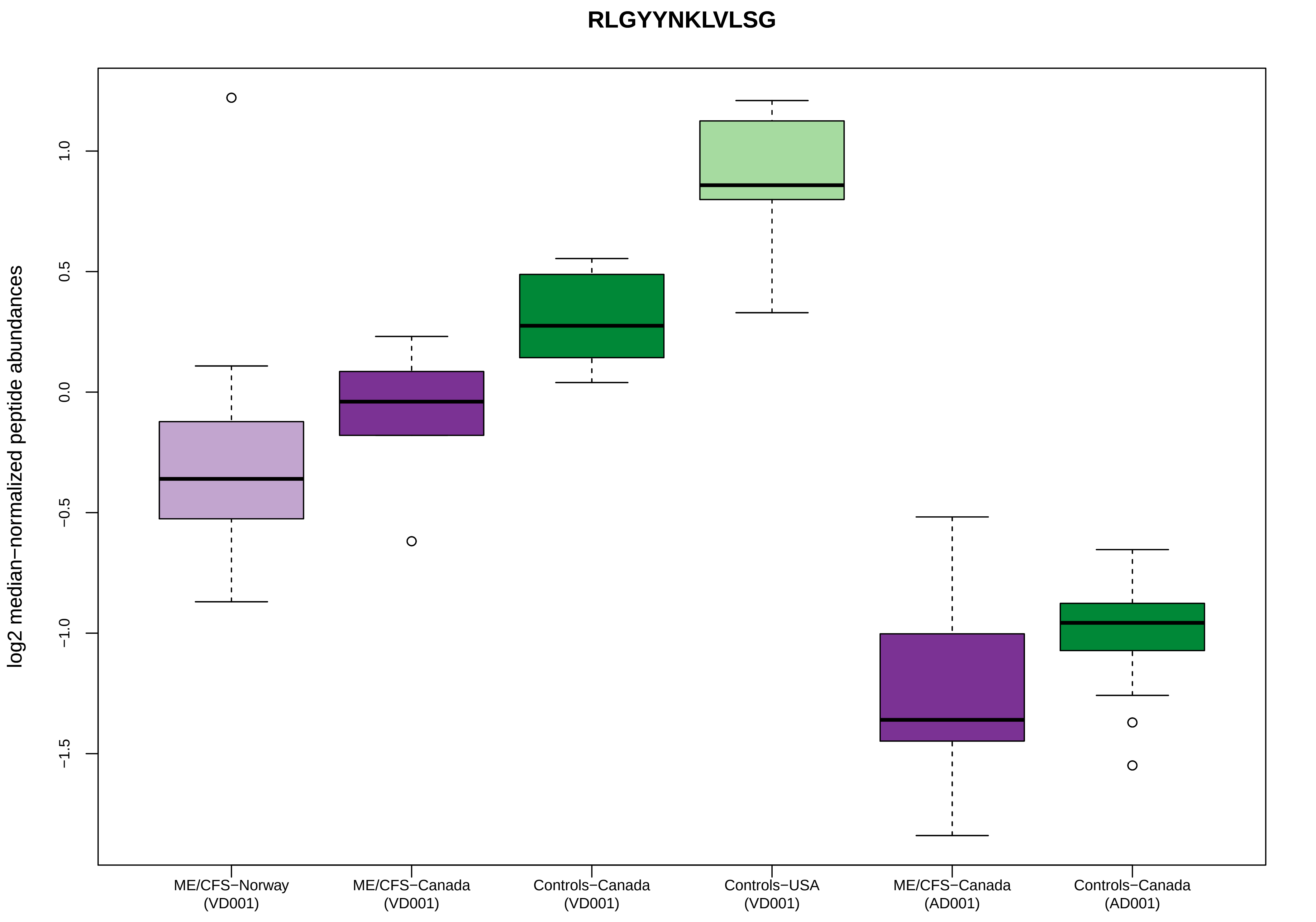

RLGYYNKLVLSG
log2 median−normalized peptide abundances
1.0
0.5
0.0
−0.5
−1.0
−1.5
ME/CFS−Norway (VD001)
ME/CFS−Canada (VD001)
Controls−Canada (VD001)
Controls−USA (VD001)
ME/CFS−Canada (AD001)
Controls−Canada (AD001)

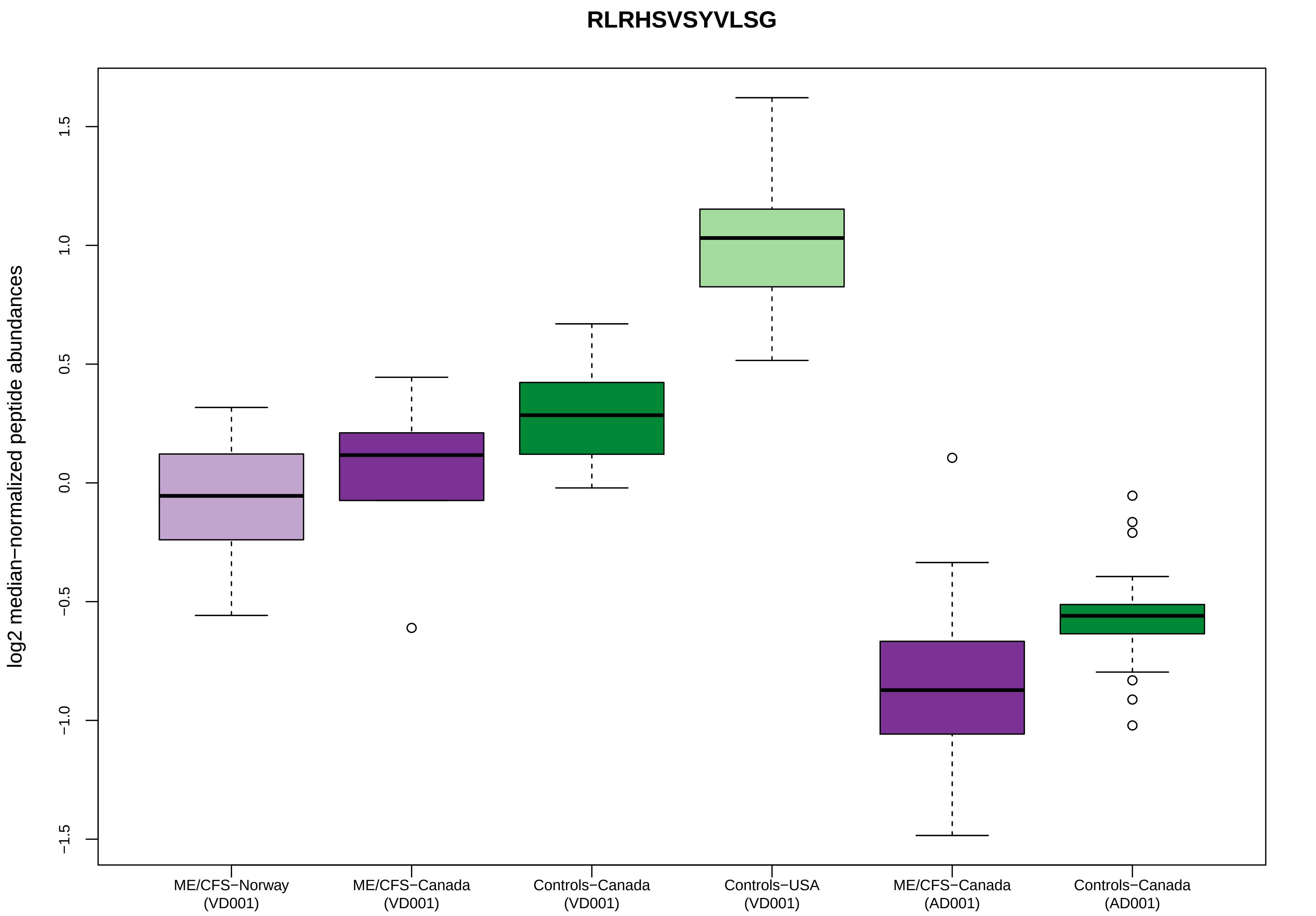
RLRHSVSYVLSG
log2 median−normalized peptide abundances
1.5
1.0
0.5
0.0
−0.5
−1.0
−1.5
ME/CFS−Norway (VD001)
ME/CFS−Canada (VD001)
Controls−Canada (VD001)
Controls−USA (VD001)
ME/CFS−Canada (AD001)
Controls−Canada (AD001)

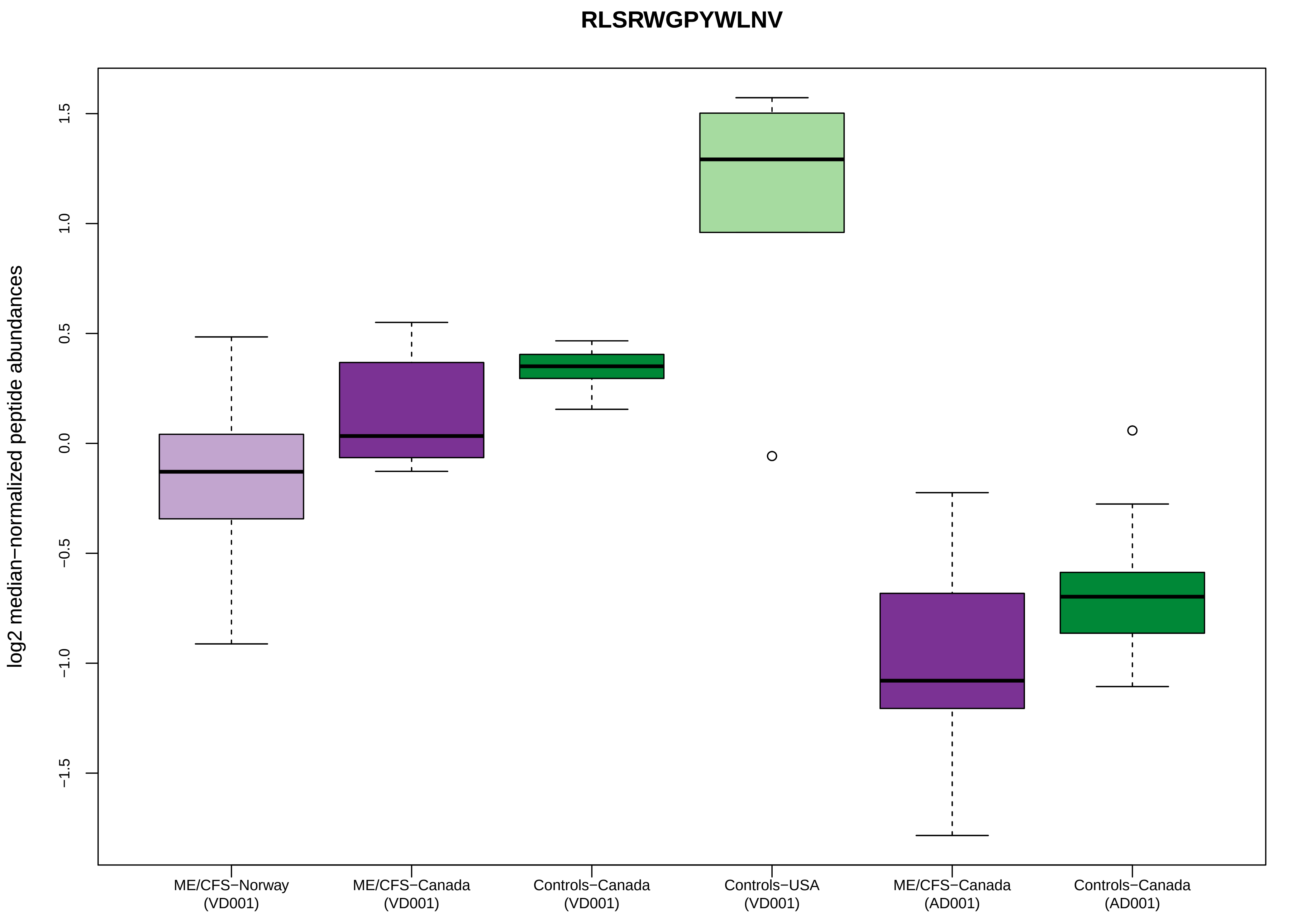
RLSRWGPYWLNV
log2 median−normalized peptide abundances
1.5
1.0
0.5
0.0
−0.5
−1.0
−1.5
ME/CFS−Norway (VD001)
ME/CFS−Canada (VD001)
Controls−Canada (VD001)
Controls−USA (VD001)
ME/CFS−Canada (AD001)
Controls−Canada (AD001)

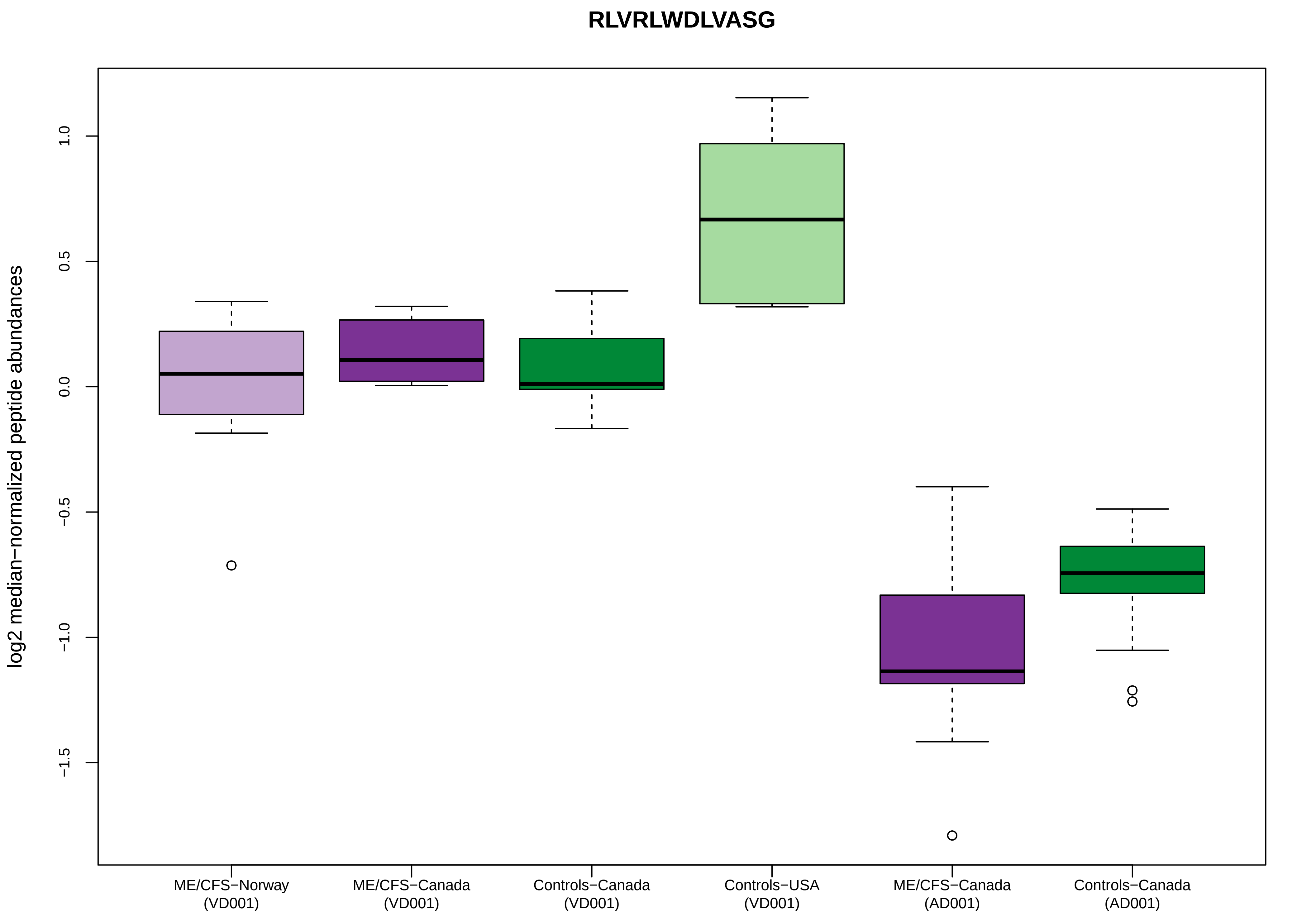
RLVRLWDLVASG
log2 median−normalized peptide abundances
1.0
0.5
0.0
−0.5
−1.0
−1.5
ME/CFS−Norway (VD001)
ME/CFS−Canada (VD001)
Controls−Canada (VD001)
Controls−USA (VD001)
ME/CFS−Canada (AD001)
Controls−Canada (AD001)

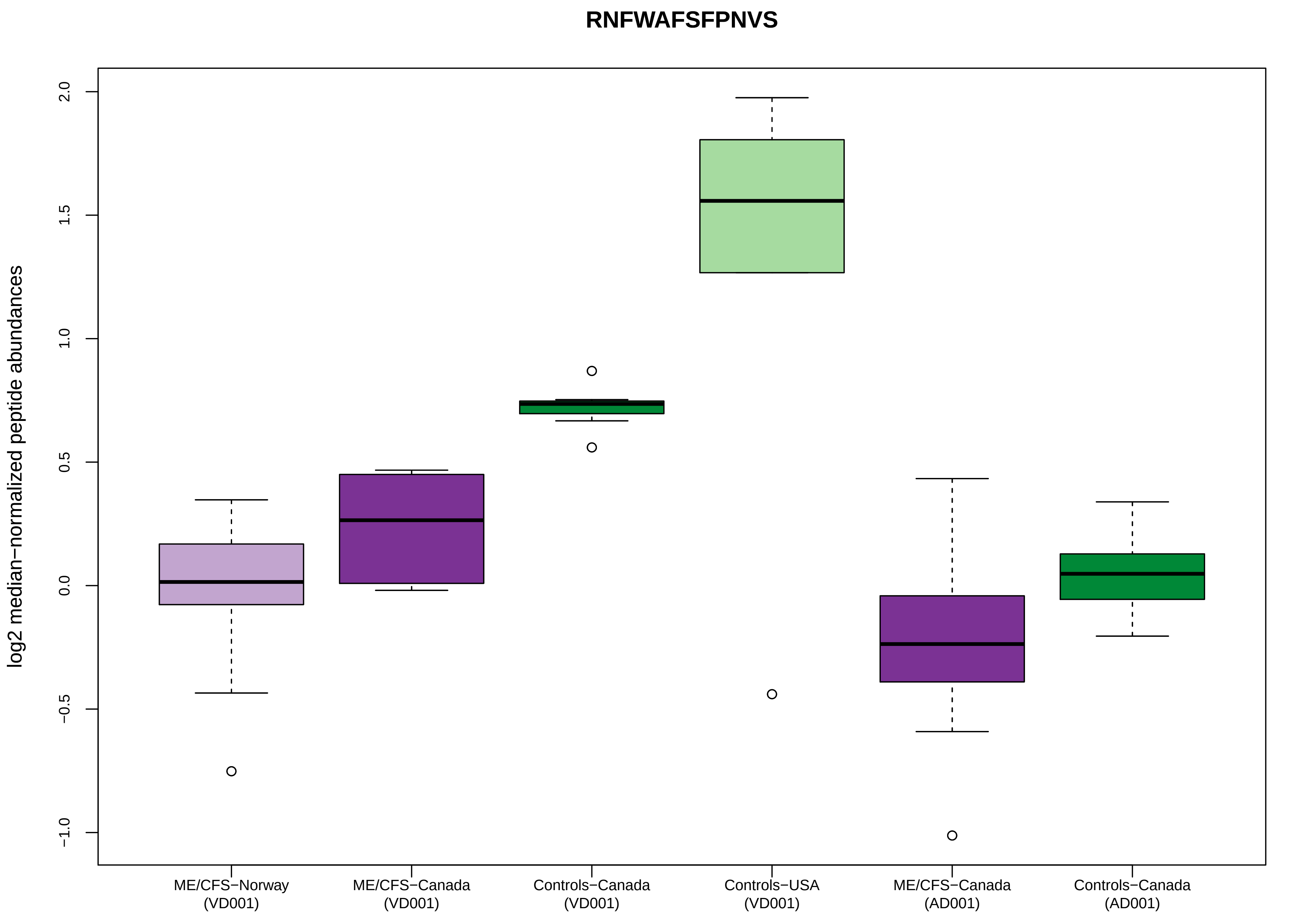
RNFWAFSFPNVS
log2 median−normalized peptide abundances
2.0
1.5
1.0
0.5
0.0
−0.5
−1.0
ME/CFS−Norway (VD001)
ME/CFS−Canada (VD001)
Controls−Canada (VD001)
Controls−USA (VD001)
ME/CFS−Canada (AD001)
Controls−Canada (AD001)

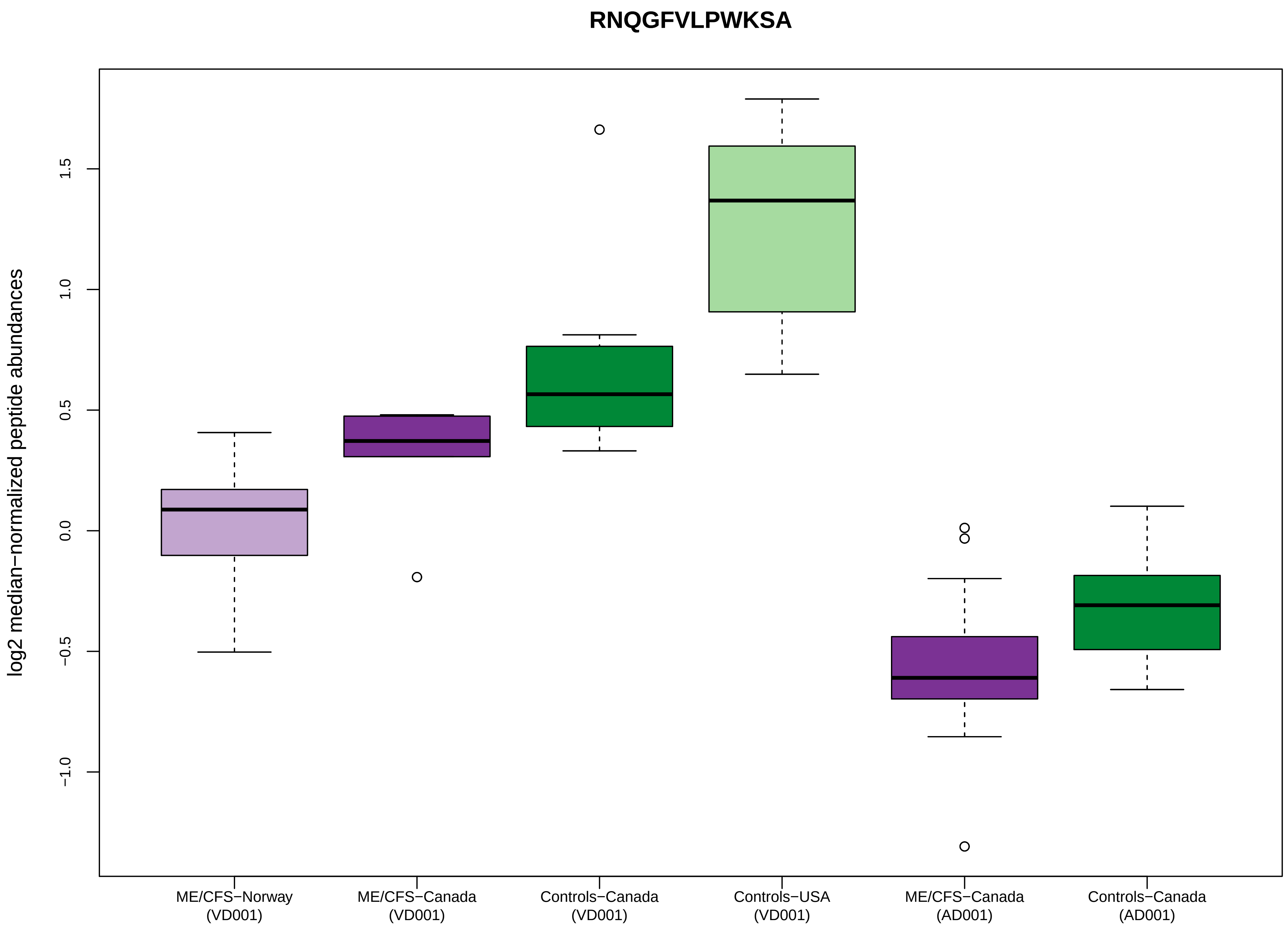
RNQGFVLPWKSA
log2 median−normalized peptide abundances
1.5
1.0
0.5
0.0
−0.5
−1.0
ME/CFS−Norway (VD001)
ME/CFS−Canada (VD001)
Controls−Canada (VD001)
Controls−USA (VD001)
ME/CFS−Canada (AD001)
Controls−Canada (AD001)

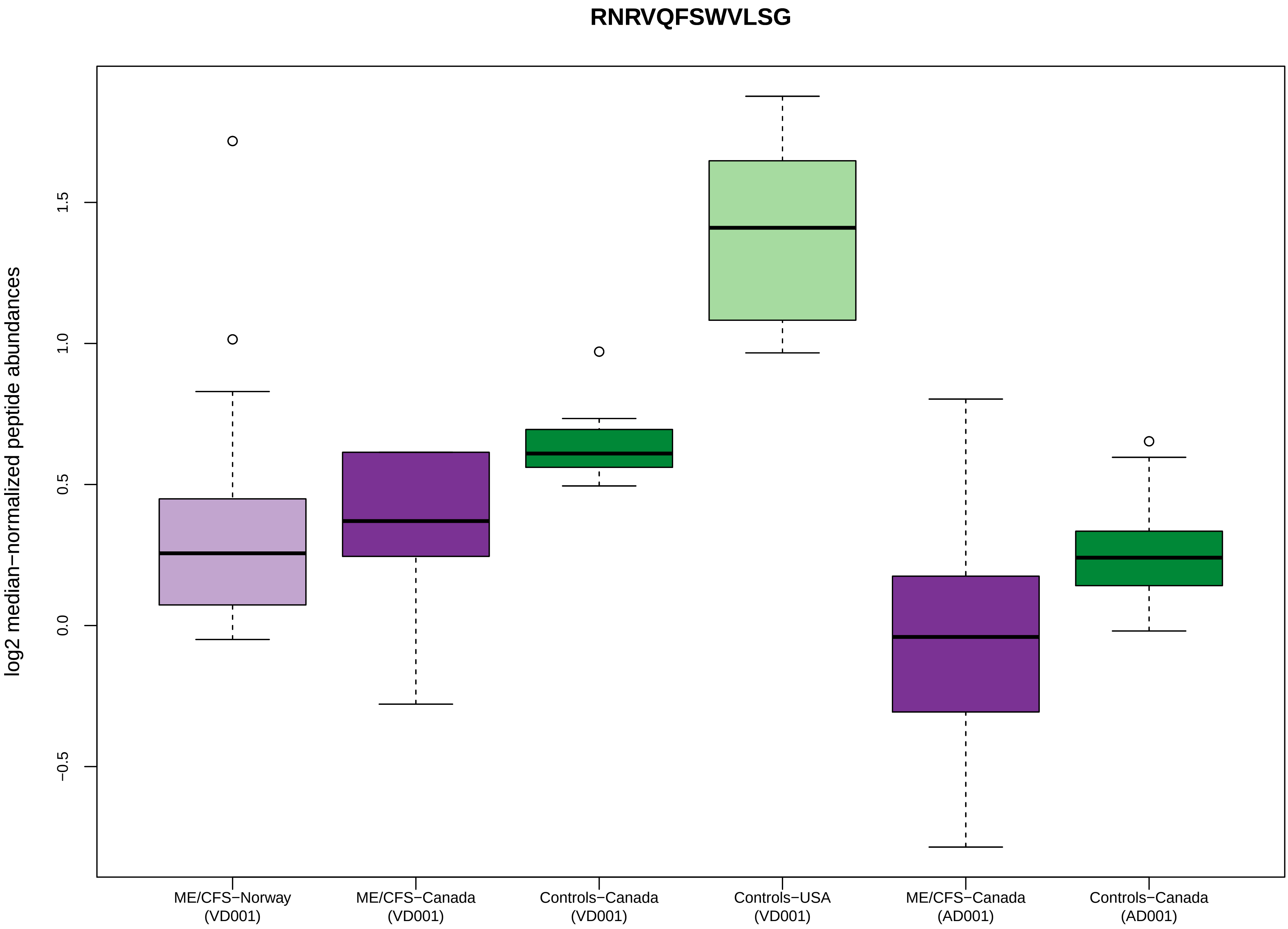
RNRVQFSWVLSG
log2 median−normalized peptide abundances
−0.5
0.0
0.5
1.0
1.5
ME/CFS−Norway (VD001)
ME/CFS−Canada (VD001)
Controls−Canada (VD001)
Controls−USA (VD001)
ME/CFS−Canada (AD001)
Controls−Canada (AD001)

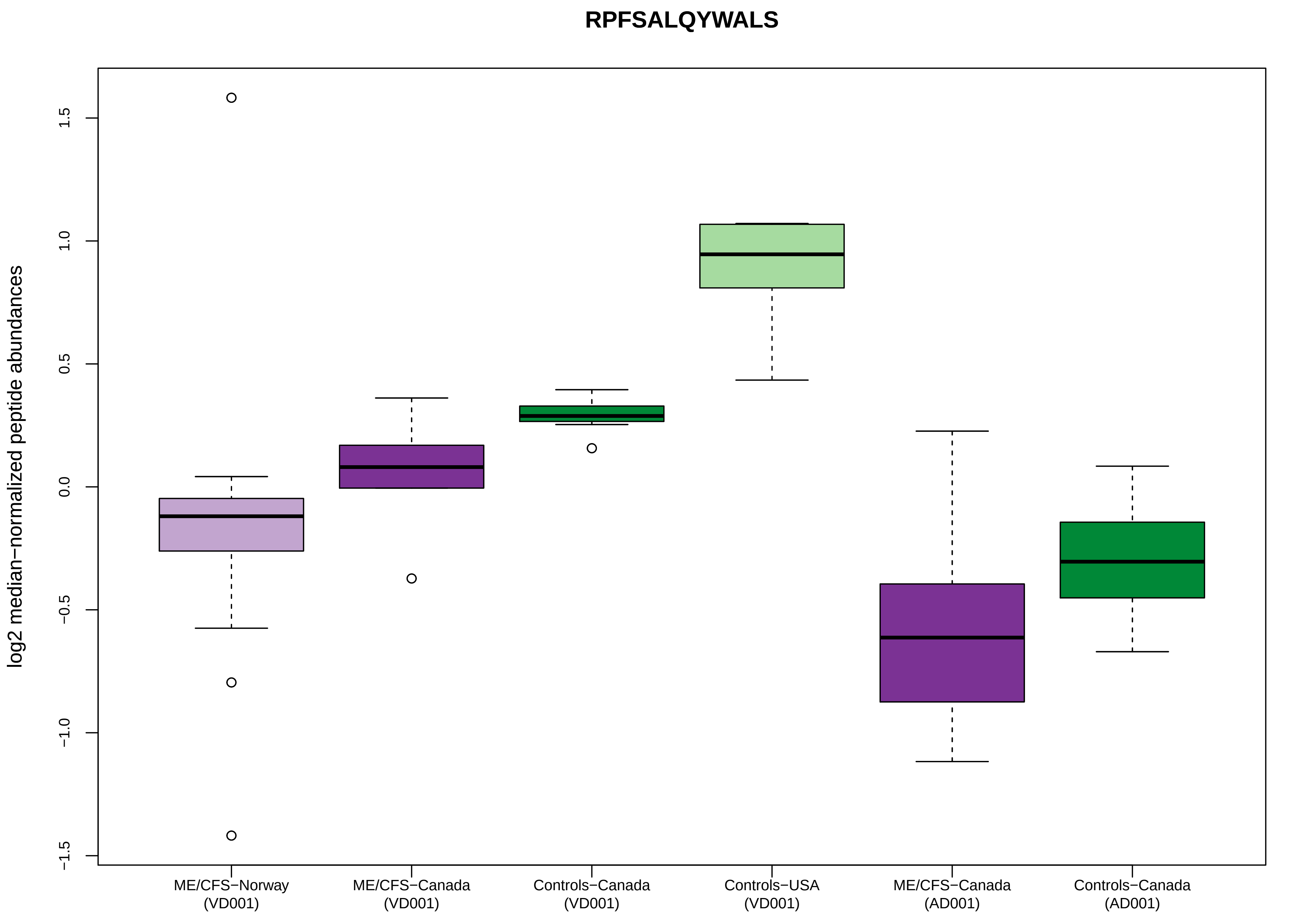
RPFSALQYWALS
log2 median−normalized peptide abundances
1.5
1.0
0.5
0.0
−0.5
−1.0
−1.5
ME/CFS−Norway (VD001)
ME/CFS−Canada (VD001)
Controls−Canada (VD001)
Controls−USA (VD001)
ME/CFS−Canada (AD001)
Controls−Canada (AD001)

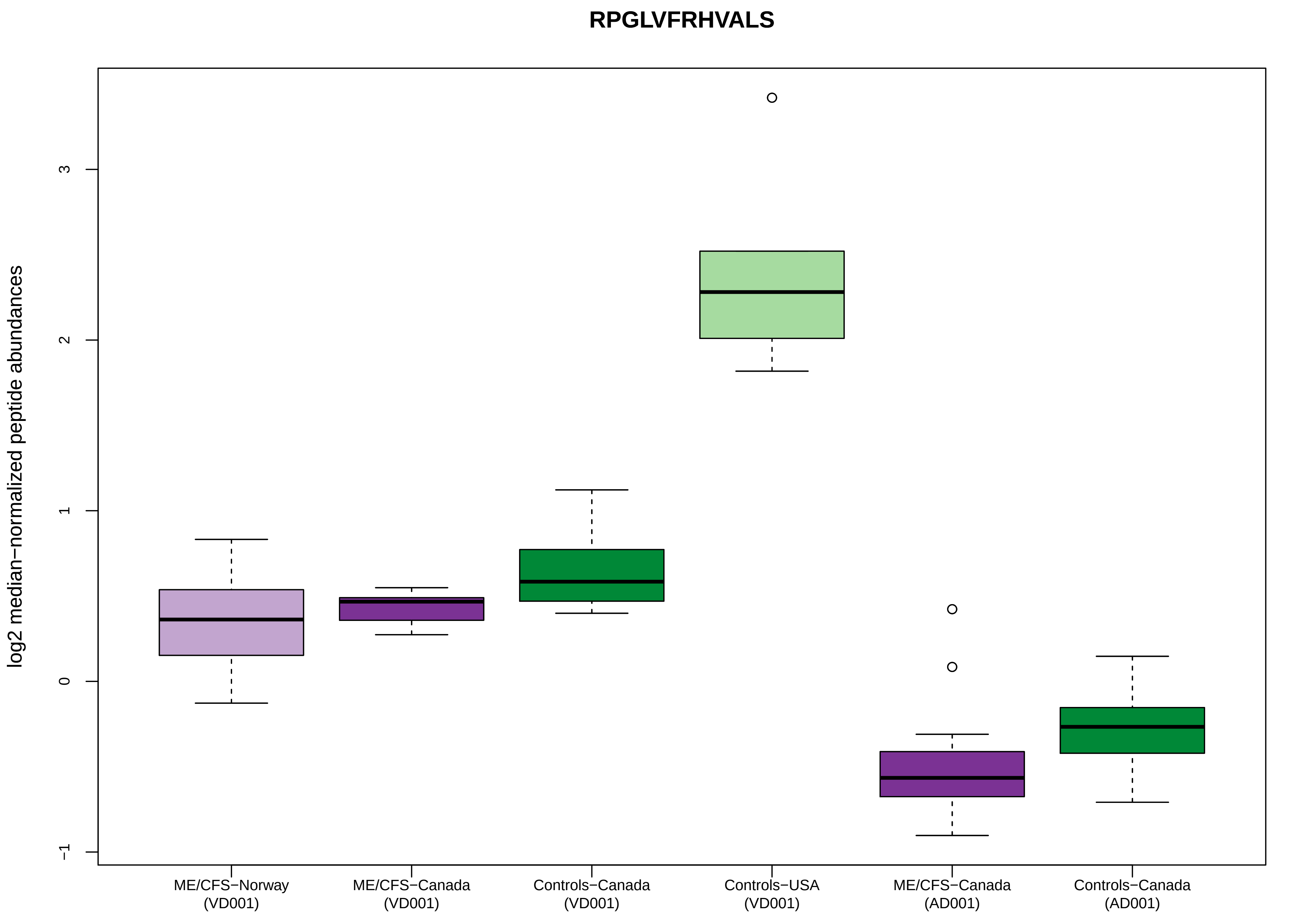
RPGLVFRHVALS
log2 median−normalized peptide abundances
−1
0
1
2
3
ME/CFS−Norway (VD001)
ME/CFS−Canada (VD001)
Controls−Canada (VD001)
Controls−USA (VD001)
ME/CFS−Canada (AD001)
Controls−Canada (AD001)

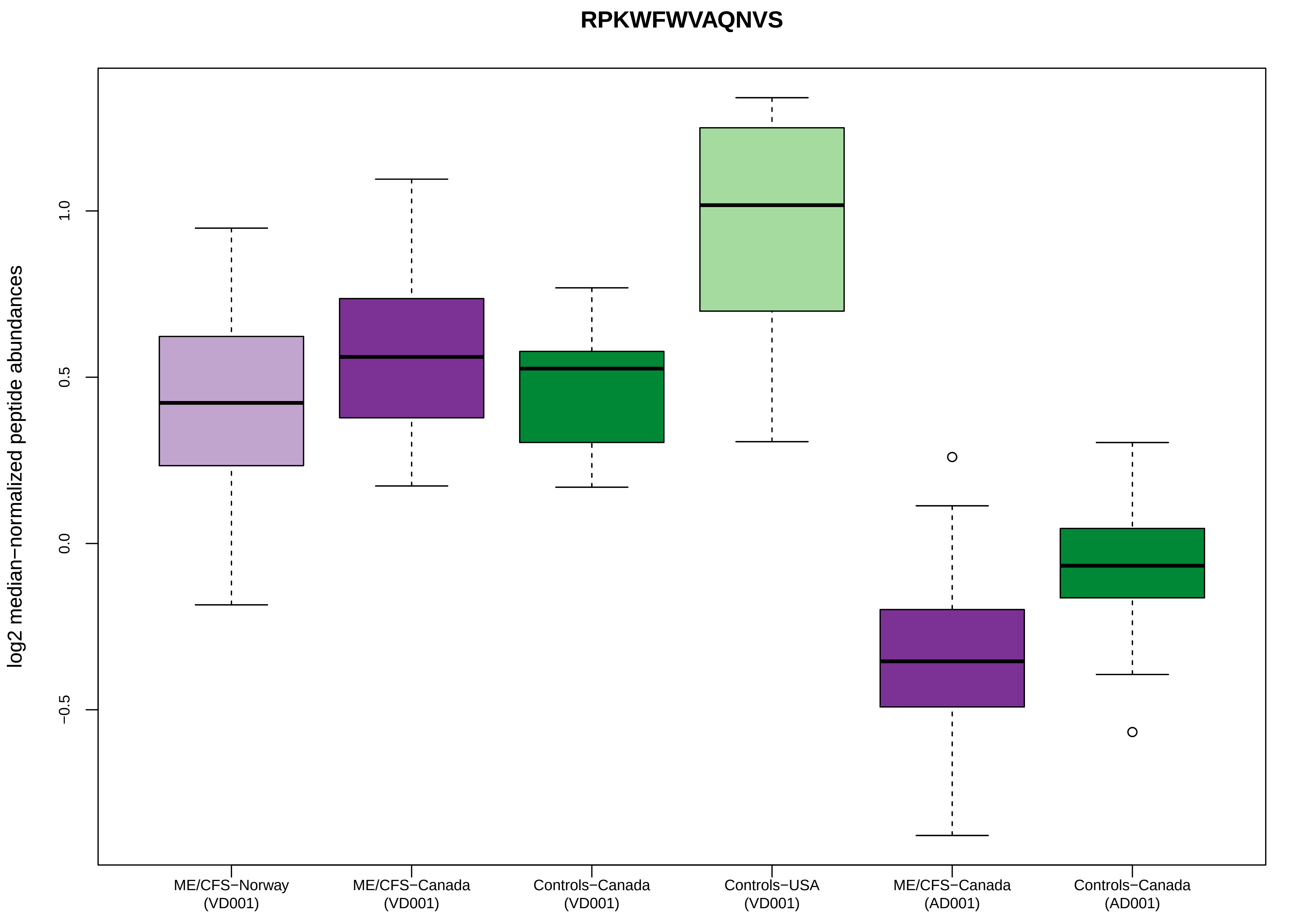
RPKWFWVAQNVS
log2 median−normalized peptide abundances
1.0
0.5
0.0
−0.5
ME/CFS−Norway (VD001)
ME/CFS−Canada (VD001)
Controls−Canada (VD001)
Controls−USA (VD001)
ME/CFS−Canada (AD001)
Controls−Canada (AD001)

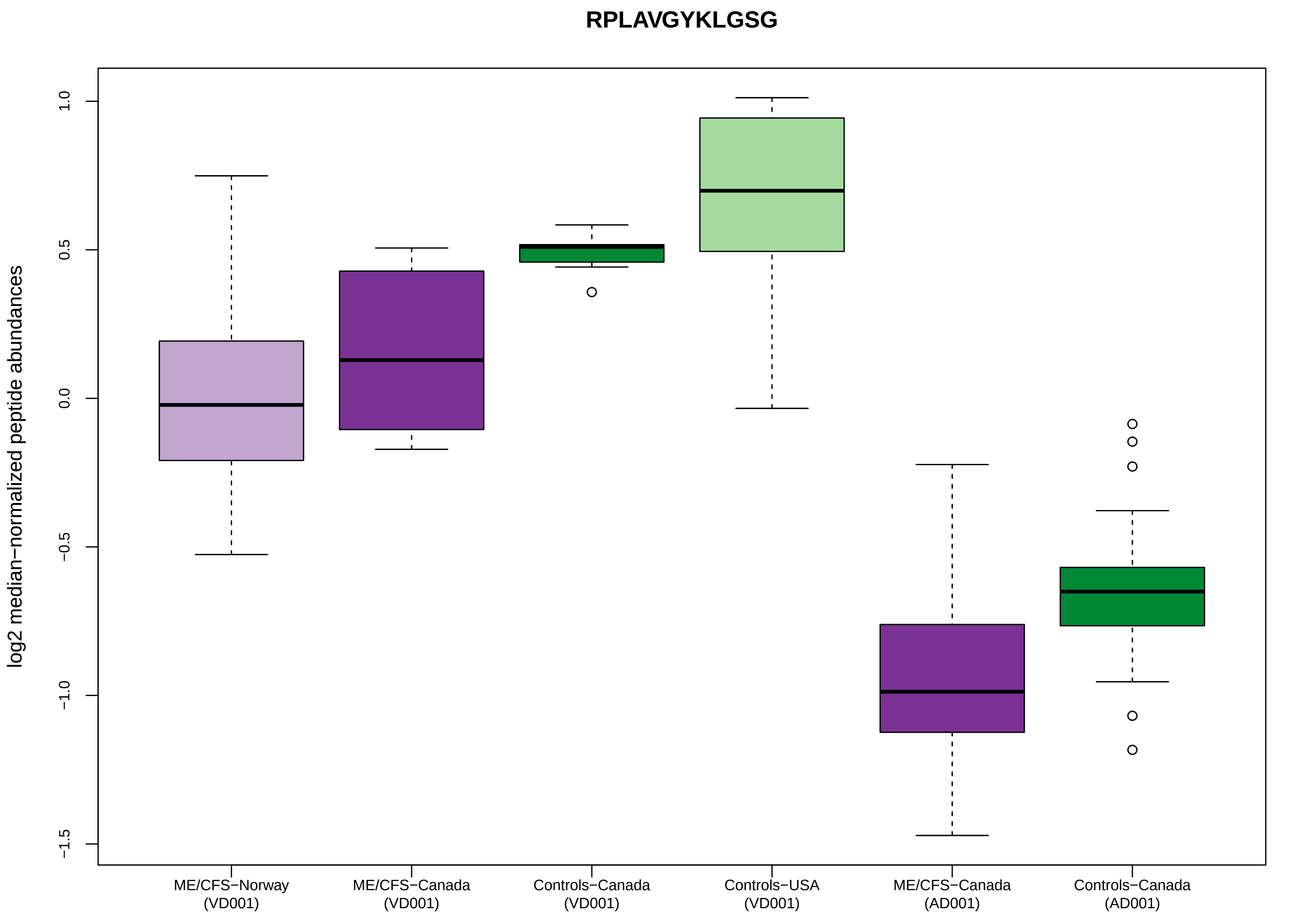
RPLAVGYKLGSG
log2 median−normalized peptide abundances
1.0
0.5
0.0
−0.5
−1.0
−1.5
ME/CFS−Norway (VD001)
ME/CFS−Canada (VD001)
Controls−Canada (VD001)
Controls−USA (VD001)
ME/CFS−Canada (AD001)
Controls−Canada (AD001)

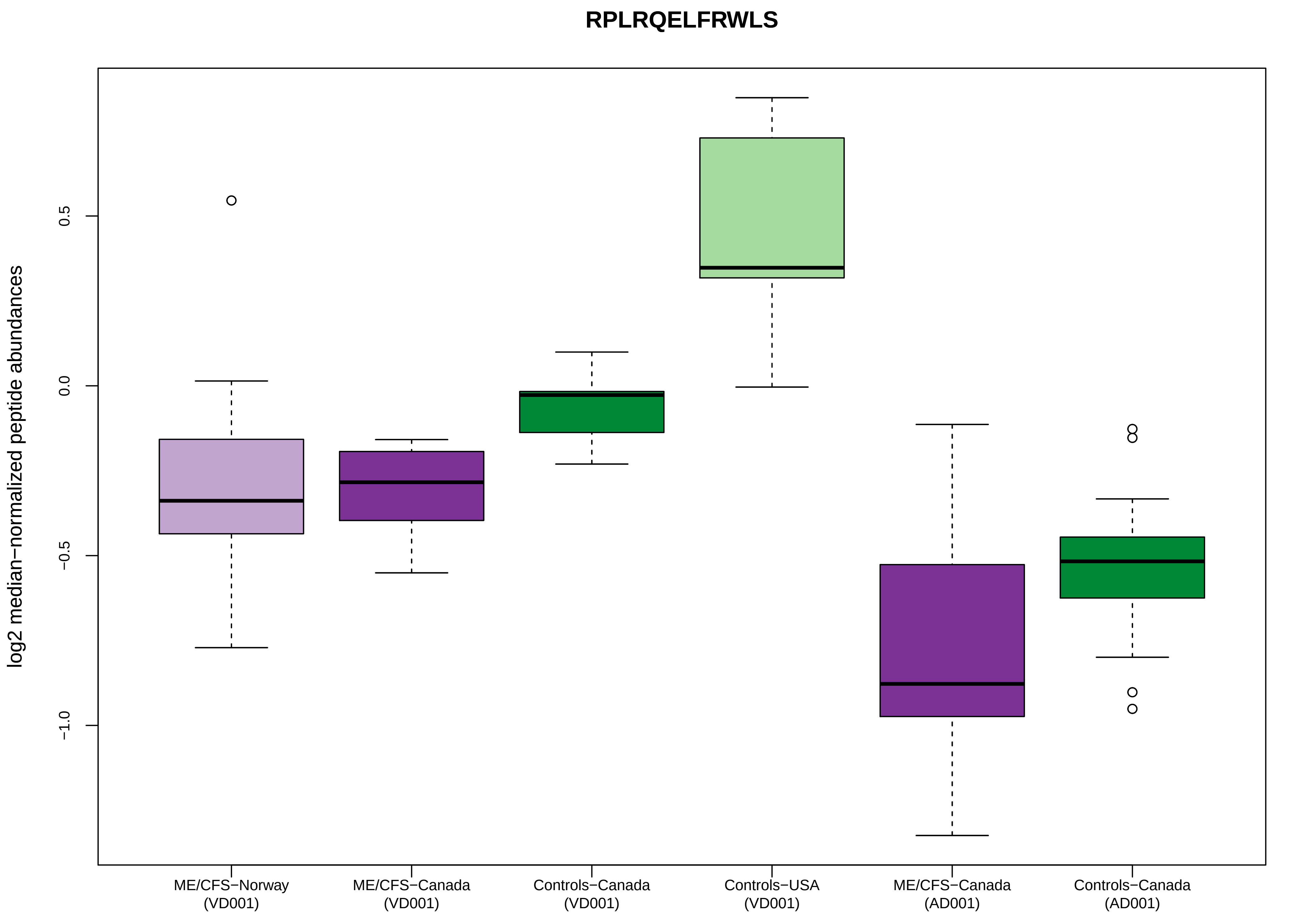
RPLRQELFRWLS
log2 median−normalized peptide abundances
0.5
0.0
−0.5
−1.0
ME/CFS−Norway (VD001)
ME/CFS−Canada (VD001)
Controls−Canada (VD001)
Controls−USA (VD001)
ME/CFS−Canada (AD001)
Controls−Canada (AD001)

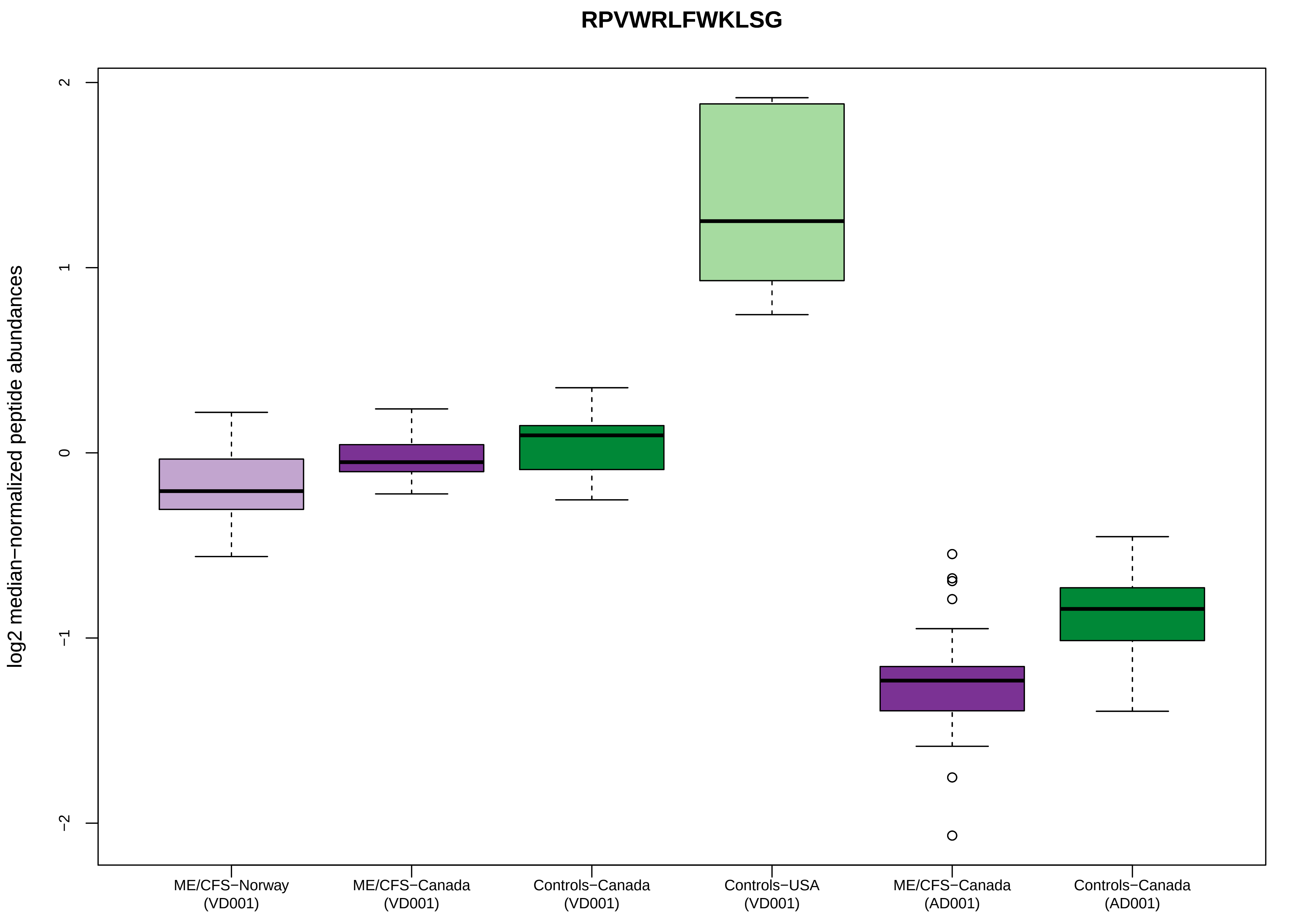
RPVWRLFWKLSG
log2 median−normalized peptide abundances
2
1
0
−1
−2
ME/CFS−Norway (VD001)
ME/CFS−Canada (VD001)
Controls−Canada (VD001)
Controls−USA (VD001)
ME/CFS−Canada (AD001)
Controls−Canada (AD001)

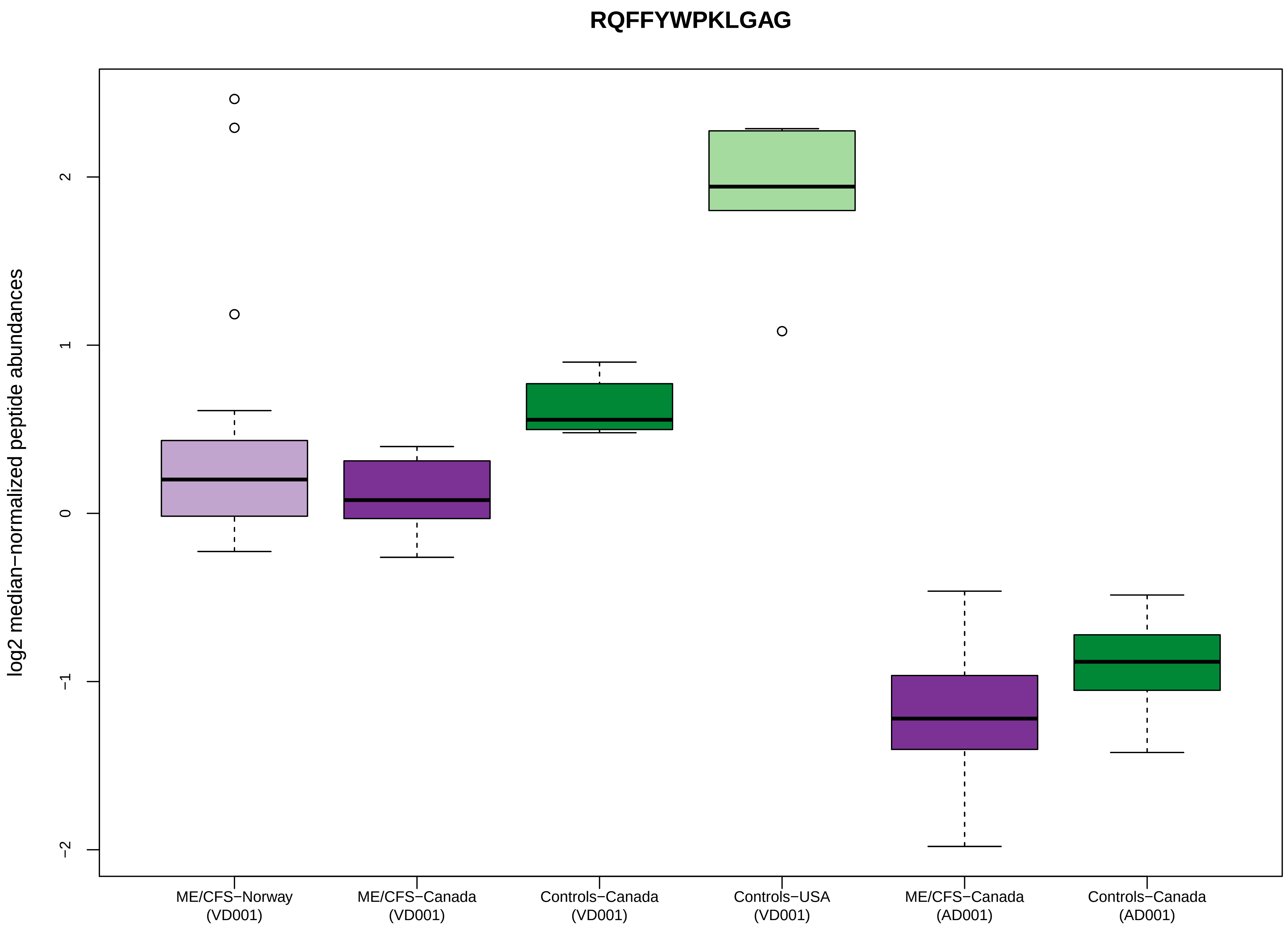

RQFFYWPKLGAG
log2 median−normalized peptide abundances
−2
−1
0
1
2
ME/CFS−Norway (VD001)
ME/CFS−Canada (VD001)
Controls−Canada (VD001)
Controls−USA (VD001)
ME/CFS−Canada (AD001)
Controls−Canada (AD001)

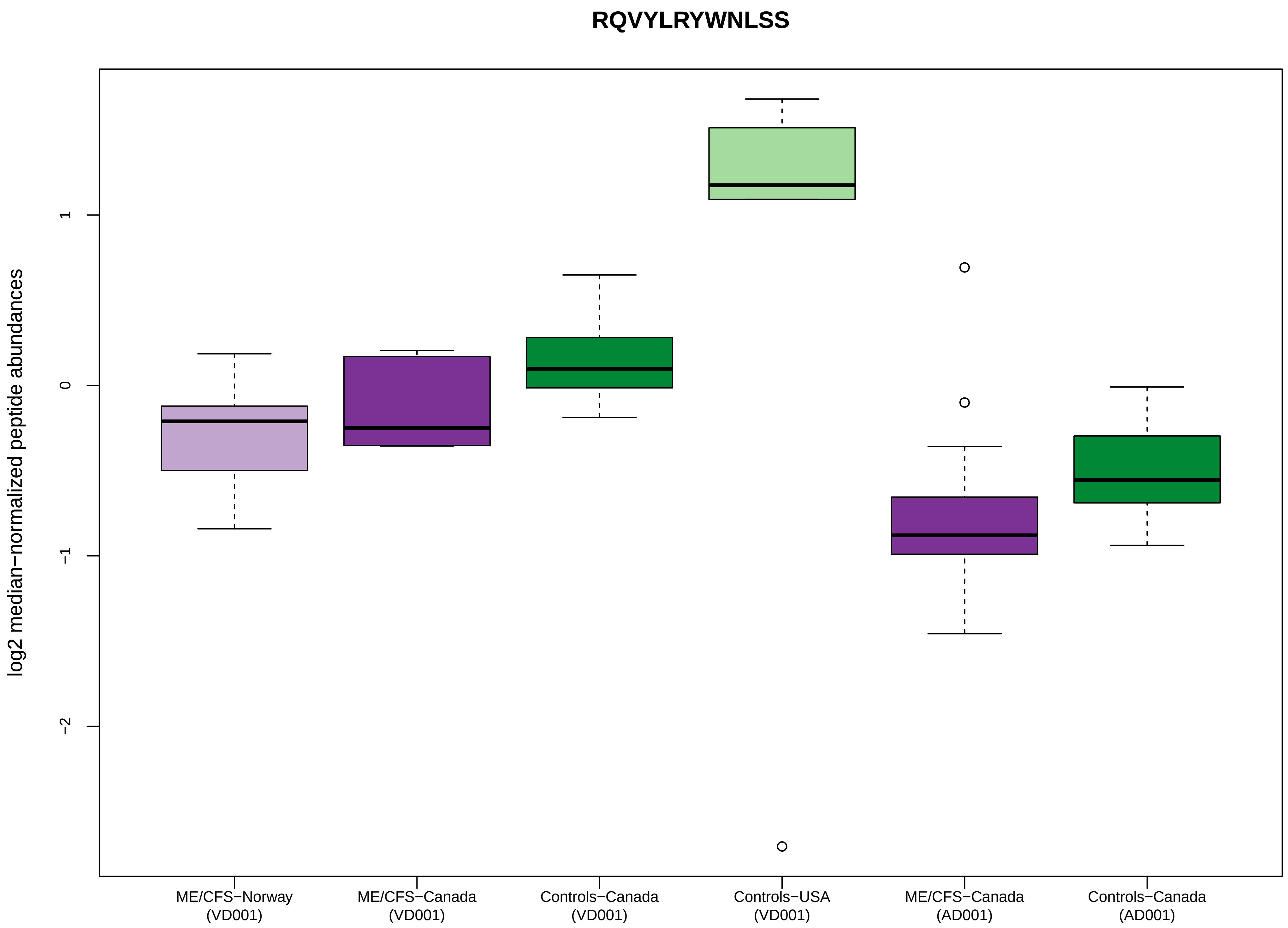

RQVYLRYWNLSS
log2 median−normalized peptide abundances
1
0
−1
−2
ME/CFS−Norway (VD001)
ME/CFS−Canada (VD001)
Controls−Canada (VD001)
Controls−USA (VD001)
ME/CFS−Canada (AD001)
Controls−Canada (AD001)

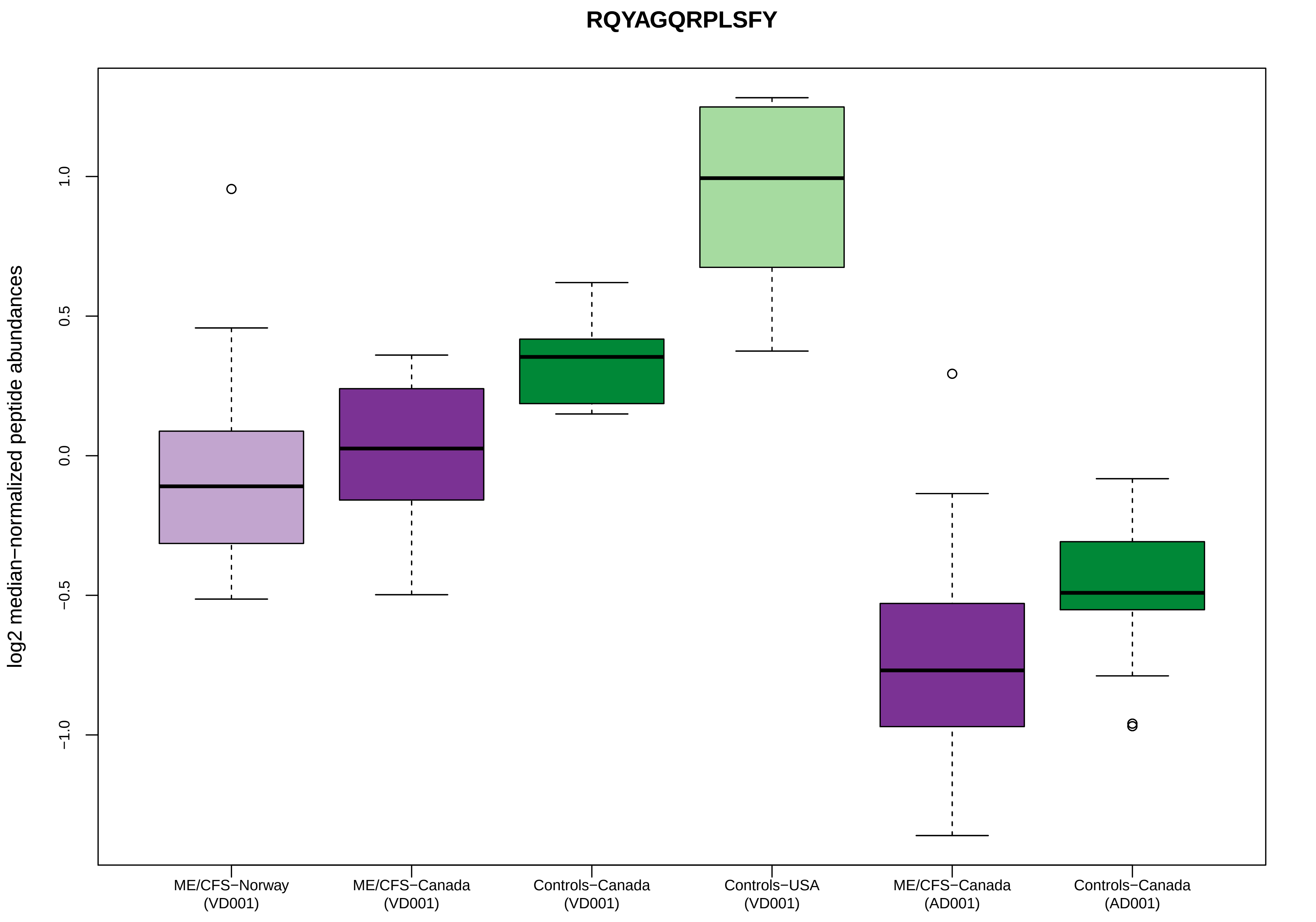
RQYAGQRPLSFY
log2 median−normalized peptide abundances
1.0
0.5
0.0
−0.5
−1.0
ME/CFS−Norway (VD001)
ME/CFS−Canada (VD001)
Controls−Canada (VD001)
Controls−USA (VD001)
ME/CFS−Canada (AD001)
Controls−Canada (AD001)

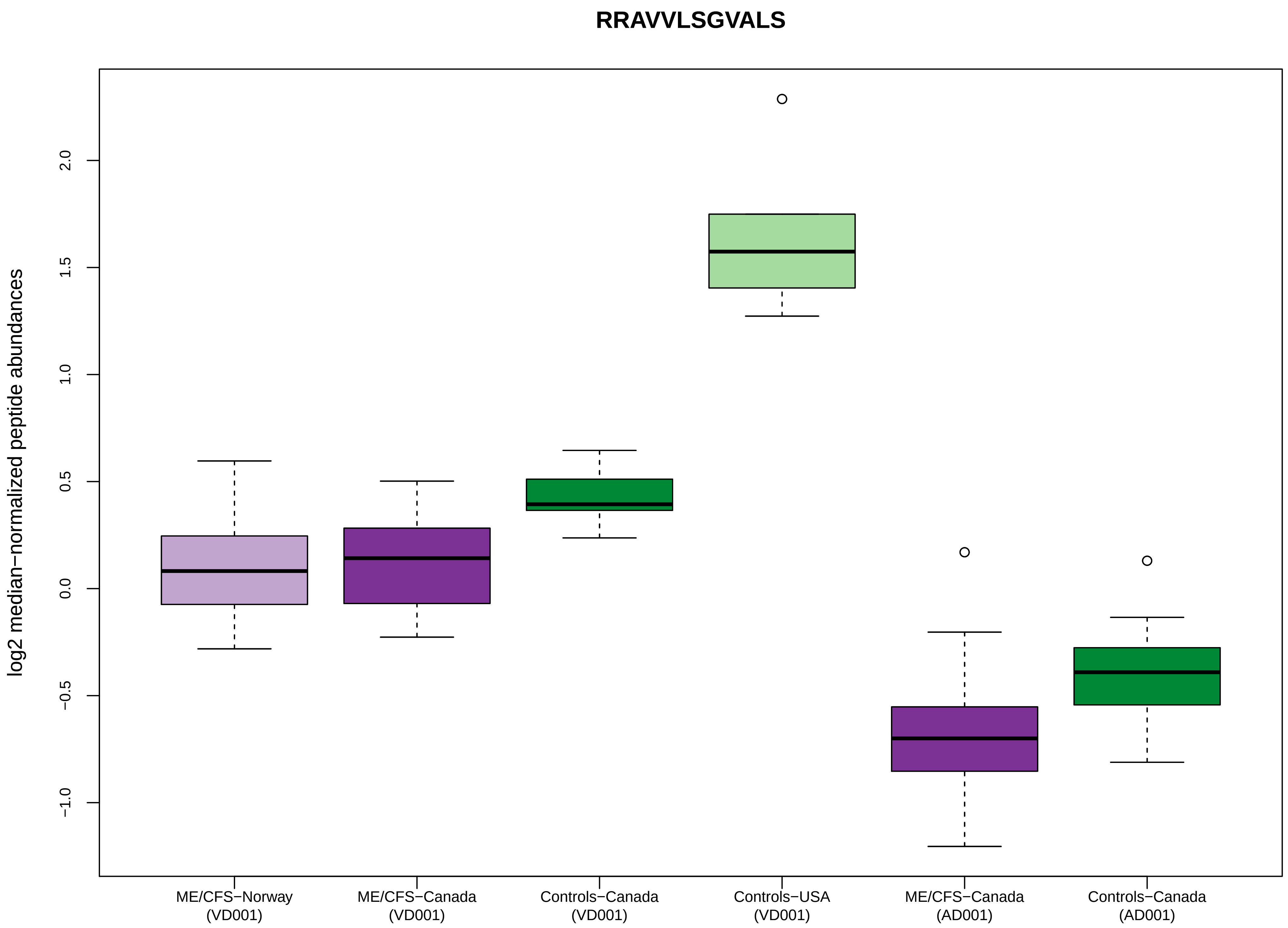
RRAVVLSGVALS
log2 median−normalized peptide abundances
2.0
1.5
1.0
0.5
0.0
−0.5
−1.0
ME/CFS−Norway (VD001)
ME/CFS−Canada (VD001)
Controls−Canada (VD001)
Controls−USA (VD001)
ME/CFS−Canada (AD001)
Controls−Canada (AD001)

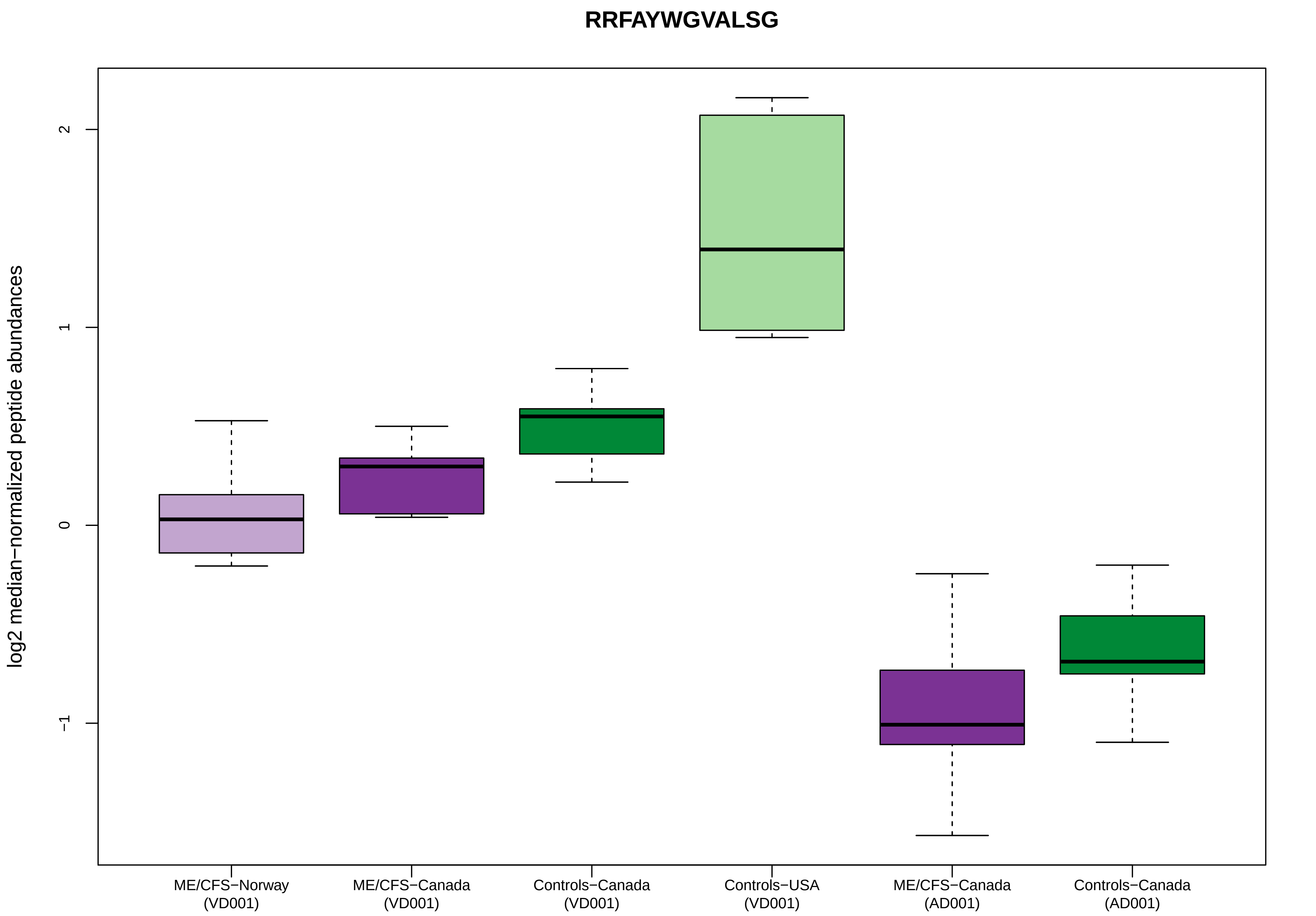

RRFAYWGVALSG
log2 median−normalized peptide abundances
2
1
0
−1
ME/CFS−Norway (VD001)
ME/CFS−Canada (VD001)
Controls−Canada (VD001)
Controls−USA (VD001)
ME/CFS−Canada (AD001)
Controls−Canada (AD001)

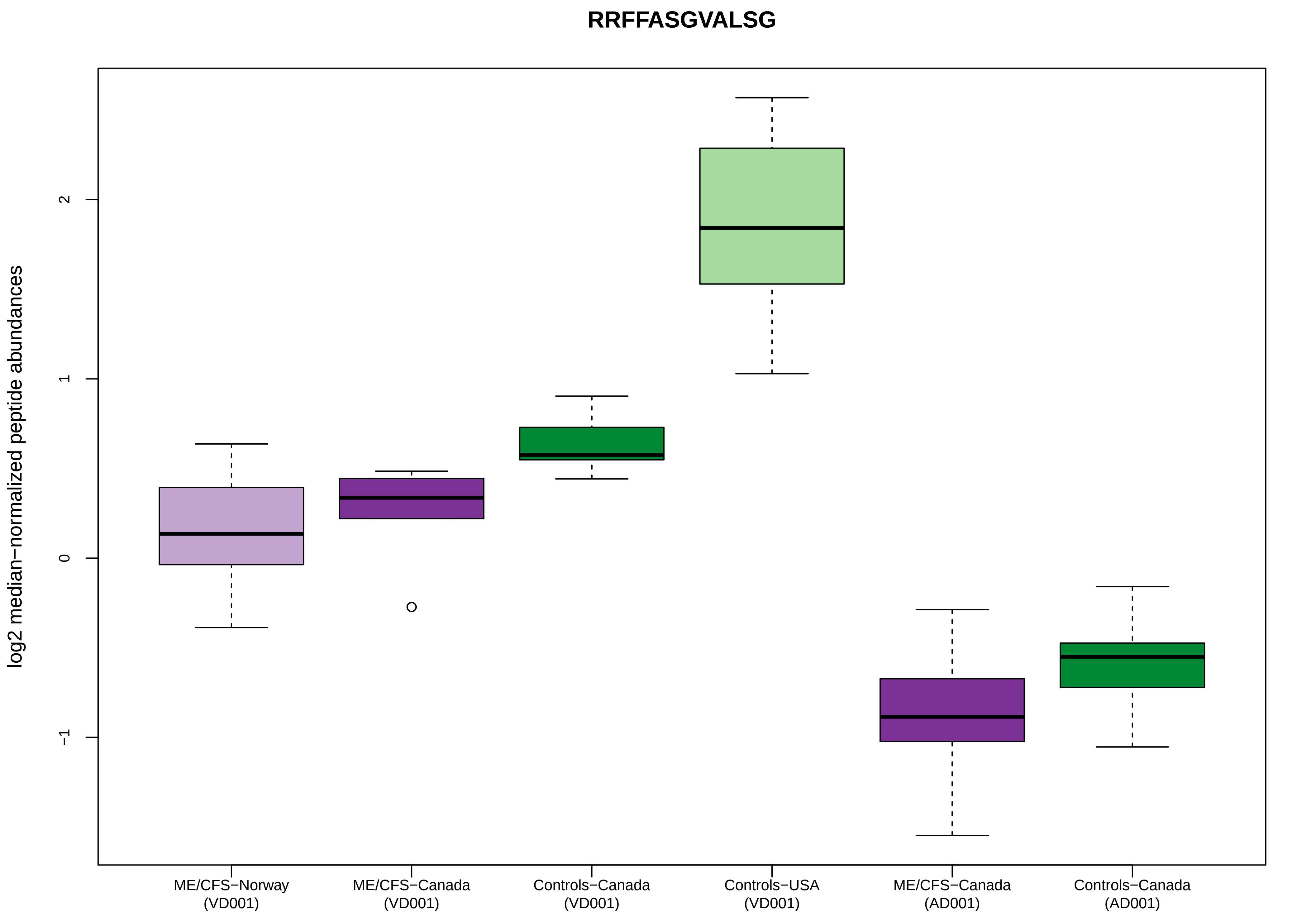

RRFFASGVALSG
log2 median−normalized peptide abundances
2
1
0
−1
ME/CFS−Norway
(VD001)
ME/CFS−Canada
(VD001)
Controls−Canada
(VD001)
Controls−USA
(VD001)
ME/CFS−Canada
(AD001)
Controls−Canada
(AD001)

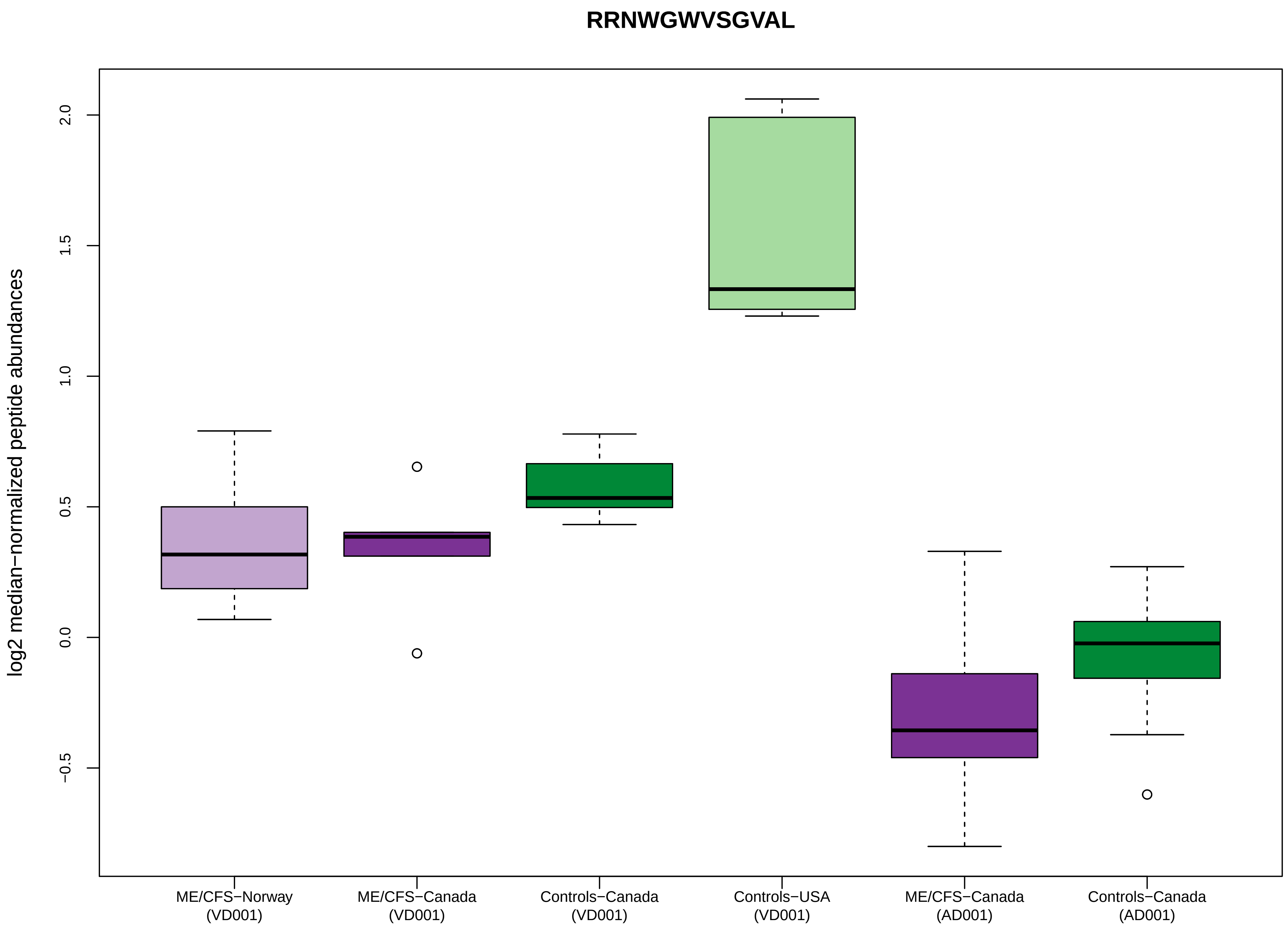
RRNWGWVSGVAL
log2 median−normalized peptide abundances
2.0
1.5
1.0
0.5
0.0
−0.5
ME/CFS−Norway (VD001)
ME/CFS−Canada (VD001)
Controls−Canada (VD001)
Controls−USA (VD001)
ME/CFS−Canada (AD001)
Controls−Canada (AD001)

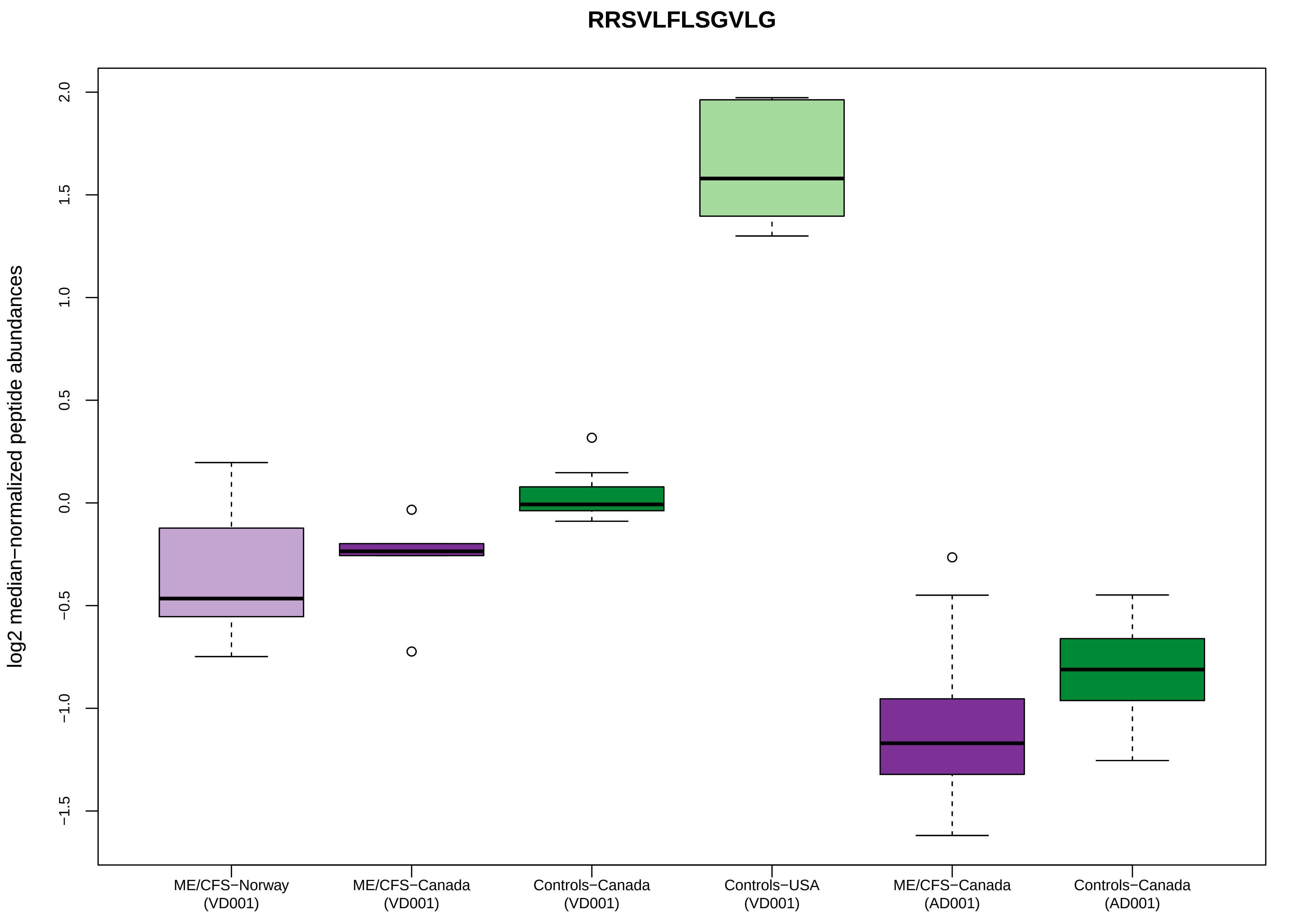

RRSVLFLSGVLG
log2 median−normalized peptide abundances
2.0
1.5
1.0
0.5
0.0
−0.5
−1.0
−1.5
ME/CFS−Norway (VD001)
ME/CFS−Canada (VD001)
Controls−Canada (VD001)
Controls−USA (VD001)
ME/CFS−Canada (AD001)
Controls−Canada (AD001)

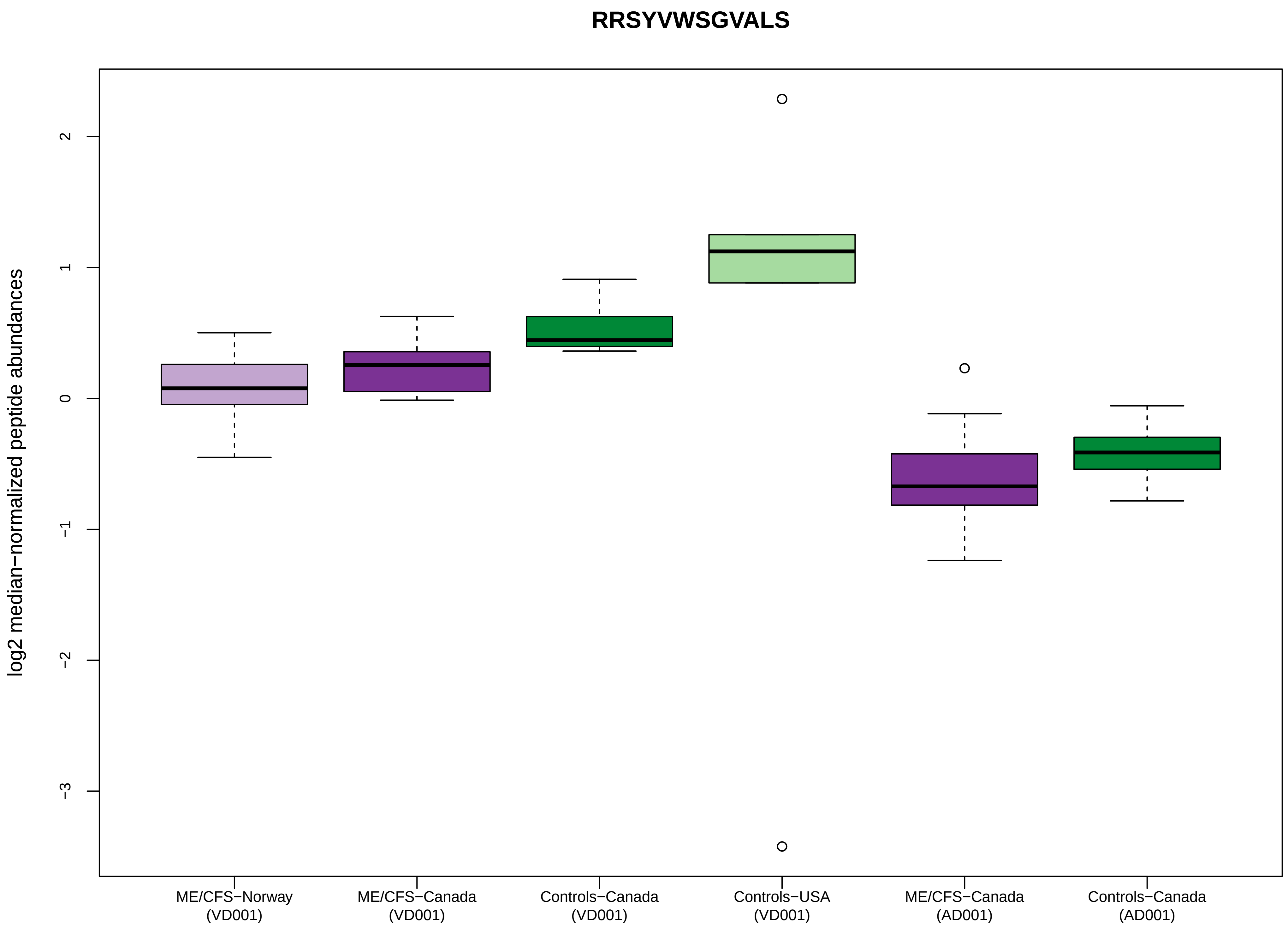

RRSYVWSGVALS
log2 median−normalized peptide abundances
2
1
0
−1
−2
−3
ME/CFS−Norway (VD001)
ME/CFS−Canada (VD001)
Controls−Canada (VD001)
Controls−USA (VD001)
ME/CFS−Canada (AD001)
Controls−Canada (AD001)

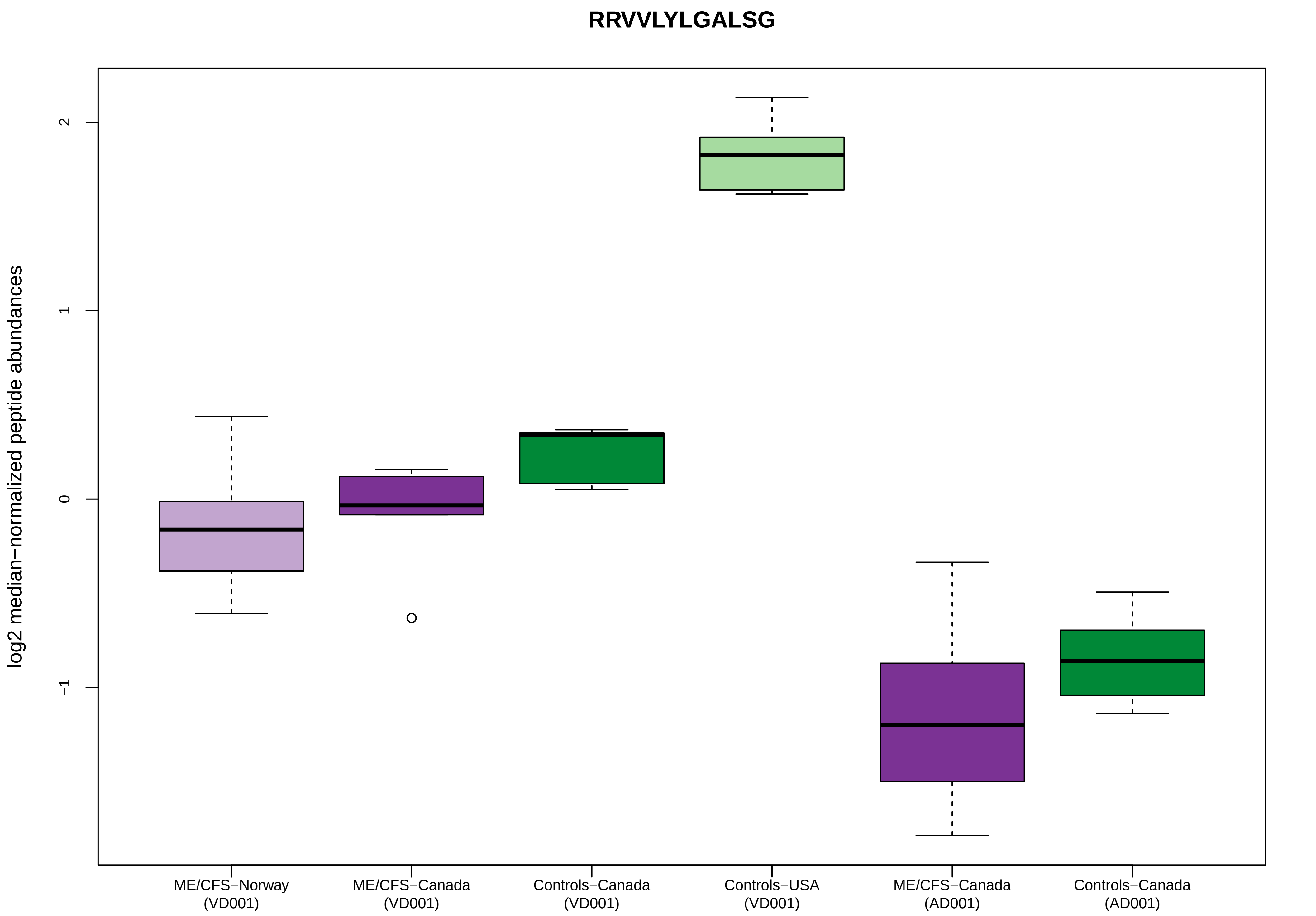

RRVVLYLGALSG
log2 median−normalized peptide abundances
2
1
0
−1
ME/CFS−Norway (VD001)
ME/CFS−Canada (VD001)
Controls−Canada (VD001)
Controls−USA (VD001)
ME/CFS−Canada (AD001)
Controls−Canada (AD001)

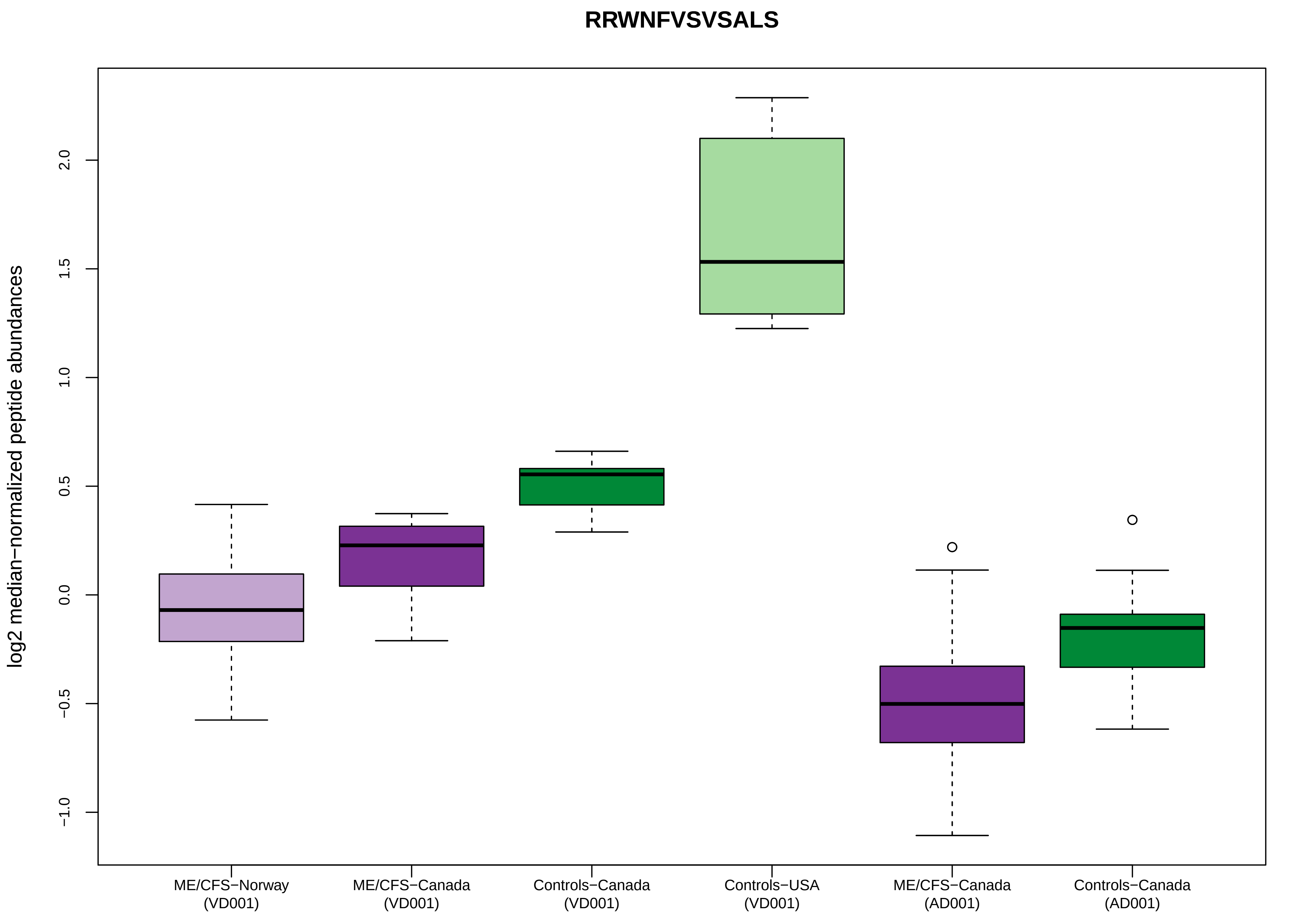
RRWNFVSVSALS
log2 median−normalized peptide abundances
2.0
1.5
1.0
0.5
0.0
−0.5
−1.0
ME/CFS−Norway (VD001)
ME/CFS−Canada (VD001)
Controls−Canada (VD001)
Controls−USA (VD001)
ME/CFS−Canada (AD001)
Controls−Canada (AD001)

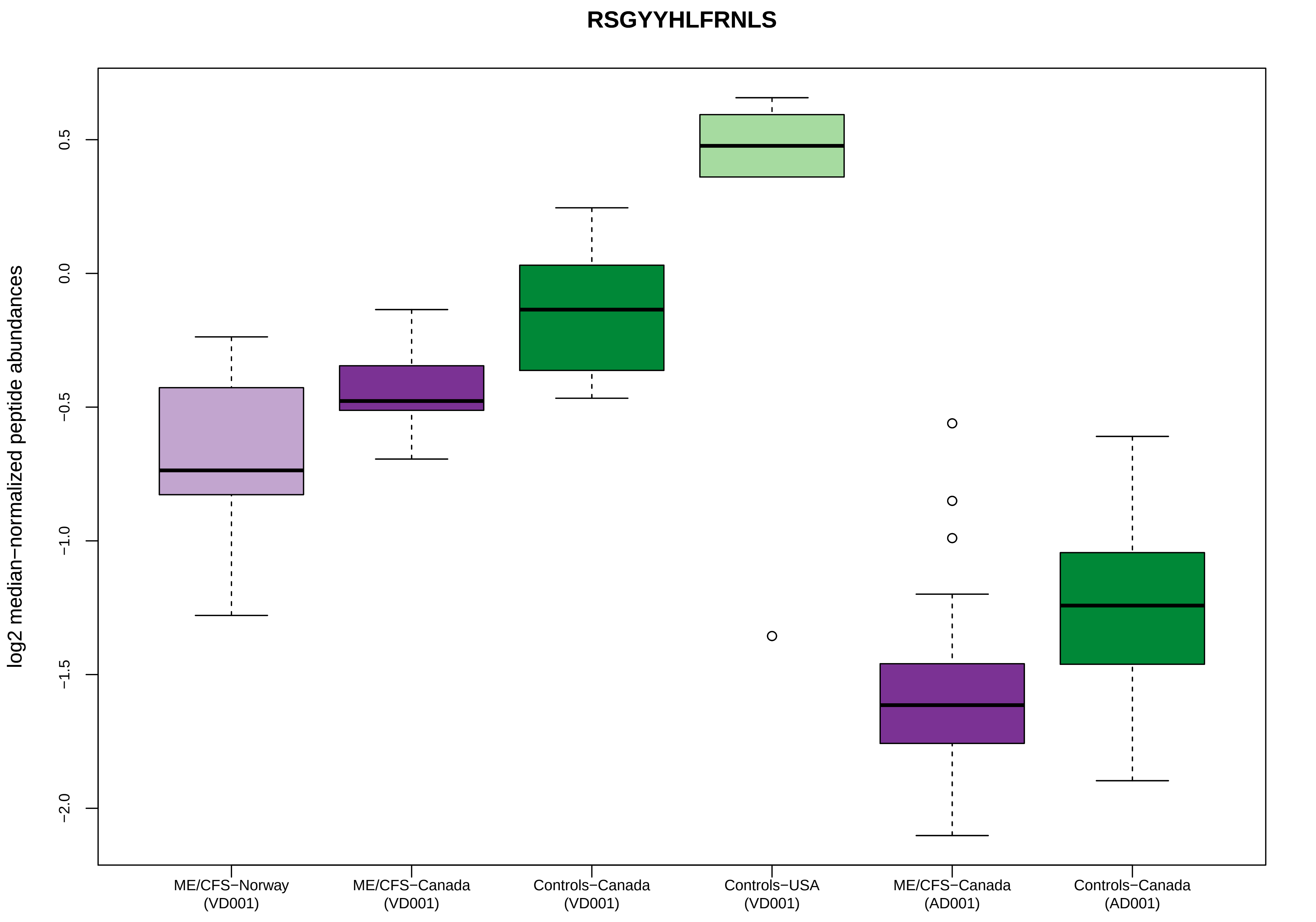

RSGYYHLFRNLS
log2 median−normalized peptide abundances
0.5
0.0
−0.5
−1.0
−1.5
−2.0
ME/CFS−Norway (VD001)
ME/CFS−Canada (VD001)
Controls−Canada (VD001)
Controls−USA (VD001)
ME/CFS−Canada (AD001)
Controls−Canada (AD001)

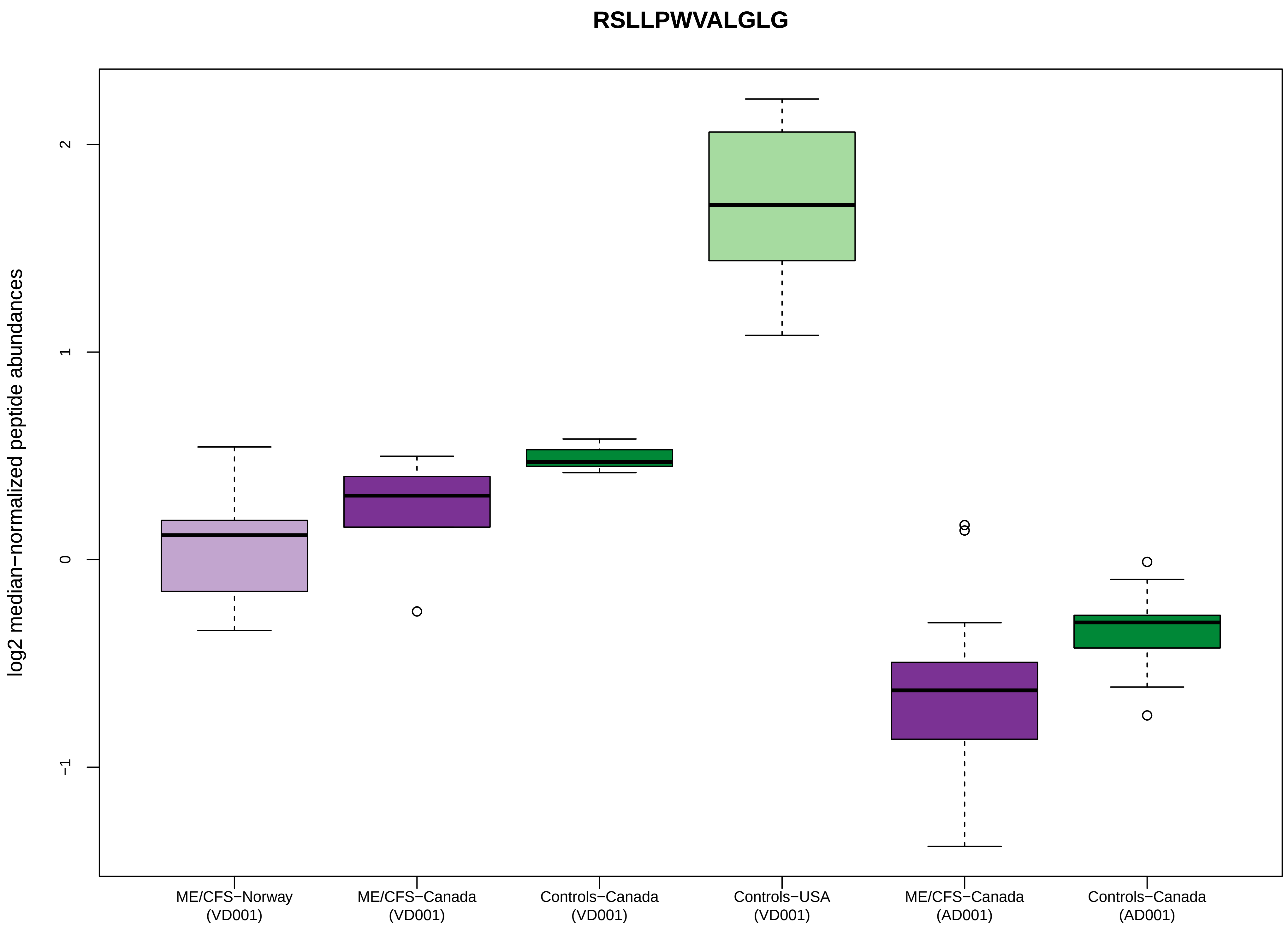

RSLLPWVALGLG
log2 median−normalized peptide abundances
2
1
0
−1
ME/CFS−Norway (VD001)
ME/CFS−Canada (VD001)
Controls−Canada (VD001)
Controls−USA (VD001)
ME/CFS−Canada (AD001)
Controls−Canada (AD001)

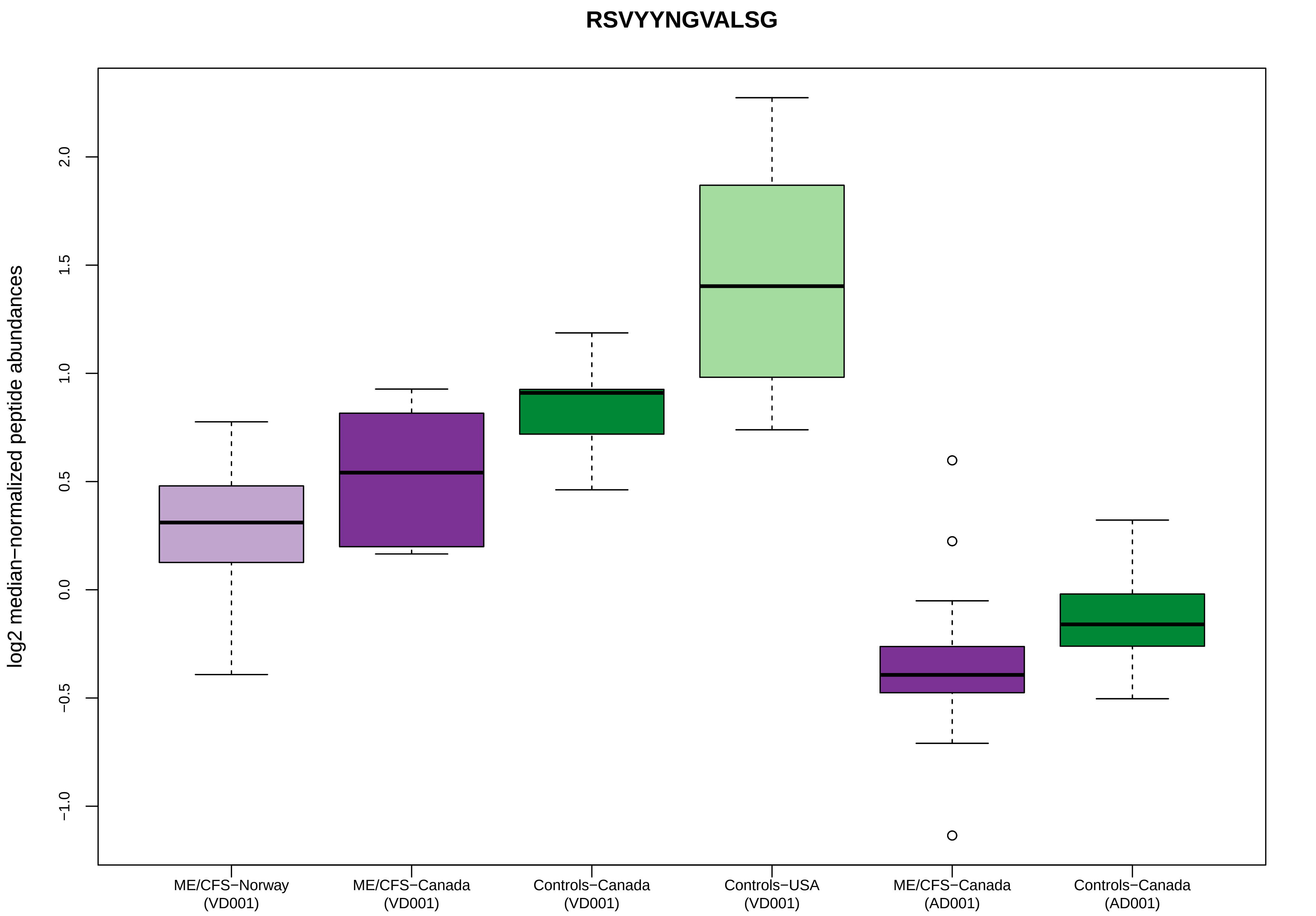

RSVYYNGVALSG
log2 median−normalized peptide abundances
−1.0
−0.5
0.0
0.5
1.0
1.5
2.0
ME/CFS−Norway (VD001)
ME/CFS−Canada (VD001)
Controls−Canada (VD001)
Controls−USA (VD001)
ME/CFS−Canada (AD001)
Controls−Canada (AD001)

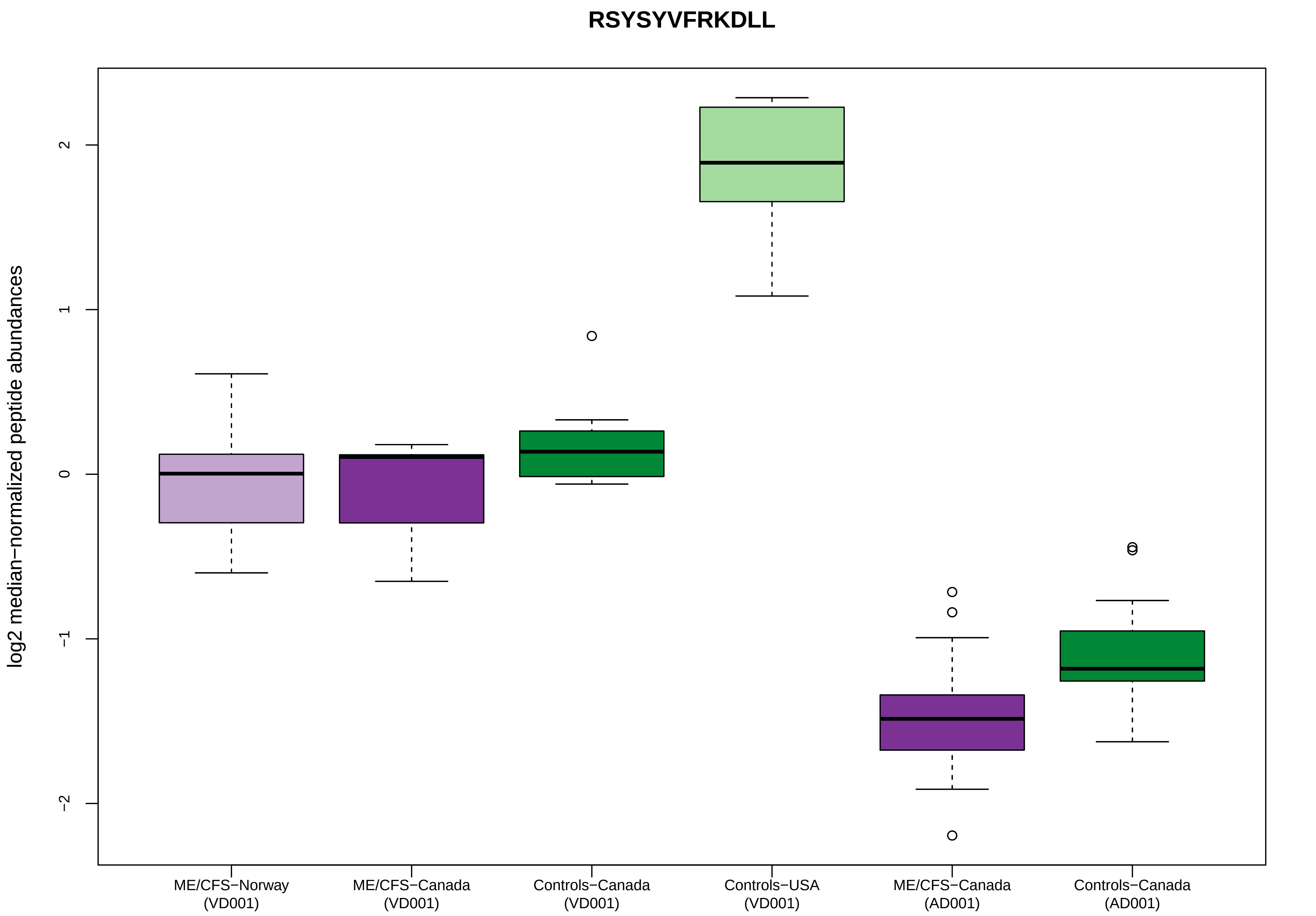
RSYSYVFRKDLL
log2 median−normalized peptide abundances
2
1
0
−1
−2
ME/CFS−Norway
(VD001)
ME/CFS−Canada
(VD001)
Controls−Canada
(VD001)
Controls−USA
(VD001)
ME/CFS−Canada
(AD001)
Controls−Canada
(AD001)

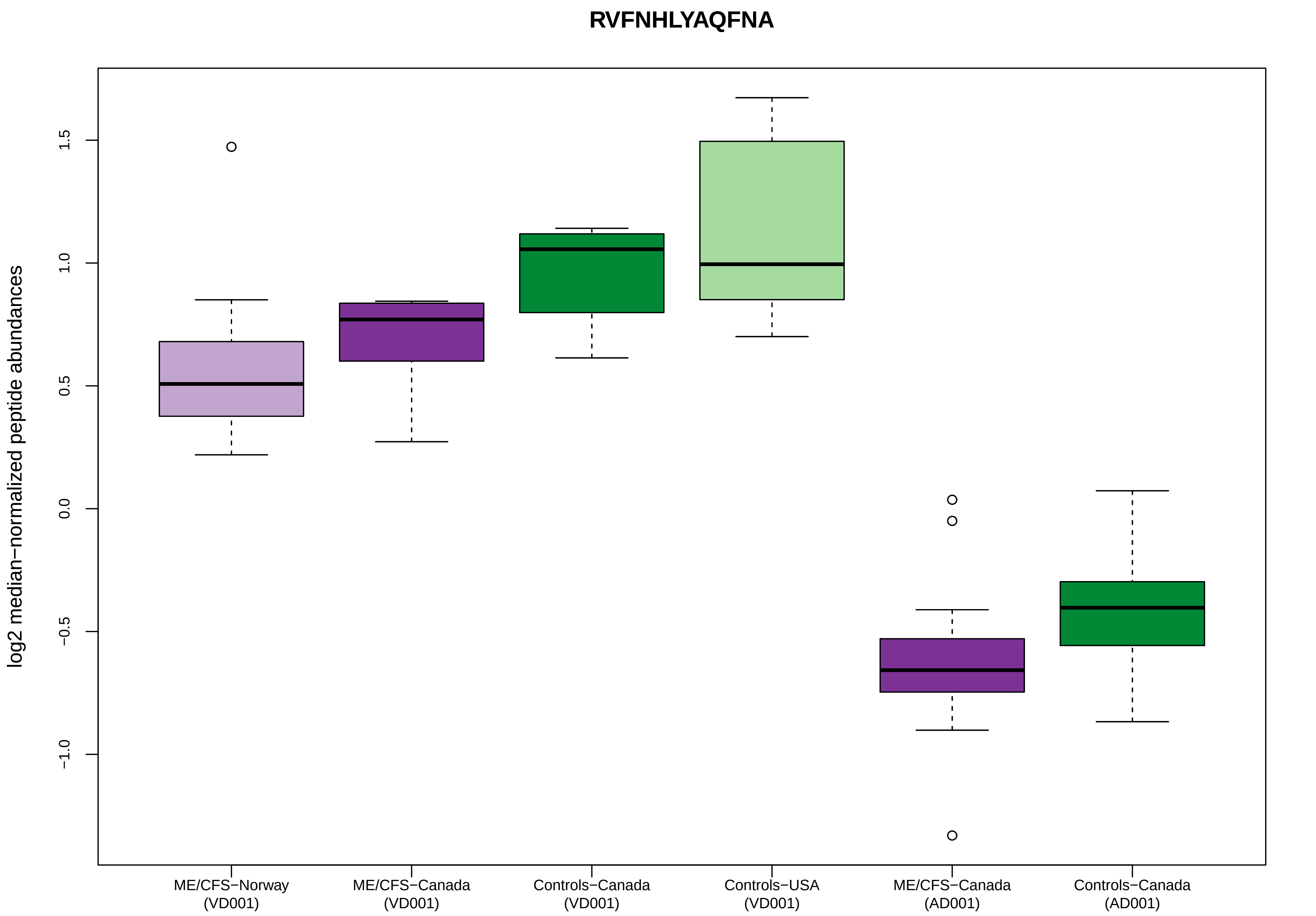

RVFNHLYAQFNA
log2 median−normalized peptide abundances
1.5
1.0
0.5
0.0
−0.5
−1.0
ME/CFS−Norway (VD001)
ME/CFS−Canada (VD001)
Controls−Canada (VD001)
Controls−USA (VD001)
ME/CFS−Canada (AD001)
Controls−Canada (AD001)

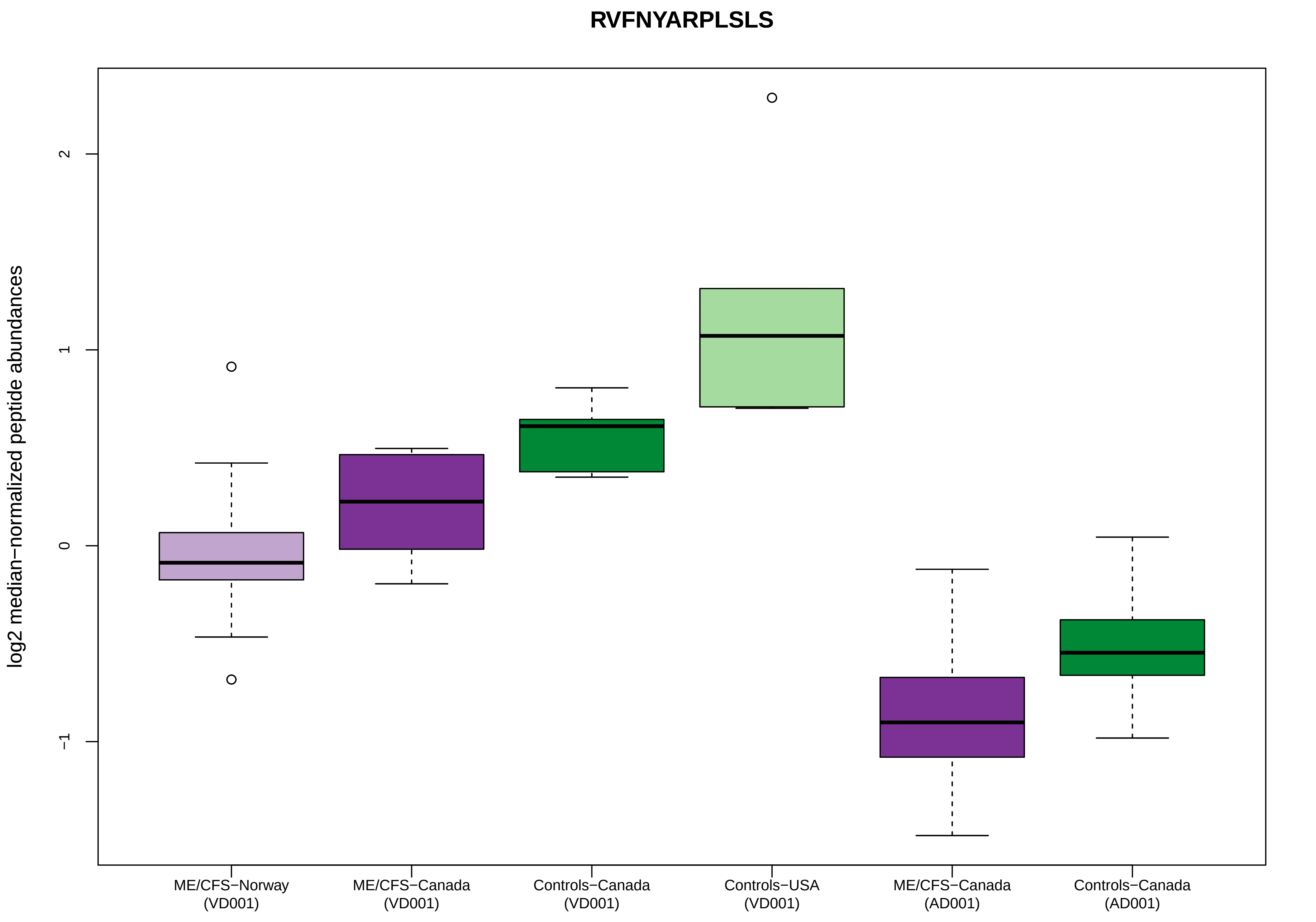
RVFNYARPLSLS
log2 median−normalized peptide abundances
2
1
0
−1
ME/CFS−Norway (VD001)
ME/CFS−Canada (VD001)
Controls−Canada (VD001)
Controls−USA (VD001)
ME/CFS−Canada (AD001)
Controls−Canada (AD001)

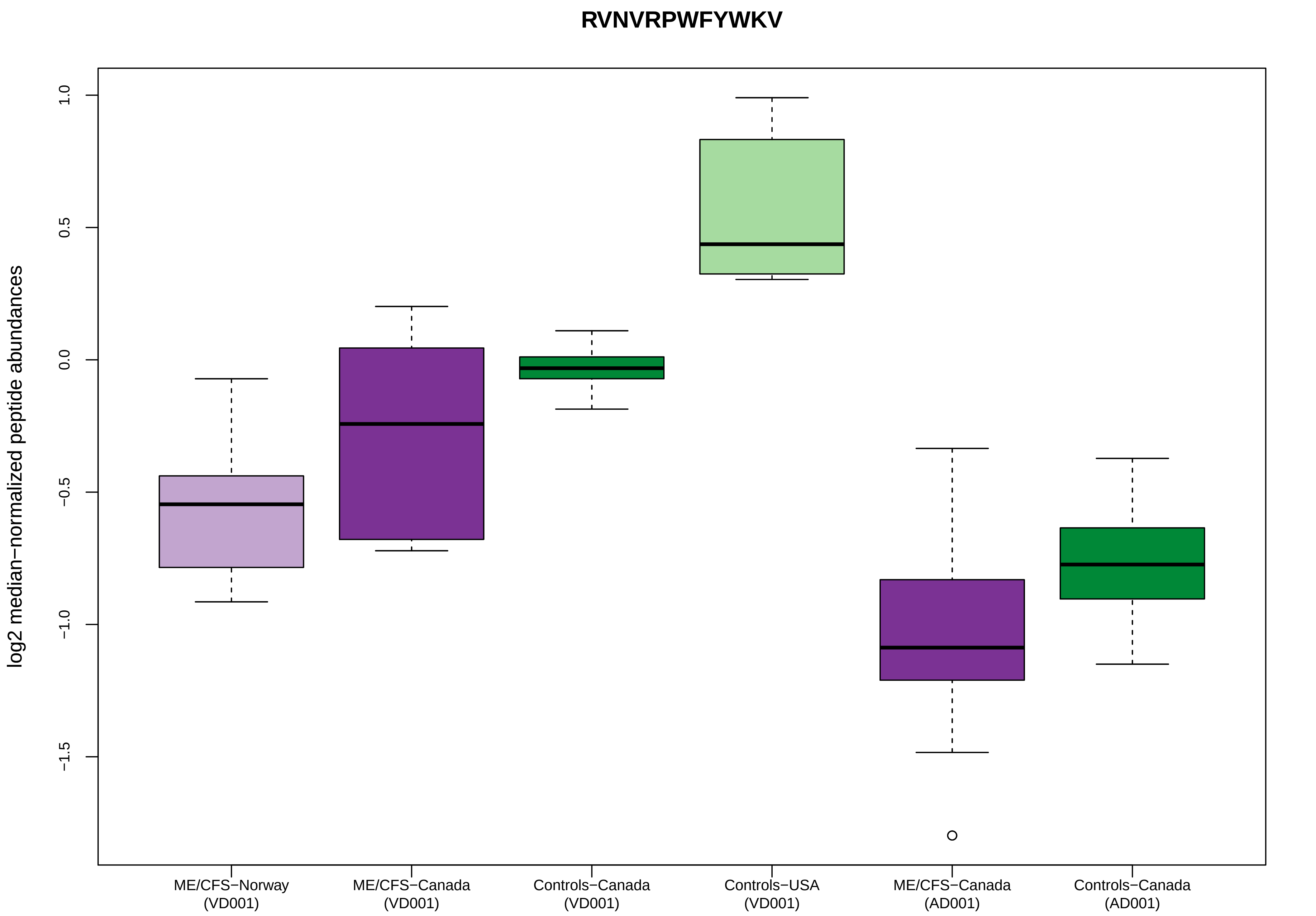
RVNVRPWFYWKV
log2 median−normalized peptide abundances
1.0
0.5
0.0
−0.5
−1.0
−1.5
ME/CFS−Norway (VD001)
ME/CFS−Canada (VD001)
Controls−Canada (VD001)
Controls−USA (VD001)
ME/CFS−Canada (AD001)
Controls−Canada (AD001)

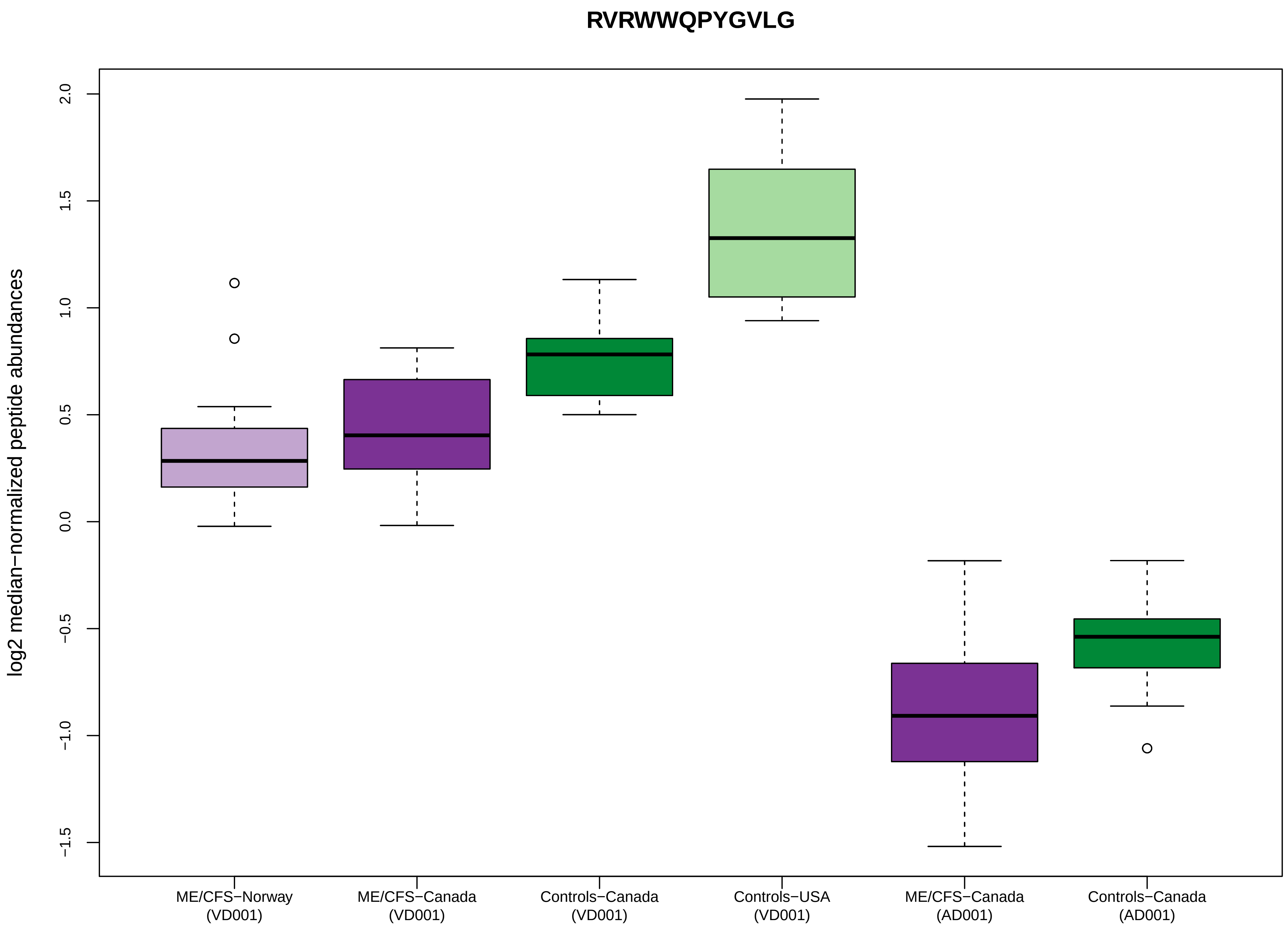
RVRWWQPYGVLG
log2 median−normalized peptide abundances
2.0
1.5
1.0
0.5
0.0
−0.5
−1.0
−1.5
ME/CFS−Norway (VD001)
ME/CFS−Canada (VD001)
Controls−Canada (VD001)
Controls−USA (VD001)
ME/CFS−Canada (AD001)
Controls−Canada (AD001)

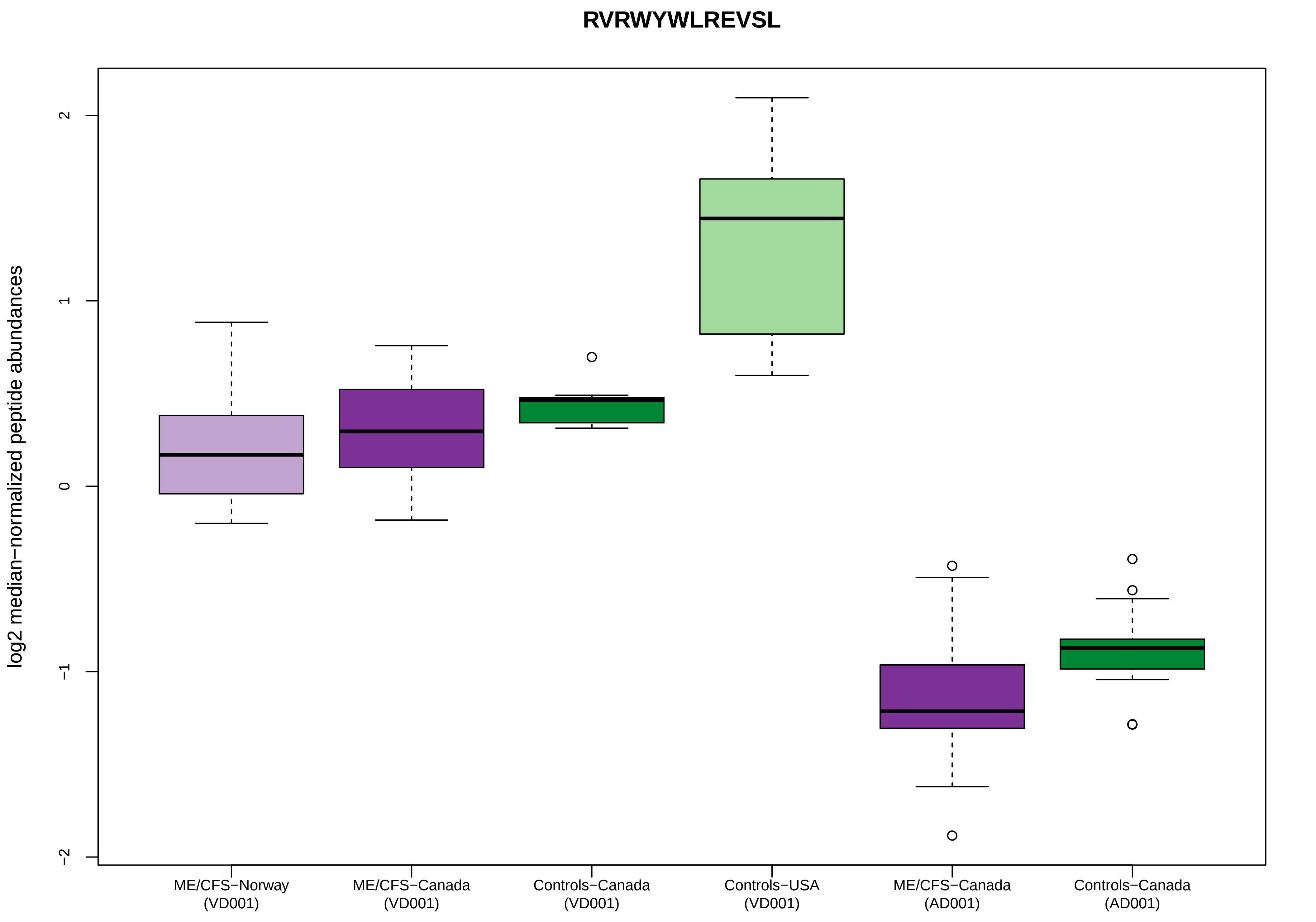
RVRWYWLREVSL
log2 median−normalized peptide abundances
2
1
0
−1
−2
ME/CFS−Norway (VD001)
ME/CFS−Canada (VD001)
Controls−Canada (VD001)
Controls−USA (VD001)
ME/CFS−Canada (AD001)
Controls−Canada (AD001)

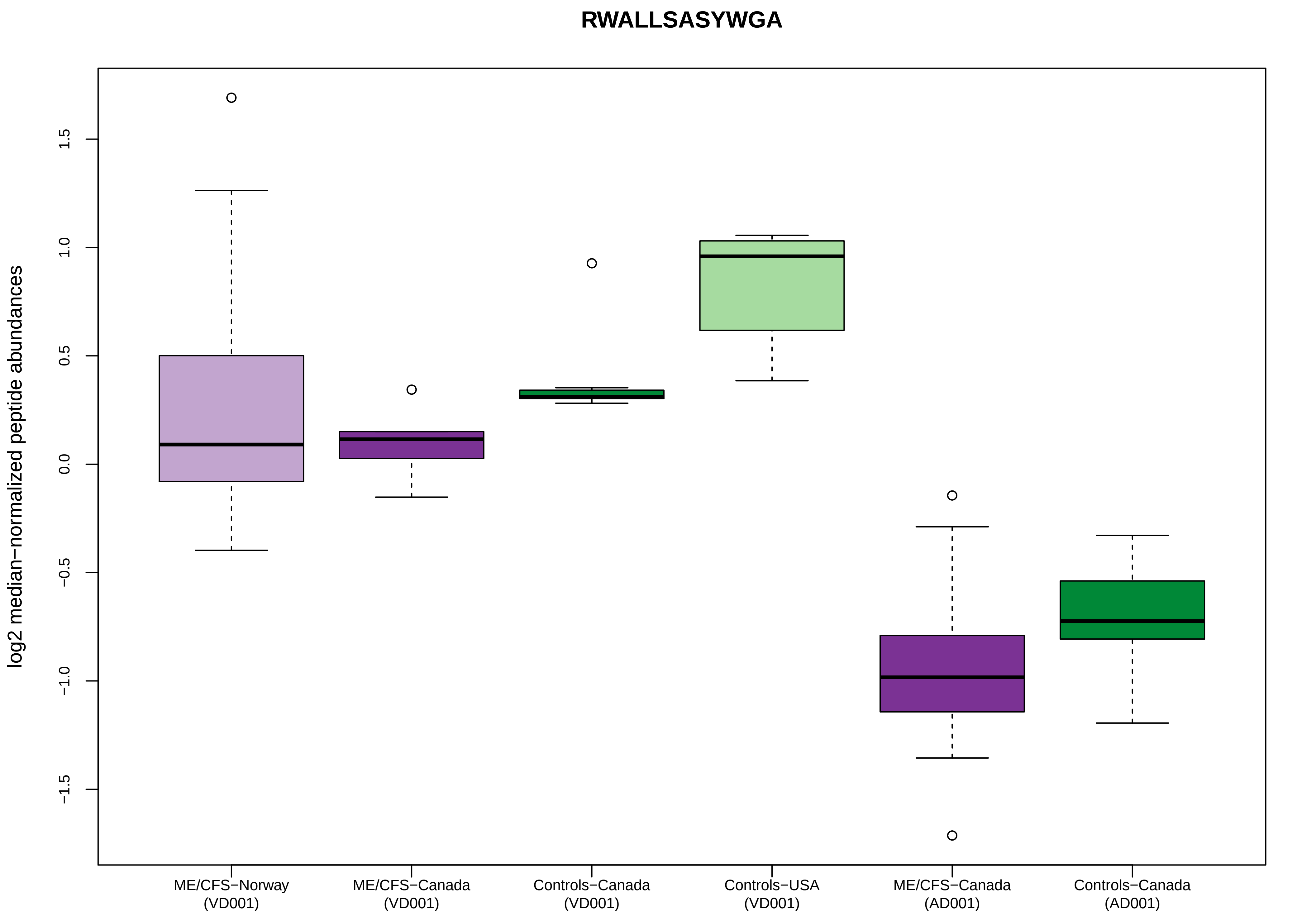
RWALLSASYWGA
log2 median−normalized peptide abundances
1.5
1.0
0.5
0.0
−0.5
−1.0
−1.5
ME/CFS−Norway (VD001)
ME/CFS−Canada (VD001)
Controls−Canada (VD001)
Controls−USA (VD001)
ME/CFS−Canada (AD001)
Controls−Canada (AD001)

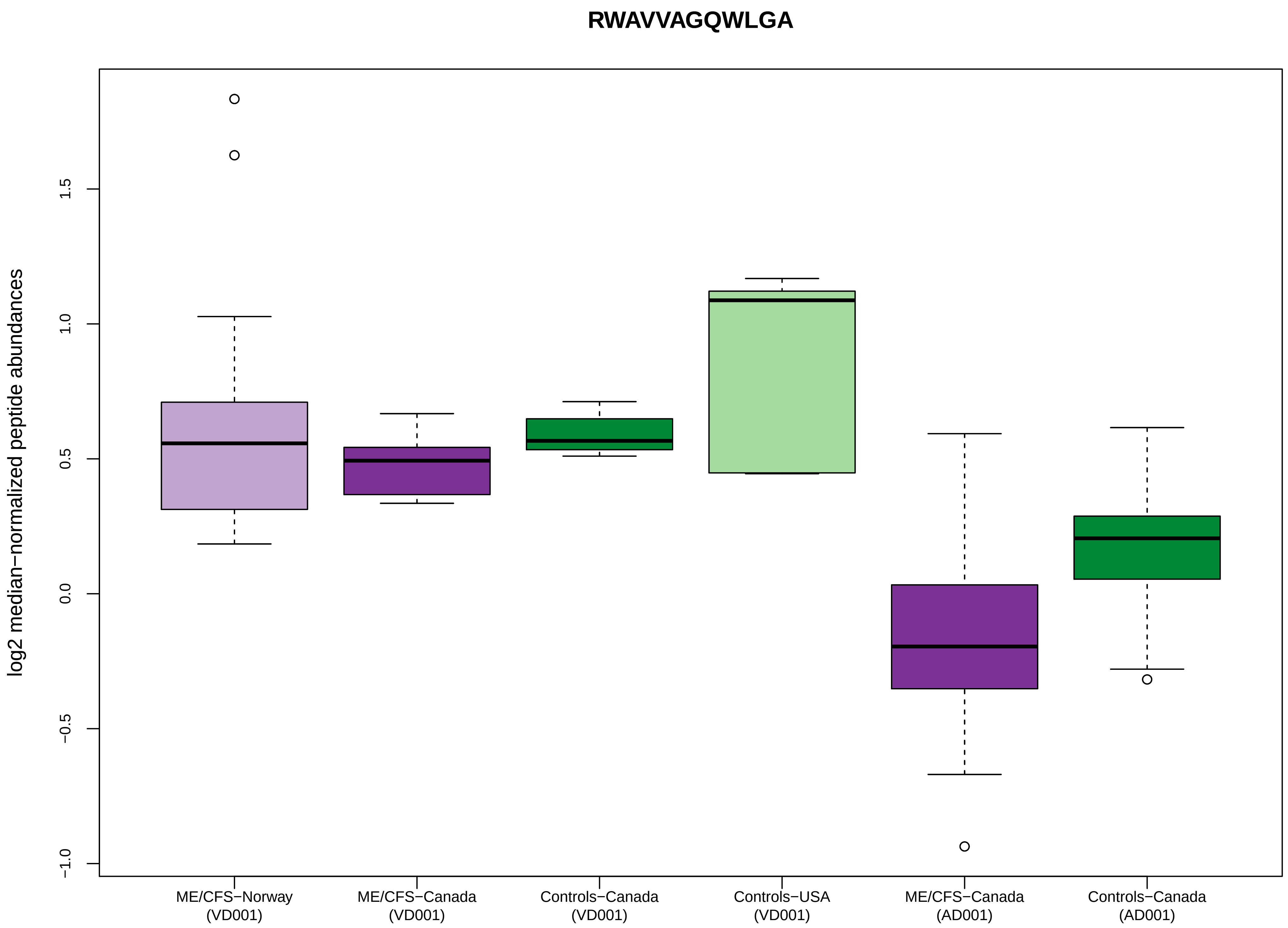

RWAVVAGQWLGA
log2 median−normalized peptide abundances
1.5
1.0
0.5
0.0
−0.5
−1.0
ME/CFS−Norway (VD001)
ME/CFS−Canada (VD001)
Controls−Canada (VD001)
Controls−USA (VD001)
ME/CFS−Canada (AD001)
Controls−Canada (AD001)

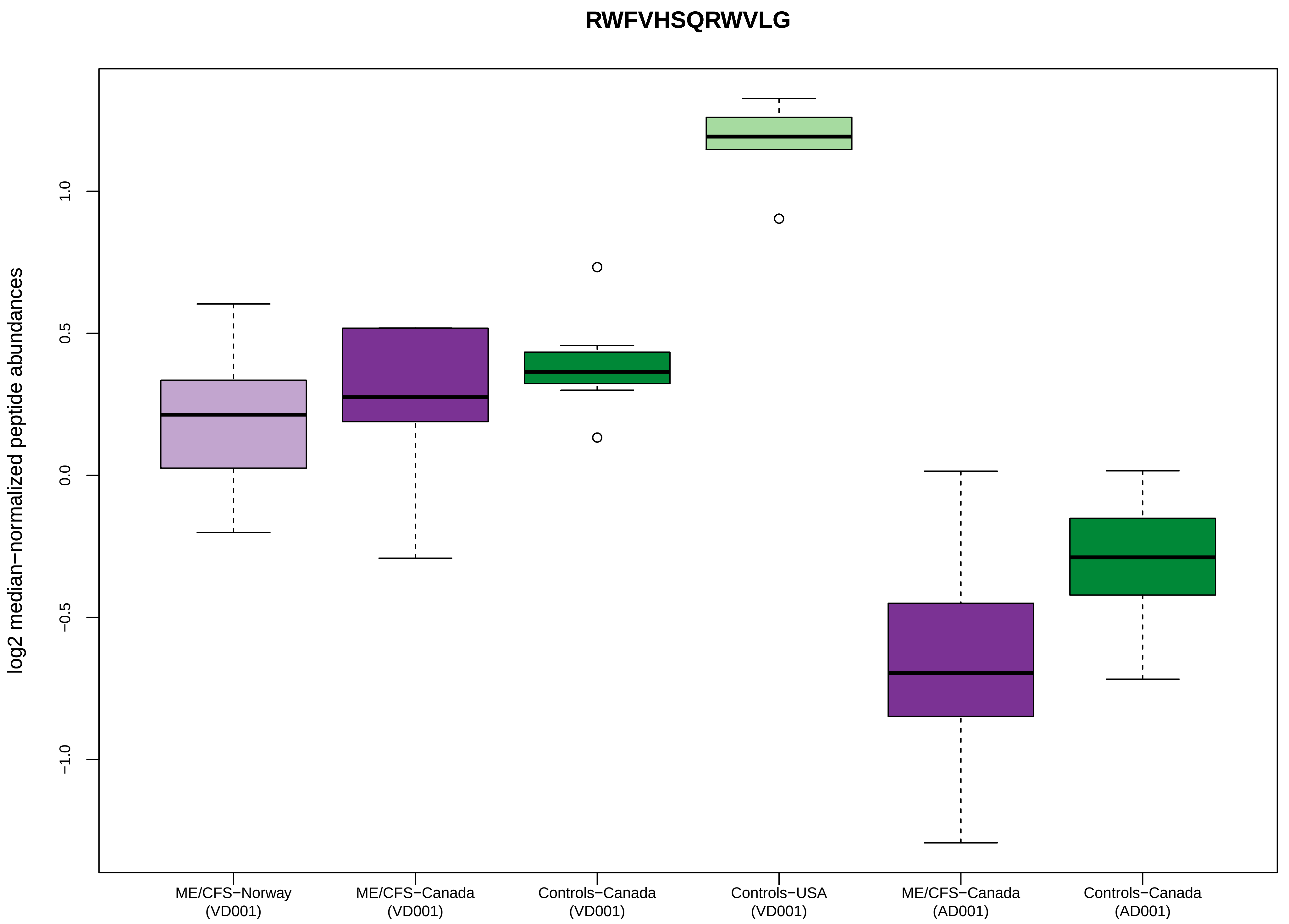
RWFVHSQRWVLG
log2 median−normalized peptide abundances
1.0
0.5
0.0
−0.5
−1.0
ME/CFS−Norway (VD001)
ME/CFS−Canada (VD001)
Controls−Canada (VD001)
Controls−USA (VD001)
ME/CFS−Canada (AD001)
Controls−Canada (AD001)

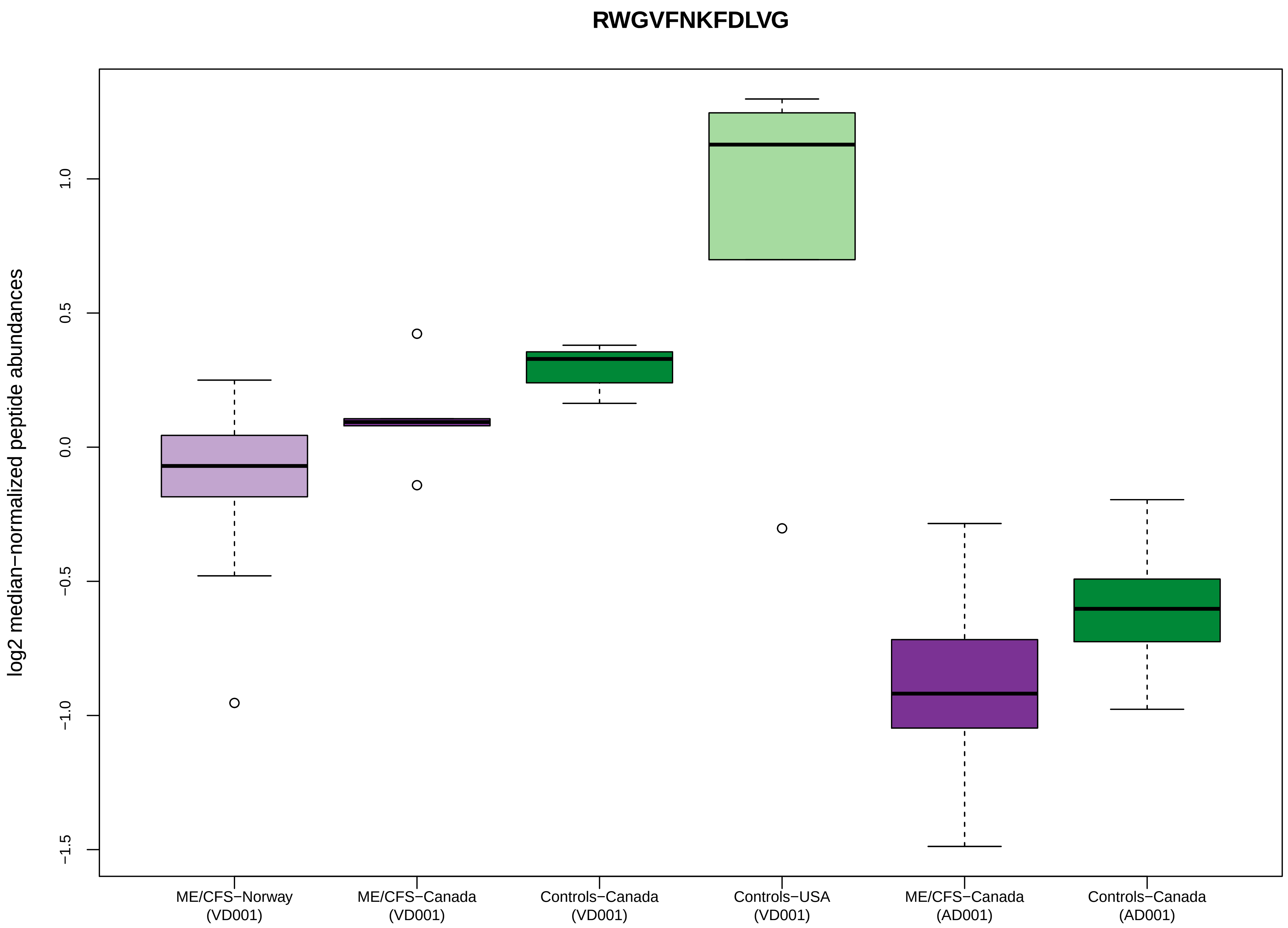

RWGVFNKFDLVG
log2 median−normalized peptide abundances
1.0
0.5
0.0
−0.5
−1.0
−1.5
ME/CFS−Norway (VD001)
ME/CFS−Canada (VD001)
Controls−Canada (VD001)
Controls−USA (VD001)
ME/CFS−Canada (AD001)
Controls−Canada (AD001)

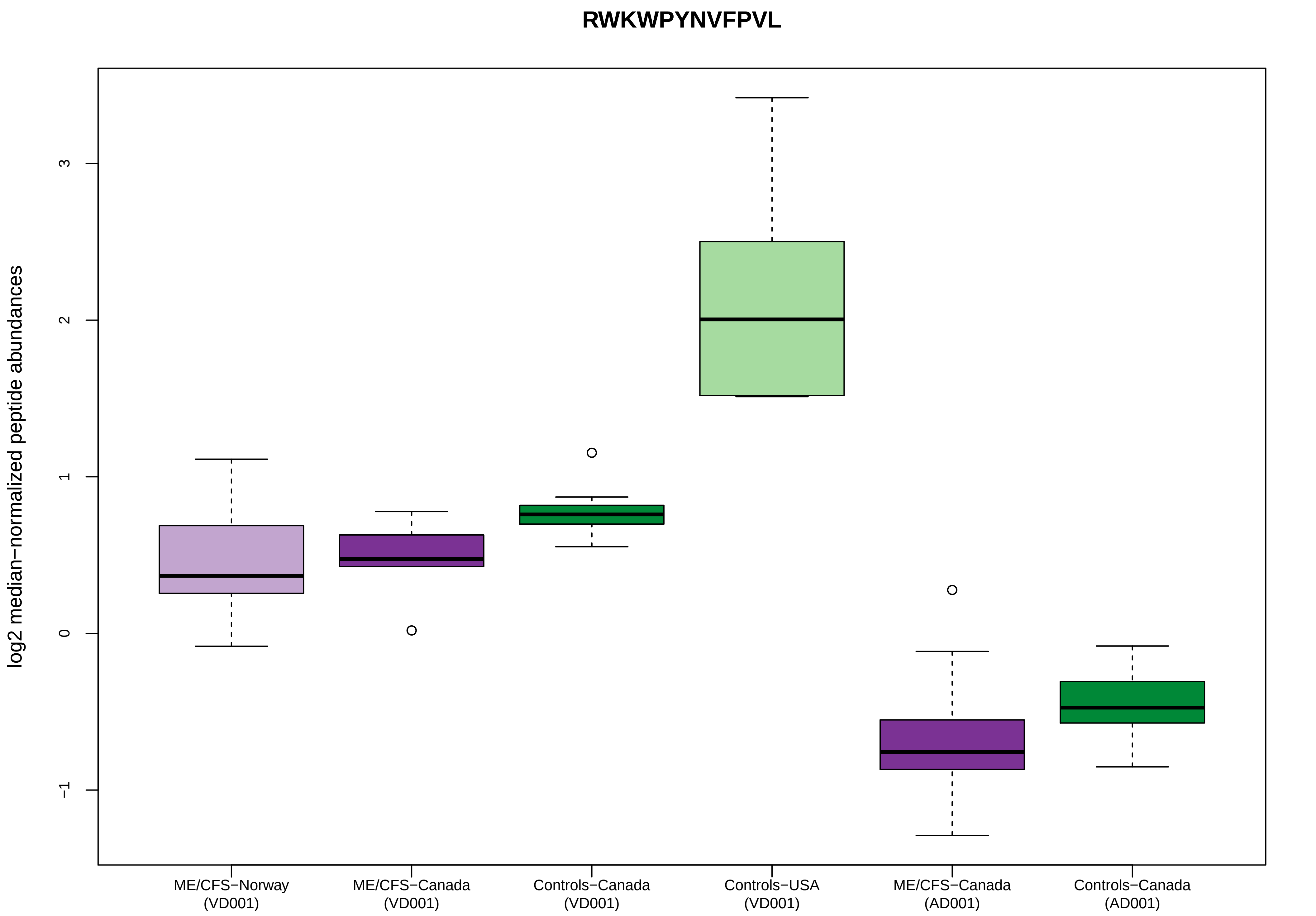
RWKWPYNVFPVL
log2 median−normalized peptide abundances
−1
0
1
2
3
ME/CFS−Norway (VD001)
ME/CFS−Canada (VD001)
Controls−Canada (VD001)
Controls−USA (VD001)
ME/CFS−Canada (AD001)
Controls−Canada (AD001)

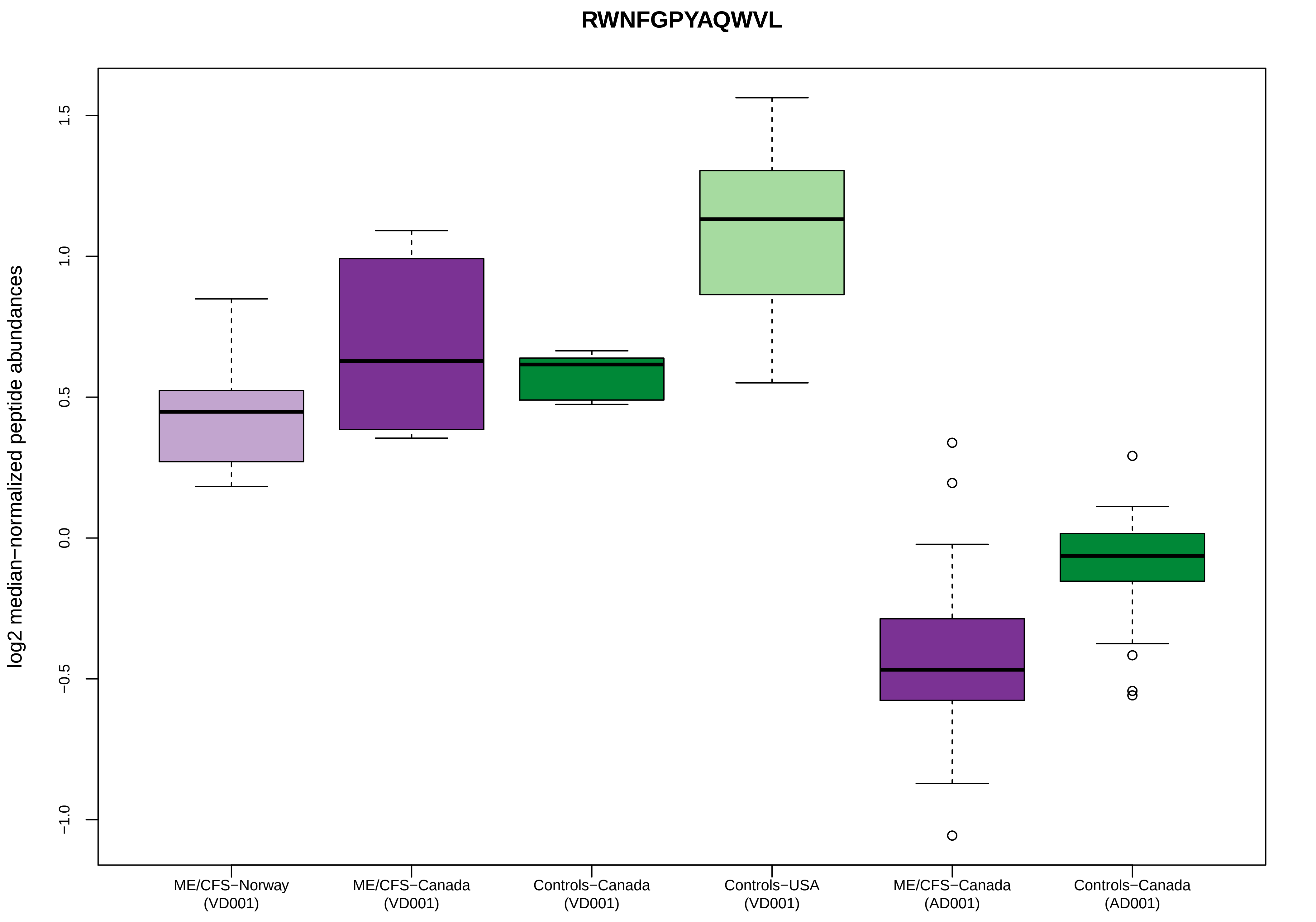
RWNFGPYAQWVL
log2 median−normalized peptide abundances
1.5
1.0
0.5
0.0
−0.5
−1.0
ME/CFS−Norway (VD001)
ME/CFS−Canada (VD001)
Controls−Canada (VD001)
Controls−USA (VD001)
ME/CFS−Canada (AD001)
Controls−Canada (AD001)

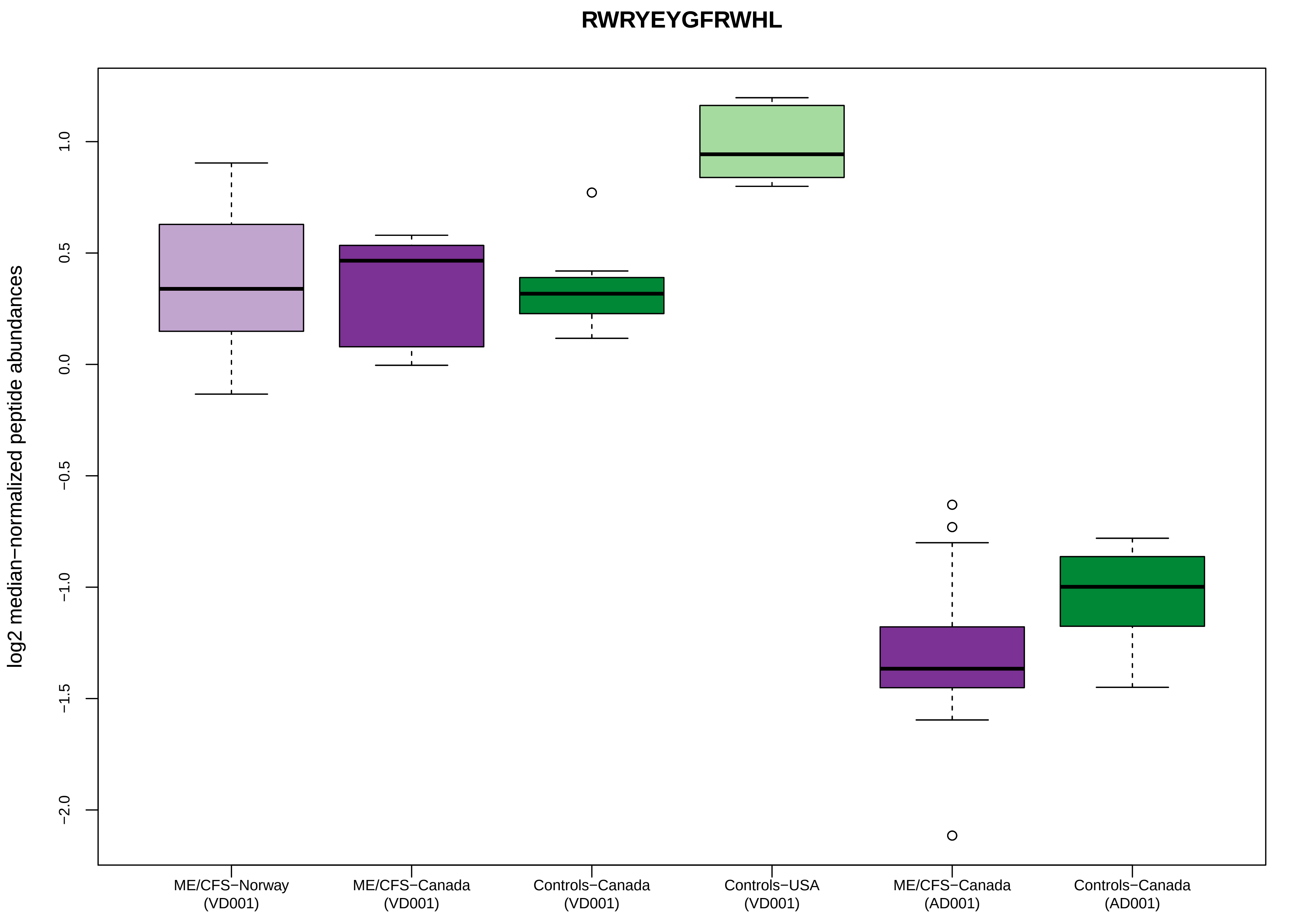

RWRYEYGFRWHL
log2 median−normalized peptide abundances
1.0
0.5
0.0
−0.5
−1.0
−1.5
−2.0
ME/CFS−Norway (VD001)
ME/CFS−Canada (VD001)
Controls−Canada (VD001)
Controls−USA (VD001)
ME/CFS−Canada (AD001)
Controls−Canada (AD001)

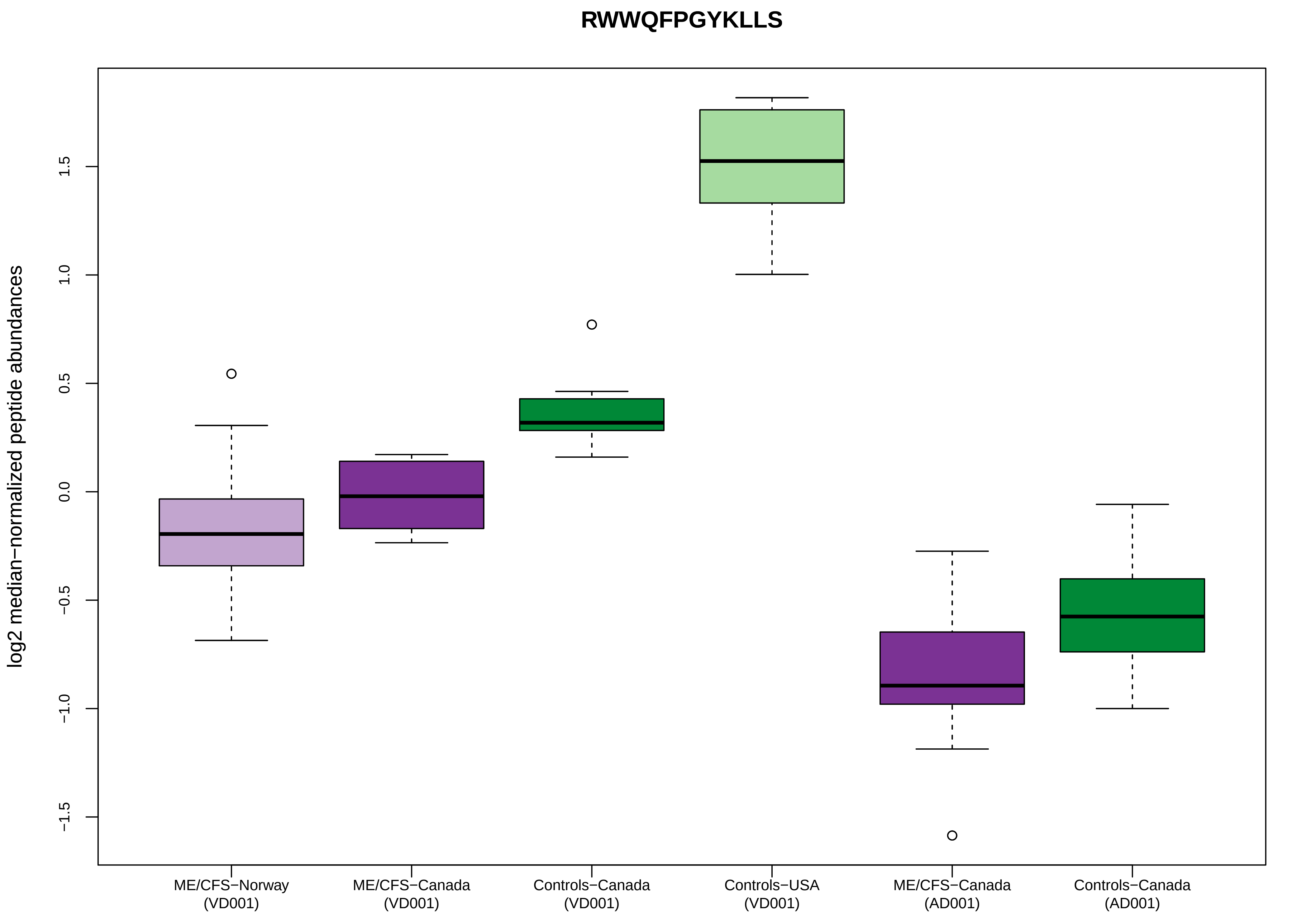
RWWQFPGYKLLS
log2 median−normalized peptide abundances
1.5
1.0
0.5
0.0
−0.5
−1.0
−1.5
ME/CFS−Norway (VD001)
ME/CFS−Canada (VD001)
Controls−Canada (VD001)
Controls−USA (VD001)
ME/CFS−Canada (AD001)
Controls−Canada (AD001)

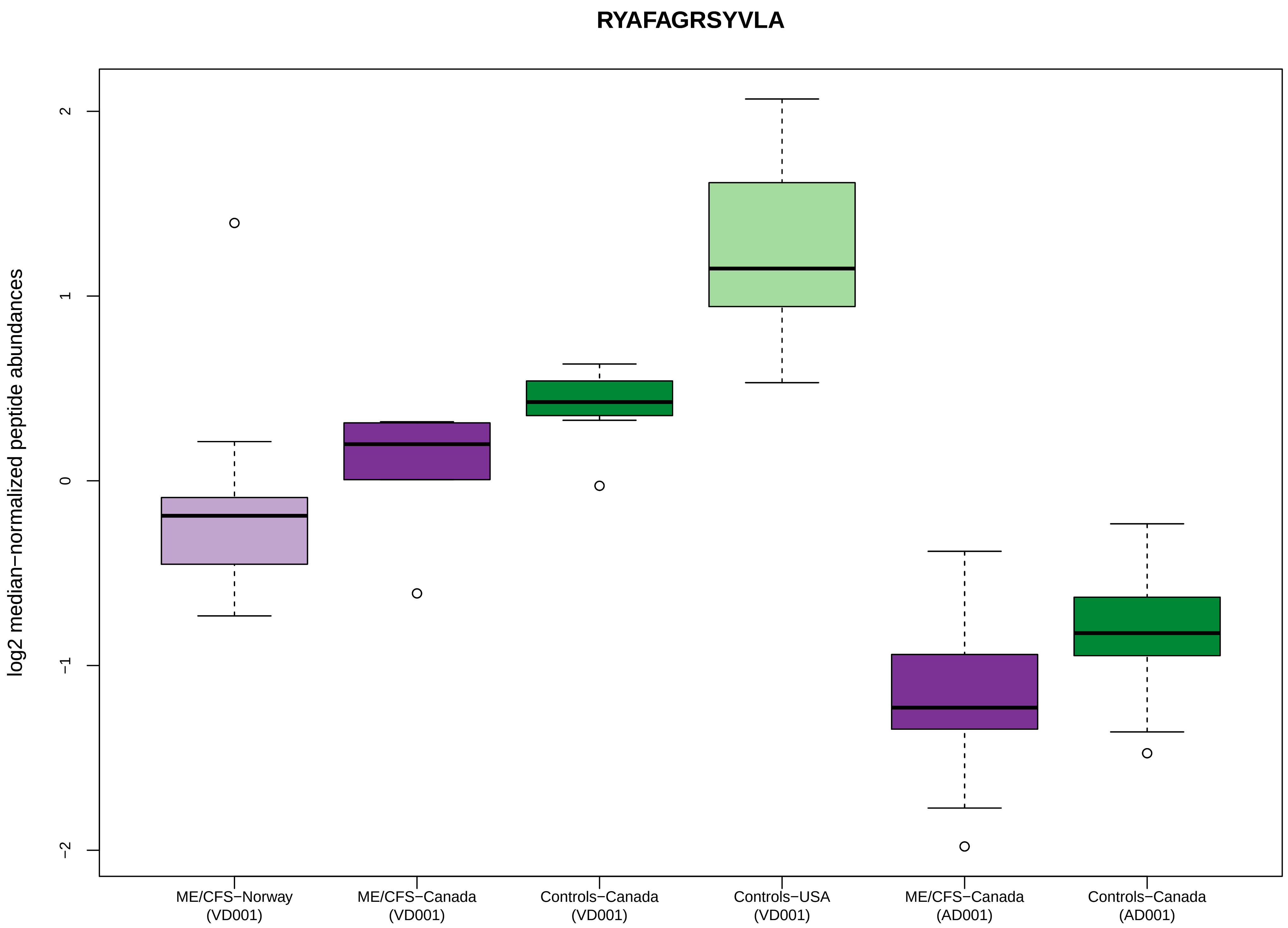
RYAFAGRSYVLA
log2 median−normalized peptide abundances
2
1
0
−1
−2
ME/CFS−Norway (VD001)
ME/CFS−Canada (VD001)
Controls−Canada (VD001)
Controls−USA (VD001)
ME/CFS−Canada (AD001)
Controls−Canada (AD001)

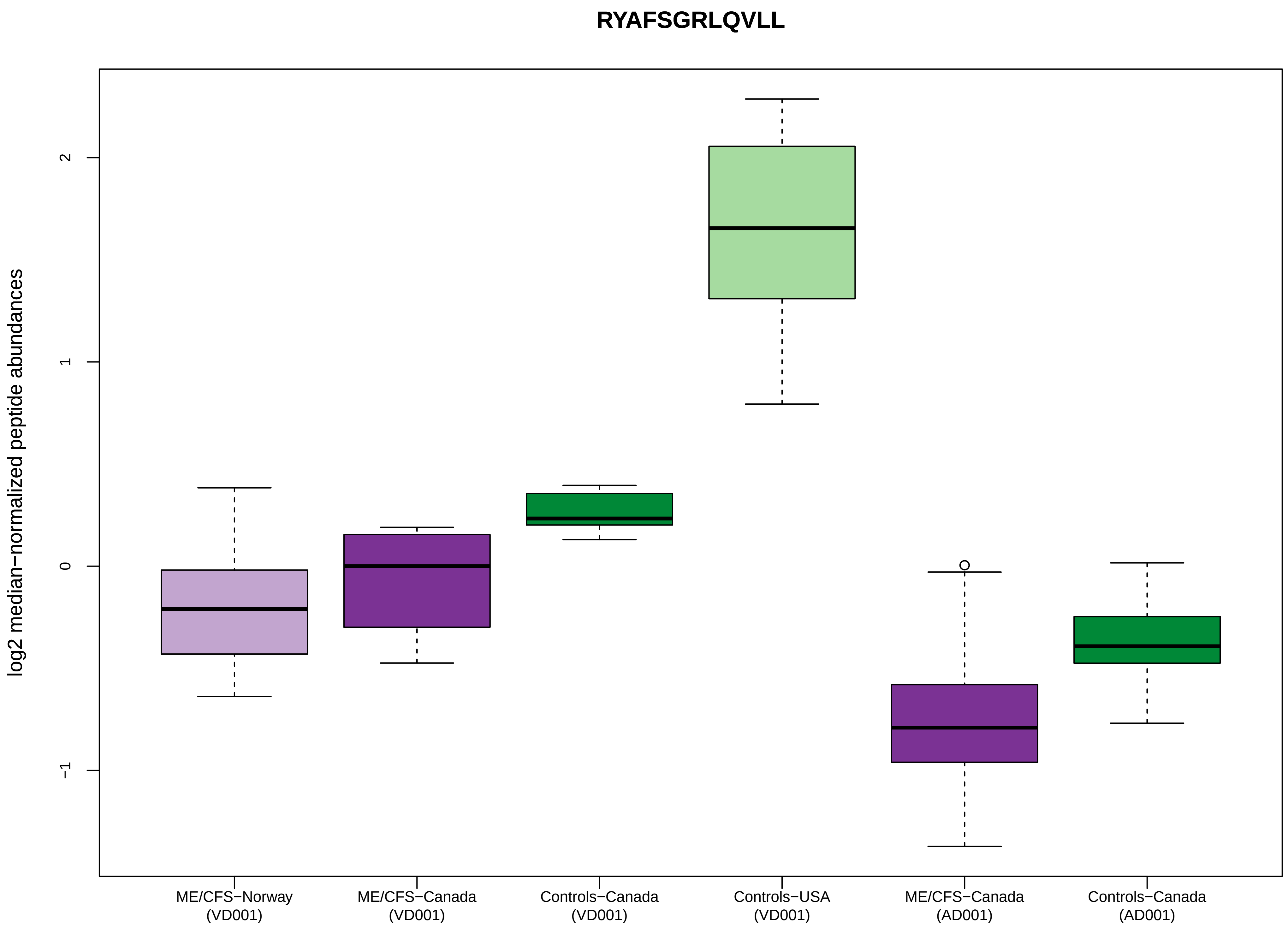

RYAFSGRLQVLL
log2 median−normalized peptide abundances
2
1
0
−1
ME/CFS−Norway (VD001)
ME/CFS−Canada (VD001)
Controls−Canada (VD001)
Controls−USA (VD001)
ME/CFS−Canada (AD001)
Controls−Canada (AD001)

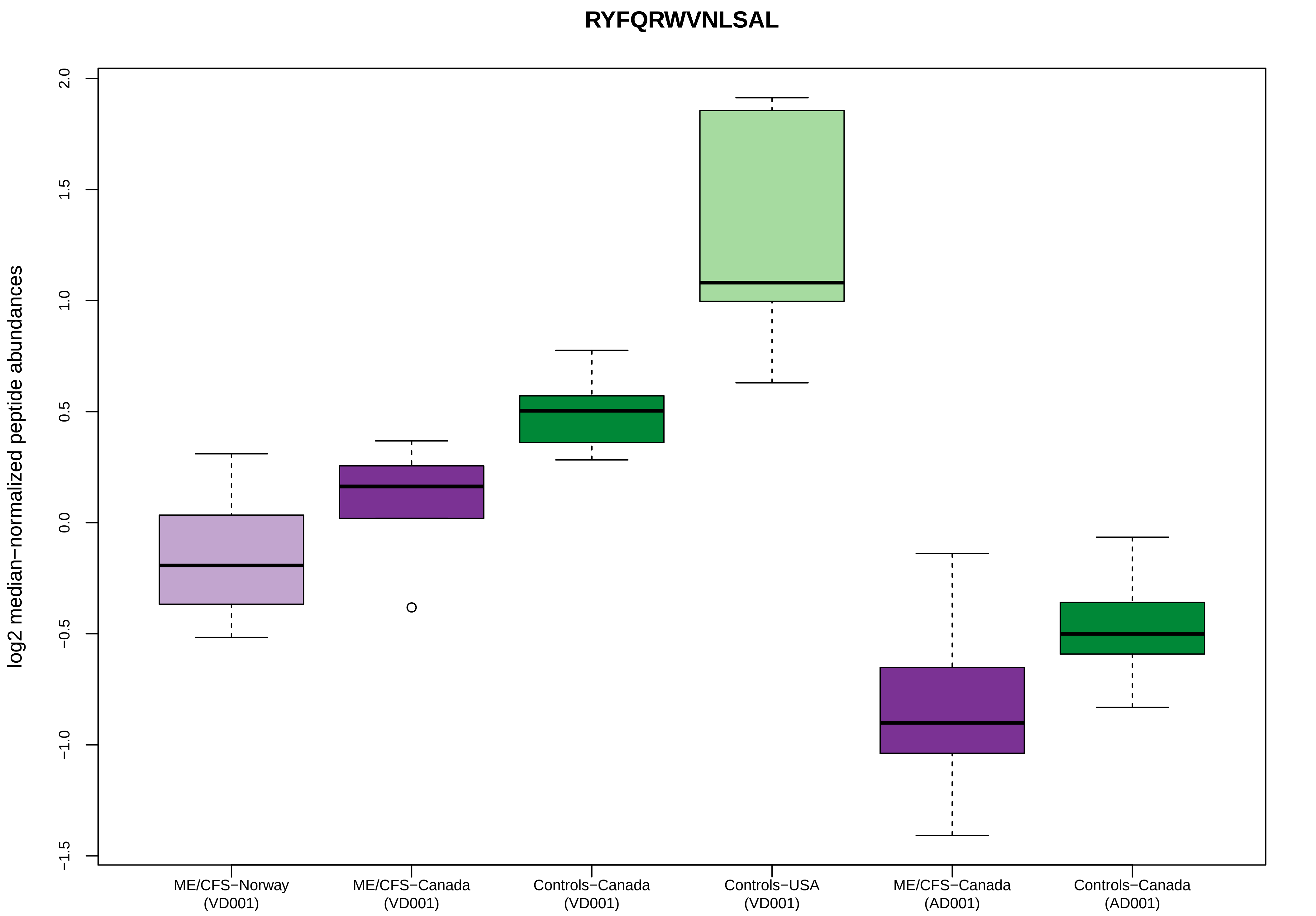

RYFQRWVNLSAL
log2 median−normalized peptide abundances
2.0
1.5
1.0
0.5
0.0
−0.5
−1.0
−1.5
ME/CFS−Norway (VD001)
ME/CFS−Canada (VD001)
Controls−Canada (VD001)
Controls−USA (VD001)
ME/CFS−Canada (AD001)
Controls−Canada (AD001)

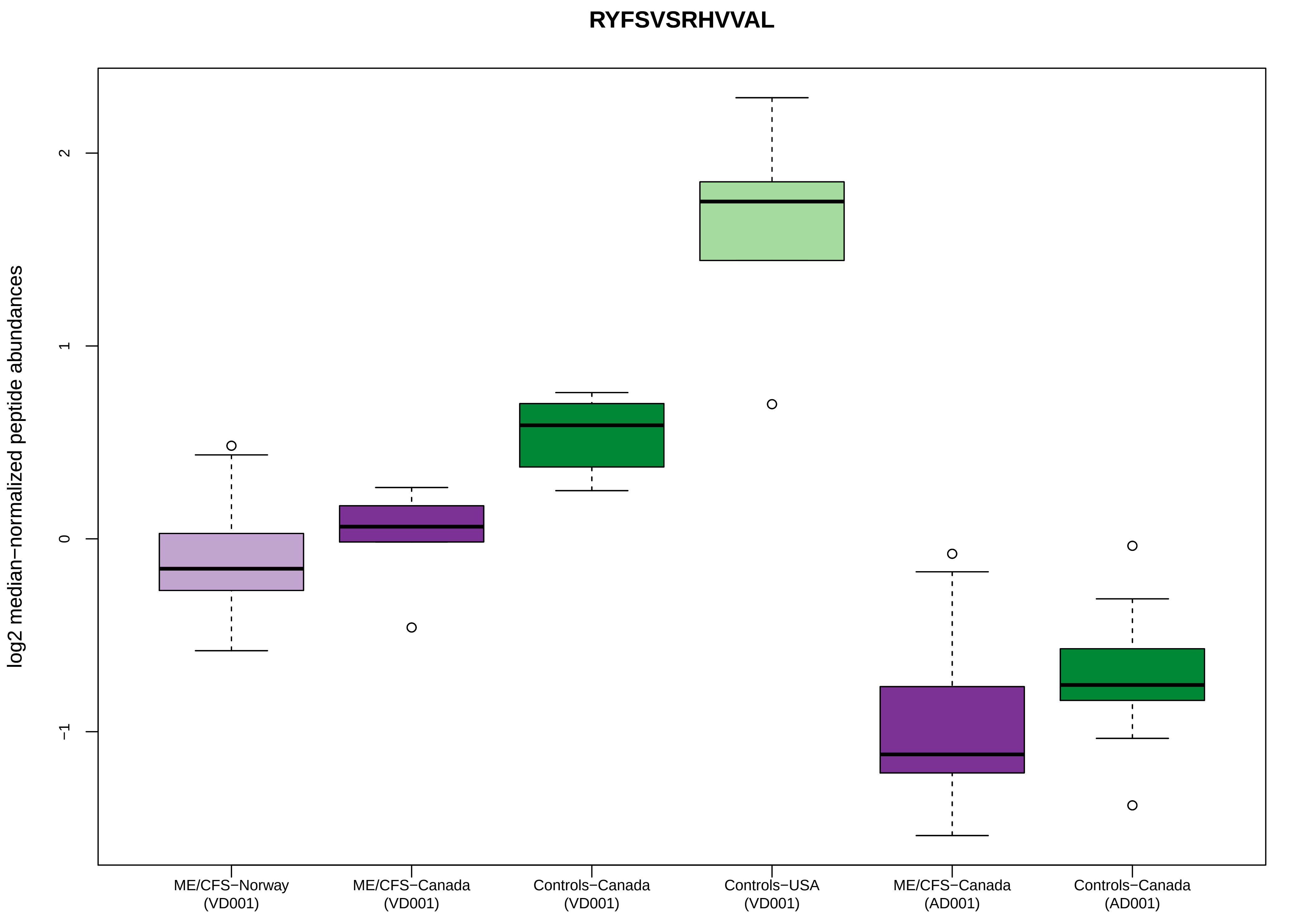
RYFSVSRHVVAL
log2 median−normalized peptide abundances
2
1
0
−1
ME/CFS−Norway (VD001)
ME/CFS−Canada (VD001)
Controls−Canada (VD001)
Controls−USA (VD001)
ME/CFS−Canada (AD001)
Controls−Canada (AD001)

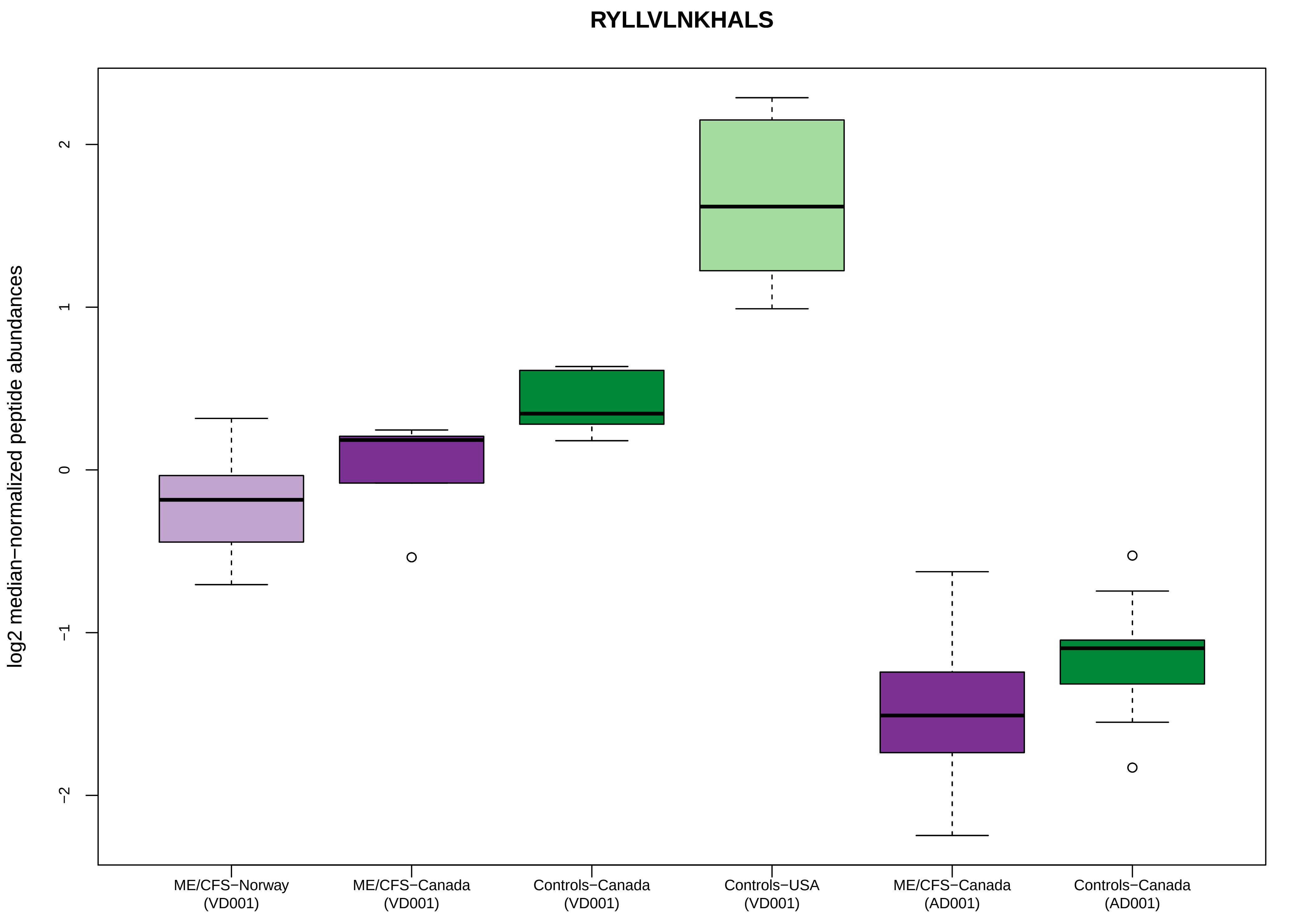
RYLLVLNKHALS
log2 median−normalized peptide abundances
2
1
0
−1
−2
ME/CFS−Norway
(VD001)
ME/CFS−Canada
(VD001)
Controls−Canada
(VD001)
Controls−USA
(VD001)
ME/CFS−Canada
(AD001)
Controls−Canada
(AD001)

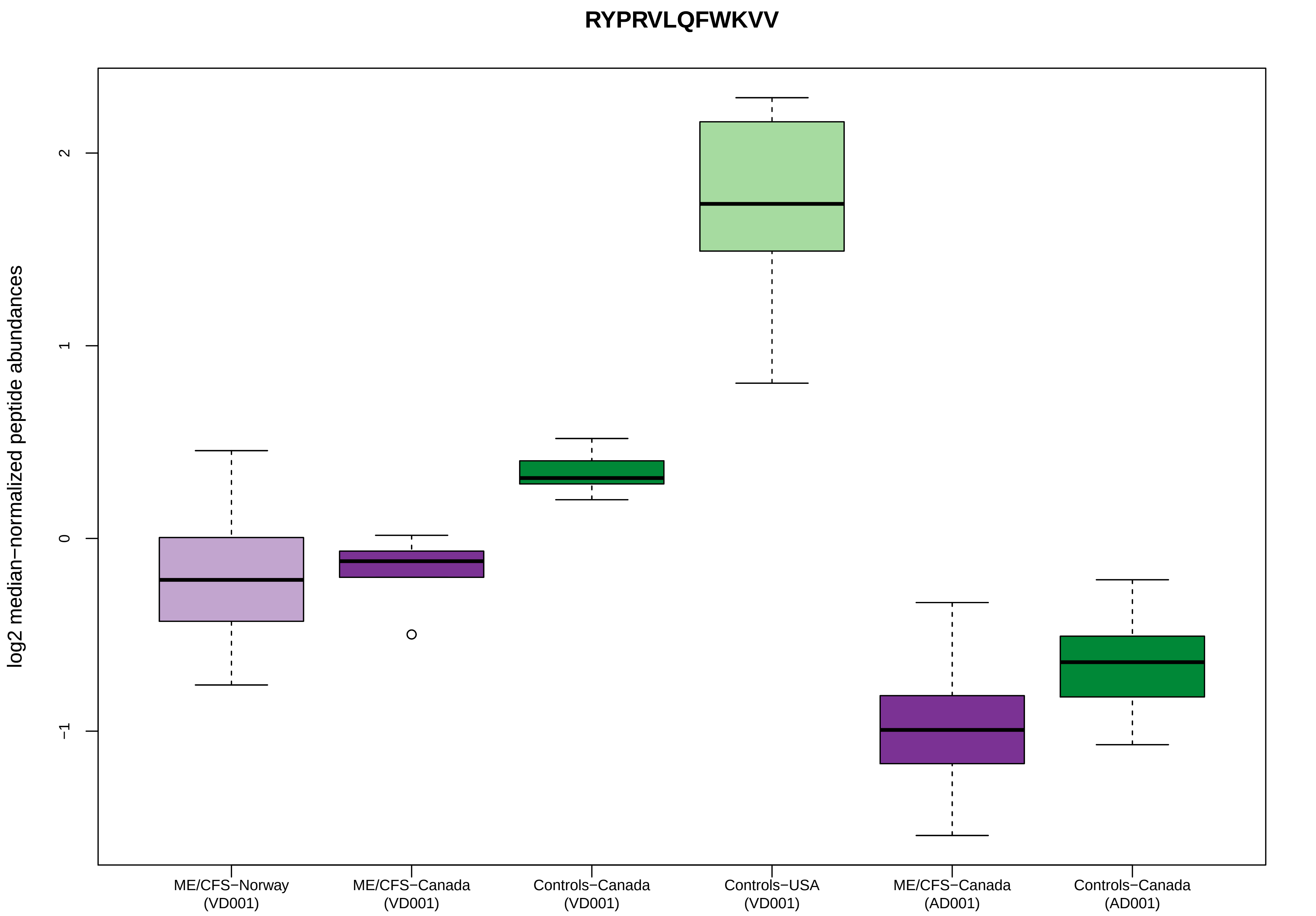

RYPRVLQFWKVV
log2 median−normalized peptide abundances
2
1
0
−1
ME/CFS−Norway (VD001)
ME/CFS−Canada (VD001)
Controls−Canada (VD001)
Controls−USA (VD001)
ME/CFS−Canada (AD001)
Controls−Canada (AD001)

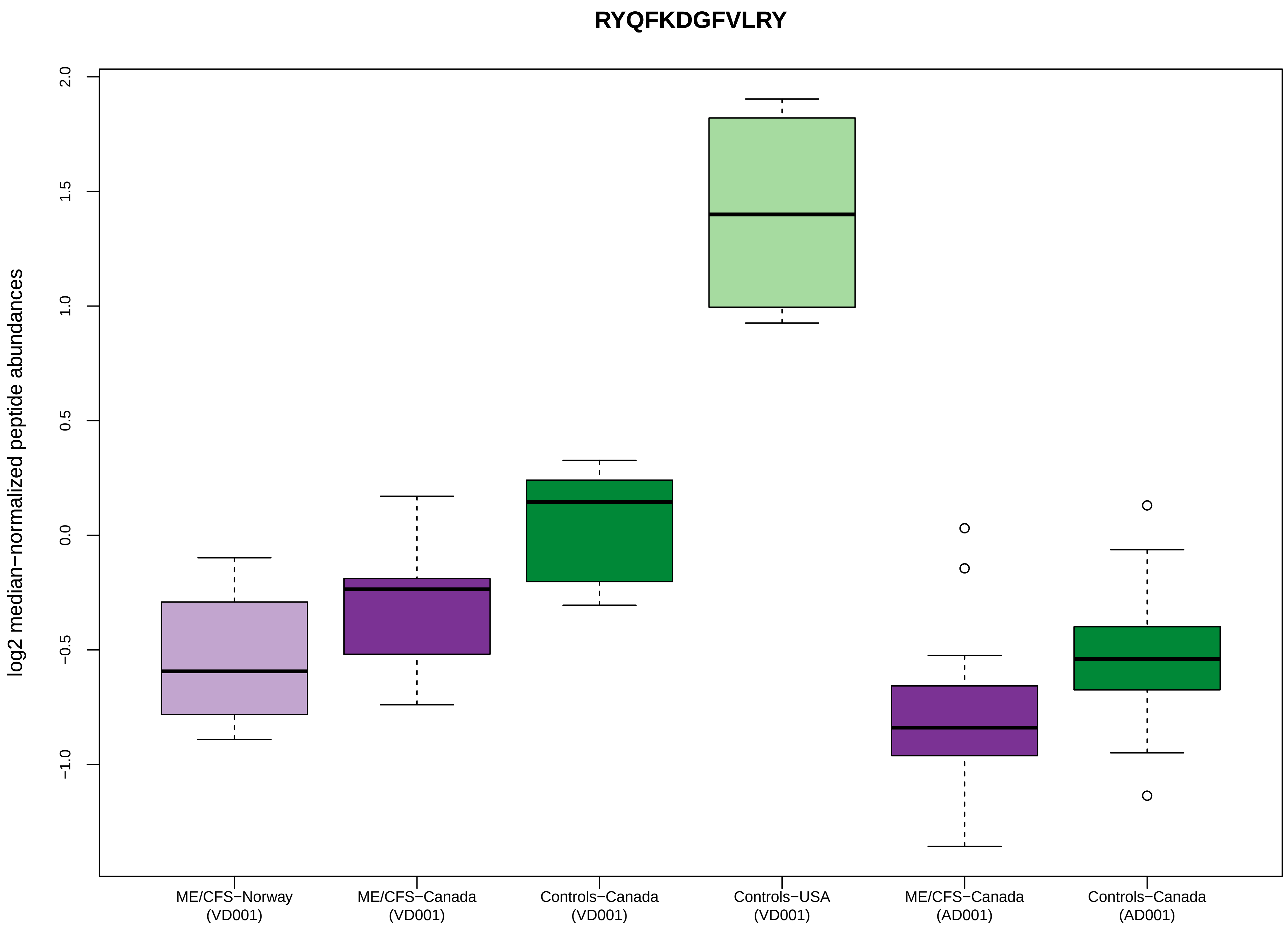

RYQFKDGFVLRY
log2 median−normalized peptide abundances
2.0
1.5
1.0
0.5
0.0
−0.5
−1.0
ME/CFS−Norway (VD001)
ME/CFS−Canada (VD001)
Controls−Canada (VD001)
Controls−USA (VD001)
ME/CFS−Canada (AD001)
Controls−Canada (AD001)

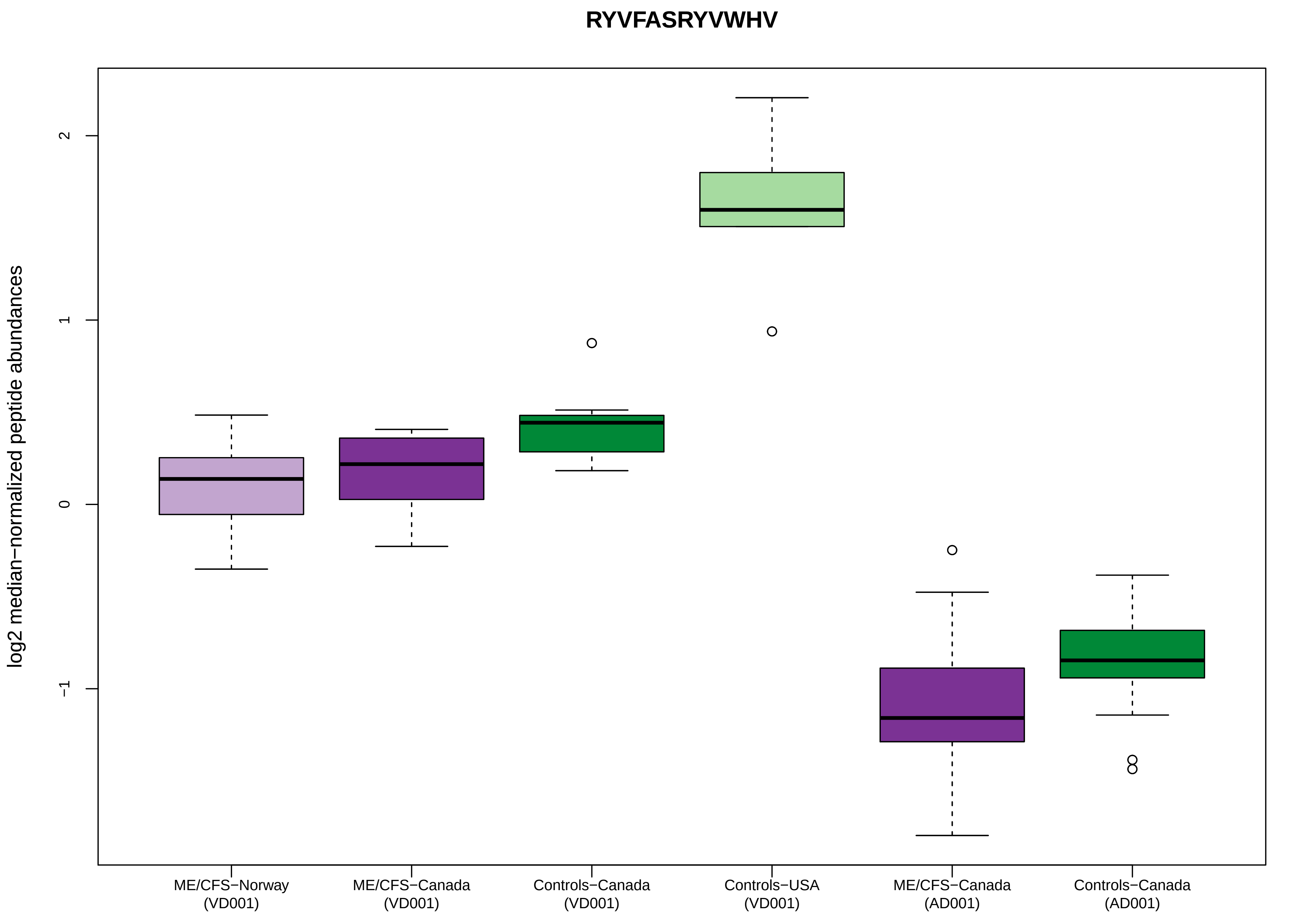

RYVFASRYVWHV
log2 median−normalized peptide abundances
2
1
0
−1
ME/CFS−Norway
(VD001)
ME/CFS−Canada
(VD001)
Controls−Canada
(VD001)
Controls−USA
(VD001)
ME/CFS−Canada
(AD001)
Controls−Canada
(AD001)

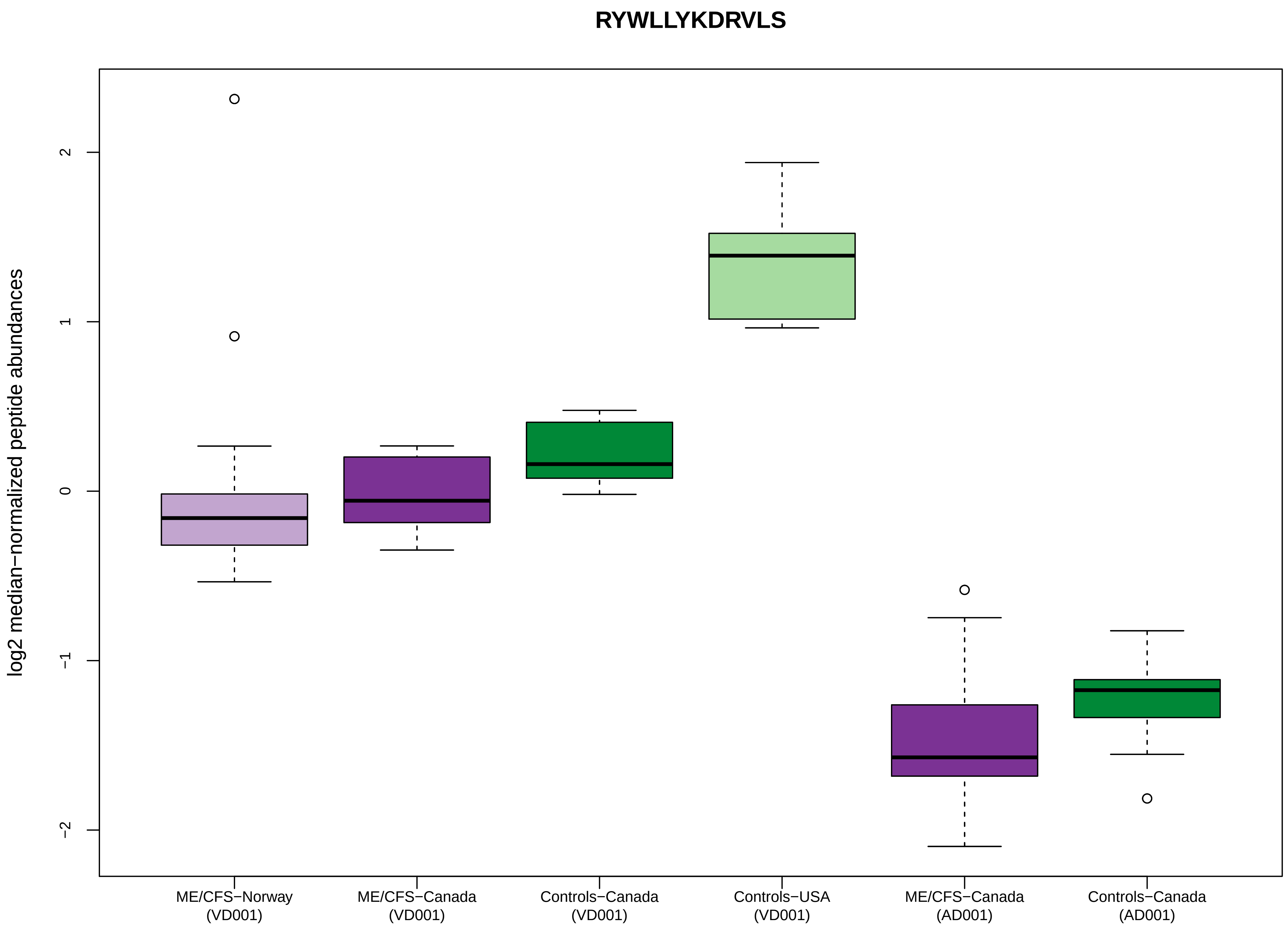

RYWLLYKDRVLS
log2 median−normalized peptide abundances
2
1
0
−1
−2
ME/CFS−Norway
(VD001)
ME/CFS−Canada
(VD001)
Controls−Canada
(VD001)
Controls−USA
(VD001)
ME/CFS−Canada
(AD001)
Controls−Canada
(AD001)

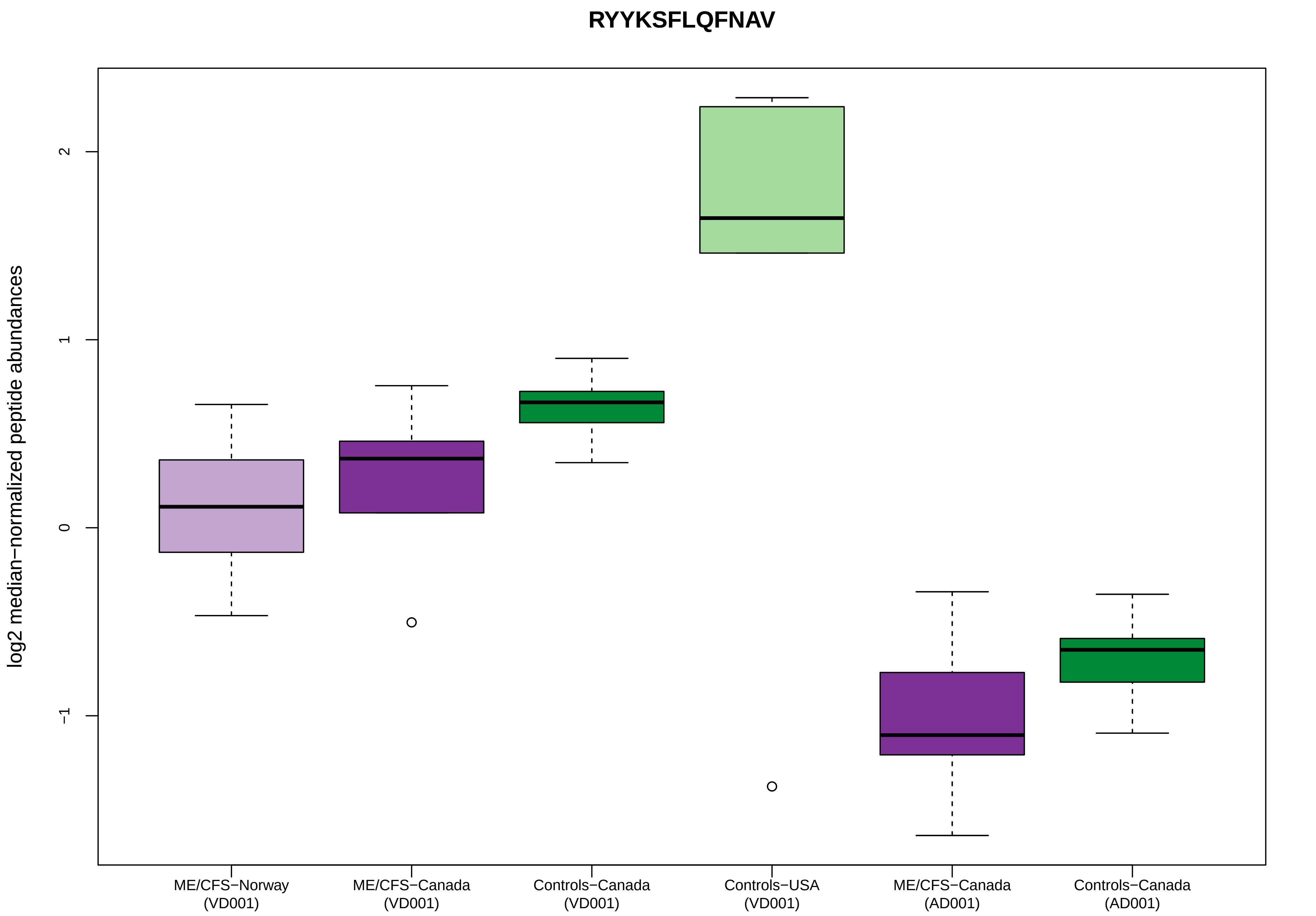
RYYKSFLQFNAV
log2 median−normalized peptide abundances
−1
0
1
2
ME/CFS−Norway (VD001)
ME/CFS−Canada (VD001)
Controls−Canada (VD001)
Controls−USA (VD001)
ME/CFS−Canada (AD001)
Controls−Canada (AD001)

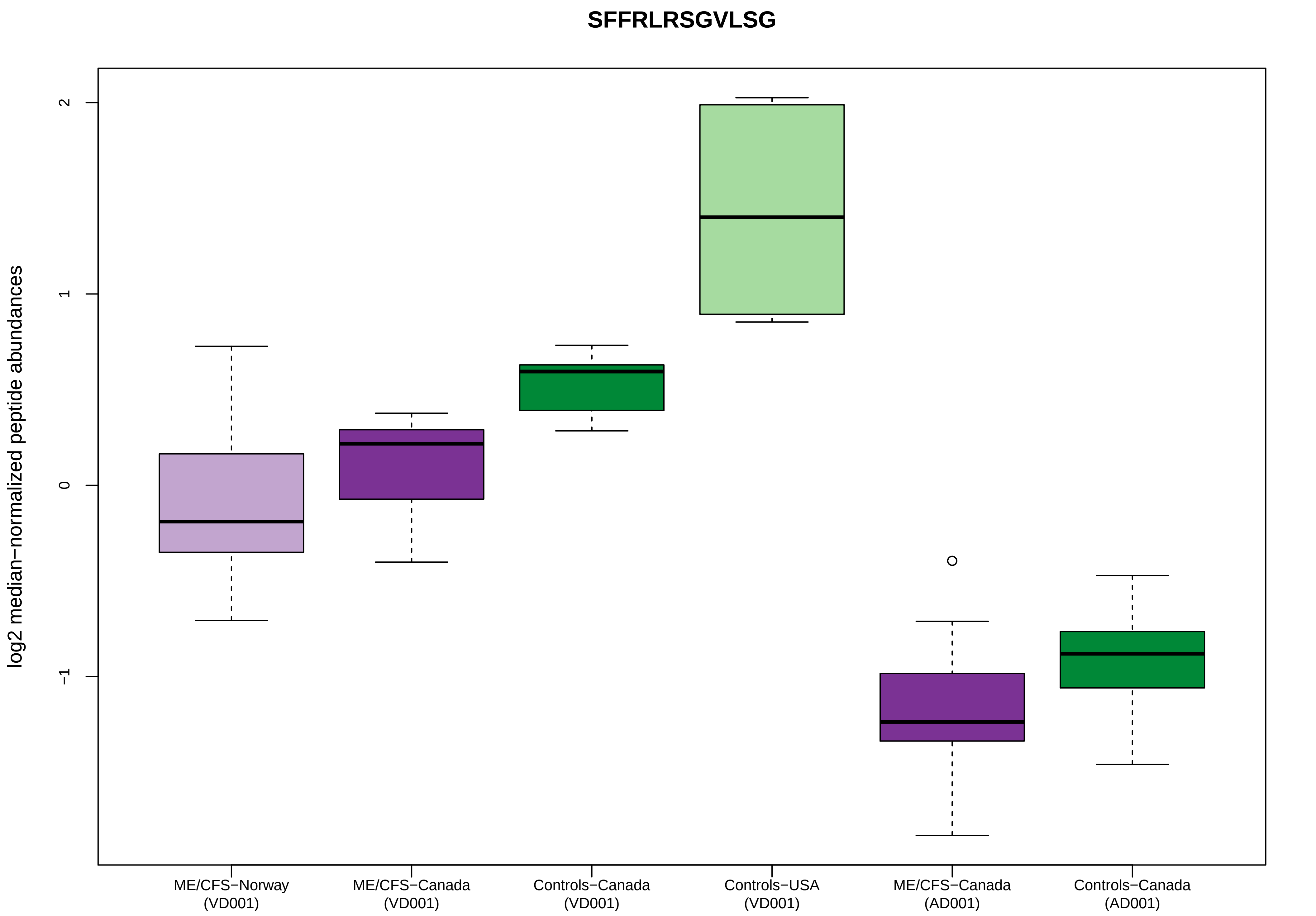
SFFRLRSGVLSG
log2 median−normalized peptide abundances
2
1
0
−1
ME/CFS−Norway (VD001)
ME/CFS−Canada (VD001)
Controls−Canada (VD001)
Controls−USA (VD001)
ME/CFS−Canada (AD001)
Controls−Canada (AD001)

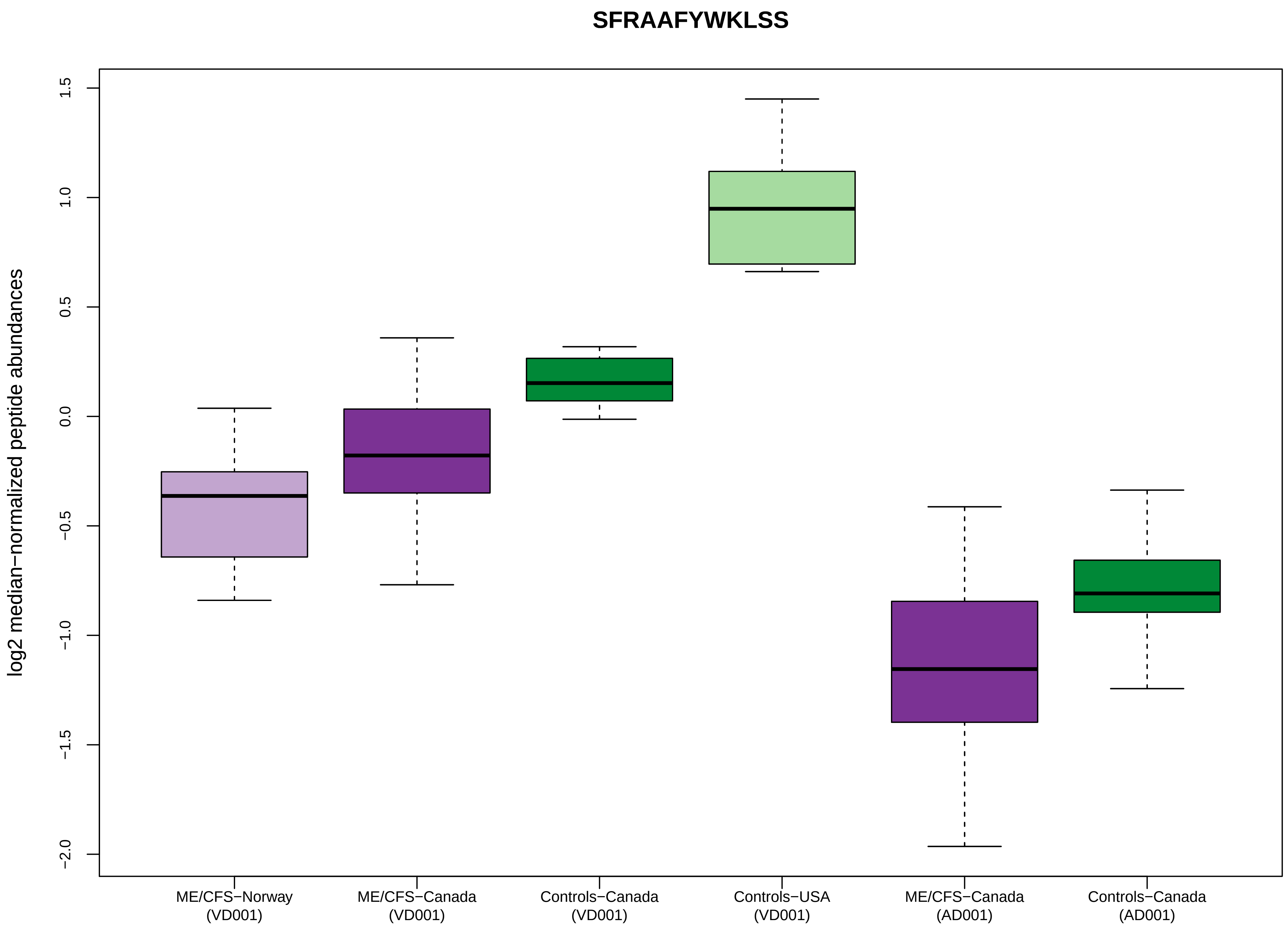

SFRAAFYWKLSS
log2 median−normalized peptide abundances
1.5
1.0
0.5
0.0
−0.5
−1.0
−1.5
−2.0
ME/CFS−Norway (VD001)
ME/CFS−Canada (VD001)
Controls−Canada (VD001)
Controls−USA (VD001)
ME/CFS−Canada (AD001)
Controls−Canada (AD001)

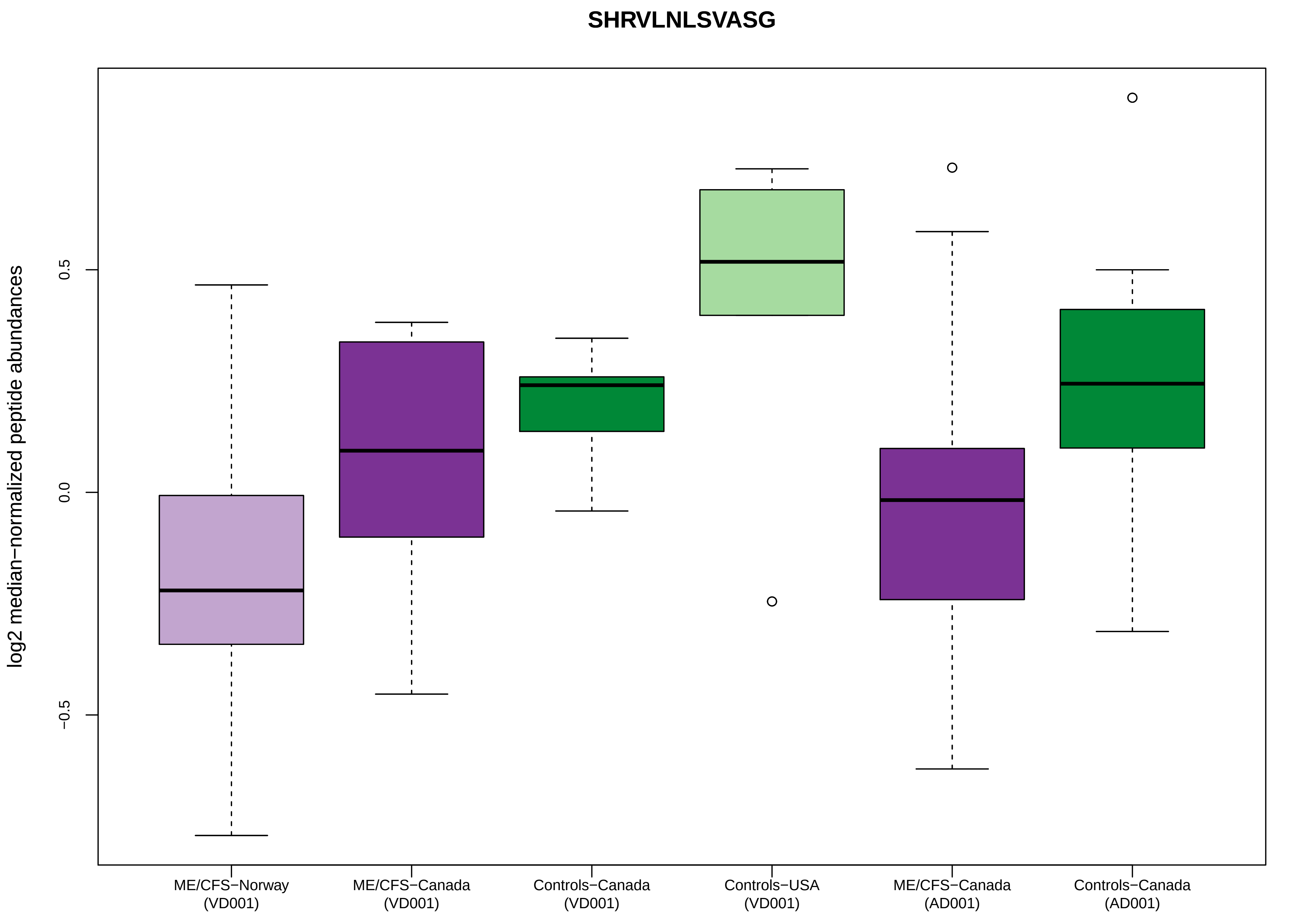

SHRVLNLSVASG
log2 median−normalized peptide abundances
0.5
0.0
−0.5
ME/CFS−Norway (VD001)
ME/CFS−Canada (VD001)
Controls−Canada (VD001)
Controls−USA (VD001)
ME/CFS−Canada (AD001)
Controls−Canada (AD001)

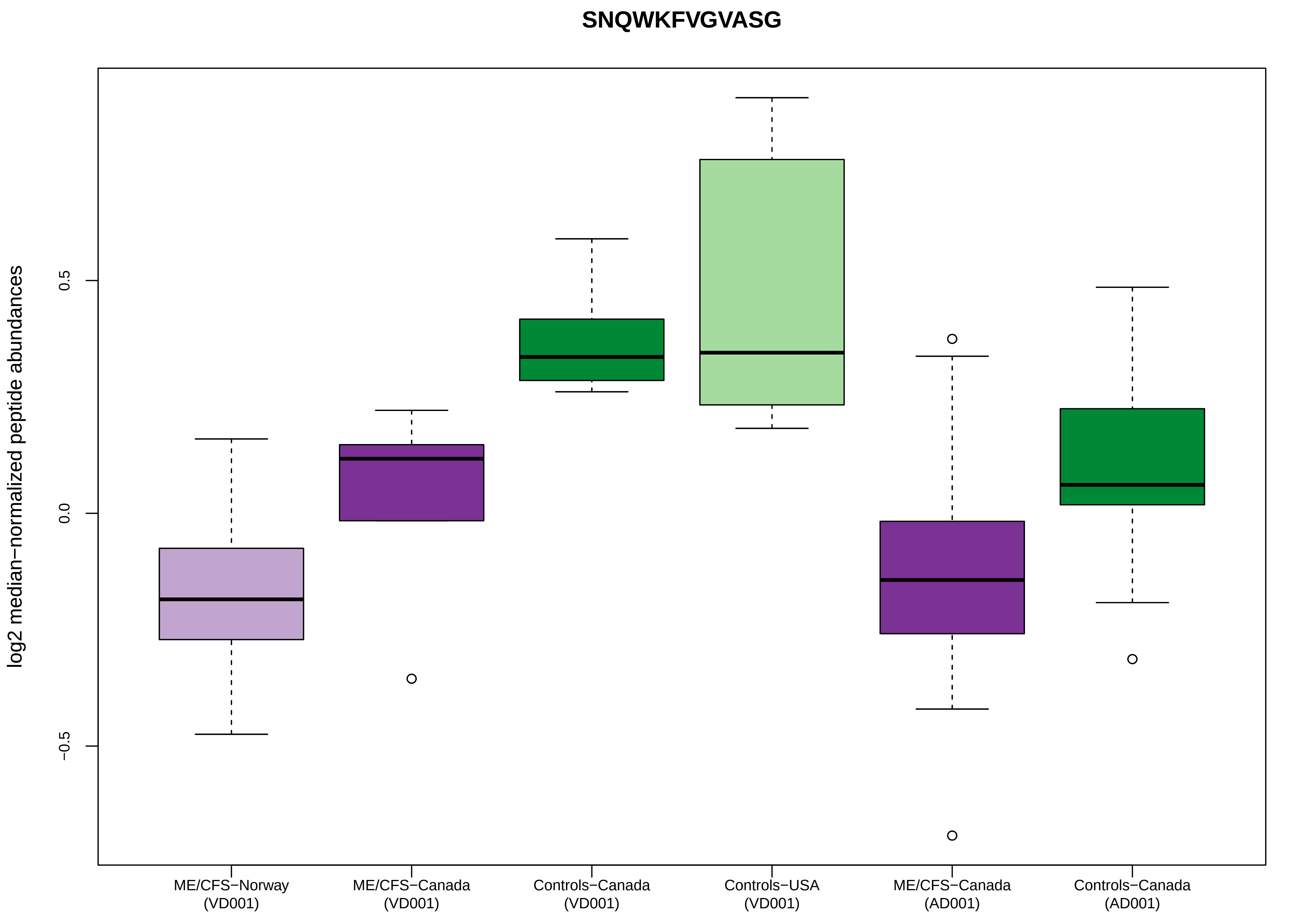

SNQWKFVGVASG
log2 median−normalized peptide abundances
0.5
0.0
−0.5
ME/CFS−Norway (VD001)
ME/CFS−Canada (VD001)
Controls−Canada (VD001)
Controls−USA (VD001)
ME/CFS−Canada (AD001)
Controls−Canada (AD001)

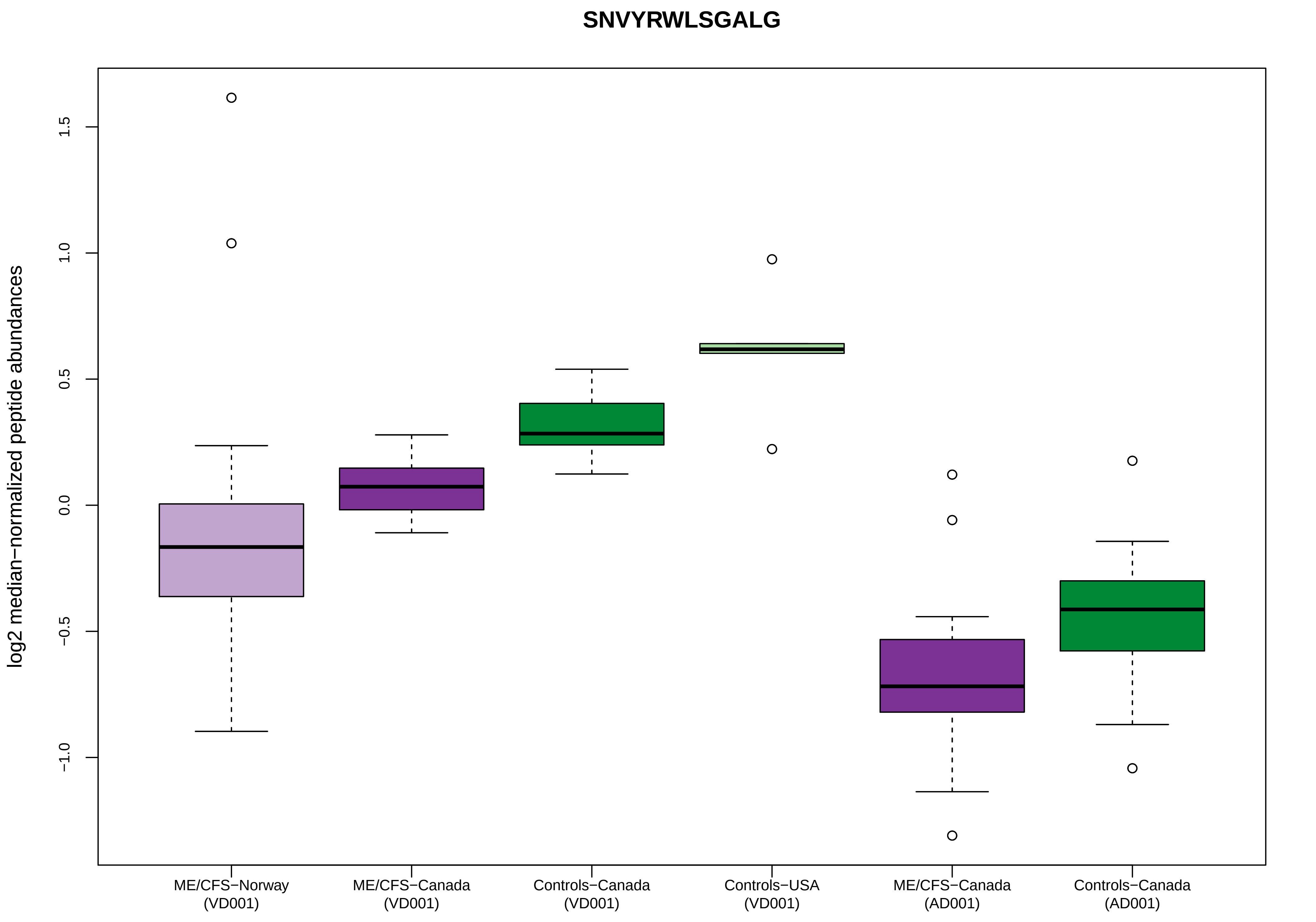
SNVYRWLSGALG
log2 median−normalized peptide abundances
1.5
1.0
0.5
0.0
−0.5
−1.0
ME/CFS−Norway (VD001)
ME/CFS−Canada (VD001)
Controls−Canada (VD001)
Controls−USA (VD001)
ME/CFS−Canada (AD001)
Controls−Canada (AD001)

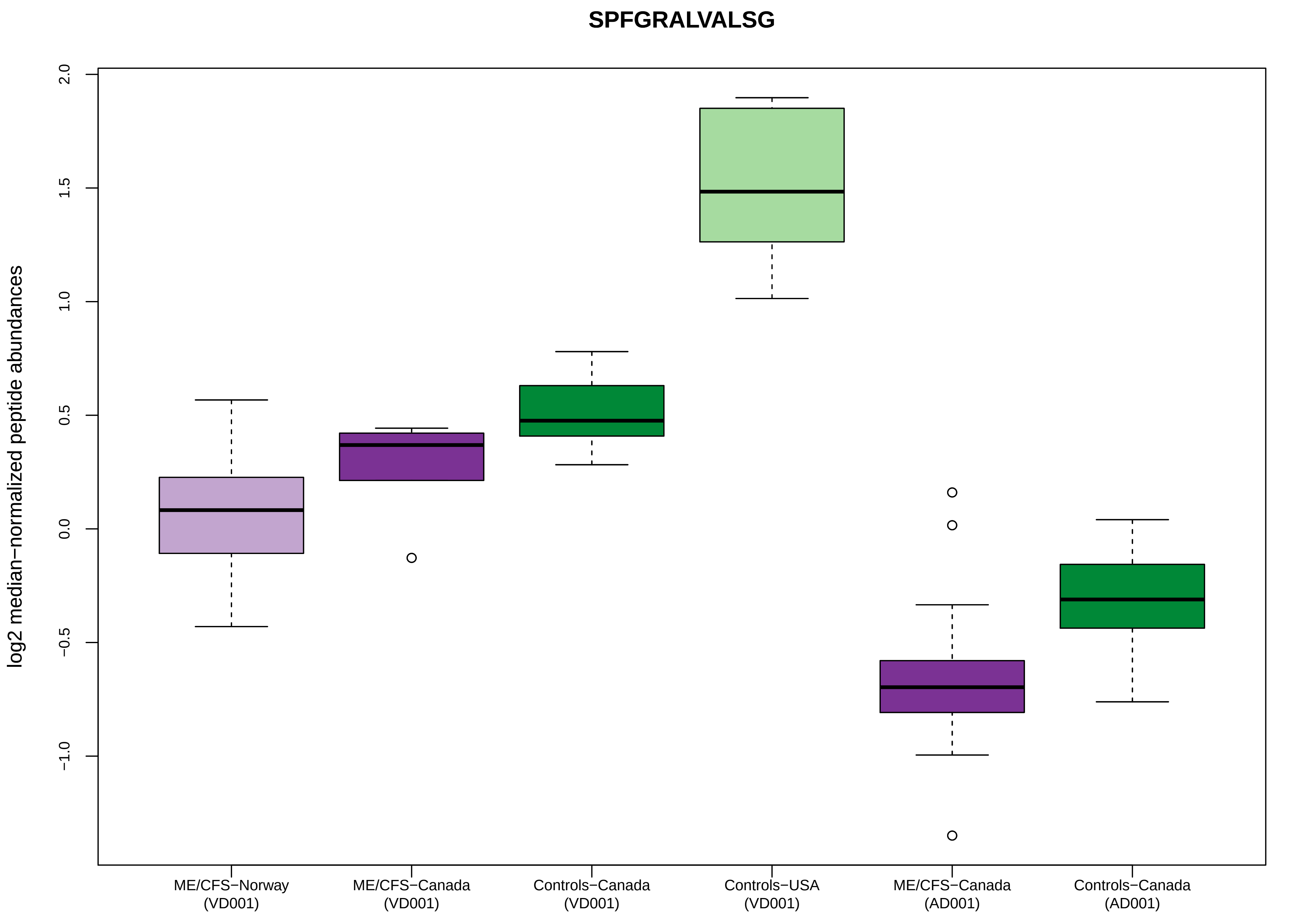

SPFGRALVALSG
log2 median−normalized peptide abundances
2.0
1.5
1.0
0.5
0.0
−0.5
−1.0
ME/CFS−Norway (VD001)
ME/CFS−Canada (VD001)
Controls−Canada (VD001)
Controls−USA (VD001)
ME/CFS−Canada (AD001)
Controls−Canada (AD001)

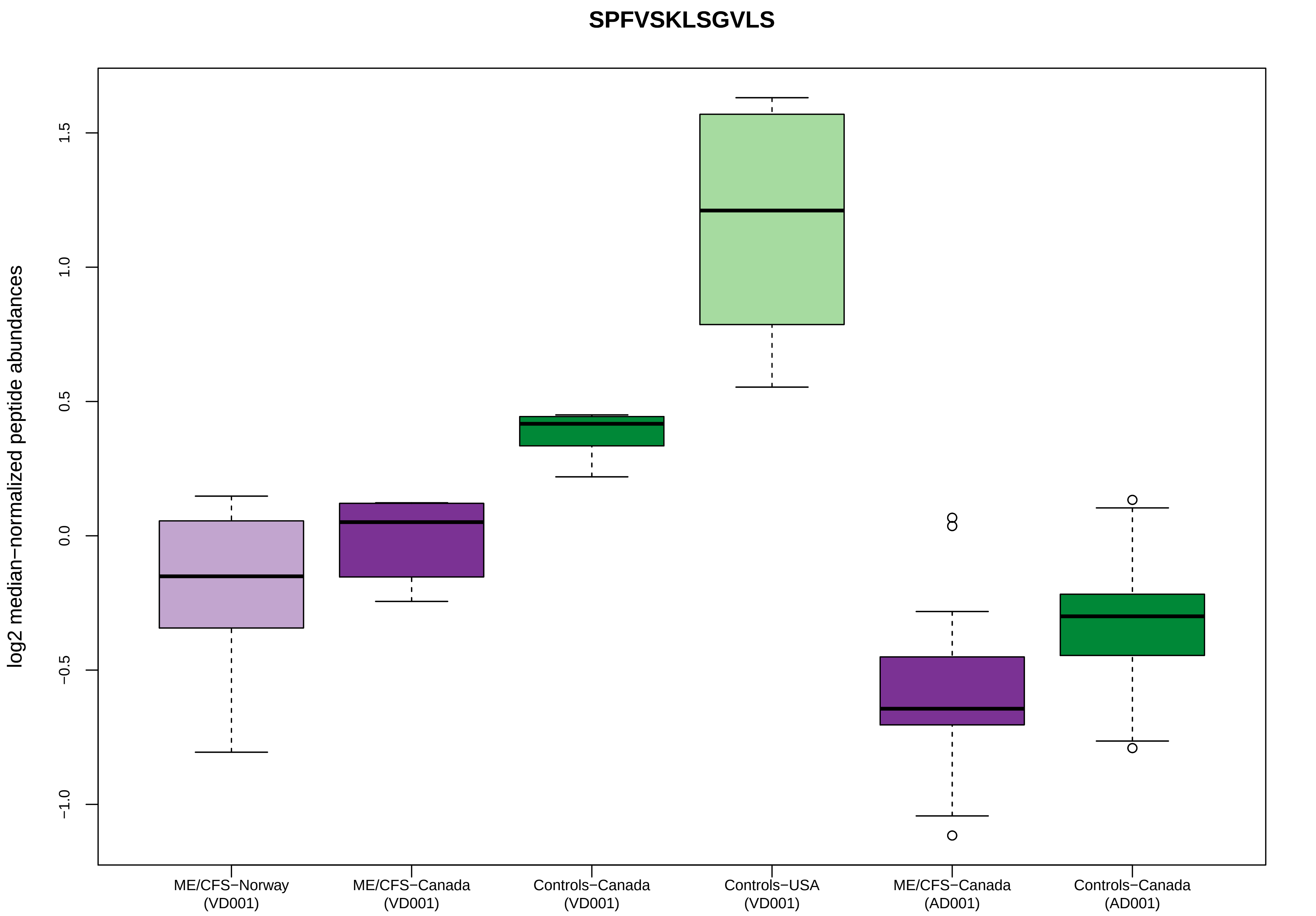

SPFVSKLSGVLS
log2 median−normalized peptide abundances
1.5
1.0
0.5
0.0
−0.5
−1.0
ME/CFS−Norway (VD001)
ME/CFS−Canada (VD001)
Controls−Canada (VD001)
Controls−USA (VD001)
ME/CFS−Canada (AD001)
Controls−Canada (AD001)

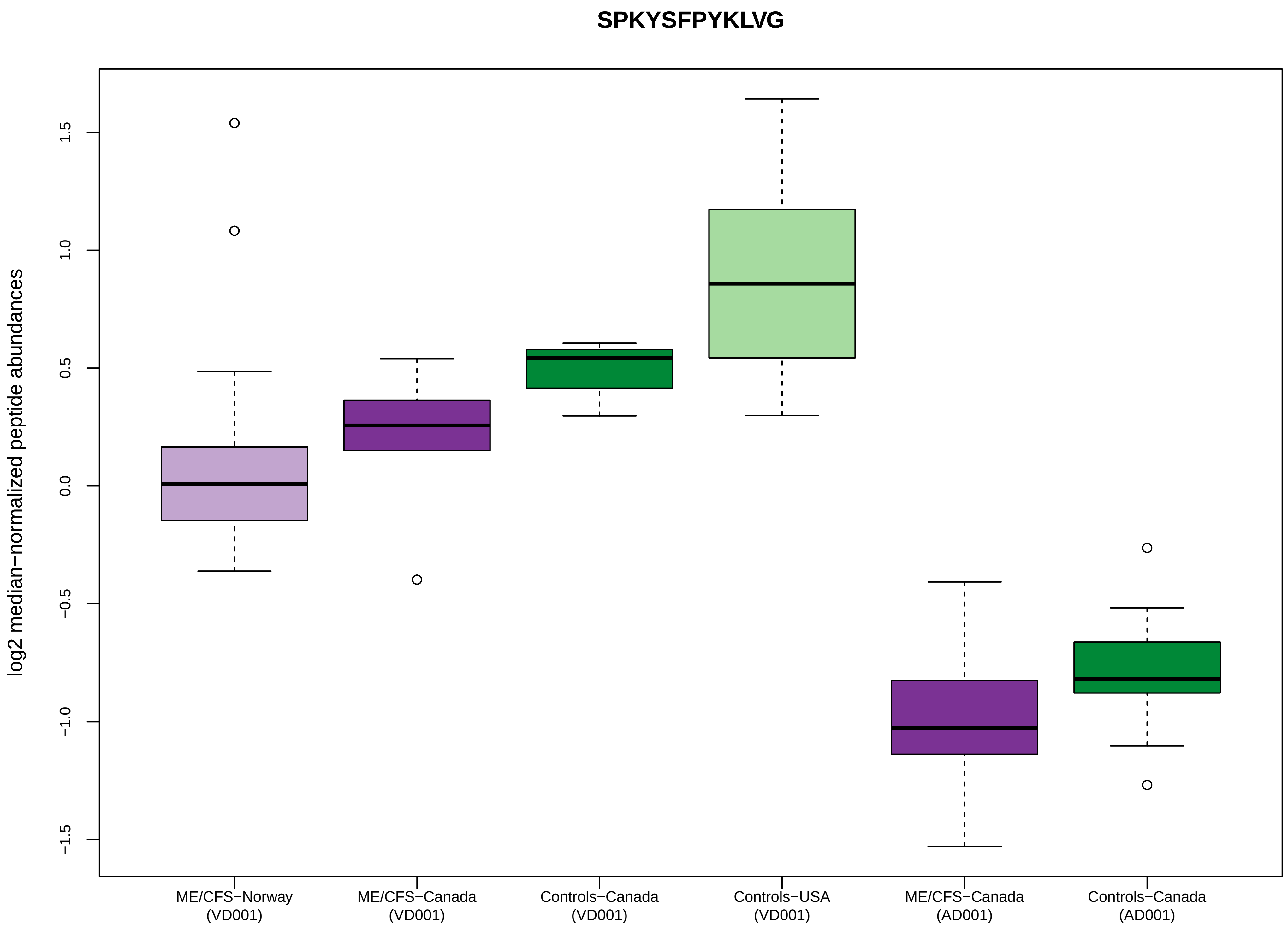

SPKYSFPYKLVG
log2 median−normalized peptide abundances
1.5
1.0
0.5
0.0
−0.5
−1.0
−1.5
ME/CFS−Norway (VD001)
ME/CFS−Canada (VD001)
Controls−Canada (VD001)
Controls−USA (VD001)
ME/CFS−Canada (AD001)
Controls−Canada (AD001)

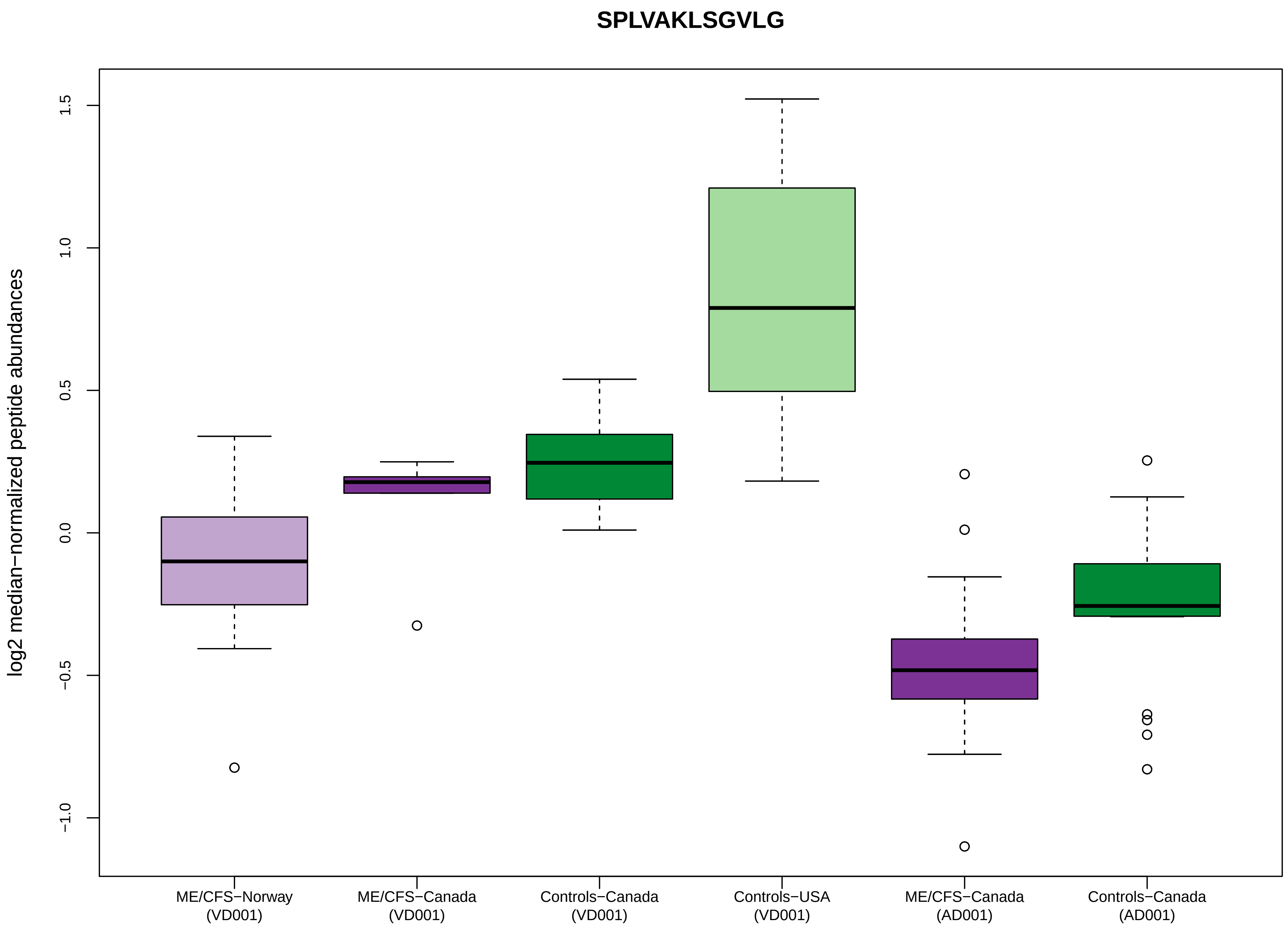
SPLVAKLSGVLG
log2 median−normalized peptide abundances
1.5
1.0
0.5
0.0
−0.5
−1.0
ME/CFS−Norway (VD001)
ME/CFS−Canada (VD001)
Controls−Canada (VD001)
Controls−USA (VD001)
ME/CFS−Canada (AD001)
Controls−Canada (AD001)

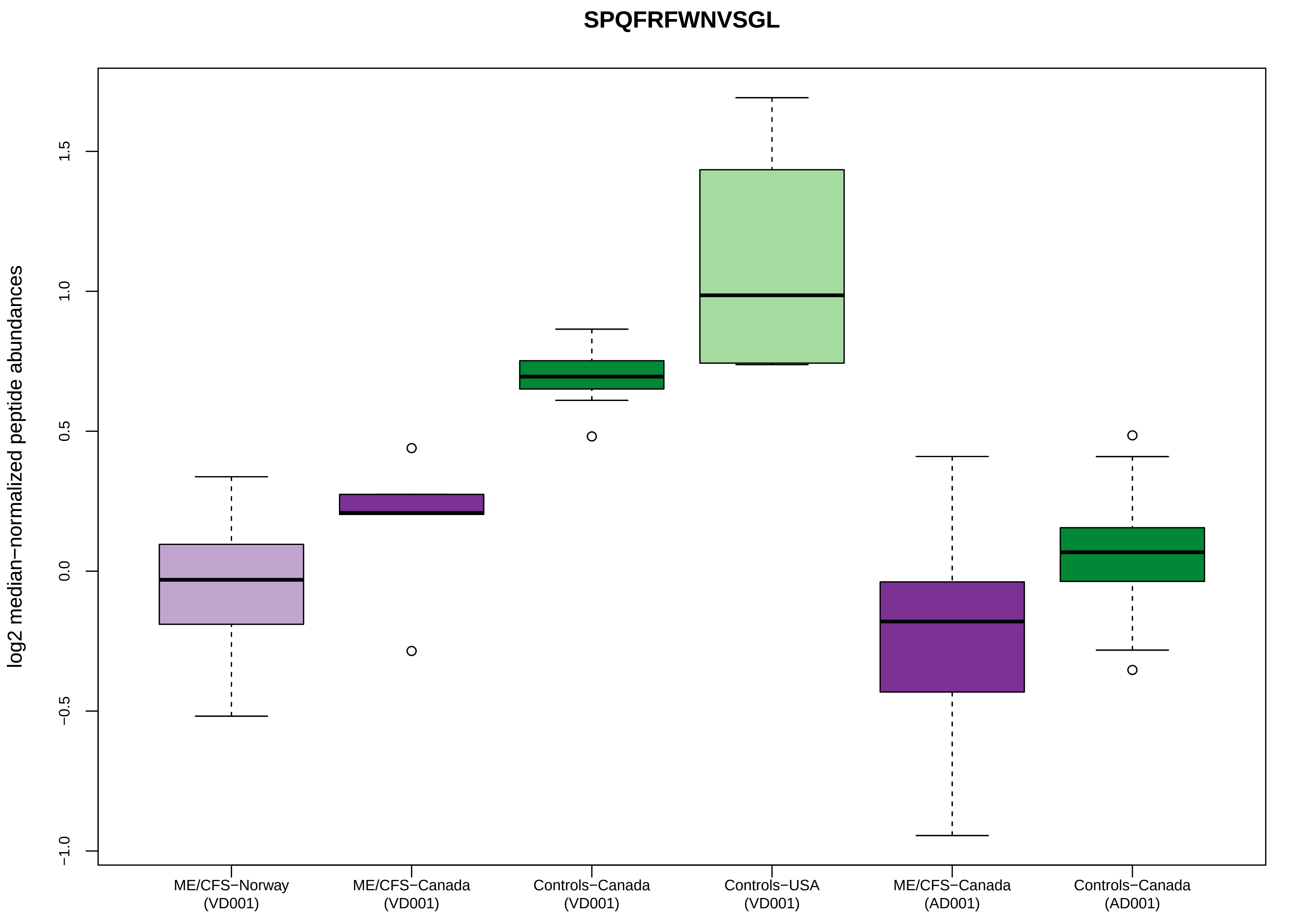
SPQFRFWNVSGL
log2 median−normalized peptide abundances
−1.0
−0.5
0.0
0.5
1.0
1.5
ME/CFS−Norway (VD001)
ME/CFS−Canada (VD001)
Controls−Canada (VD001)
Controls−USA (VD001)
ME/CFS−Canada (AD001)
Controls−Canada (AD001)

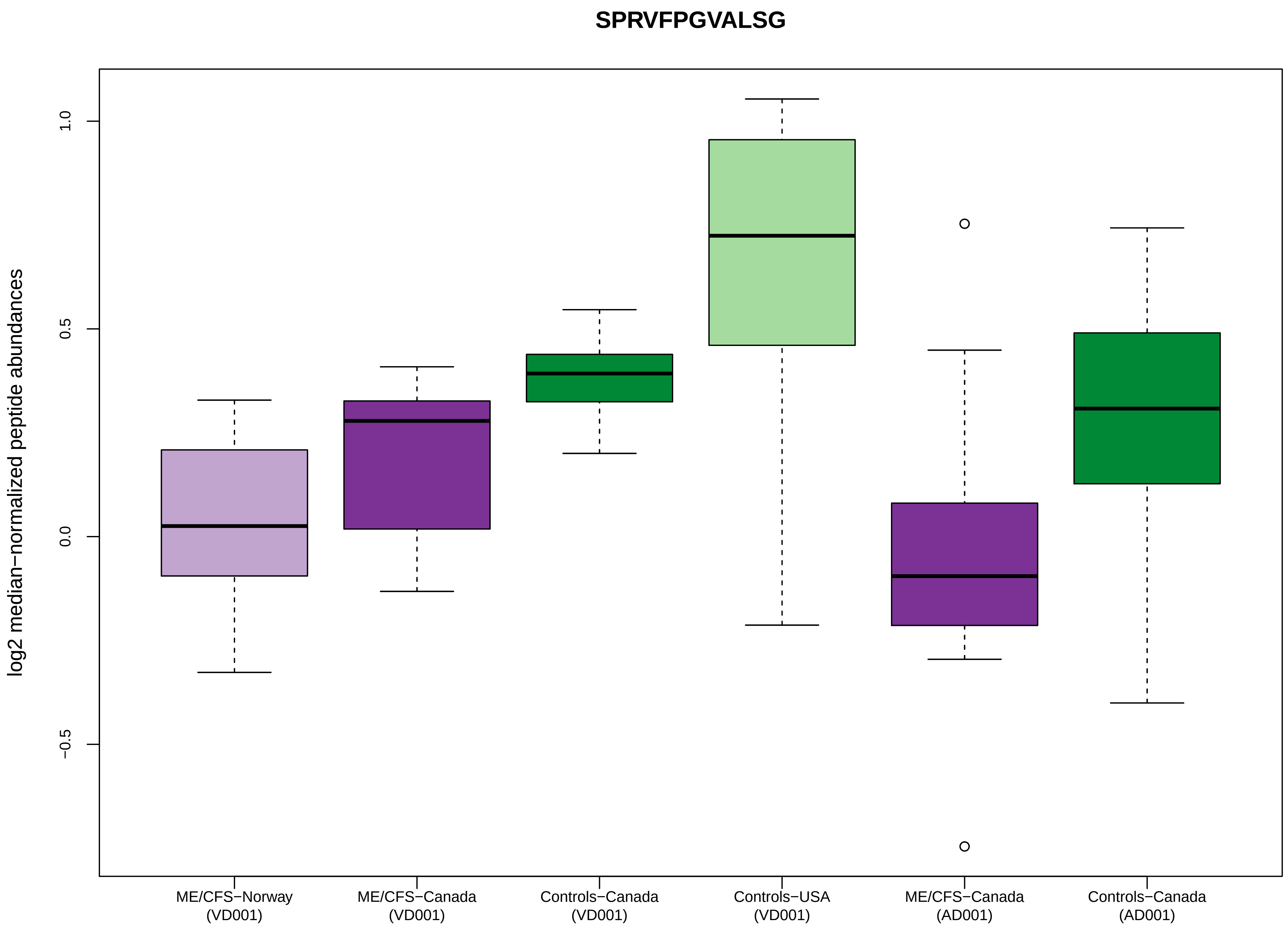

SPRVFPGVALSG
log2 median−normalized peptide abundances
1.0
0.5
0.0
−0.5
ME/CFS−Norway (VD001)
ME/CFS−Canada (VD001)
Controls−Canada (VD001)
Controls−USA (VD001)
ME/CFS−Canada (AD001)
Controls−Canada (AD001)

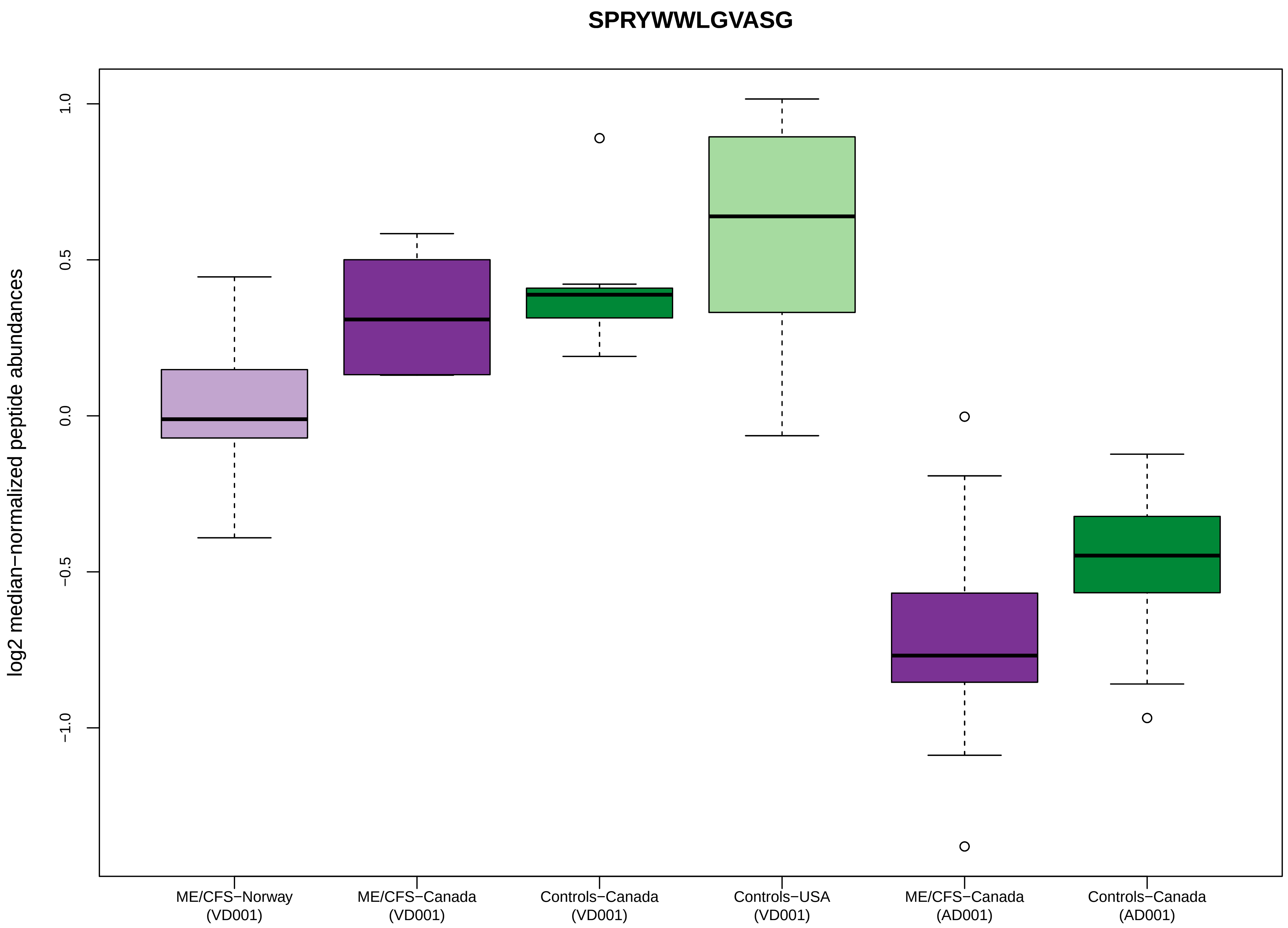
SPRYWWLGVASG
log2 median−normalized peptide abundances
1.0
0.5
0.0
−0.5
−1.0
ME/CFS−Norway (VD001)
ME/CFS−Canada (VD001)
Controls−Canada (VD001)
Controls−USA (VD001)
ME/CFS−Canada (AD001)
Controls−Canada (AD001)

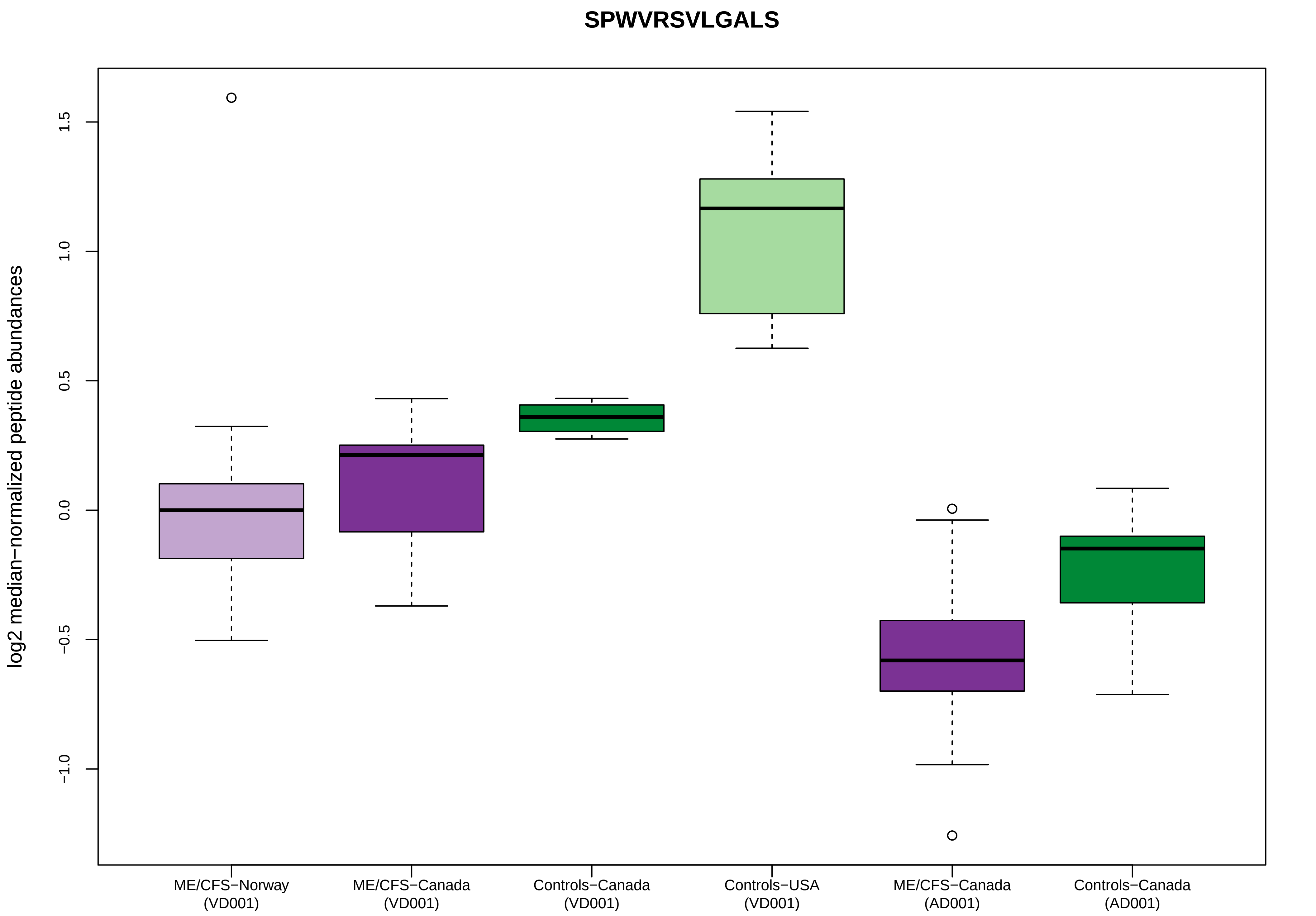
SPWVRSVLGALS
log2 median−normalized peptide abundances
1.5
1.0
0.5
0.0
−0.5
−1.0
ME/CFS−Norway (VD001)
ME/CFS−Canada (VD001)
Controls−Canada (VD001)
Controls−USA (VD001)
ME/CFS−Canada (AD001)
Controls−Canada (AD001)

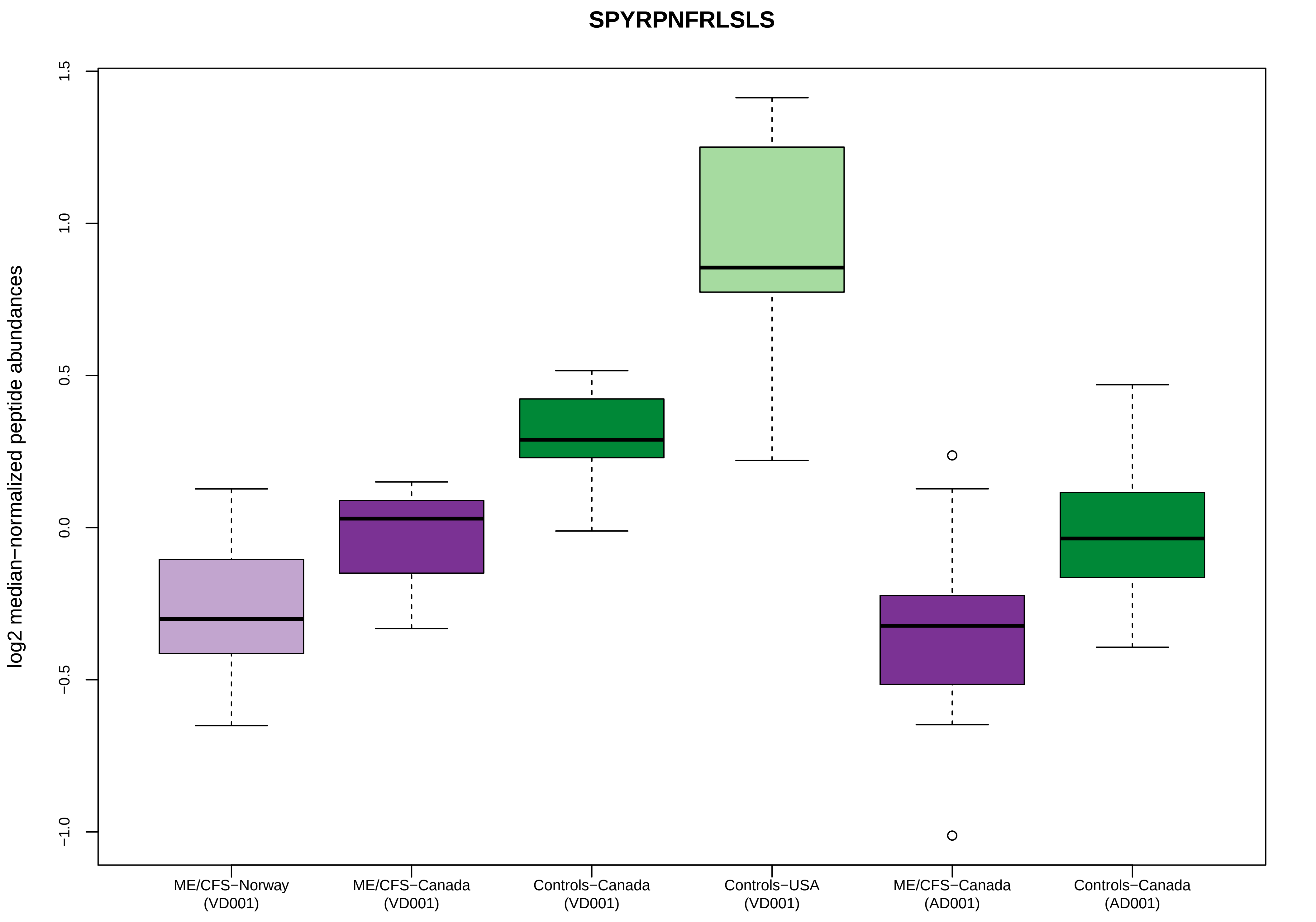
SPYRPNFRLSLS
log2 median−normalized peptide abundances
1.5
1.0
0.5
0.0
−0.5
−1.0
ME/CFS−Norway (VD001)
ME/CFS−Canada (VD001)
Controls−Canada (VD001)
Controls−USA (VD001)
ME/CFS−Canada (AD001)
Controls−Canada (AD001)

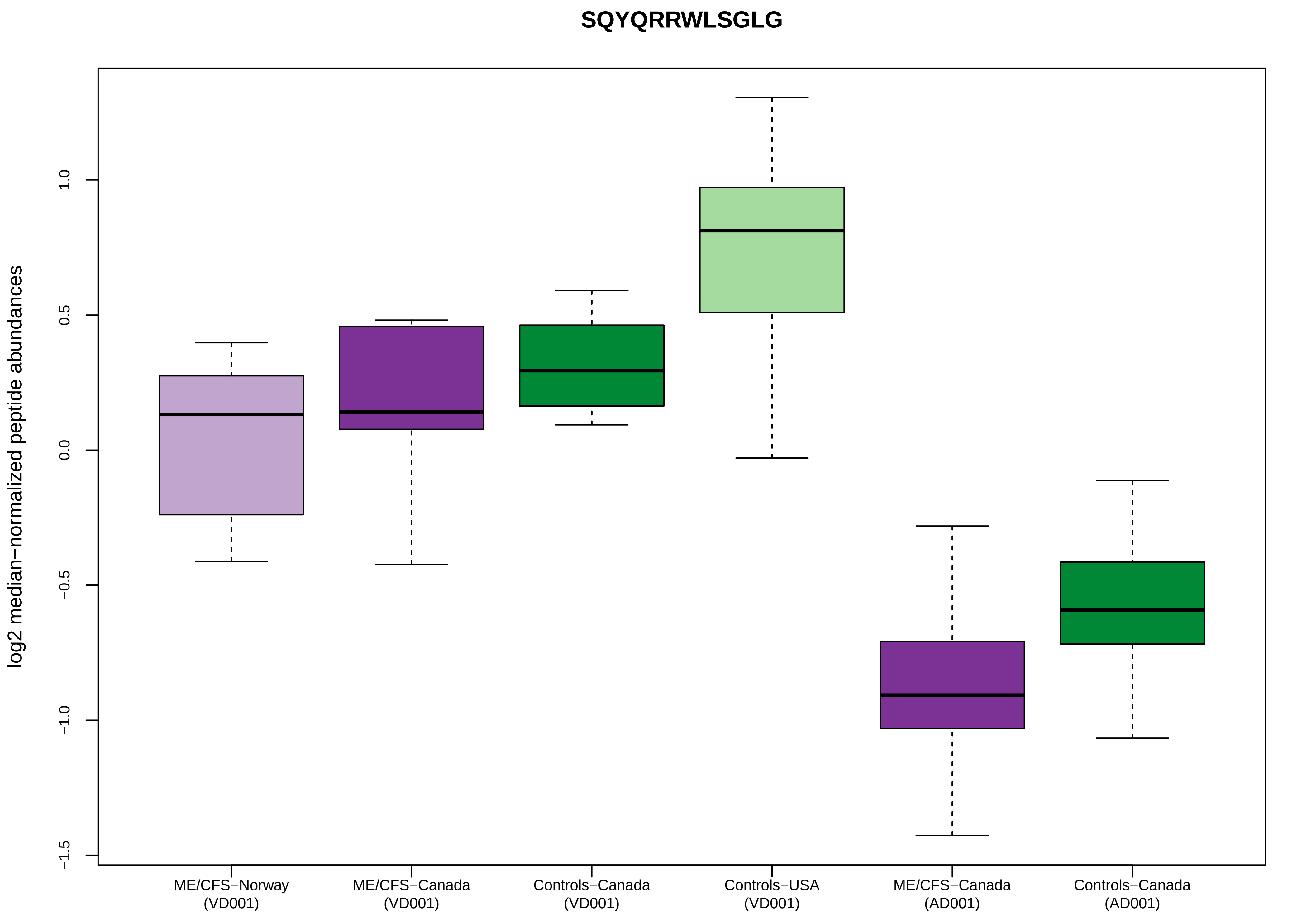
SQYQRRWLSGLG
log2 median−normalized peptide abundances
1.0
0.5
0.0
−0.5
−1.0
−1.5
ME/CFS−Norway (VD001)
ME/CFS−Canada (VD001)
Controls−Canada (VD001)
Controls−USA (VD001)
ME/CFS−Canada (AD001)
Controls−Canada (AD001)

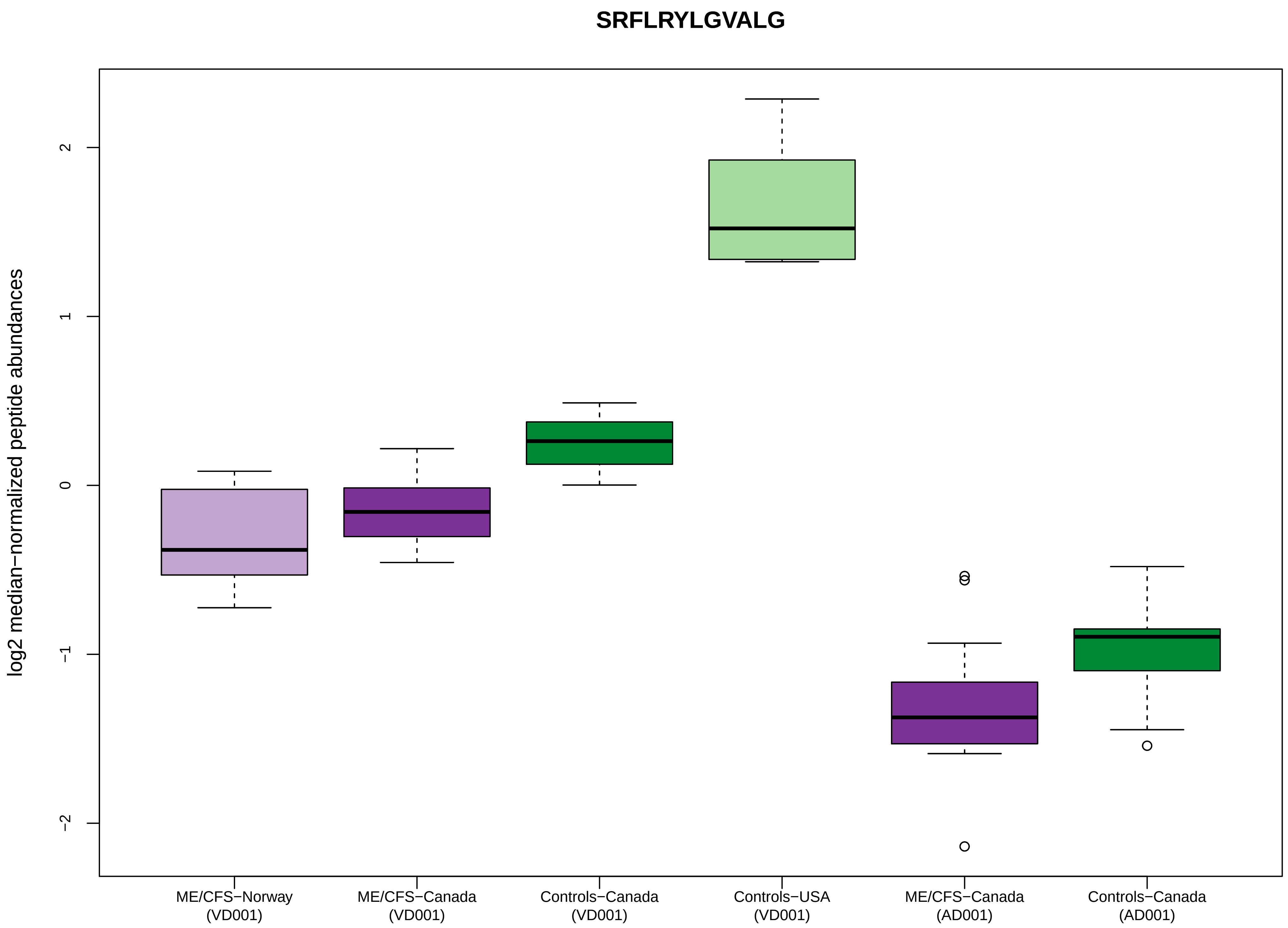

SRFLRYLGVALG
log2 median−normalized peptide abundances
2
1
0
−1
−2
ME/CFS−Norway (VD001)
ME/CFS−Canada (VD001)
Controls−Canada (VD001)
Controls−USA (VD001)
ME/CFS−Canada (AD001)
Controls−Canada (AD001)

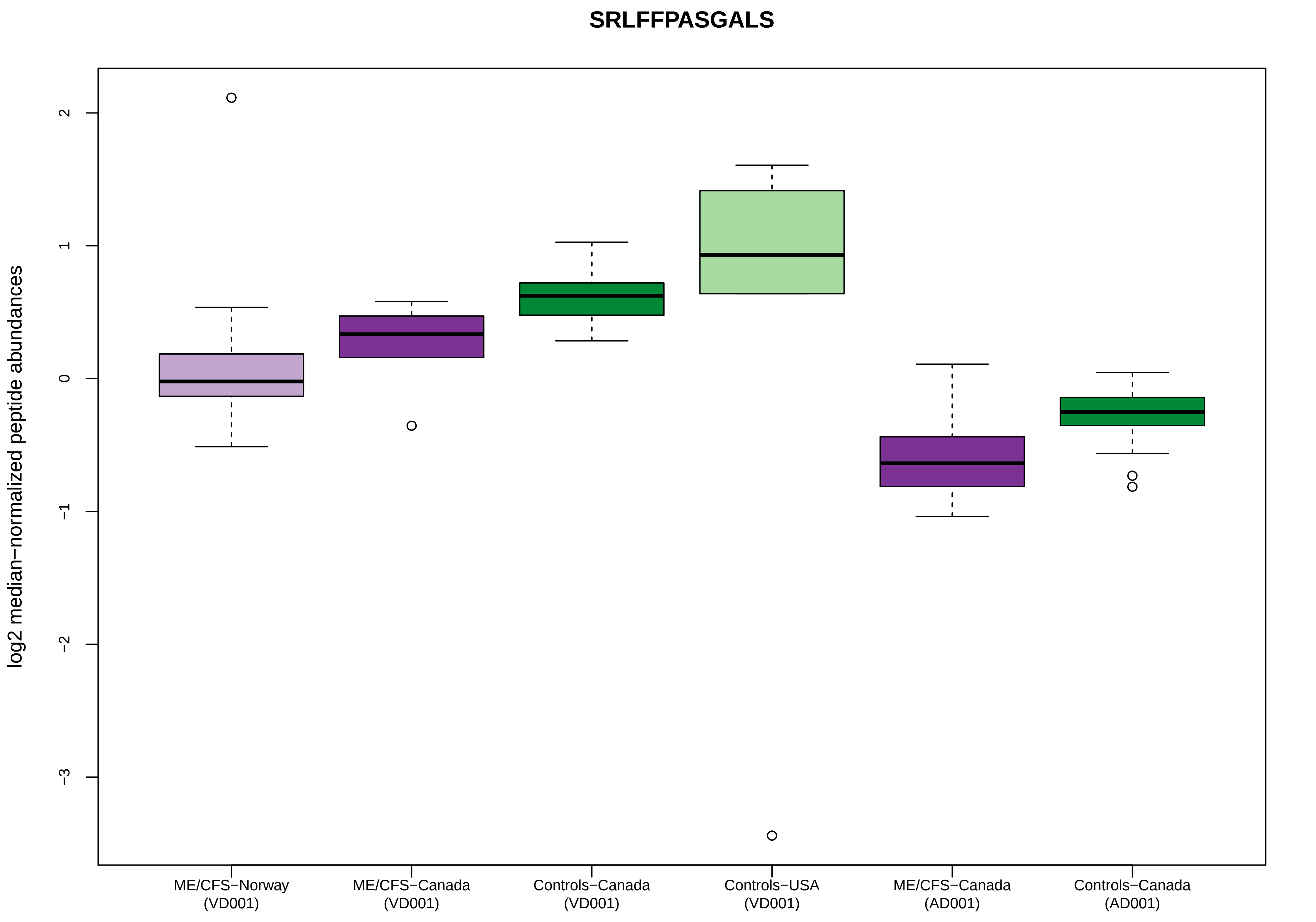
SRLFFPASGALS
log2 median−normalized peptide abundances
2
1
0
−1
−2
−3
ME/CFS−Norway (VD001)
ME/CFS−Canada (VD001)
Controls−Canada (VD001)
Controls−USA (VD001)
ME/CFS−Canada (AD001)
Controls−Canada (AD001)

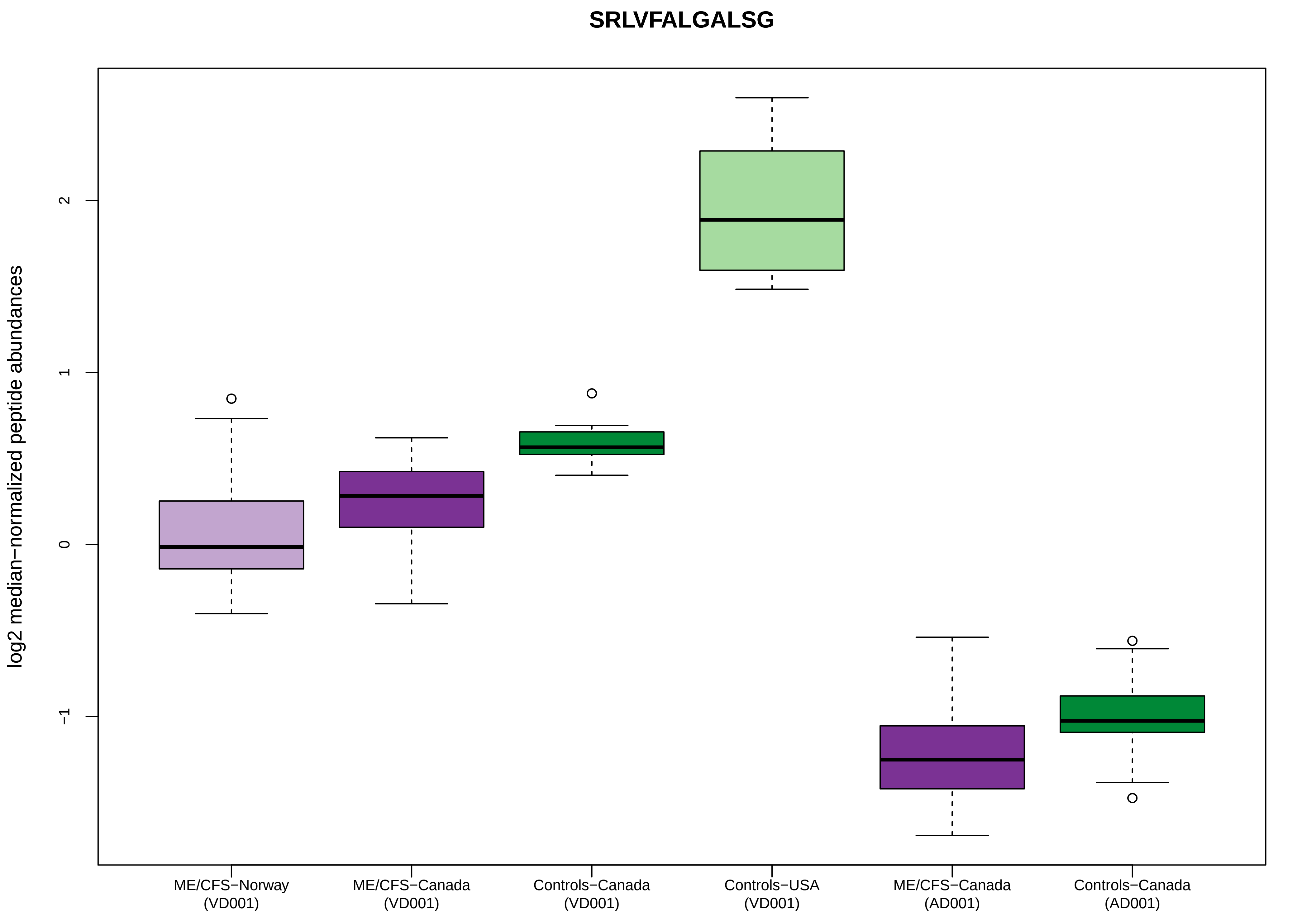

SRLVFALGALSG
log2 median−normalized peptide abundances
2
1
0
−1
ME/CFS−Norway
(VD001)
ME/CFS−Canada
(VD001)
Controls−Canada
(VD001)
Controls−USA
(VD001)
ME/CFS−Canada
(AD001)
Controls−Canada
(AD001)

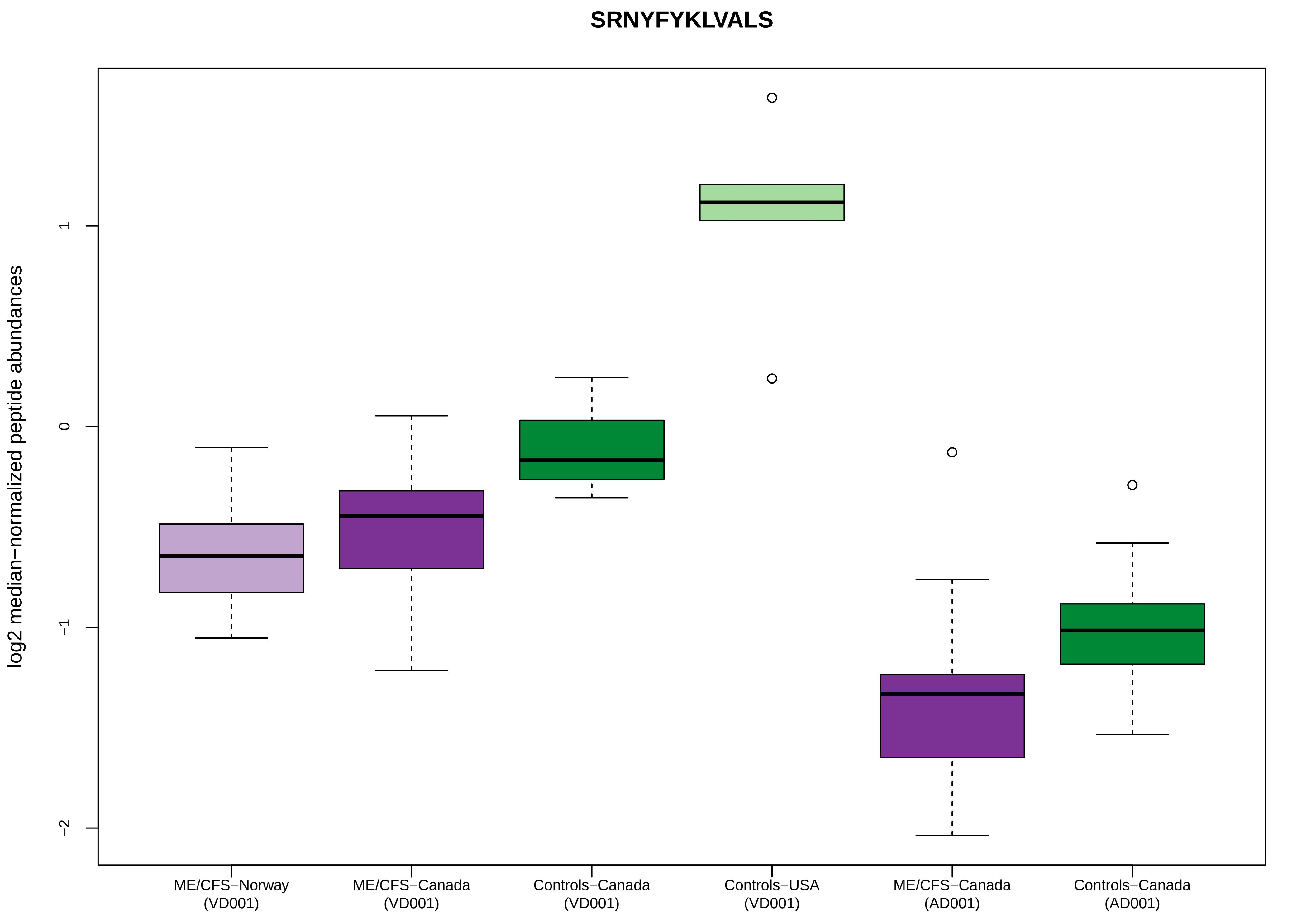

SRNYFYKLVALS
log2 median−normalized peptide abundances
1
0
−1
−2
ME/CFS−Norway (VD001)
ME/CFS−Canada (VD001)
Controls−Canada (VD001)
Controls−USA (VD001)
ME/CFS−Canada (AD001)
Controls−Canada (AD001)

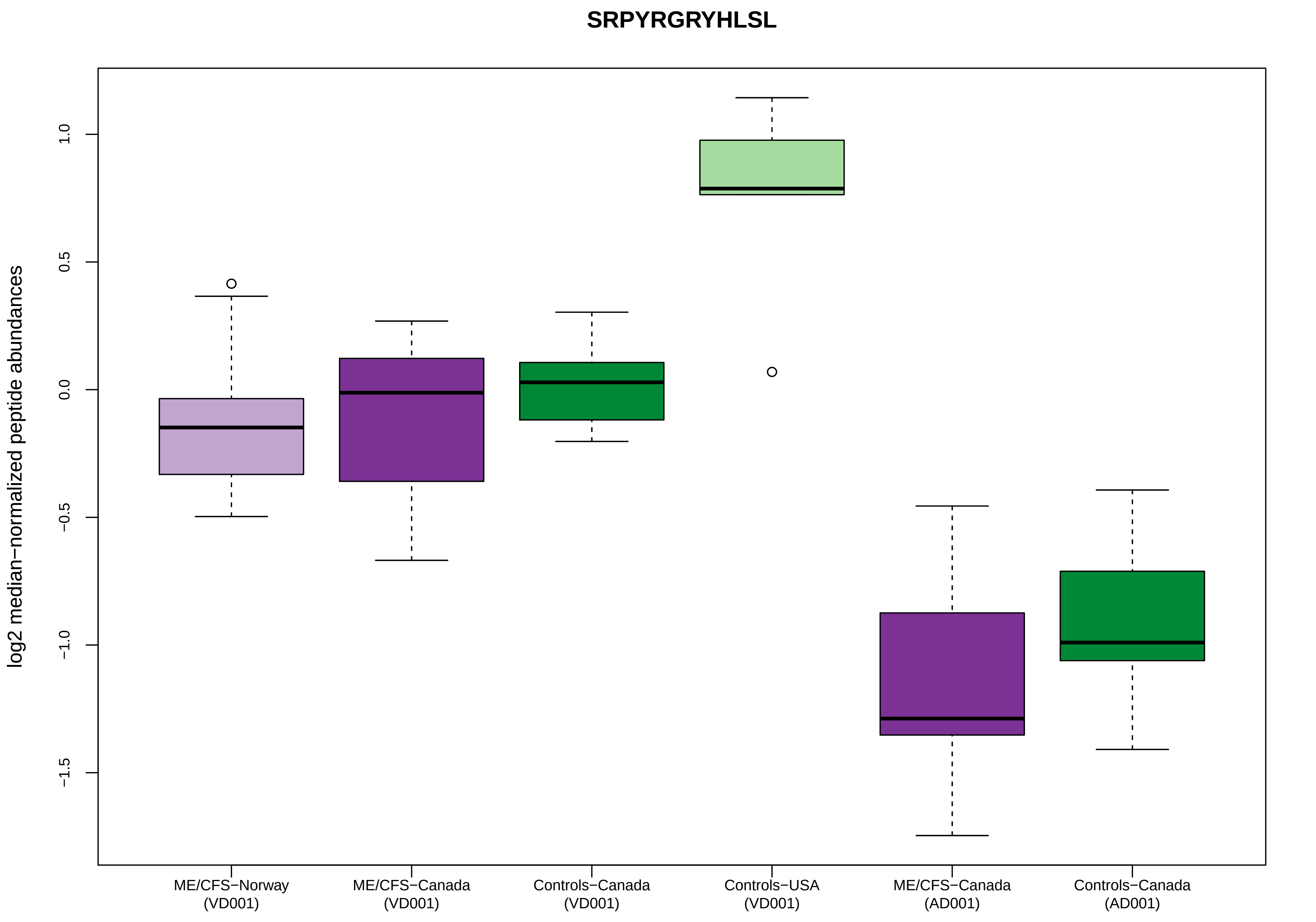
SRPYRGRYHLSL
log2 median−normalized peptide abundances
1.0
0.5
0.0
−0.5
−1.0
−1.5
ME/CFS−Norway (VD001)
ME/CFS−Canada (VD001)
Controls−Canada (VD001)
Controls−USA (VD001)
ME/CFS−Canada (AD001)
Controls−Canada (AD001)

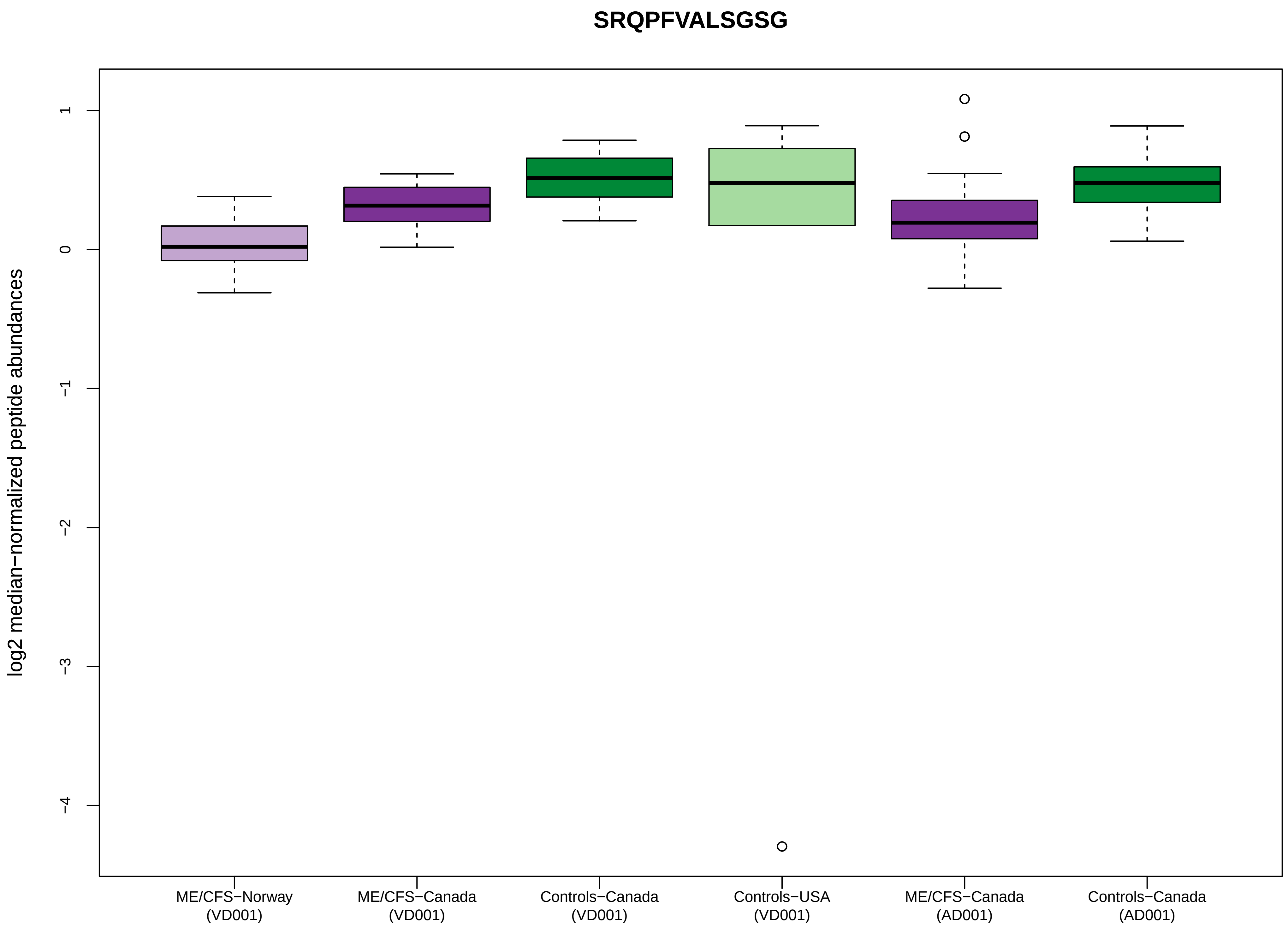
SRQPFVALSGSG
log2 median−normalized peptide abundances
1
0
−1
−2
−3
−4
ME/CFS−Norway (VD001)
ME/CFS−Canada (VD001)
Controls−Canada (VD001)
Controls−USA (VD001)
ME/CFS−Canada (AD001)
Controls−Canada (AD001)

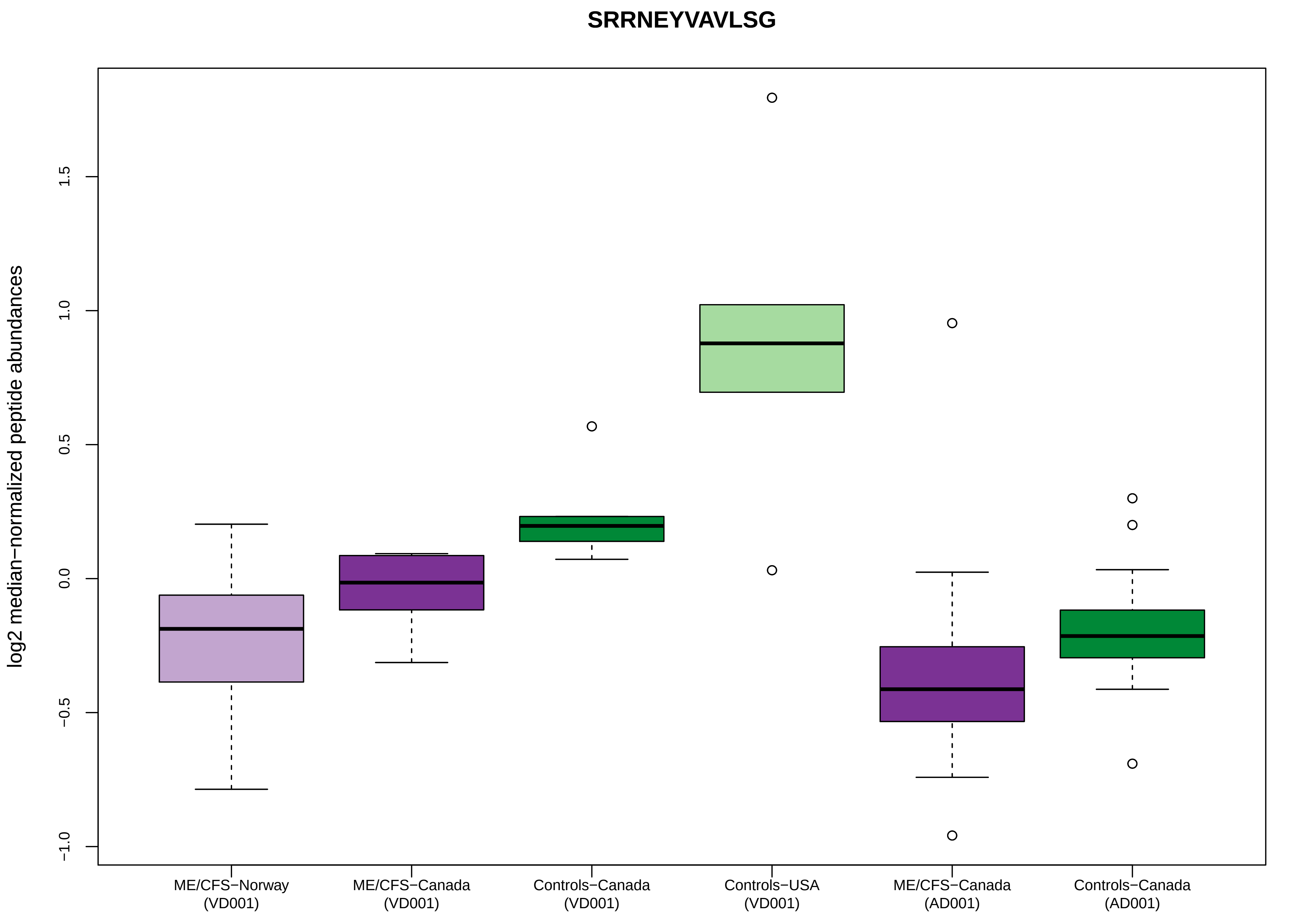
SRRNEYVAVLSG
log2 median−normalized peptide abundances
1.5
1.0
0.5
0.0
−0.5
−1.0
ME/CFS−Norway (VD001)
ME/CFS−Canada (VD001)
Controls−Canada (VD001)
Controls−USA (VD001)
ME/CFS−Canada (AD001)
Controls−Canada (AD001)

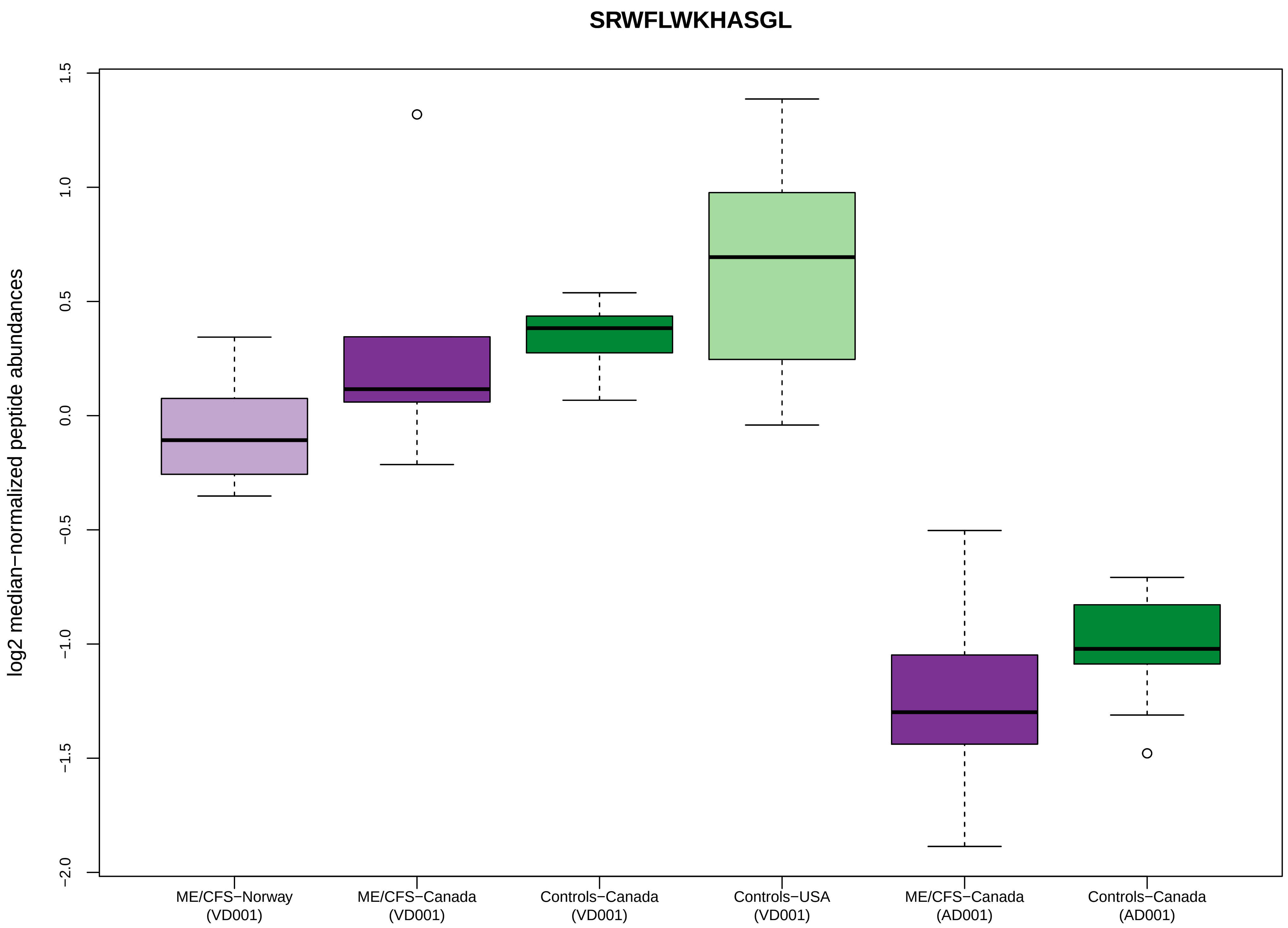
SRWFLWKHASGL
log2 median−normalized peptide abundances
1.5
1.0
0.5
0.0
−0.5
−1.0
−1.5
−2.0
ME/CFS−Norway (VD001)
ME/CFS−Canada (VD001)
Controls−Canada (VD001)
Controls−USA (VD001)
ME/CFS−Canada (AD001)
Controls−Canada (AD001)

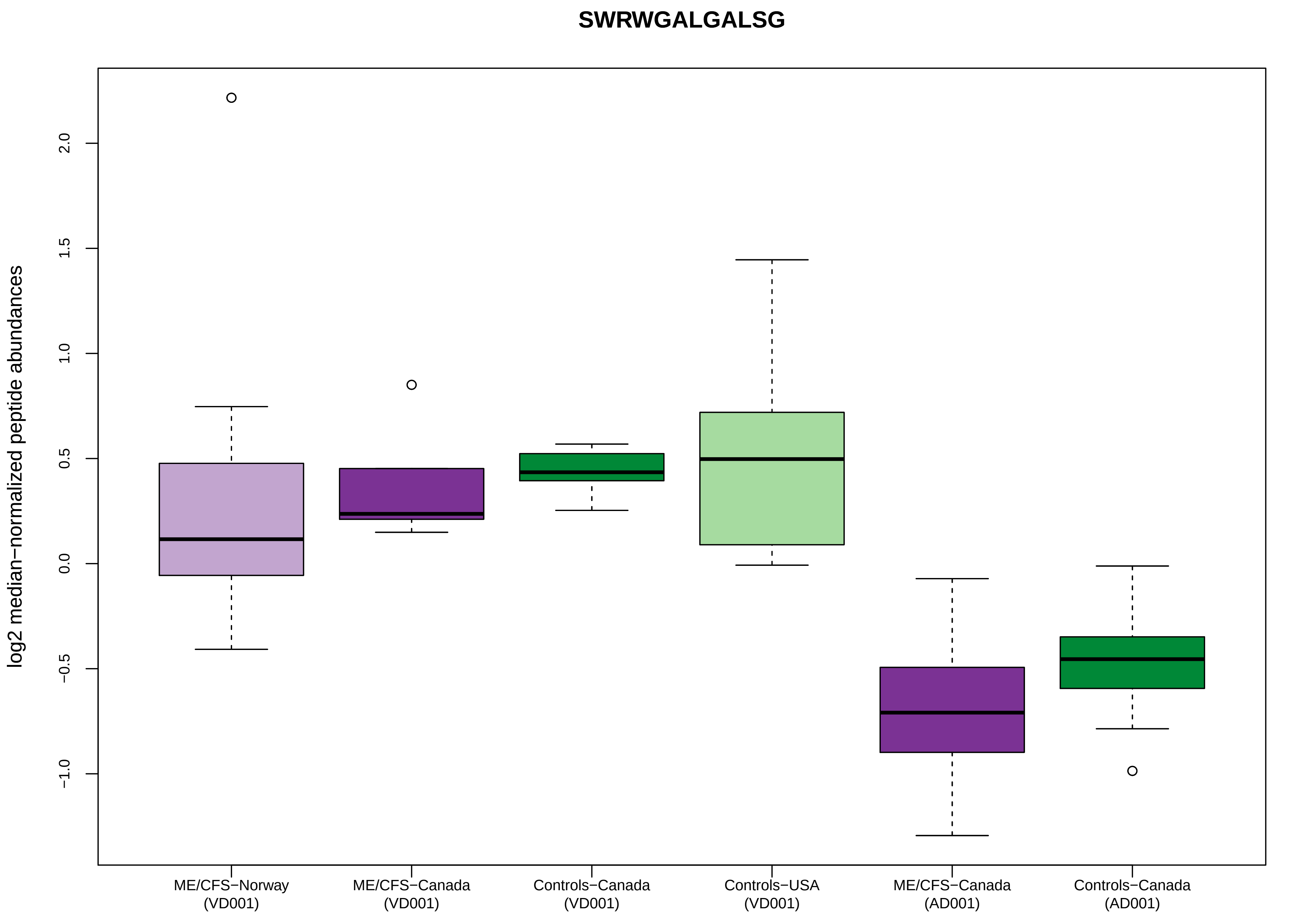

SWRWGALGALSG
log2 median−normalized peptide abundances
2.0
1.5
1.0
0.5
0.0
−0.5
−1.0
ME/CFS−Norway (VD001)
ME/CFS−Canada (VD001)
Controls−Canada (VD001)
Controls−USA (VD001)
ME/CFS−Canada (AD001)
Controls−Canada (AD001)

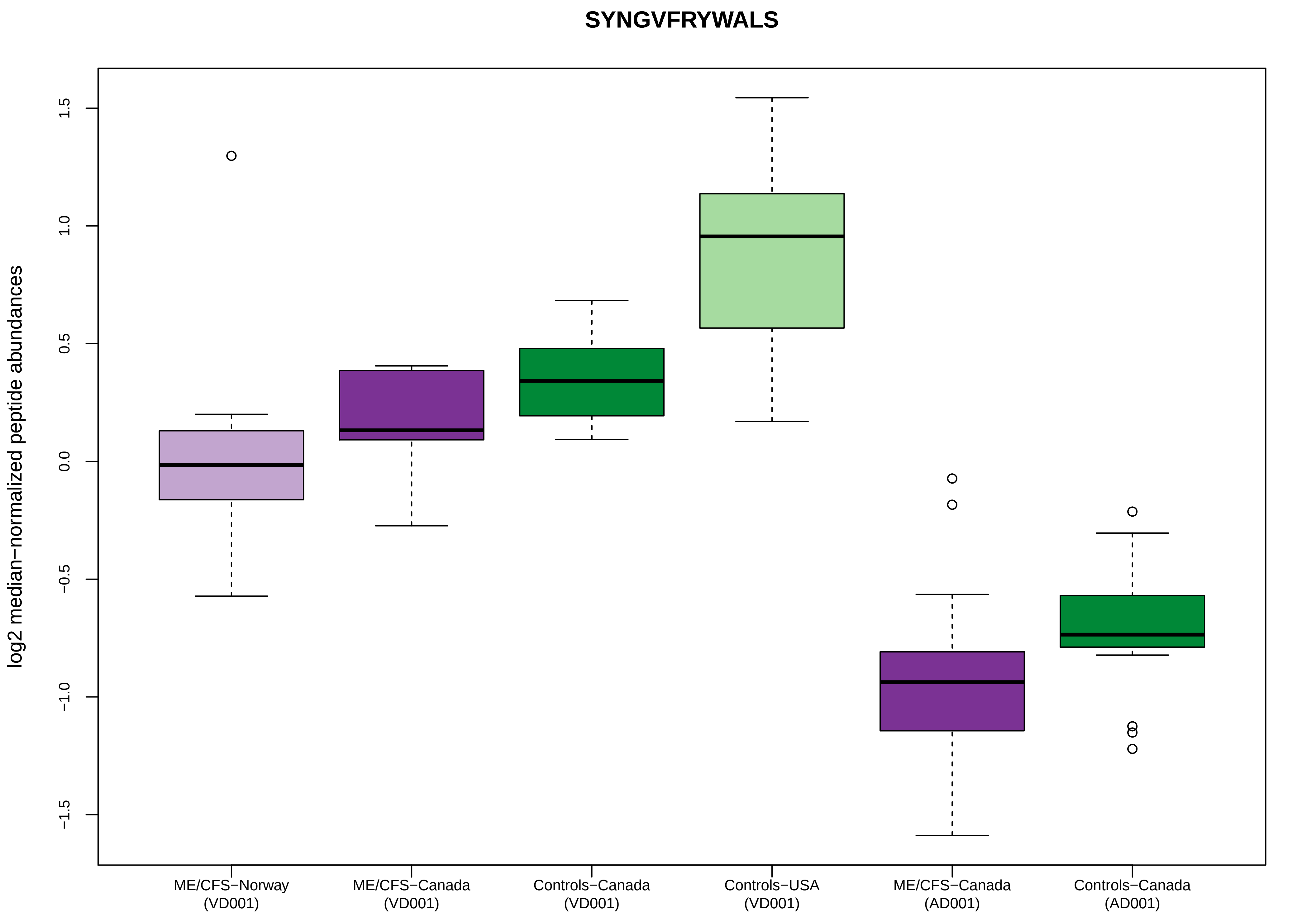

SYNGVFRYWALS
log2 median−normalized peptide abundances
1.5
1.0
0.5
0.0
−0.5
−1.0
−1.5
ME/CFS−Norway
(VD001)
ME/CFS−Canada
(VD001)
Controls−Canada
(VD001)
Controls−USA
(VD001)
ME/CFS−Canada
(AD001)
Controls−Canada
(AD001)

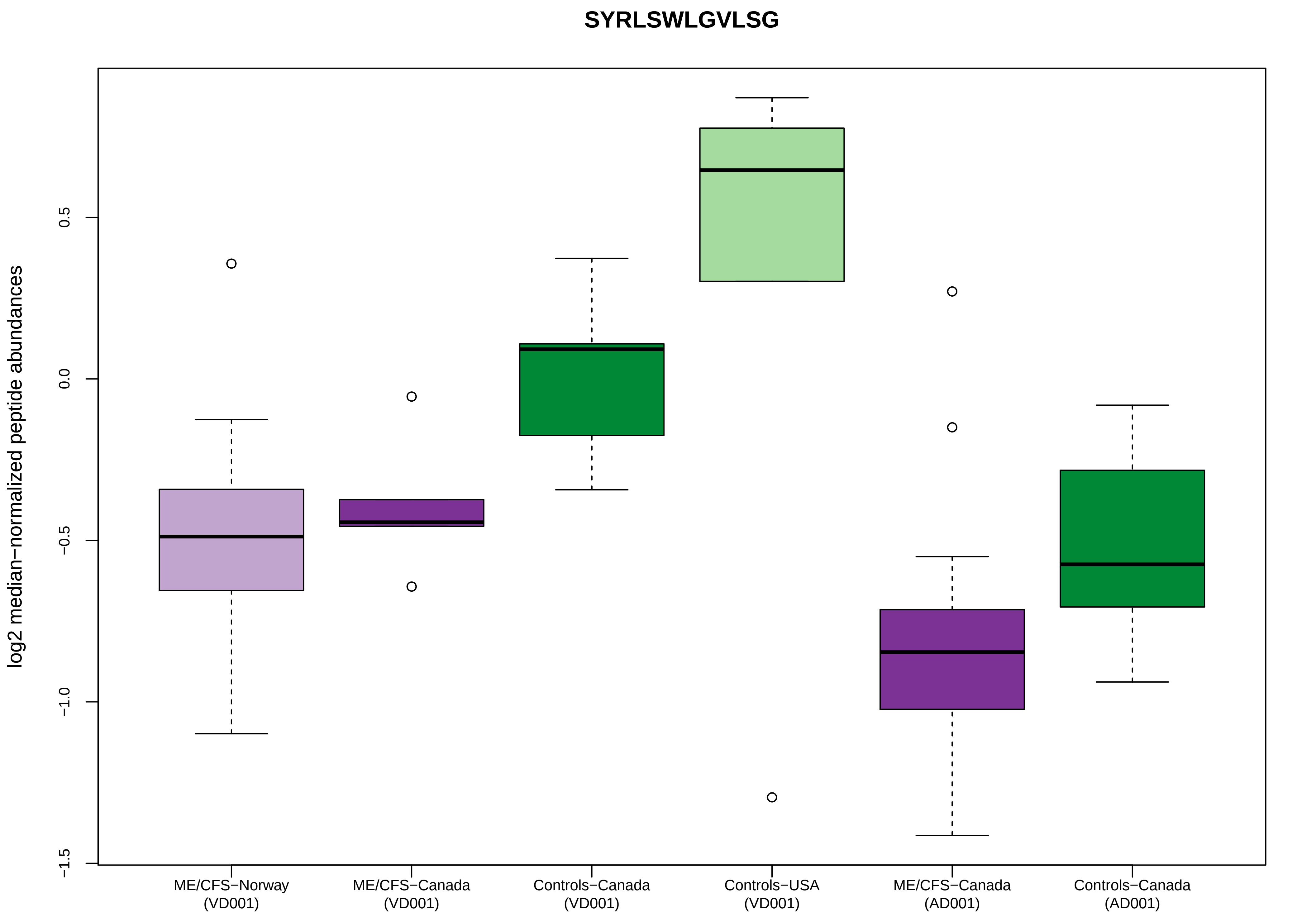

SYRLSWLGVLSG
log2 median−normalized peptide abundances
0.5
0.0
−0.5
−1.0
−1.5
ME/CFS−Norway (VD001)
ME/CFS−Canada (VD001)
Controls−Canada (VD001)
Controls−USA (VD001)
ME/CFS−Canada (AD001)
Controls−Canada (AD001)

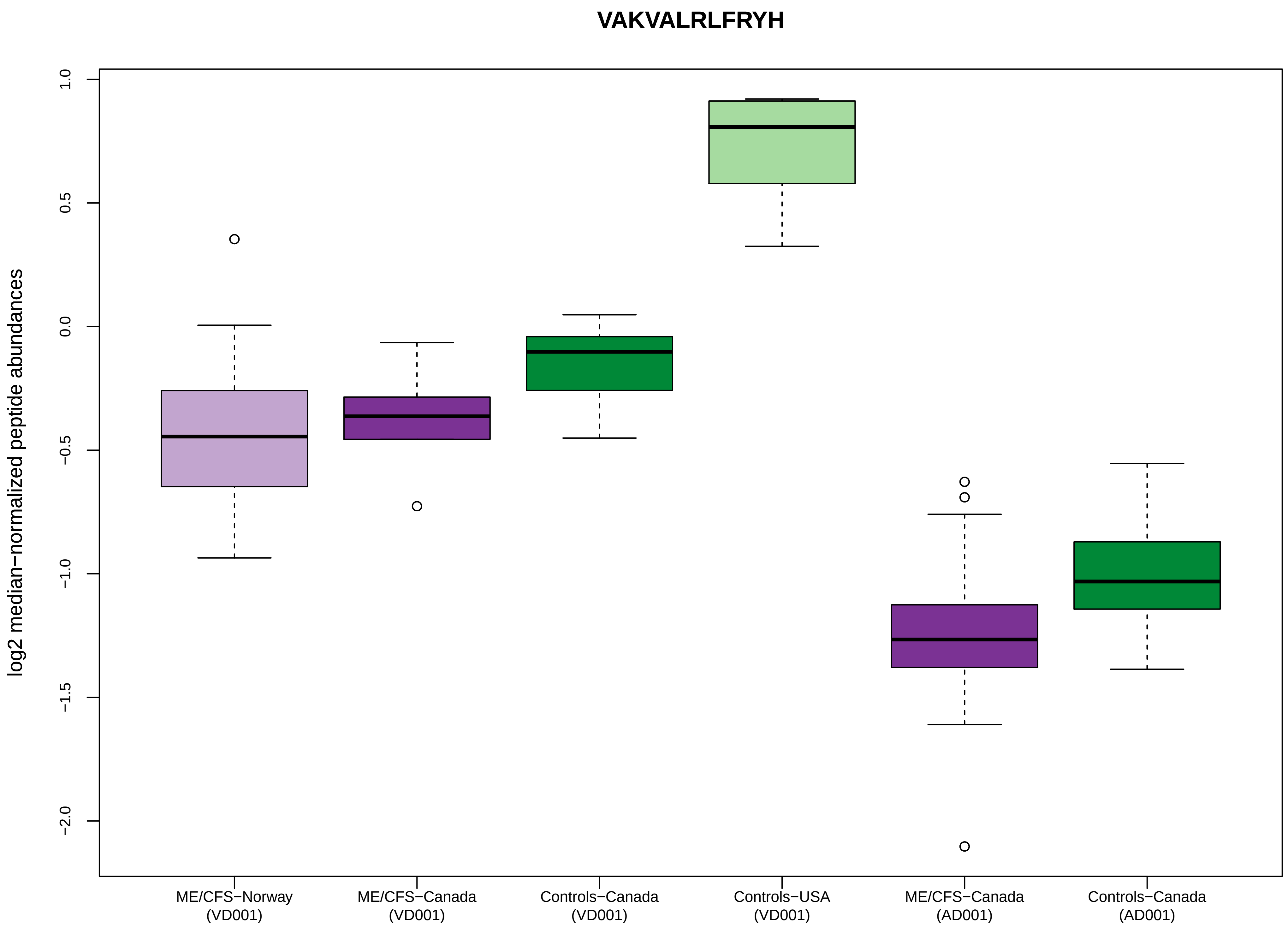

VAKVALRLFRYH
log2 median−normalized peptide abundances
1.0
0.5
0.0
−0.5
−1.0
−1.5
−2.0
ME/CFS−Norway (VD001)
ME/CFS−Canada (VD001)
Controls−Canada (VD001)
Controls−USA (VD001)
ME/CFS−Canada (AD001)
Controls−Canada (AD001)

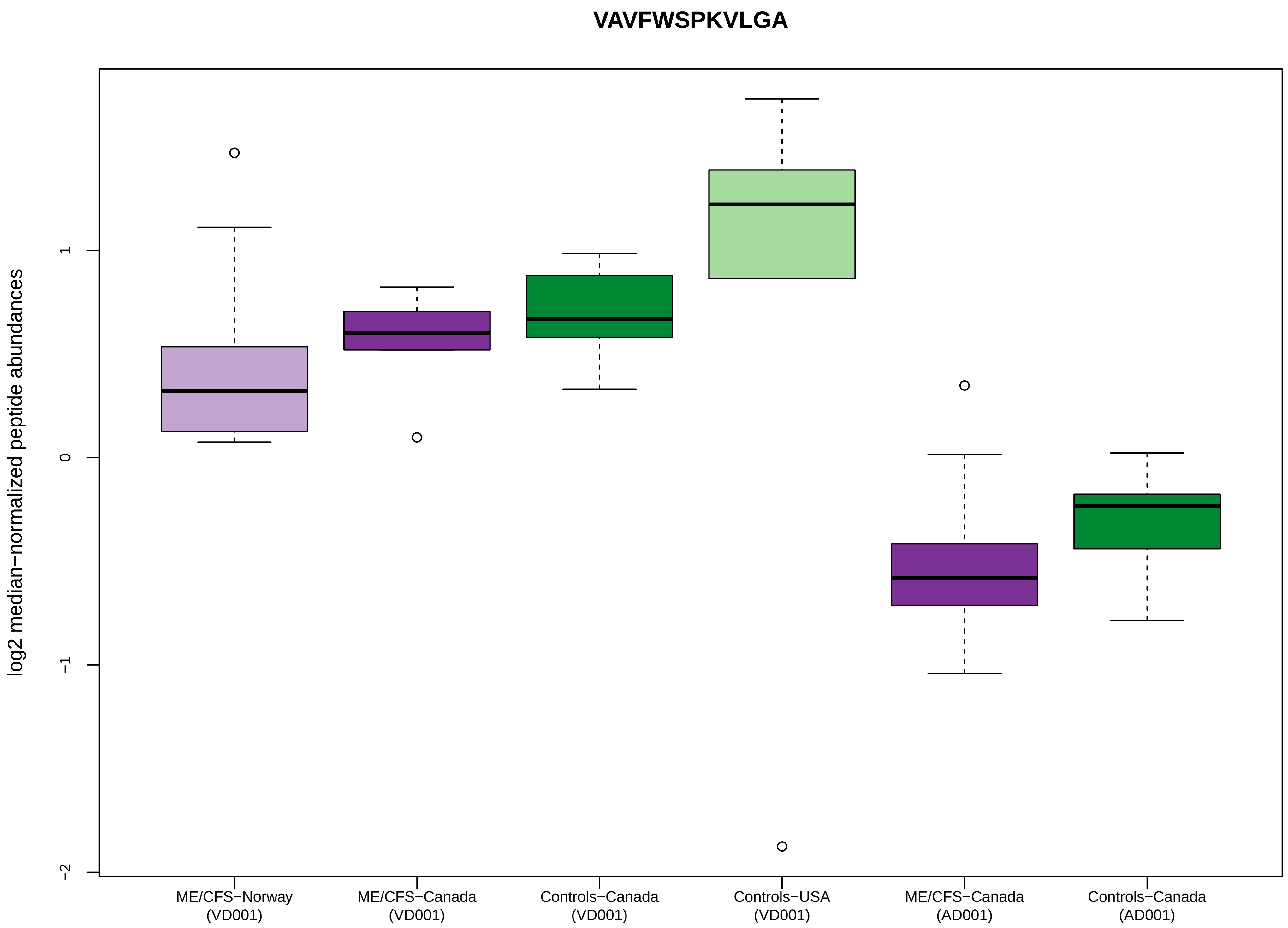
VAVFWSPKVLGA
log2 median−normalized peptide abundances
−2
−1
0
1
ME/CFS−Norway (VD001)
ME/CFS−Canada (VD001)
Controls−Canada (VD001)
Controls−USA (VD001)
ME/CFS−Canada (AD001)
Controls−Canada (AD001)

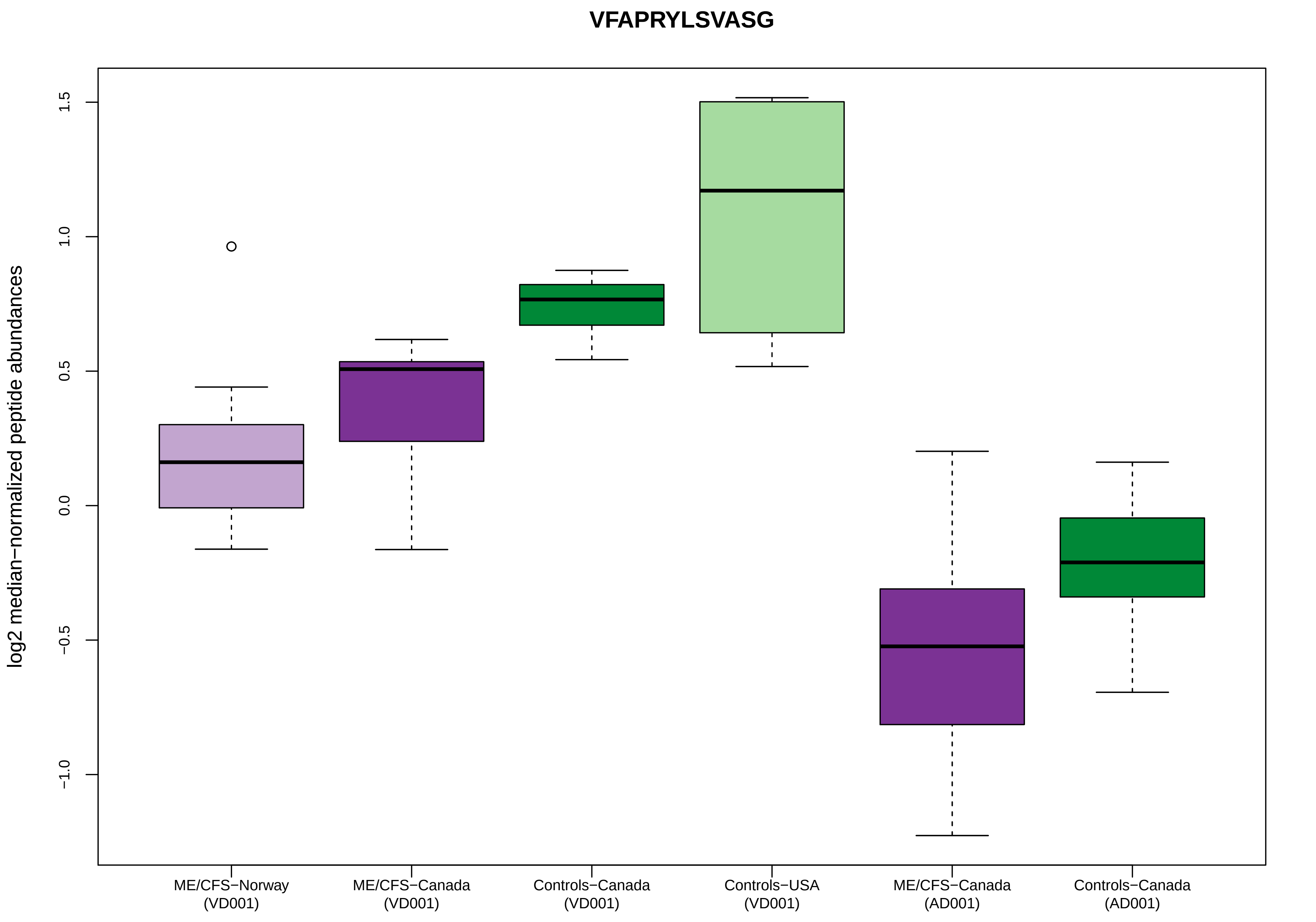

VFAPRYLSVASG
log2 median−normalized peptide abundances
1.5
1.0
0.5
0.0
−0.5
−1.0
ME/CFS−Norway (VD001)
ME/CFS−Canada (VD001)
Controls−Canada (VD001)
Controls−USA (VD001)
ME/CFS−Canada (AD001)
Controls−Canada (AD001)

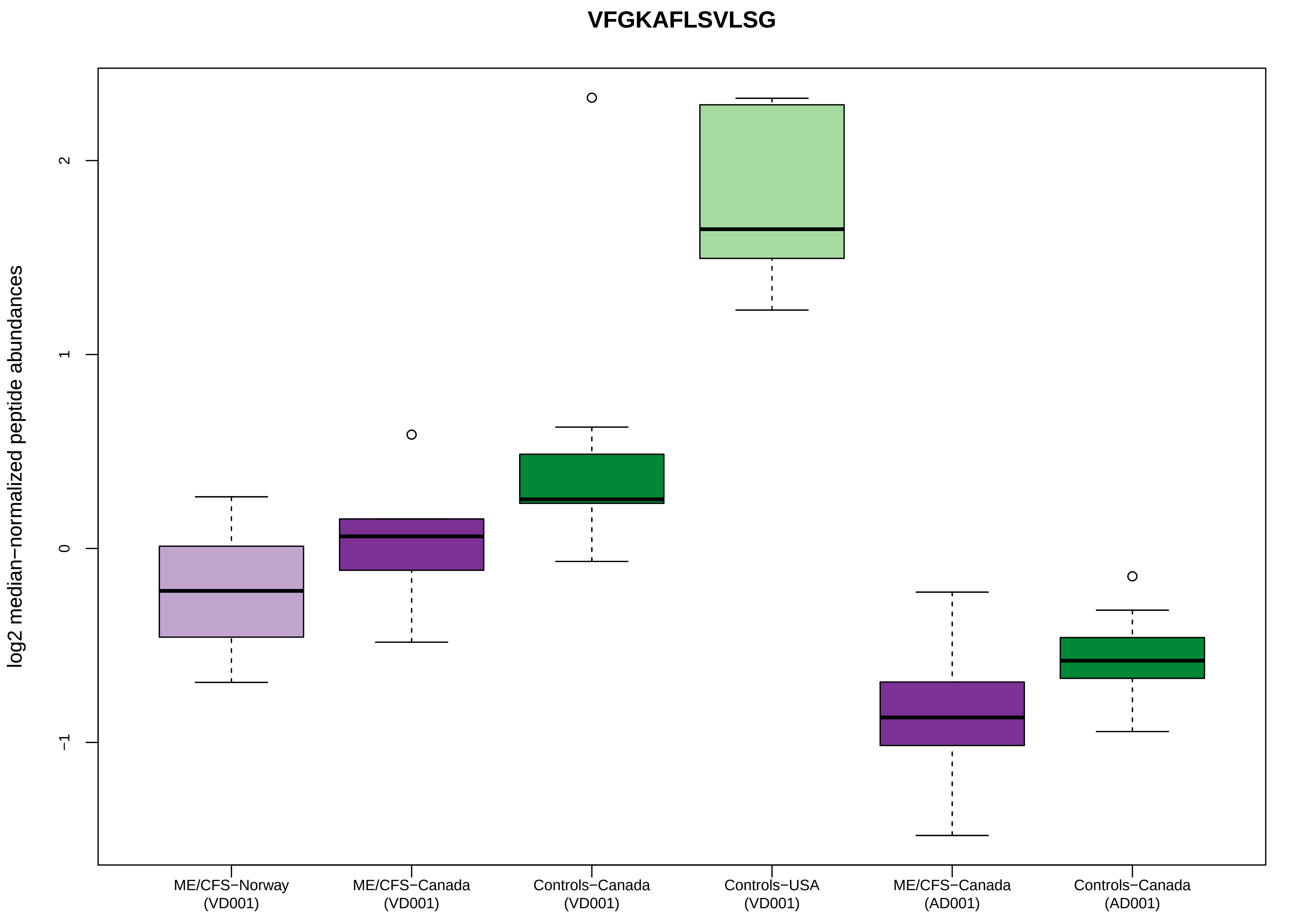

VFGKAFLSVLSG
log2 median−normalized peptide abundances
2
1
0
−1
ME/CFS−Norway (VD001)
ME/CFS−Canada (VD001)
Controls−Canada (VD001)
Controls−USA (VD001)
ME/CFS−Canada (AD001)
Controls−Canada (AD001)

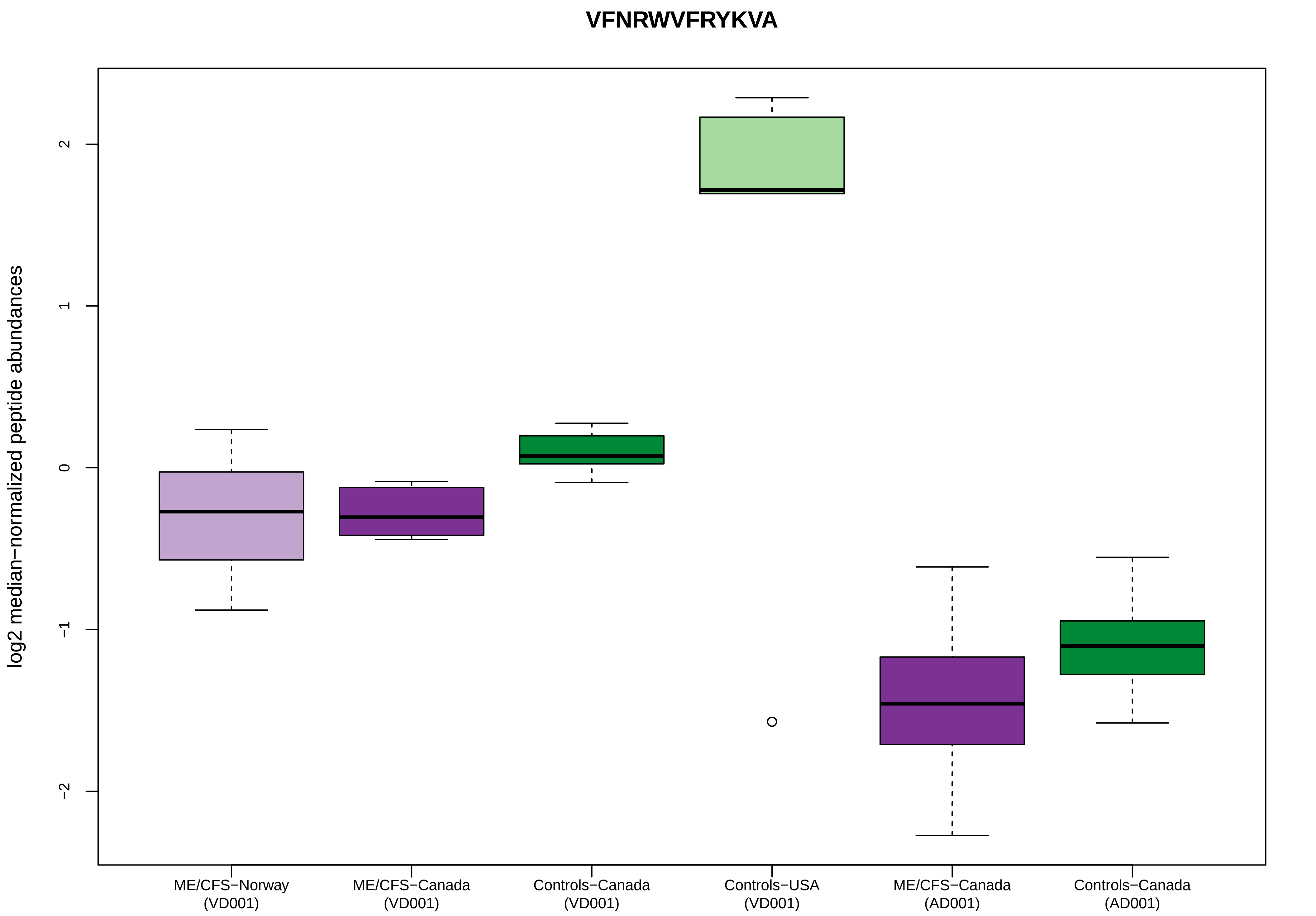
VFNRWVFRYKVA
log2 median−normalized peptide abundances
−2
−1
0
1
2
ME/CFS−Norway (VD001)
ME/CFS−Canada (VD001)
Controls−Canada (VD001)
Controls−USA (VD001)
ME/CFS−Canada (AD001)
Controls−Canada (AD001)

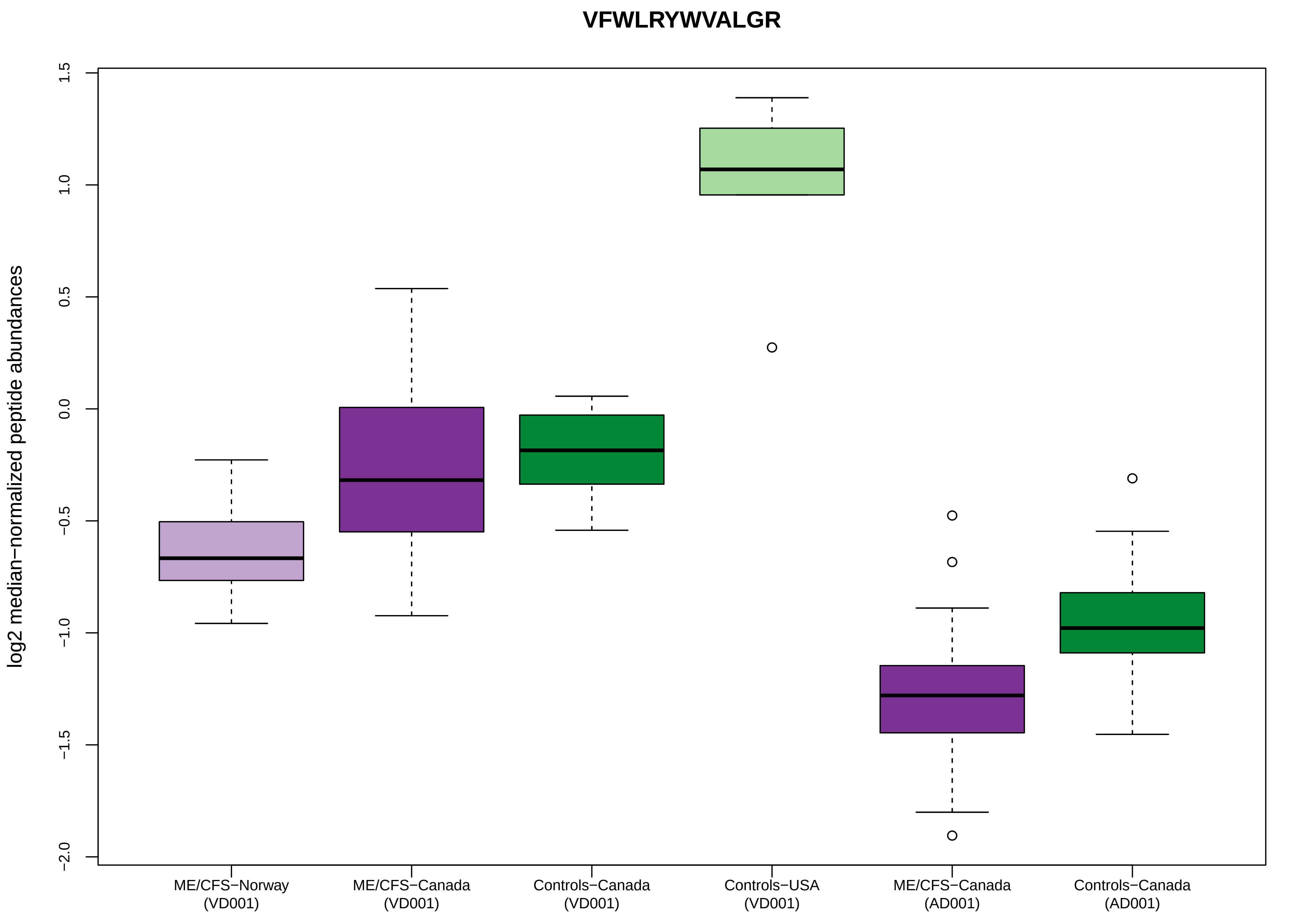
VFWLRYWVALGR
log2 median−normalized peptide abundances
1.5
1.0
0.5
0.0
−0.5
−1.0
−1.5
−2.0
ME/CFS−Norway (VD001)
ME/CFS−Canada (VD001)
Controls−Canada (VD001)
Controls−USA (VD001)
ME/CFS−Canada (AD001)
Controls−Canada (AD001)

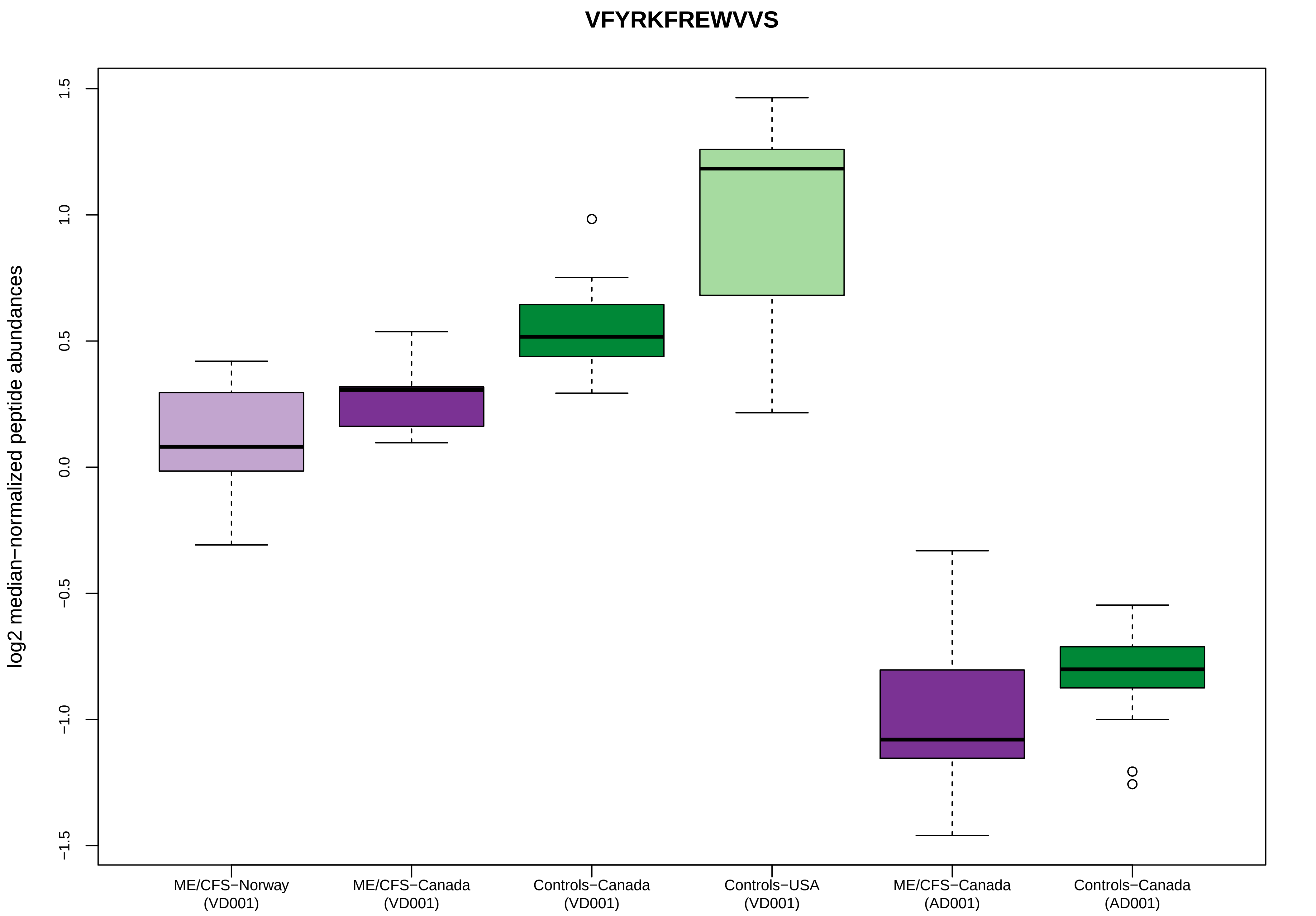

VFYRKFREWVVS
log2 median−normalized peptide abundances
1.5
1.0
0.5
0.0
−0.5
−1.0
−1.5
ME/CFS−Norway (VD001)
ME/CFS−Canada (VD001)
Controls−Canada (VD001)
Controls−USA (VD001)
ME/CFS−Canada (AD001)
Controls−Canada (AD001)

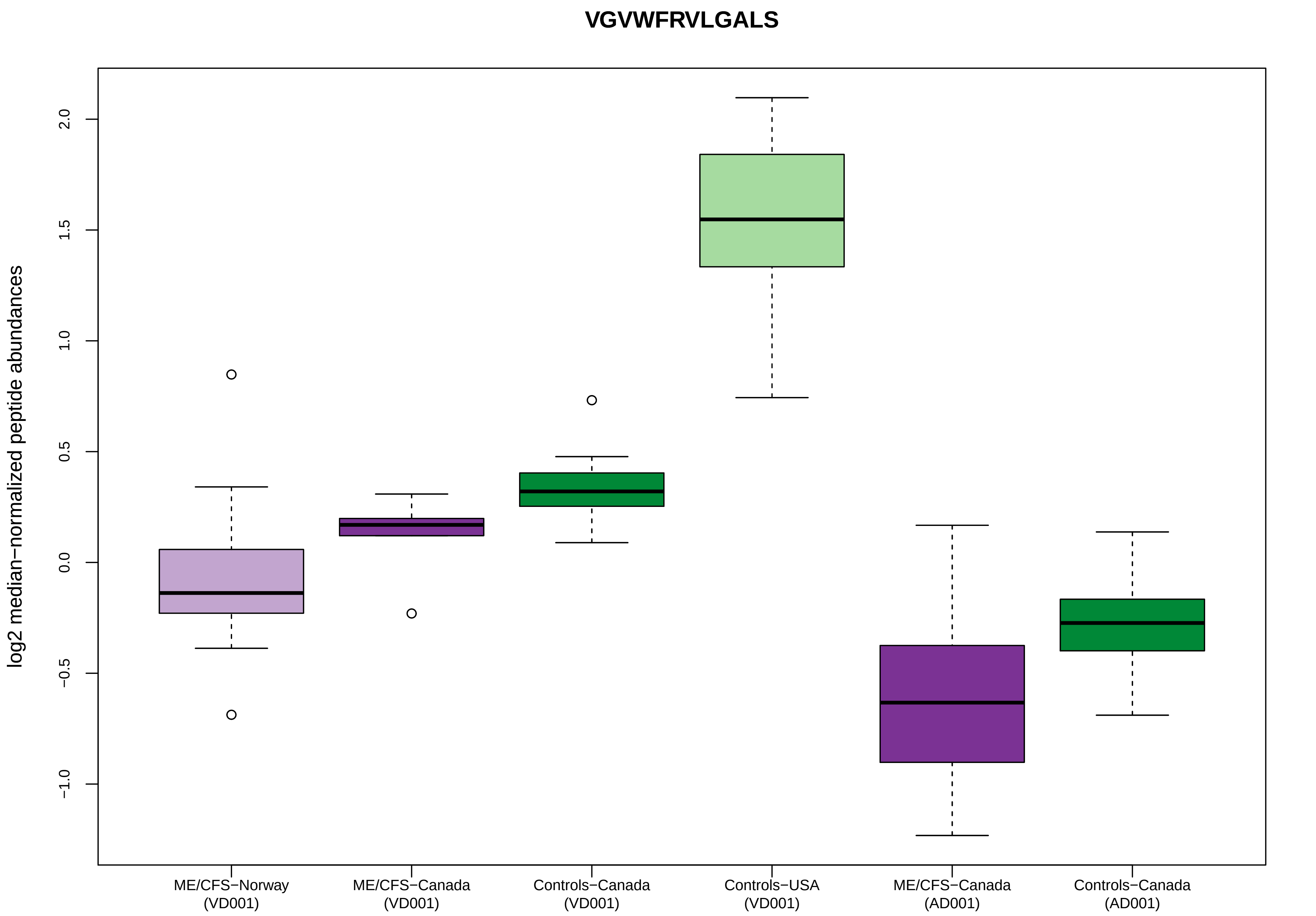
VGVWFRVLGALS
log2 median−normalized peptide abundances
2.0
1.5
1.0
0.5
0.0
−0.5
−1.0
ME/CFS−Norway (VD001)
ME/CFS−Canada (VD001)
Controls−Canada (VD001)
Controls−USA (VD001)
ME/CFS−Canada (AD001)
Controls−Canada (AD001)

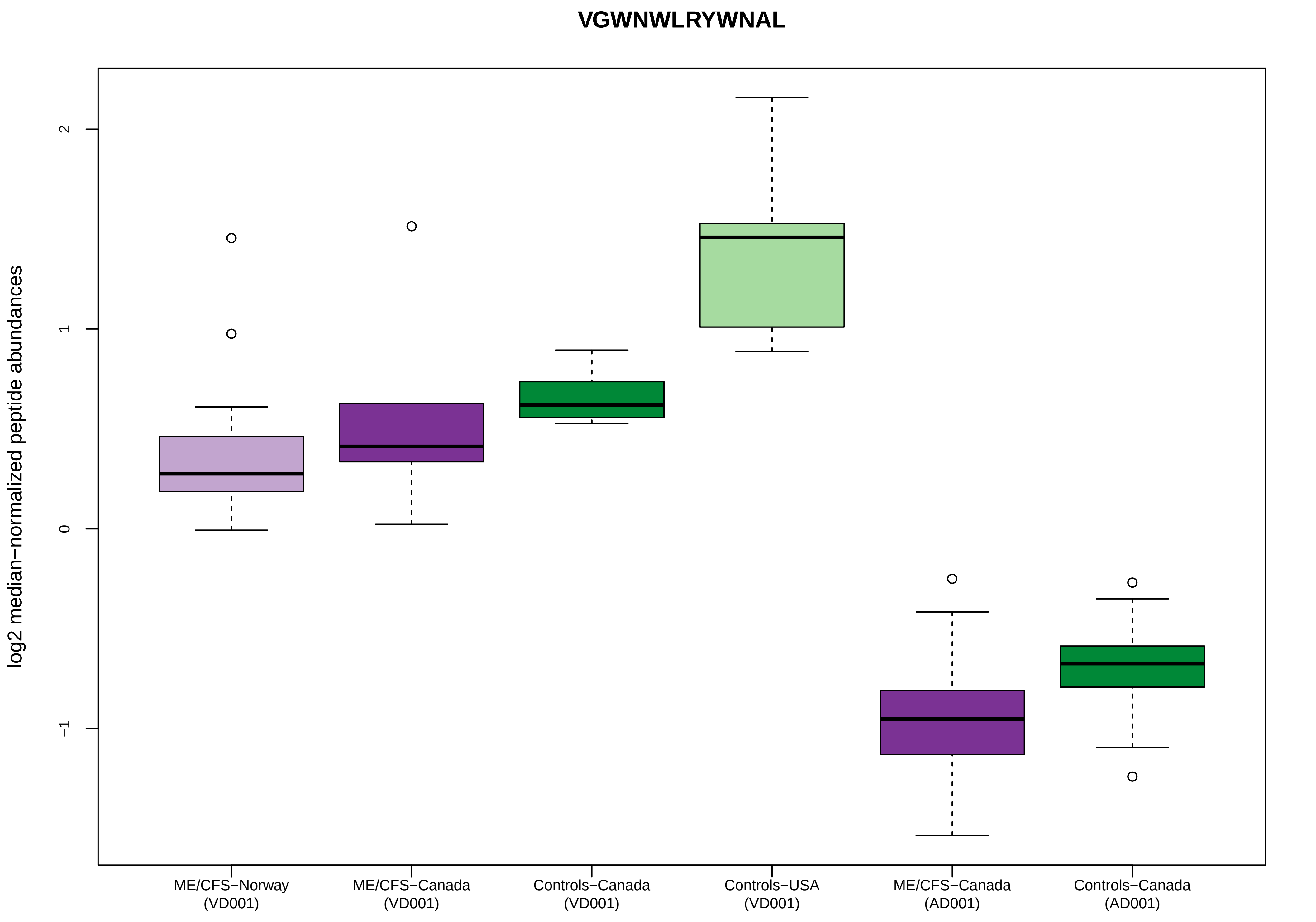

VGWNWLRYWNAL
log2 median−normalized peptide abundances
2
1
0
−1
ME/CFS−Norway (VD001)
ME/CFS−Canada (VD001)
Controls−Canada (VD001)
Controls−USA (VD001)
ME/CFS−Canada (AD001)
Controls−Canada (AD001)

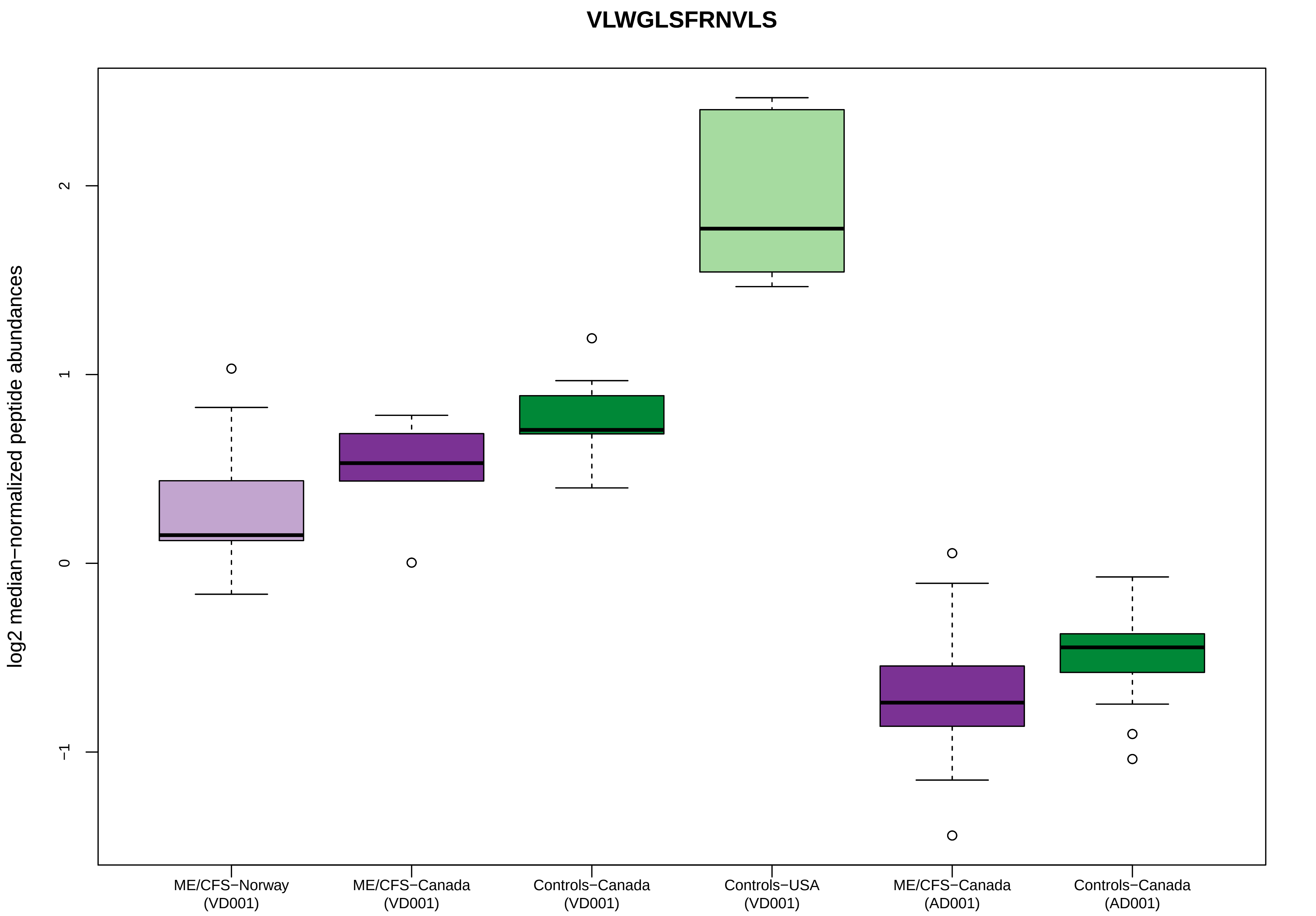
VLWGLSFRNVLS
log2 median−normalized peptide abundances
−1
0
1
2
ME/CFS−Norway (VD001)
ME/CFS−Canada (VD001)
Controls−Canada (VD001)
Controls−USA (VD001)
ME/CFS−Canada (AD001)
Controls−Canada (AD001)

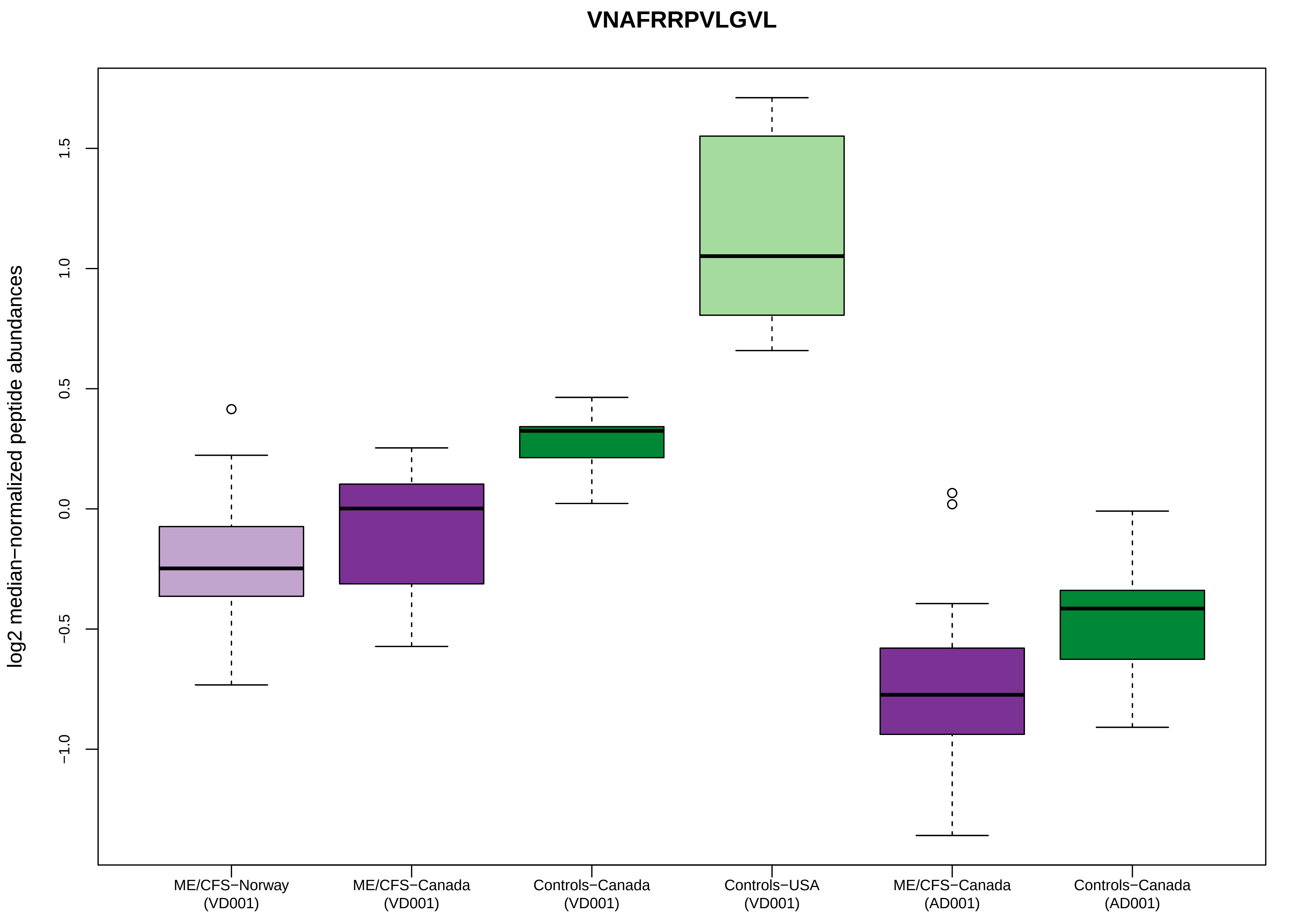

VNAFRRPVLGVL
log2 median−normalized peptide abundances
1.5
1.0
0.5
0.0
−0.5
−1.0
ME/CFS−Norway (VD001)
ME/CFS−Canada (VD001)
Controls−Canada (VD001)
Controls−USA (VD001)
ME/CFS−Canada (AD001)
Controls−Canada (AD001)

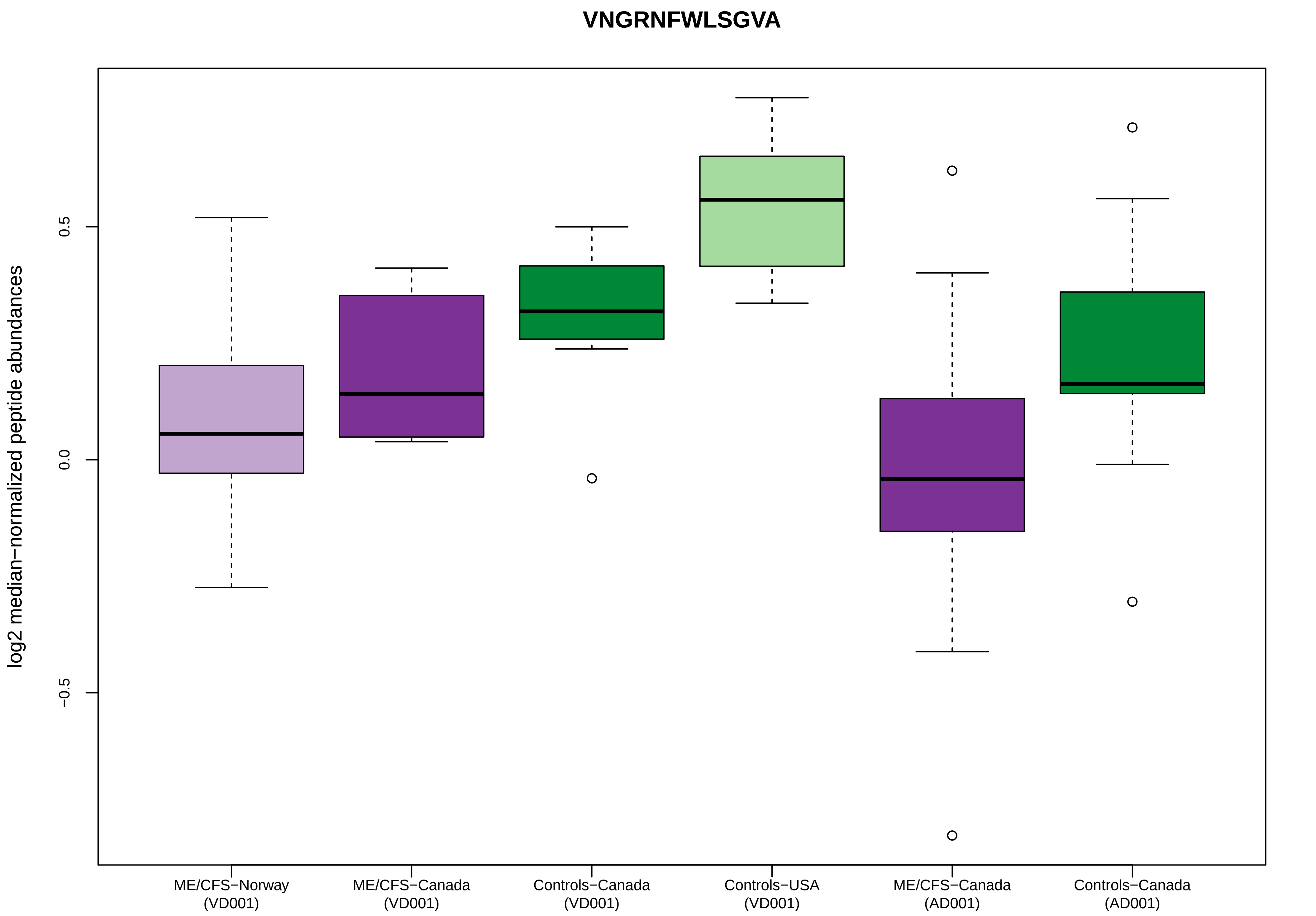

VNGRNFWLSGVA
log2 median−normalized peptide abundances
0.5
0.0
−0.5
ME/CFS−Norway
(VD001)
ME/CFS−Canada
(VD001)
Controls−Canada
(VD001)
Controls−USA
(VD001)
ME/CFS−Canada
(AD001)
Controls−Canada
(AD001)

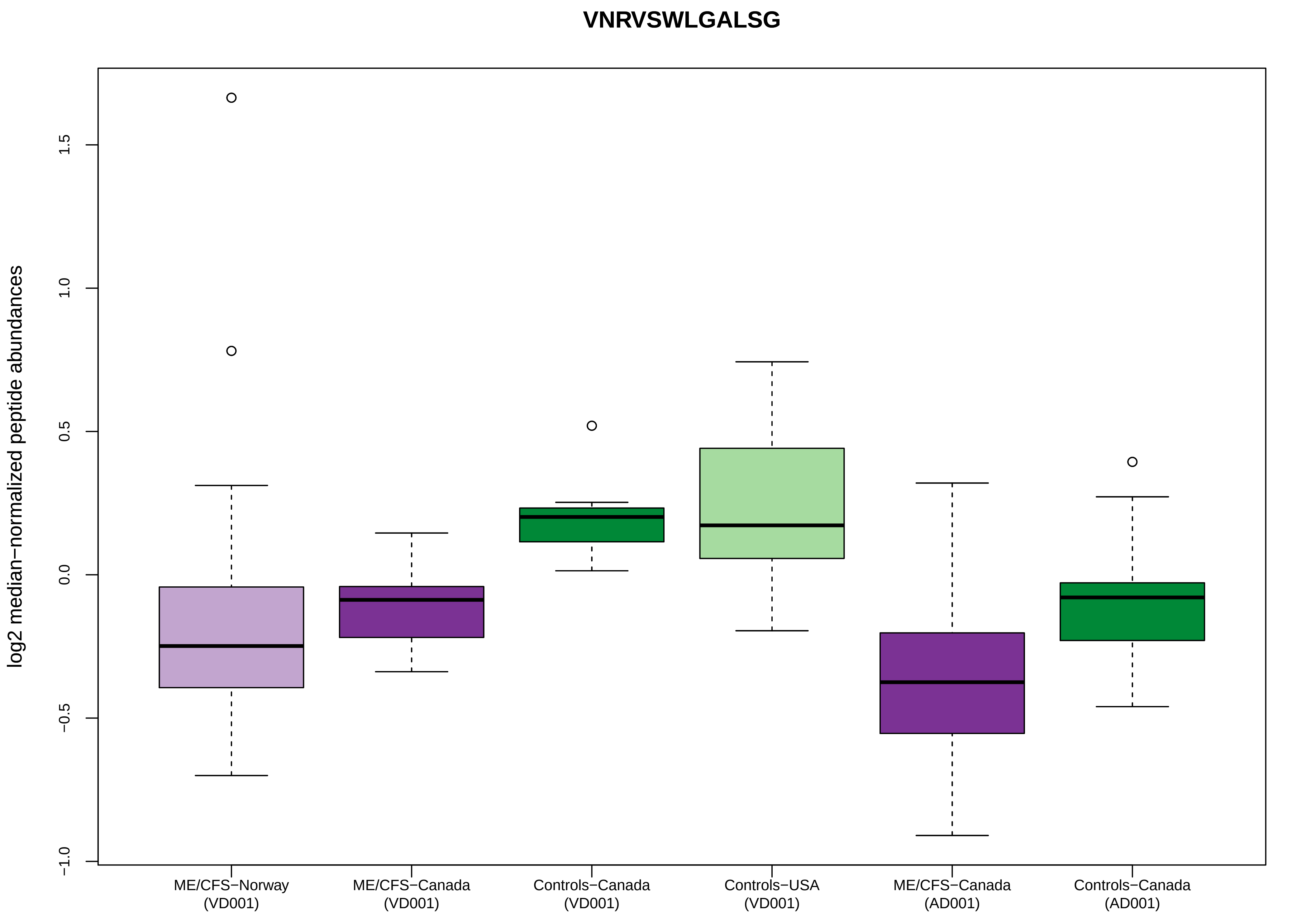
VNRVSWLGALSG
log2 median−normalized peptide abundances
1.5
1.0
0.5
0.0
−0.5
−1.0
ME/CFS−Norway (VD001)
ME/CFS−Canada (VD001)
Controls−Canada (VD001)
Controls−USA (VD001)
ME/CFS−Canada (AD001)
Controls−Canada (AD001)

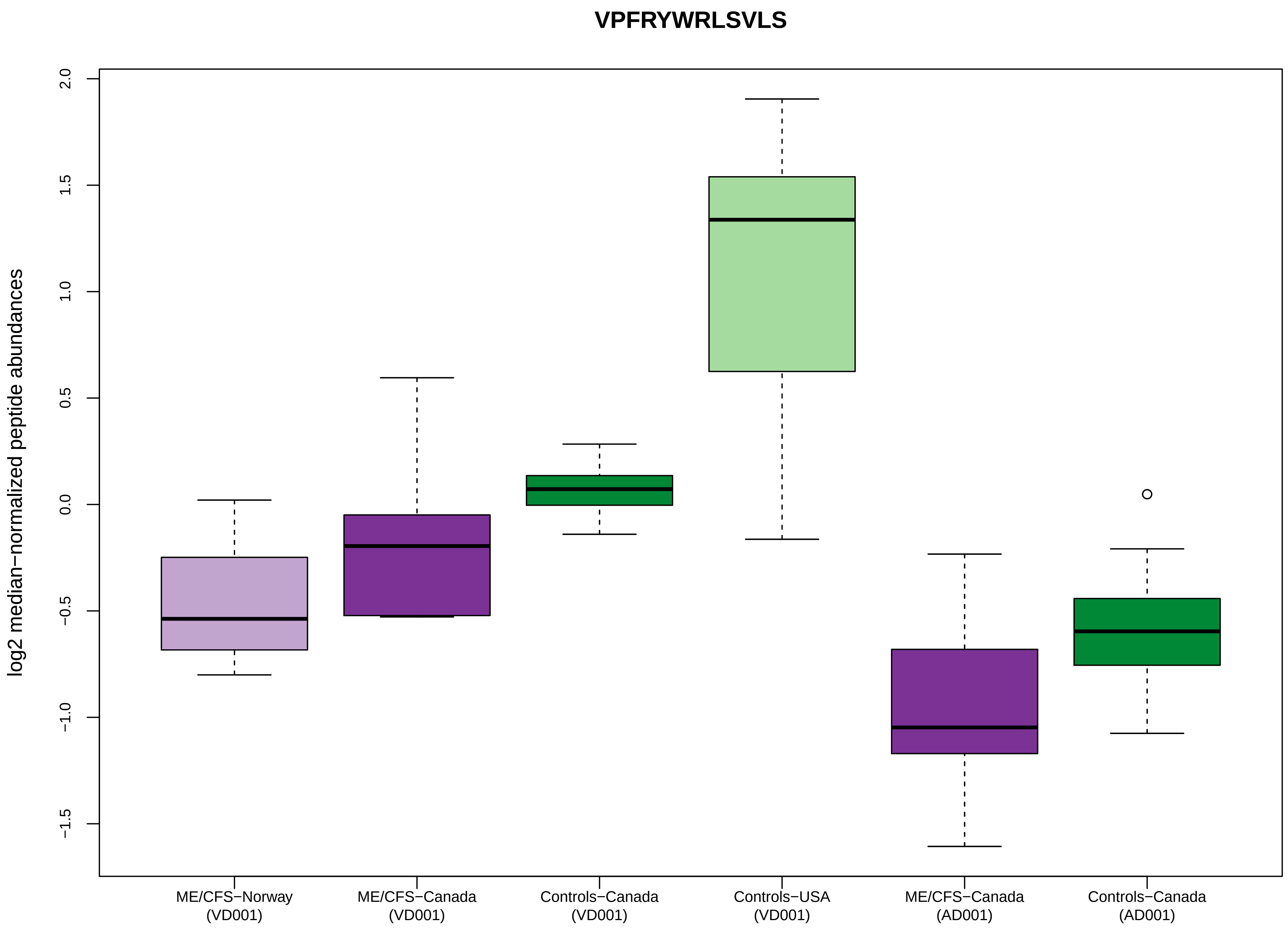

VPFRYWRLSVLS
log2 median−normalized peptide abundances
2.0
1.5
1.0
0.5
0.0
−0.5
−1.0
−1.5
ME/CFS−Norway (VD001)
ME/CFS−Canada (VD001)
Controls−Canada (VD001)
Controls−USA (VD001)
ME/CFS−Canada (AD001)
Controls−Canada (AD001)

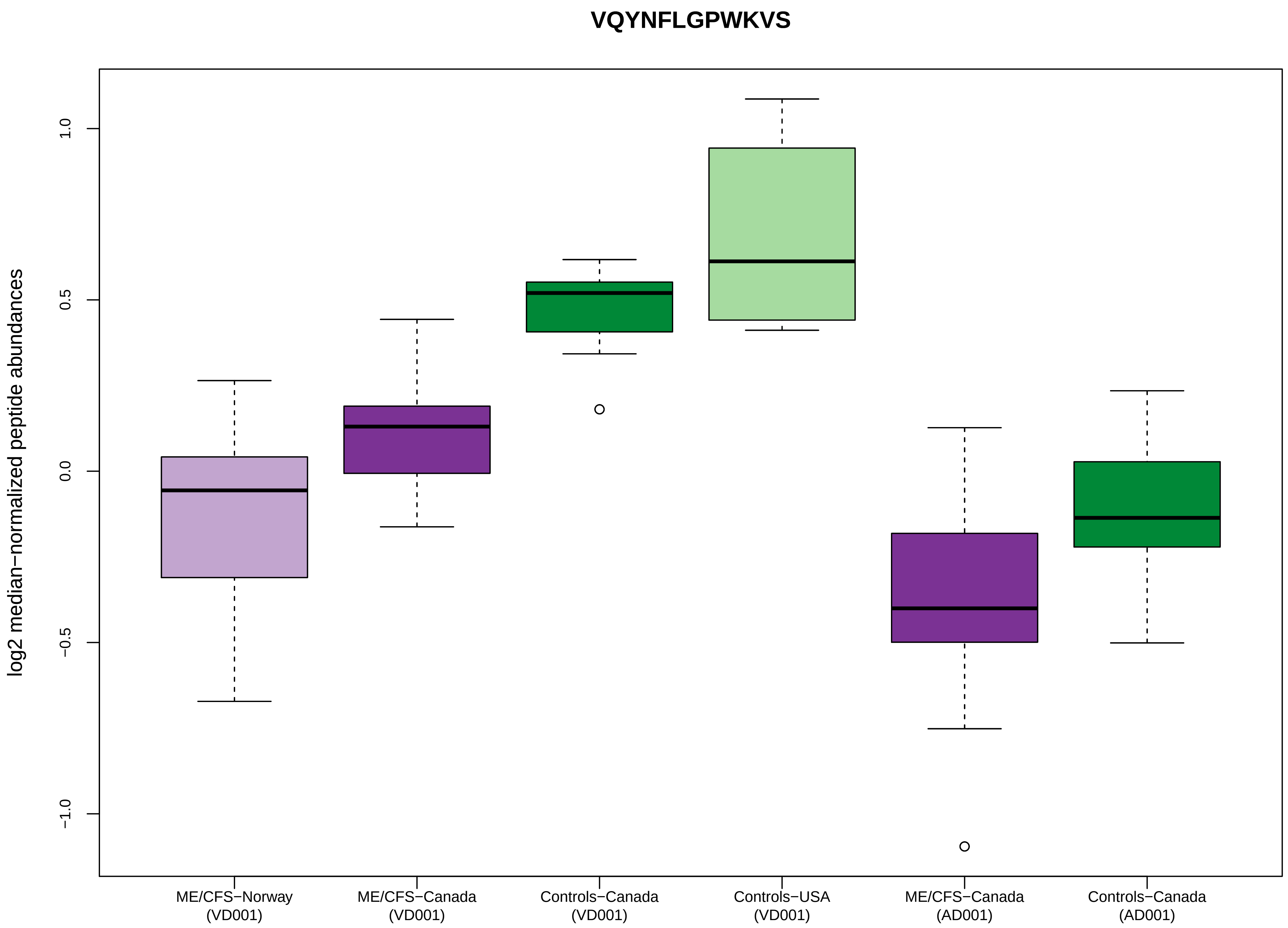
VQYNFLGPWKVS
log2 median−normalized peptide abundances
1.0
0.5
0.0
−0.5
−1.0
ME/CFS−Norway (VD001)
ME/CFS−Canada (VD001)
Controls−Canada (VD001)
Controls−USA (VD001)
ME/CFS−Canada (AD001)
Controls−Canada (AD001)

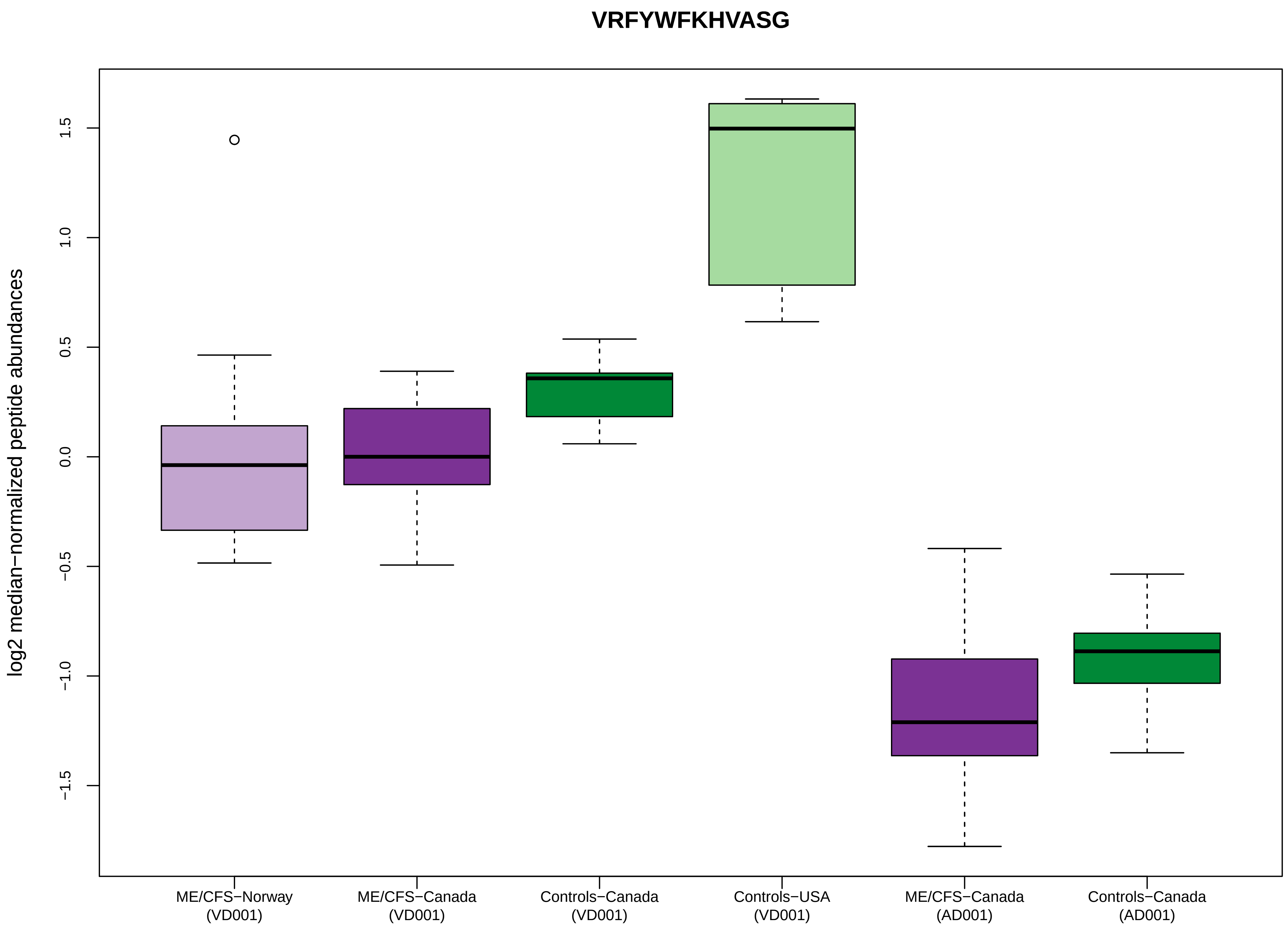
VRFYWFKHVASG
log2 median−normalized peptide abundances
1.5
1.0
0.5
0.0
−0.5
−1.0
−1.5
ME/CFS−Norway (VD001)
ME/CFS−Canada (VD001)
Controls−Canada (VD001)
Controls−USA (VD001)
ME/CFS−Canada (AD001)
Controls−Canada (AD001)

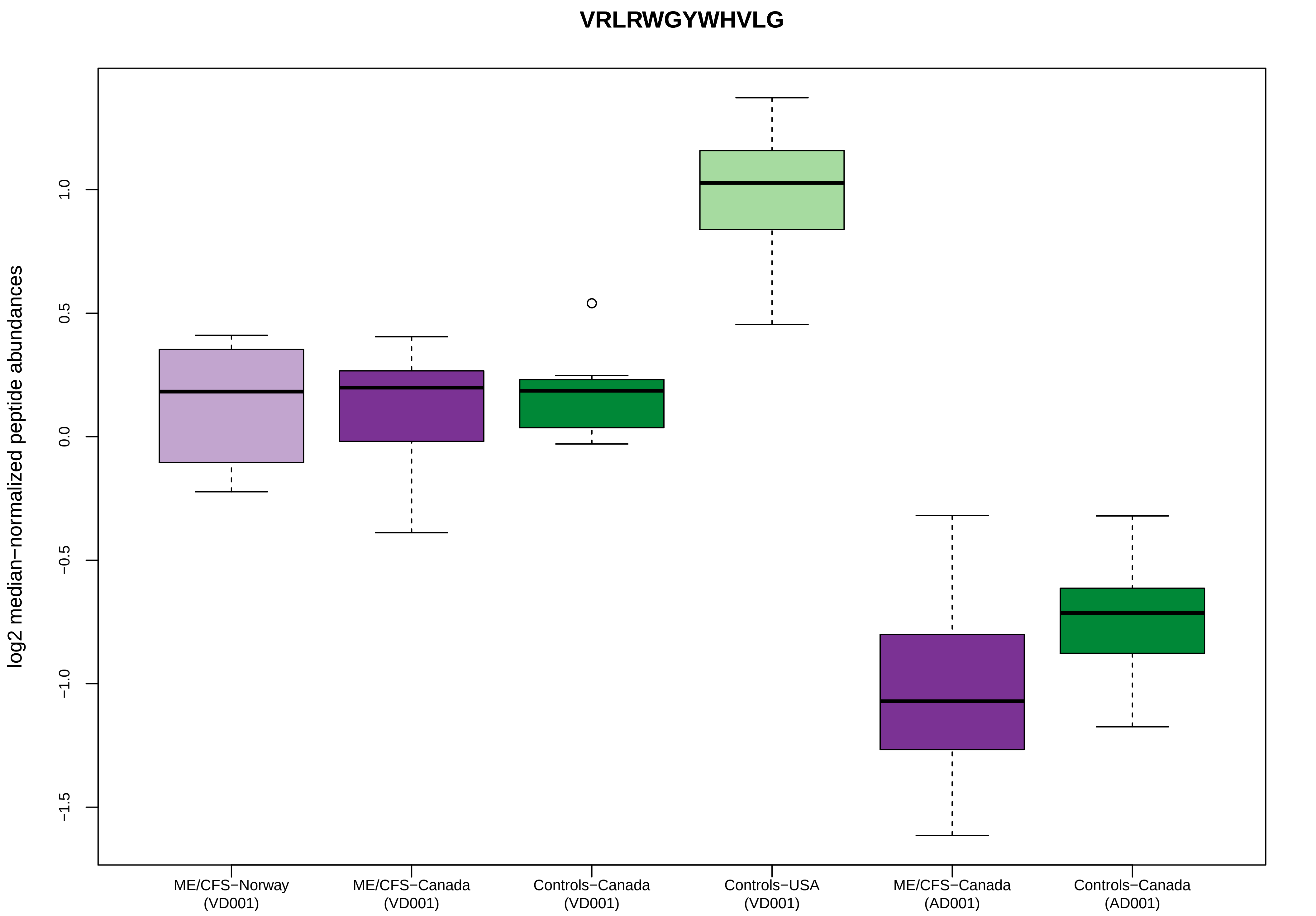

VRLRWGYWHVLG
log2 median−normalized peptide abundances
1.0
0.5
0.0
−0.5
−1.0
−1.5
ME/CFS−Norway (VD001)
ME/CFS−Canada (VD001)
Controls−Canada (VD001)
Controls−USA (VD001)
ME/CFS−Canada (AD001)
Controls−Canada (AD001)

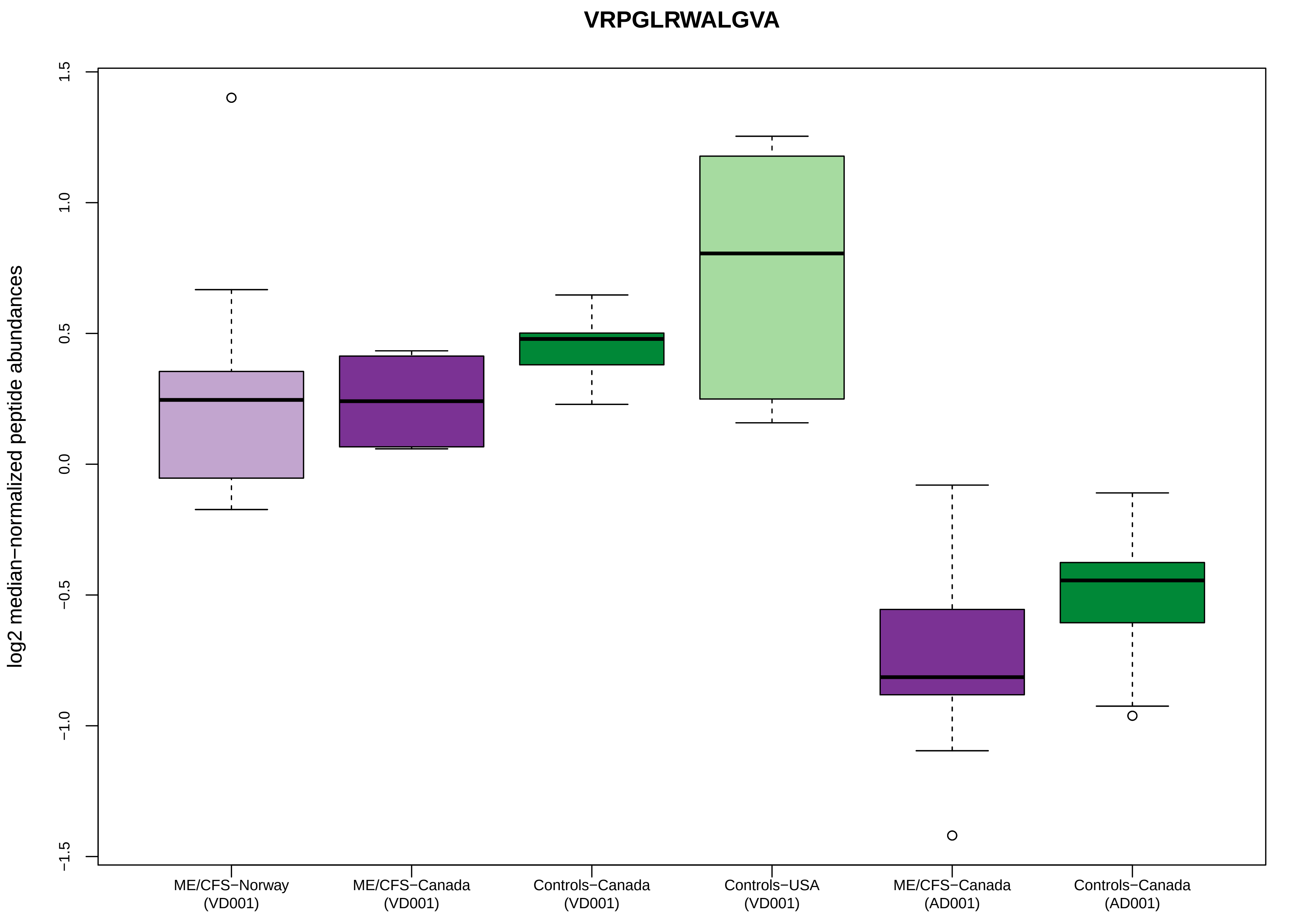
VRPGLRWALGVA
log2 median−normalized peptide abundances
1.5
1.0
0.5
0.0
−0.5
−1.0
−1.5
ME/CFS−Norway (VD001)
ME/CFS−Canada (VD001)
Controls−Canada (VD001)
Controls−USA (VD001)
ME/CFS−Canada (AD001)
Controls−Canada (AD001)

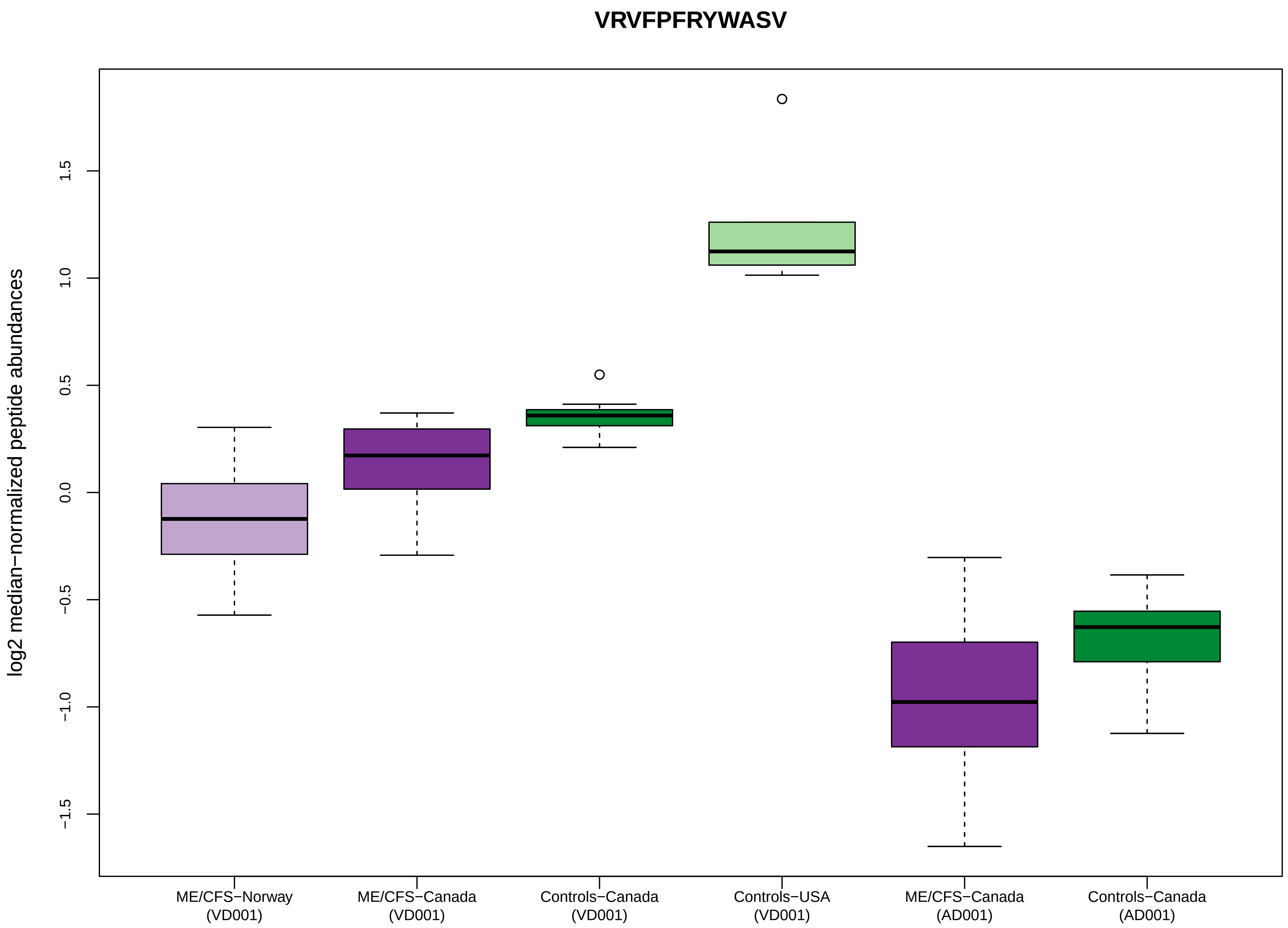
VRVFPFRYWASV
log2 median−normalized peptide abundances
1.5
1.0
0.5
0.0
−0.5
−1.0
−1.5
ME/CFS−Norway (VD001)
ME/CFS−Canada (VD001)
Controls−Canada (VD001)
Controls−USA (VD001)
ME/CFS−Canada (AD001)
Controls−Canada (AD001)

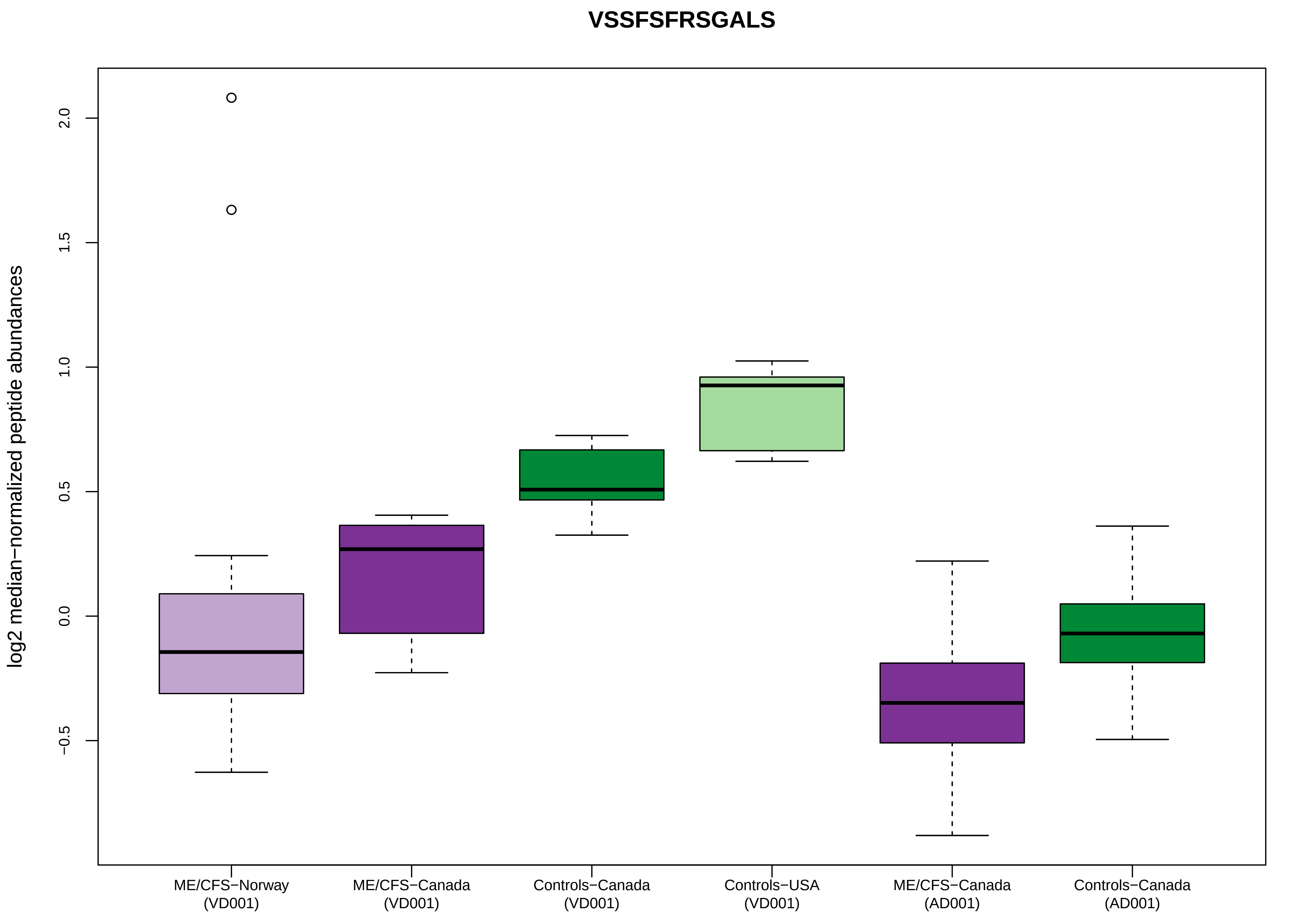

VSSFSFRSGALS
log2 median−normalized peptide abundances
2.0
1.5
1.0
0.5
0.0
−0.5
ME/CFS−Norway (VD001)
ME/CFS−Canada (VD001)
Controls−Canada (VD001)
Controls−USA (VD001)
ME/CFS−Canada (AD001)
Controls−Canada (AD001)

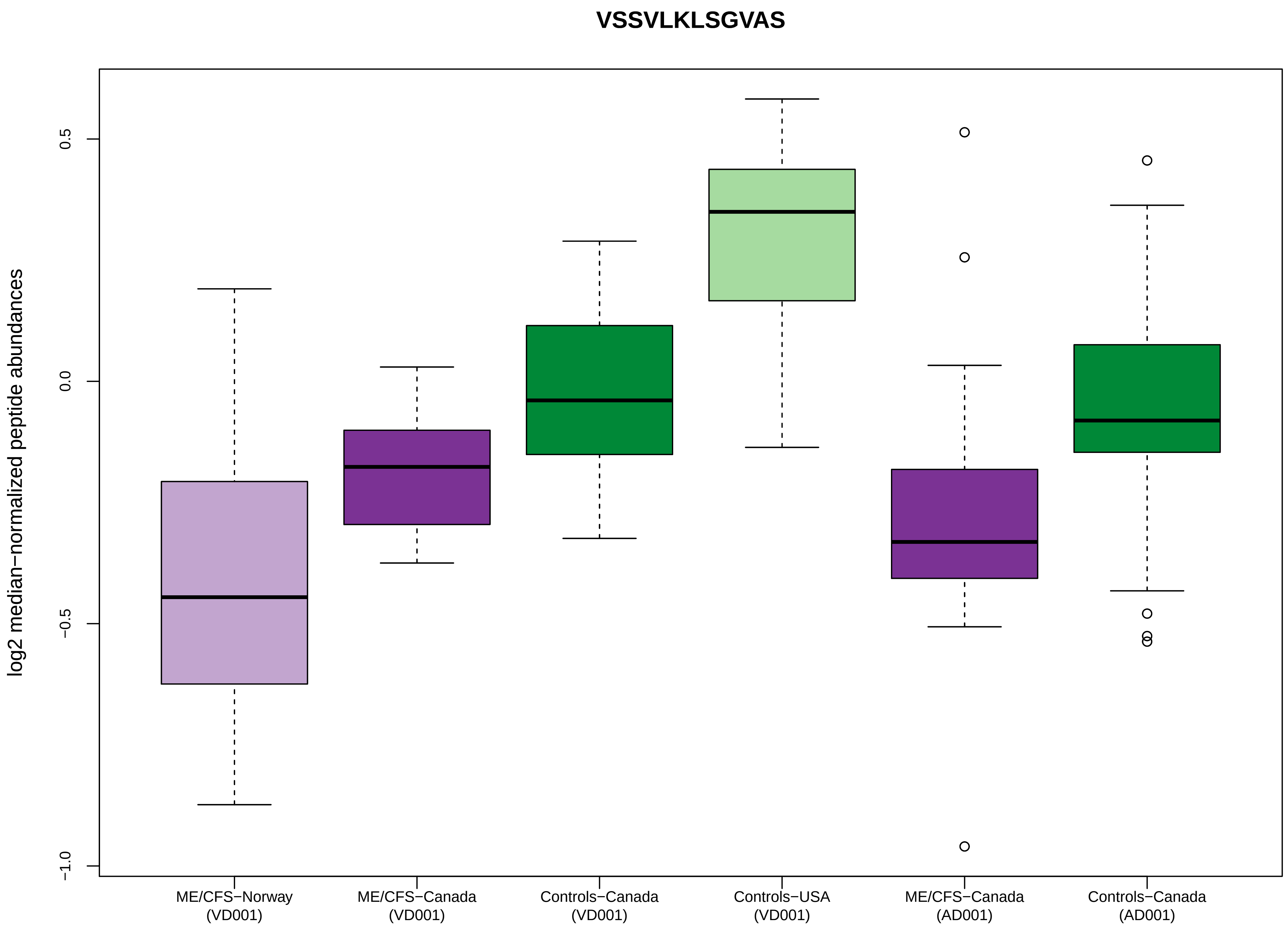

VSSVLKLSGVAS
log2 median−normalized peptide abundances
0.5
0.0
−0.5
−1.0
ME/CFS−Norway (VD001)
ME/CFS−Canada (VD001)
Controls−Canada (VD001)
Controls−USA (VD001)
ME/CFS−Canada (AD001)
Controls−Canada (AD001)

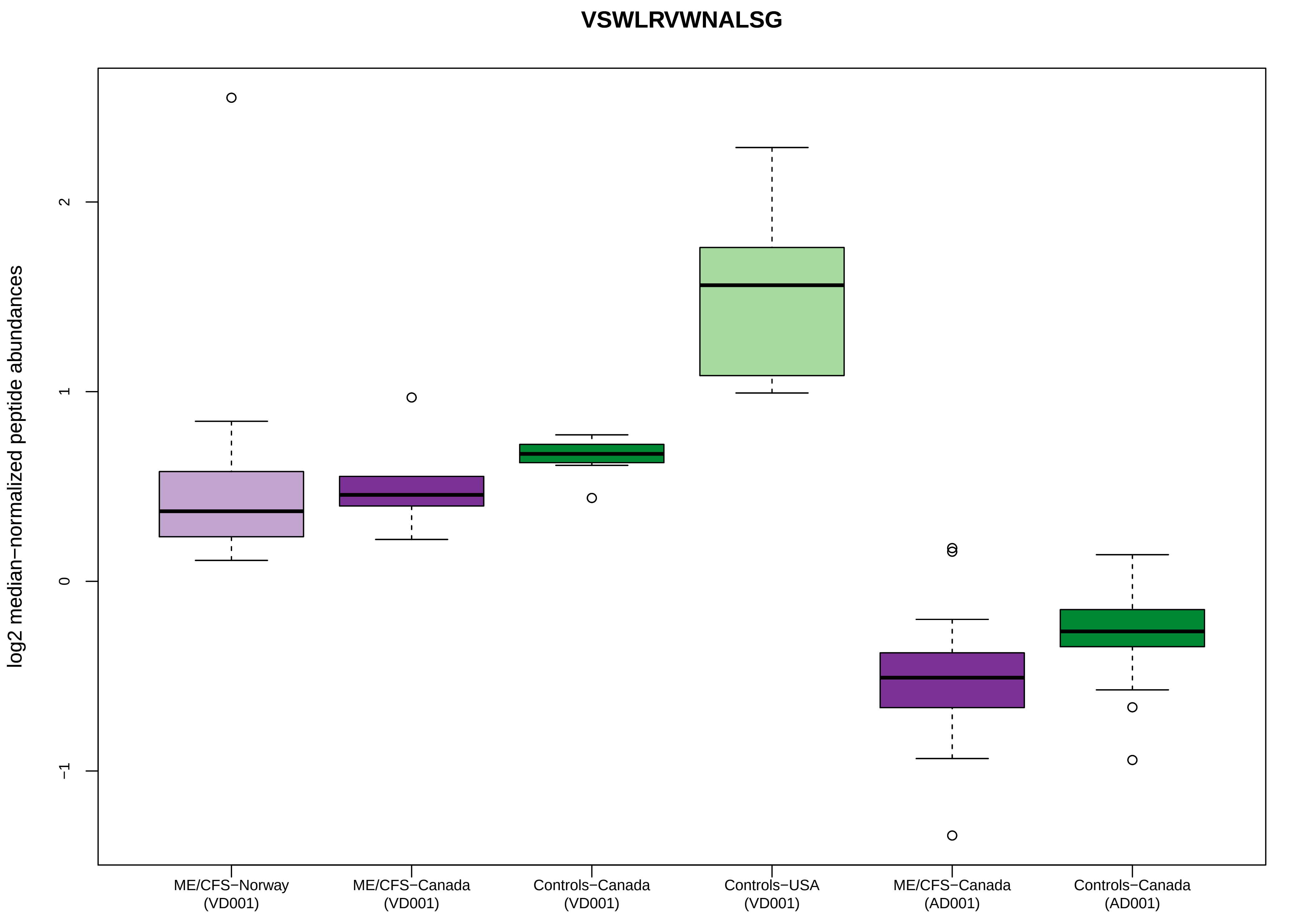

VSWLRVWNALSG
log2 median−normalized peptide abundances
2
1
0
−1
ME/CFS−Norway
(VD001)
ME/CFS−Canada
(VD001)
Controls−Canada
(VD001)
Controls−USA
(VD001)
ME/CFS−Canada
(AD001)
Controls−Canada
(AD001)

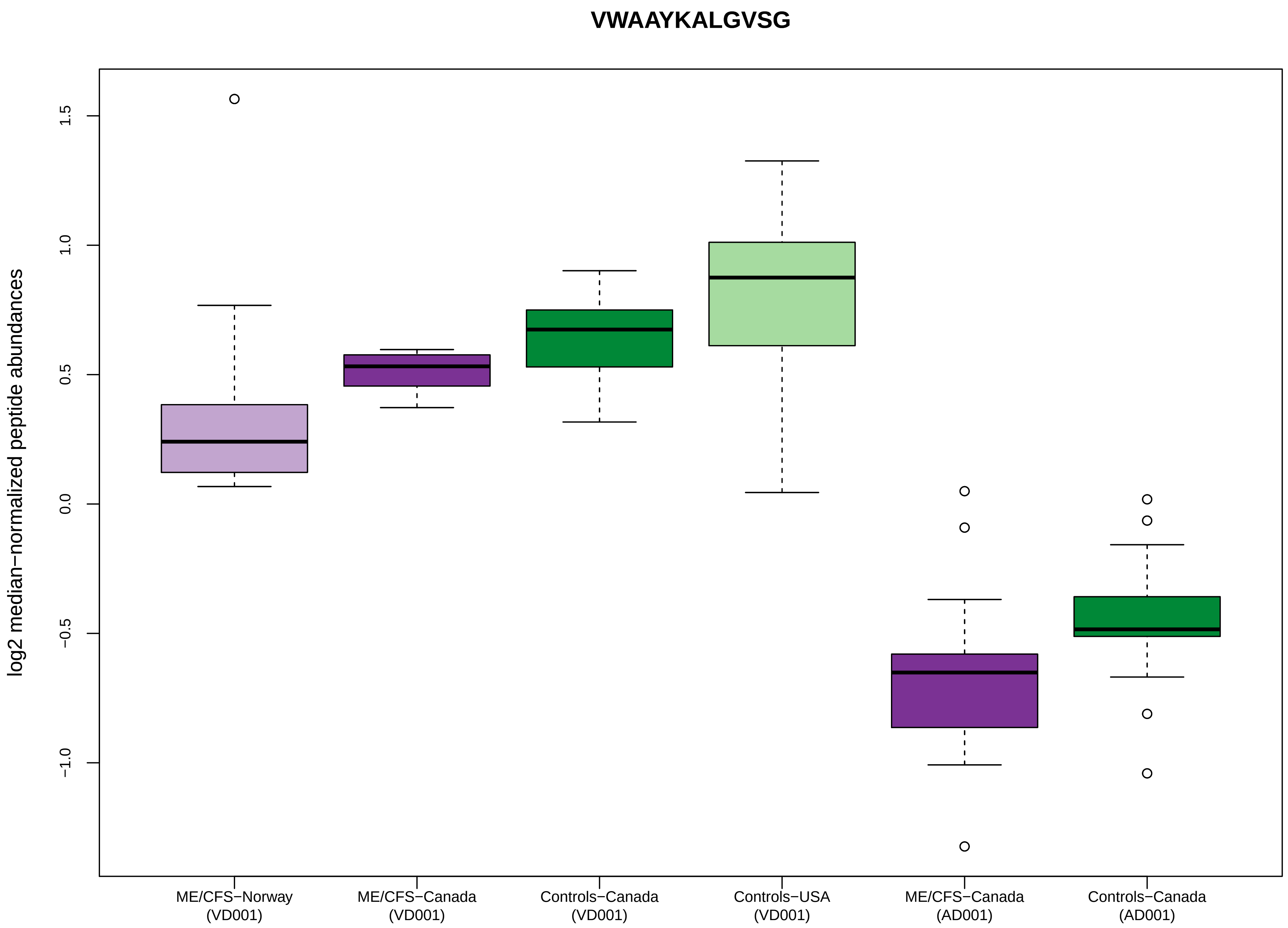

VWAAYKALGVSG
log2 median−normalized peptide abundances
1.5
1.0
0.5
0.0
−0.5
−1.0
ME/CFS−Norway (VD001)
ME/CFS−Canada (VD001)
Controls−Canada (VD001)
Controls−USA (VD001)
ME/CFS−Canada (AD001)
Controls−Canada (AD001)

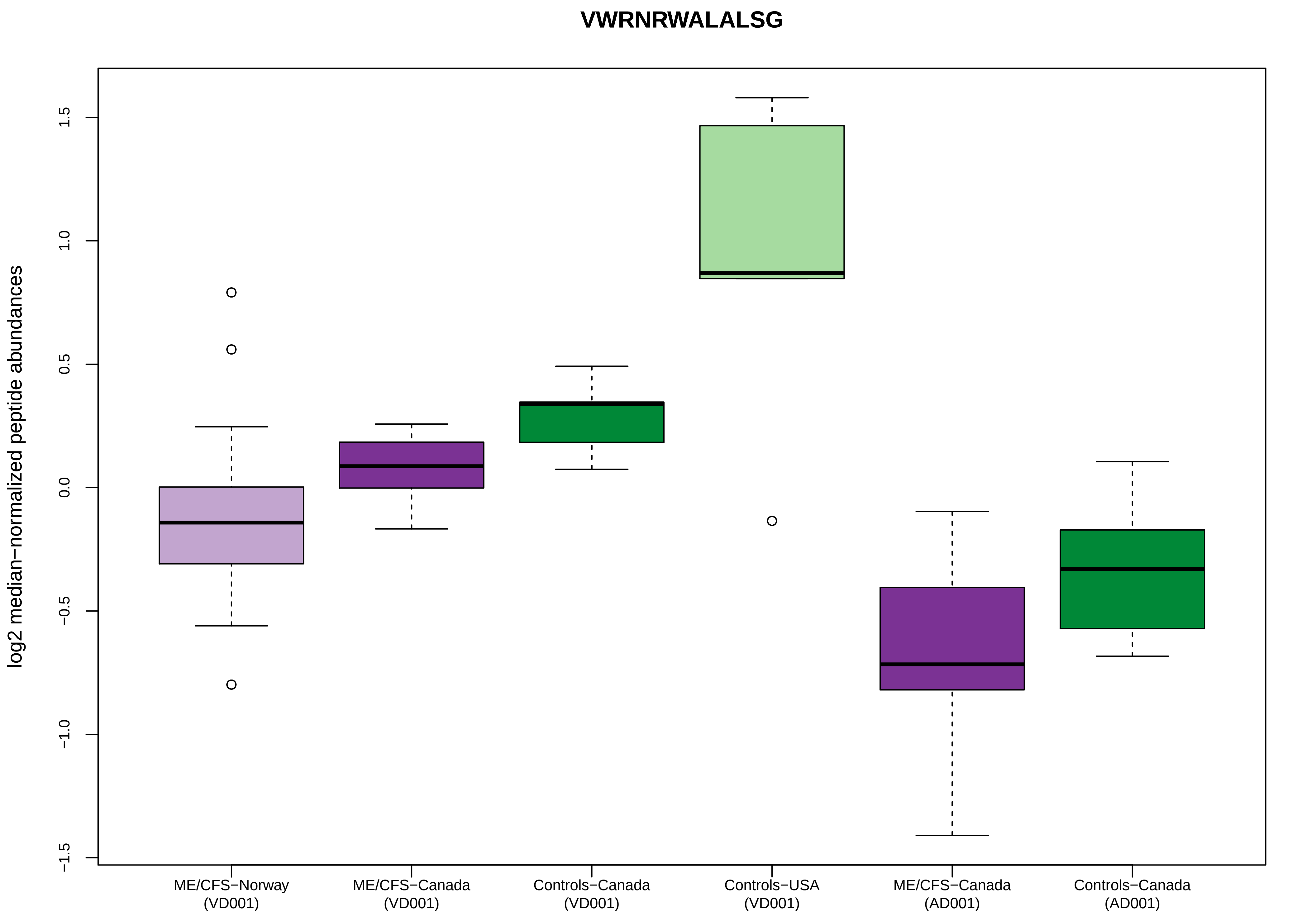
VWRNRWALALSG
log2 median−normalized peptide abundances
1.5
1.0
0.5
0.0
−0.5
−1.0
−1.5
ME/CFS−Norway (VD001)
ME/CFS−Canada (VD001)
Controls−Canada (VD001)
Controls−USA (VD001)
ME/CFS−Canada (AD001)
Controls−Canada (AD001)

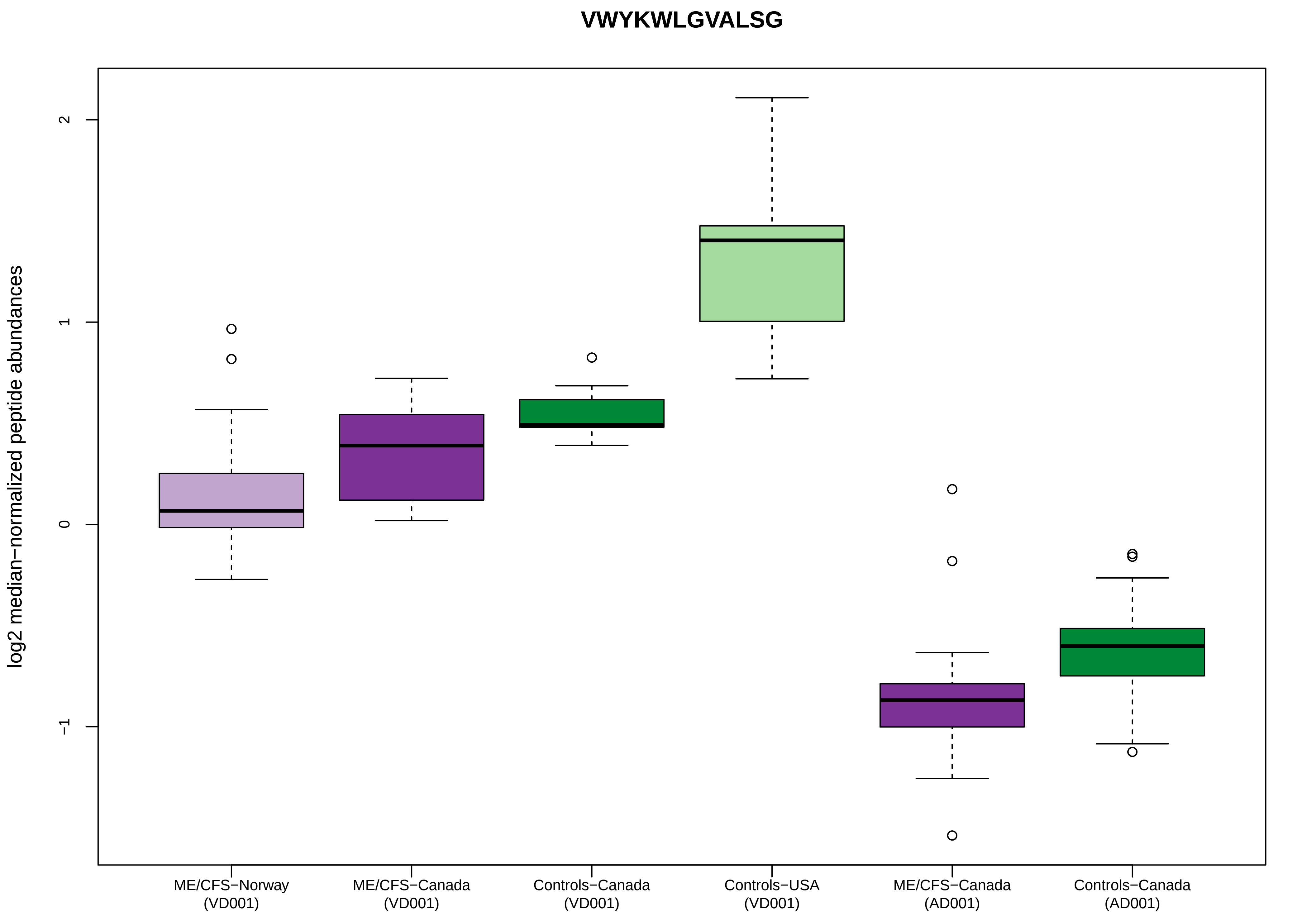
VWYKWLGVALSG
log2 median−normalized peptide abundances
2
1
0
−1
ME/CFS−Norway (VD001)
ME/CFS−Canada (VD001)
Controls−Canada (VD001)
Controls−USA (VD001)
ME/CFS−Canada (AD001)
Controls−Canada (AD001)

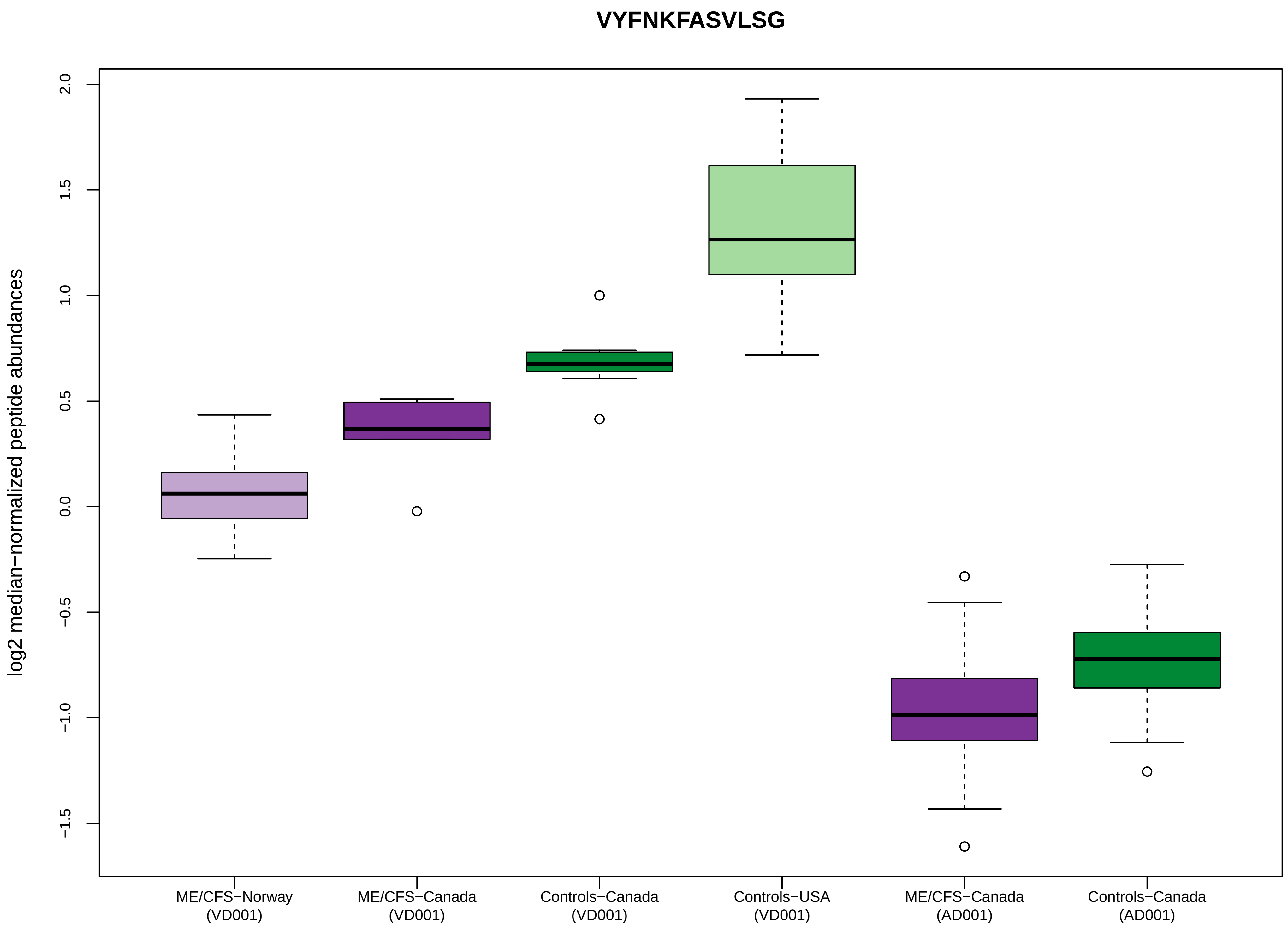

VYFNKFASVLSG
log2 median−normalized peptide abundances
2.0
1.5
1.0
0.5
0.0
−0.5
−1.0
−1.5
ME/CFS−Norway (VD001)
ME/CFS−Canada (VD001)
Controls−Canada (VD001)
Controls−USA (VD001)
ME/CFS−Canada (AD001)
Controls−Canada (AD001)

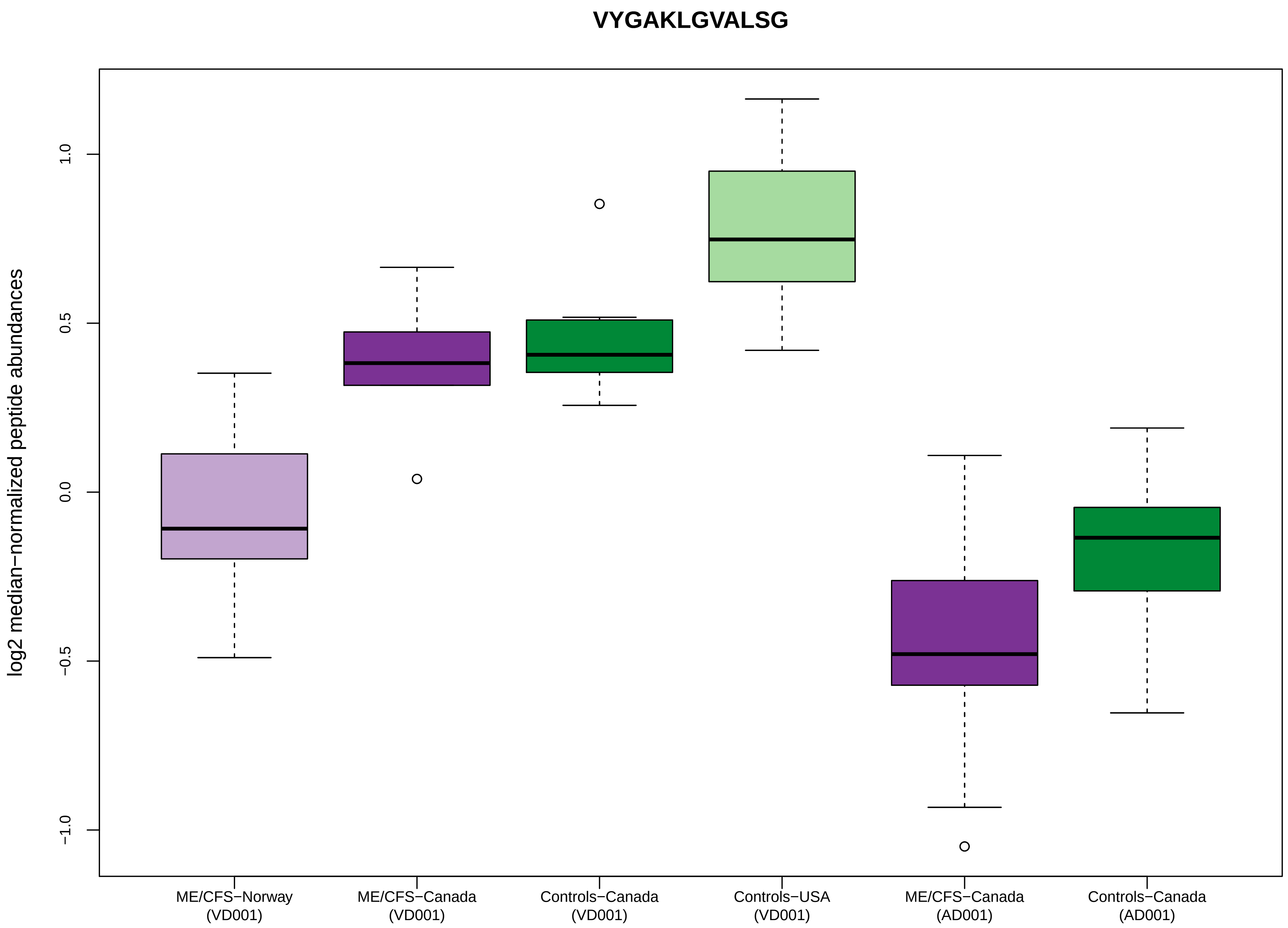
VYGAKLGVALSG
log2 median−normalized peptide abundances
1.0
0.5
0.0
−0.5
−1.0
ME/CFS−Norway (VD001)
ME/CFS−Canada (VD001)
Controls−Canada (VD001)
Controls−USA (VD001)
ME/CFS−Canada (AD001)
Controls−Canada (AD001)

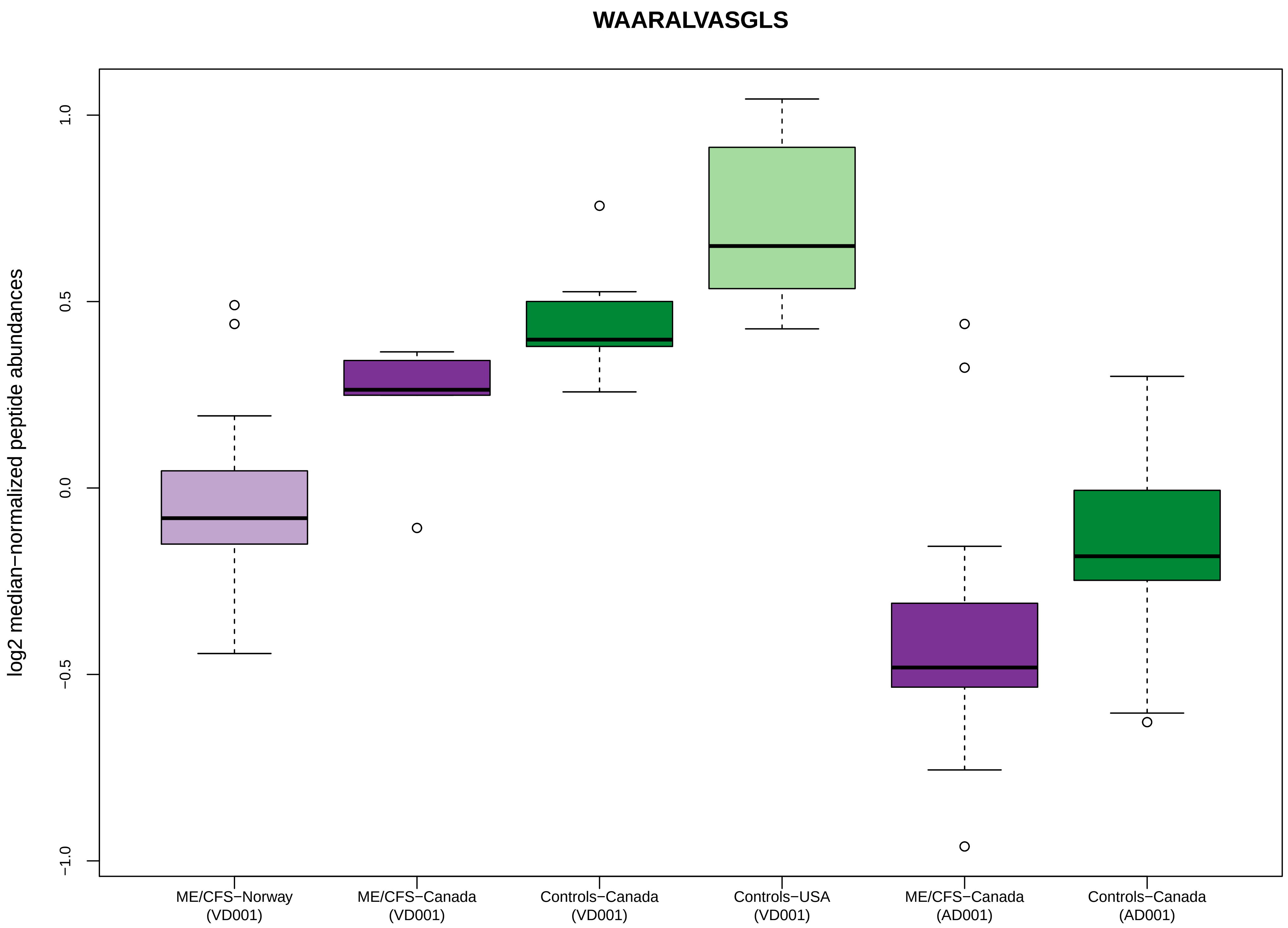

WAARALVASGLS
log2 median−normalized peptide abundances
1.0
0.5
0.0
−0.5
−1.0
ME/CFS−Norway (VD001)
ME/CFS−Canada (VD001)
Controls−Canada (VD001)
Controls−USA (VD001)
ME/CFS−Canada (AD001)
Controls−Canada (AD001)

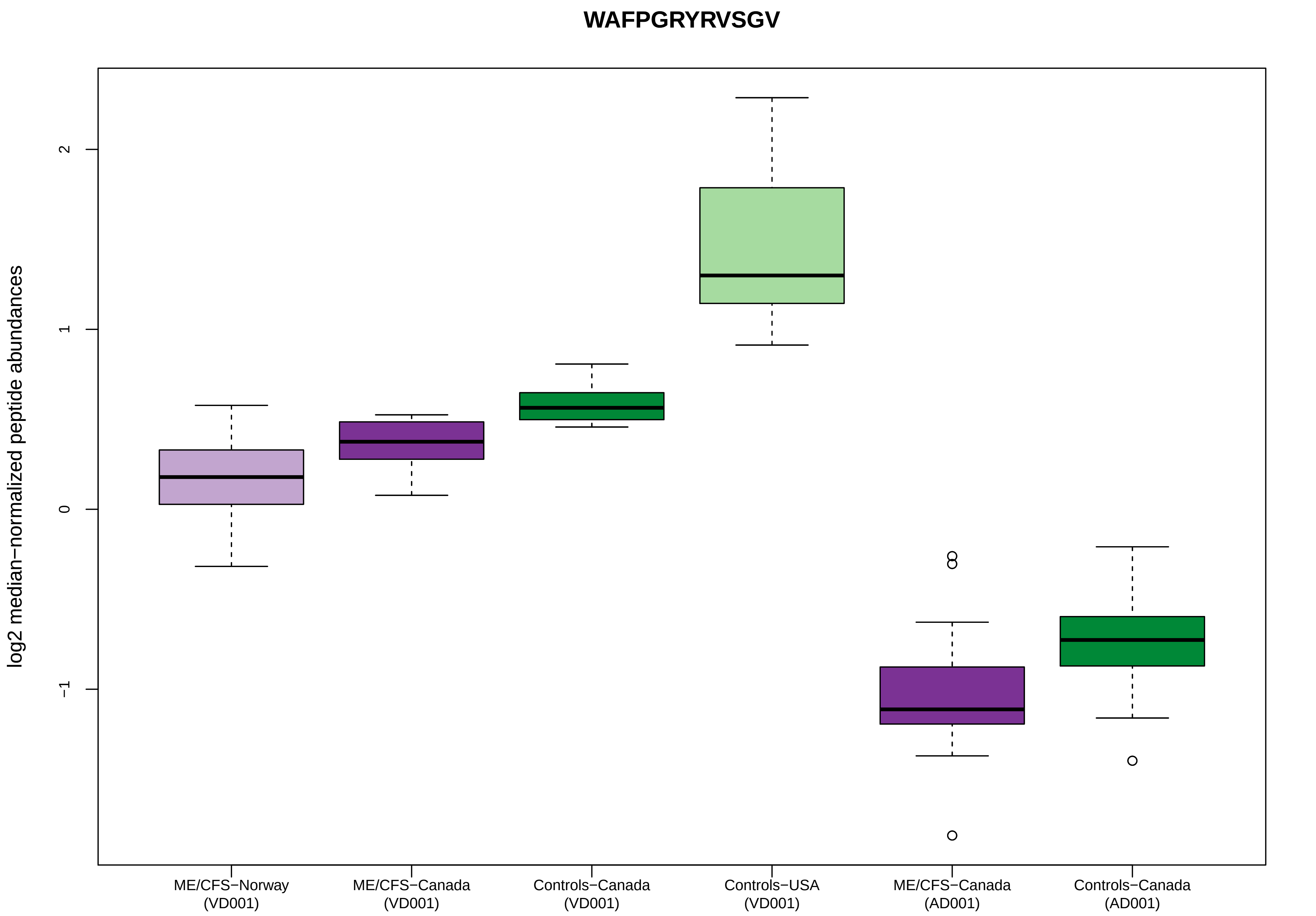

WAFPGRYRVSGV
log2 median−normalized peptide abundances
2
1
0
−1
ME/CFS−Norway (VD001)
ME/CFS−Canada (VD001)
Controls−Canada (VD001)
Controls−USA (VD001)
ME/CFS−Canada (AD001)
Controls−Canada (AD001)

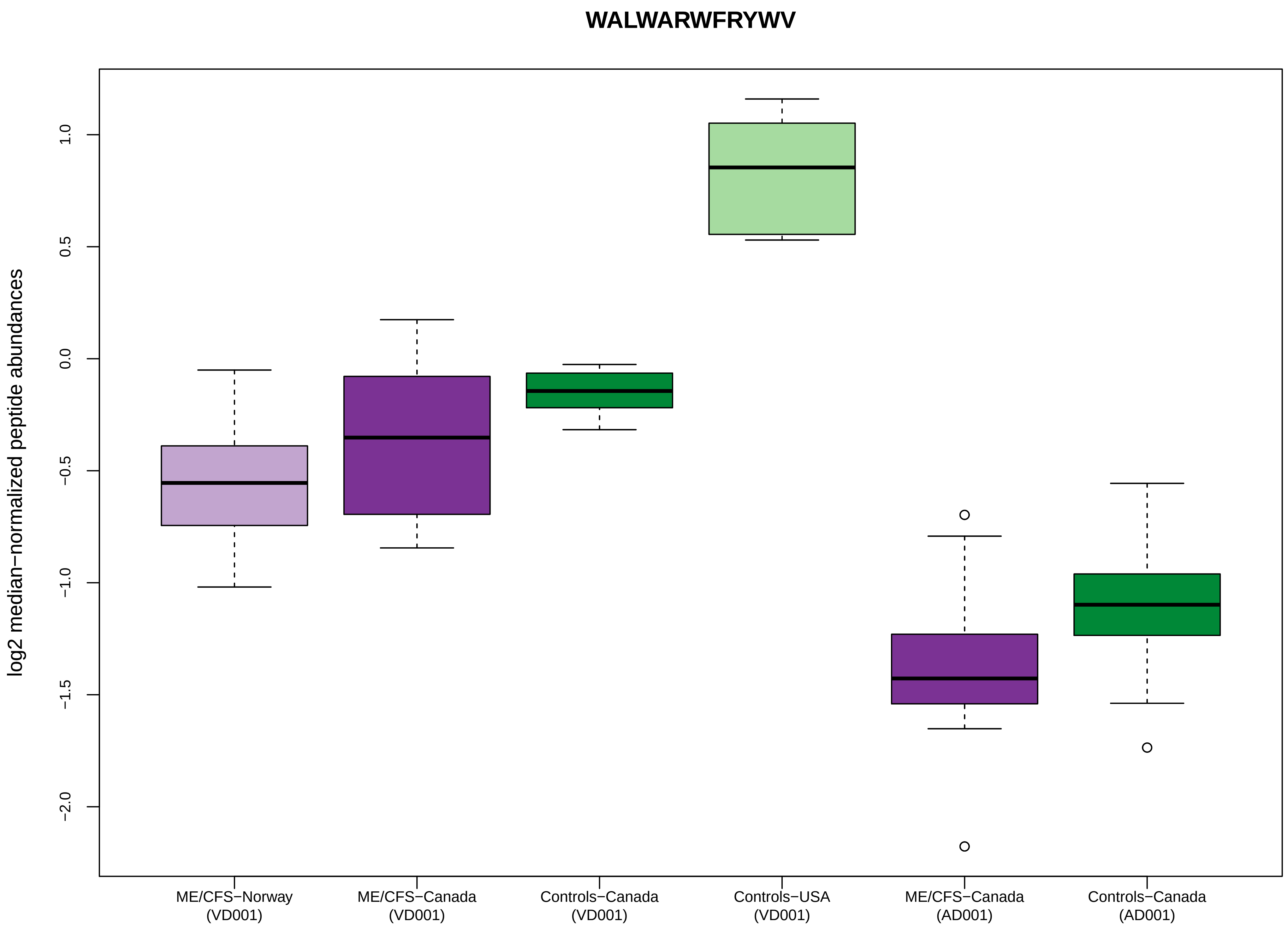

WALWARWFRYWV
log2 median−normalized peptide abundances
1.0
0.5
0.0
−0.5
−1.0
−1.5
−2.0
ME/CFS−Norway (VD001)
ME/CFS−Canada (VD001)
Controls−Canada (VD001)
Controls−USA (VD001)
ME/CFS−Canada (AD001)
Controls−Canada (AD001)

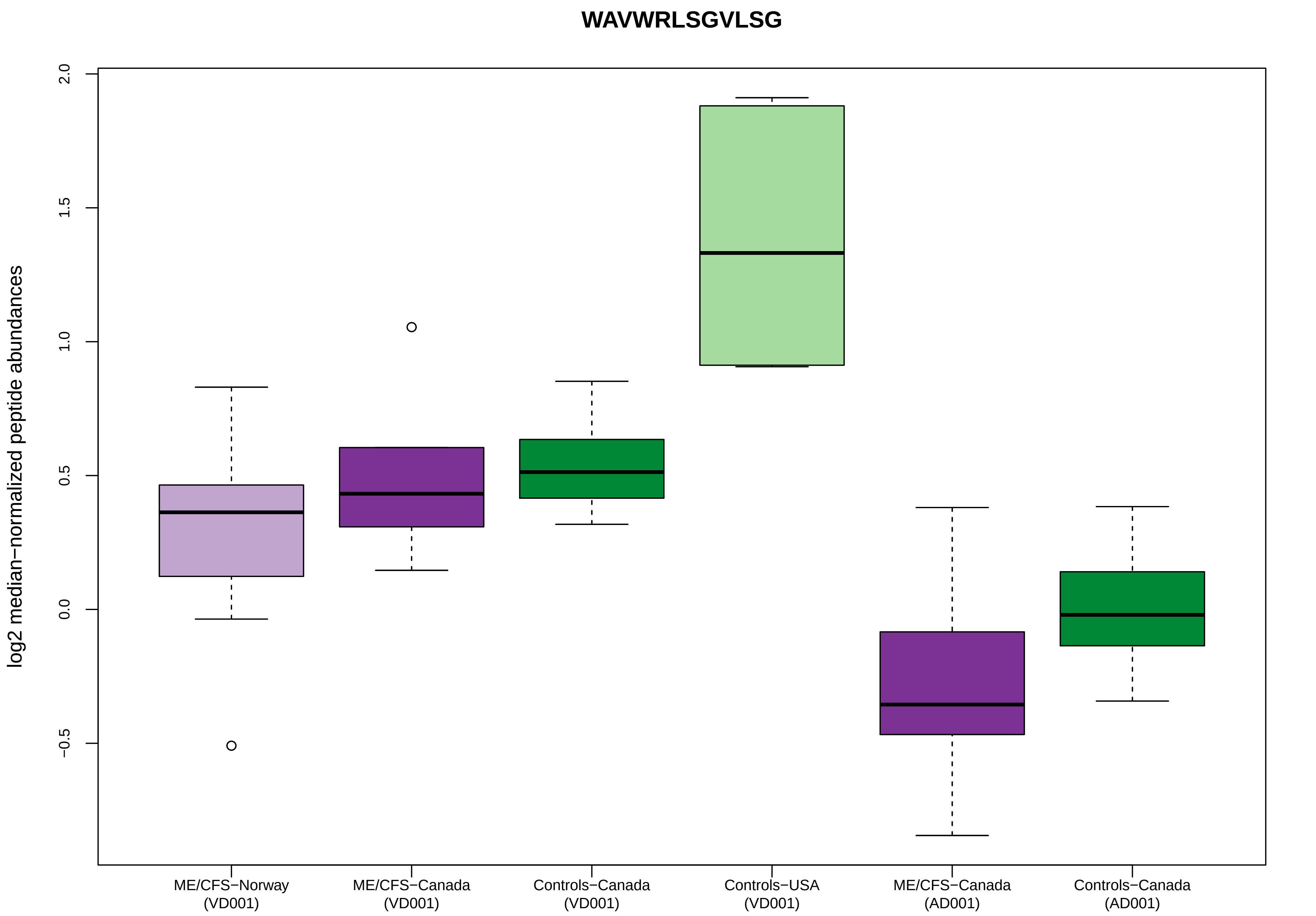
WAVWRLSGVLSG
log2 median−normalized peptide abundances
2.0
1.5
1.0
0.5
0.0
−0.5
ME/CFS−Norway (VD001)
ME/CFS−Canada (VD001)
Controls−Canada (VD001)
Controls−USA (VD001)
ME/CFS−Canada (AD001)
Controls−Canada (AD001)

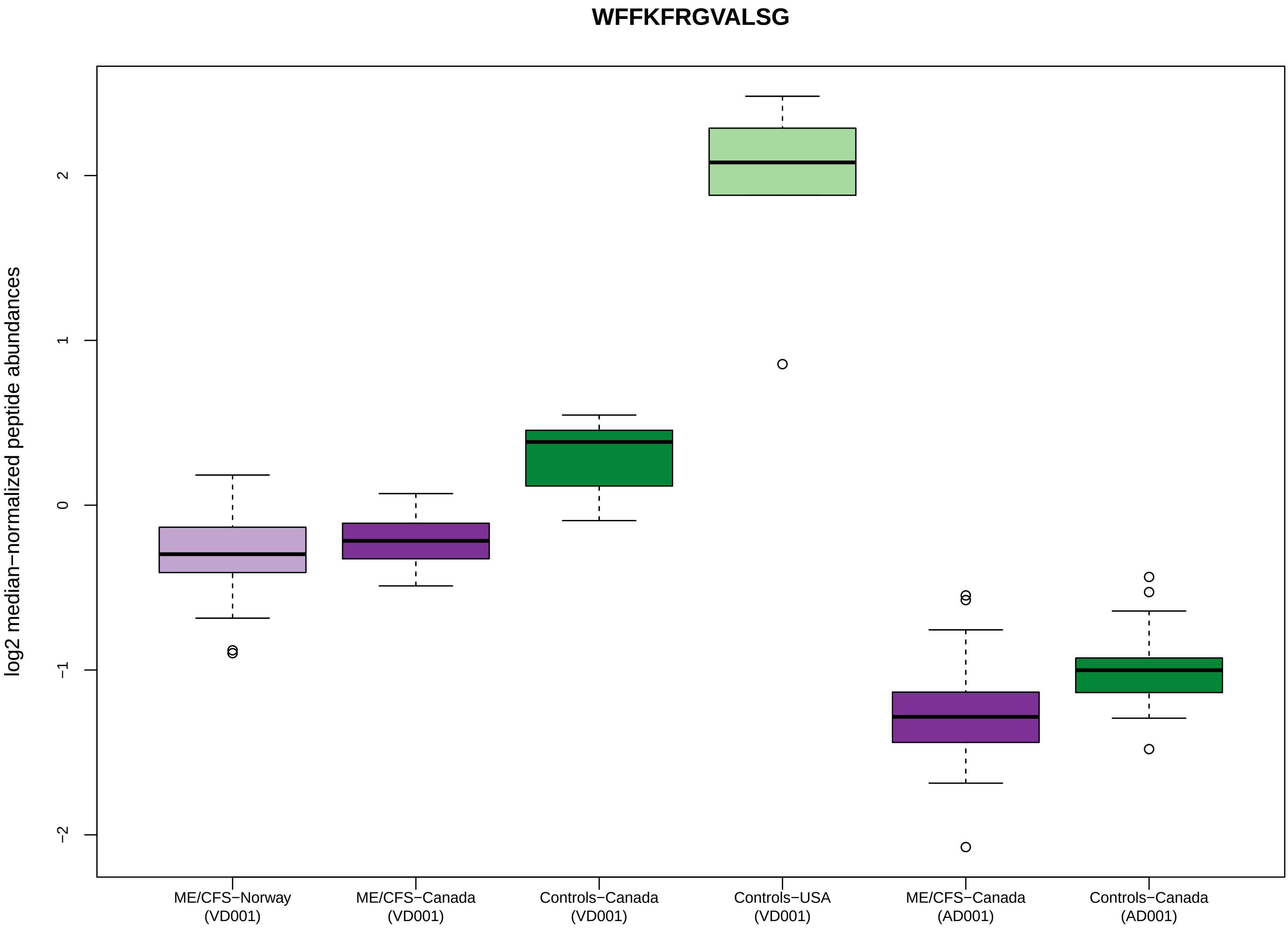
WFFKFRGVALSG
log2 median−normalized peptide abundances
2
1
0
−1
−2
ME/CFS−Norway
(VD001)
ME/CFS−Canada
(VD001)
Controls−Canada
(VD001)
Controls−USA
(VD001)
ME/CFS−Canada
(AD001)
Controls−Canada
(AD001)

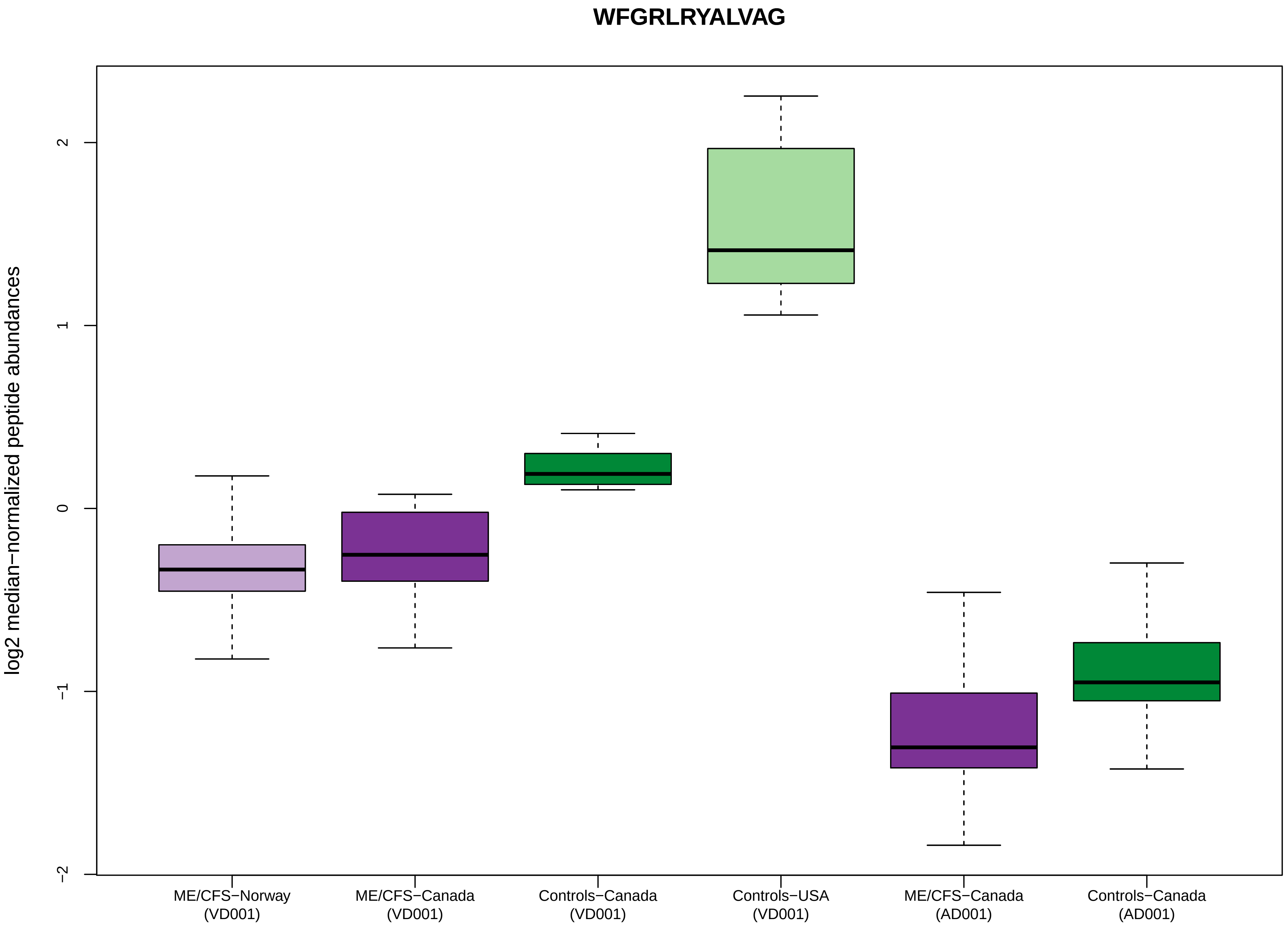

WFGRLRYALVAG
log2 median−normalized peptide abundances
2
1
0
−1
−2
ME/CFS−Norway (VD001)
ME/CFS−Canada (VD001)
Controls−Canada (VD001)
Controls−USA (VD001)
ME/CFS−Canada (AD001)
Controls−Canada (AD001)

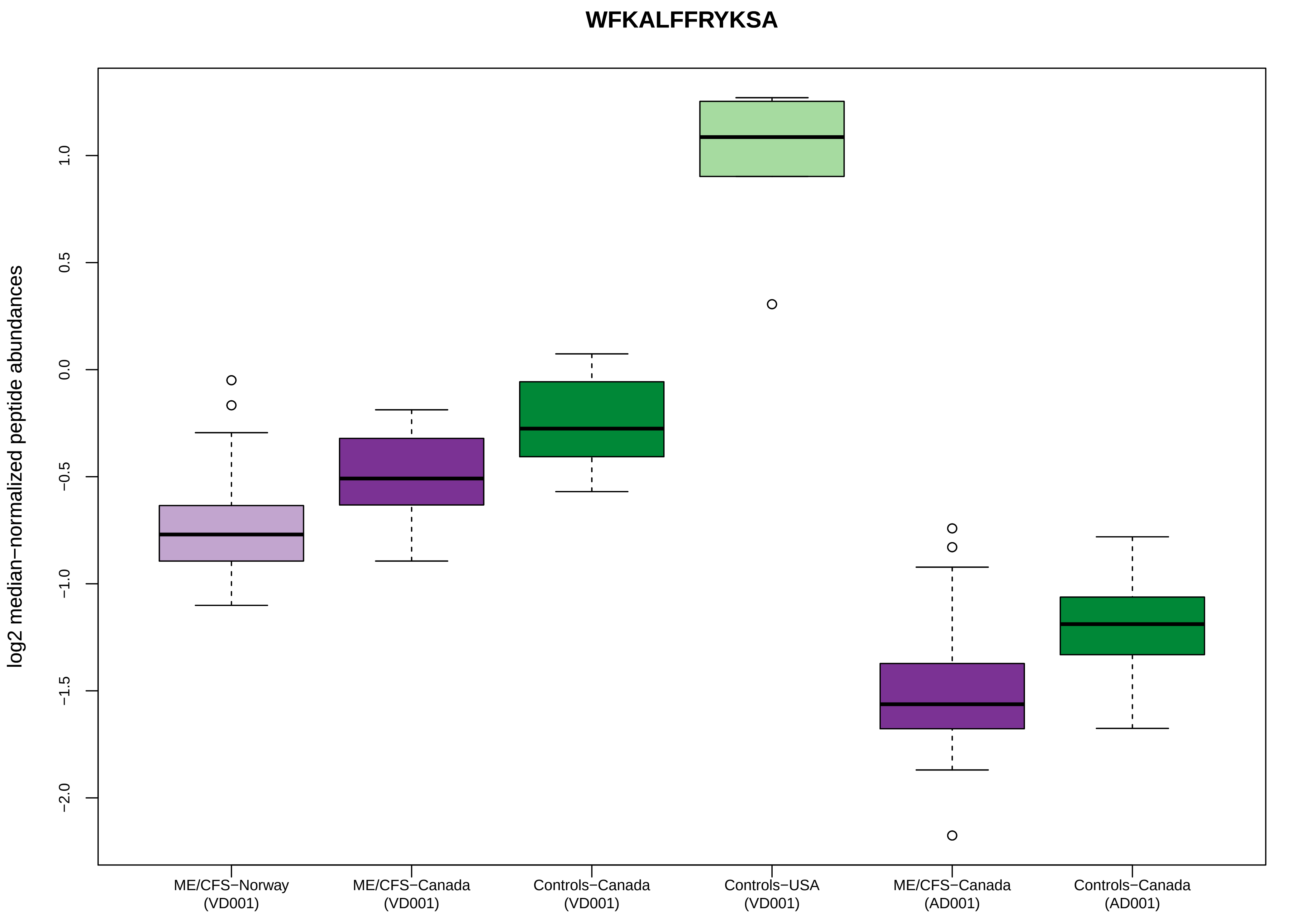

WFKALFFRYKSA
log2 median−normalized peptide abundances
1.0
0.5
0.0
−0.5
−1.0
−1.5
−2.0
ME/CFS−Norway (VD001)
ME/CFS−Canada (VD001)
Controls−Canada (VD001)
Controls−USA (VD001)
ME/CFS−Canada (AD001)
Controls−Canada (AD001)

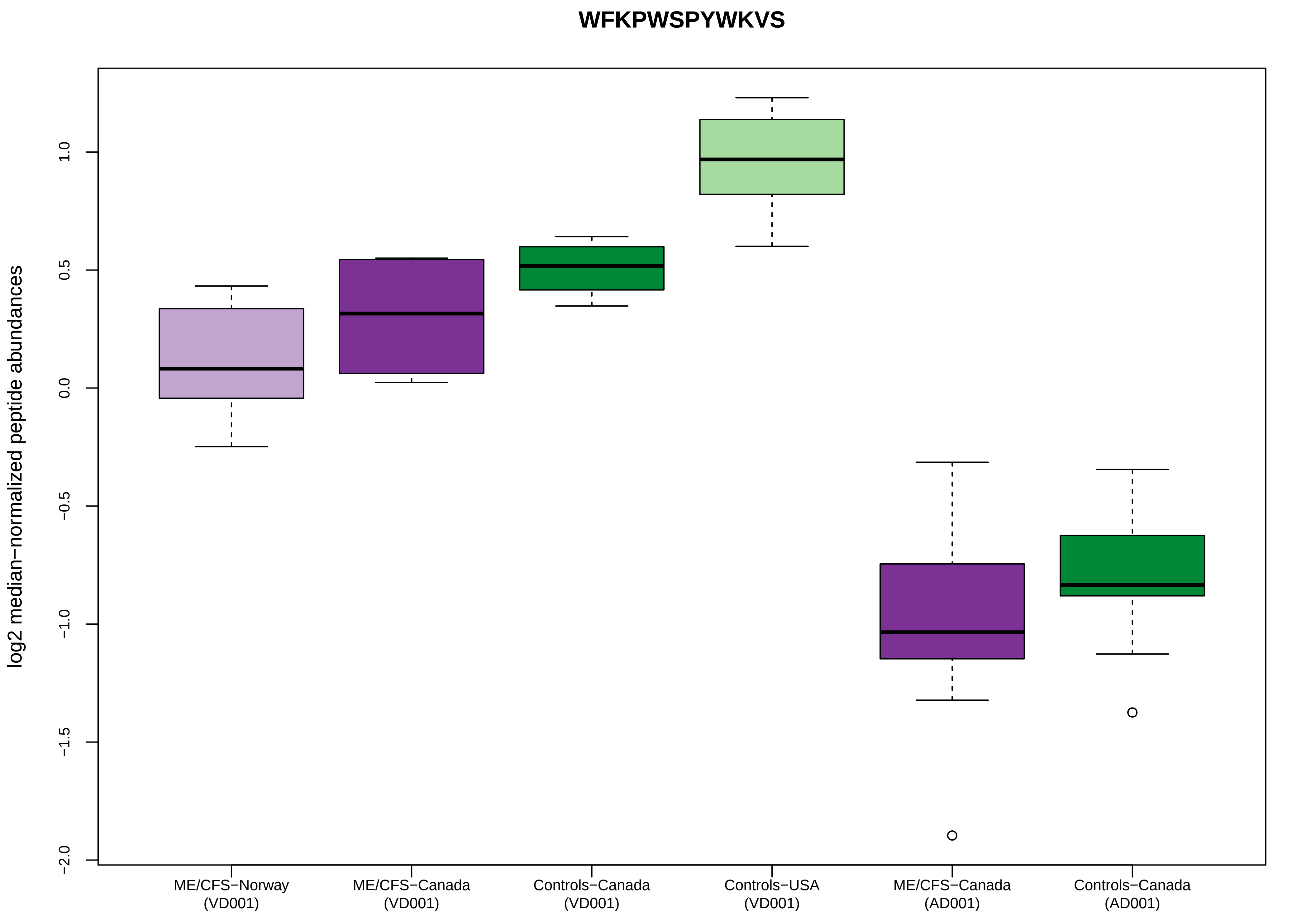
WFKPWSPYWKVS
log2 median−normalized peptide abundances
1.0
0.5
0.0
−0.5
−1.0
−1.5
−2.0
ME/CFS−Norway (VD001)
ME/CFS−Canada (VD001)
Controls−Canada (VD001)
Controls−USA (VD001)
ME/CFS−Canada (AD001)
Controls−Canada (AD001)

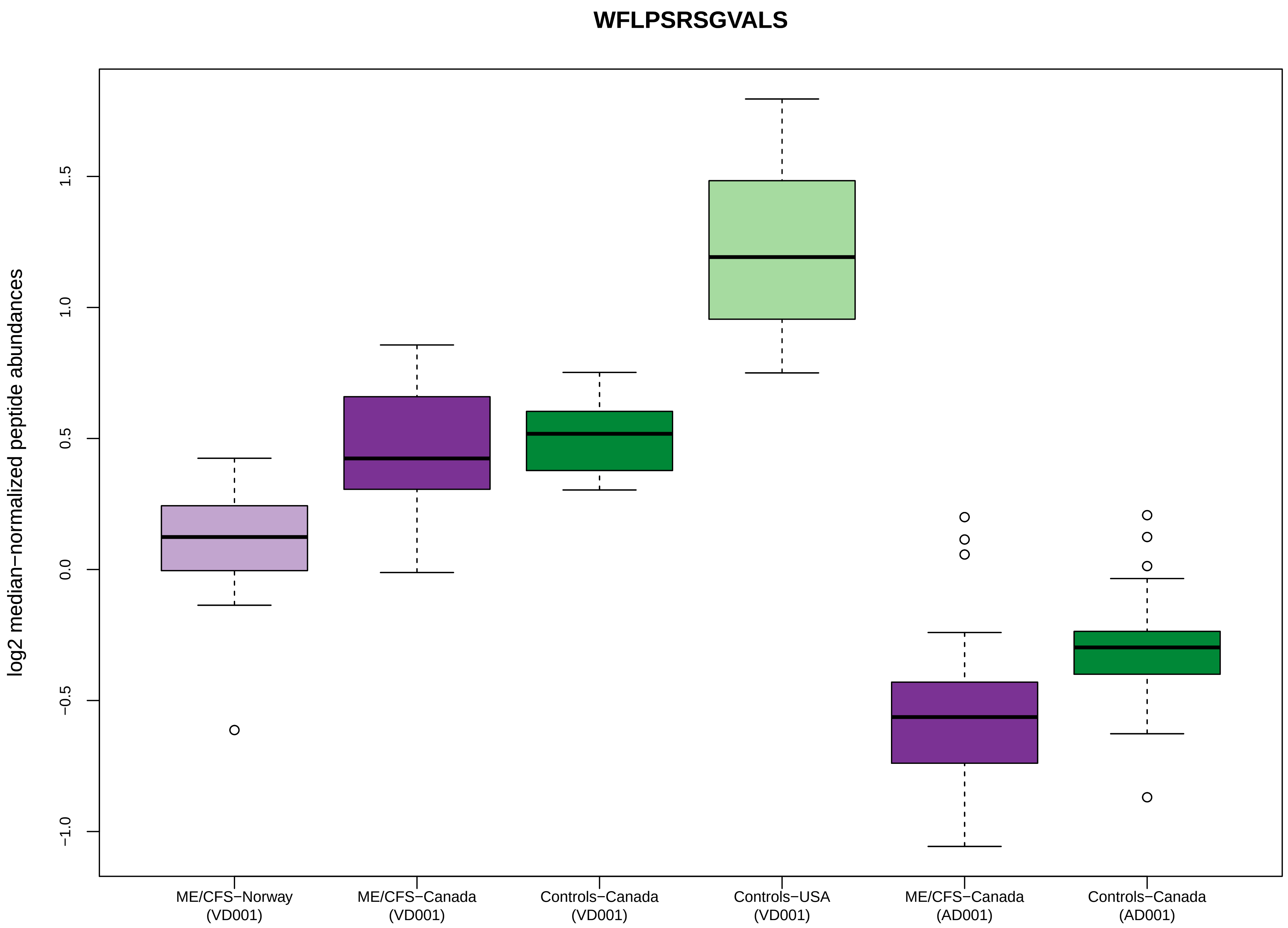

WFLPSRSGVALS
log2 median−normalized peptide abundances
1.5
1.0
0.5
0.0
−0.5
−1.0
ME/CFS−Norway (VD001)
ME/CFS−Canada (VD001)
Controls−Canada (VD001)
Controls−USA (VD001)
ME/CFS−Canada (AD001)
Controls−Canada (AD001)

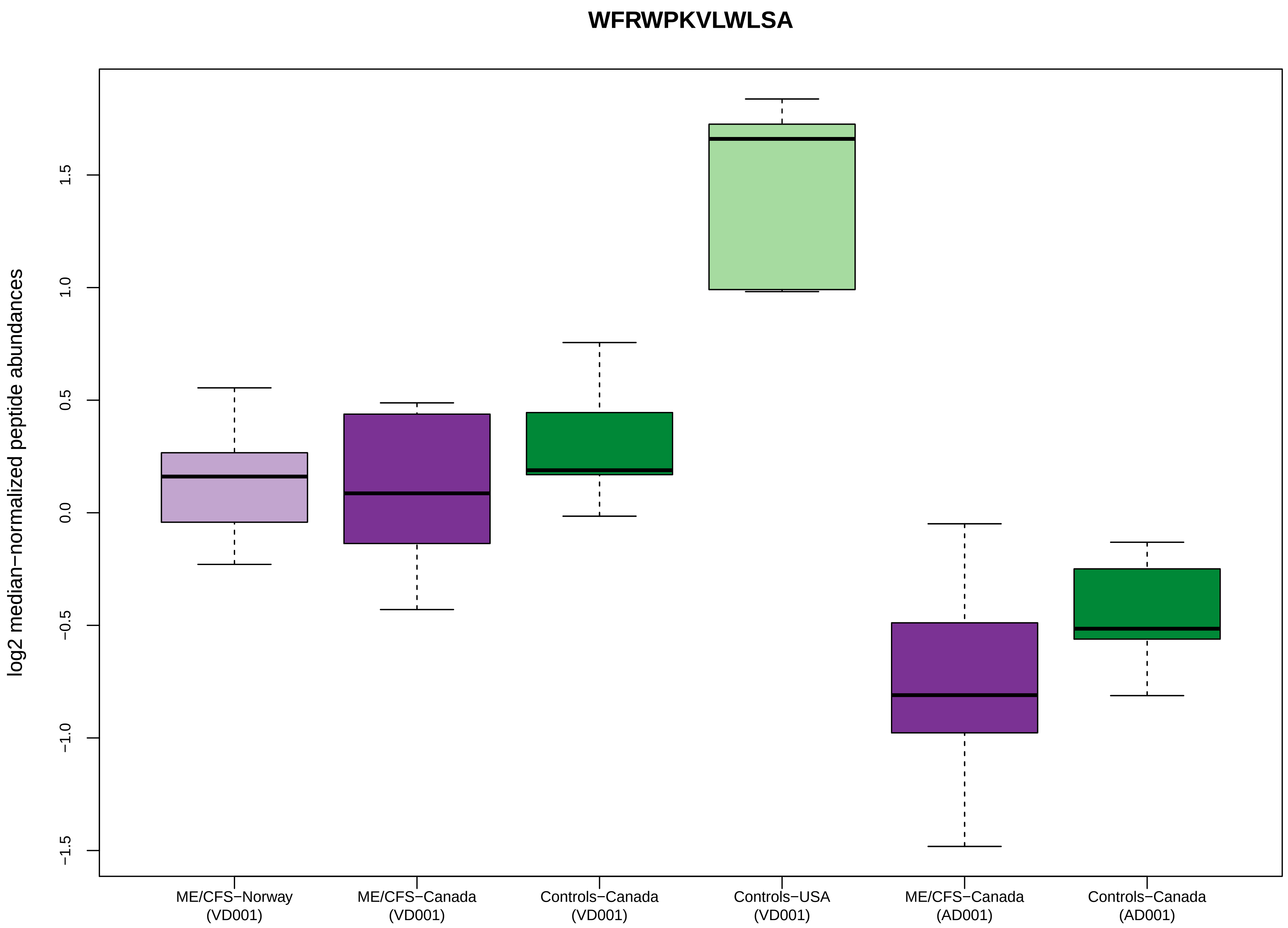
WFRWPKVLWLSA
log2 median−normalized peptide abundances
1.5
1.0
0.5
0.0
−0.5
−1.0
−1.5
ME/CFS−Norway (VD001)
ME/CFS−Canada (VD001)
Controls−Canada (VD001)
Controls−USA (VD001)
ME/CFS−Canada (AD001)
Controls−Canada (AD001)

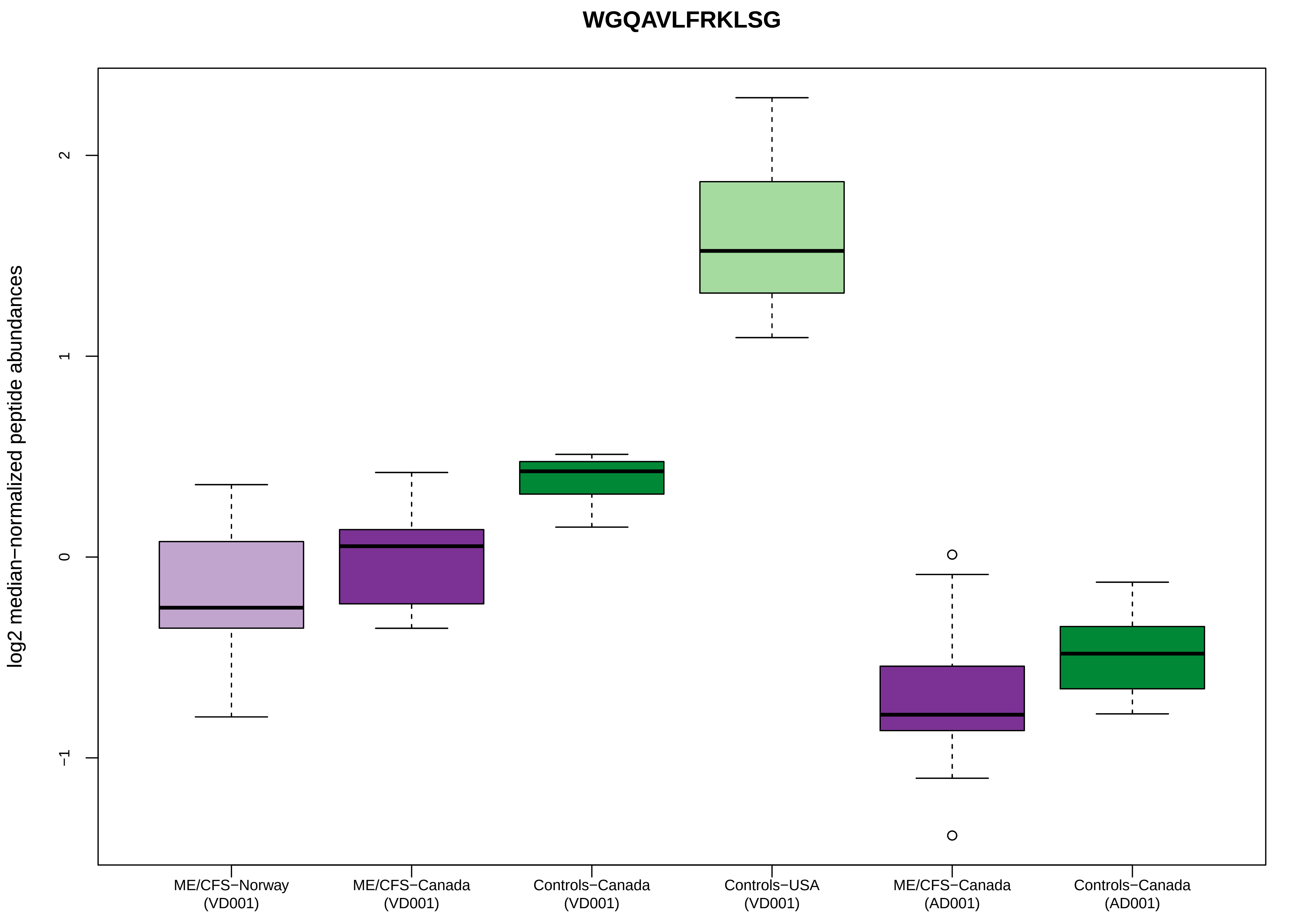

WGQAVLFRKLSG
log2 median−normalized peptide abundances
2
1
0
−1
ME/CFS−Norway (VD001)
ME/CFS−Canada (VD001)
Controls−Canada (VD001)
Controls−USA (VD001)
ME/CFS−Canada (AD001)
Controls−Canada (AD001)

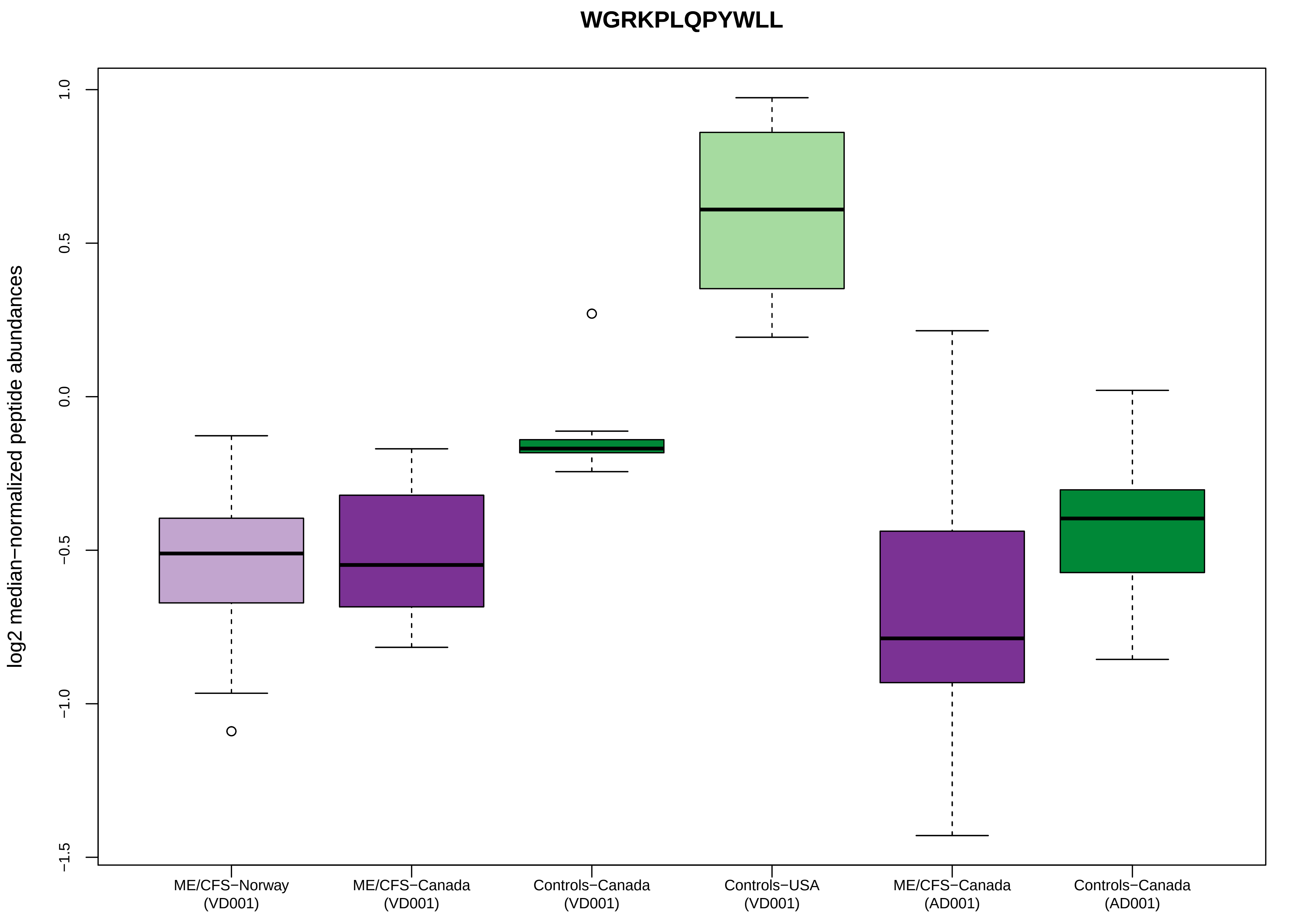

WGRKPLQPYWLL
log2 median−normalized peptide abundances
1.0
0.5
0.0
−0.5
−1.0
−1.5
ME/CFS−Norway (VD001)
ME/CFS−Canada (VD001)
Controls−Canada (VD001)
Controls−USA (VD001)
ME/CFS−Canada (AD001)
Controls−Canada (AD001)

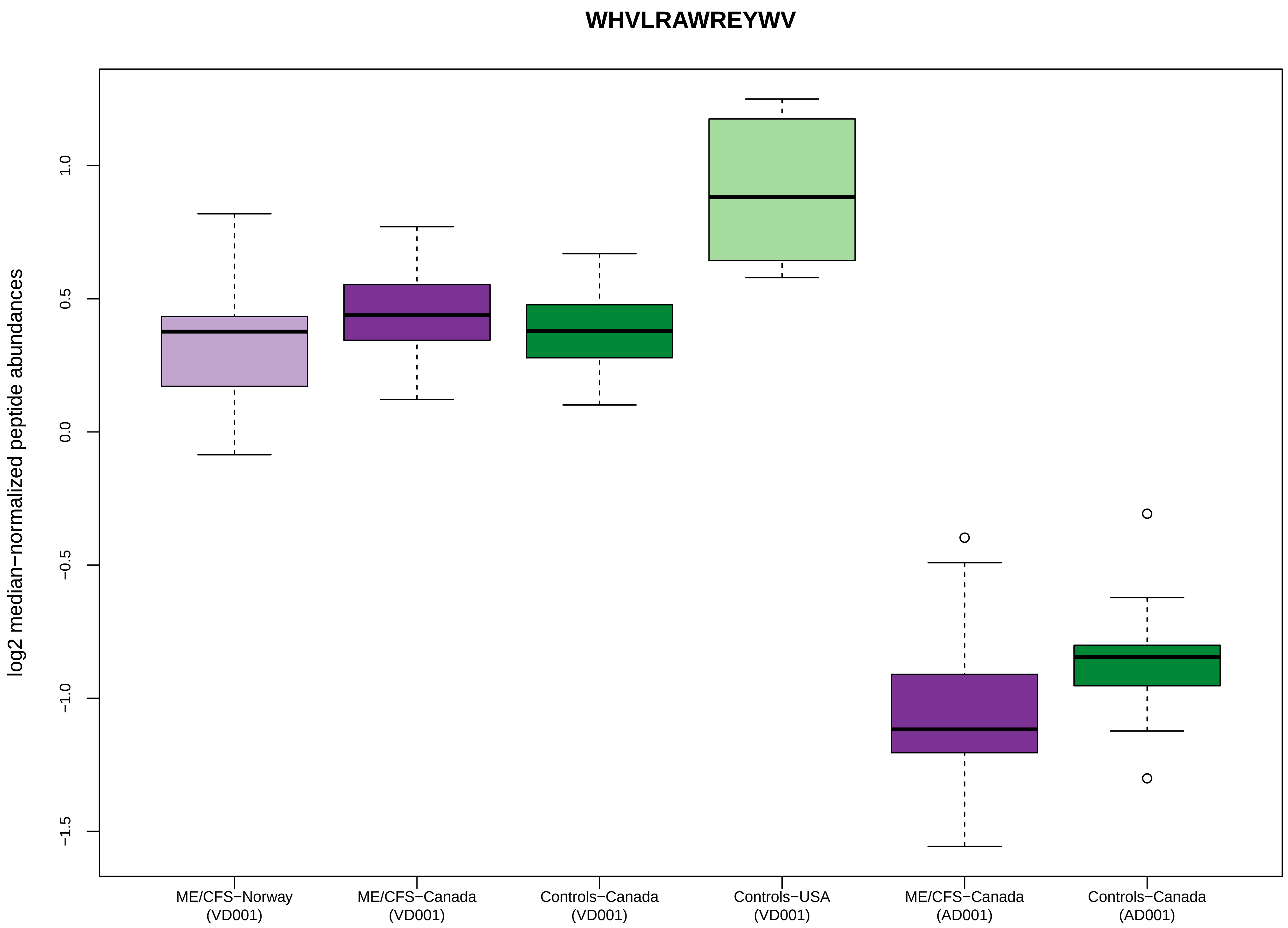

WHVLRAWREYWV
log2 median−normalized peptide abundances
1.0
0.5
0.0
−0.5
−1.0
−1.5
ME/CFS−Norway (VD001)
ME/CFS−Canada (VD001)
Controls−Canada (VD001)
Controls−USA (VD001)
ME/CFS−Canada (AD001)
Controls−Canada (AD001)

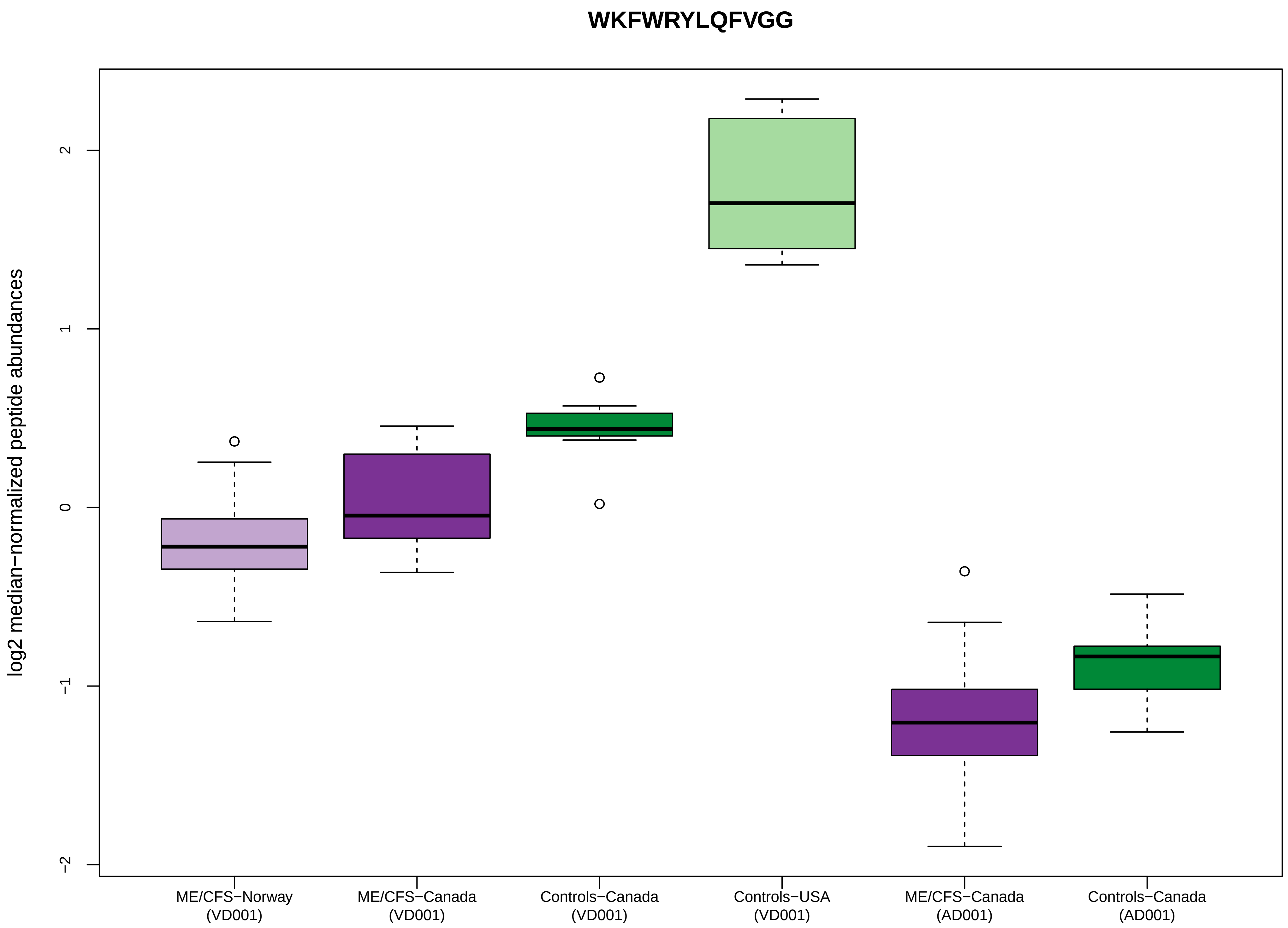

WKFWRYLQFVGG
log2 median−normalized peptide abundances
2
1
0
−1
−2
ME/CFS−Norway (VD001)
ME/CFS−Canada (VD001)
Controls−Canada (VD001)
Controls−USA (VD001)
ME/CFS−Canada (AD001)
Controls−Canada (AD001)

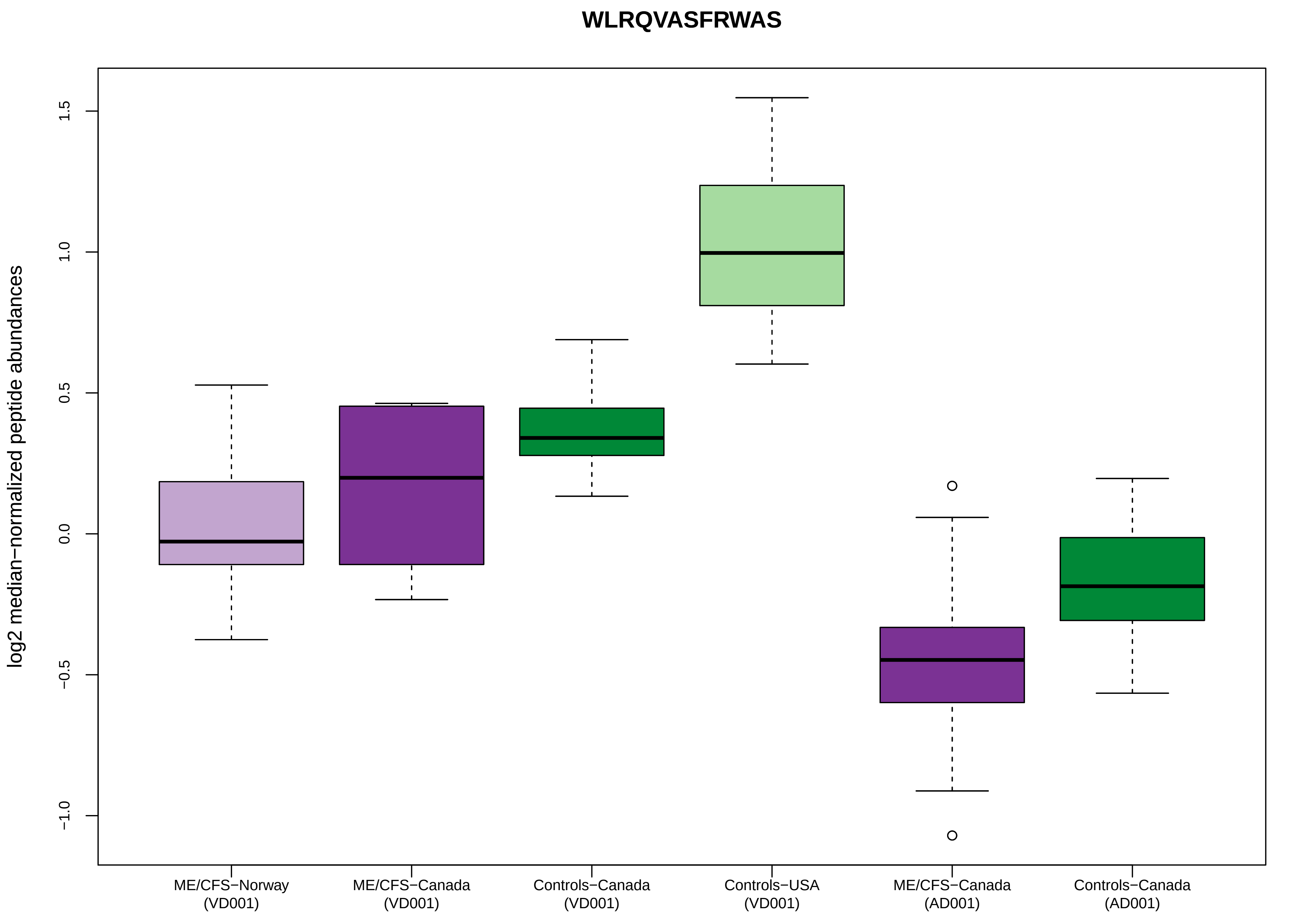

WLRQVASFRWAS
log2 median−normalized peptide abundances
1.5
1.0
0.5
0.0
−0.5
−1.0
ME/CFS−Norway (VD001)
ME/CFS−Canada (VD001)
Controls−Canada (VD001)
Controls−USA (VD001)
ME/CFS−Canada (AD001)
Controls−Canada (AD001)

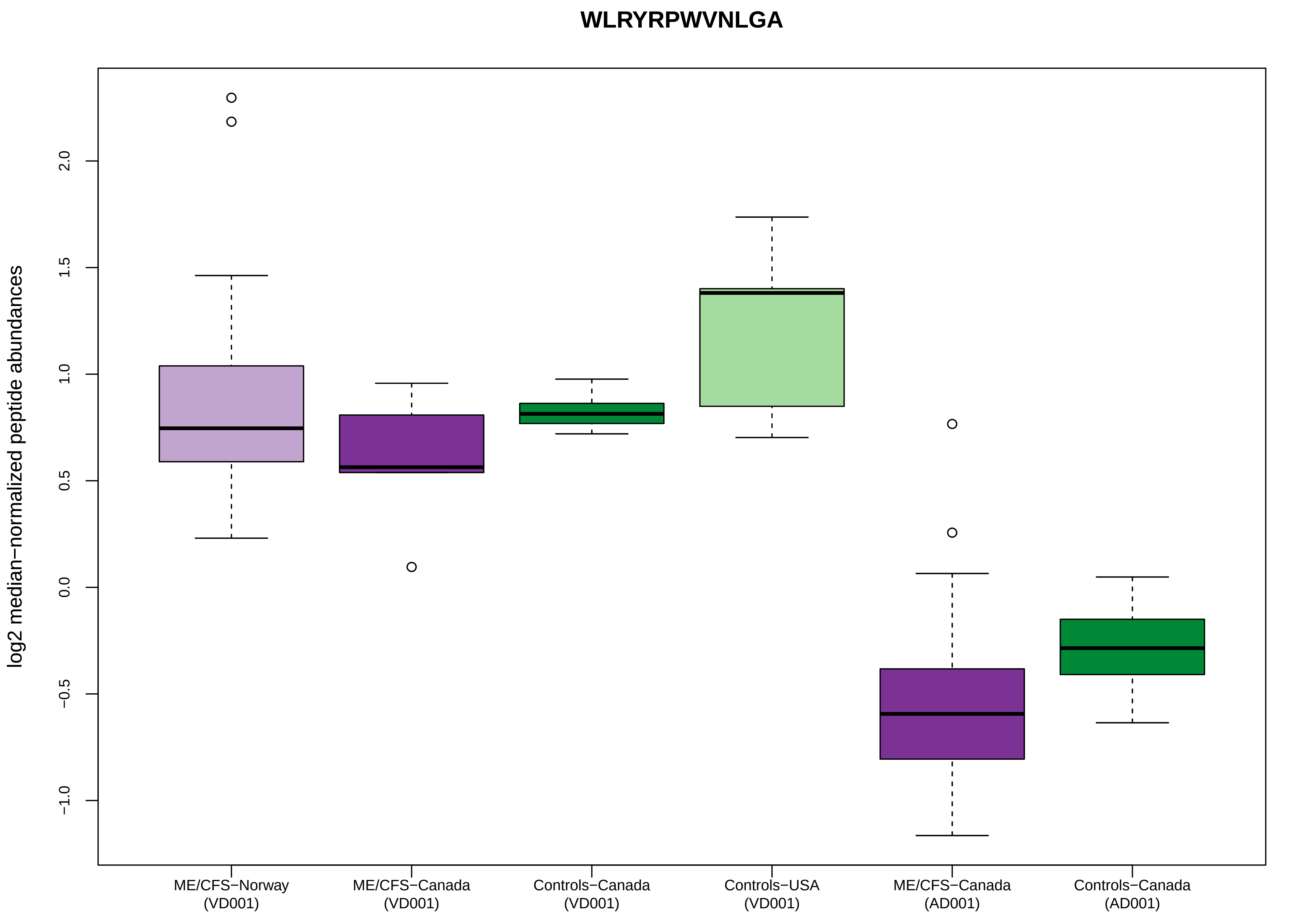
WLRYRPWVNLGA
log2 median−normalized peptide abundances
2.0
1.5
1.0
0.5
0.0
−0.5
−1.0
ME/CFS−Norway (VD001)
ME/CFS−Canada (VD001)
Controls−Canada (VD001)
Controls−USA (VD001)
ME/CFS−Canada (AD001)
Controls−Canada (AD001)

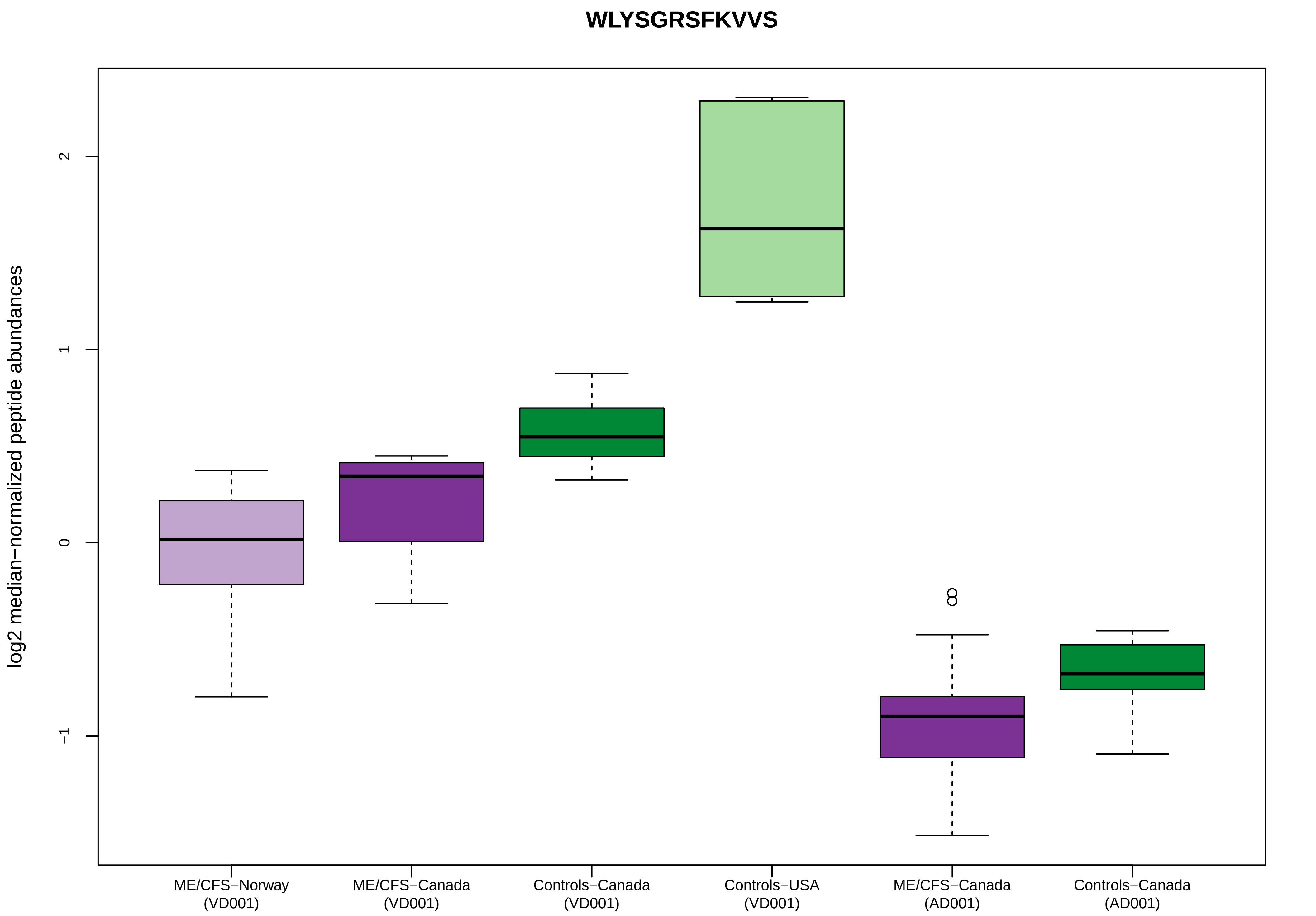
WLYSGRSFKVVS
log2 median−normalized peptide abundances
2
1
0
−1
ME/CFS−Norway (VD001)
ME/CFS−Canada (VD001)
Controls−Canada (VD001)
Controls−USA (VD001)
ME/CFS−Canada (AD001)
Controls−Canada (AD001)

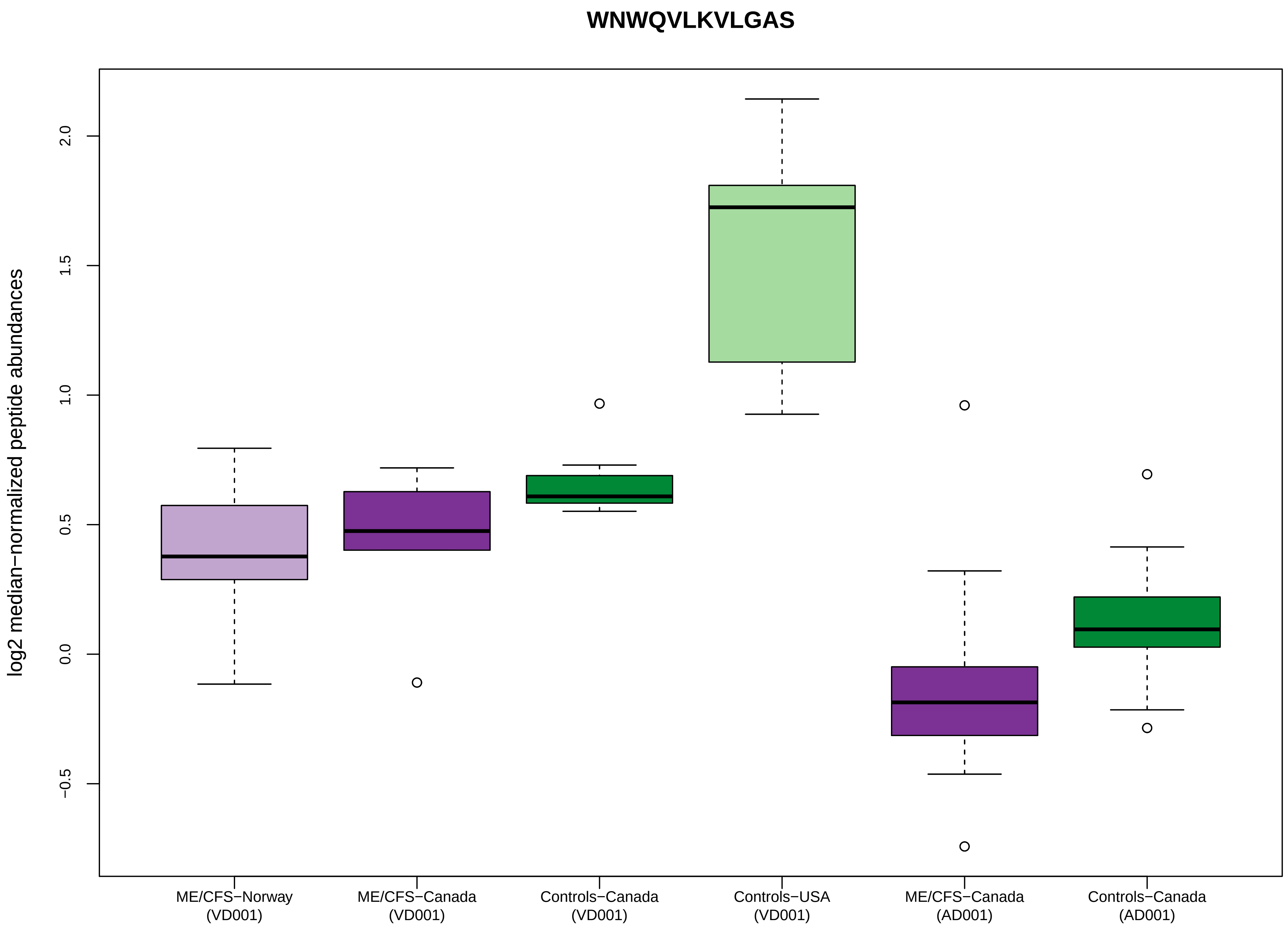

WNWQVLKVLGAS
log2 median−normalized peptide abundances
2.0
1.5
1.0
0.5
0.0
−0.5
ME/CFS−Norway (VD001)
ME/CFS−Canada (VD001)
Controls−Canada (VD001)
Controls−USA (VD001)
ME/CFS−Canada (AD001)
Controls−Canada (AD001)

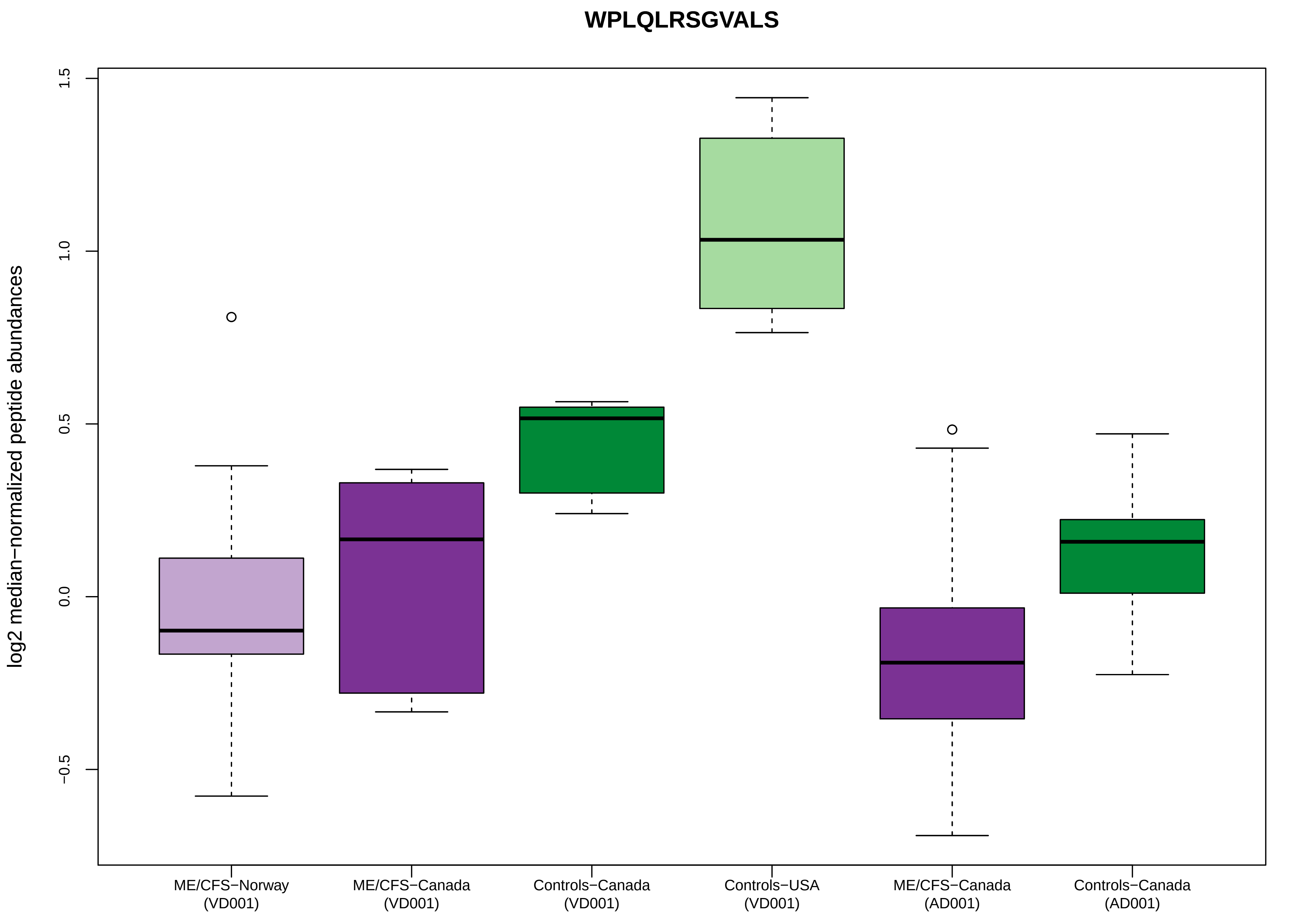

WPLQLRSGVALS
log2 median−normalized peptide abundances
1.5
1.0
0.5
0.0
−0.5
ME/CFS−Norway (VD001)
ME/CFS−Canada (VD001)
Controls−Canada (VD001)
Controls−USA (VD001)
ME/CFS−Canada (AD001)
Controls−Canada (AD001)

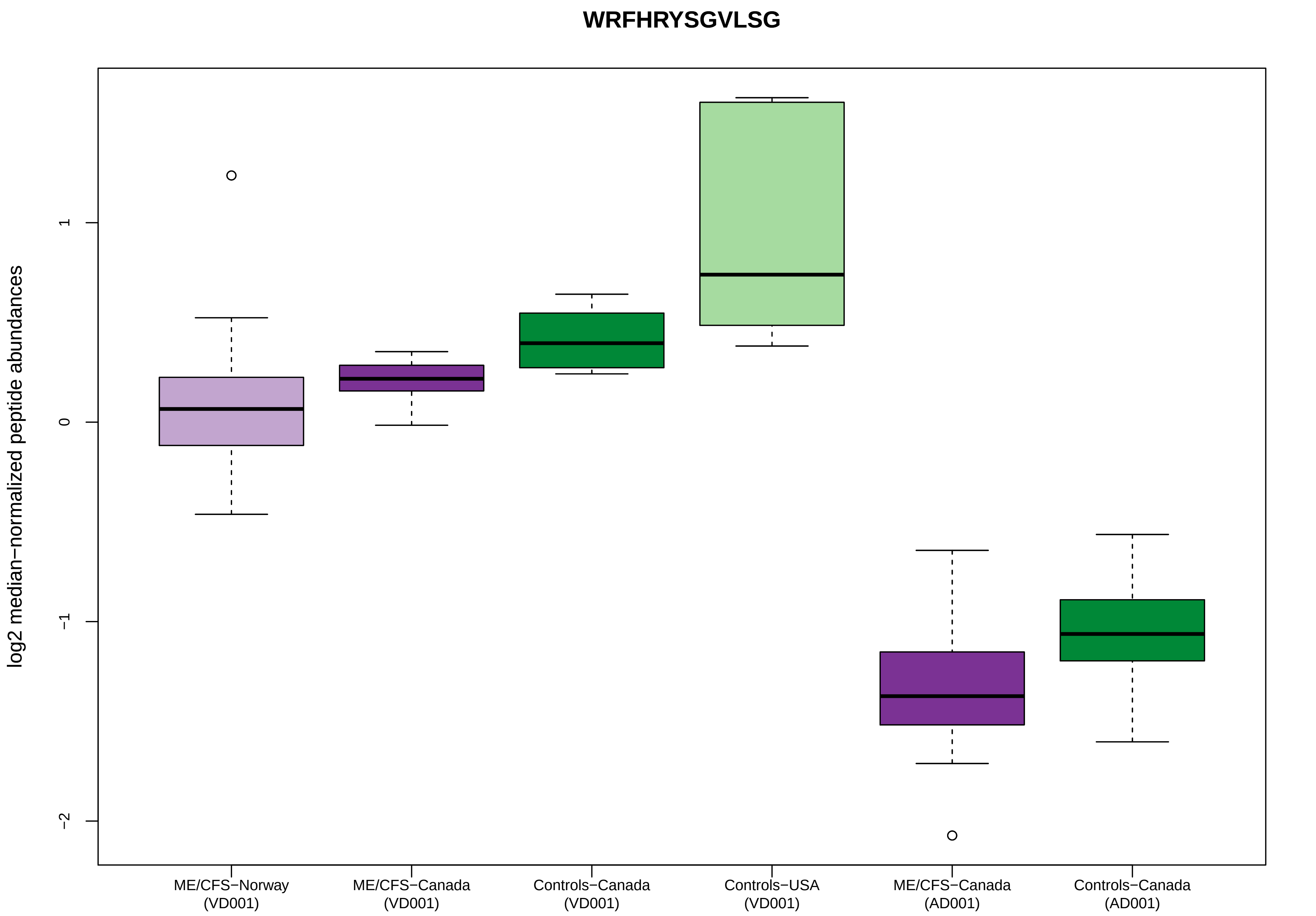

WRFHRYSGVLSG
log2 median−normalized peptide abundances
1
0
−1
−2
ME/CFS−Norway (VD001)
ME/CFS−Canada (VD001)
Controls−Canada (VD001)
Controls−USA (VD001)
ME/CFS−Canada (AD001)
Controls−Canada (AD001)

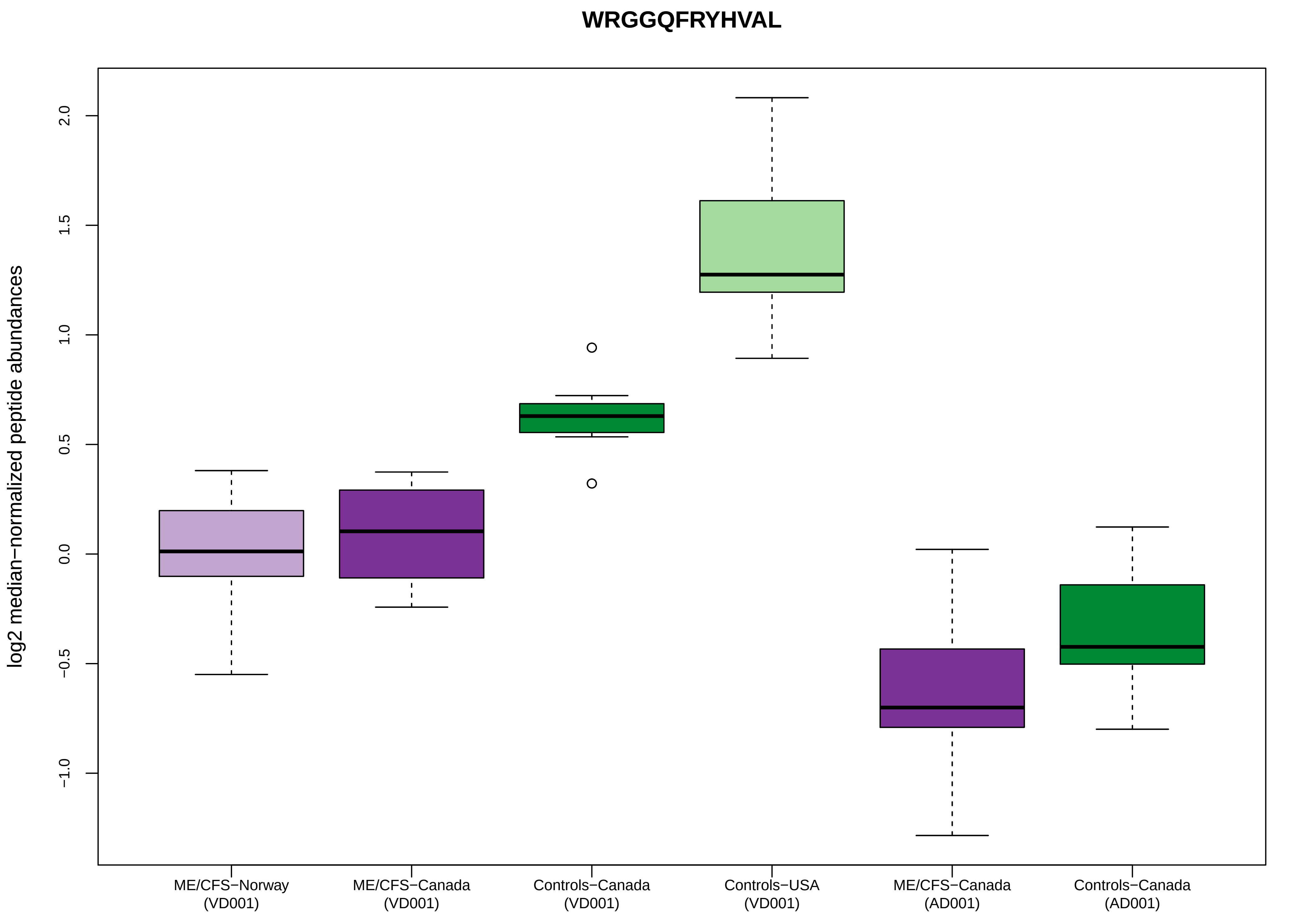

WRGGQFRYHVAL
log2 median−normalized peptide abundances
2.0
1.5
1.0
0.5
0.0
−0.5
−1.0
ME/CFS−Norway (VD001)
ME/CFS−Canada (VD001)
Controls−Canada (VD001)
Controls−USA (VD001)
ME/CFS−Canada (AD001)
Controls−Canada (AD001)

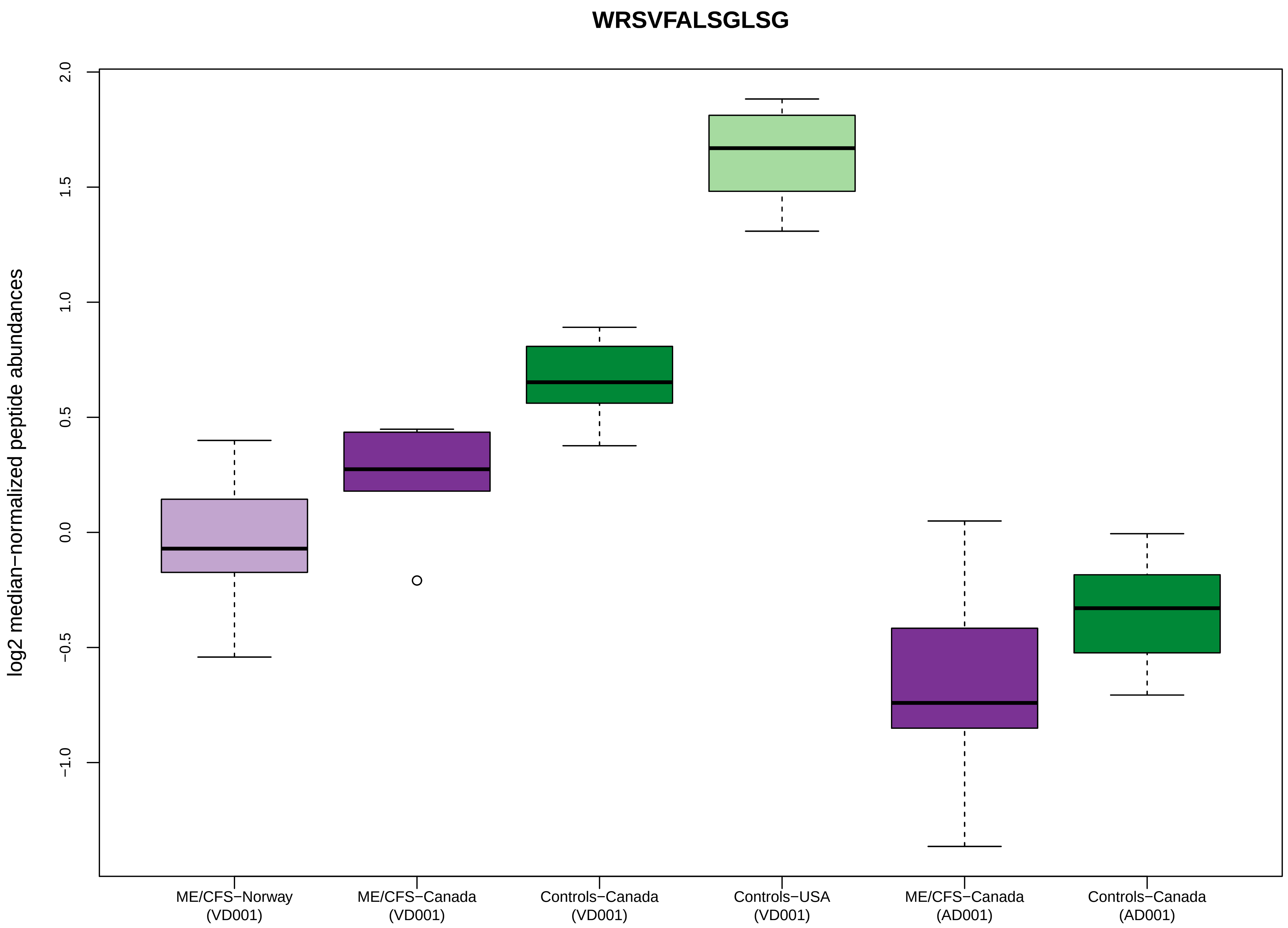

WRSVFALSGLSG
log2 median−normalized peptide abundances
2.0
1.5
1.0
0.5
0.0
−0.5
−1.0
ME/CFS−Norway (VD001)
ME/CFS−Canada (VD001)
Controls−Canada (VD001)
Controls−USA (VD001)
ME/CFS−Canada (AD001)
Controls−Canada (AD001)

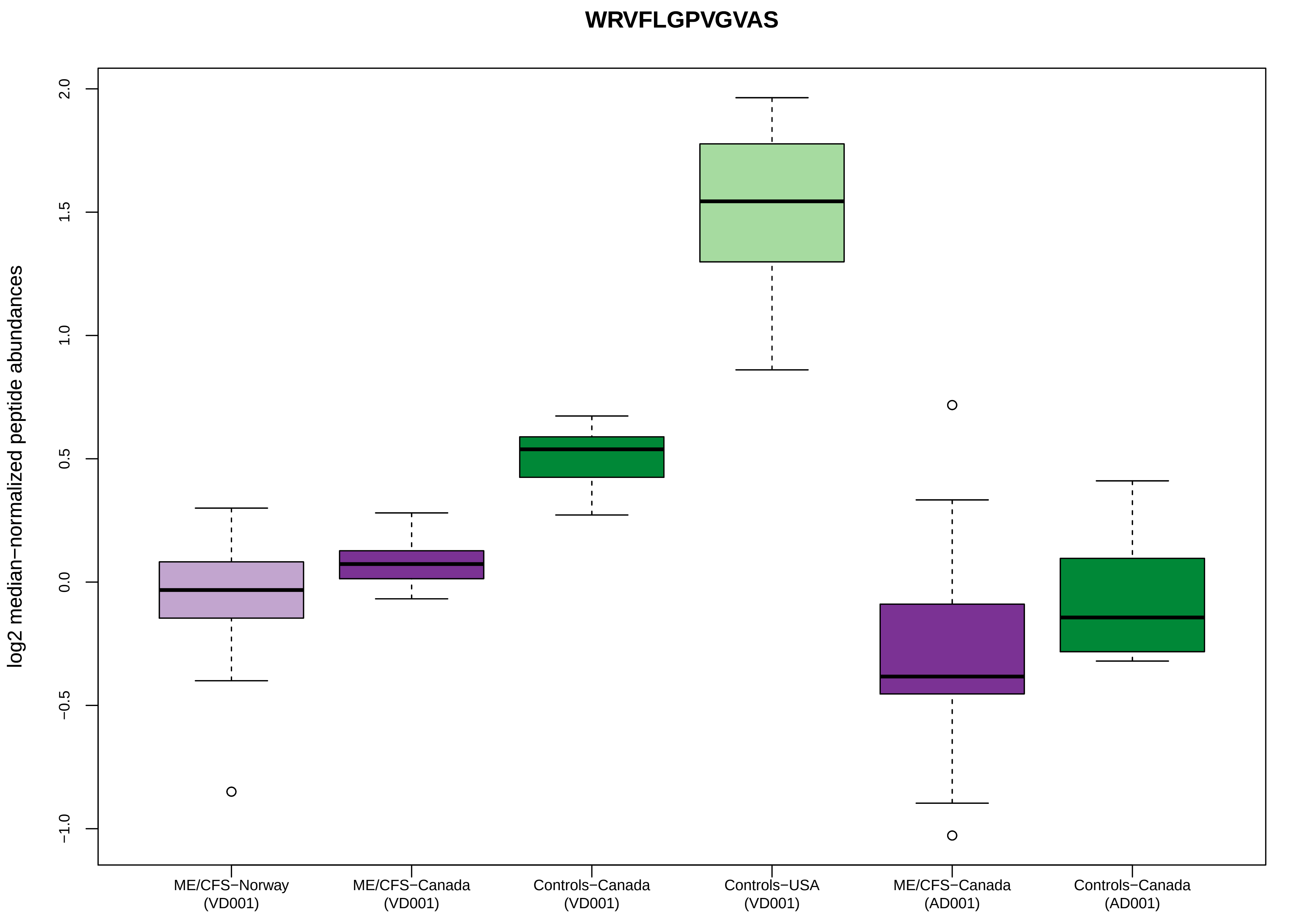

WRVFLGPVGVAS
log2 median−normalized peptide abundances
2.0
1.5
1.0
0.5
0.0
−0.5
−1.0
ME/CFS−Norway (VD001)
ME/CFS−Canada (VD001)
Controls−Canada (VD001)
Controls−USA (VD001)
ME/CFS−Canada (AD001)
Controls−Canada (AD001)

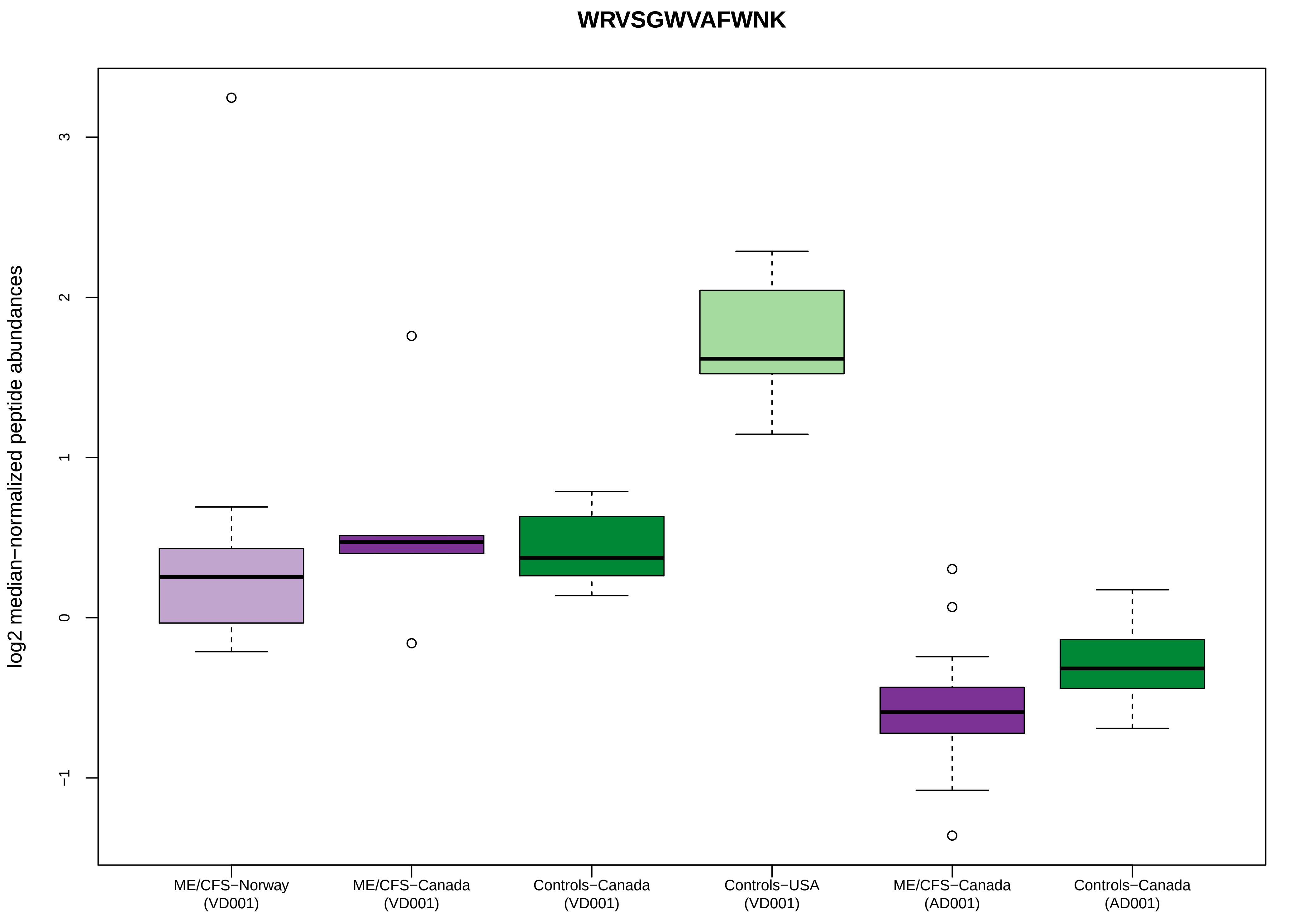
WRVSGWVAFWNK
log2 median−normalized peptide abundances
−1
0
1
2
3
ME/CFS−Norway (VD001)
ME/CFS−Canada (VD001)
Controls−Canada (VD001)
Controls−USA (VD001)
ME/CFS−Canada (AD001)
Controls−Canada (AD001)

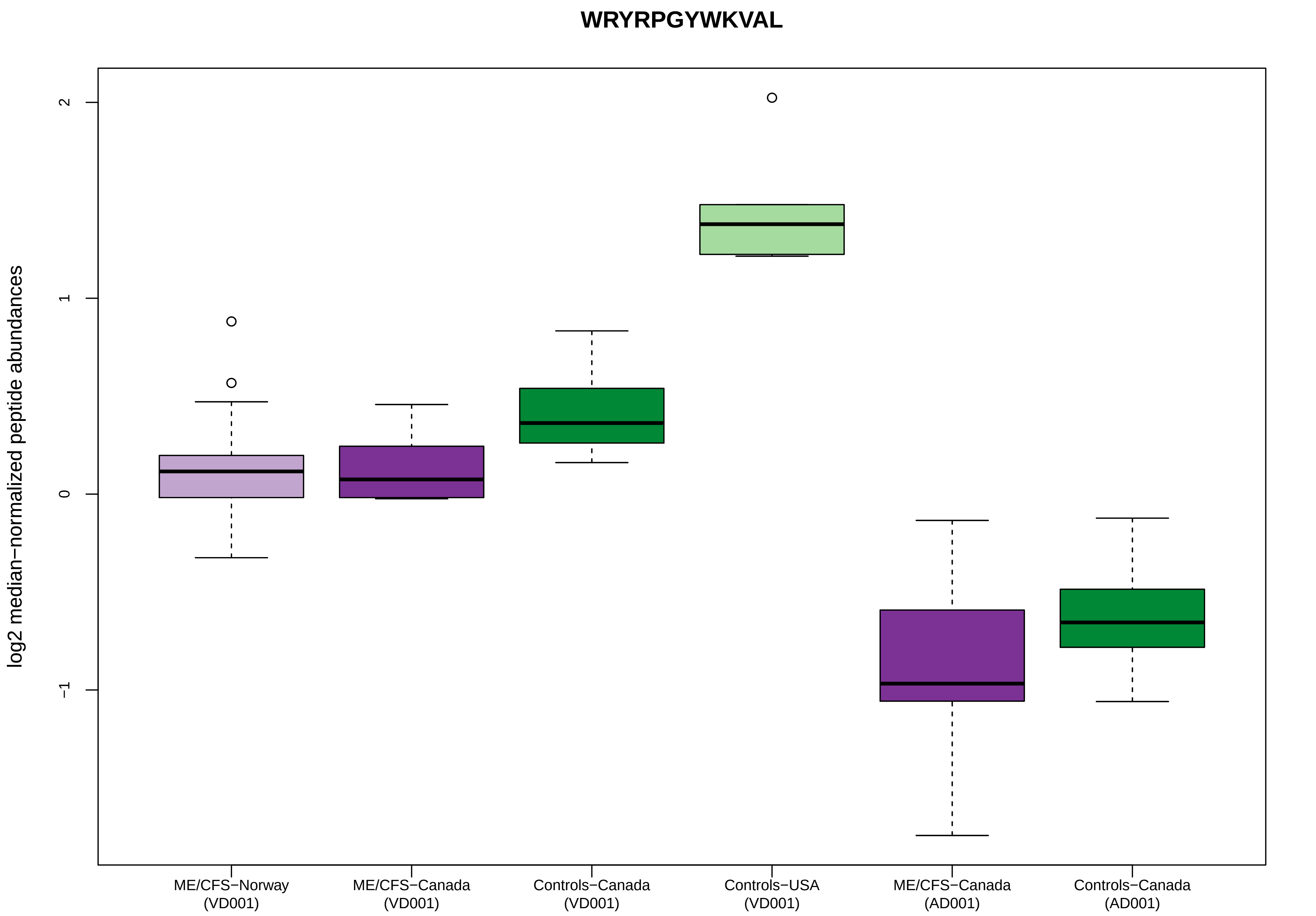
WRYRPGYWKVAL
log2 median−normalized peptide abundances
2
1
0
−1
ME/CFS−Norway
(VD001)
ME/CFS−Canada
(VD001)
Controls−Canada
(VD001)
Controls−USA
(VD001)
ME/CFS−Canada
(AD001)
Controls−Canada
(AD001)

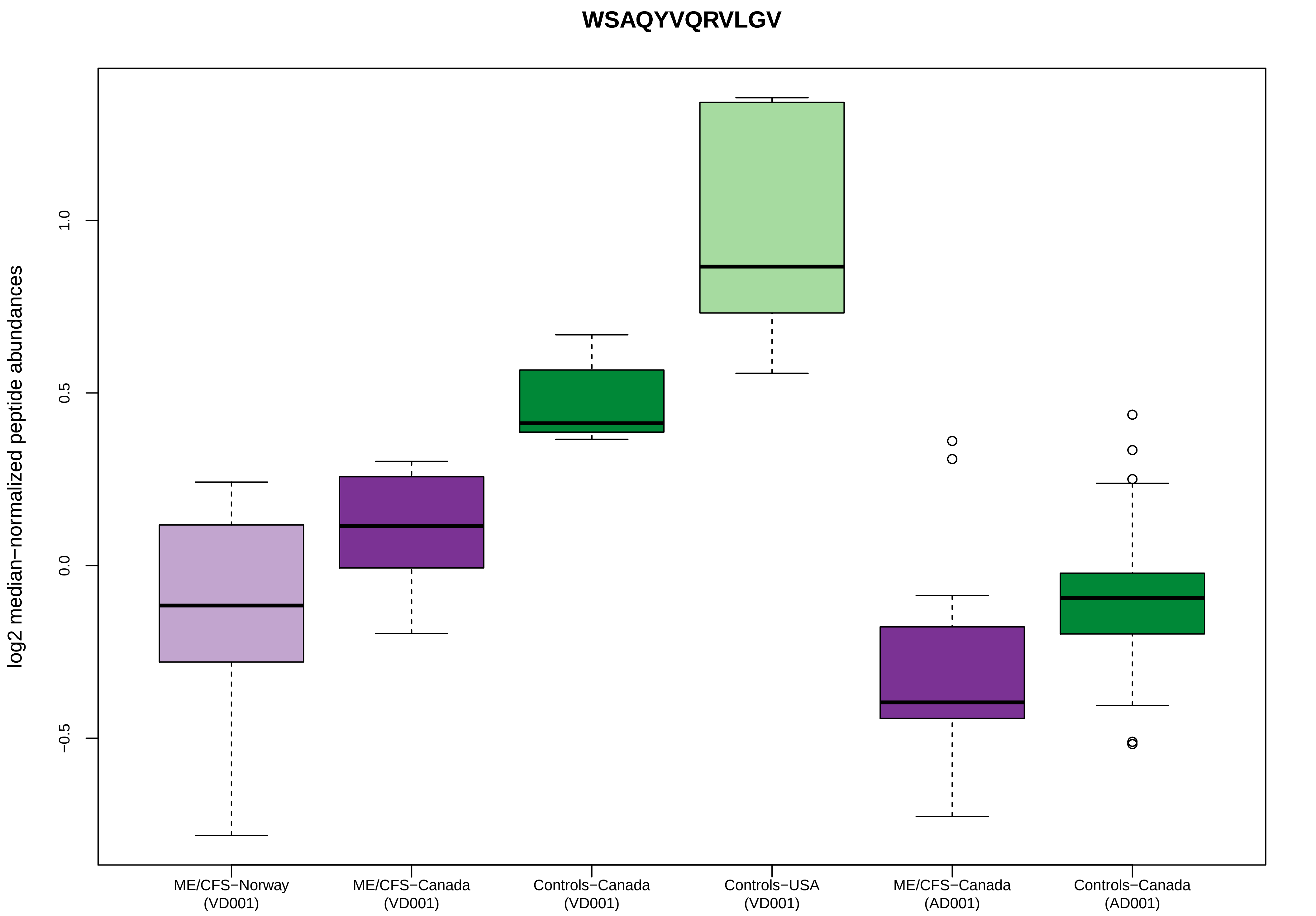

WSAQYVQRVLGV
log2 median−normalized peptide abundances
1.0
0.5
0.0
−0.5
ME/CFS−Norway (VD001)
ME/CFS−Canada (VD001)
Controls−Canada (VD001)
Controls−USA (VD001)
ME/CFS−Canada (AD001)
Controls−Canada (AD001)

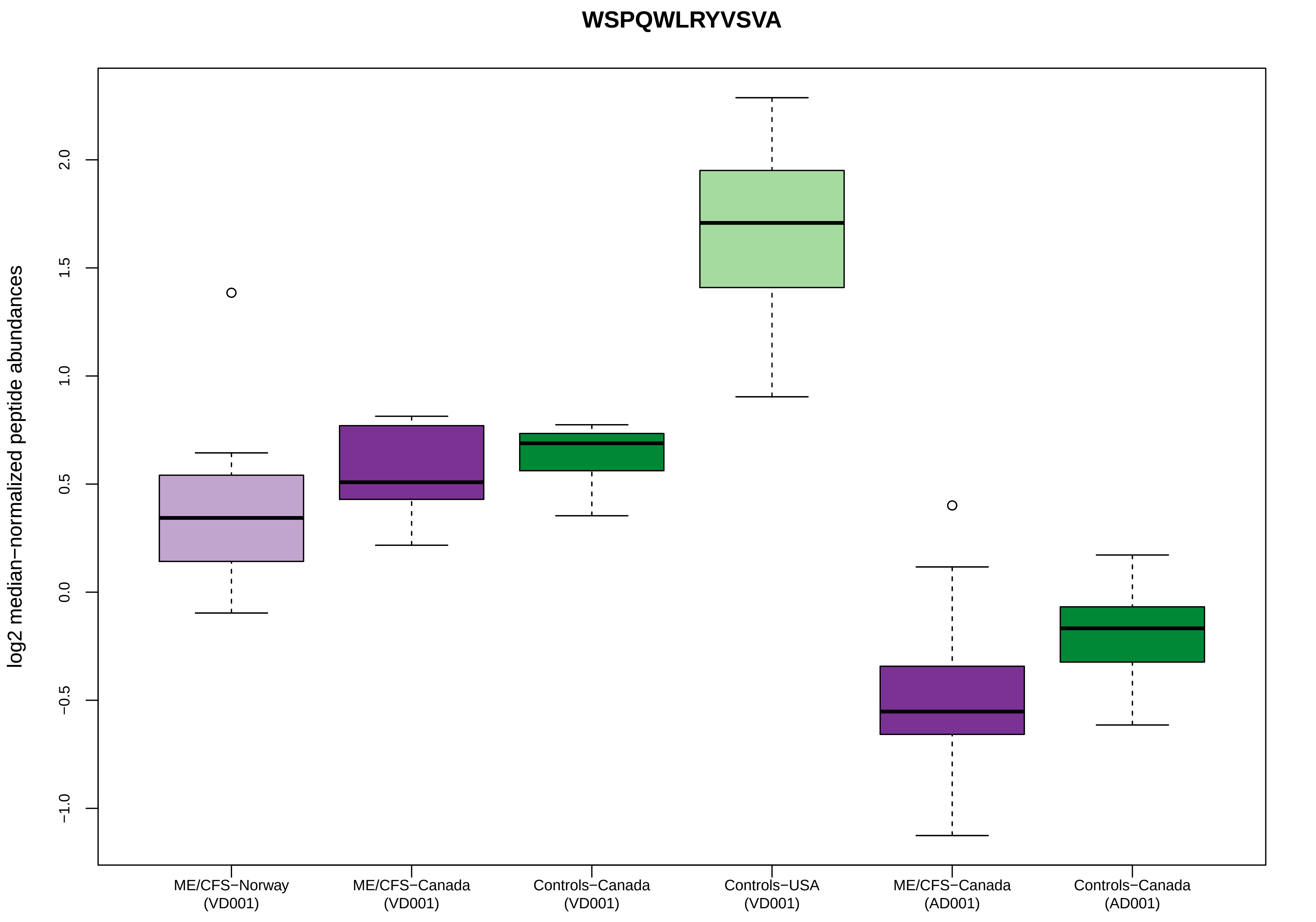

WSPQWLRYVSVA
log2 median−normalized peptide abundances
−1.0
−0.5
0.0
0.5
1.0
1.5
2.0
ME/CFS−Norway (VD001)
ME/CFS−Canada (VD001)
Controls−Canada (VD001)
Controls−USA (VD001)
ME/CFS−Canada (AD001)
Controls−Canada (AD001)

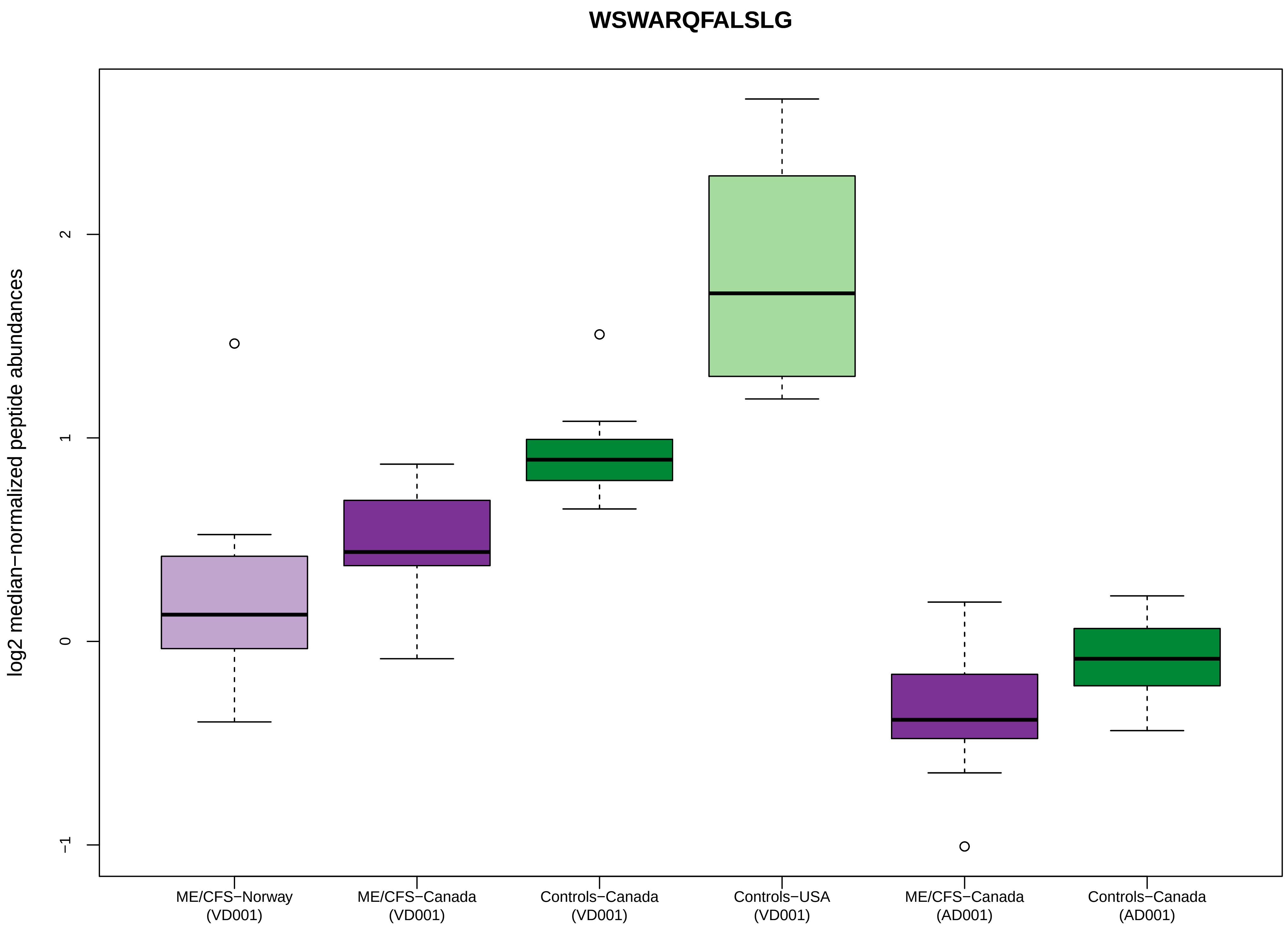

WSWARQFALSLG
log2 median−normalized peptide abundances
2
1
0
−1
ME/CFS−Norway (VD001)
ME/CFS−Canada (VD001)
Controls−Canada (VD001)
Controls−USA (VD001)
ME/CFS−Canada (AD001)
Controls−Canada (AD001)

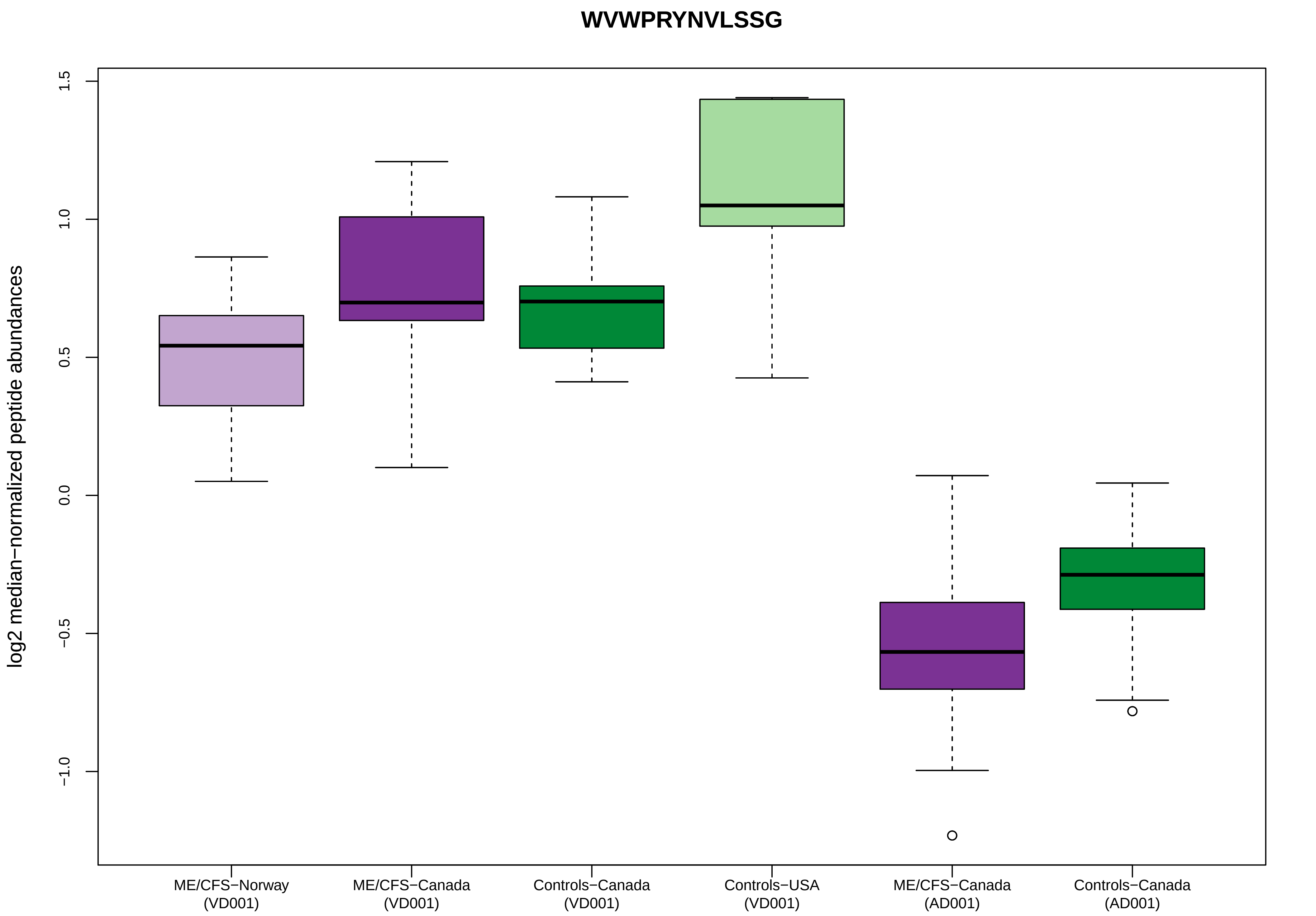
WVWPRYNVLSSG
log2 median−normalized peptide abundances
1.5
1.0
0.5
0.0
−0.5
−1.0
ME/CFS−Norway (VD001)
ME/CFS−Canada (VD001)
Controls−Canada (VD001)
Controls−USA (VD001)
ME/CFS−Canada (AD001)
Controls−Canada (AD001)

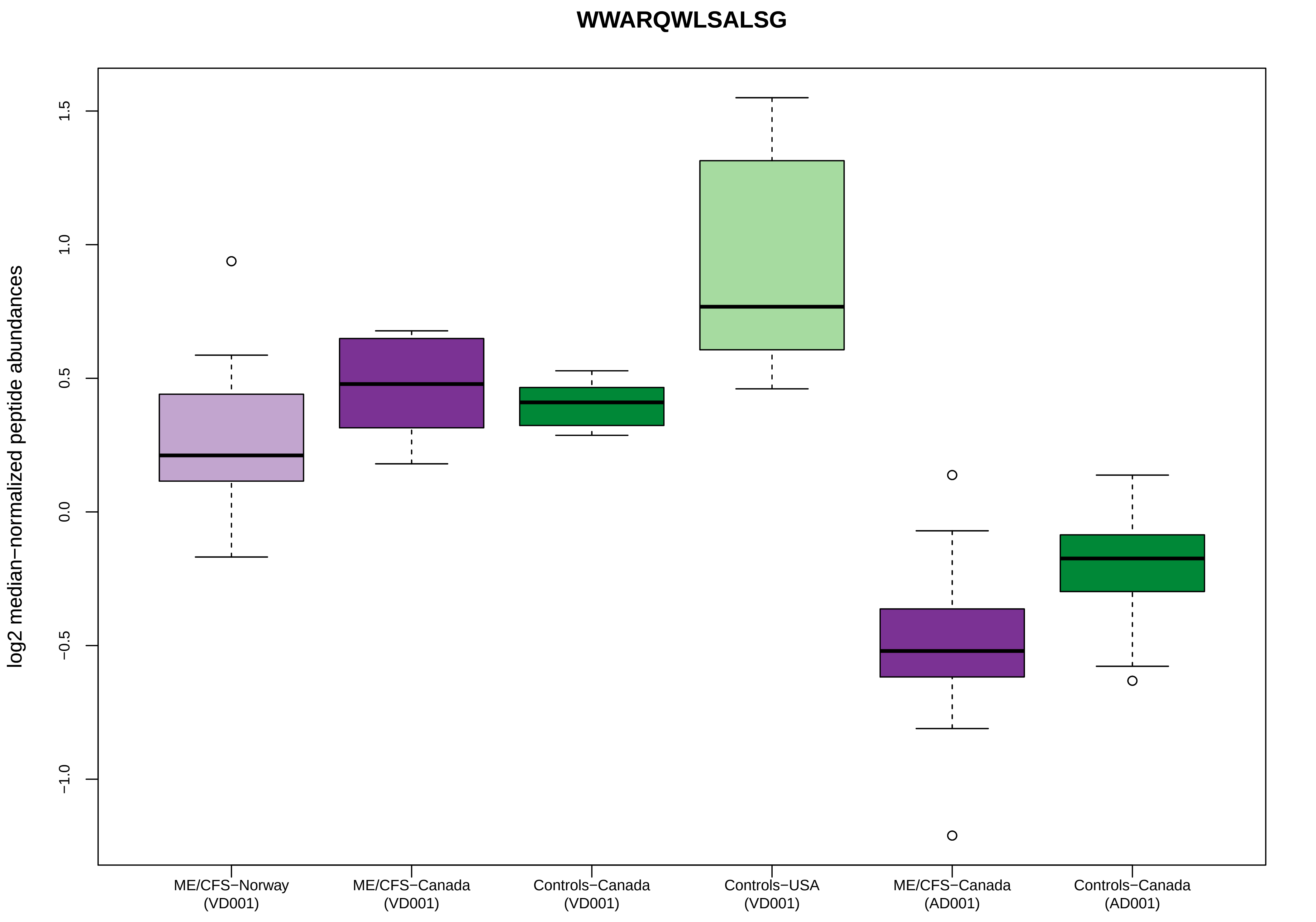

WWARQWLSALSG
log2 median−normalized peptide abundances
1.5
1.0
0.5
0.0
−0.5
−1.0
ME/CFS−Norway (VD001)
ME/CFS−Canada (VD001)
Controls−Canada (VD001)
Controls−USA (VD001)
ME/CFS−Canada (AD001)
Controls−Canada (AD001)

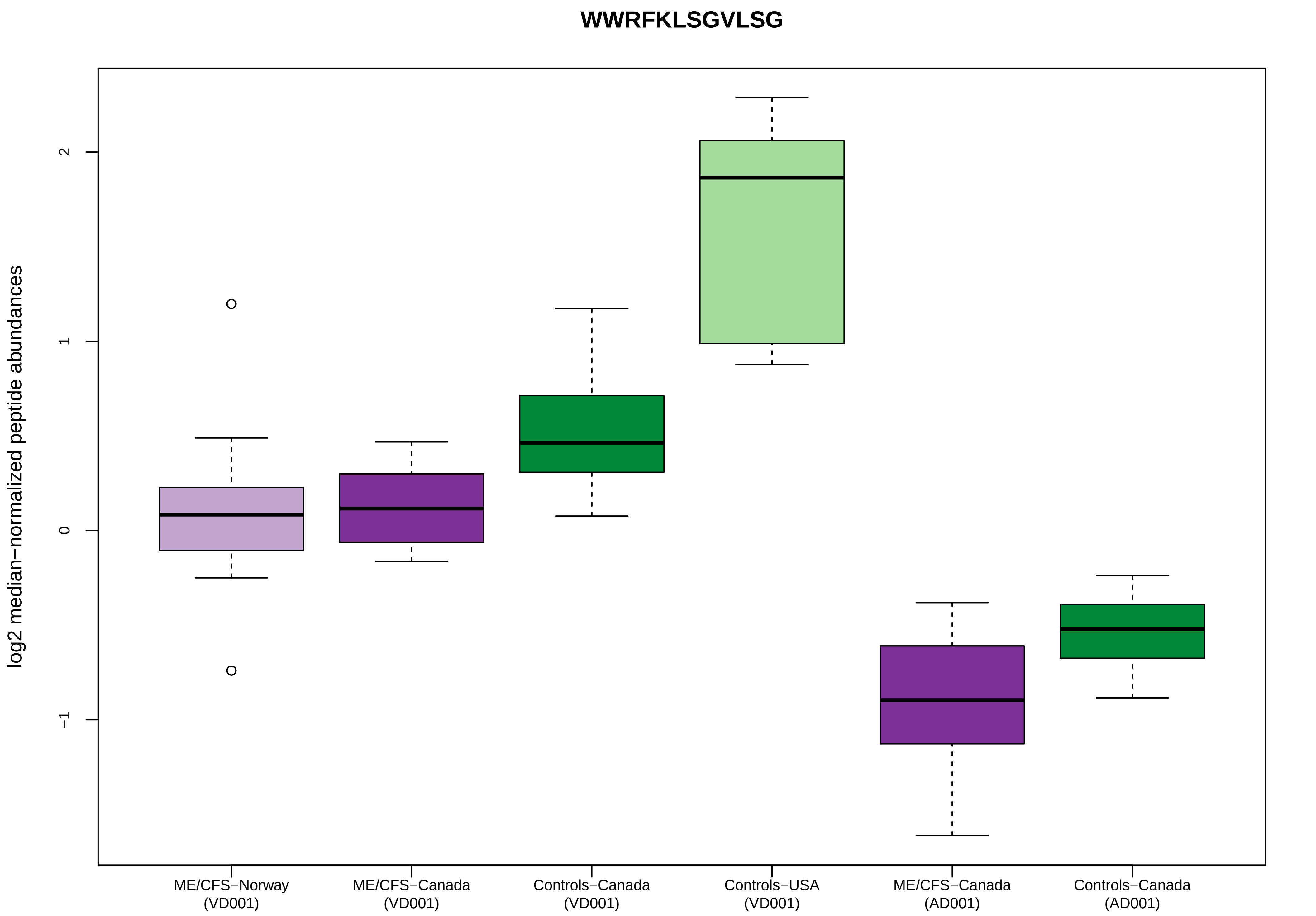

WWRFKLSGVLSG
log2 median−normalized peptide abundances
−1
0
1
2
ME/CFS−Norway (VD001)
ME/CFS−Canada (VD001)
Controls−Canada (VD001)
Controls−USA (VD001)
ME/CFS−Canada (AD001)
Controls−Canada (AD001)

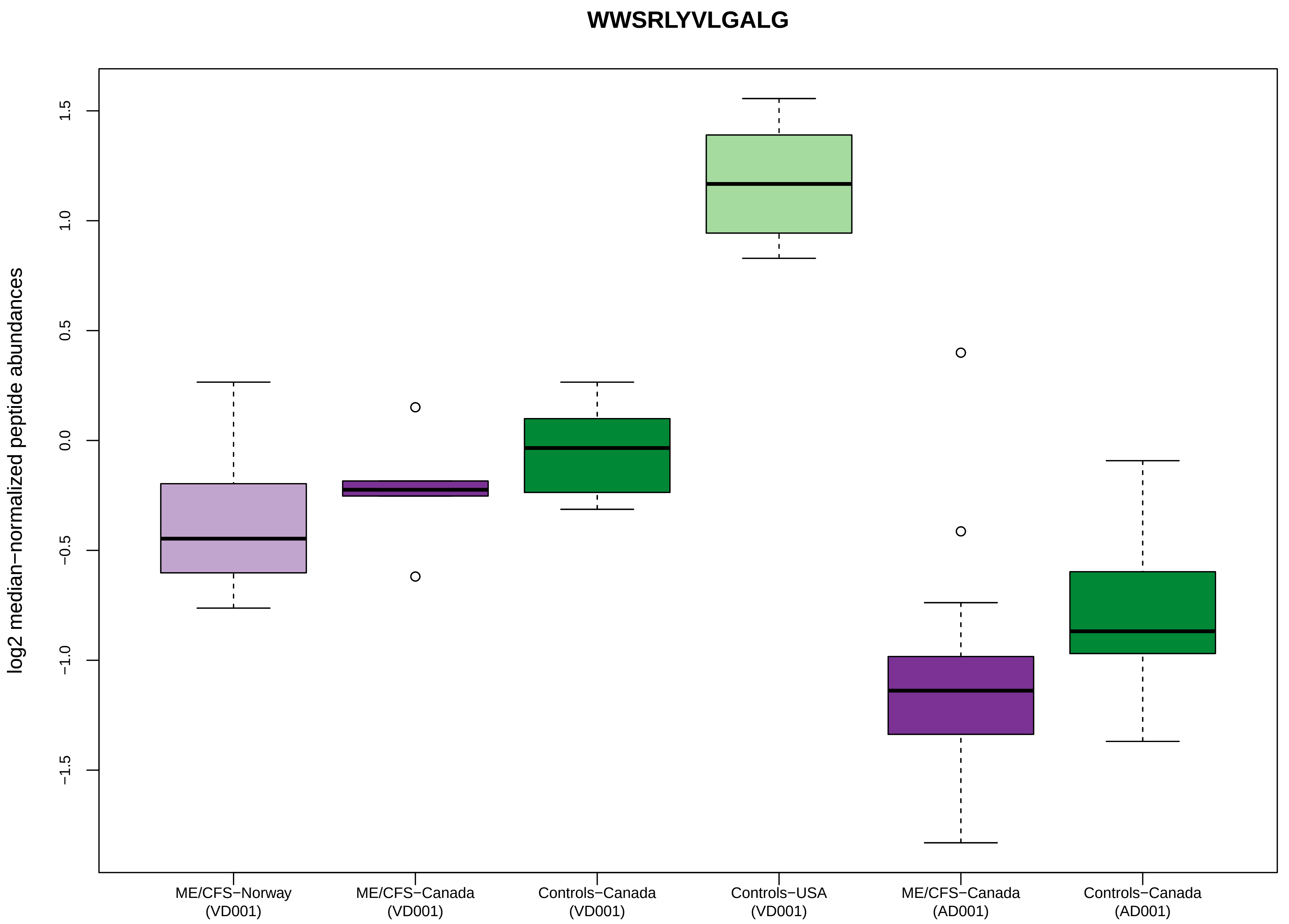

WWSRLYVLGALG
log2 median−normalized peptide abundances
1.5
1.0
0.5
0.0
−0.5
−1.0
−1.5
ME/CFS−Norway (VD001)
ME/CFS−Canada (VD001)
Controls−Canada (VD001)
Controls−USA (VD001)
ME/CFS−Canada (AD001)
Controls−Canada (AD001)

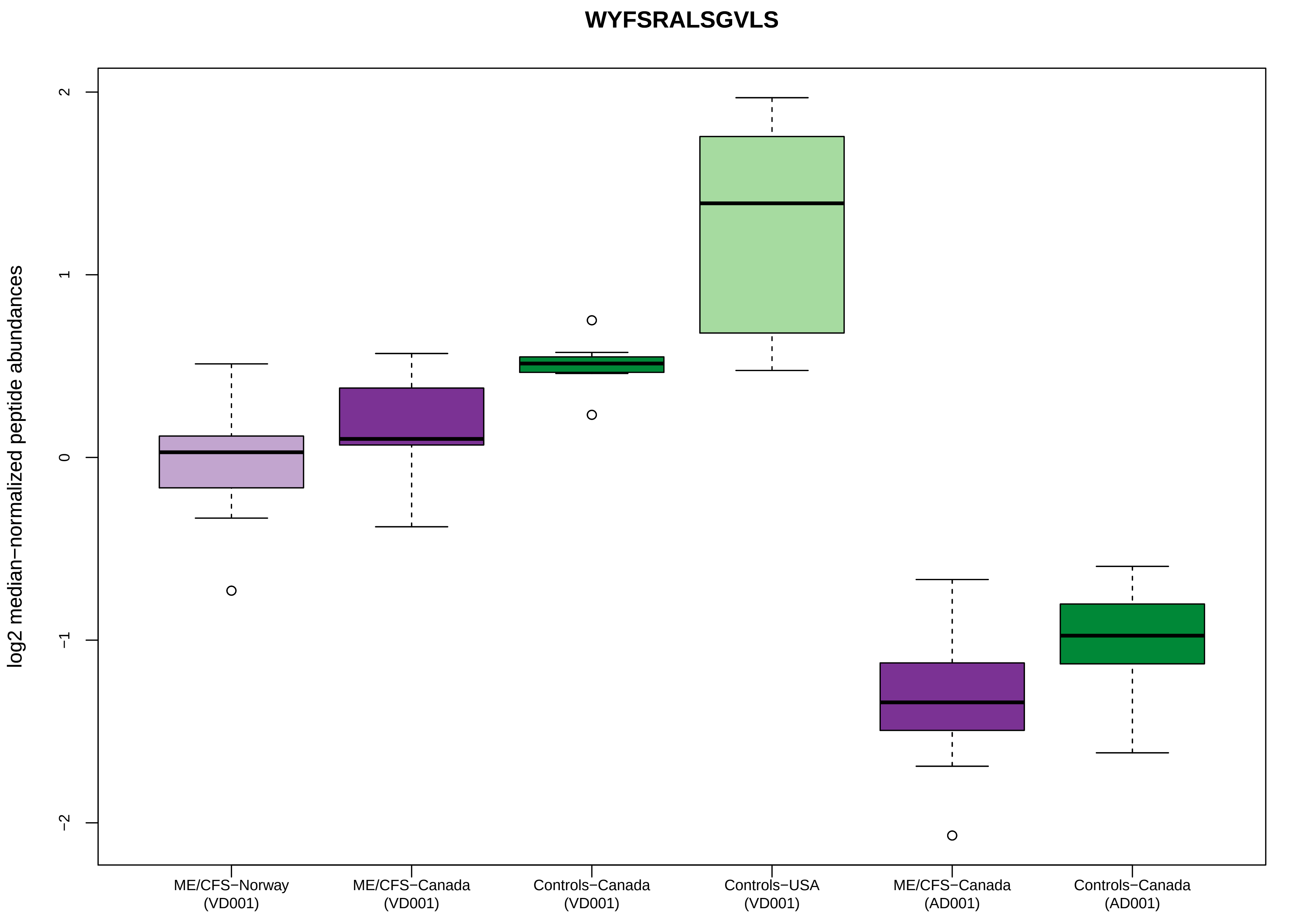

WYFSRALSGVLS
log2 median−normalized peptide abundances
2
1
0
−1
−2
ME/CFS−Norway (VD001)
ME/CFS−Canada (VD001)
Controls−Canada (VD001)
Controls−USA (VD001)
ME/CFS−Canada (AD001)
Controls−Canada (AD001)

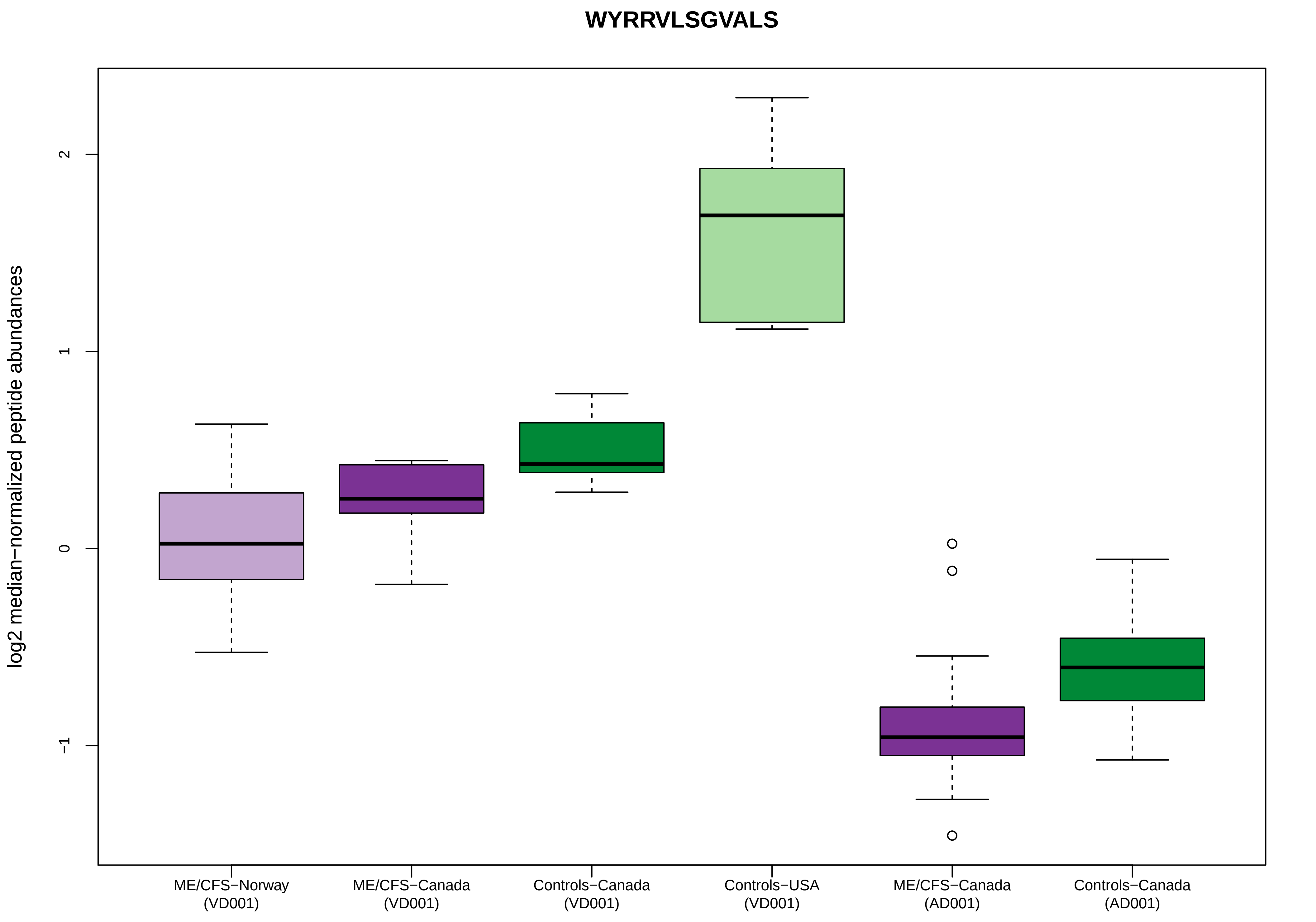
WYRRVLSGVALS
log2 median−normalized peptide abundances
2
1
0
−1
ME/CFS−Norway (VD001)
ME/CFS−Canada (VD001)
Controls−Canada (VD001)
Controls−USA (VD001)
ME/CFS−Canada (AD001)
Controls−Canada (AD001)

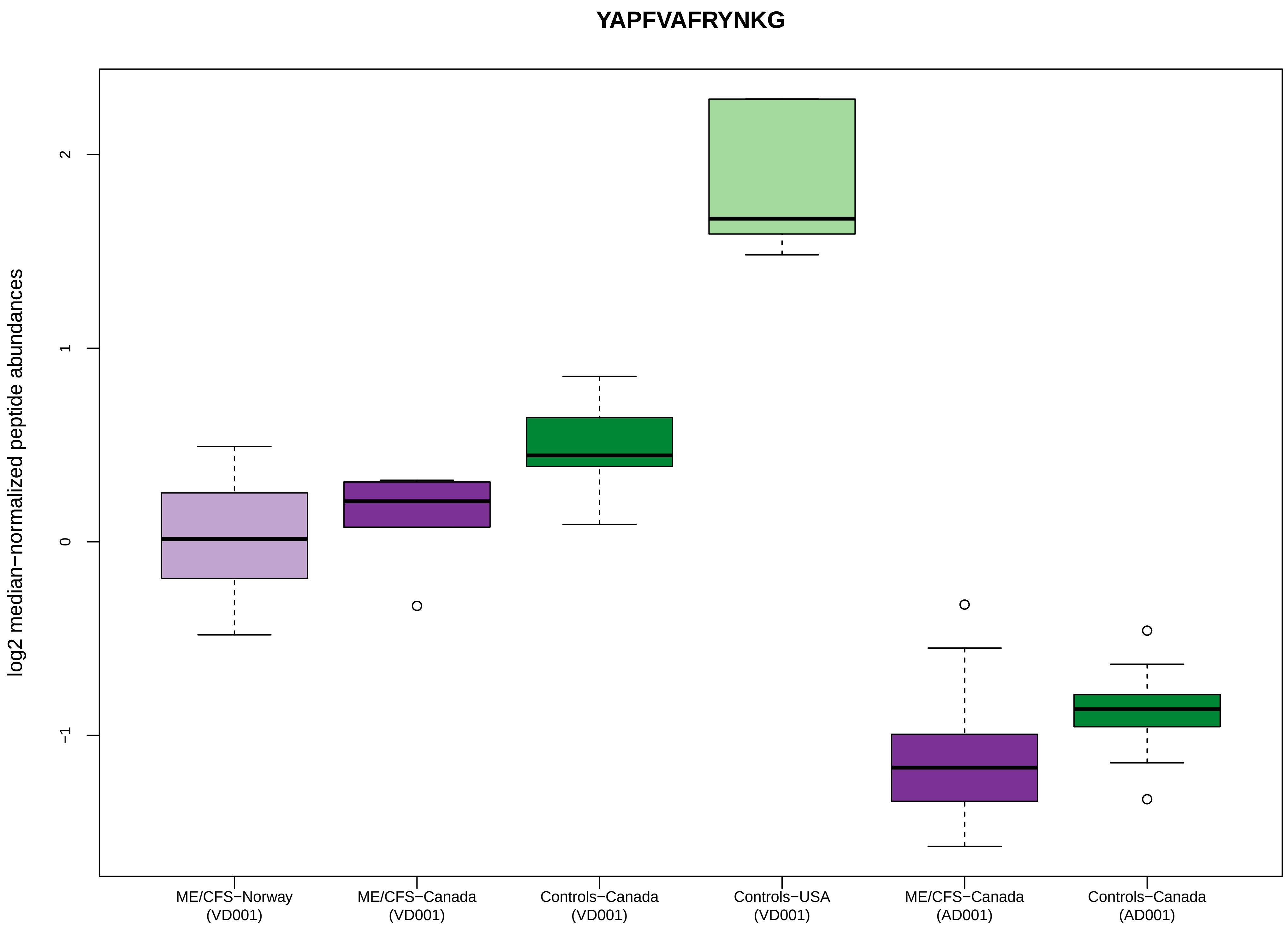

YAPFVAFRYNKG
log2 median−normalized peptide abundances
2
1
0
−1
ME/CFS−Norway (VD001)
ME/CFS−Canada (VD001)
Controls−Canada (VD001)
Controls−USA (VD001)
ME/CFS−Canada (AD001)
Controls−Canada (AD001)

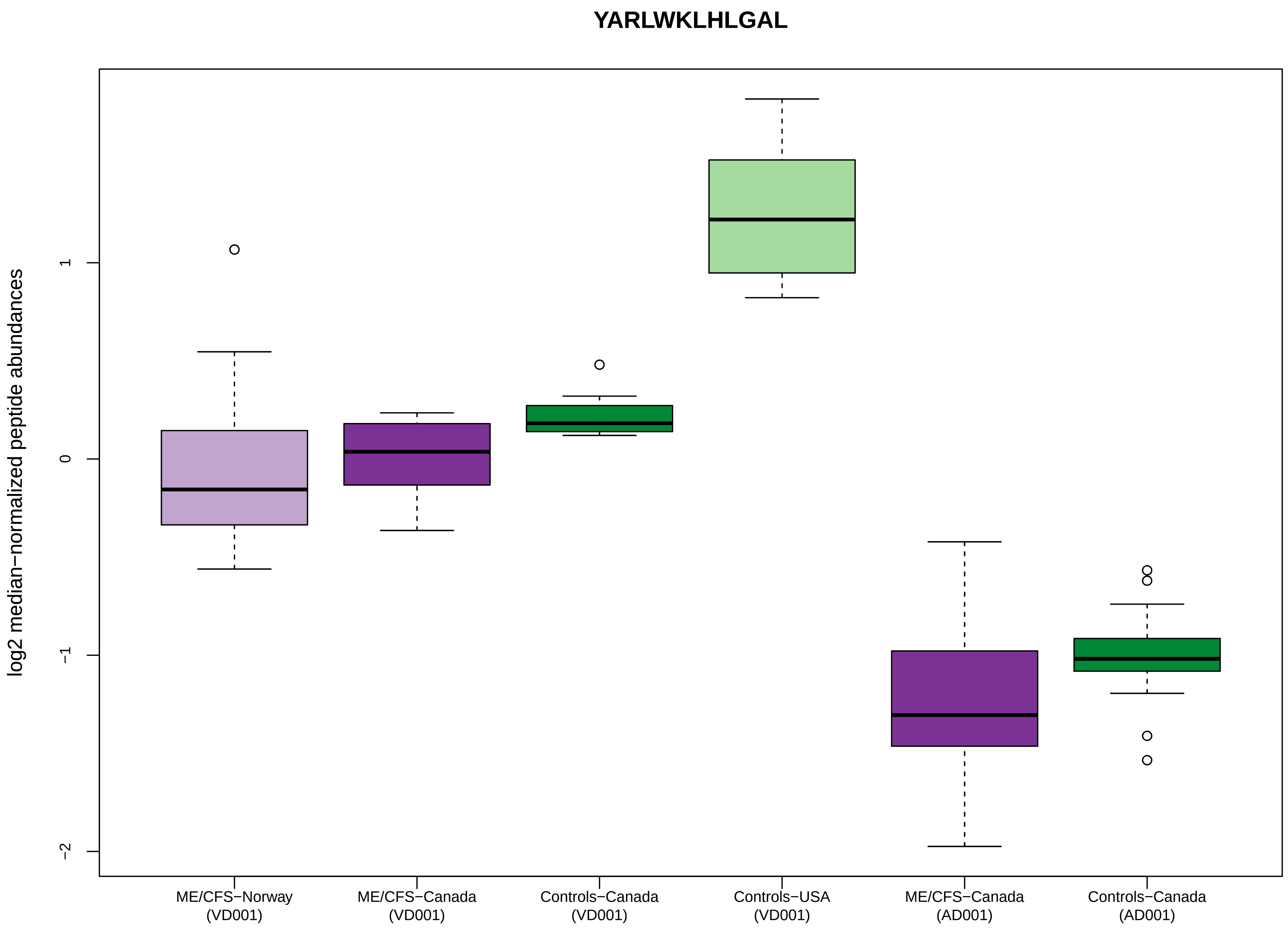
YARLWKLHLGAL
log2 median−normalized peptide abundances
1
0
−1
−2
ME/CFS−Norway (VD001)
ME/CFS−Canada (VD001)
Controls−Canada (VD001)
Controls−USA (VD001)
ME/CFS−Canada (AD001)
Controls−Canada (AD001)

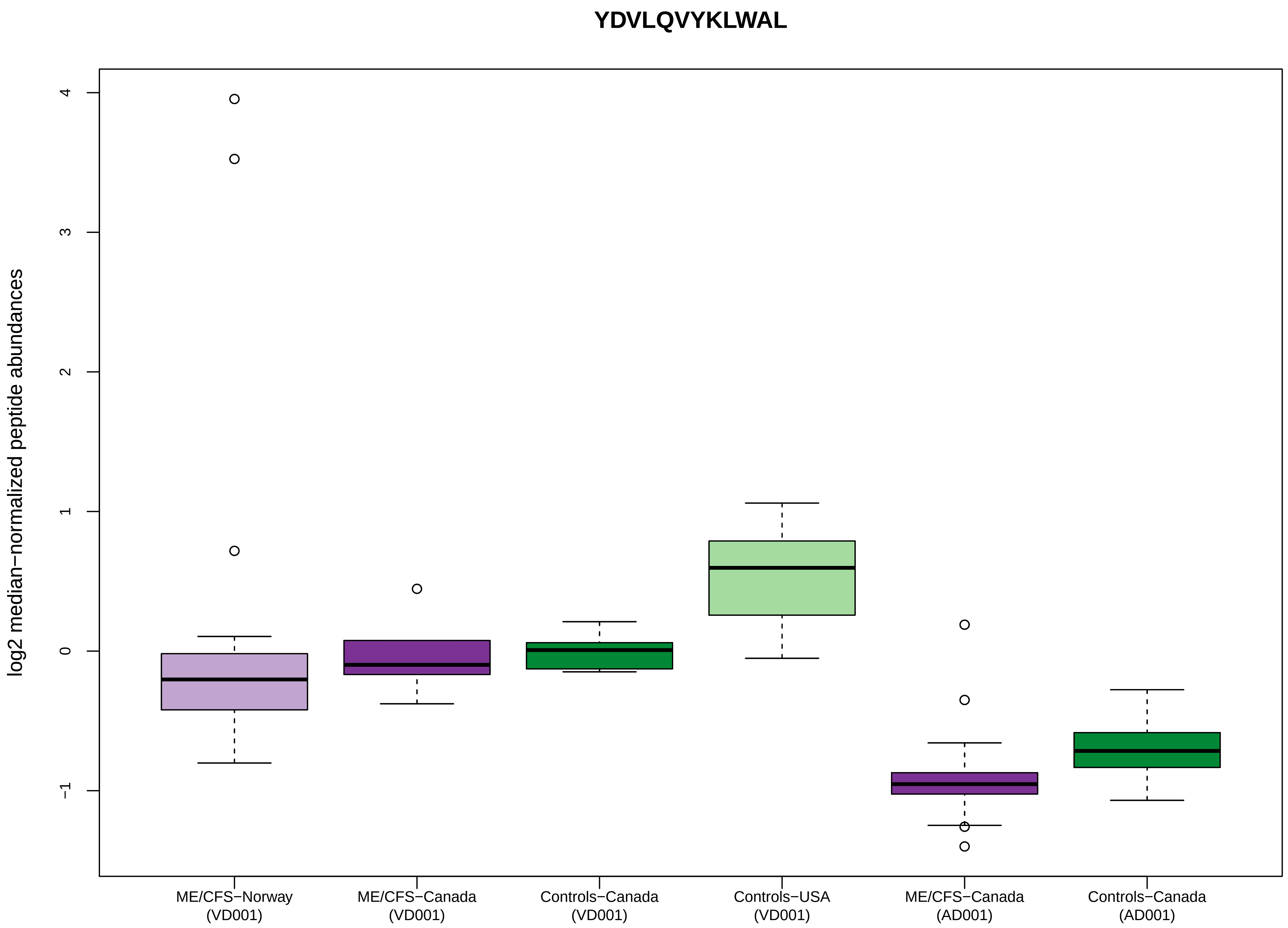
YDVLQVYKLWAL
log2 median−normalized peptide abundances
−1
0
1
2
3
4
ME/CFS−Norway (VD001)
ME/CFS−Canada (VD001)
Controls−Canada (VD001)
Controls−USA (VD001)
ME/CFS−Canada (AD001)
Controls−Canada (AD001)

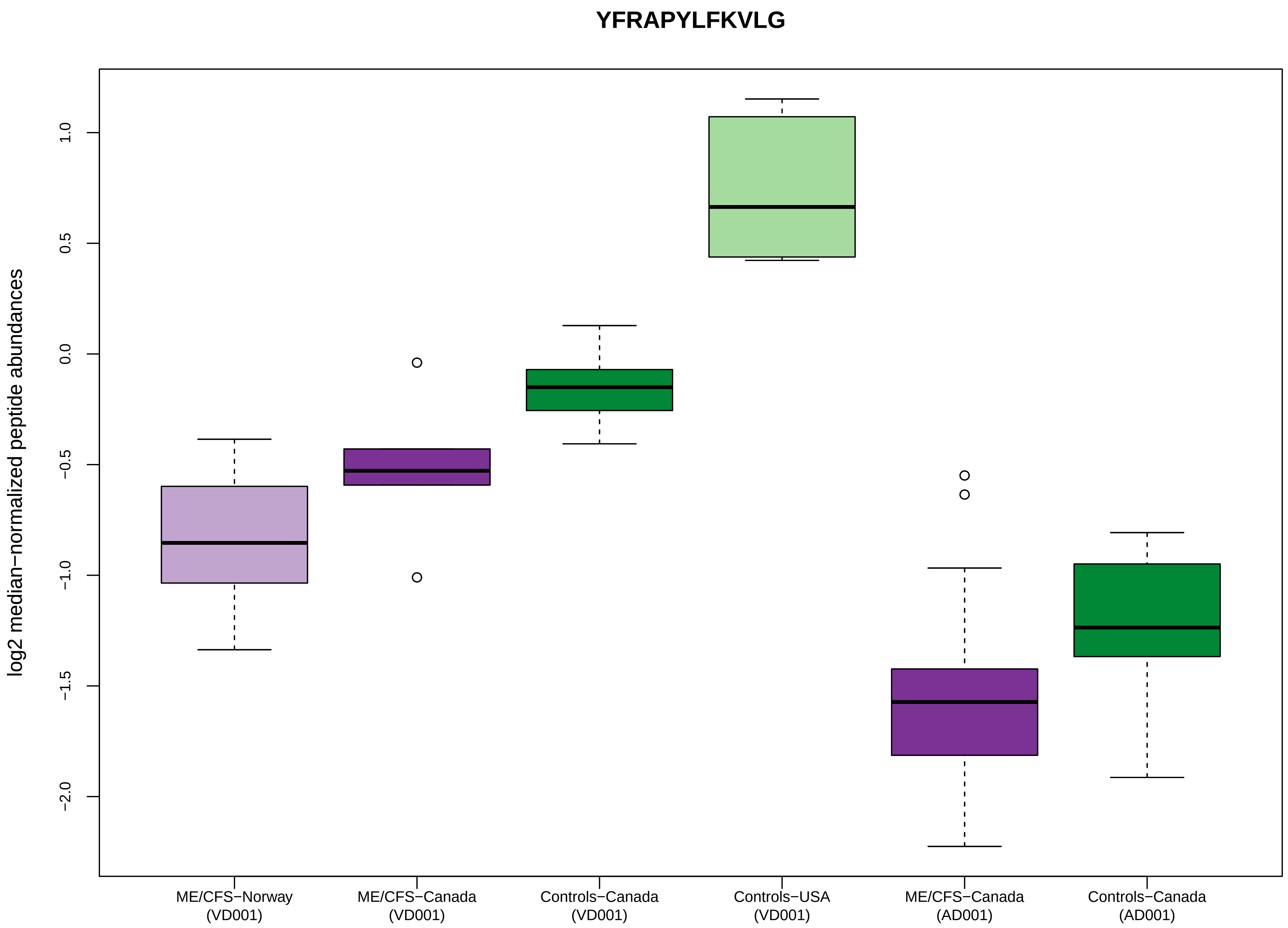

YFRAPYLFKVLG
log2 median−normalized peptide abundances
1.0
0.5
0.0
−0.5
−1.0
−1.5
−2.0
ME/CFS−Norway (VD001)
ME/CFS−Canada (VD001)
Controls−Canada (VD001)
Controls−USA (VD001)
ME/CFS−Canada (AD001)
Controls−Canada (AD001)

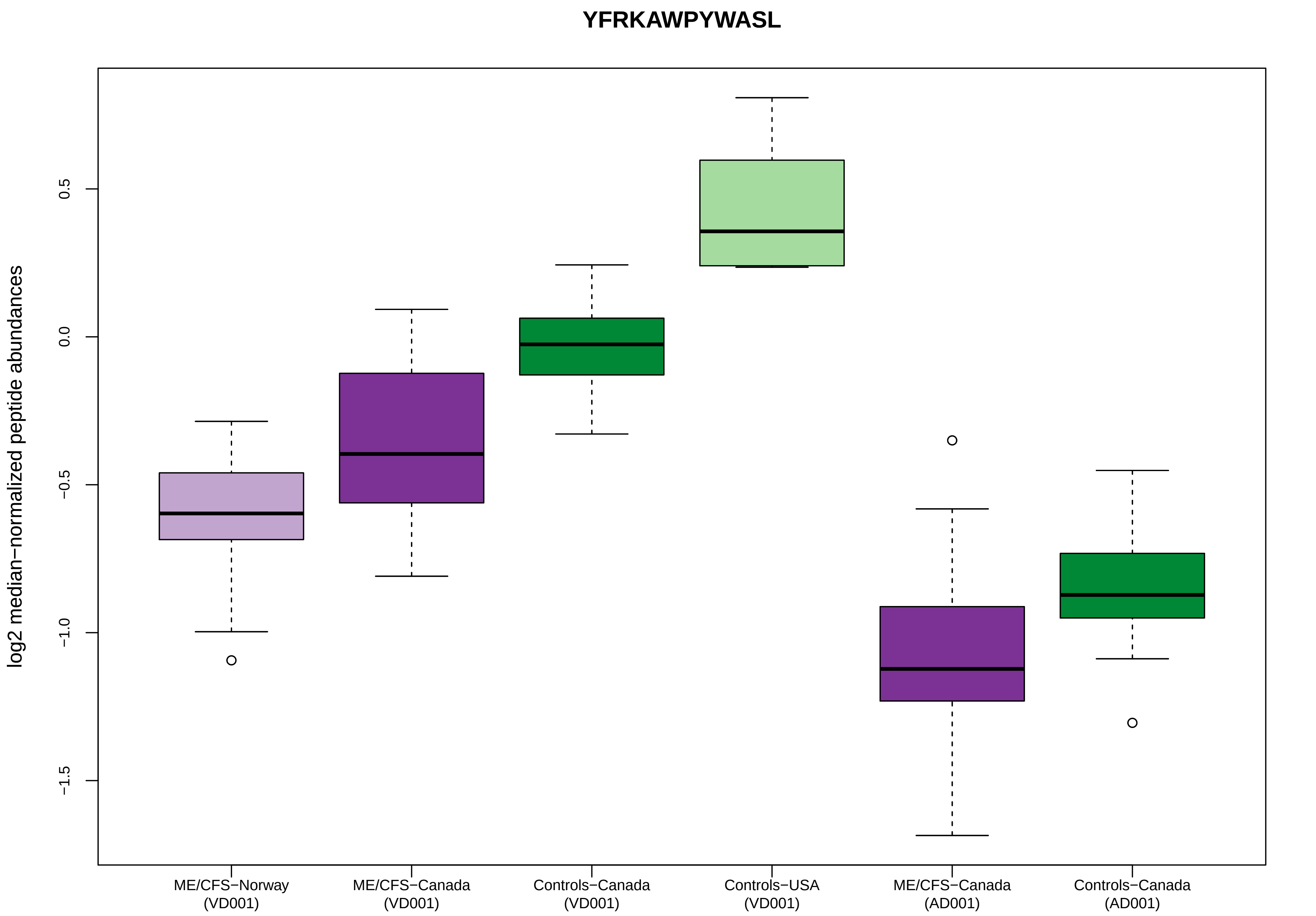

YFRKAWPYWASL
log2 median−normalized peptide abundances
0.5
0.0
−0.5
−1.0
−1.5
ME/CFS−Norway (VD001)
ME/CFS−Canada (VD001)
Controls−Canada (VD001)
Controls−USA (VD001)
ME/CFS−Canada (AD001)
Controls−Canada (AD001)

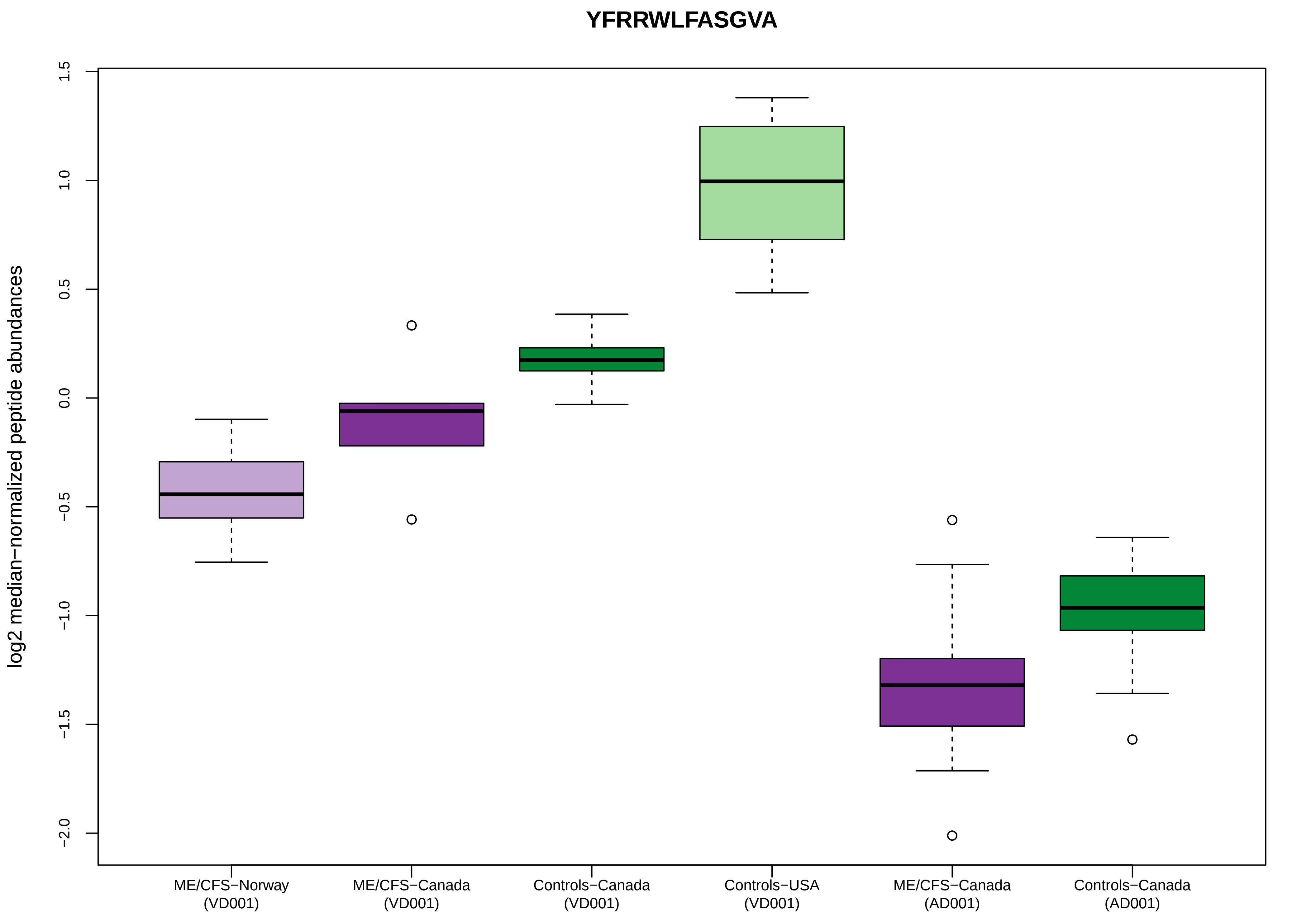
YFRRWLFASGVA
log2 median−normalized peptide abundances
1.5
1.0
0.5
0.0
−0.5
−1.0
−1.5
−2.0
ME/CFS−Norway (VD001)
ME/CFS−Canada (VD001)
Controls−Canada (VD001)
Controls−USA (VD001)
ME/CFS−Canada (AD001)
Controls−Canada (AD001)

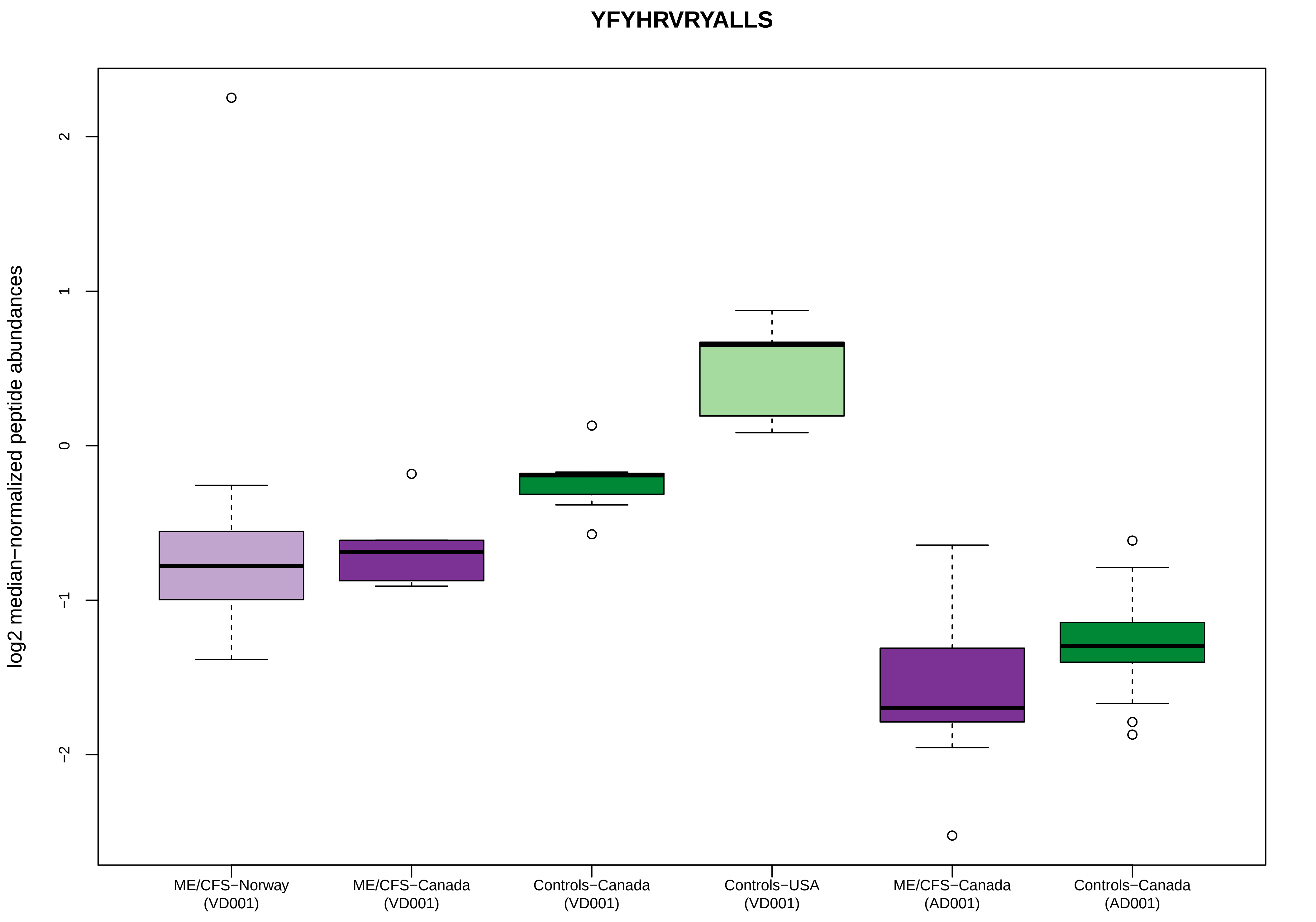

YFYHRVRYALLS
log2 median−normalized peptide abundances
2
1
0
−1
−2
ME/CFS−Norway (VD001)
ME/CFS−Canada (VD001)
Controls−Canada (VD001)
Controls−USA (VD001)
ME/CFS−Canada (AD001)
Controls−Canada (AD001)

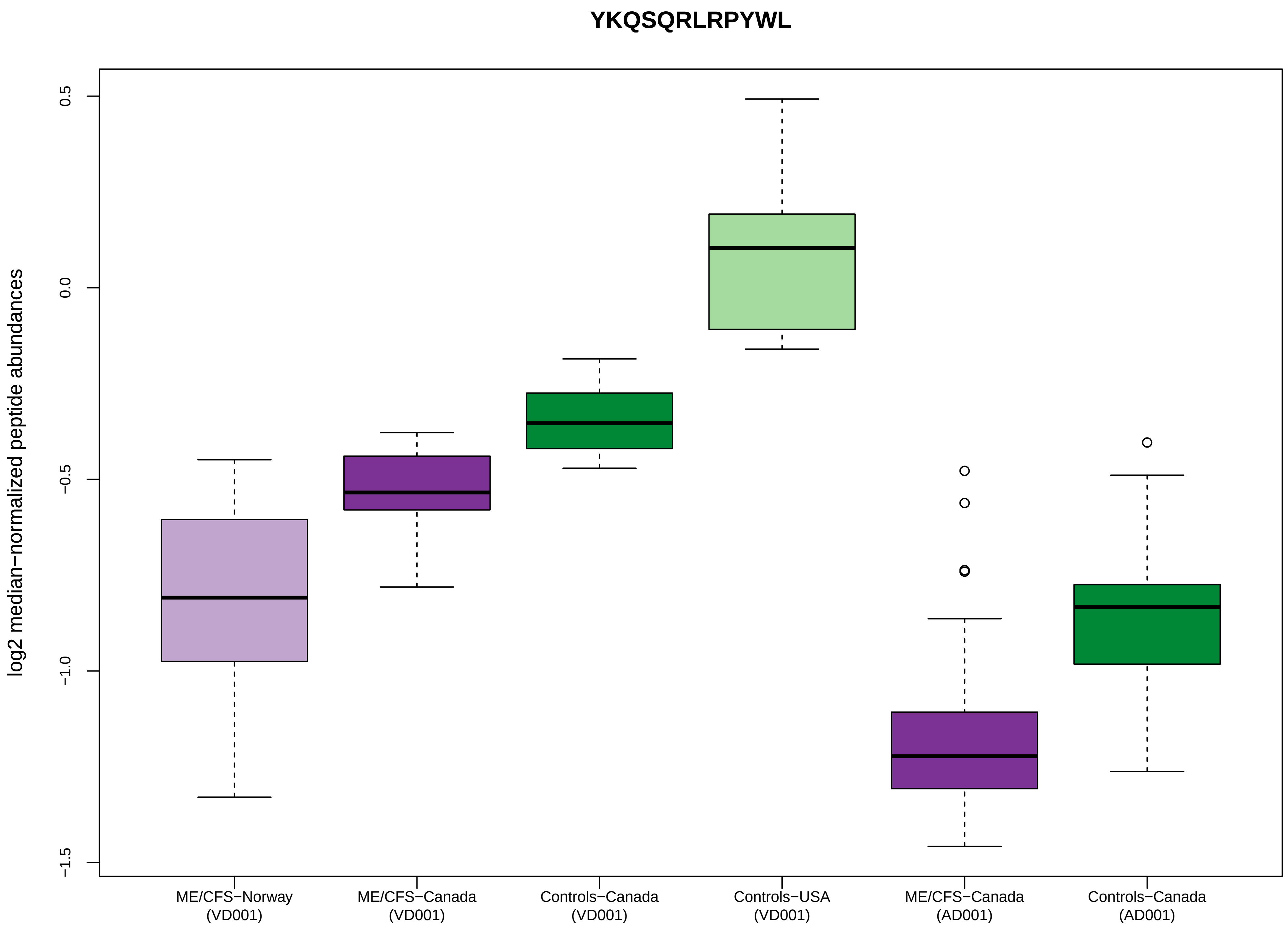

YKQSQRLRPYWL
log2 median−normalized peptide abundances
0.5
0.0
−0.5
−1.0
−1.5
ME/CFS−Norway (VD001)
ME/CFS−Canada (VD001)
Controls−Canada (VD001)
Controls−USA (VD001)
ME/CFS−Canada (AD001)
Controls−Canada (AD001)

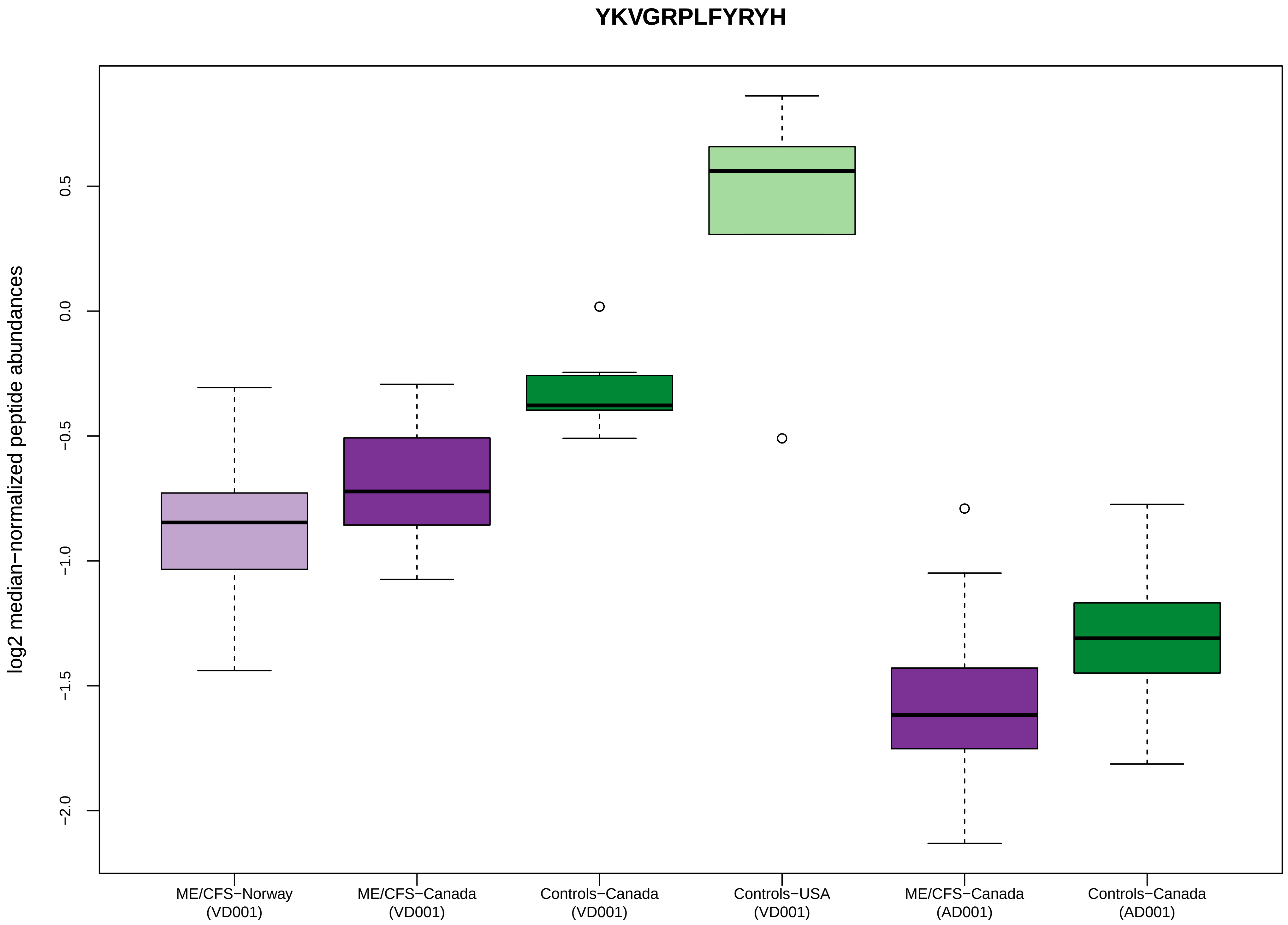

YKVGRPLFYRYH
log2 median−normalized peptide abundances
0.5
0.0
−0.5
−1.0
−1.5
−2.0
ME/CFS−Norway (VD001)
ME/CFS−Canada (VD001)
Controls−Canada (VD001)
Controls−USA (VD001)
ME/CFS−Canada (AD001)
Controls−Canada (AD001)

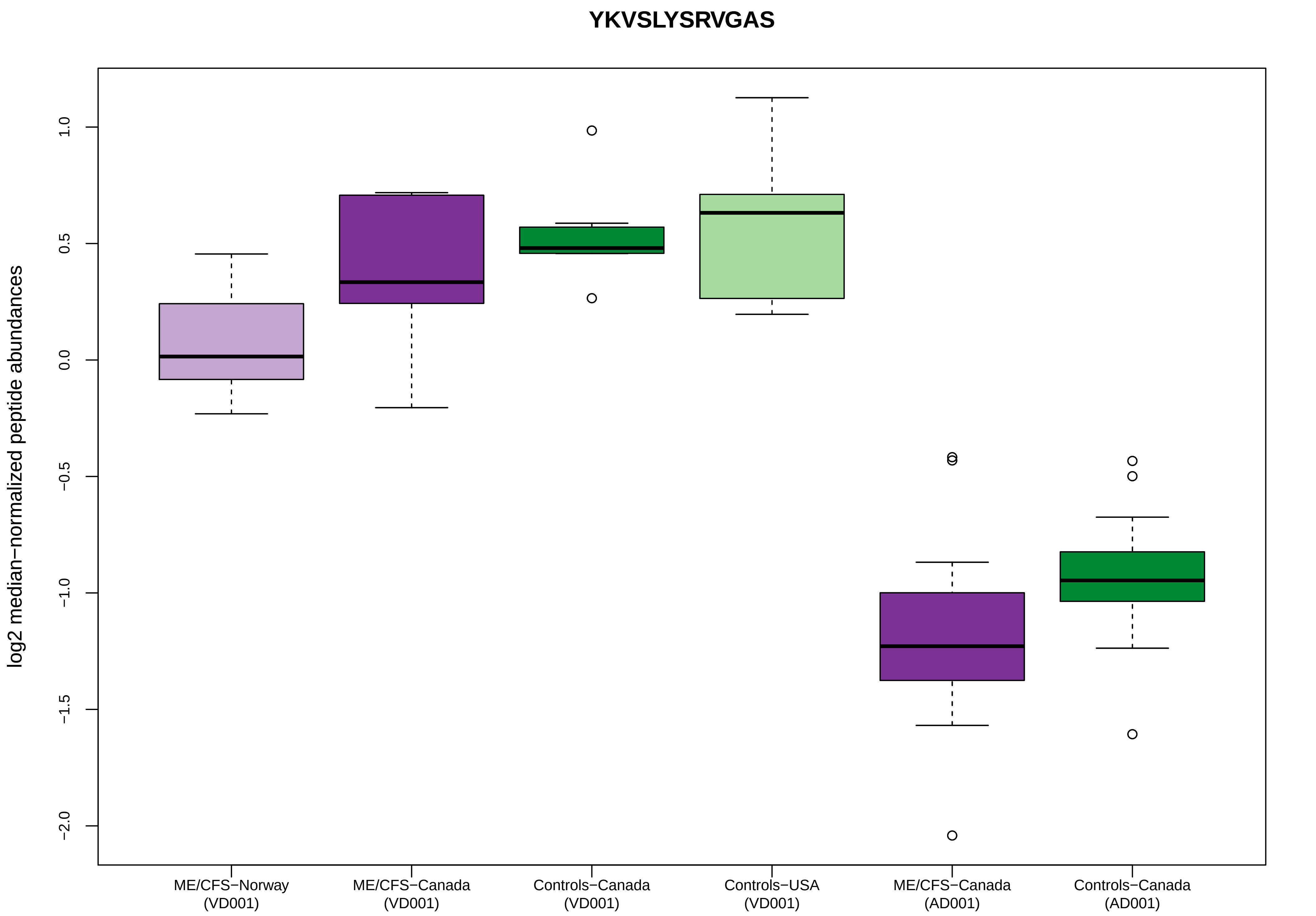
YKVSLYSRVGAS
log2 median−normalized peptide abundances
1.0
0.5
0.0
−0.5
−1.0
−1.5
−2.0
ME/CFS−Norway (VD001)
ME/CFS−Canada (VD001)
Controls−Canada (VD001)
Controls−USA (VD001)
ME/CFS−Canada (AD001)
Controls−Canada (AD001)

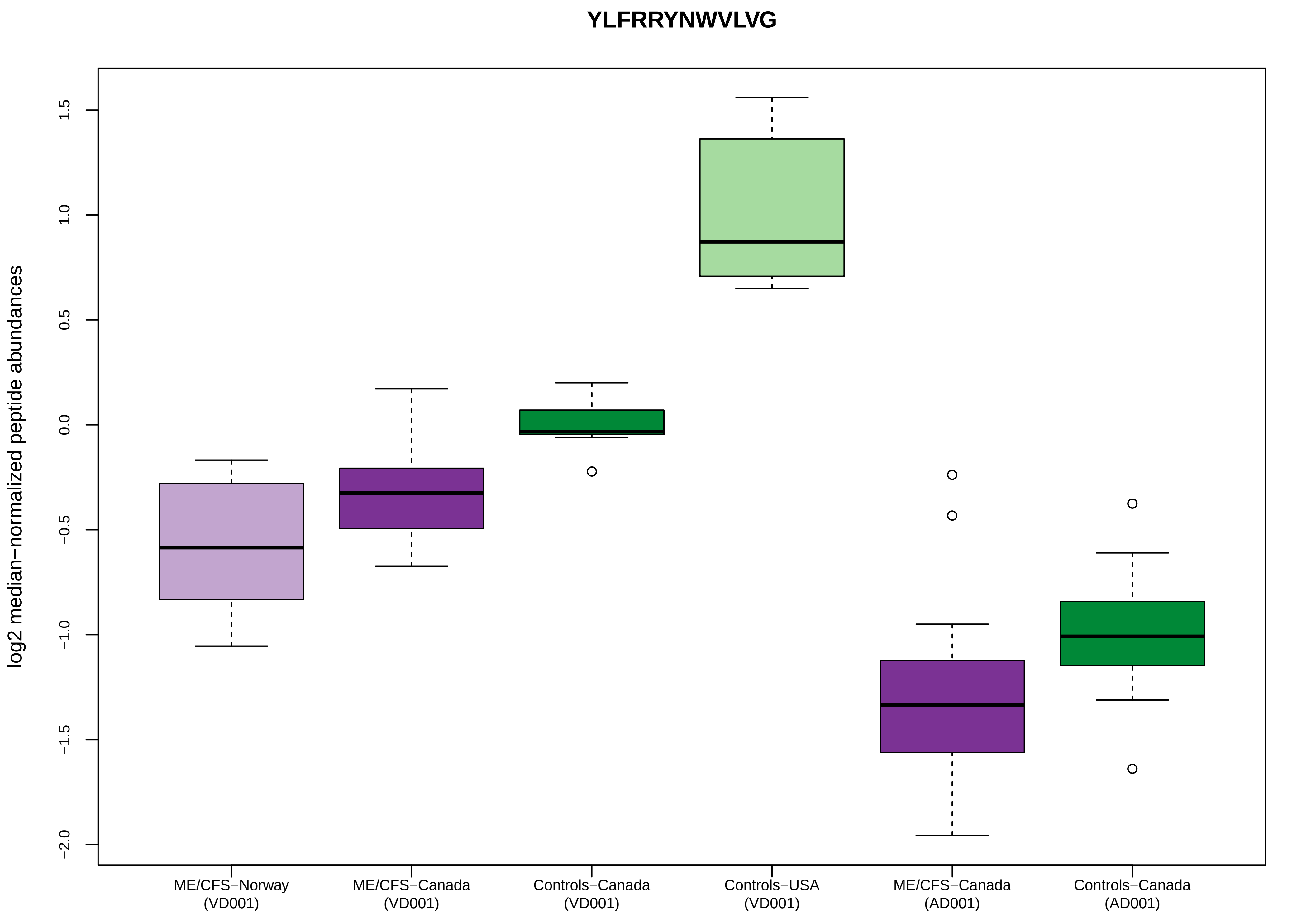

YLFRRYNWVLVG
log2 median−normalized peptide abundances
1.5
1.0
0.5
0.0
−0.5
−1.0
−1.5
−2.0
ME/CFS−Norway (VD001)
ME/CFS−Canada (VD001)
Controls−Canada (VD001)
Controls−USA (VD001)
ME/CFS−Canada (AD001)
Controls−Canada (AD001)

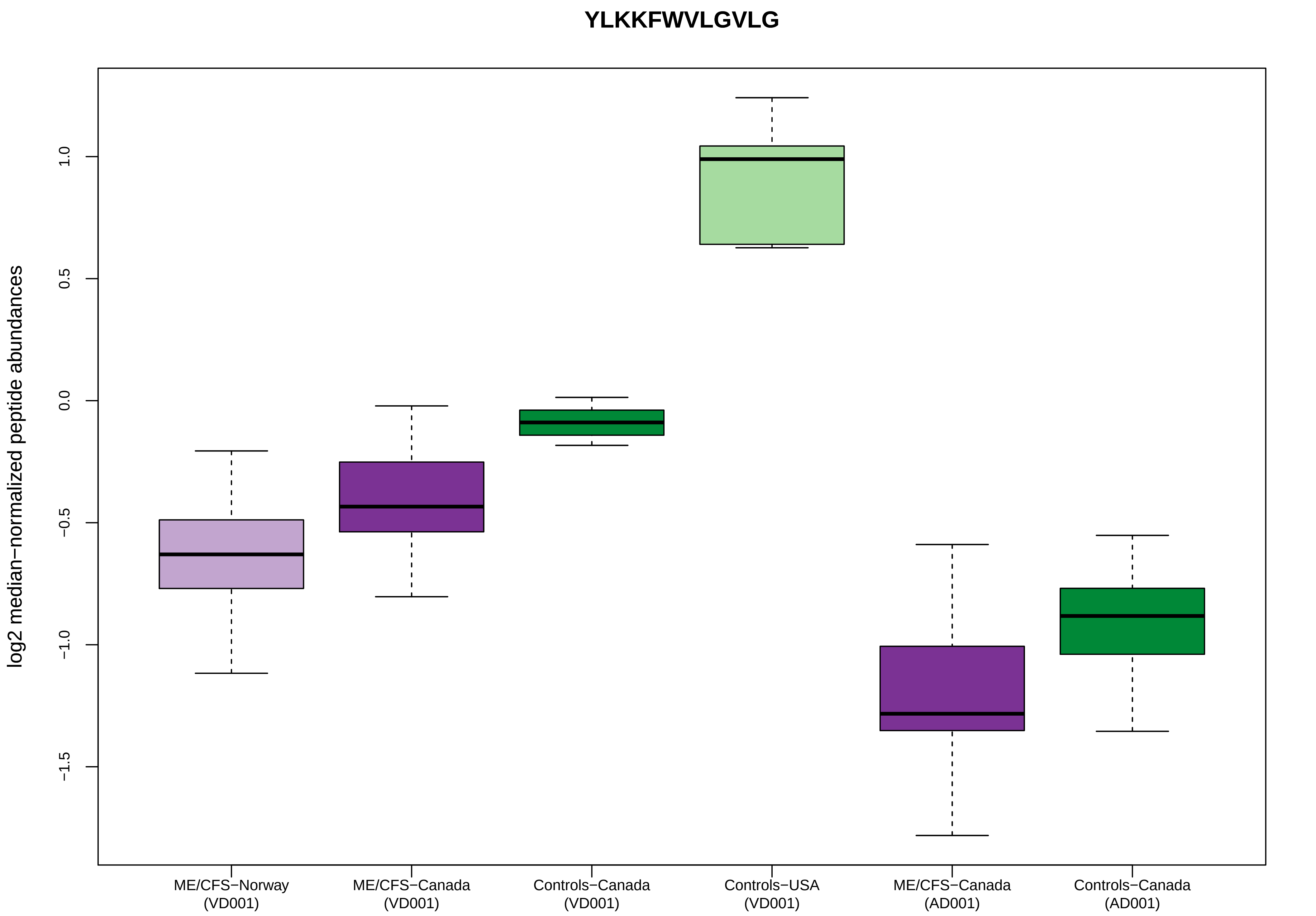

YLKKFWVLGVLG
log2 median−normalized peptide abundances
1.0
0.5
0.0
−0.5
−1.0
−1.5
ME/CFS−Norway (VD001)
ME/CFS−Canada (VD001)
Controls−Canada (VD001)
Controls−USA (VD001)
ME/CFS−Canada (AD001)
Controls−Canada (AD001)

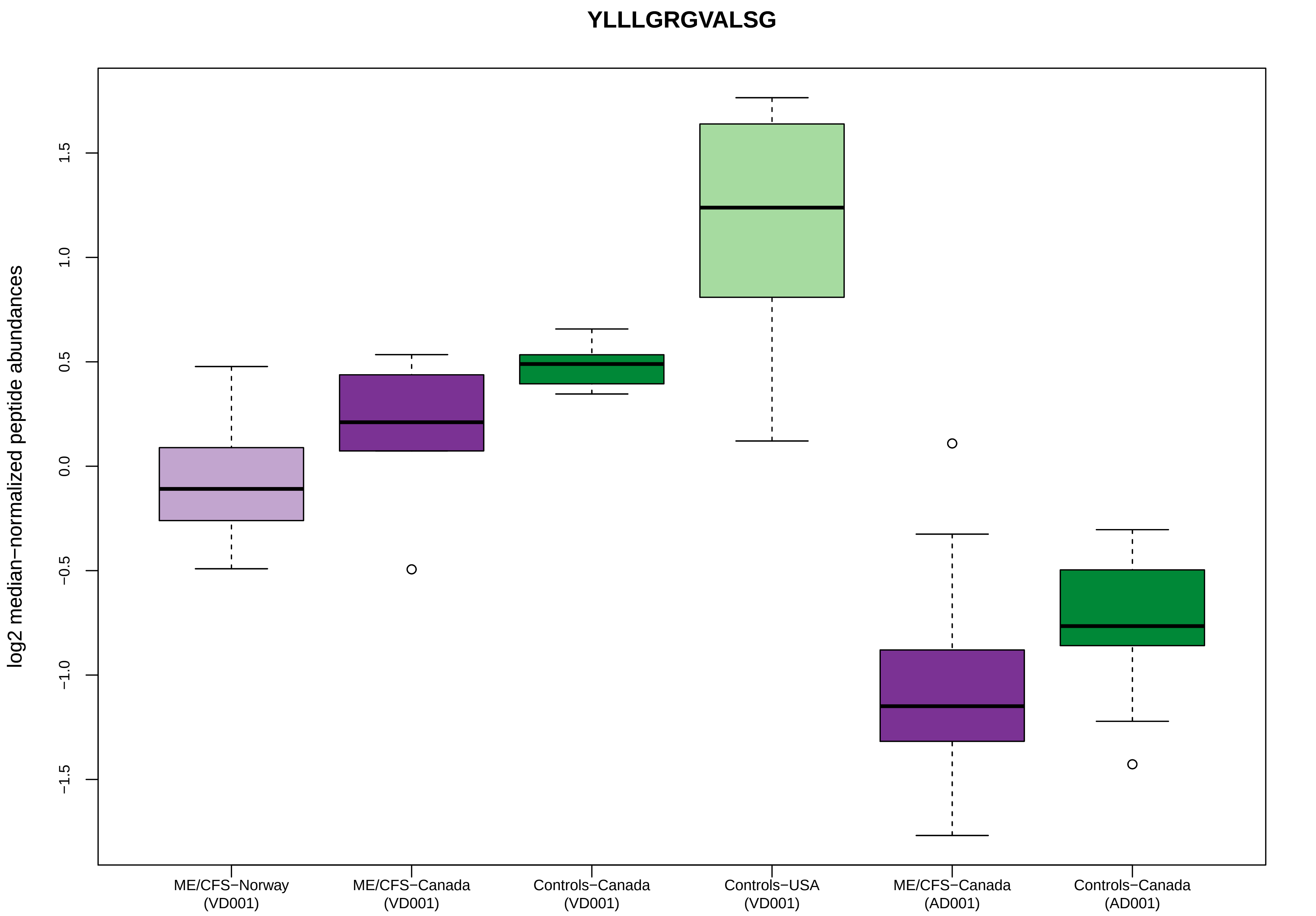

YLLLGRGVALSG
log2 median−normalized peptide abundances
−1.5
−1.0
−0.5
0.0
0.5
1.0
1.5
ME/CFS−Norway (VD001)
ME/CFS−Canada (VD001)
Controls−Canada (VD001)
Controls−USA (VD001)
ME/CFS−Canada (AD001)
Controls−Canada (AD001)

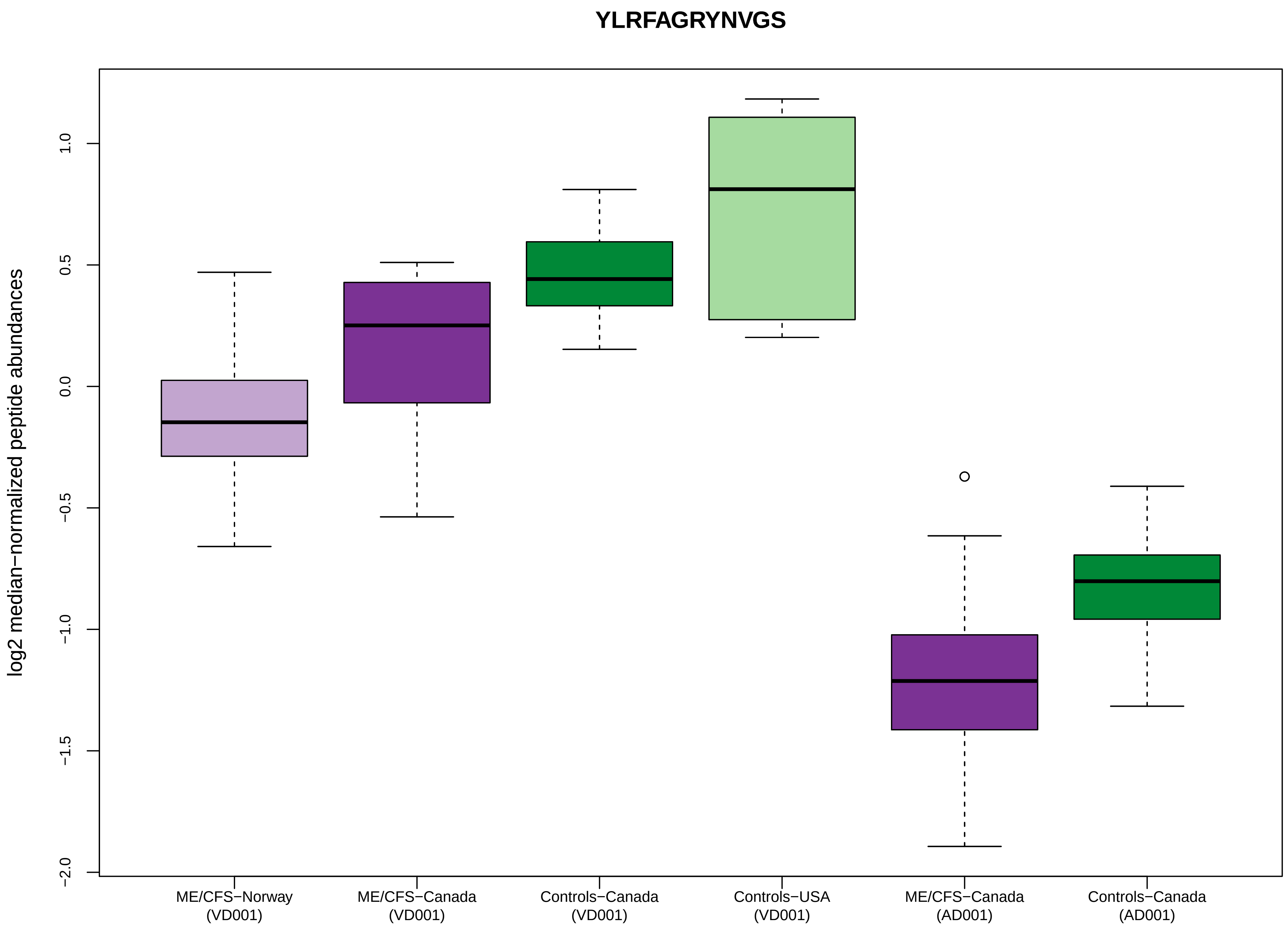
YLRFAGRYNVGS
log2 median−normalized peptide abundances
1.0
0.5
0.0
−0.5
−1.0
−1.5
−2.0
ME/CFS−Norway (VD001)
ME/CFS−Canada (VD001)
Controls−Canada (VD001)
Controls−USA (VD001)
ME/CFS−Canada (AD001)
Controls−Canada (AD001)

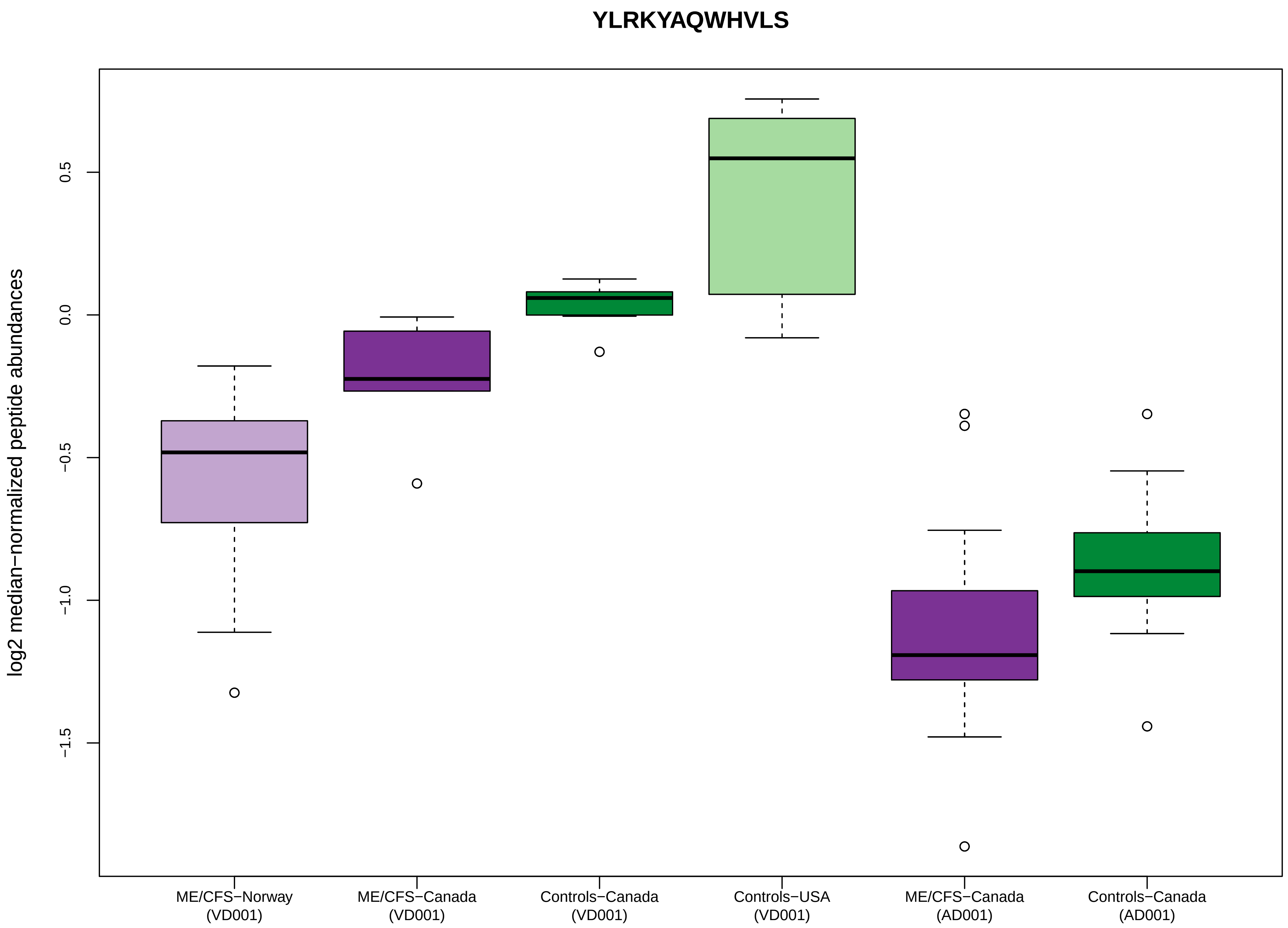
YLRKYAQWHVLS
log2 median−normalized peptide abundances
0.5
0.0
−0.5
−1.0
−1.5
ME/CFS−Norway (VD001)
ME/CFS−Canada (VD001)
Controls−Canada (VD001)
Controls−USA (VD001)
ME/CFS−Canada (AD001)
Controls−Canada (AD001)

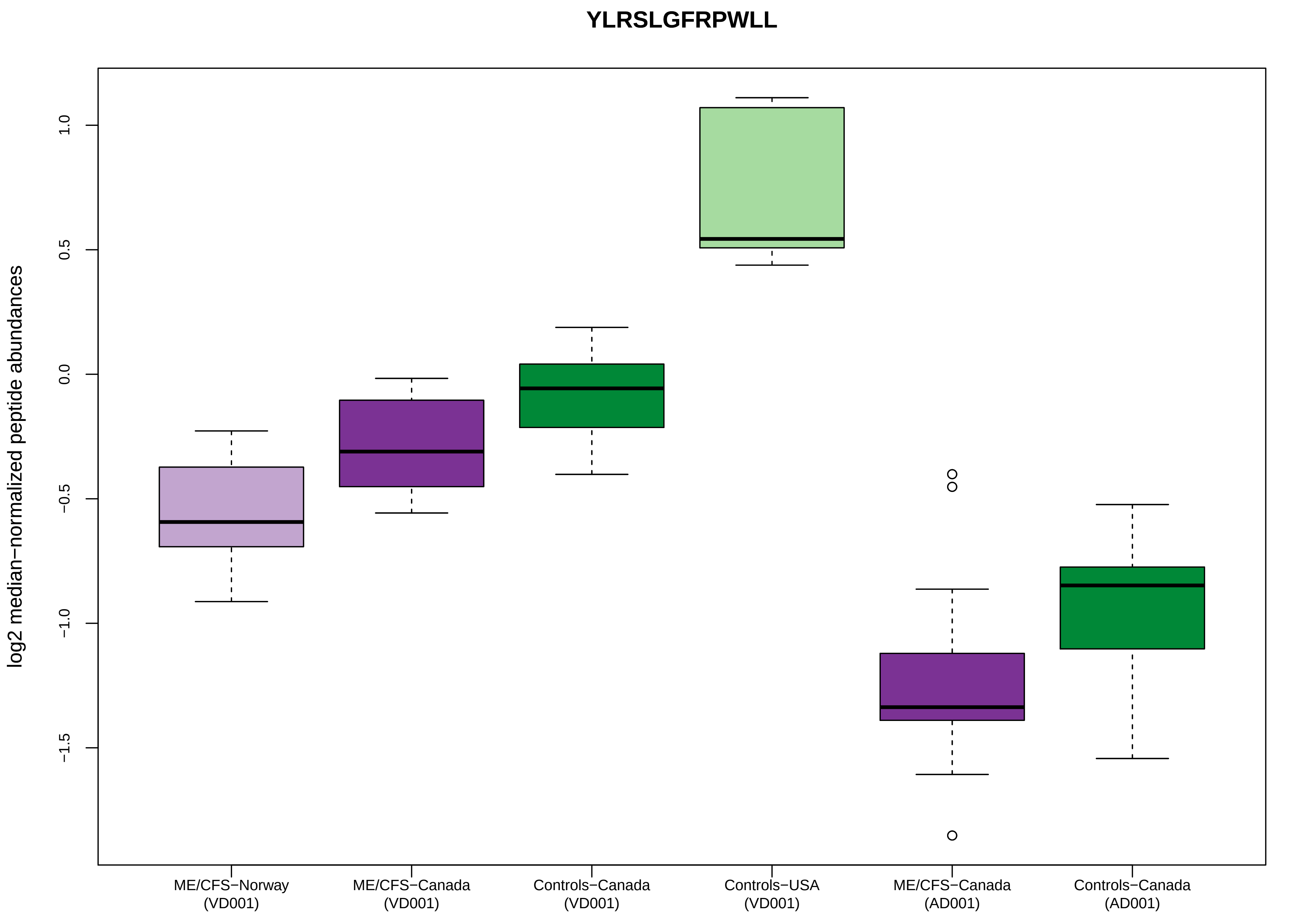

YLRSLGFRPWLL
log2 median−normalized peptide abundances
1.0
0.5
0.0
−0.5
−1.0
−1.5
ME/CFS−Norway (VD001)
ME/CFS−Canada (VD001)
Controls−Canada (VD001)
Controls−USA (VD001)
ME/CFS−Canada (AD001)
Controls−Canada (AD001)

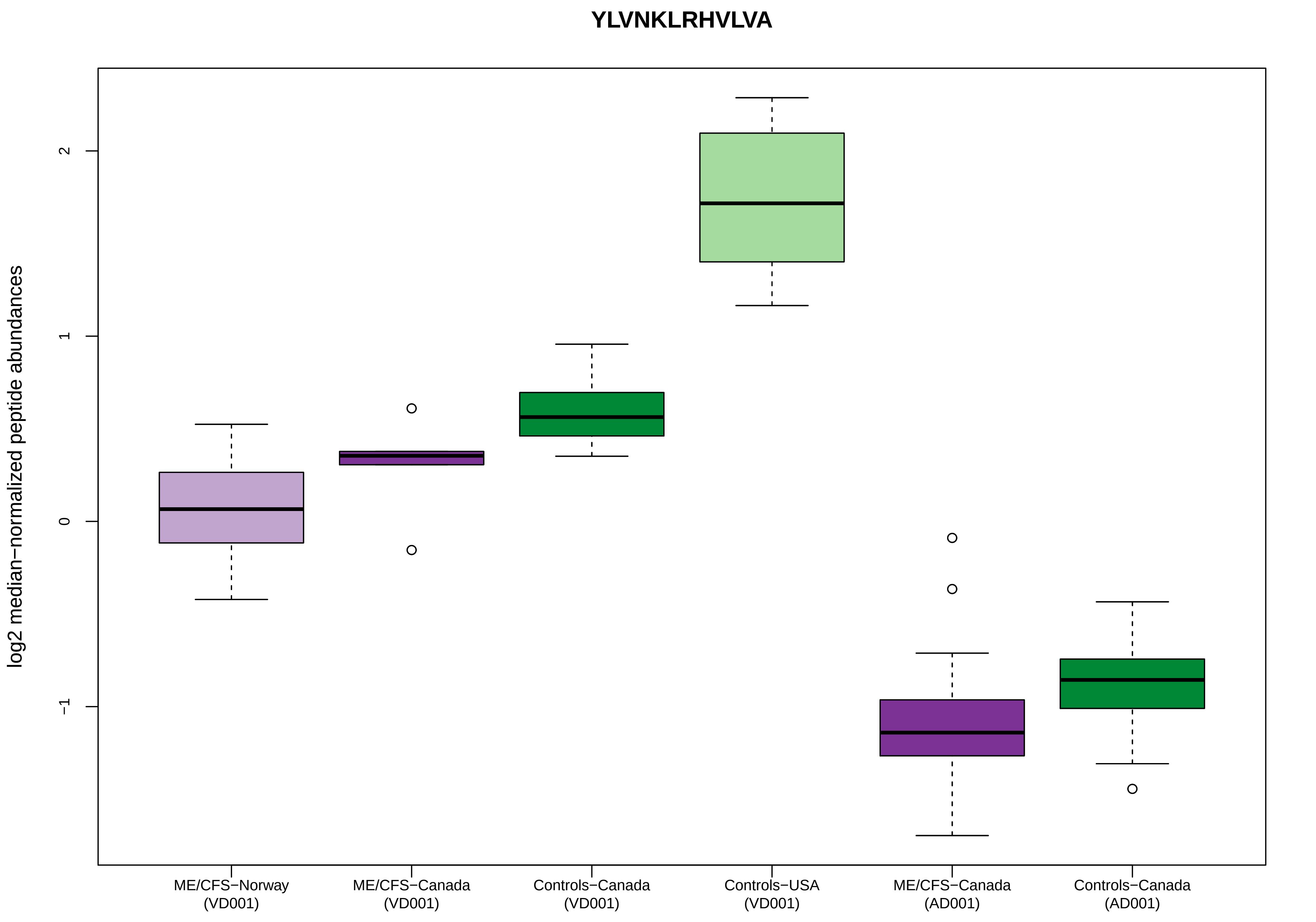
YLVNKLRHVLVA
log2 median−normalized peptide abundances
2
1
0
−1
ME/CFS−Norway (VD001)
ME/CFS−Canada (VD001)
Controls−Canada (VD001)
Controls−USA (VD001)
ME/CFS−Canada (AD001)
Controls−Canada (AD001)

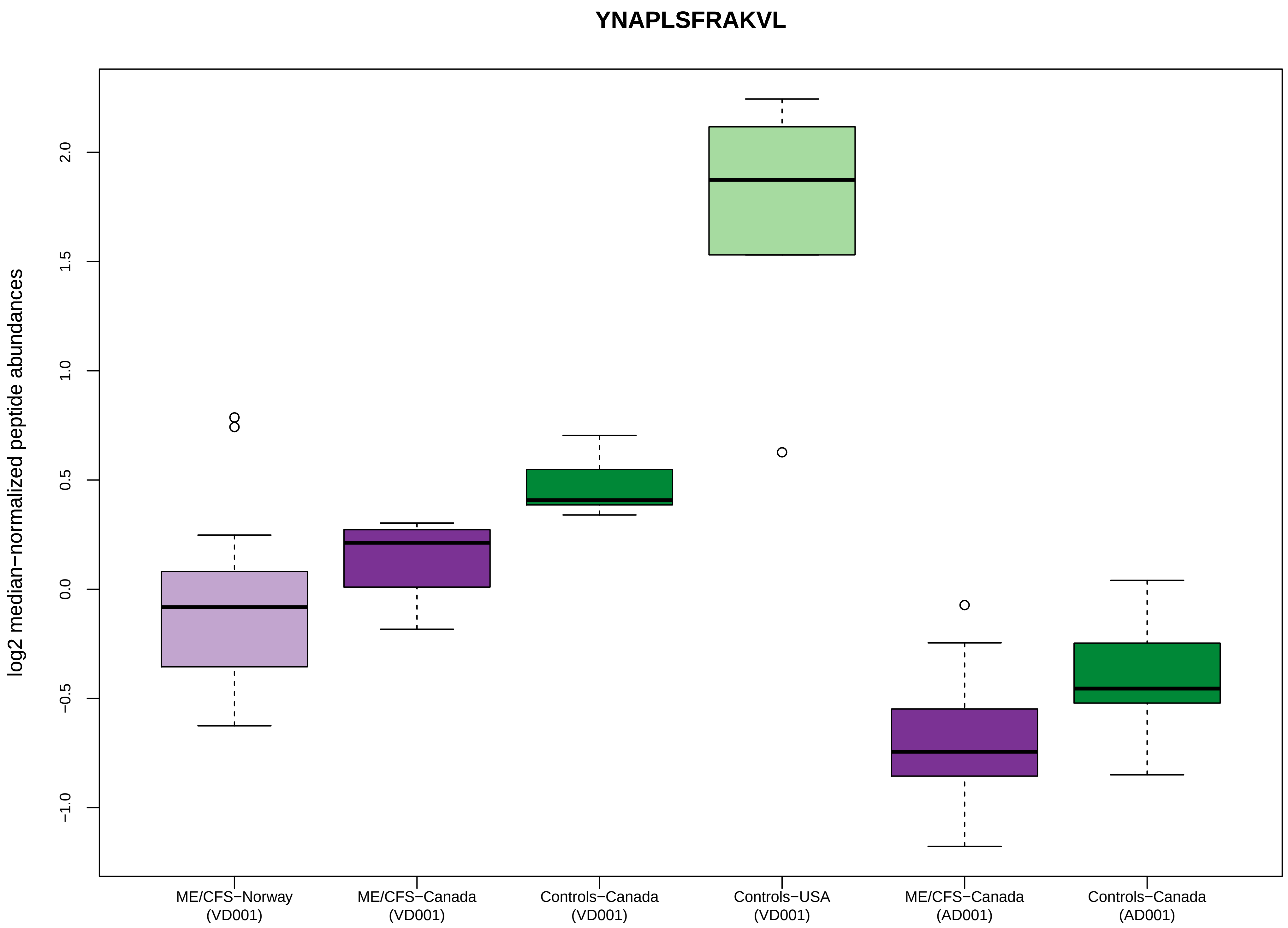
YNAPLSFRAKVL
log2 median−normalized peptide abundances
2.0
1.5
1.0
0.5
0.0
−0.5
−1.0
ME/CFS−Norway (VD001)
ME/CFS−Canada (VD001)
Controls−Canada (VD001)
Controls−USA (VD001)
ME/CFS−Canada (AD001)
Controls−Canada (AD001)

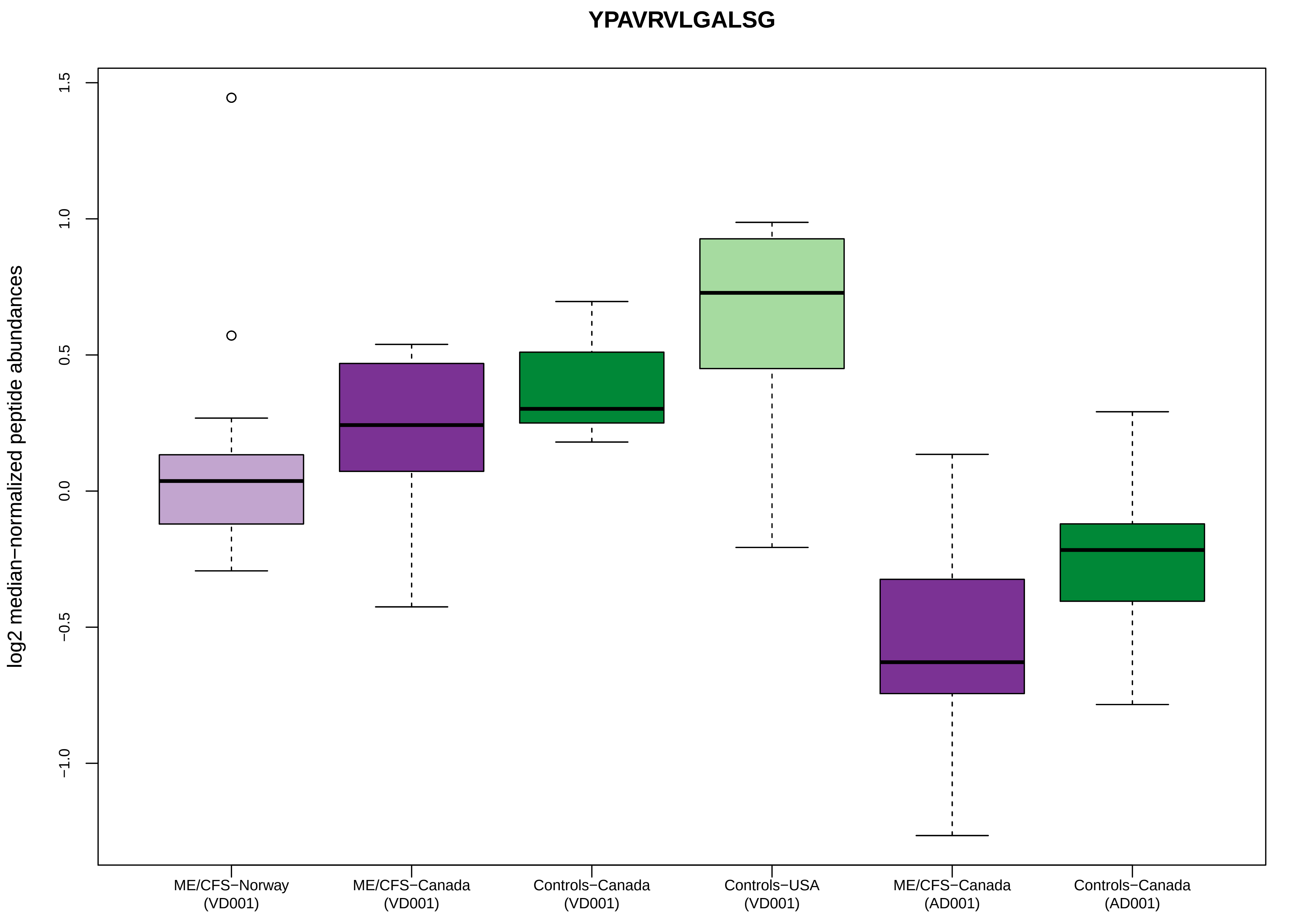

YPAVRVLGALSG
log2 median−normalized peptide abundances
1.5
1.0
0.5
0.0
−0.5
−1.0
ME/CFS−Norway (VD001)
ME/CFS−Canada (VD001)
Controls−Canada (VD001)
Controls−USA (VD001)
ME/CFS−Canada (AD001)
Controls−Canada (AD001)

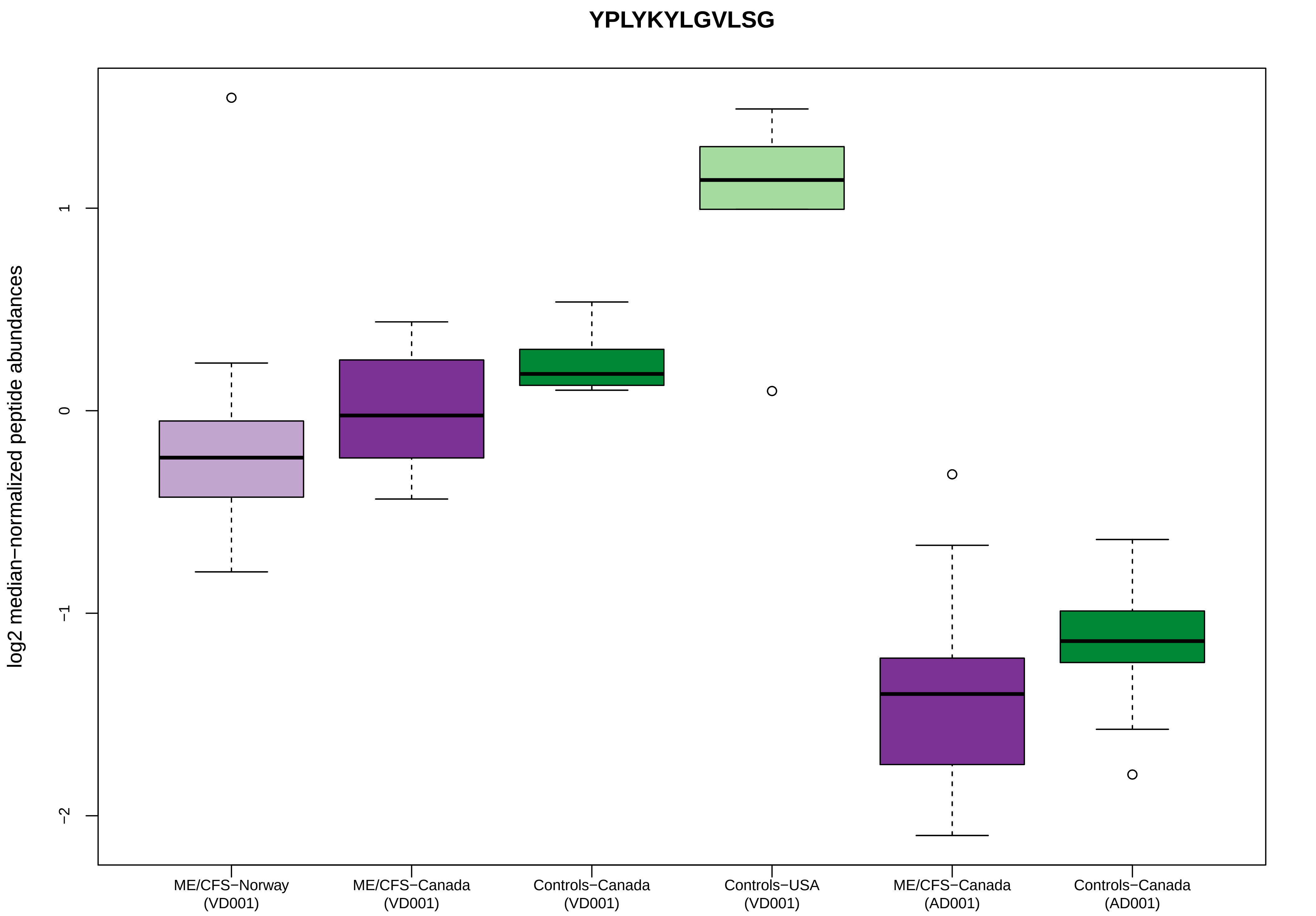

YPLYKYLGVLSG
log2 median−normalized peptide abundances
1
0
−1
−2
ME/CFS−Norway (VD001)
ME/CFS−Canada (VD001)
Controls−Canada (VD001)
Controls−USA (VD001)
ME/CFS−Canada (AD001)
Controls−Canada (AD001)

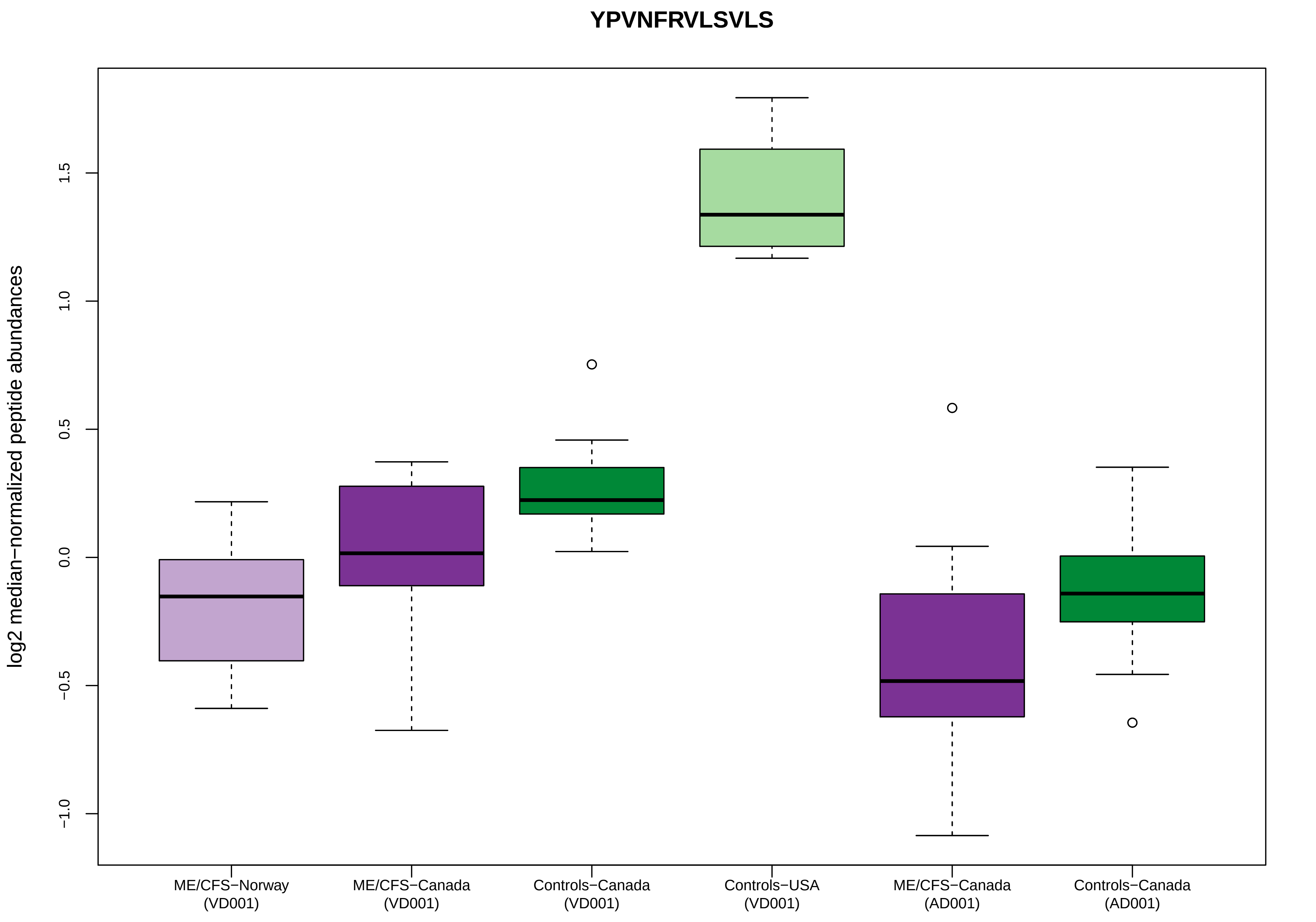

YPVNFRVLSVLS
log2 median−normalized peptide abundances
1.5
1.0
0.5
0.0
−0.5
−1.0
ME/CFS−Norway (VD001)
ME/CFS−Canada (VD001)
Controls−Canada (VD001)
Controls−USA (VD001)
ME/CFS−Canada (AD001)
Controls−Canada (AD001)

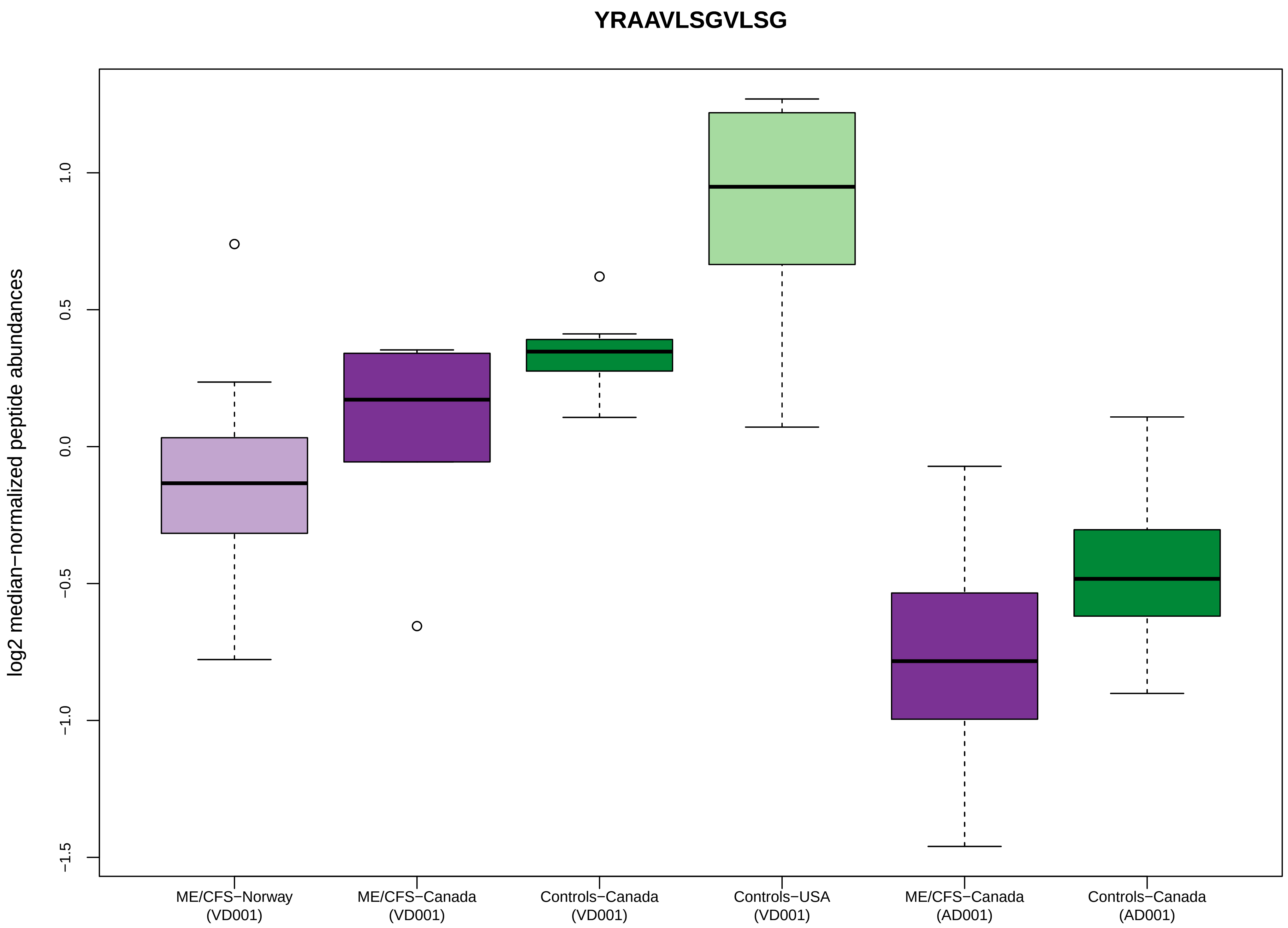

YRAAVLSGVLSG
log2 median−normalized peptide abundances
1.0
0.5
0.0
−0.5
−1.0
−1.5
ME/CFS−Norway (VD001)
ME/CFS−Canada (VD001)
Controls−Canada (VD001)
Controls−USA (VD001)
ME/CFS−Canada (AD001)
Controls−Canada (AD001)

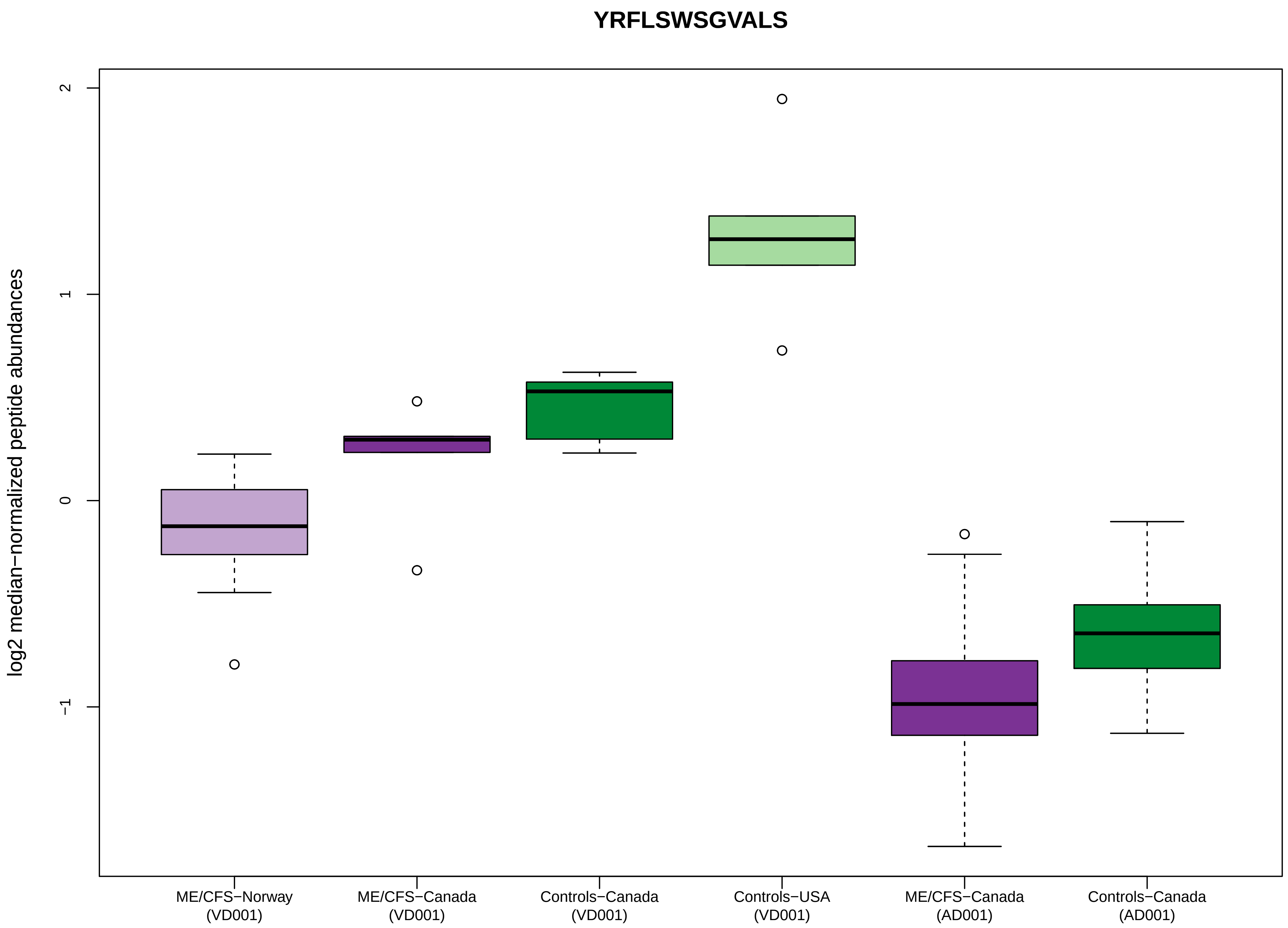

YRFLSWSGVALS
log2 median−normalized peptide abundances
2
1
0
−1
ME/CFS−Norway (VD001)
ME/CFS−Canada (VD001)
Controls−Canada (VD001)
Controls−USA (VD001)
ME/CFS−Canada (AD001)
Controls−Canada (AD001)

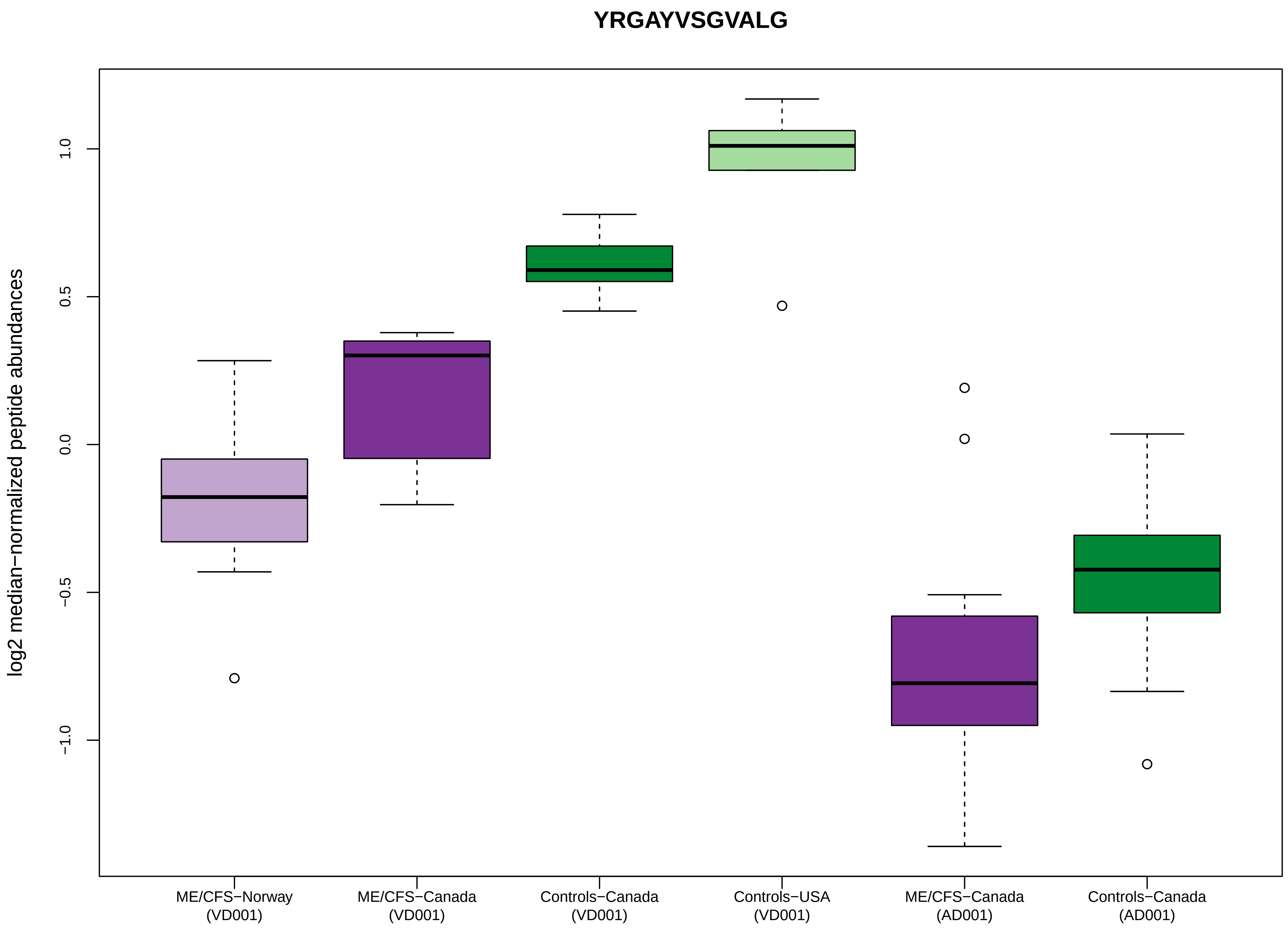
YRGAYVSGVALG
log2 median−normalized peptide abundances
1.0
0.5
0.0
−0.5
−1.0
ME/CFS−Norway (VD001)
ME/CFS−Canada (VD001)
Controls−Canada (VD001)
Controls−USA (VD001)
ME/CFS−Canada (AD001)
Controls−Canada (AD001)

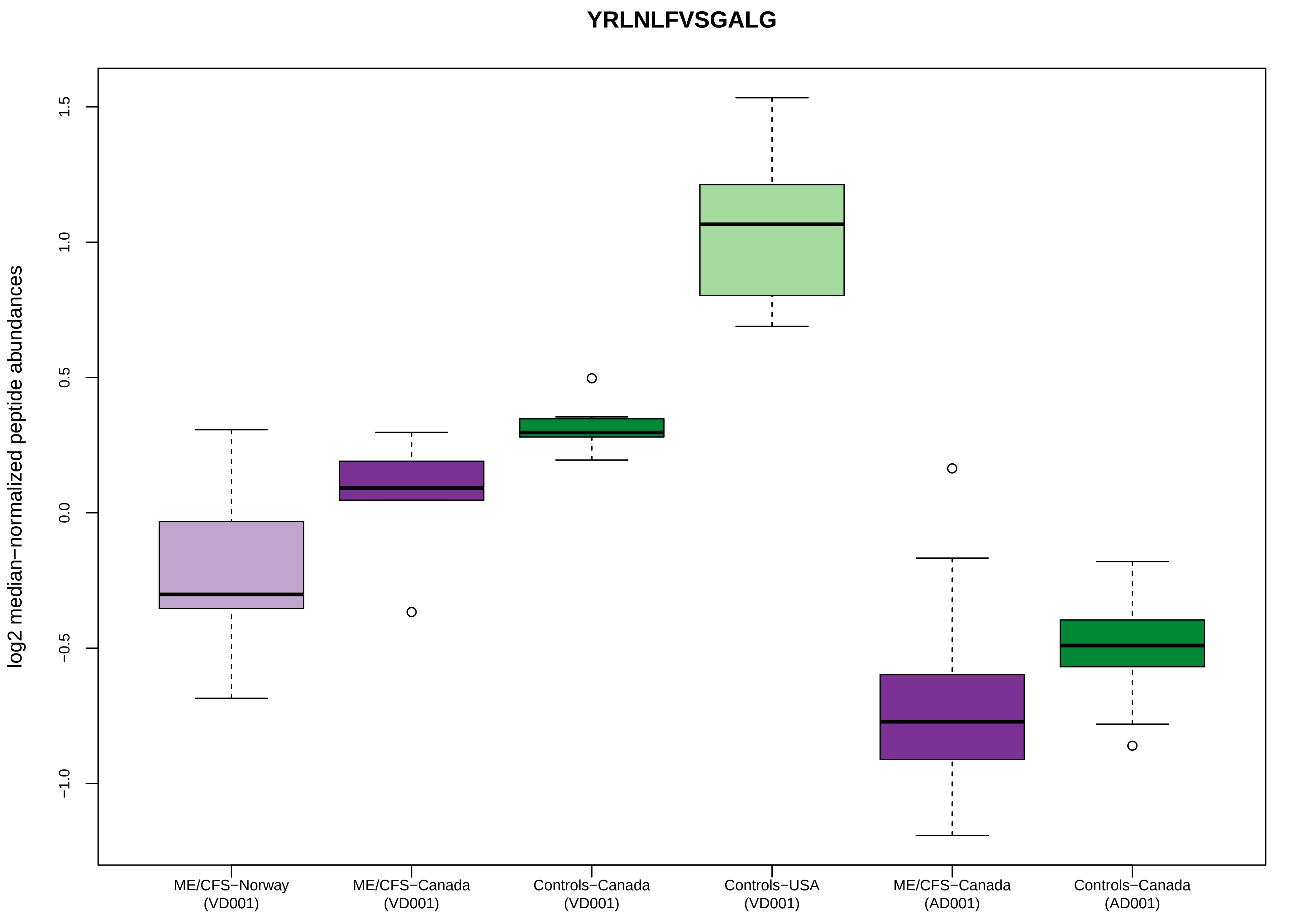
YRLNLFVSGALG
log2 median−normalized peptide abundances
1.5
1.0
0.5
0.0
−0.5
−1.0
ME/CFS−Norway (VD001)
ME/CFS−Canada (VD001)
Controls−Canada (VD001)
Controls−USA (VD001)
ME/CFS−Canada (AD001)
Controls−Canada (AD001)

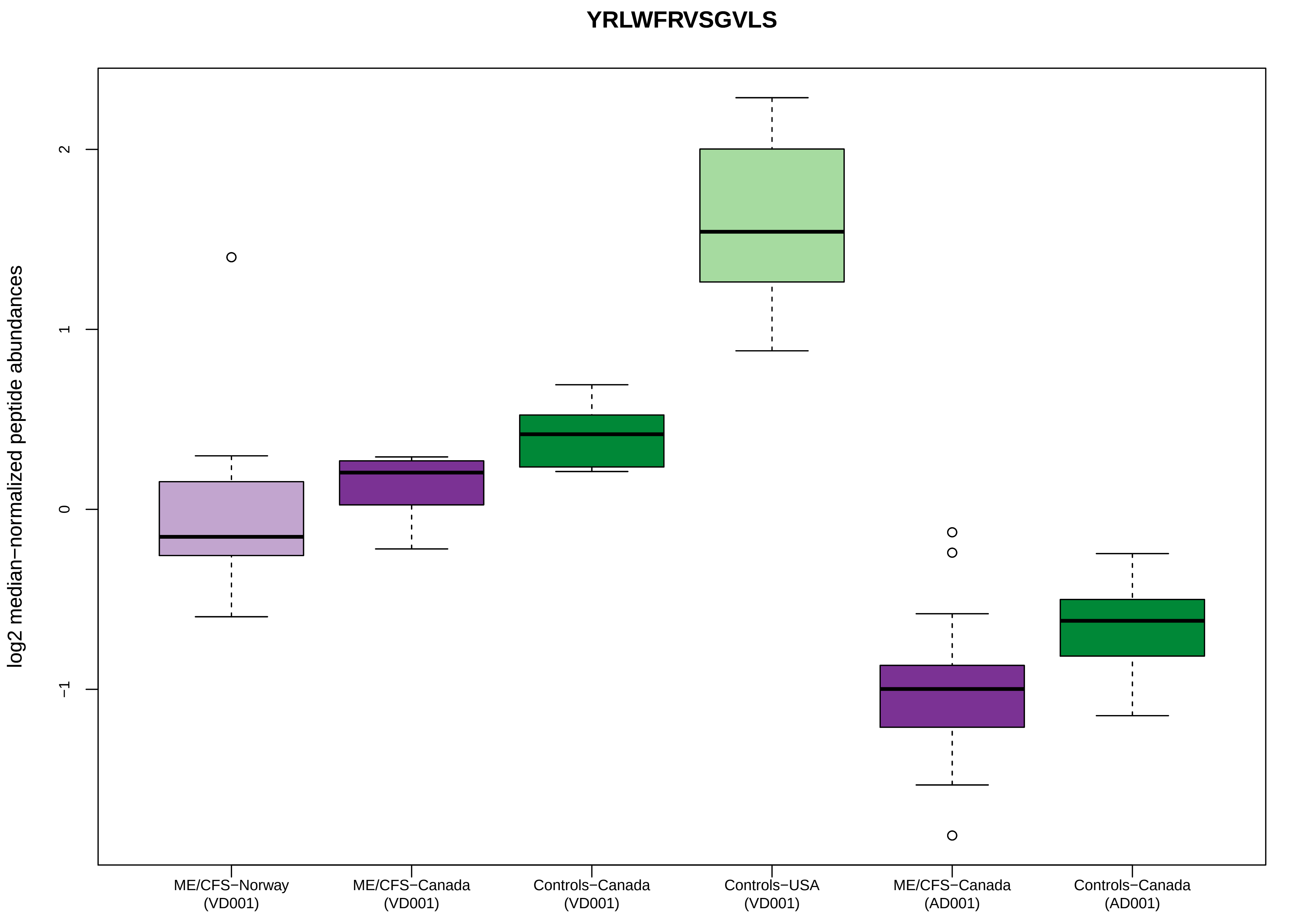

YRLWFRVSGVLS
log2 median−normalized peptide abundances
2
1
0
−1
ME/CFS−Norway (VD001)
ME/CFS−Canada (VD001)
Controls−Canada (VD001)
Controls−USA (VD001)
ME/CFS−Canada (AD001)
Controls−Canada (AD001)

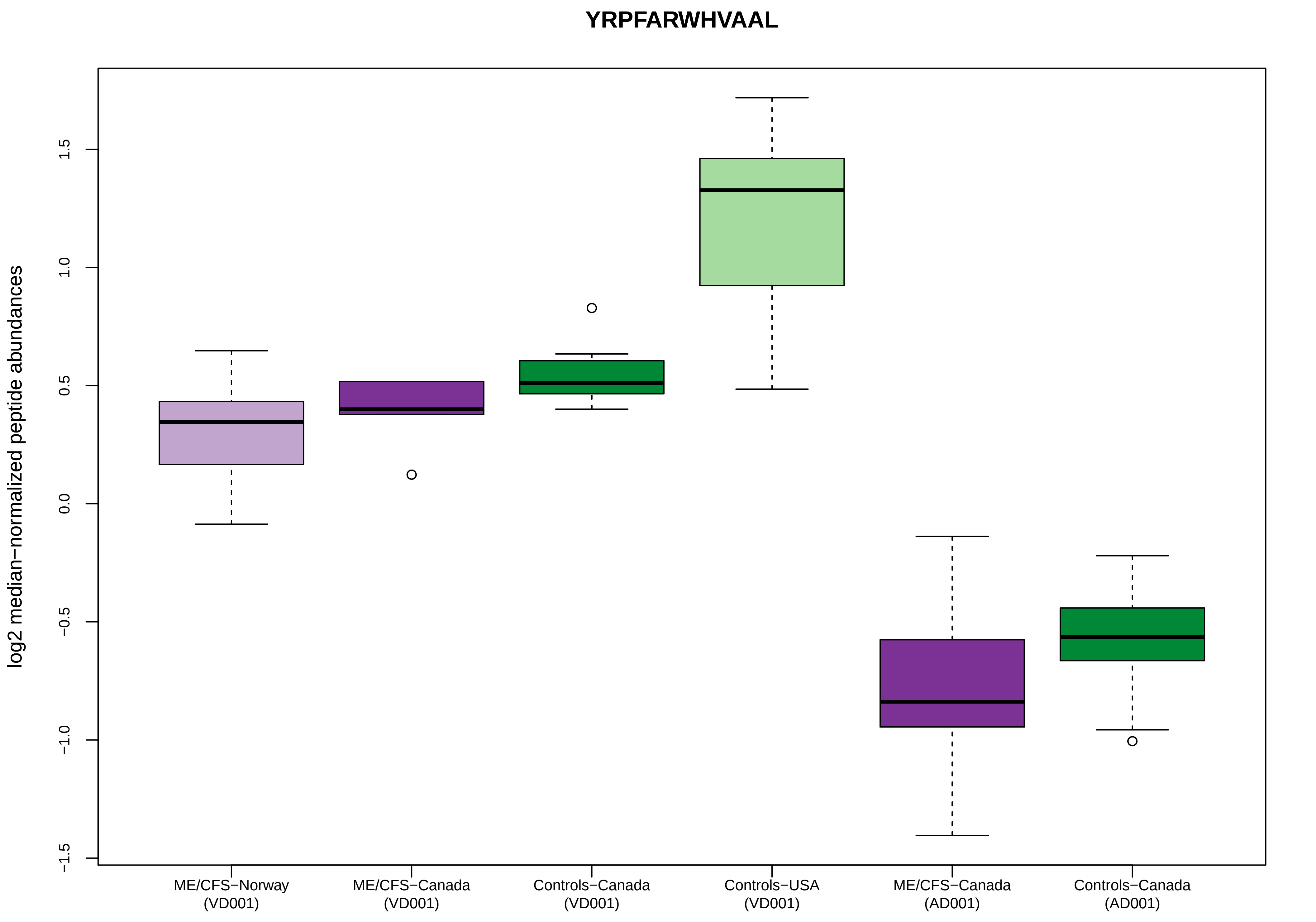

YRPFARWHVAAL
log2 median−normalized peptide abundances
1.5
1.0
0.5
0.0
−0.5
−1.0
−1.5
ME/CFS−Norway (VD001)
ME/CFS−Canada (VD001)
Controls−Canada (VD001)
Controls−USA (VD001)
ME/CFS−Canada (AD001)
Controls−Canada (AD001)

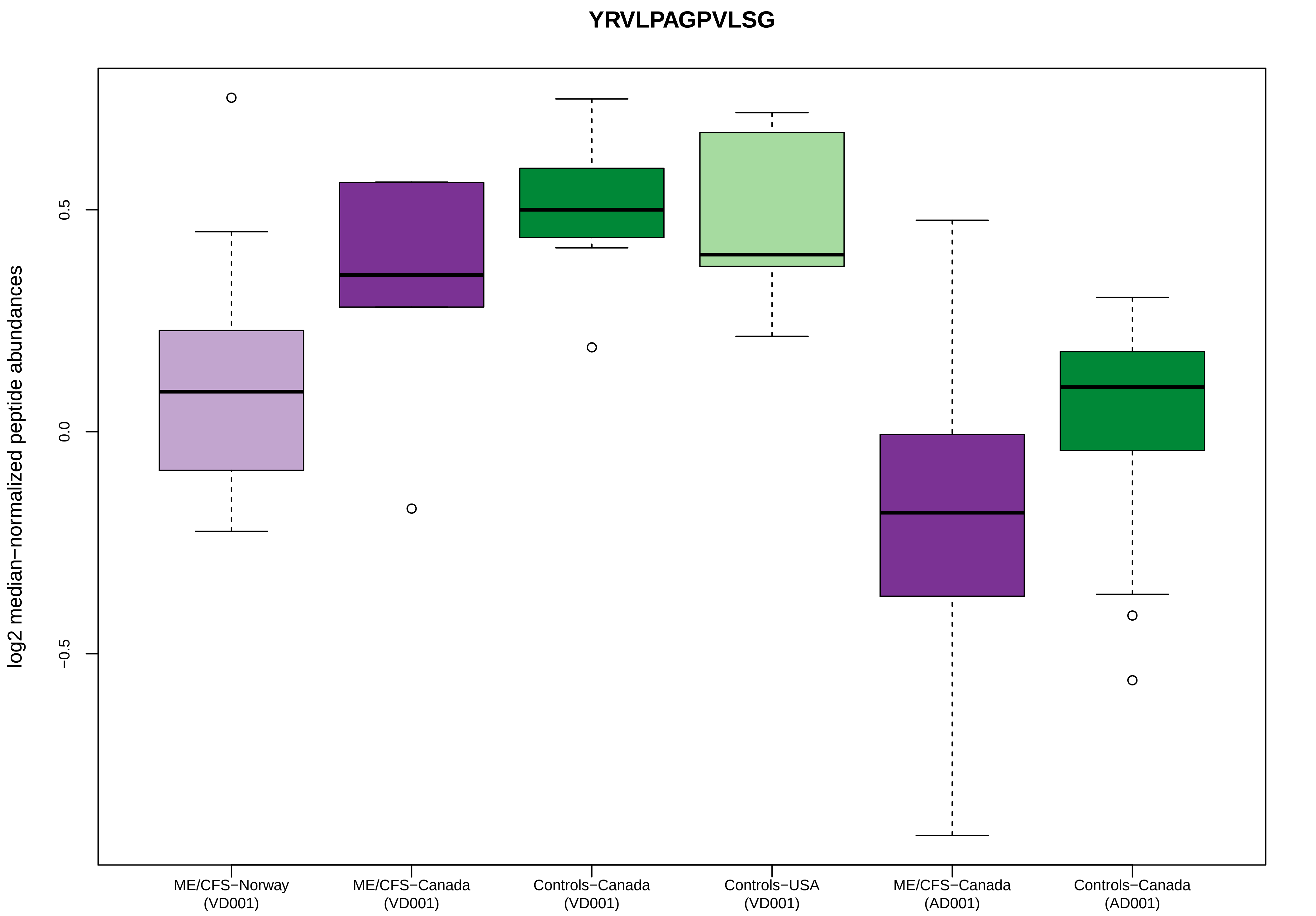
YRVLPAGPVLSG
log2 median−normalized peptide abundances
0.5
0.0
−0.5
ME/CFS−Norway (VD001)
ME/CFS−Canada (VD001)
Controls−Canada (VD001)
Controls−USA (VD001)
ME/CFS−Canada (AD001)
Controls−Canada (AD001)

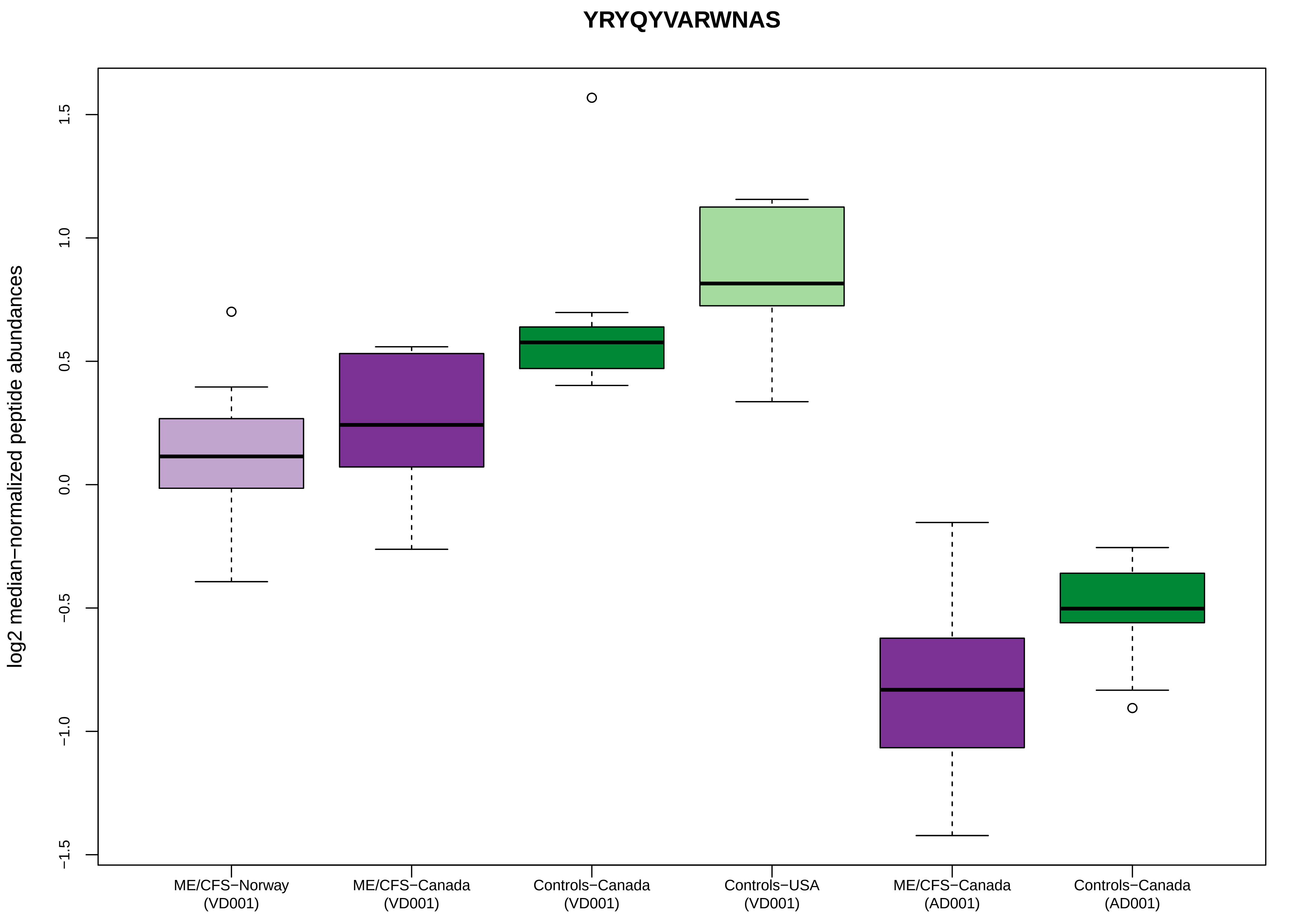

YRYQYVARWNAS
log2 median−normalized peptide abundances
1.5
1.0
0.5
0.0
−0.5
−1.0
−1.5
ME/CFS−Norway (VD001)
ME/CFS−Canada (VD001)
Controls−Canada (VD001)
Controls−USA (VD001)
ME/CFS−Canada (AD001)
Controls−Canada (AD001)

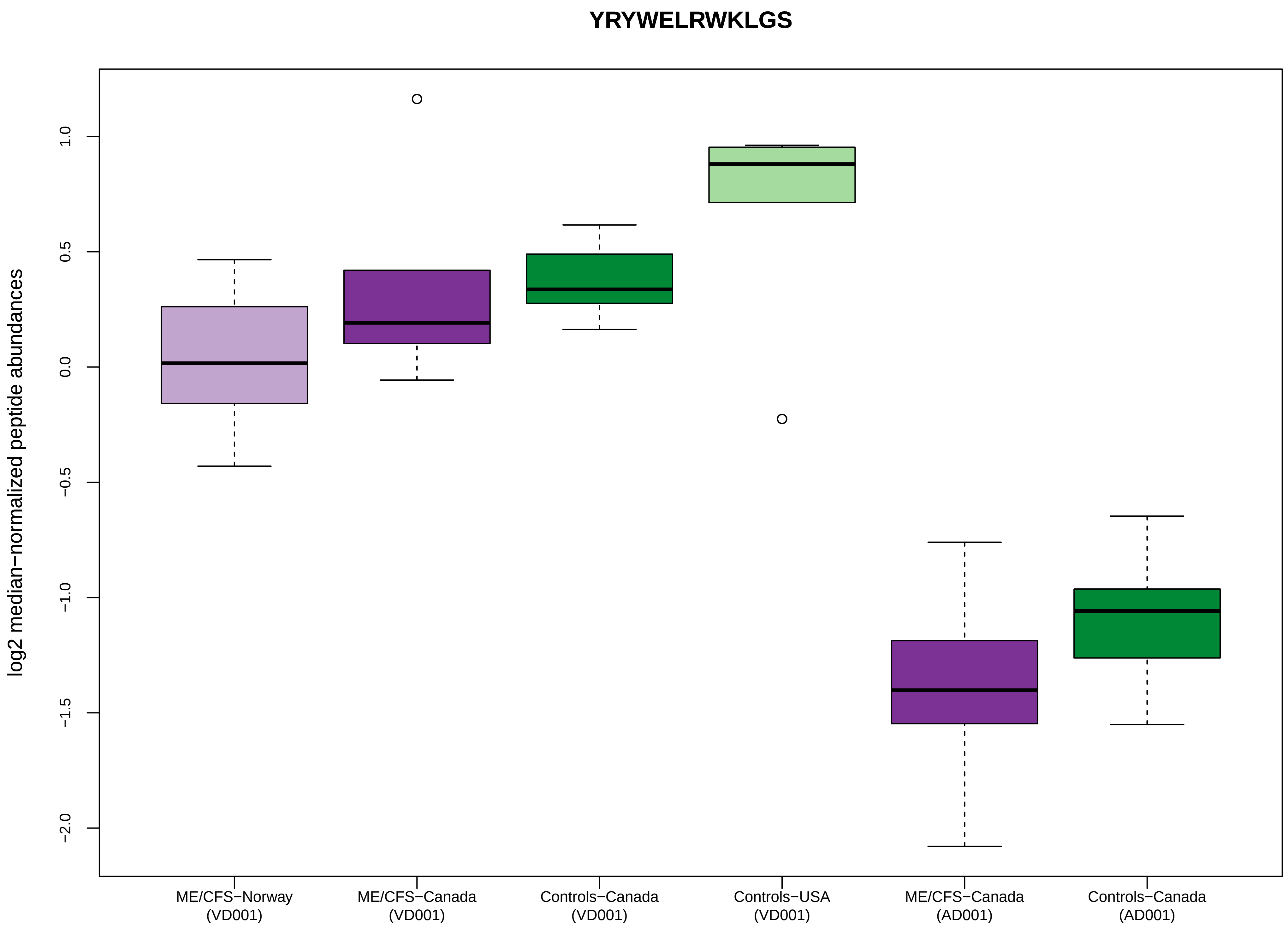

YRYWELRWKLGS
log2 median−normalized peptide abundances
1.0
0.5
0.0
−0.5
−1.0
−1.5
−2.0
ME/CFS−Norway (VD001)
ME/CFS−Canada (VD001)
Controls−Canada (VD001)
Controls−USA (VD001)
ME/CFS−Canada (AD001)
Controls−Canada (AD001)

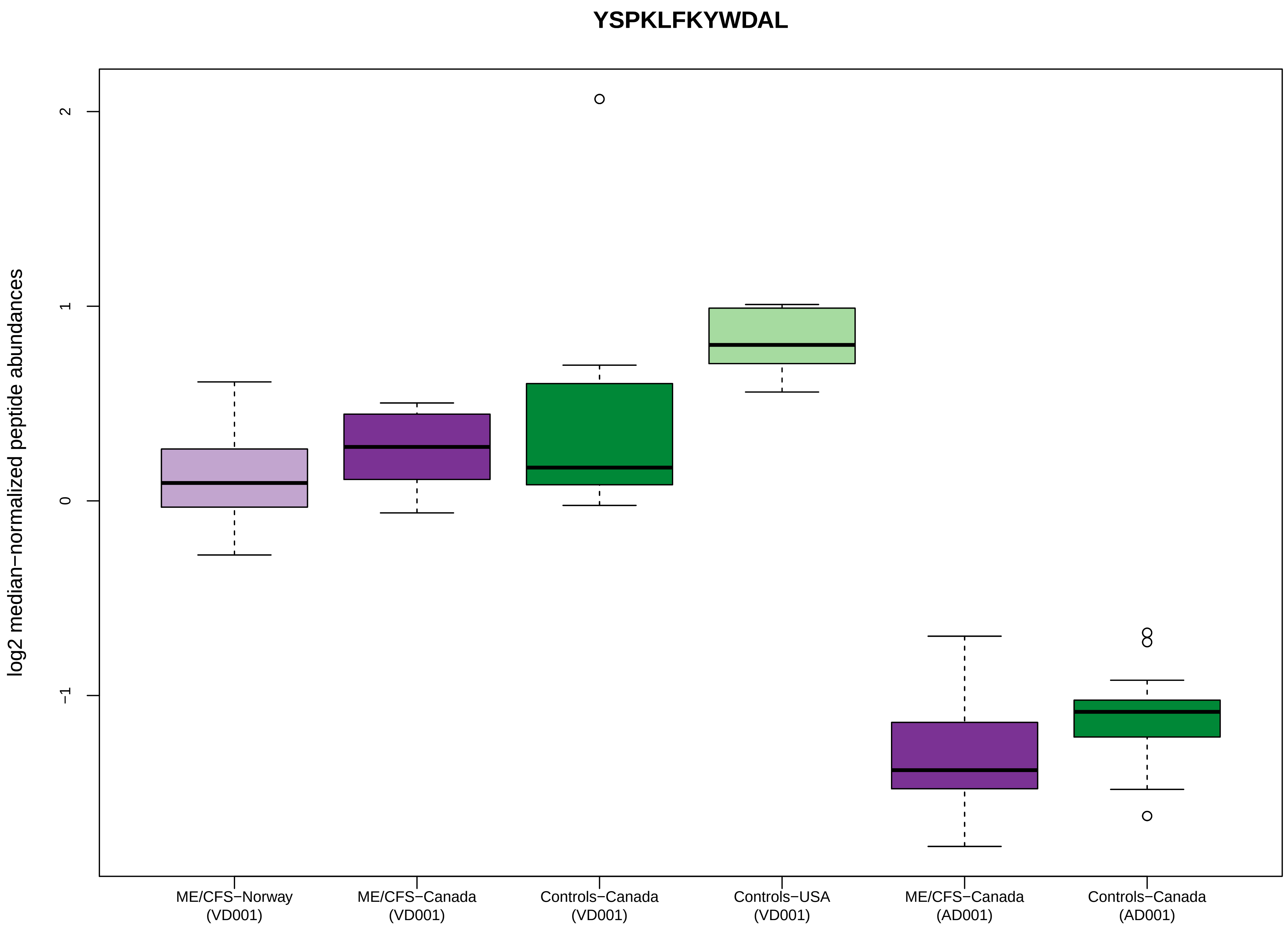

YSPKLFKYWDAL
log2 median−normalized peptide abundances
2
1
0
−1
ME/CFS−Norway (VD001)
ME/CFS−Canada (VD001)
Controls−Canada (VD001)
Controls−USA (VD001)
ME/CFS−Canada (AD001)
Controls−Canada (AD001)

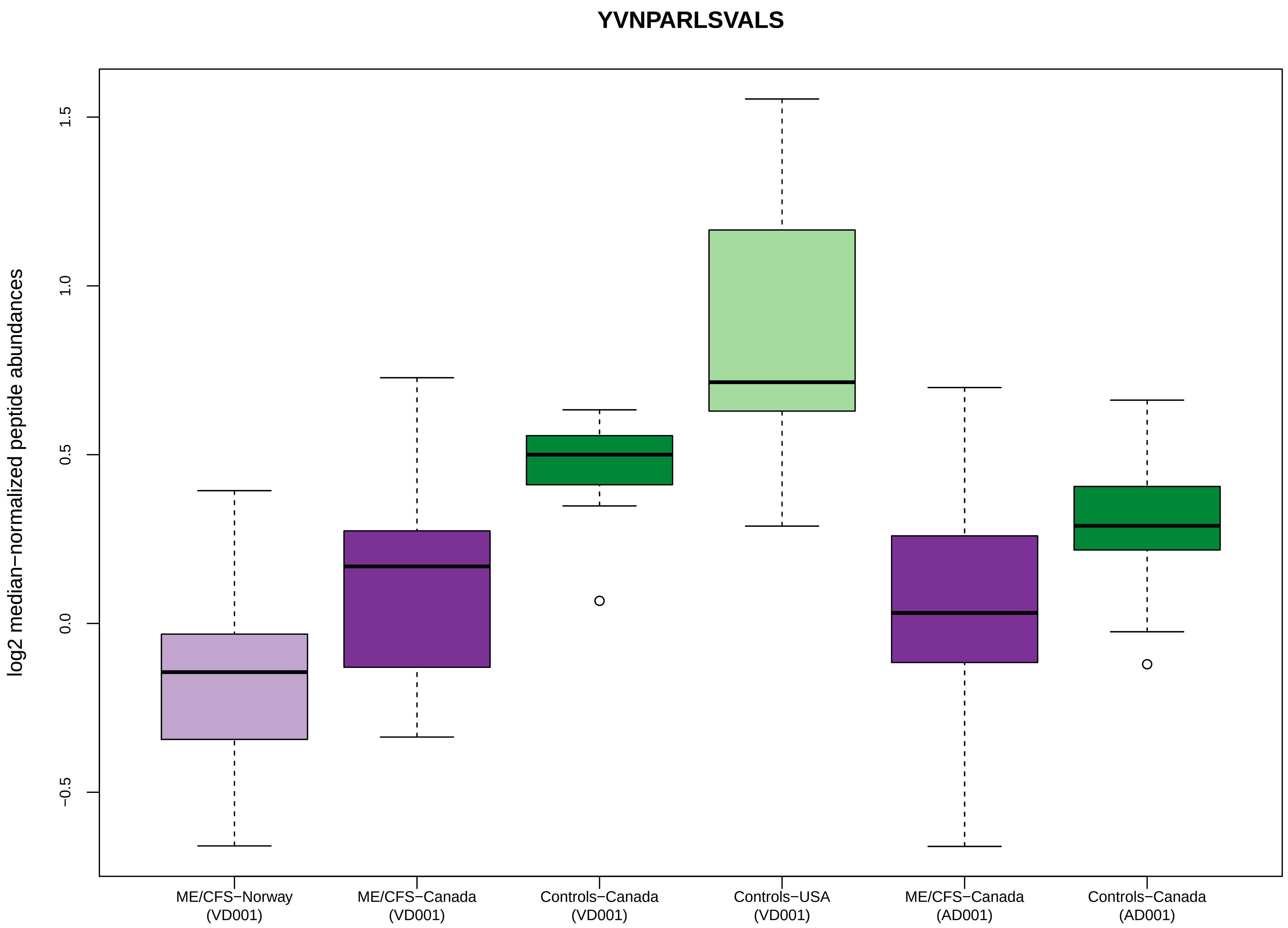

YVNPARLSVALS
log2 median−normalized peptide abundances
1.5
1.0
0.5
0.0
−0.5
ME/CFS−Norway (VD001)
ME/CFS−Canada (VD001)
Controls−Canada (VD001)
Controls−USA (VD001)
ME/CFS−Canada (AD001)
Controls−Canada (AD001)

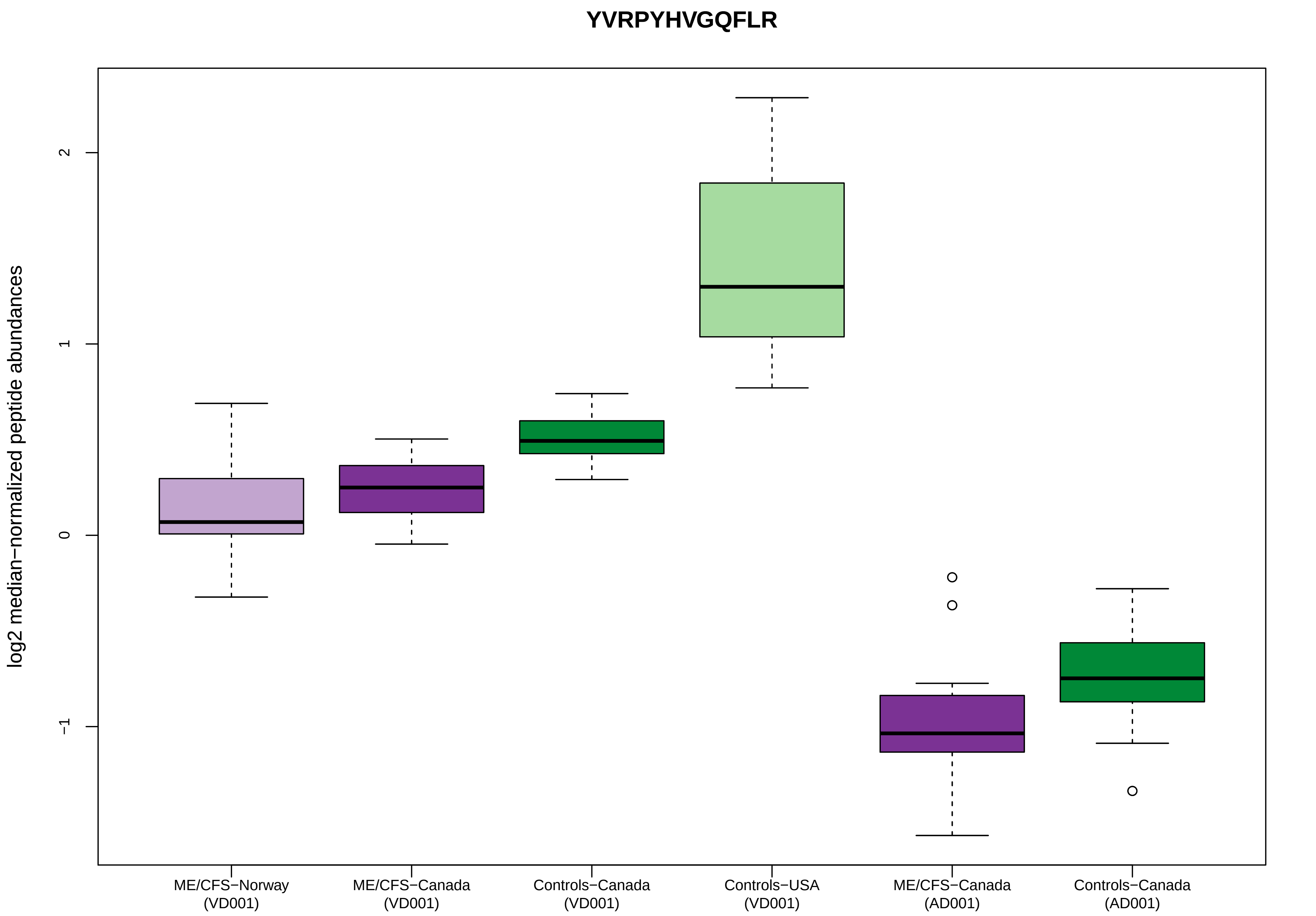

YVRPYHVGQFLR
log2 median−normalized peptide abundances
2
1
0
−1
ME/CFS−Norway (VD001)
ME/CFS−Canada (VD001)
Controls−Canada (VD001)
Controls−USA (VD001)
ME/CFS−Canada (AD001)
Controls−Canada (AD001)

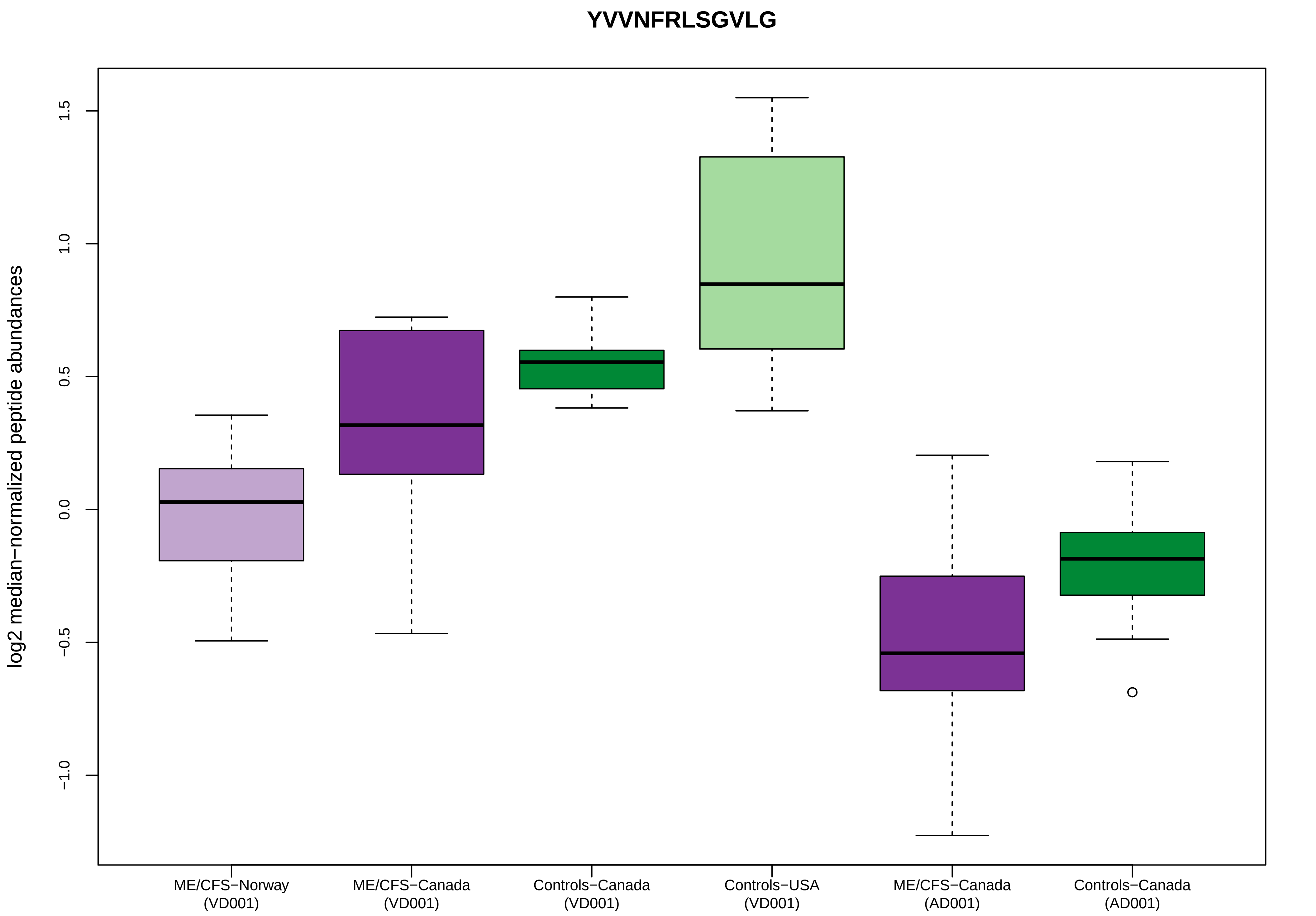

YVVNFRLSGVLG
log2 median−normalized peptide abundances
1.5
1.0
0.5
0.0
−0.5
−1.0
ME/CFS−Norway
(VD001)
ME/CFS−Canada
(VD001)
Controls−Canada
(VD001)
Controls−USA
(VD001)
ME/CFS−Canada
(AD001)
Controls−Canada
(AD001)

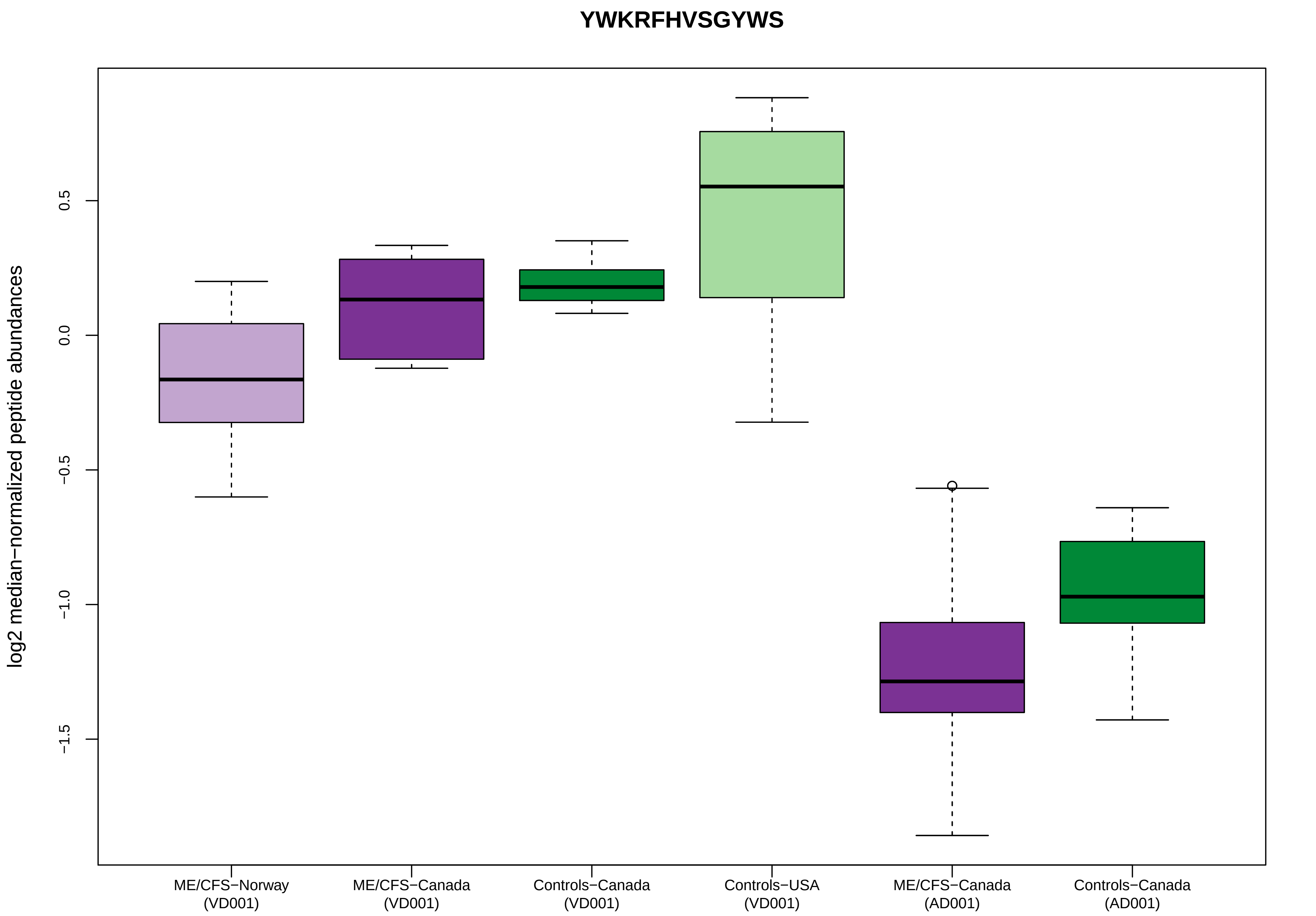

YWKRFHVSGYWS
log2 median−normalized peptide abundances
0.5
0.0
−0.5
−1.0
−1.5
ME/CFS−Norway (VD001)
ME/CFS−Canada (VD001)
Controls−Canada (VD001)
Controls−USA (VD001)
ME/CFS−Canada (AD001)
Controls−Canada (AD001)

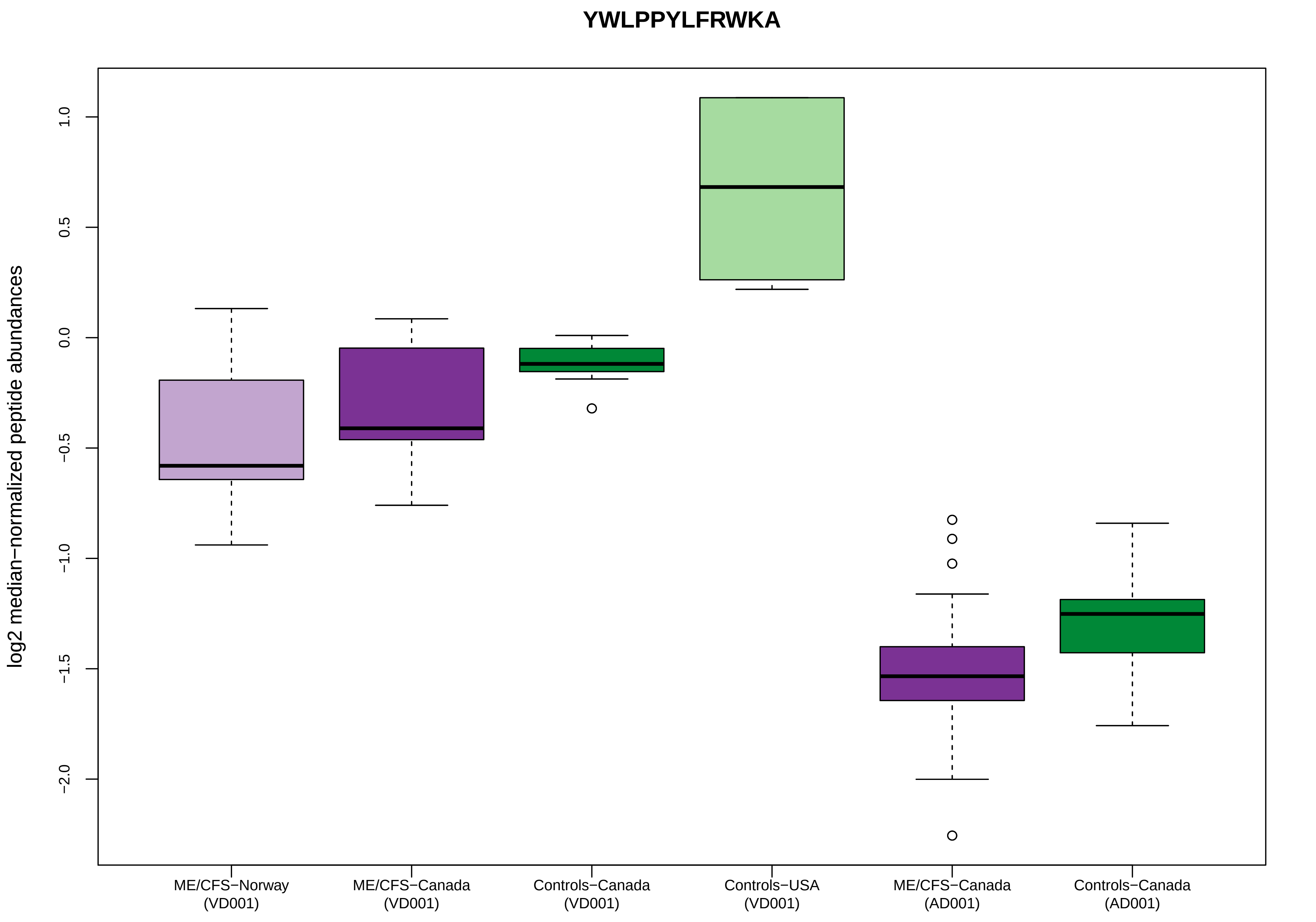

YWLPPYLFRWKA
log2 median−normalized peptide abundances
1.0
0.5
0.0
−0.5
−1.0
−1.5
−2.0
ME/CFS−Norway (VD001)
ME/CFS−Canada (VD001)
Controls−Canada (VD001)
Controls−USA (VD001)
ME/CFS−Canada (AD001)
Controls−Canada (AD001)

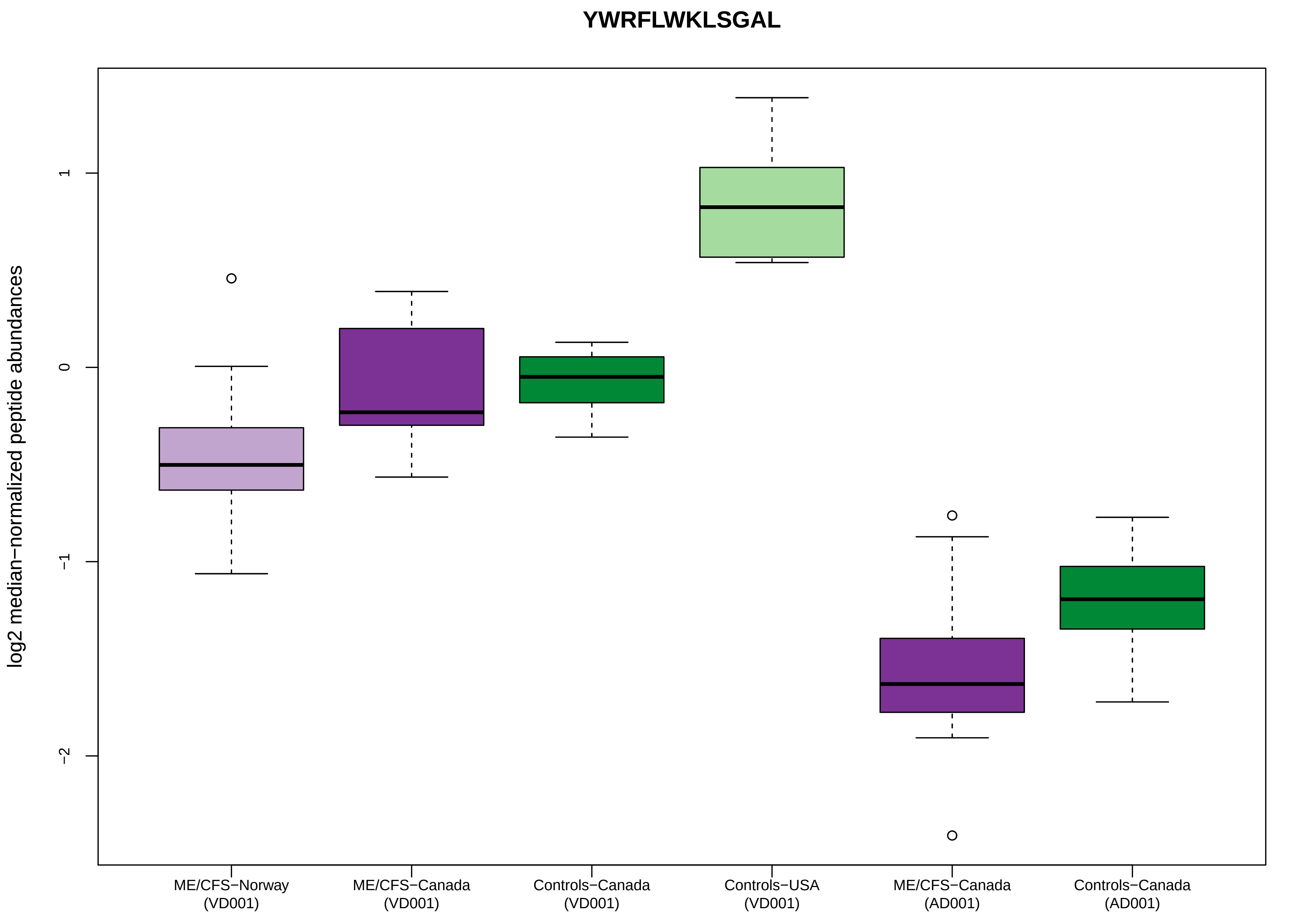
YWRFLWKLSGAL
log2 median−normalized peptide abundances
1
0
−1
−2
ME/CFS−Norway (VD001)
ME/CFS−Canada (VD001)
Controls−Canada (VD001)
Controls−USA (VD001)
ME/CFS−Canada (AD001)
Controls−Canada (AD001)

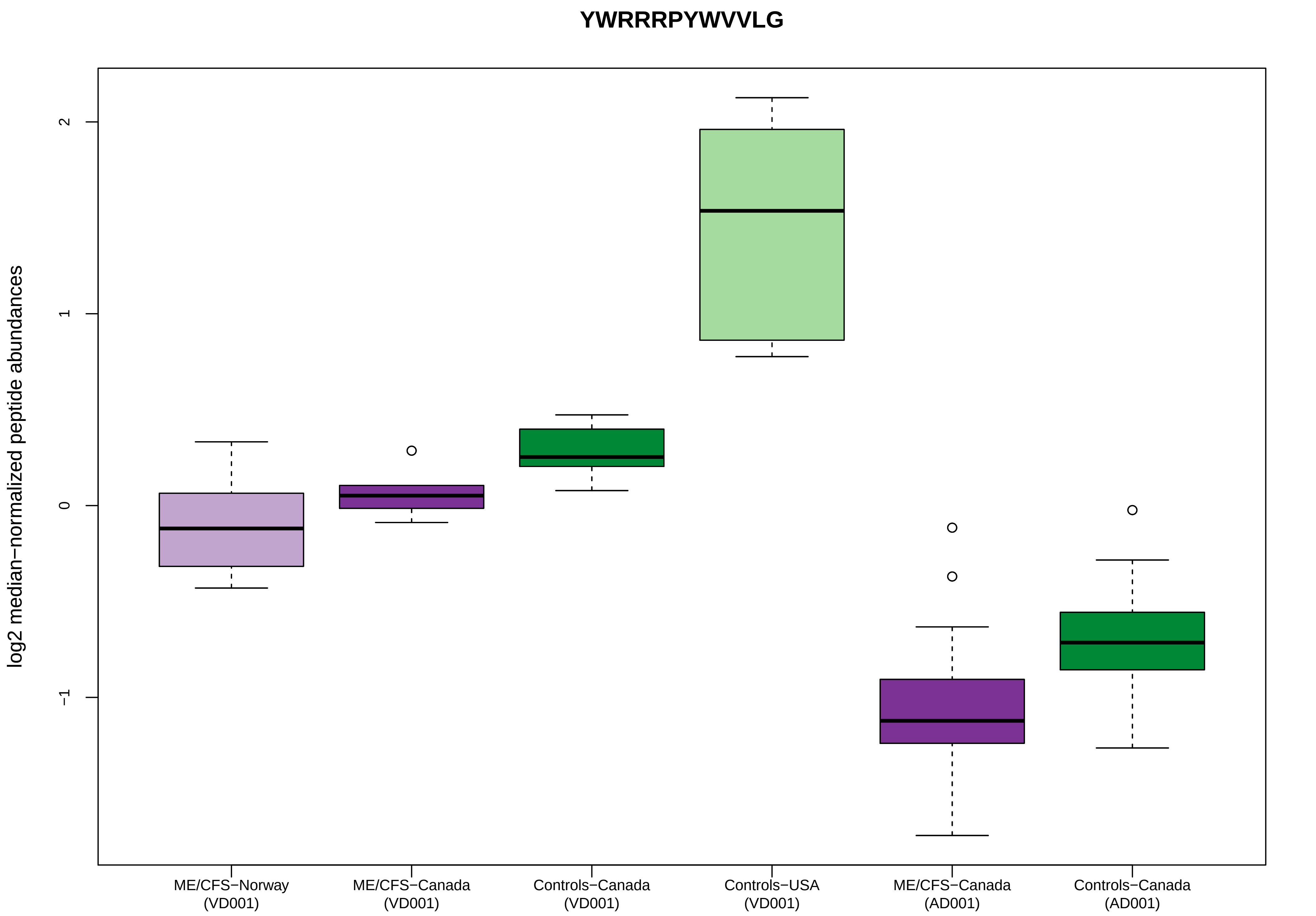
YWRRRPYWVVLG
log2 median−normalized peptide abundances
2
1
0
−1
ME/CFS−Norway (VD001)
ME/CFS−Canada (VD001)
Controls−Canada (VD001)
Controls−USA (VD001)
ME/CFS−Canada (AD001)
Controls−Canada (AD001)

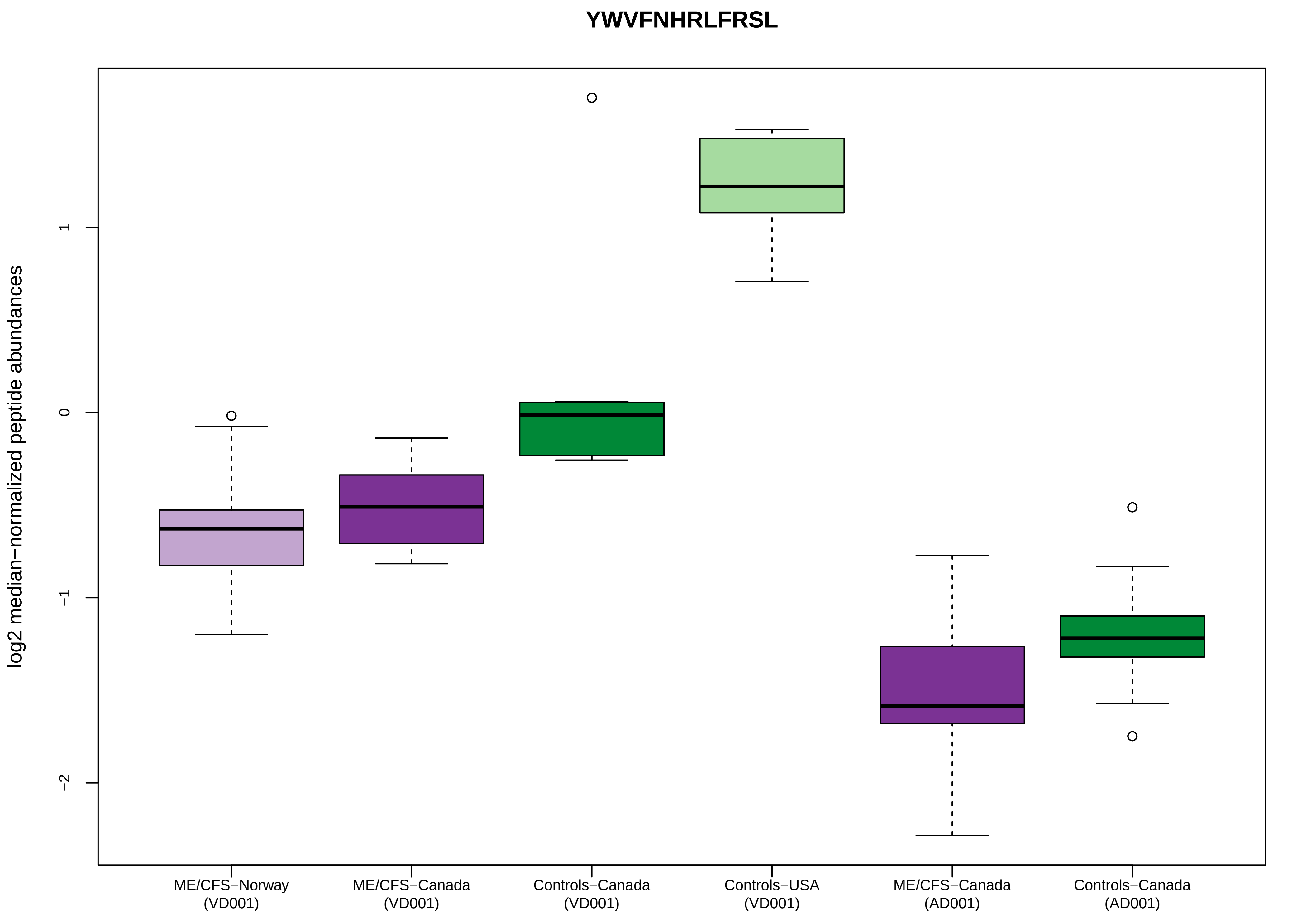

YWVFNHRLFRSL
log2 median−normalized peptide abundances
1
0
−1
−2
ME/CFS−Norway (VD001)
ME/CFS−Canada (VD001)
Controls−Canada (VD001)
Controls−USA (VD001)
ME/CFS−Canada (AD001)
Controls−Canada (AD001)

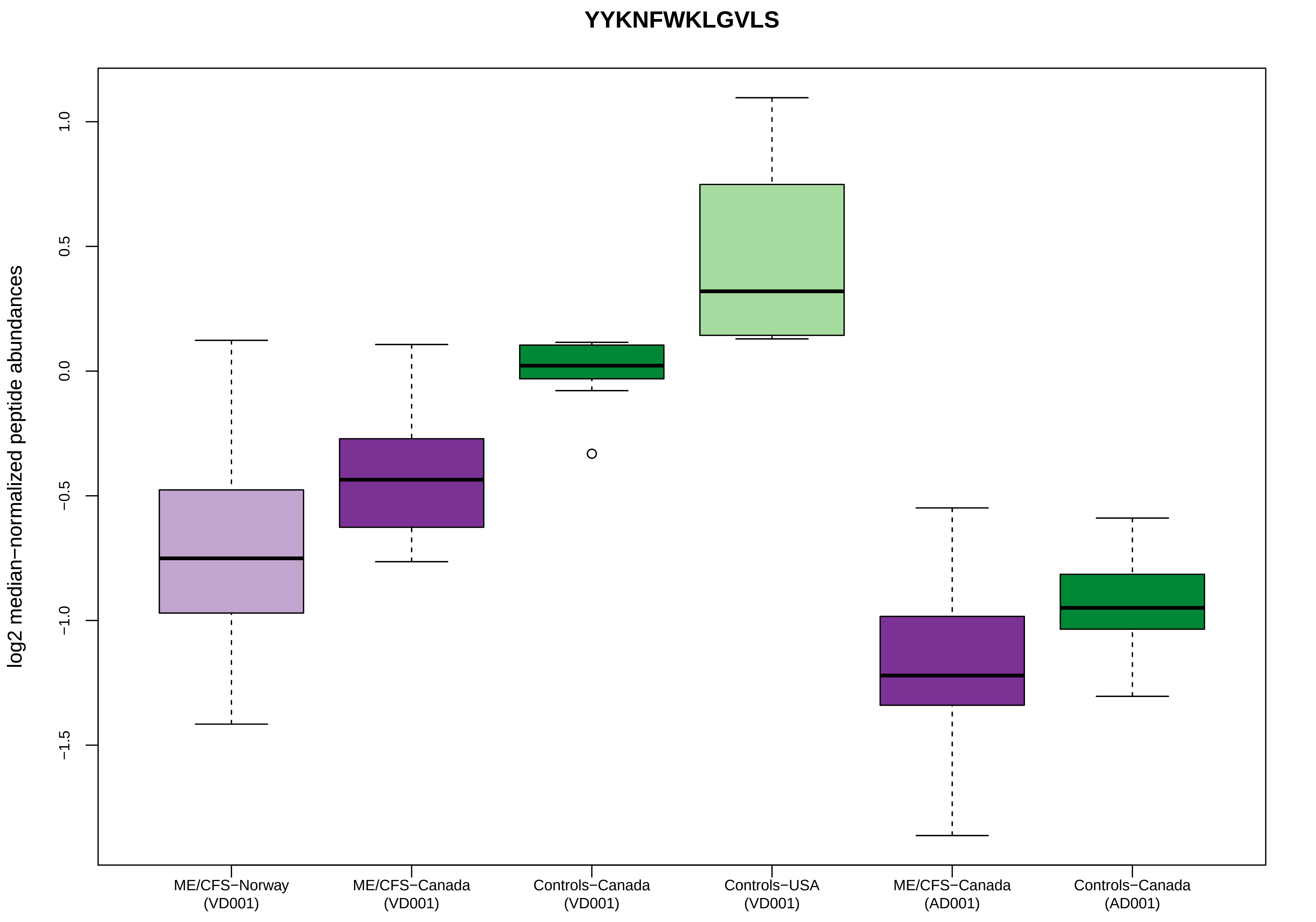

YYKNFWKLGVLS
log2 median−normalized peptide abundances
1.0
0.5
0.0
−0.5
−1.0
−1.5
ME/CFS−Norway (VD001)
ME/CFS−Canada (VD001)
Controls−Canada (VD001)
Controls−USA (VD001)
ME/CFS−Canada (AD001)
Controls−Canada (AD001)

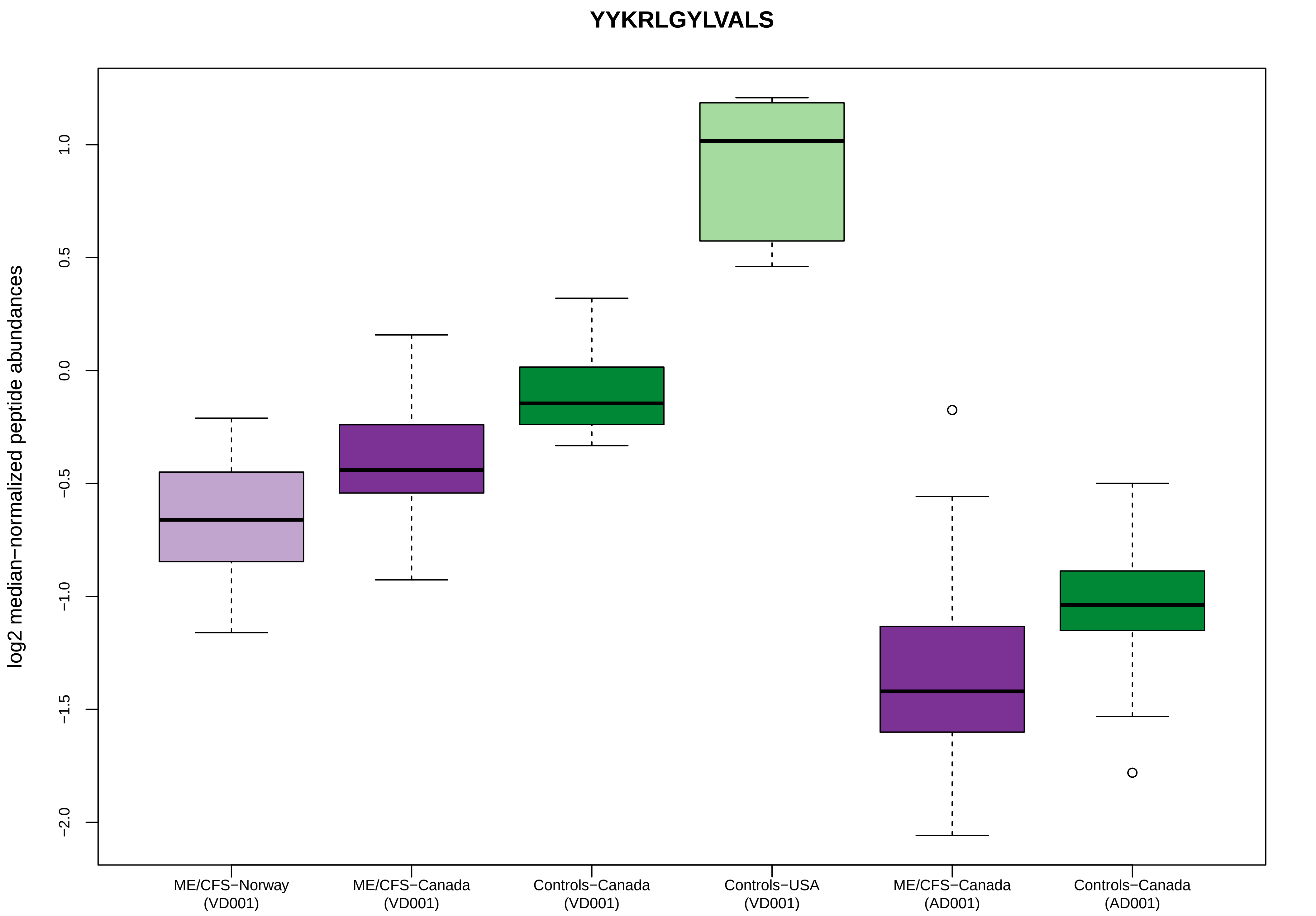
YYKRLGYLVALS
log2 median−normalized peptide abundances
1.0
0.5
0.0
−0.5
−1.0
−1.5
−2.0
ME/CFS−Norway (VD001)
ME/CFS−Canada (VD001)
Controls−Canada (VD001)
Controls−USA (VD001)
ME/CFS−Canada (AD001)
Controls−Canada (AD001)

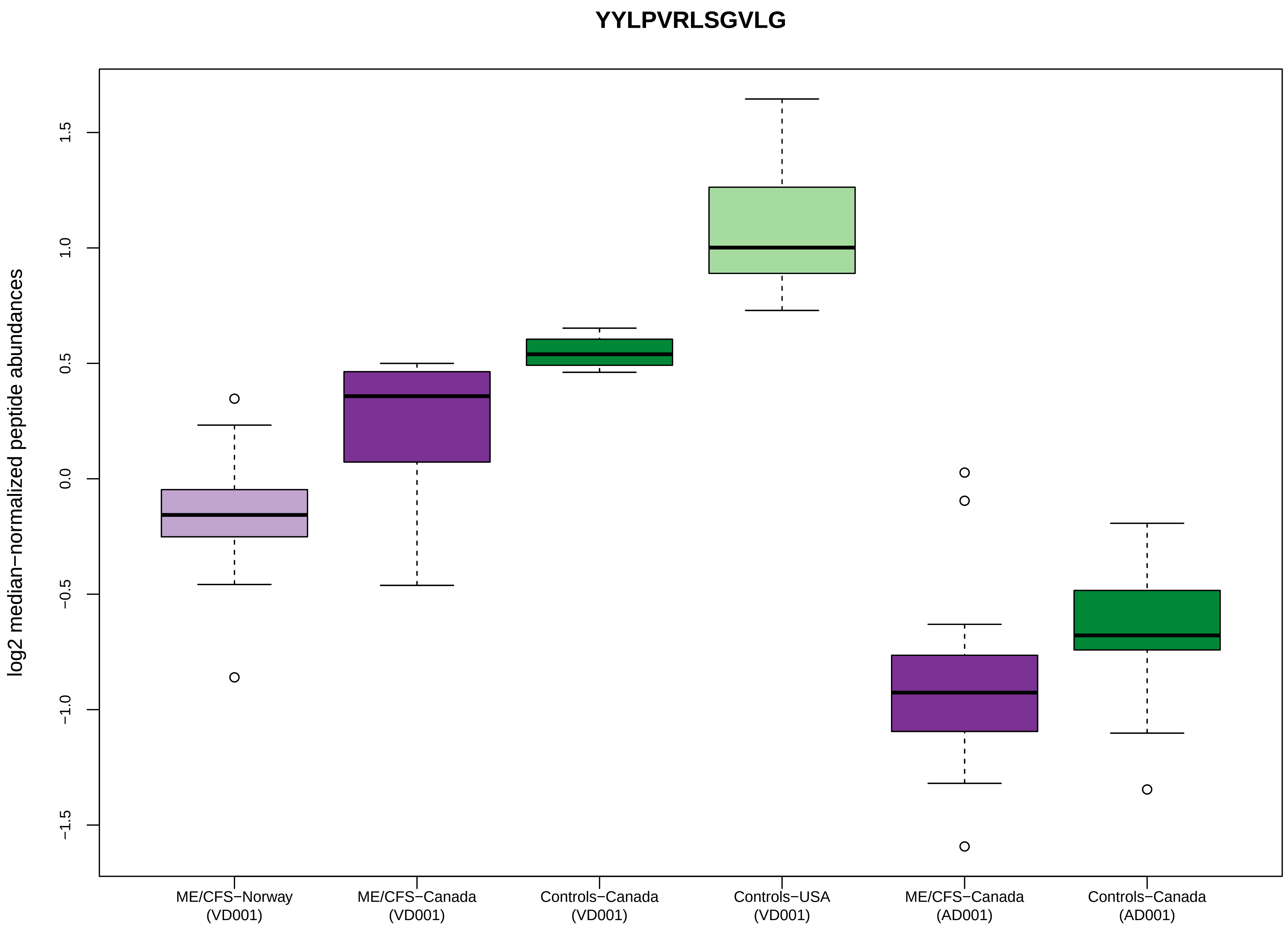

YYLPVRLSGVLG
log2 median−normalized peptide abundances
1.5
1.0
0.5
0.0
−0.5
−1.0
−1.5
ME/CFS−Norway (VD001)
ME/CFS−Canada (VD001)
Controls−Canada (VD001)
Controls−USA (VD001)
ME/CFS−Canada (AD001)
Controls−Canada (AD001)

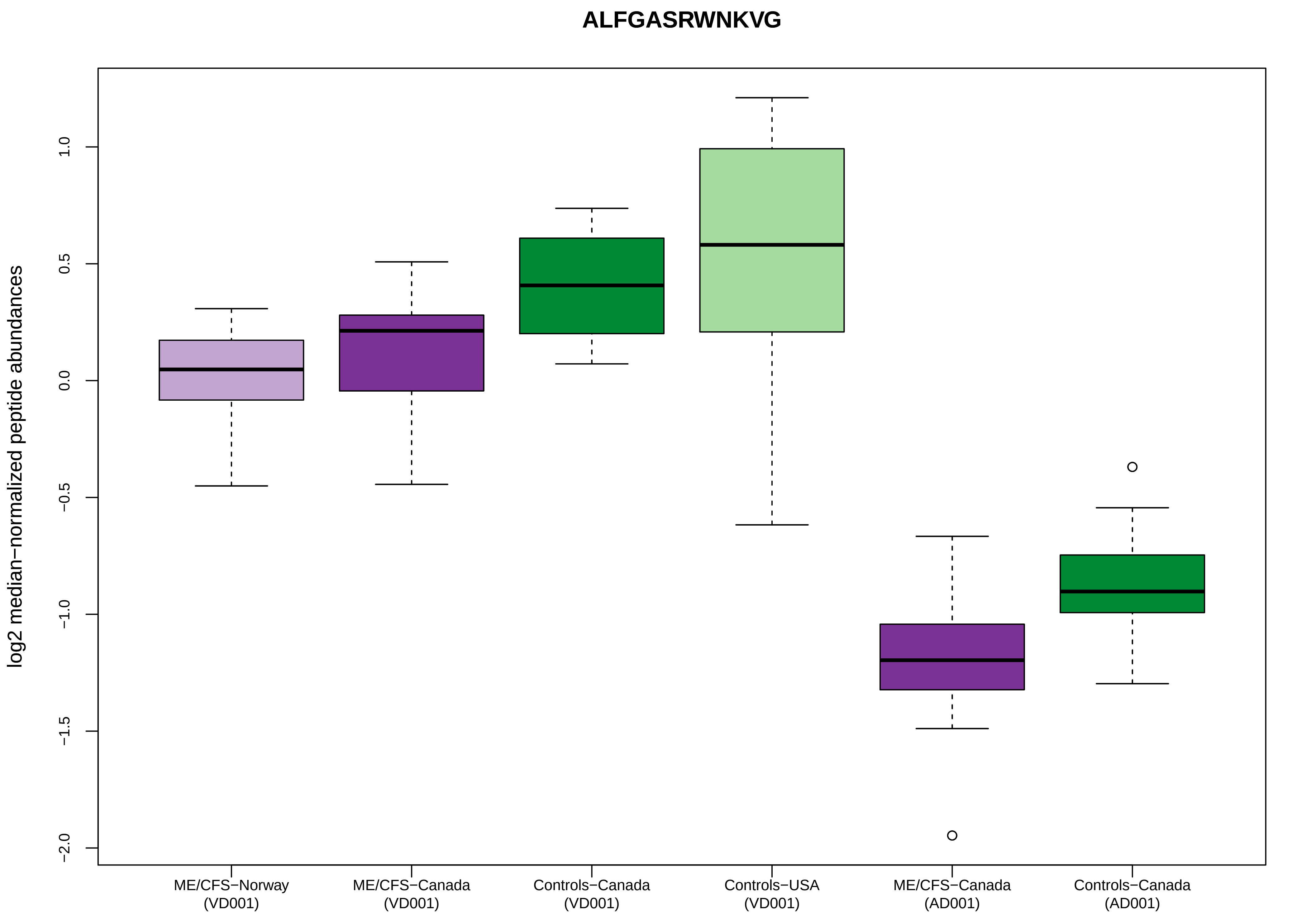
ALFGASRWNKVG
log2 median−normalized peptide abundances
1.0
0.5
0.0
−0.5
−1.0
−1.5
−2.0
ME/CFS−Norway (VD001)
ME/CFS−Canada (VD001)
Controls−Canada (VD001)
Controls−USA (VD001)
ME/CFS−Canada (AD001)
Controls−Canada (AD001)

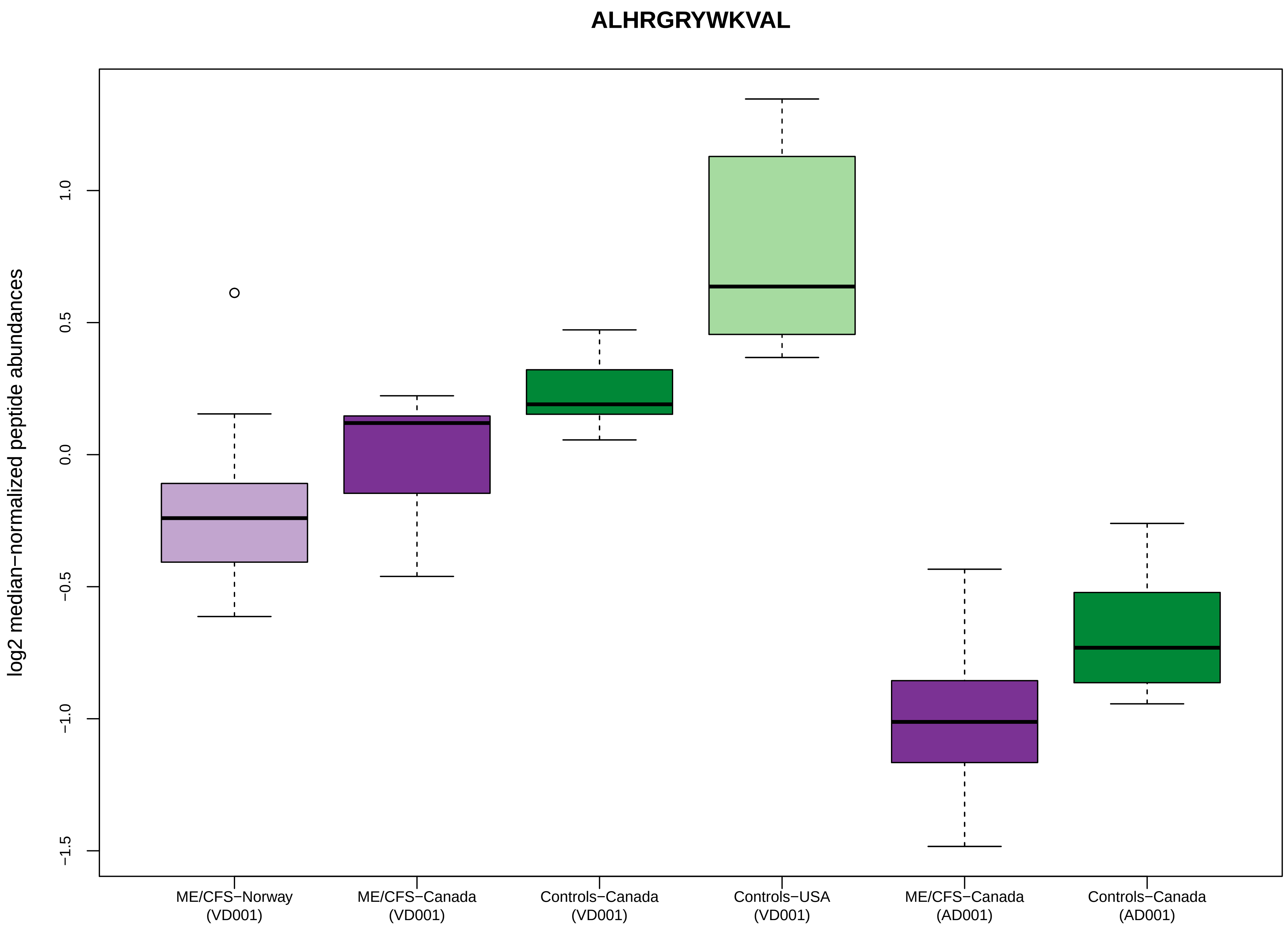

ALHRGRYWKVAL
log2 median−normalized peptide abundances
1.0
0.5
0.0
−0.5
−1.0
−1.5
ME/CFS−Norway (VD001)
ME/CFS−Canada (VD001)
Controls−Canada (VD001)
Controls−USA (VD001)
ME/CFS−Canada (AD001)
Controls−Canada (AD001)

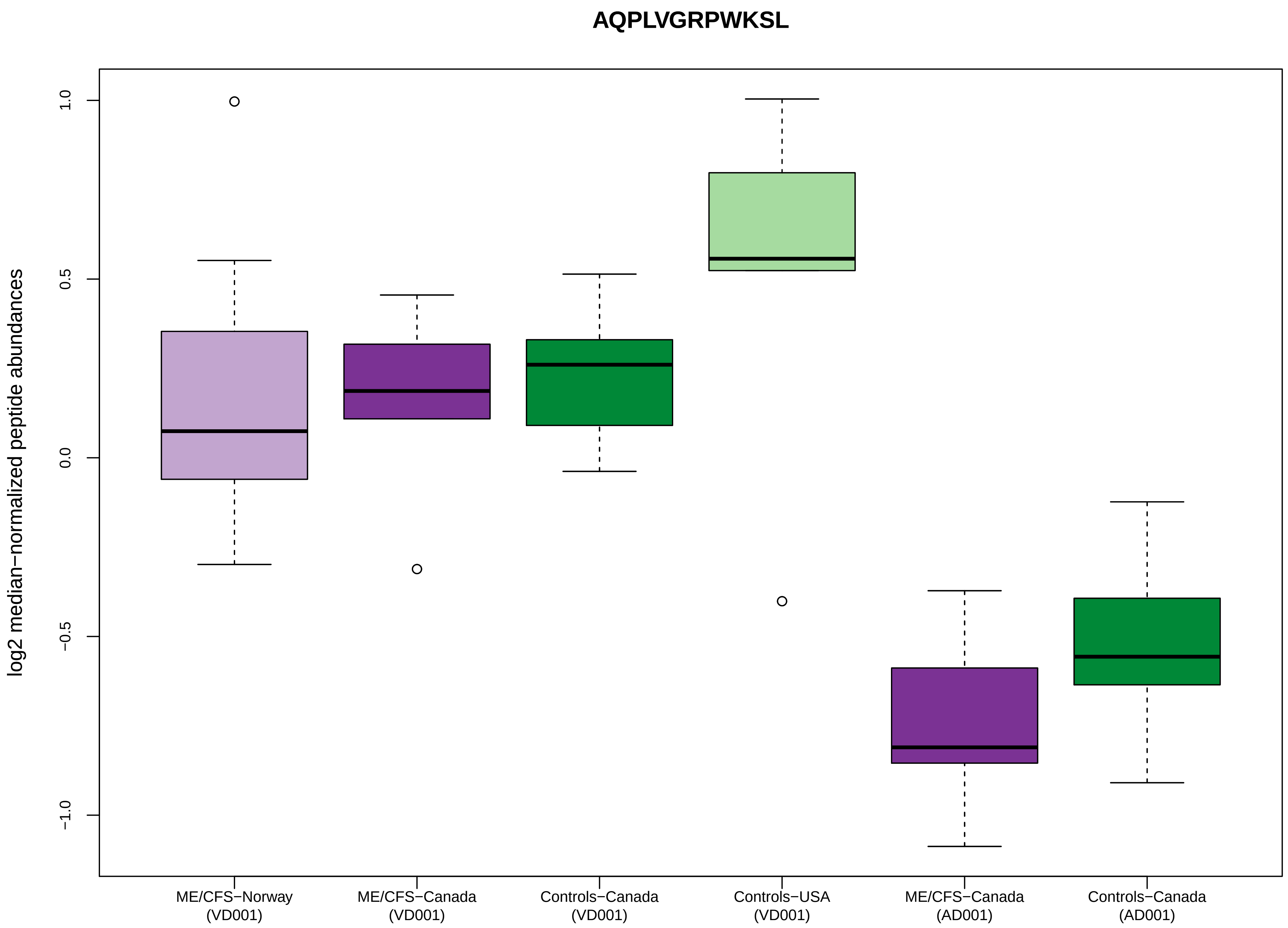

AQPLVGRPWKSL
log2 median−normalized peptide abundances
1.0
0.5
0.0
−0.5
−1.0
ME/CFS−Norway (VD001)
ME/CFS−Canada (VD001)
Controls−Canada (VD001)
Controls−USA (VD001)
ME/CFS−Canada (AD001)
Controls−Canada (AD001)

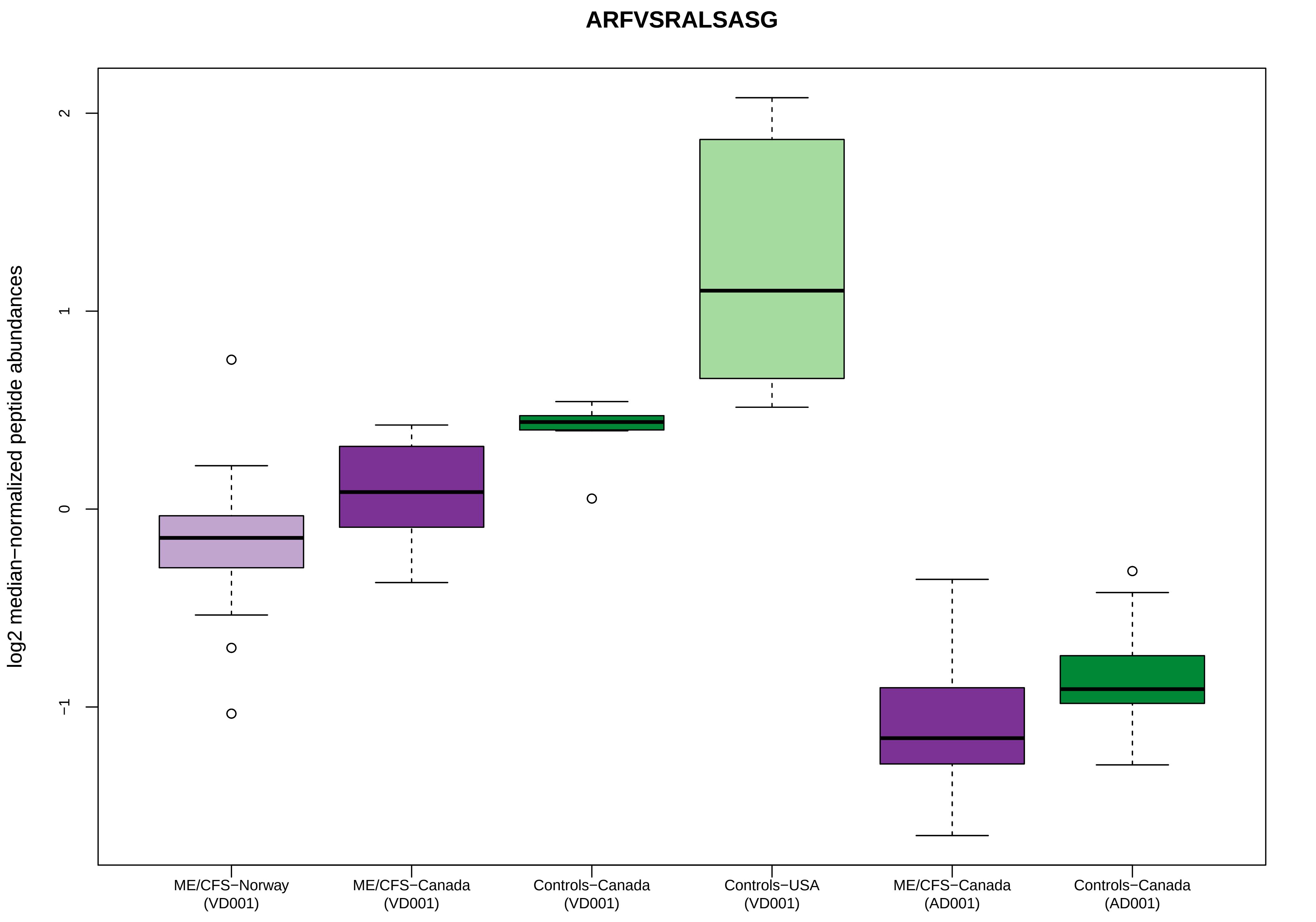

ARFVSRALSASG
log2 median−normalized peptide abundances
2
1
0
−1
ME/CFS−Norway (VD001)
ME/CFS−Canada (VD001)
Controls−Canada (VD001)
Controls−USA (VD001)
ME/CFS−Canada (AD001)
Controls−Canada (AD001)

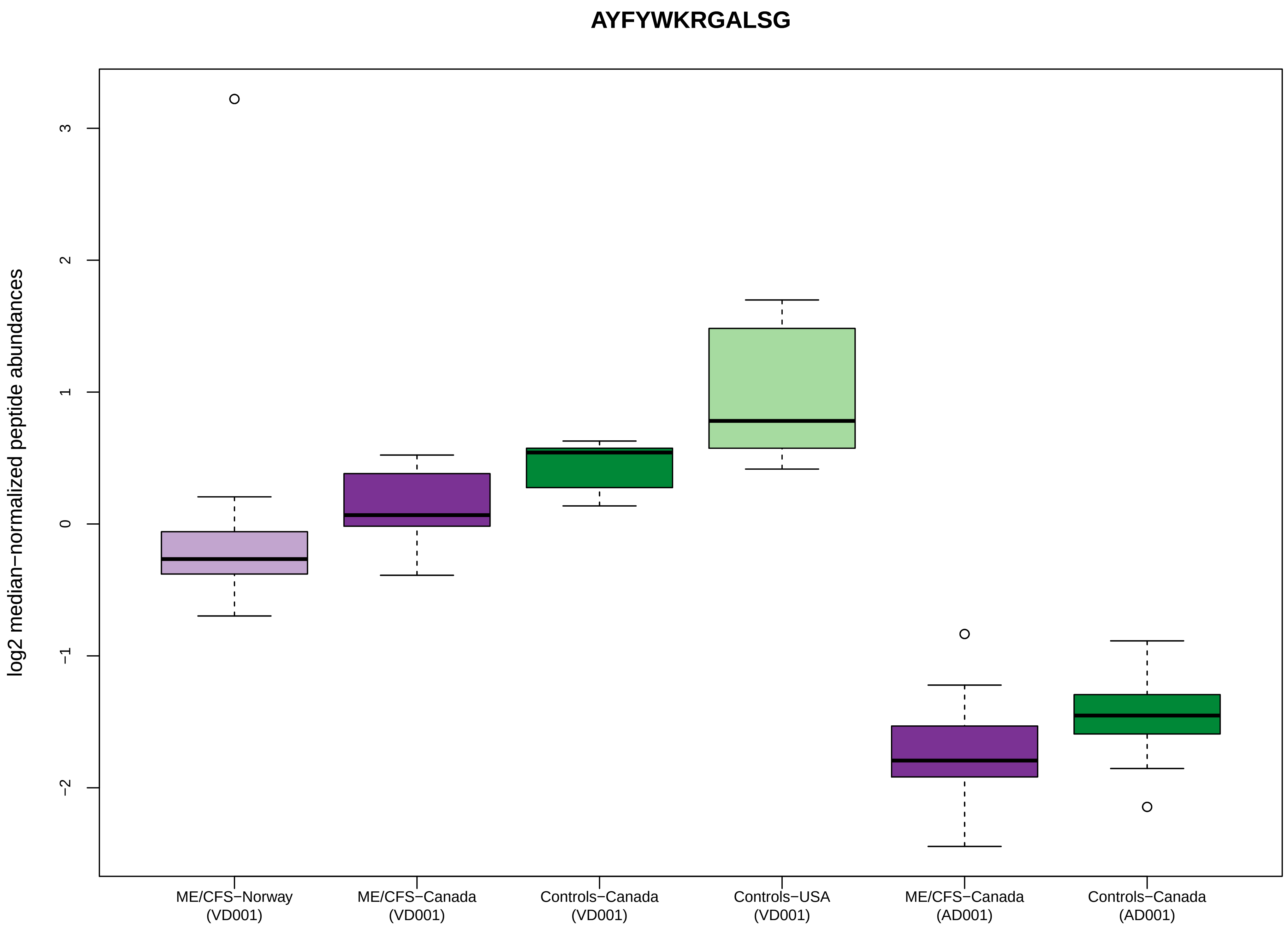

AYFYWKRGALSG
log2 median−normalized peptide abundances
3
2
1
0
−1
−2
ME/CFS−Norway (VD001)
ME/CFS−Canada (VD001)
Controls−Canada (VD001)
Controls−USA (VD001)
ME/CFS−Canada (AD001)
Controls−Canada (AD001)

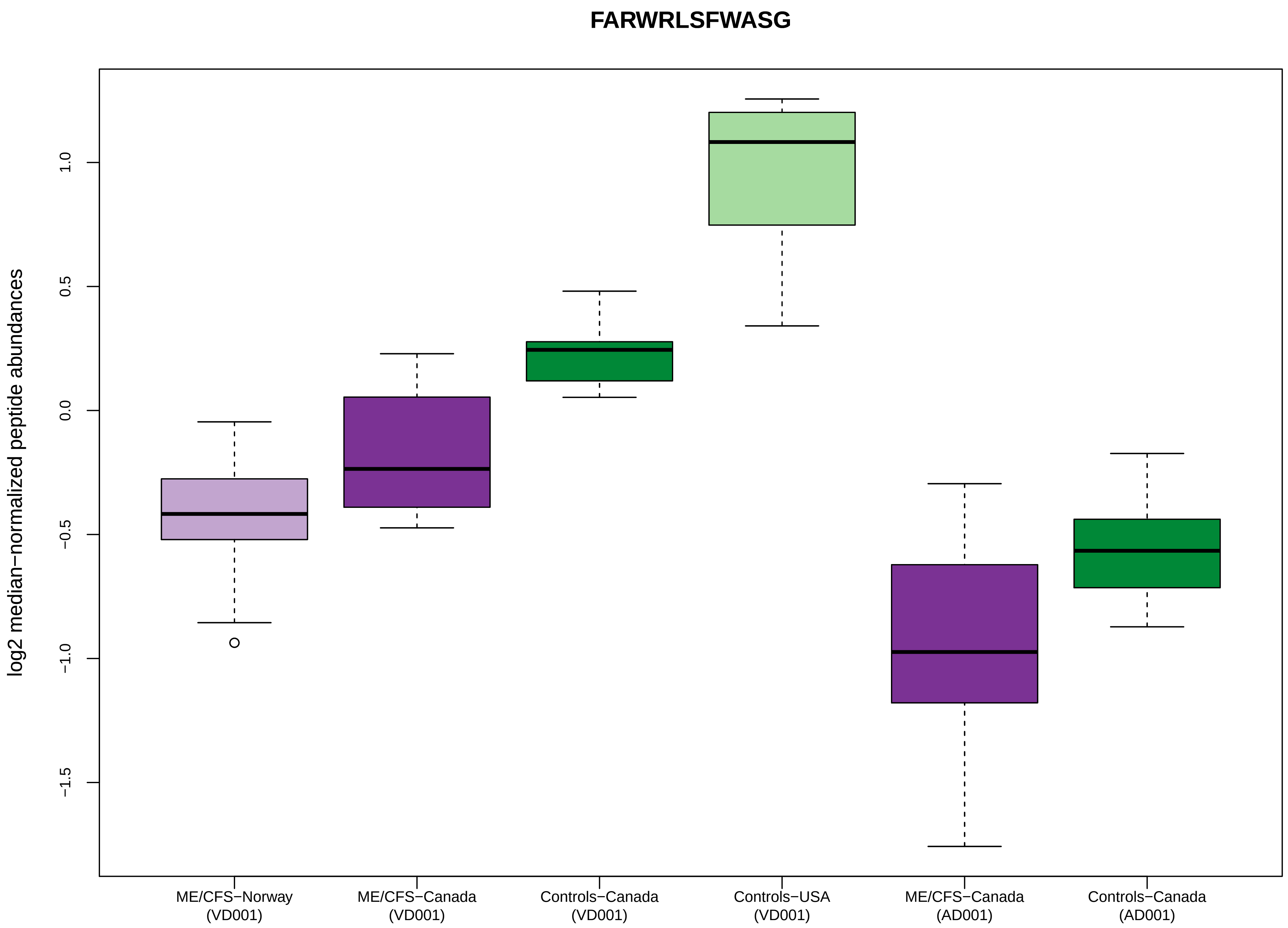

FARWRLSFWASG
log2 median−normalized peptide abundances
1.0
0.5
0.0
−0.5
−1.0
−1.5
ME/CFS−Norway (VD001)
ME/CFS−Canada (VD001)
Controls−Canada (VD001)
Controls−USA (VD001)
ME/CFS−Canada (AD001)
Controls−Canada (AD001)

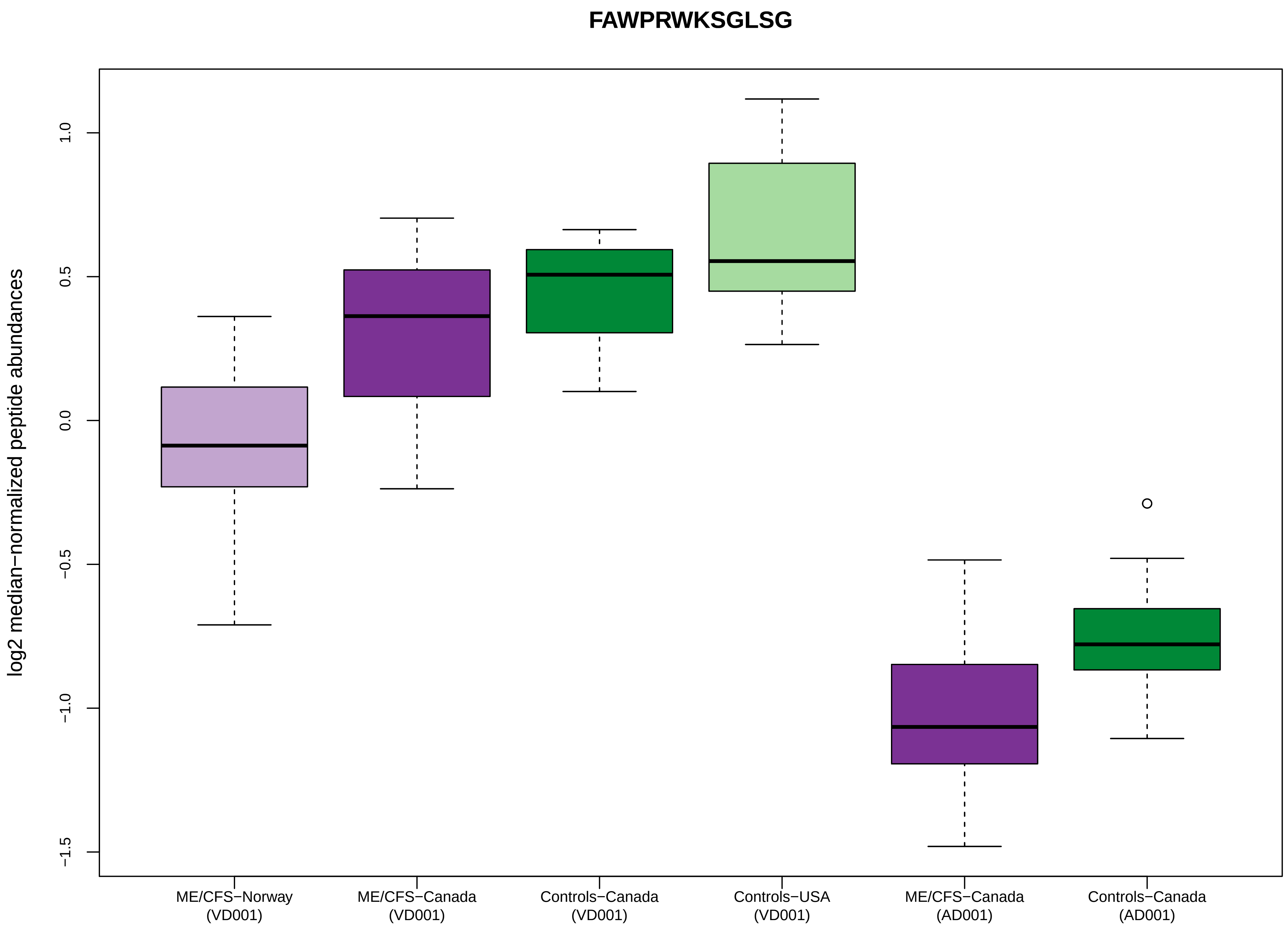
FAWPRWKSGLSG
log2 median−normalized peptide abundances
1.0
0.5
0.0
−0.5
−1.0
−1.5
ME/CFS−Norway (VD001)
ME/CFS−Canada (VD001)
Controls−Canada (VD001)
Controls−USA (VD001)
ME/CFS−Canada (AD001)
Controls−Canada (AD001)

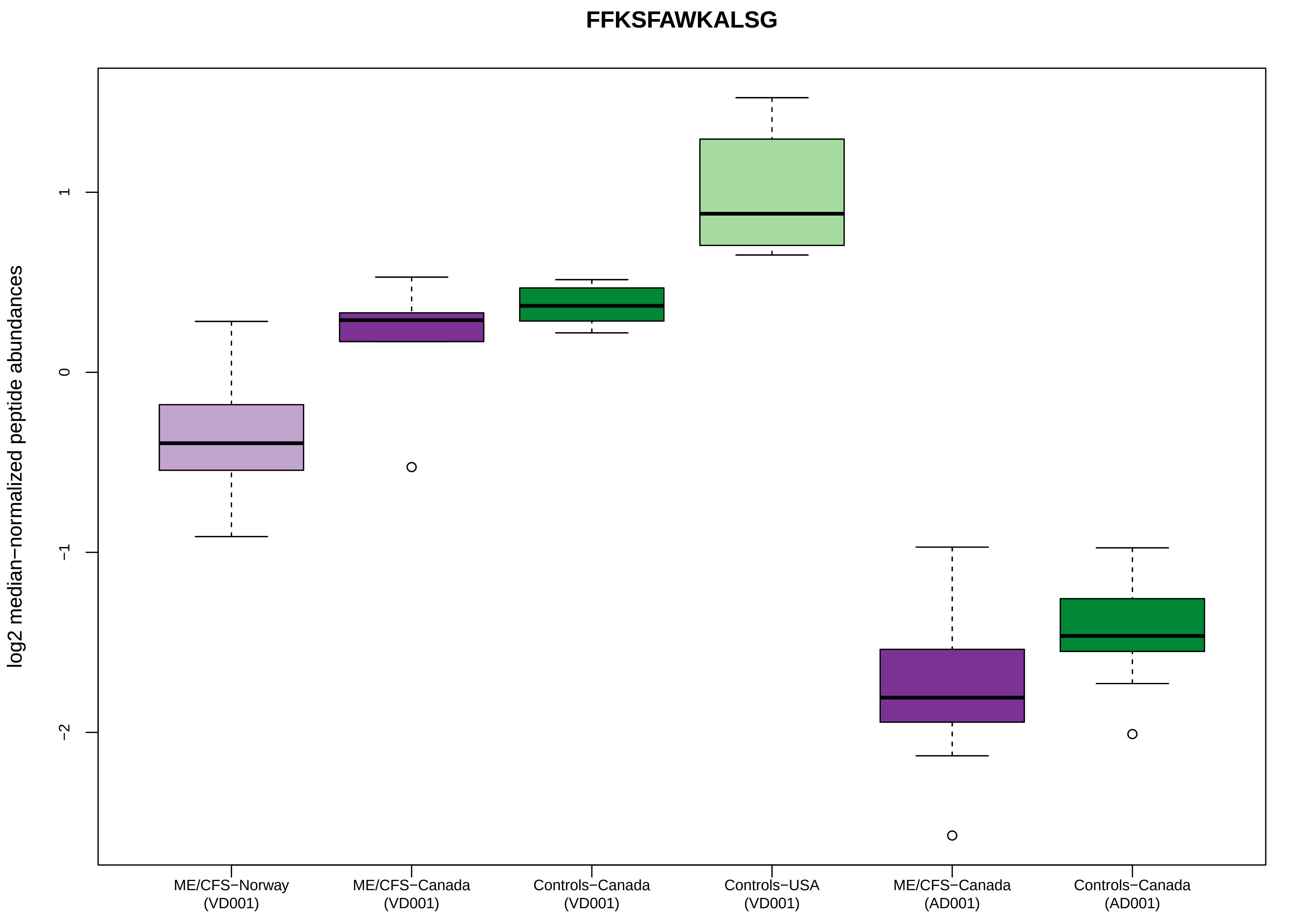

FFKSFAWKALSG
log2 median−normalized peptide abundances
1
0
−1
−2
ME/CFS−Norway (VD001)
ME/CFS−Canada (VD001)
Controls−Canada (VD001)
Controls−USA (VD001)
ME/CFS−Canada (AD001)
Controls−Canada (AD001)

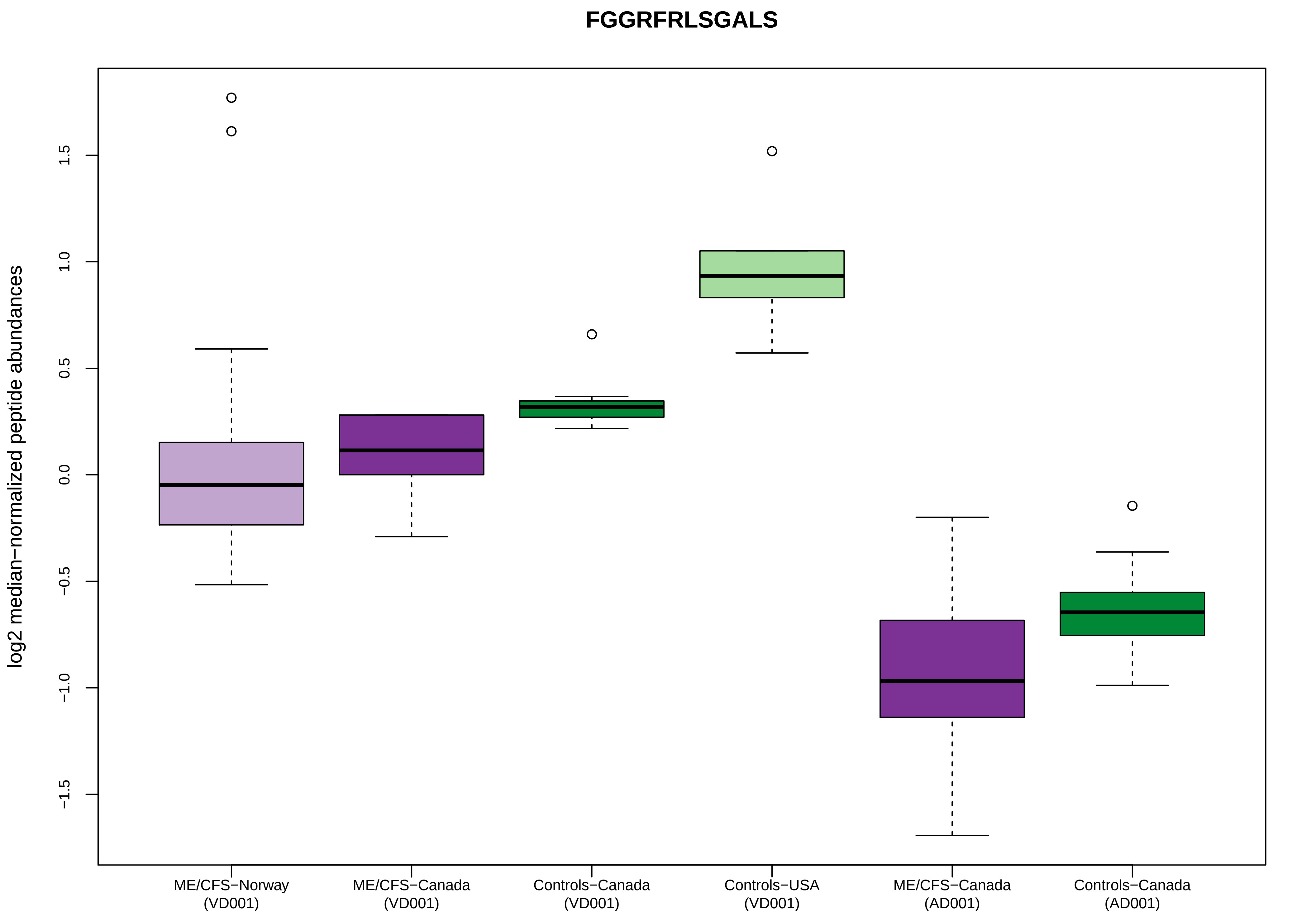
FGGRFRLSGALS
log2 median−normalized peptide abundances
1.5
1.0
0.5
0.0
−0.5
−1.0
−1.5
ME/CFS−Norway (VD001)
ME/CFS−Canada (VD001)
Controls−Canada (VD001)
Controls−USA (VD001)
ME/CFS−Canada (AD001)
Controls−Canada (AD001)

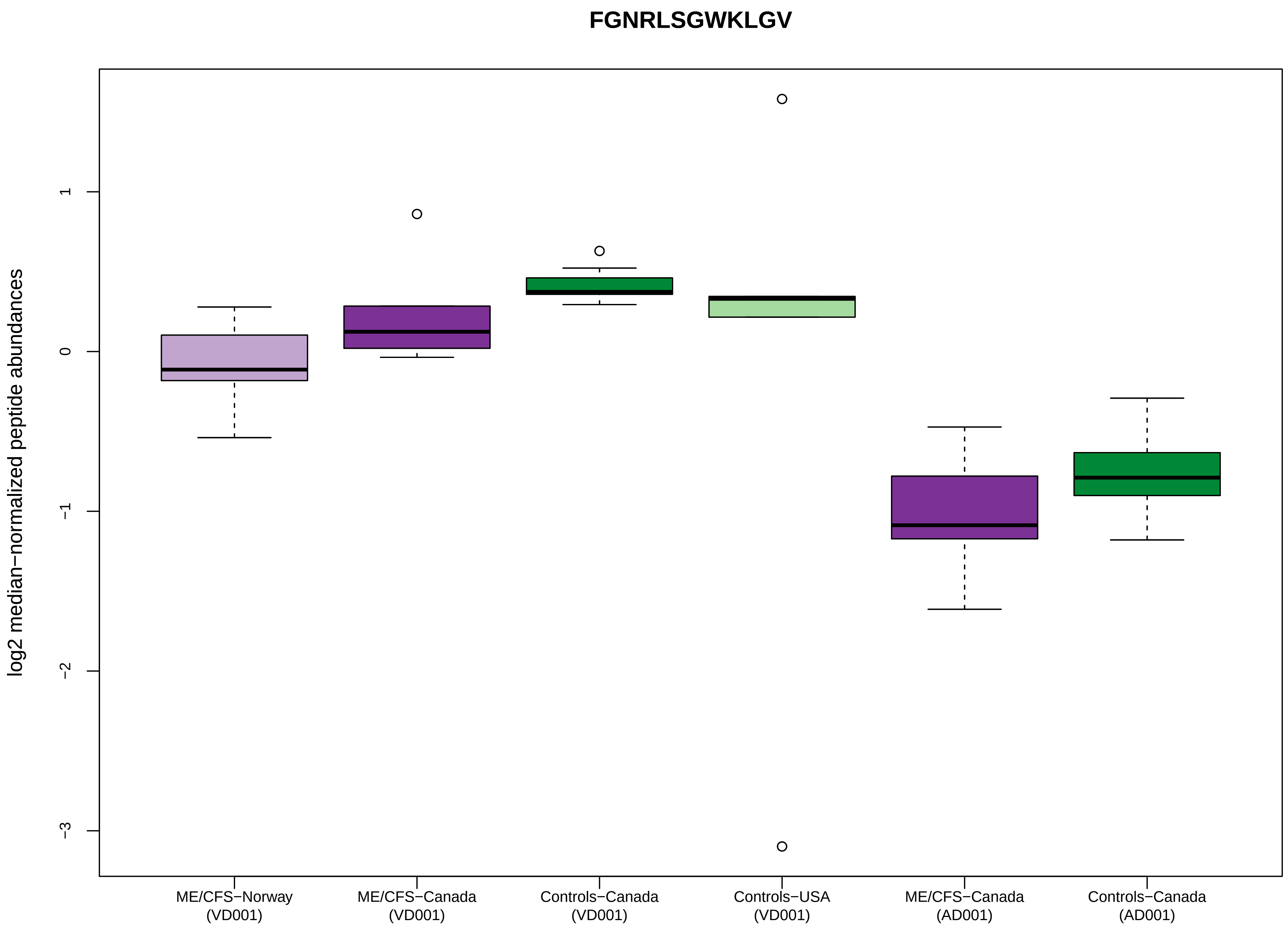

FGNRLSGWKLGV
log2 median−normalized peptide abundances
1
0
−1
−2
−3
ME/CFS−Norway (VD001)
ME/CFS−Canada (VD001)
Controls−Canada (VD001)
Controls−USA (VD001)
ME/CFS−Canada (AD001)
Controls−Canada (AD001)

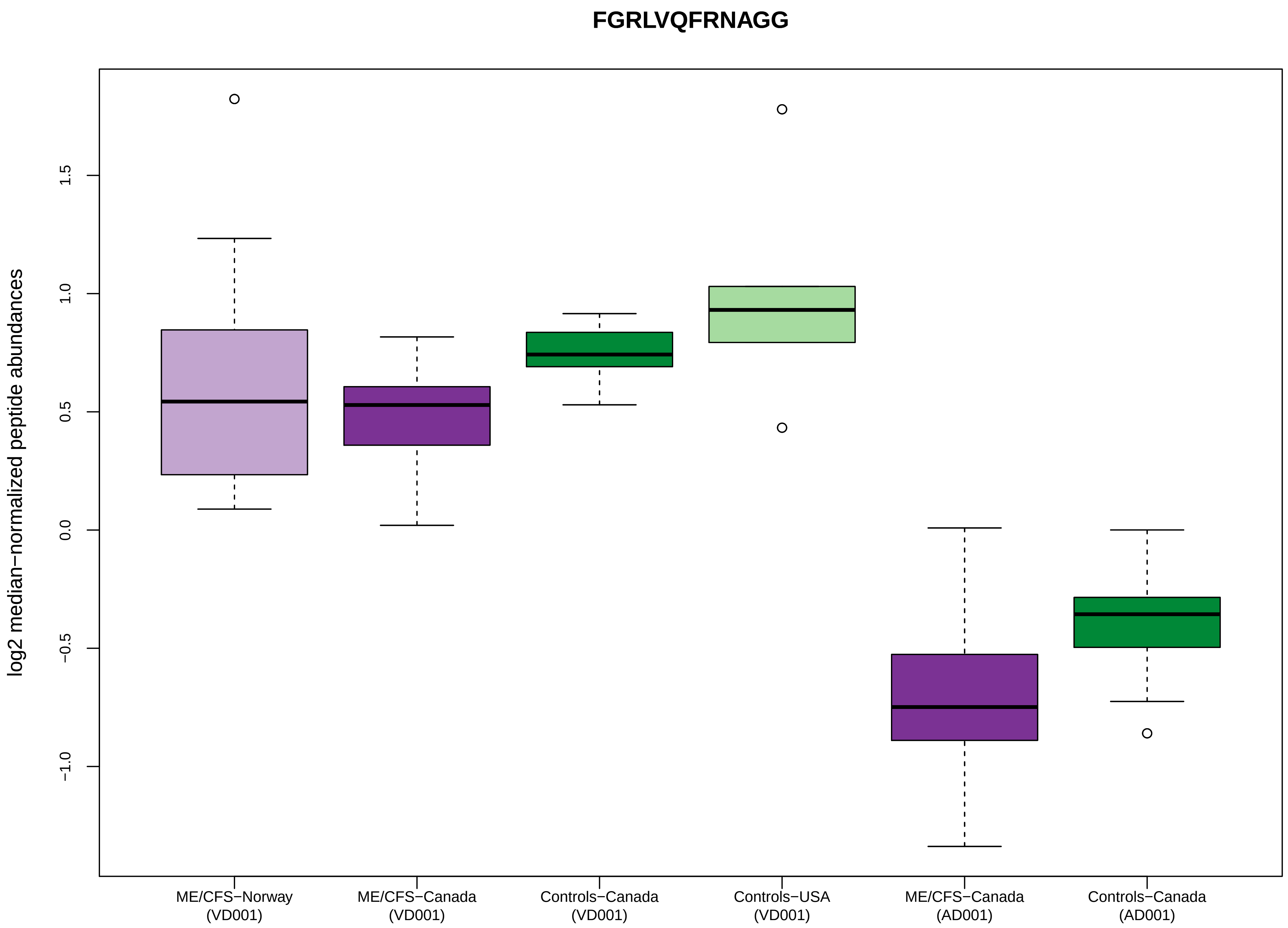

FGRLVQFRNAGG
log2 median−normalized peptide abundances
1.5
1.0
0.5
0.0
−0.5
−1.0
ME/CFS−Norway (VD001)
ME/CFS−Canada (VD001)
Controls−Canada (VD001)
Controls−USA (VD001)
ME/CFS−Canada (AD001)
Controls−Canada (AD001)

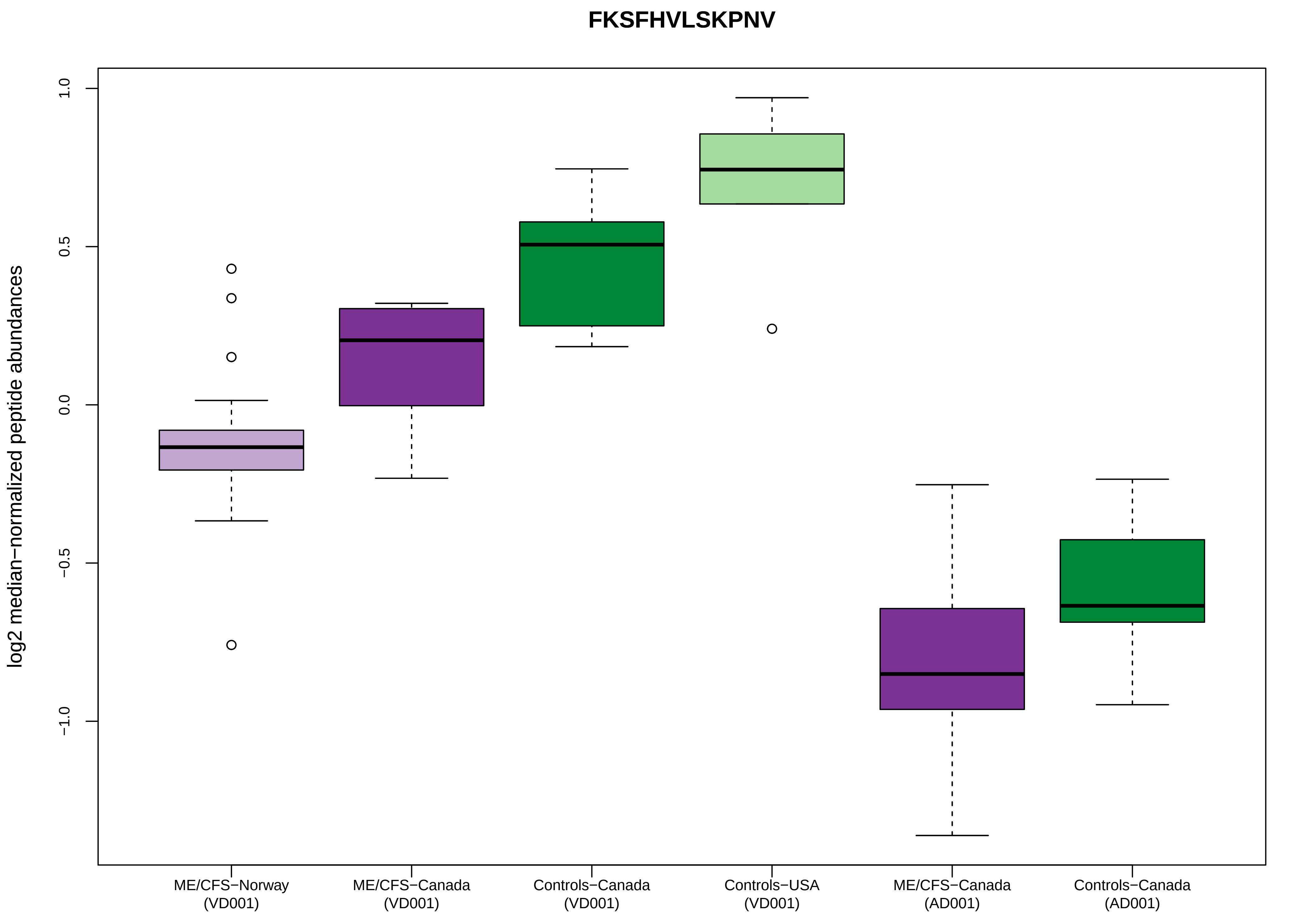

FKSFHVLSKPNV
log2 median−normalized peptide abundances
1.0
0.5
0.0
−0.5
−1.0
ME/CFS−Norway (VD001)
ME/CFS−Canada (VD001)
Controls−Canada (VD001)
Controls−USA (VD001)
ME/CFS−Canada (AD001)
Controls−Canada (AD001)

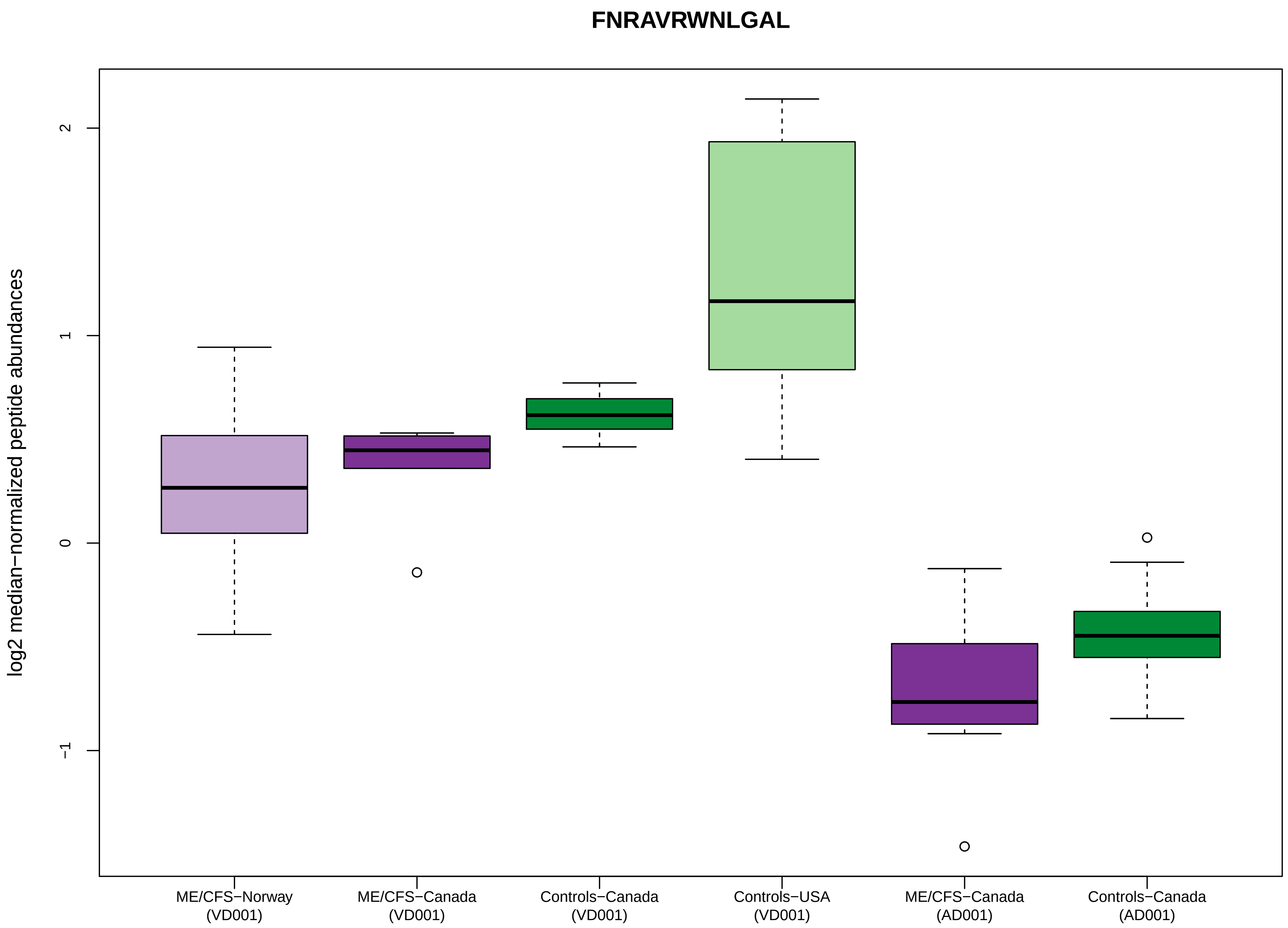

FNRAVRWNLGAL
log2 median−normalized peptide abundances
2
1
0
−1
ME/CFS−Norway (VD001)
ME/CFS−Canada (VD001)
Controls−Canada (VD001)
Controls−USA (VD001)
ME/CFS−Canada (AD001)
Controls−Canada (AD001)

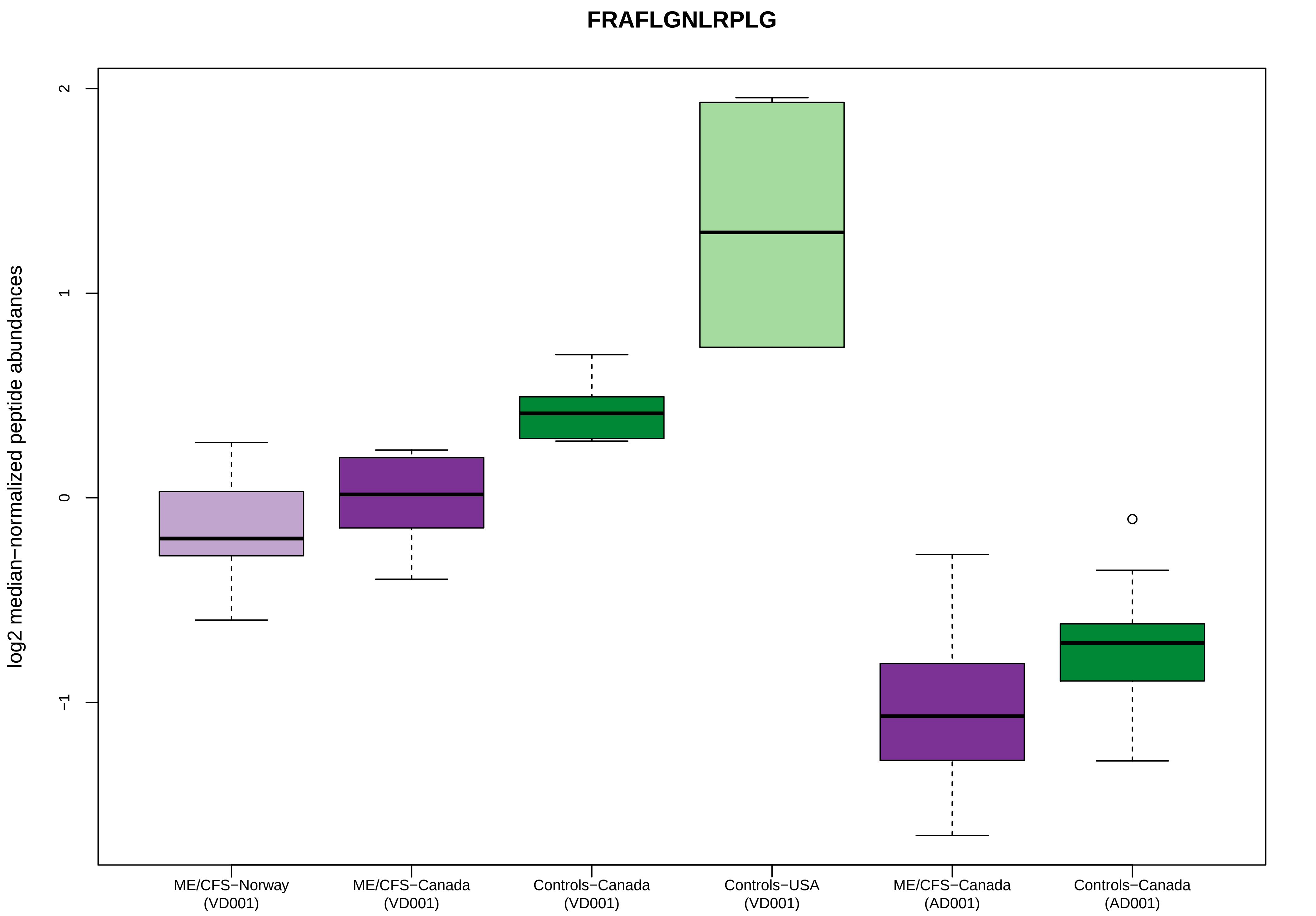

FRAFLGNLRPLG
log2 median−normalized peptide abundances
2
1
0
−1
ME/CFS−Norway (VD001)
ME/CFS−Canada (VD001)
Controls−Canada (VD001)
Controls−USA (VD001)
ME/CFS−Canada (AD001)
Controls−Canada (AD001)

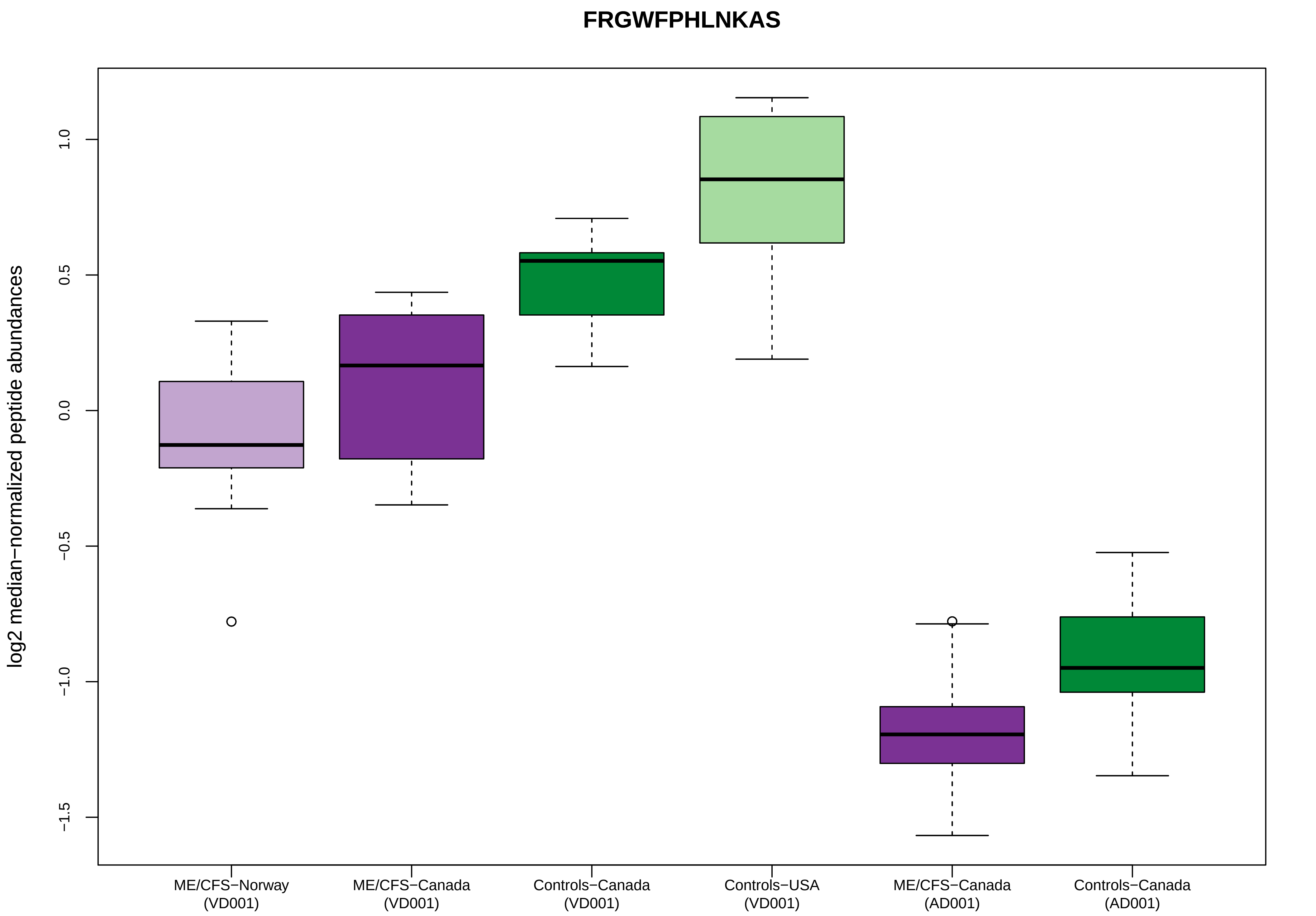

FRGWFPHLNKAS
log2 median−normalized peptide abundances
1.0
0.5
0.0
−0.5
−1.0
−1.5
ME/CFS−Norway (VD001)
ME/CFS−Canada (VD001)
Controls−Canada (VD001)
Controls−USA (VD001)
ME/CFS−Canada (AD001)
Controls−Canada (AD001)

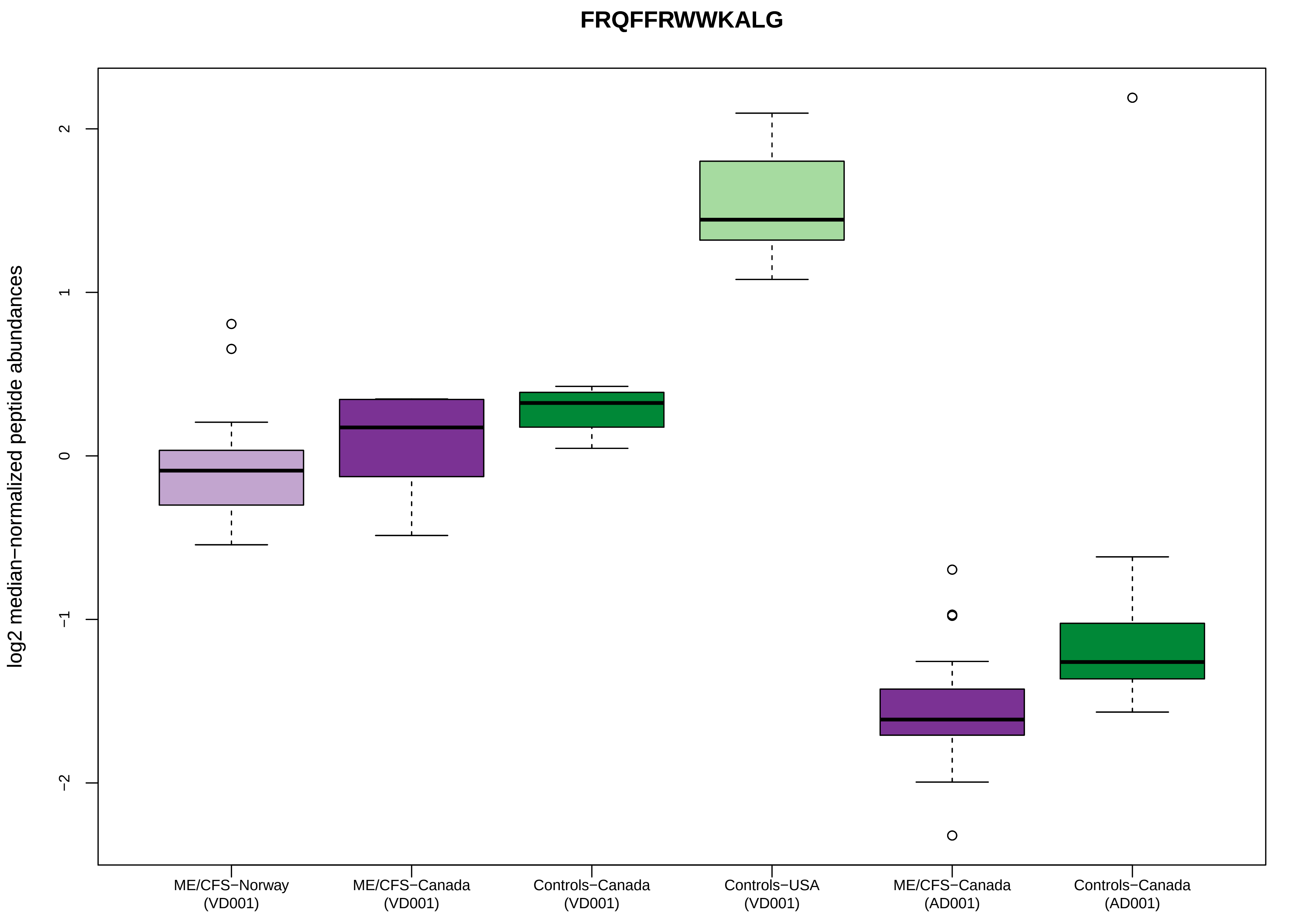

FRQFFRWWKALG
log2 median−normalized peptide abundances
2
1
0
−1
−2
ME/CFS−Norway (VD001)
ME/CFS−Canada (VD001)
Controls−Canada (VD001)
Controls−USA (VD001)
ME/CFS−Canada (AD001)
Controls−Canada (AD001)

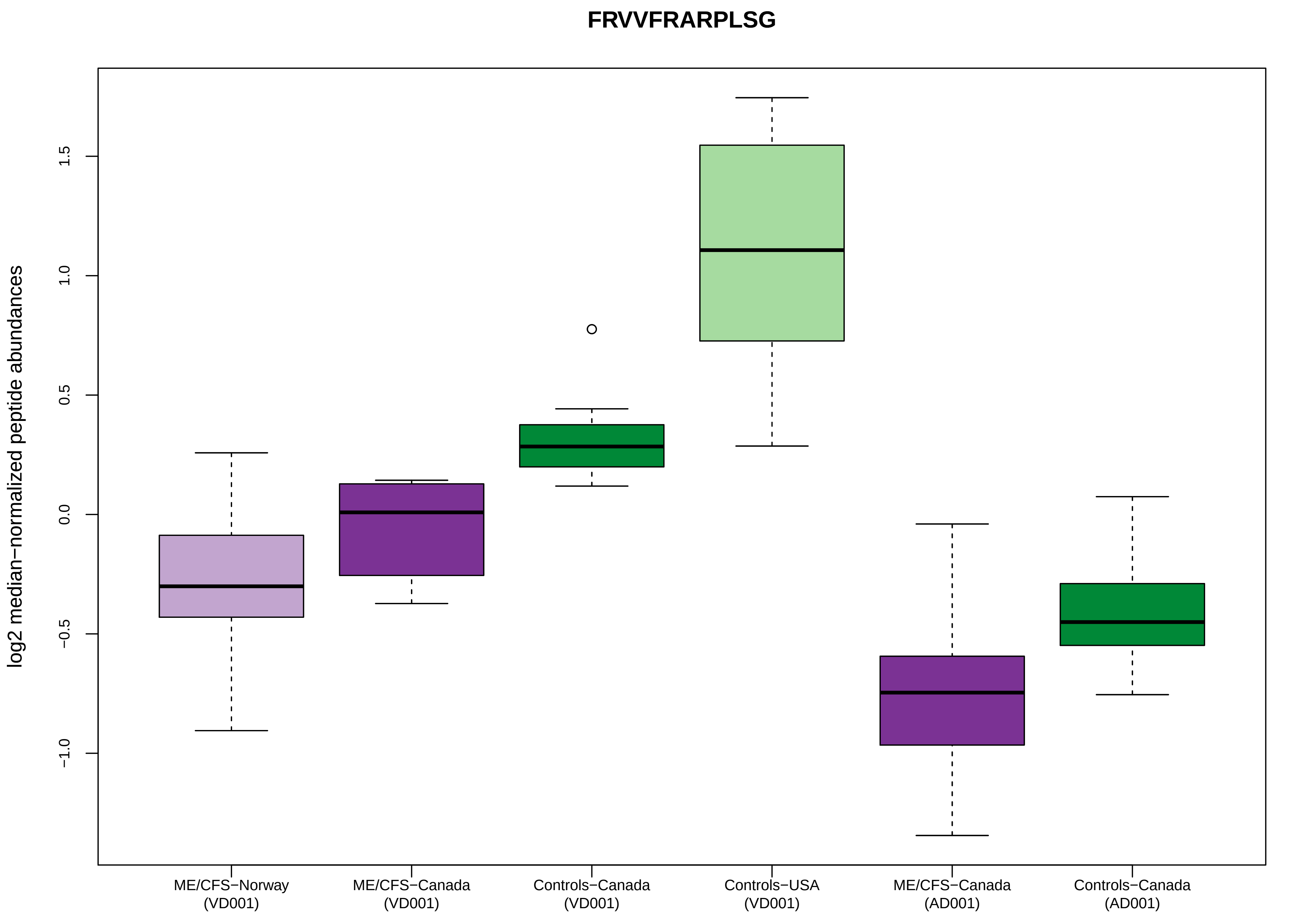
FRVVFRARPLSG
log2 median−normalized peptide abundances
1.5
1.0
0.5
0.0
−0.5
−1.0
ME/CFS−Norway (VD001)
ME/CFS−Canada (VD001)
Controls−Canada (VD001)
Controls−USA (VD001)
ME/CFS−Canada (AD001)
Controls−Canada (AD001)

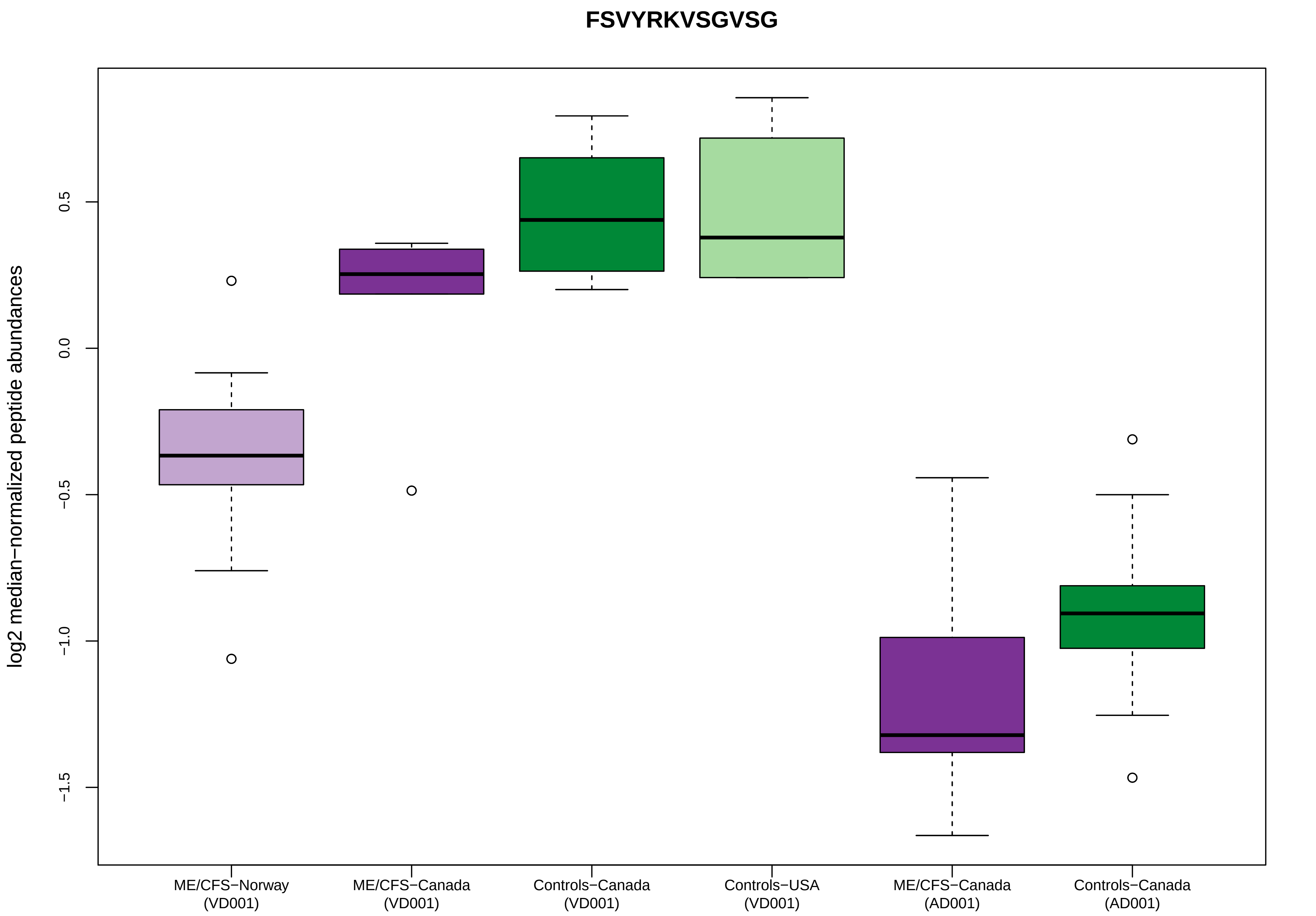

FSVYRKVSGVSG
log2 median−normalized peptide abundances
0.5
0.0
−0.5
−1.0
−1.5
ME/CFS−Norway (VD001)
ME/CFS−Canada (VD001)
Controls−Canada (VD001)
Controls−USA (VD001)
ME/CFS−Canada (AD001)
Controls−Canada (AD001)

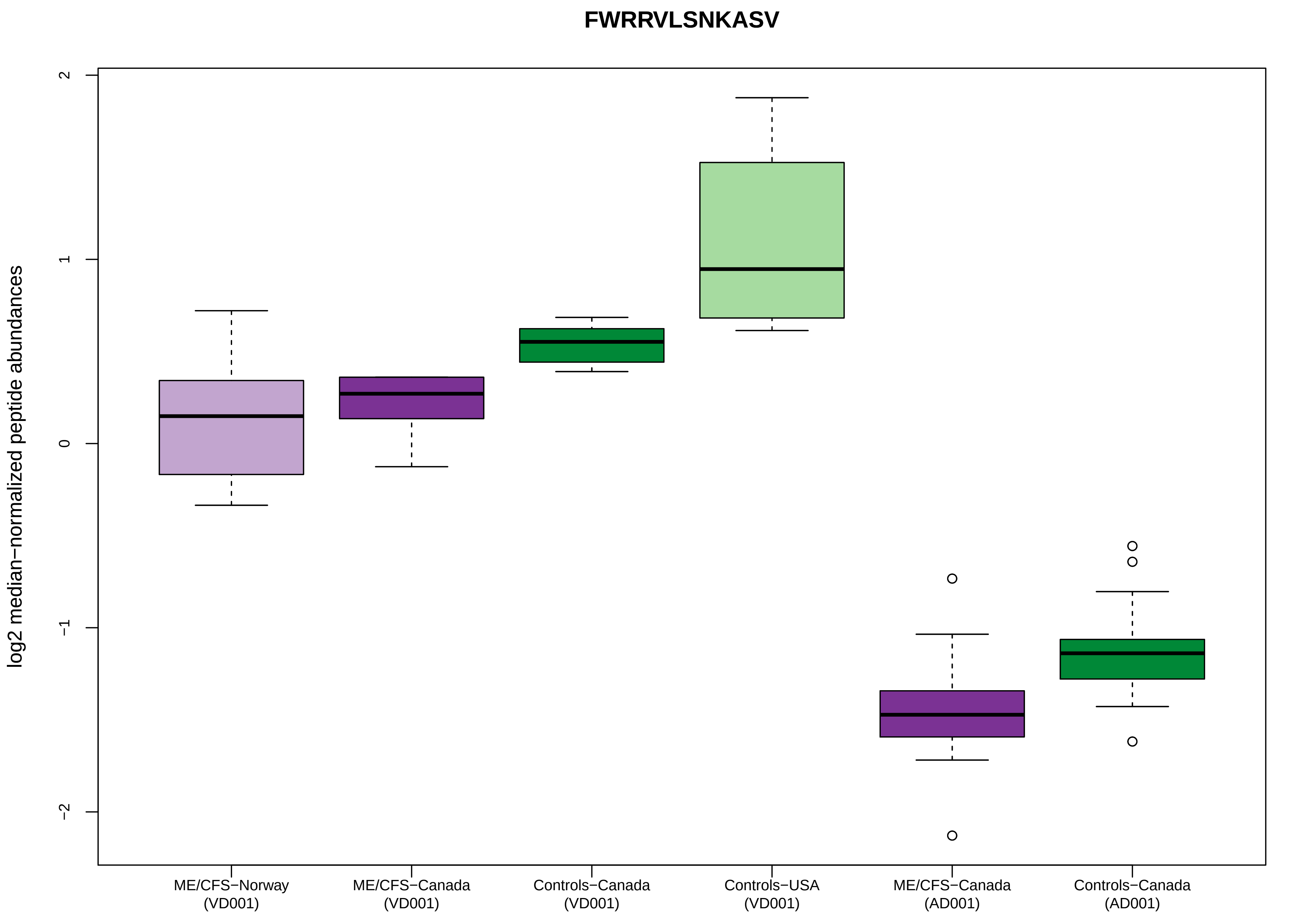

FWRRVLSNKASV
log2 median−normalized peptide abundances
2
1
0
−1
−2
ME/CFS−Norway (VD001)
ME/CFS−Canada (VD001)
Controls−Canada (VD001)
Controls−USA (VD001)
ME/CFS−Canada (AD001)
Controls−Canada (AD001)

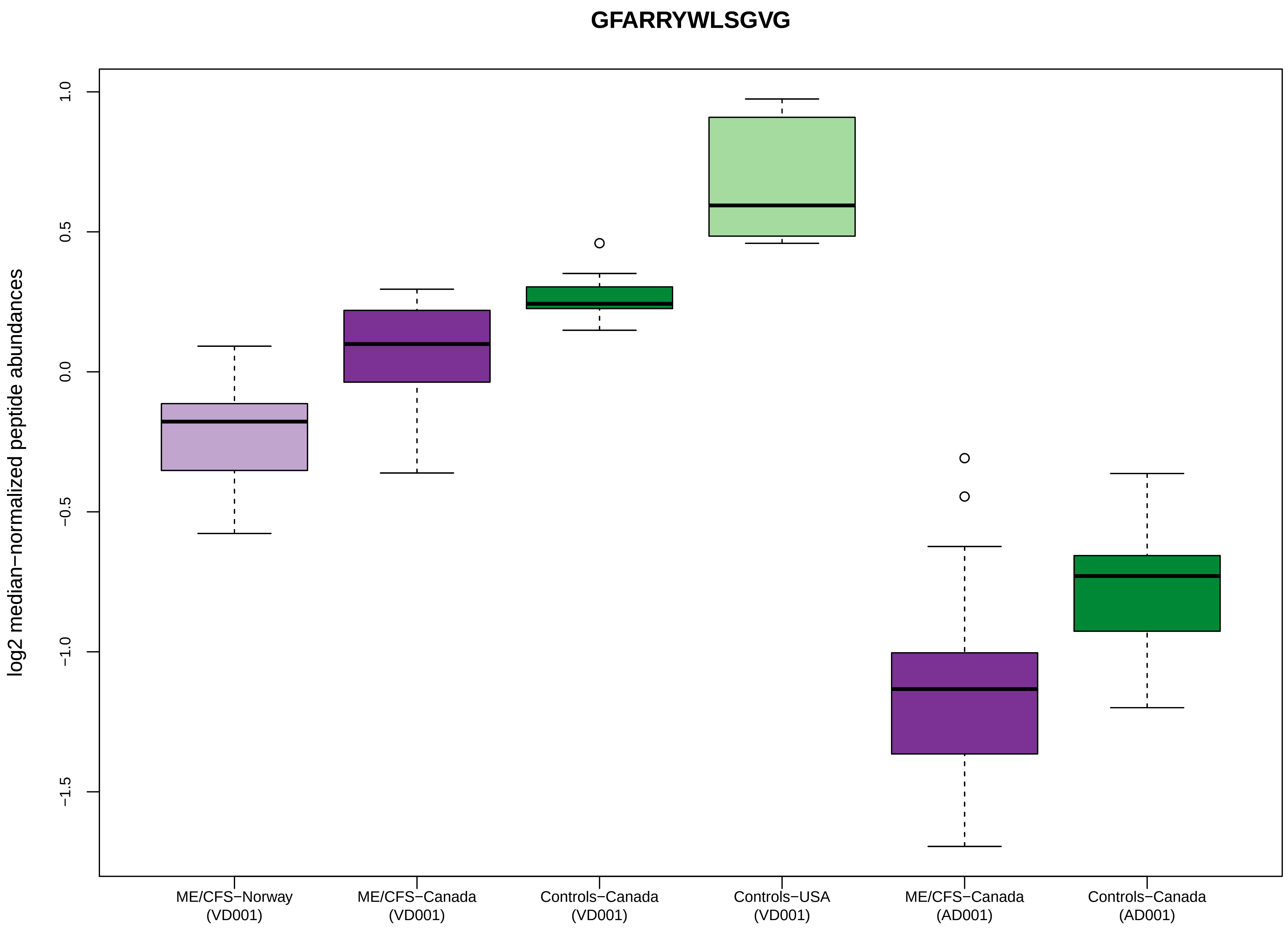

GFARRYWLSGVG
log2 median−normalized peptide abundances
1.0
0.5
0.0
−0.5
−1.0
−1.5
ME/CFS−Norway (VD001)
ME/CFS−Canada (VD001)
Controls−Canada (VD001)
Controls−USA (VD001)
ME/CFS−Canada (AD001)
Controls−Canada (AD001)

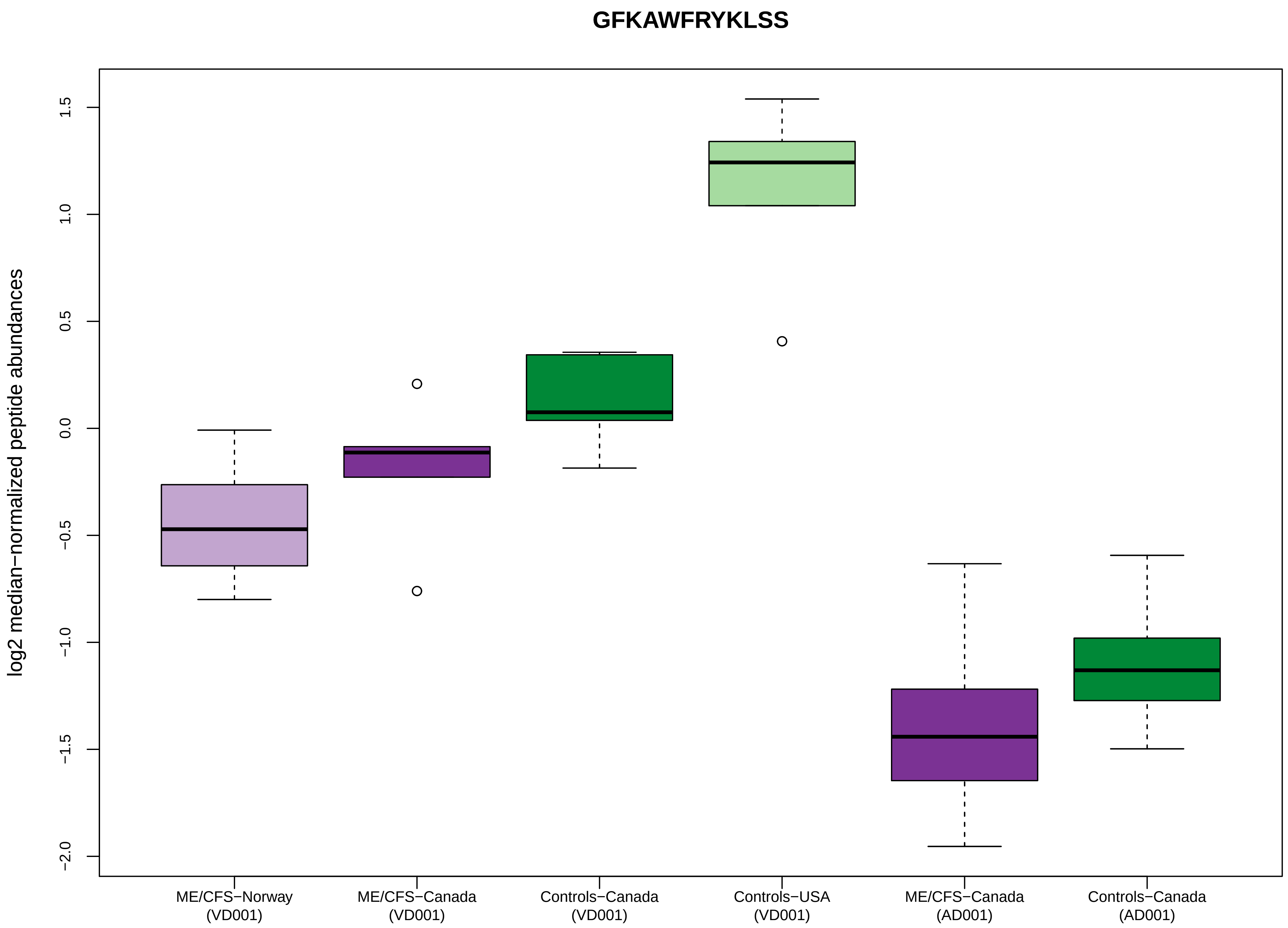

GFKAWFRYKLSS
log2 median−normalized peptide abundances
1.5
1.0
0.5
0.0
−0.5
−1.0
−1.5
−2.0
ME/CFS−Norway (VD001)
ME/CFS−Canada (VD001)
Controls−Canada (VD001)
Controls−USA (VD001)
ME/CFS−Canada (AD001)
Controls−Canada (AD001)

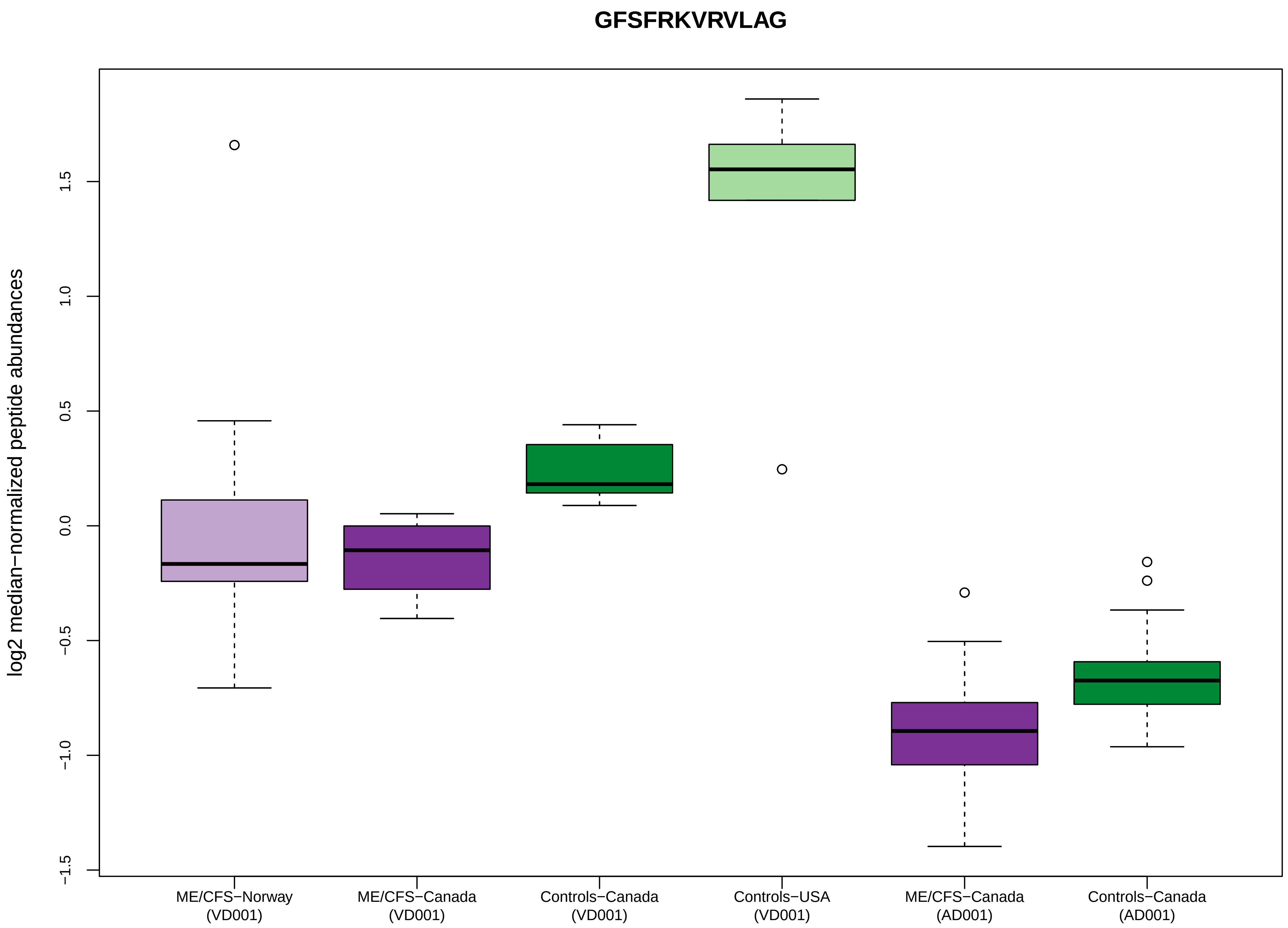

GFSFRKVRVLAG
log2 median−normalized peptide abundances
1.5
1.0
0.5
0.0
−0.5
−1.0
−1.5
ME/CFS−Norway (VD001)
ME/CFS−Canada (VD001)
Controls−Canada (VD001)
Controls−USA (VD001)
ME/CFS−Canada (AD001)
Controls−Canada (AD001)

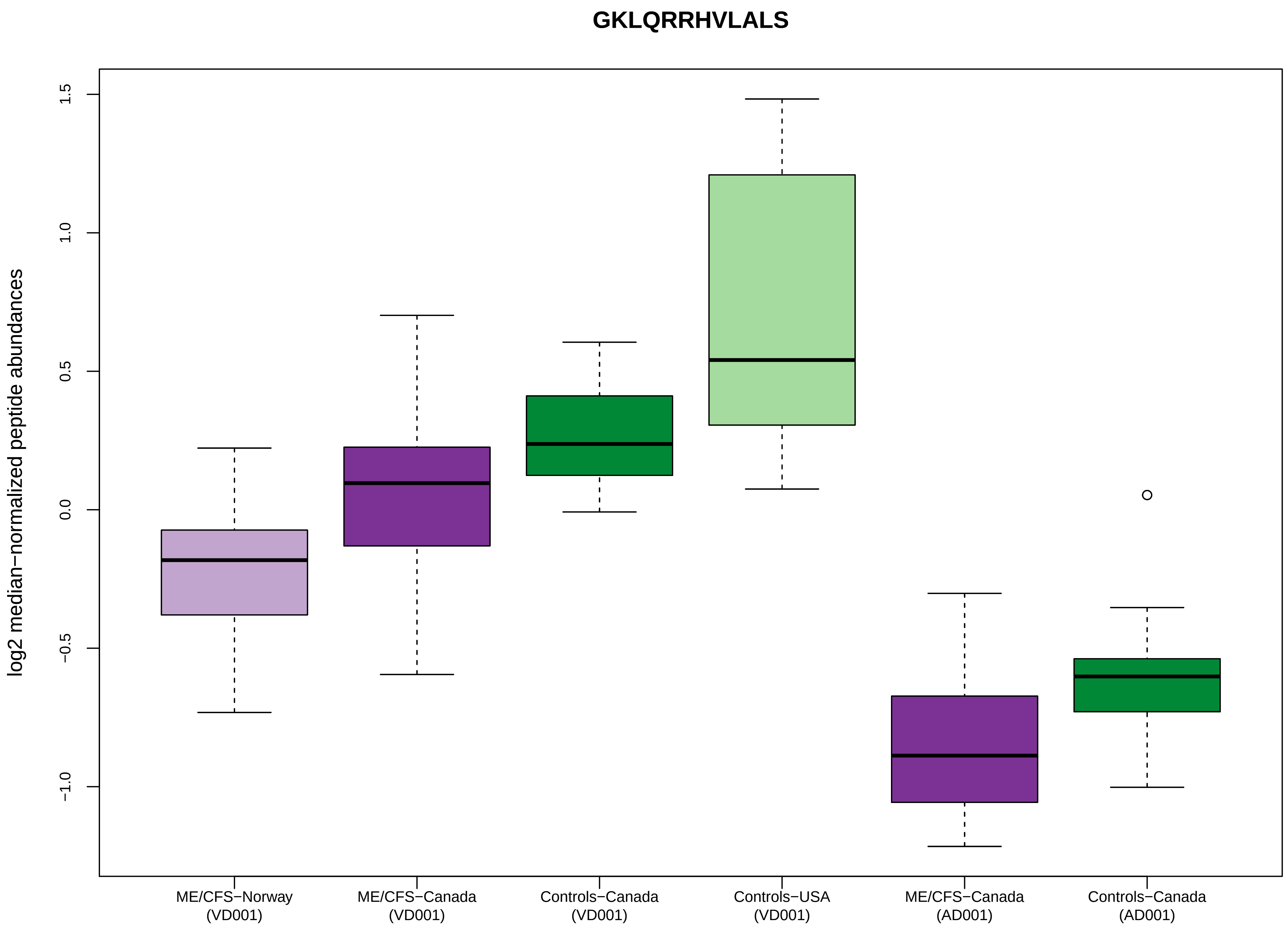

GKLQRRHVLALS
log2 median−normalized peptide abundances
1.5
1.0
0.5
0.0
−0.5
−1.0
ME/CFS−Norway (VD001)
ME/CFS−Canada (VD001)
Controls−Canada (VD001)
Controls−USA (VD001)
ME/CFS−Canada (AD001)
Controls−Canada (AD001)

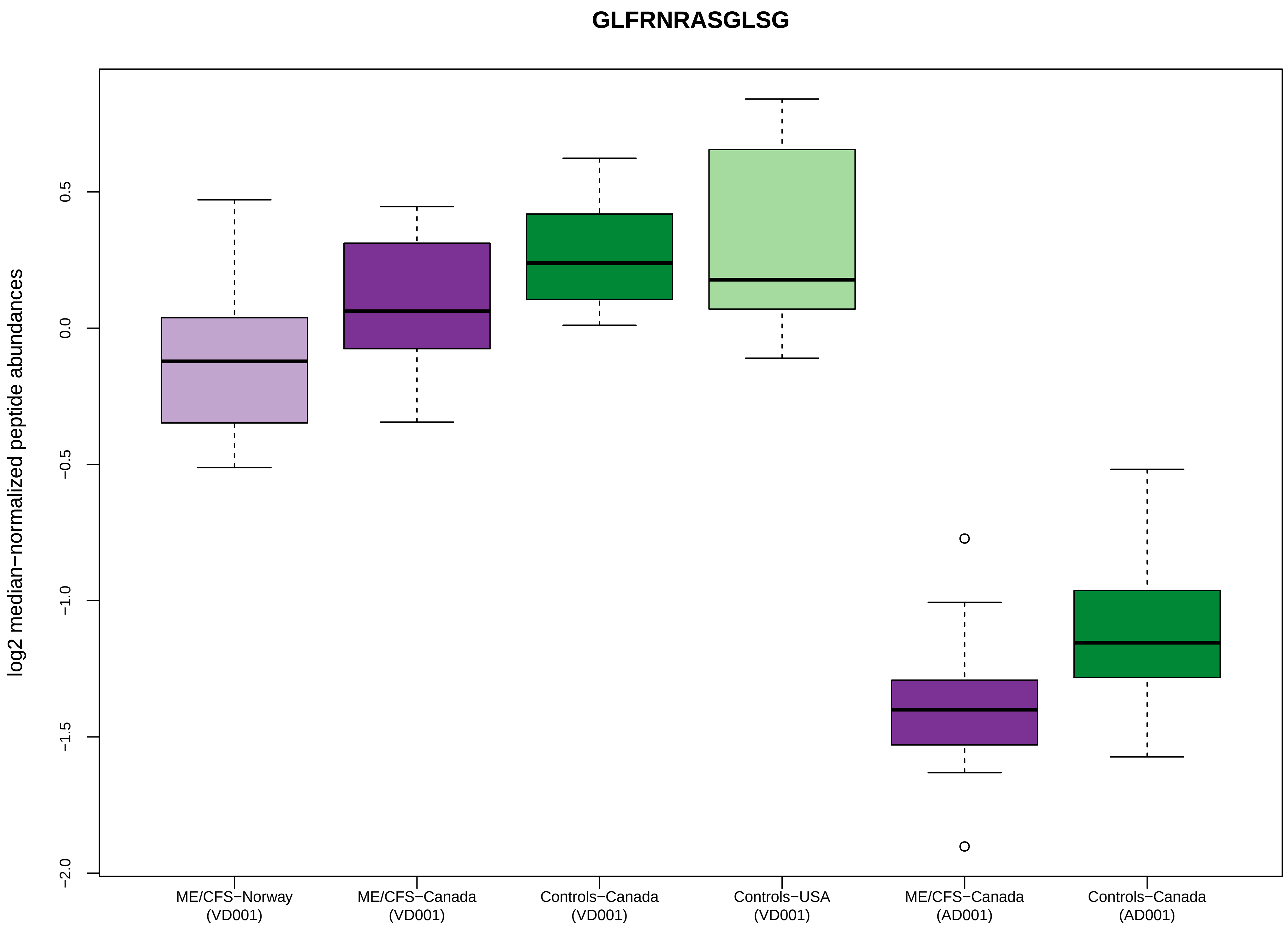

GLFRNRASGLSG
log2 median−normalized peptide abundances
0.5
0.0
−0.5
−1.0
−1.5
−2.0
ME/CFS−Norway (VD001)
ME/CFS−Canada (VD001)
Controls−Canada (VD001)
Controls−USA (VD001)
ME/CFS−Canada (AD001)
Controls−Canada (AD001)

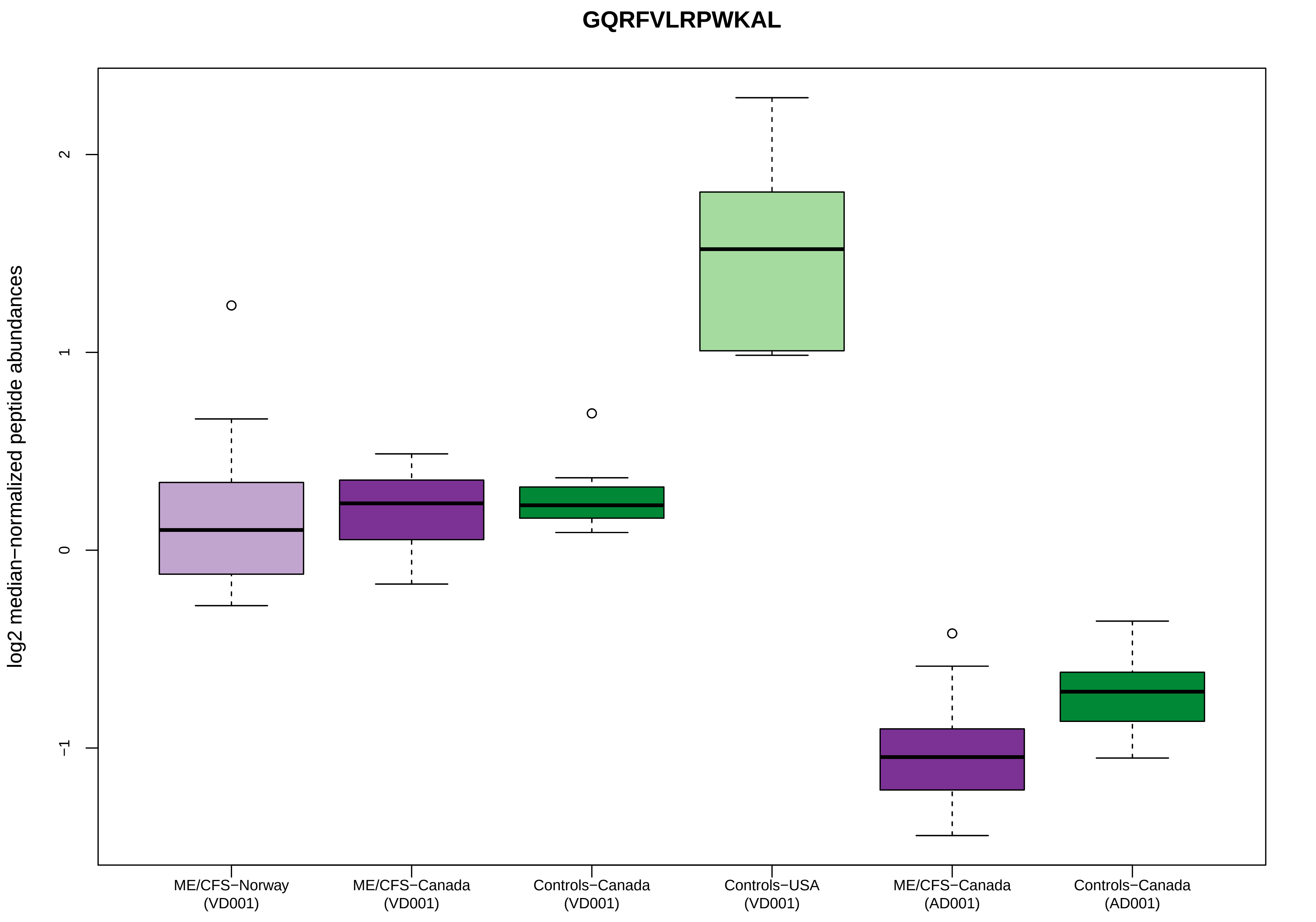
GQRFVLRPWKAL
log2 median−normalized peptide abundances
2
1
0
−1
ME/CFS−Norway (VD001)
ME/CFS−Canada (VD001)
Controls−Canada (VD001)
Controls−USA (VD001)
ME/CFS−Canada (AD001)
Controls−Canada (AD001)

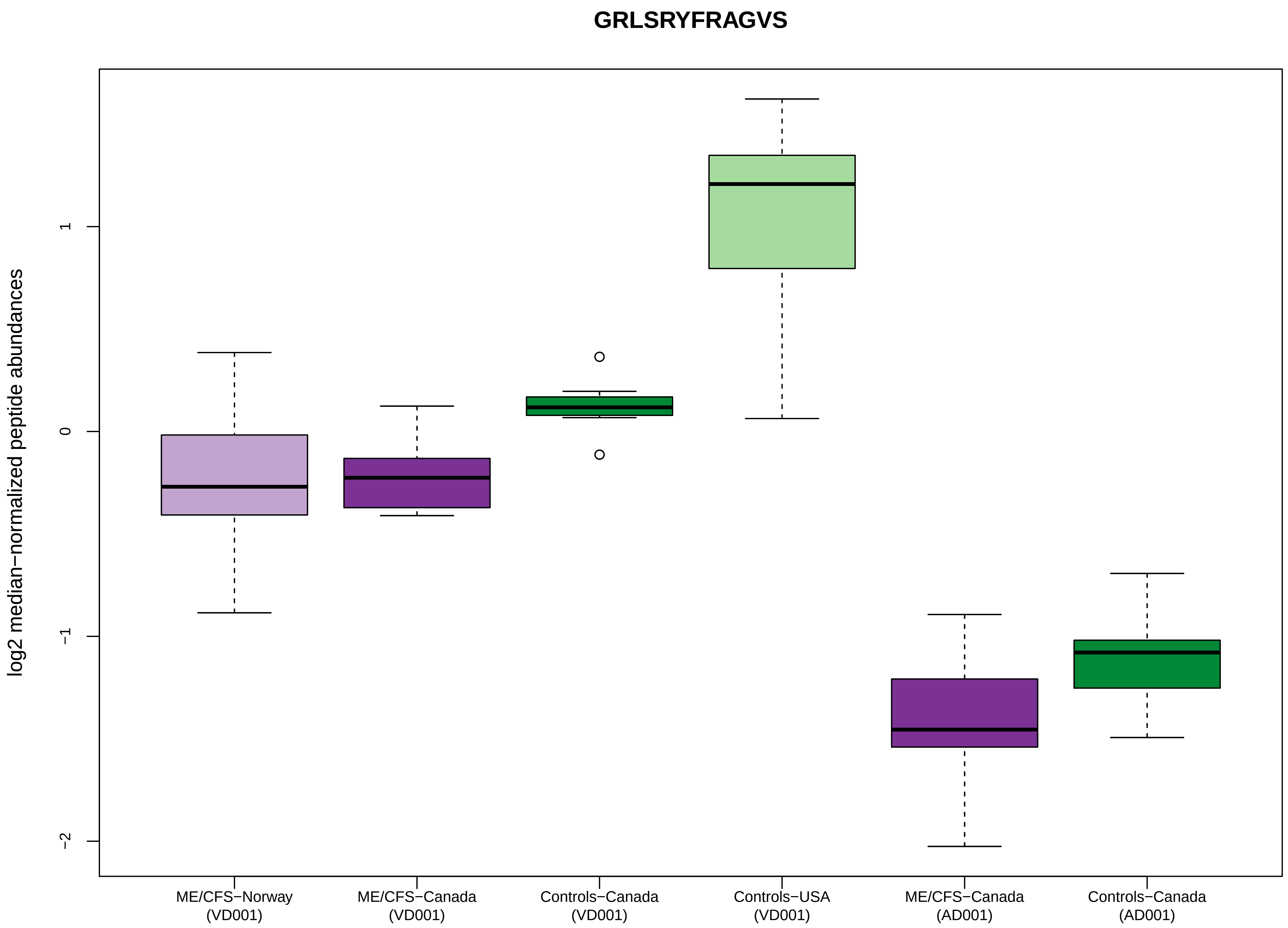
GRLSRYFRAGVS
log2 median−normalized peptide abundances
1
0
−1
−2
ME/CFS−Norway (VD001)
ME/CFS−Canada (VD001)
Controls−Canada (VD001)
Controls−USA (VD001)
ME/CFS−Canada (AD001)
Controls−Canada (AD001)

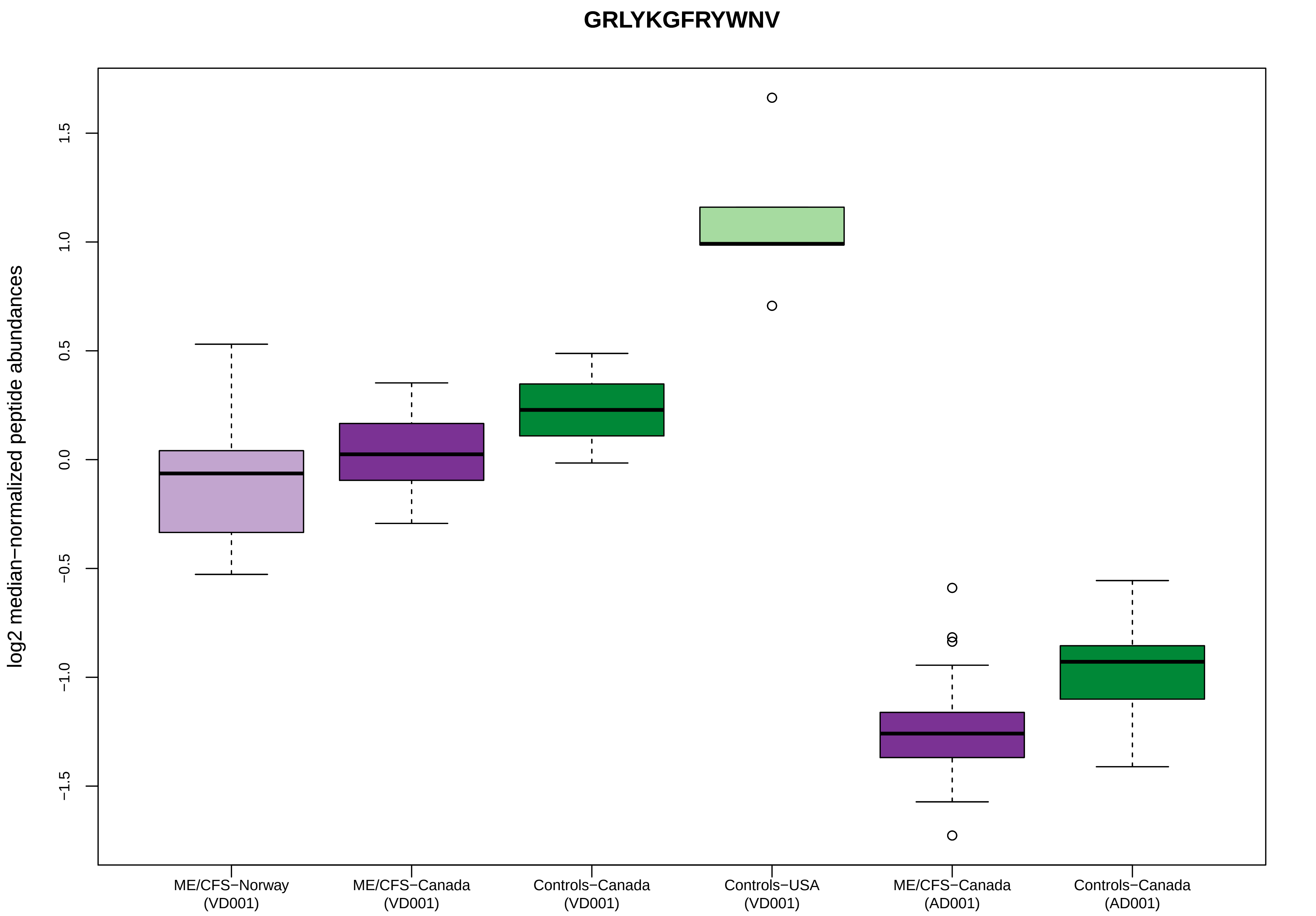
GRLYKGFRYWNV
log2 median−normalized peptide abundances
1.5
1.0
0.5
0.0
−0.5
−1.0
−1.5
ME/CFS−Norway (VD001)
ME/CFS−Canada (VD001)
Controls−Canada (VD001)
Controls−USA (VD001)
ME/CFS−Canada (AD001)
Controls−Canada (AD001)

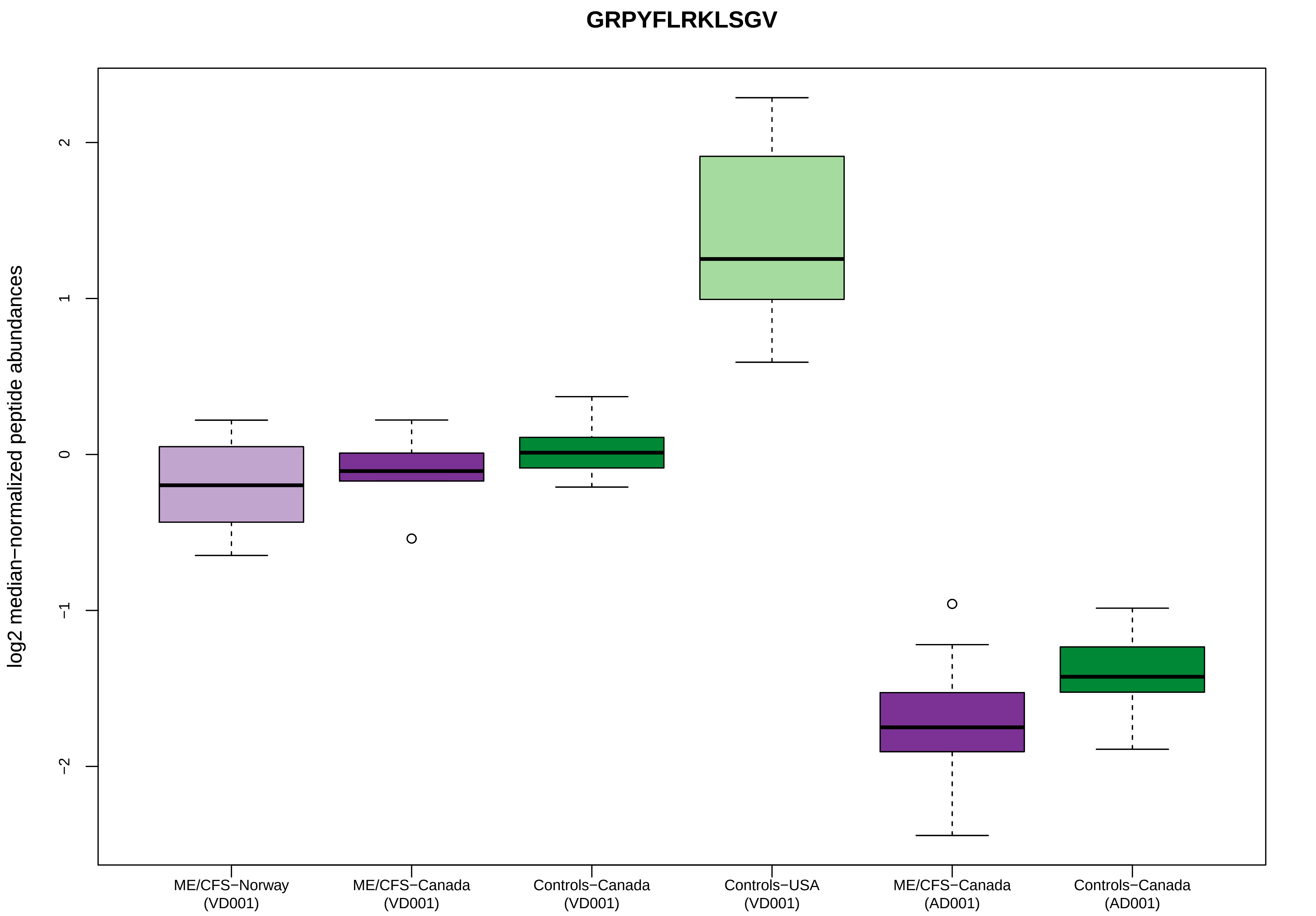
GRPYFLRKLSGV
log2 median−normalized peptide abundances
2
1
0
−1
−2
ME/CFS−Norway
(VD001)
ME/CFS−Canada
(VD001)
Controls−Canada
(VD001)
Controls−USA
(VD001)
ME/CFS−Canada
(AD001)
Controls−Canada
(AD001)

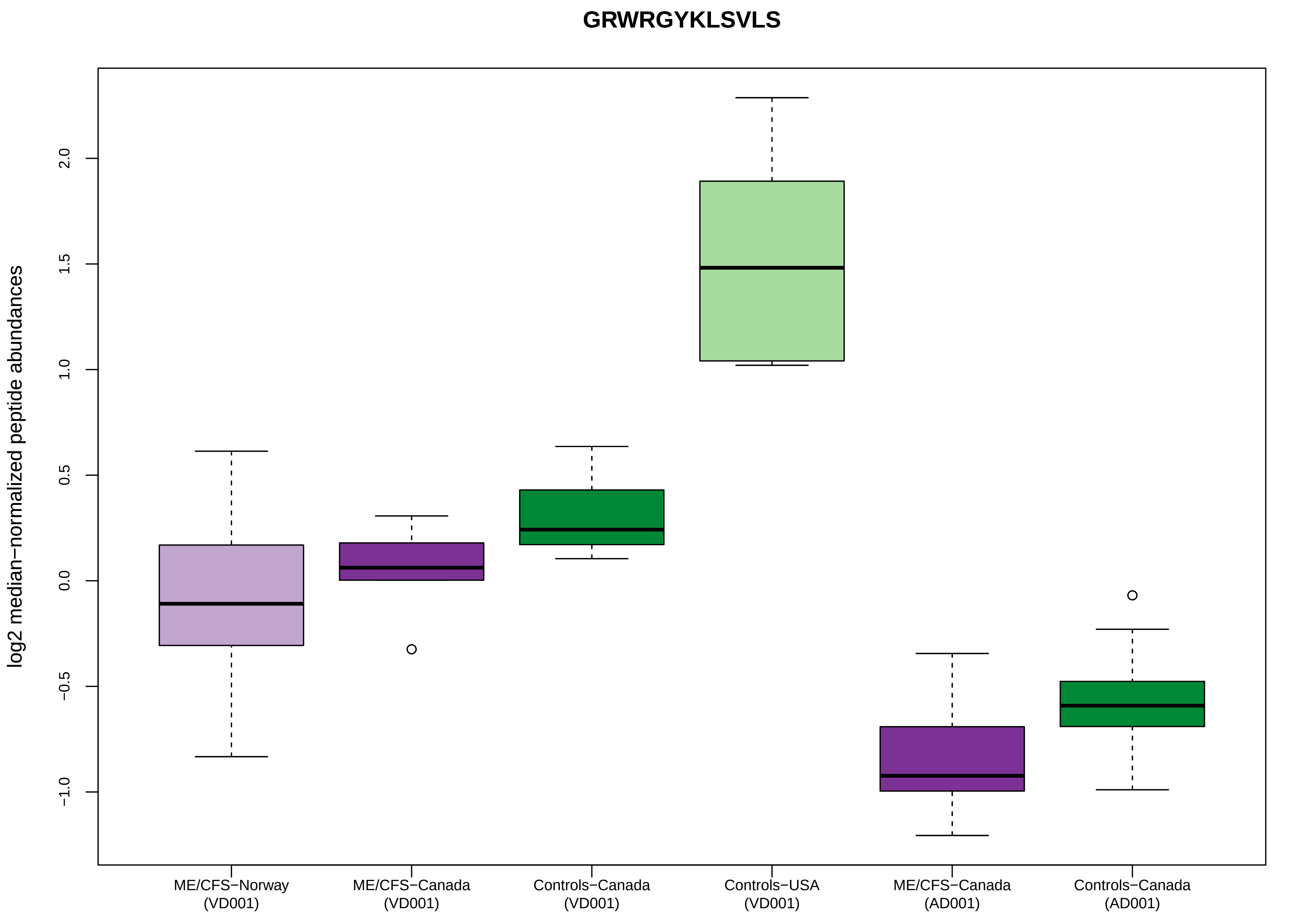
GRWRGYKLSVLS
log2 median−normalized peptide abundances
2.0
1.5
1.0
0.5
0.0
−0.5
−1.0
ME/CFS−Norway (VD001)
ME/CFS−Canada (VD001)
Controls−Canada (VD001)
Controls−USA (VD001)
ME/CFS−Canada (AD001)
Controls−Canada (AD001)

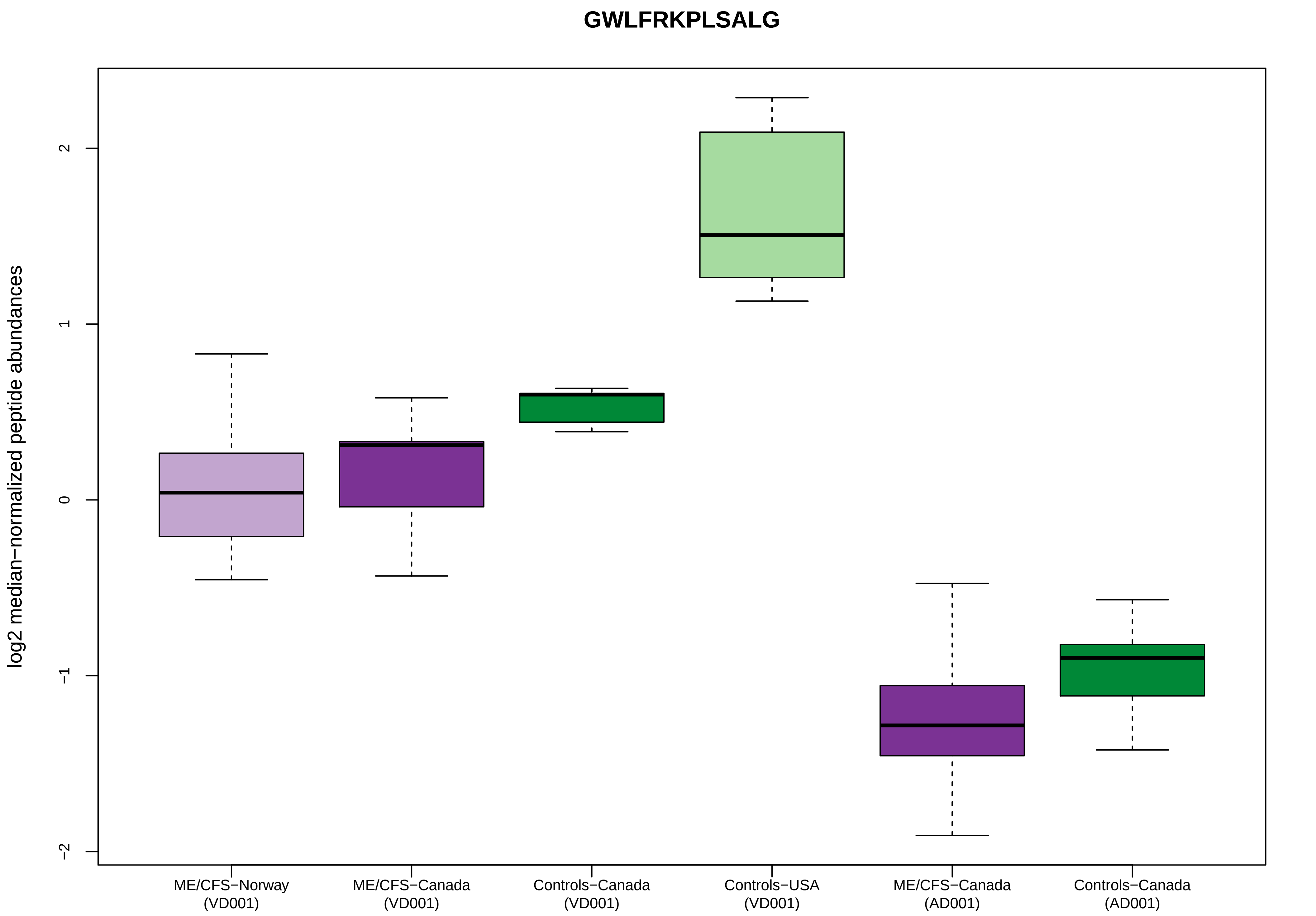

GWLFRKPLSALG
log2 median−normalized peptide abundances
2
1
0
−1
−2
ME/CFS−Norway (VD001)
ME/CFS−Canada (VD001)
Controls−Canada (VD001)
Controls−USA (VD001)
ME/CFS−Canada (AD001)
Controls−Canada (AD001)

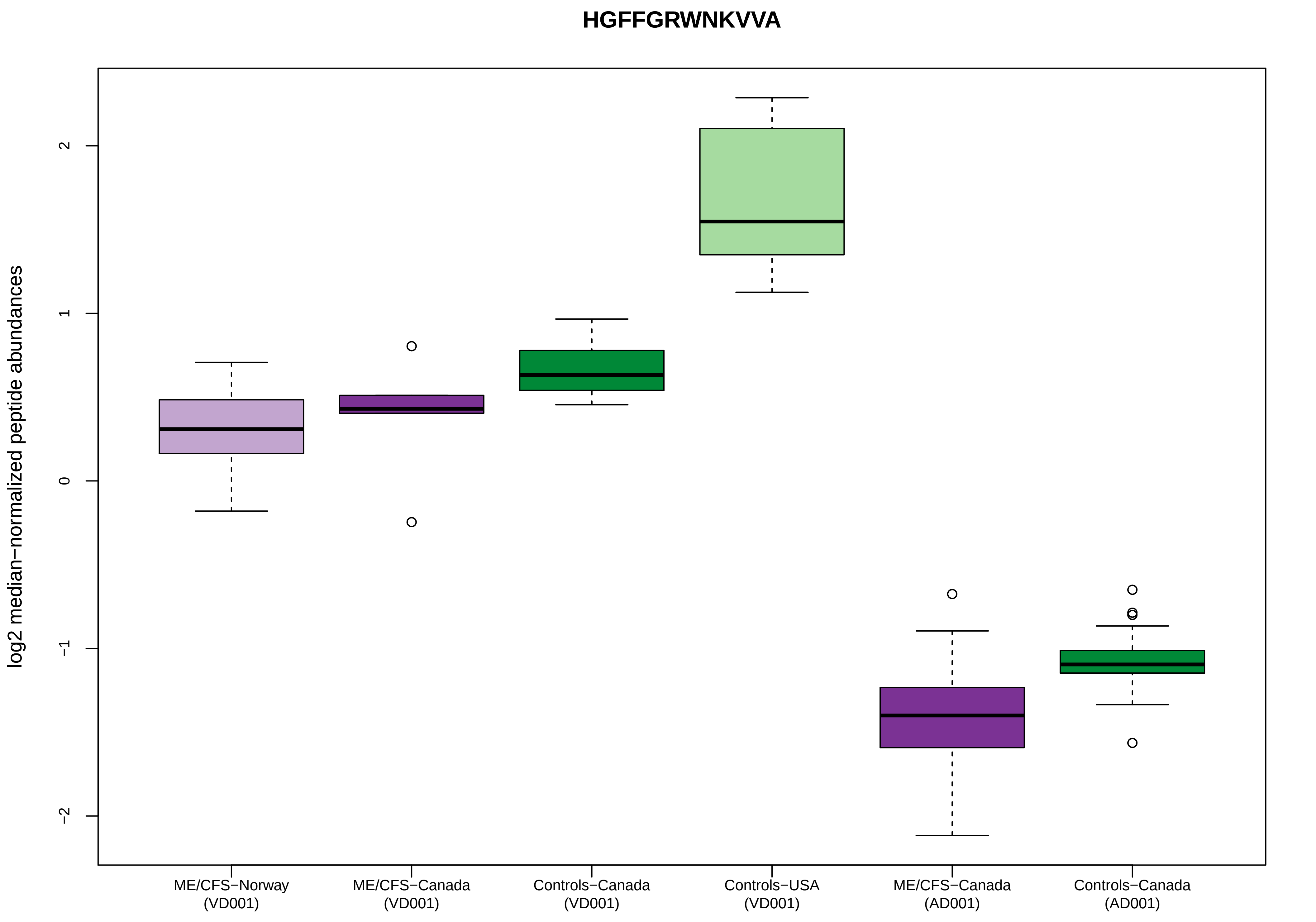
HGFFGRWNKVVA
log2 median−normalized peptide abundances
2
1
0
−1
−2
ME/CFS−Norway (VD001)
ME/CFS−Canada (VD001)
Controls−Canada (VD001)
Controls−USA (VD001)
ME/CFS−Canada (AD001)
Controls−Canada (AD001)

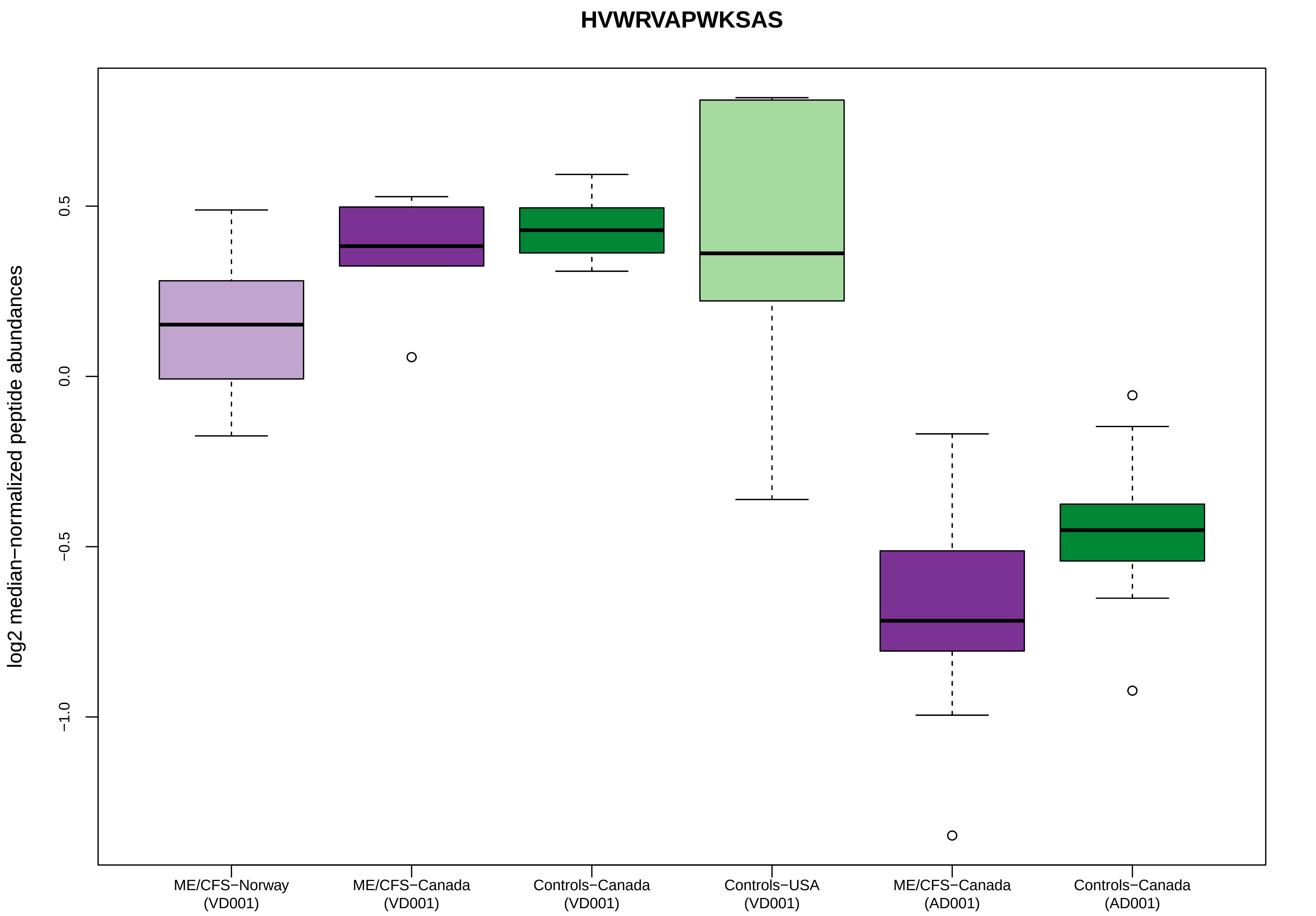

HVWRVAPWKSAS
log2 median−normalized peptide abundances
0.5
0.0
−0.5
−1.0
ME/CFS−Norway (VD001)
ME/CFS−Canada (VD001)
Controls−Canada (VD001)
Controls−USA (VD001)
ME/CFS−Canada (AD001)
Controls−Canada (AD001)

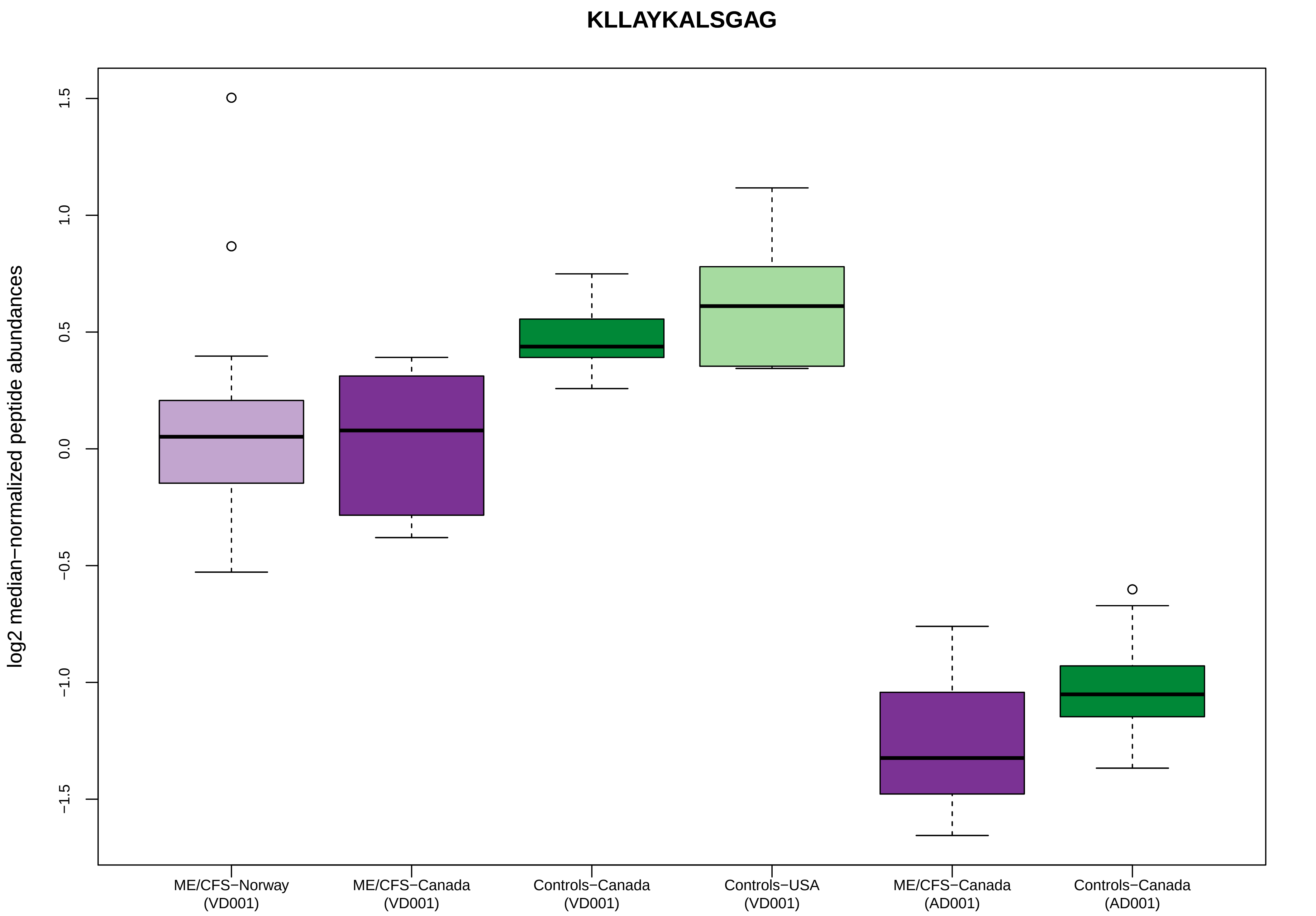
KLLAYKALSGAG
log2 median−normalized peptide abundances
1.5
1.0
0.5
0.0
−0.5
−1.0
−1.5
ME/CFS−Norway (VD001)
ME/CFS−Canada (VD001)
Controls−Canada (VD001)
Controls−USA (VD001)
ME/CFS−Canada (AD001)
Controls−Canada (AD001)

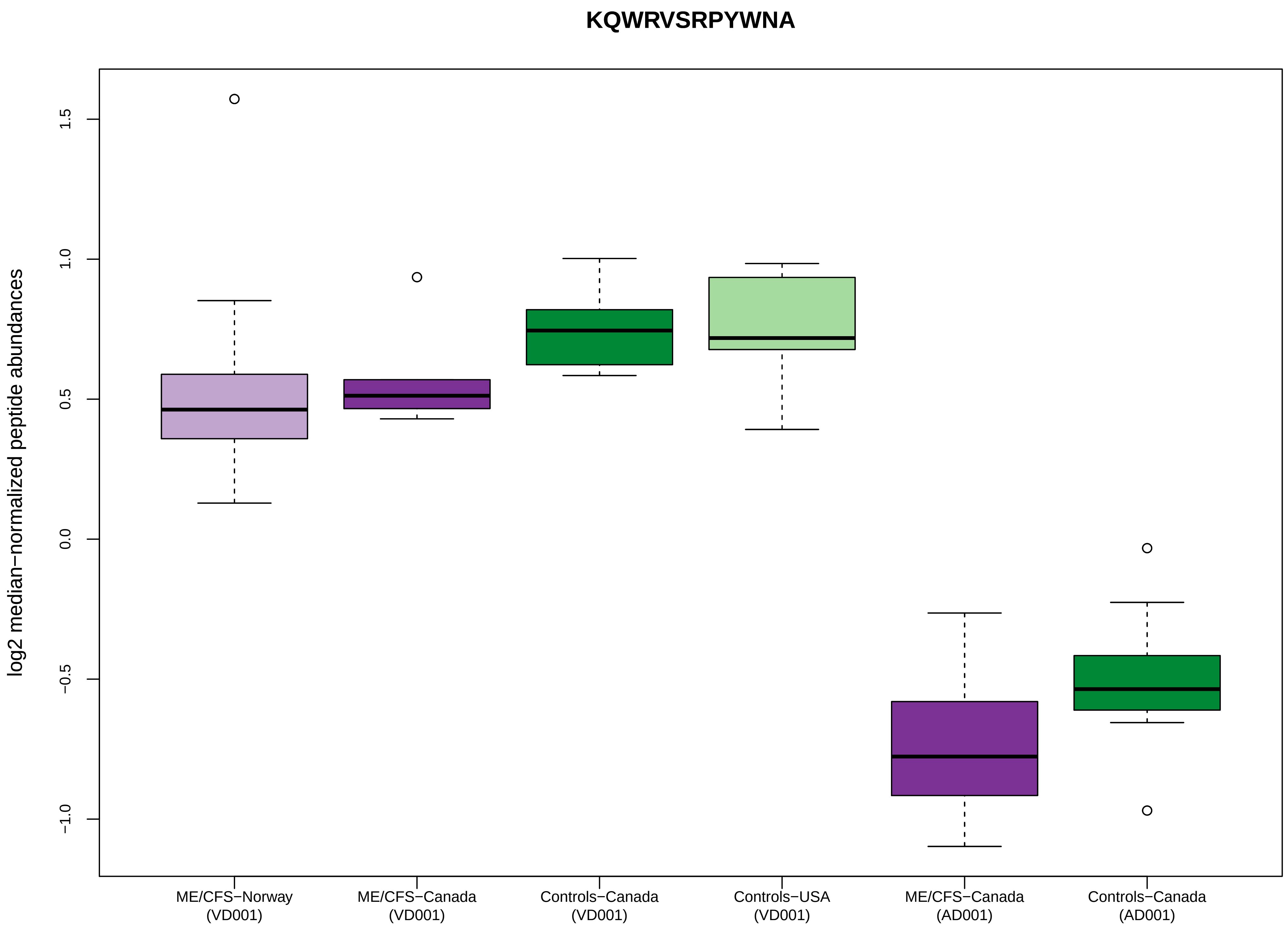

KQWRVSRPYWNA
log2 median−normalized peptide abundances
1.5
1.0
0.5
0.0
−0.5
−1.0
ME/CFS−Norway (VD001)
ME/CFS−Canada (VD001)
Controls−Canada (VD001)
Controls−USA (VD001)
ME/CFS−Canada (AD001)
Controls−Canada (AD001)

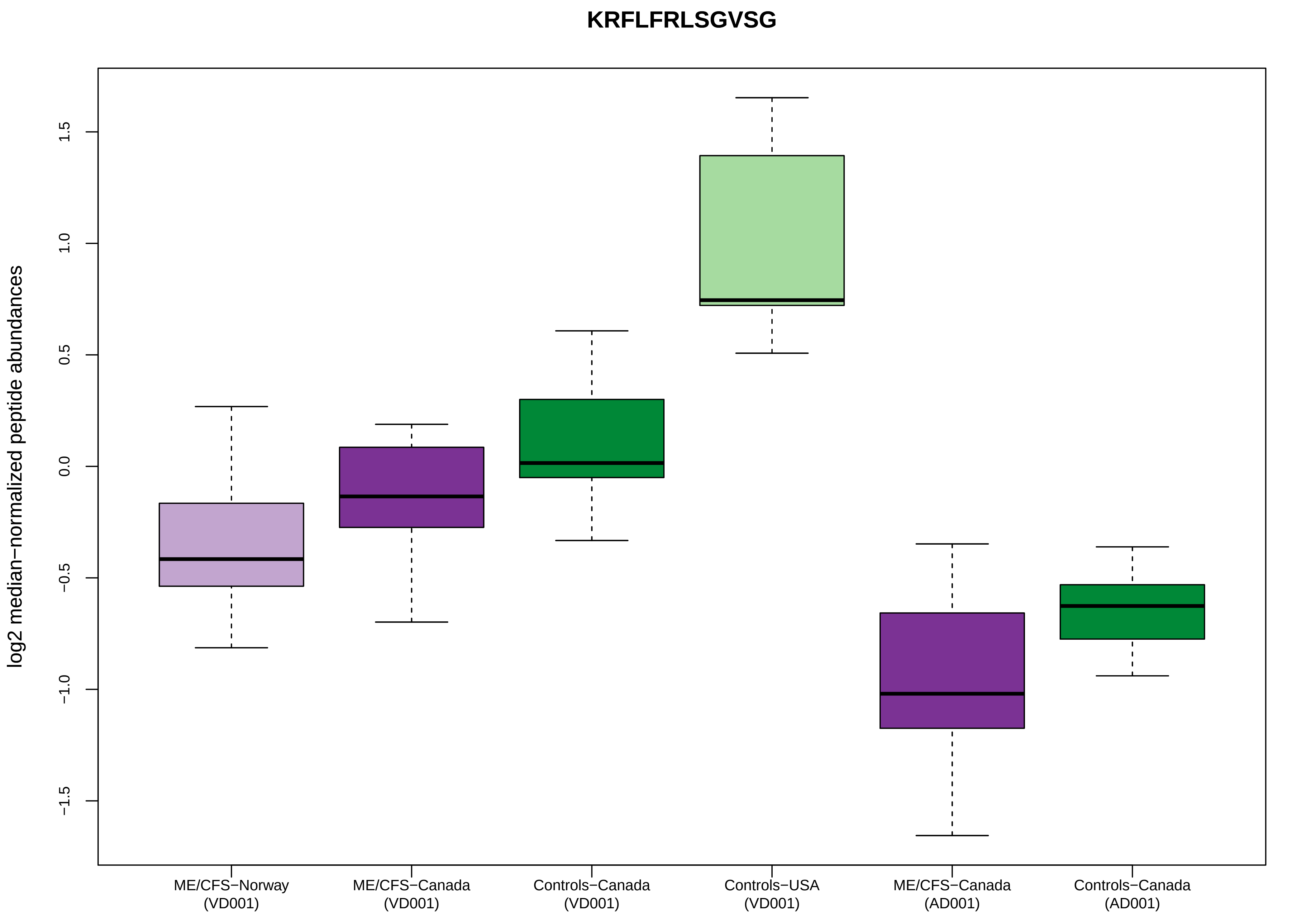
KRFLFRLSGVSG
log2 median−normalized peptide abundances
1.5
1.0
0.5
0.0
−0.5
−1.0
−1.5
ME/CFS−Norway (VD001)
ME/CFS−Canada (VD001)
Controls−Canada (VD001)
Controls−USA (VD001)
ME/CFS−Canada (AD001)
Controls−Canada (AD001)

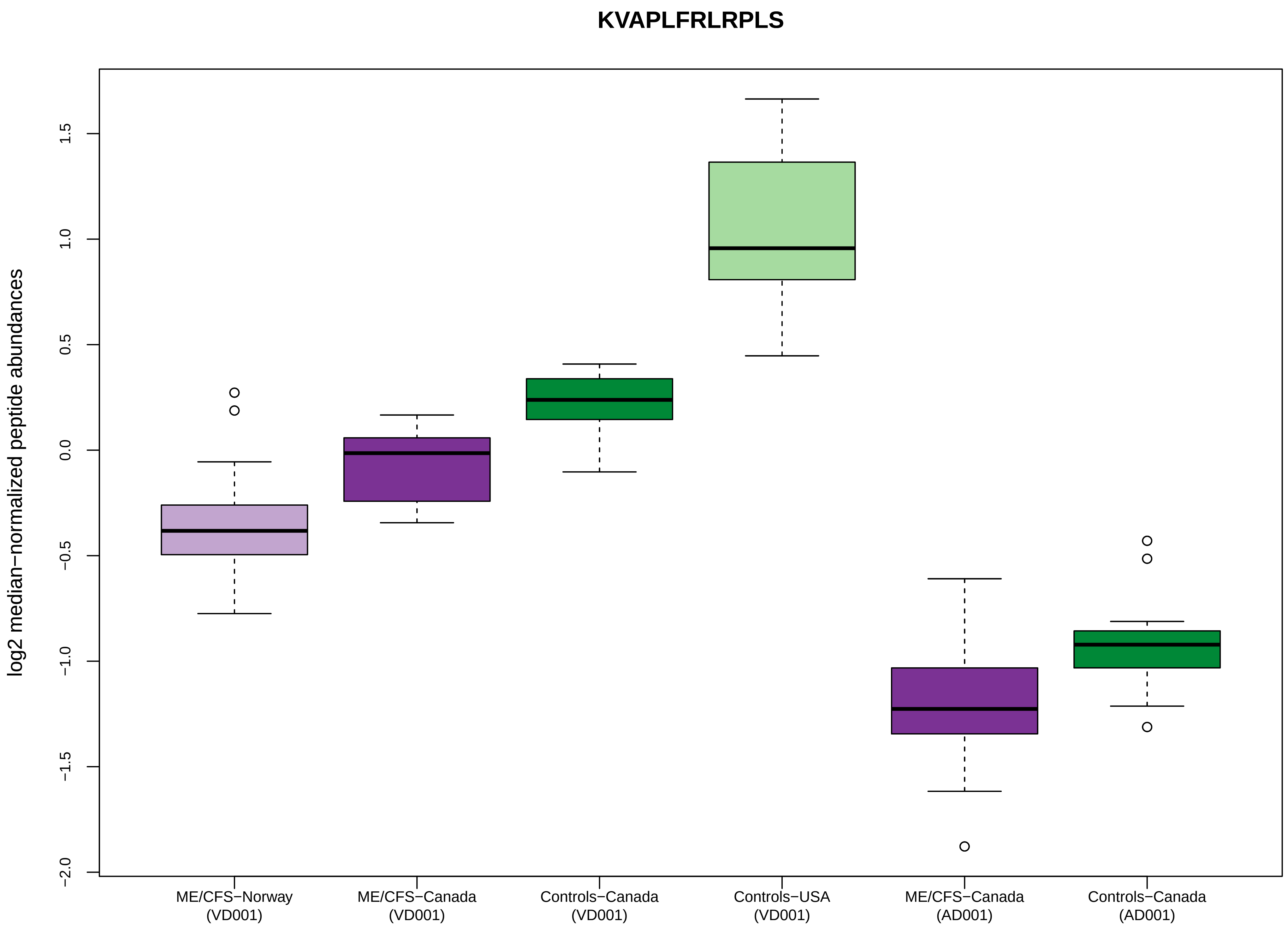

KVAPLFRLRPLS
log2 median−normalized peptide abundances
1.5
1.0
0.5
0.0
−0.5
−1.0
−1.5
−2.0
ME/CFS−Norway (VD001)
ME/CFS−Canada (VD001)
Controls−Canada (VD001)
Controls−USA (VD001)
ME/CFS−Canada (AD001)
Controls−Canada (AD001)

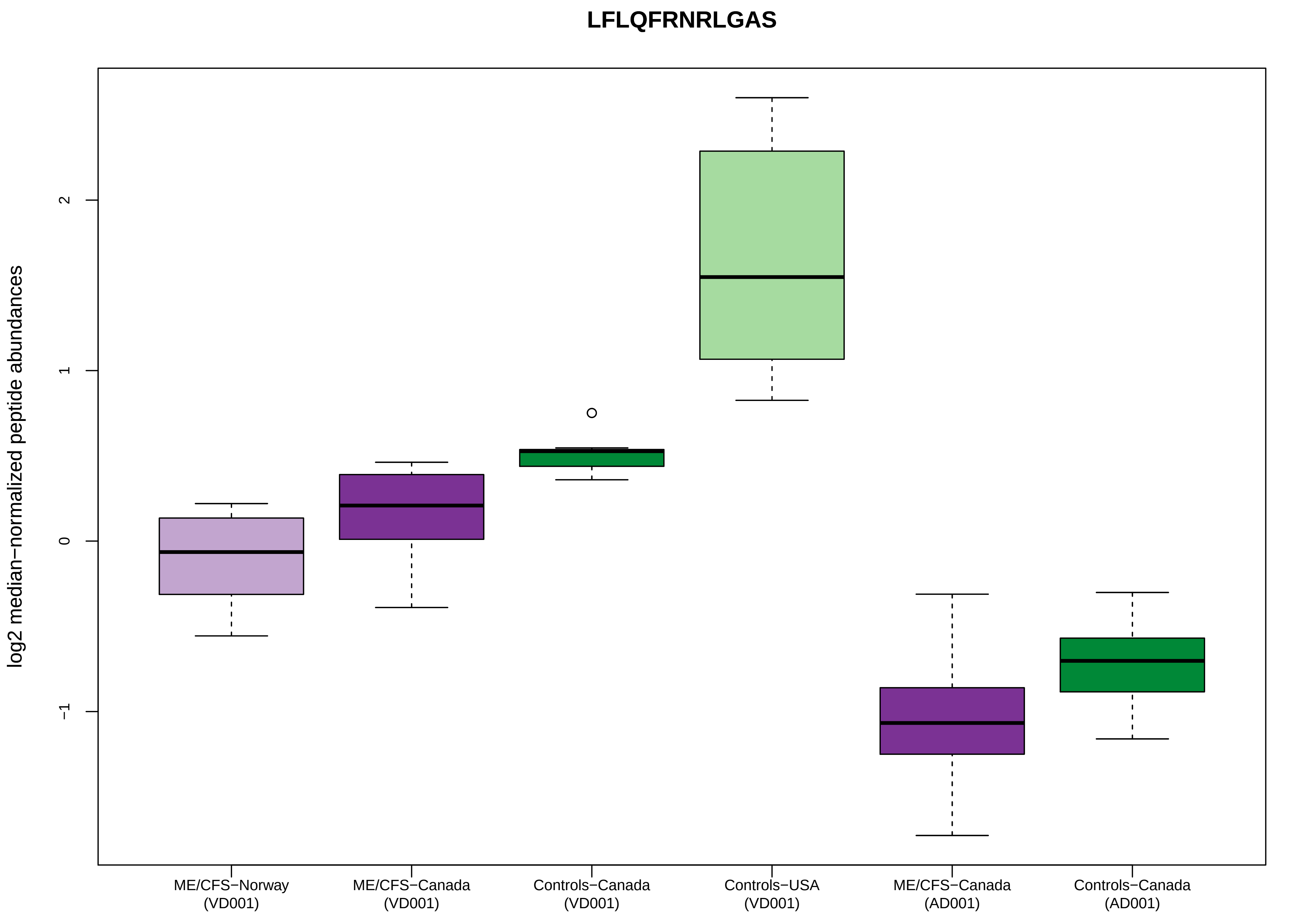

LFLQFRNRLGAS
log2 median−normalized peptide abundances
2
1
0
−1
ME/CFS−Norway (VD001)
ME/CFS−Canada (VD001)
Controls−Canada (VD001)
Controls−USA (VD001)
ME/CFS−Canada (AD001)
Controls−Canada (AD001)

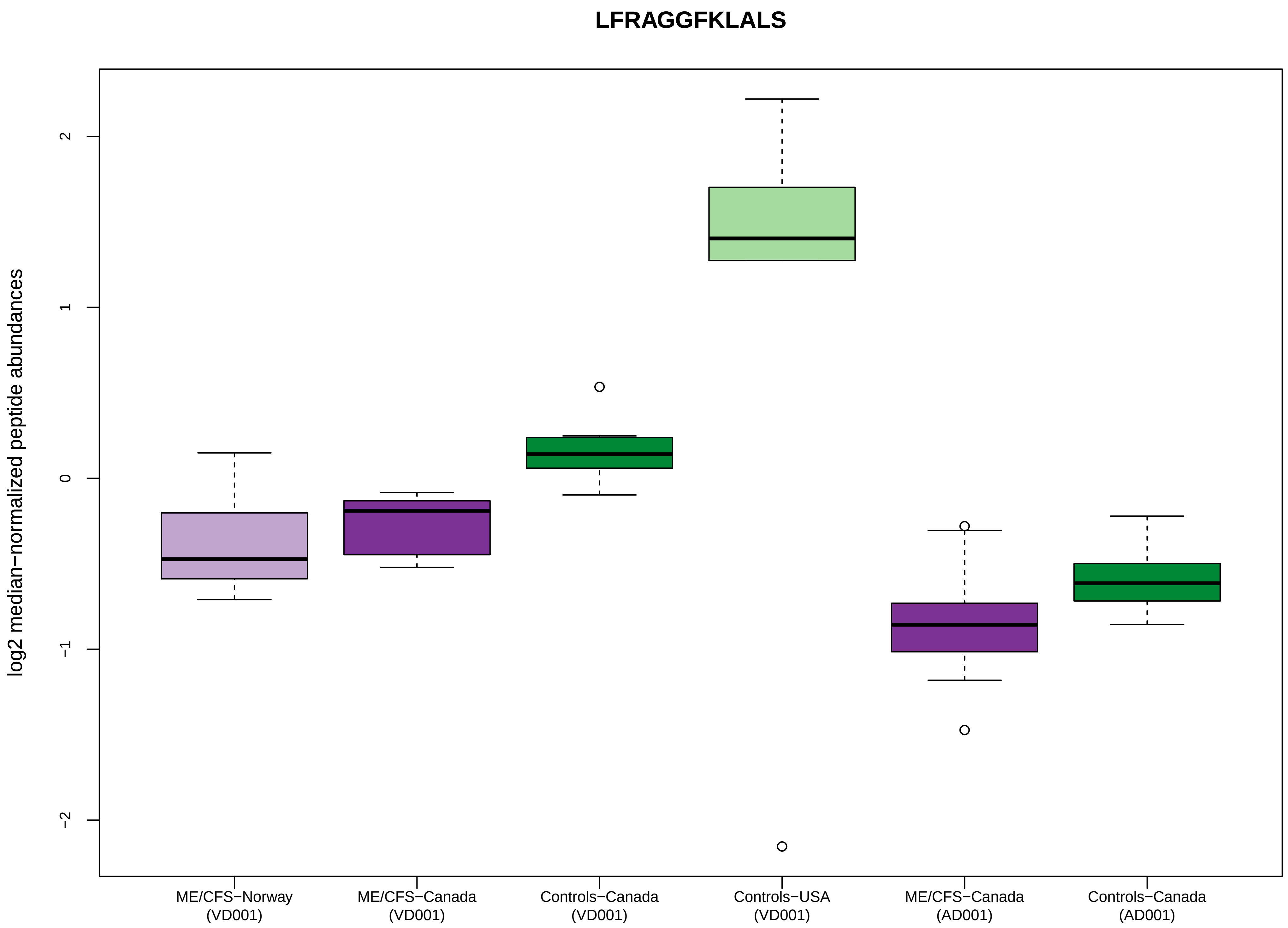

LFRAGGFKLALS
log2 median−normalized peptide abundances
−2
−1
0
1
2
ME/CFS−Norway (VD001)
ME/CFS−Canada (VD001)
Controls−Canada (VD001)
Controls−USA (VD001)
ME/CFS−Canada (AD001)
Controls−Canada (AD001)

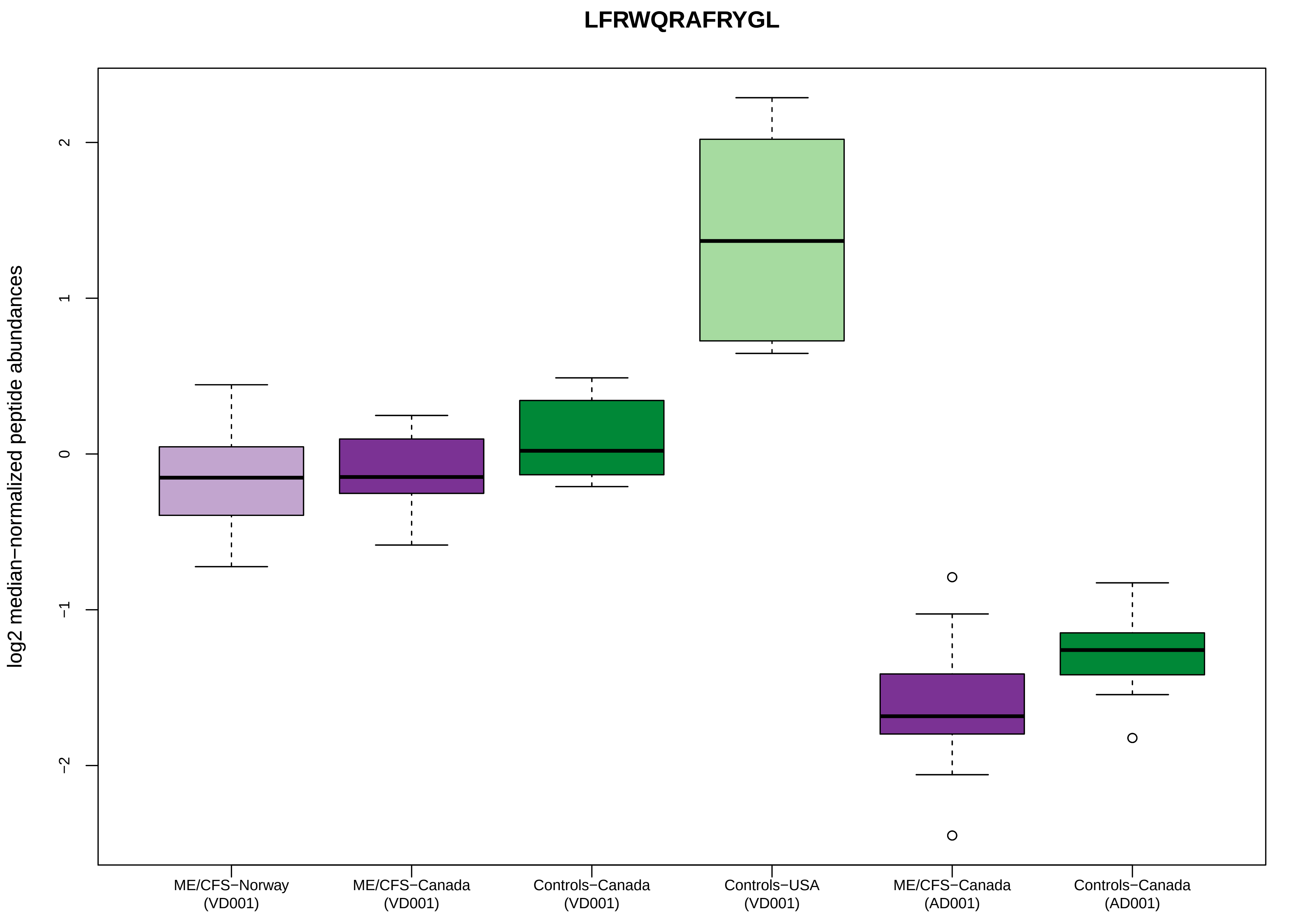
LFRWQRAFRYGL
log2 median−normalized peptide abundances
2
1
0
−1
−2
ME/CFS−Norway (VD001)
ME/CFS−Canada (VD001)
Controls−Canada (VD001)
Controls−USA (VD001)
ME/CFS−Canada (AD001)
Controls−Canada (AD001)

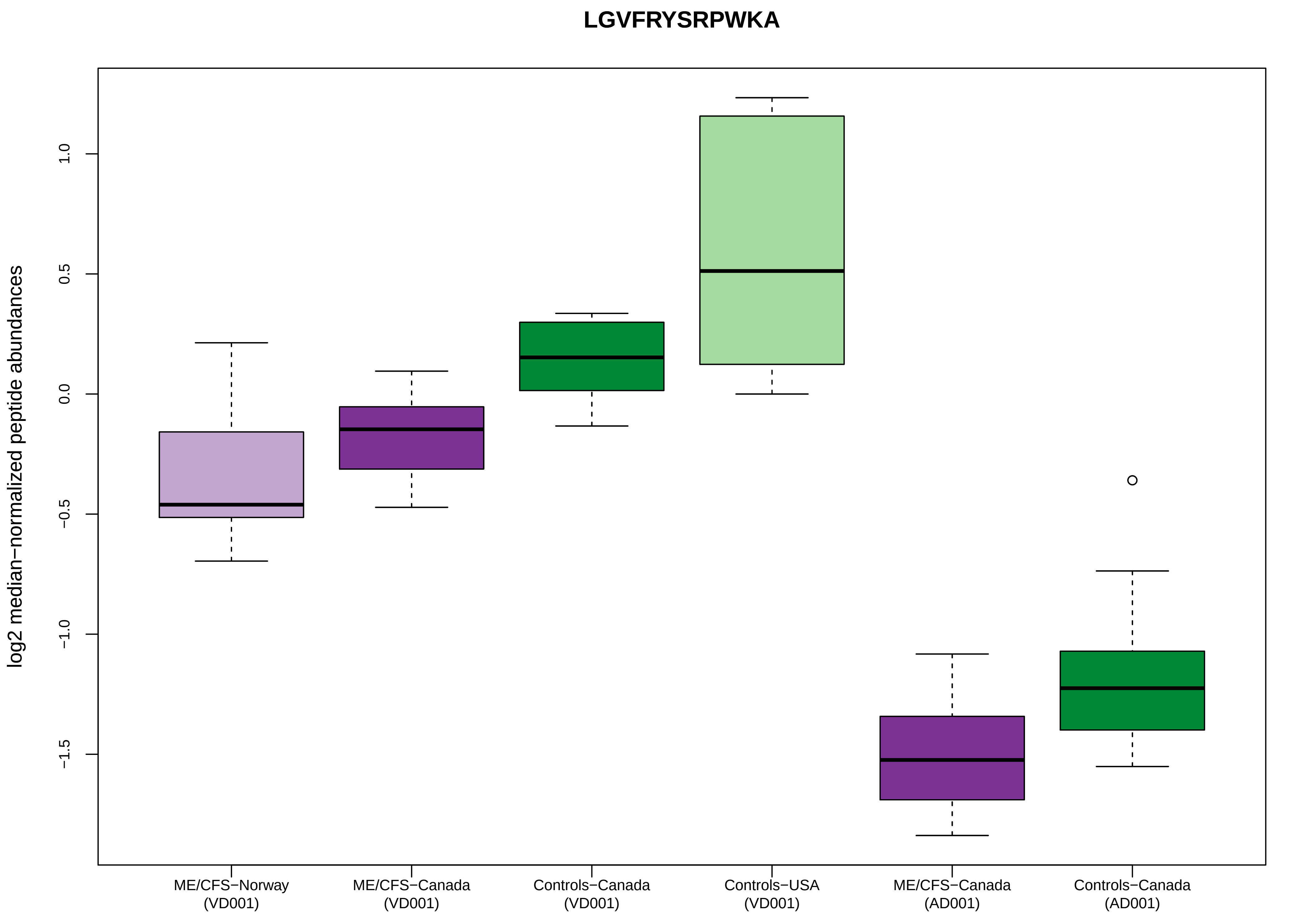
LGVFRYSRPWKA
log2 median−normalized peptide abundances
1.0
0.5
0.0
−0.5
−1.0
−1.5
ME/CFS−Norway (VD001)
ME/CFS−Canada (VD001)
Controls−Canada (VD001)
Controls−USA (VD001)
ME/CFS−Canada (AD001)
Controls−Canada (AD001)

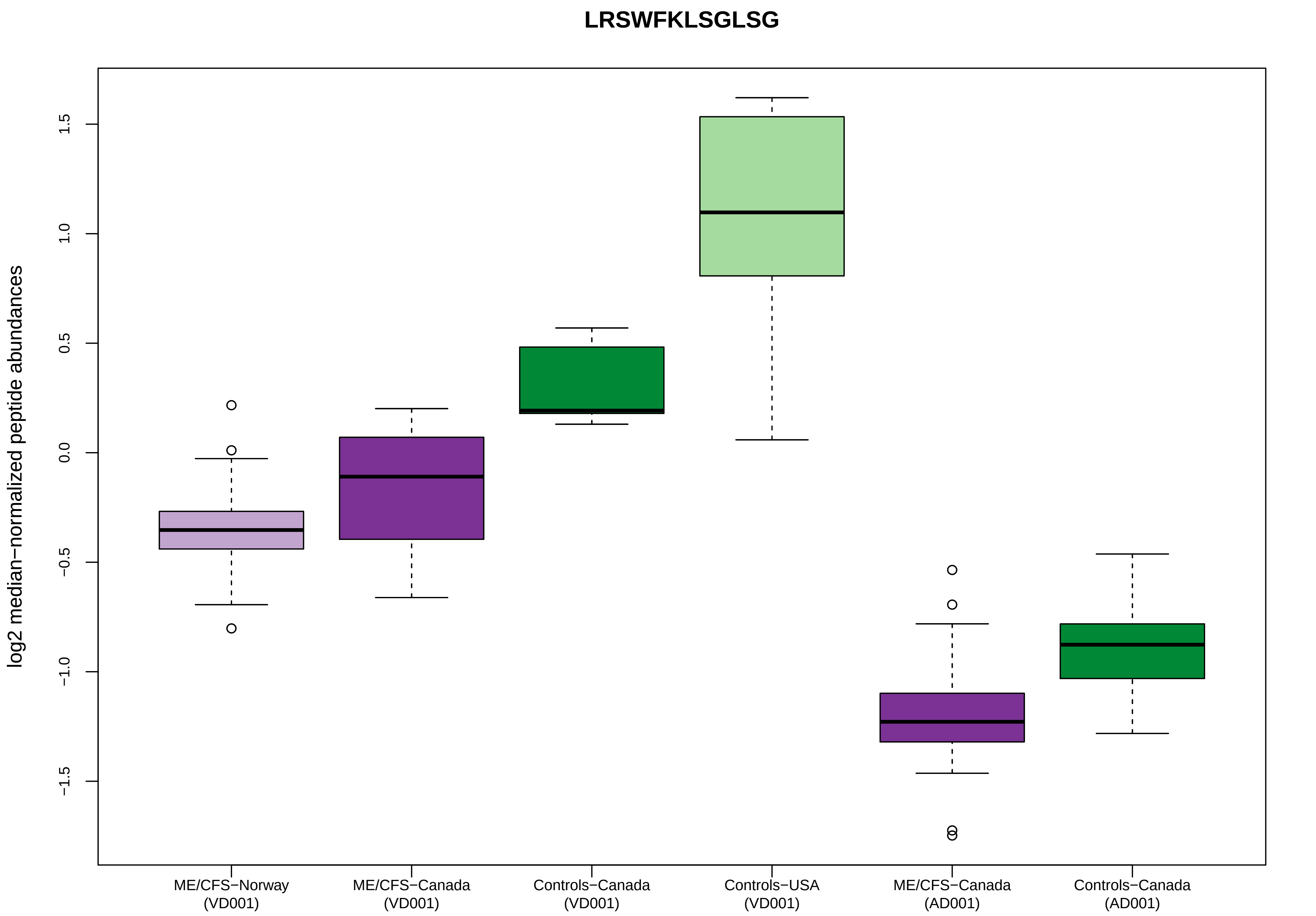

LRSWFKLSGLSG
log2 median−normalized peptide abundances
1.5
1.0
0.5
0.0
−0.5
−1.0
−1.5
ME/CFS−Norway (VD001)
ME/CFS−Canada (VD001)
Controls−Canada (VD001)
Controls−USA (VD001)
ME/CFS−Canada (AD001)
Controls−Canada (AD001)

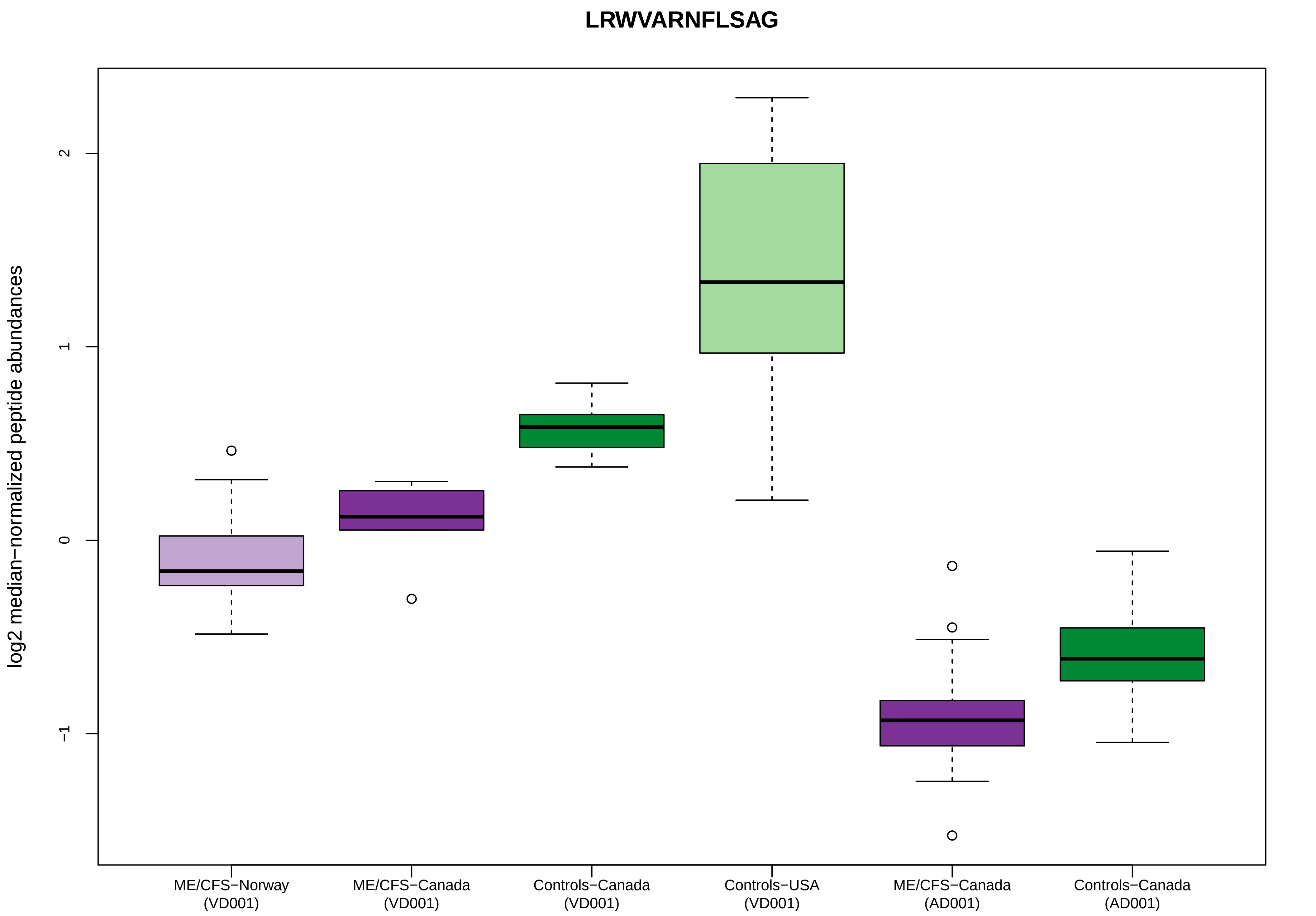

LRWVARNFLSAG
log2 median−normalized peptide abundances
2
1
0
−1
ME/CFS−Norway (VD001)
ME/CFS−Canada (VD001)
Controls−Canada (VD001)
Controls−USA (VD001)
ME/CFS−Canada (AD001)
Controls−Canada (AD001)

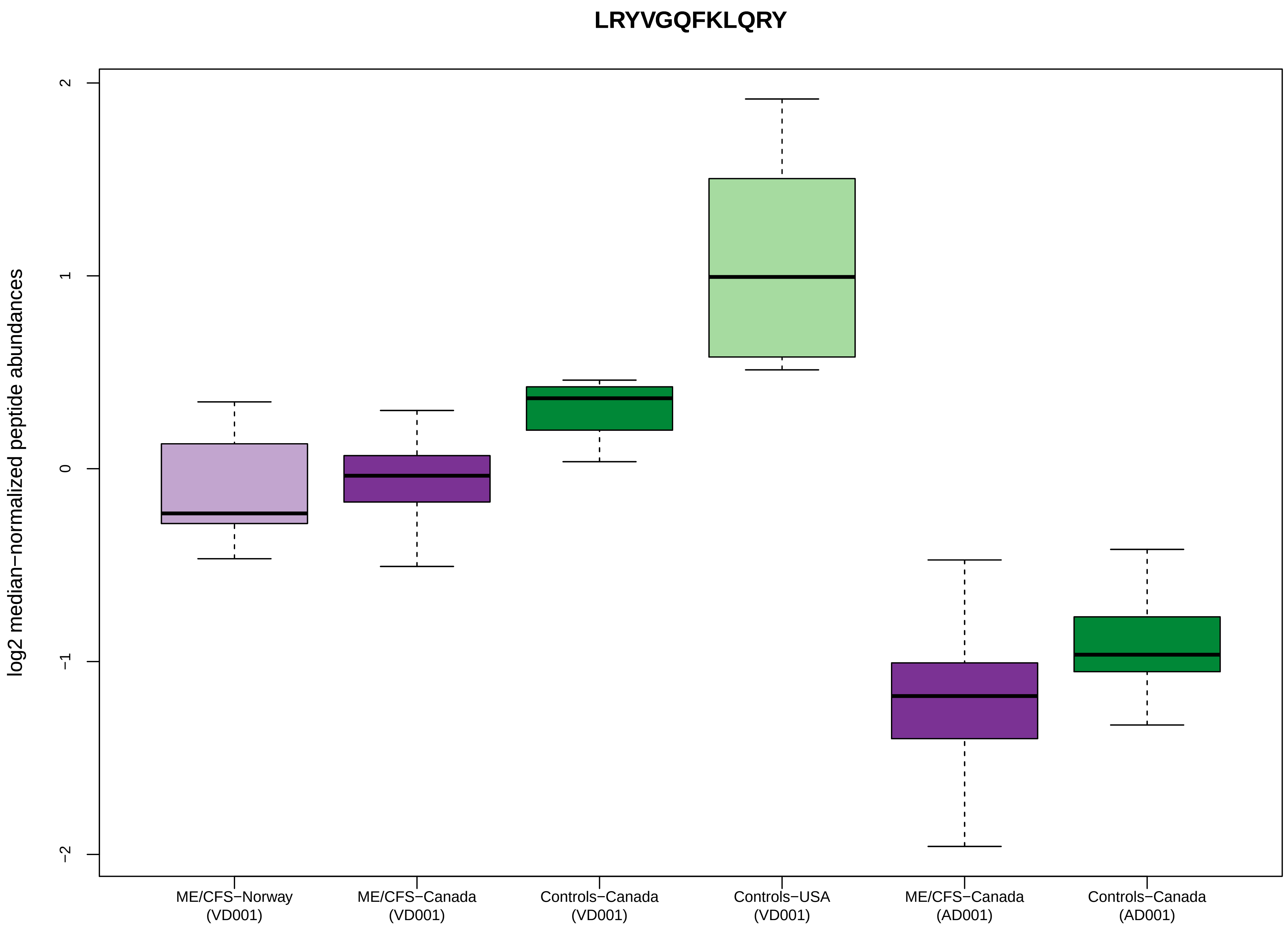

LRYVGQFKLQRY
log2 median−normalized peptide abundances
2
1
0
−1
−2
ME/CFS−Norway (VD001)
ME/CFS−Canada (VD001)
Controls−Canada (VD001)
Controls−USA (VD001)
ME/CFS−Canada (AD001)
Controls−Canada (AD001)

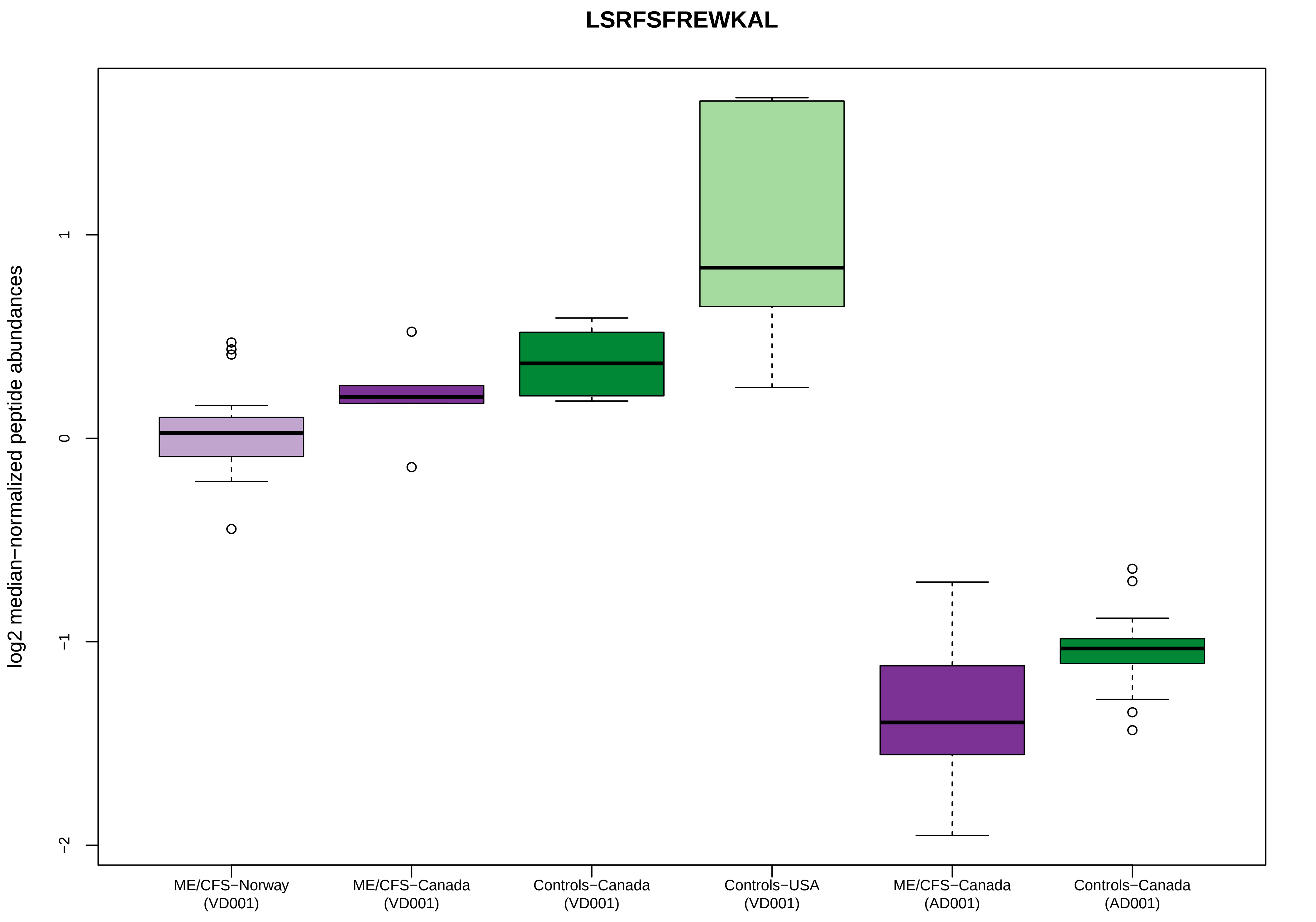

LSRFSFREWKAL
log2 median−normalized peptide abundances
1
0
−1
−2
ME/CFS−Norway (VD001)
ME/CFS−Canada (VD001)
Controls−Canada (VD001)
Controls−USA (VD001)
ME/CFS−Canada (AD001)
Controls−Canada (AD001)

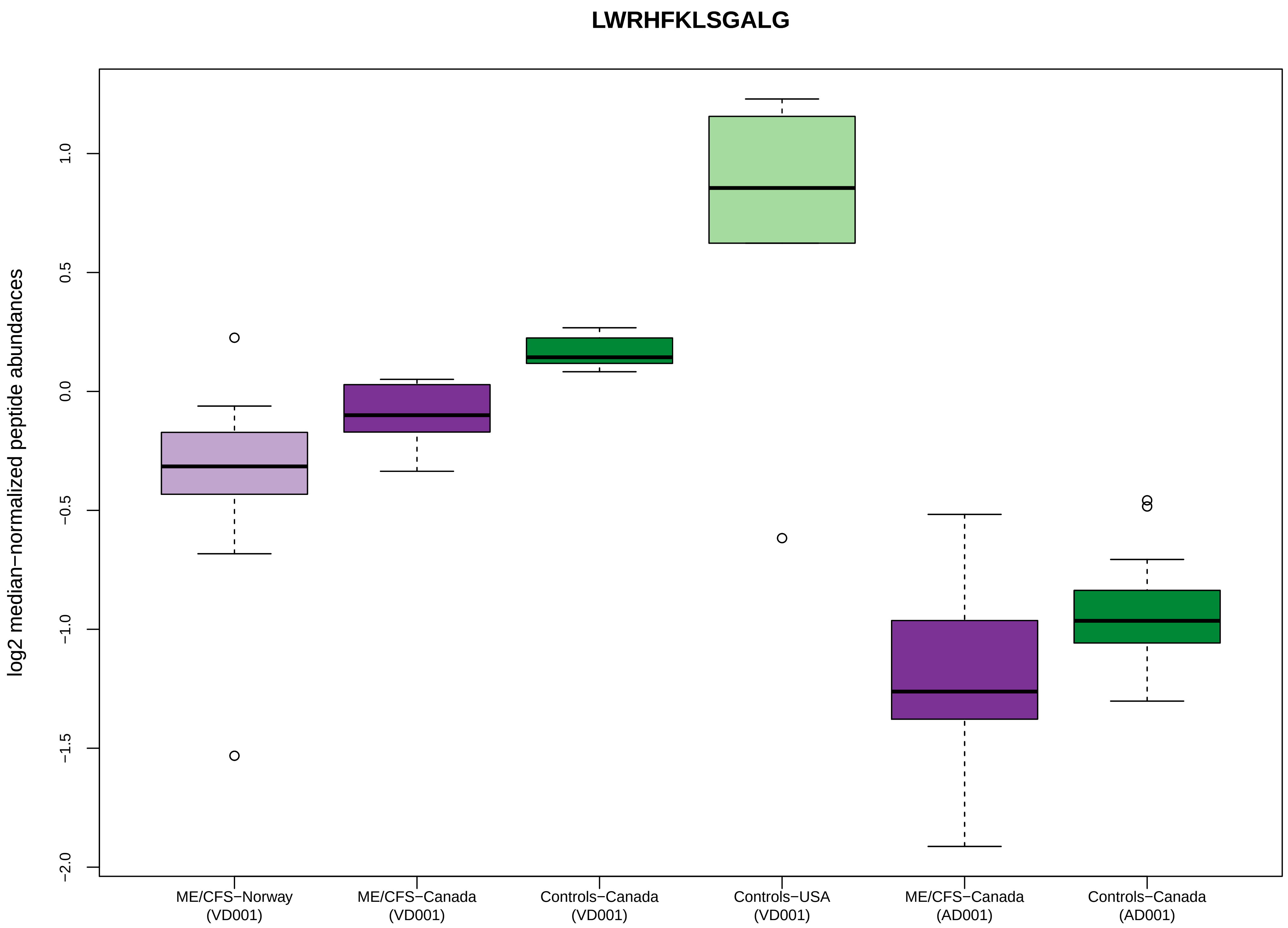
LWRHFKLSGALG
log2 median−normalized peptide abundances
1.0
0.5
0.0
−0.5
−1.0
−1.5
−2.0
ME/CFS−Norway (VD001)
ME/CFS−Canada (VD001)
Controls−Canada (VD001)
Controls−USA (VD001)
ME/CFS−Canada (AD001)
Controls−Canada (AD001)

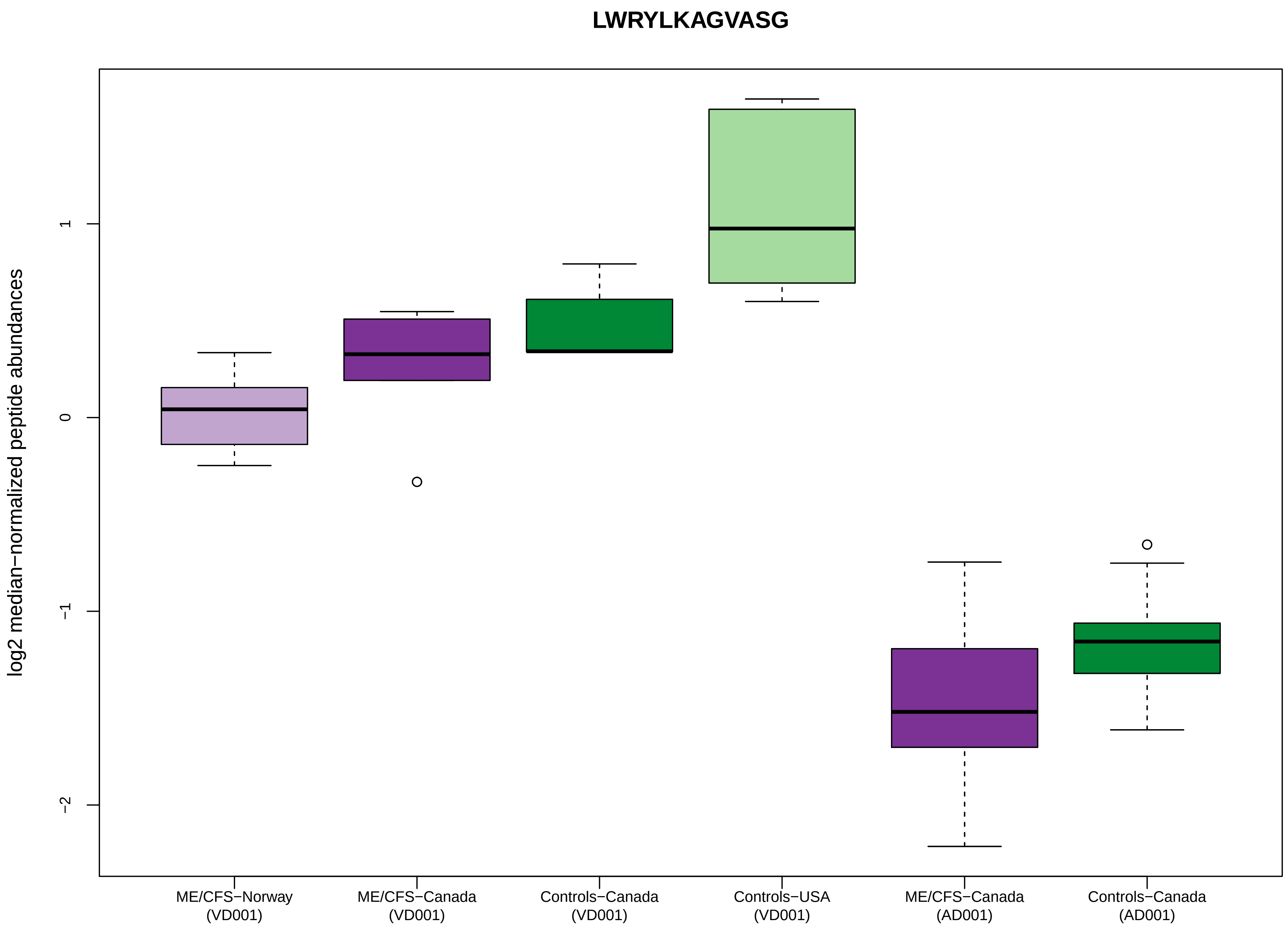

LWRYLKAGVASG
log2 median−normalized peptide abundances
1
0
−1
−2
ME/CFS−Norway (VD001)
ME/CFS−Canada (VD001)
Controls−Canada (VD001)
Controls−USA (VD001)
ME/CFS−Canada (AD001)
Controls−Canada (AD001)

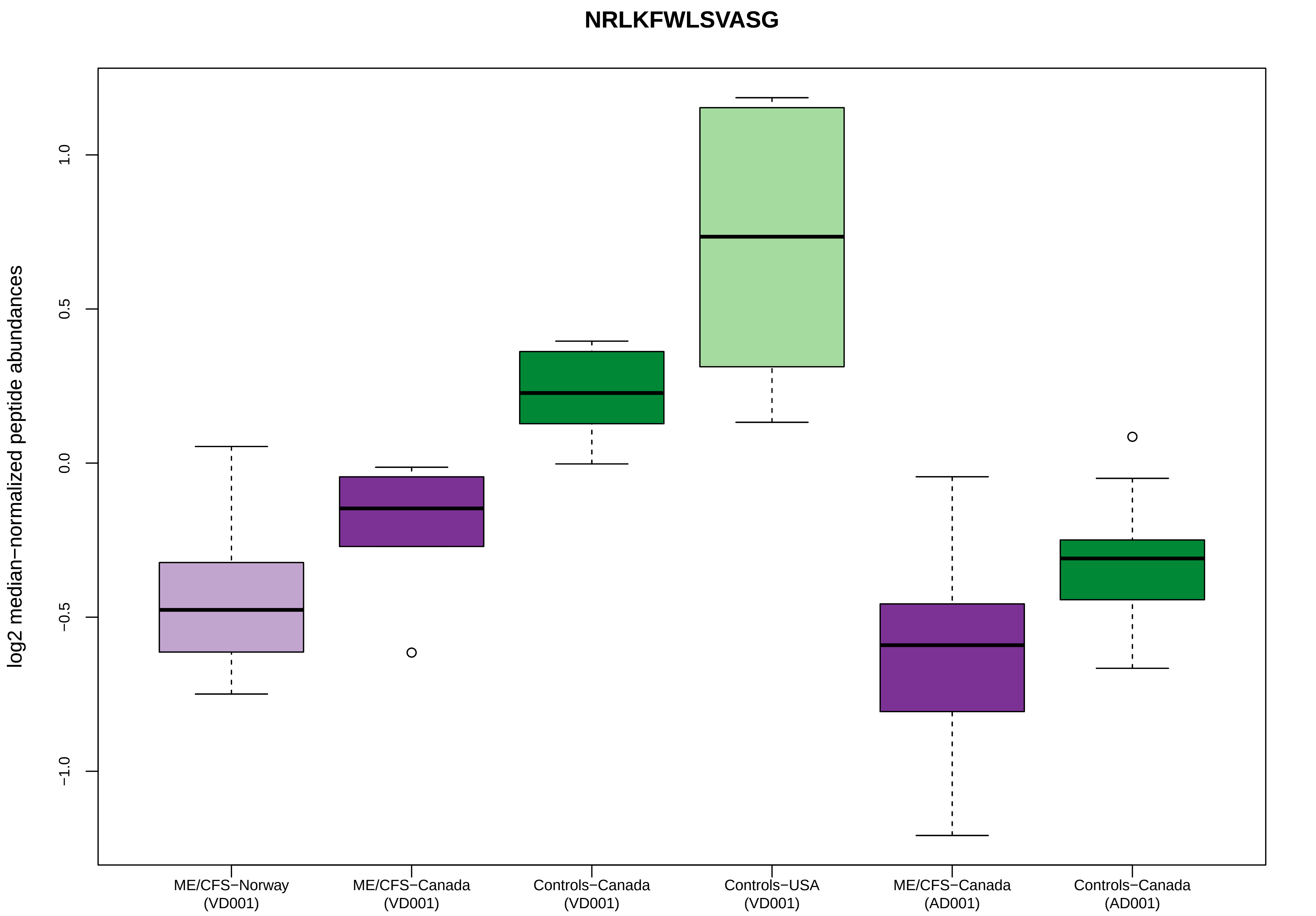

NRLKFWLSVASG
log2 median−normalized peptide abundances
1.0
0.5
0.0
−0.5
−1.0
ME/CFS−Norway (VD001)
ME/CFS−Canada (VD001)
Controls−Canada (VD001)
Controls−USA (VD001)
ME/CFS−Canada (AD001)
Controls−Canada (AD001)

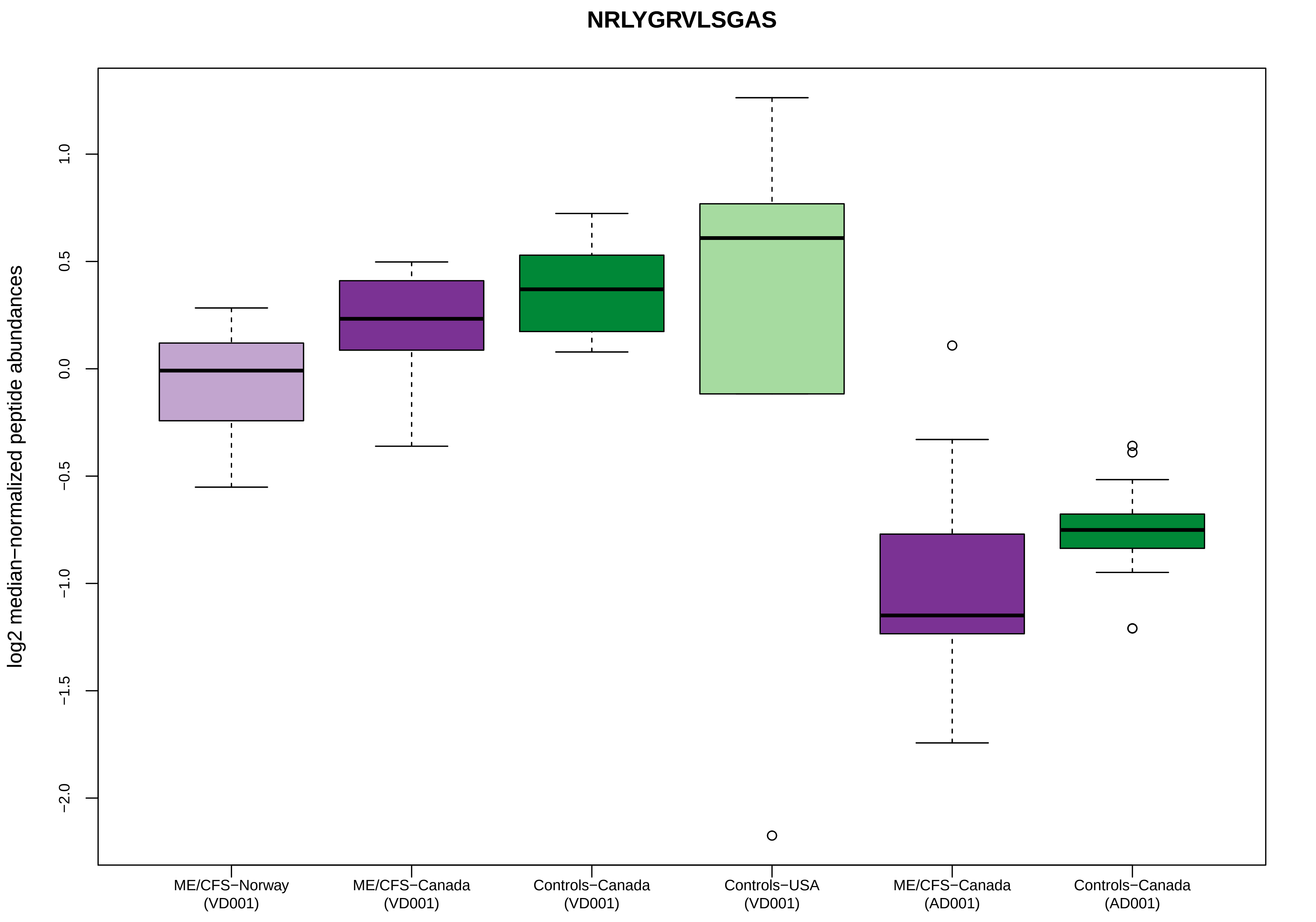

NRLYGRVLSGAS
log2 median−normalized peptide abundances
1.0
0.5
0.0
−0.5
−1.0
−1.5
−2.0
ME/CFS−Norway (VD001)
ME/CFS−Canada (VD001)
Controls−Canada (VD001)
Controls−USA (VD001)
ME/CFS−Canada (AD001)
Controls−Canada (AD001)

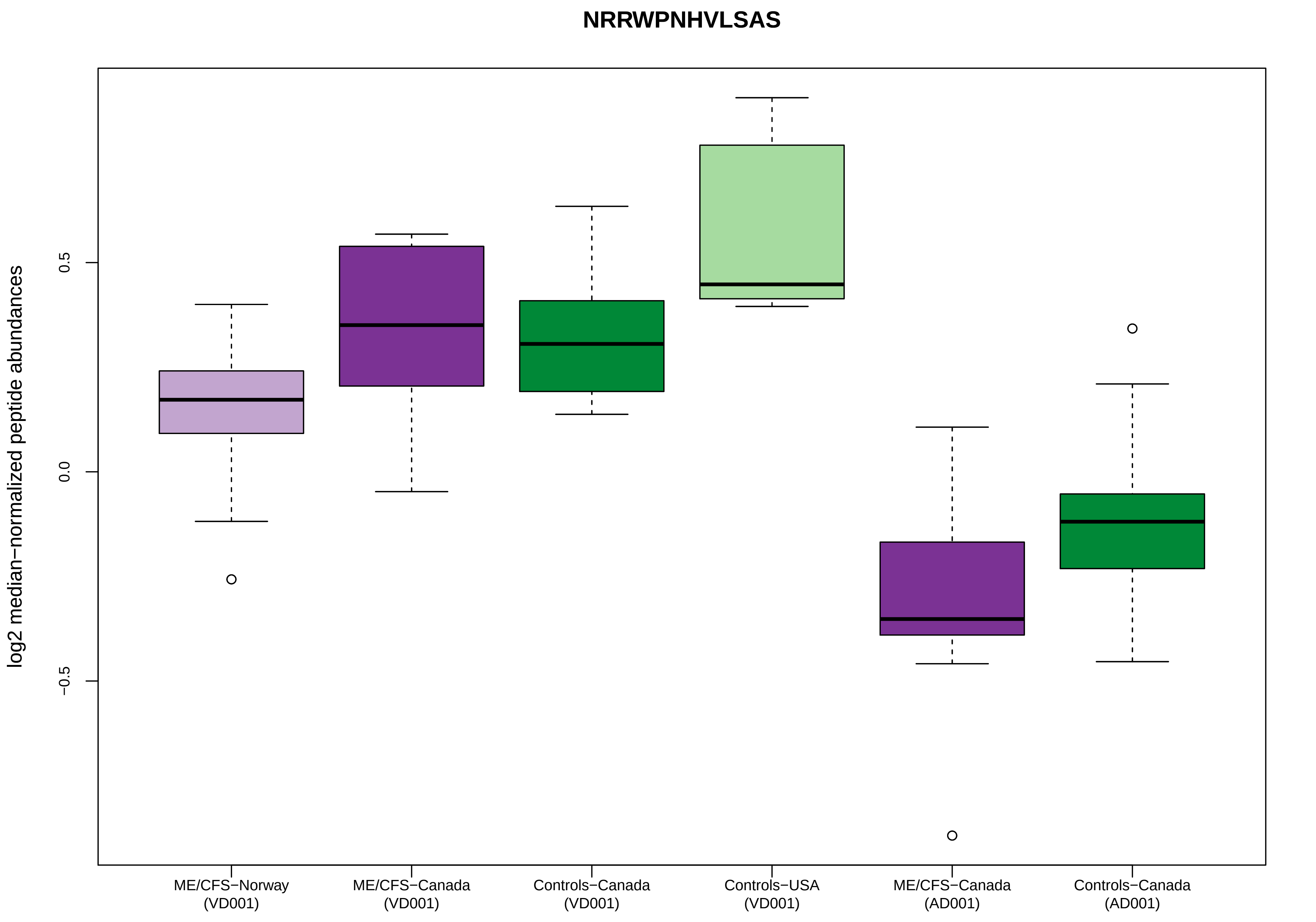
NRRWPNHVLSAS
log2 median−normalized peptide abundances
0.5
0.0
−0.5
ME/CFS−Norway (VD001)
ME/CFS−Canada (VD001)
Controls−Canada (VD001)
Controls−USA (VD001)
ME/CFS−Canada (AD001)
Controls−Canada (AD001)

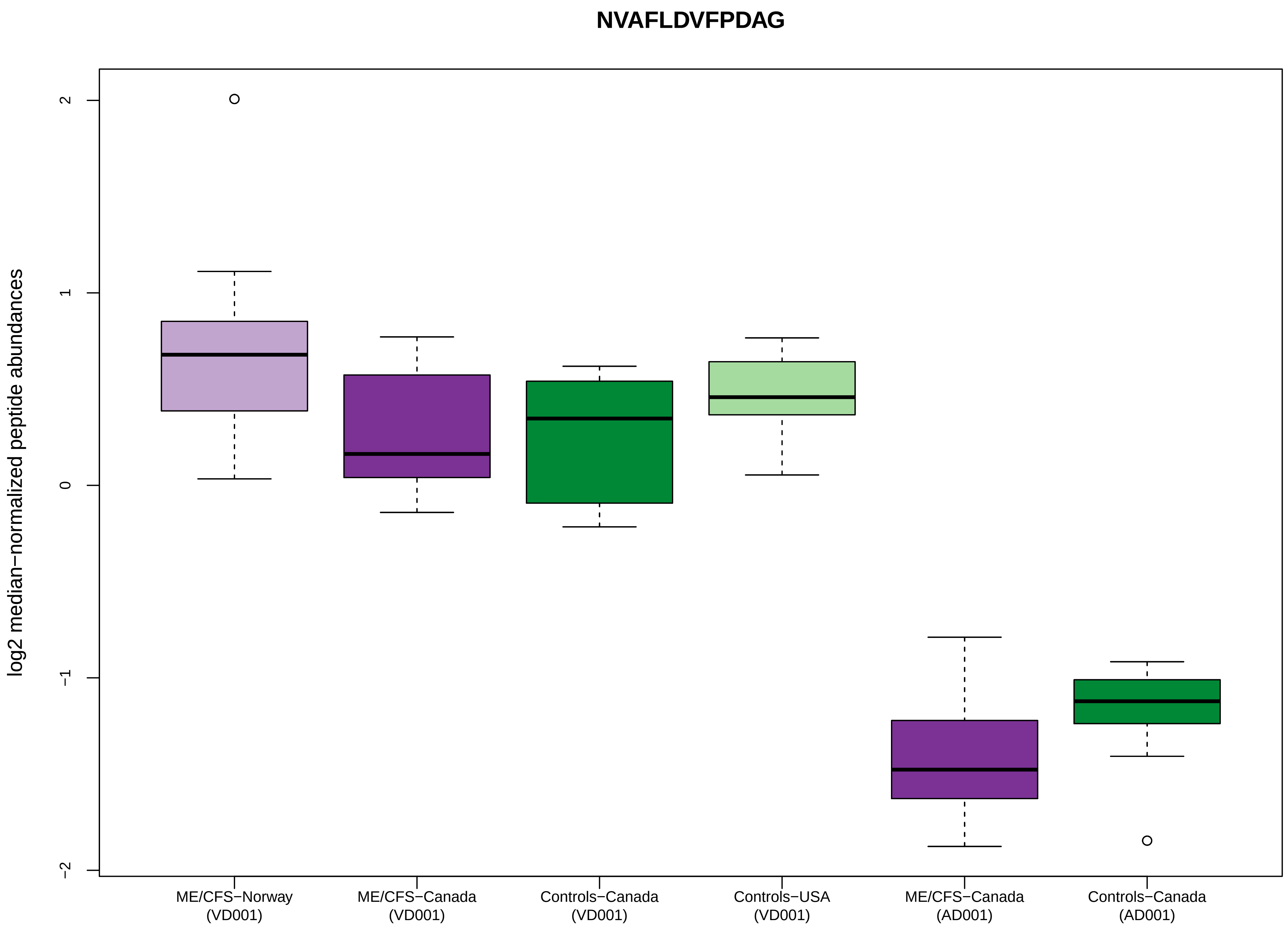

NVAFLDVFPDAG
log2 median−normalized peptide abundances
2
1
0
−1
−2
ME/CFS−Norway (VD001)
ME/CFS−Canada (VD001)
Controls−Canada (VD001)
Controls−USA (VD001)
ME/CFS−Canada (AD001)
Controls−Canada (AD001)

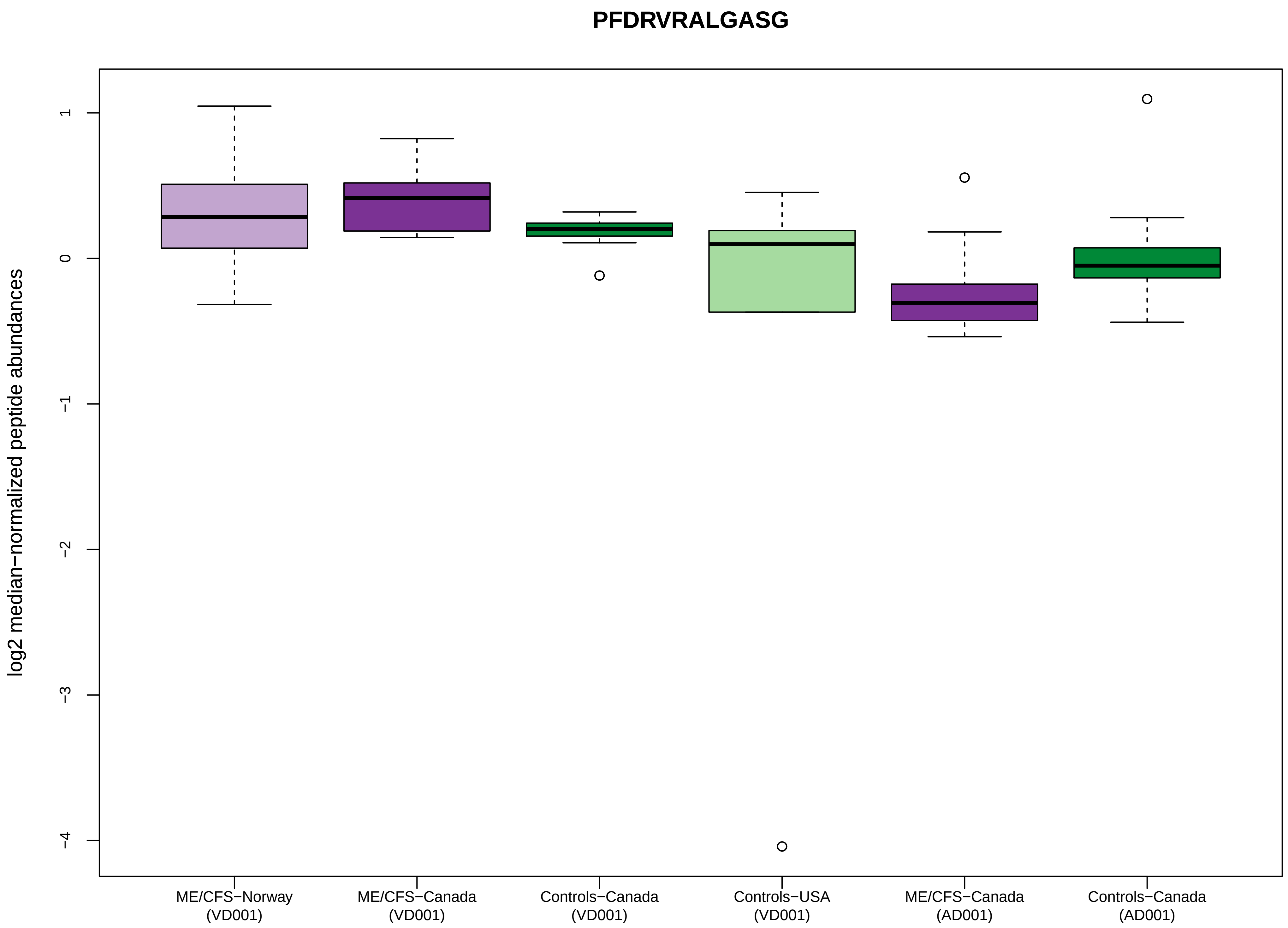

PFDRVRALGASG
log2 median−normalized peptide abundances
1
0
−1
−2
−3
−4
ME/CFS−Norway (VD001)
ME/CFS−Canada (VD001)
Controls−Canada (VD001)
Controls−USA (VD001)
ME/CFS−Canada (AD001)
Controls−Canada (AD001)

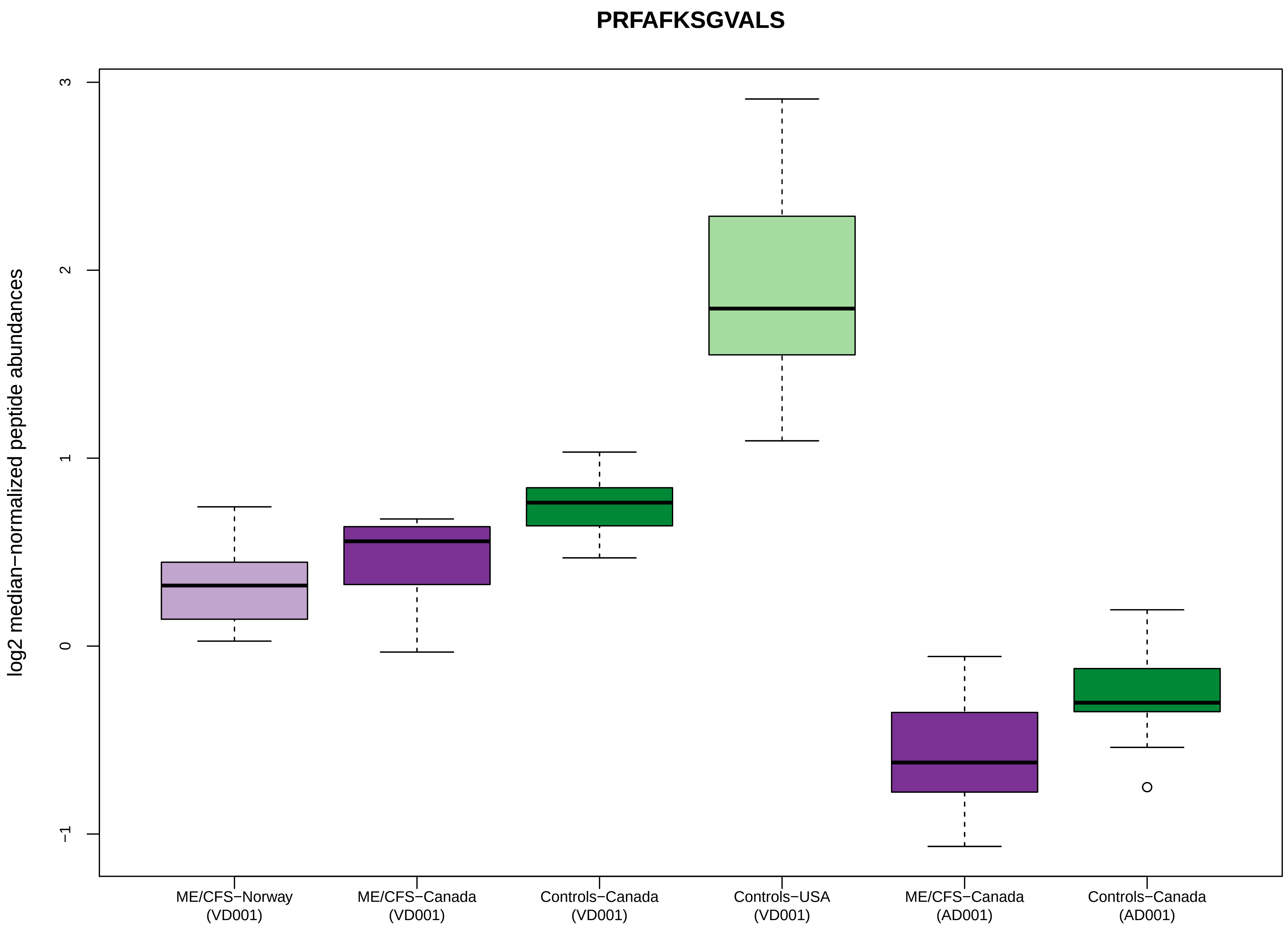
PRFAFKSGVALS
log2 median−normalized peptide abundances
3
2
1
0
−1
ME/CFS−Norway (VD001)
ME/CFS−Canada (VD001)
Controls−Canada (VD001)
Controls−USA (VD001)
ME/CFS−Canada (AD001)
Controls−Canada (AD001)

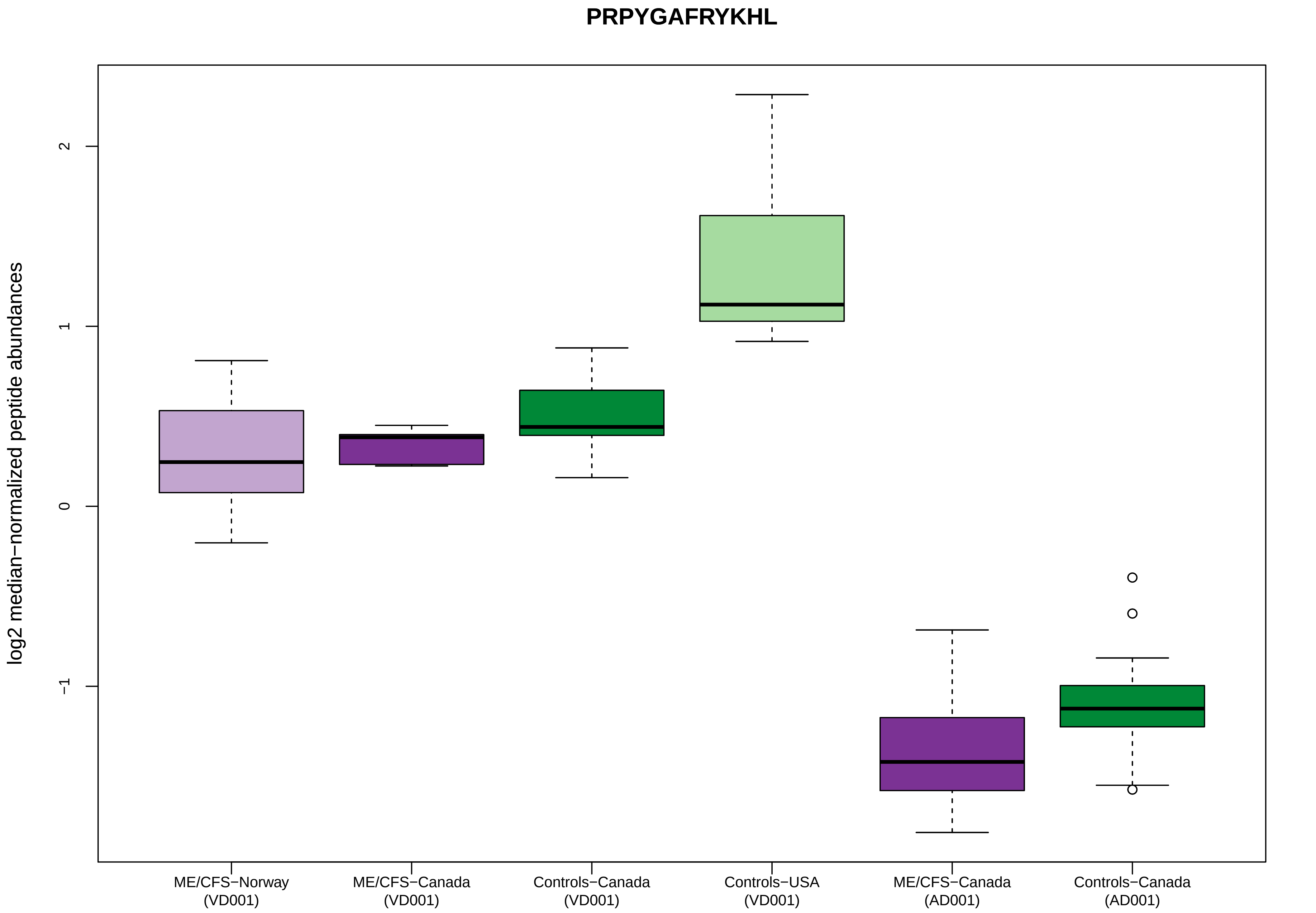
PRPYGAFRYKHL
log2 median−normalized peptide abundances
2
1
0
−1
ME/CFS−Norway (VD001)
ME/CFS−Canada (VD001)
Controls−Canada (VD001)
Controls−USA (VD001)
ME/CFS−Canada (AD001)
Controls−Canada (AD001)

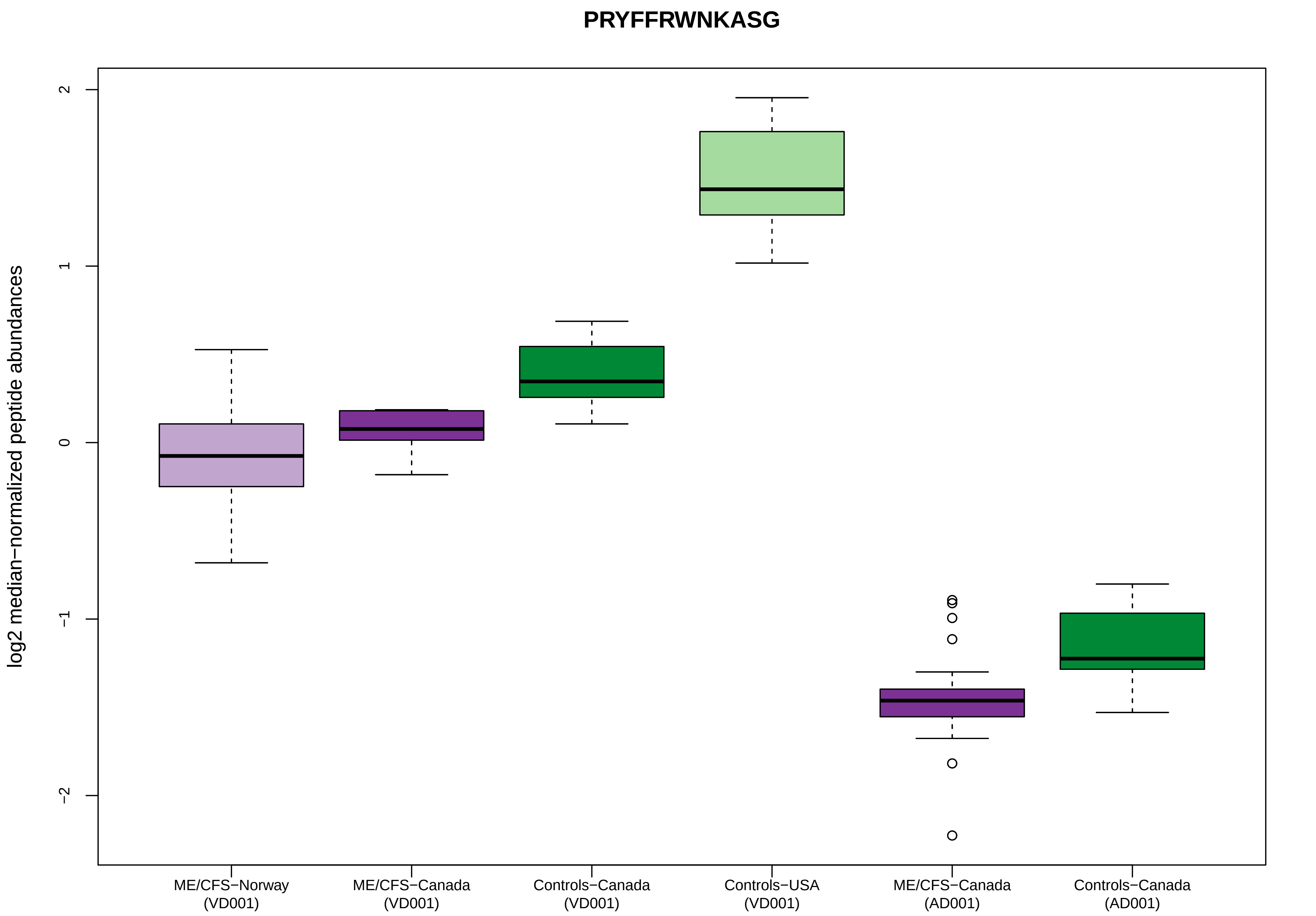
PRYFFRWNKASG
log2 median−normalized peptide abundances
2
1
0
−1
−2
ME/CFS−Norway
(VD001)
ME/CFS−Canada
(VD001)
Controls−Canada
(VD001)
Controls−USA
(VD001)
ME/CFS−Canada
(AD001)
Controls−Canada
(AD001)

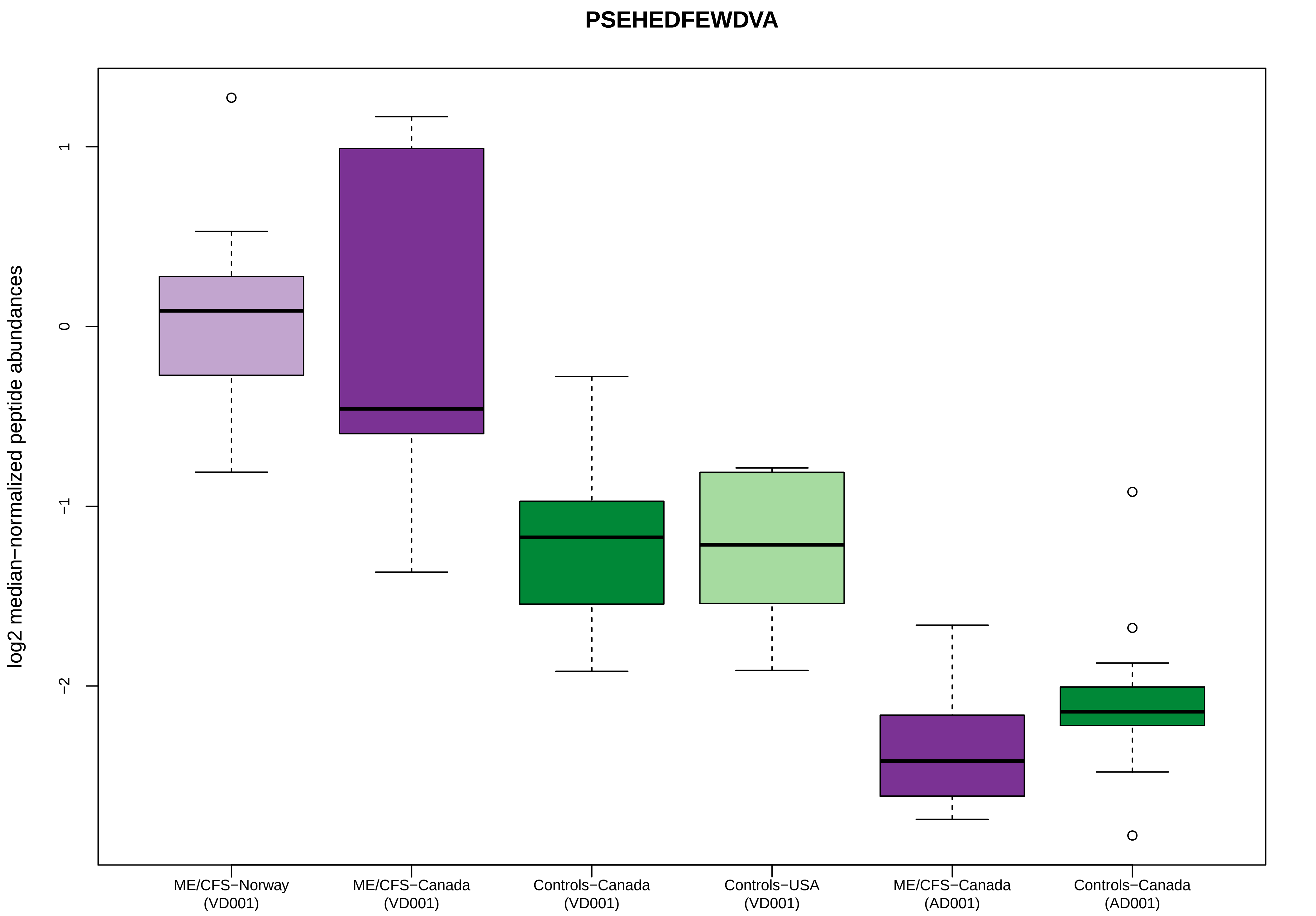
PSEHEDFEWDVA
log2 median−normalized peptide abundances
1
0
−1
−2
ME/CFS−Norway (VD001)
ME/CFS−Canada (VD001)
Controls−Canada (VD001)
Controls−USA (VD001)
ME/CFS−Canada (AD001)
Controls−Canada (AD001)

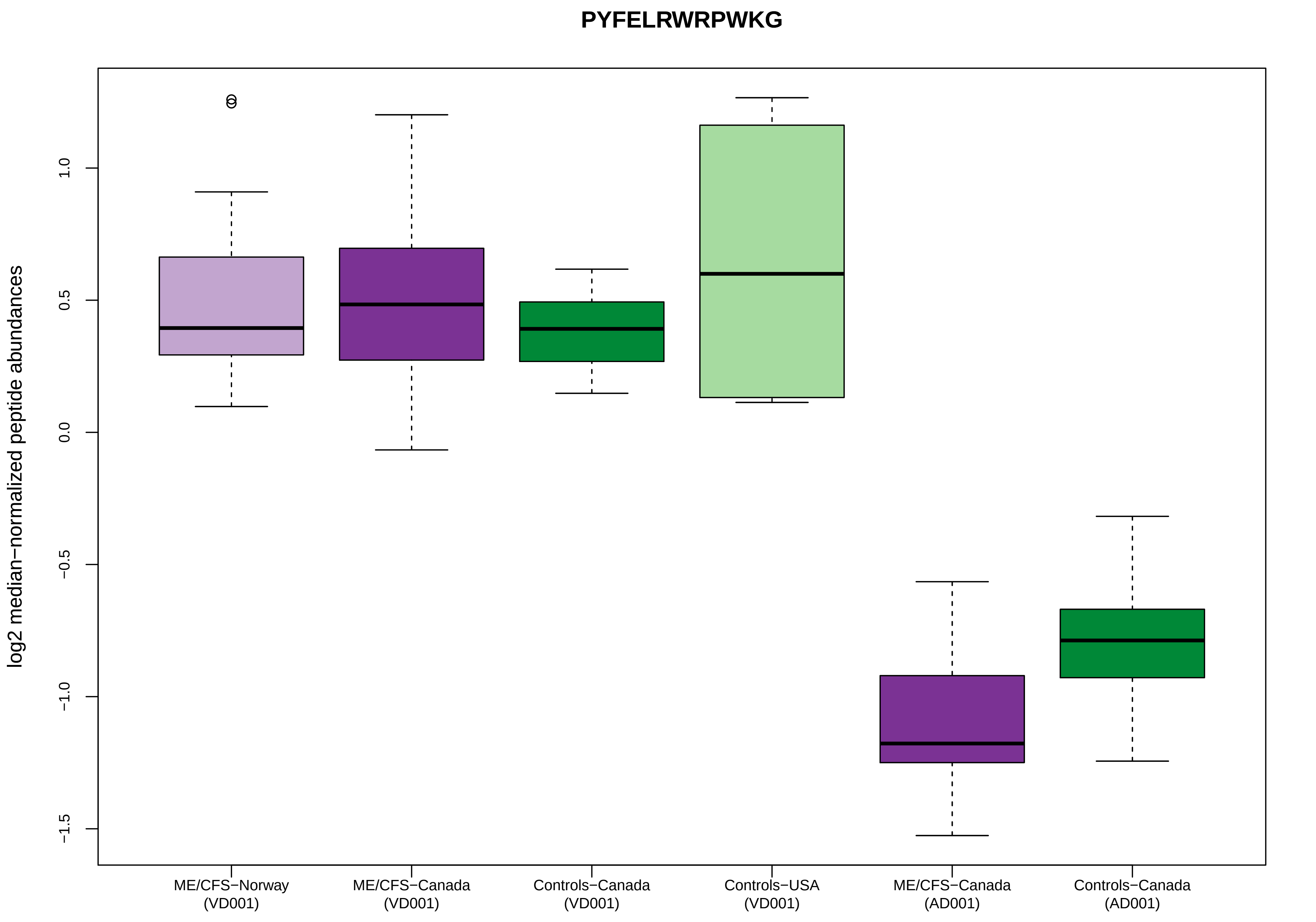
PYFELRWRPWKG
log2 median−normalized peptide abundances
1.0
0.5
0.0
−0.5
−1.0
−1.5
ME/CFS−Norway (VD001)
ME/CFS−Canada (VD001)
Controls−Canada (VD001)
Controls−USA (VD001)
ME/CFS−Canada (AD001)
Controls−Canada (AD001)

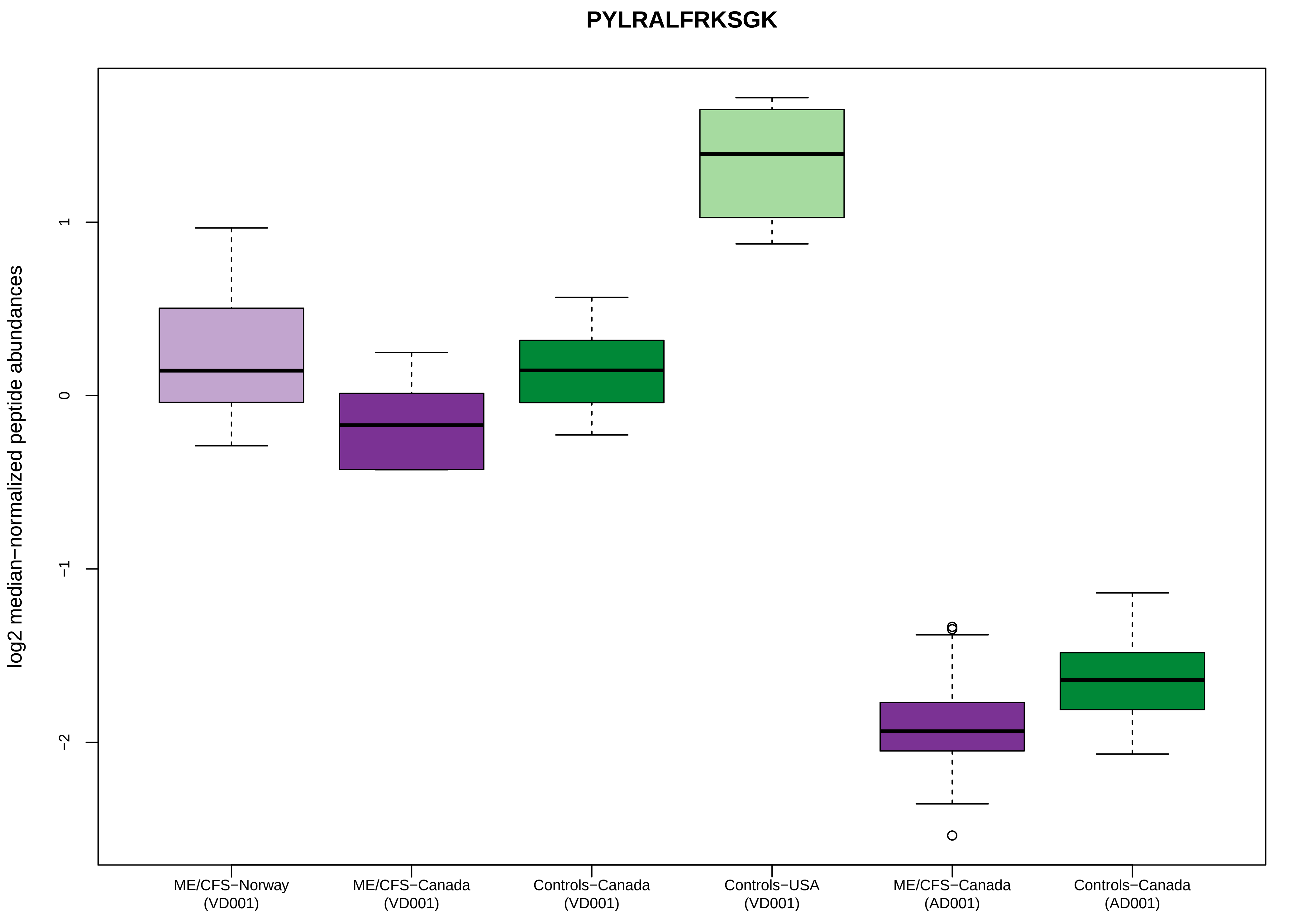
PYLRALFRKSGK
log2 median−normalized peptide abundances
1
0
−1
−2
ME/CFS−Norway (VD001)
ME/CFS−Canada (VD001)
Controls−Canada (VD001)
Controls−USA (VD001)
ME/CFS−Canada (AD001)
Controls−Canada (AD001)

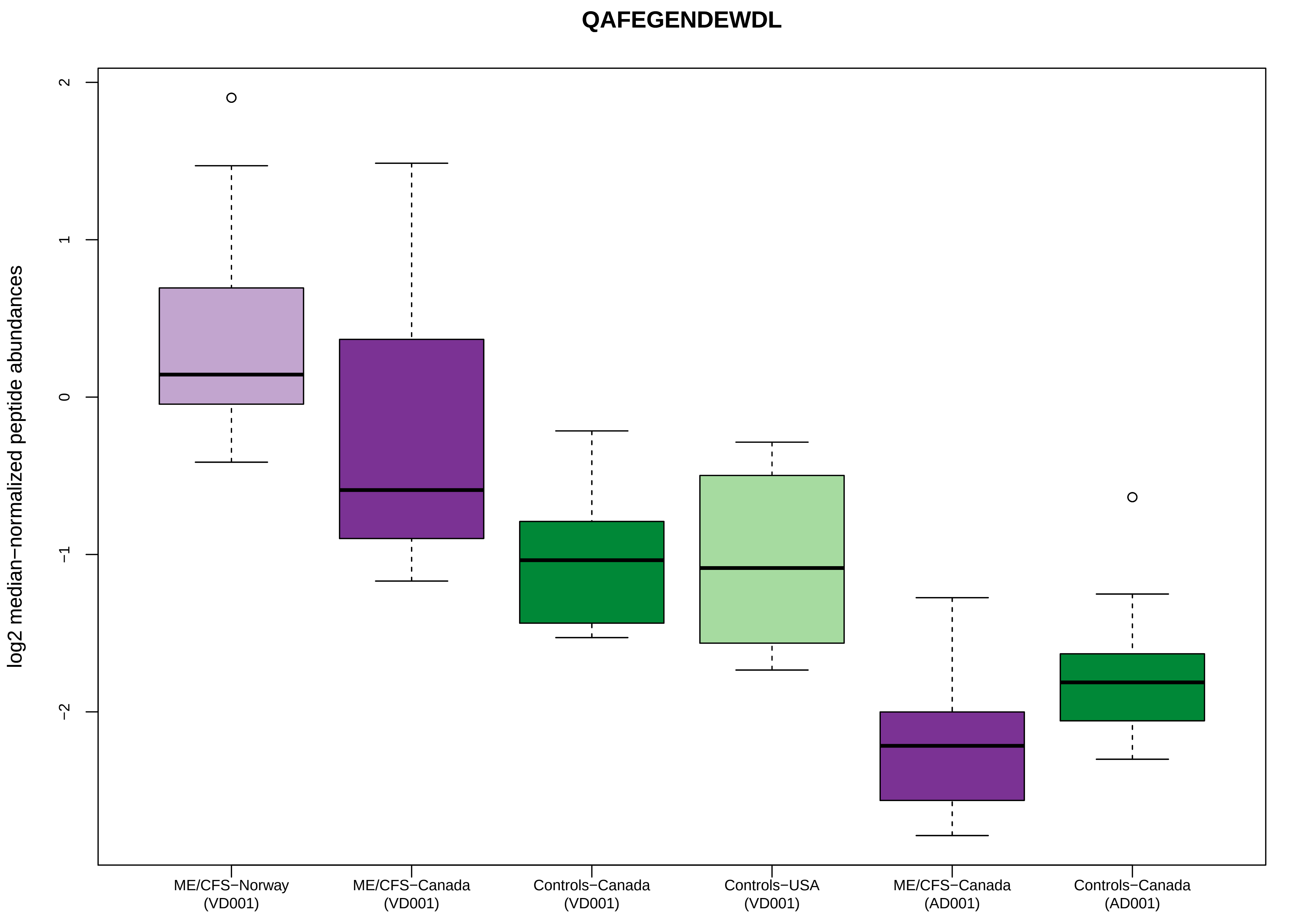
QAFEGENDEWDL
log2 median−normalized peptide abundances
2
1
0
−1
−2
ME/CFS−Norway (VD001)
ME/CFS−Canada (VD001)
Controls−Canada (VD001)
Controls−USA (VD001)
ME/CFS−Canada (AD001)
Controls−Canada (AD001)

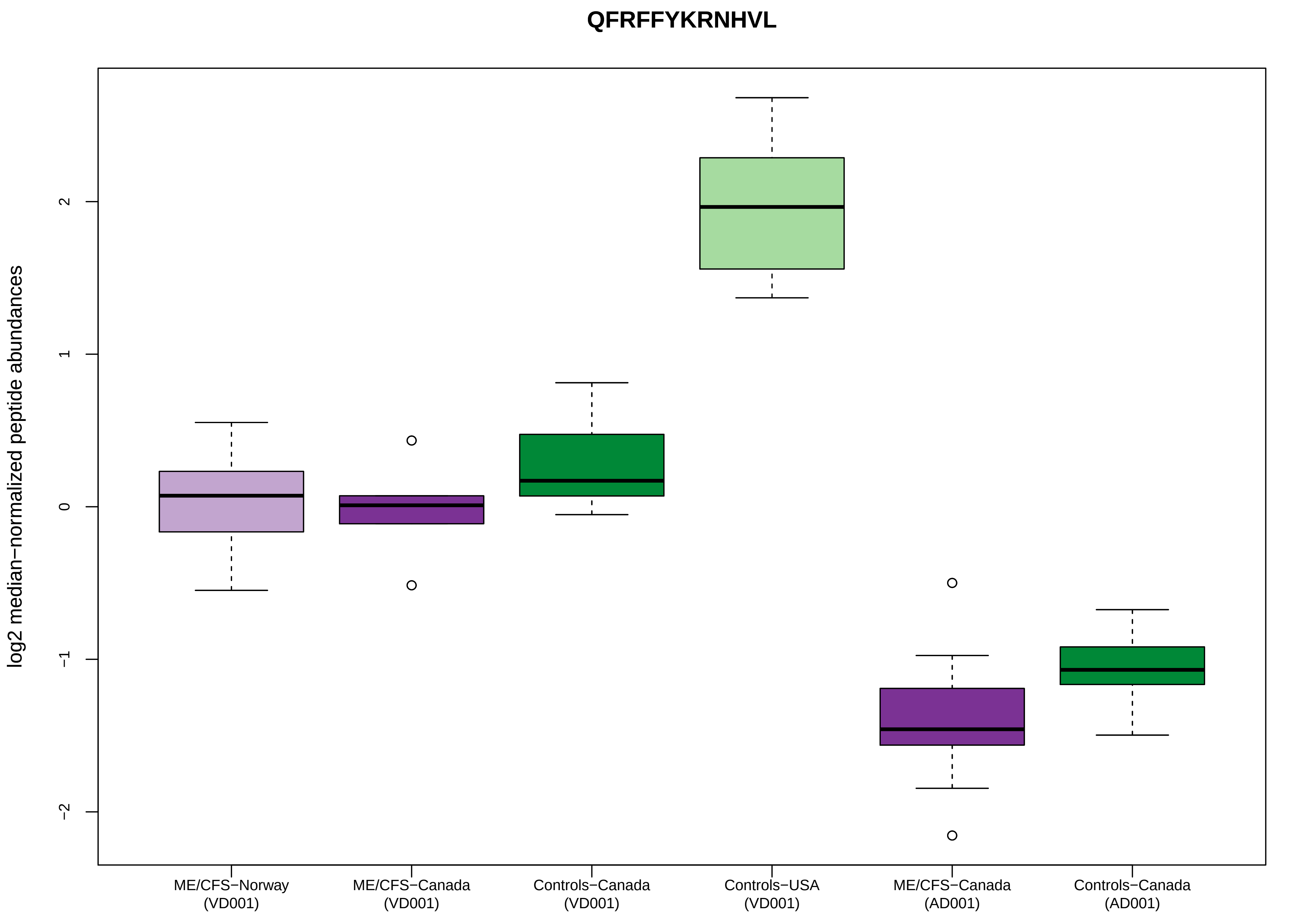
QFRFFYKRNHVL
log2 median−normalized peptide abundances
−2
−1
0
1
2
ME/CFS−Norway (VD001)
ME/CFS−Canada (VD001)
Controls−Canada (VD001)
Controls−USA (VD001)
ME/CFS−Canada (AD001)
Controls−Canada (AD001)

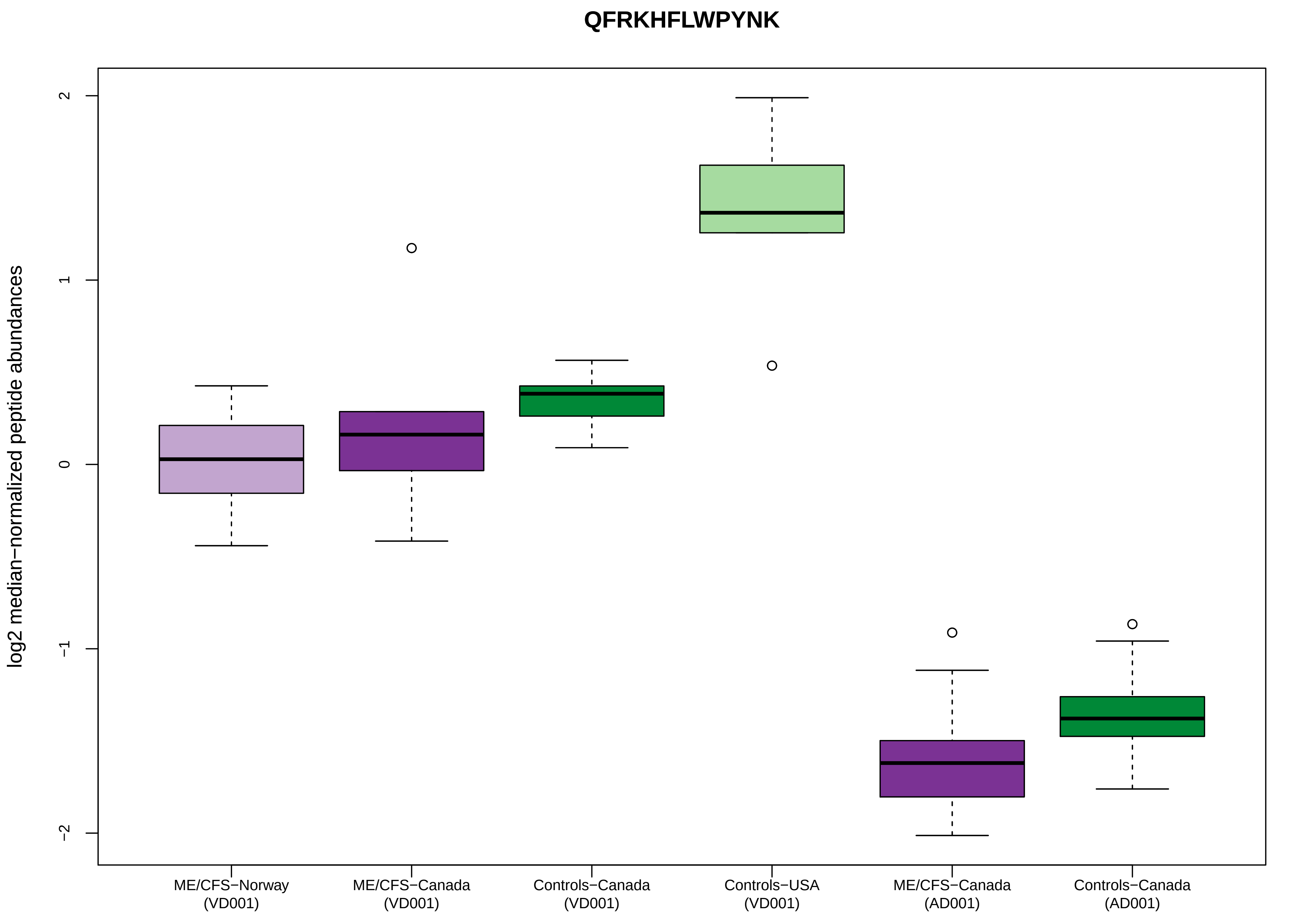

QFRKHFLWPYNK
log2 median−normalized peptide abundances
2
1
0
−1
−2
ME/CFS−Norway (VD001)
ME/CFS−Canada (VD001)
Controls−Canada (VD001)
Controls−USA (VD001)
ME/CFS−Canada (AD001)
Controls−Canada (AD001)

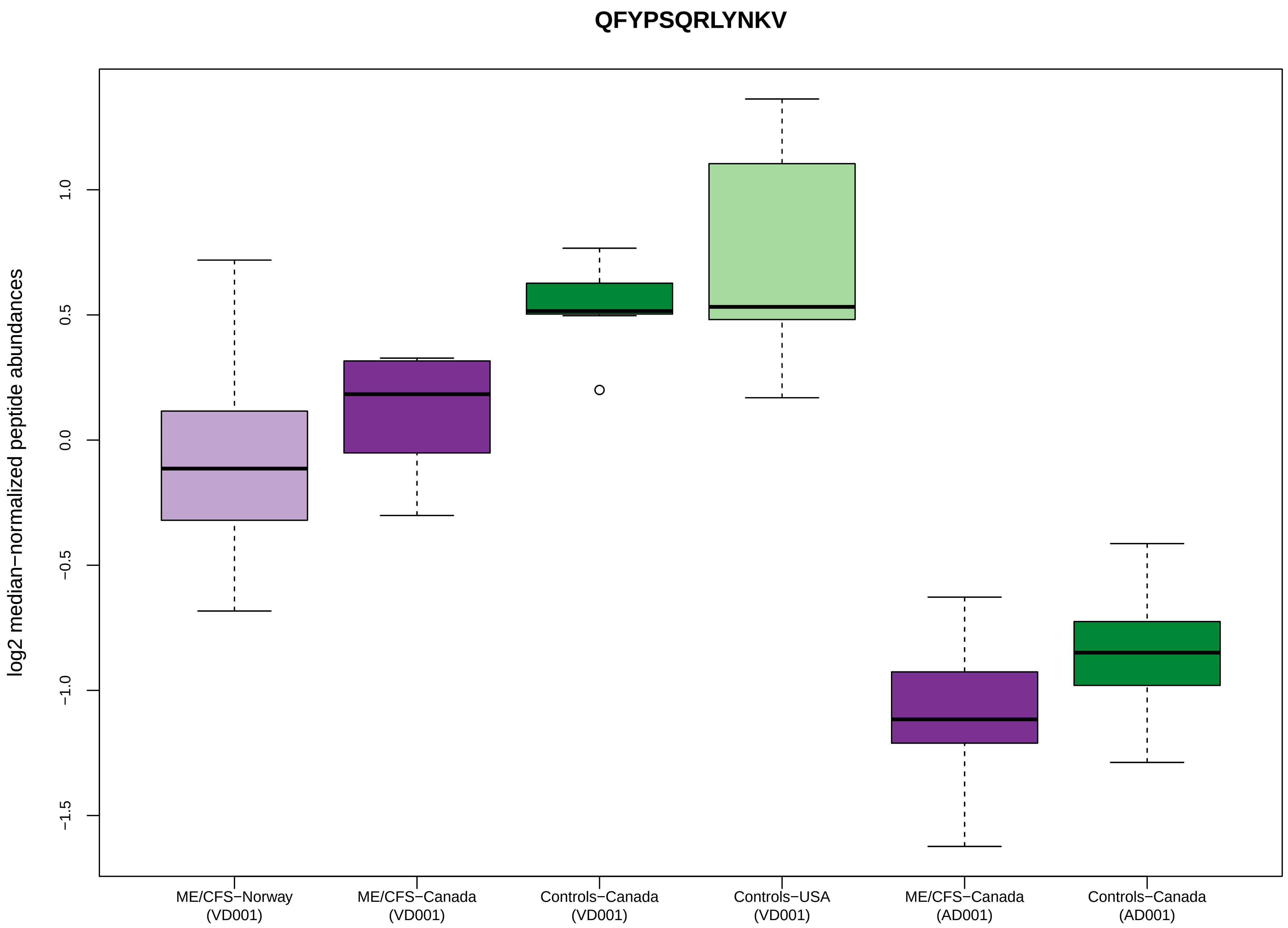

QFYPSQRLYNKV
log2 median−normalized peptide abundances
1.0
0.5
0.0
−0.5
−1.0
−1.5
ME/CFS−Norway (VD001)
ME/CFS−Canada (VD001)
Controls−Canada (VD001)
Controls−USA (VD001)
ME/CFS−Canada (AD001)
Controls−Canada (AD001)

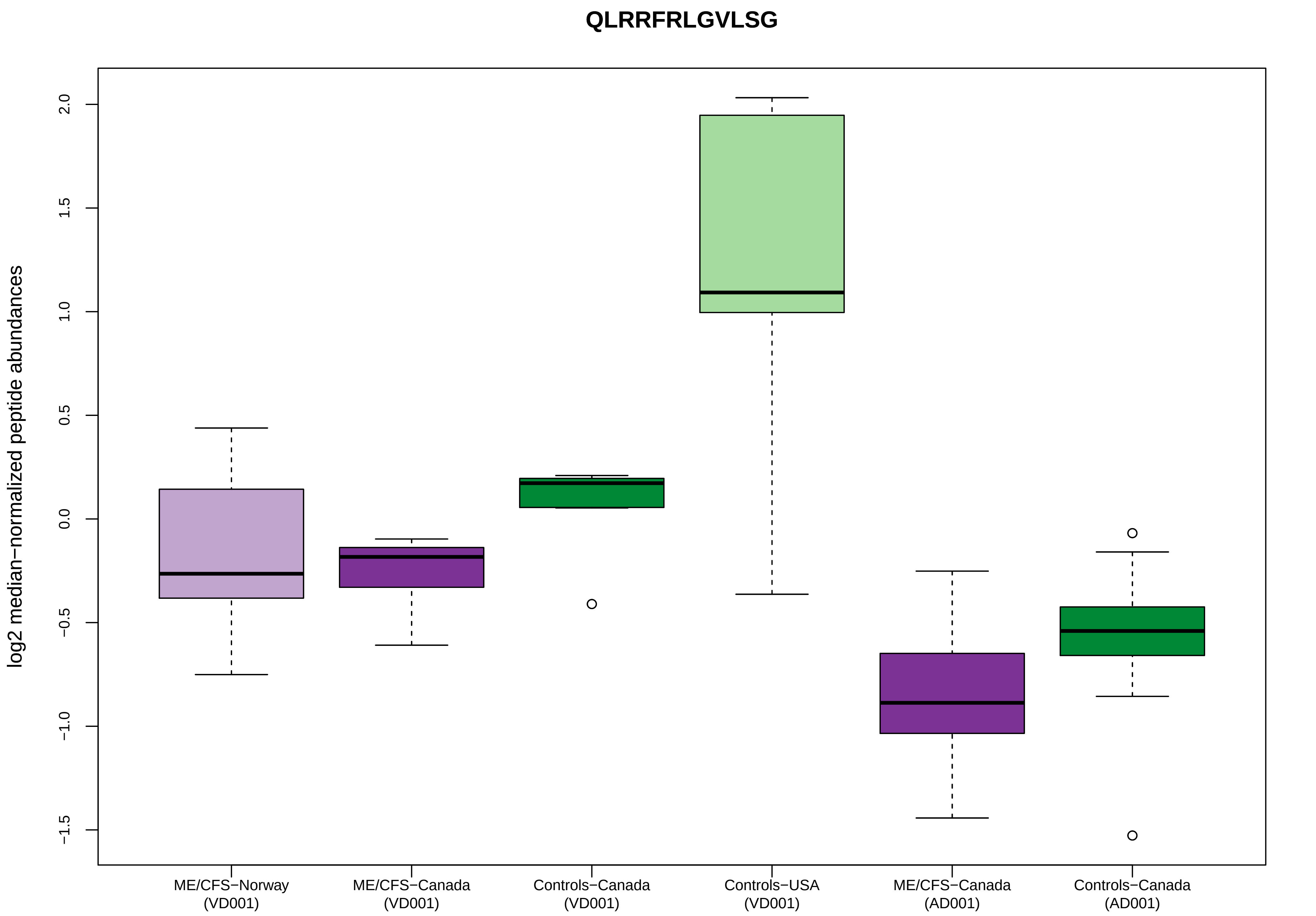

QLRRFRLGVLSG
log2 median−normalized peptide abundances
2.0
1.5
1.0
0.5
0.0
−0.5
−1.0
−1.5
ME/CFS−Norway (VD001)
ME/CFS−Canada (VD001)
Controls−Canada (VD001)
Controls−USA (VD001)
ME/CFS−Canada (AD001)
Controls−Canada (AD001)

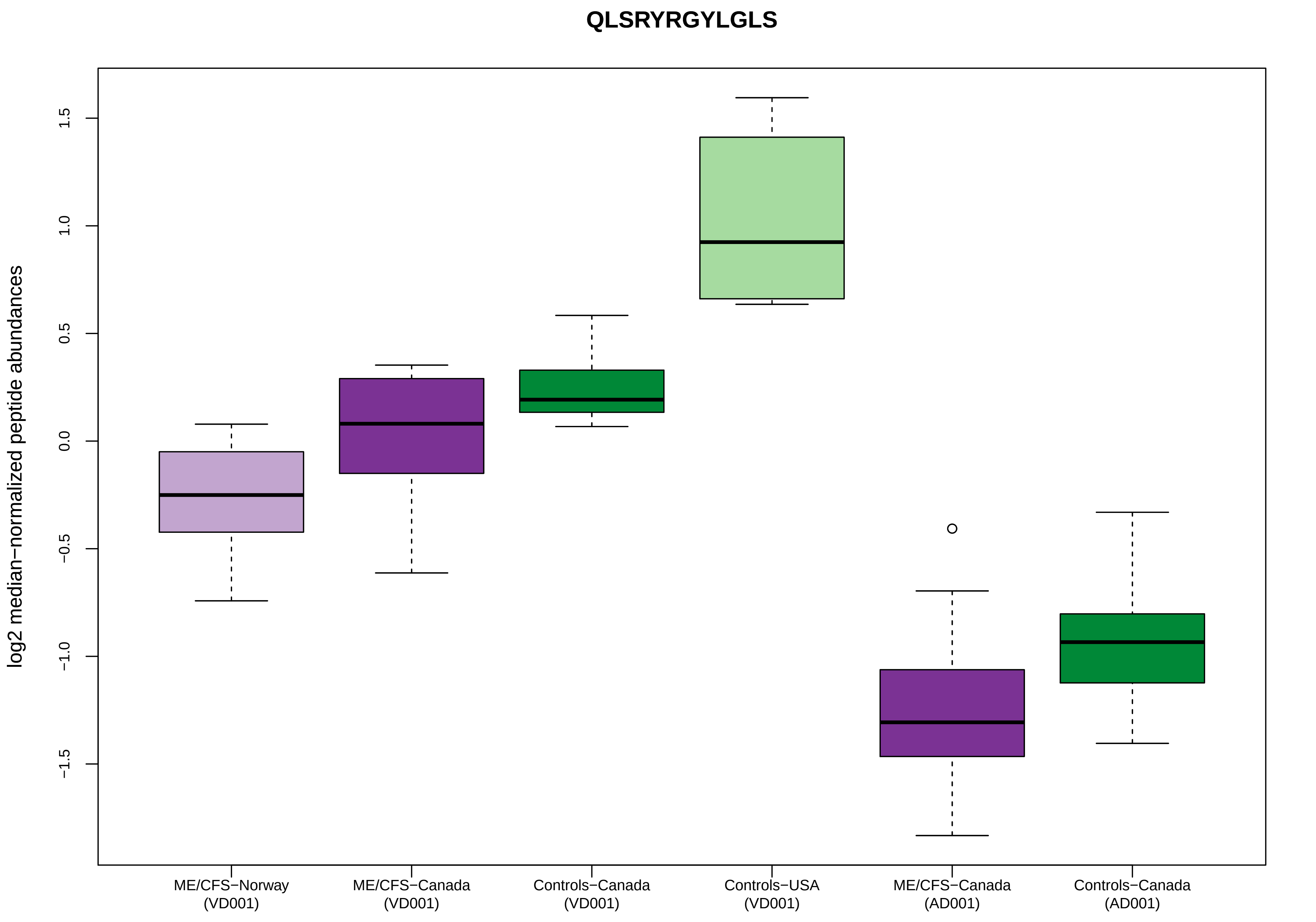
QLSRYRGYLGLS
log2 median−normalized peptide abundances
1.5
1.0
0.5
0.0
−0.5
−1.0
−1.5
ME/CFS−Norway (VD001)
ME/CFS−Canada (VD001)
Controls−Canada (VD001)
Controls−USA (VD001)
ME/CFS−Canada (AD001)
Controls−Canada (AD001)

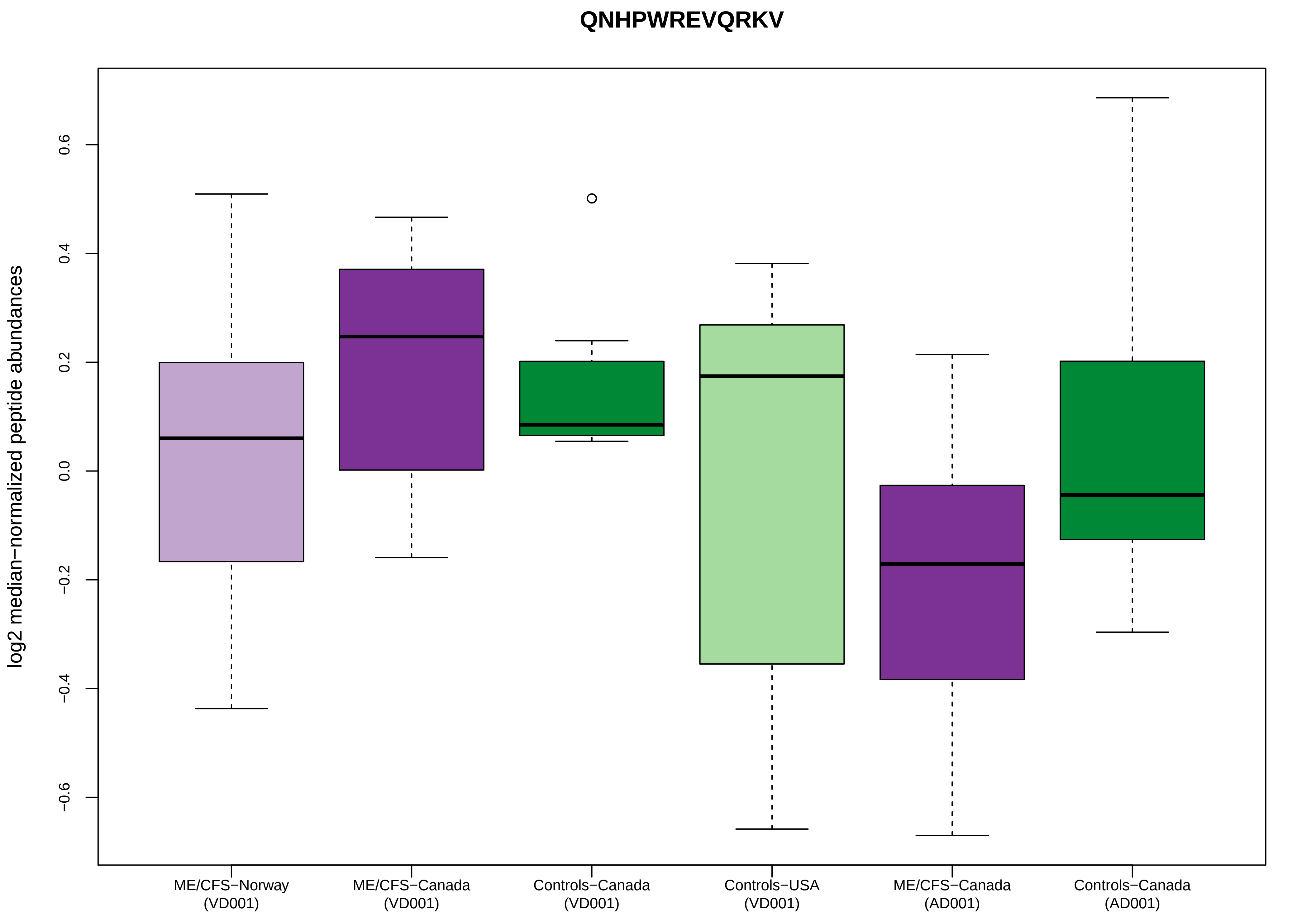

QNHPWREVQRKV
log2 median−normalized peptide abundances
0.6
0.4
0.2
0.0
−0.2
−0.4
−0.6
ME/CFS−Norway (VD001)
ME/CFS−Canada (VD001)
Controls−Canada (VD001)
Controls−USA (VD001)
ME/CFS−Canada (AD001)
Controls−Canada (AD001)

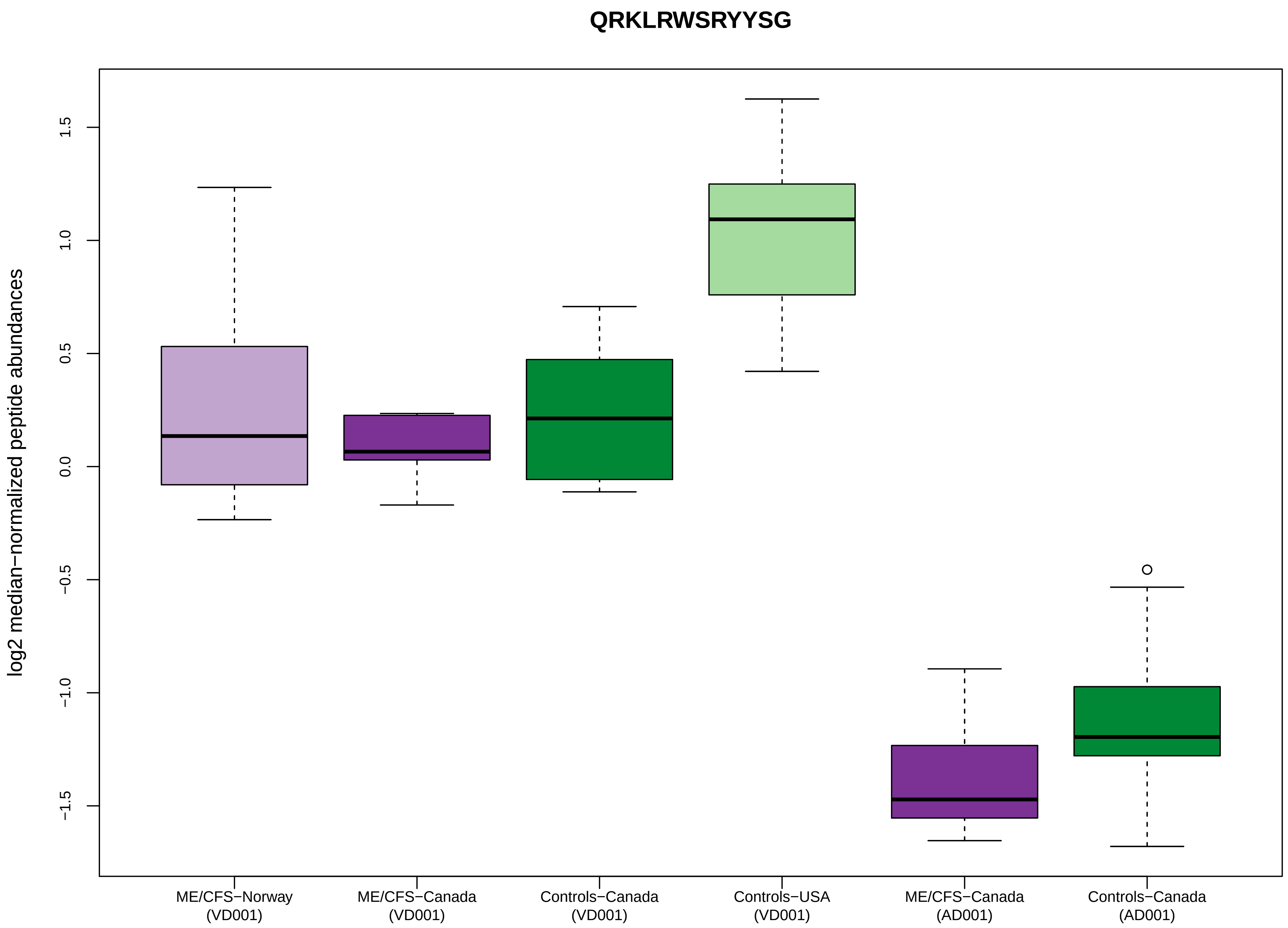

QRKLRWSRYYSG
log2 median−normalized peptide abundances
1.5
1.0
0.5
0.0
−0.5
−1.0
−1.5
ME/CFS−Norway (VD001)
ME/CFS−Canada (VD001)
Controls−Canada (VD001)
Controls−USA (VD001)
ME/CFS−Canada (AD001)
Controls−Canada (AD001)

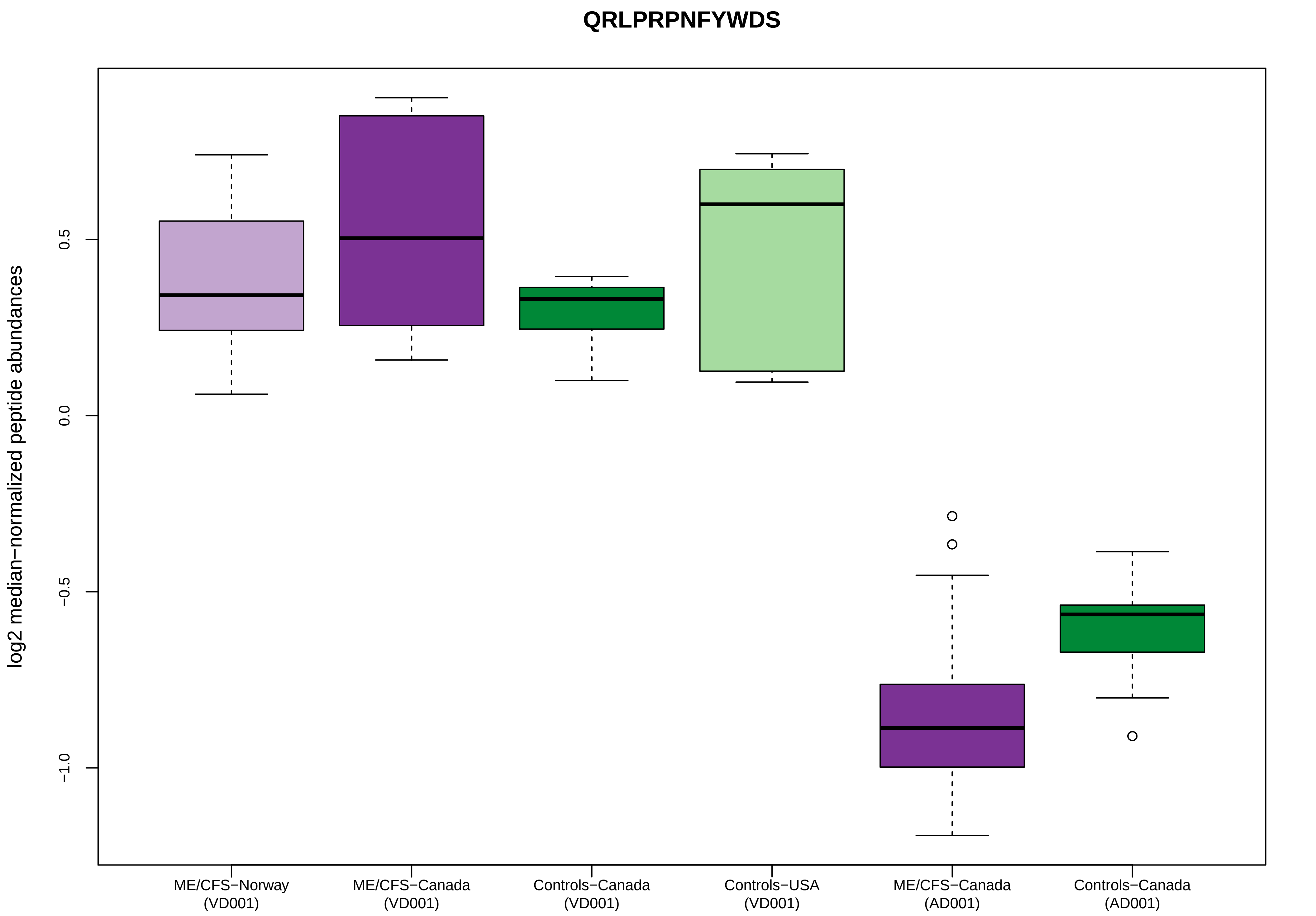
QRLPRPNFYWDS
log2 median−normalized peptide abundances
0.5
0.0
−0.5
−1.0
ME/CFS−Norway (VD001)
ME/CFS−Canada (VD001)
Controls−Canada (VD001)
Controls−USA (VD001)
ME/CFS−Canada (AD001)
Controls−Canada (AD001)

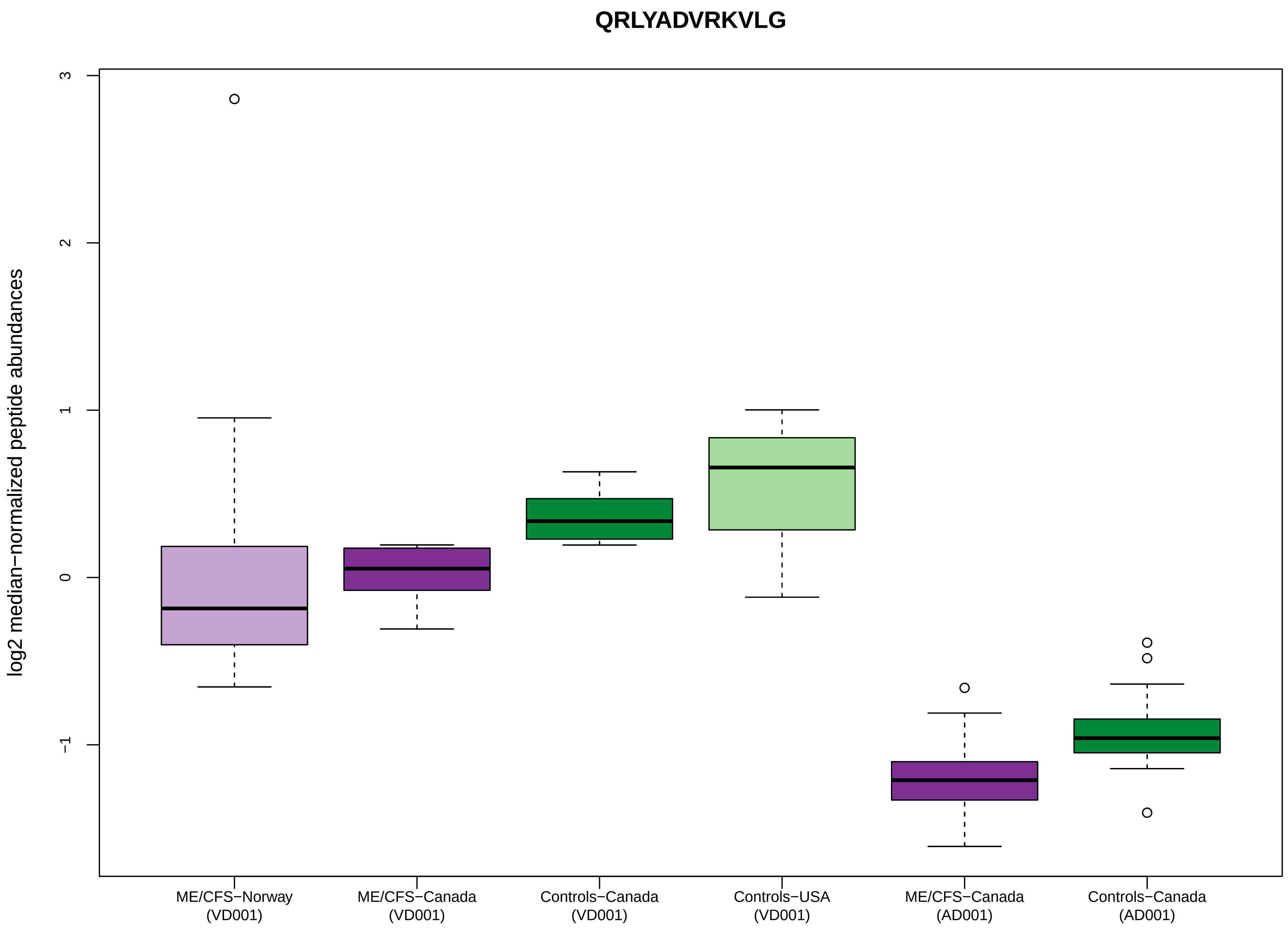
QRLYADVRKVLG
log2 median−normalized peptide abundances
3
2
1
0
−1
ME/CFS−Norway (VD001)
ME/CFS−Canada (VD001)
Controls−Canada (VD001)
Controls−USA (VD001)
ME/CFS−Canada (AD001)
Controls−Canada (AD001)

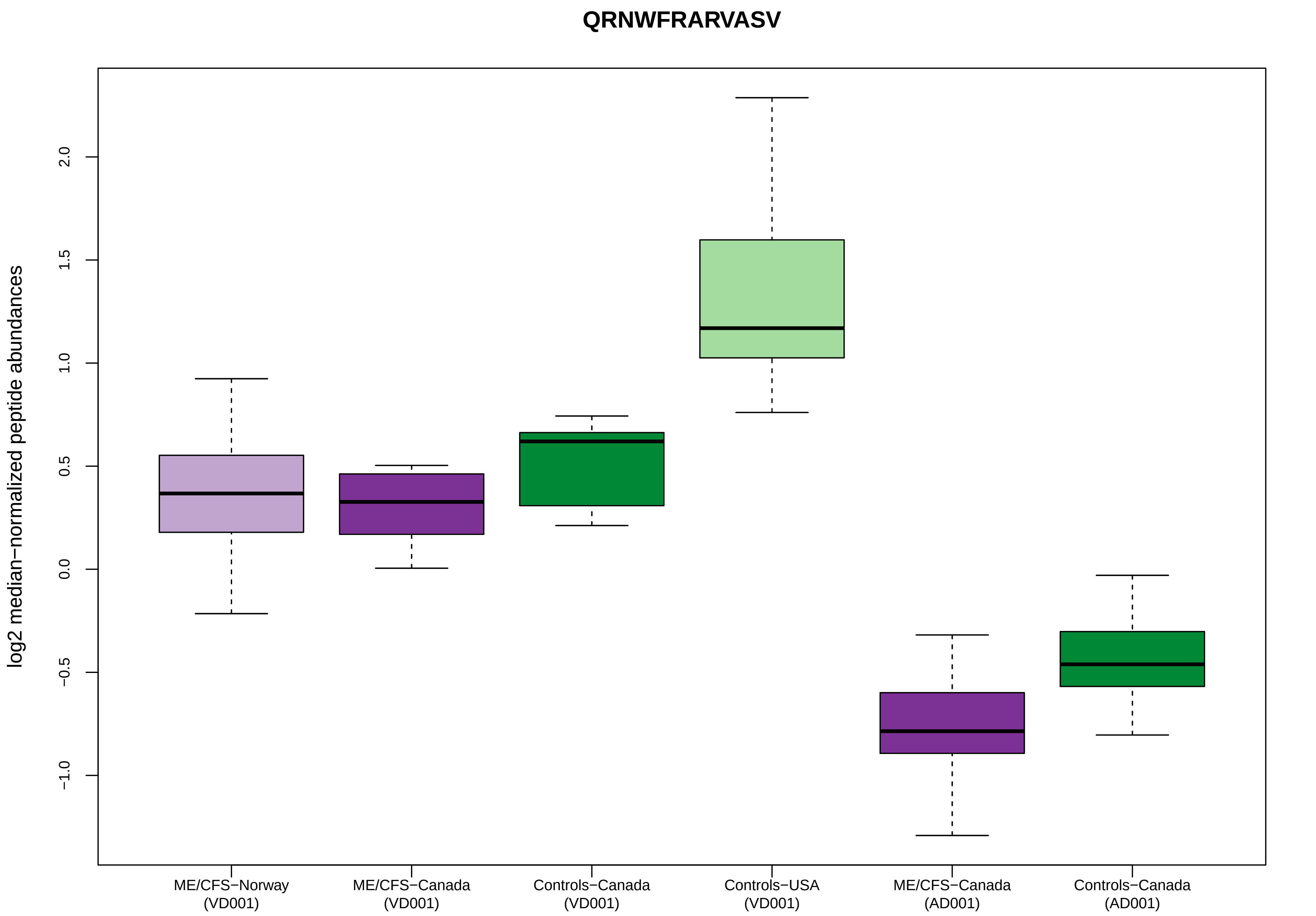

QRNWFRARVASV
log2 median−normalized peptide abundances
2.0
1.5
1.0
0.5
0.0
−0.5
−1.0
ME/CFS−Norway (VD001)
ME/CFS−Canada (VD001)
Controls−Canada (VD001)
Controls−USA (VD001)
ME/CFS−Canada (AD001)
Controls−Canada (AD001)

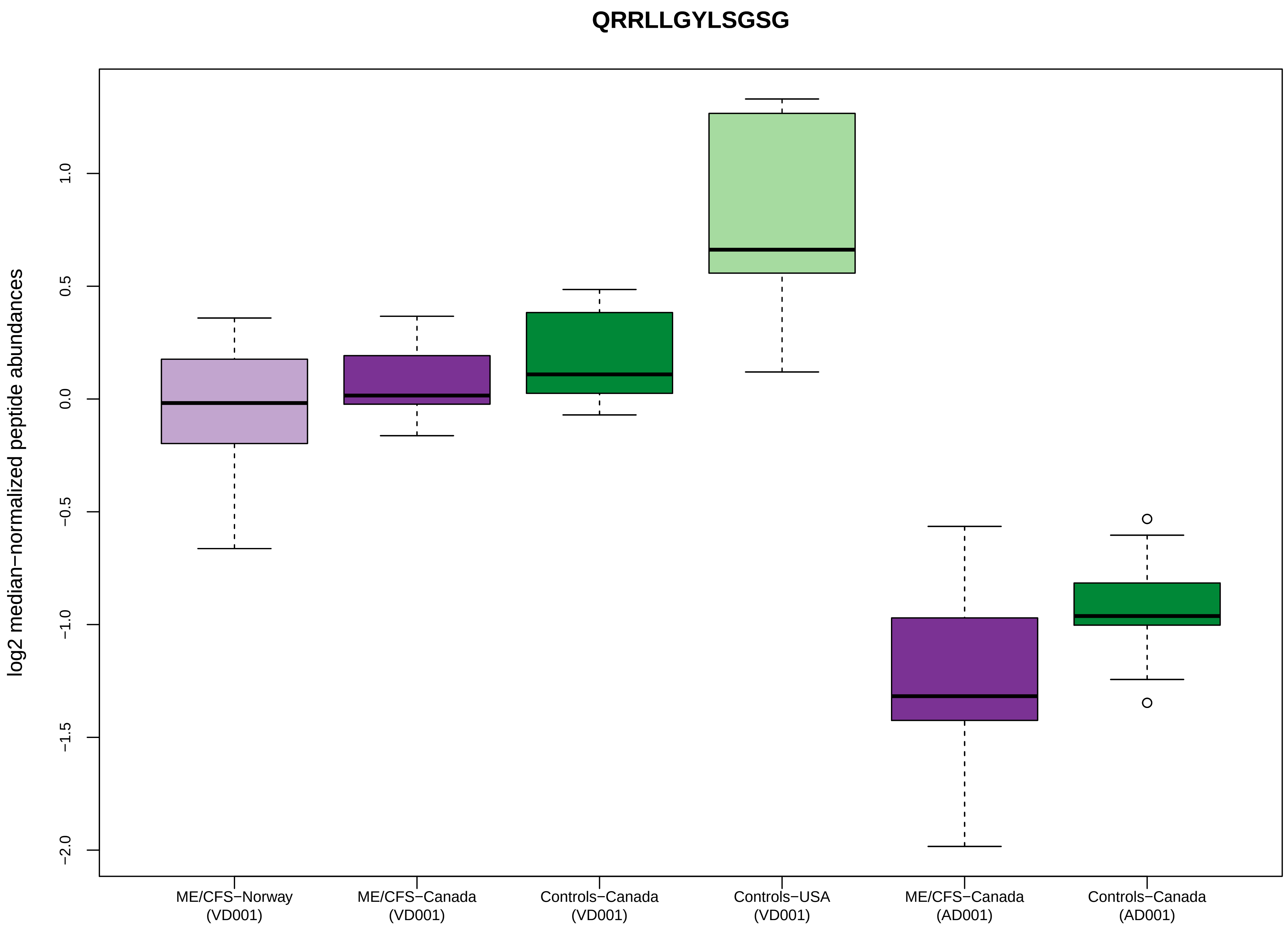
QRRLLGYLSGSG
log2 median−normalized peptide abundances
1.0
0.5
0.0
−0.5
−1.0
−1.5
−2.0
ME/CFS−Norway (VD001)
ME/CFS−Canada (VD001)
Controls−Canada (VD001)
Controls−USA (VD001)
ME/CFS−Canada (AD001)
Controls−Canada (AD001)

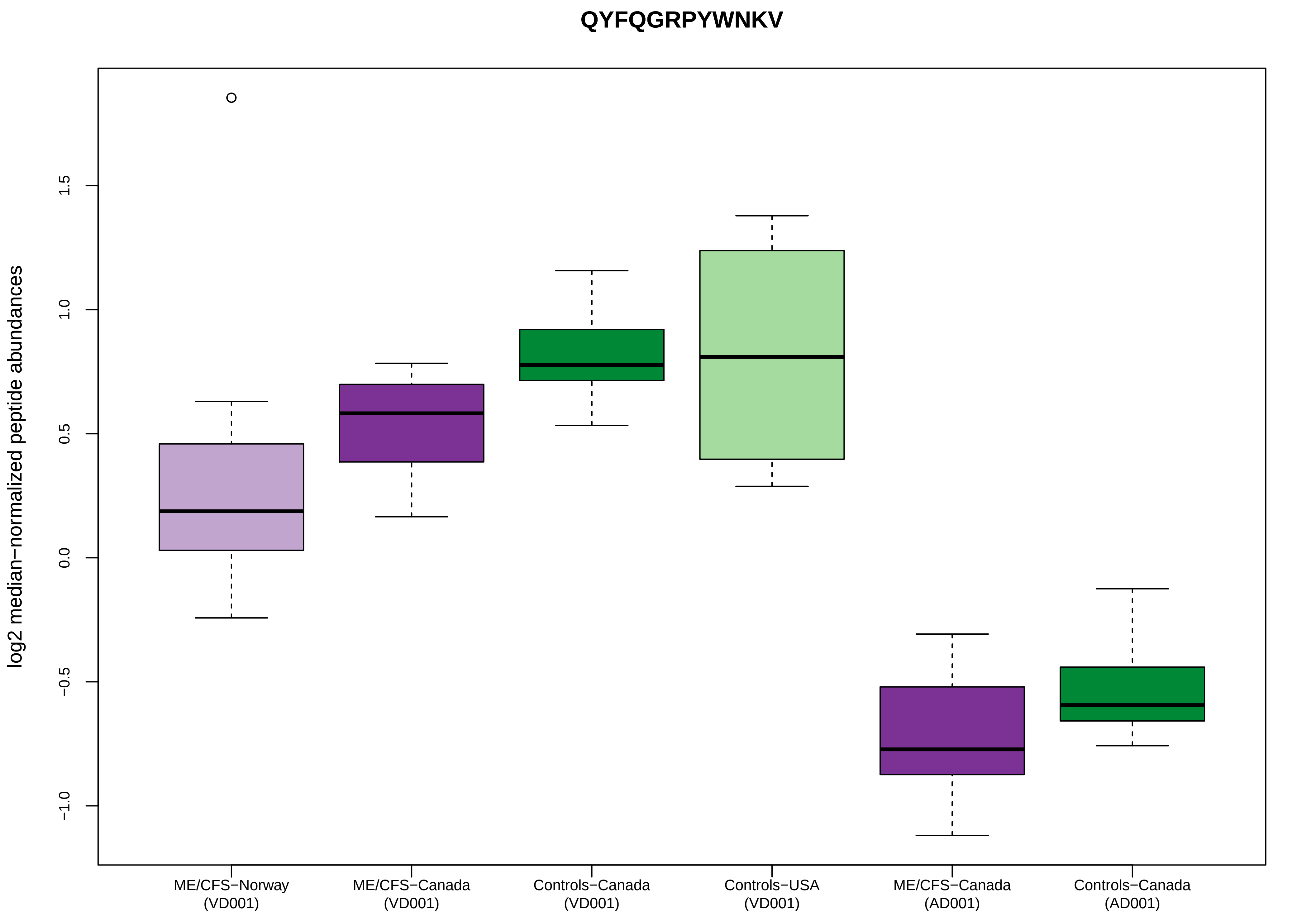

QYFQGRPYWNKV
log2 median−normalized peptide abundances
1.5
1.0
0.5
0.0
−0.5
−1.0
ME/CFS−Norway (VD001)
ME/CFS−Canada (VD001)
Controls−Canada (VD001)
Controls−USA (VD001)
ME/CFS−Canada (AD001)
Controls−Canada (AD001)

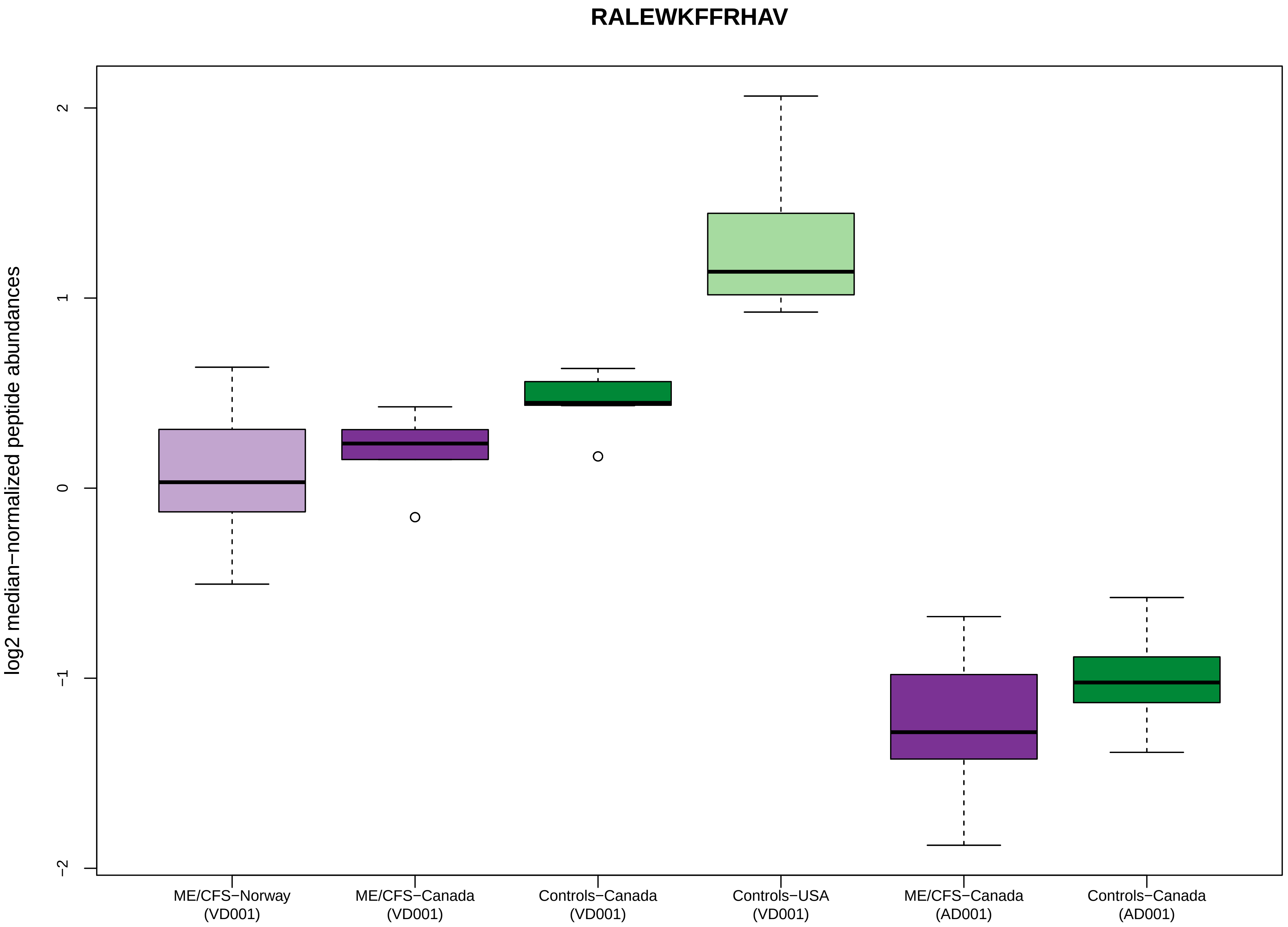

RALEWKFFRHAV
log2 median−normalized peptide abundances
2
1
0
−1
−2
ME/CFS−Norway (VD001)
ME/CFS−Canada (VD001)
Controls−Canada (VD001)
Controls−USA (VD001)
ME/CFS−Canada (AD001)
Controls−Canada (AD001)

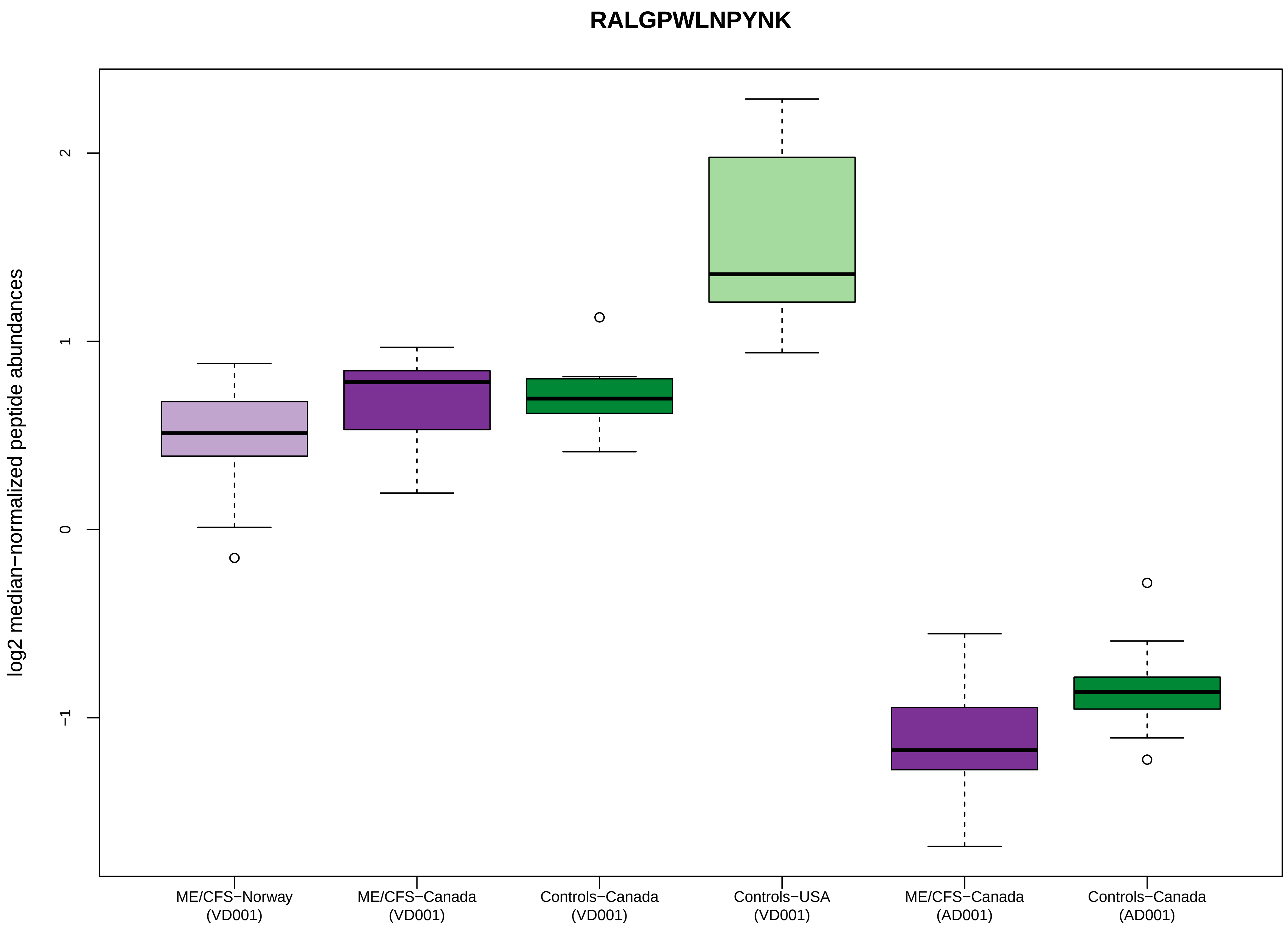

RALGPWLNPYNK
log2 median−normalized peptide abundances
2
1
0
−1
ME/CFS−Norway (VD001)
ME/CFS−Canada (VD001)
Controls−Canada (VD001)
Controls−USA (VD001)
ME/CFS−Canada (AD001)
Controls−Canada (AD001)

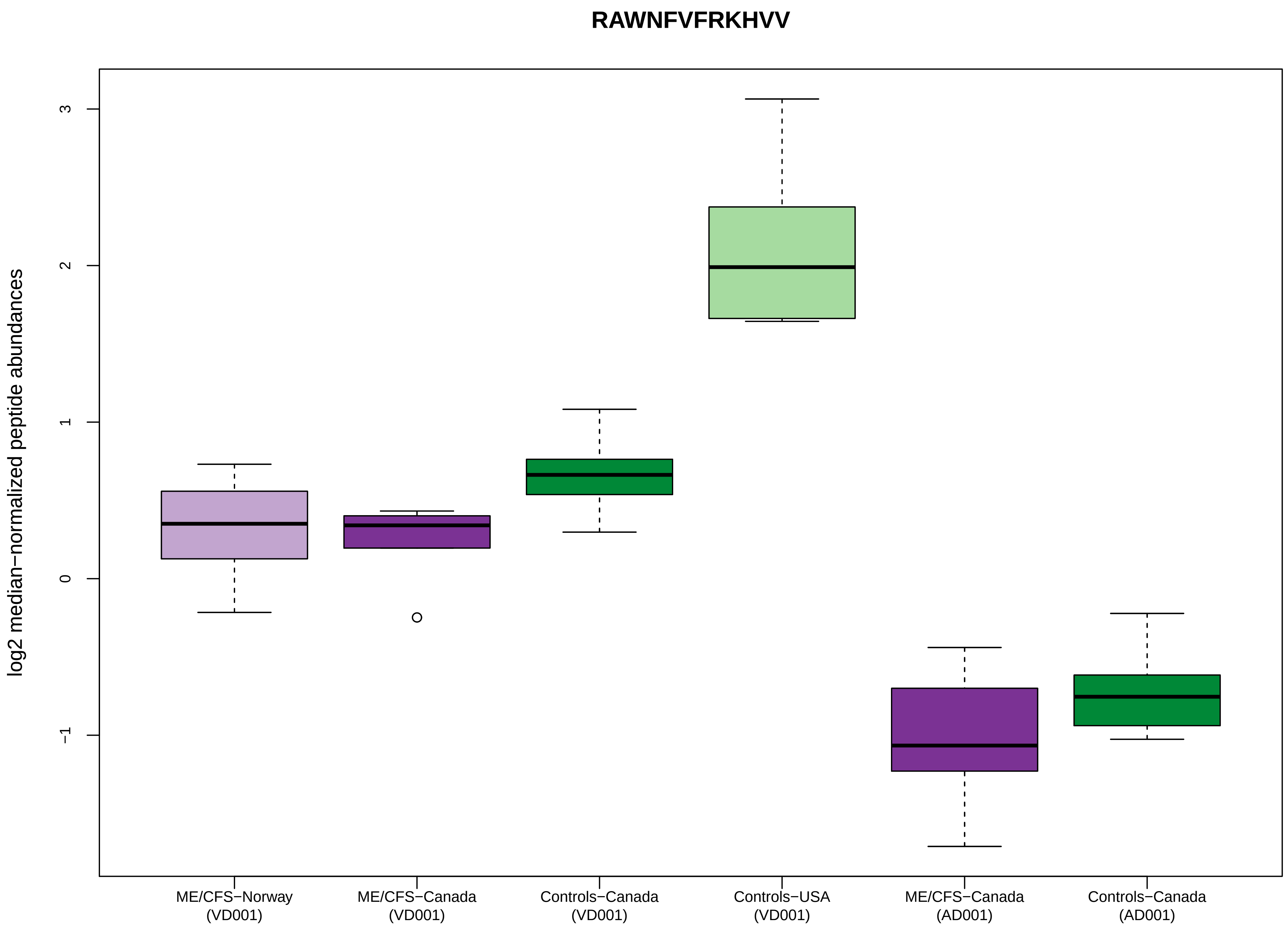

RAWNFVFRKHVV
log2 median−normalized peptide abundances
3
2
1
0
−1
ME/CFS−Norway
(VD001)
ME/CFS−Canada
(VD001)
Controls−Canada
(VD001)
Controls−USA
(VD001)
ME/CFS−Canada
(AD001)
Controls−Canada
(AD001)

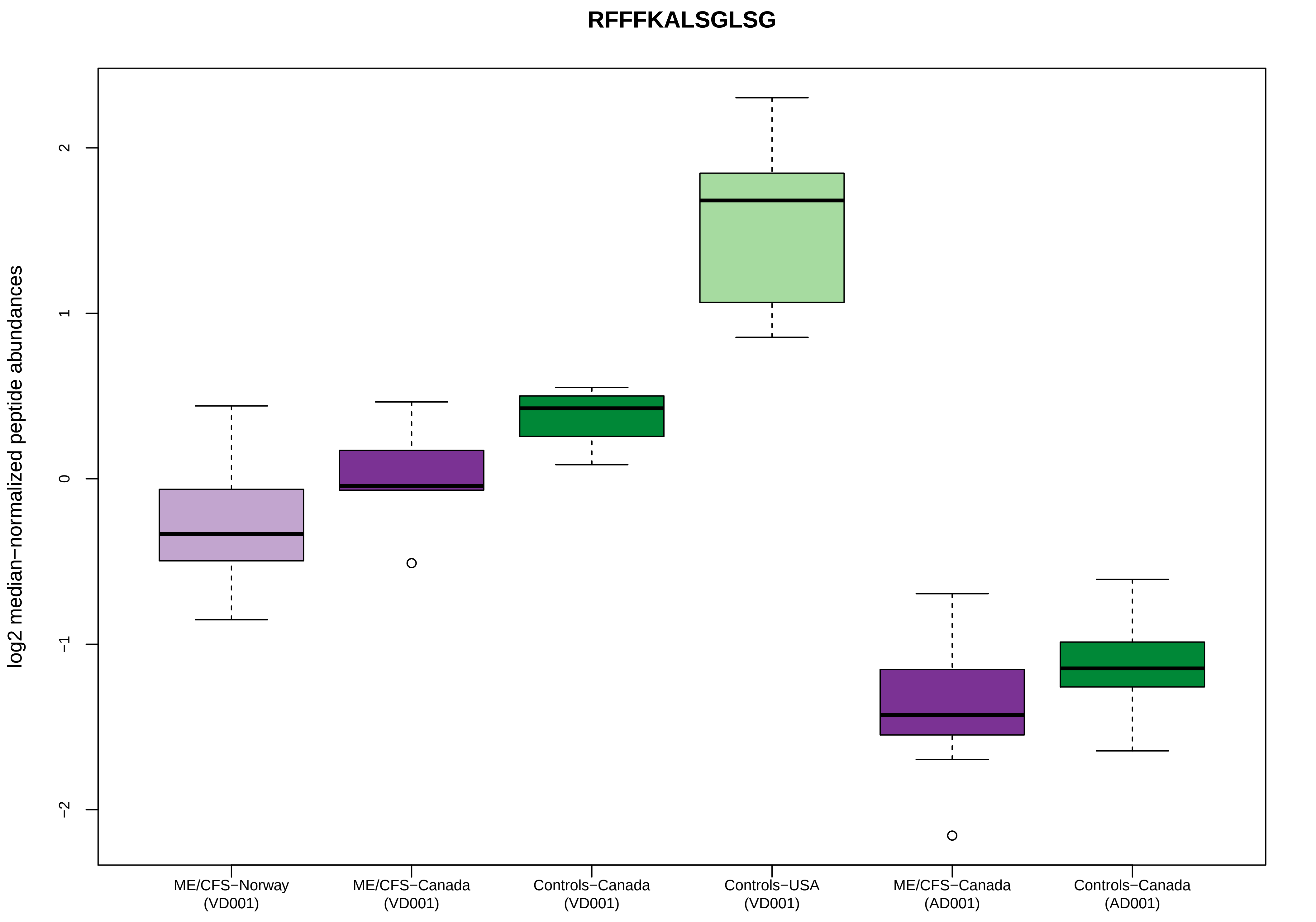
RFFFKALSGLSG
log2 median−normalized peptide abundances
2
1
0
−1
−2
ME/CFS−Norway
(VD001)
ME/CFS−Canada
(VD001)
Controls−Canada
(VD001)
Controls−USA
(VD001)
ME/CFS−Canada
(AD001)
Controls−Canada
(AD001)

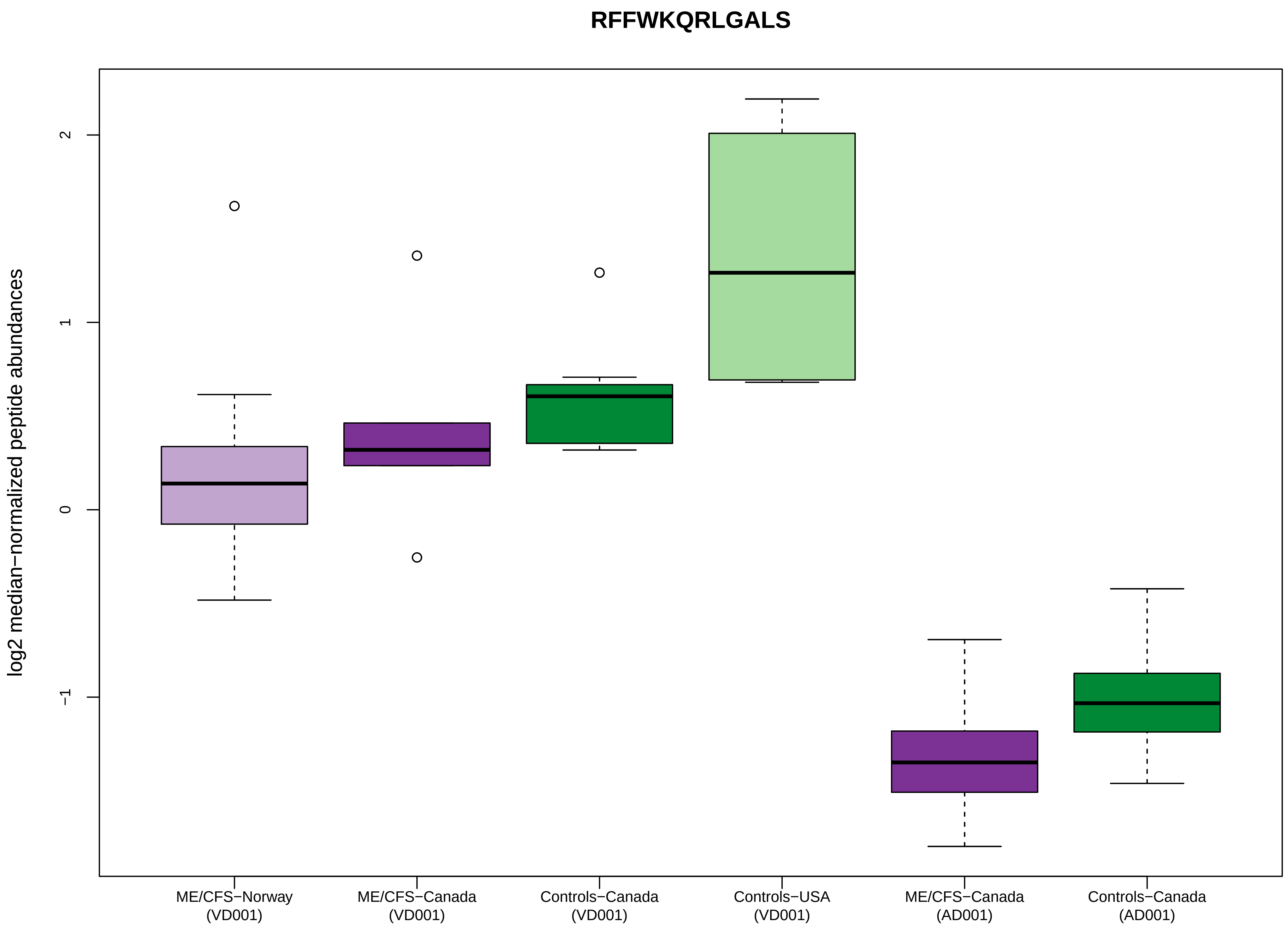

RFFWKQRLGALS
log2 median−normalized peptide abundances
2
1
0
−1
ME/CFS−Norway (VD001)
ME/CFS−Canada (VD001)
Controls−Canada (VD001)
Controls−USA (VD001)
ME/CFS−Canada (AD001)
Controls−Canada (AD001)

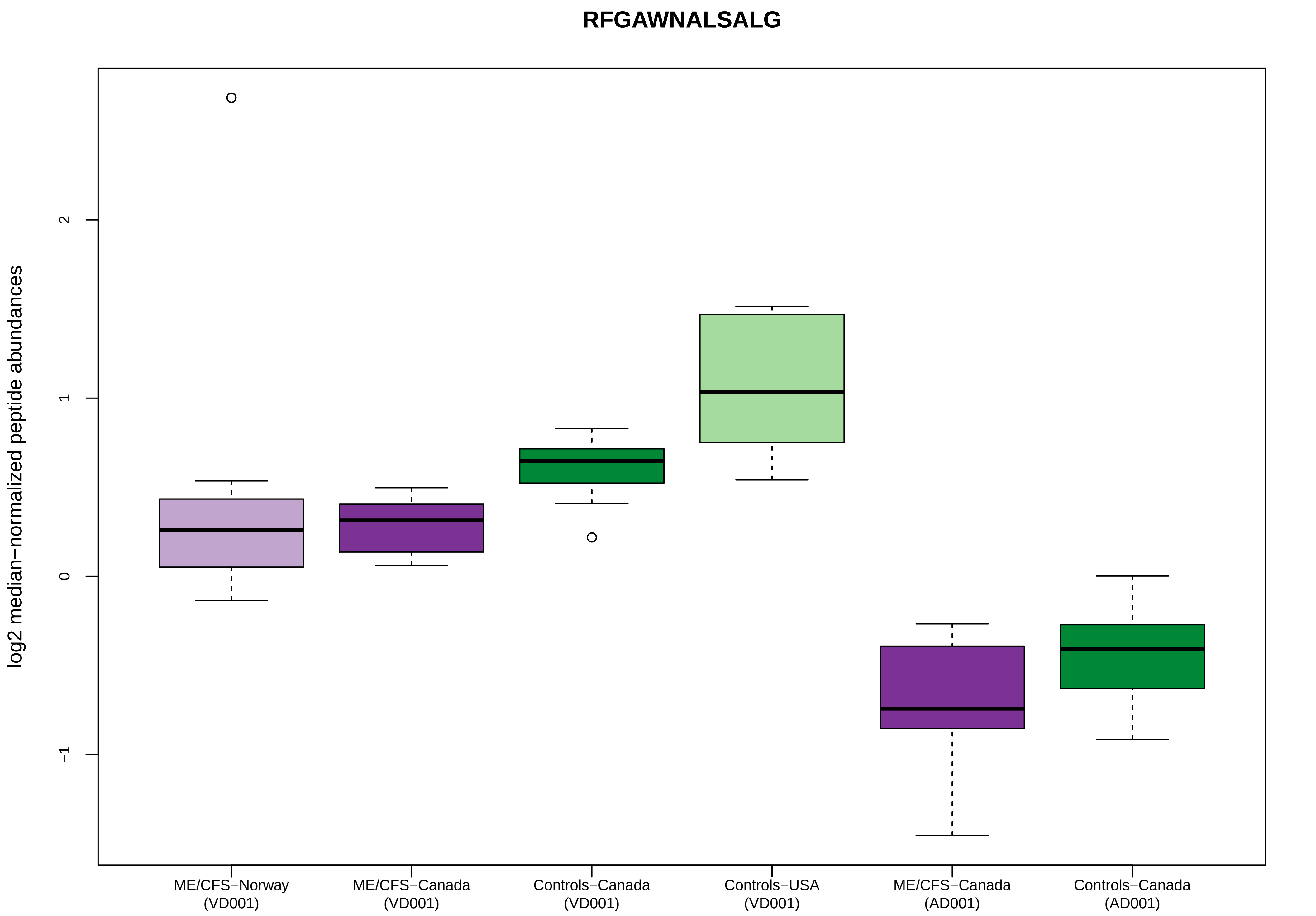

RFGAWNALSALG
log2 median−normalized peptide abundances
−1
0
1
2
ME/CFS−Norway (VD001)
ME/CFS−Canada (VD001)
Controls−Canada (VD001)
Controls−USA (VD001)
ME/CFS−Canada (AD001)
Controls−Canada (AD001)

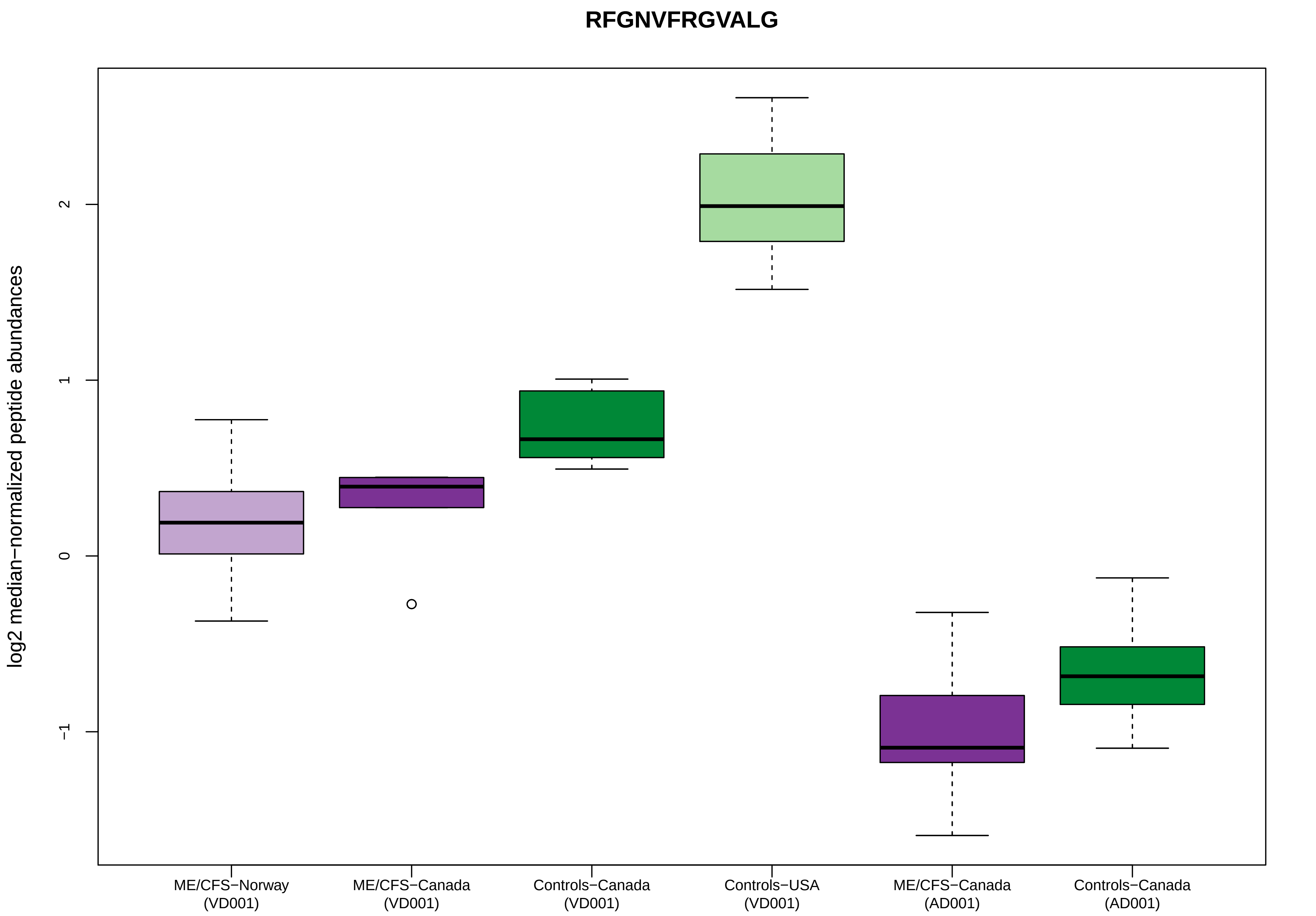

RFGNVFRGVALG
log2 median−normalized peptide abundances
2
1
0
−1
ME/CFS−Norway (VD001)
ME/CFS−Canada (VD001)
Controls−Canada (VD001)
Controls−USA (VD001)
ME/CFS−Canada (AD001)
Controls−Canada (AD001)

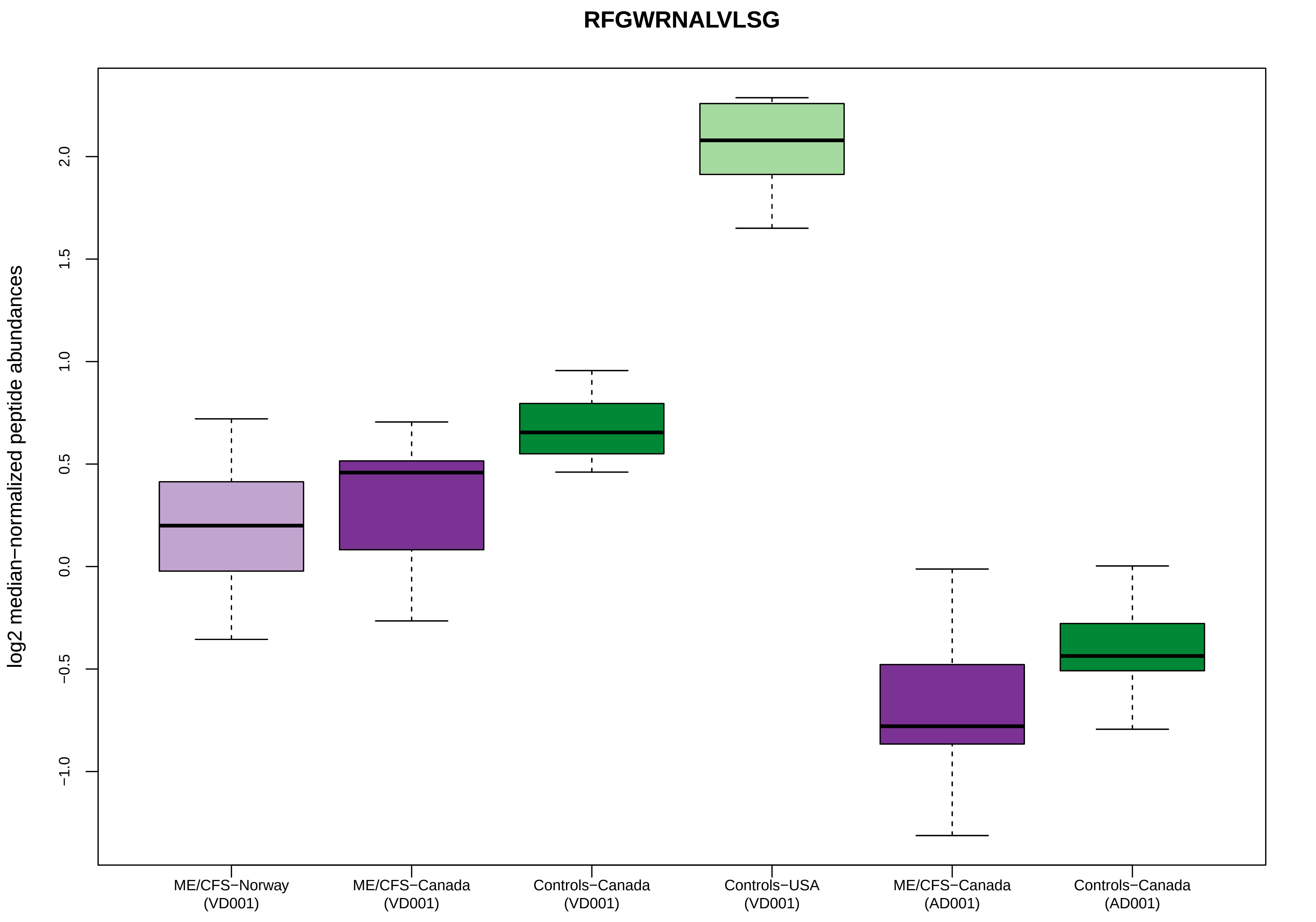

RFGWRNALVLSG
log2 median−normalized peptide abundances
2.0
1.5
1.0
0.5
0.0
−0.5
−1.0
ME/CFS−Norway (VD001)
ME/CFS−Canada (VD001)
Controls−Canada (VD001)
Controls−USA (VD001)
ME/CFS−Canada (AD001)
Controls−Canada (AD001)

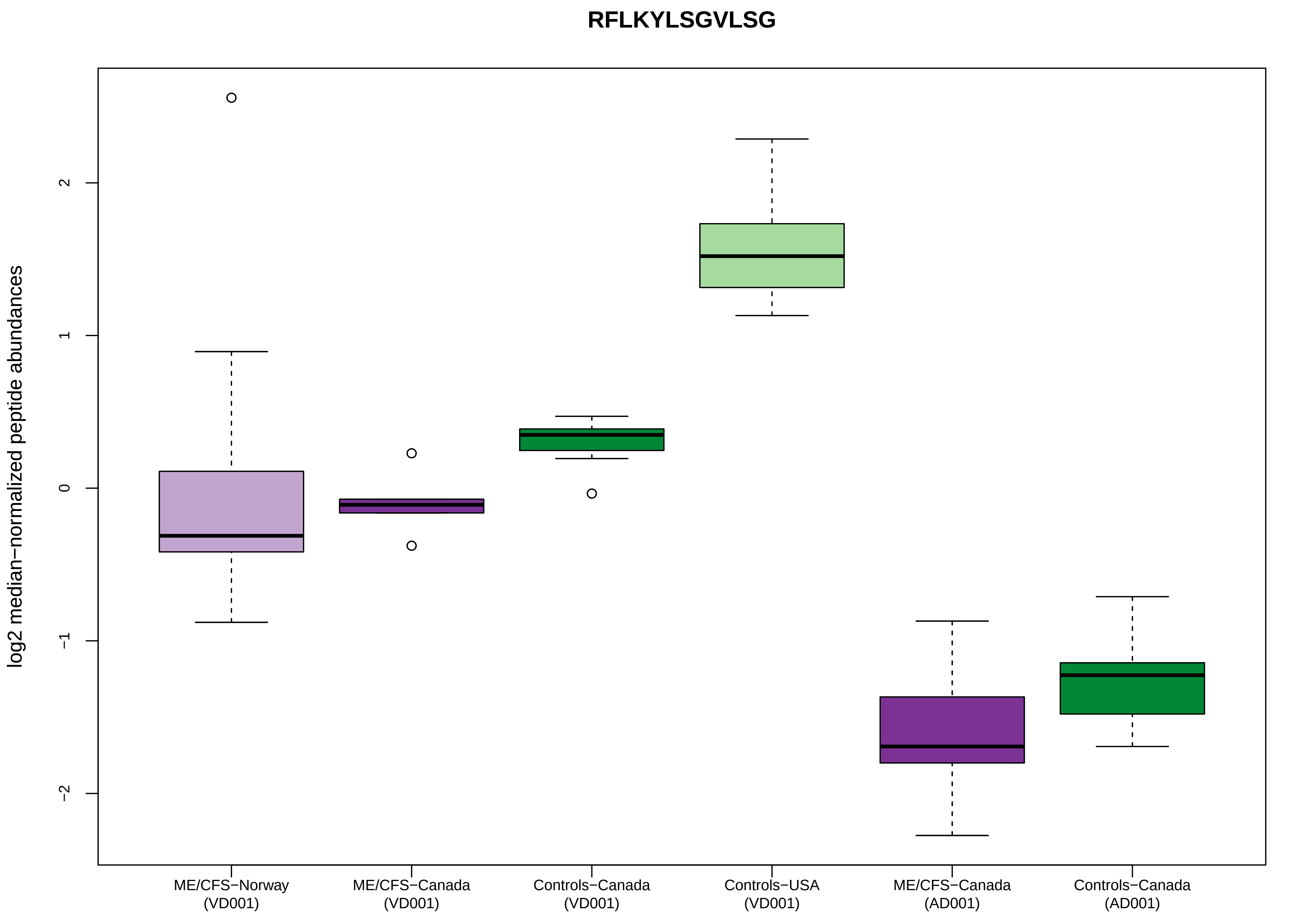

RFLKYLSGVLSG
log2 median−normalized peptide abundances
−2
−1
0
1
2
ME/CFS−Norway (VD001)
ME/CFS−Canada (VD001)
Controls−Canada (VD001)
Controls−USA (VD001)
ME/CFS−Canada (AD001)
Controls−Canada (AD001)

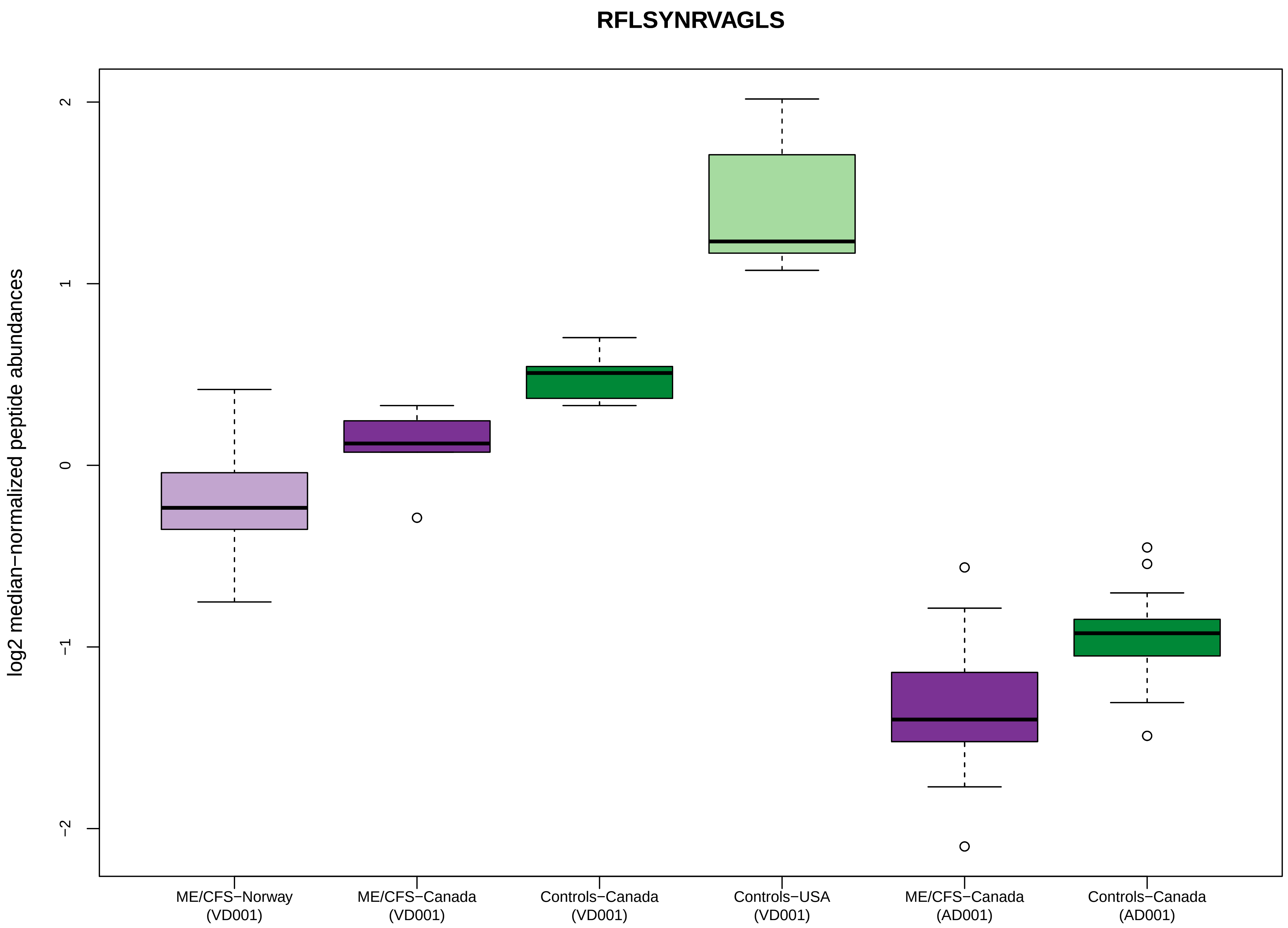

RFLSYNRVAGLS
log2 median−normalized peptide abundances
2
1
0
−1
−2
ME/CFS−Norway (VD001)
ME/CFS−Canada (VD001)
Controls−Canada (VD001)
Controls−USA (VD001)
ME/CFS−Canada (AD001)
Controls−Canada (AD001)

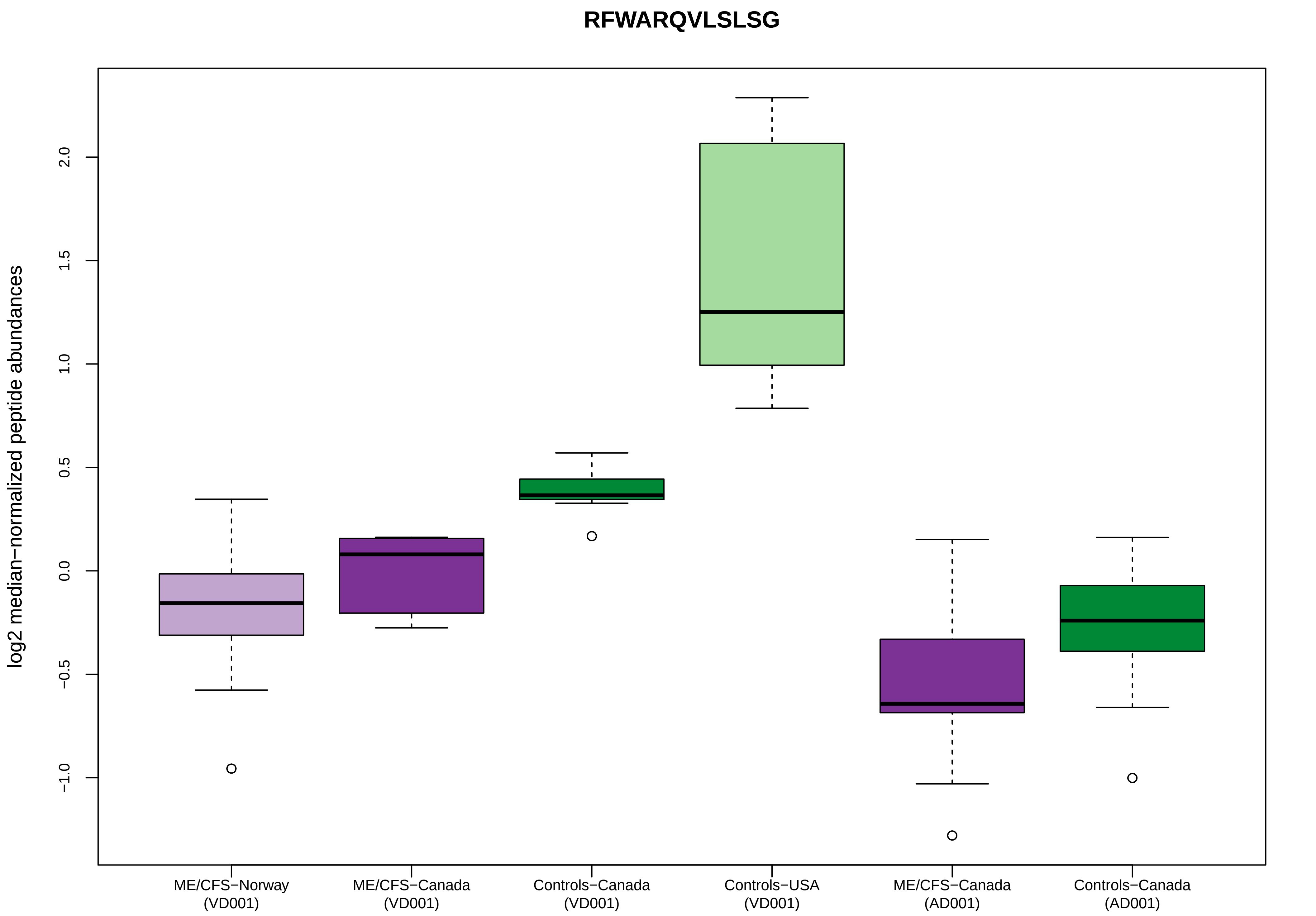

RFWARQVLSLSG
log2 median−normalized peptide abundances
−1.0
−0.5
0.0
0.5
1.0
1.5
2.0
ME/CFS−Norway (VD001)
ME/CFS−Canada (VD001)
Controls−Canada (VD001)
Controls−USA (VD001)
ME/CFS−Canada (AD001)
Controls−Canada (AD001)

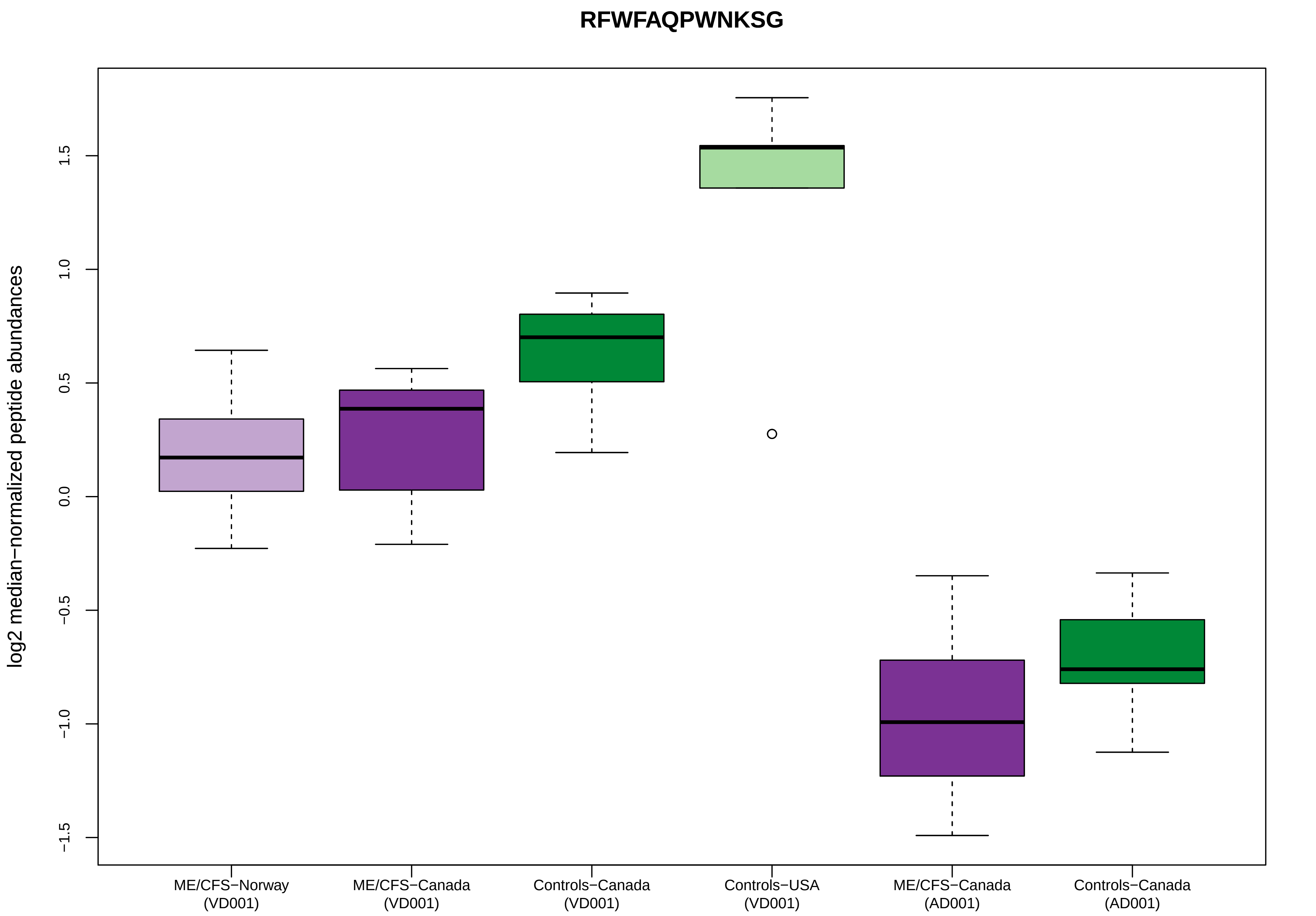

RFWFAQPWNKSG
log2 median−normalized peptide abundances
1.5
1.0
0.5
0.0
−0.5
−1.0
−1.5
ME/CFS−Norway (VD001)
ME/CFS−Canada (VD001)
Controls−Canada (VD001)
Controls−USA (VD001)
ME/CFS−Canada (AD001)
Controls−Canada (AD001)

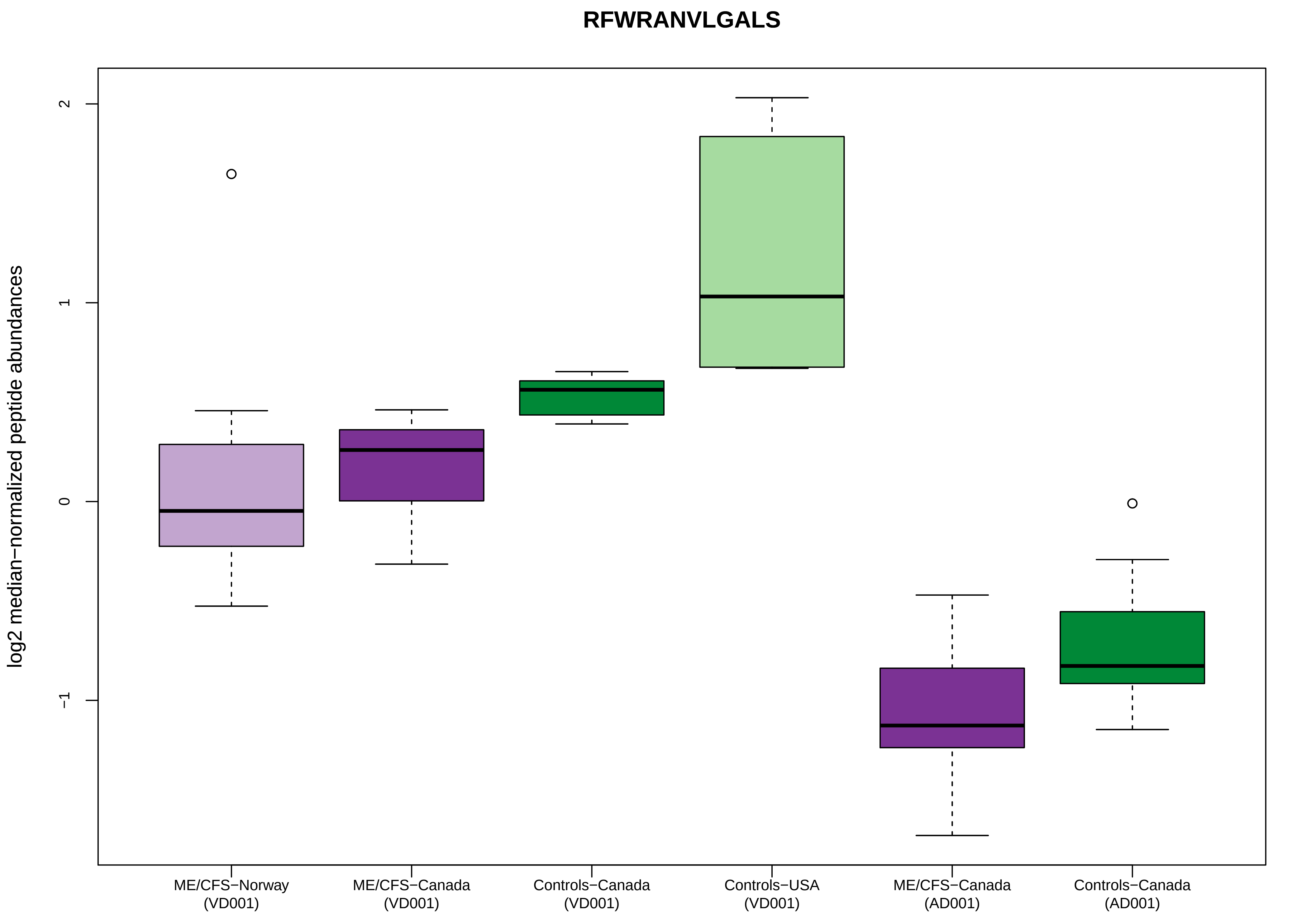
RFWRANVLGALS
log2 median−normalized peptide abundances
2
1
0
−1
ME/CFS−Norway (VD001)
ME/CFS−Canada (VD001)
Controls−Canada (VD001)
Controls−USA (VD001)
ME/CFS−Canada (AD001)
Controls−Canada (AD001)

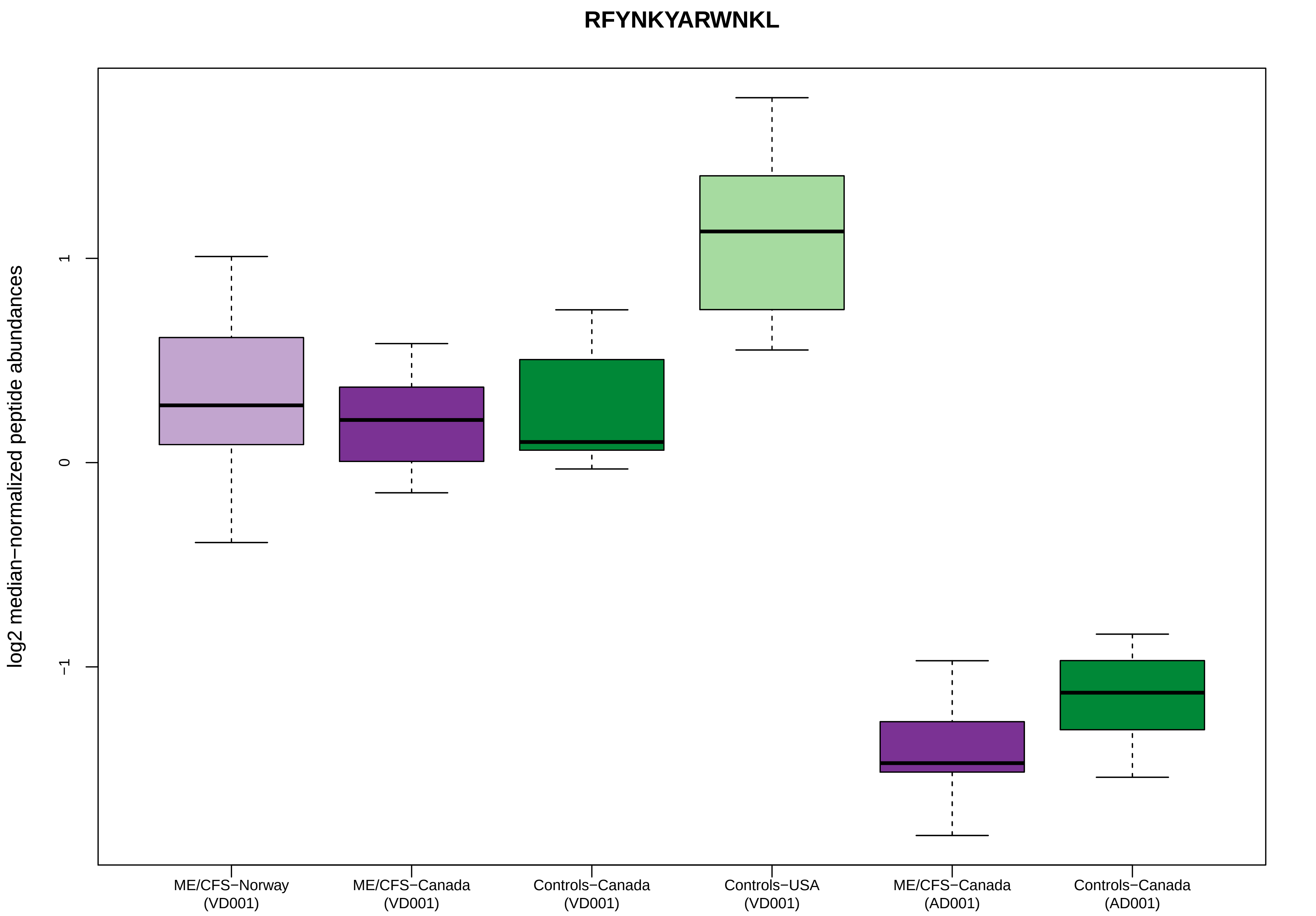
RFYNKYARWNKL
log2 median−normalized peptide abundances
1
0
−1
ME/CFS−Norway (VD001)
ME/CFS−Canada (VD001)
Controls−Canada (VD001)
Controls−USA (VD001)
ME/CFS−Canada (AD001)
Controls−Canada (AD001)

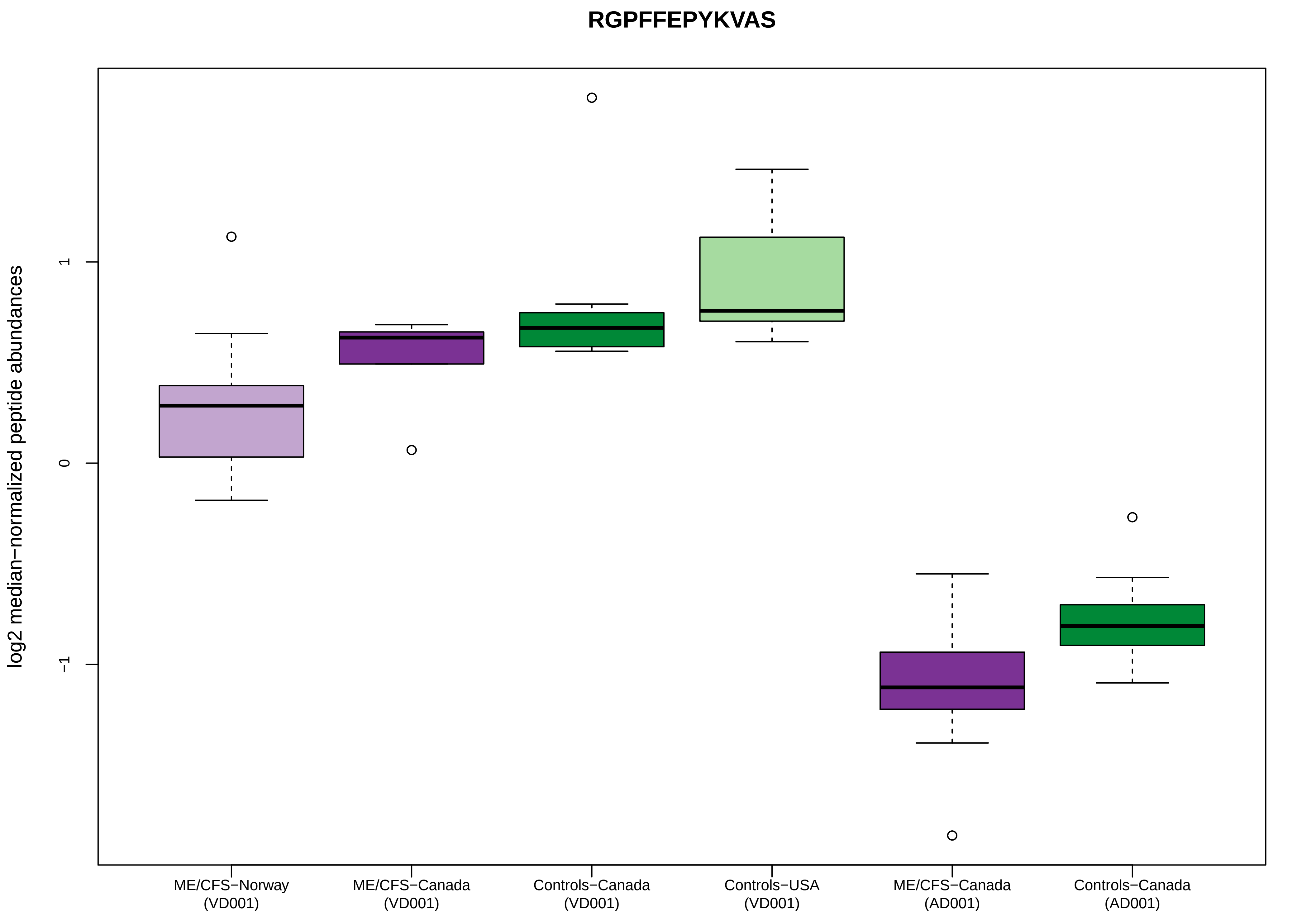
RGPFFEPYKVAS
log2 median−normalized peptide abundances
1
0
−1
ME/CFS−Norway (VD001)
ME/CFS−Canada (VD001)
Controls−Canada (VD001)
Controls−USA (VD001)
ME/CFS−Canada (AD001)
Controls−Canada (AD001)

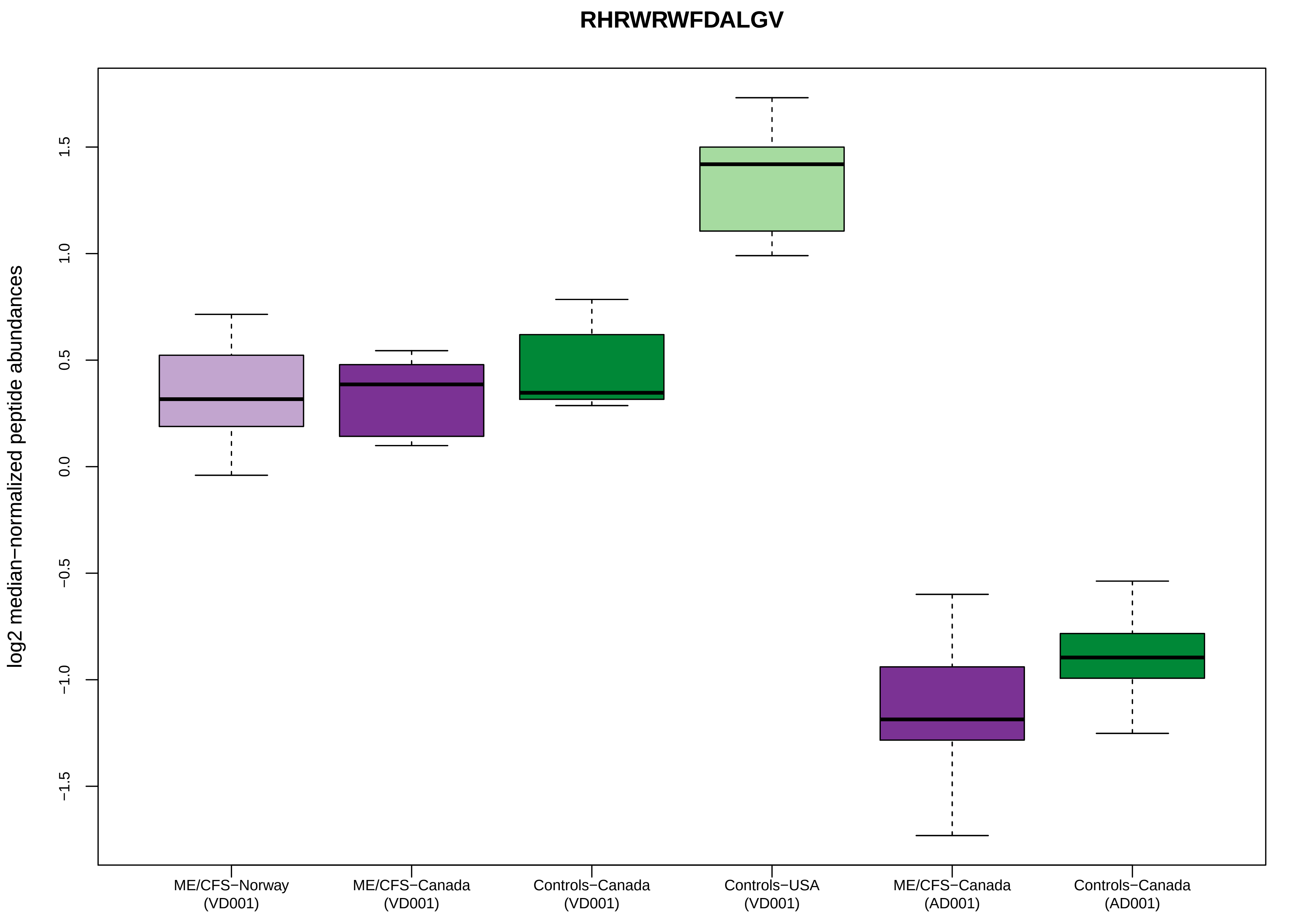

RHRWRWFDALGV
log2 median−normalized peptide abundances
1.5
1.0
0.5
0.0
−0.5
−1.0
−1.5
ME/CFS−Norway (VD001)
ME/CFS−Canada (VD001)
Controls−Canada (VD001)
Controls−USA (VD001)
ME/CFS−Canada (AD001)
Controls−Canada (AD001)

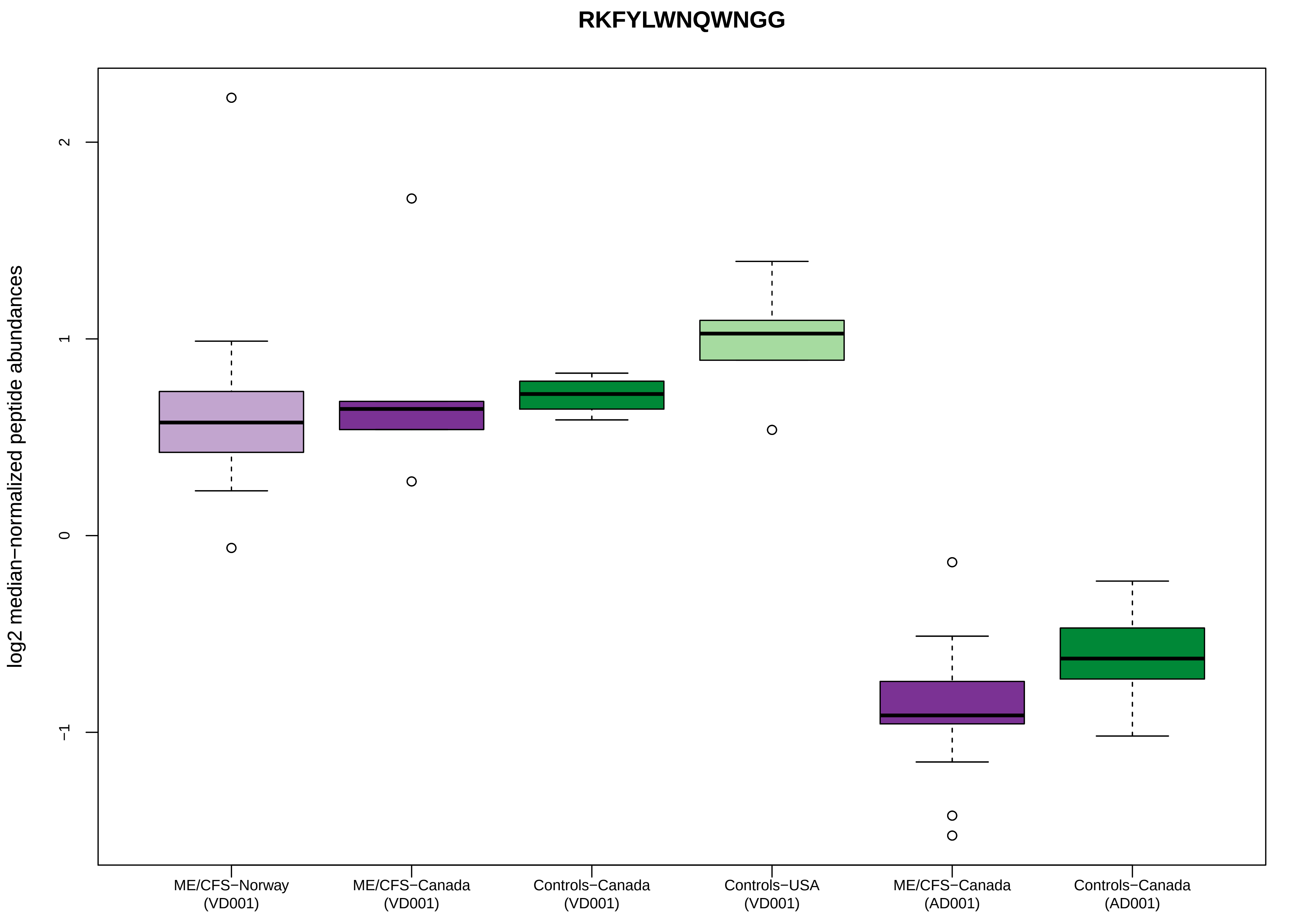
RKFYLWNQWNGG
log2 median−normalized peptide abundances
2
1
0
−1
ME/CFS−Norway
(VD001)
ME/CFS−Canada
(VD001)
Controls−Canada
(VD001)
Controls−USA
(VD001)
ME/CFS−Canada
(AD001)
Controls−Canada
(AD001)

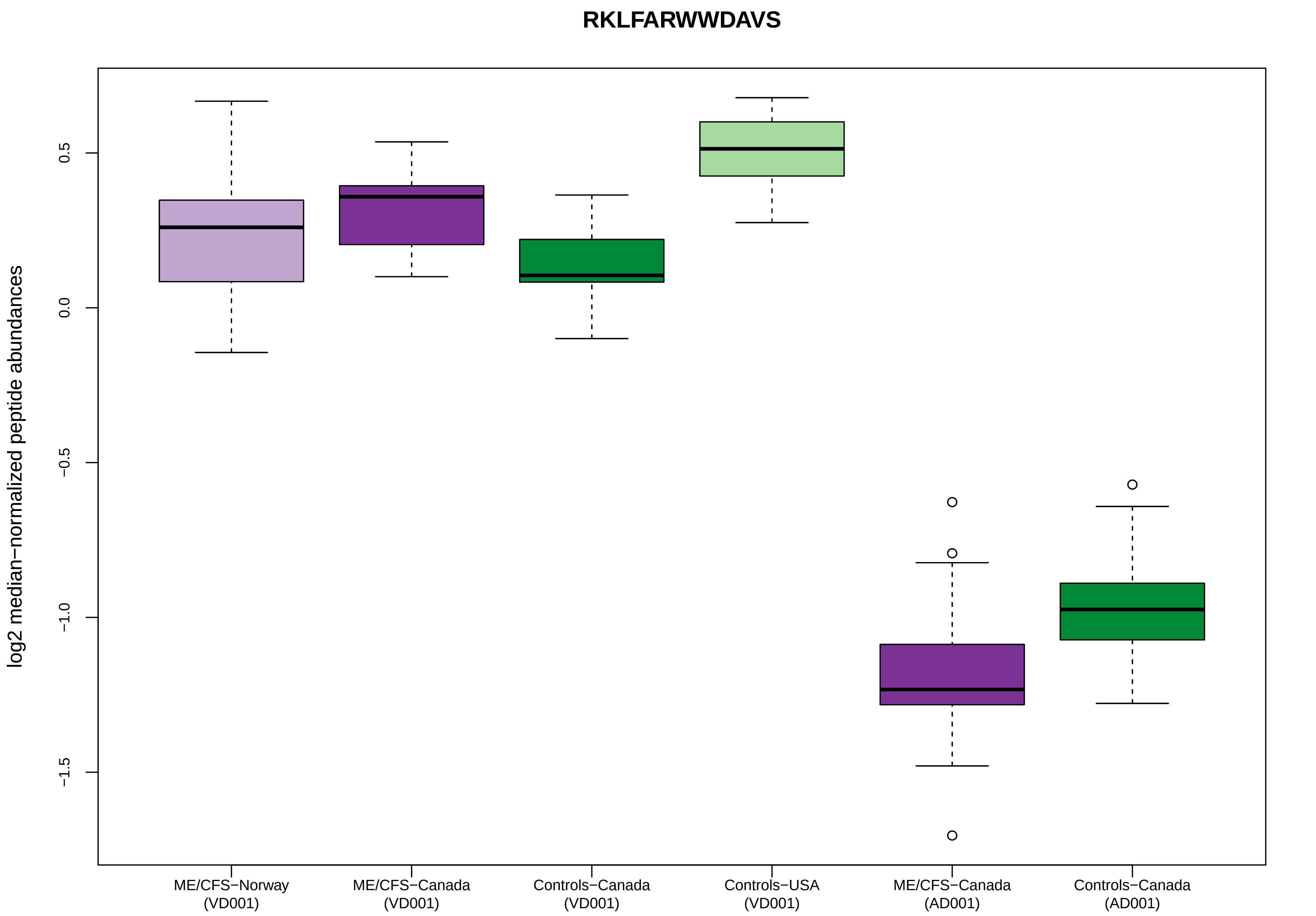
RKLFARWWDAVS
log2 median−normalized peptide abundances
0.5
0.0
−0.5
−1.0
−1.5
ME/CFS−Norway (VD001)
ME/CFS−Canada (VD001)
Controls−Canada (VD001)
Controls−USA (VD001)
ME/CFS−Canada (AD001)
Controls−Canada (AD001)

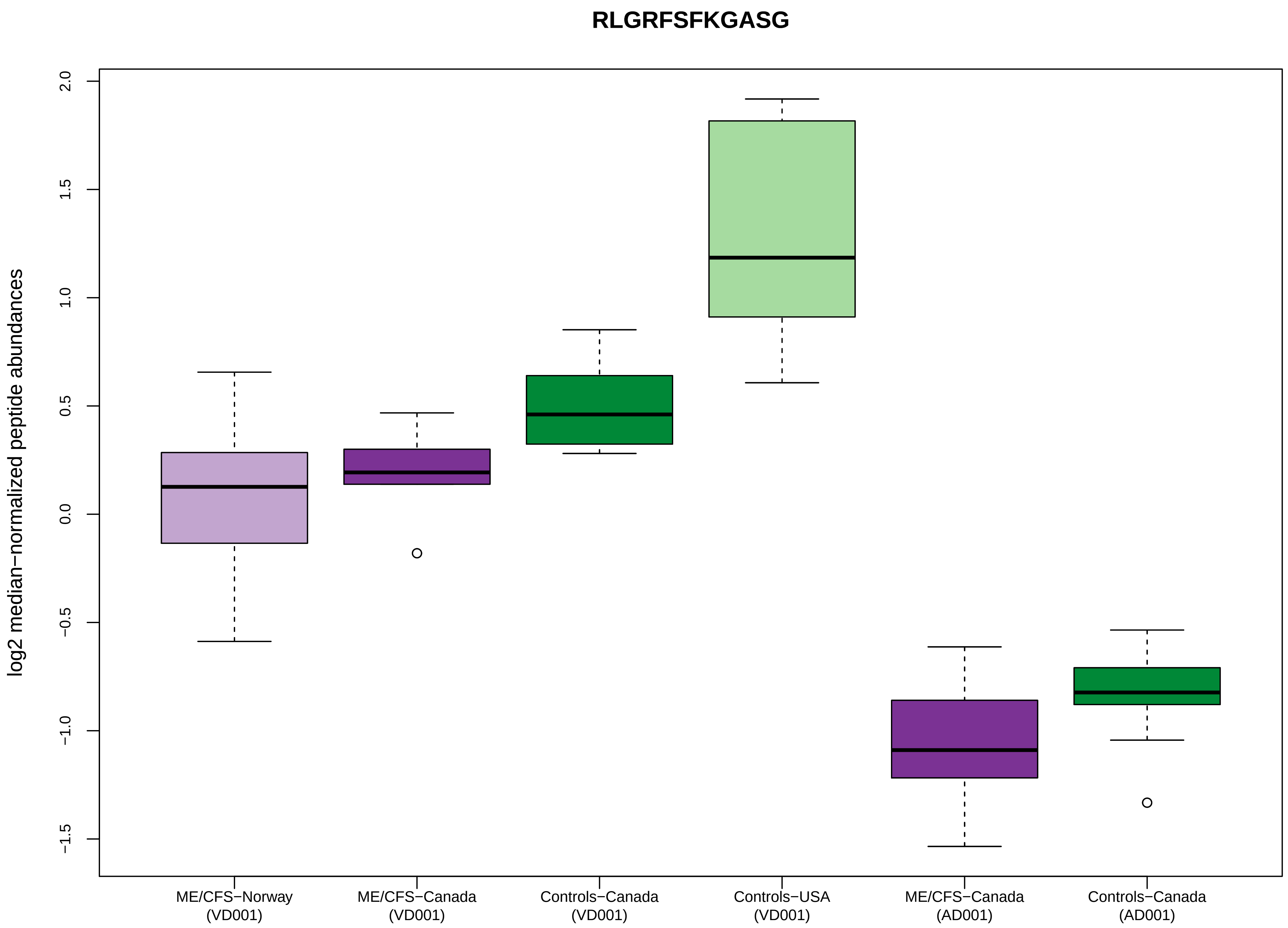

RLGRFSFKGASG
log2 median−normalized peptide abundances
2.0
1.5
1.0
0.5
0.0
−0.5
−1.0
−1.5
ME/CFS−Norway (VD001)
ME/CFS−Canada (VD001)
Controls−Canada (VD001)
Controls−USA (VD001)
ME/CFS−Canada (AD001)
Controls−Canada (AD001)

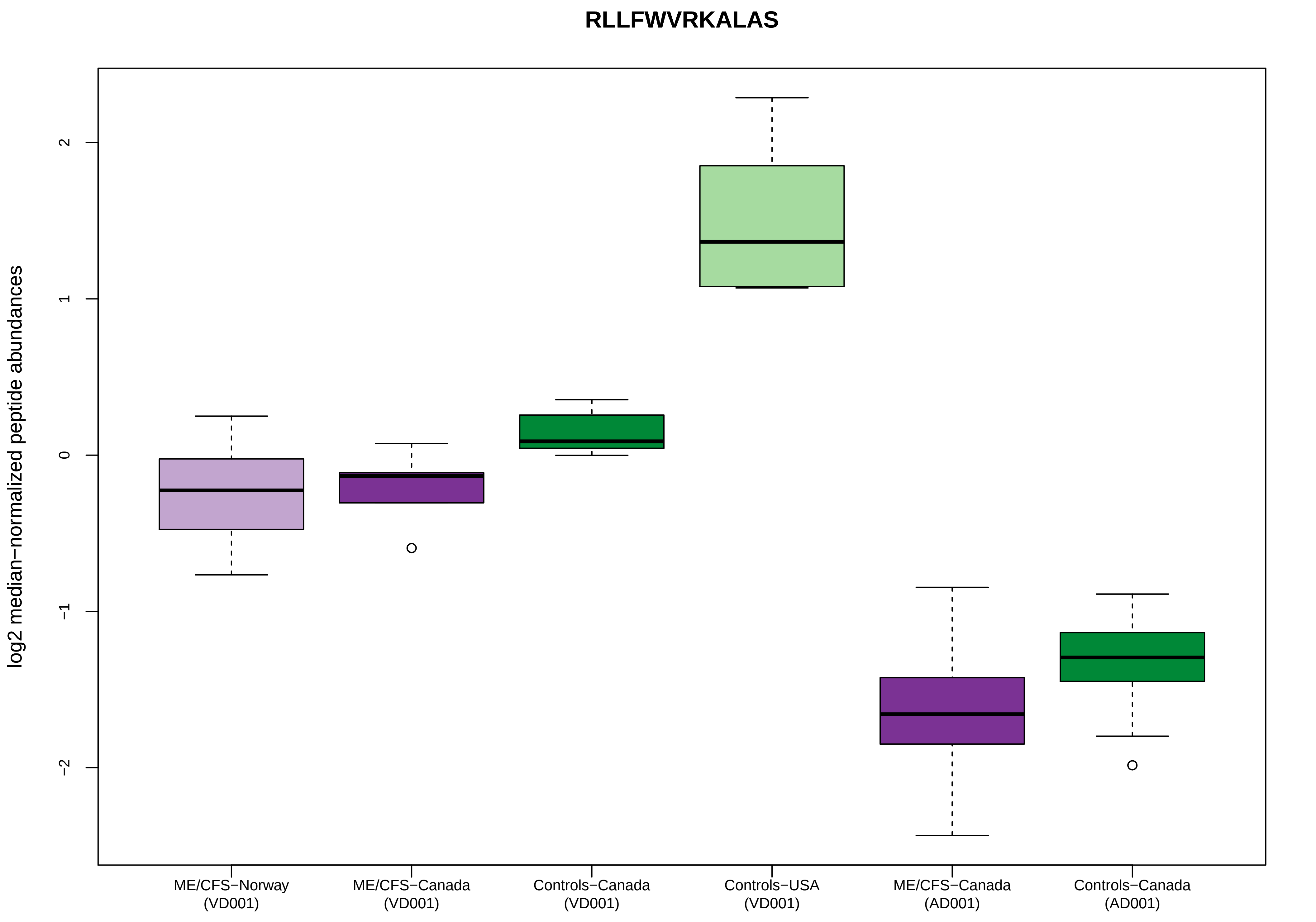
RLLFWVRKALAS
log2 median−normalized peptide abundances
2
1
0
−1
−2
ME/CFS−Norway (VD001)
ME/CFS−Canada (VD001)
Controls−Canada (VD001)
Controls−USA (VD001)
ME/CFS−Canada (AD001)
Controls−Canada (AD001)

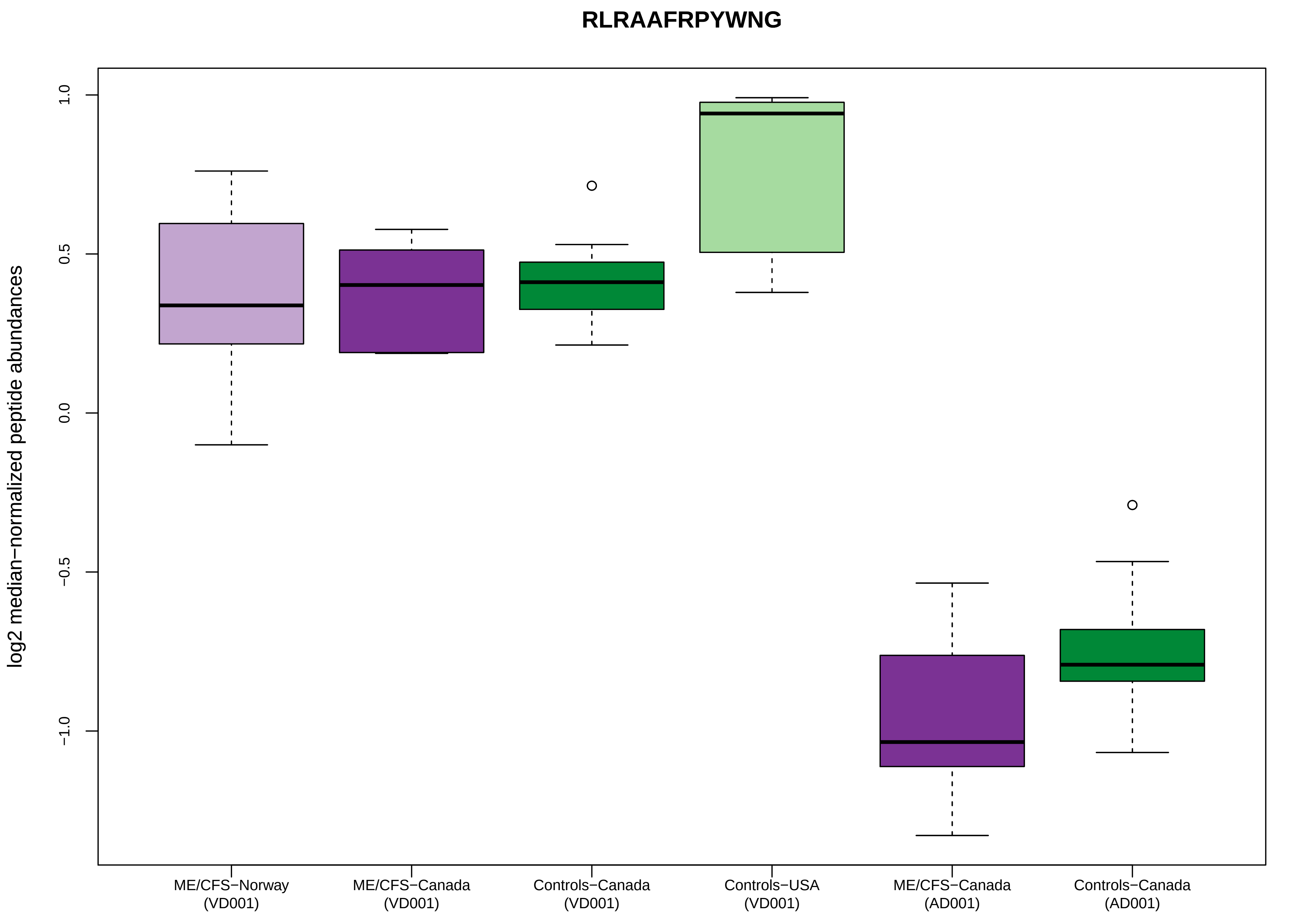

RLRAAFRPYWNG
log2 median−normalized peptide abundances
1.0
0.5
0.0
−0.5
−1.0
ME/CFS−Norway (VD001)
ME/CFS−Canada (VD001)
Controls−Canada (VD001)
Controls−USA (VD001)
ME/CFS−Canada (AD001)
Controls−Canada (AD001)

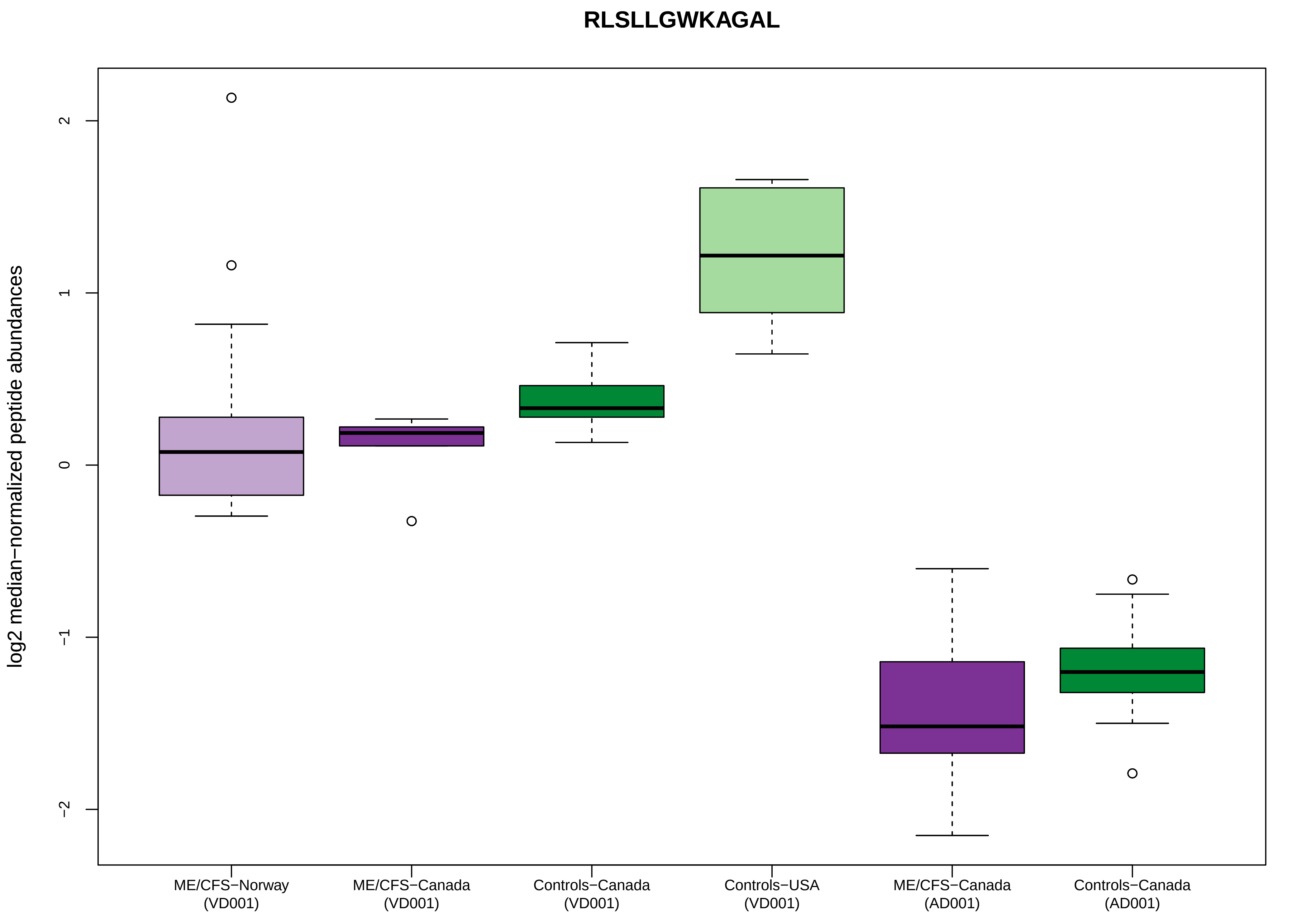

RLSLLGWKAGAL
log2 median−normalized peptide abundances
2
1
0
−1
−2
ME/CFS−Norway (VD001)
ME/CFS−Canada (VD001)
Controls−Canada (VD001)
Controls−USA (VD001)
ME/CFS−Canada (AD001)
Controls−Canada (AD001)

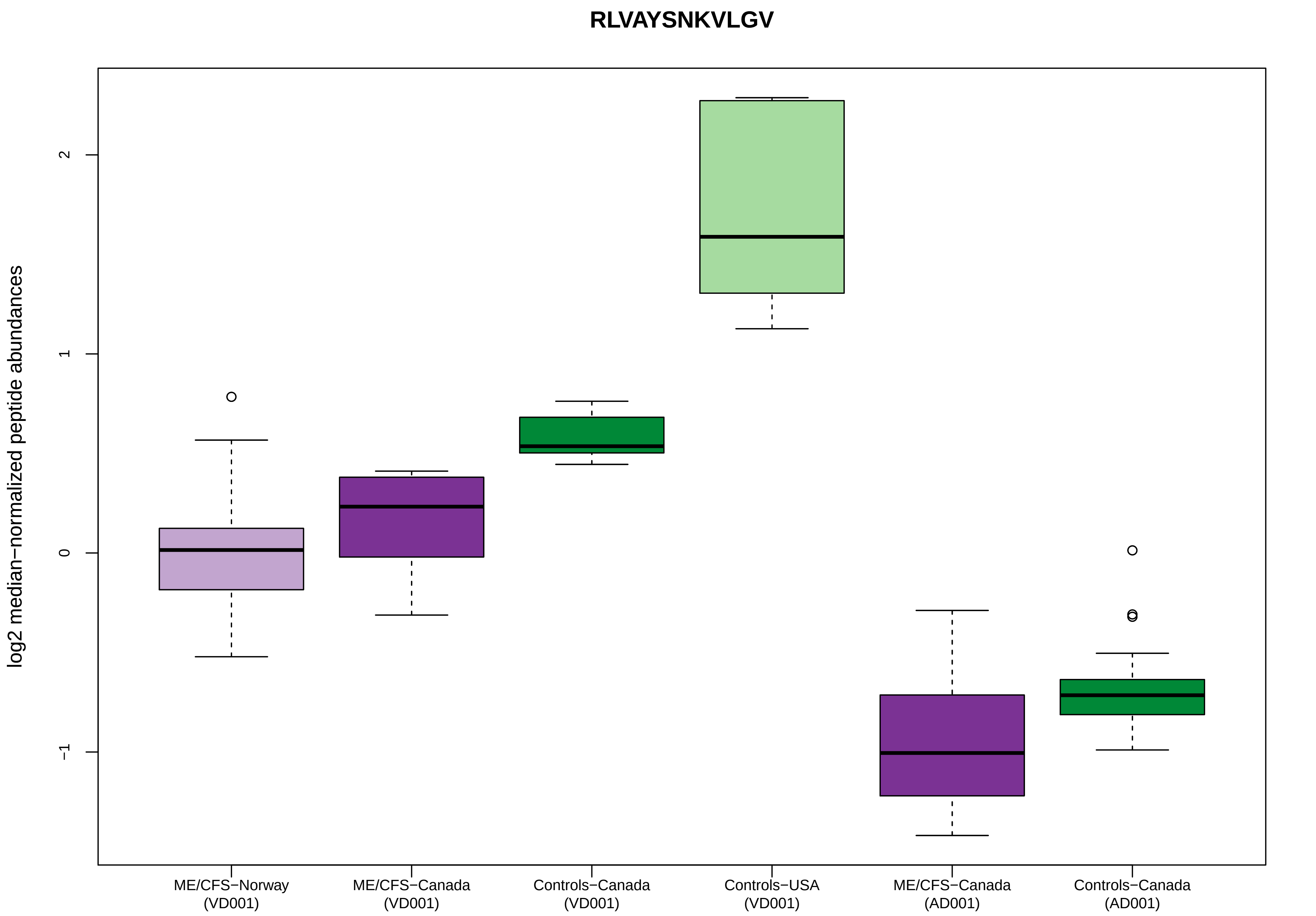
RLVAYSNKVLGV
log2 median−normalized peptide abundances
2
1
0
−1
ME/CFS−Norway (VD001)
ME/CFS−Canada (VD001)
Controls−Canada (VD001)
Controls−USA (VD001)
ME/CFS−Canada (AD001)
Controls−Canada (AD001)

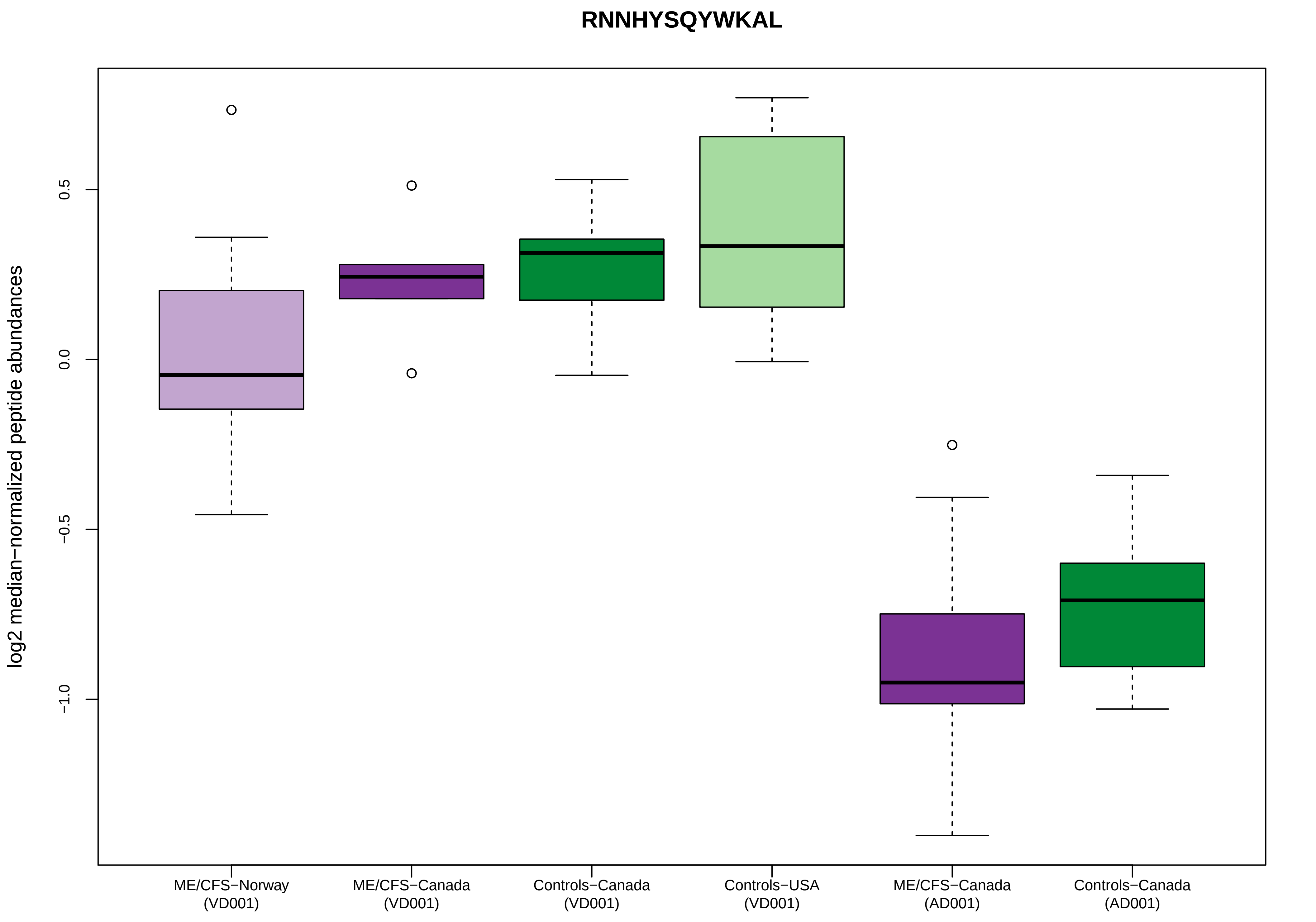
RNNHYSQYWKAL
log2 median−normalized peptide abundances
0.5
0.0
−0.5
−1.0
ME/CFS−Norway (VD001)
ME/CFS−Canada (VD001)
Controls−Canada (VD001)
Controls−USA (VD001)
ME/CFS−Canada (AD001)
Controls−Canada (AD001)

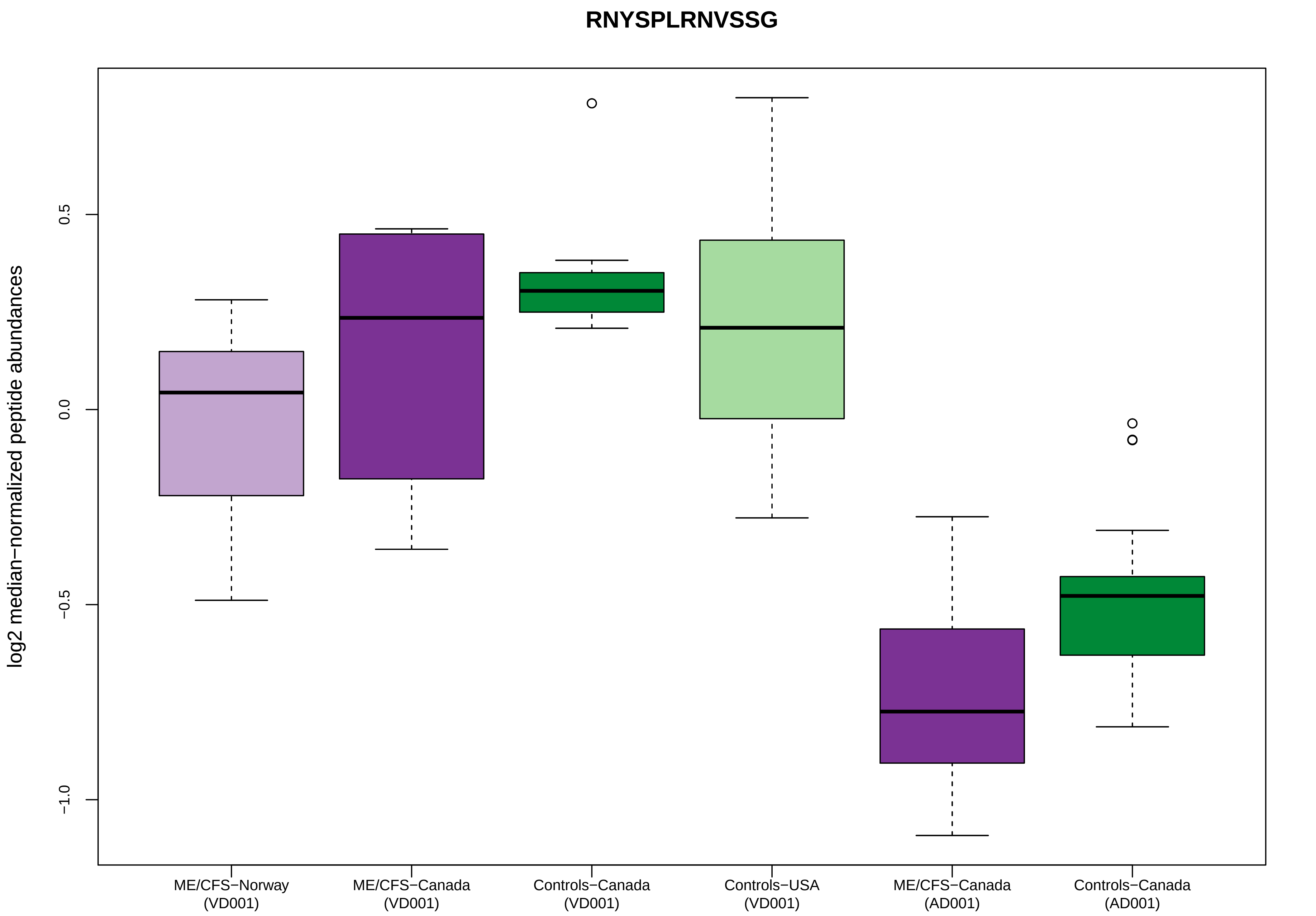
RNYSPLRNVSSG
log2 median−normalized peptide abundances
0.5
0.0
−0.5
−1.0
ME/CFS−Norway (VD001)
ME/CFS−Canada (VD001)
Controls−Canada (VD001)
Controls−USA (VD001)
ME/CFS−Canada (AD001)
Controls−Canada (AD001)

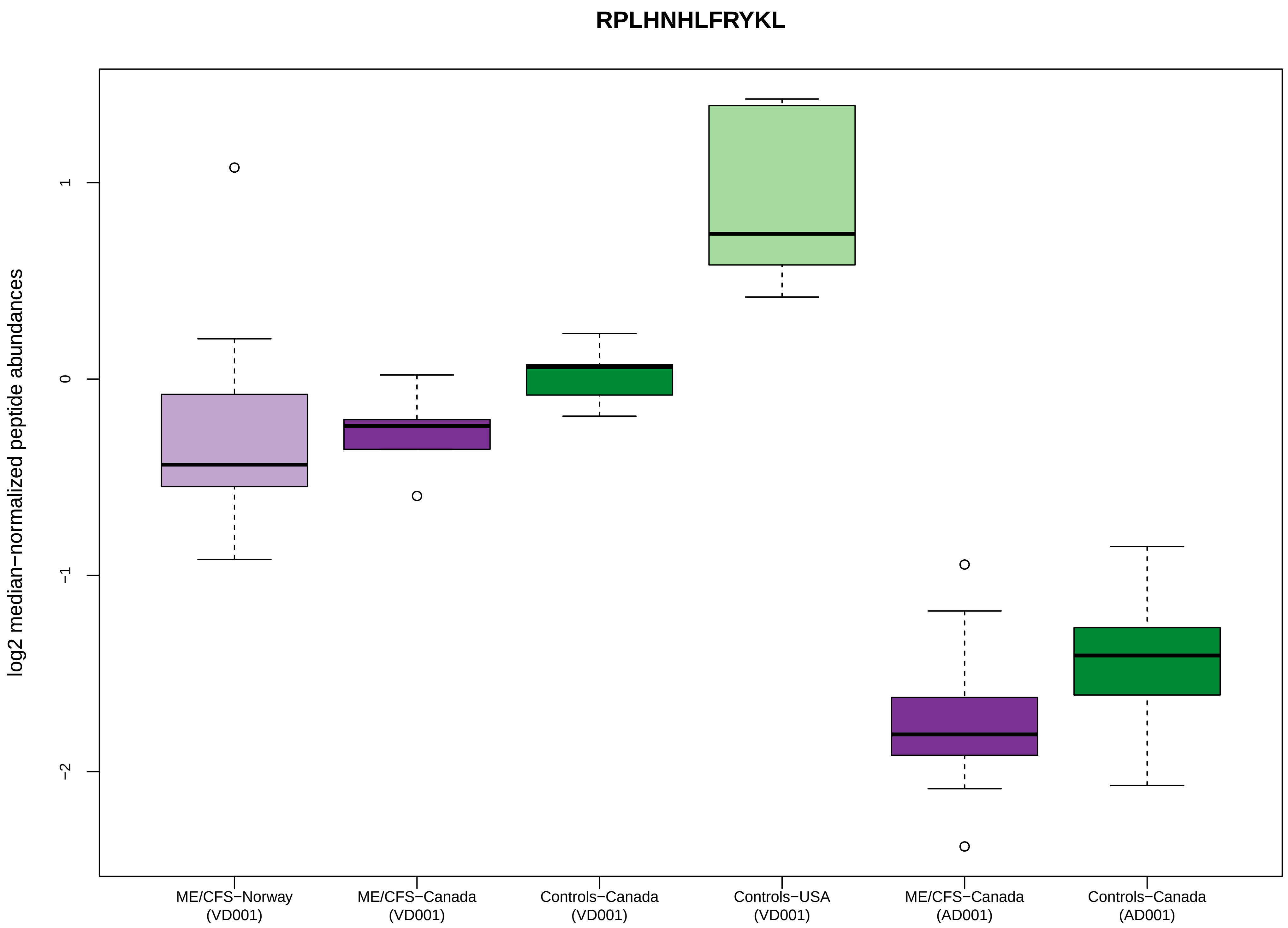

RPLHNHLFRYKL
log2 median−normalized peptide abundances
1
0
−1
−2
ME/CFS−Norway (VD001)
ME/CFS−Canada (VD001)
Controls−Canada (VD001)
Controls−USA (VD001)
ME/CFS−Canada (AD001)
Controls−Canada (AD001)

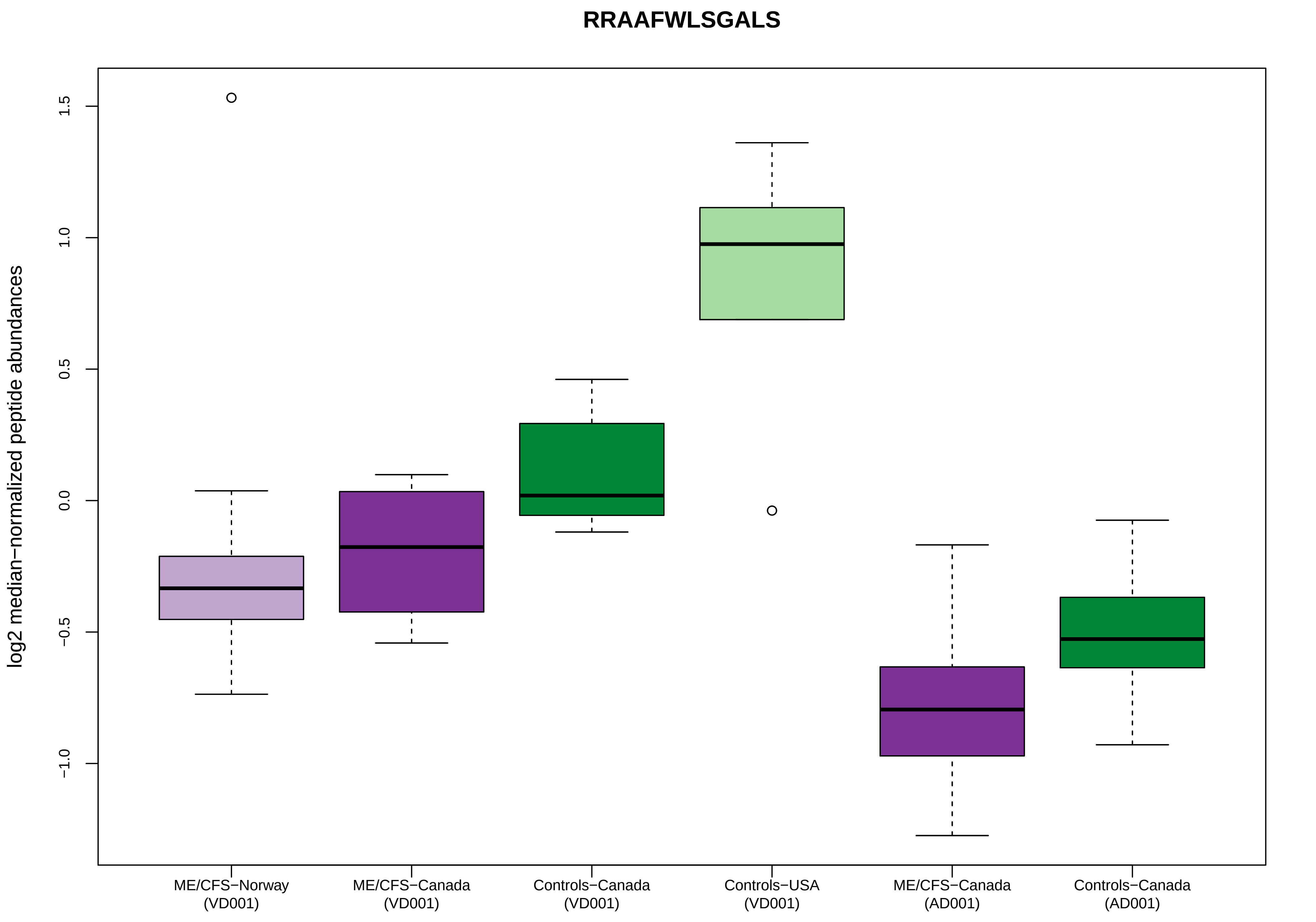

RRAAFWLSGALS
log2 median−normalized peptide abundances
1.5
1.0
0.5
0.0
−0.5
−1.0
ME/CFS−Norway (VD001)
ME/CFS−Canada (VD001)
Controls−Canada (VD001)
Controls−USA (VD001)
ME/CFS−Canada (AD001)
Controls−Canada (AD001)

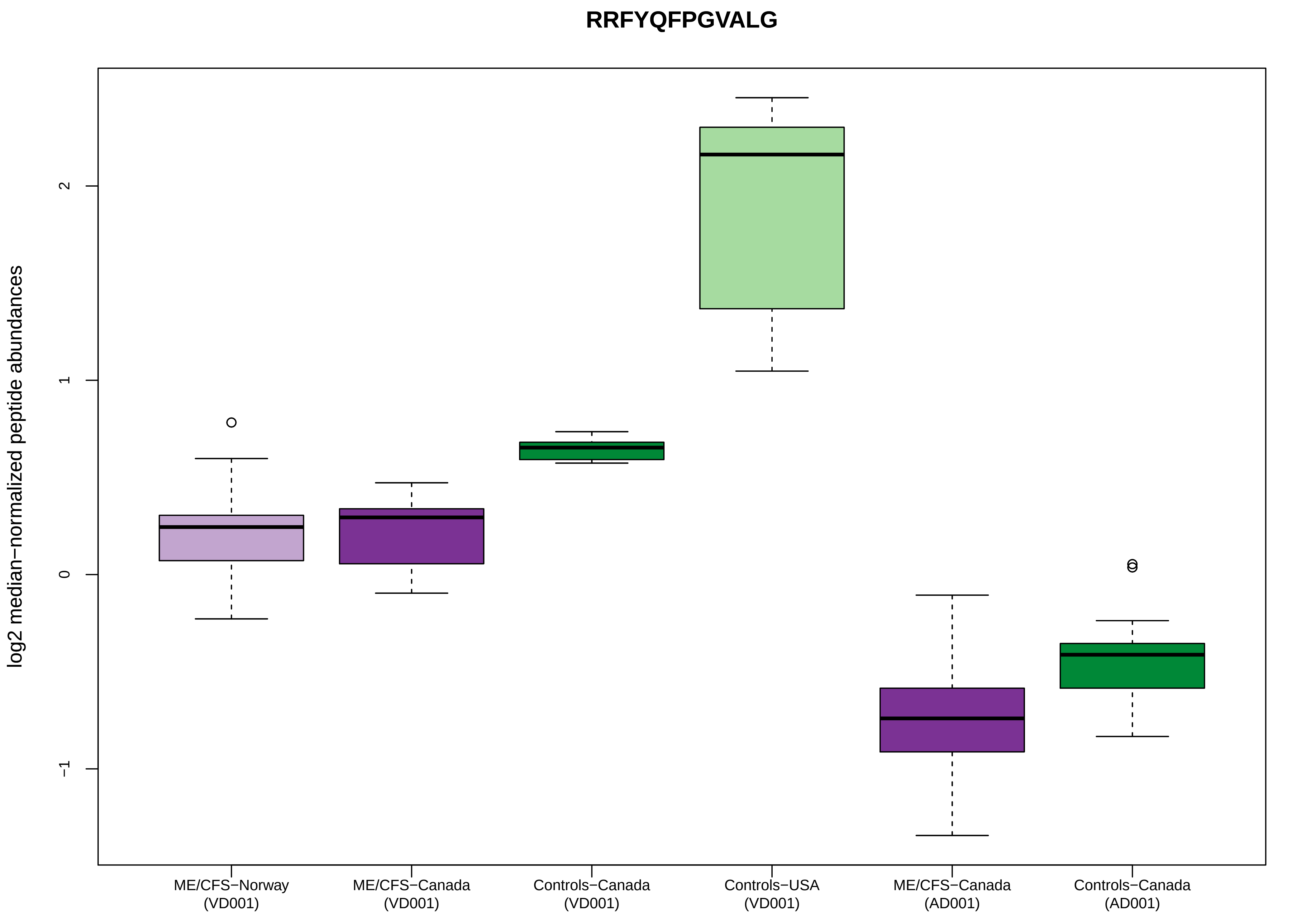
RRFYQFPGVALG
log2 median−normalized peptide abundances
2
1
0
−1
ME/CFS−Norway (VD001)
ME/CFS−Canada (VD001)
Controls−Canada (VD001)
Controls−USA (VD001)
ME/CFS−Canada (AD001)
Controls−Canada (AD001)

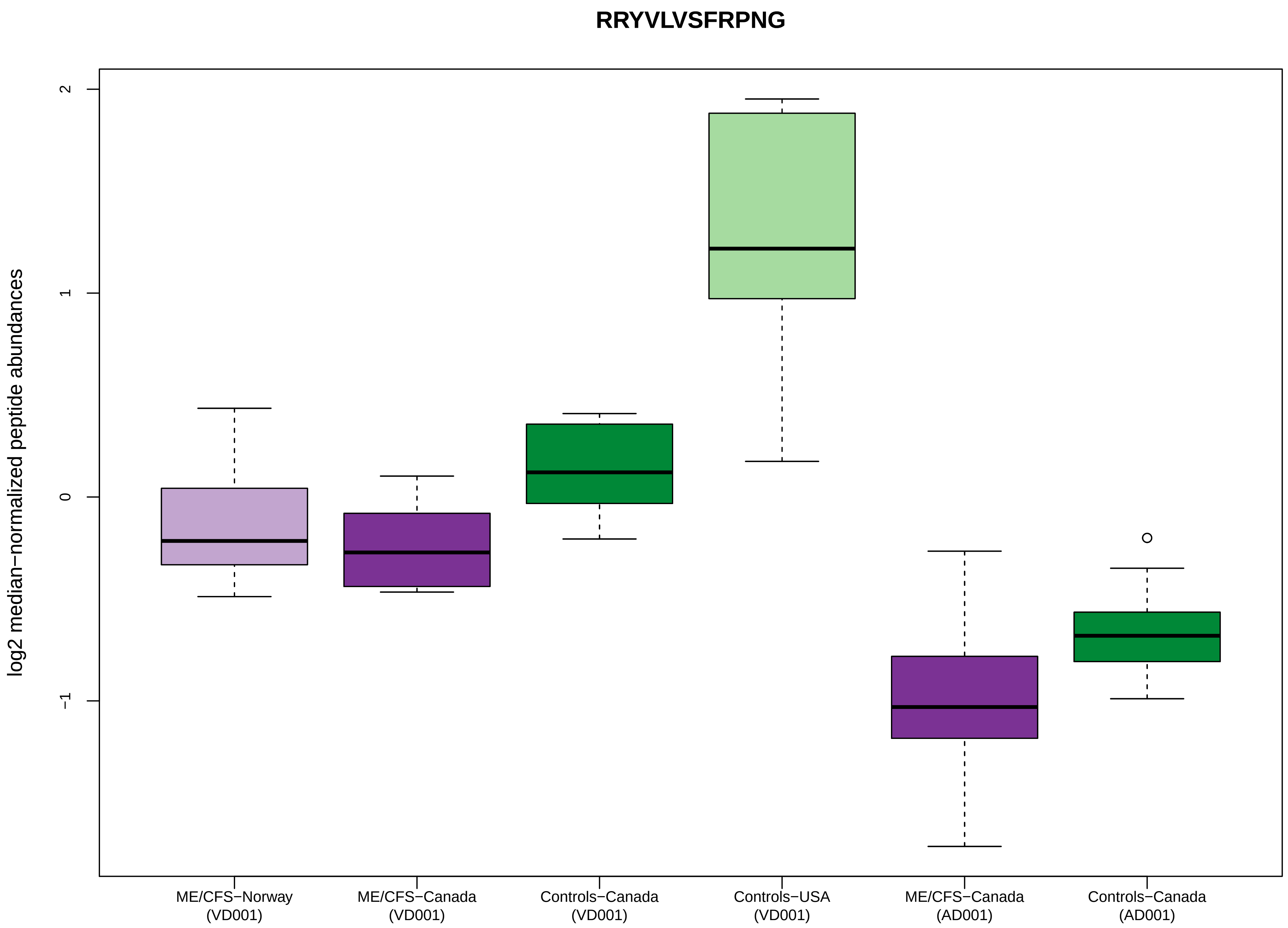

RRYVLVSFRPNG
log2 median−normalized peptide abundances
2
1
0
−1
ME/CFS−Norway (VD001)
ME/CFS−Canada (VD001)
Controls−Canada (VD001)
Controls−USA (VD001)
ME/CFS−Canada (AD001)
Controls−Canada (AD001)

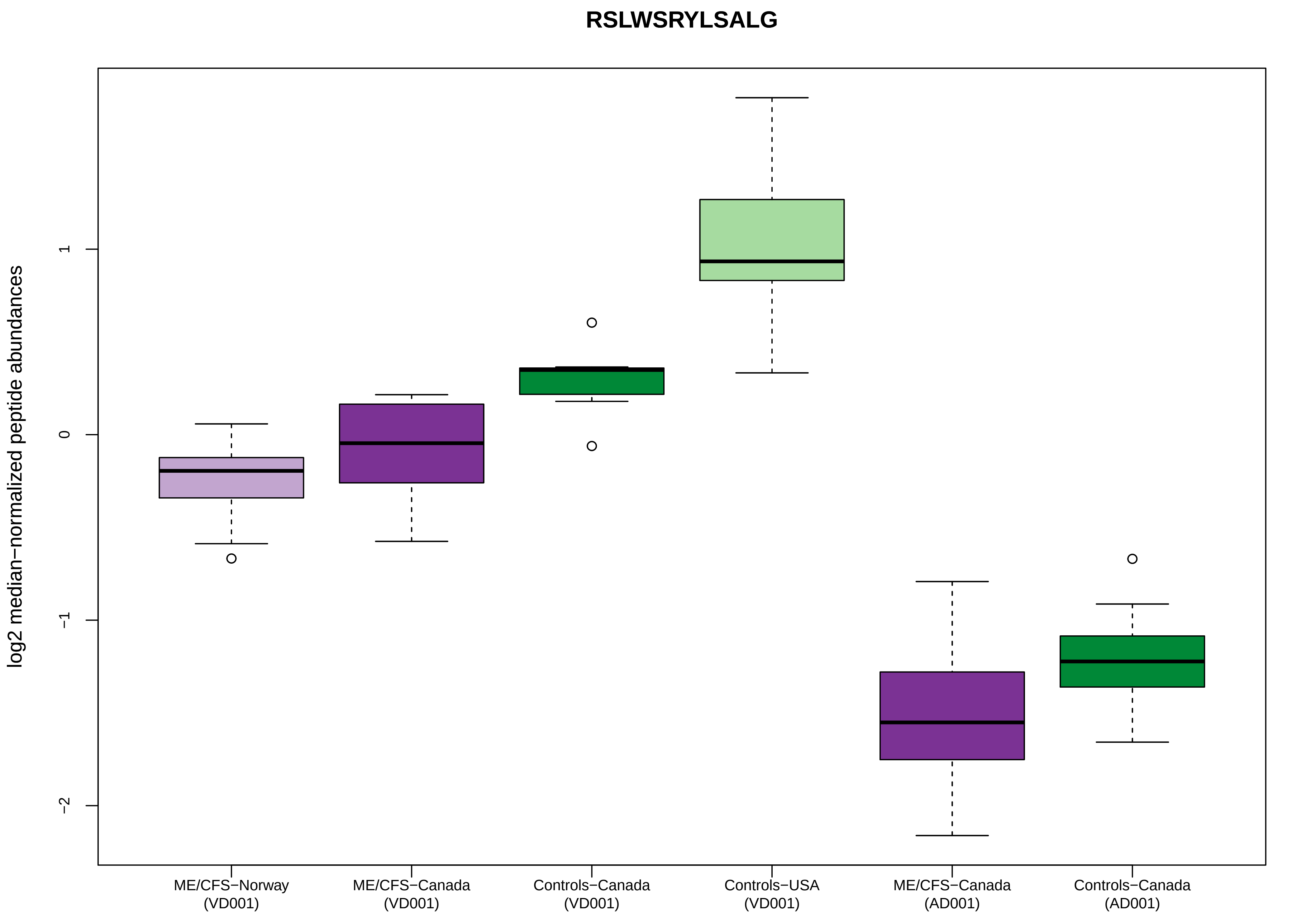

RSLWSRYLSALG
log2 median−normalized peptide abundances
1
0
−1
−2
ME/CFS−Norway (VD001)
ME/CFS−Canada (VD001)
Controls−Canada (VD001)
Controls−USA (VD001)
ME/CFS−Canada (AD001)
Controls−Canada (AD001)

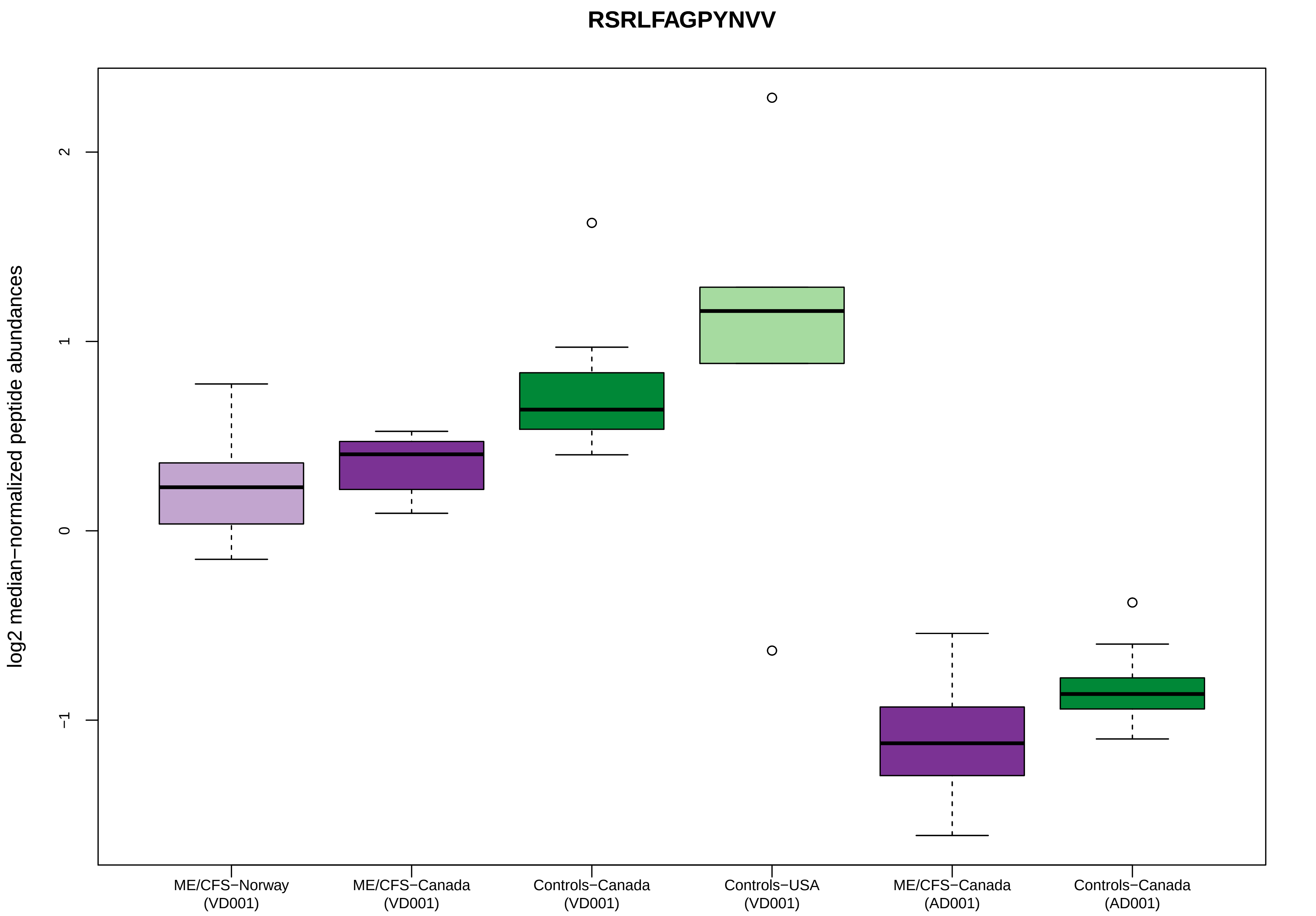
RSRLFAGPYNVV
log2 median−normalized peptide abundances
2
1
0
−1
ME/CFS−Norway (VD001)
ME/CFS−Canada (VD001)
Controls−Canada (VD001)
Controls−USA (VD001)
ME/CFS−Canada (AD001)
Controls−Canada (AD001)

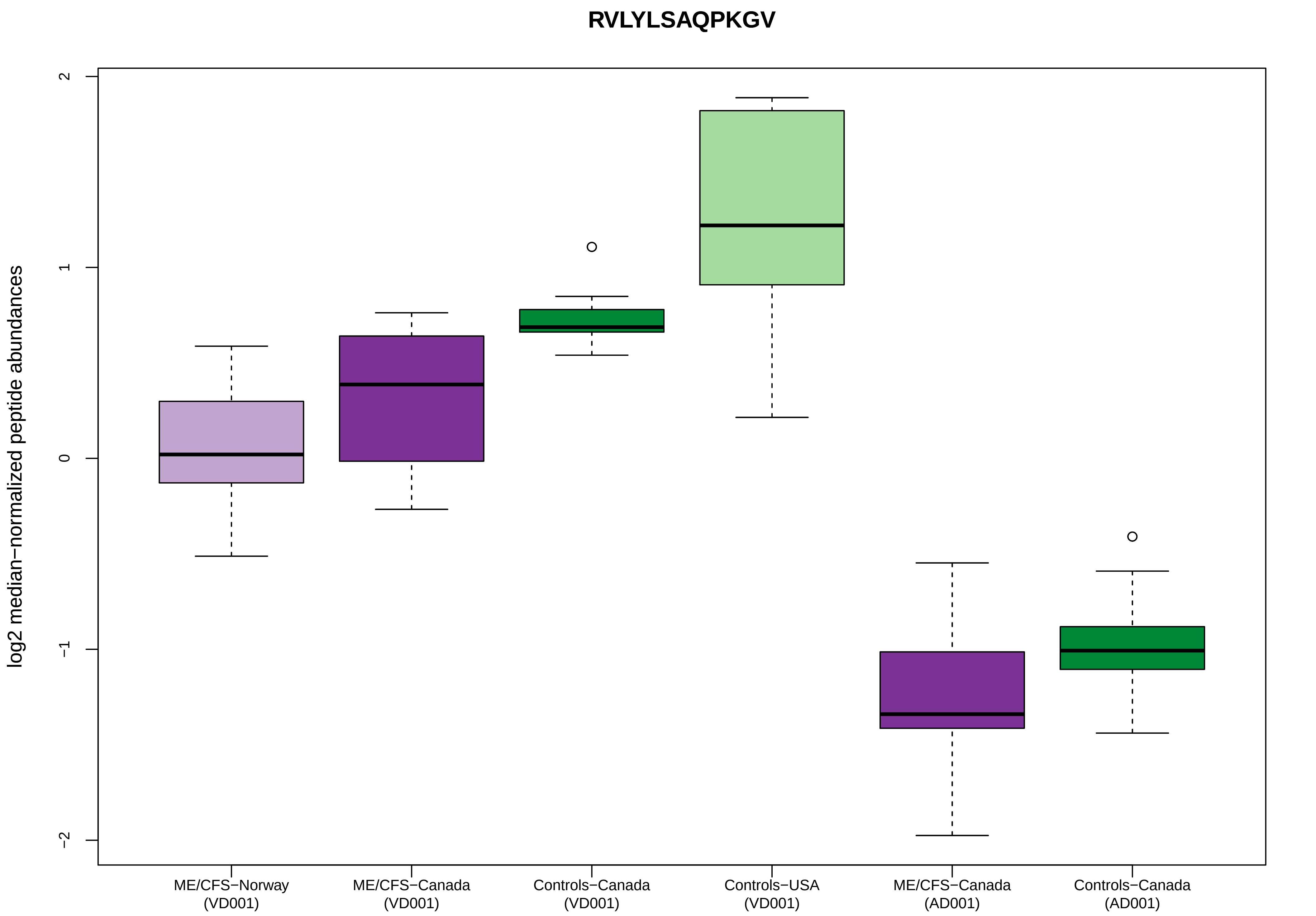
RVLYLSAQPKGV
log2 median−normalized peptide abundances
2
1
0
−1
−2
ME/CFS−Norway (VD001)
ME/CFS−Canada (VD001)
Controls−Canada (VD001)
Controls−USA (VD001)
ME/CFS−Canada (AD001)
Controls−Canada (AD001)

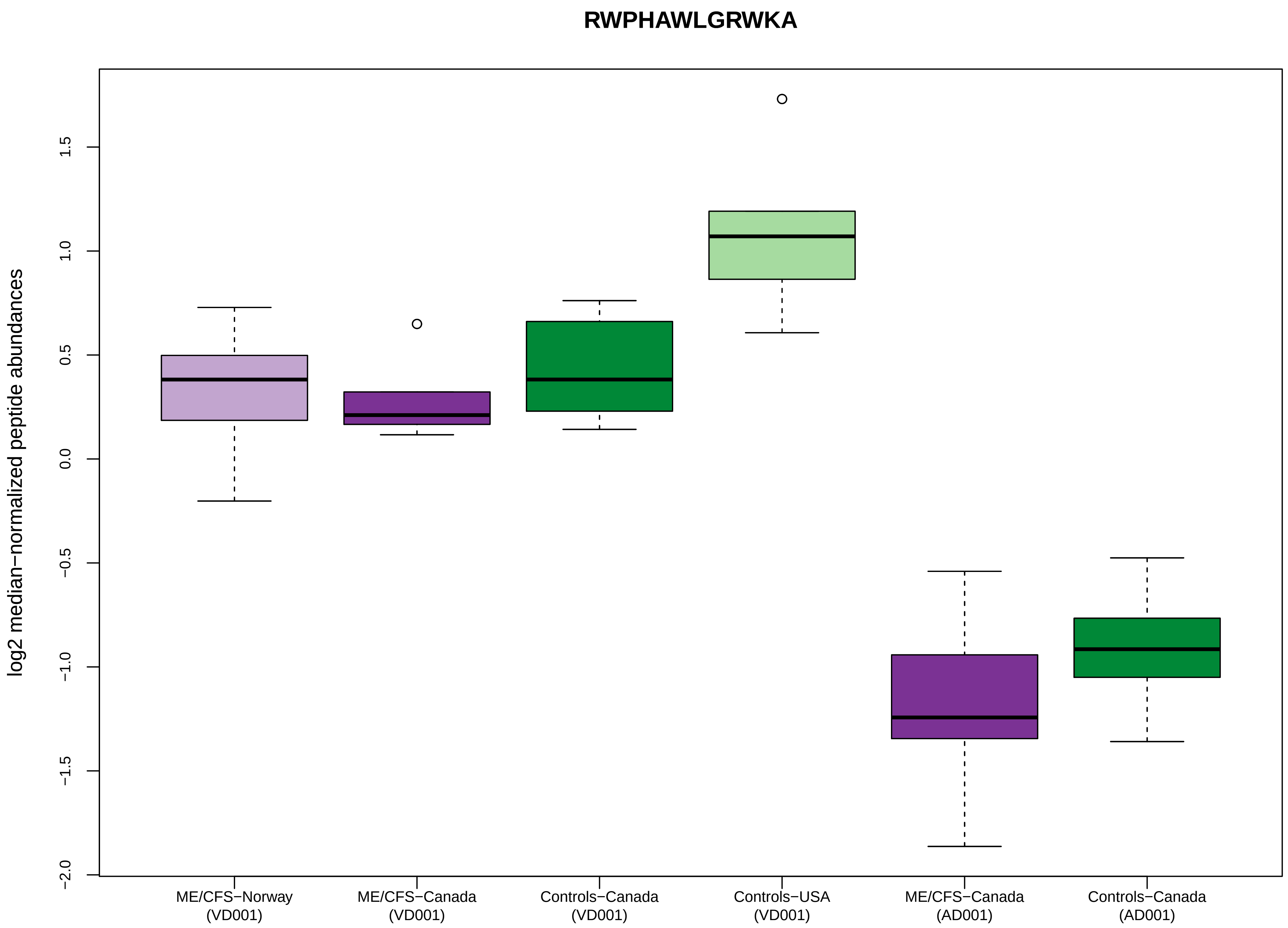

RWPHAWLGRWKA
log2 median−normalized peptide abundances
1.5
1.0
0.5
0.0
−0.5
−1.0
−1.5
−2.0
ME/CFS−Norway (VD001)
ME/CFS−Canada (VD001)
Controls−Canada (VD001)
Controls−USA (VD001)
ME/CFS−Canada (AD001)
Controls−Canada (AD001)

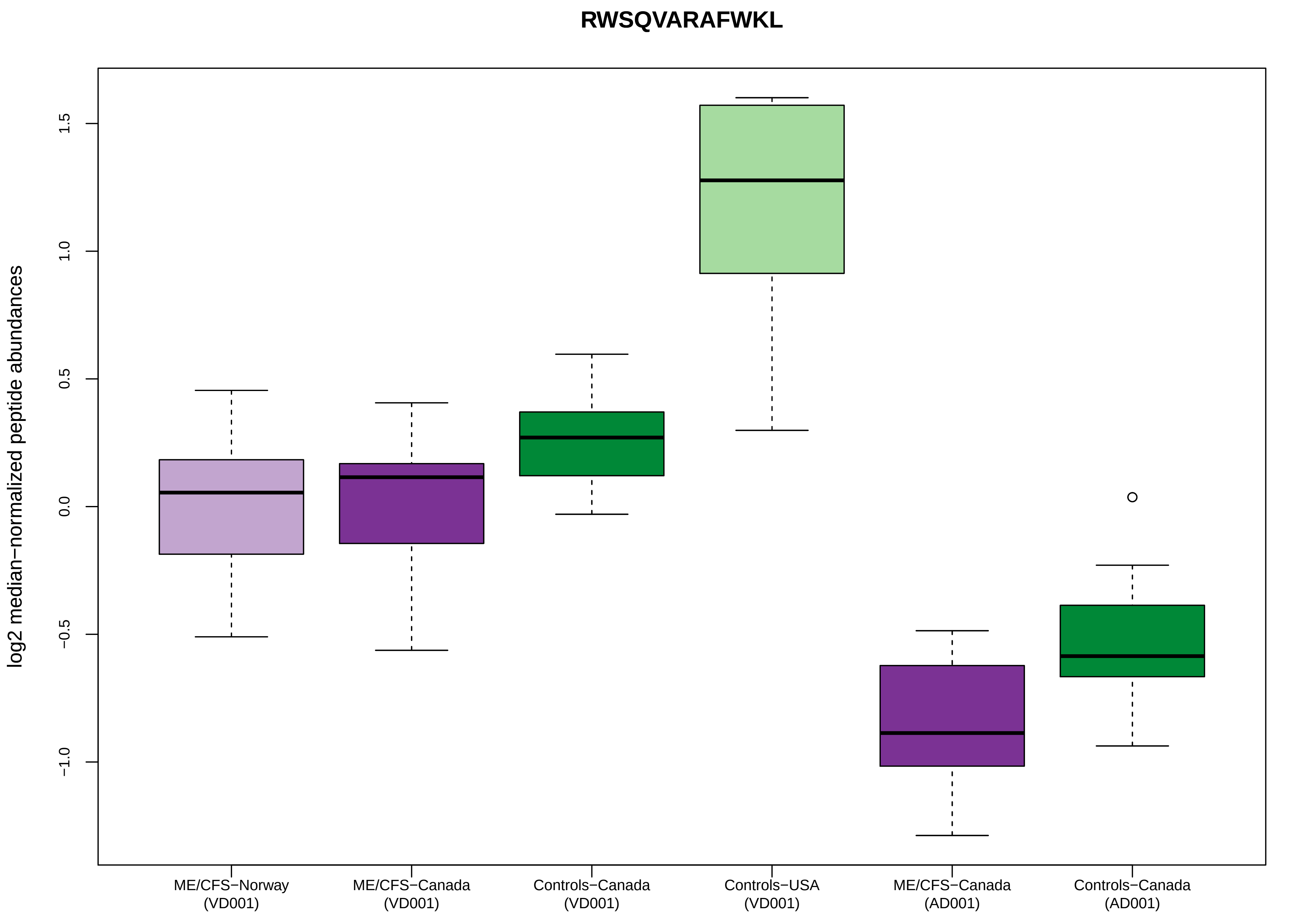
RWSQVARAFWKL
log2 median−normalized peptide abundances
−1.0
−0.5
0.0
0.5
1.0
1.5
ME/CFS−Norway (VD001)
ME/CFS−Canada (VD001)
Controls−Canada (VD001)
Controls−USA (VD001)
ME/CFS−Canada (AD001)
Controls−Canada (AD001)

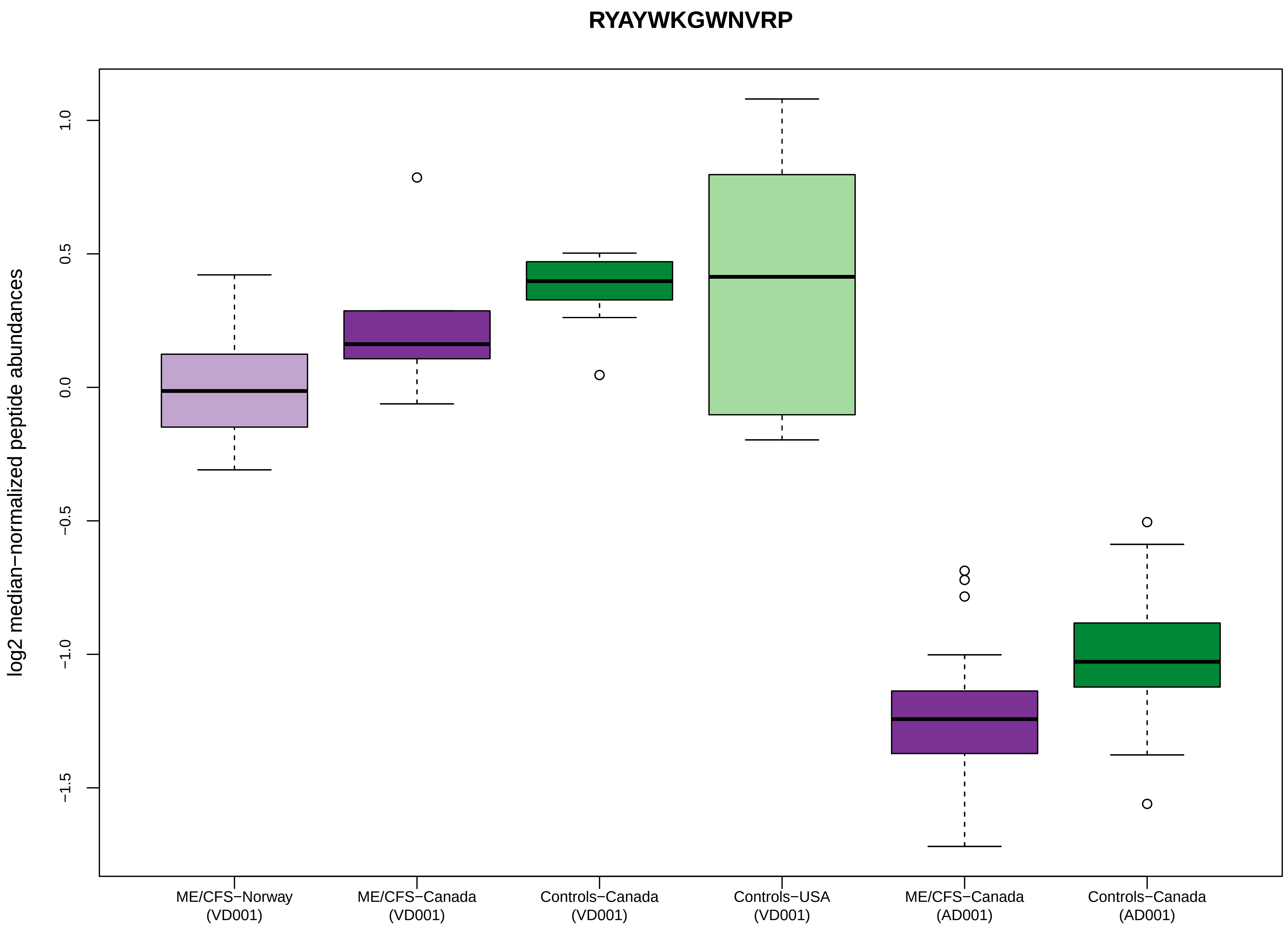

RYAYWKGWNVRP
log2 median−normalized peptide abundances
1.0
0.5
0.0
−0.5
−1.0
−1.5
ME/CFS−Norway (VD001)
ME/CFS−Canada (VD001)
Controls−Canada (VD001)
Controls−USA (VD001)
ME/CFS−Canada (AD001)
Controls−Canada (AD001)

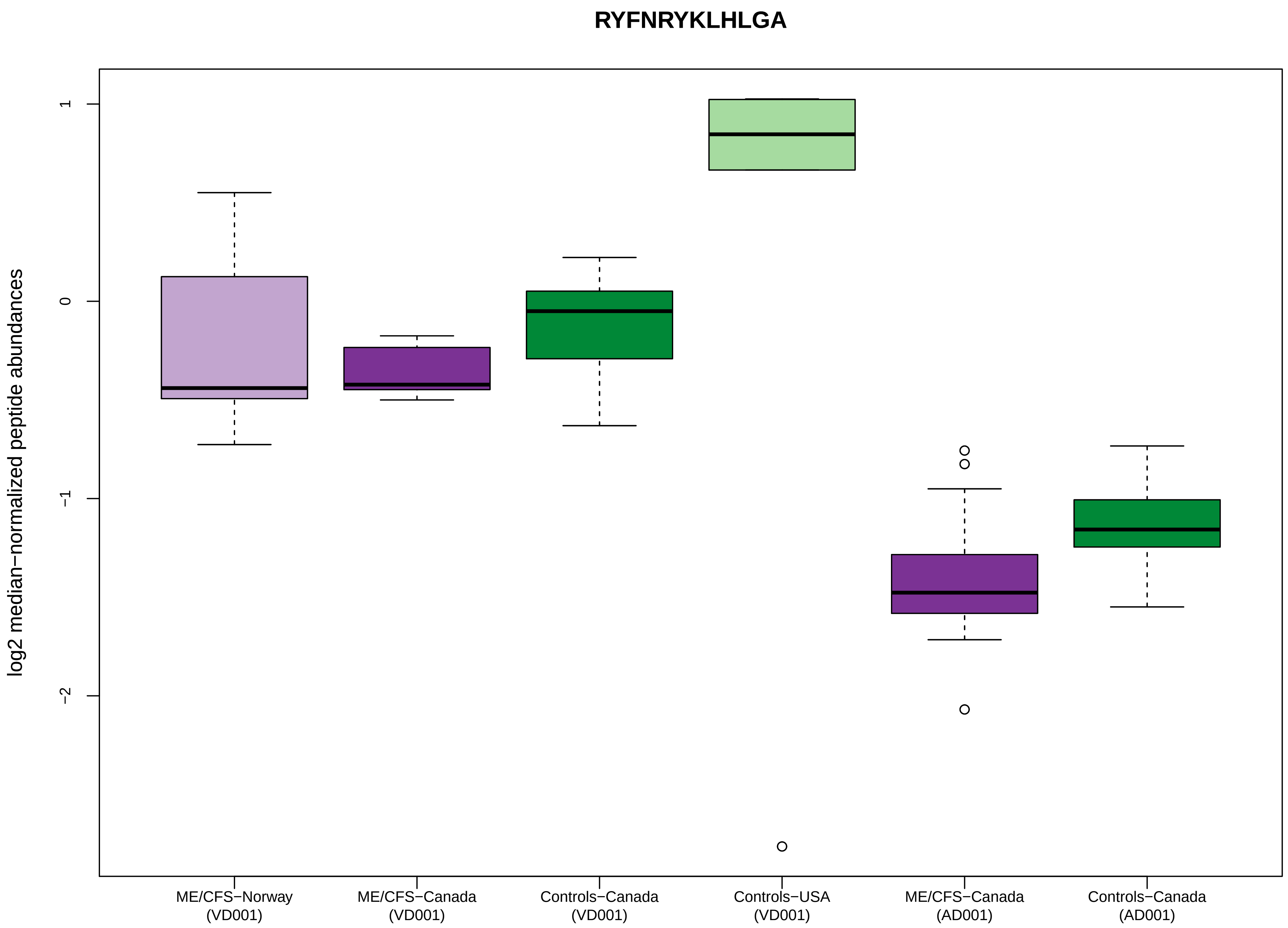
RYFNRYKLHLGA
log2 median−normalized peptide abundances
1
0
−1
−2
ME/CFS−Norway
(VD001)
ME/CFS−Canada
(VD001)
Controls−Canada
(VD001)
Controls−USA
(VD001)
ME/CFS−Canada
(AD001)
Controls−Canada
(AD001)

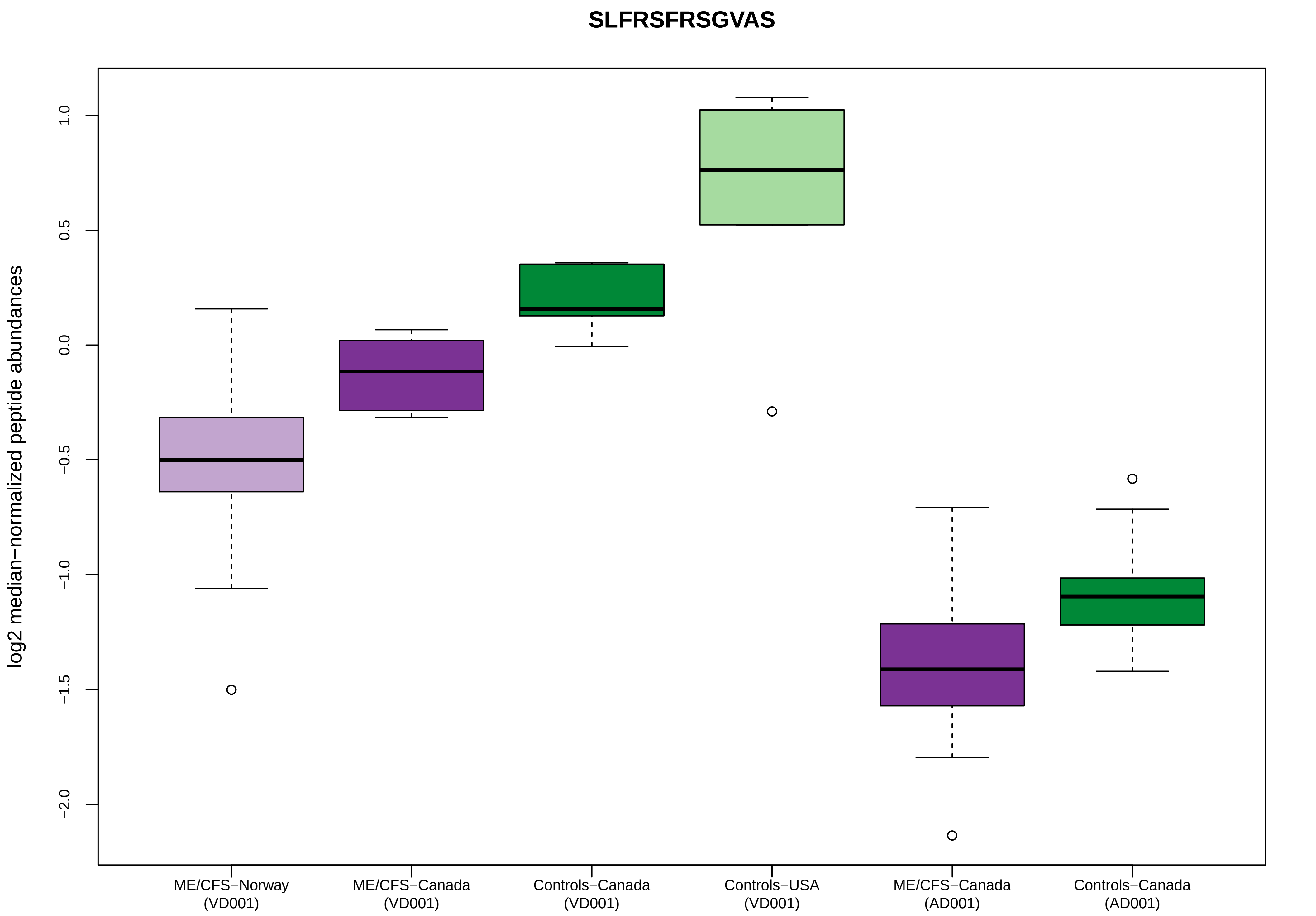

SLFRSFRSGVAS
log2 median−normalized peptide abundances
1.0
0.5
0.0
−0.5
−1.0
−1.5
−2.0
ME/CFS−Norway (VD001)
ME/CFS−Canada (VD001)
Controls−Canada (VD001)
Controls−USA (VD001)
ME/CFS−Canada (AD001)
Controls−Canada (AD001)

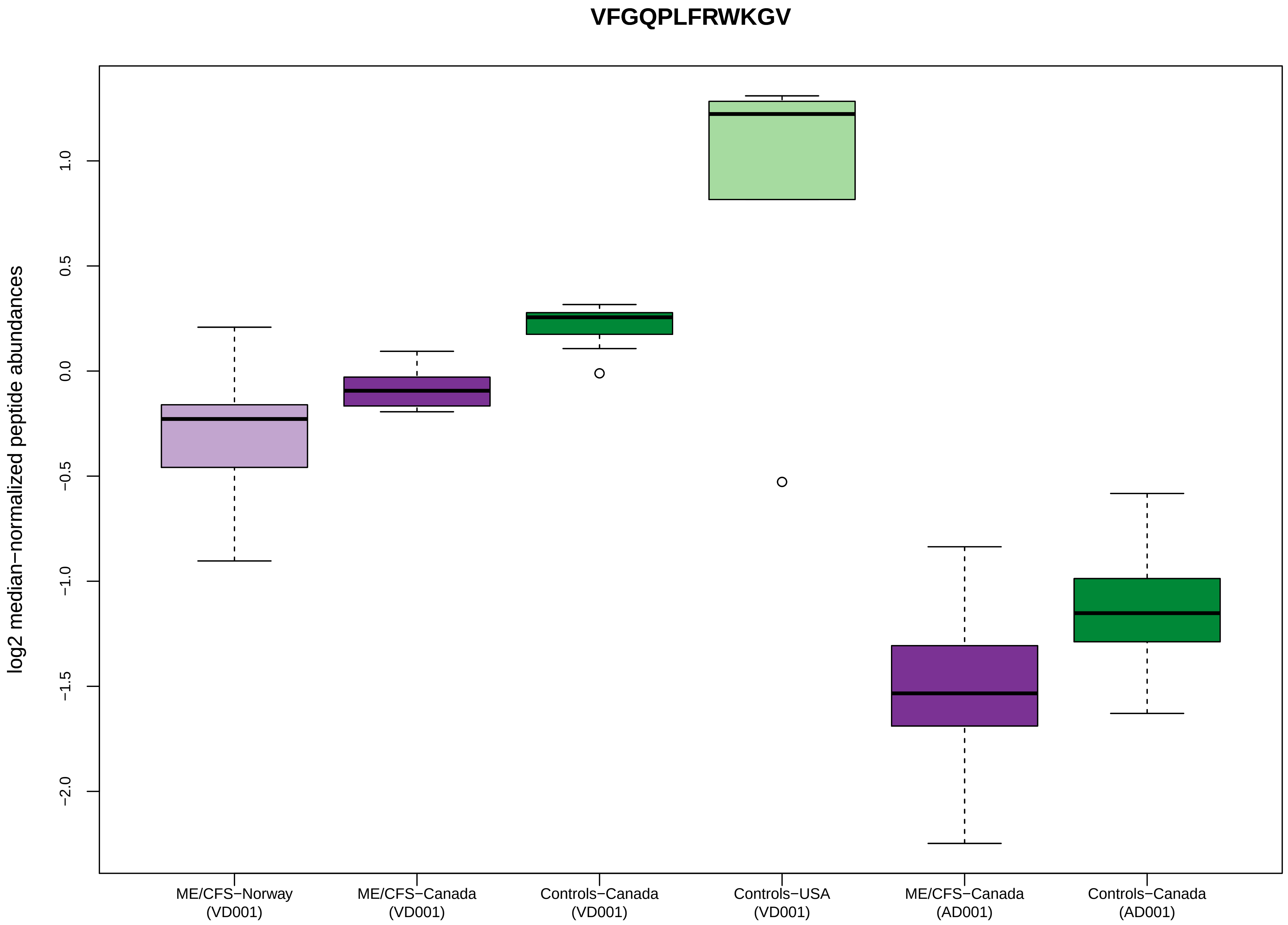
VFGQPLFRWKGV
log2 median−normalized peptide abundances
1.0
0.5
0.0
−0.5
−1.0
−1.5
−2.0
ME/CFS−Norway (VD001)
ME/CFS−Canada (VD001)
Controls−Canada (VD001)
Controls−USA (VD001)
ME/CFS−Canada (AD001)
Controls−Canada (AD001)

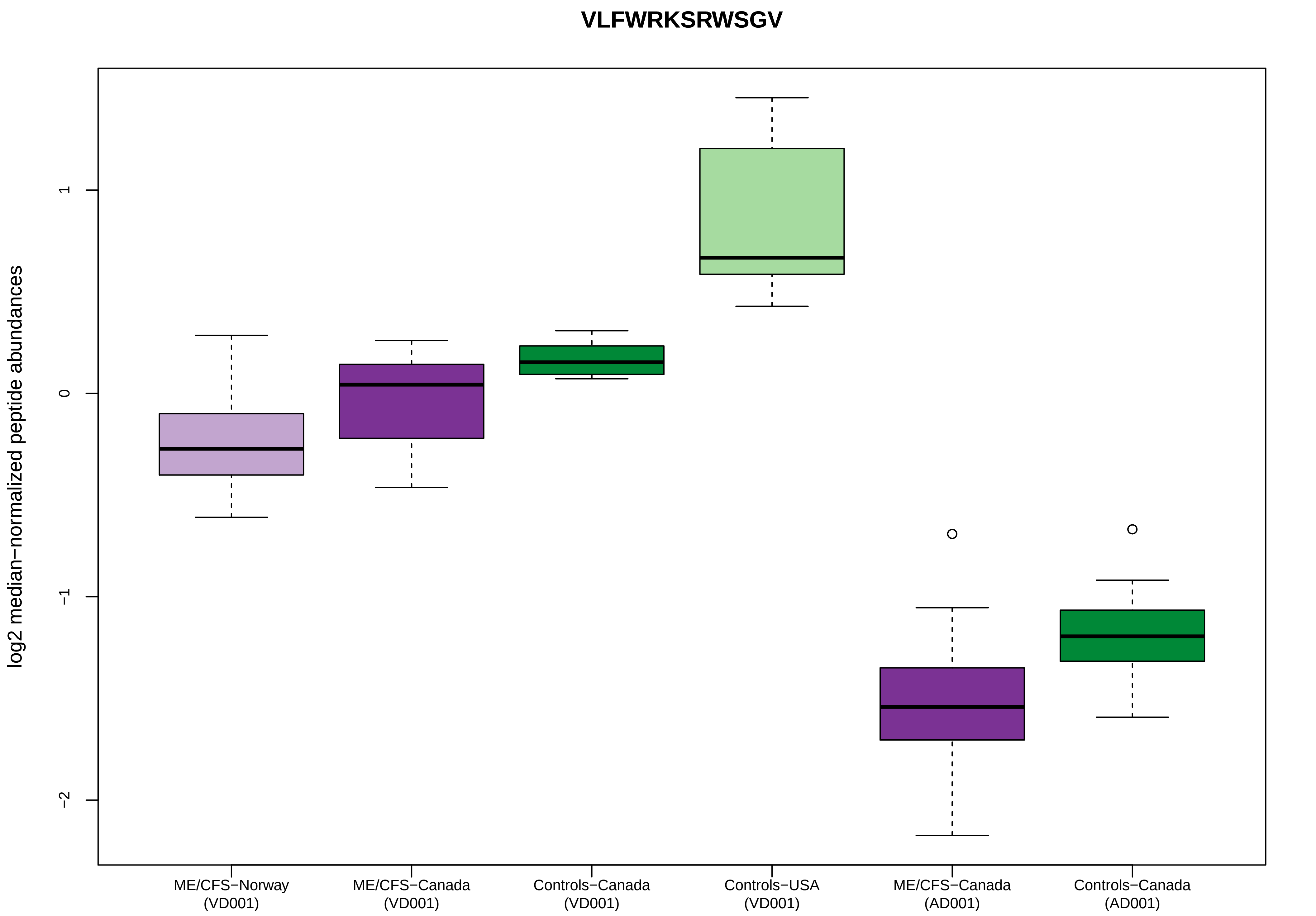

VLFWRKSRWSGV
log2 median−normalized peptide abundances
1
0
−1
−2
ME/CFS−Norway (VD001)
ME/CFS−Canada (VD001)
Controls−Canada (VD001)
Controls−USA (VD001)
ME/CFS−Canada (AD001)
Controls−Canada (AD001)

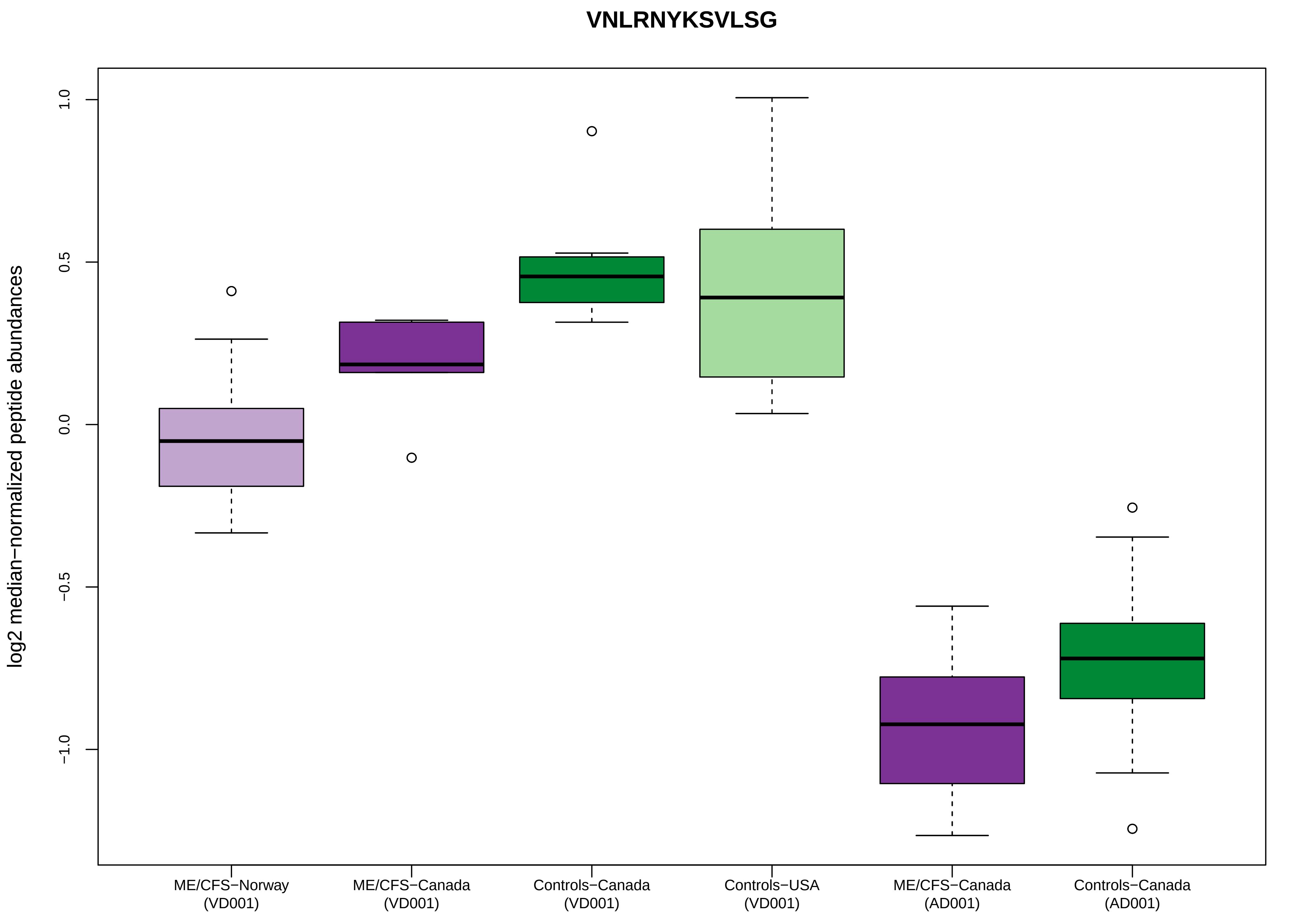
VNLRNYKSVLSG
log2 median−normalized peptide abundances
1.0
0.5
0.0
−0.5
−1.0
ME/CFS−Norway (VD001)
ME/CFS−Canada (VD001)
Controls−Canada (VD001)
Controls−USA (VD001)
ME/CFS−Canada (AD001)
Controls−Canada (AD001)

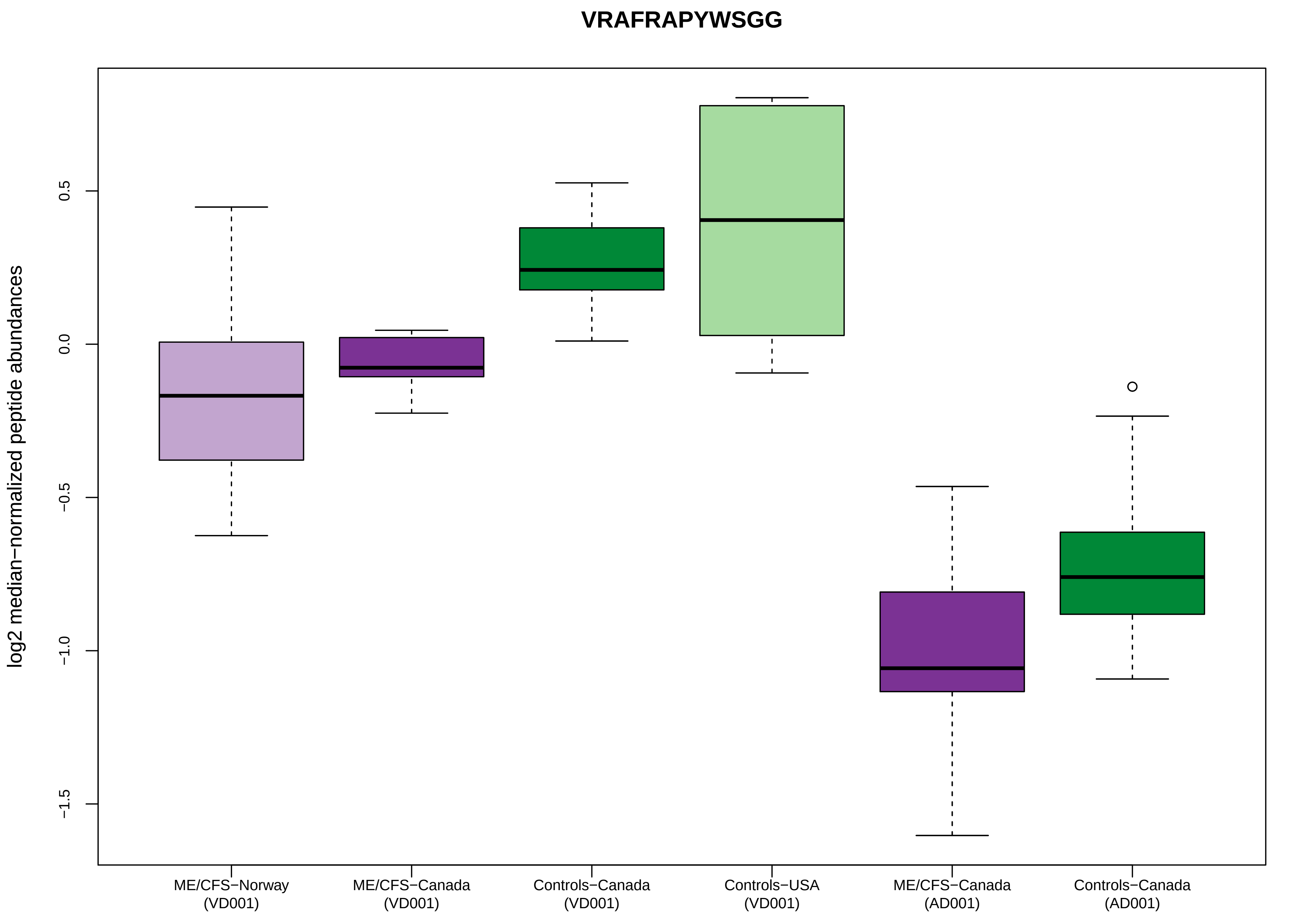
VRAFRAPYWSGG
log2 median−normalized peptide abundances
0.5
0.0
−0.5
−1.0
−1.5
ME/CFS−Norway (VD001)
ME/CFS−Canada (VD001)
Controls−Canada (VD001)
Controls−USA (VD001)
ME/CFS−Canada (AD001)
Controls−Canada (AD001)

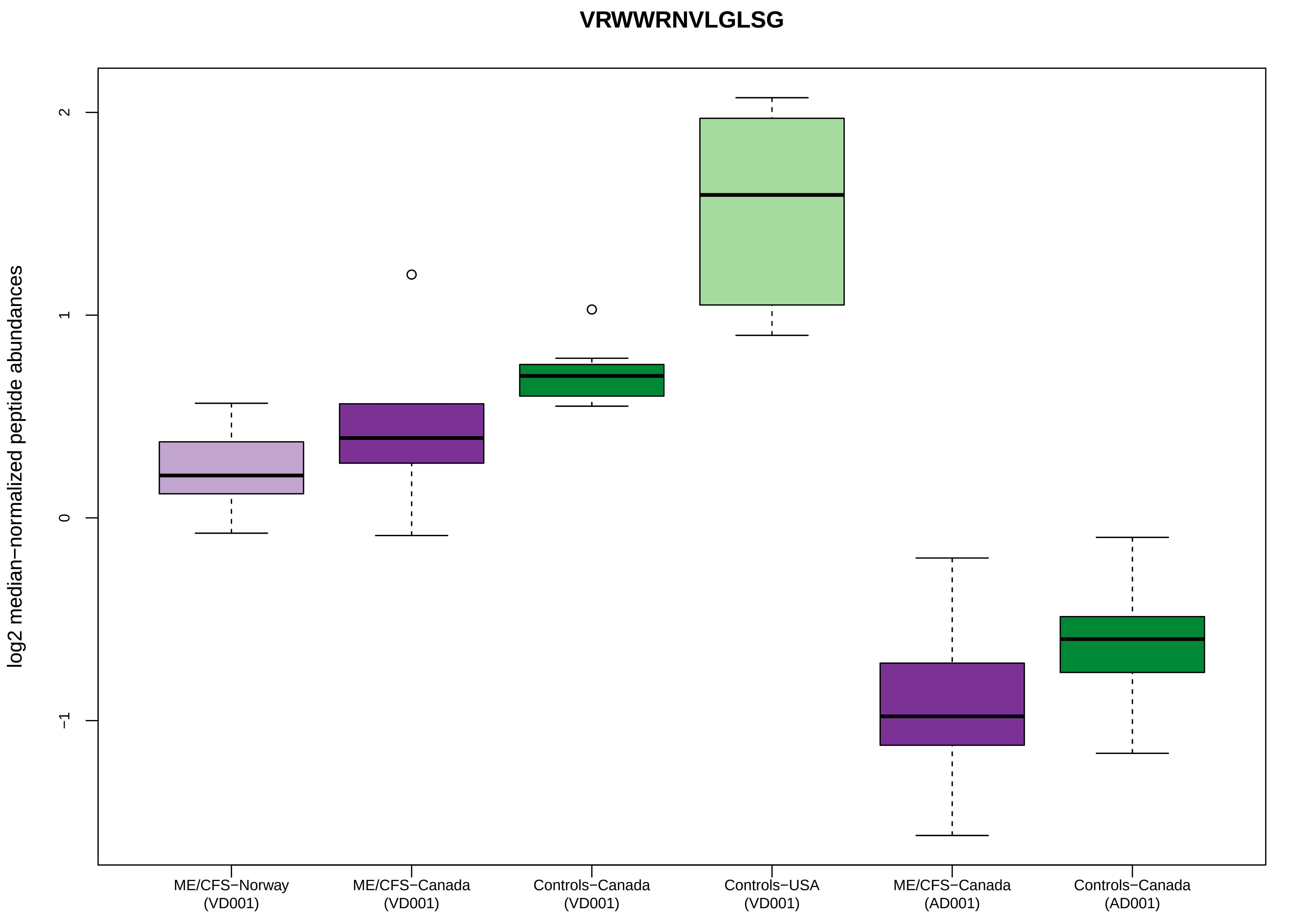

VRWWRNVLGLSG
log2 median−normalized peptide abundances
2
1
0
−1
ME/CFS−Norway (VD001)
ME/CFS−Canada (VD001)
Controls−Canada (VD001)
Controls−USA (VD001)
ME/CFS−Canada (AD001)
Controls−Canada (AD001)

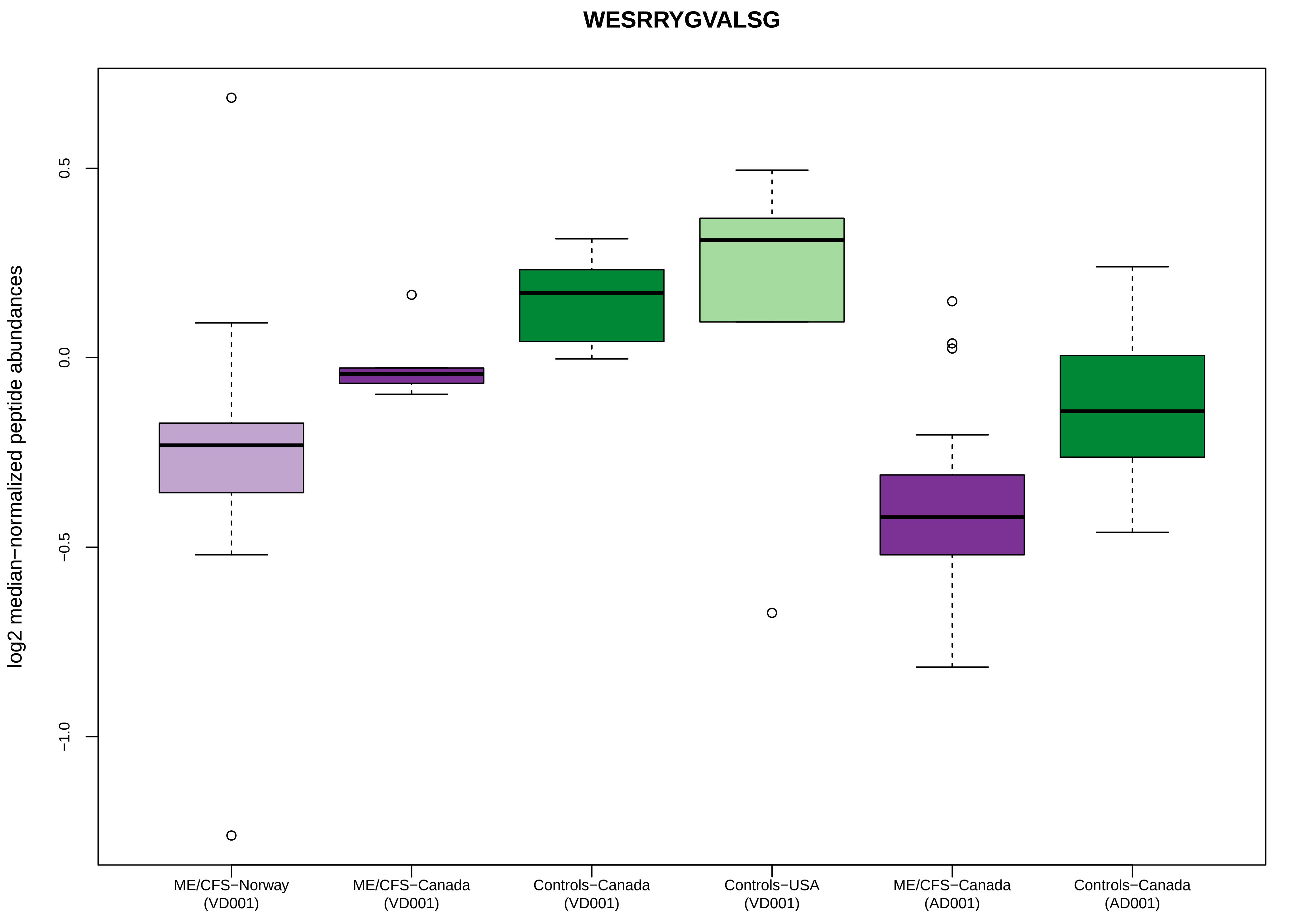
WESRRYGVALSG
log2 median−normalized peptide abundances
0.5
0.0
−0.5
−1.0
ME/CFS−Norway (VD001)
ME/CFS−Canada (VD001)
Controls−Canada (VD001)
Controls−USA (VD001)
ME/CFS−Canada (AD001)
Controls−Canada (AD001)

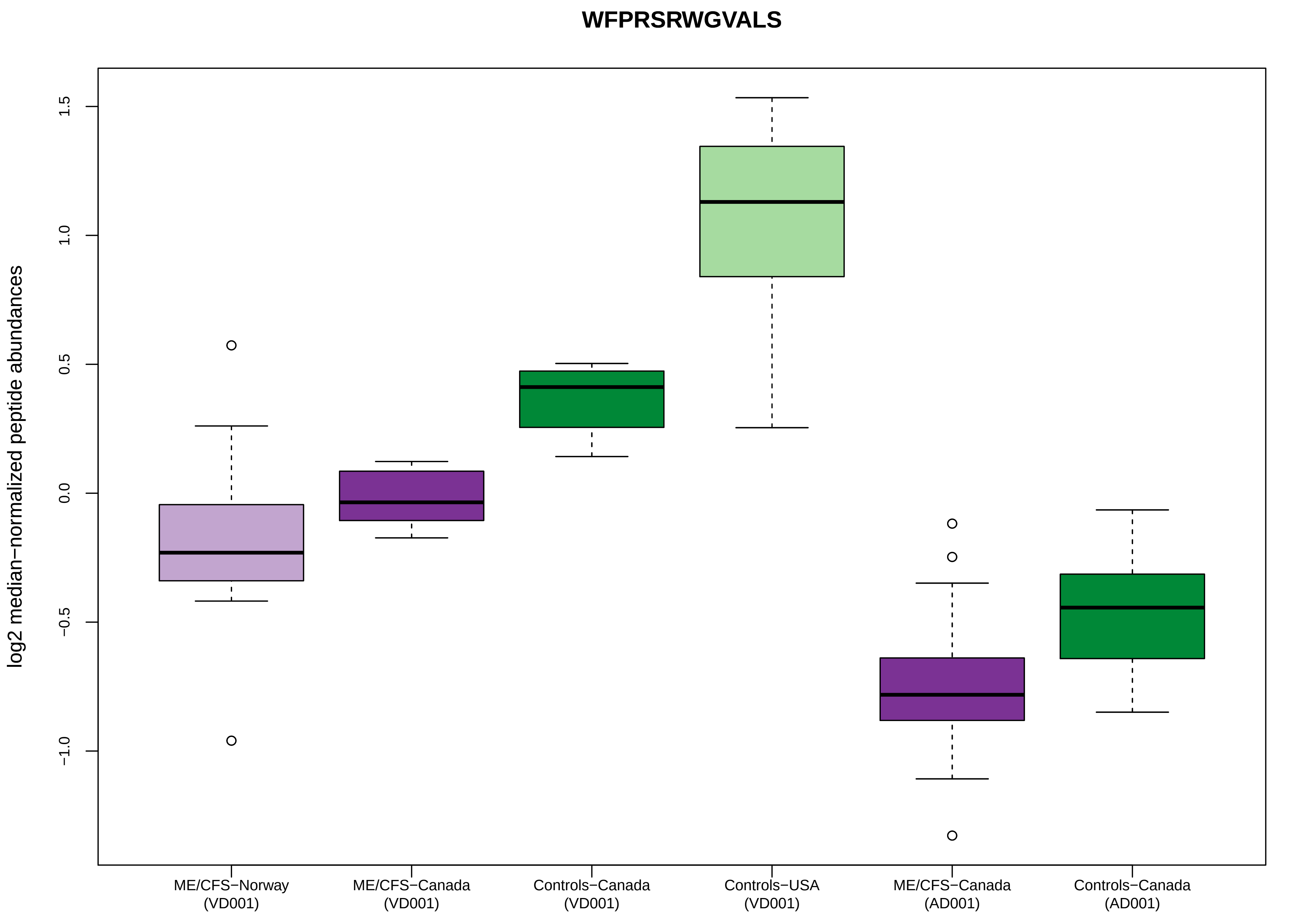
WFPRSRWGVALS
log2 median−normalized peptide abundances
1.5
1.0
0.5
0.0
−0.5
−1.0
ME/CFS−Norway (VD001)
ME/CFS−Canada (VD001)
Controls−Canada (VD001)
Controls−USA (VD001)
ME/CFS−Canada (AD001)
Controls−Canada (AD001)

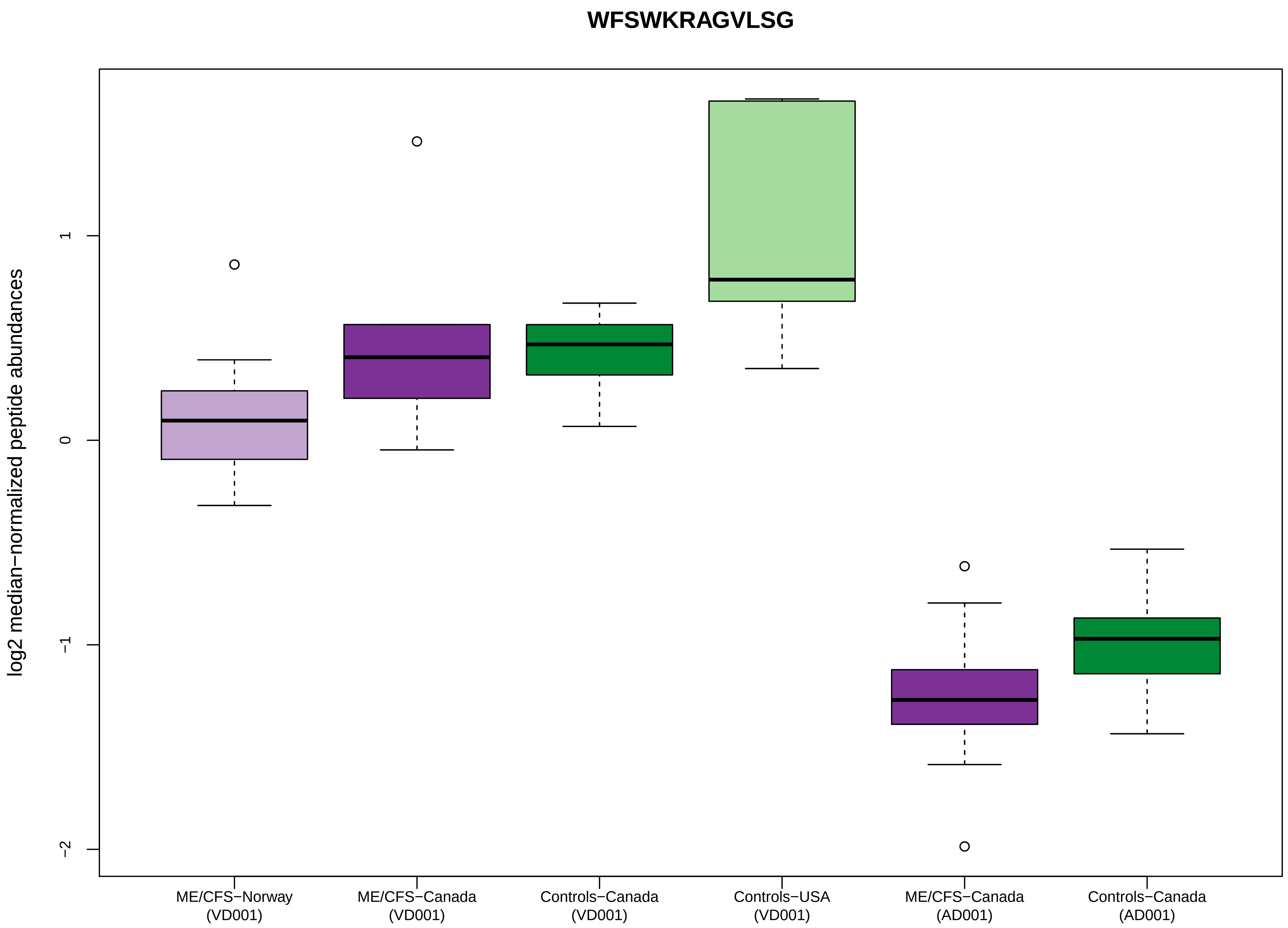
WFSWKRAGVLSG
log2 median−normalized peptide abundances
1
0
−1
−2
ME/CFS−Norway (VD001)
ME/CFS−Canada (VD001)
Controls−Canada (VD001)
Controls−USA (VD001)
ME/CFS−Canada (AD001)
Controls−Canada (AD001)

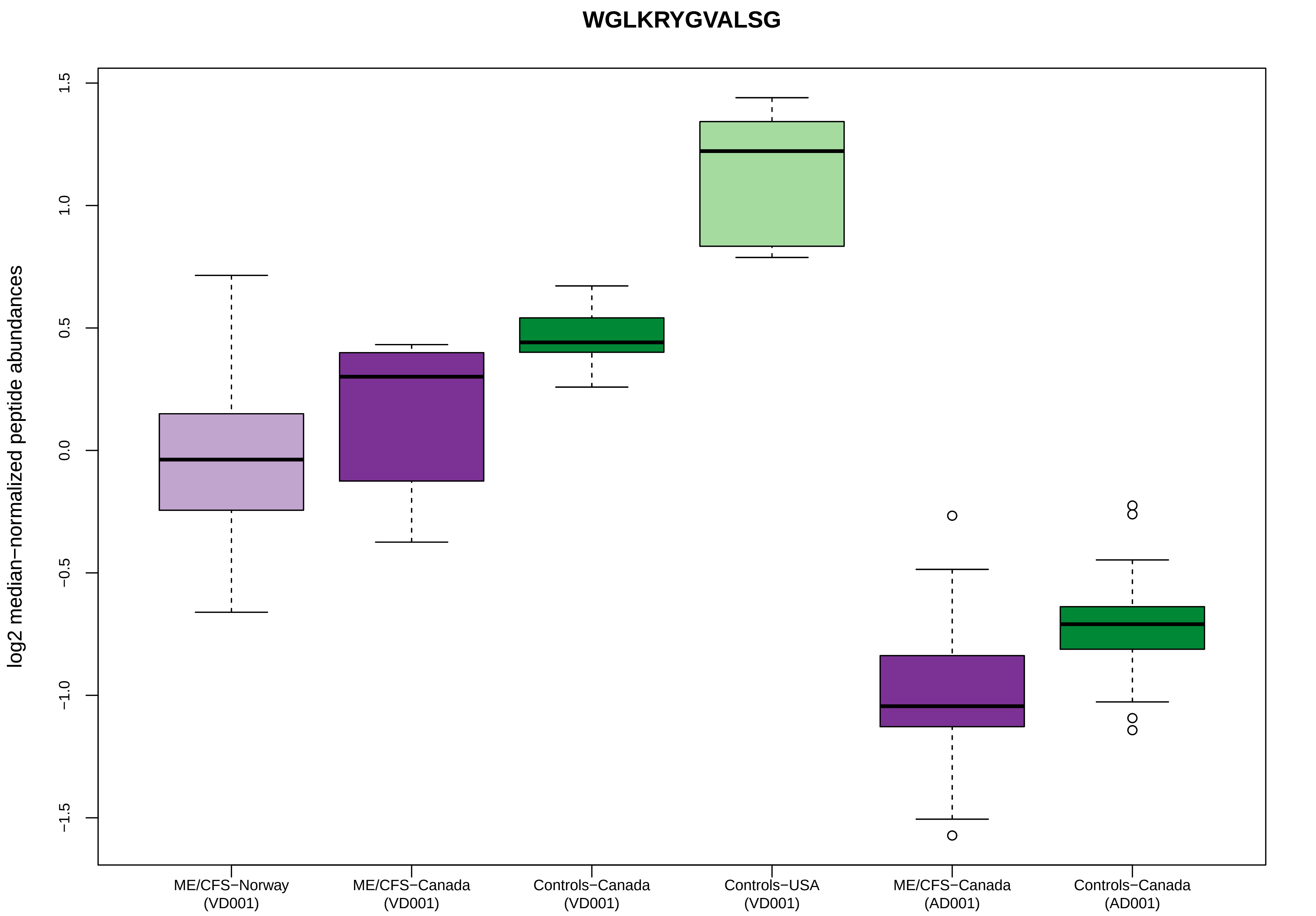
WGLKRYGVALSG
log2 median−normalized peptide abundances
1.5
1.0
0.5
0.0
−0.5
−1.0
−1.5
ME/CFS−Norway (VD001)
ME/CFS−Canada (VD001)
Controls−Canada (VD001)
Controls−USA (VD001)
ME/CFS−Canada (AD001)
Controls−Canada (AD001)

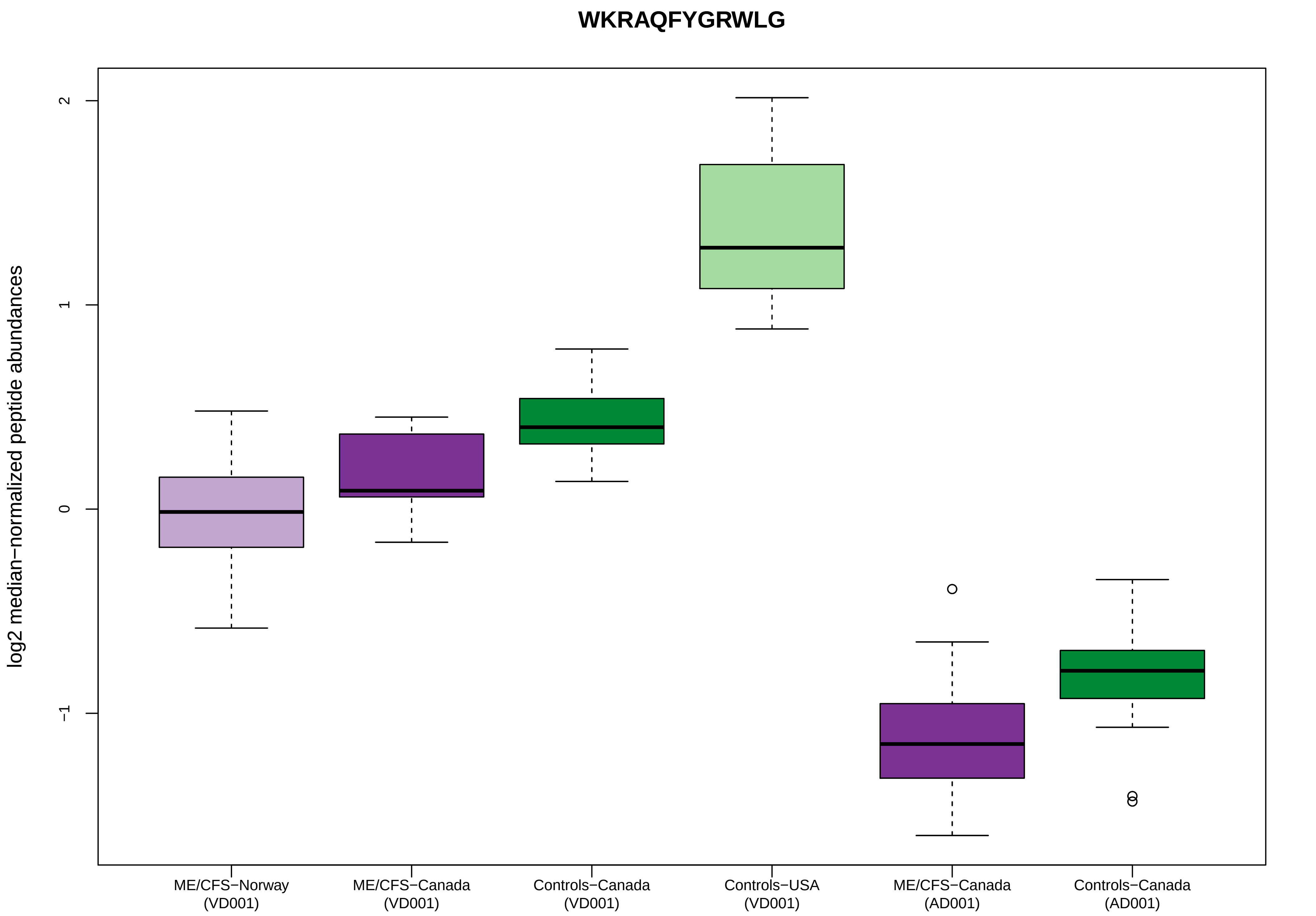
WKRAQFYGRWLG
log2 median−normalized peptide abundances
2
1
0
−1
ME/CFS−Norway
(VD001)
ME/CFS−Canada
(VD001)
Controls−Canada
(VD001)
Controls−USA
(VD001)
ME/CFS−Canada
(AD001)
Controls−Canada
(AD001)

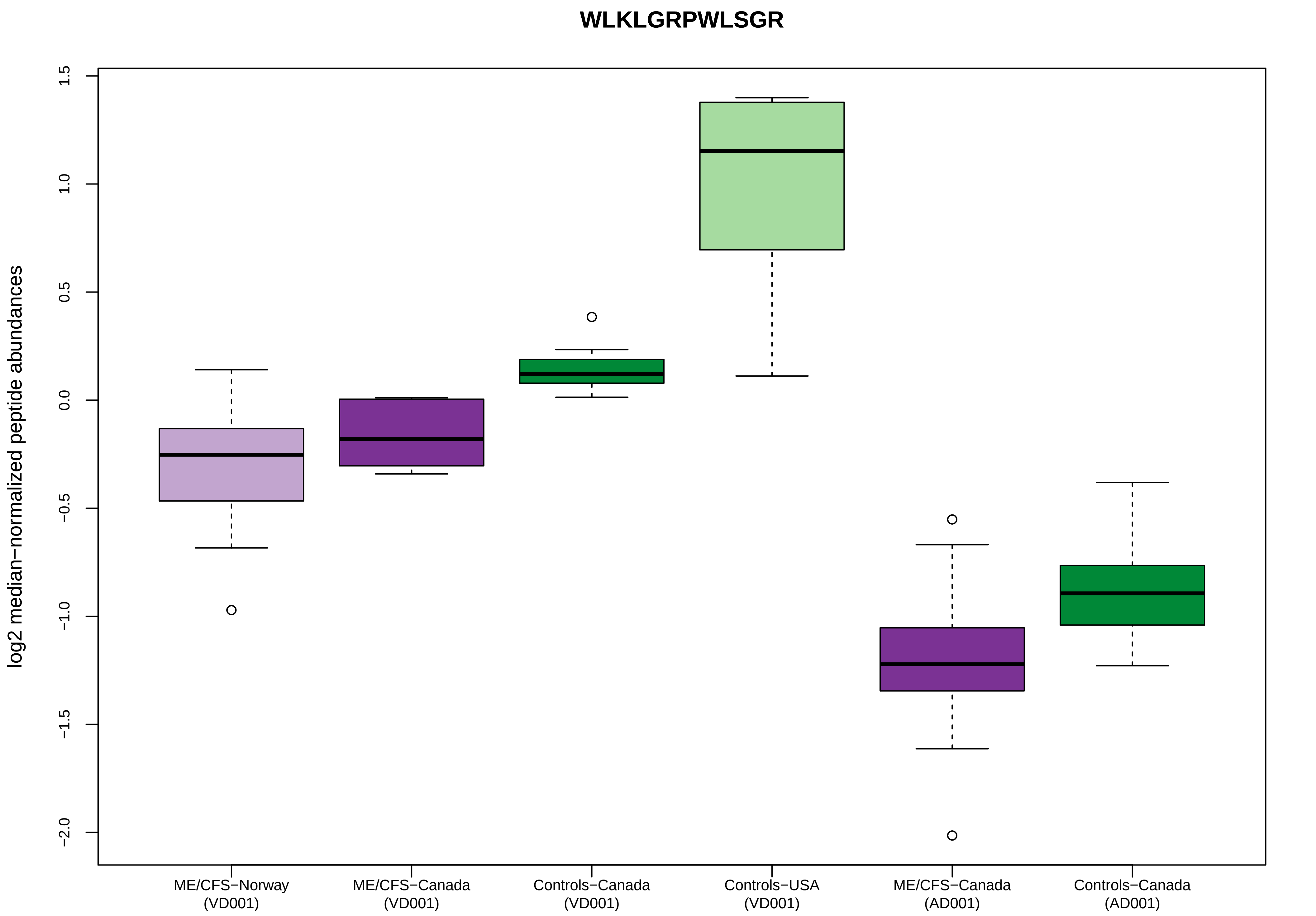

WLKLGRPWLSGR
log2 median−normalized peptide abundances
1.5
1.0
0.5
0.0
−0.5
−1.0
−1.5
−2.0
ME/CFS−Norway (VD001)
ME/CFS−Canada (VD001)
Controls−Canada (VD001)
Controls−USA (VD001)
ME/CFS−Canada (AD001)
Controls−Canada (AD001)

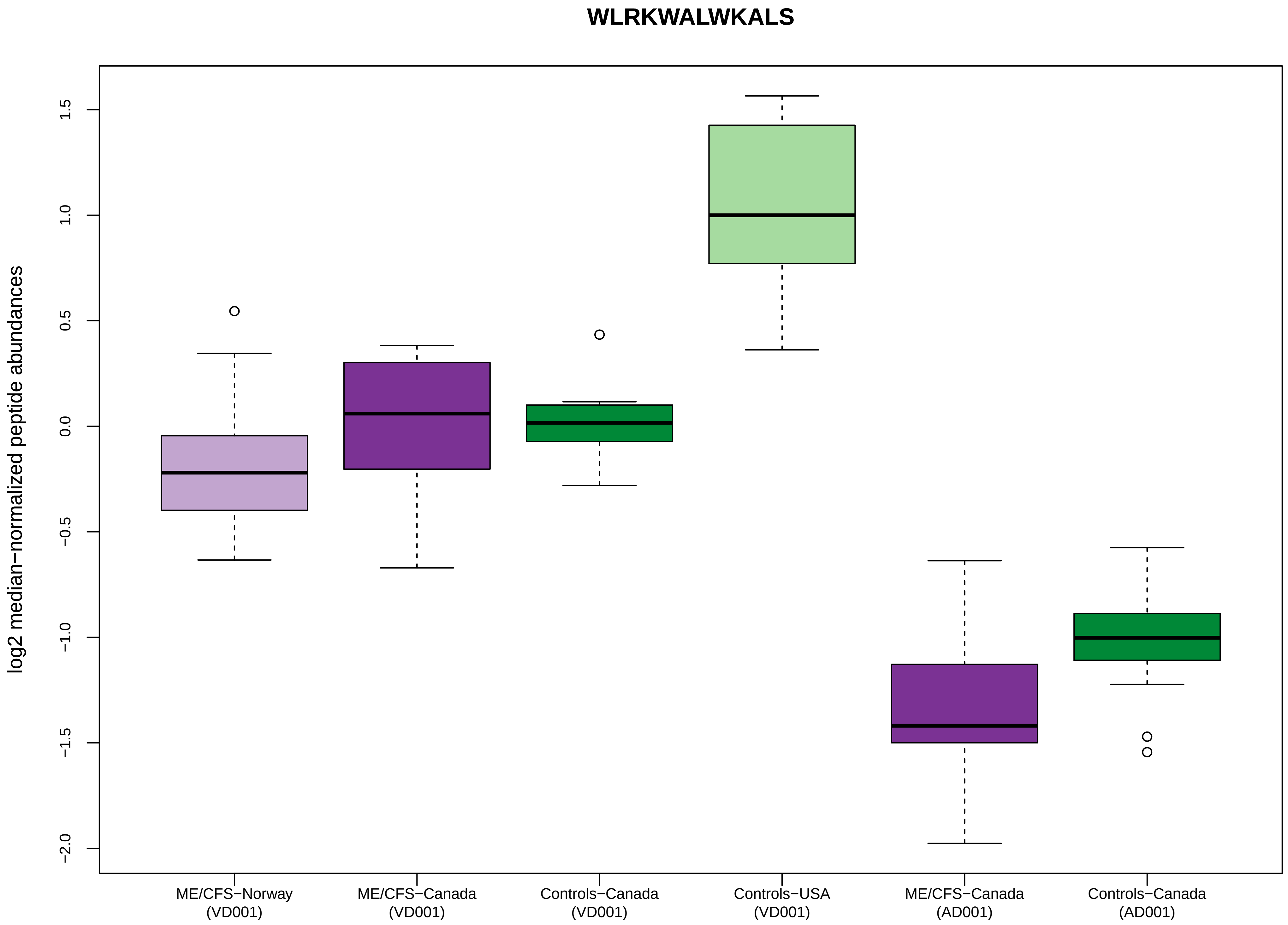

WLRKWALWKALS
log2 median−normalized peptide abundances
1.5
1.0
0.5
0.0
−0.5
−1.0
−1.5
−2.0
ME/CFS−Norway (VD001)
ME/CFS−Canada (VD001)
Controls−Canada (VD001)
Controls−USA (VD001)
ME/CFS−Canada (AD001)
Controls−Canada (AD001)

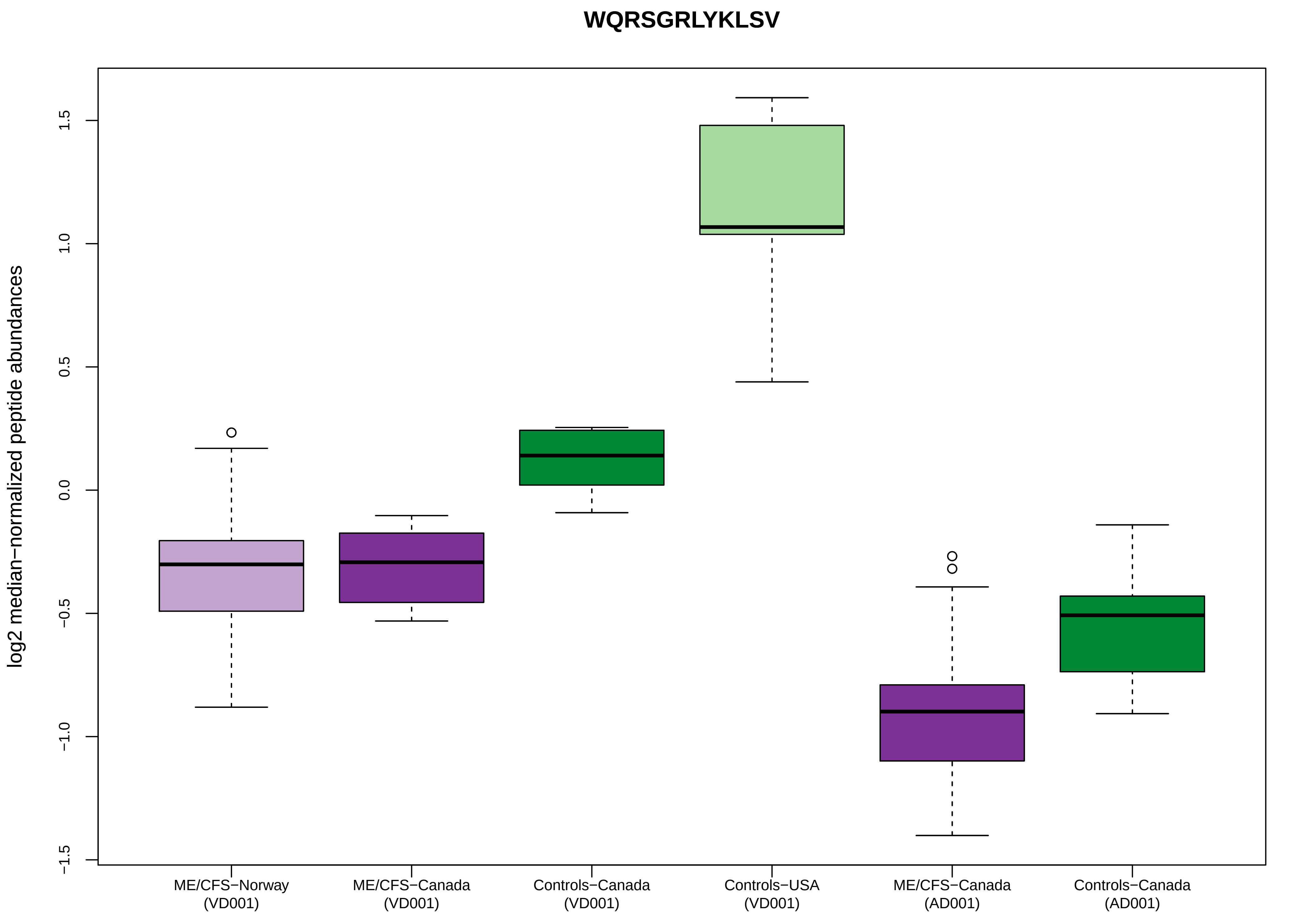
WQRSGRLYKLSV
log2 median−normalized peptide abundances
1.5
1.0
0.5
0.0
−0.5
−1.0
−1.5
ME/CFS−Norway (VD001)
ME/CFS−Canada (VD001)
Controls−Canada (VD001)
Controls−USA (VD001)
ME/CFS−Canada (AD001)
Controls−Canada (AD001)

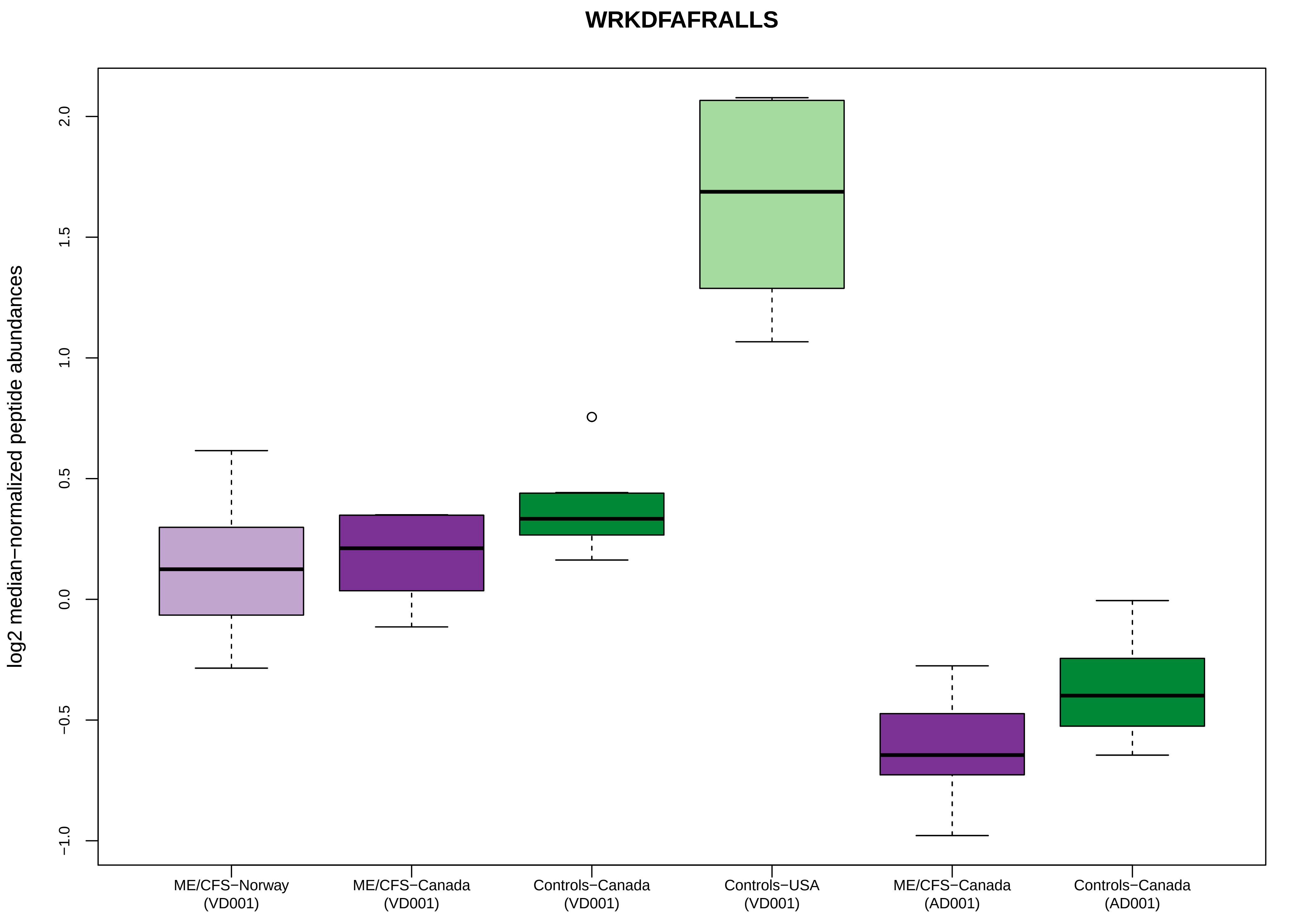
WRKDFAFRALLS
log2 median−normalized peptide abundances
2.0
1.5
1.0
0.5
0.0
−0.5
−1.0
ME/CFS−Norway (VD001)
ME/CFS−Canada (VD001)
Controls−Canada (VD001)
Controls−USA (VD001)
ME/CFS−Canada (AD001)
Controls−Canada (AD001)

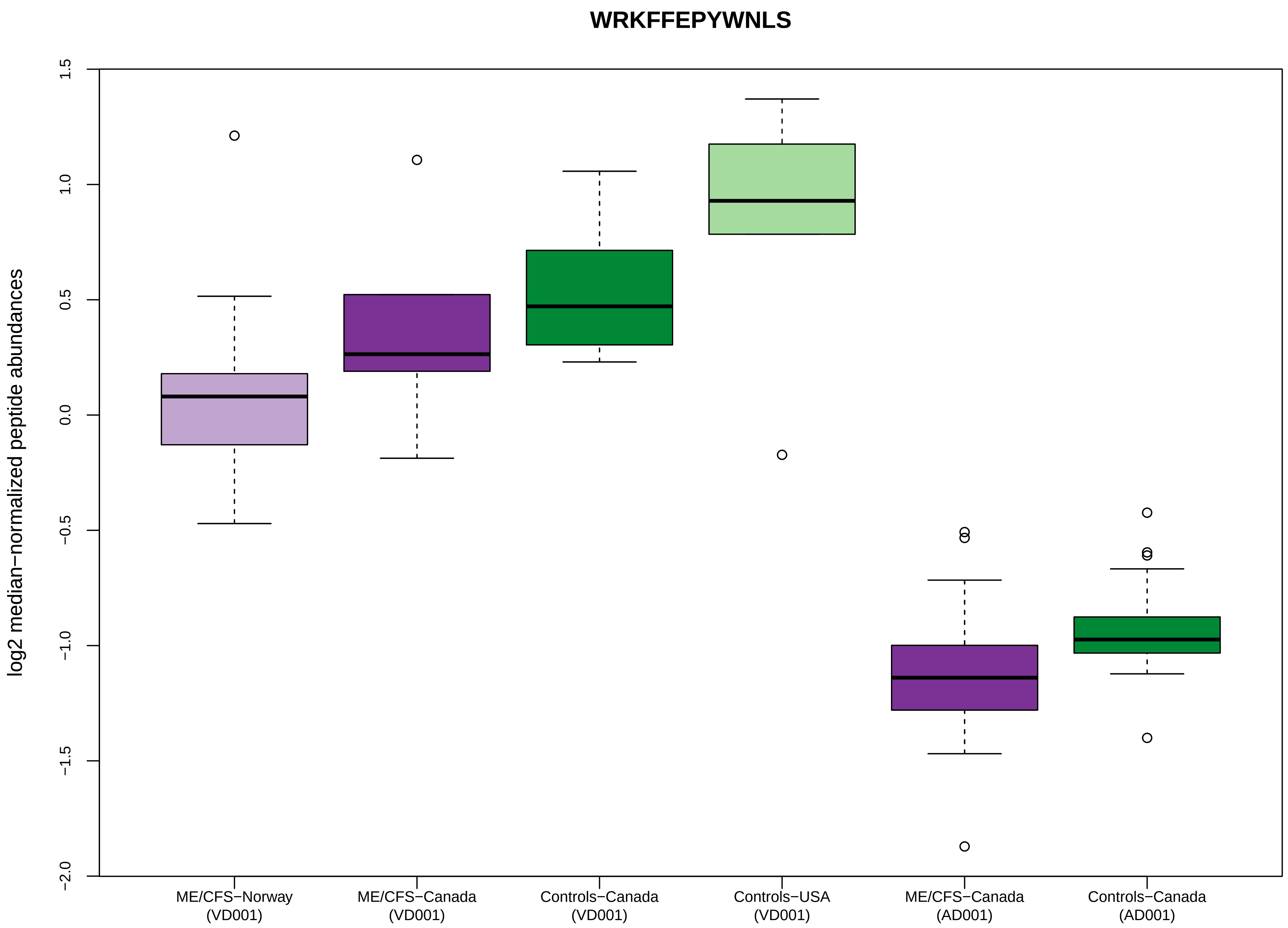
WRKFFEPYWNLS
log2 median−normalized peptide abundances
1.5
1.0
0.5
0.0
−0.5
−1.0
−1.5
−2.0
ME/CFS−Norway (VD001)
ME/CFS−Canada (VD001)
Controls−Canada (VD001)
Controls−USA (VD001)
ME/CFS−Canada (AD001)
Controls−Canada (AD001)

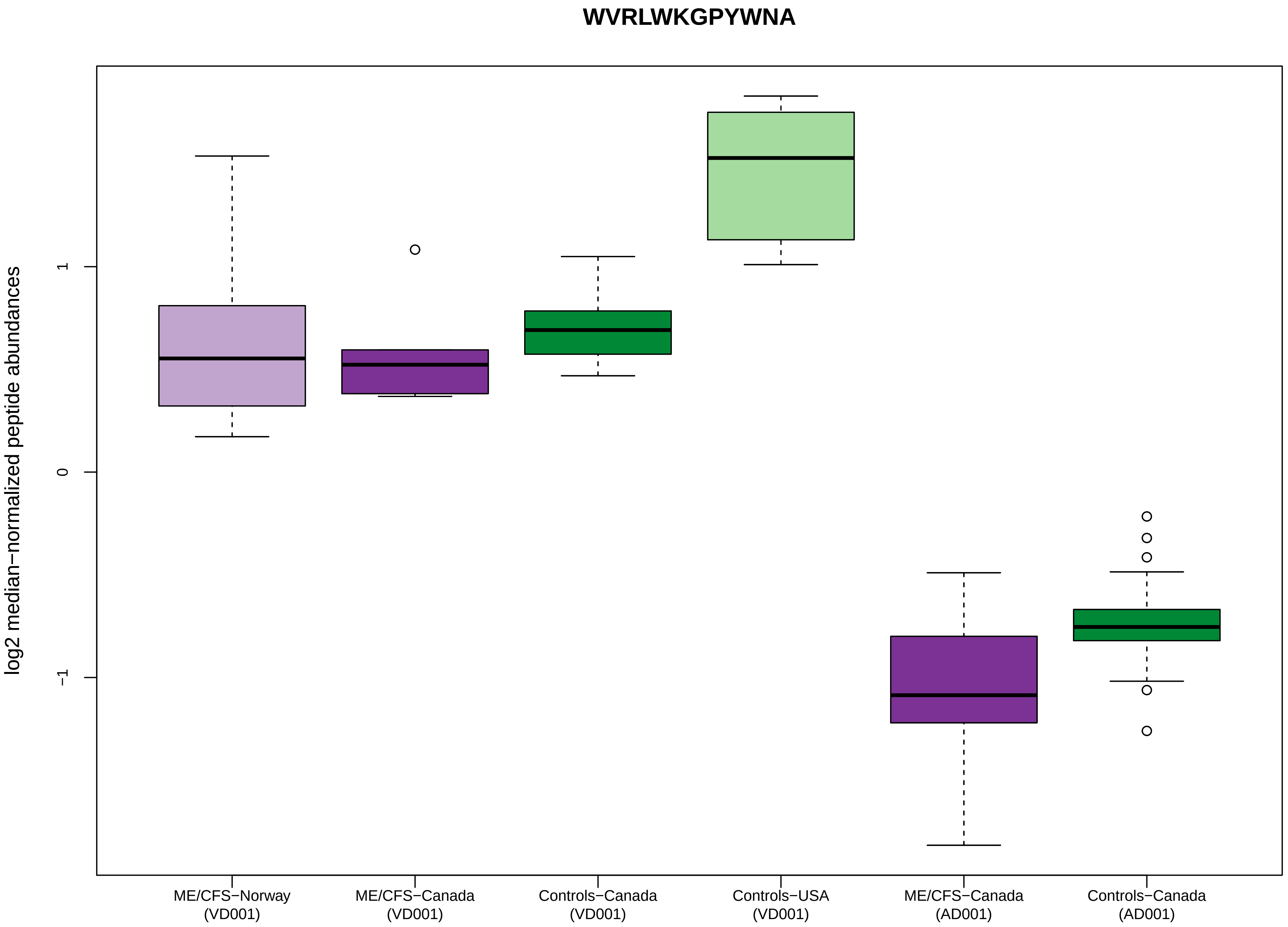
WVRLWKGPYWNA
log2 median−normalized peptide abundances
1
0
−1
ME/CFS−Norway (VD001)
ME/CFS−Canada (VD001)
Controls−Canada (VD001)
Controls−USA (VD001)
ME/CFS−Canada (AD001)
Controls−Canada (AD001)

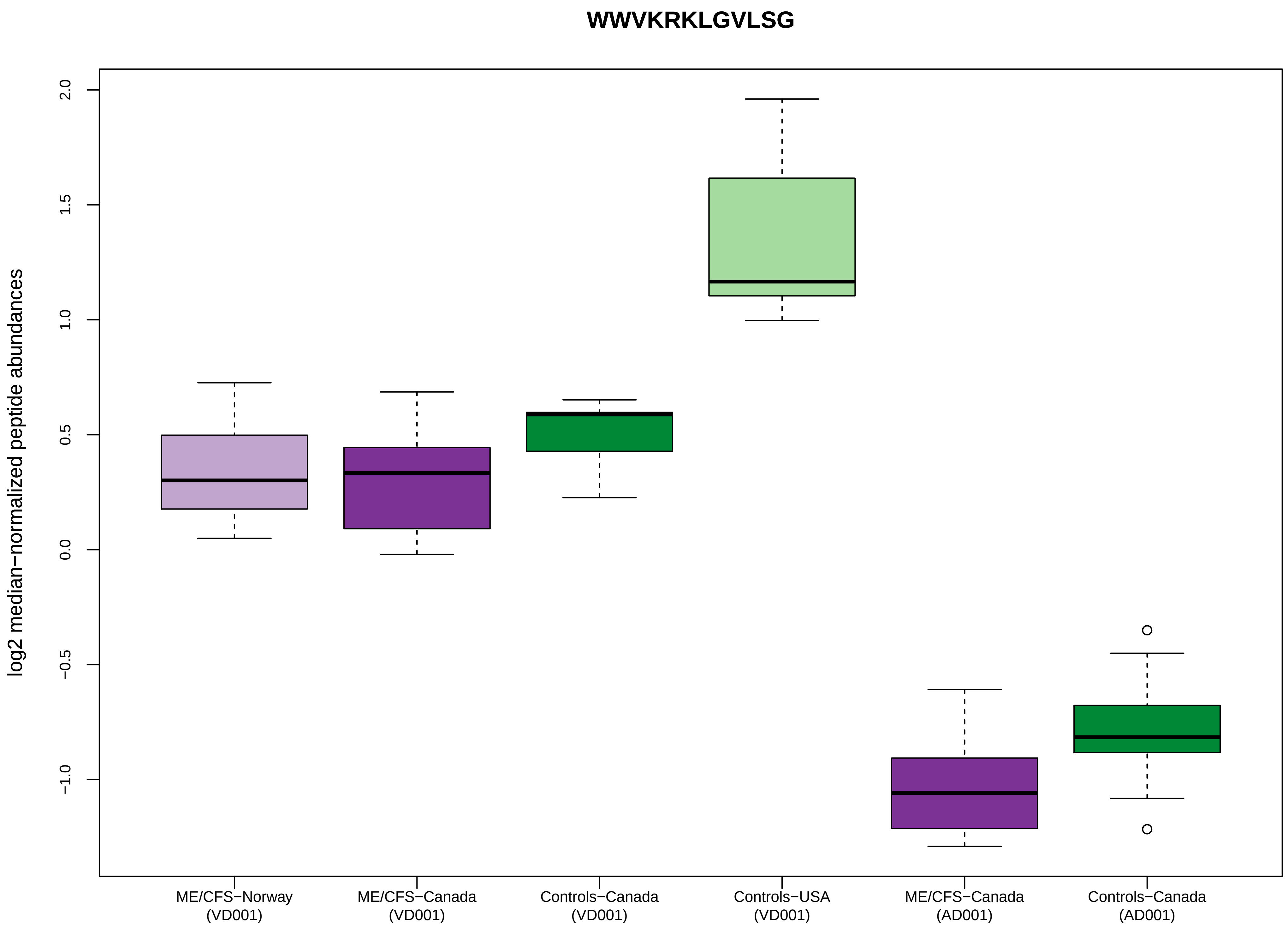
WWVKRKLGVLSG
log2 median−normalized peptide abundances
2.0
1.5
1.0
0.5
0.0
−0.5
−1.0
ME/CFS−Norway (VD001)
ME/CFS−Canada (VD001)
Controls−Canada (VD001)
Controls−USA (VD001)
ME/CFS−Canada (AD001)
Controls−Canada (AD001)

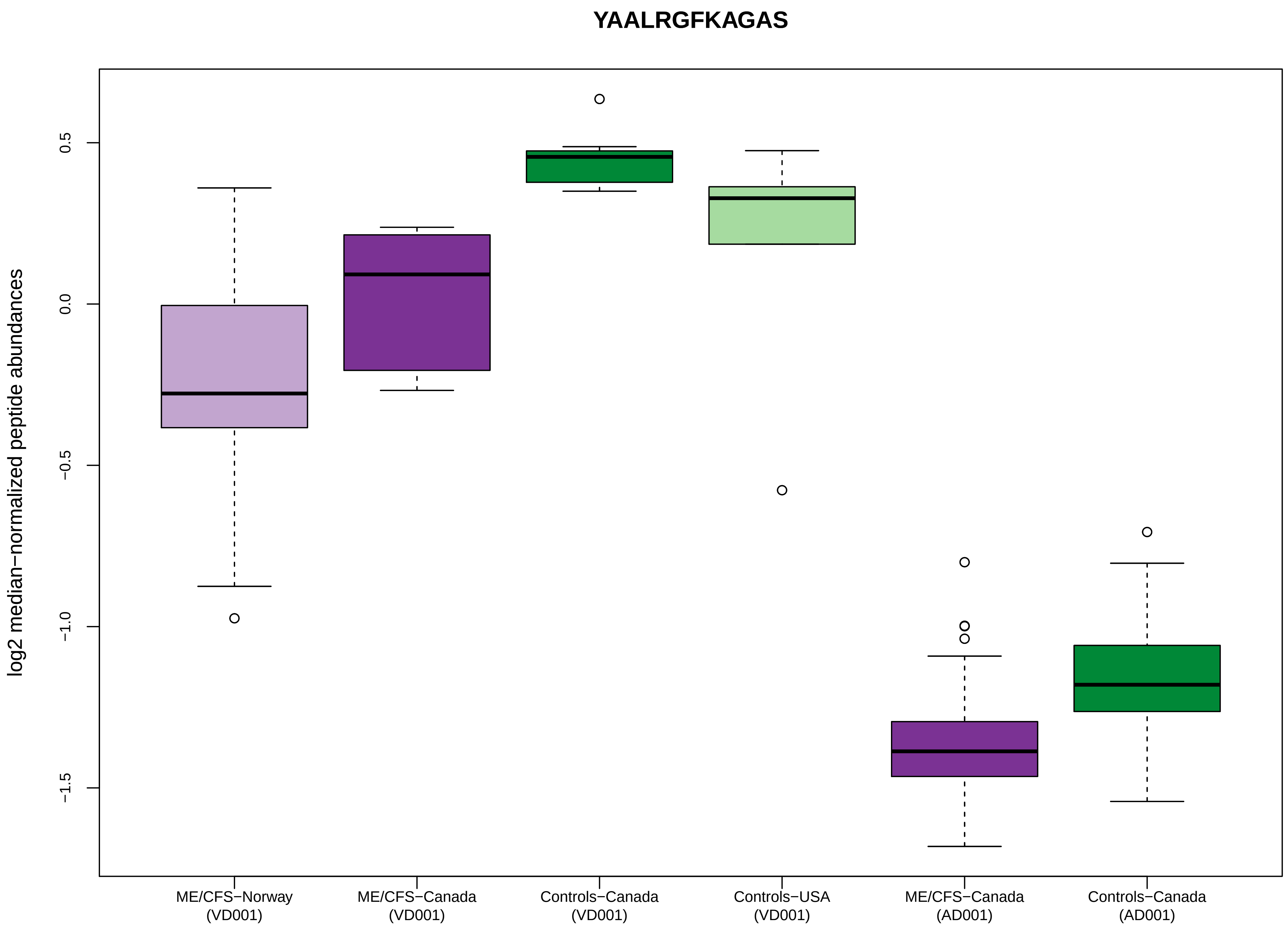

YAALRGFKAGAS
log2 median−normalized peptide abundances
0.5
0.0
−0.5
−1.0
−1.5
ME/CFS−Norway (VD001)
ME/CFS−Canada (VD001)
Controls−Canada (VD001)
Controls−USA (VD001)
ME/CFS−Canada (AD001)
Controls−Canada (AD001)

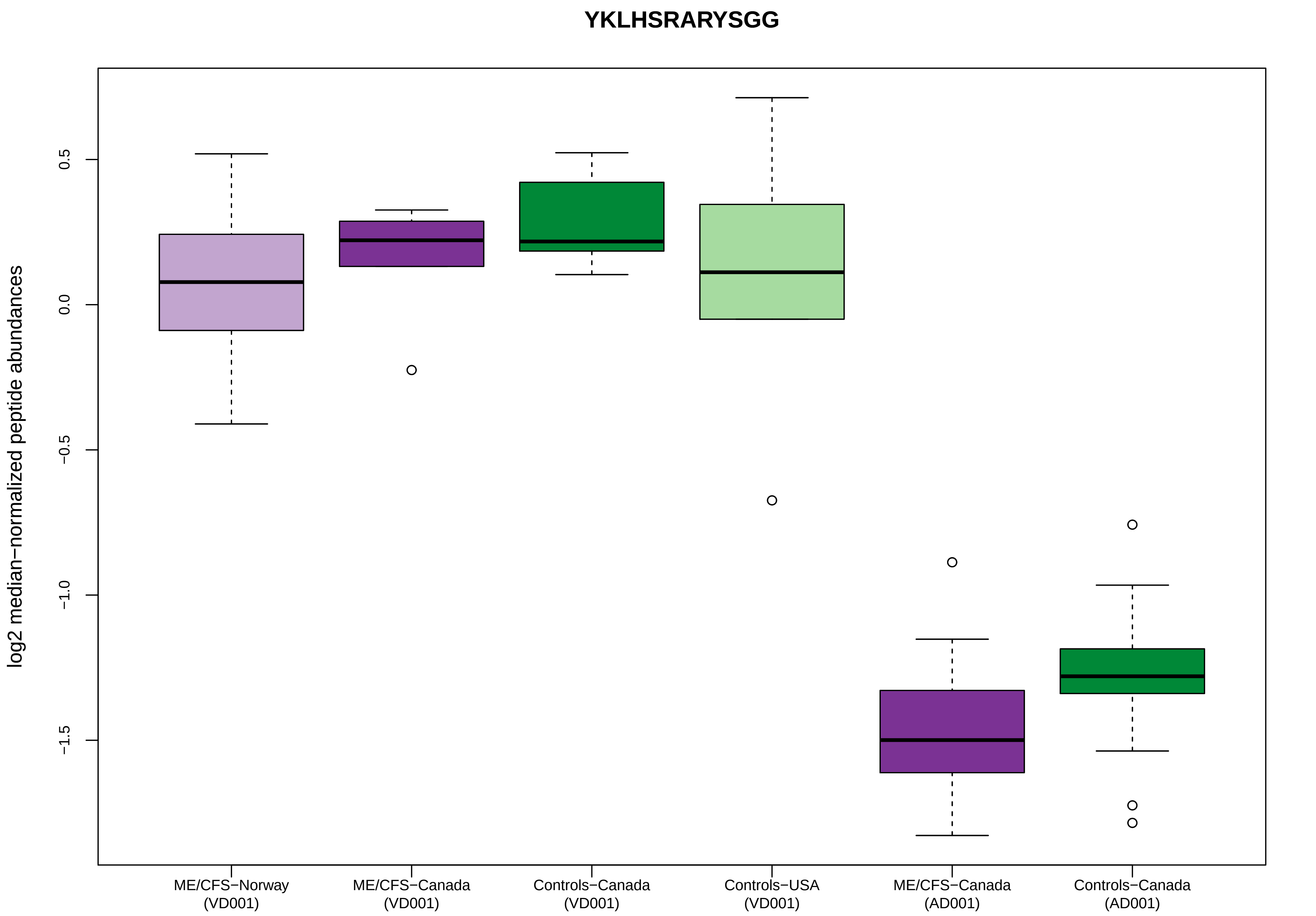

YKLHSRARYSGG
log2 median−normalized peptide abundances
0.5
0.0
−0.5
−1.0
−1.5
ME/CFS−Norway (VD001)
ME/CFS−Canada (VD001)
Controls−Canada (VD001)
Controls−USA (VD001)
ME/CFS−Canada (AD001)
Controls−Canada (AD001)

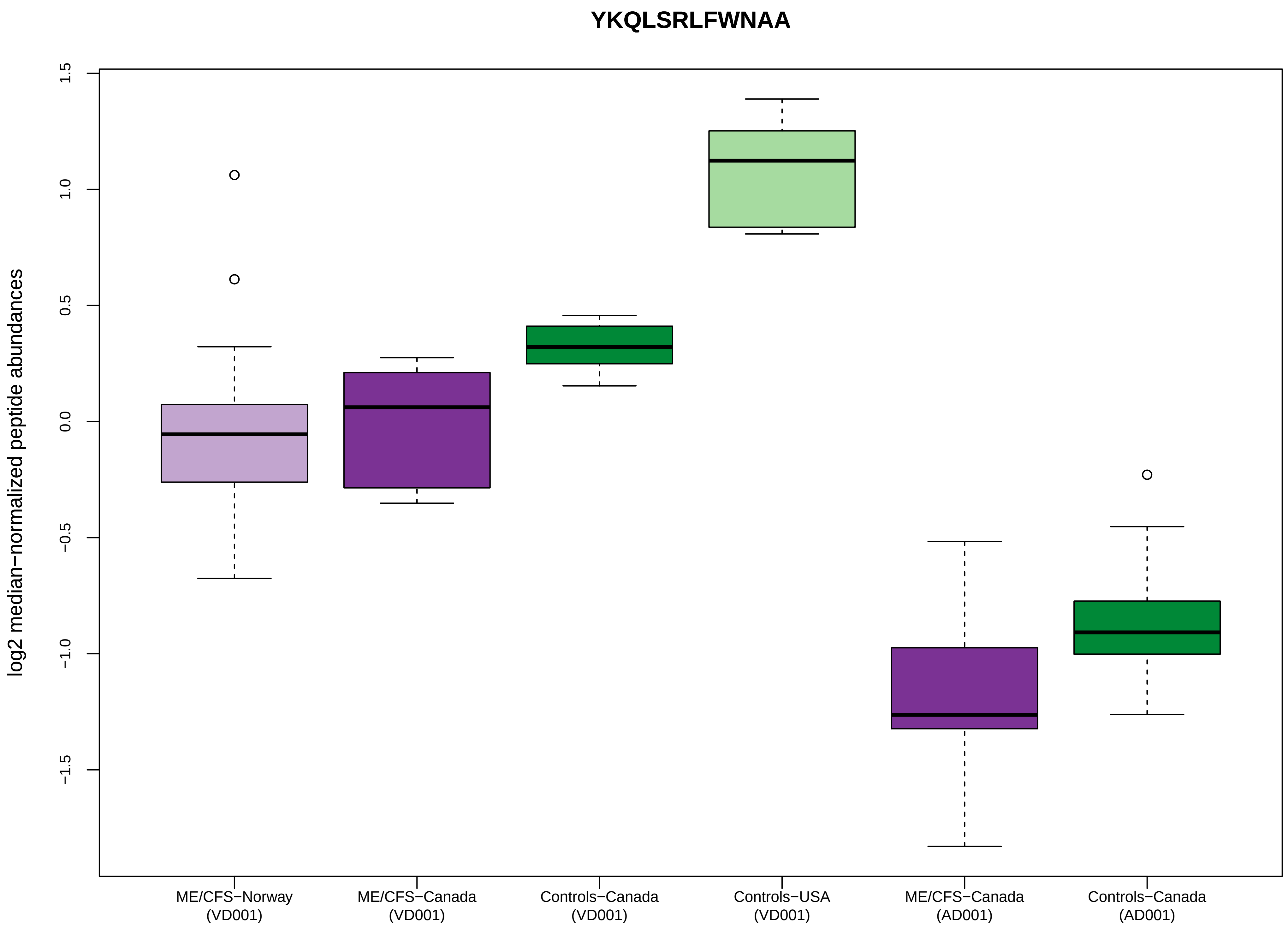
YKQLSRLFWNAA
log2 median−normalized peptide abundances
1.5
1.0
0.5
0.0
−0.5
−1.0
−1.5
ME/CFS−Norway (VD001)
ME/CFS−Canada (VD001)
Controls−Canada (VD001)
Controls−USA (VD001)
ME/CFS−Canada (AD001)
Controls−Canada (AD001)

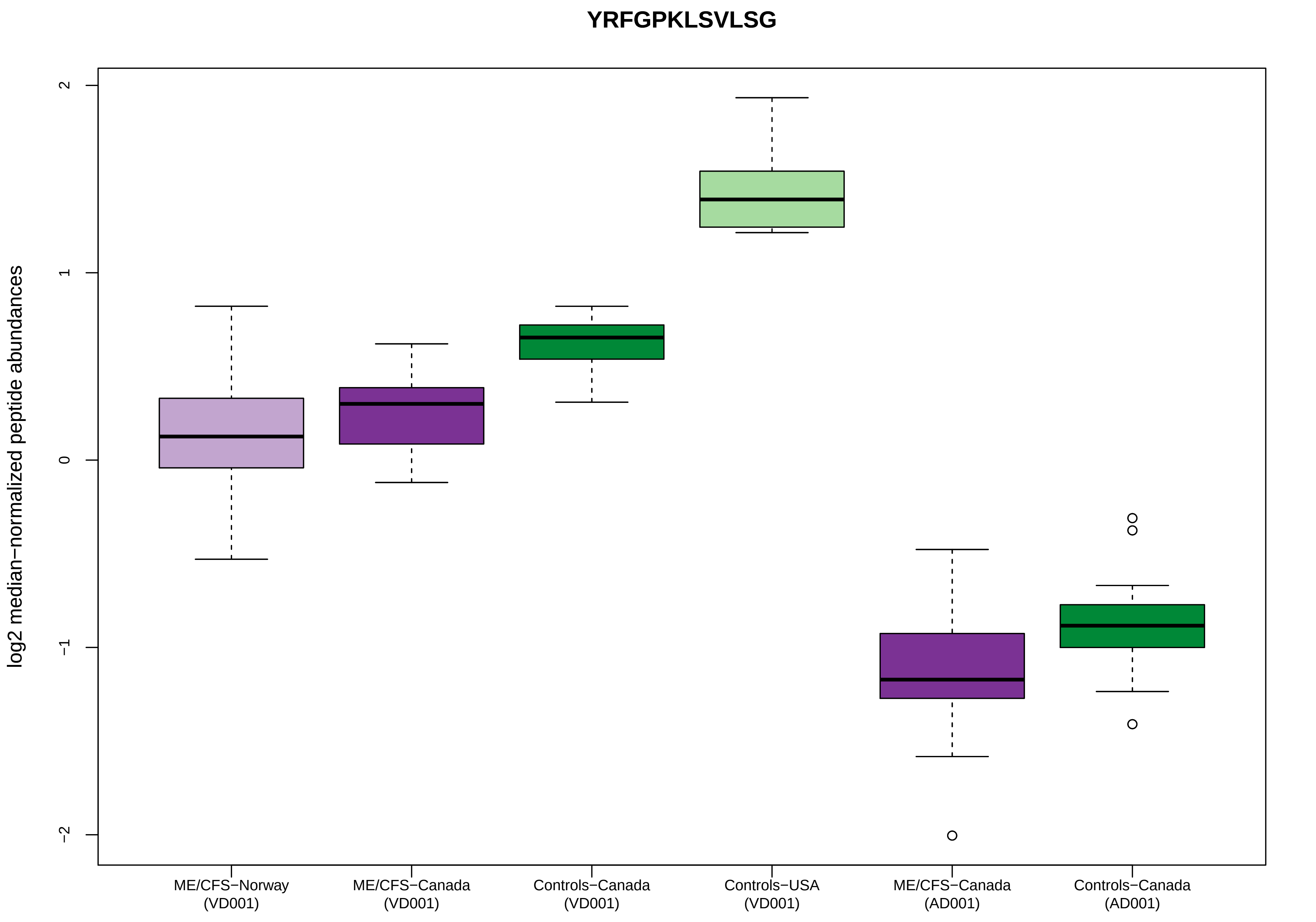

YRFGPKLSVLSG
log2 median−normalized peptide abundances
2
1
0
−1
−2
ME/CFS−Norway (VD001)
ME/CFS−Canada (VD001)
Controls−Canada (VD001)
Controls−USA (VD001)
ME/CFS−Canada (AD001)
Controls−Canada (AD001)

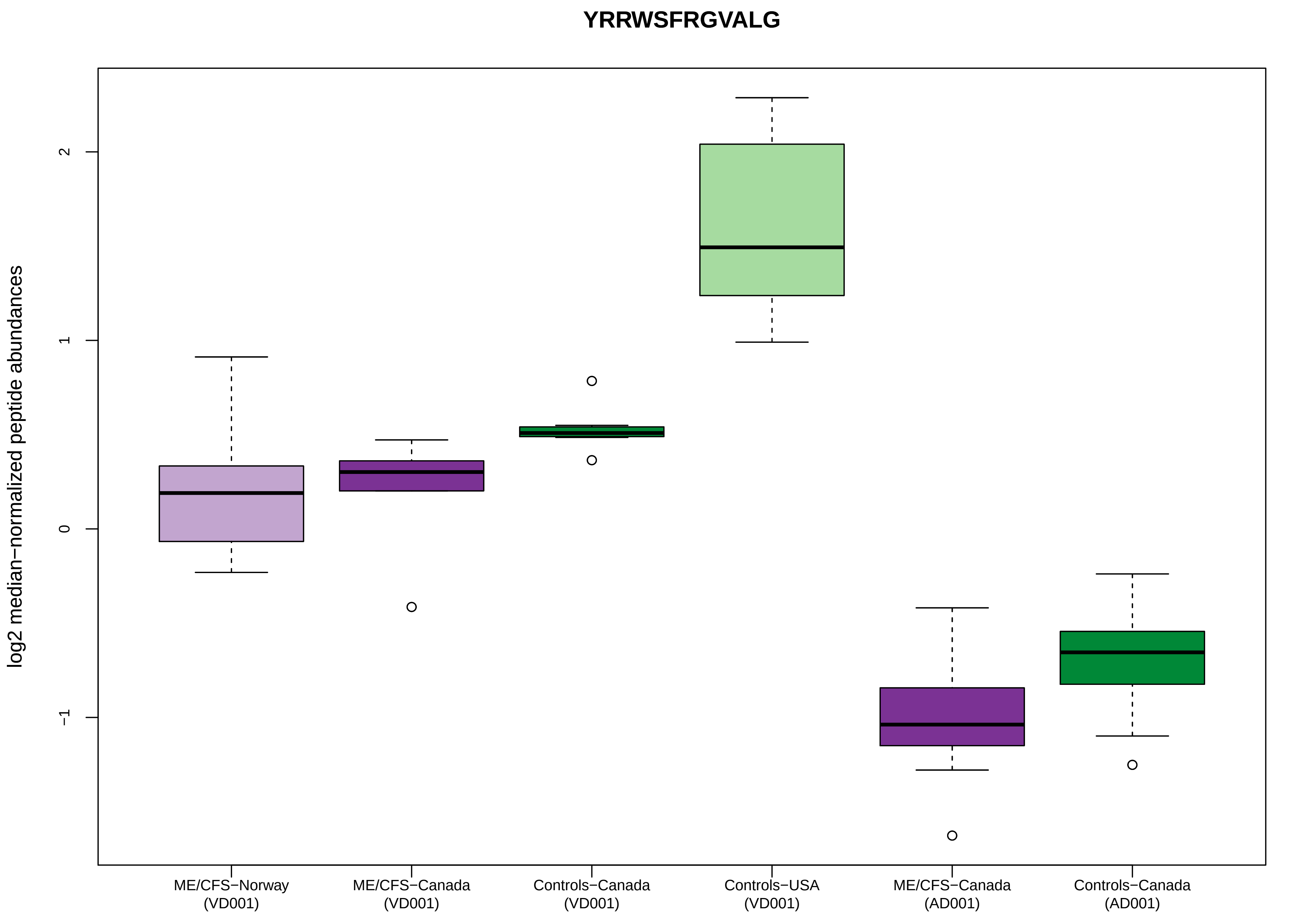

YRRWSFRGVALG
log2 median−normalized peptide abundances
2
1
0
−1
ME/CFS−Norway (VD001)
ME/CFS−Canada (VD001)
Controls−Canada (VD001)
Controls−USA (VD001)
ME/CFS−Canada (AD001)
Controls−Canada (AD001)

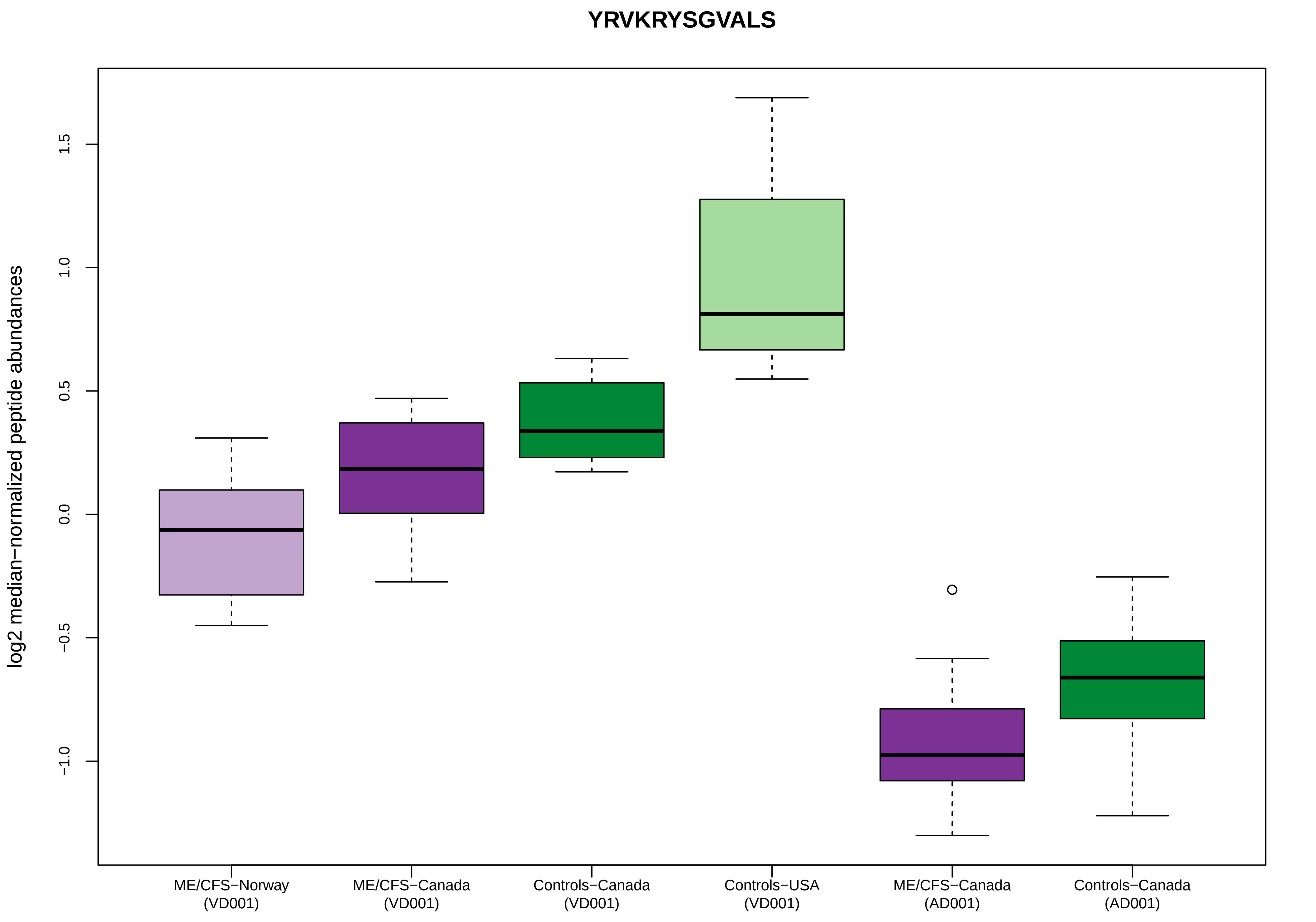

YRVKRYSGVALS
log2 median−normalized peptide abundances
1.5
1.0
0.5
0.0
−0.5
−1.0
ME/CFS−Norway (VD001)
ME/CFS−Canada (VD001)
Controls−Canada (VD001)
Controls−USA (VD001)
ME/CFS−Canada (AD001)
Controls−Canada (AD001)

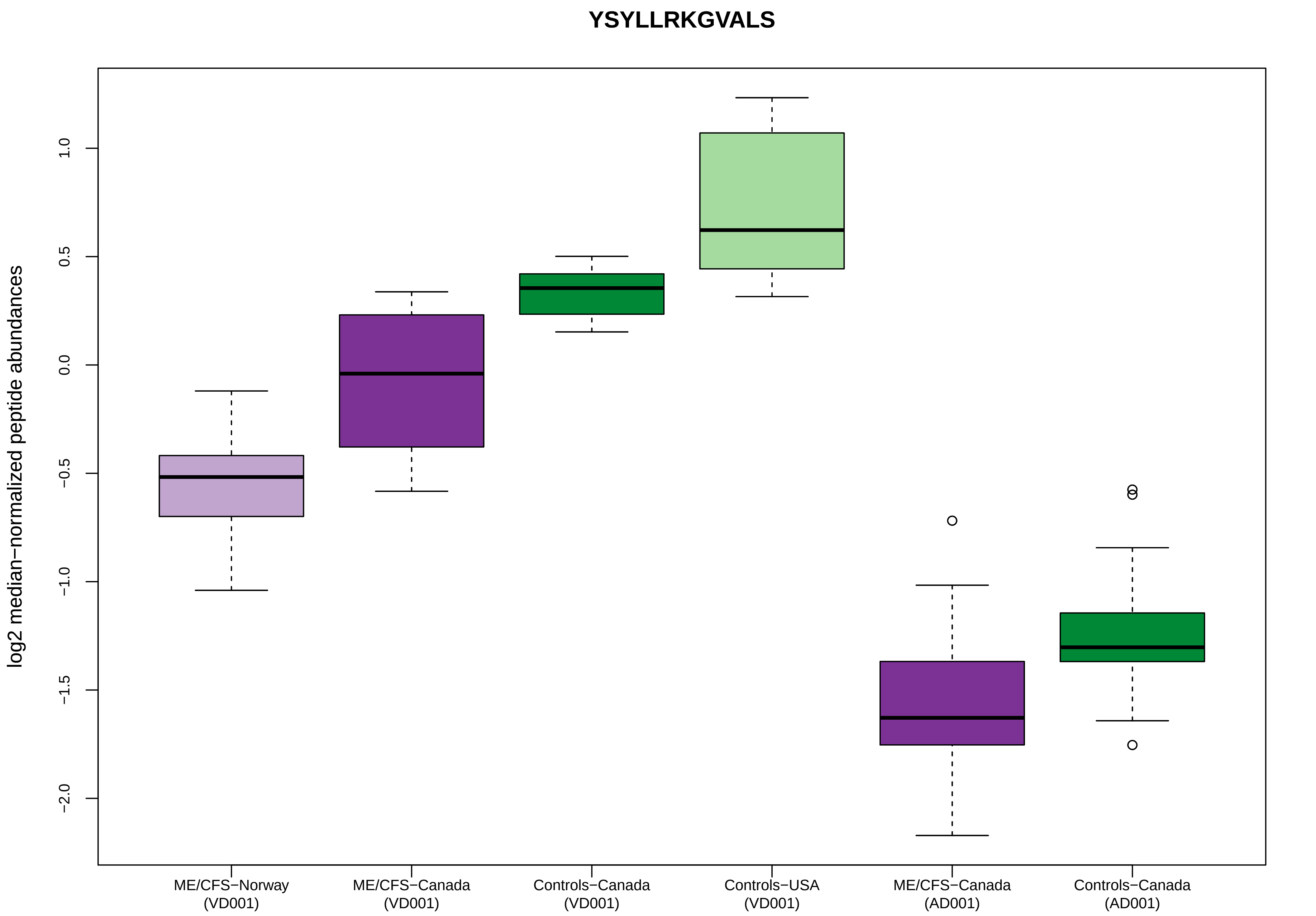
YSYLLRKGVALS
log2 median−normalized peptide abundances
1.0
0.5
0.0
−0.5
−1.0
−1.5
−2.0
ME/CFS−Norway (VD001)
ME/CFS−Canada (VD001)
Controls−Canada (VD001)
Controls−USA (VD001)
ME/CFS−Canada (AD001)
Controls−Canada (AD001)

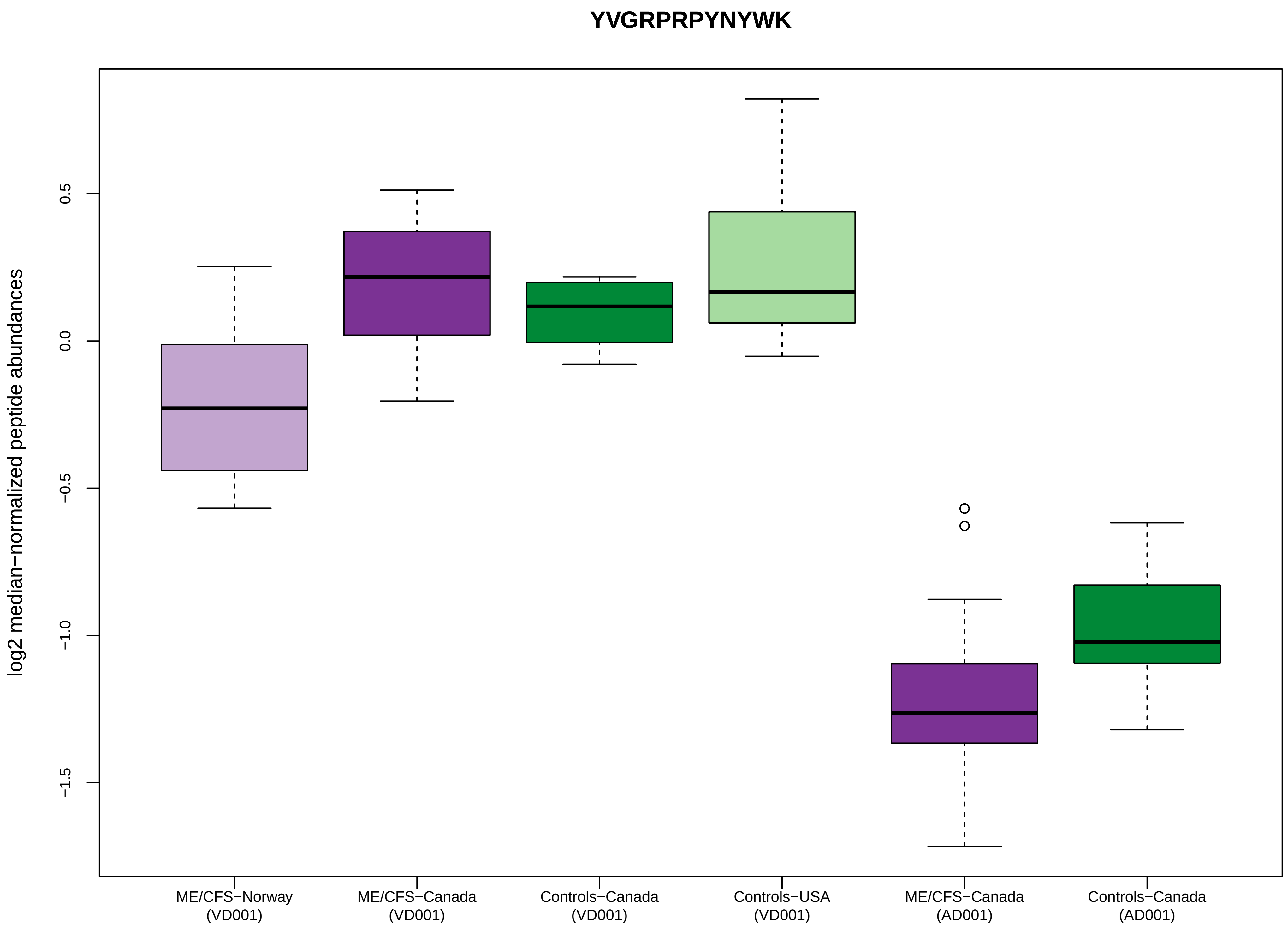

YVGRPRPYNYWK
log2 median−normalized peptide abundances
0.5
0.0
−0.5
−1.0
−1.5
ME/CFS−Norway (VD001)
ME/CFS−Canada (VD001)
Controls−Canada (VD001)
Controls−USA (VD001)
ME/CFS−Canada (AD001)
Controls−Canada (AD001)

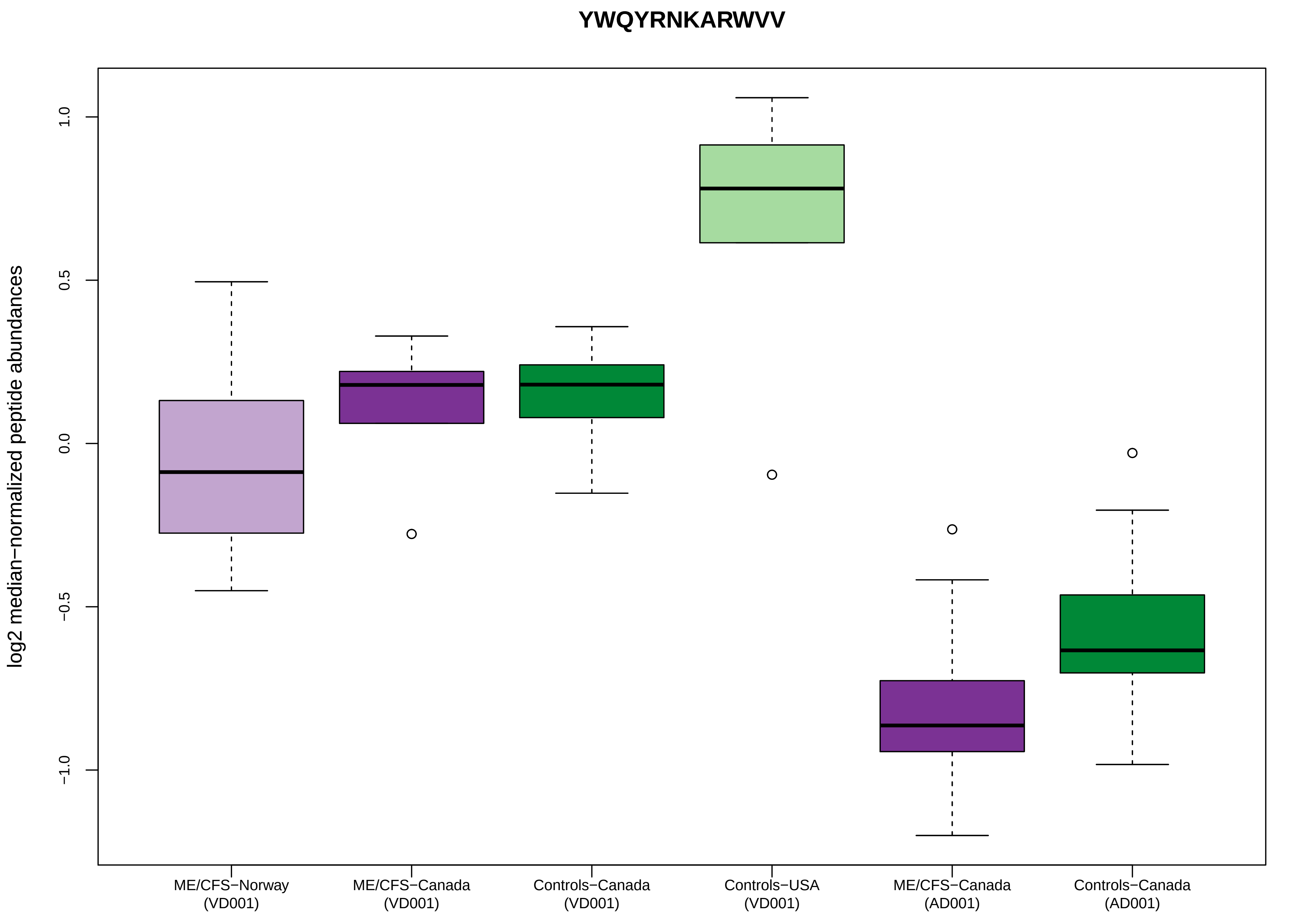
YWQYRNKARWVV
log2 median−normalized peptide abundances
1.0
0.5
0.0
−0.5
−1.0
ME/CFS−Norway (VD001)
ME/CFS−Canada (VD001)
Controls−Canada (VD001)
Controls−USA (VD001)
ME/CFS−Canada (AD001)
Controls−Canada (AD001)

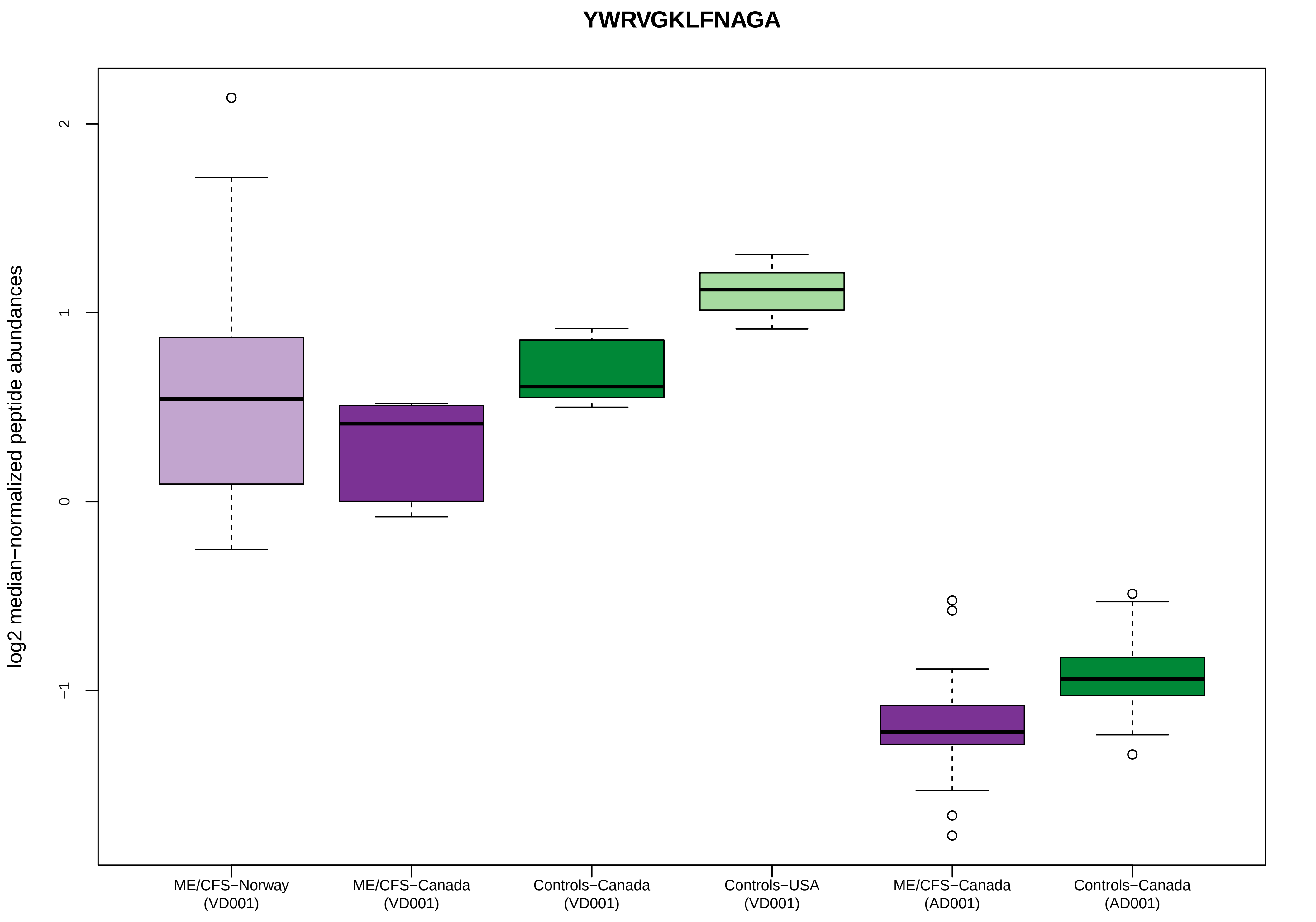

YWRVGKLFNAGA
log2 median−normalized peptide abundances
2
1
0
−1
ME/CFS−Norway (VD001)
ME/CFS−Canada (VD001)
Controls−Canada (VD001)
Controls−USA (VD001)
ME/CFS−Canada (AD001)
Controls−Canada (AD001)
