# Supplementary material for: Immunosignature Analysis of Myalgic Encephalomyelitis/Chronic Fatigue Syndrome (ME/CFS)
Source: Mol Neurobiol. 2018 Oct 8;56(6):4249–57. doi: 10.1007/s12035-018-1354-8 (PMC6505503; doi:10.1007/s12035-018-1354-8)
Supplement: Supplementary file 6 — (PDF 385 kb) [file 12035_2018_1354_MOESM6_ESM.pdf]

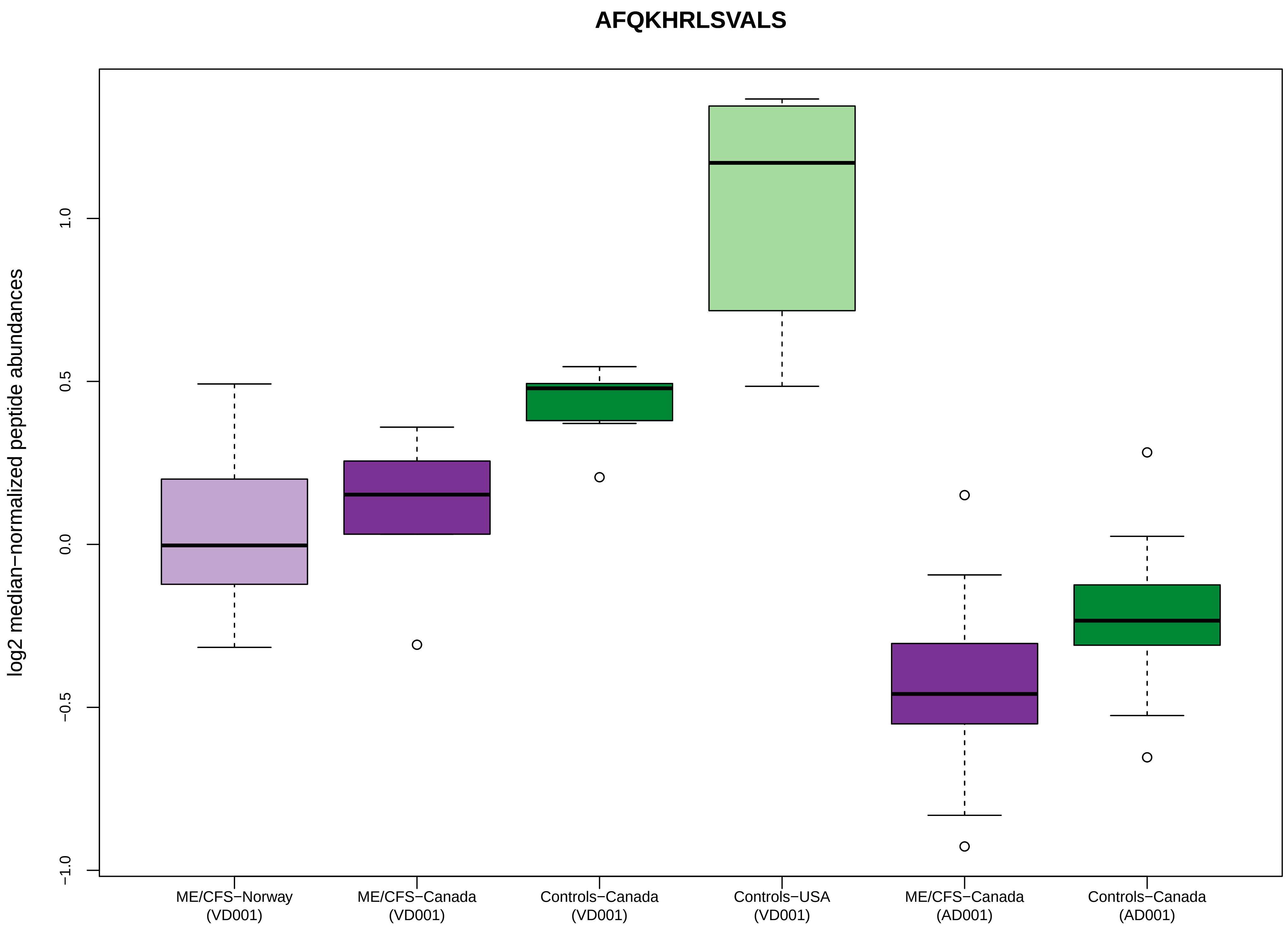

AFQKHRLSVALS
log2 median−normalized peptide abundances
1.0
0.5
0.0
−0.5
−1.0
ME/CFS−Norway (VD001)
ME/CFS−Canada (VD001)
Controls−Canada (VD001)
Controls−USA (VD001)
ME/CFS−Canada (AD001)
Controls−Canada (AD001)

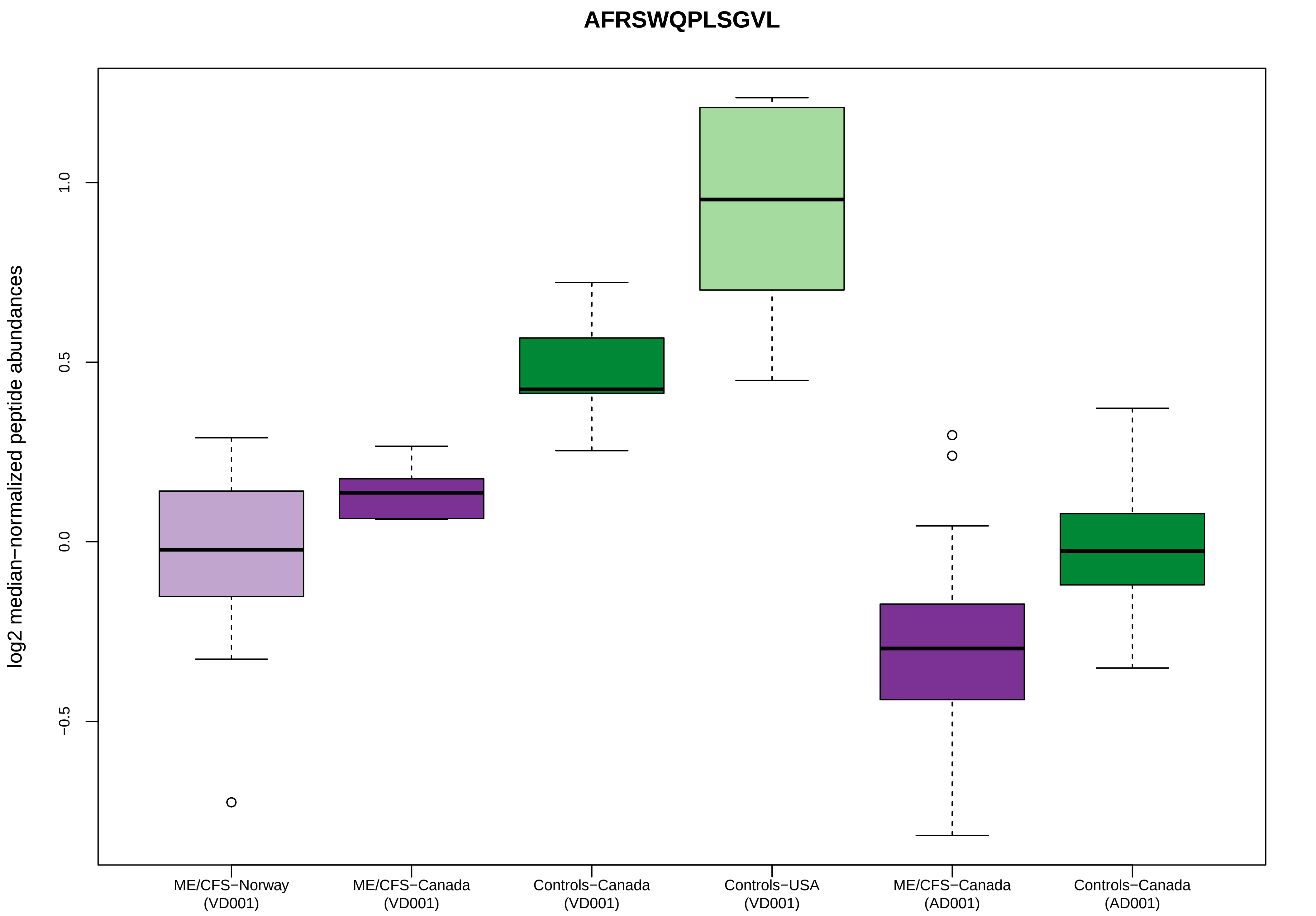

AFRSWQPLSGVL
log2 median−normalized peptide abundances
1.0
0.5
0.0
−0.5
ME/CFS−Norway (VD001)
ME/CFS−Canada (VD001)
Controls−Canada (VD001)
Controls−USA (VD001)
ME/CFS−Canada (AD001)
Controls−Canada (AD001)

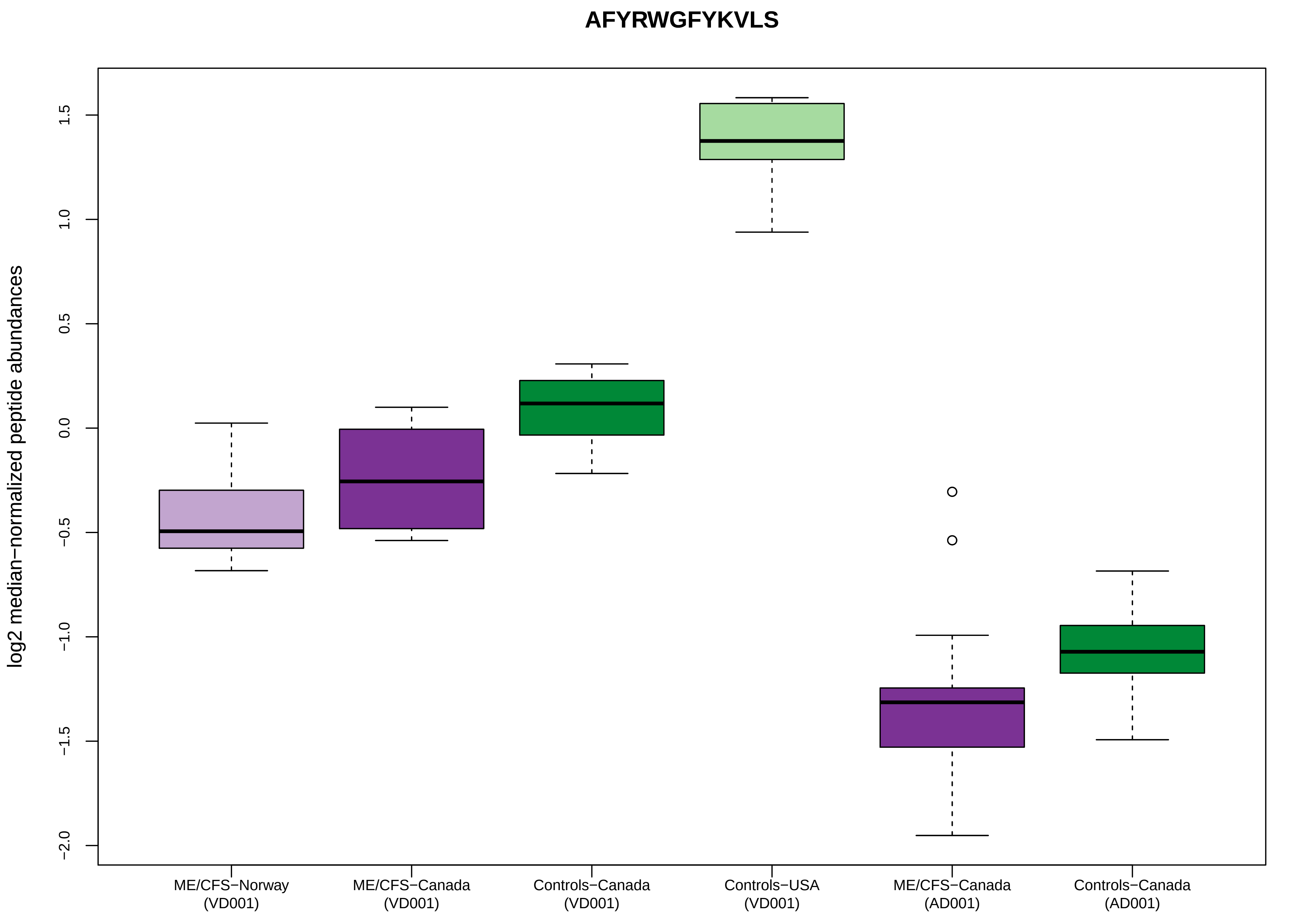
AFYRWGFYKVLS
log2 median−normalized peptide abundances
1.5
1.0
0.5
0.0
−0.5
−1.0
−1.5
−2.0
ME/CFS−Norway (VD001)
ME/CFS−Canada (VD001)
Controls−Canada (VD001)
Controls−USA (VD001)
ME/CFS−Canada (AD001)
Controls−Canada (AD001)

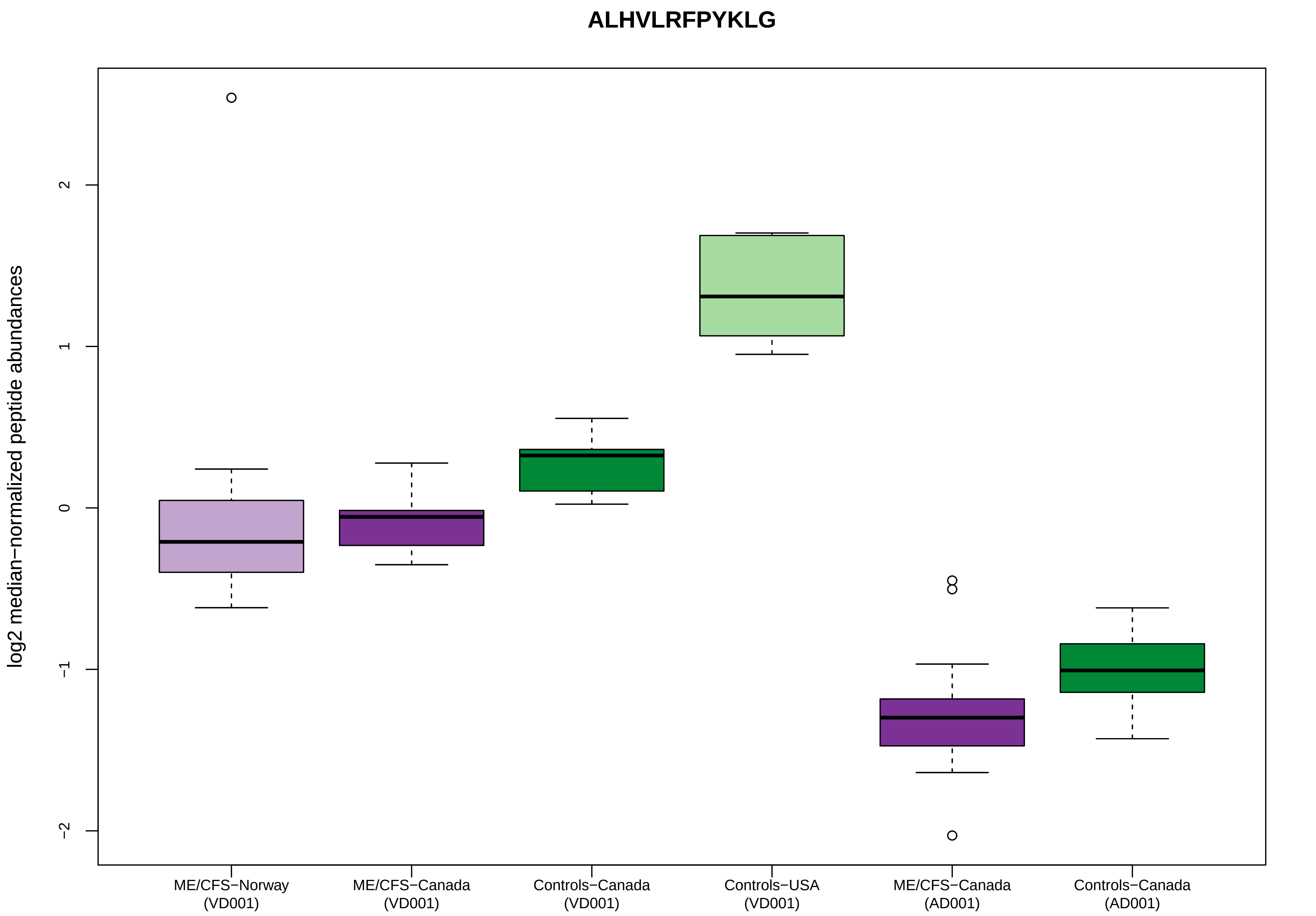

ALHVLRFPYKLG
log2 median−normalized peptide abundances
2
1
0
−1
−2
ME/CFS−Norway (VD001)
ME/CFS−Canada (VD001)
Controls−Canada (VD001)
Controls−USA (VD001)
ME/CFS−Canada (AD001)
Controls−Canada (AD001)

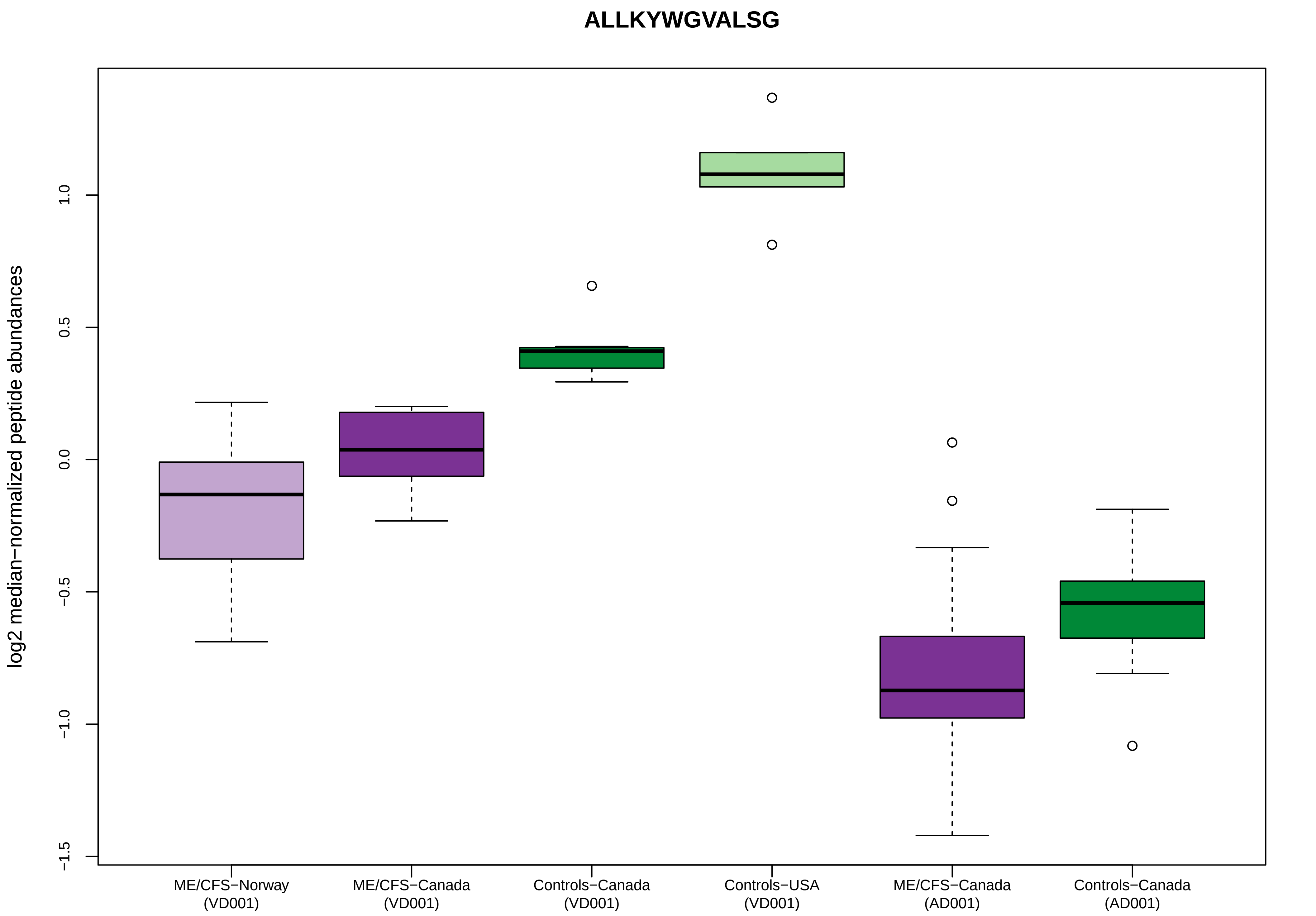

ALLKYWGVALSG
log2 median−normalized peptide abundances
1.0
0.5
0.0
−0.5
−1.0
−1.5
ME/CFS−Norway (VD001)
ME/CFS−Canada (VD001)
Controls−Canada (VD001)
Controls−USA (VD001)
ME/CFS−Canada (AD001)
Controls−Canada (AD001)

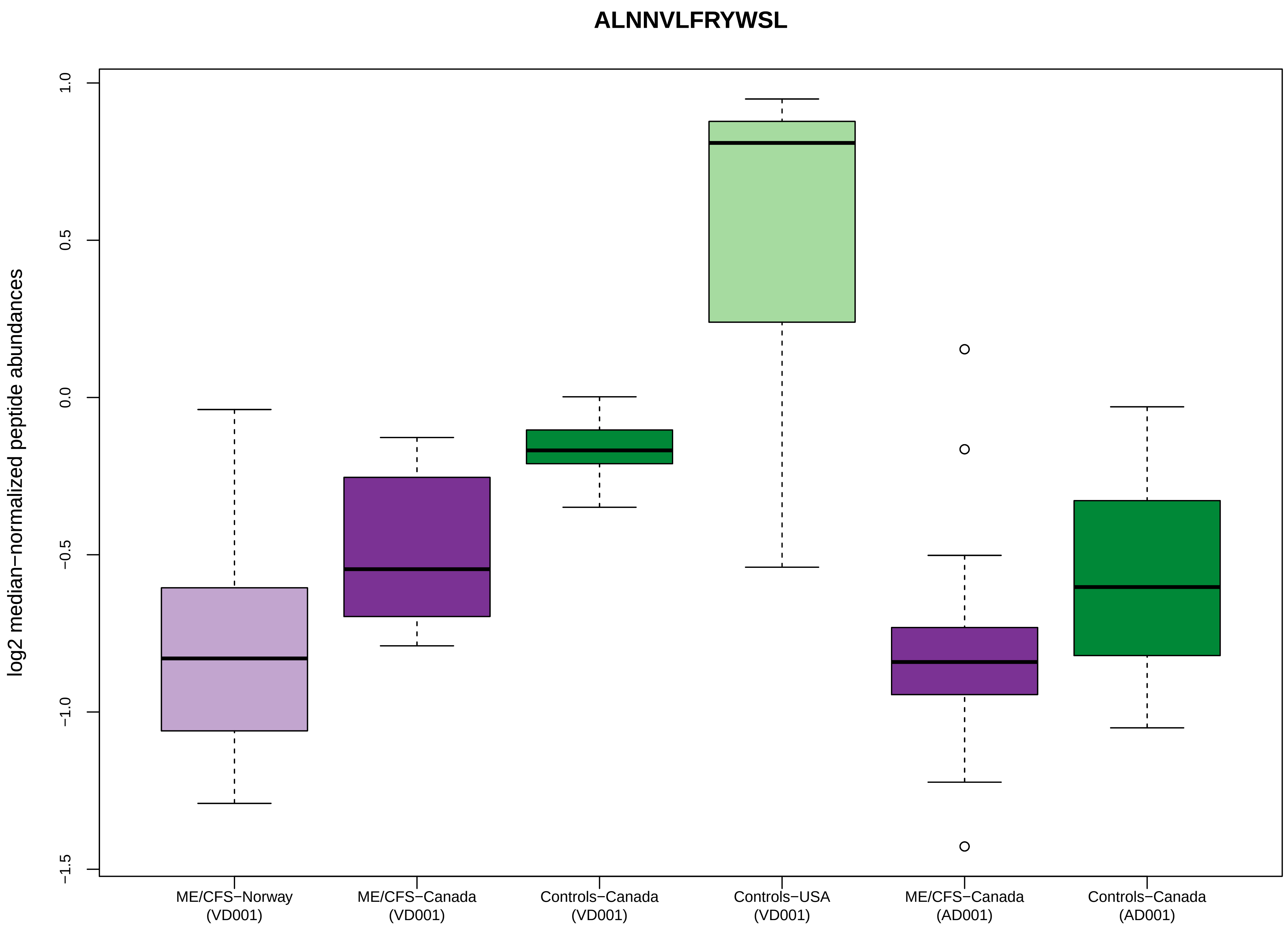
ALNNVLFRYWSL
log2 median−normalized peptide abundances
1.0
0.5
0.0
−0.5
−1.0
−1.5
ME/CFS−Norway (VD001)
ME/CFS−Canada (VD001)
Controls−Canada (VD001)
Controls−USA (VD001)
ME/CFS−Canada (AD001)
Controls−Canada (AD001)

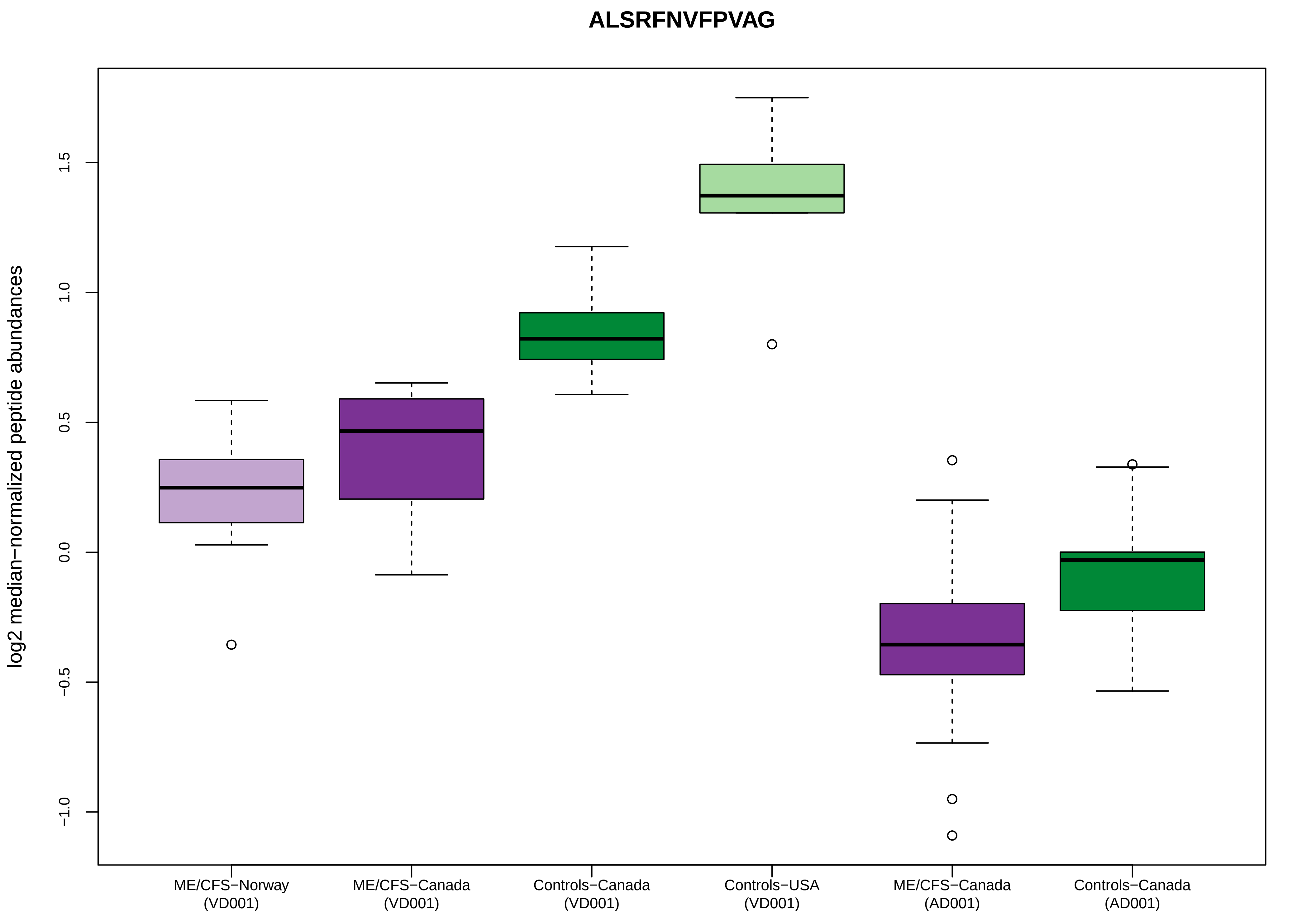
ALSRFNVFPVAG
log2 median−normalized peptide abundances
1.5
1.0
0.5
0.0
−0.5
−1.0
ME/CFS−Norway (VD001)
ME/CFS−Canada (VD001)
Controls−Canada (VD001)
Controls−USA (VD001)
ME/CFS−Canada (AD001)
Controls−Canada (AD001)

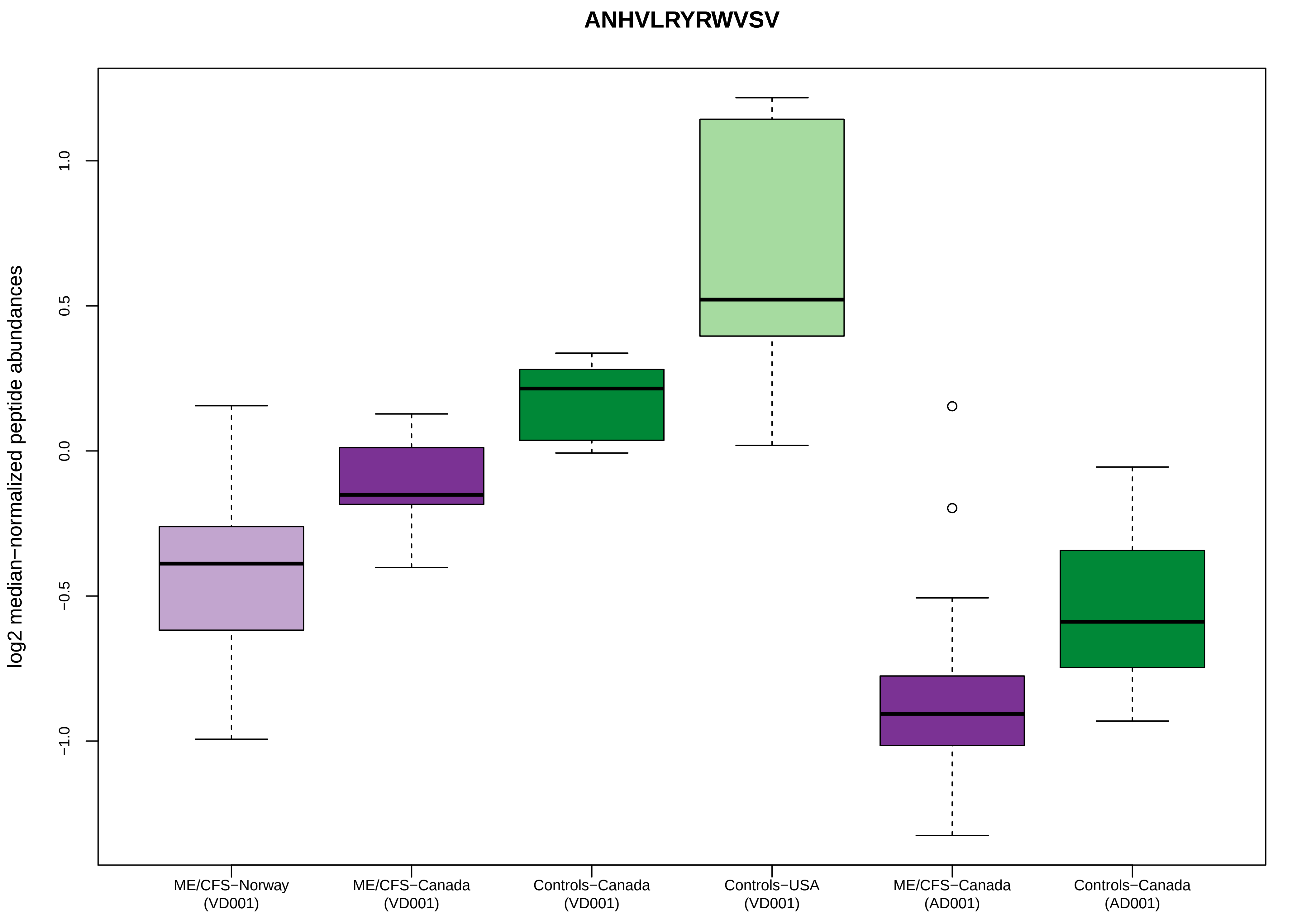
ANHVLRYRWVSV
log2 median−normalized peptide abundances
1.0
0.5
0.0
−0.5
−1.0
ME/CFS−Norway (VD001)
ME/CFS−Canada (VD001)
Controls−Canada (VD001)
Controls−USA (VD001)
ME/CFS−Canada (AD001)
Controls−Canada (AD001)

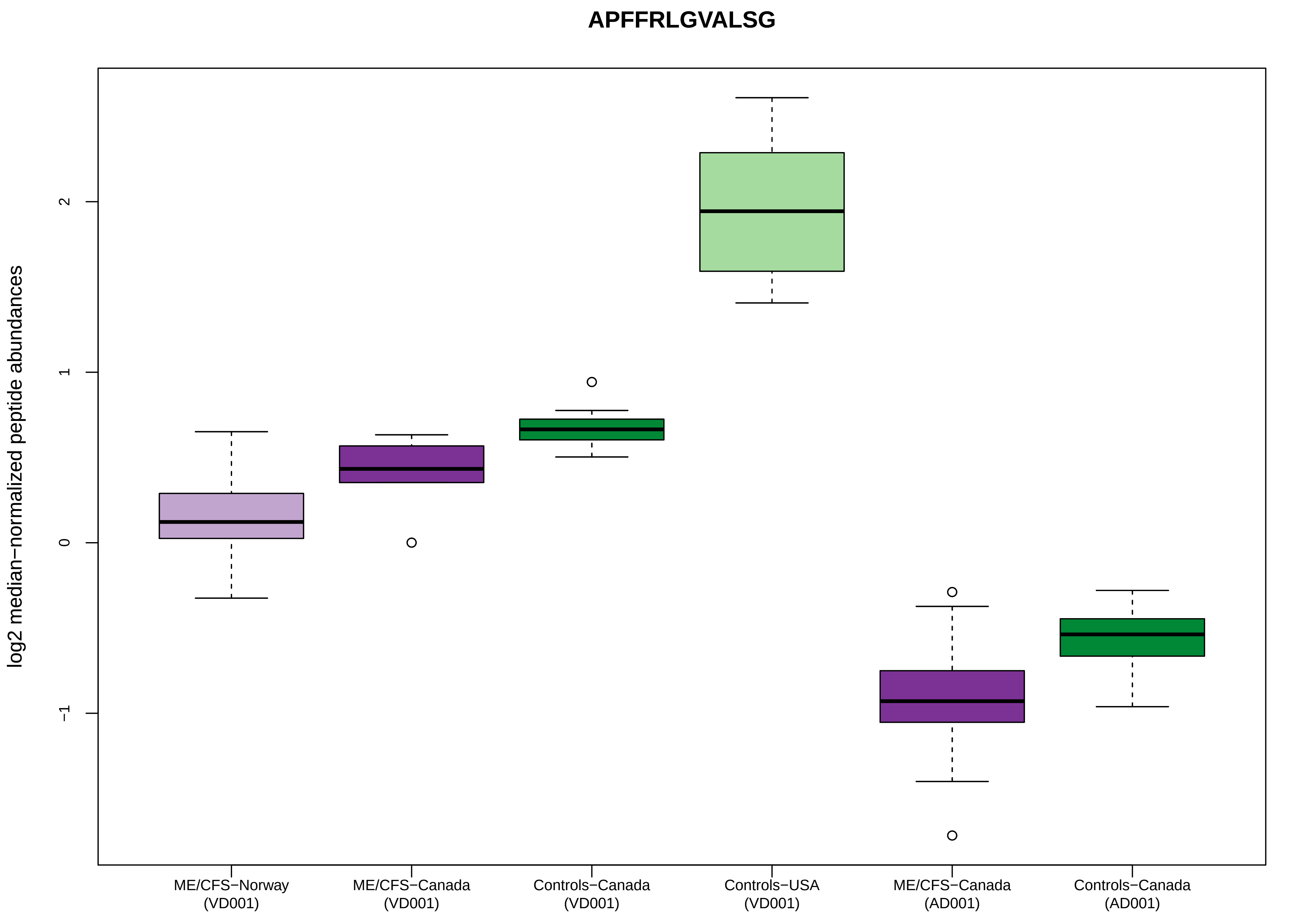

APFFRLGVALSG
log2 median−normalized peptide abundances
2
1
0
−1
ME/CFS−Norway (VD001)
ME/CFS−Canada (VD001)
Controls−Canada (VD001)
Controls−USA (VD001)
ME/CFS−Canada (AD001)
Controls−Canada (AD001)

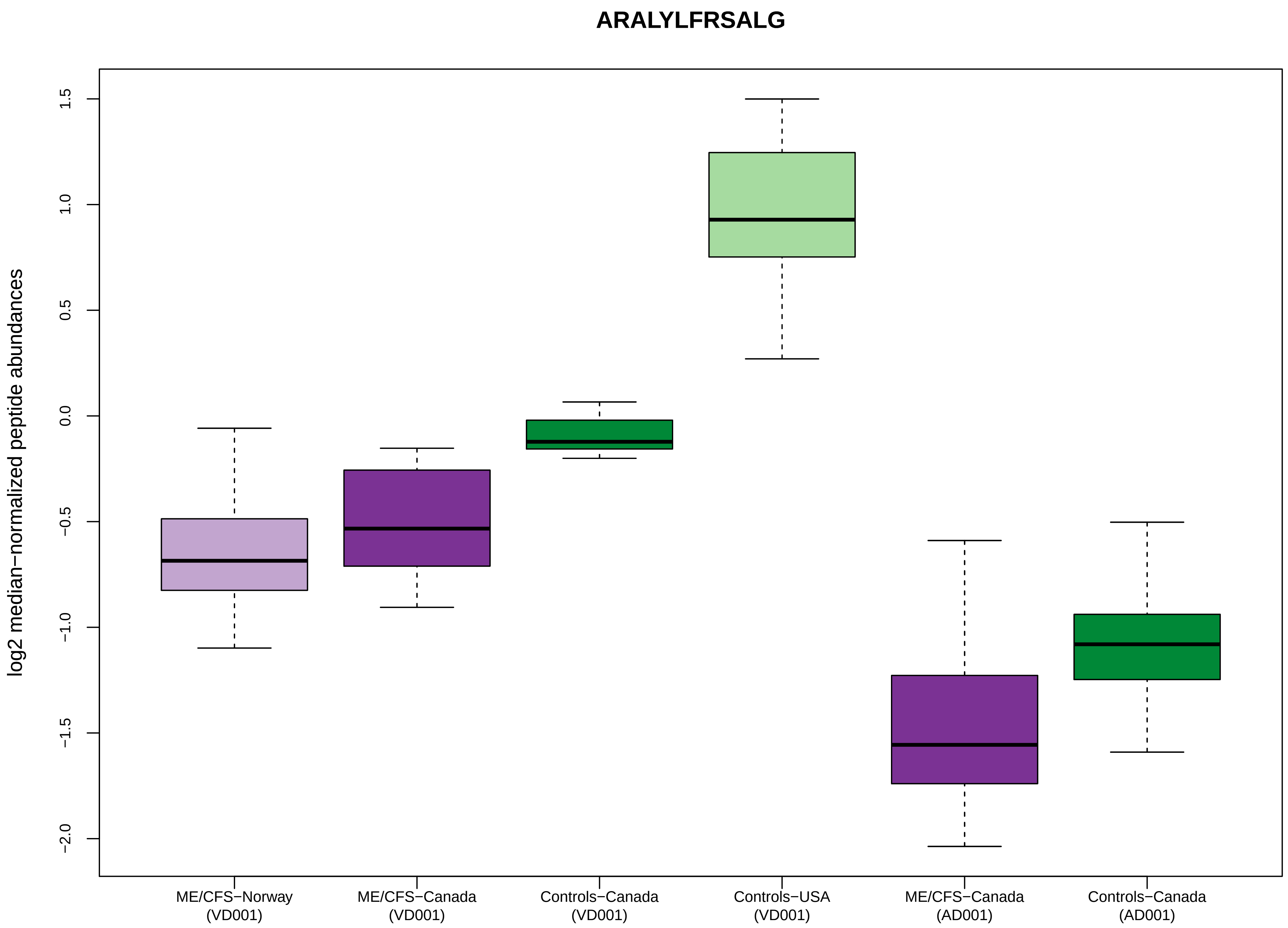
ARALYLFRSALG
log2 median−normalized peptide abundances
1.5
1.0
0.5
0.0
−0.5
−1.0
−1.5
−2.0
ME/CFS−Norway (VD001)
ME/CFS−Canada (VD001)
Controls−Canada (VD001)
Controls−USA (VD001)
ME/CFS−Canada (AD001)
Controls−Canada (AD001)

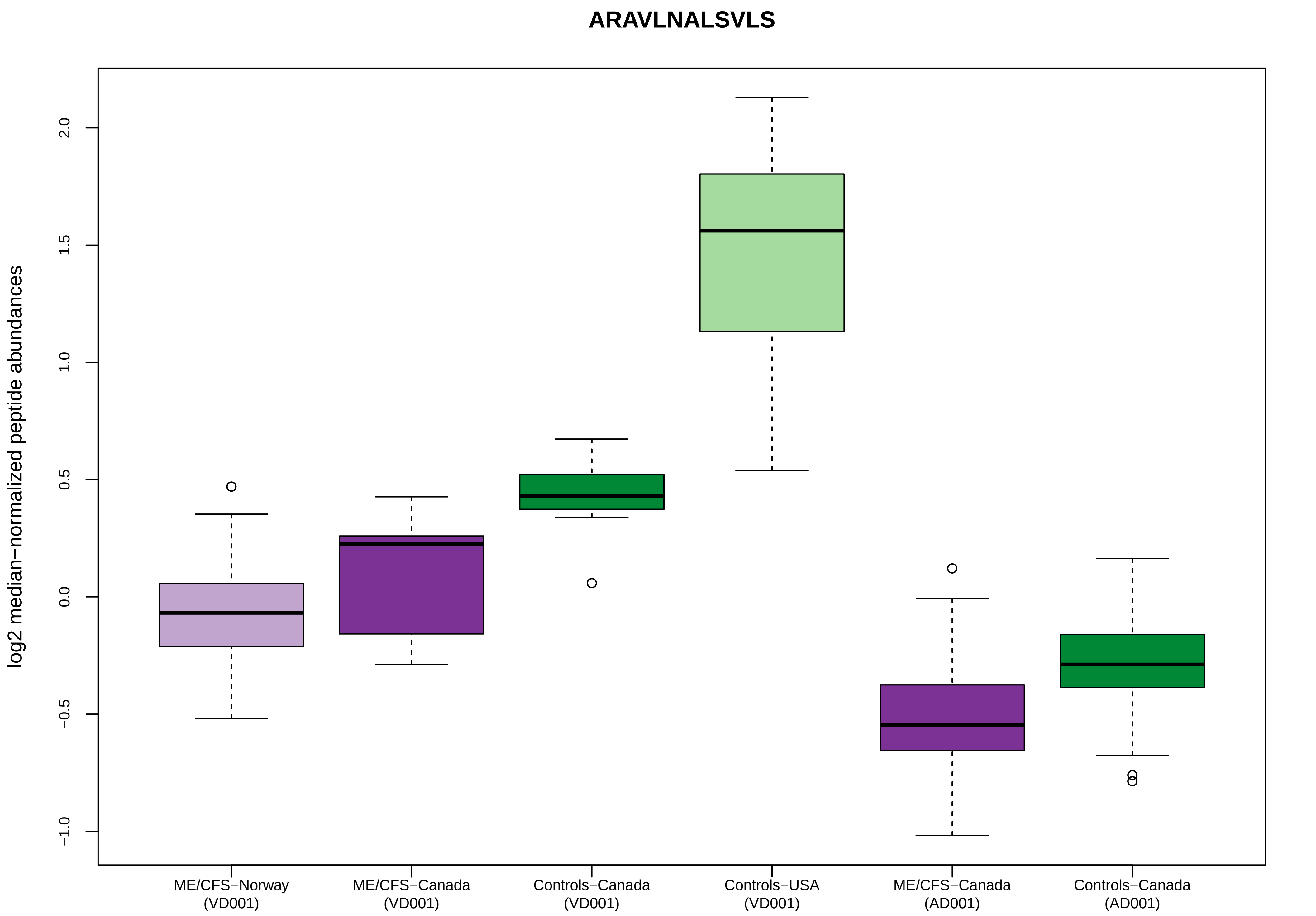
ARAVLNALSVLS
log2 median−normalized peptide abundances
2.0
1.5
1.0
0.5
0.0
−0.5
−1.0
ME/CFS−Norway (VD001)
ME/CFS−Canada (VD001)
Controls−Canada (VD001)
Controls−USA (VD001)
ME/CFS−Canada (AD001)
Controls−Canada (AD001)

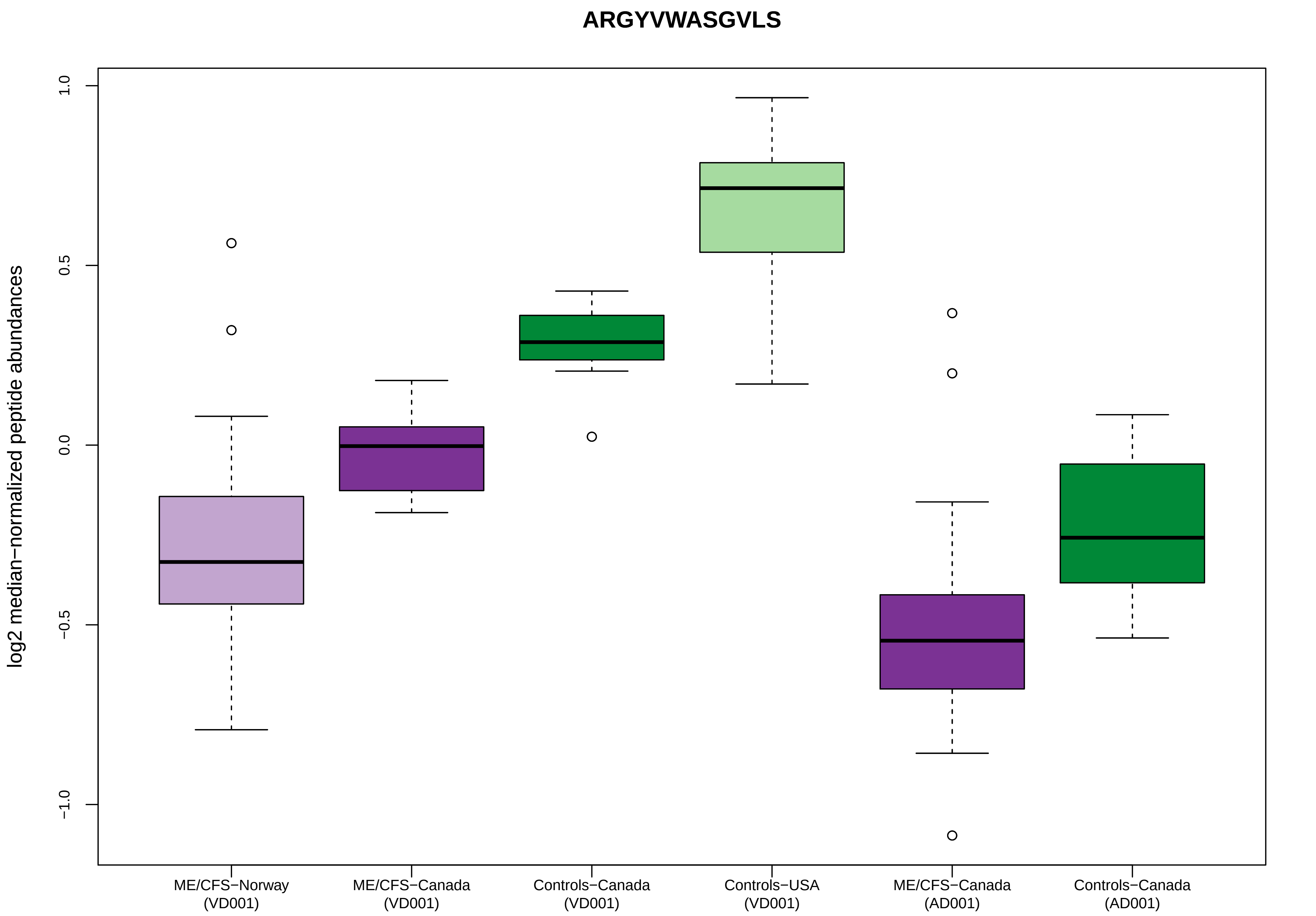
ARGYVWASGVLS
log2 median−normalized peptide abundances
1.0
0.5
0.0
−0.5
−1.0
ME/CFS−Norway (VD001)
ME/CFS−Canada (VD001)
Controls−Canada (VD001)
Controls−USA (VD001)
ME/CFS−Canada (AD001)
Controls−Canada (AD001)

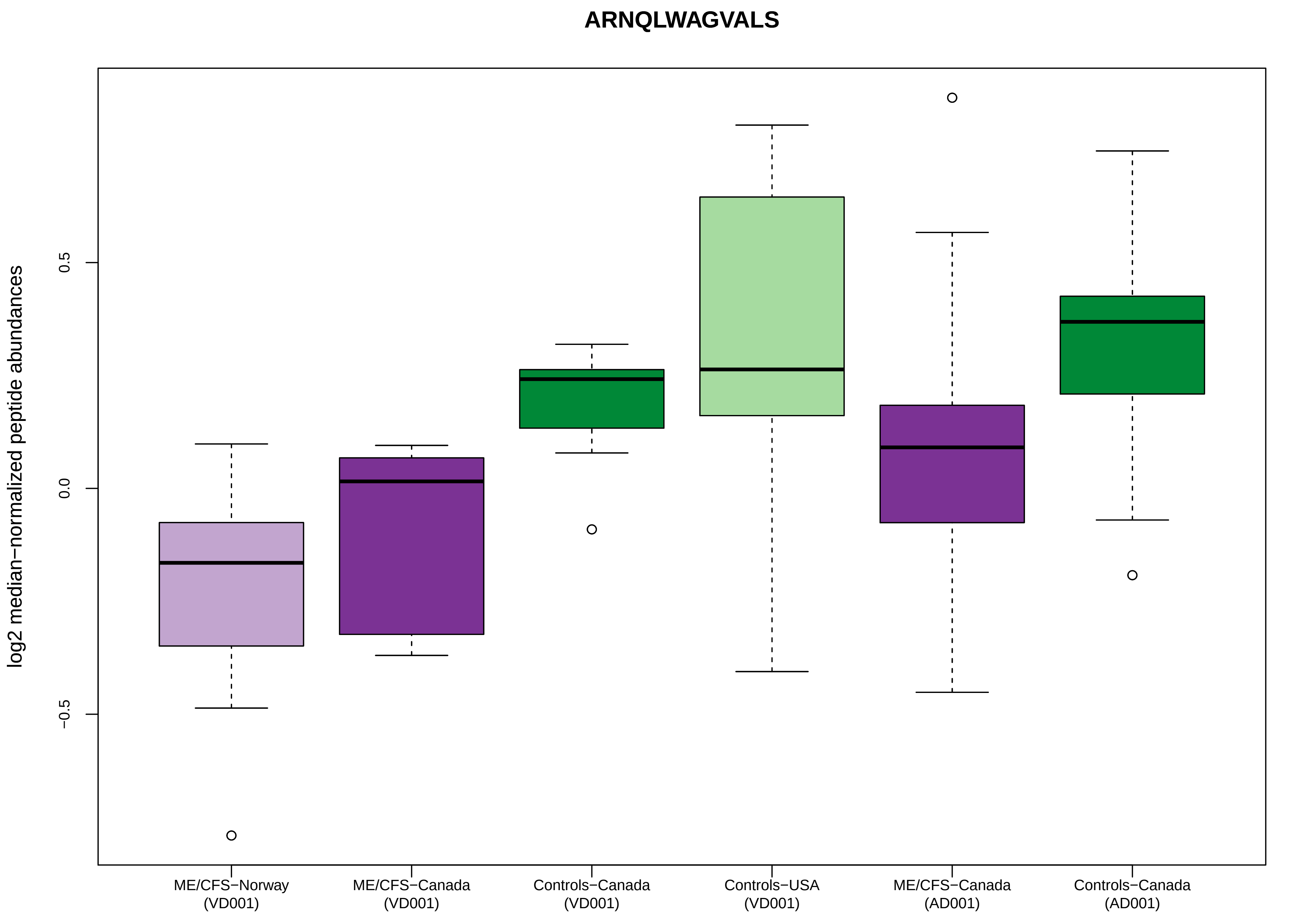
ARNQLWAGVALS
log2 median−normalized peptide abundances
0.5
0.0
−0.5
ME/CFS−Norway (VD001)
ME/CFS−Canada (VD001)
Controls−Canada (VD001)
Controls−USA (VD001)
ME/CFS−Canada (AD001)
Controls−Canada (AD001)

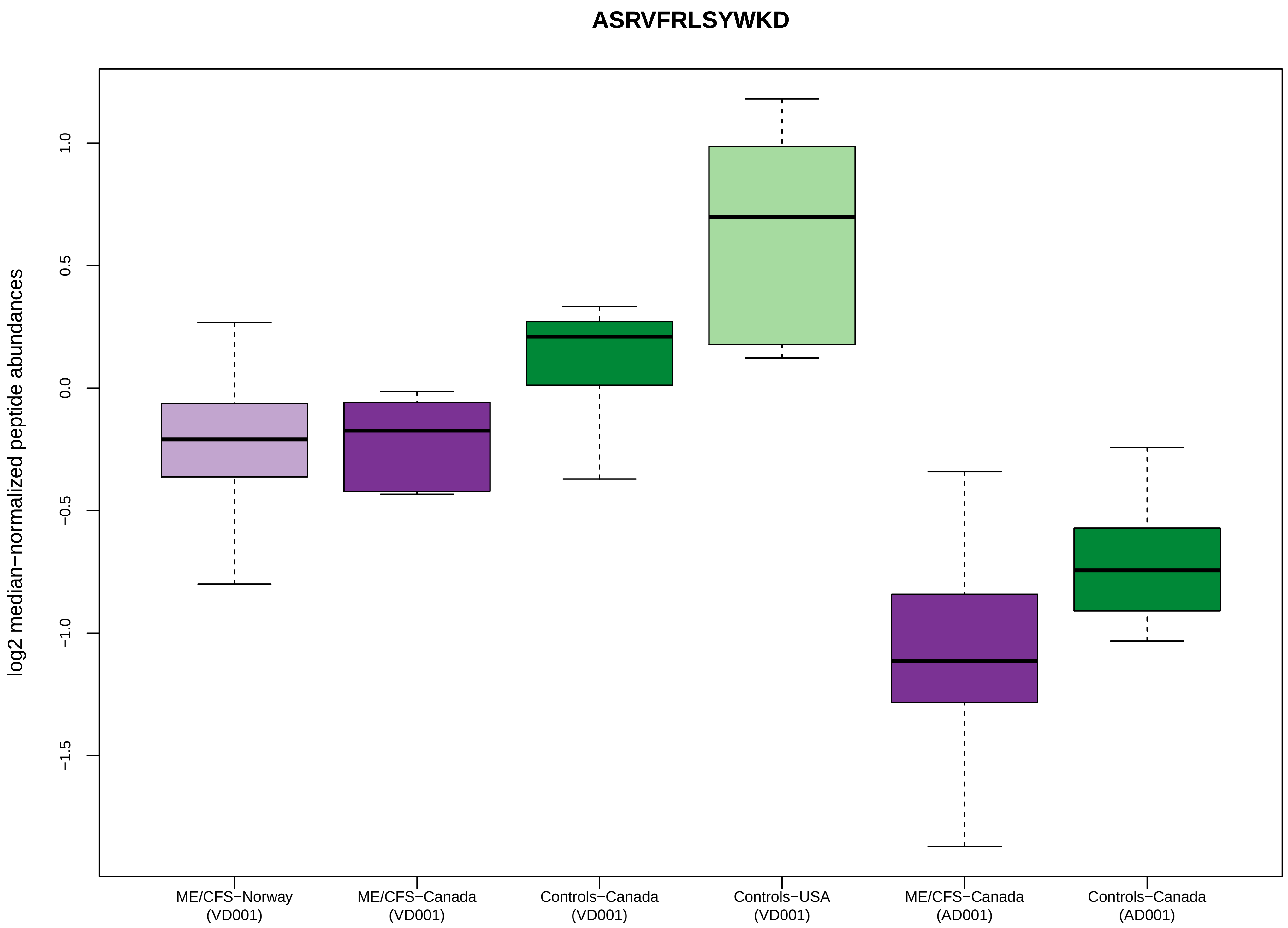
ASRVFRLSYWKD
log2 median−normalized peptide abundances
1.0
0.5
0.0
−0.5
−1.0
−1.5
ME/CFS−Norway (VD001)
ME/CFS−Canada (VD001)
Controls−Canada (VD001)
Controls−USA (VD001)
ME/CFS−Canada (AD001)
Controls−Canada (AD001)

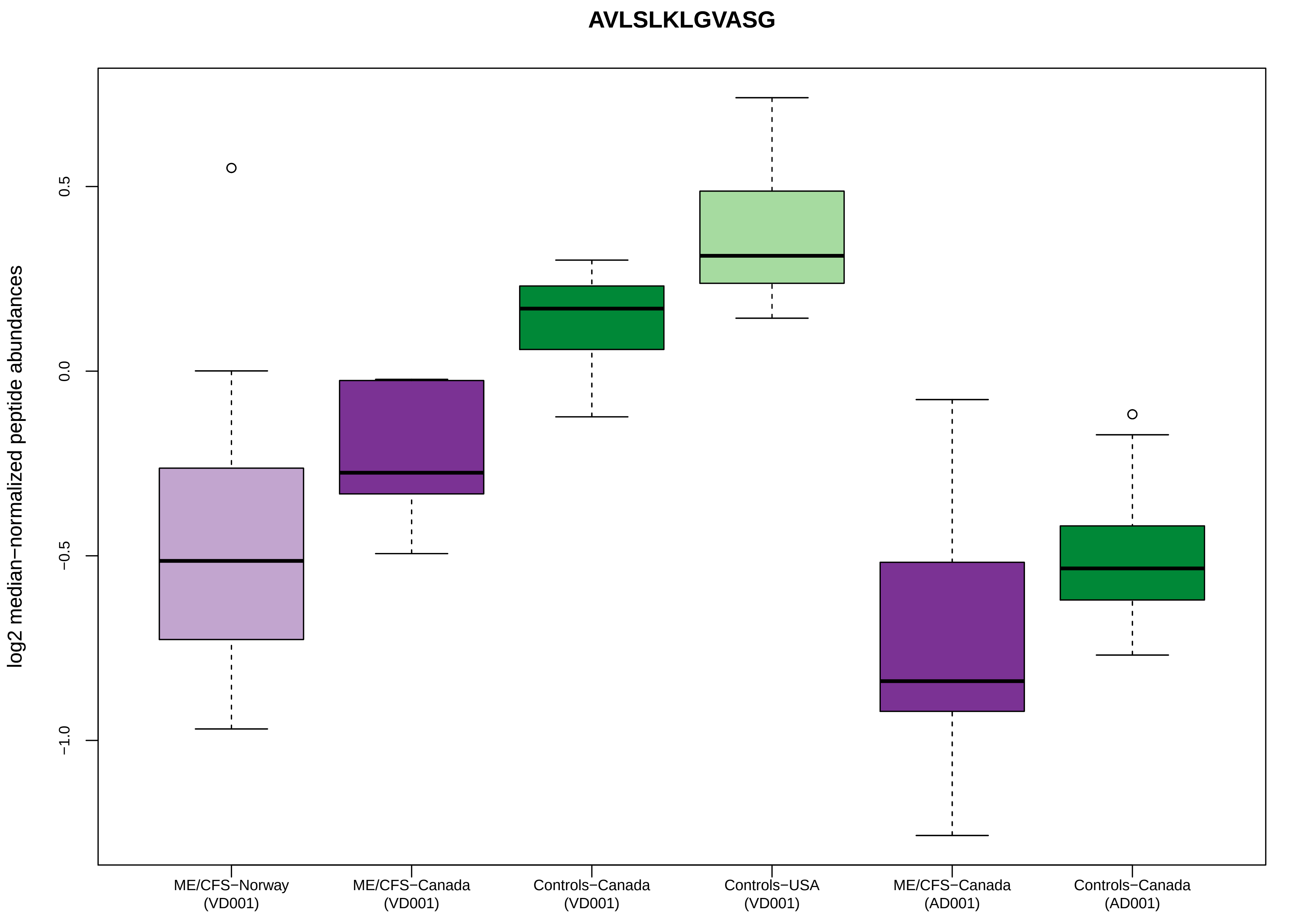
AVLSLKLGVASG
log2 median−normalized peptide abundances
0.5
0.0
−0.5
−1.0
ME/CFS−Norway (VD001)
ME/CFS−Canada (VD001)
Controls−Canada (VD001)
Controls−USA (VD001)
ME/CFS−Canada (AD001)
Controls−Canada (AD001)

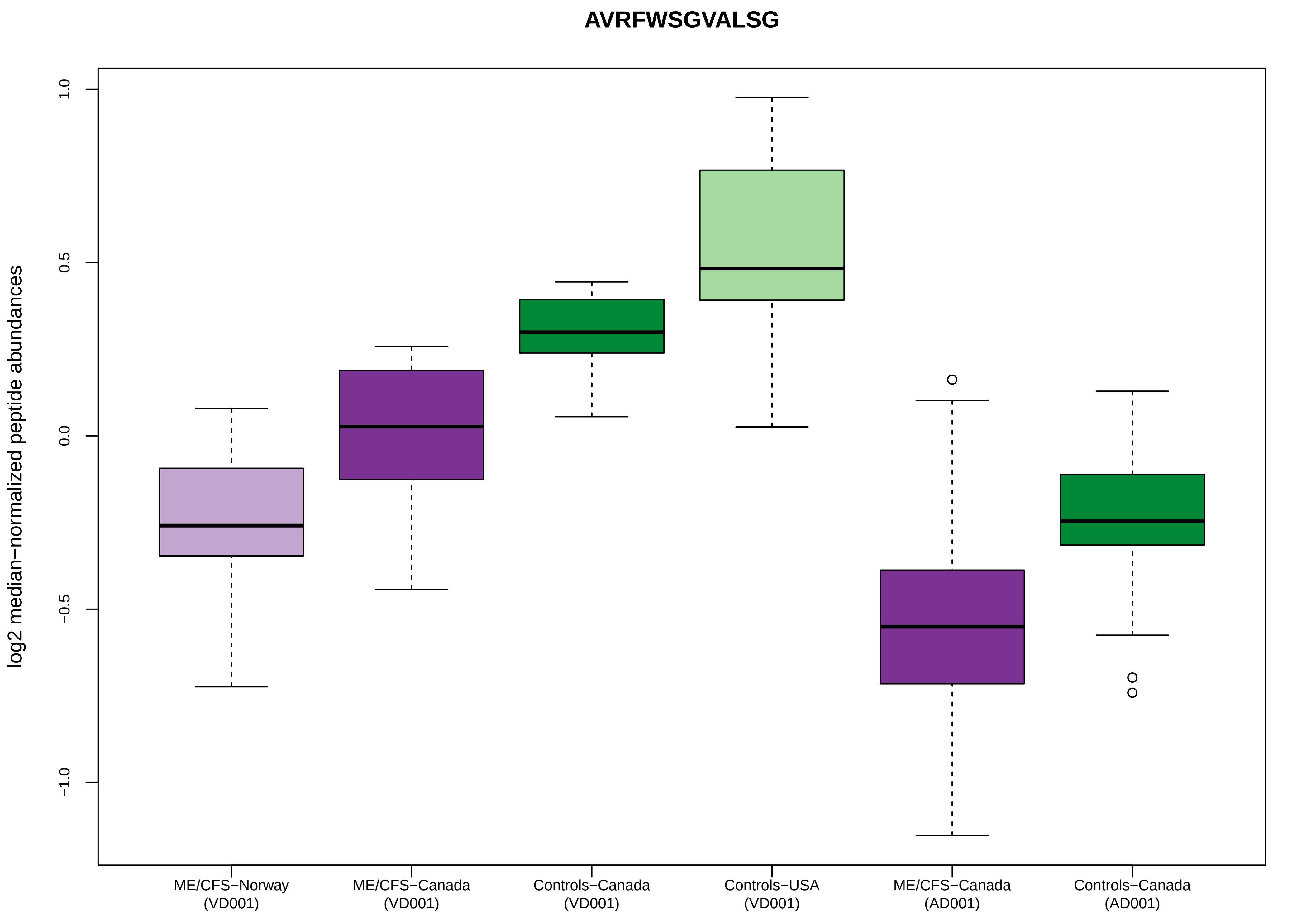

AVRFWSGVALSG
log2 median−normalized peptide abundances
1.0
0.5
0.0
−0.5
−1.0
ME/CFS−Norway
(VD001)
ME/CFS−Canada
(VD001)
Controls−Canada
(VD001)
Controls−USA
(VD001)
ME/CFS−Canada
(AD001)
Controls−Canada
(AD001)

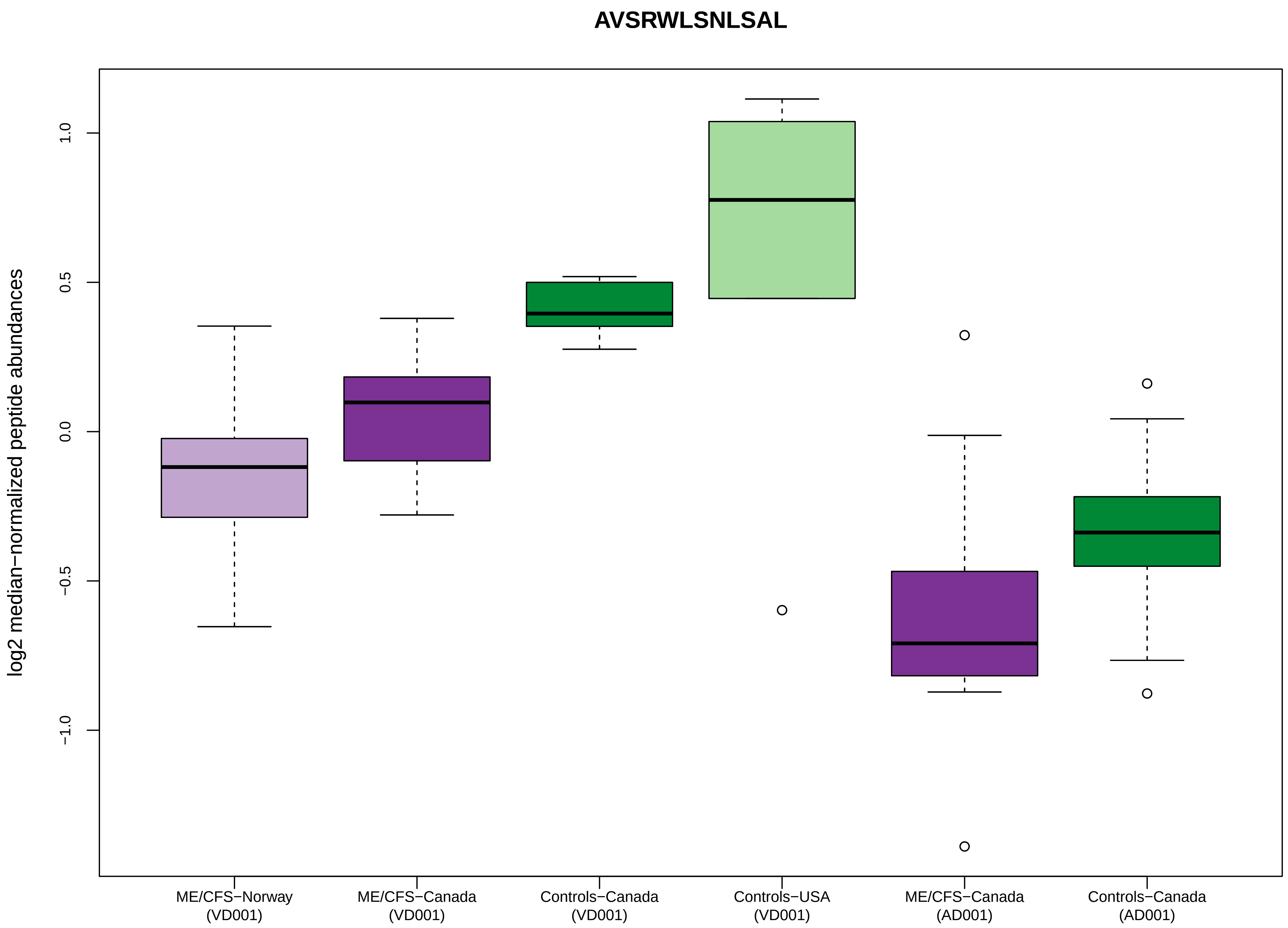

AVSRWLSNLSAL
log2 median−normalized peptide abundances
1.0
0.5
0.0
−0.5
−1.0
ME/CFS−Norway (VD001)
ME/CFS−Canada (VD001)
Controls−Canada (VD001)
Controls−USA (VD001)
ME/CFS−Canada (AD001)
Controls−Canada (AD001)

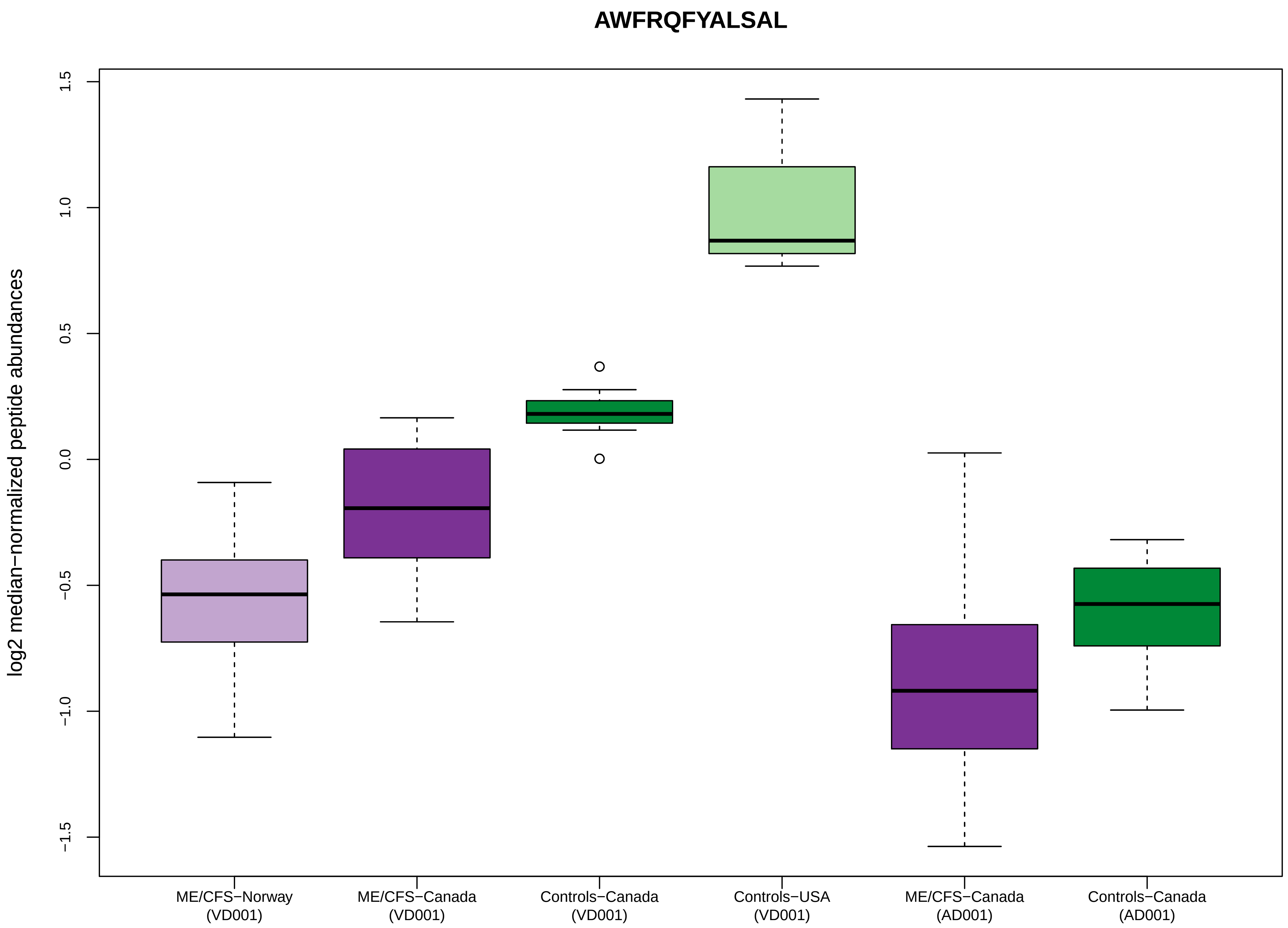

AWFRQFYALSAL
log2 median−normalized peptide abundances
1.5
1.0
0.5
0.0
−0.5
−1.0
−1.5
ME/CFS−Norway (VD001)
ME/CFS−Canada (VD001)
Controls−Canada (VD001)
Controls−USA (VD001)
ME/CFS−Canada (AD001)
Controls−Canada (AD001)

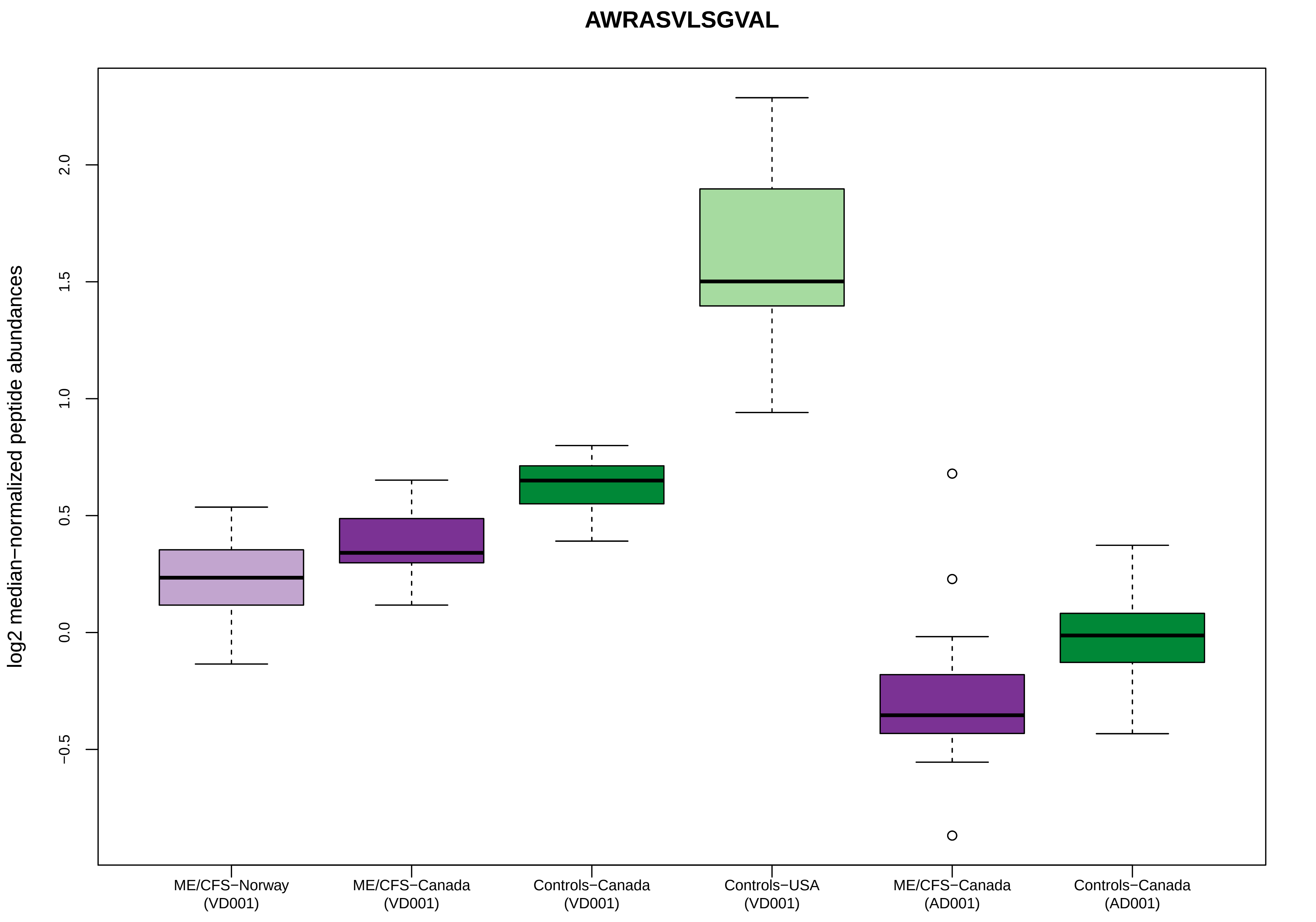
AWRASVLSGVAL
log2 median−normalized peptide abundances
−0.5
0.0
0.5
1.0
1.5
2.0
ME/CFS−Norway (VD001)
ME/CFS−Canada (VD001)
Controls−Canada (VD001)
Controls−USA (VD001)
ME/CFS−Canada (AD001)
Controls−Canada (AD001)

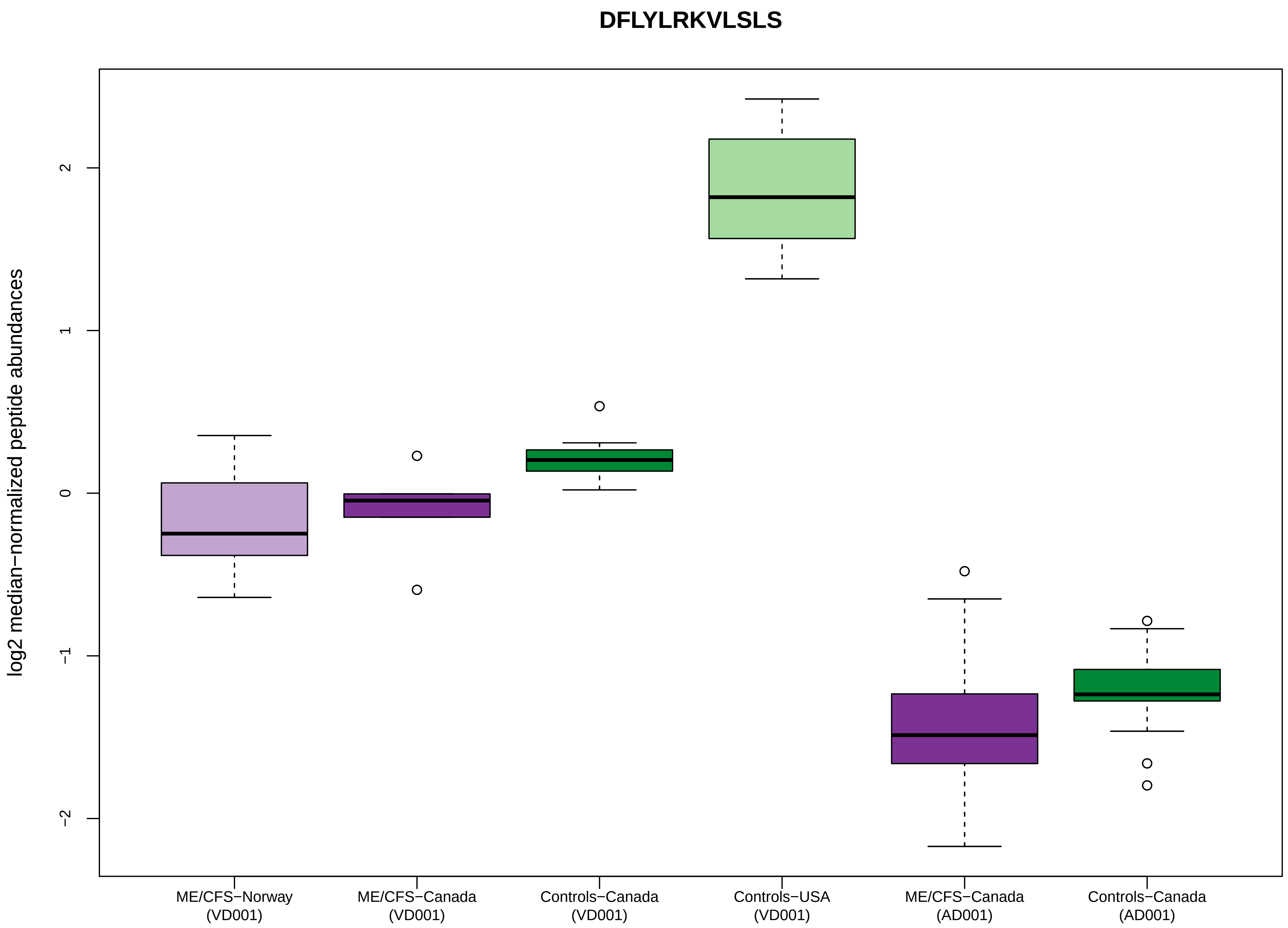
DFLYLRKVLSLS
log2 median−normalized peptide abundances
2
1
0
−1
−2
ME/CFS−Norway (VD001)
ME/CFS−Canada (VD001)
Controls−Canada (VD001)
Controls−USA (VD001)
ME/CFS−Canada (AD001)
Controls−Canada (AD001)

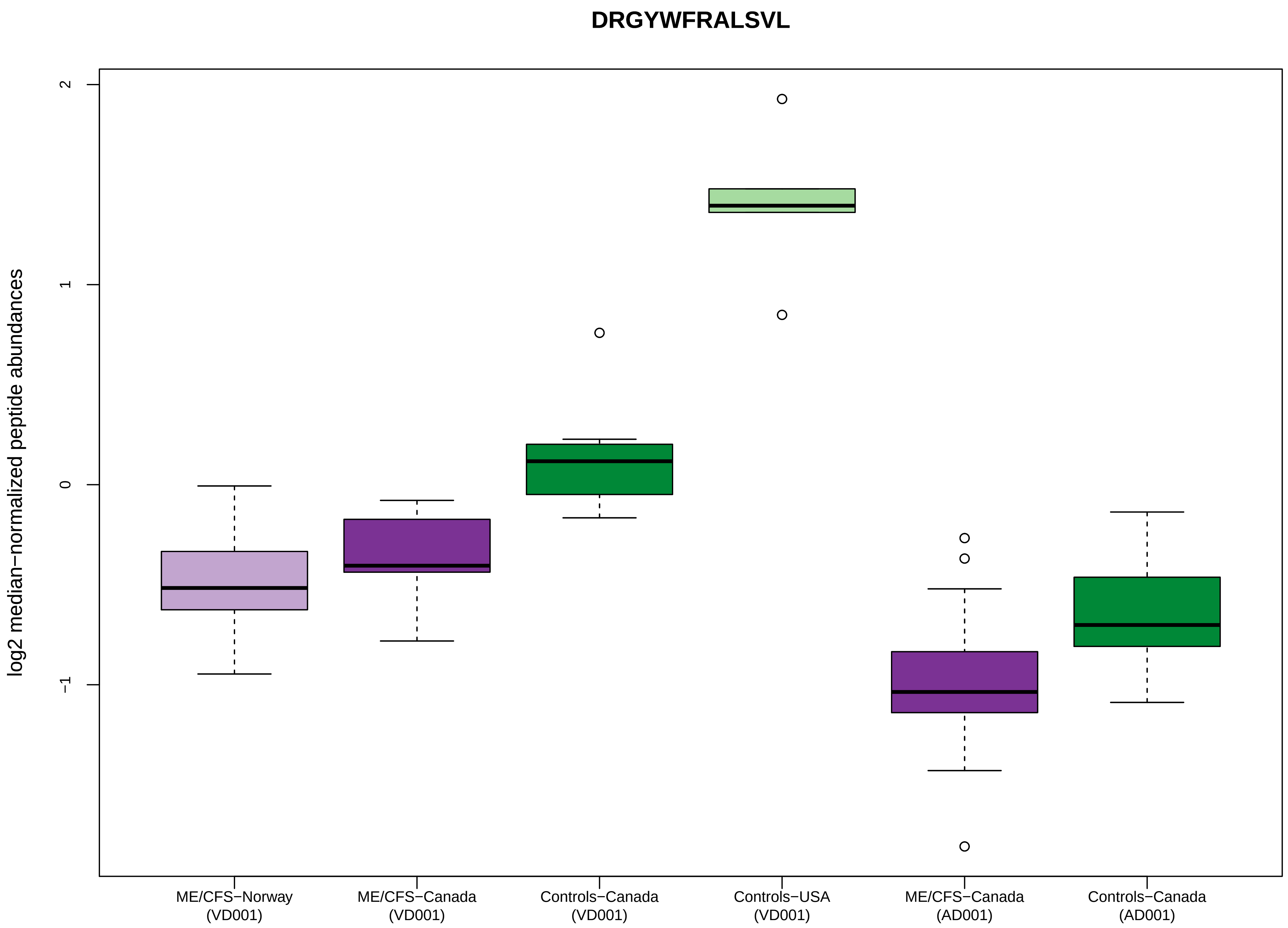
DRGYWFRALSVL
log2 median−normalized peptide abundances
2
1
0
−1
ME/CFS−Norway (VD001)
ME/CFS−Canada (VD001)
Controls−Canada (VD001)
Controls−USA (VD001)
ME/CFS−Canada (AD001)
Controls−Canada (AD001)

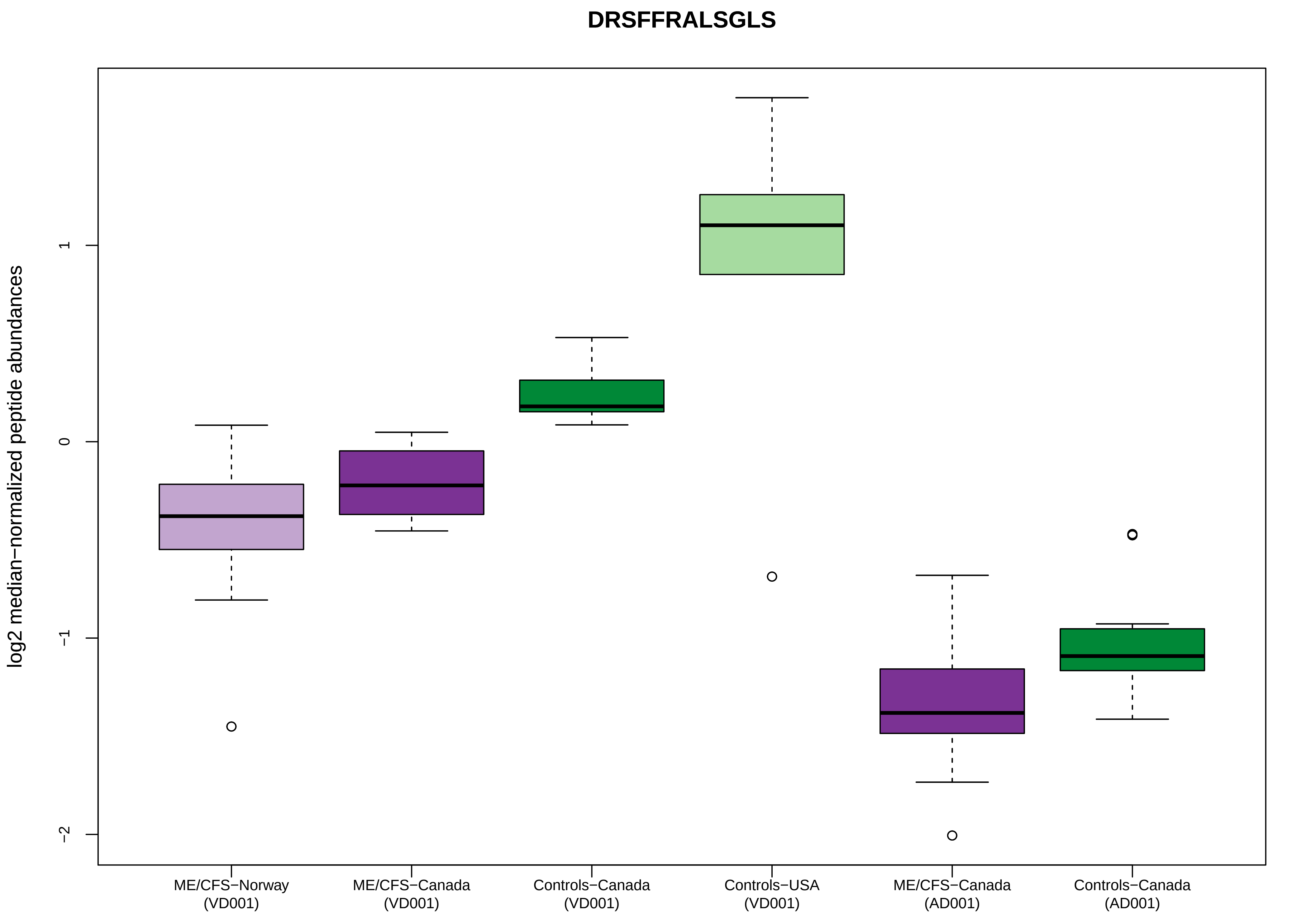
DRSFFRALSGLS
log2 median−normalized peptide abundances
1
0
−1
−2
ME/CFS−Norway (VD001)
ME/CFS−Canada (VD001)
Controls−Canada (VD001)
Controls−USA (VD001)
ME/CFS−Canada (AD001)
Controls−Canada (AD001)

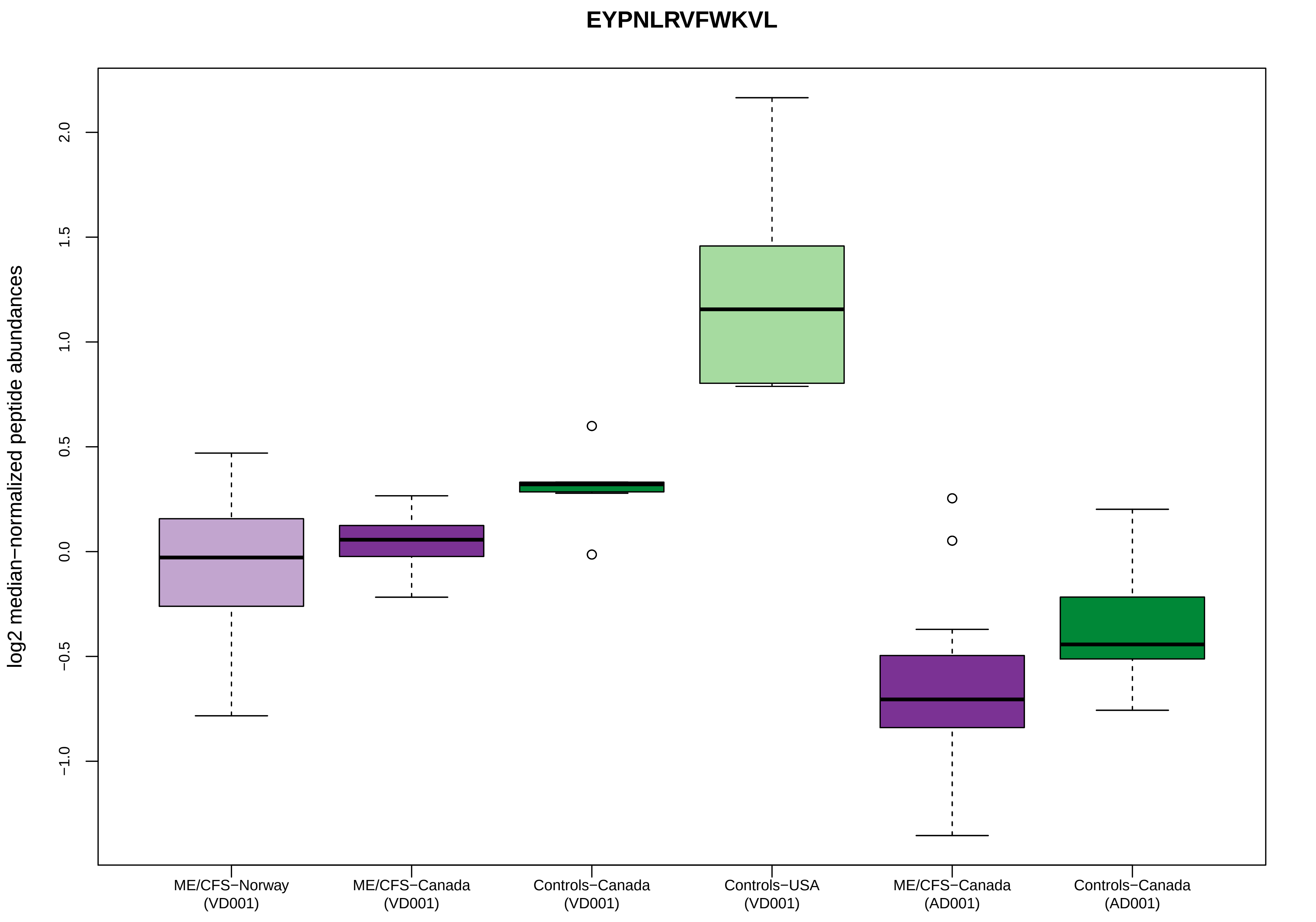
EYPNLRVFWKVL
log2 median−normalized peptide abundances
2.0
1.5
1.0
0.5
0.0
−0.5
−1.0
ME/CFS−Norway (VD001)
ME/CFS−Canada (VD001)
Controls−Canada (VD001)
Controls−USA (VD001)
ME/CFS−Canada (AD001)
Controls−Canada (AD001)

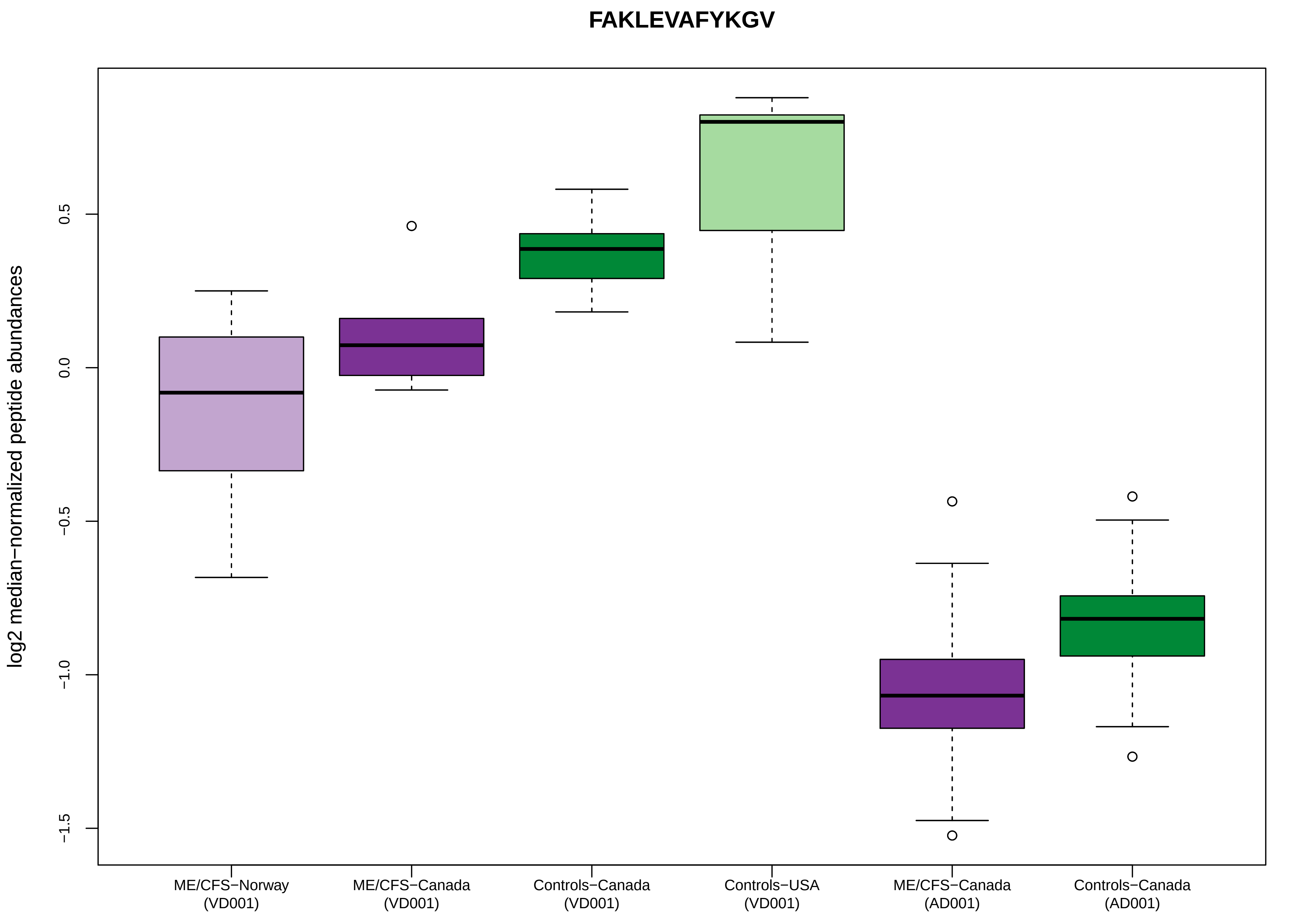
FAKLEVAFYKGV
log2 median−normalized peptide abundances
0.5
0.0
−0.5
−1.0
−1.5
ME/CFS−Norway (VD001)
ME/CFS−Canada (VD001)
Controls−Canada (VD001)
Controls−USA (VD001)
ME/CFS−Canada (AD001)
Controls−Canada (AD001)

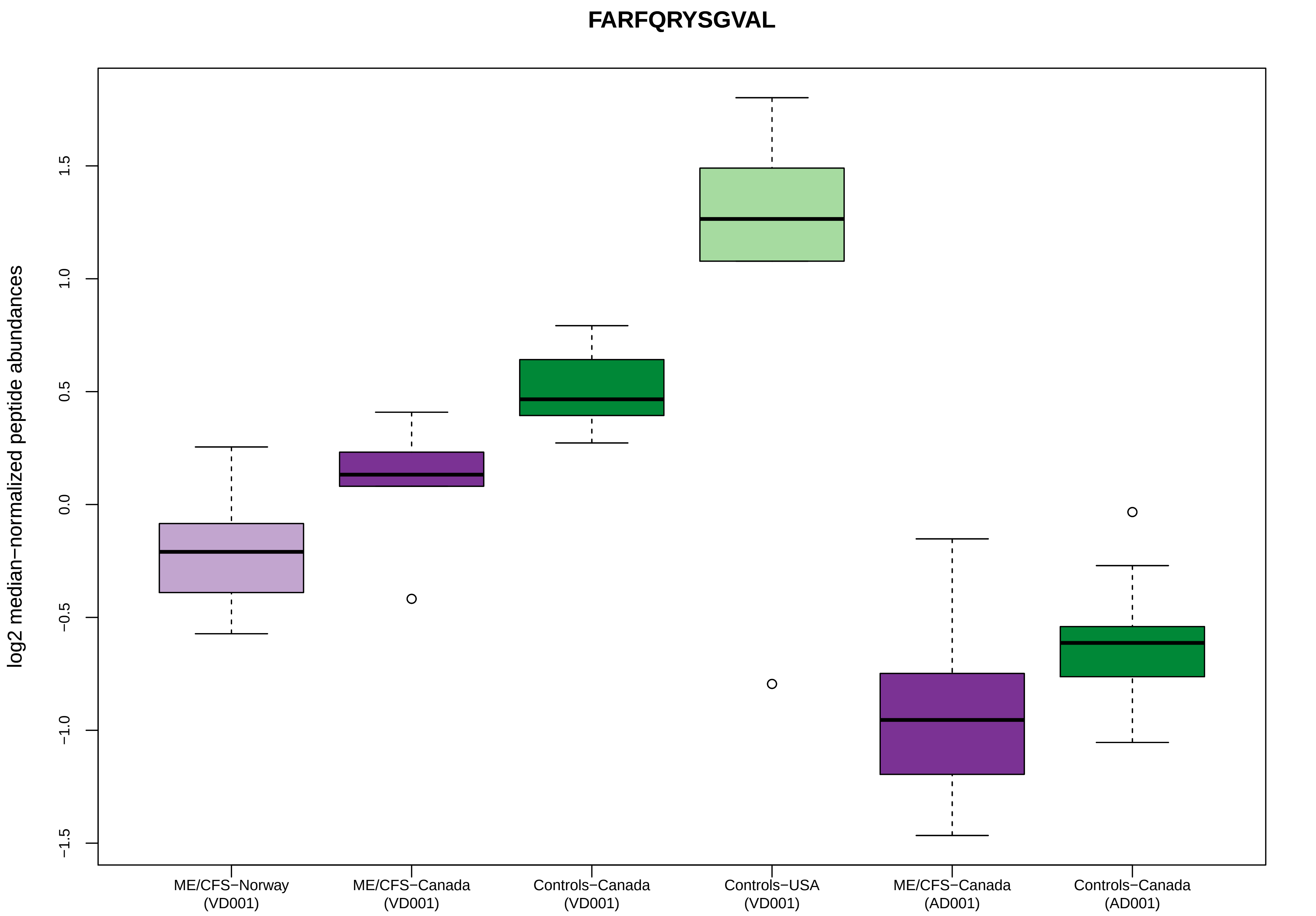
FARFQRYSGVAL
log2 median−normalized peptide abundances
1.5
1.0
0.5
0.0
−0.5
−1.0
−1.5
ME/CFS−Norway (VD001)
ME/CFS−Canada (VD001)
Controls−Canada (VD001)
Controls−USA (VD001)
ME/CFS−Canada (AD001)
Controls−Canada (AD001)

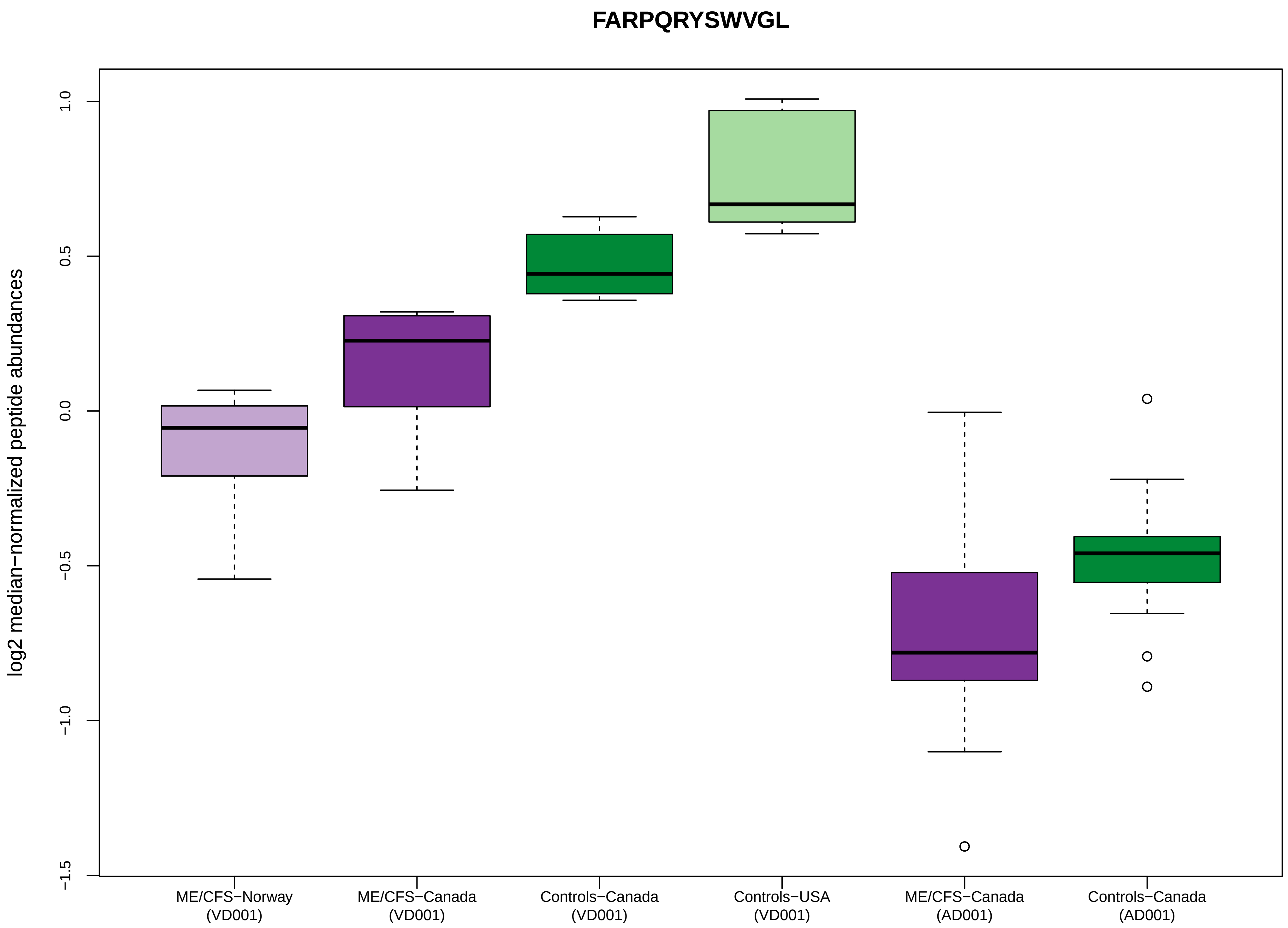

FARPQRYSWVGL
log2 median−normalized peptide abundances
1.0
0.5
0.0
−0.5
−1.0
−1.5
ME/CFS−Norway (VD001)
ME/CFS−Canada (VD001)
Controls−Canada (VD001)
Controls−USA (VD001)
ME/CFS−Canada (AD001)
Controls−Canada (AD001)

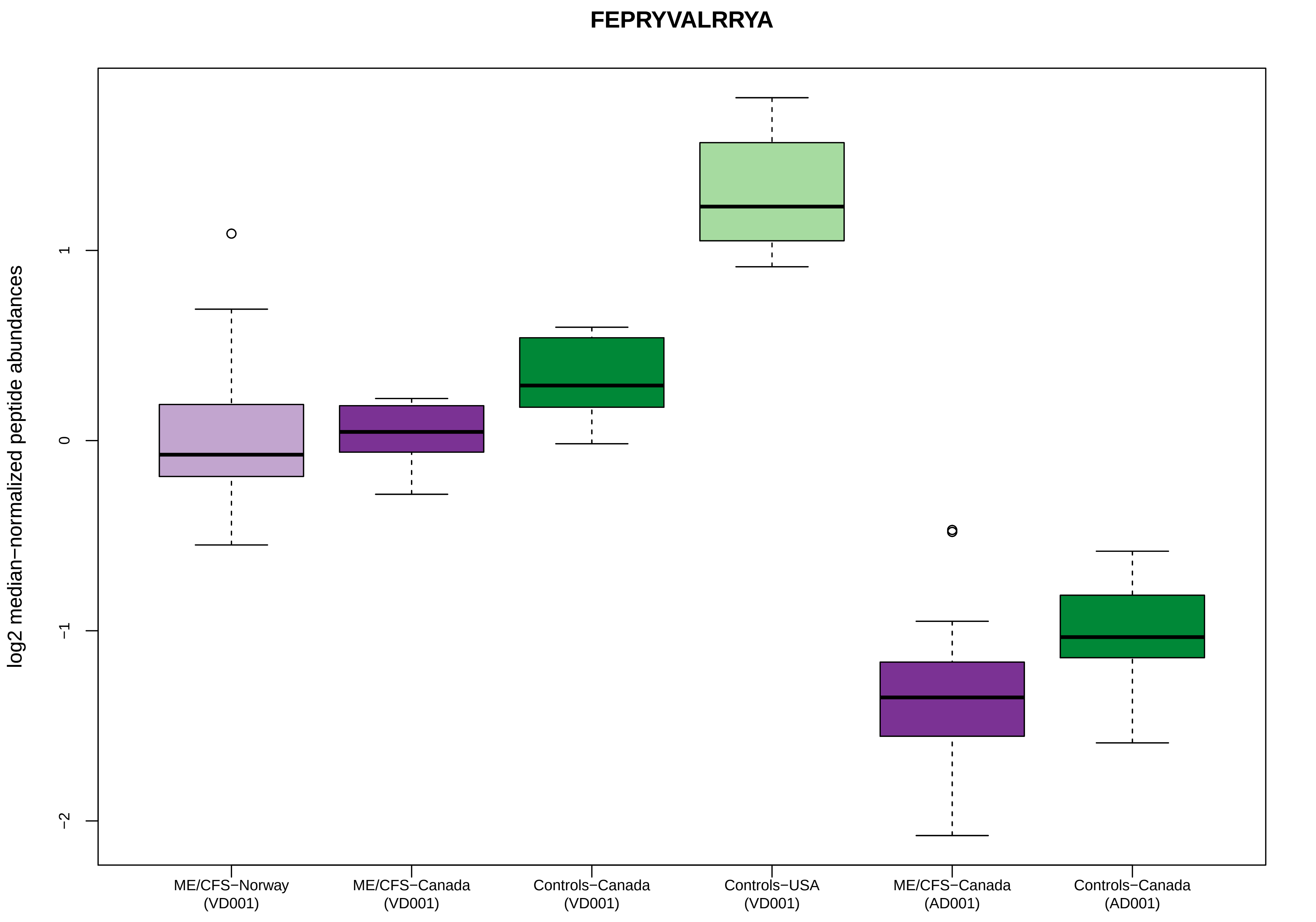

FEPRYVALRRYA
log2 median−normalized peptide abundances
1
0
−1
−2
ME/CFS−Norway (VD001)
ME/CFS−Canada (VD001)
Controls−Canada (VD001)
Controls−USA (VD001)
ME/CFS−Canada (AD001)
Controls−Canada (AD001)

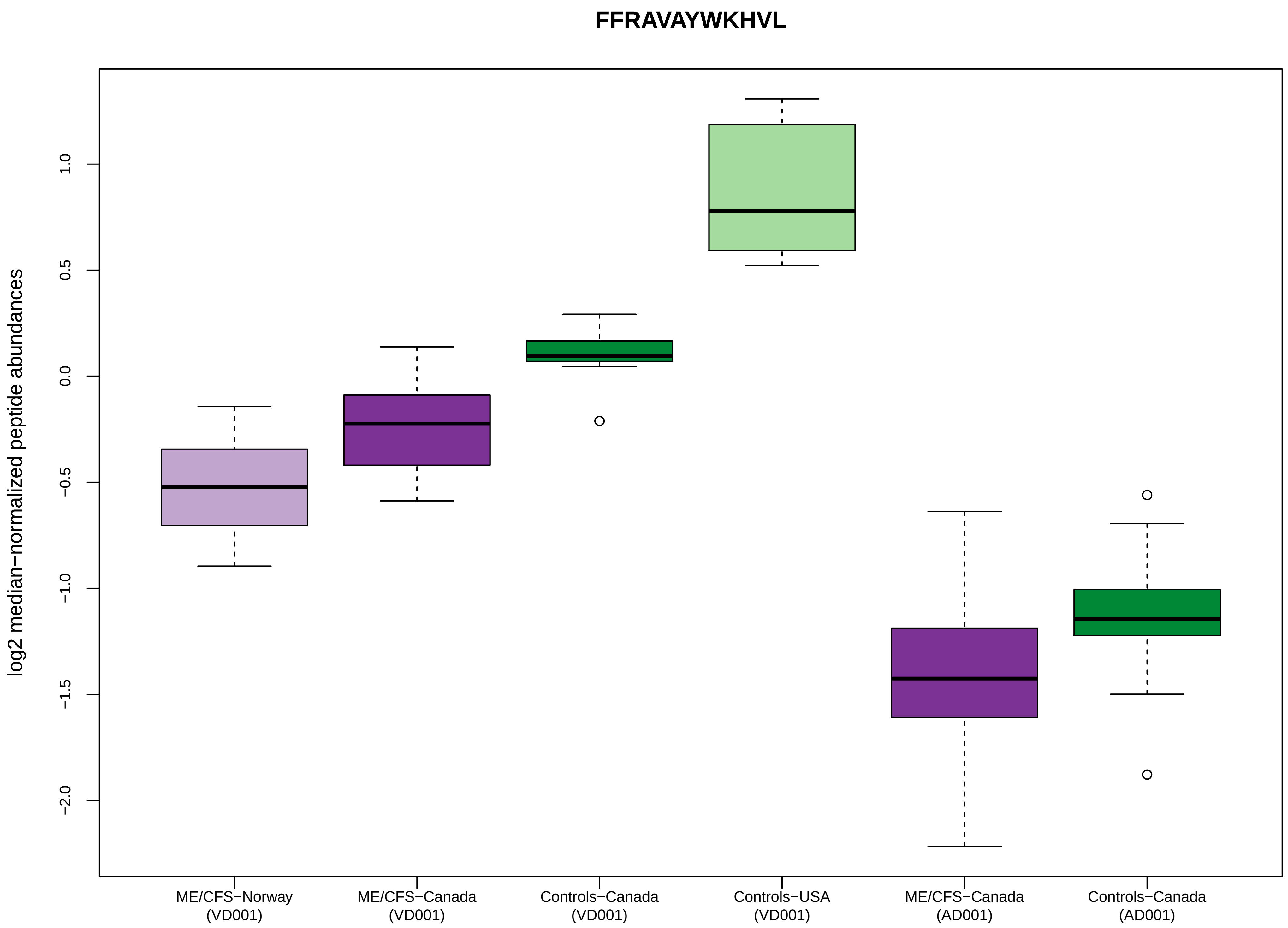
FFRAVAYWKHVL
log2 median−normalized peptide abundances
1.0
0.5
0.0
−0.5
−1.0
−1.5
−2.0
ME/CFS−Norway (VD001)
ME/CFS−Canada (VD001)
Controls−Canada (VD001)
Controls−USA (VD001)
ME/CFS−Canada (AD001)
Controls−Canada (AD001)

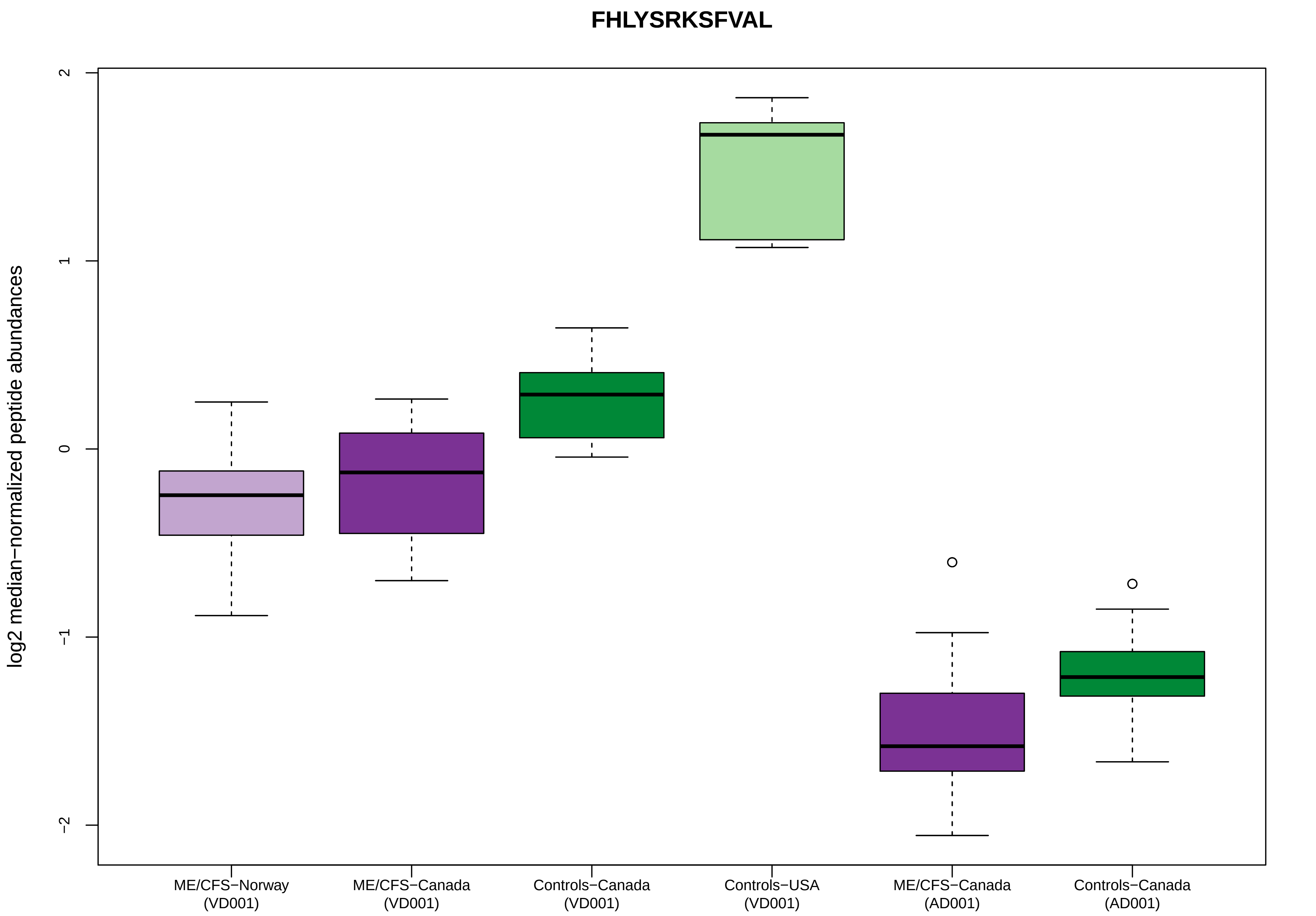

FHLYSRKSFVAL
log2 median−normalized peptide abundances
2
1
0
−1
−2
ME/CFS−Norway (VD001)
ME/CFS−Canada (VD001)
Controls−Canada (VD001)
Controls−USA (VD001)
ME/CFS−Canada (AD001)
Controls−Canada (AD001)

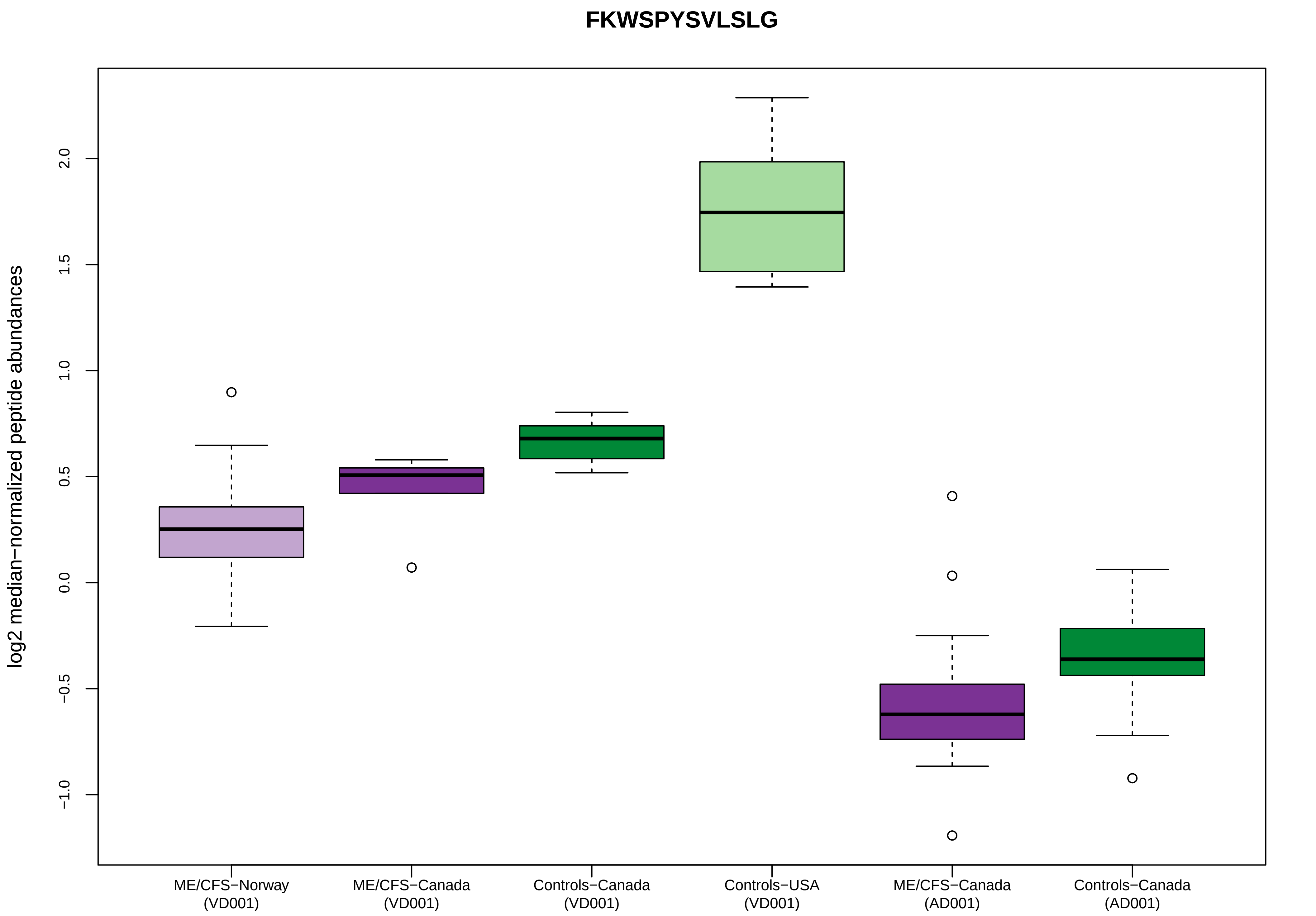
FKWSPYSVLSLG
log2 median−normalized peptide abundances
2.0
1.5
1.0
0.5
0.0
−0.5
−1.0
ME/CFS−Norway (VD001)
ME/CFS−Canada (VD001)
Controls−Canada (VD001)
Controls−USA (VD001)
ME/CFS−Canada (AD001)
Controls−Canada (AD001)

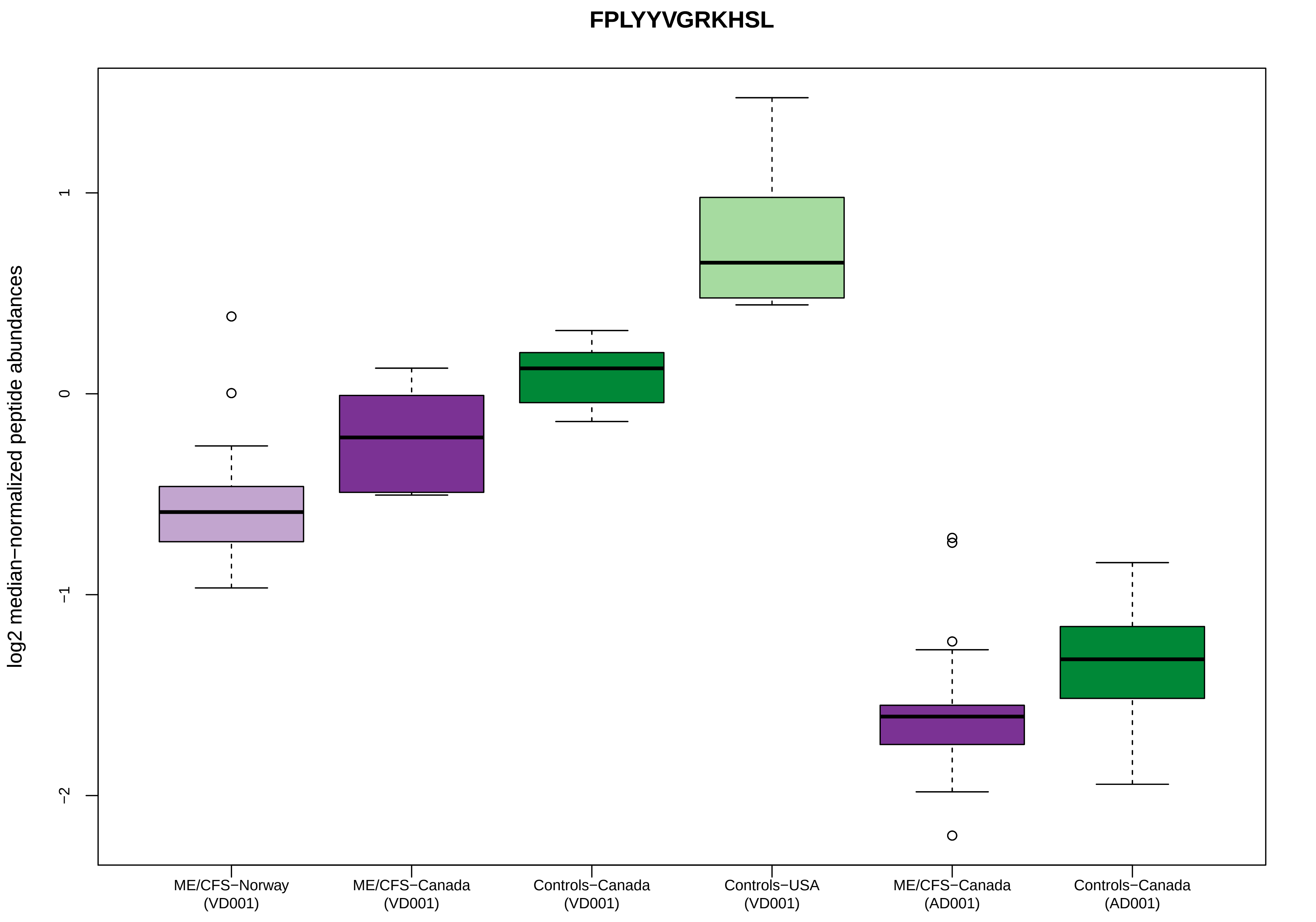
FPLYYVGRKHSL
log2 median−normalized peptide abundances
1
0
−1
−2
ME/CFS−Norway (VD001)
ME/CFS−Canada (VD001)
Controls−Canada (VD001)
Controls−USA (VD001)
ME/CFS−Canada (AD001)
Controls−Canada (AD001)

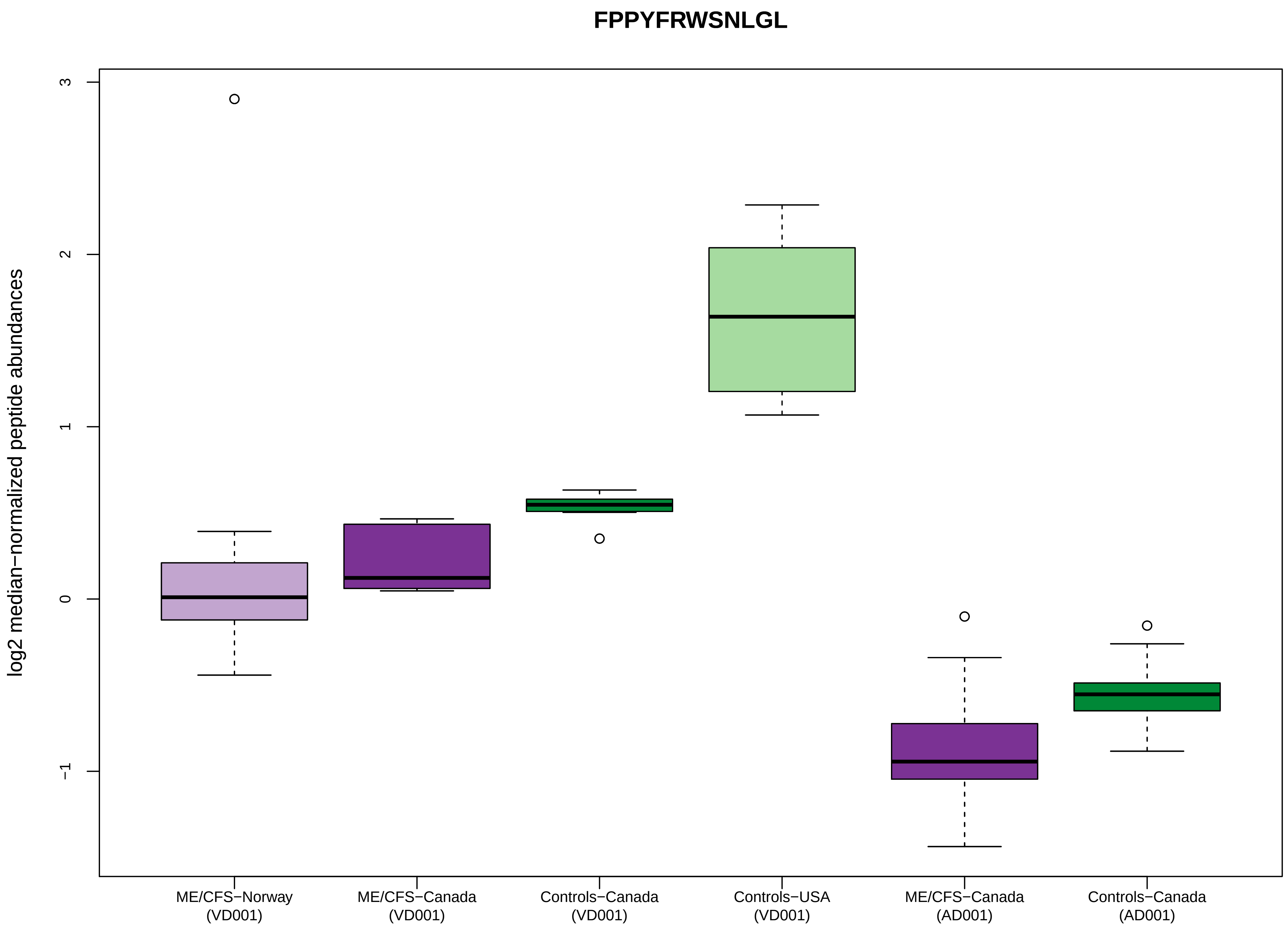
FPPYFRWSNLGL
log2 median−normalized peptide abundances
3
2
1
0
−1
ME/CFS−Norway (VD001)
ME/CFS−Canada (VD001)
Controls−Canada (VD001)
Controls−USA (VD001)
ME/CFS−Canada (AD001)
Controls−Canada (AD001)

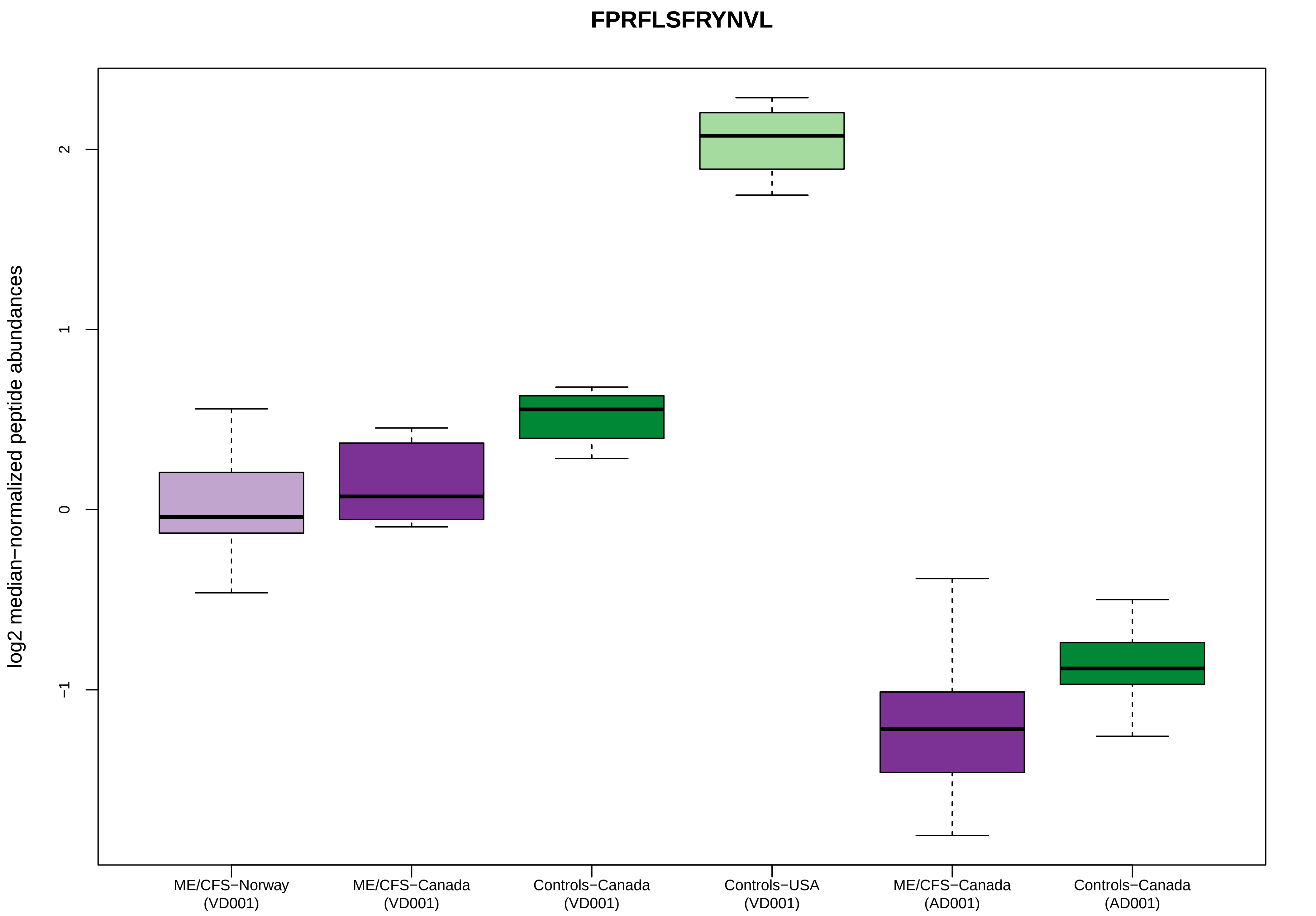

FPRFLSFRYNVL
log2 median−normalized peptide abundances
2
1
0
−1
ME/CFS−Norway (VD001)
ME/CFS−Canada (VD001)
Controls−Canada (VD001)
Controls−USA (VD001)
ME/CFS−Canada (AD001)
Controls−Canada (AD001)

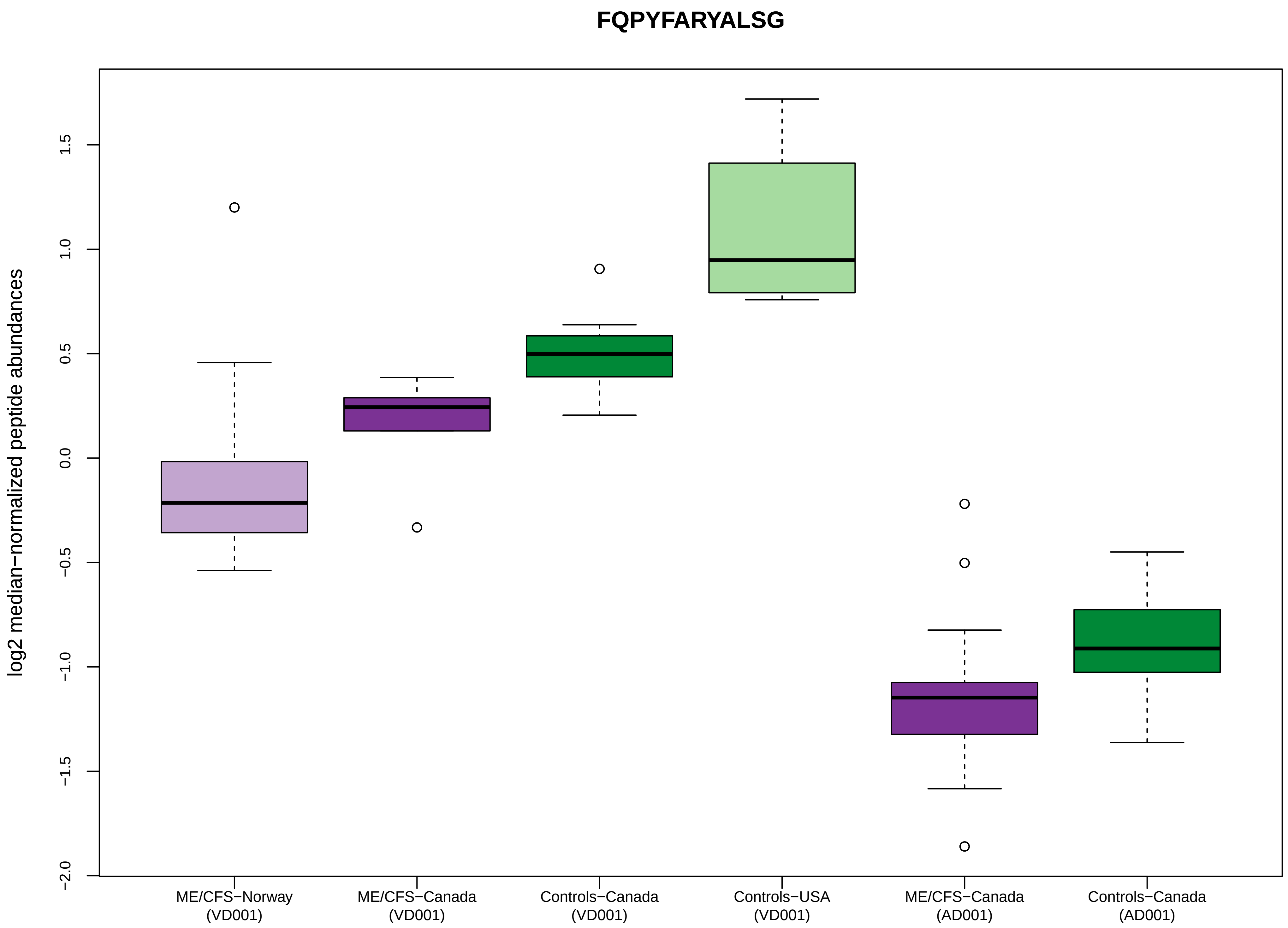

FQPYFARYALSG
log2 median−normalized peptide abundances
1.5
1.0
0.5
0.0
−0.5
−1.0
−1.5
−2.0
ME/CFS−Norway (VD001)
ME/CFS−Canada (VD001)
Controls−Canada (VD001)
Controls−USA (VD001)
ME/CFS−Canada (AD001)
Controls−Canada (AD001)

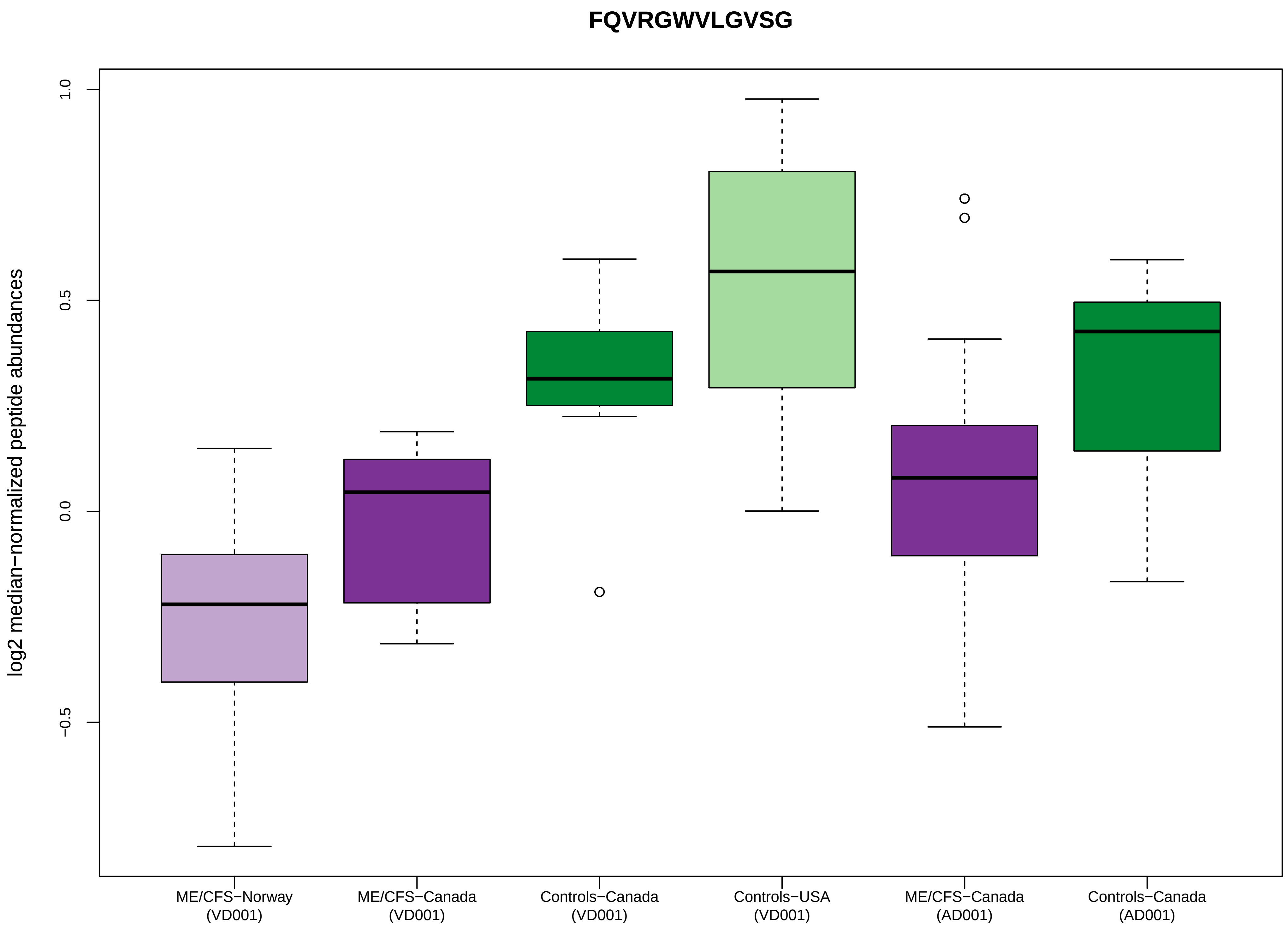

FQVRGWVLGVSG
log2 median−normalized peptide abundances
1.0
0.5
0.0
−0.5
ME/CFS−Norway (VD001)
ME/CFS−Canada (VD001)
Controls−Canada (VD001)
Controls−USA (VD001)
ME/CFS−Canada (AD001)
Controls−Canada (AD001)

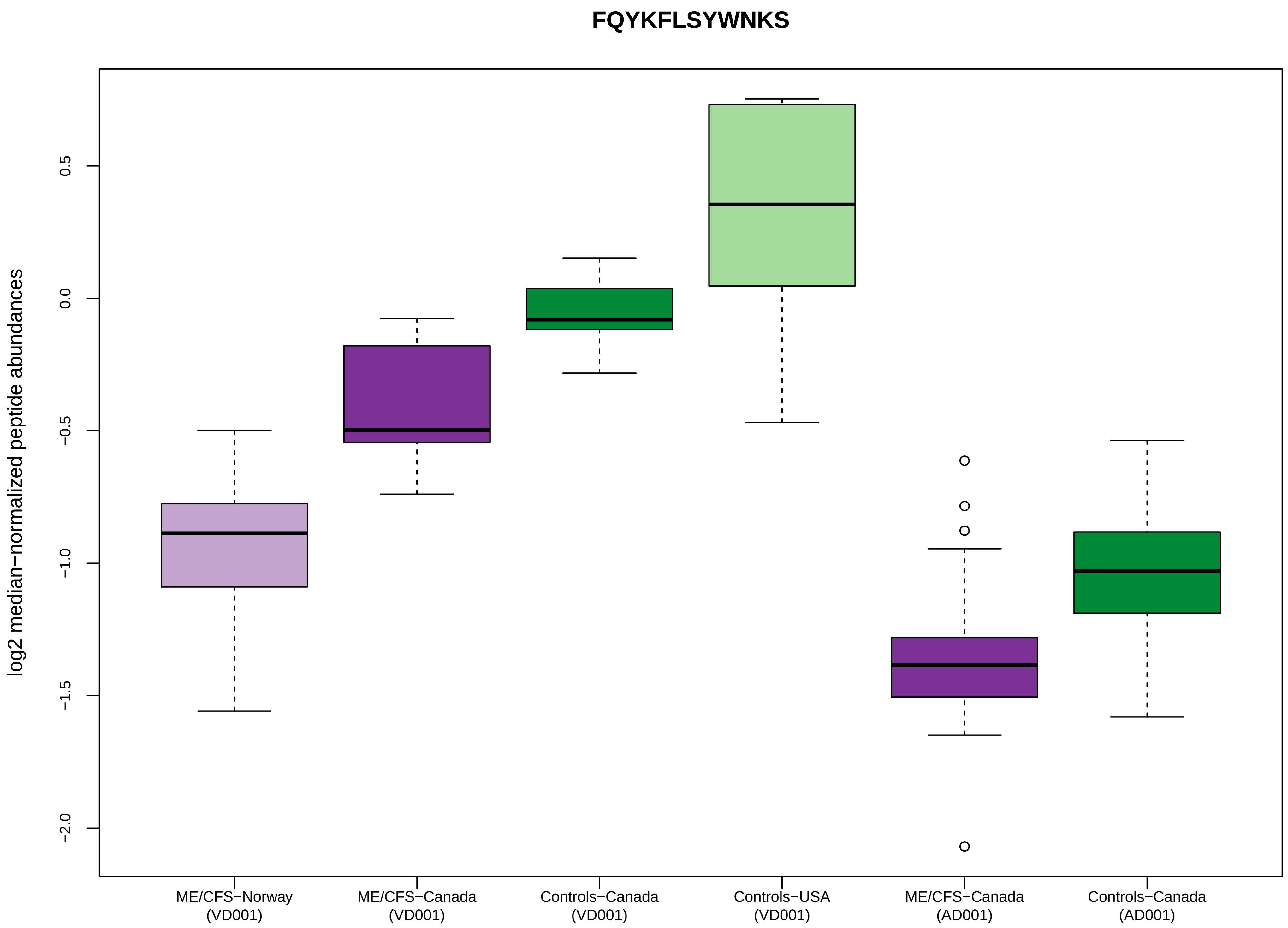
FQYKFLSYWNKS
log2 median−normalized peptide abundances
0.5
0.0
−0.5
−1.0
−1.5
−2.0
ME/CFS−Norway (VD001)
ME/CFS−Canada (VD001)
Controls−Canada (VD001)
Controls−USA (VD001)
ME/CFS−Canada (AD001)
Controls−Canada (AD001)

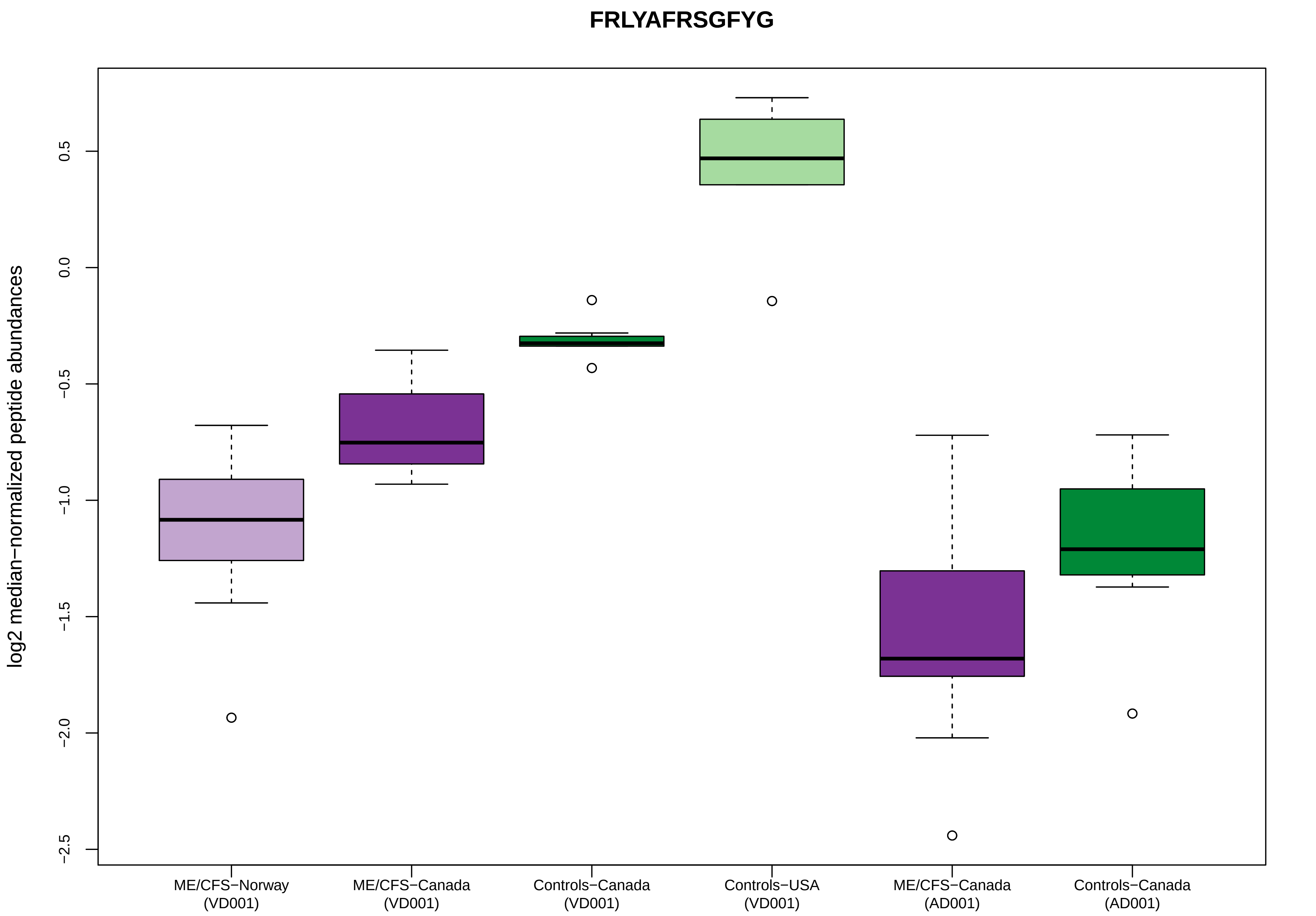

FRLYAFRSGFYG
log2 median−normalized peptide abundances
0.5
0.0
−0.5
−1.0
−1.5
−2.0
−2.5
ME/CFS−Norway (VD001)
ME/CFS−Canada (VD001)
Controls−Canada (VD001)
Controls−USA (VD001)
ME/CFS−Canada (AD001)
Controls−Canada (AD001)

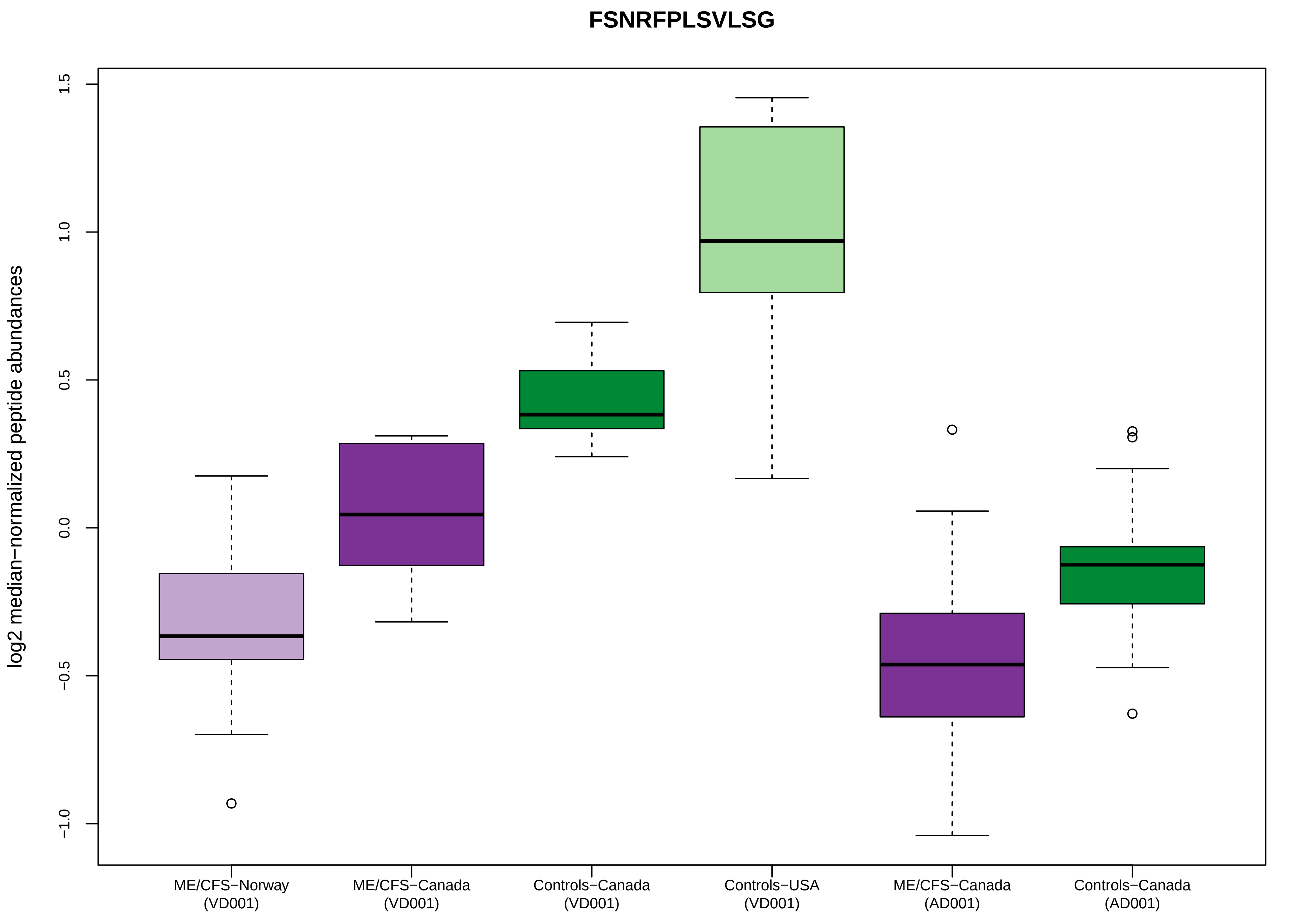
FSNRFPLSVLSG
log2 median−normalized peptide abundances
1.5
1.0
0.5
0.0
−0.5
−1.0
ME/CFS−Norway (VD001)
ME/CFS−Canada (VD001)
Controls−Canada (VD001)
Controls−USA (VD001)
ME/CFS−Canada (AD001)
Controls−Canada (AD001)

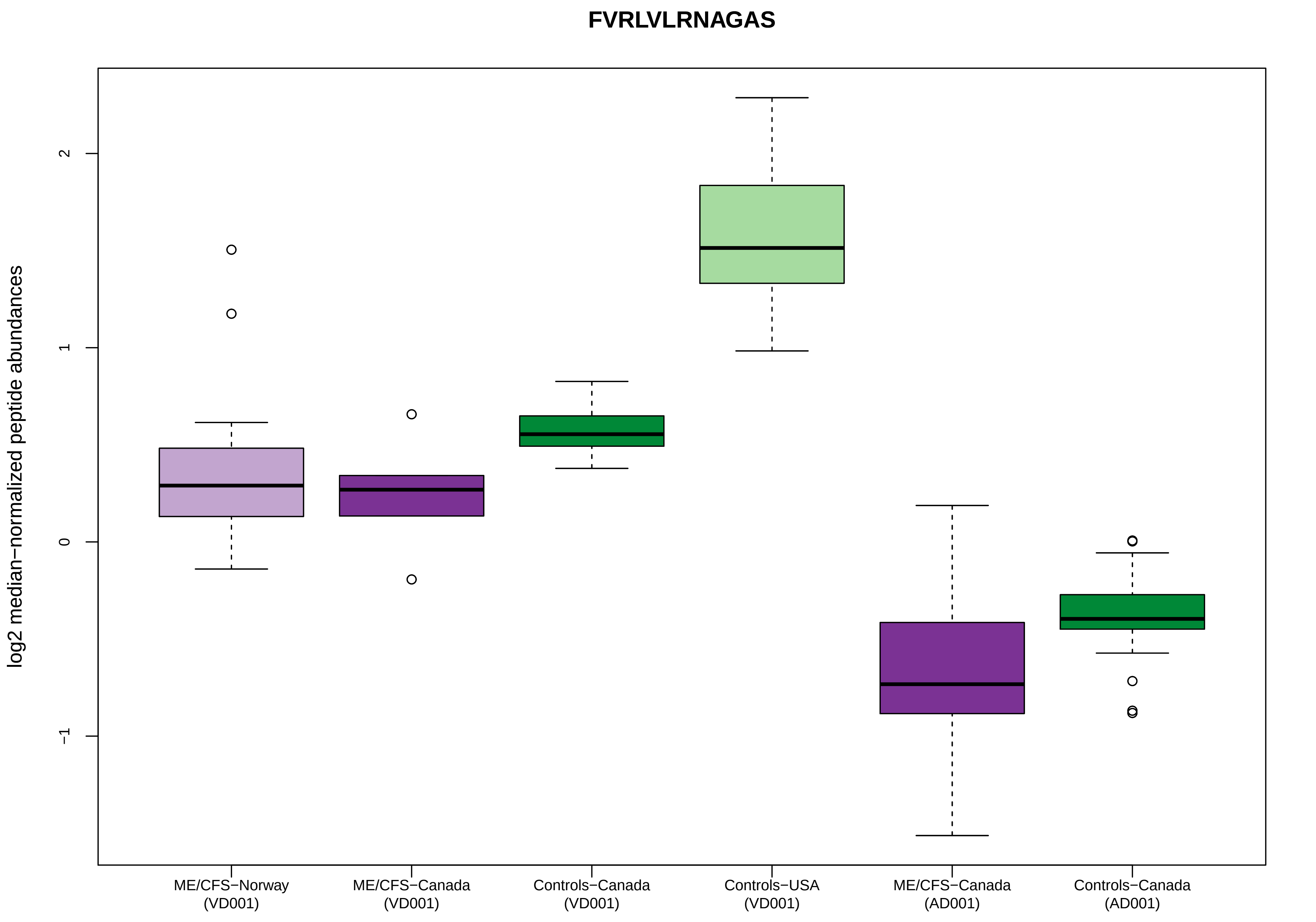

FVRLVLRNAGAS
log2 median−normalized peptide abundances
2
1
0
−1
ME/CFS−Norway (VD001)
ME/CFS−Canada (VD001)
Controls−Canada (VD001)
Controls−USA (VD001)
ME/CFS−Canada (AD001)
Controls−Canada (AD001)

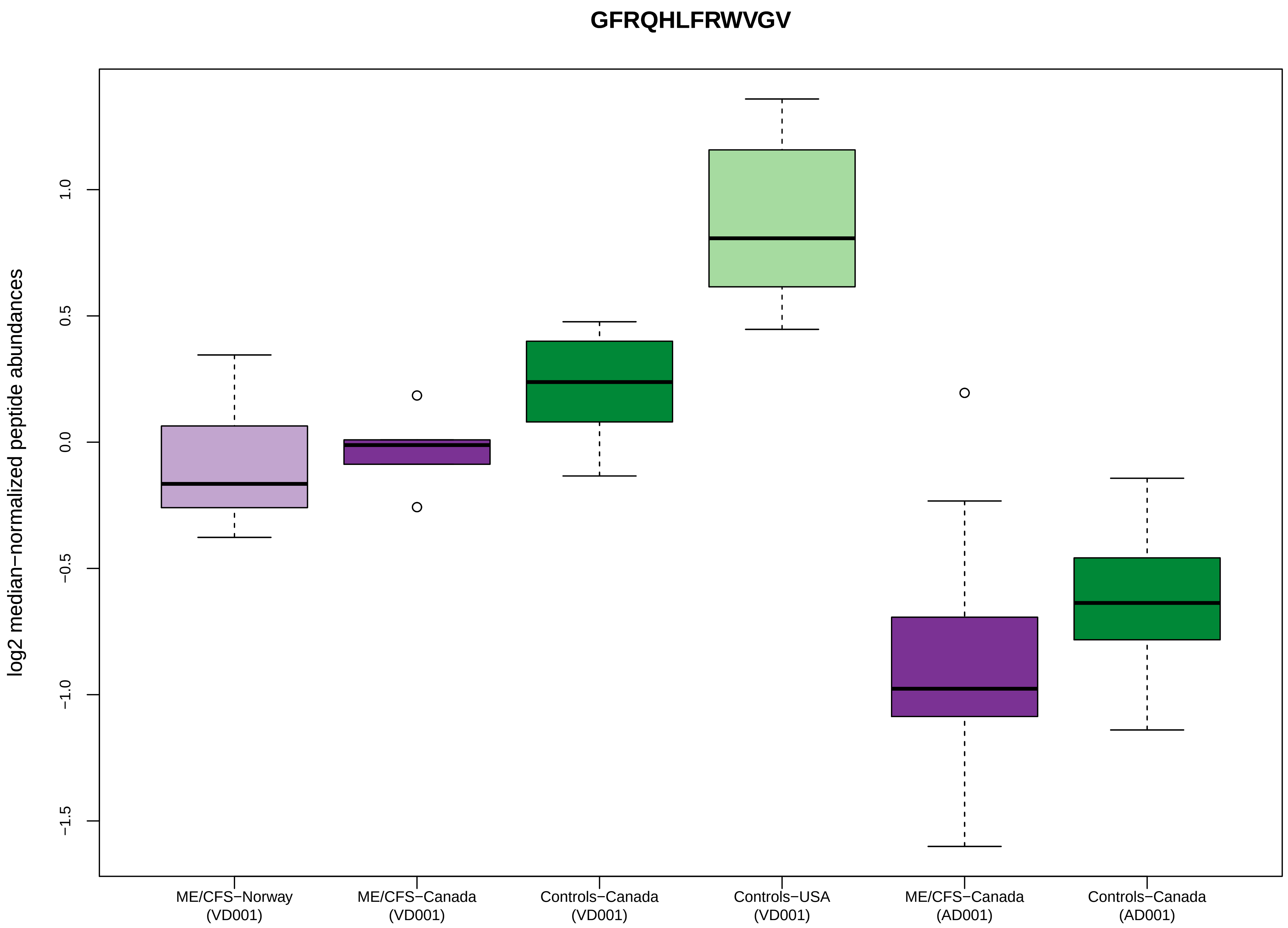

GFRQHLFRWVGV
log2 median−normalized peptide abundances
1.0
0.5
0.0
−0.5
−1.0
−1.5
ME/CFS−Norway (VD001)
ME/CFS−Canada (VD001)
Controls−Canada (VD001)
Controls−USA (VD001)
ME/CFS−Canada (AD001)
Controls−Canada (AD001)

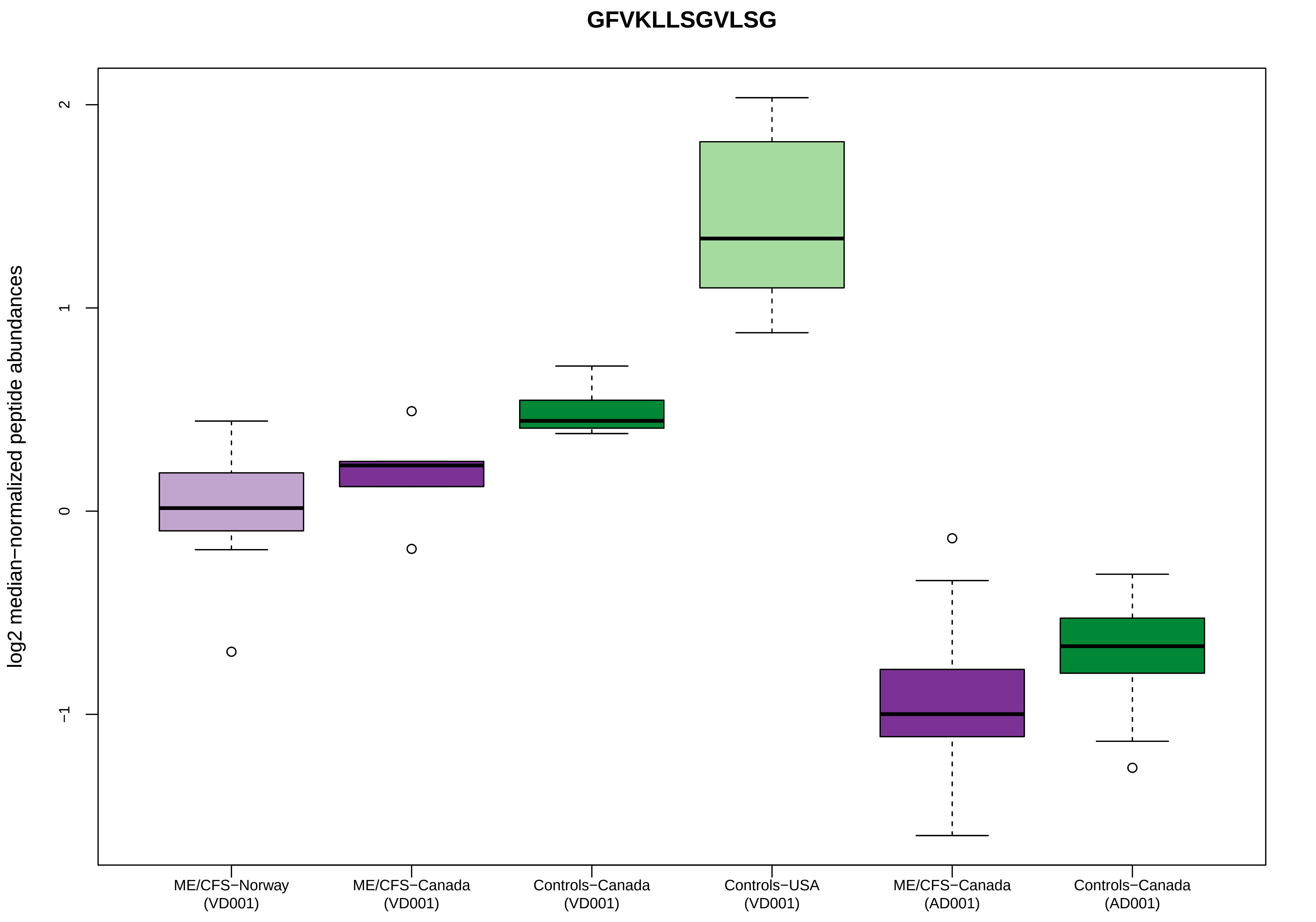
GFVKLLSGVLSG
log2 median−normalized peptide abundances
2
1
0
−1
ME/CFS−Norway (VD001)
ME/CFS−Canada (VD001)
Controls−Canada (VD001)
Controls−USA (VD001)
ME/CFS−Canada (AD001)
Controls−Canada (AD001)

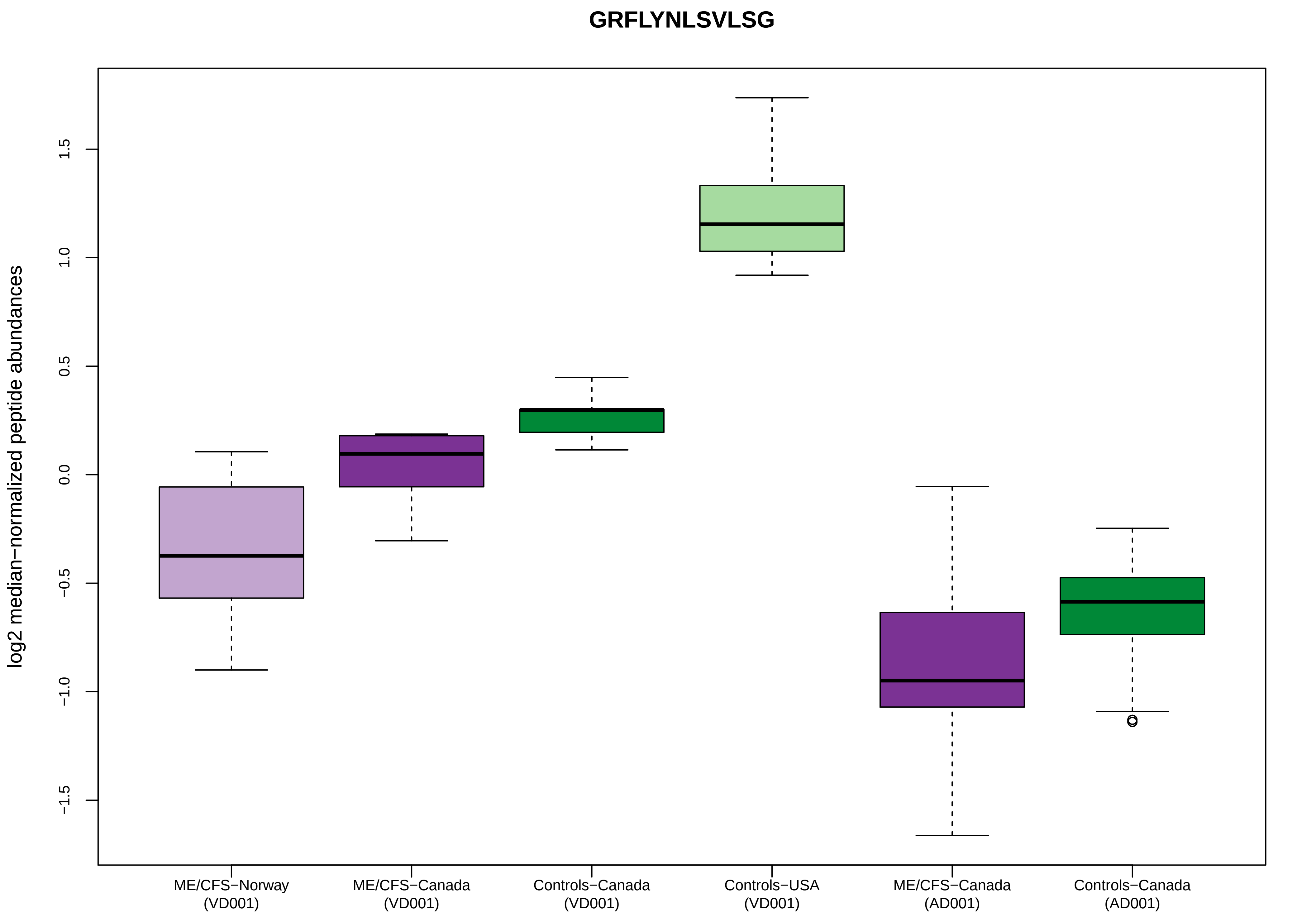

GRFLYNLSVLSG
log2 median−normalized peptide abundances
1.5
1.0
0.5
0.0
−0.5
−1.0
−1.5
ME/CFS−Norway (VD001)
ME/CFS−Canada (VD001)
Controls−Canada (VD001)
Controls−USA (VD001)
ME/CFS−Canada (AD001)
Controls−Canada (AD001)

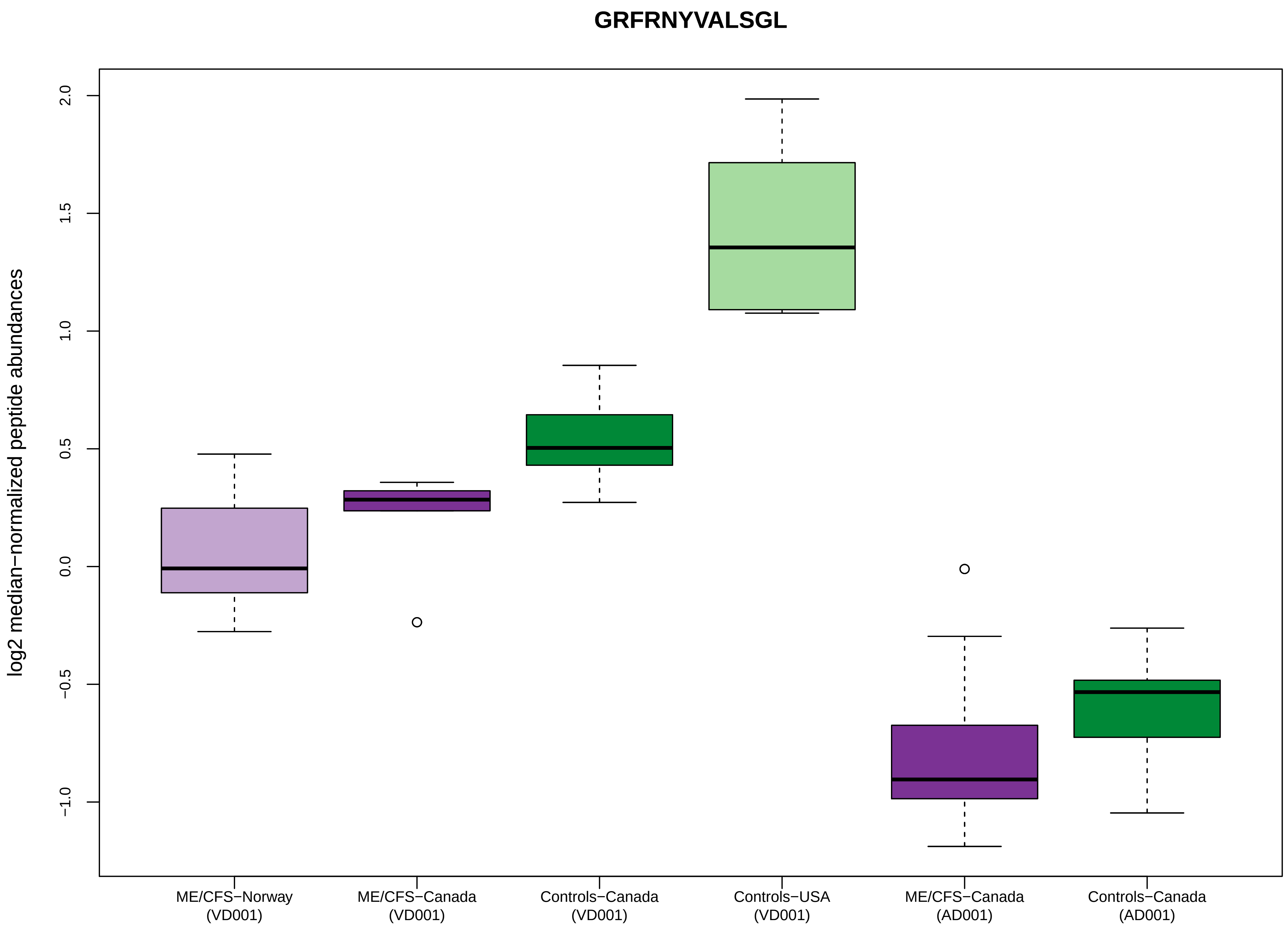

GRFRNYVALSGL
log2 median−normalized peptide abundances
2.0
1.5
1.0
0.5
0.0
−0.5
−1.0
ME/CFS−Norway (VD001)
ME/CFS−Canada (VD001)
Controls−Canada (VD001)
Controls−USA (VD001)
ME/CFS−Canada (AD001)
Controls−Canada (AD001)

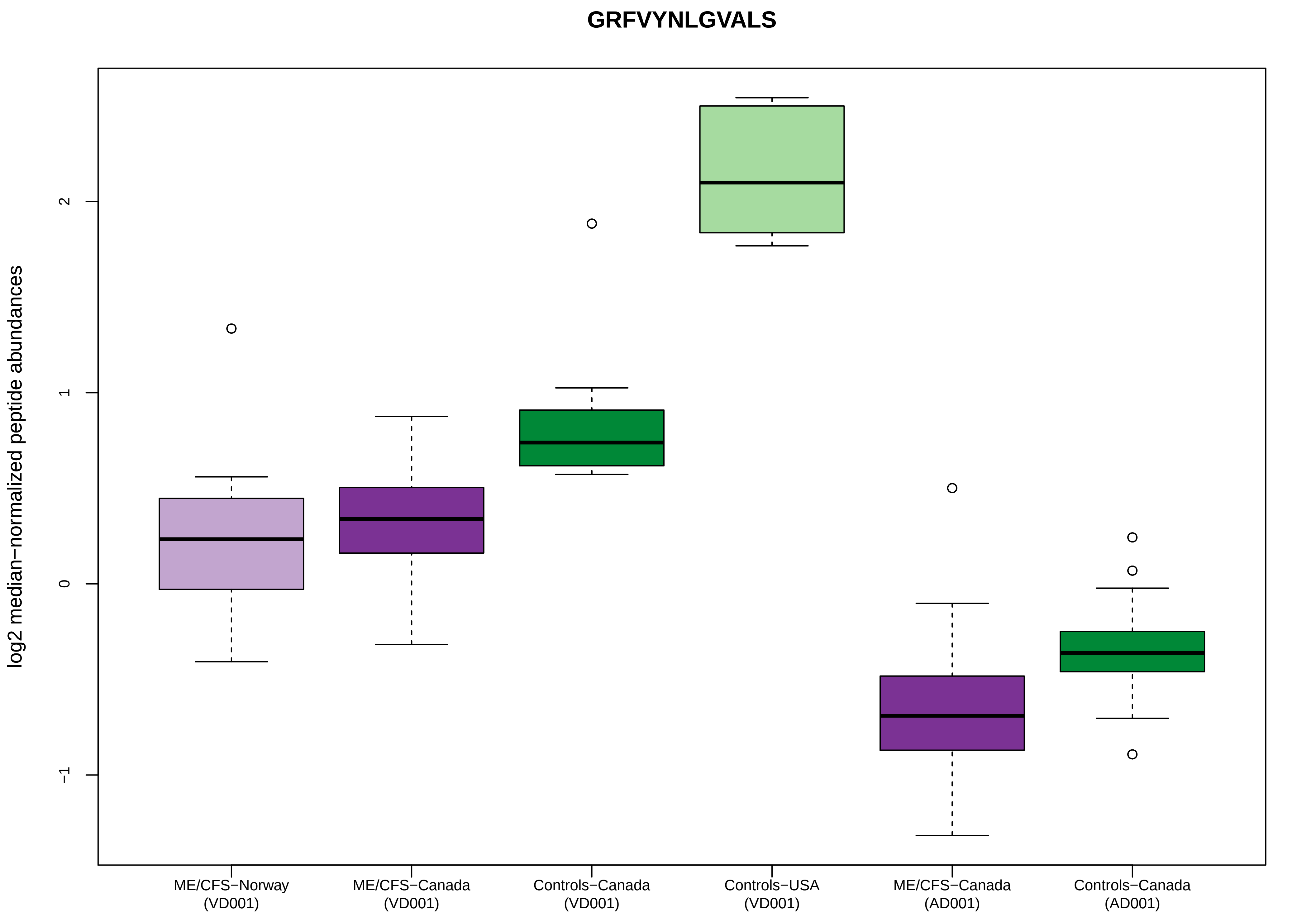

GRFVYNLGVALS
log2 median−normalized peptide abundances
−1
0
1
2
ME/CFS−Norway (VD001)
ME/CFS−Canada (VD001)
Controls−Canada (VD001)
Controls−USA (VD001)
ME/CFS−Canada (AD001)
Controls−Canada (AD001)

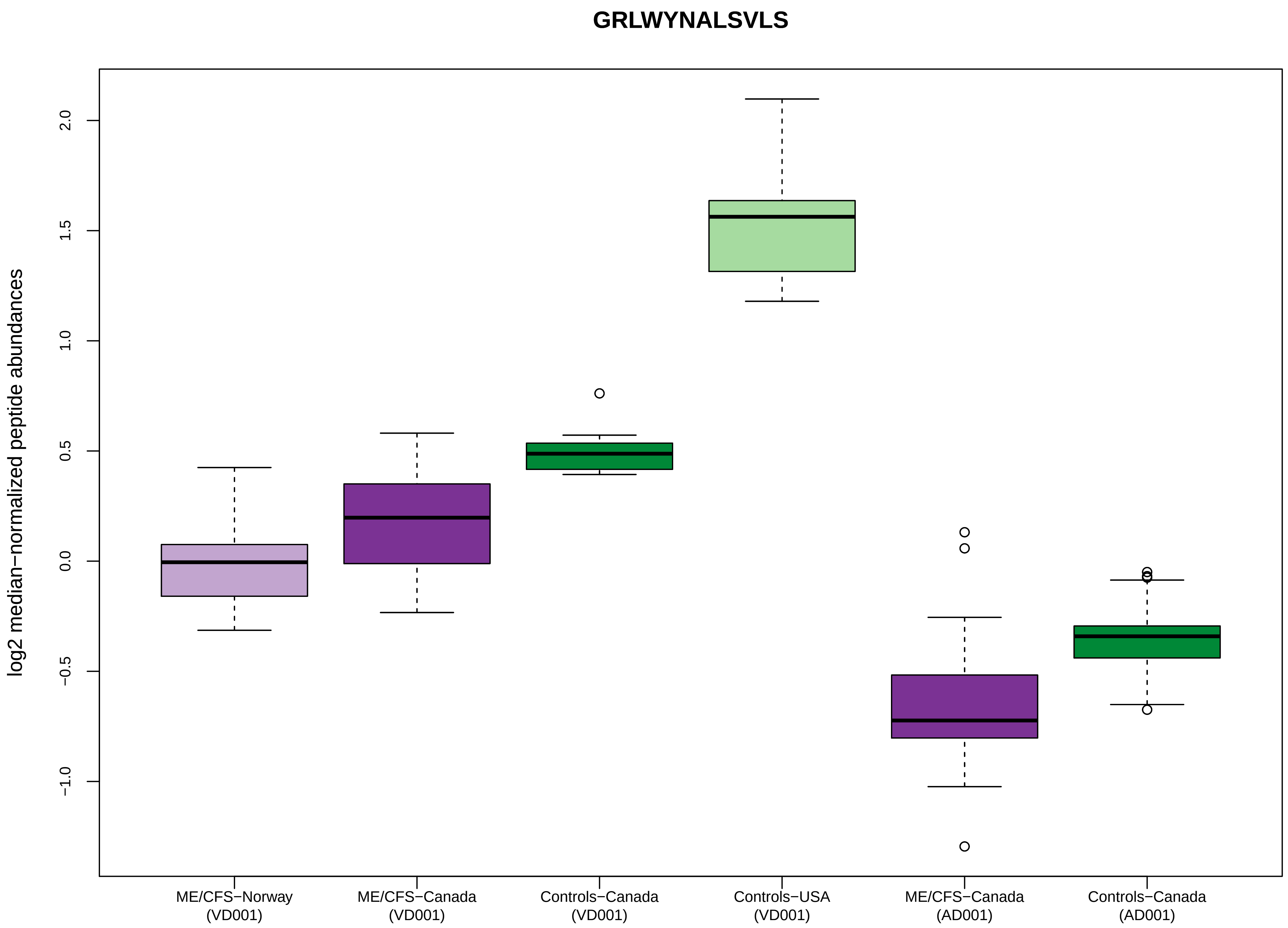
GRLWYNALSVLS
log2 median−normalized peptide abundances
2.0
1.5
1.0
0.5
0.0
−0.5
−1.0
ME/CFS−Norway (VD001)
ME/CFS−Canada (VD001)
Controls−Canada (VD001)
Controls−USA (VD001)
ME/CFS−Canada (AD001)
Controls−Canada (AD001)

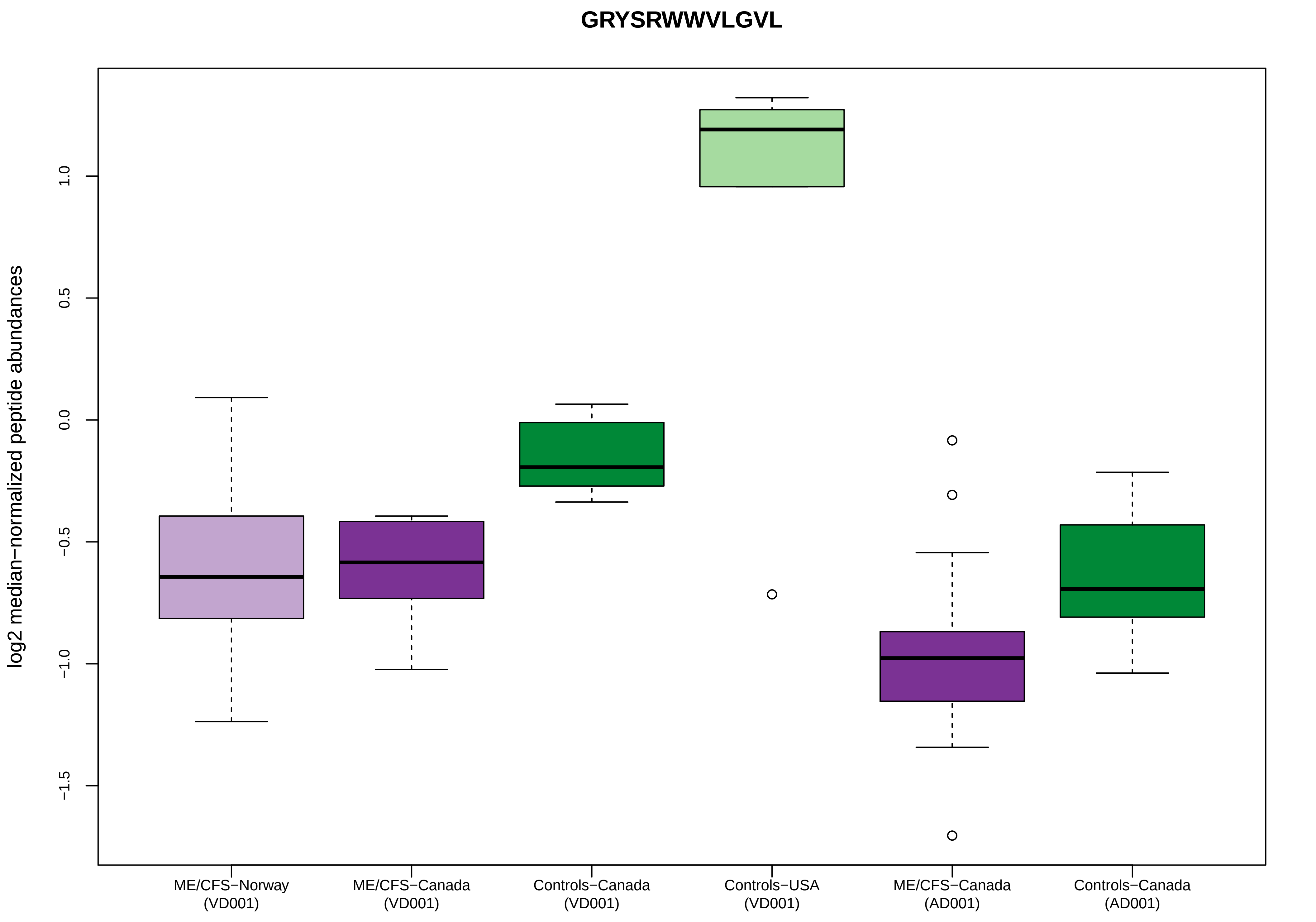
GRYSRWWVLGVL
log2 median−normalized peptide abundances
1.0
0.5
0.0
−0.5
−1.0
−1.5
ME/CFS−Norway (VD001)
ME/CFS−Canada (VD001)
Controls−Canada (VD001)
Controls−USA (VD001)
ME/CFS−Canada (AD001)
Controls−Canada (AD001)

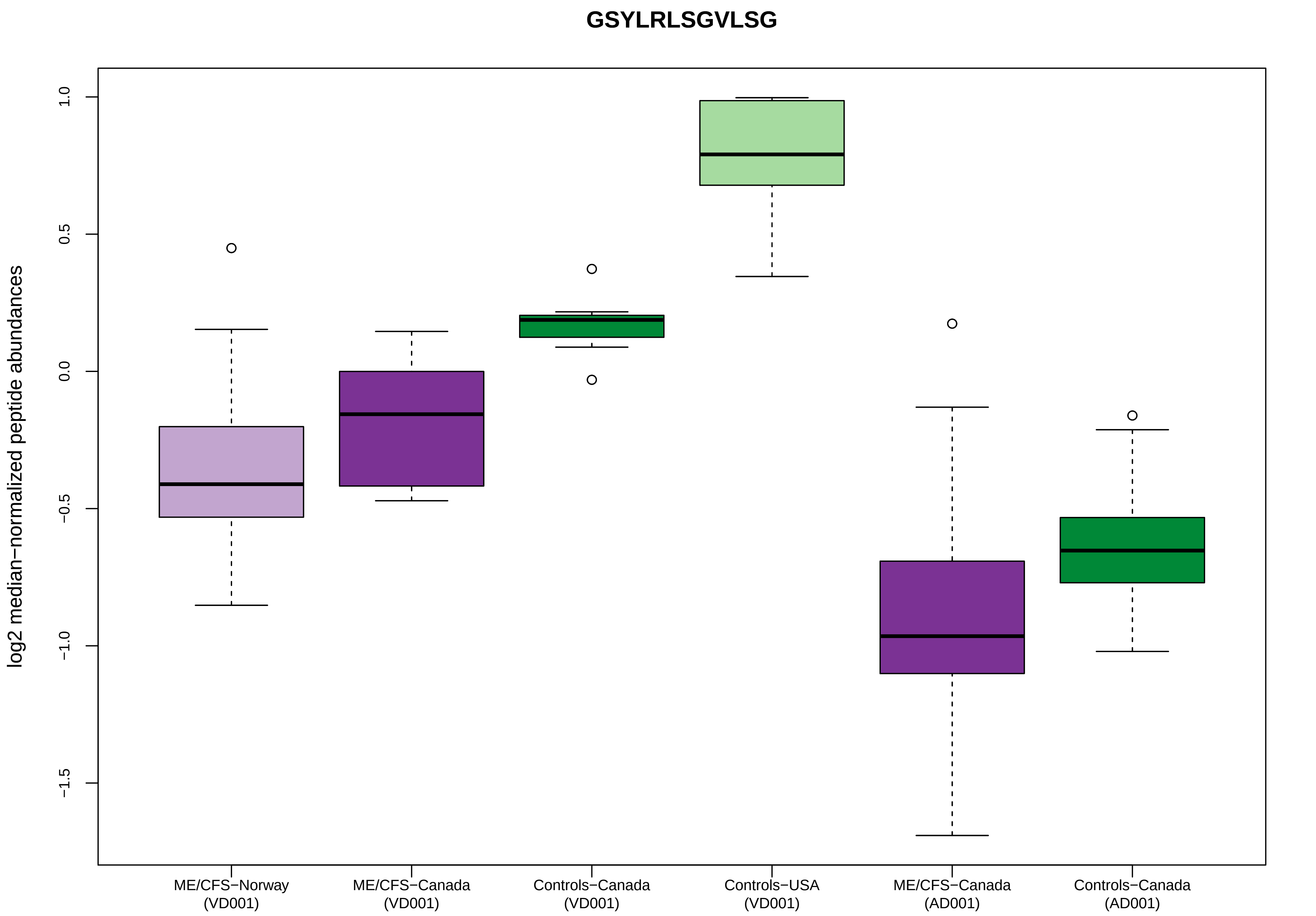

GSYLRLSGVLSG
log2 median−normalized peptide abundances
1.0
0.5
0.0
−0.5
−1.0
−1.5
ME/CFS−Norway (VD001)
ME/CFS−Canada (VD001)
Controls−Canada (VD001)
Controls−USA (VD001)
ME/CFS−Canada (AD001)
Controls−Canada (AD001)

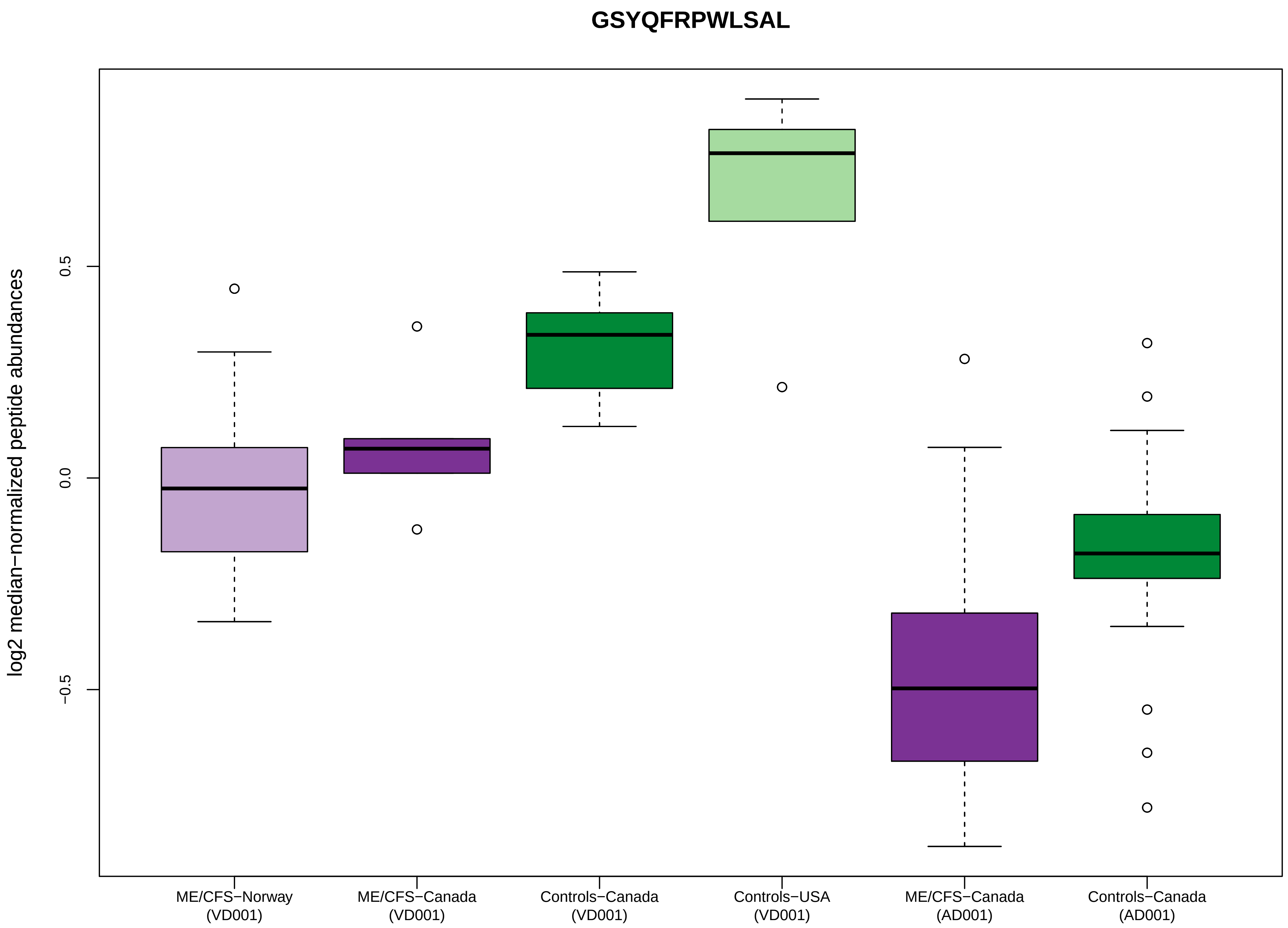
GSYQFRPWLSAL
log2 median−normalized peptide abundances
0.5
0.0
−0.5
ME/CFS−Norway (VD001)
ME/CFS−Canada (VD001)
Controls−Canada (VD001)
Controls−USA (VD001)
ME/CFS−Canada (AD001)
Controls−Canada (AD001)

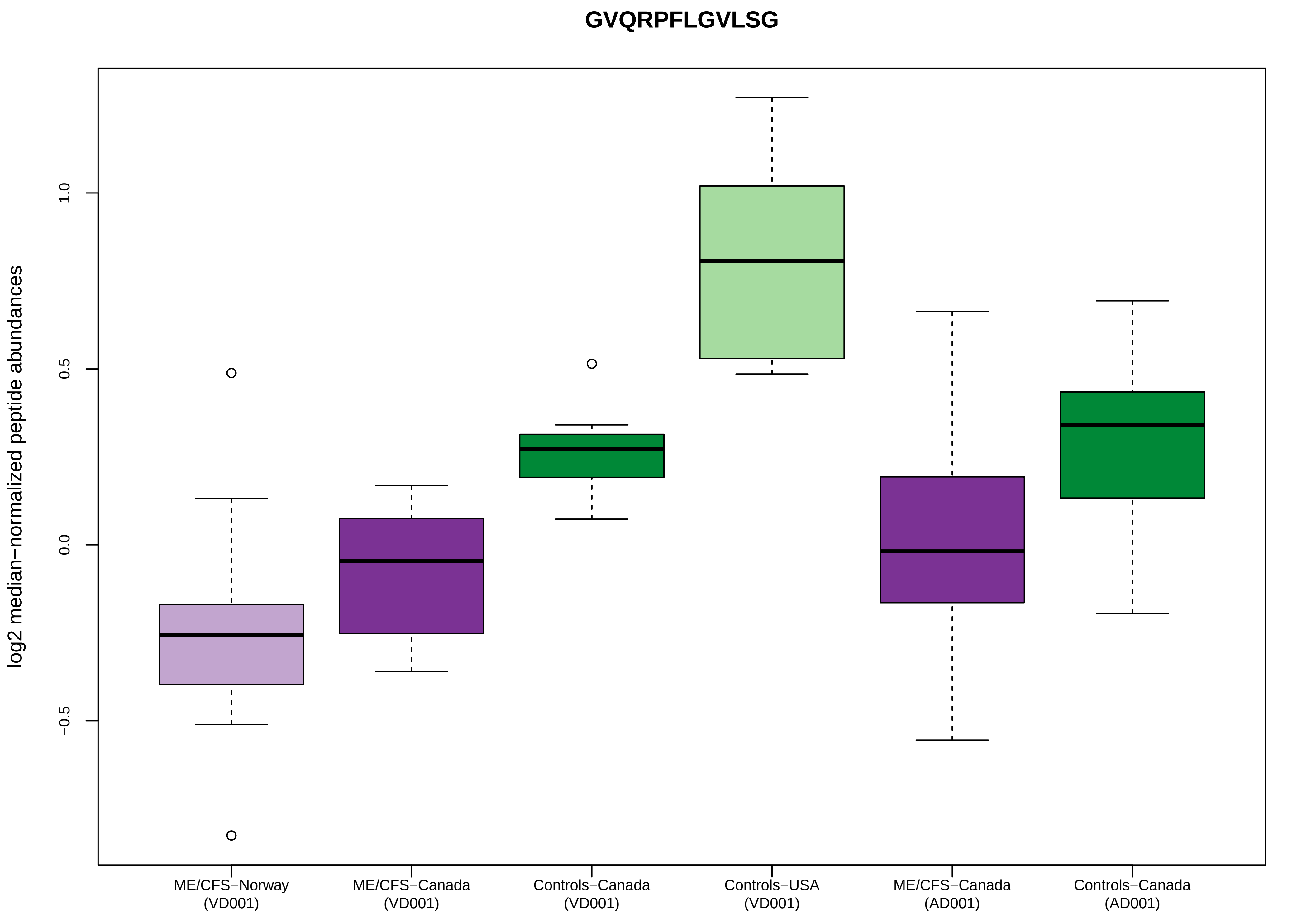

GVQRPFLGVLSG
log2 median−normalized peptide abundances
1.0
0.5
0.0
−0.5
ME/CFS−Norway (VD001)
ME/CFS−Canada (VD001)
Controls−Canada (VD001)
Controls−USA (VD001)
ME/CFS−Canada (AD001)
Controls−Canada (AD001)

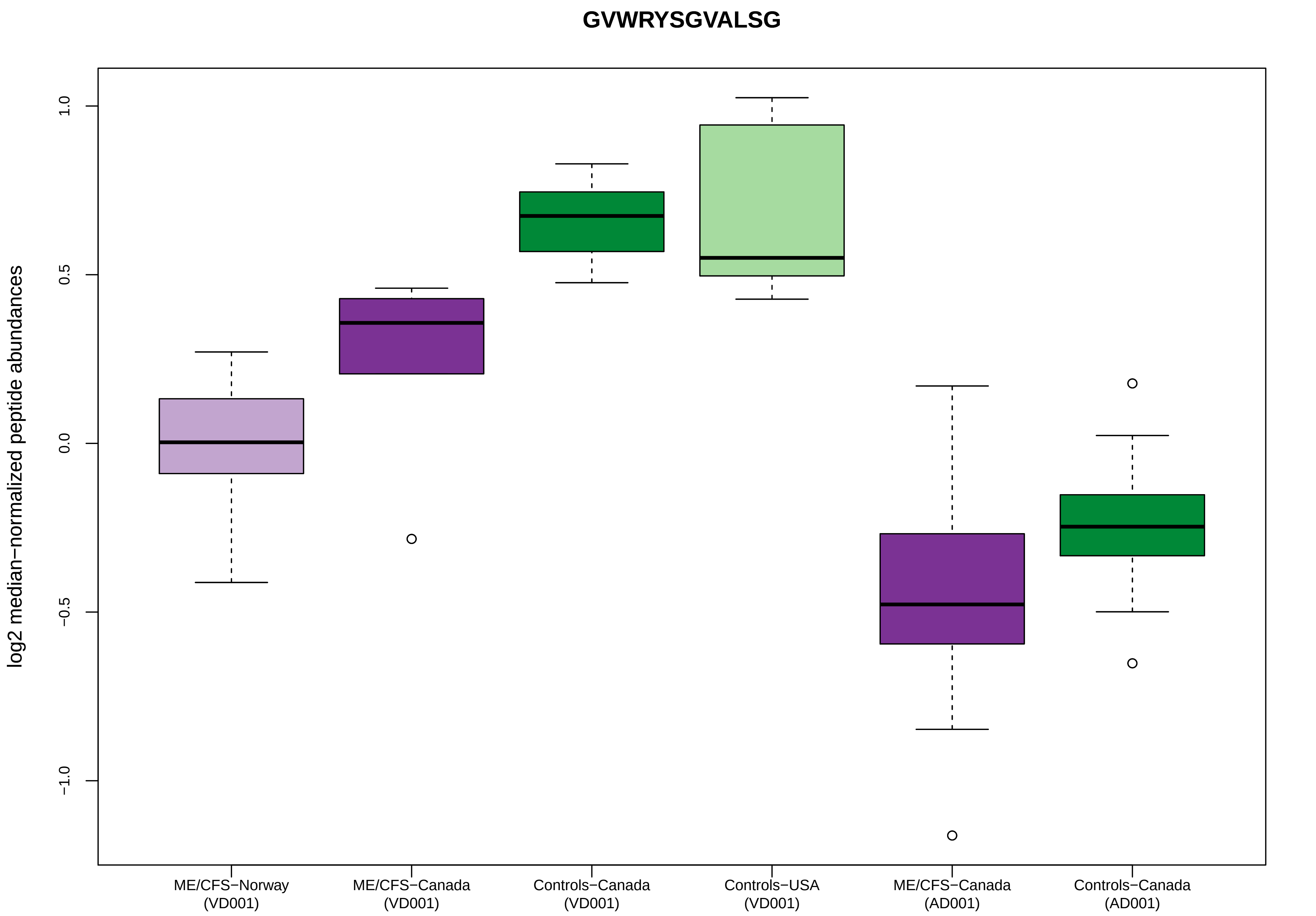
GVWRYSGVALSG
log2 median−normalized peptide abundances
1.0
0.5
0.0
−0.5
−1.0
ME/CFS−Norway (VD001)
ME/CFS−Canada (VD001)
Controls−Canada (VD001)
Controls−USA (VD001)
ME/CFS−Canada (AD001)
Controls−Canada (AD001)

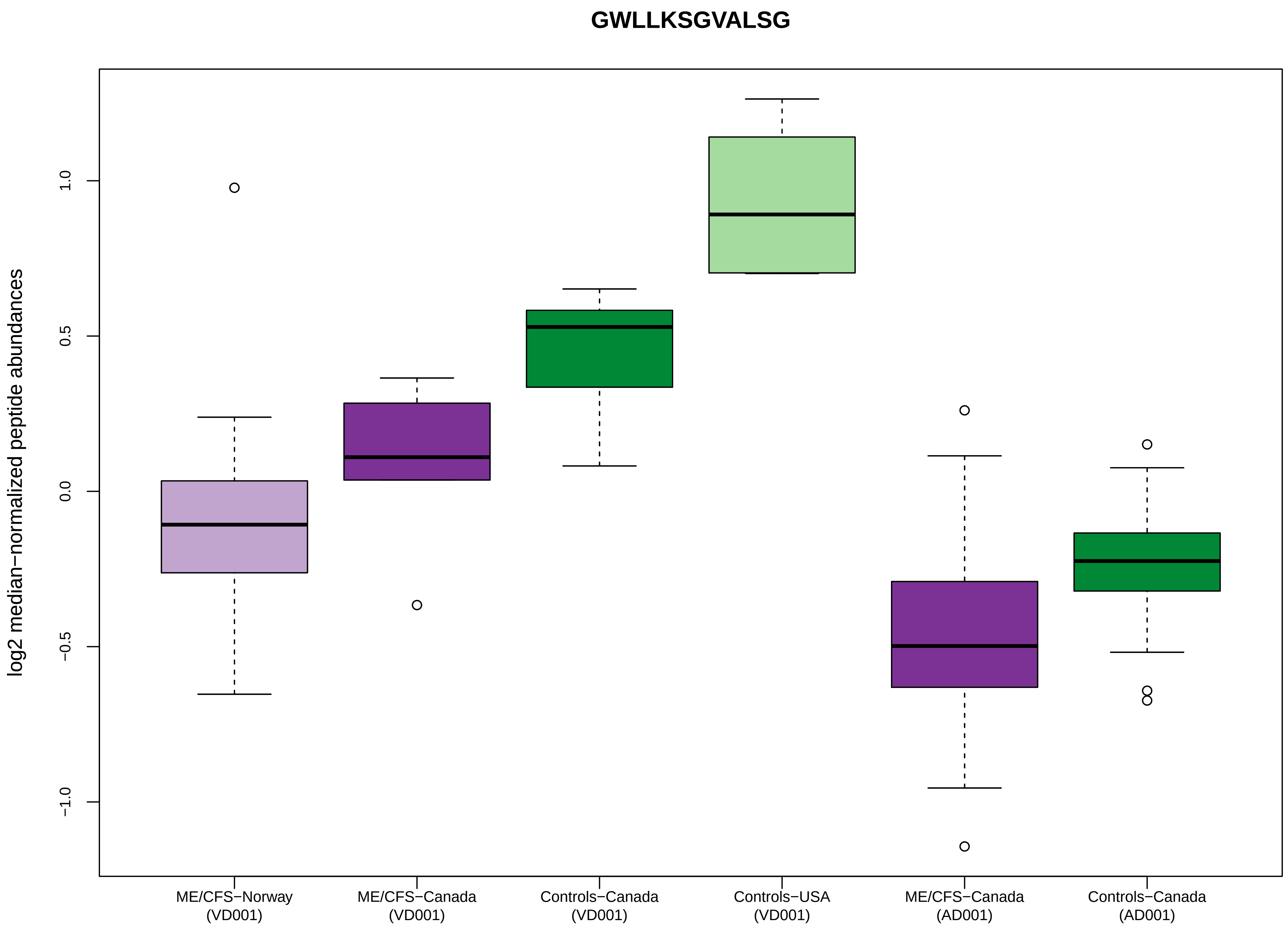
GWLLKSGVALSG
log2 median−normalized peptide abundances
1.0
0.5
0.0
−0.5
−1.0
ME/CFS−Norway (VD001)
ME/CFS−Canada (VD001)
Controls−Canada (VD001)
Controls−USA (VD001)
ME/CFS−Canada (AD001)
Controls−Canada (AD001)

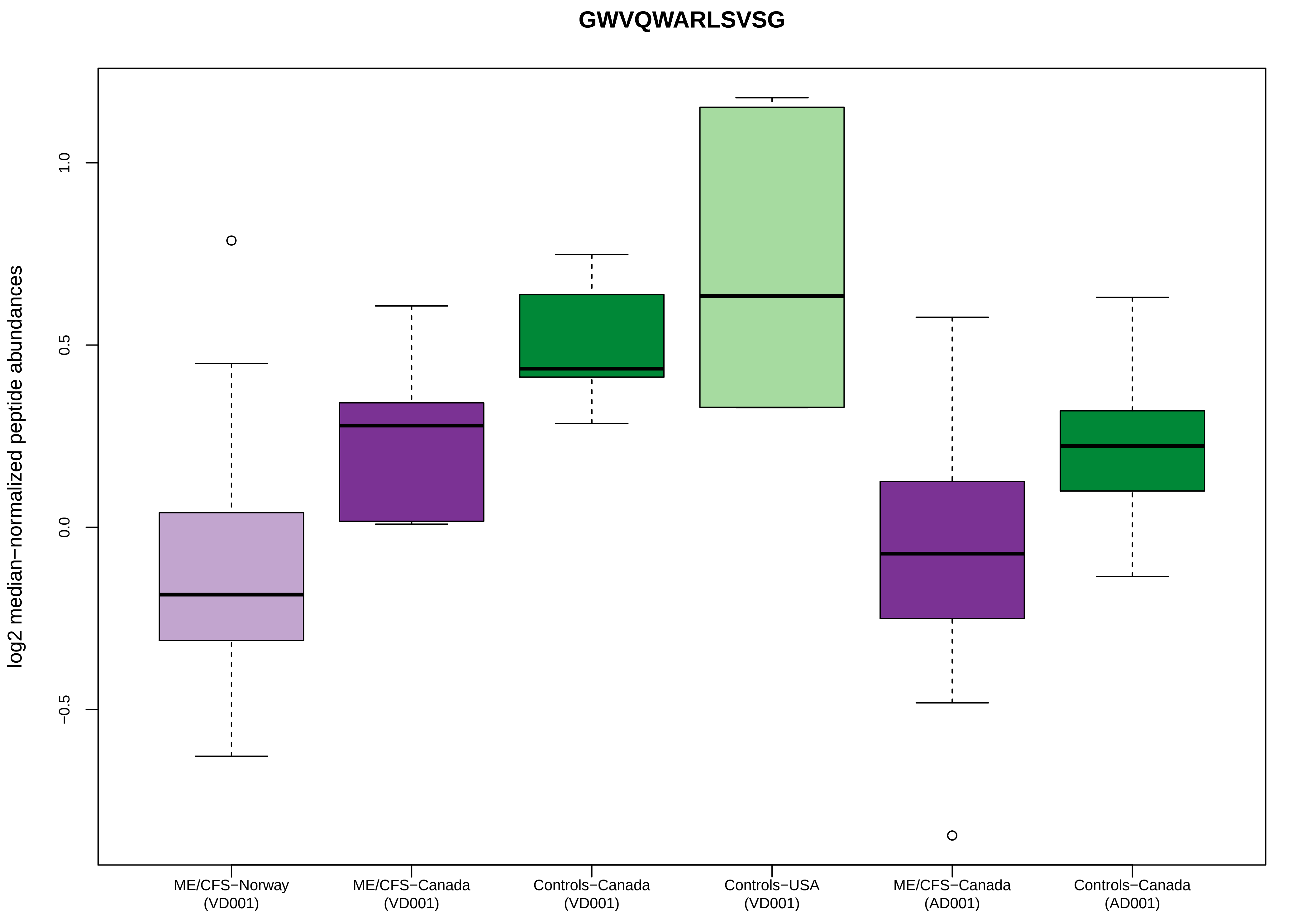

GWVQWARLSVSG
log2 median−normalized peptide abundances
1.0
0.5
0.0
−0.5
ME/CFS−Norway (VD001)
ME/CFS−Canada (VD001)
Controls−Canada (VD001)
Controls−USA (VD001)
ME/CFS−Canada (AD001)
Controls−Canada (AD001)

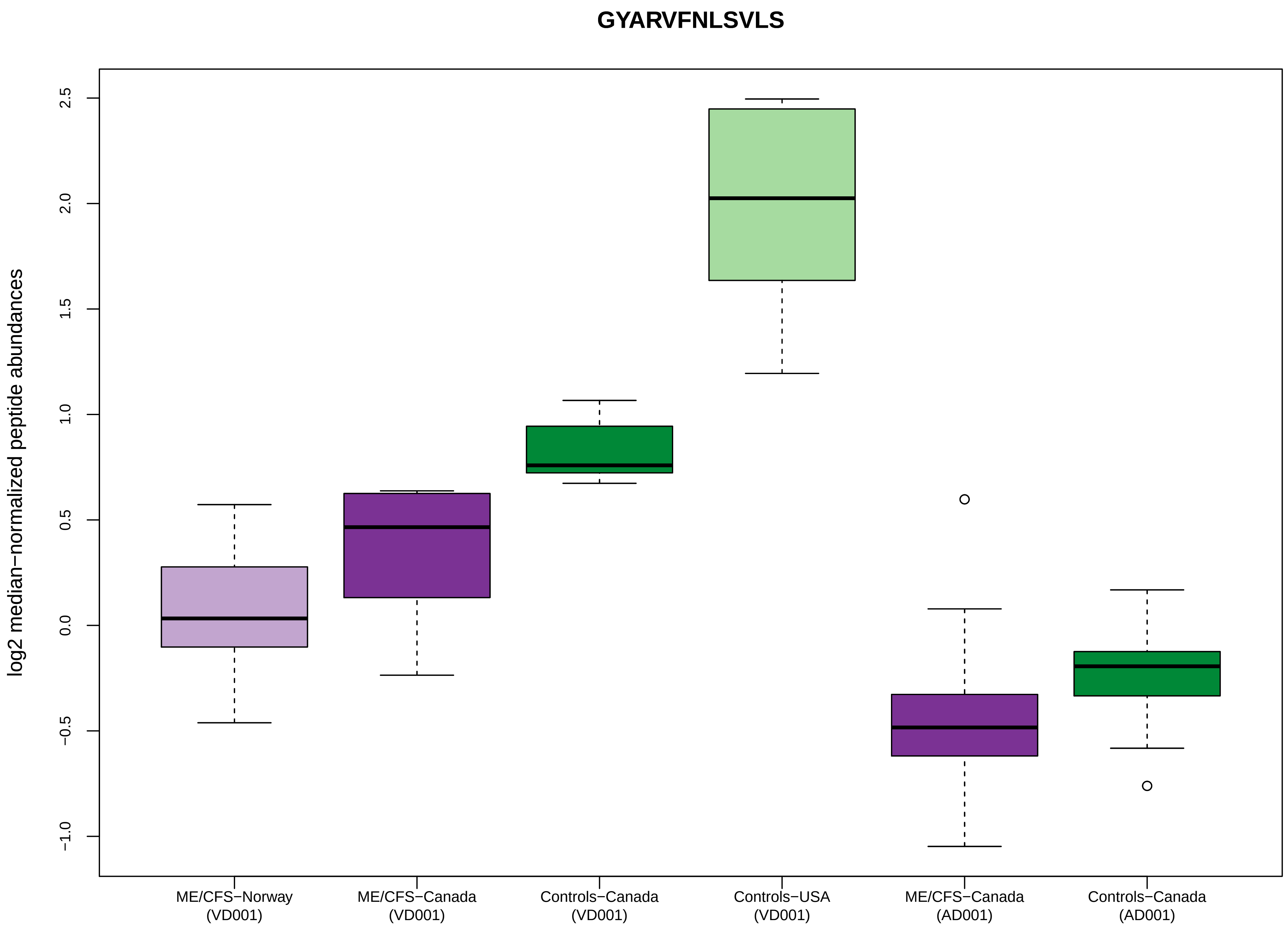

GYARVFNLSVLS
log2 median−normalized peptide abundances
2.5
2.0
1.5
1.0
0.5
0.0
−0.5
−1.0
ME/CFS−Norway (VD001)
ME/CFS−Canada (VD001)
Controls−Canada (VD001)
Controls−USA (VD001)
ME/CFS−Canada (AD001)
Controls−Canada (AD001)

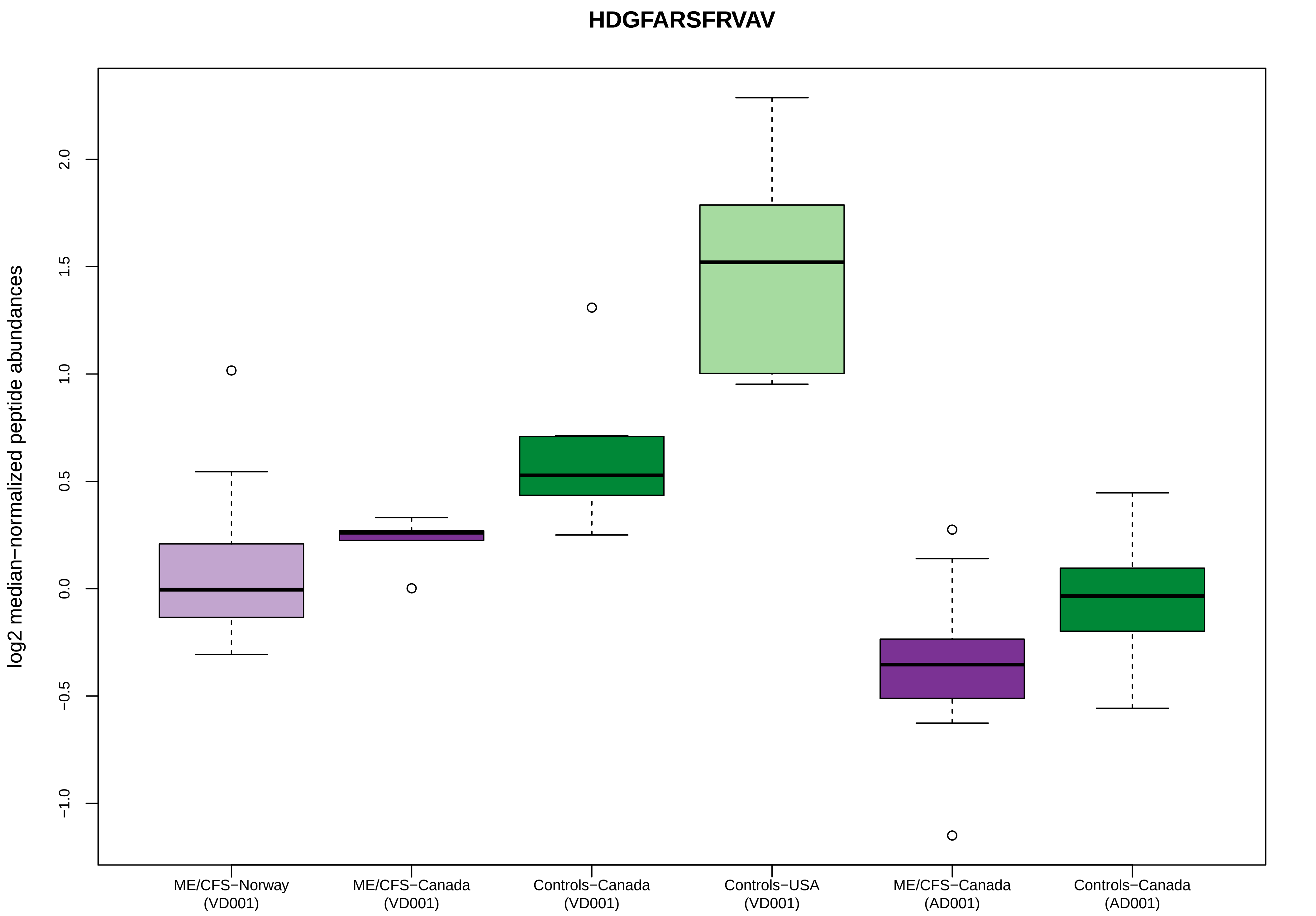
HDGFARSFRVAV
log2 median−normalized peptide abundances
2.0
1.5
1.0
0.5
0.0
−0.5
−1.0
ME/CFS−Norway (VD001)
ME/CFS−Canada (VD001)
Controls−Canada (VD001)
Controls−USA (VD001)
ME/CFS−Canada (AD001)
Controls−Canada (AD001)

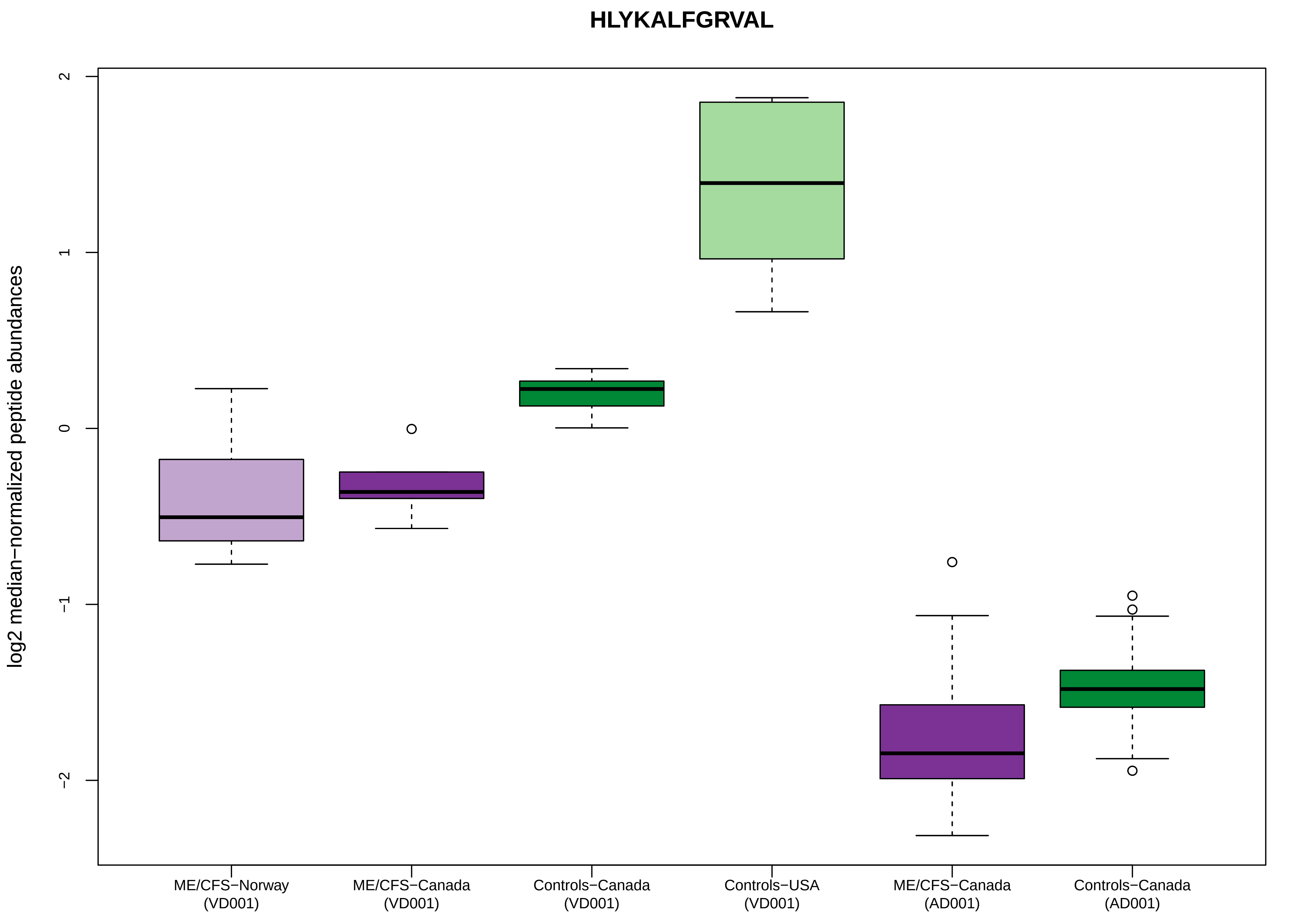

HLYKALFGRVAL
log2 median−normalized peptide abundances
2
1
0
−1
−2
ME/CFS−Norway (VD001)
ME/CFS−Canada (VD001)
Controls−Canada (VD001)
Controls−USA (VD001)
ME/CFS−Canada (AD001)
Controls−Canada (AD001)

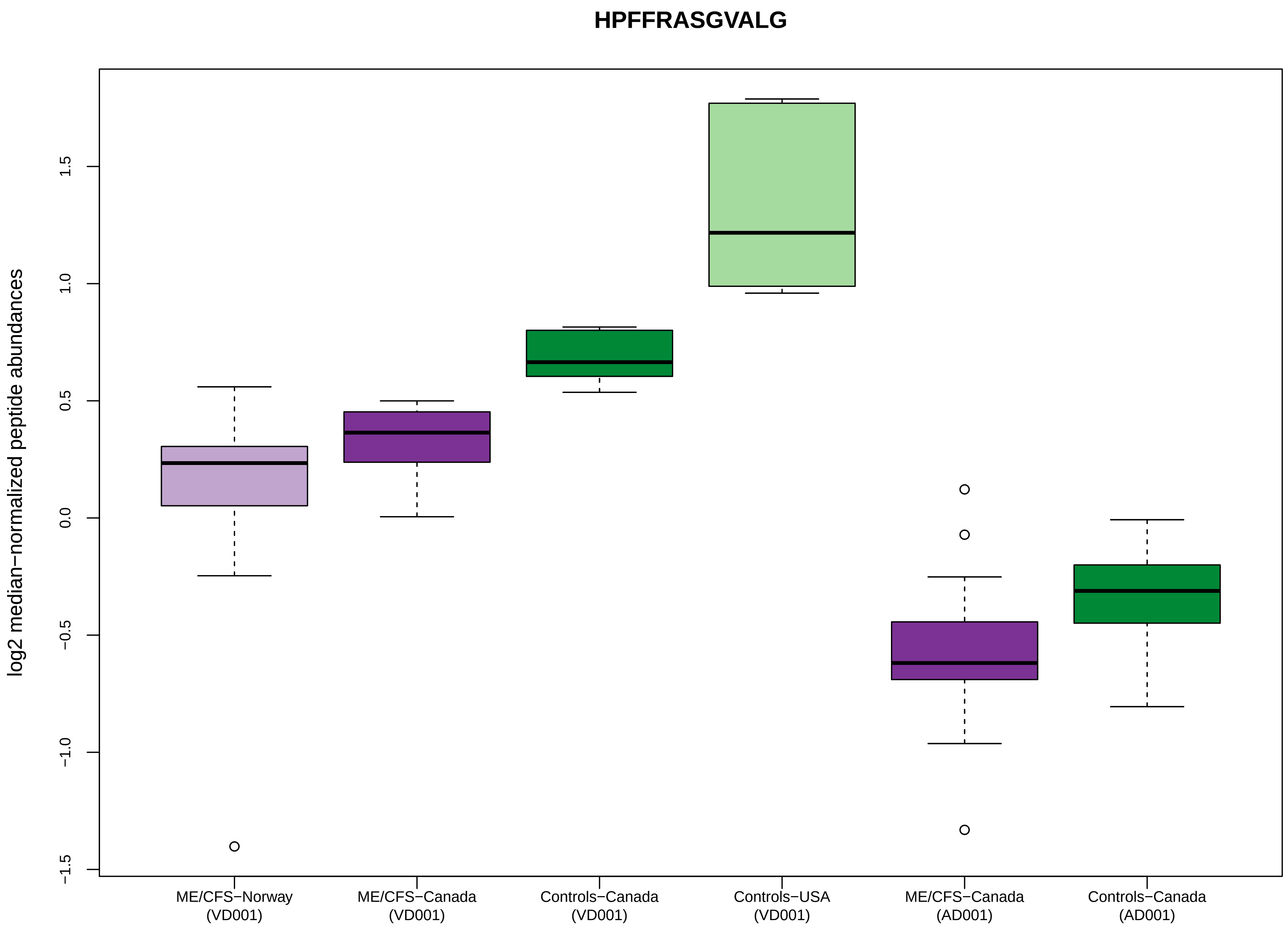

HPFFRASGVALG
log2 median−normalized peptide abundances
1.5
1.0
0.5
0.0
−0.5
−1.0
−1.5
ME/CFS−Norway (VD001)
ME/CFS−Canada (VD001)
Controls−Canada (VD001)
Controls−USA (VD001)
ME/CFS−Canada (AD001)
Controls−Canada (AD001)

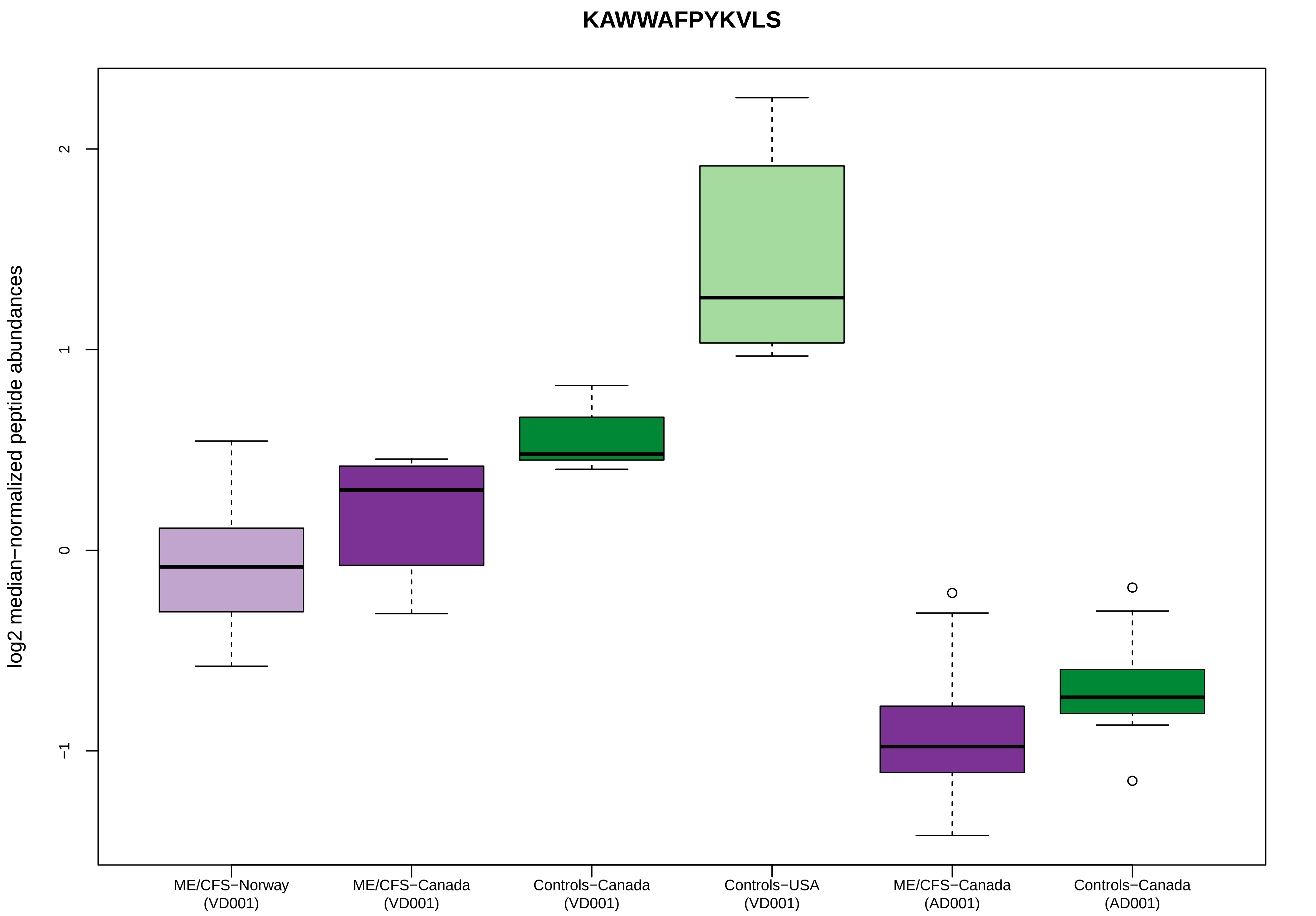

KAWWAFPYKVLS
log2 median−normalized peptide abundances
2
1
0
−1
ME/CFS−Norway (VD001)
ME/CFS−Canada (VD001)
Controls−Canada (VD001)
Controls−USA (VD001)
ME/CFS−Canada (AD001)
Controls−Canada (AD001)

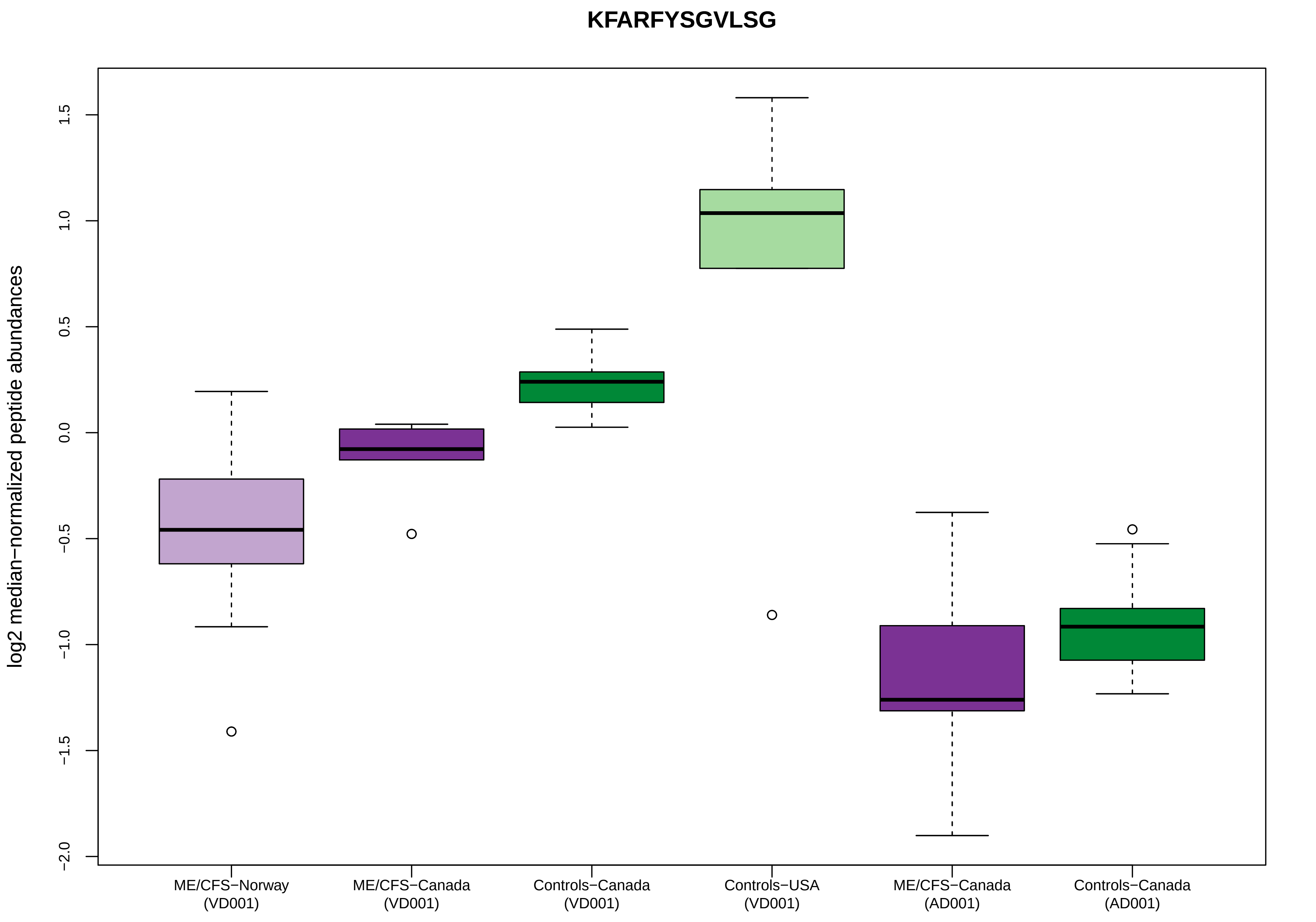
KFARFYSGVLSG
log2 median−normalized peptide abundances
1.5
1.0
0.5
0.0
−0.5
−1.0
−1.5
−2.0
ME/CFS−Norway (VD001)
ME/CFS−Canada (VD001)
Controls−Canada (VD001)
Controls−USA (VD001)
ME/CFS−Canada (AD001)
Controls−Canada (AD001)

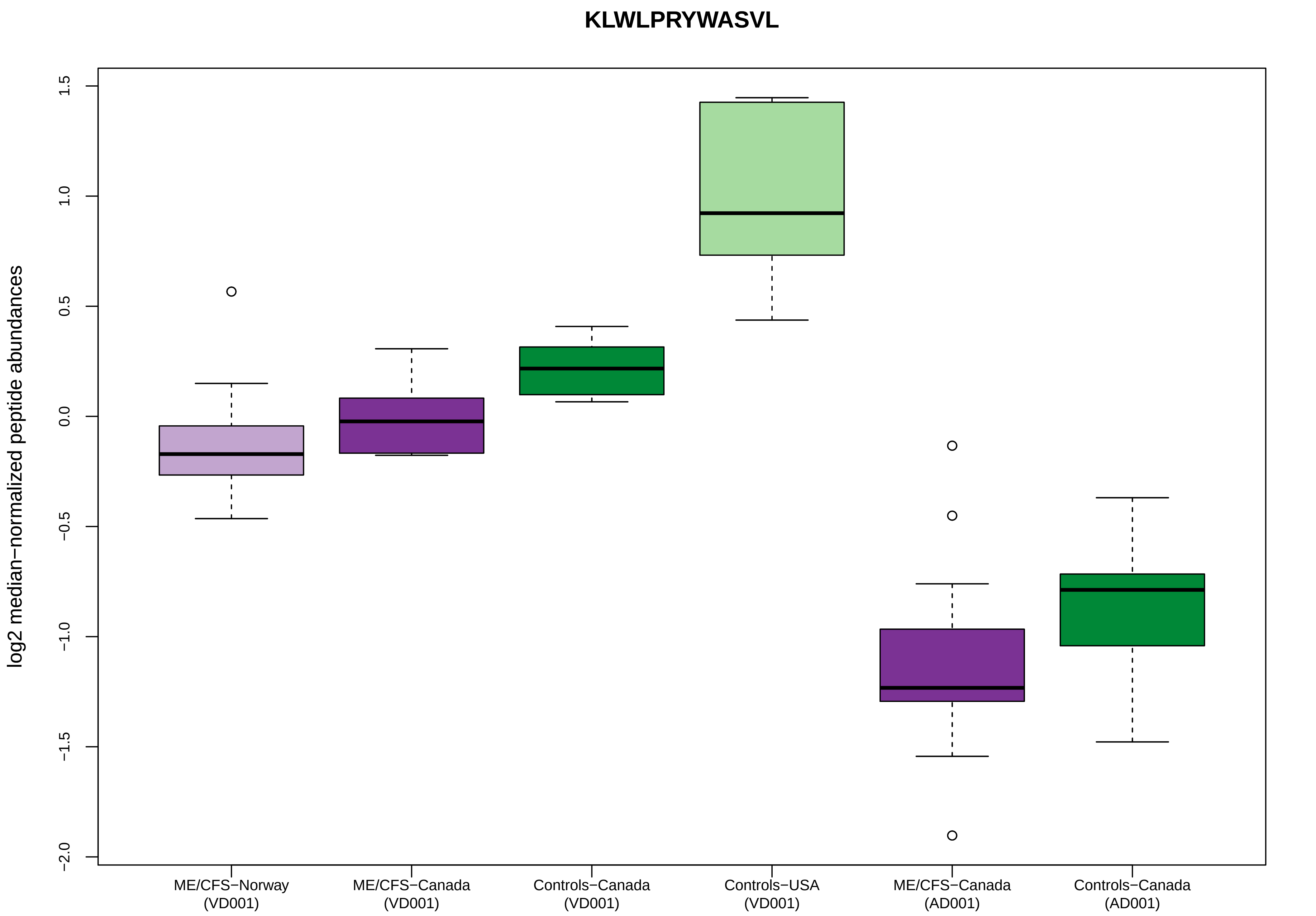
KLWLPRYWASVL
log2 median−normalized peptide abundances
1.5
1.0
0.5
0.0
−0.5
−1.0
−1.5
−2.0
ME/CFS−Norway (VD001)
ME/CFS−Canada (VD001)
Controls−Canada (VD001)
Controls−USA (VD001)
ME/CFS−Canada (AD001)
Controls−Canada (AD001)

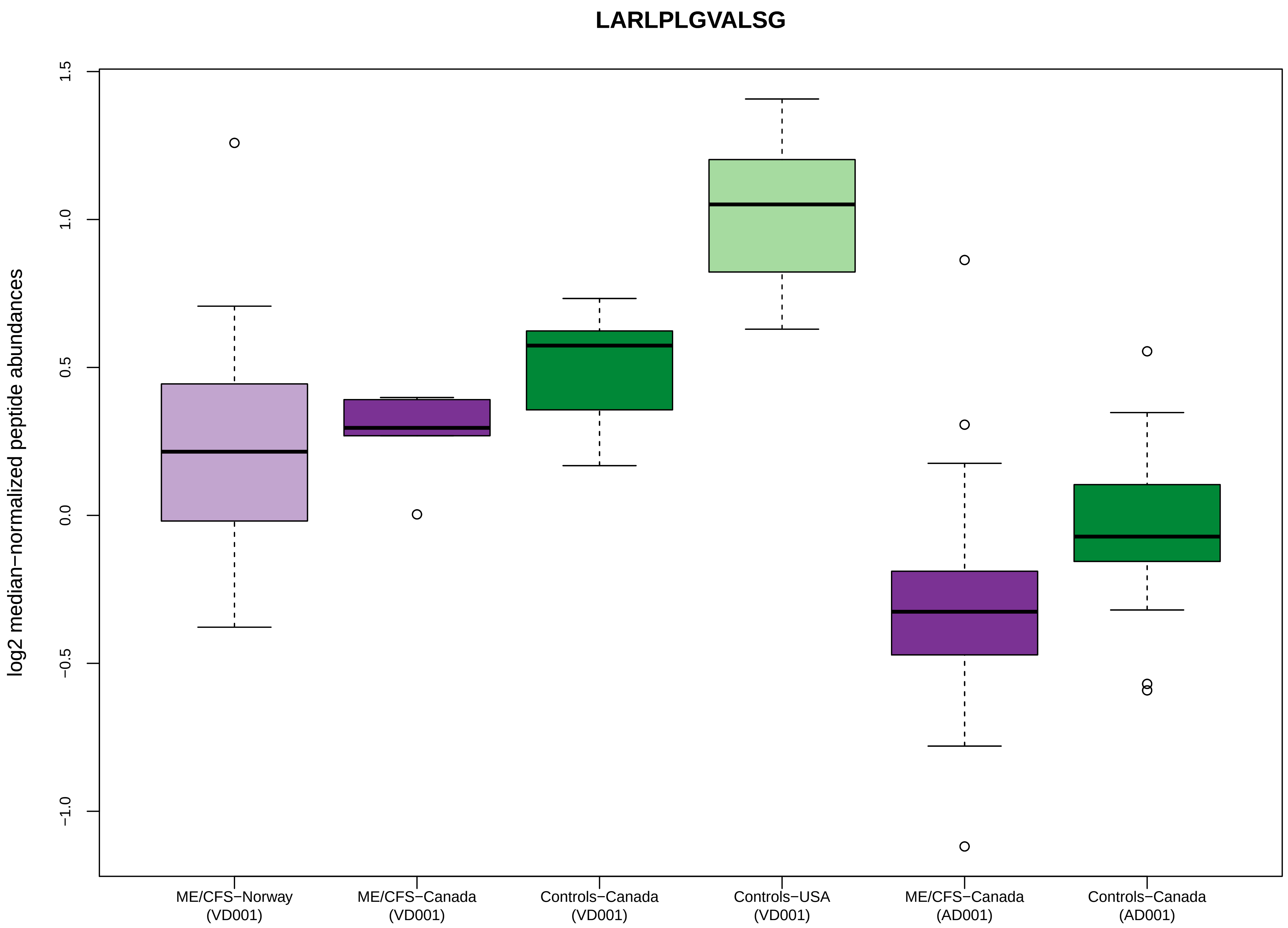
LARLPLGVALSG
log2 median−normalized peptide abundances
1.5
1.0
0.5
0.0
−0.5
−1.0
ME/CFS−Norway (VD001)
ME/CFS−Canada (VD001)
Controls−Canada (VD001)
Controls−USA (VD001)
ME/CFS−Canada (AD001)
Controls−Canada (AD001)

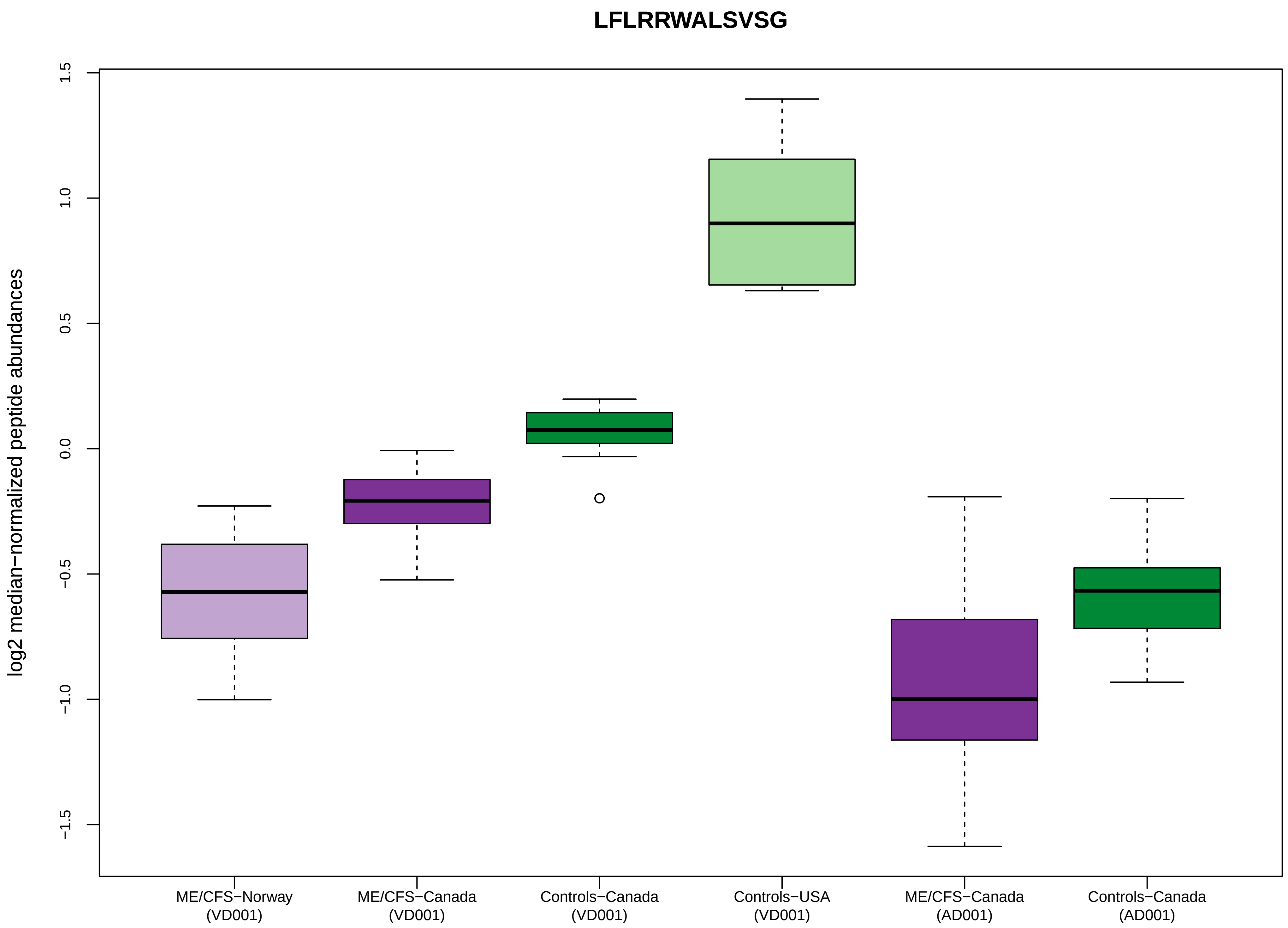
LFLRRWALSVSG
log2 median−normalized peptide abundances
1.5
1.0
0.5
0.0
−0.5
−1.0
−1.5
ME/CFS−Norway (VD001)
ME/CFS−Canada (VD001)
Controls−Canada (VD001)
Controls−USA (VD001)
ME/CFS−Canada (AD001)
Controls−Canada (AD001)

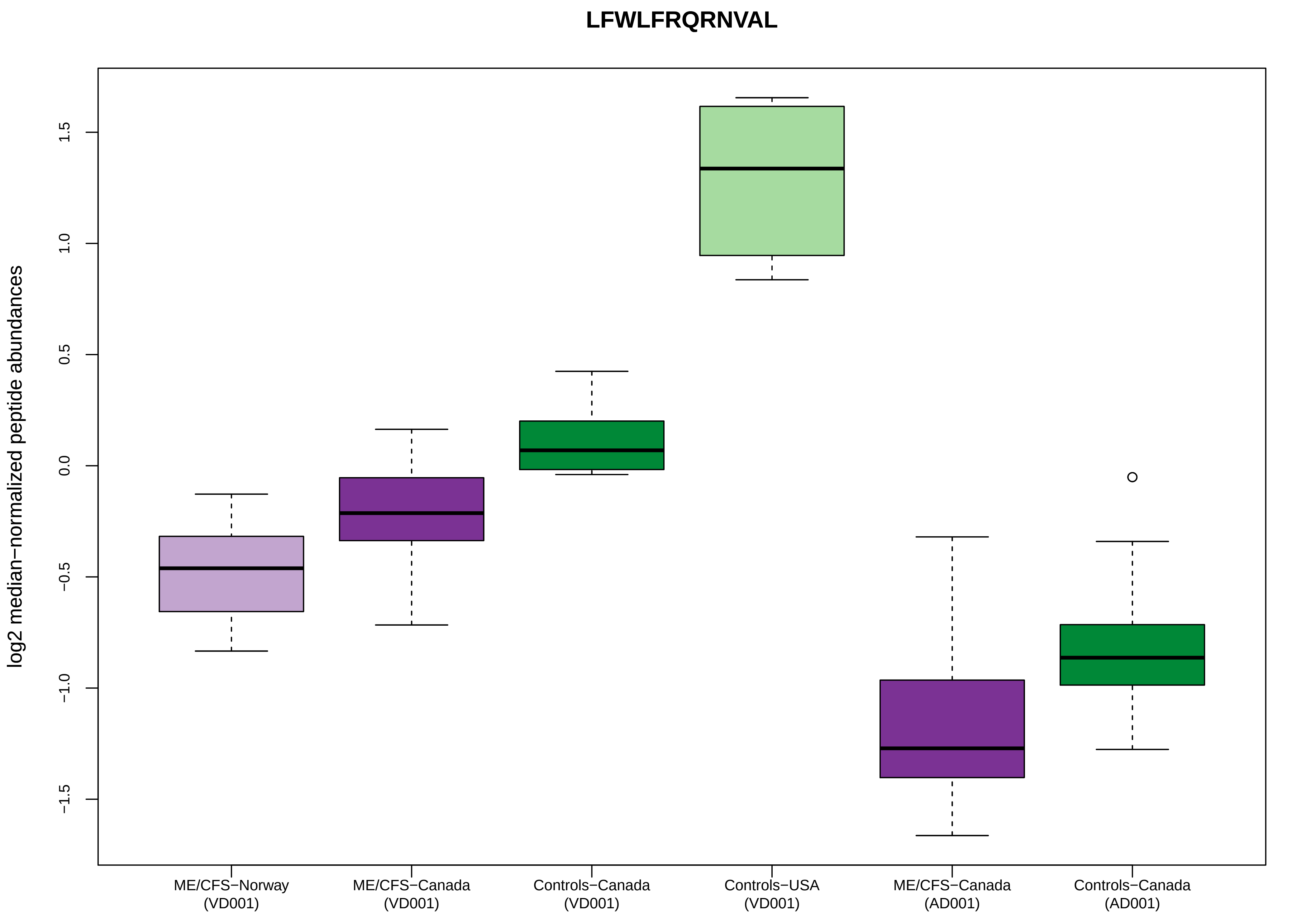

LFWLFRQRNVAL
log2 median−normalized peptide abundances
1.5
1.0
0.5
0.0
−0.5
−1.0
−1.5
ME/CFS−Norway (VD001)
ME/CFS−Canada (VD001)
Controls−Canada (VD001)
Controls−USA (VD001)
ME/CFS−Canada (AD001)
Controls−Canada (AD001)

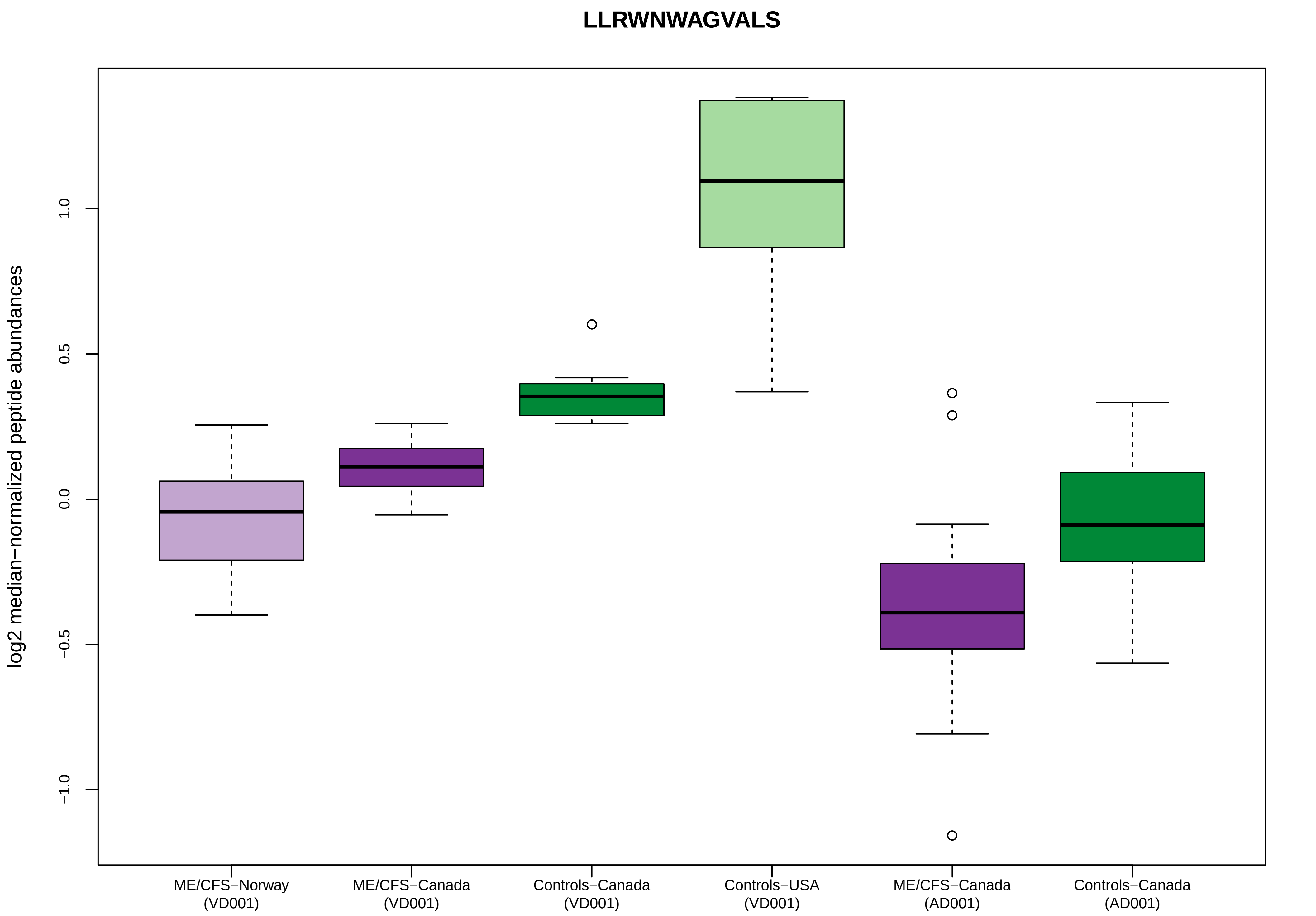

LLRWNWAGVALS
log2 median−normalized peptide abundances
1.0
0.5
0.0
−0.5
−1.0
ME/CFS−Norway (VD001)
ME/CFS−Canada (VD001)
Controls−Canada (VD001)
Controls−USA (VD001)
ME/CFS−Canada (AD001)
Controls−Canada (AD001)

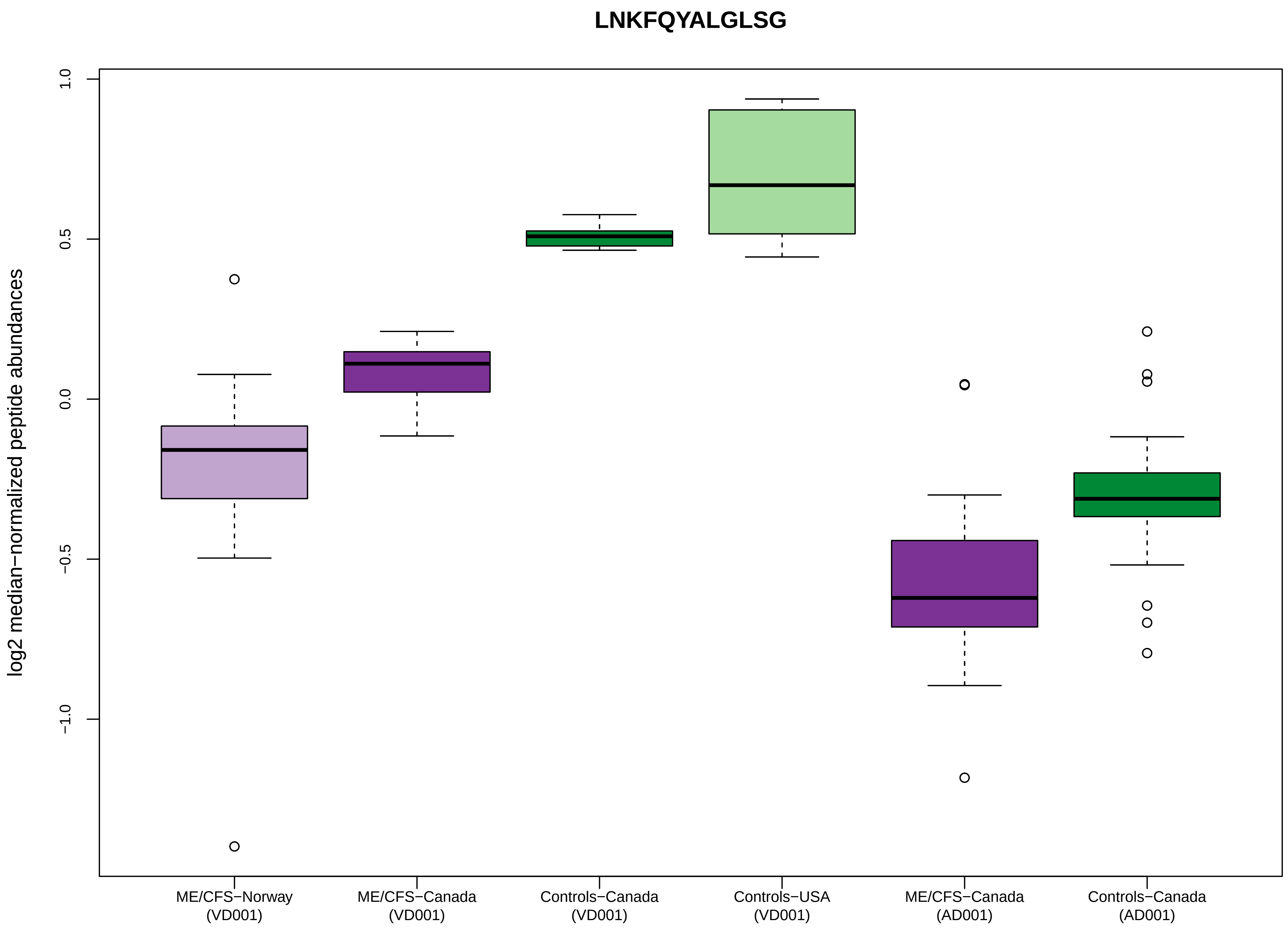
LNKFQYALGLSG
log2 median−normalized peptide abundances
1.0
0.5
0.0
−0.5
−1.0
ME/CFS−Norway (VD001)
ME/CFS−Canada (VD001)
Controls−Canada (VD001)
Controls−USA (VD001)
ME/CFS−Canada (AD001)
Controls−Canada (AD001)

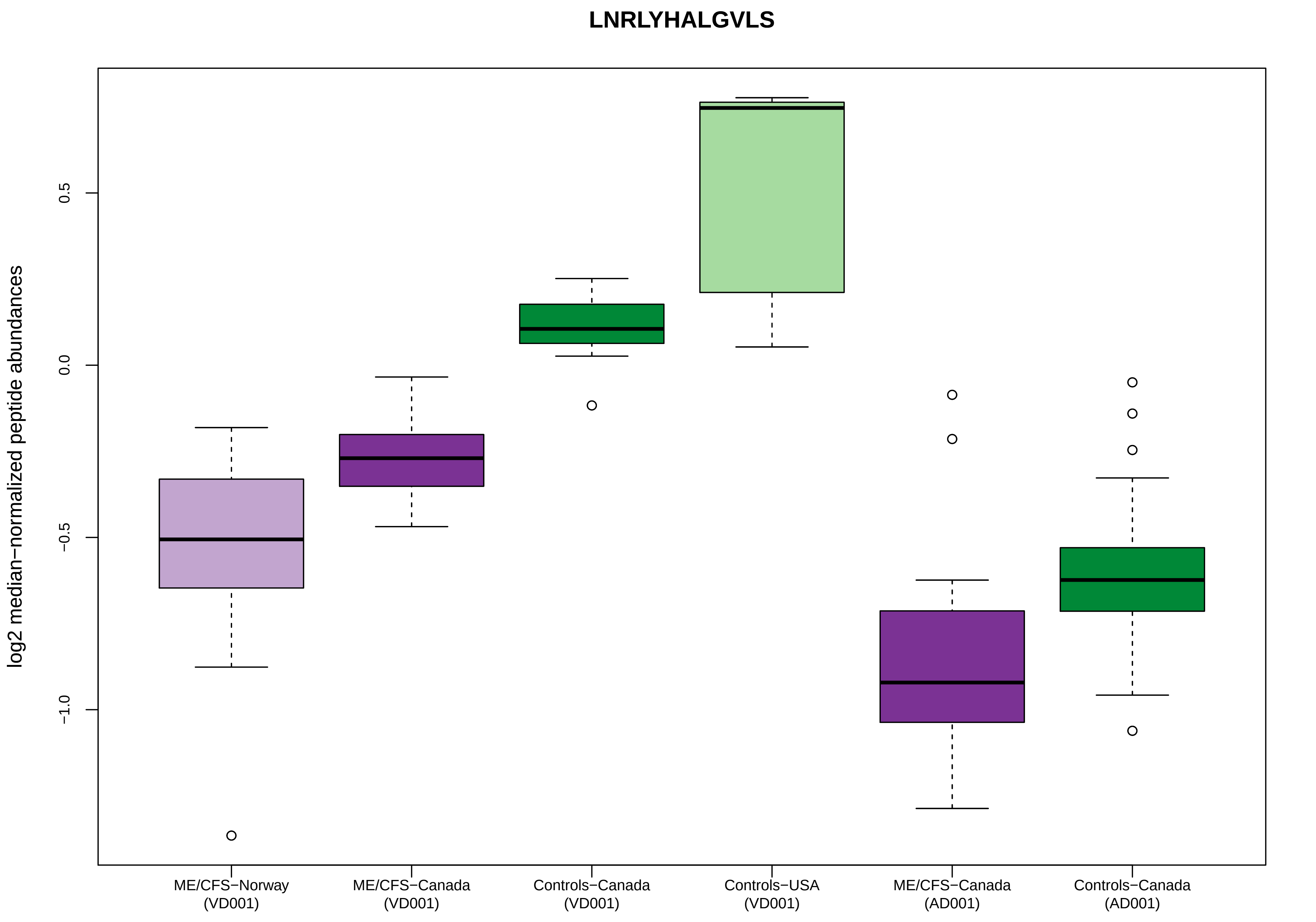

LNRLYHALGVLS
log2 median−normalized peptide abundances
0.5
0.0
−0.5
−1.0
ME/CFS−Norway (VD001)
ME/CFS−Canada (VD001)
Controls−Canada (VD001)
Controls−USA (VD001)
ME/CFS−Canada (AD001)
Controls−Canada (AD001)

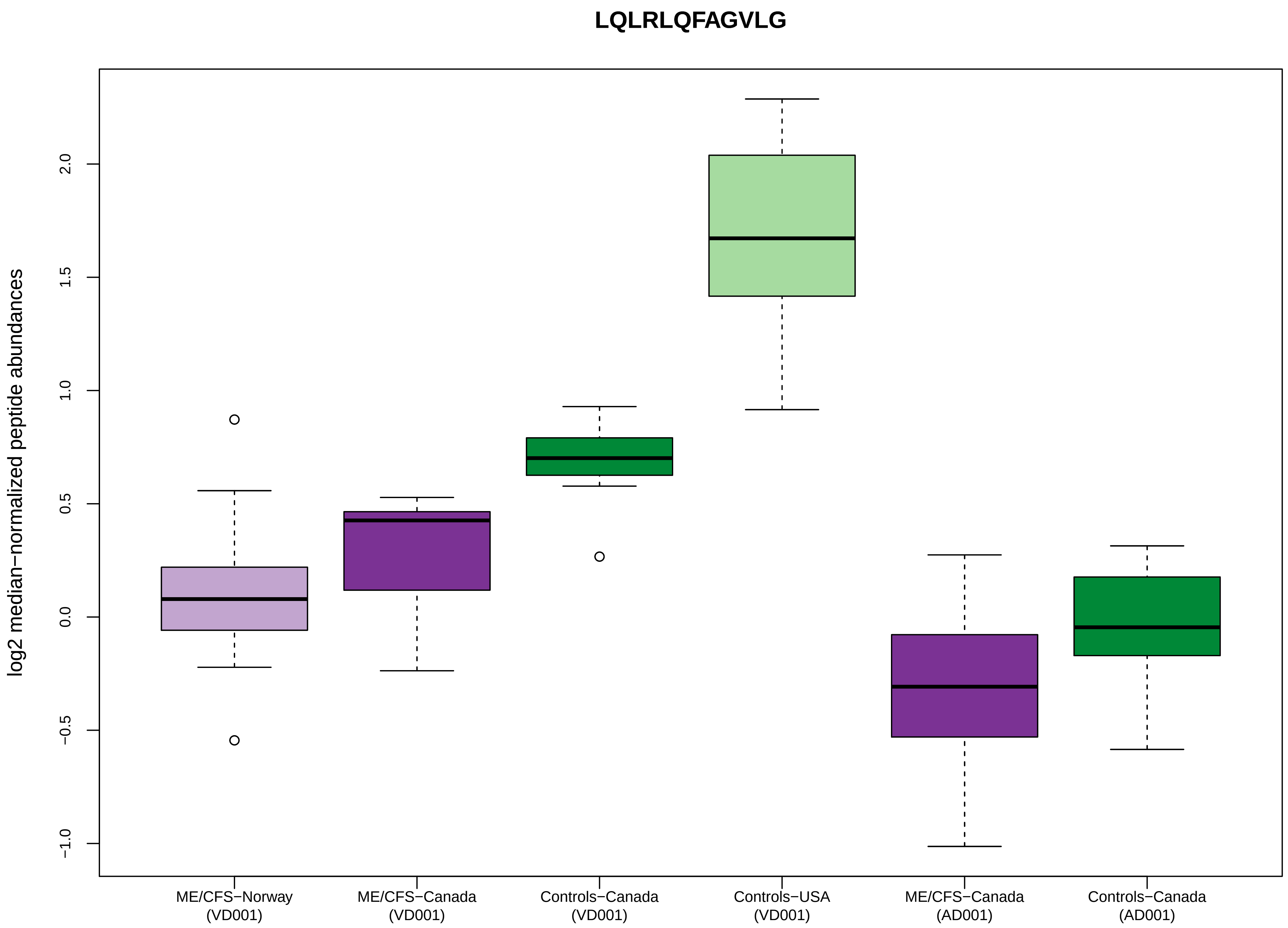

LQLRLQFAGVLG
log2 median−normalized peptide abundances
−1.0
−0.5
0.0
0.5
1.0
1.5
2.0
ME/CFS−Norway (VD001)
ME/CFS−Canada (VD001)
Controls−Canada (VD001)
Controls−USA (VD001)
ME/CFS−Canada (AD001)
Controls−Canada (AD001)

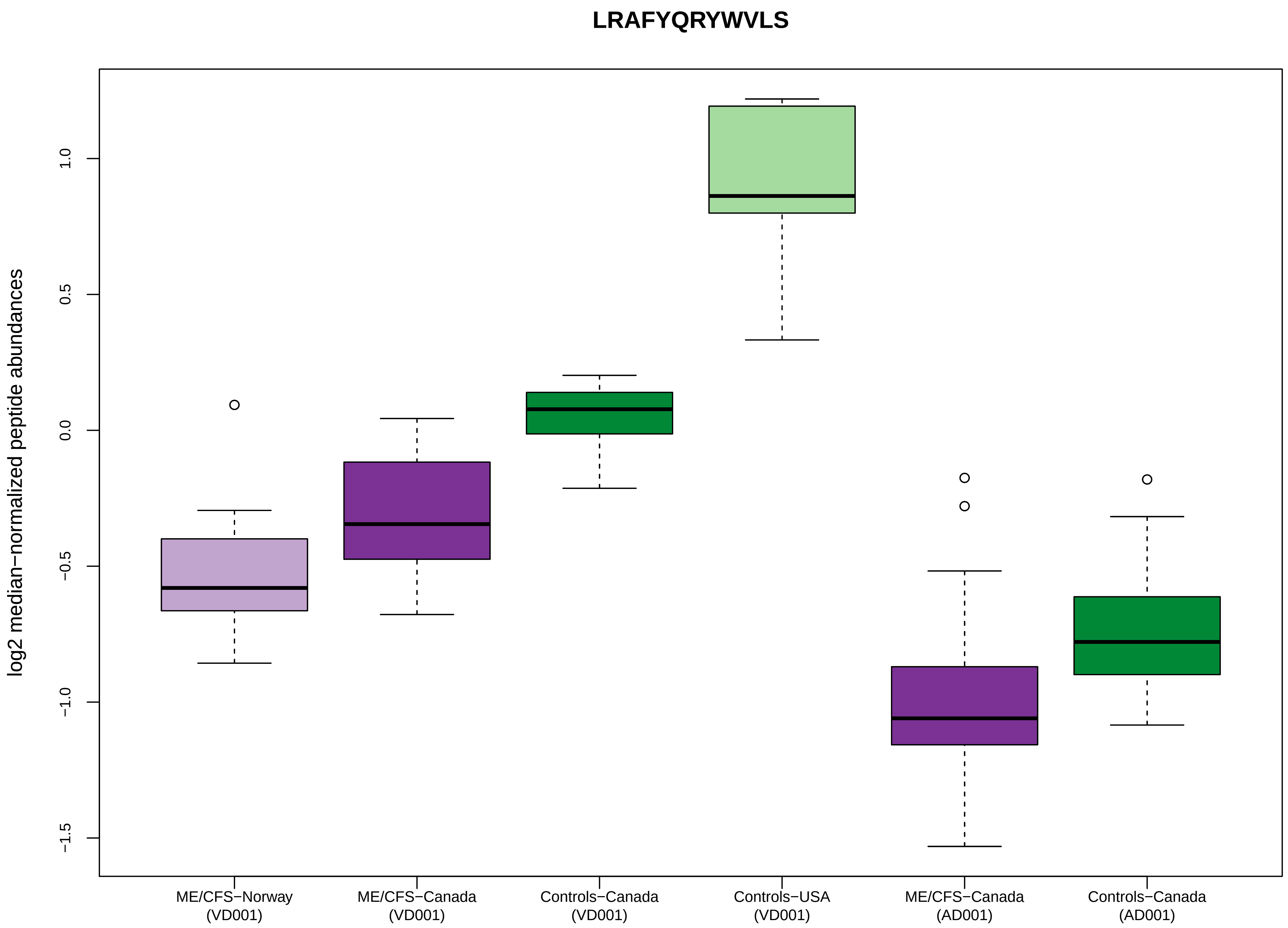
LRAFYQRYWVLS
log2 median−normalized peptide abundances
1.0
0.5
0.0
−0.5
−1.0
−1.5
ME/CFS−Norway (VD001)
ME/CFS−Canada (VD001)
Controls−Canada (VD001)
Controls−USA (VD001)
ME/CFS−Canada (AD001)
Controls−Canada (AD001)

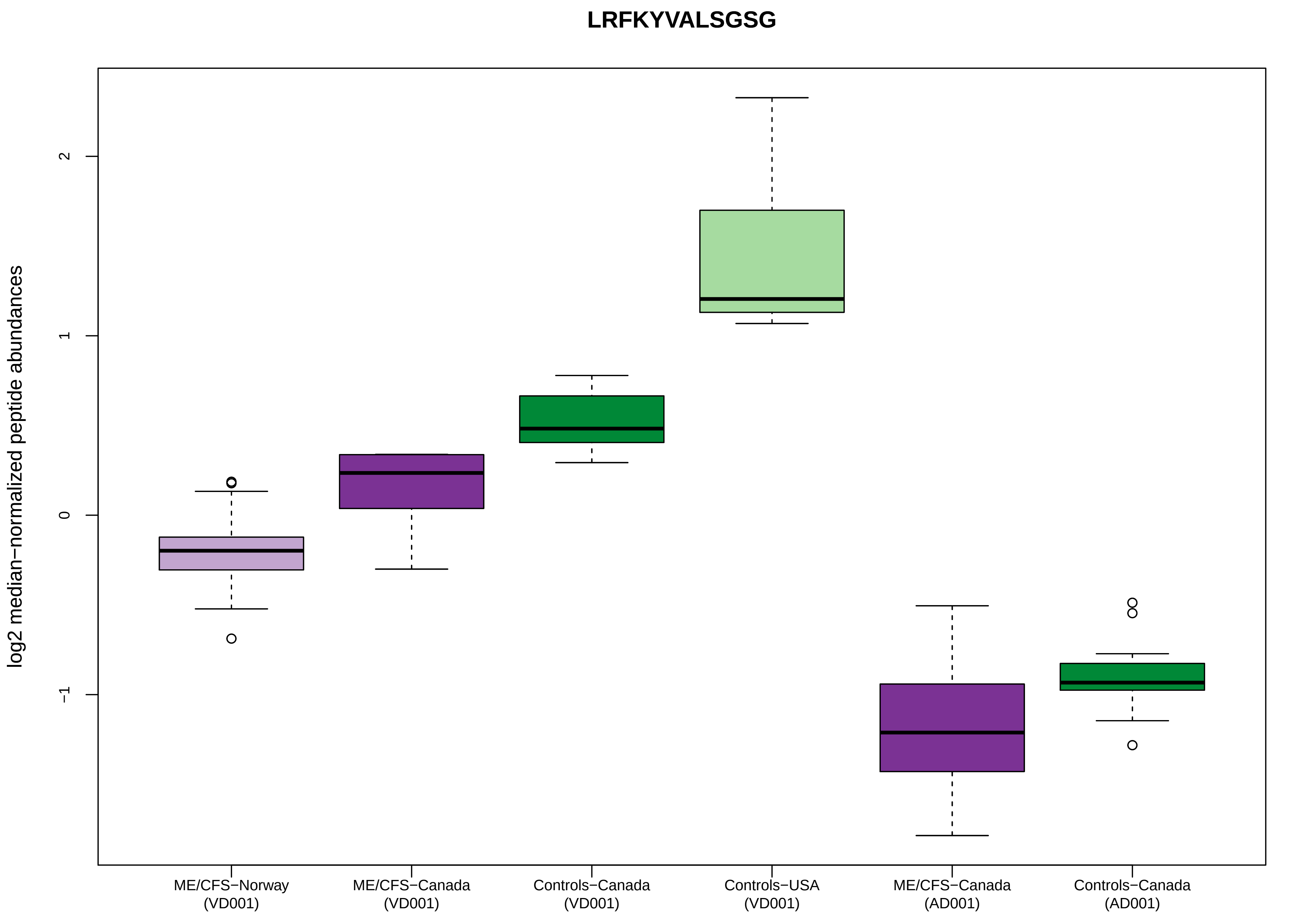
LRFKYVALSGSG
log2 median−normalized peptide abundances
2
1
0
−1
ME/CFS−Norway (VD001)
ME/CFS−Canada (VD001)
Controls−Canada (VD001)
Controls−USA (VD001)
ME/CFS−Canada (AD001)
Controls−Canada (AD001)

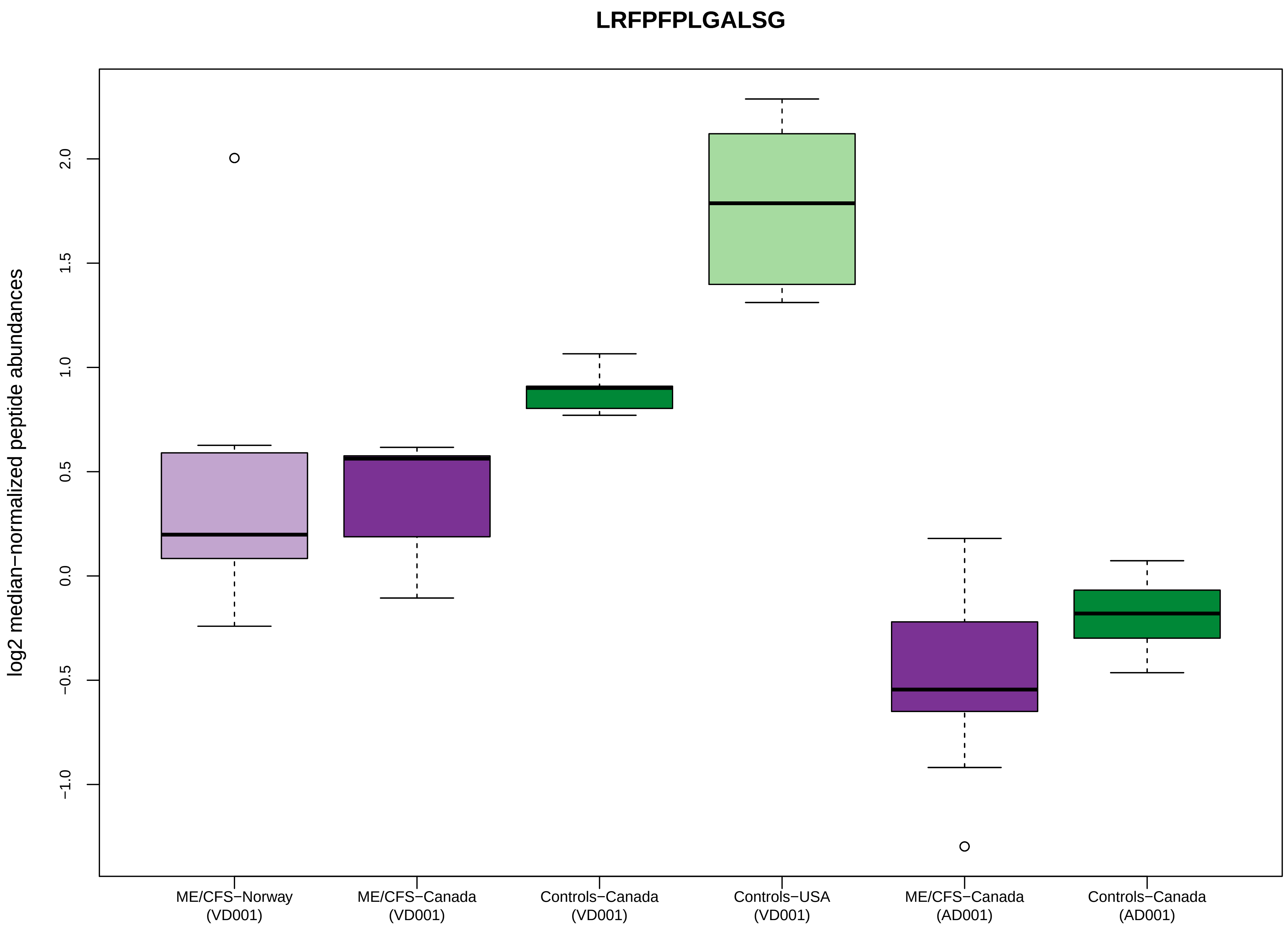

LRFPFPLGALSG
log2 median−normalized peptide abundances
2.0
1.5
1.0
0.5
0.0
−0.5
−1.0
ME/CFS−Norway (VD001)
ME/CFS−Canada (VD001)
Controls−Canada (VD001)
Controls−USA (VD001)
ME/CFS−Canada (AD001)
Controls−Canada (AD001)

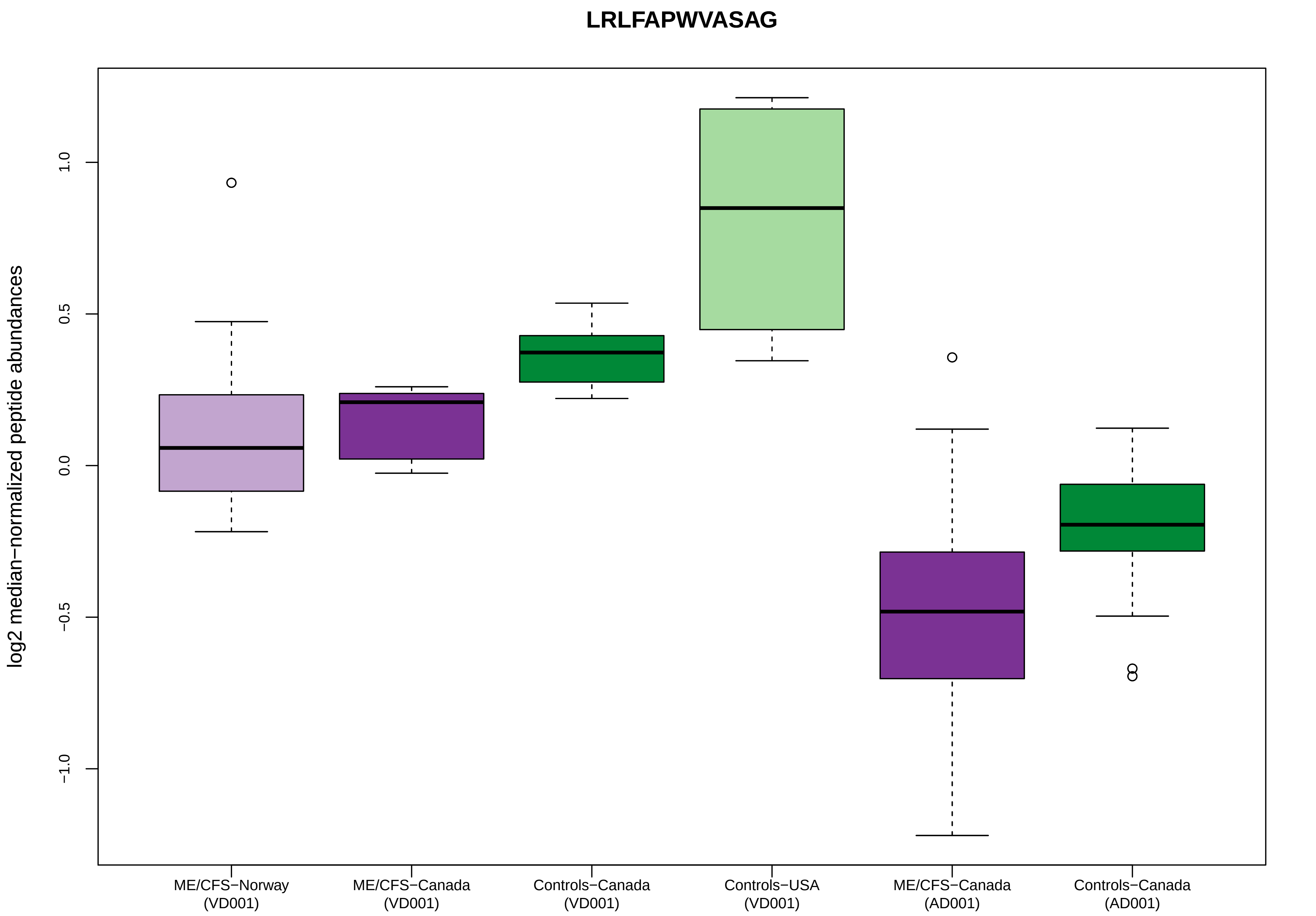
LRLFAPWVASAG
log2 median−normalized peptide abundances
1.0
0.5
0.0
−0.5
−1.0
ME/CFS−Norway (VD001)
ME/CFS−Canada (VD001)
Controls−Canada (VD001)
Controls−USA (VD001)
ME/CFS−Canada (AD001)
Controls−Canada (AD001)

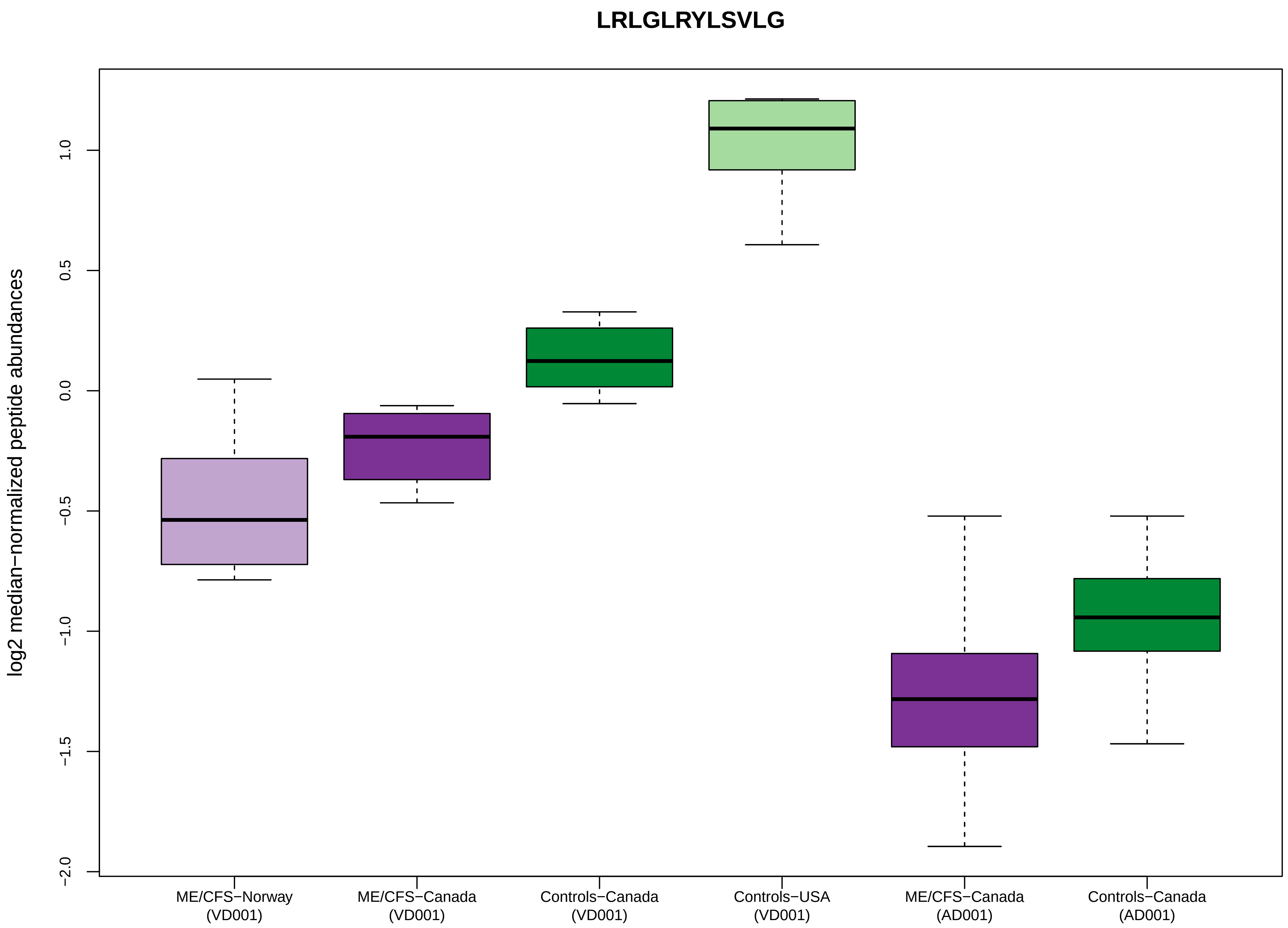

LRLGLRYLSVLG
log2 median−normalized peptide abundances
1.0
0.5
0.0
−0.5
−1.0
−1.5
−2.0
ME/CFS−Norway (VD001)
ME/CFS−Canada (VD001)
Controls−Canada (VD001)
Controls−USA (VD001)
ME/CFS−Canada (AD001)
Controls−Canada (AD001)

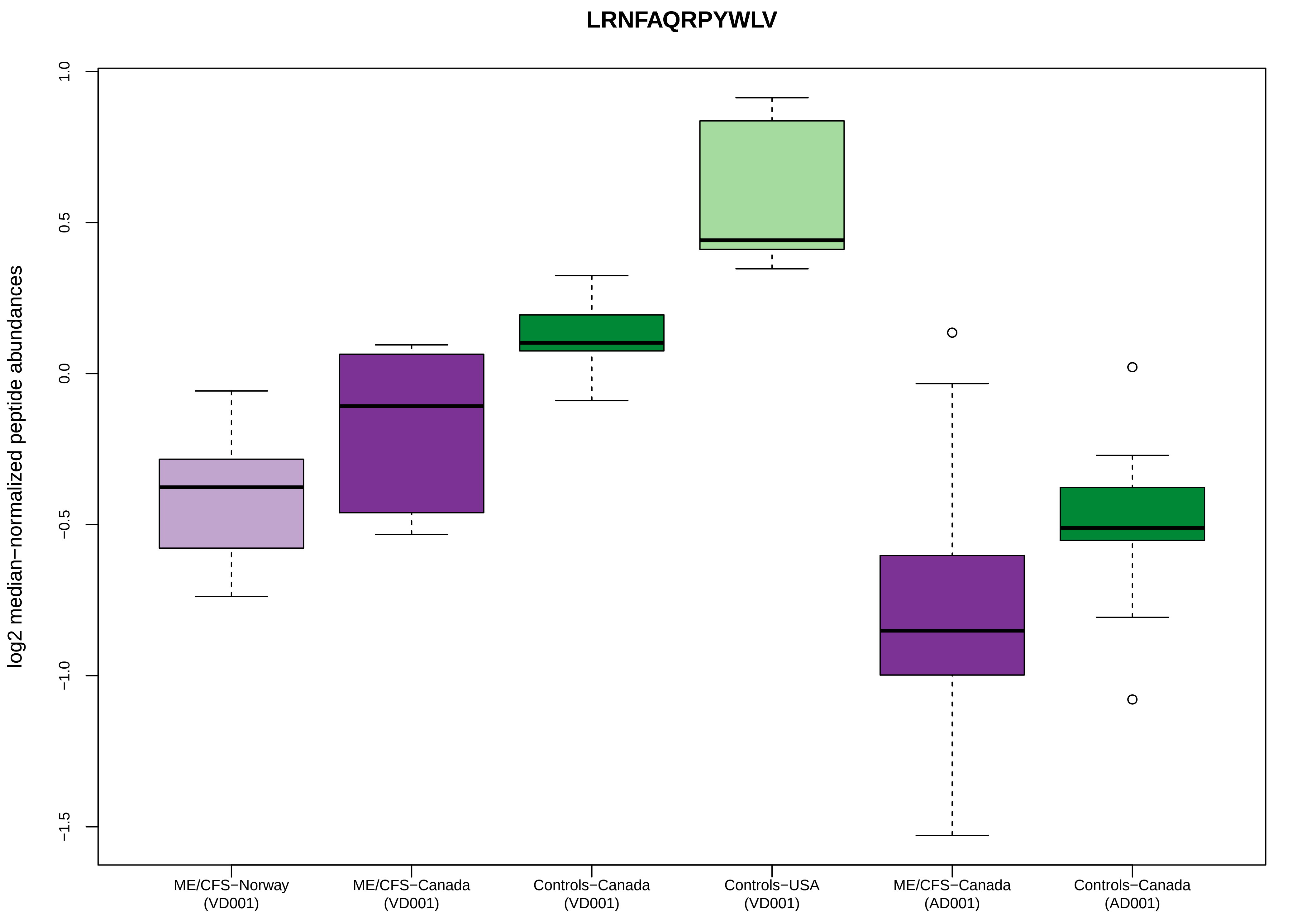
LRNFAQRPYWLV
log2 median−normalized peptide abundances
1.0
0.5
0.0
−0.5
−1.0
−1.5
ME/CFS−Norway (VD001)
ME/CFS−Canada (VD001)
Controls−Canada (VD001)
Controls−USA (VD001)
ME/CFS−Canada (AD001)
Controls−Canada (AD001)

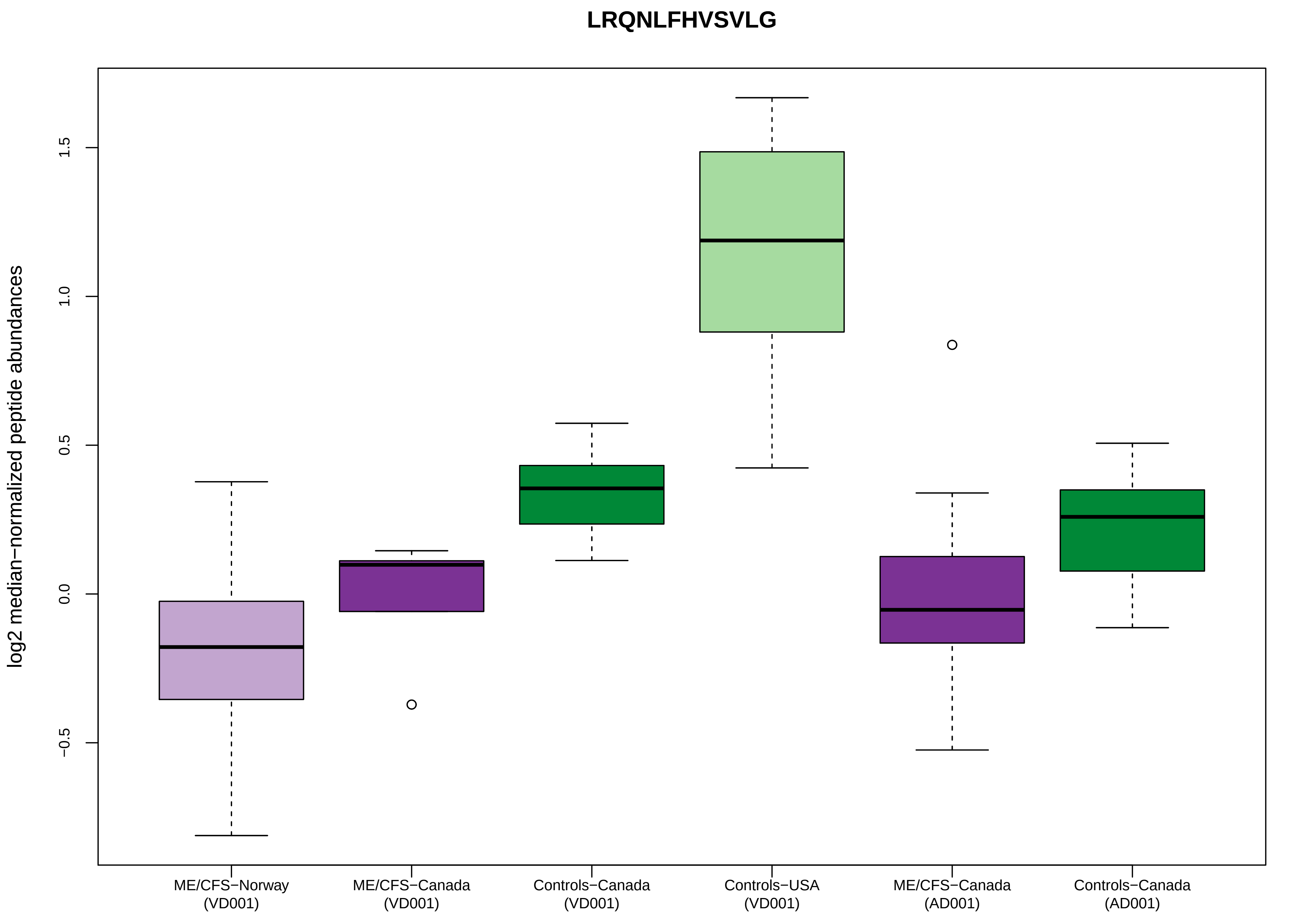

LRQNLFHVSVLG
log2 median−normalized peptide abundances
−0.5
0.0
0.5
1.0
1.5
ME/CFS−Norway (VD001)
ME/CFS−Canada (VD001)
Controls−Canada (VD001)
Controls−USA (VD001)
ME/CFS−Canada (AD001)
Controls−Canada (AD001)

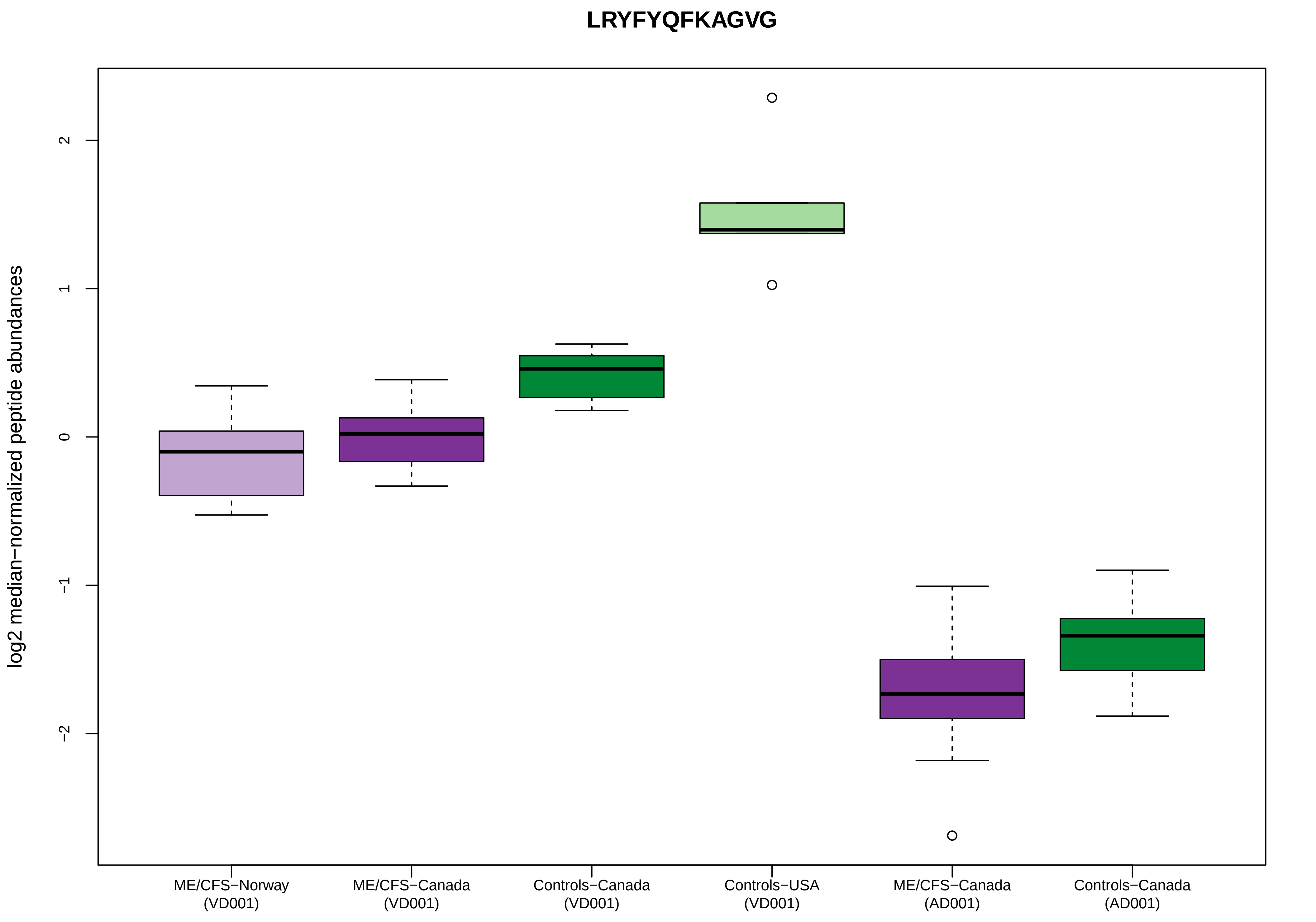

LRYFYQFKAGVG
log2 median−normalized peptide abundances
2
1
0
−1
−2
ME/CFS−Norway (VD001)
ME/CFS−Canada (VD001)
Controls−Canada (VD001)
Controls−USA (VD001)
ME/CFS−Canada (AD001)
Controls−Canada (AD001)

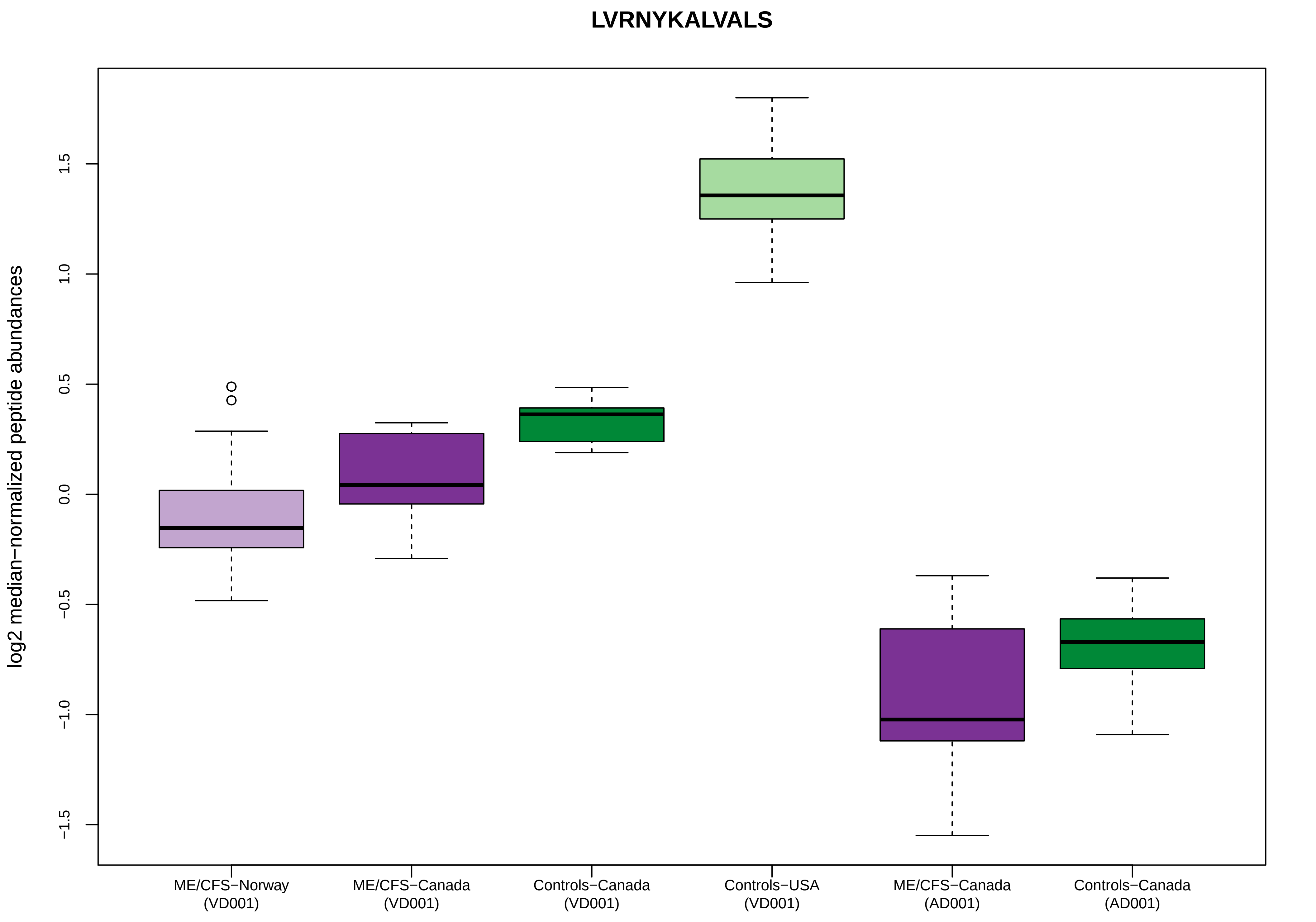

LVRNYKALVALS
log2 median−normalized peptide abundances
1.5
1.0
0.5
0.0
−0.5
−1.0
−1.5
ME/CFS−Norway (VD001)
ME/CFS−Canada (VD001)
Controls−Canada (VD001)
Controls−USA (VD001)
ME/CFS−Canada (AD001)
Controls−Canada (AD001)

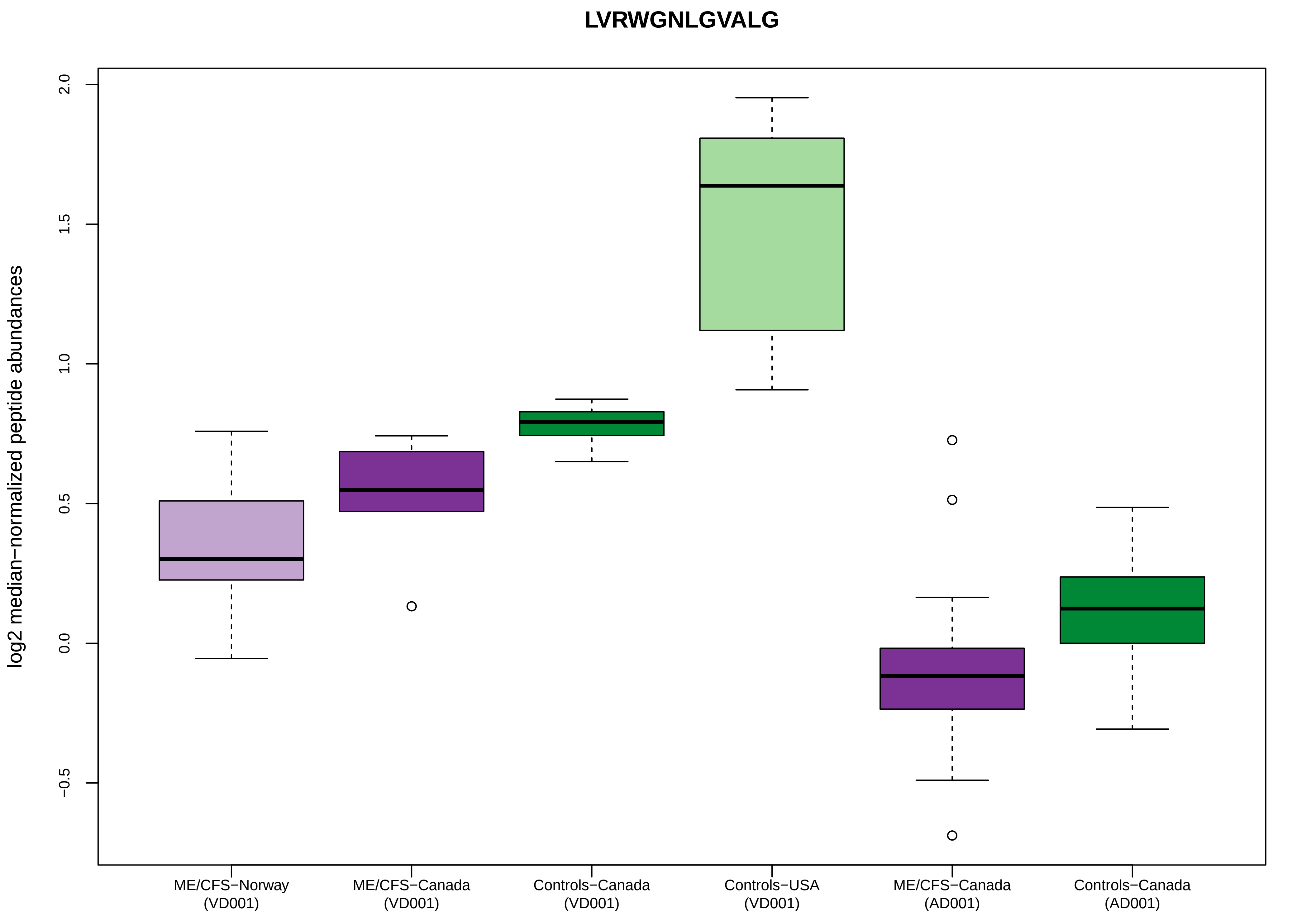
LVRWGNLGVALG
log2 median−normalized peptide abundances
2.0
1.5
1.0
0.5
0.0
−0.5
ME/CFS−Norway
(VD001)
ME/CFS−Canada
(VD001)
Controls−Canada
(VD001)
Controls−USA
(VD001)
ME/CFS−Canada
(AD001)
Controls−Canada
(AD001)

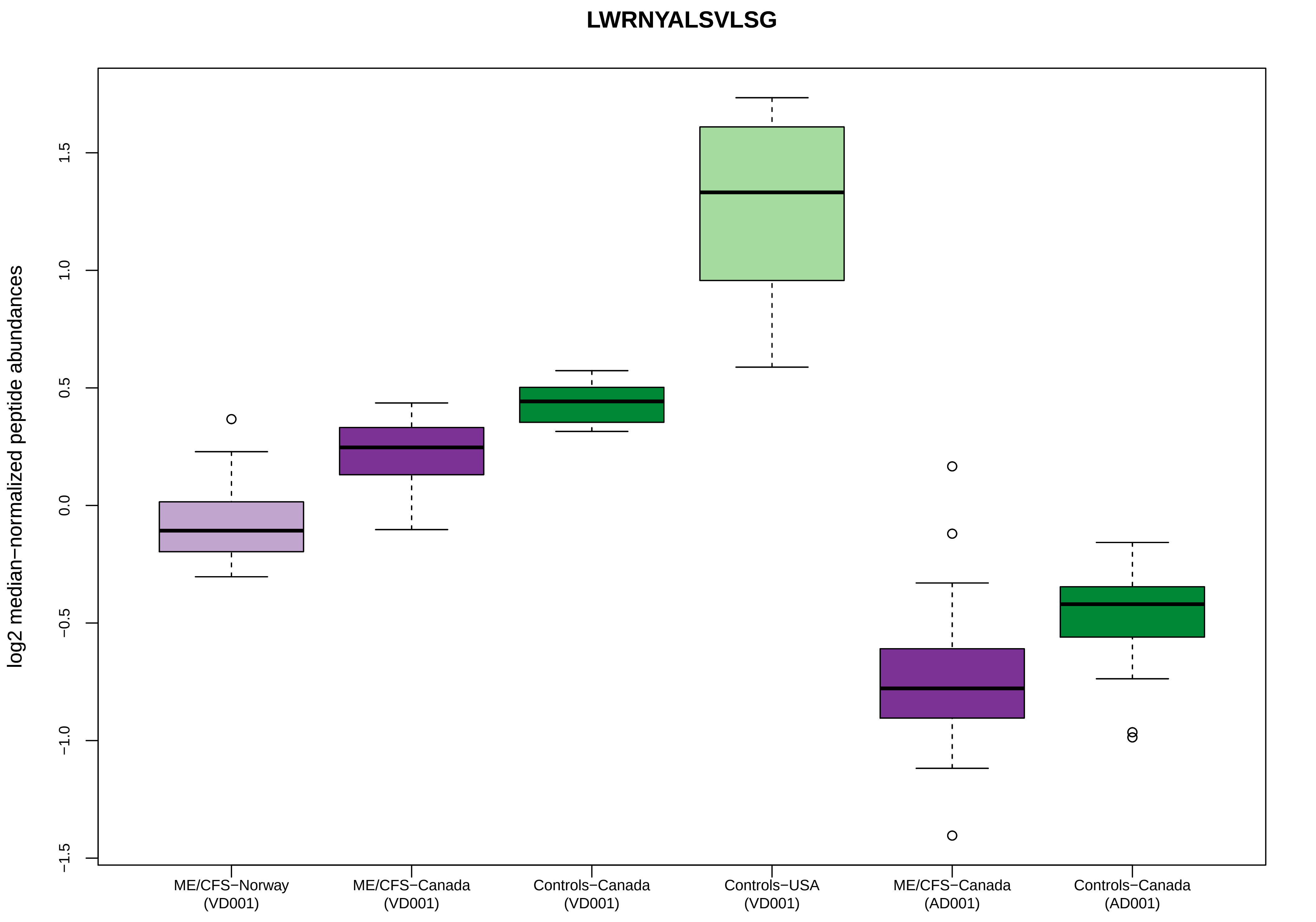

LWRNYALSVLSG
log2 median−normalized peptide abundances
1.5
1.0
0.5
0.0
−0.5
−1.0
−1.5
ME/CFS−Norway (VD001)
ME/CFS−Canada (VD001)
Controls−Canada (VD001)
Controls−USA (VD001)
ME/CFS−Canada (AD001)
Controls−Canada (AD001)

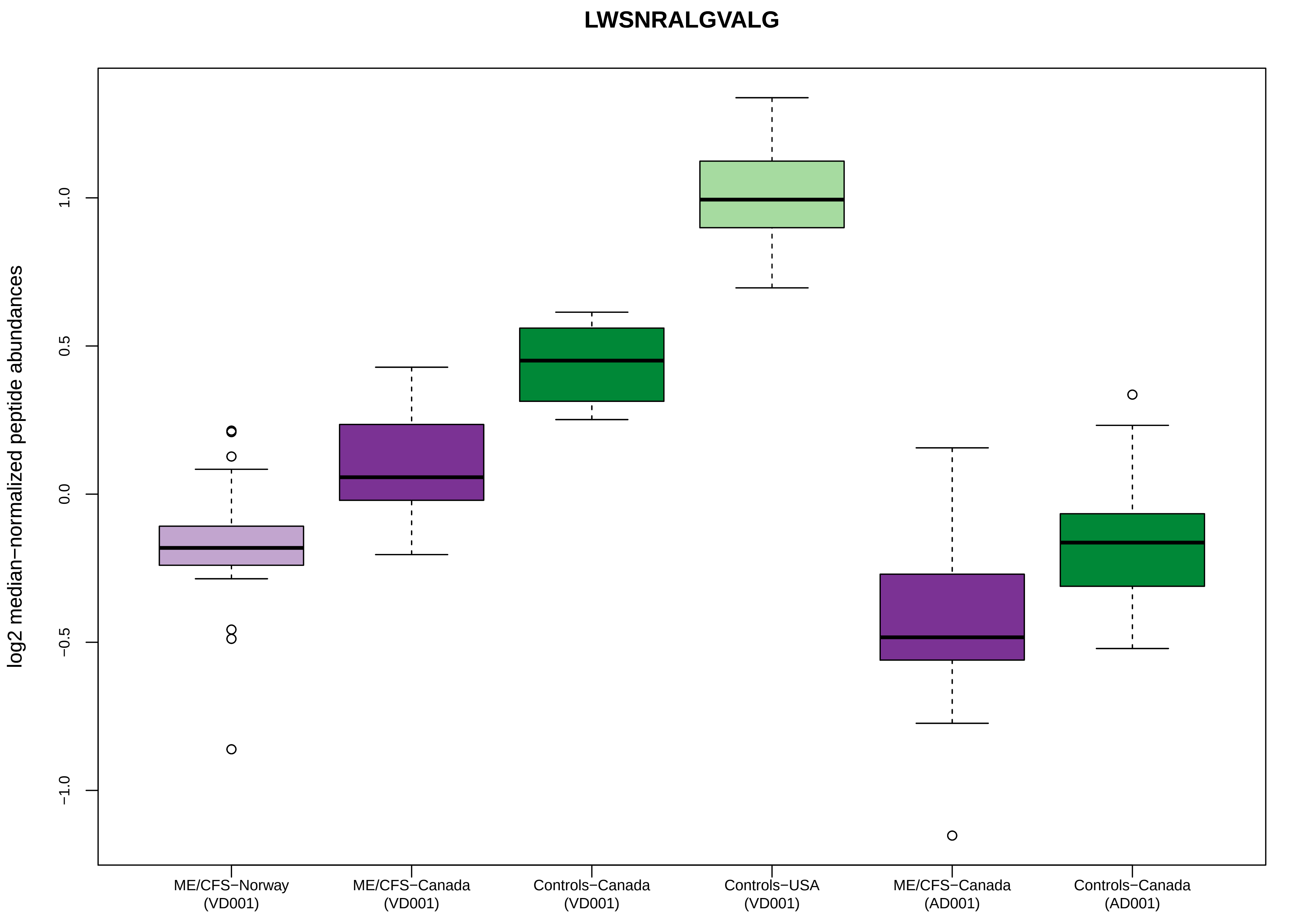

LWSNRALGVALG
log2 median−normalized peptide abundances
1.0
0.5
0.0
−0.5
−1.0
ME/CFS−Norway
(VD001)
ME/CFS−Canada
(VD001)
Controls−Canada
(VD001)
Controls−USA
(VD001)
ME/CFS−Canada
(AD001)
Controls−Canada
(AD001)

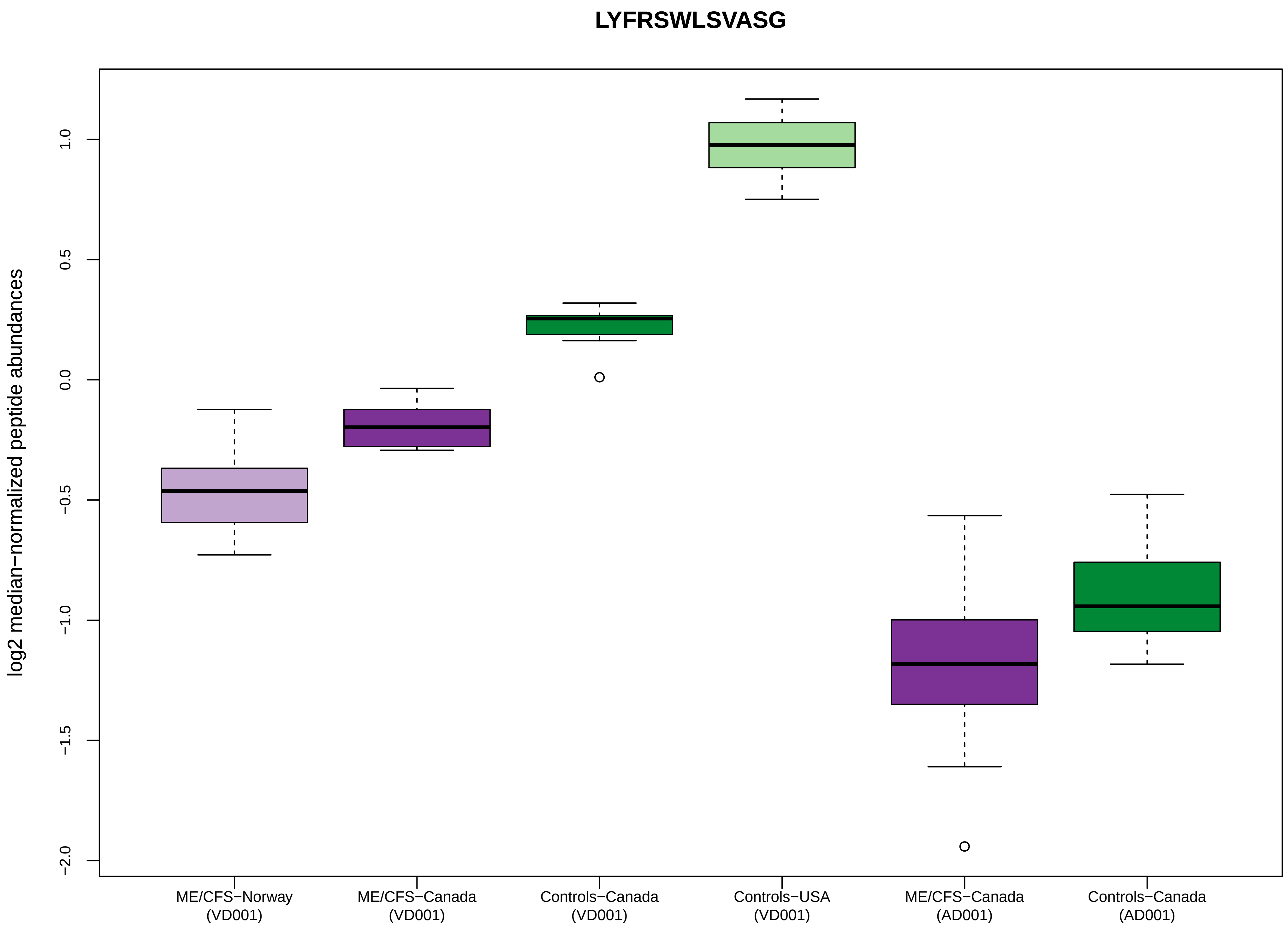

LYFRSWLSVASG
log2 median−normalized peptide abundances
1.0
0.5
0.0
−0.5
−1.0
−1.5
−2.0
ME/CFS−Norway (VD001)
ME/CFS−Canada (VD001)
Controls−Canada (VD001)
Controls−USA (VD001)
ME/CFS−Canada (AD001)
Controls−Canada (AD001)

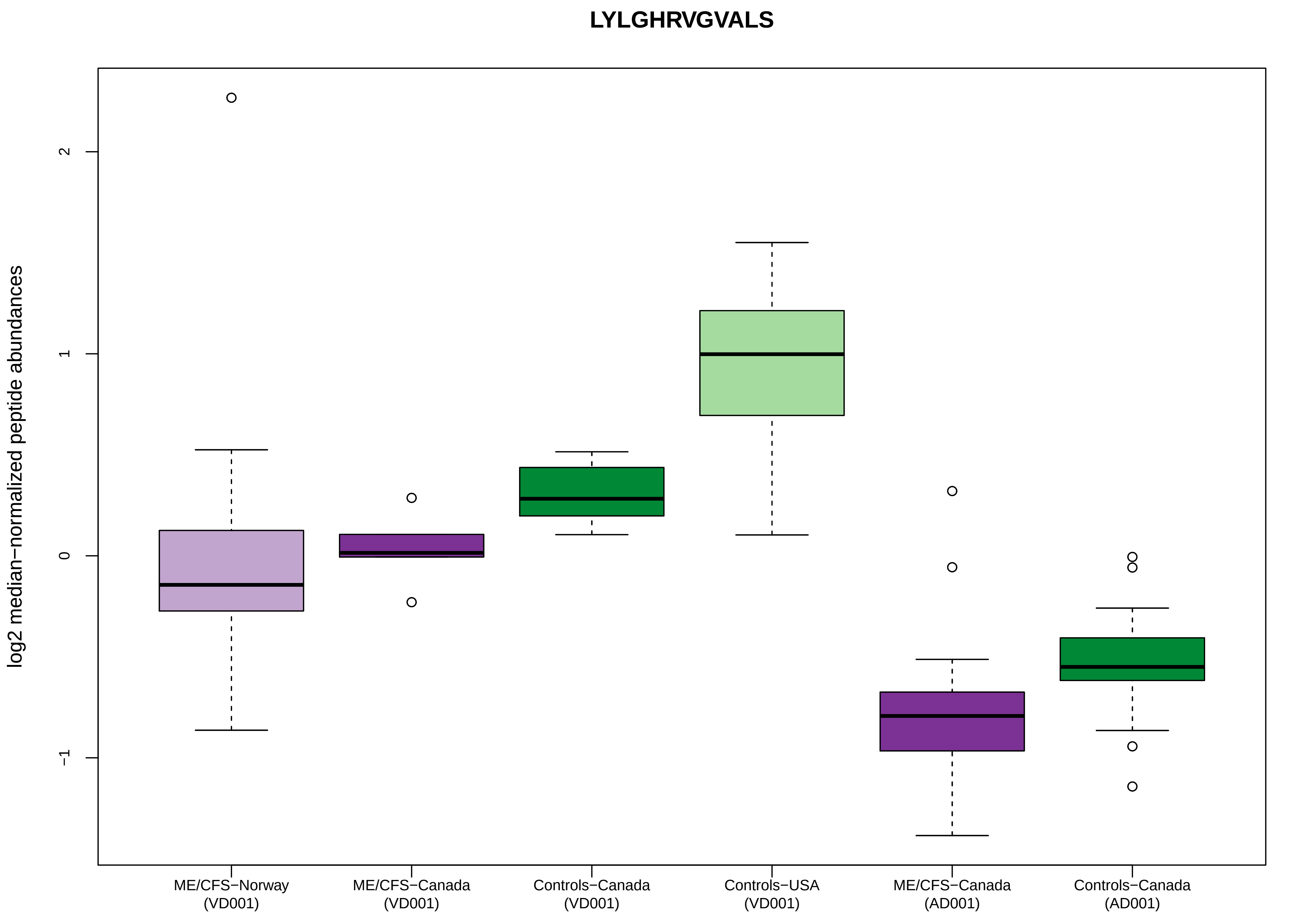

LYLGHRVGVALS
log2 median−normalized peptide abundances
2
1
0
−1
ME/CFS−Norway (VD001)
ME/CFS−Canada (VD001)
Controls−Canada (VD001)
Controls−USA (VD001)
ME/CFS−Canada (AD001)
Controls−Canada (AD001)

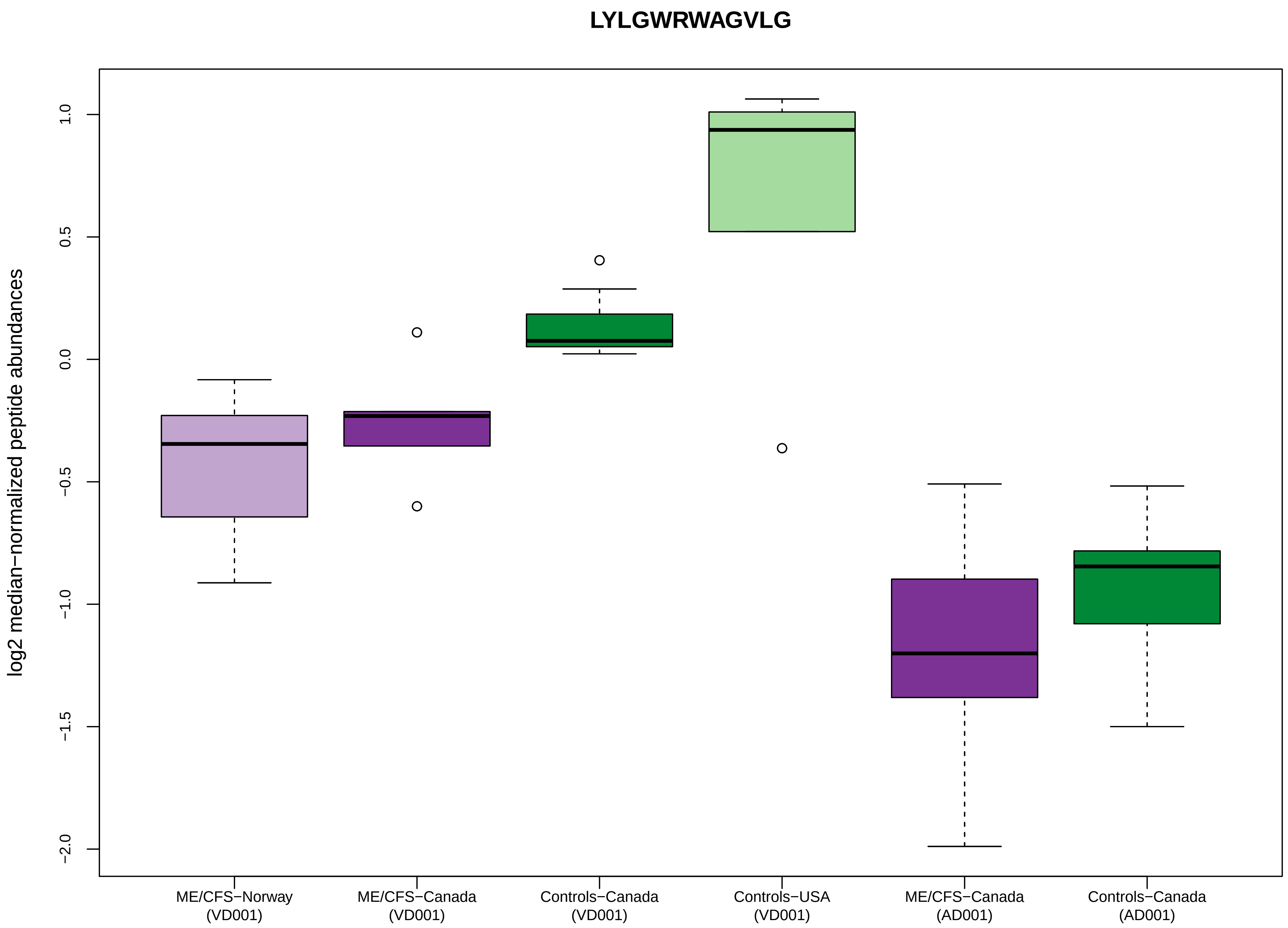

LYLGWRWAGVLG
log2 median−normalized peptide abundances
1.0
0.5
0.0
−0.5
−1.0
−1.5
−2.0
ME/CFS−Norway (VD001)
ME/CFS−Canada (VD001)
Controls−Canada (VD001)
Controls−USA (VD001)
ME/CFS−Canada (AD001)
Controls−Canada (AD001)

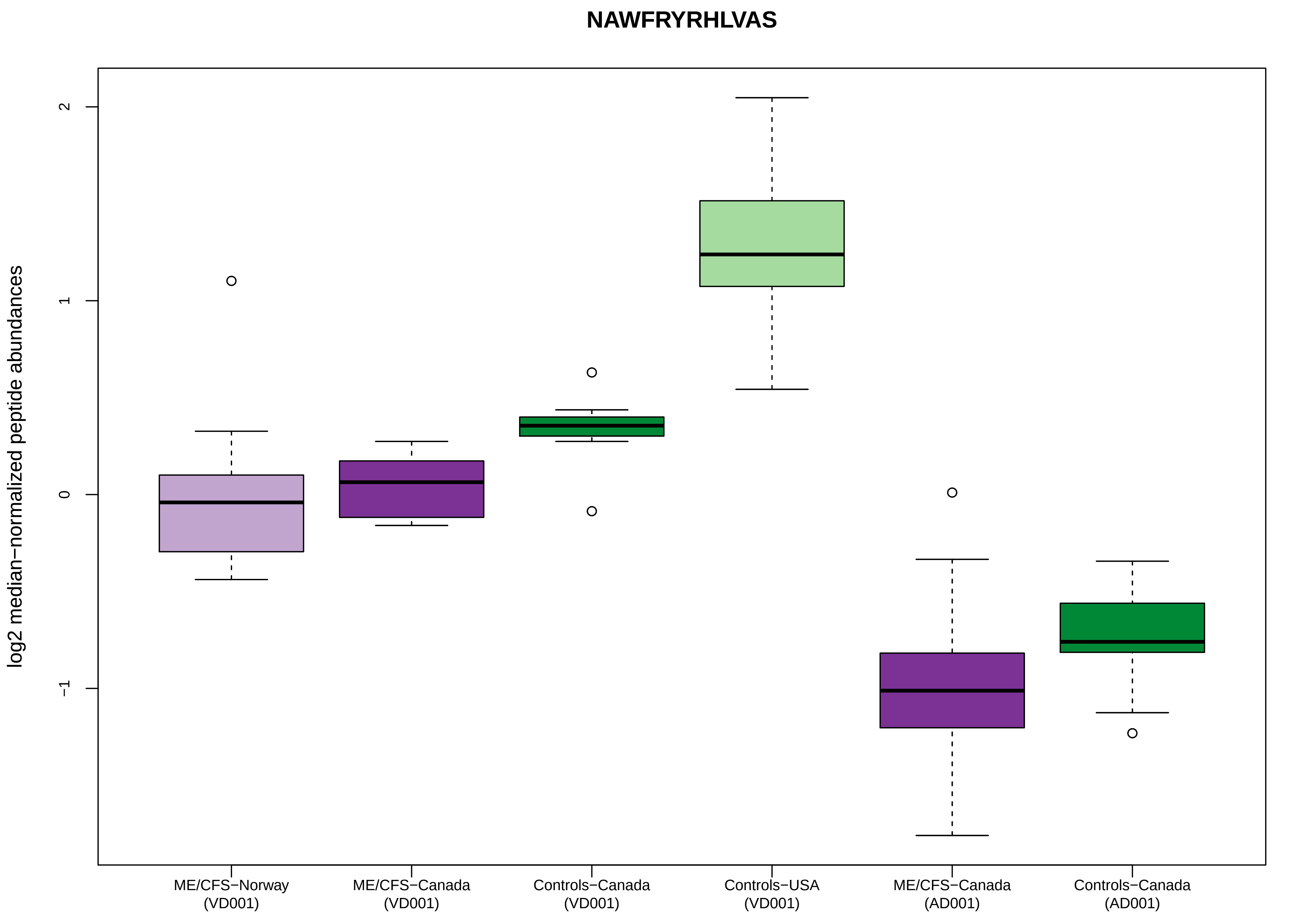
NAWFRYRHLVAS
log2 median−normalized peptide abundances
2
1
0
−1
ME/CFS−Norway (VD001)
ME/CFS−Canada (VD001)
Controls−Canada (VD001)
Controls−USA (VD001)
ME/CFS−Canada (AD001)
Controls−Canada (AD001)

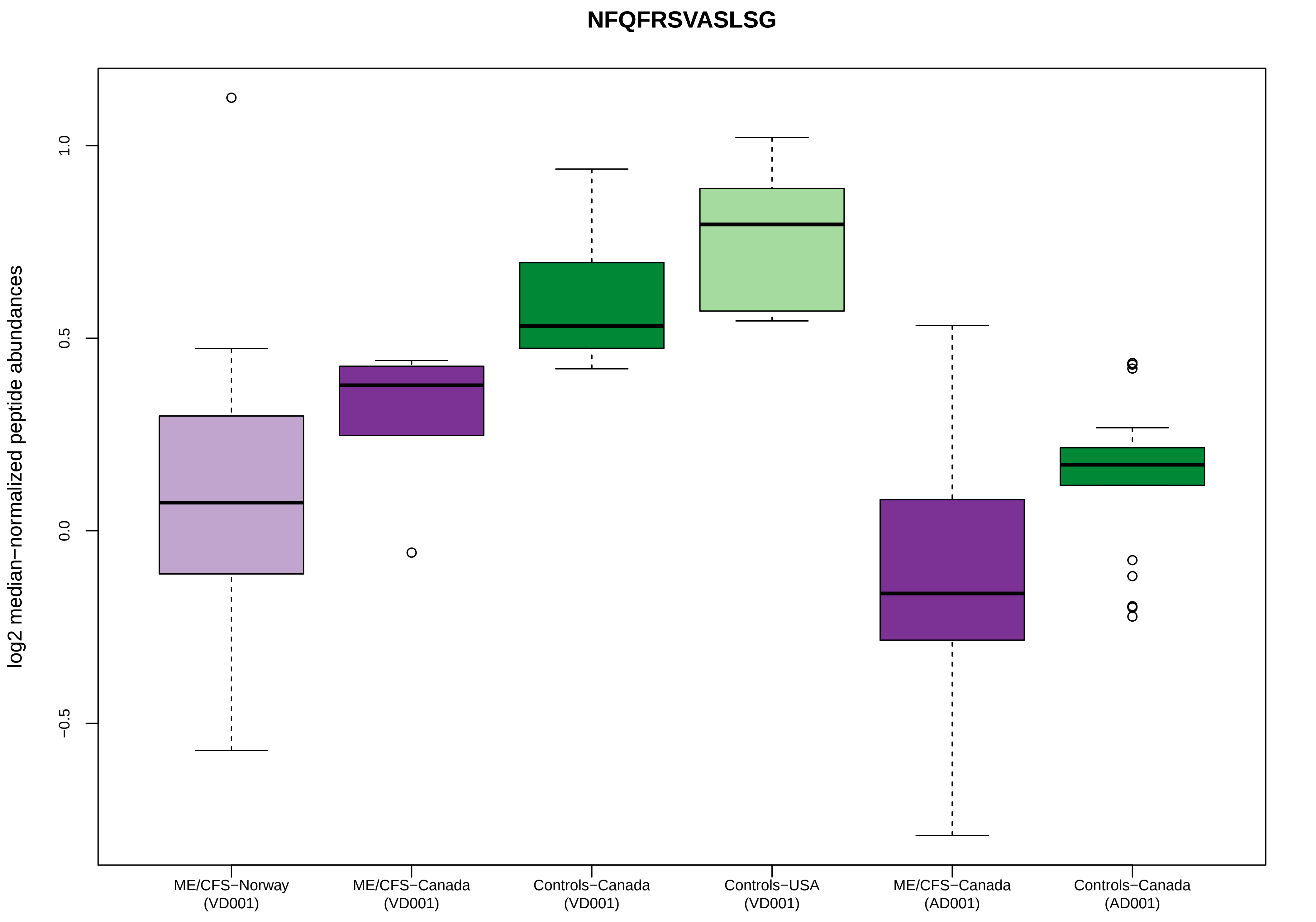

NFQFRSVASLSG
log2 median−normalized peptide abundances
1.0
0.5
0.0
−0.5
ME/CFS−Norway (VD001)
ME/CFS−Canada (VD001)
Controls−Canada (VD001)
Controls−USA (VD001)
ME/CFS−Canada (AD001)
Controls−Canada (AD001)

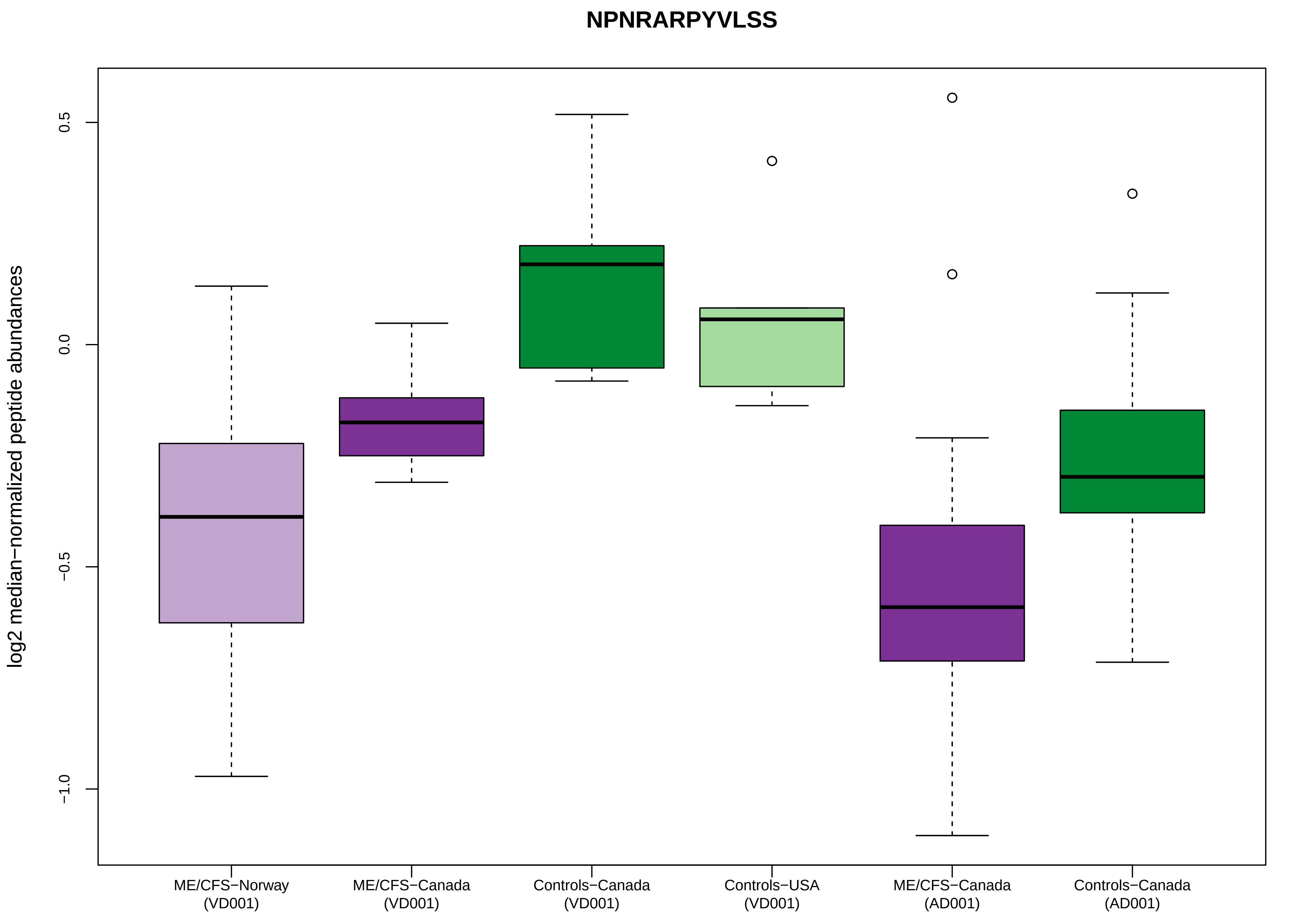

NPNRARPYVLSS
log2 median−normalized peptide abundances
0.5
0.0
−0.5
−1.0
ME/CFS−Norway (VD001)
ME/CFS−Canada (VD001)
Controls−Canada (VD001)
Controls−USA (VD001)
ME/CFS−Canada (AD001)
Controls−Canada (AD001)

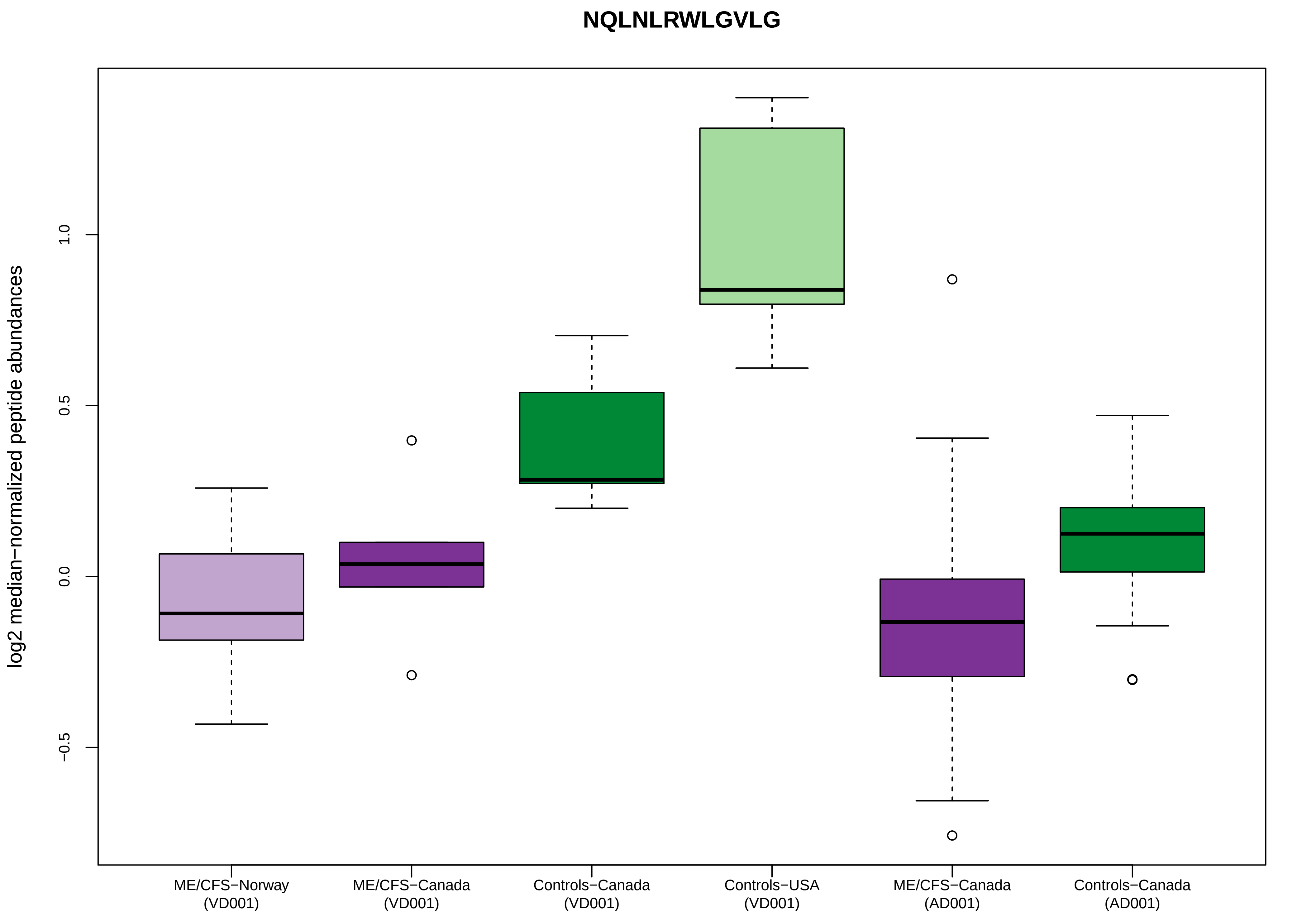

NQLNLRWLGVLG
log2 median−normalized peptide abundances
1.0
0.5
0.0
−0.5
ME/CFS−Norway (VD001)
ME/CFS−Canada (VD001)
Controls−Canada (VD001)
Controls−USA (VD001)
ME/CFS−Canada (AD001)
Controls−Canada (AD001)

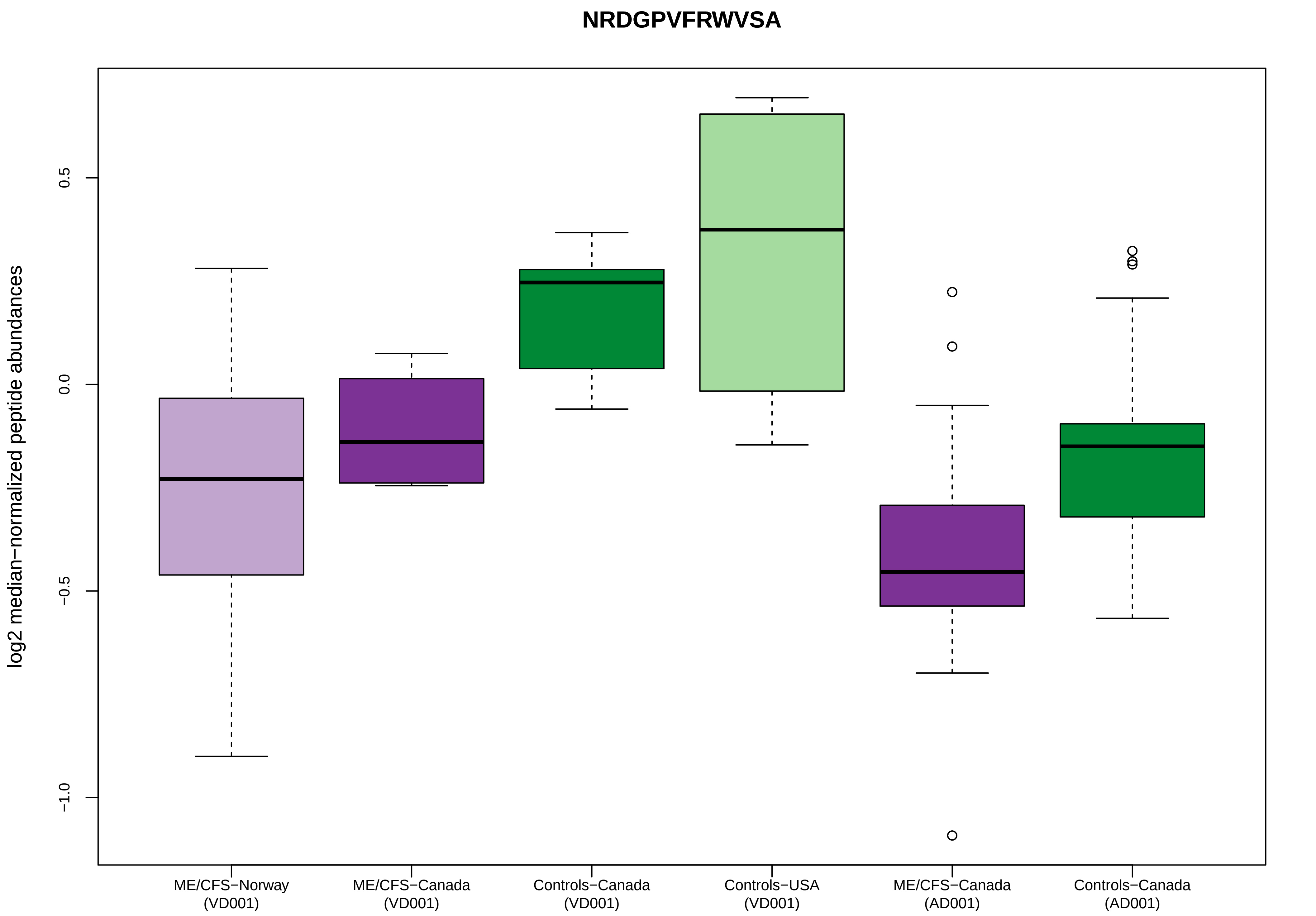
NRDGPVFRWVSA
log2 median−normalized peptide abundances
0.5
0.0
−0.5
−1.0
ME/CFS−Norway (VD001)
ME/CFS−Canada (VD001)
Controls−Canada (VD001)
Controls−USA (VD001)
ME/CFS−Canada (AD001)
Controls−Canada (AD001)

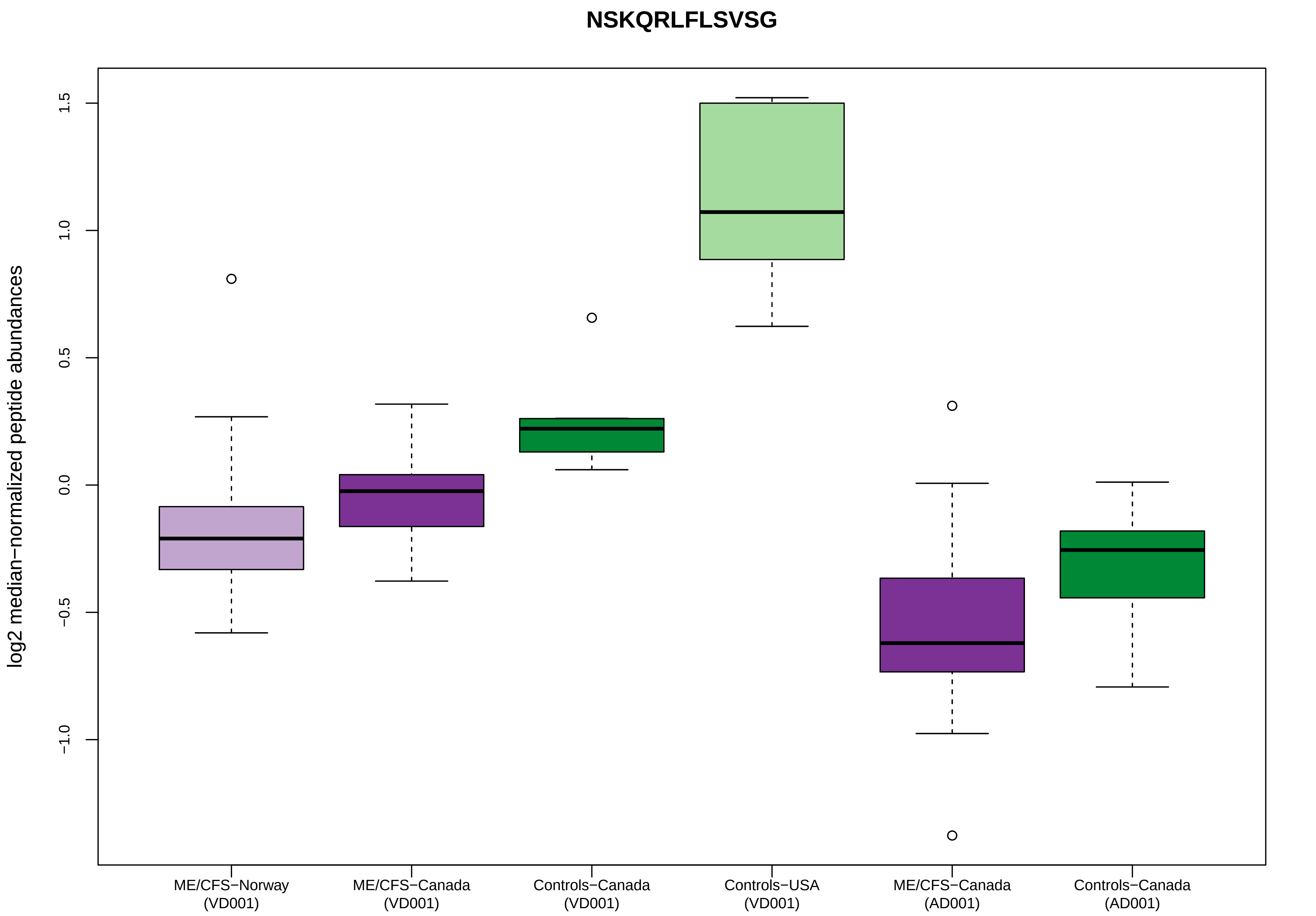
NSKQRLFLSVSG
log2 median−normalized peptide abundances
1.5
1.0
0.5
0.0
−0.5
−1.0
ME/CFS−Norway (VD001)
ME/CFS−Canada (VD001)
Controls−Canada (VD001)
Controls−USA (VD001)
ME/CFS−Canada (AD001)
Controls−Canada (AD001)

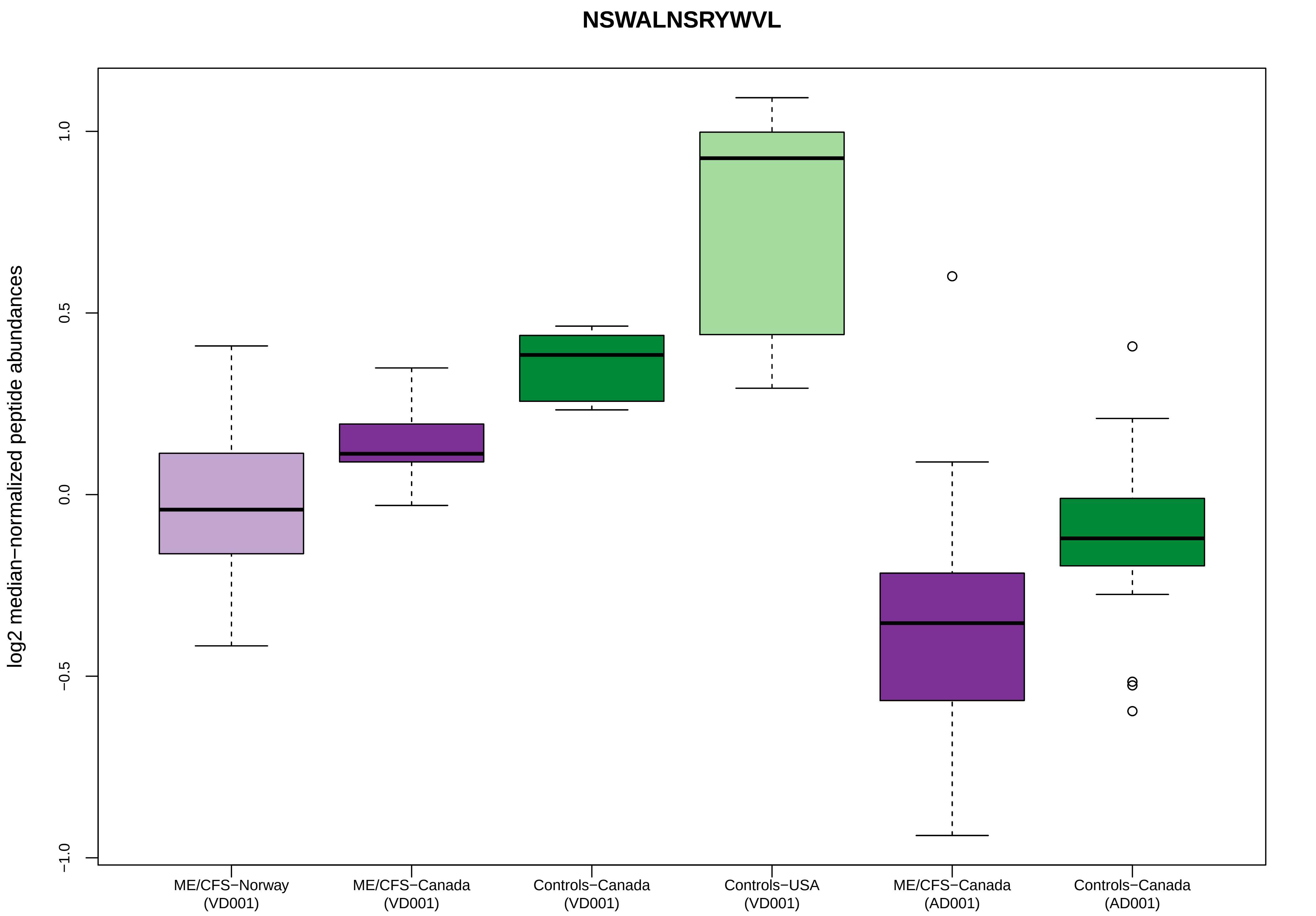
NSWALNSRYWVL
log2 median−normalized peptide abundances
1.0
0.5
0.0
−0.5
−1.0
ME/CFS−Norway (VD001)
ME/CFS−Canada (VD001)
Controls−Canada (VD001)
Controls−USA (VD001)
ME/CFS−Canada (AD001)
Controls−Canada (AD001)

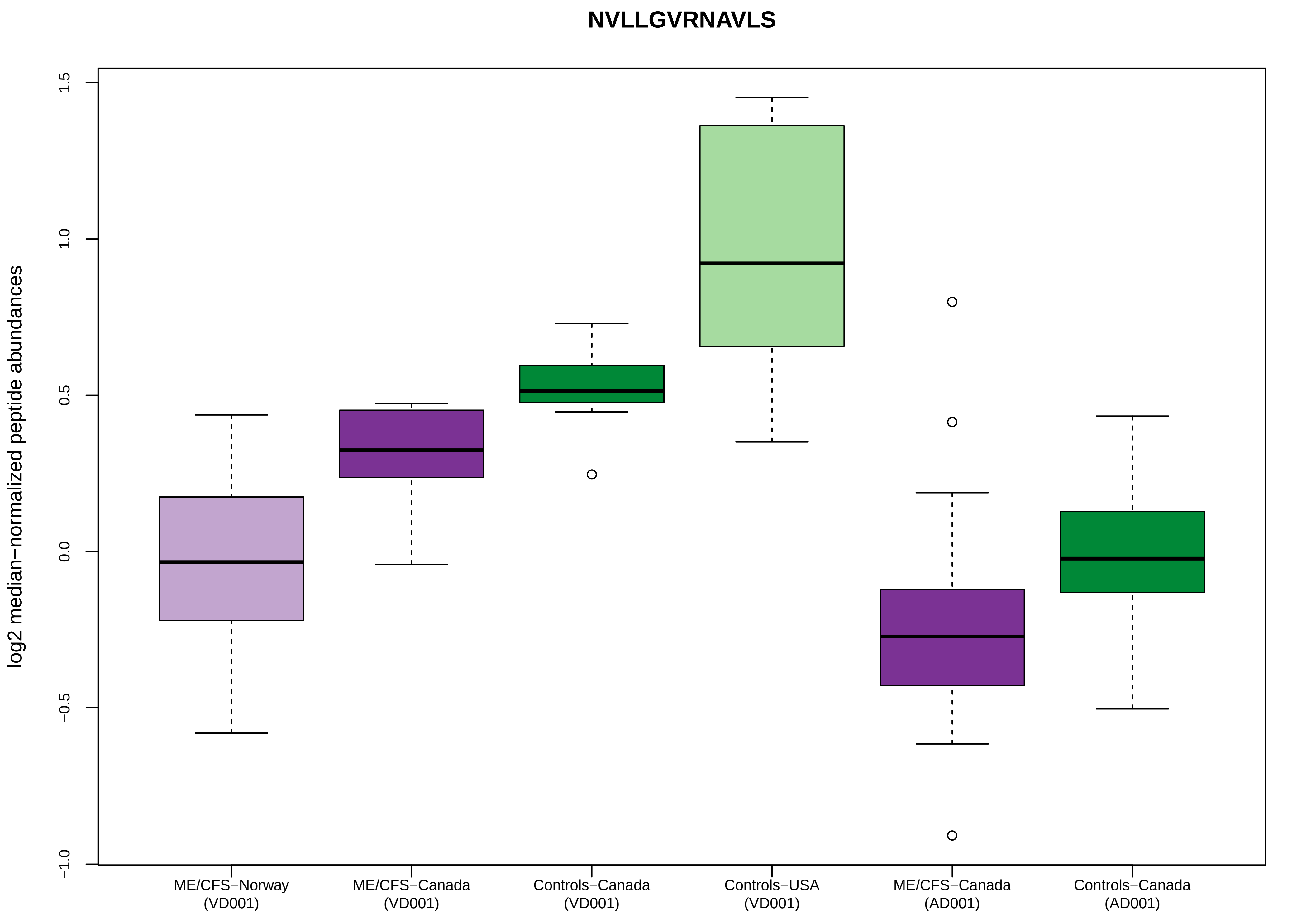

NVLLGVRNAVLS
log2 median−normalized peptide abundances
1.5
1.0
0.5
0.0
−0.5
−1.0
ME/CFS−Norway (VD001)
ME/CFS−Canada (VD001)
Controls−Canada (VD001)
Controls−USA (VD001)
ME/CFS−Canada (AD001)
Controls−Canada (AD001)

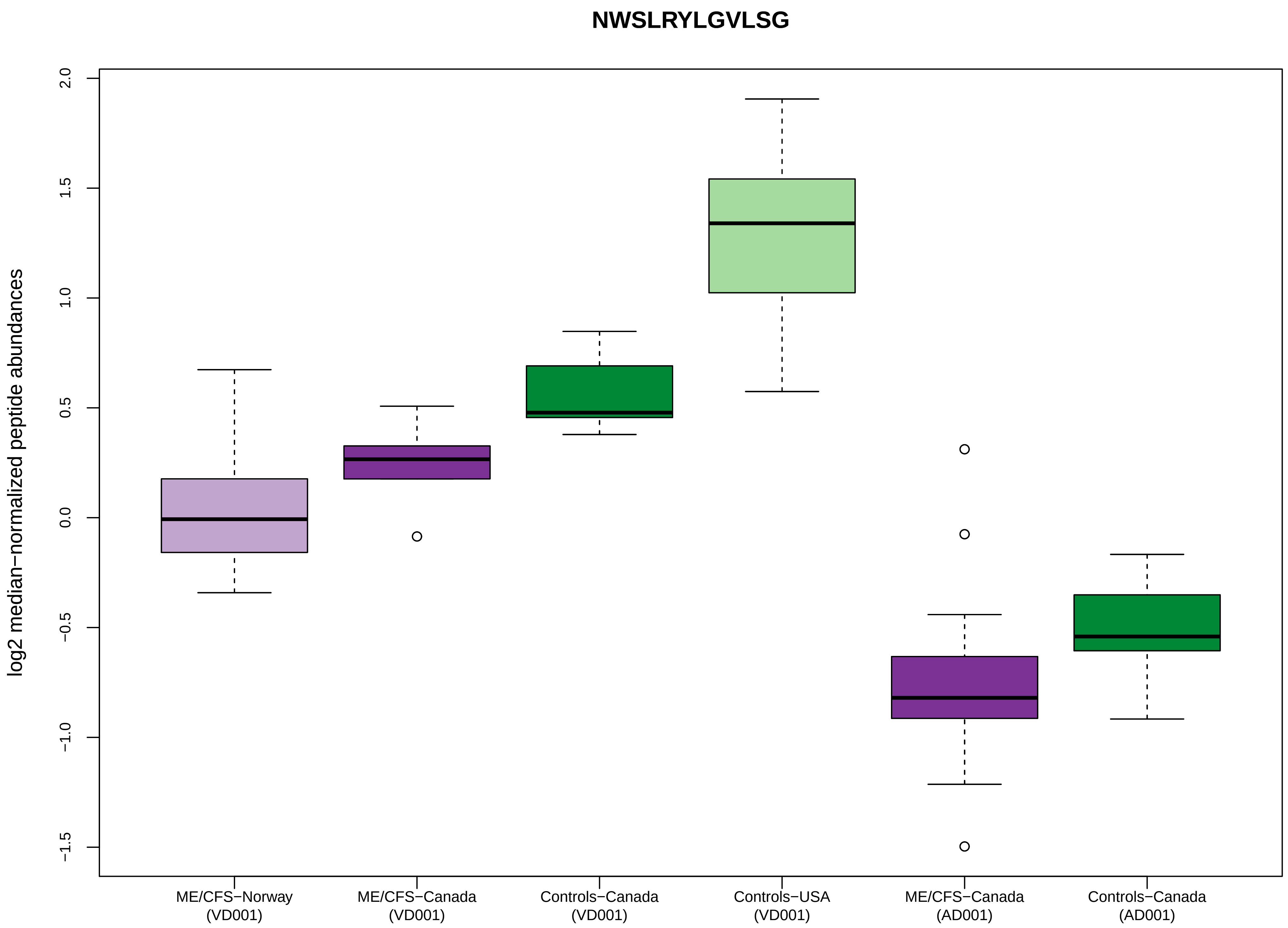
NWSLRYLGVLSG
log2 median−normalized peptide abundances
2.0
1.5
1.0
0.5
0.0
−0.5
−1.0
−1.5
ME/CFS−Norway (VD001)
ME/CFS−Canada (VD001)
Controls−Canada (VD001)
Controls−USA (VD001)
ME/CFS−Canada (AD001)
Controls−Canada (AD001)

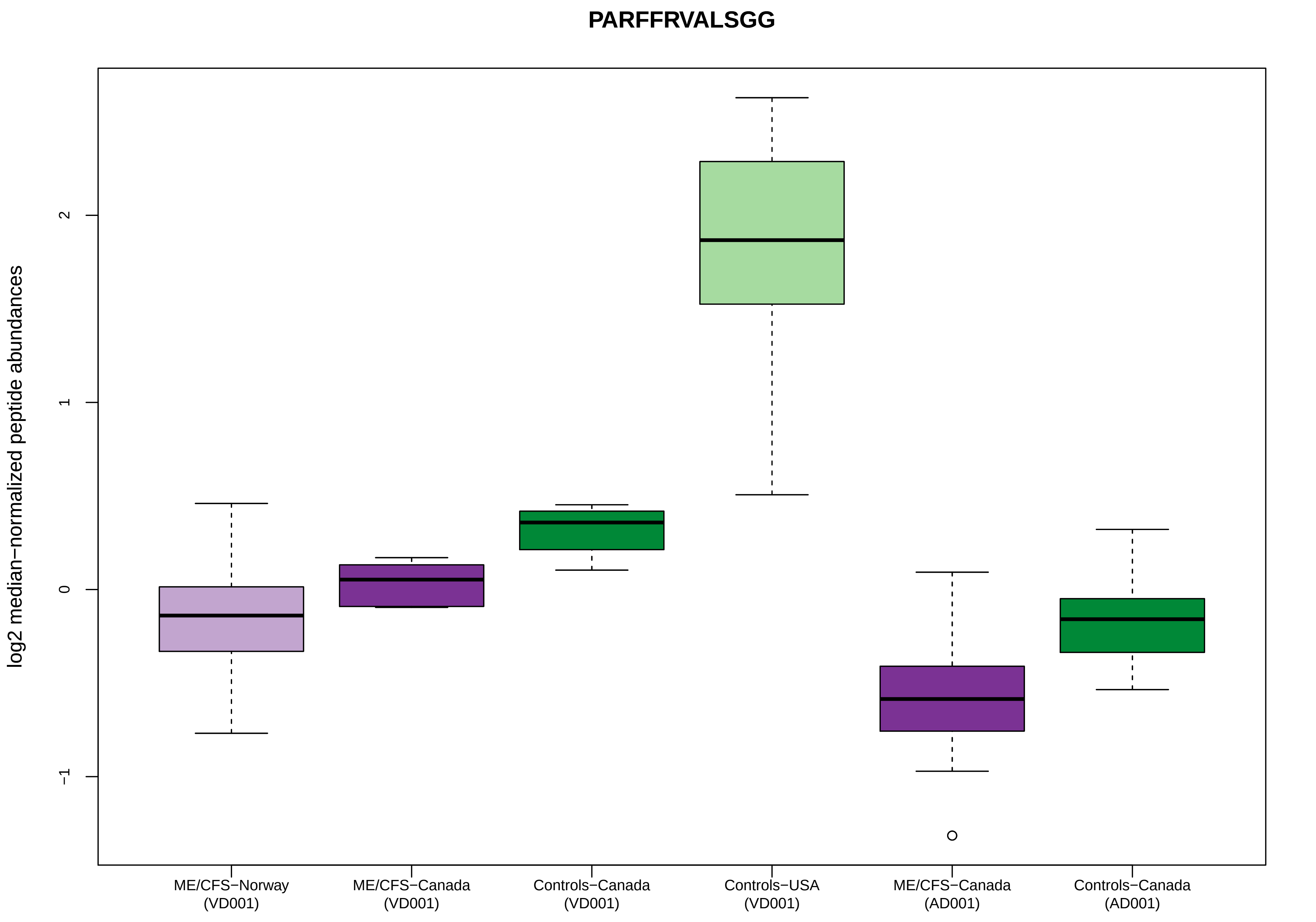
PARFFRVALSGG
log2 median−normalized peptide abundances
−1
0
1
2
ME/CFS−Norway (VD001)
ME/CFS−Canada (VD001)
Controls−Canada (VD001)
Controls−USA (VD001)
ME/CFS−Canada (AD001)
Controls−Canada (AD001)

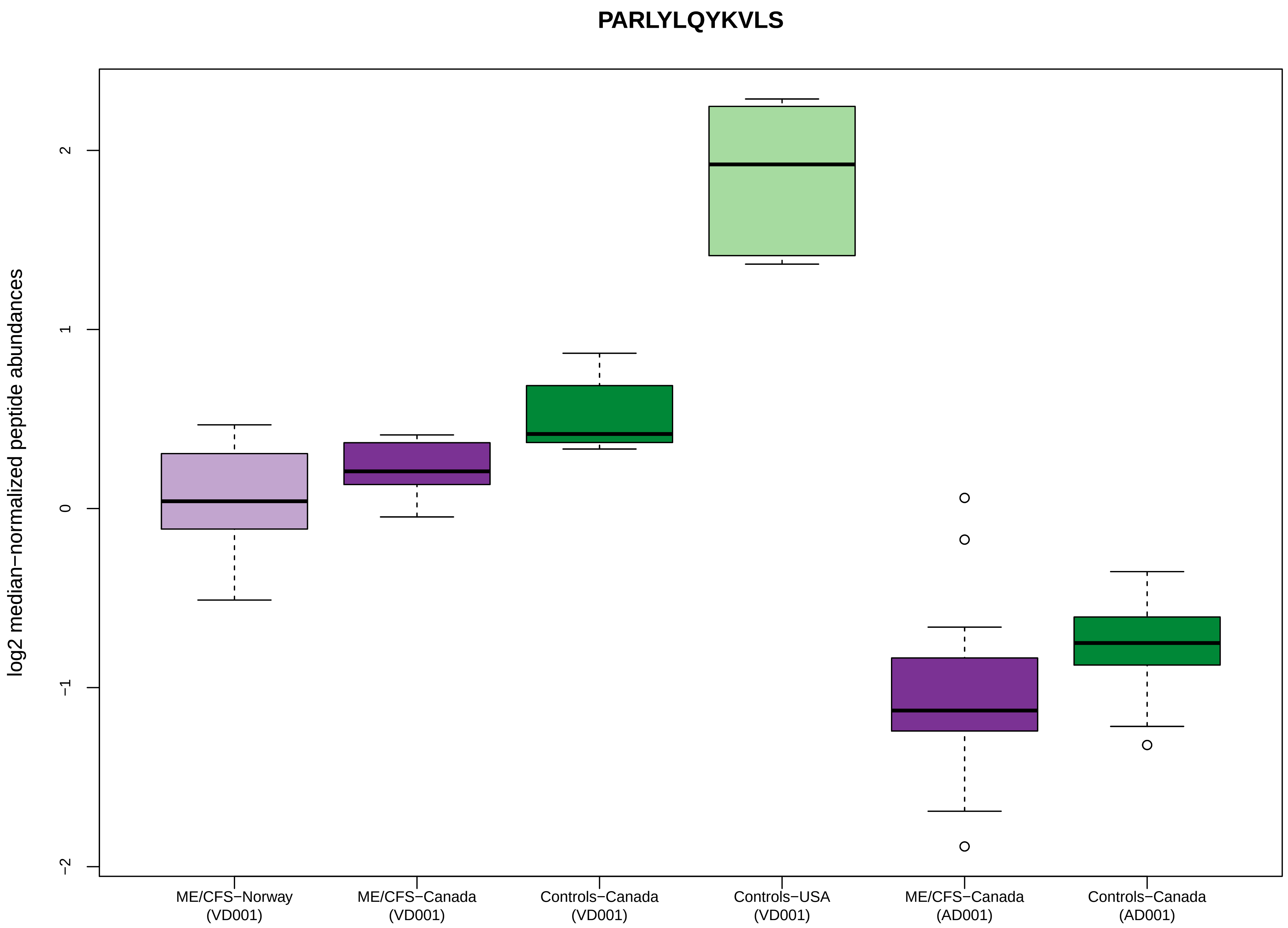
PARLYLQYKVLS
log2 median−normalized peptide abundances
2
1
0
−1
−2
ME/CFS−Norway (VD001)
ME/CFS−Canada (VD001)
Controls−Canada (VD001)
Controls−USA (VD001)
ME/CFS−Canada (AD001)
Controls−Canada (AD001)

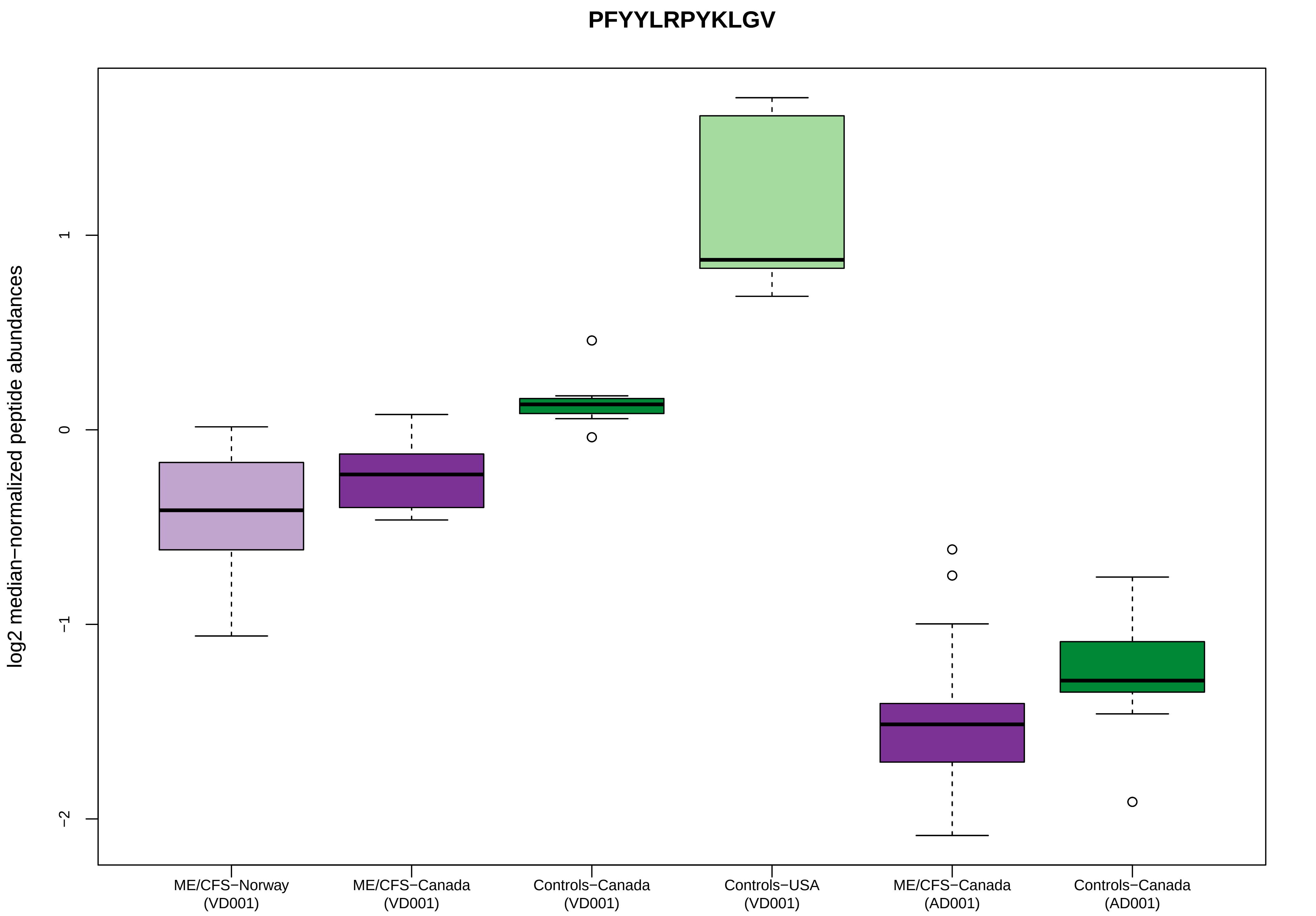

PFYYLRPYKLGV
log2 median−normalized peptide abundances
1
0
−1
−2
ME/CFS−Norway (VD001)
ME/CFS−Canada (VD001)
Controls−Canada (VD001)
Controls−USA (VD001)
ME/CFS−Canada (AD001)
Controls−Canada (AD001)

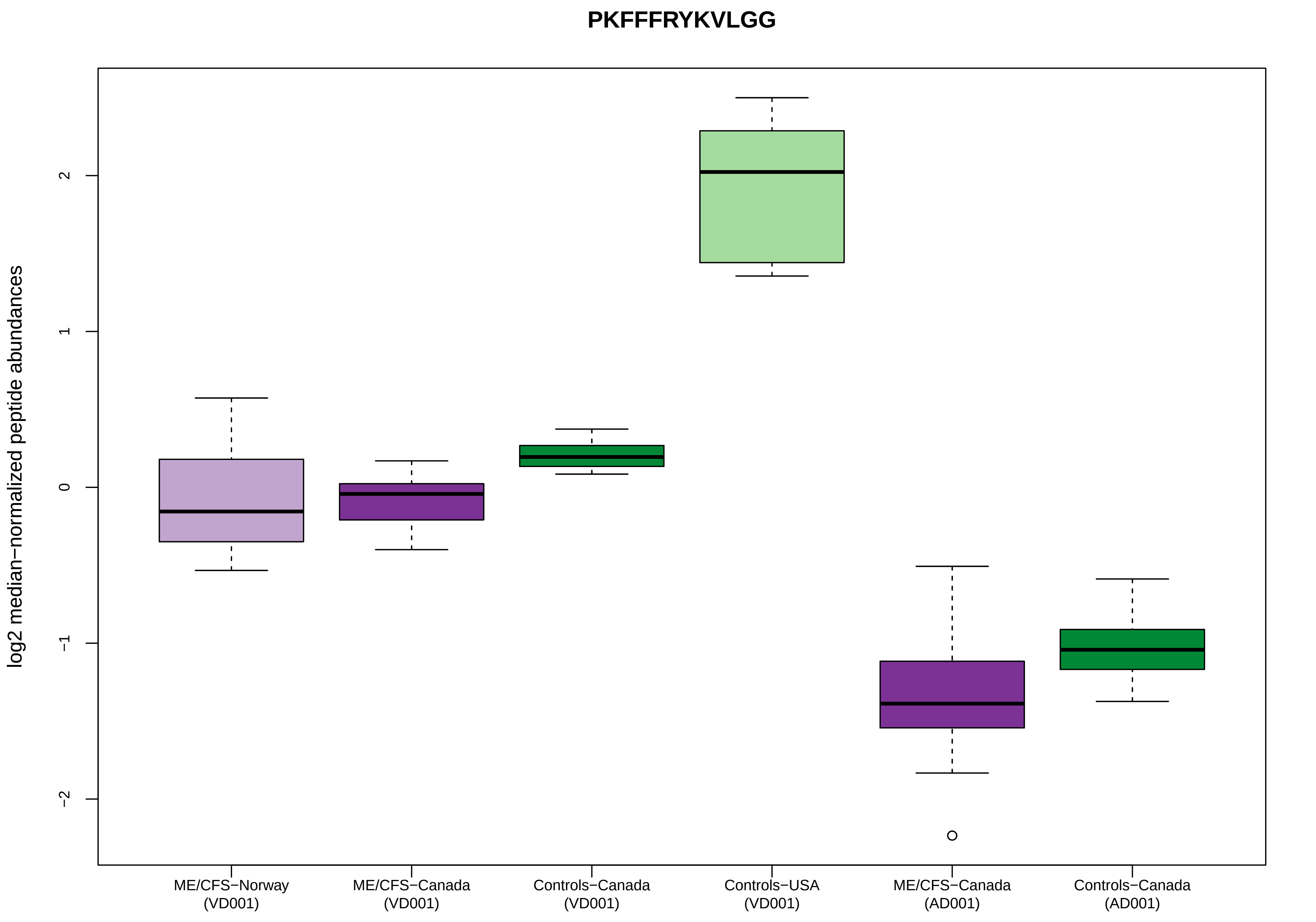
PKFFFRYKVLGG
log2 median−normalized peptide abundances
2
1
0
−1
−2
ME/CFS−Norway (VD001)
ME/CFS−Canada (VD001)
Controls−Canada (VD001)
Controls−USA (VD001)
ME/CFS−Canada (AD001)
Controls−Canada (AD001)

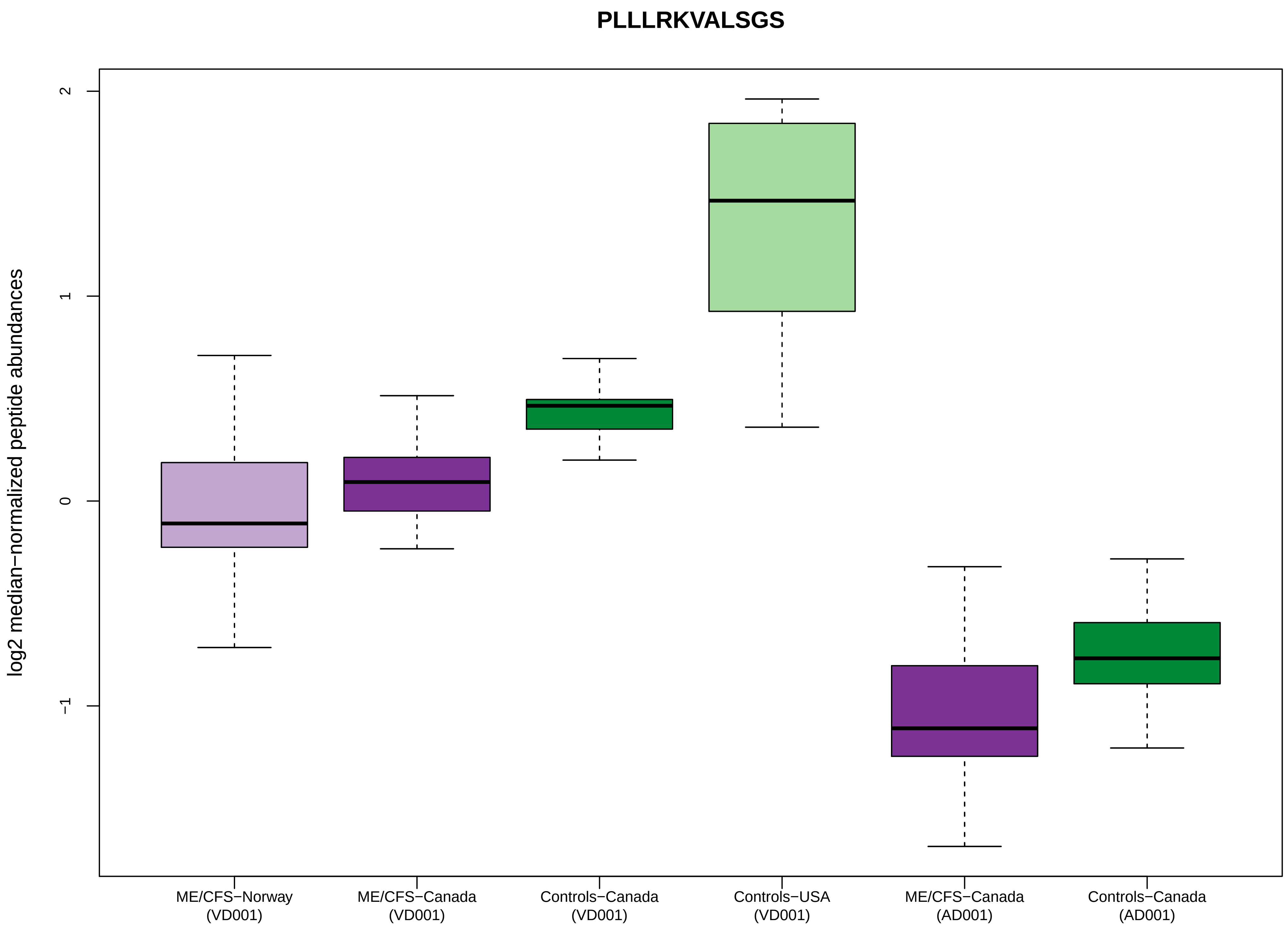

PLLLRKVALSGS
log2 median−normalized peptide abundances
2
1
0
−1
ME/CFS−Norway (VD001)
ME/CFS−Canada (VD001)
Controls−Canada (VD001)
Controls−USA (VD001)
ME/CFS−Canada (AD001)
Controls−Canada (AD001)

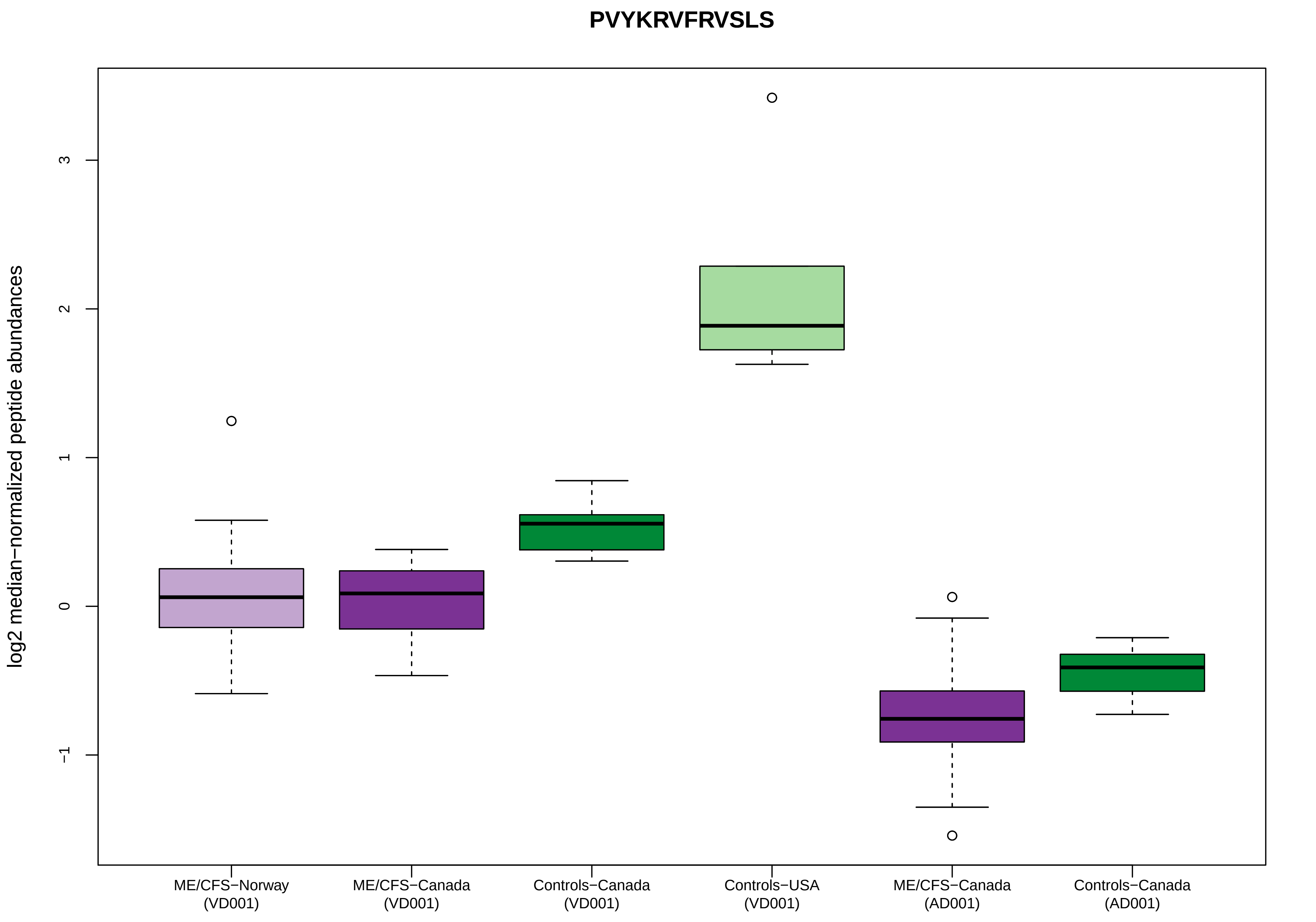
PVYKRVFRVSLS
log2 median−normalized peptide abundances
3
2
1
0
−1
ME/CFS−Norway (VD001)
ME/CFS−Canada (VD001)
Controls−Canada (VD001)
Controls−USA (VD001)
ME/CFS−Canada (AD001)
Controls−Canada (AD001)

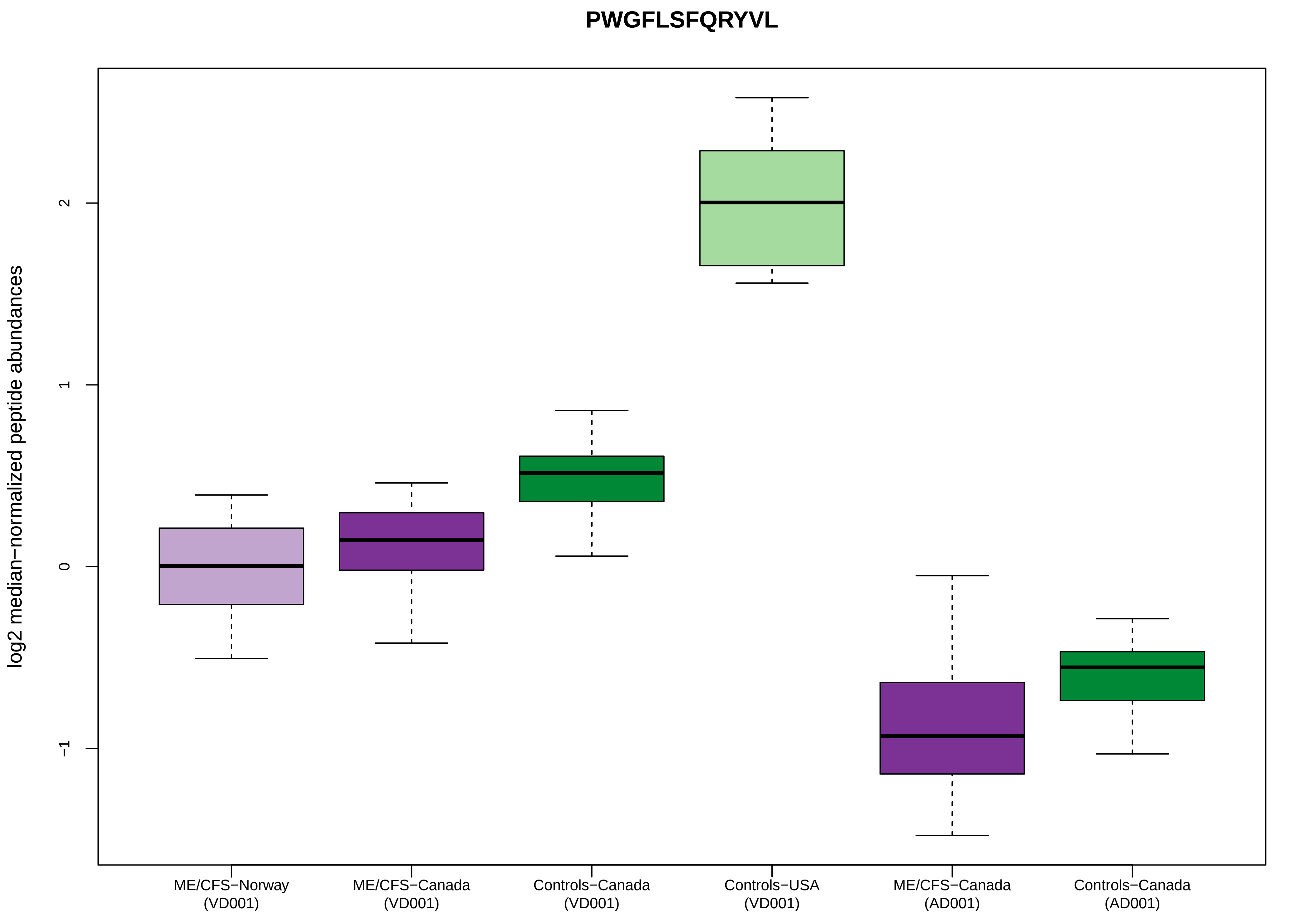
PWGFLSFQRYVL
log2 median−normalized peptide abundances
2
1
0
−1
ME/CFS−Norway (VD001)
ME/CFS−Canada (VD001)
Controls−Canada (VD001)
Controls−USA (VD001)
ME/CFS−Canada (AD001)
Controls−Canada (AD001)

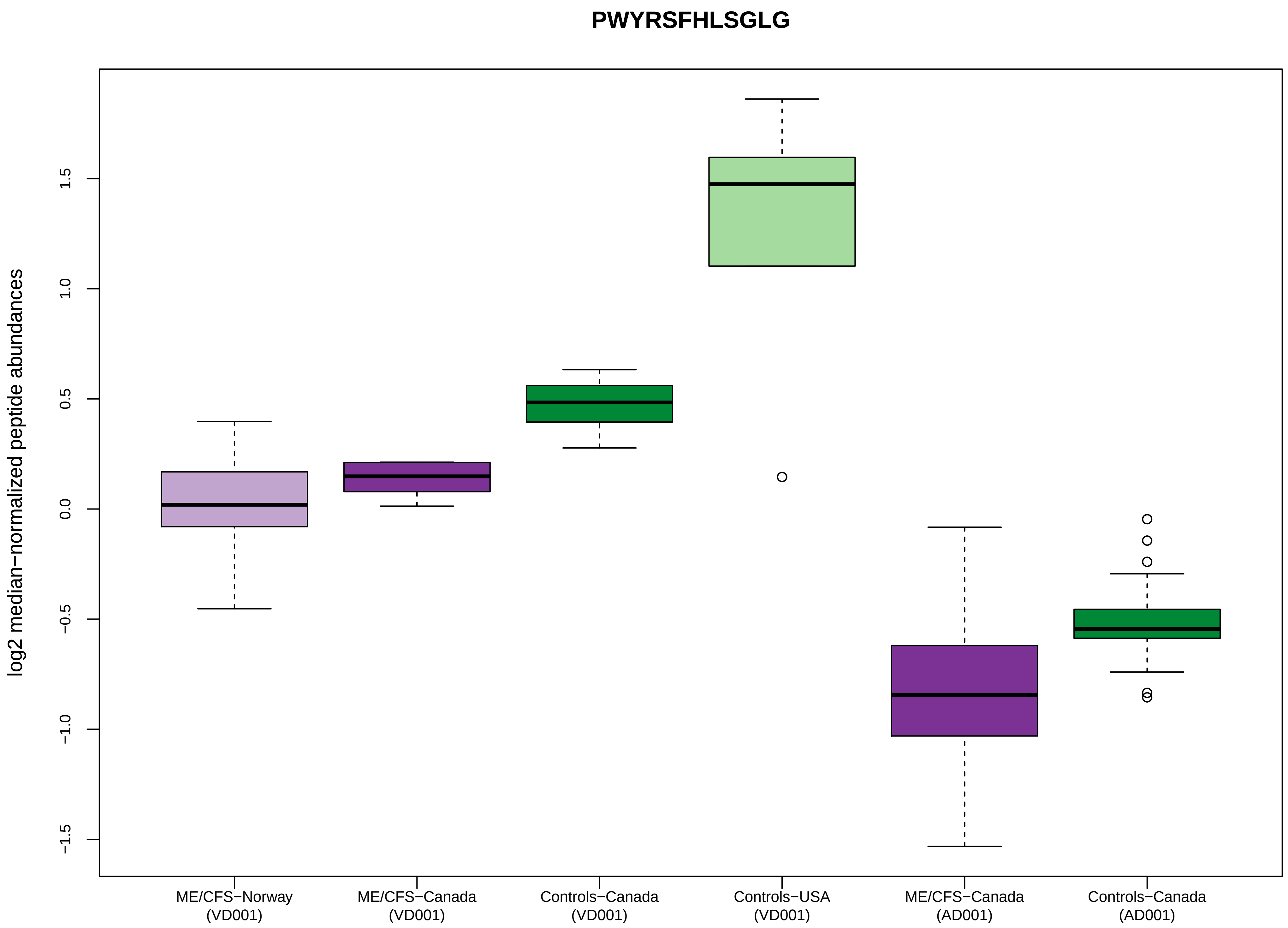
PWYRSFHLSGLG
log2 median−normalized peptide abundances
1.5
1.0
0.5
0.0
−0.5
−1.0
−1.5
ME/CFS−Norway (VD001)
ME/CFS−Canada (VD001)
Controls−Canada (VD001)
Controls−USA (VD001)
ME/CFS−Canada (AD001)
Controls−Canada (AD001)

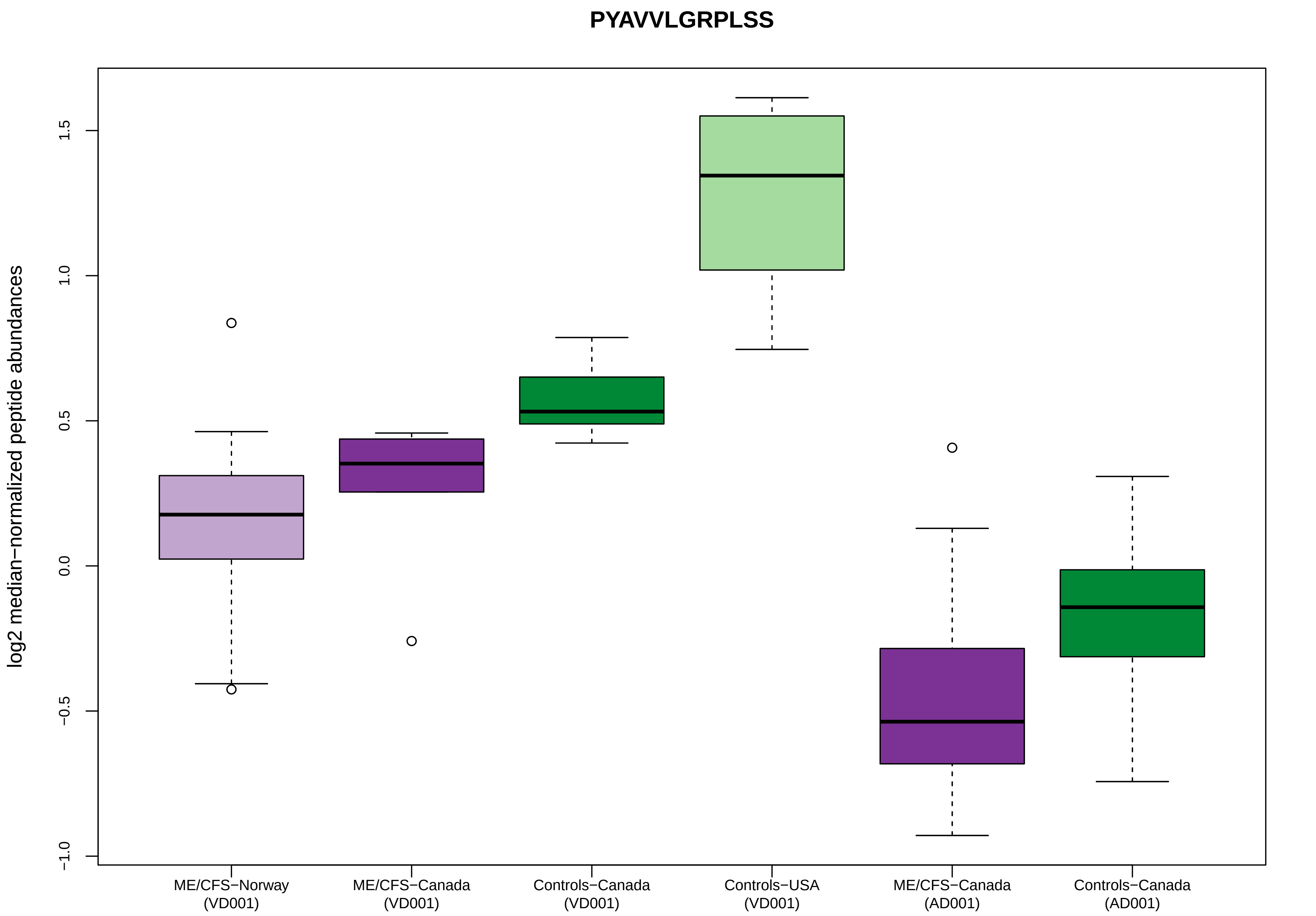

PYAVVLGRPLSS
log2 median−normalized peptide abundances
1.5
1.0
0.5
0.0
−0.5
−1.0
ME/CFS−Norway (VD001)
ME/CFS−Canada (VD001)
Controls−Canada (VD001)
Controls−USA (VD001)
ME/CFS−Canada (AD001)
Controls−Canada (AD001)

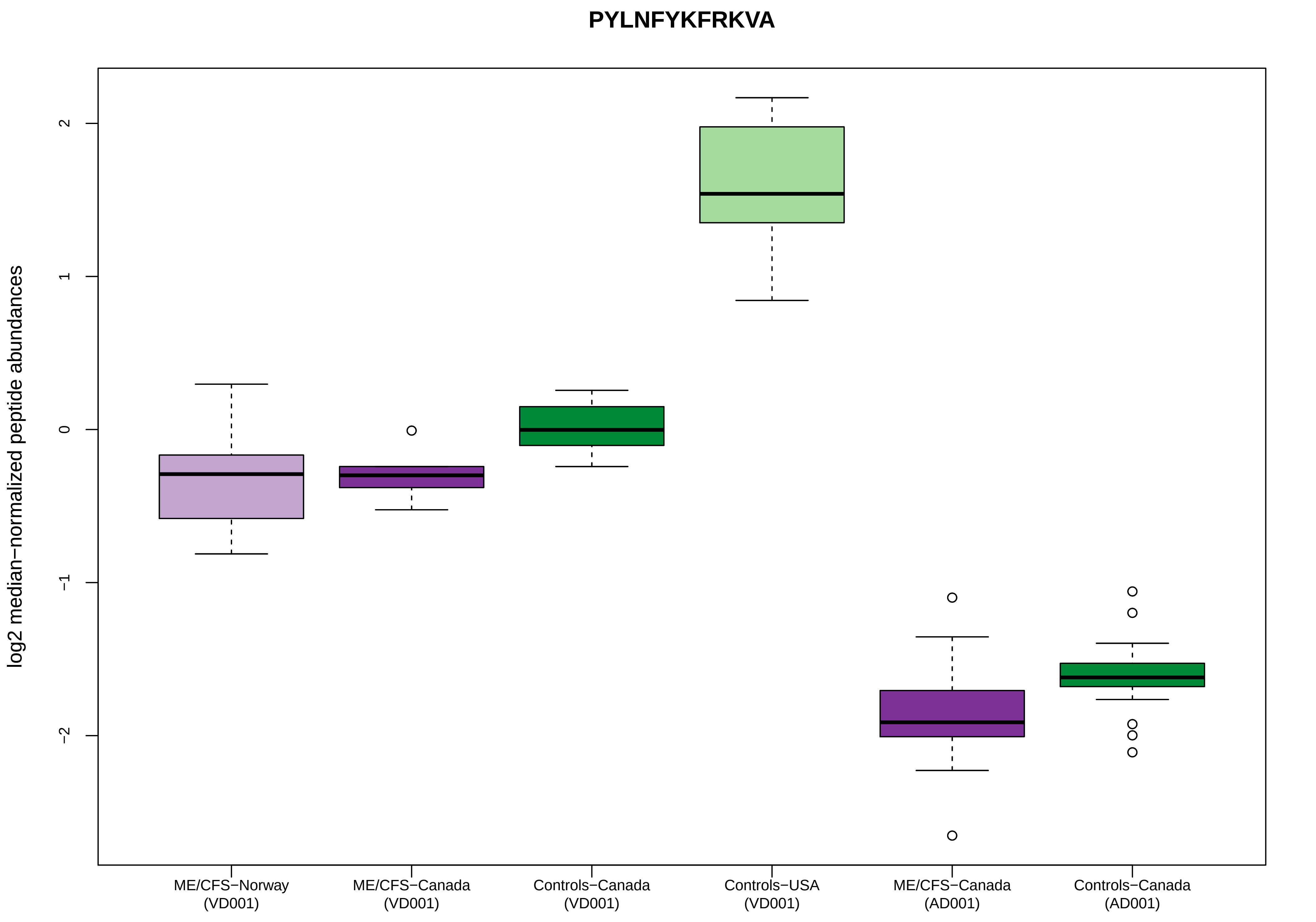
PYLNFYKFRKVA
log2 median−normalized peptide abundances
2
1
0
−1
−2
ME/CFS−Norway (VD001)
ME/CFS−Canada (VD001)
Controls−Canada (VD001)
Controls−USA (VD001)
ME/CFS−Canada (AD001)
Controls−Canada (AD001)

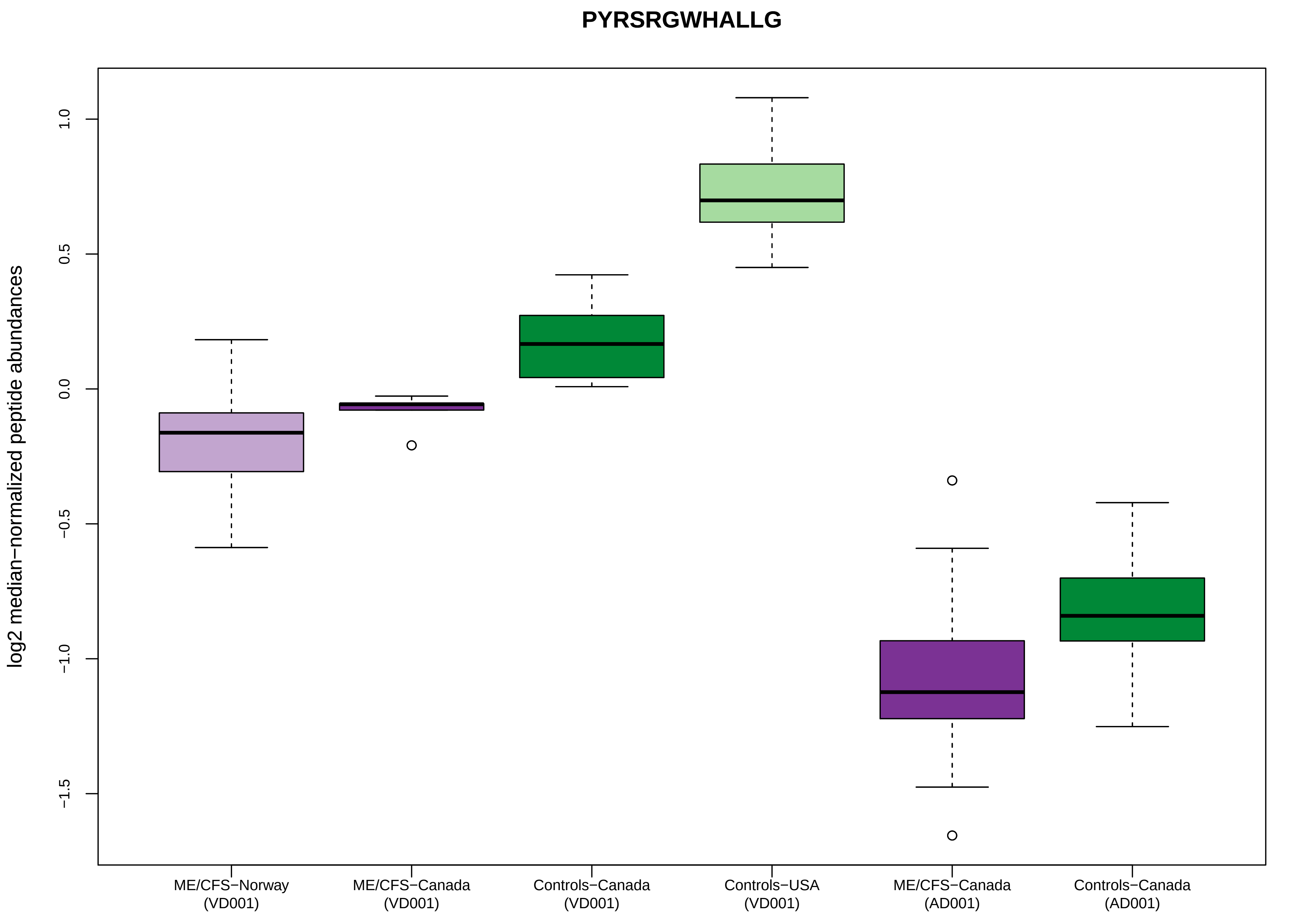

PYRSRGWHALLG
log2 median−normalized peptide abundances
1.0
0.5
0.0
−0.5
−1.0
−1.5
ME/CFS−Norway (VD001)
ME/CFS−Canada (VD001)
Controls−Canada (VD001)
Controls−USA (VD001)
ME/CFS−Canada (AD001)
Controls−Canada (AD001)

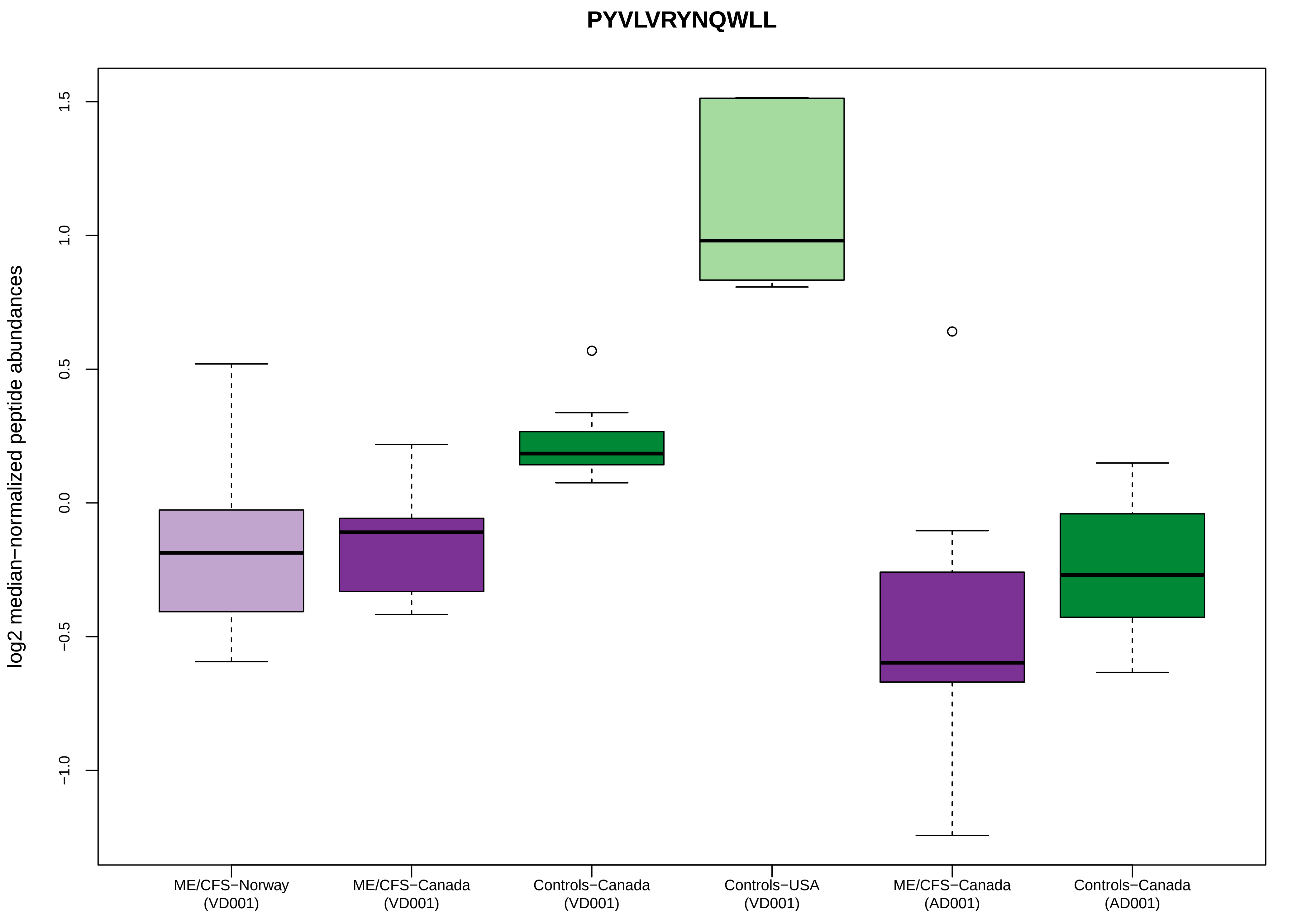
PYVLVRYNQWLL
log2 median−normalized peptide abundances
1.5
1.0
0.5
0.0
−0.5
−1.0
ME/CFS−Norway (VD001)
ME/CFS−Canada (VD001)
Controls−Canada (VD001)
Controls−USA (VD001)
ME/CFS−Canada (AD001)
Controls−Canada (AD001)

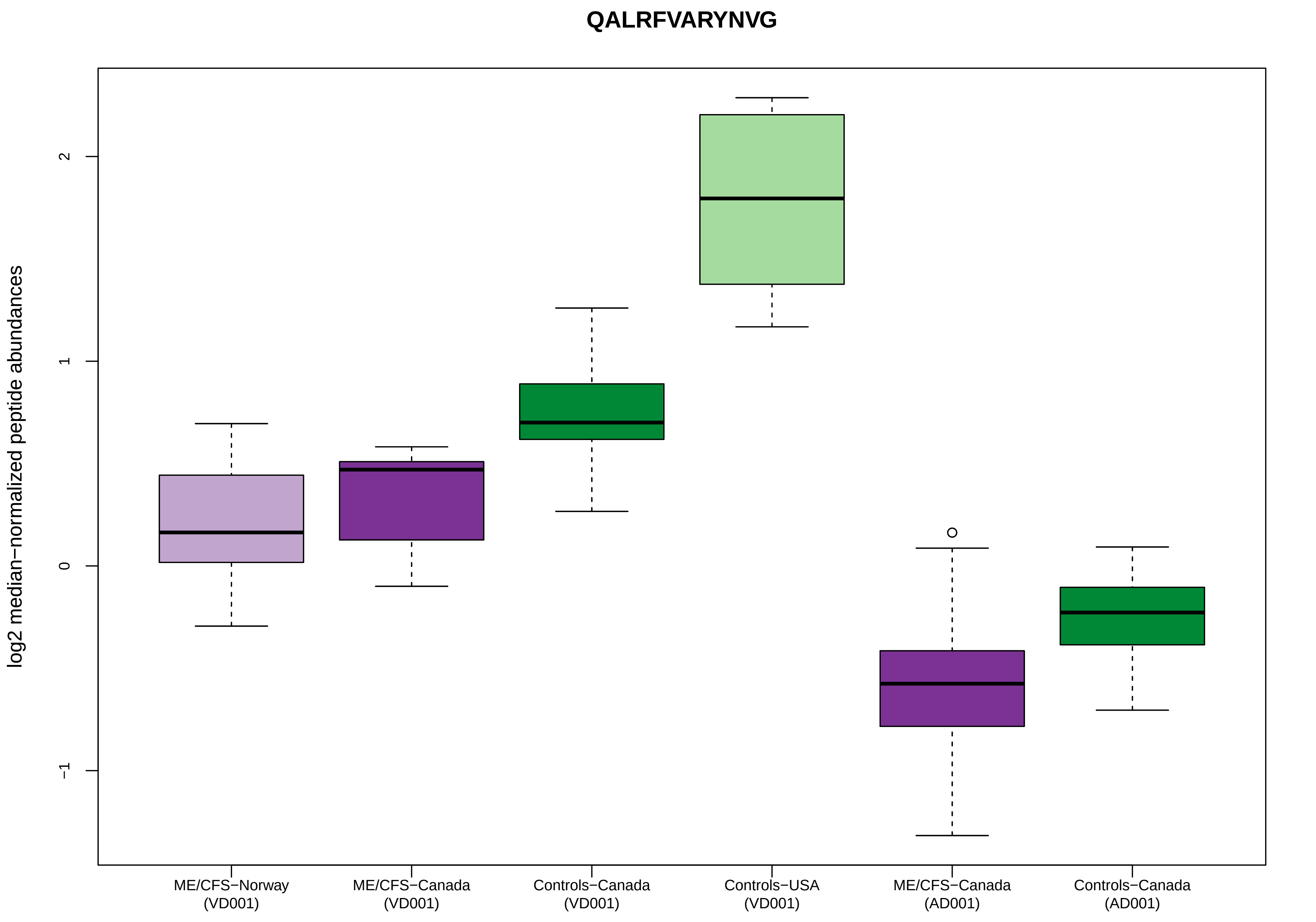

QALRFVARYNVG
log2 median−normalized peptide abundances
−1
0
1
2
ME/CFS−Norway (VD001)
ME/CFS−Canada (VD001)
Controls−Canada (VD001)
Controls−USA (VD001)
ME/CFS−Canada (AD001)
Controls−Canada (AD001)

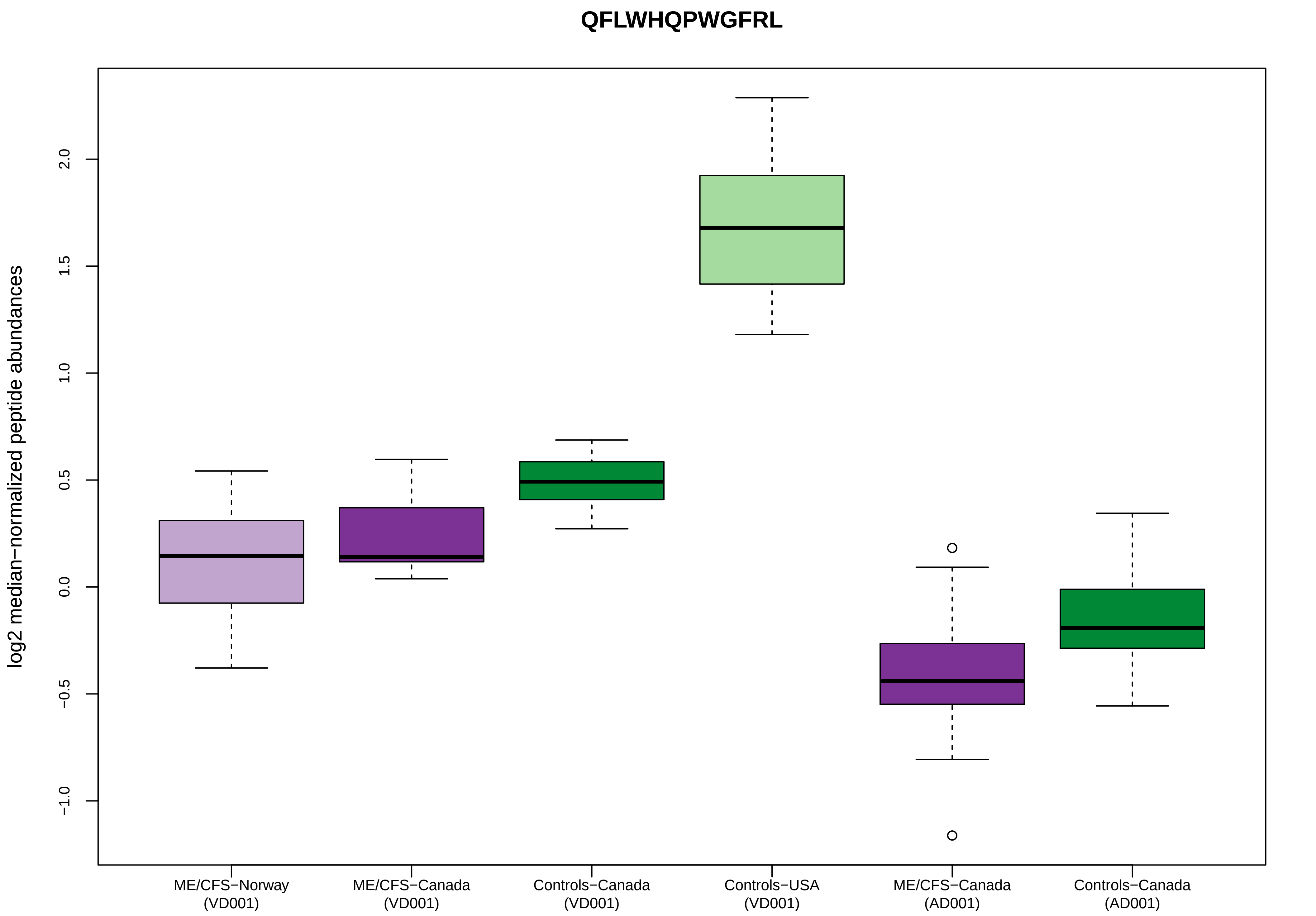

QFLWHQPWGFRL
log2 median−normalized peptide abundances
2.0
1.5
1.0
0.5
0.0
−0.5
−1.0
ME/CFS−Norway (VD001)
ME/CFS−Canada (VD001)
Controls−Canada (VD001)
Controls−USA (VD001)
ME/CFS−Canada (AD001)
Controls−Canada (AD001)

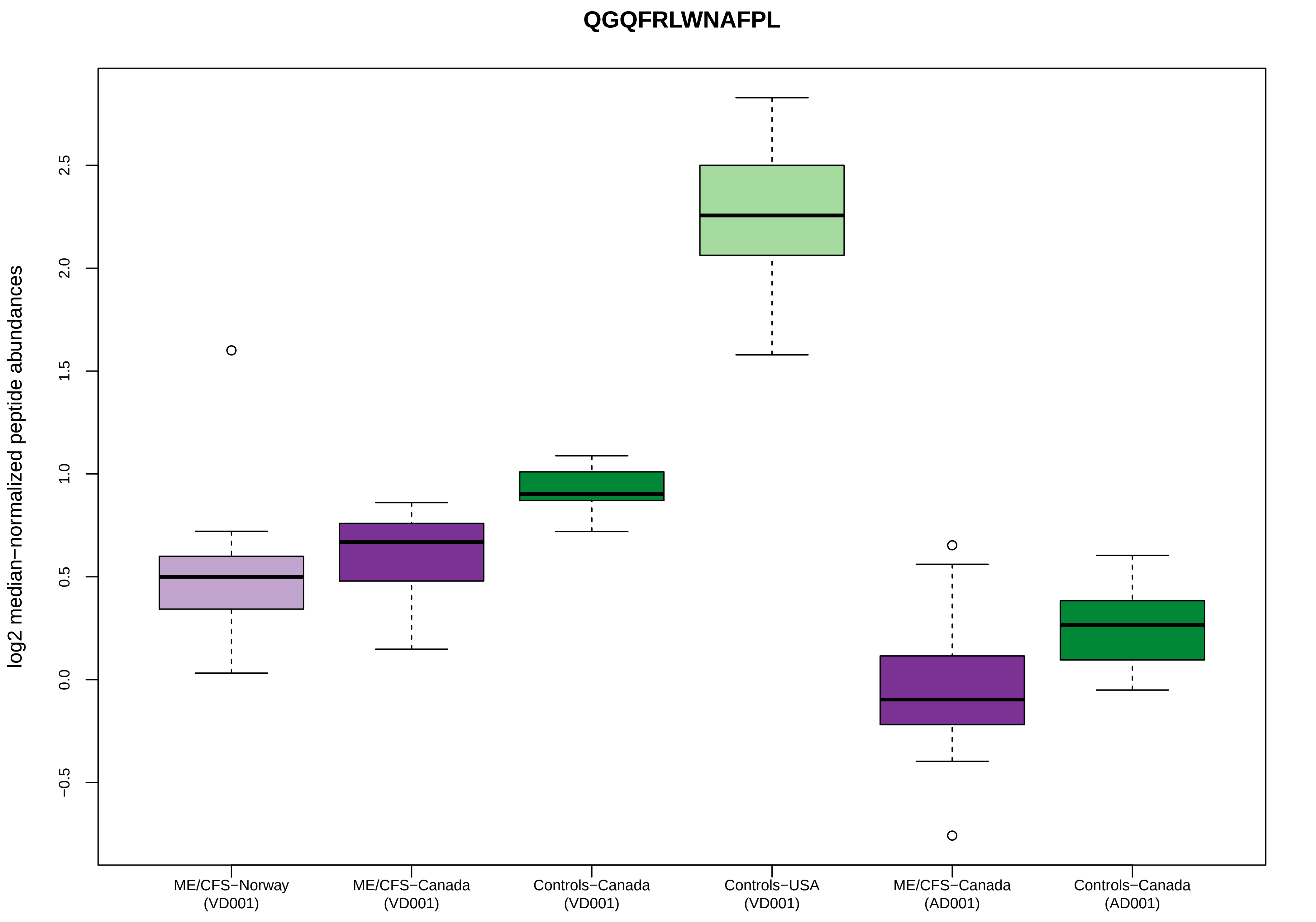

QGQFRLWNAFPL
log2 median−normalized peptide abundances
2.5
2.0
1.5
1.0
0.5
0.0
−0.5
ME/CFS−Norway (VD001)
ME/CFS−Canada (VD001)
Controls−Canada (VD001)
Controls−USA (VD001)
ME/CFS−Canada (AD001)
Controls−Canada (AD001)

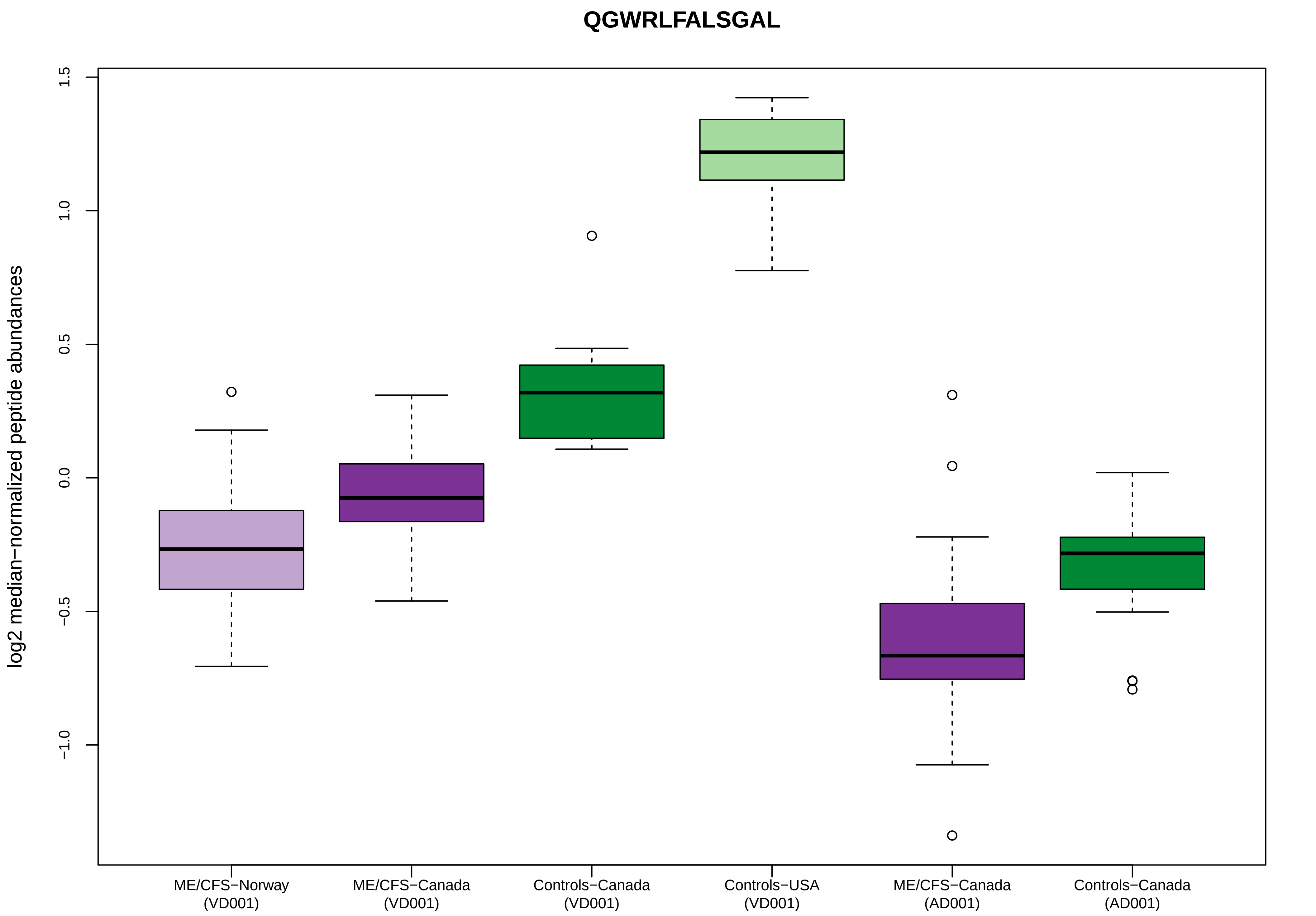
QGWRLFALSGAL
log2 median−normalized peptide abundances
1.5
1.0
0.5
0.0
−0.5
−1.0
ME/CFS−Norway (VD001)
ME/CFS−Canada (VD001)
Controls−Canada (VD001)
Controls−USA (VD001)
ME/CFS−Canada (AD001)
Controls−Canada (AD001)

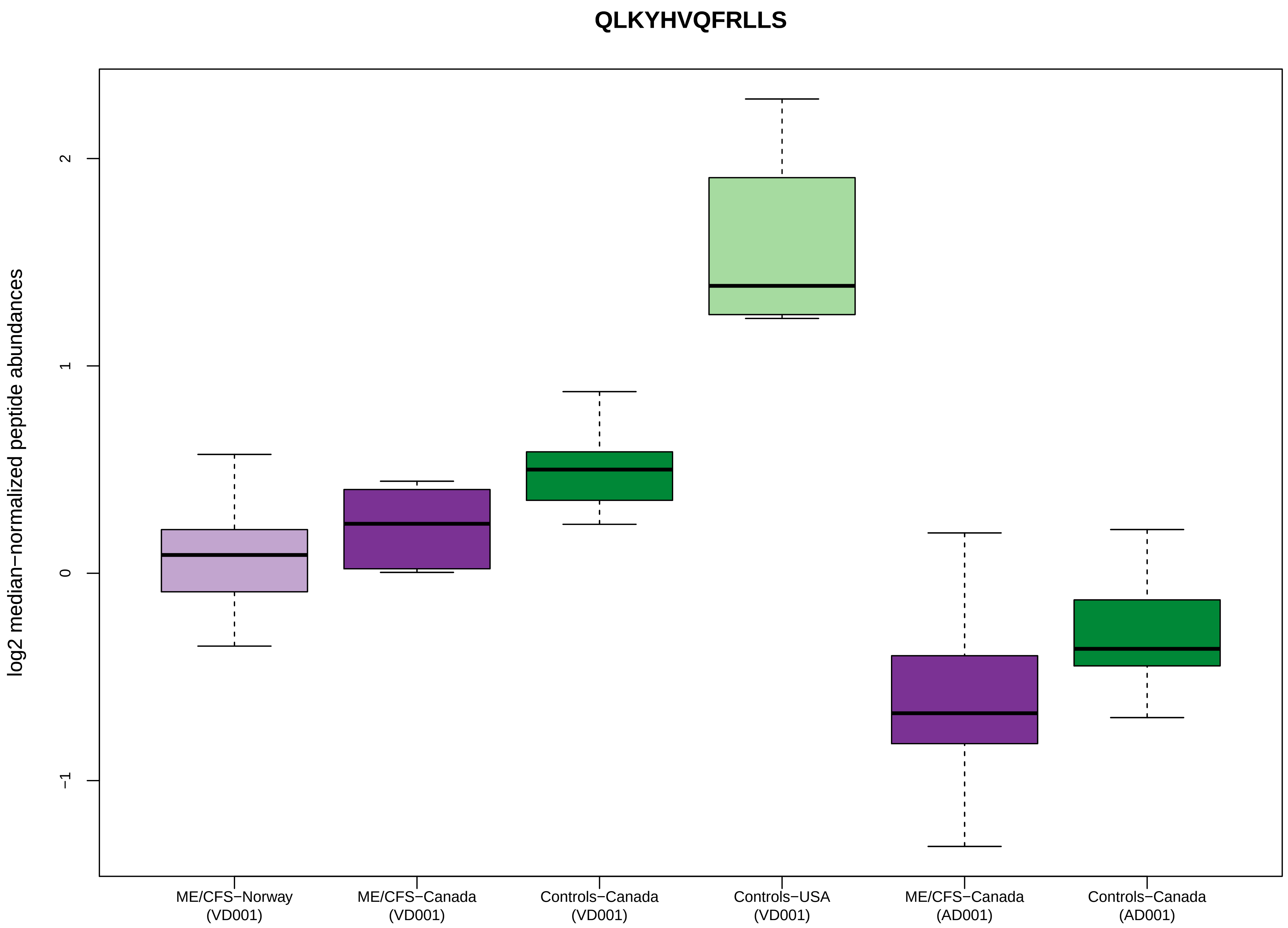
QLKYHVQFRLLS
log2 median−normalized peptide abundances
2
1
0
−1
ME/CFS−Norway (VD001)
ME/CFS−Canada (VD001)
Controls−Canada (VD001)
Controls−USA (VD001)
ME/CFS−Canada (AD001)
Controls−Canada (AD001)

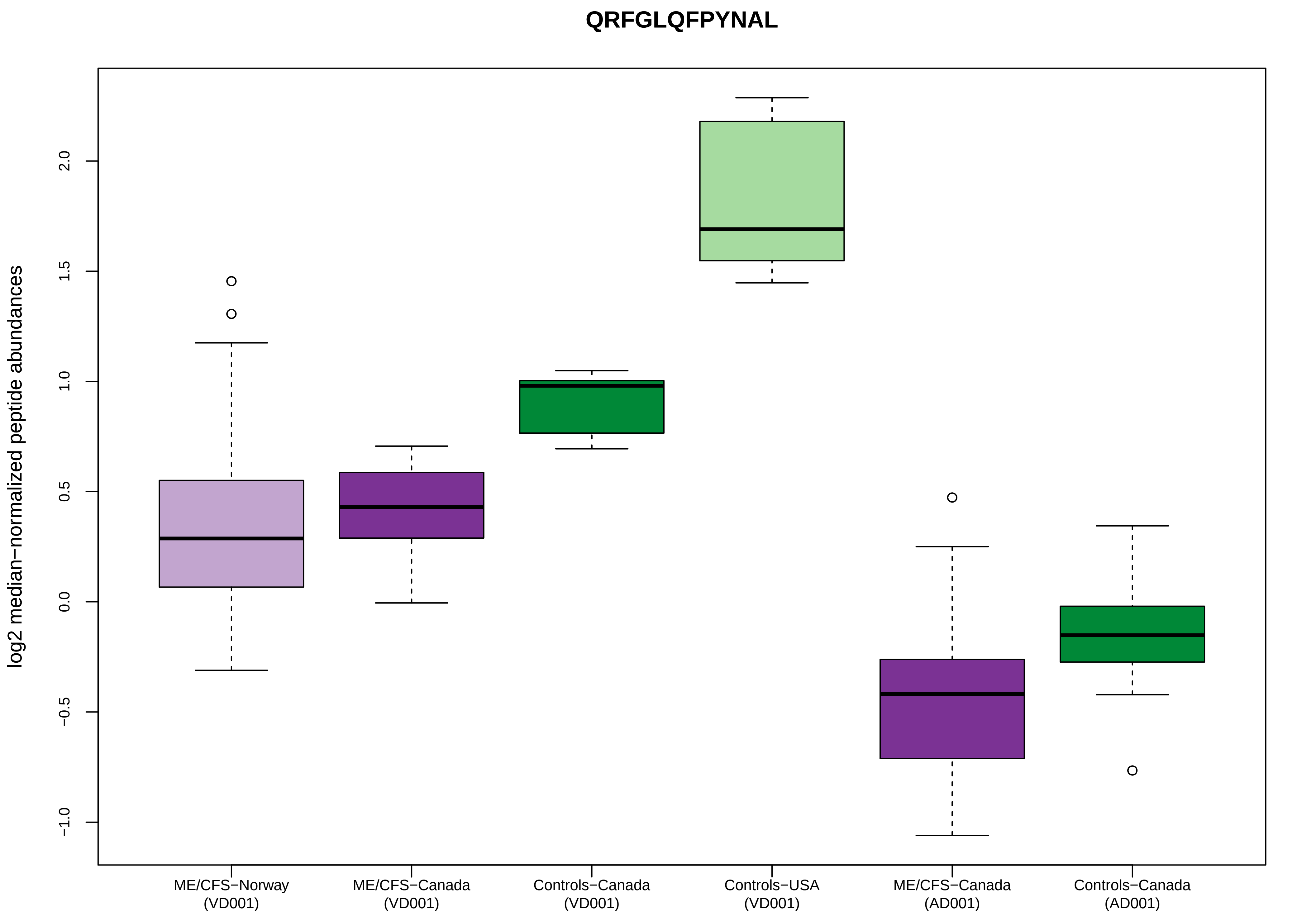
QRFGLQFPYNAL
log2 median−normalized peptide abundances
−1.0
−0.5
0.0
0.5
1.0
1.5
2.0
ME/CFS−Norway (VD001)
ME/CFS−Canada (VD001)
Controls−Canada (VD001)
Controls−USA (VD001)
ME/CFS−Canada (AD001)
Controls−Canada (AD001)

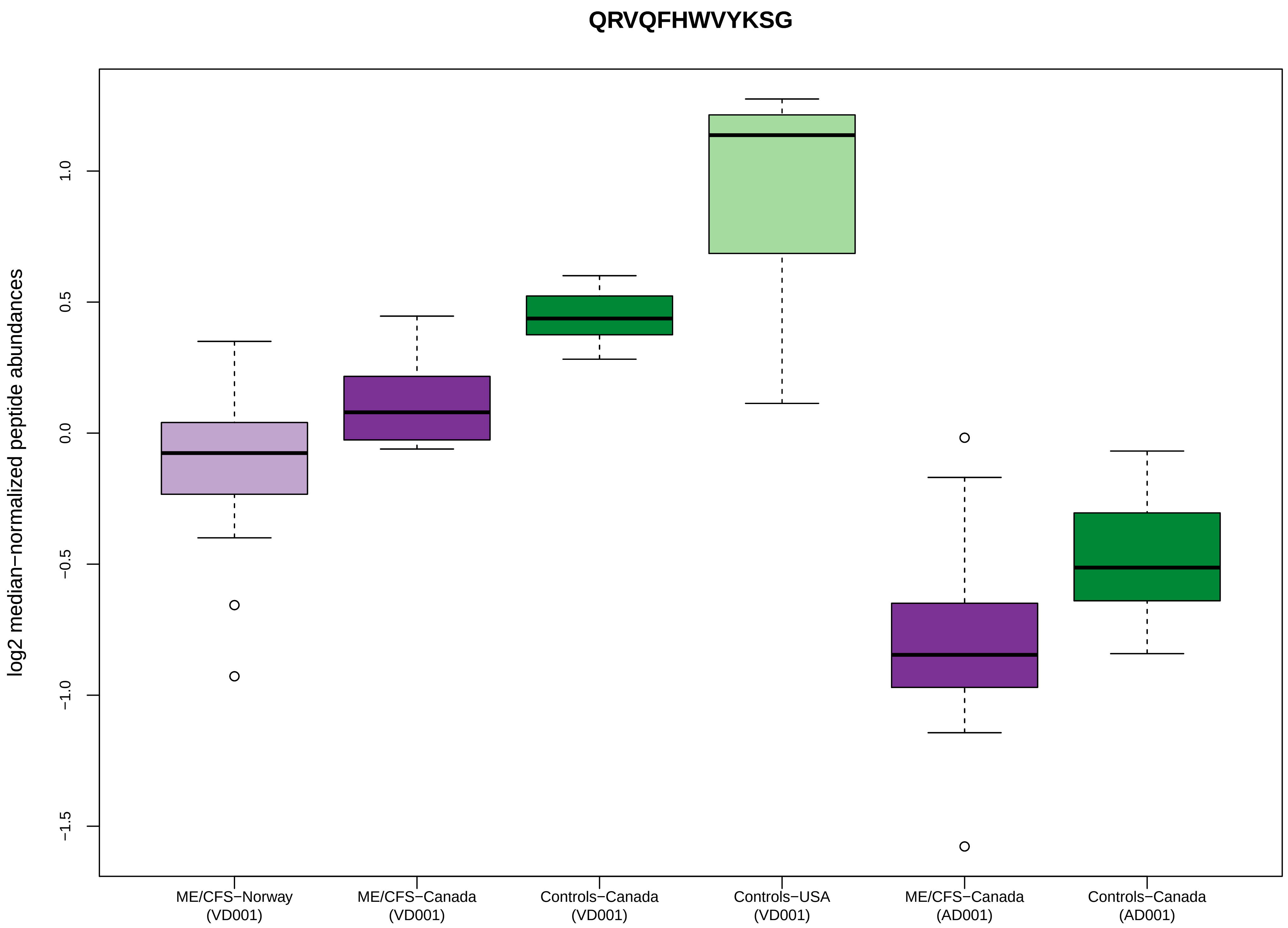

QRVQFHWVYKSG
log2 median−normalized peptide abundances
1.0
0.5
0.0
−0.5
−1.0
−1.5
ME/CFS−Norway (VD001)
ME/CFS−Canada (VD001)
Controls−Canada (VD001)
Controls−USA (VD001)
ME/CFS−Canada (AD001)
Controls−Canada (AD001)

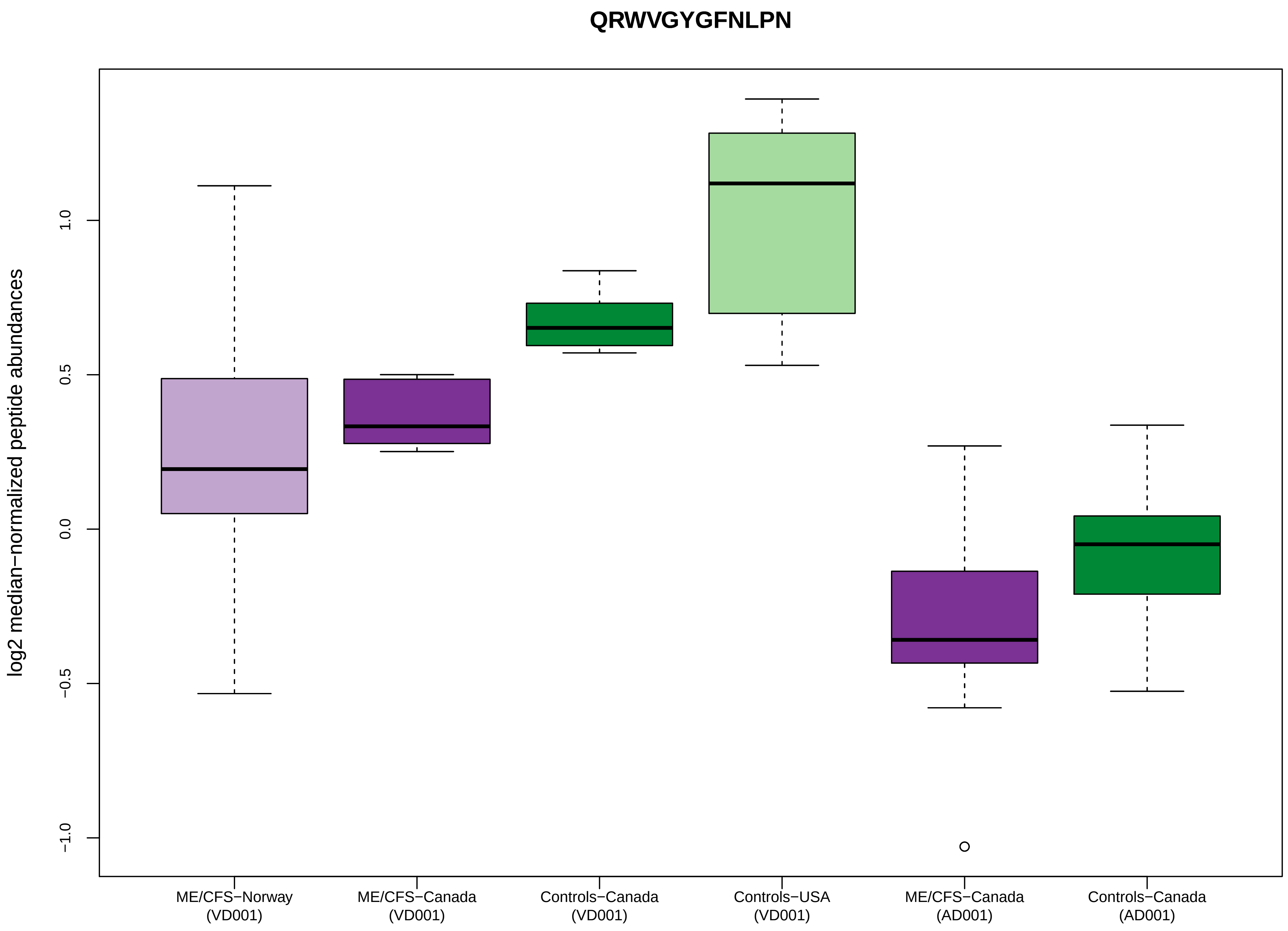

QRWVGYGFNLPN
log2 median−normalized peptide abundances
1.0
0.5
0.0
−0.5
−1.0
ME/CFS−Norway (VD001)
ME/CFS−Canada (VD001)
Controls−Canada (VD001)
Controls−USA (VD001)
ME/CFS−Canada (AD001)
Controls−Canada (AD001)

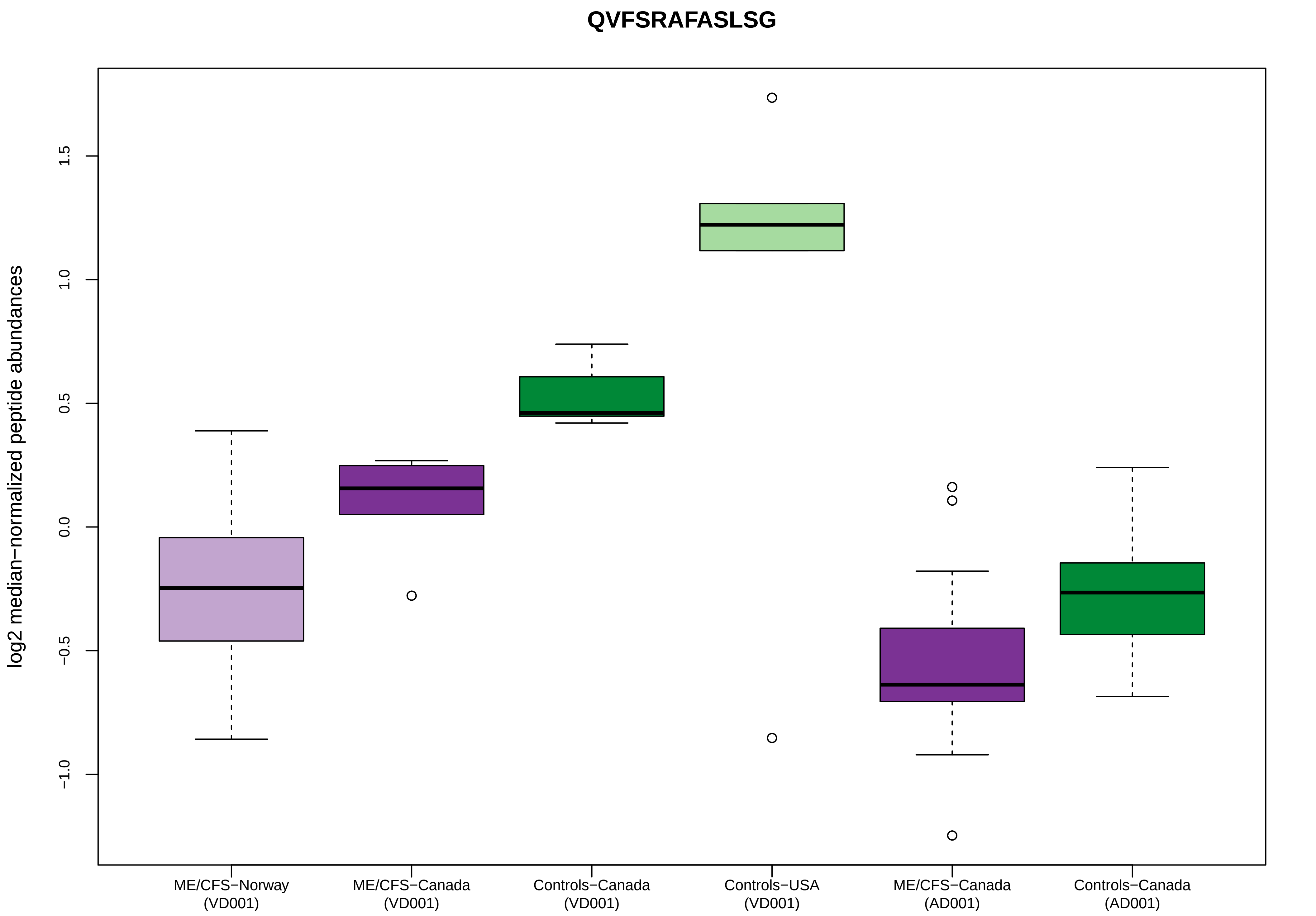
QVFSRAFASLSG
log2 median−normalized peptide abundances
1.5
1.0
0.5
0.0
−0.5
−1.0
ME/CFS−Norway (VD001)
ME/CFS−Canada (VD001)
Controls−Canada (VD001)
Controls−USA (VD001)
ME/CFS−Canada (AD001)
Controls−Canada (AD001)

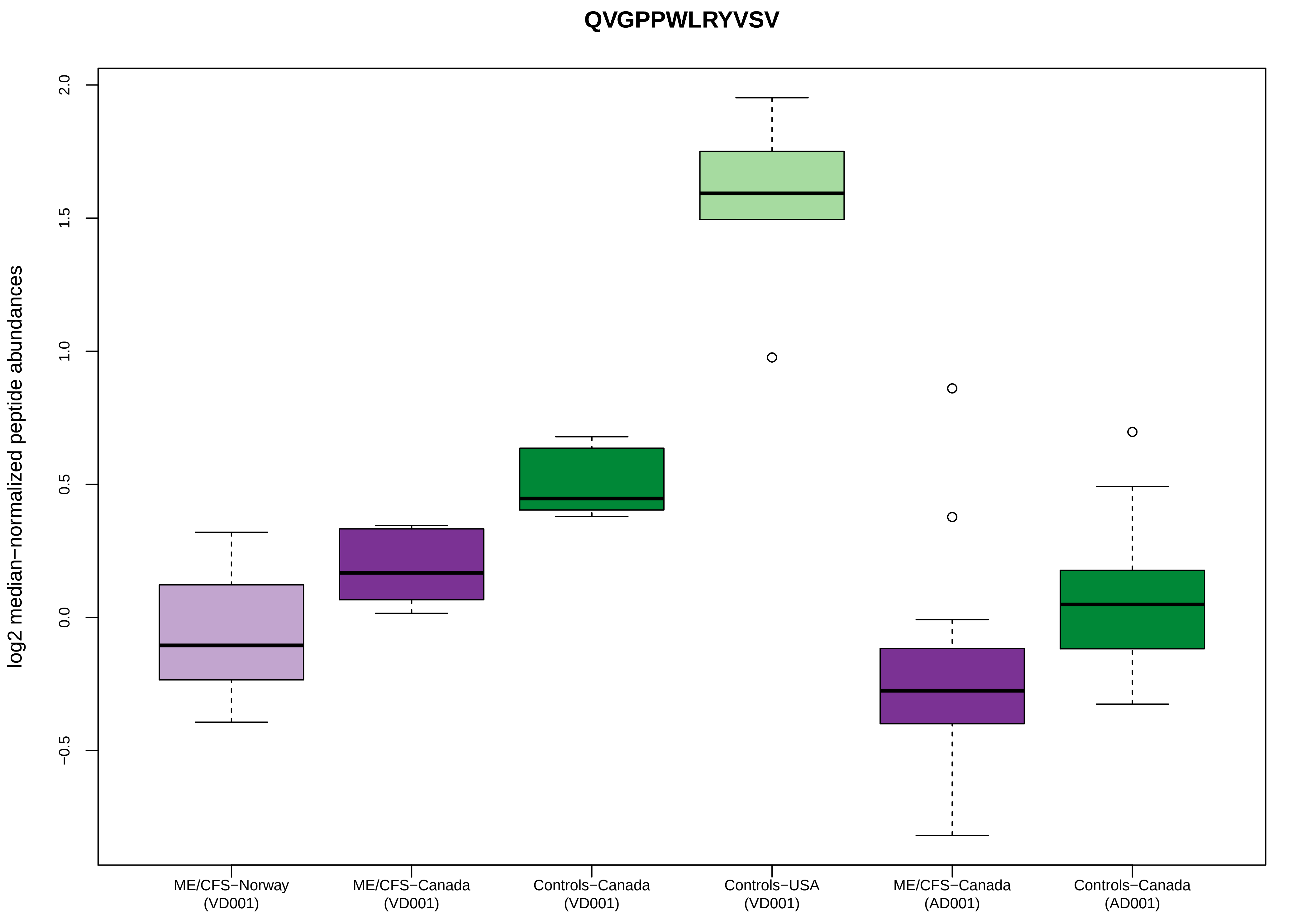

QVGPPWLRYVSV
log2 median−normalized peptide abundances
2.0
1.5
1.0
0.5
0.0
−0.5
ME/CFS−Norway (VD001)
ME/CFS−Canada (VD001)
Controls−Canada (VD001)
Controls−USA (VD001)
ME/CFS−Canada (AD001)
Controls−Canada (AD001)

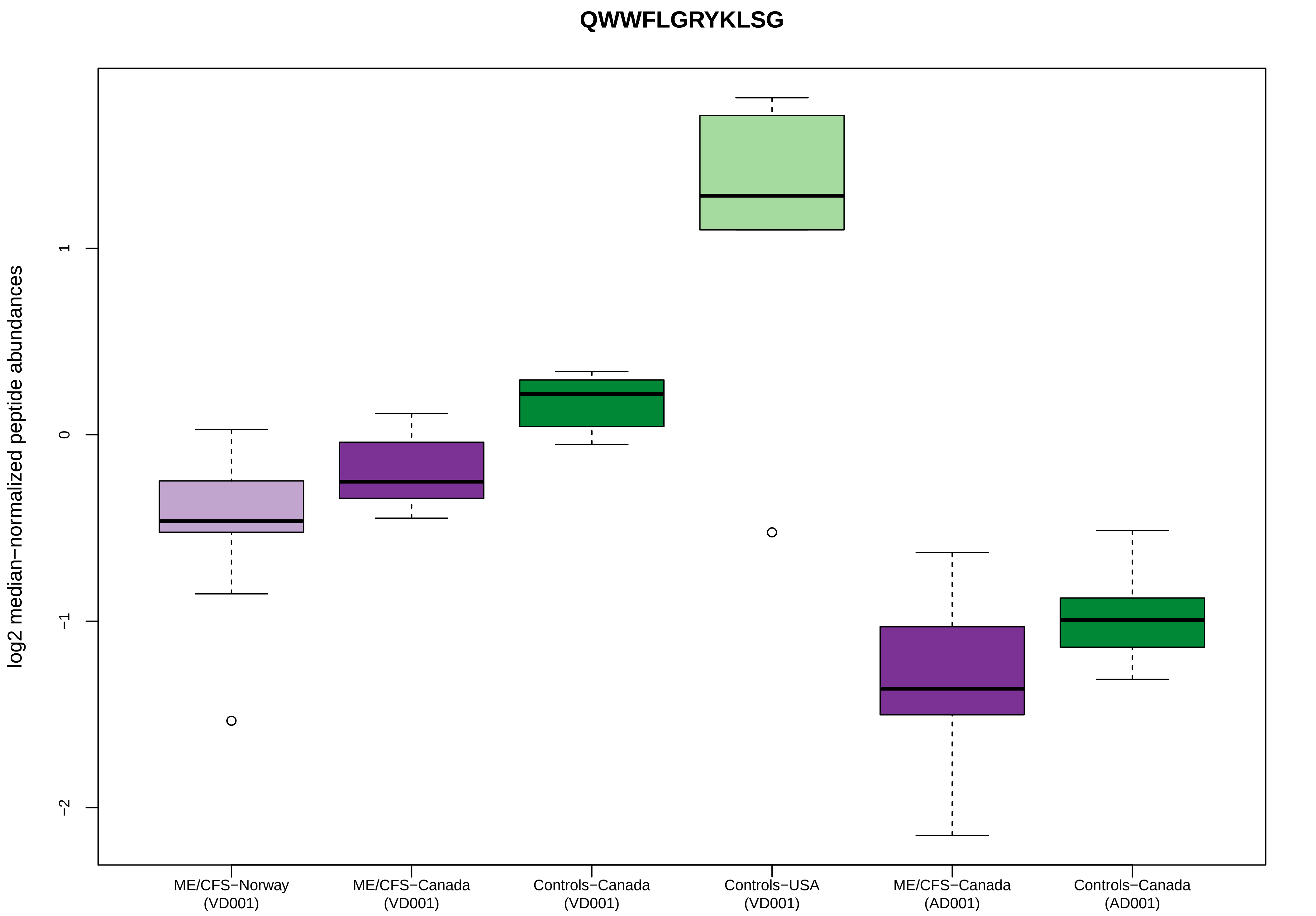
QWWFLGRYKLSG
log2 median−normalized peptide abundances
1
0
−1
−2
ME/CFS−Norway (VD001)
ME/CFS−Canada (VD001)
Controls−Canada (VD001)
Controls−USA (VD001)
ME/CFS−Canada (AD001)
Controls−Canada (AD001)

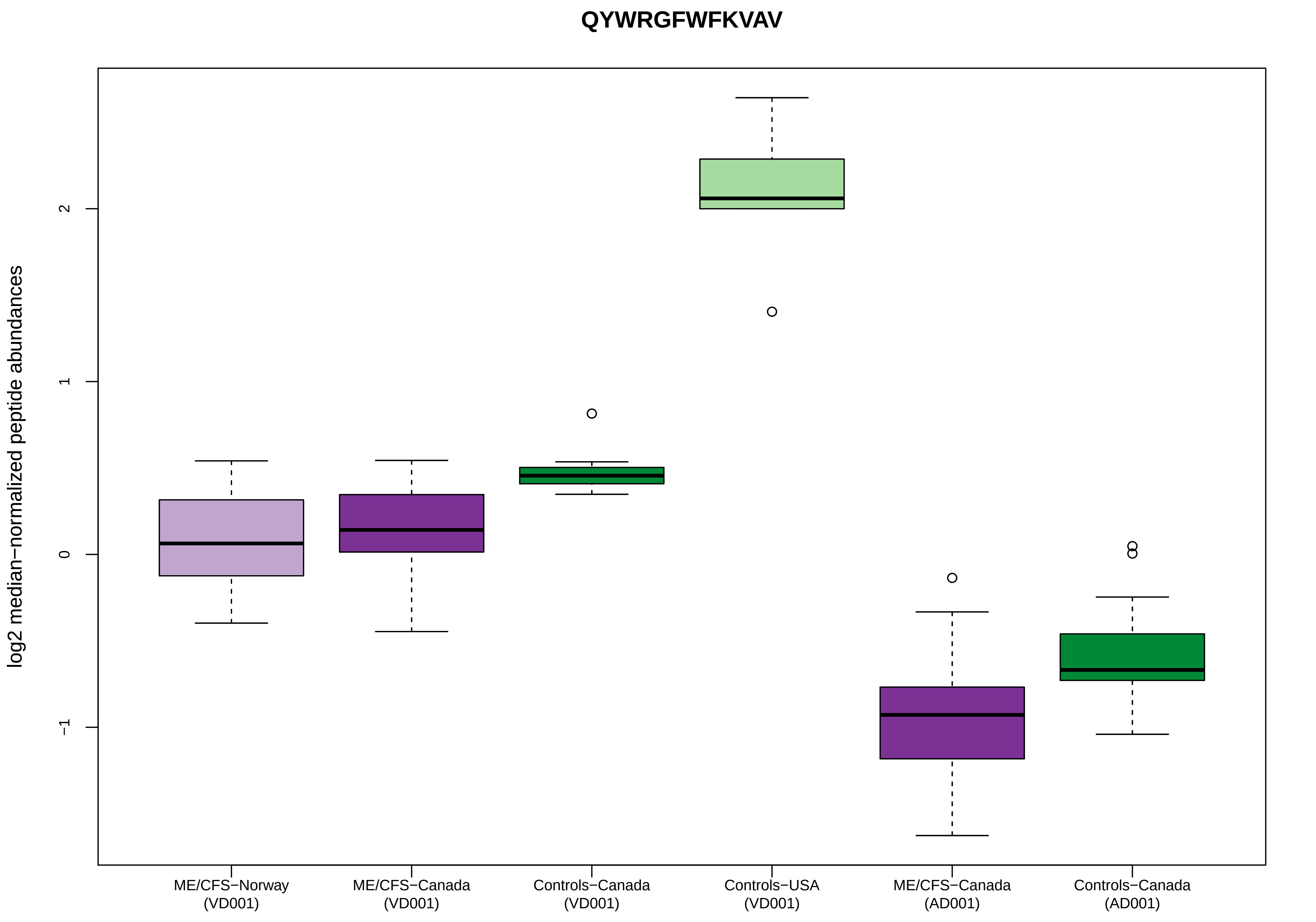
QYWRGFWFKVAV
log2 median−normalized peptide abundances
2
1
0
−1
ME/CFS−Norway (VD001)
ME/CFS−Canada (VD001)
Controls−Canada (VD001)
Controls−USA (VD001)
ME/CFS−Canada (AD001)
Controls−Canada (AD001)

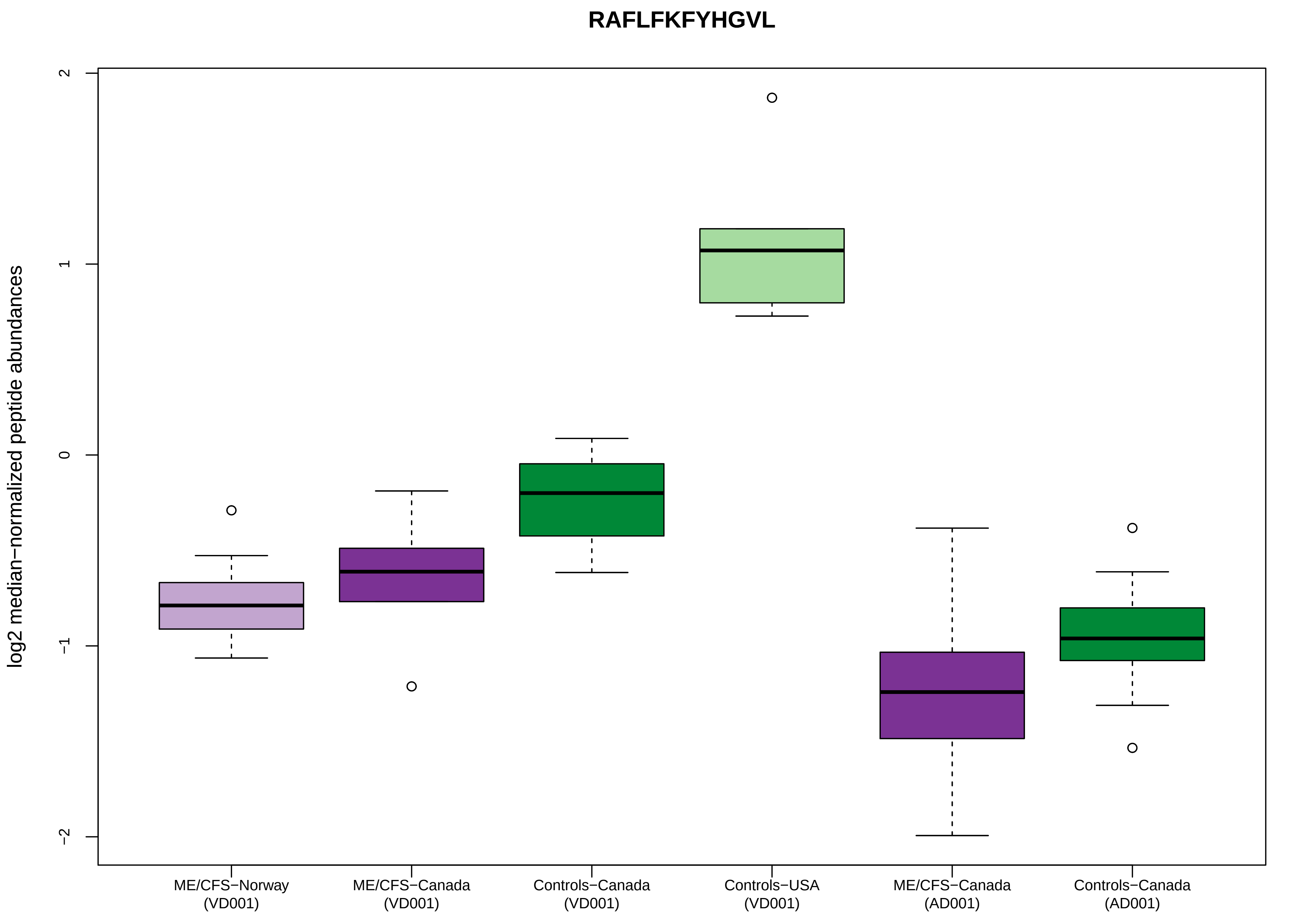

RAFLFKFYHGVL
log2 median−normalized peptide abundances
2
1
0
−1
−2
ME/CFS−Norway (VD001)
ME/CFS−Canada (VD001)
Controls−Canada (VD001)
Controls−USA (VD001)
ME/CFS−Canada (AD001)
Controls−Canada (AD001)

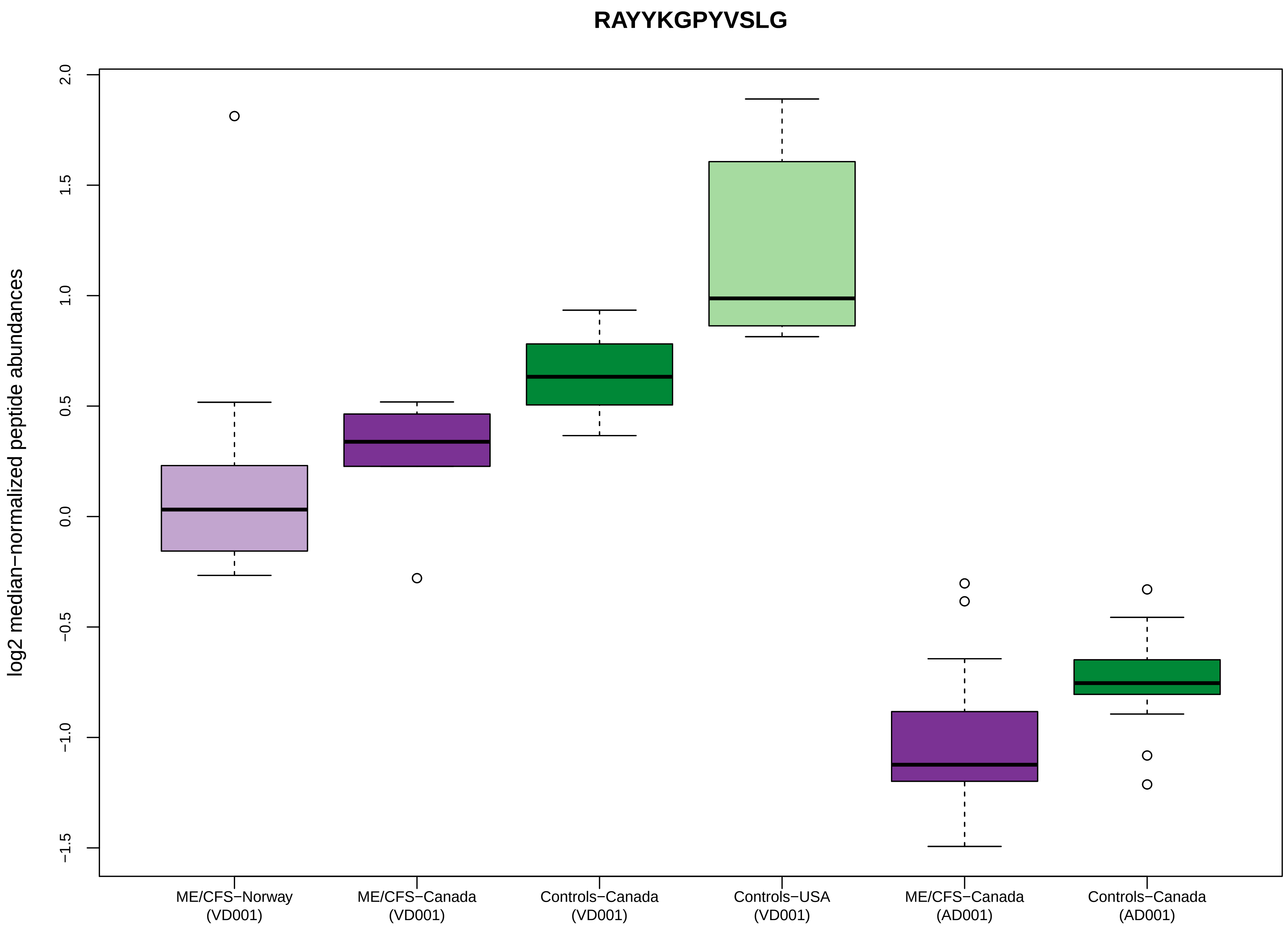
RAYYKGPYVSLG
log2 median−normalized peptide abundances
2.0
1.5
1.0
0.5
0.0
−0.5
−1.0
−1.5
ME/CFS−Norway (VD001)
ME/CFS−Canada (VD001)
Controls−Canada (VD001)
Controls−USA (VD001)
ME/CFS−Canada (AD001)
Controls−Canada (AD001)

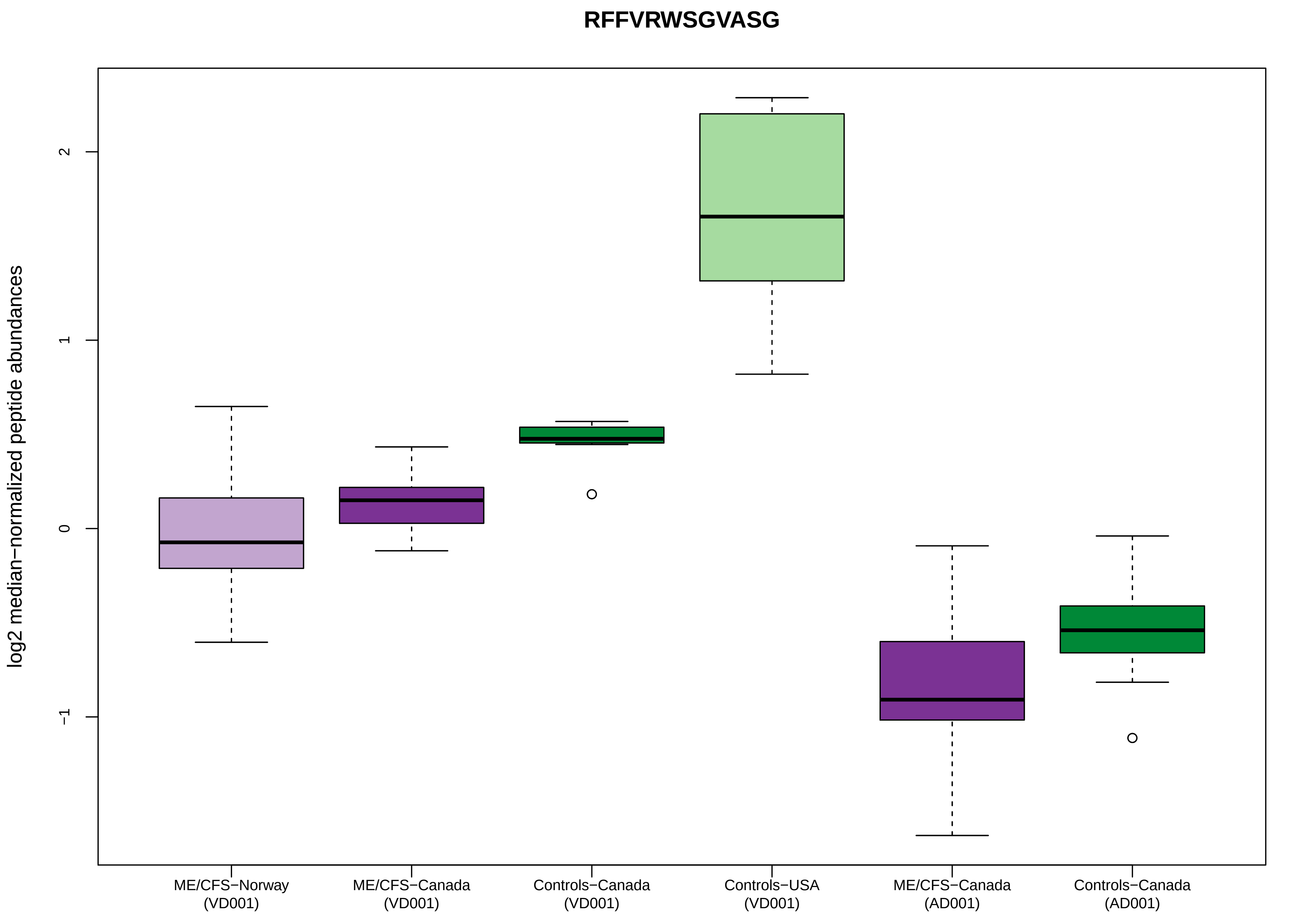
RFFVRWSGVASG
log2 median−normalized peptide abundances
2
1
0
−1
ME/CFS−Norway (VD001)
ME/CFS−Canada (VD001)
Controls−Canada (VD001)
Controls−USA (VD001)
ME/CFS−Canada (AD001)
Controls−Canada (AD001)

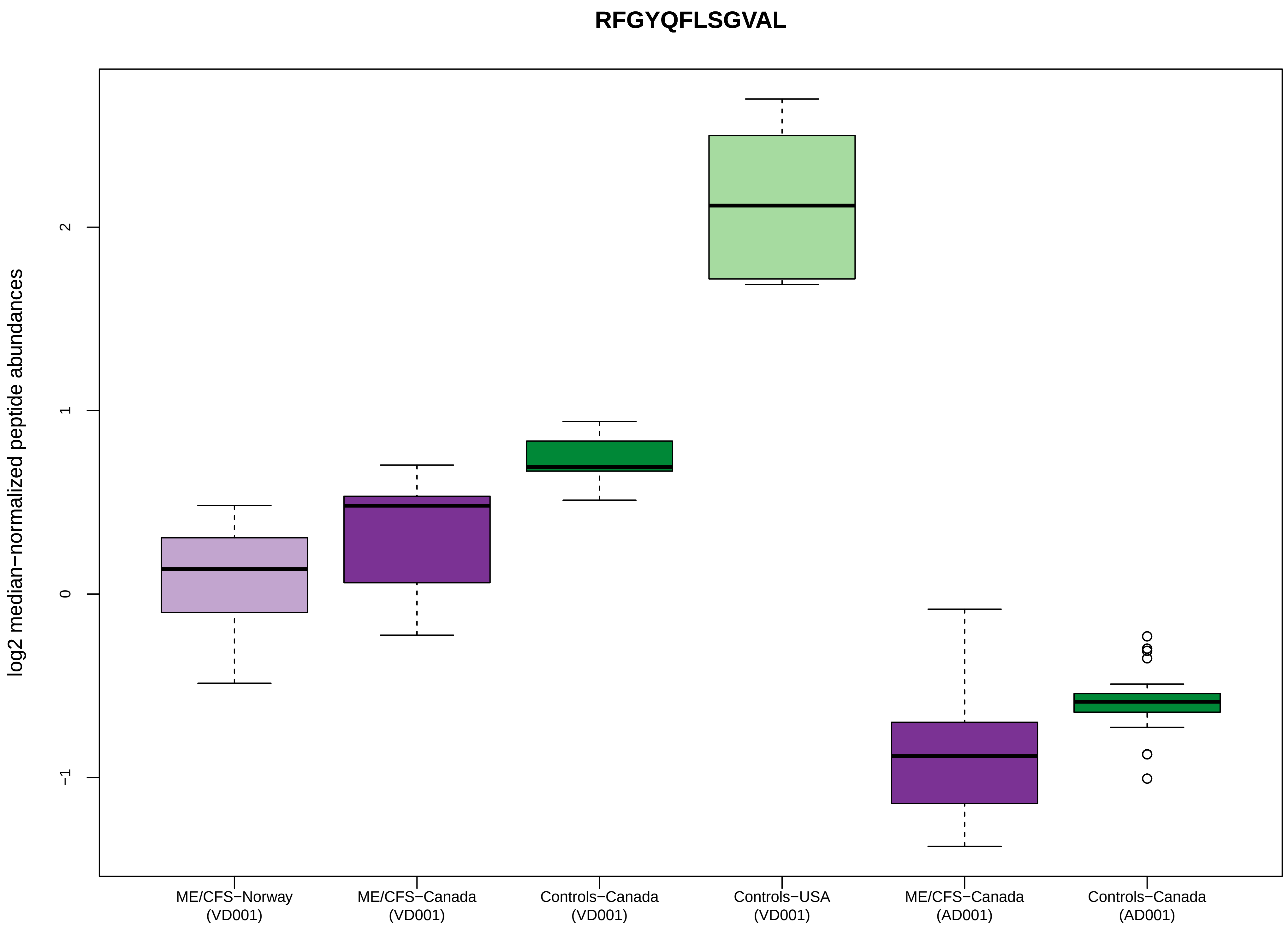
RFGYQFLSGVAL
log2 median−normalized peptide abundances
2
1
0
−1
ME/CFS−Norway (VD001)
ME/CFS−Canada (VD001)
Controls−Canada (VD001)
Controls−USA (VD001)
ME/CFS−Canada (AD001)
Controls−Canada (AD001)

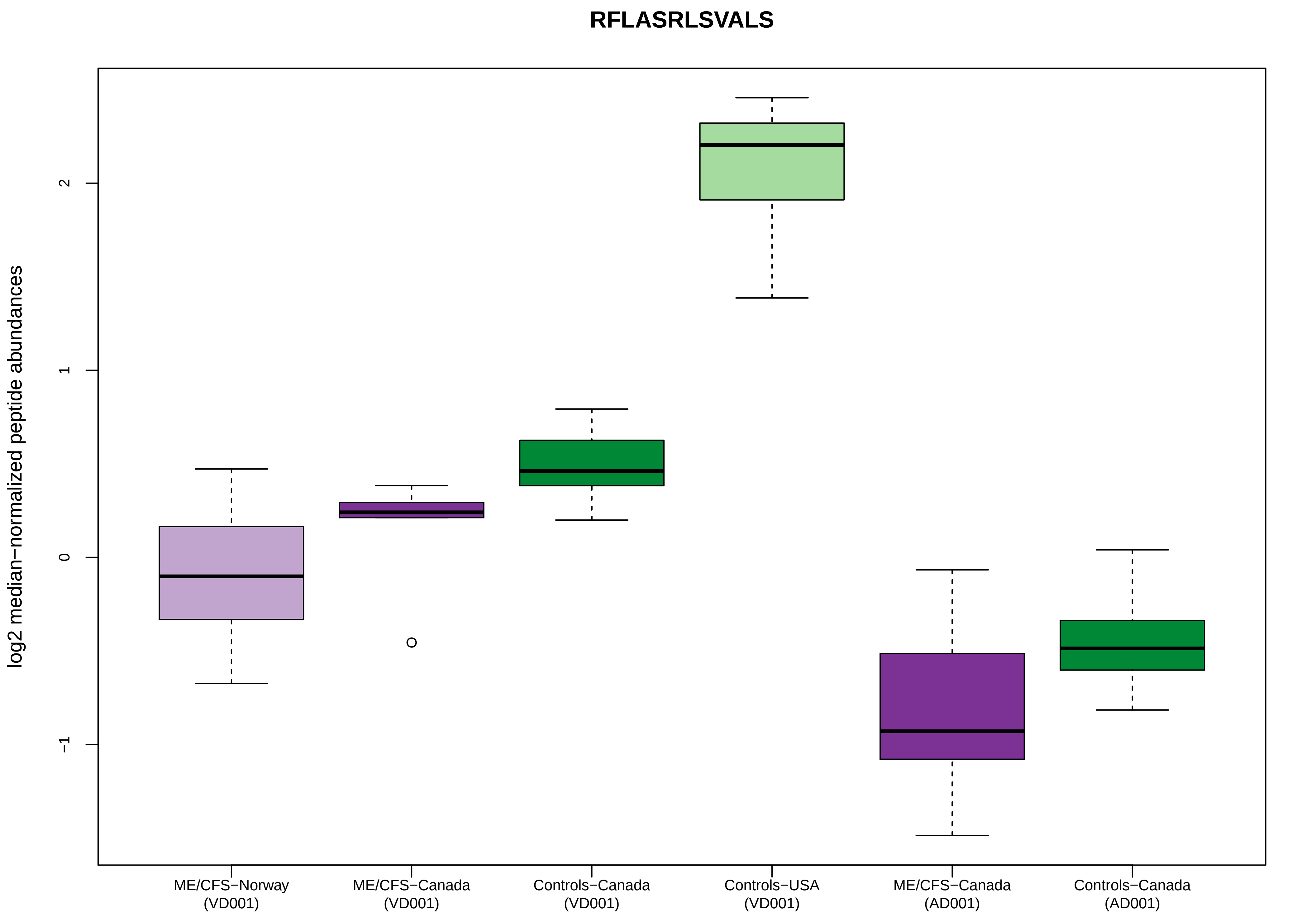

RFLASRLSVALS
log2 median−normalized peptide abundances
−1
0
1
2
ME/CFS−Norway (VD001)
ME/CFS−Canada (VD001)
Controls−Canada (VD001)
Controls−USA (VD001)
ME/CFS−Canada (AD001)
Controls−Canada (AD001)

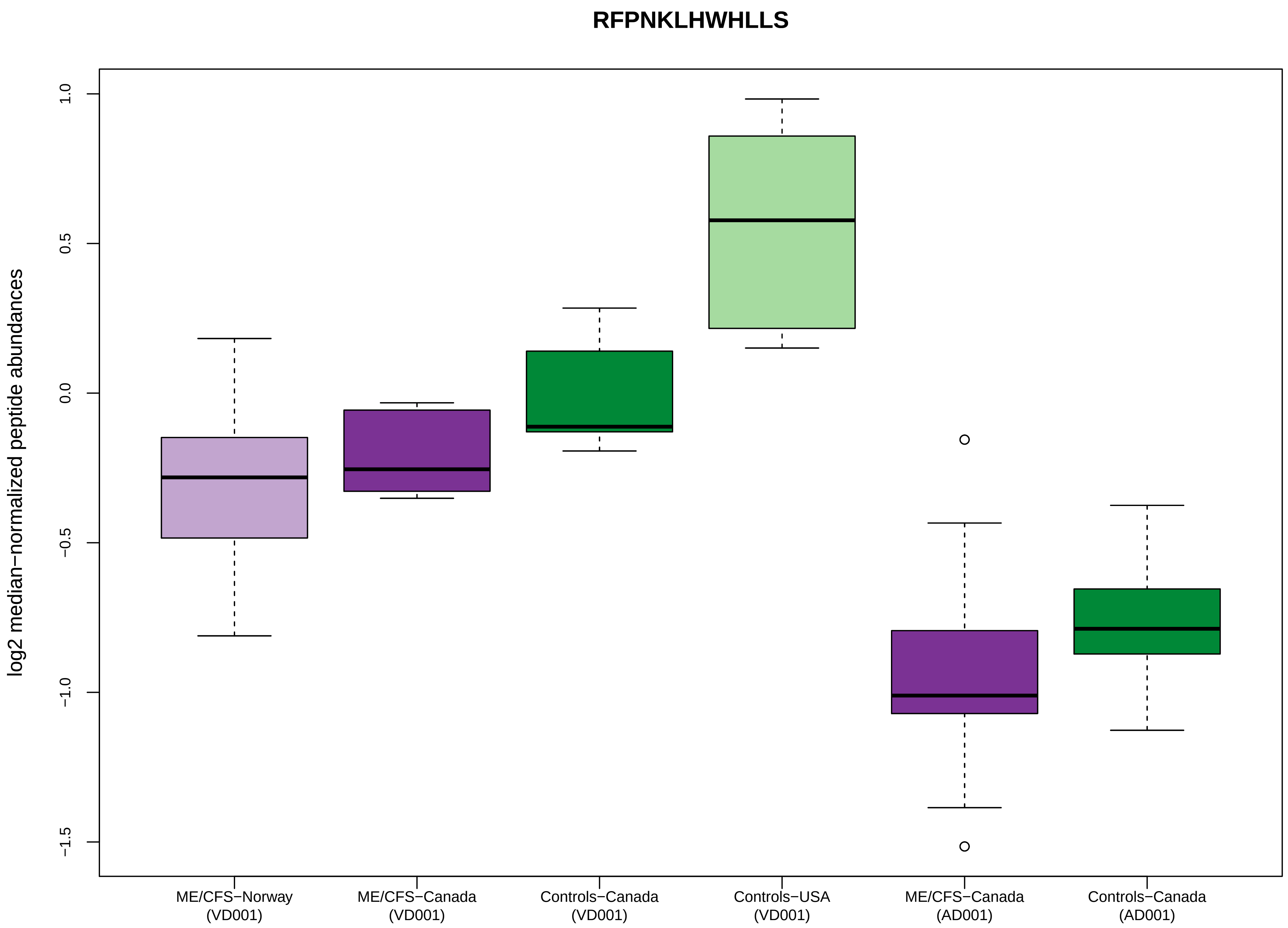

RFPNKLHWHLLS
log2 median−normalized peptide abundances
1.0
0.5
0.0
−0.5
−1.0
−1.5
ME/CFS−Norway (VD001)
ME/CFS−Canada (VD001)
Controls−Canada (VD001)
Controls−USA (VD001)
ME/CFS−Canada (AD001)
Controls−Canada (AD001)

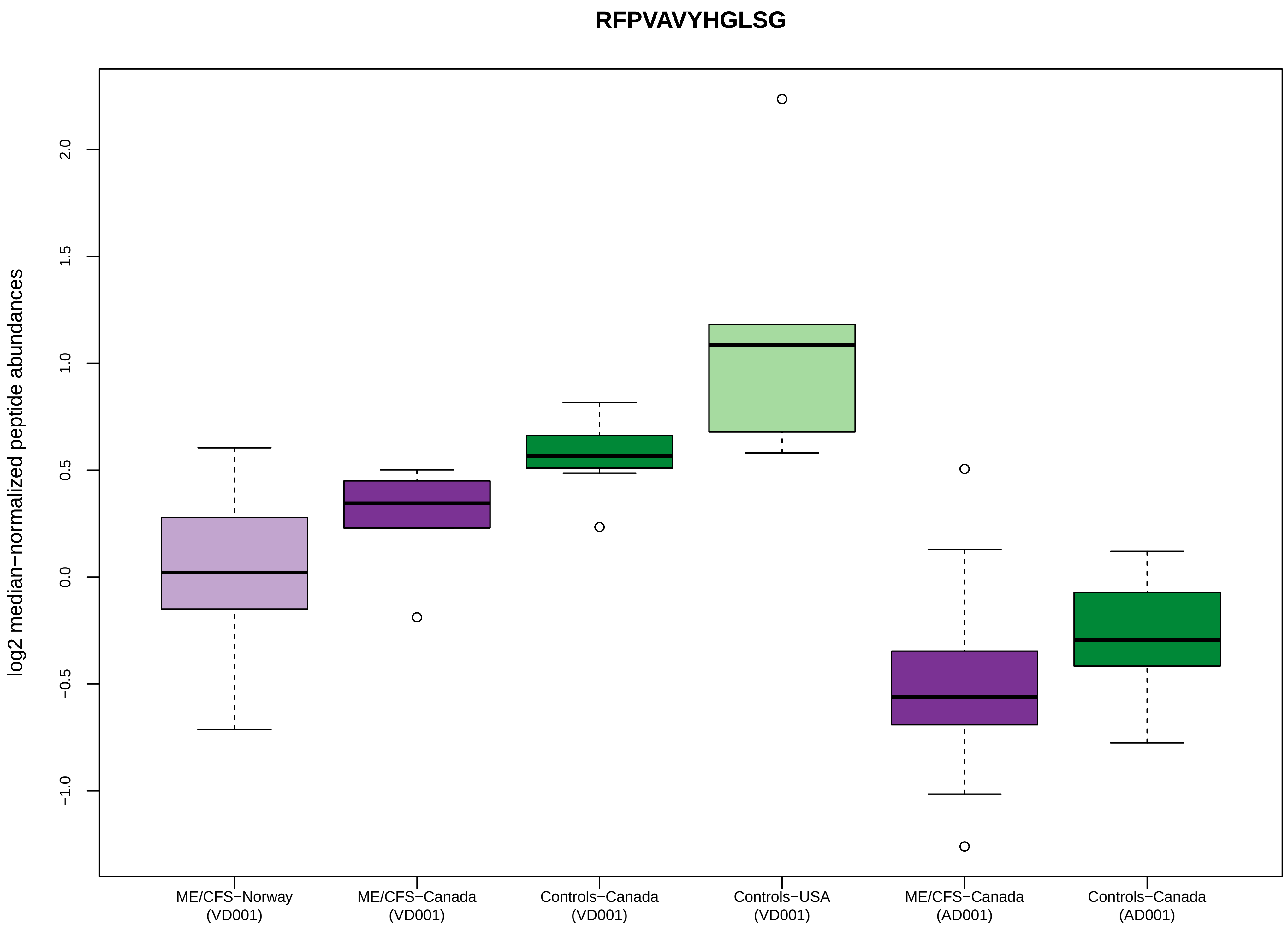

RFPVAVYHGLSG
log2 median−normalized peptide abundances
2.0
1.5
1.0
0.5
0.0
−0.5
−1.0
ME/CFS−Norway (VD001)
ME/CFS−Canada (VD001)
Controls−Canada (VD001)
Controls−USA (VD001)
ME/CFS−Canada (AD001)
Controls−Canada (AD001)

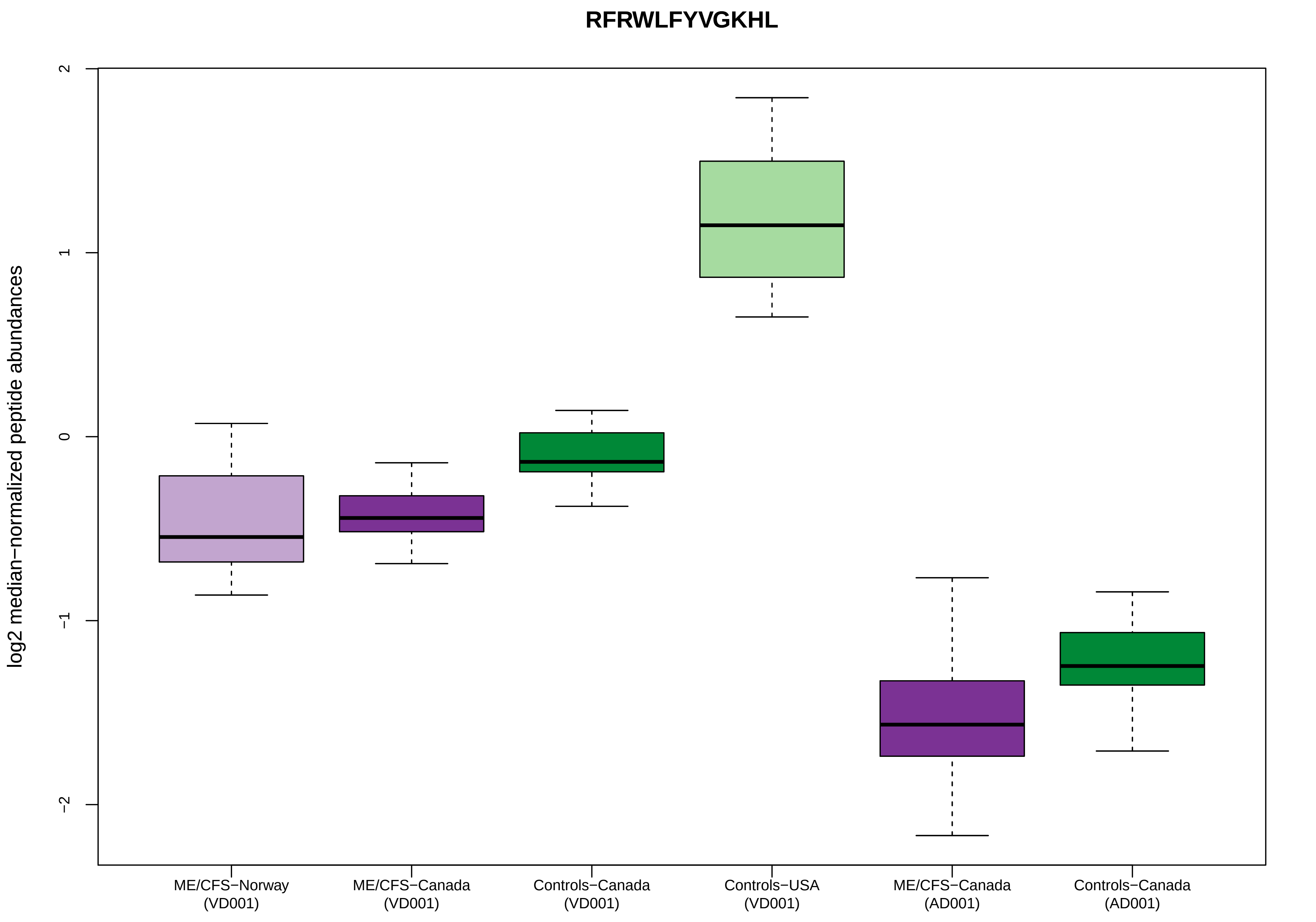
RFRWLFYVGKHL
log2 median−normalized peptide abundances
2
1
0
−1
−2
ME/CFS−Norway (VD001)
ME/CFS−Canada (VD001)
Controls−Canada (VD001)
Controls−USA (VD001)
ME/CFS−Canada (AD001)
Controls−Canada (AD001)

RFSALWALSALG

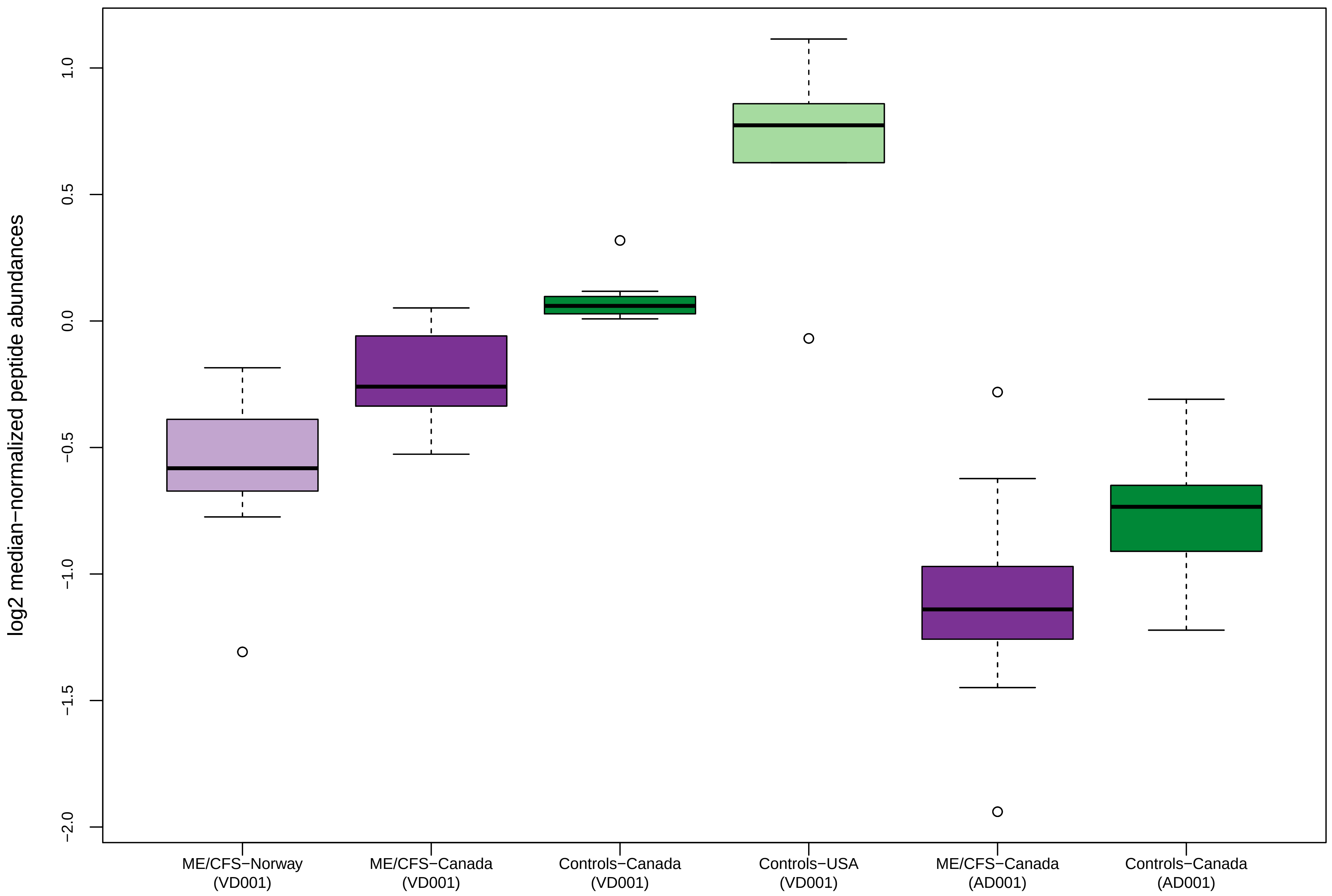

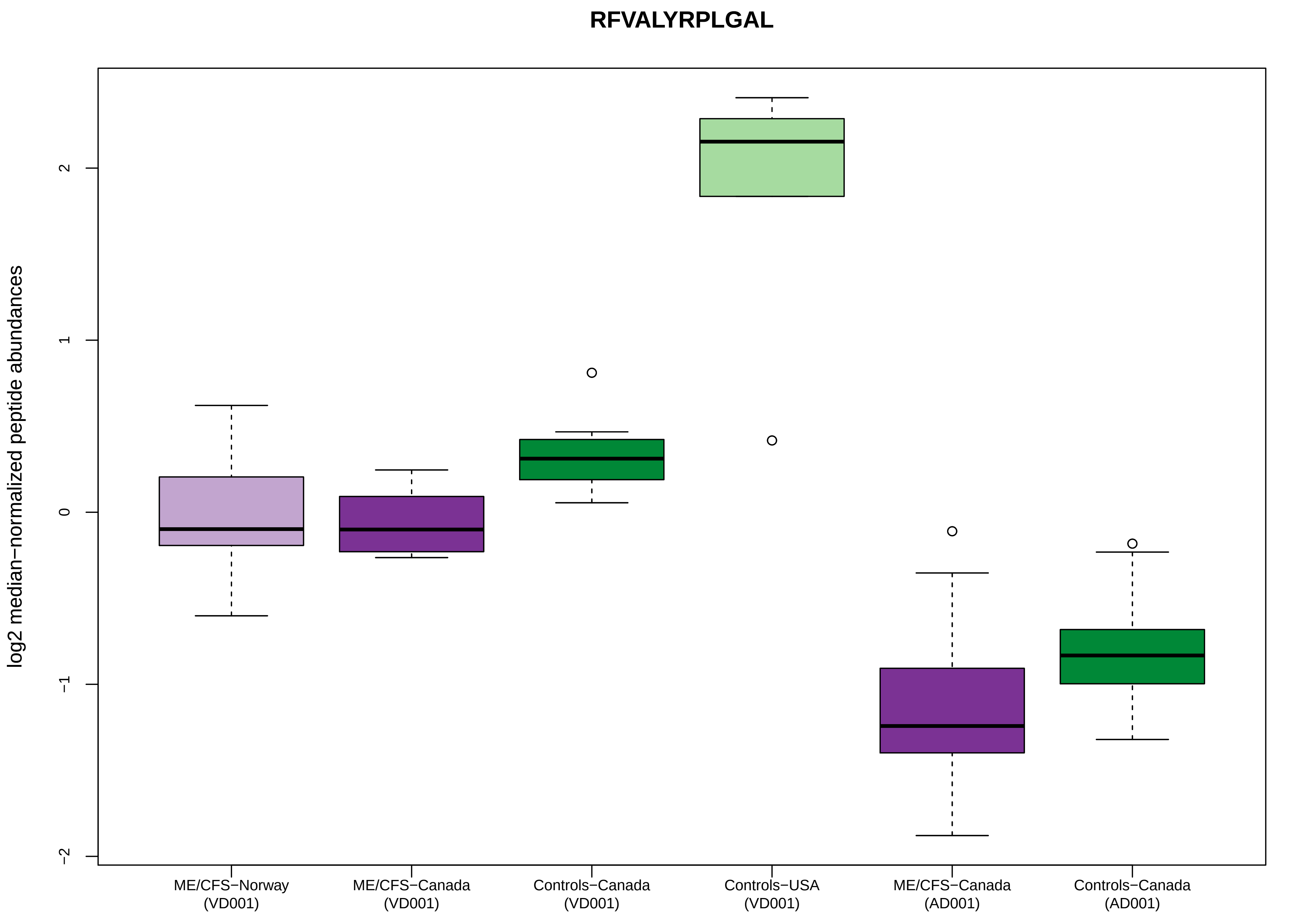

RFVALYRPLGAL
log2 median−normalized peptide abundances
2
1
0
−1
−2
ME/CFS−Norway (VD001)
ME/CFS−Canada (VD001)
Controls−Canada (VD001)
Controls−USA (VD001)
ME/CFS−Canada (AD001)
Controls−Canada (AD001)

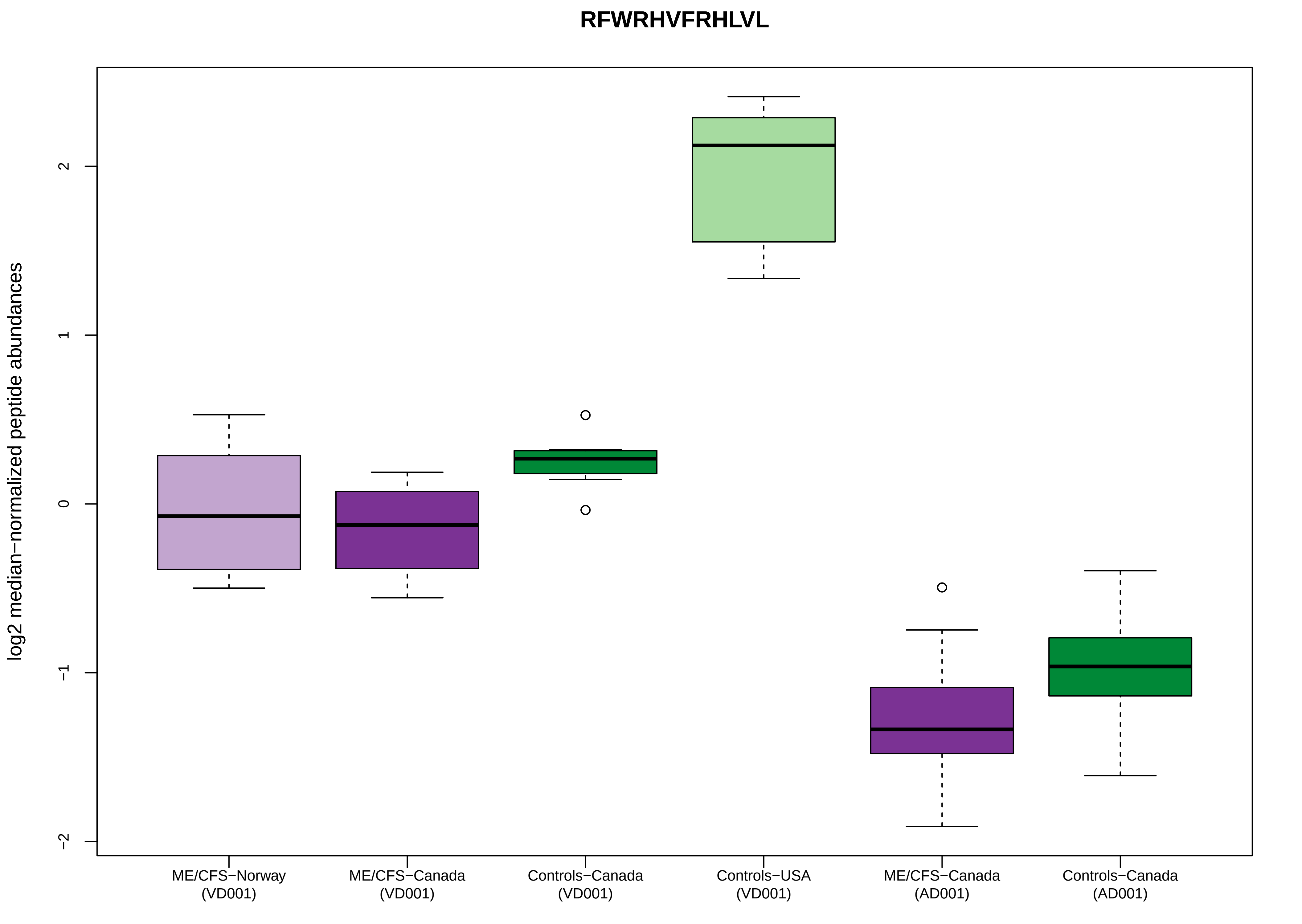

RFWRHVFRHLVL
log2 median−normalized peptide abundances
2
1
0
−1
−2
ME/CFS−Norway (VD001)
ME/CFS−Canada (VD001)
Controls−Canada (VD001)
Controls−USA (VD001)
ME/CFS−Canada (AD001)
Controls−Canada (AD001)

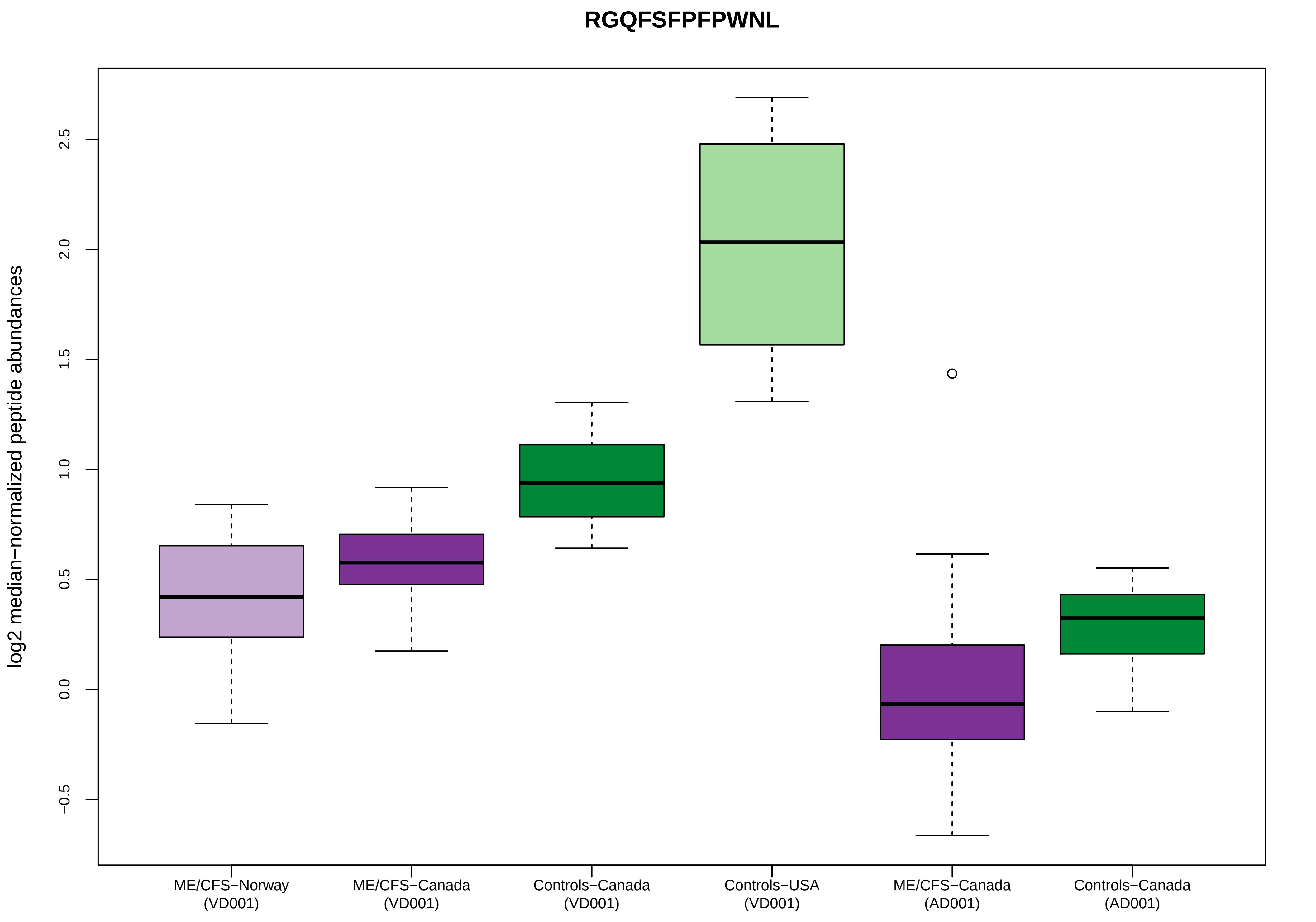
RGQFSFPFPWNL
log2 median−normalized peptide abundances
−0.5
0.0
0.5
1.0
1.5
2.0
2.5
ME/CFS−Norway (VD001)
ME/CFS−Canada (VD001)
Controls−Canada (VD001)
Controls−USA (VD001)
ME/CFS−Canada (AD001)
Controls−Canada (AD001)

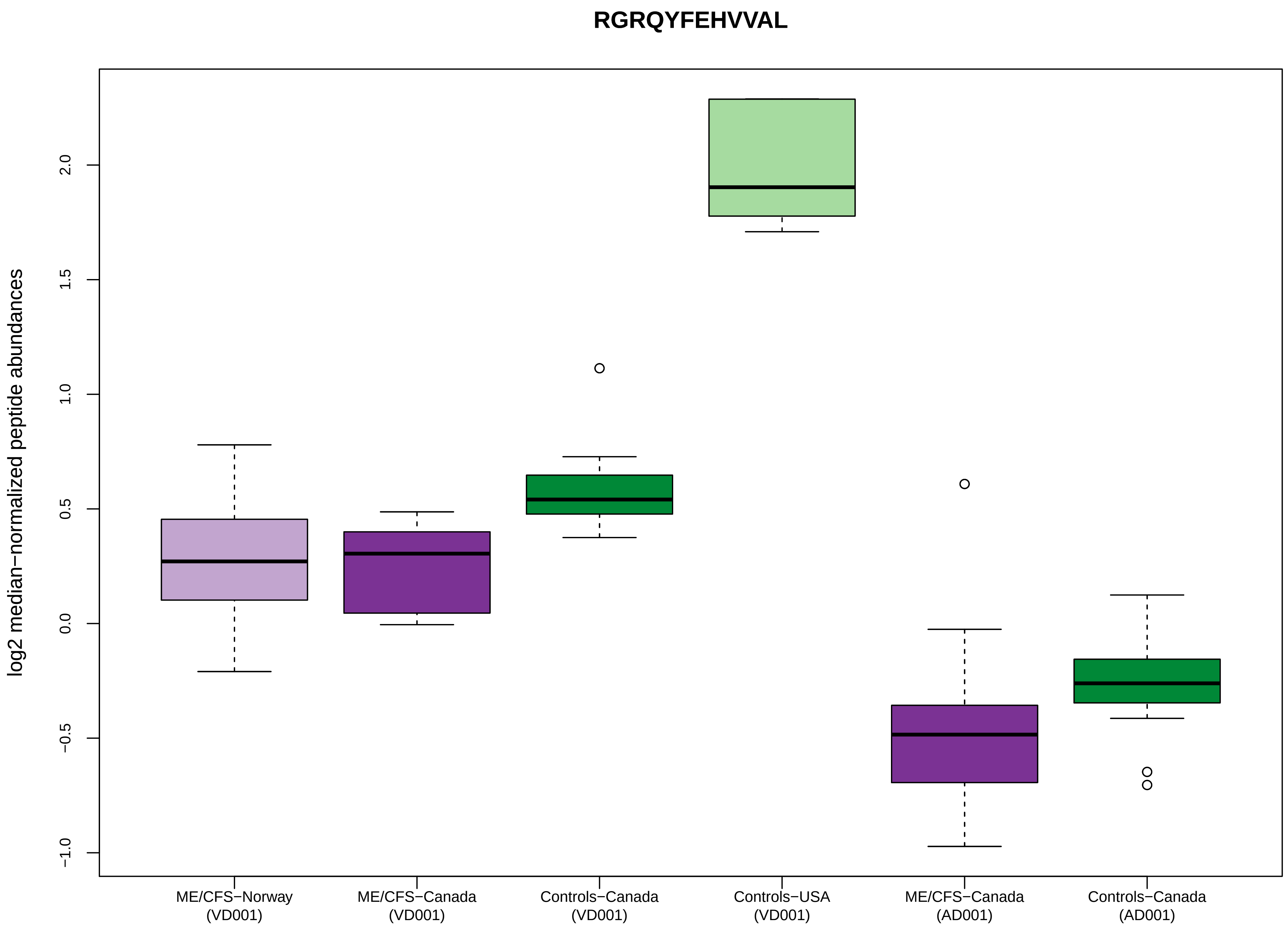

RGRQYFEHVVAL
log2 median−normalized peptide abundances
2.0
1.5
1.0
0.5
0.0
−0.5
−1.0
ME/CFS−Norway (VD001)
ME/CFS−Canada (VD001)
Controls−Canada (VD001)
Controls−USA (VD001)
ME/CFS−Canada (AD001)
Controls−Canada (AD001)

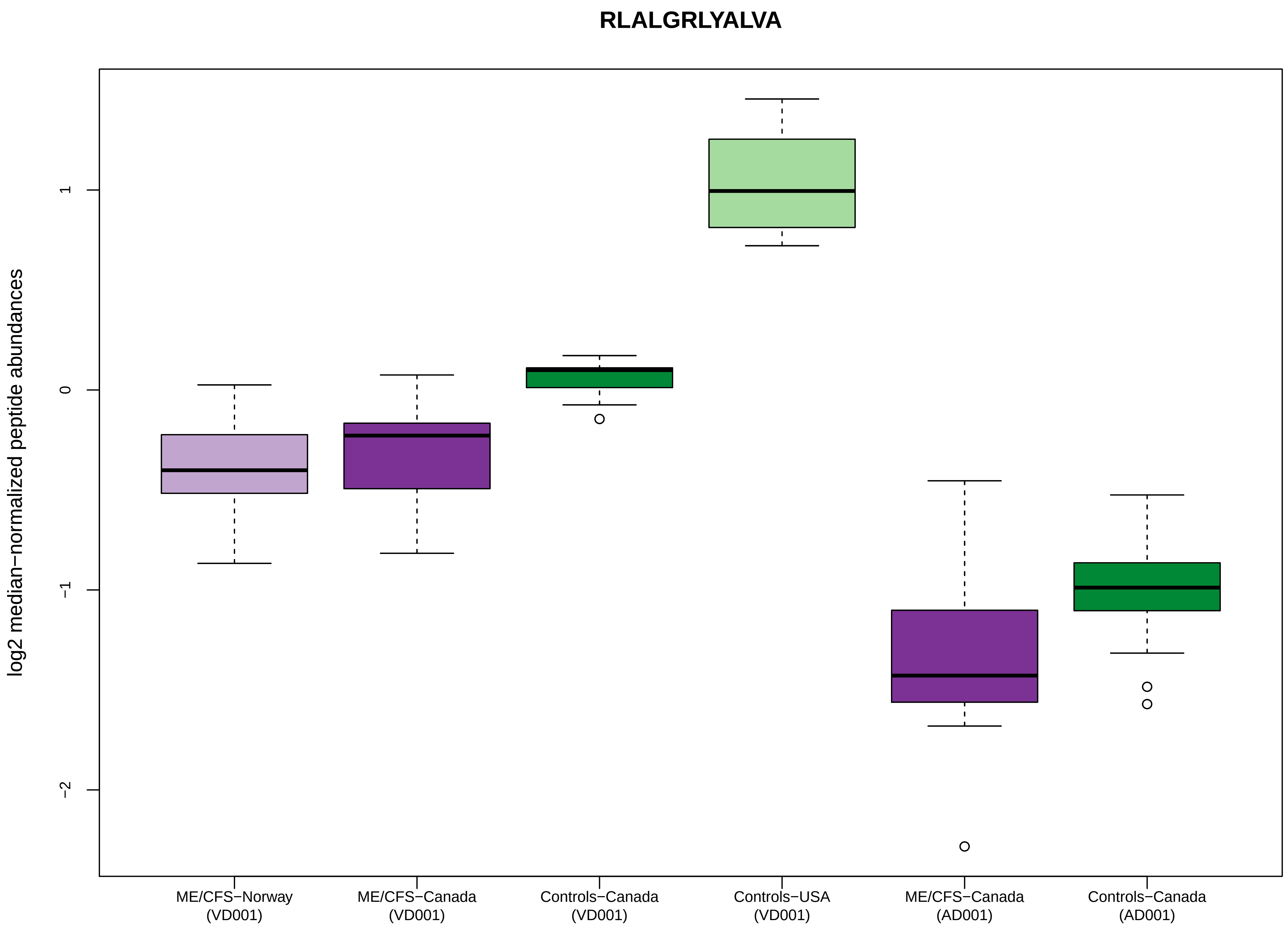

RLALGRLYALVA
log2 median−normalized peptide abundances
1
0
−1
−2
ME/CFS−Norway (VD001)
ME/CFS−Canada (VD001)
Controls−Canada (VD001)
Controls−USA (VD001)
ME/CFS−Canada (AD001)
Controls−Canada (AD001)

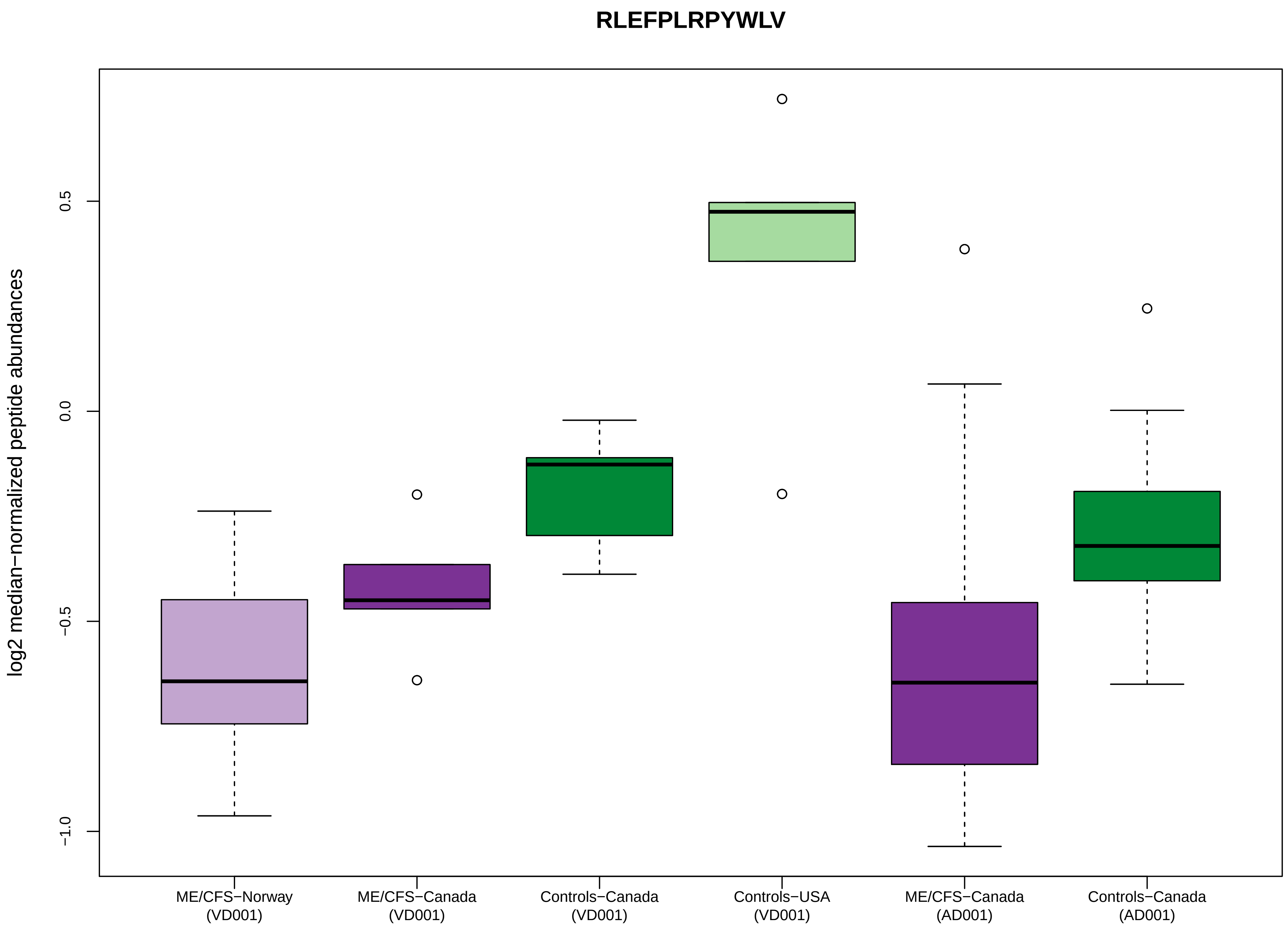

RLEFPLRPYWLV
log2 median−normalized peptide abundances
0.5
0.0
−0.5
−1.0
ME/CFS−Norway (VD001)
ME/CFS−Canada (VD001)
Controls−Canada (VD001)
Controls−USA (VD001)
ME/CFS−Canada (AD001)
Controls−Canada (AD001)

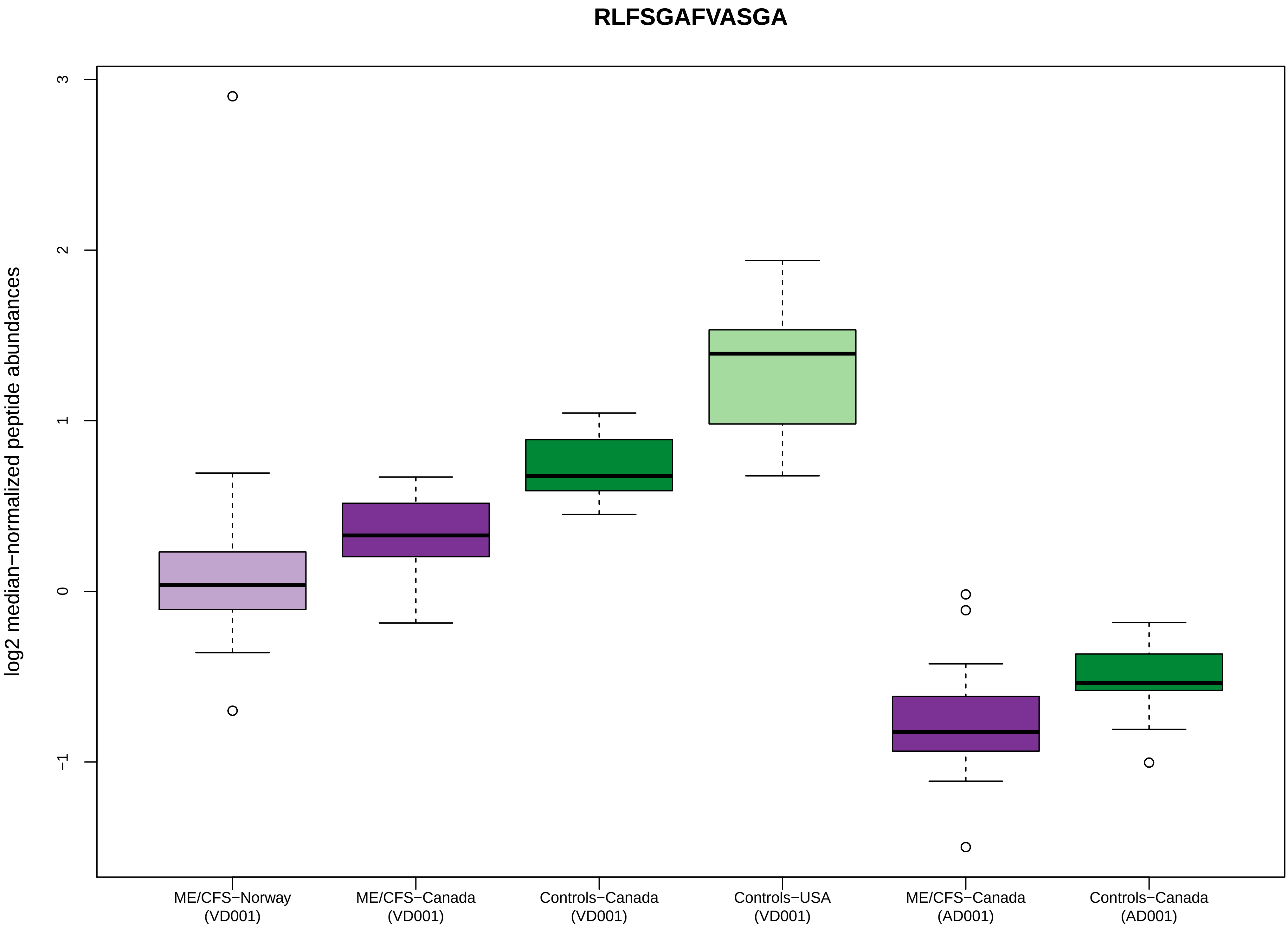
RLFSGAFVASGA
log2 median−normalized peptide abundances
3
2
1
0
−1
ME/CFS−Norway (VD001)
ME/CFS−Canada (VD001)
Controls−Canada (VD001)
Controls−USA (VD001)
ME/CFS−Canada (AD001)
Controls−Canada (AD001)

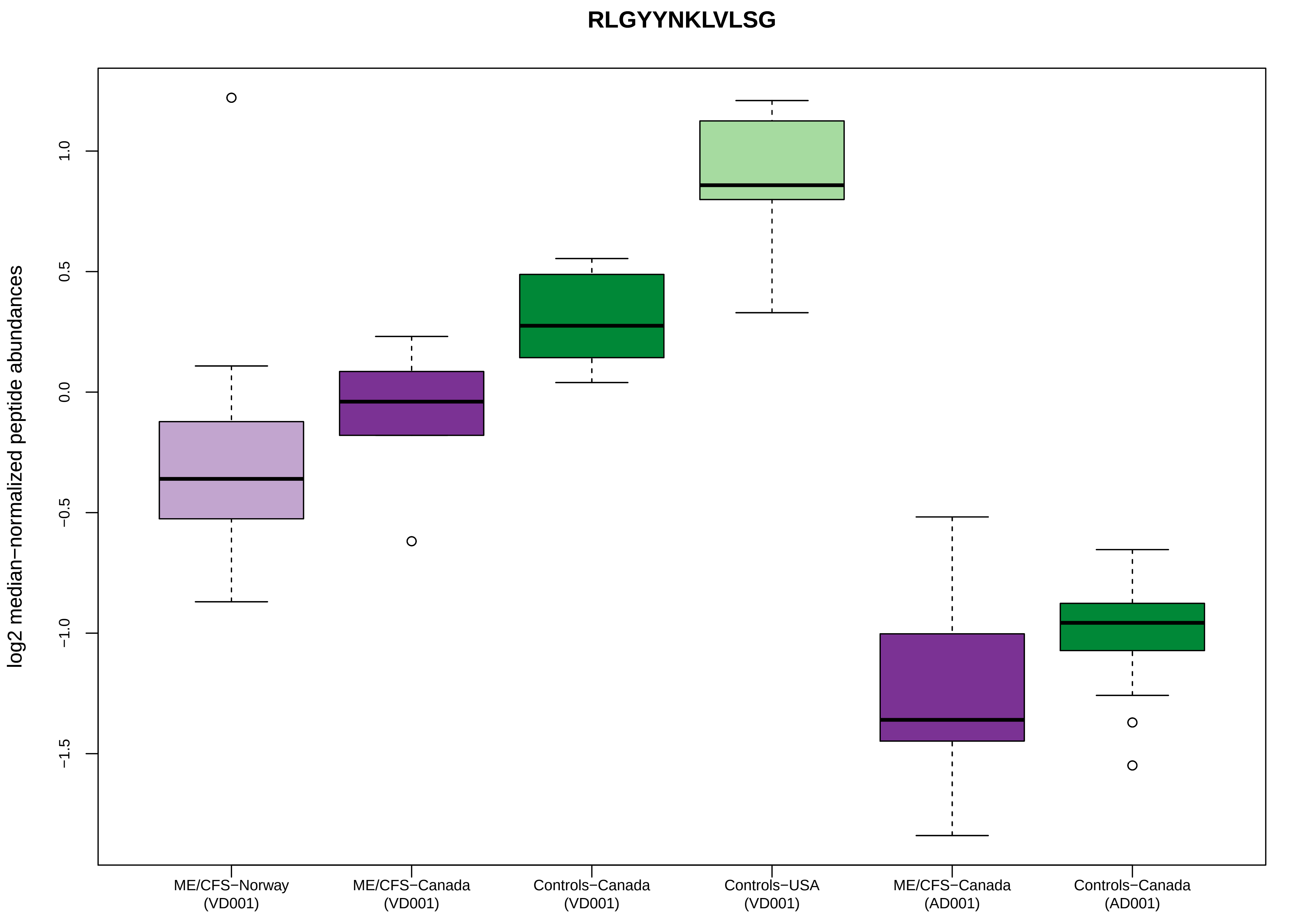
RLGYYNKLVLSG
log2 median−normalized peptide abundances
1.0
0.5
0.0
−0.5
−1.0
−1.5
ME/CFS−Norway (VD001)
ME/CFS−Canada (VD001)
Controls−Canada (VD001)
Controls−USA (VD001)
ME/CFS−Canada (AD001)
Controls−Canada (AD001)

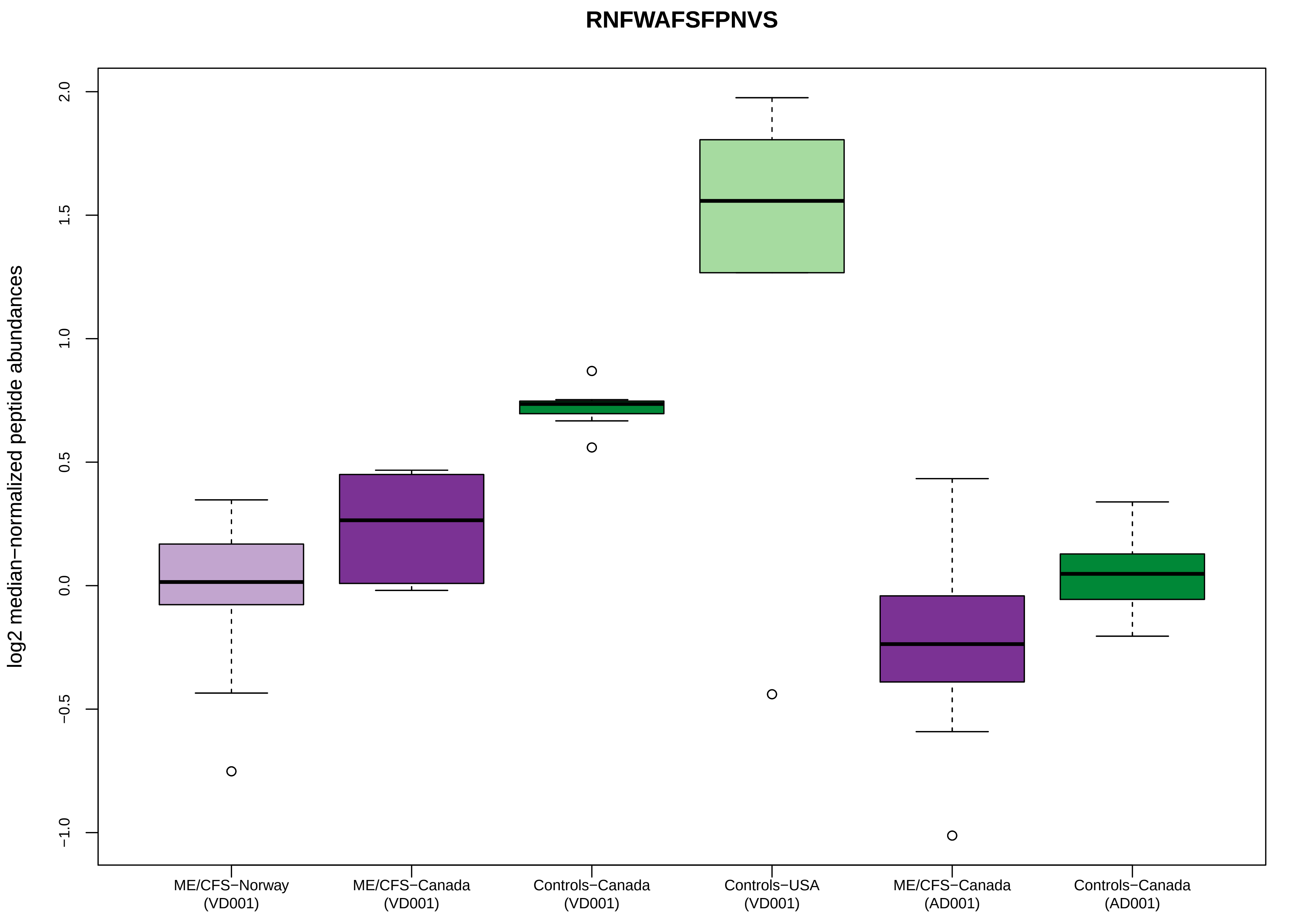

RNFWAFSFPNVS
log2 median−normalized peptide abundances
−1.0
−0.5
0.0
0.5
1.0
1.5
2.0
ME/CFS−Norway (VD001)
ME/CFS−Canada (VD001)
Controls−Canada (VD001)
Controls−USA (VD001)
ME/CFS−Canada (AD001)
Controls−Canada (AD001)

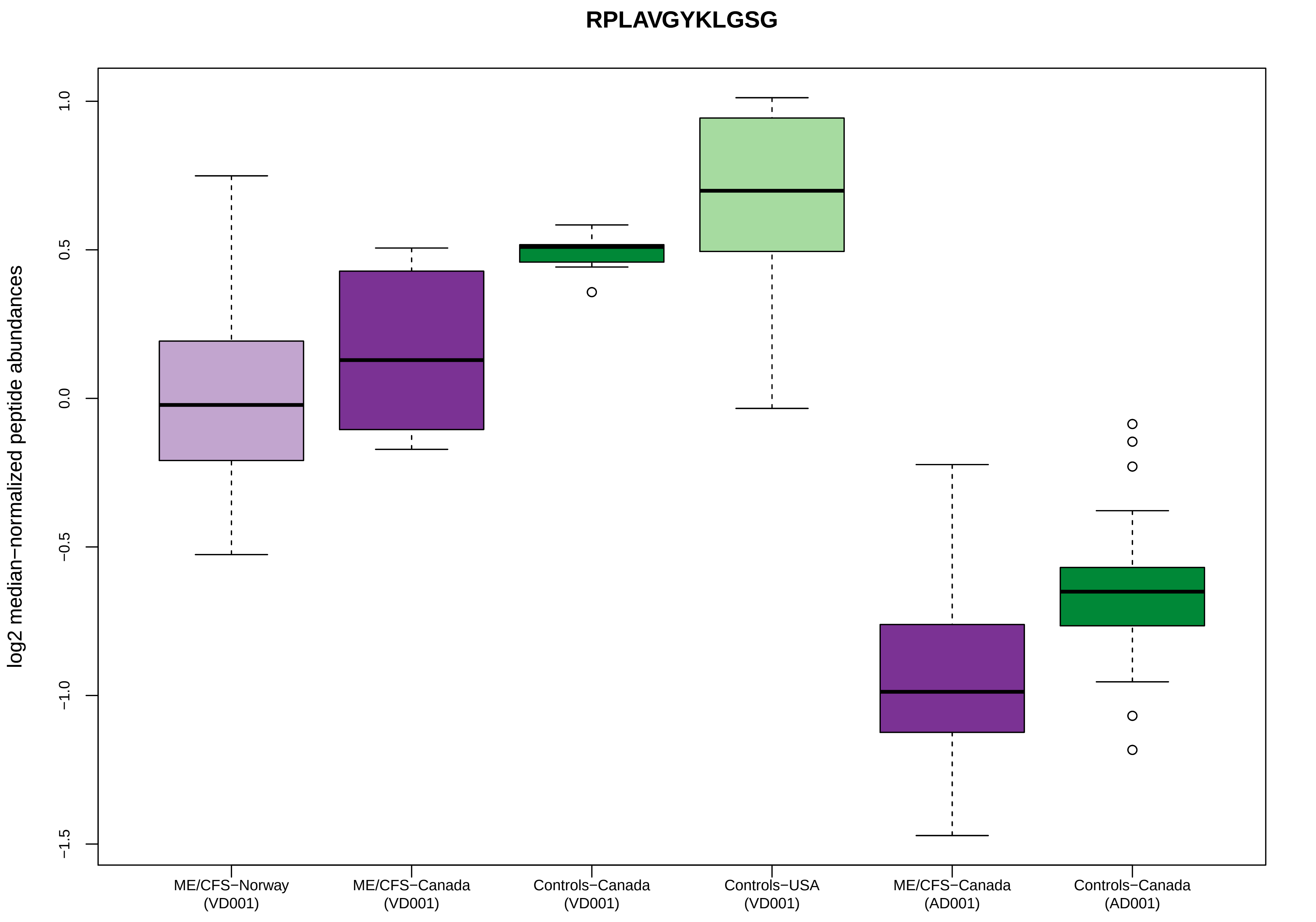

RPLAVGYKLGSG
log2 median−normalized peptide abundances
1.0
0.5
0.0
−0.5
−1.0
−1.5
ME/CFS−Norway (VD001)
ME/CFS−Canada (VD001)
Controls−Canada (VD001)
Controls−USA (VD001)
ME/CFS−Canada (AD001)
Controls−Canada (AD001)

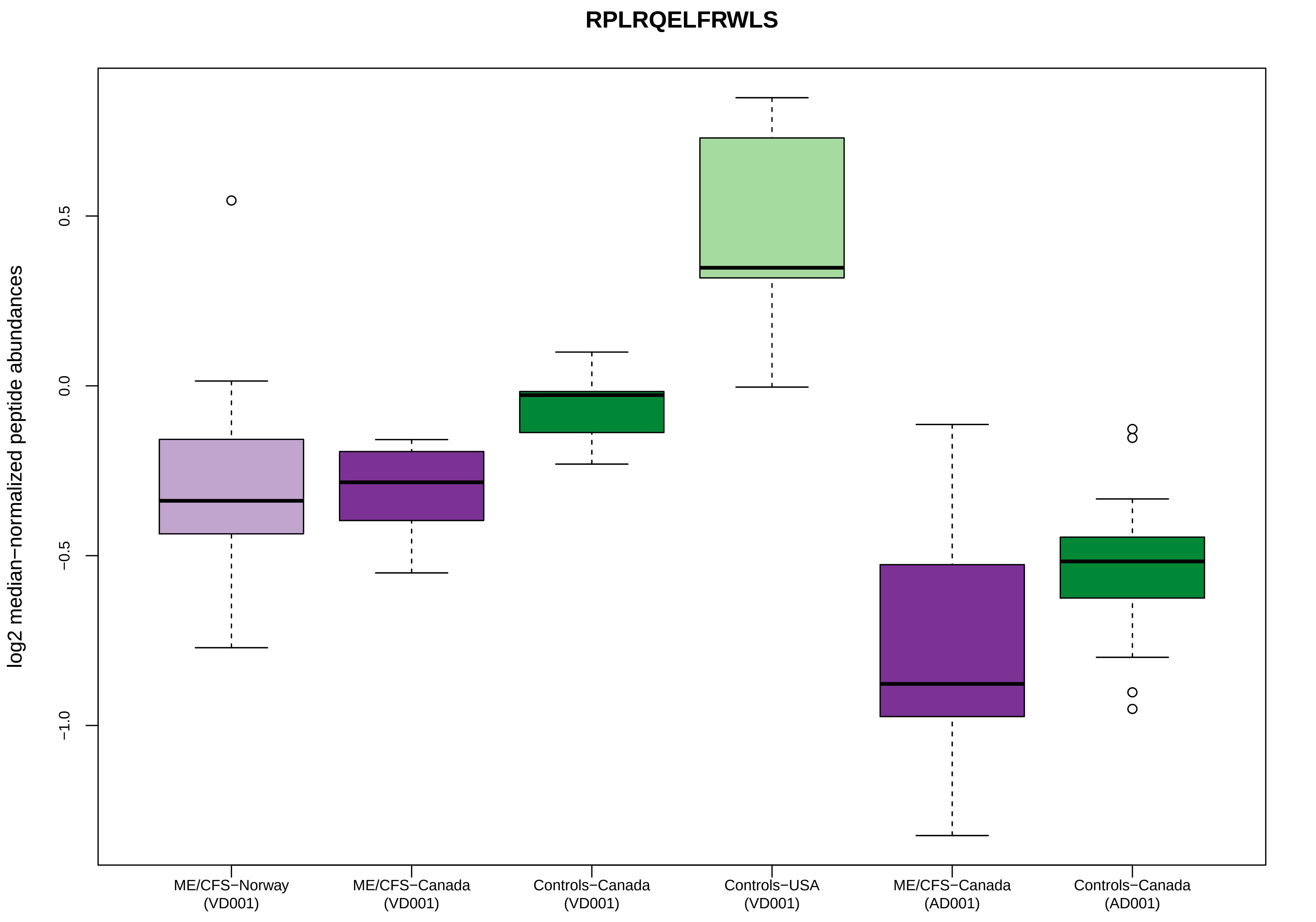
RPLRQELFRWLS
log2 median−normalized peptide abundances
0.5
0.0
−0.5
−1.0
ME/CFS−Norway (VD001)
ME/CFS−Canada (VD001)
Controls−Canada (VD001)
Controls−USA (VD001)
ME/CFS−Canada (AD001)
Controls−Canada (AD001)

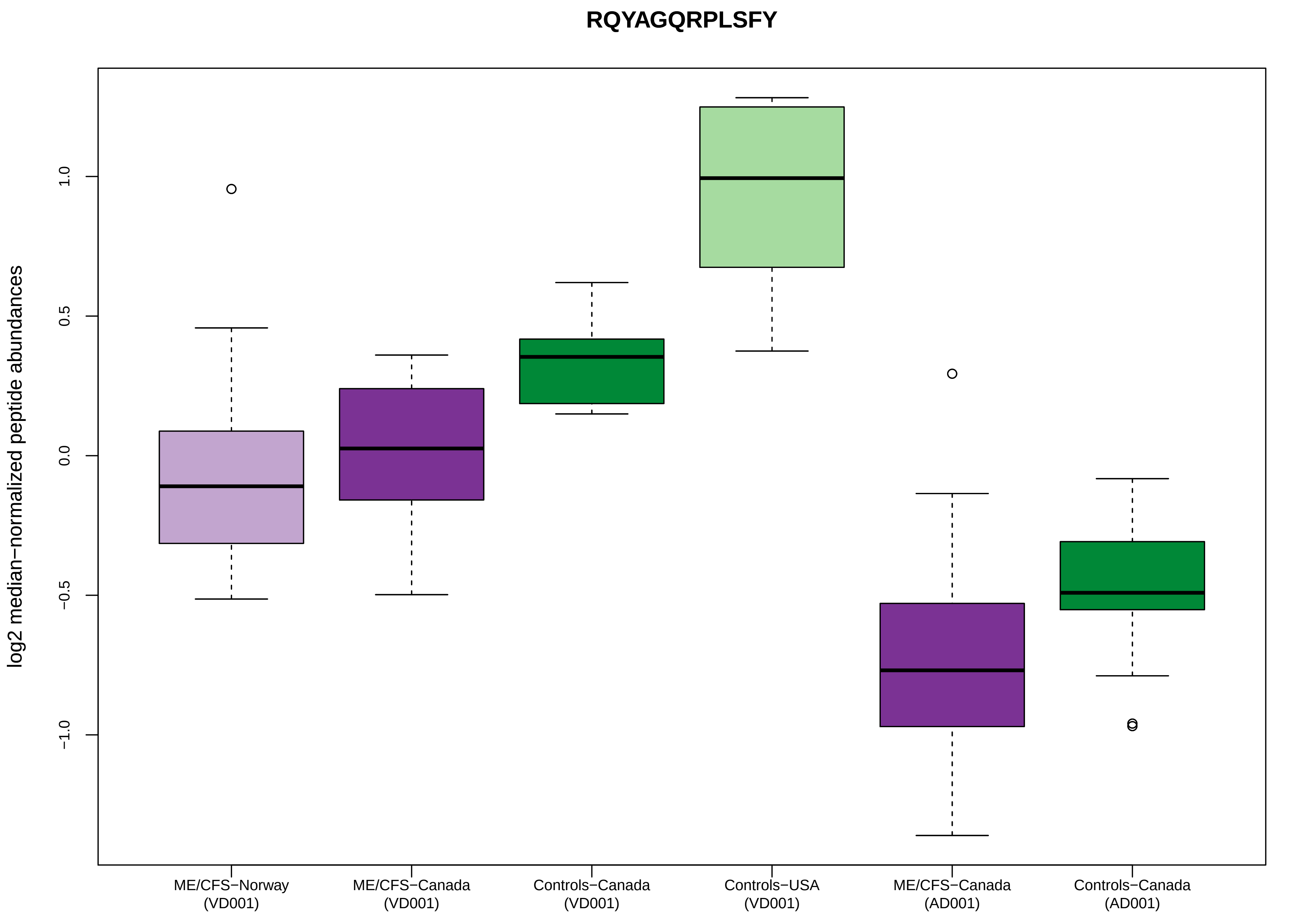
RQYAGQRPLSFY
log2 median−normalized peptide abundances
1.0
0.5
0.0
−0.5
−1.0
ME/CFS−Norway (VD001)
ME/CFS−Canada (VD001)
Controls−Canada (VD001)
Controls−USA (VD001)
ME/CFS−Canada (AD001)
Controls−Canada (AD001)

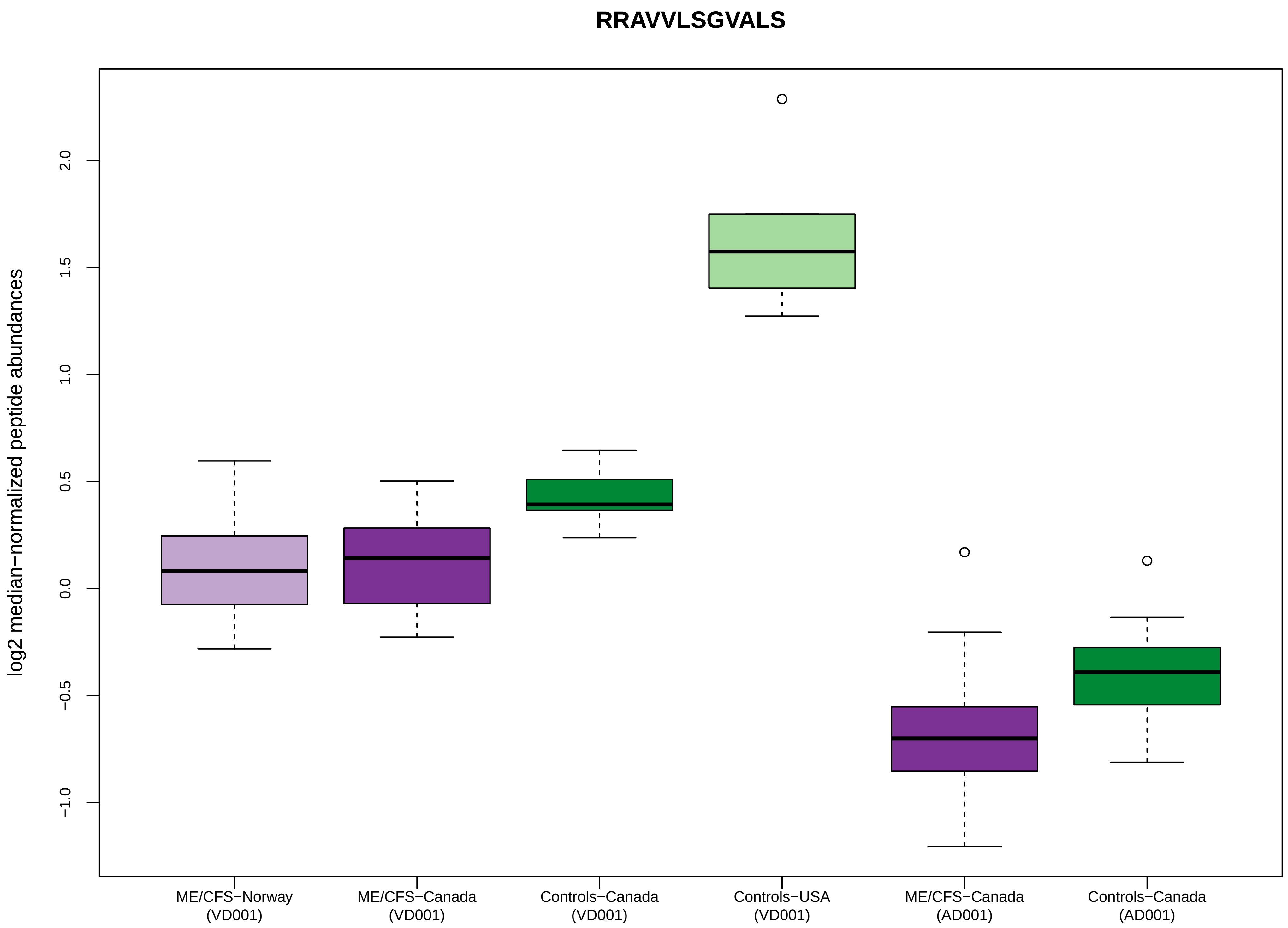
RRAVVLSGVALS
log2 median−normalized peptide abundances
2.0
1.5
1.0
0.5
0.0
−0.5
−1.0
ME/CFS−Norway (VD001)
ME/CFS−Canada (VD001)
Controls−Canada (VD001)
Controls−USA (VD001)
ME/CFS−Canada (AD001)
Controls−Canada (AD001)

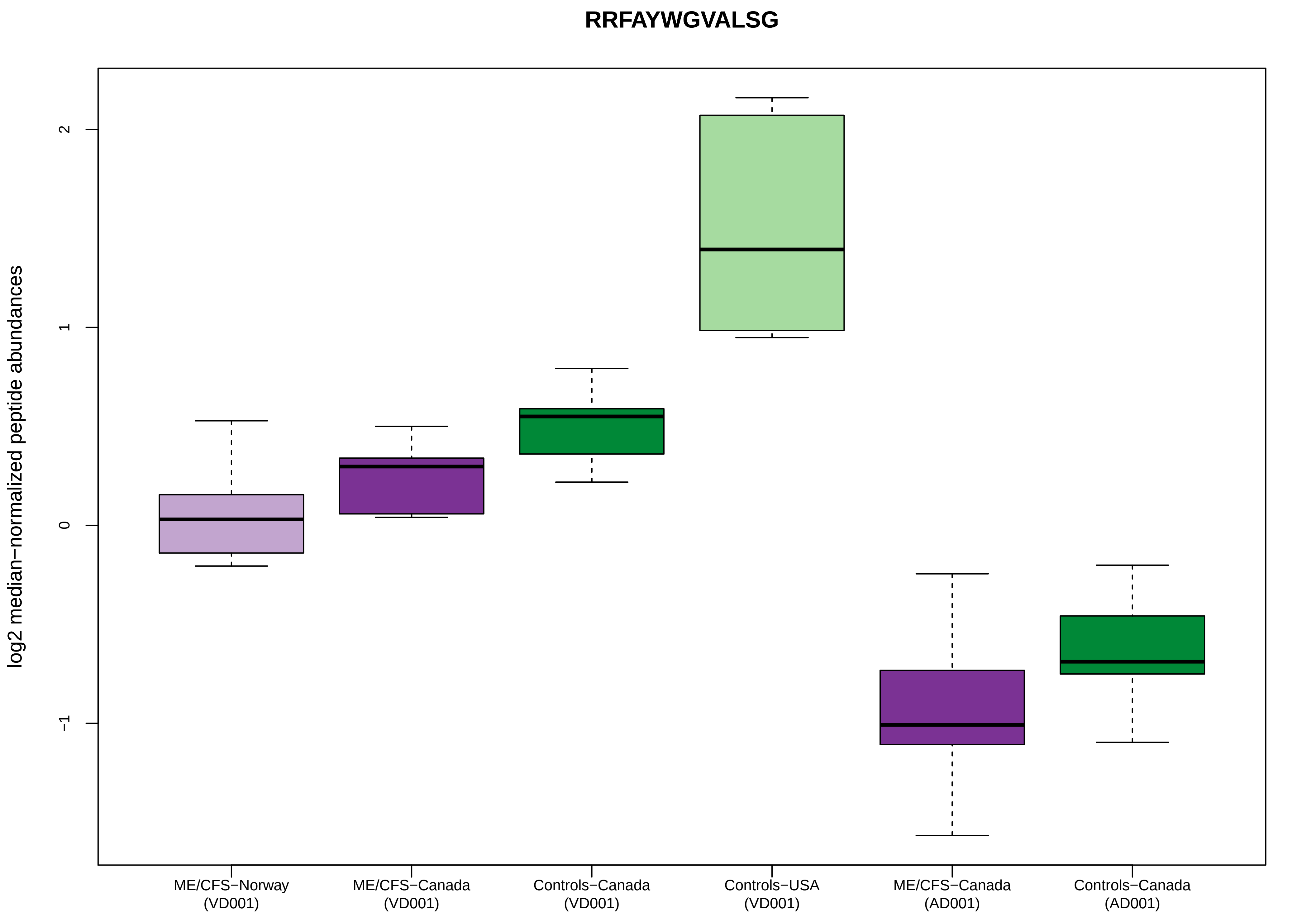

RRFAYWGVALSG
log2 median−normalized peptide abundances
2
1
0
−1
ME/CFS−Norway (VD001)
ME/CFS−Canada (VD001)
Controls−Canada (VD001)
Controls−USA (VD001)
ME/CFS−Canada (AD001)
Controls−Canada (AD001)

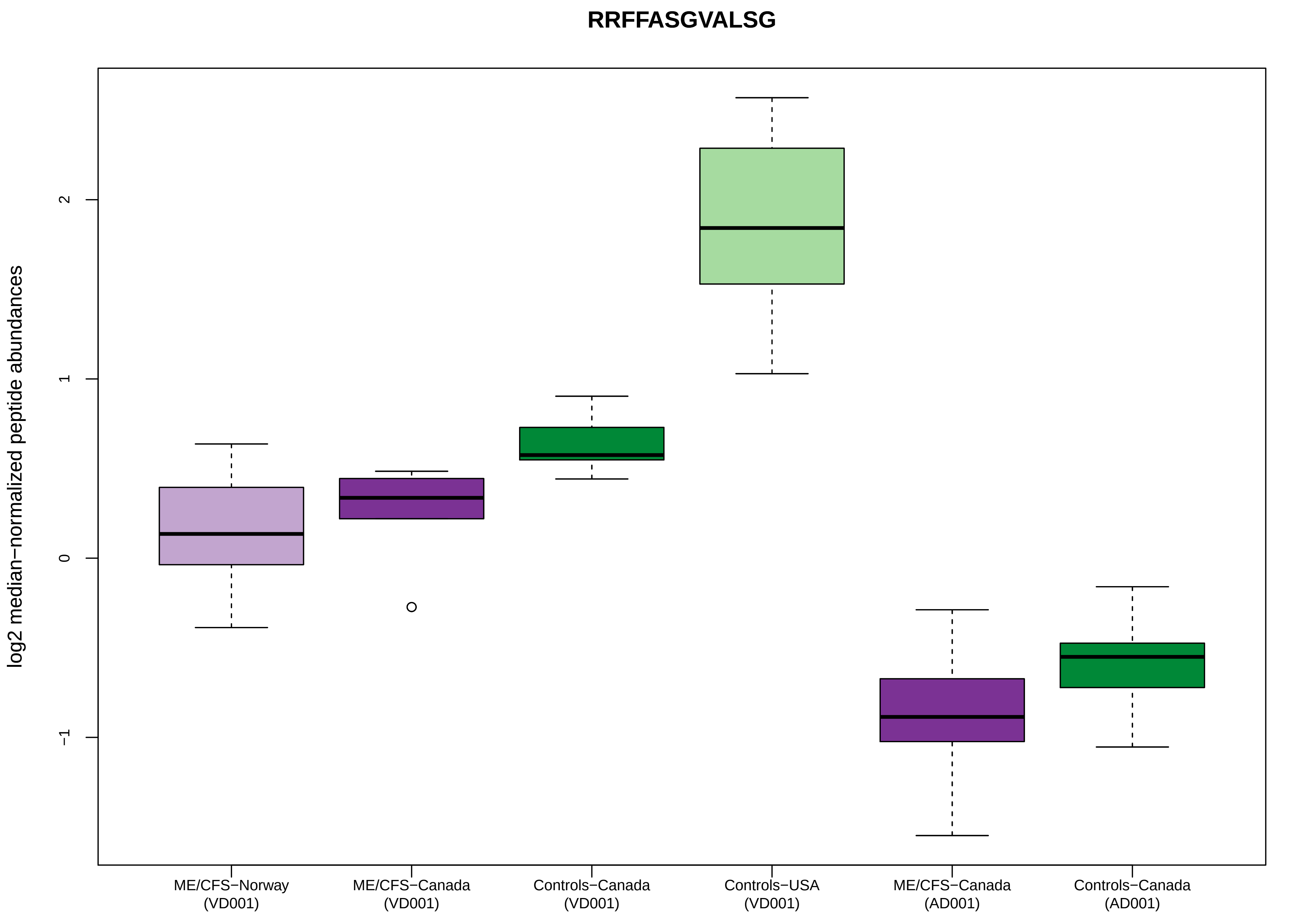

RRFFASGVALSG
log2 median−normalized peptide abundances
2
1
0
−1
ME/CFS−Norway (VD001)
ME/CFS−Canada (VD001)
Controls−Canada (VD001)
Controls−USA (VD001)
ME/CFS−Canada (AD001)
Controls−Canada (AD001)

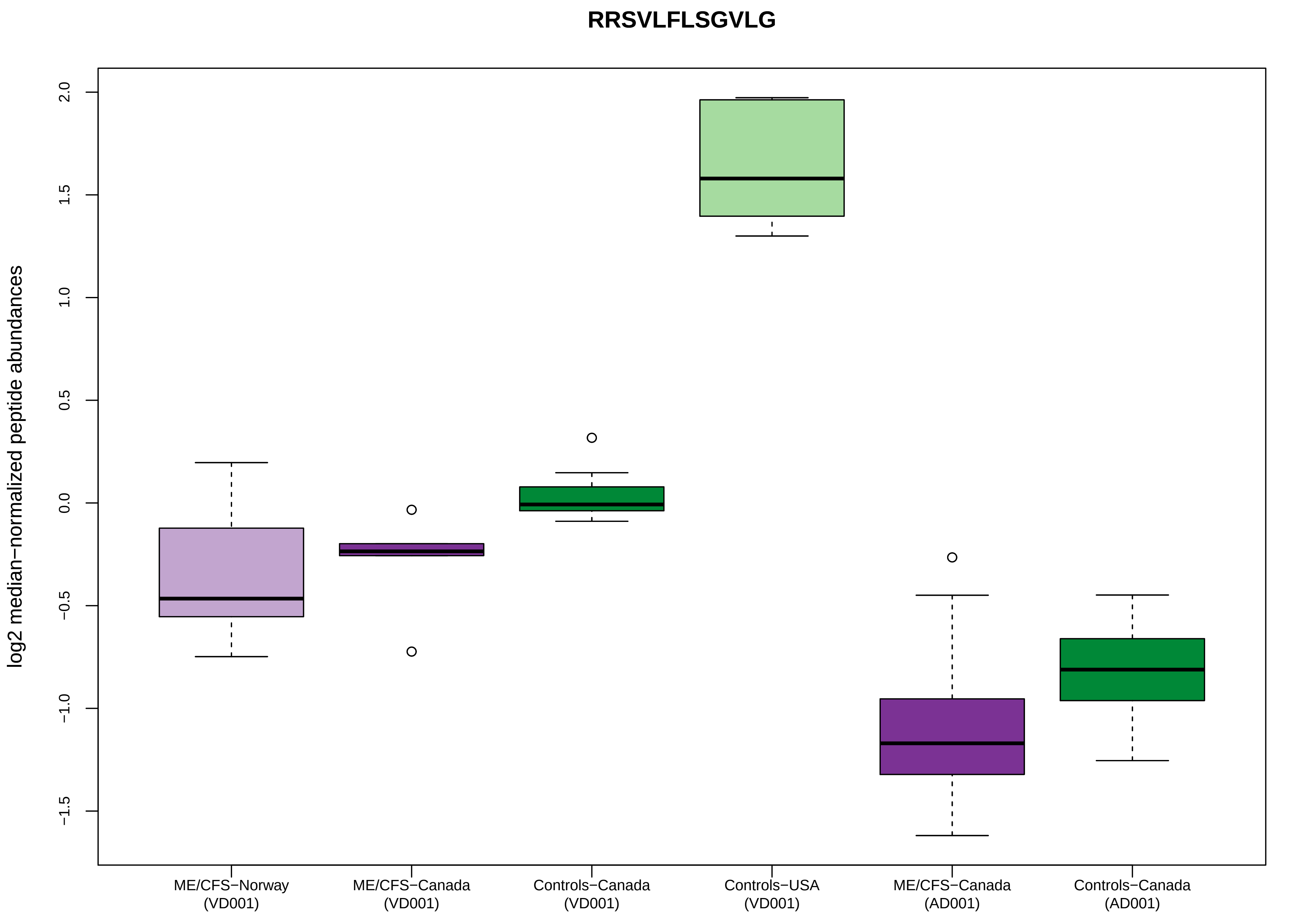
RRSVLFLSGVLG
log2 median−normalized peptide abundances
2.0
1.5
1.0
0.5
0.0
−0.5
−1.0
−1.5
ME/CFS−Norway (VD001)
ME/CFS−Canada (VD001)
Controls−Canada (VD001)
Controls−USA (VD001)
ME/CFS−Canada (AD001)
Controls−Canada (AD001)

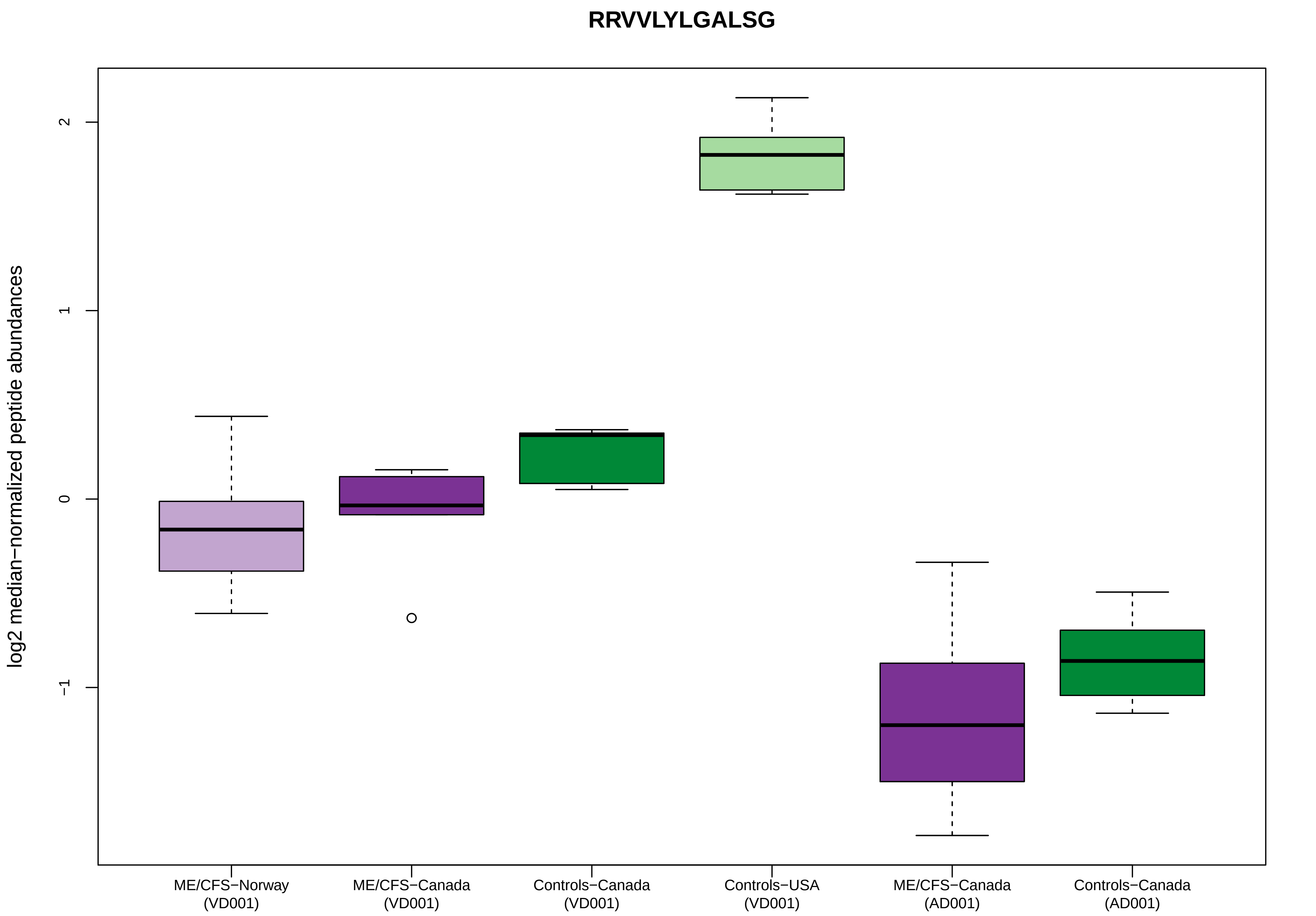

RRVVLYLGALSG
log2 median−normalized peptide abundances
2
1
0
−1
ME/CFS−Norway (VD001)
ME/CFS−Canada (VD001)
Controls−Canada (VD001)
Controls−USA (VD001)
ME/CFS−Canada (AD001)
Controls−Canada (AD001)

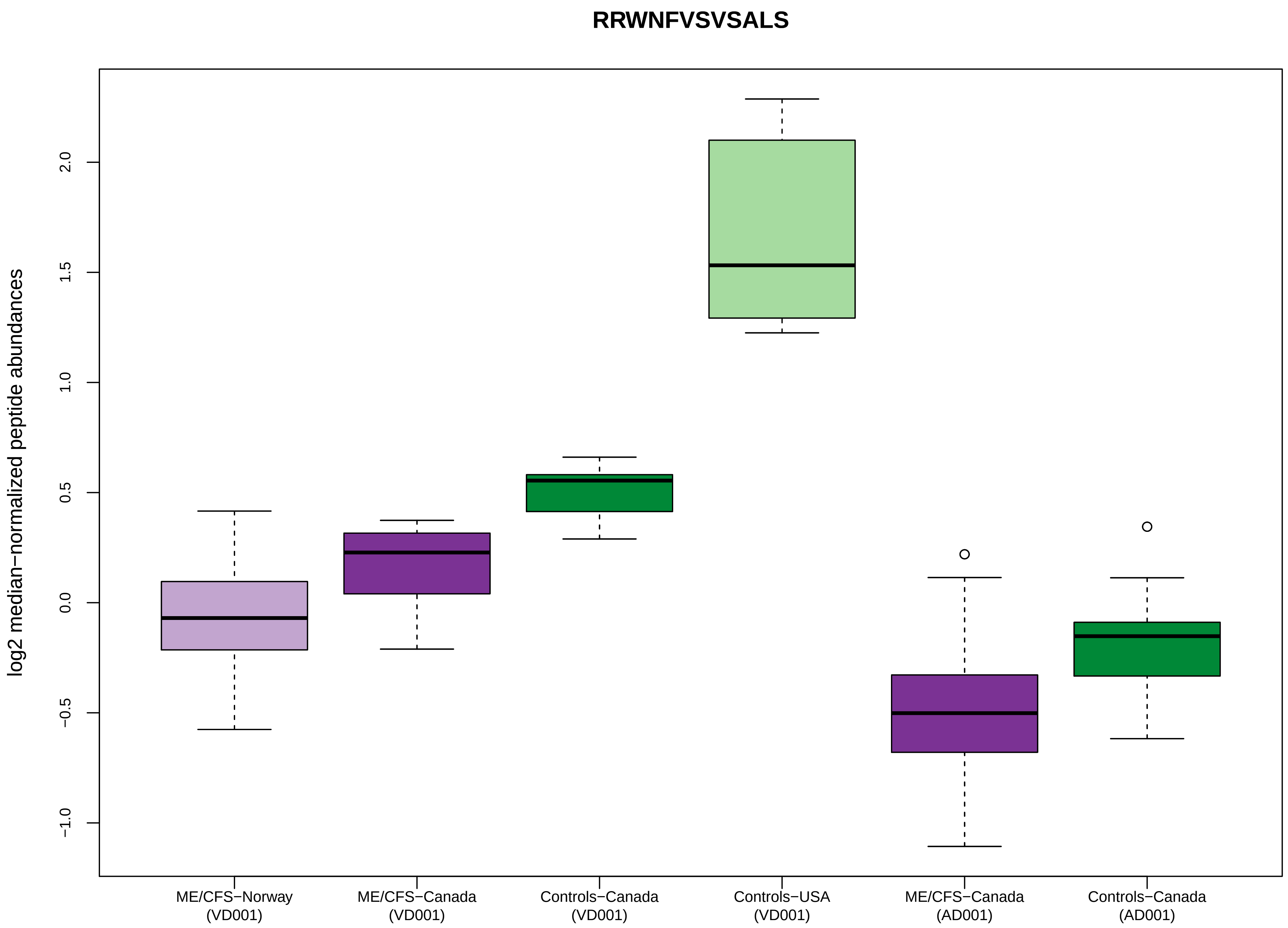

RRWNFVSVSALS
log2 median−normalized peptide abundances
2.0
1.5
1.0
0.5
0.0
−0.5
−1.0
ME/CFS−Norway (VD001)
ME/CFS−Canada (VD001)
Controls−Canada (VD001)
Controls−USA (VD001)
ME/CFS−Canada (AD001)
Controls−Canada (AD001)

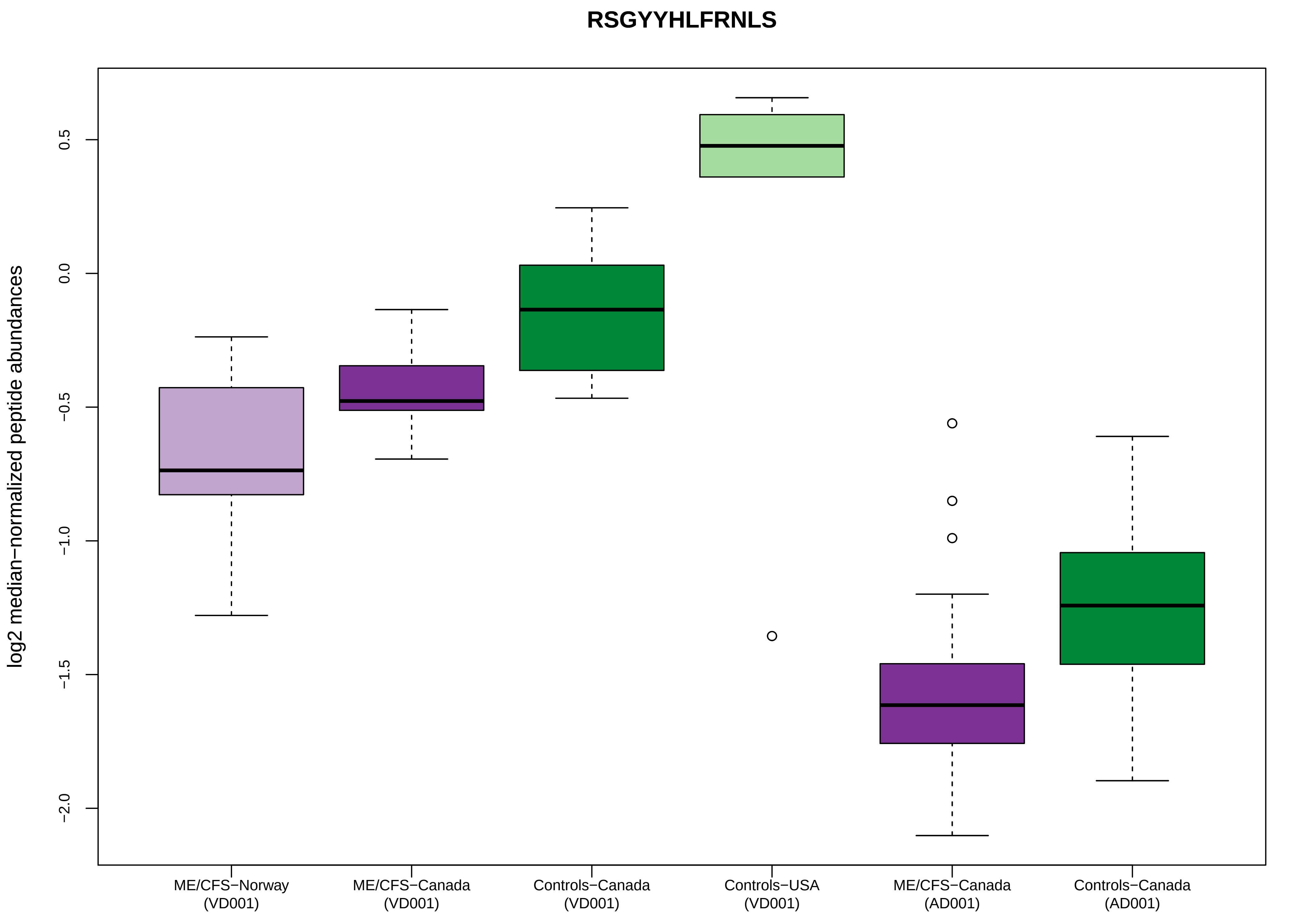

RSGYYHLFRNLS
log2 median−normalized peptide abundances
0.5
0.0
−0.5
−1.0
−1.5
−2.0
ME/CFS−Norway (VD001)
ME/CFS−Canada (VD001)
Controls−Canada (VD001)
Controls−USA (VD001)
ME/CFS−Canada (AD001)
Controls−Canada (AD001)

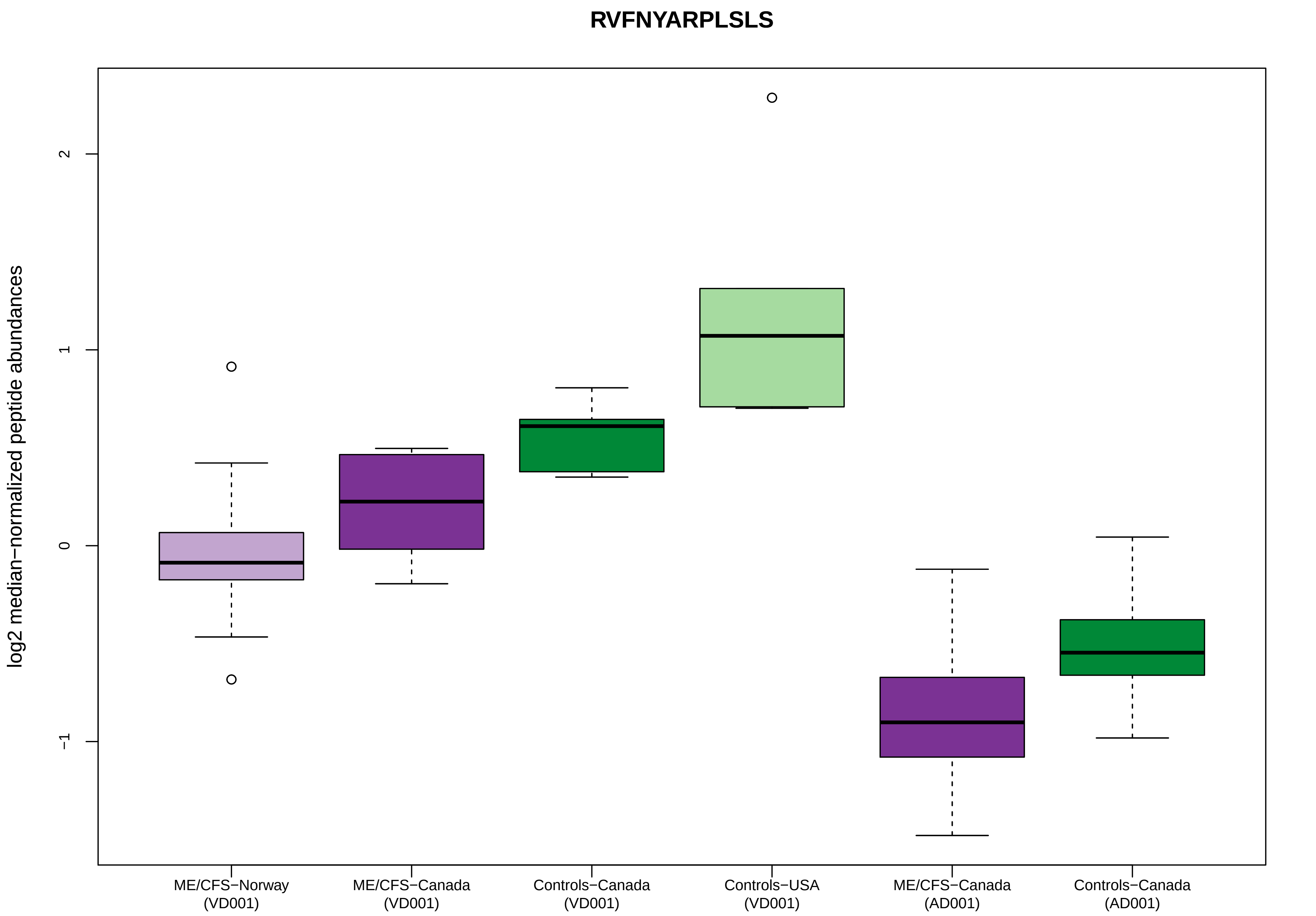

RVFNYARPLSLS
log2 median−normalized peptide abundances
2
1
0
−1
ME/CFS−Norway (VD001)
ME/CFS−Canada (VD001)
Controls−Canada (VD001)
Controls−USA (VD001)
ME/CFS−Canada (AD001)
Controls−Canada (AD001)

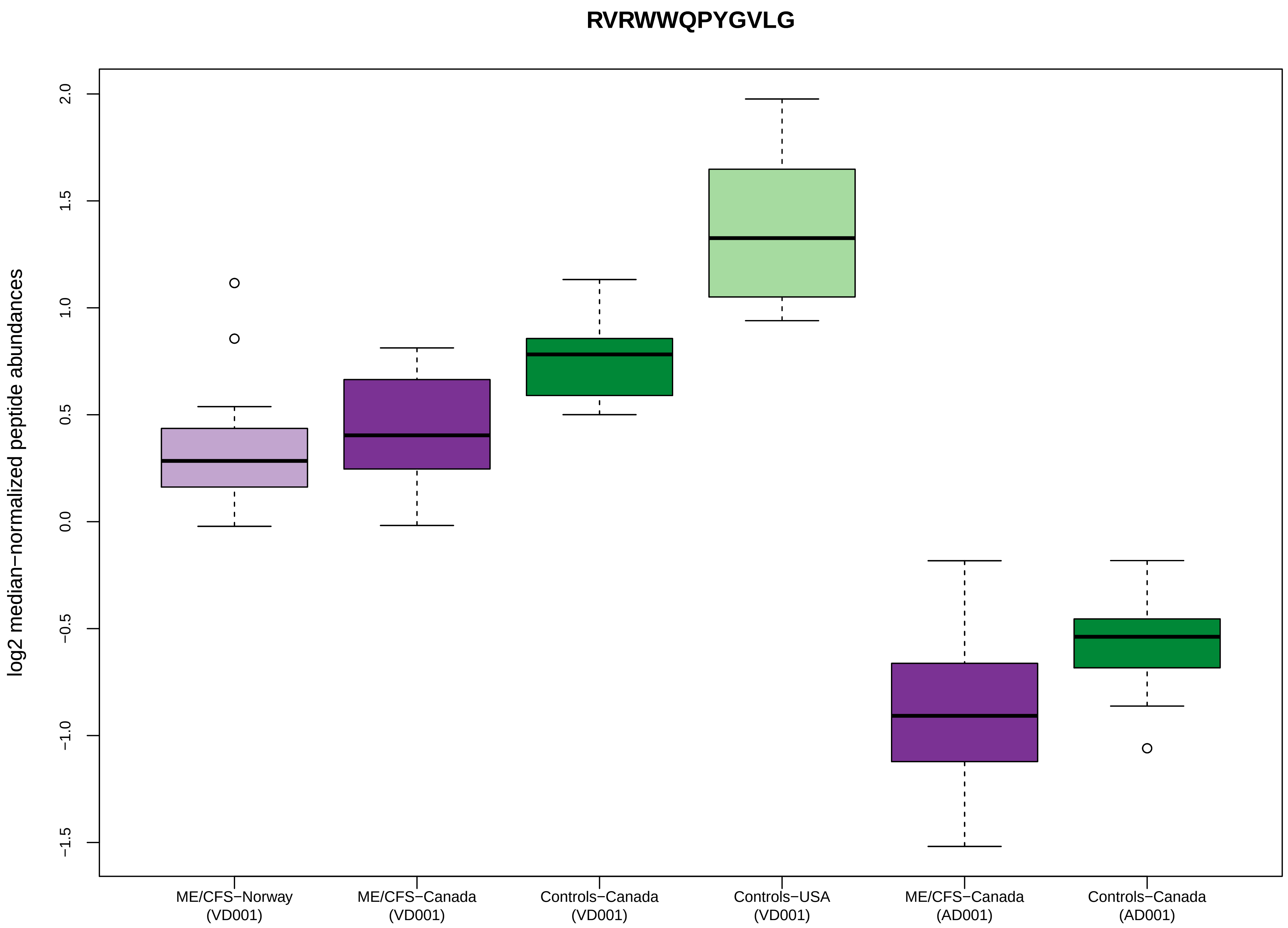

RVRWWQPYGVLG
log2 median−normalized peptide abundances
2.0
1.5
1.0
0.5
0.0
−0.5
−1.0
−1.5
ME/CFS−Norway (VD001)
ME/CFS−Canada (VD001)
Controls−Canada (VD001)
Controls−USA (VD001)
ME/CFS−Canada (AD001)
Controls−Canada (AD001)

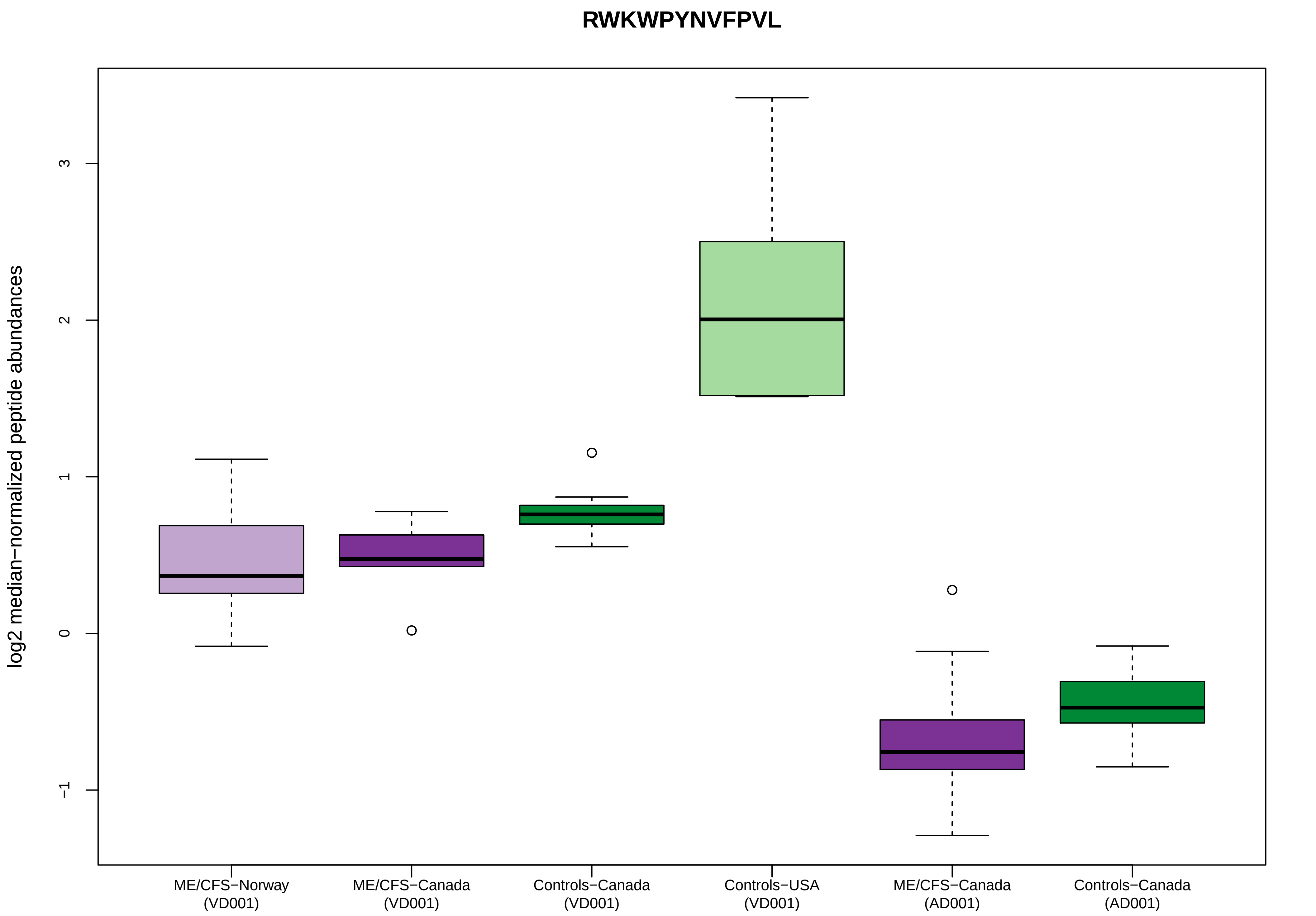

RWKWPYNVFPVL
log2 median−normalized peptide abundances
3
2
1
0
−1
ME/CFS−Norway (VD001)
ME/CFS−Canada (VD001)
Controls−Canada (VD001)
Controls−USA (VD001)
ME/CFS−Canada (AD001)
Controls−Canada (AD001)

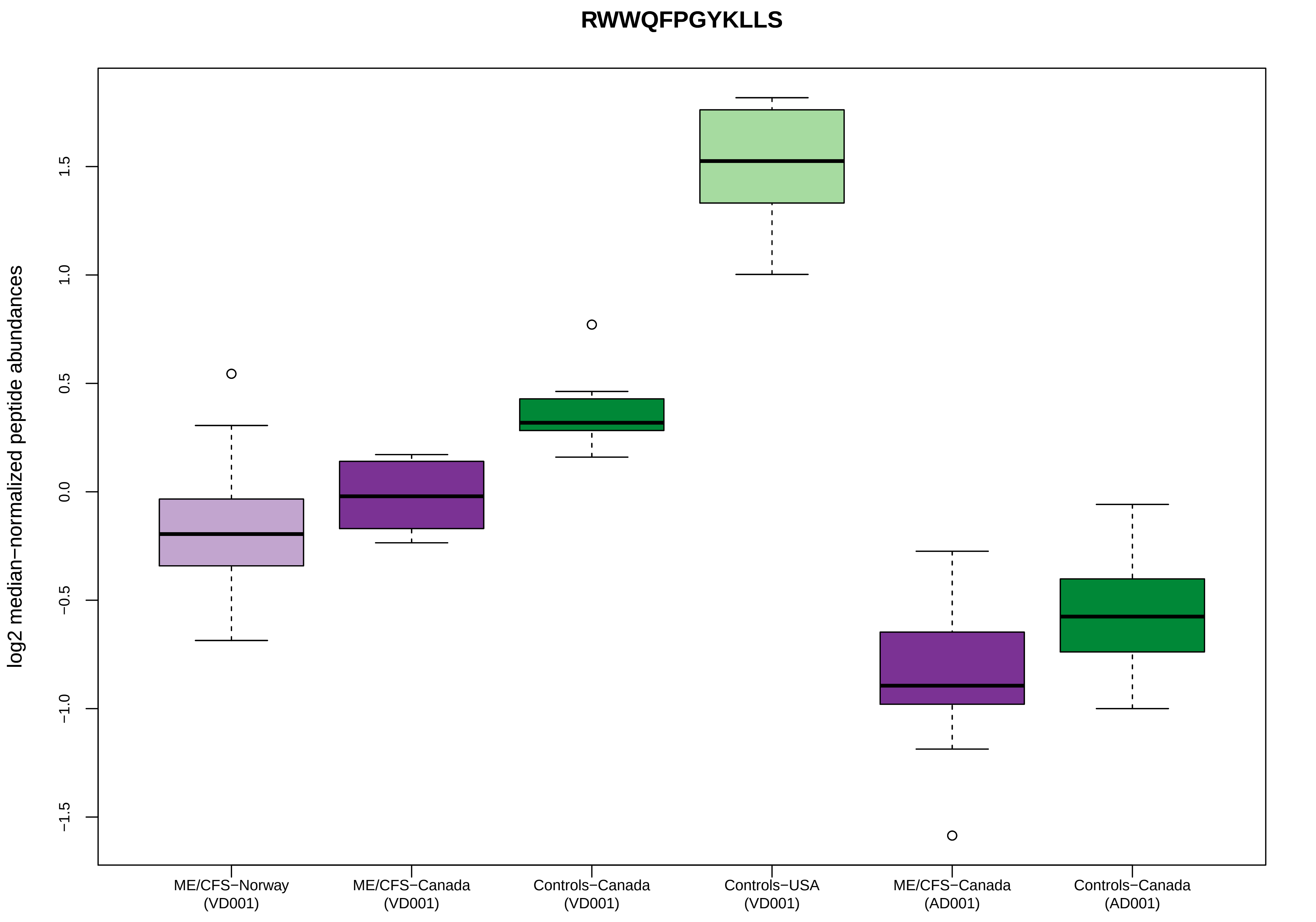
RWWQFPGYKLLS
log2 median−normalized peptide abundances
1.5
1.0
0.5
0.0
−0.5
−1.0
−1.5
ME/CFS−Norway (VD001)
ME/CFS−Canada (VD001)
Controls−Canada (VD001)
Controls−USA (VD001)
ME/CFS−Canada (AD001)
Controls−Canada (AD001)

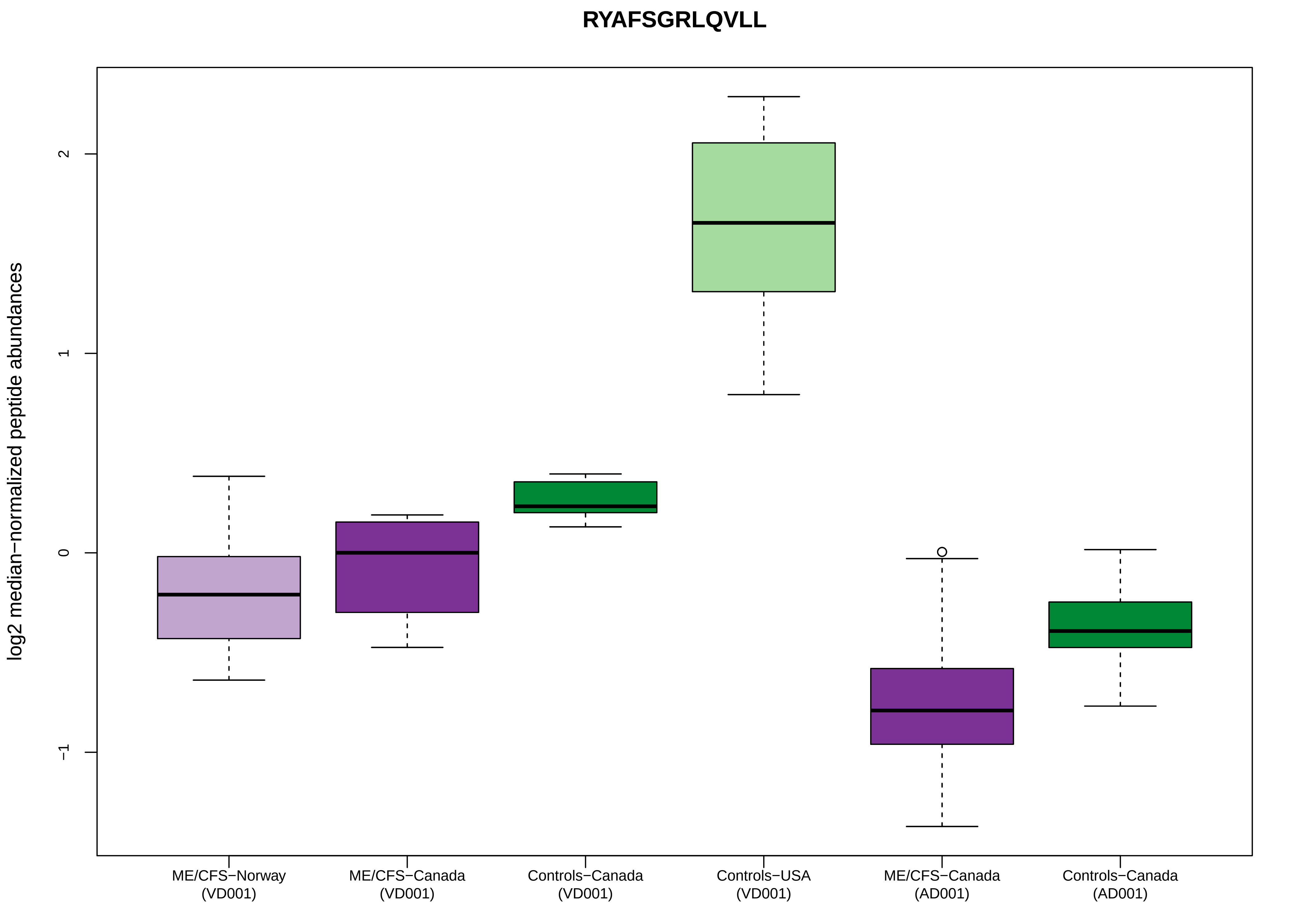

RYAFSGRLQVLL
log2 median−normalized peptide abundances
2
1
0
−1
ME/CFS−Norway (VD001)
ME/CFS−Canada (VD001)
Controls−Canada (VD001)
Controls−USA (VD001)
ME/CFS−Canada (AD001)
Controls−Canada (AD001)

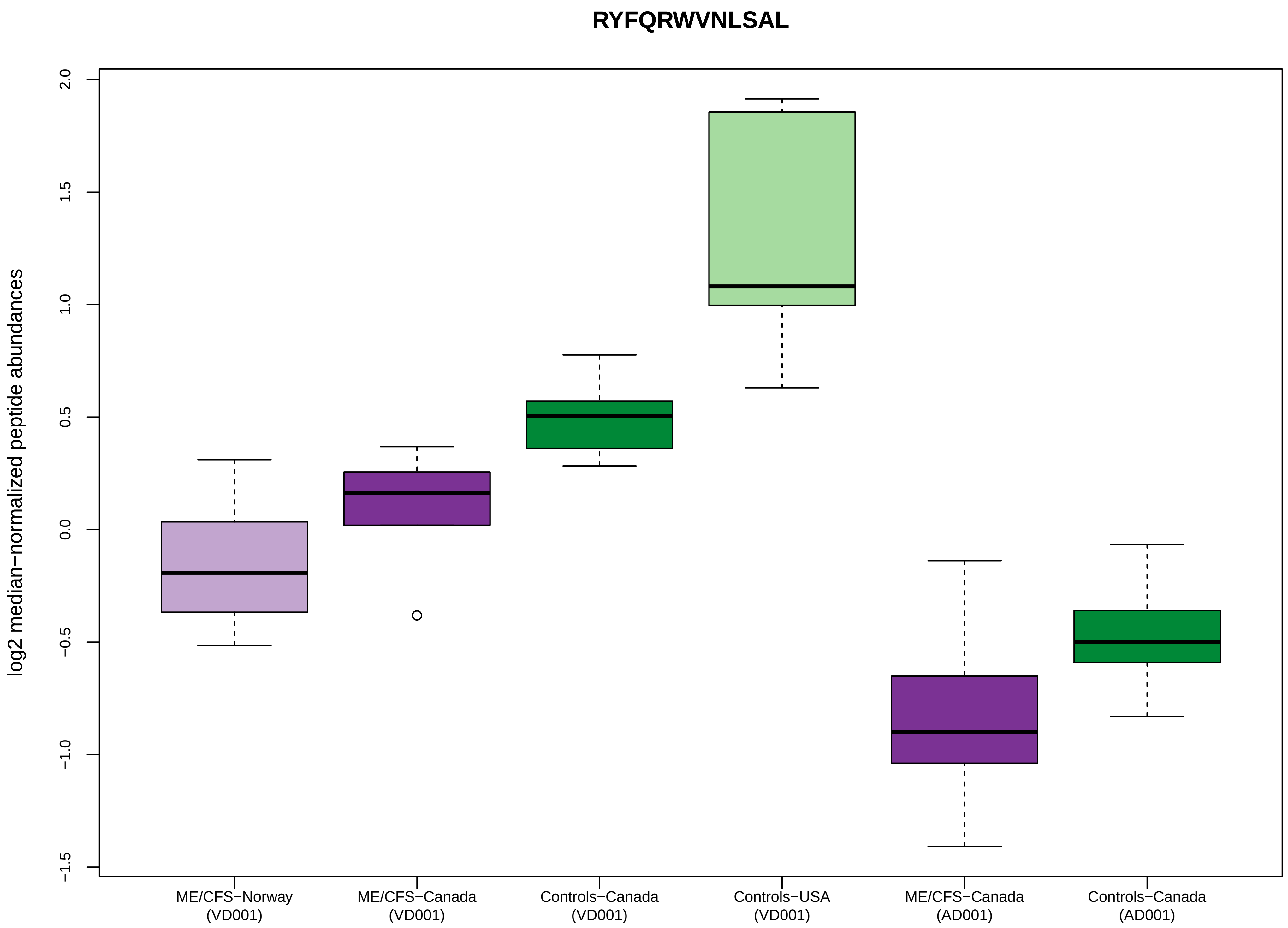
RYFQRWVNLSAL
log2 median−normalized peptide abundances
2.0
1.5
1.0
0.5
0.0
−0.5
−1.0
−1.5
ME/CFS−Norway (VD001)
ME/CFS−Canada (VD001)
Controls−Canada (VD001)
Controls−USA (VD001)
ME/CFS−Canada (AD001)
Controls−Canada (AD001)

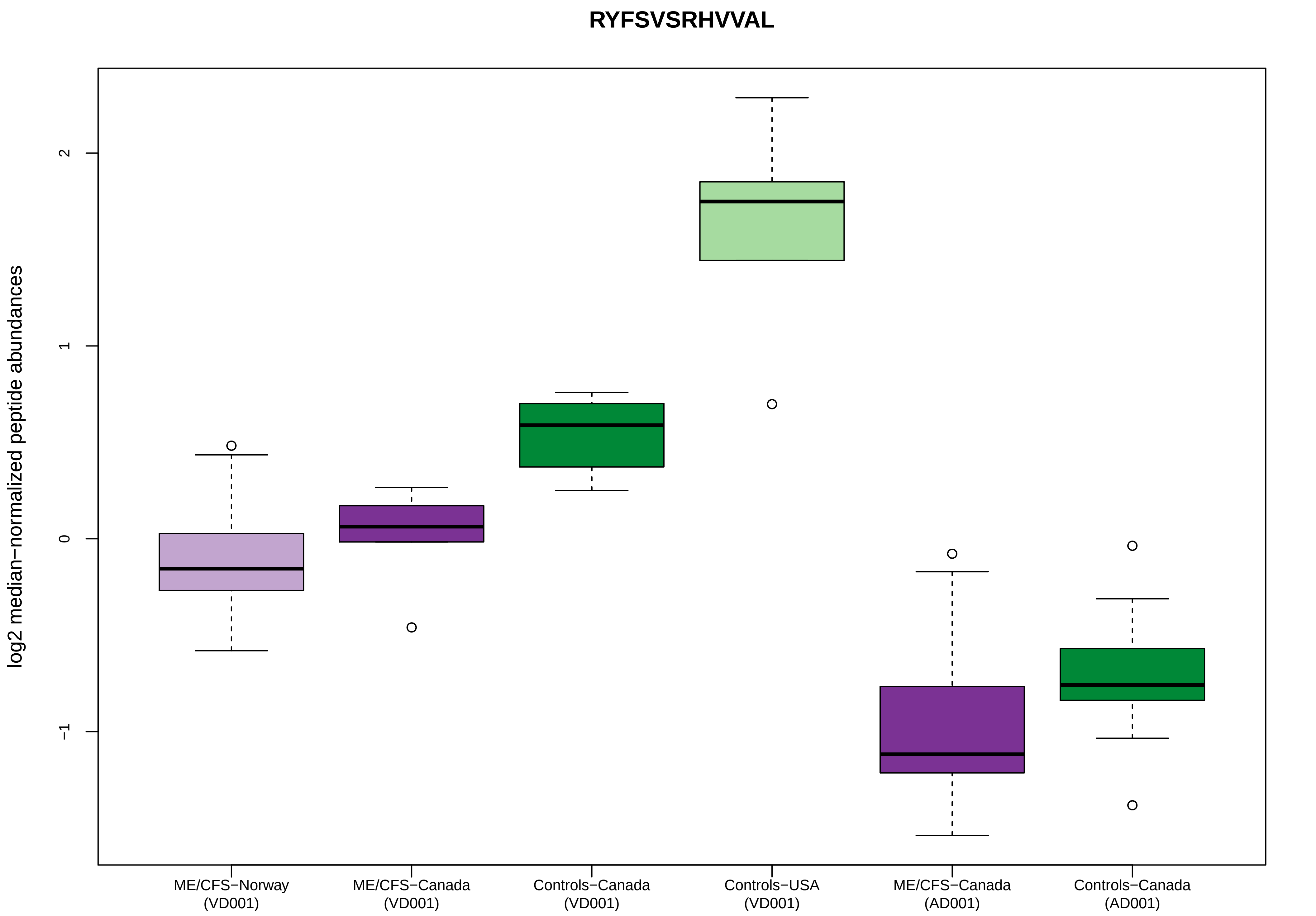

RYFSVSRHVVAL
log2 median−normalized peptide abundances
2
1
0
−1
ME/CFS−Norway (VD001)
ME/CFS−Canada (VD001)
Controls−Canada (VD001)
Controls−USA (VD001)
ME/CFS−Canada (AD001)
Controls−Canada (AD001)

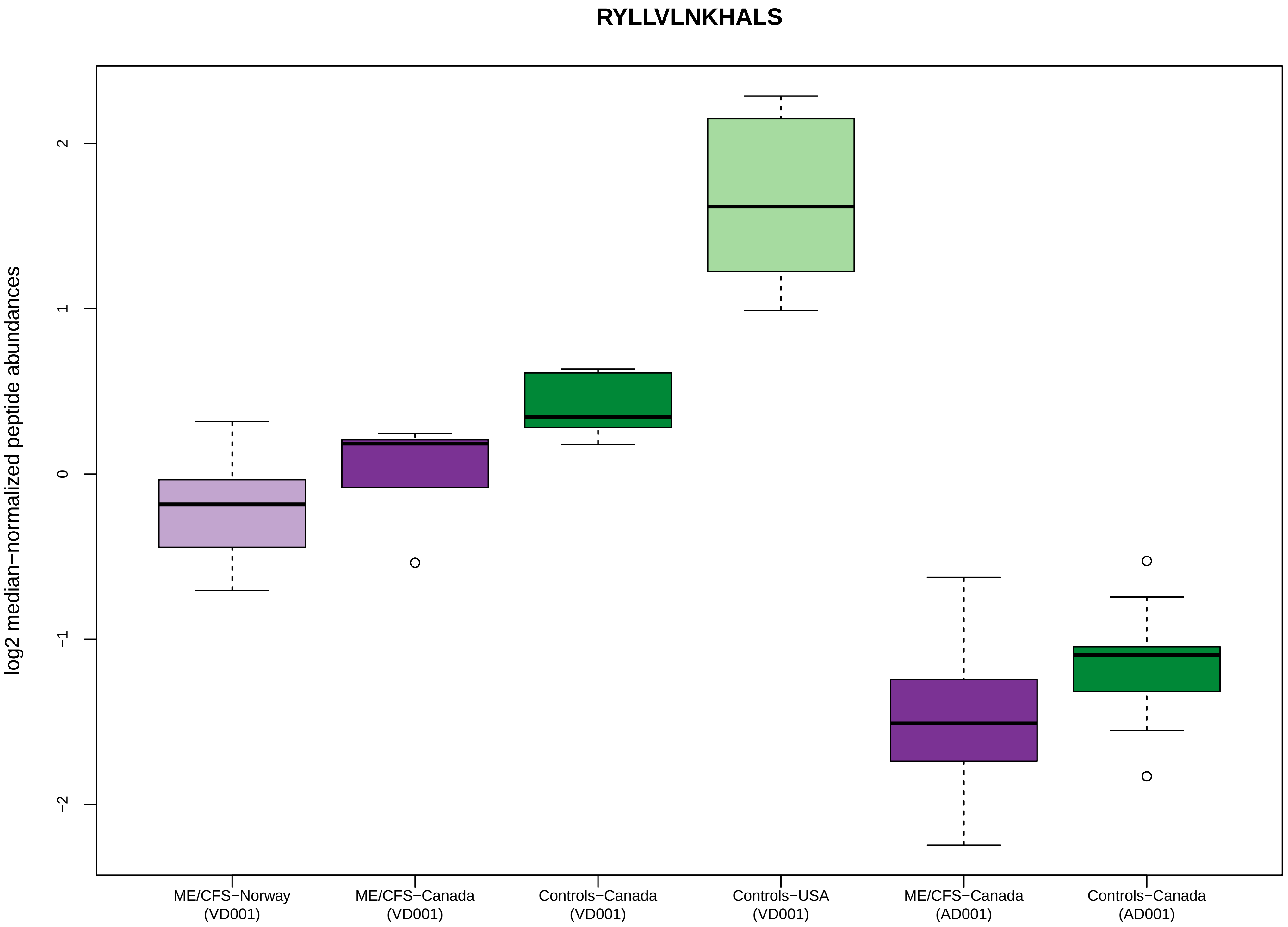
RYLLVLNKHALS
log2 median−normalized peptide abundances
2
1
0
−1
−2
ME/CFS−Norway (VD001)
ME/CFS−Canada (VD001)
Controls−Canada (VD001)
Controls−USA (VD001)
ME/CFS−Canada (AD001)
Controls−Canada (AD001)

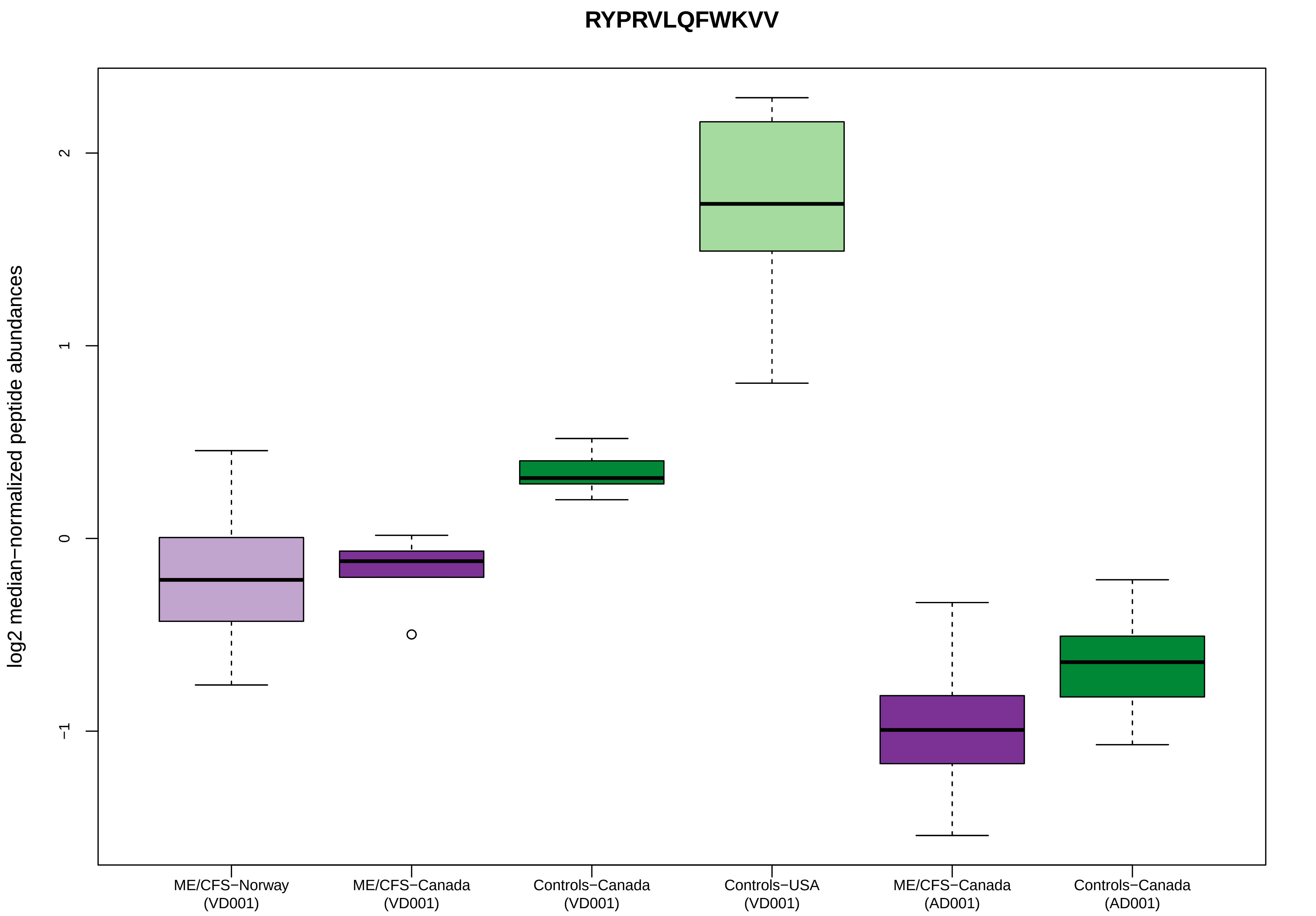
RYPRVLQFWKVV
log2 median−normalized peptide abundances
2
1
0
−1
ME/CFS−Norway (VD001)
ME/CFS−Canada (VD001)
Controls−Canada (VD001)
Controls−USA (VD001)
ME/CFS−Canada (AD001)
Controls−Canada (AD001)

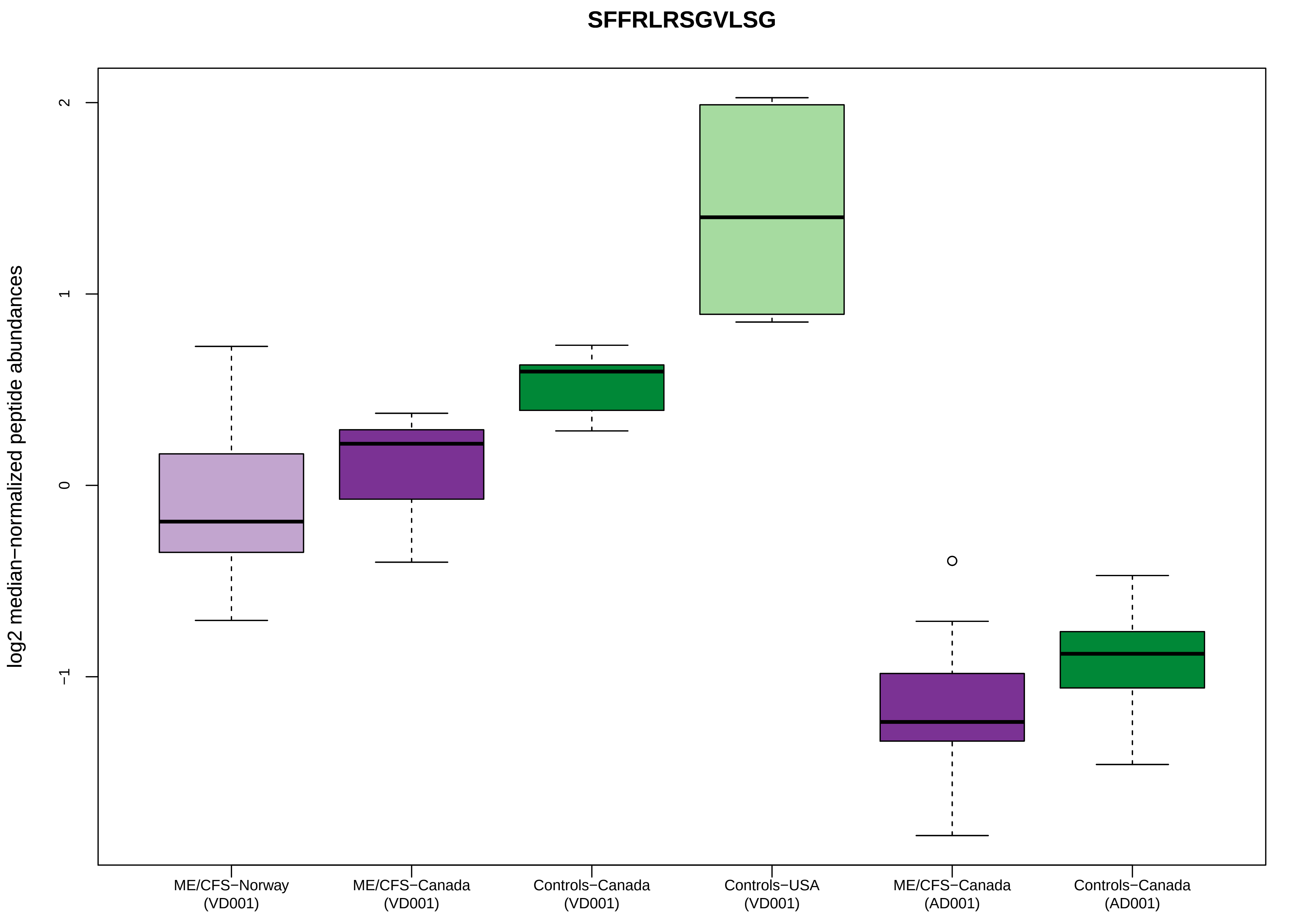
SFFRLRSGVLSG
log2 median−normalized peptide abundances
2
1
0
−1
ME/CFS−Norway (VD001)
ME/CFS−Canada (VD001)
Controls−Canada (VD001)
Controls−USA (VD001)
ME/CFS−Canada (AD001)
Controls−Canada (AD001)

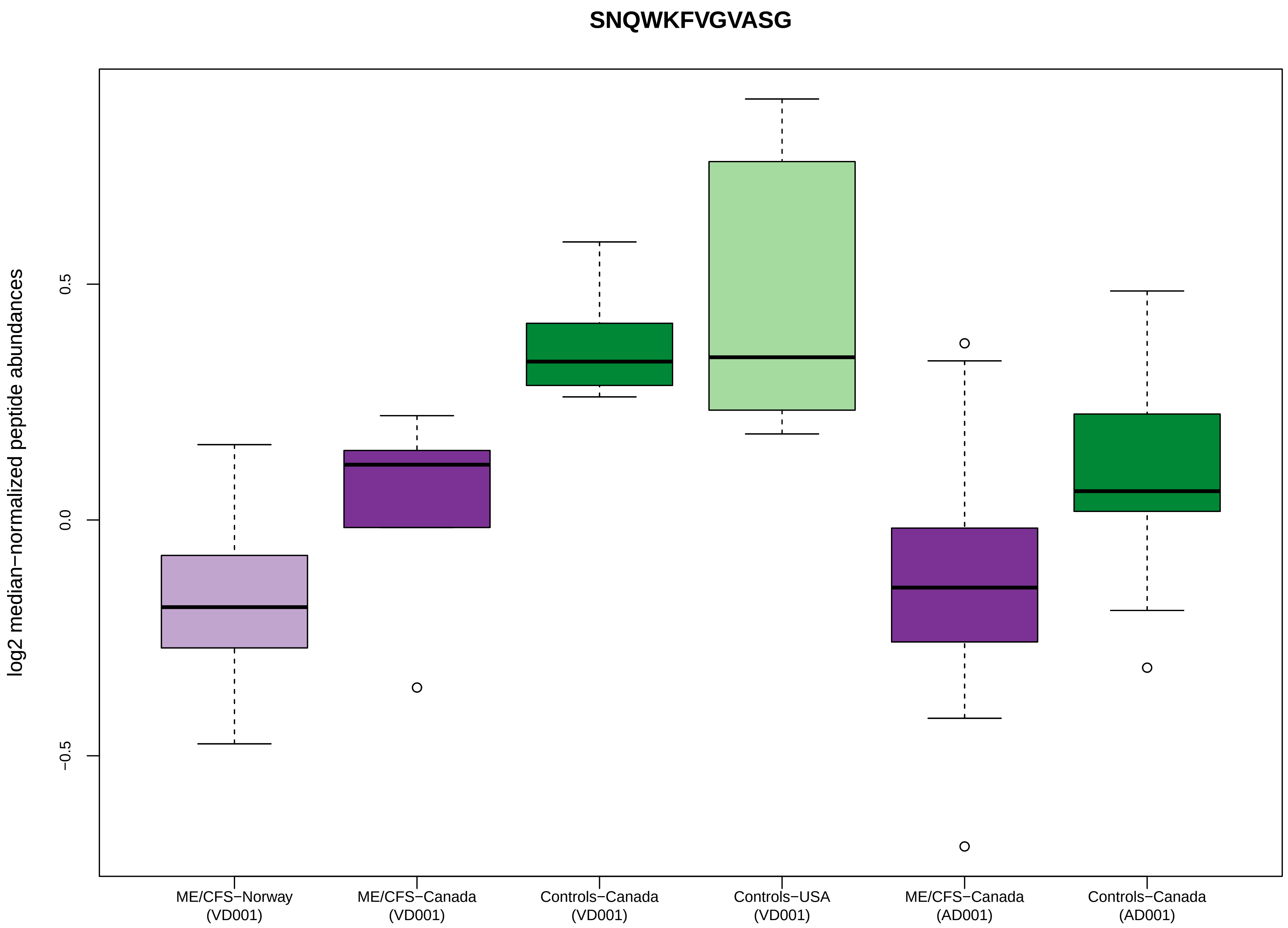

SNQWKFVGVASG
log2 median−normalized peptide abundances
0.5
0.0
−0.5
ME/CFS−Norway (VD001)
ME/CFS−Canada (VD001)
Controls−Canada (VD001)
Controls−USA (VD001)
ME/CFS−Canada (AD001)
Controls−Canada (AD001)

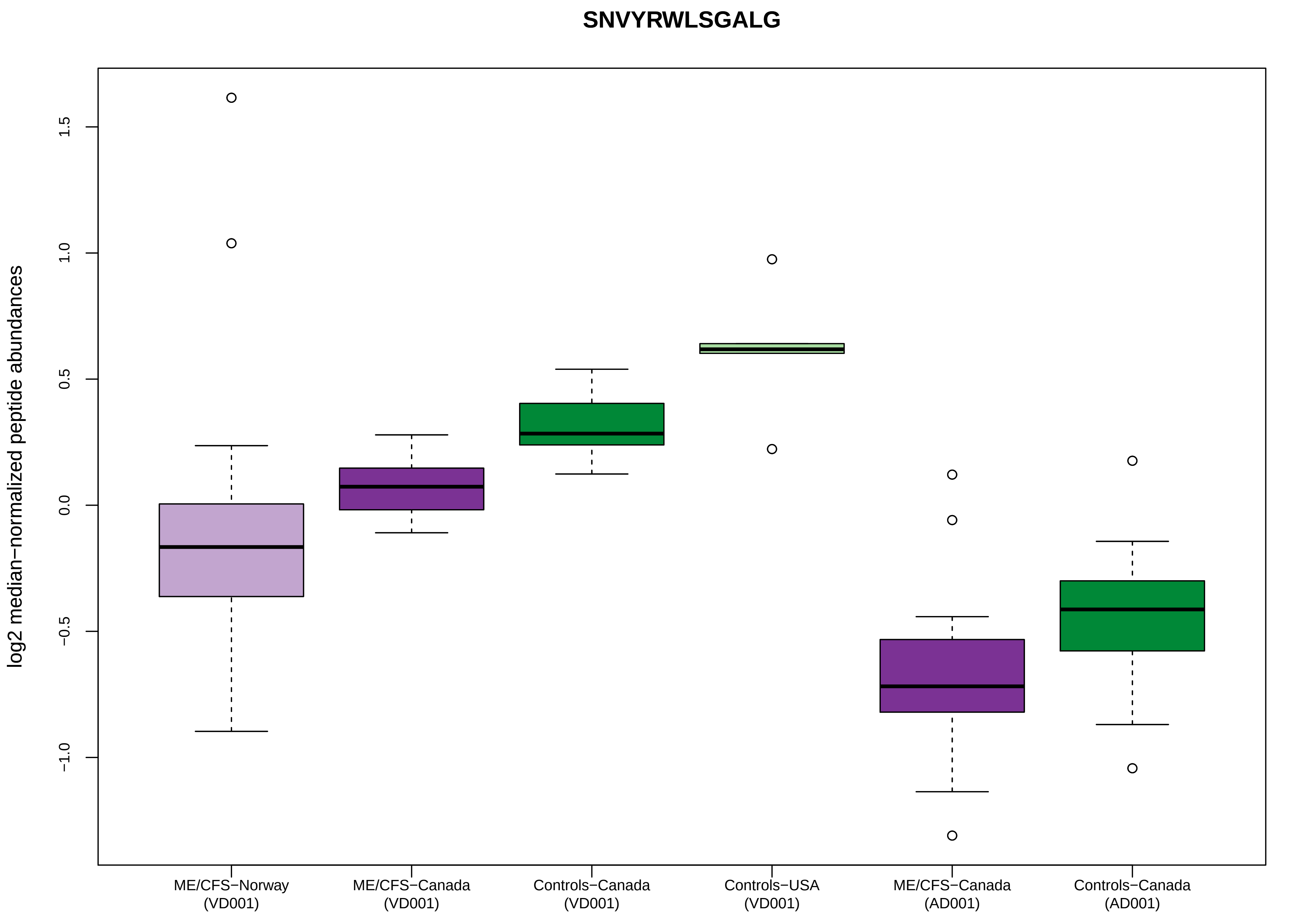

SNVYRWLSGALG
log2 median−normalized peptide abundances
1.5
1.0
0.5
0.0
−0.5
−1.0
ME/CFS−Norway (VD001)
ME/CFS−Canada (VD001)
Controls−Canada (VD001)
Controls−USA (VD001)
ME/CFS−Canada (AD001)
Controls−Canada (AD001)

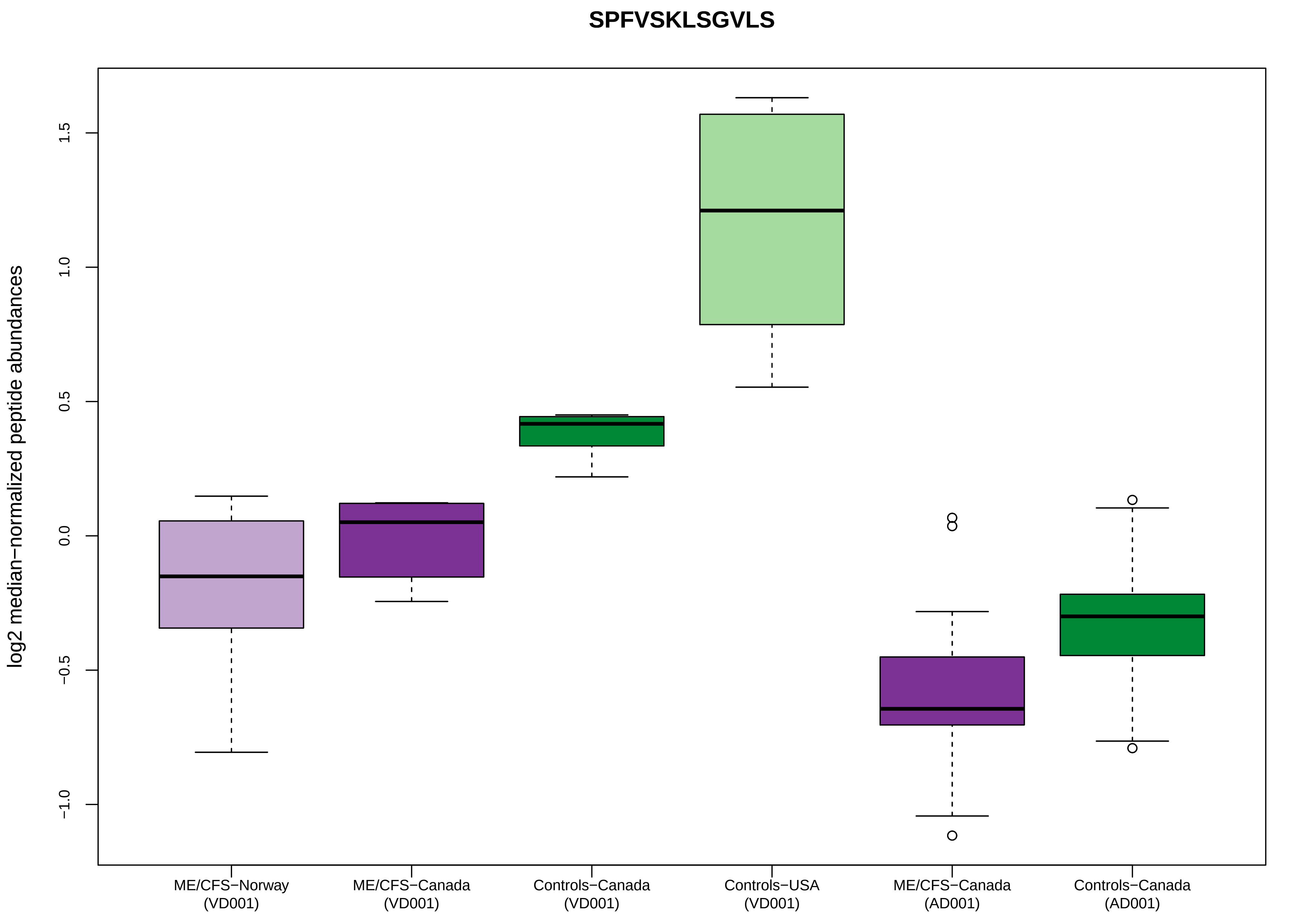

SPFVSKLSGVLS
log2 median−normalized peptide abundances
1.5
1.0
0.5
0.0
−0.5
−1.0
ME/CFS−Norway (VD001)
ME/CFS−Canada (VD001)
Controls−Canada (VD001)
Controls−USA (VD001)
ME/CFS−Canada (AD001)
Controls−Canada (AD001)

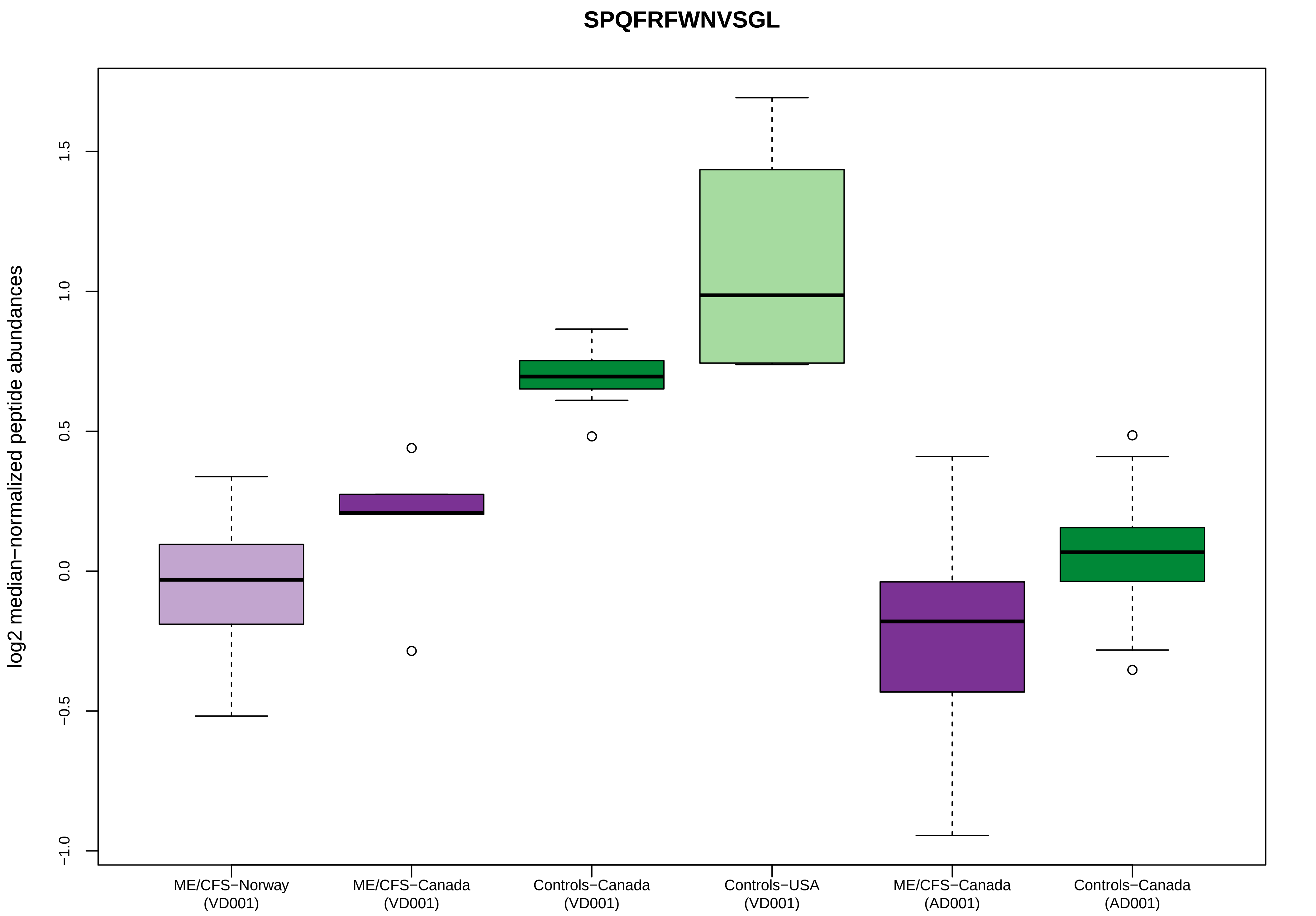
SPQFRFWNVSGL
log2 median−normalized peptide abundances
1.5
1.0
0.5
0.0
−0.5
−1.0
ME/CFS−Norway (VD001)
ME/CFS−Canada (VD001)
Controls−Canada (VD001)
Controls−USA (VD001)
ME/CFS−Canada (AD001)
Controls−Canada (AD001)

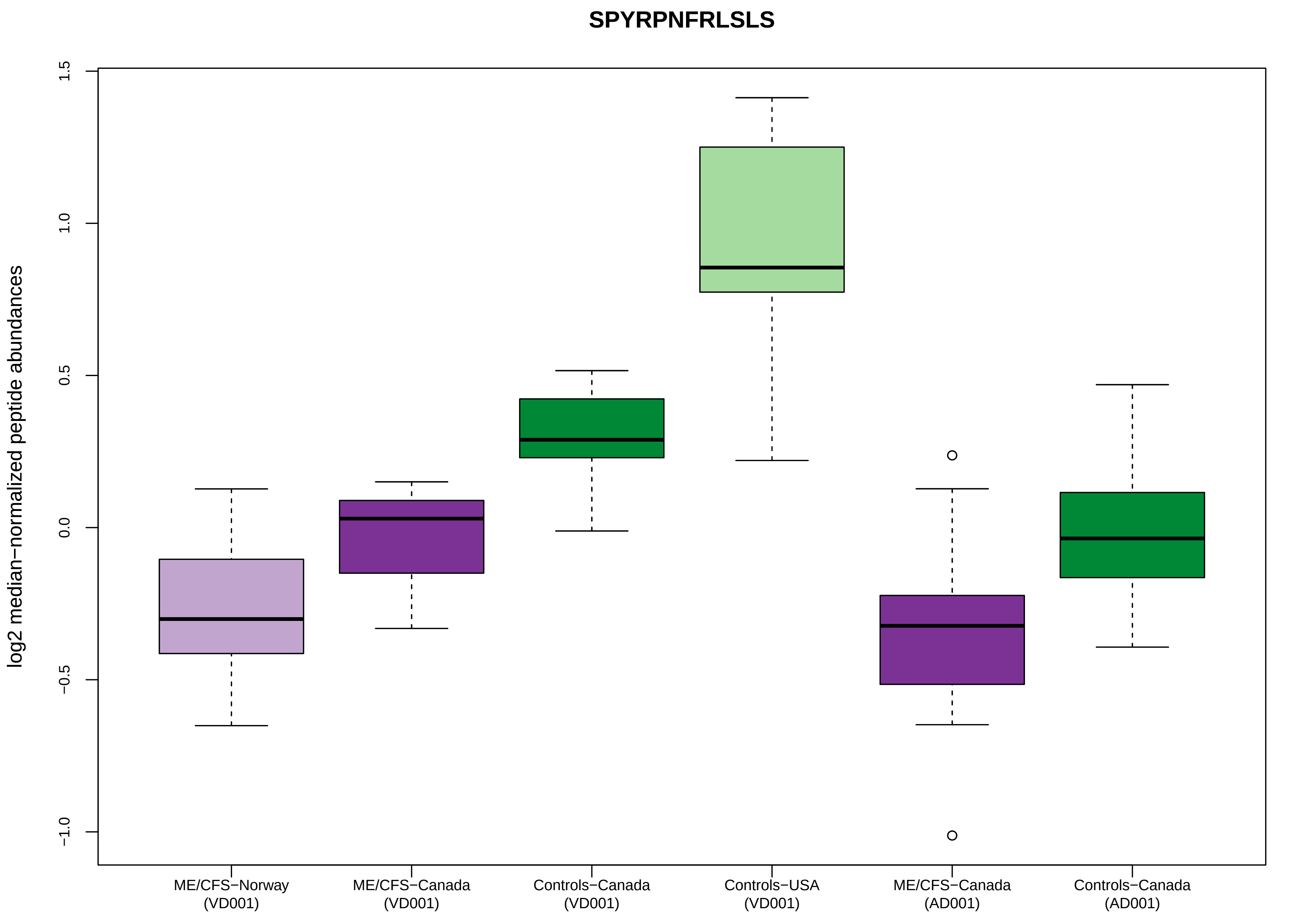
SPYRPNFRLSLS
log2 median−normalized peptide abundances
1.5
1.0
0.5
0.0
−0.5
−1.0
ME/CFS−Norway (VD001)
ME/CFS−Canada (VD001)
Controls−Canada (VD001)
Controls−USA (VD001)
ME/CFS−Canada (AD001)
Controls−Canada (AD001)

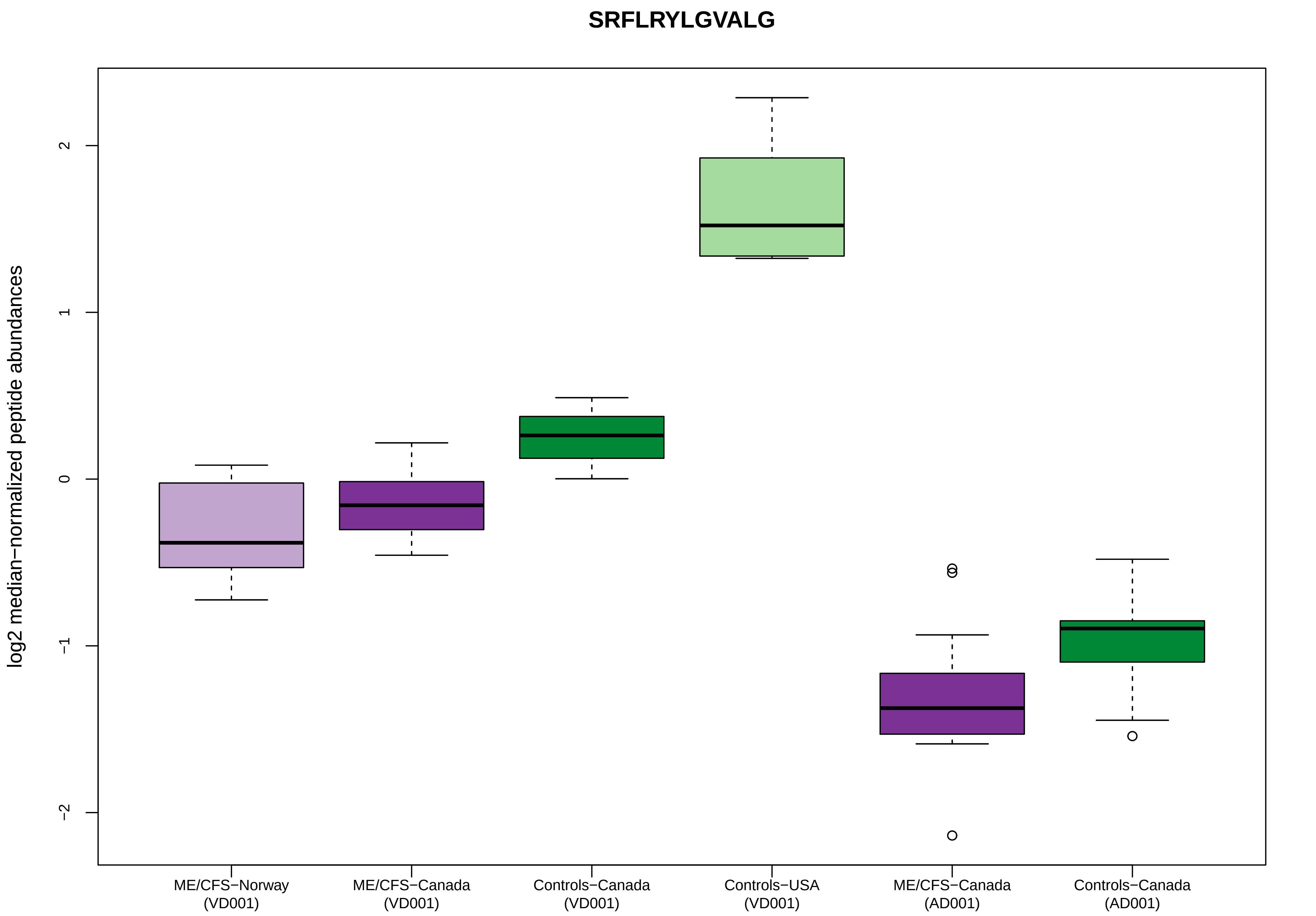
SRFLRYLGVALG
log2 median−normalized peptide abundances
−2
−1
0
1
2
ME/CFS−Norway (VD001)
ME/CFS−Canada (VD001)
Controls−Canada (VD001)
Controls−USA (VD001)
ME/CFS−Canada (AD001)
Controls−Canada (AD001)

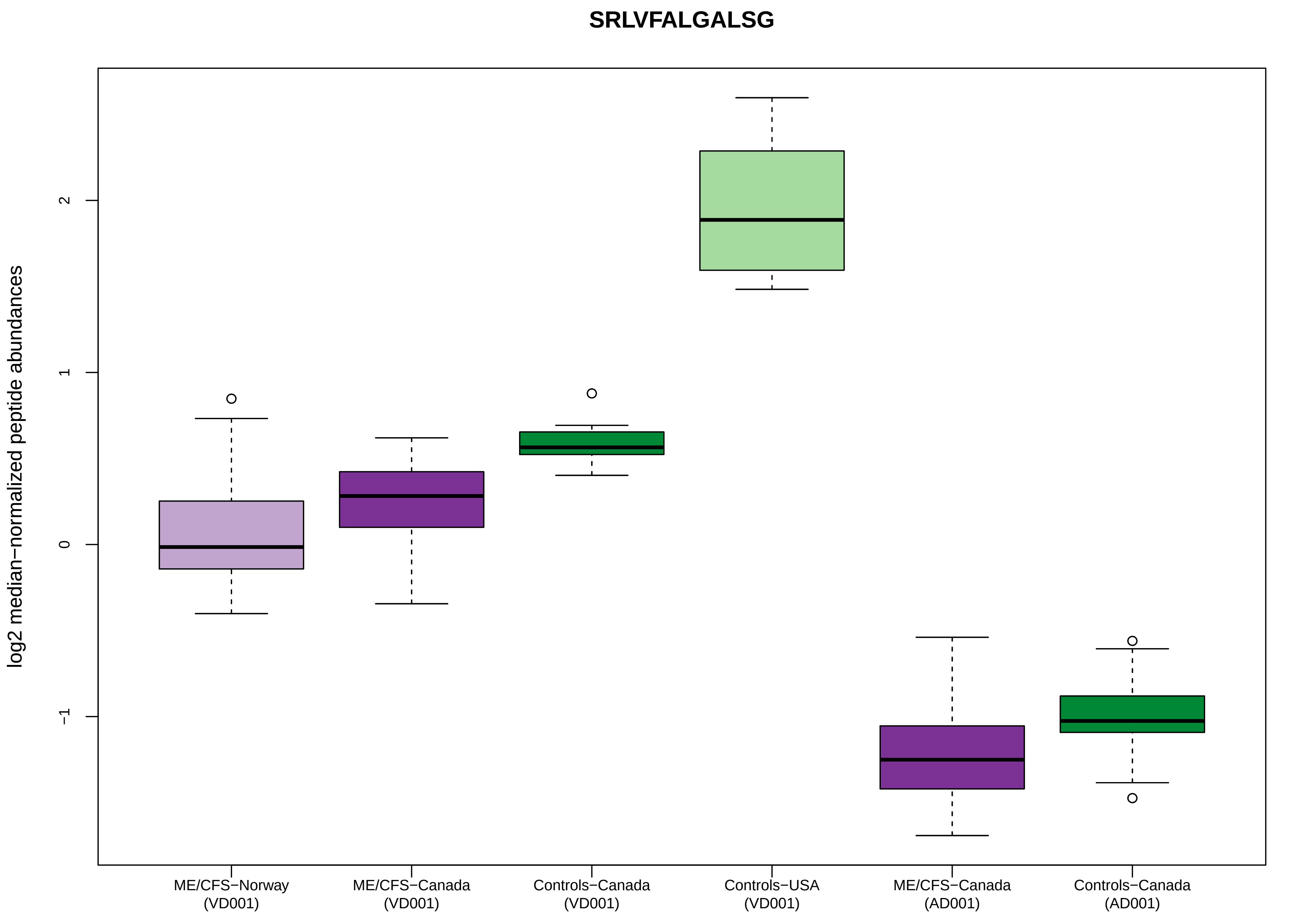
SRLVFALGALSG
log2 median−normalized peptide abundances
2
1
0
−1
ME/CFS−Norway (VD001)
ME/CFS−Canada (VD001)
Controls−Canada (VD001)
Controls−USA (VD001)
ME/CFS−Canada (AD001)
Controls−Canada (AD001)

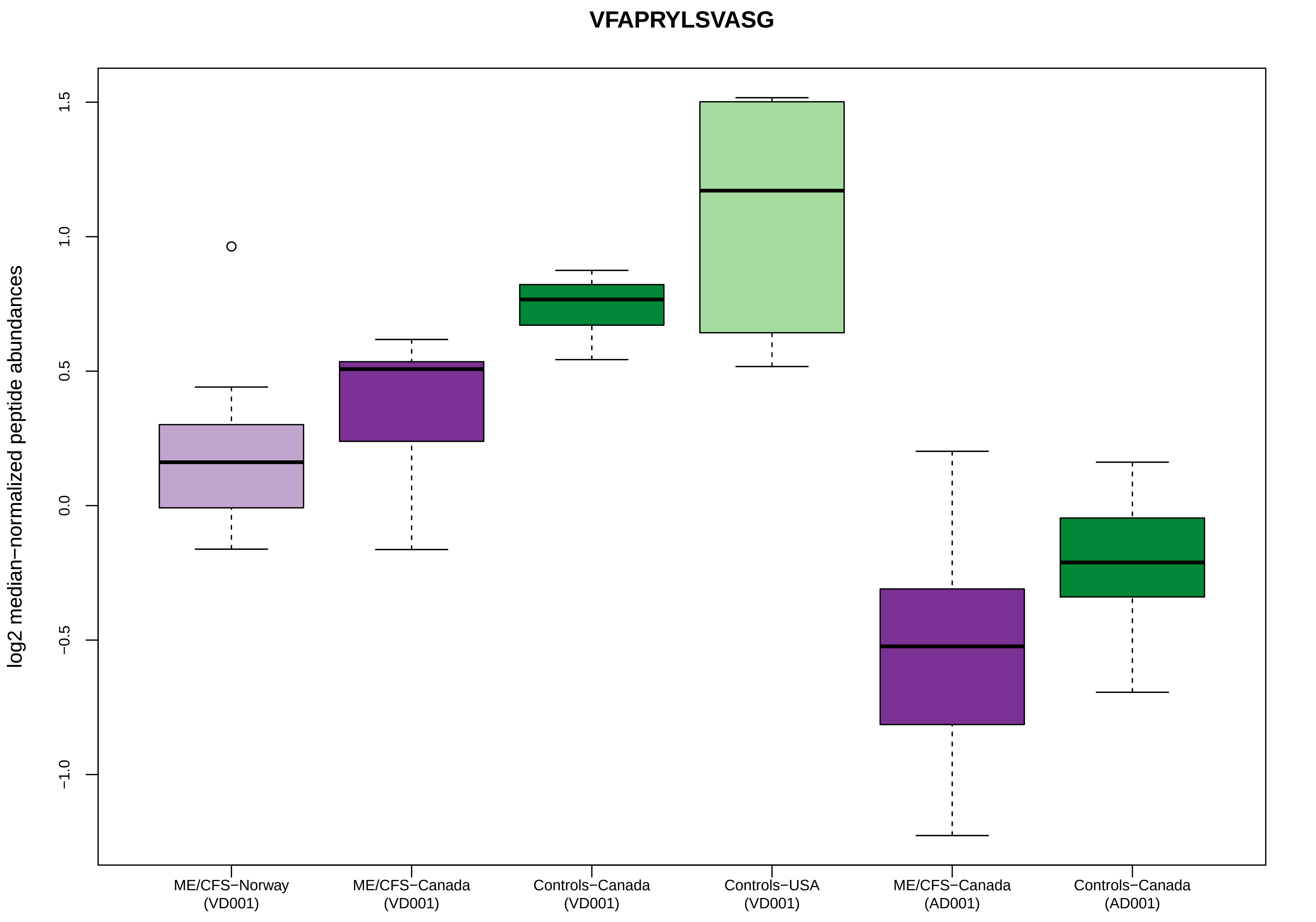
VFAPRYLSVASG
log2 median−normalized peptide abundances
−1.0
−0.5
0.0
0.5
1.0
1.5
ME/CFS−Norway (VD001)
ME/CFS−Canada (VD001)
Controls−Canada (VD001)
Controls−USA (VD001)
ME/CFS−Canada (AD001)
Controls−Canada (AD001)

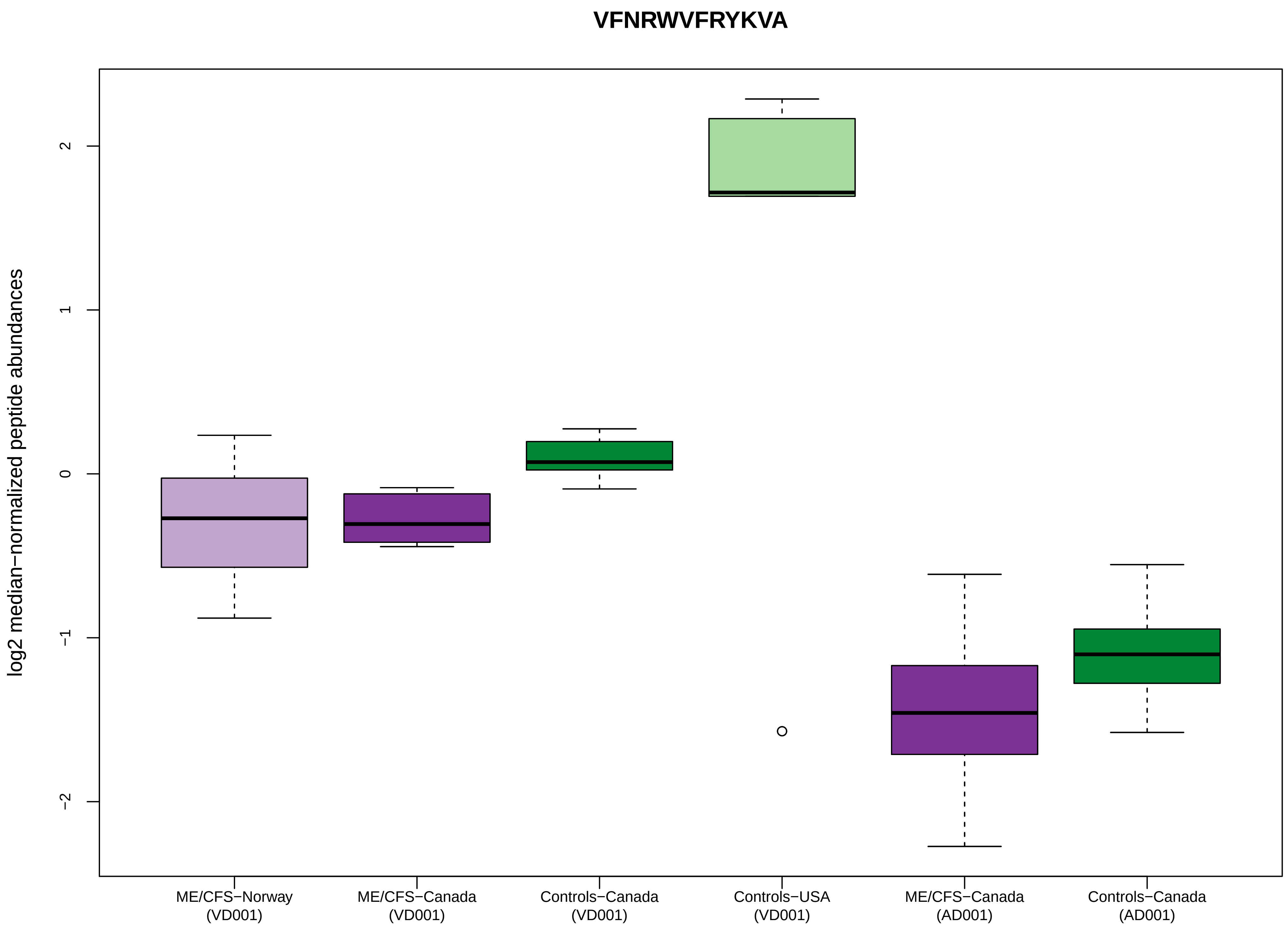

VFNRWVFRYKVA
log2 median−normalized peptide abundances
2
1
0
−1
−2
ME/CFS−Norway (VD001)
ME/CFS−Canada (VD001)
Controls−Canada (VD001)
Controls−USA (VD001)
ME/CFS−Canada (AD001)
Controls−Canada (AD001)

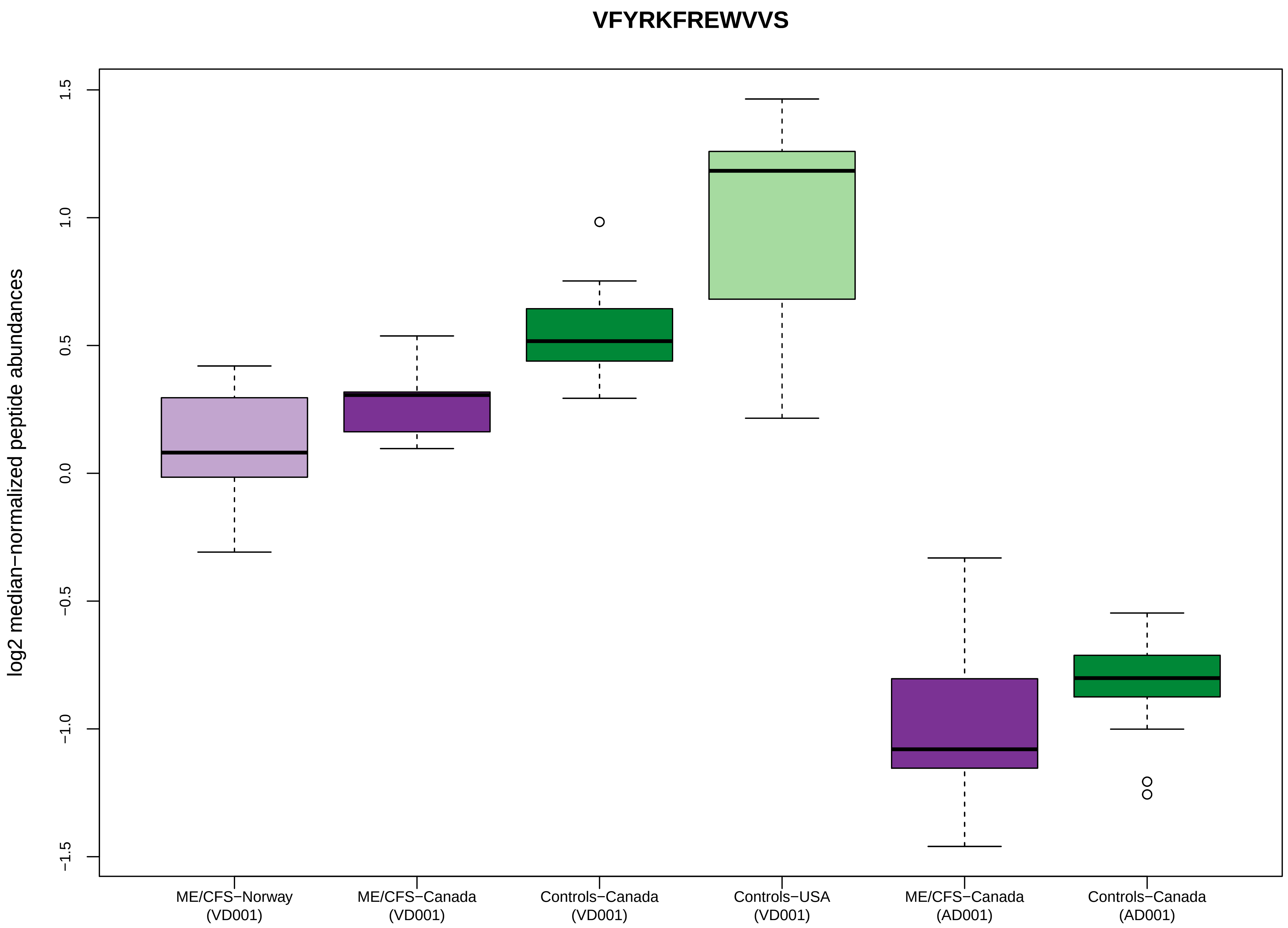

VFYRKFREWVVS
log2 median−normalized peptide abundances
1.5
1.0
0.5
0.0
−0.5
−1.0
−1.5
ME/CFS−Norway (VD001)
ME/CFS−Canada (VD001)
Controls−Canada (VD001)
Controls−USA (VD001)
ME/CFS−Canada (AD001)
Controls−Canada (AD001)

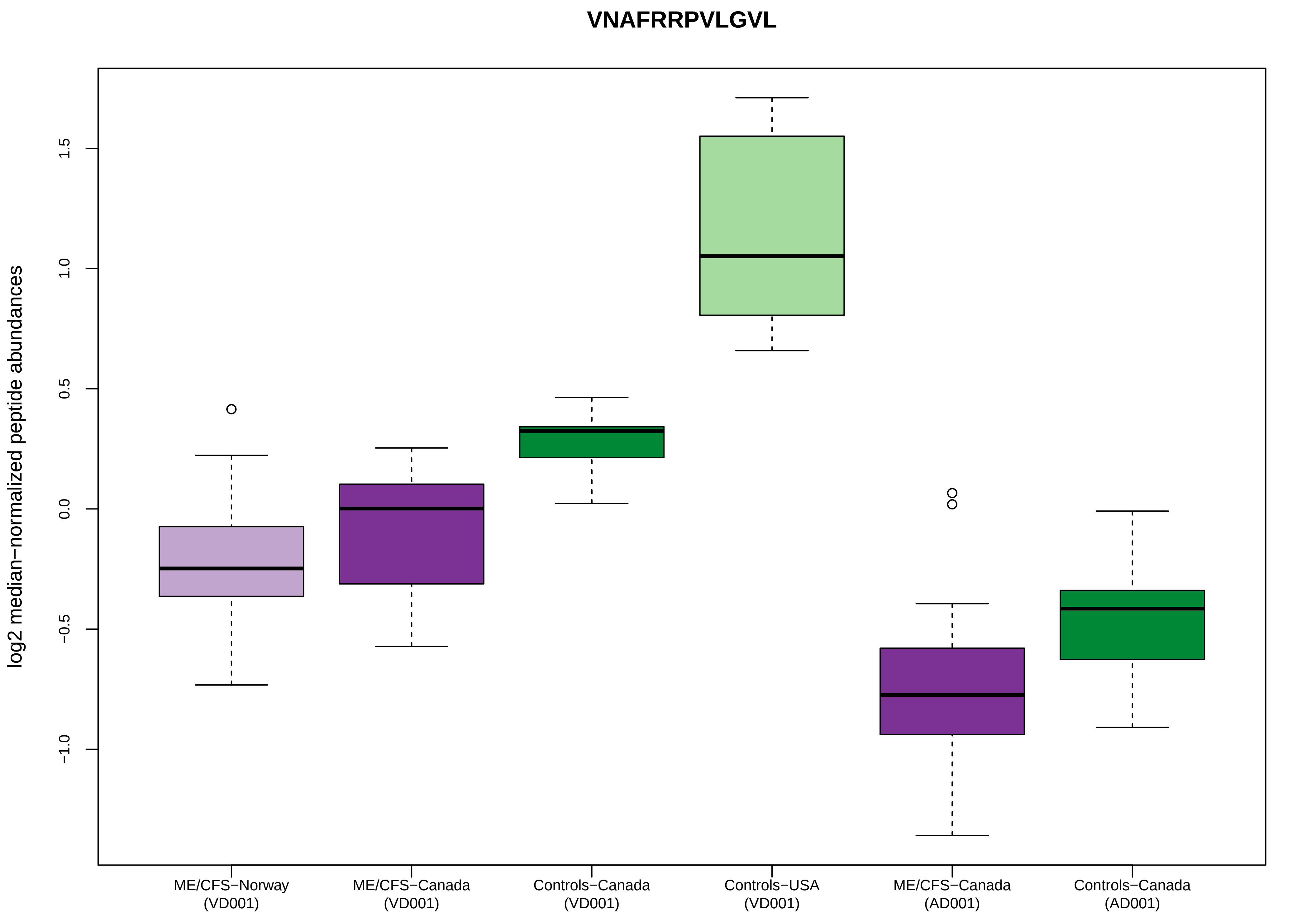

VNAFRRPVLGVL
log2 median−normalized peptide abundances
1.5
1.0
0.5
0.0
−0.5
−1.0
ME/CFS−Norway (VD001)
ME/CFS−Canada (VD001)
Controls−Canada (VD001)
Controls−USA (VD001)
ME/CFS−Canada (AD001)
Controls−Canada (AD001)

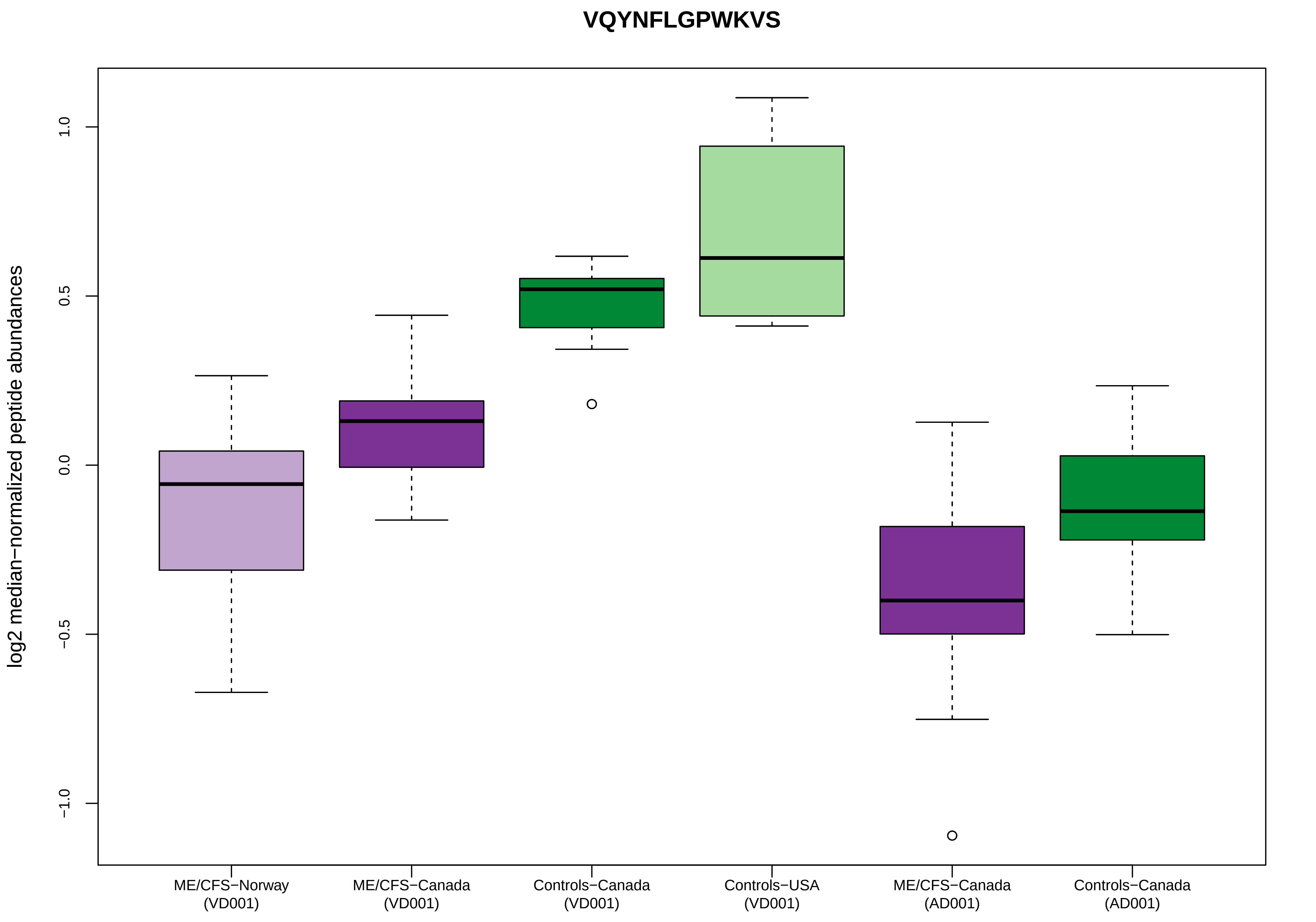
VQYNFLGPWKVS
log2 median−normalized peptide abundances
1.0
0.5
0.0
−0.5
−1.0
ME/CFS−Norway (VD001)
ME/CFS−Canada (VD001)
Controls−Canada (VD001)
Controls−USA (VD001)
ME/CFS−Canada (AD001)
Controls−Canada (AD001)

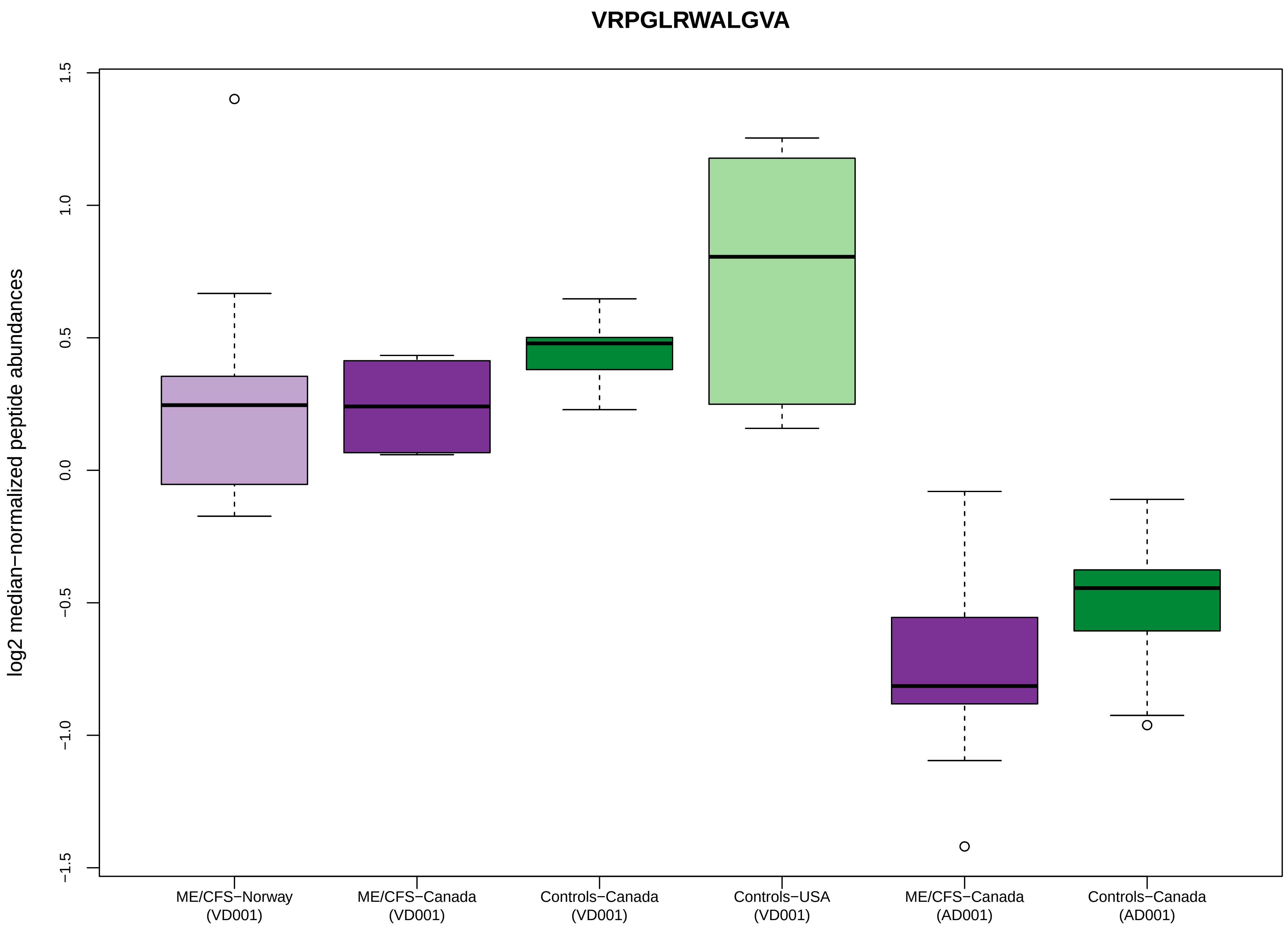
VRPGLRWALGVA
log2 median−normalized peptide abundances
1.5
1.0
0.5
0.0
−0.5
−1.0
−1.5
ME/CFS−Norway (VD001)
ME/CFS−Canada (VD001)
Controls−Canada (VD001)
Controls−USA (VD001)
ME/CFS−Canada (AD001)
Controls−Canada (AD001)

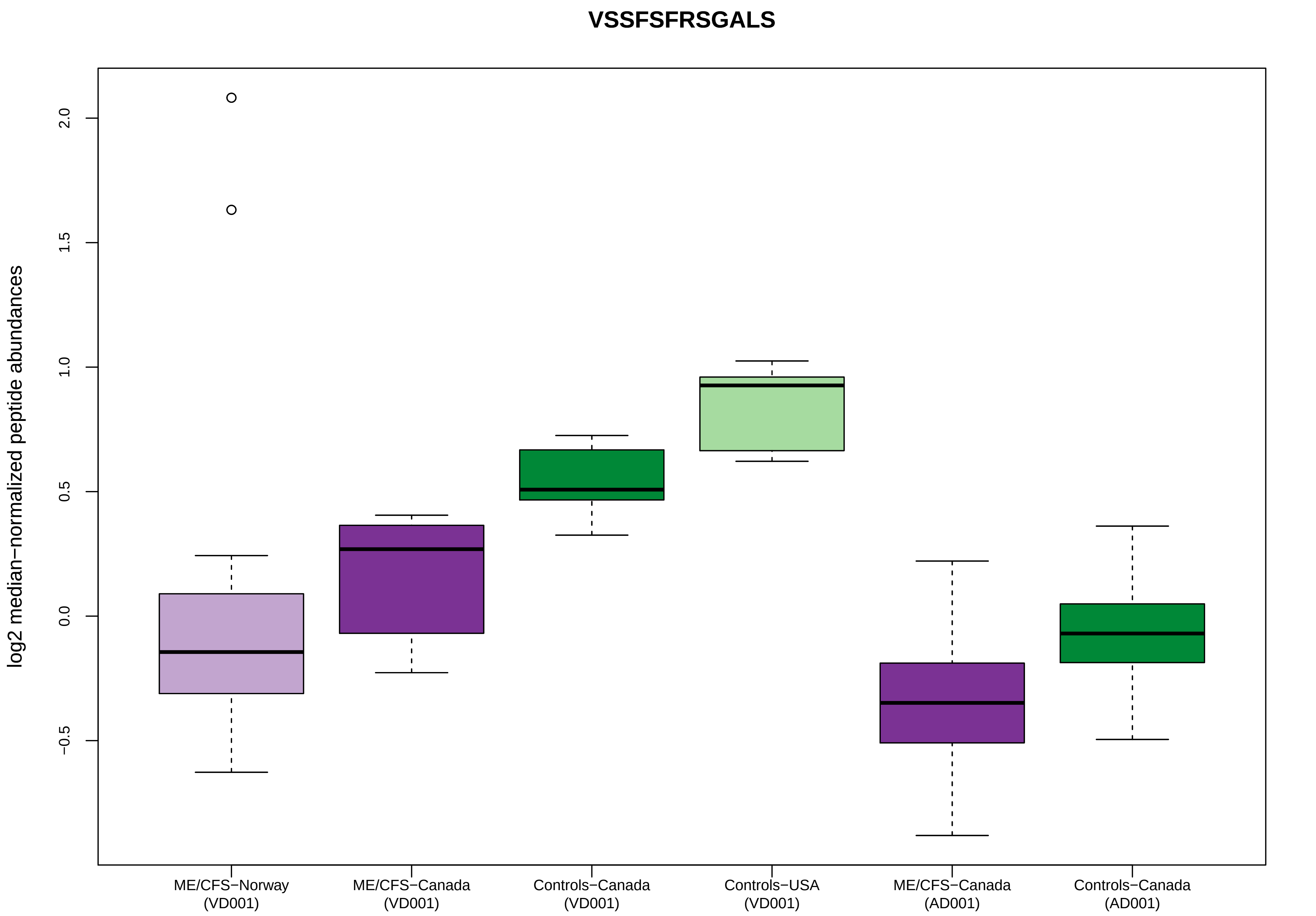

VSSFSFRSGALS
log2 median−normalized peptide abundances
2.0
1.5
1.0
0.5
0.0
−0.5
ME/CFS−Norway (VD001)
ME/CFS−Canada (VD001)
Controls−Canada (VD001)
Controls−USA (VD001)
ME/CFS−Canada (AD001)
Controls−Canada (AD001)

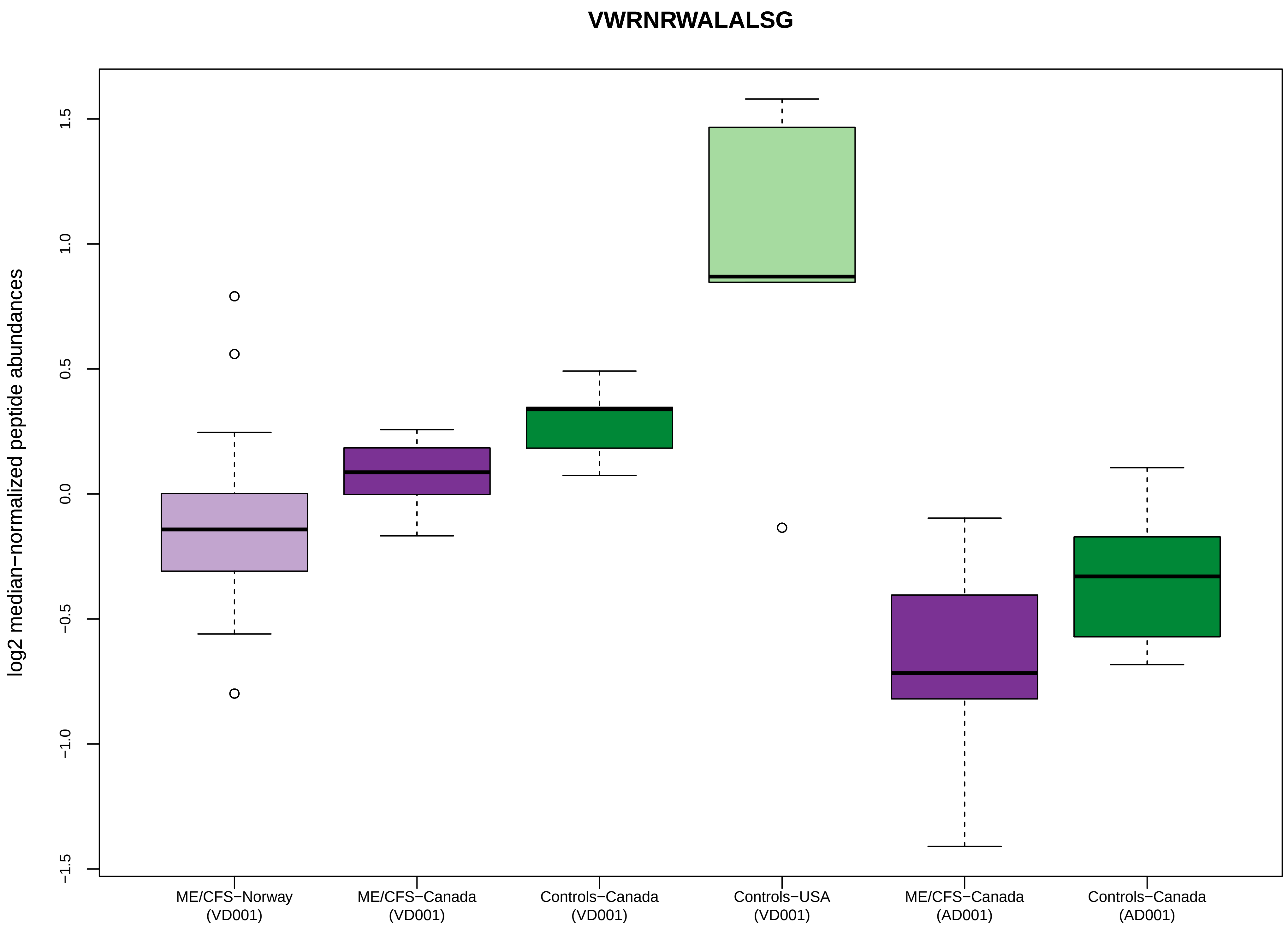

VWRNRWALALSG
log2 median−normalized peptide abundances
1.5
1.0
0.5
0.0
−0.5
−1.0
−1.5
ME/CFS−Norway (VD001)
ME/CFS−Canada (VD001)
Controls−Canada (VD001)
Controls−USA (VD001)
ME/CFS−Canada (AD001)
Controls−Canada (AD001)

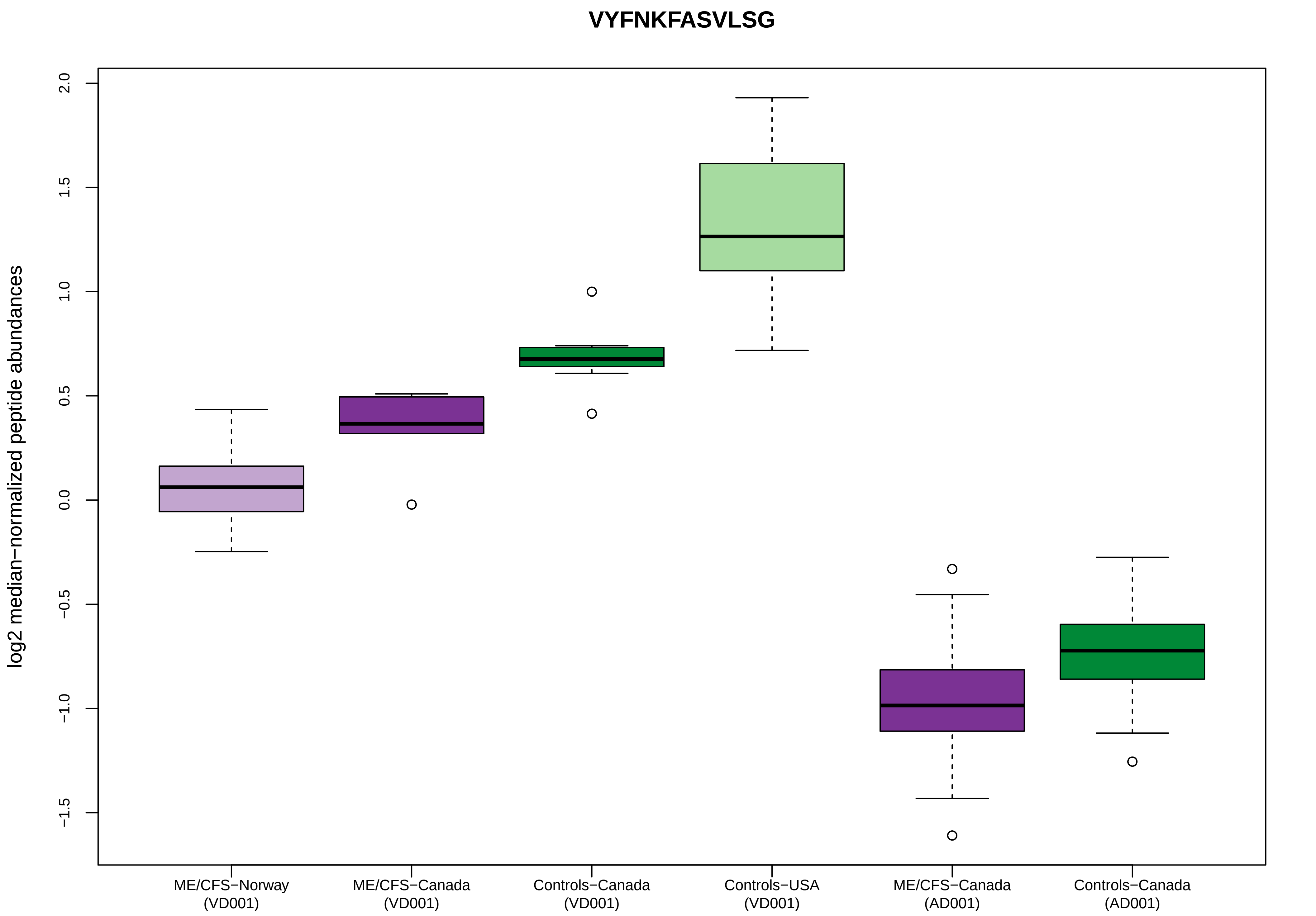

VYFNKFASVLSG
log2 median−normalized peptide abundances
2.0
1.5
1.0
0.5
0.0
−0.5
−1.0
−1.5
ME/CFS−Norway (VD001)
ME/CFS−Canada (VD001)
Controls−Canada (VD001)
Controls−USA (VD001)
ME/CFS−Canada (AD001)
Controls−Canada (AD001)

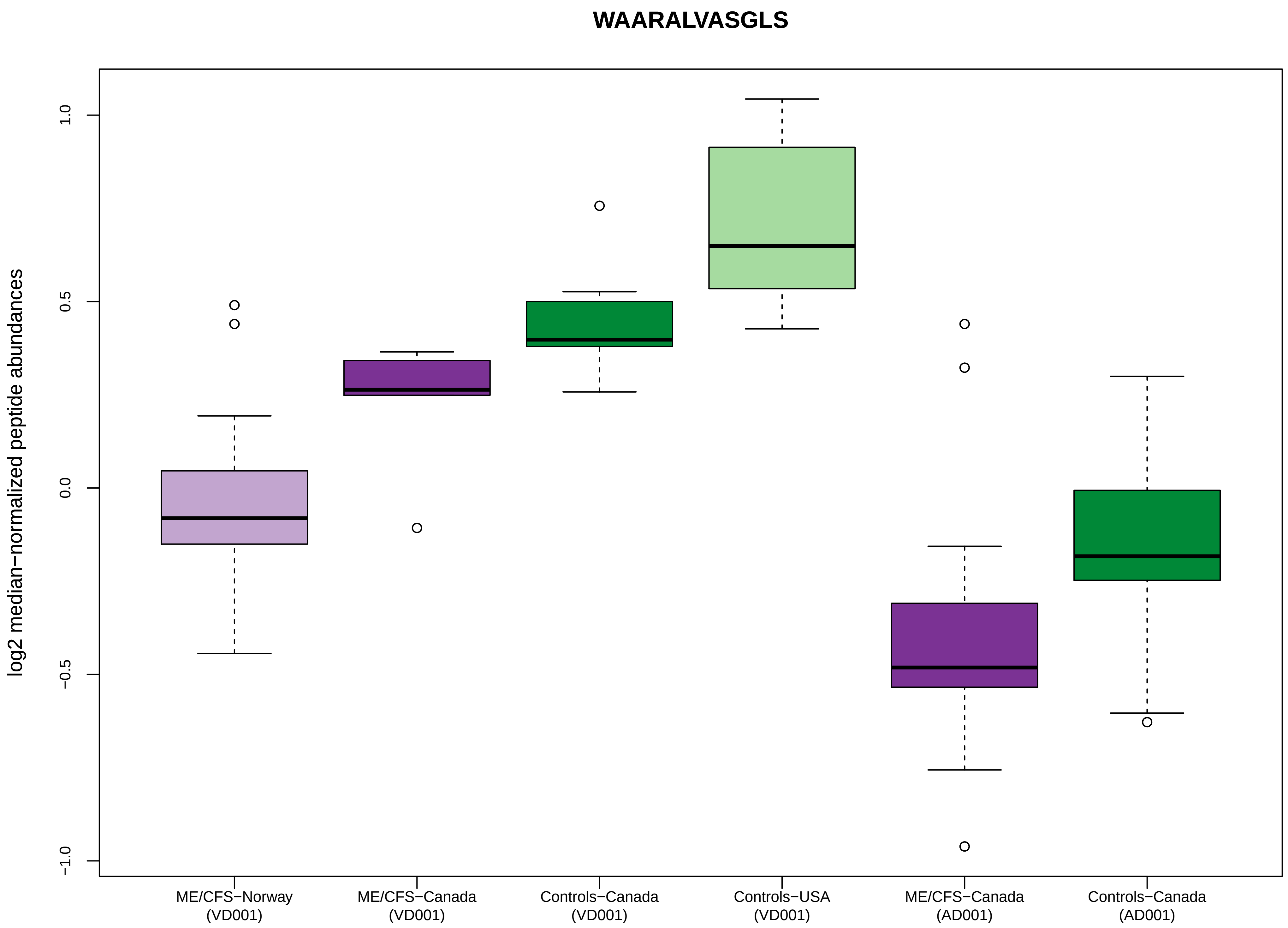

WAARALVASGLS
log2 median−normalized peptide abundances
1.0
0.5
0.0
−0.5
−1.0
ME/CFS−Norway (VD001)
ME/CFS−Canada (VD001)
Controls−Canada (VD001)
Controls−USA (VD001)
ME/CFS−Canada (AD001)
Controls−Canada (AD001)

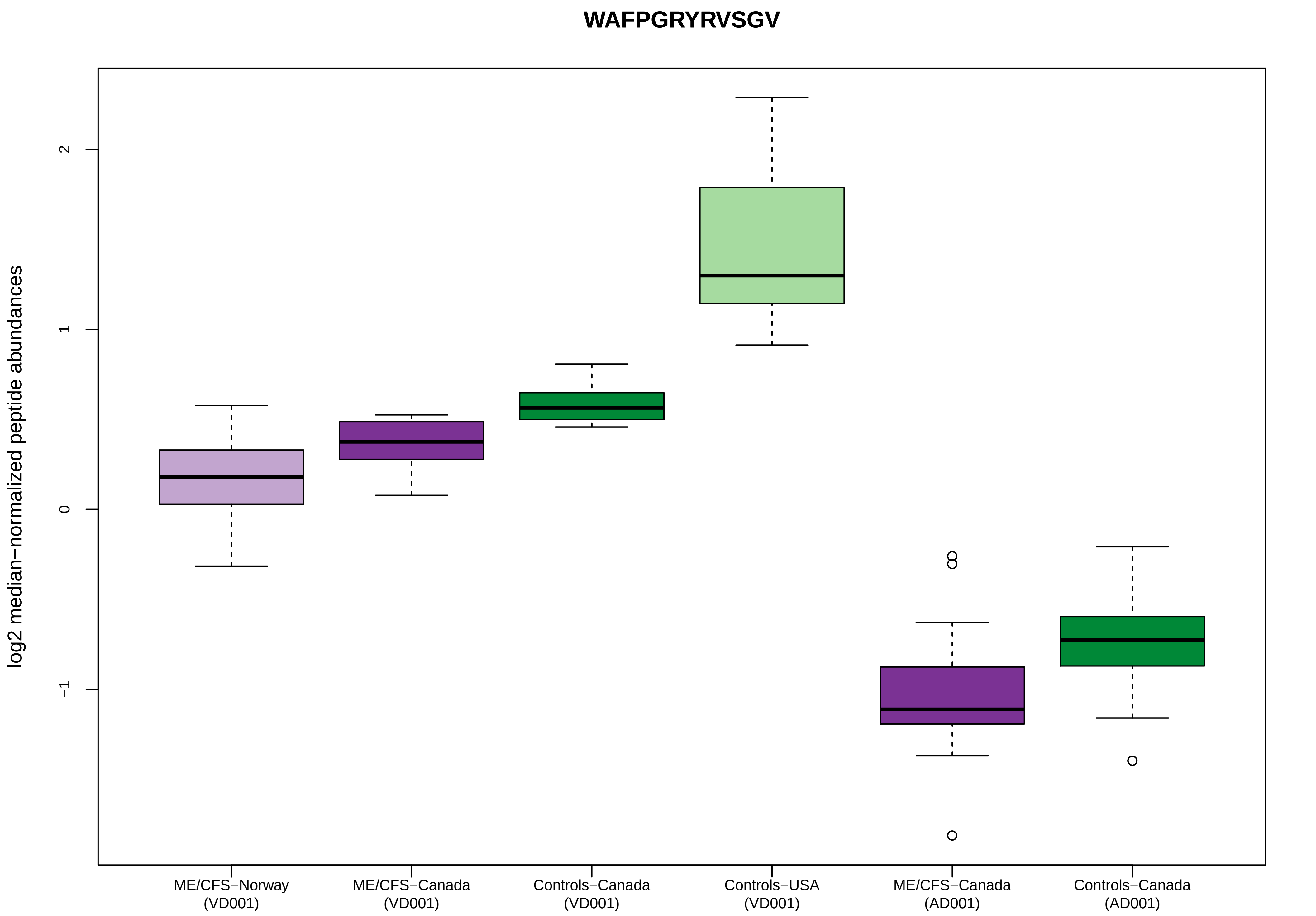
WAFPGRYRVSGV
log2 median−normalized peptide abundances
2
1
0
−1
ME/CFS−Norway (VD001)
ME/CFS−Canada (VD001)
Controls−Canada (VD001)
Controls−USA (VD001)
ME/CFS−Canada (AD001)
Controls−Canada (AD001)

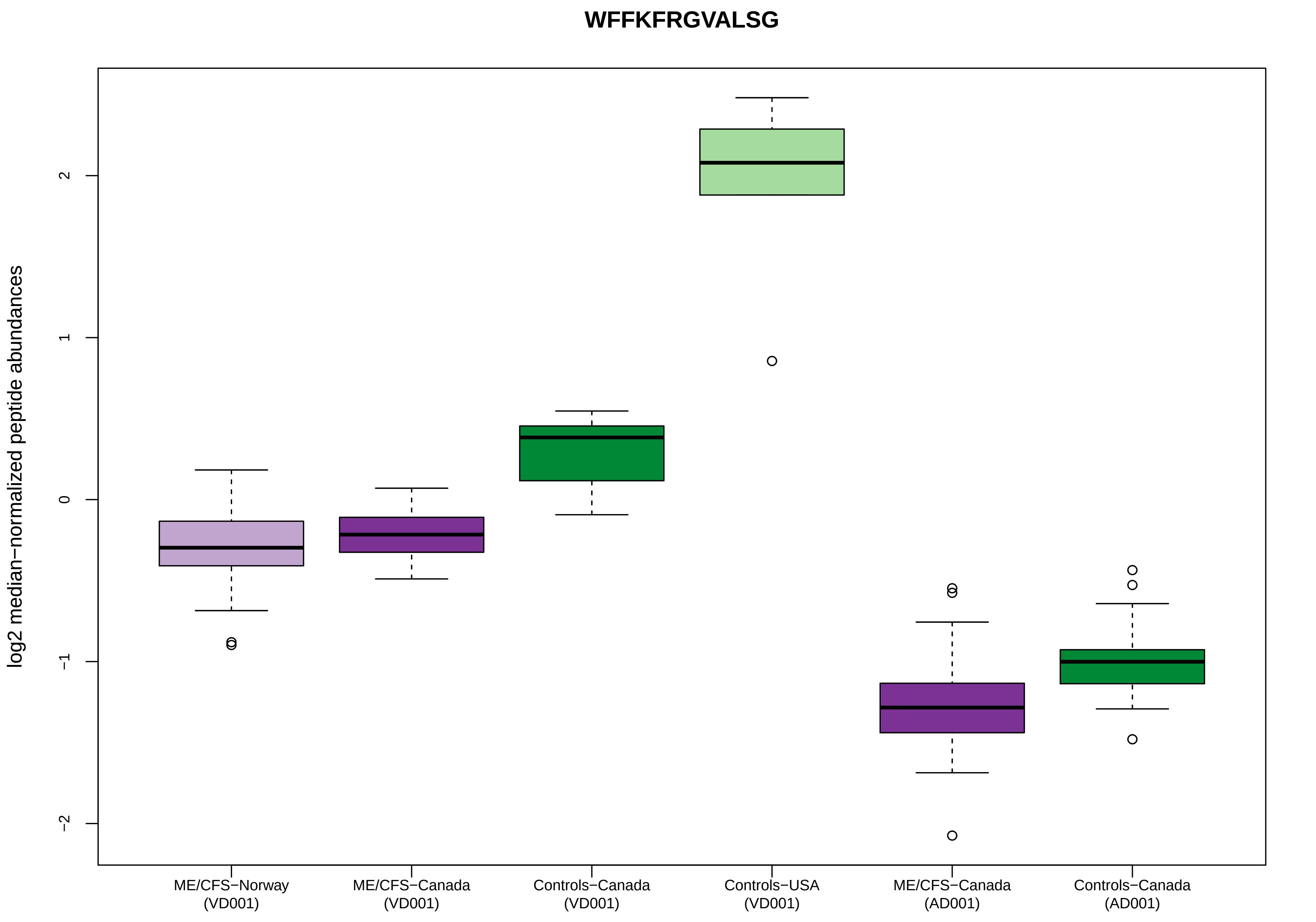
WFFKFRGVALSG
log2 median−normalized peptide abundances
−2
−1
0
1
2
ME/CFS−Norway (VD001)
ME/CFS−Canada (VD001)
Controls−Canada (VD001)
Controls−USA (VD001)
ME/CFS−Canada (AD001)
Controls−Canada (AD001)

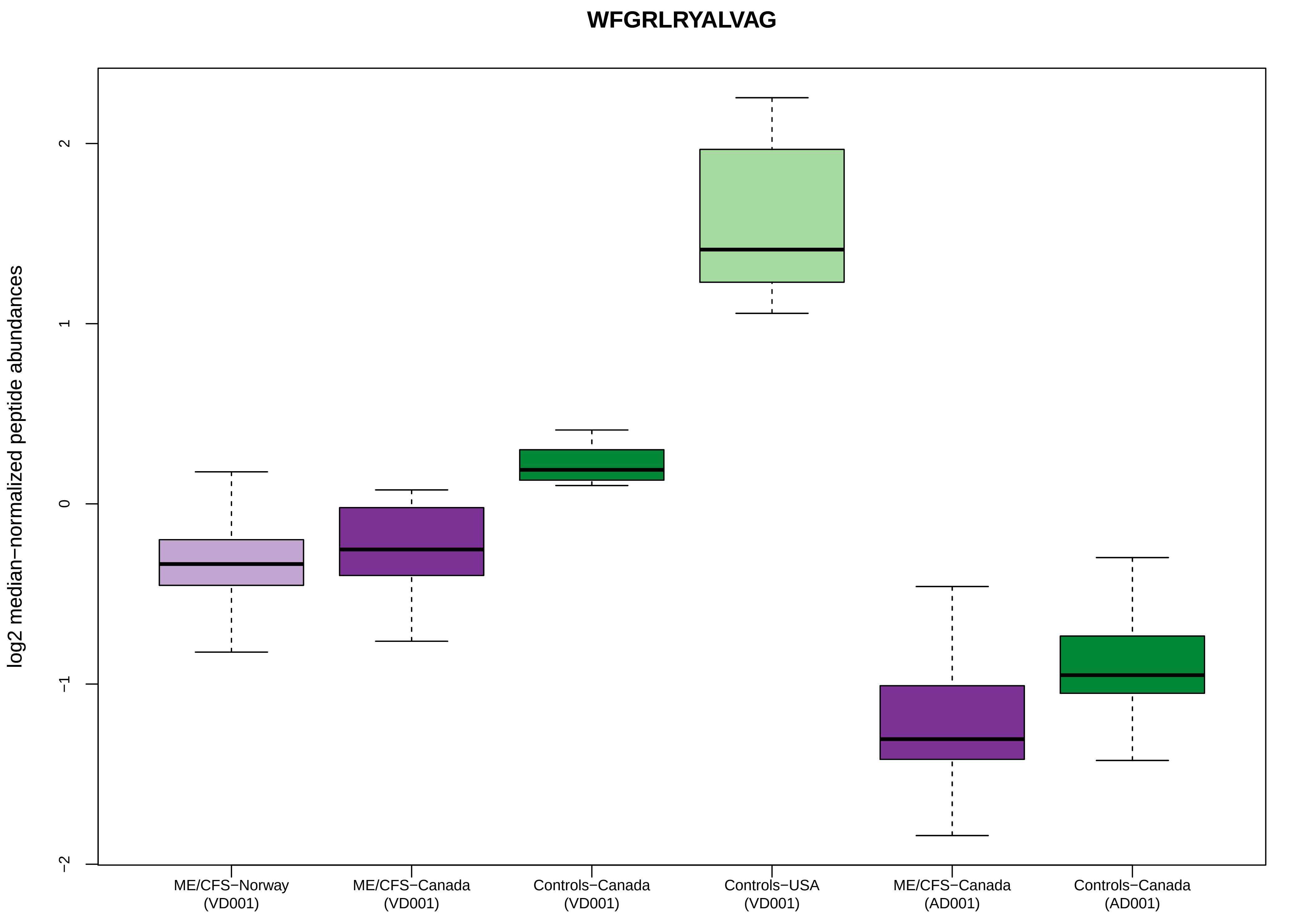
WFGRLRYALVAG
log2 median−normalized peptide abundances
2
1
0
−1
−2
ME/CFS−Norway (VD001)
ME/CFS−Canada (VD001)
Controls−Canada (VD001)
Controls−USA (VD001)
ME/CFS−Canada (AD001)
Controls−Canada (AD001)

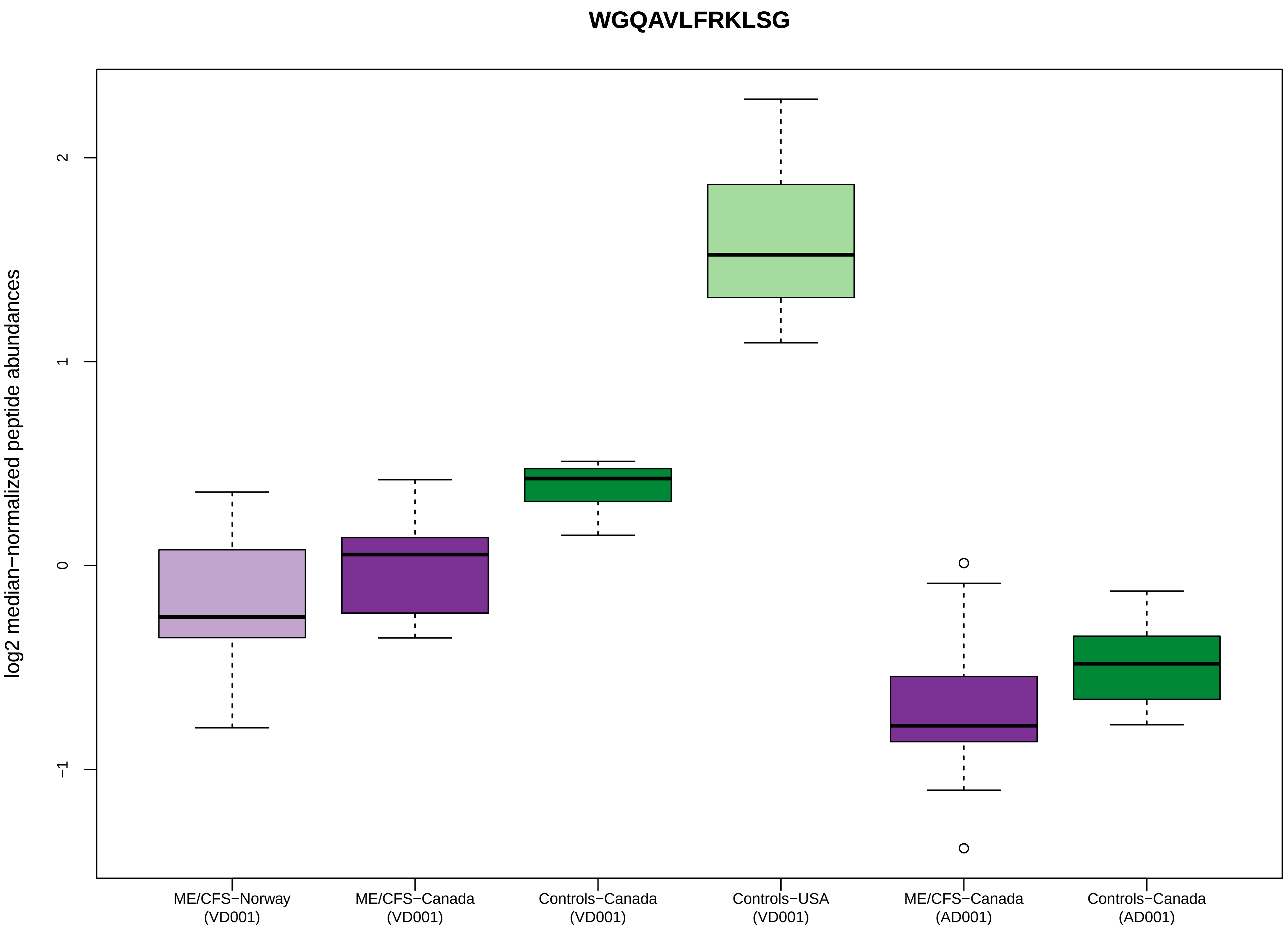
WGQAVLFRKLSG
log2 median−normalized peptide abundances
2
1
0
−1
ME/CFS−Norway (VD001)
ME/CFS−Canada (VD001)
Controls−Canada (VD001)
Controls−USA (VD001)
ME/CFS−Canada (AD001)
Controls−Canada (AD001)

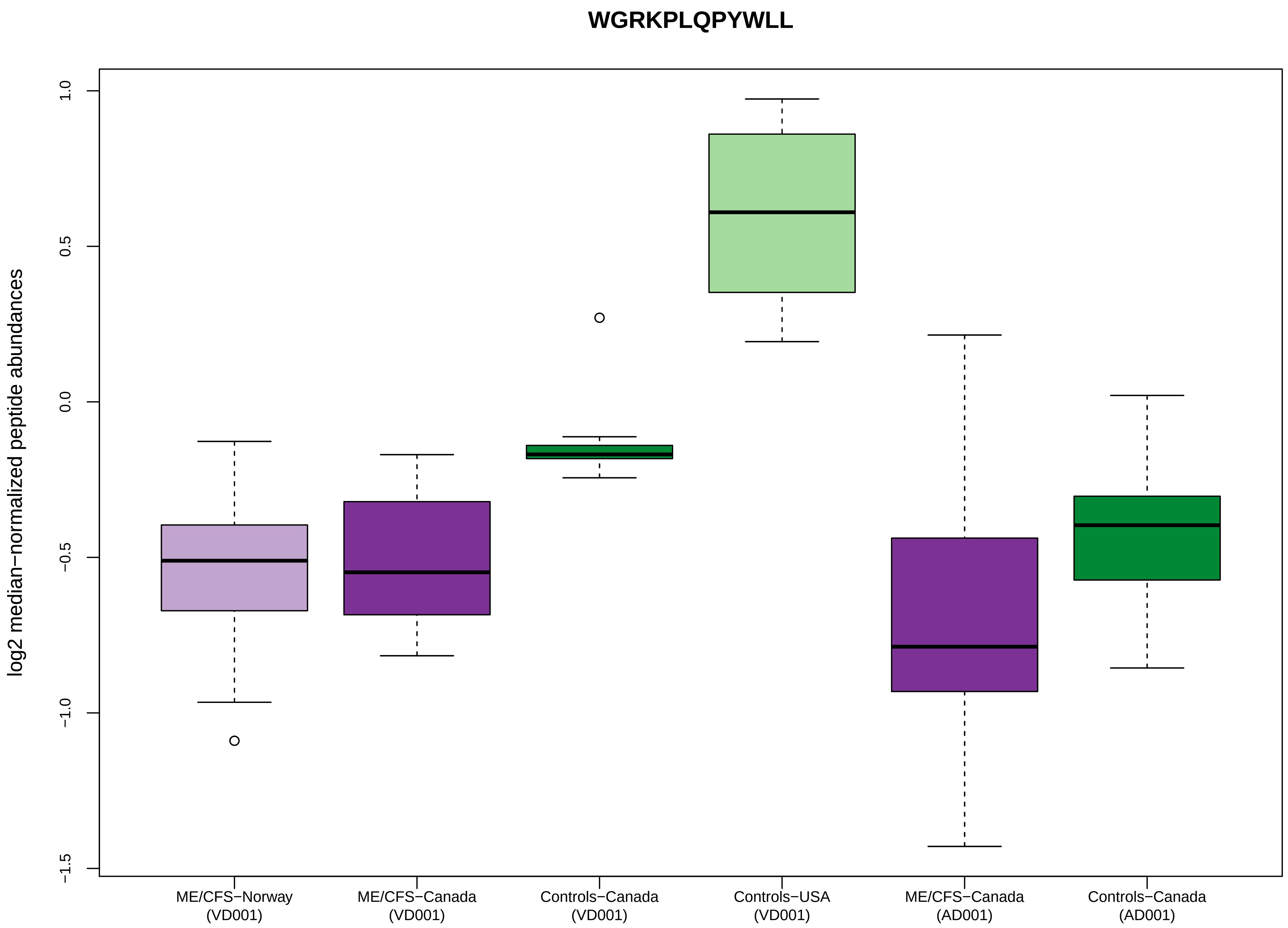

WGRKPLQPYWLL
log2 median−normalized peptide abundances
1.0
0.5
0.0
−0.5
−1.0
−1.5
ME/CFS−Norway (VD001)
ME/CFS−Canada (VD001)
Controls−Canada (VD001)
Controls−USA (VD001)
ME/CFS−Canada (AD001)
Controls−Canada (AD001)

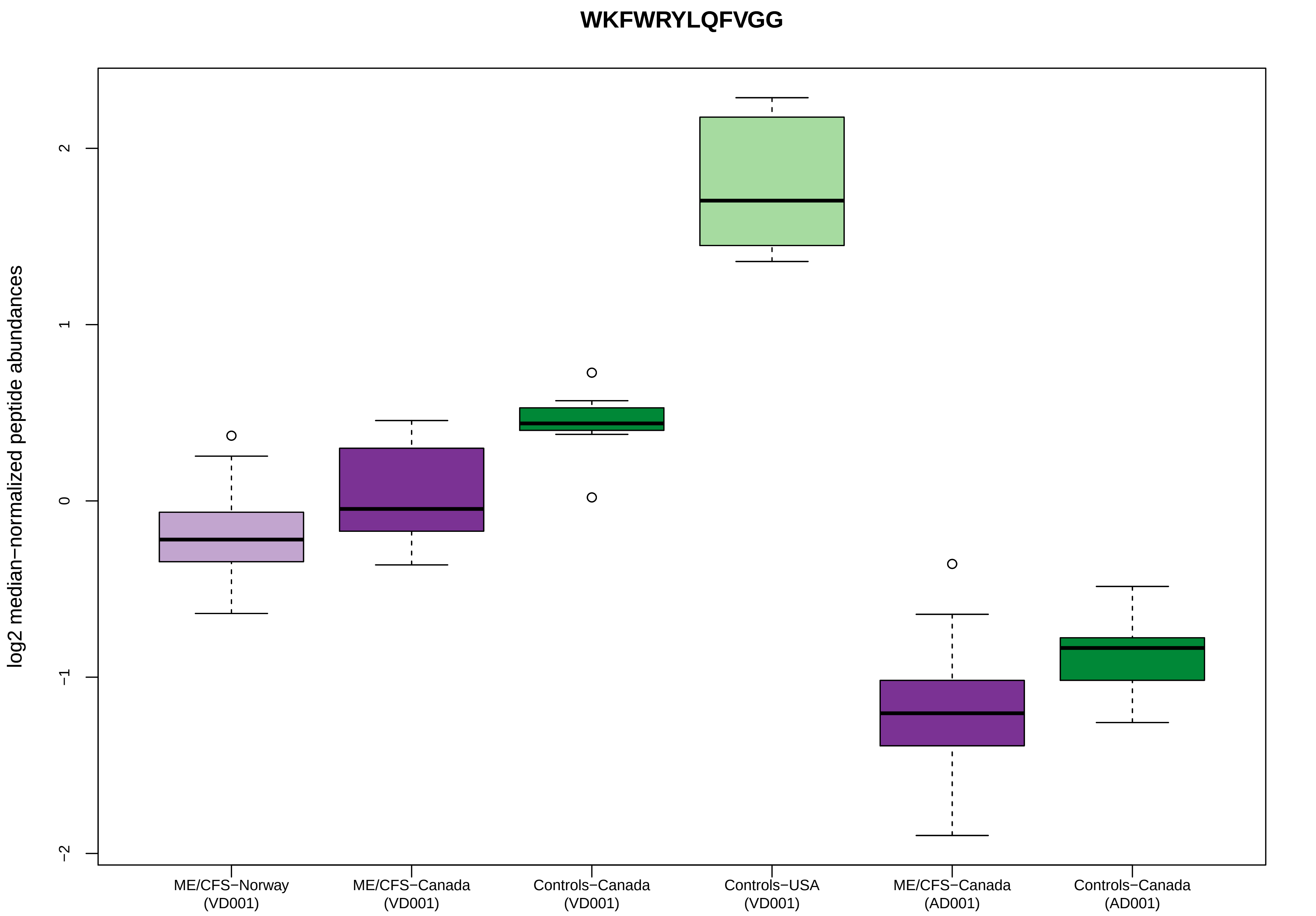

WKFWRYLQFVGG
log2 median−normalized peptide abundances
2
1
0
−1
−2
ME/CFS−Norway (VD001)
ME/CFS−Canada (VD001)
Controls−Canada (VD001)
Controls−USA (VD001)
ME/CFS−Canada (AD001)
Controls−Canada (AD001)

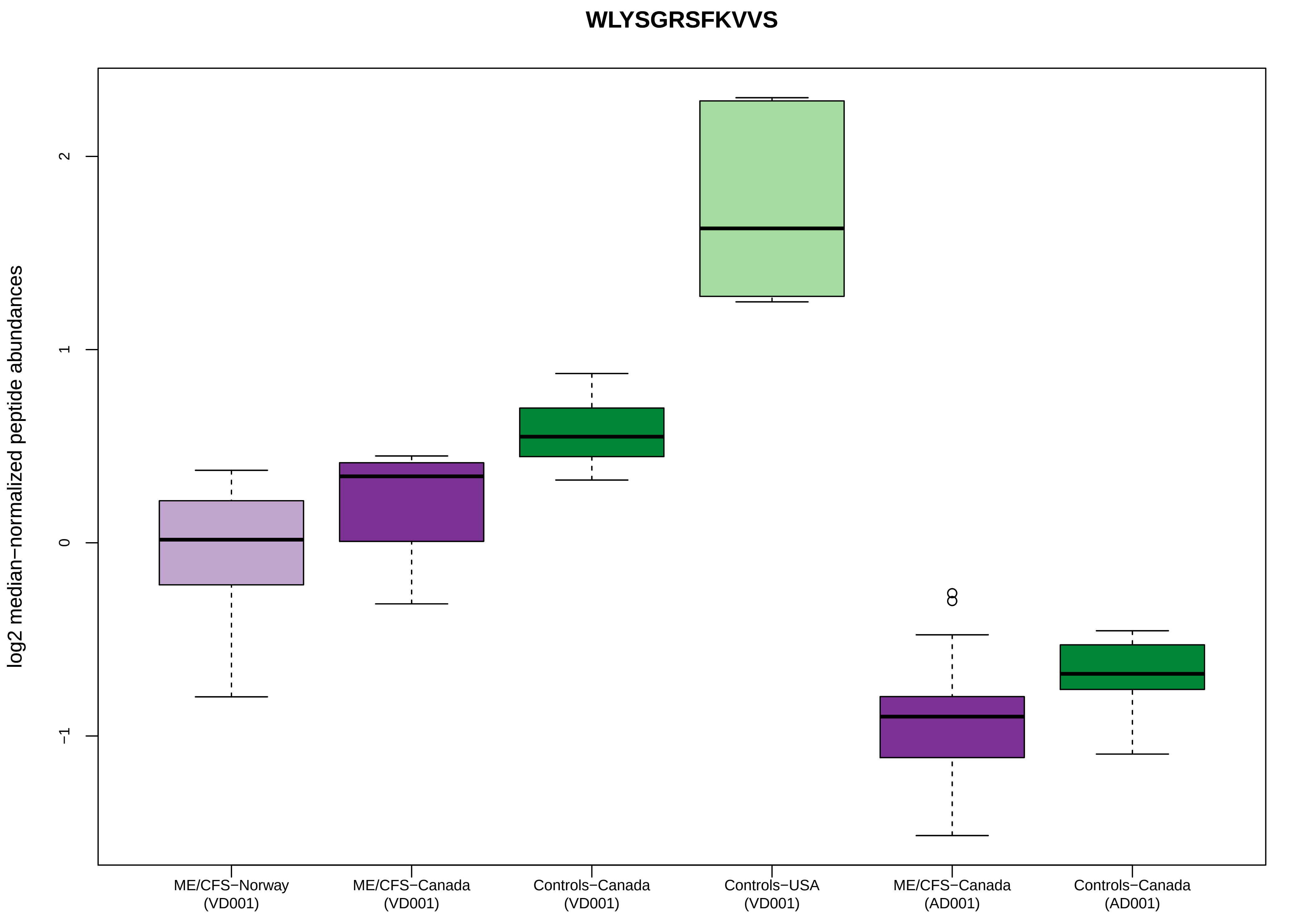
WLYSGRSFKVVS
log2 median−normalized peptide abundances
−1
0
1
2
ME/CFS−Norway (VD001)
ME/CFS−Canada (VD001)
Controls−Canada (VD001)
Controls−USA (VD001)
ME/CFS−Canada (AD001)
Controls−Canada (AD001)

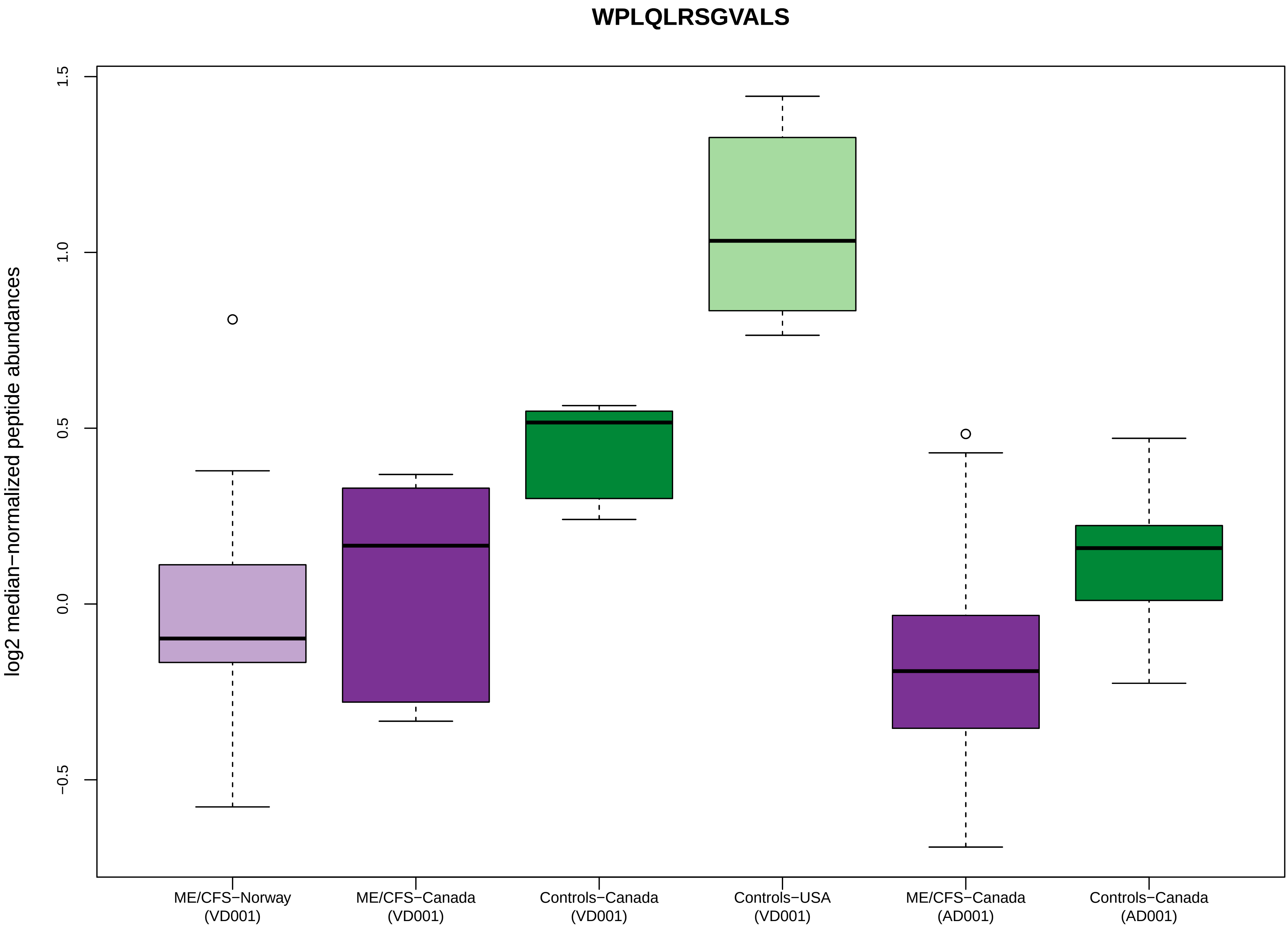
WPLQLRSGVALS
log2 median−normalized peptide abundances
1.5
1.0
0.5
0.0
−0.5
ME/CFS−Norway (VD001)
ME/CFS−Canada (VD001)
Controls−Canada (VD001)
Controls−USA (VD001)
ME/CFS−Canada (AD001)
Controls−Canada (AD001)

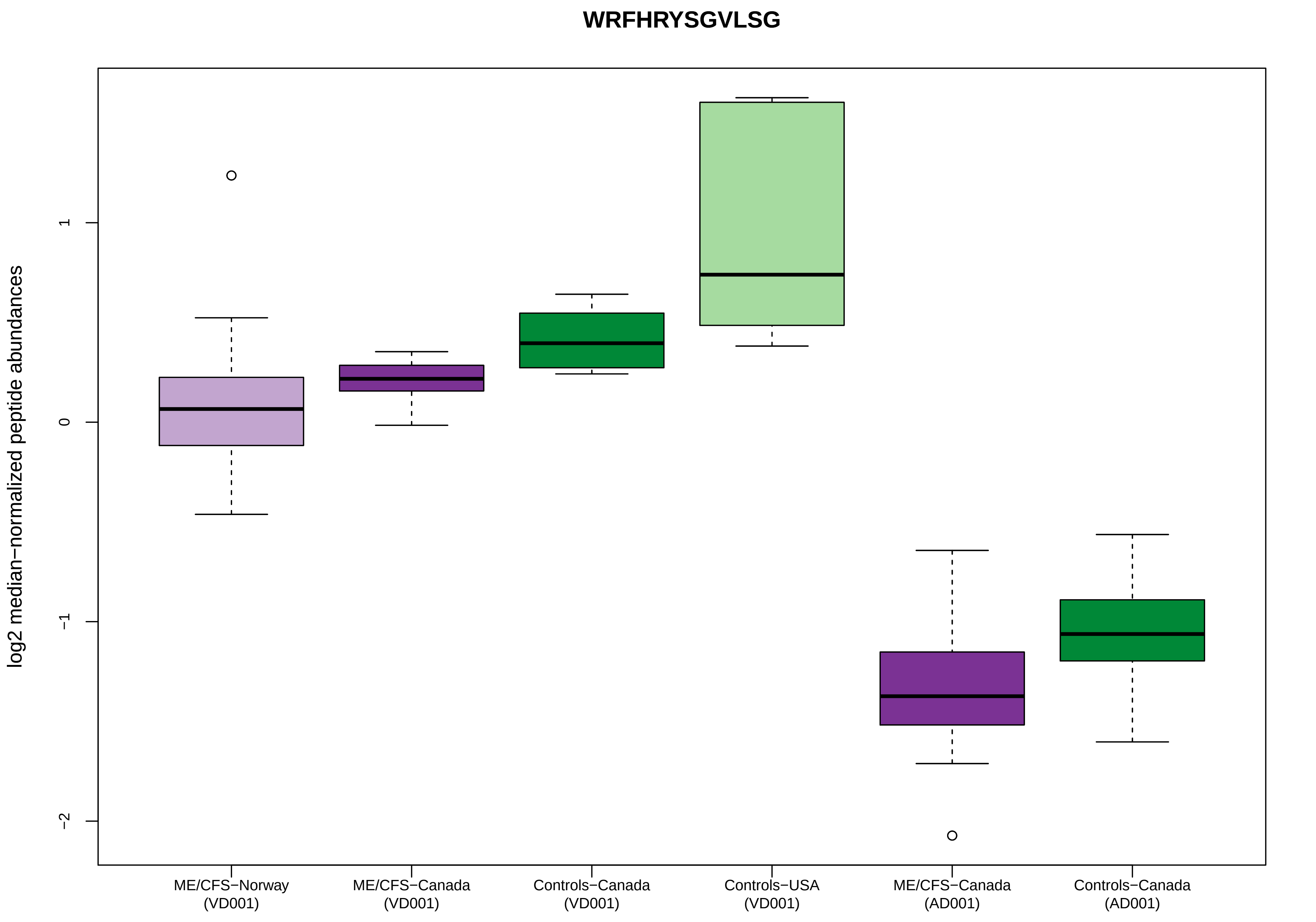

WRFHRYSGVLSG
log2 median−normalized peptide abundances
1
0
−1
−2
ME/CFS−Norway
(VD001)
ME/CFS−Canada
(VD001)
Controls−Canada
(VD001)
Controls−USA
(VD001)
ME/CFS−Canada
(AD001)
Controls−Canada
(AD001)

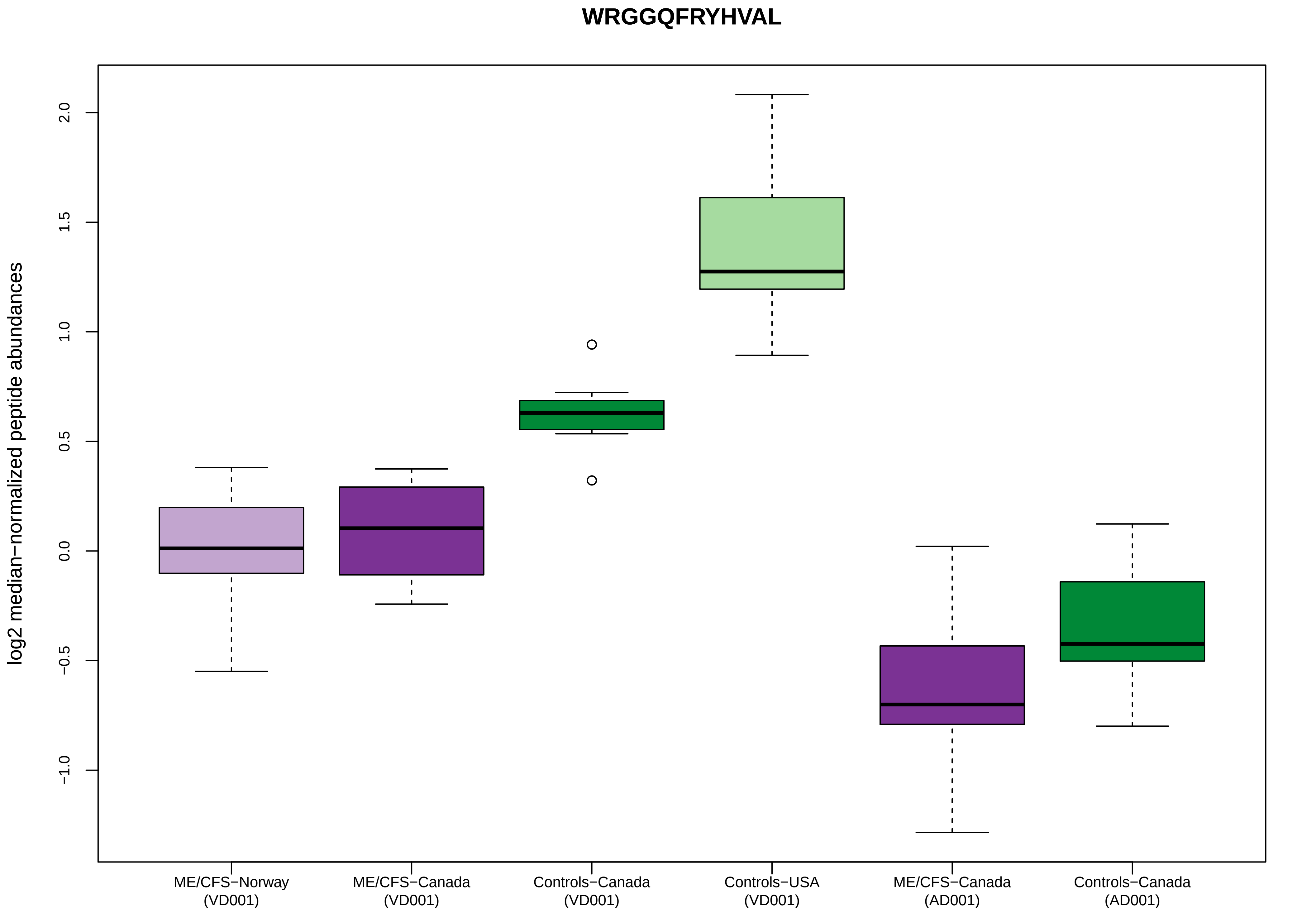

WRGGQFRYHVAL
log2 median−normalized peptide abundances
2.0
1.5
1.0
0.5
0.0
−0.5
−1.0
ME/CFS−Norway (VD001)
ME/CFS−Canada (VD001)
Controls−Canada (VD001)
Controls−USA (VD001)
ME/CFS−Canada (AD001)
Controls−Canada (AD001)

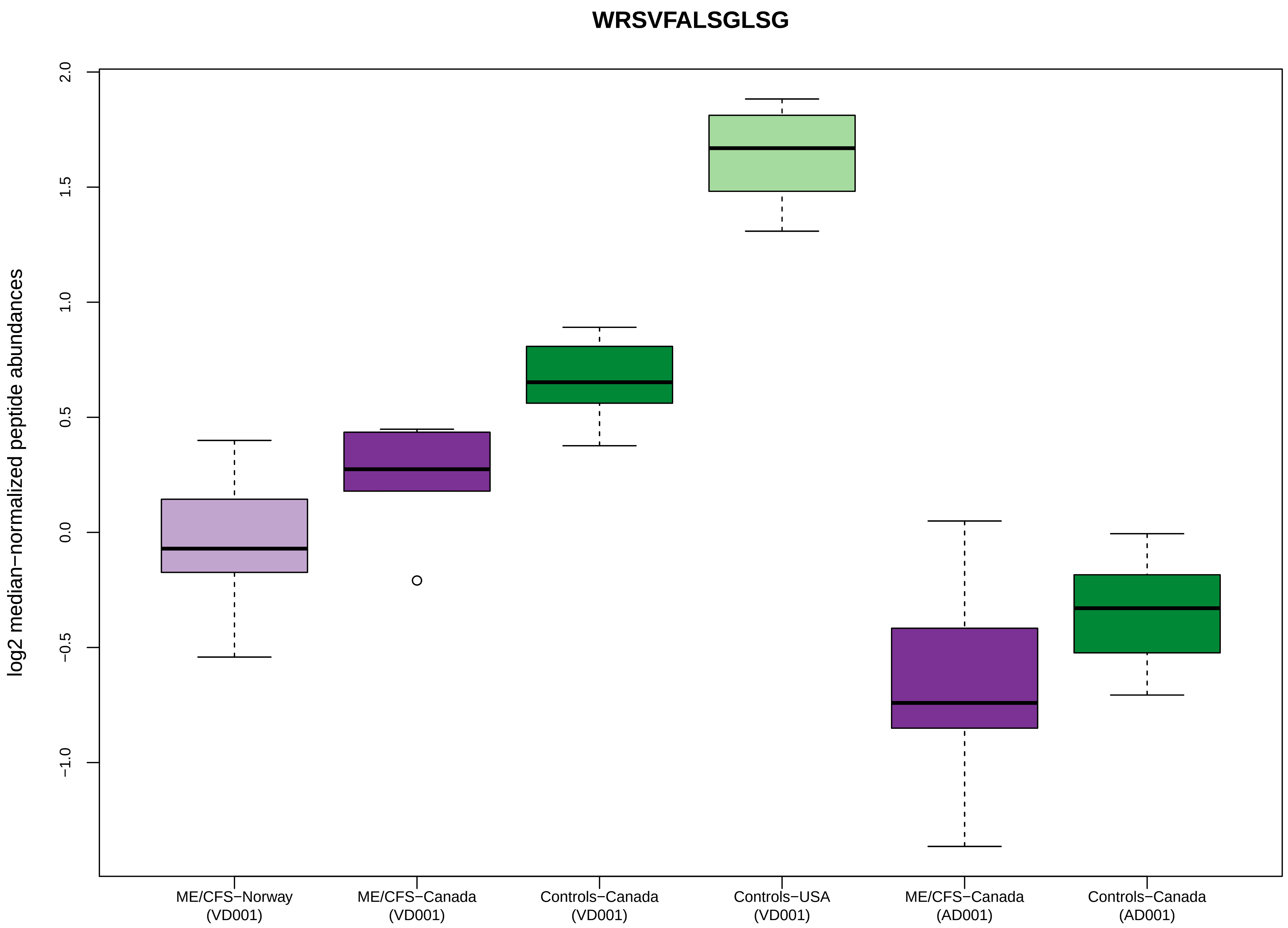

WRSVFALSGLSG
log2 median−normalized peptide abundances
2.0
1.5
1.0
0.5
0.0
−0.5
−1.0
ME/CFS−Norway (VD001)
ME/CFS−Canada (VD001)
Controls−Canada (VD001)
Controls−USA (VD001)
ME/CFS−Canada (AD001)
Controls−Canada (AD001)

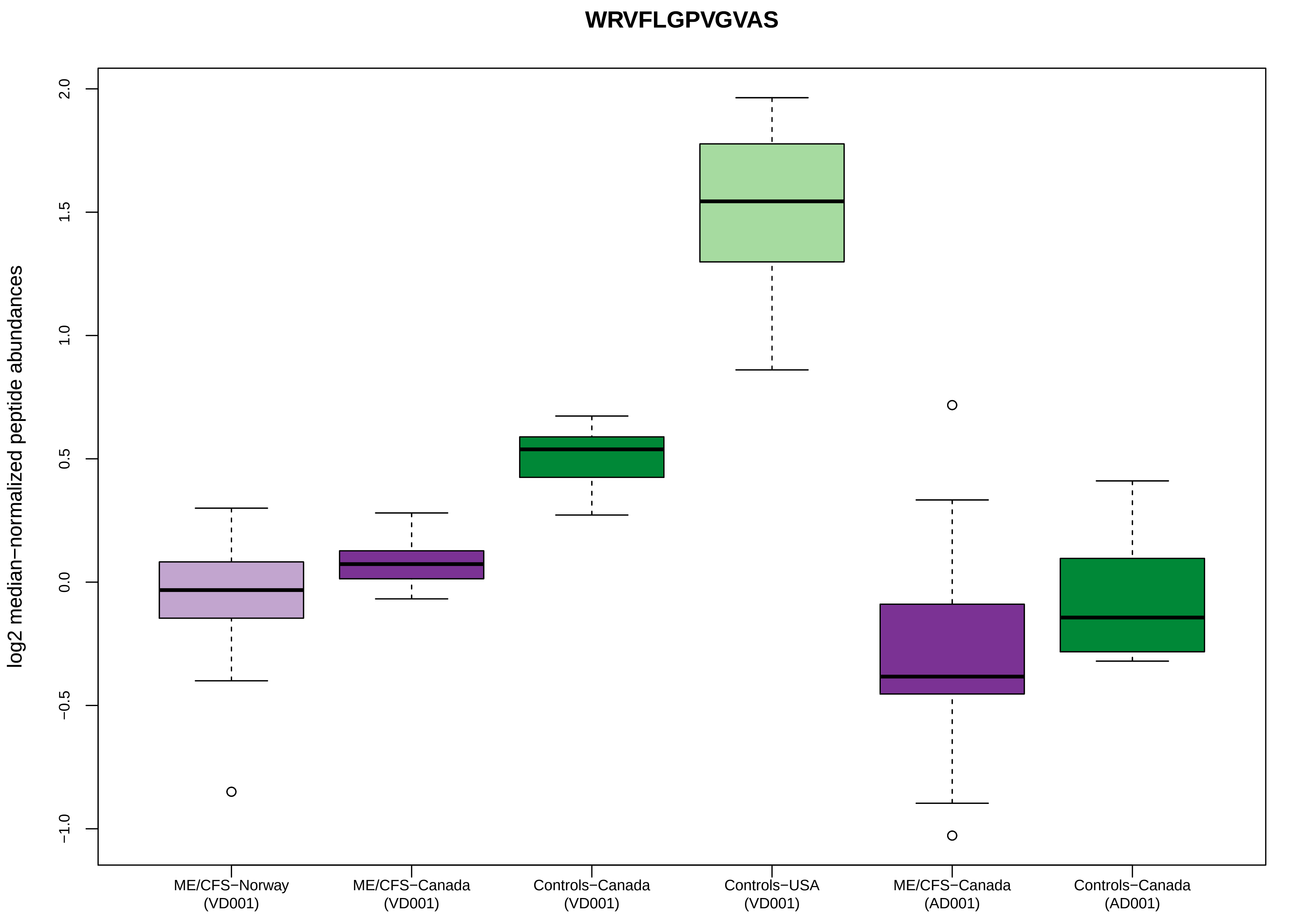
WRVFLGPVGVAS
log2 median−normalized peptide abundances
2.0
1.5
1.0
0.5
0.0
−0.5
−1.0
ME/CFS−Norway (VD001)
ME/CFS−Canada (VD001)
Controls−Canada (VD001)
Controls−USA (VD001)
ME/CFS−Canada (AD001)
Controls−Canada (AD001)

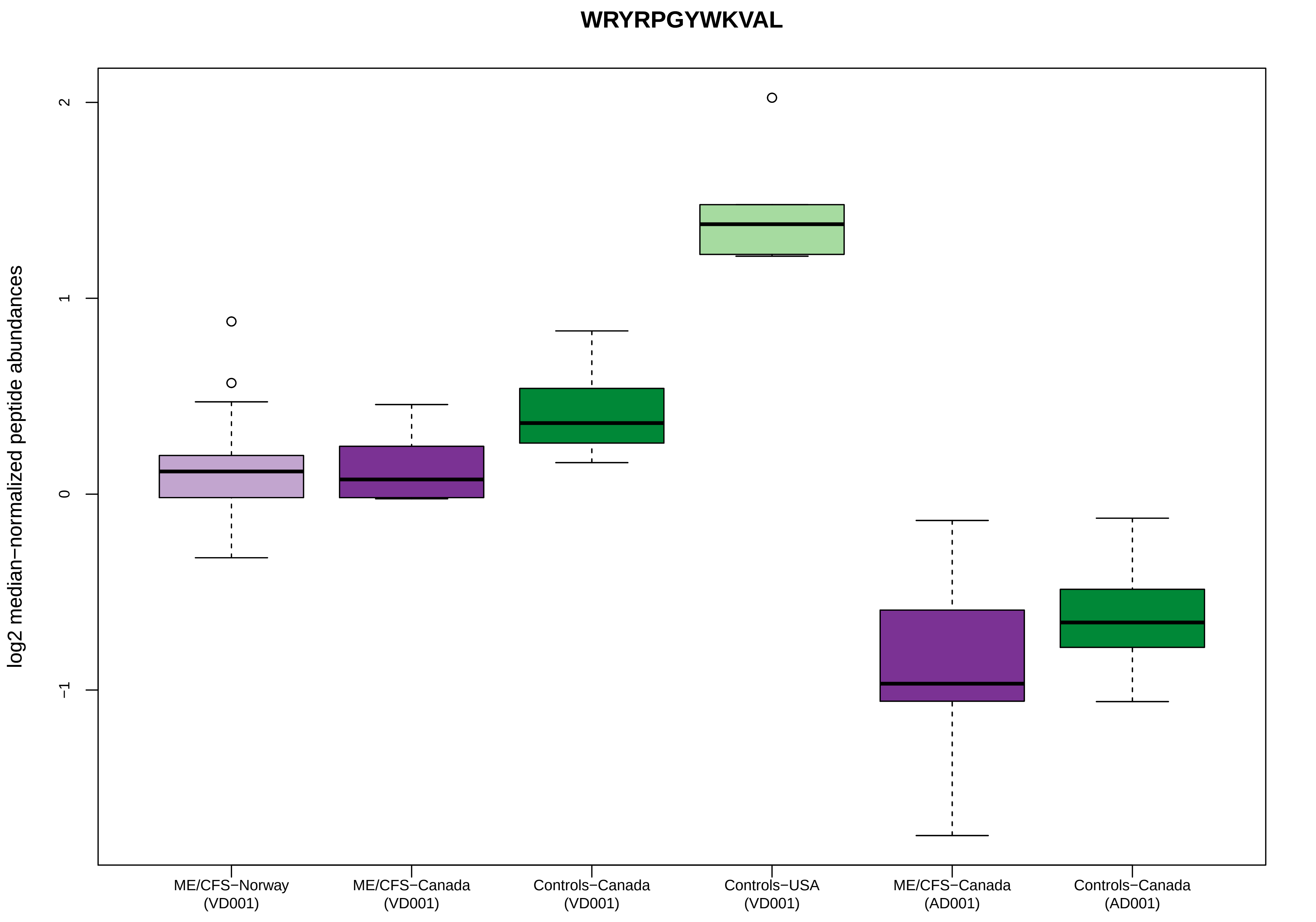
WRYRPGYWKVAL
log2 median−normalized peptide abundances
2
1
0
−1
ME/CFS−Norway (VD001)
ME/CFS−Canada (VD001)
Controls−Canada (VD001)
Controls−USA (VD001)
ME/CFS−Canada (AD001)
Controls−Canada (AD001)

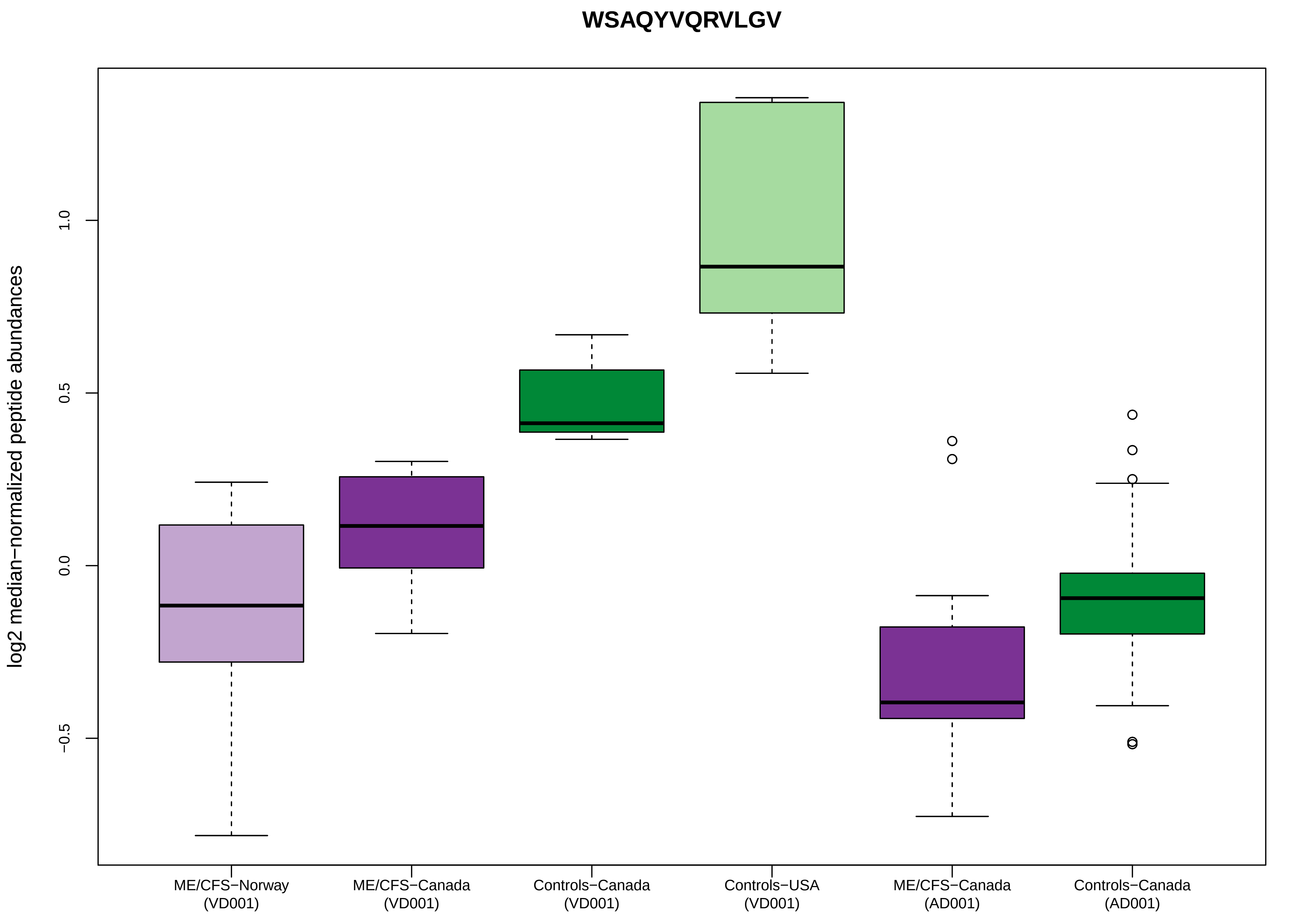

WSAQYVQRVLGV
log2 median−normalized peptide abundances
1.0
0.5
0.0
−0.5
ME/CFS−Norway (VD001)
ME/CFS−Canada (VD001)
Controls−Canada (VD001)
Controls−USA (VD001)
ME/CFS−Canada (AD001)
Controls−Canada (AD001)

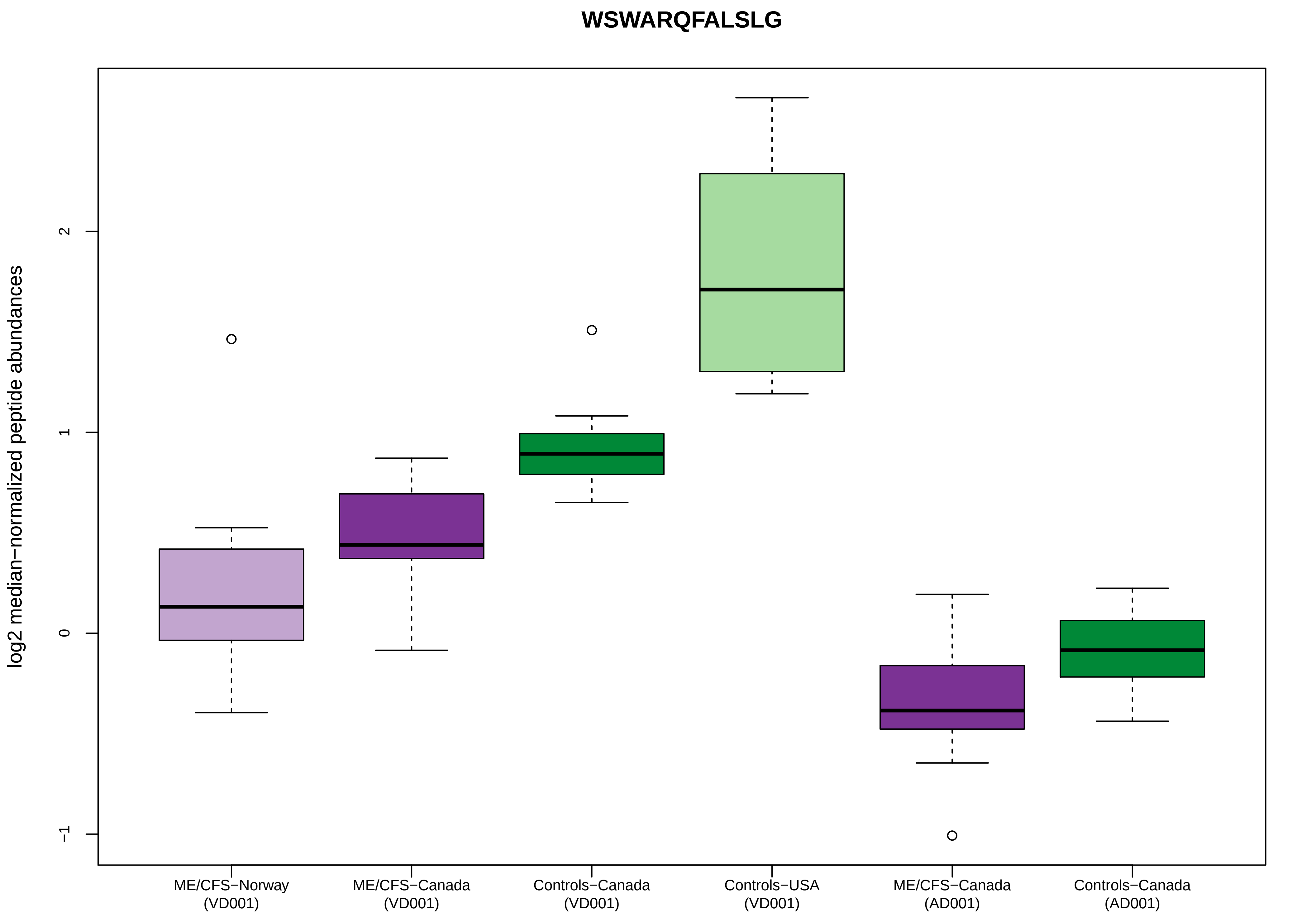

WSWARQFALSLG
log2 median−normalized peptide abundances
2
1
0
−1
ME/CFS−Norway (VD001)
ME/CFS−Canada (VD001)
Controls−Canada (VD001)
Controls−USA (VD001)
ME/CFS−Canada (AD001)
Controls−Canada (AD001)

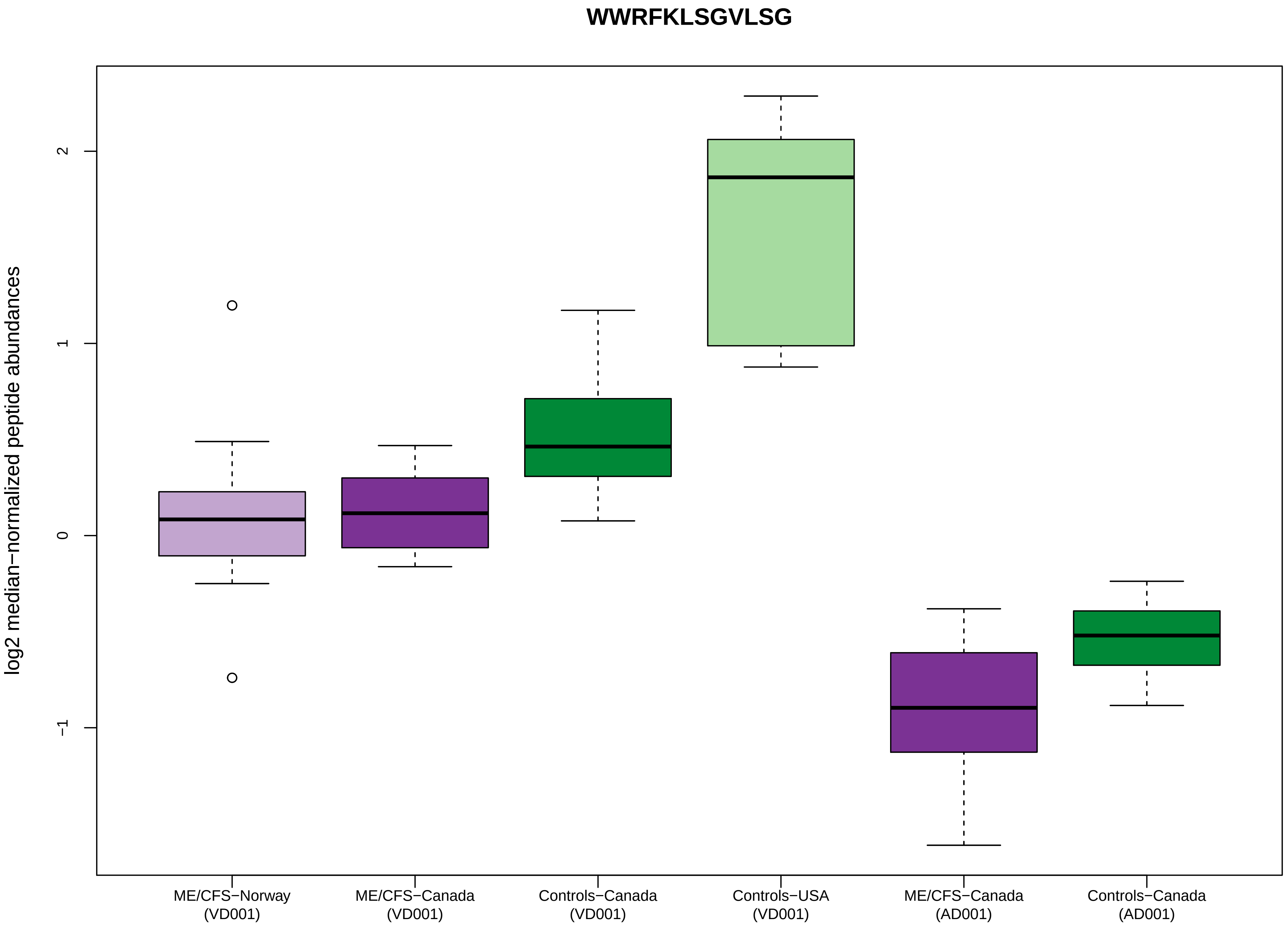

WWRFKLSGVLSG
log2 median−normalized peptide abundances
2
1
0
−1
ME/CFS−Norway (VD001)
ME/CFS−Canada (VD001)
Controls−Canada (VD001)
Controls−USA (VD001)
ME/CFS−Canada (AD001)
Controls−Canada (AD001)

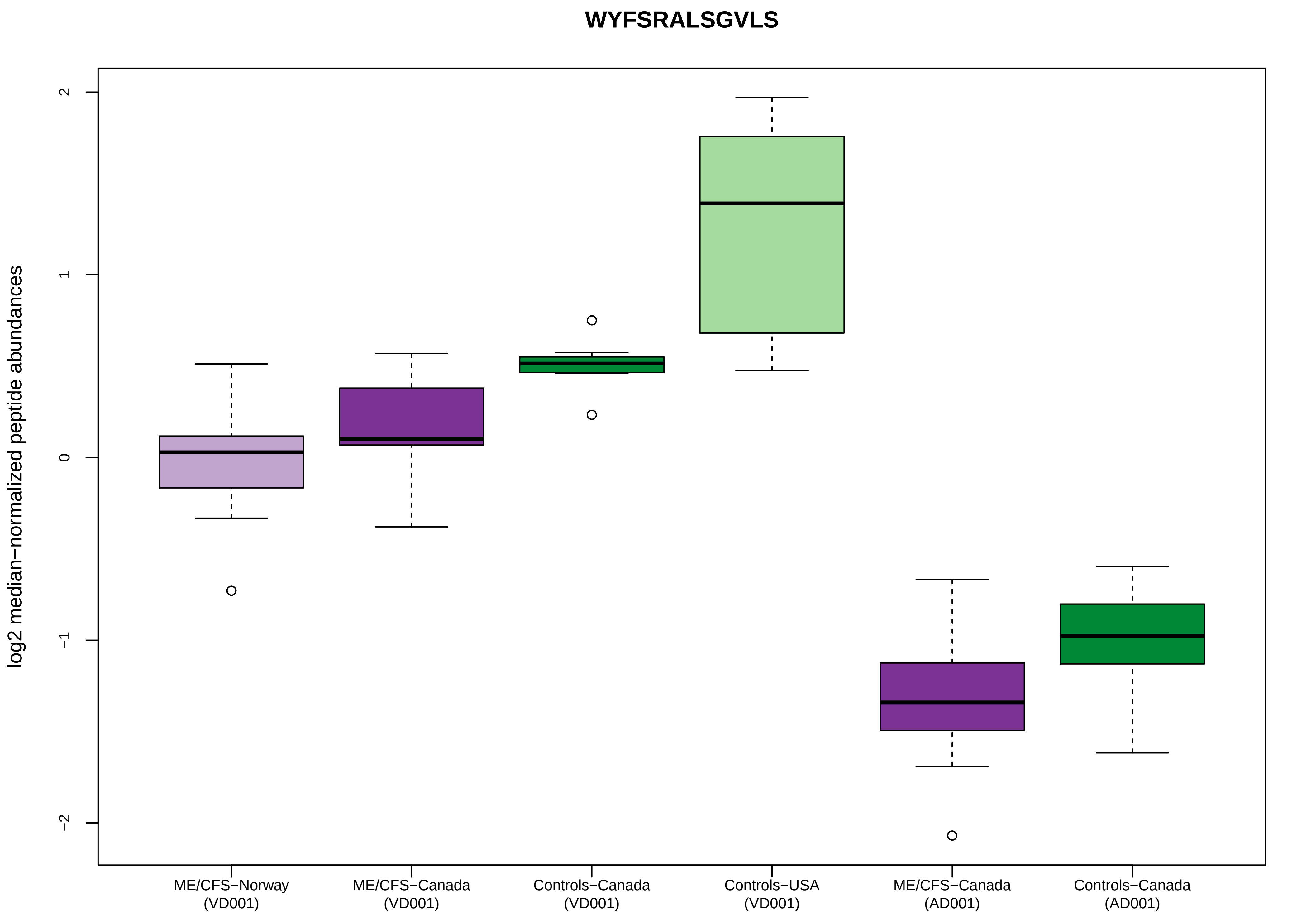
WYFSRALSGVLS
log2 median−normalized peptide abundances
2
1
0
−1
−2
ME/CFS−Norway (VD001)
ME/CFS−Canada (VD001)
Controls−Canada (VD001)
Controls−USA (VD001)
ME/CFS−Canada (AD001)
Controls−Canada (AD001)

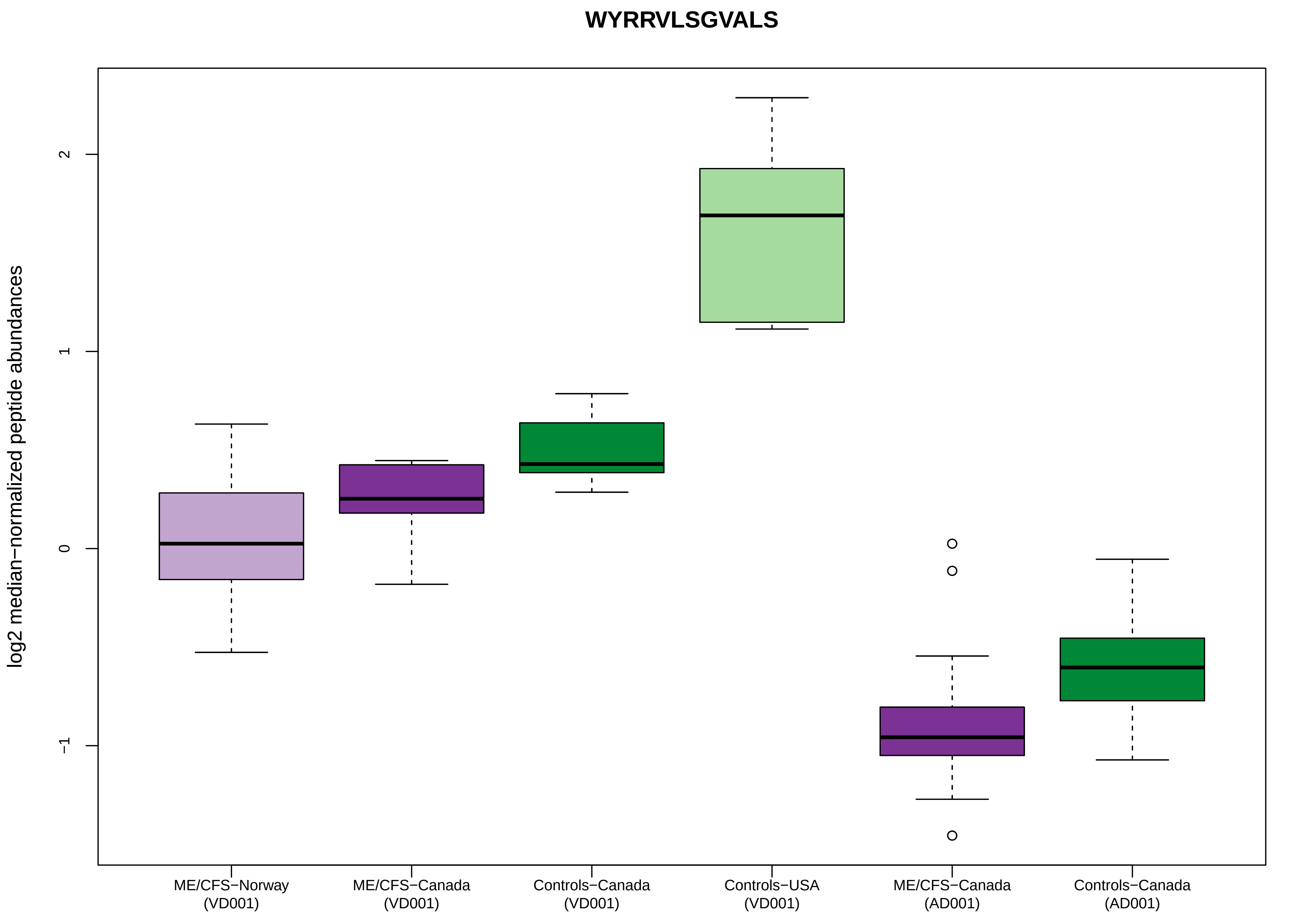

WYRRVLSGVALS
log2 median−normalized peptide abundances
2
1
0
−1
ME/CFS−Norway (VD001)
ME/CFS−Canada (VD001)
Controls−Canada (VD001)
Controls−USA (VD001)
ME/CFS−Canada (AD001)
Controls−Canada (AD001)

YAPFVAFRYNKG

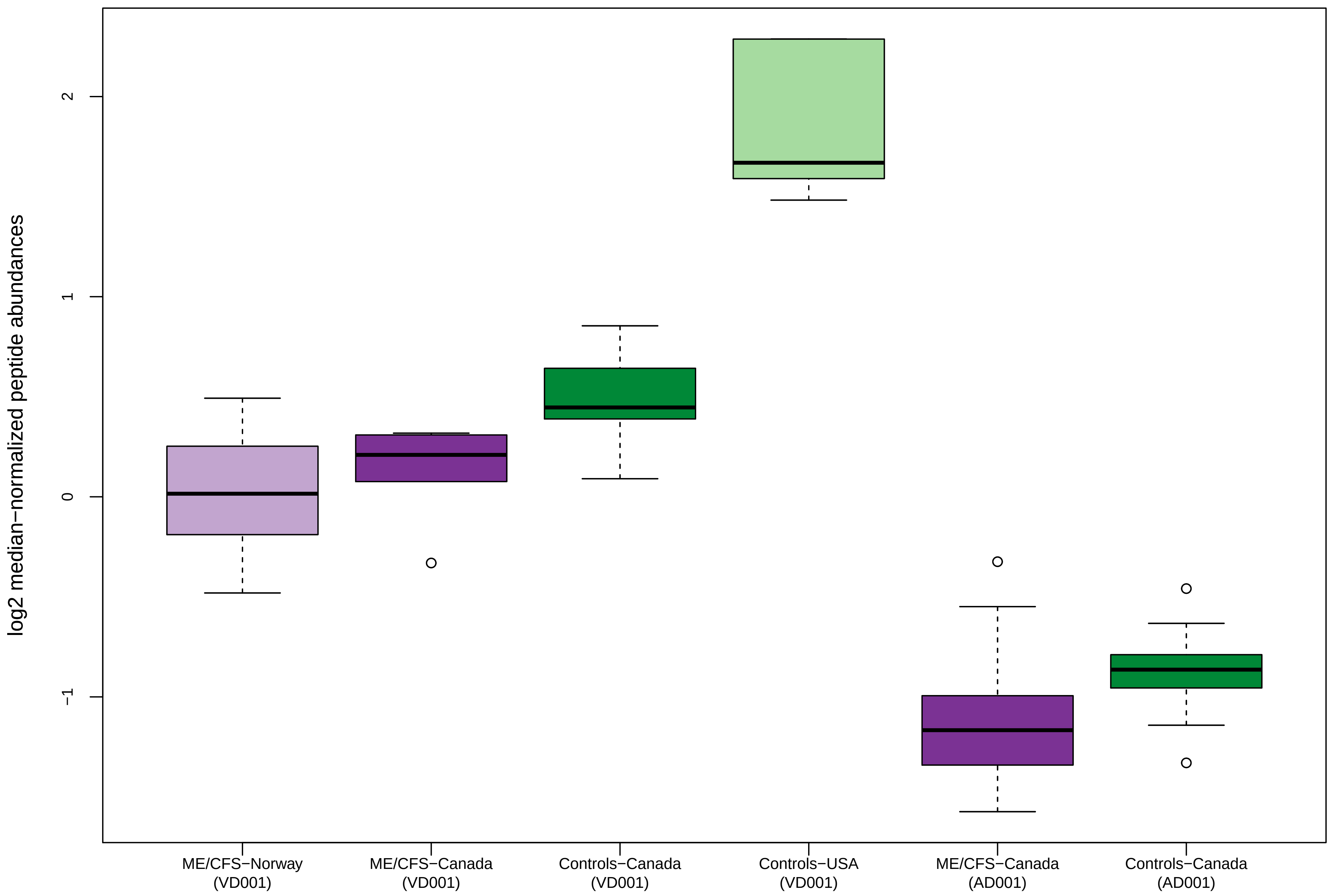

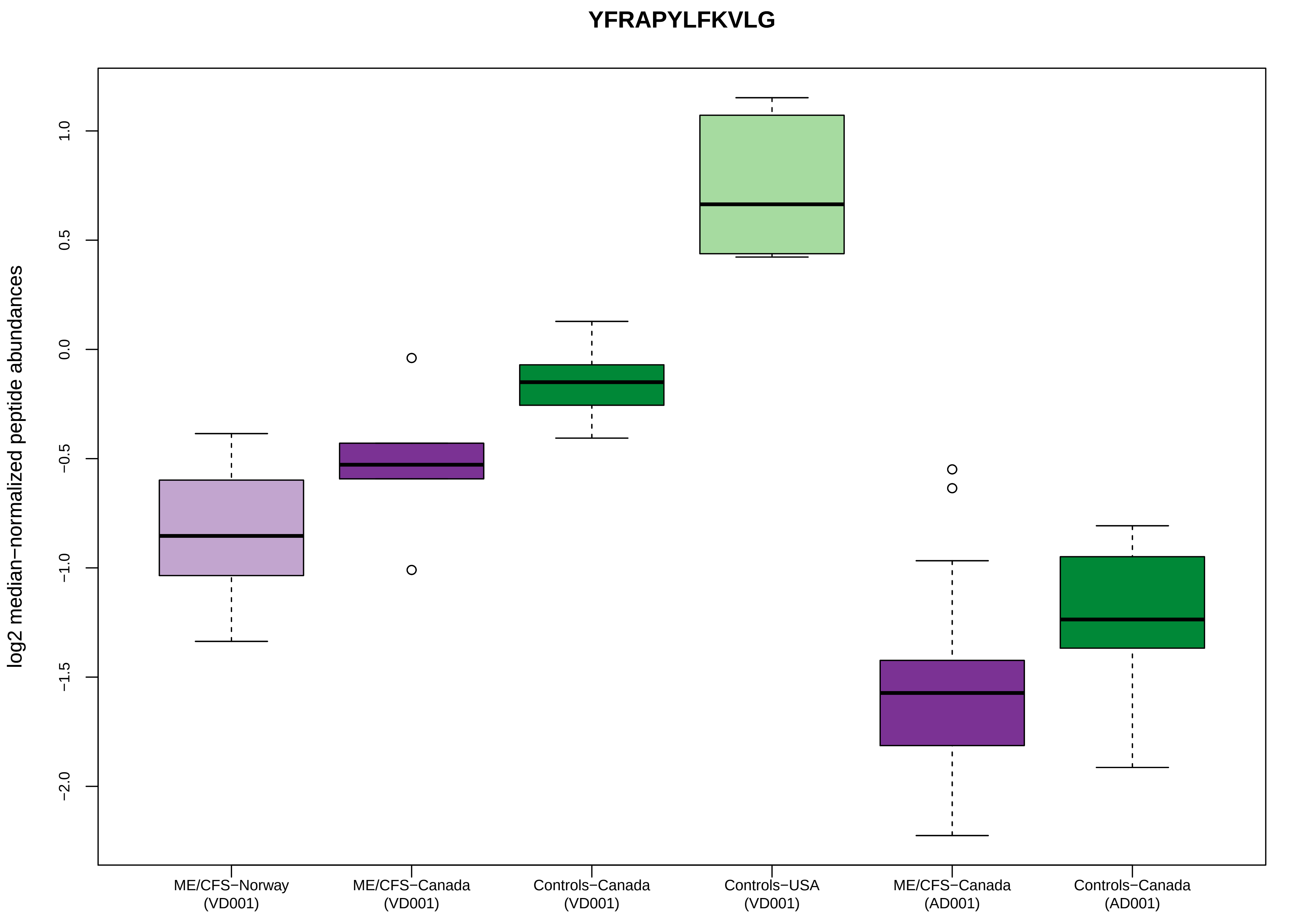

YFRAPYLFKVLG
log2 median−normalized peptide abundances
1.0
0.5
0.0
−0.5
−1.0
−1.5
−2.0
ME/CFS−Norway (VD001)
ME/CFS−Canada (VD001)
Controls−Canada (VD001)
Controls−USA (VD001)
ME/CFS−Canada (AD001)
Controls−Canada (AD001)

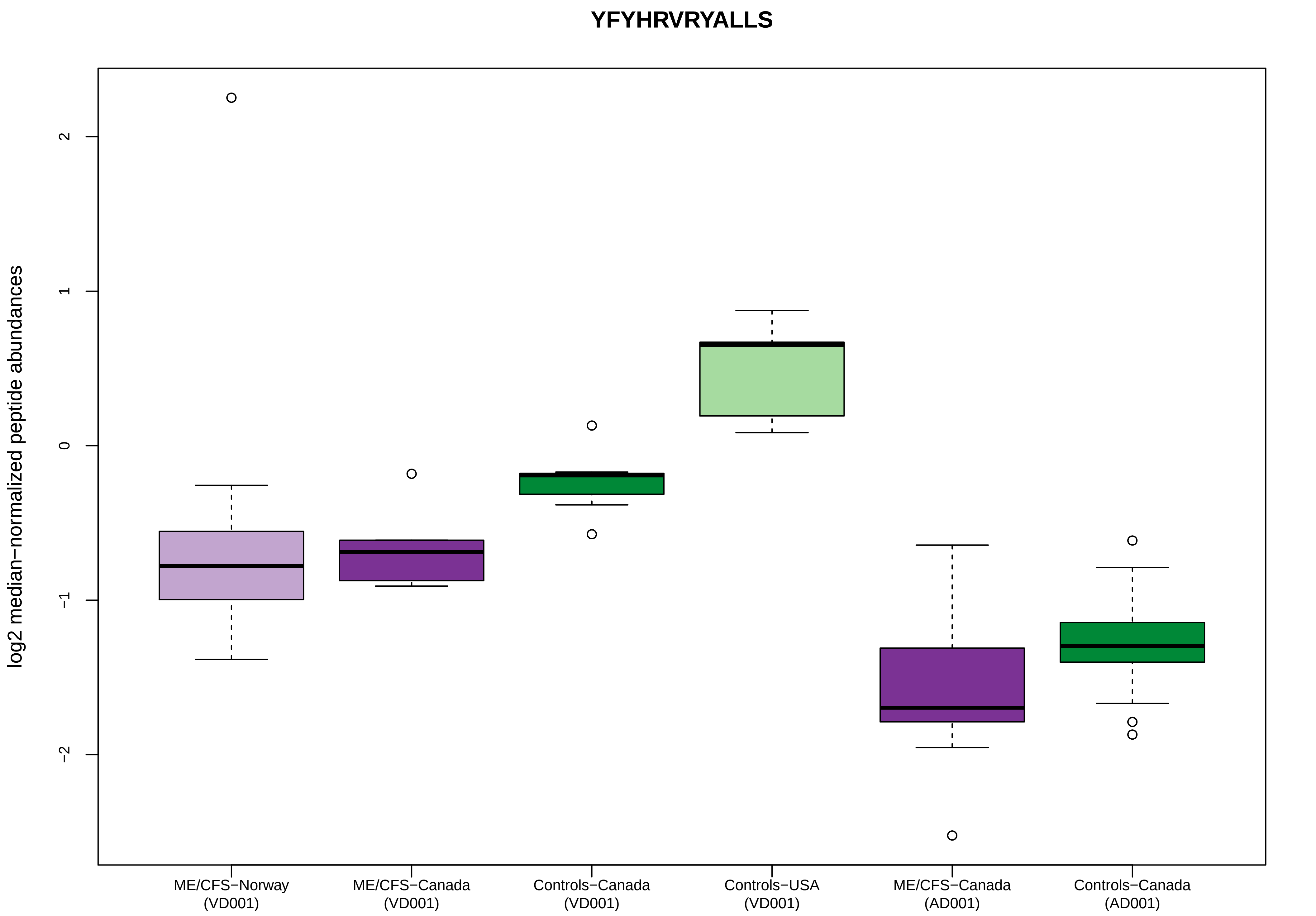
YFYHRVRYALLS
log2 median−normalized peptide abundances
2
1
0
−1
−2
ME/CFS−Norway (VD001)
ME/CFS−Canada (VD001)
Controls−Canada (VD001)
Controls−USA (VD001)
ME/CFS−Canada (AD001)
Controls−Canada (AD001)

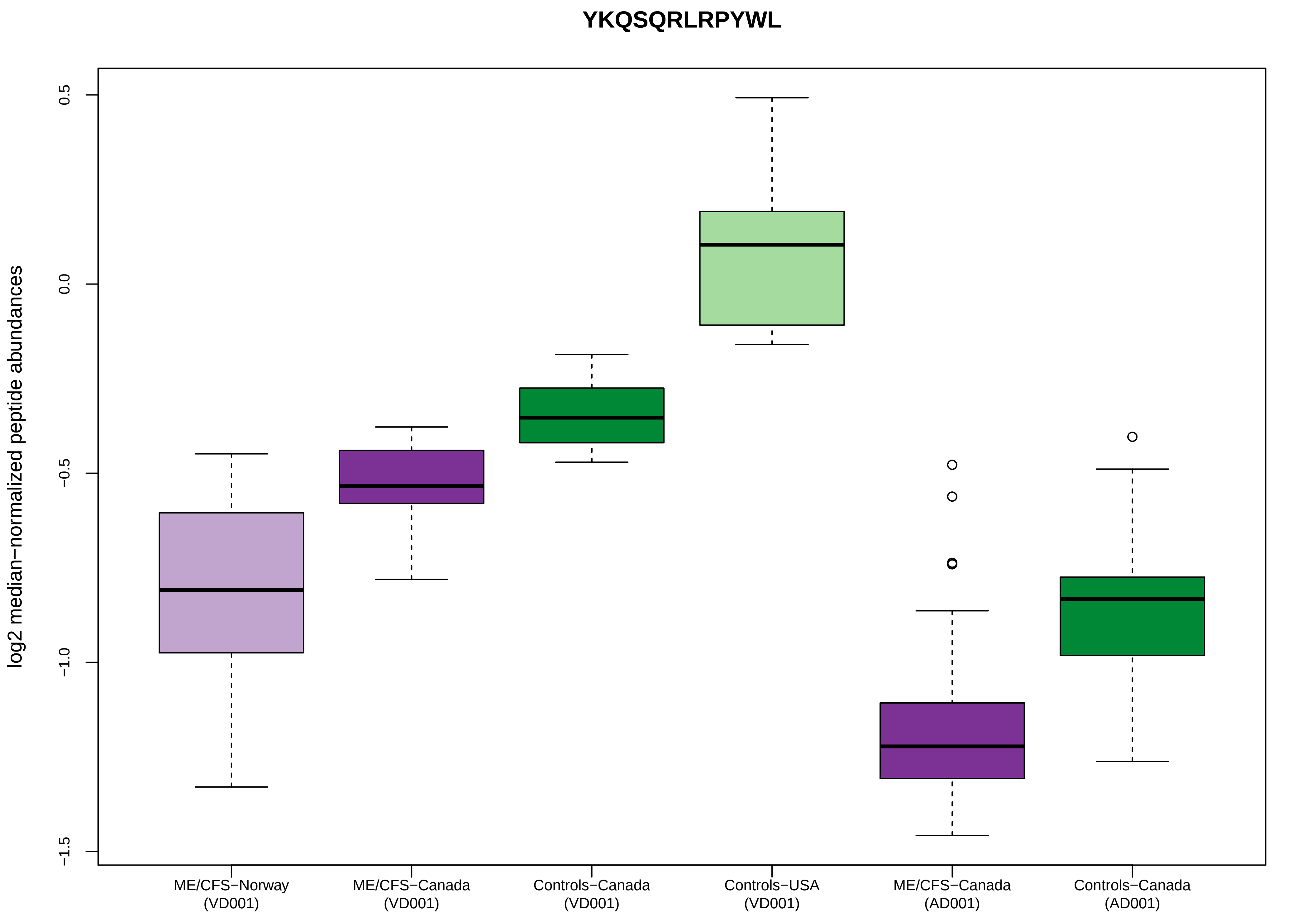
YKQSQRLRPYWL
log2 median−normalized peptide abundances
0.5
0.0
−0.5
−1.0
−1.5
ME/CFS−Norway (VD001)
ME/CFS−Canada (VD001)
Controls−Canada (VD001)
Controls−USA (VD001)
ME/CFS−Canada (AD001)
Controls−Canada (AD001)

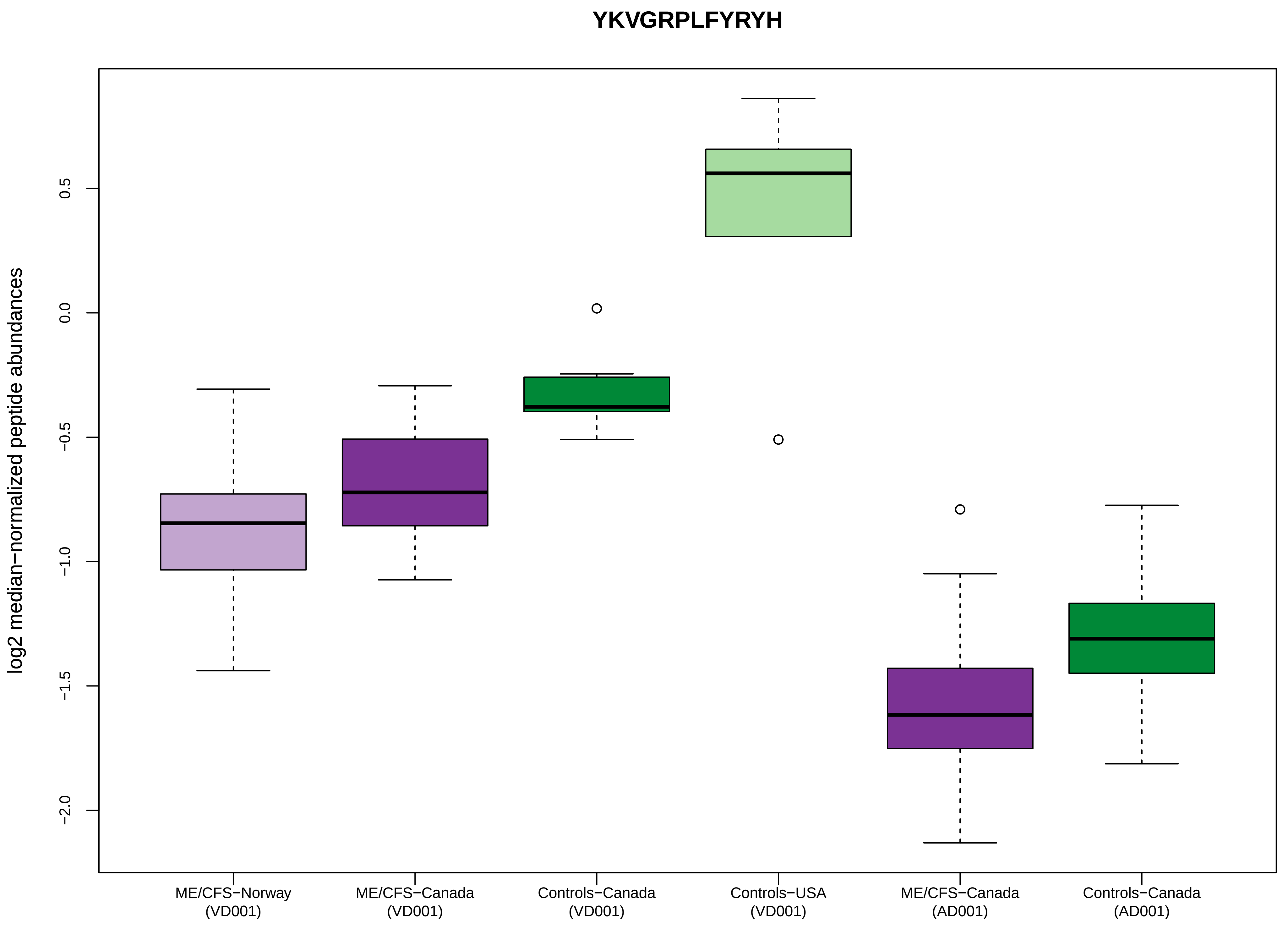
YKVGRPLFYRYH
log2 median−normalized peptide abundances
0.5
0.0
−0.5
−1.0
−1.5
−2.0
ME/CFS−Norway (VD001)
ME/CFS−Canada (VD001)
Controls−Canada (VD001)
Controls−USA (VD001)
ME/CFS−Canada (AD001)
Controls−Canada (AD001)

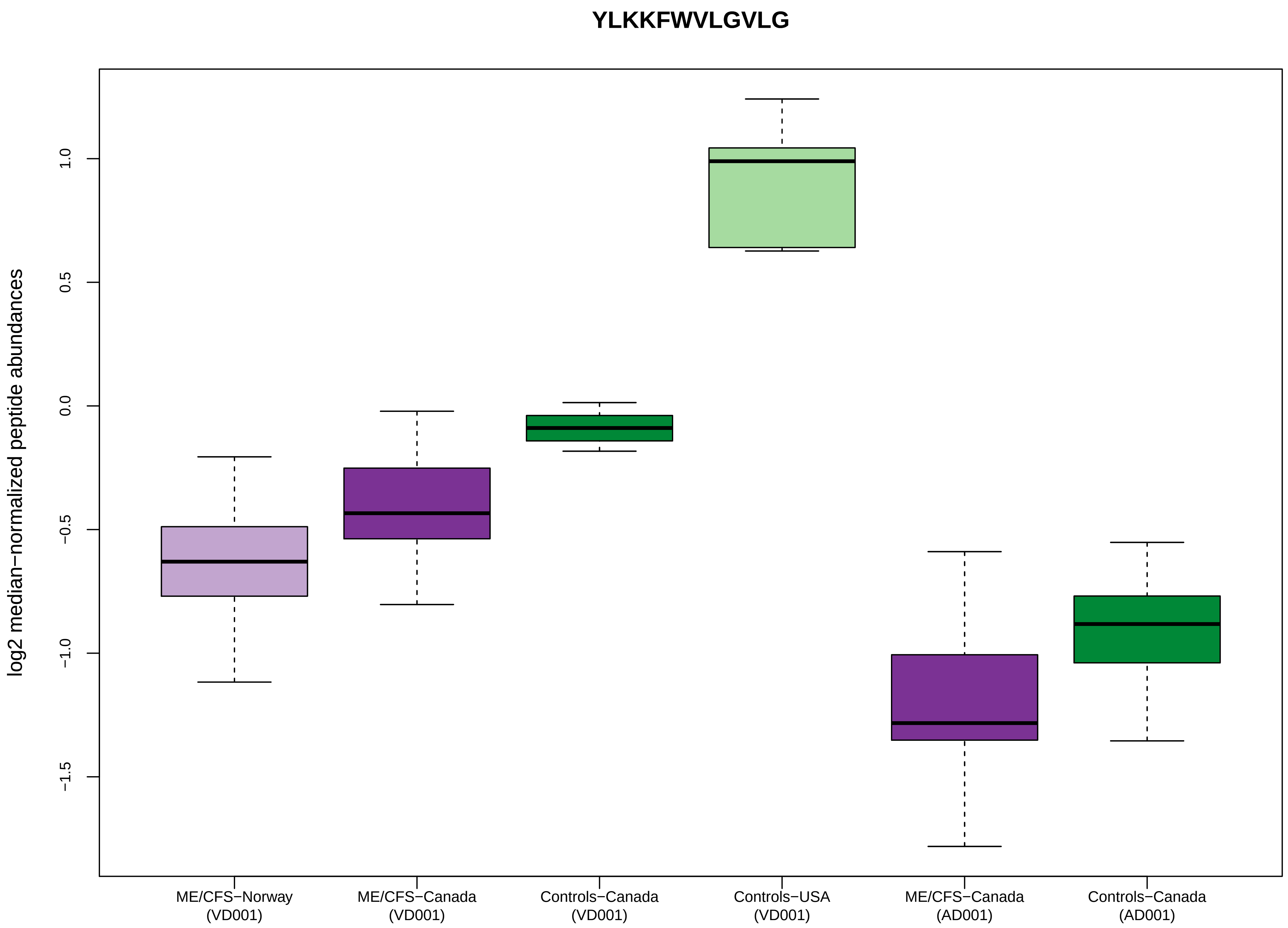
YLKKFWVLGVLG
log2 median−normalized peptide abundances
1.0
0.5
0.0
−0.5
−1.0
−1.5
ME/CFS−Norway (VD001)
ME/CFS−Canada (VD001)
Controls−Canada (VD001)
Controls−USA (VD001)
ME/CFS−Canada (AD001)
Controls−Canada (AD001)

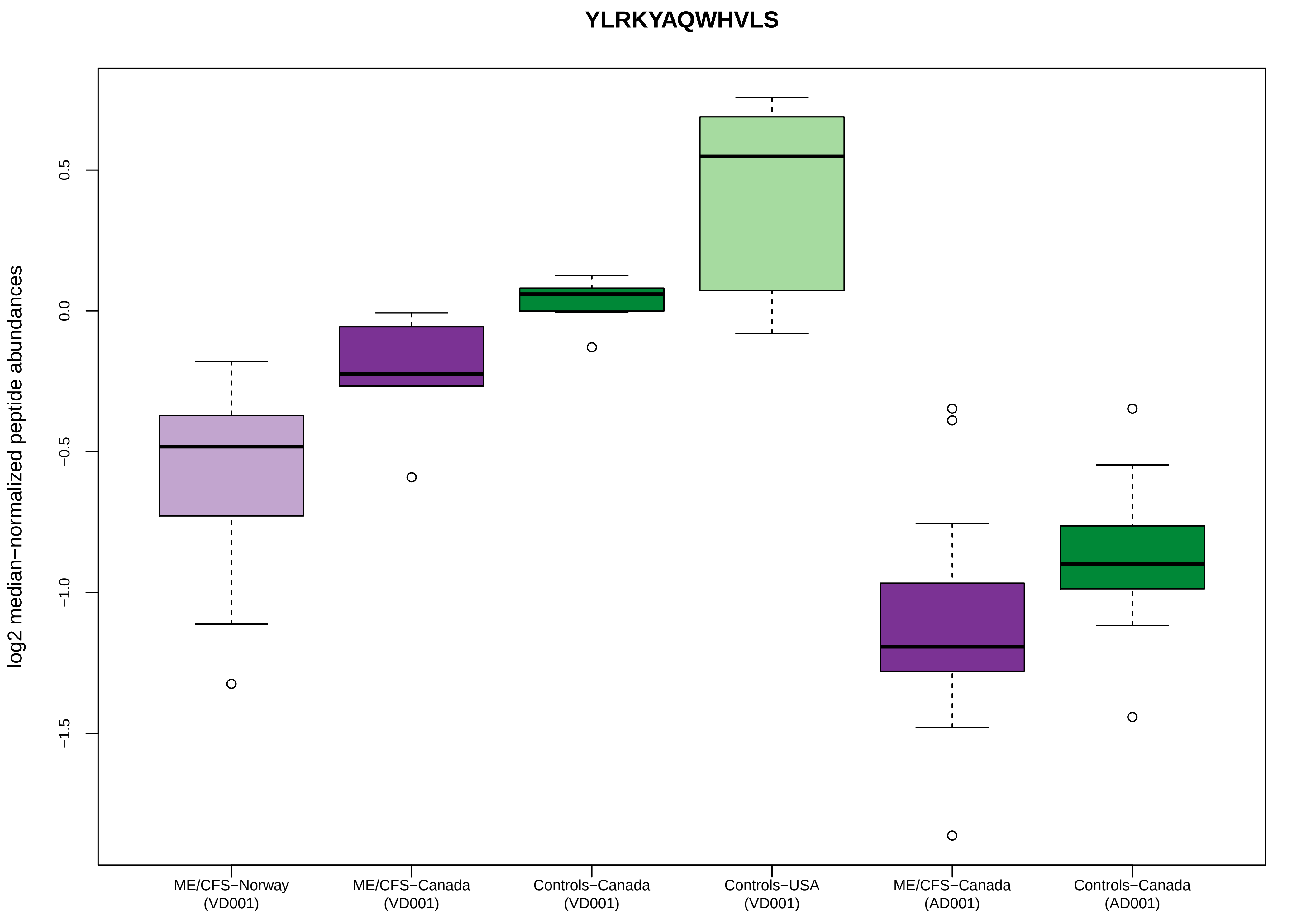

YLRKYAQWHVLS
log2 median−normalized peptide abundances
0.5
0.0
−0.5
−1.0
−1.5
ME/CFS−Norway (VD001)
ME/CFS−Canada (VD001)
Controls−Canada (VD001)
Controls−USA (VD001)
ME/CFS−Canada (AD001)
Controls−Canada (AD001)

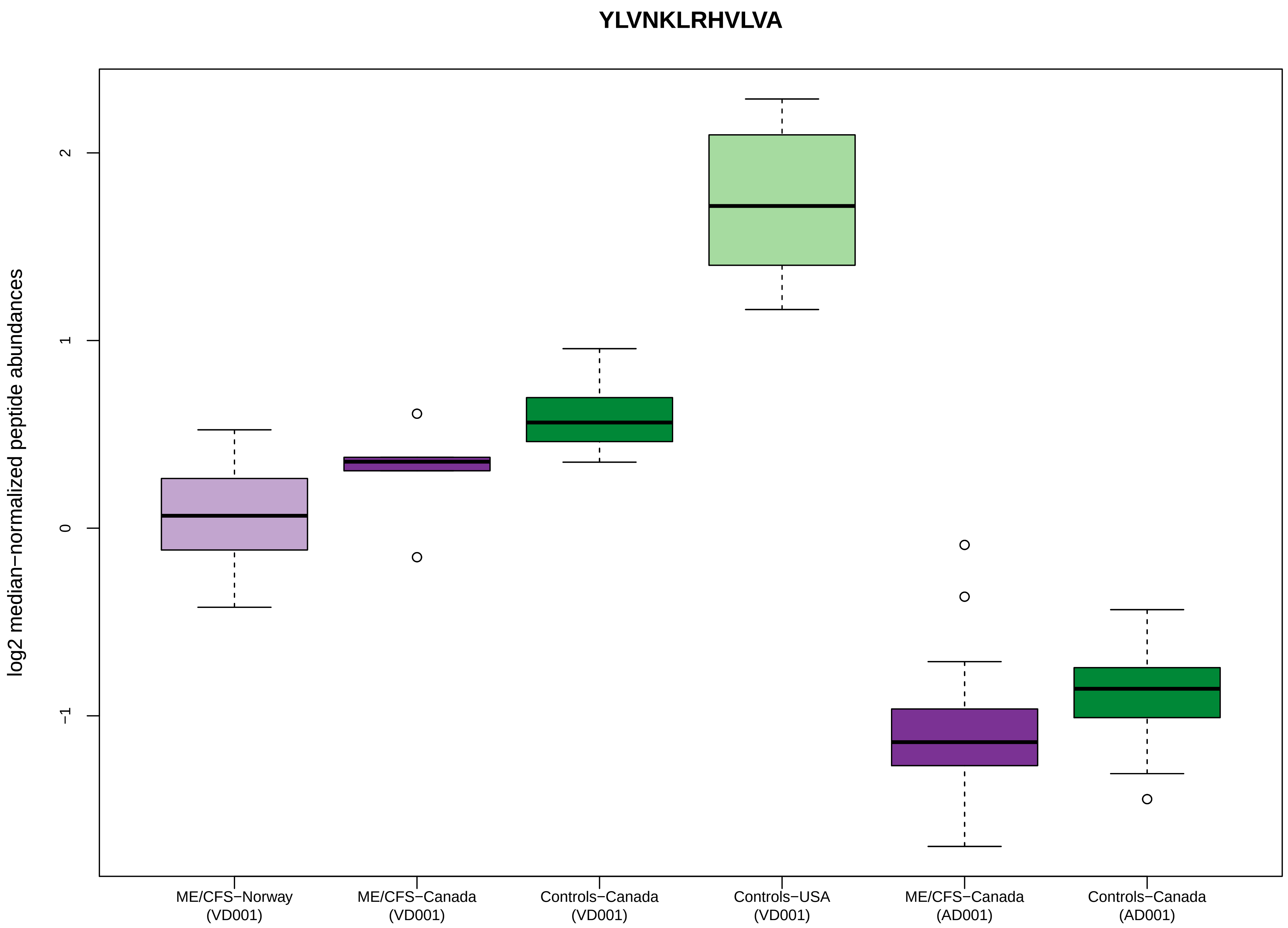
YLVNKLRHVLVA
log2 median−normalized peptide abundances
2
1
0
−1
ME/CFS−Norway (VD001)
ME/CFS−Canada (VD001)
Controls−Canada (VD001)
Controls−USA (VD001)
ME/CFS−Canada (AD001)
Controls−Canada (AD001)

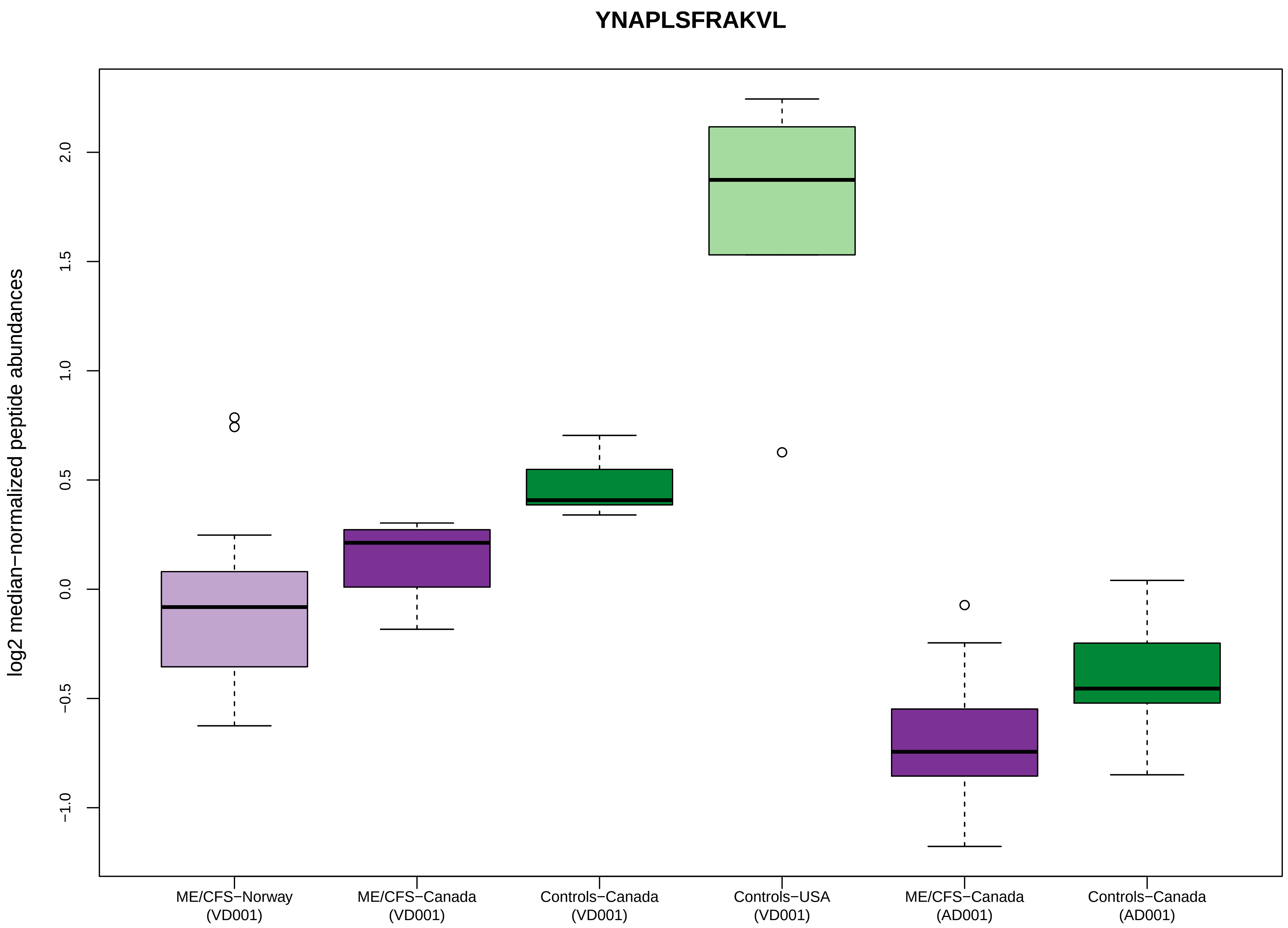

YNAPLSFRAKVL
log2 median−normalized peptide abundances
2.0
1.5
1.0
0.5
0.0
−0.5
−1.0
ME/CFS−Norway (VD001)
ME/CFS−Canada (VD001)
Controls−Canada (VD001)
Controls−USA (VD001)
ME/CFS−Canada (AD001)
Controls−Canada (AD001)

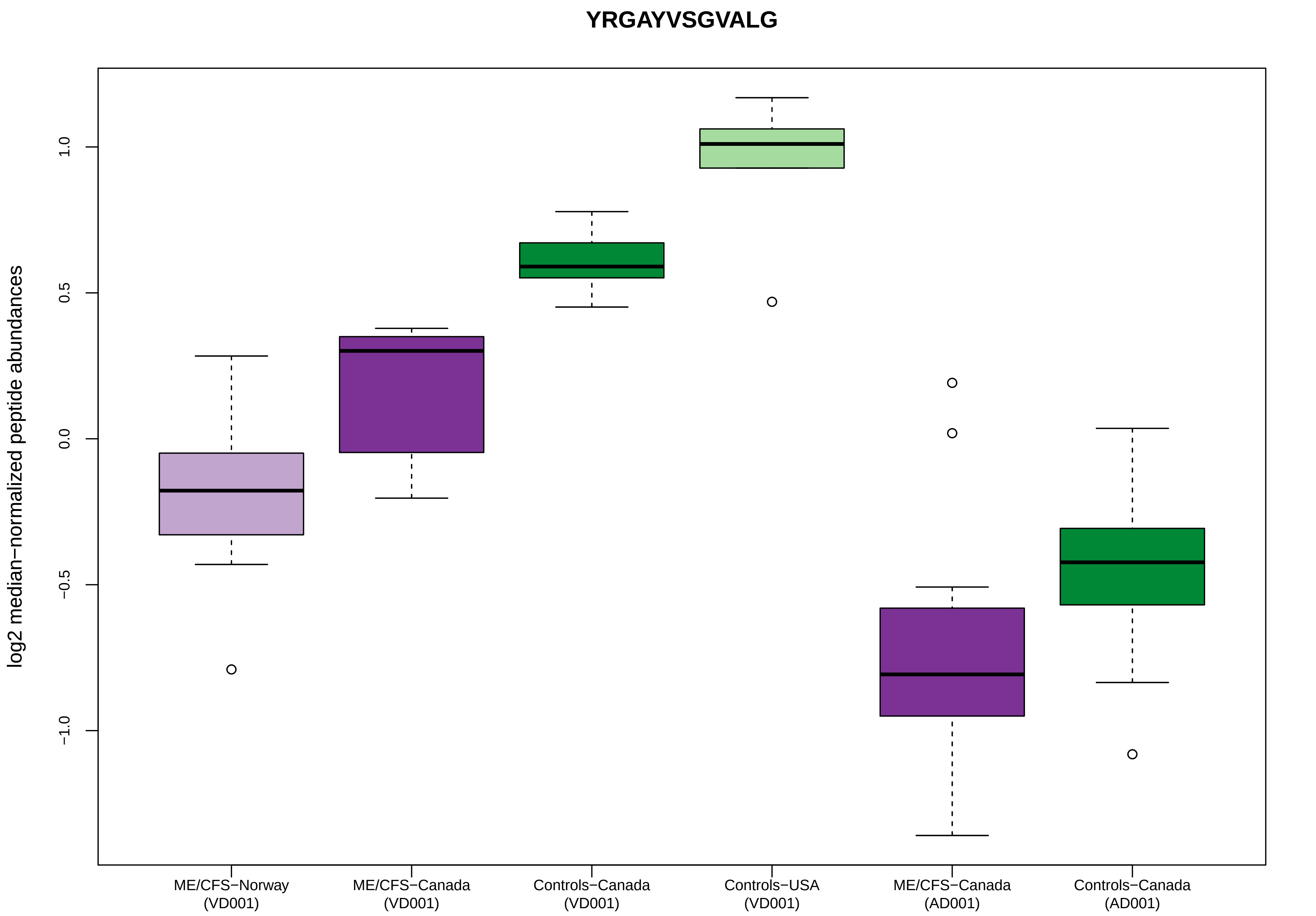

YRGAYVSGVALG
log2 median−normalized peptide abundances
1.0
0.5
0.0
−0.5
−1.0
ME/CFS−Norway (VD001)
ME/CFS−Canada (VD001)
Controls−Canada (VD001)
Controls−USA (VD001)
ME/CFS−Canada (AD001)
Controls−Canada (AD001)

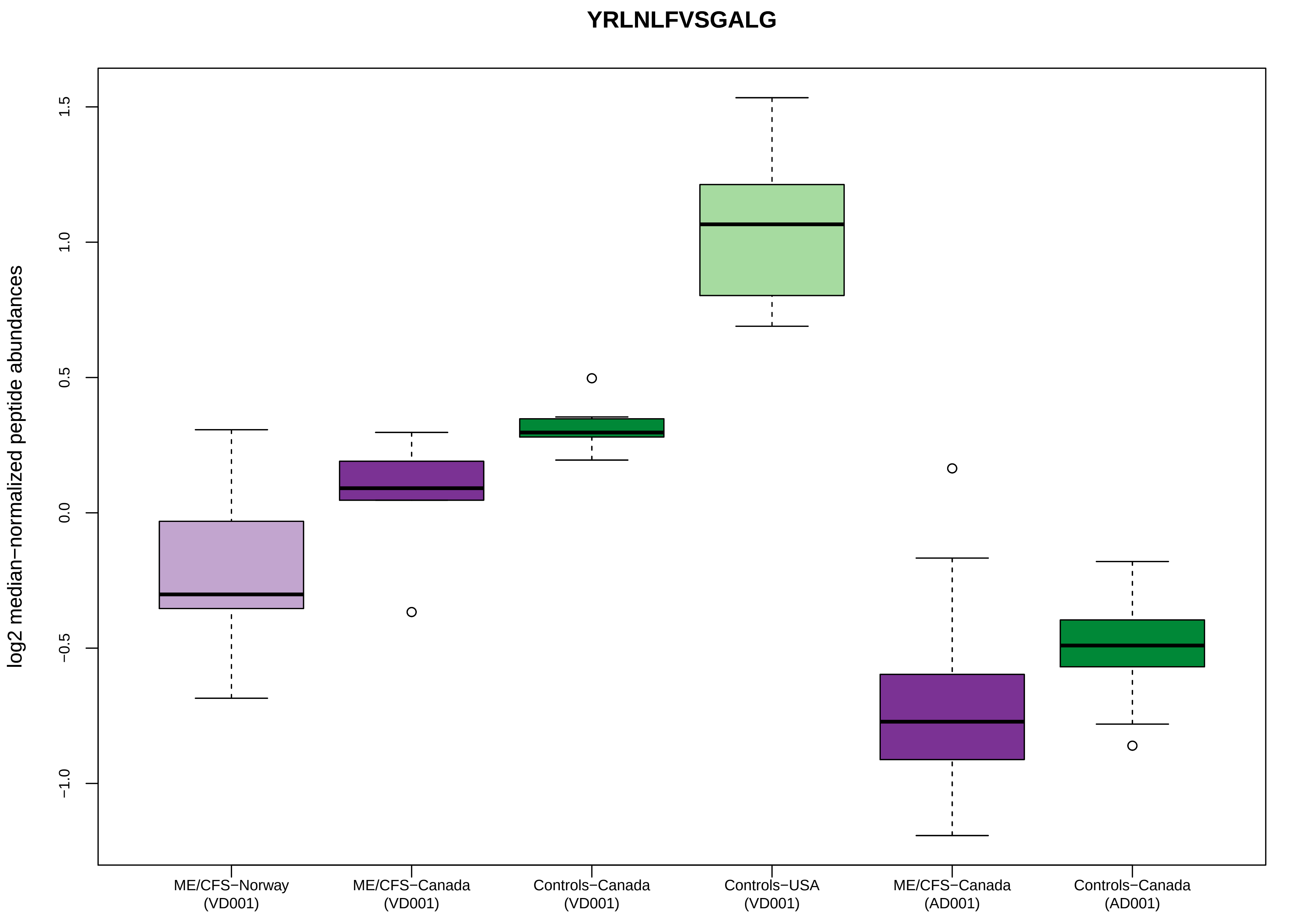
YRLNLFVSGALG
log2 median−normalized peptide abundances
1.5
1.0
0.5
0.0
−0.5
−1.0
ME/CFS−Norway (VD001)
ME/CFS−Canada (VD001)
Controls−Canada (VD001)
Controls−USA (VD001)
ME/CFS−Canada (AD001)
Controls−Canada (AD001)

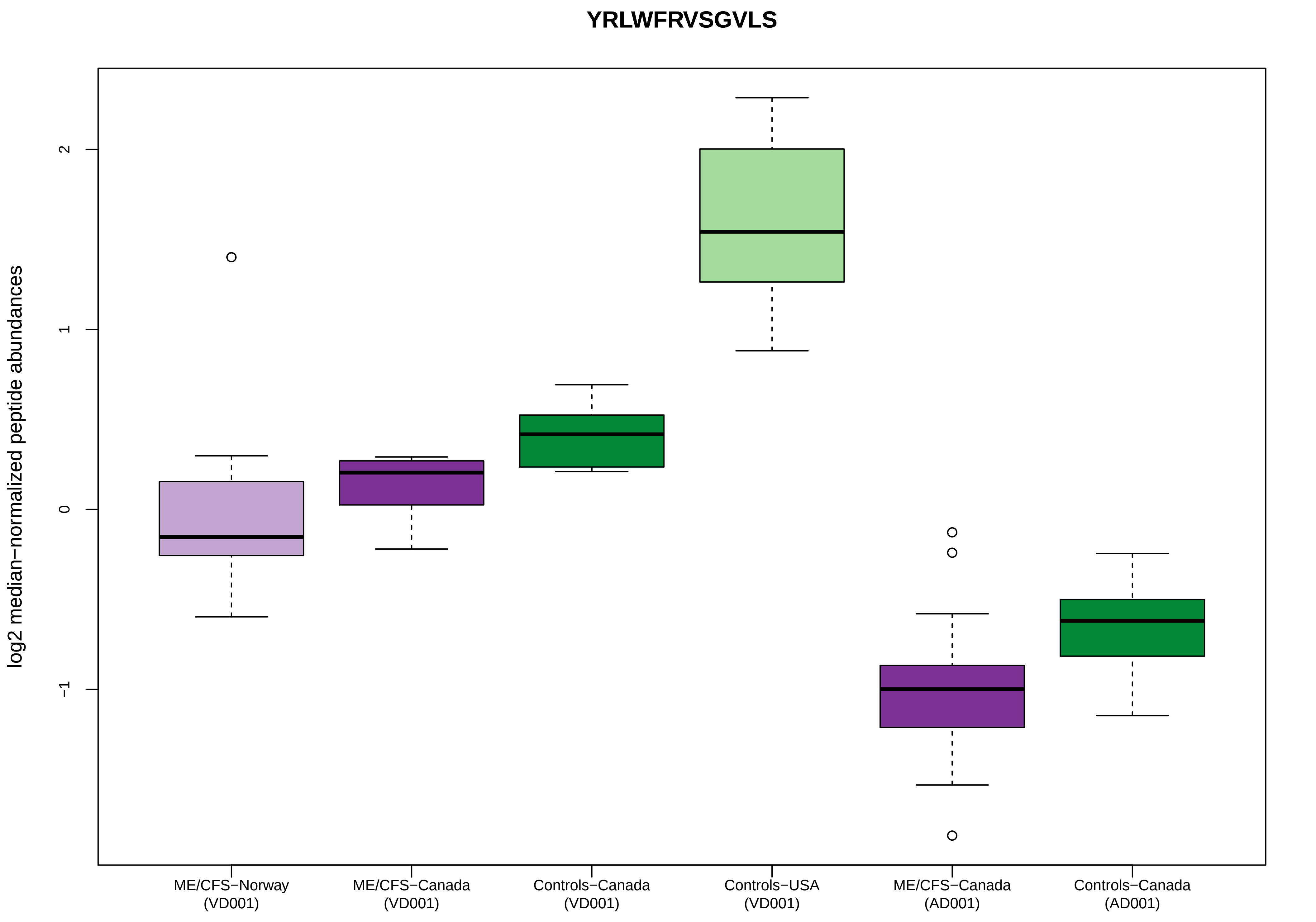
YRLWFRVSGVLS
log2 median−normalized peptide abundances
2
1
0
−1
ME/CFS−Norway (VD001)
ME/CFS−Canada (VD001)
Controls−Canada (VD001)
Controls−USA (VD001)
ME/CFS−Canada (AD001)
Controls−Canada (AD001)

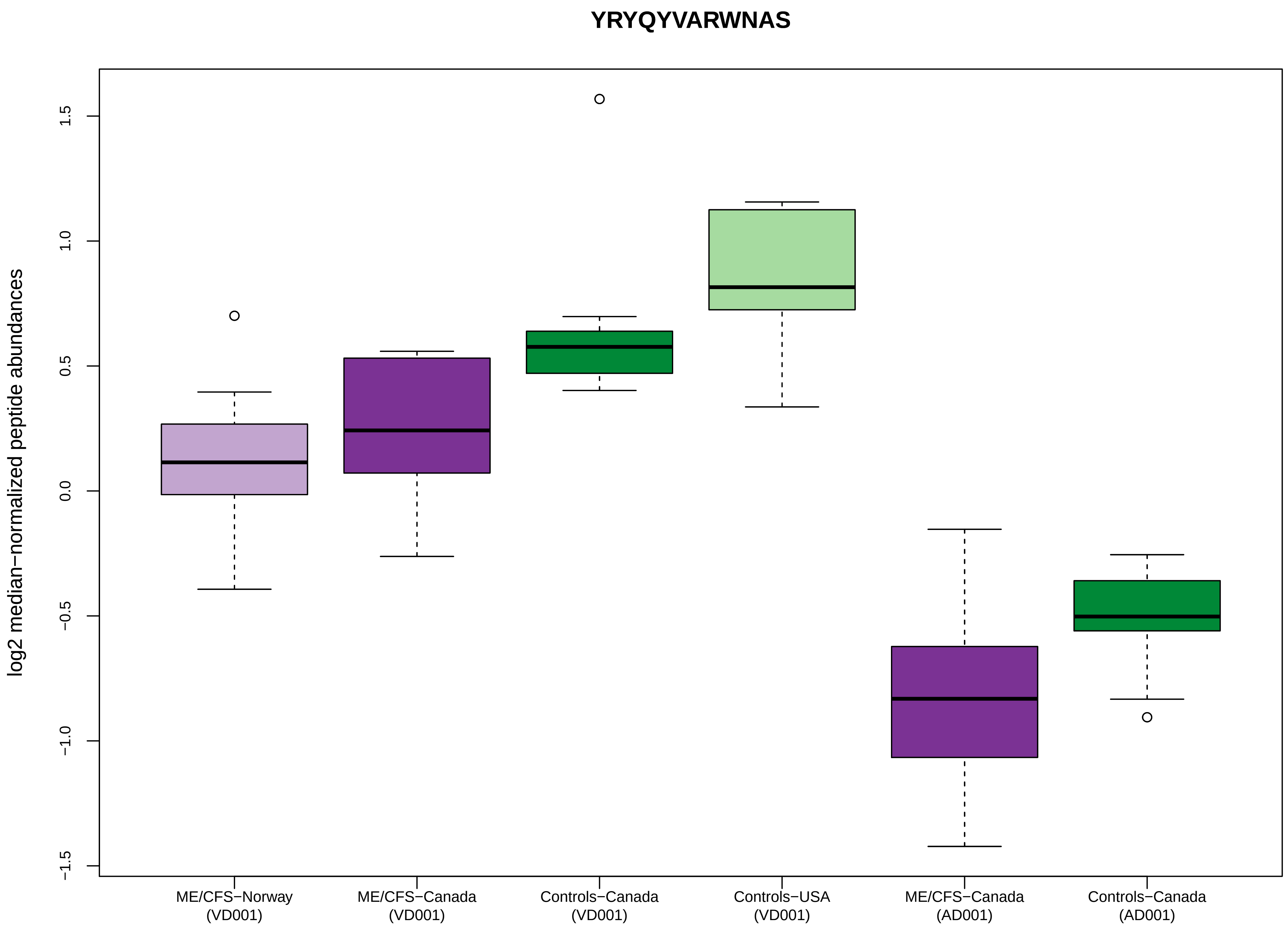
YRYQYVARWNAS
log2 median−normalized peptide abundances
1.5
1.0
0.5
0.0
−0.5
−1.0
−1.5
ME/CFS−Norway (VD001)
ME/CFS−Canada (VD001)
Controls−Canada (VD001)
Controls−USA (VD001)
ME/CFS−Canada (AD001)
Controls−Canada (AD001)

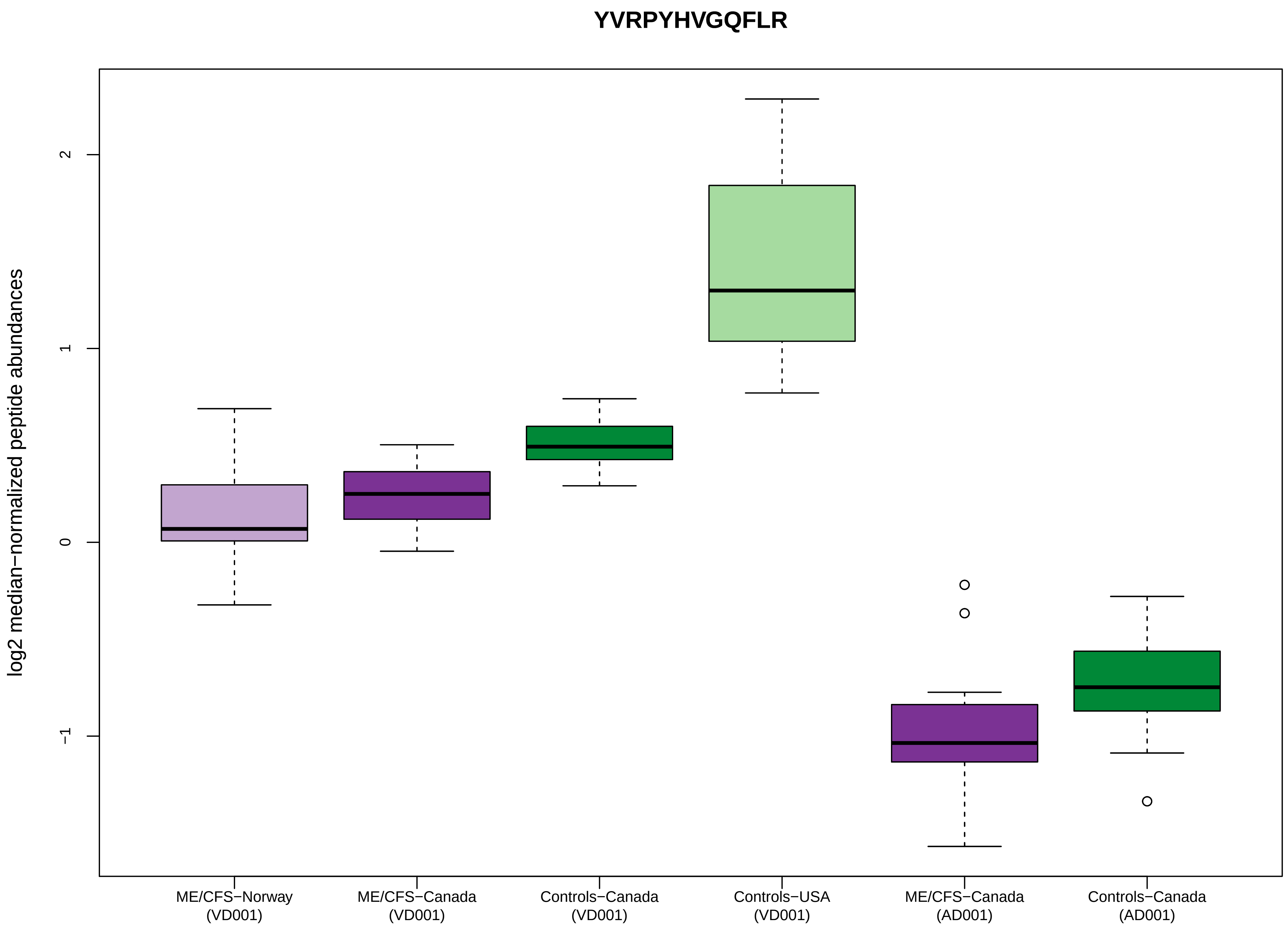

YVRPYHVGQFLR
log2 median−normalized peptide abundances
2
1
0
−1
ME/CFS−Norway (VD001)
ME/CFS−Canada (VD001)
Controls−Canada (VD001)
Controls−USA (VD001)
ME/CFS−Canada (AD001)
Controls−Canada (AD001)

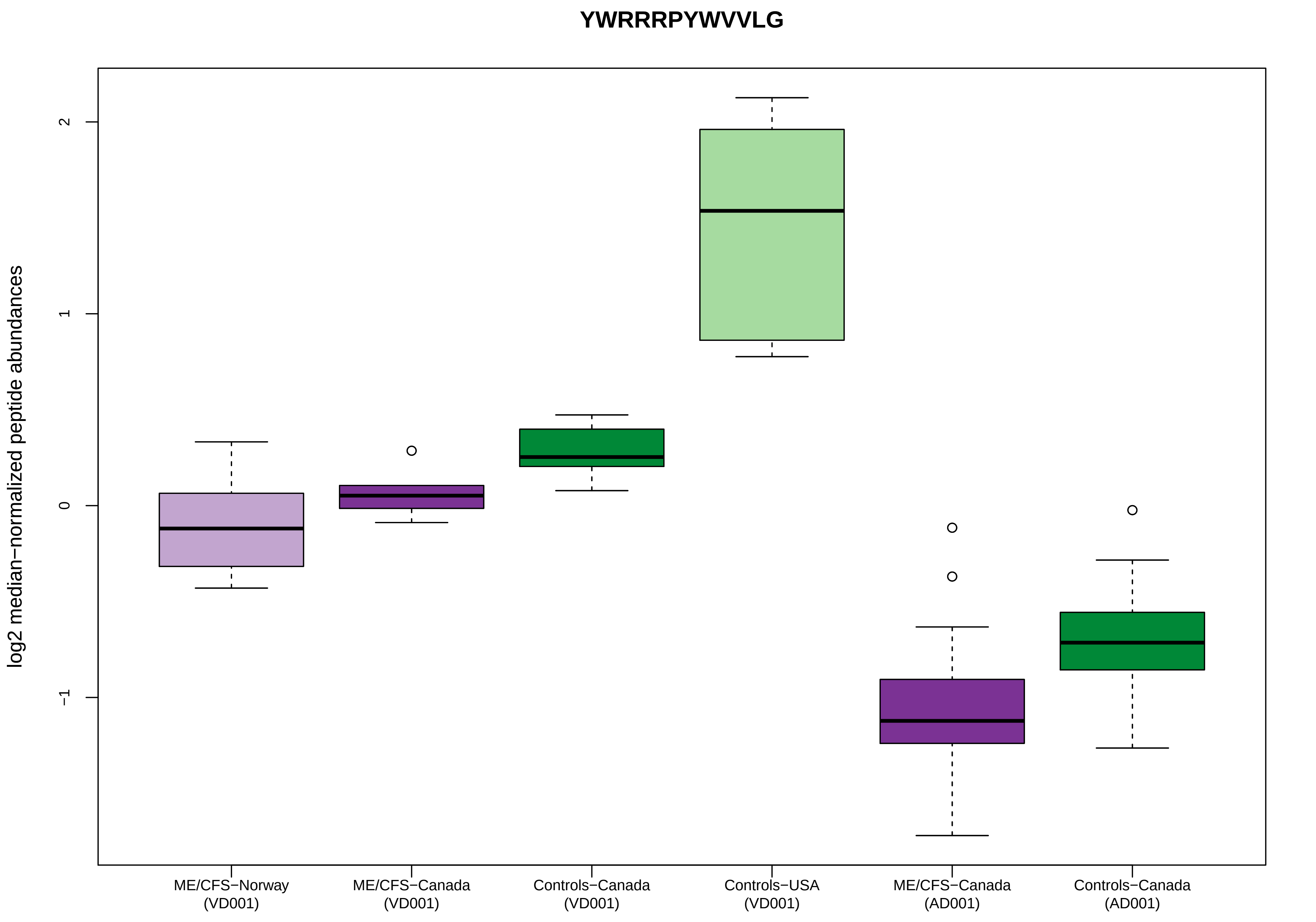
YWRRRPYWVVLG
log2 median−normalized peptide abundances
2
1
0
−1
ME/CFS−Norway (VD001)
ME/CFS−Canada (VD001)
Controls−Canada (VD001)
Controls−USA (VD001)
ME/CFS−Canada (AD001)
Controls−Canada (AD001)

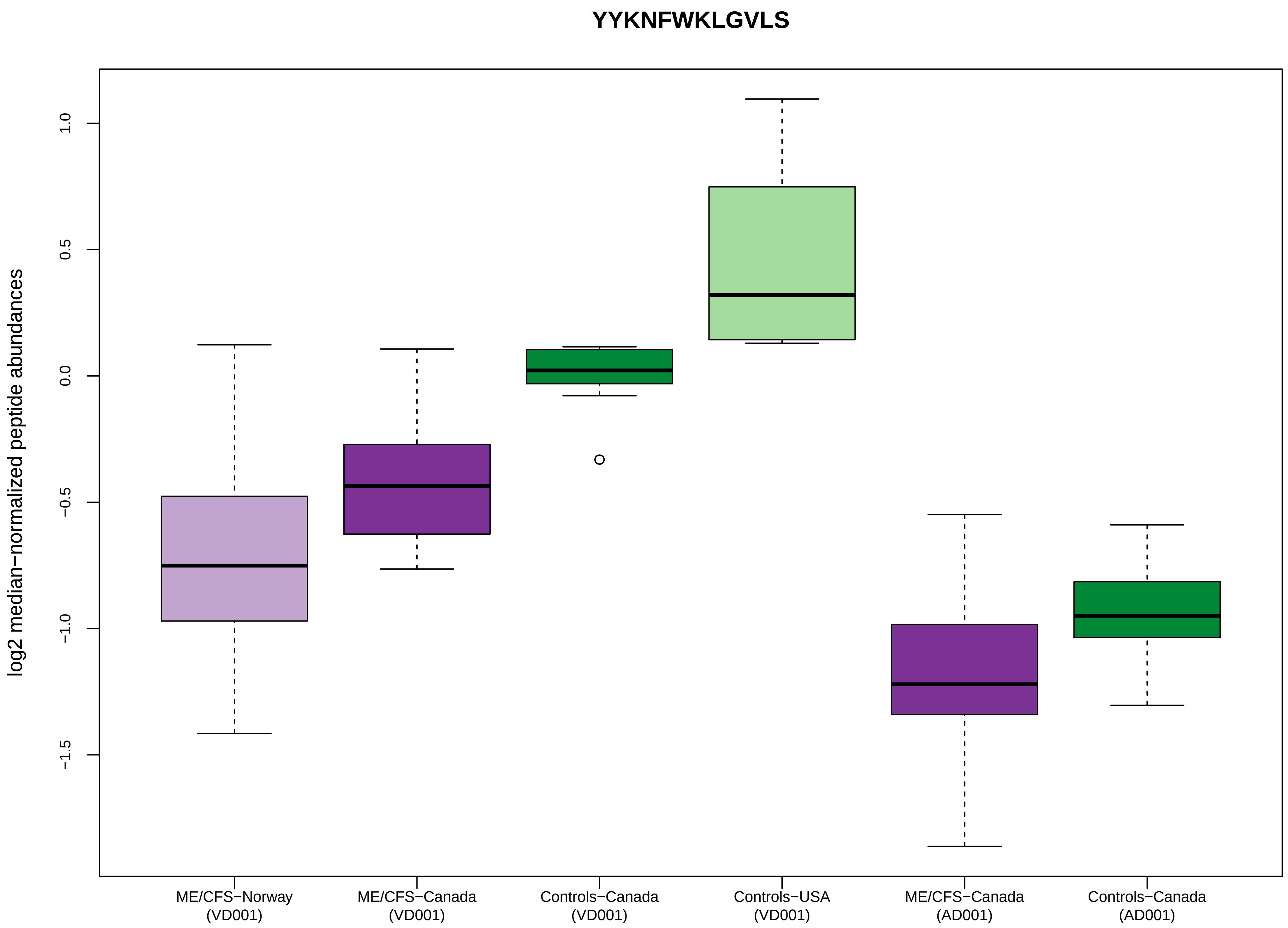
YYKNFWKLGVLS
log2 median−normalized peptide abundances
1.0
0.5
0.0
−0.5
−1.0
−1.5
ME/CFS−Norway (VD001)
ME/CFS−Canada (VD001)
Controls−Canada (VD001)
Controls−USA (VD001)
ME/CFS−Canada (AD001)
Controls−Canada (AD001)

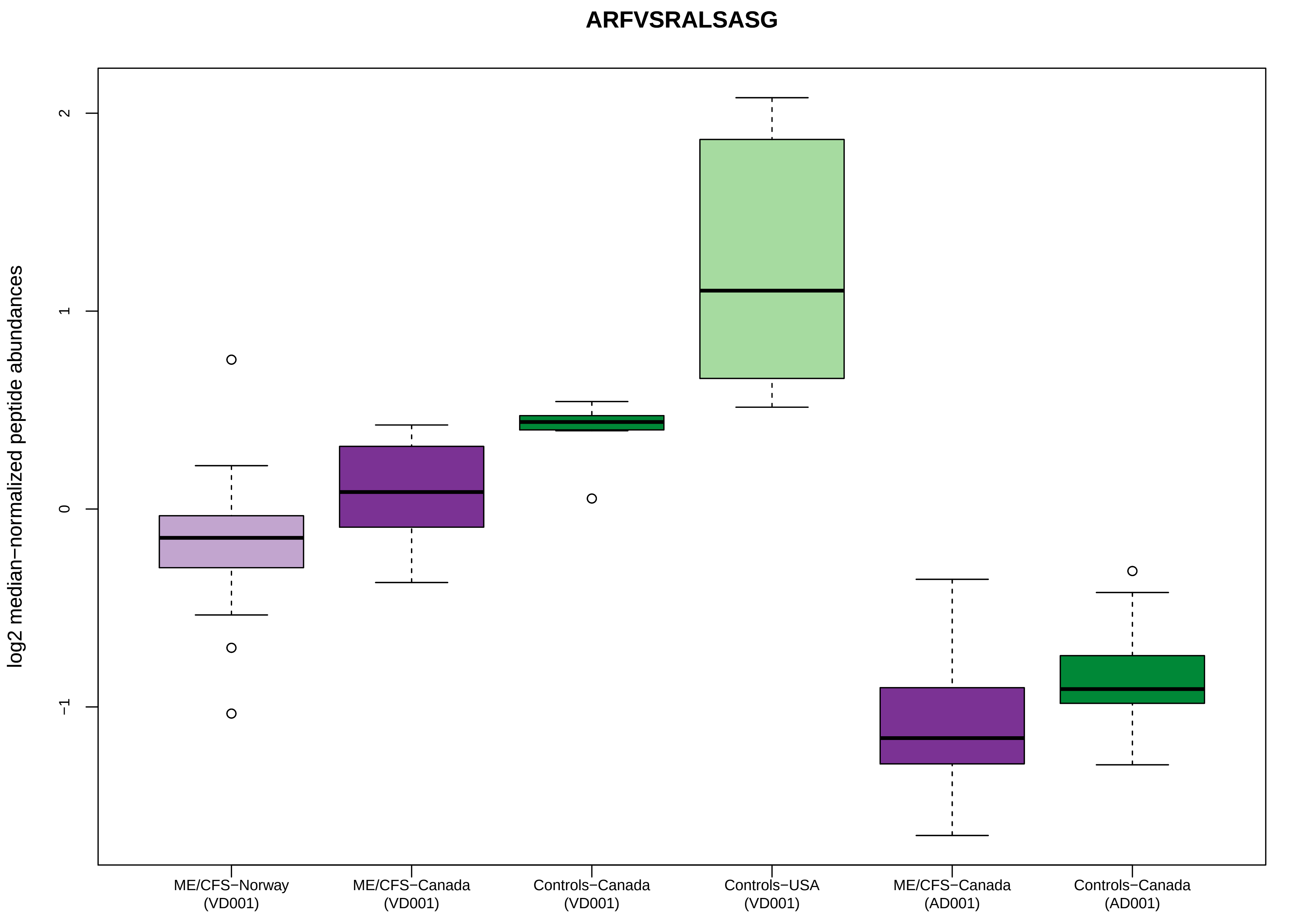
ARFVSRALSASG
log2 median−normalized peptide abundances
2
1
0
−1
ME/CFS−Norway (VD001)
ME/CFS−Canada (VD001)
Controls−Canada (VD001)
Controls−USA (VD001)
ME/CFS−Canada (AD001)
Controls−Canada (AD001)

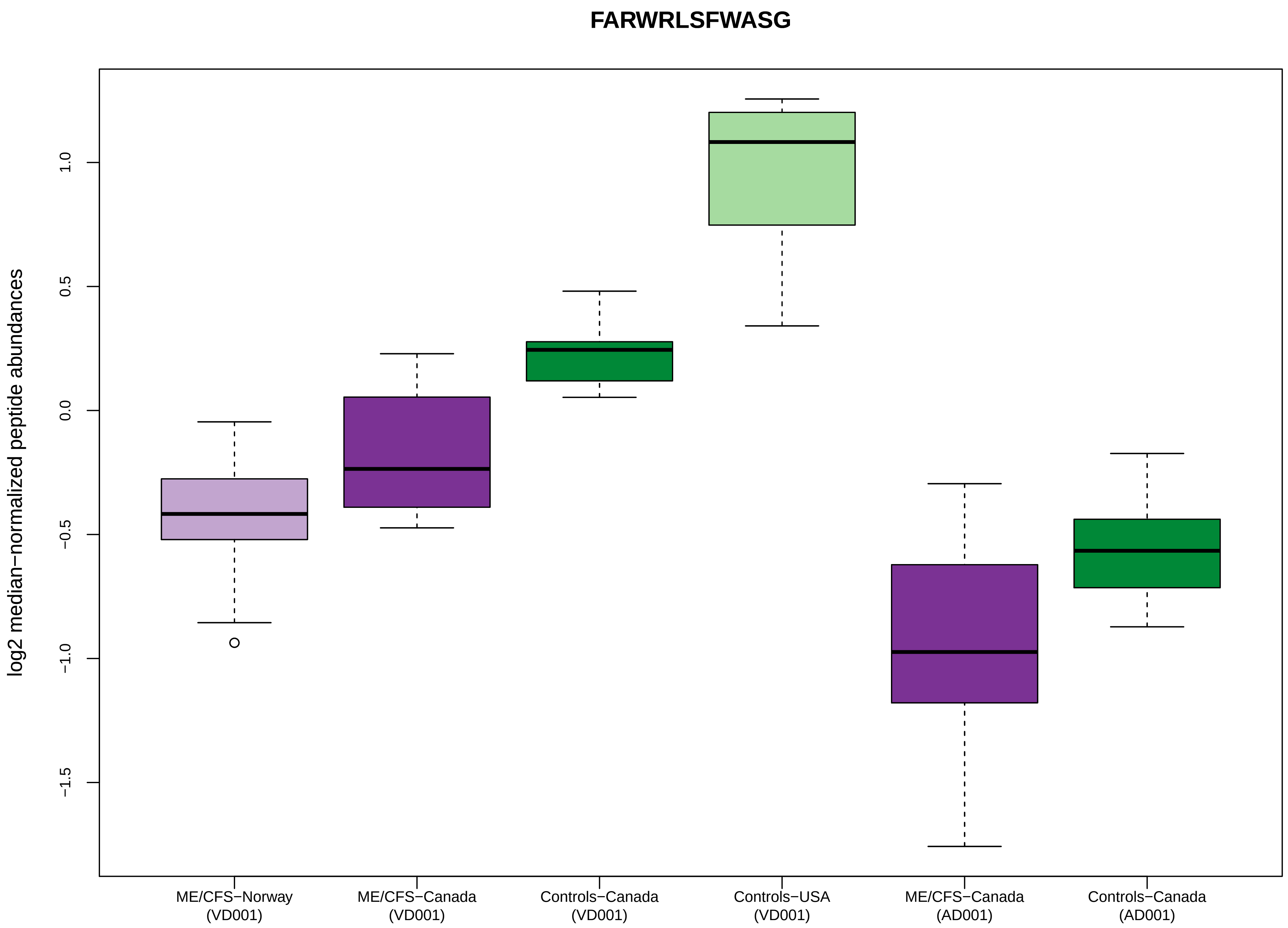

FARWRLSFWASG
log2 median−normalized peptide abundances
1.0
0.5
0.0
−0.5
−1.0
−1.5
ME/CFS−Norway (VD001)
ME/CFS−Canada (VD001)
Controls−Canada (VD001)
Controls−USA (VD001)
ME/CFS−Canada (AD001)
Controls−Canada (AD001)

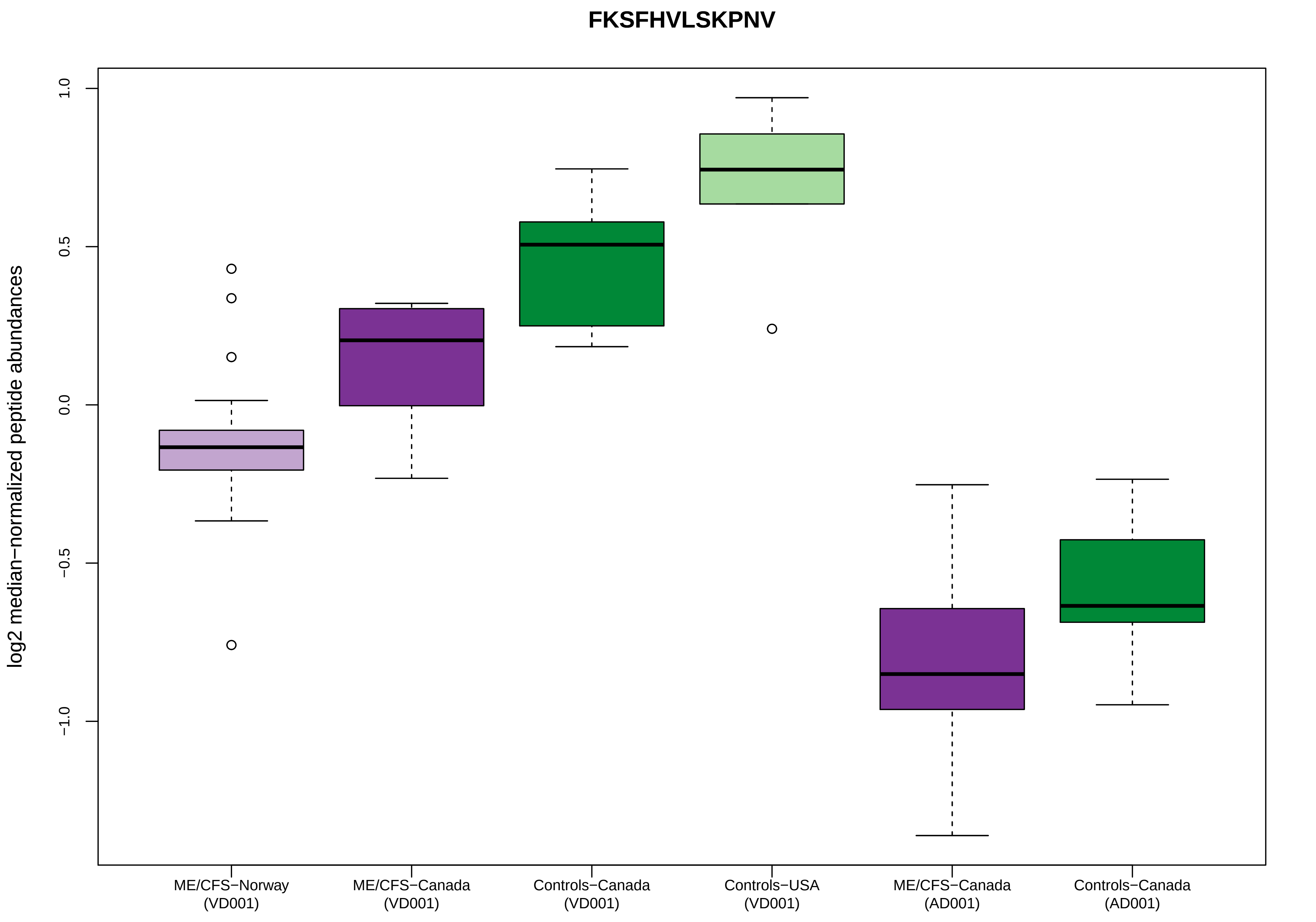
FKSFHVLSKPNV
log2 median−normalized peptide abundances
1.0
0.5
0.0
−0.5
−1.0
ME/CFS−Norway (VD001)
ME/CFS−Canada (VD001)
Controls−Canada (VD001)
Controls−USA (VD001)
ME/CFS−Canada (AD001)
Controls−Canada (AD001)

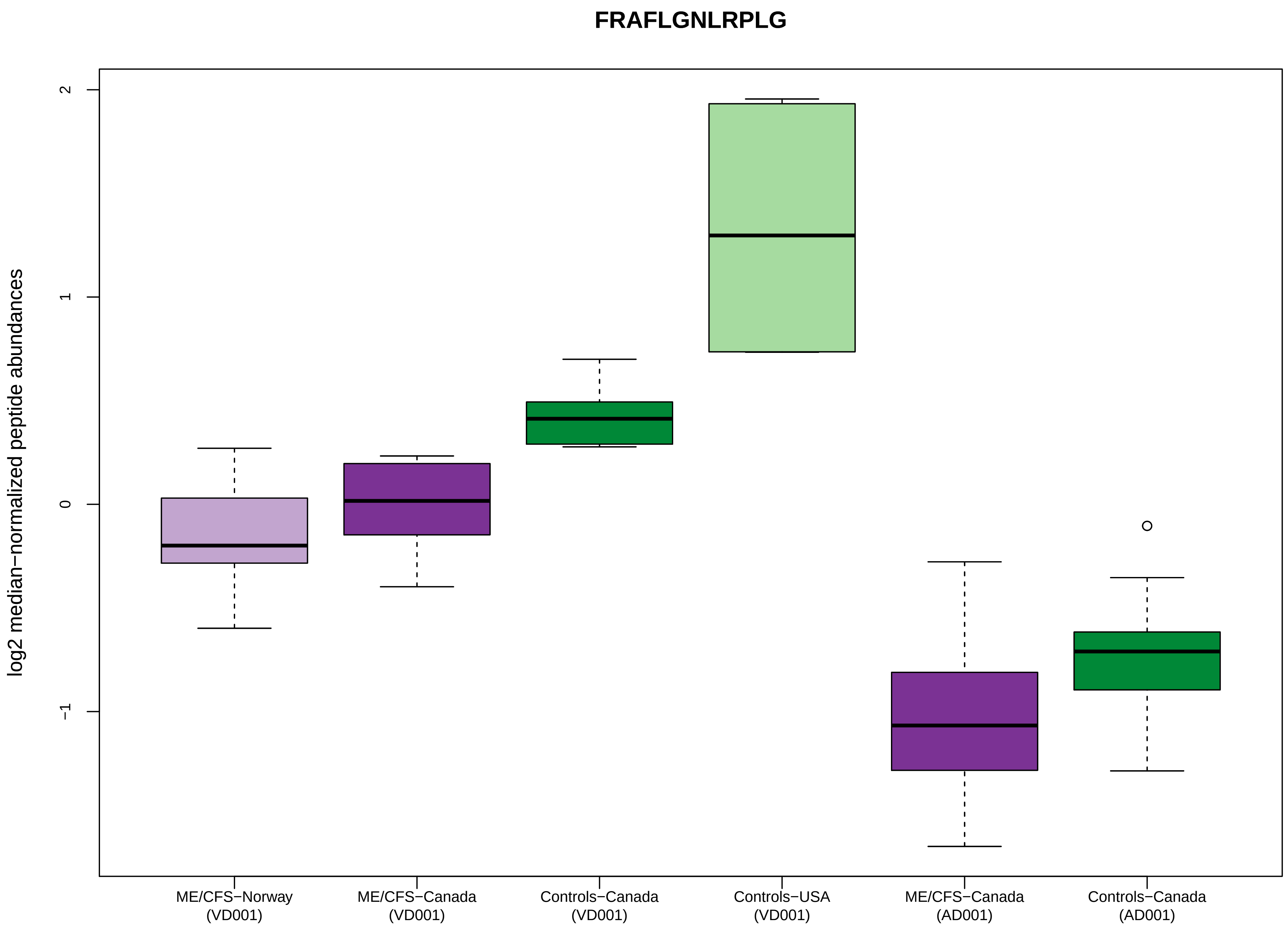
FRAFLGNLRPLG
log2 median−normalized peptide abundances
2
1
0
−1
ME/CFS−Norway (VD001)
ME/CFS−Canada (VD001)
Controls−Canada (VD001)
Controls−USA (VD001)
ME/CFS−Canada (AD001)
Controls−Canada (AD001)

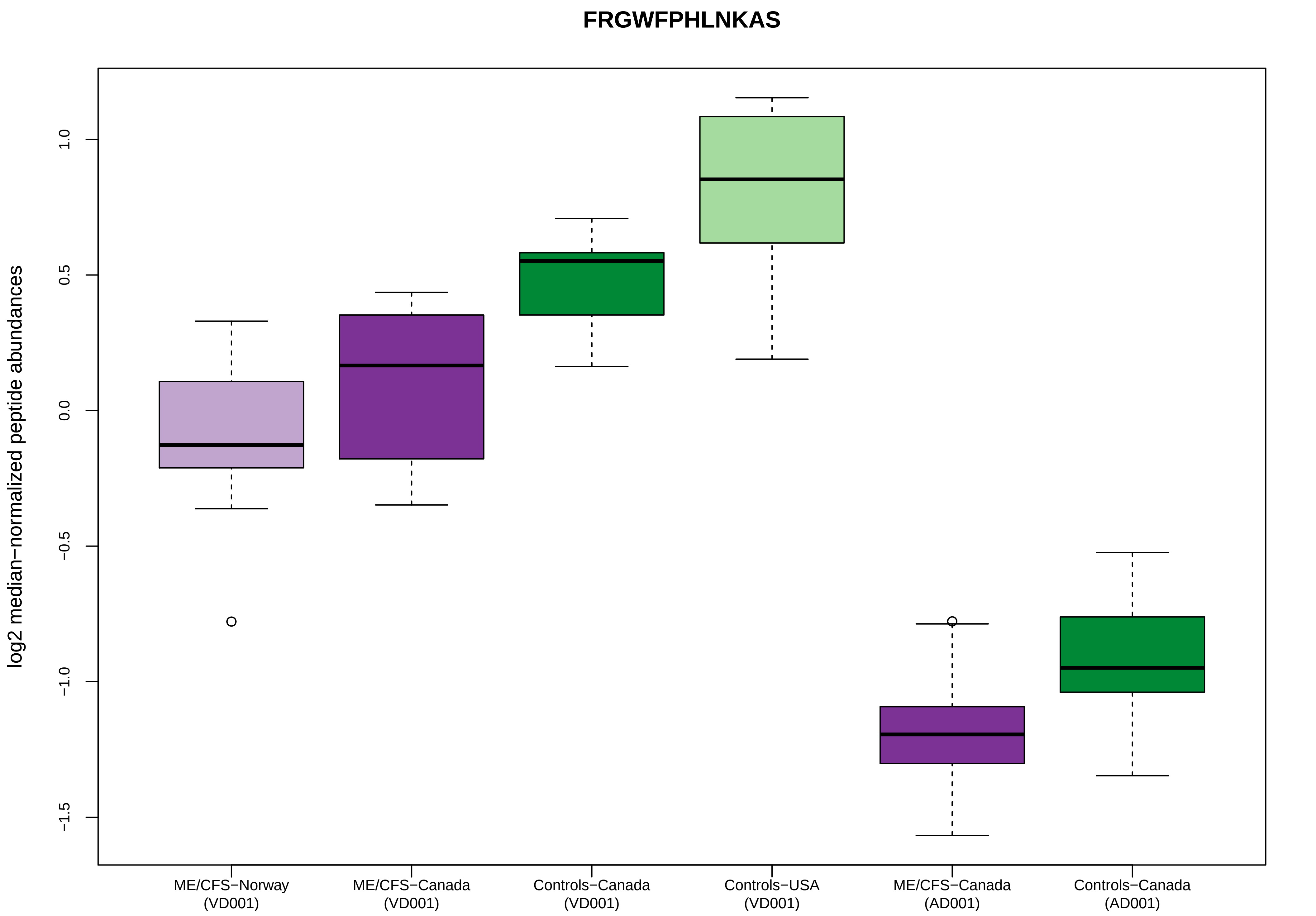
FRGWFPHLNKAS
log2 median−normalized peptide abundances
1.0
0.5
0.0
−0.5
−1.0
−1.5
ME/CFS−Norway (VD001)
ME/CFS−Canada (VD001)
Controls−Canada (VD001)
Controls−USA (VD001)
ME/CFS−Canada (AD001)
Controls−Canada (AD001)

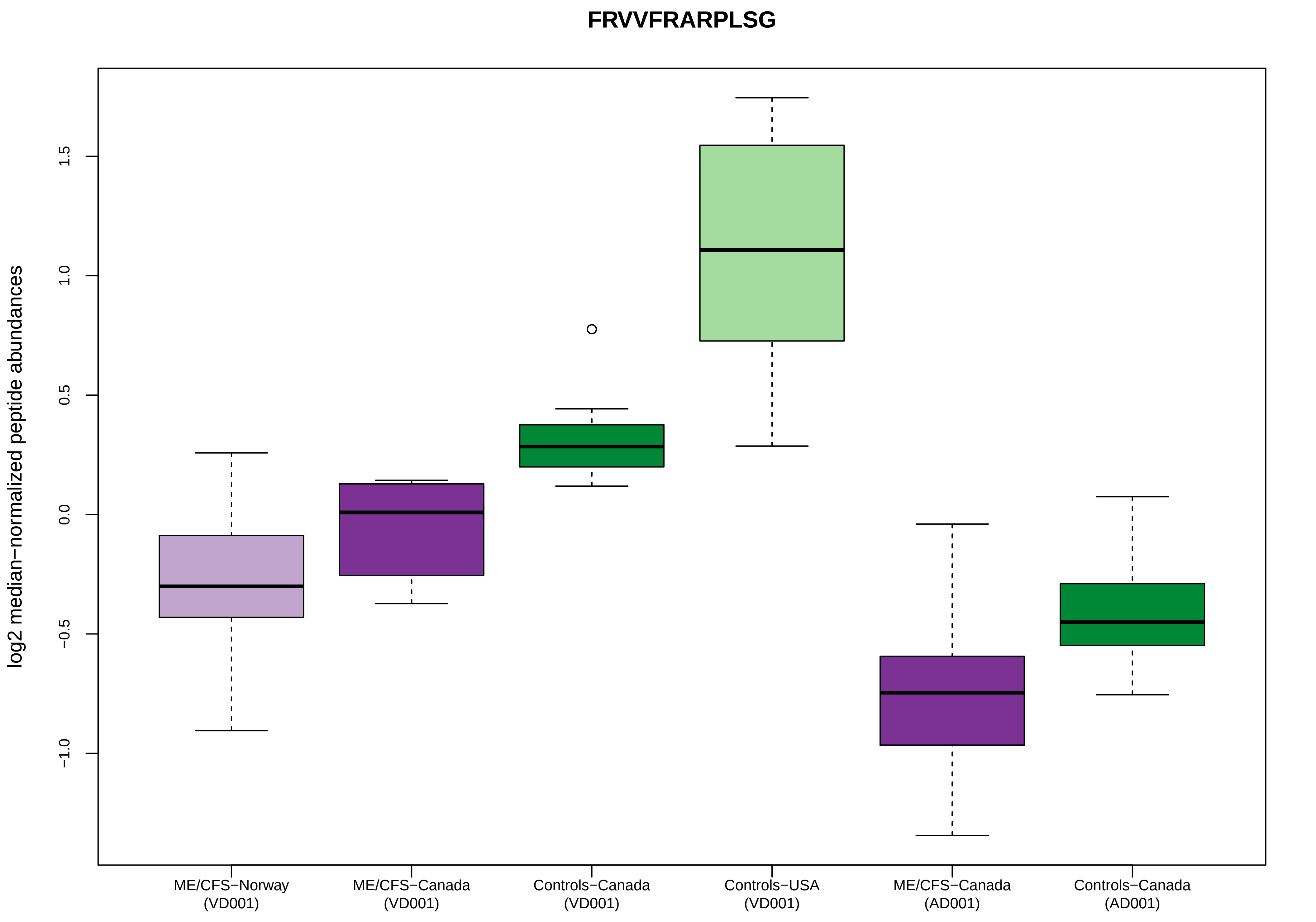
FRVVFRARPLSG
log2 median−normalized peptide abundances
1.5
1.0
0.5
0.0
−0.5
−1.0
ME/CFS−Norway (VD001)
ME/CFS−Canada (VD001)
Controls−Canada (VD001)
Controls−USA (VD001)
ME/CFS−Canada (AD001)
Controls−Canada (AD001)

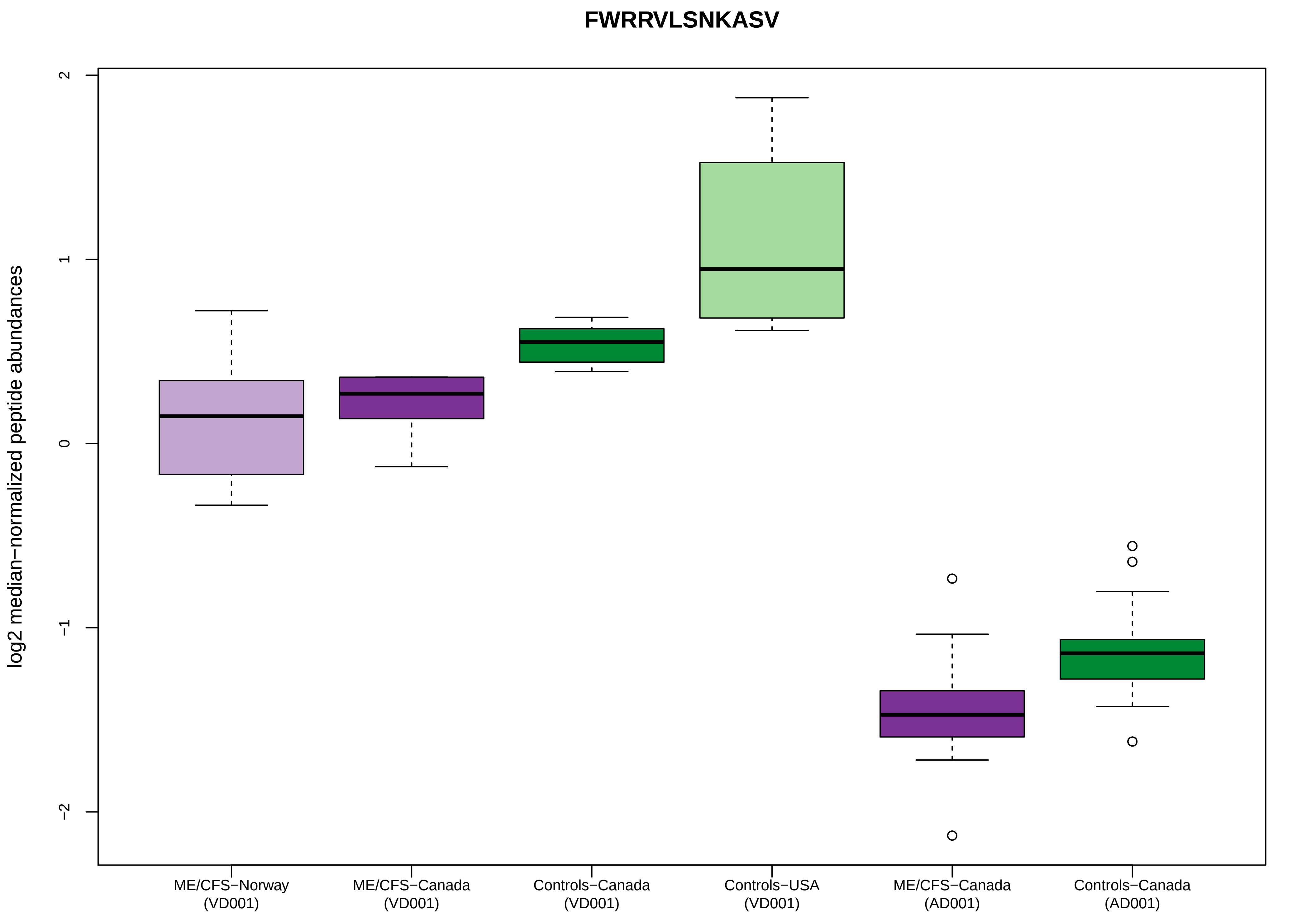
FWRRVLSNKASV
log2 median−normalized peptide abundances
2
1
0
−1
−2
ME/CFS−Norway (VD001)
ME/CFS−Canada (VD001)
Controls−Canada (VD001)
Controls−USA (VD001)
ME/CFS−Canada (AD001)
Controls−Canada (AD001)

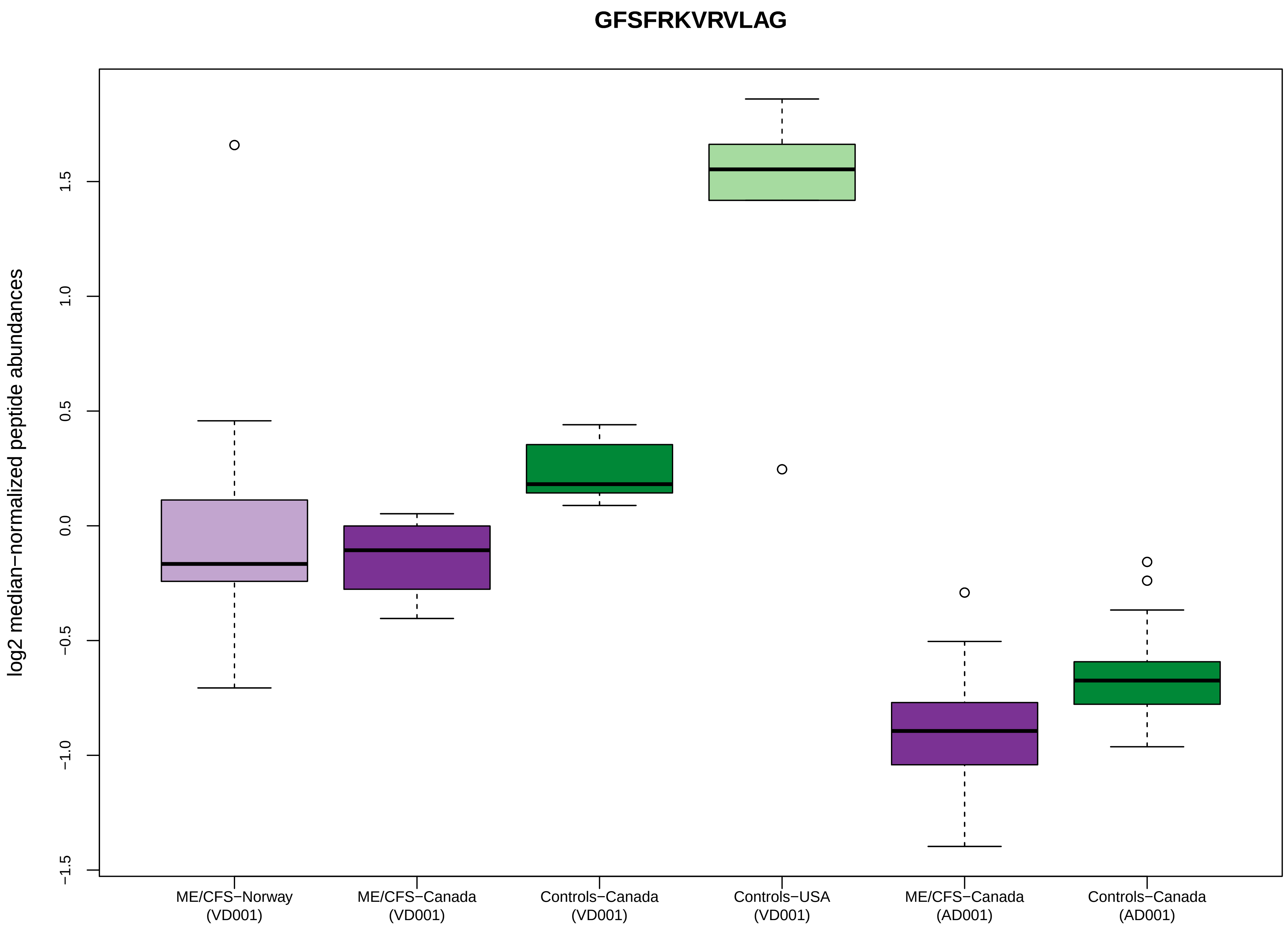
GFSFRKVRVLAG
log2 median−normalized peptide abundances
1.5
1.0
0.5
0.0
−0.5
−1.0
−1.5
ME/CFS−Norway (VD001)
ME/CFS−Canada (VD001)
Controls−Canada (VD001)
Controls−USA (VD001)
ME/CFS−Canada (AD001)
Controls−Canada (AD001)

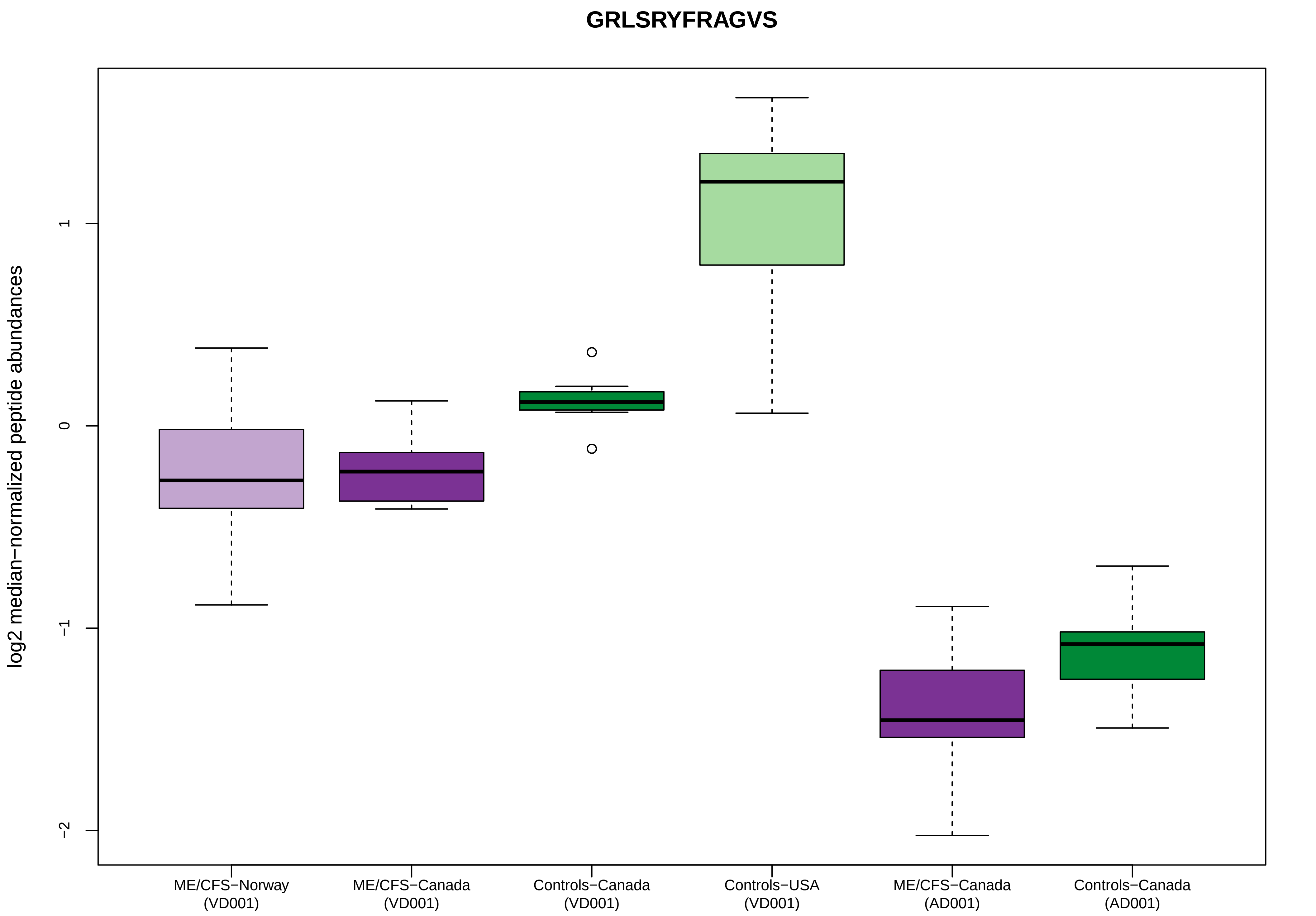

GRLSRYFRAGVS
log2 median−normalized peptide abundances
1
0
−1
−2
ME/CFS−Norway (VD001)
ME/CFS−Canada (VD001)
Controls−Canada (VD001)
Controls−USA (VD001)
ME/CFS−Canada (AD001)
Controls−Canada (AD001)

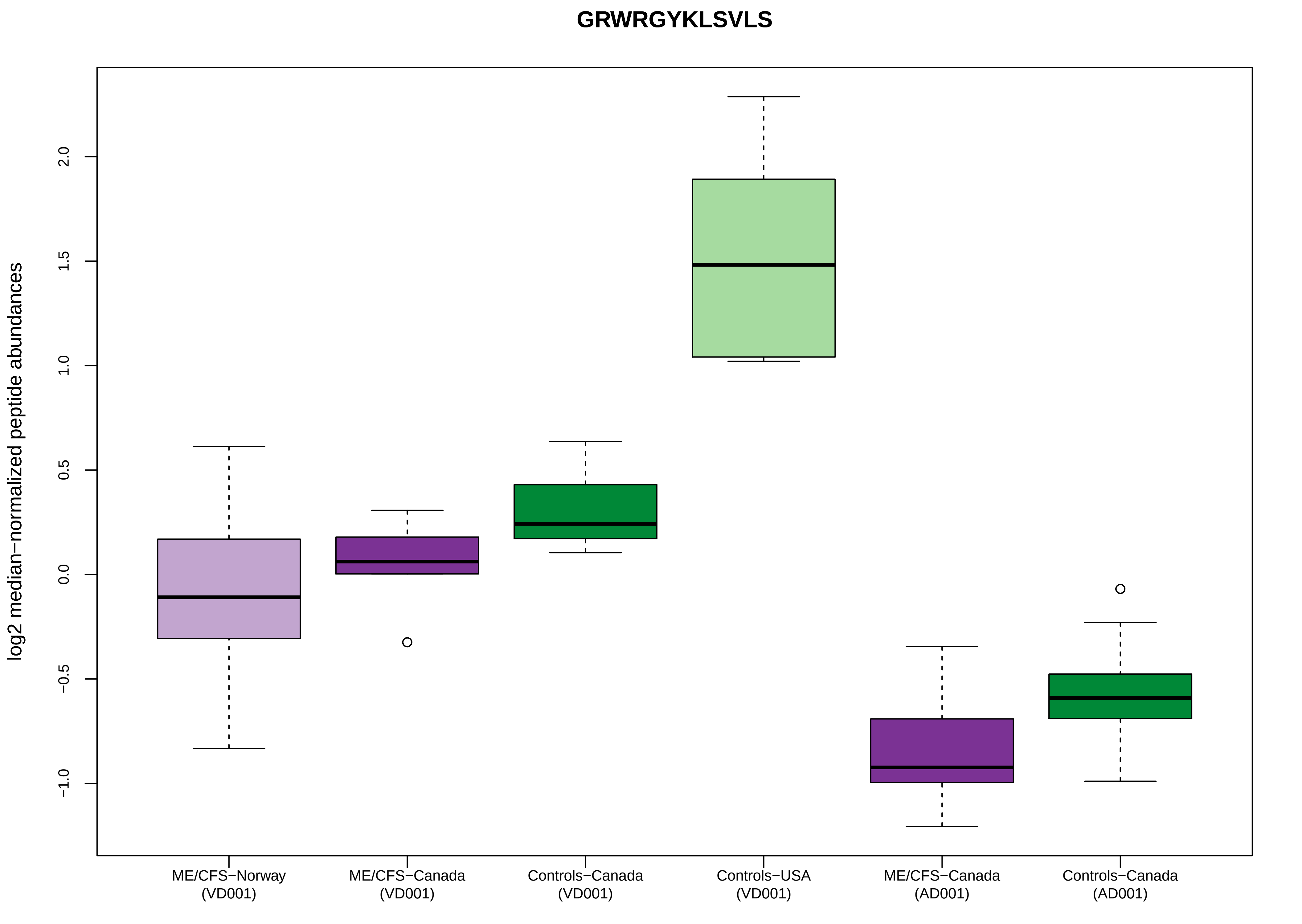

GRWRGYKLSVLS
log2 median−normalized peptide abundances
2.0
1.5
1.0
0.5
0.0
−0.5
−1.0
ME/CFS−Norway (VD001)
ME/CFS−Canada (VD001)
Controls−Canada (VD001)
Controls−USA (VD001)
ME/CFS−Canada (AD001)
Controls−Canada (AD001)

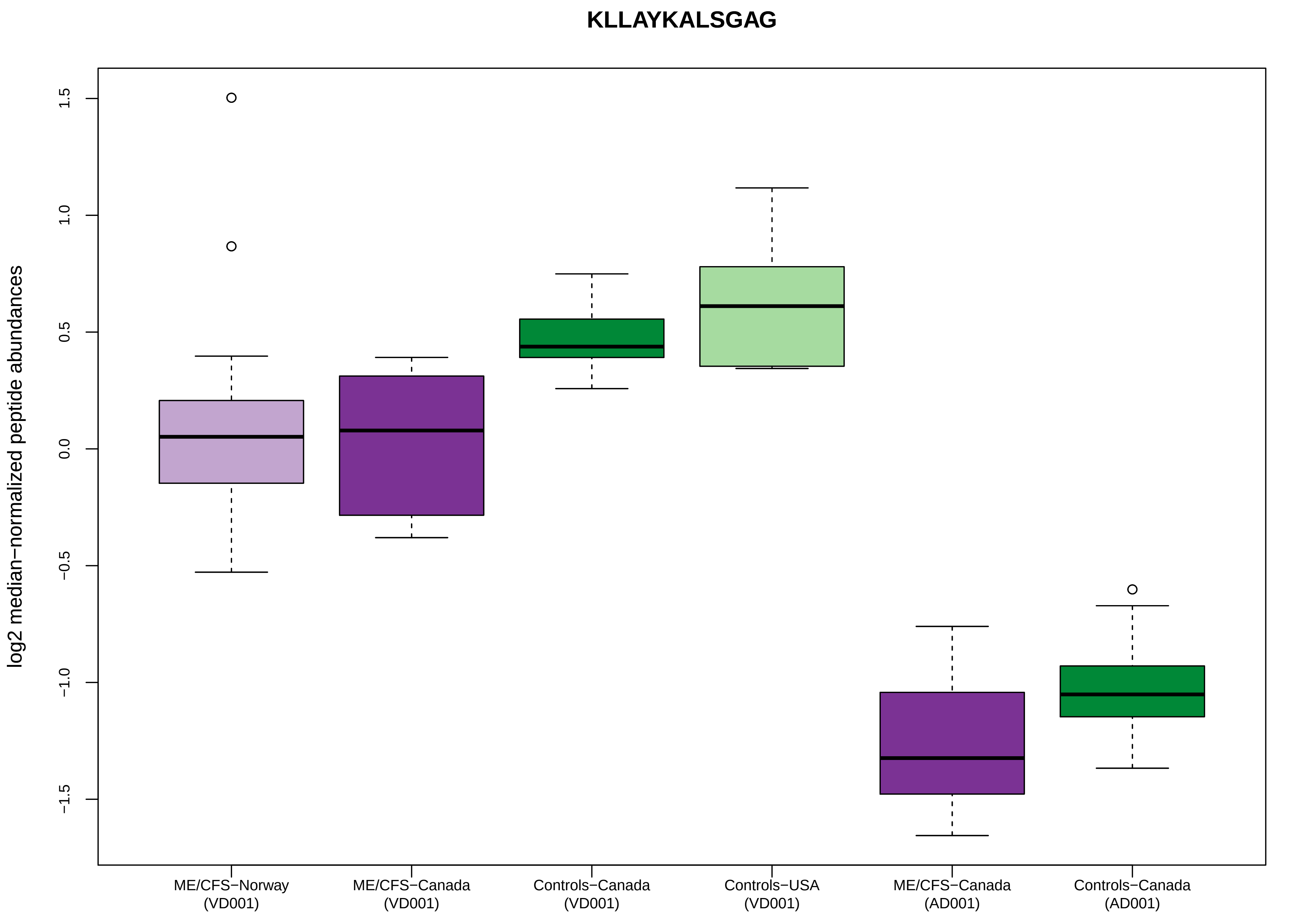

KLLAYKALSGAG
log2 median−normalized peptide abundances
1.5
1.0
0.5
0.0
−0.5
−1.0
−1.5
ME/CFS−Norway (VD001)
ME/CFS−Canada (VD001)
Controls−Canada (VD001)
Controls−USA (VD001)
ME/CFS−Canada (AD001)
Controls−Canada (AD001)

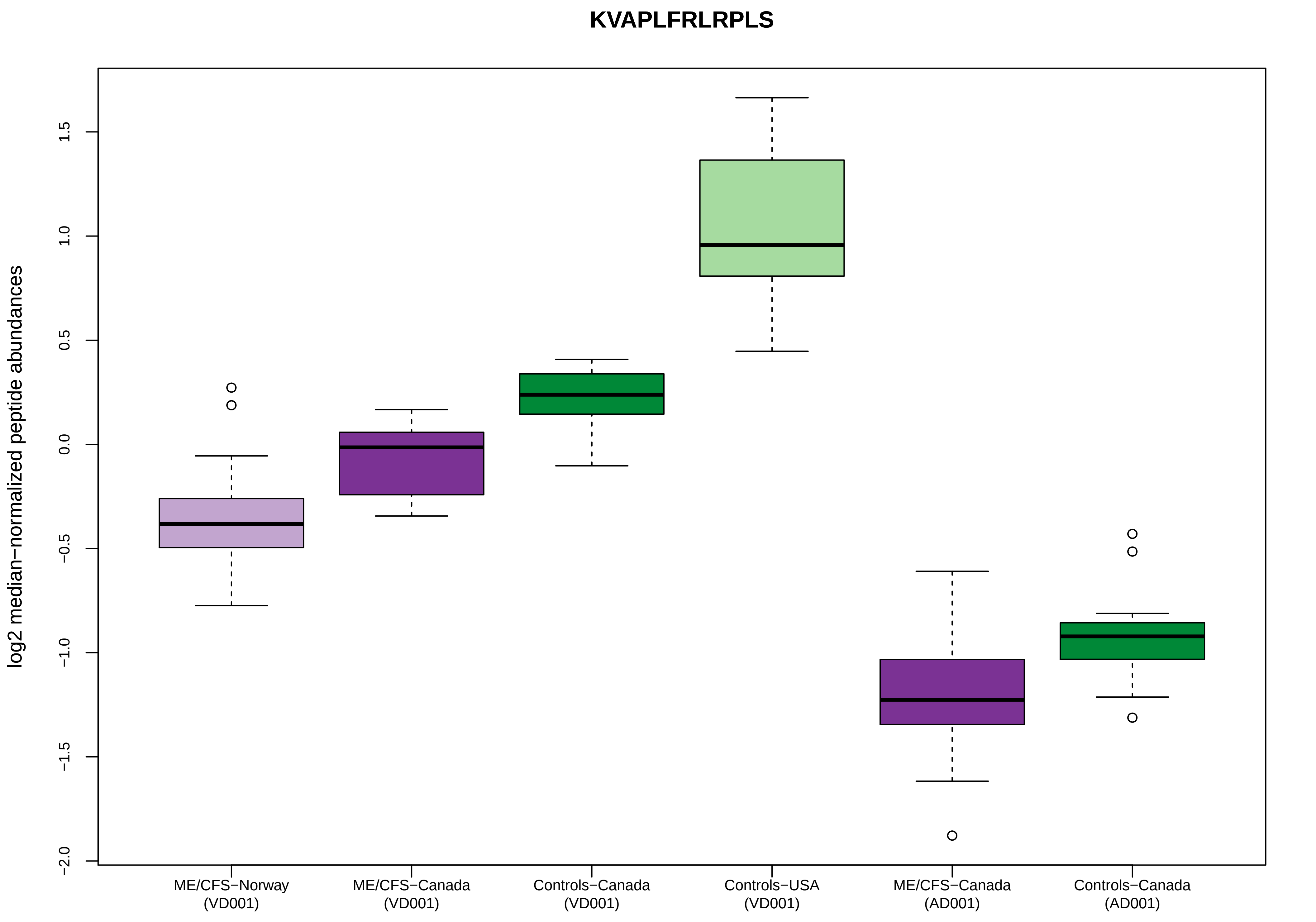

KVAPLFRLRPLS
log2 median−normalized peptide abundances
1.5
1.0
0.5
0.0
−0.5
−1.0
−1.5
−2.0
ME/CFS−Norway (VD001)
ME/CFS−Canada (VD001)
Controls−Canada (VD001)
Controls−USA (VD001)
ME/CFS−Canada (AD001)
Controls−Canada (AD001)

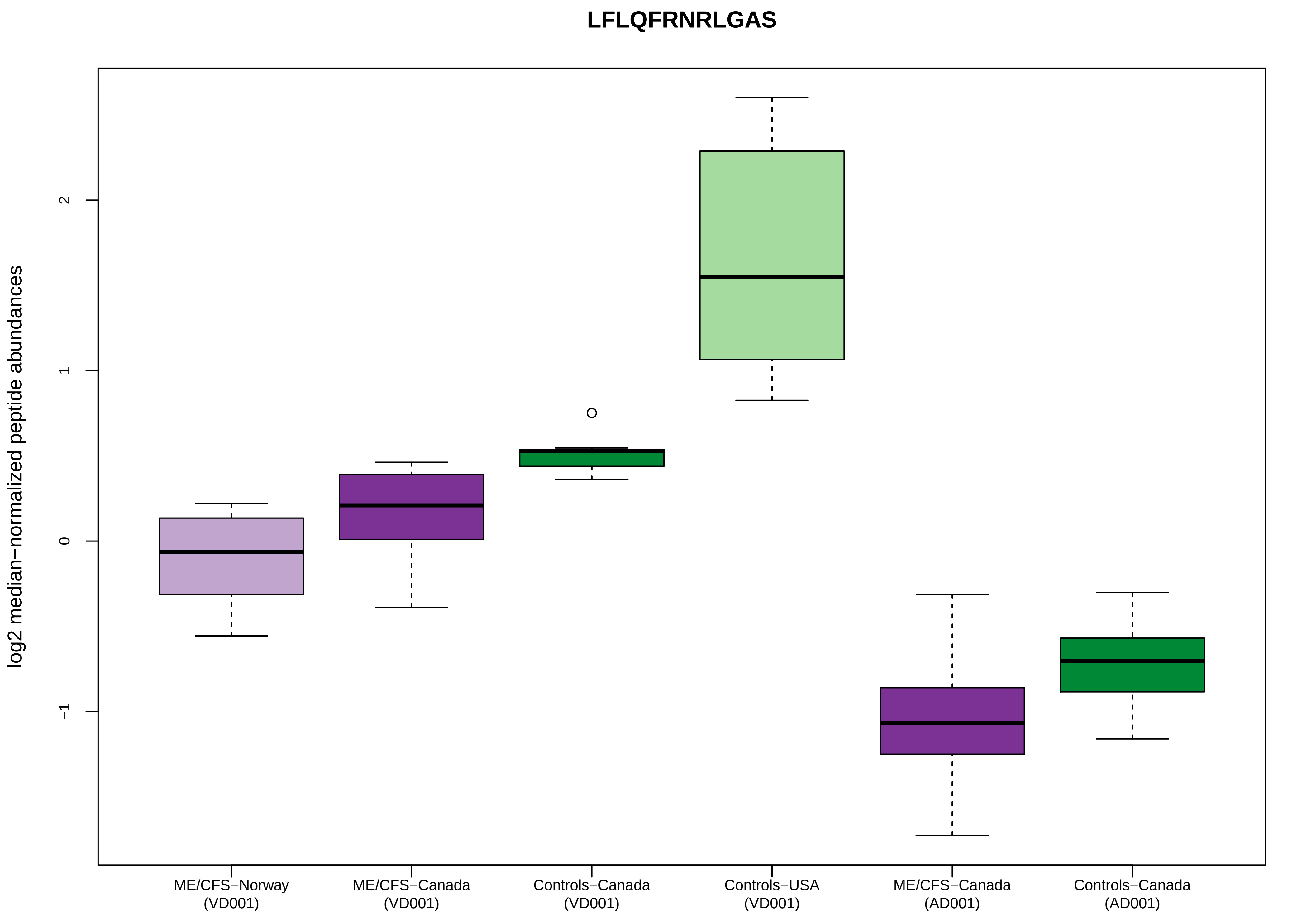

LFLQFRNRLGAS
log2 median−normalized peptide abundances
2
1
0
−1
ME/CFS−Norway (VD001)
ME/CFS−Canada (VD001)
Controls−Canada (VD001)
Controls−USA (VD001)
ME/CFS−Canada (AD001)
Controls−Canada (AD001)

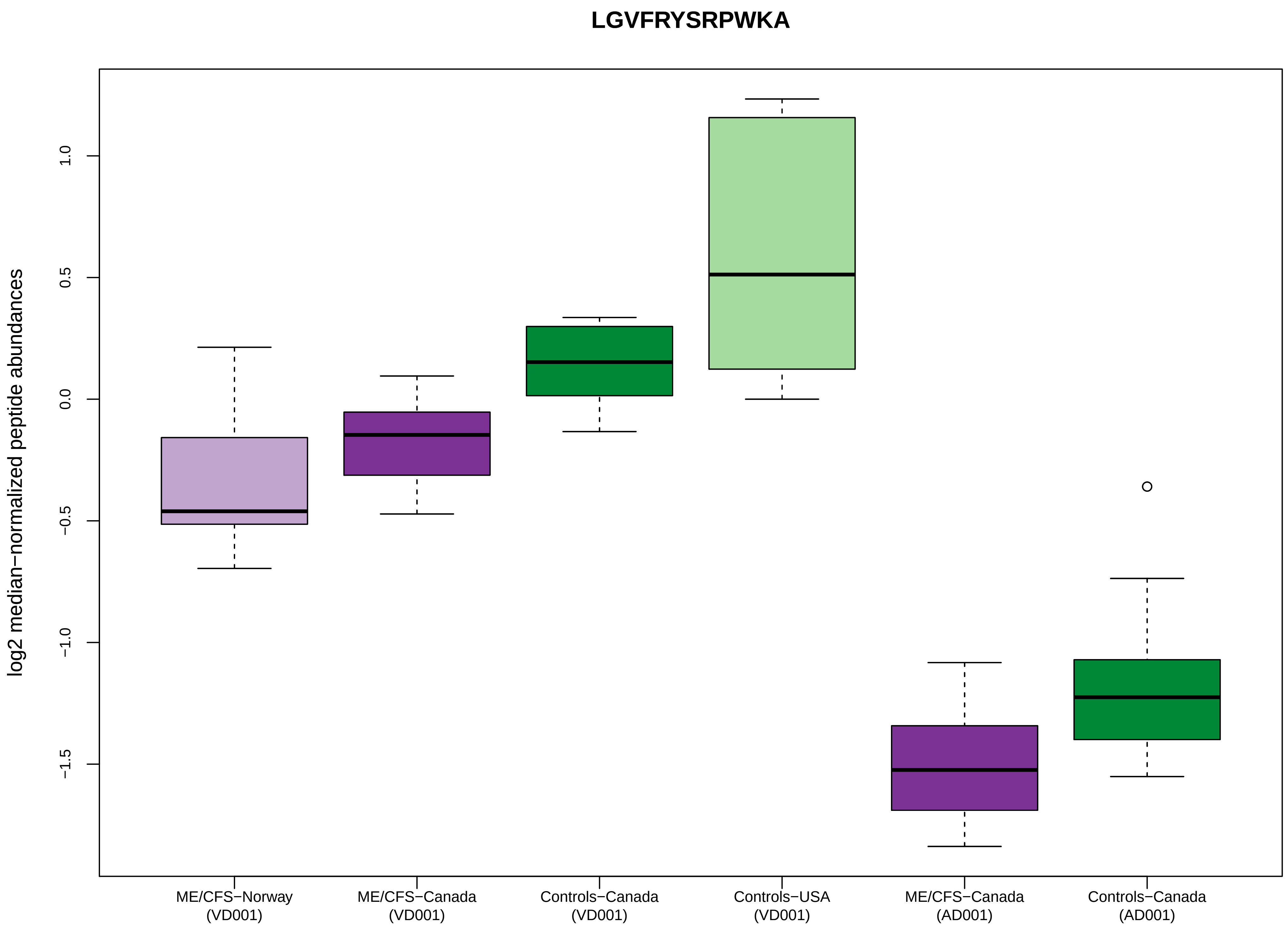

LGVFRYSRPWKA
log2 median−normalized peptide abundances
1.0
0.5
0.0
−0.5
−1.0
−1.5
ME/CFS−Norway (VD001)
ME/CFS−Canada (VD001)
Controls−Canada (VD001)
Controls−USA (VD001)
ME/CFS−Canada (AD001)
Controls−Canada (AD001)

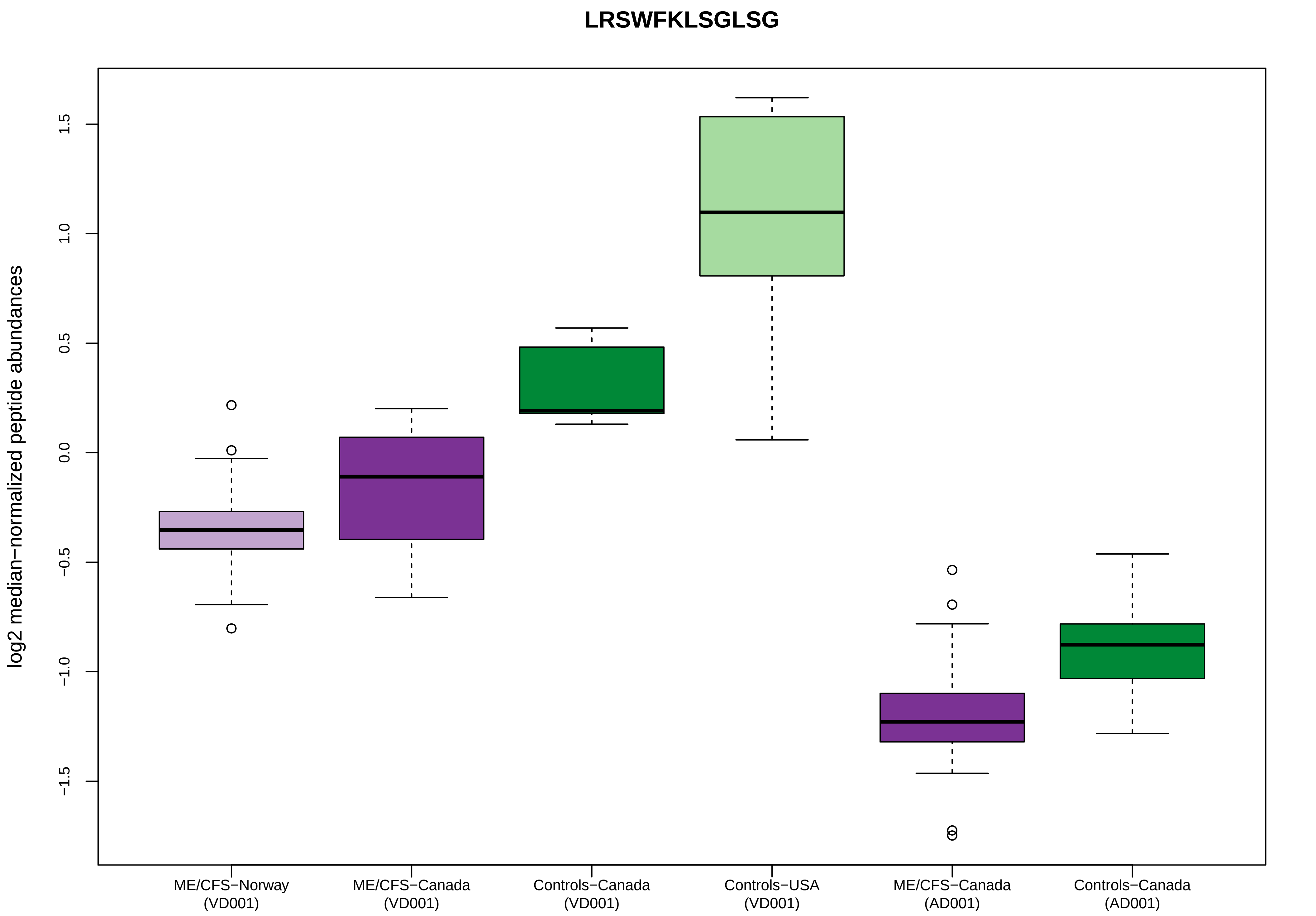
LRSWFKLSGLSG
log2 median−normalized peptide abundances
1.5
1.0
0.5
0.0
−0.5
−1.0
−1.5
ME/CFS−Norway (VD001)
ME/CFS−Canada (VD001)
Controls−Canada (VD001)
Controls−USA (VD001)
ME/CFS−Canada (AD001)
Controls−Canada (AD001)

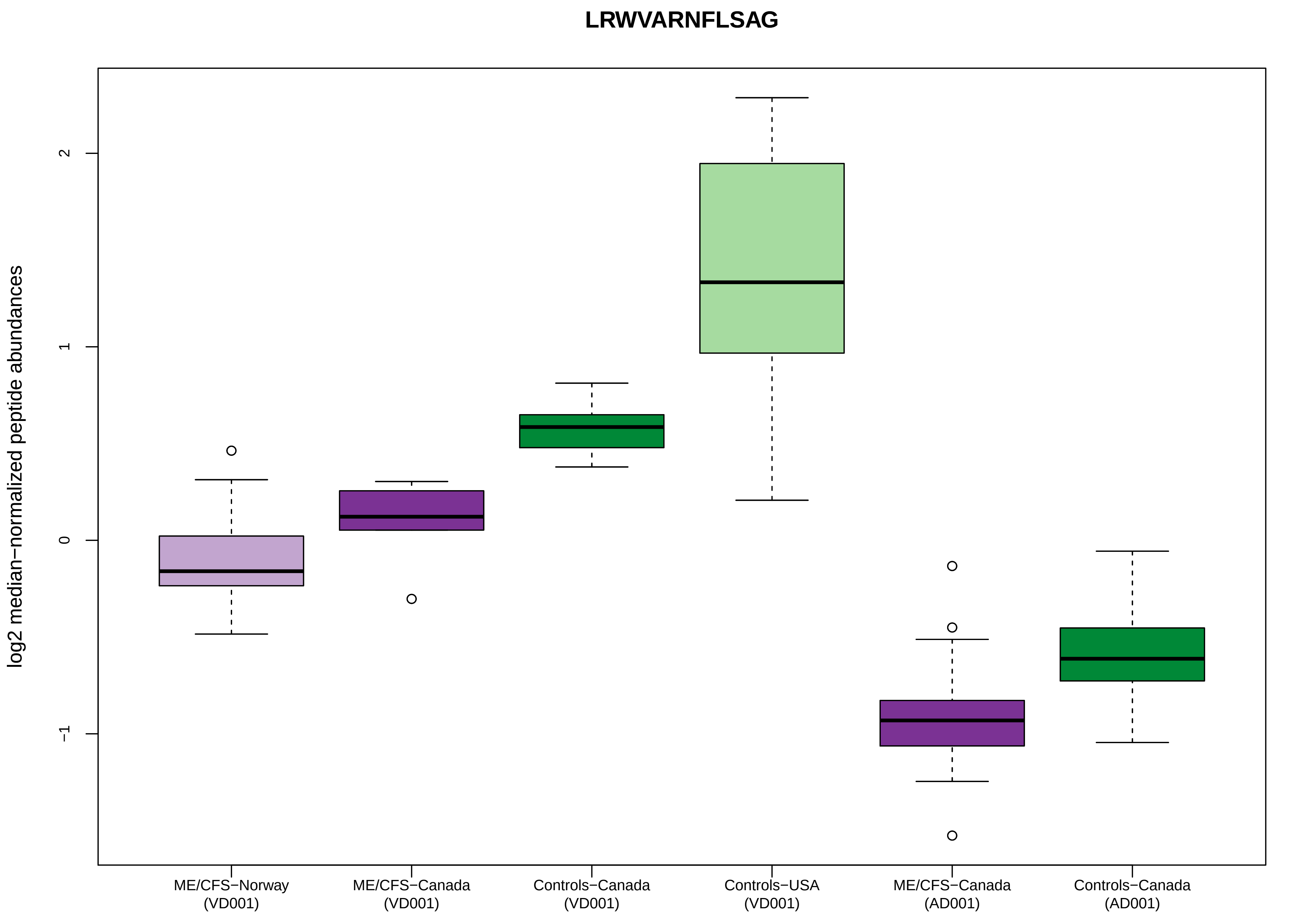

LRWVARNFLSAG
log2 median−normalized peptide abundances
2
1
0
−1
ME/CFS−Norway (VD001)
ME/CFS−Canada (VD001)
Controls−Canada (VD001)
Controls−USA (VD001)
ME/CFS−Canada (AD001)
Controls−Canada (AD001)

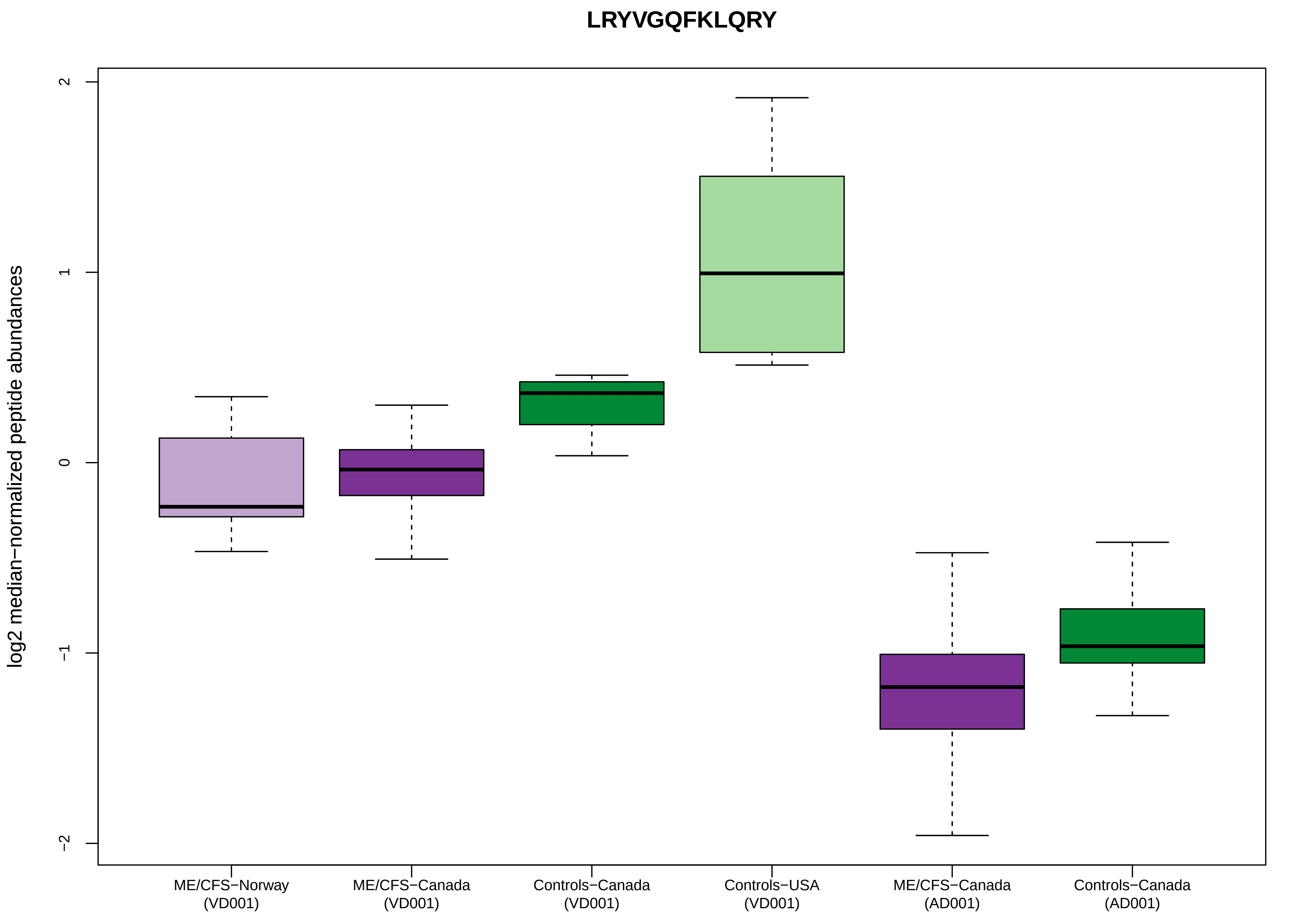

LRYVGQFKLQRY
log2 median−normalized peptide abundances
2
1
0
−1
−2
ME/CFS−Norway (VD001)
ME/CFS−Canada (VD001)
Controls−Canada (VD001)
Controls−USA (VD001)
ME/CFS−Canada (AD001)
Controls−Canada (AD001)

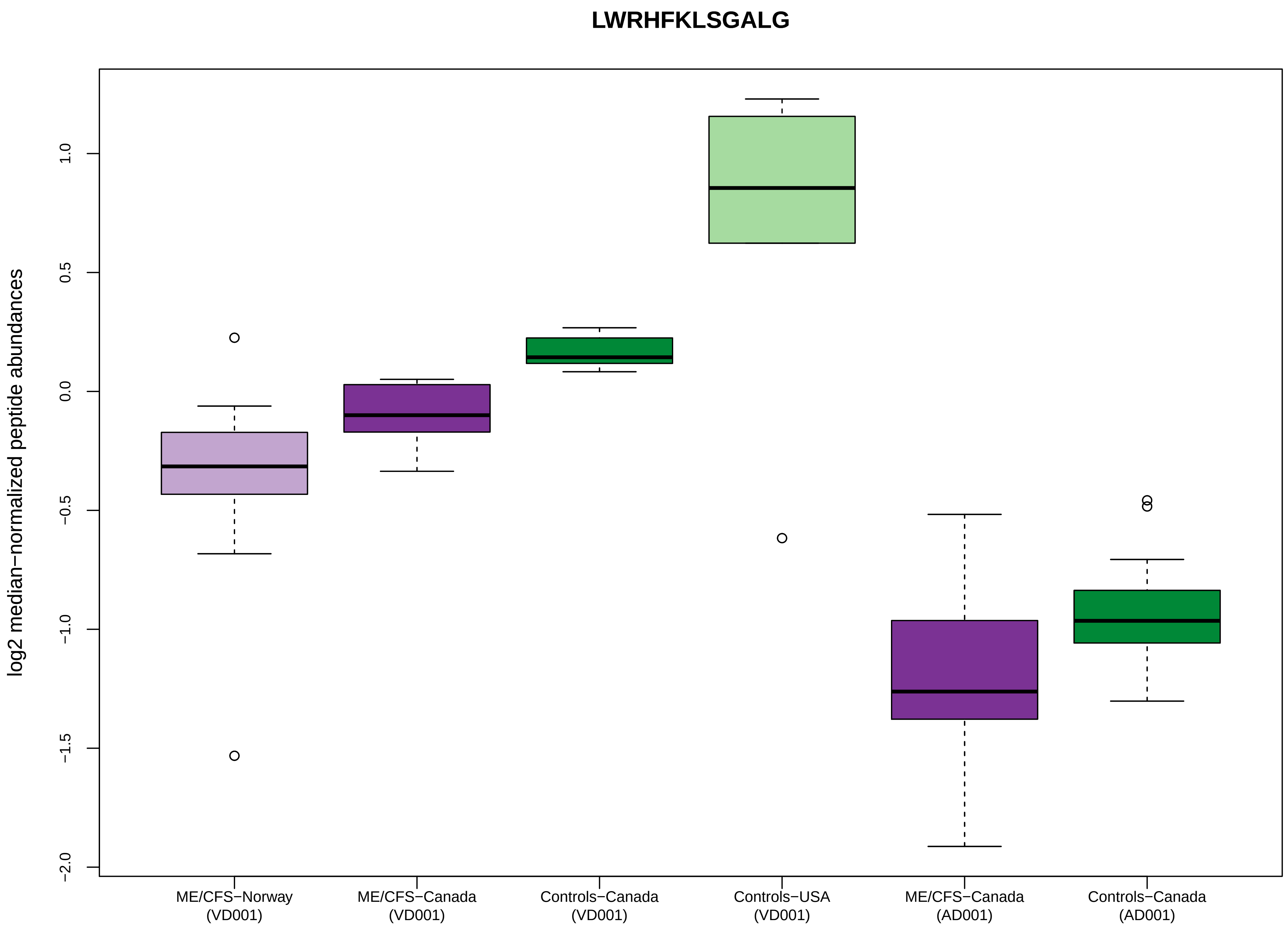

LWRHFKLSGALG
log2 median−normalized peptide abundances
1.0
0.5
0.0
−0.5
−1.0
−1.5
−2.0
ME/CFS−Norway (VD001)
ME/CFS−Canada (VD001)
Controls−Canada (VD001)
Controls−USA (VD001)
ME/CFS−Canada (AD001)
Controls−Canada (AD001)

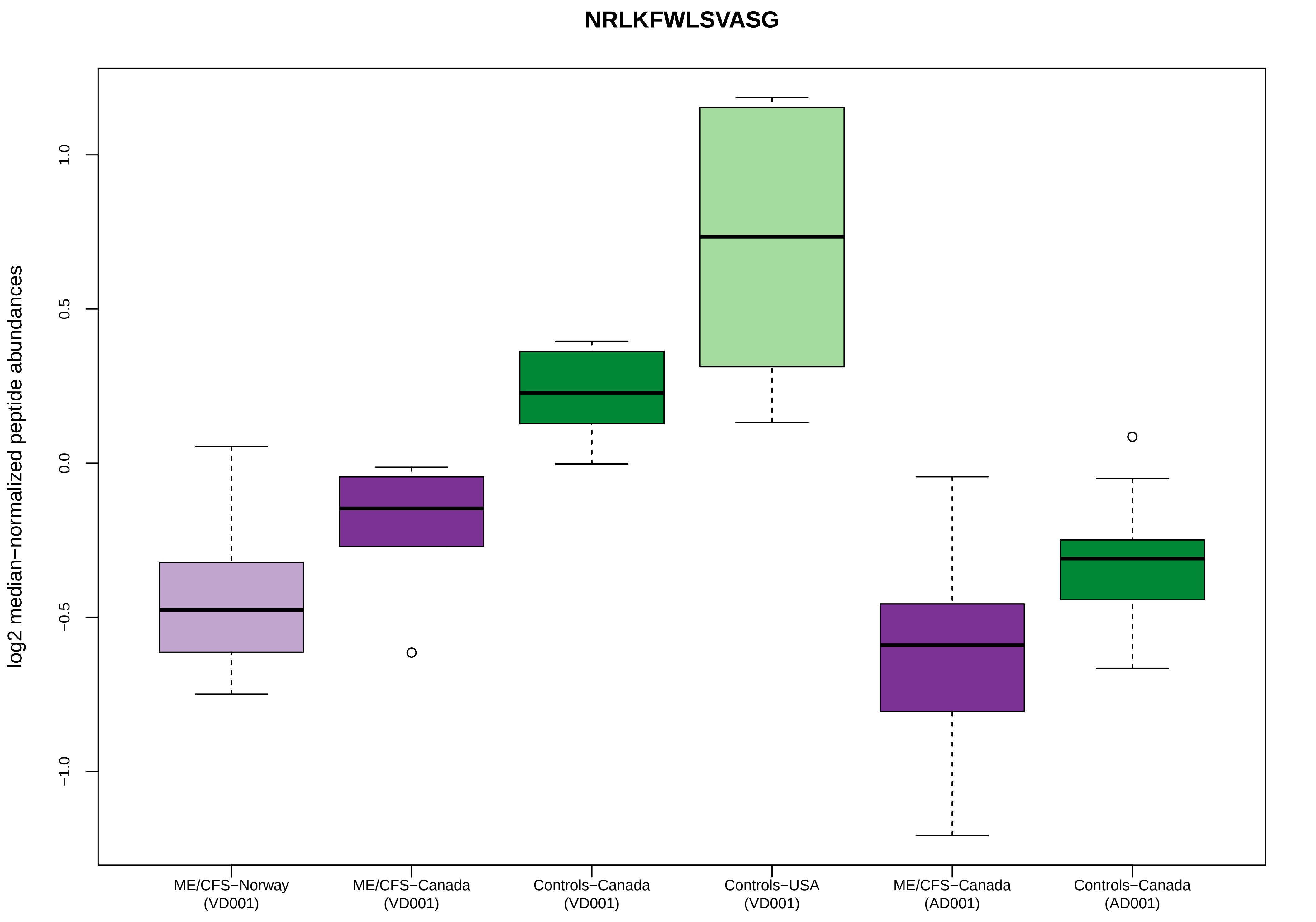
NRLKFWLSVASG
log2 median−normalized peptide abundances
1.0
0.5
0.0
−0.5
−1.0
ME/CFS−Norway (VD001)
ME/CFS−Canada (VD001)
Controls−Canada (VD001)
Controls−USA (VD001)
ME/CFS−Canada (AD001)
Controls−Canada (AD001)

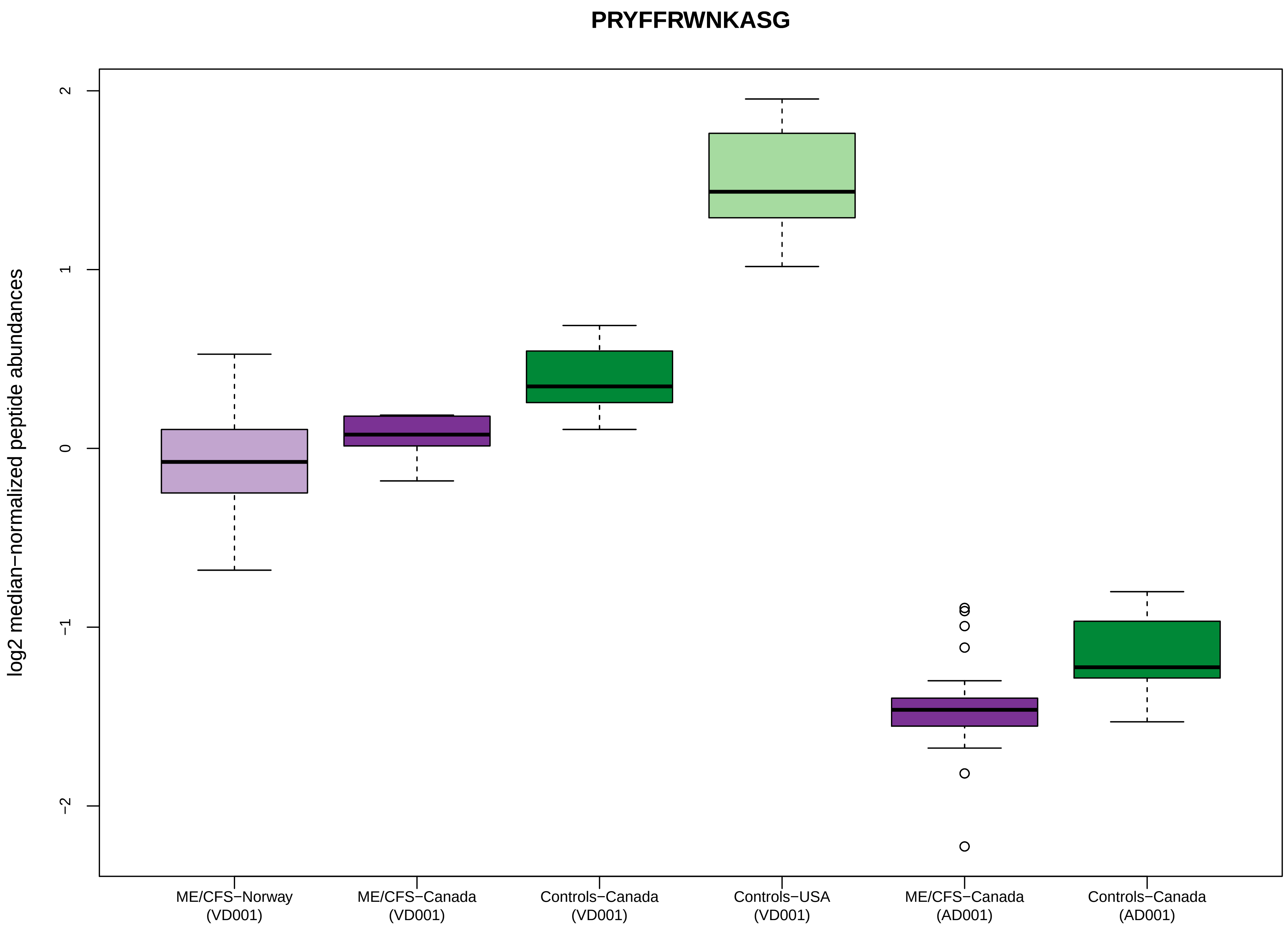
PRYFFRWNKASG
log2 median−normalized peptide abundances
2
1
0
−1
−2
ME/CFS−Norway (VD001)
ME/CFS−Canada (VD001)
Controls−Canada (VD001)
Controls−USA (VD001)
ME/CFS−Canada (AD001)
Controls−Canada (AD001)

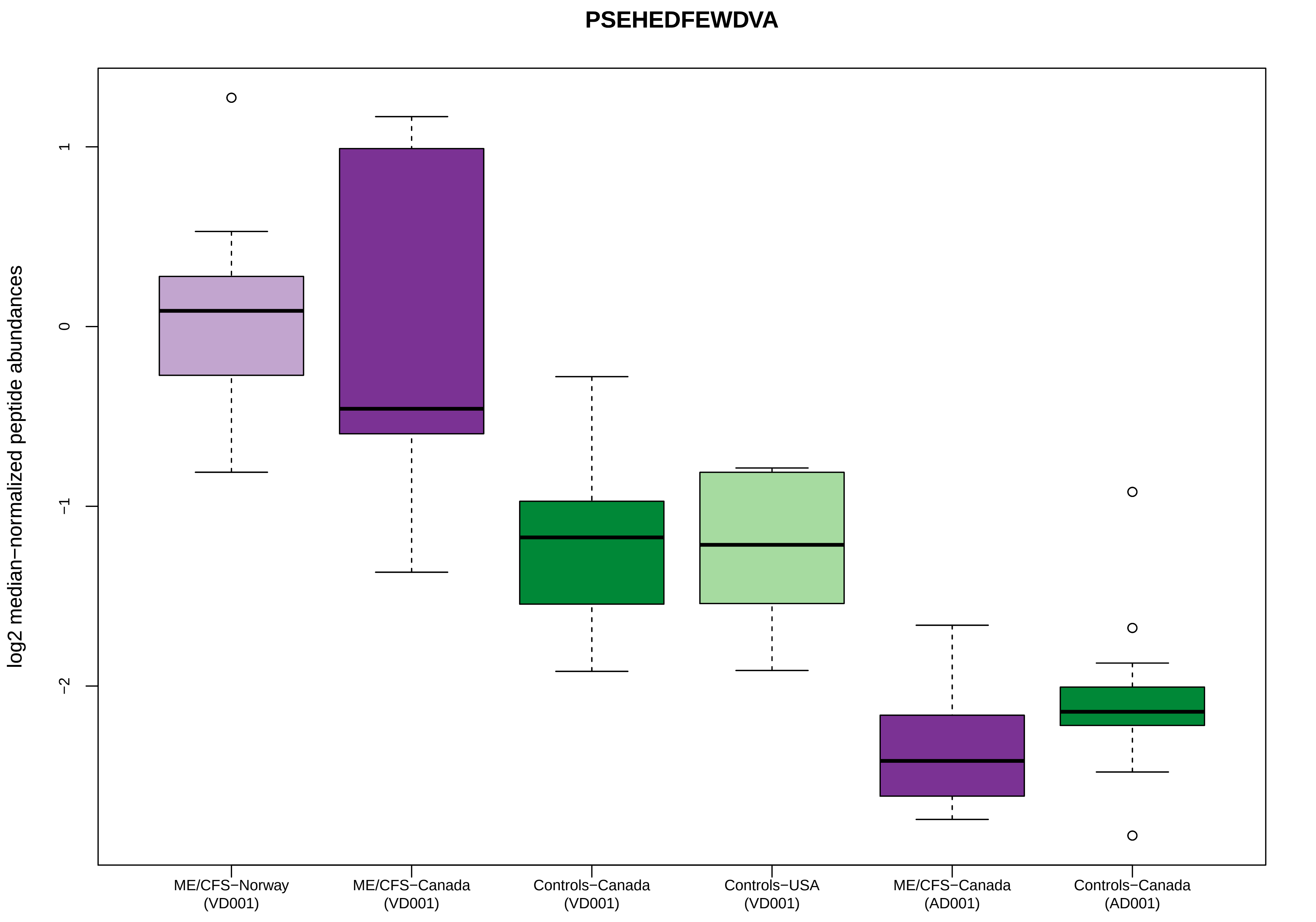
PSEHEDFEWDVA
log2 median−normalized peptide abundances
1
0
−1
−2
ME/CFS−Norway (VD001)
ME/CFS−Canada (VD001)
Controls−Canada (VD001)
Controls−USA (VD001)
ME/CFS−Canada (AD001)
Controls−Canada (AD001)

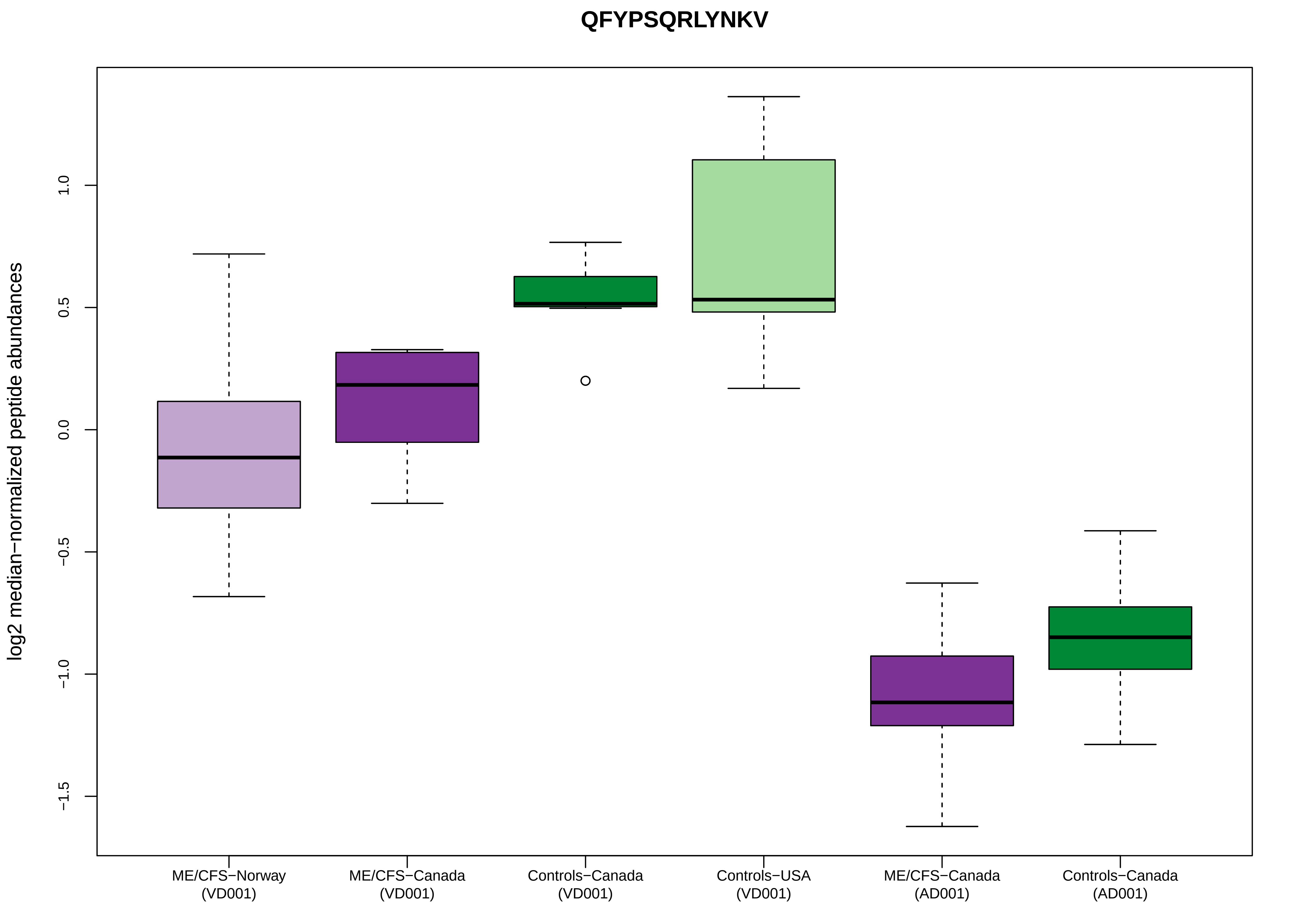
QFYPSQRLYNKV
log2 median−normalized peptide abundances
1.0
0.5
0.0
−0.5
−1.0
−1.5
ME/CFS−Norway (VD001)
ME/CFS−Canada (VD001)
Controls−Canada (VD001)
Controls−USA (VD001)
ME/CFS−Canada (AD001)
Controls−Canada (AD001)

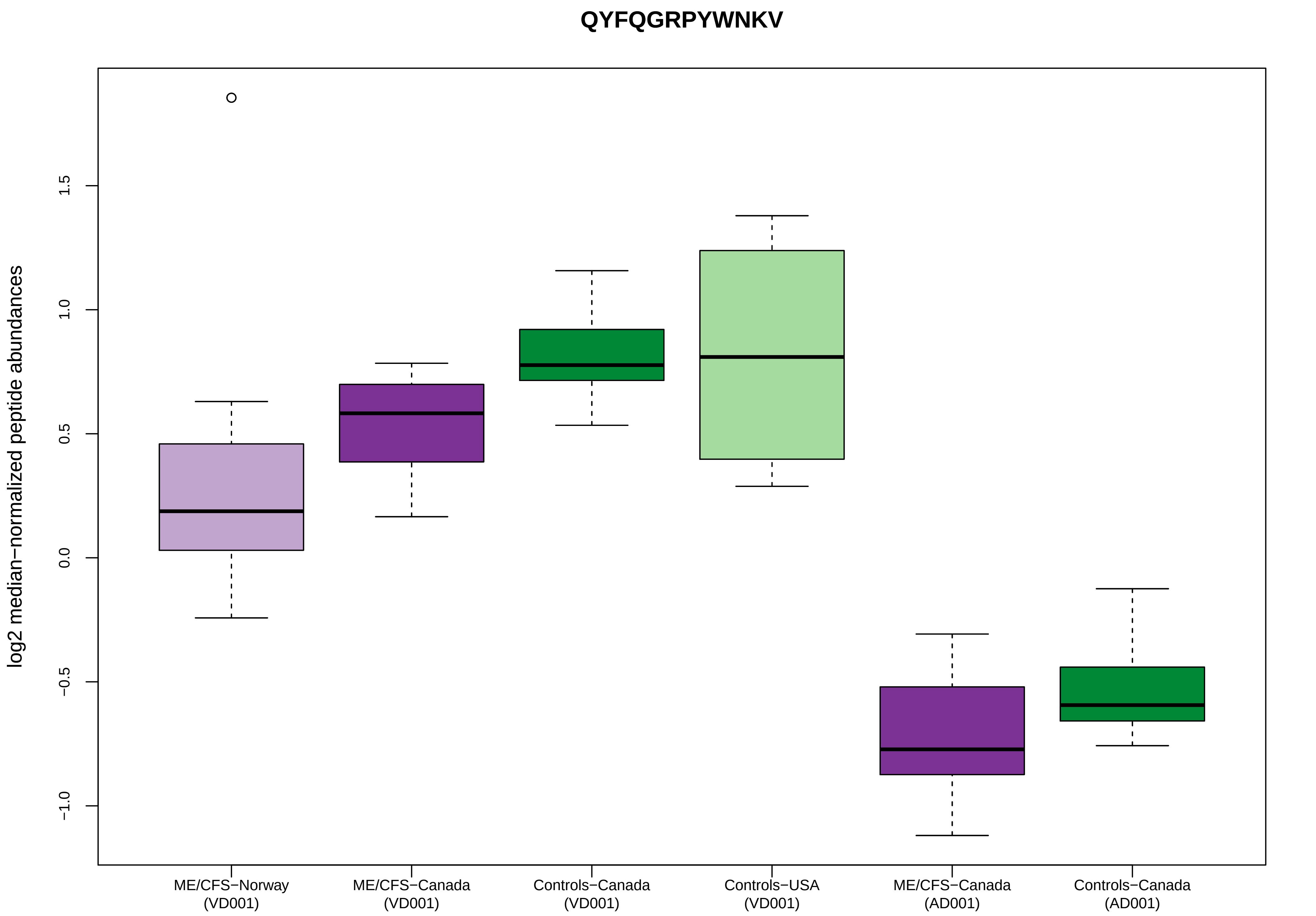
QYFQGRPYWNKV
log2 median−normalized peptide abundances
1.5
1.0
0.5
0.0
−0.5
−1.0
ME/CFS−Norway (VD001)
ME/CFS−Canada (VD001)
Controls−Canada (VD001)
Controls−USA (VD001)
ME/CFS−Canada (AD001)
Controls−Canada (AD001)

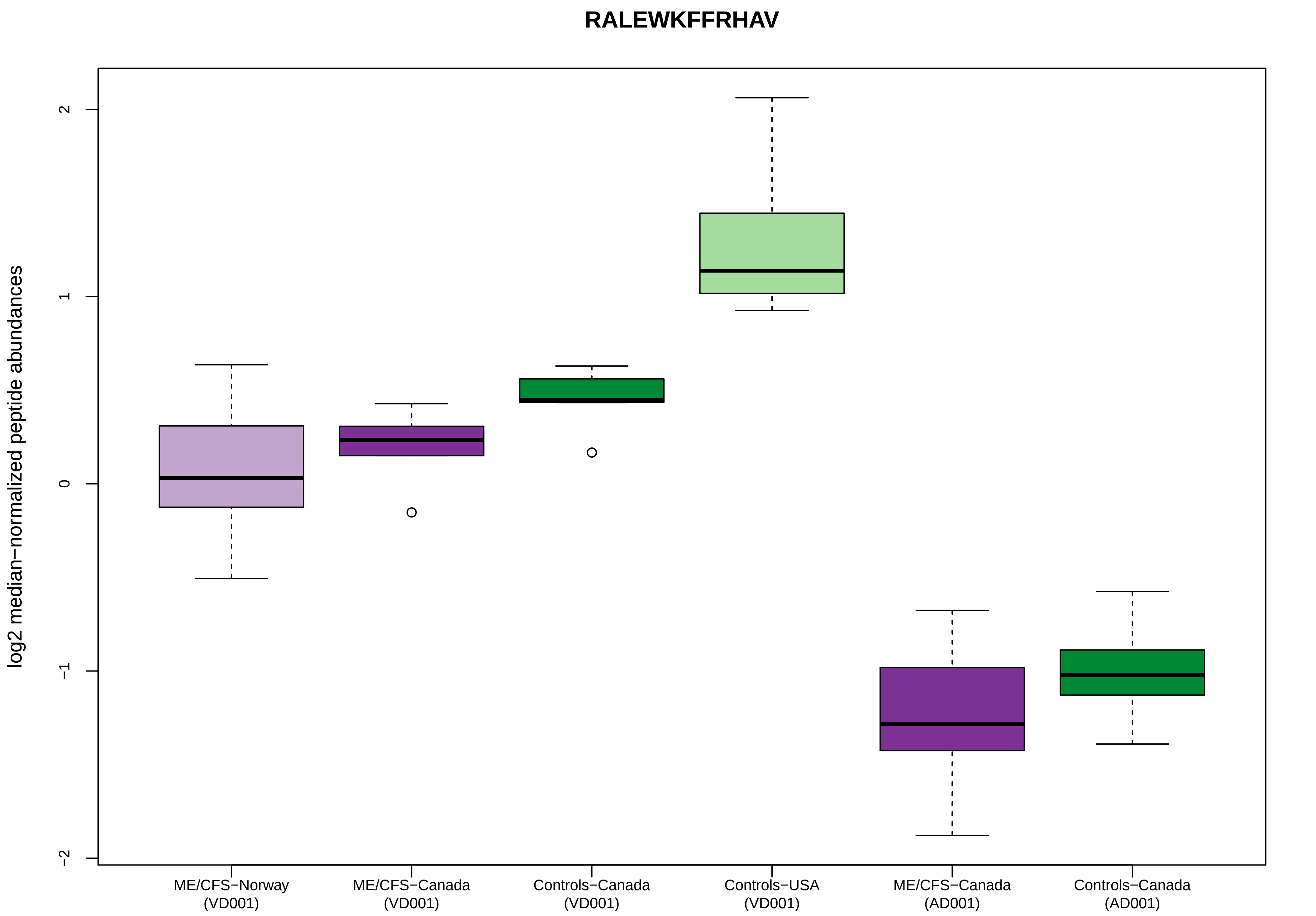

RALEWKFFRHAV
log2 median−normalized peptide abundances
2
1
0
−1
−2
ME/CFS−Norway (VD001)
ME/CFS−Canada (VD001)
Controls−Canada (VD001)
Controls−USA (VD001)
ME/CFS−Canada (AD001)
Controls−Canada (AD001)

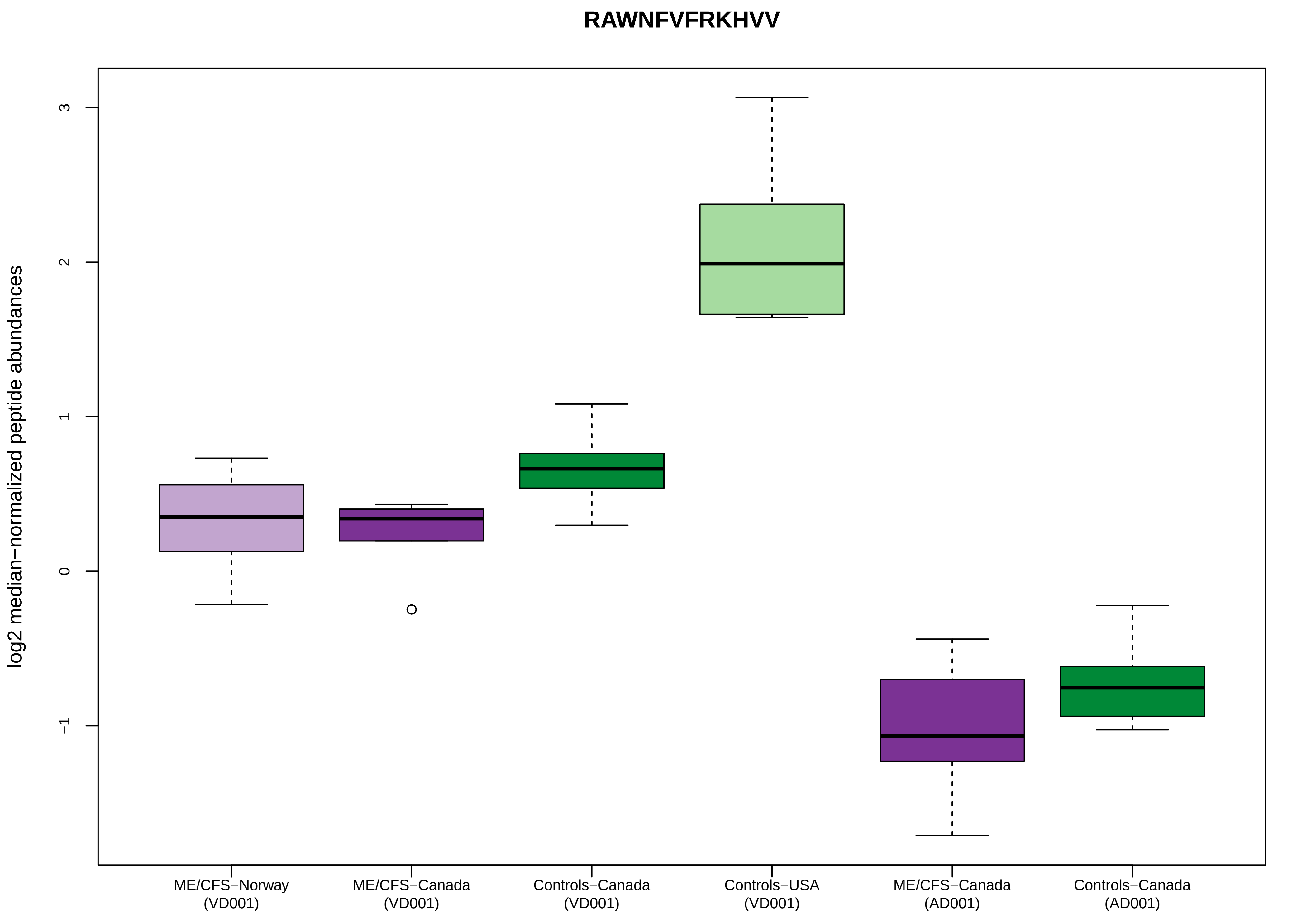
RAWNFVFRKHVV
log2 median−normalized peptide abundances
3
2
1
0
−1
ME/CFS−Norway (VD001)
ME/CFS−Canada (VD001)
Controls−Canada (VD001)
Controls−USA (VD001)
ME/CFS−Canada (AD001)
Controls−Canada (AD001)

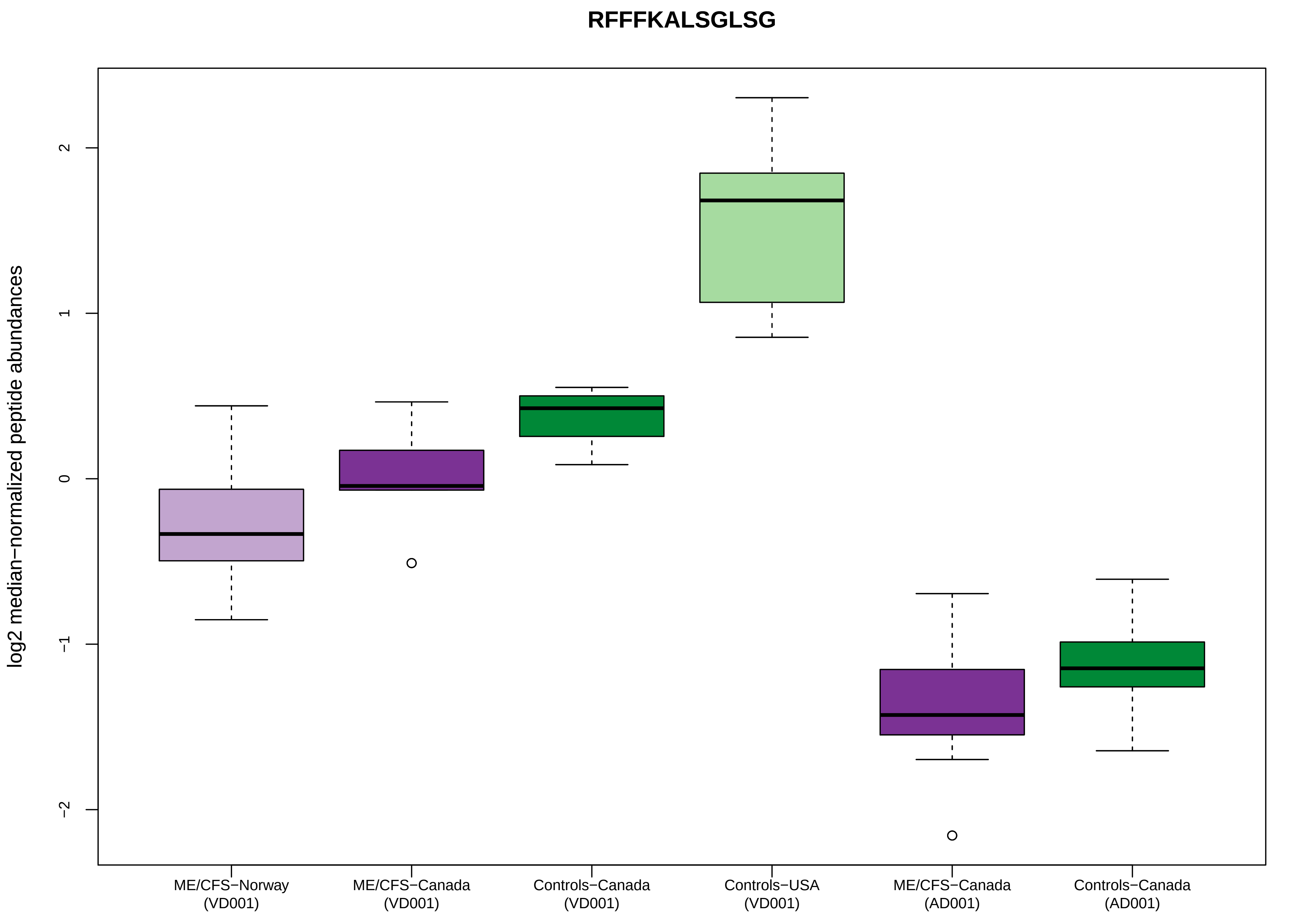

RFFFKALSGLSG
log2 median−normalized peptide abundances
2
1
0
−1
−2
ME/CFS−Norway (VD001)
ME/CFS−Canada (VD001)
Controls−Canada (VD001)
Controls−USA (VD001)
ME/CFS−Canada (AD001)
Controls−Canada (AD001)

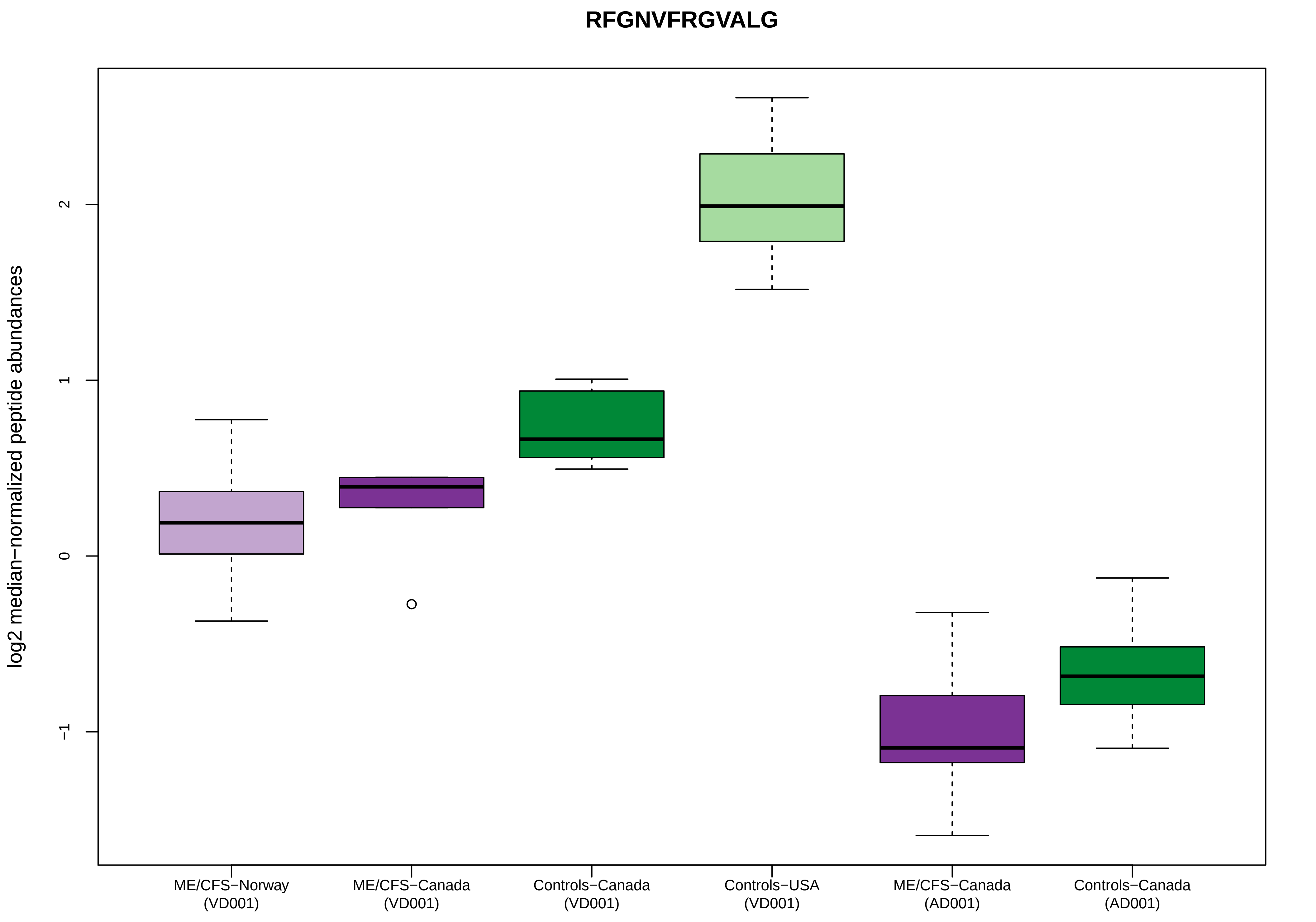
RFGNVFRGVALG
log2 median−normalized peptide abundances
2
1
0
−1
ME/CFS−Norway (VD001)
ME/CFS−Canada (VD001)
Controls−Canada (VD001)
Controls−USA (VD001)
ME/CFS−Canada (AD001)
Controls−Canada (AD001)

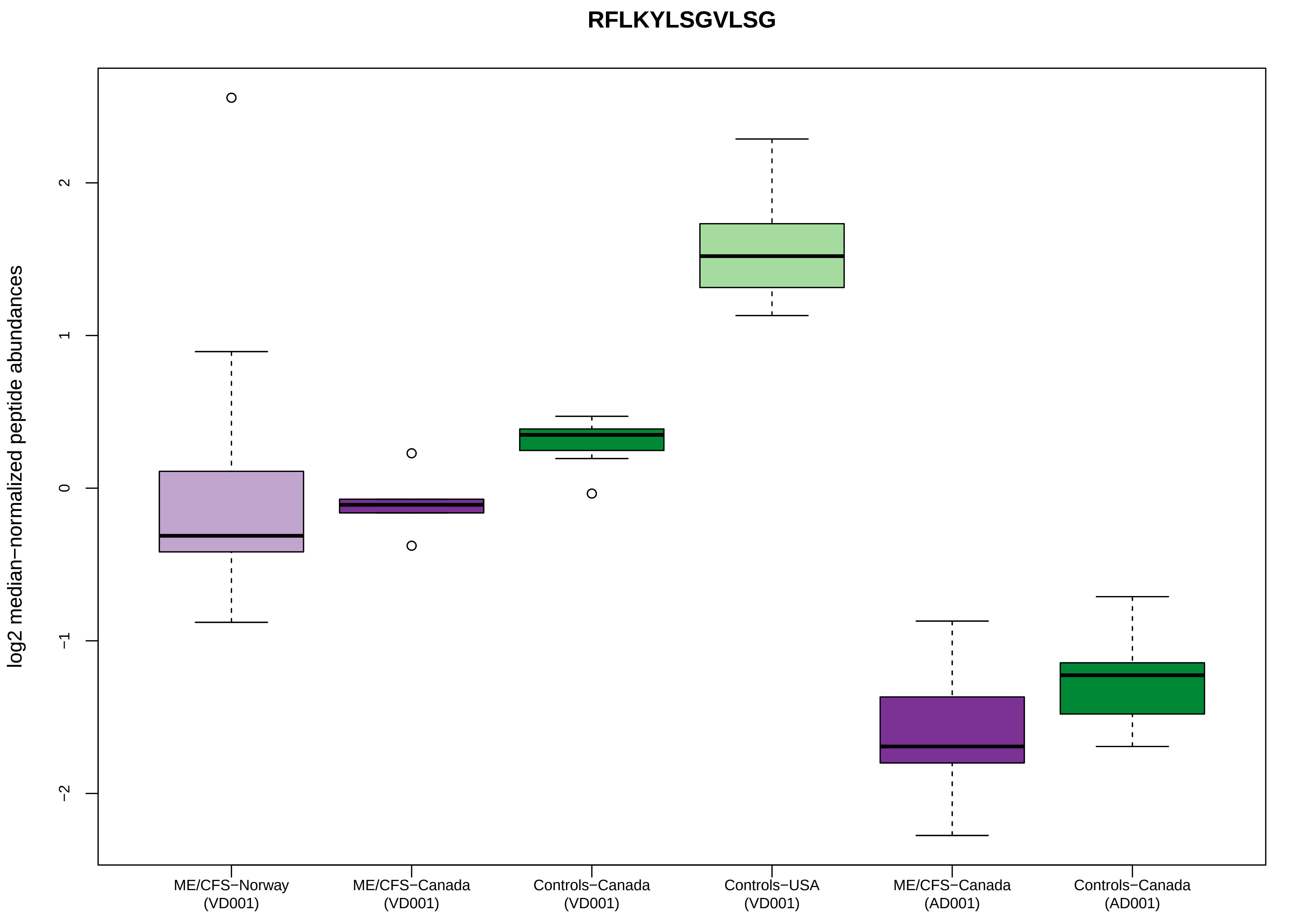

RFLKYLSGVLSG
log2 median−normalized peptide abundances
2
1
0
−1
−2
ME/CFS−Norway (VD001)
ME/CFS−Canada (VD001)
Controls−Canada (VD001)
Controls−USA (VD001)
ME/CFS−Canada (AD001)
Controls−Canada (AD001)

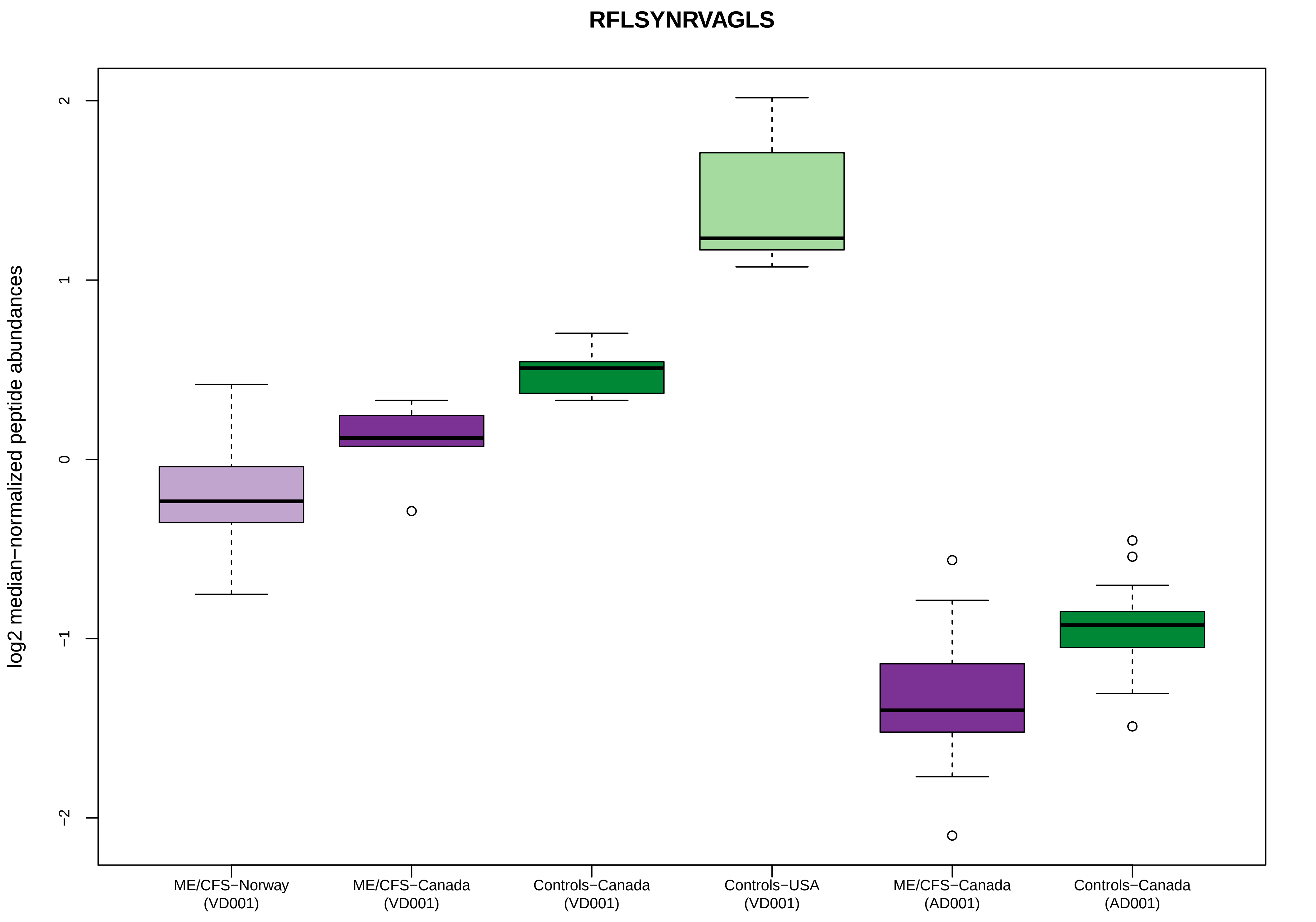

RFLSYNRVAGLS
log2 median−normalized peptide abundances
2
1
0
−1
−2
ME/CFS−Norway (VD001)
ME/CFS−Canada (VD001)
Controls−Canada (VD001)
Controls−USA (VD001)
ME/CFS−Canada (AD001)
Controls−Canada (AD001)

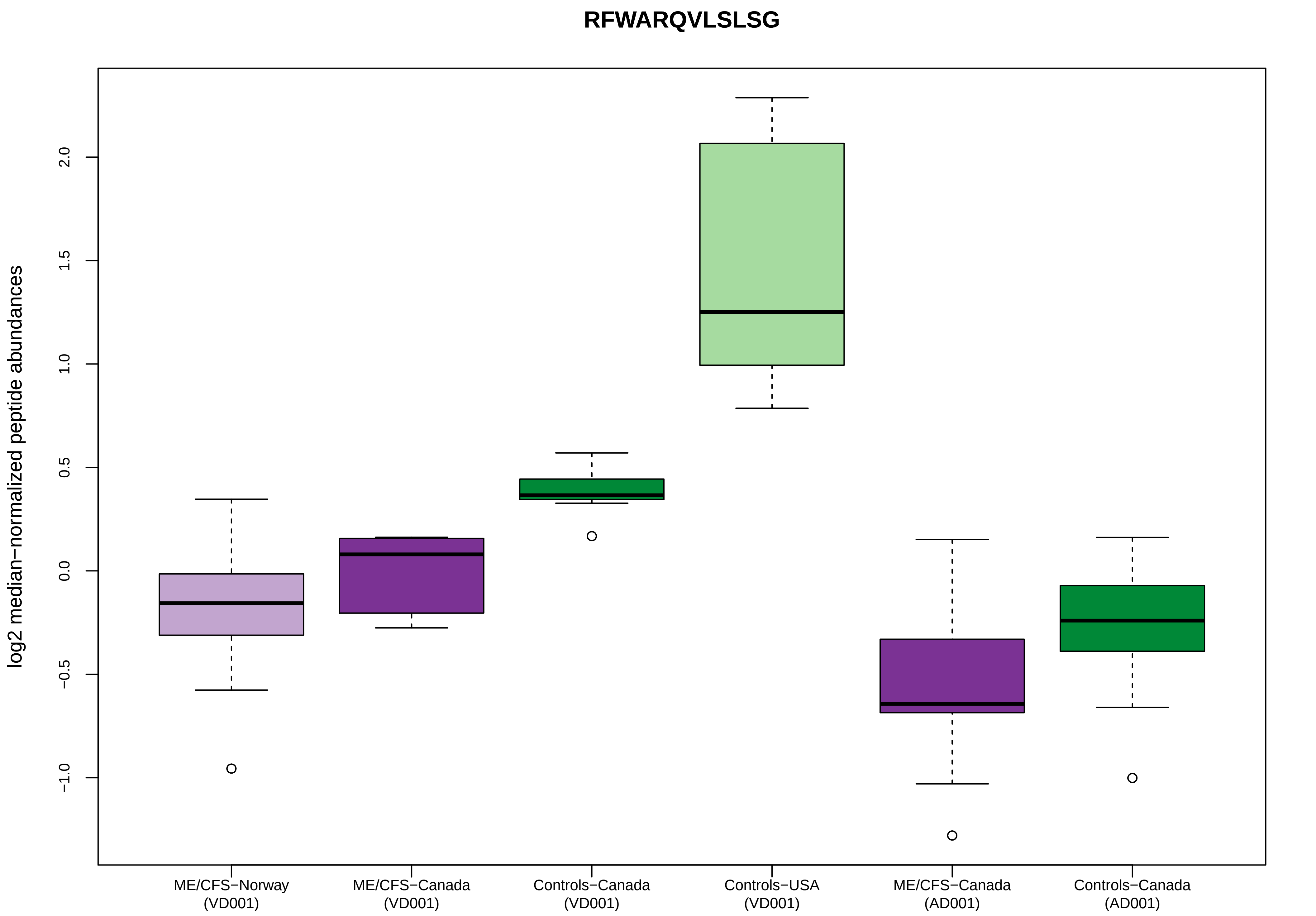
RFWARQVLSLSG
log2 median−normalized peptide abundances
2.0
1.5
1.0
0.5
0.0
−0.5
−1.0
ME/CFS−Norway (VD001)
ME/CFS−Canada (VD001)
Controls−Canada (VD001)
Controls−USA (VD001)
ME/CFS−Canada (AD001)
Controls−Canada (AD001)

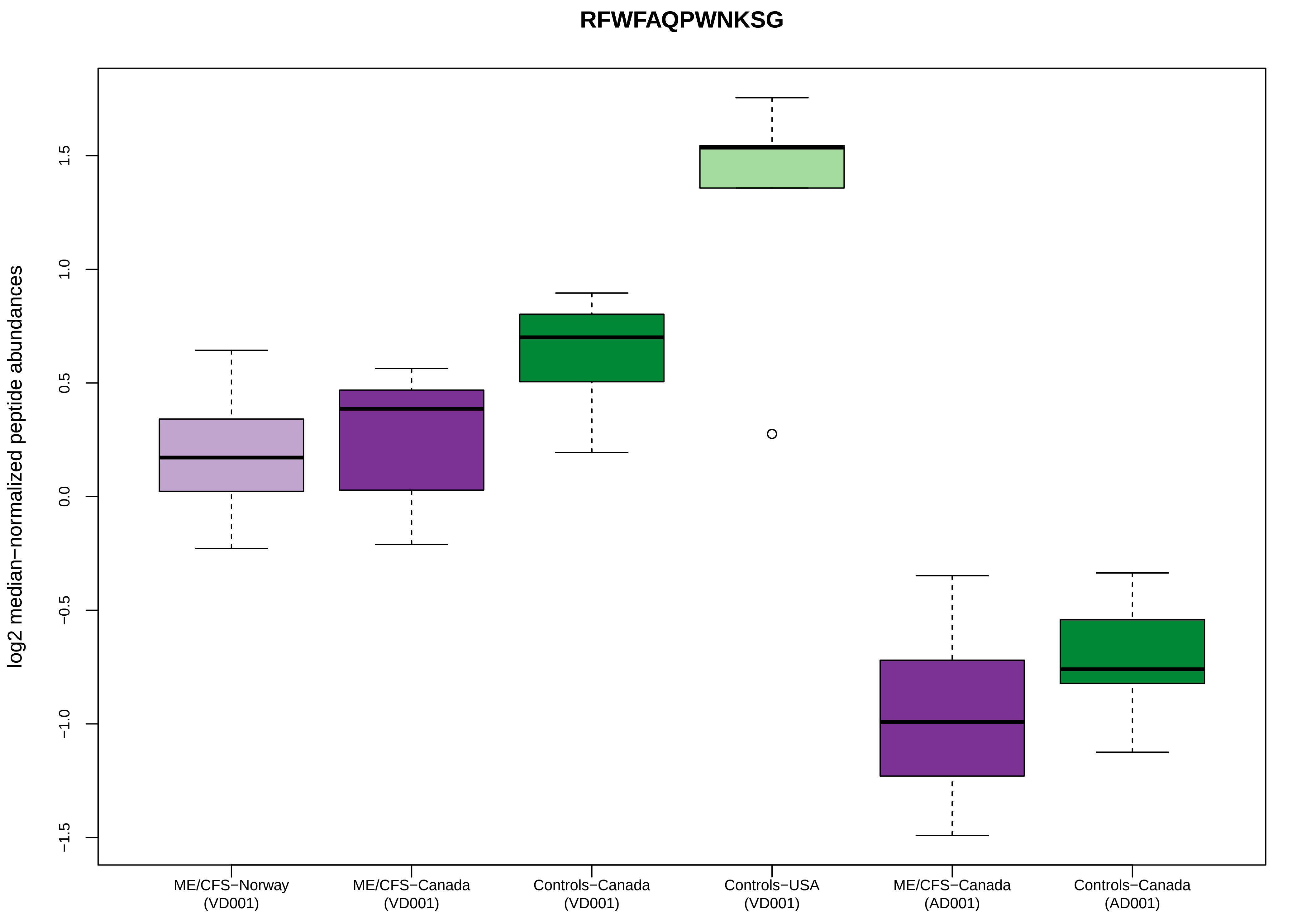
RFWFAQPWNKSG
log2 median−normalized peptide abundances
1.5
1.0
0.5
0.0
−0.5
−1.0
−1.5
ME/CFS−Norway (VD001)
ME/CFS−Canada (VD001)
Controls−Canada (VD001)
Controls−USA (VD001)
ME/CFS−Canada (AD001)
Controls−Canada (AD001)

RFWRANVLGALS

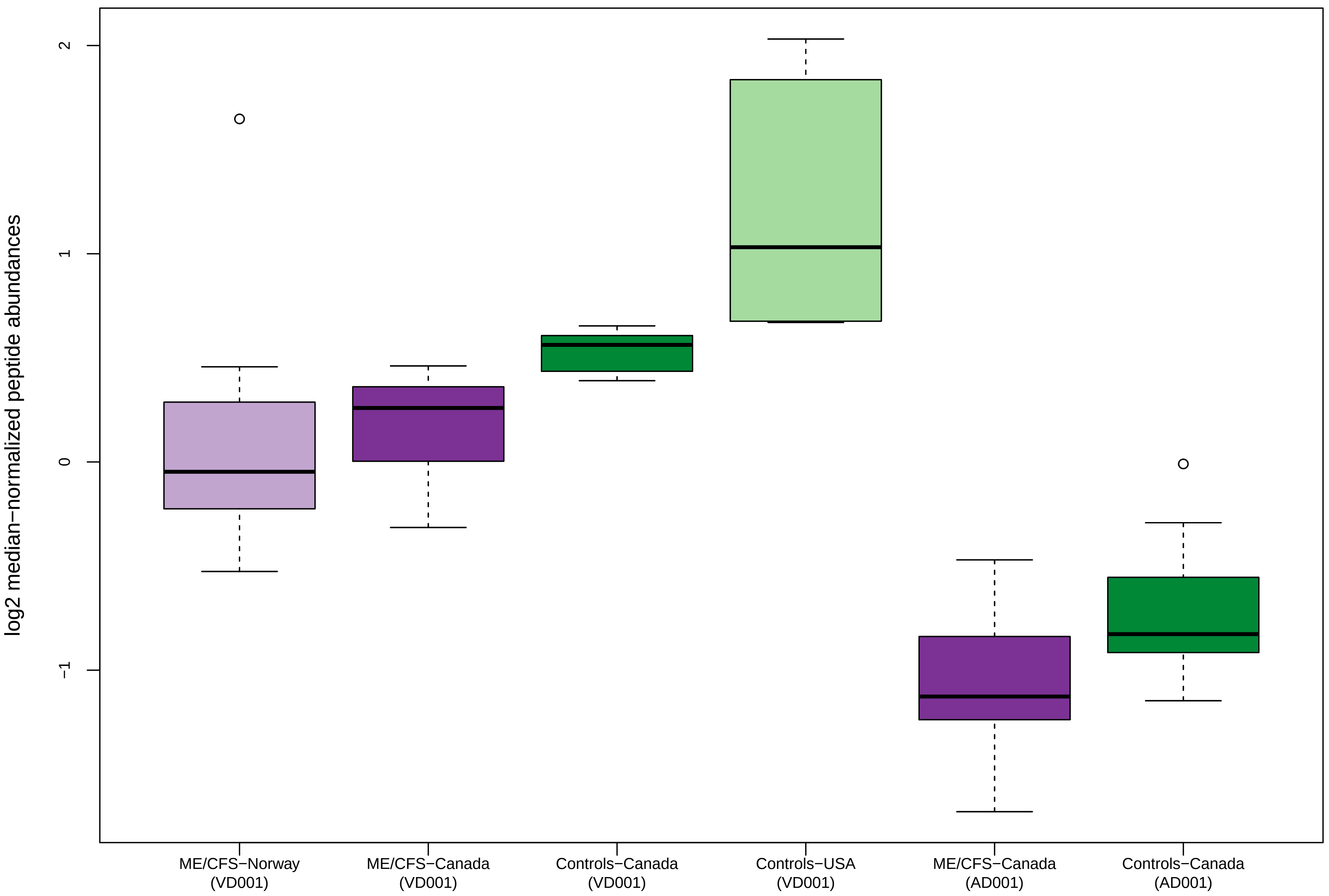

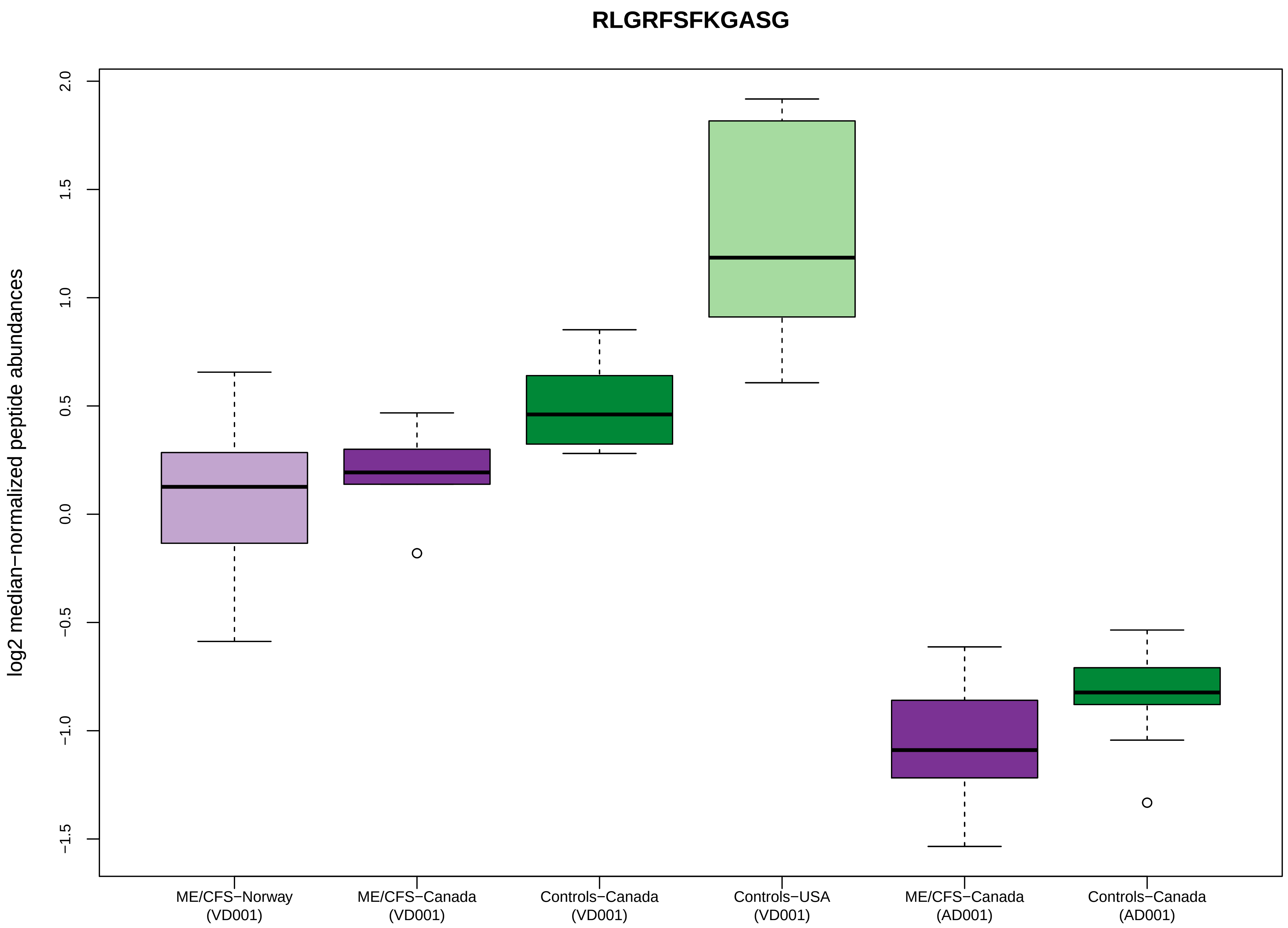

RLGRFSFKGASG
log2 median−normalized peptide abundances
2.0
1.5
1.0
0.5
0.0
−0.5
−1.0
−1.5
ME/CFS−Norway (VD001)
ME/CFS−Canada (VD001)
Controls−Canada (VD001)
Controls−USA (VD001)
ME/CFS−Canada (AD001)
Controls−Canada (AD001)

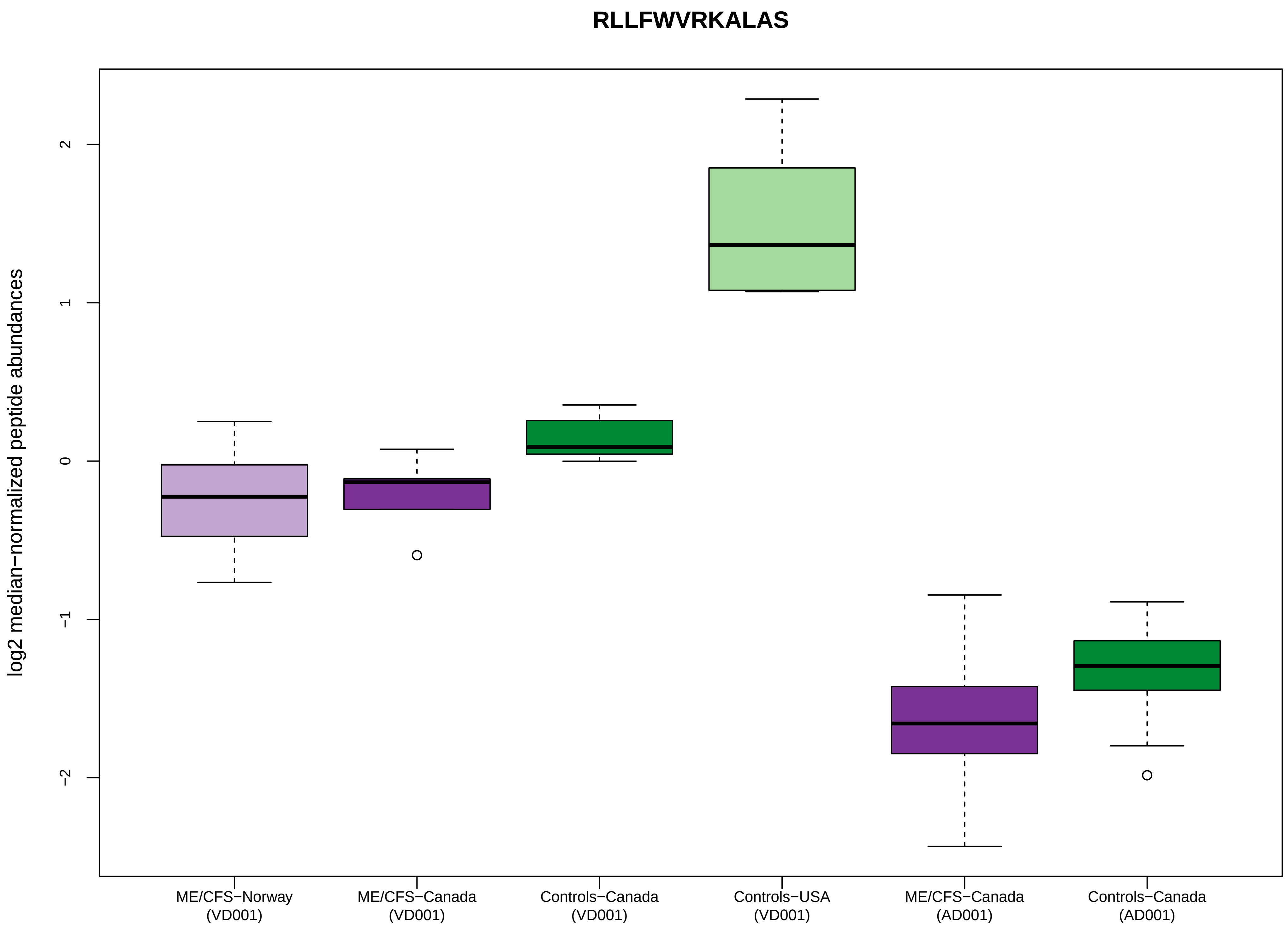

RLLFWVRKALAS
log2 median−normalized peptide abundances
2
1
0
−1
−2
ME/CFS−Norway (VD001)
ME/CFS−Canada (VD001)
Controls−Canada (VD001)
Controls−USA (VD001)
ME/CFS−Canada (AD001)
Controls−Canada (AD001)

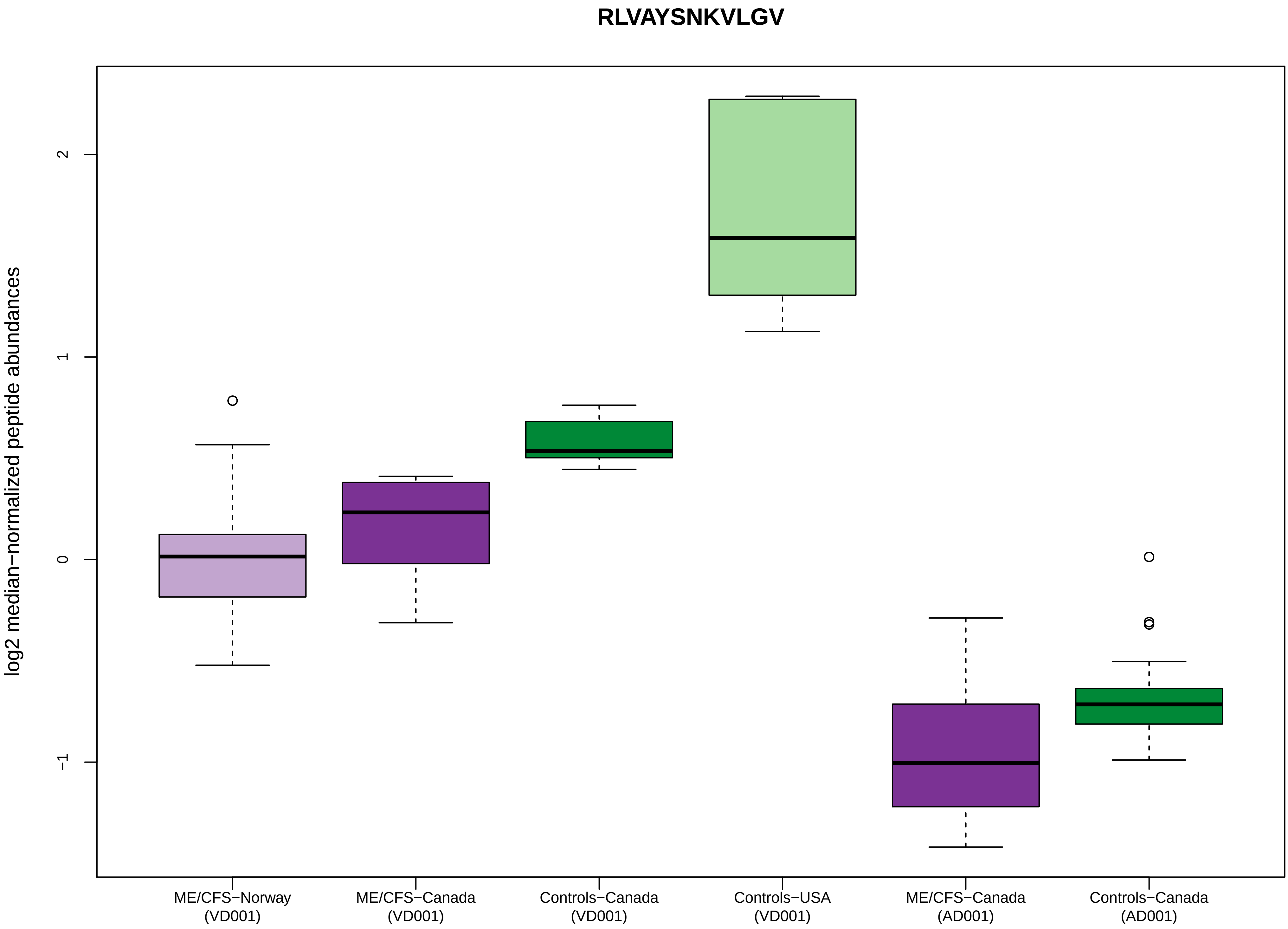

RLVAYSNKVLGV
log2 median−normalized peptide abundances
2
1
0
−1
ME/CFS−Norway (VD001)
ME/CFS−Canada (VD001)
Controls−Canada (VD001)
Controls−USA (VD001)
ME/CFS−Canada (AD001)
Controls−Canada (AD001)

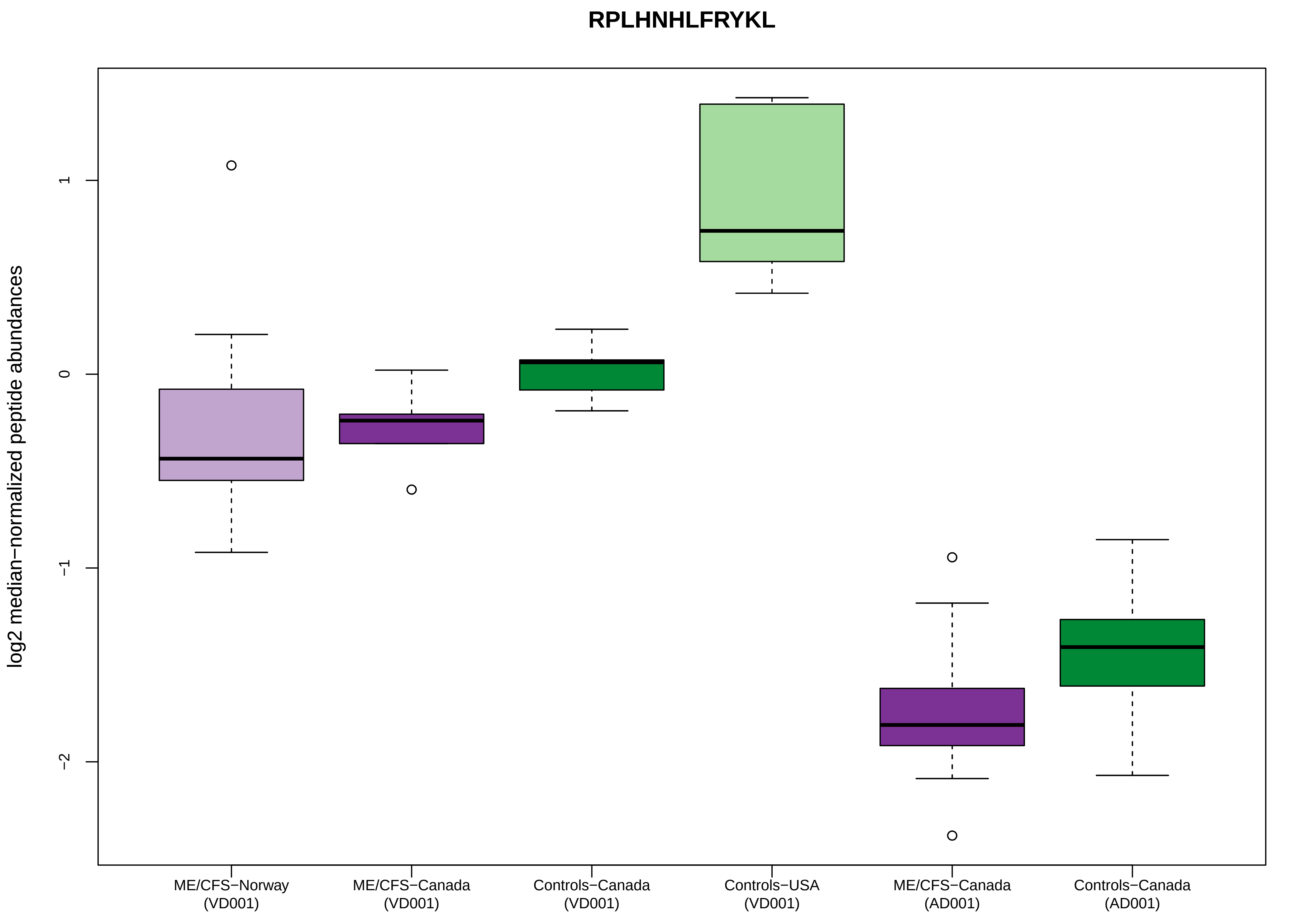
RPLHNHLFRYKL
log2 median−normalized peptide abundances
1
0
−1
−2
ME/CFS−Norway (VD001)
ME/CFS−Canada (VD001)
Controls−Canada (VD001)
Controls−USA (VD001)
ME/CFS−Canada (AD001)
Controls−Canada (AD001)

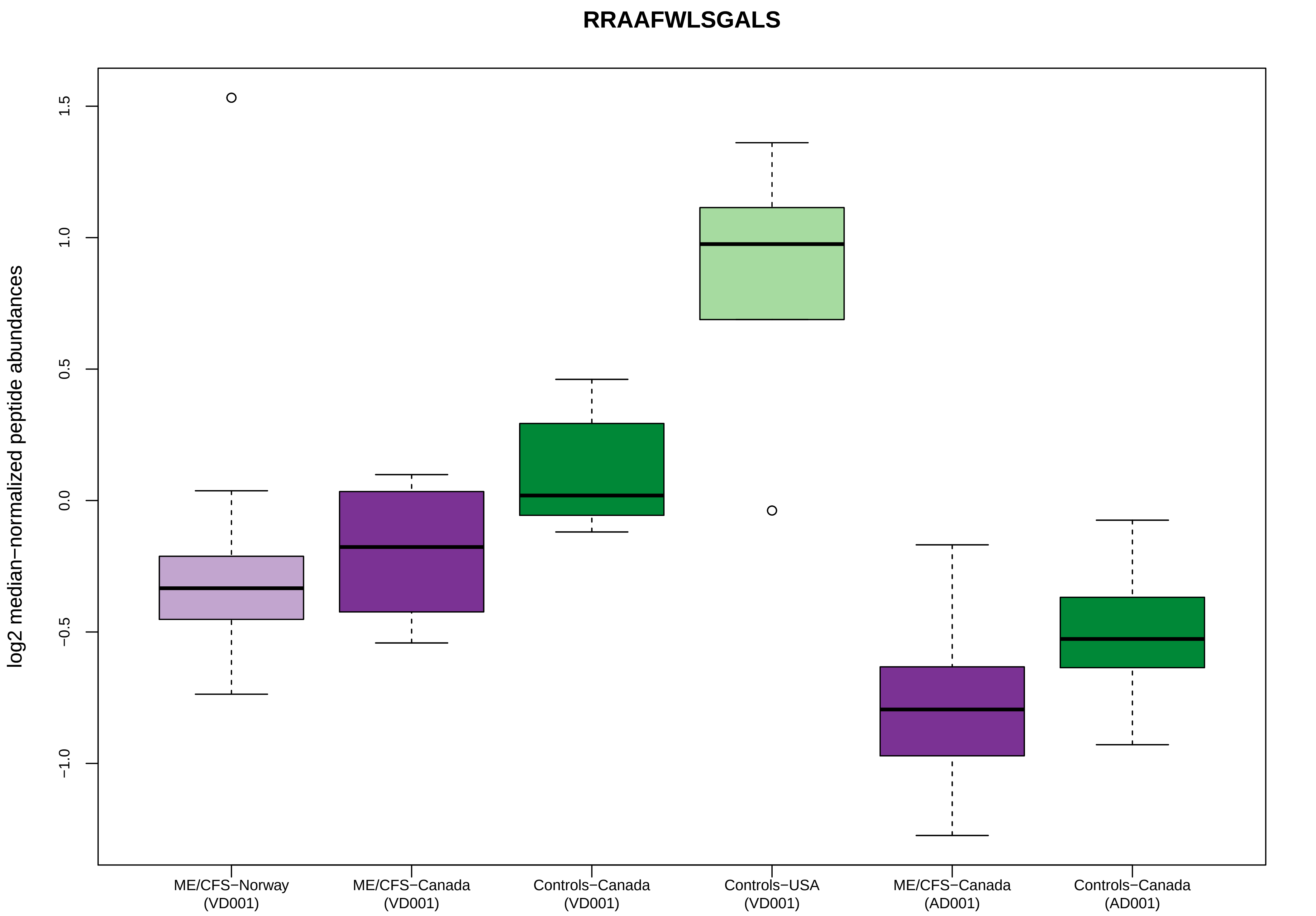
RRAAFWLSGALS
log2 median−normalized peptide abundances
1.5
1.0
0.5
0.0
−0.5
−1.0
ME/CFS−Norway (VD001)
ME/CFS−Canada (VD001)
Controls−Canada (VD001)
Controls−USA (VD001)
ME/CFS−Canada (AD001)
Controls−Canada (AD001)

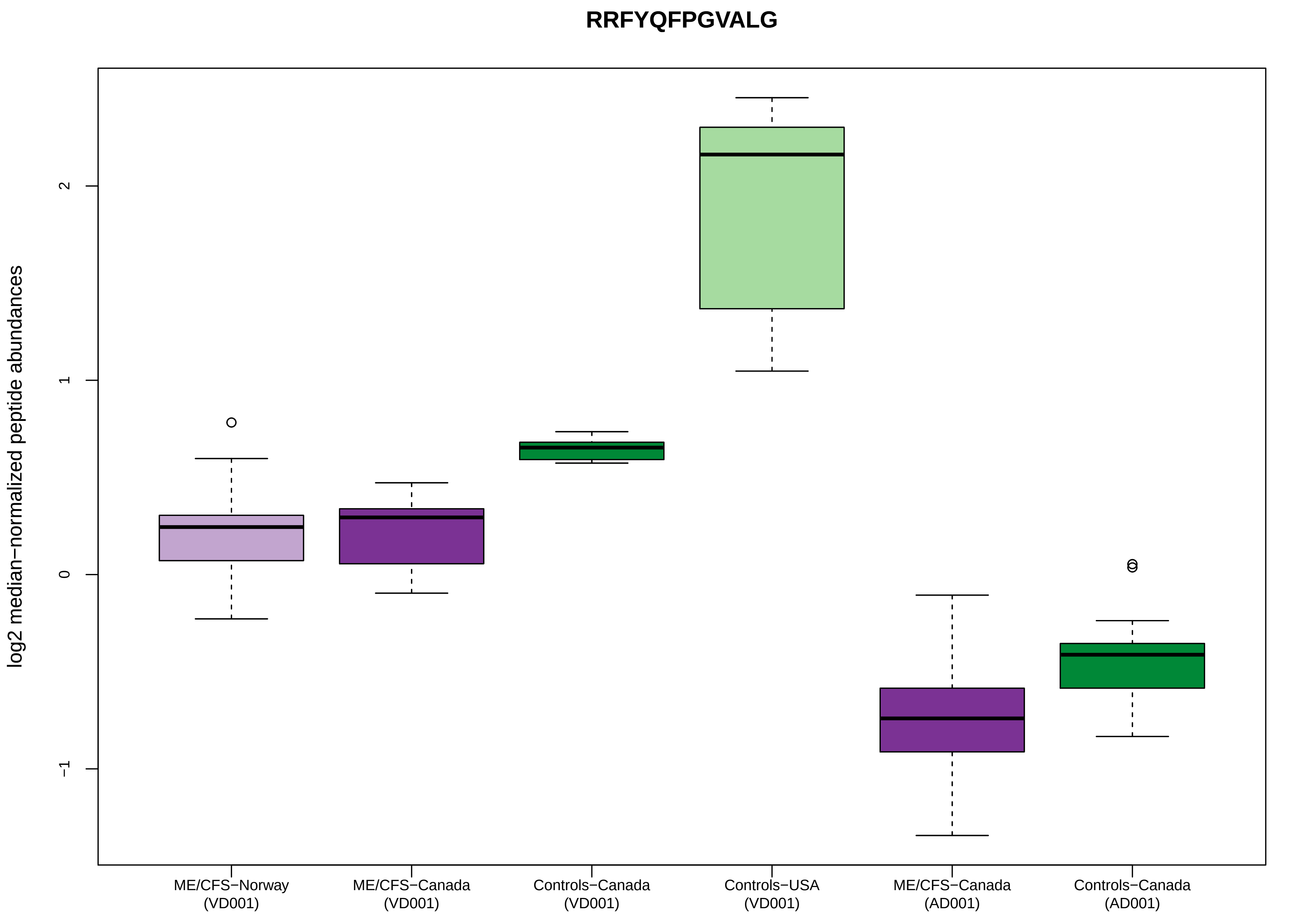
RRFYQFPGVALG
log2 median−normalized peptide abundances
2
1
0
−1
ME/CFS−Norway (VD001)
ME/CFS−Canada (VD001)
Controls−Canada (VD001)
Controls−USA (VD001)
ME/CFS−Canada (AD001)
Controls−Canada (AD001)

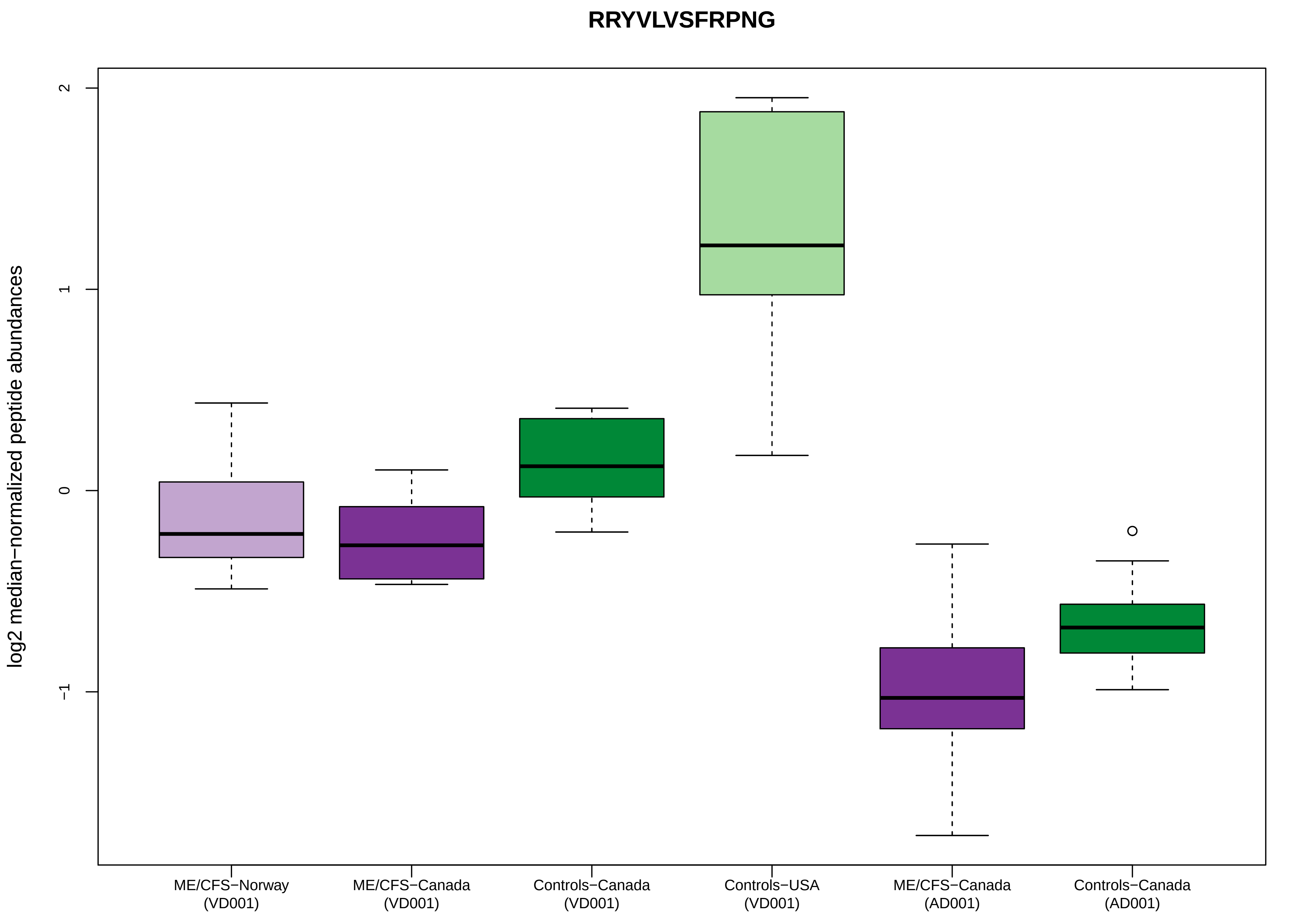

RRYVLVSFRPNG
log2 median−normalized peptide abundances
2
1
0
−1
ME/CFS−Norway (VD001)
ME/CFS−Canada (VD001)
Controls−Canada (VD001)
Controls−USA (VD001)
ME/CFS−Canada (AD001)
Controls−Canada (AD001)

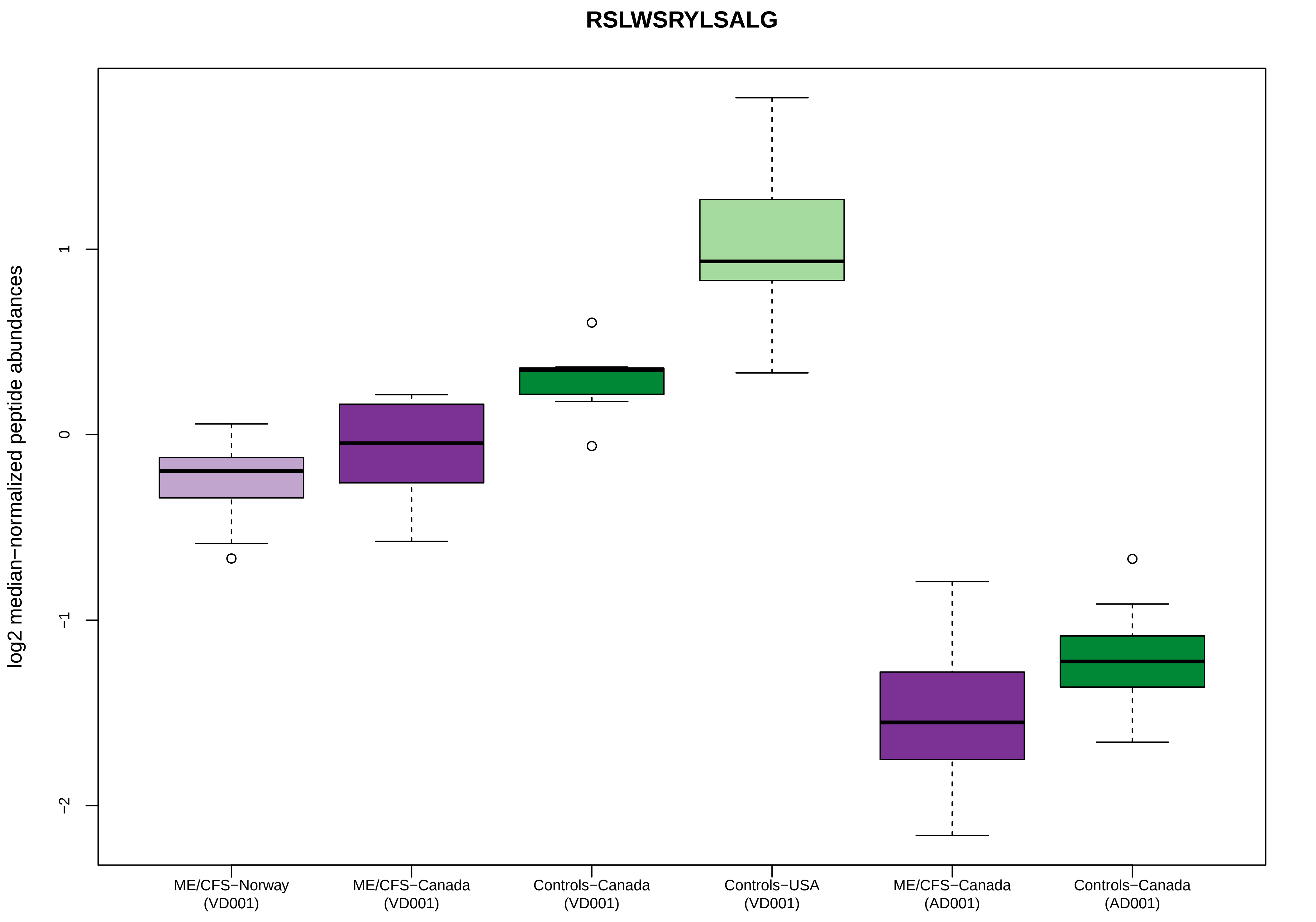
RSLWSRYLSALG
log2 median−normalized peptide abundances
1
0
−1
−2
ME/CFS−Norway (VD001)
ME/CFS−Canada (VD001)
Controls−Canada (VD001)
Controls−USA (VD001)
ME/CFS−Canada (AD001)
Controls−Canada (AD001)

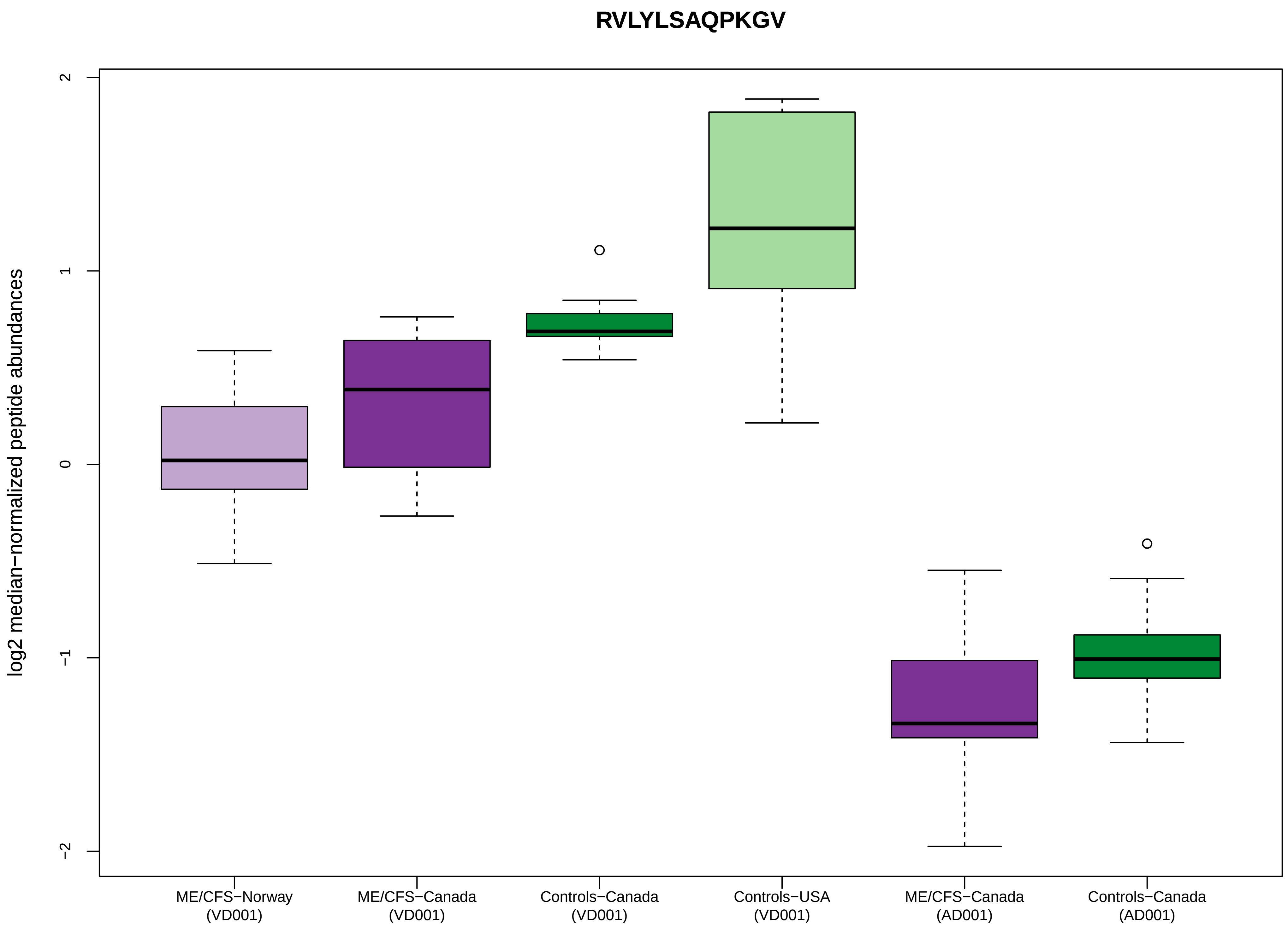
RVLYLSAQPKGV
log2 median−normalized peptide abundances
2
1
0
−1
−2
ME/CFS−Norway (VD001)
ME/CFS−Canada (VD001)
Controls−Canada (VD001)
Controls−USA (VD001)
ME/CFS−Canada (AD001)
Controls−Canada (AD001)

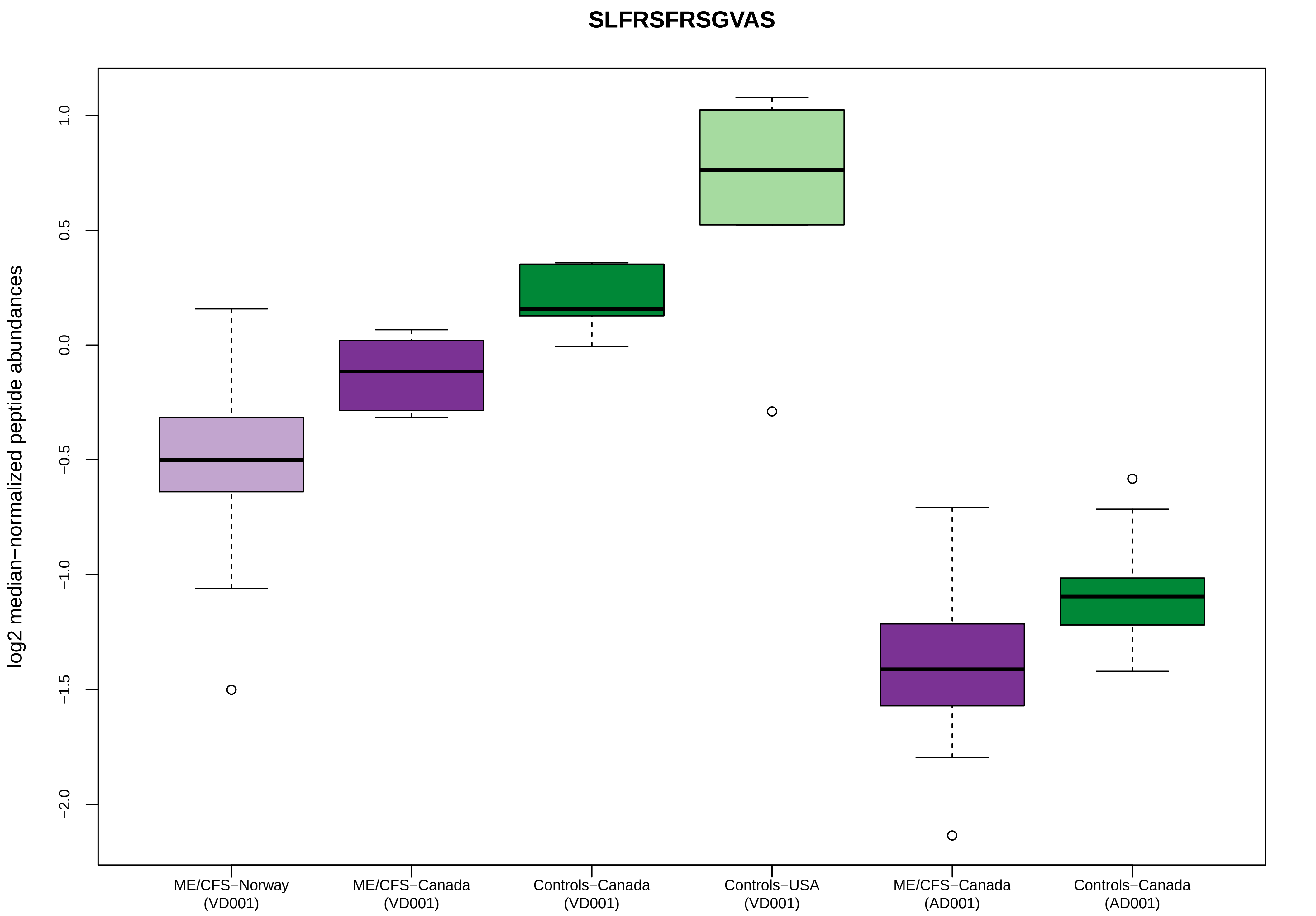

SLFRSFRSGVAS
log2 median−normalized peptide abundances
1.0
0.5
0.0
−0.5
−1.0
−1.5
−2.0
ME/CFS−Norway (VD001)
ME/CFS−Canada (VD001)
Controls−Canada (VD001)
Controls−USA (VD001)
ME/CFS−Canada (AD001)
Controls−Canada (AD001)

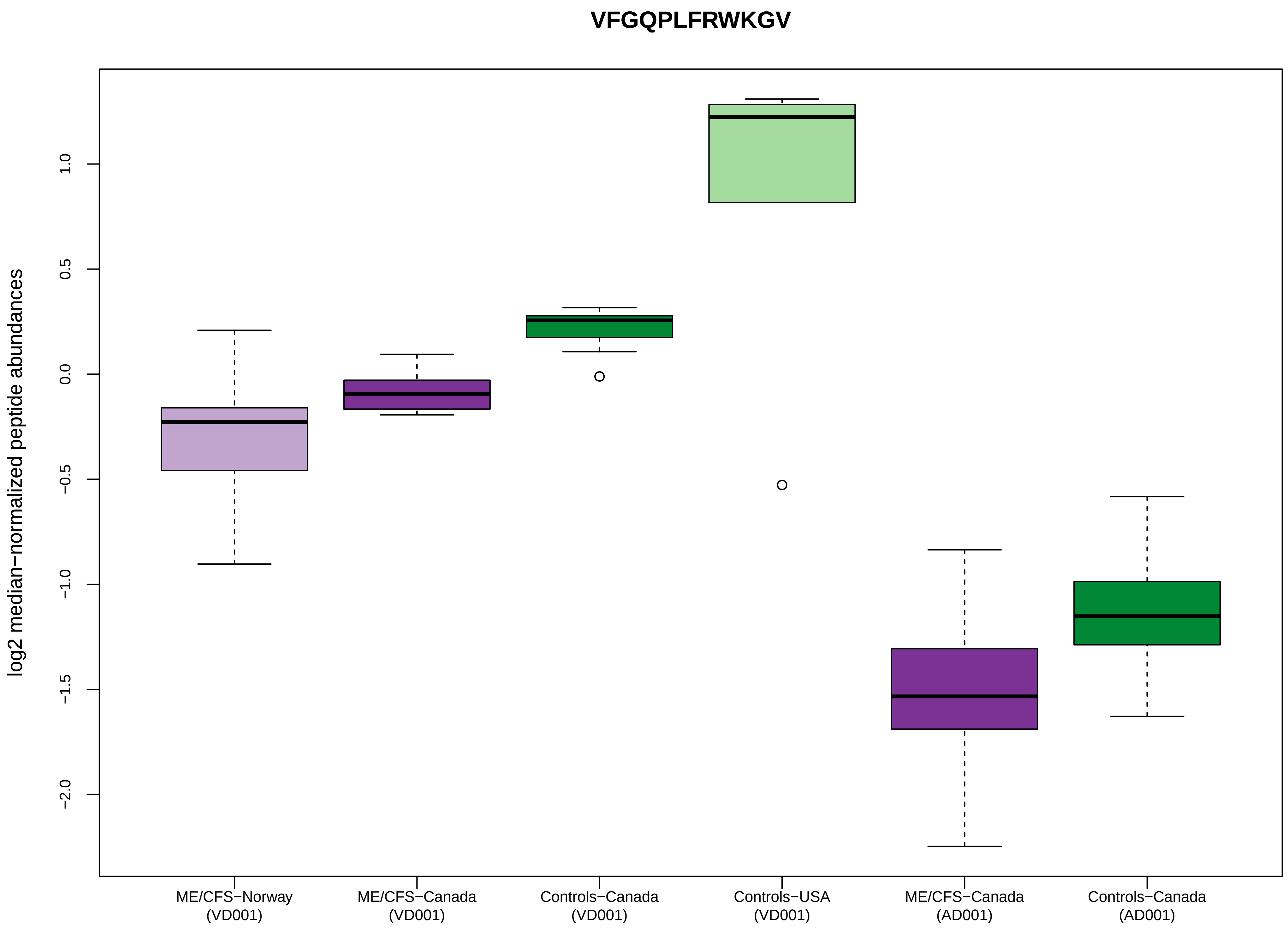
VFGQPLFRWKGV
log2 median−normalized peptide abundances
1.0
0.5
0.0
−0.5
−1.0
−1.5
−2.0
ME/CFS−Norway (VD001)
ME/CFS−Canada (VD001)
Controls−Canada (VD001)
Controls−USA (VD001)
ME/CFS−Canada (AD001)
Controls−Canada (AD001)

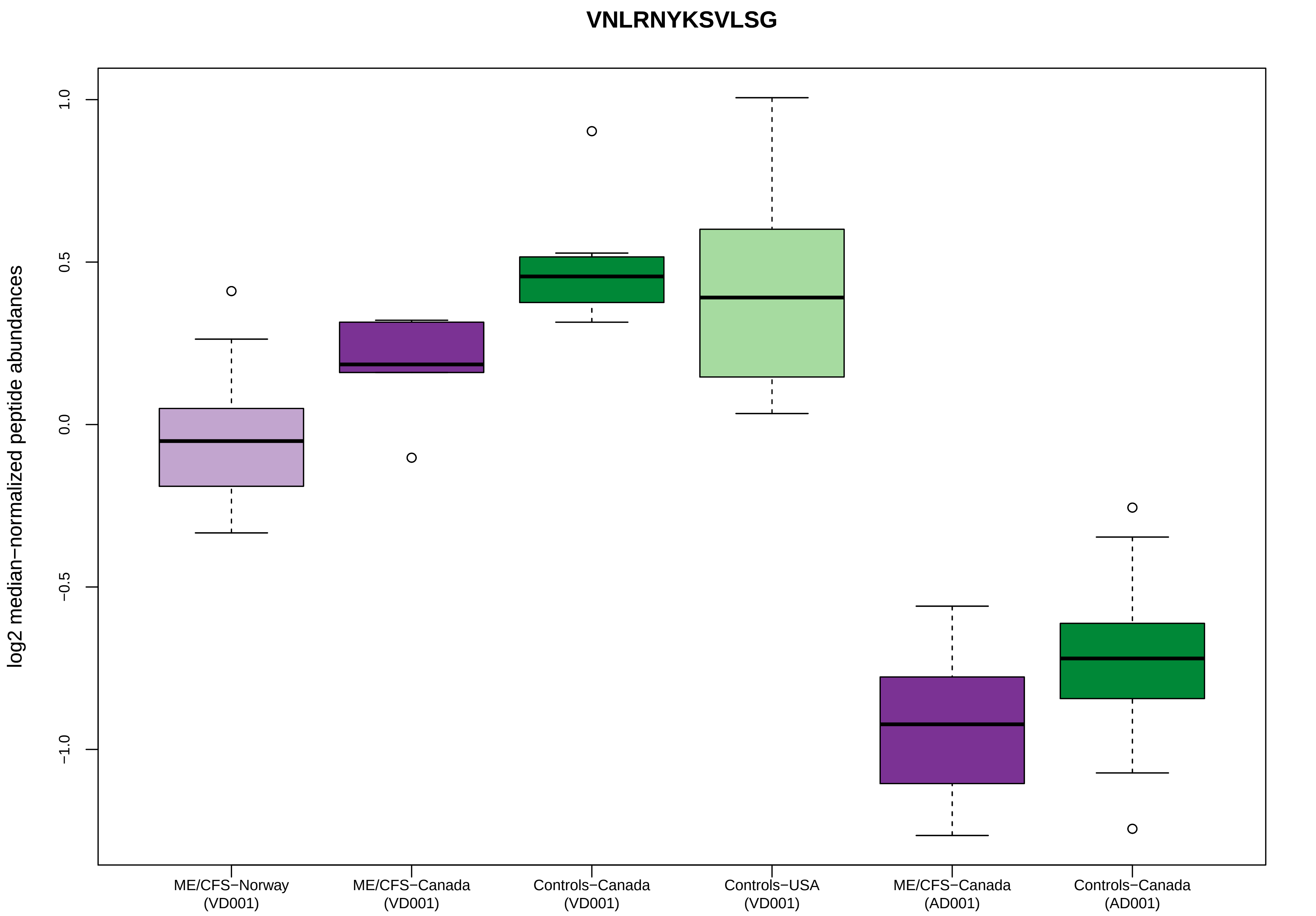
VNLRNYKSVLSG
log2 median−normalized peptide abundances
1.0
0.5
0.0
−0.5
−1.0
ME/CFS−Norway (VD001)
ME/CFS−Canada (VD001)
Controls−Canada (VD001)
Controls−USA (VD001)
ME/CFS−Canada (AD001)
Controls−Canada (AD001)

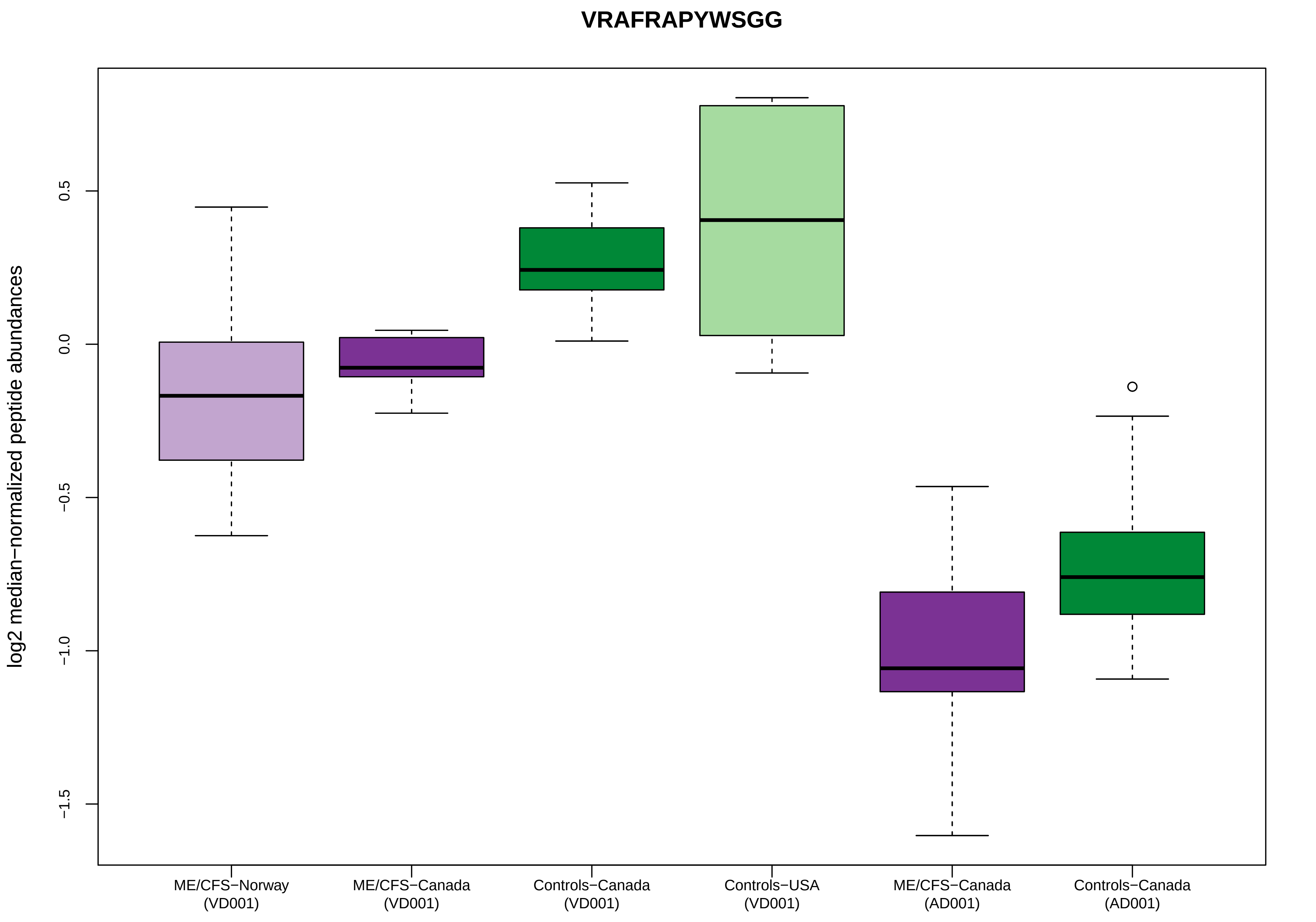

VRAFRAPYWSGG
log2 median−normalized peptide abundances
0.5
0.0
−0.5
−1.0
−1.5
ME/CFS−Norway (VD001)
ME/CFS−Canada (VD001)
Controls−Canada (VD001)
Controls−USA (VD001)
ME/CFS−Canada (AD001)
Controls−Canada (AD001)

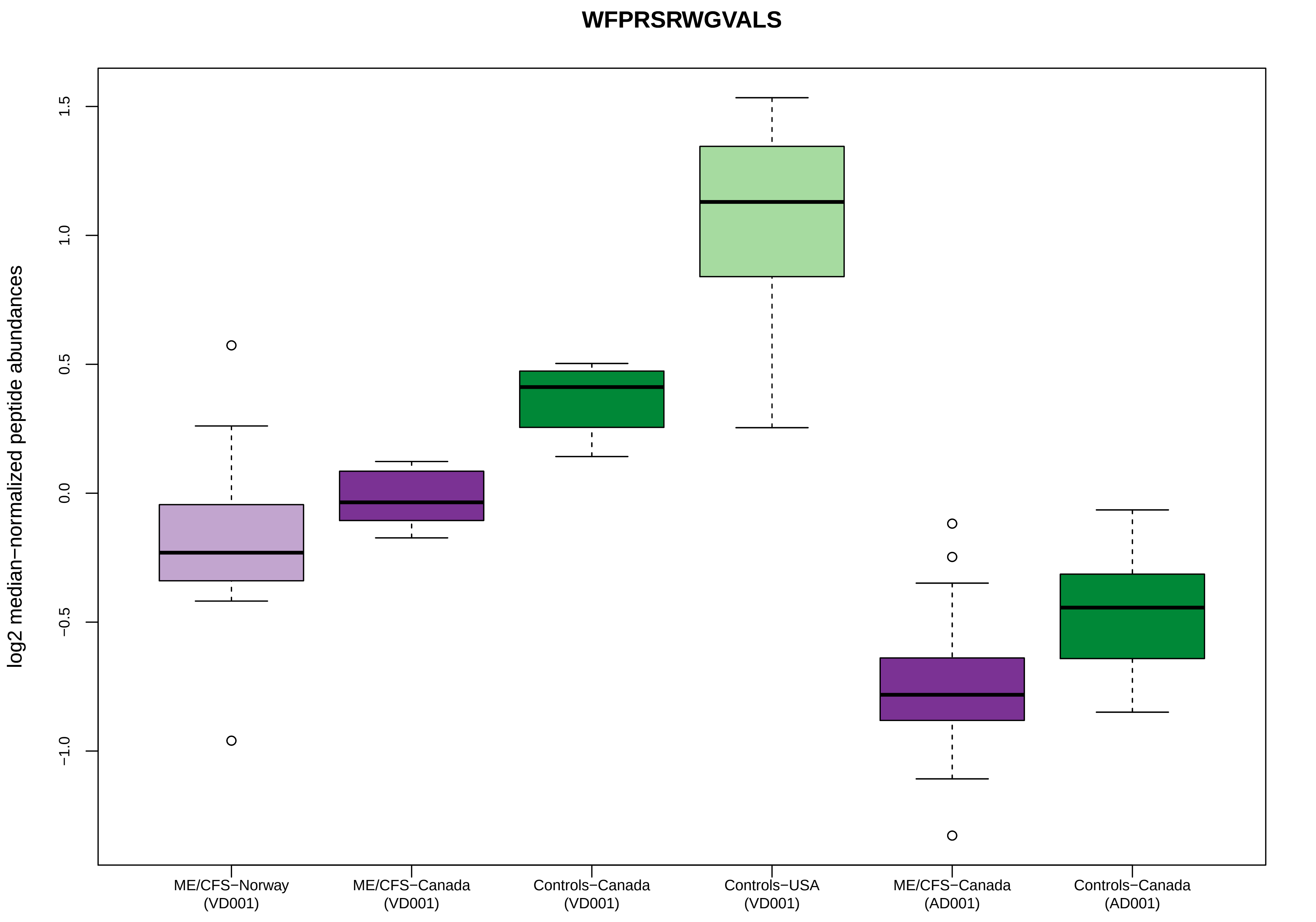

WFPRSRWGVALS
log2 median−normalized peptide abundances
1.5
1.0
0.5
0.0
−0.5
−1.0
ME/CFS−Norway (VD001)
ME/CFS−Canada (VD001)
Controls−Canada (VD001)
Controls−USA (VD001)
ME/CFS−Canada (AD001)
Controls−Canada (AD001)

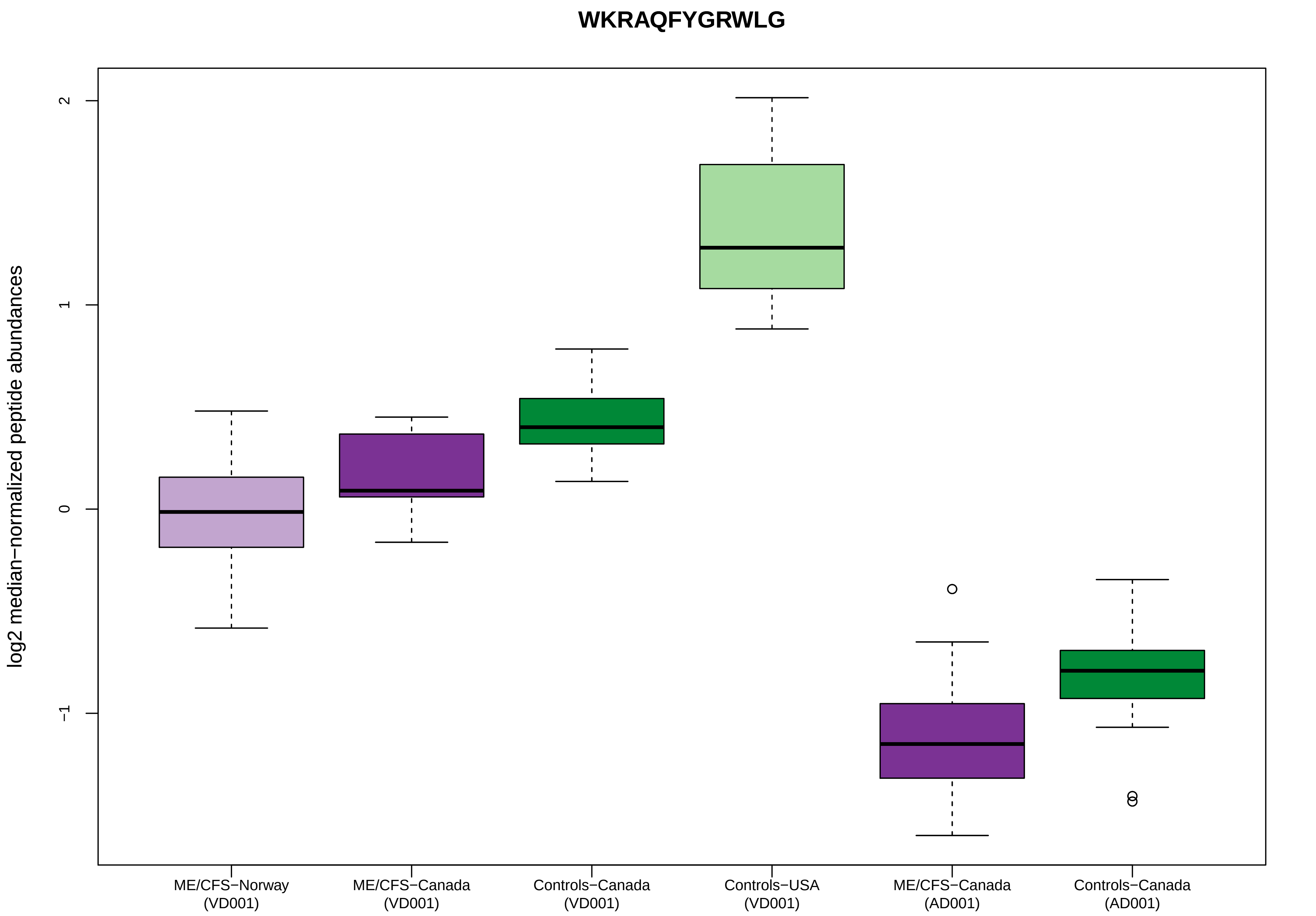
WKRAQFYGRWLG
log2 median−normalized peptide abundances
2
1
0
−1
ME/CFS−Norway (VD001)
ME/CFS−Canada (VD001)
Controls−Canada (VD001)
Controls−USA (VD001)
ME/CFS−Canada (AD001)
Controls−Canada (AD001)

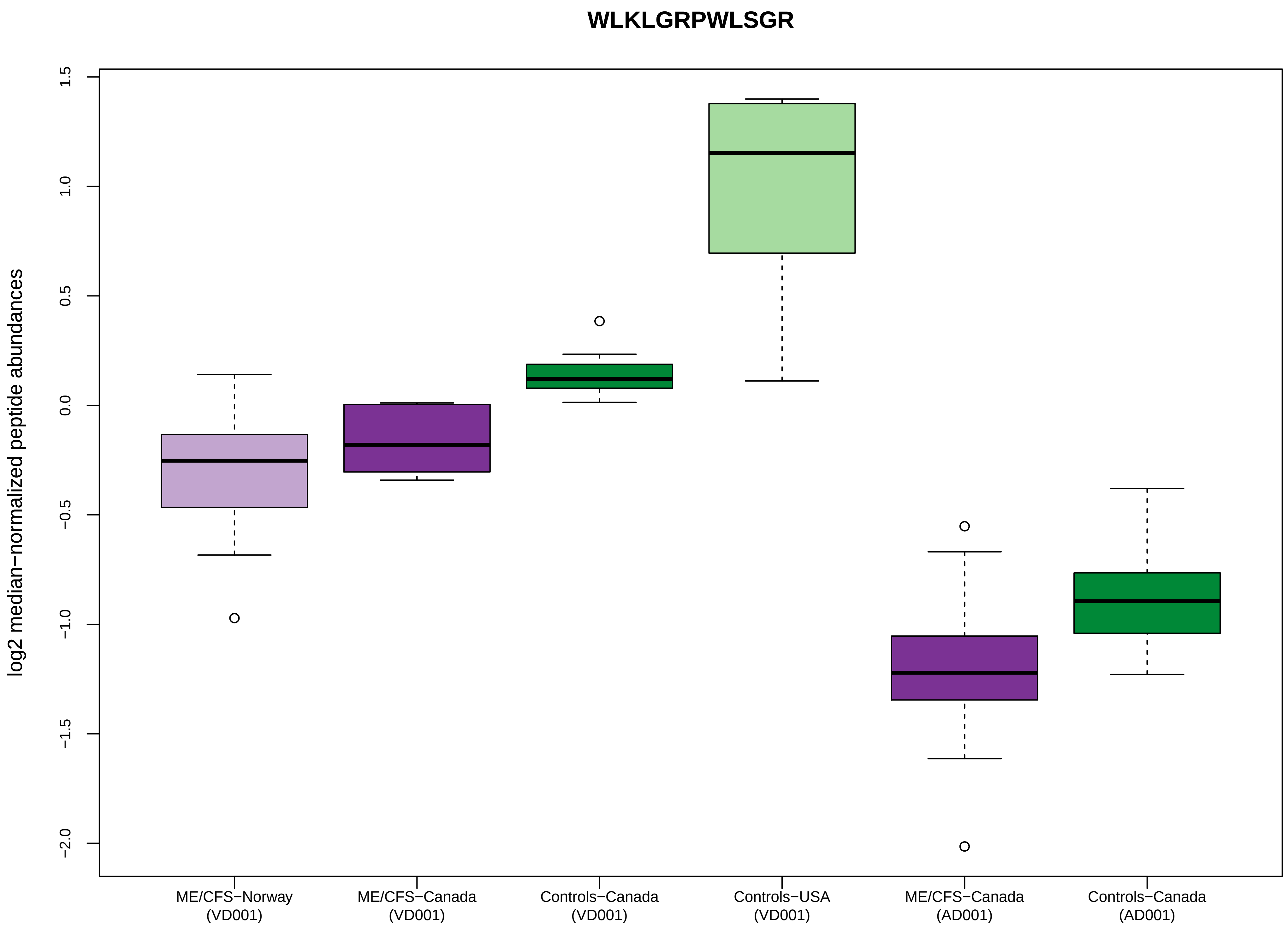

WLKLGRPWLSGR
log2 median−normalized peptide abundances
1.5
1.0
0.5
0.0
−0.5
−1.0
−1.5
−2.0
ME/CFS−Norway (VD001)
ME/CFS−Canada (VD001)
Controls−Canada (VD001)
Controls−USA (VD001)
ME/CFS−Canada (AD001)
Controls−Canada (AD001)

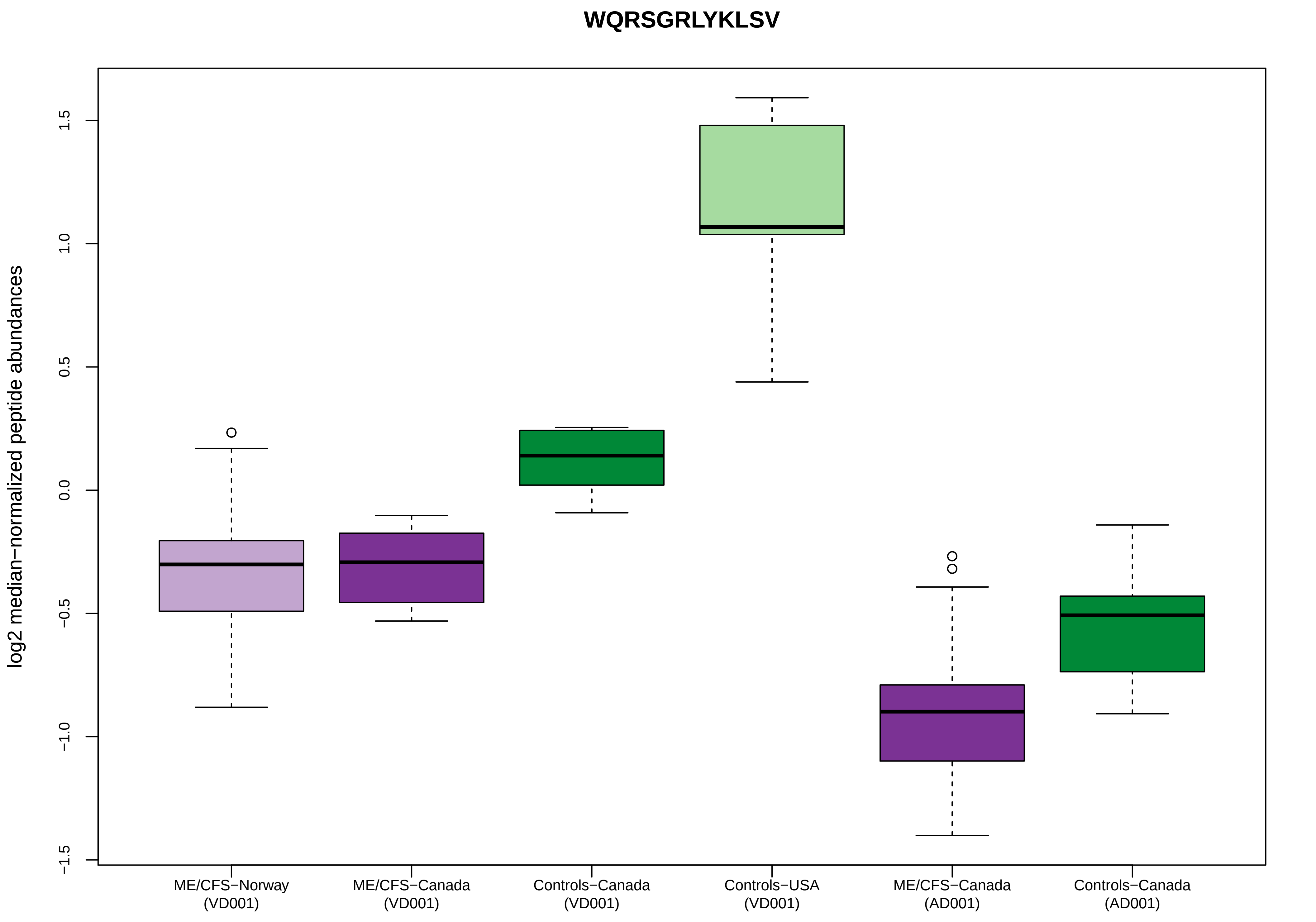
WQRSGRLYKLSV
log2 median−normalized peptide abundances
1.5
1.0
0.5
0.0
−0.5
−1.0
−1.5
ME/CFS−Norway (VD001)
ME/CFS−Canada (VD001)
Controls−Canada (VD001)
Controls−USA (VD001)
ME/CFS−Canada (AD001)
Controls−Canada (AD001)

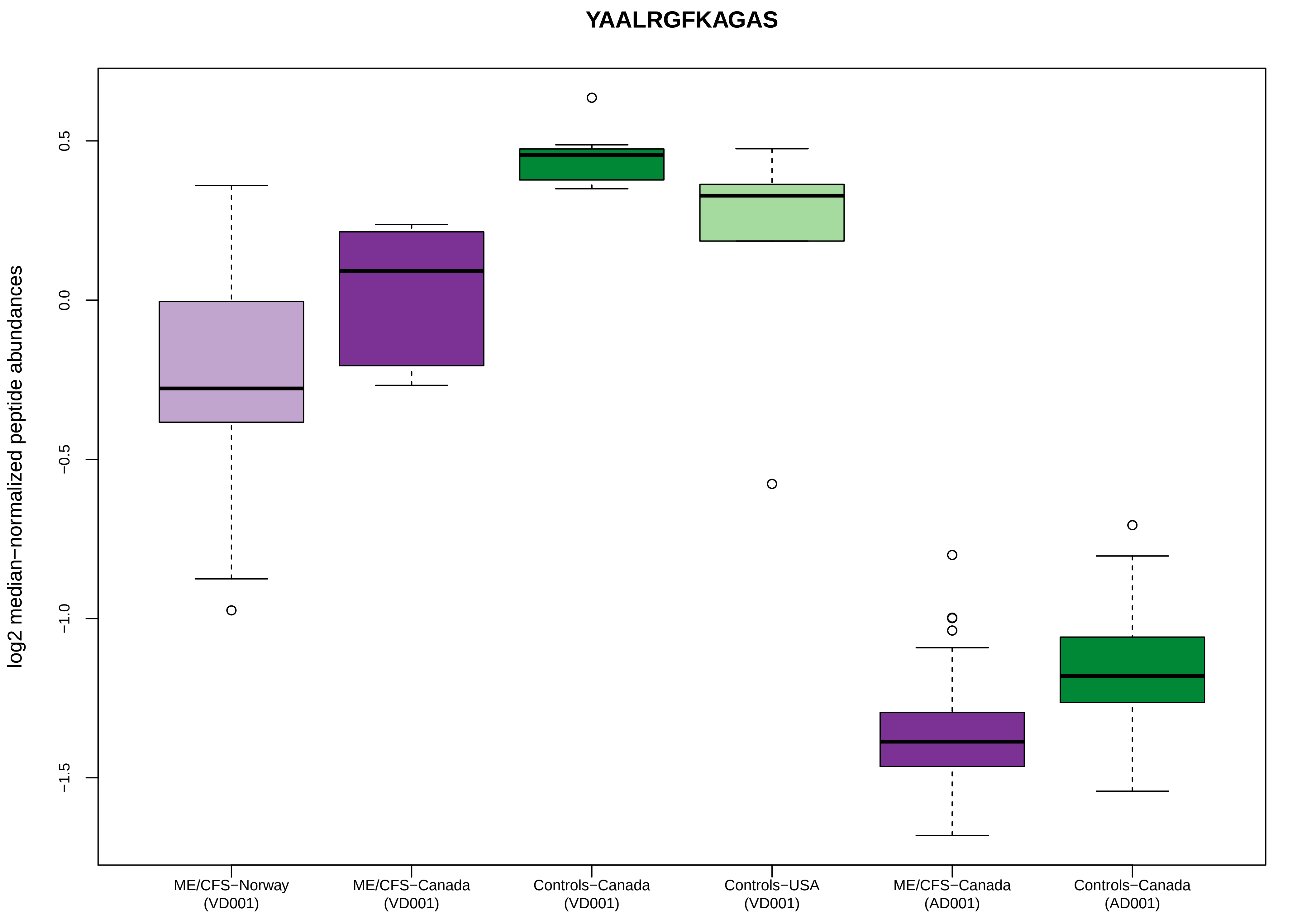
YAALRGFKAGAS
log2 median−normalized peptide abundances
0.5
0.0
−0.5
−1.0
−1.5
ME/CFS−Norway (VD001)
ME/CFS−Canada (VD001)
Controls−Canada (VD001)
Controls−USA (VD001)
ME/CFS−Canada (AD001)
Controls−Canada (AD001)

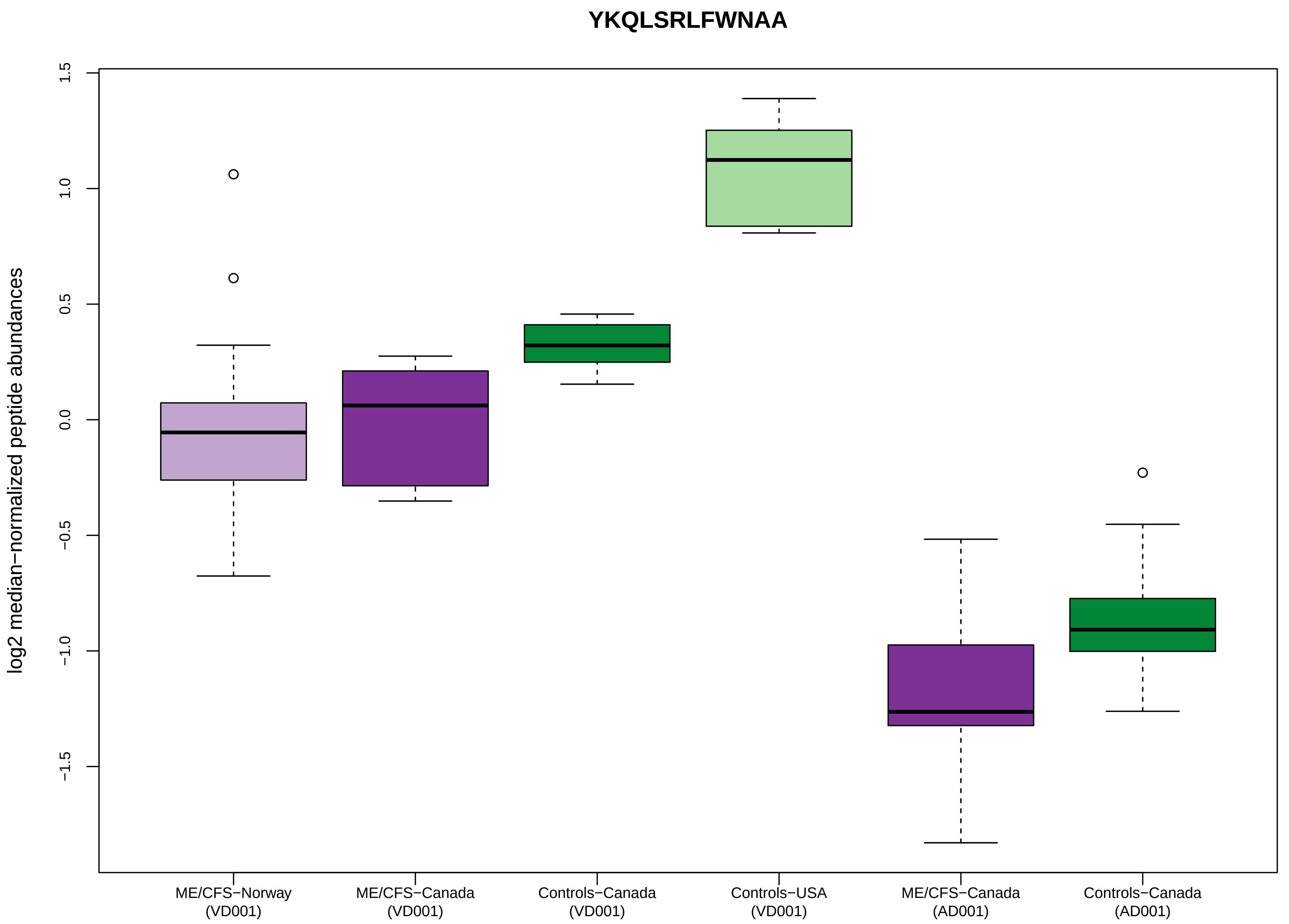
YKQLSRLFWNAA
log2 median−normalized peptide abundances
1.5
1.0
0.5
0.0
−0.5
−1.0
−1.5
ME/CFS−Norway (VD001)
ME/CFS−Canada (VD001)
Controls−Canada (VD001)
Controls−USA (VD001)
ME/CFS−Canada (AD001)
Controls−Canada (AD001)

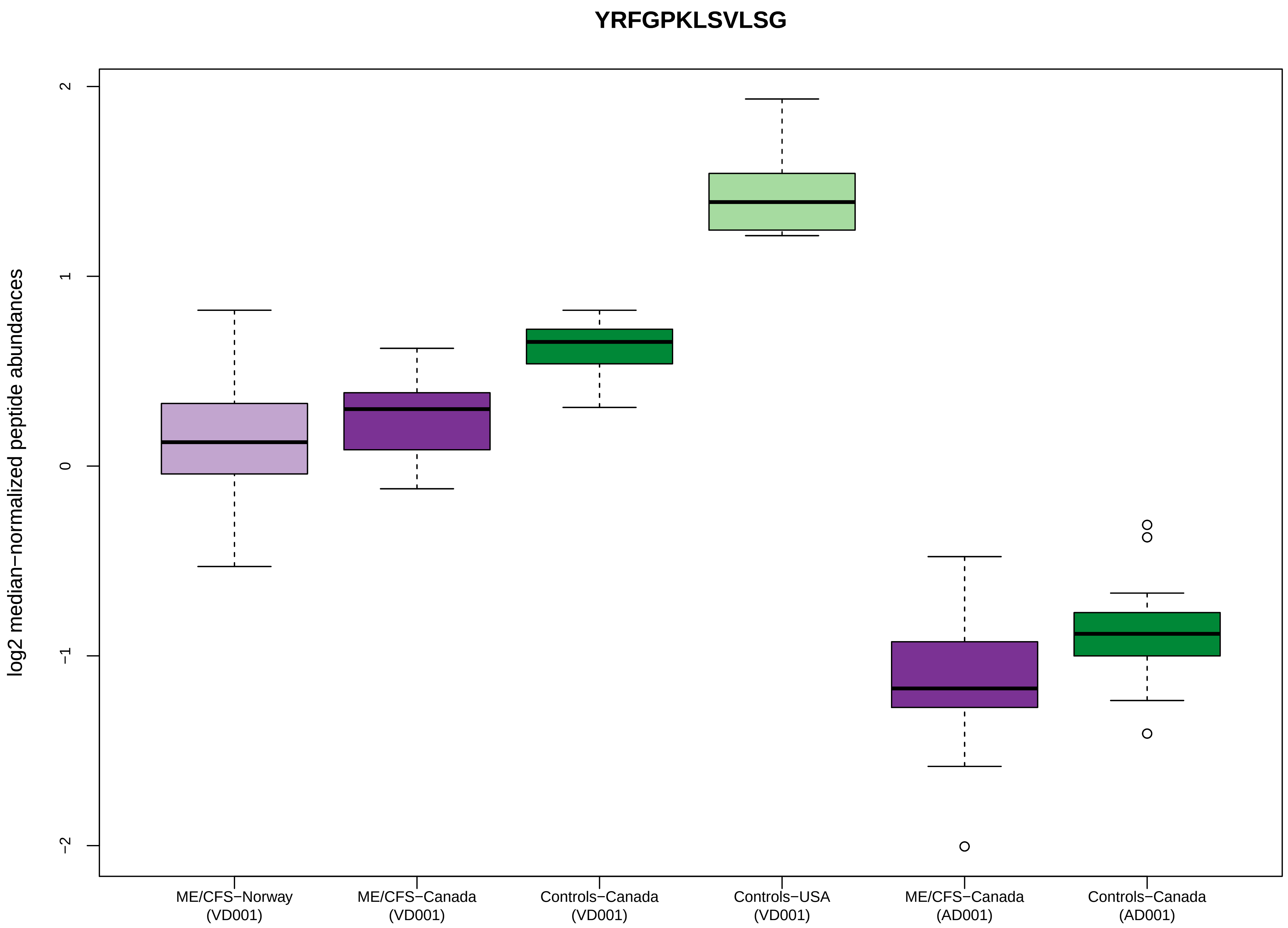
YRFGPKLSVLSG
log2 median−normalized peptide abundances
2
1
0
−1
−2
ME/CFS−Norway (VD001)
ME/CFS−Canada (VD001)
Controls−Canada (VD001)
Controls−USA (VD001)
ME/CFS−Canada (AD001)
Controls−Canada (AD001)

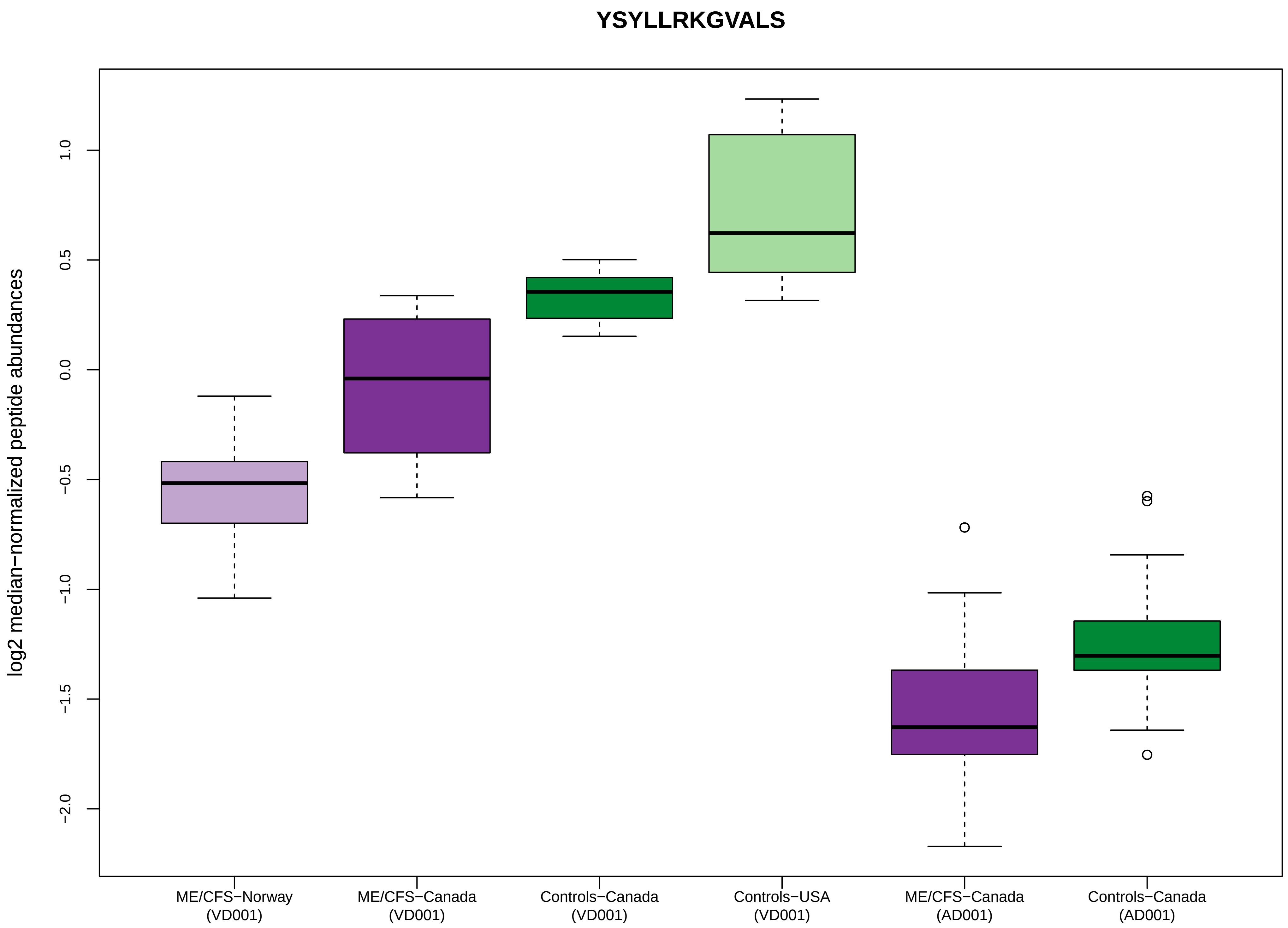
YSYLLRKGVALS
log2 median−normalized peptide abundances
1.0
0.5
0.0
−0.5
−1.0
−1.5
−2.0
ME/CFS−Norway (VD001)
ME/CFS−Canada (VD001)
Controls−Canada (VD001)
Controls−USA (VD001)
ME/CFS−Canada (AD001)
Controls−Canada (AD001)
