# Supplementary figures and images for: Immunosignature Analysis of Myalgic Encephalomyelitis/Chronic Fatigue Syndrome (ME/CFS)
Source: Mol Neurobiol. 2018 Oct 8;56(6):4249–57. doi: 10.1007/s12035-018-1354-8 (PMC6505503; doi:10.1007/s12035-018-1354-8)

Sample Correlation Boxplots (99 samples; 85987 features; pearson correlation)

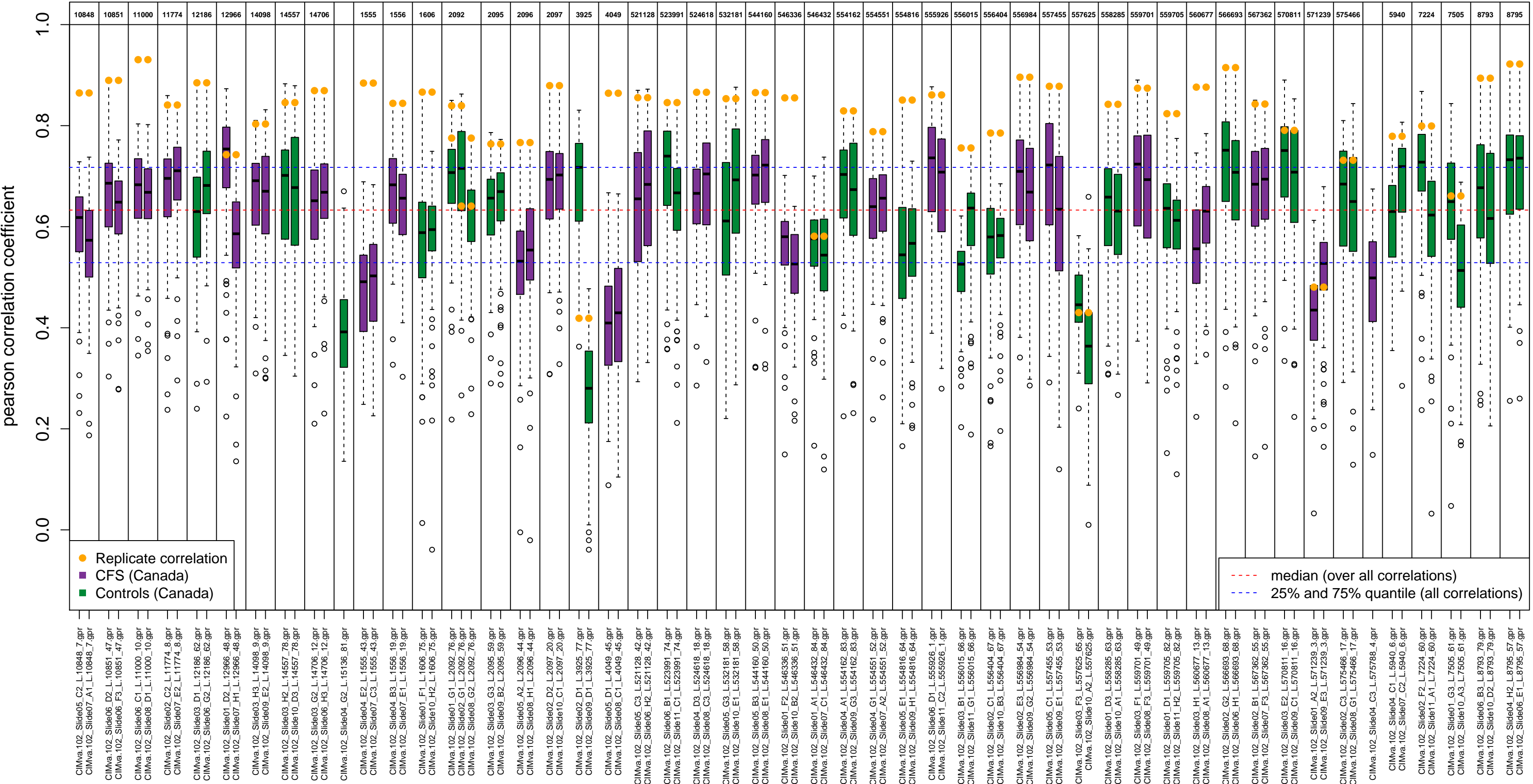

Supplement: Supplementary file 7 — (PDF 19 kb) [file 12035_2018_1354_MOESM7_ESM.pdf]

Sample Correlation Boxplots (94 samples; 111876 features; pearson correlation)

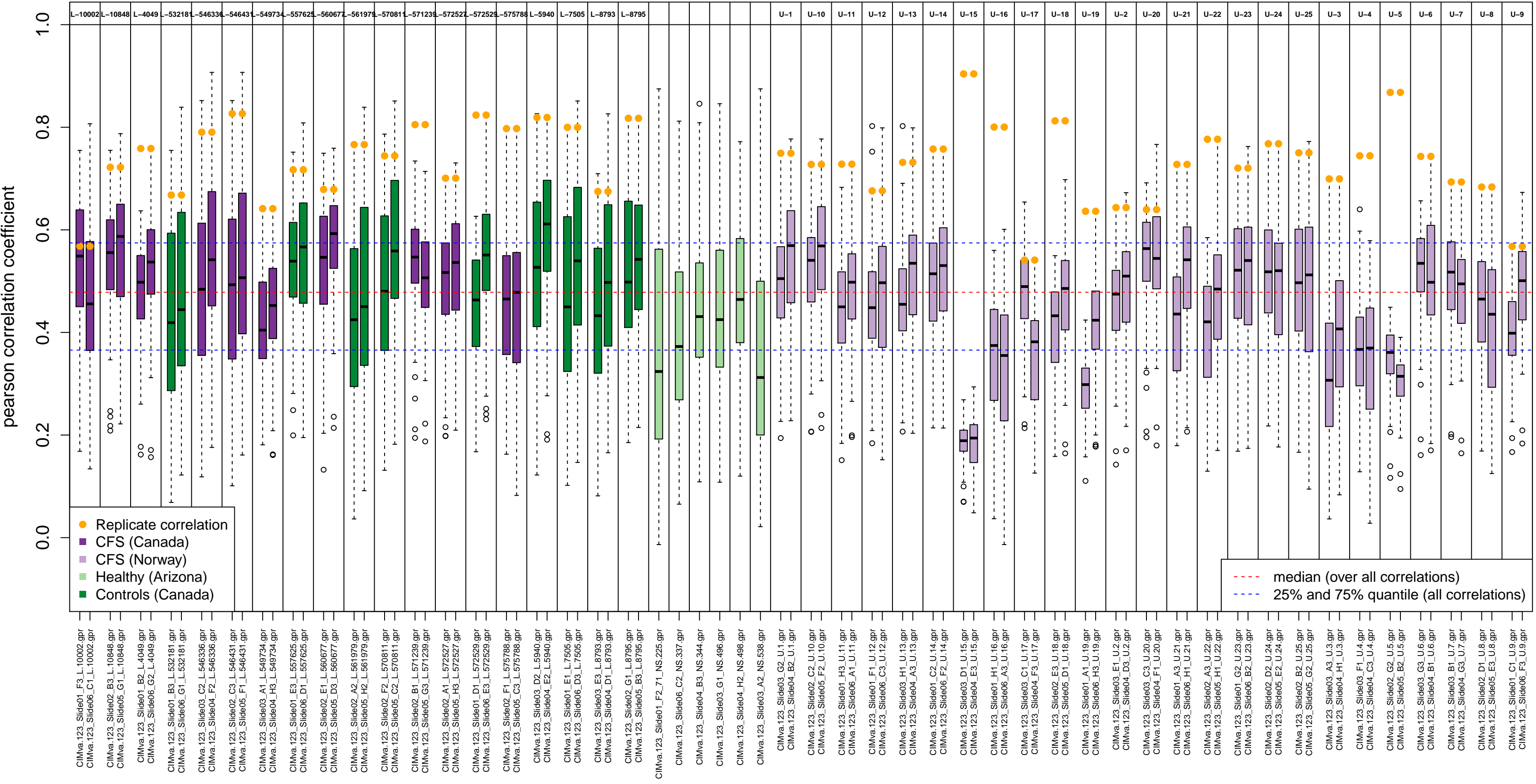

Supplement: Supplementary file 10 — (PDF 16 kb) [file 12035_2018_1354_MOESM10_ESM.pdf]

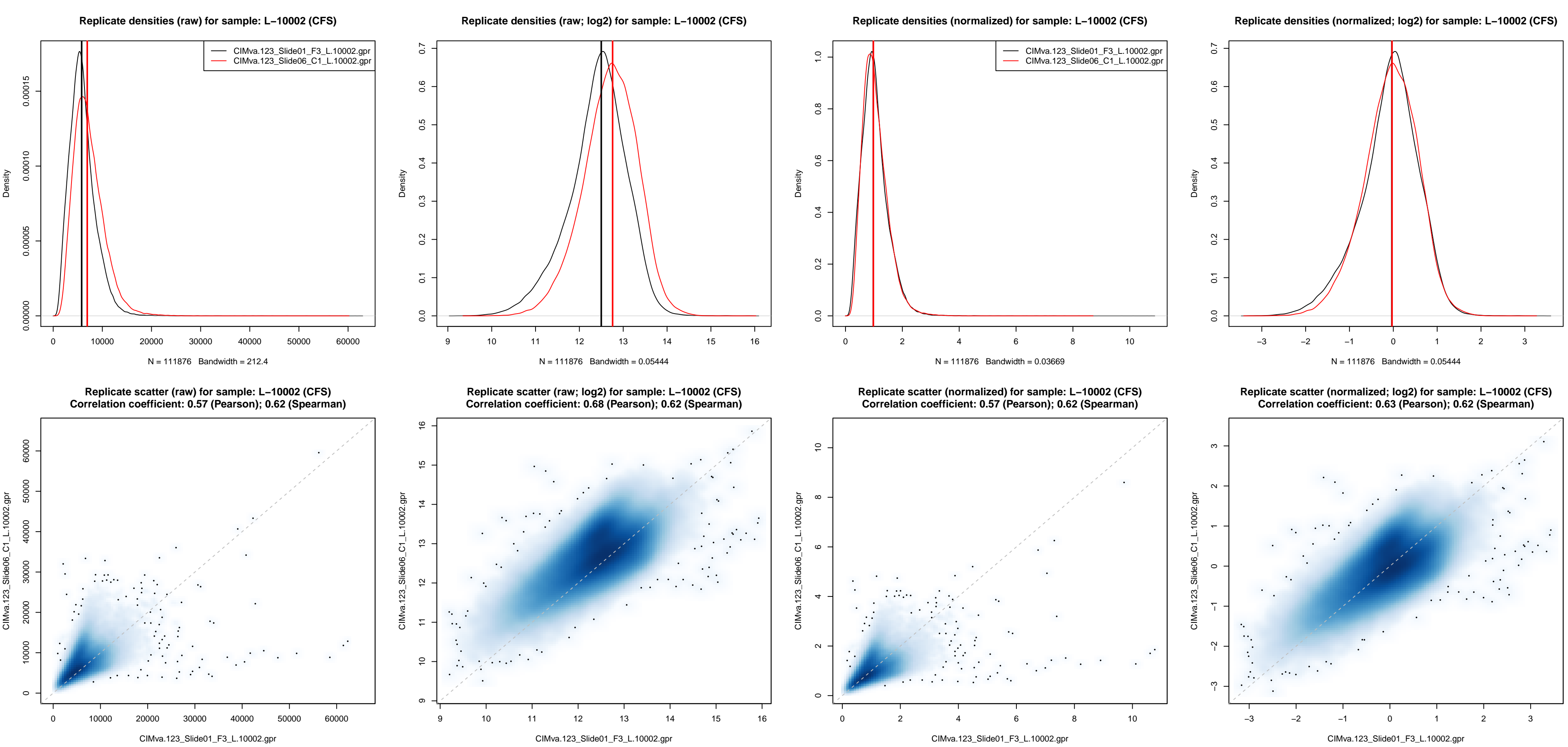

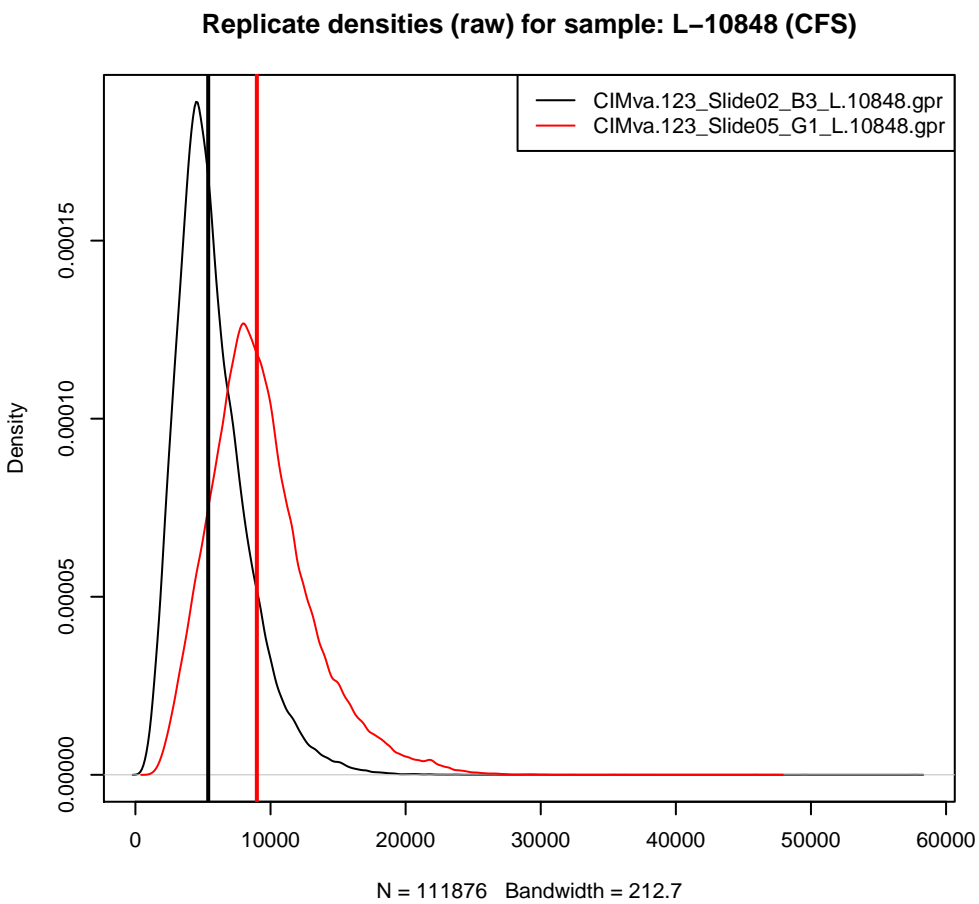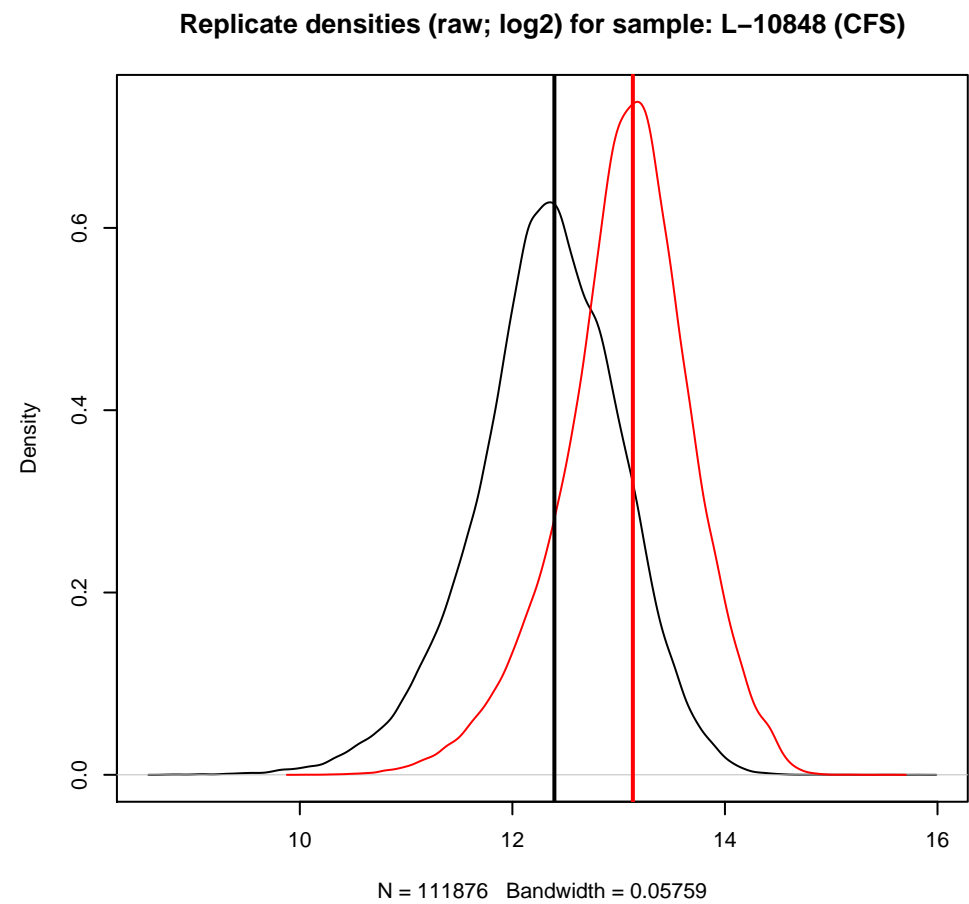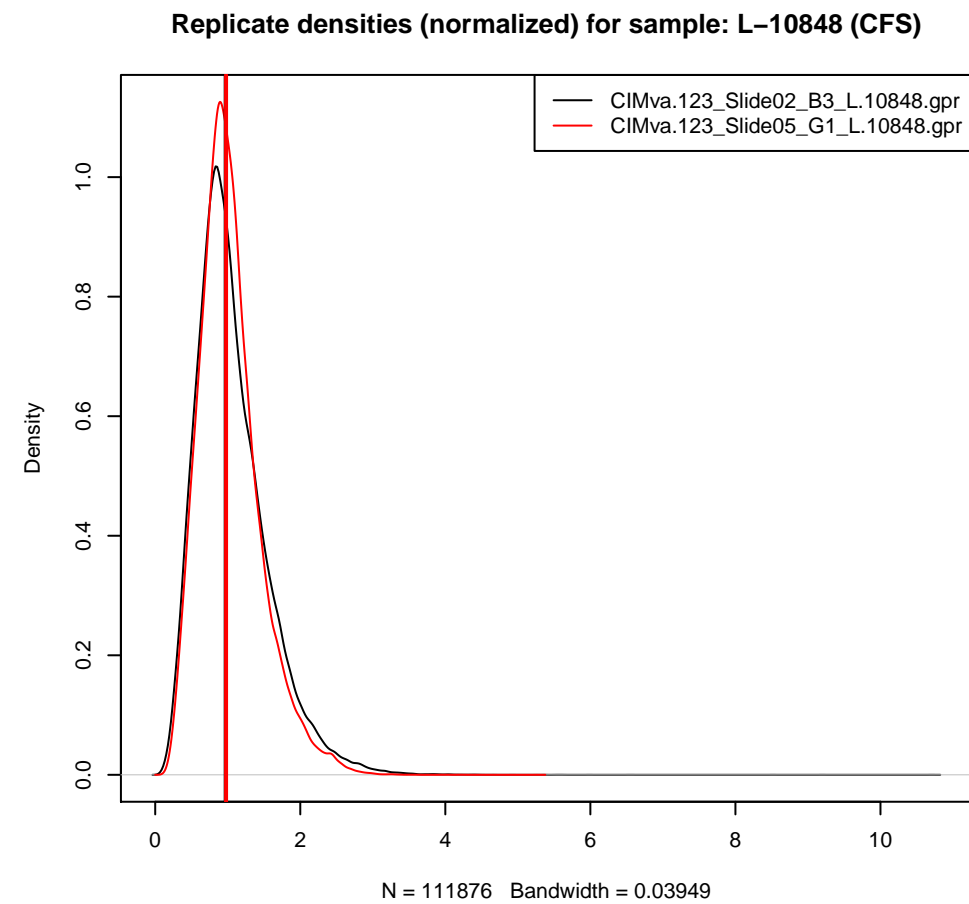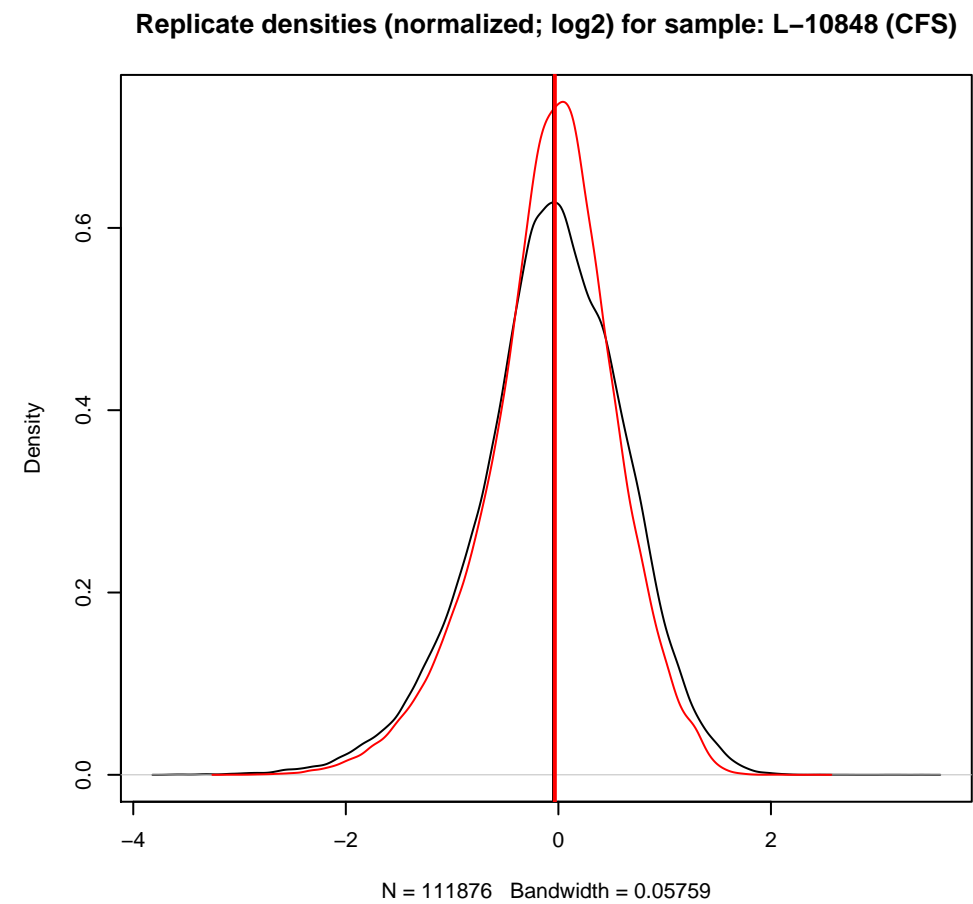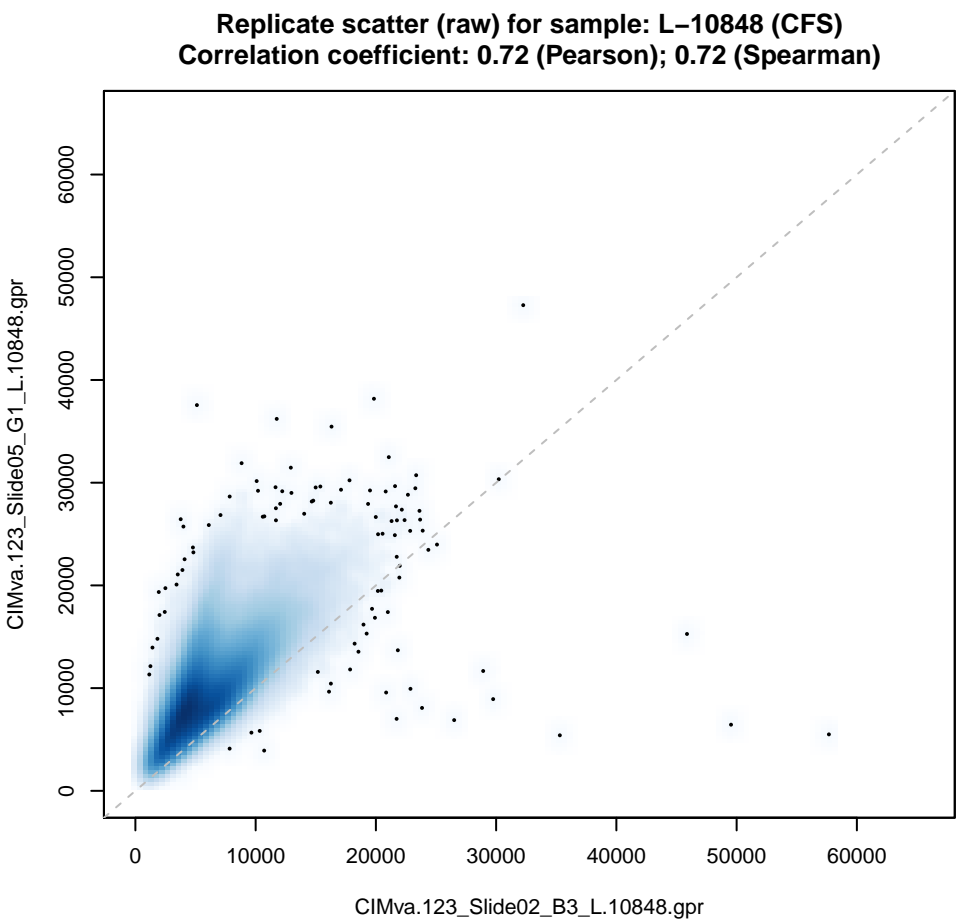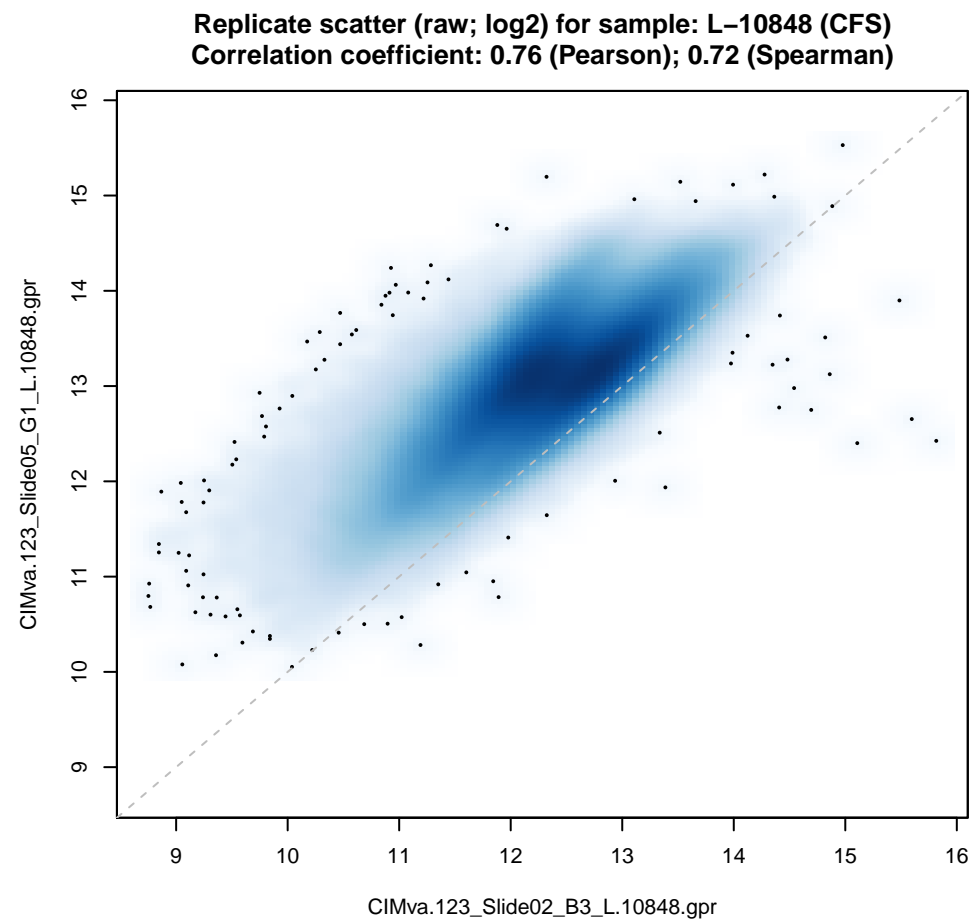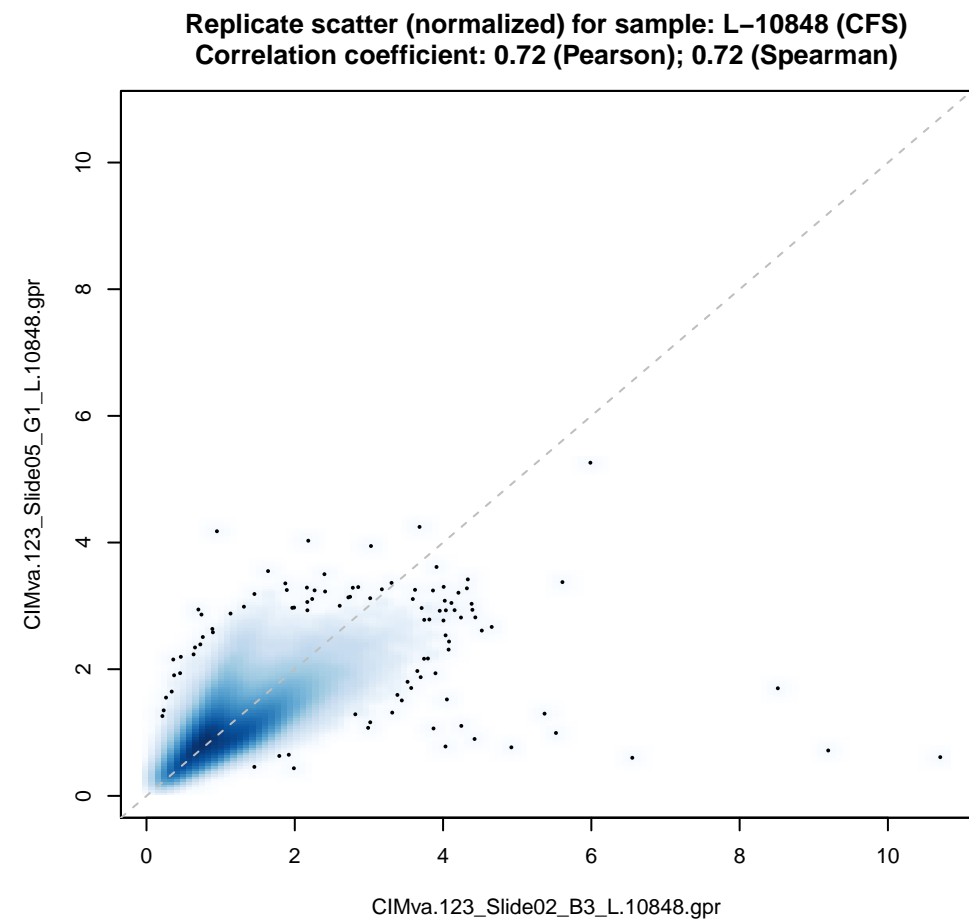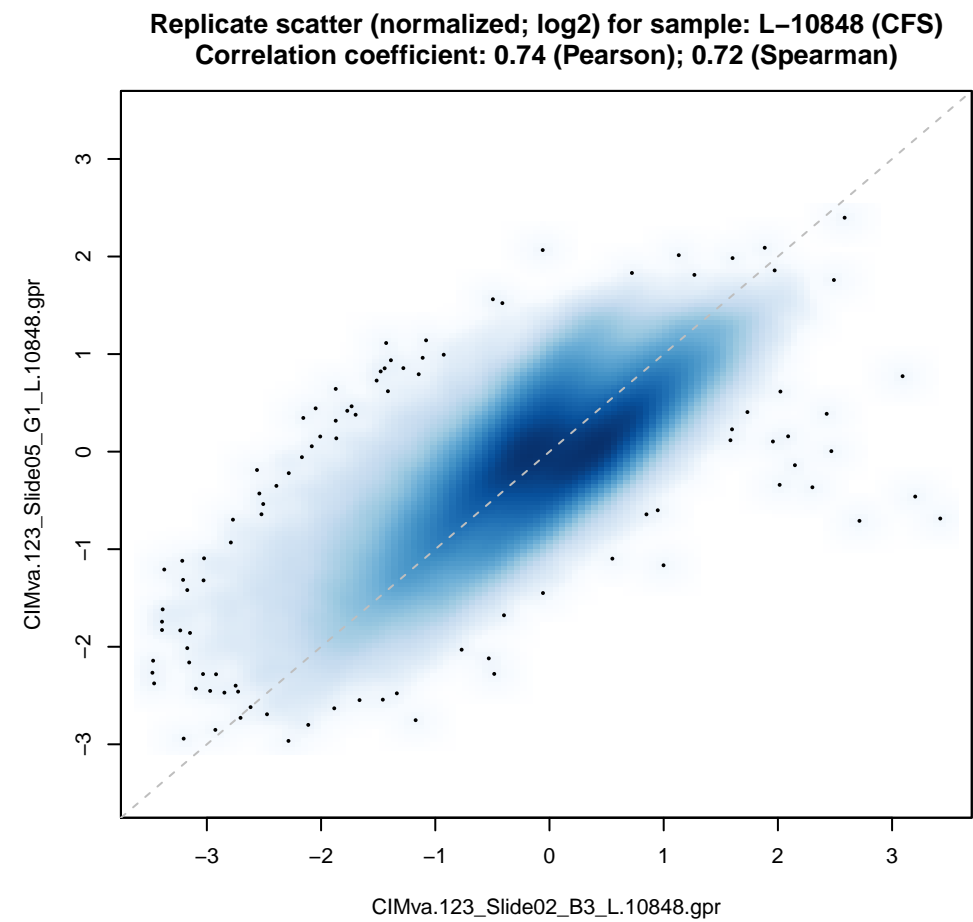

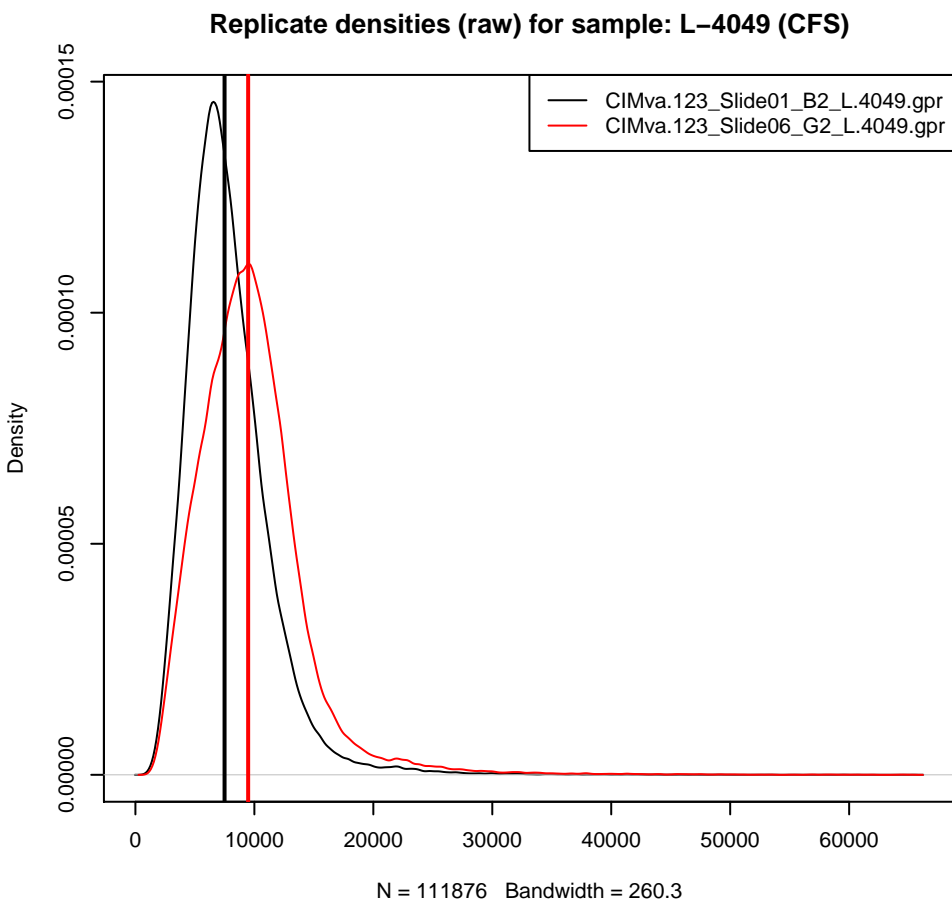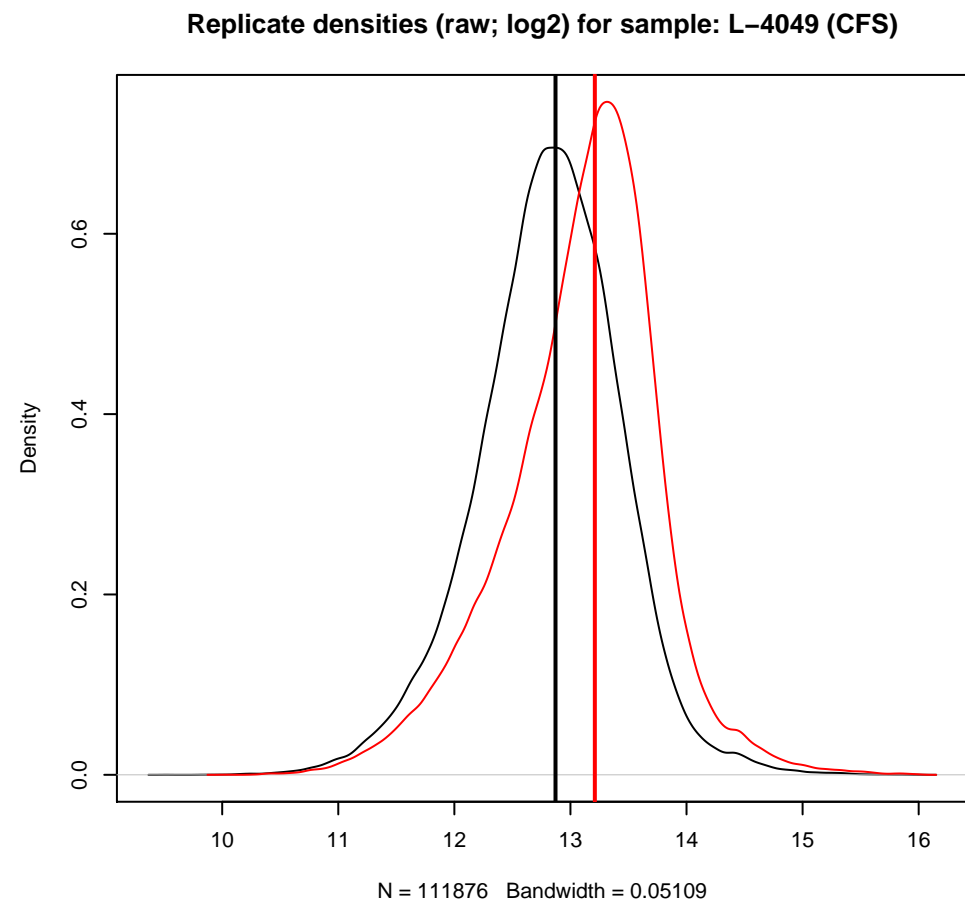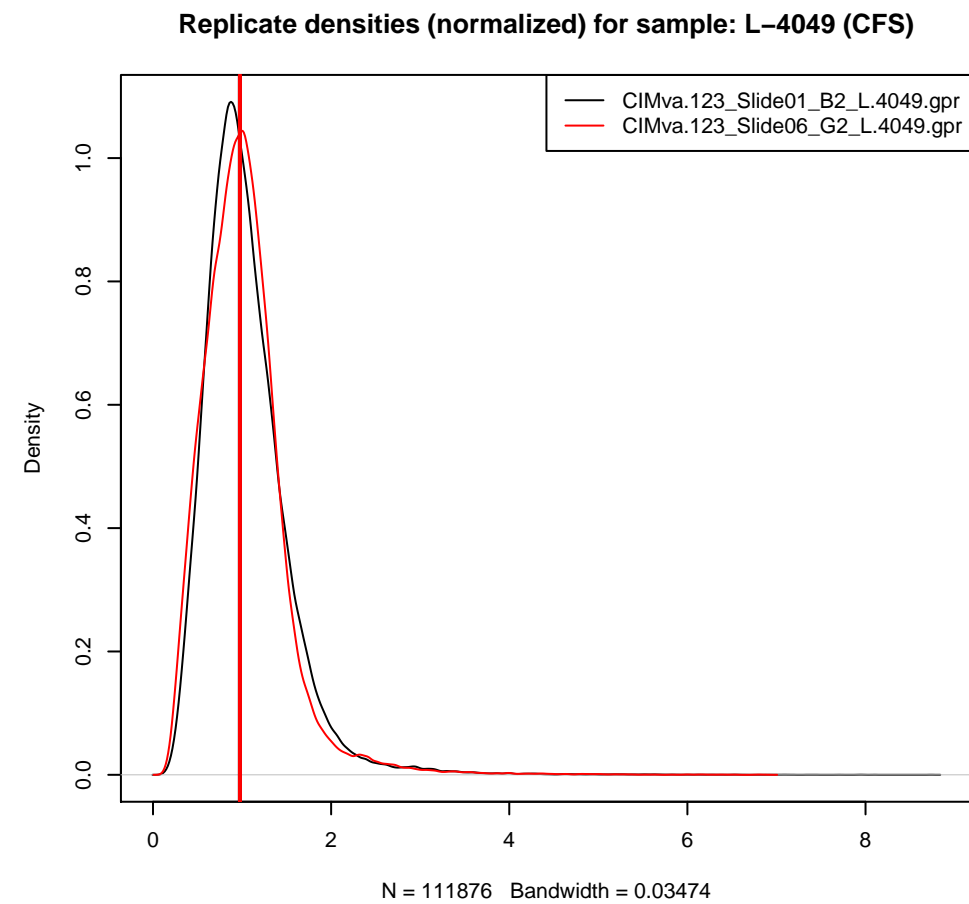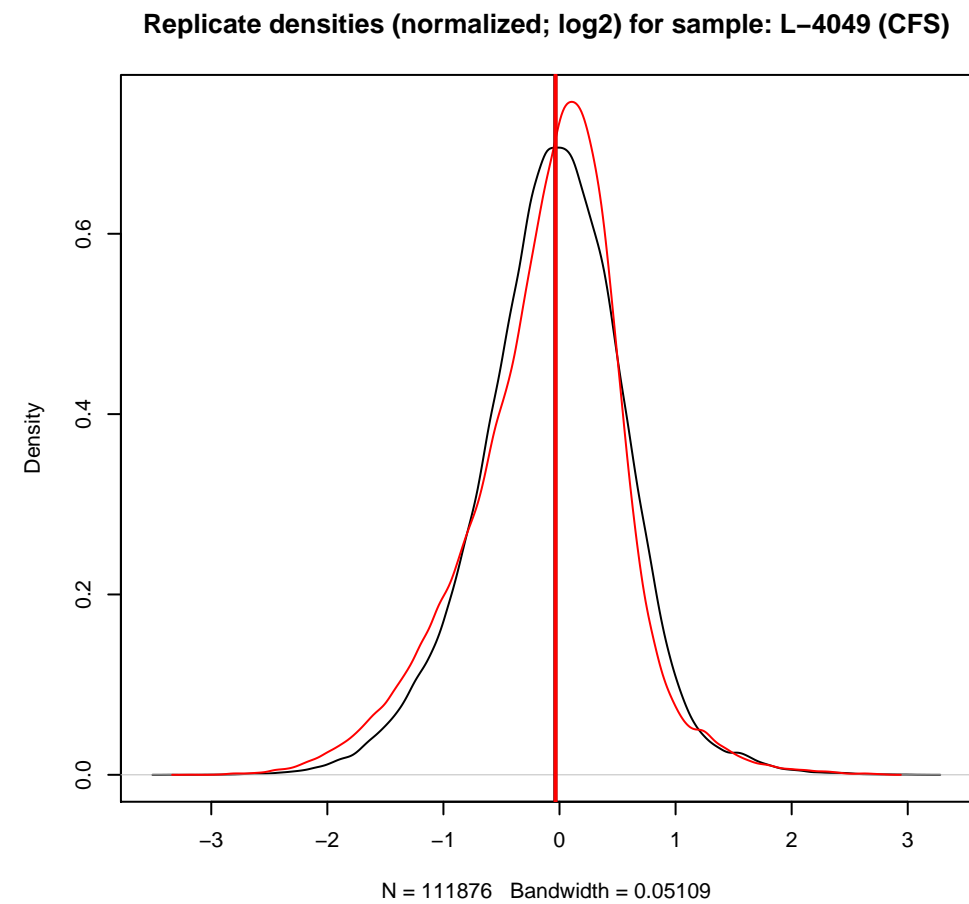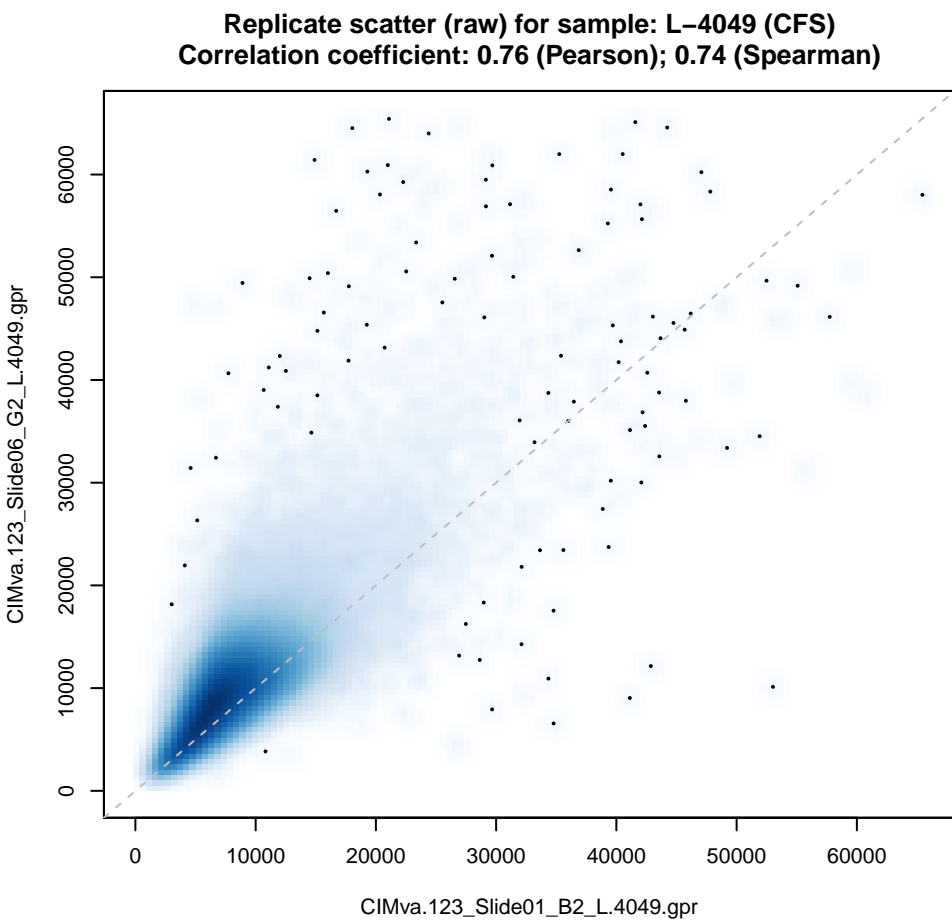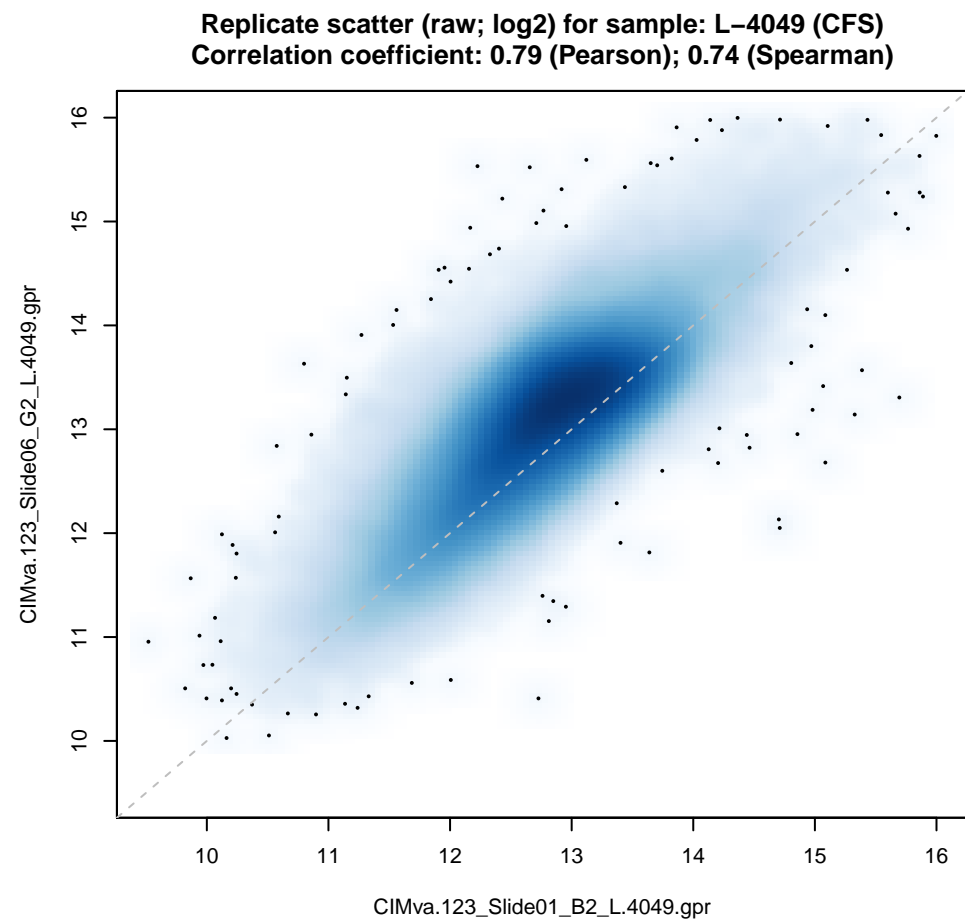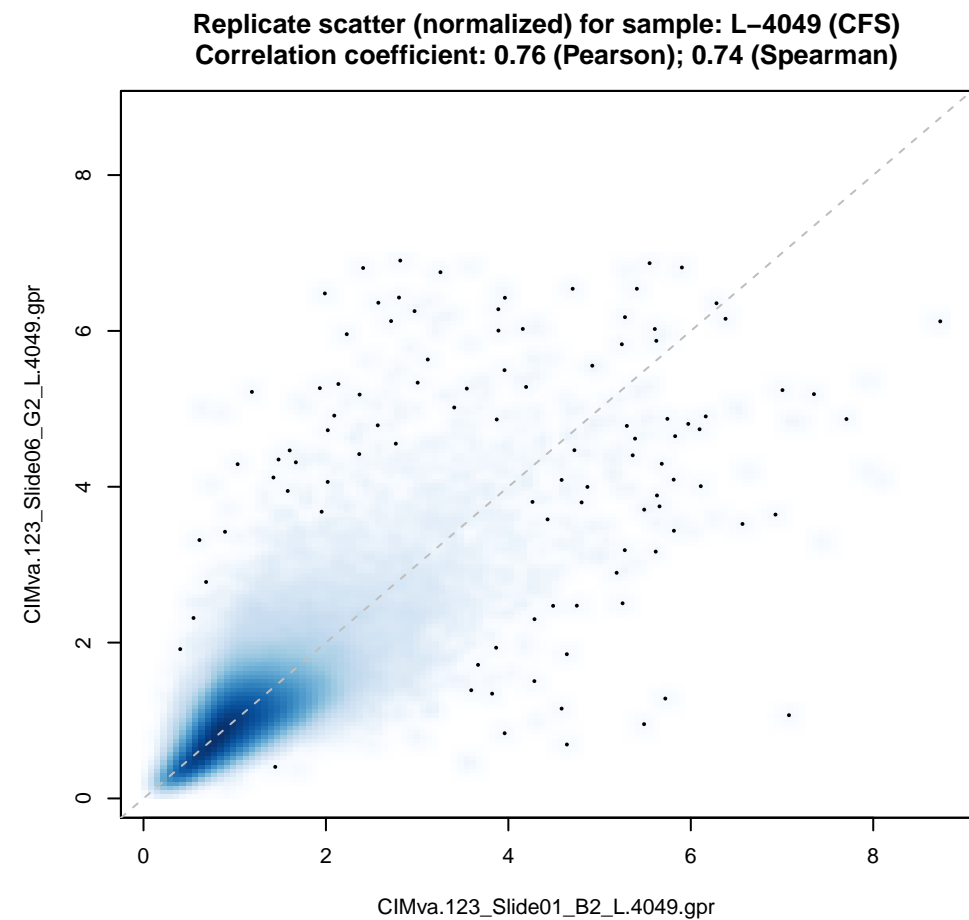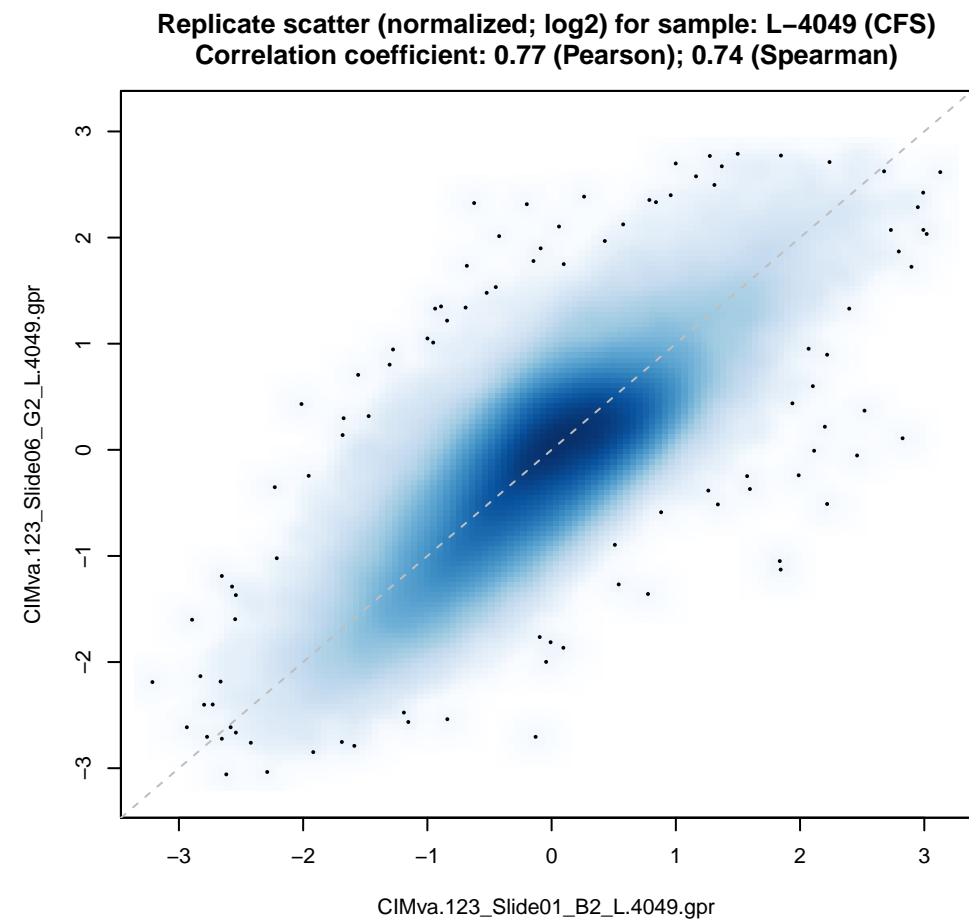

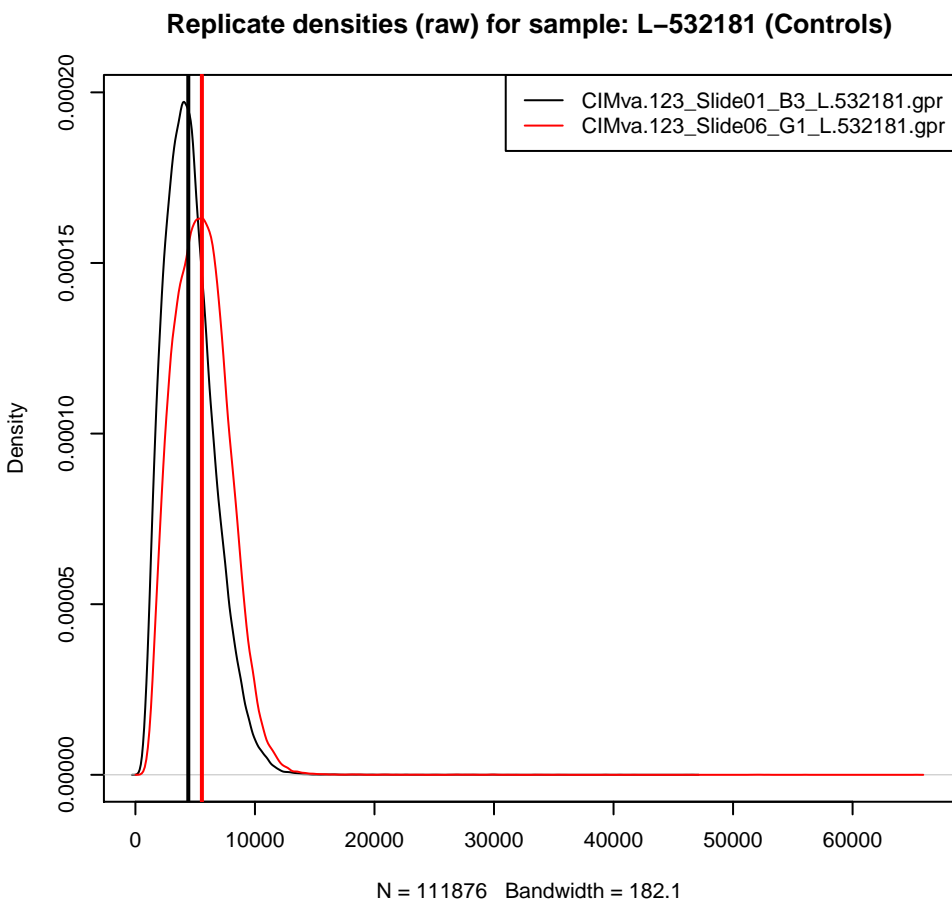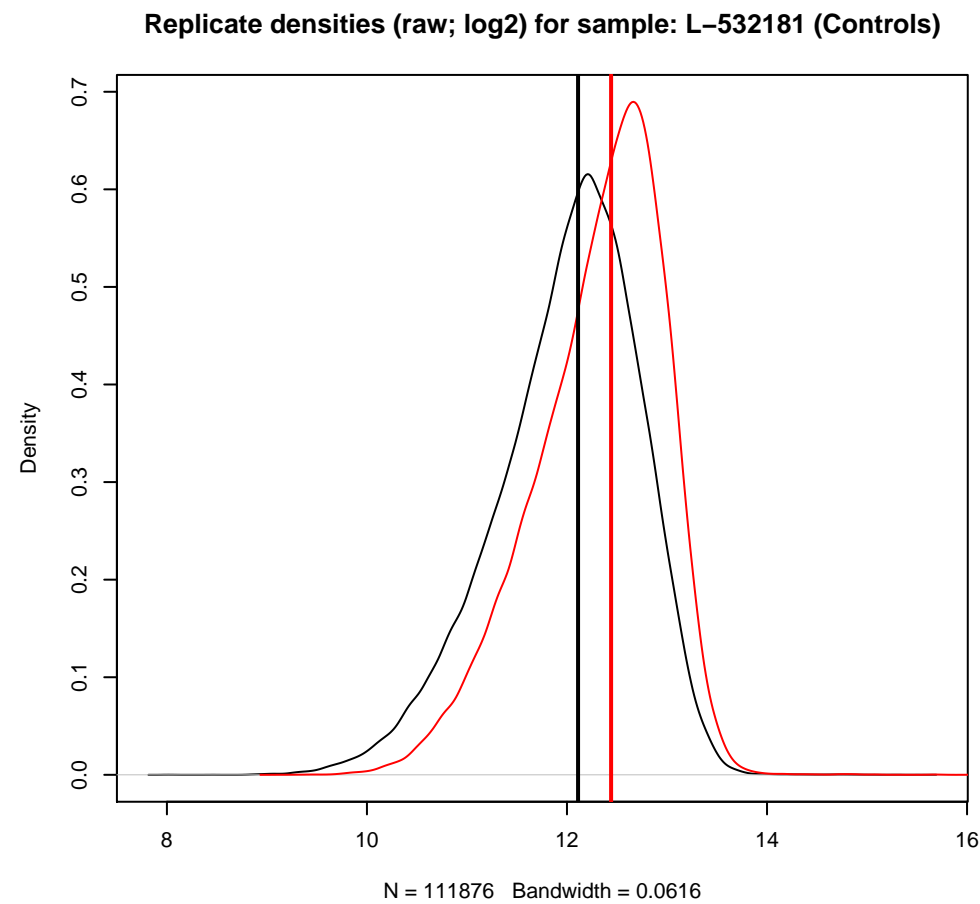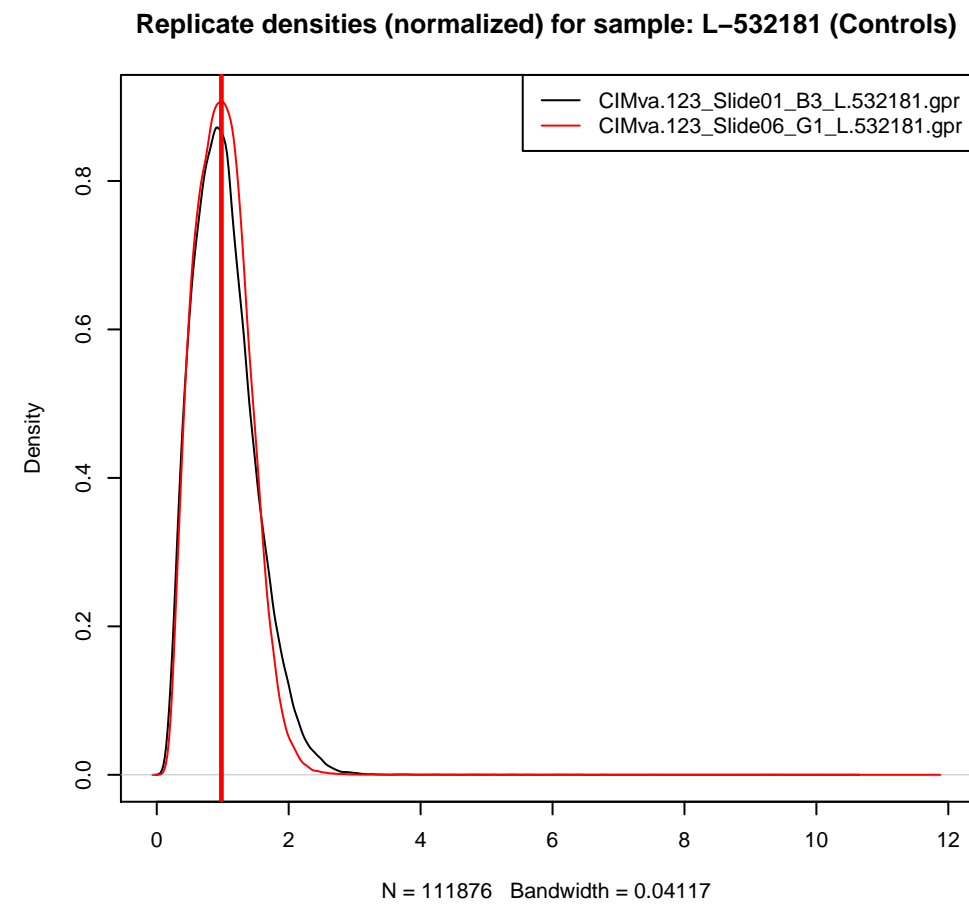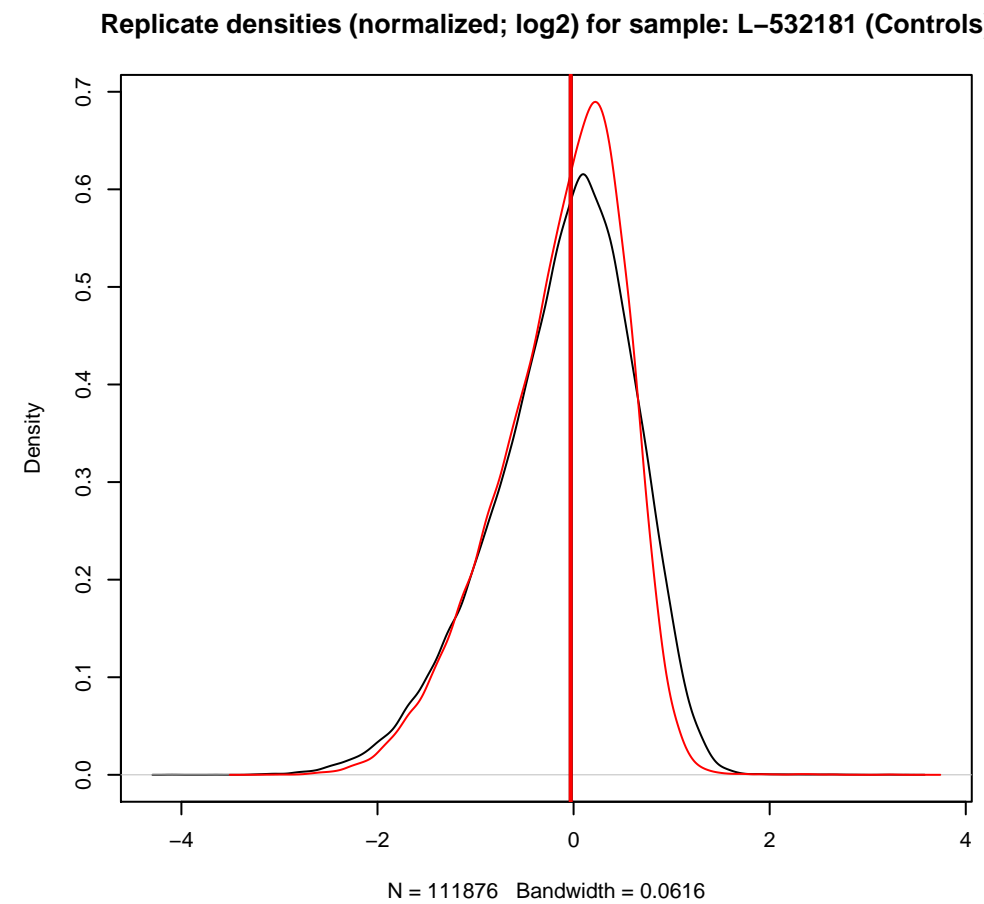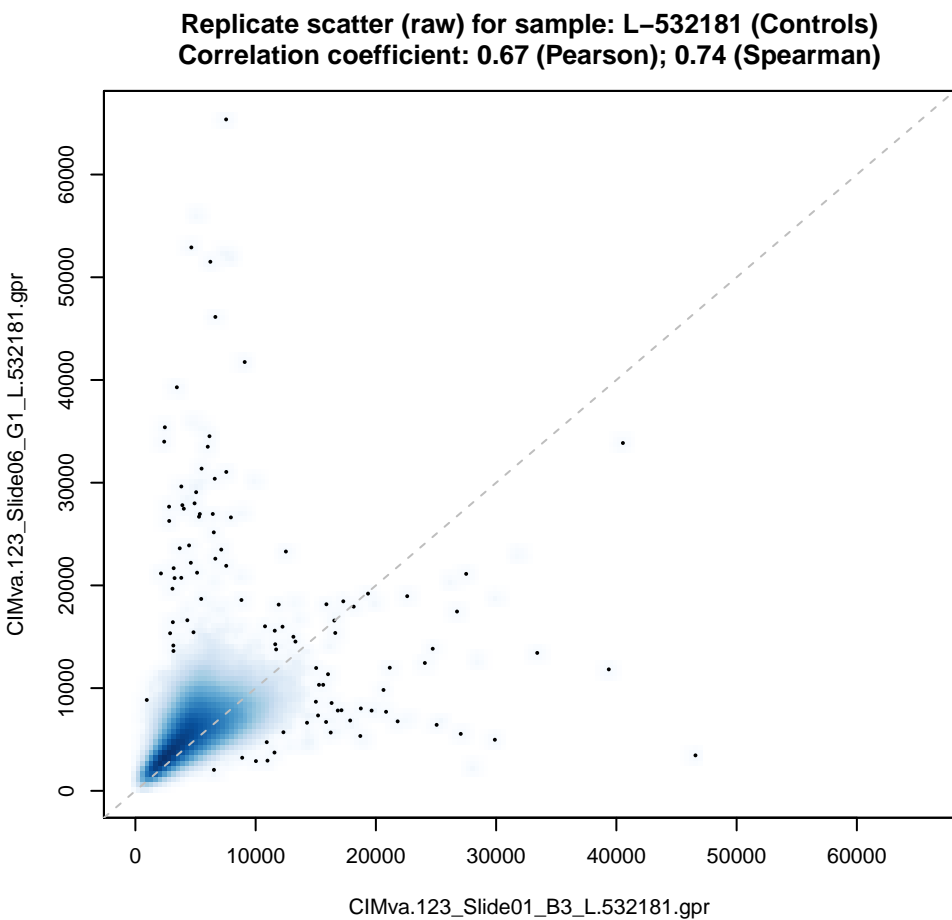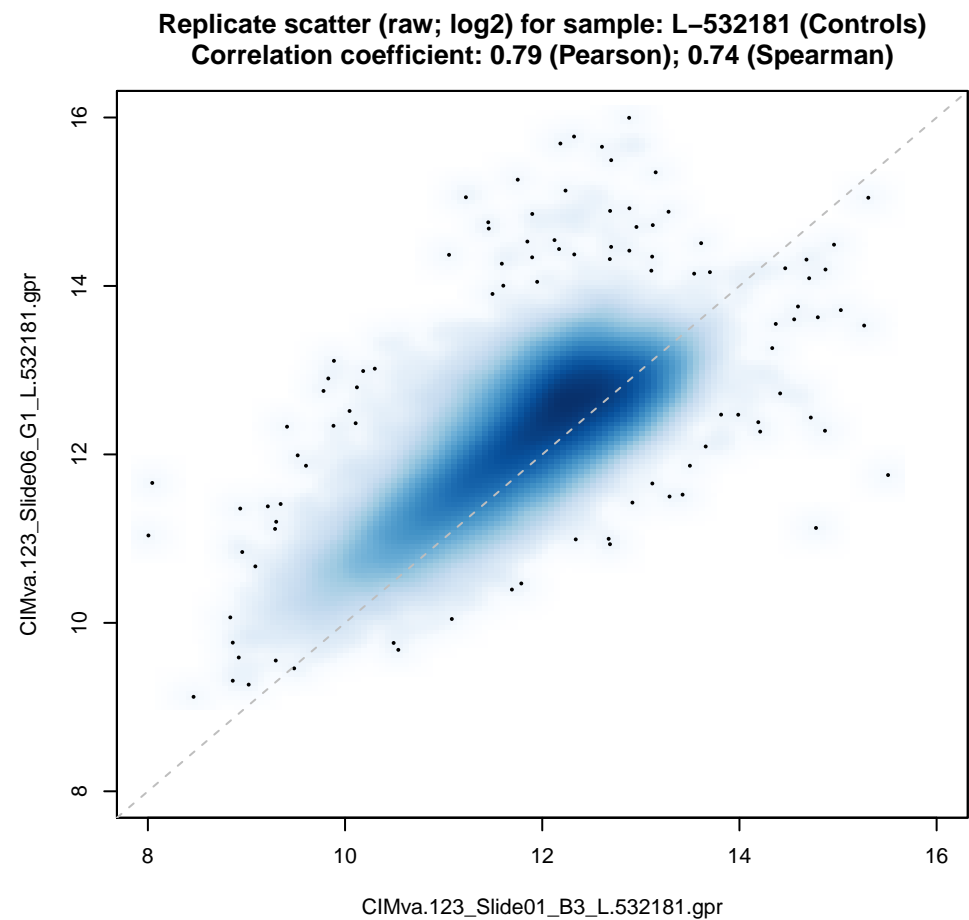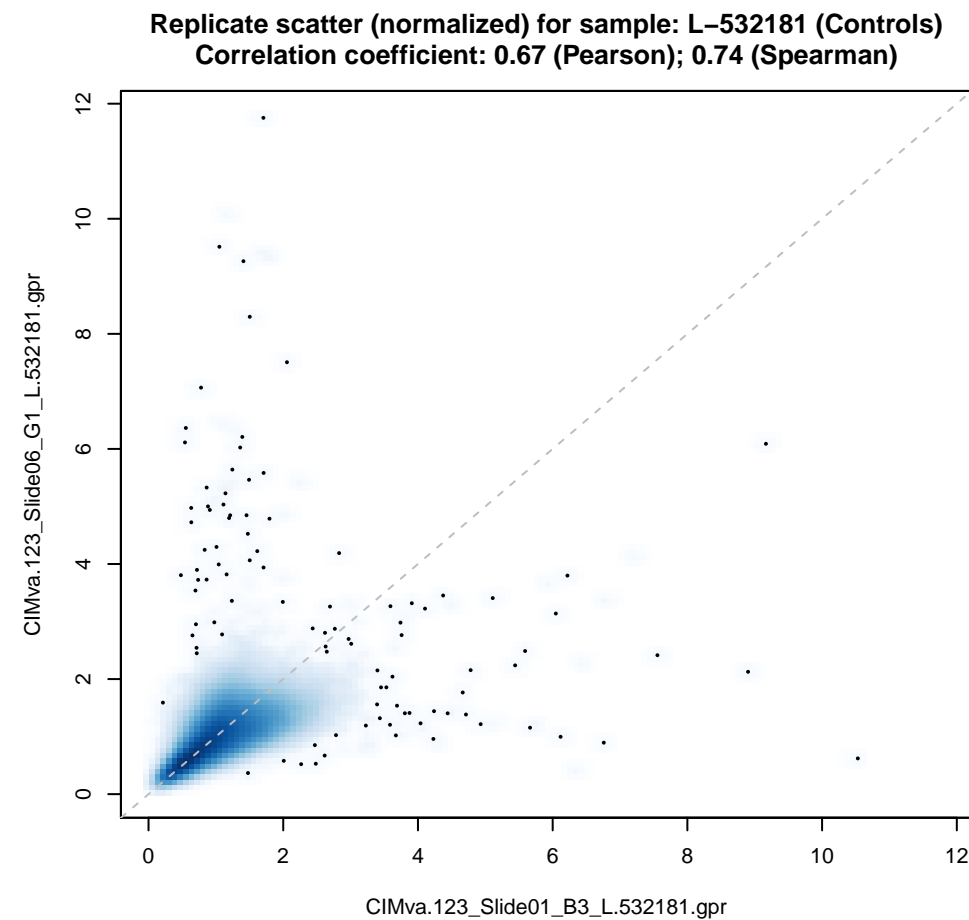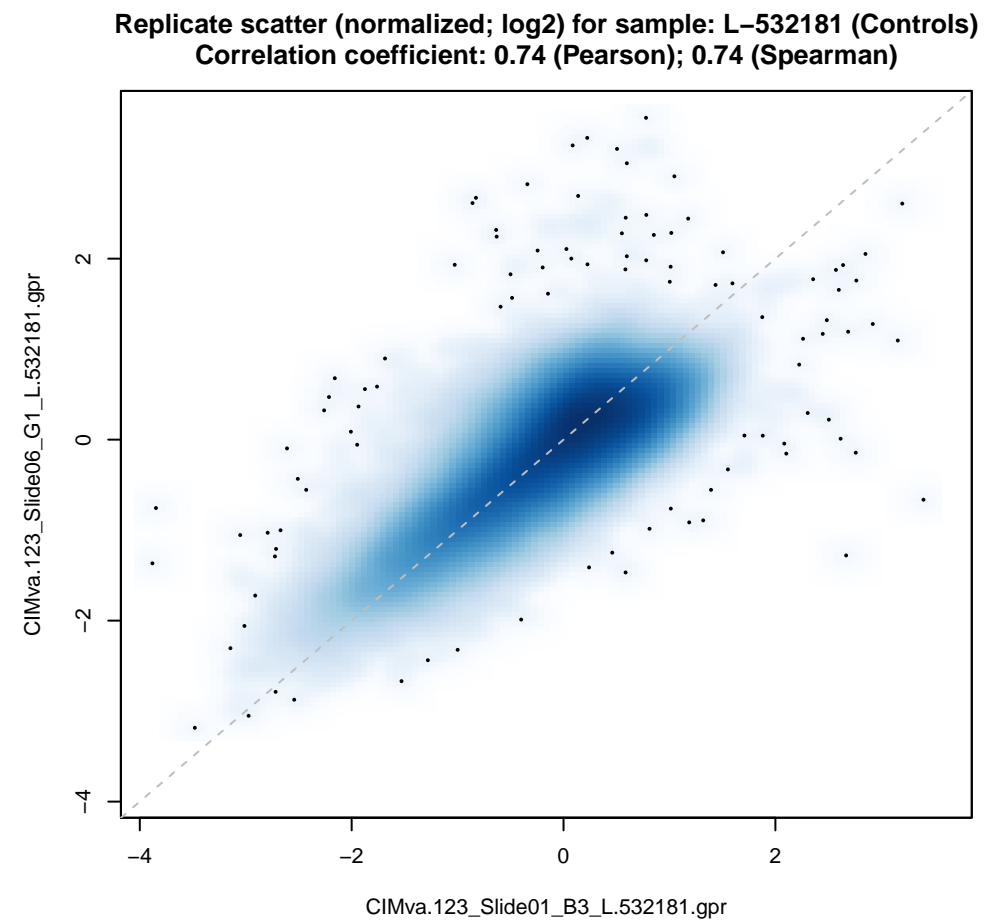

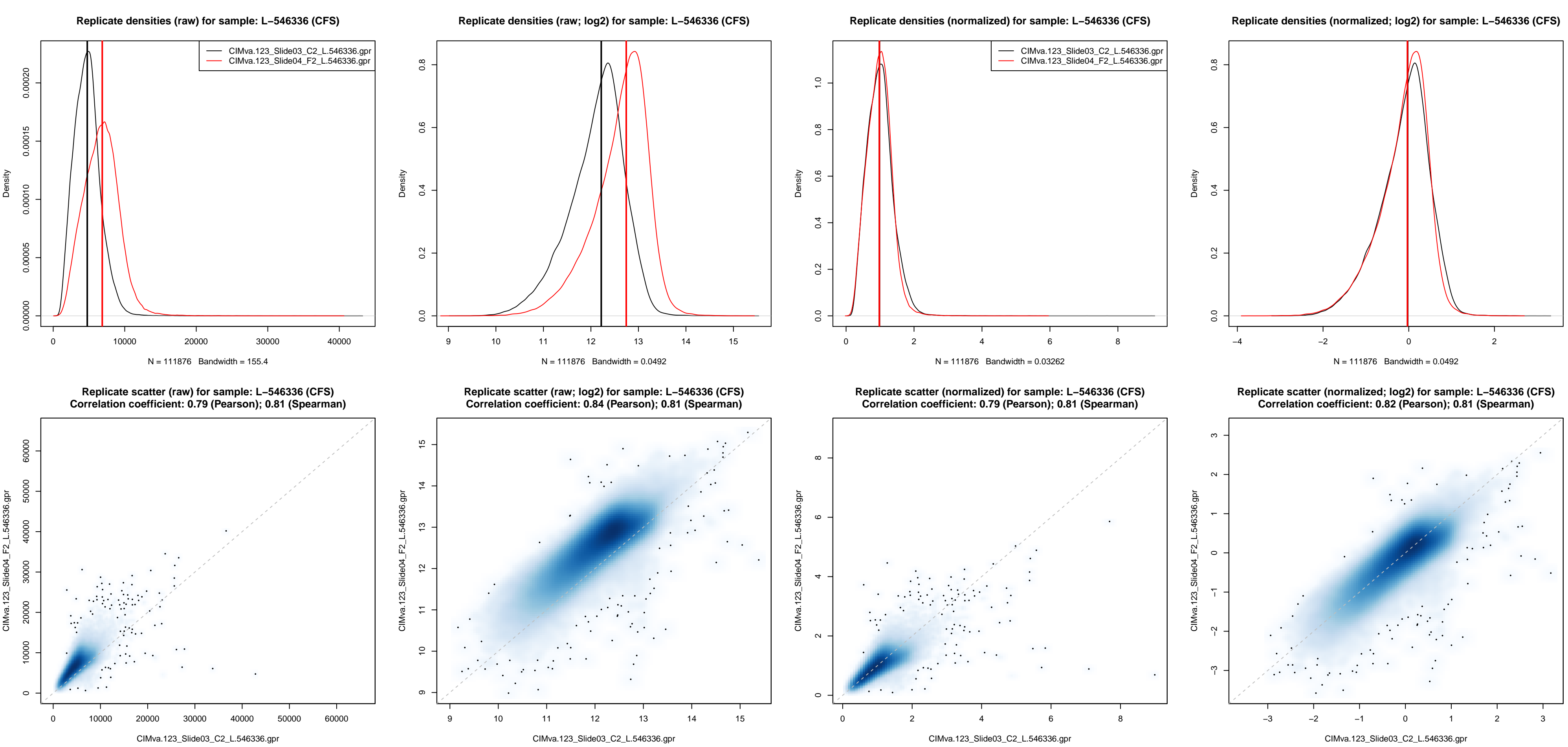

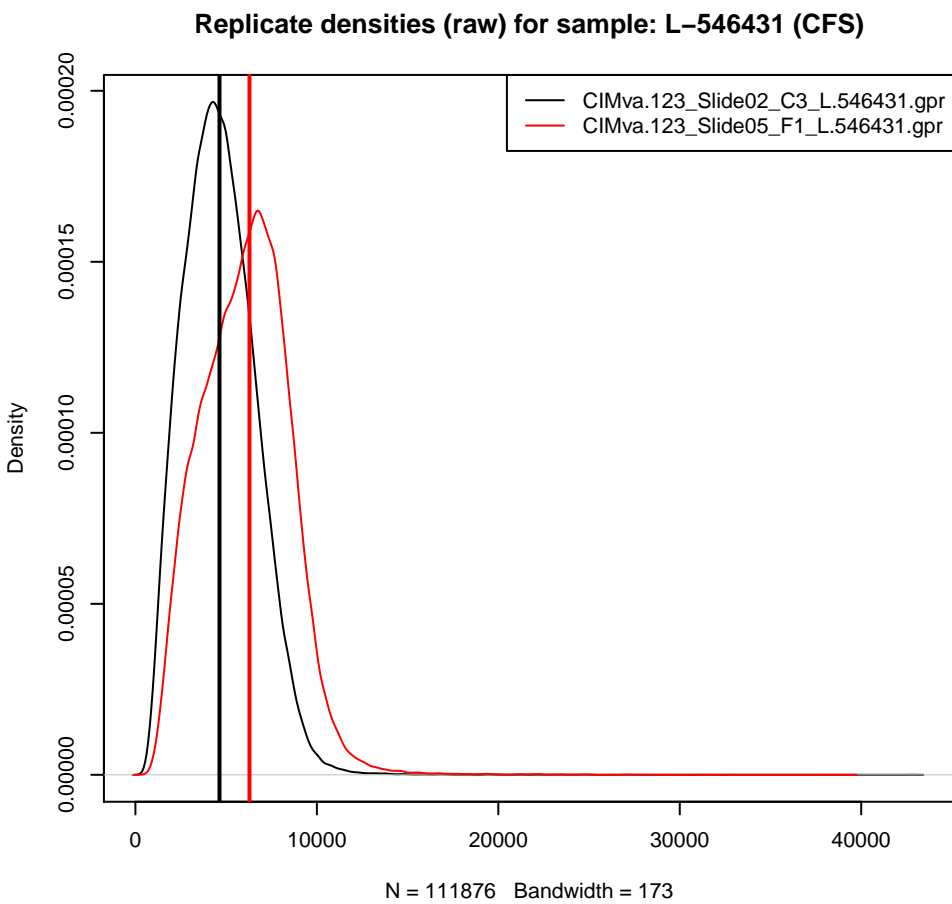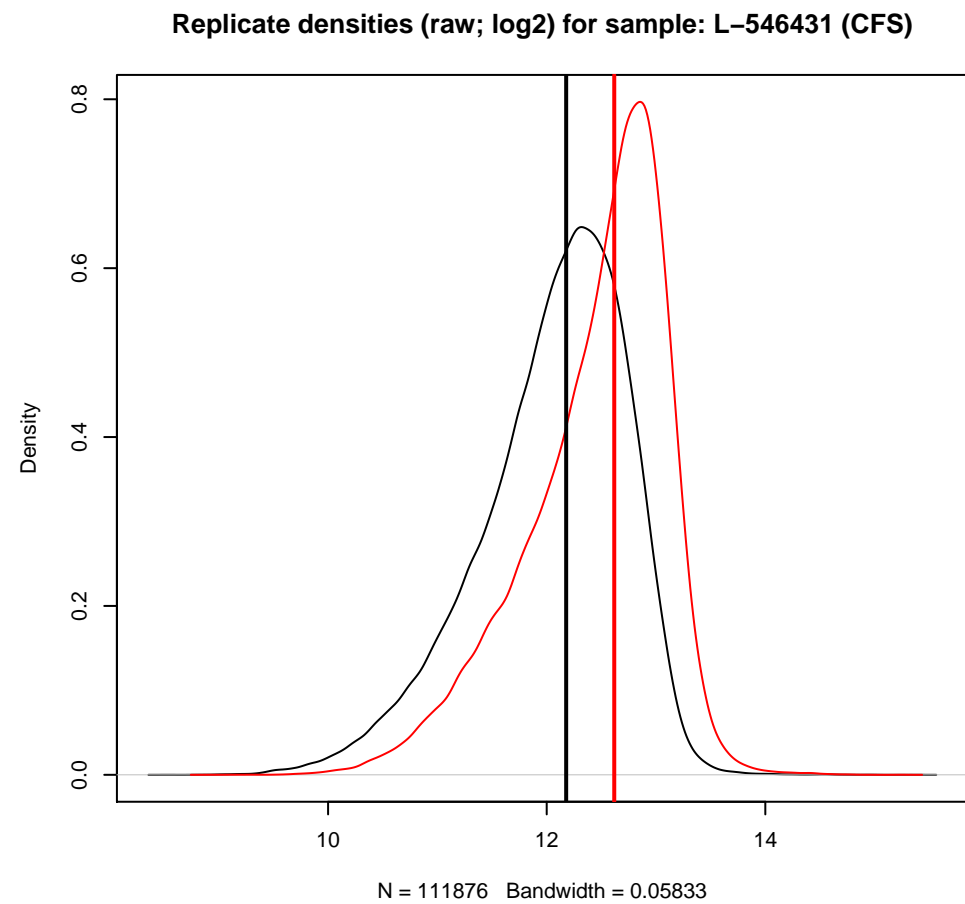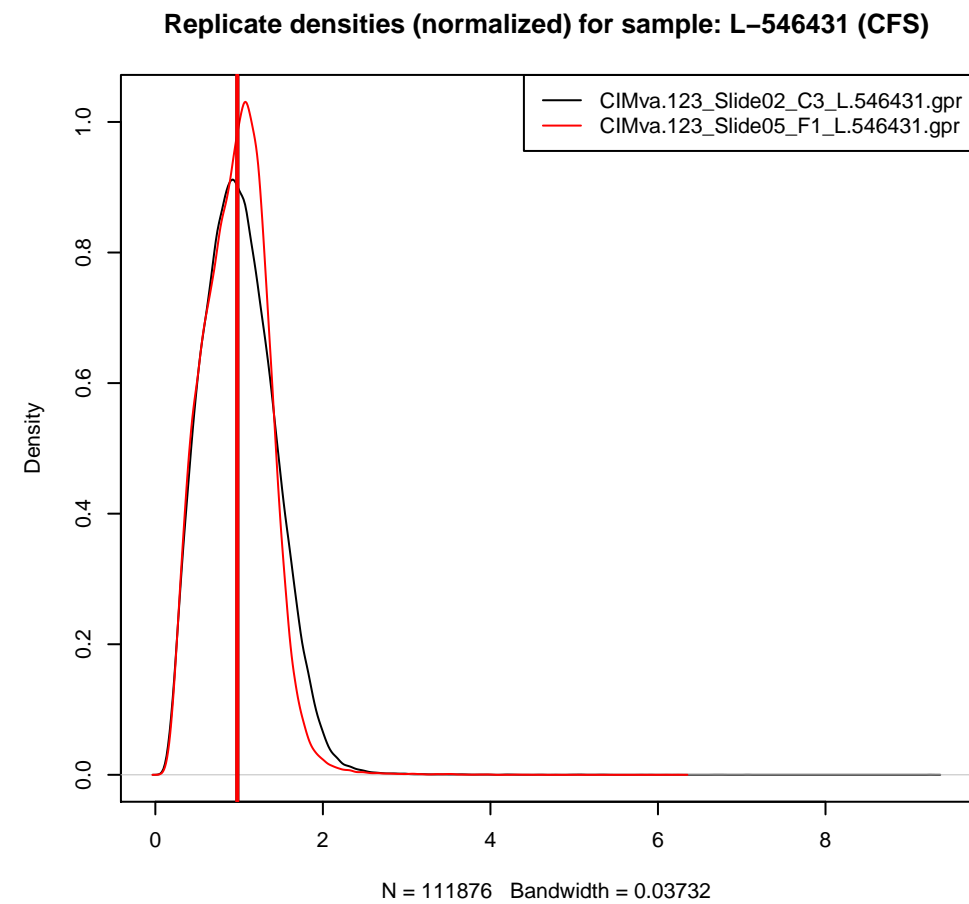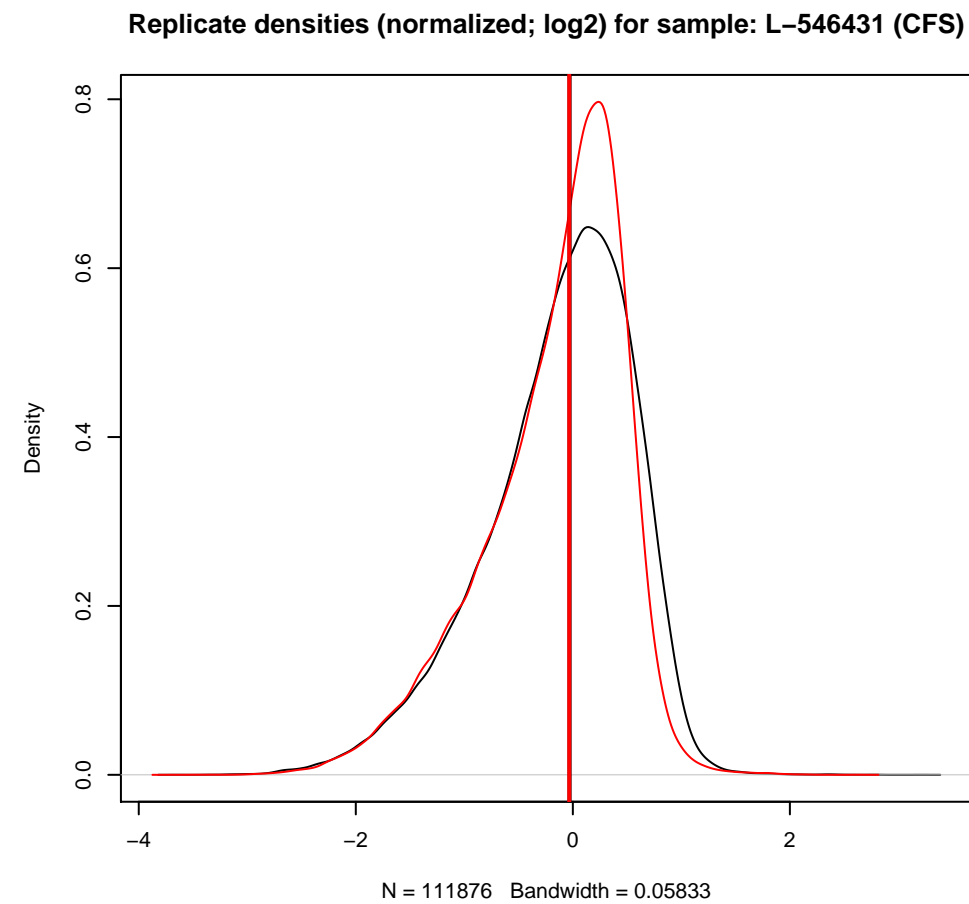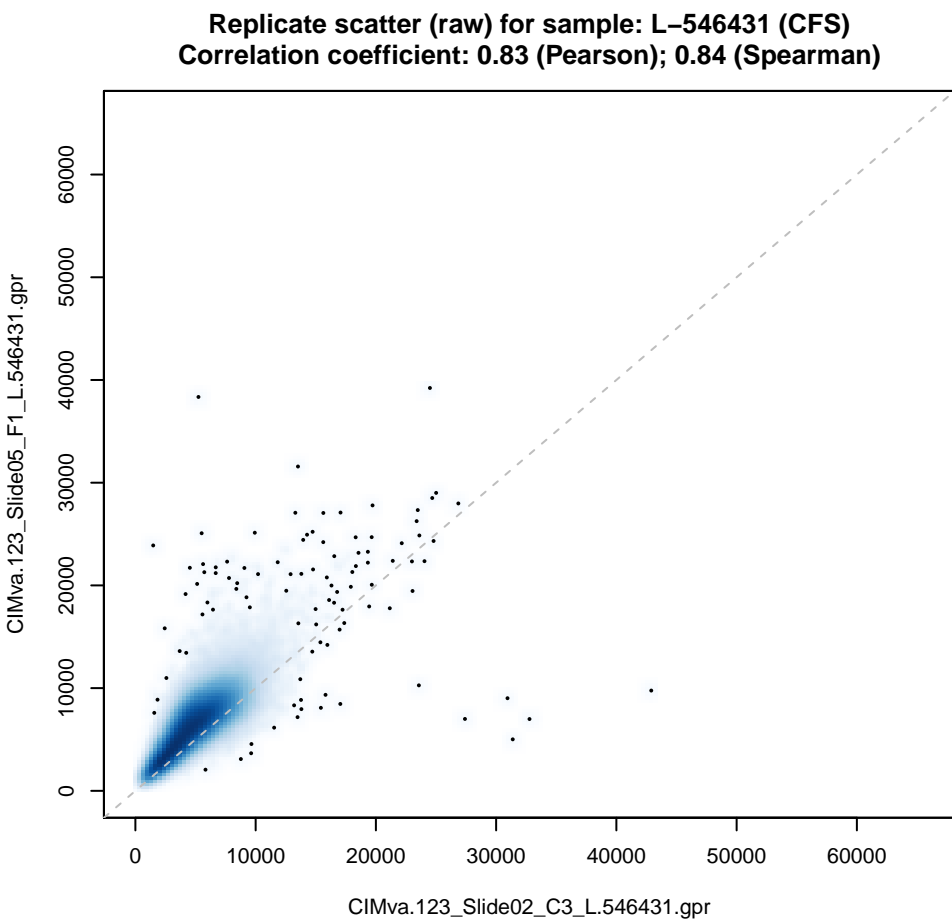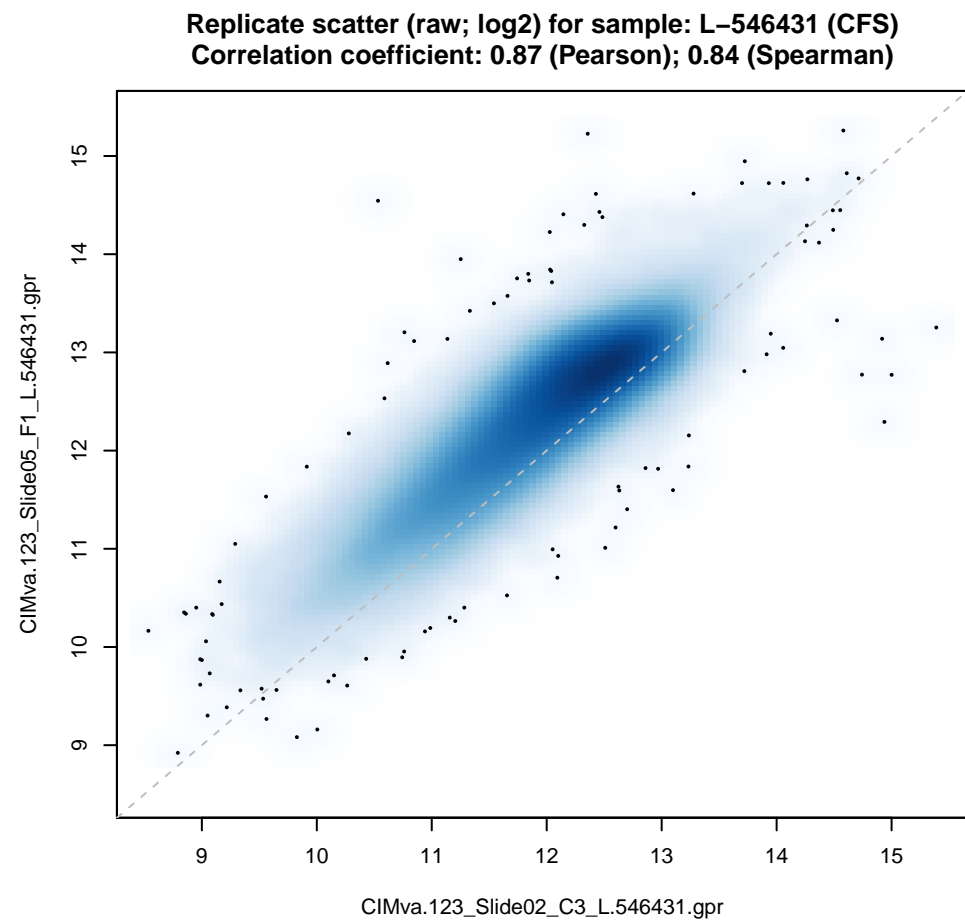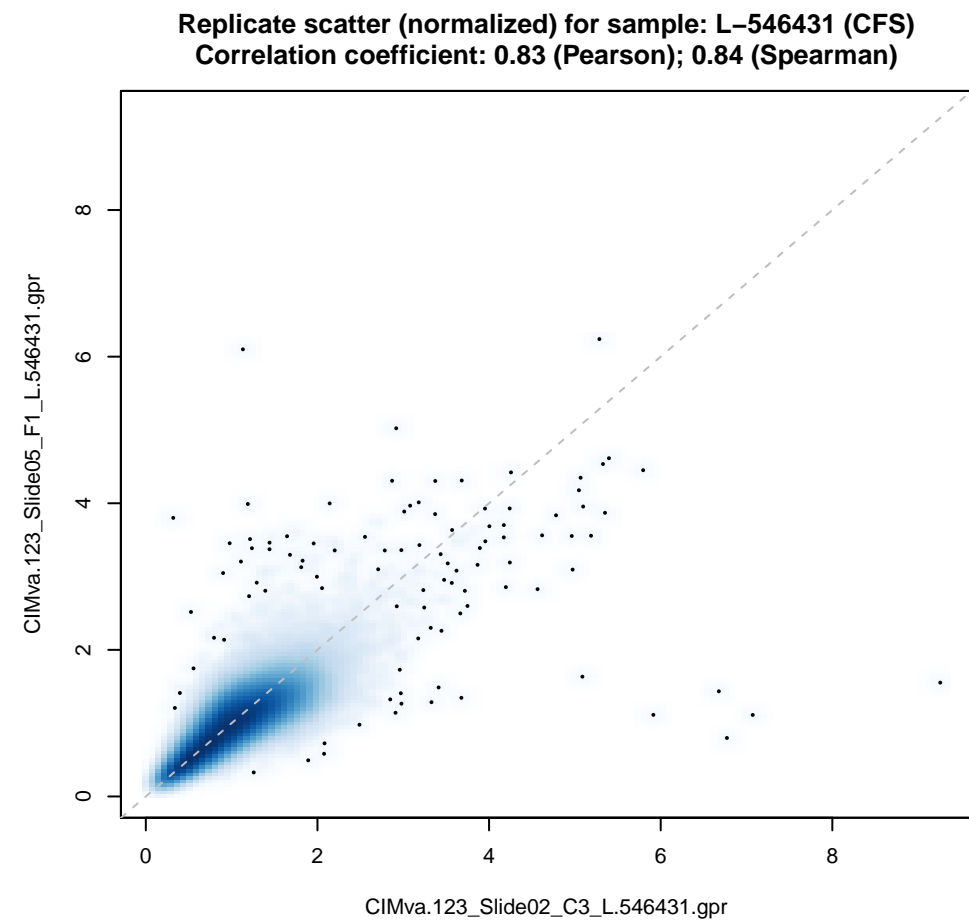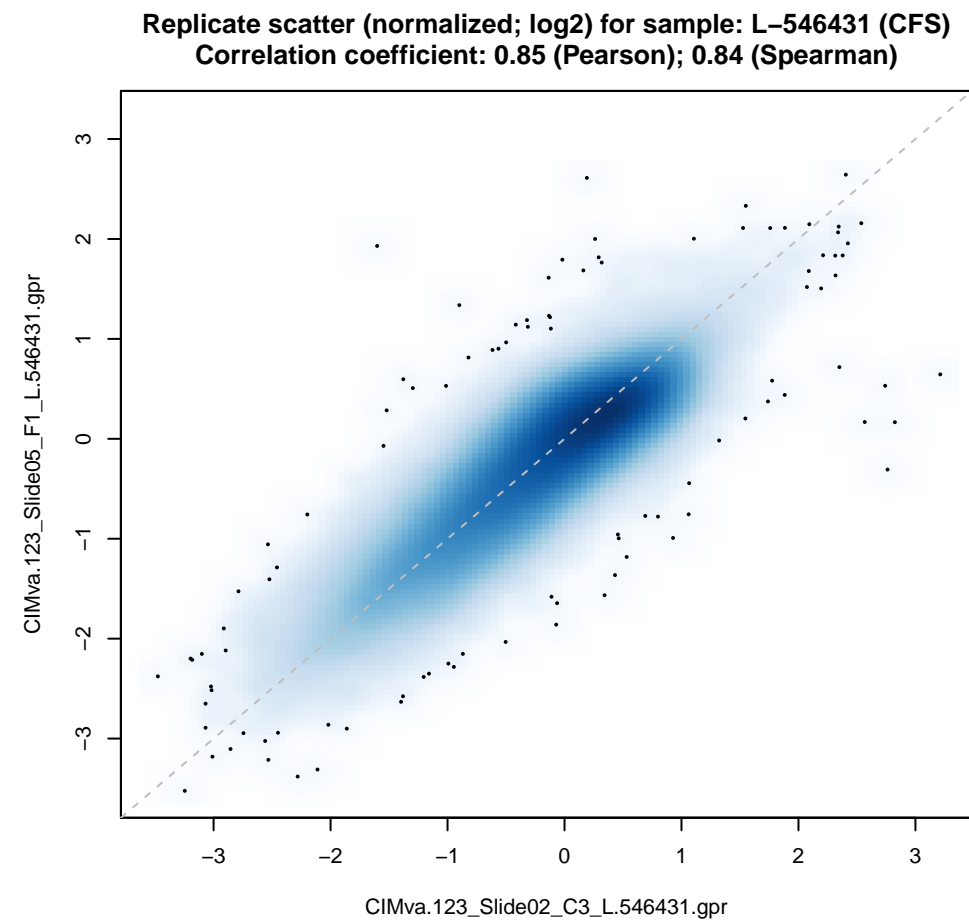

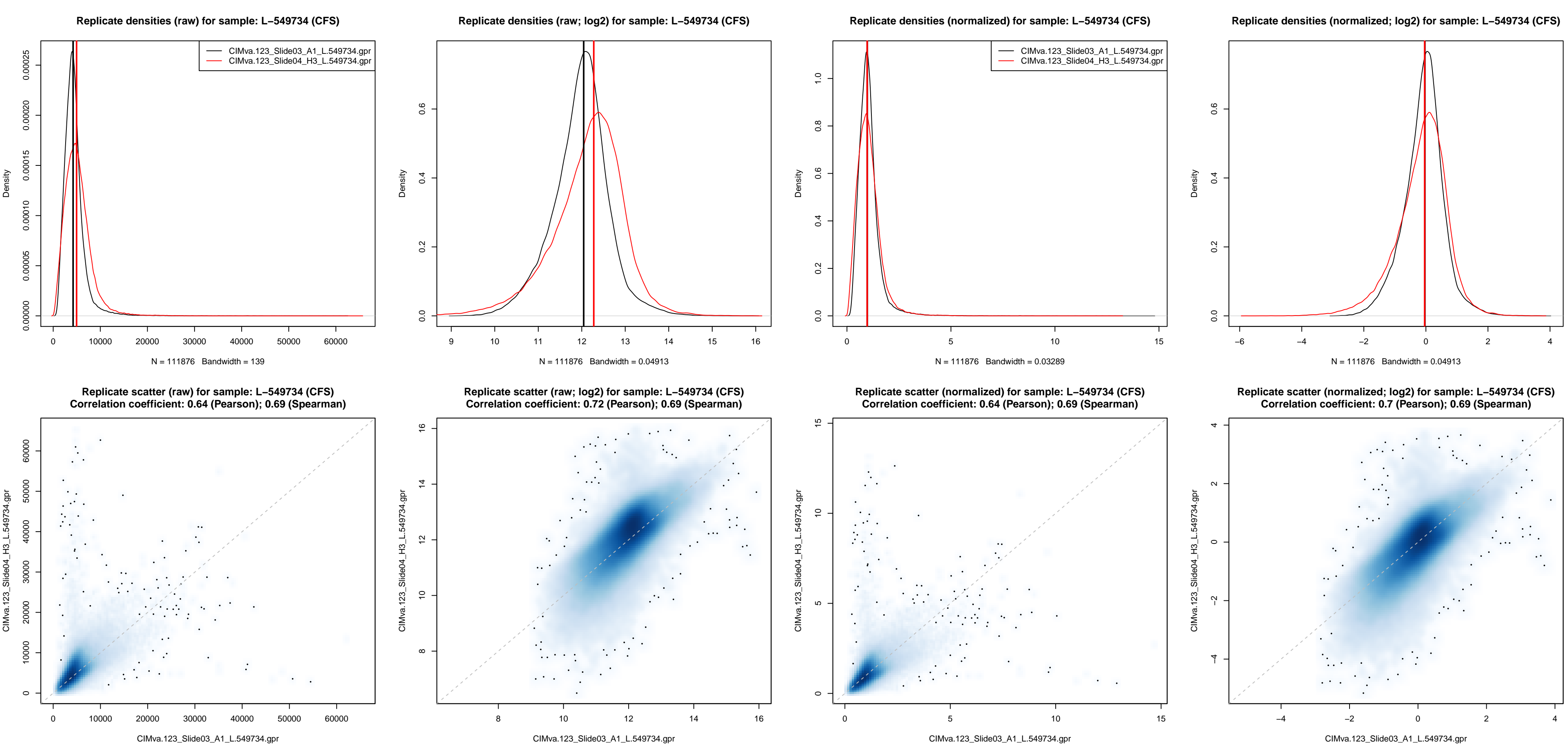

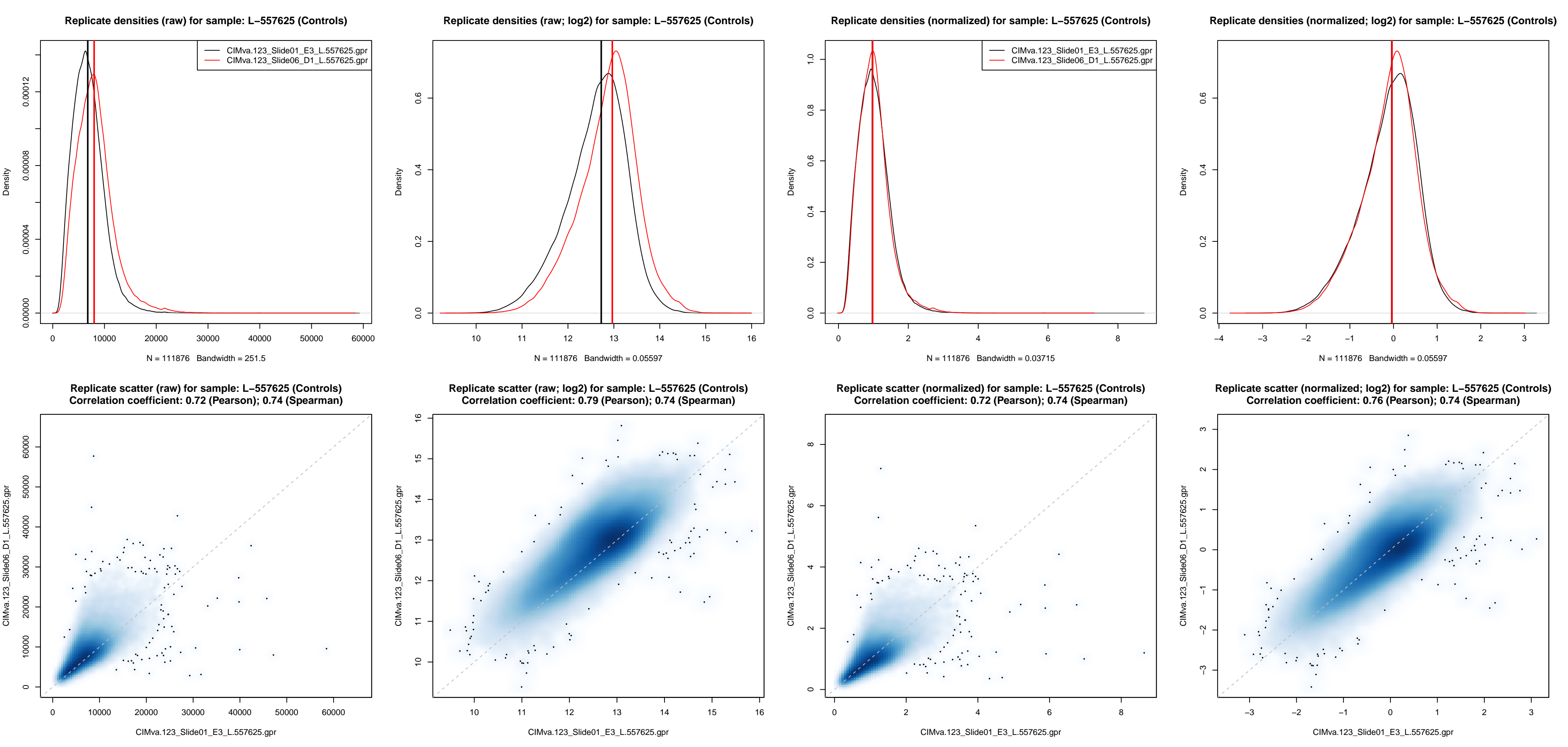

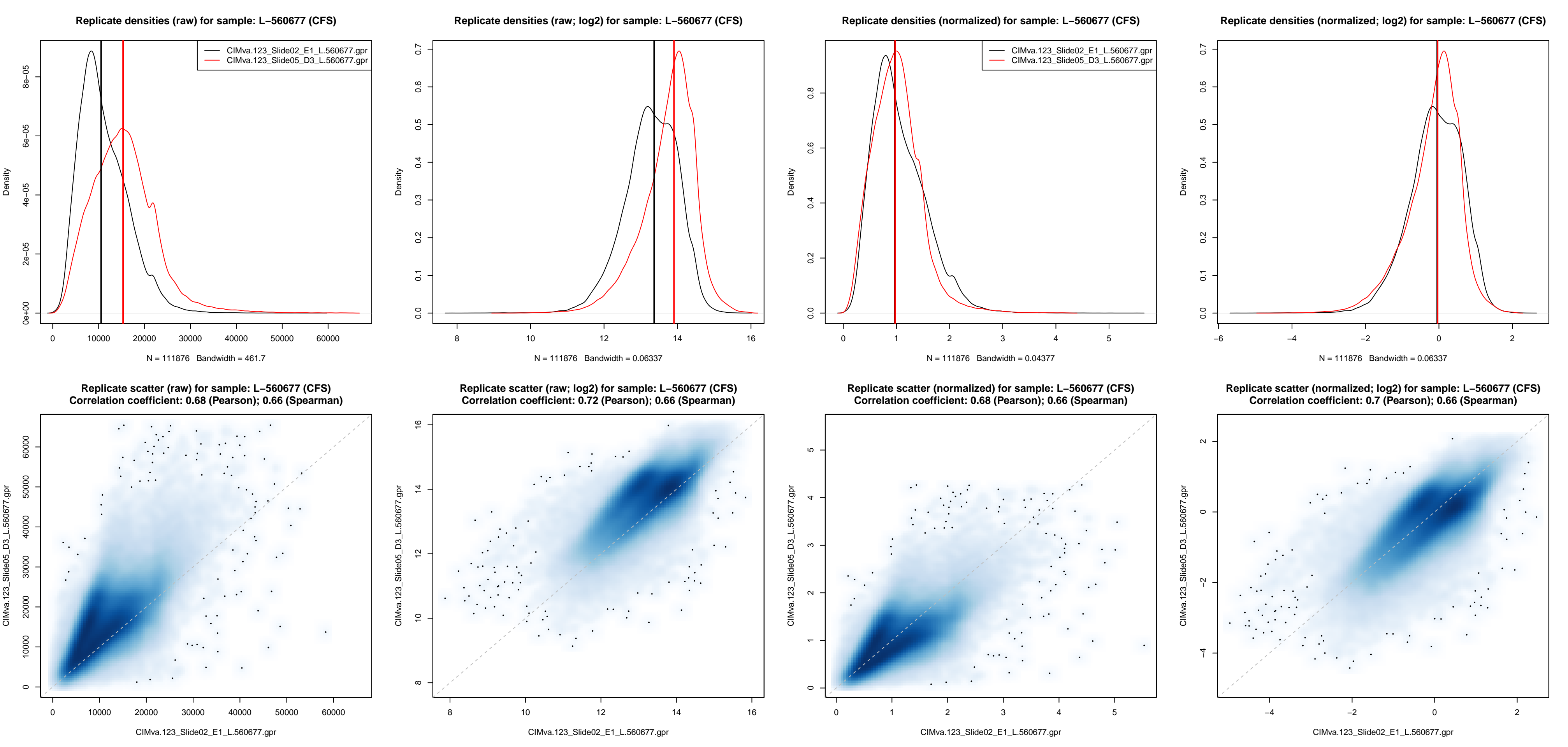

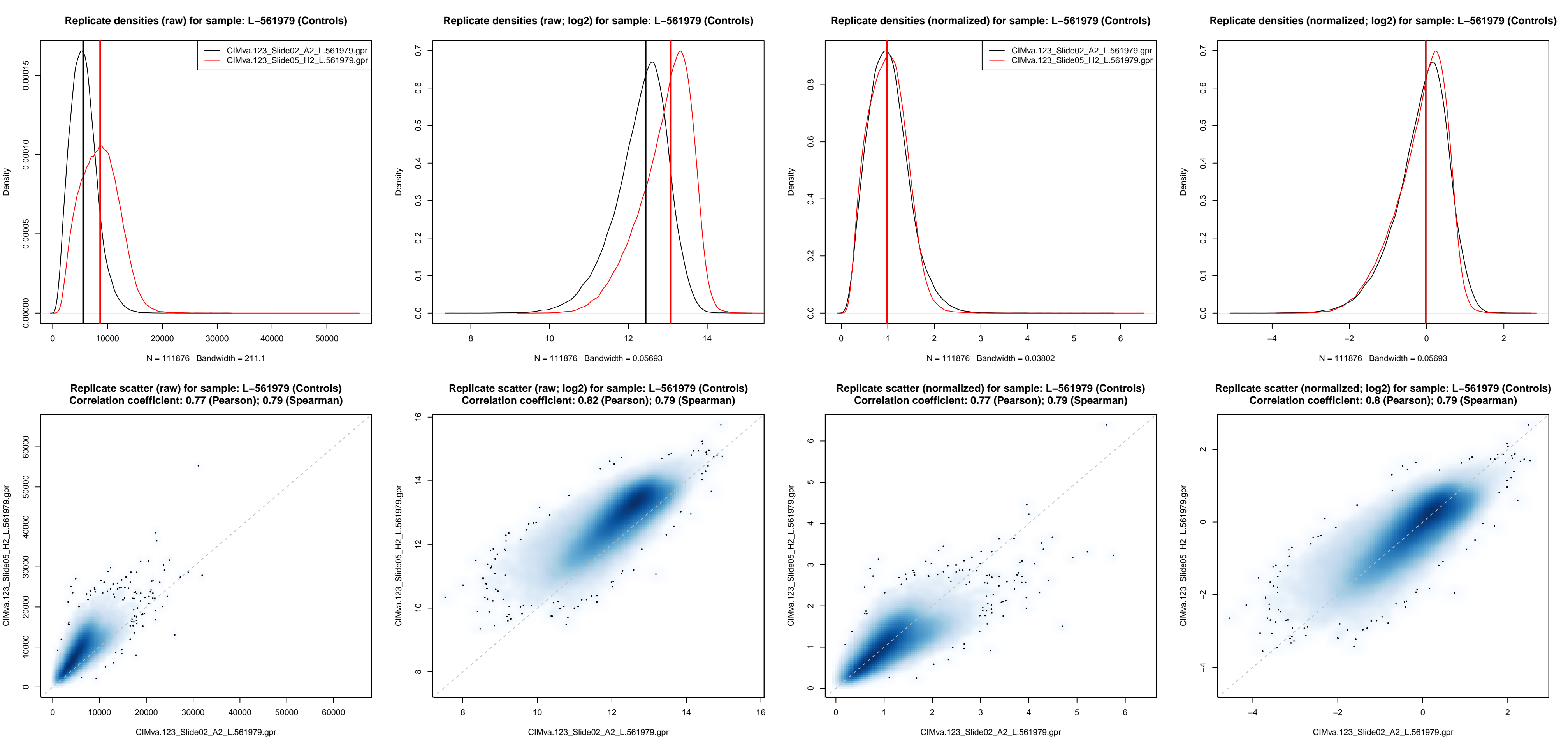

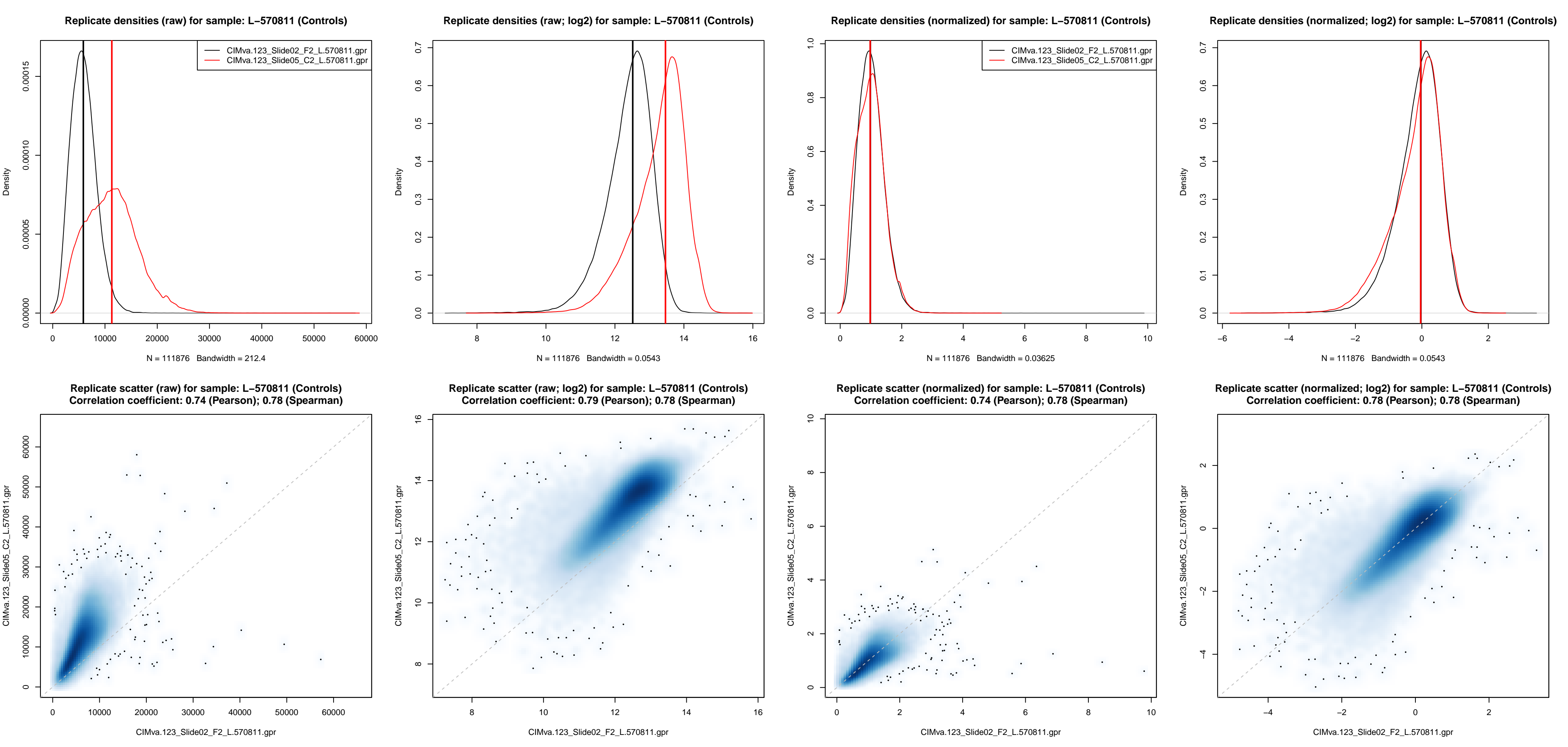

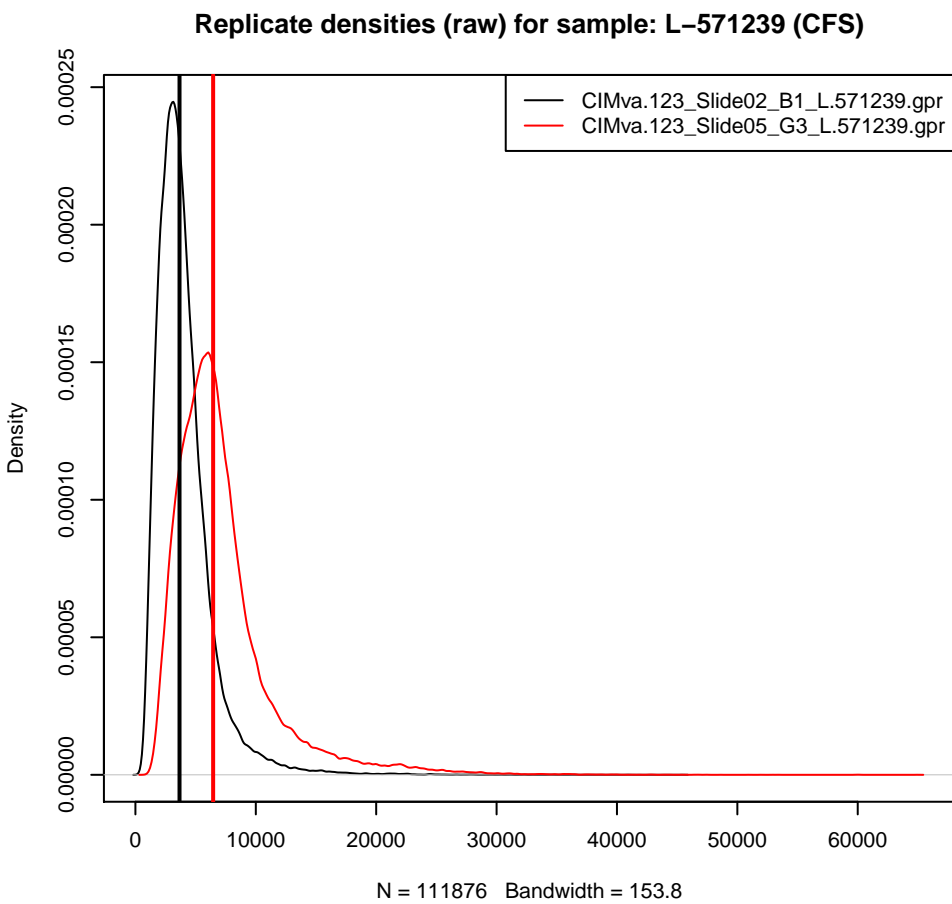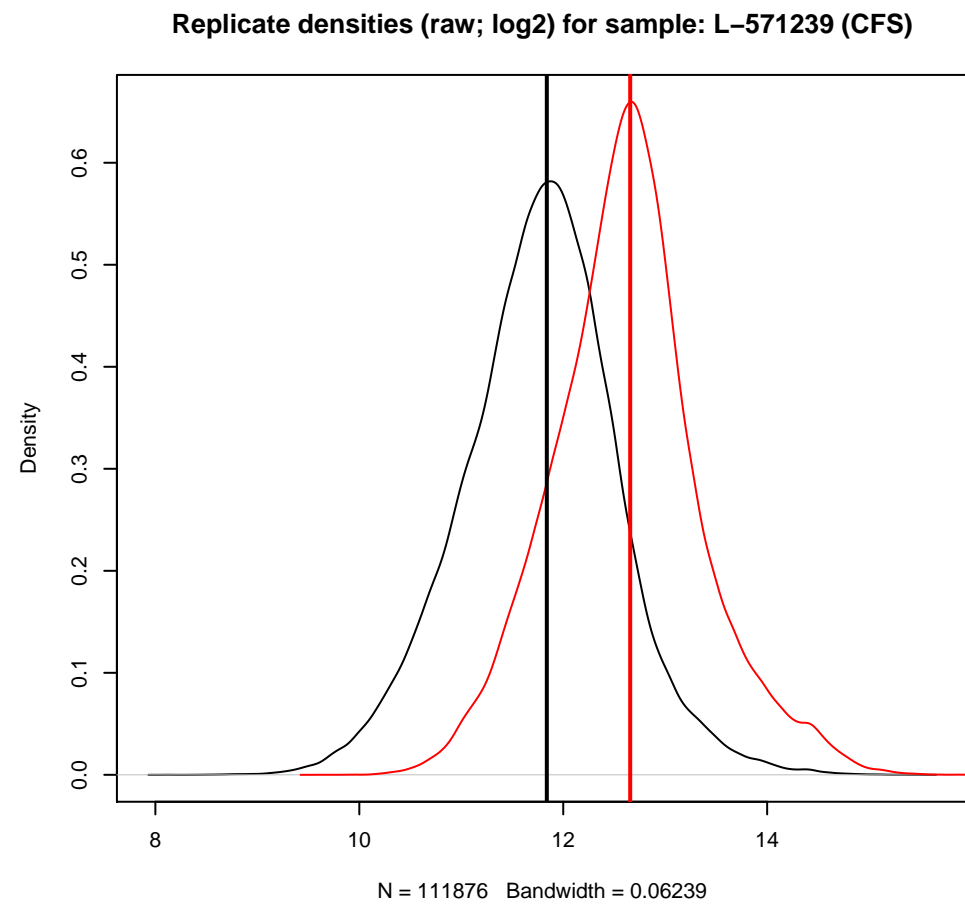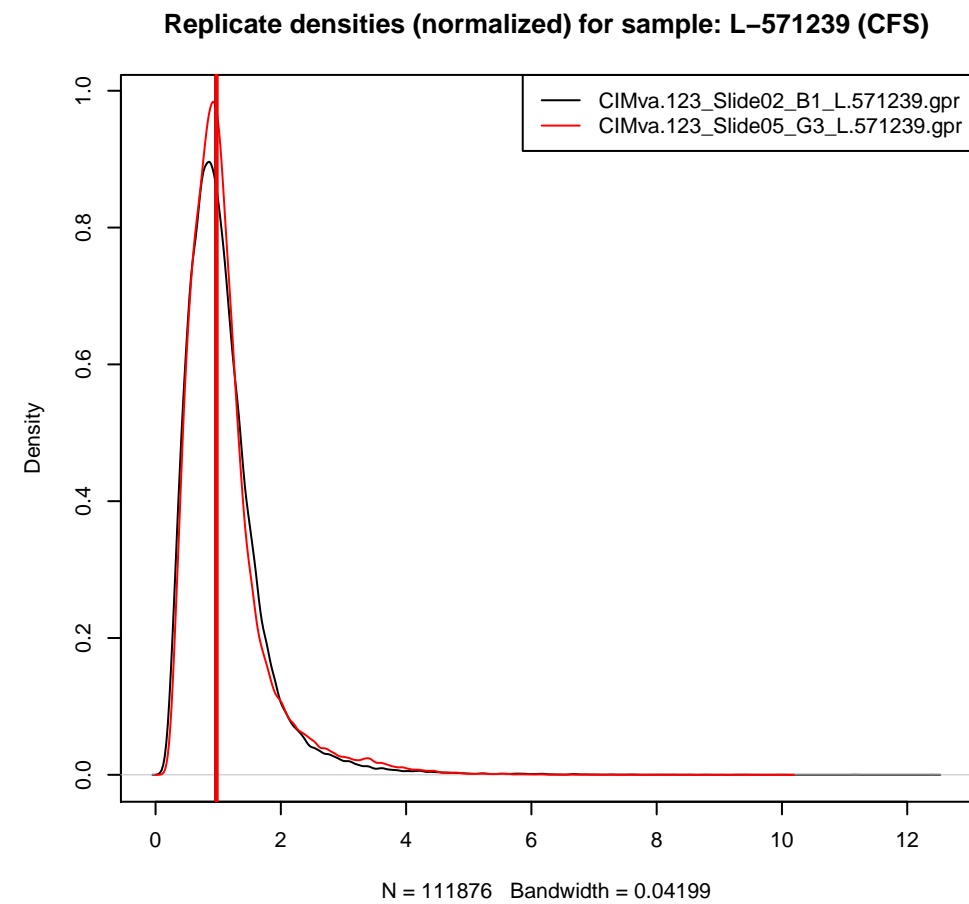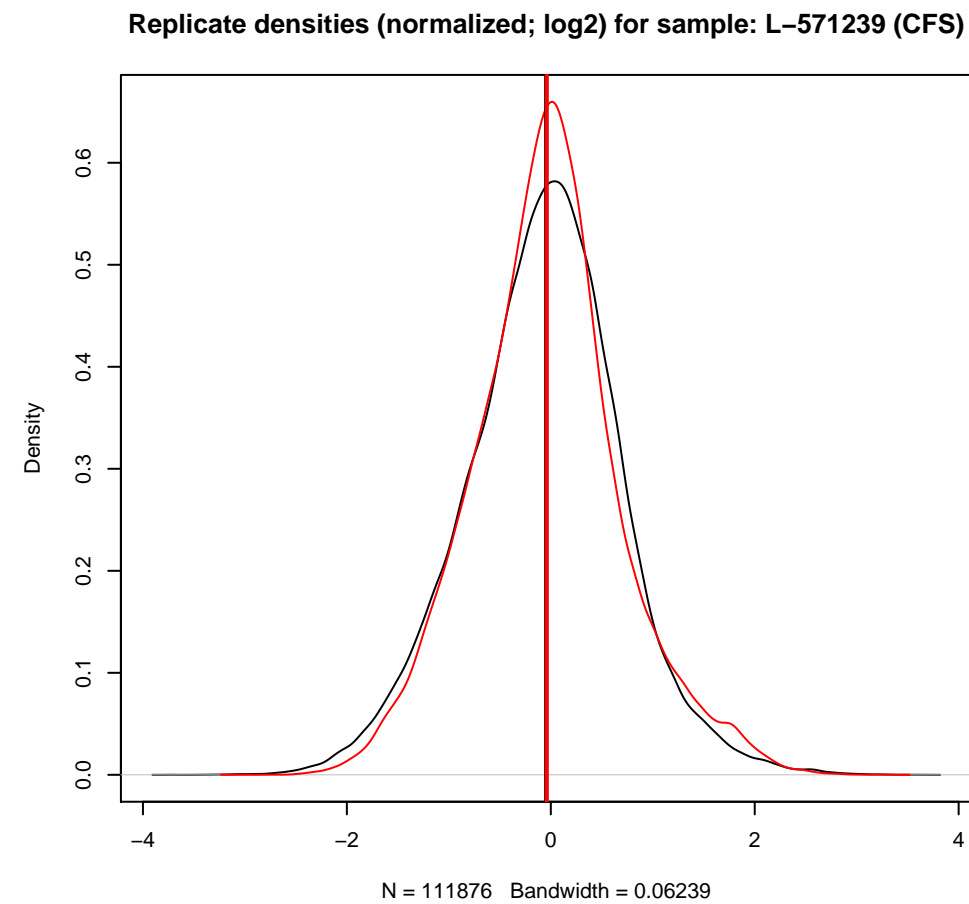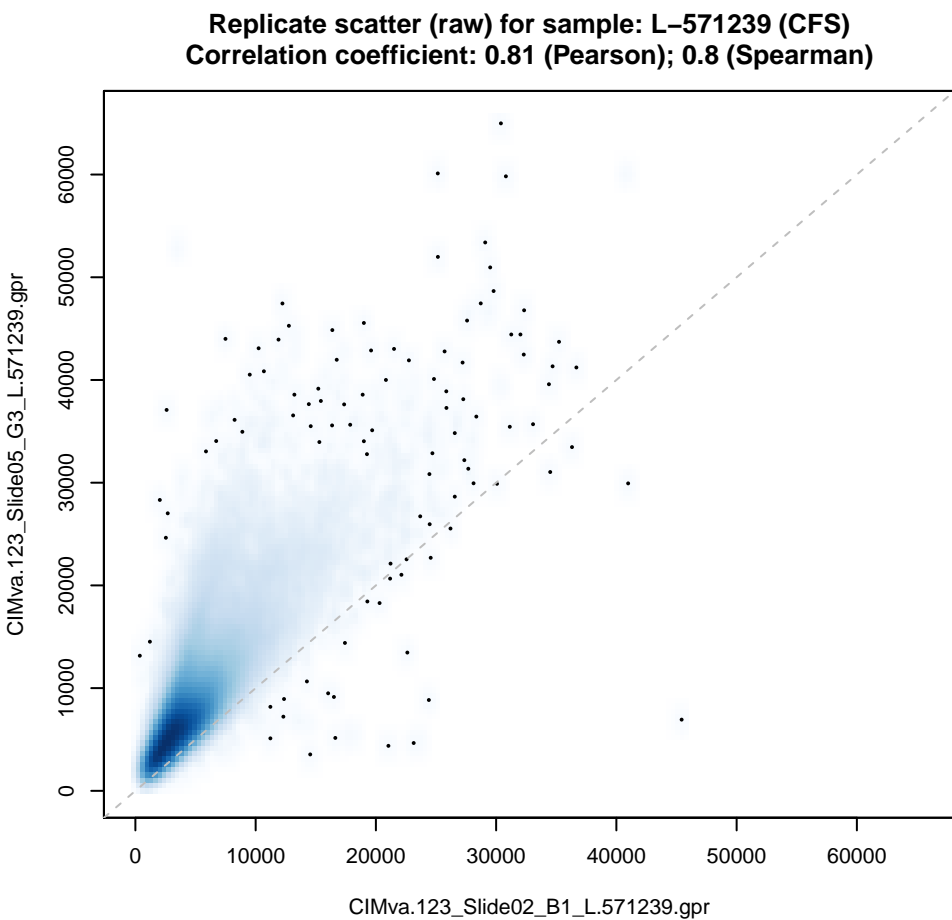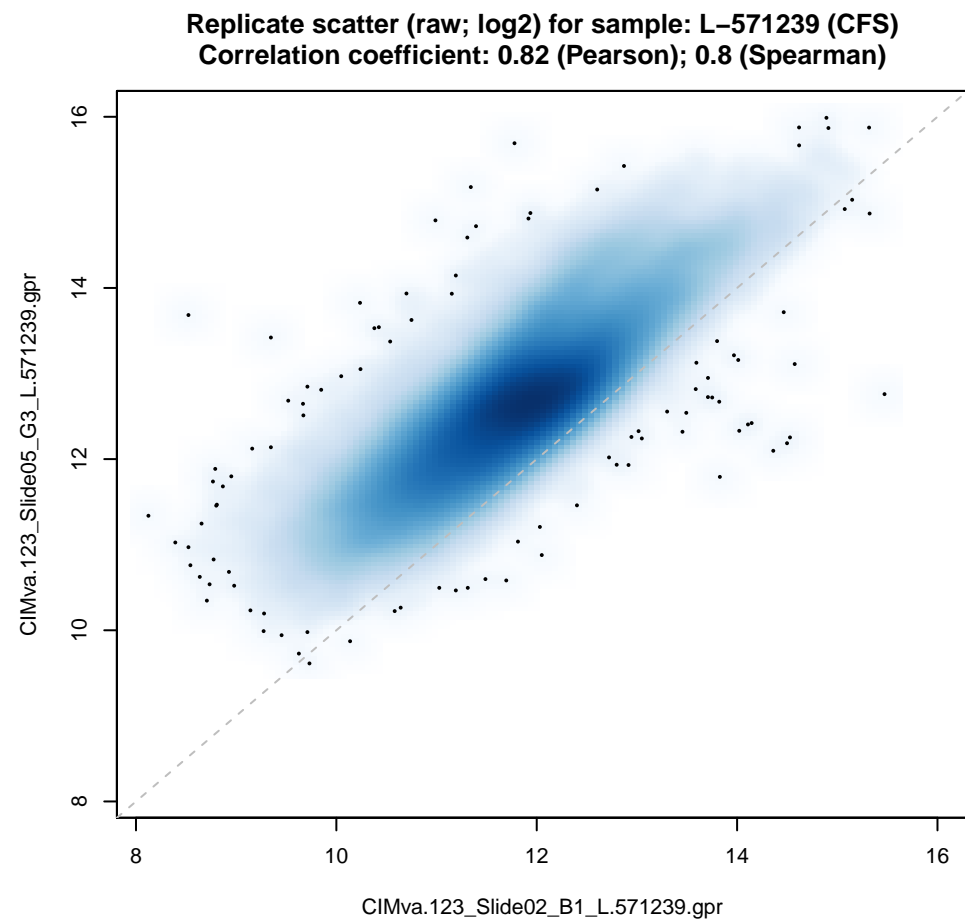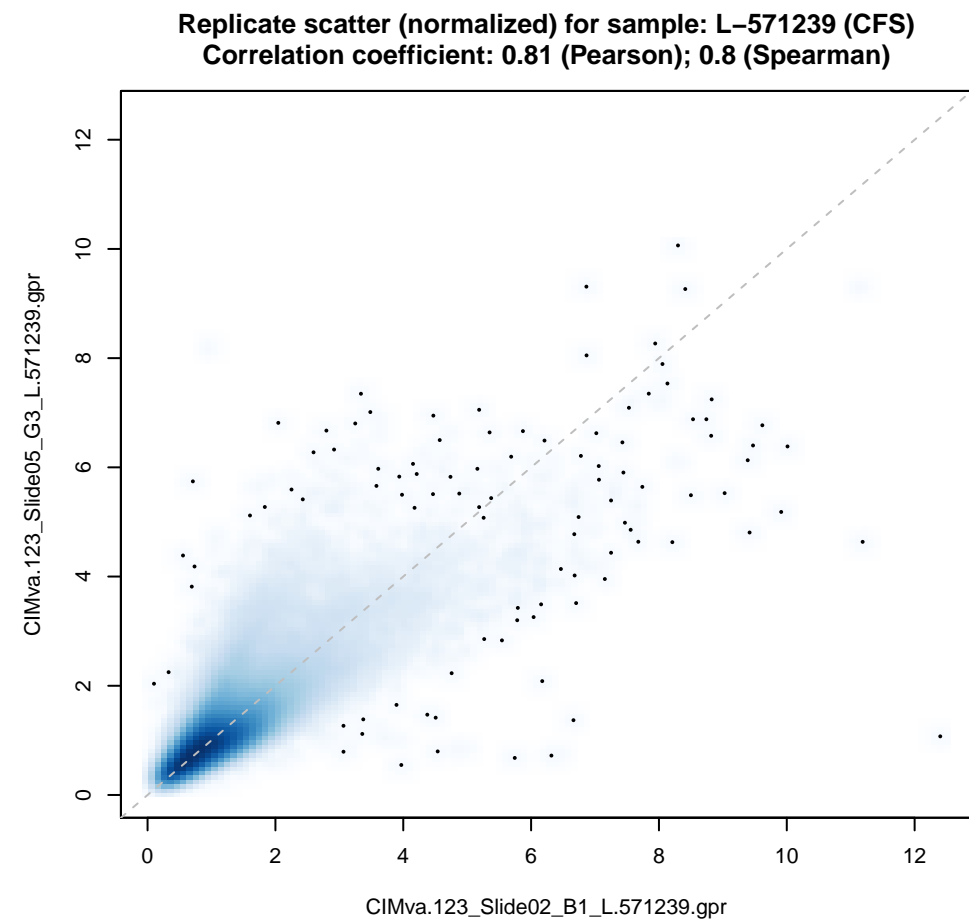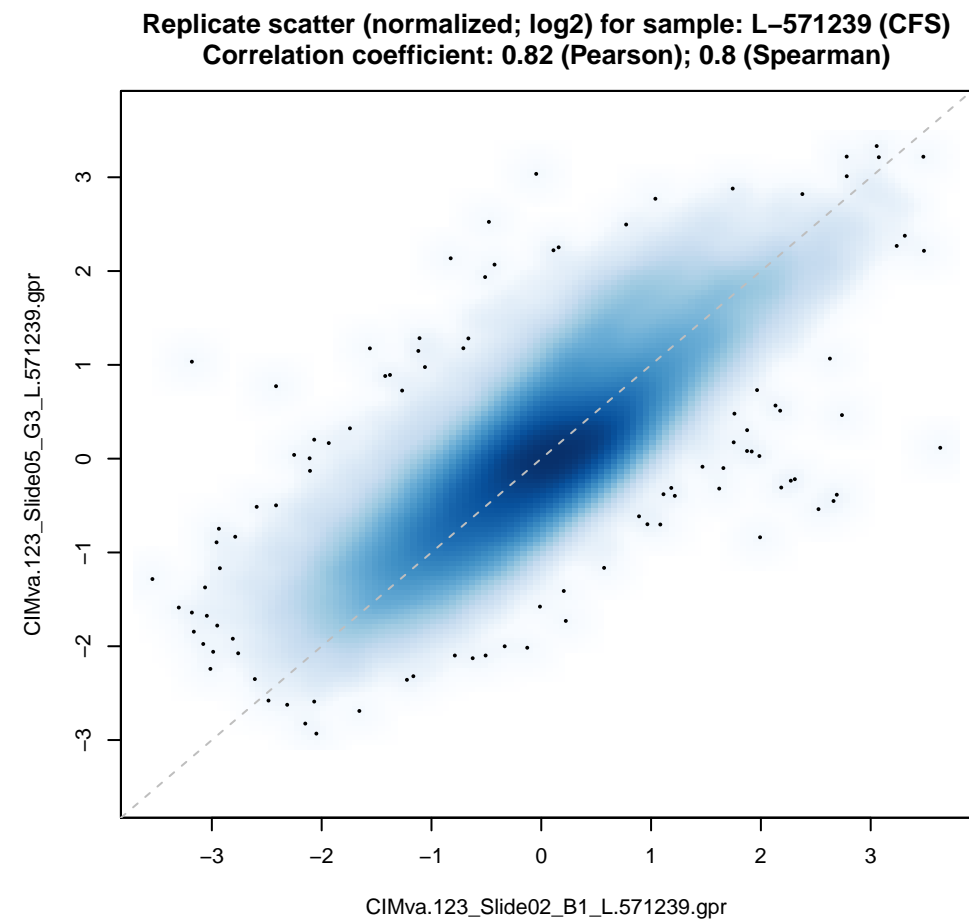

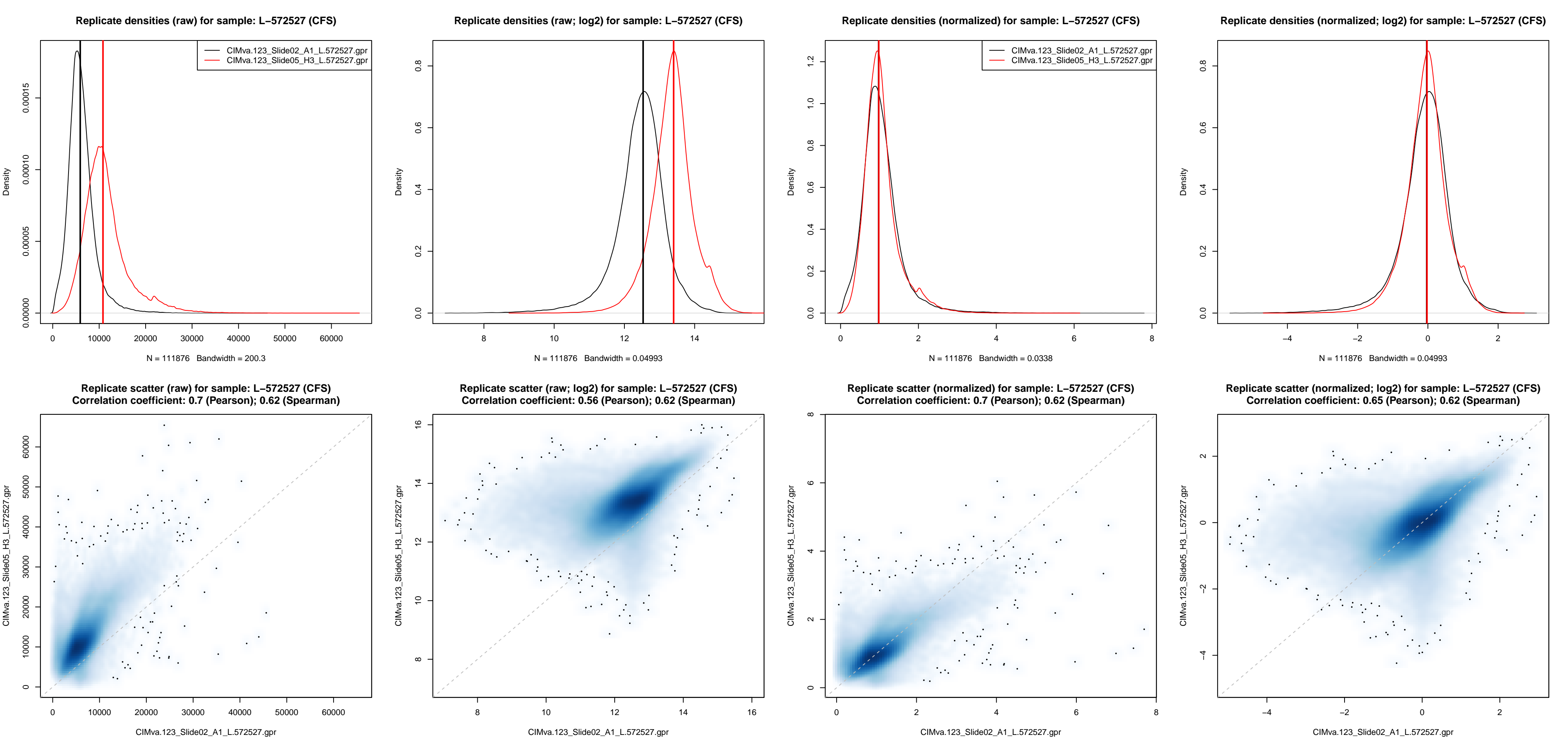

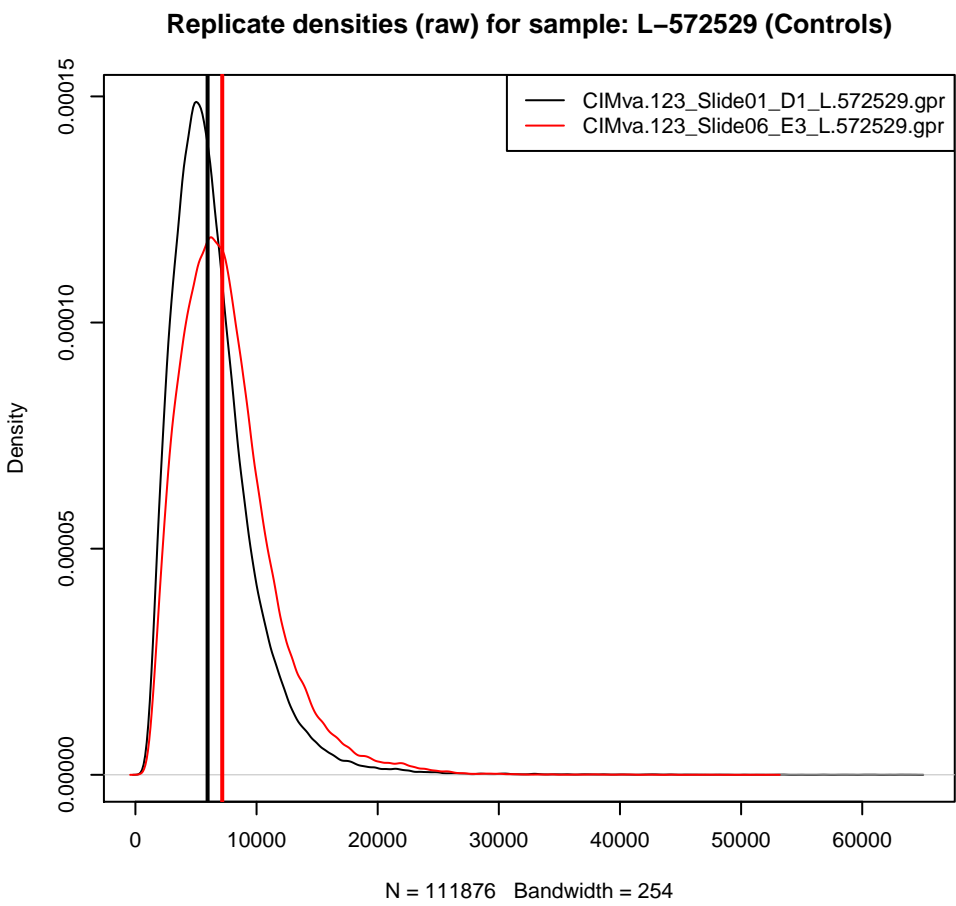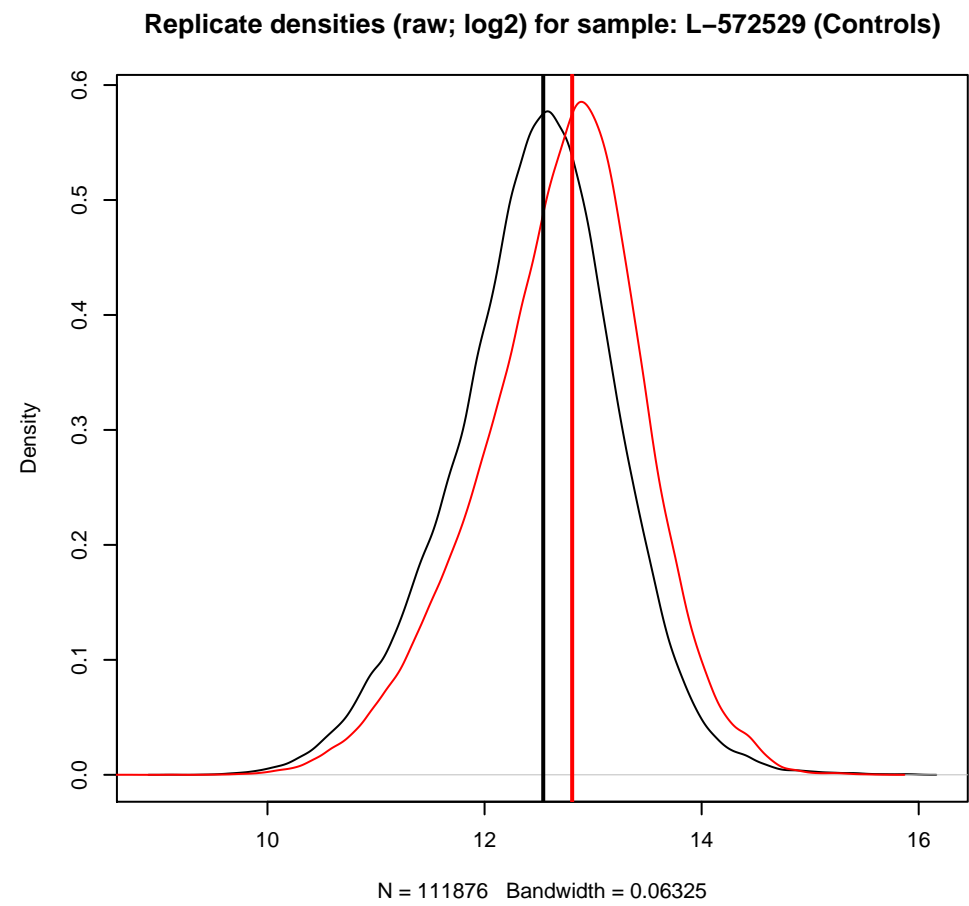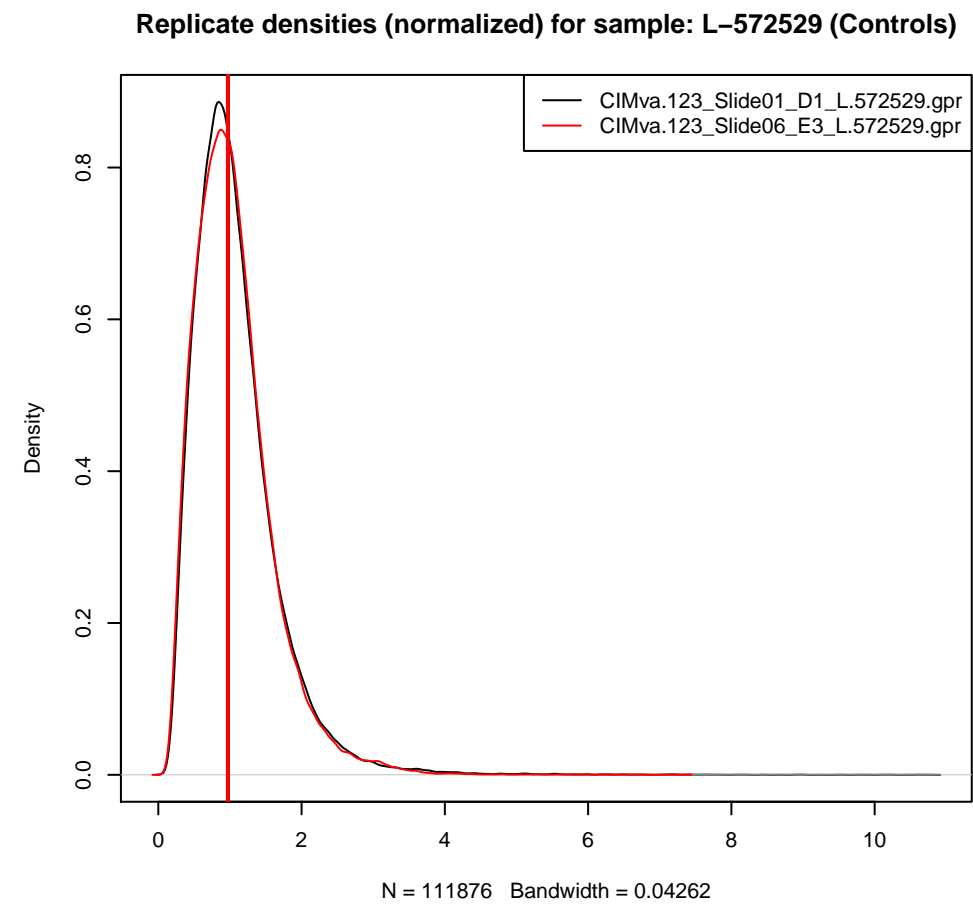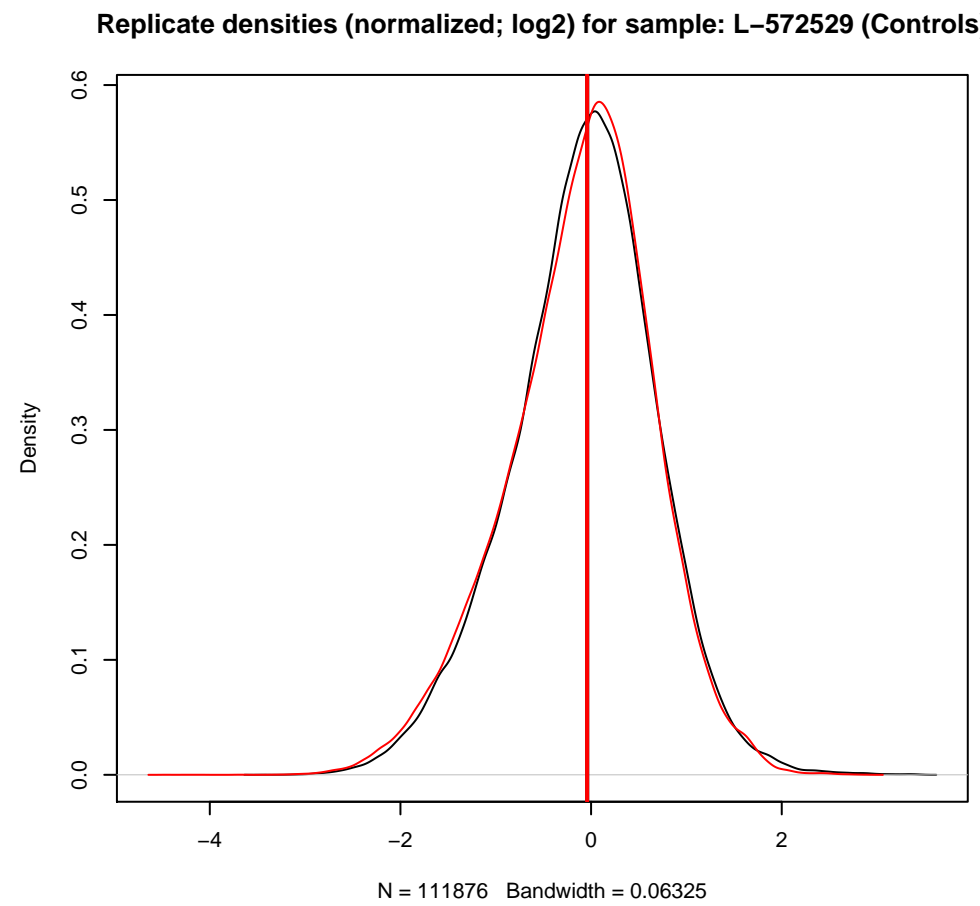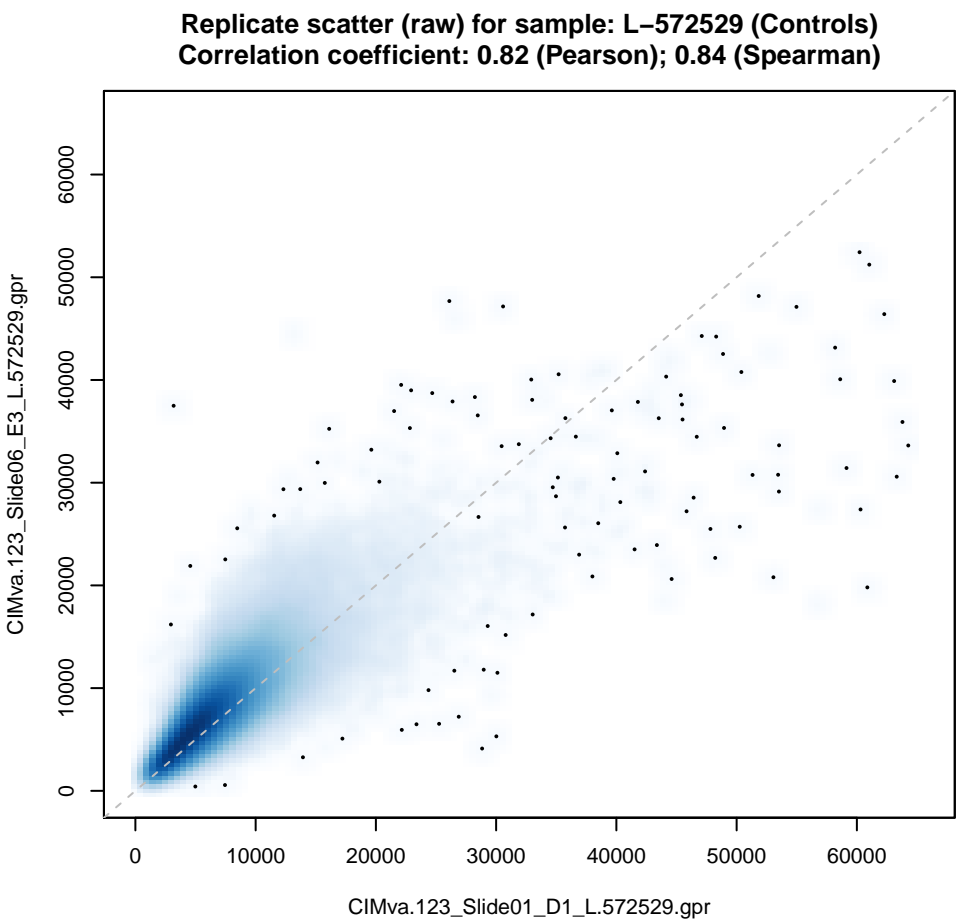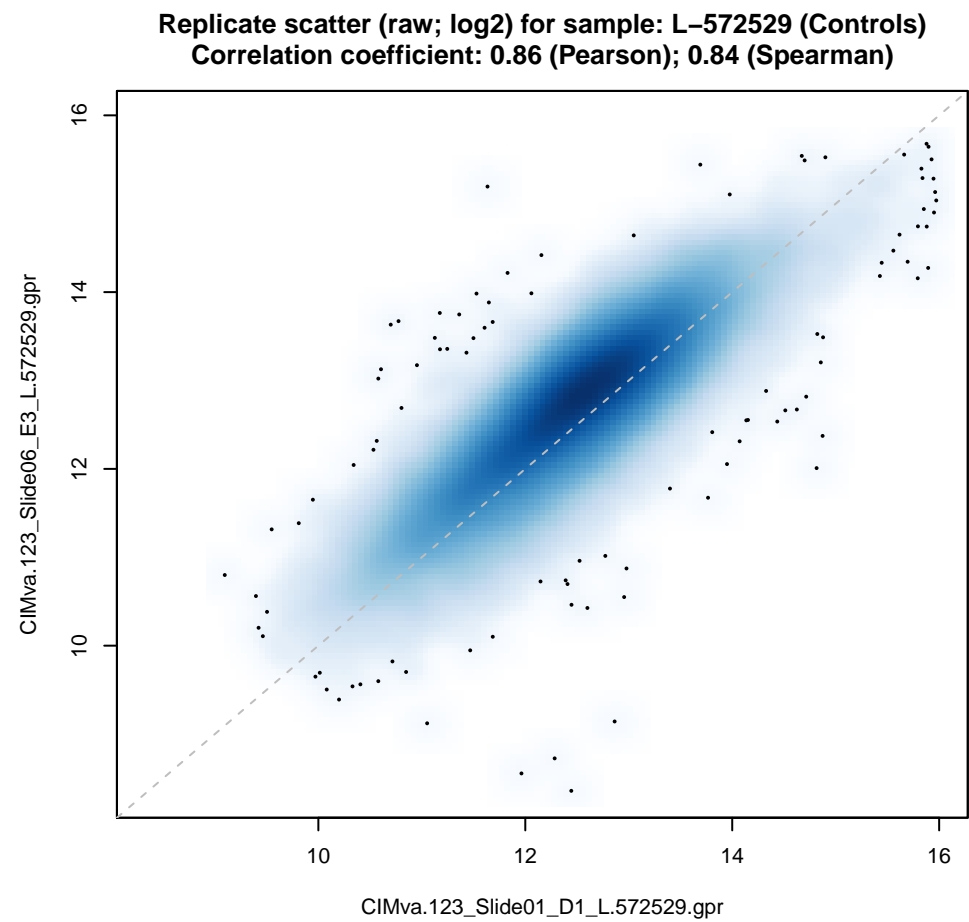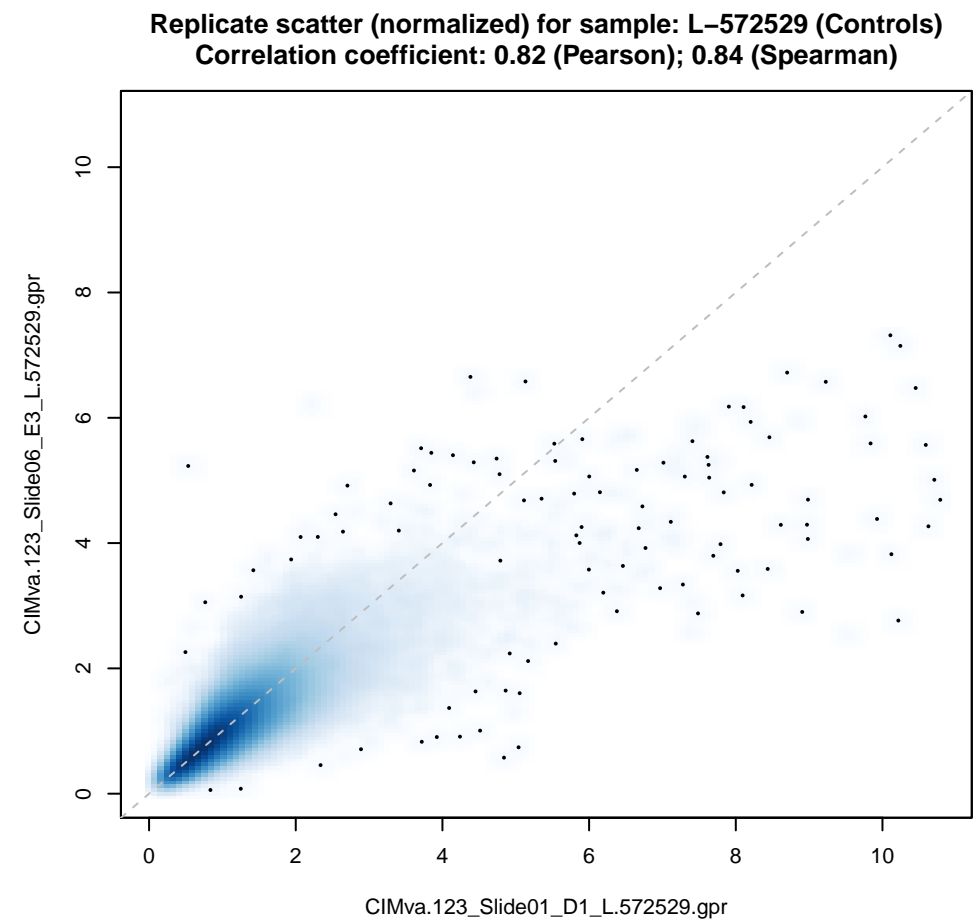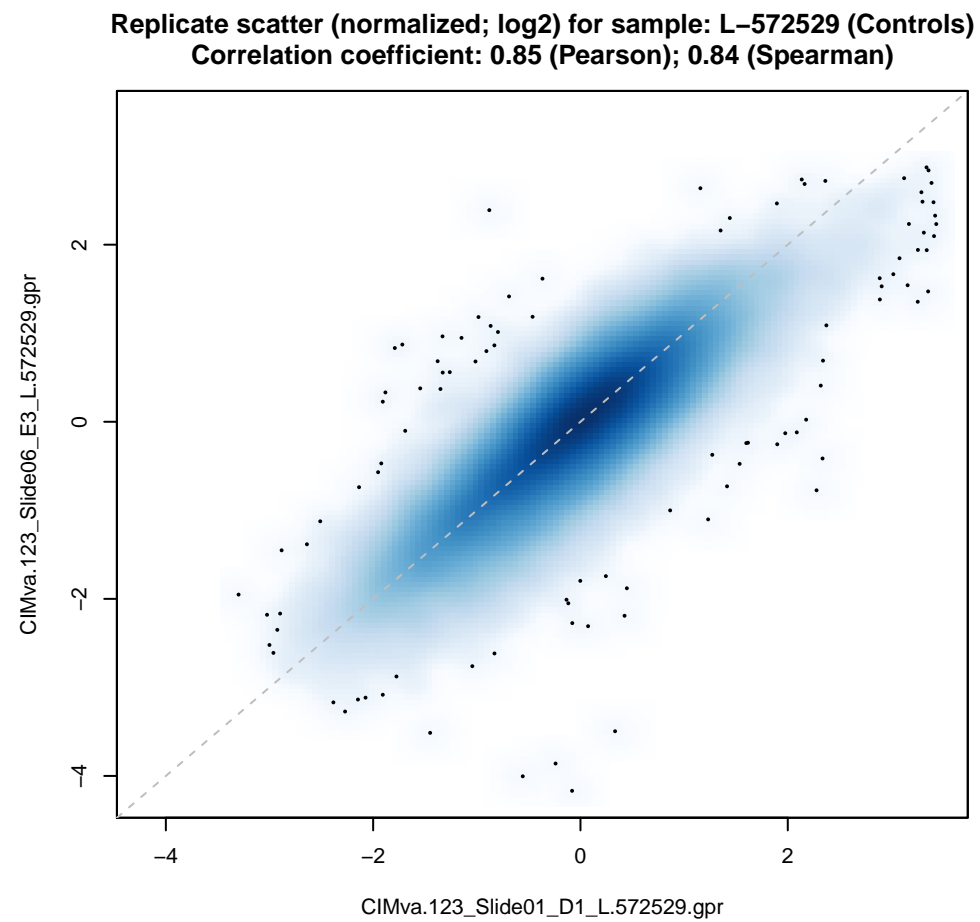

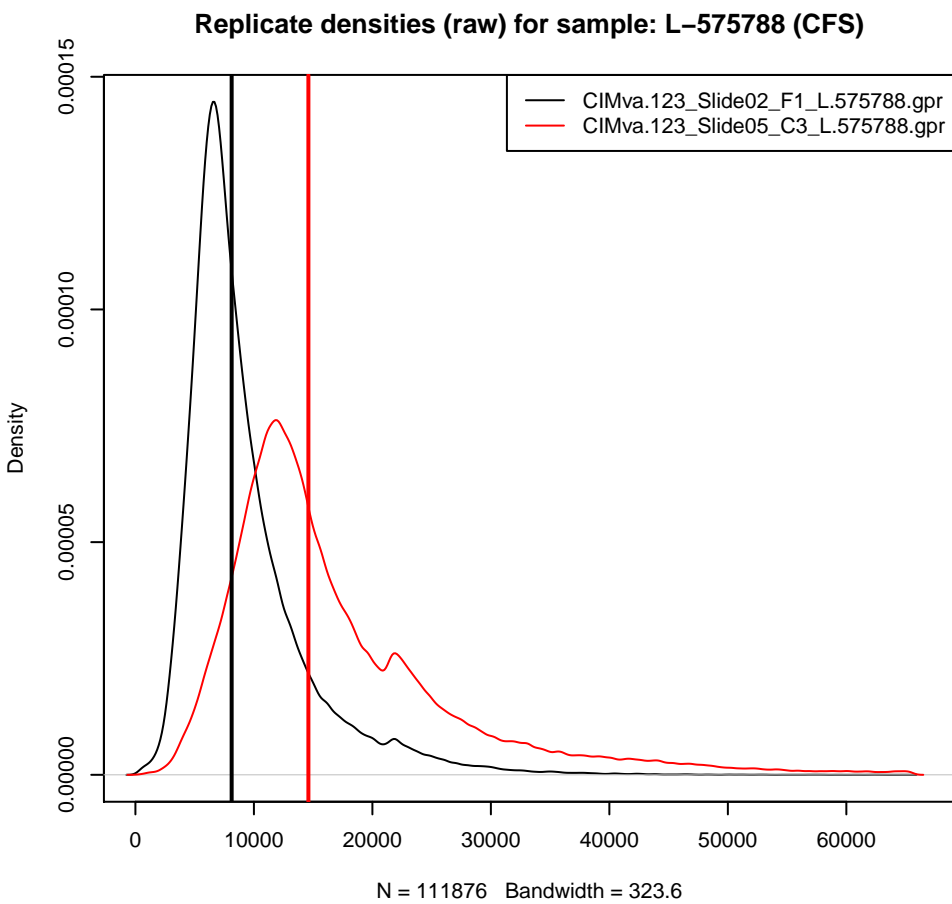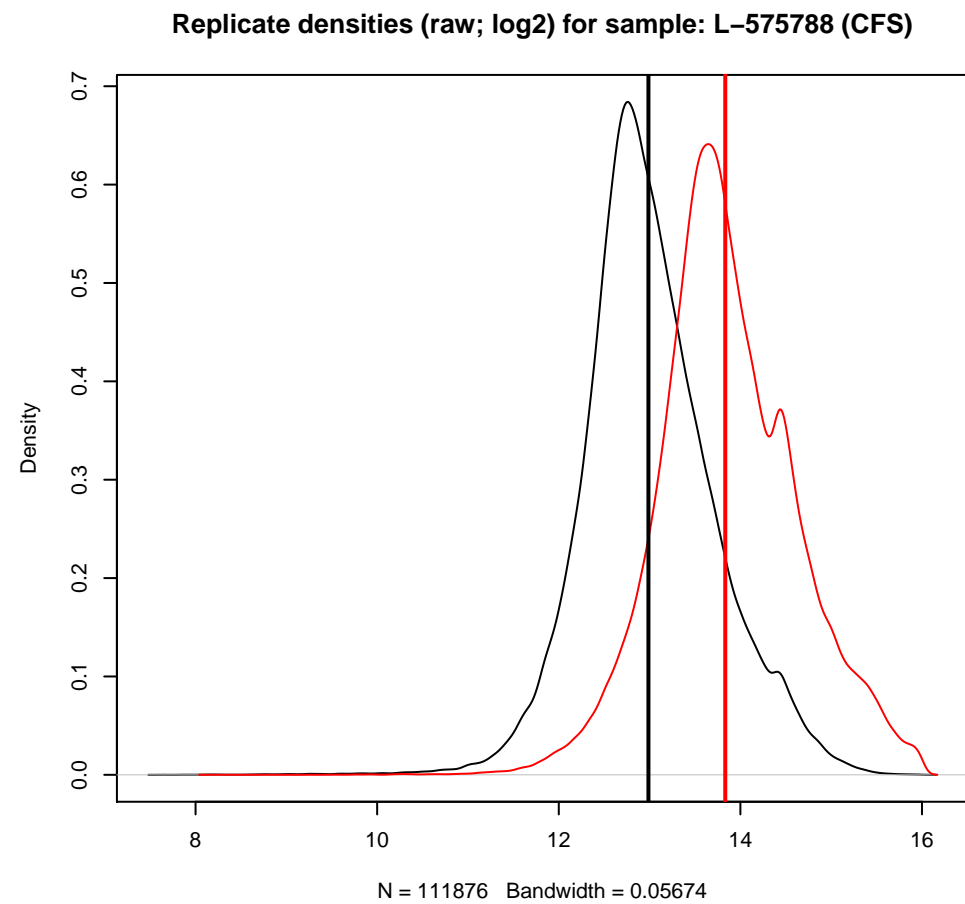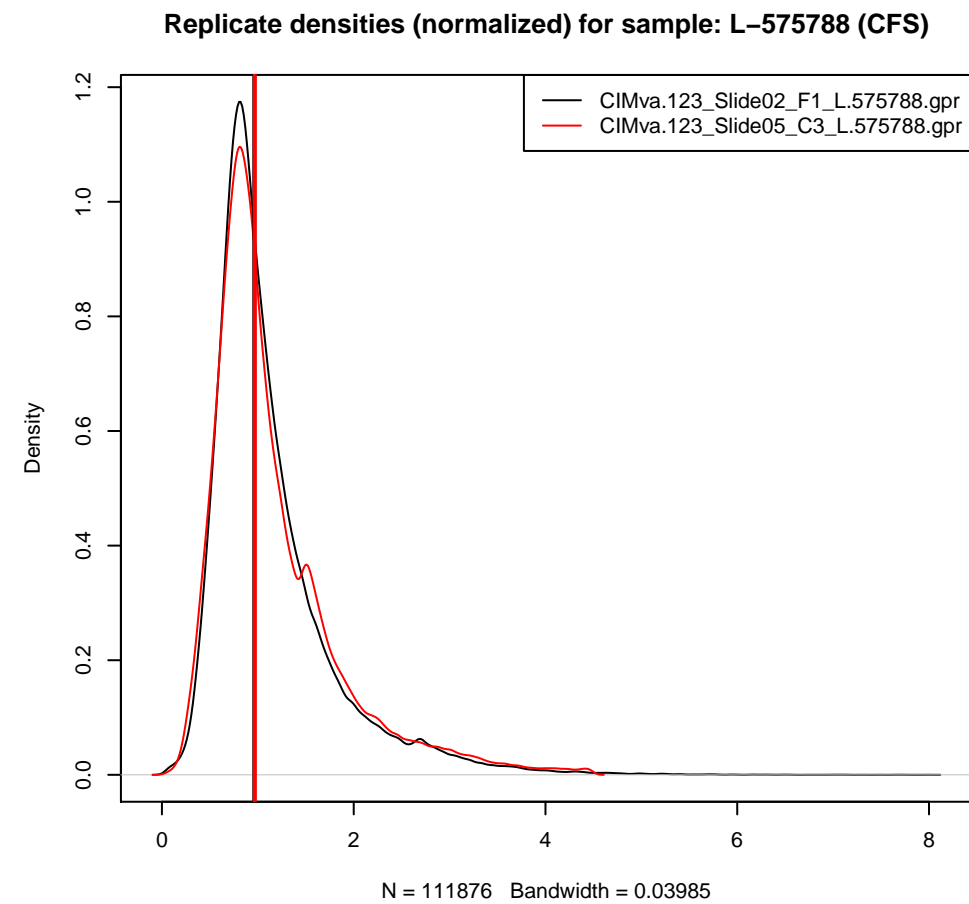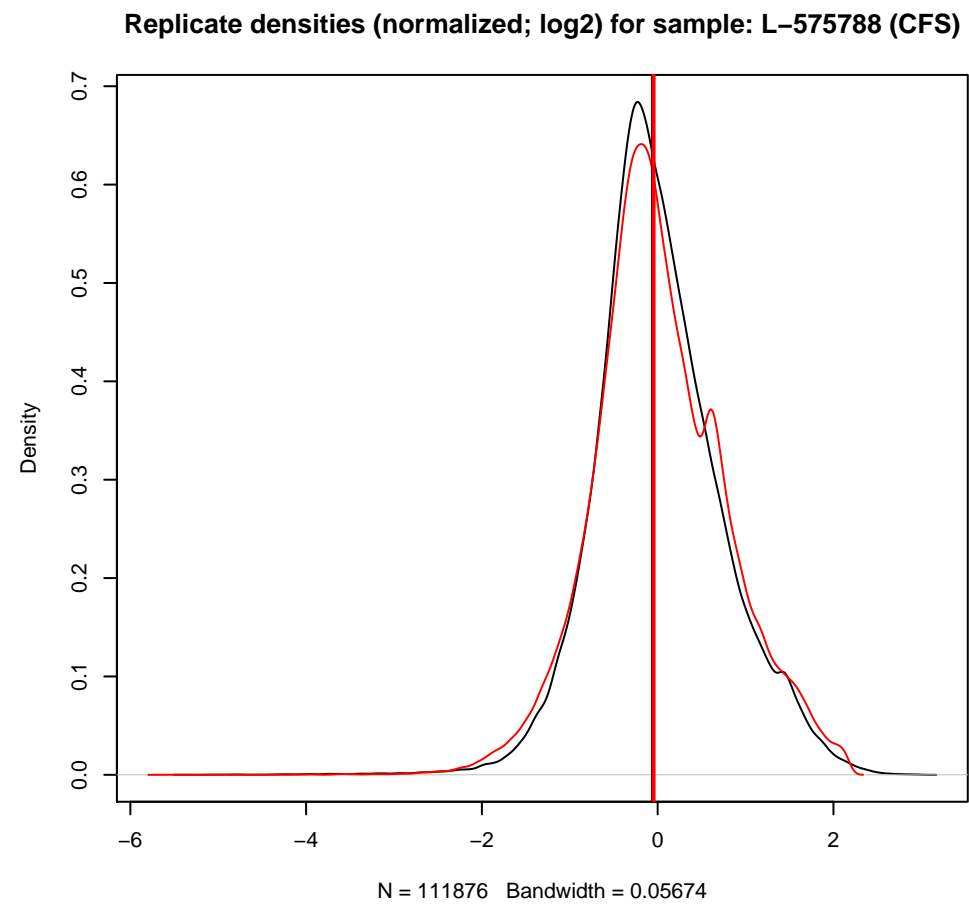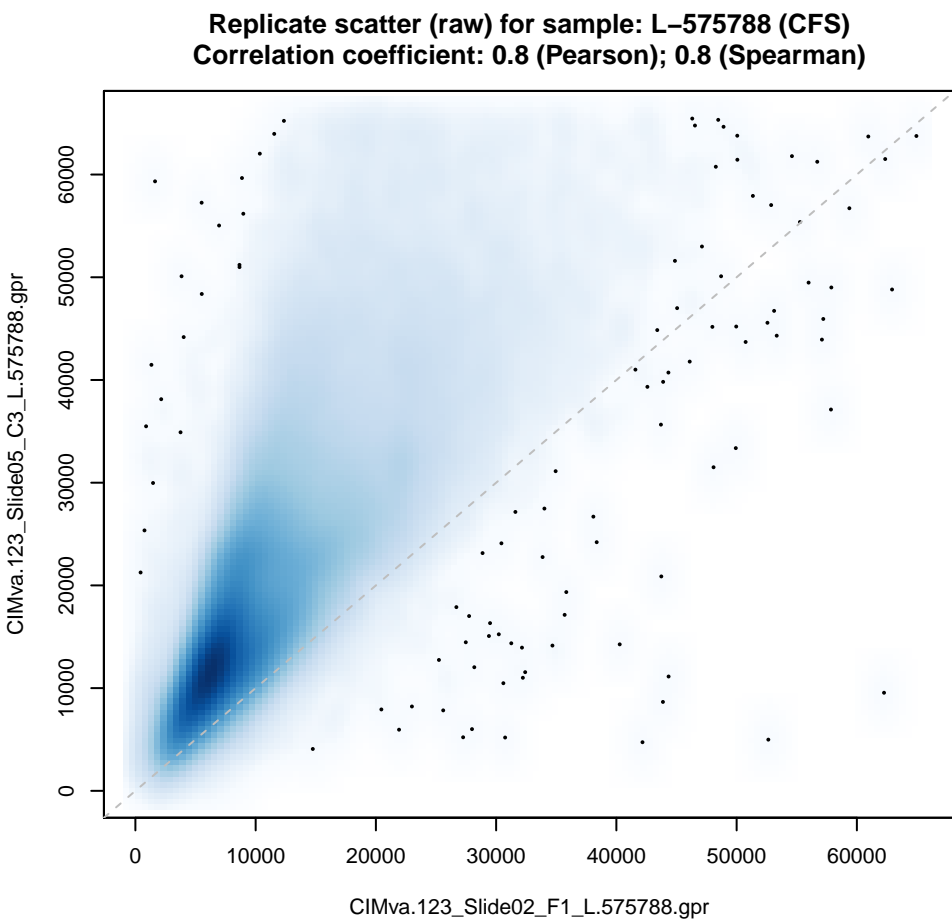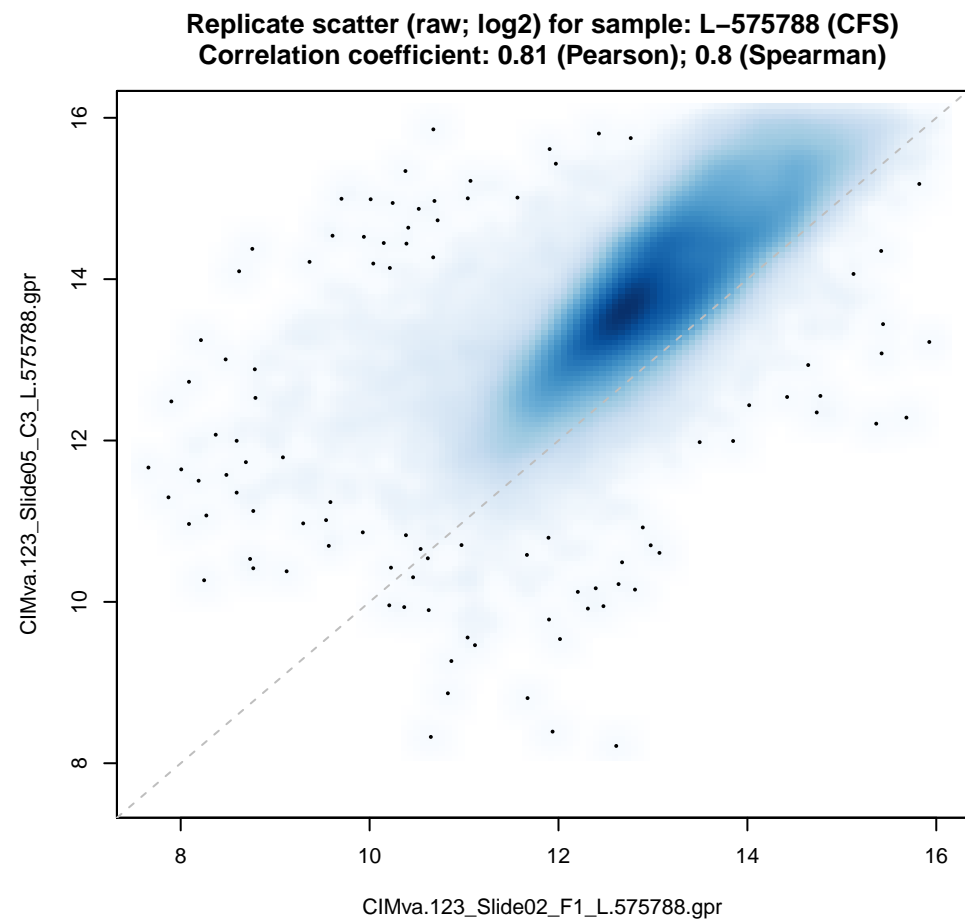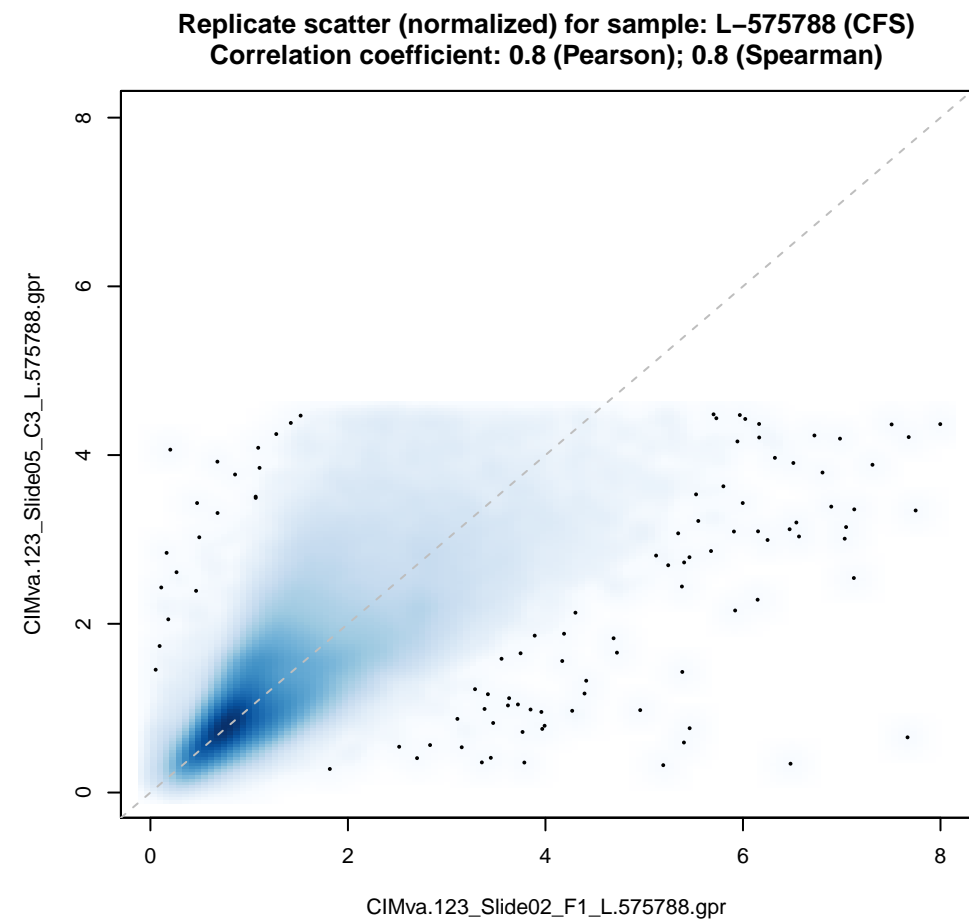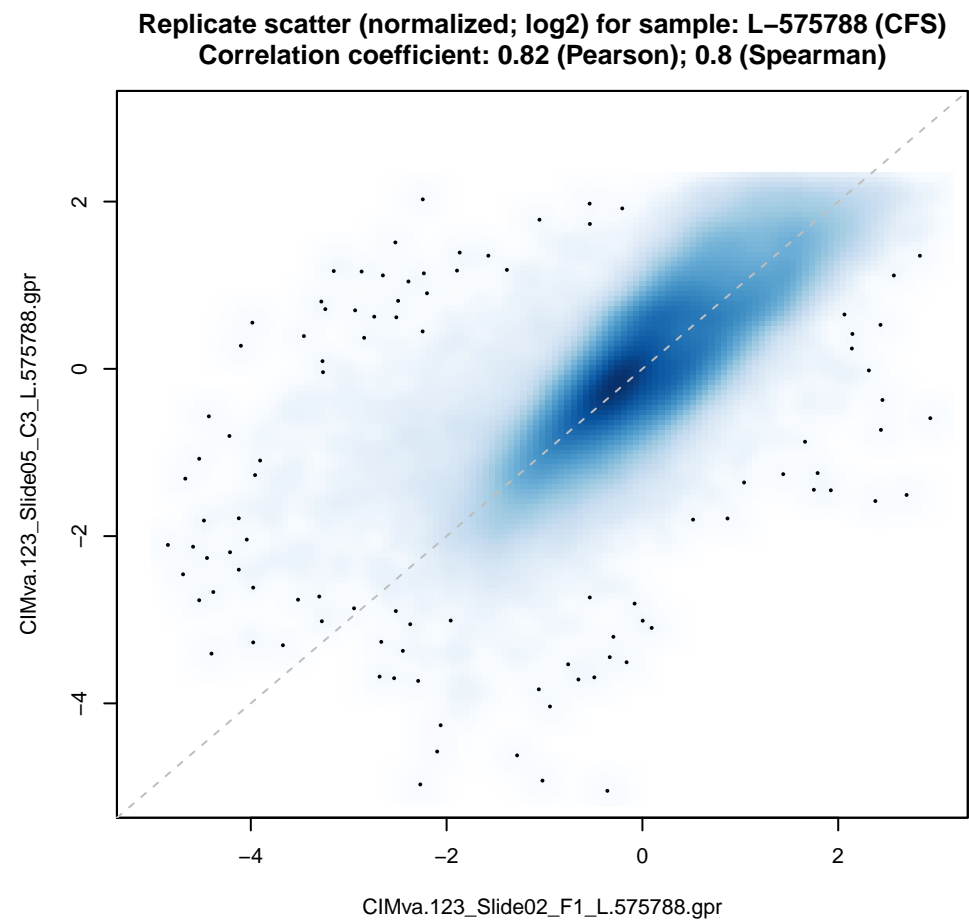

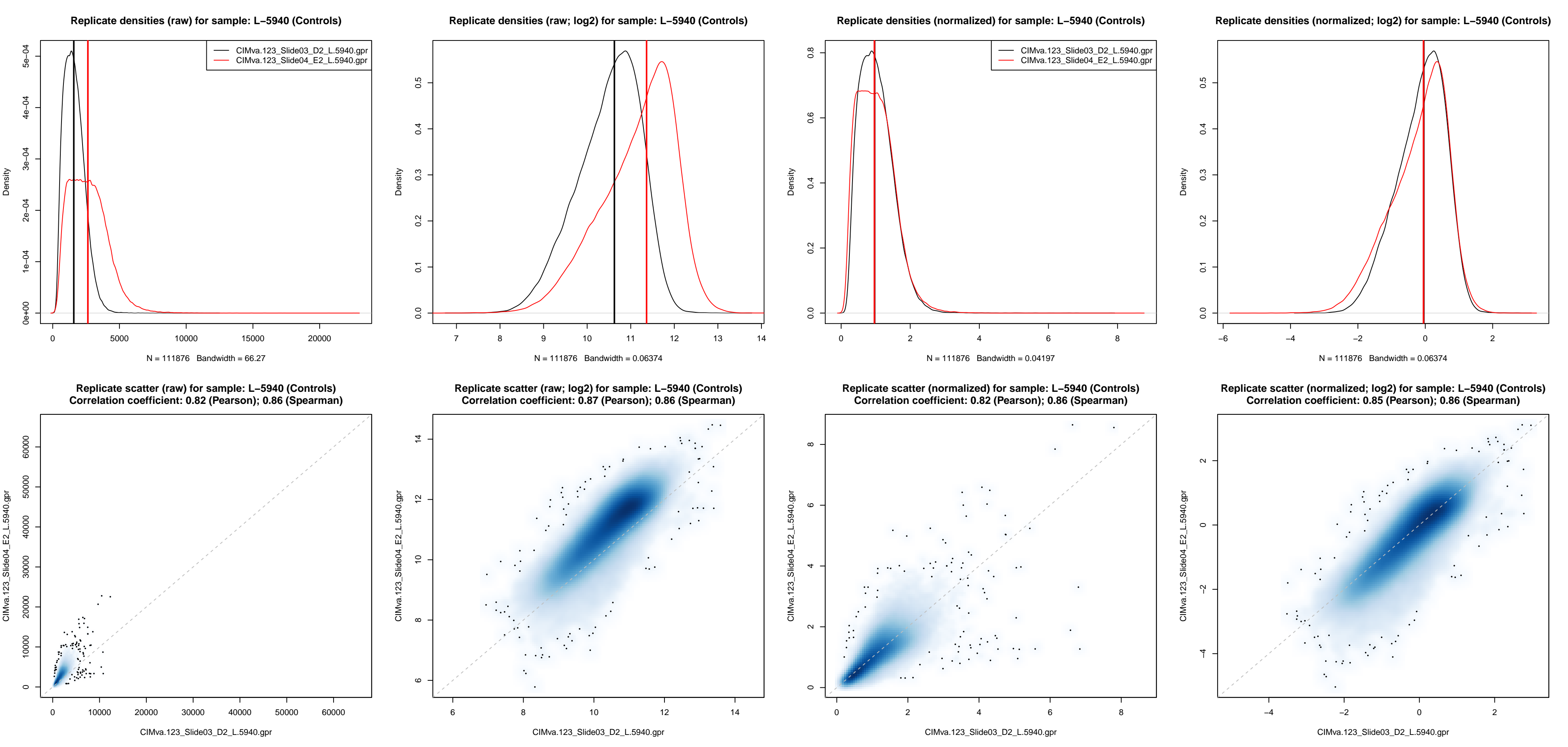

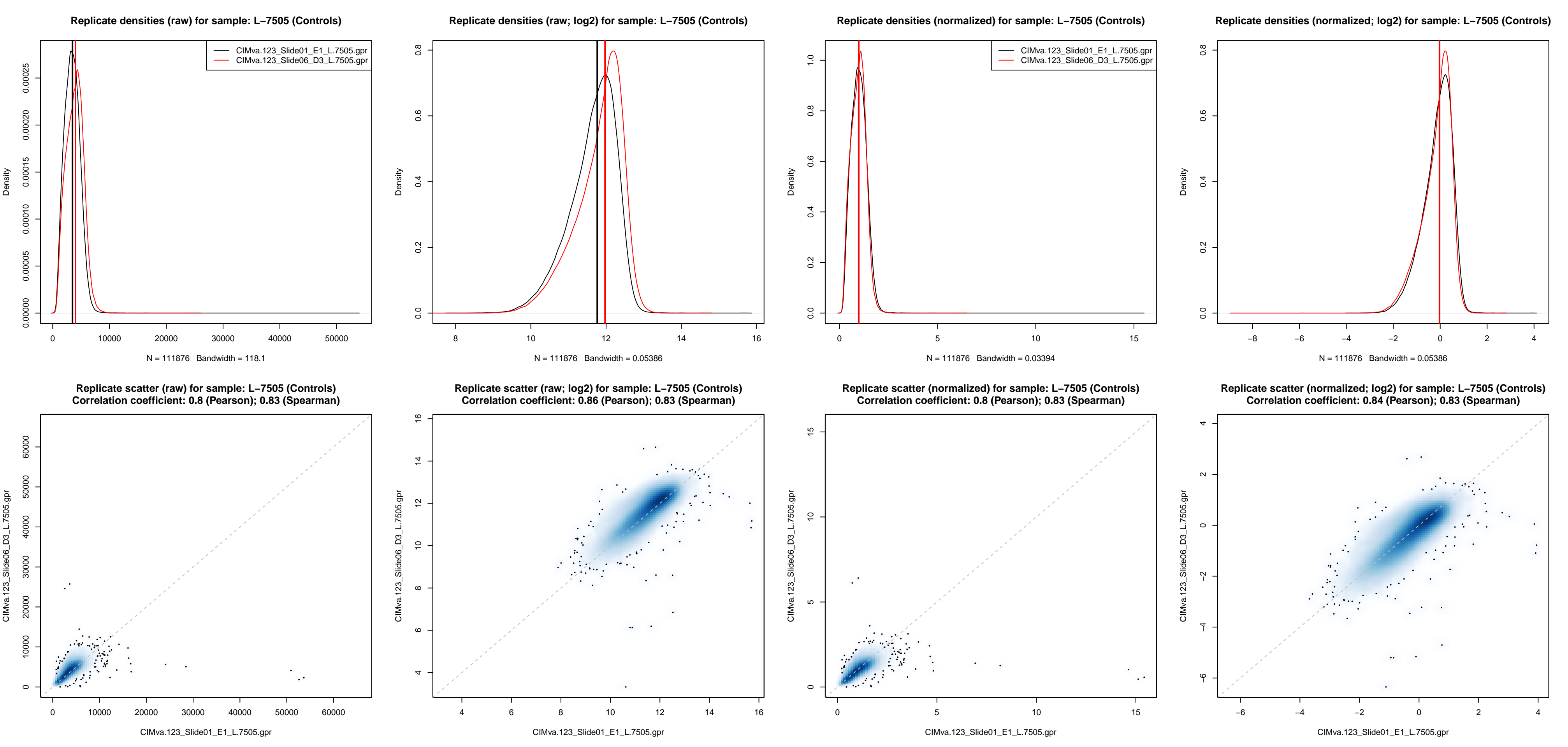

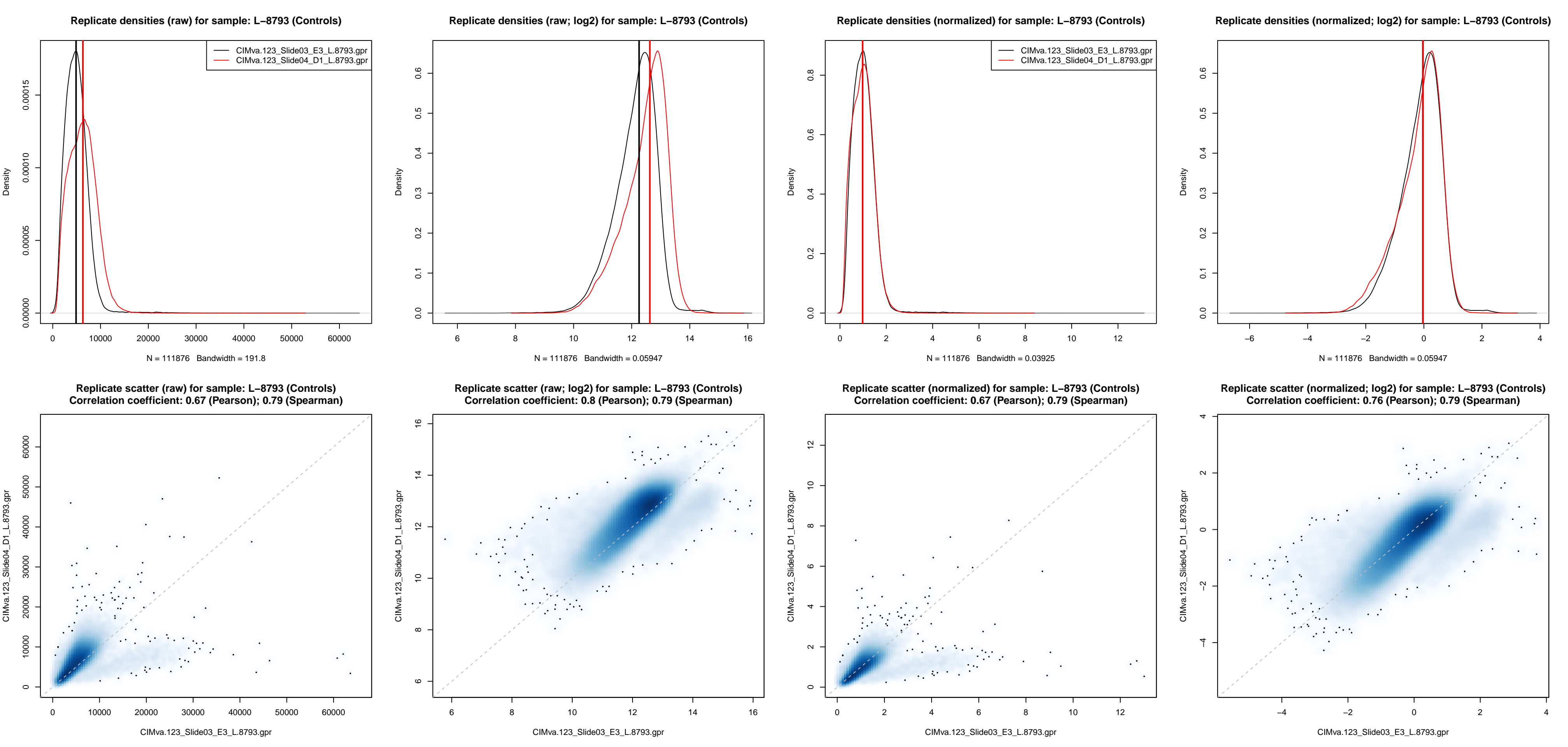

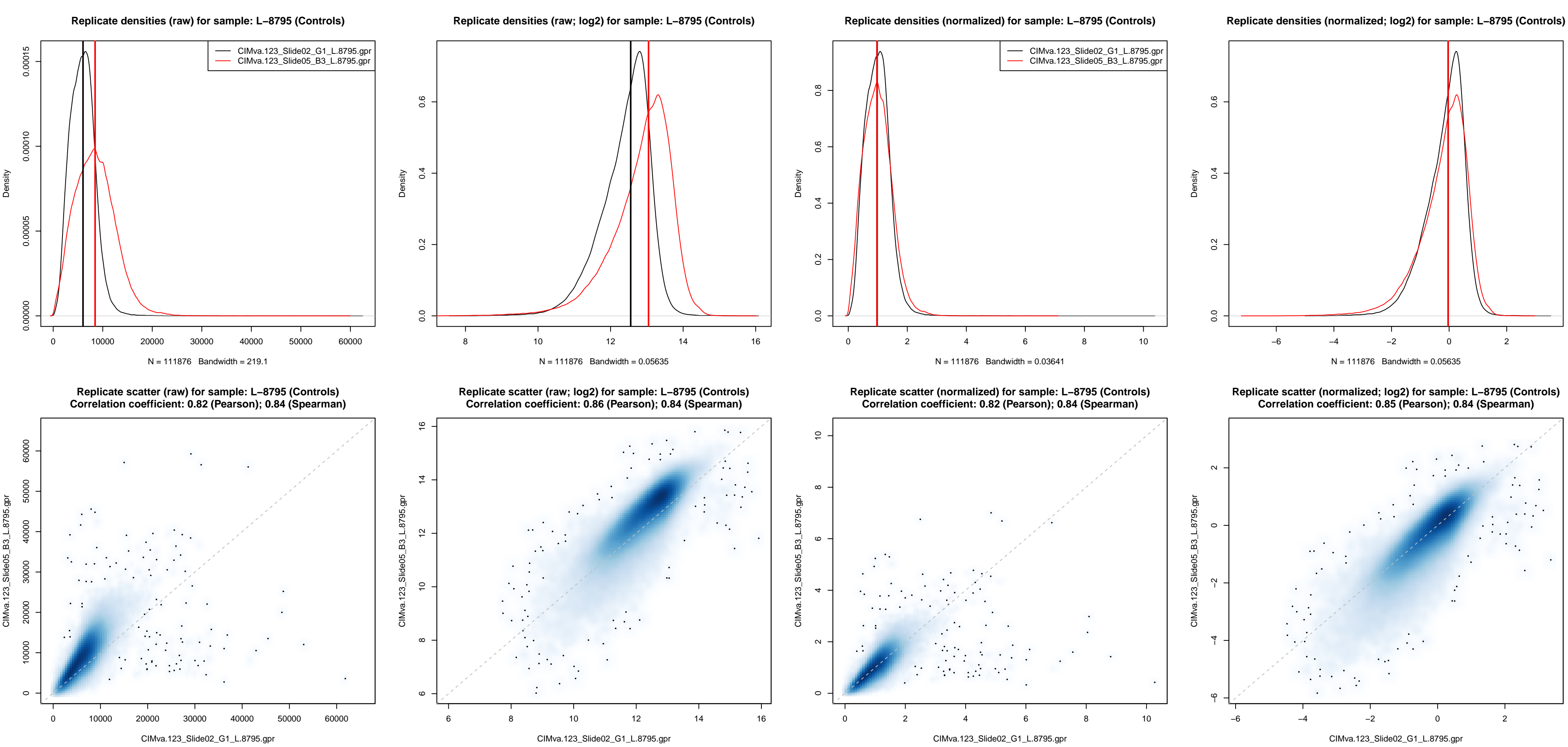

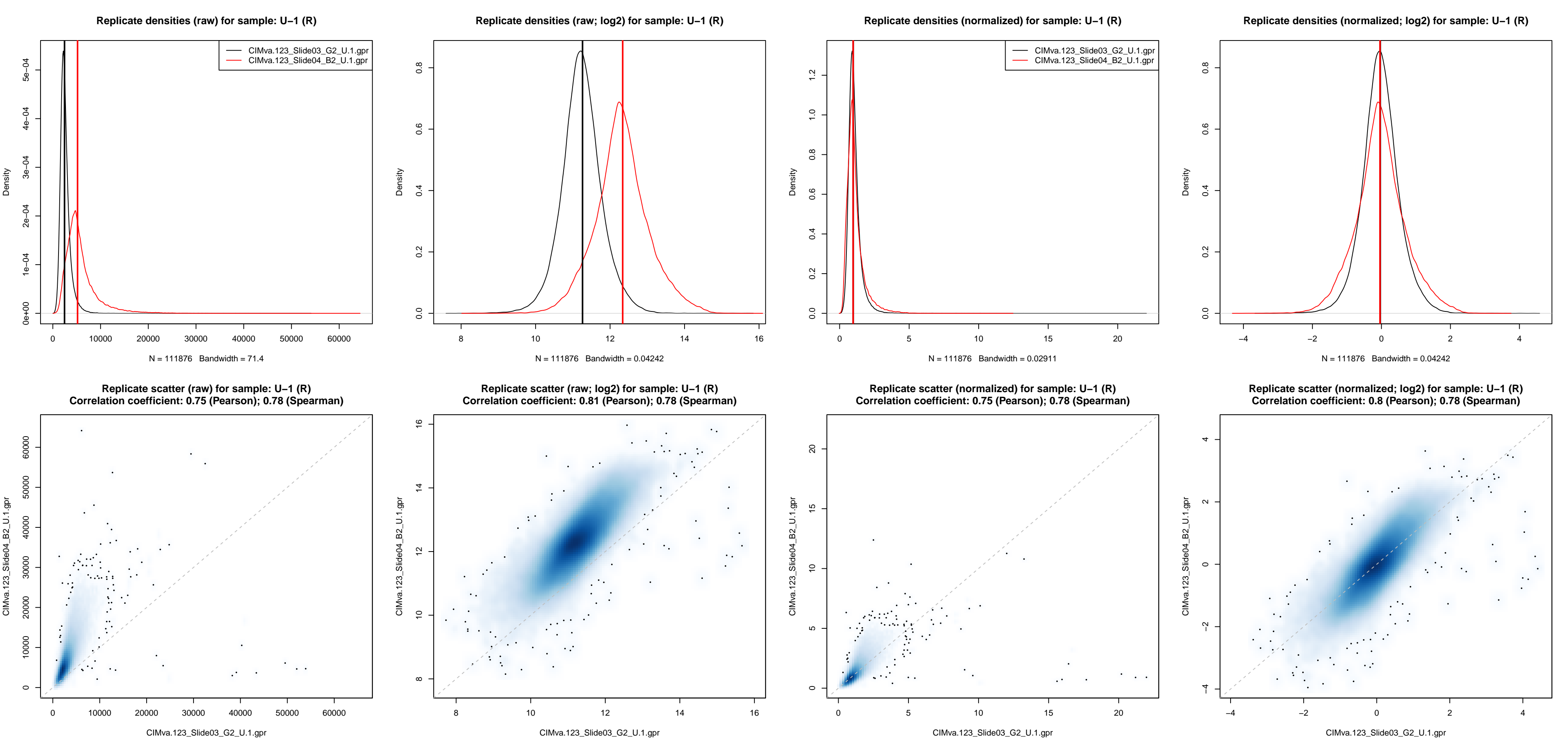

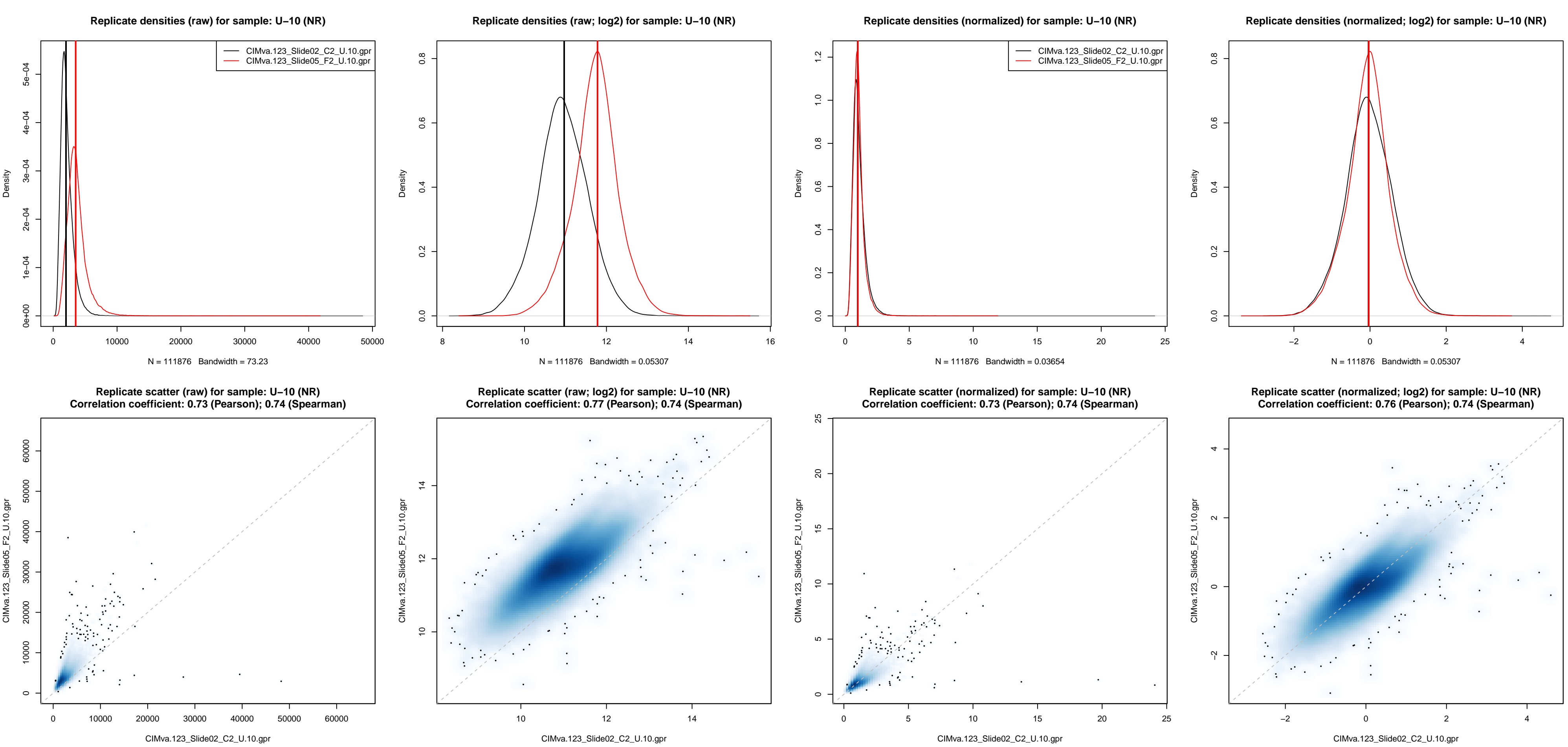

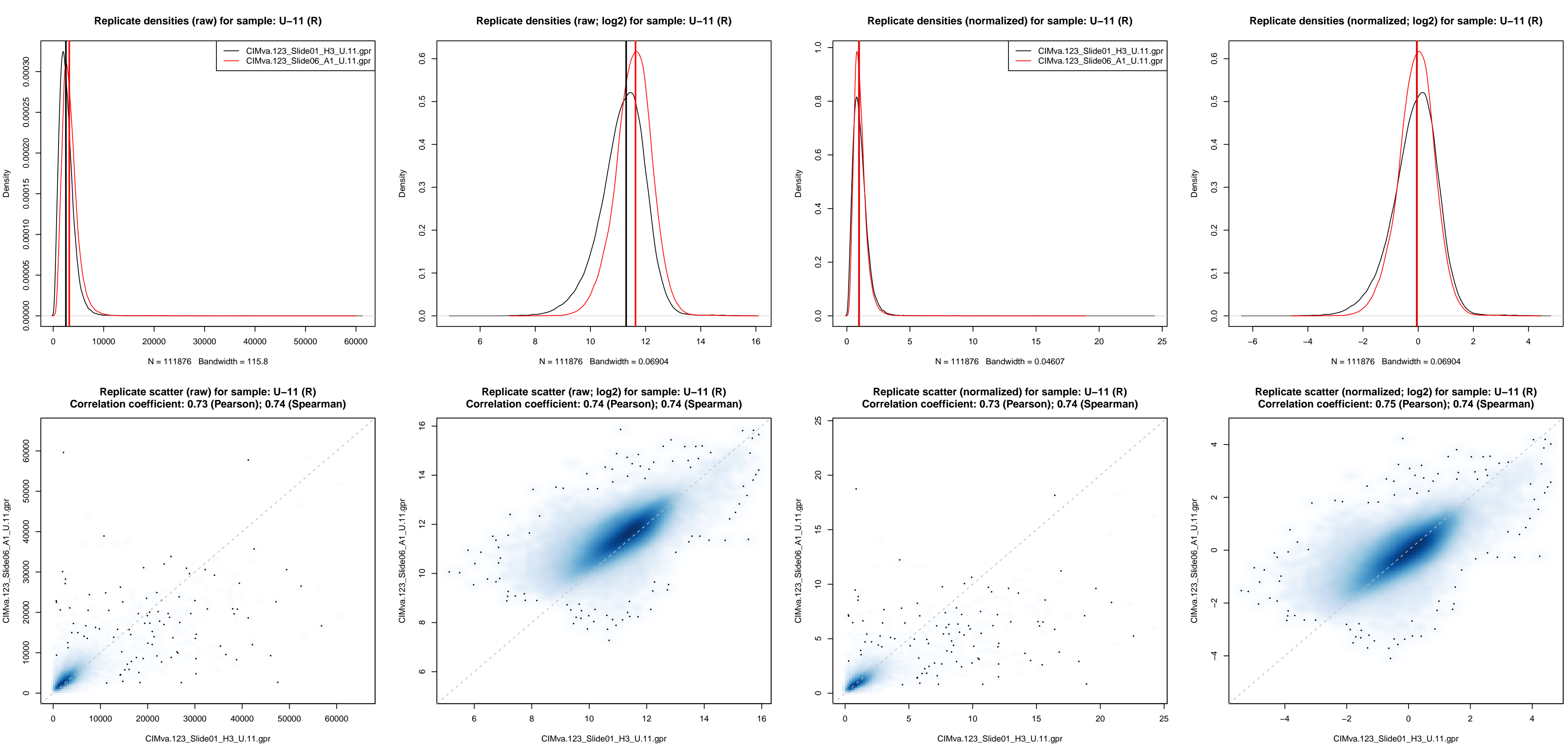

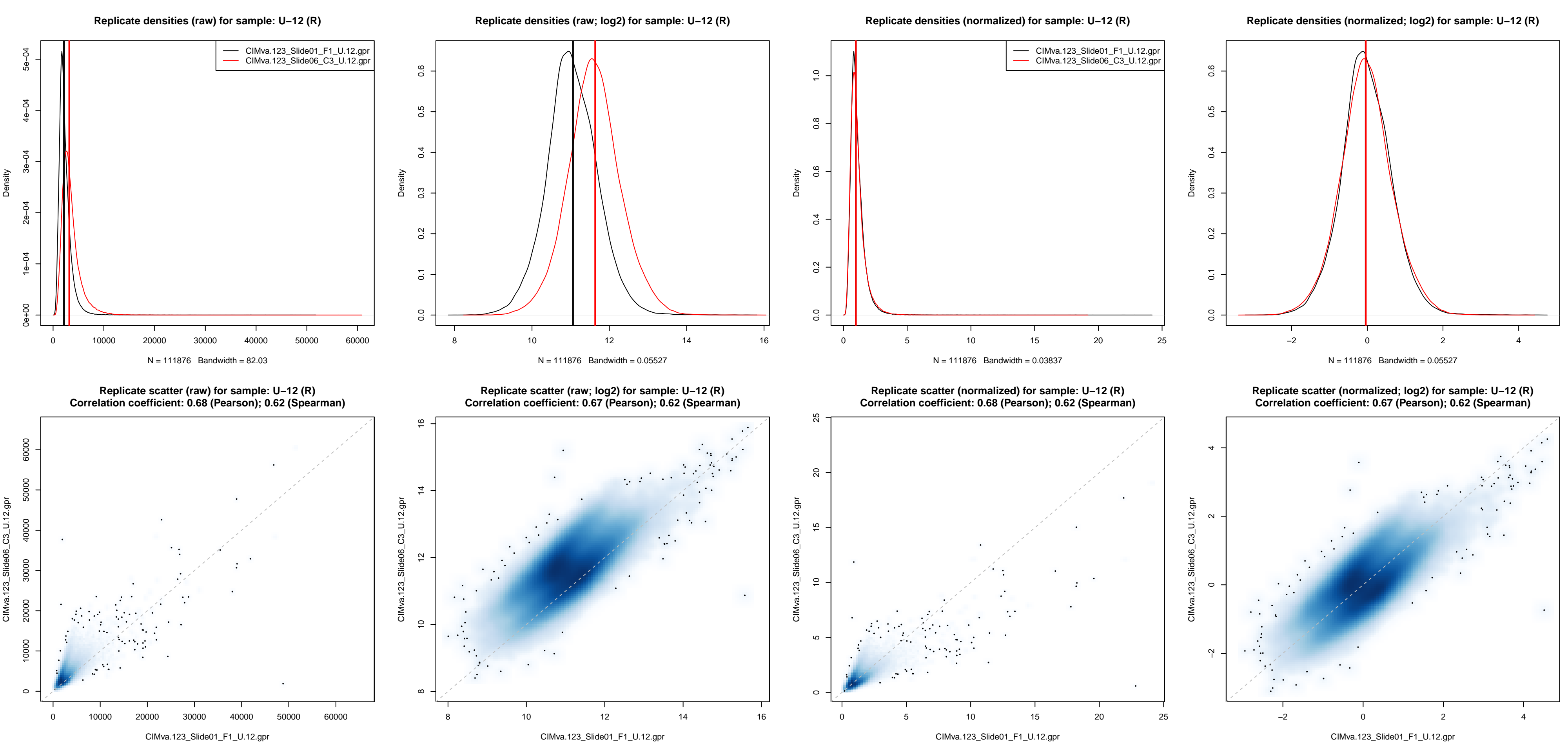

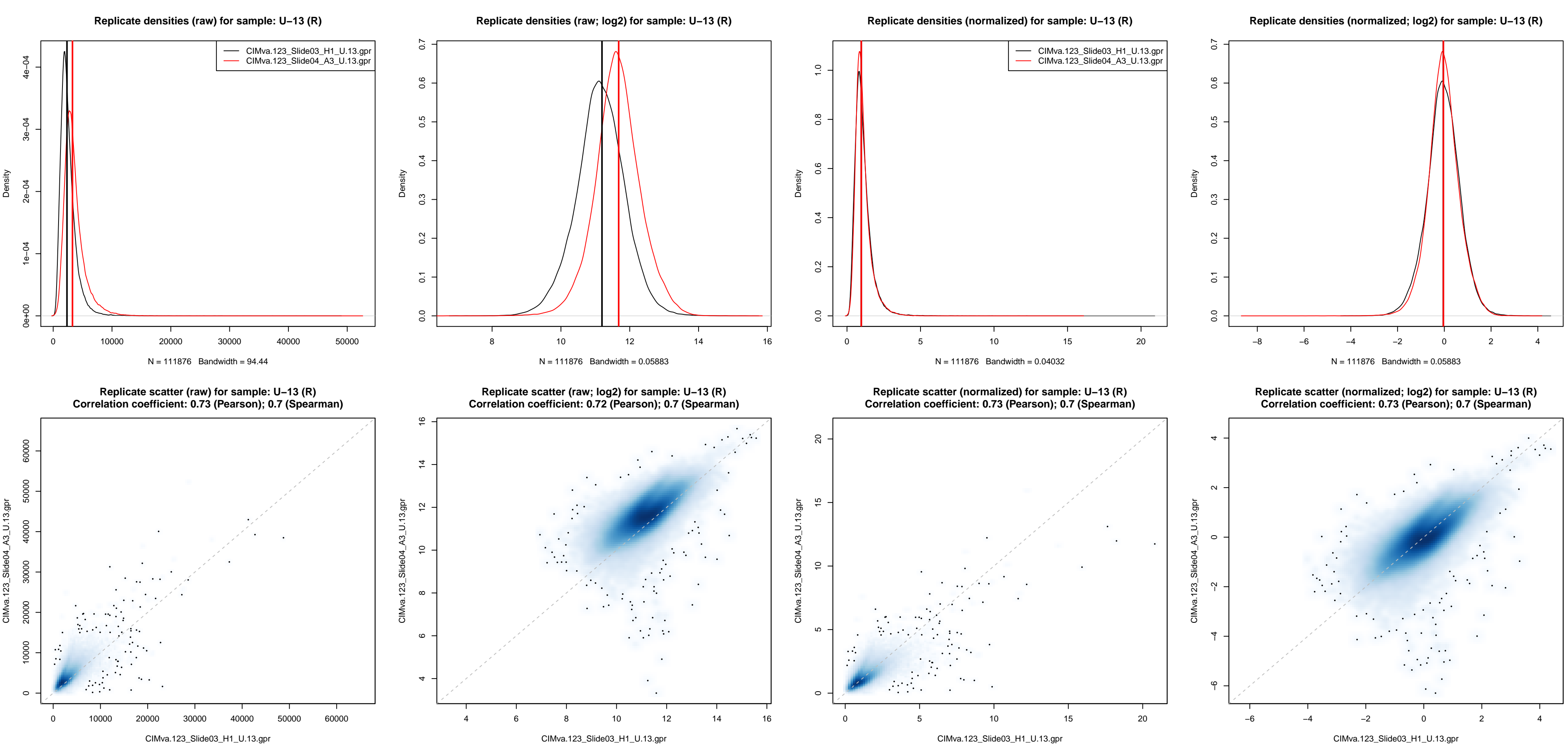

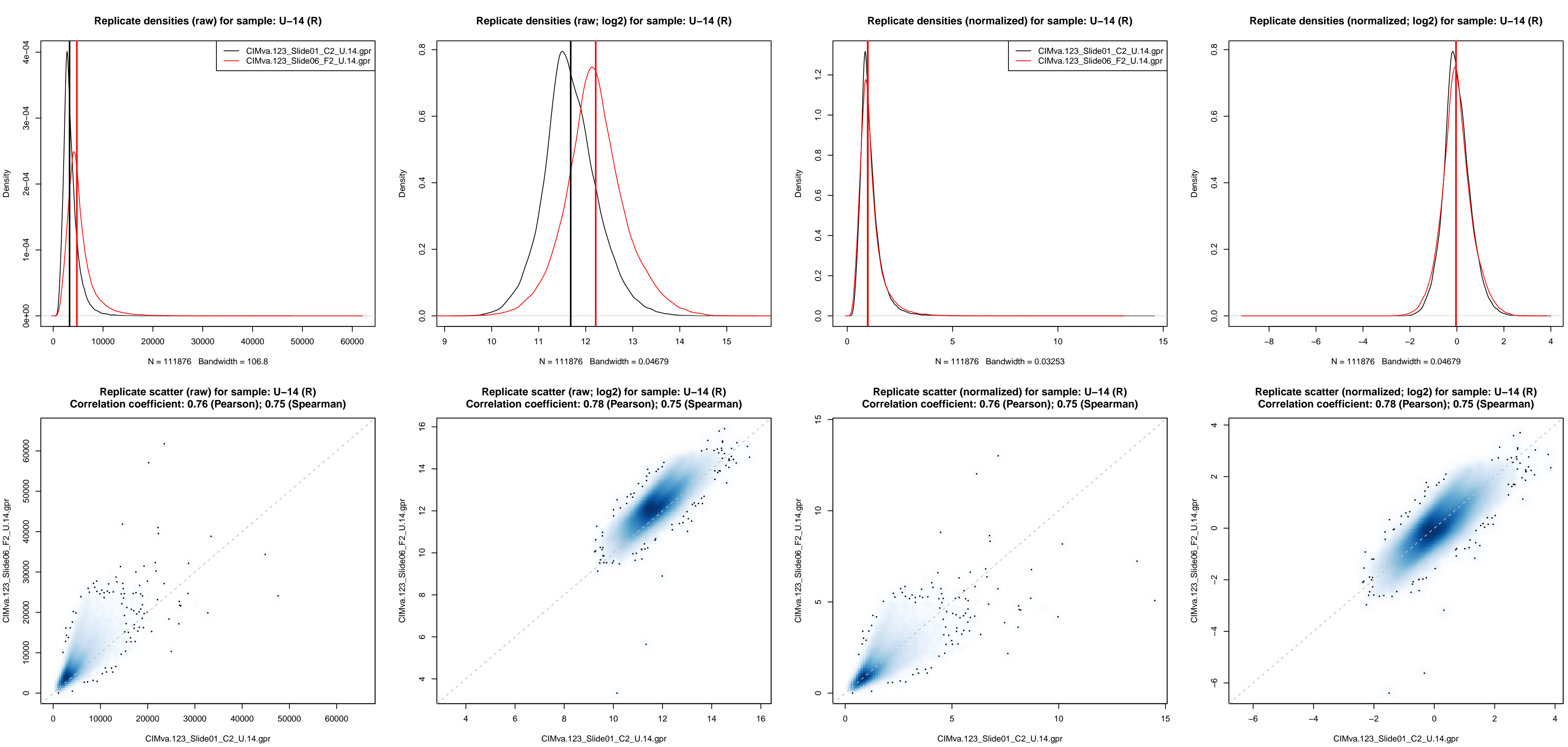

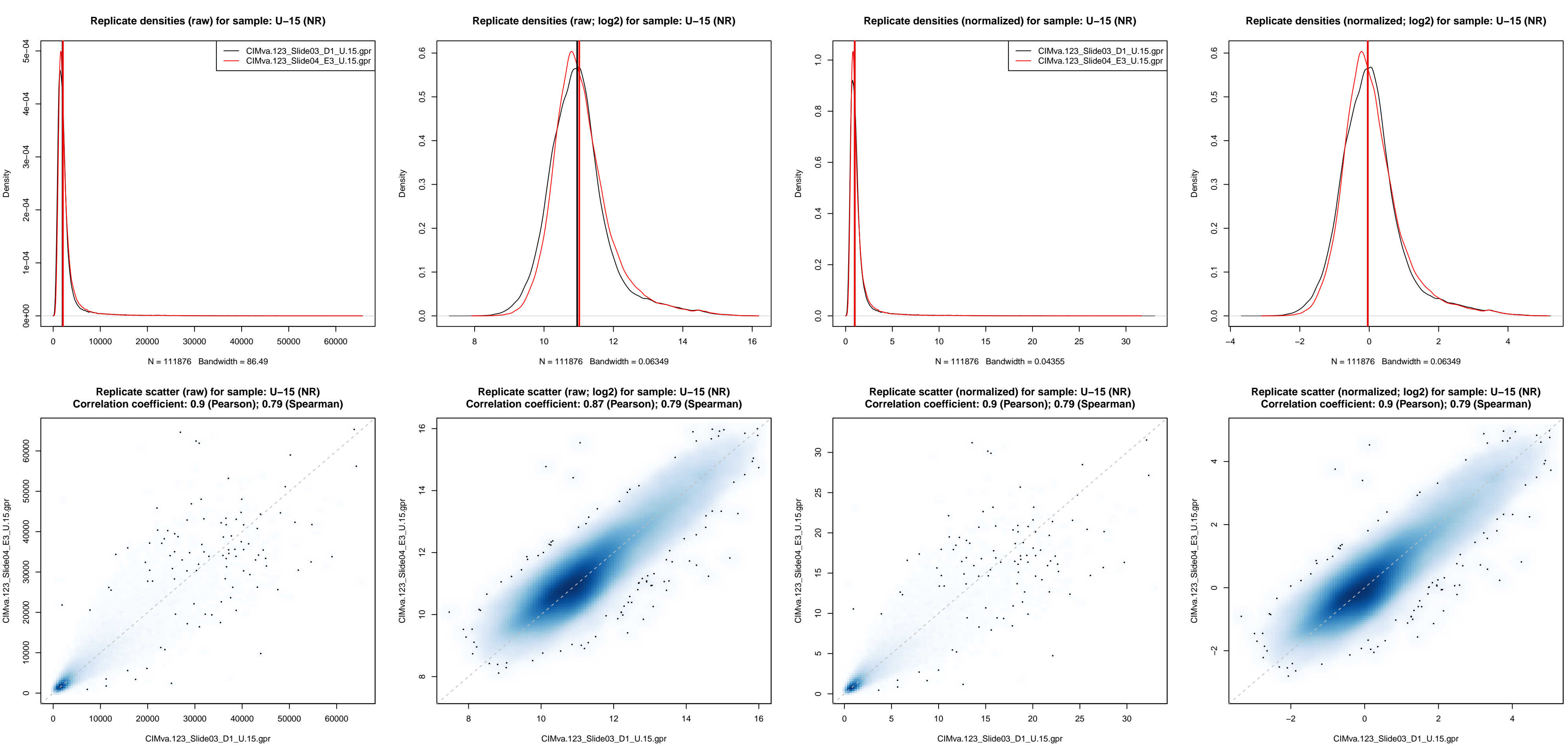

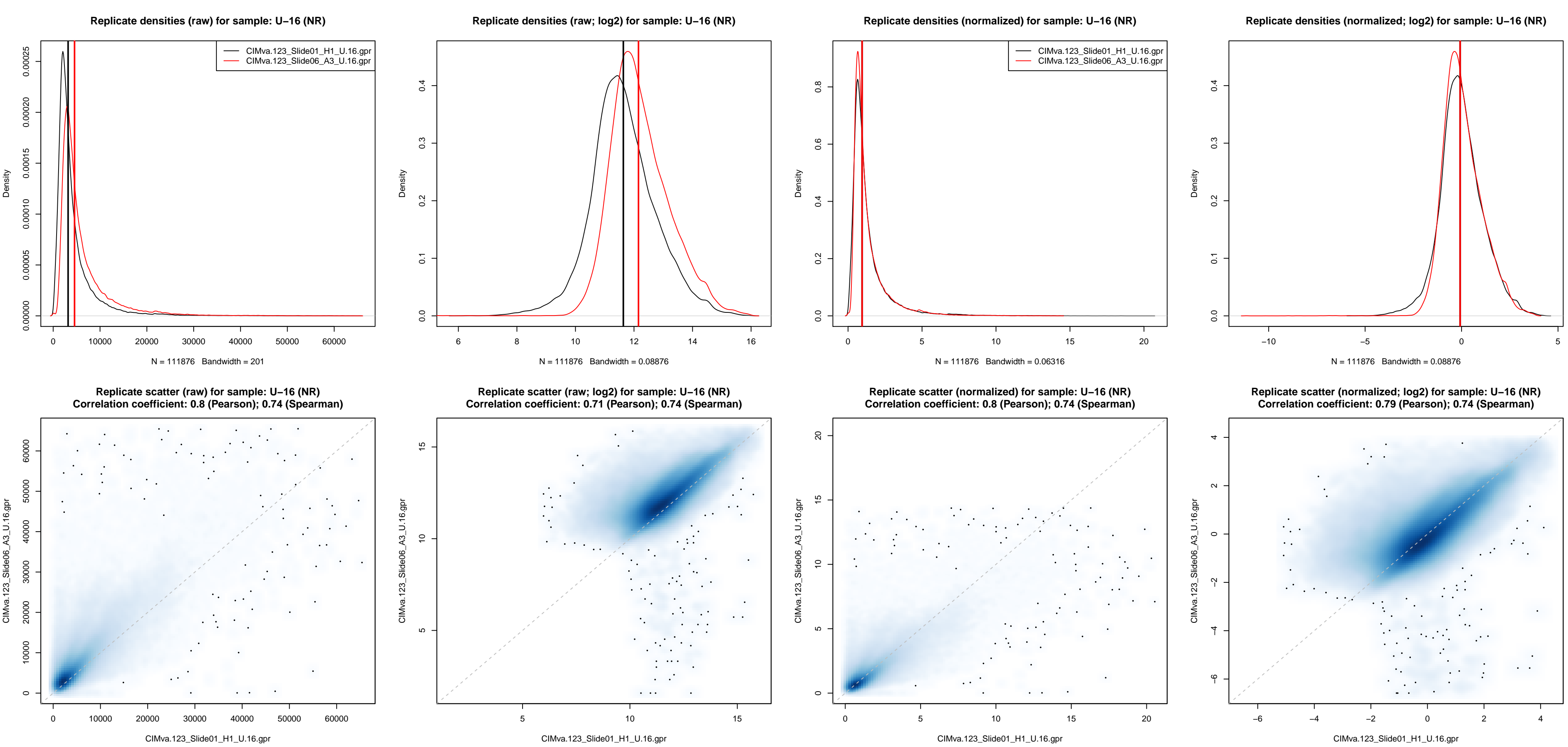

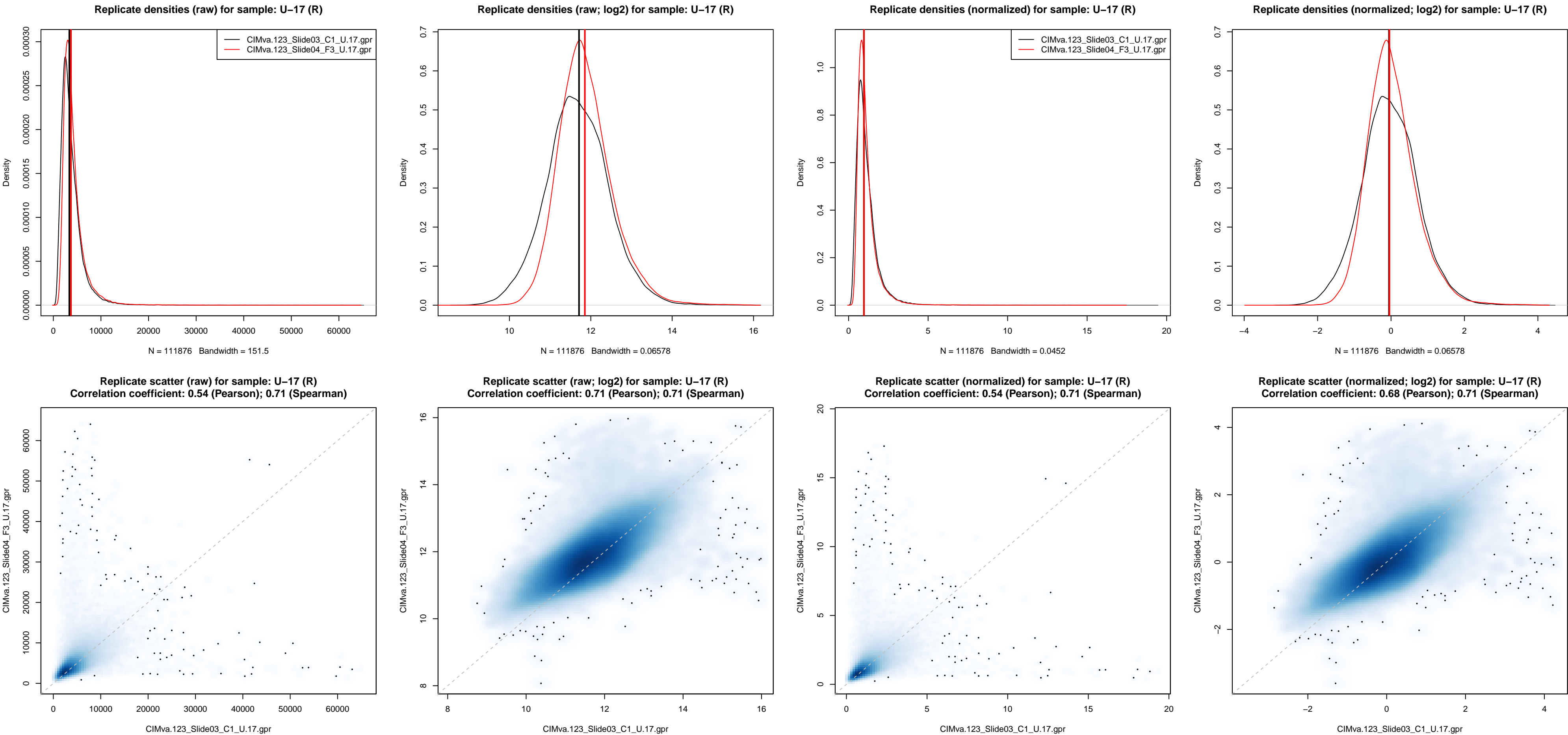

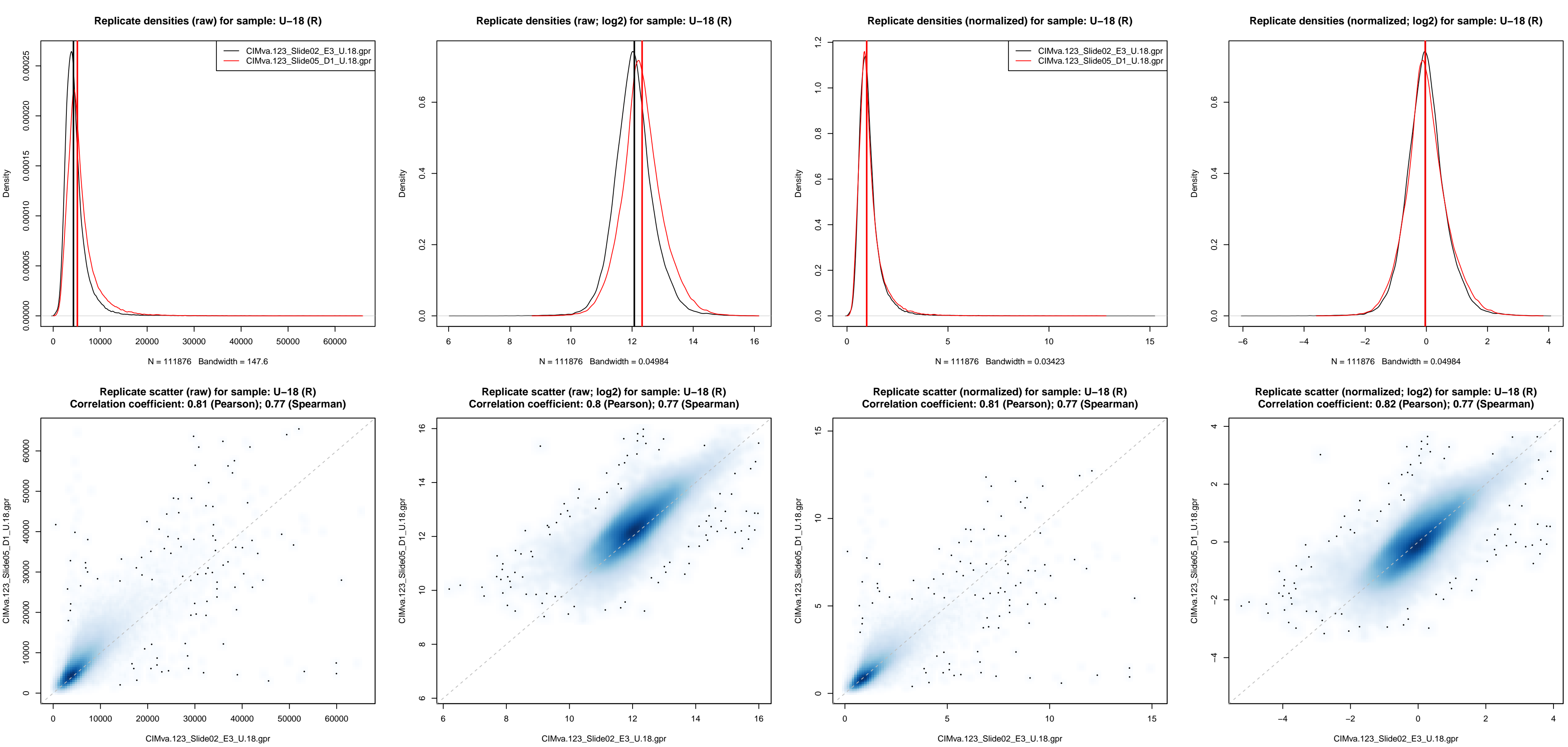

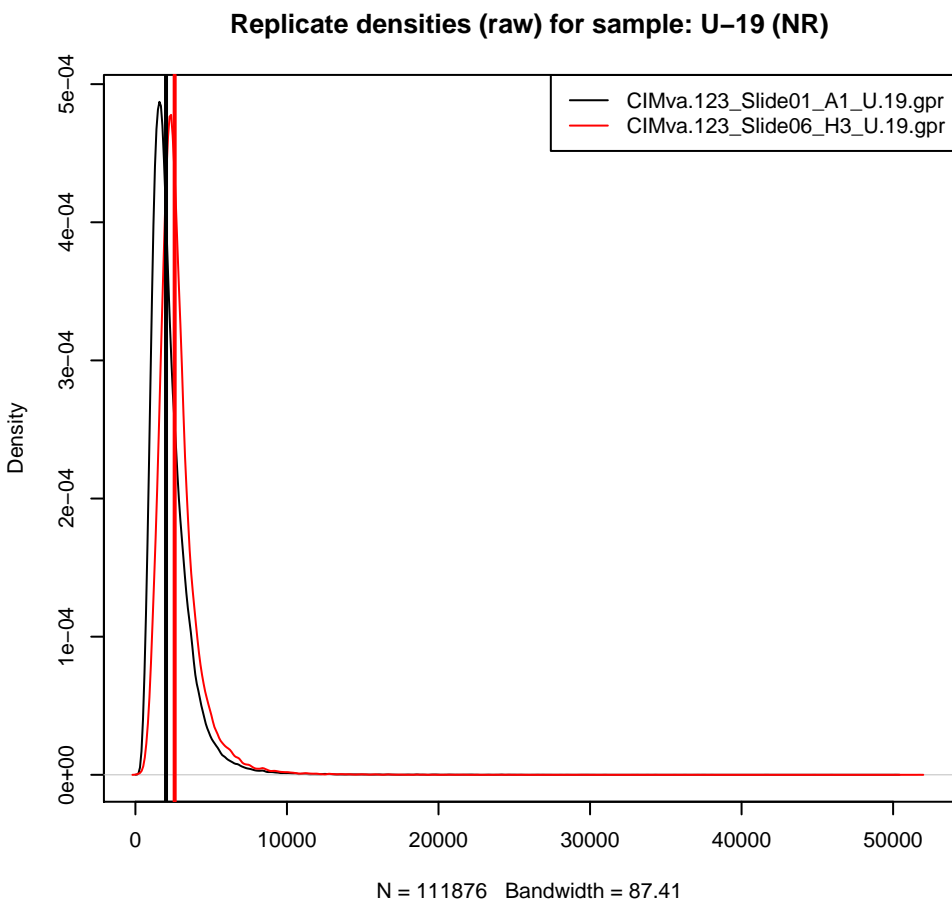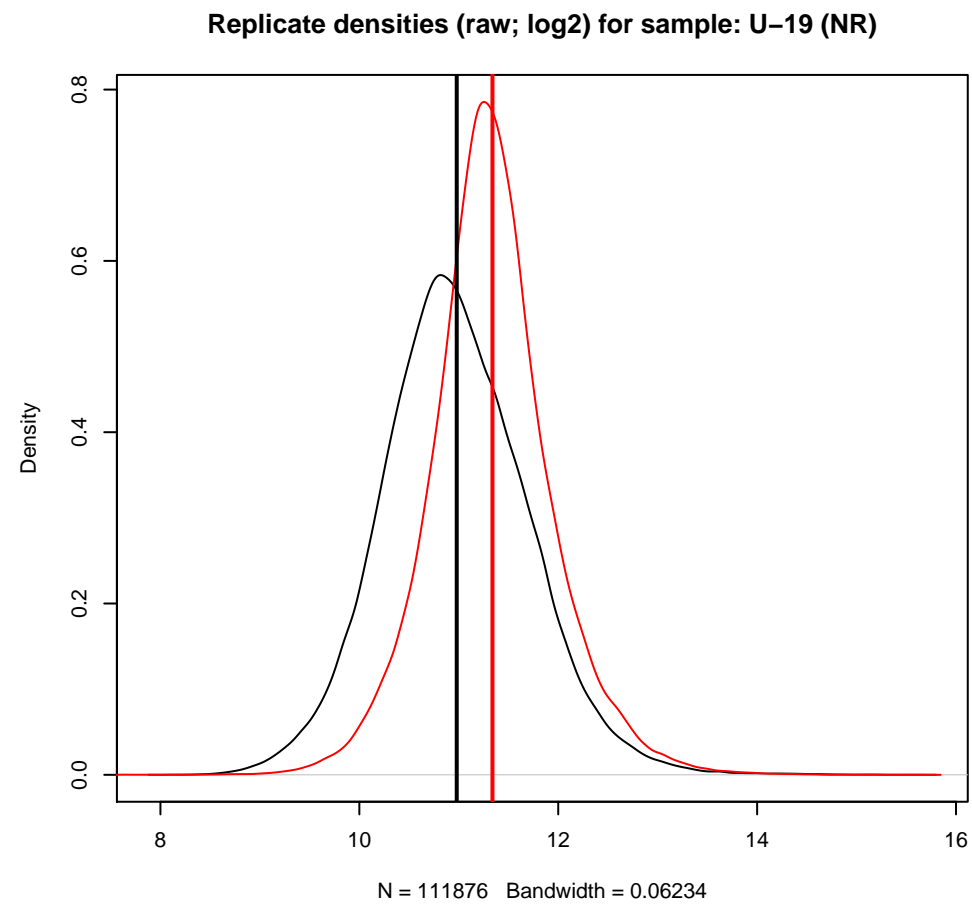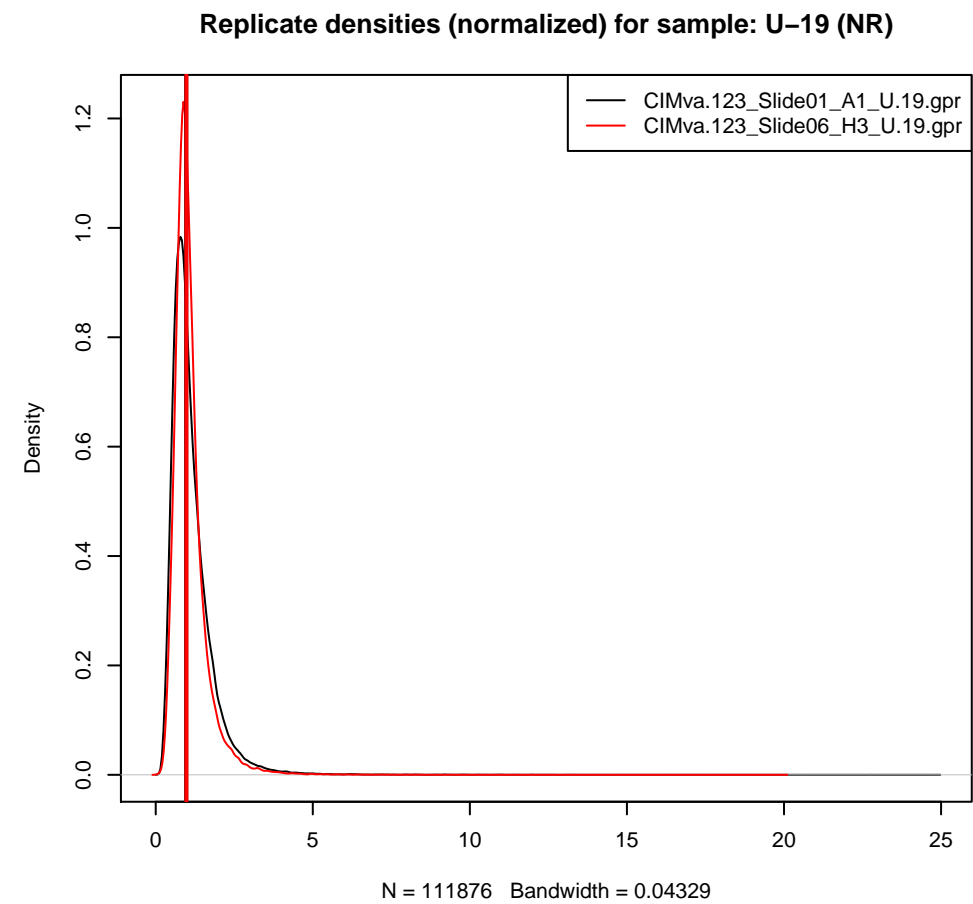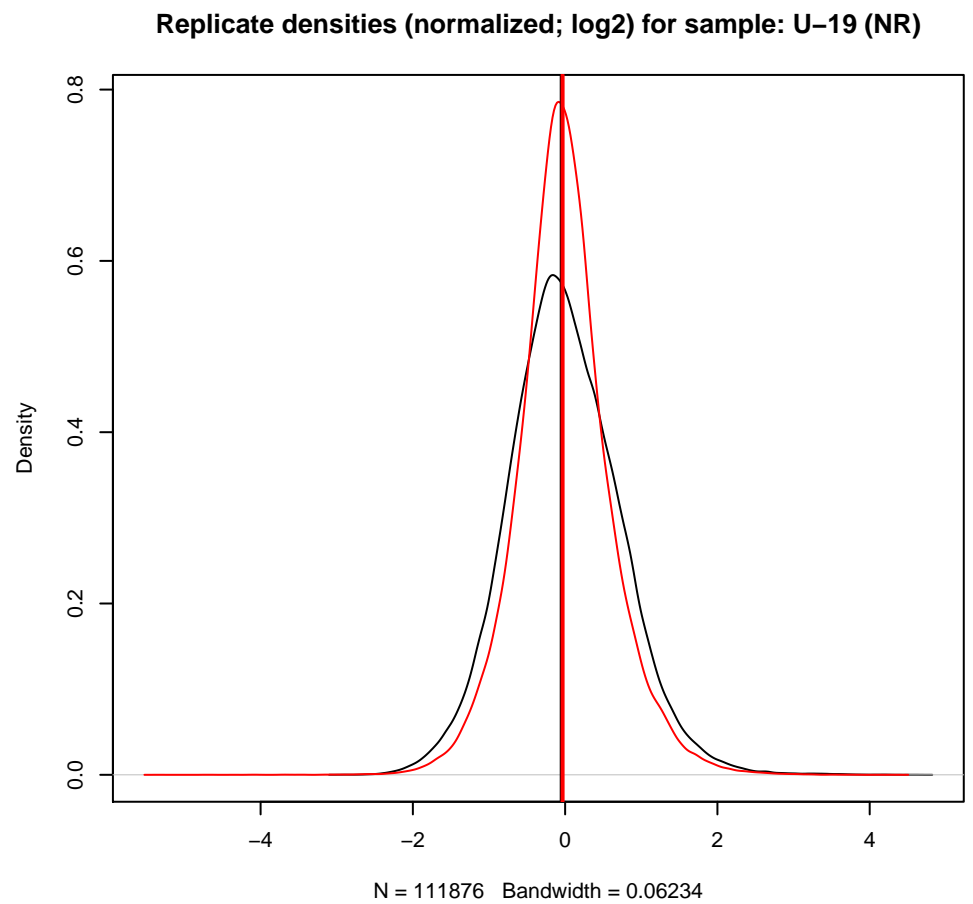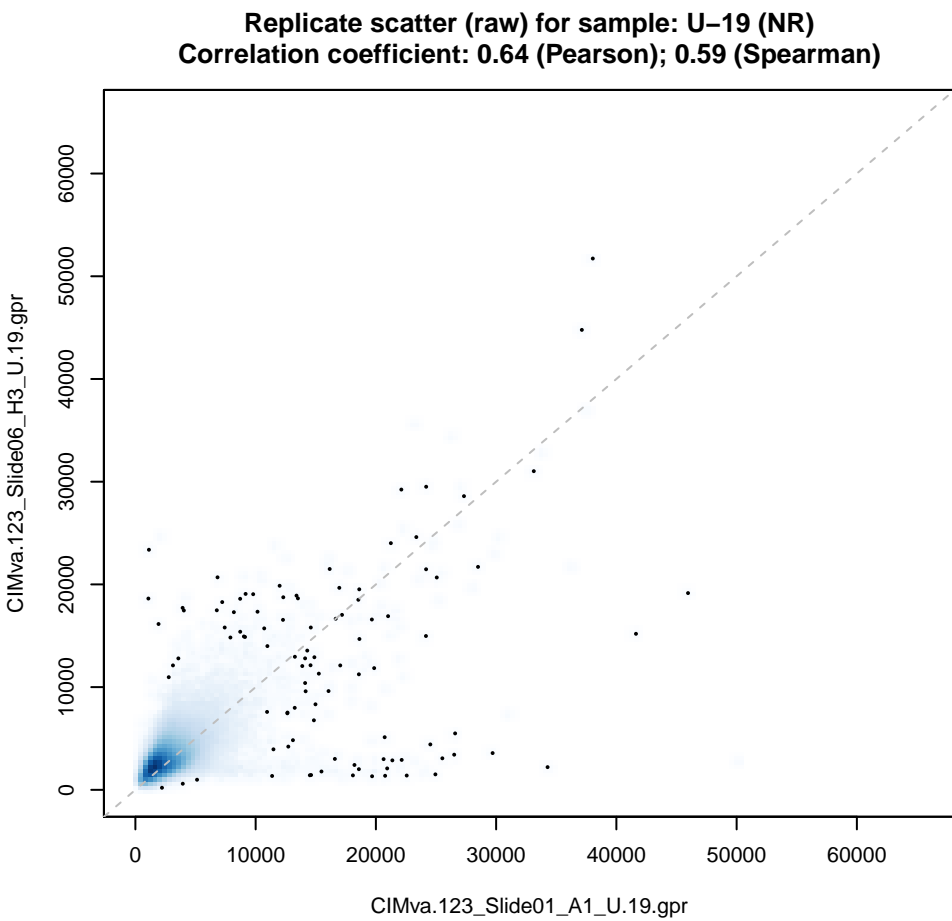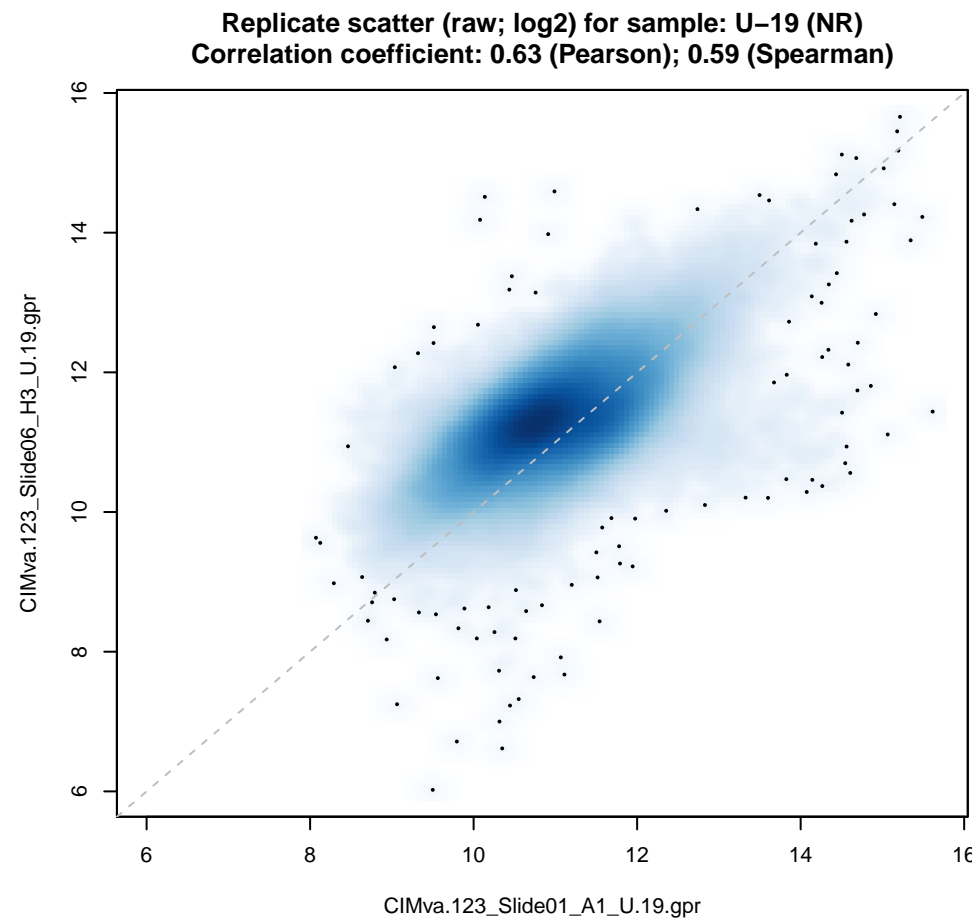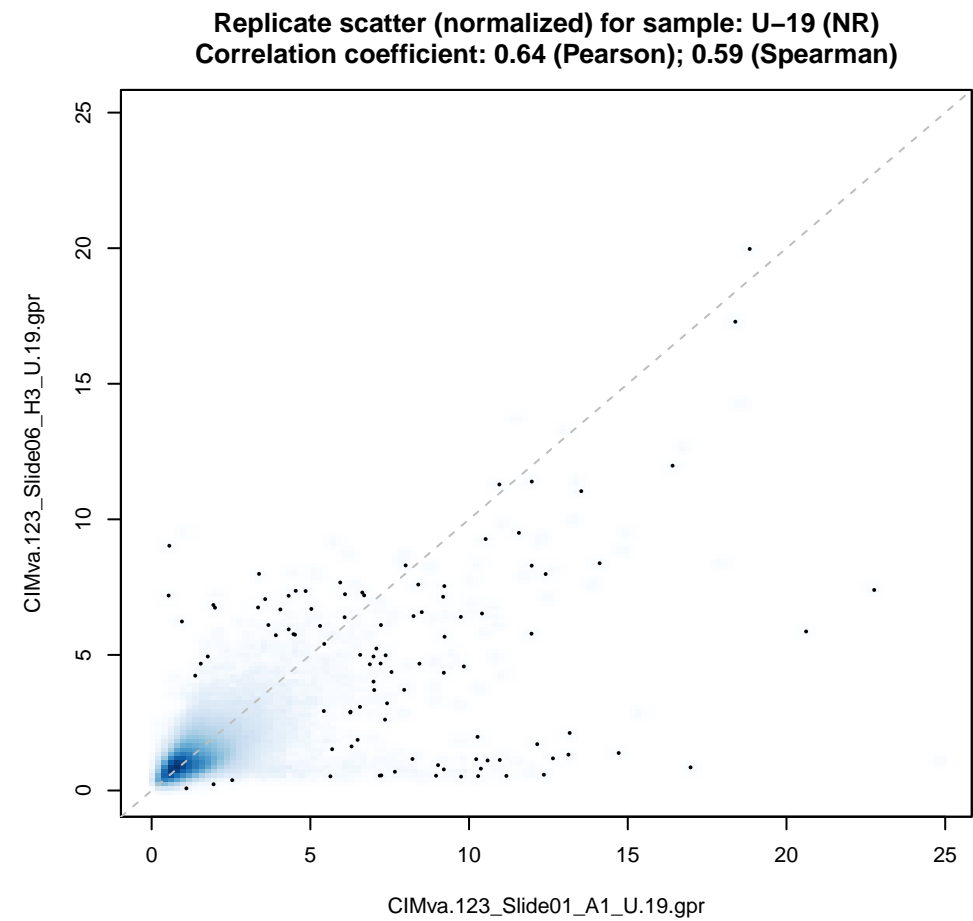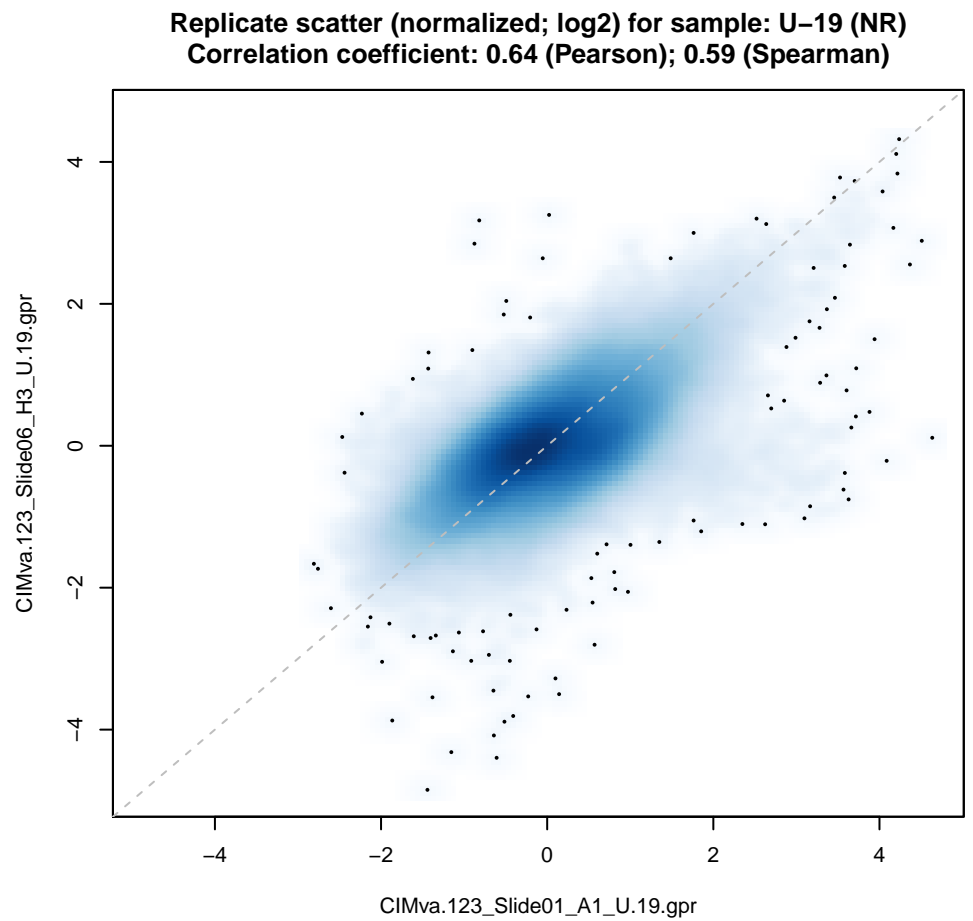

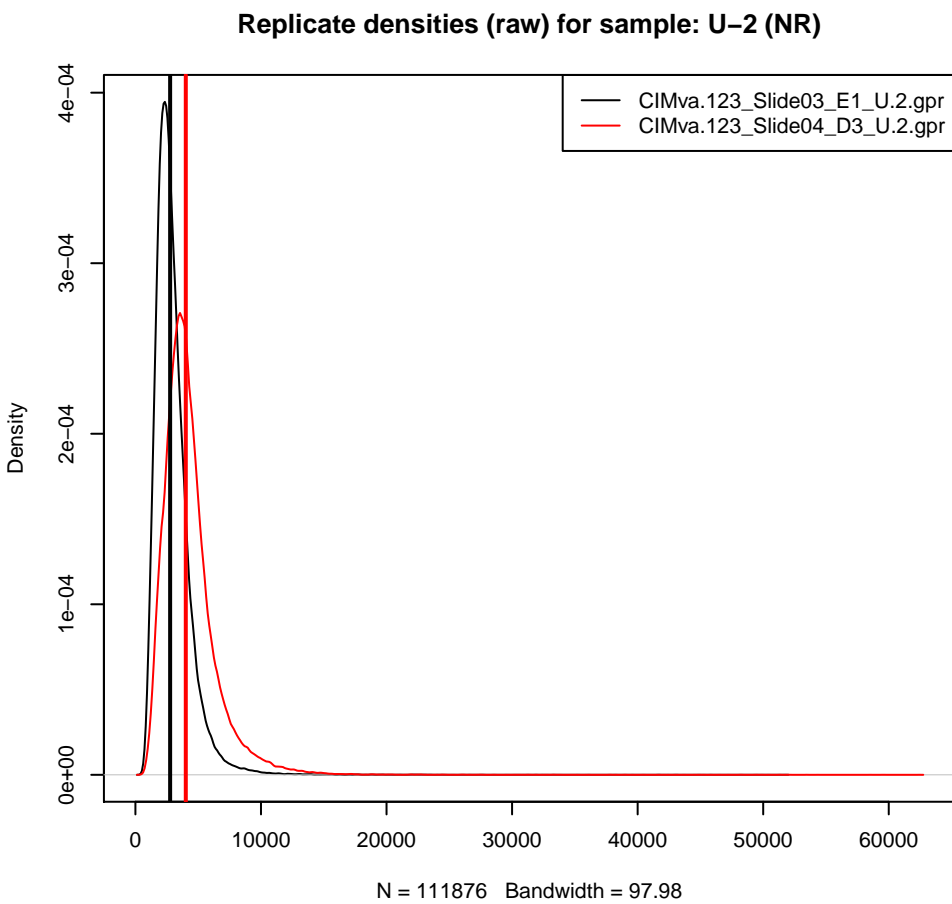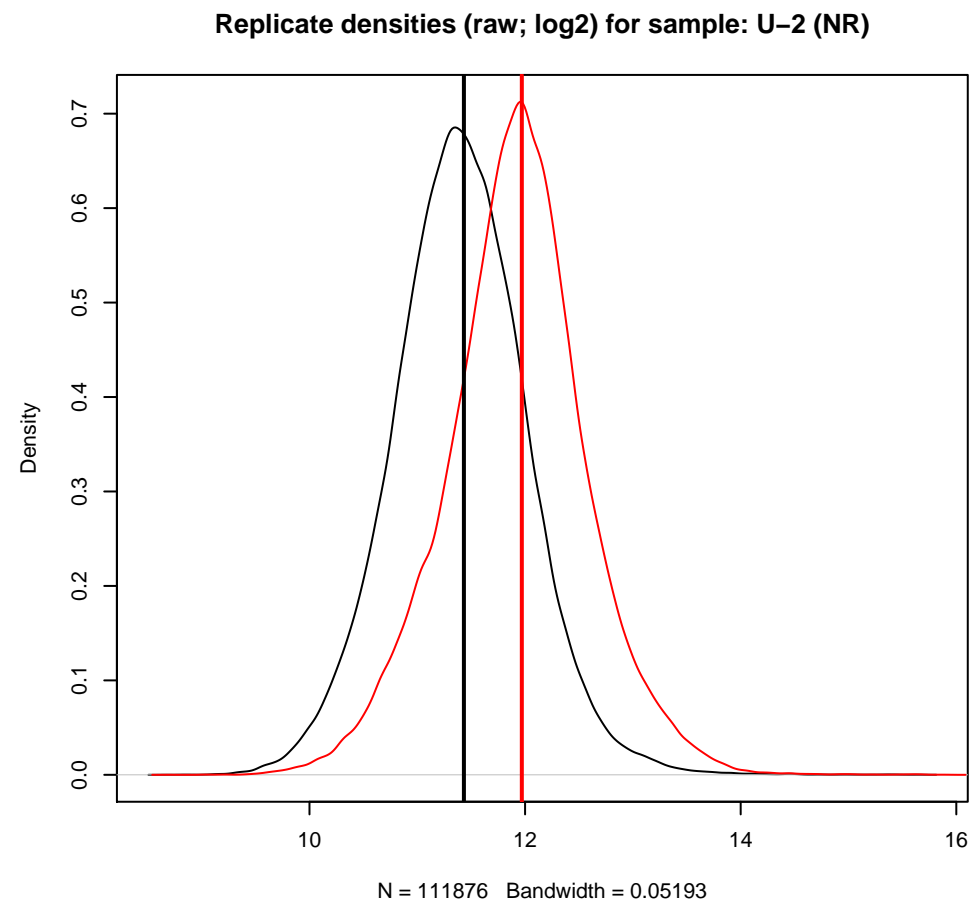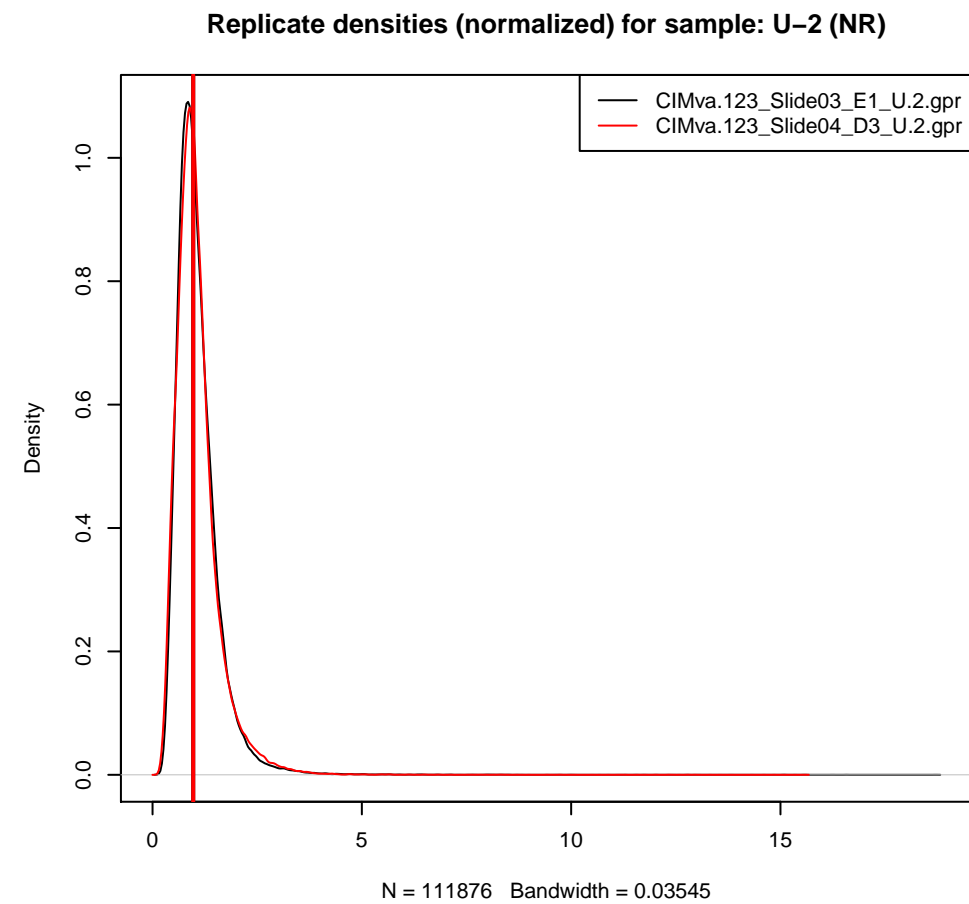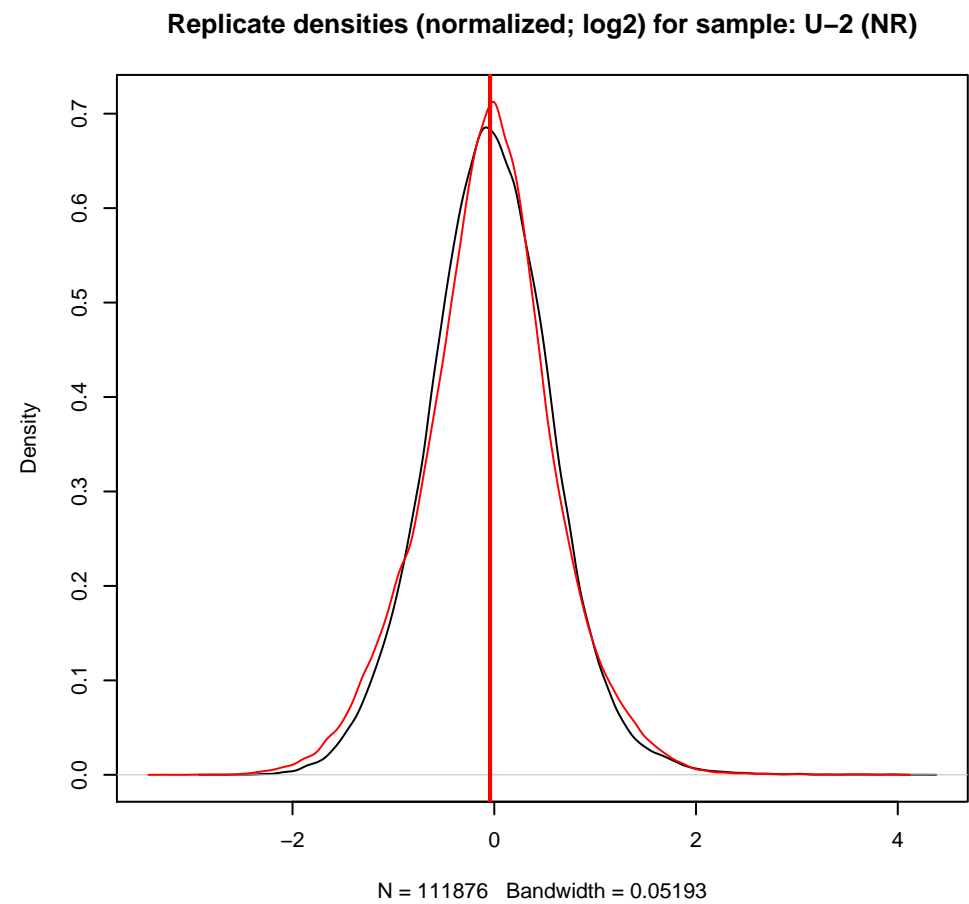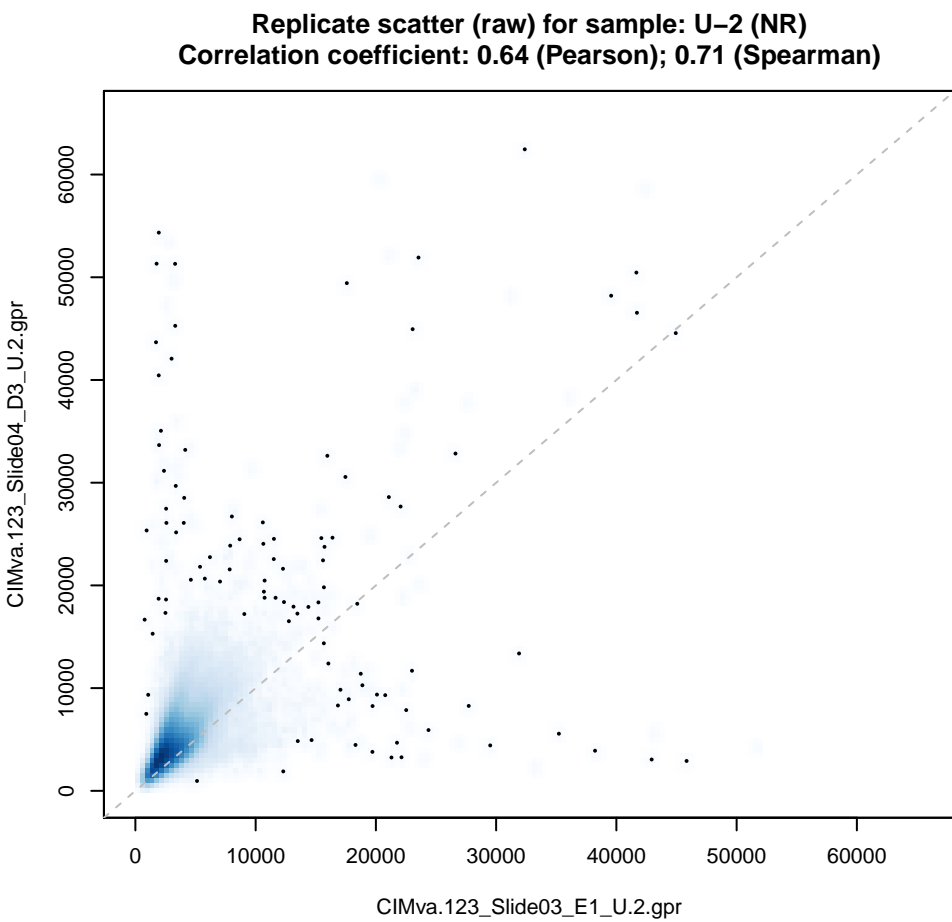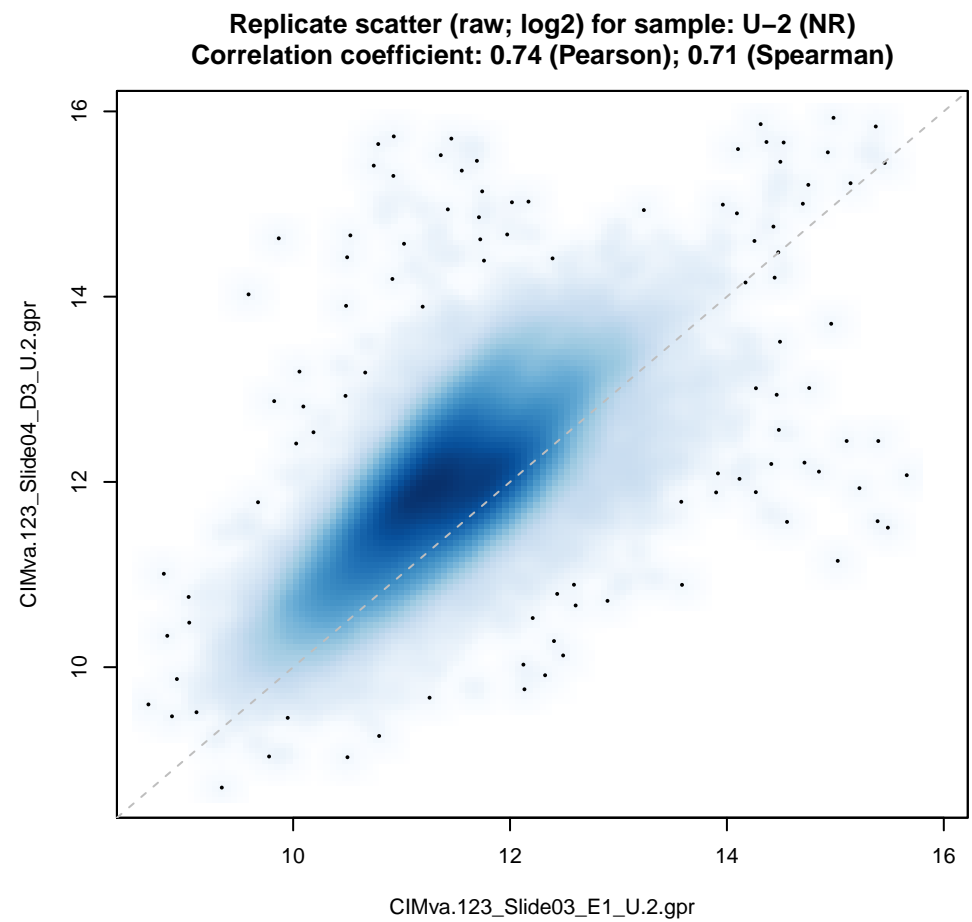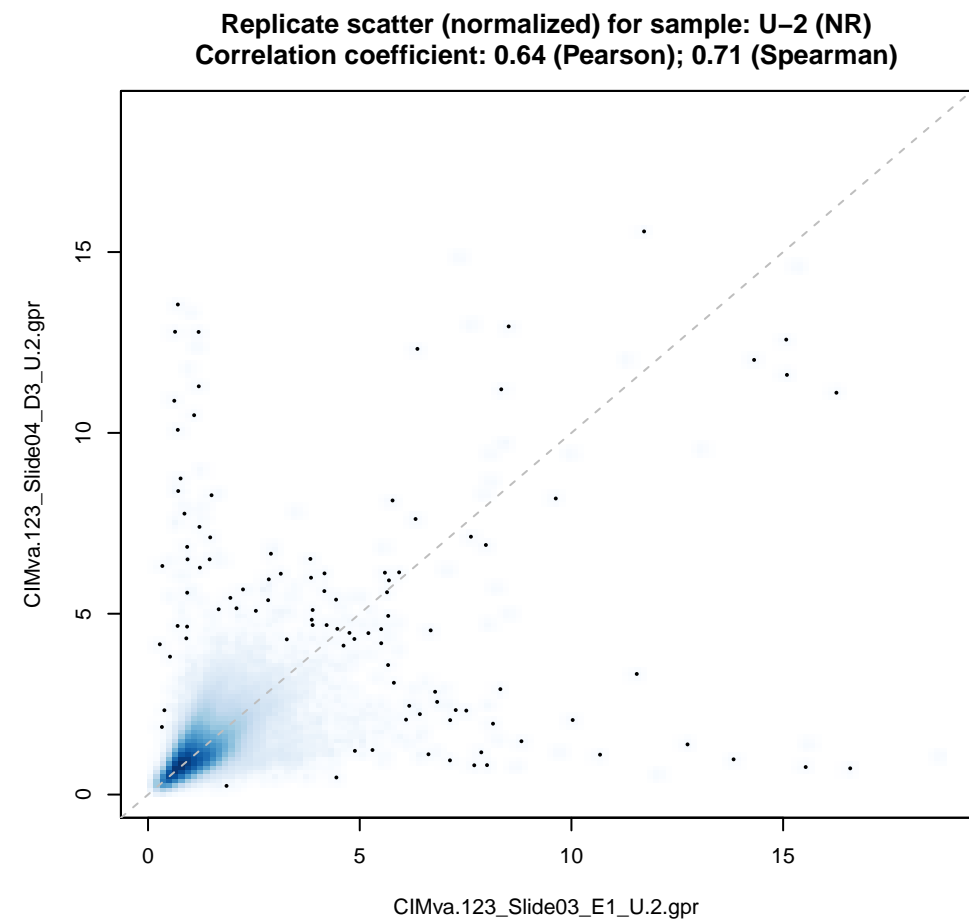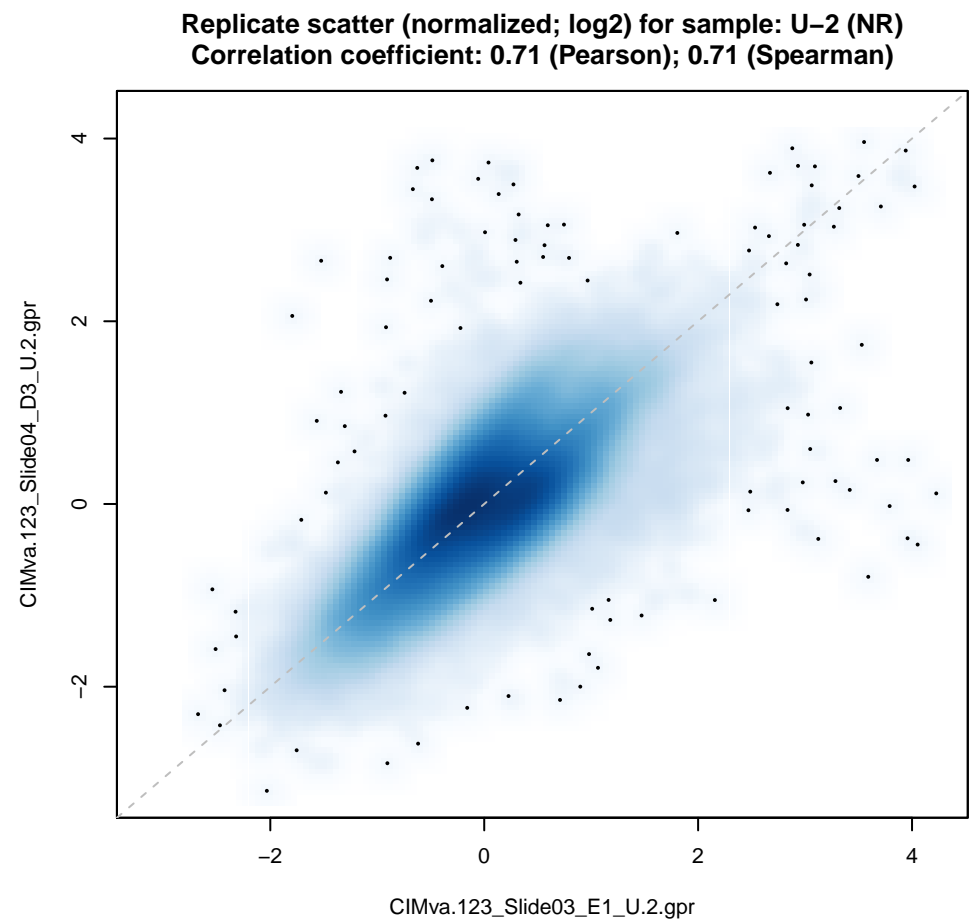

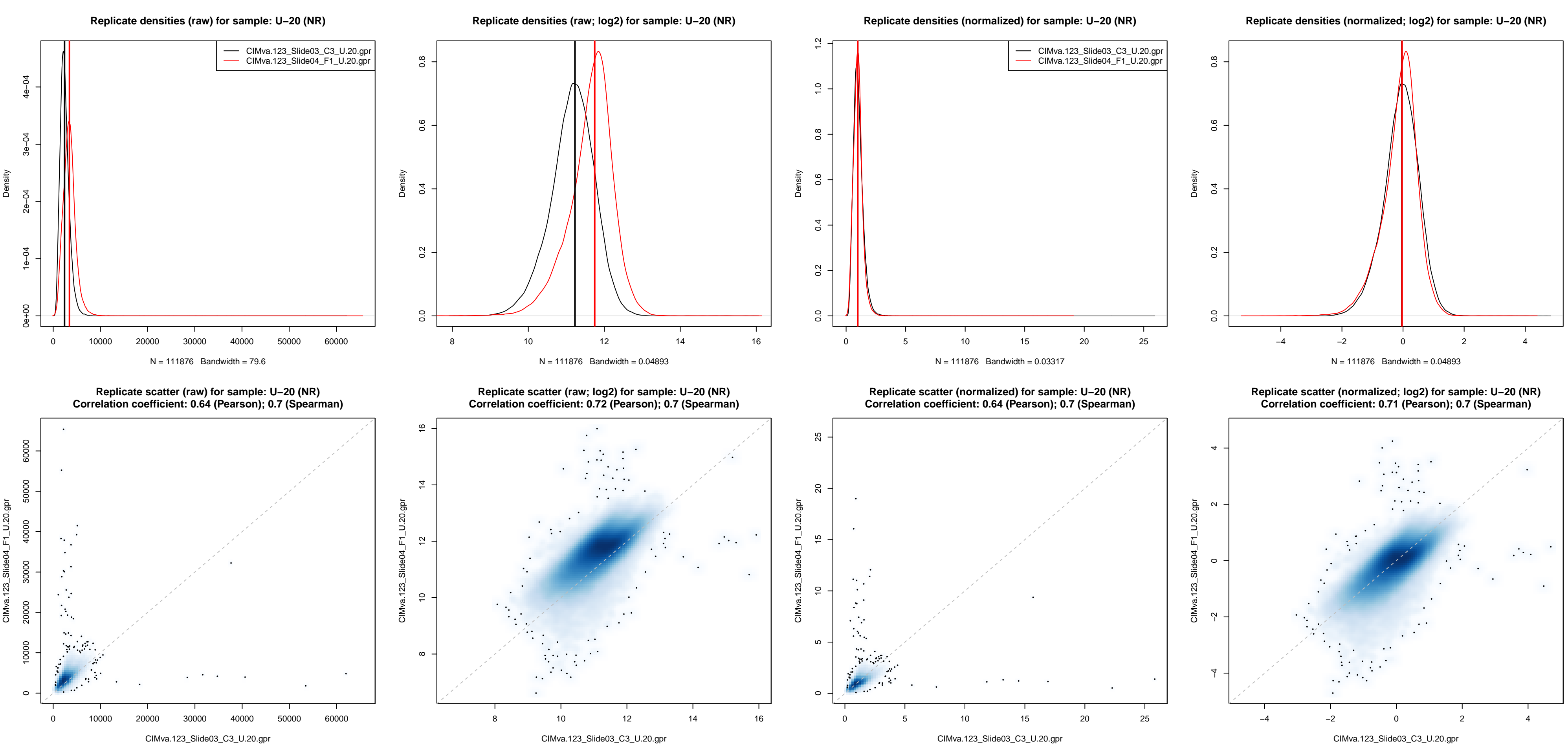

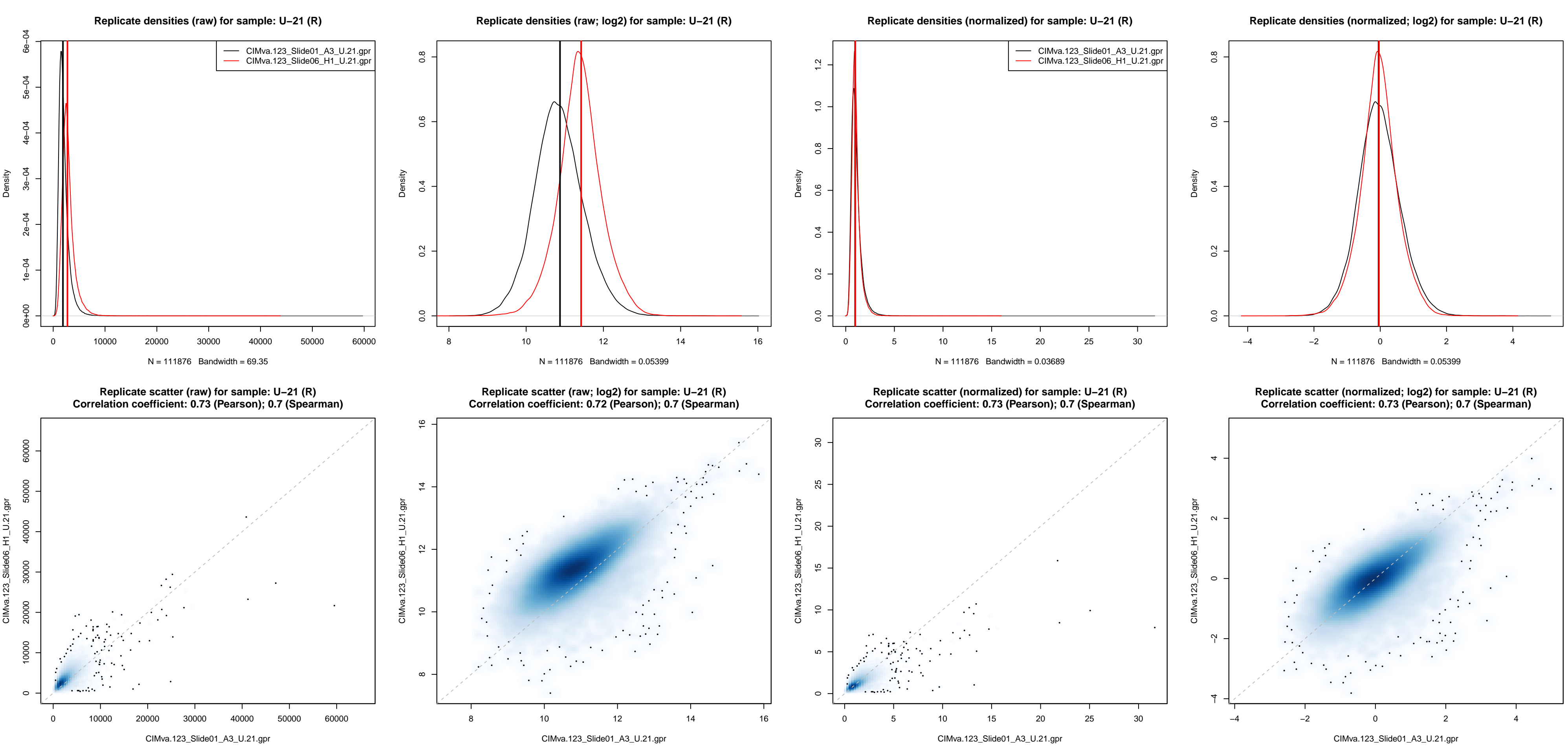

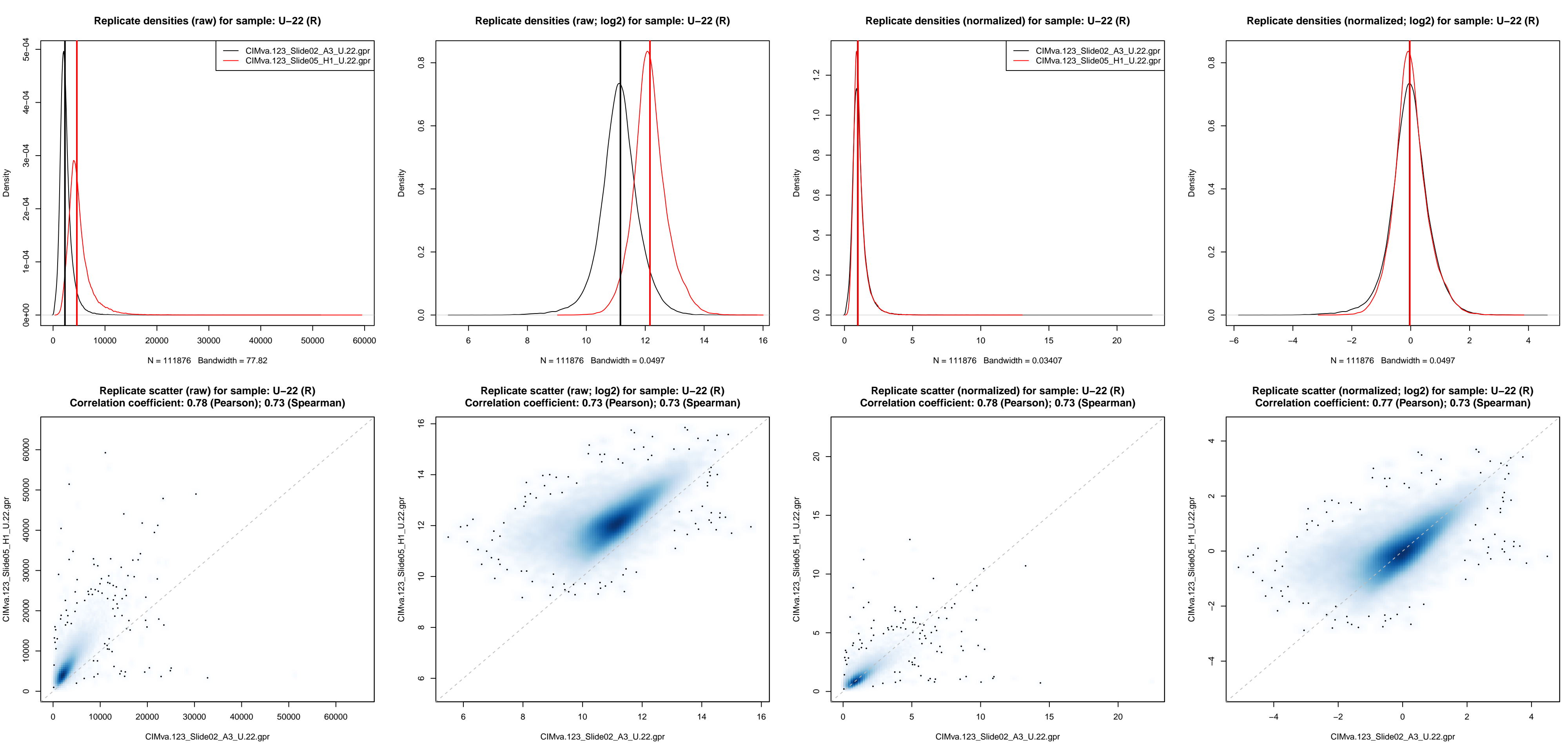

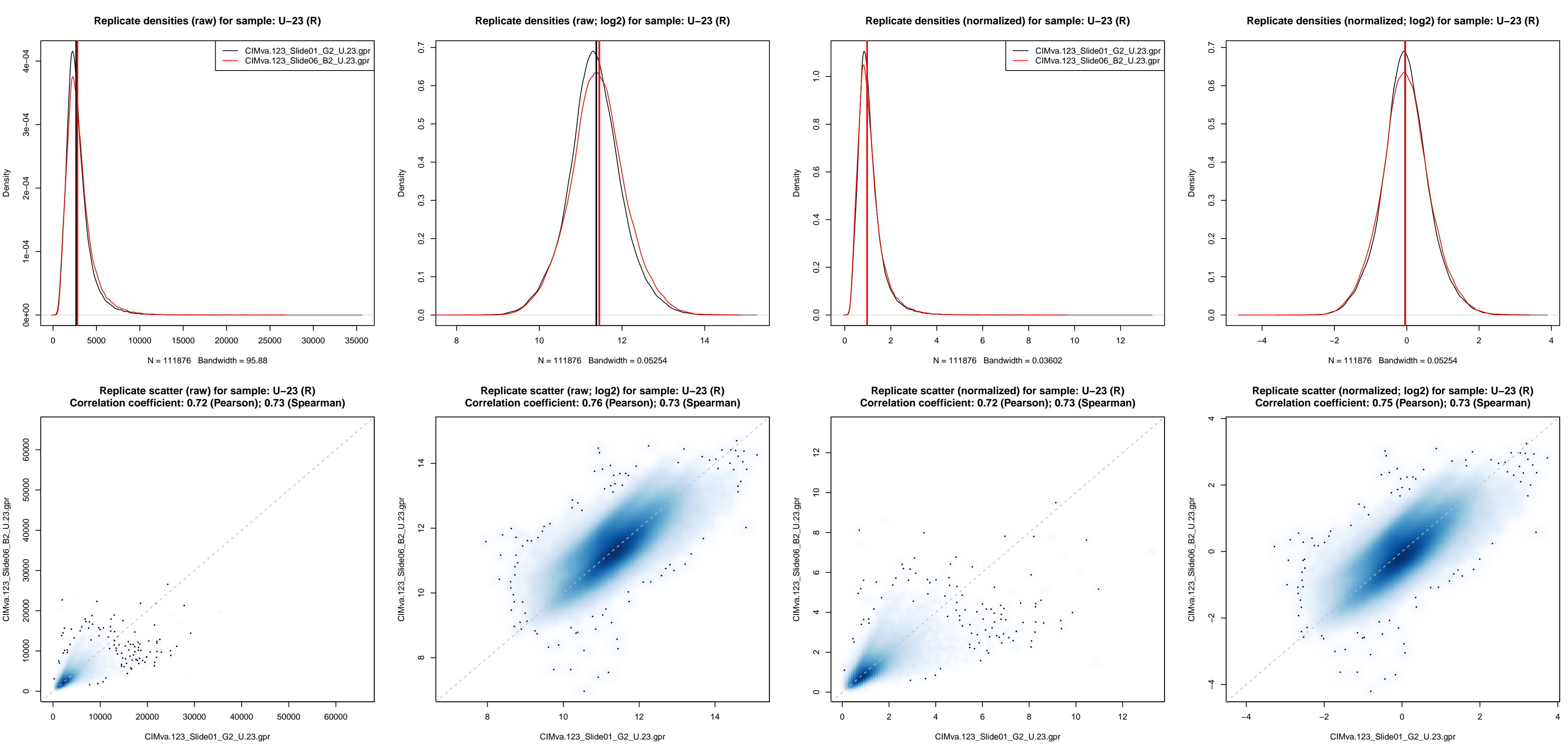

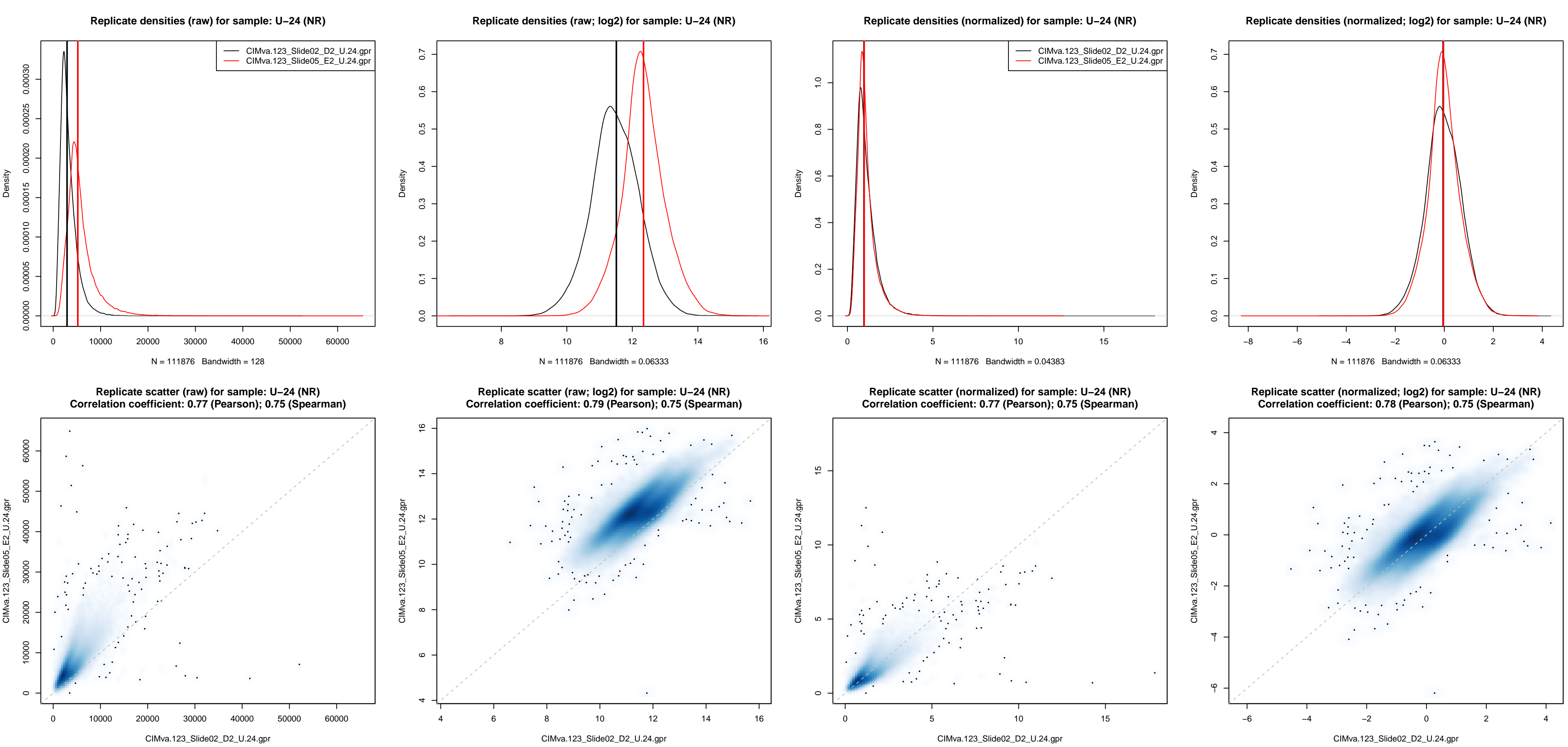

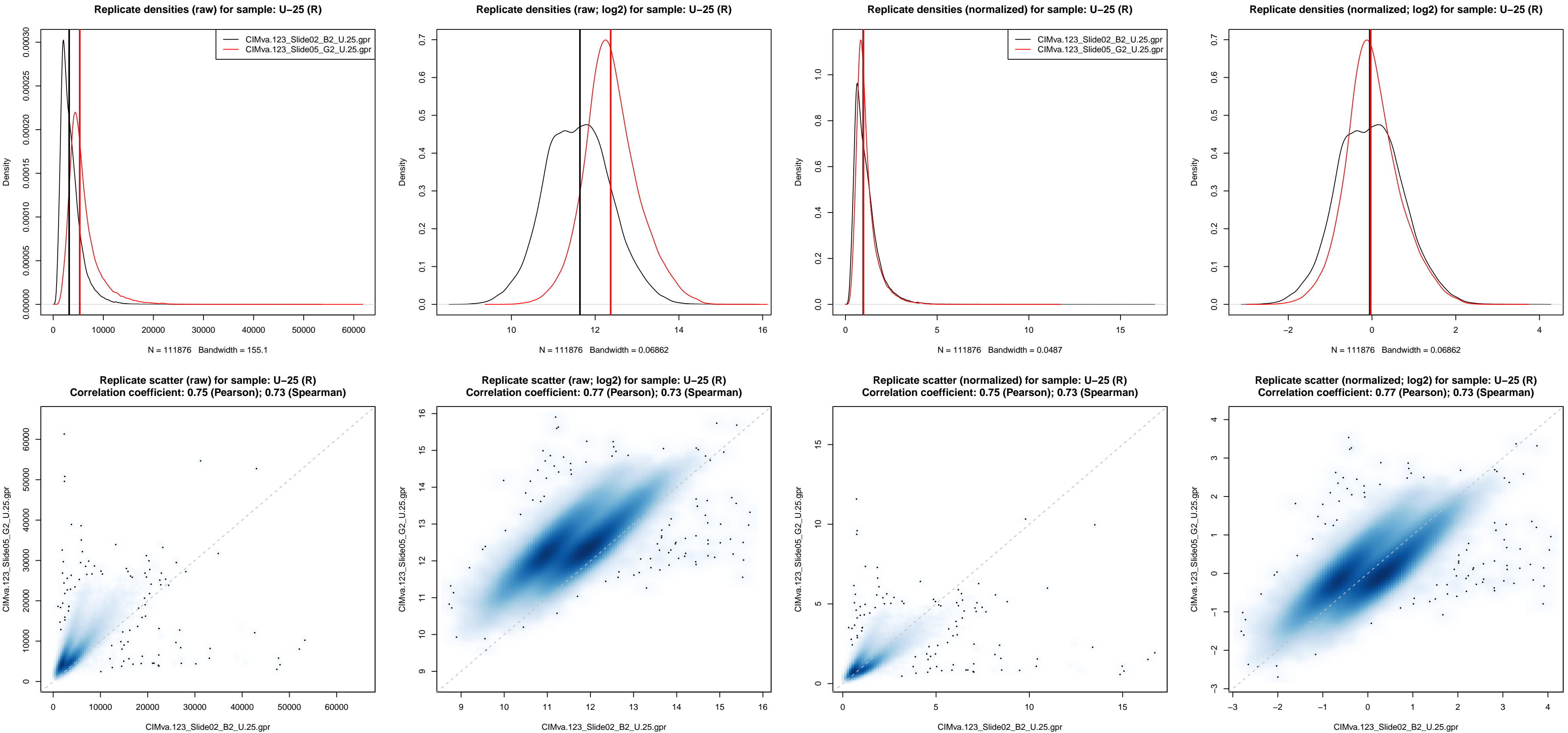

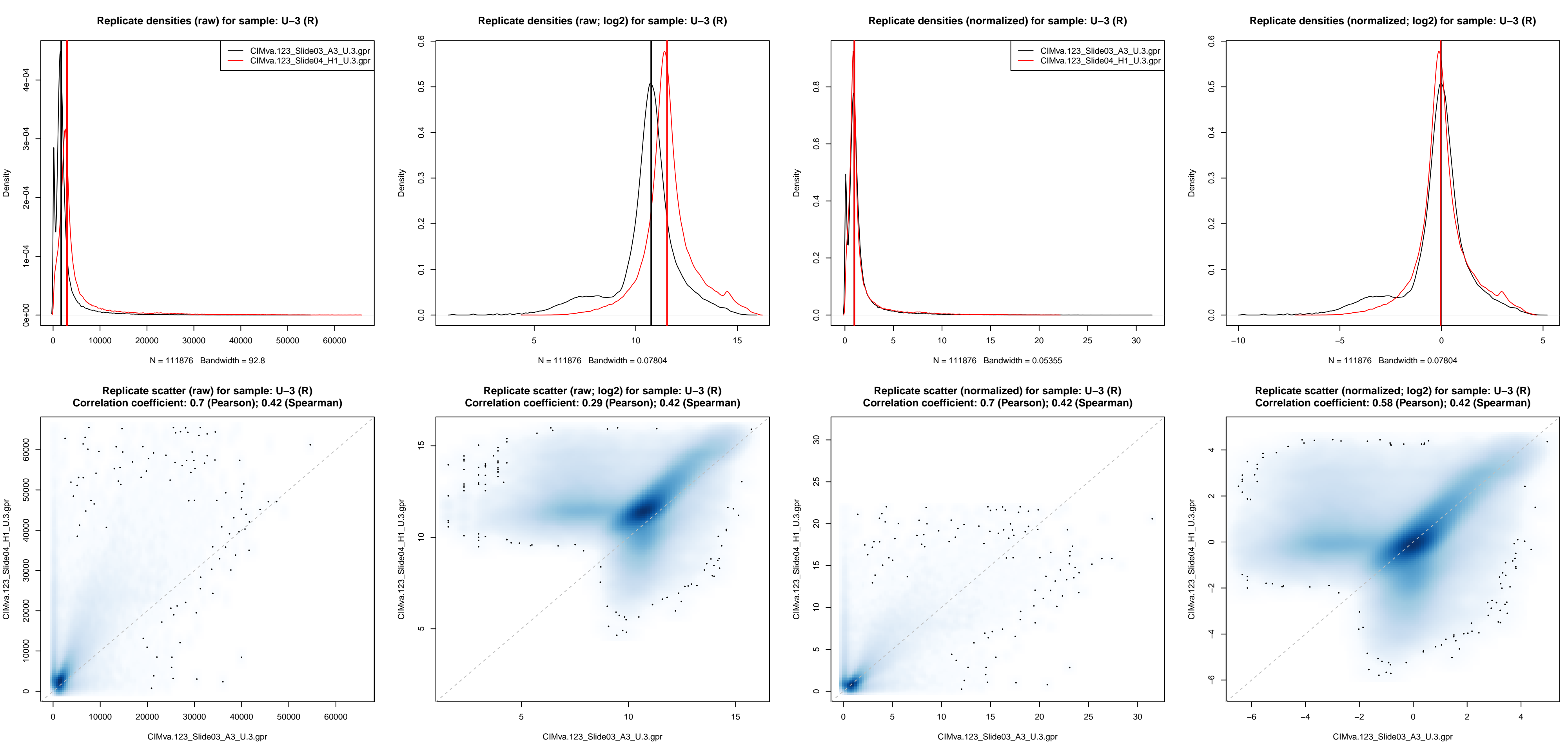

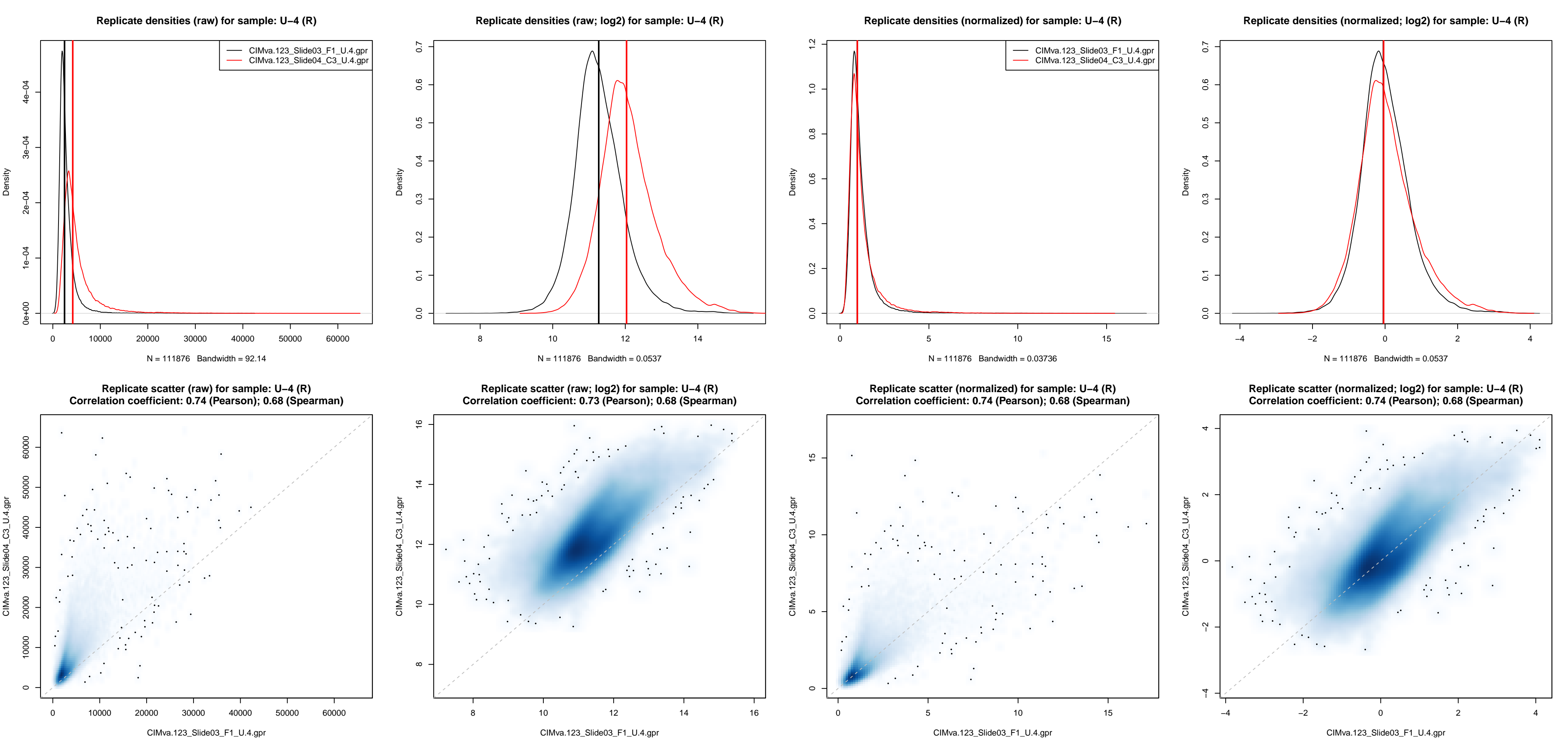

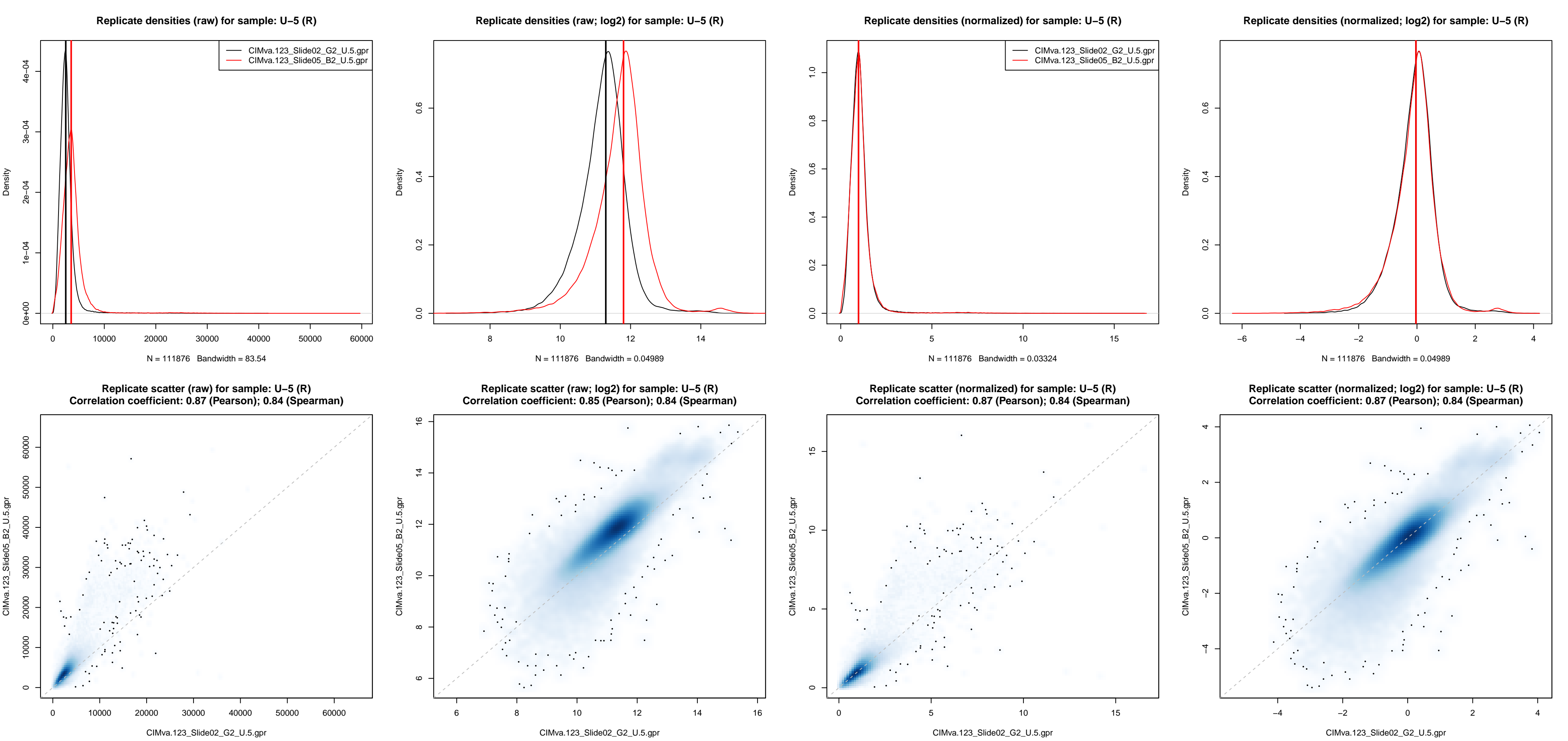

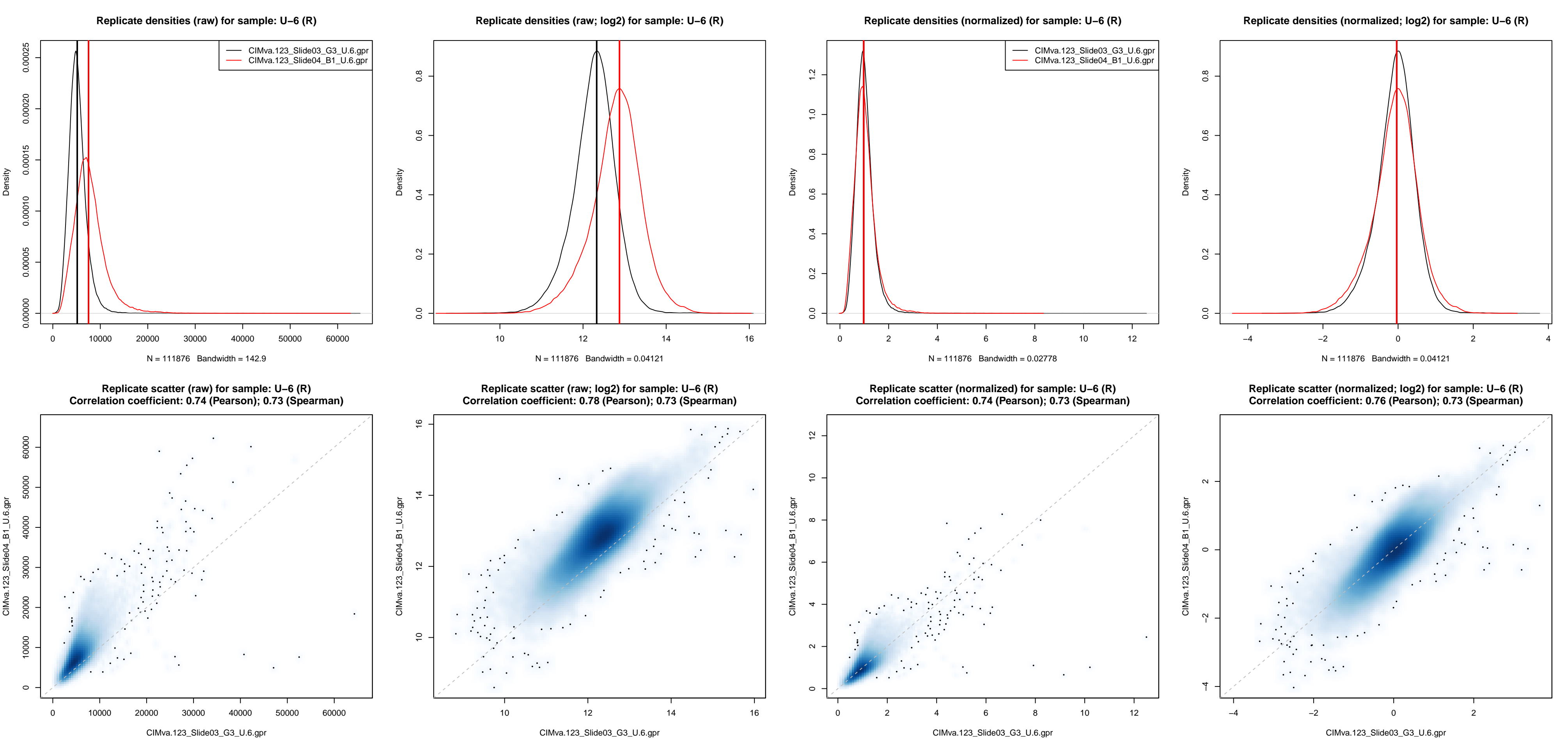

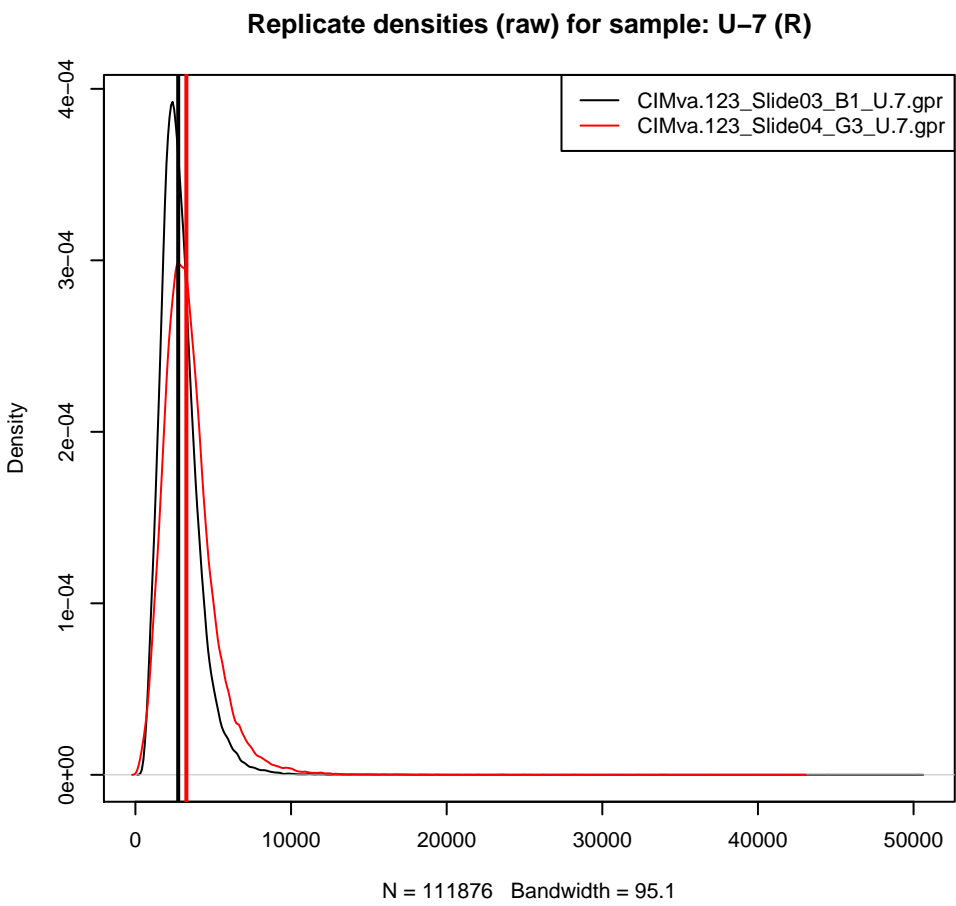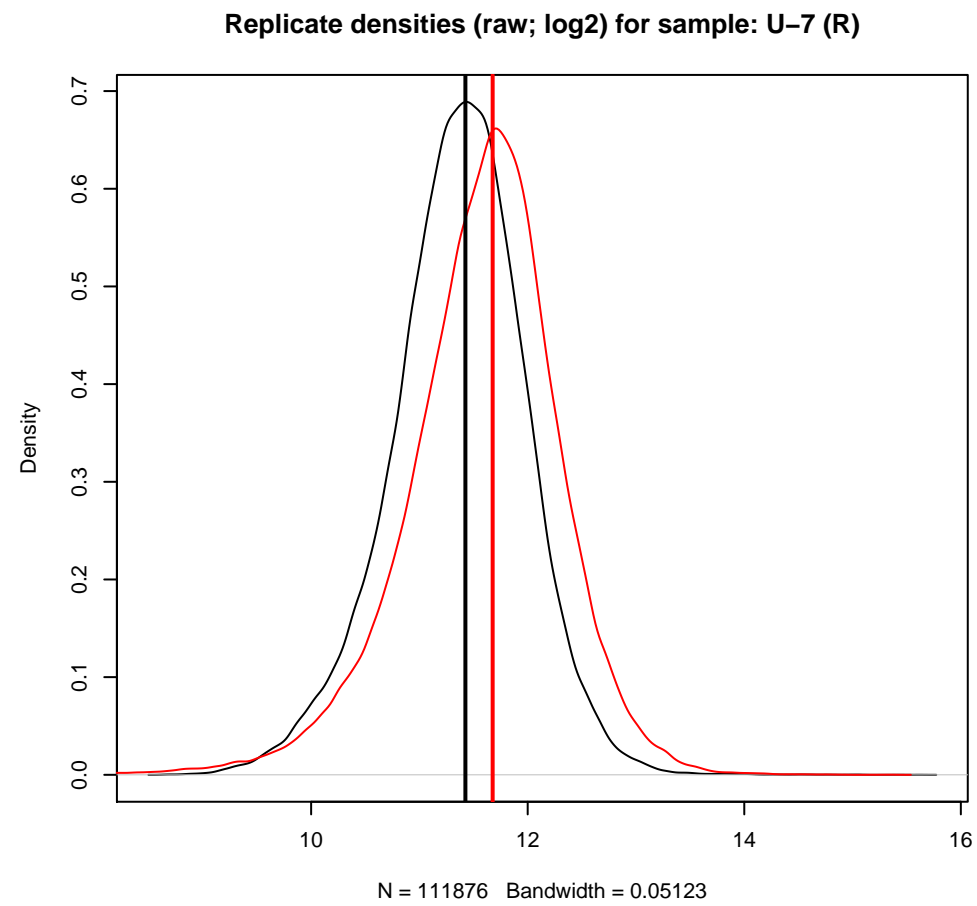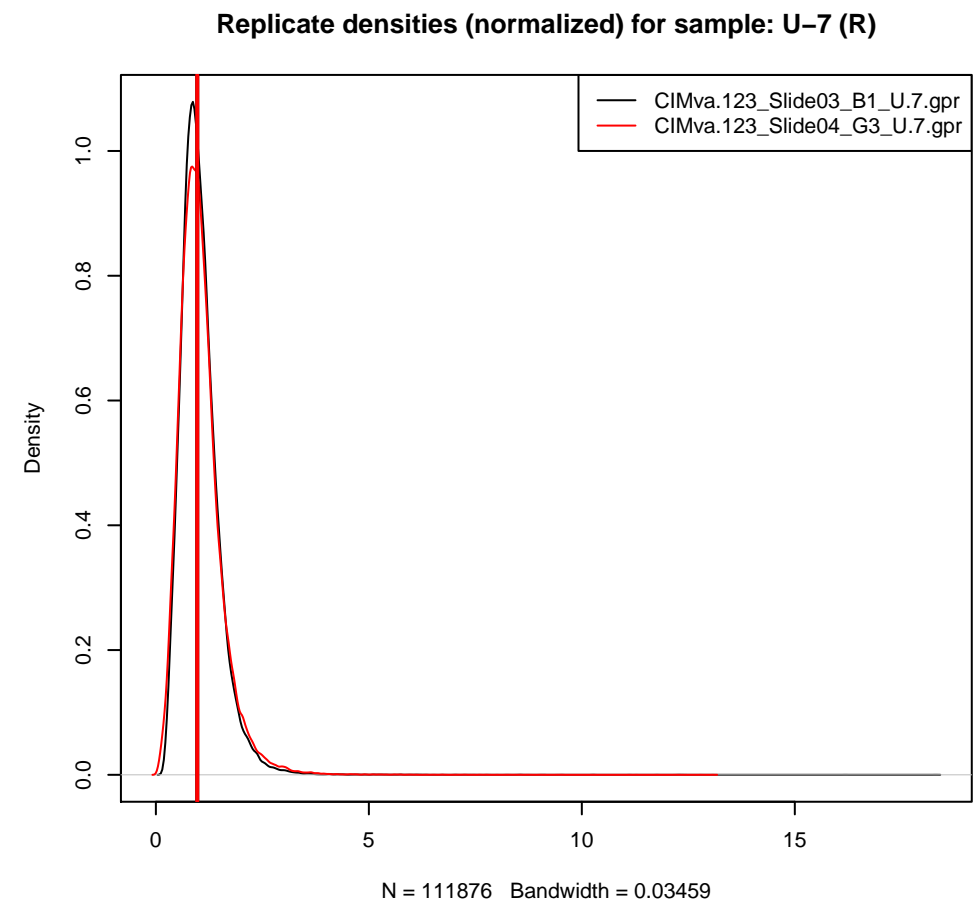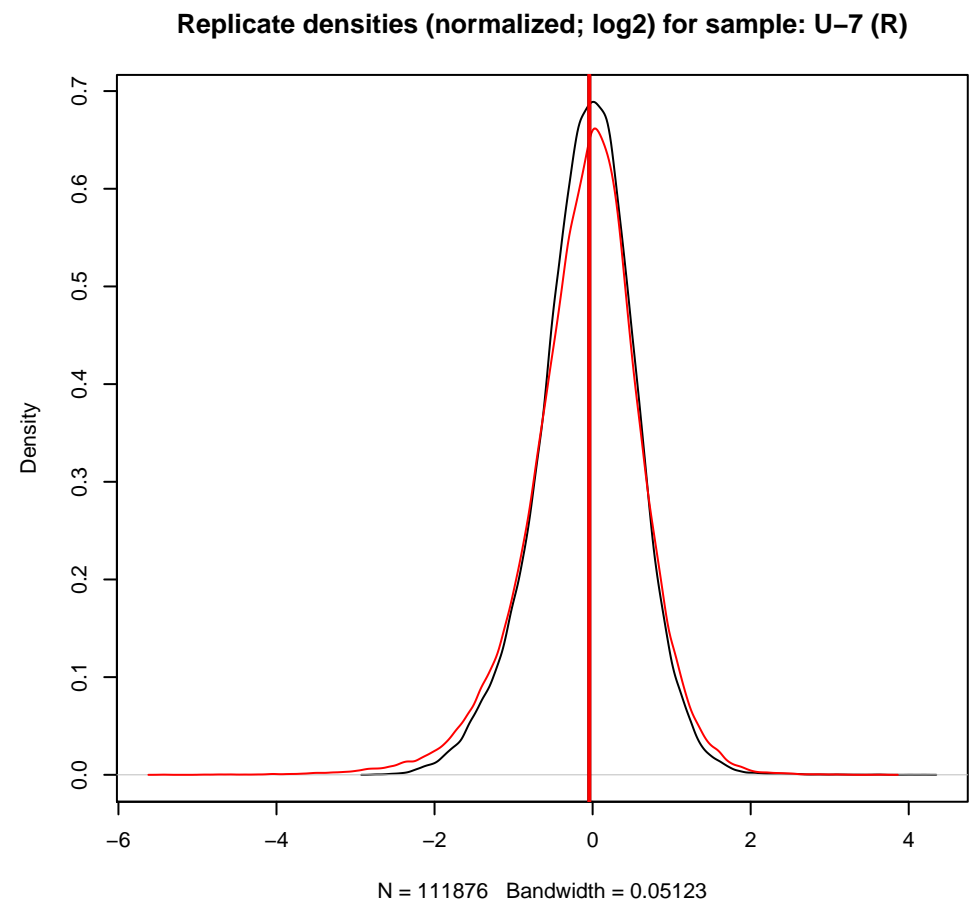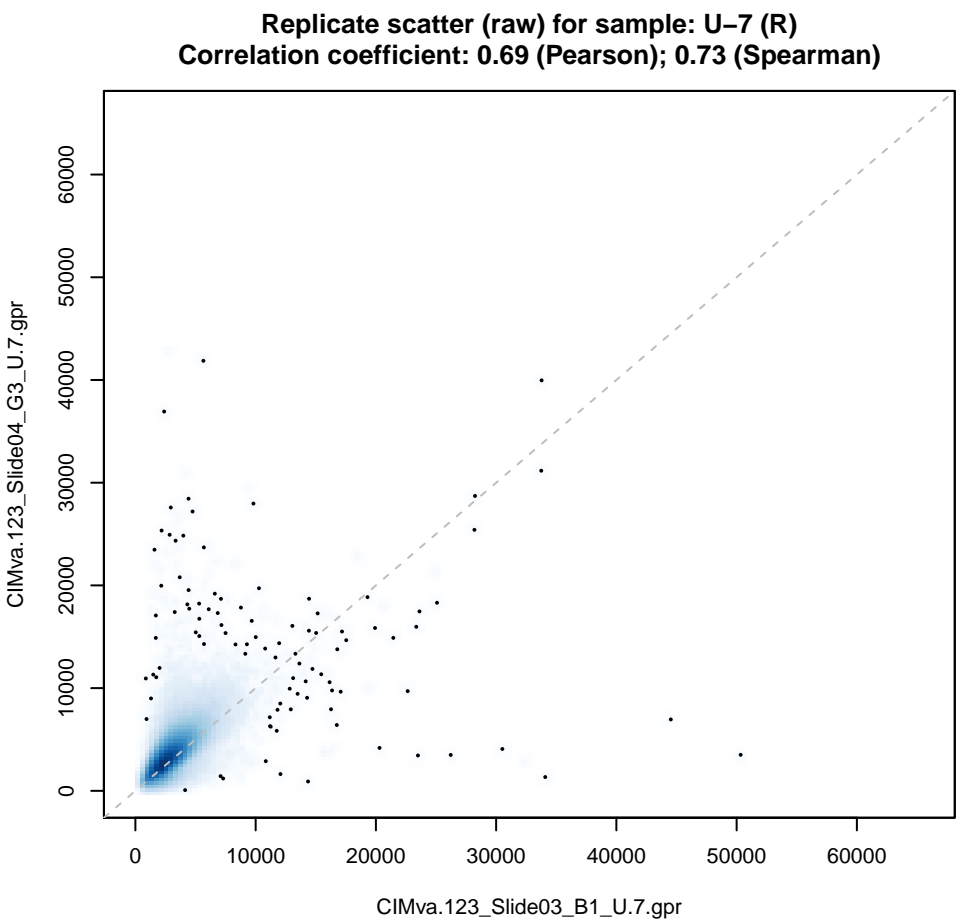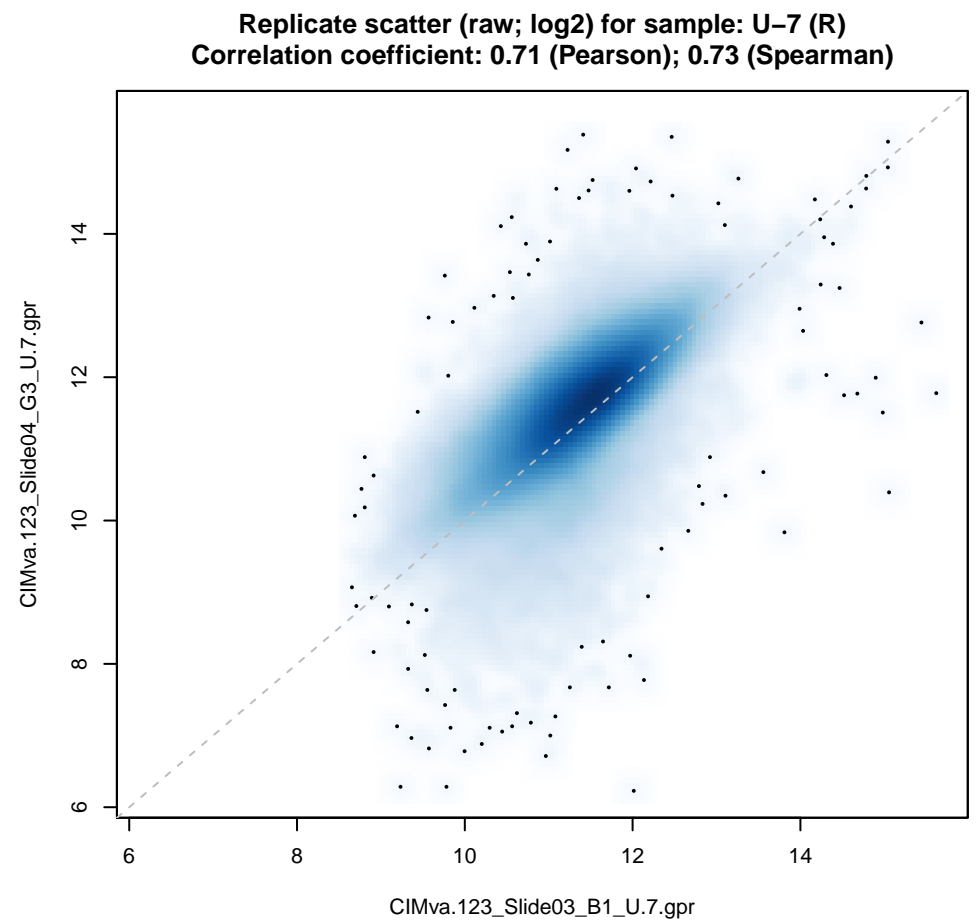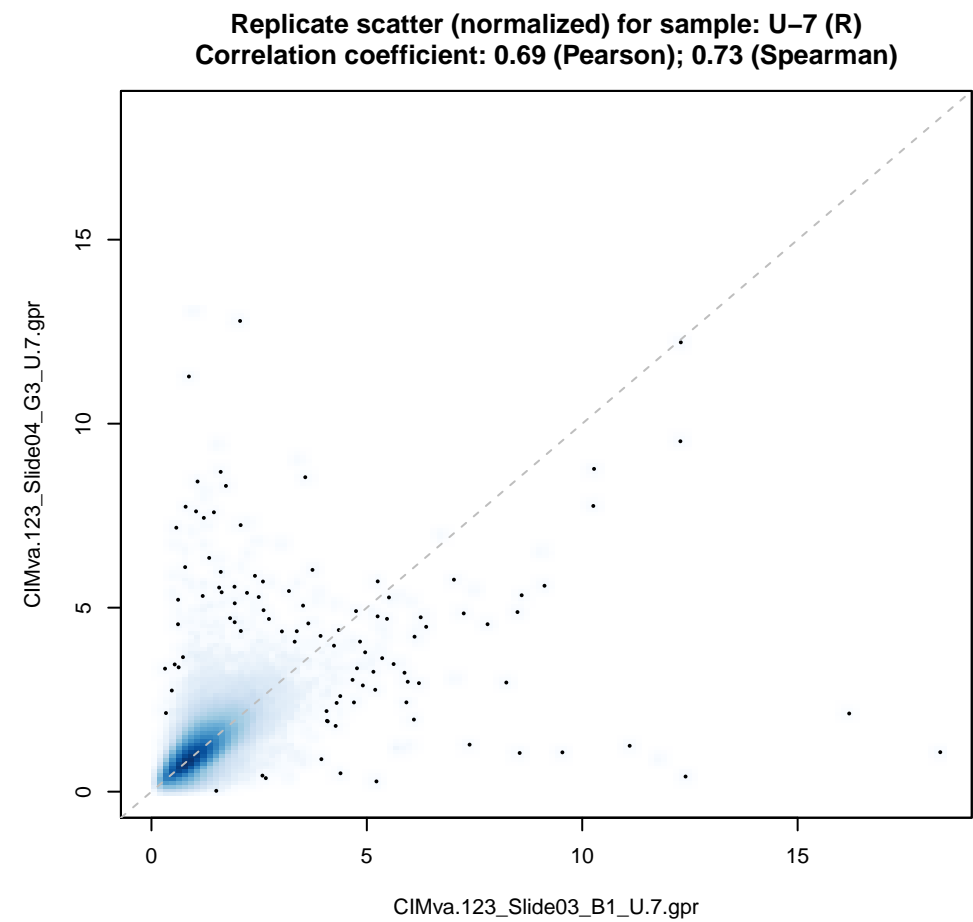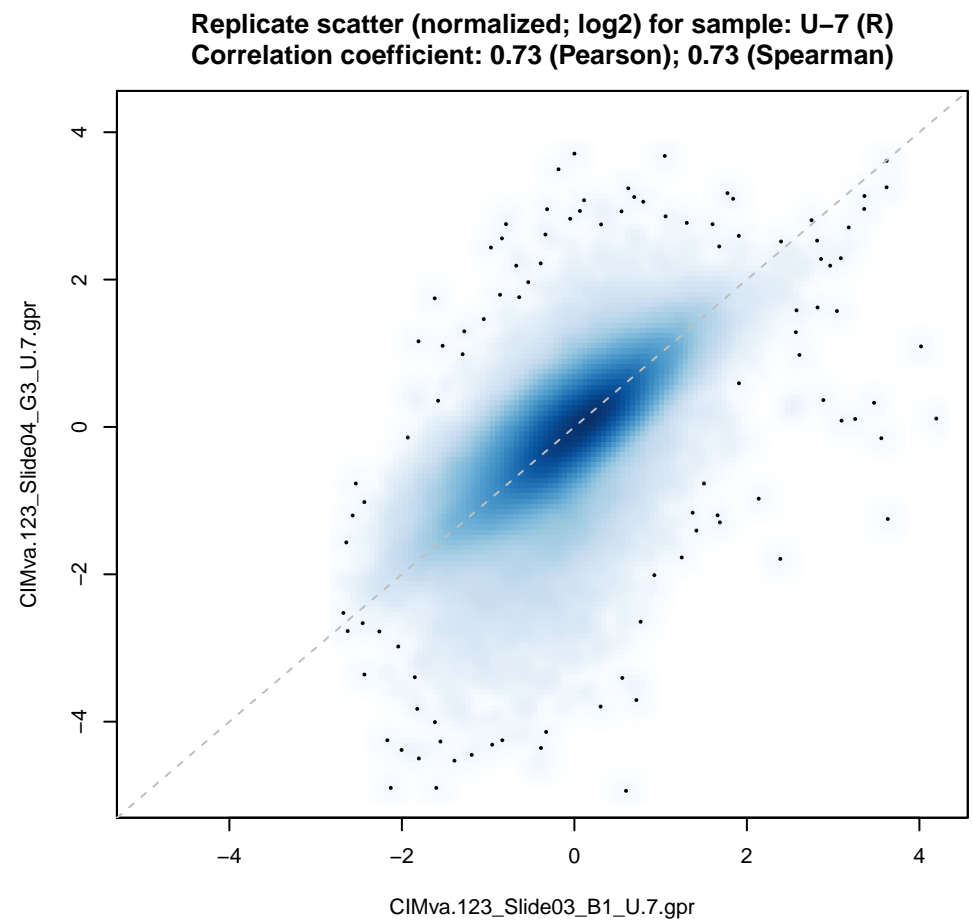

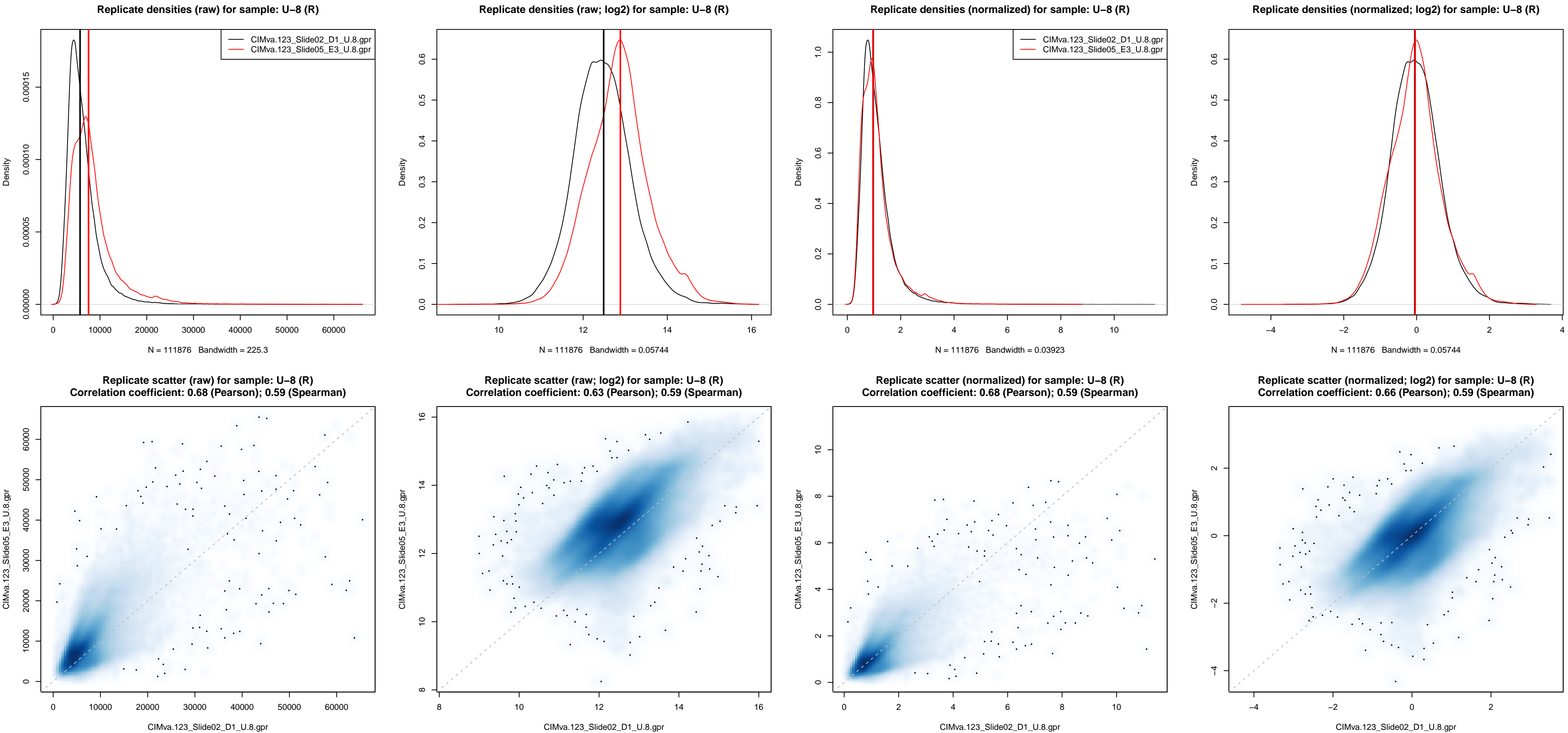

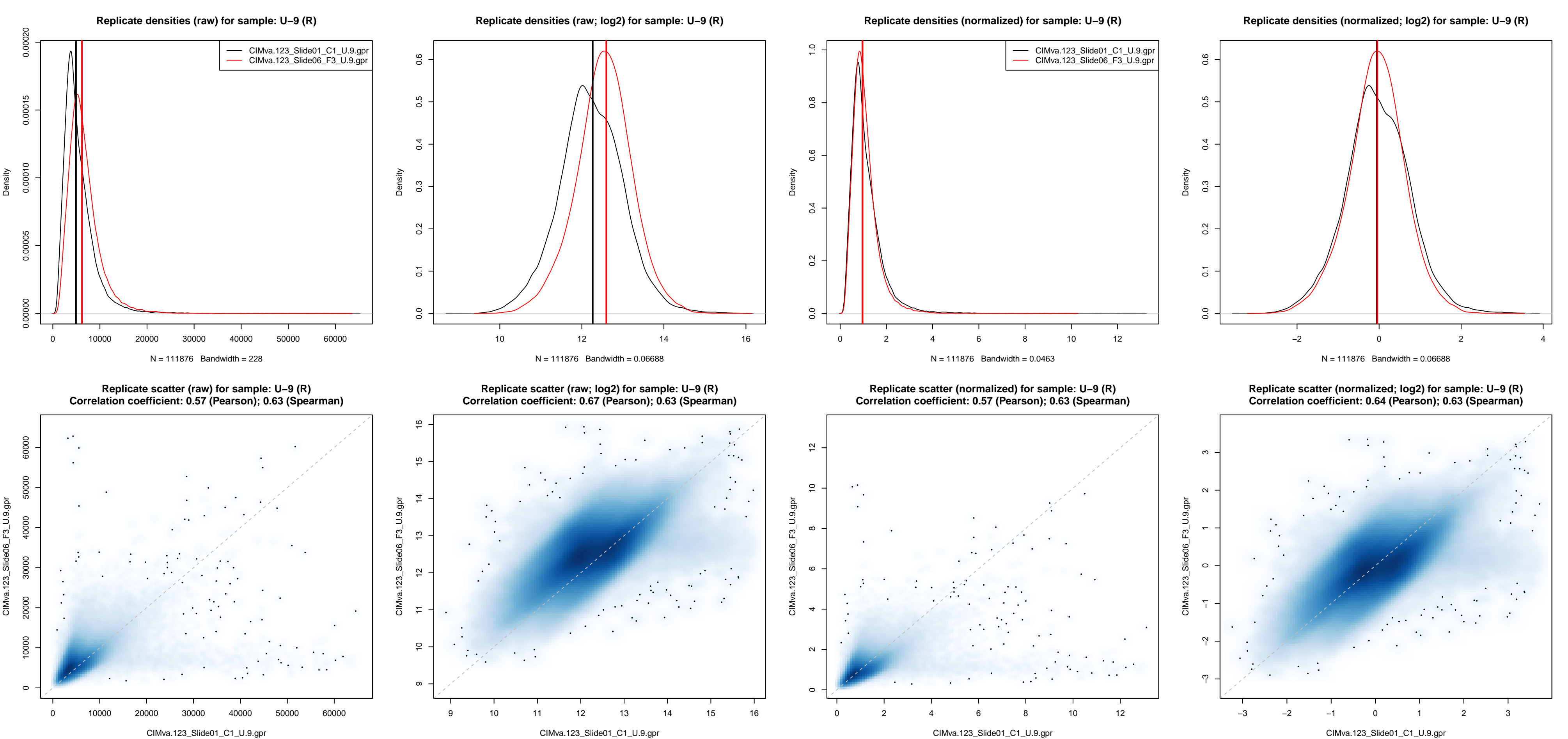

Supplement: Supplementary file 12 — (PDF 12693 kb) [file 12035_2018_1354_MOESM12_ESM.pdf]
