# Supplementary material for: Immunosignature Analysis of Myalgic Encephalomyelitis/Chronic Fatigue Syndrome (ME/CFS)
Source: Mol Neurobiol. 2018 Oct 8;56(6):4249–57. doi: 10.1007/s12035-018-1354-8 (PMC6505503; doi:10.1007/s12035-018-1354-8)
Supplement: Supplementary file 9 — (PDF 14873 kb) [file 12035_2018_1354_MOESM9_ESM.pdf]

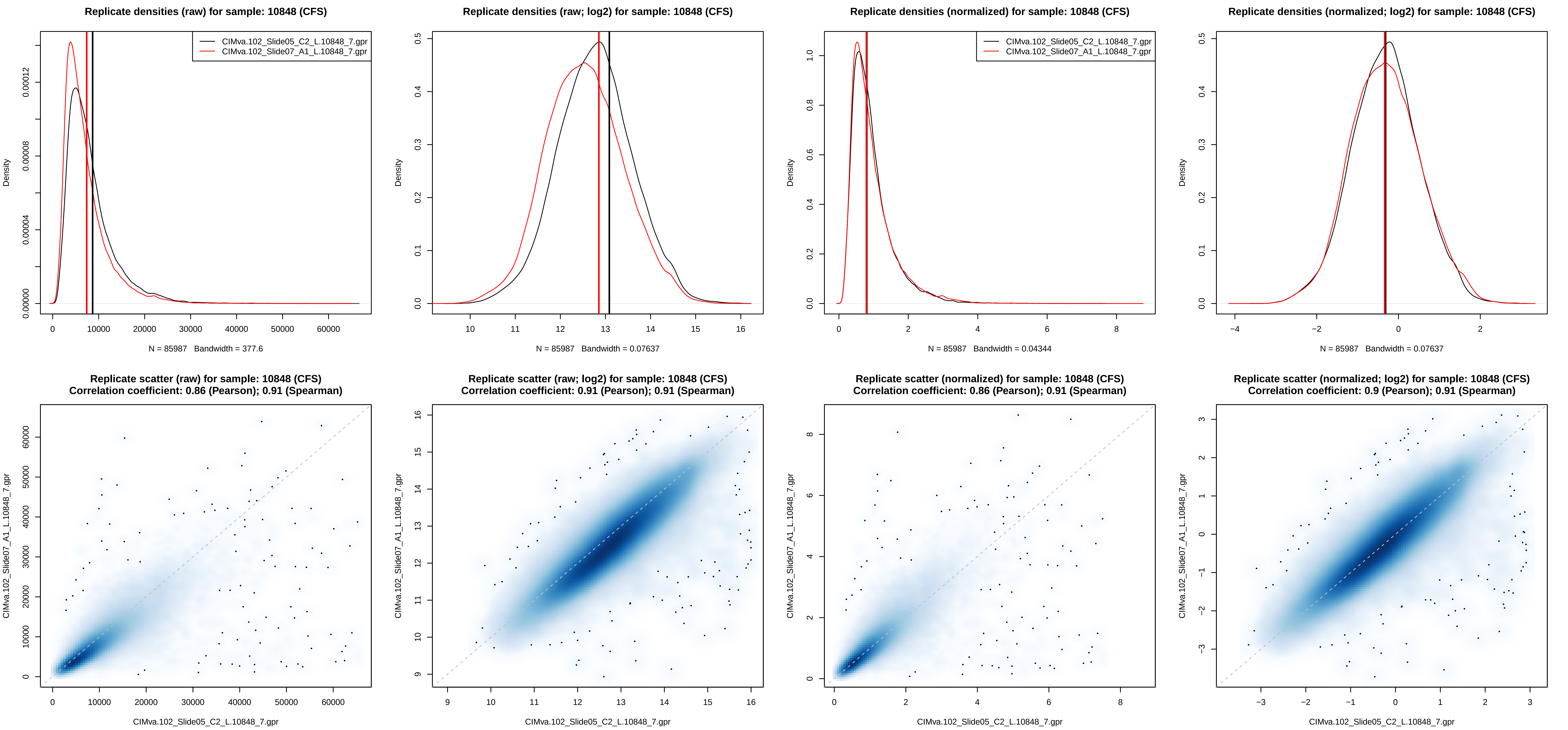
Replicate densities (raw) for sample: 10848 (CFS)
CIMva.102_Slide05_C2_L.10848_7.gpr
CIMva.102_Slide07_A1_L.10848_7.gpr
Density
N = 85987 Bandwidth = 377.6
Replicate densities (raw; log2) for sample: 10848 (CFS)
Density
N = 85987 Bandwidth = 0.07637
Replicate densities (normalized) for sample: 10848 (CFS)
CIMva.102_Slide05_C2_L.10848_7.gpr
CIMva.102_Slide07_A1_L.10848_7.gpr
Density
N = 85987 Bandwidth = 0.04344
Replicate densities (normalized; log2) for sample: 10848 (CFS)
Density
N = 85987 Bandwidth = 0.07637
Replicate scatter (raw) for sample: 10848 (CFS)
Correlation coefficient: 0.86 (Pearson); 0.91 (Spearman)
CIMva.102_Slide07_A1_L.10848_7.gpr
CIMva.102_Slide05_C2_L.10848_7.gpr
Replicate scatter (raw; log2) for sample: 10848 (CFS)
Correlation coefficient: 0.91 (Pearson); 0.91 (Spearman)
CIMva.102_Slide07_A1_L.10848_7.gpr
CIMva.102_Slide05_C2_L.10848_7.gpr
Replicate scatter (normalized) for sample: 10848 (CFS)
Correlation coefficient: 0.86 (Pearson); 0.91 (Spearman)
CIMva.102_Slide07_A1_L.10848_7.gpr
CIMva.102_Slide05_C2_L.10848_7.gpr
Replicate scatter (normalized; log2) for sample: 10848 (CFS)
Correlation coefficient: 0.9 (Pearson); 0.91 (Spearman)
CIMva.102_Slide07_A1_L.10848_7.gpr
CIMva.102_Slide05_C2_L.10848_7.gpr

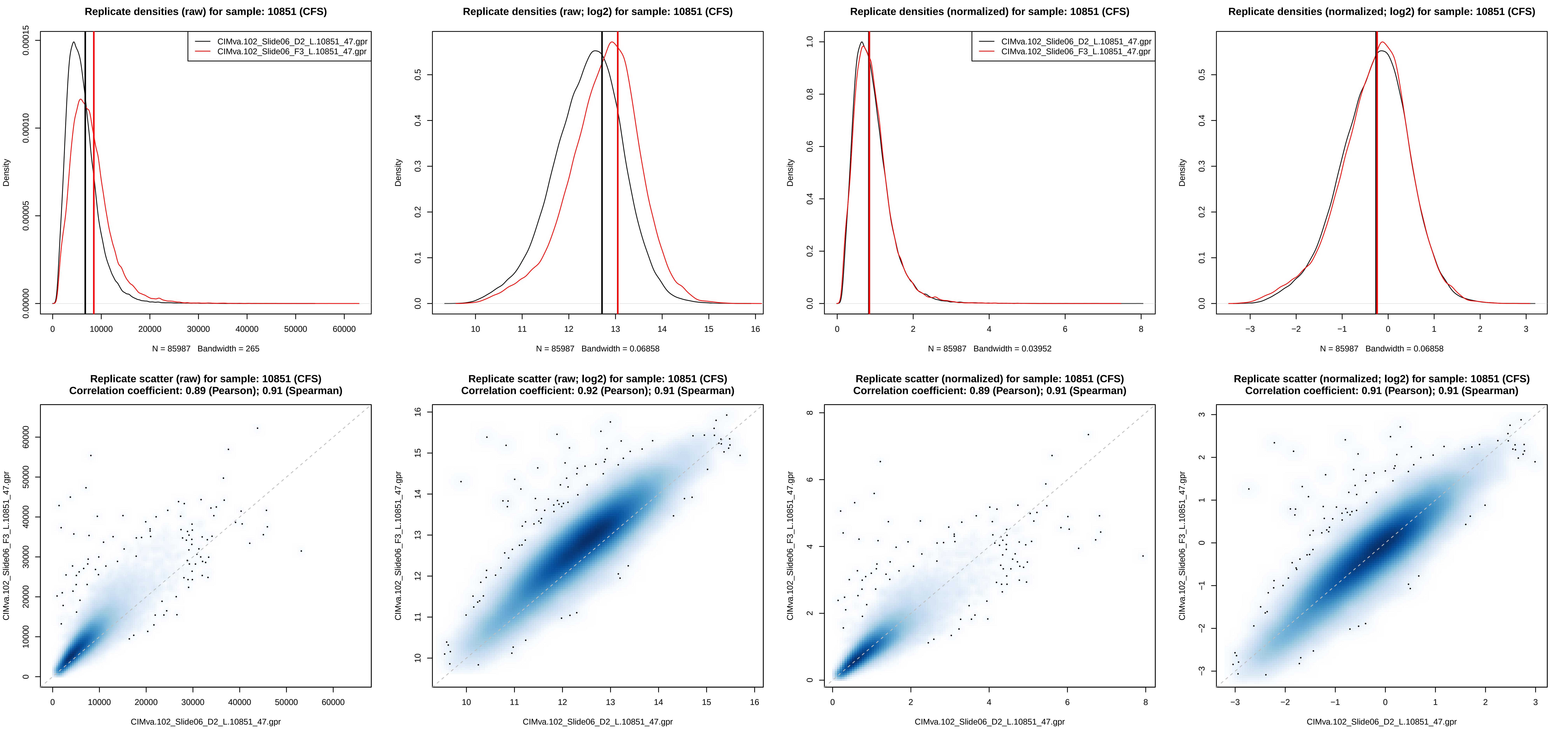
Replicate densities (raw) for sample: 10851 (CFS)
CIMva.102_Slide06_D2_L.10851_47.gpr
CIMva.102_Slide06_F3_L.10851_47.gpr
Density
N = 85987 Bandwidth = 265
Replicate densities (raw; log2) for sample: 10851 (CFS)
Density
N = 85987 Bandwidth = 0.06858
Replicate densities (normalized) for sample: 10851 (CFS)
CIMva.102_Slide06_D2_L.10851_47.gpr
CIMva.102_Slide06_F3_L.10851_47.gpr
Density
N = 85987 Bandwidth = 0.03952
Replicate densities (normalized; log2) for sample: 10851 (CFS)
Density
N = 85987 Bandwidth = 0.06858
Replicate scatter (raw) for sample: 10851 (CFS)
Correlation coefficient: 0.89 (Pearson); 0.91 (Spearman)
CIMva.102_Slide06_F3_L.10851_47.gpr
CIMva.102_Slide06_D2_L.10851_47.gpr
Replicate scatter (raw; log2) for sample: 10851 (CFS)
Correlation coefficient: 0.92 (Pearson); 0.91 (Spearman)
CIMva.102_Slide06_F3_L.10851_47.gpr
CIMva.102_Slide06_D2_L.10851_47.gpr
Replicate scatter (normalized) for sample: 10851 (CFS)
Correlation coefficient: 0.89 (Pearson); 0.91 (Spearman)
CIMva.102_Slide06_F3_L.10851_47.gpr
CIMva.102_Slide06_D2_L.10851_47.gpr
Replicate scatter (normalized; log2) for sample: 10851 (CFS)
Correlation coefficient: 0.91 (Pearson); 0.91 (Spearman)
CIMva.102_Slide06_F3_L.10851_47.gpr
CIMva.102_Slide06_D2_L.10851_47.gpr

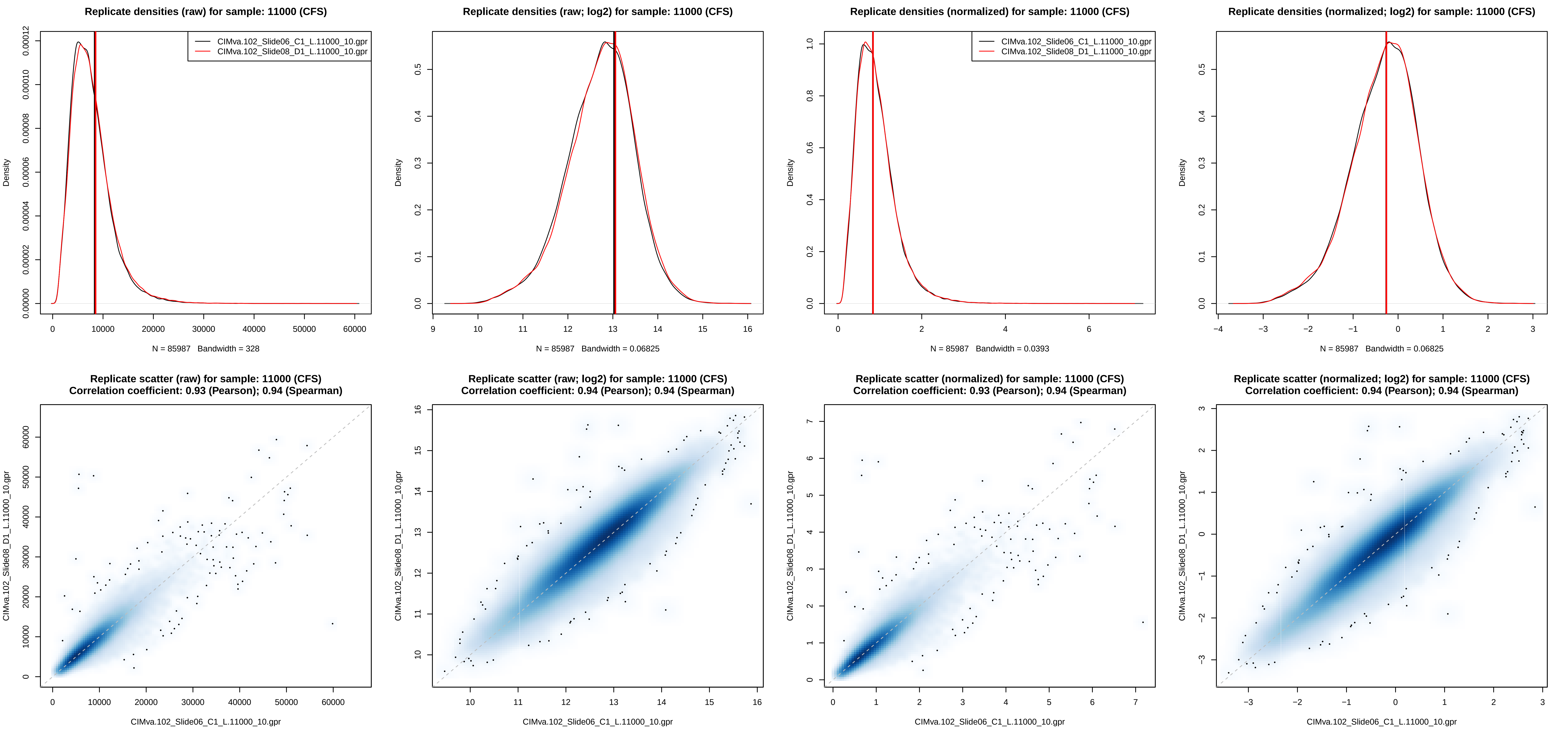
Replicate densities (raw) for sample: 11000 (CFS)
CIMva.102_Slide06_C1_L.11000_10.gpr
CIMva.102_Slide08_D1_L.11000_10.gpr
Density
N = 85987 Bandwidth = 328
Replicate densities (raw; log2) for sample: 11000 (CFS)
Density
N = 85987 Bandwidth = 0.06825
Replicate densities (normalized) for sample: 11000 (CFS)
CIMva.102_Slide06_C1_L.11000_10.gpr
CIMva.102_Slide08_D1_L.11000_10.gpr
Density
N = 85987 Bandwidth = 0.0393
Replicate densities (normalized; log2) for sample: 11000 (CFS)
Density
N = 85987 Bandwidth = 0.06825
Replicate scatter (raw) for sample: 11000 (CFS)
Correlation coefficient: 0.93 (Pearson); 0.94 (Spearman)
CIMva.102_Slide08_D1_L.11000_10.gpr
CIMva.102_Slide06_C1_L.11000_10.gpr
Replicate scatter (raw; log2) for sample: 11000 (CFS)
Correlation coefficient: 0.94 (Pearson); 0.94 (Spearman)
CIMva.102_Slide08_D1_L.11000_10.gpr
CIMva.102_Slide06_C1_L.11000_10.gpr
Replicate scatter (normalized) for sample: 11000 (CFS)
Correlation coefficient: 0.93 (Pearson); 0.94 (Spearman)
CIMva.102_Slide08_D1_L.11000_10.gpr
CIMva.102_Slide06_C1_L.11000_10.gpr
Replicate scatter (normalized; log2) for sample: 11000 (CFS)
Correlation coefficient: 0.94 (Pearson); 0.94 (Spearman)
CIMva.102_Slide08_D1_L.11000_10.gpr
CIMva.102_Slide06_C1_L.11000_10.gpr

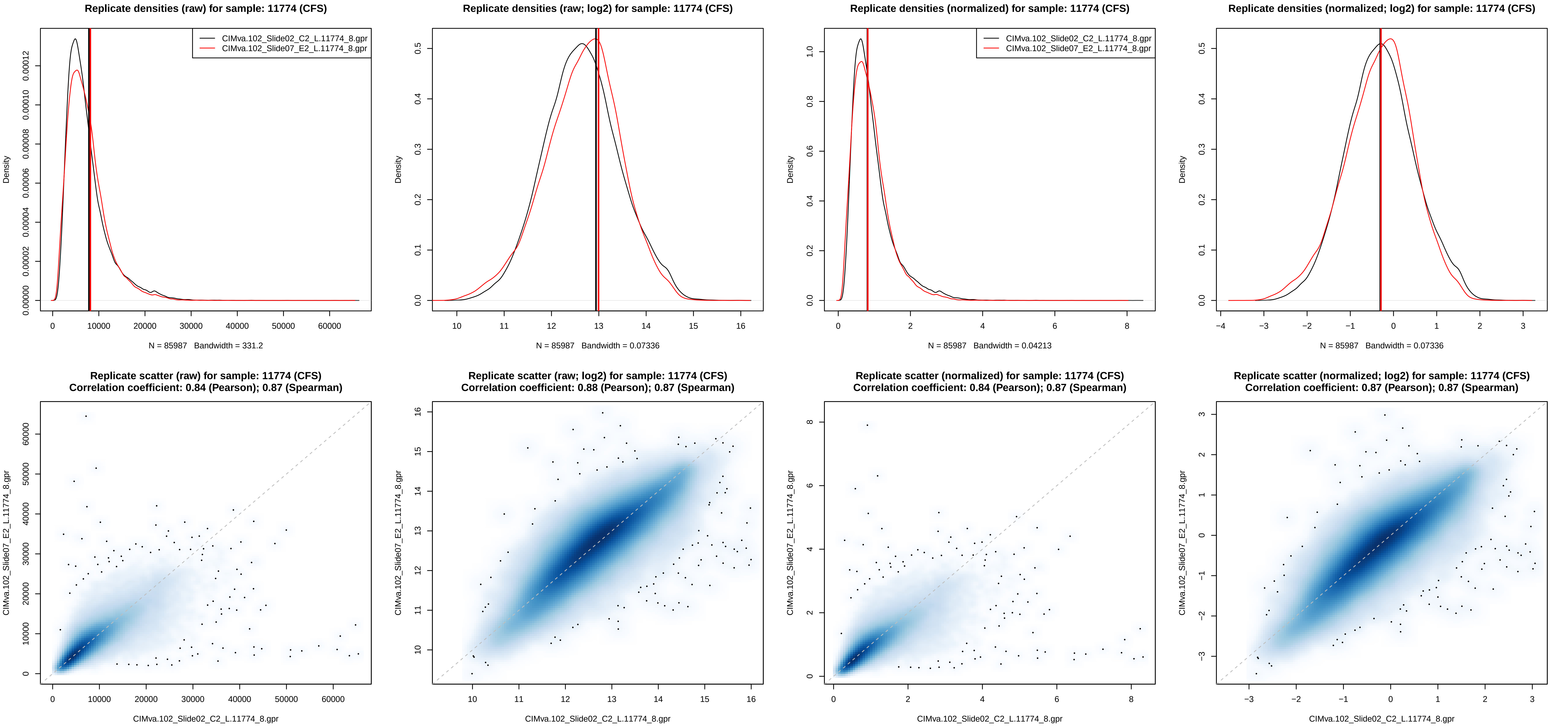
Replicate densities (raw) for sample: 11774 (CFS)
CIMva.102_Slide02_C2_L.11774_8.gpr
CIMva.102_Slide07_E2_L.11774_8.gpr
Density
N = 85987 Bandwidth = 331.2
Replicate densities (raw; log2) for sample: 11774 (CFS)
Density
N = 85987 Bandwidth = 0.07336
Replicate densities (normalized) for sample: 11774 (CFS)
CIMva.102_Slide02_C2_L.11774_8.gpr
CIMva.102_Slide07_E2_L.11774_8.gpr
Density
N = 85987 Bandwidth = 0.04213
Replicate densities (normalized; log2) for sample: 11774 (CFS)
Density
N = 85987 Bandwidth = 0.07336
Replicate scatter (raw) for sample: 11774 (CFS)
Correlation coefficient: 0.84 (Pearson); 0.87 (Spearman)
CIMva.102_Slide07_E2_L.11774_8.gpr
CIMva.102_Slide02_C2_L.11774_8.gpr
Replicate scatter (raw; log2) for sample: 11774 (CFS)
Correlation coefficient: 0.88 (Pearson); 0.87 (Spearman)
CIMva.102_Slide07_E2_L.11774_8.gpr
CIMva.102_Slide02_C2_L.11774_8.gpr
Replicate scatter (normalized) for sample: 11774 (CFS)
Correlation coefficient: 0.84 (Pearson); 0.87 (Spearman)
CIMva.102_Slide07_E2_L.11774_8.gpr
CIMva.102_Slide02_C2_L.11774_8.gpr
Replicate scatter (normalized; log2) for sample: 11774 (CFS)
Correlation coefficient: 0.87 (Pearson); 0.87 (Spearman)
CIMva.102_Slide07_E2_L.11774_8.gpr
CIMva.102_Slide02_C2_L.11774_8.gpr

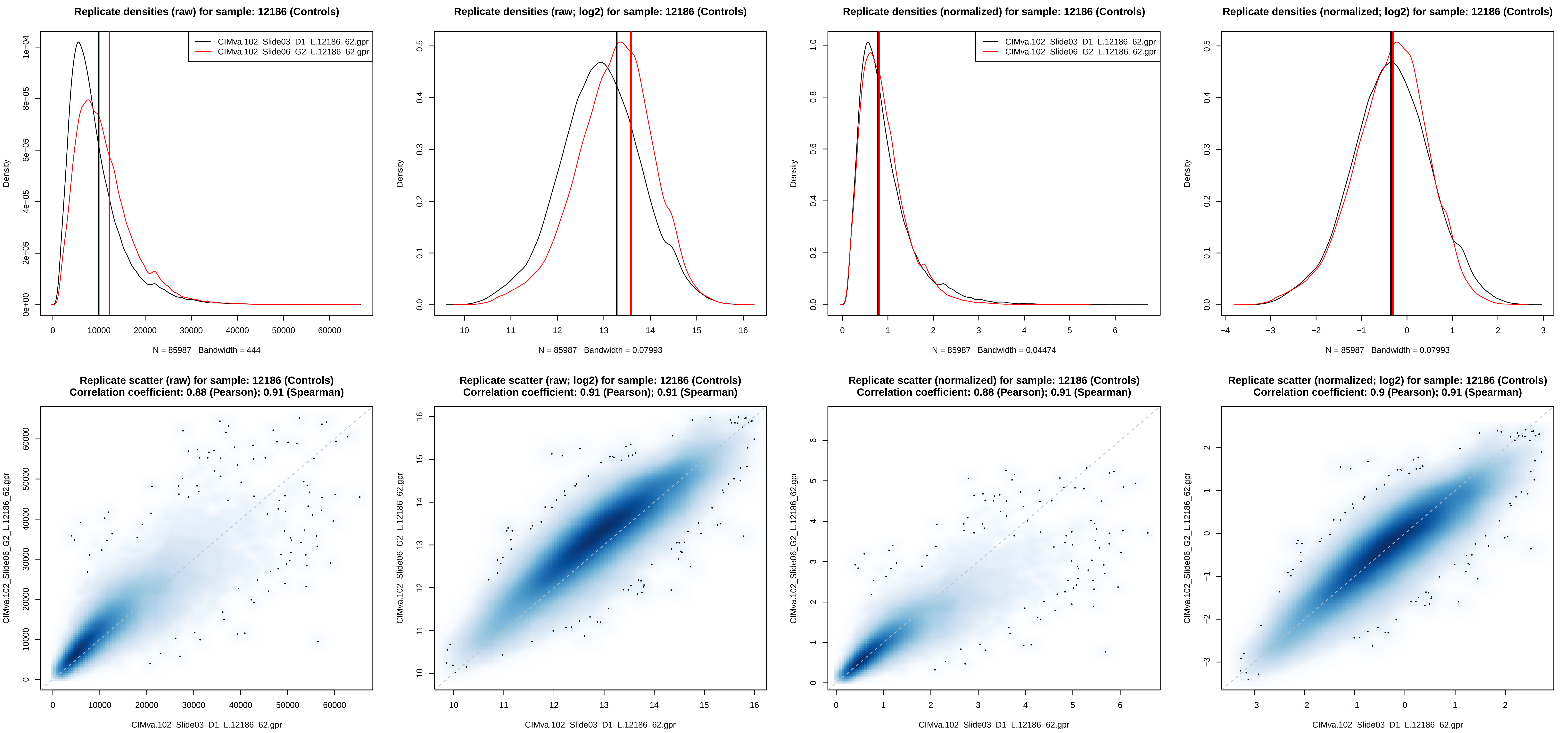
Replicate densities (raw) for sample: 12186 (Controls)
CIMva.102_Slide03_D1_L.12186_62.gpr
CIMva.102_Slide06_G2_L.12186_62.gpr
Density
N = 85987 Bandwidth = 444
Replicate densities (raw; log2) for sample: 12186 (Controls)
Density
N = 85987 Bandwidth = 0.07993
Replicate densities (normalized) for sample: 12186 (Controls)
CIMva.102_Slide03_D1_L.12186_62.gpr
CIMva.102_Slide06_G2_L.12186_62.gpr
Density
N = 85987 Bandwidth = 0.04474
Replicate densities (normalized; log2) for sample: 12186 (Controls)
Density
N = 85987 Bandwidth = 0.07993
Replicate scatter (raw) for sample: 12186 (Controls)
Correlation coefficient: 0.88 (Pearson); 0.91 (Spearman)
CIMva.102_Slide06_G2_L.12186_62.gpr
CIMva.102_Slide03_D1_L.12186_62.gpr
Replicate scatter (raw; log2) for sample: 12186 (Controls)
Correlation coefficient: 0.91 (Pearson); 0.91 (Spearman)
CIMva.102_Slide06_G2_L.12186_62.gpr
CIMva.102_Slide03_D1_L.12186_62.gpr
Replicate scatter (normalized) for sample: 12186 (Controls)
Correlation coefficient: 0.88 (Pearson); 0.91 (Spearman)
CIMva.102_Slide06_G2_L.12186_62.gpr
CIMva.102_Slide03_D1_L.12186_62.gpr
Replicate scatter (normalized; log2) for sample: 12186 (Controls)
Correlation coefficient: 0.9 (Pearson); 0.91 (Spearman)
CIMva.102_Slide06_G2_L.12186_62.gpr
CIMva.102_Slide03_D1_L.12186_62.gpr

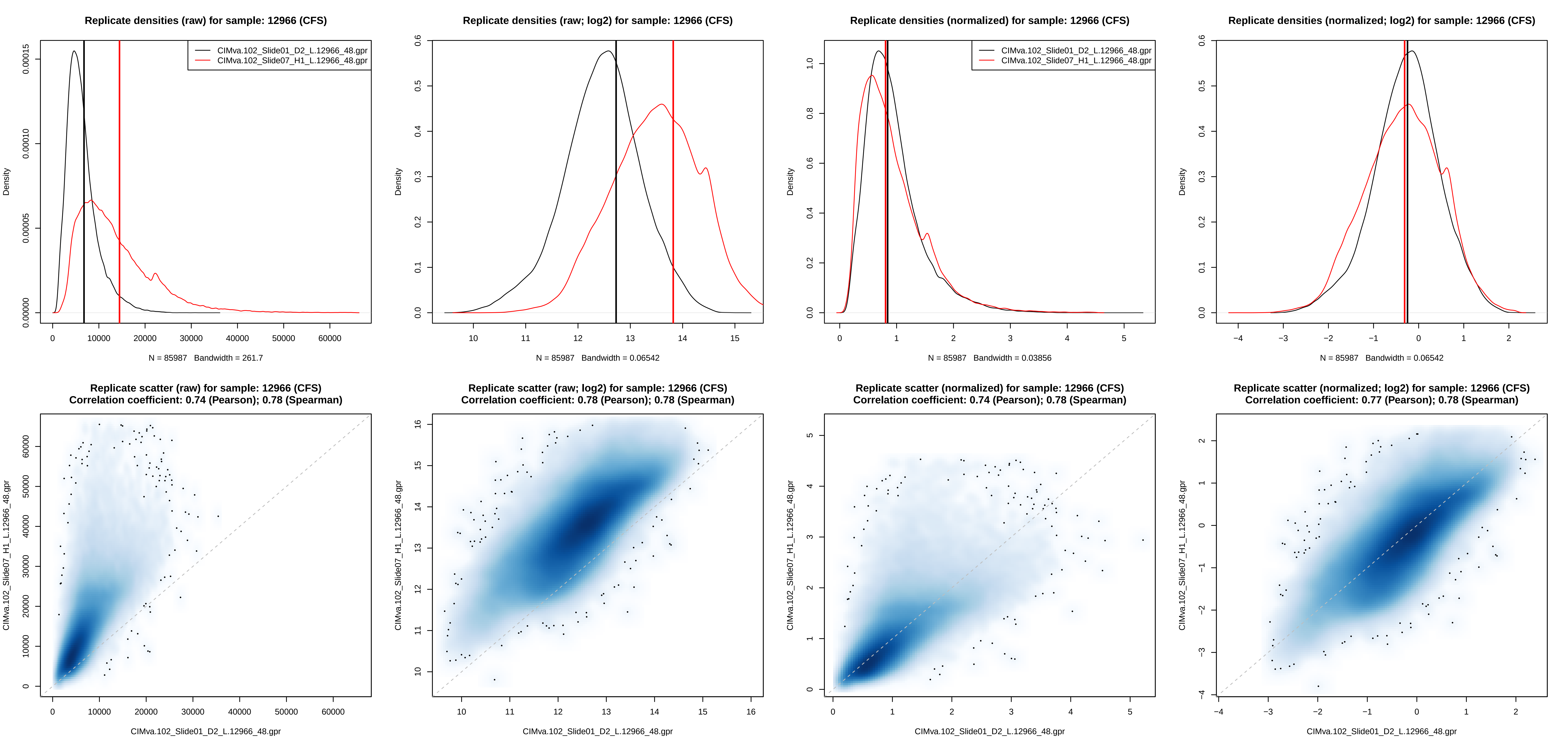
Replicate densities (raw) for sample: 12966 (CFS)
CIMva.102_Slide01_D2_L.12966_48.gpr
CIMva.102_Slide07_H1_L.12966_48.gpr
Density
N = 85987 Bandwidth = 261.7
Replicate densities (raw; log2) for sample: 12966 (CFS)
Density
N = 85987 Bandwidth = 0.06542
Replicate densities (normalized) for sample: 12966 (CFS)
CIMva.102_Slide01_D2_L.12966_48.gpr
CIMva.102_Slide07_H1_L.12966_48.gpr
Density
N = 85987 Bandwidth = 0.03856
Replicate densities (normalized; log2) for sample: 12966 (CFS)
Density
N = 85987 Bandwidth = 0.06542
Replicate scatter (raw) for sample: 12966 (CFS)
Correlation coefficient: 0.74 (Pearson); 0.78 (Spearman)
CIMva.102_Slide07_H1_L.12966_48.gpr
CIMva.102_Slide01_D2_L.12966_48.gpr
Replicate scatter (raw; log2) for sample: 12966 (CFS)
Correlation coefficient: 0.78 (Pearson); 0.78 (Spearman)
CIMva.102_Slide07_H1_L.12966_48.gpr
CIMva.102_Slide01_D2_L.12966_48.gpr
Replicate scatter (normalized) for sample: 12966 (CFS)
Correlation coefficient: 0.74 (Pearson); 0.78 (Spearman)
CIMva.102_Slide07_H1_L.12966_48.gpr
CIMva.102_Slide01_D2_L.12966_48.gpr
Replicate scatter (normalized; log2) for sample: 12966 (CFS)
Correlation coefficient: 0.77 (Pearson); 0.78 (Spearman)
CIMva.102_Slide07_H1_L.12966_48.gpr
CIMva.102_Slide01_D2_L.12966_48.gpr

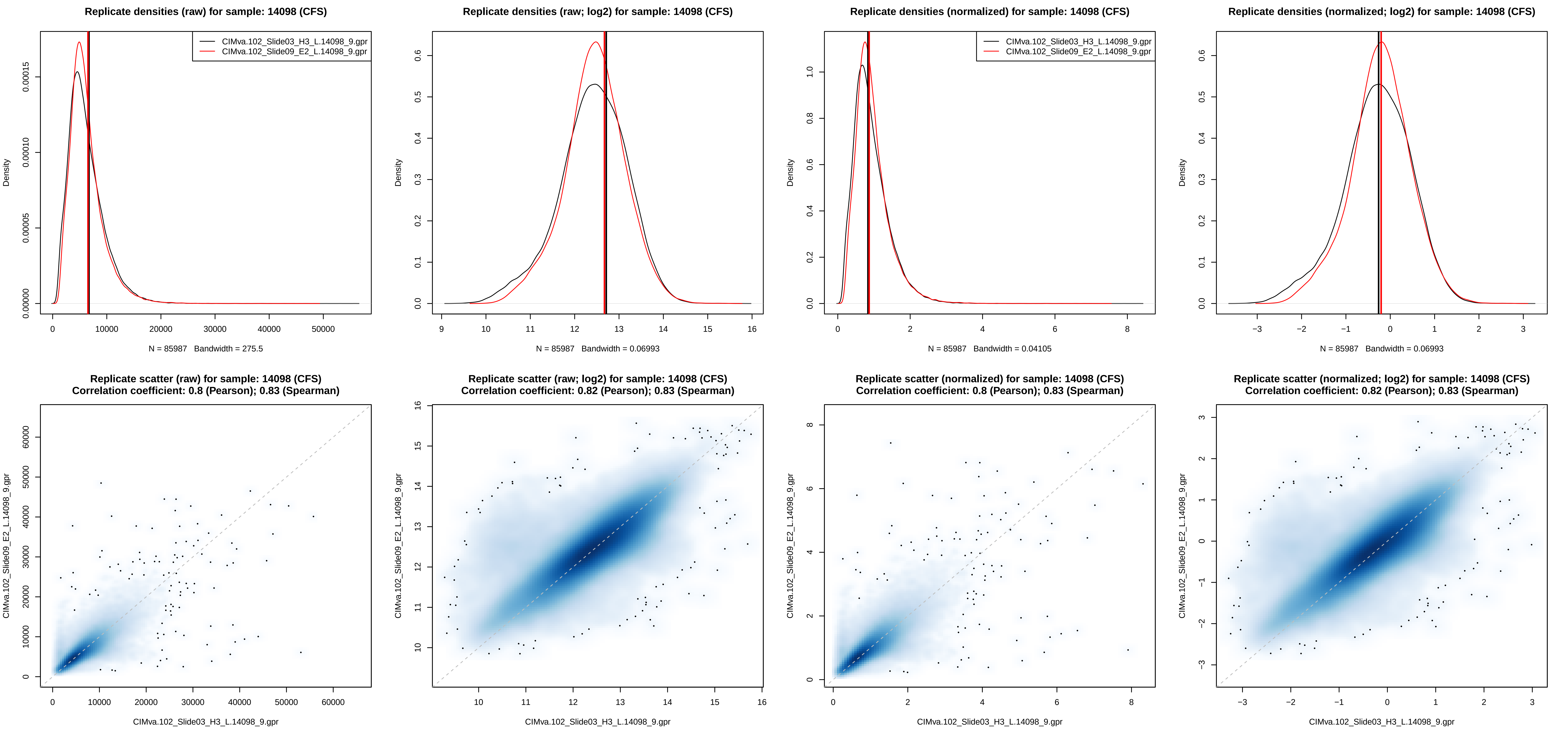

Replicate densities (raw) for sample: 14098 (CFS)
CIMva.102_Slide03_H3_L.14098_9.gpr
CIMva.102_Slide09_E2_L.14098_9.gpr
Density
N = 85987 Bandwidth = 275.5
Replicate densities (raw; log2) for sample: 14098 (CFS)
Density
N = 85987 Bandwidth = 0.06993
Replicate densities (normalized) for sample: 14098 (CFS)
CIMva.102_Slide03_H3_L.14098_9.gpr
CIMva.102_Slide09_E2_L.14098_9.gpr
Density
N = 85987 Bandwidth = 0.04105
Replicate densities (normalized; log2) for sample: 14098 (CFS)
Density
N = 85987 Bandwidth = 0.06993
Replicate scatter (raw) for sample: 14098 (CFS)
Correlation coefficient: 0.8 (Pearson); 0.83 (Spearman)
CIMva.102_Slide09_E2_L.14098_9.gpr
CIMva.102_Slide03_H3_L.14098_9.gpr
Replicate scatter (raw; log2) for sample: 14098 (CFS)
Correlation coefficient: 0.82 (Pearson); 0.83 (Spearman)
CIMva.102_Slide09_E2_L.14098_9.gpr
CIMva.102_Slide03_H3_L.14098_9.gpr
Replicate scatter (normalized) for sample: 14098 (CFS)
Correlation coefficient: 0.8 (Pearson); 0.83 (Spearman)
CIMva.102_Slide09_E2_L.14098_9.gpr
CIMva.102_Slide03_H3_L.14098_9.gpr
Replicate scatter (normalized; log2) for sample: 14098 (CFS)
Correlation coefficient: 0.82 (Pearson); 0.83 (Spearman)
CIMva.102_Slide09_E2_L.14098_9.gpr
CIMva.102_Slide03_H3_L.14098_9.gpr

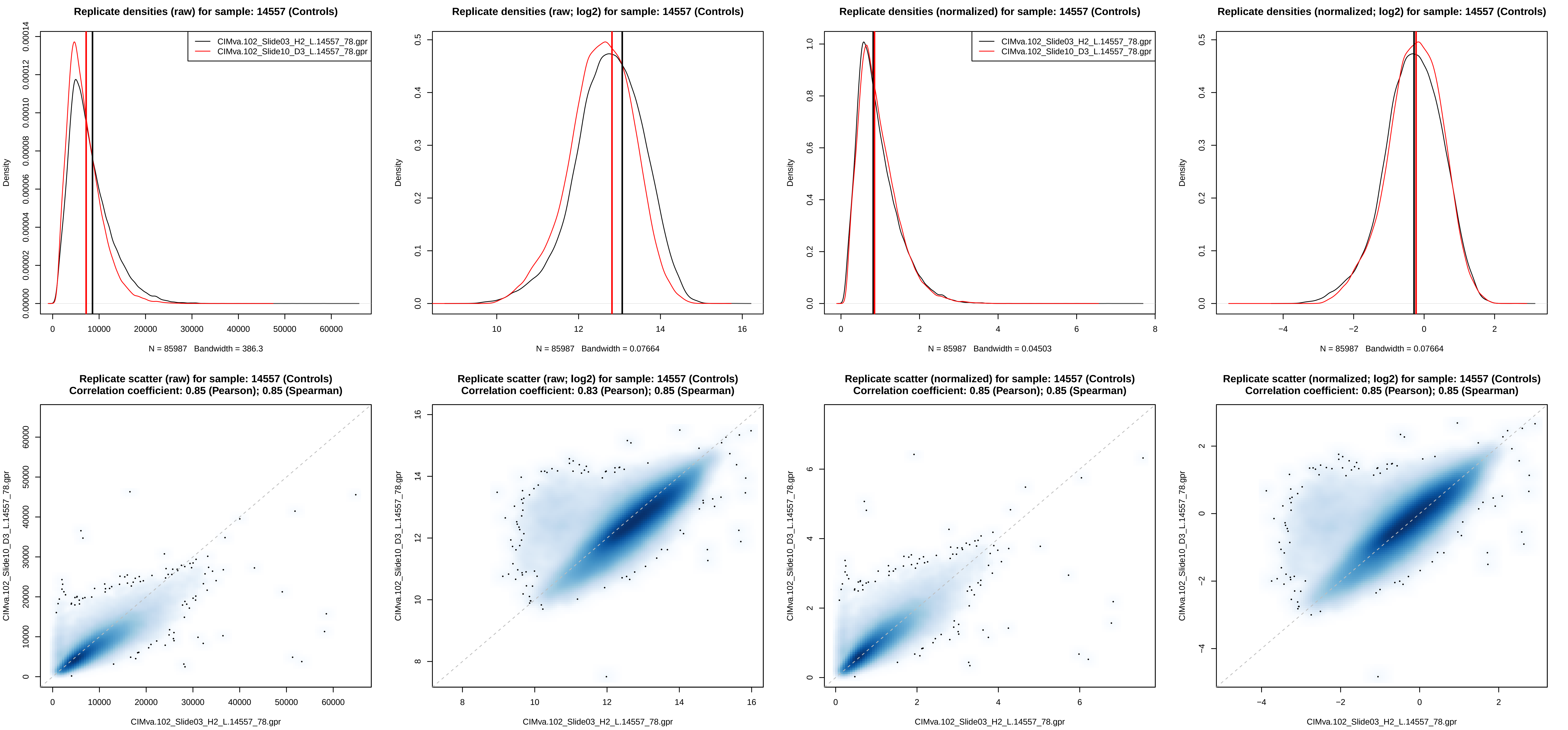
Replicate densities (raw) for sample: 14557 (Controls)
CIMva.102_Slide03_H2_L.14557_78.gpr
CIMva.102_Slide10_D3_L.14557_78.gpr
Density
N = 85987 Bandwidth = 386.3
Replicate densities (raw; log2) for sample: 14557 (Controls)
Density
N = 85987 Bandwidth = 0.07664
Replicate densities (normalized) for sample: 14557 (Controls)
CIMva.102_Slide03_H2_L.14557_78.gpr
CIMva.102_Slide10_D3_L.14557_78.gpr
Density
N = 85987 Bandwidth = 0.04503
Replicate densities (normalized; log2) for sample: 14557 (Controls)
Density
N = 85987 Bandwidth = 0.07664
Replicate scatter (raw) for sample: 14557 (Controls)
Correlation coefficient: 0.85 (Pearson); 0.85 (Spearman)
CIMva.102_Slide10_D3_L.14557_78.gpr
CIMva.102_Slide03_H2_L.14557_78.gpr
Replicate scatter (raw; log2) for sample: 14557 (Controls)
Correlation coefficient: 0.83 (Pearson); 0.85 (Spearman)
CIMva.102_Slide10_D3_L.14557_78.gpr
CIMva.102_Slide03_H2_L.14557_78.gpr
Replicate scatter (normalized) for sample: 14557 (Controls)
Correlation coefficient: 0.85 (Pearson); 0.85 (Spearman)
CIMva.102_Slide10_D3_L.14557_78.gpr
CIMva.102_Slide03_H2_L.14557_78.gpr
Replicate scatter (normalized; log2) for sample: 14557 (Controls)
Correlation coefficient: 0.85 (Pearson); 0.85 (Spearman)
CIMva.102_Slide10_D3_L.14557_78.gpr
CIMva.102_Slide03_H2_L.14557_78.gpr

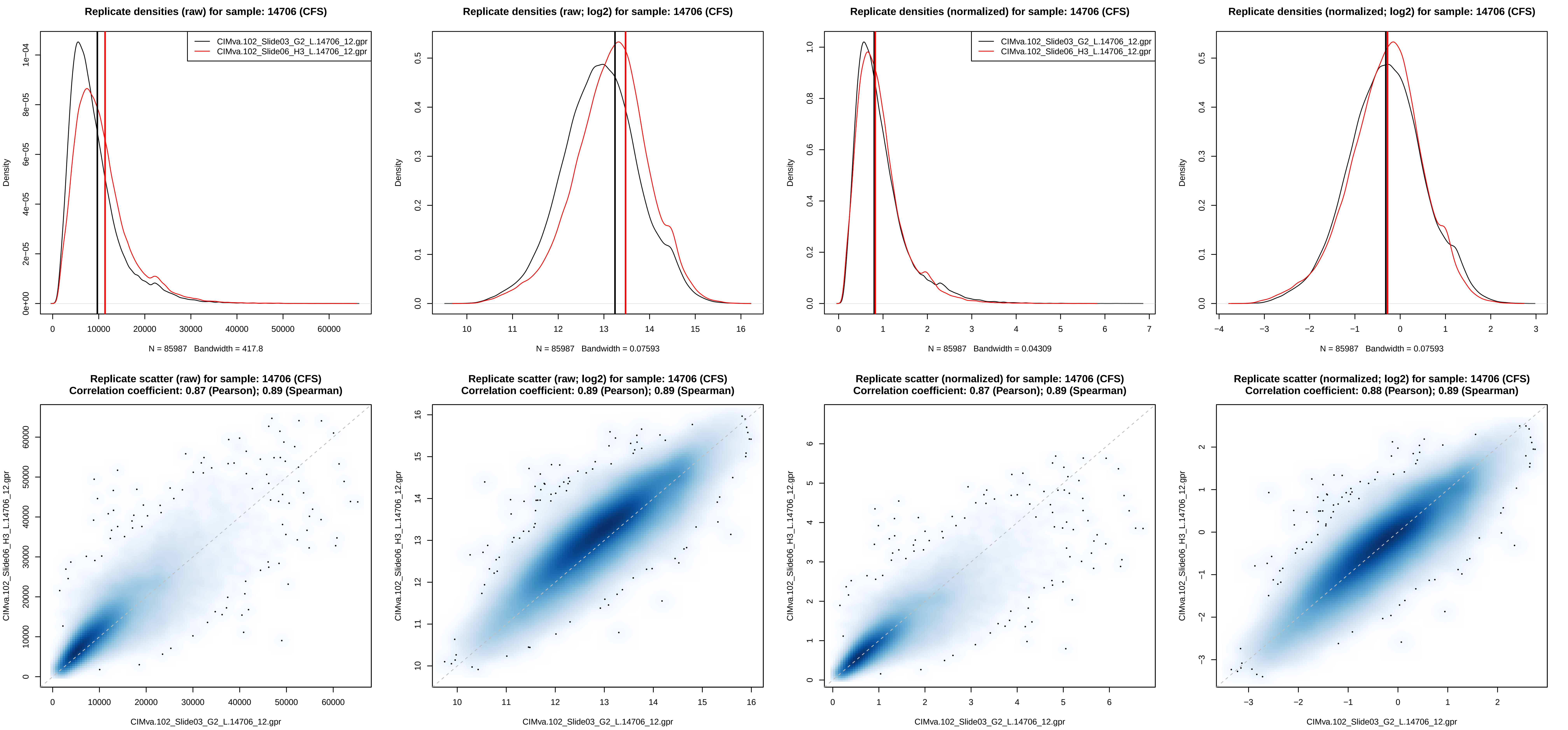

Replicate densities (raw) for sample: 14706 (CFS)
Replicate densities (raw; log2) for sample: 14706 (CFS)
Replicate densities (normalized) for sample: 14706 (CFS)
Replicate densities (normalized; log2) for sample: 14706 (CFS)
CIMva.102_Slide03_G2_L.14706_12.gpr
CIMva.102_Slide06_H3_L.14706_12.gpr
Density
N = 85987 Bandwidth = 417.8
N = 85987 Bandwidth = 0.07593
N = 85987 Bandwidth = 0.04309
N = 85987 Bandwidth = 0.07593
Replicate scatter (raw) for sample: 14706 (CFS)
Correlation coefficient: 0.87 (Pearson); 0.89 (Spearman)
Replicate scatter (raw; log2) for sample: 14706 (CFS)
Correlation coefficient: 0.89 (Pearson); 0.89 (Spearman)
Replicate scatter (normalized) for sample: 14706 (CFS)
Correlation coefficient: 0.87 (Pearson); 0.89 (Spearman)
Replicate scatter (normalized; log2) for sample: 14706 (CFS)
Correlation coefficient: 0.88 (Pearson); 0.89 (Spearman)
CIMva.102_Slide03_G2_L.14706_12.gpr
CIMva.102_Slide06_H3_L.14706_12.gpr

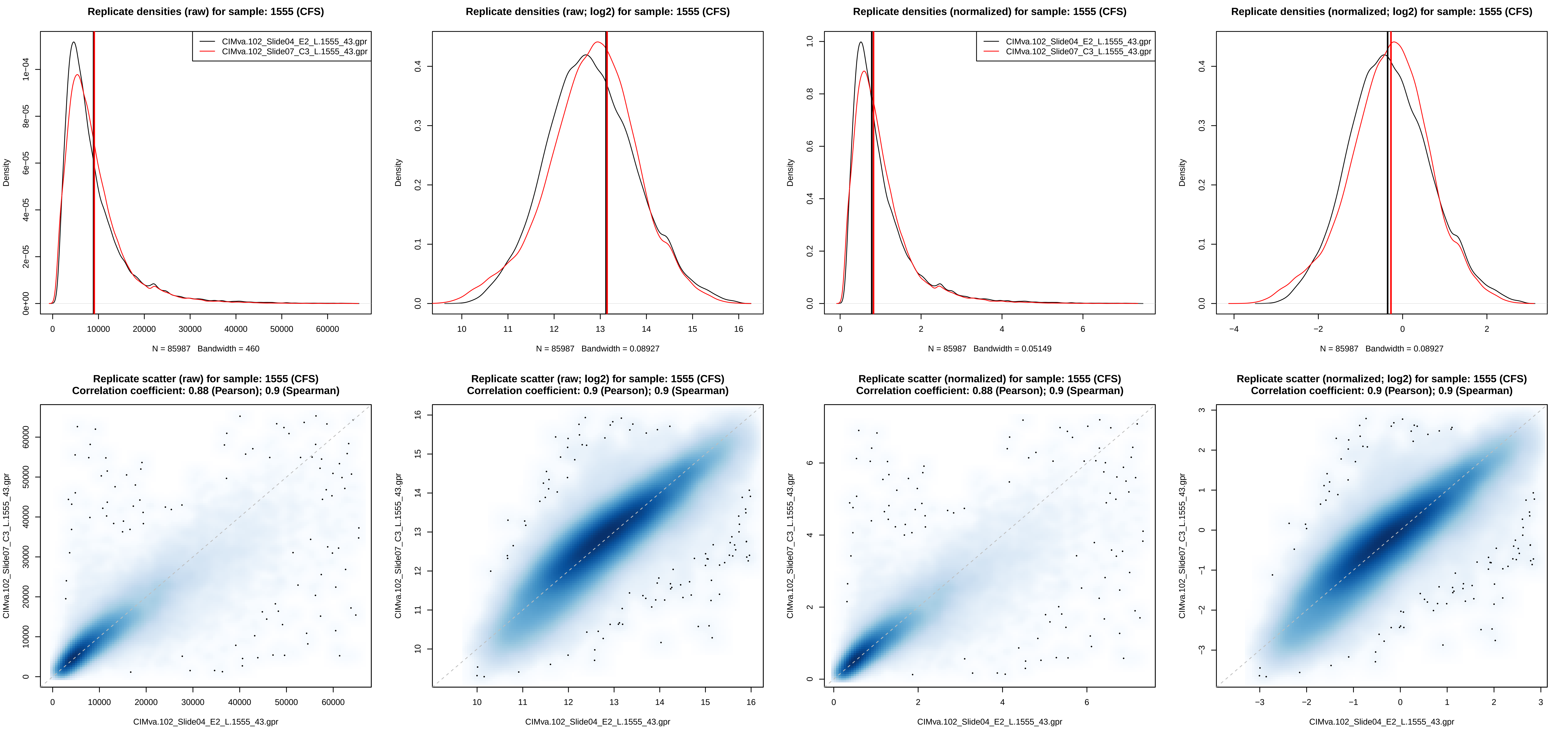

Replicate densities (raw) for sample: 1555 (CFS)
CIMva.102_Slide04_E2_L.1555_43.gpr
CIMva.102_Slide07_C3_L.1555_43.gpr
Density
N = 85987 Bandwidth = 460
Replicate densities (raw; log2) for sample: 1555 (CFS)
Density
N = 85987 Bandwidth = 0.08927
Replicate densities (normalized) for sample: 1555 (CFS)
CIMva.102_Slide04_E2_L.1555_43.gpr
CIMva.102_Slide07_C3_L.1555_43.gpr
Density
N = 85987 Bandwidth = 0.05149
Replicate densities (normalized; log2) for sample: 1555 (CFS)
Density
N = 85987 Bandwidth = 0.08927
Replicate scatter (raw) for sample: 1555 (CFS)
Correlation coefficient: 0.88 (Pearson); 0.9 (Spearman)
CIMva.102_Slide04_E2_L.1555_43.gpr
CIMva.102_Slide07_C3_L.1555_43.gpr
Replicate scatter (raw; log2) for sample: 1555 (CFS)
Correlation coefficient: 0.9 (Pearson); 0.9 (Spearman)
CIMva.102_Slide04_E2_L.1555_43.gpr
CIMva.102_Slide07_C3_L.1555_43.gpr
Replicate scatter (normalized) for sample: 1555 (CFS)
Correlation coefficient: 0.88 (Pearson); 0.9 (Spearman)
CIMva.102_Slide04_E2_L.1555_43.gpr
CIMva.102_Slide07_C3_L.1555_43.gpr
Replicate scatter (normalized; log2) for sample: 1555 (CFS)
Correlation coefficient: 0.9 (Pearson); 0.9 (Spearman)
CIMva.102_Slide04_E2_L.1555_43.gpr
CIMva.102_Slide07_C3_L.1555_43.gpr

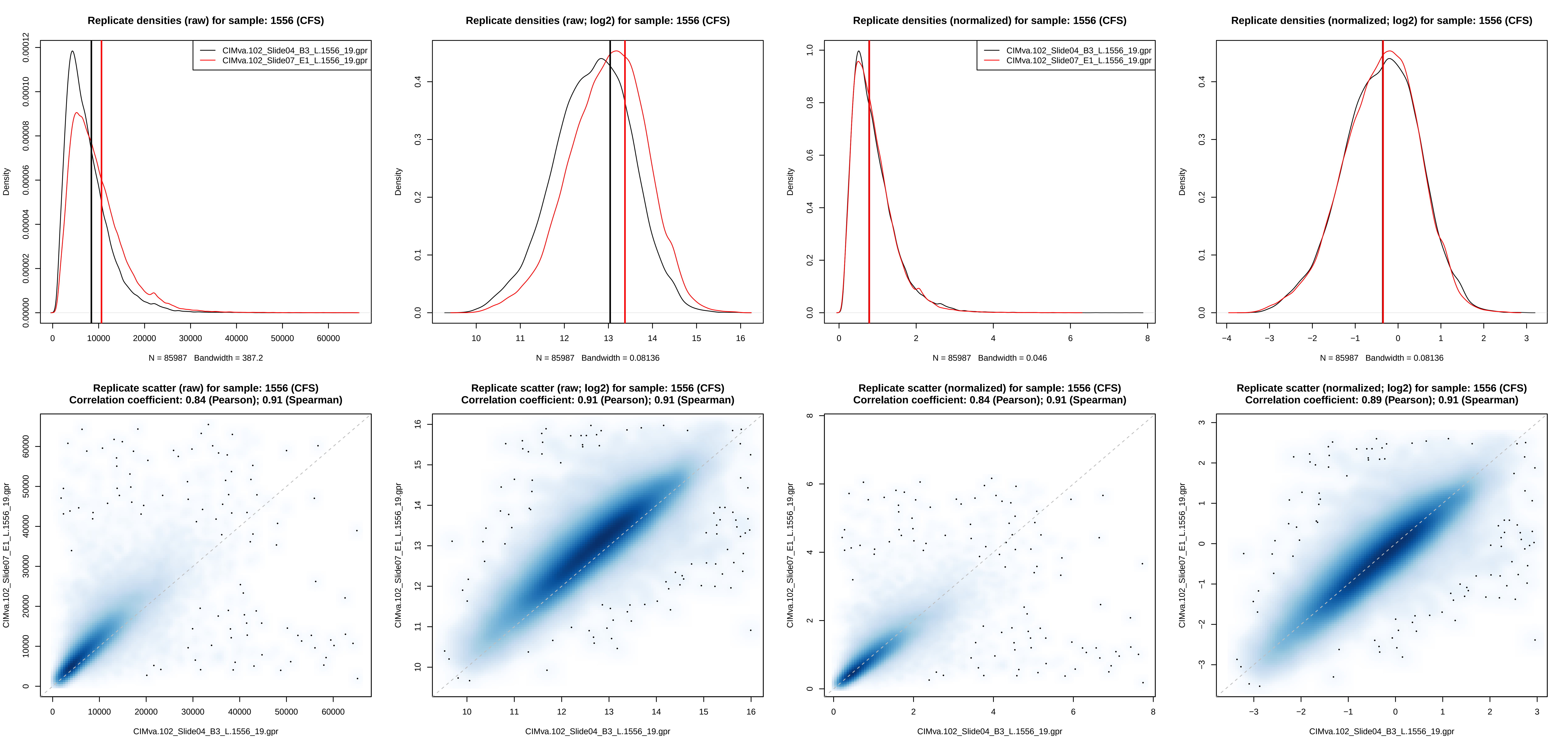
Replicate densities (raw) for sample: 1556 (CFS)
CIMva.102_Slide04_B3_L.1556_19.gpr
CIMva.102_Slide07_E1_L.1556_19.gpr
Density
N = 85987 Bandwidth = 387.2
Replicate densities (raw; log2) for sample: 1556 (CFS)
Density
N = 85987 Bandwidth = 0.08136
Replicate densities (normalized) for sample: 1556 (CFS)
CIMva.102_Slide04_B3_L.1556_19.gpr
CIMva.102_Slide07_E1_L.1556_19.gpr
Density
N = 85987 Bandwidth = 0.046
Replicate densities (normalized; log2) for sample: 1556 (CFS)
Density
N = 85987 Bandwidth = 0.08136
Replicate scatter (raw) for sample: 1556 (CFS)
Correlation coefficient: 0.84 (Pearson); 0.91 (Spearman)
CIMva.102_Slide07_E1_L.1556_19.gpr
CIMva.102_Slide04_B3_L.1556_19.gpr
Replicate scatter (raw; log2) for sample: 1556 (CFS)
Correlation coefficient: 0.91 (Pearson); 0.91 (Spearman)
CIMva.102_Slide07_E1_L.1556_19.gpr
CIMva.102_Slide04_B3_L.1556_19.gpr
Replicate scatter (normalized) for sample: 1556 (CFS)
Correlation coefficient: 0.84 (Pearson); 0.91 (Spearman)
CIMva.102_Slide07_E1_L.1556_19.gpr
CIMva.102_Slide04_B3_L.1556_19.gpr
Replicate scatter (normalized; log2) for sample: 1556 (CFS)
Correlation coefficient: 0.89 (Pearson); 0.91 (Spearman)
CIMva.102_Slide07_E1_L.1556_19.gpr
CIMva.102_Slide04_B3_L.1556_19.gpr

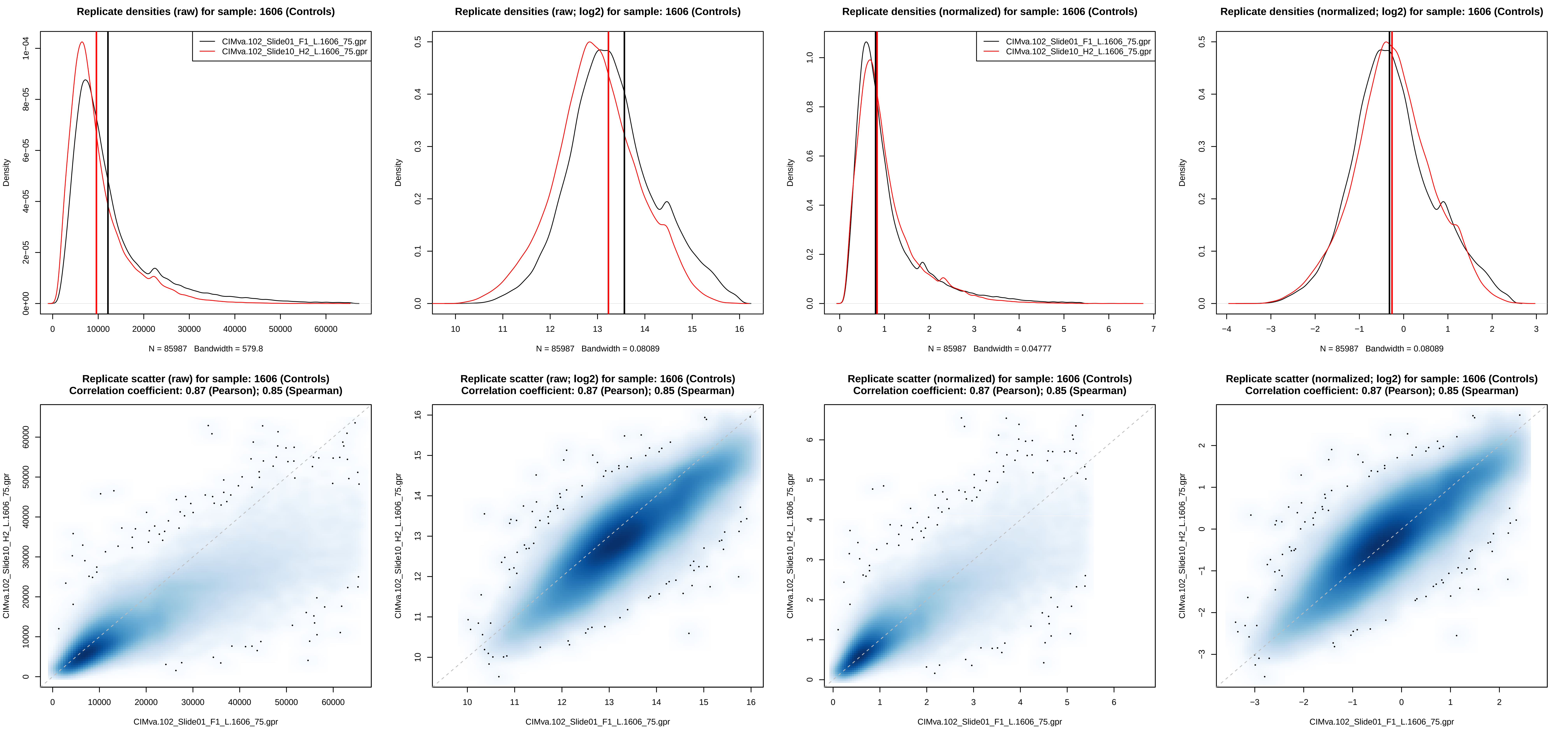
Replicate densities (raw) for sample: 1606 (Controls)
CIMva.102_Slide01_F1_L.1606_75.gpr
CIMva.102_Slide10_H2_L.1606_75.gpr
Density
N = 85987 Bandwidth = 579.8
Replicate densities (raw; log2) for sample: 1606 (Controls)
N = 85987 Bandwidth = 0.08089
Replicate densities (normalized) for sample: 1606 (Controls)
N = 85987 Bandwidth = 0.04777
Replicate densities (normalized; log2) for sample: 1606 (Controls)
N = 85987 Bandwidth = 0.08089
Replicate scatter (raw) for sample: 1606 (Controls)
Correlation coefficient: 0.87 (Pearson); 0.85 (Spearman)
Replicate scatter (raw; log2) for sample: 1606 (Controls)
Correlation coefficient: 0.87 (Pearson); 0.85 (Spearman)
Replicate scatter (normalized) for sample: 1606 (Controls)
Correlation coefficient: 0.87 (Pearson); 0.85 (Spearman)
Replicate scatter (normalized; log2) for sample: 1606 (Controls)
Correlation coefficient: 0.87 (Pearson); 0.85 (Spearman)
CIMva.102_Slide01_F1_L.1606_75.gpr
CIMva.102_Slide10_H2_L.1606_75.gpr

Replicate densities (raw) for sample: 2092 (Controls)

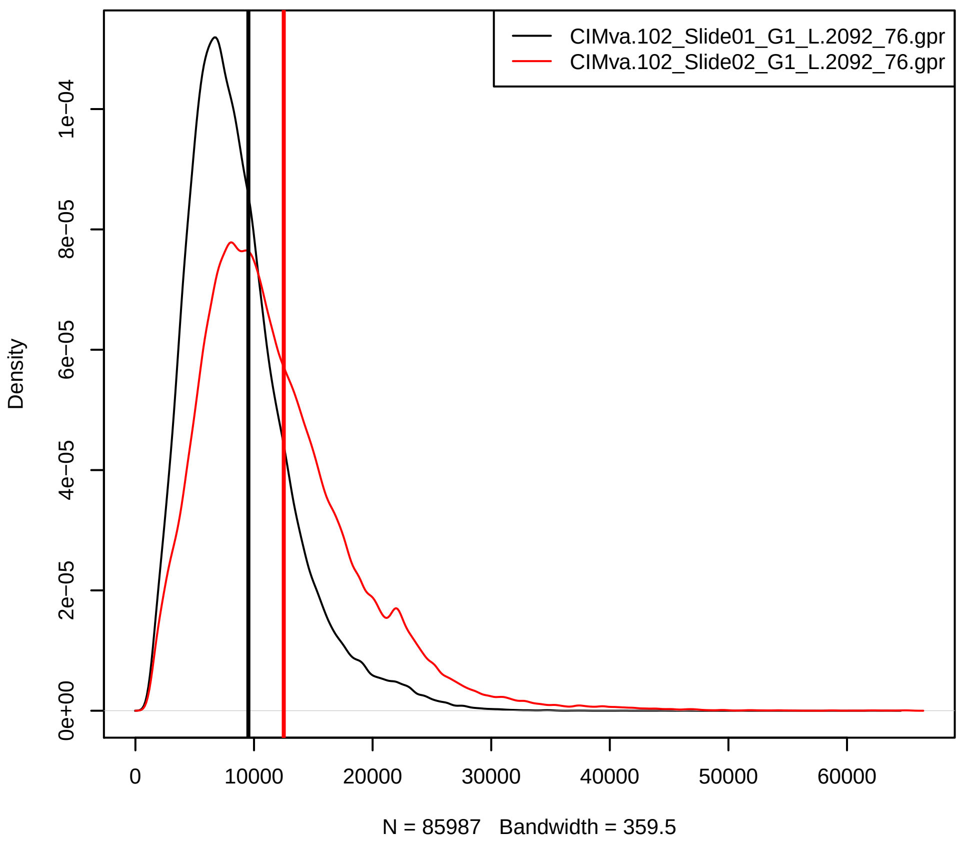

Replicate densities (raw; log2) for sample: 2092 (Controls)

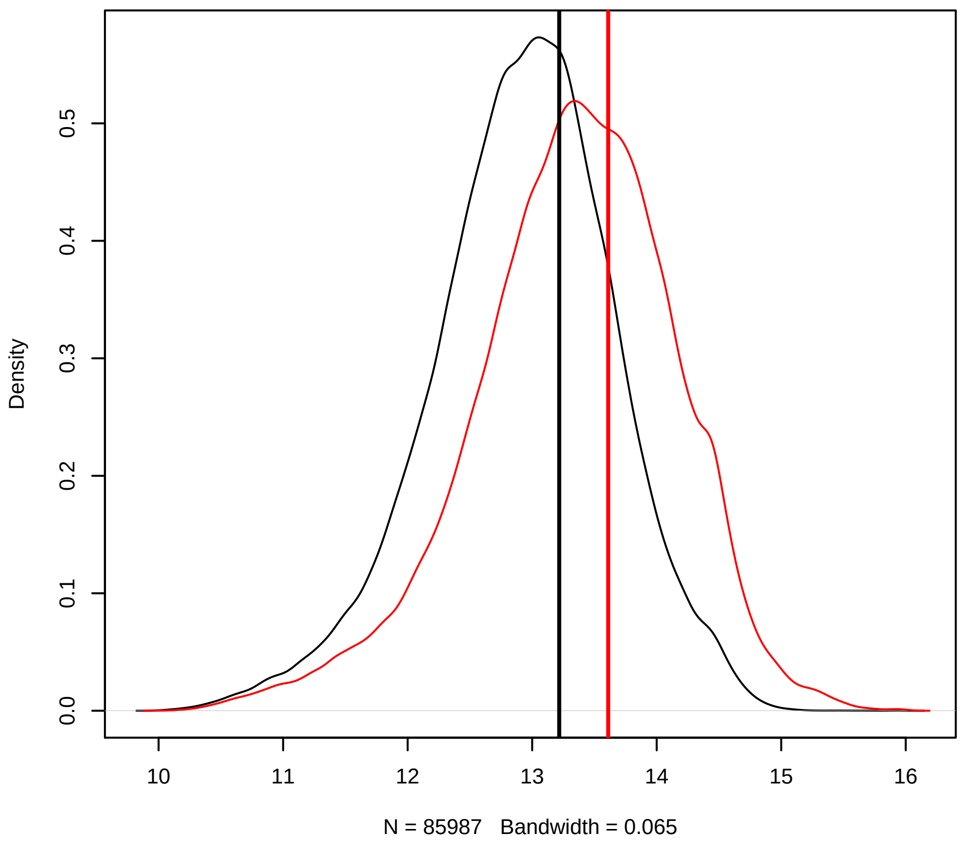

Replicate densities (normalized) for sample: 2092 (Controls)

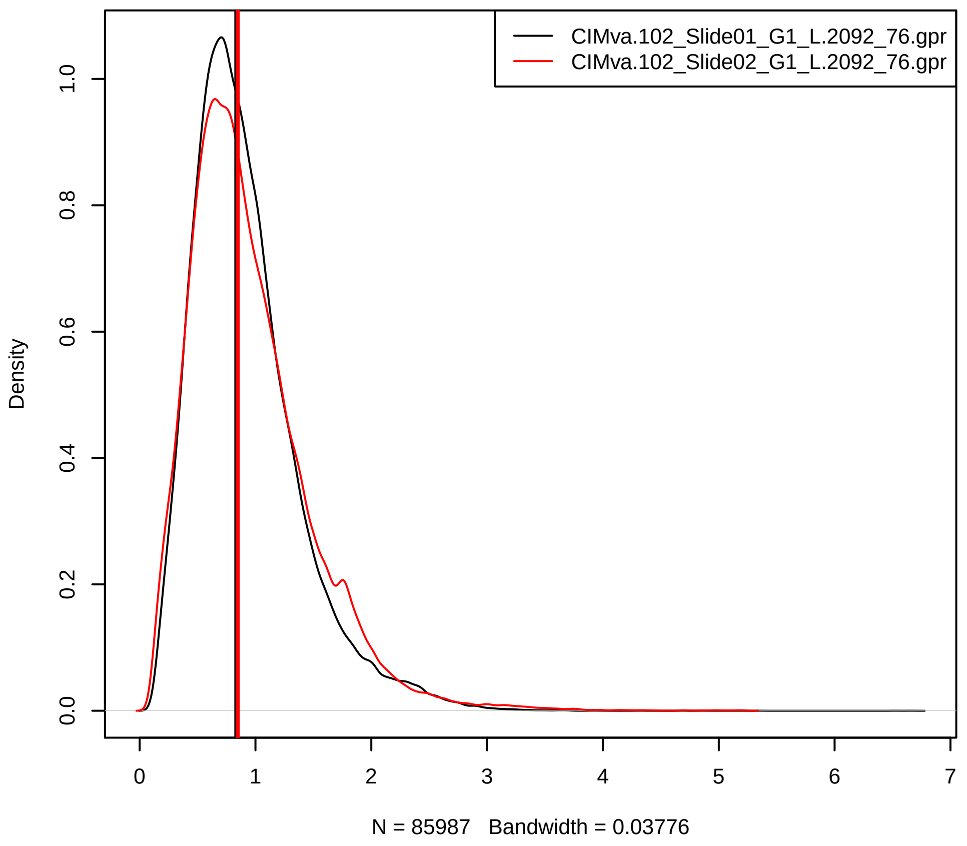

Replicate densities (normalized; log2) for sample: 2092 (Controls)

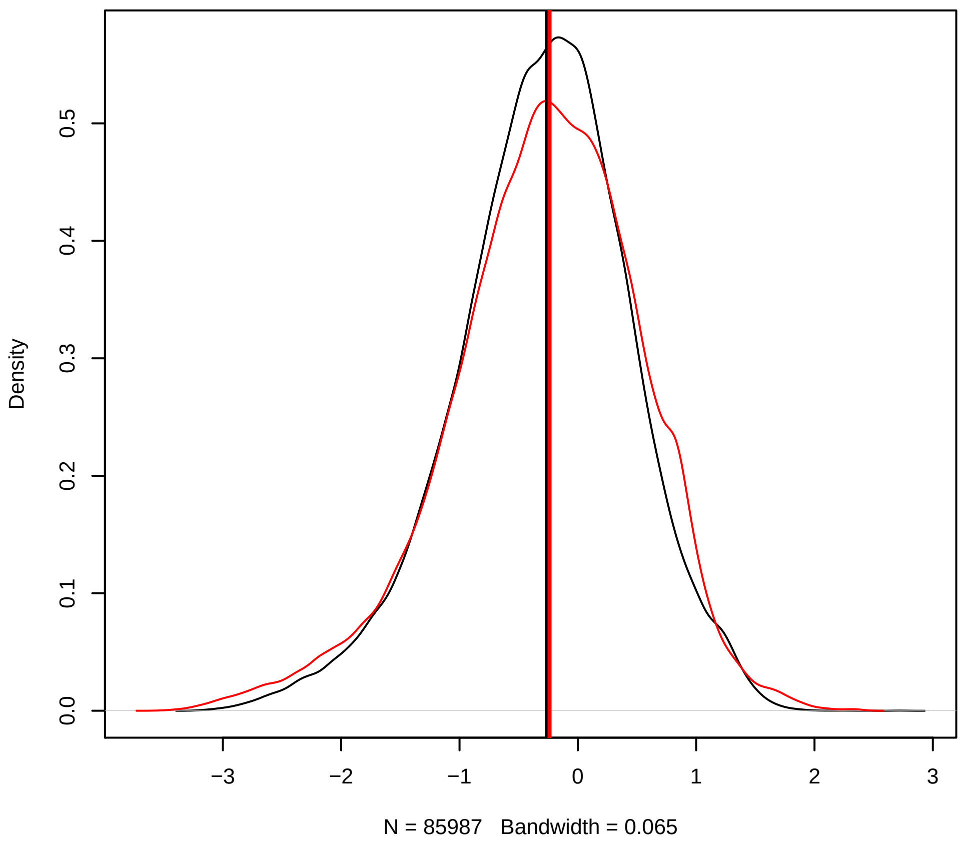

Replicate scatter (raw) for sample: 2092 (Controls)
Correlation coefficient: 0.84 (Pearson); 0.87 (Spearman)

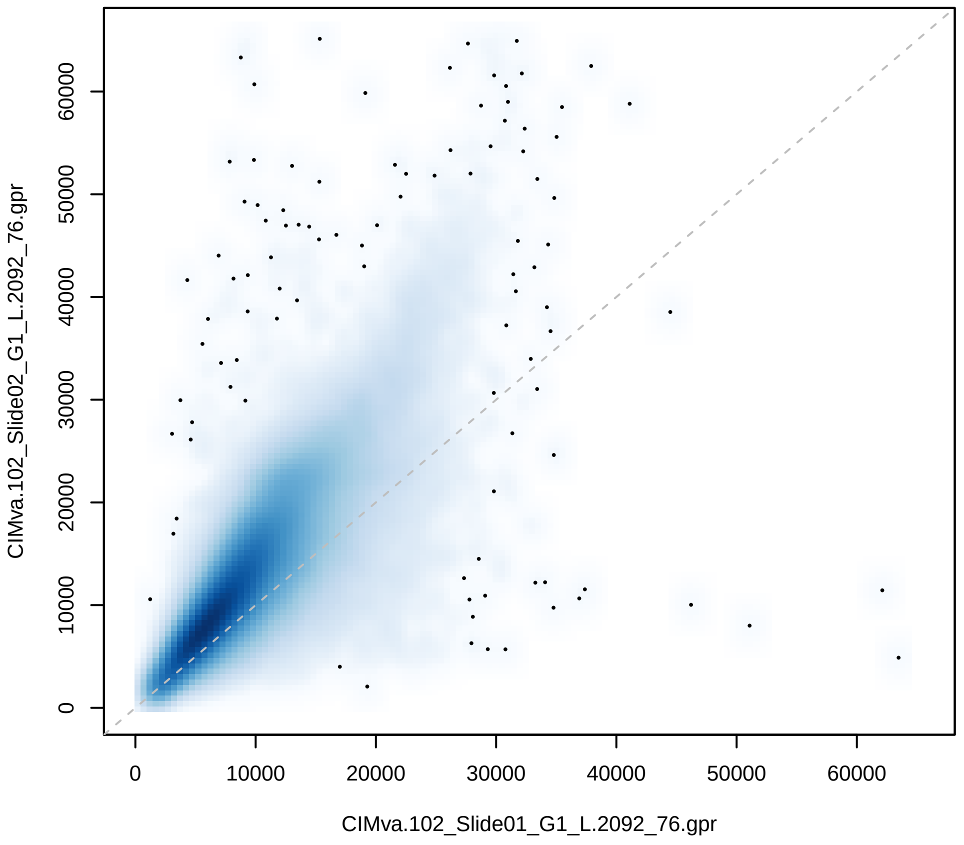

Replicate scatter (raw; log2) for sample: 2092 (Controls)
Correlation coefficient: 0.88 (Pearson); 0.87 (Spearman)

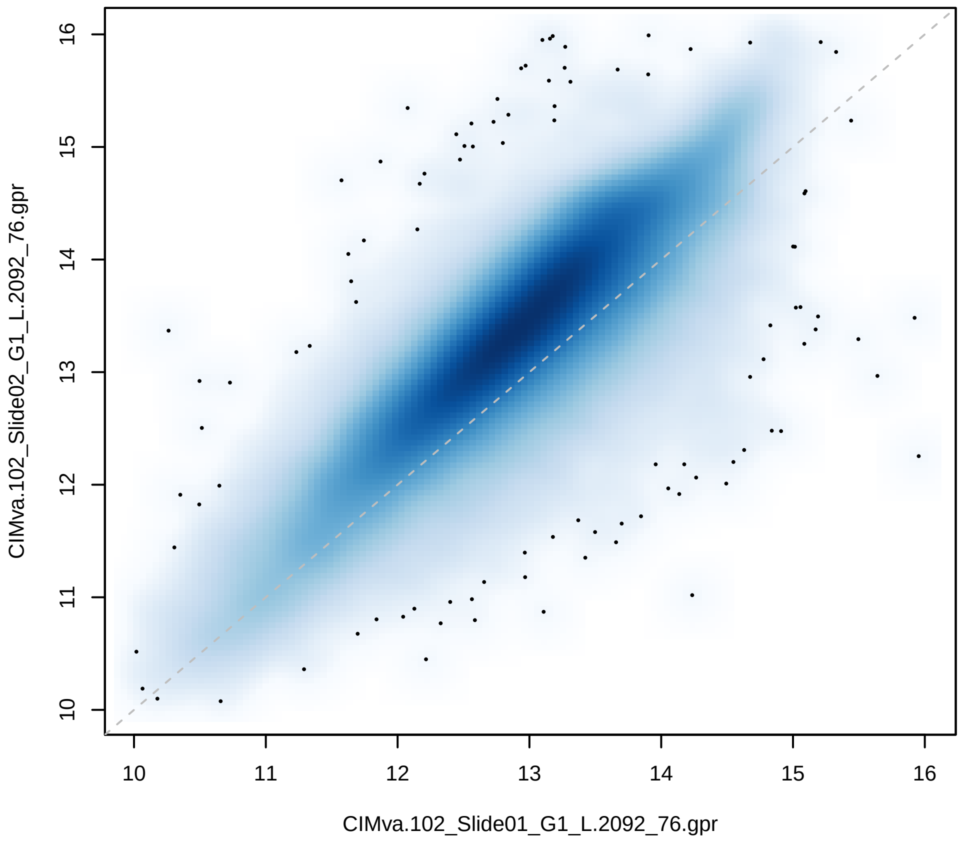

Replicate scatter (normalized) for sample: 2092 (Controls)
Correlation coefficient: 0.84 (Pearson); 0.87 (Spearman)

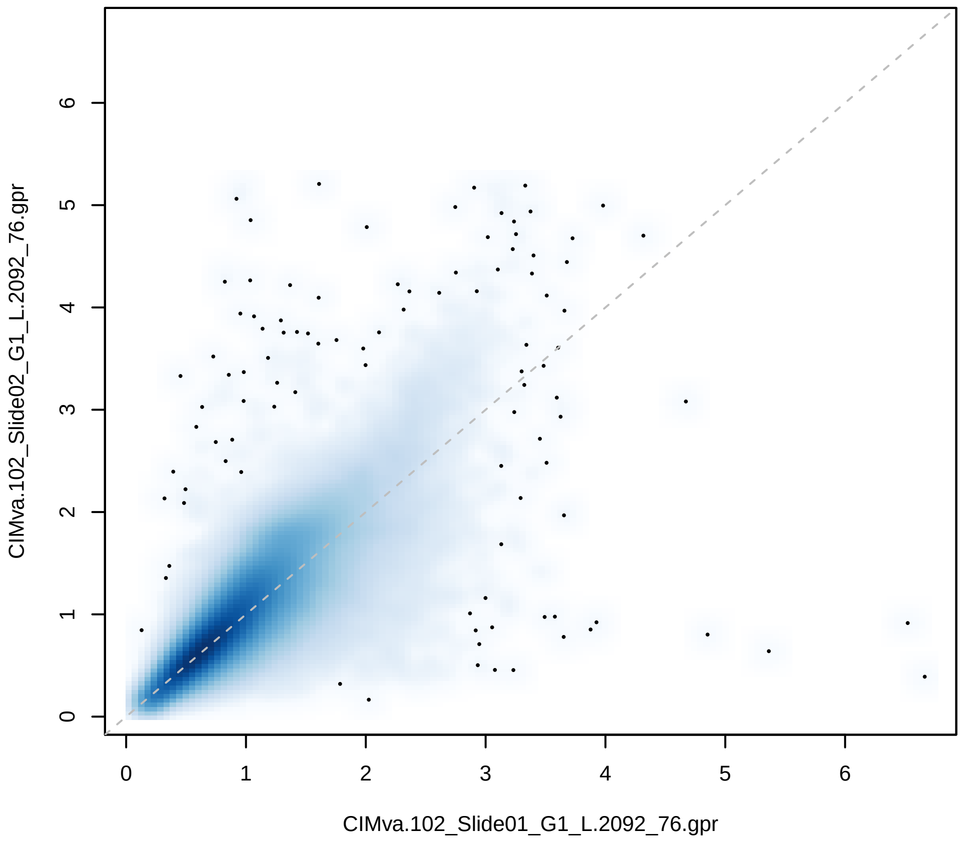

Replicate scatter (normalized; log2) for sample: 2092 (Controls)
Correlation coefficient: 0.86 (Pearson); 0.87 (Spearman)

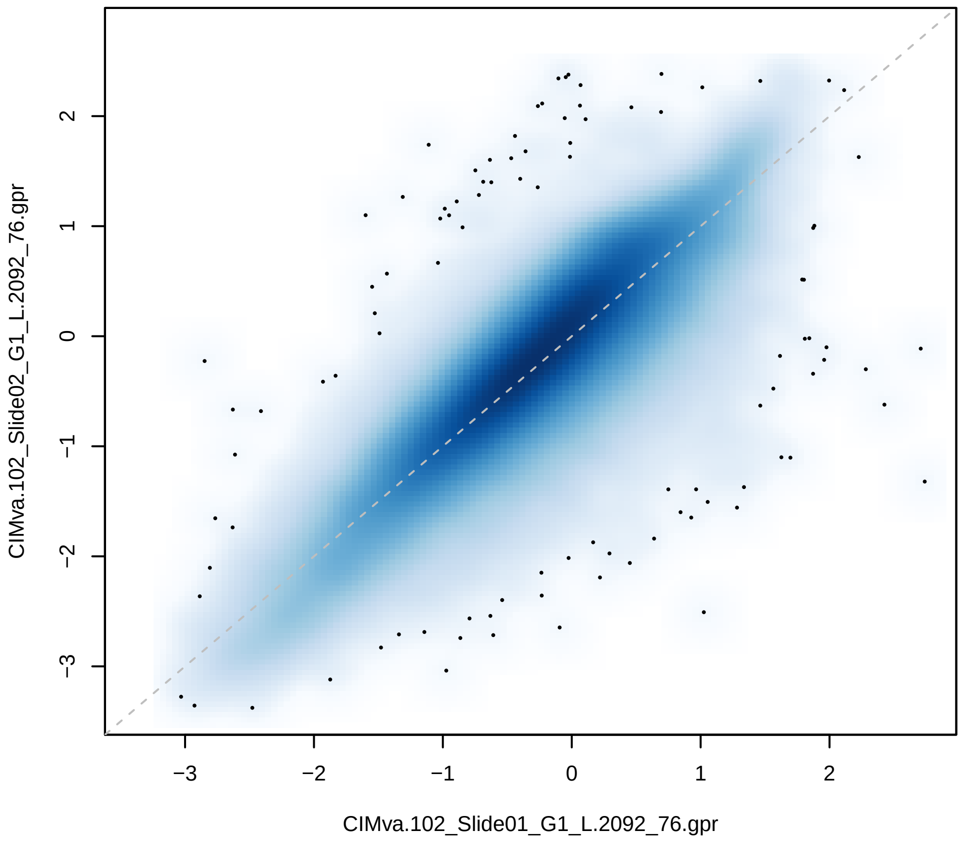

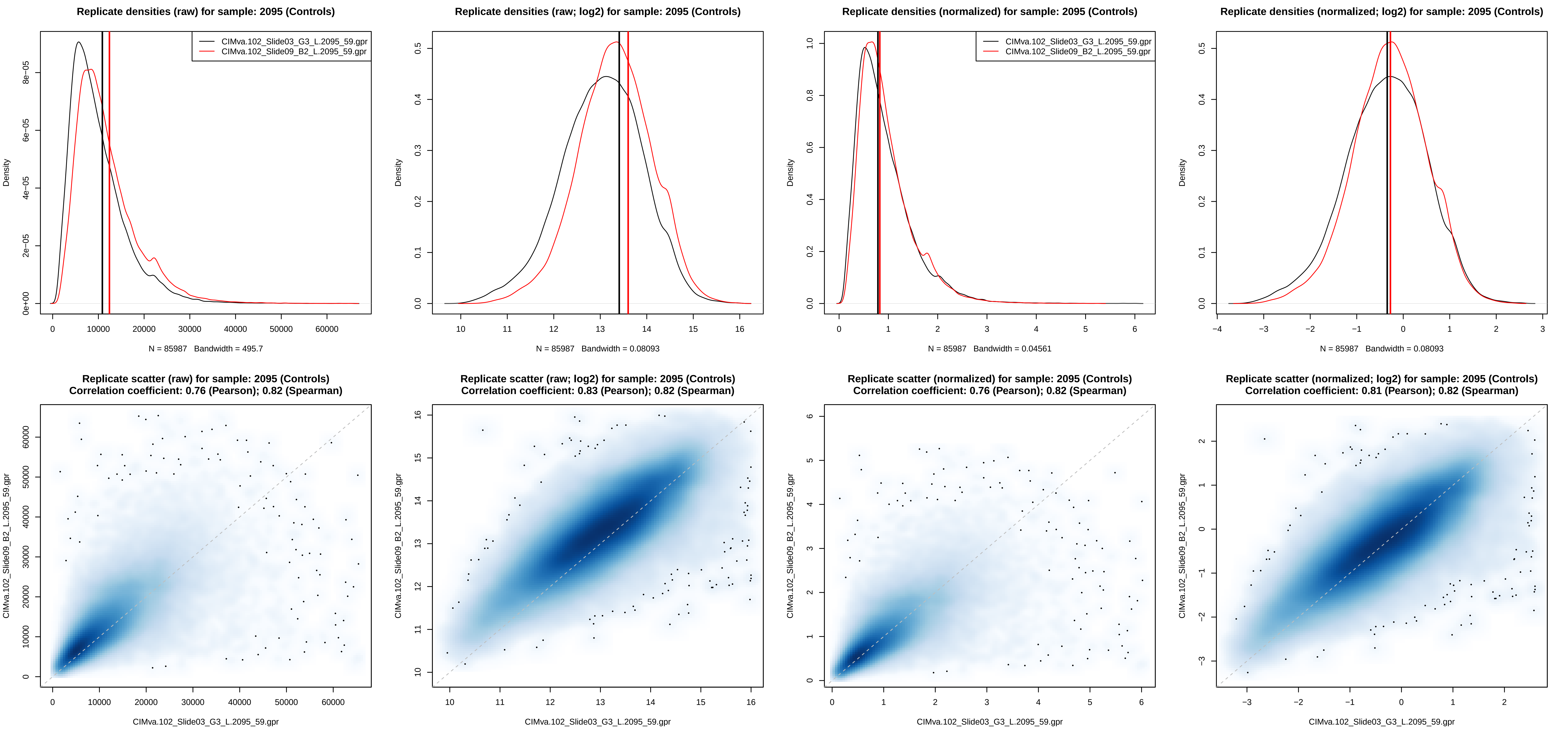
Replicate densities (raw) for sample: 2095 (Controls)
CIMva.102_Slide03_G3_L.2095_59.gpr
CIMva.102_Slide09_B2_L.2095_59.gpr
Density
N = 85987 Bandwidth = 495.7
Replicate densities (raw; log2) for sample: 2095 (Controls)
Density
N = 85987 Bandwidth = 0.08093
Replicate densities (normalized) for sample: 2095 (Controls)
CIMva.102_Slide03_G3_L.2095_59.gpr
CIMva.102_Slide09_B2_L.2095_59.gpr
Density
N = 85987 Bandwidth = 0.04561
Replicate densities (normalized; log2) for sample: 2095 (Controls)
Density
N = 85987 Bandwidth = 0.08093
Replicate scatter (raw) for sample: 2095 (Controls)
Correlation coefficient: 0.76 (Pearson); 0.82 (Spearman)
CIMva.102_Slide09_B2_L.2095_59.gpr
CIMva.102_Slide03_G3_L.2095_59.gpr
Replicate scatter (raw; log2) for sample: 2095 (Controls)
Correlation coefficient: 0.83 (Pearson); 0.82 (Spearman)
CIMva.102_Slide09_B2_L.2095_59.gpr
CIMva.102_Slide03_G3_L.2095_59.gpr
Replicate scatter (normalized) for sample: 2095 (Controls)
Correlation coefficient: 0.76 (Pearson); 0.82 (Spearman)
CIMva.102_Slide09_B2_L.2095_59.gpr
CIMva.102_Slide03_G3_L.2095_59.gpr
Replicate scatter (normalized; log2) for sample: 2095 (Controls)
Correlation coefficient: 0.81 (Pearson); 0.82 (Spearman)
CIMva.102_Slide09_B2_L.2095_59.gpr
CIMva.102_Slide03_G3_L.2095_59.gpr

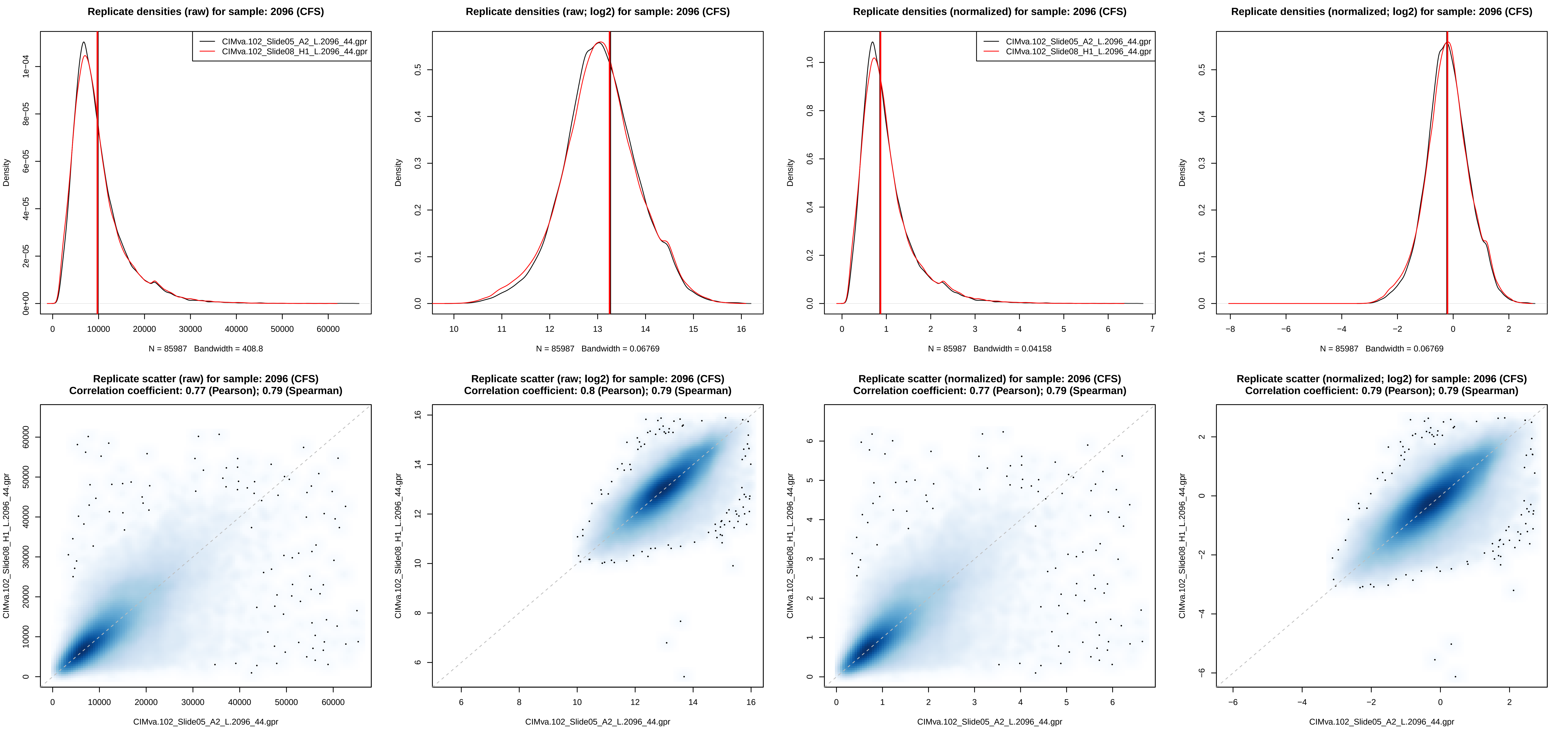
Replicate densities (raw) for sample: 2096 (CFS)
CIMva.102_Slide05_A2_L.2096_44.gpr
CIMva.102_Slide08_H1_L.2096_44.gpr
Density
N = 85987 Bandwidth = 408.8
Replicate densities (raw; log2) for sample: 2096 (CFS)
Density
N = 85987 Bandwidth = 0.06769
Replicate densities (normalized) for sample: 2096 (CFS)
CIMva.102_Slide05_A2_L.2096_44.gpr
CIMva.102_Slide08_H1_L.2096_44.gpr
Density
N = 85987 Bandwidth = 0.04158
Replicate densities (normalized; log2) for sample: 2096 (CFS)
Density
N = 85987 Bandwidth = 0.06769
Replicate scatter (raw) for sample: 2096 (CFS)
Correlation coefficient: 0.77 (Pearson); 0.79 (Spearman)
CIMva.102_Slide05_A2_L.2096_44.gpr
CIMva.102_Slide08_H1_L.2096_44.gpr
Replicate scatter (raw; log2) for sample: 2096 (CFS)
Correlation coefficient: 0.8 (Pearson); 0.79 (Spearman)
CIMva.102_Slide05_A2_L.2096_44.gpr
CIMva.102_Slide08_H1_L.2096_44.gpr
Replicate scatter (normalized) for sample: 2096 (CFS)
Correlation coefficient: 0.77 (Pearson); 0.79 (Spearman)
CIMva.102_Slide05_A2_L.2096_44.gpr
CIMva.102_Slide08_H1_L.2096_44.gpr
Replicate scatter (normalized; log2) for sample: 2096 (CFS)
Correlation coefficient: 0.79 (Pearson); 0.79 (Spearman)
CIMva.102_Slide05_A2_L.2096_44.gpr
CIMva.102_Slide08_H1_L.2096_44.gpr

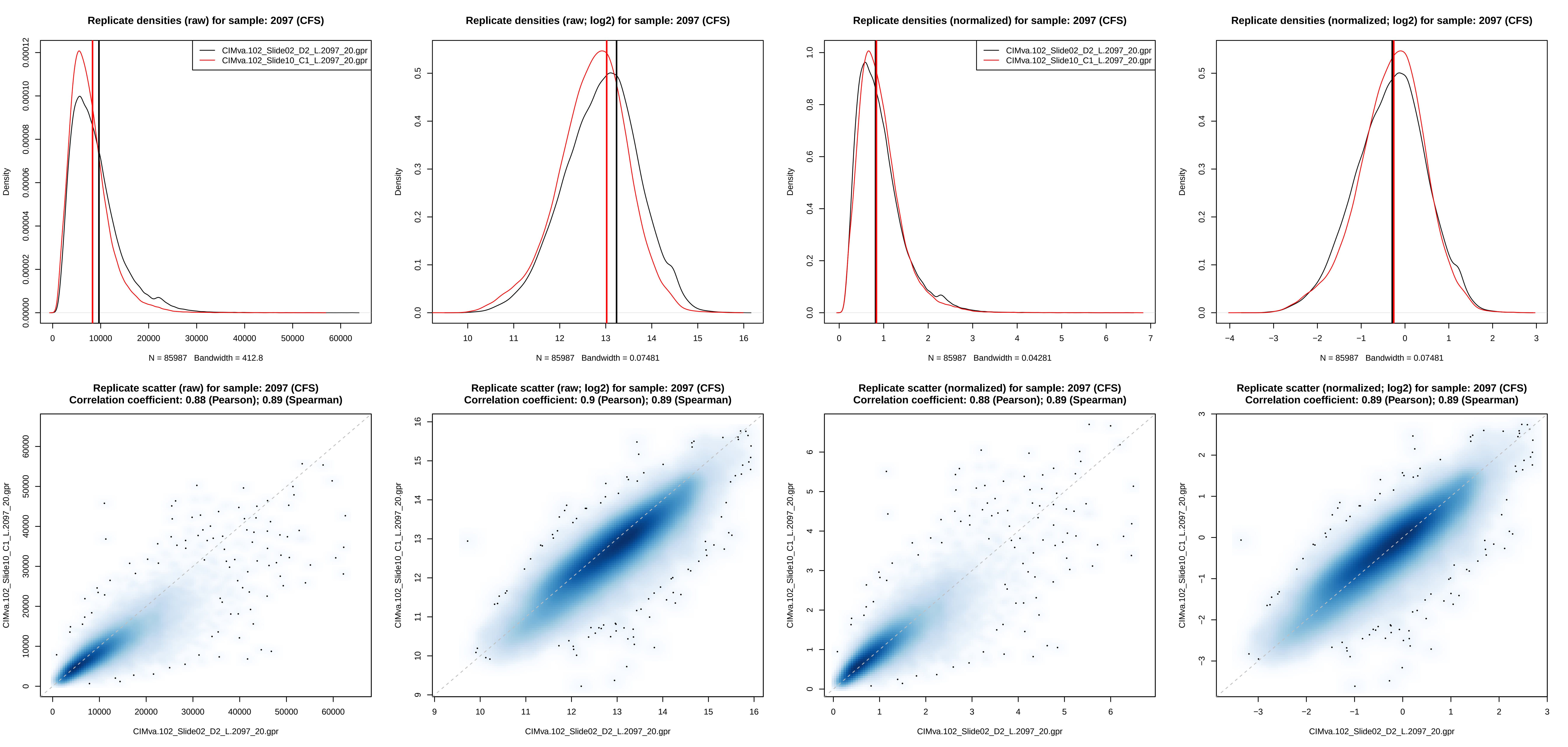
Replicate densities (raw) for sample: 2097 (CFS)
CIMva.102_Slide02_D2_L.2097_20.gpr
CIMva.102_Slide10_C1_L.2097_20.gpr
Density
N = 85987 Bandwidth = 412.8
Replicate densities (raw; log2) for sample: 2097 (CFS)
Density
N = 85987 Bandwidth = 0.07481
Replicate densities (normalized) for sample: 2097 (CFS)
CIMva.102_Slide02_D2_L.2097_20.gpr
CIMva.102_Slide10_C1_L.2097_20.gpr
Density
N = 85987 Bandwidth = 0.04281
Replicate densities (normalized; log2) for sample: 2097 (CFS)
Density
N = 85987 Bandwidth = 0.07481
Replicate scatter (raw) for sample: 2097 (CFS)
Correlation coefficient: 0.88 (Pearson); 0.89 (Spearman)
CIMva.102_Slide10_C1_L.2097_20.gpr
CIMva.102_Slide02_D2_L.2097_20.gpr
Replicate scatter (raw; log2) for sample: 2097 (CFS)
Correlation coefficient: 0.9 (Pearson); 0.89 (Spearman)
CIMva.102_Slide10_C1_L.2097_20.gpr
CIMva.102_Slide02_D2_L.2097_20.gpr
Replicate scatter (normalized) for sample: 2097 (CFS)
Correlation coefficient: 0.88 (Pearson); 0.89 (Spearman)
CIMva.102_Slide10_C1_L.2097_20.gpr
CIMva.102_Slide02_D2_L.2097_20.gpr
Replicate scatter (normalized; log2) for sample: 2097 (CFS)
Correlation coefficient: 0.89 (Pearson); 0.89 (Spearman)
CIMva.102_Slide10_C1_L.2097_20.gpr
CIMva.102_Slide02_D2_L.2097_20.gpr

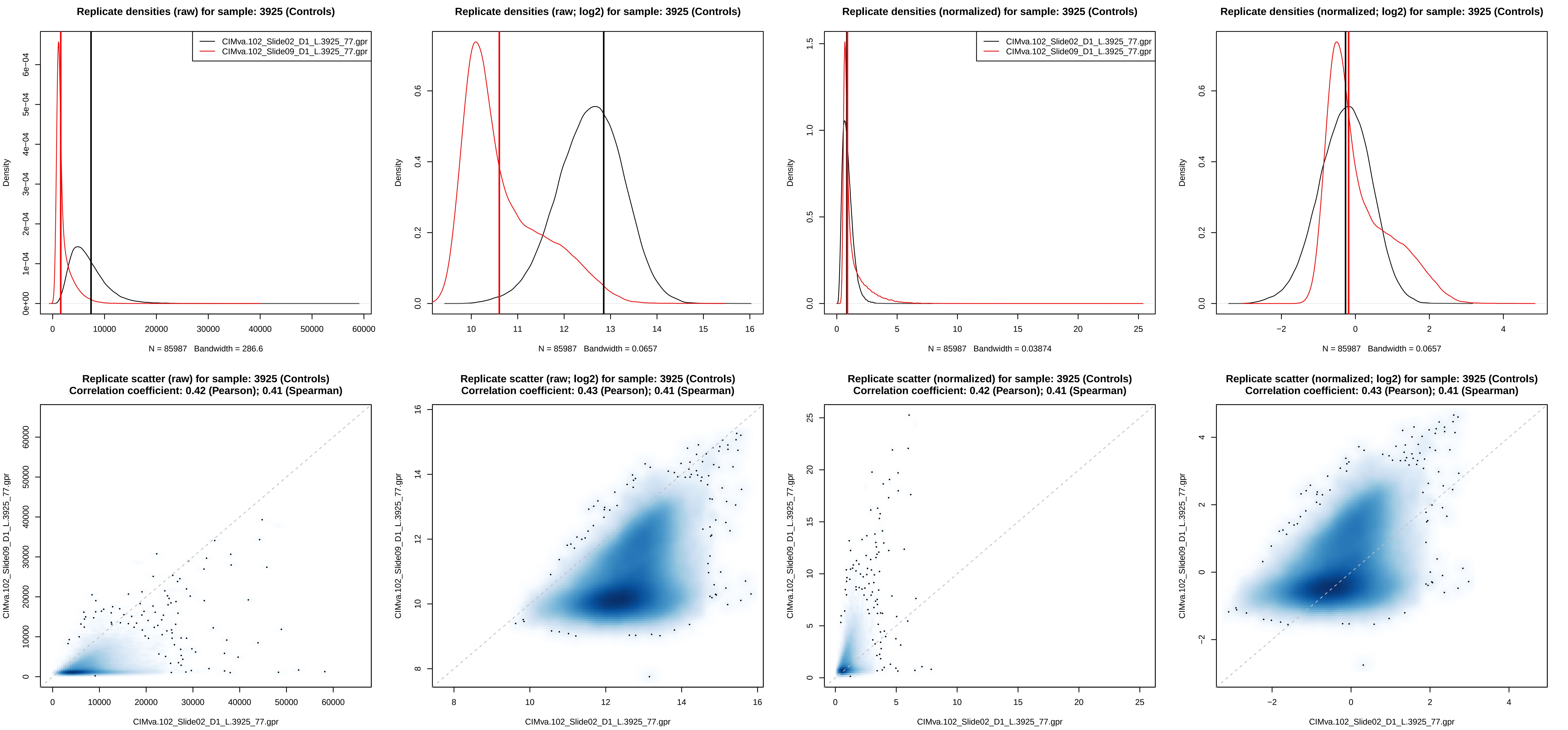

Replicate densities (raw) for sample: 3925 (Controls)
CIMva.102_Slide02_D1_L.3925_77.gpr
CIMva.102_Slide09_D1_L.3925_77.gpr
Density
0e+00
1e−04
2e−04
3e−04
4e−04
5e−04
6e−04
0
10000
20000
30000
40000
50000
60000
N = 85987 Bandwidth = 286.6
Replicate densities (raw; log2) for sample: 3925 (Controls)
Density
0.0
0.2
0.4
0.6
10
11
12
13
14
15
16
N = 85987 Bandwidth = 0.0657
Replicate densities (normalized) for sample: 3925 (Controls)
CIMva.102_Slide02_D1_L.3925_77.gpr
CIMva.102_Slide09_D1_L.3925_77.gpr
Density
0.0
0.5
1.0
1.5
0
5
10
15
20
25
N = 85987 Bandwidth = 0.03874
Replicate densities (normalized; log2) for sample: 3925 (Controls)
Density
0.0
0.2
0.4
0.6
−2
0
2
4
N = 85987 Bandwidth = 0.0657
Replicate scatter (raw) for sample: 3925 (Controls)
Correlation coefficient: 0.42 (Pearson); 0.41 (Spearman)
CIMva.102_Slide09_D1_L.3925_77.gpr
CIMva.102_Slide02_D1_L.3925_77.gpr
0
10000
20000
30000
40000
50000
60000
Replicate scatter (raw; log2) for sample: 3925 (Controls)
Correlation coefficient: 0.43 (Pearson); 0.41 (Spearman)
CIMva.102_Slide09_D1_L.3925_77.gpr
CIMva.102_Slide02_D1_L.3925_77.gpr
8
10
12
14
16
Replicate scatter (normalized) for sample: 3925 (Controls)
Correlation coefficient: 0.42 (Pearson); 0.41 (Spearman)
CIMva.102_Slide09_D1_L.3925_77.gpr
CIMva.102_Slide02_D1_L.3925_77.gpr
0
5
10
15
20
25
Replicate scatter (normalized; log2) for sample: 3925 (Controls)
Correlation coefficient: 0.43 (Pearson); 0.41 (Spearman)
CIMva.102_Slide09_D1_L.3925_77.gpr
CIMva.102_Slide02_D1_L.3925_77.gpr
−2
0
2
4

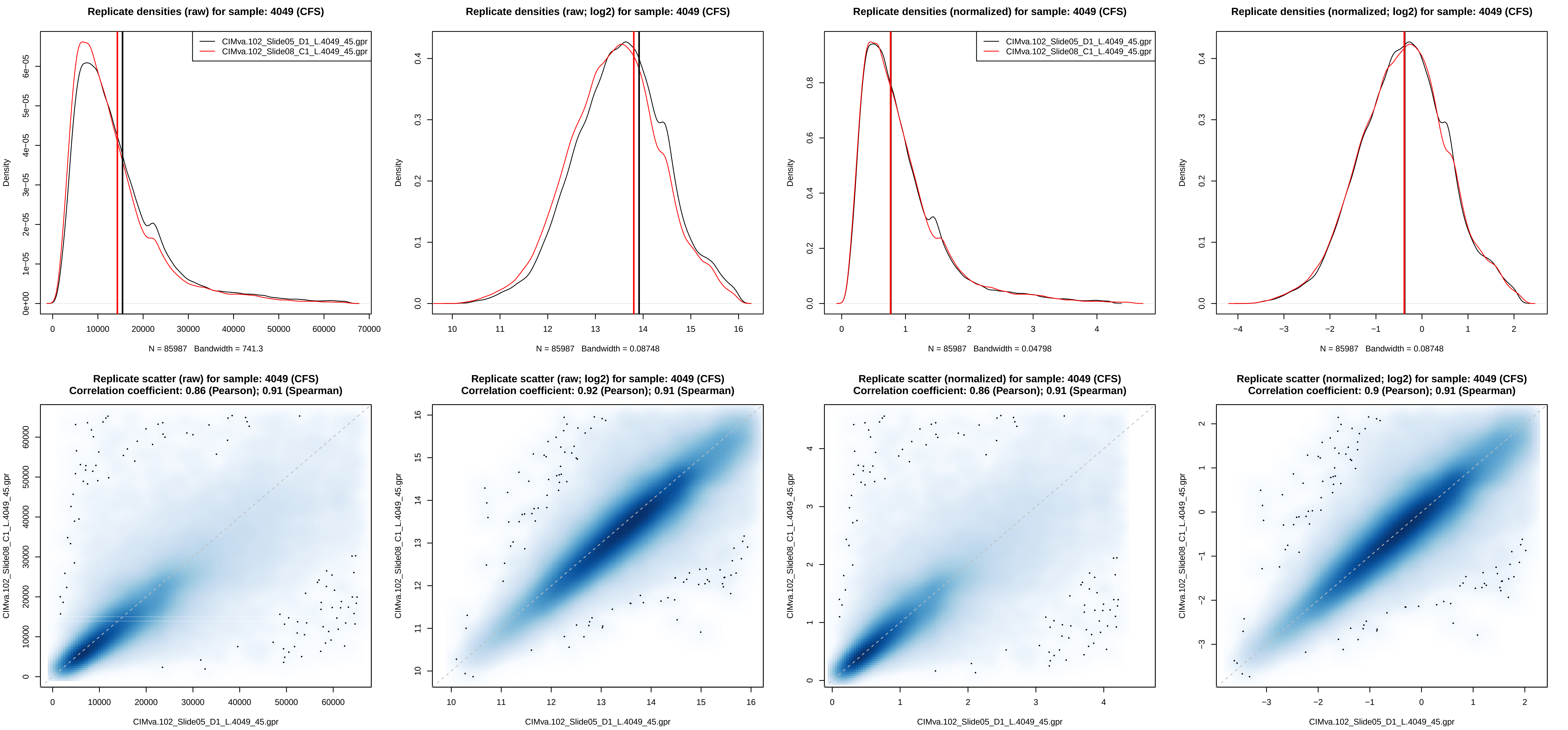
Replicate densities (raw) for sample: 4049 (CFS)
CIMva.102_Slide05_D1_L.4049_45.gpr
CIMva.102_Slide08_C1_L.4049_45.gpr
Density
N = 85987 Bandwidth = 741.3
Replicate densities (raw; log2) for sample: 4049 (CFS)
Density
N = 85987 Bandwidth = 0.08748
Replicate densities (normalized) for sample: 4049 (CFS)
CIMva.102_Slide05_D1_L.4049_45.gpr
CIMva.102_Slide08_C1_L.4049_45.gpr
Density
N = 85987 Bandwidth = 0.04798
Replicate densities (normalized; log2) for sample: 4049 (CFS)
Density
N = 85987 Bandwidth = 0.08748
Replicate scatter (raw) for sample: 4049 (CFS)
Correlation coefficient: 0.86 (Pearson); 0.91 (Spearman)
CIMva.102_Slide08_C1_L.4049_45.gpr
CIMva.102_Slide05_D1_L.4049_45.gpr
Replicate scatter (raw; log2) for sample: 4049 (CFS)
Correlation coefficient: 0.92 (Pearson); 0.91 (Spearman)
CIMva.102_Slide08_C1_L.4049_45.gpr
CIMva.102_Slide05_D1_L.4049_45.gpr
Replicate scatter (normalized) for sample: 4049 (CFS)
Correlation coefficient: 0.86 (Pearson); 0.91 (Spearman)
CIMva.102_Slide08_C1_L.4049_45.gpr
CIMva.102_Slide05_D1_L.4049_45.gpr
Replicate scatter (normalized; log2) for sample: 4049 (CFS)
Correlation coefficient: 0.9 (Pearson); 0.91 (Spearman)
CIMva.102_Slide08_C1_L.4049_45.gpr
CIMva.102_Slide05_D1_L.4049_45.gpr

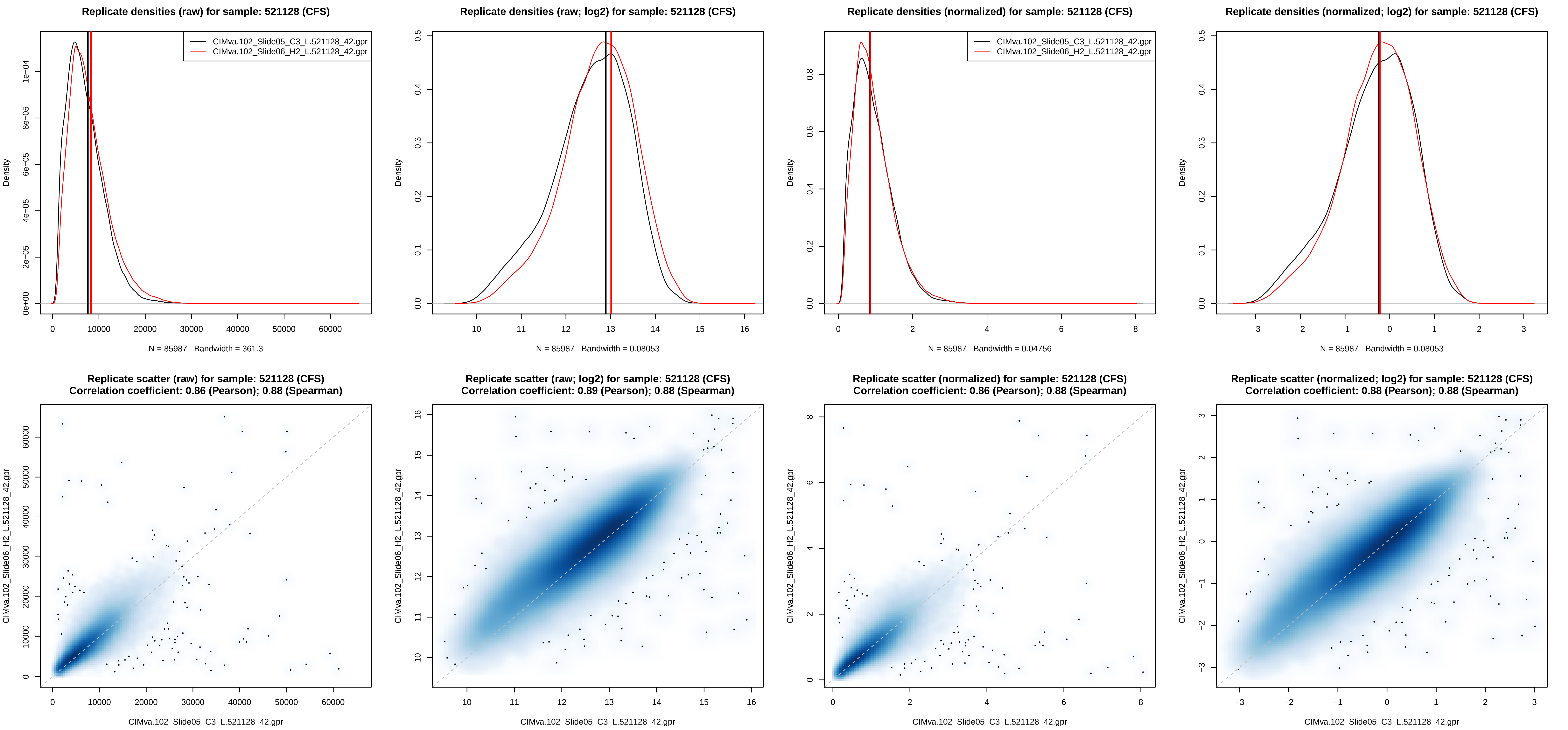

Replicate densities (raw) for sample: 521128 (CFS)
CIMva.102_Slide05_C3_L.521128_42.gpr
CIMva.102_Slide06_H2_L.521128_42.gpr
Density
N = 85987 Bandwidth = 361.3
Replicate densities (raw; log2) for sample: 521128 (CFS)
Density
N = 85987 Bandwidth = 0.08053
Replicate densities (normalized) for sample: 521128 (CFS)
CIMva.102_Slide05_C3_L.521128_42.gpr
CIMva.102_Slide06_H2_L.521128_42.gpr
Density
N = 85987 Bandwidth = 0.04756
Replicate densities (normalized; log2) for sample: 521128 (CFS)
Density
N = 85987 Bandwidth = 0.08053
Replicate scatter (raw) for sample: 521128 (CFS)
Correlation coefficient: 0.86 (Pearson); 0.88 (Spearman)
CIMva.102_Slide06_H2_L.521128_42.gpr
CIMva.102_Slide05_C3_L.521128_42.gpr
Replicate scatter (raw; log2) for sample: 521128 (CFS)
Correlation coefficient: 0.89 (Pearson); 0.88 (Spearman)
CIMva.102_Slide06_H2_L.521128_42.gpr
CIMva.102_Slide05_C3_L.521128_42.gpr
Replicate scatter (normalized) for sample: 521128 (CFS)
Correlation coefficient: 0.86 (Pearson); 0.88 (Spearman)
CIMva.102_Slide06_H2_L.521128_42.gpr
CIMva.102_Slide05_C3_L.521128_42.gpr
Replicate scatter (normalized; log2) for sample: 521128 (CFS)
Correlation coefficient: 0.88 (Pearson); 0.88 (Spearman)
CIMva.102_Slide06_H2_L.521128_42.gpr
CIMva.102_Slide05_C3_L.521128_42.gpr

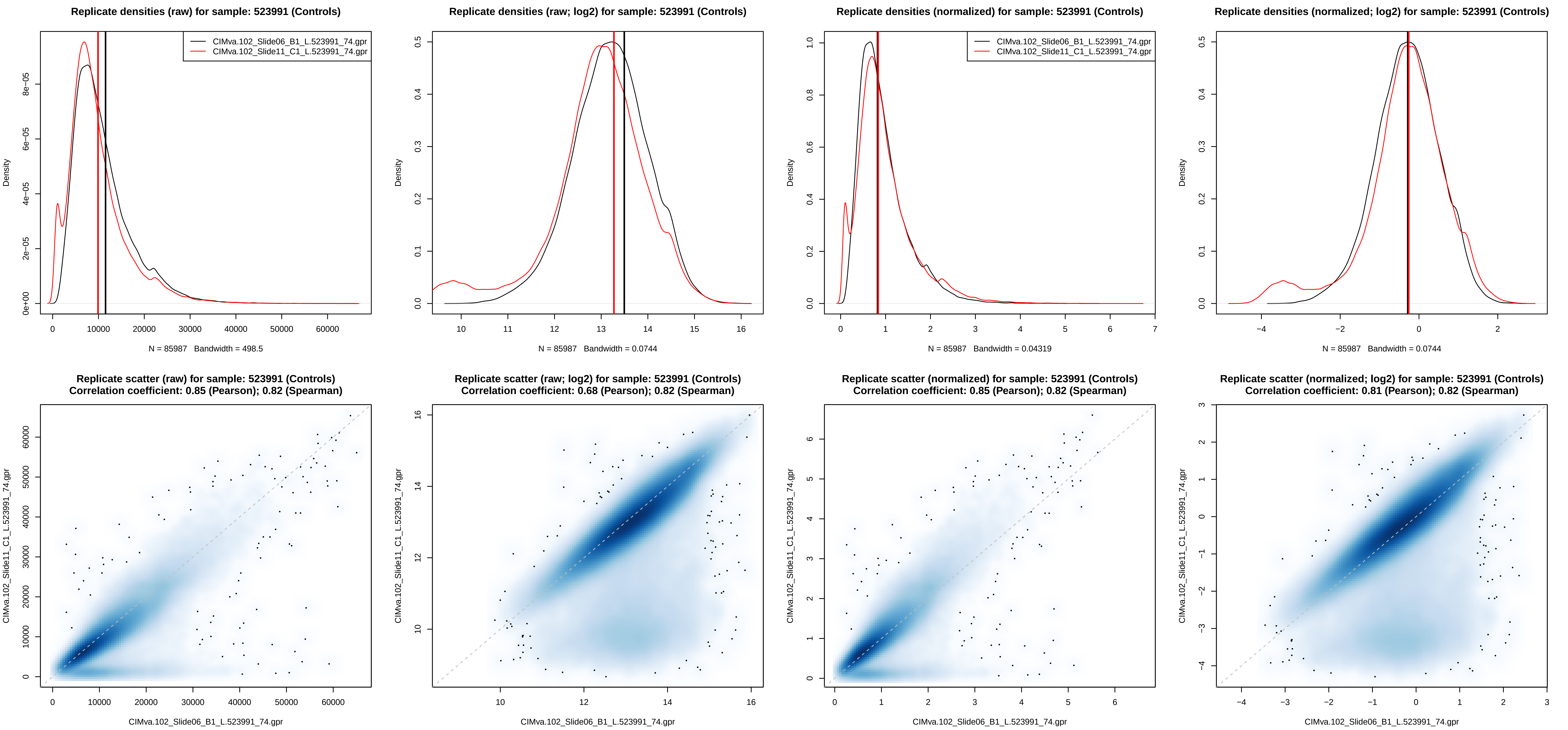
Replicate densities (raw) for sample: 523991 (Controls)
CIMva.102_Slide06_B1_L.523991_74.gpr
CIMva.102_Slide11_C1_L.523991_74.gpr
Density
N = 85987 Bandwidth = 498.5
Replicate densities (raw; log2) for sample: 523991 (Controls)
Density
N = 85987 Bandwidth = 0.0744
Replicate densities (normalized) for sample: 523991 (Controls)
CIMva.102_Slide06_B1_L.523991_74.gpr
CIMva.102_Slide11_C1_L.523991_74.gpr
Density
N = 85987 Bandwidth = 0.04319
Replicate densities (normalized; log2) for sample: 523991 (Controls)
Density
N = 85987 Bandwidth = 0.0744
Replicate scatter (raw) for sample: 523991 (Controls)
Correlation coefficient: 0.85 (Pearson); 0.82 (Spearman)
CIMva.102_Slide11_C1_L.523991_74.gpr
CIMva.102_Slide06_B1_L.523991_74.gpr
Replicate scatter (raw; log2) for sample: 523991 (Controls)
Correlation coefficient: 0.68 (Pearson); 0.82 (Spearman)
CIMva.102_Slide11_C1_L.523991_74.gpr
CIMva.102_Slide06_B1_L.523991_74.gpr
Replicate scatter (normalized) for sample: 523991 (Controls)
Correlation coefficient: 0.85 (Pearson); 0.82 (Spearman)
CIMva.102_Slide11_C1_L.523991_74.gpr
CIMva.102_Slide06_B1_L.523991_74.gpr
Replicate scatter (normalized; log2) for sample: 523991 (Controls)
Correlation coefficient: 0.81 (Pearson); 0.82 (Spearman)
CIMva.102_Slide11_C1_L.523991_74.gpr
CIMva.102_Slide06_B1_L.523991_74.gpr

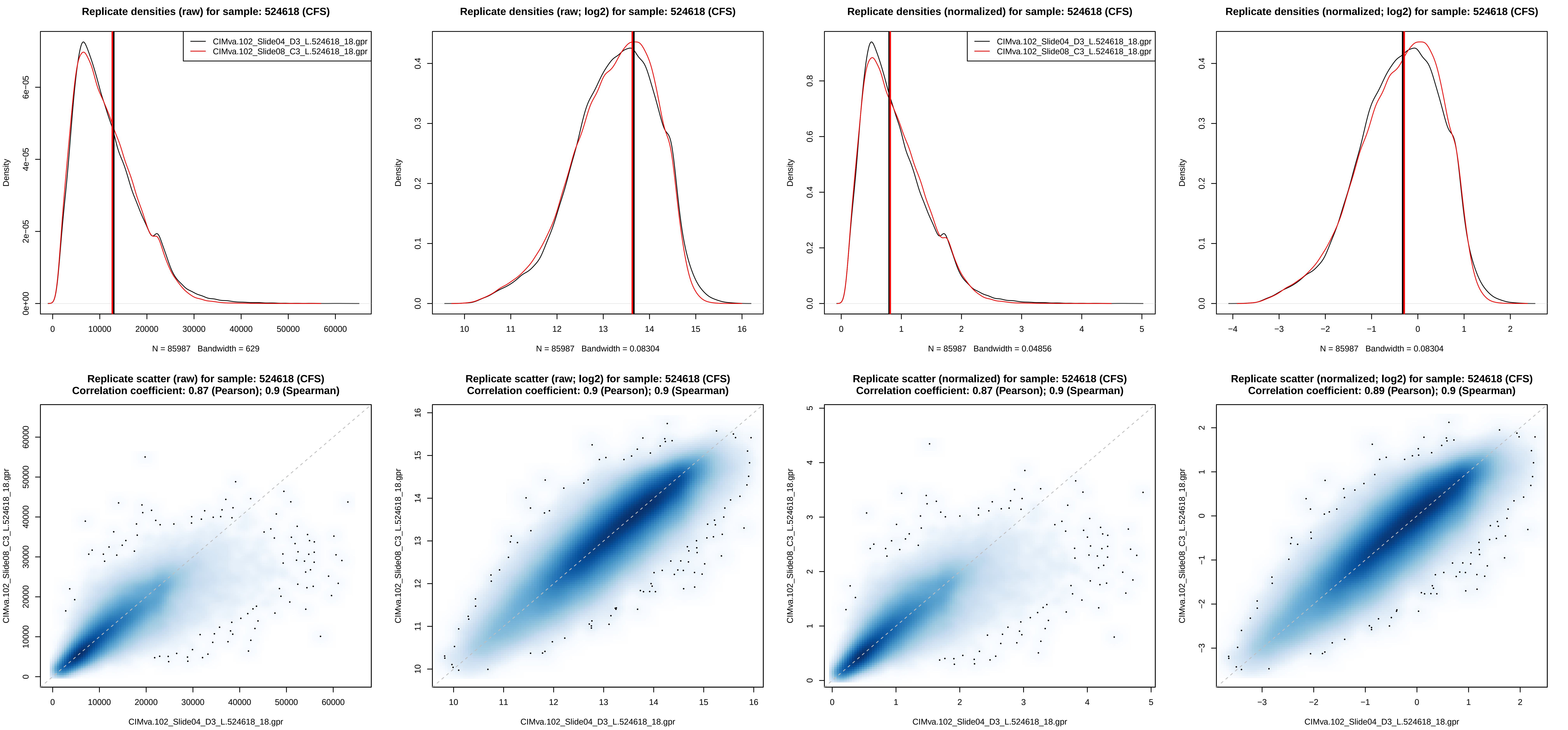

Replicate densities (raw) for sample: 524618 (CFS)
CIMva.102_Slide04_D3_L.524618_18.gpr
CIMva.102_Slide08_C3_L.524618_18.gpr
Density
N = 85987   Bandwidth = 629
Replicate densities (raw; log2) for sample: 524618 (CFS)
Density
N = 85987   Bandwidth = 0.08304
Replicate densities (normalized) for sample: 524618 (CFS)
CIMva.102_Slide04_D3_L.524618_18.gpr
CIMva.102_Slide08_C3_L.524618_18.gpr
Density
N = 85987   Bandwidth = 0.04856
Replicate densities (normalized; log2) for sample: 524618 (CFS)
Density
N = 85987   Bandwidth = 0.08304
Replicate scatter (raw) for sample: 524618 (CFS)
Correlation coefficient: 0.87 (Pearson); 0.9 (Spearman)
CIMva.102_Slide08_C3_L.524618_18.gpr
CIMva.102_Slide04_D3_L.524618_18.gpr
Replicate scatter (raw; log2) for sample: 524618 (CFS)
Correlation coefficient: 0.9 (Pearson); 0.9 (Spearman)
CIMva.102_Slide08_C3_L.524618_18.gpr
CIMva.102_Slide04_D3_L.524618_18.gpr
Replicate scatter (normalized) for sample: 524618 (CFS)
Correlation coefficient: 0.87 (Pearson); 0.9 (Spearman)
CIMva.102_Slide08_C3_L.524618_18.gpr
CIMva.102_Slide04_D3_L.524618_18.gpr
Replicate scatter (normalized; log2) for sample: 524618 (CFS)
Correlation coefficient: 0.89 (Pearson); 0.9 (Spearman)
CIMva.102_Slide08_C3_L.524618_18.gpr
CIMva.102_Slide04_D3_L.524618_18.gpr

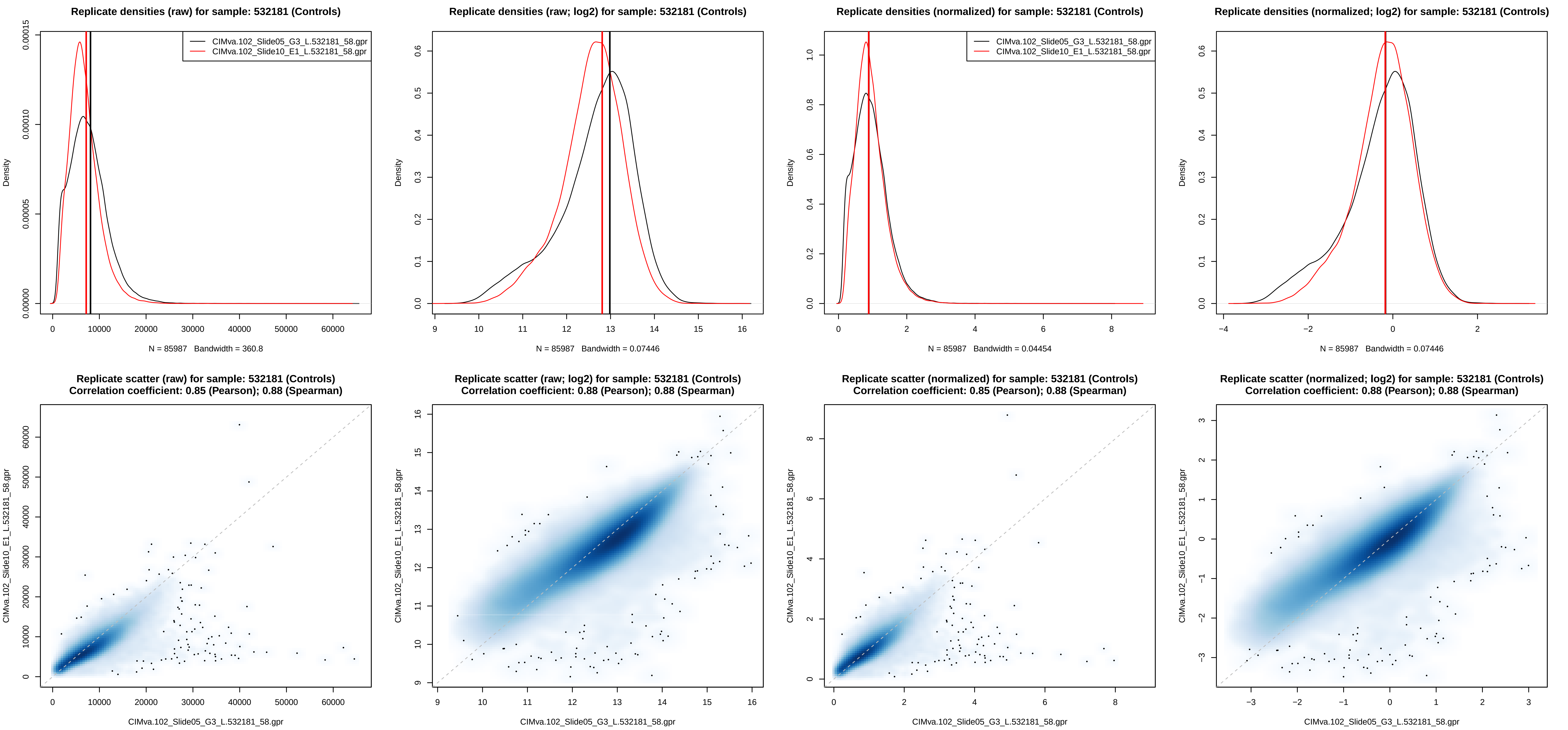
Replicate densities (raw) for sample: 532181 (Controls)
CIMva.102_Slide05_G3_L.532181_58.gpr
CIMva.102_Slide10_E1_L.532181_58.gpr
Density
N = 85987 Bandwidth = 360.8
Replicate densities (raw; log2) for sample: 532181 (Controls)
Density
N = 85987 Bandwidth = 0.07446
Replicate densities (normalized) for sample: 532181 (Controls)
CIMva.102_Slide05_G3_L.532181_58.gpr
CIMva.102_Slide10_E1_L.532181_58.gpr
Density
N = 85987 Bandwidth = 0.04454
Replicate densities (normalized; log2) for sample: 532181 (Controls)
Density
N = 85987 Bandwidth = 0.07446
Replicate scatter (raw) for sample: 532181 (Controls)
Correlation coefficient: 0.85 (Pearson); 0.88 (Spearman)
CIMva.102_Slide10_E1_L.532181_58.gpr
CIMva.102_Slide05_G3_L.532181_58.gpr
Replicate scatter (raw; log2) for sample: 532181 (Controls)
Correlation coefficient: 0.88 (Pearson); 0.88 (Spearman)
CIMva.102_Slide10_E1_L.532181_58.gpr
CIMva.102_Slide05_G3_L.532181_58.gpr
Replicate scatter (normalized) for sample: 532181 (Controls)
Correlation coefficient: 0.85 (Pearson); 0.88 (Spearman)
CIMva.102_Slide10_E1_L.532181_58.gpr
CIMva.102_Slide05_G3_L.532181_58.gpr
Replicate scatter (normalized; log2) for sample: 532181 (Controls)
Correlation coefficient: 0.88 (Pearson); 0.88 (Spearman)
CIMva.102_Slide10_E1_L.532181_58.gpr
CIMva.102_Slide05_G3_L.532181_58.gpr

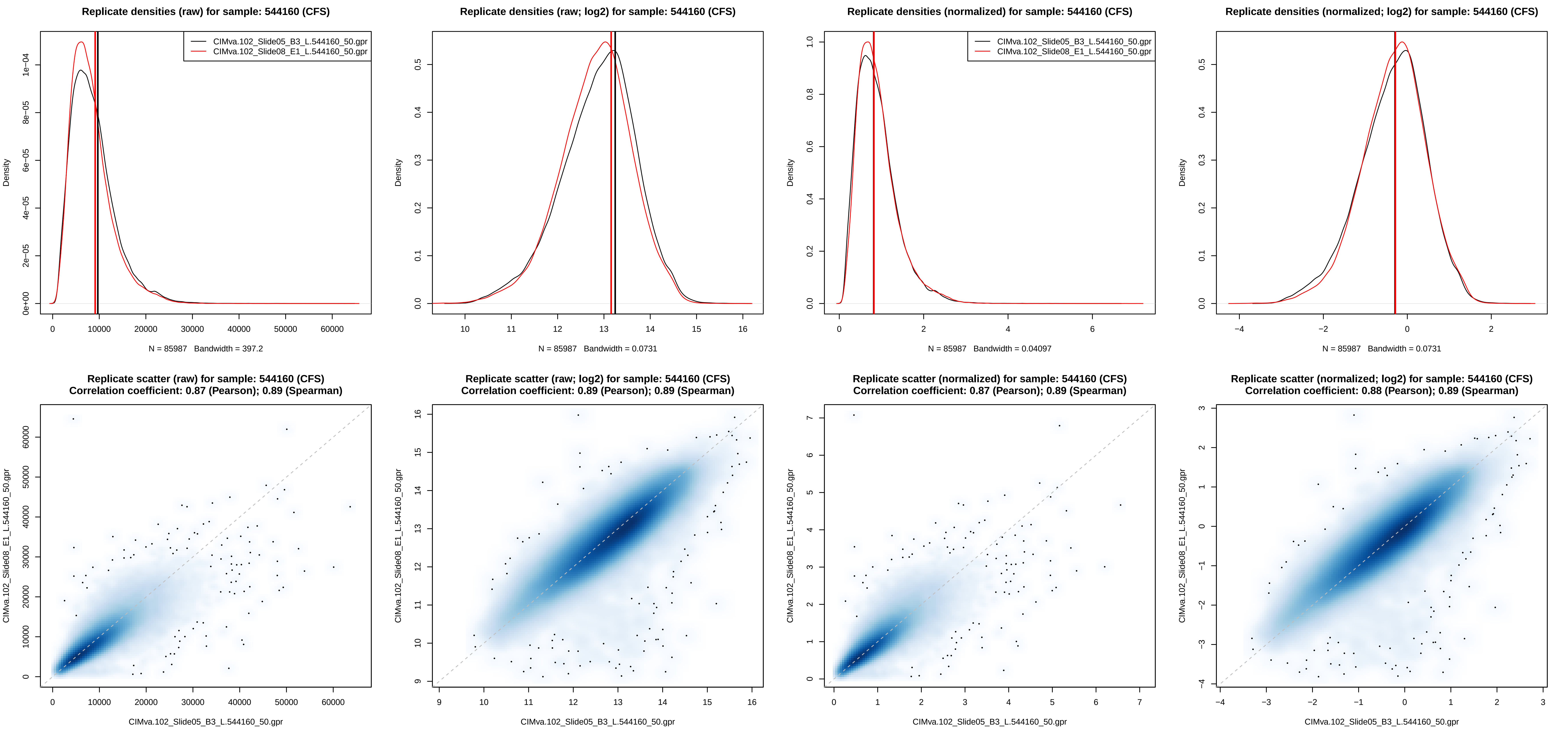

Replicate densities (raw) for sample: 544160 (CFS)
CIMva.102_Slide05_B3_L.544160_50.gpr
CIMva.102_Slide08_E1_L.544160_50.gpr
Density
N = 85987 Bandwidth = 397.2
Replicate densities (raw; log2) for sample: 544160 (CFS)
Density
N = 85987 Bandwidth = 0.0731
Replicate densities (normalized) for sample: 544160 (CFS)
CIMva.102_Slide05_B3_L.544160_50.gpr
CIMva.102_Slide08_E1_L.544160_50.gpr
Density
N = 85987 Bandwidth = 0.04097
Replicate densities (normalized; log2) for sample: 544160 (CFS)
Density
N = 85987 Bandwidth = 0.0731
Replicate scatter (raw) for sample: 544160 (CFS)
Correlation coefficient: 0.87 (Pearson); 0.89 (Spearman)
CIMva.102_Slide08_E1_L.544160_50.gpr
CIMva.102_Slide05_B3_L.544160_50.gpr
Replicate scatter (raw; log2) for sample: 544160 (CFS)
Correlation coefficient: 0.89 (Pearson); 0.89 (Spearman)
CIMva.102_Slide08_E1_L.544160_50.gpr
CIMva.102_Slide05_B3_L.544160_50.gpr
Replicate scatter (normalized) for sample: 544160 (CFS)
Correlation coefficient: 0.87 (Pearson); 0.89 (Spearman)
CIMva.102_Slide08_E1_L.544160_50.gpr
CIMva.102_Slide05_B3_L.544160_50.gpr
Replicate scatter (normalized; log2) for sample: 544160 (CFS)
Correlation coefficient: 0.88 (Pearson); 0.89 (Spearman)
CIMva.102_Slide08_E1_L.544160_50.gpr
CIMva.102_Slide05_B3_L.544160_50.gpr

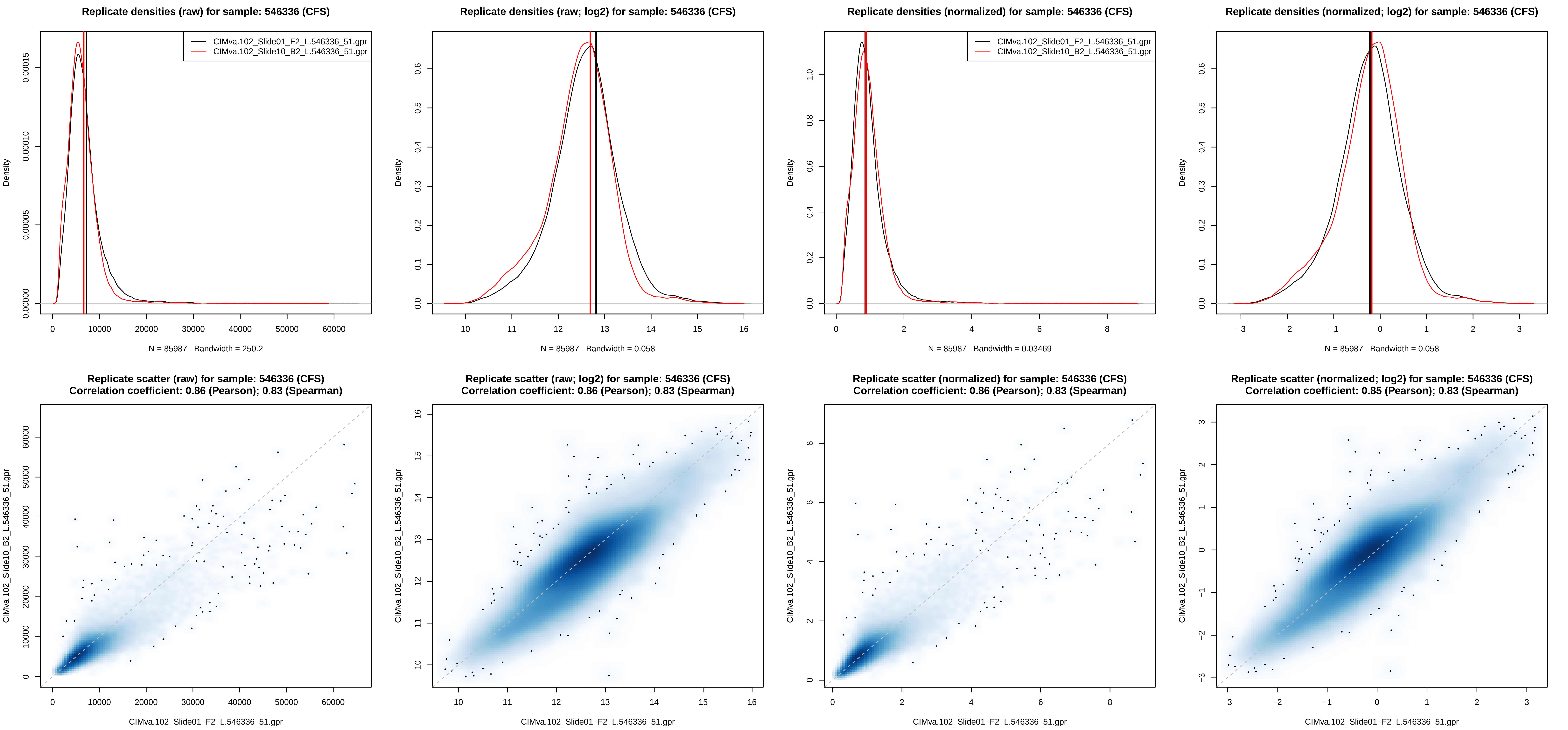

Replicate densities (raw) for sample: 546336 (CFS)
CIMva.102_Slide01_F2_L.546336_51.gpr
CIMva.102_Slide10_B2_L.546336_51.gpr
Density
N = 85987 Bandwidth = 250.2
Replicate densities (raw; log2) for sample: 546336 (CFS)
Density
N = 85987 Bandwidth = 0.058
Replicate densities (normalized) for sample: 546336 (CFS)
CIMva.102_Slide01_F2_L.546336_51.gpr
CIMva.102_Slide10_B2_L.546336_51.gpr
Density
N = 85987 Bandwidth = 0.03469
Replicate densities (normalized; log2) for sample: 546336 (CFS)
Density
N = 85987 Bandwidth = 0.058
Replicate scatter (raw) for sample: 546336 (CFS)
Correlation coefficient: 0.86 (Pearson); 0.83 (Spearman)
CIMva.102_Slide10_B2_L.546336_51.gpr
CIMva.102_Slide01_F2_L.546336_51.gpr
Replicate scatter (raw; log2) for sample: 546336 (CFS)
Correlation coefficient: 0.86 (Pearson); 0.83 (Spearman)
CIMva.102_Slide10_B2_L.546336_51.gpr
CIMva.102_Slide01_F2_L.546336_51.gpr
Replicate scatter (normalized) for sample: 546336 (CFS)
Correlation coefficient: 0.86 (Pearson); 0.83 (Spearman)
CIMva.102_Slide10_B2_L.546336_51.gpr
CIMva.102_Slide01_F2_L.546336_51.gpr
Replicate scatter (normalized; log2) for sample: 546336 (CFS)
Correlation coefficient: 0.85 (Pearson); 0.83 (Spearman)
CIMva.102_Slide10_B2_L.546336_51.gpr
CIMva.102_Slide01_F2_L.546336_51.gpr

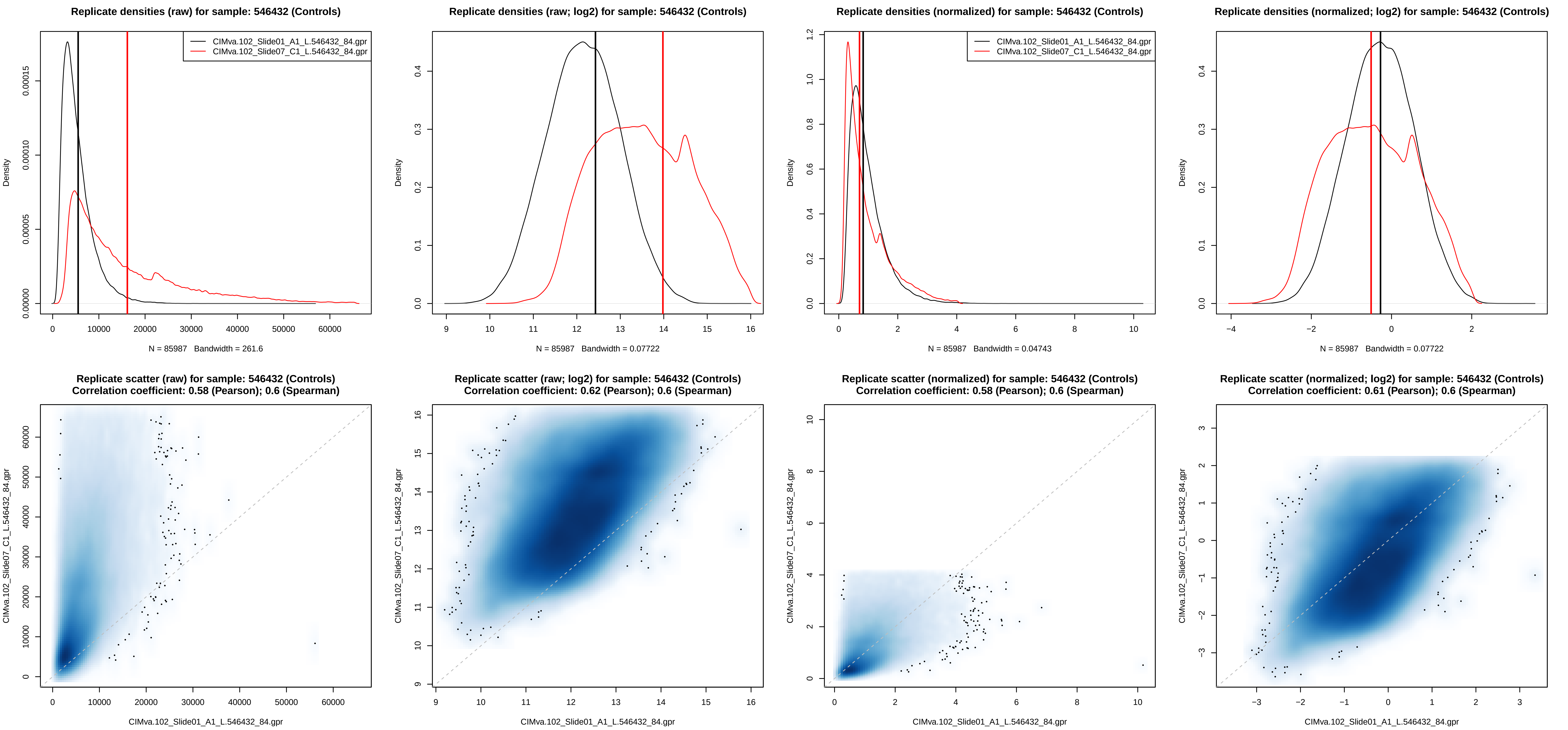

Replicate densities (raw) for sample: 546432 (Controls)
CIMva.102_Slide01_A1_L.546432_84.gpr
CIMva.102_Slide07_C1_L.546432_84.gpr
Density
N = 85987 Bandwidth = 261.6
Replicate densities (raw; log2) for sample: 546432 (Controls)
Density
N = 85987 Bandwidth = 0.07722
Replicate densities (normalized) for sample: 546432 (Controls)
CIMva.102_Slide01_A1_L.546432_84.gpr
CIMva.102_Slide07_C1_L.546432_84.gpr
Density
N = 85987 Bandwidth = 0.04743
Replicate densities (normalized; log2) for sample: 546432 (Controls)
Density
N = 85987 Bandwidth = 0.07722
Replicate scatter (raw) for sample: 546432 (Controls)
Correlation coefficient: 0.58 (Pearson); 0.6 (Spearman)
CIMva.102_Slide07_C1_L.546432_84.gpr
CIMva.102_Slide01_A1_L.546432_84.gpr
Replicate scatter (raw; log2) for sample: 546432 (Controls)
Correlation coefficient: 0.62 (Pearson); 0.6 (Spearman)
CIMva.102_Slide07_C1_L.546432_84.gpr
CIMva.102_Slide01_A1_L.546432_84.gpr
Replicate scatter (normalized) for sample: 546432 (Controls)
Correlation coefficient: 0.58 (Pearson); 0.6 (Spearman)
CIMva.102_Slide07_C1_L.546432_84.gpr
CIMva.102_Slide01_A1_L.546432_84.gpr
Replicate scatter (normalized; log2) for sample: 546432 (Controls)
Correlation coefficient: 0.61 (Pearson); 0.6 (Spearman)
CIMva.102_Slide07_C1_L.546432_84.gpr
CIMva.102_Slide01_A1_L.546432_84.gpr

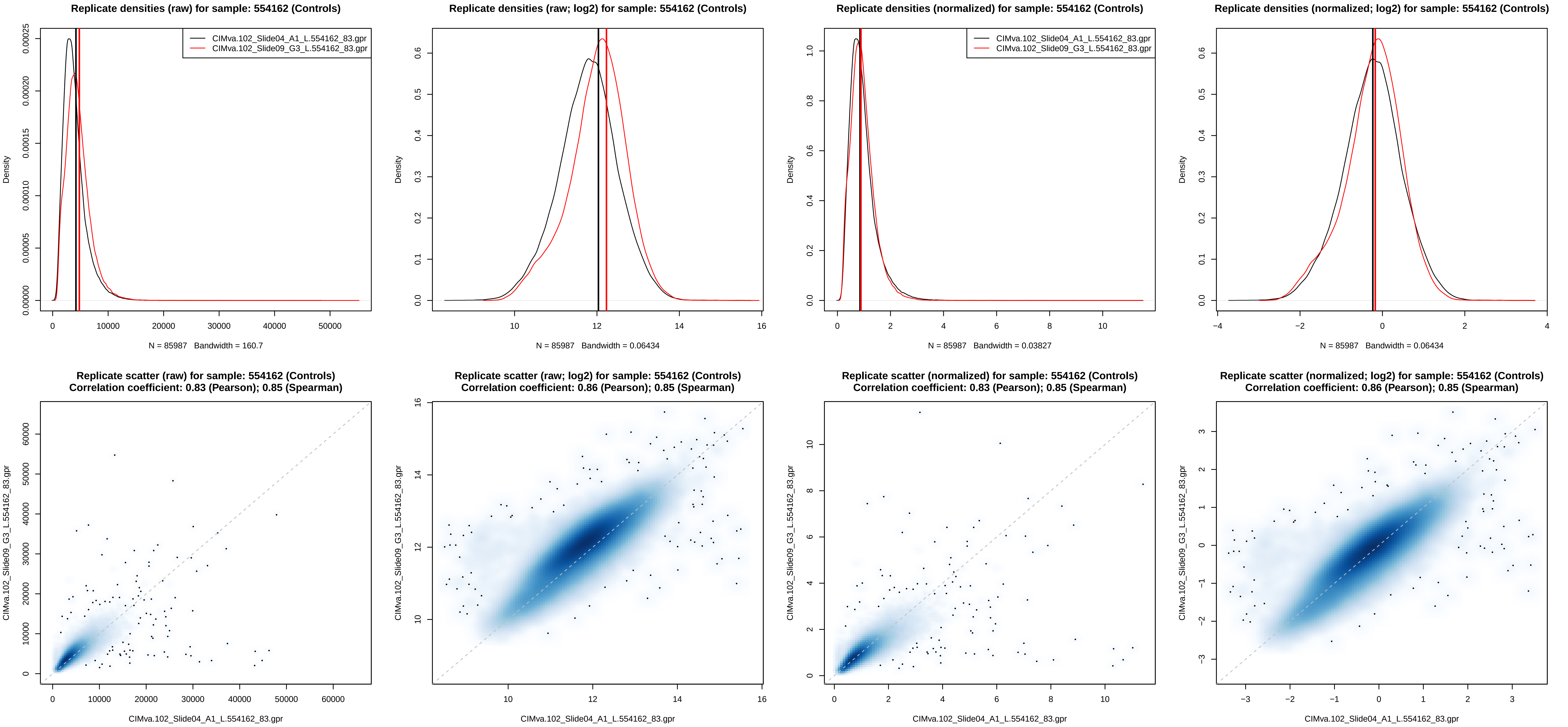

Replicate densities (raw) for sample: 554162 (Controls)
CIMva.102_Slide04_A1_L.554162_83.gpr
CIMva.102_Slide09_G3_L.554162_83.gpr
Density
N = 85987 Bandwidth = 160.7
Replicate densities (raw; log2) for sample: 554162 (Controls)
Density
N = 85987 Bandwidth = 0.06434
Replicate densities (normalized) for sample: 554162 (Controls)
CIMva.102_Slide04_A1_L.554162_83.gpr
CIMva.102_Slide09_G3_L.554162_83.gpr
Density
N = 85987 Bandwidth = 0.03827
Replicate densities (normalized; log2) for sample: 554162 (Controls)
Density
N = 85987 Bandwidth = 0.06434
Replicate scatter (raw) for sample: 554162 (Controls)
Correlation coefficient: 0.83 (Pearson); 0.85 (Spearman)
CIMva.102_Slide09_G3_L.554162_83.gpr
CIMva.102_Slide04_A1_L.554162_83.gpr
Replicate scatter (raw; log2) for sample: 554162 (Controls)
Correlation coefficient: 0.86 (Pearson); 0.85 (Spearman)
CIMva.102_Slide09_G3_L.554162_83.gpr
CIMva.102_Slide04_A1_L.554162_83.gpr
Replicate scatter (normalized) for sample: 554162 (Controls)
Correlation coefficient: 0.83 (Pearson); 0.85 (Spearman)
CIMva.102_Slide09_G3_L.554162_83.gpr
CIMva.102_Slide04_A1_L.554162_83.gpr
Replicate scatter (normalized; log2) for sample: 554162 (Controls)
Correlation coefficient: 0.86 (Pearson); 0.85 (Spearman)
CIMva.102_Slide09_G3_L.554162_83.gpr
CIMva.102_Slide04_A1_L.554162_83.gpr

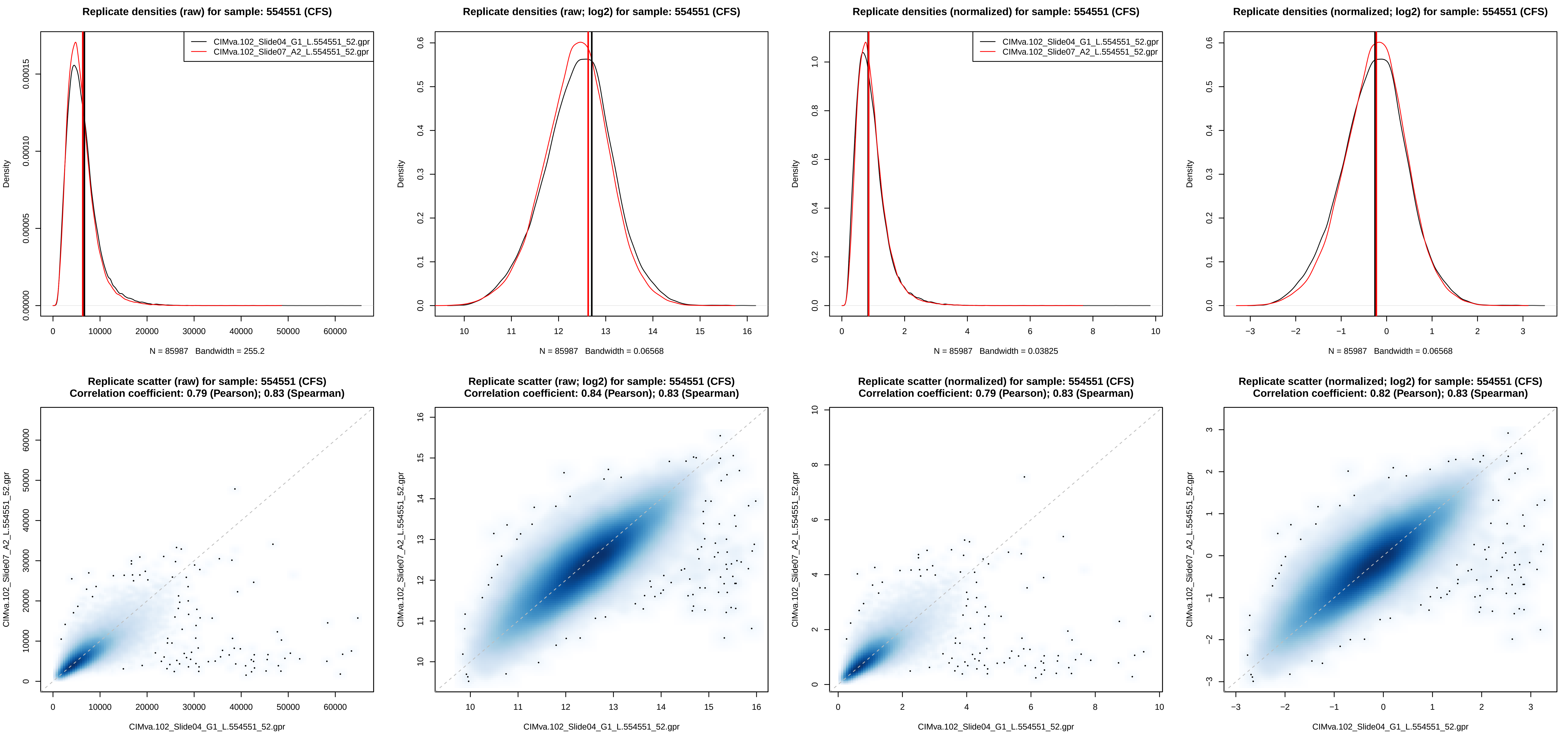
Replicate densities (raw) for sample: 554551 (CFS)
CIMva.102_Slide04_G1_L.554551_52.gpr
CIMva.102_Slide07_A2_L.554551_52.gpr
Density
N = 85987 Bandwidth = 255.2
Replicate densities (raw; log2) for sample: 554551 (CFS)
Density
N = 85987 Bandwidth = 0.06568
Replicate densities (normalized) for sample: 554551 (CFS)
CIMva.102_Slide04_G1_L.554551_52.gpr
CIMva.102_Slide07_A2_L.554551_52.gpr
Density
N = 85987 Bandwidth = 0.03825
Replicate densities (normalized; log2) for sample: 554551 (CFS)
Density
N = 85987 Bandwidth = 0.06568
Replicate scatter (raw) for sample: 554551 (CFS)
Correlation coefficient: 0.79 (Pearson); 0.83 (Spearman)
CIMva.102_Slide07_A2_L.554551_52.gpr
CIMva.102_Slide04_G1_L.554551_52.gpr
Replicate scatter (raw; log2) for sample: 554551 (CFS)
Correlation coefficient: 0.84 (Pearson); 0.83 (Spearman)
CIMva.102_Slide07_A2_L.554551_52.gpr
CIMva.102_Slide04_G1_L.554551_52.gpr
Replicate scatter (normalized) for sample: 554551 (CFS)
Correlation coefficient: 0.79 (Pearson); 0.83 (Spearman)
CIMva.102_Slide07_A2_L.554551_52.gpr
CIMva.102_Slide04_G1_L.554551_52.gpr
Replicate scatter (normalized; log2) for sample: 554551 (CFS)
Correlation coefficient: 0.82 (Pearson); 0.83 (Spearman)
CIMva.102_Slide07_A2_L.554551_52.gpr
CIMva.102_Slide04_G1_L.554551_52.gpr

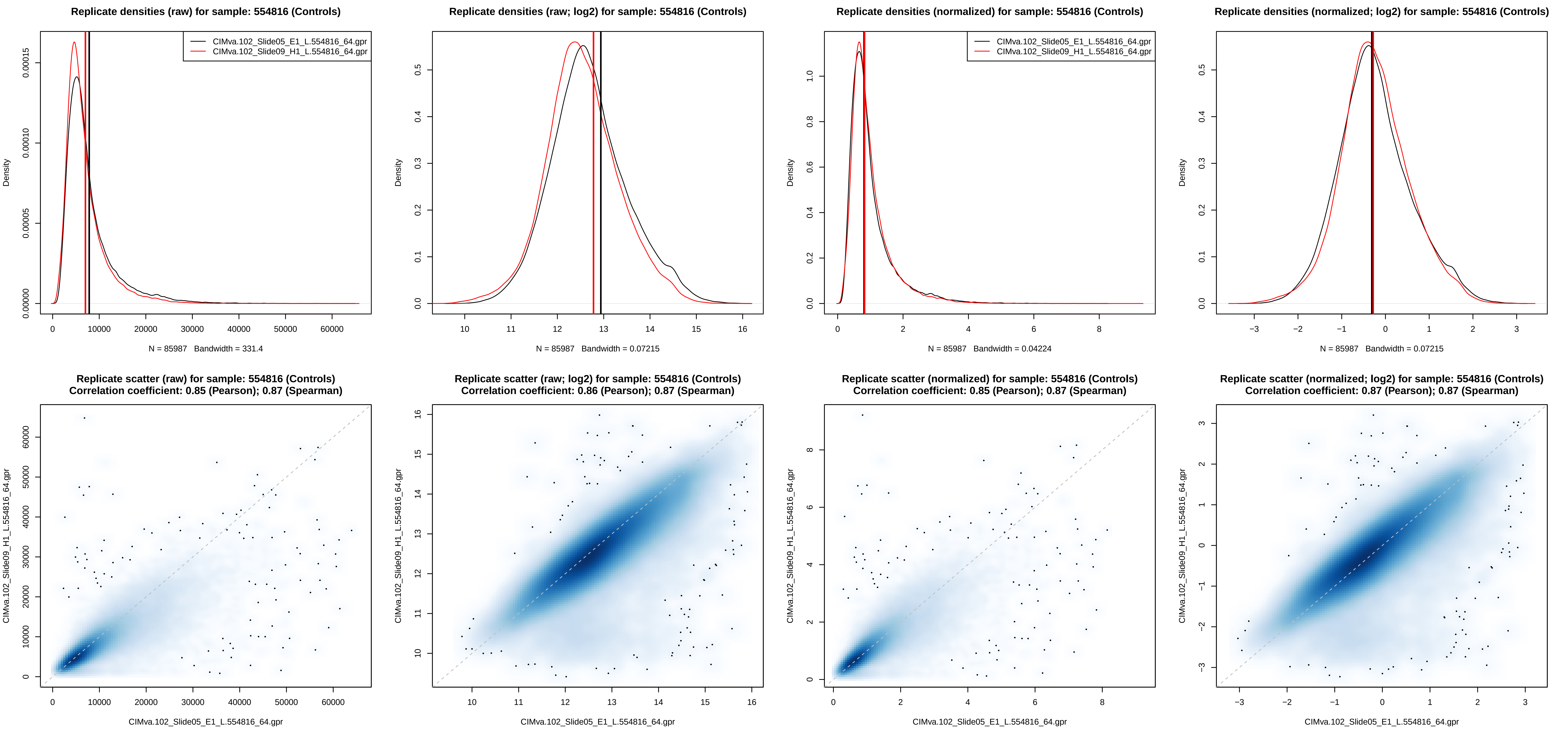
Replicate densities (raw) for sample: 554816 (Controls)
CIMva.102_Slide05_E1_L.554816_64.gpr
CIMva.102_Slide09_H1_L.554816_64.gpr
Density
N = 85987 Bandwidth = 331.4
Replicate densities (raw; log2) for sample: 554816 (Controls)
Density
N = 85987 Bandwidth = 0.07215
Replicate densities (normalized) for sample: 554816 (Controls)
CIMva.102_Slide05_E1_L.554816_64.gpr
CIMva.102_Slide09_H1_L.554816_64.gpr
Density
N = 85987 Bandwidth = 0.04224
Replicate densities (normalized; log2) for sample: 554816 (Controls)
Density
N = 85987 Bandwidth = 0.07215
Replicate scatter (raw) for sample: 554816 (Controls)
Correlation coefficient: 0.85 (Pearson); 0.87 (Spearman)
CIMva.102_Slide09_H1_L.554816_64.gpr
CIMva.102_Slide05_E1_L.554816_64.gpr
Replicate scatter (raw; log2) for sample: 554816 (Controls)
Correlation coefficient: 0.86 (Pearson); 0.87 (Spearman)
CIMva.102_Slide09_H1_L.554816_64.gpr
CIMva.102_Slide05_E1_L.554816_64.gpr
Replicate scatter (normalized) for sample: 554816 (Controls)
Correlation coefficient: 0.85 (Pearson); 0.87 (Spearman)
CIMva.102_Slide09_H1_L.554816_64.gpr
CIMva.102_Slide05_E1_L.554816_64.gpr
Replicate scatter (normalized; log2) for sample: 554816 (Controls)
Correlation coefficient: 0.87 (Pearson); 0.87 (Spearman)
CIMva.102_Slide09_H1_L.554816_64.gpr
CIMva.102_Slide05_E1_L.554816_64.gpr

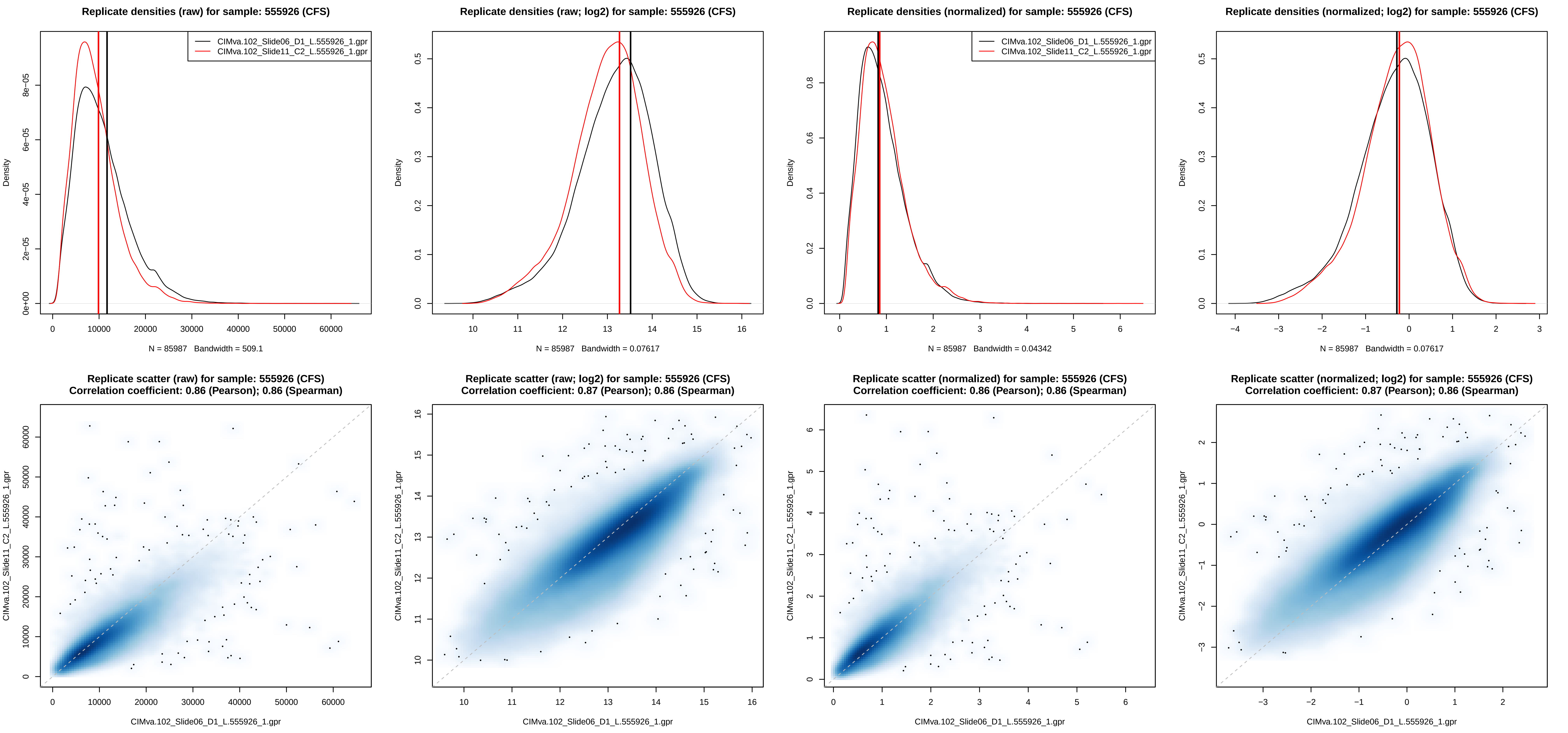
Replicate densities (raw) for sample: 555926 (CFS)
CIMva.102_Slide06_D1_L.555926_1.gpr
CIMva.102_Slide11_C2_L.555926_1.gpr
Density
N = 85987 Bandwidth = 509.1
Replicate densities (raw; log2) for sample: 555926 (CFS)
Density
N = 85987 Bandwidth = 0.07617
Replicate densities (normalized) for sample: 555926 (CFS)
CIMva.102_Slide06_D1_L.555926_1.gpr
CIMva.102_Slide11_C2_L.555926_1.gpr
Density
N = 85987 Bandwidth = 0.04342
Replicate densities (normalized; log2) for sample: 555926 (CFS)
Density
N = 85987 Bandwidth = 0.07617
Replicate scatter (raw) for sample: 555926 (CFS)
Correlation coefficient: 0.86 (Pearson); 0.86 (Spearman)
CIMva.102_Slide11_C2_L.555926_1.gpr
CIMva.102_Slide06_D1_L.555926_1.gpr
Replicate scatter (raw; log2) for sample: 555926 (CFS)
Correlation coefficient: 0.87 (Pearson); 0.86 (Spearman)
CIMva.102_Slide11_C2_L.555926_1.gpr
CIMva.102_Slide06_D1_L.555926_1.gpr
Replicate scatter (normalized) for sample: 555926 (CFS)
Correlation coefficient: 0.86 (Pearson); 0.86 (Spearman)
CIMva.102_Slide11_C2_L.555926_1.gpr
CIMva.102_Slide06_D1_L.555926_1.gpr
Replicate scatter (normalized; log2) for sample: 555926 (CFS)
Correlation coefficient: 0.87 (Pearson); 0.86 (Spearman)
CIMva.102_Slide11_C2_L.555926_1.gpr
CIMva.102_Slide06_D1_L.555926_1.gpr

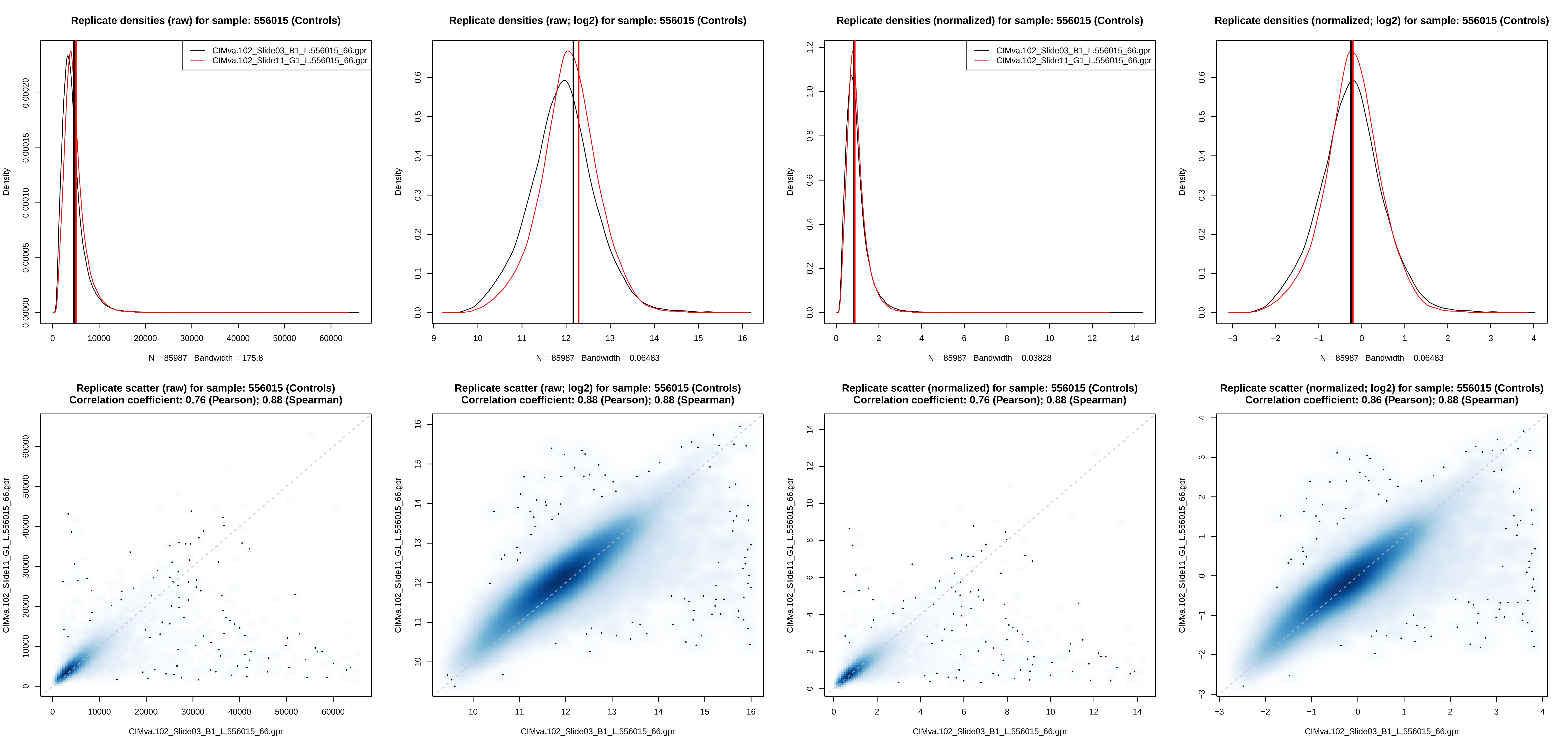
Replicate densities (raw) for sample: 556015 (Controls)
CIMva.102_Slide03_B1_L.556015_66.gpr
CIMva.102_Slide11_G1_L.556015_66.gpr
Density
N = 85987 Bandwidth = 175.8
Replicate densities (raw; log2) for sample: 556015 (Controls)
Density
N = 85987 Bandwidth = 0.06483
Replicate densities (normalized) for sample: 556015 (Controls)
CIMva.102_Slide03_B1_L.556015_66.gpr
CIMva.102_Slide11_G1_L.556015_66.gpr
Density
N = 85987 Bandwidth = 0.03828
Replicate densities (normalized; log2) for sample: 556015 (Controls)
Density
N = 85987 Bandwidth = 0.06483
Replicate scatter (raw) for sample: 556015 (Controls)
Correlation coefficient: 0.76 (Pearson); 0.88 (Spearman)
CIMva.102_Slide11_G1_L.556015_66.gpr
CIMva.102_Slide03_B1_L.556015_66.gpr
Replicate scatter (raw; log2) for sample: 556015 (Controls)
Correlation coefficient: 0.88 (Pearson); 0.88 (Spearman)
CIMva.102_Slide11_G1_L.556015_66.gpr
CIMva.102_Slide03_B1_L.556015_66.gpr
Replicate scatter (normalized) for sample: 556015 (Controls)
Correlation coefficient: 0.76 (Pearson); 0.88 (Spearman)
CIMva.102_Slide11_G1_L.556015_66.gpr
CIMva.102_Slide03_B1_L.556015_66.gpr
Replicate scatter (normalized; log2) for sample: 556015 (Controls)
Correlation coefficient: 0.86 (Pearson); 0.88 (Spearman)
CIMva.102_Slide11_G1_L.556015_66.gpr
CIMva.102_Slide03_B1_L.556015_66.gpr

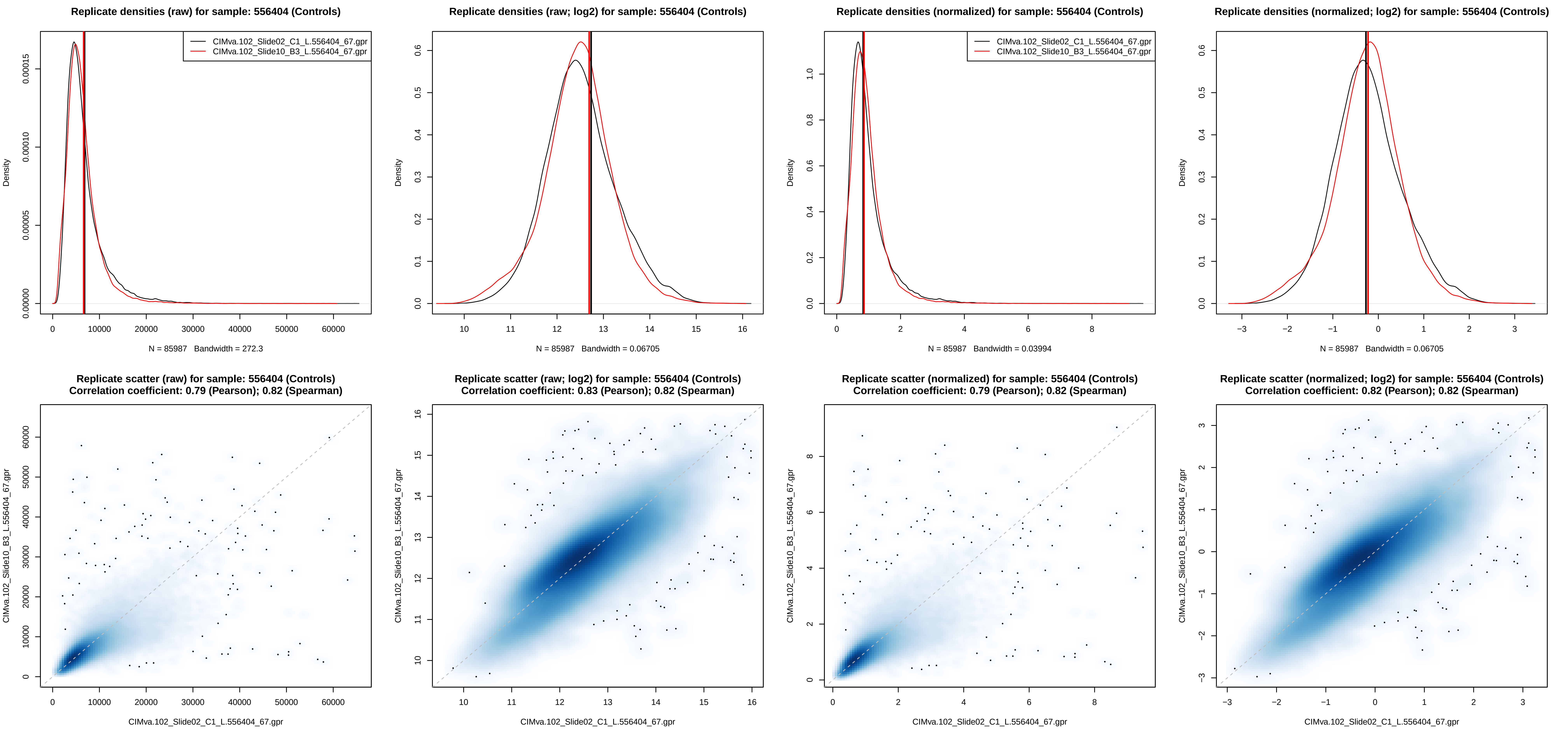
Replicate densities (raw) for sample: 556404 (Controls)
CIMva.102_Slide02_C1_L.556404_67.gpr
CIMva.102_Slide10_B3_L.556404_67.gpr
Density
N = 85987 Bandwidth = 272.3
Replicate densities (raw; log2) for sample: 556404 (Controls)
Density
N = 85987 Bandwidth = 0.06705
Replicate densities (normalized) for sample: 556404 (Controls)
CIMva.102_Slide02_C1_L.556404_67.gpr
CIMva.102_Slide10_B3_L.556404_67.gpr
Density
N = 85987 Bandwidth = 0.03994
Replicate densities (normalized; log2) for sample: 556404 (Controls)
Density
N = 85987 Bandwidth = 0.06705
Replicate scatter (raw) for sample: 556404 (Controls)
Correlation coefficient: 0.79 (Pearson); 0.82 (Spearman)
CIMva.102_Slide10_B3_L.556404_67.gpr
CIMva.102_Slide02_C1_L.556404_67.gpr
Replicate scatter (raw; log2) for sample: 556404 (Controls)
Correlation coefficient: 0.83 (Pearson); 0.82 (Spearman)
CIMva.102_Slide10_B3_L.556404_67.gpr
CIMva.102_Slide02_C1_L.556404_67.gpr
Replicate scatter (normalized) for sample: 556404 (Controls)
Correlation coefficient: 0.79 (Pearson); 0.82 (Spearman)
CIMva.102_Slide10_B3_L.556404_67.gpr
CIMva.102_Slide02_C1_L.556404_67.gpr
Replicate scatter (normalized; log2) for sample: 556404 (Controls)
Correlation coefficient: 0.82 (Pearson); 0.82 (Spearman)
CIMva.102_Slide10_B3_L.556404_67.gpr
CIMva.102_Slide02_C1_L.556404_67.gpr

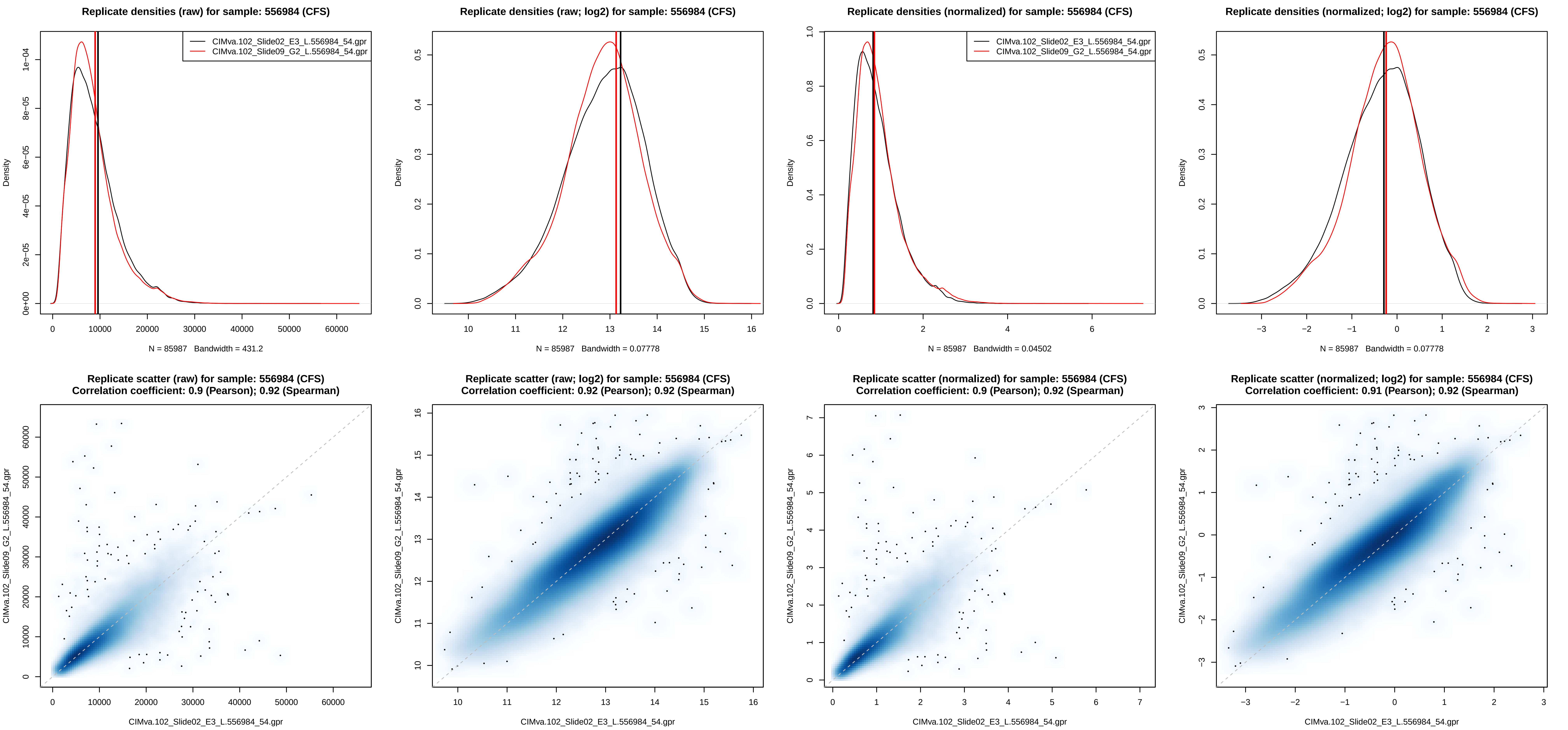
Replicate densities (raw) for sample: 556984 (CFS)
CIMva.102_Slide02_E3_L.556984_54.gpr
CIMva.102_Slide09_G2_L.556984_54.gpr
Density
N = 85987 Bandwidth = 431.2
Replicate densities (raw; log2) for sample: 556984 (CFS)
Density
N = 85987 Bandwidth = 0.07778
Replicate densities (normalized) for sample: 556984 (CFS)
CIMva.102_Slide02_E3_L.556984_54.gpr
CIMva.102_Slide09_G2_L.556984_54.gpr
Density
N = 85987 Bandwidth = 0.04502
Replicate densities (normalized; log2) for sample: 556984 (CFS)
Density
N = 85987 Bandwidth = 0.07778
Replicate scatter (raw) for sample: 556984 (CFS)
Correlation coefficient: 0.9 (Pearson); 0.92 (Spearman)
CIMva.102_Slide09_G2_L.556984_54.gpr
CIMva.102_Slide02_E3_L.556984_54.gpr
Replicate scatter (raw; log2) for sample: 556984 (CFS)
Correlation coefficient: 0.92 (Pearson); 0.92 (Spearman)
CIMva.102_Slide09_G2_L.556984_54.gpr
CIMva.102_Slide02_E3_L.556984_54.gpr
Replicate scatter (normalized) for sample: 556984 (CFS)
Correlation coefficient: 0.9 (Pearson); 0.92 (Spearman)
CIMva.102_Slide09_G2_L.556984_54.gpr
CIMva.102_Slide02_E3_L.556984_54.gpr
Replicate scatter (normalized; log2) for sample: 556984 (CFS)
Correlation coefficient: 0.91 (Pearson); 0.92 (Spearman)
CIMva.102_Slide09_G2_L.556984_54.gpr
CIMva.102_Slide02_E3_L.556984_54.gpr

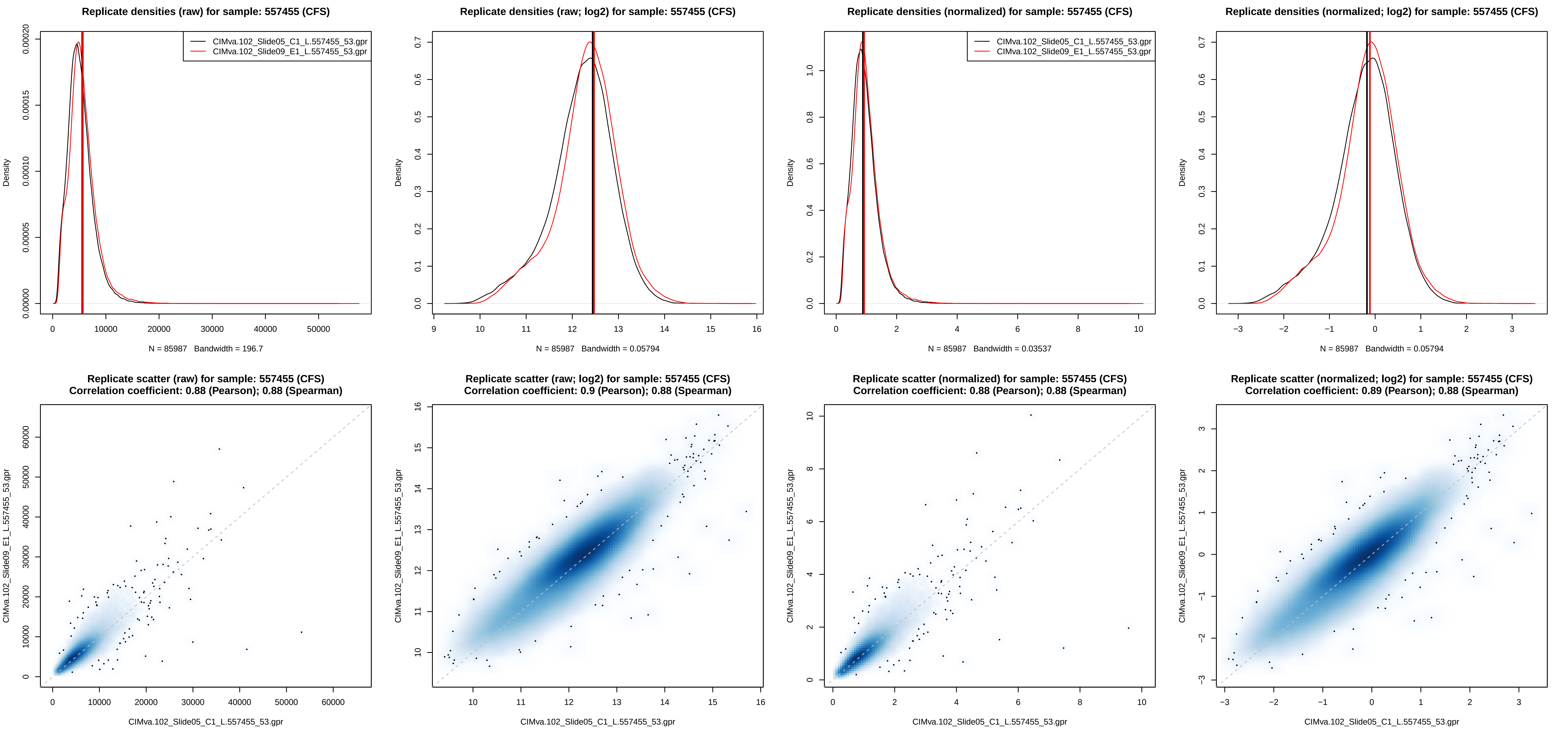
Replicate densities (raw) for sample: 557455 (CFS)
CIMva.102_Slide05_C1_L.557455_53.gpr
CIMva.102_Slide09_E1_L.557455_53.gpr
Density
N = 85987 Bandwidth = 196.7
Replicate densities (raw; log2) for sample: 557455 (CFS)
Density
N = 85987 Bandwidth = 0.05794
Replicate densities (normalized) for sample: 557455 (CFS)
CIMva.102_Slide05_C1_L.557455_53.gpr
CIMva.102_Slide09_E1_L.557455_53.gpr
Density
N = 85987 Bandwidth = 0.03537
Replicate densities (normalized; log2) for sample: 557455 (CFS)
Density
N = 85987 Bandwidth = 0.05794
Replicate scatter (raw) for sample: 557455 (CFS)
Correlation coefficient: 0.88 (Pearson); 0.88 (Spearman)
CIMva.102_Slide09_E1_L.557455_53.gpr
CIMva.102_Slide05_C1_L.557455_53.gpr
Replicate scatter (raw; log2) for sample: 557455 (CFS)
Correlation coefficient: 0.9 (Pearson); 0.88 (Spearman)
CIMva.102_Slide09_E1_L.557455_53.gpr
CIMva.102_Slide05_C1_L.557455_53.gpr
Replicate scatter (normalized) for sample: 557455 (CFS)
Correlation coefficient: 0.88 (Pearson); 0.88 (Spearman)
CIMva.102_Slide09_E1_L.557455_53.gpr
CIMva.102_Slide05_C1_L.557455_53.gpr
Replicate scatter (normalized; log2) for sample: 557455 (CFS)
Correlation coefficient: 0.89 (Pearson); 0.88 (Spearman)
CIMva.102_Slide09_E1_L.557455_53.gpr
CIMva.102_Slide05_C1_L.557455_53.gpr

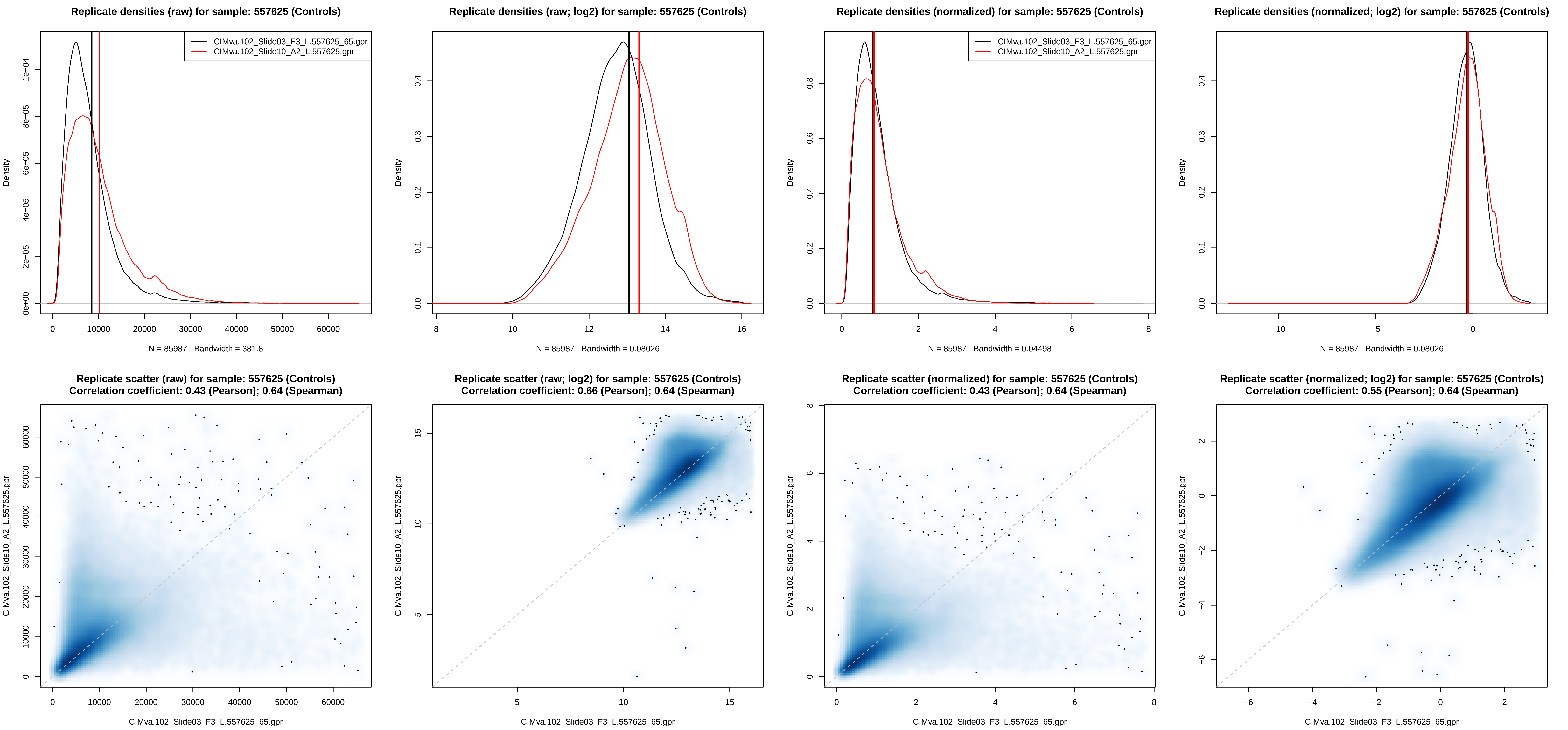
Replicate densities (raw) for sample: 557625 (Controls)
CIMva.102_Slide03_F3_L.557625_65.gpr
CIMva.102_Slide10_A2_L.557625.gpr
Density
N = 85987 Bandwidth = 381.8
Replicate densities (raw; log2) for sample: 557625 (Controls)
Density
N = 85987 Bandwidth = 0.08026
Replicate densities (normalized) for sample: 557625 (Controls)
CIMva.102_Slide03_F3_L.557625_65.gpr
CIMva.102_Slide10_A2_L.557625.gpr
Density
N = 85987 Bandwidth = 0.04498
Replicate densities (normalized; log2) for sample: 557625 (Controls)
Density
N = 85987 Bandwidth = 0.08026
Replicate scatter (raw) for sample: 557625 (Controls)
Correlation coefficient: 0.43 (Pearson); 0.64 (Spearman)
CIMva.102_Slide10_A2_L.557625.gpr
CIMva.102_Slide03_F3_L.557625_65.gpr
Replicate scatter (raw; log2) for sample: 557625 (Controls)
Correlation coefficient: 0.66 (Pearson); 0.64 (Spearman)
CIMva.102_Slide10_A2_L.557625.gpr
CIMva.102_Slide03_F3_L.557625_65.gpr
Replicate scatter (normalized) for sample: 557625 (Controls)
Correlation coefficient: 0.43 (Pearson); 0.64 (Spearman)
CIMva.102_Slide10_A2_L.557625.gpr
CIMva.102_Slide03_F3_L.557625_65.gpr
Replicate scatter (normalized; log2) for sample: 557625 (Controls)
Correlation coefficient: 0.55 (Pearson); 0.64 (Spearman)
CIMva.102_Slide10_A2_L.557625.gpr
CIMva.102_Slide03_F3_L.557625_65.gpr

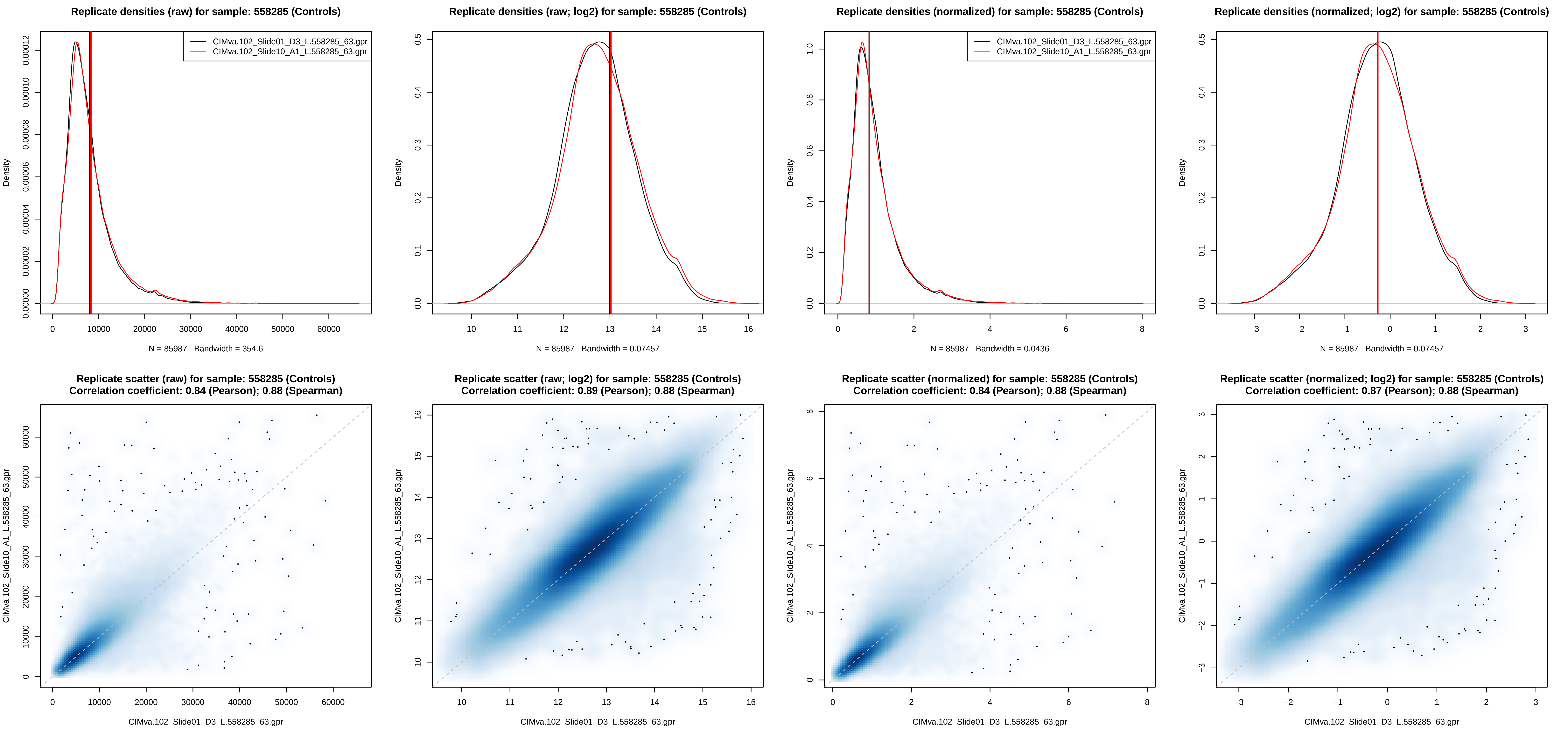
Replicate densities (raw) for sample: 558285 (Controls)
CIMva.102_Slide01_D3_L.558285_63.gpr
CIMva.102_Slide10_A1_L.558285_63.gpr
Density
N = 85987 Bandwidth = 354.6
Replicate densities (raw; log2) for sample: 558285 (Controls)
Density
N = 85987 Bandwidth = 0.07457
Replicate densities (normalized) for sample: 558285 (Controls)
CIMva.102_Slide01_D3_L.558285_63.gpr
CIMva.102_Slide10_A1_L.558285_63.gpr
Density
N = 85987 Bandwidth = 0.0436
Replicate densities (normalized; log2) for sample: 558285 (Controls)
Density
N = 85987 Bandwidth = 0.07457
Replicate scatter (raw) for sample: 558285 (Controls)
Correlation coefficient: 0.84 (Pearson); 0.88 (Spearman)
CIMva.102_Slide10_A1_L.558285_63.gpr
CIMva.102_Slide01_D3_L.558285_63.gpr
Replicate scatter (raw; log2) for sample: 558285 (Controls)
Correlation coefficient: 0.89 (Pearson); 0.88 (Spearman)
CIMva.102_Slide10_A1_L.558285_63.gpr
CIMva.102_Slide01_D3_L.558285_63.gpr
Replicate scatter (normalized) for sample: 558285 (Controls)
Correlation coefficient: 0.84 (Pearson); 0.88 (Spearman)
CIMva.102_Slide10_A1_L.558285_63.gpr
CIMva.102_Slide01_D3_L.558285_63.gpr
Replicate scatter (normalized; log2) for sample: 558285 (Controls)
Correlation coefficient: 0.87 (Pearson); 0.88 (Spearman)
CIMva.102_Slide10_A1_L.558285_63.gpr
CIMva.102_Slide01_D3_L.558285_63.gpr

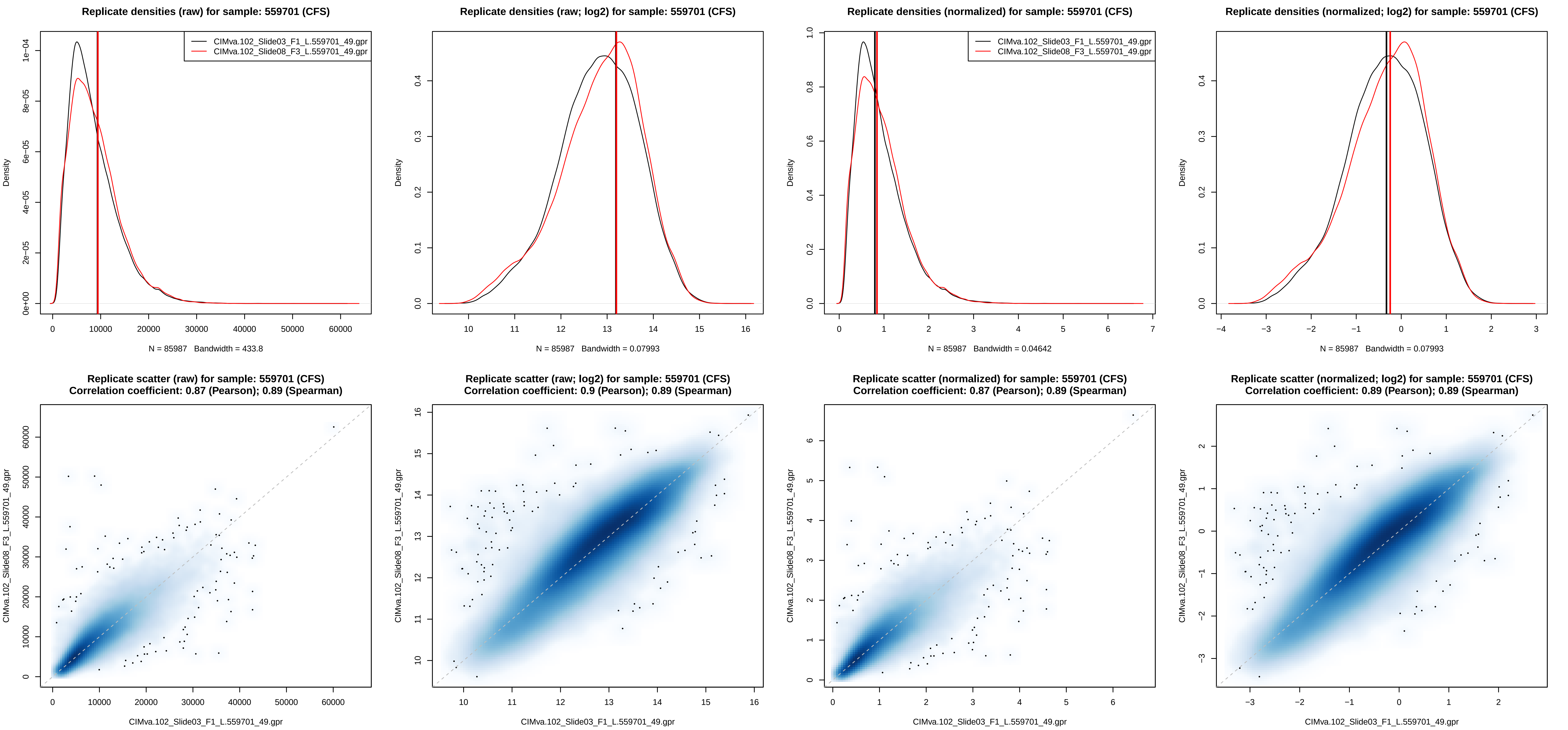
Replicate densities (raw) for sample: 559701 (CFS)
CIMva.102_Slide03_F1_L.559701_49.gpr
CIMva.102_Slide08_F3_L.559701_49.gpr
Density
N = 85987 Bandwidth = 433.8
Replicate densities (raw; log2) for sample: 559701 (CFS)
Density
N = 85987 Bandwidth = 0.07993
Replicate densities (normalized) for sample: 559701 (CFS)
CIMva.102_Slide03_F1_L.559701_49.gpr
CIMva.102_Slide08_F3_L.559701_49.gpr
Density
N = 85987 Bandwidth = 0.04642
Replicate densities (normalized; log2) for sample: 559701 (CFS)
Density
N = 85987 Bandwidth = 0.07993
Replicate scatter (raw) for sample: 559701 (CFS)
Correlation coefficient: 0.87 (Pearson); 0.89 (Spearman)
CIMva.102_Slide08_F3_L.559701_49.gpr
CIMva.102_Slide03_F1_L.559701_49.gpr
Replicate scatter (raw; log2) for sample: 559701 (CFS)
Correlation coefficient: 0.9 (Pearson); 0.89 (Spearman)
CIMva.102_Slide08_F3_L.559701_49.gpr
CIMva.102_Slide03_F1_L.559701_49.gpr
Replicate scatter (normalized) for sample: 559701 (CFS)
Correlation coefficient: 0.87 (Pearson); 0.89 (Spearman)
CIMva.102_Slide08_F3_L.559701_49.gpr
CIMva.102_Slide03_F1_L.559701_49.gpr
Replicate scatter (normalized; log2) for sample: 559701 (CFS)
Correlation coefficient: 0.89 (Pearson); 0.89 (Spearman)
CIMva.102_Slide08_F3_L.559701_49.gpr
CIMva.102_Slide03_F1_L.559701_49.gpr

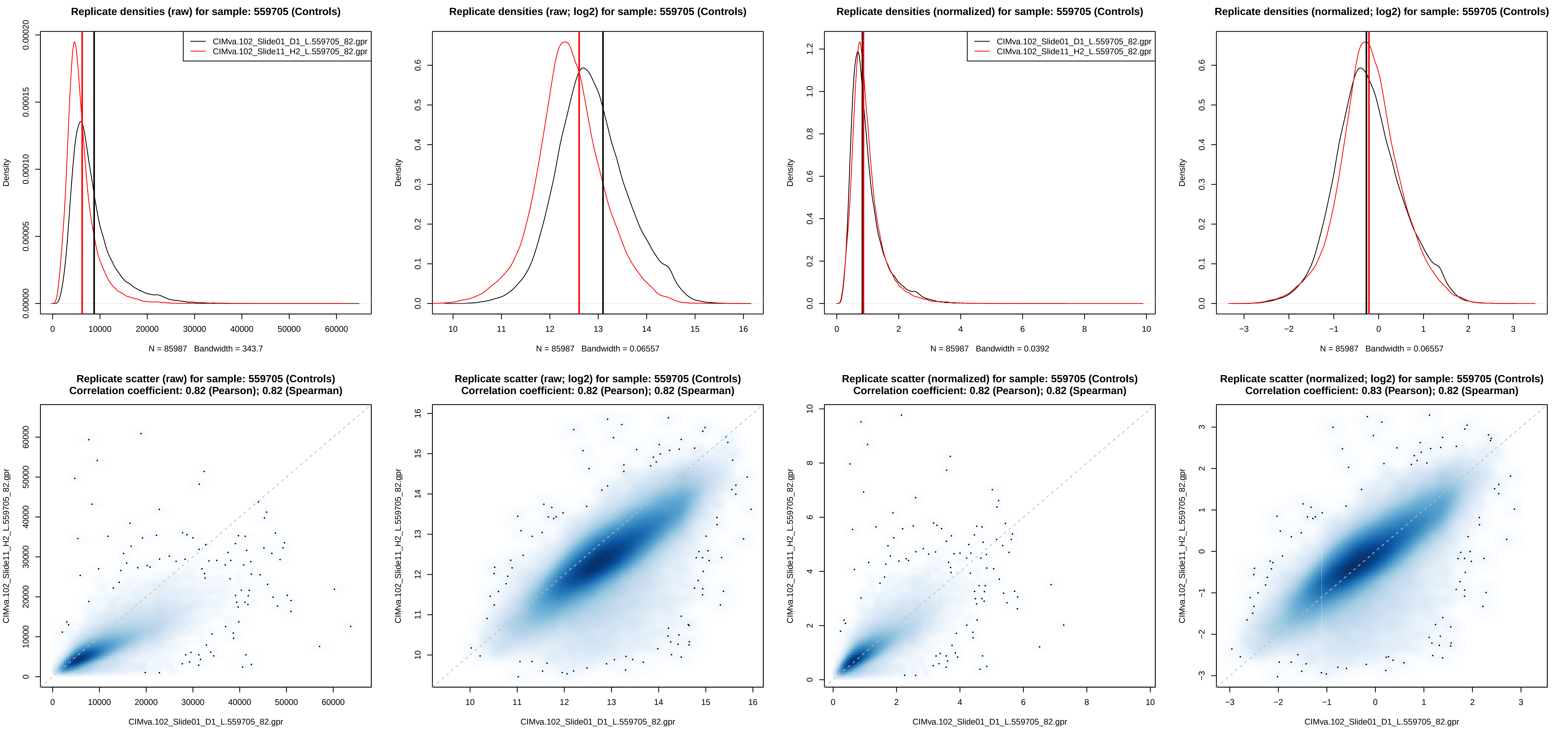
Replicate densities (raw) for sample: 559705 (Controls)
CIMva.102_Slide01_D1_L.559705_82.gpr
CIMva.102_Slide11_H2_L.559705_82.gpr
Density
N = 85987 Bandwidth = 343.7
Replicate densities (raw; log2) for sample: 559705 (Controls)
Density
N = 85987 Bandwidth = 0.06557
Replicate densities (normalized) for sample: 559705 (Controls)
CIMva.102_Slide01_D1_L.559705_82.gpr
CIMva.102_Slide11_H2_L.559705_82.gpr
Density
N = 85987 Bandwidth = 0.0392
Replicate densities (normalized; log2) for sample: 559705 (Controls)
Density
N = 85987 Bandwidth = 0.06557
Replicate scatter (raw) for sample: 559705 (Controls)
Correlation coefficient: 0.82 (Pearson); 0.82 (Spearman)
CIMva.102_Slide11_H2_L.559705_82.gpr
CIMva.102_Slide01_D1_L.559705_82.gpr
Replicate scatter (raw; log2) for sample: 559705 (Controls)
Correlation coefficient: 0.82 (Pearson); 0.82 (Spearman)
CIMva.102_Slide11_H2_L.559705_82.gpr
CIMva.102_Slide01_D1_L.559705_82.gpr
Replicate scatter (normalized) for sample: 559705 (Controls)
Correlation coefficient: 0.82 (Pearson); 0.82 (Spearman)
CIMva.102_Slide11_H2_L.559705_82.gpr
CIMva.102_Slide01_D1_L.559705_82.gpr
Replicate scatter (normalized; log2) for sample: 559705 (Controls)
Correlation coefficient: 0.83 (Pearson); 0.82 (Spearman)
CIMva.102_Slide11_H2_L.559705_82.gpr
CIMva.102_Slide01_D1_L.559705_82.gpr

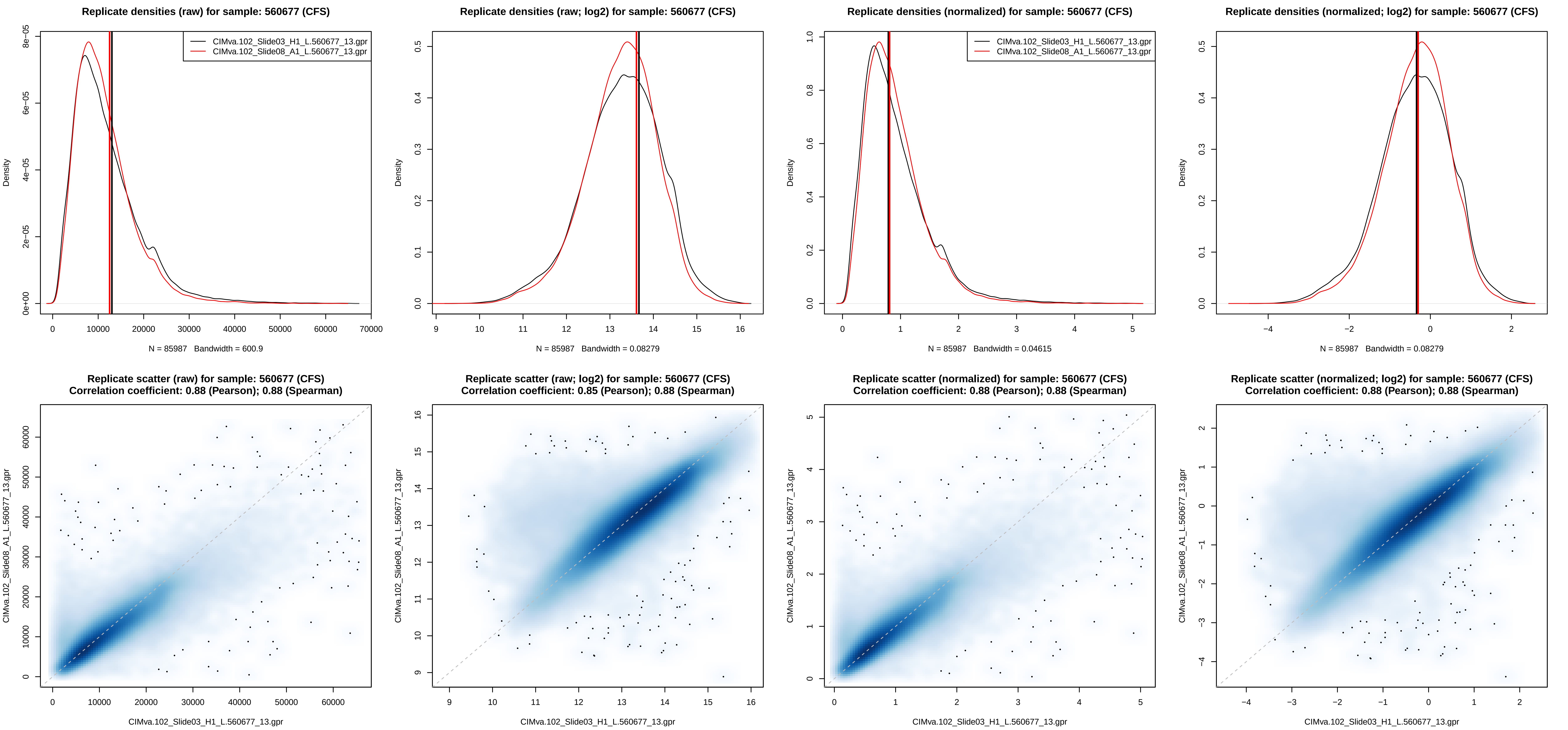
Replicate densities (raw) for sample: 560677 (CFS)
CIMva.102_Slide03_H1_L.560677_13.gpr
CIMva.102_Slide08_A1_L.560677_13.gpr
Density
N = 85987 Bandwidth = 600.9
Replicate densities (raw; log2) for sample: 560677 (CFS)
Density
N = 85987 Bandwidth = 0.08279
Replicate densities (normalized) for sample: 560677 (CFS)
CIMva.102_Slide03_H1_L.560677_13.gpr
CIMva.102_Slide08_A1_L.560677_13.gpr
Density
N = 85987 Bandwidth = 0.04615
Replicate densities (normalized; log2) for sample: 560677 (CFS)
Density
N = 85987 Bandwidth = 0.08279
Replicate scatter (raw) for sample: 560677 (CFS)
Correlation coefficient: 0.88 (Pearson); 0.88 (Spearman)
CIMva.102_Slide08_A1_L.560677_13.gpr
CIMva.102_Slide03_H1_L.560677_13.gpr
Replicate scatter (raw; log2) for sample: 560677 (CFS)
Correlation coefficient: 0.85 (Pearson); 0.88 (Spearman)
CIMva.102_Slide08_A1_L.560677_13.gpr
CIMva.102_Slide03_H1_L.560677_13.gpr
Replicate scatter (normalized) for sample: 560677 (CFS)
Correlation coefficient: 0.88 (Pearson); 0.88 (Spearman)
CIMva.102_Slide08_A1_L.560677_13.gpr
CIMva.102_Slide03_H1_L.560677_13.gpr
Replicate scatter (normalized; log2) for sample: 560677 (CFS)
Correlation coefficient: 0.88 (Pearson); 0.88 (Spearman)
CIMva.102_Slide08_A1_L.560677_13.gpr
CIMva.102_Slide03_H1_L.560677_13.gpr

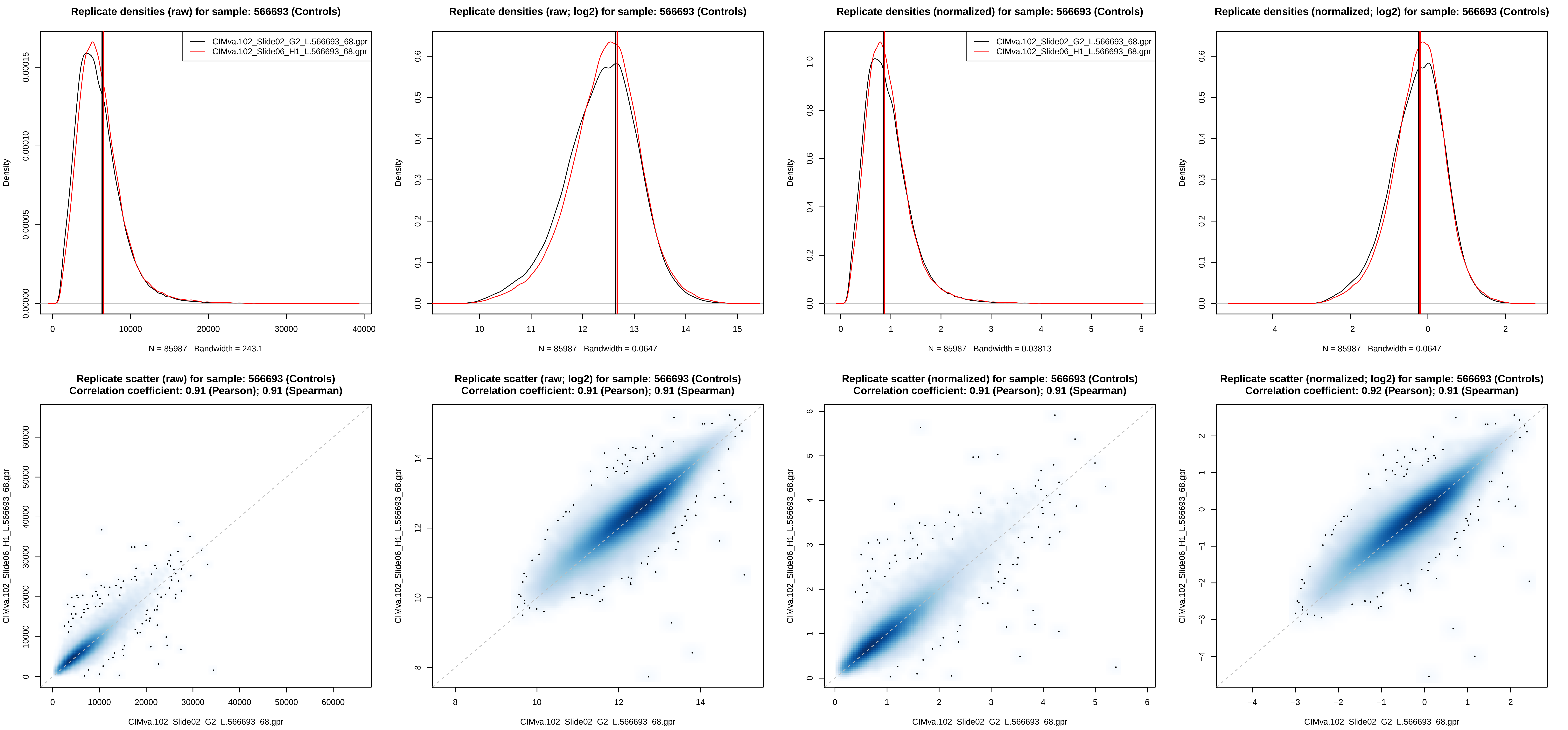
Replicate densities (raw) for sample: 566693 (Controls)
CIMva.102_Slide02_G2_L.566693_68.gpr
CIMva.102_Slide06_H1_L.566693_68.gpr
Density
N = 85987 Bandwidth = 243.1
Replicate densities (raw; log2) for sample: 566693 (Controls)
Density
N = 85987 Bandwidth = 0.0647
Replicate densities (normalized) for sample: 566693 (Controls)
CIMva.102_Slide02_G2_L.566693_68.gpr
CIMva.102_Slide06_H1_L.566693_68.gpr
Density
N = 85987 Bandwidth = 0.03813
Replicate densities (normalized; log2) for sample: 566693 (Controls)
Density
N = 85987 Bandwidth = 0.0647
Replicate scatter (raw) for sample: 566693 (Controls)
Correlation coefficient: 0.91 (Pearson); 0.91 (Spearman)
CIMva.102_Slide06_H1_L.566693_68.gpr
CIMva.102_Slide02_G2_L.566693_68.gpr
Replicate scatter (raw; log2) for sample: 566693 (Controls)
Correlation coefficient: 0.91 (Pearson); 0.91 (Spearman)
CIMva.102_Slide06_H1_L.566693_68.gpr
CIMva.102_Slide02_G2_L.566693_68.gpr
Replicate scatter (normalized) for sample: 566693 (Controls)
Correlation coefficient: 0.91 (Pearson); 0.91 (Spearman)
CIMva.102_Slide06_H1_L.566693_68.gpr
CIMva.102_Slide02_G2_L.566693_68.gpr
Replicate scatter (normalized; log2) for sample: 566693 (Controls)
Correlation coefficient: 0.92 (Pearson); 0.91 (Spearman)
CIMva.102_Slide06_H1_L.566693_68.gpr
CIMva.102_Slide02_G2_L.566693_68.gpr

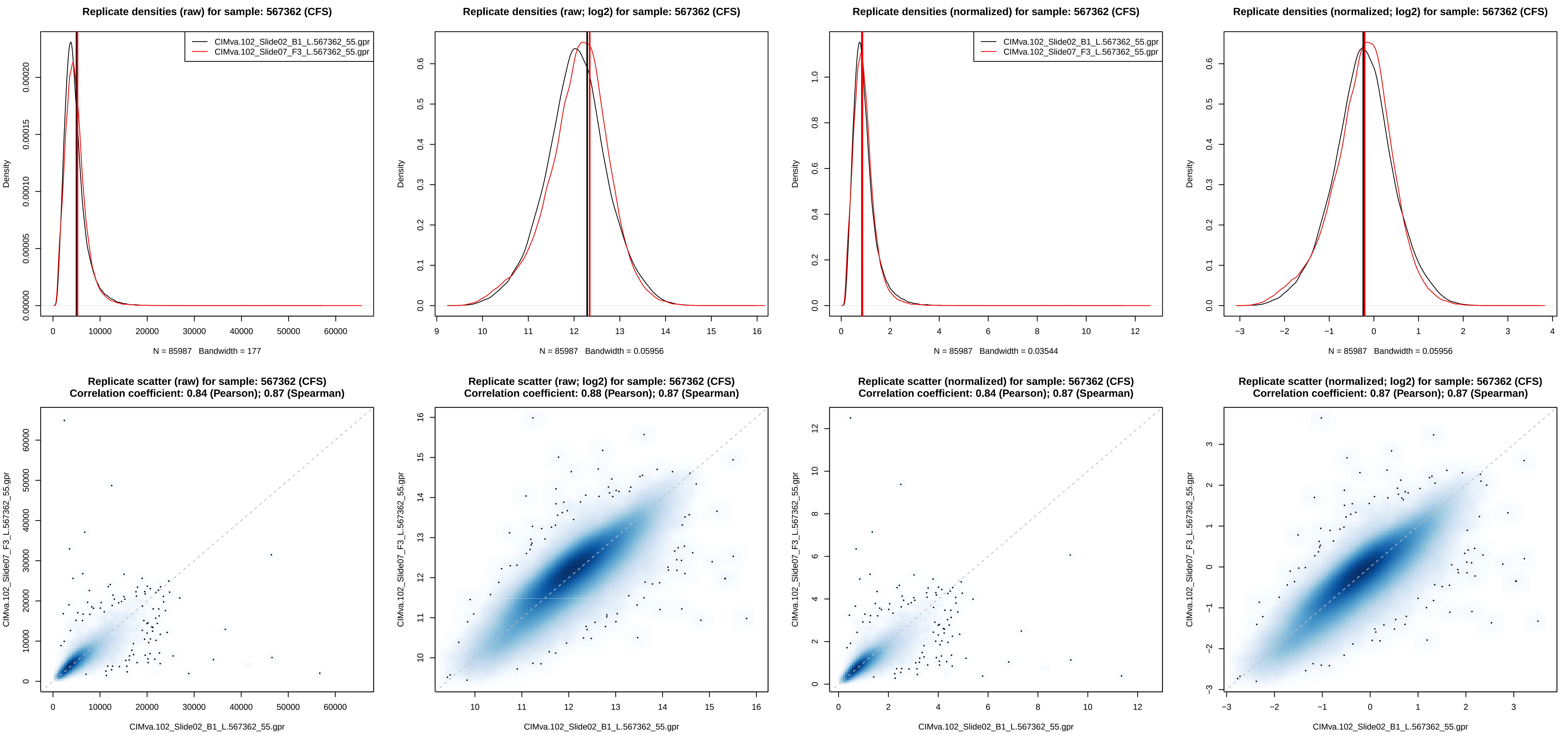
Replicate densities (raw) for sample: 567362 (CFS)
CIMva.102_Slide02_B1_L.567362_55.gpr
CIMva.102_Slide07_F3_L.567362_55.gpr
Density
N = 85987 Bandwidth = 177
Replicate densities (raw; log2) for sample: 567362 (CFS)
Density
N = 85987 Bandwidth = 0.05956
Replicate densities (normalized) for sample: 567362 (CFS)
CIMva.102_Slide02_B1_L.567362_55.gpr
CIMva.102_Slide07_F3_L.567362_55.gpr
Density
N = 85987 Bandwidth = 0.03544
Replicate densities (normalized; log2) for sample: 567362 (CFS)
Density
N = 85987 Bandwidth = 0.05956
Replicate scatter (raw) for sample: 567362 (CFS)
Correlation coefficient: 0.84 (Pearson); 0.87 (Spearman)
CIMva.102_Slide07_F3_L.567362_55.gpr
CIMva.102_Slide02_B1_L.567362_55.gpr
Replicate scatter (raw; log2) for sample: 567362 (CFS)
Correlation coefficient: 0.88 (Pearson); 0.87 (Spearman)
CIMva.102_Slide07_F3_L.567362_55.gpr
CIMva.102_Slide02_B1_L.567362_55.gpr
Replicate scatter (normalized) for sample: 567362 (CFS)
Correlation coefficient: 0.84 (Pearson); 0.87 (Spearman)
CIMva.102_Slide07_F3_L.567362_55.gpr
CIMva.102_Slide02_B1_L.567362_55.gpr
Replicate scatter (normalized; log2) for sample: 567362 (CFS)
Correlation coefficient: 0.87 (Pearson); 0.87 (Spearman)
CIMva.102_Slide07_F3_L.567362_55.gpr
CIMva.102_Slide02_B1_L.567362_55.gpr

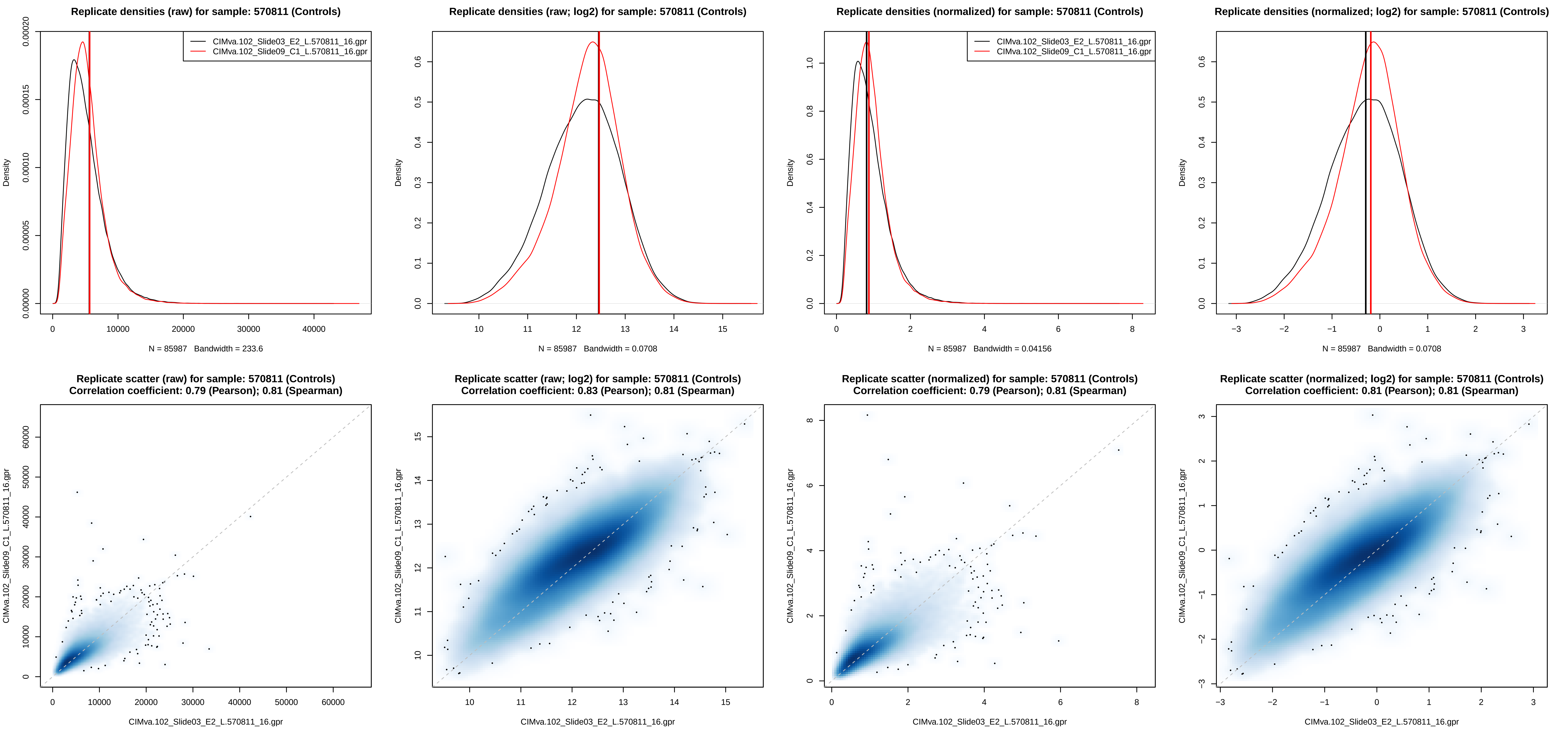
Replicate densities (raw) for sample: 570811 (Controls)
CIMva.102_Slide03_E2_L.570811_16.gpr
CIMva.102_Slide09_C1_L.570811_16.gpr
Density
N = 85987 Bandwidth = 233.6
Replicate densities (raw; log2) for sample: 570811 (Controls)
Density
N = 85987 Bandwidth = 0.0708
Replicate densities (normalized) for sample: 570811 (Controls)
CIMva.102_Slide03_E2_L.570811_16.gpr
CIMva.102_Slide09_C1_L.570811_16.gpr
Density
N = 85987 Bandwidth = 0.04156
Replicate densities (normalized; log2) for sample: 570811 (Controls)
Density
N = 85987 Bandwidth = 0.0708
Replicate scatter (raw) for sample: 570811 (Controls)
Correlation coefficient: 0.79 (Pearson); 0.81 (Spearman)
CIMva.102_Slide09_C1_L.570811_16.gpr
CIMva.102_Slide03_E2_L.570811_16.gpr
Replicate scatter (raw; log2) for sample: 570811 (Controls)
Correlation coefficient: 0.83 (Pearson); 0.81 (Spearman)
CIMva.102_Slide09_C1_L.570811_16.gpr
CIMva.102_Slide03_E2_L.570811_16.gpr
Replicate scatter (normalized) for sample: 570811 (Controls)
Correlation coefficient: 0.79 (Pearson); 0.81 (Spearman)
CIMva.102_Slide09_C1_L.570811_16.gpr
CIMva.102_Slide03_E2_L.570811_16.gpr
Replicate scatter (normalized; log2) for sample: 570811 (Controls)
Correlation coefficient: 0.81 (Pearson); 0.81 (Spearman)
CIMva.102_Slide09_C1_L.570811_16.gpr
CIMva.102_Slide03_E2_L.570811_16.gpr

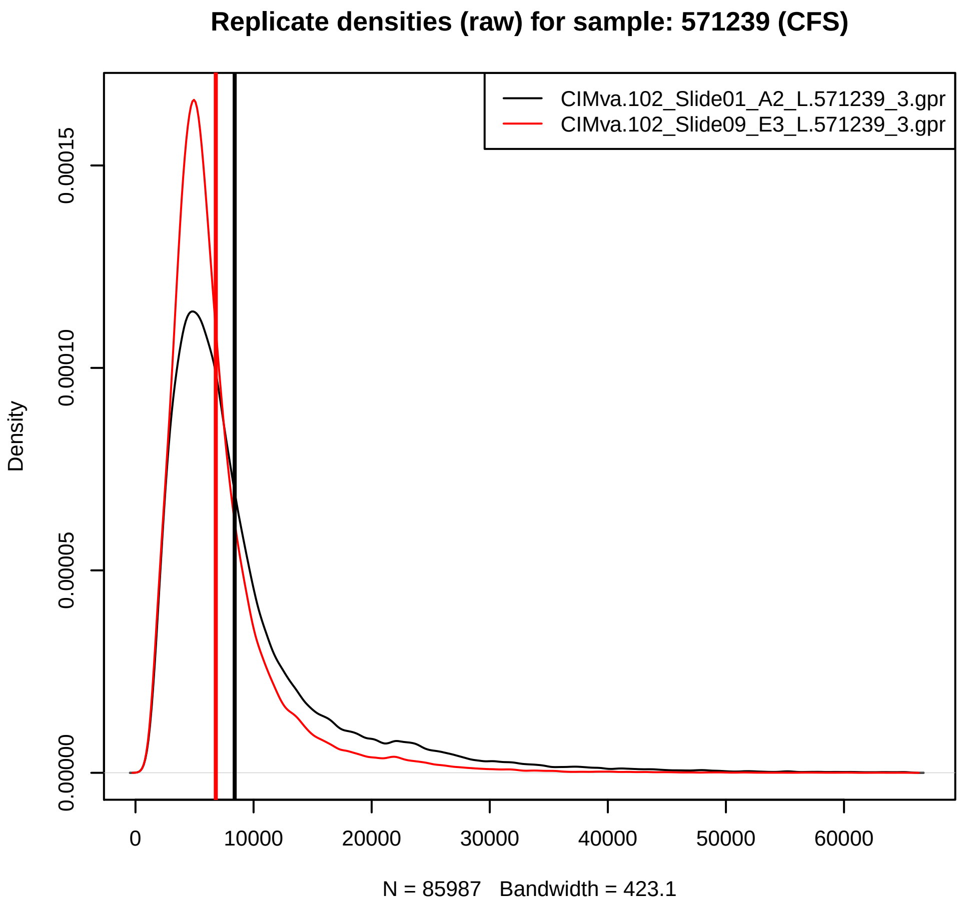
Replicate densities (raw) for sample: 571239 (CFS)
CIMva.102_Slide01_A2_L.571239_3.gpr
CIMva.102_Slide09_E3_L.571239_3.gpr
Density
N = 85987 Bandwidth = 423.1

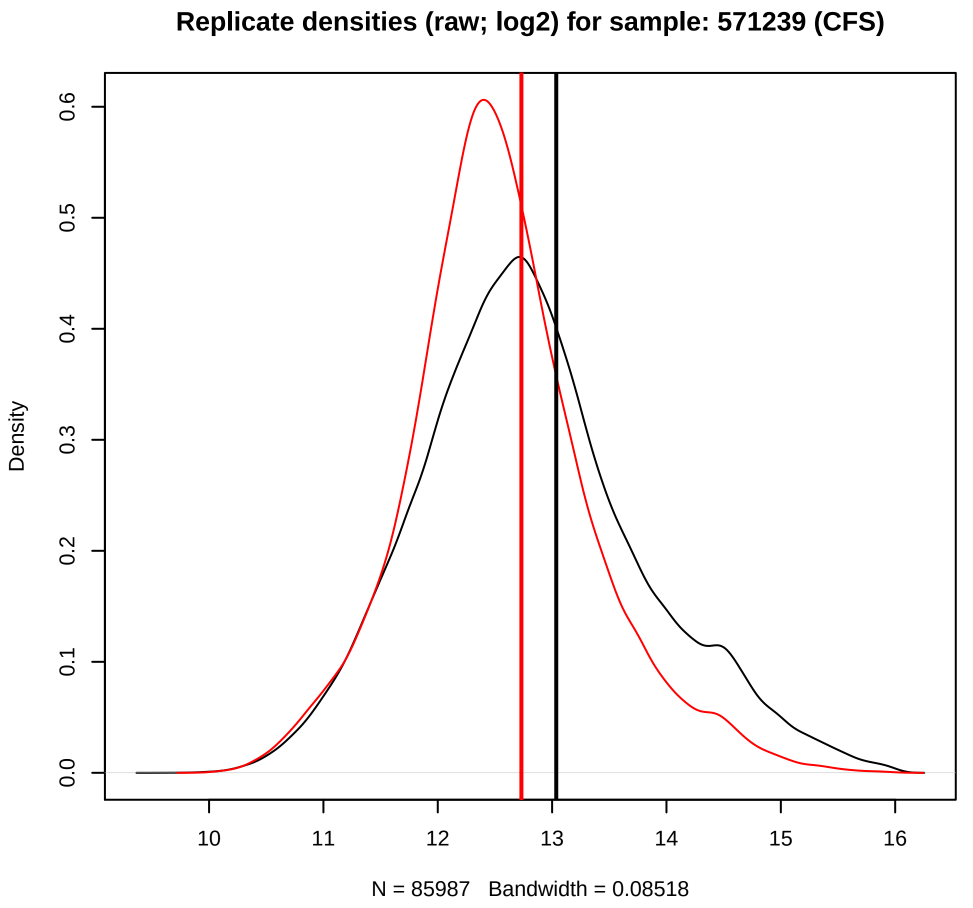
Replicate densities (raw; log2) for sample: 571239 (CFS)
Density
N = 85987 Bandwidth = 0.08518

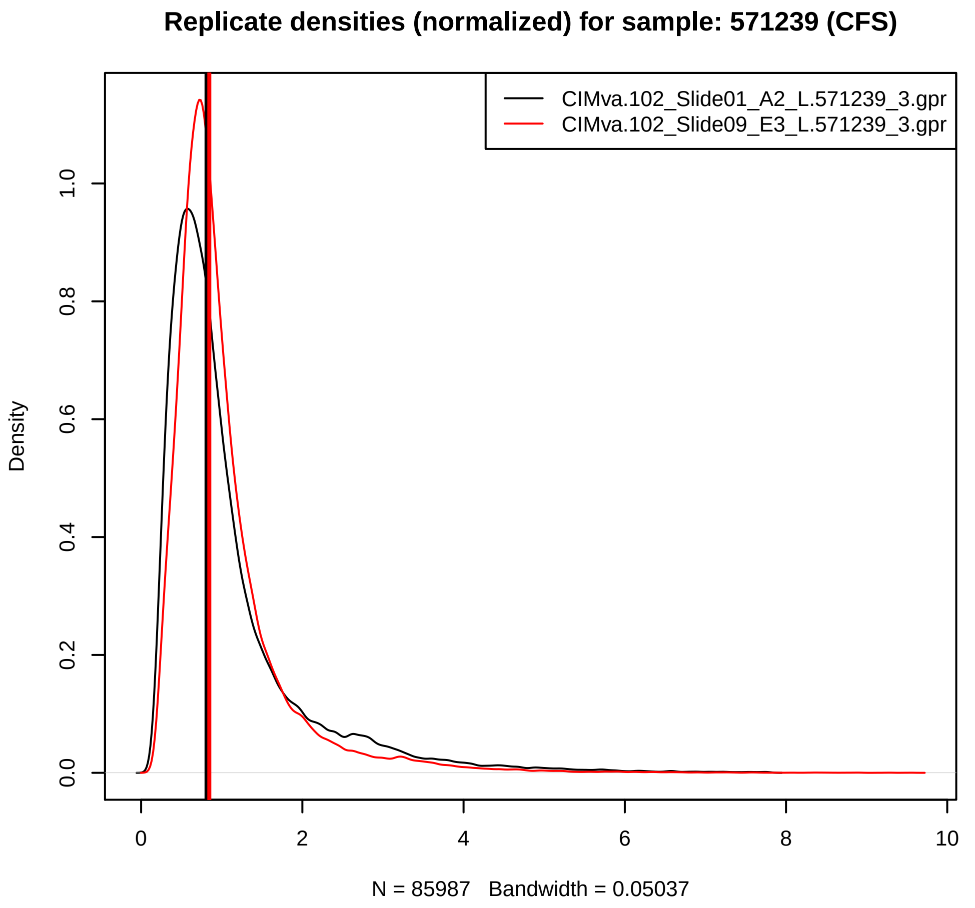
Replicate densities (normalized) for sample: 571239 (CFS)
CIMva.102_Slide01_A2_L.571239_3.gpr
CIMva.102_Slide09_E3_L.571239_3.gpr
Density
N = 85987 Bandwidth = 0.05037

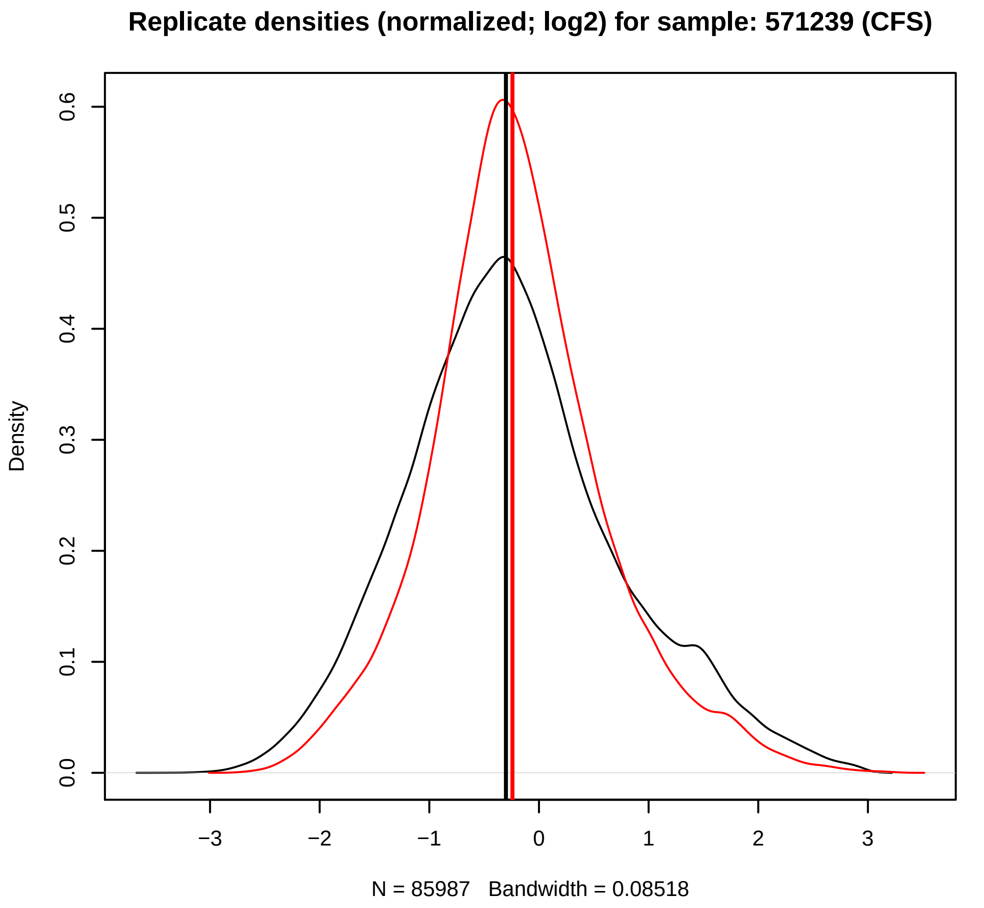
Replicate densities (normalized; log2) for sample: 571239 (CFS)
Density
N = 85987 Bandwidth = 0.08518

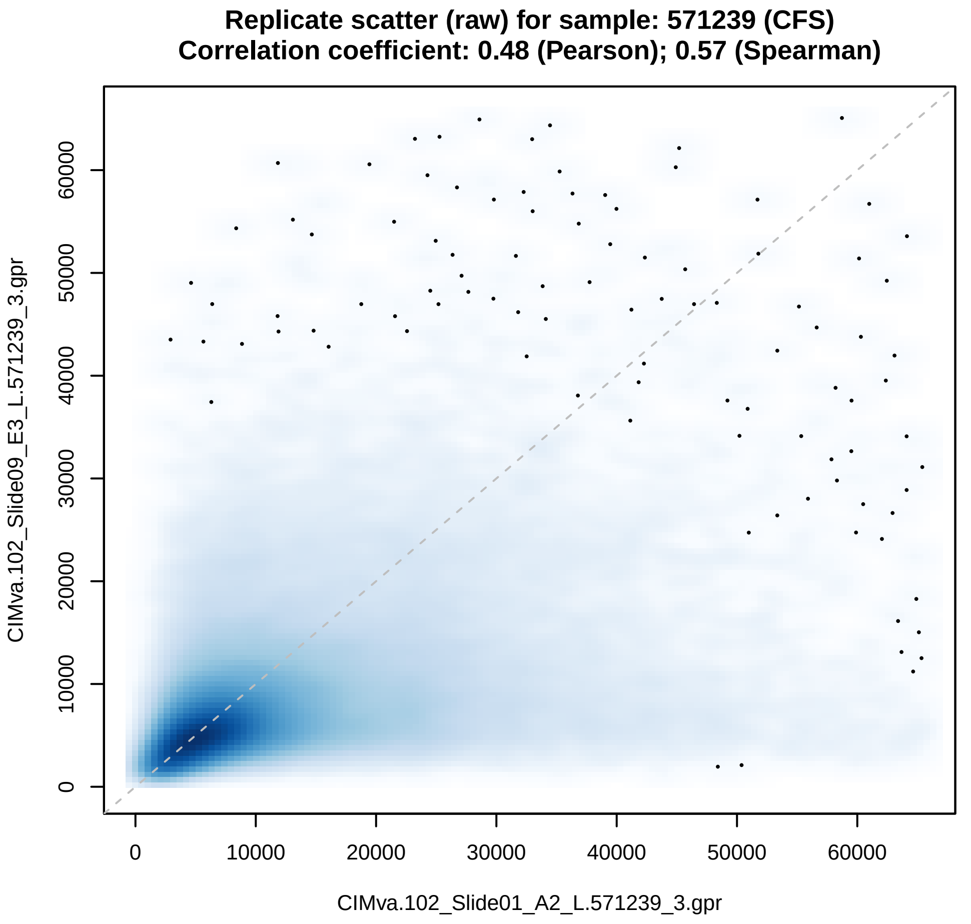
Replicate scatter (raw) for sample: 571239 (CFS)
Correlation coefficient: 0.48 (Pearson); 0.57 (Spearman)
CIMva.102_Slide09_E3_L.571239_3.gpr
CIMva.102_Slide01_A2_L.571239_3.gpr

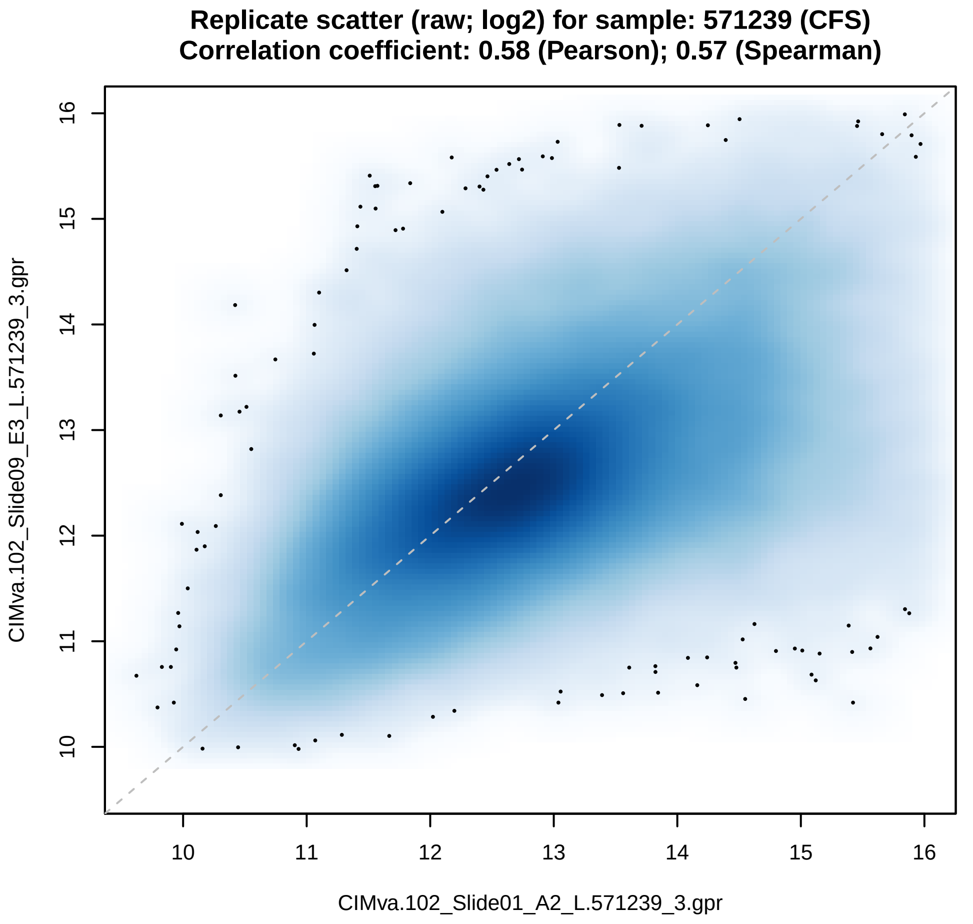
Replicate scatter (raw; log2) for sample: 571239 (CFS)
Correlation coefficient: 0.58 (Pearson); 0.57 (Spearman)
CIMva.102_Slide09_E3_L.571239_3.gpr
CIMva.102_Slide01_A2_L.571239_3.gpr

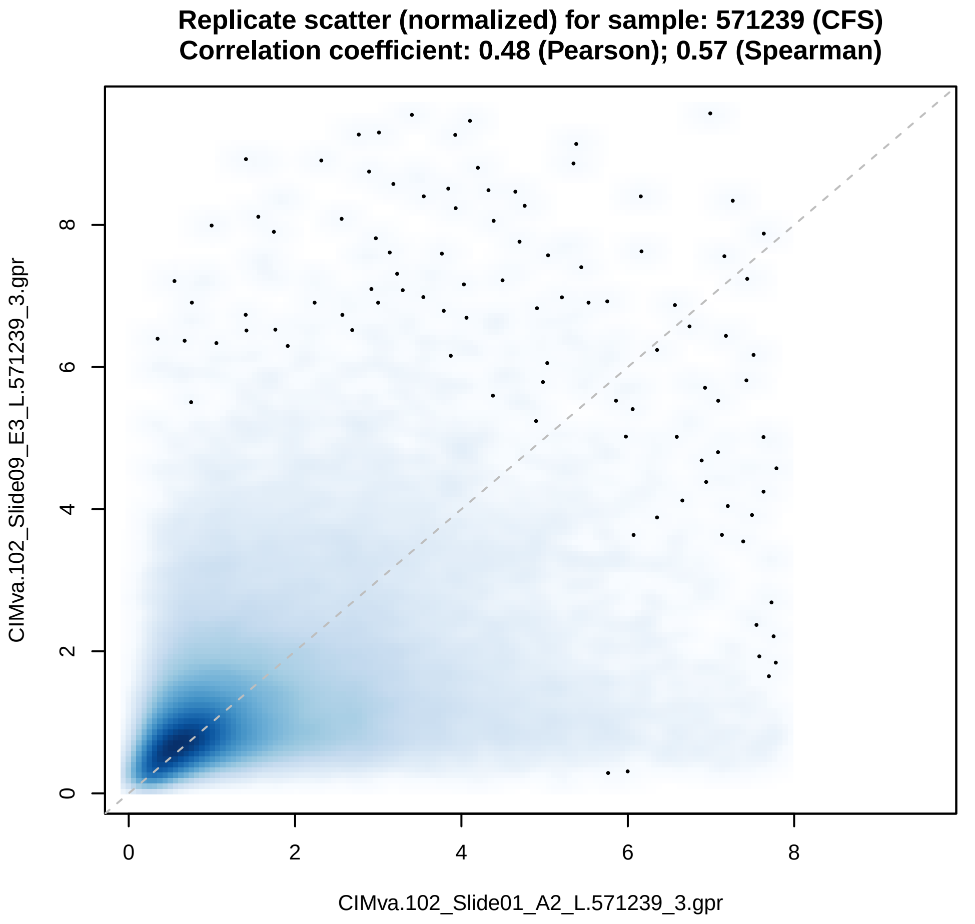
Replicate scatter (normalized) for sample: 571239 (CFS)
Correlation coefficient: 0.48 (Pearson); 0.57 (Spearman)
CIMva.102_Slide09_E3_L.571239_3.gpr
CIMva.102_Slide01_A2_L.571239_3.gpr

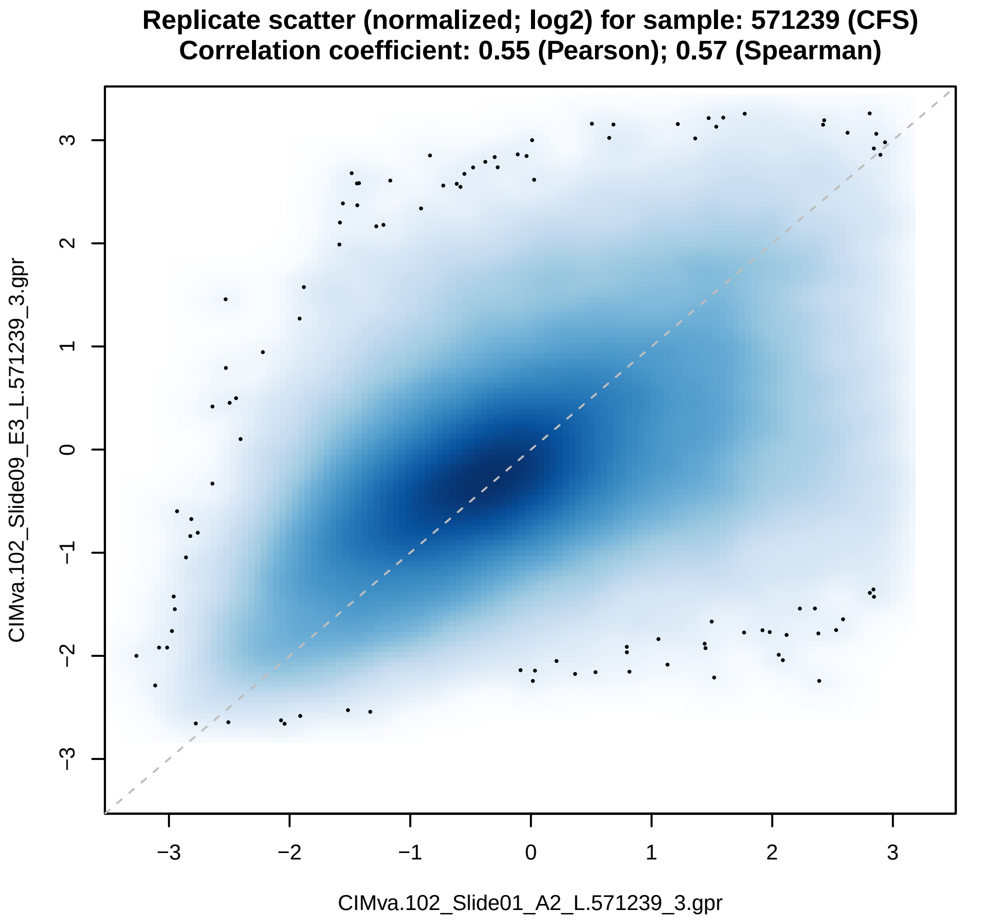
Replicate scatter (normalized; log2) for sample: 571239 (CFS)
Correlation coefficient: 0.55 (Pearson); 0.57 (Spearman)
CIMva.102_Slide09_E3_L.571239_3.gpr
CIMva.102_Slide01_A2_L.571239_3.gpr

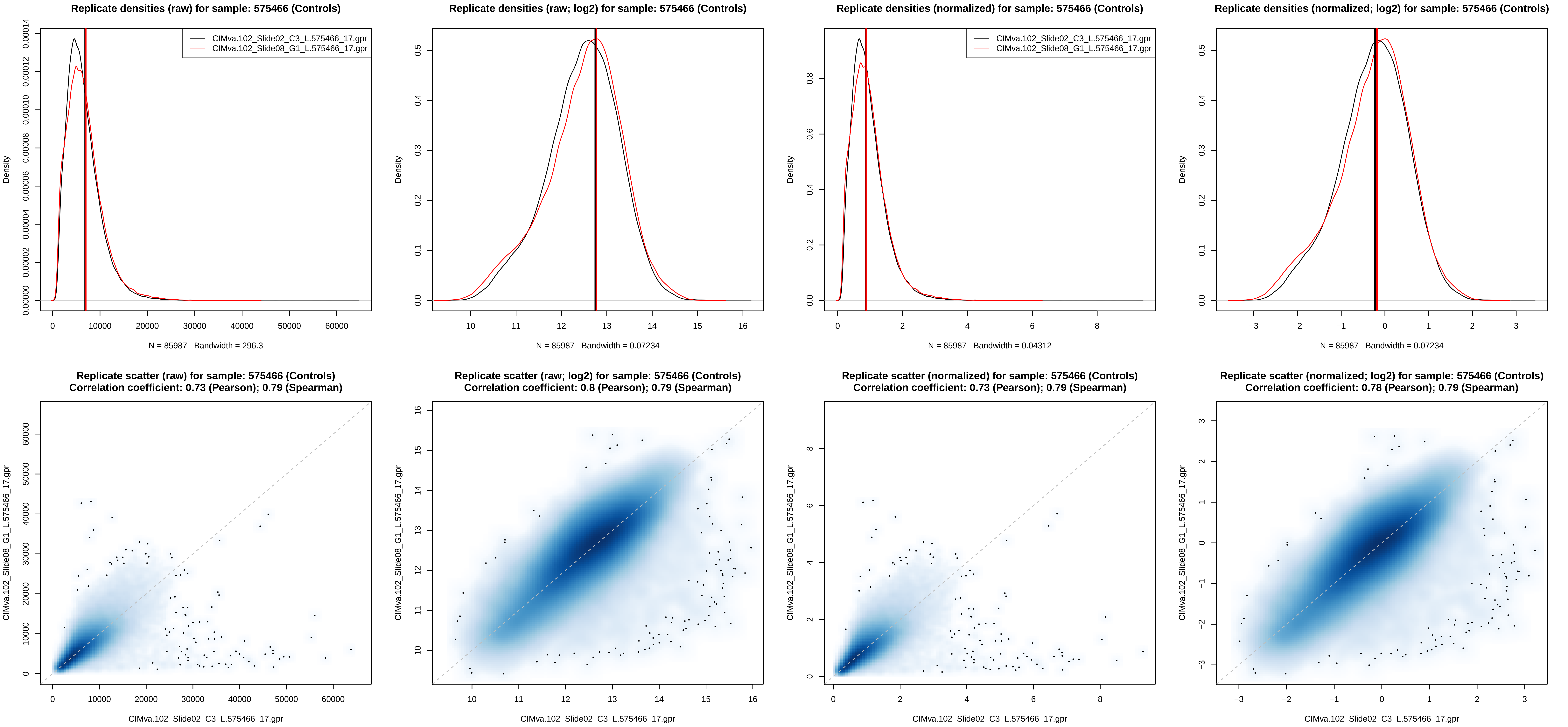
Replicate densities (raw) for sample: 575466 (Controls)
CIMva.102_Slide02_C3_L.575466_17.gpr
CIMva.102_Slide08_G1_L.575466_17.gpr
Density
N = 85987 Bandwidth = 296.3
Replicate densities (raw; log2) for sample: 575466 (Controls)
Density
N = 85987 Bandwidth = 0.07234
Replicate densities (normalized) for sample: 575466 (Controls)
CIMva.102_Slide02_C3_L.575466_17.gpr
CIMva.102_Slide08_G1_L.575466_17.gpr
Density
N = 85987 Bandwidth = 0.04312
Replicate densities (normalized; log2) for sample: 575466 (Controls)
Density
N = 85987 Bandwidth = 0.07234
Replicate scatter (raw) for sample: 575466 (Controls)
Correlation coefficient: 0.73 (Pearson); 0.79 (Spearman)
CIMva.102_Slide08_G1_L.575466_17.gpr
CIMva.102_Slide02_C3_L.575466_17.gpr
Replicate scatter (raw; log2) for sample: 575466 (Controls)
Correlation coefficient: 0.8 (Pearson); 0.79 (Spearman)
CIMva.102_Slide08_G1_L.575466_17.gpr
CIMva.102_Slide02_C3_L.575466_17.gpr
Replicate scatter (normalized) for sample: 575466 (Controls)
Correlation coefficient: 0.73 (Pearson); 0.79 (Spearman)
CIMva.102_Slide08_G1_L.575466_17.gpr
CIMva.102_Slide02_C3_L.575466_17.gpr
Replicate scatter (normalized; log2) for sample: 575466 (Controls)
Correlation coefficient: 0.78 (Pearson); 0.79 (Spearman)
CIMva.102_Slide08_G1_L.575466_17.gpr
CIMva.102_Slide02_C3_L.575466_17.gpr

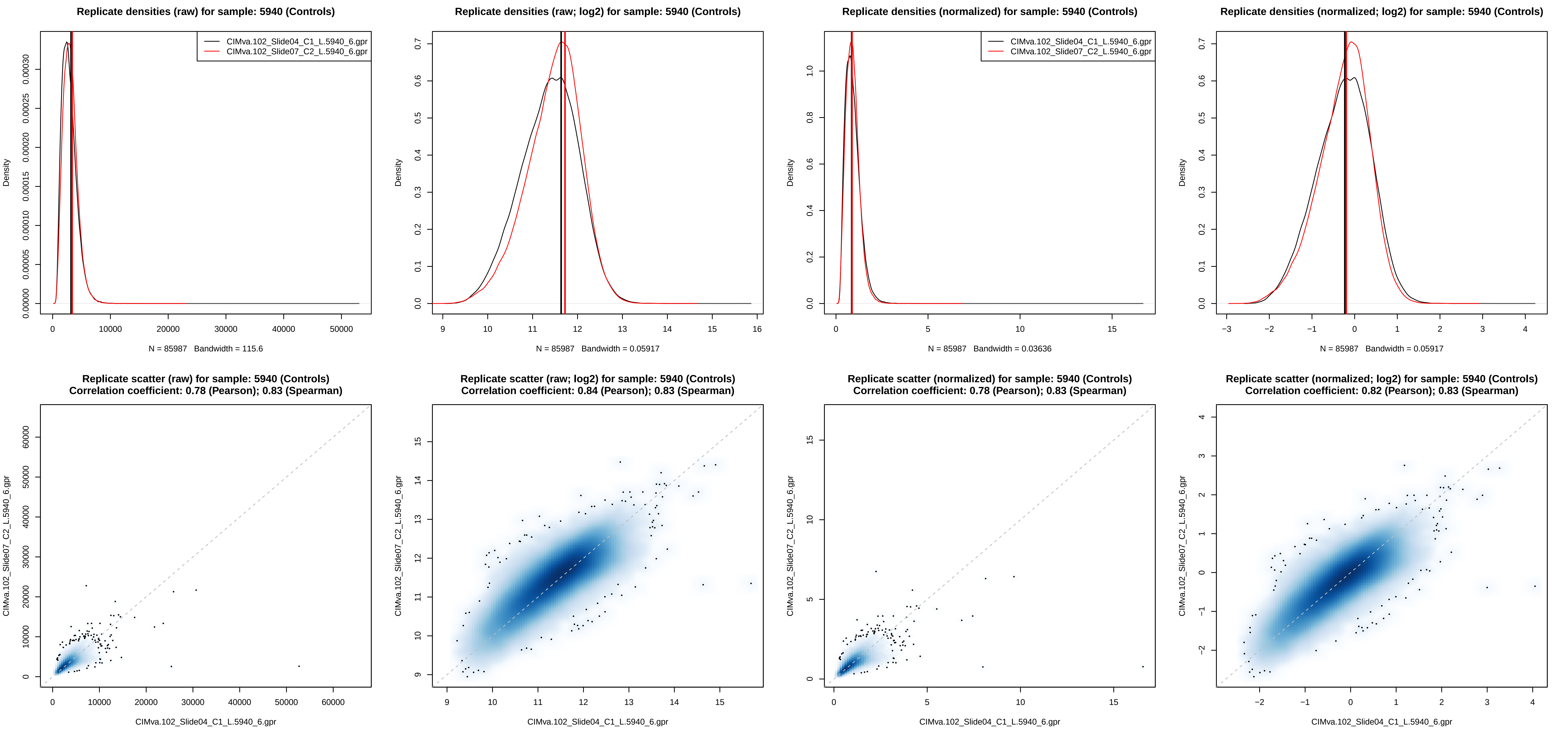

Replicate densities (raw) for sample: 5940 (Controls)
CIMva.102_Slide04_C1_L.5940_6.gpr
CIMva.102_Slide07_C2_L.5940_6.gpr
Density
N = 85987 Bandwidth = 115.6
Replicate densities (raw; log2) for sample: 5940 (Controls)
Density
N = 85987 Bandwidth = 0.05917
Replicate densities (normalized) for sample: 5940 (Controls)
CIMva.102_Slide04_C1_L.5940_6.gpr
CIMva.102_Slide07_C2_L.5940_6.gpr
Density
N = 85987 Bandwidth = 0.03636
Replicate densities (normalized; log2) for sample: 5940 (Controls)
Density
N = 85987 Bandwidth = 0.05917
Replicate scatter (raw) for sample: 5940 (Controls)
Correlation coefficient: 0.78 (Pearson); 0.83 (Spearman)
CIMva.102_Slide07_C2_L.5940_6.gpr
CIMva.102_Slide04_C1_L.5940_6.gpr
Replicate scatter (raw; log2) for sample: 5940 (Controls)
Correlation coefficient: 0.84 (Pearson); 0.83 (Spearman)
CIMva.102_Slide07_C2_L.5940_6.gpr
CIMva.102_Slide04_C1_L.5940_6.gpr
Replicate scatter (normalized) for sample: 5940 (Controls)
Correlation coefficient: 0.78 (Pearson); 0.83 (Spearman)
CIMva.102_Slide07_C2_L.5940_6.gpr
CIMva.102_Slide04_C1_L.5940_6.gpr
Replicate scatter (normalized; log2) for sample: 5940 (Controls)
Correlation coefficient: 0.82 (Pearson); 0.83 (Spearman)
CIMva.102_Slide07_C2_L.5940_6.gpr
CIMva.102_Slide04_C1_L.5940_6.gpr

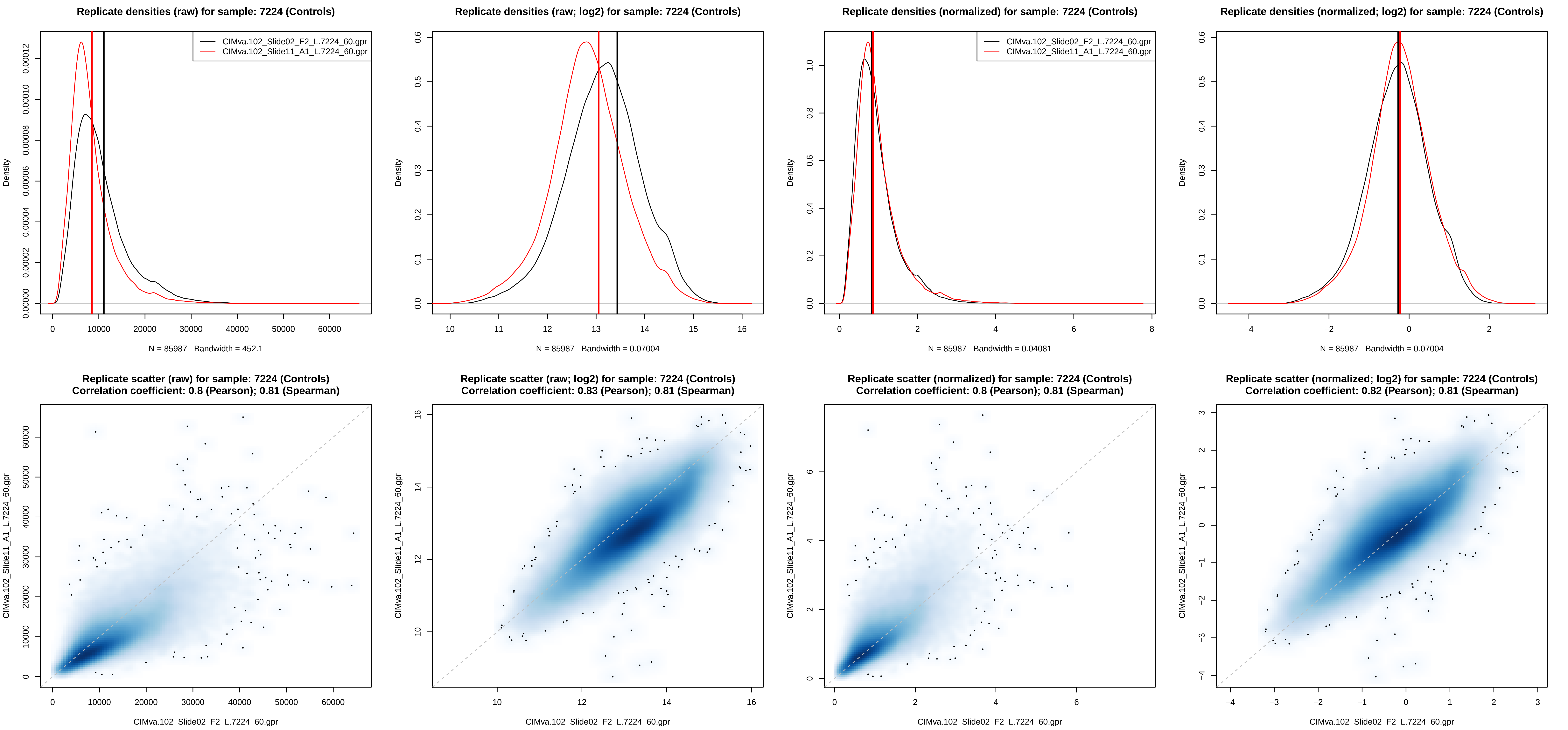
Replicate densities (raw) for sample: 7224 (Controls)
CIMva.102_Slide02_F2_L.7224_60.gpr
CIMva.102_Slide11_A1_L.7224_60.gpr
Density
N = 85987 Bandwidth = 452.1
Replicate densities (raw; log2) for sample: 7224 (Controls)
Density
N = 85987 Bandwidth = 0.07004
Replicate densities (normalized) for sample: 7224 (Controls)
CIMva.102_Slide02_F2_L.7224_60.gpr
CIMva.102_Slide11_A1_L.7224_60.gpr
Density
N = 85987 Bandwidth = 0.04081
Replicate densities (normalized; log2) for sample: 7224 (Controls)
Density
N = 85987 Bandwidth = 0.07004
Replicate scatter (raw) for sample: 7224 (Controls)
Correlation coefficient: 0.8 (Pearson); 0.81 (Spearman)
CIMva.102_Slide11_A1_L.7224_60.gpr
CIMva.102_Slide02_F2_L.7224_60.gpr
Replicate scatter (raw; log2) for sample: 7224 (Controls)
Correlation coefficient: 0.83 (Pearson); 0.81 (Spearman)
CIMva.102_Slide11_A1_L.7224_60.gpr
CIMva.102_Slide02_F2_L.7224_60.gpr
Replicate scatter (normalized) for sample: 7224 (Controls)
Correlation coefficient: 0.8 (Pearson); 0.81 (Spearman)
CIMva.102_Slide11_A1_L.7224_60.gpr
CIMva.102_Slide02_F2_L.7224_60.gpr
Replicate scatter (normalized; log2) for sample: 7224 (Controls)
Correlation coefficient: 0.82 (Pearson); 0.81 (Spearman)
CIMva.102_Slide11_A1_L.7224_60.gpr
CIMva.102_Slide02_F2_L.7224_60.gpr

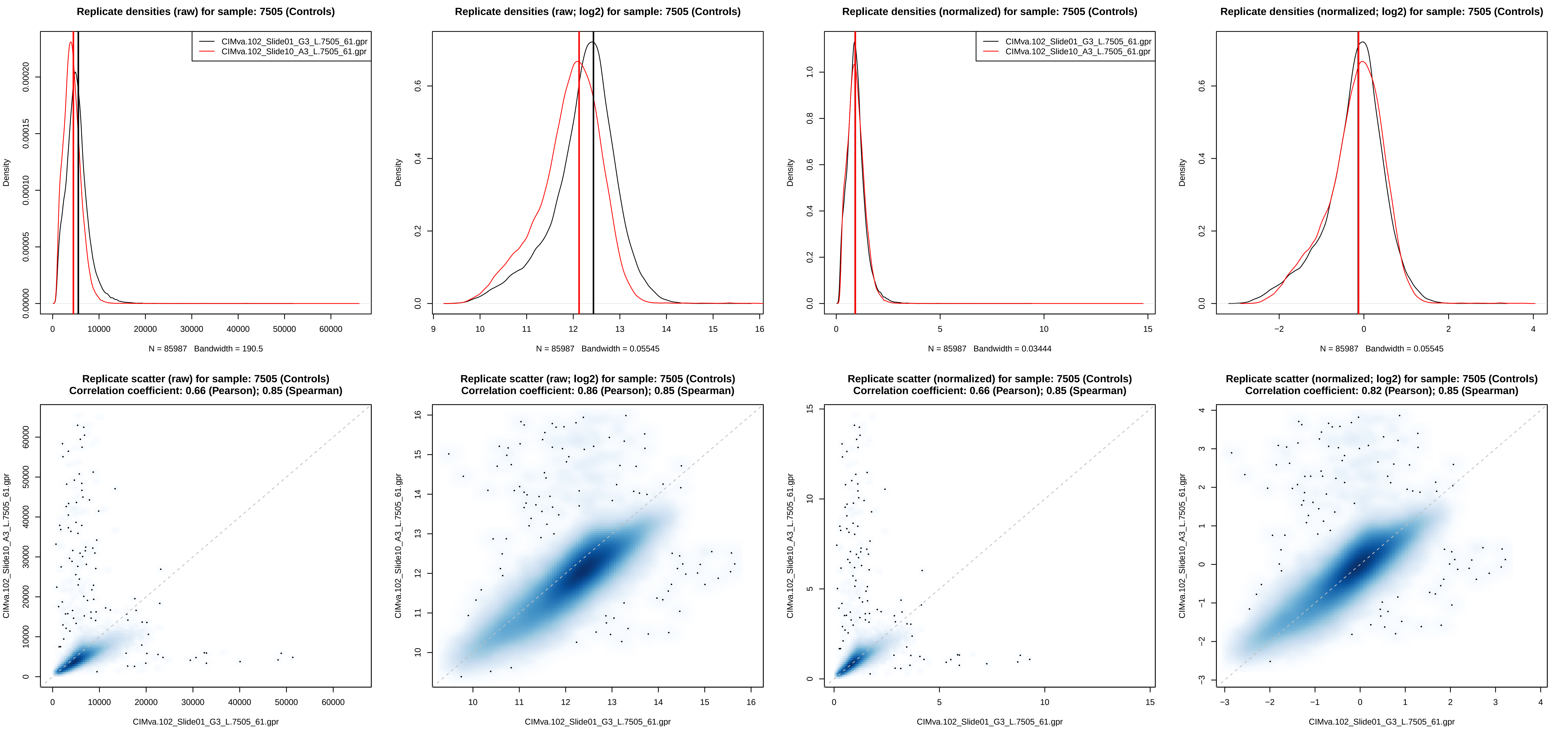
Replicate densities (raw) for sample: 7505 (Controls)
CIMva.102_Slide01_G3_L.7505_61.gpr
CIMva.102_Slide10_A3_L.7505_61.gpr
Density
N = 85987 Bandwidth = 190.5
Replicate densities (raw; log2) for sample: 7505 (Controls)
Density
N = 85987 Bandwidth = 0.05545
Replicate densities (normalized) for sample: 7505 (Controls)
CIMva.102_Slide01_G3_L.7505_61.gpr
CIMva.102_Slide10_A3_L.7505_61.gpr
Density
N = 85987 Bandwidth = 0.03444
Replicate densities (normalized; log2) for sample: 7505 (Controls)
Density
N = 85987 Bandwidth = 0.05545
Replicate scatter (raw) for sample: 7505 (Controls)
Correlation coefficient: 0.66 (Pearson); 0.85 (Spearman)
CIMva.102_Slide10_A3_L.7505_61.gpr
CIMva.102_Slide01_G3_L.7505_61.gpr
Replicate scatter (raw; log2) for sample: 7505 (Controls)
Correlation coefficient: 0.86 (Pearson); 0.85 (Spearman)
CIMva.102_Slide10_A3_L.7505_61.gpr
CIMva.102_Slide01_G3_L.7505_61.gpr
Replicate scatter (normalized) for sample: 7505 (Controls)
Correlation coefficient: 0.66 (Pearson); 0.85 (Spearman)
CIMva.102_Slide10_A3_L.7505_61.gpr
CIMva.102_Slide01_G3_L.7505_61.gpr
Replicate scatter (normalized; log2) for sample: 7505 (Controls)
Correlation coefficient: 0.82 (Pearson); 0.85 (Spearman)
CIMva.102_Slide10_A3_L.7505_61.gpr
CIMva.102_Slide01_G3_L.7505_61.gpr

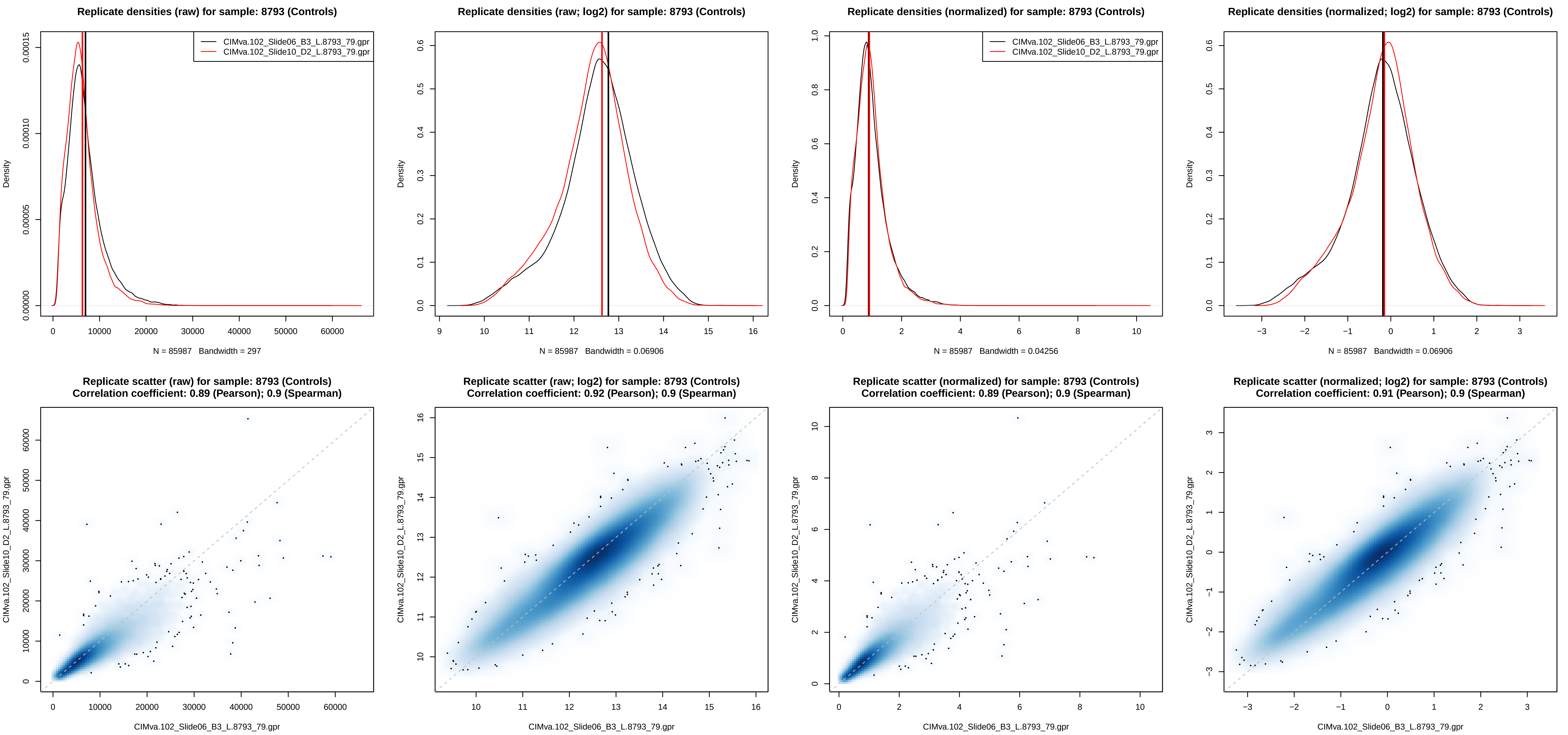

Replicate densities (raw) for sample: 8793 (Controls)
CIMva.102_Slide06_B3_L.8793_79.gpr
CIMva.102_Slide10_D2_L.8793_79.gpr
Density
N = 85987 Bandwidth = 297
Replicate densities (raw; log2) for sample: 8793 (Controls)
Density
N = 85987 Bandwidth = 0.06906
Replicate densities (normalized) for sample: 8793 (Controls)
CIMva.102_Slide06_B3_L.8793_79.gpr
CIMva.102_Slide10_D2_L.8793_79.gpr
Density
N = 85987 Bandwidth = 0.04256
Replicate densities (normalized; log2) for sample: 8793 (Controls)
Density
N = 85987 Bandwidth = 0.06906
Replicate scatter (raw) for sample: 8793 (Controls)
Correlation coefficient: 0.89 (Pearson); 0.9 (Spearman)
CIMva.102_Slide10_D2_L.8793_79.gpr
CIMva.102_Slide06_B3_L.8793_79.gpr
Replicate scatter (raw; log2) for sample: 8793 (Controls)
Correlation coefficient: 0.92 (Pearson); 0.9 (Spearman)
CIMva.102_Slide10_D2_L.8793_79.gpr
CIMva.102_Slide06_B3_L.8793_79.gpr
Replicate scatter (normalized) for sample: 8793 (Controls)
Correlation coefficient: 0.89 (Pearson); 0.9 (Spearman)
CIMva.102_Slide10_D2_L.8793_79.gpr
CIMva.102_Slide06_B3_L.8793_79.gpr
Replicate scatter (normalized; log2) for sample: 8793 (Controls)
Correlation coefficient: 0.91 (Pearson); 0.9 (Spearman)
CIMva.102_Slide10_D2_L.8793_79.gpr
CIMva.102_Slide06_B3_L.8793_79.gpr

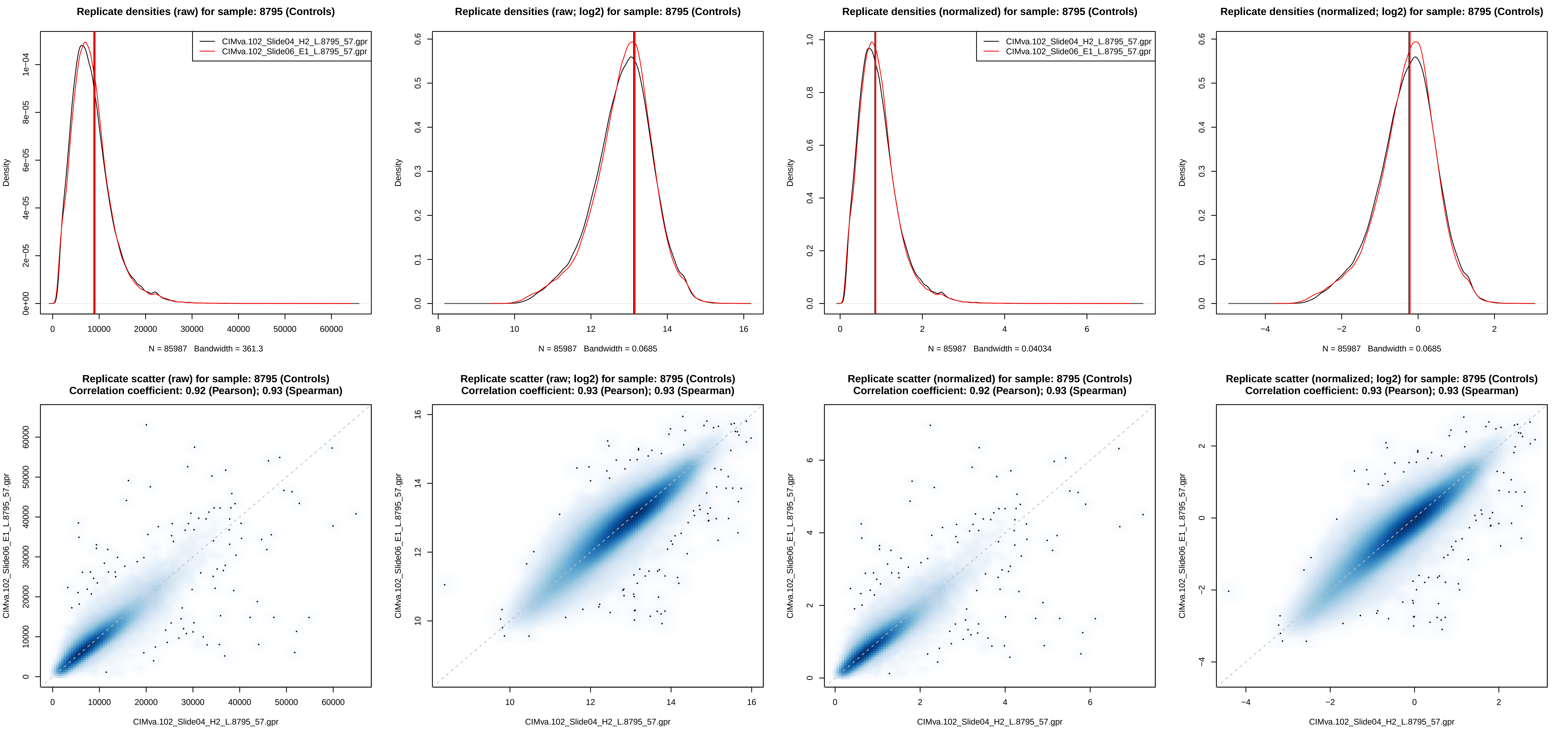
Replicate densities (raw) for sample: 8795 (Controls)
CIMva.102_Slide04_H2_L.8795_57.gpr
CIMva.102_Slide06_E1_L.8795_57.gpr
Density
N = 85987 Bandwidth = 361.3
Replicate densities (raw; log2) for sample: 8795 (Controls)
Density
N = 85987 Bandwidth = 0.0685
Replicate densities (normalized) for sample: 8795 (Controls)
CIMva.102_Slide04_H2_L.8795_57.gpr
CIMva.102_Slide06_E1_L.8795_57.gpr
Density
N = 85987 Bandwidth = 0.04034
Replicate densities (normalized; log2) for sample: 8795 (Controls)
Density
N = 85987 Bandwidth = 0.0685
Replicate scatter (raw) for sample: 8795 (Controls)
Correlation coefficient: 0.92 (Pearson); 0.93 (Spearman)
CIMva.102_Slide06_E1_L.8795_57.gpr
CIMva.102_Slide04_H2_L.8795_57.gpr
Replicate scatter (raw; log2) for sample: 8795 (Controls)
Correlation coefficient: 0.93 (Pearson); 0.93 (Spearman)
CIMva.102_Slide06_E1_L.8795_57.gpr
CIMva.102_Slide04_H2_L.8795_57.gpr
Replicate scatter (normalized) for sample: 8795 (Controls)
Correlation coefficient: 0.92 (Pearson); 0.93 (Spearman)
CIMva.102_Slide06_E1_L.8795_57.gpr
CIMva.102_Slide04_H2_L.8795_57.gpr
Replicate scatter (normalized; log2) for sample: 8795 (Controls)
Correlation coefficient: 0.93 (Pearson); 0.93 (Spearman)
CIMva.102_Slide06_E1_L.8795_57.gpr
CIMva.102_Slide04_H2_L.8795_57.gpr
